# Supplementary material for: Coherent poly propagation materials with 3-dimensional photonic control over visible light
Source: PLoS One. 2019 Oct 17;14(10):e0223715. doi: 10.1371/journal.pone.0223715 (PMC6797174; doi:10.1371/journal.pone.0223715)
Supplement: S1 Table — Spreadsheet generated graph at bottom of table. (PDF) [file pone.0223715.s010.pdf]

Wavelength Scan Report - Specimen 1

Company	Complete Consulting Services LLC		
Operator	Dr. Michelle R. Stem	Test Time	2/13/2017 1:21
Document No.	WLS1702130001_1	Print Date	9/6/2019 20:38
Start wavelength	1100nm	Number of data	7801
End wavelength	320nm	K1	0.99
Unit		K0	0
Interval	0.1nm	K2	0
Calculate the Conc.	No	K3	0
Modification time	2/13/2017 1:21	Access time	2/13/2017 1:21
Remark	S02a011 - Specimen 1		

Scan Data

No.	Wavelength	Abs1	Abs2	Abs3	Abs4	Visible
1	1100	1.4255	0.2302	0.2254	0.7577	0
2	1099.9	1.4252	0.2291	0.2252	0.7578	0
3	1099.8	1.4241	0.2285	0.2252	0.7577	0
4	1099.7	1.4246	0.2277	0.2251	0.7579	0
5	1099.6	1.4241	0.2266	0.2248	0.7574	0
6	1099.5	1.4236	0.2255	0.2242	0.7571	0
7	1099.4	1.4236	0.2248	0.2244	0.7573	0
8	1099.3	1.4234	0.224	0.224	0.7571	0
9	1099.2	1.4227	0.2228	0.2238	0.757	0
10	1099.1	1.4228	0.222	0.2235	0.7566	0
11	1099	1.4229	0.2216	0.2233	0.7572	0
12	1098.9	1.4226	0.2207	0.2229	0.7572	0
13	1098.8	1.4225	0.2202	0.2224	0.7571	0
14	1098.7	1.4222	0.2194	0.2216	0.7569	0
15	1098.6	1.4218	0.2192	0.2211	0.757	0
16	1098.5	1.4224	0.219	0.2209	0.7573	0
17	1098.4	1.4224	0.2187	0.2203	0.7572	0
18	1098.3	1.4226	0.2185	0.2203	0.7576	0
19	1098.2	1.4229	0.2178	0.2198	0.7575	0
20	1098.1	1.4224	0.2172	0.2193	0.7576	0
21	1098	1.4225	0.2171	0.2192	0.7576	0
22	1097.9	1.4229	0.2168	0.219	0.758	0
23	1097.8	1.4233	0.2165	0.2188	0.7579	0
24	1097.7	1.4226	0.216	0.2186	0.7575	0
25	1097.6	1.423	0.2158	0.2186	0.7577	0
26	1097.5	1.4228	0.2156	0.2184	0.7575	0
27	1097.4	1.4223	0.2151	0.2178	0.7567	0
28	1097.3	1.4223	0.215	0.218	0.7572	0
29	1097.2	1.4224	0.2142	0.2174	0.7569	0

30	1097.1	1.4225	0.2138	0.2172	0.7567	0
31	1097	1.4226	0.2136	0.2171	0.7569	0
32	1096.9	1.4227	0.2133	0.2173	0.7569	0
33	1096.8	1.423	0.2131	0.2171	0.757	0
34	1096.7	1.4235	0.2131	0.2176	0.7574	0
35	1096.6	1.4242	0.2132	0.2176	0.7582	0
36	1096.5	1.4245	0.2134	0.2176	0.7586	0
37	1096.4	1.4253	0.2134	0.2177	0.7586	0
38	1096.3	1.4256	0.2134	0.2179	0.7592	0
39	1096.2	1.4253	0.2132	0.2181	0.7589	0
40	1096.1	1.4257	0.213	0.2182	0.7592	0
41	1096	1.425	0.2129	0.2183	0.759	0
42	1095.9	1.4252	0.2128	0.2182	0.7586	0
43	1095.8	1.4245	0.2128	0.2182	0.7585	0
44	1095.7	1.4243	0.2127	0.2182	0.7579	0
45	1095.6	1.4241	0.2124	0.2179	0.757	0
46	1095.5	1.4233	0.212	0.2178	0.7562	0
47	1095.4	1.423	0.2114	0.2173	0.7559	0
48	1095.3	1.4224	0.2112	0.2171	0.7552	0
49	1095.2	1.4218	0.2107	0.217	0.7549	0
50	1095.1	1.4221	0.2105	0.2168	0.7549	0
51	1095	1.4213	0.2099	0.2165	0.7543	0
52	1094.9	1.4216	0.2097	0.2164	0.7545	0
53	1094.8	1.4231	0.2098	0.2166	0.7548	0
54	1094.7	1.4234	0.2098	0.2166	0.7556	0
55	1094.6	1.4244	0.2096	0.2165	0.7562	0
56	1094.5	1.4256	0.2104	0.2172	0.7576	0
57	1094.4	1.4274	0.2108	0.2176	0.7593	0
58	1094.3	1.4288	0.2114	0.2182	0.761	0
59	1094.2	1.4312	0.2116	0.2188	0.7628	0
60	1094.1	1.4325	0.2123	0.2194	0.7644	0
61	1094	1.4348	0.2134	0.2203	0.7664	0
62	1093.9	1.4361	0.2135	0.2205	0.7676	0
63	1093.8	1.4377	0.2142	0.2213	0.769	0
64	1093.7	1.4384	0.2147	0.2216	0.7701	0
65	1093.6	1.4391	0.2151	0.222	0.7704	0
66	1093.5	1.4395	0.2149	0.2223	0.7708	0
67	1093.4	1.4393	0.2154	0.2226	0.7711	0
68	1093.3	1.4399	0.2153	0.2226	0.7708	0
69	1093.2	1.439	0.2154	0.2226	0.7703	0
70	1093.1	1.4386	0.2154	0.2225	0.7696	0
71	1093	1.4378	0.2149	0.2223	0.7684	0
72	1092.9	1.4362	0.2143	0.2219	0.767	0

73	1092.8	1.4345	0.2139	0.2213	0.7656	0
74	1092.7	1.4334	0.2132	0.2208	0.7635	0
75	1092.6	1.4315	0.2124	0.2201	0.7614	0
76	1092.5	1.4288	0.2117	0.2195	0.7596	0
77	1092.4	1.4272	0.2107	0.2189	0.7572	0
78	1092.3	1.4252	0.2099	0.2183	0.7551	0
79	1092.2	1.4223	0.209	0.2174	0.7529	0
80	1092.1	1.4201	0.2081	0.2166	0.7504	0
81	1092	1.4177	0.2074	0.216	0.7485	0
82	1091.9	1.4161	0.2063	0.2151	0.7464	0
83	1091.8	1.4137	0.2056	0.2143	0.7441	0
84	1091.7	1.4115	0.2044	0.213	0.742	0
85	1091.6	1.4096	0.2034	0.2126	0.7397	0
86	1091.5	1.4079	0.2027	0.2118	0.738	0
87	1091.4	1.4062	0.2018	0.2112	0.7361	0
88	1091.3	1.4046	0.2009	0.2101	0.7345	0
89	1091.2	1.4033	0.2006	0.21	0.7329	0
90	1091.1	1.4008	0.1993	0.2091	0.7311	0
91	1091	1.3992	0.1987	0.2085	0.7297	0
92	1090.9	1.3985	0.198	0.2077	0.7282	0
93	1090.8	1.397	0.1971	0.2071	0.7271	0
94	1090.7	1.3962	0.1969	0.2068	0.7261	0
95	1090.6	1.3949	0.196	0.2063	0.7247	0
96	1090.5	1.3943	0.1959	0.2059	0.7238	0
97	1090.4	1.3932	0.1954	0.2056	0.7228	0
98	1090.3	1.3921	0.1949	0.2051	0.7219	0
99	1090.2	1.3917	0.1945	0.2046	0.7212	0
100	1090.1	1.3911	0.1943	0.2046	0.7208	0
101	1090	1.3903	0.1943	0.2043	0.7201	0
102	1089.9	1.3893	0.1939	0.2043	0.7194	0
103	1089.8	1.3887	0.1934	0.2037	0.7188	0
104	1089.7	1.3888	0.1931	0.2035	0.7185	0
105	1089.6	1.3889	0.1929	0.2037	0.7183	0
106	1089.5	1.3884	0.1929	0.2034	0.7179	0
107	1089.4	1.3877	0.1925	0.203	0.7172	0
108	1089.3	1.3873	0.1924	0.2029	0.7171	0
109	1089.2	1.387	0.1922	0.2027	0.7169	0
110	1089.1	1.3865	0.1921	0.2025	0.7162	0
111	1089	1.3862	0.1919	0.2026	0.7158	0
112	1088.9	1.3856	0.1914	0.2023	0.7154	0
113	1088.8	1.3854	0.1912	0.2019	0.7151	0
114	1088.7	1.3853	0.1914	0.2023	0.715	0
115	1088.6	1.3852	0.1912	0.2021	0.7148	0

116	1088.5	1.3854	0.1912	0.202	0.7146	0
117	1088.4	1.3848	0.191	0.2019	0.7146	0
118	1088.3	1.3848	0.191	0.2017	0.7145	0
119	1088.2	1.3848	0.1909	0.2017	0.7144	0
120	1088.1	1.385	0.1906	0.2014	0.7143	0
121	1088	1.3851	0.1909	0.2017	0.7143	0
122	1087.9	1.3844	0.1903	0.2012	0.7141	0
123	1087.8	1.3846	0.1907	0.2013	0.7144	0
124	1087.7	1.3852	0.1908	0.2016	0.7143	0
125	1087.6	1.3845	0.1906	0.2014	0.7146	0
126	1087.5	1.3843	0.1906	0.2013	0.7143	0
127	1087.4	1.3843	0.1902	0.2012	0.7142	0
128	1087.3	1.3846	0.1907	0.2014	0.7147	0
129	1087.2	1.3845	0.1905	0.2014	0.7142	0
130	1087.1	1.3852	0.1902	0.2014	0.7143	0
131	1087	1.3848	0.1906	0.2013	0.7144	0
132	1086.9	1.384	0.1907	0.2012	0.7142	0
133	1086.8	1.3845	0.1903	0.2013	0.7144	0
134	1086.7	1.385	0.1905	0.2014	0.7144	0
135	1086.6	1.3849	0.1906	0.2014	0.7143	0
136	1086.5	1.3847	0.1904	0.2013	0.7143	0
137	1086.4	1.3847	0.1901	0.2012	0.7141	0
138	1086.3	1.3849	0.1901	0.2011	0.714	0
139	1086.2	1.3849	0.1903	0.201	0.7141	0
140	1086.1	1.3848	0.19	0.2009	0.7141	0
141	1086	1.3842	0.1901	0.201	0.7141	0
142	1085.9	1.3847	0.1899	0.2012	0.714	0
143	1085.8	1.385	0.1903	0.2011	0.7142	0
144	1085.7	1.3846	0.1904	0.2011	0.7141	0
145	1085.6	1.3849	0.1899	0.201	0.7137	0
146	1085.5	1.385	0.1902	0.2012	0.7139	0
147	1085.4	1.385	0.1899	0.2012	0.7139	0
148	1085.3	1.3854	0.1904	0.2013	0.7139	0
149	1085.2	1.3857	0.1904	0.2013	0.7141	0
150	1085.1	1.3854	0.1901	0.2013	0.7143	0
151	1085	1.3855	0.1902	0.2013	0.7142	0
152	1084.9	1.3853	0.1903	0.2011	0.7142	0
153	1084.8	1.386	0.1902	0.2013	0.7143	0
154	1084.7	1.3859	0.1907	0.2017	0.7147	0
155	1084.6	1.3864	0.1906	0.2016	0.7145	0
156	1084.5	1.3856	0.1904	0.2013	0.7146	0
157	1084.4	1.3864	0.1906	0.2016	0.7147	0
158	1084.3	1.3871	0.1907	0.2016	0.7148	0

159	1084.2	1.3864	0.1905	0.2015	0.7147	0
160	1084.1	1.3864	0.1905	0.2019	0.7147	0
161	1084	1.3866	0.1905	0.2018	0.7149	0
162	1083.9	1.3869	0.1905	0.2015	0.7149	0
163	1083.8	1.3869	0.1906	0.2018	0.7154	0
164	1083.7	1.3876	0.1906	0.202	0.7154	0
165	1083.6	1.3874	0.1908	0.2021	0.7153	0
166	1083.5	1.3878	0.1909	0.2023	0.7157	0
167	1083.4	1.3881	0.191	0.2022	0.7158	0
168	1083.3	1.3879	0.191	0.2021	0.7156	0
169	1083.2	1.3881	0.1909	0.2021	0.7154	0
170	1083.1	1.3882	0.1908	0.2019	0.7154	0
171	1083	1.3881	0.1909	0.2022	0.7157	0
172	1082.9	1.3884	0.1912	0.2019	0.7157	0
173	1082.8	1.3887	0.1909	0.2023	0.7158	0
174	1082.7	1.3888	0.1909	0.202	0.7156	0
175	1082.6	1.3883	0.1908	0.2021	0.716	0
176	1082.5	1.389	0.1912	0.2023	0.7161	0
177	1082.4	1.3889	0.1913	0.2024	0.7163	0
178	1082.3	1.3891	0.1913	0.2024	0.7161	0
179	1082.2	1.3889	0.191	0.2021	0.7158	0
180	1082.1	1.389	0.1909	0.202	0.7158	0
181	1082	1.3891	0.1905	0.202	0.7158	0
182	1081.9	1.3897	0.1911	0.2023	0.7162	0
183	1081.8	1.3894	0.1912	0.2024	0.7161	0
184	1081.7	1.3891	0.1911	0.2021	0.7164	0
185	1081.6	1.3896	0.1913	0.2027	0.7168	0
186	1081.5	1.3899	0.1913	0.2024	0.7168	0
187	1081.4	1.39	0.1914	0.2024	0.7168	0
188	1081.3	1.3902	0.1914	0.2024	0.717	0
189	1081.2	1.3901	0.1915	0.2025	0.7167	0
190	1081.1	1.3901	0.1913	0.2028	0.7166	0
191	1081	1.3902	0.1914	0.2027	0.7169	0
192	1080.9	1.3907	0.1914	0.2028	0.7169	0
193	1080.8	1.3906	0.1916	0.2026	0.7173	0
194	1080.7	1.3907	0.1914	0.2023	0.7173	0
195	1080.6	1.3908	0.1915	0.2024	0.7172	0
196	1080.5	1.3907	0.1913	0.2025	0.717	0
197	1080.4	1.3907	0.1913	0.2024	0.7172	0
198	1080.3	1.3908	0.1912	0.2024	0.717	0
199	1080.2	1.391	0.1914	0.2026	0.7171	0
200	1080.1	1.3917	0.1912	0.2026	0.7173	0
201	1080	1.3911	0.1914	0.2026	0.7172	0

202	1079.9	1.3912	0.1912	0.2024	0.7175	0
203	1079.8	1.3915	0.1917	0.2026	0.7175	0
204	1079.7	1.3915	0.1917	0.2026	0.7175	0
205	1079.6	1.391	0.1916	0.2025	0.7172	0
206	1079.5	1.3911	0.1914	0.2025	0.7173	0
207	1079.4	1.3911	0.1909	0.2023	0.7173	0
208	1079.3	1.3917	0.1914	0.2023	0.7173	0
209	1079.2	1.3915	0.1916	0.2024	0.7174	0
210	1079.1	1.3917	0.1912	0.2025	0.7175	0
211	1079	1.3914	0.1913	0.2025	0.7173	0
212	1078.9	1.3912	0.1915	0.2026	0.7178	0
213	1078.8	1.3913	0.1915	0.2027	0.7177	0
214	1078.7	1.391	0.1911	0.2024	0.7175	0
215	1078.6	1.3911	0.1913	0.2023	0.7177	0
216	1078.5	1.391	0.1913	0.2022	0.7173	0
217	1078.4	1.3907	0.1908	0.2018	0.7171	0
218	1078.3	1.3911	0.1909	0.2021	0.7172	0
219	1078.2	1.3908	0.1909	0.2024	0.7175	0
220	1078.1	1.391	0.1911	0.2022	0.7175	0
221	1078	1.3911	0.1912	0.2023	0.7177	0
222	1077.9	1.3913	0.1913	0.2024	0.7177	0
223	1077.8	1.3913	0.1909	0.2022	0.7175	0
224	1077.7	1.3913	0.1912	0.2026	0.7181	0
225	1077.6	1.3911	0.1911	0.2026	0.718	0
226	1077.5	1.391	0.1907	0.2021	0.7175	0
227	1077.4	1.3914	0.1908	0.2023	0.7178	0
228	1077.3	1.3914	0.1914	0.2025	0.7179	0
229	1077.2	1.3915	0.1914	0.2025	0.718	0
230	1077.1	1.3913	0.1914	0.2026	0.718	0
231	1077	1.3921	0.1912	0.2025	0.7181	0
232	1076.9	1.3915	0.1913	0.2021	0.7181	0
233	1076.8	1.3915	0.191	0.2019	0.7176	0
234	1076.7	1.3915	0.1913	0.2023	0.7179	0
235	1076.6	1.3913	0.1912	0.2023	0.718	0
236	1076.5	1.3918	0.1912	0.2024	0.7182	0
237	1076.4	1.3917	0.1913	0.2023	0.7179	0
238	1076.3	1.3911	0.1911	0.2022	0.7181	0
239	1076.2	1.3914	0.1909	0.2024	0.7182	0
240	1076.1	1.392	0.191	0.2021	0.7179	0
241	1076	1.3927	0.191	0.2024	0.7183	0
242	1075.9	1.3923	0.1913	0.2024	0.7184	0
243	1075.8	1.3922	0.1911	0.202	0.7181	0
244	1075.7	1.392	0.1912	0.2024	0.7183	0

245	1075.6	1.3926	0.191	0.2023	0.7186	0
246	1075.5	1.3923	0.1913	0.2024	0.7185	0
247	1075.4	1.3924	0.1913	0.2022	0.7184	0
248	1075.3	1.3924	0.1909	0.2024	0.7184	0
249	1075.2	1.3925	0.191	0.2022	0.7182	0
250	1075.1	1.3925	0.1911	0.2021	0.7184	0
251	1075	1.3927	0.1915	0.2025	0.7186	0
252	1074.9	1.3926	0.1915	0.2025	0.7186	0
253	1074.8	1.3927	0.1915	0.202	0.7183	0
254	1074.7	1.3929	0.1914	0.2024	0.7186	0
255	1074.6	1.3931	0.1916	0.2023	0.7186	0
256	1074.5	1.3936	0.1917	0.2024	0.7187	0
257	1074.4	1.3935	0.1916	0.2024	0.7189	0
258	1074.3	1.3937	0.1914	0.2022	0.719	0
259	1074.2	1.3937	0.1916	0.2025	0.7194	0
260	1074.1	1.3936	0.1917	0.2028	0.7193	0
261	1074	1.3937	0.1918	0.2027	0.7194	0
262	1073.9	1.3944	0.1915	0.2028	0.7191	0
263	1073.8	1.3943	0.1914	0.2025	0.7192	0
264	1073.7	1.3944	0.1919	0.2028	0.7196	0
265	1073.6	1.394	0.1917	0.2027	0.7192	0
266	1073.5	1.3946	0.1919	0.2029	0.7195	0
267	1073.4	1.3949	0.1921	0.2029	0.7199	0
268	1073.3	1.3946	0.1921	0.2032	0.7198	0
269	1073.2	1.3946	0.192	0.2031	0.7196	0
270	1073.1	1.3951	0.1922	0.2031	0.72	0
271	1073	1.3953	0.1921	0.203	0.7199	0
272	1072.9	1.3955	0.1922	0.2034	0.7202	0
273	1072.8	1.3954	0.1922	0.2033	0.7201	0
274	1072.7	1.3956	0.1922	0.2033	0.7204	0
275	1072.6	1.3962	0.1921	0.2032	0.7203	0
276	1072.5	1.3959	0.1924	0.2031	0.7204	0
277	1072.4	1.396	0.1924	0.2035	0.7207	0
278	1072.3	1.3963	0.1927	0.2034	0.7206	0
279	1072.2	1.3965	0.1925	0.2032	0.7206	0
280	1072.1	1.3966	0.1924	0.2031	0.7205	0
281	1072	1.3966	0.1925	0.2035	0.7208	0
282	1071.9	1.3962	0.1925	0.2033	0.7207	0
283	1071.8	1.3964	0.1925	0.2034	0.7207	0
284	1071.7	1.3968	0.1926	0.2037	0.7208	0
285	1071.6	1.3973	0.1928	0.2036	0.721	0
286	1071.5	1.3969	0.1925	0.2035	0.7208	0
287	1071.4	1.3977	0.1927	0.2038	0.7214	0

288	1071.3	1.397	0.1926	0.2037	0.7214	0
289	1071.2	1.397	0.1924	0.2033	0.7209	0
290	1071.1	1.3973	0.1927	0.2036	0.721	0
291	1071	1.3976	0.1929	0.2035	0.7212	0
292	1070.9	1.3979	0.1928	0.2037	0.7214	0
293	1070.8	1.3976	0.1926	0.2038	0.7216	0
294	1070.7	1.3978	0.1927	0.2036	0.7215	0
295	1070.6	1.3978	0.1931	0.2041	0.7217	0
296	1070.5	1.398	0.1929	0.204	0.7217	0
297	1070.4	1.3984	0.1932	0.2042	0.722	0
298	1070.3	1.3982	0.193	0.2038	0.7217	0
299	1070.2	1.398	0.1929	0.2039	0.7218	0
300	1070.1	1.3981	0.1928	0.2038	0.722	0
301	1070	1.3984	0.1929	0.204	0.7221	0
302	1069.9	1.398	0.1929	0.2037	0.7218	0
303	1069.8	1.3978	0.1926	0.2038	0.7218	0
304	1069.7	1.3985	0.1933	0.2039	0.7222	0
305	1069.6	1.399	0.193	0.2041	0.722	0
306	1069.5	1.3984	0.1929	0.2039	0.7221	0
307	1069.4	1.3989	0.1929	0.2041	0.7223	0
308	1069.3	1.3985	0.1928	0.204	0.7219	0
309	1069.2	1.3985	0.1931	0.204	0.7222	0
310	1069.1	1.3987	0.1932	0.2041	0.7221	0
311	1069	1.3989	0.1932	0.2044	0.7224	0
312	1068.9	1.3987	0.1929	0.2042	0.7222	0
313	1068.8	1.3987	0.193	0.2043	0.7224	0
314	1068.7	1.3988	0.1931	0.2045	0.7228	0
315	1068.6	1.3995	0.1935	0.2047	0.7228	0
316	1068.5	1.3994	0.1934	0.2047	0.7229	0
317	1068.4	1.399	0.1929	0.2042	0.7225	0
318	1068.3	1.399	0.1929	0.2042	0.7226	0
319	1068.2	1.3984	0.193	0.2039	0.7222	0
320	1068.1	1.3989	0.1932	0.2043	0.7227	0
321	1068	1.399	0.1933	0.2045	0.7227	0
322	1067.9	1.3991	0.1931	0.2043	0.7225	0
323	1067.8	1.3989	0.1932	0.2041	0.7225	0
324	1067.7	1.3988	0.1929	0.2041	0.7226	0
325	1067.6	1.3989	0.193	0.2039	0.7224	0
326	1067.5	1.399	0.1931	0.2041	0.7227	0
327	1067.4	1.3991	0.1932	0.2039	0.7227	0
328	1067.3	1.399	0.1932	0.2041	0.7228	0
329	1067.2	1.3992	0.1933	0.2039	0.7227	0
330	1067.1	1.3997	0.1931	0.2039	0.7224	0

331	1067	1.3991	0.1932	0.2039	0.7228	0
332	1066.9	1.3997	0.1931	0.2041	0.7228	0
333	1066.8	1.3994	0.1935	0.2042	0.7225	0
334	1066.7	1.3997	0.1933	0.2041	0.7226	0
335	1066.6	1.3995	0.1929	0.2037	0.7226	0
336	1066.5	1.399	0.1929	0.2038	0.7226	0
337	1066.4	1.3989	0.193	0.204	0.7228	0
338	1066.3	1.3992	0.1931	0.2042	0.7232	0
339	1066.2	1.3988	0.1931	0.2041	0.7227	0
340	1066.1	1.3995	0.1929	0.2039	0.7228	0
341	1066	1.3996	0.1931	0.2041	0.723	0
342	1065.9	1.3995	0.1928	0.204	0.7229	0
343	1065.8	1.3991	0.1931	0.2042	0.7235	0
344	1065.7	1.3993	0.1932	0.2044	0.7232	0
345	1065.6	1.3995	0.193	0.2043	0.7232	0
346	1065.5	1.3996	0.1935	0.2047	0.7234	0
347	1065.4	1.3996	0.1931	0.2041	0.7232	0
348	1065.3	1.3996	0.1934	0.2045	0.7234	0
349	1065.2	1.4002	0.1931	0.204	0.7232	0
350	1065.1	1.4002	0.1933	0.2042	0.7235	0
351	1065	1.4005	0.1933	0.2042	0.7234	0
352	1064.9	1.4003	0.1933	0.2044	0.7234	0
353	1064.8	1.4004	0.1935	0.2046	0.7238	0
354	1064.7	1.4004	0.1936	0.2047	0.7236	0
355	1064.6	1.4002	0.1935	0.2046	0.724	0
356	1064.5	1.4007	0.1936	0.2044	0.7239	0
357	1064.4	1.4005	0.1938	0.2044	0.7238	0
358	1064.3	1.4005	0.1934	0.2045	0.724	0
359	1064.2	1.4002	0.1932	0.2043	0.7238	0
360	1064.1	1.401	0.1936	0.2044	0.7238	0
361	1064	1.4016	0.1935	0.2046	0.7242	0
362	1063.9	1.4008	0.1932	0.2043	0.724	0
363	1063.8	1.4011	0.1938	0.2045	0.7242	0
364	1063.7	1.4018	0.1936	0.2046	0.7244	0
365	1063.6	1.4017	0.1938	0.2044	0.7245	0
366	1063.5	1.4018	0.1937	0.2046	0.7244	0
367	1063.4	1.4022	0.1939	0.2047	0.7245	0
368	1063.3	1.4028	0.1937	0.2046	0.7248	0
369	1063.2	1.4023	0.1937	0.2046	0.7246	0
370	1063.1	1.4024	0.194	0.2047	0.7246	0
371	1063	1.4021	0.1938	0.2048	0.7247	0
372	1062.9	1.402	0.1937	0.2047	0.7247	0
373	1062.8	1.4026	0.194	0.2045	0.7246	0

374	1062.7	1.4027	0.1937	0.2045	0.7247	0
375	1062.6	1.403	0.1941	0.2049	0.7247	0
376	1062.5	1.4024	0.194	0.2049	0.7247	0
377	1062.4	1.403	0.1941	0.2049	0.7248	0
378	1062.3	1.4026	0.1939	0.2048	0.7251	0
379	1062.2	1.4032	0.194	0.2048	0.7251	0
380	1062.1	1.4027	0.1939	0.2049	0.725	0
381	1062	1.4028	0.1941	0.205	0.7251	0
382	1061.9	1.4022	0.1938	0.2046	0.7248	0
383	1061.8	1.4035	0.1939	0.2048	0.7252	0
384	1061.7	1.4031	0.1938	0.205	0.7252	0
385	1061.6	1.4032	0.194	0.2048	0.7251	0
386	1061.5	1.4039	0.1943	0.205	0.7255	0
387	1061.4	1.404	0.1944	0.2049	0.7255	0
388	1061.3	1.4036	0.1945	0.2048	0.7254	0
389	1061.2	1.4036	0.1941	0.2049	0.7251	0
390	1061.1	1.4037	0.1942	0.205	0.7251	0
391	1061	1.4044	0.1941	0.205	0.7252	0
392	1060.9	1.404	0.1941	0.205	0.7257	0
393	1060.8	1.4043	0.1945	0.2053	0.7257	0
394	1060.7	1.4048	0.1943	0.2051	0.7258	0
395	1060.6	1.4054	0.1943	0.2053	0.7258	0
396	1060.5	1.4045	0.1944	0.2053	0.7255	0
397	1060.4	1.4047	0.1946	0.2058	0.7263	0
398	1060.3	1.4051	0.1945	0.2053	0.726	0
399	1060.2	1.4053	0.1947	0.2054	0.7261	0
400	1060.1	1.405	0.195	0.2057	0.7264	0
401	1060	1.4056	0.1947	0.2059	0.7266	0
402	1059.9	1.4055	0.1951	0.206	0.7268	0
403	1059.8	1.406	0.195	0.206	0.727	0
404	1059.7	1.4064	0.1948	0.206	0.7266	0
405	1059.6	1.4059	0.1948	0.2059	0.7266	0
406	1059.5	1.4058	0.1948	0.2058	0.7266	0
407	1059.4	1.4059	0.1948	0.2058	0.7265	0
408	1059.3	1.4063	0.1946	0.2058	0.7266	0
409	1059.2	1.4059	0.195	0.2058	0.7266	0
410	1059.1	1.4061	0.1948	0.2057	0.7269	0
411	1059	1.4059	0.1949	0.2056	0.7268	0
412	1058.9	1.406	0.1951	0.2059	0.7268	0
413	1058.8	1.406	0.1949	0.2061	0.7269	0
414	1058.7	1.4067	0.1949	0.2063	0.7272	0
415	1058.6	1.406	0.1951	0.206	0.7272	0
416	1058.5	1.4062	0.1949	0.206	0.7268	0

417	1058.4	1.4064	0.195	0.2061	0.7271	0
418	1058.3	1.4062	0.1952	0.2061	0.727	0
419	1058.2	1.4069	0.1953	0.2065	0.7273	0
420	1058.1	1.4061	0.195	0.2059	0.727	0
421	1058	1.4069	0.195	0.2059	0.7269	0
422	1057.9	1.4067	0.1951	0.2062	0.7273	0
423	1057.8	1.4066	0.195	0.2059	0.7269	0
424	1057.7	1.4068	0.1947	0.2058	0.7271	0
425	1057.6	1.4069	0.1951	0.2061	0.7275	0
426	1057.5	1.407	0.195	0.2061	0.7271	0
427	1057.4	1.4069	0.1947	0.2061	0.7273	0
428	1057.3	1.4071	0.1953	0.2061	0.7275	0
429	1057.2	1.4064	0.1949	0.2061	0.7271	0
430	1057.1	1.4074	0.195	0.2062	0.7273	0
431	1057	1.4063	0.1951	0.2057	0.7272	0
432	1056.9	1.4069	0.1953	0.2061	0.7275	0
433	1056.8	1.4077	0.195	0.2061	0.7273	0
434	1056.7	1.4071	0.1949	0.2062	0.7276	0
435	1056.6	1.4072	0.1952	0.2061	0.7276	0
436	1056.5	1.4075	0.1954	0.2063	0.7276	0
437	1056.4	1.4075	0.1953	0.2064	0.7278	0
438	1056.3	1.4077	0.1952	0.2062	0.7277	0
439	1056.2	1.407	0.1953	0.2064	0.7278	0
440	1056.1	1.4076	0.1948	0.2062	0.7277	0
441	1056	1.4071	0.1952	0.2063	0.7278	0
442	1055.9	1.4075	0.1952	0.2064	0.7279	0
443	1055.8	1.4078	0.1952	0.2062	0.7278	0
444	1055.7	1.408	0.1956	0.2066	0.7278	0
445	1055.6	1.4082	0.1955	0.2063	0.7278	0
446	1055.5	1.4081	0.1954	0.2064	0.728	0
447	1055.4	1.408	0.1955	0.2065	0.7281	0
448	1055.3	1.4082	0.1954	0.2066	0.7285	0
449	1055.2	1.4086	0.1957	0.2066	0.7284	0
450	1055.1	1.4089	0.1956	0.2066	0.7284	0
451	1055	1.4086	0.1957	0.207	0.7287	0
452	1054.9	1.409	0.1957	0.207	0.7287	0
453	1054.8	1.409	0.1955	0.2066	0.7284	0
454	1054.7	1.4086	0.1956	0.2066	0.7286	0
455	1054.6	1.4093	0.1953	0.2066	0.7286	0
456	1054.5	1.4098	0.1957	0.2068	0.7286	0
457	1054.4	1.4094	0.1956	0.2065	0.7288	0
458	1054.3	1.409	0.1958	0.2068	0.7289	0
459	1054.2	1.4094	0.1958	0.2069	0.729	0

460	1054.1	1.4095	0.1959	0.207	0.7289	0
461	1054	1.4099	0.196	0.2071	0.7292	0
462	1053.9	1.4099	0.1961	0.2072	0.7292	0
463	1053.8	1.4104	0.1961	0.2073	0.7293	0
464	1053.7	1.4105	0.1965	0.2076	0.7296	0
465	1053.6	1.4105	0.1962	0.2072	0.7293	0
466	1053.5	1.4101	0.1961	0.207	0.7294	0
467	1053.4	1.411	0.1961	0.2075	0.7297	0
468	1053.3	1.4106	0.1965	0.2075	0.7295	0
469	1053.2	1.411	0.1964	0.2074	0.7296	0
470	1053.1	1.4107	0.1961	0.2073	0.7295	0
471	1053	1.4112	0.1963	0.2072	0.7298	0
472	1052.9	1.4111	0.1961	0.2075	0.73	0
473	1052.8	1.411	0.196	0.2072	0.7299	0
474	1052.7	1.4117	0.1965	0.2077	0.73	0
475	1052.6	1.4116	0.1963	0.2075	0.73	0
476	1052.5	1.4113	0.1965	0.2078	0.7302	0
477	1052.4	1.4115	0.1964	0.2075	0.7303	0
478	1052.3	1.4113	0.1964	0.2072	0.7301	0
479	1052.2	1.4116	0.1963	0.2072	0.7302	0
480	1052.1	1.4117	0.1964	0.2075	0.7305	0
481	1052	1.4117	0.1965	0.2076	0.7303	0
482	1051.9	1.4121	0.1966	0.2078	0.7304	0
483	1051.8	1.4119	0.1968	0.2076	0.7303	0
484	1051.7	1.4127	0.1967	0.2075	0.7304	0
485	1051.6	1.4125	0.1964	0.2075	0.7303	0
486	1051.5	1.4118	0.1967	0.2076	0.7304	0
487	1051.4	1.412	0.1966	0.2074	0.7304	0
488	1051.3	1.4121	0.1966	0.2077	0.7305	0
489	1051.2	1.4119	0.1966	0.2076	0.7306	0
490	1051.1	1.412	0.1963	0.2073	0.7303	0
491	1051	1.4125	0.1965	0.2075	0.7307	0
492	1050.9	1.412	0.1963	0.2076	0.7305	0
493	1050.8	1.4123	0.1964	0.2075	0.7306	0
494	1050.7	1.4124	0.1963	0.2076	0.7308	0
495	1050.6	1.4125	0.1961	0.2073	0.7306	0
496	1050.5	1.4126	0.1965	0.2076	0.7309	0
497	1050.4	1.4121	0.1967	0.2077	0.7307	0
498	1050.3	1.4126	0.1967	0.208	0.731	0
499	1050.2	1.4126	0.1965	0.2076	0.7306	0
500	1050.1	1.4126	0.1967	0.2074	0.731	0
501	1050	1.4122	0.1966	0.2077	0.7309	0
502	1049.9	1.4124	0.1966	0.2075	0.7308	0

503	1049.8	1.4129	0.1967	0.2075	0.7311	0
504	1049.7	1.4133	0.1967	0.2077	0.731	0
505	1049.6	1.4129	0.1969	0.2078	0.7311	0
506	1049.5	1.4132	0.1969	0.2077	0.7311	0
507	1049.4	1.4124	0.1964	0.2076	0.7309	0
508	1049.3	1.4123	0.1964	0.2074	0.731	0
509	1049.2	1.4128	0.1965	0.2076	0.7312	0
510	1049.1	1.4133	0.1969	0.2078	0.7311	0
511	1049	1.4134	0.1968	0.2078	0.7311	0
512	1048.9	1.4129	0.1966	0.2076	0.7312	0
513	1048.8	1.4131	0.1965	0.2074	0.7309	0
514	1048.7	1.4133	0.1966	0.2075	0.7311	0
515	1048.6	1.4133	0.1966	0.2075	0.7312	0
516	1048.5	1.4138	0.1969	0.2079	0.7315	0
517	1048.4	1.4133	0.1968	0.2079	0.7316	0
518	1048.3	1.4133	0.1969	0.2078	0.7315	0
519	1048.2	1.4135	0.1967	0.2076	0.7315	0
520	1048.1	1.4136	0.1968	0.2077	0.7316	0
521	1048	1.414	0.1968	0.2076	0.7314	0
522	1047.9	1.4135	0.1968	0.2078	0.7313	0
523	1047.8	1.4134	0.1968	0.2077	0.7315	0
524	1047.7	1.4131	0.1967	0.2074	0.7314	0
525	1047.6	1.414	0.1967	0.2077	0.7316	0
526	1047.5	1.4137	0.197	0.2079	0.7317	0
527	1047.4	1.4141	0.1972	0.2081	0.732	0
528	1047.3	1.4138	0.1971	0.2078	0.7318	0
529	1047.2	1.4135	0.1971	0.2077	0.732	0
530	1047.1	1.4145	0.1976	0.2082	0.7322	0
531	1047	1.4147	0.1972	0.2083	0.7322	0
532	1046.9	1.4146	0.1974	0.2083	0.7323	0
533	1046.8	1.4141	0.1972	0.2083	0.7322	0
534	1046.7	1.4146	0.1975	0.2084	0.7325	0
535	1046.6	1.4149	0.1972	0.2085	0.7326	0
536	1046.5	1.4145	0.1973	0.2082	0.7323	0
537	1046.4	1.4148	0.1973	0.2082	0.7322	0
538	1046.3	1.4149	0.1976	0.2084	0.7325	0
539	1046.2	1.4153	0.1976	0.2085	0.7327	0
540	1046.1	1.4152	0.1978	0.2084	0.7326	0
541	1046	1.4152	0.1978	0.2087	0.7326	0
542	1045.9	1.4154	0.1976	0.2086	0.7327	0
543	1045.8	1.4154	0.1978	0.2088	0.7329	0
544	1045.7	1.4156	0.1975	0.2087	0.7327	0
545	1045.6	1.4151	0.1977	0.2086	0.7328	0

546	1045.5	1.4157	0.1975	0.2086	0.7327	0
547	1045.4	1.4159	0.1978	0.2089	0.7331	0
548	1045.3	1.4163	0.1982	0.2089	0.7331	0
549	1045.2	1.4161	0.1976	0.2087	0.733	0
550	1045.1	1.4165	0.1977	0.2091	0.7334	0
551	1045	1.4165	0.198	0.2092	0.7334	0
552	1044.9	1.4169	0.1981	0.2094	0.7334	0
553	1044.8	1.4166	0.1979	0.2092	0.7334	0
554	1044.7	1.4167	0.1982	0.2088	0.7336	0
555	1044.6	1.4162	0.1982	0.209	0.7337	0
556	1044.5	1.4167	0.1981	0.2088	0.7335	0
557	1044.4	1.4166	0.198	0.2091	0.7337	0
558	1044.3	1.4169	0.1979	0.2088	0.7335	0
559	1044.2	1.4171	0.1983	0.2093	0.7338	0
560	1044.1	1.4171	0.1979	0.2089	0.7334	0
561	1044	1.4178	0.1981	0.2093	0.7338	0
562	1043.9	1.4175	0.1979	0.2089	0.7337	0
563	1043.8	1.418	0.1981	0.2091	0.7337	0
564	1043.7	1.4171	0.198	0.2089	0.7336	0
565	1043.6	1.418	0.1981	0.2091	0.7335	0
566	1043.5	1.4182	0.1982	0.2093	0.7339	0
567	1043.4	1.418	0.1981	0.2094	0.7341	0
568	1043.3	1.4183	0.1986	0.2098	0.7345	0
569	1043.2	1.4185	0.1984	0.2096	0.7343	0
570	1043.1	1.4183	0.1985	0.2096	0.7344	0
571	1043	1.4183	0.1984	0.2093	0.7342	0
572	1042.9	1.4183	0.1985	0.2097	0.7341	0
573	1042.8	1.4185	0.1986	0.2096	0.7341	0
574	1042.7	1.4184	0.1986	0.2095	0.7342	0
575	1042.6	1.4188	0.1985	0.2096	0.7346	0
576	1042.5	1.419	0.1988	0.2094	0.7345	0
577	1042.4	1.4188	0.1985	0.2096	0.7343	0
578	1042.3	1.4192	0.1988	0.2098	0.7348	0
579	1042.2	1.4189	0.1988	0.2097	0.7347	0
580	1042.1	1.419	0.1986	0.2096	0.7346	0
581	1042	1.4193	0.1987	0.2097	0.7349	0
582	1041.9	1.4188	0.199	0.2099	0.7349	0
583	1041.8	1.419	0.1989	0.2097	0.7348	0
584	1041.7	1.419	0.1984	0.2095	0.7347	0
585	1041.6	1.4193	0.1985	0.2094	0.7345	0
586	1041.5	1.4196	0.1987	0.21	0.7347	0
587	1041.4	1.4193	0.1986	0.2098	0.7347	0
588	1041.3	1.4191	0.1986	0.2092	0.7345	0

589	1041.2	1.4194	0.1988	0.2094	0.7349	0
590	1041.1	1.4192	0.1985	0.2096	0.7351	0
591	1041	1.4197	0.1987	0.2096	0.7348	0
592	1040.9	1.4196	0.1988	0.2098	0.7353	0
593	1040.8	1.4194	0.1988	0.21	0.7352	0
594	1040.7	1.4194	0.1989	0.2098	0.7349	0
595	1040.6	1.42	0.199	0.21	0.7354	0
596	1040.5	1.4194	0.1989	0.21	0.7354	0
597	1040.4	1.42	0.1991	0.2101	0.7353	0
598	1040.3	1.4203	0.1989	0.2099	0.7353	0
599	1040.2	1.42	0.1989	0.21	0.7355	0
600	1040.1	1.4198	0.1991	0.2099	0.7353	0
601	1040	1.4199	0.1989	0.2099	0.7355	0
602	1039.9	1.4197	0.1987	0.2098	0.7353	0
603	1039.8	1.4198	0.199	0.2101	0.7353	0
604	1039.7	1.4192	0.1987	0.2099	0.7353	0
605	1039.6	1.4197	0.1987	0.2098	0.7353	0
606	1039.5	1.4201	0.1988	0.2102	0.7358	0
607	1039.4	1.4204	0.199	0.2101	0.7355	0
608	1039.3	1.4201	0.1989	0.21	0.7359	0
609	1039.2	1.4195	0.1989	0.21	0.7356	0
610	1039.1	1.4197	0.199	0.2099	0.7358	0
611	1039	1.4199	0.1992	0.2102	0.7359	0
612	1038.9	1.4196	0.199	0.2098	0.7358	0
613	1038.8	1.4197	0.1987	0.2099	0.7357	0
614	1038.7	1.4199	0.1993	0.2101	0.7358	0
615	1038.6	1.4194	0.1992	0.2099	0.7353	0
616	1038.5	1.4203	0.1993	0.2101	0.7356	0
617	1038.4	1.4196	0.1987	0.2098	0.7356	0
618	1038.3	1.4197	0.1988	0.21	0.7357	0
619	1038.2	1.42	0.1989	0.2099	0.7358	0
620	1038.1	1.4199	0.1992	0.21	0.7358	0
621	1038	1.4198	0.199	0.2099	0.7358	0
622	1037.9	1.4194	0.1987	0.2098	0.7356	0
623	1037.8	1.4197	0.1988	0.2099	0.7358	0
624	1037.7	1.42	0.199	0.2098	0.7359	0
625	1037.6	1.4197	0.199	0.2097	0.7358	0
626	1037.5	1.4199	0.199	0.2098	0.7356	0
627	1037.4	1.4197	0.199	0.2098	0.7359	0
628	1037.3	1.4203	0.1991	0.2099	0.736	0
629	1037.2	1.4197	0.1989	0.2097	0.736	0
630	1037.1	1.4202	0.1991	0.2093	0.736	0
631	1037	1.4202	0.1993	0.21	0.7361	0

632	1036.9	1.4203	0.1992	0.2098	0.7358	0
633	1036.8	1.4206	0.1992	0.2101	0.736	0
634	1036.7	1.4205	0.1996	0.2104	0.7367	0
635	1036.6	1.4205	0.1992	0.21	0.7363	0
636	1036.5	1.4207	0.1993	0.2101	0.7364	0
637	1036.4	1.4204	0.1992	0.2098	0.7364	0
638	1036.3	1.4209	0.1991	0.21	0.7363	0
639	1036.2	1.4212	0.1992	0.21	0.7366	0
640	1036.1	1.4214	0.1994	0.2103	0.7366	0
641	1036	1.4213	0.1994	0.2103	0.7368	0
642	1035.9	1.4211	0.1994	0.2102	0.7366	0
643	1035.8	1.4213	0.1991	0.2102	0.7368	0
644	1035.7	1.4219	0.1991	0.21	0.7367	0
645	1035.6	1.4217	0.1993	0.21	0.7368	0
646	1035.5	1.4219	0.1994	0.2099	0.7368	0
647	1035.4	1.4213	0.1995	0.2095	0.7369	0
648	1035.3	1.4219	0.1995	0.2094	0.737	0
649	1035.2	1.4221	0.1993	0.2095	0.737	0
650	1035.1	1.4221	0.1991	0.2092	0.7369	0
651	1035	1.4221	0.1994	0.2092	0.7371	0
652	1034.9	1.4227	0.1995	0.2091	0.7372	0
653	1034.8	1.4227	0.1997	0.2093	0.7372	0
654	1034.7	1.4226	0.1998	0.2093	0.7376	0
655	1034.6	1.4231	0.2	0.2096	0.7378	0
656	1034.5	1.4235	0.2002	0.2097	0.7381	0
657	1034.4	1.4235	0.2001	0.2094	0.738	0
658	1034.3	1.423	0.2	0.2092	0.7377	0
659	1034.2	1.4235	0.1999	0.2091	0.7378	0
660	1034.1	1.4237	0.1999	0.2092	0.7379	0
661	1034	1.4235	0.2	0.2092	0.7378	0
662	1033.9	1.4237	0.2003	0.2094	0.738	0
663	1033.8	1.4233	0.2	0.2092	0.738	0
664	1033.7	1.4237	0.2001	0.2091	0.7383	0
665	1033.6	1.4239	0.2006	0.2093	0.7385	0
666	1033.5	1.4238	0.2003	0.2091	0.7383	0
667	1033.4	1.4242	0.2002	0.2089	0.7382	0
668	1033.3	1.4243	0.2005	0.2092	0.7389	0
669	1033.2	1.4244	0.2006	0.2092	0.7381	0
670	1033.1	1.4244	0.2003	0.2091	0.7387	0
671	1033	1.4247	0.2005	0.2093	0.7387	0
672	1032.9	1.425	0.2008	0.2096	0.7389	0
673	1032.8	1.425	0.2007	0.2093	0.7389	0
674	1032.7	1.4249	0.2009	0.2093	0.739	0

675	1032.6	1.4249	0.2007	0.2094	0.7389	0
676	1032.5	1.425	0.2007	0.209	0.7389	0
677	1032.4	1.425	0.2007	0.2091	0.739	0
678	1032.3	1.4251	0.2011	0.2093	0.739	0
679	1032.2	1.4255	0.2009	0.2094	0.7392	0
680	1032.1	1.4251	0.2007	0.2092	0.739	0
681	1032	1.4254	0.2008	0.2091	0.7391	0
682	1031.9	1.4256	0.2008	0.2094	0.7392	0
683	1031.8	1.4255	0.2011	0.2093	0.7393	0
684	1031.7	1.426	0.2011	0.2093	0.7394	0
685	1031.6	1.4263	0.2012	0.2093	0.7394	0
686	1031.5	1.4257	0.2011	0.2091	0.7396	0
687	1031.4	1.4262	0.2012	0.2094	0.7396	0
688	1031.3	1.4265	0.2014	0.2096	0.7399	0
689	1031.2	1.4261	0.2013	0.2095	0.7397	0
690	1031.1	1.4264	0.2013	0.2091	0.7398	0
691	1031	1.426	0.201	0.2091	0.7397	0
692	1030.9	1.4261	0.2011	0.2089	0.7397	0
693	1030.8	1.4266	0.2013	0.209	0.7398	0
694	1030.7	1.4264	0.2013	0.2089	0.7398	0
695	1030.6	1.4266	0.2013	0.2089	0.74	0
696	1030.5	1.4264	0.201	0.2089	0.7397	0
697	1030.4	1.4262	0.2013	0.2088	0.74	0
698	1030.3	1.4261	0.2011	0.2086	0.7398	0
699	1030.2	1.4262	0.2012	0.2087	0.7399	0
700	1030.1	1.4263	0.2012	0.2088	0.7398	0
701	1030	1.4264	0.2013	0.2089	0.7401	0
702	1029.9	1.4264	0.2014	0.2088	0.74	0
703	1029.8	1.4261	0.2011	0.2088	0.74	0
704	1029.7	1.4263	0.201	0.2087	0.7399	0
705	1029.6	1.4262	0.2013	0.2088	0.74	0
706	1029.5	1.4261	0.2011	0.2088	0.74	0
707	1029.4	1.4262	0.2014	0.2089	0.7402	0
708	1029.3	1.4259	0.2013	0.209	0.7403	0
709	1029.2	1.4265	0.2013	0.2089	0.74	0
710	1029.1	1.4265	0.2011	0.2087	0.7402	0
711	1029	1.426	0.2008	0.2085	0.7398	0
712	1028.9	1.4262	0.2009	0.2084	0.7398	0
713	1028.8	1.4258	0.2008	0.2083	0.7399	0
714	1028.7	1.4257	0.201	0.2088	0.7401	0
715	1028.6	1.426	0.2009	0.2087	0.7402	0
716	1028.5	1.4259	0.2008	0.2086	0.7399	0
717	1028.4	1.4265	0.2011	0.2088	0.7404	0

718	1028.3	1.4262	0.2007	0.2082	0.74	0
719	1028.2	1.4264	0.2009	0.2086	0.7403	0
720	1028.1	1.4259	0.2006	0.2082	0.7402	0
721	1028	1.4263	0.2009	0.2086	0.7402	0
722	1027.9	1.4256	0.2006	0.2084	0.7399	0
723	1027.8	1.426	0.2009	0.2082	0.74	0
724	1027.7	1.4259	0.2007	0.2082	0.74	0
725	1027.6	1.4263	0.2009	0.2081	0.7401	0
726	1027.5	1.4265	0.2013	0.2085	0.7404	0
727	1027.4	1.426	0.2008	0.2083	0.74	0
728	1027.3	1.426	0.201	0.2082	0.7399	0
729	1027.2	1.4257	0.2013	0.2083	0.74	0
730	1027.1	1.426	0.201	0.2081	0.7399	0
731	1027	1.4263	0.2013	0.2083	0.7405	0
732	1026.9	1.4261	0.201	0.2083	0.7403	0
733	1026.8	1.426	0.201	0.2084	0.7404	0
734	1026.7	1.4262	0.2007	0.2081	0.7401	0
735	1026.6	1.4258	0.2009	0.2079	0.7403	0
736	1026.5	1.426	0.2009	0.2082	0.7403	0
737	1026.4	1.4261	0.2007	0.2079	0.74	0
738	1026.3	1.4259	0.2009	0.2079	0.7403	0
739	1026.2	1.4264	0.2008	0.2082	0.7406	0
740	1026.1	1.4267	0.2012	0.2085	0.7407	0
741	1026	1.4268	0.2012	0.2083	0.7405	0
742	1025.9	1.427	0.2012	0.2083	0.7405	0
743	1025.8	1.4266	0.2013	0.2087	0.7408	0
744	1025.7	1.4266	0.2013	0.2084	0.7407	0
745	1025.6	1.4272	0.2012	0.2082	0.7407	0
746	1025.5	1.4267	0.2012	0.2083	0.7406	0
747	1025.4	1.4273	0.2016	0.2084	0.7409	0
748	1025.3	1.4278	0.2016	0.2087	0.741	0
749	1025.2	1.4276	0.2015	0.2087	0.7409	0
750	1025.1	1.4278	0.2017	0.2089	0.7414	0
751	1025	1.4276	0.2017	0.2087	0.7412	0
752	1024.9	1.4277	0.2015	0.2086	0.7413	0
753	1024.8	1.4278	0.2015	0.2085	0.7414	0
754	1024.7	1.4279	0.2016	0.2087	0.7413	0
755	1024.6	1.4278	0.2018	0.2088	0.7414	0
756	1024.5	1.4284	0.2018	0.2091	0.7415	0
757	1024.4	1.4285	0.2021	0.2091	0.7417	0
758	1024.3	1.4285	0.2022	0.2093	0.7419	0
759	1024.2	1.4293	0.2022	0.2091	0.7417	0
760	1024.1	1.4292	0.202	0.2093	0.7419	0

761	1024	1.429	0.202	0.209	0.7416	0
762	1023.9	1.4288	0.2019	0.209	0.7418	0
763	1023.8	1.4297	0.2025	0.2095	0.742	0
764	1023.7	1.4297	0.2025	0.2095	0.7424	0
765	1023.6	1.4302	0.2027	0.2098	0.7426	0
766	1023.5	1.4299	0.2025	0.2095	0.7425	0
767	1023.4	1.43	0.2025	0.2097	0.7425	0
768	1023.3	1.4302	0.2027	0.2096	0.7427	0
769	1023.2	1.4302	0.2026	0.2095	0.7427	0
770	1023.1	1.4305	0.2027	0.2097	0.7429	0
771	1023	1.4307	0.2027	0.2097	0.7431	0
772	1022.9	1.4304	0.2025	0.2096	0.7427	0
773	1022.8	1.4304	0.2024	0.2096	0.7427	0
774	1022.7	1.4304	0.2023	0.2096	0.7428	0
775	1022.6	1.4305	0.2025	0.2097	0.7429	0
776	1022.5	1.4312	0.2028	0.2101	0.7435	0
777	1022.4	1.4311	0.2031	0.2101	0.743	0
778	1022.3	1.4314	0.2033	0.2103	0.7436	0
779	1022.2	1.4312	0.203	0.2099	0.7432	0
780	1022.1	1.4309	0.2031	0.21	0.7433	0
781	1022	1.431	0.2029	0.21	0.7433	0
782	1021.9	1.4311	0.2029	0.21	0.7432	0
783	1021.8	1.4311	0.2029	0.21	0.7435	0
784	1021.7	1.431	0.2029	0.2099	0.7433	0
785	1021.6	1.431	0.2029	0.2099	0.7435	0
786	1021.5	1.431	0.2027	0.2098	0.7429	0
787	1021.4	1.4316	0.2029	0.21	0.7433	0
788	1021.3	1.4312	0.2028	0.21	0.7433	0
789	1021.2	1.4316	0.203	0.21	0.7433	0
790	1021.1	1.4319	0.203	0.2101	0.7431	0
791	1021	1.4314	0.2029	0.21	0.7434	0
792	1020.9	1.4317	0.2031	0.2101	0.7436	0
793	1020.8	1.4316	0.2033	0.2102	0.7434	0
794	1020.7	1.4317	0.2031	0.2101	0.7433	0
795	1020.6	1.4316	0.203	0.21	0.7435	0
796	1020.5	1.4319	0.2031	0.2101	0.7436	0
797	1020.4	1.4319	0.2029	0.2102	0.7435	0
798	1020.3	1.4314	0.2028	0.2098	0.7433	0
799	1020.2	1.432	0.203	0.2098	0.7435	0
800	1020.1	1.4318	0.2031	0.2101	0.7438	0
801	1020	1.4324	0.2031	0.2101	0.7439	0
802	1019.9	1.4321	0.2033	0.2101	0.7438	0
803	1019.8	1.4318	0.203	0.21	0.7435	0

804	1019.7	1.4319	0.2031	0.21	0.7436	0
805	1019.6	1.4317	0.2029	0.2101	0.7438	0
806	1019.5	1.4321	0.2033	0.2103	0.7443	0
807	1019.4	1.4317	0.203	0.2098	0.7436	0
808	1019.3	1.4317	0.2029	0.2099	0.744	0
809	1019.2	1.4316	0.2029	0.2098	0.7437	0
810	1019.1	1.4319	0.203	0.21	0.7438	0
811	1019	1.4315	0.2029	0.21	0.7437	0
812	1018.9	1.4319	0.2028	0.2099	0.7438	0
813	1018.8	1.4315	0.203	0.2096	0.7439	0
814	1018.7	1.4318	0.203	0.2099	0.7441	0
815	1018.6	1.432	0.2031	0.21	0.7438	0
816	1018.5	1.4322	0.203	0.21	0.744	0
817	1018.4	1.432	0.2029	0.21	0.7438	0
818	1018.3	1.4317	0.203	0.2101	0.7442	0
819	1018.2	1.4319	0.2029	0.21	0.7441	0
820	1018.1	1.4318	0.203	0.2099	0.744	0
821	1018	1.4315	0.203	0.2098	0.7438	0
822	1017.9	1.4314	0.2026	0.2098	0.7438	0
823	1017.8	1.4321	0.2032	0.21	0.7443	0
824	1017.7	1.432	0.2032	0.2103	0.7443	0
825	1017.6	1.432	0.203	0.21	0.7442	0
826	1017.5	1.4317	0.2031	0.2096	0.744	0
827	1017.4	1.4316	0.2029	0.2097	0.744	0
828	1017.3	1.4315	0.2028	0.2097	0.7442	0
829	1017.2	1.4319	0.2031	0.21	0.7444	0
830	1017.1	1.4322	0.2032	0.2101	0.7442	0
831	1017	1.4324	0.203	0.2099	0.7443	0
832	1016.9	1.4319	0.203	0.2097	0.7443	0
833	1016.8	1.4317	0.2025	0.2093	0.7439	0
834	1016.7	1.4314	0.2026	0.2095	0.7439	0
835	1016.6	1.4316	0.203	0.2098	0.7442	0
836	1016.5	1.432	0.2026	0.2097	0.7442	0
837	1016.4	1.432	0.2026	0.2098	0.7442	0
838	1016.3	1.4319	0.2027	0.2096	0.7443	0
839	1016.2	1.4319	0.203	0.2096	0.7441	0
840	1016.1	1.4322	0.2031	0.2101	0.7445	0
841	1016	1.4327	0.2035	0.2103	0.7449	0
842	1015.9	1.4326	0.2035	0.2108	0.745	0
843	1015.8	1.4326	0.2033	0.2103	0.7448	0
844	1015.7	1.4321	0.2029	0.21	0.7445	0
845	1015.6	1.4323	0.203	0.2098	0.7445	0
846	1015.5	1.4326	0.2032	0.2101	0.7447	0

847	1015.4	1.4324	0.2031	0.2103	0.7447	0
848	1015.3	1.4321	0.2032	0.2102	0.7447	0
849	1015.2	1.4321	0.2033	0.2103	0.7448	0
850	1015.1	1.4324	0.2036	0.2104	0.7451	0
851	1015	1.433	0.2035	0.2102	0.7448	0
852	1014.9	1.4333	0.2035	0.2103	0.745	0
853	1014.8	1.4325	0.2033	0.2102	0.7451	0
854	1014.7	1.4335	0.2036	0.2103	0.7451	0
855	1014.6	1.4339	0.2036	0.2106	0.7454	0
856	1014.5	1.4329	0.2034	0.2103	0.745	0
857	1014.4	1.4337	0.2033	0.2104	0.7452	0
858	1014.3	1.4341	0.2034	0.2106	0.7454	0
859	1014.2	1.4336	0.2037	0.2109	0.7454	0
860	1014.1	1.4334	0.2039	0.2107	0.7455	0
861	1014	1.4336	0.2037	0.2108	0.7456	0
862	1013.9	1.4335	0.2039	0.2108	0.7458	0
863	1013.8	1.4341	0.2038	0.2107	0.7458	0
864	1013.7	1.4345	0.204	0.2109	0.7458	0
865	1013.6	1.4344	0.204	0.211	0.746	0
866	1013.5	1.4345	0.2039	0.2109	0.7458	0
867	1013.4	1.4345	0.2041	0.2112	0.746	0
868	1013.3	1.4337	0.2042	0.211	0.7459	0
869	1013.2	1.4345	0.2041	0.211	0.7459	0
870	1013.1	1.4343	0.2041	0.2111	0.7462	0
871	1013	1.4346	0.204	0.2113	0.7462	0
872	1012.9	1.4349	0.2043	0.2113	0.7462	0
873	1012.8	1.4357	0.2043	0.211	0.7465	0
874	1012.7	1.435	0.2042	0.2111	0.7465	0
875	1012.6	1.4358	0.2043	0.2111	0.7465	0
876	1012.5	1.4351	0.2043	0.2113	0.7463	0
877	1012.4	1.4359	0.2045	0.2114	0.7467	0
878	1012.3	1.4353	0.2047	0.2114	0.7467	0
879	1012.2	1.436	0.2047	0.2114	0.7469	0
880	1012.1	1.4356	0.2043	0.2113	0.7465	0
881	1012	1.435	0.2045	0.2116	0.7468	0
882	1011.9	1.4362	0.2047	0.2112	0.7469	0
883	1011.8	1.4359	0.2047	0.2115	0.7469	0
884	1011.7	1.4362	0.2048	0.2114	0.747	0
885	1011.6	1.436	0.2047	0.2115	0.7469	0
886	1011.5	1.4367	0.2047	0.2118	0.7471	0
887	1011.4	1.4366	0.2046	0.2118	0.7472	0
888	1011.3	1.4367	0.2048	0.2117	0.7468	0
889	1011.2	1.4364	0.2048	0.2117	0.747	0

890	1011.1	1.4361	0.2047	0.2116	0.7472	0
891	1011	1.4372	0.2047	0.2118	0.7474	0
892	1010.9	1.4364	0.2048	0.2116	0.7473	0
893	1010.8	1.4365	0.2046	0.2116	0.7472	0
894	1010.7	1.4362	0.2048	0.2116	0.747	0
895	1010.6	1.4365	0.2049	0.2117	0.7471	0
896	1010.5	1.4372	0.205	0.2118	0.7474	0
897	1010.4	1.437	0.2049	0.212	0.7475	0
898	1010.3	1.437	0.2049	0.2116	0.7476	0
899	1010.2	1.4369	0.205	0.212	0.7474	0
900	1010.1	1.4362	0.205	0.212	0.7478	0
901	1010	1.4367	0.2049	0.2118	0.7473	0
902	1009.9	1.4372	0.2053	0.2122	0.748	0
903	1009.8	1.4366	0.205	0.2119	0.7476	0
904	1009.7	1.4362	0.2046	0.2117	0.7476	0
905	1009.6	1.4368	0.2051	0.2117	0.7477	0
906	1009.5	1.4367	0.205	0.2119	0.7476	0
907	1009.4	1.4367	0.2049	0.2116	0.7477	0
908	1009.3	1.4368	0.2048	0.2117	0.7475	0
909	1009.2	1.4365	0.2048	0.2119	0.7475	0
910	1009.1	1.4373	0.2048	0.212	0.7479	0
911	1009	1.4371	0.2051	0.2121	0.7479	0
912	1008.9	1.4368	0.2052	0.2121	0.7479	0
913	1008.8	1.437	0.2052	0.212	0.7481	0
914	1008.7	1.4371	0.2052	0.2122	0.7481	0
915	1008.6	1.4371	0.2051	0.212	0.7481	0
916	1008.5	1.4364	0.2053	0.2121	0.7481	0
917	1008.4	1.4372	0.2049	0.2118	0.748	0
918	1008.3	1.4371	0.2051	0.2123	0.7479	0
919	1008.2	1.4372	0.2051	0.2121	0.748	0
920	1008.1	1.4368	0.2051	0.2118	0.7482	0
921	1008	1.4365	0.2052	0.2121	0.7482	0
922	1007.9	1.437	0.2048	0.2118	0.748	0
923	1007.8	1.4366	0.2051	0.2118	0.7481	0
924	1007.7	1.4371	0.2051	0.2121	0.7483	0
925	1007.6	1.4374	0.2048	0.2118	0.7481	0
926	1007.5	1.4367	0.205	0.2122	0.7482	0
927	1007.4	1.4373	0.2052	0.2123	0.7484	0
928	1007.3	1.4371	0.205	0.2123	0.7484	0
929	1007.2	1.4367	0.205	0.2121	0.7483	0
930	1007.1	1.4366	0.2049	0.2119	0.7482	0
931	1007	1.4363	0.205	0.212	0.7482	0
932	1006.9	1.4371	0.2051	0.2122	0.7482	0

933	1006.8	1.4372	0.2051	0.2121	0.7483	0
934	1006.7	1.4367	0.2049	0.2116	0.7481	0
935	1006.6	1.4373	0.2047	0.2121	0.748	0
936	1006.5	1.4371	0.2048	0.2119	0.7481	0
937	1006.4	1.4371	0.2048	0.2119	0.748	0
938	1006.3	1.4368	0.2051	0.212	0.7482	0
939	1006.2	1.4371	0.2051	0.2121	0.7486	0
940	1006.1	1.4373	0.2052	0.2121	0.7485	0
941	1006	1.4367	0.205	0.2119	0.7482	0
942	1005.9	1.4369	0.205	0.2119	0.7482	0
943	1005.8	1.4368	0.205	0.2121	0.7485	0
944	1005.7	1.4374	0.205	0.2122	0.7484	0
945	1005.6	1.4371	0.2051	0.2123	0.7485	0
946	1005.5	1.4372	0.2051	0.2122	0.7485	0
947	1005.4	1.4374	0.2051	0.2122	0.7487	0
948	1005.3	1.4376	0.2053	0.2123	0.7487	0
949	1005.2	1.4368	0.2051	0.2123	0.7488	0
950	1005.1	1.438	0.2051	0.2124	0.7489	0
951	1005	1.4381	0.2052	0.2122	0.7488	0
952	1004.9	1.4375	0.2054	0.2123	0.7489	0
953	1004.8	1.4379	0.2052	0.212	0.7486	0
954	1004.7	1.4378	0.2052	0.2122	0.749	0
955	1004.6	1.438	0.2055	0.2123	0.7494	0
956	1004.5	1.4378	0.2055	0.2126	0.7492	0
957	1004.4	1.4377	0.2054	0.2122	0.7487	0
958	1004.3	1.4381	0.2054	0.2125	0.7492	0
959	1004.2	1.4384	0.2056	0.2126	0.7492	0
960	1004.1	1.4389	0.2054	0.2124	0.7489	0
961	1004	1.4386	0.2055	0.2125	0.7491	0
962	1003.9	1.4384	0.2054	0.2123	0.7492	0
963	1003.8	1.4387	0.2056	0.2125	0.7496	0
964	1003.7	1.4388	0.2056	0.2126	0.7494	0
965	1003.6	1.439	0.2056	0.2124	0.7492	0
966	1003.5	1.4392	0.2058	0.2128	0.7491	0
967	1003.4	1.4393	0.206	0.2134	0.7497	0
968	1003.3	1.4391	0.2057	0.2128	0.7495	0
969	1003.2	1.4394	0.2058	0.2127	0.7498	0
970	1003.1	1.4392	0.2059	0.2129	0.7499	0
971	1003	1.4397	0.2063	0.2133	0.7501	0
972	1002.9	1.439	0.2058	0.2127	0.7499	0
973	1002.8	1.4393	0.2059	0.2131	0.75	0
974	1002.7	1.4387	0.2056	0.2128	0.7496	0
975	1002.6	1.4395	0.2058	0.2129	0.7499	0

976	1002.5	1.4391	0.2057	0.2127	0.75	0
977	1002.4	1.4397	0.2061	0.2131	0.7501	0
978	1002.3	1.4393	0.206	0.2131	0.7501	0
979	1002.2	1.4393	0.206	0.213	0.7502	0
980	1002.1	1.4394	0.2059	0.2131	0.7501	0
981	1002	1.44	0.2058	0.213	0.7501	0
982	1001.9	1.44	0.2058	0.2129	0.75	0
983	1001.8	1.4399	0.206	0.213	0.7502	0
984	1001.7	1.4397	0.2061	0.2132	0.7504	0
985	1001.6	1.4402	0.206	0.2131	0.7503	0
986	1001.5	1.4399	0.206	0.213	0.7504	0
987	1001.4	1.4399	0.2058	0.213	0.7501	0
988	1001.3	1.4403	0.2061	0.2131	0.7505	0
989	1001.2	1.4405	0.2062	0.2134	0.7505	0
990	1001.1	1.4402	0.2062	0.2132	0.7504	0
991	1001	1.4405	0.2063	0.2135	0.7508	0
992	1000.9	1.4407	0.2062	0.2134	0.7505	0
993	1000.8	1.4402	0.2062	0.2133	0.7506	0
994	1000.7	1.4408	0.2061	0.2133	0.7506	0
995	1000.6	1.4405	0.2062	0.2134	0.7508	0
996	1000.5	1.4405	0.2061	0.2133	0.7507	0
997	1000.4	1.441	0.2063	0.2139	0.7513	0
998	1000.3	1.4415	0.2063	0.2136	0.7508	0
999	1000.2	1.4406	0.2062	0.2136	0.7508	0
1000	1000.1	1.4411	0.2067	0.2138	0.7514	0
1001	1000	1.441	0.2063	0.2137	0.7512	0
1002	999.9	1.4411	0.2063	0.2134	0.7512	0
1003	999.8	1.4414	0.2065	0.2137	0.7513	0
1004	999.7	1.4412	0.2067	0.2138	0.7512	0
1005	999.6	1.4418	0.2067	0.2138	0.7514	0
1006	999.5	1.4407	0.2063	0.2137	0.7512	0
1007	999.4	1.4409	0.2064	0.2138	0.7514	0
1008	999.3	1.441	0.2064	0.2135	0.7514	0
1009	999.2	1.4416	0.2064	0.2136	0.7514	0
1010	999.1	1.4418	0.2066	0.2136	0.7515	0
1011	999	1.4414	0.2067	0.2139	0.7515	0
1012	998.9	1.4416	0.2067	0.214	0.7516	0
1013	998.8	1.4417	0.2063	0.2138	0.7515	0
1014	998.7	1.4417	0.2064	0.214	0.7513	0
1015	998.6	1.4409	0.206	0.2135	0.751	0
1016	998.5	1.4414	0.2065	0.2138	0.7512	0
1017	998.4	1.4419	0.2062	0.2137	0.7514	0
1018	998.3	1.4417	0.2066	0.2138	0.7516	0

1019	998.2	1.4418	0.2065	0.2138	0.7513	0
1020	998.1	1.4414	0.2068	0.2136	0.7515	0
1021	998	1.4414	0.2063	0.2136	0.7514	0
1022	997.9	1.4409	0.2064	0.2136	0.7512	0
1023	997.8	1.4415	0.2064	0.2135	0.7513	0
1024	997.7	1.4412	0.2065	0.2135	0.7514	0
1025	997.6	1.4417	0.2062	0.2135	0.7513	0
1026	997.5	1.4412	0.2061	0.2134	0.7514	0
1027	997.4	1.4416	0.2065	0.2136	0.7516	0
1028	997.3	1.4414	0.2061	0.2132	0.7513	0
1029	997.2	1.4414	0.2066	0.2136	0.7516	0
1030	997.1	1.442	0.2066	0.2138	0.7518	0
1031	997	1.4416	0.2064	0.2136	0.7518	0
1032	996.9	1.441	0.2061	0.2136	0.7515	0
1033	996.8	1.4419	0.2064	0.2139	0.7519	0
1034	996.7	1.442	0.2065	0.2139	0.7518	0
1035	996.6	1.4422	0.2066	0.2139	0.752	0
1036	996.5	1.4421	0.2065	0.2136	0.7518	0
1037	996.4	1.4418	0.2066	0.2137	0.7519	0
1038	996.3	1.442	0.2065	0.214	0.752	0
1039	996.2	1.4427	0.2065	0.2141	0.7521	0
1040	996.1	1.442	0.2068	0.2142	0.7522	0
1041	996	1.4419	0.2067	0.2139	0.7521	0
1042	995.9	1.4416	0.2067	0.2142	0.7521	0
1043	995.8	1.4418	0.2068	0.2142	0.7522	0
1044	995.7	1.4423	0.2069	0.2141	0.7525	0
1045	995.6	1.4419	0.2066	0.2141	0.7521	0
1046	995.5	1.4421	0.2068	0.2144	0.7521	0
1047	995.4	1.4426	0.2068	0.2141	0.7522	0
1048	995.3	1.4423	0.2068	0.214	0.7521	0
1049	995.2	1.4422	0.2066	0.214	0.752	0
1050	995.1	1.4423	0.2065	0.2139	0.7521	0
1051	995	1.4426	0.2066	0.214	0.7522	0
1052	994.9	1.4419	0.2069	0.2143	0.7525	0
1053	994.8	1.442	0.2069	0.2142	0.7523	0
1054	994.7	1.4425	0.2067	0.2142	0.7524	0
1055	994.6	1.4423	0.2069	0.2142	0.7526	0
1056	994.5	1.4425	0.2071	0.2145	0.7525	0
1057	994.4	1.4424	0.2069	0.2143	0.7525	0
1058	994.3	1.4425	0.2069	0.2141	0.7524	0
1059	994.2	1.4433	0.2071	0.2145	0.7527	0
1060	994.1	1.443	0.2069	0.2145	0.7528	0
1061	994	1.4431	0.2068	0.2144	0.7526	0

1062	993.9	1.4433	0.207	0.2146	0.7529	0
1063	993.8	1.4432	0.2068	0.214	0.7525	0
1064	993.7	1.443	0.2071	0.2143	0.753	0
1065	993.6	1.4426	0.207	0.2142	0.7529	0
1066	993.5	1.4432	0.2071	0.2143	0.7531	0
1067	993.4	1.4429	0.207	0.2144	0.7531	0
1068	993.3	1.4435	0.2071	0.2144	0.7531	0
1069	993.2	1.4435	0.2068	0.2141	0.7528	0
1070	993.1	1.4434	0.2072	0.2145	0.7534	0
1071	993	1.4434	0.2072	0.2145	0.7529	0
1072	992.9	1.4435	0.2071	0.2145	0.7529	0
1073	992.8	1.4435	0.2075	0.2149	0.7534	0
1074	992.7	1.444	0.2073	0.2148	0.7534	0
1075	992.6	1.4435	0.2072	0.2146	0.7533	0
1076	992.5	1.4443	0.2074	0.2149	0.7535	0
1077	992.4	1.4439	0.2074	0.2149	0.7535	0
1078	992.3	1.4443	0.2071	0.2147	0.7534	0
1079	992.2	1.4439	0.2071	0.2147	0.7533	0
1080	992.1	1.444	0.207	0.2146	0.7533	0
1081	992	1.4441	0.2072	0.2147	0.7535	0
1082	991.9	1.4439	0.2075	0.2149	0.7536	0
1083	991.8	1.4446	0.207	0.215	0.7538	0
1084	991.7	1.4442	0.207	0.2151	0.7537	0
1085	991.6	1.4443	0.2073	0.215	0.7536	0
1086	991.5	1.4442	0.2073	0.2147	0.7536	0
1087	991.4	1.4445	0.2075	0.215	0.7538	0
1088	991.3	1.4442	0.2075	0.215	0.7538	0
1089	991.2	1.4447	0.2075	0.2149	0.7538	0
1090	991.1	1.4444	0.2075	0.2152	0.7539	0
1091	991	1.4448	0.2073	0.2148	0.7539	0
1092	990.9	1.4443	0.2073	0.2146	0.7537	0
1093	990.8	1.4446	0.2074	0.2151	0.7541	0
1094	990.7	1.4445	0.2073	0.2147	0.7539	0
1095	990.6	1.4441	0.2071	0.2148	0.7539	0
1096	990.5	1.444	0.2071	0.2147	0.7538	0
1097	990.4	1.4443	0.2072	0.2149	0.754	0
1098	990.3	1.4448	0.2073	0.2149	0.7539	0
1099	990.2	1.444	0.2074	0.215	0.7542	0
1100	990.1	1.4448	0.2076	0.2152	0.7543	0
1101	990	1.4453	0.2077	0.2151	0.7543	0
1102	989.9	1.445	0.2076	0.2153	0.7544	0
1103	989.8	1.4448	0.2074	0.2151	0.7541	0
1104	989.7	1.445	0.2077	0.2151	0.7543	0

1105	989.6	1.4446	0.2075	0.215	0.7541	0
1106	989.5	1.4451	0.2074	0.2151	0.7543	0
1107	989.4	1.4447	0.2073	0.2151	0.7542	0
1108	989.3	1.4447	0.2072	0.215	0.7541	0
1109	989.2	1.4445	0.2077	0.2153	0.7543	0
1110	989.1	1.4457	0.2078	0.2154	0.7544	0
1111	989	1.4452	0.2075	0.2153	0.7544	0
1112	988.9	1.4452	0.2075	0.2151	0.7542	0
1113	988.8	1.4452	0.2074	0.215	0.7544	0
1114	988.7	1.4451	0.2078	0.2156	0.7546	0
1115	988.6	1.4453	0.2081	0.2156	0.7546	0
1116	988.5	1.4454	0.208	0.2154	0.755	0
1117	988.4	1.4454	0.2079	0.2154	0.755	0
1118	988.3	1.4453	0.2078	0.2151	0.7548	0
1119	988.2	1.4454	0.2079	0.2153	0.7545	0
1120	988.1	1.4455	0.2078	0.2154	0.7548	0
1121	988	1.4456	0.2077	0.2153	0.7547	0
1122	987.9	1.4451	0.2076	0.2152	0.7546	0
1123	987.8	1.4456	0.2076	0.2151	0.7547	0
1124	987.7	1.4456	0.2077	0.2149	0.7546	0
1125	987.6	1.4455	0.2078	0.2154	0.7549	0
1126	987.5	1.4457	0.2076	0.2149	0.7545	0
1127	987.4	1.4457	0.208	0.2153	0.7547	0
1128	987.3	1.4456	0.2078	0.2151	0.7546	0
1129	987.2	1.4459	0.2078	0.2152	0.7551	0
1130	987.1	1.4459	0.208	0.2152	0.7551	0
1131	987	1.4456	0.2081	0.2154	0.755	0
1132	986.9	1.446	0.2078	0.2152	0.755	0
1133	986.8	1.4463	0.208	0.2154	0.7551	0
1134	986.7	1.4463	0.2079	0.2154	0.7553	0
1135	986.6	1.4458	0.2079	0.2152	0.7549	0
1136	986.5	1.4462	0.2082	0.2156	0.7554	0
1137	986.4	1.4462	0.2081	0.2154	0.7552	0
1138	986.3	1.4458	0.208	0.2156	0.7554	0
1139	986.2	1.446	0.2081	0.2157	0.7554	0
1140	986.1	1.4464	0.2081	0.2156	0.755	0
1141	986	1.4467	0.2081	0.2156	0.7554	0
1142	985.9	1.4467	0.2082	0.2157	0.7553	0
1143	985.8	1.4464	0.2082	0.2157	0.7556	0
1144	985.7	1.4465	0.2081	0.2157	0.7557	0
1145	985.6	1.4462	0.2084	0.2158	0.7557	0
1146	985.5	1.447	0.2081	0.2157	0.7557	0
1147	985.4	1.4468	0.2083	0.2157	0.7555	0

1148	985.3	1.4465	0.2083	0.2158	0.7558	0
1149	985.2	1.4471	0.2083	0.2159	0.7556	0
1150	985.1	1.4471	0.2083	0.2158	0.7558	0
1151	985	1.447	0.208	0.2156	0.7555	0
1152	984.9	1.4467	0.2079	0.2158	0.7556	0
1153	984.8	1.4474	0.2083	0.2159	0.7558	0
1154	984.7	1.4469	0.2086	0.216	0.7561	0
1155	984.6	1.4472	0.2084	0.2159	0.756	0
1156	984.5	1.4483	0.2084	0.216	0.7561	0
1157	984.4	1.447	0.2083	0.2159	0.756	0
1158	984.3	1.4471	0.2085	0.2159	0.7561	0
1159	984.2	1.447	0.2085	0.2161	0.7562	0
1160	984.1	1.4478	0.2083	0.216	0.7561	0
1161	984	1.4475	0.2084	0.2161	0.7562	0
1162	983.9	1.4481	0.2085	0.2162	0.7565	0
1163	983.8	1.4475	0.2082	0.216	0.7562	0
1164	983.7	1.4477	0.2086	0.2158	0.756	0
1165	983.6	1.448	0.2087	0.2164	0.7566	0
1166	983.5	1.4486	0.2089	0.2164	0.7566	0
1167	983.4	1.448	0.2088	0.2161	0.7563	0
1168	983.3	1.4476	0.2085	0.216	0.7562	0
1169	983.2	1.4483	0.2087	0.2163	0.7566	0
1170	983.1	1.4486	0.2087	0.2164	0.7566	0
1171	983	1.4487	0.2088	0.2167	0.7567	0
1172	982.9	1.4485	0.2088	0.2165	0.7566	0
1173	982.8	1.4486	0.2088	0.2162	0.7567	0
1174	982.7	1.4483	0.2089	0.2164	0.7569	0
1175	982.6	1.448	0.2089	0.2166	0.757	0
1176	982.5	1.4484	0.2088	0.2165	0.7568	0
1177	982.4	1.4485	0.2089	0.2164	0.7564	0
1178	982.3	1.4487	0.209	0.2167	0.7569	0
1179	982.2	1.4482	0.2089	0.2165	0.7571	0
1180	982.1	1.4482	0.2088	0.2163	0.7568	0
1181	982	1.4486	0.2087	0.2162	0.7566	0
1182	981.9	1.4488	0.2088	0.2164	0.7569	0
1183	981.8	1.4486	0.2091	0.2166	0.7571	0
1184	981.7	1.4486	0.2091	0.2164	0.7572	0
1185	981.6	1.4488	0.2087	0.2161	0.7568	0
1186	981.5	1.4486	0.2091	0.2164	0.7569	0
1187	981.4	1.4492	0.2091	0.2164	0.7571	0
1188	981.3	1.4491	0.209	0.2162	0.7569	0
1189	981.2	1.4481	0.2086	0.2162	0.7568	0
1190	981.1	1.4484	0.2088	0.2163	0.757	0

1191	981	1.449	0.209	0.2166	0.7573	0
1192	980.9	1.4491	0.2091	0.2166	0.7574	0
1193	980.8	1.4484	0.2088	0.2164	0.7569	0
1194	980.7	1.449	0.2089	0.2164	0.7571	0
1195	980.6	1.4487	0.2089	0.2166	0.7572	0
1196	980.5	1.4492	0.2087	0.2165	0.7574	0
1197	980.4	1.4485	0.2087	0.2163	0.7571	0
1198	980.3	1.4483	0.209	0.2165	0.7571	0
1199	980.2	1.449	0.209	0.2169	0.7574	0
1200	980.1	1.4485	0.209	0.2167	0.7574	0
1201	980	1.449	0.2092	0.2168	0.7574	0
1202	979.9	1.449	0.2091	0.2166	0.7574	0
1203	979.8	1.4486	0.2091	0.2167	0.7575	0
1204	979.7	1.4485	0.2091	0.2164	0.7574	0
1205	979.6	1.449	0.2091	0.2164	0.7573	0
1206	979.5	1.4489	0.2089	0.2166	0.7574	0
1207	979.4	1.449	0.2089	0.2166	0.7572	0
1208	979.3	1.448	0.2087	0.2165	0.7571	0
1209	979.2	1.4482	0.2089	0.2165	0.7574	0
1210	979.1	1.4481	0.2088	0.2167	0.7575	0
1211	979	1.4486	0.2088	0.2165	0.757	0
1212	978.9	1.4486	0.2089	0.2166	0.7575	0
1213	978.8	1.4486	0.2086	0.2165	0.7572	0
1214	978.7	1.4486	0.2089	0.2164	0.7573	0
1215	978.6	1.4485	0.2088	0.2162	0.7572	0
1216	978.5	1.4481	0.2088	0.2162	0.7574	0
1217	978.4	1.4484	0.2085	0.216	0.7573	0
1218	978.3	1.4486	0.209	0.2161	0.7573	0
1219	978.2	1.4481	0.2087	0.216	0.7572	0
1220	978.1	1.4485	0.209	0.2165	0.7575	0
1221	978	1.4487	0.2086	0.2161	0.7572	0
1222	977.9	1.4481	0.2087	0.216	0.757	0
1223	977.8	1.4486	0.2087	0.2162	0.7571	0
1224	977.7	1.4474	0.2085	0.2163	0.7572	0
1225	977.6	1.4485	0.2087	0.2163	0.7574	0
1226	977.5	1.4485	0.2087	0.2163	0.7574	0
1227	977.4	1.4486	0.2087	0.2164	0.7573	0
1228	977.3	1.4481	0.2085	0.2163	0.7573	0
1229	977.2	1.4485	0.2085	0.2163	0.7575	0
1230	977.1	1.4481	0.2085	0.2161	0.7574	0
1231	977	1.4487	0.2086	0.2165	0.7574	0
1232	976.9	1.4482	0.2083	0.2164	0.7573	0
1233	976.8	1.4489	0.2087	0.2164	0.7576	0

1234	976.7	1.4491	0.209	0.2164	0.7575	0
1235	976.6	1.4493	0.209	0.2168	0.7578	0
1236	976.5	1.4486	0.2087	0.2162	0.7575	0
1237	976.4	1.4486	0.2085	0.2163	0.7576	0
1238	976.3	1.4487	0.2086	0.2163	0.7576	0
1239	976.2	1.4489	0.2086	0.2164	0.7575	0
1240	976.1	1.4487	0.209	0.2168	0.7579	0
1241	976	1.4494	0.2086	0.2163	0.7577	0
1242	975.9	1.4492	0.2088	0.2166	0.7579	0
1243	975.8	1.4499	0.209	0.2168	0.7583	0
1244	975.7	1.4495	0.2089	0.2166	0.7582	0
1245	975.6	1.4495	0.2091	0.2165	0.7579	0
1246	975.5	1.4497	0.2092	0.2167	0.758	0
1247	975.4	1.4498	0.2091	0.2169	0.7583	0
1248	975.3	1.4496	0.2091	0.2168	0.7584	0
1249	975.2	1.45	0.2091	0.2169	0.7584	0
1250	975.1	1.4504	0.2096	0.2172	0.7585	0
1251	975	1.4508	0.2092	0.2169	0.7585	0
1252	974.9	1.4504	0.2092	0.2171	0.7584	0
1253	974.8	1.4499	0.209	0.2168	0.7583	0
1254	974.7	1.4502	0.2094	0.2173	0.7586	0
1255	974.6	1.4508	0.2094	0.2172	0.7587	0
1256	974.5	1.4511	0.2096	0.2175	0.7589	0
1257	974.4	1.4512	0.2097	0.2175	0.7589	0
1258	974.3	1.4511	0.2097	0.2173	0.7588	0
1259	974.2	1.451	0.2101	0.2176	0.759	0
1260	974.1	1.4511	0.2098	0.2177	0.759	0
1261	974	1.452	0.2098	0.2177	0.7591	0
1262	973.9	1.4515	0.2094	0.2174	0.7589	0
1263	973.8	1.4518	0.2099	0.2175	0.7592	0
1264	973.7	1.4515	0.21	0.2177	0.7593	0
1265	973.6	1.452	0.21	0.2176	0.7592	0
1266	973.5	1.4521	0.2099	0.2176	0.7592	0
1267	973.4	1.4523	0.21	0.2178	0.7595	0
1268	973.3	1.4524	0.2101	0.2179	0.7596	0
1269	973.2	1.4525	0.2102	0.2179	0.7597	0
1270	973.1	1.4527	0.2101	0.218	0.7596	0
1271	973	1.4528	0.2103	0.2182	0.7598	0
1272	972.9	1.4527	0.2101	0.2181	0.7596	0
1273	972.8	1.4524	0.2101	0.2181	0.7597	0
1274	972.7	1.4524	0.2102	0.2178	0.7596	0
1275	972.6	1.4526	0.2102	0.2183	0.7598	0
1276	972.5	1.4531	0.2102	0.2182	0.7597	0

1277	972.4	1.4526	0.2102	0.218	0.7596	0
1278	972.3	1.4527	0.2104	0.2181	0.7597	0
1279	972.2	1.4527	0.2105	0.2181	0.7596	0
1280	972.1	1.4527	0.2103	0.2181	0.7598	0
1281	972	1.453	0.2104	0.2182	0.7599	0
1282	971.9	1.4532	0.2108	0.2184	0.7604	0
1283	971.8	1.4539	0.2108	0.2186	0.7605	0
1284	971.7	1.4538	0.2107	0.2183	0.7602	0
1285	971.6	1.4538	0.2107	0.2185	0.7603	0
1286	971.5	1.4537	0.2105	0.2184	0.7602	0
1287	971.4	1.4538	0.2105	0.2184	0.7602	0
1288	971.3	1.4534	0.2106	0.2182	0.7603	0
1289	971.2	1.4533	0.2105	0.2182	0.7602	0
1290	971.1	1.4532	0.2103	0.218	0.7601	0
1291	971	1.4537	0.2104	0.2179	0.7601	0
1292	970.9	1.4536	0.2104	0.218	0.76	0
1293	970.8	1.4533	0.2104	0.218	0.7602	0
1294	970.7	1.4534	0.2106	0.2182	0.7602	0
1295	970.6	1.4535	0.2107	0.2186	0.7602	0
1296	970.5	1.4536	0.2107	0.2183	0.7604	0
1297	970.4	1.4531	0.2104	0.2184	0.7603	0
1298	970.3	1.4535	0.2105	0.2182	0.7603	0
1299	970.2	1.4535	0.2104	0.2184	0.7603	0
1300	970.1	1.4532	0.2103	0.2185	0.7607	0
1301	970	1.4536	0.2105	0.2181	0.7603	0
1302	969.9	1.453	0.2106	0.2183	0.76	0
1303	969.8	1.4532	0.2103	0.2183	0.7602	0
1304	969.7	1.4535	0.2103	0.2182	0.7602	0
1305	969.6	1.4536	0.2104	0.2182	0.7603	0
1306	969.5	1.4535	0.2105	0.2183	0.7603	0
1307	969.4	1.4526	0.2103	0.2183	0.7603	0
1308	969.3	1.4534	0.2103	0.2181	0.7601	0
1309	969.2	1.4535	0.2102	0.2182	0.7603	0
1310	969.1	1.4534	0.2102	0.218	0.7601	0
1311	969	1.453	0.2101	0.218	0.76	0
1312	968.9	1.4528	0.21	0.218	0.7601	0
1313	968.8	1.4533	0.2101	0.218	0.7601	0
1314	968.7	1.4531	0.2103	0.2182	0.7601	0
1315	968.6	1.4525	0.2103	0.2181	0.76	0
1316	968.5	1.4525	0.2102	0.218	0.7602	0
1317	968.4	1.453	0.2102	0.218	0.7601	0
1318	968.3	1.453	0.2102	0.2178	0.7601	0
1319	968.2	1.4531	0.2102	0.2182	0.7604	0

1320	968.1	1.4529	0.2101	0.2179	0.7601	0
1321	968	1.4531	0.2104	0.2182	0.7604	0
1322	967.9	1.4529	0.2106	0.2183	0.7605	0
1323	967.8	1.4524	0.21	0.2178	0.7601	0
1324	967.7	1.4523	0.21	0.2177	0.7601	0
1325	967.6	1.4525	0.21	0.2179	0.7603	0
1326	967.5	1.4526	0.2099	0.2176	0.7599	0
1327	967.4	1.4522	0.2099	0.2176	0.7601	0
1328	967.3	1.4527	0.2099	0.2176	0.7599	0
1329	967.2	1.4521	0.2097	0.2174	0.7596	0
1330	967.1	1.4527	0.2098	0.2175	0.7599	0
1331	967	1.4522	0.2098	0.2175	0.7599	0
1332	966.9	1.4526	0.2098	0.2176	0.76	0
1333	966.8	1.4523	0.2098	0.2177	0.7602	0
1334	966.7	1.4524	0.21	0.2177	0.7599	0
1335	966.6	1.4529	0.2099	0.2177	0.7601	0
1336	966.5	1.4527	0.2102	0.218	0.7605	0
1337	966.4	1.4526	0.2101	0.2179	0.7604	0
1338	966.3	1.4522	0.21	0.2178	0.7604	0
1339	966.2	1.4526	0.2101	0.2179	0.7602	0
1340	966.1	1.4528	0.21	0.218	0.7604	0
1341	966	1.4533	0.2101	0.218	0.7605	0
1342	965.9	1.4533	0.21	0.2179	0.7605	0
1343	965.8	1.4525	0.2101	0.2179	0.7604	0
1344	965.7	1.4529	0.21	0.2179	0.7603	0
1345	965.6	1.4529	0.21	0.2178	0.7602	0
1346	965.5	1.453	0.21	0.2181	0.7605	0
1347	965.4	1.4536	0.2102	0.218	0.7606	0
1348	965.3	1.453	0.21	0.2178	0.7605	0
1349	965.2	1.4533	0.2102	0.2182	0.7606	0
1350	965.1	1.4535	0.2104	0.218	0.7608	0
1351	965	1.4536	0.2105	0.2183	0.7608	0
1352	964.9	1.4536	0.21	0.2182	0.7607	0
1353	964.8	1.4543	0.2106	0.2184	0.7609	0
1354	964.7	1.4545	0.2108	0.2185	0.7611	0
1355	964.6	1.4543	0.211	0.2188	0.7612	0
1356	964.5	1.4546	0.2107	0.2186	0.7611	0
1357	964.4	1.4549	0.2107	0.2188	0.7614	0
1358	964.3	1.4555	0.2111	0.2188	0.7612	0
1359	964.2	1.4551	0.2108	0.2187	0.7614	0
1360	964.1	1.4551	0.211	0.2186	0.7612	0
1361	964	1.4551	0.2107	0.2185	0.7614	0
1362	963.9	1.4552	0.211	0.2187	0.7612	0

1363	963.8	1.4555	0.2111	0.219	0.7615	0
1364	963.7	1.4556	0.2113	0.2191	0.7615	0
1365	963.6	1.4559	0.2114	0.2193	0.7617	0
1366	963.5	1.4559	0.2113	0.2193	0.7619	0
1367	963.4	1.4561	0.2111	0.2194	0.7621	0
1368	963.3	1.456	0.2112	0.219	0.762	0
1369	963.2	1.4561	0.2115	0.2193	0.7622	0
1370	963.1	1.4566	0.2112	0.2193	0.7619	0
1371	963	1.4561	0.2113	0.2192	0.7618	0
1372	962.9	1.4563	0.2114	0.2194	0.7622	0
1373	962.8	1.457	0.2117	0.2194	0.7624	0
1374	962.7	1.4566	0.2115	0.2198	0.7624	0
1375	962.6	1.4566	0.2116	0.2195	0.7623	0
1376	962.5	1.4572	0.2118	0.2196	0.7626	0
1377	962.4	1.4566	0.2116	0.2195	0.7623	0
1378	962.3	1.4567	0.2115	0.2195	0.7624	0
1379	962.2	1.4568	0.2116	0.2196	0.7624	0
1380	962.1	1.4577	0.2116	0.2195	0.7623	0
1381	962	1.4571	0.2118	0.2199	0.7625	0
1382	961.9	1.4576	0.2121	0.2198	0.7626	0
1383	961.8	1.4577	0.2119	0.2199	0.7628	0
1384	961.7	1.4576	0.212	0.2197	0.7627	0
1385	961.6	1.4576	0.2118	0.2198	0.7626	0
1386	961.5	1.4576	0.2119	0.2196	0.7627	0
1387	961.4	1.4573	0.2119	0.2198	0.7628	0
1388	961.3	1.4576	0.2118	0.2197	0.7628	0
1389	961.2	1.4577	0.2117	0.2197	0.7628	0
1390	961.1	1.4588	0.2121	0.2199	0.763	0
1391	961	1.458	0.2122	0.22	0.7631	0
1392	960.9	1.458	0.2122	0.22	0.763	0
1393	960.8	1.4576	0.212	0.2201	0.763	0
1394	960.7	1.458	0.2121	0.2201	0.7631	0
1395	960.6	1.4579	0.2119	0.2199	0.7627	0
1396	960.5	1.458	0.212	0.2201	0.7628	0
1397	960.4	1.4586	0.2119	0.2202	0.7632	0
1398	960.3	1.4582	0.2121	0.2202	0.7634	0
1399	960.2	1.4582	0.212	0.2202	0.7628	0
1400	960.1	1.4577	0.212	0.22	0.7628	0
1401	960	1.458	0.2118	0.2199	0.7628	0
1402	959.9	1.458	0.2117	0.22	0.763	0
1403	959.8	1.4579	0.2117	0.2201	0.7628	0
1404	959.7	1.4579	0.2117	0.2198	0.763	0
1405	959.6	1.4581	0.212	0.2201	0.763	0

1406	959.5	1.4582	0.2117	0.2198	0.7626	0
1407	959.4	1.4578	0.2117	0.2197	0.7626	0
1408	959.3	1.458	0.212	0.2199	0.763	0
1409	959.2	1.4581	0.2121	0.2201	0.763	0
1410	959.1	1.4577	0.2123	0.2199	0.763	0
1411	959	1.458	0.2121	0.22	0.7629	0
1412	958.9	1.4579	0.212	0.2199	0.763	0
1413	958.8	1.4579	0.2121	0.2199	0.763	0
1414	958.7	1.4584	0.2121	0.2202	0.7629	0
1415	958.6	1.4578	0.2118	0.2199	0.7628	0
1416	958.5	1.4585	0.2122	0.2199	0.7632	0
1417	958.4	1.4579	0.212	0.22	0.7631	0
1418	958.3	1.4574	0.2118	0.2198	0.763	0
1419	958.2	1.4572	0.2117	0.2197	0.7628	0
1420	958.1	1.4575	0.2116	0.2197	0.7625	0
1421	958	1.4574	0.2115	0.2194	0.7627	0
1422	957.9	1.4575	0.2116	0.2195	0.7629	0
1423	957.8	1.4575	0.2116	0.2197	0.7627	0
1424	957.7	1.457	0.2116	0.2194	0.7625	0
1425	957.6	1.457	0.2115	0.2195	0.7626	0
1426	957.5	1.4575	0.2117	0.2195	0.7624	0
1427	957.4	1.4571	0.2119	0.2195	0.7626	0
1428	957.3	1.457	0.2115	0.2194	0.7625	0
1429	957.2	1.4573	0.2114	0.2194	0.7623	0
1430	957.1	1.4573	0.2118	0.2195	0.7624	0
1431	957	1.457	0.2118	0.2196	0.7625	0
1432	956.9	1.457	0.2116	0.2195	0.7627	0
1433	956.8	1.457	0.2115	0.2199	0.7626	0
1434	956.7	1.4569	0.2114	0.2196	0.7625	0
1435	956.6	1.4574	0.2114	0.2196	0.7624	0
1436	956.5	1.4575	0.2116	0.2197	0.7626	0
1437	956.4	1.4571	0.2116	0.2199	0.7628	0
1438	956.3	1.4577	0.2118	0.2199	0.7626	0
1439	956.2	1.4575	0.2117	0.2196	0.7625	0
1440	956.1	1.4577	0.2119	0.22	0.7627	0
1441	956	1.4576	0.2117	0.2197	0.7625	0
1442	955.9	1.4571	0.2117	0.2199	0.7625	0
1443	955.8	1.4575	0.2116	0.2196	0.7626	0
1444	955.7	1.4577	0.2117	0.2196	0.7626	0
1445	955.6	1.4576	0.2116	0.2196	0.7625	0
1446	955.5	1.4575	0.2116	0.2195	0.7624	0
1447	955.4	1.4573	0.2118	0.2198	0.7627	0
1448	955.3	1.4573	0.2118	0.2199	0.7624	0

1449	955.2	1.4578	0.2117	0.2198	0.7623	0
1450	955.1	1.458	0.2114	0.2194	0.7623	0
1451	955	1.4577	0.2116	0.2195	0.7623	0
1452	954.9	1.4576	0.2113	0.2195	0.7621	0
1453	954.8	1.4578	0.2116	0.2196	0.7622	0
1454	954.7	1.4575	0.2119	0.2197	0.7624	0
1455	954.6	1.4585	0.2119	0.2197	0.7624	0
1456	954.5	1.458	0.2119	0.2196	0.7622	0
1457	954.4	1.4582	0.2119	0.2198	0.7625	0
1458	954.3	1.4584	0.2121	0.2199	0.7627	0
1459	954.2	1.4583	0.2118	0.2198	0.7625	0
1460	954.1	1.4582	0.2118	0.2198	0.7624	0
1461	954	1.4585	0.212	0.2203	0.7627	0
1462	953.9	1.4588	0.2119	0.2198	0.7625	0
1463	953.8	1.4585	0.2122	0.2199	0.7625	0
1464	953.7	1.4591	0.212	0.2201	0.7627	0
1465	953.6	1.4589	0.2124	0.2202	0.7628	0
1466	953.5	1.4594	0.2124	0.2204	0.7629	0
1467	953.4	1.4595	0.2121	0.2203	0.7629	0
1468	953.3	1.4595	0.2123	0.2203	0.7628	0
1469	953.2	1.4597	0.2124	0.2206	0.7629	0
1470	953.1	1.4602	0.2125	0.2205	0.763	0
1471	953	1.4599	0.2124	0.2205	0.7629	0
1472	952.9	1.4599	0.2125	0.2206	0.7627	0
1473	952.8	1.4599	0.2125	0.2205	0.7627	0
1474	952.7	1.4605	0.2125	0.2206	0.7628	0
1475	952.6	1.4601	0.2127	0.2207	0.7629	0
1476	952.5	1.4606	0.2126	0.2206	0.7629	0
1477	952.4	1.4611	0.2126	0.2208	0.7629	0
1478	952.3	1.4601	0.2125	0.2206	0.7628	0
1479	952.2	1.4608	0.2126	0.2208	0.7631	0
1480	952.1	1.4609	0.213	0.2209	0.763	0
1481	952	1.4609	0.2127	0.2209	0.7631	0
1482	951.9	1.4611	0.213	0.221	0.7632	0
1483	951.8	1.4616	0.2131	0.2212	0.7631	0
1484	951.7	1.4616	0.2131	0.2211	0.7633	0
1485	951.6	1.4614	0.2132	0.2213	0.7633	0
1486	951.5	1.4607	0.213	0.2211	0.763	0
1487	951.4	1.4616	0.2129	0.221	0.7632	0
1488	951.3	1.4617	0.2132	0.2211	0.7631	0
1489	951.2	1.4612	0.2128	0.2211	0.763	0
1490	951.1	1.4612	0.2132	0.2211	0.763	0
1491	951	1.4613	0.2131	0.2212	0.7628	0

1492	950.9	1.4618	0.213	0.221	0.7631	0
1493	950.8	1.462	0.2132	0.2211	0.763	0
1494	950.7	1.4616	0.2133	0.2214	0.7632	0
1495	950.6	1.4619	0.2131	0.2212	0.763	0
1496	950.5	1.462	0.2133	0.2211	0.7629	0
1497	950.4	1.4618	0.2134	0.2215	0.7633	0
1498	950.3	1.4622	0.2133	0.2215	0.7632	0
1499	950.2	1.4621	0.2134	0.2215	0.7631	0
1500	950.1	1.4616	0.2133	0.2212	0.7629	0
1501	950	1.4621	0.2133	0.2215	0.7629	0
1502	949.9	1.4622	0.2135	0.2215	0.7631	0
1503	949.8	1.4616	0.2134	0.2214	0.7629	0
1504	949.7	1.4616	0.2133	0.2214	0.763	0
1505	949.6	1.4615	0.2134	0.2212	0.7628	0
1506	949.5	1.4615	0.2134	0.2215	0.763	0
1507	949.4	1.4621	0.2134	0.2215	0.763	0
1508	949.3	1.4617	0.2135	0.2217	0.7632	0
1509	949.2	1.4615	0.2133	0.2215	0.7629	0
1510	949.1	1.4619	0.2134	0.2213	0.7628	0
1511	949	1.4619	0.2132	0.2214	0.763	0
1512	948.9	1.4615	0.2135	0.2215	0.7629	0
1513	948.8	1.4616	0.2135	0.2215	0.7629	0
1514	948.7	1.4617	0.2131	0.2212	0.7627	0
1515	948.6	1.4615	0.2132	0.2211	0.7626	0
1516	948.5	1.4617	0.2134	0.2212	0.7625	0
1517	948.4	1.4617	0.2132	0.2213	0.7627	0
1518	948.3	1.4613	0.2134	0.2213	0.7626	0
1519	948.2	1.4618	0.2133	0.2212	0.7626	0
1520	948.1	1.4617	0.2133	0.2212	0.7627	0
1521	948	1.4618	0.2134	0.2216	0.763	0
1522	947.9	1.4618	0.2133	0.2213	0.7626	0
1523	947.8	1.4616	0.2131	0.2212	0.7626	0
1524	947.7	1.4611	0.2132	0.2211	0.7625	0
1525	947.6	1.4611	0.213	0.2212	0.7625	0
1526	947.5	1.4615	0.213	0.2212	0.7625	0
1527	947.4	1.4615	0.213	0.2211	0.7622	0
1528	947.3	1.4614	0.213	0.221	0.7625	0
1529	947.2	1.4608	0.2128	0.2209	0.7623	0
1530	947.1	1.4609	0.2129	0.2209	0.7623	0
1531	947	1.4604	0.213	0.2209	0.7622	0
1532	946.9	1.4604	0.213	0.2211	0.7622	0
1533	946.8	1.4615	0.2132	0.2214	0.7625	0
1534	946.7	1.461	0.2131	0.2213	0.7623	0

1535	946.6	1.4611	0.2133	0.2213	0.7625	0
1536	946.5	1.4608	0.2127	0.221	0.7621	0
1537	946.4	1.4608	0.213	0.2211	0.7621	0
1538	946.3	1.4612	0.2131	0.2207	0.7623	0
1539	946.2	1.4606	0.2128	0.2208	0.7619	0
1540	946.1	1.4613	0.2128	0.221	0.7621	0
1541	946	1.4605	0.213	0.2212	0.7623	0
1542	945.9	1.4615	0.2133	0.2212	0.7625	0
1543	945.8	1.4609	0.2132	0.2212	0.7624	0
1544	945.7	1.4615	0.2131	0.2212	0.7624	0
1545	945.6	1.4615	0.2132	0.2212	0.7625	0
1546	945.5	1.4613	0.2131	0.2211	0.7624	0
1547	945.4	1.4613	0.2131	0.2212	0.7624	0
1548	945.3	1.4615	0.2129	0.2209	0.7621	0
1549	945.2	1.4612	0.2131	0.221	0.7621	0
1550	945.1	1.4617	0.2131	0.2209	0.762	0
1551	945	1.4617	0.2132	0.2212	0.762	0
1552	944.9	1.4612	0.213	0.2211	0.7623	0
1553	944.8	1.4618	0.2132	0.2213	0.7623	0
1554	944.7	1.4615	0.2135	0.2214	0.7624	0
1555	944.6	1.4621	0.2134	0.2215	0.7627	0
1556	944.5	1.4615	0.2133	0.2212	0.7626	0
1557	944.4	1.4616	0.2132	0.2213	0.7624	0
1558	944.3	1.4615	0.2132	0.2212	0.7625	0
1559	944.2	1.462	0.2132	0.2213	0.7623	0
1560	944.1	1.4622	0.2134	0.2214	0.7625	0
1561	944	1.4623	0.2136	0.2215	0.7625	0
1562	943.9	1.4627	0.2136	0.2214	0.7626	0
1563	943.8	1.4621	0.2133	0.2214	0.7625	0
1564	943.7	1.4626	0.2134	0.2214	0.7626	0
1565	943.6	1.463	0.2135	0.2215	0.7626	0
1566	943.5	1.4626	0.2135	0.2215	0.7627	0
1567	943.4	1.463	0.2133	0.2215	0.7626	0
1568	943.3	1.4626	0.2136	0.2215	0.7626	0
1569	943.2	1.4624	0.2137	0.2217	0.7624	0
1570	943.1	1.4633	0.2138	0.2218	0.7627	0
1571	943	1.4629	0.2137	0.2218	0.7626	0
1572	942.9	1.4636	0.2138	0.222	0.7628	0
1573	942.8	1.4634	0.2138	0.2219	0.7627	0
1574	942.7	1.4634	0.2139	0.2217	0.7627	0
1575	942.6	1.4634	0.2137	0.2217	0.7625	0
1576	942.5	1.4634	0.2137	0.2218	0.7627	0
1577	942.4	1.4634	0.2135	0.2218	0.7626	0

1578	942.3	1.463	0.2136	0.2218	0.7627	0
1579	942.2	1.4636	0.2138	0.2218	0.7627	0
1580	942.1	1.4638	0.214	0.2221	0.7626	0
1581	942	1.4638	0.2139	0.222	0.7628	0
1582	941.9	1.4638	0.2139	0.222	0.7627	0
1583	941.8	1.465	0.2143	0.2222	0.7631	0
1584	941.7	1.4649	0.2142	0.2223	0.7629	0
1585	941.6	1.4642	0.2143	0.2223	0.7631	0
1586	941.5	1.4647	0.2142	0.2223	0.763	0
1587	941.4	1.4648	0.2143	0.2224	0.7634	0
1588	941.3	1.4643	0.2142	0.2223	0.7631	0
1589	941.2	1.4641	0.214	0.2222	0.7628	0
1590	941.1	1.4642	0.2141	0.2222	0.7631	0
1591	941	1.465	0.2142	0.2224	0.7631	0
1592	940.9	1.4641	0.2142	0.2224	0.7633	0
1593	940.8	1.4647	0.2144	0.2225	0.7635	0
1594	940.7	1.4646	0.2141	0.2224	0.7633	0
1595	940.6	1.4652	0.2143	0.2227	0.7634	0
1596	940.5	1.4642	0.2142	0.2224	0.7632	0
1597	940.4	1.4645	0.2142	0.2223	0.7632	0
1598	940.3	1.4649	0.2141	0.2222	0.7632	0
1599	940.2	1.4651	0.2143	0.2224	0.7633	0
1600	940.1	1.4653	0.2146	0.2229	0.7634	0
1601	940	1.4652	0.2143	0.2226	0.7632	0
1602	939.9	1.4652	0.2144	0.2226	0.7632	0
1603	939.8	1.4647	0.2142	0.2226	0.7632	0
1604	939.7	1.4651	0.2141	0.2225	0.7631	0
1605	939.6	1.4653	0.2145	0.2224	0.7634	0
1606	939.5	1.4655	0.2148	0.2229	0.7637	0
1607	939.4	1.4658	0.2148	0.2228	0.7636	0
1608	939.3	1.4648	0.2147	0.2227	0.7635	0
1609	939.2	1.4653	0.2144	0.2227	0.7633	0
1610	939.1	1.4655	0.2148	0.2229	0.7636	0
1611	939	1.4656	0.2149	0.223	0.7639	0
1612	938.9	1.4653	0.2147	0.2227	0.7636	0
1613	938.8	1.4652	0.2145	0.2228	0.7634	0
1614	938.7	1.4652	0.2148	0.2228	0.7633	0
1615	938.6	1.4656	0.2147	0.2227	0.7634	0
1616	938.5	1.466	0.2144	0.2228	0.7635	0
1617	938.4	1.4656	0.2146	0.2228	0.7636	0
1618	938.3	1.4655	0.2147	0.2229	0.7636	0
1619	938.2	1.4653	0.2144	0.2227	0.7634	0
1620	938.1	1.4654	0.2146	0.2229	0.7635	0

1621	938	1.4653	0.2145	0.2228	0.7633	0
1622	937.9	1.4647	0.2145	0.2226	0.7634	0
1623	937.8	1.4657	0.2148	0.2229	0.7635	0
1624	937.7	1.4655	0.2147	0.2226	0.7634	0
1625	937.6	1.4656	0.2148	0.2228	0.7633	0
1626	937.5	1.466	0.2146	0.2227	0.7635	0
1627	937.4	1.4654	0.2147	0.2226	0.7634	0
1628	937.3	1.4655	0.2146	0.2229	0.7633	0
1629	937.2	1.4656	0.2148	0.2229	0.7637	0
1630	937.1	1.4654	0.2147	0.2227	0.7636	0
1631	937	1.4657	0.2146	0.2226	0.7634	0
1632	936.9	1.4655	0.2144	0.2226	0.7633	0
1633	936.8	1.4658	0.2146	0.2229	0.7635	0
1634	936.7	1.4658	0.2148	0.2228	0.7635	0
1635	936.6	1.466	0.2144	0.2224	0.7631	0
1636	936.5	1.4662	0.2145	0.2228	0.7634	0
1637	936.4	1.4657	0.2145	0.2227	0.7636	0
1638	936.3	1.4656	0.2144	0.2227	0.7635	0
1639	936.2	1.4659	0.2148	0.2229	0.7637	0
1640	936.1	1.4658	0.2147	0.2228	0.7636	0
1641	936	1.4653	0.2146	0.2229	0.7635	0
1642	935.9	1.4658	0.2146	0.2228	0.7635	0
1643	935.8	1.4655	0.2143	0.2225	0.7633	0
1644	935.7	1.4659	0.2145	0.2225	0.7633	0
1645	935.6	1.4656	0.2145	0.2226	0.7634	0
1646	935.5	1.4662	0.2148	0.2229	0.7636	0
1647	935.4	1.4662	0.2149	0.223	0.7634	0
1648	935.3	1.4662	0.215	0.223	0.7634	0
1649	935.2	1.4659	0.2148	0.2226	0.7634	0
1650	935.1	1.4664	0.2147	0.2227	0.7633	0
1651	935	1.4665	0.2147	0.2229	0.7634	0
1652	934.9	1.4665	0.2149	0.2229	0.7634	0
1653	934.8	1.4661	0.215	0.2229	0.7634	0
1654	934.7	1.4661	0.2151	0.2228	0.7636	0
1655	934.6	1.4664	0.2149	0.2229	0.7635	0
1656	934.5	1.4666	0.2149	0.223	0.7635	0
1657	934.4	1.467	0.2151	0.2229	0.7636	0
1658	934.3	1.4665	0.2147	0.2228	0.7636	0
1659	934.2	1.4665	0.2149	0.2228	0.7634	0
1660	934.1	1.4669	0.215	0.223	0.7636	0
1661	934	1.467	0.215	0.2231	0.7637	0
1662	933.9	1.467	0.2148	0.2231	0.7635	0
1663	933.8	1.4666	0.215	0.2231	0.7638	0

1664	933.7	1.467	0.2149	0.2231	0.7636	0
1665	933.6	1.4675	0.2151	0.2233	0.7636	0
1666	933.5	1.4671	0.215	0.2232	0.7637	0
1667	933.4	1.4673	0.2151	0.2234	0.7638	0
1668	933.3	1.4677	0.2151	0.2234	0.7638	0
1669	933.2	1.4676	0.2156	0.2237	0.7642	0
1670	933.1	1.4679	0.2154	0.2235	0.7638	0
1671	933	1.4681	0.2156	0.2237	0.7641	0
1672	932.9	1.4672	0.2153	0.2237	0.7643	0
1673	932.8	1.468	0.2155	0.2235	0.764	0
1674	932.7	1.4681	0.2154	0.2235	0.7639	0
1675	932.6	1.4678	0.2157	0.2237	0.7643	0
1676	932.5	1.468	0.2153	0.2234	0.764	0
1677	932.4	1.4686	0.2155	0.2236	0.7642	0
1678	932.3	1.4682	0.2157	0.2237	0.7642	0
1679	932.2	1.4678	0.2155	0.2237	0.764	0
1680	932.1	1.4683	0.2157	0.2239	0.7643	0
1681	932	1.4678	0.2157	0.2239	0.7642	0
1682	931.9	1.4683	0.2157	0.224	0.7642	0
1683	931.8	1.4673	0.2155	0.2238	0.7643	0
1684	931.7	1.4682	0.2154	0.2237	0.7642	0
1685	931.6	1.4688	0.2158	0.2239	0.7643	0
1686	931.5	1.4686	0.216	0.2242	0.7646	0
1687	931.4	1.4683	0.2159	0.2239	0.7644	0
1688	931.3	1.4685	0.2159	0.2241	0.7647	0
1689	931.2	1.4684	0.2158	0.2238	0.7645	0
1690	931.1	1.4684	0.2159	0.2238	0.7646	0
1691	931	1.4684	0.2157	0.224	0.7644	0
1692	930.9	1.4681	0.2155	0.2238	0.7642	0
1693	930.8	1.4685	0.2154	0.2237	0.7642	0
1694	930.7	1.4687	0.2156	0.2239	0.7643	0
1695	930.6	1.4685	0.2156	0.2238	0.7643	0
1696	930.5	1.4682	0.2156	0.2238	0.7643	0
1697	930.4	1.4689	0.2159	0.2241	0.7646	0
1698	930.3	1.4687	0.2157	0.2239	0.7644	0
1699	930.2	1.4689	0.2157	0.2236	0.7641	0
1700	930.1	1.4679	0.2153	0.2236	0.7642	0
1701	930	1.4685	0.2156	0.2235	0.7641	0
1702	929.9	1.468	0.2155	0.2237	0.7642	0
1703	929.8	1.4679	0.2155	0.2236	0.764	0
1704	929.7	1.4684	0.2154	0.2238	0.7641	0
1705	929.6	1.4684	0.2156	0.2238	0.7641	0
1706	929.5	1.4684	0.2155	0.2238	0.7642	0

1707	929.4	1.4682	0.2158	0.2239	0.7644	0
1708	929.3	1.4685	0.2157	0.2239	0.7644	0
1709	929.2	1.4689	0.2156	0.224	0.7644	0
1710	929.1	1.468	0.2155	0.2239	0.7642	0
1711	929	1.469	0.2157	0.2239	0.7644	0
1712	928.9	1.4687	0.2154	0.2236	0.7641	0
1713	928.8	1.4683	0.2156	0.2239	0.7642	0
1714	928.7	1.4688	0.2157	0.2239	0.7643	0
1715	928.6	1.4689	0.2158	0.2238	0.7644	0
1716	928.5	1.4684	0.216	0.2242	0.7643	0
1717	928.4	1.4686	0.2155	0.2237	0.7641	0
1718	928.3	1.4687	0.2157	0.2241	0.7644	0
1719	928.2	1.4689	0.2158	0.2241	0.7646	0
1720	928.1	1.4689	0.2158	0.2242	0.7648	0
1721	928	1.4693	0.2158	0.2242	0.7646	0
1722	927.9	1.4689	0.2158	0.2243	0.7646	0
1723	927.8	1.4694	0.2159	0.2243	0.7645	0
1724	927.7	1.4694	0.216	0.2242	0.7645	0
1725	927.6	1.4683	0.2157	0.2242	0.7644	0
1726	927.5	1.4688	0.2159	0.2244	0.7645	0
1727	927.4	1.4688	0.216	0.2244	0.7649	0
1728	927.3	1.4686	0.2157	0.2241	0.7645	0
1729	927.2	1.4692	0.2158	0.2243	0.7644	0
1730	927.1	1.469	0.2156	0.2241	0.7644	0
1731	927	1.4688	0.2159	0.2242	0.7648	0
1732	926.9	1.4694	0.216	0.2244	0.7649	0
1733	926.8	1.4694	0.2162	0.2246	0.7648	0
1734	926.7	1.4693	0.216	0.2244	0.7648	0
1735	926.6	1.4692	0.216	0.2243	0.7647	0
1736	926.5	1.4693	0.2161	0.2244	0.7648	0
1737	926.4	1.4696	0.2165	0.2249	0.7652	0
1738	926.3	1.47	0.2164	0.2247	0.7651	0
1739	926.2	1.4695	0.2163	0.2246	0.7652	0
1740	926.1	1.4693	0.216	0.2244	0.765	0
1741	926	1.4697	0.2161	0.2243	0.7648	0
1742	925.9	1.4696	0.2159	0.2242	0.7647	0
1743	925.8	1.4697	0.2163	0.2247	0.765	0
1744	925.7	1.4702	0.2162	0.2246	0.7651	0
1745	925.6	1.47	0.2165	0.2249	0.7654	0
1746	925.5	1.4699	0.2164	0.2246	0.7652	0
1747	925.4	1.4699	0.2163	0.2246	0.7652	0
1748	925.3	1.4693	0.2164	0.2246	0.7652	0
1749	925.2	1.4704	0.2164	0.2247	0.7655	0

1750	925.1	1.4707	0.2163	0.2246	0.7654	0
1751	925	1.47	0.216	0.2244	0.765	0
1752	924.9	1.4701	0.216	0.2246	0.765	0
1753	924.8	1.4702	0.2164	0.2248	0.7652	0
1754	924.7	1.4698	0.2163	0.2246	0.7651	0
1755	924.6	1.4703	0.2164	0.2247	0.7652	0
1756	924.5	1.4703	0.2164	0.2248	0.7652	0
1757	924.4	1.4702	0.2164	0.2247	0.7654	0
1758	924.3	1.4705	0.2162	0.2246	0.7651	0
1759	924.2	1.4706	0.2162	0.2246	0.7652	0
1760	924.1	1.4706	0.2165	0.2247	0.7651	0
1761	924	1.4701	0.2163	0.2247	0.7652	0
1762	923.9	1.4708	0.2165	0.2249	0.7654	0
1763	923.8	1.471	0.2164	0.2246	0.7652	0
1764	923.7	1.4707	0.2166	0.2248	0.7654	0
1765	923.6	1.4709	0.2169	0.2251	0.7655	0
1766	923.5	1.4714	0.2169	0.2253	0.7658	0
1767	923.4	1.4716	0.2166	0.2248	0.7655	0
1768	923.3	1.4713	0.2168	0.225	0.7657	0
1769	923.2	1.4711	0.2167	0.2249	0.7656	0
1770	923.1	1.4717	0.2165	0.2251	0.7653	0
1771	923	1.4714	0.217	0.2249	0.7653	0
1772	922.9	1.4717	0.2169	0.2251	0.7657	0
1773	922.8	1.4713	0.2169	0.2251	0.7658	0
1774	922.7	1.4712	0.2168	0.2251	0.7656	0
1775	922.6	1.4717	0.2168	0.2253	0.7658	0
1776	922.5	1.4719	0.2171	0.2254	0.7658	0
1777	922.4	1.4714	0.2171	0.2252	0.7659	0
1778	922.3	1.4721	0.2169	0.2253	0.7659	0
1779	922.2	1.4716	0.2169	0.2252	0.7656	0
1780	922.1	1.4715	0.2168	0.2251	0.7657	0
1781	922	1.4712	0.217	0.2253	0.7657	0
1782	921.9	1.4716	0.2169	0.2255	0.7659	0
1783	921.8	1.4719	0.2169	0.2252	0.7656	0
1784	921.7	1.4709	0.2169	0.2252	0.7657	0
1785	921.6	1.4715	0.217	0.2254	0.766	0
1786	921.5	1.4714	0.2169	0.2252	0.7659	0
1787	921.4	1.4719	0.217	0.2255	0.766	0
1788	921.3	1.4719	0.2171	0.2254	0.7661	0
1789	921.2	1.4714	0.217	0.2256	0.7659	0
1790	921.1	1.4719	0.2172	0.2255	0.766	0
1791	921	1.4719	0.2171	0.2256	0.766	0
1792	920.9	1.4711	0.2169	0.2253	0.7657	0

1793	920.8	1.4719	0.2167	0.2253	0.7658	0
1794	920.7	1.4719	0.2171	0.2254	0.766	0
1795	920.6	1.4719	0.2169	0.2254	0.7659	0
1796	920.5	1.4715	0.217	0.2256	0.766	0
1797	920.4	1.4719	0.217	0.2254	0.7663	0
1798	920.3	1.4717	0.217	0.2254	0.766	0
1799	920.2	1.4713	0.2169	0.2255	0.7661	0
1800	920.1	1.4718	0.217	0.2255	0.7663	0
1801	920	1.4711	0.2169	0.2252	0.7658	0
1802	919.9	1.4716	0.2168	0.2252	0.7659	0
1803	919.8	1.4714	0.2168	0.2254	0.7658	0
1804	919.7	1.4715	0.2167	0.2255	0.7661	0
1805	919.6	1.4719	0.2169	0.2252	0.7658	0
1806	919.5	1.472	0.2171	0.2256	0.7662	0
1807	919.4	1.4713	0.2169	0.2253	0.7658	0
1808	919.3	1.4716	0.2168	0.2254	0.7659	0
1809	919.2	1.4716	0.2168	0.2251	0.7659	0
1810	919.1	1.4716	0.2169	0.2253	0.7661	0
1811	919	1.4719	0.2168	0.2253	0.766	0
1812	918.9	1.4717	0.2166	0.2251	0.7657	0
1813	918.8	1.4717	0.2168	0.2252	0.7659	0
1814	918.7	1.4716	0.2167	0.2252	0.7656	0
1815	918.6	1.4711	0.2167	0.225	0.766	0
1816	918.5	1.4713	0.2166	0.225	0.7658	0
1817	918.4	1.4713	0.2169	0.2251	0.7656	0
1818	918.3	1.4713	0.2167	0.2249	0.7658	0
1819	918.2	1.4714	0.2167	0.2252	0.7656	0
1820	918.1	1.4713	0.2168	0.2252	0.7658	0
1821	918	1.4717	0.2168	0.2251	0.7658	0
1822	917.9	1.4715	0.2165	0.2251	0.7656	0
1823	917.8	1.4711	0.2166	0.2251	0.7656	0
1824	917.7	1.4718	0.2169	0.2254	0.766	0
1825	917.6	1.4717	0.2169	0.2256	0.7661	0
1826	917.5	1.4716	0.2167	0.2252	0.766	0
1827	917.4	1.4714	0.2166	0.2251	0.7659	0
1828	917.3	1.4711	0.2168	0.2252	0.766	0
1829	917.2	1.4711	0.2168	0.2253	0.766	0
1830	917.1	1.4711	0.2169	0.2255	0.7661	0
1831	917	1.4711	0.2168	0.2256	0.7662	0
1832	916.9	1.4714	0.2169	0.2253	0.7659	0
1833	916.8	1.4724	0.217	0.2254	0.7661	0
1834	916.7	1.4715	0.2171	0.2254	0.7662	0
1835	916.6	1.4719	0.2169	0.2255	0.7664	0

1836	916.5	1.4717	0.2168	0.2251	0.766	0
1837	916.4	1.4721	0.2169	0.2252	0.7659	0
1838	916.3	1.4722	0.217	0.2253	0.7661	0
1839	916.2	1.4719	0.2172	0.2255	0.7663	0
1840	916.1	1.4719	0.2171	0.2256	0.7665	0
1841	916	1.4714	0.2171	0.2257	0.7664	0
1842	915.9	1.4718	0.217	0.2256	0.7664	0
1843	915.8	1.4724	0.2173	0.2257	0.7667	0
1844	915.7	1.4722	0.2171	0.2255	0.7663	0
1845	915.6	1.4724	0.2172	0.2256	0.7667	0
1846	915.5	1.4728	0.2173	0.2258	0.7668	0
1847	915.4	1.4729	0.2175	0.2258	0.7666	0
1848	915.3	1.4727	0.2173	0.2256	0.7664	0
1849	915.2	1.4722	0.2172	0.2256	0.7665	0
1850	915.1	1.4722	0.2172	0.2258	0.7666	0
1851	915	1.4729	0.2175	0.226	0.7668	0
1852	914.9	1.4733	0.2174	0.226	0.7668	0
1853	914.8	1.473	0.2176	0.2261	0.7668	0
1854	914.7	1.4731	0.2177	0.2263	0.767	0
1855	914.6	1.474	0.2177	0.226	0.767	0
1856	914.5	1.473	0.2177	0.2265	0.7668	0
1857	914.4	1.4729	0.2177	0.2261	0.7668	0
1858	914.3	1.4733	0.2174	0.2261	0.767	0
1859	914.2	1.474	0.2178	0.2262	0.767	0
1860	914.1	1.4733	0.2176	0.2261	0.7667	0
1861	914	1.4733	0.2178	0.2263	0.7671	0
1862	913.9	1.4733	0.2177	0.2261	0.7668	0
1863	913.8	1.4732	0.2176	0.2262	0.767	0
1864	913.7	1.4738	0.2176	0.2262	0.7671	0
1865	913.6	1.4733	0.2178	0.2263	0.767	0
1866	913.5	1.4743	0.2177	0.2262	0.7672	0
1867	913.4	1.4739	0.218	0.2263	0.7674	0
1868	913.3	1.4738	0.2179	0.2263	0.7672	0
1869	913.2	1.4737	0.2178	0.2263	0.7673	0
1870	913.1	1.4738	0.2181	0.2263	0.7672	0
1871	913	1.4742	0.2179	0.2264	0.7673	0
1872	912.9	1.4742	0.2179	0.2264	0.7671	0
1873	912.8	1.4742	0.218	0.2264	0.7673	0
1874	912.7	1.4746	0.2181	0.2263	0.7675	0
1875	912.6	1.4743	0.2182	0.2267	0.7677	0
1876	912.5	1.4747	0.2183	0.2269	0.7676	0
1877	912.4	1.4743	0.2182	0.2267	0.7675	0
1878	912.3	1.4741	0.2181	0.2267	0.7674	0

1879	912.2	1.4742	0.2184	0.2269	0.7677	0
1880	912.1	1.4747	0.2183	0.2271	0.7678	0
1881	912	1.4749	0.2185	0.2271	0.7678	0
1882	911.9	1.4749	0.2185	0.2274	0.7677	0
1883	911.8	1.4743	0.2184	0.2271	0.7678	0
1884	911.7	1.4753	0.2186	0.2272	0.7681	0
1885	911.6	1.4751	0.2183	0.2272	0.7679	0
1886	911.5	1.4747	0.2184	0.227	0.768	0
1887	911.4	1.4746	0.2184	0.2271	0.7681	0
1888	911.3	1.4751	0.2184	0.2271	0.768	0
1889	911.2	1.4747	0.2185	0.2271	0.768	0
1890	911.1	1.475	0.2186	0.2271	0.7682	0
1891	911	1.4742	0.2184	0.2269	0.7679	0
1892	910.9	1.4749	0.2188	0.227	0.768	0
1893	910.8	1.4752	0.2184	0.227	0.7682	0
1894	910.7	1.4745	0.2184	0.2267	0.7679	0
1895	910.6	1.475	0.2183	0.2268	0.7679	0
1896	910.5	1.475	0.2185	0.227	0.768	0
1897	910.4	1.4749	0.2182	0.2268	0.7681	0
1898	910.3	1.4748	0.2184	0.2268	0.7679	0
1899	910.2	1.4749	0.2184	0.2269	0.7681	0
1900	910.1	1.4746	0.2181	0.2267	0.7678	0
1901	910	1.475	0.2181	0.2268	0.7678	0
1902	909.9	1.4746	0.2183	0.2269	0.7678	0
1903	909.8	1.4747	0.2184	0.2269	0.768	0
1904	909.7	1.4751	0.2185	0.2272	0.7682	0
1905	909.6	1.4748	0.2183	0.2268	0.7679	0
1906	909.5	1.4747	0.2181	0.2269	0.7679	0
1907	909.4	1.4746	0.2182	0.2267	0.768	0
1908	909.3	1.4745	0.2181	0.2269	0.768	0
1909	909.2	1.475	0.2182	0.227	0.768	0
1910	909.1	1.4749	0.2183	0.2268	0.7678	0
1911	909	1.4738	0.2182	0.2266	0.768	0
1912	908.9	1.4746	0.218	0.2266	0.768	0
1913	908.8	1.4751	0.2181	0.2268	0.7681	0
1914	908.7	1.4745	0.2181	0.2266	0.768	0
1915	908.6	1.4744	0.2181	0.2268	0.7677	0
1916	908.5	1.4743	0.218	0.2266	0.7681	0
1917	908.4	1.4747	0.218	0.2265	0.768	0
1918	908.3	1.4742	0.2181	0.2267	0.7679	0
1919	908.2	1.474	0.2179	0.2266	0.7676	0
1920	908.1	1.4738	0.2177	0.2264	0.7676	0
1921	908	1.4739	0.218	0.2265	0.7679	0

1922	907.9	1.4743	0.2179	0.2265	0.7681	0
1923	907.8	1.4748	0.218	0.2266	0.768	0
1924	907.7	1.4747	0.218	0.2266	0.7681	0
1925	907.6	1.4747	0.2181	0.2266	0.768	0
1926	907.5	1.4745	0.218	0.2263	0.7679	0
1927	907.4	1.4745	0.218	0.2266	0.768	0
1928	907.3	1.4738	0.2178	0.2263	0.7678	0
1929	907.2	1.4742	0.2177	0.2264	0.7677	0
1930	907.1	1.4742	0.2179	0.2264	0.7676	0
1931	907	1.474	0.2177	0.2264	0.7677	0
1932	906.9	1.4746	0.2178	0.2264	0.7681	0
1933	906.8	1.4747	0.2182	0.2266	0.7683	0
1934	906.7	1.4747	0.2183	0.2268	0.7683	0
1935	906.6	1.4743	0.2185	0.2267	0.7683	0
1936	906.5	1.475	0.2179	0.2265	0.7682	0
1937	906.4	1.4746	0.2182	0.2267	0.7681	0
1938	906.3	1.4744	0.218	0.2266	0.7679	0
1939	906.2	1.4743	0.218	0.2267	0.7681	0
1940	906.1	1.4748	0.2179	0.2266	0.7681	0
1941	906	1.4747	0.218	0.2266	0.7682	0
1942	905.9	1.4743	0.2181	0.2267	0.7682	0
1943	905.8	1.4749	0.2182	0.2268	0.7681	0
1944	905.7	1.4746	0.2182	0.2266	0.7682	0
1945	905.6	1.4749	0.2185	0.2268	0.7684	0
1946	905.5	1.4753	0.2183	0.227	0.7685	0
1947	905.4	1.4749	0.2184	0.2272	0.7687	0
1948	905.3	1.4753	0.2183	0.227	0.7686	0
1949	905.2	1.4757	0.2184	0.227	0.7685	0
1950	905.1	1.4757	0.2185	0.227	0.7686	0
1951	905	1.4757	0.2185	0.227	0.7686	0
1952	904.9	1.4752	0.2187	0.2271	0.7686	0
1953	904.8	1.4753	0.2187	0.2273	0.7687	0
1954	904.7	1.4755	0.2187	0.2274	0.7688	0
1955	904.6	1.4759	0.2187	0.2274	0.7689	0
1956	904.5	1.476	0.2189	0.2274	0.7691	0
1957	904.4	1.4763	0.2188	0.2273	0.7689	0
1958	904.3	1.4759	0.2189	0.2274	0.7691	0
1959	904.2	1.4761	0.219	0.2275	0.7694	0
1960	904.1	1.4761	0.2193	0.2275	0.7693	0
1961	904	1.4765	0.2191	0.2277	0.7691	0
1962	903.9	1.4767	0.2191	0.2279	0.7695	0
1963	903.8	1.4766	0.2192	0.2278	0.7694	0
1964	903.7	1.4766	0.2192	0.2279	0.7692	0

1965	903.6	1.477	0.2191	0.2278	0.7695	0
1966	903.5	1.4767	0.2193	0.2279	0.7696	0
1967	903.4	1.4772	0.2193	0.2279	0.7697	0
1968	903.3	1.4771	0.2194	0.2279	0.7697	0
1969	903.2	1.4776	0.2194	0.2281	0.7697	0
1970	903.1	1.4777	0.2195	0.228	0.77	0
1971	903	1.4775	0.2193	0.2278	0.7696	0
1972	902.9	1.4775	0.2195	0.228	0.7697	0
1973	902.8	1.4776	0.2195	0.2282	0.77	0
1974	902.7	1.4771	0.2196	0.2284	0.7698	0
1975	902.6	1.4775	0.2195	0.2281	0.7699	0
1976	902.5	1.4774	0.2196	0.228	0.7699	0
1977	902.4	1.4773	0.2195	0.2282	0.7699	0
1978	902.3	1.4779	0.2197	0.2283	0.7699	0
1979	902.2	1.4779	0.2197	0.2283	0.7702	0
1980	902.1	1.4773	0.2196	0.2283	0.77	0
1981	902	1.4781	0.2195	0.2282	0.7697	0
1982	901.9	1.4781	0.2197	0.2283	0.77	0
1983	901.8	1.4778	0.22	0.2286	0.7704	0
1984	901.7	1.4783	0.2198	0.2286	0.7704	0
1985	901.6	1.4784	0.2197	0.2283	0.7702	0
1986	901.5	1.478	0.2197	0.2283	0.7702	0
1987	901.4	1.4777	0.2199	0.2285	0.7704	0
1988	901.3	1.4782	0.2198	0.2288	0.7705	0
1989	901.2	1.4783	0.2195	0.2283	0.7701	0
1990	901.1	1.4779	0.2198	0.2286	0.7702	0
1991	901	1.4781	0.2195	0.2284	0.7702	0
1992	900.9	1.4781	0.2196	0.2283	0.7704	0
1993	900.8	1.4775	0.2195	0.2284	0.7702	0
1994	900.7	1.4784	0.2197	0.2284	0.7701	0
1995	900.6	1.4783	0.2195	0.2284	0.77	0
1996	900.5	1.4785	0.2198	0.2285	0.7701	0
1997	900.4	1.4782	0.2196	0.2283	0.7702	0
1998	900.3	1.4781	0.2196	0.2283	0.77	0
1999	900.2	1.4786	0.2196	0.2283	0.7703	0
2000	900.1	1.4782	0.2196	0.2285	0.7704	0
2001	900	1.4783	0.2199	0.2286	0.7705	0
2002	899.9	1.4785	0.2197	0.2283	0.7704	0
2003	899.8	1.478	0.2197	0.2284	0.7703	0
2004	899.7	1.4779	0.2197	0.2285	0.7704	0
2005	899.6	1.4784	0.22	0.2286	0.7704	0
2006	899.5	1.4777	0.2198	0.2284	0.7703	0
2007	899.4	1.4785	0.2198	0.2282	0.7703	0

2008	899.3	1.4785	0.2195	0.2284	0.7702	0
2009	899.2	1.4784	0.2195	0.2283	0.7703	0
2010	899.1	1.4778	0.2196	0.2284	0.7704	0
2011	899	1.4778	0.2196	0.2284	0.7706	0
2012	898.9	1.4778	0.2196	0.2286	0.7707	0
2013	898.8	1.4787	0.22	0.2285	0.7707	0
2014	898.7	1.4781	0.2198	0.2283	0.7704	0
2015	898.6	1.4774	0.2197	0.2283	0.7703	0
2016	898.5	1.4773	0.2196	0.2282	0.7703	0
2017	898.4	1.4779	0.2199	0.2284	0.7707	0
2018	898.3	1.4773	0.2199	0.2285	0.7704	0
2019	898.2	1.4786	0.2198	0.2284	0.7703	0
2020	898.1	1.4785	0.2196	0.2286	0.7704	0
2021	898	1.4777	0.2195	0.2281	0.7703	0
2022	897.9	1.4772	0.2194	0.228	0.7702	0
2023	897.8	1.4778	0.2196	0.2282	0.7707	0
2024	897.7	1.4779	0.2193	0.2279	0.7705	0
2025	897.6	1.4774	0.2192	0.2279	0.7705	0
2026	897.5	1.4778	0.2194	0.2279	0.7703	0
2027	897.4	1.4777	0.2194	0.2281	0.7702	0
2028	897.3	1.4777	0.2194	0.2283	0.7705	0
2029	897.2	1.4782	0.2195	0.2284	0.7705	0
2030	897.1	1.4775	0.2194	0.2281	0.7704	0
2031	897	1.4778	0.2194	0.2283	0.7704	0
2032	896.9	1.4775	0.2192	0.2279	0.7701	0
2033	896.8	1.4771	0.2192	0.228	0.7701	0
2034	896.7	1.4771	0.2193	0.228	0.7704	0
2035	896.6	1.4782	0.2196	0.2281	0.7705	0
2036	896.5	1.4776	0.2194	0.2281	0.7704	0
2037	896.4	1.4779	0.2195	0.2281	0.7703	0
2038	896.3	1.4778	0.2194	0.2282	0.7703	0
2039	896.2	1.4772	0.2193	0.228	0.7702	0
2040	896.1	1.4771	0.2192	0.228	0.7702	0
2041	896	1.4776	0.2192	0.228	0.7703	0
2042	895.9	1.4782	0.2195	0.2281	0.7707	0
2043	895.8	1.4779	0.2192	0.2279	0.7705	0
2044	895.7	1.4777	0.2196	0.2284	0.7704	0
2045	895.6	1.4776	0.2196	0.2283	0.7702	0
2046	895.5	1.4779	0.2194	0.2282	0.7704	0
2047	895.4	1.4774	0.2193	0.2281	0.7703	0
2048	895.3	1.4778	0.2194	0.2282	0.7705	0
2049	895.2	1.4777	0.2193	0.2281	0.7703	0
2050	895.1	1.4777	0.2194	0.2283	0.7704	0

2051	895	1.4782	0.2194	0.2284	0.7706	0
2052	894.9	1.4783	0.2196	0.2284	0.7709	0
2053	894.8	1.4778	0.2197	0.2284	0.7708	0
2054	894.7	1.4783	0.2197	0.2285	0.7709	0
2055	894.6	1.4776	0.2194	0.2283	0.7708	0
2056	894.5	1.4786	0.2194	0.2282	0.7708	0
2057	894.4	1.478	0.2194	0.2284	0.7708	0
2058	894.3	1.478	0.2196	0.2282	0.7705	0
2059	894.2	1.478	0.2196	0.2283	0.7707	0
2060	894.1	1.478	0.2196	0.2284	0.7707	0
2061	894	1.479	0.2198	0.2284	0.7709	0
2062	893.9	1.4785	0.2197	0.2286	0.7712	0
2063	893.8	1.4786	0.2198	0.2288	0.771	0
2064	893.7	1.4792	0.2199	0.2288	0.7712	0
2065	893.6	1.4794	0.2204	0.229	0.7715	0
2066	893.5	1.4793	0.2202	0.229	0.7714	0
2067	893.4	1.4794	0.2204	0.2292	0.7716	0
2068	893.3	1.4793	0.2203	0.2292	0.7718	0
2069	893.2	1.4793	0.2204	0.2291	0.7716	0
2070	893.1	1.4796	0.2203	0.229	0.7717	0
2071	893	1.4792	0.2203	0.2292	0.7717	0
2072	892.9	1.4798	0.2203	0.2293	0.7719	0
2073	892.8	1.4796	0.2203	0.2291	0.7718	0
2074	892.7	1.4797	0.2205	0.2293	0.7715	0
2075	892.6	1.4797	0.2205	0.2295	0.772	0
2076	892.5	1.4802	0.2206	0.2295	0.7721	0
2077	892.4	1.4802	0.2208	0.2295	0.772	0
2078	892.3	1.4798	0.2208	0.2296	0.7721	0
2079	892.2	1.4802	0.2208	0.2295	0.772	0
2080	892.1	1.4802	0.2211	0.2297	0.7723	0
2081	892	1.4797	0.2208	0.2296	0.7725	0
2082	891.9	1.4806	0.2207	0.2296	0.7725	0
2083	891.8	1.4807	0.221	0.2297	0.7725	0
2084	891.7	1.4806	0.2209	0.2298	0.7726	0
2085	891.6	1.4806	0.2207	0.2297	0.7723	0
2086	891.5	1.4806	0.221	0.2298	0.7726	0
2087	891.4	1.4815	0.2211	0.2299	0.7724	0
2088	891.3	1.4814	0.221	0.2298	0.7723	0
2089	891.2	1.4809	0.2211	0.2296	0.7726	0
2090	891.1	1.4812	0.2209	0.2296	0.7726	0
2091	891	1.4805	0.2209	0.2296	0.7723	0
2092	890.9	1.4811	0.221	0.2297	0.7725	0
2093	890.8	1.4806	0.221	0.2299	0.7725	0

2094	890.7	1.4805	0.2209	0.2299	0.7724	0
2095	890.6	1.481	0.2211	0.2299	0.7726	0
2096	890.5	1.4813	0.221	0.2298	0.7727	0
2097	890.4	1.4813	0.221	0.2298	0.7726	0
2098	890.3	1.4811	0.2209	0.2298	0.7724	0
2099	890.2	1.4811	0.221	0.2298	0.7727	0
2100	890.1	1.4813	0.2211	0.2301	0.7728	0
2101	890	1.4806	0.2211	0.23	0.7728	0
2102	889.9	1.481	0.2211	0.23	0.7727	0
2103	889.8	1.4812	0.2214	0.2302	0.7728	0
2104	889.7	1.4811	0.2214	0.2301	0.7726	0
2105	889.6	1.4814	0.2212	0.2301	0.7729	0
2106	889.5	1.4814	0.2211	0.2301	0.773	0
2107	889.4	1.4812	0.2215	0.23	0.773	0
2108	889.3	1.4805	0.2211	0.2299	0.7728	0
2109	889.2	1.4809	0.221	0.2298	0.7728	0
2110	889.1	1.4813	0.221	0.2299	0.7726	0
2111	889	1.4813	0.2211	0.2299	0.7726	0
2112	888.9	1.4815	0.2211	0.2303	0.7732	0
2113	888.8	1.4816	0.2212	0.23	0.7729	0
2114	888.7	1.4811	0.221	0.2299	0.7727	0
2115	888.6	1.481	0.2212	0.23	0.7728	0
2116	888.5	1.4814	0.2209	0.2299	0.7729	0
2117	888.4	1.481	0.2214	0.2301	0.7729	0
2118	888.3	1.4807	0.2211	0.2299	0.7728	0
2119	888.2	1.4811	0.2212	0.2299	0.7728	0
2120	888.1	1.4806	0.2213	0.2302	0.7729	0
2121	888	1.4803	0.2208	0.2296	0.7727	0
2122	887.9	1.4806	0.2208	0.2295	0.7726	0
2123	887.8	1.4808	0.2207	0.2293	0.7725	0
2124	887.7	1.4805	0.2211	0.2295	0.7725	0
2125	887.6	1.4805	0.2208	0.2297	0.7728	0
2126	887.5	1.4809	0.2209	0.2298	0.7728	0
2127	887.4	1.4809	0.2211	0.2298	0.7726	0
2128	887.3	1.4808	0.2209	0.2297	0.7726	0
2129	887.2	1.4805	0.2208	0.2294	0.7725	0
2130	887.1	1.4804	0.2206	0.2295	0.7726	0
2131	887	1.4803	0.2207	0.2295	0.7725	0
2132	886.9	1.4808	0.221	0.2296	0.7726	0
2133	886.8	1.4802	0.2208	0.2295	0.7725	0
2134	886.7	1.4806	0.2207	0.2296	0.7726	0
2135	886.6	1.4806	0.2208	0.2296	0.773	0
2136	886.5	1.4809	0.2207	0.2293	0.7728	0

2137	886.4	1.4805	0.2209	0.2297	0.7728	0
2138	886.3	1.4809	0.2207	0.2294	0.7729	0
2139	886.2	1.4807	0.2206	0.2296	0.7729	0
2140	886.1	1.4801	0.2207	0.2295	0.7726	0
2141	886	1.4805	0.2208	0.2296	0.7727	0
2142	885.9	1.4812	0.2207	0.2297	0.7731	0
2143	885.8	1.4806	0.2209	0.2296	0.7729	0
2144	885.7	1.4804	0.2205	0.2295	0.7727	0
2145	885.6	1.4806	0.2209	0.2298	0.7729	0
2146	885.5	1.4809	0.221	0.2296	0.7729	0
2147	885.4	1.4812	0.2208	0.2297	0.7725	0
2148	885.3	1.4807	0.2208	0.2295	0.7728	0
2149	885.2	1.4812	0.2208	0.2296	0.7728	0
2150	885.1	1.4813	0.2211	0.2297	0.7731	0
2151	885	1.4807	0.2212	0.2298	0.7732	0
2152	884.9	1.4811	0.221	0.2297	0.7732	0
2153	884.8	1.481	0.221	0.2296	0.7729	0
2154	884.7	1.4812	0.2212	0.23	0.7733	0
2155	884.6	1.4811	0.2211	0.23	0.7732	0
2156	884.5	1.4815	0.2212	0.2299	0.7735	0
2157	884.4	1.4812	0.2214	0.2301	0.7736	0
2158	884.3	1.4814	0.2212	0.2299	0.7735	0
2159	884.2	1.4815	0.2213	0.2299	0.7734	0
2160	884.1	1.4814	0.2212	0.2298	0.7735	0
2161	884	1.481	0.2213	0.23	0.7738	0
2162	883.9	1.4818	0.2211	0.2299	0.7736	0
2163	883.8	1.4822	0.2212	0.23	0.7736	0
2164	883.7	1.4822	0.2214	0.2301	0.7738	0
2165	883.6	1.4816	0.2212	0.23	0.7737	0
2166	883.5	1.4822	0.2213	0.2301	0.7738	0
2167	883.4	1.4822	0.2214	0.2303	0.7736	0
2168	883.3	1.4817	0.2215	0.2302	0.7738	0
2169	883.2	1.4817	0.2217	0.2301	0.7738	0
2170	883.1	1.4818	0.2216	0.2305	0.774	0
2171	883	1.4822	0.2217	0.2305	0.7738	0
2172	882.9	1.482	0.2215	0.2305	0.7739	0
2173	882.8	1.4815	0.2216	0.2305	0.7741	0
2174	882.7	1.4819	0.2213	0.2304	0.7739	0
2175	882.6	1.4824	0.2217	0.2306	0.7741	0
2176	882.5	1.4821	0.2218	0.2309	0.7742	0
2177	882.4	1.4826	0.2219	0.2307	0.7743	0
2178	882.3	1.4825	0.2219	0.2308	0.7742	0
2179	882.2	1.4829	0.2218	0.2307	0.7743	0

2180	882.1	1.4822	0.2215	0.2307	0.7741	0
2181	882	1.4826	0.2216	0.2305	0.7741	0
2182	881.9	1.4828	0.2217	0.2308	0.7743	0
2183	881.8	1.4821	0.2216	0.2305	0.7744	0
2184	881.7	1.4827	0.2219	0.2307	0.7745	0
2185	881.6	1.4823	0.2222	0.231	0.7744	0
2186	881.5	1.4826	0.2219	0.2308	0.7745	0
2187	881.4	1.4826	0.222	0.2308	0.7745	0
2188	881.3	1.4826	0.222	0.2309	0.7746	0
2189	881.2	1.4831	0.2221	0.231	0.7747	0
2190	881.1	1.4828	0.2219	0.2307	0.7745	0
2191	881	1.4828	0.2219	0.2308	0.7744	0
2192	880.9	1.4827	0.2218	0.2307	0.7743	0
2193	880.8	1.4829	0.222	0.231	0.7746	0
2194	880.7	1.4833	0.2222	0.2309	0.7749	0
2195	880.6	1.4832	0.2222	0.2309	0.7747	0
2196	880.5	1.4831	0.2221	0.2309	0.7747	0
2197	880.4	1.4836	0.2221	0.231	0.7747	0
2198	880.3	1.4835	0.222	0.2309	0.7752	0
2199	880.2	1.484	0.2221	0.2311	0.7752	0
2200	880.1	1.4837	0.2219	0.2308	0.7748	0
2201	880	1.4833	0.2223	0.2309	0.7749	0
2202	879.9	1.4833	0.2223	0.2311	0.775	0
2203	879.8	1.4836	0.2222	0.231	0.7748	0
2204	879.7	1.483	0.2221	0.231	0.7748	0
2205	879.6	1.483	0.222	0.2309	0.7748	0
2206	879.5	1.4833	0.222	0.2309	0.7746	0
2207	879.4	1.4832	0.2218	0.2309	0.7748	0
2208	879.3	1.4831	0.222	0.2307	0.7749	0
2209	879.2	1.4833	0.222	0.231	0.7749	0
2210	879.1	1.4843	0.2223	0.2312	0.7752	0
2211	879	1.4837	0.2222	0.2311	0.7751	0
2212	878.9	1.4831	0.2222	0.2311	0.7752	0
2213	878.8	1.4836	0.2221	0.2311	0.7753	0
2214	878.7	1.483	0.2223	0.2311	0.7751	0
2215	878.6	1.4833	0.2221	0.231	0.7747	0
2216	878.5	1.4833	0.222	0.2311	0.7751	0
2217	878.4	1.4835	0.2219	0.2308	0.7749	0
2218	878.3	1.4835	0.222	0.2309	0.7751	0
2219	878.2	1.4835	0.222	0.231	0.775	0
2220	878.1	1.4831	0.2222	0.2312	0.7753	0
2221	878	1.483	0.2222	0.2312	0.7752	0
2222	877.9	1.4834	0.2223	0.2313	0.7751	0

2223	877.8	1.4833	0.2221	0.2312	0.775	0
2224	877.7	1.4838	0.2223	0.2311	0.7753	0
2225	877.6	1.4839	0.2225	0.2313	0.7756	0
2226	877.5	1.4832	0.2224	0.2312	0.7755	0
2227	877.4	1.4835	0.2221	0.2312	0.7752	0
2228	877.3	1.484	0.2223	0.2311	0.7755	0
2229	877.2	1.4833	0.2221	0.231	0.7753	0
2230	877.1	1.4833	0.2221	0.231	0.7752	0
2231	877	1.4834	0.2218	0.2307	0.775	0
2232	876.9	1.4835	0.222	0.2311	0.775	0
2233	876.8	1.483	0.222	0.2311	0.7751	0
2234	876.7	1.4839	0.2221	0.2309	0.7753	0
2235	876.6	1.4834	0.2222	0.2309	0.7753	0
2236	876.5	1.4835	0.2223	0.2312	0.7756	0
2237	876.4	1.4832	0.222	0.2309	0.7754	0
2238	876.3	1.4833	0.2221	0.231	0.7753	0
2239	876.2	1.4838	0.2223	0.2311	0.7753	0
2240	876.1	1.4837	0.2223	0.231	0.7754	0
2241	876	1.4836	0.2223	0.2312	0.7754	0
2242	875.9	1.4837	0.2223	0.2311	0.7756	0
2243	875.8	1.4836	0.2221	0.2311	0.7754	0
2244	875.7	1.4836	0.2224	0.2312	0.7759	0
2245	875.6	1.484	0.2224	0.2312	0.7757	0
2246	875.5	1.4838	0.2222	0.231	0.7754	0
2247	875.4	1.4836	0.2221	0.231	0.7755	0
2248	875.3	1.4837	0.2222	0.2311	0.7756	0
2249	875.2	1.4842	0.2224	0.2311	0.7757	0
2250	875.1	1.4846	0.2221	0.231	0.7754	0
2251	875	1.4839	0.2221	0.231	0.7754	0
2252	874.9	1.484	0.2224	0.2312	0.7757	0
2253	874.8	1.4835	0.2223	0.2312	0.7756	0
2254	874.7	1.4835	0.2224	0.2313	0.7758	0
2255	874.6	1.4845	0.2225	0.2312	0.7759	0
2256	874.5	1.484	0.2224	0.2313	0.776	0
2257	874.4	1.484	0.2223	0.2314	0.7759	0
2258	874.3	1.4844	0.2224	0.2313	0.7759	0
2259	874.2	1.4844	0.2225	0.2315	0.776	0
2260	874.1	1.4844	0.2224	0.2314	0.7758	0
2261	874	1.4844	0.2225	0.2313	0.7758	0
2262	873.9	1.485	0.2227	0.2314	0.776	0
2263	873.8	1.4845	0.2227	0.2316	0.776	0
2264	873.7	1.485	0.2226	0.2317	0.7762	0
2265	873.6	1.4849	0.2227	0.2317	0.7762	0

2266	873.5	1.4848	0.2226	0.2317	0.7761	0
2267	873.4	1.4853	0.2225	0.2317	0.7763	0
2268	873.3	1.4853	0.2227	0.2317	0.7764	0
2269	873.2	1.4847	0.2227	0.2316	0.7763	0
2270	873.1	1.4851	0.2225	0.2317	0.7765	0
2271	873	1.485	0.2226	0.2315	0.7761	0
2272	872.9	1.485	0.2226	0.2316	0.776	0
2273	872.8	1.4849	0.2225	0.2315	0.7762	0
2274	872.7	1.4859	0.2225	0.2315	0.7763	0
2275	872.6	1.4849	0.2226	0.2316	0.7763	0
2276	872.5	1.4854	0.2227	0.2316	0.7764	0
2277	872.4	1.485	0.2228	0.2318	0.7768	0
2278	872.3	1.4853	0.2227	0.2316	0.7766	0
2279	872.2	1.485	0.2231	0.2319	0.7768	0
2280	872.1	1.4853	0.2229	0.2318	0.7766	0
2281	872	1.4854	0.2229	0.2319	0.7768	0
2282	871.9	1.4854	0.2228	0.2321	0.7767	0
2283	871.8	1.4858	0.223	0.2322	0.7768	0
2284	871.7	1.4852	0.2229	0.232	0.7767	0
2285	871.6	1.4857	0.223	0.232	0.7766	0
2286	871.5	1.4857	0.223	0.2319	0.777	0
2287	871.4	1.4845	0.223	0.2319	0.7768	0
2288	871.3	1.4854	0.223	0.2318	0.7768	0
2289	871.2	1.4855	0.2231	0.232	0.7765	0
2290	871.1	1.4855	0.2231	0.232	0.7769	0
2291	871	1.4861	0.2232	0.2322	0.7771	0
2292	870.9	1.4854	0.2229	0.2322	0.777	0
2293	870.8	1.4857	0.223	0.232	0.7768	0
2294	870.7	1.4853	0.223	0.2321	0.7768	0
2295	870.6	1.4857	0.223	0.2319	0.7771	0
2296	870.5	1.4851	0.2231	0.232	0.777	0
2297	870.4	1.4855	0.223	0.232	0.777	0
2298	870.3	1.4858	0.223	0.2318	0.7771	0
2299	870.2	1.4858	0.2231	0.2318	0.777	0
2300	870.1	1.4859	0.2231	0.2321	0.777	0
2301	870	1.4859	0.2232	0.2323	0.7772	0
2302	869.9	1.4862	0.2231	0.232	0.777	0
2303	869.8	1.4854	0.2228	0.2317	0.777	0
2304	869.7	1.486	0.2229	0.232	0.777	0
2305	869.6	1.4855	0.223	0.2321	0.7769	0
2306	869.5	1.4854	0.223	0.2319	0.7771	0
2307	869.4	1.4848	0.2229	0.2319	0.7769	0
2308	869.3	1.4858	0.2229	0.2318	0.7771	0

2309	869.2	1.4853	0.223	0.2319	0.7772	0
2310	869.1	1.4856	0.2229	0.2318	0.7772	0
2311	869	1.4855	0.2228	0.2318	0.7769	0
2312	868.9	1.4854	0.2228	0.2319	0.777	0
2313	868.8	1.486	0.223	0.2319	0.7773	0
2314	868.7	1.4854	0.2229	0.2321	0.7772	0
2315	868.6	1.4853	0.2229	0.2321	0.7773	0
2316	868.5	1.4852	0.223	0.2319	0.7773	0
2317	868.4	1.4853	0.2231	0.2321	0.7773	0
2318	868.3	1.4857	0.2231	0.232	0.7773	0
2319	868.2	1.4857	0.2233	0.2321	0.7773	0
2320	868.1	1.4853	0.2232	0.2321	0.7772	0
2321	868	1.4857	0.2231	0.2322	0.7774	0
2322	867.9	1.4859	0.2233	0.2324	0.7777	0
2323	867.8	1.4863	0.2234	0.2323	0.7778	0
2324	867.7	1.4855	0.223	0.232	0.7773	0
2325	867.6	1.4867	0.2234	0.2325	0.7777	0
2326	867.5	1.4859	0.2231	0.2322	0.7774	0
2327	867.4	1.4858	0.2229	0.2321	0.7773	0
2328	867.3	1.4858	0.2231	0.2322	0.7773	0
2329	867.2	1.4857	0.223	0.2321	0.7774	0
2330	867.1	1.4857	0.223	0.2321	0.7774	0
2331	867	1.4858	0.2232	0.2322	0.7774	0
2332	866.9	1.4863	0.2232	0.2323	0.7777	0
2333	866.8	1.4856	0.2233	0.2322	0.7778	0
2334	866.7	1.4852	0.2234	0.2324	0.7777	0
2335	866.6	1.4857	0.2232	0.2324	0.7779	0
2336	866.5	1.4858	0.2233	0.2326	0.7781	0
2337	866.4	1.486	0.2232	0.2321	0.7778	0
2338	866.3	1.4861	0.2233	0.2323	0.7779	0
2339	866.2	1.486	0.2233	0.2322	0.7779	0
2340	866.1	1.4866	0.2233	0.2323	0.7779	0
2341	866	1.4861	0.2234	0.2323	0.7781	0
2342	865.9	1.4865	0.2233	0.2323	0.7779	0
2343	865.8	1.4865	0.2234	0.2324	0.7782	0
2344	865.7	1.4861	0.2235	0.2325	0.7782	0
2345	865.6	1.4868	0.2235	0.2329	0.7785	0
2346	865.5	1.4873	0.2237	0.2329	0.7786	0
2347	865.4	1.4872	0.2237	0.2327	0.7784	0
2348	865.3	1.4871	0.2236	0.2327	0.7784	0
2349	865.2	1.487	0.2236	0.2327	0.7783	0
2350	865.1	1.487	0.2237	0.2326	0.7784	0
2351	865	1.487	0.2236	0.2327	0.7784	0

2352	864.9	1.4874	0.2236	0.2327	0.7782	0
2353	864.8	1.4869	0.2236	0.2327	0.7783	0
2354	864.7	1.488	0.2237	0.2327	0.7786	0
2355	864.6	1.4874	0.2237	0.2328	0.7787	0
2356	864.5	1.488	0.2239	0.233	0.7788	0
2357	864.4	1.4869	0.2236	0.233	0.7787	0
2358	864.3	1.487	0.2239	0.233	0.7786	0
2359	864.2	1.4875	0.2238	0.2331	0.7786	0
2360	864.1	1.4875	0.224	0.233	0.7787	0
2361	864	1.488	0.224	0.2329	0.7789	0
2362	863.9	1.488	0.2239	0.2331	0.7788	0
2363	863.8	1.4891	0.224	0.2329	0.7791	0
2364	863.7	1.4887	0.2241	0.2332	0.7791	0
2365	863.6	1.4886	0.2242	0.2332	0.7793	0
2366	863.5	1.488	0.224	0.233	0.779	0
2367	863.4	1.4886	0.2243	0.2331	0.7791	0
2368	863.3	1.4887	0.2244	0.2333	0.7794	0
2369	863.2	1.4891	0.2241	0.2333	0.7793	0
2370	863.1	1.4886	0.2242	0.2333	0.7791	0
2371	863	1.4891	0.2241	0.2332	0.7794	0
2372	862.9	1.4891	0.2242	0.2334	0.7793	0
2373	862.8	1.4883	0.2239	0.2331	0.7791	0
2374	862.7	1.4887	0.2239	0.2329	0.779	0
2375	862.6	1.4888	0.2242	0.2331	0.7791	0
2376	862.5	1.4891	0.2244	0.2335	0.7795	0
2377	862.4	1.4891	0.2245	0.2335	0.7796	0
2378	862.3	1.4886	0.2245	0.2335	0.7796	0
2379	862.2	1.4888	0.2242	0.2332	0.7796	0
2380	862.1	1.4886	0.2239	0.2331	0.7793	0
2381	862	1.4888	0.2243	0.2334	0.7796	0
2382	861.9	1.4899	0.2242	0.2336	0.7795	0
2383	861.8	1.4893	0.2245	0.2334	0.7796	0
2384	861.7	1.4893	0.2244	0.2334	0.7796	0
2385	861.6	1.4897	0.2244	0.2333	0.7797	0
2386	861.5	1.4897	0.2244	0.2333	0.7795	0
2387	861.4	1.4891	0.2244	0.2334	0.7794	0
2388	861.3	1.4897	0.2243	0.2336	0.7795	0
2389	861.2	1.4895	0.2241	0.2332	0.7794	0
2390	861.1	1.4896	0.2244	0.2335	0.7797	0
2391	861	1.4892	0.2245	0.2334	0.7798	0
2392	860.9	1.4891	0.2244	0.2333	0.7798	0
2393	860.8	1.4897	0.2246	0.2335	0.7799	0
2394	860.7	1.4895	0.2244	0.2334	0.78	0

2395	860.6	1.4895	0.2244	0.2333	0.7796	0
2396	860.5	1.4899	0.2244	0.2333	0.7796	0
2397	860.4	1.4899	0.2245	0.2336	0.7797	0
2398	860.3	1.4892	0.2243	0.2332	0.7797	0
2399	860.2	1.4893	0.2244	0.2333	0.7798	0
2400	860.1	1.4903	0.2245	0.2335	0.7799	0
2401	860	1.4902	0.2244	0.2332	0.7797	0
2402	859.9	1.4893	0.2245	0.2334	0.78	0
2403	859.8	1.4898	0.2244	0.2333	0.7802	0
2404	859.7	1.4891	0.2243	0.2334	0.7801	0
2405	859.6	1.4896	0.2244	0.2335	0.7799	0
2406	859.5	1.4896	0.2245	0.2334	0.7801	0
2407	859.4	1.4896	0.2245	0.2335	0.7799	0
2408	859.3	1.4891	0.2246	0.2334	0.78	0
2409	859.2	1.489	0.2246	0.2333	0.7801	0
2410	859.1	1.4893	0.2243	0.2332	0.7797	0
2411	859	1.4893	0.2244	0.2332	0.78	0
2412	858.9	1.4887	0.2244	0.2331	0.7798	0
2413	858.8	1.4893	0.2245	0.2332	0.7799	0
2414	858.7	1.4891	0.2242	0.2331	0.7799	0
2415	858.6	1.4891	0.2242	0.2331	0.78	0
2416	858.5	1.4892	0.2243	0.2334	0.7802	0
2417	858.4	1.4895	0.2242	0.2333	0.7802	0
2418	858.3	1.489	0.2242	0.2332	0.78	0
2419	858.2	1.4893	0.2241	0.233	0.7799	0
2420	858.1	1.4888	0.2243	0.2331	0.7799	0
2421	858	1.4886	0.224	0.2327	0.7797	0
2422	857.9	1.4886	0.224	0.233	0.7799	0
2423	857.8	1.4893	0.2241	0.2332	0.78	0
2424	857.7	1.4895	0.2244	0.2332	0.7803	0
2425	857.6	1.4897	0.224	0.2331	0.7802	0
2426	857.5	1.489	0.2239	0.2329	0.7799	0
2427	857.4	1.4891	0.224	0.233	0.7802	0
2428	857.3	1.4886	0.2242	0.2332	0.7801	0
2429	857.2	1.4886	0.2241	0.2331	0.7801	0
2430	857.1	1.4892	0.2242	0.2333	0.7801	0
2431	857	1.489	0.2242	0.233	0.78	0
2432	856.9	1.4894	0.2241	0.233	0.78	0
2433	856.8	1.4884	0.2241	0.233	0.78	0
2434	856.7	1.489	0.2241	0.2331	0.7802	0
2435	856.6	1.4892	0.2243	0.2333	0.7804	0
2436	856.5	1.489	0.2242	0.2331	0.7802	0
2437	856.4	1.4893	0.224	0.2328	0.7802	0

2438	856.3	1.4889	0.2241	0.233	0.78	0
2439	856.2	1.4891	0.2241	0.2333	0.7804	0
2440	856.1	1.4897	0.2244	0.2334	0.7808	0
2441	856	1.4893	0.2245	0.2334	0.7808	0
2442	855.9	1.4894	0.2246	0.2335	0.7808	0
2443	855.8	1.4903	0.2246	0.2334	0.7804	0
2444	855.7	1.4898	0.2244	0.2334	0.7808	0
2445	855.6	1.4899	0.2246	0.2335	0.781	0
2446	855.5	1.4901	0.2247	0.2337	0.781	0
2447	855.4	1.4902	0.2249	0.2338	0.7815	0
2448	855.3	1.4902	0.2251	0.2337	0.7811	0
2449	855.2	1.4901	0.2247	0.2336	0.7812	0
2450	855.1	1.4913	0.2249	0.2339	0.7813	0
2451	855	1.4907	0.2247	0.2339	0.7813	0
2452	854.9	1.4902	0.2248	0.2338	0.7814	0
2453	854.8	1.4902	0.2248	0.2338	0.7813	0
2454	854.7	1.4901	0.2246	0.2336	0.7811	0
2455	854.6	1.4908	0.2248	0.2338	0.7813	0
2456	854.5	1.4911	0.2247	0.2337	0.7812	0
2457	854.4	1.4913	0.2249	0.2338	0.7813	0
2458	854.3	1.4903	0.225	0.2339	0.7814	0
2459	854.2	1.4915	0.225	0.234	0.7815	0
2460	854.1	1.4916	0.225	0.2339	0.7819	0
2461	854	1.491	0.2251	0.234	0.7817	0
2462	853.9	1.4917	0.2253	0.2343	0.7816	0
2463	853.8	1.4916	0.2252	0.2341	0.7818	0
2464	853.7	1.4918	0.2254	0.2341	0.782	0
2465	853.6	1.4913	0.2253	0.2342	0.7818	0
2466	853.5	1.4919	0.2254	0.2344	0.7819	0
2467	853.4	1.4918	0.2253	0.2342	0.782	0
2468	853.3	1.4924	0.2254	0.2342	0.7822	0
2469	853.2	1.4927	0.2256	0.2346	0.7824	0
2470	853.1	1.4932	0.2257	0.2345	0.7823	0
2471	853	1.492	0.2255	0.2342	0.7823	0
2472	852.9	1.4921	0.2256	0.2343	0.7822	0
2473	852.8	1.4932	0.2257	0.2344	0.7823	0
2474	852.7	1.4924	0.2253	0.234	0.7821	0
2475	852.6	1.4925	0.2255	0.2342	0.7822	0
2476	852.5	1.493	0.2255	0.2342	0.7822	0
2477	852.4	1.4926	0.2257	0.2343	0.7824	0
2478	852.3	1.4926	0.2254	0.2342	0.7824	0
2479	852.2	1.4931	0.2255	0.2343	0.7824	0
2480	852.1	1.492	0.2255	0.2342	0.7825	0

2481	852	1.4926	0.2255	0.2344	0.7825	0
2482	851.9	1.4927	0.2256	0.2343	0.7825	0
2483	851.8	1.4922	0.2258	0.2344	0.7826	0
2484	851.7	1.4927	0.2257	0.2344	0.7827	0
2485	851.6	1.4933	0.2257	0.2346	0.7828	0
2486	851.5	1.4922	0.2256	0.2343	0.7827	0
2487	851.4	1.4927	0.2256	0.2345	0.7825	0
2488	851.3	1.4933	0.2259	0.2345	0.7828	0
2489	851.2	1.4927	0.2256	0.2344	0.7825	0
2490	851.1	1.4927	0.2256	0.2345	0.7825	0
2491	851	1.4927	0.2255	0.2345	0.7828	0
2492	850.9	1.4933	0.2257	0.2345	0.7826	0
2493	850.8	1.4933	0.2257	0.2346	0.7827	0
2494	850.7	1.4927	0.2257	0.2343	0.7828	0
2495	850.6	1.4933	0.2257	0.2344	0.783	0
2496	850.5	1.4936	0.2256	0.2343	0.7827	0
2497	850.4	1.4922	0.2257	0.2344	0.7828	0
2498	850.3	1.4928	0.2258	0.2347	0.7829	0
2499	850.2	1.4927	0.2259	0.2345	0.783	0
2500	850.1	1.4927	0.2258	0.2345	0.7828	0
2501	850	1.4921	0.2256	0.2344	0.7829	0
2502	849.9	1.4421	0.2226	0.2312	0.7677	0
2503	849.8	1.4412	0.2224	0.2312	0.7679	0
2504	849.7	1.4417	0.2224	0.2312	0.7677	0
2505	849.6	1.4413	0.2225	0.2313	0.7679	0
2506	849.5	1.442	0.2224	0.2312	0.7677	0
2507	849.4	1.4417	0.2226	0.2312	0.7678	0
2508	849.3	1.4416	0.2224	0.2312	0.7678	0
2509	849.2	1.4408	0.2226	0.2313	0.7682	0
2510	849.1	1.4416	0.2226	0.2311	0.7677	0
2511	849	1.4416	0.2226	0.2311	0.7678	0
2512	848.9	1.4415	0.2224	0.2311	0.7676	0
2513	848.8	1.4409	0.2222	0.2308	0.7674	0
2514	848.7	1.4409	0.2222	0.2309	0.7677	0
2515	848.6	1.4409	0.2223	0.2309	0.7676	0
2516	848.5	1.4409	0.2223	0.2308	0.7677	0
2517	848.4	1.4408	0.2222	0.2308	0.7676	0
2518	848.3	1.4404	0.2221	0.2309	0.7676	0
2519	848.2	1.4412	0.2221	0.2308	0.7675	0
2520	848.1	1.4408	0.2222	0.2308	0.7677	0
2521	848	1.4414	0.2224	0.231	0.7679	0
2522	847.9	1.4418	0.2224	0.231	0.7676	0
2523	847.8	1.4416	0.2222	0.2308	0.7676	0

2524	847.7	1.4411	0.2222	0.2308	0.7675	0
2525	847.6	1.4408	0.2222	0.2308	0.7676	0
2526	847.5	1.4414	0.2224	0.231	0.7679	0
2527	847.4	1.4414	0.2224	0.2311	0.7677	0
2528	847.3	1.4417	0.2223	0.2309	0.7678	0
2529	847.2	1.4412	0.222	0.2309	0.7678	0
2530	847.1	1.4407	0.222	0.2308	0.7675	0
2531	847	1.4408	0.2222	0.2309	0.7677	0
2532	846.9	1.4405	0.2221	0.2308	0.768	0
2533	846.8	1.4418	0.2222	0.2309	0.7678	0
2534	846.7	1.4411	0.2224	0.231	0.7681	0
2535	846.6	1.4417	0.2224	0.2312	0.7683	0
2536	846.5	1.4411	0.2222	0.231	0.768	0
2537	846.4	1.4417	0.2221	0.2307	0.768	0
2538	846.3	1.4414	0.2222	0.2309	0.7679	0
2539	846.2	1.4416	0.2223	0.231	0.7683	0
2540	846.1	1.4415	0.2223	0.2309	0.7678	0
2541	846	1.4412	0.2223	0.231	0.7682	0
2542	845.9	1.4411	0.2223	0.2309	0.7679	0
2543	845.8	1.4416	0.2223	0.231	0.768	0
2544	845.7	1.4417	0.2223	0.2311	0.7683	0
2545	845.6	1.4418	0.2223	0.2311	0.7681	0
2546	845.5	1.4418	0.2223	0.231	0.768	0
2547	845.4	1.4419	0.2225	0.2312	0.7683	0
2548	845.3	1.442	0.2225	0.2313	0.7684	0
2549	845.2	1.442	0.2225	0.2313	0.7684	0
2550	845.1	1.4426	0.2225	0.2313	0.7686	0
2551	845	1.4423	0.2227	0.2314	0.7687	0
2552	844.9	1.4419	0.2227	0.2314	0.7687	0
2553	844.8	1.4421	0.2227	0.2316	0.7689	0
2554	844.7	1.4425	0.2227	0.2315	0.7689	0
2555	844.6	1.4426	0.2227	0.2315	0.7689	0
2556	844.5	1.4422	0.2227	0.2316	0.7689	0
2557	844.4	1.4432	0.2229	0.2317	0.7691	0
2558	844.3	1.4429	0.2228	0.2317	0.7692	0
2559	844.2	1.4429	0.2229	0.2316	0.7692	0
2560	844.1	1.4429	0.2228	0.2314	0.769	0
2561	844	1.4429	0.2232	0.2319	0.7695	0
2562	843.9	1.4432	0.223	0.2319	0.7696	0
2563	843.8	1.4439	0.2233	0.2321	0.7698	0
2564	843.7	1.4436	0.2233	0.232	0.7698	0
2565	843.6	1.4437	0.2233	0.2322	0.7698	0
2566	843.5	1.4444	0.2236	0.2324	0.7698	0

2567	843.4	1.444	0.2236	0.2323	0.7698	0
2568	843.3	1.4445	0.2235	0.2324	0.77	0
2569	843.2	1.4445	0.2236	0.2324	0.77	0
2570	843.1	1.4451	0.2237	0.2325	0.7704	0
2571	843	1.4449	0.2237	0.2327	0.7704	0
2572	842.9	1.4451	0.2239	0.2327	0.7706	0
2573	842.8	1.4442	0.2239	0.2327	0.7704	0
2574	842.7	1.4447	0.2241	0.2327	0.7707	0
2575	842.6	1.4445	0.2236	0.2326	0.7703	0
2576	842.5	1.4447	0.2238	0.2327	0.7705	0
2577	842.4	1.4452	0.2238	0.2327	0.7706	0
2578	842.3	1.4444	0.2239	0.2327	0.7707	0
2579	842.2	1.4448	0.2238	0.2327	0.7704	0
2580	842.1	1.4445	0.2238	0.2327	0.7706	0
2581	842	1.4453	0.2238	0.2325	0.7709	0
2582	841.9	1.4453	0.2237	0.2325	0.7705	0
2583	841.8	1.4456	0.224	0.2327	0.7707	0
2584	841.7	1.4455	0.2238	0.2327	0.7707	0
2585	841.6	1.4451	0.2239	0.2326	0.7707	0
2586	841.5	1.4452	0.2238	0.2327	0.771	0
2587	841.4	1.4448	0.2238	0.2327	0.7708	0
2588	841.3	1.4452	0.2238	0.2326	0.7707	0
2589	841.2	1.4452	0.2238	0.2324	0.7709	0
2590	841.1	1.4458	0.2239	0.2328	0.7709	0
2591	841	1.4455	0.224	0.2328	0.7708	0
2592	840.9	1.4457	0.2242	0.233	0.7712	0
2593	840.8	1.4452	0.2242	0.2329	0.7706	0
2594	840.7	1.4451	0.2238	0.2328	0.7708	0
2595	840.6	1.4456	0.2239	0.2326	0.7709	0
2596	840.5	1.4445	0.2239	0.2329	0.7712	0
2597	840.4	1.4448	0.2238	0.2327	0.7707	0
2598	840.3	1.4453	0.2239	0.2329	0.771	0
2599	840.2	1.4455	0.2236	0.2325	0.7707	0
2600	840.1	1.4449	0.2238	0.2327	0.771	0
2601	840	1.4458	0.2239	0.2329	0.7709	0
2602	839.9	1.4455	0.2239	0.2329	0.771	0
2603	839.8	1.445	0.2238	0.2327	0.7711	0
2604	839.7	1.4456	0.224	0.2329	0.7711	0
2605	839.6	1.4451	0.2239	0.2327	0.7708	0
2606	839.5	1.4452	0.2239	0.2327	0.771	0
2607	839.4	1.4452	0.2239	0.2327	0.7712	0
2608	839.3	1.4447	0.2237	0.2326	0.7709	0
2609	839.2	1.4448	0.2237	0.2327	0.7711	0

2610	839.1	1.4449	0.2238	0.2328	0.771	0
2611	839	1.4453	0.2238	0.2327	0.7712	0
2612	838.9	1.445	0.2238	0.2328	0.7711	0
2613	838.8	1.4451	0.2237	0.2328	0.7713	0
2614	838.7	1.4447	0.2239	0.2328	0.7711	0
2615	838.6	1.4453	0.2236	0.2326	0.7709	0
2616	838.5	1.445	0.2237	0.2326	0.7711	0
2617	838.4	1.4445	0.2236	0.2325	0.7709	0
2618	838.3	1.4446	0.2236	0.2324	0.771	0
2619	838.2	1.4446	0.2235	0.2324	0.7711	0
2620	838.1	1.4446	0.2236	0.2324	0.7709	0
2621	838	1.4442	0.2235	0.2324	0.7709	0
2622	837.9	1.4443	0.2236	0.2325	0.771	0
2623	837.8	1.4445	0.2238	0.2326	0.7713	0
2624	837.7	1.4441	0.2237	0.2325	0.7713	0
2625	837.6	1.4448	0.2235	0.2324	0.771	0
2626	837.5	1.4452	0.2233	0.2323	0.771	0
2627	837.4	1.445	0.2235	0.2325	0.7712	0
2628	837.3	1.4445	0.2235	0.2323	0.7712	0
2629	837.2	1.4444	0.2235	0.2323	0.7711	0
2630	837.1	1.4446	0.2235	0.2323	0.7711	0
2631	837	1.4451	0.2235	0.2324	0.7711	0
2632	836.9	1.4447	0.2236	0.2325	0.7715	0
2633	836.8	1.4447	0.2235	0.2322	0.7714	0
2634	836.7	1.4443	0.2235	0.2323	0.771	0
2635	836.6	1.445	0.2236	0.2326	0.7714	0
2636	836.5	1.4451	0.2238	0.2325	0.7714	0
2637	836.4	1.4449	0.2233	0.2324	0.771	0
2638	836.3	1.4451	0.2236	0.2325	0.7712	0
2639	836.2	1.4448	0.2237	0.2325	0.7713	0
2640	836.1	1.4449	0.2238	0.2325	0.7714	0
2641	836	1.4445	0.2238	0.2325	0.7715	0
2642	835.9	1.4451	0.2236	0.2326	0.7715	0
2643	835.8	1.4446	0.2235	0.2324	0.7715	0
2644	835.7	1.4448	0.2237	0.2326	0.7717	0
2645	835.6	1.4445	0.2237	0.2324	0.7716	0
2646	835.5	1.4446	0.2238	0.2325	0.7717	0
2647	835.4	1.4449	0.2235	0.2323	0.7716	0
2648	835.3	1.445	0.2238	0.2323	0.7716	0
2649	835.2	1.4448	0.2237	0.2326	0.7717	0
2650	835.1	1.4449	0.2237	0.2325	0.7717	0
2651	835	1.4451	0.2238	0.2327	0.7719	0
2652	834.9	1.4451	0.2238	0.2326	0.7715	0

2653	834.8	1.4451	0.2237	0.2326	0.7719	0
2654	834.7	1.4453	0.2239	0.2327	0.7717	0
2655	834.6	1.4451	0.2239	0.2329	0.772	0
2656	834.5	1.4451	0.2238	0.2327	0.7719	0
2657	834.4	1.4452	0.2239	0.2327	0.772	0
2658	834.3	1.4454	0.224	0.2327	0.7721	0
2659	834.2	1.4459	0.2239	0.2327	0.7722	0
2660	834.1	1.446	0.2238	0.2328	0.7722	0
2661	834	1.4462	0.2241	0.233	0.7722	0
2662	833.9	1.4461	0.2243	0.2331	0.7725	0
2663	833.8	1.4467	0.2244	0.2332	0.7728	0
2664	833.7	1.4464	0.2244	0.2333	0.7726	0
2665	833.6	1.4467	0.2246	0.2335	0.7729	0
2666	833.5	1.4463	0.2245	0.2333	0.7729	0
2667	833.4	1.4472	0.2245	0.2335	0.7733	0
2668	833.3	1.4465	0.2245	0.2335	0.773	0
2669	833.2	1.4467	0.2247	0.2335	0.773	0
2670	833.1	1.4478	0.2248	0.2336	0.7734	0
2671	833	1.4474	0.2246	0.2336	0.7731	0
2672	832.9	1.4471	0.2247	0.2336	0.7732	0
2673	832.8	1.4473	0.2247	0.2337	0.7734	0
2674	832.7	1.447	0.2248	0.2337	0.7734	0
2675	832.6	1.4476	0.2249	0.2337	0.7736	0
2676	832.5	1.4478	0.2249	0.2338	0.7735	0
2677	832.4	1.447	0.2248	0.2338	0.7738	0
2678	832.3	1.4471	0.225	0.2339	0.7735	0
2679	832.2	1.4478	0.2251	0.234	0.7737	0
2680	832.1	1.4473	0.225	0.2339	0.7738	0
2681	832	1.4479	0.2251	0.234	0.774	0
2682	831.9	1.4481	0.2252	0.2339	0.7738	0
2683	831.8	1.4478	0.2253	0.234	0.7739	0
2684	831.7	1.4479	0.2252	0.234	0.774	0
2685	831.6	1.4481	0.2253	0.2342	0.774	0
2686	831.5	1.4482	0.2253	0.2342	0.7739	0
2687	831.4	1.4482	0.2253	0.2341	0.7742	0
2688	831.3	1.4483	0.2252	0.2341	0.7741	0
2689	831.2	1.4485	0.2253	0.2343	0.7743	0
2690	831.1	1.4479	0.2251	0.2339	0.774	0
2691	831	1.4482	0.2253	0.2341	0.7742	0
2692	830.9	1.4483	0.2254	0.2342	0.7742	0
2693	830.8	1.4487	0.2252	0.2341	0.7741	0
2694	830.7	1.4484	0.2253	0.2341	0.7743	0
2695	830.6	1.4485	0.2252	0.2341	0.7744	0

2696	830.5	1.4483	0.2253	0.2342	0.7742	0
2697	830.4	1.4489	0.2255	0.2343	0.7743	0
2698	830.3	1.4485	0.2253	0.2342	0.7744	0
2699	830.2	1.4486	0.2253	0.2341	0.7744	0
2700	830.1	1.4487	0.2253	0.2341	0.7744	0
2701	830	1.4488	0.2253	0.2341	0.7745	0
2702	829.9	1.4484	0.2253	0.2339	0.7743	0
2703	829.8	1.4477	0.2254	0.2341	0.7743	0
2704	829.7	1.4487	0.2254	0.2342	0.7745	0
2705	829.6	1.4487	0.2252	0.2341	0.7743	0
2706	829.5	1.4487	0.2253	0.234	0.7744	0
2707	829.4	1.4484	0.2254	0.2341	0.7745	0
2708	829.3	1.4489	0.2252	0.234	0.7744	0
2709	829.2	1.448	0.2251	0.2339	0.7744	0
2710	829.1	1.448	0.225	0.2338	0.7742	0
2711	829	1.4482	0.2251	0.2339	0.7742	0
2712	828.9	1.4488	0.2253	0.2341	0.7746	0
2713	828.8	1.4488	0.2252	0.2338	0.7743	0
2714	828.7	1.4489	0.2253	0.234	0.7743	0
2715	828.6	1.4482	0.2252	0.2339	0.7744	0
2716	828.5	1.448	0.2252	0.2341	0.7747	0
2717	828.4	1.448	0.2252	0.234	0.7745	0
2718	828.3	1.4484	0.225	0.2339	0.7745	0
2719	828.2	1.4485	0.225	0.2339	0.7744	0
2720	828.1	1.4482	0.2251	0.234	0.7746	0
2721	828	1.4484	0.2252	0.2341	0.7745	0
2722	827.9	1.448	0.2252	0.234	0.7745	0
2723	827.8	1.4481	0.225	0.234	0.7744	0
2724	827.7	1.4478	0.2251	0.2341	0.7745	0
2725	827.6	1.4478	0.2251	0.2339	0.7745	0
2726	827.5	1.4479	0.225	0.234	0.7744	0
2727	827.4	1.4485	0.2251	0.2341	0.7745	0
2728	827.3	1.4483	0.2252	0.2342	0.7747	0
2729	827.2	1.4482	0.225	0.2341	0.7746	0
2730	827.1	1.4482	0.225	0.234	0.7745	0
2731	827	1.4478	0.2249	0.2337	0.7746	0
2732	826.9	1.4484	0.225	0.2339	0.7744	0
2733	826.8	1.4481	0.2248	0.2338	0.7745	0
2734	826.7	1.4483	0.225	0.2339	0.7747	0
2735	826.6	1.4481	0.2251	0.2341	0.7752	0
2736	826.5	1.4481	0.225	0.2338	0.7751	0
2737	826.4	1.4482	0.225	0.234	0.7749	0
2738	826.3	1.4482	0.225	0.234	0.7746	0

2739	826.2	1.448	0.225	0.2341	0.7749	0
2740	826.1	1.4481	0.2251	0.234	0.775	0
2741	826	1.4482	0.225	0.234	0.7746	0
2742	825.9	1.448	0.2251	0.2342	0.7749	0
2743	825.8	1.4485	0.2252	0.2341	0.7748	0
2744	825.7	1.4483	0.2248	0.2337	0.7743	0
2745	825.6	1.4486	0.2251	0.234	0.7745	0
2746	825.5	1.4482	0.2249	0.2338	0.775	0
2747	825.4	1.4485	0.2251	0.234	0.7749	0
2748	825.3	1.4482	0.225	0.234	0.7751	0
2749	825.2	1.4487	0.2251	0.234	0.775	0
2750	825.1	1.4485	0.2252	0.2342	0.775	0
2751	825	1.4486	0.2251	0.2341	0.7747	0
2752	824.9	1.4488	0.2253	0.2342	0.775	0
2753	824.8	1.4488	0.2252	0.234	0.7751	0
2754	824.7	1.449	0.2252	0.2342	0.7751	0
2755	824.6	1.4489	0.2254	0.2344	0.7752	0
2756	824.5	1.4489	0.2252	0.2342	0.7753	0
2757	824.4	1.4487	0.2254	0.2344	0.7752	0
2758	824.3	1.4492	0.2253	0.2344	0.7752	0
2759	824.2	1.449	0.2253	0.2343	0.7752	0
2760	824.1	1.4488	0.2254	0.2345	0.7755	0
2761	824	1.449	0.2255	0.2345	0.7756	0
2762	823.9	1.4495	0.2255	0.2346	0.7757	0
2763	823.8	1.4491	0.2255	0.2345	0.7758	0
2764	823.7	1.4492	0.2254	0.2343	0.7755	0
2765	823.6	1.4495	0.2254	0.2345	0.7755	0
2766	823.5	1.4497	0.2256	0.2346	0.7756	0
2767	823.4	1.4496	0.2259	0.2348	0.7758	0
2768	823.3	1.4498	0.2257	0.2349	0.776	0
2769	823.2	1.4499	0.226	0.2349	0.7761	0
2770	823.1	1.4501	0.2258	0.2348	0.7761	0
2771	823	1.4501	0.2258	0.2348	0.7761	0
2772	822.9	1.4498	0.2257	0.2347	0.7761	0
2773	822.8	1.4501	0.2258	0.2349	0.7761	0
2774	822.7	1.4503	0.2259	0.2349	0.7763	0
2775	822.6	1.4501	0.226	0.235	0.7762	0
2776	822.5	1.4507	0.2261	0.235	0.7764	0
2777	822.4	1.4503	0.226	0.235	0.7762	0
2778	822.3	1.45	0.2258	0.2349	0.7761	0
2779	822.2	1.4502	0.2259	0.235	0.7764	0
2780	822.1	1.4504	0.226	0.235	0.7765	0
2781	822	1.4501	0.226	0.2349	0.7765	0

2782	821.9	1.4499	0.2261	0.235	0.7765	0
2783	821.8	1.4501	0.2262	0.2351	0.7766	0
2784	821.7	1.4507	0.226	0.2353	0.7766	0
2785	821.6	1.4498	0.2259	0.235	0.7766	0
2786	821.5	1.4506	0.2262	0.2352	0.7769	0
2787	821.4	1.4504	0.2263	0.2353	0.7768	0
2788	821.3	1.4505	0.2263	0.2353	0.7768	0
2789	821.2	1.4506	0.2261	0.2352	0.7768	0
2790	821.1	1.4504	0.2262	0.2352	0.7768	0
2791	821	1.4505	0.2263	0.2351	0.7768	0
2792	820.9	1.4506	0.2261	0.2352	0.7769	0
2793	820.8	1.4509	0.2262	0.2353	0.777	0
2794	820.7	1.4507	0.2264	0.2353	0.7771	0
2795	820.6	1.45	0.2264	0.2353	0.7772	0
2796	820.5	1.4508	0.2262	0.2352	0.7771	0
2797	820.4	1.4509	0.2263	0.2351	0.7769	0
2798	820.3	1.4506	0.2262	0.2352	0.777	0
2799	820.2	1.4513	0.2263	0.2354	0.777	0
2800	820.1	1.4505	0.2262	0.2352	0.777	0
2801	820	1.4513	0.2264	0.2355	0.7775	0
2802	819.9	1.4508	0.2262	0.2351	0.777	0
2803	819.8	1.4506	0.2262	0.2352	0.7772	0
2804	819.7	1.4507	0.2261	0.2352	0.7772	0
2805	819.6	1.4505	0.2262	0.2354	0.7773	0
2806	819.5	1.4507	0.2263	0.2353	0.7774	0
2807	819.4	1.451	0.2265	0.2354	0.7776	0
2808	819.3	1.451	0.2264	0.2354	0.7776	0
2809	819.2	1.451	0.2262	0.2352	0.7773	0
2810	819.1	1.4516	0.2262	0.2353	0.7772	0
2811	819	1.4513	0.2262	0.2353	0.7774	0
2812	818.9	1.4514	0.2263	0.2352	0.7774	0
2813	818.8	1.4511	0.2262	0.2353	0.7773	0
2814	818.7	1.4513	0.2264	0.2352	0.7774	0
2815	818.6	1.451	0.2262	0.2353	0.7774	0
2816	818.5	1.4508	0.2265	0.2354	0.7775	0
2817	818.4	1.451	0.2265	0.2354	0.7774	0
2818	818.3	1.4507	0.2264	0.2354	0.7775	0
2819	818.2	1.451	0.226	0.2351	0.7774	0
2820	818.1	1.4511	0.2261	0.2352	0.7774	0
2821	818	1.451	0.2262	0.2351	0.7774	0
2822	817.9	1.451	0.2263	0.2352	0.7775	0
2823	817.8	1.4505	0.2262	0.2353	0.7776	0
2824	817.7	1.451	0.2264	0.2354	0.7777	0

2825	817.6	1.4512	0.2263	0.2354	0.7776	0
2826	817.5	1.4513	0.2264	0.2355	0.7778	0
2827	817.4	1.452	0.2265	0.2355	0.7778	0
2828	817.3	1.4513	0.2267	0.2355	0.7779	0
2829	817.2	1.4514	0.2263	0.2354	0.7778	0
2830	817.1	1.451	0.2263	0.2353	0.7776	0
2831	817	1.4507	0.2262	0.2352	0.7777	0
2832	816.9	1.4516	0.2262	0.2353	0.7777	0
2833	816.8	1.451	0.2263	0.2353	0.7777	0
2834	816.7	1.4516	0.2263	0.2352	0.7777	0
2835	816.6	1.4517	0.2263	0.2353	0.778	0
2836	816.5	1.4514	0.2263	0.2352	0.7779	0
2837	816.4	1.4516	0.2264	0.2354	0.7778	0
2838	816.3	1.4513	0.2263	0.2353	0.7778	0
2839	816.2	1.451	0.2261	0.2352	0.7777	0
2840	816.1	1.4517	0.2265	0.2354	0.7779	0
2841	816	1.4516	0.2262	0.2351	0.7778	0
2842	815.9	1.4516	0.2264	0.2354	0.7781	0
2843	815.8	1.4514	0.2265	0.2354	0.7781	0
2844	815.7	1.4515	0.2265	0.2353	0.778	0
2845	815.6	1.4516	0.2264	0.2353	0.7779	0
2846	815.5	1.4513	0.2263	0.2352	0.7781	0
2847	815.4	1.4511	0.2263	0.2353	0.7781	0
2848	815.3	1.4512	0.2264	0.2352	0.7782	0
2849	815.2	1.4514	0.2265	0.2353	0.7782	0
2850	815.1	1.4516	0.2265	0.2353	0.7783	0
2851	815	1.4511	0.2262	0.2352	0.7781	0
2852	814.9	1.4506	0.2264	0.2353	0.7783	0
2853	814.8	1.4515	0.2262	0.2352	0.7783	0
2854	814.7	1.4514	0.2265	0.2353	0.7783	0
2855	814.6	1.4511	0.2263	0.2354	0.7783	0
2856	814.5	1.4517	0.2264	0.2355	0.7782	0
2857	814.4	1.451	0.2264	0.2355	0.7782	0
2858	814.3	1.452	0.2264	0.2355	0.7783	0
2859	814.2	1.4517	0.2265	0.2354	0.7783	0
2860	814.1	1.452	0.2266	0.2356	0.7783	0
2861	814	1.4517	0.2266	0.2356	0.7784	0
2862	813.9	1.4521	0.2268	0.2357	0.7786	0
2863	813.8	1.4522	0.2267	0.2357	0.7786	0
2864	813.7	1.4521	0.2269	0.2358	0.7786	0
2865	813.6	1.4524	0.2271	0.236	0.7788	0
2866	813.5	1.452	0.2267	0.236	0.7787	0
2867	813.4	1.4522	0.2269	0.236	0.7788	0

2868	813.3	1.4521	0.2269	0.236	0.7789	0
2869	813.2	1.4522	0.227	0.236	0.7792	0
2870	813.1	1.4524	0.227	0.236	0.779	0
2871	813	1.4526	0.2271	0.236	0.7791	0
2872	812.9	1.4531	0.2269	0.236	0.7791	0
2873	812.8	1.4528	0.227	0.2359	0.7791	0
2874	812.7	1.4531	0.2272	0.236	0.7793	0
2875	812.6	1.4529	0.2272	0.236	0.7793	0
2876	812.5	1.4531	0.2272	0.236	0.7792	0
2877	812.4	1.4528	0.227	0.236	0.7792	0
2878	812.3	1.4526	0.2271	0.2361	0.7795	0
2879	812.2	1.4531	0.227	0.2361	0.7792	0
2880	812.1	1.4529	0.2271	0.2361	0.7794	0
2881	812	1.4528	0.2273	0.2363	0.7796	0
2882	811.9	1.4534	0.2272	0.2363	0.7795	0
2883	811.8	1.4531	0.2273	0.2362	0.7794	0
2884	811.7	1.4532	0.2271	0.2361	0.7794	0
2885	811.6	1.4534	0.2272	0.2362	0.7794	0
2886	811.5	1.4529	0.2272	0.2365	0.7795	0
2887	811.4	1.4531	0.2273	0.2363	0.7795	0
2888	811.3	1.4532	0.2273	0.2364	0.7798	0
2889	811.2	1.4531	0.2273	0.2364	0.7797	0
2890	811.1	1.453	0.2276	0.2365	0.78	0
2891	811	1.4535	0.2274	0.2365	0.7799	0
2892	810.9	1.4532	0.2274	0.2365	0.7799	0
2893	810.8	1.4534	0.2272	0.2364	0.7801	0
2894	810.7	1.4534	0.2272	0.2364	0.7798	0
2895	810.6	1.4533	0.2273	0.2363	0.7798	0
2896	810.5	1.4534	0.2274	0.2364	0.7799	0
2897	810.4	1.4536	0.2274	0.2364	0.7799	0
2898	810.3	1.4528	0.2274	0.2362	0.7799	0
2899	810.2	1.4535	0.2274	0.2364	0.7798	0
2900	810.1	1.4533	0.2275	0.2364	0.7799	0
2901	810	1.4534	0.2274	0.2362	0.7801	0
2902	809.9	1.4535	0.2272	0.2361	0.7797	0
2903	809.8	1.4532	0.2272	0.2362	0.7797	0
2904	809.7	1.4535	0.2273	0.2363	0.78	0
2905	809.6	1.4532	0.2274	0.2362	0.7801	0
2906	809.5	1.4531	0.2274	0.2364	0.7804	0
2907	809.4	1.4536	0.2273	0.2364	0.7801	0
2908	809.3	1.4534	0.2274	0.2364	0.7802	0
2909	809.2	1.4536	0.2275	0.2365	0.78	0
2910	809.1	1.4539	0.2275	0.2365	0.7801	0

2911	809	1.4539	0.2274	0.2364	0.78	0
2912	808.9	1.4538	0.2275	0.2365	0.7802	0
2913	808.8	1.4535	0.2274	0.2364	0.7803	0
2914	808.7	1.4541	0.2275	0.2365	0.7805	0
2915	808.6	1.4538	0.2273	0.2365	0.7802	0
2916	808.5	1.4536	0.2275	0.2364	0.7802	0
2917	808.4	1.4536	0.2273	0.2363	0.7801	0
2918	808.3	1.4535	0.2274	0.2364	0.7802	0
2919	808.2	1.4533	0.2273	0.2364	0.7807	0
2920	808.1	1.4536	0.2274	0.2366	0.7805	0
2921	808	1.4537	0.2275	0.2363	0.7803	0
2922	807.9	1.4538	0.2275	0.2364	0.7805	0
2923	807.8	1.4535	0.2273	0.2363	0.7804	0
2924	807.7	1.4536	0.2273	0.2362	0.7801	0
2925	807.6	1.4538	0.2272	0.2364	0.7804	0
2926	807.5	1.4535	0.2273	0.2362	0.7803	0
2927	807.4	1.4534	0.2273	0.2363	0.7805	0
2928	807.3	1.4533	0.2274	0.2366	0.7806	0
2929	807.2	1.4535	0.2275	0.2365	0.7805	0
2930	807.1	1.4533	0.2275	0.2364	0.7806	0
2931	807	1.4539	0.2275	0.2366	0.7806	0
2932	806.9	1.4532	0.2275	0.2366	0.7809	0
2933	806.8	1.4537	0.2274	0.2364	0.7806	0
2934	806.7	1.4536	0.2276	0.2366	0.7807	0
2935	806.6	1.4539	0.2276	0.2368	0.7809	0
2936	806.5	1.4535	0.2274	0.2364	0.7807	0
2937	806.4	1.4541	0.2275	0.2366	0.781	0
2938	806.3	1.4538	0.2274	0.2363	0.7808	0
2939	806.2	1.4536	0.2274	0.2365	0.7808	0
2940	806.1	1.4542	0.2275	0.2367	0.7807	0
2941	806	1.454	0.2275	0.2365	0.7808	0
2942	805.9	1.4542	0.2275	0.2365	0.7807	0
2943	805.8	1.454	0.2276	0.2366	0.7811	0
2944	805.7	1.4541	0.2275	0.2364	0.7809	0
2945	805.6	1.4541	0.2277	0.2367	0.7806	0
2946	805.5	1.4541	0.2275	0.2364	0.7809	0
2947	805.4	1.4539	0.2275	0.2364	0.7811	0
2948	805.3	1.4542	0.2277	0.2366	0.7813	0
2949	805.2	1.4544	0.2276	0.2367	0.781	0
2950	805.1	1.4544	0.2278	0.2369	0.7813	0
2951	805	1.4544	0.2278	0.2366	0.7811	0
2952	804.9	1.454	0.2279	0.2368	0.7813	0
2953	804.8	1.4545	0.2278	0.237	0.7815	0

2954	804.7	1.4544	0.228	0.2369	0.7813	0
2955	804.6	1.4549	0.2279	0.2369	0.7814	0
2956	804.5	1.4549	0.228	0.2371	0.7818	0
2957	804.4	1.4546	0.228	0.237	0.7815	0
2958	804.3	1.4549	0.2281	0.2371	0.782	0
2959	804.2	1.4551	0.2281	0.2372	0.7819	0
2960	804.1	1.4549	0.2283	0.2371	0.7818	0
2961	804	1.4553	0.2283	0.2372	0.782	0
2962	803.9	1.4552	0.2284	0.2374	0.7821	0
2963	803.8	1.4549	0.2284	0.2373	0.7821	0
2964	803.7	1.4551	0.2284	0.2373	0.782	0
2965	803.6	1.4553	0.2285	0.2374	0.7822	0
2966	803.5	1.4555	0.2283	0.2375	0.7819	0
2967	803.4	1.4552	0.2283	0.2374	0.7818	0
2968	803.3	1.455	0.2284	0.2374	0.782	0
2969	803.2	1.4556	0.2284	0.2375	0.7821	0
2970	803.1	1.4549	0.2283	0.2376	0.7821	0
2971	803	1.4556	0.2286	0.2374	0.7823	0
2972	802.9	1.4555	0.2284	0.2375	0.7825	0
2973	802.8	1.4557	0.2284	0.2375	0.7824	0
2974	802.7	1.4554	0.2283	0.2375	0.7822	0
2975	802.6	1.4556	0.2285	0.2375	0.7824	0
2976	802.5	1.4556	0.2288	0.2378	0.7828	0
2977	802.4	1.4552	0.2285	0.2376	0.7825	0
2978	802.3	1.4554	0.2285	0.2378	0.7826	0
2979	802.2	1.4552	0.2285	0.2377	0.7828	0
2980	802.1	1.4559	0.2288	0.2378	0.7826	0
2981	802	1.456	0.2287	0.2377	0.7826	0
2982	801.9	1.456	0.2289	0.2381	0.7828	0
2983	801.8	1.4559	0.2287	0.2377	0.7827	0
2984	801.7	1.4561	0.2287	0.2376	0.7827	0
2985	801.6	1.4559	0.2287	0.2377	0.7829	0
2986	801.5	1.4564	0.2287	0.2377	0.7827	0
2987	801.4	1.4554	0.2286	0.2377	0.7828	0
2988	801.3	1.4561	0.2287	0.2376	0.7829	0
2989	801.2	1.4563	0.2289	0.2378	0.7832	0
2990	801.1	1.4561	0.2288	0.2379	0.7828	0
2991	801	1.4562	0.2289	0.2377	0.7827	0
2992	800.9	1.4559	0.2285	0.2377	0.7829	0
2993	800.8	1.4559	0.2287	0.2376	0.7826	0
2994	800.7	1.4559	0.2288	0.2378	0.7828	0
2995	800.6	1.4556	0.2289	0.2378	0.7826	0
2996	800.5	1.4557	0.2287	0.2376	0.7827	0

2997	800.4	1.4557	0.2288	0.2379	0.783	0
2998	800.3	1.4562	0.2287	0.2378	0.7827	0
2999	800.2	1.4558	0.2287	0.2376	0.7828	0
3000	800.1	1.4557	0.2286	0.2377	0.7828	0
3001	800	1.4561	0.2286	0.2375	0.7828	0
3002	799.9	1.4559	0.2285	0.2375	0.7827	0
3003	799.8	1.4562	0.2287	0.2376	0.783	0
3004	799.7	1.4556	0.2287	0.2378	0.7828	0
3005	799.6	1.4556	0.2286	0.2375	0.7828	0
3006	799.5	1.4556	0.2287	0.2378	0.7832	0
3007	799.4	1.4559	0.229	0.2378	0.7832	0
3008	799.3	1.4563	0.2286	0.2377	0.783	0
3009	799.2	1.4556	0.2286	0.2375	0.783	0
3010	799.1	1.4555	0.2287	0.2376	0.7832	0
3011	799	1.4557	0.2287	0.2376	0.7832	0
3012	798.9	1.4557	0.2284	0.2374	0.783	0
3013	798.8	1.4561	0.2284	0.2374	0.7829	0
3014	798.7	1.4555	0.2284	0.2374	0.7828	0
3015	798.6	1.4558	0.2284	0.2374	0.783	0
3016	798.5	1.4555	0.2284	0.2373	0.7829	0
3017	798.4	1.4557	0.2284	0.2374	0.7831	0
3018	798.3	1.4554	0.2283	0.2372	0.783	0
3019	798.2	1.4555	0.2282	0.2372	0.7828	0
3020	798.1	1.4554	0.2282	0.2374	0.7831	0
3021	798	1.4556	0.2283	0.2372	0.783	0
3022	797.9	1.4557	0.2282	0.2372	0.7828	0
3023	797.8	1.4563	0.2282	0.2371	0.783	0
3024	797.7	1.4557	0.2282	0.2372	0.783	0
3025	797.6	1.4555	0.2282	0.2371	0.783	0
3026	797.5	1.4553	0.2282	0.2372	0.783	0
3027	797.4	1.4556	0.2283	0.2372	0.783	0
3028	797.3	1.4556	0.2281	0.2371	0.7832	0
3029	797.2	1.4556	0.2284	0.2373	0.7832	0
3030	797.1	1.4557	0.2283	0.2374	0.7832	0
3031	797	1.4559	0.2282	0.2372	0.7832	0
3032	796.9	1.4554	0.2283	0.2376	0.7834	0
3033	796.8	1.456	0.2284	0.2373	0.7836	0
3034	796.7	1.4558	0.2283	0.2373	0.7832	0
3035	796.6	1.4553	0.2285	0.2374	0.7832	0
3036	796.5	1.4558	0.2284	0.2373	0.7833	0
3037	796.4	1.4555	0.2282	0.2374	0.7834	0
3038	796.3	1.4551	0.2284	0.2374	0.7834	0
3039	796.2	1.4562	0.2286	0.2375	0.7835	0

3040	796.1	1.4561	0.2286	0.2377	0.7837	0
3041	796	1.4557	0.2284	0.2374	0.7837	0
3042	795.9	1.4565	0.2286	0.2376	0.7836	0
3043	795.8	1.4559	0.2287	0.2376	0.7836	0
3044	795.7	1.4562	0.2288	0.2376	0.7838	0
3045	795.6	1.4566	0.229	0.2379	0.7839	0
3046	795.5	1.4566	0.2288	0.2377	0.7837	0
3047	795.4	1.4561	0.2288	0.2376	0.7839	0
3048	795.3	1.4561	0.2291	0.238	0.784	0
3049	795.2	1.4566	0.229	0.238	0.7841	0
3050	795.1	1.4565	0.2289	0.238	0.7841	0
3051	795	1.4568	0.2291	0.238	0.7839	0
3052	794.9	1.4566	0.2291	0.238	0.784	0
3053	794.8	1.4568	0.2292	0.2381	0.7843	0
3054	794.7	1.4574	0.2292	0.238	0.7844	0
3055	794.6	1.4574	0.2292	0.2383	0.7844	0
3056	794.5	1.4568	0.2292	0.2381	0.7844	0
3057	794.4	1.4574	0.2294	0.2382	0.7845	0
3058	794.3	1.4577	0.2294	0.2383	0.7846	0
3059	794.2	1.4571	0.2294	0.2383	0.7844	0
3060	794.1	1.4578	0.2294	0.2383	0.7845	0
3061	794	1.4577	0.2295	0.2384	0.7849	0
3062	793.9	1.4575	0.2295	0.2386	0.7846	0
3063	793.8	1.4577	0.2296	0.2386	0.7846	0
3064	793.7	1.458	0.2298	0.2387	0.7847	0
3065	793.6	1.4579	0.2298	0.2387	0.785	0
3066	793.5	1.4574	0.2299	0.2388	0.785	0
3067	793.4	1.4579	0.2297	0.2388	0.7849	0
3068	793.3	1.4582	0.2297	0.2388	0.7851	0
3069	793.2	1.4581	0.2299	0.2389	0.7852	0
3070	793.1	1.4583	0.2298	0.2389	0.7853	0
3071	793	1.4581	0.2299	0.2387	0.7851	0
3072	792.9	1.4582	0.2298	0.2388	0.785	0
3073	792.8	1.4578	0.2299	0.2389	0.7852	0
3074	792.7	1.4579	0.2298	0.239	0.7852	0
3075	792.6	1.4584	0.2298	0.2389	0.7854	0
3076	792.5	1.4581	0.2298	0.2387	0.7852	0
3077	792.4	1.4585	0.2301	0.2389	0.7854	0
3078	792.3	1.4584	0.2301	0.2391	0.7855	0
3079	792.2	1.4581	0.2299	0.2389	0.7853	0
3080	792.1	1.4588	0.23	0.2391	0.7856	0
3081	792	1.4582	0.2301	0.239	0.7853	0
3082	791.9	1.4583	0.2296	0.2389	0.7852	0

3083	791.8	1.4581	0.2299	0.239	0.7854	0
3084	791.7	1.4583	0.2299	0.2391	0.7853	0
3085	791.6	1.4584	0.2302	0.239	0.7854	0
3086	791.5	1.4587	0.2302	0.2391	0.7855	0
3087	791.4	1.4586	0.2301	0.239	0.7858	0
3088	791.3	1.4587	0.23	0.2389	0.7856	0
3089	791.2	1.4584	0.2301	0.2389	0.7855	0
3090	791.1	1.4586	0.23	0.2391	0.7856	0
3091	791	1.4583	0.2298	0.2388	0.7855	0
3092	790.9	1.459	0.2299	0.2389	0.7857	0
3093	790.8	1.4584	0.23	0.239	0.7856	0
3094	790.7	1.4586	0.2302	0.239	0.7858	0
3095	790.6	1.4583	0.23	0.2389	0.7856	0
3096	790.5	1.4584	0.23	0.2389	0.7854	0
3097	790.4	1.4583	0.2299	0.2389	0.7855	0
3098	790.3	1.4589	0.2301	0.2391	0.7858	0
3099	790.2	1.459	0.2301	0.239	0.7856	0
3100	790.1	1.4585	0.2301	0.2391	0.7856	0
3101	790	1.4586	0.2301	0.239	0.7857	0
3102	789.9	1.4581	0.2302	0.239	0.7858	0
3103	789.8	1.4586	0.2299	0.239	0.7856	0
3104	789.7	1.4588	0.2302	0.2391	0.7857	0
3105	789.6	1.4586	0.23	0.239	0.7857	0
3106	789.5	1.4586	0.23	0.239	0.7857	0
3107	789.4	1.458	0.23	0.2388	0.7857	0
3108	789.3	1.4585	0.2297	0.2387	0.7855	0
3109	789.2	1.4582	0.2297	0.2386	0.7855	0
3110	789.1	1.4584	0.2299	0.2387	0.7857	0
3111	789	1.4584	0.2296	0.2386	0.7855	0
3112	788.9	1.4583	0.2298	0.2386	0.7856	0
3113	788.8	1.458	0.2296	0.2386	0.7856	0
3114	788.7	1.4577	0.2295	0.2385	0.7854	0
3115	788.6	1.4575	0.2296	0.2385	0.7854	0
3116	788.5	1.4578	0.2296	0.2386	0.7855	0
3117	788.4	1.4576	0.2296	0.2386	0.7855	0
3118	788.3	1.4574	0.2297	0.2387	0.7856	0
3119	788.2	1.4578	0.2295	0.2385	0.7855	0
3120	788.1	1.4577	0.2296	0.2386	0.7854	0
3121	788	1.4582	0.2295	0.2386	0.7857	0
3122	787.9	1.458	0.2297	0.2384	0.7856	0
3123	787.8	1.4578	0.2296	0.2385	0.7857	0
3124	787.7	1.4576	0.2298	0.2385	0.7857	0
3125	787.6	1.4579	0.2293	0.2381	0.7853	0

3126	787.5	1.4576	0.2293	0.2382	0.7853	0
3127	787.4	1.4575	0.2294	0.2382	0.7854	0
3128	787.3	1.4577	0.2294	0.2382	0.7855	0
3129	787.2	1.4572	0.2295	0.2382	0.7854	0
3130	787.1	1.4574	0.2294	0.2383	0.7855	0
3131	787	1.4572	0.2294	0.2383	0.7854	0
3132	786.9	1.4575	0.2294	0.2383	0.7858	0
3133	786.8	1.4577	0.2295	0.2385	0.7857	0
3134	786.7	1.4575	0.2296	0.2383	0.7855	0
3135	786.6	1.458	0.2295	0.2384	0.7859	0
3136	786.5	1.4575	0.2295	0.2383	0.7856	0
3137	786.4	1.4579	0.2295	0.2382	0.7855	0
3138	786.3	1.4575	0.2295	0.2383	0.7856	0
3139	786.2	1.4574	0.2296	0.2385	0.7858	0
3140	786.1	1.4583	0.2295	0.2384	0.7855	0
3141	786	1.4579	0.2294	0.2383	0.7857	0
3142	785.9	1.4578	0.2295	0.2385	0.7857	0
3143	785.8	1.4581	0.2295	0.2384	0.7856	0
3144	785.7	1.4578	0.2294	0.2383	0.7857	0
3145	785.6	1.4581	0.2295	0.2383	0.7859	0
3146	785.5	1.4577	0.2294	0.2381	0.7856	0
3147	785.4	1.4587	0.2297	0.2387	0.7861	0
3148	785.3	1.4584	0.2298	0.2384	0.786	0
3149	785.2	1.4583	0.2298	0.2386	0.7861	0
3150	785.1	1.4583	0.23	0.2387	0.7861	0
3151	785	1.4588	0.2299	0.2386	0.7861	0
3152	784.9	1.458	0.2298	0.2386	0.7862	0
3153	784.8	1.4581	0.2299	0.2386	0.7861	0
3154	784.7	1.4582	0.2299	0.2389	0.7864	0
3155	784.6	1.458	0.2298	0.2389	0.7865	0
3156	784.5	1.4588	0.2299	0.2387	0.7864	0
3157	784.4	1.4588	0.2302	0.239	0.7865	0
3158	784.3	1.4595	0.2304	0.2391	0.7866	0
3159	784.2	1.4587	0.2303	0.2391	0.7866	0
3160	784.1	1.4588	0.2302	0.239	0.7866	0
3161	784	1.4588	0.2303	0.2392	0.7865	0
3162	783.9	1.4594	0.2303	0.2392	0.7867	0
3163	783.8	1.4597	0.2305	0.2392	0.7866	0
3164	783.7	1.4595	0.2305	0.2394	0.7866	0
3165	783.6	1.4595	0.2305	0.2394	0.787	0
3166	783.5	1.4597	0.2307	0.2395	0.7869	0
3167	783.4	1.4596	0.2306	0.2396	0.787	0
3168	783.3	1.4597	0.2305	0.2395	0.7871	0

3169	783.2	1.4596	0.2306	0.2396	0.7871	0
3170	783.1	1.4599	0.2308	0.2397	0.7871	0
3171	783	1.4592	0.2305	0.2395	0.787	0
3172	782.9	1.4593	0.2307	0.2396	0.7873	0
3173	782.8	1.4595	0.2309	0.2398	0.7872	0
3174	782.7	1.4593	0.2307	0.2398	0.7872	0
3175	782.6	1.4596	0.2309	0.2398	0.7873	0
3176	782.5	1.4599	0.2311	0.24	0.7875	0
3177	782.4	1.4601	0.2309	0.24	0.7877	0
3178	782.3	1.4599	0.231	0.2401	0.7875	0
3179	782.2	1.4597	0.231	0.24	0.7876	0
3180	782.1	1.46	0.2311	0.2401	0.7876	0
3181	782	1.4601	0.231	0.2399	0.7875	0
3182	781.9	1.46	0.2311	0.2402	0.7875	0
3183	781.8	1.46	0.231	0.24	0.7875	0
3184	781.7	1.4599	0.2311	0.2401	0.7874	0
3185	781.6	1.4596	0.2309	0.24	0.7875	0
3186	781.5	1.4599	0.231	0.24	0.7876	0
3187	781.4	1.4596	0.2309	0.24	0.7874	0
3188	781.3	1.4598	0.2309	0.2399	0.7875	0
3189	781.2	1.4596	0.2309	0.2401	0.7875	0
3190	781.1	1.4598	0.231	0.2399	0.7875	0
3191	781	1.4598	0.2312	0.2402	0.7877	0
3192	780.9	1.46	0.2312	0.2403	0.7877	0
3193	780.8	1.4598	0.2312	0.2403	0.788	0
3194	780.7	1.4599	0.2311	0.2403	0.7877	0
3195	780.6	1.4597	0.2311	0.2402	0.7879	0
3196	780.5	1.4595	0.2312	0.2401	0.7877	0
3197	780.4	1.4597	0.2311	0.2402	0.7877	0
3198	780.3	1.4606	0.2311	0.2403	0.7877	0
3199	780.2	1.4605	0.2313	0.2401	0.7881	0
3200	780.1	1.4603	0.2312	0.2402	0.7879	0
3201	780	1.46	0.231	0.2403	0.788	0
3202	779.9	1.4602	0.2311	0.2402	0.7879	0
3203	779.8	1.4608	0.2312	0.2403	0.788	0
3204	779.7	1.4604	0.2313	0.2404	0.7881	0
3205	779.6	1.4599	0.2311	0.2401	0.7881	0
3206	779.5	1.46	0.2312	0.2401	0.7879	0
3207	779.4	1.4601	0.2311	0.24	0.7879	0
3208	779.3	1.4603	0.231	0.2398	0.7879	0
3209	779.2	1.46	0.2308	0.2399	0.7876	0
3210	779.1	1.46	0.231	0.24	0.788	0
3211	779	1.4604	0.2312	0.2401	0.7878	0

3212	778.9	1.4602	0.2309	0.2399	0.7879	0
3213	778.8	1.46	0.2309	0.2399	0.7878	0
3214	778.7	1.46	0.2309	0.2398	0.7877	0
3215	778.6	1.4605	0.2308	0.2397	0.7877	0
3216	778.5	1.4599	0.2308	0.2397	0.7878	0
3217	778.4	1.4594	0.2308	0.2399	0.7878	0
3218	778.3	1.4599	0.2308	0.2397	0.7878	0
3219	778.2	1.4598	0.2308	0.24	0.788	0
3220	778.1	1.4599	0.2309	0.2399	0.7879	0
3221	778	1.4596	0.2307	0.2396	0.7881	0
3222	777.9	1.4597	0.2306	0.2397	0.7879	0
3223	777.8	1.46	0.2308	0.2399	0.7879	0
3224	777.7	1.4601	0.2308	0.2398	0.788	0
3225	777.6	1.4601	0.2308	0.2397	0.788	0
3226	777.5	1.4597	0.2308	0.2396	0.788	0
3227	777.4	1.4597	0.2307	0.2396	0.7879	0
3228	777.3	1.4596	0.2307	0.2398	0.7881	0
3229	777.2	1.46	0.2307	0.2397	0.7879	0
3230	777.1	1.4595	0.2306	0.2396	0.7878	0
3231	777	1.4593	0.2305	0.2396	0.7878	0
3232	776.9	1.4596	0.2303	0.2396	0.7878	0
3233	776.8	1.4596	0.2305	0.2395	0.7878	0
3234	776.7	1.4597	0.2304	0.2396	0.7878	0
3235	776.6	1.4591	0.2304	0.2394	0.7878	0
3236	776.5	1.4597	0.2305	0.2395	0.788	0
3237	776.4	1.4595	0.2306	0.2395	0.7879	0
3238	776.3	1.4598	0.2306	0.2397	0.7882	0
3239	776.2	1.4596	0.2305	0.2396	0.7877	0
3240	776.1	1.4598	0.2308	0.2397	0.7881	0
3241	776	1.4596	0.2306	0.2398	0.7881	0
3242	775.9	1.4597	0.2306	0.2396	0.788	0
3243	775.8	1.4595	0.2306	0.2396	0.7881	0
3244	775.7	1.4594	0.2306	0.2396	0.788	0
3245	775.6	1.4596	0.2306	0.2396	0.7881	0
3246	775.5	1.4597	0.2307	0.2395	0.7882	0
3247	775.4	1.4596	0.2308	0.2396	0.7881	0
3248	775.3	1.46	0.2307	0.2395	0.7881	0
3249	775.2	1.4601	0.2309	0.2398	0.7883	0
3250	775.1	1.4597	0.2307	0.2395	0.7881	0
3251	775	1.46	0.2307	0.2396	0.7881	0
3252	774.9	1.4601	0.231	0.2398	0.7885	0
3253	774.8	1.46	0.2311	0.24	0.7885	0
3254	774.7	1.4605	0.2311	0.2399	0.7885	0

3255	774.6	1.4603	0.2311	0.24	0.7886	0
3256	774.5	1.4602	0.2311	0.24	0.7887	0
3257	774.4	1.4604	0.2312	0.24	0.7887	0
3258	774.3	1.4602	0.2311	0.24	0.7886	0
3259	774.2	1.4601	0.2312	0.24	0.7887	0
3260	774.1	1.4603	0.2312	0.2401	0.7886	0
3261	774	1.4606	0.2314	0.2402	0.7887	0
3262	773.9	1.4604	0.2314	0.2401	0.7888	0
3263	773.8	1.4604	0.2315	0.2402	0.7889	0
3264	773.7	1.4605	0.2312	0.2401	0.7887	0
3265	773.6	1.4608	0.2314	0.2403	0.7888	0
3266	773.5	1.4607	0.2316	0.2403	0.7887	0
3267	773.4	1.461	0.2316	0.2405	0.7891	0
3268	773.3	1.4605	0.2317	0.2406	0.7892	0
3269	773.2	1.4608	0.2317	0.2406	0.7893	0
3270	773.1	1.4606	0.2316	0.2406	0.7892	0
3271	773	1.4613	0.2317	0.2408	0.7893	0
3272	772.9	1.461	0.2317	0.2405	0.7893	0
3273	772.8	1.4606	0.232	0.2407	0.7893	0
3274	772.7	1.4614	0.232	0.241	0.7896	0
3275	772.6	1.4619	0.232	0.241	0.7896	0
3276	772.5	1.4614	0.2321	0.241	0.7895	0
3277	772.4	1.4614	0.2322	0.2411	0.7898	0
3278	772.3	1.4613	0.2323	0.2411	0.7899	0
3279	772.2	1.4613	0.2321	0.241	0.7896	0
3280	772.1	1.4615	0.2323	0.2411	0.79	0
3281	772	1.4613	0.2322	0.2411	0.79	0
3282	771.9	1.4613	0.2323	0.2412	0.79	0
3283	771.8	1.4617	0.2322	0.2412	0.7899	0
3284	771.7	1.4618	0.2325	0.2413	0.7902	0
3285	771.6	1.4618	0.2324	0.2413	0.7898	0
3286	771.5	1.4614	0.2324	0.2413	0.7902	0
3287	771.4	1.4616	0.2326	0.2414	0.7903	0
3288	771.3	1.4614	0.2324	0.2413	0.7901	0
3289	771.2	1.4615	0.2324	0.2412	0.79	0
3290	771.1	1.4617	0.2323	0.2414	0.7901	0
3291	771	1.4619	0.2324	0.2414	0.7898	0
3292	770.9	1.4617	0.2324	0.2414	0.7901	0
3293	770.8	1.4619	0.2325	0.2413	0.7902	0
3294	770.7	1.462	0.2324	0.2413	0.7902	0
3295	770.6	1.4623	0.2326	0.2414	0.7903	0
3296	770.5	1.4621	0.2325	0.2413	0.7902	0
3297	770.4	1.4619	0.2326	0.2414	0.7901	0

3298	770.3	1.4621	0.2325	0.2414	0.7904	0
3299	770.2	1.4619	0.2324	0.2413	0.7904	0
3300	770.1	1.4621	0.2325	0.2413	0.7902	0
3301	770	1.462	0.2325	0.2414	0.7903	0
3302	769.9	1.4623	0.2328	0.2417	0.7903	0
3303	769.8	1.4619	0.2325	0.2413	0.7902	0
3304	769.7	1.4621	0.2325	0.2413	0.7903	0
3305	769.6	1.4618	0.2325	0.2414	0.7905	0
3306	769.5	1.4615	0.2324	0.2413	0.7905	0
3307	769.4	1.4618	0.2324	0.2413	0.7904	0
3308	769.3	1.4616	0.2325	0.2412	0.7904	0
3309	769.2	1.4618	0.2326	0.2412	0.7903	0
3310	769.1	1.4614	0.2322	0.2412	0.7902	0
3311	769	1.462	0.2324	0.2413	0.7905	0
3312	768.9	1.4619	0.2325	0.2413	0.7905	0
3313	768.8	1.4618	0.2326	0.2414	0.7906	0
3314	768.7	1.4618	0.2326	0.2412	0.7905	0
3315	768.6	1.4619	0.2326	0.2412	0.7905	0
3316	768.5	1.4614	0.2325	0.2413	0.7906	0
3317	768.4	1.4618	0.2324	0.241	0.7903	0
3318	768.3	1.4623	0.2324	0.2411	0.7905	0
3319	768.2	1.4617	0.2324	0.241	0.7905	0
3320	768.1	1.4618	0.2324	0.241	0.7905	0
3321	768	1.4619	0.2323	0.241	0.7903	0
3322	767.9	1.4619	0.2321	0.2409	0.7903	0
3323	767.8	1.4616	0.2321	0.2409	0.7902	0
3324	767.7	1.4617	0.2323	0.2411	0.7903	0
3325	767.6	1.4618	0.2322	0.2409	0.7904	0
3326	767.5	1.4618	0.2323	0.2409	0.7903	0
3327	767.4	1.4617	0.2323	0.241	0.7903	0
3328	767.3	1.4613	0.2321	0.2407	0.7903	0
3329	767.2	1.4619	0.2322	0.2408	0.7904	0
3330	767.1	1.4617	0.2322	0.2408	0.7904	0
3331	767	1.4619	0.2322	0.2409	0.7905	0
3332	766.9	1.4617	0.2323	0.2409	0.7907	0
3333	766.8	1.4618	0.2322	0.2408	0.7905	0
3334	766.7	1.4617	0.2323	0.2409	0.7906	0
3335	766.6	1.4617	0.2322	0.2409	0.7906	0
3336	766.5	1.4618	0.2322	0.2408	0.7906	0
3337	766.4	1.462	0.2322	0.2409	0.7906	0
3338	766.3	1.4617	0.2321	0.2407	0.7905	0
3339	766.2	1.4618	0.2321	0.2408	0.7904	0
3340	766.1	1.4613	0.2322	0.2408	0.7905	0

3341	766	1.4617	0.2322	0.2407	0.7905	0
3342	765.9	1.4615	0.2321	0.2407	0.7906	0
3343	765.8	1.4617	0.232	0.2408	0.7905	0
3344	765.7	1.4619	0.2322	0.2408	0.7907	0
3345	765.6	1.4618	0.2323	0.2408	0.7908	0
3346	765.5	1.4615	0.2323	0.2408	0.7909	0
3347	765.4	1.4616	0.2322	0.2408	0.7907	0
3348	765.3	1.4615	0.2321	0.2408	0.7906	0
3349	765.2	1.4617	0.2323	0.2409	0.7909	0
3350	765.1	1.4615	0.2323	0.241	0.791	0
3351	765	1.4616	0.2321	0.2408	0.7907	0
3352	764.9	1.4618	0.2322	0.2409	0.7909	0
3353	764.8	1.4619	0.2322	0.241	0.7909	0
3354	764.7	1.4617	0.2322	0.2409	0.7907	0
3355	764.6	1.4621	0.2325	0.2412	0.7911	0
3356	764.5	1.462	0.2326	0.2413	0.7912	0
3357	764.4	1.4622	0.2326	0.2414	0.7912	0
3358	764.3	1.4622	0.2326	0.2411	0.7912	0
3359	764.2	1.4624	0.2325	0.2412	0.7911	0
3360	764.1	1.4621	0.2324	0.2409	0.791	0
3361	764	1.462	0.2326	0.2412	0.7912	0
3362	763.9	1.4621	0.2325	0.2412	0.7913	0
3363	763.8	1.462	0.2325	0.2413	0.7913	0
3364	763.7	1.4622	0.2325	0.2412	0.7914	0
3365	763.6	1.4621	0.2328	0.2414	0.7914	0
3366	763.5	1.4623	0.2328	0.2415	0.7917	0
3367	763.4	1.4626	0.2327	0.2415	0.7915	0
3368	763.3	1.4624	0.2328	0.2415	0.7918	0
3369	763.2	1.4627	0.233	0.2417	0.7917	0
3370	763.1	1.4626	0.233	0.2417	0.7914	0
3371	763	1.4627	0.2329	0.2416	0.7915	0
3372	762.9	1.4626	0.2331	0.2417	0.7918	0
3373	762.8	1.4621	0.233	0.2417	0.7917	0
3374	762.7	1.4624	0.2331	0.2419	0.7918	0
3375	762.6	1.4625	0.2331	0.2419	0.7917	0
3376	762.5	1.463	0.2331	0.2418	0.7918	0
3377	762.4	1.4628	0.2331	0.2418	0.7916	0
3378	762.3	1.4629	0.2331	0.2418	0.7916	0
3379	762.2	1.4627	0.2331	0.2418	0.7916	0
3380	762.1	1.4625	0.2329	0.2417	0.7917	0
3381	762	1.4629	0.2332	0.2419	0.792	0
3382	761.9	1.4625	0.2329	0.2418	0.7919	0
3383	761.8	1.4628	0.2331	0.2418	0.7919	0

3384	761.7	1.4623	0.2331	0.2419	0.7919	0
3385	761.6	1.4624	0.2332	0.2419	0.792	0
3386	761.5	1.4626	0.2333	0.242	0.792	0
3387	761.4	1.4625	0.2333	0.2421	0.792	0
3388	761.3	1.4626	0.2333	0.2421	0.7922	0
3389	761.2	1.463	0.2333	0.2419	0.7921	0
3390	761.1	1.4629	0.2333	0.2421	0.7924	0
3391	761	1.4623	0.2333	0.242	0.7923	0
3392	760.9	1.4624	0.2331	0.2419	0.7921	0
3393	760.8	1.4625	0.2334	0.2419	0.7921	0
3394	760.7	1.4624	0.2334	0.242	0.7923	0
3395	760.6	1.4629	0.2334	0.242	0.7919	0
3396	760.5	1.463	0.2333	0.2419	0.7921	0
3397	760.4	1.4631	0.2333	0.242	0.7922	0
3398	760.3	1.4627	0.2333	0.242	0.7924	0
3399	760.2	1.4629	0.2334	0.2421	0.7923	0
3400	760.1	1.4631	0.2336	0.242	0.7924	0
3401	760	1.4631	0.2337	0.2422	0.7926	0
3402	759.9	1.4629	0.2335	0.2421	0.7926	0
3403	759.8	1.4627	0.2335	0.2423	0.7924	0
3404	759.7	1.4629	0.2335	0.2423	0.7923	0
3405	759.6	1.4634	0.2334	0.2421	0.7924	0
3406	759.5	1.4629	0.2336	0.2422	0.7925	0
3407	759.4	1.4626	0.2332	0.2419	0.7923	0
3408	759.3	1.4629	0.2335	0.2422	0.7925	0
3409	759.2	1.4627	0.2333	0.242	0.7924	0
3410	759.1	1.4626	0.2335	0.2421	0.7925	0
3411	759	1.4631	0.2335	0.2421	0.7924	0
3412	758.9	1.4629	0.2334	0.2422	0.7926	0
3413	758.8	1.4628	0.2336	0.2422	0.7924	0
3414	758.7	1.463	0.2336	0.2422	0.7927	0
3415	758.6	1.4632	0.2335	0.2422	0.7927	0
3416	758.5	1.4628	0.2334	0.2422	0.7926	0
3417	758.4	1.4629	0.2334	0.242	0.7927	0
3418	758.3	1.4631	0.2334	0.2421	0.7927	0
3419	758.2	1.4627	0.2334	0.2421	0.7927	0
3420	758.1	1.463	0.2333	0.2421	0.7927	0
3421	758	1.4628	0.2333	0.242	0.7926	0
3422	757.9	1.4631	0.2333	0.2421	0.7929	0
3423	757.8	1.4628	0.2334	0.242	0.7927	0
3424	757.7	1.463	0.2334	0.2421	0.7927	0
3425	757.6	1.4634	0.2333	0.2422	0.7927	0
3426	757.5	1.4627	0.2331	0.2419	0.7926	0

3427	757.4	1.463	0.2334	0.242	0.7927	0
3428	757.3	1.4632	0.2334	0.2421	0.7928	0
3429	757.2	1.4629	0.2332	0.2421	0.7926	0
3430	757.1	1.4627	0.2332	0.2419	0.7927	0
3431	757	1.4628	0.2332	0.2418	0.7924	0
3432	756.9	1.4629	0.2334	0.2421	0.7927	0
3433	756.8	1.4632	0.2332	0.2422	0.7928	0
3434	756.7	1.4629	0.2333	0.242	0.7928	0
3435	756.6	1.4632	0.2334	0.2421	0.793	0
3436	756.5	1.4632	0.2334	0.242	0.7929	0
3437	756.4	1.4633	0.2333	0.242	0.7928	0
3438	756.3	1.4632	0.2334	0.2421	0.7929	0
3439	756.2	1.4631	0.2334	0.2422	0.7927	0
3440	756.1	1.4632	0.2334	0.2422	0.793	0
3441	756	1.4631	0.2335	0.2422	0.7929	0
3442	755.9	1.4631	0.2336	0.2423	0.7928	0
3443	755.8	1.4634	0.2336	0.2423	0.7931	0
3444	755.7	1.4635	0.2337	0.2423	0.7932	0
3445	755.6	1.4631	0.2336	0.2423	0.7931	0
3446	755.5	1.4635	0.2337	0.2425	0.7933	0
3447	755.4	1.4631	0.2334	0.2421	0.7931	0
3448	755.3	1.4634	0.2336	0.2424	0.7932	0
3449	755.2	1.4637	0.2337	0.2425	0.7931	0
3450	755.1	1.4631	0.2336	0.2425	0.7931	0
3451	755	1.4634	0.2335	0.2422	0.7932	0
3452	754.9	1.4632	0.2335	0.2423	0.793	0
3453	754.8	1.463	0.2332	0.2422	0.7931	0
3454	754.7	1.4632	0.2336	0.2423	0.7933	0
3455	754.6	1.4632	0.2338	0.2427	0.7933	0
3456	754.5	1.4634	0.2339	0.2428	0.7934	0
3457	754.4	1.4632	0.2339	0.2426	0.7934	0
3458	754.3	1.4632	0.2338	0.2425	0.7934	0
3459	754.2	1.4635	0.2338	0.2426	0.7935	0
3460	754.1	1.4635	0.2338	0.2426	0.7935	0
3461	754	1.4634	0.234	0.2426	0.7938	0
3462	753.9	1.464	0.234	0.2427	0.7937	0
3463	753.8	1.4638	0.234	0.2427	0.7937	0
3464	753.7	1.4637	0.2341	0.2429	0.7938	0
3465	753.6	1.4639	0.2341	0.243	0.7938	0
3466	753.5	1.4638	0.2342	0.243	0.7937	0
3467	753.4	1.4634	0.234	0.2427	0.7936	0
3468	753.3	1.4638	0.2341	0.2428	0.7937	0
3469	753.2	1.4633	0.2342	0.2428	0.7936	0

3470	753.1	1.4639	0.2341	0.243	0.7937	0
3471	753	1.4633	0.2341	0.2429	0.7937	0
3472	752.9	1.4634	0.2341	0.2429	0.7937	0
3473	752.8	1.4635	0.2341	0.2428	0.7936	0
3474	752.7	1.464	0.2343	0.2429	0.7937	0
3475	752.6	1.4637	0.2342	0.243	0.7939	0
3476	752.5	1.4642	0.2343	0.2431	0.794	0
3477	752.4	1.4642	0.2342	0.2431	0.7939	0
3478	752.3	1.4639	0.2341	0.2429	0.7938	0
3479	752.2	1.4638	0.2343	0.2428	0.7939	0
3480	752.1	1.4636	0.2342	0.2429	0.794	0
3481	752	1.464	0.2341	0.2429	0.7938	0
3482	751.9	1.4635	0.2341	0.2431	0.7941	0
3483	751.8	1.4639	0.2342	0.2431	0.7939	0
3484	751.7	1.4639	0.2343	0.2432	0.794	0
3485	751.6	1.464	0.2344	0.2431	0.7939	0
3486	751.5	1.4641	0.2343	0.2431	0.794	0
3487	751.4	1.4639	0.2344	0.2432	0.7942	0
3488	751.3	1.4637	0.2343	0.2433	0.7941	0
3489	751.2	1.4639	0.2344	0.2431	0.794	0
3490	751.1	1.4636	0.2343	0.243	0.794	0
3491	751	1.4636	0.2343	0.243	0.794	0
3492	750.9	1.4641	0.2343	0.2431	0.794	0
3493	750.8	1.4637	0.2342	0.2431	0.7943	0
3494	750.7	1.4641	0.2343	0.2431	0.7941	0
3495	750.6	1.4639	0.2344	0.2431	0.7944	0
3496	750.5	1.4637	0.2343	0.2431	0.7943	0
3497	750.4	1.464	0.2344	0.2432	0.7944	0
3498	750.3	1.464	0.2343	0.2432	0.7943	0
3499	750.2	1.4641	0.2341	0.2432	0.7942	0
3500	750.1	1.4639	0.2343	0.2432	0.7944	0
3501	750	1.464	0.2342	0.2431	0.7942	0
3502	749.9	1.4638	0.2343	0.2432	0.7944	0
3503	749.8	1.4636	0.2342	0.2431	0.7942	0
3504	749.7	1.4638	0.2342	0.2432	0.794	0
3505	749.6	1.4636	0.2343	0.2432	0.7943	0
3506	749.5	1.4643	0.2342	0.243	0.7944	0
3507	749.4	1.4637	0.2342	0.2429	0.7944	0
3508	749.3	1.4637	0.2343	0.2431	0.7944	0
3509	749.2	1.4642	0.2344	0.2432	0.7946	0
3510	749.1	1.4638	0.2343	0.243	0.7945	0
3511	749	1.464	0.2341	0.2428	0.7942	0
3512	748.9	1.4639	0.2342	0.2429	0.7943	0

3513	748.8	1.4638	0.2342	0.243	0.7945	0
3514	748.7	1.4635	0.2344	0.2432	0.7947	0
3515	748.6	1.4642	0.2343	0.2431	0.7946	0
3516	748.5	1.4639	0.2342	0.2431	0.7946	0
3517	748.4	1.4641	0.2343	0.2432	0.7944	0
3518	748.3	1.4641	0.2343	0.2431	0.7944	0
3519	748.2	1.4638	0.2344	0.2432	0.7947	0
3520	748.1	1.4639	0.2345	0.2434	0.7947	0
3521	748	1.4639	0.2342	0.2431	0.7946	0
3522	747.9	1.4638	0.2344	0.2433	0.7947	0
3523	747.8	1.4637	0.2342	0.2431	0.7945	0
3524	747.7	1.4639	0.2343	0.2432	0.7946	0
3525	747.6	1.4638	0.2344	0.2433	0.7948	0
3526	747.5	1.4638	0.2344	0.2433	0.7947	0
3527	747.4	1.4643	0.2346	0.2432	0.7946	0
3528	747.3	1.464	0.2343	0.2432	0.7947	0
3529	747.2	1.4643	0.2344	0.2432	0.7949	0
3530	747.1	1.4644	0.2343	0.2433	0.7948	0
3531	747	1.4637	0.2342	0.2431	0.7946	0
3532	746.9	1.4639	0.2344	0.243	0.7948	0
3533	746.8	1.4645	0.2346	0.2433	0.7949	0
3534	746.7	1.4646	0.2345	0.2433	0.7949	0
3535	746.6	1.464	0.2344	0.2433	0.7947	0
3536	746.5	1.4642	0.2344	0.2432	0.795	0
3537	746.4	1.4643	0.2344	0.2432	0.7951	0
3538	746.3	1.4638	0.2342	0.2429	0.7945	0
3539	746.2	1.4642	0.2344	0.2431	0.795	0
3540	746.1	1.4642	0.2346	0.2434	0.7952	0
3541	746	1.464	0.2346	0.2434	0.7952	0
3542	745.9	1.4638	0.2347	0.2434	0.7952	0
3543	745.8	1.4642	0.2346	0.2435	0.7951	0
3544	745.7	1.4644	0.2347	0.2435	0.7952	0
3545	745.6	1.4642	0.2348	0.2435	0.7951	0
3546	745.5	1.4643	0.2347	0.2435	0.7952	0
3547	745.4	1.464	0.2345	0.2432	0.795	0
3548	745.3	1.4638	0.2346	0.2434	0.7952	0
3549	745.2	1.4641	0.2348	0.2435	0.7951	0
3550	745.1	1.4641	0.2347	0.2434	0.7953	0
3551	745	1.4641	0.235	0.2436	0.7953	0
3552	744.9	1.4646	0.2351	0.2438	0.7954	0
3553	744.8	1.4643	0.235	0.2436	0.7953	0
3554	744.7	1.4644	0.2348	0.2436	0.7952	0
3555	744.6	1.465	0.2352	0.2438	0.7956	0

3556	744.5	1.465	0.235	0.2437	0.7958	0
3557	744.4	1.4645	0.235	0.2438	0.7957	0
3558	744.3	1.4646	0.2349	0.2437	0.7955	0
3559	744.2	1.4648	0.2352	0.2438	0.7955	0
3560	744.1	1.4649	0.2351	0.2439	0.7955	0
3561	744	1.4651	0.2351	0.2439	0.7954	0
3562	743.9	1.4647	0.2353	0.2441	0.7956	0
3563	743.8	1.4646	0.2352	0.2442	0.7958	0
3564	743.7	1.4646	0.2354	0.2441	0.7957	0
3565	743.6	1.4653	0.2353	0.2441	0.7956	0
3566	743.5	1.4649	0.2356	0.2442	0.7957	0
3567	743.4	1.4654	0.2355	0.2442	0.7959	0
3568	743.3	1.4652	0.2354	0.2442	0.7959	0
3569	743.2	1.4649	0.2353	0.2441	0.7959	0
3570	743.1	1.4652	0.2356	0.2443	0.7959	0
3571	743	1.4647	0.2357	0.2446	0.7961	0
3572	742.9	1.4648	0.2356	0.2444	0.7961	0
3573	742.8	1.4652	0.2356	0.2445	0.7961	0
3574	742.7	1.4653	0.2355	0.2445	0.7961	0
3575	742.6	1.4654	0.2356	0.2444	0.7962	0
3576	742.5	1.4653	0.2356	0.2445	0.7963	0
3577	742.4	1.465	0.2357	0.2445	0.796	0
3578	742.3	1.4651	0.2356	0.2444	0.7961	0
3579	742.2	1.4652	0.2356	0.2444	0.7962	0
3580	742.1	1.4652	0.2356	0.2444	0.796	0
3581	742	1.465	0.2357	0.2444	0.7959	0
3582	741.9	1.4656	0.2355	0.2444	0.7962	0
3583	741.8	1.4655	0.2356	0.2444	0.7963	0
3584	741.7	1.465	0.2357	0.2444	0.7961	0
3585	741.6	1.4652	0.2358	0.2445	0.7963	0
3586	741.5	1.4649	0.2356	0.2443	0.7962	0
3587	741.4	1.4652	0.2355	0.2444	0.7962	0
3588	741.3	1.4654	0.2357	0.2444	0.7963	0
3589	741.2	1.465	0.2356	0.2443	0.7962	0
3590	741.1	1.4652	0.2357	0.2444	0.7962	0
3591	741	1.4652	0.2356	0.2443	0.7963	0
3592	740.9	1.4651	0.2356	0.2445	0.7962	0
3593	740.8	1.465	0.2357	0.2445	0.7965	0
3594	740.7	1.465	0.2357	0.2445	0.7964	0
3595	740.6	1.4651	0.2357	0.2445	0.7965	0
3596	740.5	1.4654	0.2357	0.2442	0.7965	0
3597	740.4	1.4655	0.2357	0.2444	0.7966	0
3598	740.3	1.4652	0.2355	0.2443	0.7962	0

3599	740.2	1.4655	0.2354	0.2443	0.7962	0
3600	740.1	1.4648	0.2355	0.2445	0.7963	0
3601	740	1.4649	0.2354	0.2445	0.7965	0
3602	739.9	1.4647	0.2353	0.2443	0.7964	0
3603	739.8	1.4646	0.2354	0.2442	0.7963	0
3604	739.7	1.4651	0.2354	0.2443	0.7962	0
3605	739.6	1.4651	0.2353	0.2443	0.7964	0
3606	739.5	1.4645	0.2353	0.2442	0.7961	0
3607	739.4	1.4653	0.2354	0.2442	0.7963	0
3608	739.3	1.4651	0.2354	0.2442	0.7963	0
3609	739.2	1.4648	0.2353	0.2442	0.7964	0
3610	739.1	1.4649	0.2354	0.2442	0.7963	0
3611	739	1.4649	0.2353	0.2442	0.7964	0
3612	738.9	1.465	0.2353	0.2441	0.7962	0
3613	738.8	1.465	0.2352	0.244	0.7962	0
3614	738.7	1.4649	0.2354	0.2443	0.7966	0
3615	738.6	1.4649	0.2353	0.2441	0.7964	0
3616	738.5	1.4652	0.2355	0.2443	0.7965	0
3617	738.4	1.4651	0.2352	0.244	0.7964	0
3618	738.3	1.4651	0.2352	0.2442	0.7963	0
3619	738.2	1.4652	0.2353	0.2441	0.7963	0
3620	738.1	1.4647	0.2351	0.2441	0.7964	0
3621	738	1.4649	0.2353	0.2441	0.7964	0
3622	737.9	1.465	0.2353	0.2442	0.7966	0
3623	737.8	1.465	0.2352	0.2441	0.7964	0
3624	737.7	1.4653	0.2352	0.2442	0.7964	0
3625	737.6	1.4651	0.235	0.244	0.7965	0
3626	737.5	1.4649	0.2352	0.2441	0.7964	0
3627	737.4	1.4647	0.2352	0.2439	0.7964	0
3628	737.3	1.4649	0.2351	0.2439	0.7964	0
3629	737.2	1.4651	0.235	0.244	0.7964	0
3630	737.1	1.4653	0.2353	0.2442	0.7963	0
3631	737	1.4647	0.2352	0.244	0.7963	0
3632	736.9	1.4652	0.2353	0.2441	0.7966	0
3633	736.8	1.4648	0.2354	0.2442	0.7965	0
3634	736.7	1.4655	0.2353	0.2442	0.7967	0
3635	736.6	1.4647	0.2354	0.2443	0.7965	0
3636	736.5	1.4654	0.2355	0.2442	0.7967	0
3637	736.4	1.4652	0.2355	0.2443	0.7967	0
3638	736.3	1.4653	0.2355	0.2443	0.7965	0
3639	736.2	1.4655	0.2356	0.2444	0.7968	0
3640	736.1	1.4653	0.2356	0.2445	0.7969	0
3641	736	1.4654	0.2358	0.2445	0.797	0

3642	735.9	1.4655	0.2357	0.2445	0.7969	0
3643	735.8	1.4654	0.2356	0.2443	0.7967	0
3644	735.7	1.4648	0.2356	0.2446	0.797	0
3645	735.6	1.4652	0.2358	0.2447	0.7969	0
3646	735.5	1.4651	0.2357	0.2444	0.7968	0
3647	735.4	1.465	0.2356	0.2445	0.7969	0
3648	735.3	1.4652	0.2358	0.2447	0.7969	0
3649	735.2	1.465	0.2357	0.2446	0.7971	0
3650	735.1	1.4652	0.2359	0.2447	0.7972	0
3651	735	1.4655	0.2358	0.2447	0.7971	0
3652	734.9	1.4654	0.2359	0.2447	0.7973	0
3653	734.8	1.4654	0.2358	0.2447	0.7971	0
3654	734.7	1.4654	0.2358	0.2447	0.7971	0
3655	734.6	1.4657	0.236	0.2449	0.7973	0
3656	734.5	1.4655	0.236	0.2448	0.7971	0
3657	734.4	1.4657	0.2362	0.2451	0.7972	0
3658	734.3	1.4655	0.2362	0.245	0.7973	0
3659	734.2	1.4654	0.236	0.2449	0.797	0
3660	734.1	1.4656	0.2361	0.2449	0.7973	0
3661	734	1.4658	0.2363	0.2451	0.7973	0
3662	733.9	1.4656	0.2362	0.2452	0.7974	0
3663	733.8	1.4657	0.2362	0.2451	0.7973	0
3664	733.7	1.466	0.2366	0.2453	0.7977	0
3665	733.6	1.4659	0.2363	0.2452	0.7976	0
3666	733.5	1.4658	0.2364	0.2453	0.7977	0
3667	733.4	1.466	0.2365	0.2455	0.7977	0
3668	733.3	1.4662	0.2367	0.2456	0.7979	0
3669	733.2	1.4663	0.2366	0.2456	0.798	0
3670	733.1	1.4661	0.2365	0.2455	0.7978	0
3671	733	1.466	0.2366	0.2456	0.7977	0
3672	732.9	1.4666	0.2369	0.2457	0.798	0
3673	732.8	1.4664	0.2368	0.2457	0.7982	0
3674	732.7	1.4663	0.2369	0.2456	0.7981	0
3675	732.6	1.4662	0.2368	0.2457	0.7982	0
3676	732.5	1.4661	0.237	0.2458	0.7981	0
3677	732.4	1.4664	0.2369	0.2458	0.7979	0
3678	732.3	1.4659	0.237	0.2457	0.798	0
3679	732.2	1.4662	0.2369	0.2456	0.7982	0
3680	732.1	1.4659	0.2368	0.2456	0.798	0
3681	732	1.4663	0.2367	0.2457	0.7981	0
3682	731.9	1.4667	0.2369	0.2457	0.7981	0
3683	731.8	1.4662	0.2369	0.2458	0.7982	0
3684	731.7	1.4662	0.2369	0.2457	0.7982	0

3685	731.6	1.4666	0.2371	0.2458	0.7983	0
3686	731.5	1.4664	0.237	0.2457	0.7982	0
3687	731.4	1.4666	0.2372	0.2459	0.7986	0
3688	731.3	1.4663	0.237	0.2459	0.7983	0
3689	731.2	1.4667	0.2369	0.2459	0.7983	0
3690	731.1	1.4662	0.2367	0.2457	0.798	0
3691	731	1.4665	0.2369	0.2458	0.7982	0
3692	730.9	1.4665	0.237	0.2459	0.7983	0
3693	730.8	1.4666	0.2368	0.2458	0.7984	0
3694	730.7	1.4667	0.2371	0.2459	0.7985	0
3695	730.6	1.4667	0.237	0.2459	0.7985	0
3696	730.5	1.4664	0.2369	0.2458	0.7984	0
3697	730.4	1.4665	0.2366	0.2456	0.7981	0
3698	730.3	1.4667	0.2369	0.2456	0.798	0
3699	730.2	1.4665	0.2368	0.2458	0.7983	0
3700	730.1	1.4663	0.237	0.2459	0.7984	0
3701	730	1.467	0.2369	0.2458	0.7986	0
3702	729.9	1.4666	0.2369	0.2458	0.7983	0
3703	729.8	1.4663	0.2367	0.2456	0.7981	0
3704	729.7	1.4659	0.2365	0.2455	0.7982	0
3705	729.6	1.4665	0.2366	0.2456	0.7983	0
3706	729.5	1.4661	0.2366	0.2456	0.7982	0
3707	729.4	1.466	0.2366	0.2454	0.7981	0
3708	729.3	1.4661	0.2366	0.2455	0.7982	0
3709	729.2	1.4664	0.2365	0.2455	0.7982	0
3710	729.1	1.466	0.2367	0.2456	0.7983	0
3711	729	1.4663	0.2364	0.2455	0.7983	0
3712	728.9	1.4663	0.2366	0.2454	0.7983	0
3713	728.8	1.4663	0.2366	0.2453	0.7983	0
3714	728.7	1.4658	0.2363	0.2452	0.798	0
3715	728.6	1.4663	0.2365	0.2453	0.798	0
3716	728.5	1.466	0.2364	0.2453	0.798	0
3717	728.4	1.4657	0.2363	0.2452	0.7981	0
3718	728.3	1.4661	0.2364	0.2452	0.7981	0
3719	728.2	1.4662	0.2366	0.2453	0.7982	0
3720	728.1	1.4663	0.2365	0.2453	0.7981	0
3721	728	1.4659	0.2364	0.2452	0.7981	0
3722	727.9	1.4662	0.2363	0.2452	0.7982	0
3723	727.8	1.4659	0.2363	0.2451	0.7981	0
3724	727.7	1.466	0.2362	0.2451	0.7981	0
3725	727.6	1.4664	0.2365	0.2452	0.7981	0
3726	727.5	1.4661	0.2364	0.2452	0.7981	0
3727	727.4	1.4661	0.2363	0.2451	0.798	0

3728	727.3	1.4658	0.2363	0.2451	0.7981	0
3729	727.2	1.4659	0.2363	0.2452	0.7981	0
3730	727.1	1.4659	0.2363	0.2452	0.7982	0
3731	727	1.4657	0.2363	0.2452	0.7982	0
3732	726.9	1.4657	0.2363	0.2451	0.7983	0
3733	726.8	1.4658	0.2363	0.2451	0.7981	0
3734	726.7	1.4658	0.2363	0.2451	0.7981	0
3735	726.6	1.4659	0.2364	0.2452	0.7982	0
3736	726.5	1.4655	0.2363	0.2451	0.7982	0
3737	726.4	1.4657	0.2364	0.2452	0.7981	0
3738	726.3	1.4663	0.2364	0.2451	0.7983	0
3739	726.2	1.466	0.2363	0.2451	0.7981	0
3740	726.1	1.4659	0.2365	0.2452	0.7983	0
3741	726	1.4659	0.2363	0.2451	0.7984	0
3742	725.9	1.4654	0.2363	0.245	0.7982	0
3743	725.8	1.4659	0.2364	0.2451	0.7982	0
3744	725.7	1.4657	0.2363	0.2451	0.7982	0
3745	725.6	1.4661	0.2365	0.2452	0.7983	0
3746	725.5	1.4661	0.2365	0.2452	0.7984	0
3747	725.4	1.4655	0.2364	0.2452	0.7982	0
3748	725.3	1.4663	0.2366	0.2453	0.7984	0
3749	725.2	1.4661	0.2365	0.2454	0.7984	0
3750	725.1	1.4663	0.2368	0.2456	0.7986	0
3751	725	1.4663	0.2371	0.2458	0.7989	0
3752	724.9	1.4662	0.2368	0.2455	0.7988	0
3753	724.8	1.4662	0.2368	0.2456	0.7988	0
3754	724.7	1.4663	0.2367	0.2456	0.7988	0
3755	724.6	1.4662	0.2371	0.2458	0.7992	0
3756	724.5	1.4663	0.2371	0.2459	0.7989	0
3757	724.4	1.4666	0.2371	0.2458	0.7991	0
3758	724.3	1.4667	0.2371	0.246	0.799	0
3759	724.2	1.4669	0.2373	0.246	0.799	0
3760	724.1	1.4667	0.2374	0.2461	0.7991	0
3761	724	1.4668	0.2373	0.2462	0.799	0
3762	723.9	1.4665	0.2373	0.246	0.7989	0
3763	723.8	1.4669	0.2374	0.2462	0.799	0
3764	723.7	1.4672	0.2376	0.2464	0.7993	0
3765	723.6	1.4667	0.2377	0.2465	0.7995	0
3766	723.5	1.4669	0.2377	0.2464	0.7992	0
3767	723.4	1.4671	0.2378	0.2466	0.7996	0
3768	723.3	1.4675	0.2379	0.2466	0.7995	0
3769	723.2	1.4676	0.2379	0.2468	0.7995	0
3770	723.1	1.4673	0.2378	0.2465	0.7994	0

3771	723	1.4672	0.2379	0.2467	0.7996	0
3772	722.9	1.4672	0.238	0.2467	0.7997	0
3773	722.8	1.4672	0.238	0.2468	0.7998	0
3774	722.7	1.4671	0.2378	0.2465	0.7996	0
3775	722.6	1.4676	0.238	0.2467	0.7996	0
3776	722.5	1.4673	0.238	0.2467	0.7997	0
3777	722.4	1.4677	0.238	0.2467	0.7994	0
3778	722.3	1.4677	0.238	0.2467	0.7997	0
3779	722.2	1.4679	0.2382	0.2469	0.7997	0
3780	722.1	1.4679	0.2381	0.2469	0.7999	0
3781	722	1.4677	0.2382	0.2469	0.8	0
3782	721.9	1.4676	0.2379	0.2468	0.7998	0
3783	721.8	1.4676	0.2381	0.2468	0.7999	0
3784	721.7	1.4681	0.2382	0.2471	0.8001	0
3785	721.6	1.4676	0.2383	0.247	0.8001	0
3786	721.5	1.4677	0.2381	0.2469	0.8	0
3787	721.4	1.4679	0.2382	0.247	0.8001	0
3788	721.3	1.4673	0.2382	0.2469	0.8001	0
3789	721.2	1.468	0.2383	0.2472	0.8002	0
3790	721.1	1.468	0.2384	0.2471	0.8004	0
3791	721	1.4679	0.2383	0.2469	0.8001	0
3792	720.9	1.4677	0.2381	0.247	0.8001	0
3793	720.8	1.4679	0.2382	0.2469	0.7998	0
3794	720.7	1.4676	0.2381	0.2469	0.8001	0
3795	720.6	1.4679	0.2381	0.2469	0.8	0
3796	720.5	1.4678	0.2384	0.2471	0.8003	0
3797	720.4	1.4678	0.2382	0.2471	0.8003	0
3798	720.3	1.468	0.2382	0.247	0.8001	0
3799	720.2	1.4679	0.2383	0.2471	0.8004	0
3800	720.1	1.468	0.2382	0.2469	0.8001	0
3801	720	1.4675	0.2382	0.2471	0.8	0
3802	719.9	1.4678	0.2383	0.2471	0.8001	0
3803	719.8	1.4679	0.2384	0.2471	0.8002	0
3804	719.7	1.4678	0.2383	0.2471	0.8003	0
3805	719.6	1.4678	0.2382	0.2469	0.8003	0
3806	719.5	1.4674	0.2382	0.2468	0.8002	0
3807	719.4	1.4676	0.2381	0.2468	0.8001	0
3808	719.3	1.4678	0.2381	0.2469	0.8002	0
3809	719.2	1.4679	0.2382	0.247	0.8005	0
3810	719.1	1.4674	0.2381	0.2467	0.8002	0
3811	719	1.4676	0.2379	0.2465	0.8	0
3812	718.9	1.4678	0.2381	0.2468	0.8003	0
3813	718.8	1.4681	0.2381	0.2468	0.8003	0

3814	718.7	1.4678	0.2382	0.2469	0.8003	0
3815	718.6	1.4678	0.2381	0.2468	0.8001	0
3816	718.5	1.4676	0.2378	0.2466	0.8002	0
3817	718.4	1.4675	0.2378	0.2466	0.8003	0
3818	718.3	1.4672	0.2378	0.2465	0.8001	0
3819	718.2	1.4673	0.2379	0.2466	0.8	0
3820	718.1	1.4677	0.2379	0.2467	0.8001	0
3821	718	1.4672	0.2378	0.2466	0.8003	0
3822	717.9	1.4678	0.2378	0.2466	0.8002	0
3823	717.8	1.4675	0.2378	0.2466	0.8003	0
3824	717.7	1.4676	0.2379	0.2466	0.8003	0
3825	717.6	1.4675	0.2378	0.2465	0.8	0
3826	717.5	1.4676	0.2377	0.2464	0.8	0
3827	717.4	1.4673	0.2376	0.2463	0.8	0
3828	717.3	1.4674	0.2378	0.2464	0.8004	0
3829	717.2	1.4671	0.2377	0.2466	0.8003	0
3830	717.1	1.4675	0.2379	0.2465	0.8001	0
3831	717	1.4671	0.2376	0.2464	0.8003	0
3832	716.9	1.4676	0.2376	0.2462	0.8001	0
3833	716.8	1.4673	0.2377	0.2464	0.8002	0
3834	716.7	1.4672	0.2376	0.2463	0.8003	0
3835	716.6	1.467	0.2375	0.2462	0.8002	0
3836	716.5	1.4674	0.2375	0.2462	0.8001	0
3837	716.4	1.4673	0.2377	0.2463	0.8002	0
3838	716.3	1.4673	0.2376	0.2464	0.8004	0
3839	716.2	1.4676	0.2376	0.2463	0.8	0
3840	716.1	1.4672	0.2376	0.2462	0.8	0
3841	716	1.4669	0.2376	0.2462	0.8002	0
3842	715.9	1.4675	0.2376	0.2462	0.8	0
3843	715.8	1.4672	0.2376	0.2462	0.8003	0
3844	715.7	1.4674	0.2378	0.2464	0.8003	0
3845	715.6	1.4677	0.2378	0.2464	0.8004	0
3846	715.5	1.4673	0.2376	0.2463	0.8003	0
3847	715.4	1.4673	0.2378	0.2463	0.8003	0
3848	715.3	1.4676	0.2377	0.2463	0.8002	0
3849	715.2	1.4676	0.2378	0.2463	0.8004	0
3850	715.1	1.4677	0.2378	0.2465	0.8007	0
3851	715	1.4677	0.2379	0.2466	0.8005	0
3852	714.9	1.4675	0.2379	0.2467	0.8005	0
3853	714.8	1.4677	0.2378	0.2465	0.8006	0
3854	714.7	1.4674	0.2379	0.2465	0.8006	0
3855	714.6	1.4678	0.238	0.2467	0.8007	0
3856	714.5	1.4679	0.2381	0.2467	0.8008	0

3857	714.4	1.4672	0.2381	0.2468	0.8008	0
3858	714.3	1.4679	0.2382	0.2469	0.8009	0
3859	714.2	1.4677	0.2384	0.247	0.8011	0
3860	714.1	1.468	0.2384	0.247	0.8011	0
3861	714	1.4679	0.2383	0.2469	0.801	0
3862	713.9	1.4675	0.2383	0.2469	0.8009	0
3863	713.8	1.4678	0.2382	0.2468	0.8009	0
3864	713.7	1.4679	0.2382	0.247	0.801	0
3865	713.6	1.468	0.2384	0.2471	0.801	0
3866	713.5	1.4678	0.2385	0.2472	0.8009	0
3867	713.4	1.4681	0.2386	0.2474	0.8011	0
3868	713.3	1.4686	0.2388	0.2475	0.8015	0
3869	713.2	1.4682	0.2387	0.2475	0.8014	0
3870	713.1	1.4682	0.2386	0.2475	0.8011	0
3871	713	1.4682	0.2389	0.2475	0.8011	0
3872	712.9	1.4686	0.239	0.2476	0.8014	0
3873	712.8	1.4683	0.2389	0.2477	0.8014	0
3874	712.7	1.4686	0.239	0.2477	0.8014	0
3875	712.6	1.4682	0.239	0.2477	0.8015	0
3876	712.5	1.4682	0.2388	0.2476	0.8018	0
3877	712.4	1.4684	0.2388	0.2476	0.8013	0
3878	712.3	1.4688	0.2389	0.2477	0.8015	0
3879	712.2	1.4685	0.239	0.2477	0.8018	0
3880	712.1	1.4689	0.2392	0.2478	0.8019	0
3881	712	1.4682	0.2391	0.2479	0.8018	0
3882	711.9	1.4689	0.2393	0.248	0.8018	0
3883	711.8	1.4686	0.2393	0.2482	0.8018	0
3884	711.7	1.4689	0.2393	0.2481	0.802	0
3885	711.6	1.4689	0.2393	0.2481	0.802	0
3886	711.5	1.4688	0.2393	0.2482	0.802	0
3887	711.4	1.4686	0.2394	0.2481	0.8021	0
3888	711.3	1.4688	0.2394	0.248	0.8019	0
3889	711.2	1.4687	0.2392	0.2481	0.802	0
3890	711.1	1.4687	0.2395	0.2481	0.8021	0
3891	711	1.469	0.2395	0.2481	0.8021	0
3892	710.9	1.4687	0.2394	0.2481	0.8021	0
3893	710.8	1.4689	0.2394	0.2482	0.802	0
3894	710.7	1.4686	0.2394	0.2481	0.8021	0
3895	710.6	1.469	0.2397	0.2484	0.8022	0
3896	710.5	1.4689	0.2395	0.2482	0.8019	0
3897	710.4	1.4689	0.2396	0.2482	0.8021	0
3898	710.3	1.4693	0.2398	0.2484	0.8022	0
3899	710.2	1.4689	0.2396	0.2484	0.8023	0

3900	710.1	1.4691	0.2397	0.2483	0.8023	0
3901	710	1.4688	0.2396	0.2485	0.8024	0
3902	709.9	1.469	0.2397	0.2484	0.8022	0
3903	709.8	1.4693	0.2397	0.2485	0.8024	0
3904	709.7	1.4689	0.2397	0.2484	0.8022	0
3905	709.6	1.4691	0.2395	0.2483	0.8024	0
3906	709.5	1.4691	0.2397	0.2482	0.8023	0
3907	709.4	1.4692	0.2394	0.2482	0.8023	0
3908	709.3	1.4692	0.2395	0.2485	0.8021	0
3909	709.2	1.4691	0.2395	0.2483	0.8022	0
3910	709.1	1.4688	0.2395	0.2483	0.8023	0
3911	709	1.4691	0.2397	0.2484	0.8025	0
3912	708.9	1.4691	0.2395	0.2483	0.8024	0
3913	708.8	1.4688	0.2394	0.2481	0.8023	0
3914	708.7	1.4691	0.2395	0.2482	0.8023	0
3915	708.6	1.4692	0.2396	0.2485	0.8025	0
3916	708.5	1.469	0.2393	0.2481	0.8024	0
3917	708.4	1.4689	0.2395	0.2482	0.8024	0
3918	708.3	1.4692	0.2395	0.2482	0.8024	0
3919	708.2	1.4694	0.2394	0.2481	0.8024	0
3920	708.1	1.4691	0.2395	0.2483	0.8024	0
3921	708	1.4692	0.2393	0.2481	0.8022	0
3922	707.9	1.4689	0.2394	0.2481	0.8026	0
3923	707.8	1.4688	0.2394	0.248	0.8025	0
3924	707.7	1.469	0.2395	0.2481	0.8026	0
3925	707.6	1.4693	0.2395	0.2481	0.8026	0
3926	707.5	1.4688	0.2393	0.2481	0.8026	0
3927	707.4	1.469	0.2392	0.248	0.8025	0
3928	707.3	1.469	0.2394	0.2481	0.8027	0
3929	707.2	1.4691	0.2392	0.2479	0.8026	0
3930	707.1	1.4687	0.2392	0.248	0.8024	0
3931	707	1.4687	0.2393	0.248	0.8025	0
3932	706.9	1.4689	0.2394	0.248	0.8024	0
3933	706.8	1.4689	0.2393	0.2479	0.8027	0
3934	706.7	1.4688	0.2394	0.2479	0.8026	0
3935	706.6	1.4693	0.2391	0.248	0.8024	0
3936	706.5	1.4689	0.2392	0.2479	0.8026	0
3937	706.4	1.4691	0.2392	0.2478	0.8025	0
3938	706.3	1.469	0.2392	0.2478	0.8025	0
3939	706.2	1.469	0.2392	0.248	0.8026	0
3940	706.1	1.4686	0.2392	0.248	0.8028	0
3941	706	1.4685	0.2392	0.2478	0.8025	0
3942	705.9	1.4691	0.2392	0.2478	0.8027	0

3943	705.8	1.4689	0.2392	0.2478	0.8026	0
3944	705.7	1.4691	0.2391	0.2479	0.8027	0
3945	705.6	1.4688	0.2392	0.2479	0.8026	0
3946	705.5	1.4687	0.2392	0.2479	0.8024	0
3947	705.4	1.4686	0.2391	0.2478	0.8024	0
3948	705.3	1.469	0.2393	0.248	0.8026	0
3949	705.2	1.4689	0.2394	0.2482	0.8025	0
3950	705.1	1.4692	0.2395	0.2482	0.8028	0
3951	705	1.469	0.2392	0.2481	0.8027	0
3952	704.9	1.4693	0.2393	0.2481	0.8027	0
3953	704.8	1.469	0.2395	0.2482	0.8027	0
3954	704.7	1.4685	0.2394	0.2482	0.8028	0
3955	704.6	1.4691	0.2394	0.2482	0.8028	0
3956	704.5	1.4692	0.2395	0.2483	0.803	0
3957	704.4	1.4695	0.2396	0.2485	0.8032	0
3958	704.3	1.4693	0.2396	0.2482	0.8031	0
3959	704.2	1.4693	0.2398	0.2484	0.8032	0
3960	704.1	1.4691	0.2396	0.2484	0.8032	0
3961	704	1.4692	0.2398	0.2485	0.8034	0
3962	703.9	1.4697	0.2397	0.2486	0.8033	0
3963	703.8	1.4693	0.2399	0.2485	0.8034	0
3964	703.7	1.4692	0.2398	0.2485	0.8034	0
3965	703.6	1.4694	0.2397	0.2485	0.8035	0
3966	703.5	1.4693	0.2398	0.2487	0.8033	0
3967	703.4	1.4692	0.2397	0.2484	0.8033	0
3968	703.3	1.4696	0.24	0.2488	0.8036	0
3969	703.2	1.4692	0.2401	0.2486	0.8036	0
3970	703.1	1.4693	0.24	0.2486	0.8036	0
3971	703	1.4694	0.2401	0.2489	0.8035	0
3972	702.9	1.4698	0.2401	0.2487	0.8036	0
3973	702.8	1.47	0.24	0.2489	0.8037	0
3974	702.7	1.4696	0.2401	0.2487	0.8036	0
3975	702.6	1.4698	0.2399	0.2488	0.8036	0
3976	702.5	1.47	0.24	0.2487	0.8034	0
3977	702.4	1.47	0.2402	0.249	0.804	0
3978	702.3	1.4698	0.2401	0.2488	0.8036	0
3979	702.2	1.4697	0.2401	0.2487	0.8036	0
3980	702.1	1.4699	0.2401	0.2488	0.8036	0
3981	702	1.4704	0.2403	0.2491	0.8039	0
3982	701.9	1.4702	0.2403	0.2491	0.8038	0
3983	701.8	1.4704	0.2403	0.249	0.8038	0
3984	701.7	1.4703	0.2404	0.2491	0.8039	0
3985	701.6	1.4702	0.2404	0.2491	0.8038	0

3986	701.5	1.4706	0.2406	0.2493	0.8041	0
3987	701.4	1.4703	0.2405	0.2493	0.8039	0
3988	701.3	1.4702	0.2405	0.2491	0.8039	0
3989	701.2	1.4701	0.2406	0.2493	0.804	0
3990	701.1	1.4701	0.2406	0.2492	0.8042	0
3991	701	1.4703	0.2407	0.2492	0.8042	0
3992	700.9	1.4702	0.2407	0.2493	0.8044	0
3993	700.8	1.4707	0.2407	0.2494	0.8043	0
3994	700.7	1.4705	0.2405	0.2493	0.8042	0
3995	700.6	1.47	0.2407	0.2492	0.8043	0
3996	700.5	1.4702	0.2405	0.2493	0.8043	0
3997	700.4	1.4702	0.2405	0.2495	0.8044	0
3998	700.3	1.4704	0.2407	0.2495	0.8043	0
3999	700.2	1.4703	0.2408	0.2496	0.8044	0
4000	700.1	1.4701	0.2406	0.2495	0.8043	0
4001	700	1.4707	0.2407	0.2495	0.8045	0
4002	699.9	1.4705	0.2408	0.2495	0.8045	0
4003	699.8	1.4701	0.2407	0.2494	0.8044	0
4004	699.7	1.4703	0.2408	0.2494	0.8044	0
4005	699.6	1.4702	0.2409	0.2497	0.8046	0
4006	699.5	1.4703	0.2409	0.2495	0.8046	0
4007	699.4	1.4705	0.2409	0.2495	0.8045	0
4008	699.3	1.47	0.2408	0.2495	0.8045	0
4009	699.2	1.4701	0.2407	0.2495	0.8046	0
4010	699.1	1.4703	0.2407	0.2495	0.8044	0
4011	699	1.4706	0.2408	0.2496	0.8047	0
4012	698.9	1.4707	0.2408	0.2497	0.8046	0
4013	698.8	1.4702	0.2408	0.2495	0.8046	0
4014	698.7	1.4704	0.2409	0.2496	0.8049	0
4015	698.6	1.4701	0.2407	0.2495	0.8045	0
4016	698.5	1.4701	0.2406	0.2493	0.8045	0
4017	698.4	1.4704	0.2406	0.2495	0.8046	0
4018	698.3	1.4706	0.2407	0.2494	0.8046	0
4019	698.2	1.4704	0.2407	0.2495	0.8046	0
4020	698.1	1.4706	0.2407	0.2494	0.8048	0
4021	698	1.4701	0.2408	0.2494	0.8047	0
4022	697.9	1.4706	0.2407	0.2493	0.8048	0
4023	697.8	1.4702	0.2405	0.2493	0.8046	0
4024	697.7	1.4701	0.2405	0.2492	0.8044	0
4025	697.6	1.4696	0.2405	0.2492	0.8044	0
4026	697.5	1.4702	0.2405	0.2492	0.8046	0
4027	697.4	1.4707	0.2406	0.2493	0.8047	0
4028	697.3	1.4699	0.2406	0.2493	0.8049	0

4029	697.2	1.47	0.2405	0.2493	0.8048	0
4030	697.1	1.4705	0.2405	0.2493	0.8047	0
4031	697	1.4706	0.2406	0.2492	0.8048	0
4032	696.9	1.47	0.2405	0.2492	0.8045	0
4033	696.8	1.47	0.2407	0.2494	0.8046	0
4034	696.7	1.4702	0.2407	0.2495	0.8047	0
4035	696.6	1.4706	0.2406	0.2493	0.8047	0
4036	696.5	1.4705	0.2407	0.2493	0.8047	0
4037	696.4	1.4701	0.2407	0.2495	0.805	0
4038	696.3	1.4702	0.2407	0.2495	0.805	0
4039	696.2	1.4704	0.2406	0.2494	0.8048	0
4040	696.1	1.4708	0.2406	0.2494	0.8049	0
4041	696	1.47	0.2408	0.2494	0.8049	0
4042	695.9	1.4705	0.2408	0.2494	0.805	0
4043	695.8	1.4703	0.2408	0.2494	0.8049	0
4044	695.7	1.4705	0.2406	0.2495	0.805	0
4045	695.6	1.4707	0.2409	0.2495	0.8051	0
4046	695.5	1.4705	0.2408	0.2494	0.8052	0
4047	695.4	1.4703	0.2408	0.2495	0.805	0
4048	695.3	1.4704	0.2408	0.2495	0.8049	0
4049	695.2	1.4707	0.2409	0.2496	0.805	0
4050	695.1	1.4705	0.2409	0.2495	0.8052	0
4051	695	1.4707	0.2409	0.2495	0.8053	0
4052	694.9	1.4705	0.2409	0.2495	0.8054	0
4053	694.8	1.4708	0.2409	0.2496	0.8055	0
4054	694.7	1.4709	0.2408	0.2496	0.8055	0
4055	694.6	1.4711	0.2411	0.2498	0.8054	0
4056	694.5	1.4709	0.241	0.2497	0.8052	0
4057	694.4	1.4706	0.241	0.2495	0.8054	0
4058	694.3	1.4709	0.241	0.2498	0.8054	0
4059	694.2	1.4707	0.2409	0.2497	0.8054	0
4060	694.1	1.471	0.2413	0.2499	0.8056	0
4061	694	1.4708	0.2411	0.25	0.8056	0
4062	693.9	1.471	0.2412	0.2498	0.8057	0
4063	693.8	1.4708	0.241	0.2499	0.8056	0
4064	693.7	1.4712	0.2412	0.2499	0.8056	0
4065	693.6	1.4713	0.2414	0.2501	0.8058	0
4066	693.5	1.4713	0.2412	0.25	0.8057	0
4067	693.4	1.4708	0.2413	0.25	0.8056	0
4068	693.3	1.4713	0.2413	0.2501	0.8058	0
4069	693.2	1.4713	0.2414	0.2503	0.8057	0
4070	693.1	1.4715	0.2416	0.2502	0.8061	0
4071	693	1.4715	0.2413	0.2502	0.8059	0

4072	692.9	1.471	0.2413	0.2502	0.806	0
4073	692.8	1.4712	0.2414	0.2502	0.8059	0
4074	692.7	1.4716	0.2415	0.2502	0.806	0
4075	692.6	1.4715	0.2417	0.2503	0.8058	0
4076	692.5	1.4713	0.2416	0.2503	0.8059	0
4077	692.4	1.4711	0.2415	0.2503	0.8059	0
4078	692.3	1.4714	0.2417	0.2504	0.8062	0
4079	692.2	1.4715	0.2418	0.2504	0.8064	0
4080	692.1	1.4716	0.2418	0.2505	0.806	0
4081	692	1.4718	0.2417	0.2505	0.8061	0
4082	691.9	1.4712	0.2416	0.2504	0.8062	0
4083	691.8	1.4716	0.2418	0.2504	0.8063	0
4084	691.7	1.4714	0.2416	0.2504	0.8063	0
4085	691.6	1.4716	0.2418	0.2506	0.8063	0
4086	691.5	1.4718	0.2419	0.2507	0.8065	0
4087	691.4	1.4715	0.2417	0.2507	0.8063	0
4088	691.3	1.4717	0.2418	0.2505	0.8063	0
4089	691.2	1.4714	0.2417	0.2506	0.8062	0
4090	691.1	1.4718	0.2418	0.2506	0.8066	0
4091	691	1.4719	0.2417	0.2504	0.8066	0
4092	690.9	1.4715	0.2419	0.2507	0.8064	0
4093	690.8	1.4717	0.2419	0.2507	0.8065	0
4094	690.7	1.4718	0.2418	0.2508	0.8065	0
4095	690.6	1.4721	0.2418	0.2506	0.8066	0
4096	690.5	1.4718	0.2416	0.2503	0.8064	0
4097	690.4	1.4716	0.2417	0.2505	0.8064	0
4098	690.3	1.4717	0.2417	0.2504	0.8062	0
4099	690.2	1.4722	0.2417	0.2506	0.8066	0
4100	690.1	1.472	0.2419	0.2506	0.8066	0
4101	690	1.4719	0.2416	0.2503	0.8064	0
4102	689.9	1.472	0.2417	0.2503	0.8063	0
4103	689.8	1.4713	0.2414	0.2503	0.8061	0
4104	689.7	1.4715	0.2417	0.2504	0.8065	0
4105	689.6	1.4716	0.2417	0.2505	0.8065	0
4106	689.5	1.4714	0.2416	0.2504	0.8065	0
4107	689.4	1.4715	0.2416	0.2505	0.8066	0
4108	689.3	1.4716	0.2416	0.2504	0.8066	0
4109	689.2	1.4715	0.2416	0.2502	0.8067	0
4110	689.1	1.4713	0.2415	0.2502	0.8068	0
4111	689	1.4715	0.2417	0.2504	0.8066	0
4112	688.9	1.4715	0.2416	0.2503	0.8066	0
4113	688.8	1.4716	0.2417	0.2503	0.8066	0
4114	688.7	1.4711	0.2417	0.2504	0.8068	0

4115	688.6	1.4715	0.2417	0.2504	0.8067	0
4116	688.5	1.4716	0.2416	0.2504	0.8068	0
4117	688.4	1.4714	0.2417	0.2503	0.8066	0
4118	688.3	1.4715	0.2417	0.2503	0.8068	0
4119	688.2	1.4715	0.2416	0.2504	0.8066	0
4120	688.1	1.4717	0.2417	0.2504	0.8068	0
4121	688	1.4716	0.2419	0.2505	0.807	0
4122	687.9	1.4719	0.2417	0.2504	0.8067	0
4123	687.8	1.4719	0.2417	0.2503	0.8068	0
4124	687.7	1.4717	0.2418	0.2504	0.8068	0
4125	687.6	1.4719	0.2418	0.2505	0.8068	0
4126	687.5	1.4713	0.2418	0.2505	0.8067	0
4127	687.4	1.4717	0.2418	0.2506	0.8071	0
4128	687.3	1.4719	0.2419	0.2505	0.8071	0
4129	687.2	1.4715	0.2418	0.2504	0.8068	0
4130	687.1	1.4717	0.2419	0.2506	0.807	0
4131	687	1.4714	0.2418	0.2505	0.8069	0
4132	686.9	1.4717	0.2418	0.2505	0.8071	0
4133	686.8	1.4714	0.242	0.2507	0.8071	0
4134	686.7	1.4719	0.242	0.2508	0.8073	0
4135	686.6	1.4715	0.2418	0.2506	0.8071	0
4136	686.5	1.472	0.242	0.2507	0.8071	0
4137	686.4	1.4717	0.242	0.2507	0.8072	0
4138	686.3	1.4715	0.242	0.2508	0.8073	0
4139	686.2	1.4721	0.2418	0.2507	0.8071	0
4140	686.1	1.4716	0.2418	0.2507	0.8073	0
4141	686	1.4719	0.2421	0.2509	0.8076	0
4142	685.9	1.4719	0.2422	0.251	0.8073	0
4143	685.8	1.472	0.2421	0.2509	0.8072	0
4144	685.7	1.472	0.2422	0.2507	0.8073	0
4145	685.6	1.472	0.2423	0.2511	0.8076	0
4146	685.5	1.4724	0.2423	0.2509	0.8073	0
4147	685.4	1.4726	0.2423	0.2511	0.8075	0
4148	685.3	1.4723	0.2423	0.2512	0.8075	0
4149	685.2	1.4723	0.2422	0.2511	0.8073	0
4150	685.1	1.4724	0.2424	0.2511	0.8075	0
4151	685	1.4721	0.2421	0.251	0.8073	0
4152	684.9	1.4722	0.2423	0.2509	0.8075	0
4153	684.8	1.4724	0.2424	0.2511	0.8075	0
4154	684.7	1.4722	0.2424	0.2511	0.8078	0
4155	684.6	1.4722	0.2424	0.2511	0.8074	0
4156	684.5	1.4721	0.2424	0.2512	0.8077	0
4157	684.4	1.4726	0.2424	0.2513	0.8078	0

4158	684.3	1.4727	0.2425	0.2515	0.8077	0
4159	684.2	1.4724	0.2425	0.2513	0.8078	0
4160	684.1	1.4726	0.2426	0.2513	0.8079	0
4161	684	1.4728	0.2426	0.2514	0.808	0
4162	683.9	1.4728	0.2426	0.2513	0.8077	0
4163	683.8	1.4727	0.2427	0.2514	0.8079	0
4164	683.7	1.4728	0.2427	0.2515	0.8082	0
4165	683.6	1.4729	0.243	0.2516	0.8081	0
4166	683.5	1.4729	0.2426	0.2516	0.8079	0
4167	683.4	1.4731	0.2429	0.2517	0.8083	0
4168	683.3	1.473	0.243	0.2518	0.8083	0
4169	683.2	1.4727	0.243	0.2517	0.8084	0
4170	683.1	1.4724	0.2429	0.2518	0.8084	0
4171	683	1.4729	0.243	0.2518	0.8084	0
4172	682.9	1.4734	0.243	0.2518	0.8087	0
4173	682.8	1.4728	0.243	0.2519	0.8085	0
4174	682.7	1.4733	0.2431	0.2519	0.8085	0
4175	682.6	1.4731	0.2431	0.252	0.8085	0
4176	682.5	1.4728	0.2431	0.2519	0.8086	0
4177	682.4	1.4733	0.2432	0.252	0.8085	0
4178	682.3	1.4734	0.2432	0.252	0.8085	0
4179	682.2	1.4732	0.2433	0.2519	0.8088	0
4180	682.1	1.4727	0.2432	0.2521	0.8087	0
4181	682	1.4734	0.2431	0.2521	0.8085	0
4182	681.9	1.4731	0.2432	0.2521	0.8084	0
4183	681.8	1.4732	0.2432	0.2521	0.8085	0
4184	681.7	1.4729	0.2431	0.2519	0.8086	0
4185	681.6	1.4733	0.243	0.2519	0.8086	0
4186	681.5	1.4733	0.2432	0.2521	0.8087	0
4187	681.4	1.4733	0.2433	0.2521	0.8087	0
4188	681.3	1.4732	0.2429	0.2518	0.8085	0
4189	681.2	1.4734	0.2431	0.2521	0.8086	0
4190	681.1	1.4729	0.2431	0.2521	0.8087	0
4191	681	1.4729	0.243	0.2518	0.8087	0
4192	680.9	1.4727	0.2431	0.2521	0.8087	0
4193	680.8	1.4729	0.2431	0.2519	0.8088	0
4194	680.7	1.4729	0.2431	0.252	0.8088	0
4195	680.6	1.4727	0.2431	0.252	0.8087	0
4196	680.5	1.4732	0.2432	0.252	0.8089	0
4197	680.4	1.4728	0.243	0.2518	0.8088	0
4198	680.3	1.4727	0.2432	0.2519	0.8089	0
4199	680.2	1.4727	0.2431	0.252	0.8089	0
4200	680.1	1.4731	0.2431	0.2518	0.8088	0

4201	680	1.4727	0.2428	0.2518	0.8088	0
4202	679.9	1.4729	0.2431	0.2519	0.8086	0
4203	679.8	1.473	0.243	0.2518	0.8087	0
4204	679.7	1.4727	0.2432	0.2519	0.8088	0
4205	679.6	1.4726	0.2429	0.2518	0.8088	0
4206	679.5	1.4728	0.2429	0.2518	0.8089	0
4207	679.4	1.4725	0.2428	0.2516	0.8089	0
4208	679.3	1.4727	0.243	0.2517	0.8087	0
4209	679.2	1.4724	0.2429	0.2518	0.8089	0
4210	679.1	1.4727	0.2428	0.2517	0.8087	0
4211	679	1.4727	0.2428	0.2517	0.8085	0
4212	678.9	1.4728	0.2428	0.2514	0.8086	0
4213	678.8	1.4725	0.2428	0.2516	0.8087	0
4214	678.7	1.4723	0.2428	0.2514	0.8088	0
4215	678.6	1.4727	0.2429	0.2515	0.8088	0
4216	678.5	1.4724	0.2427	0.2516	0.8087	0
4217	678.4	1.4725	0.2427	0.2515	0.8087	0
4218	678.3	1.4723	0.2426	0.2517	0.8088	0
4219	678.2	1.4724	0.2427	0.2514	0.8089	0
4220	678.1	1.4718	0.2427	0.2514	0.8089	0
4221	678	1.4722	0.2426	0.2514	0.8089	0
4222	677.9	1.4724	0.2427	0.2515	0.8091	0
4223	677.8	1.4724	0.2427	0.2516	0.8089	0
4224	677.7	1.4723	0.2427	0.2516	0.8091	0
4225	677.6	1.4727	0.2428	0.2515	0.8092	0
4226	677.5	1.4724	0.2427	0.2514	0.8088	0
4227	677.4	1.4728	0.2427	0.2515	0.809	0
4228	677.3	1.4723	0.2428	0.2515	0.8091	0
4229	677.2	1.4728	0.2428	0.2515	0.8089	0
4230	677.1	1.4728	0.2427	0.2514	0.809	0
4231	677	1.4725	0.2427	0.2514	0.809	0
4232	676.9	1.4727	0.2427	0.2517	0.8091	0
4233	676.8	1.4724	0.2428	0.2514	0.809	0
4234	676.7	1.4726	0.2428	0.2516	0.8088	0
4235	676.6	1.4731	0.2428	0.2518	0.8089	0
4236	676.5	1.4729	0.2429	0.2519	0.8093	0
4237	676.4	1.473	0.2429	0.2518	0.8091	0
4238	676.3	1.4731	0.2428	0.2517	0.8092	0
4239	676.2	1.4733	0.2431	0.2519	0.8093	0
4240	676.1	1.4727	0.2431	0.2519	0.8093	0
4241	676	1.4729	0.2431	0.2522	0.8094	0
4242	675.9	1.4727	0.2431	0.2519	0.8093	0
4243	675.8	1.4731	0.2431	0.2519	0.8096	0

4244	675.7	1.4732	0.2431	0.2519	0.8093	0
4245	675.6	1.473	0.243	0.2519	0.8093	0
4246	675.5	1.4732	0.2432	0.252	0.8095	0
4247	675.4	1.4732	0.2431	0.2519	0.8097	0
4248	675.3	1.4731	0.2433	0.2521	0.8096	0
4249	675.2	1.4733	0.2433	0.2522	0.8098	0
4250	675.1	1.4732	0.2433	0.2521	0.8098	0
4251	675	1.4739	0.2433	0.2522	0.8096	0
4252	674.9	1.4734	0.2435	0.2523	0.8098	0
4253	674.8	1.4735	0.2435	0.2522	0.8098	0
4254	674.7	1.4734	0.2435	0.2524	0.8098	0
4255	674.6	1.474	0.2436	0.2524	0.8101	0
4256	674.5	1.4737	0.2436	0.2524	0.8098	0
4257	674.4	1.474	0.2437	0.2526	0.8102	0
4258	674.3	1.4737	0.2438	0.2527	0.81	0
4259	674.2	1.4744	0.2439	0.2527	0.8102	0
4260	674.1	1.4738	0.2439	0.2528	0.8103	0
4261	674	1.4737	0.2441	0.2529	0.8102	0
4262	673.9	1.4742	0.2441	0.253	0.8102	0
4263	673.8	1.474	0.2441	0.2532	0.8104	0
4264	673.7	1.4743	0.2442	0.2531	0.8104	0
4265	673.6	1.4744	0.2443	0.2531	0.8104	0
4266	673.5	1.4738	0.2442	0.2531	0.8103	0
4267	673.4	1.4738	0.2441	0.2529	0.8102	0
4268	673.3	1.4741	0.2442	0.2529	0.8104	0
4269	673.2	1.4746	0.2443	0.2531	0.8105	0
4270	673.1	1.4745	0.2442	0.2532	0.8106	0
4271	673	1.474	0.2444	0.2532	0.8105	0
4272	672.9	1.4744	0.2443	0.2532	0.8105	0
4273	672.8	1.4746	0.2444	0.2533	0.8108	0
4274	672.7	1.4746	0.2445	0.2533	0.8108	0
4275	672.6	1.4747	0.2444	0.2533	0.8109	0
4276	672.5	1.4746	0.2446	0.2534	0.811	0
4277	672.4	1.475	0.2444	0.2534	0.8107	0
4278	672.3	1.4748	0.2444	0.2534	0.8109	0
4279	672.2	1.475	0.2444	0.2533	0.8109	0
4280	672.1	1.4752	0.2446	0.2534	0.811	0
4281	672	1.4746	0.2445	0.2533	0.8108	0
4282	671.9	1.4751	0.2445	0.2534	0.8109	0
4283	671.8	1.4751	0.2447	0.2537	0.8109	0
4284	671.7	1.4748	0.2447	0.2534	0.8111	0
4285	671.6	1.4749	0.2444	0.2533	0.8108	0
4286	671.5	1.475	0.2445	0.2533	0.8112	0

4287	671.4	1.475	0.2447	0.2535	0.8111	0
4288	671.3	1.4748	0.2446	0.2534	0.811	0
4289	671.2	1.475	0.2446	0.2534	0.8111	0
4290	671.1	1.4751	0.2445	0.2533	0.8111	0
4291	671	1.4746	0.2445	0.2534	0.8113	0
4292	670.9	1.4751	0.2446	0.2532	0.811	0
4293	670.8	1.475	0.2446	0.2534	0.8111	0
4294	670.7	1.4749	0.2446	0.2535	0.8111	0
4295	670.6	1.4747	0.2447	0.2535	0.8113	0
4296	670.5	1.475	0.2446	0.2536	0.8113	0
4297	670.4	1.4749	0.2444	0.2534	0.811	0
4298	670.3	1.4748	0.2444	0.2533	0.8109	0
4299	670.2	1.4742	0.2443	0.2533	0.8111	0
4300	670.1	1.4744	0.2443	0.2533	0.811	0
4301	670	1.4751	0.2447	0.2535	0.8112	0
4302	669.9	1.4748	0.2444	0.2532	0.8114	0
4303	669.8	1.4746	0.2443	0.2532	0.8112	0
4304	669.7	1.4745	0.2445	0.2533	0.8113	0
4305	669.6	1.4746	0.2442	0.2532	0.8111	0
4306	669.5	1.4745	0.2444	0.2533	0.8111	0
4307	669.4	1.475	0.2442	0.253	0.8111	0
4308	669.3	1.4745	0.2443	0.2531	0.8111	0
4309	669.2	1.4743	0.2443	0.253	0.8111	0
4310	669.1	1.4744	0.2442	0.253	0.8111	0
4311	669	1.4742	0.2441	0.2528	0.811	0
4312	668.9	1.4745	0.2443	0.253	0.8112	0
4313	668.8	1.4744	0.2443	0.2529	0.8113	0
4314	668.7	1.4743	0.2439	0.2527	0.811	0
4315	668.6	1.4742	0.2439	0.2527	0.811	0
4316	668.5	1.4744	0.2439	0.2527	0.8109	0
4317	668.4	1.4747	0.2441	0.2528	0.8112	0
4318	668.3	1.4737	0.2438	0.2527	0.8111	0
4319	668.2	1.4743	0.2439	0.2527	0.8111	0
4320	668.1	1.474	0.2437	0.2524	0.8109	0
4321	668	1.4742	0.2438	0.2526	0.8111	0
4322	667.9	1.4741	0.2438	0.2526	0.8108	0
4323	667.8	1.474	0.2438	0.2526	0.8109	0
4324	667.7	1.4742	0.2438	0.2526	0.811	0
4325	667.6	1.474	0.2437	0.2526	0.8111	0
4326	667.5	1.4738	0.2437	0.2525	0.8109	0
4327	667.4	1.4733	0.2436	0.2524	0.8113	0
4328	667.3	1.4743	0.2436	0.2525	0.8111	0
4329	667.2	1.474	0.2435	0.2525	0.811	0

4330	667.1	1.4739	0.2436	0.2524	0.8109	0
4331	667	1.4742	0.2437	0.2525	0.8112	0
4332	666.9	1.4741	0.2438	0.2527	0.8113	0
4333	666.8	1.474	0.2438	0.2525	0.8113	0
4334	666.7	1.4741	0.2436	0.2525	0.8111	0
4335	666.6	1.4739	0.2435	0.2525	0.8109	0
4336	666.5	1.4734	0.2435	0.2523	0.811	0
4337	666.4	1.4741	0.2438	0.2525	0.8113	0
4338	666.3	1.4744	0.2436	0.2525	0.8111	0
4339	666.2	1.4738	0.2436	0.2524	0.8112	0
4340	666.1	1.4737	0.2436	0.2526	0.8111	0
4341	666	1.4739	0.2436	0.2526	0.8112	0
4342	665.9	1.4739	0.2438	0.2525	0.8113	0
4343	665.8	1.4739	0.244	0.2528	0.8117	0
4344	665.7	1.474	0.2437	0.2526	0.8113	0
4345	665.6	1.4744	0.244	0.2529	0.8117	0
4346	665.5	1.4742	0.2439	0.2526	0.8113	0
4347	665.4	1.4744	0.2437	0.2526	0.8115	0
4348	665.3	1.4743	0.2439	0.2527	0.8116	0
4349	665.2	1.4745	0.2439	0.2527	0.8114	0
4350	665.1	1.4741	0.2437	0.2526	0.8115	0
4351	665	1.4745	0.2439	0.2528	0.8116	0
4352	664.9	1.4749	0.244	0.2529	0.8119	0
4353	664.8	1.4752	0.2441	0.253	0.8119	0
4354	664.7	1.4746	0.244	0.253	0.8116	0
4355	664.6	1.4747	0.2442	0.2531	0.8117	0
4356	664.5	1.475	0.2444	0.2532	0.812	0
4357	664.4	1.475	0.2446	0.2533	0.812	0
4358	664.3	1.4756	0.2444	0.2533	0.812	0
4359	664.2	1.4756	0.2445	0.2533	0.8122	0
4360	664.1	1.4756	0.2448	0.2534	0.8121	0
4361	664	1.4757	0.2449	0.2536	0.8124	0
4362	663.9	1.4755	0.2448	0.2536	0.8123	0
4363	663.8	1.4755	0.2448	0.2536	0.8123	0
4364	663.7	1.4758	0.245	0.2536	0.8126	0
4365	663.6	1.4753	0.2448	0.2535	0.8122	0
4366	663.5	1.4757	0.245	0.2538	0.8125	0
4367	663.4	1.4758	0.2453	0.2539	0.8127	0
4368	663.3	1.4757	0.2451	0.2539	0.8127	0
4369	663.2	1.4759	0.2452	0.2539	0.8126	0
4370	663.1	1.4763	0.2452	0.254	0.8128	0
4371	663	1.4764	0.2452	0.2542	0.8129	0
4372	662.9	1.4767	0.2454	0.2543	0.8131	0

4373	662.8	1.4763	0.2454	0.2543	0.8128	0
4374	662.7	1.4762	0.2455	0.2543	0.813	0
4375	662.6	1.4764	0.2454	0.2543	0.8129	0
4376	662.5	1.4764	0.2454	0.2543	0.8131	0
4377	662.4	1.4764	0.2456	0.2545	0.8132	0
4378	662.3	1.4767	0.2454	0.2544	0.8131	0
4379	662.2	1.4766	0.2454	0.2544	0.8131	0
4380	662.1	1.4769	0.2455	0.2544	0.8133	0
4381	662	1.4761	0.2455	0.2544	0.8132	0
4382	661.9	1.4764	0.2454	0.2544	0.8132	0
4383	661.8	1.4765	0.2455	0.2547	0.8133	0
4384	661.7	1.4768	0.2459	0.2548	0.8133	0
4385	661.6	1.4763	0.2457	0.2547	0.8133	0
4386	661.5	1.4764	0.2458	0.2547	0.8135	0
4387	661.4	1.4764	0.2459	0.2547	0.8135	0
4388	661.3	1.4765	0.2458	0.2546	0.8134	0
4389	661.2	1.4765	0.2458	0.2547	0.8135	0
4390	661.1	1.4767	0.2457	0.2546	0.8134	0
4391	661	1.4763	0.2456	0.2546	0.8133	0
4392	660.9	1.4769	0.2456	0.2545	0.8133	0
4393	660.8	1.477	0.2458	0.2546	0.8135	0
4394	660.7	1.4766	0.2457	0.2548	0.8135	0
4395	660.6	1.477	0.2455	0.2545	0.813	0
4396	660.5	1.4768	0.2457	0.2548	0.8134	0
4397	660.4	1.4763	0.2457	0.2546	0.8135	0
4398	660.3	1.477	0.2456	0.2546	0.8137	0
4399	660.2	1.477	0.2456	0.2546	0.8135	0
4400	660.1	1.4772	0.2455	0.2546	0.8134	0
4401	660	1.4766	0.2457	0.2547	0.8137	0
4402	659.9	1.4768	0.2456	0.2546	0.8136	0
4403	659.8	1.4768	0.2458	0.2547	0.8137	0
4404	659.7	1.4764	0.2456	0.2547	0.8136	0
4405	659.6	1.4764	0.2457	0.2547	0.8137	0
4406	659.5	1.4766	0.2455	0.2546	0.8138	0
4407	659.4	1.477	0.2456	0.2546	0.8134	0
4408	659.3	1.4768	0.2455	0.2545	0.8135	0
4409	659.2	1.4764	0.2455	0.2546	0.8134	0
4410	659.1	1.4764	0.2455	0.2544	0.8135	0
4411	659	1.4767	0.2454	0.2544	0.8136	0
4412	658.9	1.4767	0.2455	0.2544	0.8135	0
4413	658.8	1.477	0.2454	0.2543	0.8136	0
4414	658.7	1.4767	0.2456	0.2544	0.8137	0
4415	658.6	1.4765	0.2453	0.2544	0.8135	0

4416	658.5	1.4764	0.2452	0.2543	0.8137	0
4417	658.4	1.4772	0.2452	0.2542	0.8136	0
4418	658.3	1.4763	0.2451	0.2541	0.8136	0
4419	658.2	1.4762	0.2453	0.2541	0.8136	0
4420	658.1	1.4767	0.2453	0.2544	0.8137	0
4421	658	1.4763	0.2453	0.2543	0.8137	0
4422	657.9	1.476	0.245	0.2541	0.8133	0
4423	657.8	1.4765	0.2451	0.2542	0.8135	0
4424	657.7	1.4765	0.2451	0.2541	0.8136	0
4425	657.6	1.4761	0.2452	0.2541	0.8135	0
4426	657.5	1.4759	0.2451	0.2539	0.8136	0
4427	657.4	1.4759	0.2449	0.254	0.8137	0
4428	657.3	1.4762	0.245	0.254	0.8133	0
4429	657.2	1.4757	0.2448	0.2538	0.8133	0
4430	657.1	1.4756	0.2448	0.2538	0.8133	0
4431	657	1.4758	0.2449	0.2539	0.8137	0
4432	656.9	1.4762	0.245	0.254	0.8137	0
4433	656.8	1.476	0.2448	0.2537	0.8135	0
4434	656.7	1.4757	0.245	0.2538	0.8136	0
4435	656.6	1.4761	0.2448	0.2538	0.8137	0
4436	656.5	1.4756	0.245	0.2537	0.8136	0
4437	656.4	1.4757	0.245	0.2538	0.8137	0
4438	656.3	1.4761	0.2448	0.2541	0.8138	0
4439	656.2	1.4761	0.2449	0.254	0.8138	0
4440	656.1	1.476	0.2449	0.254	0.8136	0
4441	656	1.476	0.2449	0.2537	0.8136	0
4442	655.9	1.4761	0.2449	0.254	0.8139	0
4443	655.8	1.4765	0.2449	0.254	0.8137	0
4444	655.7	1.4762	0.2451	0.254	0.814	0
4445	655.6	1.4759	0.2452	0.254	0.8142	0
4446	655.5	1.4759	0.2452	0.254	0.814	0
4447	655.4	1.4765	0.2451	0.2537	0.8137	0
4448	655.3	1.4758	0.245	0.2542	0.8139	0
4449	655.2	1.4763	0.2451	0.2541	0.8141	0
4450	655.1	1.4763	0.2452	0.254	0.8142	0
4451	655	1.4767	0.2452	0.2541	0.814	0
4452	654.9	1.4764	0.2453	0.2544	0.8142	0
4453	654.8	1.4765	0.2455	0.2543	0.8143	0
4454	654.7	1.4769	0.2453	0.2544	0.8144	0
4455	654.6	1.4762	0.2454	0.2543	0.8142	0
4456	654.5	1.4767	0.2455	0.2545	0.8144	0
4457	654.4	1.4767	0.2456	0.2545	0.8143	0
4458	654.3	1.4768	0.2456	0.2546	0.8144	0

4459	654.2	1.4772	0.2456	0.2545	0.8143	0
4460	654.1	1.4768	0.2456	0.2545	0.8145	0
4461	654	1.4768	0.2456	0.2545	0.8146	0
4462	653.9	1.4769	0.2456	0.2545	0.8145	0
4463	653.8	1.4773	0.2456	0.2546	0.8144	0
4464	653.7	1.4774	0.2457	0.2548	0.8146	0
4465	653.6	1.4774	0.2456	0.2547	0.8146	0
4466	653.5	1.4776	0.2459	0.2549	0.8149	0
4467	653.4	1.4771	0.2457	0.2546	0.8147	0
4468	653.3	1.4774	0.2461	0.255	0.8149	0
4469	653.2	1.4775	0.2462	0.2549	0.8151	0
4470	653.1	1.4779	0.246	0.255	0.8151	0
4471	653	1.4772	0.246	0.255	0.8148	0
4472	652.9	1.4773	0.2461	0.2551	0.8152	0
4473	652.8	1.4777	0.2461	0.2552	0.8151	0
4474	652.7	1.4781	0.2462	0.2551	0.8151	0
4475	652.6	1.4778	0.2461	0.2552	0.8152	0
4476	652.5	1.4775	0.2463	0.2551	0.8153	0
4477	652.4	1.478	0.2462	0.2553	0.8153	0
4478	652.3	1.478	0.2462	0.2553	0.8153	0
4479	652.2	1.4789	0.2465	0.2555	0.8154	0
4480	652.1	1.4778	0.2463	0.2553	0.8153	0
4481	652	1.4787	0.2463	0.2554	0.8154	0
4482	651.9	1.4788	0.2465	0.2556	0.8156	0
4483	651.8	1.4789	0.2465	0.2557	0.8158	0
4484	651.7	1.4786	0.2466	0.2556	0.8156	0
4485	651.6	1.479	0.2466	0.2555	0.8156	0
4486	651.5	1.4793	0.2468	0.256	0.8162	0
4487	651.4	1.4786	0.2467	0.256	0.8158	0
4488	651.3	1.478	0.2465	0.2556	0.8159	0
4489	651.2	1.4785	0.2467	0.2557	0.816	0
4490	651.1	1.4789	0.2466	0.2557	0.8158	0
4491	651	1.4791	0.2467	0.256	0.8159	0
4492	650.9	1.4789	0.2468	0.256	0.816	0
4493	650.8	1.4787	0.247	0.2561	0.8162	0
4494	650.7	1.4788	0.2468	0.256	0.8158	0
4495	650.6	1.479	0.2468	0.2559	0.816	0
4496	650.5	1.479	0.2467	0.2559	0.8158	0
4497	650.4	1.4788	0.2469	0.256	0.8161	0
4498	650.3	1.4784	0.2468	0.2558	0.8159	0
4499	650.2	1.479	0.2469	0.2558	0.816	0
4500	650.1	1.479	0.2469	0.2557	0.8161	0
4501	650	1.4786	0.2468	0.2558	0.8161	0

4502	649.9	1.4786	0.2467	0.2557	0.8161	0
4503	649.8	1.4788	0.2467	0.2558	0.816	0
4504	649.7	1.4788	0.2468	0.2558	0.8162	0
4505	649.6	1.4789	0.2469	0.2557	0.8162	0
4506	649.5	1.479	0.2468	0.2557	0.8163	0
4507	649.4	1.4787	0.2468	0.2559	0.8162	0
4508	649.3	1.4791	0.2467	0.2558	0.8163	0
4509	649.2	1.4788	0.2468	0.2557	0.8163	0
4510	649.1	1.4788	0.2467	0.2556	0.8161	0
4511	649	1.479	0.2467	0.2558	0.8164	0
4512	648.9	1.4783	0.2467	0.2557	0.8164	0
4513	648.8	1.4787	0.2466	0.2557	0.816	0
4514	648.7	1.4788	0.2466	0.2555	0.8162	0
4515	648.6	1.4786	0.2467	0.2557	0.8162	0
4516	648.5	1.4788	0.2465	0.2555	0.8161	0
4517	648.4	1.4785	0.2465	0.2555	0.8161	0
4518	648.3	1.479	0.2465	0.2555	0.8161	0
4519	648.2	1.4791	0.2466	0.2555	0.8165	0
4520	648.1	1.4791	0.2467	0.2553	0.8165	0
4521	648	1.4789	0.2465	0.2554	0.8163	0
4522	647.9	1.4786	0.2466	0.2554	0.8165	0
4523	647.8	1.4786	0.2464	0.2554	0.8166	0
4524	647.7	1.4787	0.2464	0.2555	0.8165	0
4525	647.6	1.4785	0.2465	0.2555	0.8164	0
4526	647.5	1.4784	0.2464	0.2552	0.8163	0
4527	647.4	1.4789	0.2461	0.2553	0.8164	0
4528	647.3	1.4787	0.2465	0.2554	0.8163	0
4529	647.2	1.4788	0.2463	0.2554	0.8166	0
4530	647.1	1.4788	0.2463	0.2554	0.8165	0
4531	647	1.4783	0.2461	0.2551	0.8162	0
4532	646.9	1.4786	0.2464	0.2553	0.8164	0
4533	646.8	1.4787	0.2462	0.2552	0.8163	0
4534	646.7	1.478	0.2462	0.2552	0.8164	0
4535	646.6	1.4785	0.2462	0.2552	0.8164	0
4536	646.5	1.4785	0.2463	0.2551	0.8164	0
4537	646.4	1.4783	0.2463	0.2551	0.8165	0
4538	646.3	1.4787	0.2462	0.2551	0.8164	0
4539	646.2	1.4789	0.2464	0.2553	0.8165	0
4540	646.1	1.4787	0.2465	0.2553	0.8167	0
4541	646	1.4788	0.2462	0.2553	0.8166	0
4542	645.9	1.4793	0.2463	0.2554	0.8166	0
4543	645.8	1.4789	0.2463	0.2552	0.8167	0
4544	645.7	1.4784	0.2463	0.2552	0.8169	0

4545	645.6	1.4788	0.2464	0.2553	0.8166	0
4546	645.5	1.479	0.2463	0.2554	0.8167	0
4547	645.4	1.4786	0.2463	0.2552	0.8168	0
4548	645.3	1.4788	0.2463	0.2553	0.8171	0
4549	645.2	1.479	0.2464	0.2554	0.8169	0
4550	645.1	1.4787	0.2463	0.2554	0.8168	0
4551	645	1.4789	0.2465	0.2553	0.8168	0
4552	644.9	1.4786	0.2463	0.2554	0.8169	0
4553	644.8	1.4787	0.2463	0.2553	0.8171	0
4554	644.7	1.4782	0.2464	0.2554	0.8169	0
4555	644.6	1.4787	0.2465	0.2553	0.8169	0
4556	644.5	1.4787	0.2463	0.2555	0.8169	0
4557	644.4	1.4789	0.2465	0.2554	0.8171	0
4558	644.3	1.4788	0.2468	0.2556	0.8174	0
4559	644.2	1.4782	0.2466	0.2556	0.817	0
4560	644.1	1.4789	0.2467	0.2558	0.8174	0
4561	644	1.4793	0.2467	0.256	0.8174	0
4562	643.9	1.4794	0.2468	0.2557	0.8176	0
4563	643.8	1.4789	0.2471	0.256	0.8175	0
4564	643.7	1.4794	0.2467	0.256	0.8175	0
4565	643.6	1.4797	0.247	0.2561	0.8176	0
4566	643.5	1.4793	0.2467	0.2559	0.8175	0
4567	643.4	1.4793	0.247	0.2562	0.8177	0
4568	643.3	1.4798	0.2469	0.256	0.8176	0
4569	643.2	1.4797	0.2472	0.2561	0.8177	0
4570	643.1	1.4798	0.247	0.256	0.8179	0
4571	643	1.4803	0.2469	0.2561	0.8179	0
4572	642.9	1.4798	0.2472	0.2561	0.818	0
4573	642.8	1.4796	0.2473	0.2564	0.818	0
4574	642.7	1.4801	0.2471	0.2564	0.818	0
4575	642.6	1.4799	0.2472	0.2563	0.8179	0
4576	642.5	1.4799	0.2472	0.2561	0.8178	0
4577	642.4	1.4798	0.2472	0.2562	0.818	0
4578	642.3	1.4801	0.2472	0.2564	0.8182	0
4579	642.2	1.4799	0.2474	0.2565	0.8184	0
4580	642.1	1.4808	0.2474	0.2566	0.8182	0
4581	642	1.4801	0.2473	0.2564	0.8181	0
4582	641.9	1.4803	0.2473	0.2564	0.8182	0
4583	641.8	1.4805	0.2474	0.2563	0.8181	0
4584	641.7	1.4807	0.2474	0.2564	0.8184	0
4585	641.6	1.4807	0.2476	0.2567	0.8186	0
4586	641.5	1.4804	0.2476	0.2566	0.8183	0
4587	641.4	1.4805	0.2474	0.2564	0.8186	0

4588	641.3	1.4803	0.2475	0.2565	0.8185	0
4589	641.2	1.4801	0.2474	0.2565	0.8184	0
4590	641.1	1.4804	0.2476	0.2566	0.8185	0
4591	641	1.4802	0.2475	0.2567	0.8184	0
4592	640.9	1.4804	0.2476	0.2568	0.8185	0
4593	640.8	1.4802	0.2475	0.2567	0.8187	0
4594	640.7	1.4808	0.2476	0.2568	0.8186	0
4595	640.6	1.4812	0.2478	0.2568	0.8189	0
4596	640.5	1.4808	0.2476	0.2568	0.8187	0
4597	640.4	1.4814	0.2477	0.2567	0.8186	0
4598	640.3	1.4805	0.2477	0.2567	0.8188	0
4599	640.2	1.4814	0.2478	0.2569	0.8189	0
4600	640.1	1.4813	0.2478	0.2569	0.8191	0
4601	640	1.481	0.2477	0.2568	0.8189	0
4602	639.9	1.4808	0.2476	0.2568	0.819	0
4603	639.8	1.481	0.2477	0.2568	0.819	0
4604	639.7	1.4812	0.2478	0.2568	0.8188	0
4605	639.6	1.4809	0.2476	0.2567	0.8189	0
4606	639.5	1.4816	0.2479	0.2569	0.8192	0
4607	639.4	1.481	0.2477	0.2568	0.819	0
4608	639.3	1.481	0.2479	0.2569	0.8192	0
4609	639.2	1.481	0.2477	0.2569	0.8192	0
4610	639.1	1.4815	0.2478	0.2569	0.8192	0
4611	639	1.4813	0.2476	0.2568	0.8192	0
4612	638.9	1.4811	0.2475	0.2568	0.8192	0
4613	638.8	1.4808	0.2476	0.2566	0.8191	0
4614	638.7	1.4809	0.2479	0.257	0.8194	0
4615	638.6	1.481	0.2479	0.257	0.8191	0
4616	638.5	1.4811	0.2476	0.2568	0.8193	0
4617	638.4	1.481	0.2477	0.2567	0.8192	0
4618	638.3	1.4813	0.2478	0.2568	0.8194	0
4619	638.2	1.4811	0.2479	0.2569	0.8196	0
4620	638.1	1.4813	0.2479	0.2571	0.8195	0
4621	638	1.4817	0.2477	0.2569	0.8195	0
4622	637.9	1.4814	0.2477	0.2566	0.8196	0
4623	637.8	1.4814	0.2476	0.2567	0.8196	0
4624	637.7	1.4812	0.2479	0.2567	0.8197	0
4625	637.6	1.4815	0.2478	0.2567	0.8199	0
4626	637.5	1.4817	0.2478	0.2567	0.8198	0
4627	637.4	1.4815	0.2478	0.2566	0.8196	0
4628	637.3	1.4813	0.2477	0.2567	0.8196	0
4629	637.2	1.4815	0.2477	0.2567	0.8195	0
4630	637.1	1.4814	0.2478	0.2566	0.8195	0

4631	637	1.4811	0.2476	0.2565	0.8195	0
4632	636.9	1.4814	0.2477	0.2565	0.8197	0
4633	636.8	1.4815	0.2477	0.2566	0.8197	0
4634	636.7	1.4818	0.2478	0.2568	0.8199	0
4635	636.6	1.4816	0.2477	0.2567	0.82	0
4636	636.5	1.4814	0.2477	0.2567	0.8201	0
4637	636.4	1.4815	0.2476	0.2565	0.8195	0
4638	636.3	1.481	0.2476	0.2566	0.8198	0
4639	636.2	1.4816	0.2476	0.2566	0.8197	0
4640	636.1	1.4816	0.2478	0.2568	0.8201	0
4641	636	1.4817	0.2477	0.2568	0.8202	0
4642	635.9	1.4815	0.2475	0.2568	0.8199	0
4643	635.8	1.4817	0.2477	0.2568	0.8203	0
4644	635.7	1.4815	0.2476	0.2566	0.82	0
4645	635.6	1.4814	0.2476	0.2567	0.8199	0
4646	635.5	1.4815	0.2476	0.2565	0.8201	0
4647	635.4	1.4814	0.2476	0.2564	0.8201	0
4648	635.3	1.4813	0.2476	0.2566	0.82	0
4649	635.2	1.4819	0.2476	0.2567	0.8199	0
4650	635.1	1.4814	0.2477	0.2567	0.8201	0
4651	635	1.4817	0.2477	0.257	0.8203	0
4652	634.9	1.4819	0.2478	0.2569	0.8203	0
4653	634.8	1.4824	0.2479	0.2566	0.8202	0
4654	634.7	1.4819	0.2477	0.2568	0.8205	0
4655	634.6	1.4821	0.2481	0.2569	0.8205	0
4656	634.5	1.4822	0.2479	0.2568	0.8205	0
4657	634.4	1.4821	0.2479	0.2569	0.8205	0
4658	634.3	1.4823	0.2478	0.2568	0.8207	0
4659	634.2	1.4818	0.2477	0.2567	0.8204	0
4660	634.1	1.4825	0.2478	0.2569	0.8206	0
4661	634	1.4824	0.248	0.2569	0.8208	0
4662	633.9	1.4821	0.2478	0.2568	0.8206	0
4663	633.8	1.4821	0.2479	0.257	0.8208	0
4664	633.7	1.4827	0.248	0.257	0.8207	0
4665	633.6	1.4825	0.248	0.2569	0.8207	0
4666	633.5	1.482	0.248	0.257	0.8207	0
4667	633.4	1.4826	0.2481	0.2571	0.8209	0
4668	633.3	1.4829	0.248	0.2572	0.8209	0
4669	633.2	1.4824	0.2481	0.2573	0.821	0
4670	633.1	1.4826	0.2482	0.2571	0.8209	0
4671	633	1.4824	0.2481	0.2571	0.8211	0
4672	632.9	1.4826	0.2482	0.2572	0.8209	0
4673	632.8	1.4828	0.248	0.2572	0.8211	0

4674	632.7	1.4825	0.2484	0.2574	0.8213	0
4675	632.6	1.4826	0.2482	0.2573	0.8213	0
4676	632.5	1.4828	0.2481	0.2571	0.8213	0
4677	632.4	1.4827	0.2485	0.2571	0.8211	0
4678	632.3	1.4831	0.2484	0.2572	0.8212	0
4679	632.2	1.4829	0.2482	0.2572	0.8211	0
4680	632.1	1.4828	0.2482	0.2572	0.8212	0
4681	632	1.4833	0.2483	0.2572	0.8213	0
4682	631.9	1.4827	0.2481	0.257	0.8211	0
4683	631.8	1.483	0.2483	0.2572	0.8211	0
4684	631.7	1.4832	0.2486	0.2575	0.8214	0
4685	631.6	1.483	0.2485	0.2574	0.8215	0
4686	631.5	1.4832	0.2484	0.2573	0.8215	0
4687	631.4	1.4831	0.2485	0.2576	0.8218	0
4688	631.3	1.4834	0.2484	0.2576	0.8215	0
4689	631.2	1.4835	0.2484	0.2575	0.8218	0
4690	631.1	1.4829	0.2483	0.2573	0.8216	0
4691	631	1.4832	0.2484	0.2573	0.8217	0
4692	630.9	1.4831	0.2483	0.2574	0.8217	0
4693	630.8	1.4833	0.2482	0.2574	0.8216	0
4694	630.7	1.4829	0.2484	0.2575	0.8219	0
4695	630.6	1.4828	0.2484	0.2575	0.8219	0
4696	630.5	1.483	0.2484	0.2574	0.8219	0
4697	630.4	1.4829	0.2483	0.2572	0.822	0
4698	630.3	1.4834	0.2482	0.2573	0.822	0
4699	630.2	1.4833	0.2482	0.2572	0.8219	0
4700	630.1	1.4833	0.2483	0.2573	0.8221	0
4701	630	1.4835	0.2482	0.2573	0.822	0
4702	629.9	1.4829	0.2481	0.2571	0.8221	0
4703	629.8	1.4829	0.2482	0.2573	0.8221	0
4704	629.7	1.483	0.2481	0.2572	0.822	0
4705	629.6	1.4826	0.2481	0.2572	0.8222	0
4706	629.5	1.4829	0.2482	0.2572	0.8222	0
4707	629.4	1.483	0.2481	0.257	0.8222	0
4708	629.3	1.483	0.2481	0.2571	0.8221	0
4709	629.2	1.4833	0.2483	0.2572	0.8226	0
4710	629.1	1.4831	0.2481	0.2571	0.8223	0
4711	629	1.4833	0.248	0.2571	0.8221	0
4712	628.9	1.4832	0.2481	0.2572	0.8224	0
4713	628.8	1.483	0.248	0.2571	0.8223	0
4714	628.7	1.4834	0.2481	0.2572	0.8222	0
4715	628.6	1.4829	0.2481	0.2572	0.8224	0
4716	628.5	1.4834	0.248	0.2572	0.8224	0

4717	628.4	1.4833	0.2479	0.257	0.8225	0
4718	628.3	1.4832	0.2479	0.257	0.8225	0
4719	628.2	1.483	0.2478	0.2568	0.8225	0
4720	628.1	1.4833	0.2479	0.2572	0.8225	0
4721	628	1.4833	0.2481	0.257	0.8224	0
4722	627.9	1.4836	0.2481	0.2571	0.8226	0
4723	627.8	1.4832	0.2481	0.2572	0.8227	0
4724	627.7	1.4835	0.2482	0.2572	0.8229	0
4725	627.6	1.483	0.2482	0.2571	0.8228	0
4726	627.5	1.4833	0.248	0.2571	0.823	0
4727	627.4	1.4836	0.2481	0.2571	0.8228	0
4728	627.3	1.4834	0.248	0.2571	0.8228	0
4729	627.2	1.4835	0.2481	0.2573	0.823	0
4730	627.1	1.4833	0.2482	0.2571	0.8232	0
4731	627	1.4831	0.2481	0.2571	0.8228	0
4732	626.9	1.4834	0.2481	0.2571	0.8229	0
4733	626.8	1.4836	0.2481	0.2571	0.8229	0
4734	626.7	1.4837	0.2484	0.2571	0.823	0
4735	626.6	1.484	0.2484	0.2573	0.8232	0
4736	626.5	1.4839	0.2484	0.2574	0.8235	0
4737	626.4	1.4841	0.2485	0.2573	0.8234	0
4738	626.3	1.4841	0.2484	0.2575	0.8235	0
4739	626.2	1.484	0.2483	0.2575	0.8233	0
4740	626.1	1.4843	0.2485	0.2574	0.8231	0
4741	626	1.4842	0.2485	0.2575	0.8232	0
4742	625.9	1.4837	0.2485	0.2574	0.8234	0
4743	625.8	1.484	0.2484	0.2575	0.8236	0
4744	625.7	1.4843	0.2487	0.2576	0.8236	0
4745	625.6	1.4842	0.2486	0.2575	0.8236	0
4746	625.5	1.4841	0.2486	0.2576	0.8234	0
4747	625.4	1.484	0.2484	0.2574	0.8237	0
4748	625.3	1.4838	0.2483	0.2574	0.8238	0
4749	625.2	1.4841	0.2484	0.2573	0.8236	0
4750	625.1	1.484	0.2485	0.2575	0.8239	0
4751	625	1.4843	0.2486	0.2574	0.8238	0
4752	624.9	1.4841	0.2488	0.2576	0.8241	0
4753	624.8	1.4843	0.2487	0.2576	0.8241	0
4754	624.7	1.4844	0.2485	0.2574	0.8241	0
4755	624.6	1.4844	0.2487	0.2576	0.8242	0
4756	624.5	1.4847	0.2487	0.2577	0.8243	0
4757	624.4	1.4846	0.2487	0.2575	0.8242	0
4758	624.3	1.4847	0.2489	0.2578	0.8244	0
4759	624.2	1.4854	0.249	0.2577	0.8246	0

4760	624.1	1.4852	0.249	0.2579	0.8245	0
4761	624	1.4848	0.2488	0.2577	0.8246	0
4762	623.9	1.4852	0.249	0.258	0.8248	0
4763	623.8	1.4851	0.249	0.2579	0.8246	0
4764	623.7	1.4851	0.2492	0.258	0.8247	0
4765	623.6	1.485	0.249	0.2579	0.8247	0
4766	623.5	1.4852	0.2491	0.2579	0.8246	0
4767	623.4	1.4848	0.2491	0.2579	0.8247	0
4768	623.3	1.485	0.2492	0.2579	0.8246	0
4769	623.2	1.485	0.2492	0.2578	0.8249	0
4770	623.1	1.4854	0.2492	0.2581	0.825	0
4771	623	1.4852	0.2492	0.258	0.8251	0
4772	622.9	1.4854	0.2491	0.2579	0.8251	0
4773	622.8	1.4857	0.2491	0.2579	0.825	0
4774	622.7	1.4852	0.2489	0.2579	0.8252	0
4775	622.6	1.4855	0.2492	0.2579	0.8252	0
4776	622.5	1.4851	0.2491	0.258	0.8254	0
4777	622.4	1.4853	0.2493	0.258	0.8253	0
4778	622.3	1.4852	0.2491	0.2581	0.8252	0
4779	622.2	1.4851	0.249	0.258	0.8252	0
4780	622.1	1.4847	0.2491	0.2581	0.8254	0
4781	622	1.4854	0.2493	0.2581	0.8256	0
4782	621.9	1.4856	0.2492	0.2581	0.8255	0
4783	621.8	1.4851	0.2491	0.2581	0.8256	0
4784	621.7	1.4856	0.2493	0.258	0.8256	0
4785	621.6	1.4855	0.2491	0.2582	0.8256	0
4786	621.5	1.4854	0.2492	0.2581	0.8256	0
4787	621.4	1.4853	0.2494	0.258	0.8255	0
4788	621.3	1.4853	0.2493	0.2581	0.8259	0
4789	621.2	1.4851	0.2491	0.2579	0.8257	0
4790	621.1	1.4847	0.249	0.258	0.8256	0
4791	621	1.485	0.2491	0.258	0.8259	0
4792	620.9	1.4855	0.2492	0.258	0.8257	0
4793	620.8	1.4854	0.2491	0.2579	0.8259	0
4794	620.7	1.4852	0.249	0.2579	0.8257	0
4795	620.6	1.4851	0.2489	0.2579	0.8255	0
4796	620.5	1.4849	0.2489	0.2578	0.8259	0
4797	620.4	1.4848	0.2488	0.2578	0.8257	0
4798	620.3	1.485	0.2488	0.2576	0.8258	0
4799	620.2	1.4851	0.2488	0.2576	0.8258	0
4800	620.1	1.4848	0.2487	0.2576	0.8259	0
4801	620	1.4853	0.2488	0.2576	0.8257	0
4802	619.9	1.4847	0.2487	0.2578	0.8259	0

4803	619.8	1.4848	0.2485	0.2574	0.8257	0
4804	619.7	1.485	0.2486	0.2574	0.8258	0
4805	619.6	1.4843	0.2487	0.2576	0.826	0
4806	619.5	1.4849	0.2485	0.2574	0.826	0
4807	619.4	1.4847	0.2487	0.2573	0.8261	0
4808	619.3	1.4846	0.2484	0.2572	0.826	0
4809	619.2	1.4845	0.2485	0.2573	0.826	0
4810	619.1	1.4845	0.2485	0.2573	0.8262	0
4811	619	1.4846	0.2484	0.2573	0.8261	0
4812	618.9	1.4842	0.2483	0.2572	0.8262	0
4813	618.8	1.484	0.2481	0.2571	0.8262	0
4814	618.7	1.4846	0.2482	0.2571	0.826	0
4815	618.6	1.4843	0.2483	0.2573	0.8262	0
4816	618.5	1.4841	0.2481	0.2572	0.8264	0
4817	618.4	1.4841	0.2483	0.257	0.826	0
4818	618.3	1.4839	0.2482	0.257	0.8265	0
4819	618.2	1.4842	0.248	0.2569	0.8261	0
4820	618.1	1.4844	0.2482	0.257	0.8264	0
4821	618	1.484	0.2482	0.2568	0.8263	0
4822	617.9	1.4838	0.2478	0.2569	0.8263	0
4823	617.8	1.4844	0.248	0.2567	0.8263	0
4824	617.7	1.4842	0.248	0.2565	0.8263	0
4825	617.6	1.4842	0.248	0.2567	0.8266	0
4826	617.5	1.4844	0.2479	0.2567	0.8265	0
4827	617.4	1.4841	0.2481	0.2567	0.8263	0
4828	617.3	1.4837	0.2479	0.2566	0.8265	0
4829	617.2	1.484	0.2476	0.2564	0.8267	0
4830	617.1	1.4839	0.2477	0.2566	0.8267	0
4831	617	1.4838	0.2477	0.2565	0.8268	0
4832	616.9	1.4836	0.2475	0.2566	0.8265	0
4833	616.8	1.4837	0.2478	0.2565	0.8266	0
4834	616.7	1.4839	0.2478	0.2566	0.8267	0
4835	616.6	1.4838	0.2478	0.2567	0.8268	0
4836	616.5	1.4837	0.2478	0.2566	0.8269	0
4837	616.4	1.484	0.2477	0.2565	0.827	0
4838	616.3	1.484	0.2478	0.2567	0.8272	0
4839	616.2	1.4839	0.2479	0.2568	0.8271	0
4840	616.1	1.4842	0.2481	0.2567	0.8274	0
4841	616	1.4841	0.2479	0.2569	0.8275	0
4842	615.9	1.4844	0.2481	0.257	0.8274	0
4843	615.8	1.4843	0.2481	0.257	0.8275	0
4844	615.7	1.4841	0.2479	0.2568	0.8275	0
4845	615.6	1.4844	0.248	0.2569	0.8274	0

4846	615.5	1.4843	0.2481	0.2569	0.8274	0
4847	615.4	1.4845	0.2481	0.2568	0.8274	0
4848	615.3	1.4841	0.2481	0.2569	0.8274	0
4849	615.2	1.4847	0.2481	0.2571	0.8278	0
4850	615.1	1.4849	0.2482	0.2569	0.8279	0
4851	615	1.4845	0.2481	0.257	0.8278	0
4852	614.9	1.4847	0.2483	0.257	0.8278	0
4853	614.8	1.4847	0.2482	0.2571	0.8278	0
4854	614.7	1.4849	0.2482	0.2572	0.828	0
4855	614.6	1.4848	0.2482	0.2573	0.8281	0
4856	614.5	1.4845	0.2482	0.2571	0.8279	0
4857	614.4	1.4848	0.2483	0.2572	0.8279	0
4858	614.3	1.4848	0.2484	0.2572	0.8282	0
4859	614.2	1.4844	0.2485	0.2574	0.8282	0
4860	614.1	1.485	0.2486	0.2575	0.8285	0
4861	614	1.4848	0.2485	0.2574	0.8285	0
4862	613.9	1.485	0.2486	0.2574	0.8285	0
4863	613.8	1.4851	0.2488	0.2576	0.8285	0
4864	613.7	1.4855	0.2489	0.2577	0.8288	0
4865	613.6	1.4851	0.249	0.2576	0.8287	0
4866	613.5	1.4851	0.2487	0.2576	0.8289	0
4867	613.4	1.4849	0.2486	0.2574	0.8286	0
4868	613.3	1.4852	0.2488	0.2576	0.8288	0
4869	613.2	1.4853	0.2489	0.2576	0.8289	0
4870	613.1	1.485	0.2489	0.2577	0.8291	0
4871	613	1.4854	0.249	0.2578	0.829	0
4872	612.9	1.4852	0.249	0.2577	0.8292	0
4873	612.8	1.485	0.2487	0.2577	0.8291	0
4874	612.7	1.4855	0.2488	0.2577	0.8294	0
4875	612.6	1.4855	0.2491	0.2578	0.8294	0
4876	612.5	1.485	0.2491	0.2579	0.8296	0
4877	612.4	1.485	0.2489	0.2577	0.8293	0
4878	612.3	1.4851	0.2488	0.2577	0.8293	0
4879	612.2	1.4852	0.2489	0.2577	0.8294	0
4880	612.1	1.4855	0.249	0.2578	0.8297	0
4881	612	1.4857	0.2491	0.2578	0.8297	0
4882	611.9	1.4854	0.249	0.2577	0.83	0
4883	611.8	1.4855	0.2489	0.2576	0.8299	0
4884	611.7	1.4856	0.2488	0.2576	0.8296	0
4885	611.6	1.4854	0.2488	0.2577	0.8297	0
4886	611.5	1.4852	0.2488	0.2576	0.8299	0
4887	611.4	1.4852	0.2487	0.2575	0.8299	0
4888	611.3	1.485	0.2487	0.2575	0.8298	0

4889	611.2	1.4846	0.2486	0.2574	0.8294	0
4890	611.1	1.4849	0.2487	0.2576	0.8295	0
4891	611	1.485	0.2486	0.2575	0.8299	0
4892	610.9	1.485	0.2486	0.2576	0.8299	0
4893	610.8	1.4845	0.2486	0.2574	0.83	0
4894	610.7	1.4852	0.2485	0.2574	0.8298	0
4895	610.6	1.4846	0.2485	0.2573	0.83	0
4896	610.5	1.4846	0.2484	0.2571	0.8298	0
4897	610.4	1.4847	0.2484	0.2574	0.8302	0
4898	610.3	1.484	0.2483	0.2571	0.8298	0
4899	610.2	1.4848	0.2484	0.2573	0.8301	0
4900	610.1	1.4848	0.2484	0.2572	0.83	0
4901	610	1.4846	0.2484	0.2575	0.8301	0
4902	609.9	1.4849	0.2483	0.2572	0.83	0
4903	609.8	1.4844	0.2481	0.2569	0.8299	0
4904	609.7	1.4841	0.2482	0.2571	0.8299	0
4905	609.6	1.4841	0.2482	0.2568	0.8299	0
4906	609.5	1.4842	0.2481	0.2569	0.8303	0
4907	609.4	1.4841	0.248	0.2569	0.8302	0
4908	609.3	1.4838	0.248	0.2569	0.8302	0
4909	609.2	1.4838	0.2479	0.2568	0.8302	0
4910	609.1	1.4838	0.2478	0.2567	0.8303	0
4911	609	1.4838	0.2479	0.2566	0.8302	0
4912	608.9	1.4834	0.2478	0.2566	0.8303	0
4913	608.8	1.4833	0.2477	0.2564	0.8301	0
4914	608.7	1.4836	0.2476	0.2565	0.8303	0
4915	608.6	1.4836	0.2477	0.2563	0.8302	0
4916	608.5	1.4835	0.2477	0.2563	0.8302	0
4917	608.4	1.4835	0.2477	0.2564	0.8305	0
4918	608.3	1.4831	0.2475	0.2562	0.8305	0
4919	608.2	1.483	0.2475	0.2561	0.8304	0
4920	608.1	1.483	0.2476	0.2562	0.8304	0
4921	608	1.4829	0.2474	0.2561	0.8305	0
4922	607.9	1.4828	0.2473	0.256	0.8305	0
4923	607.8	1.4832	0.2476	0.256	0.8305	0
4924	607.7	1.4828	0.2474	0.2559	0.8304	0
4925	607.6	1.4827	0.2474	0.2559	0.8304	0
4926	607.5	1.4824	0.2471	0.2557	0.8305	0
4927	607.4	1.4827	0.2472	0.2557	0.8305	0
4928	607.3	1.4822	0.2471	0.2556	0.8304	0
4929	607.2	1.4825	0.2473	0.2556	0.8305	0
4930	607.1	1.4819	0.247	0.2556	0.8303	0
4931	607	1.4815	0.2469	0.2555	0.8304	0

4932	606.9	1.4817	0.2469	0.2555	0.8306	0
4933	606.8	1.4818	0.2469	0.2555	0.8306	0
4934	606.7	1.482	0.2469	0.2555	0.8306	0
4935	606.6	1.4819	0.2472	0.2554	0.8307	0
4936	606.5	1.4821	0.247	0.2555	0.8308	0
4937	606.4	1.482	0.247	0.2555	0.8307	0
4938	606.3	1.4824	0.2469	0.2555	0.8308	0
4939	606.2	1.4819	0.247	0.2555	0.8308	0
4940	606.1	1.4818	0.2471	0.2556	0.831	0
4941	606	1.4819	0.247	0.2556	0.8311	0
4942	605.9	1.481	0.247	0.2555	0.831	0
4943	605.8	1.4812	0.247	0.2557	0.8309	0
4944	605.7	1.4819	0.2471	0.2556	0.8311	0
4945	605.6	1.4813	0.2471	0.2555	0.8311	0
4946	605.5	1.482	0.2469	0.2555	0.8311	0
4947	605.4	1.4818	0.2471	0.2556	0.8312	0
4948	605.3	1.4821	0.2471	0.2555	0.8313	0
4949	605.2	1.4818	0.247	0.2554	0.8313	0
4950	605.1	1.4824	0.2471	0.2556	0.8316	0
4951	605	1.4817	0.2471	0.2557	0.8315	0
4952	604.9	1.4818	0.2474	0.2558	0.8314	0
4953	604.8	1.4816	0.2473	0.2558	0.8315	0
4954	604.7	1.482	0.2473	0.2561	0.8317	0
4955	604.6	1.4823	0.2473	0.2561	0.8319	0
4956	604.5	1.4816	0.2472	0.2559	0.8317	0
4957	604.4	1.4819	0.2474	0.2558	0.8315	0
4958	604.3	1.482	0.2475	0.2561	0.8319	0
4959	604.2	1.482	0.2476	0.256	0.832	0
4960	604.1	1.482	0.2476	0.2561	0.832	0
4961	604	1.4819	0.2475	0.2562	0.8321	0
4962	603.9	1.482	0.2477	0.2562	0.832	0
4963	603.8	1.482	0.2477	0.2564	0.8323	0
4964	603.7	1.4823	0.2478	0.2563	0.8324	0
4965	603.6	1.4822	0.2479	0.2566	0.8324	0
4966	603.5	1.4825	0.2478	0.2564	0.8324	0
4967	603.4	1.4824	0.2479	0.2565	0.8326	0
4968	603.3	1.4824	0.248	0.2565	0.8325	0
4969	603.2	1.4826	0.248	0.2564	0.8326	0
4970	603.1	1.4818	0.2481	0.2566	0.8326	0
4971	603	1.4821	0.2481	0.2568	0.8327	0
4972	602.9	1.4823	0.2481	0.2569	0.8326	0
4973	602.8	1.4818	0.2482	0.2566	0.8326	0
4974	602.7	1.4819	0.2482	0.2567	0.8326	0

4975	602.6	1.4825	0.2485	0.2567	0.8328	0
4976	602.5	1.482	0.2485	0.2568	0.8327	0
4977	602.4	1.4824	0.2483	0.2567	0.8328	0
4978	602.3	1.4822	0.2483	0.2567	0.833	0
4979	602.2	1.4823	0.2482	0.2567	0.8328	0
4980	602.1	1.4817	0.2481	0.2567	0.8329	0
4981	602	1.4823	0.2485	0.2568	0.8329	0
4982	601.9	1.4823	0.2483	0.2568	0.8329	0
4983	601.8	1.4817	0.2482	0.2568	0.833	0
4984	601.7	1.4818	0.2483	0.2568	0.8331	0
4985	601.6	1.482	0.2483	0.2568	0.8332	0
4986	601.5	1.4819	0.2483	0.2567	0.833	0
4987	601.4	1.4817	0.2485	0.2569	0.8331	0
4988	601.3	1.4818	0.2485	0.257	0.833	0
4989	601.2	1.4812	0.2485	0.2568	0.8331	0
4990	601.1	1.4817	0.2484	0.2568	0.8331	0
4991	601	1.4821	0.2484	0.2567	0.833	0
4992	600.9	1.4818	0.2486	0.2568	0.8331	0
4993	600.8	1.4814	0.2484	0.2568	0.833	0
4994	600.7	1.4815	0.2484	0.2567	0.8333	0
4995	600.6	1.4811	0.2484	0.2568	0.8332	0
4996	600.5	1.4814	0.2484	0.2568	0.8331	0
4997	600.4	1.4818	0.2486	0.257	0.8335	0
4998	600.3	1.4813	0.2484	0.2568	0.8333	0
4999	600.2	1.4816	0.2483	0.2567	0.8332	0
5000	600.1	1.481	0.2485	0.257	0.8333	0
5001	600	1.4812	0.2484	0.2567	0.8334	0
5002	599.9	1.481	0.2485	0.257	0.8337	0
5003	599.8	1.4815	0.2484	0.2569	0.8337	0
5004	599.7	1.4807	0.2483	0.2568	0.8335	0
5005	599.6	1.4812	0.2482	0.2566	0.8335	0
5006	599.5	1.4811	0.2484	0.2567	0.8333	0
5007	599.4	1.4813	0.2482	0.2566	0.8331	0
5008	599.3	1.4808	0.2481	0.2565	0.8334	0
5009	599.2	1.4807	0.2481	0.2565	0.8334	0
5010	599.1	1.4808	0.2479	0.2563	0.8332	0
5011	599	1.4804	0.248	0.2564	0.8333	0
5012	598.9	1.4806	0.248	0.2566	0.8331	0
5013	598.8	1.4804	0.2477	0.2564	0.8332	0
5014	598.7	1.4807	0.248	0.2564	0.8334	0
5015	598.6	1.4805	0.2478	0.2563	0.833	0
5016	598.5	1.4798	0.2477	0.2561	0.833	0
5017	598.4	1.48	0.2477	0.2562	0.8334	0

5018	598.3	1.4796	0.2477	0.256	0.8332	0
5019	598.2	1.4797	0.2476	0.256	0.8332	0
5020	598.1	1.4793	0.2476	0.256	0.8333	0
5021	598	1.4793	0.2473	0.2558	0.8329	0
5022	597.9	1.4788	0.2474	0.2559	0.8331	0
5023	597.8	1.479	0.2473	0.2557	0.833	0
5024	597.7	1.4792	0.2474	0.2558	0.8331	0
5025	597.6	1.4793	0.2473	0.2558	0.8331	0
5026	597.5	1.4791	0.2472	0.2558	0.833	0
5027	597.4	1.479	0.2473	0.2558	0.8332	0
5028	597.3	1.4785	0.2473	0.2558	0.8332	0
5029	597.2	1.4786	0.2474	0.2557	0.8333	0
5030	597.1	1.4787	0.2473	0.2556	0.8331	0
5031	597	1.4785	0.2474	0.2555	0.8331	0
5032	596.9	1.4782	0.2473	0.2556	0.8331	0
5033	596.8	1.4782	0.2473	0.2556	0.8329	0
5034	596.7	1.4783	0.2472	0.2554	0.8331	0
5035	596.6	1.4785	0.2473	0.2556	0.8331	0
5036	596.5	1.4786	0.2474	0.2555	0.8331	0
5037	596.4	1.4783	0.2475	0.2555	0.8332	0
5038	596.3	1.4784	0.2472	0.2556	0.8333	0
5039	596.2	1.4784	0.2473	0.2557	0.8331	0
5040	596.1	1.478	0.2472	0.2555	0.833	0
5041	596	1.4778	0.2472	0.2555	0.8331	0
5042	595.9	1.4778	0.2474	0.2557	0.8333	0
5043	595.8	1.4774	0.2471	0.2556	0.833	0
5044	595.7	1.4771	0.2473	0.2556	0.8334	0
5045	595.6	1.4775	0.2474	0.2556	0.8333	0
5046	595.5	1.4773	0.2472	0.2554	0.8332	0
5047	595.4	1.4782	0.2473	0.2556	0.8332	0
5048	595.3	1.4777	0.2473	0.2556	0.8333	0
5049	595.2	1.4773	0.2474	0.2557	0.8335	0
5050	595.1	1.4773	0.2476	0.2558	0.8334	0
5051	595	1.4775	0.2473	0.2557	0.8332	0
5052	594.9	1.4773	0.2474	0.2559	0.8334	0
5053	594.8	1.4775	0.2474	0.2558	0.8335	0
5054	594.7	1.4778	0.2474	0.2557	0.8333	0
5055	594.6	1.4774	0.2477	0.2557	0.8333	0
5056	594.5	1.4775	0.2479	0.2562	0.8335	0
5057	594.4	1.4774	0.2477	0.2562	0.8335	0
5058	594.3	1.4771	0.2476	0.256	0.8335	0
5059	594.2	1.477	0.2476	0.2559	0.8333	0
5060	594.1	1.4777	0.2477	0.2559	0.8334	0

5061	594	1.4773	0.2479	0.2561	0.8335	0
5062	593.9	1.478	0.2479	0.2562	0.8334	0
5063	593.8	1.4779	0.2479	0.2562	0.8336	0
5064	593.7	1.4779	0.2482	0.2562	0.8335	0
5065	593.6	1.4774	0.248	0.2563	0.8335	0
5066	593.5	1.4776	0.248	0.2563	0.8334	0
5067	593.4	1.4776	0.2481	0.2565	0.8338	0
5068	593.3	1.4775	0.2482	0.2564	0.8338	0
5069	593.2	1.4774	0.2482	0.2564	0.8335	0
5070	593.1	1.4777	0.2484	0.2564	0.8338	0
5071	593	1.4776	0.2484	0.2565	0.8336	0
5072	592.9	1.4779	0.2484	0.2565	0.8338	0
5073	592.8	1.4781	0.2487	0.2568	0.8338	0
5074	592.7	1.478	0.2487	0.2567	0.8338	0
5075	592.6	1.4782	0.2487	0.2567	0.8339	0
5076	592.5	1.4776	0.2485	0.2567	0.8337	0
5077	592.4	1.4772	0.2485	0.2567	0.8338	0
5078	592.3	1.4777	0.2487	0.2567	0.8339	0
5079	592.2	1.4772	0.2487	0.2567	0.8339	0
5080	592.1	1.4776	0.2486	0.2569	0.834	0
5081	592	1.4776	0.2488	0.257	0.834	0
5082	591.9	1.4781	0.2488	0.2571	0.8341	0
5083	591.8	1.4772	0.2488	0.2569	0.8337	0
5084	591.7	1.4772	0.2489	0.2572	0.8339	0
5085	591.6	1.4772	0.249	0.2572	0.834	0
5086	591.5	1.4776	0.2491	0.2572	0.8339	0
5087	591.4	1.4772	0.249	0.2574	0.834	0
5088	591.3	1.4771	0.2489	0.2573	0.8338	0
5089	591.2	1.477	0.2489	0.2572	0.8337	0
5090	591.1	1.4769	0.2489	0.2571	0.8335	0
5091	591	1.4773	0.2488	0.2571	0.8336	0
5092	590.9	1.4773	0.2489	0.2572	0.8335	0
5093	590.8	1.4766	0.2491	0.2572	0.8336	0
5094	590.7	1.477	0.2492	0.2573	0.8339	0
5095	590.6	1.4775	0.2492	0.2575	0.834	0
5096	590.5	1.4771	0.2491	0.2575	0.8343	0
5097	590.4	1.4768	0.2492	0.2576	0.8341	0
5098	590.3	1.4776	0.2491	0.2575	0.834	0
5099	590.2	1.4772	0.2493	0.2574	0.834	0
5100	590.1	1.4771	0.249	0.2574	0.8339	0
5101	590	1.4767	0.249	0.2573	0.834	0
5102	589.9	1.4769	0.2491	0.2574	0.8339	0
5103	589.8	1.477	0.2492	0.2573	0.8339	0

5104	589.7	1.4765	0.2493	0.2572	0.8337	0
5105	589.6	1.4766	0.249	0.2574	0.8337	0
5106	589.5	1.4766	0.249	0.2574	0.8337	0
5107	589.4	1.4767	0.2489	0.2572	0.8339	0
5108	589.3	1.4764	0.249	0.2575	0.8337	0
5109	589.2	1.4764	0.2491	0.2573	0.8336	0
5110	589.1	1.4762	0.2492	0.2574	0.8338	0
5111	589	1.4766	0.249	0.2571	0.8334	0
5112	588.9	1.4762	0.2489	0.257	0.8335	0
5113	588.8	1.476	0.2488	0.2573	0.8337	0
5114	588.7	1.4757	0.2489	0.2573	0.8337	0
5115	588.6	1.4755	0.2489	0.2573	0.8335	0
5116	588.5	1.4757	0.2489	0.2573	0.8337	0
5117	588.4	1.4755	0.2488	0.2573	0.8339	0
5118	588.3	1.475	0.2486	0.2571	0.8334	0
5119	588.2	1.4753	0.2487	0.2569	0.8335	0
5120	588.1	1.4749	0.2486	0.2568	0.8333	0
5121	588	1.4747	0.2486	0.2569	0.8332	0
5122	587.9	1.4754	0.2486	0.257	0.8333	0
5123	587.8	1.4749	0.2487	0.2572	0.8336	0
5124	587.7	1.4746	0.2488	0.257	0.8333	0
5125	587.6	1.4748	0.2485	0.257	0.8334	0
5126	587.5	1.4743	0.2485	0.257	0.8334	0
5127	587.4	1.4741	0.2486	0.2569	0.8333	0
5128	587.3	1.4744	0.2486	0.257	0.8334	0
5129	587.2	1.4742	0.2484	0.2569	0.8331	0
5130	587.1	1.474	0.2483	0.2567	0.8333	0
5131	587	1.4733	0.2482	0.2567	0.8328	0
5132	586.9	1.4737	0.2483	0.2568	0.833	0
5133	586.8	1.4741	0.2485	0.2567	0.8331	0
5134	586.7	1.4736	0.2484	0.257	0.8333	0
5135	586.6	1.4735	0.2485	0.2569	0.8334	0
5136	586.5	1.4738	0.2483	0.2569	0.8332	0
5137	586.4	1.4742	0.2485	0.2568	0.8333	0
5138	586.3	1.4738	0.2486	0.2569	0.8333	0
5139	586.2	1.4737	0.2486	0.2572	0.8333	0
5140	586.1	1.474	0.2484	0.257	0.8334	0
5141	586	1.4728	0.2483	0.2567	0.8332	0
5142	585.9	1.4738	0.2484	0.257	0.833	0
5143	585.8	1.4736	0.2484	0.2567	0.833	0
5144	585.7	1.4732	0.2485	0.2568	0.8331	0
5145	585.6	1.4732	0.2484	0.257	0.8331	0
5146	585.5	1.4735	0.2484	0.2568	0.8332	0

5147	585.4	1.473	0.2485	0.2569	0.8334	0
5148	585.3	1.4732	0.2486	0.2571	0.8333	0
5149	585.2	1.4735	0.2486	0.257	0.833	0
5150	585.1	1.4724	0.2484	0.257	0.8331	0
5151	585	1.4733	0.2485	0.2569	0.8331	0
5152	584.9	1.538	0.2638	0.2725	0.8565	0
5153	584.8	1.5376	0.264	0.2728	0.8567	0
5154	584.7	1.5373	0.264	0.273	0.8571	0
5155	584.6	1.5385	0.264	0.2729	0.857	0
5156	584.5	1.5382	0.2641	0.273	0.8571	0
5157	584.4	1.5378	0.2644	0.2731	0.8571	0
5158	584.3	1.5384	0.2644	0.2733	0.8568	0
5159	584.2	1.5387	0.2643	0.2731	0.857	0
5160	584.1	1.5391	0.2643	0.2732	0.8571	0
5161	584	1.5378	0.2643	0.2732	0.857	0
5162	583.9	1.5393	0.2643	0.2732	0.8571	0
5163	583.8	1.5381	0.2646	0.2733	0.8572	0
5164	583.7	1.5387	0.2645	0.2732	0.857	0
5165	583.6	1.5394	0.2647	0.2734	0.8573	0
5166	583.5	1.5403	0.2647	0.2735	0.8574	0
5167	583.4	1.5393	0.2648	0.2736	0.8575	0
5168	583.3	1.5402	0.2648	0.2738	0.8576	0
5169	583.2	1.5403	0.2649	0.2737	0.8576	0
5170	583.1	1.5397	0.265	0.2737	0.8575	0
5171	583	1.5409	0.2649	0.2739	0.8577	0
5172	582.9	1.5403	0.2648	0.2737	0.8574	0
5173	582.8	1.5416	0.2649	0.2738	0.8574	0
5174	582.7	1.5413	0.2651	0.2741	0.8577	0
5175	582.6	1.541	0.265	0.2741	0.8577	0
5176	582.5	1.5416	0.2651	0.2739	0.8576	0
5177	582.4	1.5423	0.265	0.2741	0.8576	0
5178	582.3	1.5423	0.2651	0.2743	0.8579	0
5179	582.2	1.5424	0.2653	0.2741	0.858	0
5180	582.1	1.5424	0.2653	0.2742	0.8578	0
5181	582	1.5425	0.2652	0.2743	0.8579	0
5182	581.9	1.5426	0.2651	0.2743	0.8578	0
5183	581.8	1.5429	0.2651	0.2742	0.8579	0
5184	581.7	1.5433	0.2654	0.2745	0.8582	0
5185	581.6	1.5427	0.2653	0.2745	0.8581	0
5186	581.5	1.543	0.2652	0.2743	0.8578	0
5187	581.4	1.5433	0.2652	0.2742	0.8581	0
5188	581.3	1.5428	0.2653	0.2743	0.8582	0
5189	581.2	1.5431	0.2654	0.2745	0.8586	0

5190	581.1	1.5434	0.2654	0.2744	0.8583	0
5191	581	1.5429	0.2654	0.2746	0.8582	0
5192	580.9	1.5439	0.2653	0.2746	0.858	0
5193	580.8	1.5432	0.2653	0.2745	0.8585	0
5194	580.7	1.5434	0.2654	0.2744	0.8587	0
5195	580.6	1.5436	0.2652	0.2748	0.859	0
5196	580.5	1.5437	0.2655	0.2747	0.8589	0
5197	580.4	1.5439	0.2654	0.2748	0.8589	0
5198	580.3	1.543	0.2652	0.2745	0.8588	0
5199	580.2	1.5441	0.2656	0.2747	0.8593	0
5200	580.1	1.544	0.2654	0.2746	0.8591	0
5201	580	1.5443	0.2656	0.2748	0.8592	0
5202	579.9	1.5442	0.2655	0.2748	0.8594	0
5203	579.8	1.5442	0.2654	0.2747	0.8594	0
5204	579.7	1.5443	0.2655	0.2746	0.8594	0
5205	579.6	1.5443	0.2655	0.2746	0.8596	0
5206	579.5	1.5442	0.2656	0.2748	0.8597	0
5207	579.4	1.5441	0.2655	0.2746	0.8594	0
5208	579.3	1.5452	0.2657	0.2748	0.8596	0
5209	579.2	1.5443	0.2657	0.2748	0.86	0
5210	579.1	1.5444	0.2657	0.2748	0.8599	0
5211	579	1.5436	0.2659	0.2748	0.8601	0
5212	578.9	1.5444	0.2659	0.2747	0.8602	0
5213	578.8	1.545	0.266	0.2746	0.8598	0
5214	578.7	1.545	0.2658	0.2748	0.8599	0
5215	578.6	1.545	0.2657	0.2747	0.8602	0
5216	578.5	1.544	0.2658	0.2746	0.8602	0
5217	578.4	1.5456	0.2657	0.2747	0.8603	0
5218	578.3	1.5457	0.2658	0.2748	0.8604	0
5219	578.2	1.5448	0.2658	0.2747	0.8603	0
5220	578.1	1.5439	0.2658	0.2747	0.8602	0
5221	578	1.5445	0.2657	0.2747	0.8604	0
5222	577.9	1.5443	0.2658	0.2745	0.8605	0
5223	577.8	1.5443	0.2658	0.2747	0.8605	0
5224	577.7	1.5436	0.2659	0.2748	0.8608	0
5225	577.6	1.5458	0.2657	0.2747	0.8608	0
5226	577.5	1.5457	0.2659	0.2748	0.8608	0
5227	577.4	1.5447	0.2655	0.2746	0.8606	0
5228	577.3	1.5454	0.2658	0.2749	0.8605	0
5229	577.2	1.5445	0.2658	0.2746	0.8607	0
5230	577.1	1.5452	0.2659	0.2748	0.8609	0
5231	577	1.5457	0.2657	0.2746	0.8606	0
5232	576.9	1.5457	0.2658	0.2747	0.8607	0

5233	576.8	1.5449	0.2659	0.2749	0.8612	0
5234	576.7	1.5447	0.2658	0.2747	0.8611	0
5235	576.6	1.5453	0.2658	0.2749	0.8611	0
5236	576.5	1.5451	0.2657	0.2749	0.8612	0
5237	576.4	1.545	0.2658	0.2748	0.8608	0
5238	576.3	1.545	0.2657	0.2751	0.8614	0
5239	576.2	1.5464	0.2658	0.275	0.8613	0
5240	576.1	1.5455	0.2658	0.275	0.8612	0
5241	576	1.5455	0.266	0.2749	0.8611	0
5242	575.9	1.5453	0.266	0.275	0.8617	0
5243	575.8	1.5452	0.2661	0.2749	0.8618	0
5244	575.7	1.5464	0.2659	0.2747	0.8619	0
5245	575.6	1.5455	0.2657	0.2747	0.8616	0
5246	575.5	1.5454	0.2658	0.2748	0.8619	0
5247	575.4	1.5459	0.2658	0.2748	0.8616	0
5248	575.3	1.5459	0.2658	0.2748	0.8615	0
5249	575.2	1.545	0.2658	0.2748	0.8614	0
5250	575.1	1.5456	0.2659	0.2749	0.8617	0
5251	575	1.5461	0.2659	0.2748	0.8617	0
5252	574.9	1.5467	0.2658	0.275	0.8618	0
5253	574.8	1.5459	0.2661	0.2751	0.8619	0
5254	574.7	1.5466	0.2661	0.2752	0.862	0
5255	574.6	1.5472	0.266	0.2751	0.862	0
5256	574.5	1.5471	0.2661	0.275	0.8623	0
5257	574.4	1.547	0.2662	0.2752	0.8623	0
5258	574.3	1.5467	0.2662	0.2751	0.8622	0
5259	574.2	1.5465	0.2661	0.2752	0.8624	0
5260	574.1	1.5465	0.2662	0.2752	0.8624	0
5261	574	1.5471	0.2663	0.2754	0.8625	0
5262	573.9	1.5463	0.2664	0.2753	0.8625	0
5263	573.8	1.5463	0.2665	0.2755	0.8629	0
5264	573.7	1.5469	0.2666	0.2757	0.8627	0
5265	573.6	1.5465	0.2665	0.2755	0.8629	0
5266	573.5	1.5472	0.2665	0.2755	0.863	0
5267	573.4	1.547	0.2665	0.2755	0.863	0
5268	573.3	1.5469	0.2667	0.2755	0.8631	0
5269	573.2	1.5467	0.2667	0.2756	0.863	0
5270	573.1	1.5473	0.2666	0.2756	0.863	0
5271	573	1.547	0.2665	0.2755	0.863	0
5272	572.9	1.5469	0.2665	0.2755	0.8633	0
5273	572.8	1.5469	0.2667	0.2756	0.8634	0
5274	572.7	1.5475	0.2667	0.2756	0.8637	0
5275	572.6	1.5488	0.2668	0.2759	0.8637	0

5276	572.5	1.5479	0.2667	0.2758	0.8636	0
5277	572.4	1.5478	0.2667	0.2758	0.8638	0
5278	572.3	1.547	0.2668	0.2757	0.8639	0
5279	572.2	1.5483	0.2669	0.2758	0.8639	0
5280	572.1	1.5473	0.2668	0.2758	0.8638	0
5281	572	1.5473	0.2671	0.2758	0.8641	0
5282	571.9	1.5485	0.267	0.2756	0.864	0
5283	571.8	1.5484	0.2668	0.2757	0.8637	0
5284	571.7	1.5483	0.2671	0.2759	0.864	0
5285	571.6	1.5488	0.2668	0.2757	0.8642	0
5286	571.5	1.5472	0.267	0.2758	0.8644	0
5287	571.4	1.5485	0.2668	0.2757	0.8645	0
5288	571.3	1.5484	0.2668	0.2758	0.8641	0
5289	571.2	1.5483	0.267	0.2758	0.8647	0
5290	571.1	1.5481	0.2668	0.276	0.8646	0
5291	571	1.5481	0.267	0.276	0.865	0
5292	570.9	1.5485	0.2668	0.2759	0.8649	0
5293	570.8	1.5477	0.2669	0.276	0.8649	0
5294	570.7	1.5489	0.2668	0.2759	0.8647	0
5295	570.6	1.5494	0.2668	0.2759	0.8647	0
5296	570.5	1.5487	0.267	0.2758	0.8649	0
5297	570.4	1.5485	0.2668	0.2758	0.8649	0
5298	570.3	1.5483	0.2669	0.276	0.8652	0
5299	570.2	1.5481	0.2667	0.276	0.8651	0
5300	570.1	1.5482	0.2668	0.2761	0.8654	0
5301	570	1.5494	0.2669	0.276	0.8651	0
5302	569.9	1.5493	0.267	0.276	0.8653	0
5303	569.8	1.5485	0.267	0.276	0.8657	0
5304	569.7	1.5489	0.2669	0.2757	0.8655	0
5305	569.6	1.5481	0.2668	0.2759	0.8658	0
5306	569.5	1.5487	0.2667	0.2758	0.8653	0
5307	569.4	1.5486	0.2668	0.2758	0.8656	0
5308	569.3	1.5485	0.2669	0.2759	0.8655	0
5309	569.2	1.5477	0.2669	0.276	0.8659	0
5310	569.1	1.5482	0.2668	0.2759	0.8657	0
5311	569	1.548	0.2669	0.2758	0.8656	0
5312	568.9	1.5486	0.2669	0.2758	0.8659	0
5313	568.8	1.5486	0.267	0.276	0.8657	0
5314	568.7	1.5493	0.2669	0.2759	0.8659	0
5315	568.6	1.5492	0.267	0.276	0.866	0
5316	568.5	1.5497	0.2669	0.276	0.866	0
5317	568.4	1.5503	0.267	0.2759	0.8664	0
5318	568.3	1.5501	0.267	0.2759	0.8661	0

5319	568.2	1.5491	0.2669	0.2759	0.8663	0
5320	568.1	1.5484	0.267	0.276	0.8662	0
5321	568	1.5498	0.267	0.276	0.8663	0
5322	567.9	1.5496	0.2671	0.2762	0.8667	0
5323	567.8	1.5495	0.2672	0.276	0.8664	0
5324	567.7	1.5494	0.2671	0.2761	0.8668	0
5325	567.6	1.5493	0.267	0.2762	0.8667	0
5326	567.5	1.5498	0.2672	0.276	0.8666	0
5327	567.4	1.5489	0.2671	0.2761	0.867	0
5328	567.3	1.5489	0.2672	0.2763	0.8671	0
5329	567.2	1.5501	0.2673	0.2761	0.867	0
5330	567.1	1.5493	0.2672	0.2759	0.8669	0
5331	567	1.5498	0.2672	0.276	0.8671	0
5332	566.9	1.5497	0.2673	0.2762	0.8674	0
5333	566.8	1.5496	0.2672	0.2761	0.8673	0
5334	566.7	1.5502	0.2673	0.2761	0.8675	0
5335	566.6	1.55	0.2671	0.2762	0.8672	0
5336	566.5	1.5499	0.2674	0.2763	0.8677	0
5337	566.4	1.5498	0.2674	0.2763	0.8678	0
5338	566.3	1.551	0.2674	0.2761	0.8678	0
5339	566.2	1.5502	0.2673	0.2762	0.868	0
5340	566.1	1.5507	0.2674	0.2764	0.868	0
5341	566	1.5505	0.2676	0.2762	0.8681	0
5342	565.9	1.5505	0.2675	0.2766	0.8681	0
5343	565.8	1.5512	0.2677	0.2766	0.8683	0
5344	565.7	1.5502	0.2677	0.2766	0.8682	0
5345	565.6	1.5515	0.2677	0.2766	0.8683	0
5346	565.5	1.5507	0.2676	0.2765	0.8684	0
5347	565.4	1.5512	0.2677	0.2766	0.8685	0
5348	565.3	1.5503	0.2677	0.2765	0.8686	0
5349	565.2	1.551	0.2678	0.2767	0.8687	0
5350	565.1	1.5508	0.2678	0.2767	0.8688	0
5351	565	1.5514	0.268	0.2769	0.8689	0
5352	564.9	1.5514	0.2681	0.2768	0.8689	0
5353	564.8	1.5512	0.2681	0.2768	0.8692	0
5354	564.7	1.5518	0.2682	0.277	0.8693	0
5355	564.6	1.5523	0.2681	0.2769	0.8692	0
5356	564.5	1.5521	0.2679	0.277	0.8691	0
5357	564.4	1.5513	0.2681	0.277	0.8694	0
5358	564.3	1.5511	0.2679	0.277	0.8693	0
5359	564.2	1.5517	0.2681	0.2773	0.8695	0
5360	564.1	1.5517	0.2683	0.2773	0.8697	0
5361	564	1.5528	0.2682	0.2772	0.8696	0

5362	563.9	1.552	0.2681	0.2774	0.8695	0
5363	563.8	1.5525	0.2682	0.2772	0.8699	0
5364	563.7	1.5524	0.2683	0.2772	0.8701	0
5365	563.6	1.5523	0.2684	0.2773	0.87	0
5366	563.5	1.5529	0.2685	0.2773	0.8704	0
5367	563.4	1.5528	0.2687	0.2776	0.8702	0
5368	563.3	1.5526	0.2685	0.2774	0.8701	0
5369	563.2	1.5532	0.2685	0.2775	0.8703	0
5370	563.1	1.5524	0.2686	0.2774	0.8704	0
5371	563	1.5535	0.2687	0.2774	0.8708	0
5372	562.9	1.5533	0.2686	0.2776	0.8705	0
5373	562.8	1.5524	0.2686	0.2774	0.8706	0
5374	562.7	1.5523	0.2686	0.2774	0.8709	0
5375	562.6	1.5527	0.2685	0.2775	0.8707	0
5376	562.5	1.5527	0.2686	0.2776	0.871	0
5377	562.4	1.5532	0.2686	0.2775	0.8713	0
5378	562.3	1.5523	0.2687	0.2776	0.871	0
5379	562.2	1.5534	0.2687	0.2775	0.8712	0
5380	562.1	1.5532	0.2686	0.2775	0.8709	0
5381	562	1.5532	0.2687	0.2777	0.8713	0
5382	561.9	1.553	0.2687	0.2776	0.8712	0
5383	561.8	1.5534	0.2686	0.2774	0.8711	0
5384	561.7	1.5539	0.2687	0.2776	0.8715	0
5385	561.6	1.5531	0.2687	0.2778	0.8717	0
5386	561.5	1.5527	0.2684	0.2773	0.8715	0
5387	561.4	1.5532	0.2685	0.2774	0.8715	0
5388	561.3	1.5524	0.2686	0.2775	0.8717	0
5389	561.2	1.5536	0.2686	0.2776	0.8717	0
5390	561.1	1.5534	0.2687	0.2776	0.8716	0
5391	561	1.5526	0.2686	0.2775	0.8719	0
5392	560.9	1.5523	0.2687	0.2774	0.872	0
5393	560.8	1.554	0.2684	0.2773	0.8719	0
5394	560.7	1.5539	0.2686	0.2774	0.8717	0
5395	560.6	1.5536	0.2686	0.2772	0.8719	0
5396	560.5	1.5534	0.2684	0.2772	0.8722	0
5397	560.4	1.5533	0.2684	0.2773	0.872	0
5398	560.3	1.5537	0.2682	0.2772	0.8724	0
5399	560.2	1.553	0.2684	0.2771	0.8719	0
5400	560.1	1.5534	0.2682	0.2771	0.8718	0
5401	560	1.5538	0.2683	0.2771	0.8721	0
5402	559.9	1.553	0.2682	0.2769	0.8724	0
5403	559.8	1.5534	0.2682	0.2769	0.8718	0
5404	559.7	1.5528	0.2685	0.2771	0.8721	0

5405	559.6	1.5532	0.2683	0.277	0.8723	0
5406	559.5	1.5529	0.268	0.2768	0.8722	0
5407	559.4	1.5534	0.2681	0.2767	0.8724	0
5408	559.3	1.5532	0.2679	0.2769	0.8722	0
5409	559.2	1.5536	0.2679	0.2766	0.8721	0
5410	559.1	1.5535	0.2681	0.2766	0.8724	0
5411	559	1.5534	0.2681	0.2766	0.8725	0
5412	558.9	1.5532	0.2681	0.2768	0.8726	0
5413	558.8	1.5524	0.2681	0.2768	0.8727	0
5414	558.7	1.5528	0.268	0.2765	0.8728	0
5415	558.6	1.5533	0.2678	0.2765	0.8729	0
5416	558.5	1.5543	0.2678	0.2764	0.8727	0
5417	558.4	1.5522	0.2678	0.2765	0.8729	0
5418	558.3	1.5528	0.2678	0.2766	0.8729	0
5419	558.2	1.5534	0.2678	0.2768	0.8732	0
5420	558.1	1.5537	0.2678	0.2766	0.8731	0
5421	558	1.5535	0.2677	0.2764	0.8731	0
5422	557.9	1.5534	0.2678	0.2766	0.8733	0
5423	557.8	1.5539	0.2677	0.2765	0.8733	0
5424	557.7	1.553	0.2675	0.2763	0.8734	0
5425	557.6	1.5535	0.2676	0.2764	0.8734	0
5426	557.5	1.5534	0.2677	0.2763	0.8734	0
5427	557.4	1.5538	0.2676	0.2763	0.8736	0
5428	557.3	1.5537	0.2678	0.2764	0.8737	0
5429	557.2	1.5535	0.2676	0.2764	0.8736	0
5430	557.1	1.5541	0.2679	0.2766	0.8739	0
5431	557	1.5539	0.2679	0.2766	0.8741	0
5432	556.9	1.5538	0.2678	0.2766	0.8741	0
5433	556.8	1.5536	0.2678	0.2765	0.8741	0
5434	556.7	1.5534	0.2679	0.2766	0.8742	0
5435	556.6	1.5539	0.2677	0.2767	0.8741	0
5436	556.5	1.5537	0.2678	0.2766	0.8743	0
5437	556.4	1.5536	0.2679	0.2768	0.8745	0
5438	556.3	1.554	0.2679	0.2766	0.8743	0
5439	556.2	1.5546	0.2683	0.2768	0.8748	0
5440	556.1	1.5544	0.2683	0.2769	0.875	0
5441	556	1.5543	0.2684	0.277	0.8752	0
5442	555.9	1.554	0.2682	0.2769	0.8749	0
5443	555.8	1.5544	0.2683	0.2767	0.8748	0
5444	555.7	1.5549	0.2683	0.277	0.8751	0
5445	555.6	1.5547	0.2684	0.2772	0.8753	0
5446	555.5	1.5545	0.2682	0.2769	0.8753	0
5447	555.4	1.555	0.2684	0.2772	0.8753	0

5448	555.3	1.5554	0.2684	0.2773	0.8753	0
5449	555.2	1.5554	0.2687	0.2775	0.8755	0
5450	555.1	1.5549	0.2682	0.2772	0.8758	0
5451	555	1.5555	0.2687	0.2773	0.8757	0
5452	554.9	1.5559	0.2689	0.2774	0.8759	0
5453	554.8	1.555	0.2686	0.2774	0.8761	0
5454	554.7	1.5561	0.2688	0.2775	0.8763	0
5455	554.6	1.5559	0.2687	0.2774	0.8762	0
5456	554.5	1.5557	0.269	0.2774	0.8761	0
5457	554.4	1.5555	0.269	0.2776	0.8764	0
5458	554.3	1.5557	0.2687	0.2774	0.8765	0
5459	554.2	1.5567	0.2689	0.2775	0.8763	0
5460	554.1	1.5565	0.269	0.2776	0.8766	0
5461	554	1.5562	0.2691	0.2777	0.8765	0
5462	553.9	1.556	0.269	0.2777	0.8767	0
5463	553.8	1.5559	0.2692	0.278	0.8769	0
5464	553.7	1.557	0.2693	0.278	0.8772	0
5465	553.6	1.556	0.2692	0.2782	0.8772	0
5466	553.5	1.557	0.2693	0.2781	0.8773	0
5467	553.4	1.5562	0.2693	0.2785	0.8775	0
5468	553.3	1.5566	0.2696	0.2784	0.8779	0
5469	553.2	1.5568	0.2695	0.2785	0.8777	0
5470	553.1	1.557	0.2695	0.2784	0.8777	0
5471	553	1.5562	0.2695	0.2784	0.8777	0
5472	552.9	1.5571	0.2695	0.2784	0.8777	0
5473	552.8	1.5562	0.2696	0.2782	0.8779	0
5474	552.7	1.5565	0.2695	0.2783	0.8782	0
5475	552.6	1.5568	0.2696	0.2783	0.8782	0
5476	552.5	1.5577	0.2696	0.2784	0.8784	0
5477	552.4	1.5574	0.2696	0.2784	0.8784	0
5478	552.3	1.557	0.2695	0.2783	0.8782	0
5479	552.2	1.5571	0.2696	0.2782	0.8785	0
5480	552.1	1.5574	0.2698	0.2784	0.8784	0
5481	552	1.5577	0.2696	0.2783	0.8786	0
5482	551.9	1.558	0.2696	0.2785	0.879	0
5483	551.8	1.5576	0.2697	0.2785	0.8788	0
5484	551.7	1.5578	0.2696	0.2785	0.8788	0
5485	551.6	1.5574	0.2697	0.2784	0.8787	0
5486	551.5	1.5575	0.2697	0.2783	0.8789	0
5487	551.4	1.5577	0.2696	0.2783	0.8787	0
5488	551.3	1.5573	0.2696	0.2783	0.8788	0
5489	551.2	1.5575	0.2698	0.2785	0.879	0
5490	551.1	1.5571	0.2698	0.2781	0.8789	0

5491	551	1.5568	0.2697	0.2784	0.8791	0
5492	550.9	1.5576	0.2699	0.2784	0.8794	0
5493	550.8	1.5585	0.2701	0.2784	0.8792	0
5494	550.7	1.5584	0.2698	0.2783	0.8796	0
5495	550.6	1.558	0.2698	0.2784	0.8792	0
5496	550.5	1.5574	0.2696	0.2782	0.8793	0
5497	550.4	1.5581	0.2696	0.2782	0.8796	0
5498	550.3	1.5584	0.2698	0.2784	0.8798	0
5499	550.2	1.5579	0.2697	0.2784	0.8798	0
5500	550.1	1.5585	0.2696	0.2782	0.8796	0
5501	550	1.558	0.2694	0.2782	0.8794	0
5502	549.9	1.5581	0.2695	0.2782	0.8796	0
5503	549.8	1.5582	0.2695	0.2782	0.8798	0
5504	549.7	1.5582	0.2694	0.2781	0.8799	0
5505	549.6	1.5571	0.2695	0.2781	0.8799	0
5506	549.5	1.5576	0.2693	0.2782	0.8799	0
5507	549.4	1.5582	0.2692	0.278	0.88	0
5508	549.3	1.5572	0.2694	0.2781	0.8801	0
5509	549.2	1.5583	0.2693	0.2779	0.8802	0
5510	549.1	1.5573	0.2693	0.2781	0.8804	0
5511	549	1.5577	0.269	0.2781	0.8802	0
5512	548.9	1.5571	0.269	0.2777	0.8803	0
5513	548.8	1.5579	0.2691	0.2779	0.8802	0
5514	548.7	1.5579	0.2691	0.2778	0.8807	0
5515	548.6	1.5579	0.2692	0.2779	0.8806	0
5516	548.5	1.5571	0.269	0.2776	0.8808	0
5517	548.4	1.5577	0.2691	0.2778	0.8807	0
5518	548.3	1.5576	0.2691	0.2776	0.8806	0
5519	548.2	1.5575	0.2689	0.2775	0.8807	0
5520	548.1	1.5569	0.2689	0.2776	0.8805	0
5521	548	1.5573	0.2688	0.2775	0.8804	0
5522	547.9	1.5573	0.269	0.2775	0.8809	0
5523	547.8	1.5573	0.2689	0.2776	0.881	0
5524	547.7	1.5576	0.2689	0.2773	0.8807	0
5525	547.6	1.5576	0.2688	0.2775	0.8812	0
5526	547.5	1.5574	0.2687	0.2774	0.8808	0
5527	547.4	1.5579	0.2689	0.2775	0.8811	0
5528	547.3	1.5584	0.2688	0.2774	0.8812	0
5529	547.2	1.5577	0.2689	0.2774	0.8812	0
5530	547.1	1.5581	0.269	0.2775	0.8812	0
5531	547	1.5578	0.2689	0.2773	0.8811	0
5532	546.9	1.5577	0.2689	0.2772	0.8814	0
5533	546.8	1.5582	0.2692	0.2774	0.8814	0

5534	546.7	1.5581	0.2692	0.2776	0.8818	0
5535	546.6	1.5578	0.2692	0.2775	0.8817	0
5536	546.5	1.5576	0.2692	0.2775	0.8819	0
5537	546.4	1.5579	0.2692	0.2775	0.8815	0
5538	546.3	1.5576	0.2693	0.2774	0.8817	0
5539	546.2	1.5586	0.2693	0.2777	0.8817	0
5540	546.1	1.5578	0.2693	0.2779	0.882	0
5541	546	1.5581	0.2693	0.2778	0.8819	0
5542	545.9	1.5584	0.2693	0.2776	0.8823	0
5543	545.8	1.558	0.2693	0.2776	0.8821	0
5544	545.7	1.5582	0.2691	0.2773	0.8821	0
5545	545.6	1.5586	0.2694	0.2776	0.8823	0
5546	545.5	1.559	0.2695	0.2779	0.8826	0
5547	545.4	1.5592	0.2696	0.2779	0.8825	0
5548	545.3	1.5588	0.2695	0.2779	0.8824	0
5549	545.2	1.559	0.2694	0.2779	0.8827	0
5550	545.1	1.5598	0.2696	0.278	0.8827	0
5551	545	1.5601	0.2697	0.2781	0.883	0
5552	544.9	1.5597	0.2698	0.2782	0.8829	0
5553	544.8	1.5605	0.2699	0.2781	0.8831	0
5554	544.7	1.56	0.2698	0.2782	0.883	0
5555	544.6	1.5596	0.2701	0.2782	0.883	0
5556	544.5	1.5603	0.2699	0.2783	0.883	0
5557	544.4	1.5606	0.2703	0.2784	0.8836	0
5558	544.3	1.5601	0.2704	0.2785	0.8837	0
5559	544.2	1.5603	0.2704	0.2785	0.8837	0
5560	544.1	1.5605	0.2707	0.2788	0.8837	0
5561	544	1.5606	0.2707	0.2788	0.8839	0
5562	543.9	1.5614	0.2709	0.2791	0.8843	0
5563	543.8	1.5621	0.2709	0.2792	0.884	0
5564	543.7	1.5616	0.2711	0.2794	0.8843	0
5565	543.6	1.5612	0.2713	0.2797	0.8844	0
5566	543.5	1.5624	0.2714	0.2798	0.8846	0
5567	543.4	1.5618	0.2713	0.2796	0.8846	0
5568	543.3	1.5618	0.2713	0.2797	0.8845	0
5569	543.2	1.562	0.2715	0.2799	0.8848	0
5570	543.1	1.5625	0.2717	0.2799	0.8849	0
5571	543	1.5625	0.2717	0.28	0.8849	0
5572	542.9	1.563	0.2717	0.2799	0.8851	0
5573	542.8	1.5619	0.2718	0.2798	0.8851	0
5574	542.7	1.5631	0.2721	0.2799	0.8853	0
5575	542.6	1.5631	0.2721	0.2801	0.8854	0
5576	542.5	1.5625	0.2722	0.2802	0.8854	0

5577	542.4	1.563	0.2721	0.2803	0.8856	0
5578	542.3	1.5636	0.2723	0.2805	0.8857	0
5579	542.2	1.5636	0.2724	0.2805	0.8855	0
5580	542.1	1.5642	0.2724	0.2805	0.8856	0
5581	542	1.5641	0.2724	0.2806	0.886	0
5582	541.9	1.564	0.2724	0.2805	0.886	0
5583	541.8	1.5639	0.2725	0.2805	0.8861	0
5584	541.7	1.5639	0.2726	0.2807	0.8861	0
5585	541.6	1.5637	0.2725	0.2806	0.8864	0
5586	541.5	1.5642	0.2725	0.2806	0.8863	0
5587	541.4	1.5642	0.2727	0.2806	0.8861	0
5588	541.3	1.5642	0.2726	0.2807	0.8863	0
5589	541.2	1.5645	0.2726	0.2806	0.8863	0
5590	541.1	1.5645	0.2727	0.281	0.8865	0
5591	541	1.5644	0.2726	0.2806	0.8867	0
5592	540.9	1.5635	0.2726	0.2808	0.8866	0
5593	540.8	1.5653	0.2727	0.2808	0.8862	0
5594	540.7	1.5652	0.2727	0.2808	0.8867	0
5595	540.6	1.565	0.2727	0.2808	0.8867	0
5596	540.5	1.5642	0.2729	0.281	0.8866	0
5597	540.4	1.5654	0.2731	0.281	0.8865	0
5598	540.3	1.5646	0.273	0.281	0.8866	0
5599	540.2	1.565	0.2732	0.2811	0.8869	0
5600	540.1	1.5641	0.273	0.281	0.8869	0
5601	540	1.5646	0.273	0.281	0.8869	0
5602	539.9	1.5644	0.2731	0.2811	0.8869	0
5603	539.8	1.5657	0.2732	0.2812	0.8873	0
5604	539.7	1.5661	0.2733	0.2811	0.8872	0
5605	539.6	1.565	0.2731	0.281	0.8871	0
5606	539.5	1.5656	0.2729	0.281	0.887	0
5607	539.4	1.5654	0.2731	0.281	0.887	0
5608	539.3	1.5652	0.2729	0.2809	0.8871	0
5609	539.2	1.565	0.2727	0.2809	0.8869	0
5610	539.1	1.5654	0.2728	0.2809	0.8872	0
5611	539	1.5652	0.2727	0.2812	0.8871	0
5612	538.9	1.5657	0.2728	0.2812	0.8871	0
5613	538.8	1.5656	0.2728	0.281	0.8871	0
5614	538.7	1.566	0.2727	0.2811	0.8873	0
5615	538.6	1.5658	0.2727	0.2812	0.8877	0
5616	538.5	1.5656	0.2728	0.2812	0.8871	0
5617	538.4	1.5647	0.2727	0.281	0.8873	0
5618	538.3	1.5652	0.2728	0.281	0.8872	0
5619	538.2	1.565	0.2726	0.2811	0.8874	0

5620	538.1	1.5648	0.2727	0.2812	0.8876	0
5621	538	1.5647	0.2727	0.2812	0.8877	0
5622	537.9	1.5644	0.2727	0.281	0.8878	0
5623	537.8	1.5656	0.2725	0.2807	0.8873	0
5624	537.7	1.5654	0.2726	0.2806	0.8875	0
5625	537.6	1.5651	0.2724	0.2804	0.8873	0
5626	537.5	1.5658	0.2726	0.2808	0.8877	0
5627	537.4	1.5663	0.2727	0.2807	0.8876	0
5628	537.3	1.5652	0.2724	0.2805	0.8876	0
5629	537.2	1.5657	0.2724	0.2805	0.8879	0
5630	537.1	1.5656	0.2726	0.2806	0.8879	0
5631	537	1.5647	0.2726	0.2807	0.8876	0
5632	536.9	1.5643	0.2723	0.2805	0.8875	0
5633	536.8	1.5655	0.2724	0.2805	0.8877	0
5634	536.7	1.5653	0.2726	0.2805	0.8877	0
5635	536.6	1.5652	0.2725	0.2806	0.8878	0
5636	536.5	1.5657	0.2724	0.2805	0.8876	0
5637	536.4	1.5656	0.2726	0.2807	0.888	0
5638	536.3	1.5654	0.2726	0.2808	0.8881	0
5639	536.2	1.5651	0.2726	0.2809	0.8881	0
5640	536.1	1.5655	0.2726	0.2809	0.8878	0
5641	536	1.5659	0.2724	0.2806	0.8881	0
5642	535.9	1.5658	0.2725	0.2808	0.8877	0
5643	535.8	1.5656	0.2727	0.2809	0.8879	0
5644	535.7	1.5653	0.2727	0.281	0.888	0
5645	535.6	1.5651	0.2728	0.2809	0.8882	0
5646	535.5	1.5656	0.2727	0.2808	0.8884	0
5647	535.4	1.5645	0.2725	0.2808	0.8882	0
5648	535.3	1.5657	0.2727	0.2808	0.8881	0
5649	535.2	1.5661	0.2724	0.2807	0.888	0
5650	535.1	1.5652	0.2726	0.2809	0.8882	0
5651	535	1.5657	0.2728	0.281	0.8885	0
5652	534.9	1.5661	0.2726	0.2809	0.8885	0
5653	534.8	1.566	0.2728	0.281	0.8887	0
5654	534.7	1.5658	0.273	0.281	0.889	0
5655	534.6	1.5662	0.2731	0.2809	0.8886	0
5656	534.5	1.566	0.273	0.281	0.8889	0
5657	534.4	1.5665	0.273	0.2812	0.889	0
5658	534.3	1.567	0.2729	0.281	0.8887	0
5659	534.2	1.5675	0.2732	0.2811	0.8892	0
5660	534.1	1.5666	0.2733	0.2812	0.889	0
5661	534	1.5655	0.2733	0.2812	0.889	0
5662	533.9	1.567	0.2735	0.2816	0.8893	0

5663	533.8	1.5667	0.2734	0.2817	0.8895	0
5664	533.7	1.5681	0.2735	0.2819	0.8896	0
5665	533.6	1.5678	0.2735	0.2817	0.8896	0
5666	533.5	1.5675	0.2736	0.2817	0.8899	0
5667	533.4	1.5675	0.2739	0.2821	0.89	0
5668	533.3	1.5664	0.2738	0.2821	0.8898	0
5669	533.2	1.567	0.2741	0.282	0.89	0
5670	533.1	1.5667	0.2741	0.282	0.8901	0
5671	533	1.5679	0.2739	0.2818	0.8898	0
5672	532.9	1.5678	0.274	0.2819	0.8903	0
5673	532.8	1.5669	0.2742	0.2822	0.8904	0
5674	532.7	1.5682	0.2741	0.2821	0.8901	0
5675	532.6	1.5687	0.2742	0.2821	0.8902	0
5676	532.5	1.5676	0.274	0.282	0.89	0
5677	532.4	1.5682	0.274	0.282	0.89	0
5678	532.3	1.5682	0.2743	0.2823	0.8907	0
5679	532.2	1.5672	0.2742	0.2823	0.8905	0
5680	532.1	1.5686	0.2745	0.2824	0.8907	0
5681	532	1.5685	0.2744	0.2824	0.8908	0
5682	531.9	1.569	0.2745	0.2823	0.8906	0
5683	531.8	1.5681	0.2746	0.2823	0.8908	0
5684	531.7	1.5687	0.2745	0.2824	0.8912	0
5685	531.6	1.5695	0.2746	0.2825	0.8909	0
5686	531.5	1.5687	0.2748	0.2828	0.8911	0
5687	531.4	1.5694	0.2749	0.2828	0.8912	0
5688	531.3	1.5684	0.275	0.2828	0.8912	0
5689	531.2	1.5682	0.2746	0.2827	0.891	0
5690	531.1	1.569	0.2748	0.2829	0.8913	0
5691	531	1.5687	0.2746	0.2824	0.8912	0
5692	530.9	1.5687	0.2747	0.2828	0.8917	0
5693	530.8	1.5678	0.2747	0.2828	0.8915	0
5694	530.7	1.5692	0.2746	0.2826	0.8913	0
5695	530.6	1.5682	0.2747	0.2826	0.8917	0
5696	530.5	1.5689	0.2747	0.2827	0.8918	0
5697	530.4	1.5687	0.2745	0.2825	0.8911	0
5698	530.3	1.5687	0.2746	0.2827	0.8915	0
5699	530.2	1.5686	0.2747	0.2827	0.8918	0
5700	530.1	1.5684	0.2747	0.2826	0.8919	0
5701	530	1.569	0.2745	0.2825	0.8918	0
5702	529.9	1.5681	0.2745	0.2825	0.8916	0
5703	529.8	1.5688	0.2747	0.2826	0.8919	0
5704	529.7	1.5687	0.2747	0.2826	0.892	0
5705	529.6	1.5678	0.2746	0.2827	0.8919	0

5706	529.5	1.5685	0.2746	0.2828	0.8921	0
5707	529.4	1.5683	0.2744	0.2826	0.8917	0
5708	529.3	1.5681	0.2743	0.2823	0.8917	0
5709	529.2	1.568	0.2743	0.2824	0.8918	0
5710	529.1	1.5679	0.2741	0.2825	0.892	0
5711	529	1.5679	0.2742	0.2825	0.8922	0
5712	528.9	1.5688	0.2743	0.2824	0.8921	0
5713	528.8	1.567	0.2741	0.2822	0.892	0
5714	528.7	1.5669	0.2741	0.2824	0.892	0
5715	528.6	1.567	0.274	0.2821	0.892	0
5716	528.5	1.5678	0.2739	0.2818	0.8918	0
5717	528.4	1.5671	0.2738	0.2819	0.892	0
5718	528.3	1.5663	0.2737	0.2819	0.892	0
5719	528.2	1.5673	0.2737	0.2819	0.8919	0
5720	528.1	1.5666	0.2735	0.2816	0.8916	0
5721	528	1.566	0.2733	0.2814	0.8917	0
5722	527.9	1.5655	0.2731	0.2814	0.8918	0
5723	527.8	1.5667	0.2732	0.2813	0.8916	0
5724	527.7	1.5653	0.273	0.2812	0.8916	0
5725	527.6	1.5658	0.2729	0.2812	0.8916	0
5726	527.5	1.5661	0.2727	0.281	0.8916	0
5727	527.4	1.5648	0.2726	0.2808	0.8914	0
5728	527.3	1.5654	0.2727	0.2808	0.8915	0
5729	527.2	1.565	0.2725	0.2806	0.8912	0
5730	527.1	1.5648	0.2725	0.2808	0.8916	0
5731	527	1.5645	0.2723	0.2807	0.8915	0
5732	526.9	1.5642	0.2722	0.2804	0.8913	0
5733	526.8	1.5647	0.2718	0.2802	0.8913	0
5734	526.7	1.5646	0.272	0.2802	0.8912	0
5735	526.6	1.5653	0.2719	0.2804	0.891	0
5736	526.5	1.5624	0.2717	0.2801	0.8915	0
5737	526.4	1.5641	0.2718	0.2801	0.8915	0
5738	526.3	1.5639	0.2717	0.28	0.8916	0
5739	526.2	1.5637	0.2716	0.2799	0.8914	0
5740	526.1	1.5643	0.2716	0.28	0.8912	0
5741	526	1.5643	0.2719	0.2801	0.8914	0
5742	525.9	1.5642	0.2716	0.28	0.8918	0
5743	525.8	1.5648	0.2718	0.2801	0.8916	0
5744	525.7	1.5638	0.272	0.2801	0.8916	0
5745	525.6	1.5646	0.2722	0.2802	0.8924	0
5746	525.5	1.5645	0.2719	0.2802	0.892	0
5747	525.4	1.565	0.2718	0.2801	0.892	0
5748	525.3	1.5639	0.2719	0.28	0.8923	0

5749	525.2	1.5655	0.272	0.2801	0.8922	0
5750	525.1	1.5653	0.272	0.2802	0.8925	0
5751	525	1.5642	0.272	0.2802	0.8927	0
5752	524.9	1.564	0.2719	0.2803	0.8926	0
5753	524.8	1.5653	0.2718	0.2801	0.892	0
5754	524.7	1.5643	0.2719	0.2801	0.8924	0
5755	524.6	1.565	0.272	0.2803	0.8926	0
5756	524.5	1.5647	0.272	0.2803	0.8927	0
5757	524.4	1.5663	0.2721	0.2803	0.8929	0
5758	524.3	1.5643	0.2723	0.2804	0.8927	0
5759	524.2	1.565	0.2722	0.2805	0.893	0
5760	524.1	1.5656	0.2723	0.2807	0.8934	0
5761	524	1.5653	0.2723	0.2805	0.8934	0
5762	523.9	1.5642	0.2724	0.2806	0.8936	0
5763	523.8	1.5656	0.2724	0.2805	0.8938	0
5764	523.7	1.5654	0.2722	0.2806	0.894	0
5765	523.6	1.5661	0.2726	0.2807	0.8934	0
5766	523.5	1.5659	0.2727	0.281	0.894	0
5767	523.4	1.5655	0.2727	0.2808	0.8941	0
5768	523.3	1.5671	0.2727	0.2809	0.8941	0
5769	523.2	1.5668	0.2726	0.2808	0.8944	0
5770	523.1	1.5666	0.2729	0.2809	0.8945	0
5771	523	1.5663	0.2728	0.2813	0.8945	0
5772	522.9	1.5676	0.2728	0.281	0.8942	0
5773	522.8	1.5655	0.2728	0.281	0.8946	0
5774	522.7	1.5662	0.2728	0.2812	0.8948	0
5775	522.6	1.5669	0.273	0.2814	0.8948	0
5776	522.5	1.5667	0.2731	0.2814	0.8952	0
5777	522.4	1.5664	0.2731	0.2813	0.8952	0
5778	522.3	1.5659	0.2731	0.2813	0.8954	0
5779	522.2	1.5674	0.2731	0.2812	0.8952	0
5780	522.1	1.567	0.2732	0.2813	0.8948	0
5781	522	1.5669	0.2732	0.2815	0.8951	0
5782	521.9	1.5665	0.2732	0.2815	0.8952	0
5783	521.8	1.568	0.273	0.2813	0.8955	0
5784	521.7	1.5676	0.2731	0.2815	0.8956	0
5785	521.6	1.5672	0.273	0.2812	0.8953	0
5786	521.5	1.5679	0.2733	0.2815	0.8957	0
5787	521.4	1.5667	0.2733	0.2815	0.8954	0
5788	521.3	1.5673	0.2732	0.2816	0.8957	0
5789	521.2	1.567	0.2734	0.2816	0.8956	0
5790	521.1	1.5674	0.2733	0.2816	0.8955	0
5791	521	1.5662	0.2733	0.2816	0.8957	0

5792	520.9	1.5676	0.2735	0.2818	0.8959	0
5793	520.8	1.5673	0.2732	0.2816	0.8958	0
5794	520.7	1.567	0.2732	0.2814	0.896	0
5795	520.6	1.5676	0.2731	0.2817	0.8961	0
5796	520.5	1.5672	0.2732	0.2816	0.896	0
5797	520.4	1.5677	0.2731	0.2816	0.8962	0
5798	520.3	1.5674	0.2732	0.2815	0.8966	0
5799	520.2	1.567	0.273	0.2816	0.8968	0
5800	520.1	1.5668	0.273	0.2816	0.8967	0
5801	520	1.5682	0.2733	0.2816	0.8966	0
5802	519.9	1.567	0.2734	0.2816	0.8966	0
5803	519.8	1.5675	0.2735	0.2815	0.897	0
5804	519.7	1.5671	0.2733	0.2816	0.8968	0
5805	519.6	1.567	0.2735	0.2817	0.897	0
5806	519.5	1.5674	0.2733	0.2814	0.8969	0
5807	519.4	1.5679	0.2733	0.2816	0.897	0
5808	519.3	1.5675	0.2734	0.2814	0.8968	0
5809	519.2	1.5682	0.2735	0.2818	0.8973	0
5810	519.1	1.5669	0.2733	0.2816	0.8973	0
5811	519	1.5674	0.2733	0.2814	0.8971	0
5812	518.9	1.567	0.2731	0.2816	0.8971	0
5813	518.8	1.5676	0.2732	0.2815	0.8974	0
5814	518.7	1.5672	0.2731	0.2813	0.8974	0
5815	518.6	1.5678	0.2732	0.2815	0.8975	0
5816	518.5	1.5667	0.2732	0.2815	0.8978	0
5817	518.4	1.5662	0.273	0.2814	0.8979	0
5818	518.3	1.5677	0.2731	0.2814	0.8974	0
5819	518.2	1.5672	0.273	0.2814	0.8975	0
5820	518.1	1.567	0.2732	0.2814	0.8977	0
5821	518	1.5675	0.2731	0.2813	0.8975	0
5822	517.9	1.5672	0.273	0.2813	0.8979	0
5823	517.8	1.5669	0.2731	0.2813	0.8982	0
5824	517.7	1.5675	0.2732	0.2812	0.8983	0
5825	517.6	1.5681	0.2731	0.2813	0.8981	0
5826	517.5	1.5668	0.2731	0.2815	0.8984	0
5827	517.4	1.5673	0.273	0.2812	0.8989	0
5828	517.3	1.5669	0.2731	0.2811	0.8988	0
5829	517.2	1.5666	0.2732	0.2813	0.8984	0
5830	517.1	1.5663	0.2732	0.2816	0.8986	0
5831	517	1.5677	0.2731	0.2811	0.8984	0
5832	516.9	1.5674	0.2729	0.2809	0.8984	0
5833	516.8	1.567	0.273	0.2812	0.8984	0
5834	516.7	1.5686	0.2728	0.281	0.8987	0

5835	516.6	1.5674	0.273	0.281	0.8987	0
5836	516.5	1.566	0.2729	0.2811	0.8988	0
5837	516.4	1.5676	0.2729	0.281	0.8985	0
5838	516.3	1.5682	0.273	0.281	0.8988	0
5839	516.2	1.5679	0.273	0.2811	0.8991	0
5840	516.1	1.5685	0.2731	0.2811	0.8991	0
5841	516	1.5672	0.2731	0.2812	0.899	0
5842	515.9	1.5678	0.2728	0.2809	0.8994	0
5843	515.8	1.5683	0.273	0.2812	0.8991	0
5844	515.7	1.568	0.2728	0.2811	0.8996	0
5845	515.6	1.5687	0.273	0.2811	0.8995	0
5846	515.5	1.5683	0.2731	0.2811	0.8995	0
5847	515.4	1.568	0.2728	0.2811	0.8994	0
5848	515.3	1.5687	0.2731	0.2812	0.8997	0
5849	515.2	1.5674	0.2732	0.2813	0.8998	0
5850	515.1	1.5671	0.2735	0.2813	0.8999	0
5851	515	1.5677	0.2732	0.2814	0.9	0
5852	514.9	1.5673	0.273	0.2813	0.9	0
5853	514.8	1.5688	0.273	0.2811	0.9002	0
5854	514.7	1.5685	0.273	0.2812	0.9001	0
5855	514.6	1.5683	0.2732	0.2815	0.9005	0
5856	514.5	1.5699	0.2732	0.2814	0.9004	0
5857	514.4	1.5685	0.2731	0.2813	0.9001	0
5858	514.3	1.5691	0.2732	0.2815	0.9005	0
5859	514.2	1.5679	0.2733	0.2815	0.901	0
5860	514.1	1.5695	0.2734	0.2815	0.9007	0
5861	514	1.5682	0.2733	0.2814	0.9007	0
5862	513.9	1.5697	0.2732	0.2814	0.9007	0
5863	513.8	1.5693	0.2734	0.2814	0.9006	0
5864	513.7	1.5693	0.2736	0.2818	0.9011	0
5865	513.6	1.568	0.2736	0.2819	0.9011	0
5866	513.5	1.5696	0.2738	0.2818	0.9011	0
5867	513.4	1.5692	0.2736	0.2817	0.9011	0
5868	513.3	1.5698	0.2736	0.2817	0.9012	0
5869	513.2	1.5694	0.2735	0.2817	0.901	0
5870	513.1	1.5692	0.2737	0.2819	0.9014	0
5871	513	1.5698	0.2738	0.2819	0.9013	0
5872	512.9	1.5684	0.2737	0.2818	0.9012	0
5873	512.8	1.5701	0.2737	0.2818	0.9014	0
5874	512.7	1.5687	0.2734	0.2819	0.9014	0
5875	512.6	1.5703	0.2736	0.2818	0.9015	0
5876	512.5	1.569	0.2737	0.2819	0.9019	0
5877	512.4	1.5696	0.2737	0.2818	0.9023	0

5878	512.3	1.5693	0.2739	0.282	0.9024	0
5879	512.2	1.5711	0.2738	0.2821	0.9023	0
5880	512.1	1.5697	0.2737	0.2822	0.9022	0
5881	512	1.5683	0.2737	0.282	0.9022	0
5882	511.9	1.5698	0.2738	0.282	0.9022	0
5883	511.8	1.5704	0.2739	0.2819	0.902	0
5884	511.7	1.5712	0.2739	0.282	0.9026	0
5885	511.6	1.5699	0.2737	0.2822	0.9028	0
5886	511.5	1.5706	0.2737	0.2821	0.9029	0
5887	511.4	1.5692	0.2739	0.2822	0.9025	0
5888	511.3	1.5697	0.2737	0.282	0.9025	0
5889	511.2	1.5704	0.2738	0.282	0.9026	0
5890	511.1	1.5711	0.2738	0.2819	0.9029	0
5891	511	1.5697	0.2738	0.282	0.9027	0
5892	510.9	1.5704	0.2739	0.282	0.9028	0
5893	510.8	1.5699	0.2738	0.282	0.9032	0
5894	510.7	1.5694	0.2738	0.2818	0.9032	0
5895	510.6	1.5691	0.2737	0.2817	0.9028	0
5896	510.5	1.5698	0.2738	0.2817	0.9031	0
5897	510.4	1.5704	0.2736	0.2818	0.9035	0
5898	510.3	1.5701	0.2738	0.2817	0.9033	0
5899	510.2	1.5696	0.2737	0.2822	0.9033	0
5900	510.1	1.5694	0.2737	0.282	0.9035	0
5901	510	1.5709	0.2735	0.282	0.9036	0
5902	509.9	1.5695	0.2736	0.2818	0.9034	0
5903	509.8	1.5691	0.2734	0.2817	0.9032	0
5904	509.7	1.5696	0.2736	0.2815	0.9037	0
5905	509.6	1.5693	0.2735	0.2817	0.904	0
5906	509.5	1.5699	0.2734	0.2816	0.9036	0
5907	509.4	1.5696	0.2734	0.2817	0.9037	0
5908	509.3	1.5711	0.2734	0.2815	0.9037	0
5909	509.2	1.5697	0.2734	0.2815	0.9037	0
5910	509.1	1.5704	0.2734	0.2816	0.9038	0
5911	509	1.57	0.2733	0.2814	0.9038	0
5912	508.9	1.5706	0.2734	0.2815	0.9037	0
5913	508.8	1.5692	0.2735	0.2816	0.9037	0
5914	508.7	1.5698	0.2732	0.2814	0.9039	0
5915	508.6	1.5695	0.2734	0.2814	0.9036	0
5916	508.5	1.57	0.2732	0.2813	0.9038	0
5917	508.4	1.5696	0.2733	0.2814	0.9038	0
5918	508.3	1.5703	0.2733	0.2815	0.9042	0
5919	508.2	1.5688	0.2732	0.2814	0.9039	0
5920	508.1	1.5705	0.2732	0.2814	0.9038	0

5921	508	1.5702	0.2733	0.2814	0.9042	0
5922	507.9	1.5698	0.2732	0.2814	0.9044	0
5923	507.8	1.5704	0.2732	0.2814	0.9042	0
5924	507.7	1.5702	0.2732	0.2816	0.9046	0
5925	507.6	1.5717	0.2733	0.2814	0.9044	0
5926	507.5	1.5704	0.2732	0.2814	0.9047	0
5927	507.4	1.572	0.2731	0.2813	0.9047	0
5928	507.3	1.5707	0.2733	0.2813	0.9046	0
5929	507.2	1.5704	0.2732	0.2815	0.9045	0
5930	507.1	1.5711	0.2733	0.2814	0.9053	0
5931	507	1.5716	0.2734	0.2812	0.9051	0
5932	506.9	1.5703	0.2733	0.2814	0.9052	0
5933	506.8	1.5699	0.2732	0.2812	0.9048	0
5934	506.7	1.5715	0.2732	0.2813	0.9049	0
5935	506.6	1.5703	0.2732	0.2814	0.9053	0
5936	506.5	1.5699	0.2734	0.2816	0.9051	0
5937	506.4	1.5706	0.2736	0.2816	0.9055	0
5938	506.3	1.5715	0.2738	0.282	0.906	0
5939	506.2	1.5722	0.2737	0.2818	0.9055	0
5940	506.1	1.5718	0.2739	0.2817	0.9055	0
5941	506	1.5713	0.2737	0.2817	0.9059	0
5942	505.9	1.5719	0.2734	0.2815	0.9061	0
5943	505.8	1.5716	0.2737	0.2816	0.9058	0
5944	505.7	1.5714	0.2736	0.2817	0.9065	0
5945	505.6	1.5721	0.2738	0.2817	0.9062	0
5946	505.5	1.5717	0.2738	0.2818	0.9065	0
5947	505.4	1.5734	0.2736	0.2819	0.9064	0
5948	505.3	1.5732	0.2738	0.2821	0.9066	0
5949	505.2	1.5728	0.2741	0.2821	0.9069	0
5950	505.1	1.5715	0.2741	0.2822	0.9065	0
5951	505	1.5722	0.2744	0.2822	0.9069	0
5952	504.9	1.5718	0.2741	0.282	0.9068	0
5953	504.8	1.5724	0.274	0.2822	0.9066	0
5954	504.7	1.5721	0.2742	0.2822	0.9069	0
5955	504.6	1.5729	0.2743	0.2823	0.9071	0
5956	504.5	1.5737	0.2742	0.2825	0.9075	0
5957	504.4	1.5723	0.2742	0.2825	0.9074	0
5958	504.3	1.5729	0.2741	0.2825	0.9071	0
5959	504.2	1.5724	0.274	0.2822	0.9073	0
5960	504.1	1.5733	0.2742	0.2825	0.9081	0
5961	504	1.573	0.2744	0.2826	0.9078	0
5962	503.9	1.5748	0.2744	0.2828	0.9079	0
5963	503.8	1.5735	0.2746	0.2829	0.9081	0

5964	503.7	1.5742	0.2747	0.2832	0.9081	0
5965	503.6	1.5748	0.2745	0.2828	0.9078	0
5966	503.5	1.5743	0.2743	0.2827	0.9083	0
5967	503.4	1.5741	0.2746	0.2827	0.9084	0
5968	503.3	1.5738	0.2747	0.2829	0.9084	0
5969	503.2	1.5745	0.2747	0.283	0.9086	0
5970	503.1	1.5741	0.2747	0.2829	0.9086	0
5971	503	1.5749	0.2748	0.283	0.9085	0
5972	502.9	1.5735	0.2748	0.2829	0.9086	0
5973	502.8	1.5741	0.2746	0.2829	0.9087	0
5974	502.7	1.5749	0.2745	0.2828	0.9086	0
5975	502.6	1.5745	0.2747	0.2828	0.9087	0
5976	502.5	1.5743	0.2745	0.2829	0.9089	0
5977	502.4	1.5738	0.2745	0.283	0.9091	0
5978	502.3	1.5736	0.2748	0.2831	0.9092	0
5979	502.2	1.5754	0.2746	0.2832	0.9093	0
5980	502.1	1.575	0.2746	0.2829	0.9096	0
5981	502	1.5736	0.2745	0.2829	0.9092	0
5982	501.9	1.5744	0.2745	0.2829	0.9094	0
5983	501.8	1.574	0.2746	0.2829	0.9095	0
5984	501.7	1.5737	0.2746	0.2829	0.9094	0
5985	501.6	1.5755	0.2746	0.2828	0.9096	0
5986	501.5	1.5741	0.2745	0.2827	0.9095	0
5987	501.4	1.575	0.2749	0.283	0.91	0
5988	501.3	1.5746	0.2748	0.2828	0.9098	0
5989	501.2	1.5742	0.2747	0.2828	0.9099	0
5990	501.1	1.5738	0.2745	0.2825	0.9097	0
5991	501	1.5746	0.2748	0.2828	0.9101	0
5992	500.9	1.5732	0.2746	0.2828	0.91	0
5993	500.8	1.5739	0.2745	0.2824	0.9101	0
5994	500.7	1.5746	0.2745	0.2827	0.9104	0
5995	500.6	1.5742	0.2745	0.2826	0.9103	0
5996	500.5	1.575	0.2743	0.2826	0.9103	0
5997	500.4	1.5747	0.2743	0.2828	0.9107	0
5998	500.3	1.5744	0.2743	0.2826	0.9103	0
5999	500.2	1.574	0.2743	0.2824	0.9107	0
6000	500.1	1.5736	0.2742	0.2824	0.9105	0
6001	500	1.5733	0.2742	0.2825	0.9108	0
6002	499.9	1.5739	0.2742	0.2823	0.9104	0
6003	499.8	1.5725	0.2741	0.2822	0.911	0
6004	499.7	1.5744	0.2741	0.2826	0.911	0
6005	499.6	1.5729	0.2741	0.2823	0.9106	0
6006	499.5	1.5736	0.2739	0.2822	0.9109	0

6007	499.4	1.5744	0.2741	0.2822	0.9111	0
6008	499.3	1.5751	0.2742	0.2822	0.9112	0
6009	499.2	1.5747	0.2742	0.2822	0.9108	0
6010	499.1	1.5733	0.2742	0.2824	0.9109	0
6011	499	1.5739	0.2739	0.2821	0.911	0
6012	498.9	1.5736	0.274	0.2823	0.9111	0
6013	498.8	1.5744	0.2739	0.2823	0.9113	0
6014	498.7	1.5728	0.2738	0.2821	0.911	0
6015	498.6	1.5736	0.274	0.2822	0.9112	0
6016	498.5	1.5731	0.2738	0.2821	0.9112	0
6017	498.4	1.5738	0.2736	0.2817	0.9108	0
6018	498.3	1.5745	0.2736	0.2818	0.9111	0
6019	498.2	1.573	0.2737	0.282	0.9112	0
6020	498.1	1.5736	0.2734	0.2817	0.9112	0
6021	498	1.5733	0.2734	0.2818	0.9115	0
6022	497.9	1.574	0.2734	0.2818	0.9119	0
6023	497.8	1.5736	0.2733	0.2816	0.9117	0
6024	497.7	1.5732	0.2733	0.2817	0.9116	0
6025	497.6	1.574	0.2733	0.2818	0.9117	0
6026	497.5	1.5737	0.2735	0.2818	0.912	0
6027	497.4	1.5744	0.2732	0.2818	0.9117	0
6028	497.3	1.574	0.2733	0.2819	0.9123	0
6029	497.2	1.5737	0.2732	0.2818	0.9117	0
6030	497.1	1.5733	0.2737	0.2819	0.9121	0
6031	497	1.5739	0.2736	0.2818	0.9123	0
6032	496.9	1.5735	0.2734	0.2819	0.9122	0
6033	496.8	1.5743	0.2734	0.2819	0.9123	0
6034	496.7	1.5739	0.2735	0.2817	0.9122	0
6035	496.6	1.5736	0.2735	0.2819	0.9124	0
6036	496.5	1.5744	0.2736	0.282	0.9128	0
6037	496.4	1.573	0.2737	0.2819	0.9127	0
6038	496.3	1.5737	0.2736	0.2821	0.9133	0
6039	496.2	1.5744	0.2738	0.282	0.9129	0
6040	496.1	1.5753	0.2739	0.2821	0.9136	0
6041	496	1.5748	0.2737	0.282	0.9131	0
6042	495.9	1.5746	0.274	0.2822	0.9132	0
6043	495.8	1.5753	0.274	0.2822	0.9132	0
6044	495.7	1.575	0.2741	0.2823	0.9134	0
6045	495.6	1.5735	0.274	0.2823	0.913	0
6046	495.5	1.5753	0.274	0.2822	0.9134	0
6047	495.4	1.5738	0.274	0.2824	0.9132	0
6048	495.3	1.5757	0.2741	0.2825	0.9136	0
6049	495.2	1.5754	0.274	0.2826	0.914	0

6050	495.1	1.575	0.2741	0.2827	0.9141	0
6051	495	1.577	0.2742	0.2826	0.9143	0
6052	494.9	1.5743	0.2742	0.2829	0.9142	0
6053	494.8	1.5762	0.2742	0.2827	0.9143	0
6054	494.7	1.5759	0.2745	0.2828	0.9143	0
6055	494.6	1.5766	0.2744	0.2829	0.9146	0
6056	494.5	1.5763	0.2745	0.283	0.9145	0
6057	494.4	1.5759	0.2746	0.283	0.9146	0
6058	494.3	1.5767	0.2746	0.283	0.915	0
6059	494.2	1.5764	0.2749	0.283	0.9152	0
6060	494.1	1.5762	0.275	0.2835	0.9147	0
6061	494	1.5781	0.275	0.2834	0.9156	0
6062	493.9	1.5775	0.2748	0.2832	0.915	0
6063	493.8	1.5773	0.275	0.2833	0.9155	0
6064	493.7	1.577	0.2752	0.2835	0.9157	0
6065	493.6	1.5778	0.2751	0.2835	0.9156	0
6066	493.5	1.5774	0.2752	0.2837	0.916	0
6067	493.4	1.5771	0.2754	0.2837	0.916	0
6068	493.3	1.5778	0.2752	0.2837	0.9159	0
6069	493.2	1.5785	0.2752	0.2839	0.9161	0
6070	493.1	1.5782	0.2754	0.2839	0.9163	0
6071	493	1.5779	0.2751	0.2839	0.9162	0
6072	492.9	1.5798	0.2754	0.2838	0.9163	0
6073	492.8	1.5772	0.2755	0.2839	0.9168	0
6074	492.7	1.5778	0.2752	0.2838	0.9167	0
6075	492.6	1.5776	0.2752	0.2839	0.9165	0
6076	492.5	1.5783	0.2754	0.2839	0.9166	0
6077	492.4	1.5781	0.2755	0.2841	0.9171	0
6078	492.3	1.579	0.2756	0.2842	0.9171	0
6079	492.2	1.5774	0.2755	0.2841	0.9166	0
6080	492.1	1.5782	0.2756	0.2841	0.9173	0
6081	492	1.579	0.2755	0.2842	0.9172	0
6082	491.9	1.5786	0.2758	0.284	0.9179	0
6083	491.8	1.5783	0.2756	0.2839	0.9173	0
6084	491.7	1.5791	0.2756	0.2841	0.9172	0
6085	491.6	1.5776	0.2755	0.2842	0.9176	0
6086	491.5	1.5772	0.2755	0.2842	0.9174	0
6087	491.4	1.5792	0.2755	0.2839	0.9176	0
6088	491.3	1.5778	0.2757	0.2841	0.9177	0
6089	491.2	1.5786	0.2755	0.2841	0.918	0
6090	491.1	1.5794	0.2755	0.284	0.9179	0
6091	491	1.5778	0.2753	0.2841	0.918	0
6092	490.9	1.5776	0.2756	0.2842	0.9181	0

6093	490.8	1.5782	0.2755	0.284	0.9178	0
6094	490.7	1.5792	0.2755	0.284	0.9181	0
6095	490.6	1.5788	0.2755	0.284	0.9182	0
6096	490.5	1.5808	0.2753	0.284	0.9181	0
6097	490.4	1.5804	0.2754	0.284	0.918	0
6098	490.3	1.5799	0.2752	0.2838	0.918	0
6099	490.2	1.5797	0.2751	0.284	0.9177	0
6100	490.1	1.5793	0.2752	0.2839	0.9184	0
6101	490	1.5778	0.2753	0.2836	0.9186	0
6102	489.9	1.5786	0.2751	0.2834	0.9188	0
6103	489.8	1.5782	0.275	0.2833	0.9188	0
6104	489.7	1.579	0.275	0.2836	0.9187	0
6105	489.6	1.5787	0.275	0.2834	0.9187	0
6106	489.5	1.5772	0.2751	0.2835	0.9186	0
6107	489.4	1.5792	0.2749	0.2833	0.9185	0
6108	489.3	1.5788	0.2748	0.2833	0.9186	0
6109	489.2	1.5773	0.2747	0.2833	0.9186	0
6110	489.1	1.5792	0.2748	0.2831	0.9189	0
6111	489	1.5788	0.2748	0.2831	0.919	0
6112	488.9	1.5784	0.2746	0.2831	0.9186	0
6113	488.8	1.5793	0.2746	0.283	0.9188	0
6114	488.7	1.5789	0.2745	0.2829	0.919	0
6115	488.6	1.5785	0.2744	0.2827	0.9188	0
6116	488.5	1.5793	0.2743	0.2828	0.9187	0
6117	488.4	1.5778	0.2743	0.2827	0.9189	0
6118	488.3	1.5775	0.2744	0.2827	0.9194	0
6119	488.2	1.5769	0.2742	0.2825	0.9194	0
6120	488.1	1.5767	0.2743	0.2826	0.9196	0
6121	488	1.5774	0.2744	0.2825	0.9195	0
6122	487.9	1.5784	0.2743	0.2824	0.9192	0
6123	487.8	1.5779	0.274	0.2824	0.919	0
6124	487.7	1.5788	0.274	0.2824	0.9197	0
6125	487.6	1.5784	0.2741	0.2824	0.9196	0
6126	487.5	1.5792	0.2742	0.2824	0.9198	0
6127	487.4	1.5776	0.2741	0.2822	0.9193	0
6128	487.3	1.5773	0.274	0.2822	0.9198	0
6129	487.2	1.5781	0.274	0.2822	0.9199	0
6130	487.1	1.5776	0.2739	0.2822	0.9203	0
6131	487	1.5785	0.2742	0.2822	0.9202	0
6132	486.9	1.5781	0.2742	0.2822	0.9206	0
6133	486.8	1.5778	0.2742	0.2823	0.9203	0
6134	486.7	1.5774	0.2741	0.2823	0.9202	0
6135	486.6	1.5782	0.2738	0.2821	0.9205	0

6136	486.5	1.5778	0.2739	0.2822	0.9201	0
6137	486.4	1.5774	0.2739	0.2822	0.9203	0
6138	486.3	1.577	0.2738	0.2825	0.9202	0
6139	486.2	1.5779	0.2739	0.2823	0.9201	0
6140	486.1	1.5787	0.274	0.2822	0.9202	0
6141	486	1.5772	0.274	0.2824	0.9205	0
6142	485.9	1.5792	0.2739	0.2822	0.9208	0
6143	485.8	1.5788	0.2737	0.2821	0.9207	0
6144	485.7	1.5784	0.2739	0.2822	0.9209	0
6145	485.6	1.5781	0.2741	0.2823	0.9211	0
6146	485.5	1.5777	0.2741	0.2826	0.9213	0
6147	485.4	1.5796	0.2742	0.2823	0.921	0
6148	485.3	1.5782	0.2744	0.2825	0.9213	0
6149	485.2	1.579	0.2744	0.2827	0.9215	0
6150	485.1	1.5786	0.2744	0.2828	0.9213	0
6151	485	1.5795	0.2746	0.2827	0.9218	0
6152	484.9	1.5792	0.2747	0.2831	0.9218	0
6153	484.8	1.5799	0.2747	0.2828	0.9215	0
6154	484.7	1.5784	0.2747	0.2831	0.9218	0
6155	484.6	1.5794	0.2749	0.2831	0.9223	0
6156	484.5	1.5801	0.2748	0.2831	0.9221	0
6157	484.4	1.5798	0.2748	0.2831	0.9223	0
6158	484.3	1.5807	0.275	0.2833	0.9231	0
6159	484.2	1.5804	0.275	0.2837	0.9228	0
6160	484.1	1.5799	0.2752	0.2834	0.9229	0
6161	484	1.5796	0.2753	0.2835	0.9231	0
6162	483.9	1.5817	0.275	0.2833	0.923	0
6163	483.8	1.5814	0.2753	0.2837	0.9235	0
6164	483.7	1.5812	0.2755	0.284	0.9232	0
6165	483.6	1.5807	0.2757	0.2839	0.9233	0
6166	483.5	1.5805	0.2756	0.2841	0.9234	0
6167	483.4	1.5814	0.2758	0.2841	0.9241	0
6168	483.3	1.5822	0.2758	0.284	0.9237	0
6169	483.2	1.5818	0.2758	0.2843	0.9241	0
6170	483.1	1.5815	0.2759	0.2842	0.9241	0
6171	483	1.5824	0.2758	0.2843	0.924	0
6172	482.9	1.5819	0.2759	0.2841	0.9241	0
6173	482.8	1.5828	0.276	0.2844	0.9246	0
6174	482.7	1.5813	0.2761	0.2845	0.9245	0
6175	482.6	1.581	0.2761	0.2846	0.9248	0
6176	482.5	1.5831	0.2761	0.2843	0.925	0
6177	482.4	1.5814	0.2761	0.2843	0.9251	0
6178	482.3	1.5835	0.2762	0.2846	0.925	0

6179	482.2	1.5819	0.2761	0.2845	0.9252	0
6180	482.1	1.5816	0.2761	0.2847	0.9251	0
6181	482	1.5825	0.2762	0.2846	0.9259	0
6182	481.9	1.582	0.2762	0.2844	0.9254	0
6183	481.8	1.5843	0.2763	0.2845	0.9254	0
6184	481.7	1.5825	0.2763	0.2847	0.9255	0
6185	481.6	1.5847	0.2763	0.2847	0.9254	0
6186	481.5	1.583	0.2763	0.2845	0.9259	0
6187	481.4	1.5826	0.2762	0.2846	0.9257	0
6188	481.3	1.5835	0.2764	0.285	0.9256	0
6189	481.2	1.5833	0.2766	0.2849	0.9262	0
6190	481.1	1.5855	0.2764	0.2847	0.9255	0
6191	481	1.5837	0.2764	0.2846	0.9254	0
6192	480.9	1.5834	0.2765	0.2845	0.9262	0
6193	480.8	1.5831	0.2765	0.2846	0.9264	0
6194	480.7	1.5839	0.2763	0.2844	0.926	0
6195	480.6	1.5847	0.2764	0.2846	0.9261	0
6196	480.5	1.5831	0.2763	0.2845	0.9266	0
6197	480.4	1.584	0.2765	0.2846	0.9268	0
6198	480.3	1.5848	0.2763	0.2845	0.9266	0
6199	480.2	1.5844	0.2762	0.2844	0.927	0
6200	480.1	1.5842	0.2763	0.2846	0.9273	0
6201	480	1.5837	0.2762	0.2845	0.9269	0
6202	479.9	1.5833	0.2762	0.2843	0.927	0
6203	479.8	1.583	0.2762	0.2845	0.9273	0
6204	479.7	1.5838	0.2762	0.2842	0.9268	0
6205	479.6	1.5823	0.2765	0.2843	0.9275	0
6206	479.5	1.5845	0.2763	0.2842	0.9273	0
6207	479.4	1.584	0.276	0.2842	0.9266	0
6208	479.3	1.5835	0.2759	0.2841	0.9269	0
6209	479.2	1.5832	0.2761	0.2842	0.9273	0
6210	479.1	1.5827	0.2759	0.2838	0.9271	0
6211	479	1.5849	0.2757	0.2838	0.9274	0
6212	478.9	1.5832	0.2759	0.2839	0.9268	0
6213	478.8	1.5843	0.2759	0.284	0.9274	0
6214	478.7	1.5824	0.2759	0.2838	0.9274	0
6215	478.6	1.5832	0.2757	0.2835	0.927	0
6216	478.5	1.5829	0.2757	0.2836	0.9278	0
6217	478.4	1.5825	0.2756	0.2834	0.9274	0
6218	478.3	1.5834	0.2754	0.2835	0.9273	0
6219	478.2	1.5818	0.2756	0.2834	0.9275	0
6220	478.1	1.5827	0.2754	0.2835	0.9277	0
6221	478	1.5822	0.2754	0.2832	0.9276	0

6222	477.9	1.5831	0.275	0.2832	0.9273	0
6223	477.8	1.5827	0.2752	0.2831	0.927	0
6224	477.7	1.5824	0.2751	0.2833	0.9279	0
6225	477.6	1.5819	0.2751	0.2832	0.9277	0
6226	477.5	1.5815	0.2749	0.2831	0.9273	0
6227	477.4	1.5825	0.2749	0.283	0.9275	0
6228	477.3	1.5808	0.2748	0.2828	0.928	0
6229	477.2	1.5816	0.2749	0.2828	0.9278	0
6230	477.1	1.5813	0.2749	0.2828	0.9277	0
6231	477	1.5835	0.2748	0.2828	0.9279	0
6232	476.9	1.5816	0.2746	0.2827	0.9274	0
6233	476.8	1.5827	0.2746	0.2829	0.9277	0
6234	476.7	1.5811	0.2747	0.283	0.9278	0
6235	476.6	1.5819	0.2746	0.2826	0.9284	0
6236	476.5	1.5829	0.2747	0.2828	0.9287	0
6237	476.4	1.5826	0.2746	0.2827	0.9283	0
6238	476.3	1.5795	0.2746	0.2827	0.9286	0
6239	476.2	1.5818	0.2747	0.2828	0.9288	0
6240	476.1	1.5828	0.2748	0.2827	0.9284	0
6241	476	1.5824	0.2747	0.2827	0.9286	0
6242	475.9	1.5832	0.2745	0.2827	0.929	0
6243	475.8	1.5843	0.2748	0.283	0.9286	0
6244	475.7	1.5825	0.2749	0.2829	0.9289	0
6245	475.6	1.5833	0.2747	0.2828	0.9289	0
6246	475.5	1.5816	0.2747	0.2829	0.9294	0
6247	475.4	1.5839	0.2747	0.2829	0.929	0
6248	475.3	1.5835	0.2747	0.2828	0.9293	0
6249	475.2	1.5831	0.2747	0.2829	0.9297	0
6250	475.1	1.5826	0.2748	0.2828	0.9298	0
6251	475	1.5836	0.2749	0.2829	0.9298	0
6252	474.9	1.5846	0.2747	0.2829	0.9294	0
6253	474.8	1.5828	0.2748	0.283	0.9296	0
6254	474.7	1.5824	0.275	0.283	0.9298	0
6255	474.6	1.5819	0.2749	0.2827	0.9299	0
6256	474.5	1.5829	0.2749	0.283	0.9298	0
6257	474.4	1.5839	0.2751	0.2832	0.93	0
6258	474.3	1.5848	0.2749	0.283	0.9305	0
6259	474.2	1.5843	0.275	0.2832	0.9304	0
6260	474.1	1.584	0.2754	0.2836	0.9307	0
6261	474	1.585	0.2754	0.2834	0.9309	0
6262	473.9	1.5859	0.2752	0.2834	0.9307	0
6263	473.8	1.5841	0.2753	0.2836	0.9309	0
6264	473.7	1.5837	0.2755	0.2837	0.9314	0

6265	473.6	1.5861	0.2756	0.2837	0.9313	0
6266	473.5	1.5841	0.2755	0.2837	0.9317	0
6267	473.4	1.5836	0.2755	0.2836	0.9318	0
6268	473.3	1.5847	0.2757	0.2838	0.9318	0
6269	473.2	1.5843	0.2758	0.284	0.9314	0
6270	473.1	1.5852	0.2758	0.2839	0.9318	0
6271	473	1.5848	0.276	0.2841	0.9321	0
6272	472.9	1.5858	0.276	0.2841	0.9316	0
6273	472.8	1.5854	0.2763	0.2844	0.9322	0
6274	472.7	1.5848	0.2762	0.284	0.9326	0
6275	472.6	1.5859	0.2762	0.2841	0.9331	0
6276	472.5	1.5868	0.2761	0.2842	0.9326	0
6277	472.4	1.5878	0.2763	0.2844	0.9325	0
6278	472.3	1.5844	0.2764	0.2844	0.9329	0
6279	472.2	1.584	0.2763	0.2845	0.9331	0
6280	472.1	1.5848	0.2764	0.2844	0.9328	0
6281	472	1.5858	0.2763	0.2844	0.9328	0
6282	471.9	1.5854	0.2764	0.2844	0.933	0
6283	471.8	1.5864	0.2765	0.2847	0.9329	0
6284	471.7	1.5873	0.2764	0.2844	0.9333	0
6285	471.6	1.5869	0.2766	0.2845	0.9335	0
6286	471.5	1.585	0.2764	0.2844	0.934	0
6287	471.4	1.586	0.2766	0.2846	0.9339	0
6288	471.3	1.5869	0.2765	0.2843	0.9334	0
6289	471.2	1.5877	0.2764	0.2841	0.9337	0
6290	471.1	1.5873	0.2764	0.2842	0.9336	0
6291	471	1.5869	0.2764	0.2844	0.9335	0
6292	470.9	1.5865	0.2766	0.2847	0.9345	0
6293	470.8	1.5874	0.2765	0.2845	0.9341	0
6294	470.7	1.5871	0.2767	0.2846	0.9342	0
6295	470.6	1.5881	0.2767	0.2847	0.9347	0
6296	470.5	1.5876	0.2767	0.2847	0.9349	0
6297	470.4	1.587	0.2766	0.2848	0.9346	0
6298	470.3	1.5879	0.2766	0.2847	0.9344	0
6299	470.2	1.5875	0.2766	0.2846	0.935	0
6300	470.1	1.5872	0.2767	0.2847	0.9349	0
6301	470	1.588	0.2766	0.2847	0.9353	0
6302	469.9	1.589	0.2765	0.2847	0.9352	0
6303	469.8	1.5871	0.2764	0.2845	0.9353	0
6304	469.7	1.5881	0.2765	0.2846	0.9352	0
6305	469.6	1.589	0.2766	0.2844	0.935	0
6306	469.5	1.587	0.2763	0.2845	0.9351	0
6307	469.4	1.5879	0.2763	0.2844	0.9352	0

6308	469.3	1.5889	0.2765	0.2845	0.9347	0
6309	469.2	1.5885	0.2766	0.2846	0.9353	0
6310	469.1	1.588	0.2764	0.2845	0.9357	0
6311	469	1.5874	0.2764	0.2845	0.9355	0
6312	468.9	1.5871	0.2766	0.2847	0.9362	0
6313	468.8	1.5879	0.2765	0.2845	0.9359	0
6314	468.7	1.589	0.2765	0.2846	0.9358	0
6315	468.6	1.5869	0.2763	0.2845	0.9351	0
6316	468.5	1.5879	0.2762	0.2844	0.9361	0
6317	468.4	1.5874	0.2762	0.284	0.9361	0
6318	468.3	1.587	0.2762	0.2842	0.936	0
6319	468.2	1.5881	0.2763	0.2845	0.9364	0
6320	468.1	1.5859	0.2763	0.2844	0.9357	0
6321	468	1.5884	0.2762	0.2844	0.9362	0
6322	467.9	1.588	0.2764	0.2842	0.9364	0
6323	467.8	1.586	0.2761	0.2841	0.9362	0
6324	467.7	1.5885	0.276	0.2842	0.9364	0
6325	467.6	1.5879	0.2758	0.2841	0.9365	0
6326	467.5	1.586	0.2758	0.2841	0.9364	0
6327	467.4	1.5885	0.2759	0.2839	0.9366	0
6328	467.3	1.5879	0.2759	0.2838	0.9367	0
6329	467.2	1.5861	0.276	0.284	0.9367	0
6330	467.1	1.5871	0.276	0.2839	0.9368	0
6331	467	1.5865	0.2758	0.284	0.9363	0
6332	466.9	1.5874	0.2757	0.2839	0.9371	0
6333	466.8	1.5869	0.2756	0.2837	0.9366	0
6334	466.7	1.5865	0.2757	0.284	0.9368	0
6335	466.6	1.5875	0.2757	0.2837	0.937	0
6336	466.5	1.587	0.2757	0.2835	0.9368	0
6337	466.4	1.5865	0.2757	0.2835	0.9366	0
6338	466.3	1.5859	0.2756	0.2836	0.9368	0
6339	466.2	1.5871	0.2757	0.2838	0.9374	0
6340	466.1	1.5868	0.2756	0.2839	0.9371	0
6341	466	1.5878	0.2758	0.2838	0.9373	0
6342	465.9	1.5858	0.2757	0.2839	0.9375	0
6343	465.8	1.5867	0.2755	0.2835	0.9372	0
6344	465.7	1.5877	0.2757	0.2835	0.9377	0
6345	465.6	1.5857	0.2756	0.2836	0.9372	0
6346	465.5	1.5866	0.2757	0.2837	0.9377	0
6347	465.4	1.5877	0.2754	0.2836	0.9375	0
6348	465.3	1.5856	0.2753	0.2834	0.938	0
6349	465.2	1.5866	0.2754	0.2836	0.9378	0
6350	465.1	1.5862	0.2757	0.2834	0.9381	0

6351	465	1.5872	0.2757	0.2836	0.9383	0
6352	464.9	1.5882	0.2758	0.2833	0.9384	0
6353	464.8	1.5877	0.2756	0.2834	0.9383	0
6354	464.7	1.5872	0.2757	0.2836	0.9388	0
6355	464.6	1.5882	0.2756	0.2834	0.9389	0
6356	464.5	1.5862	0.2759	0.2838	0.9388	0
6357	464.4	1.5887	0.2758	0.2836	0.9386	0
6358	464.3	1.5881	0.2758	0.2836	0.9387	0
6359	464.2	1.5876	0.2758	0.2838	0.9389	0
6360	464.1	1.5871	0.2761	0.2839	0.9391	0
6361	464	1.5881	0.2761	0.284	0.9385	0
6362	463.9	1.5874	0.276	0.2837	0.9392	0
6363	463.8	1.5854	0.2761	0.2839	0.9391	0
6364	463.7	1.5879	0.2761	0.2839	0.9392	0
6365	463.6	1.5889	0.2758	0.284	0.9397	0
6366	463.5	1.5884	0.276	0.284	0.9392	0
6367	463.4	1.5893	0.2762	0.284	0.9399	0
6368	463.3	1.5888	0.2761	0.2841	0.9402	0
6369	463.2	1.5881	0.276	0.2841	0.9405	0
6370	463.1	1.5877	0.2764	0.2842	0.9401	0
6371	463	1.5886	0.2763	0.2843	0.9402	0
6372	462.9	1.5912	0.2763	0.2844	0.941	0
6373	462.8	1.5874	0.2763	0.2843	0.9404	0
6374	462.7	1.5884	0.2764	0.2841	0.9405	0
6375	462.6	1.5895	0.2765	0.2842	0.9411	0
6376	462.5	1.5888	0.2766	0.2841	0.9411	0
6377	462.4	1.5897	0.2763	0.2843	0.9412	0
6378	462.3	1.5892	0.2763	0.2844	0.941	0
6379	462.2	1.5887	0.2765	0.2845	0.9408	0
6380	462.1	1.5913	0.2766	0.2843	0.9413	0
6381	462	1.5889	0.2766	0.2843	0.9409	0
6382	461.9	1.5899	0.2766	0.2845	0.941	0
6383	461.8	1.591	0.2768	0.2845	0.9416	0
6384	461.7	1.5902	0.2769	0.2843	0.9419	0
6385	461.6	1.5896	0.2768	0.2843	0.9416	0
6386	461.5	1.5923	0.2768	0.2844	0.9414	0
6387	461.4	1.5883	0.2767	0.2842	0.9414	0
6388	461.3	1.5909	0.2766	0.2842	0.9418	0
6389	461.2	1.5885	0.2765	0.2843	0.9418	0
6390	461.1	1.5897	0.2768	0.2844	0.9421	0
6391	461	1.5906	0.2766	0.2845	0.9422	0
6392	460.9	1.5917	0.2767	0.2844	0.9423	0
6393	460.8	1.591	0.2766	0.2847	0.9424	0

6394	460.7	1.5904	0.2765	0.2847	0.9425	0
6395	460.6	1.5913	0.2769	0.2846	0.9429	0
6396	460.5	1.5906	0.2767	0.2843	0.9429	0
6397	460.4	1.5898	0.2766	0.2841	0.9428	0
6398	460.3	1.5892	0.2767	0.2844	0.9427	0
6399	460.2	1.5902	0.2766	0.2845	0.9427	0
6400	460.1	1.5896	0.2766	0.2847	0.9428	0
6401	460	1.5889	0.2767	0.2844	0.9433	0
6402	459.9	1.5897	0.2767	0.2845	0.9432	0
6403	459.8	1.5909	0.2768	0.2846	0.9434	0
6404	459.7	1.5901	0.2767	0.2847	0.9434	0
6405	459.6	1.5895	0.2768	0.2849	0.9436	0
6406	459.5	1.5904	0.2769	0.2847	0.9436	0
6407	459.4	1.5896	0.2765	0.2845	0.9438	0
6408	459.3	1.5905	0.2766	0.2845	0.9443	0
6409	459.2	1.5916	0.2768	0.2847	0.9441	0
6410	459.1	1.5927	0.2768	0.2848	0.9438	0
6411	459	1.5919	0.2767	0.2848	0.9438	0
6412	458.9	1.5928	0.2766	0.2847	0.9438	0
6413	458.8	1.5904	0.2767	0.2847	0.9438	0
6414	458.7	1.5897	0.2766	0.2847	0.9442	0
6415	458.6	1.5894	0.2767	0.2849	0.9441	0
6416	458.5	1.5908	0.2766	0.2849	0.9446	0
6417	458.4	1.59	0.2767	0.2848	0.9442	0
6418	458.3	1.5903	0.2767	0.2848	0.9445	0
6419	458.2	1.5897	0.2766	0.2847	0.9445	0
6420	458.1	1.5909	0.2766	0.2846	0.9449	0
6421	458	1.5903	0.2766	0.2845	0.9447	0
6422	457.9	1.5905	0.2765	0.2844	0.9444	0
6423	457.8	1.5907	0.2765	0.2842	0.9442	0
6424	457.7	1.59	0.2764	0.2844	0.9449	0
6425	457.6	1.5905	0.2766	0.2847	0.945	0
6426	457.5	1.5918	0.2766	0.2846	0.9452	0
6427	457.4	1.5911	0.2765	0.2845	0.9452	0
6428	457.3	1.5903	0.2768	0.2844	0.9449	0
6429	457.2	1.5896	0.2766	0.2845	0.9449	0
6430	457.1	1.5908	0.2765	0.2845	0.9455	0
6431	457	1.5912	0.2765	0.2843	0.9455	0
6432	456.9	1.5904	0.2765	0.2843	0.9455	0
6433	456.8	1.5896	0.2766	0.2843	0.9454	0
6434	456.7	1.59	0.2766	0.2843	0.9451	0
6435	456.6	1.5905	0.2768	0.2845	0.9453	0
6436	456.5	1.5897	0.2767	0.2847	0.9458	0

6437	456.4	1.5911	0.2766	0.2843	0.9457	0
6438	456.3	1.5914	0.2766	0.2844	0.9462	0
6439	456.2	1.5908	0.2766	0.2846	0.9463	0
6440	456.1	1.591	0.2766	0.2844	0.9459	0
6441	456	1.5914	0.2767	0.2846	0.9465	0
6442	455.9	1.5927	0.2765	0.2845	0.9463	0
6443	455.8	1.5919	0.2765	0.2846	0.9465	0
6444	455.7	1.5912	0.2766	0.2846	0.9463	0
6445	455.6	1.5914	0.2764	0.2844	0.9464	0
6446	455.5	1.5921	0.2767	0.2847	0.9467	0
6447	455.4	1.5913	0.2768	0.2846	0.9467	0
6448	455.3	1.5916	0.2766	0.2845	0.9471	0
6449	455.2	1.5919	0.2766	0.2844	0.9471	0
6450	455.1	1.5913	0.2767	0.2846	0.9472	0
6451	455	1.5916	0.2768	0.2842	0.9471	0
6452	454.9	1.592	0.2768	0.2845	0.9469	0
6453	454.8	1.5912	0.2768	0.2846	0.9474	0
6454	454.7	1.5904	0.2768	0.2845	0.9474	0
6455	454.6	1.5918	0.2767	0.2841	0.9476	0
6456	454.5	1.5922	0.2769	0.2843	0.9479	0
6457	454.4	1.5926	0.2769	0.2844	0.9474	0
6458	454.3	1.5929	0.2768	0.2844	0.9479	0
6459	454.2	1.5922	0.2767	0.2845	0.9479	0
6460	454.1	1.5915	0.2769	0.2844	0.9482	0
6461	454	1.5918	0.2769	0.2844	0.9481	0
6462	453.9	1.591	0.2769	0.2844	0.9484	0
6463	453.8	1.5926	0.2771	0.2845	0.9482	0
6464	453.7	1.5918	0.277	0.2845	0.9482	0
6465	453.6	1.5921	0.277	0.2846	0.9484	0
6466	453.5	1.5913	0.2771	0.2844	0.9486	0
6467	453.4	1.5917	0.2772	0.2845	0.9487	0
6468	453.3	1.592	0.2771	0.2845	0.9489	0
6469	453.2	1.5935	0.2772	0.2847	0.9488	0
6470	453.1	1.5927	0.2774	0.2847	0.9491	0
6471	453	1.592	0.2772	0.2847	0.9491	0
6472	452.9	1.5911	0.2772	0.2848	0.9493	0
6473	452.8	1.5926	0.2769	0.2847	0.9493	0
6474	452.7	1.5919	0.2773	0.2846	0.9497	0
6475	452.6	1.5922	0.2775	0.2843	0.9496	0
6476	452.5	1.5915	0.2777	0.2849	0.9494	0
6477	452.4	1.5929	0.2775	0.2847	0.9501	0
6478	452.3	1.5933	0.2776	0.285	0.9499	0
6479	452.2	1.5936	0.2773	0.2849	0.9497	0

6480	452.1	1.5928	0.2773	0.2849	0.95	0
6481	452	1.5943	0.2774	0.2847	0.9502	0
6482	451.9	1.5935	0.2774	0.285	0.9502	0
6483	451.8	1.5938	0.2773	0.285	0.9505	0
6484	451.7	1.5929	0.2773	0.2848	0.9504	0
6485	451.6	1.5944	0.2773	0.2848	0.9504	0
6486	451.5	1.5937	0.2775	0.2848	0.9506	0
6487	451.4	1.5929	0.2774	0.2848	0.9509	0
6488	451.3	1.5945	0.2775	0.285	0.9507	0
6489	451.2	1.5936	0.2774	0.2851	0.9509	0
6490	451.1	1.5927	0.2774	0.285	0.9508	0
6491	451	1.5942	0.2772	0.285	0.9513	0
6492	450.9	1.5933	0.2772	0.285	0.9511	0
6493	450.8	1.5935	0.2771	0.2846	0.9513	0
6494	450.7	1.595	0.2774	0.2847	0.9515	0
6495	450.6	1.5919	0.2772	0.2847	0.9516	0
6496	450.5	1.5946	0.2769	0.2846	0.9518	0
6497	450.4	1.5925	0.2773	0.2844	0.9514	0
6498	450.3	1.5952	0.277	0.2845	0.9516	0
6499	450.2	1.5931	0.2772	0.2845	0.951	0
6500	450.1	1.5947	0.2774	0.2847	0.9516	0
6501	450	1.5938	0.2773	0.2846	0.9515	0
6502	449.9	1.5348	0.2591	0.2664	0.9186	0
6503	449.8	1.5343	0.259	0.2664	0.9189	0
6504	449.7	1.5345	0.2588	0.2663	0.9189	0
6505	449.6	1.5348	0.2588	0.2664	0.919	0
6506	449.5	1.535	0.2589	0.2665	0.9191	0
6507	449.4	1.5345	0.2589	0.2663	0.9192	0
6508	449.3	1.5348	0.2591	0.2665	0.9194	0
6509	449.2	1.5352	0.259	0.2666	0.9195	0
6510	449.1	1.5335	0.259	0.2664	0.9192	0
6511	449	1.5347	0.259	0.2664	0.9194	0
6512	448.9	1.5349	0.259	0.2663	0.9198	0
6513	448.8	1.5343	0.2589	0.2665	0.9197	0
6514	448.7	1.5346	0.2589	0.2665	0.92	0
6515	448.6	1.534	0.2588	0.2664	0.9198	0
6516	448.5	1.5342	0.2588	0.2666	0.9198	0
6517	448.4	1.5353	0.259	0.2664	0.9205	0
6518	448.3	1.5354	0.2588	0.2662	0.9199	0
6519	448.2	1.5347	0.2588	0.2662	0.9198	0
6520	448.1	1.534	0.2587	0.2662	0.9202	0
6521	448	1.5344	0.2587	0.2664	0.9208	0
6522	447.9	1.5346	0.2589	0.2663	0.9204	0

6523	447.8	1.5348	0.2588	0.2664	0.9206	0
6524	447.7	1.5342	0.259	0.2667	0.9209	0
6525	447.6	1.535	0.2588	0.2664	0.9209	0
6526	447.5	1.5353	0.2588	0.2663	0.9208	0
6527	447.4	1.5362	0.2588	0.2665	0.9211	0
6528	447.3	1.5362	0.2587	0.2662	0.9212	0
6529	447.2	1.5365	0.2588	0.2664	0.921	0
6530	447.1	1.5342	0.2589	0.2665	0.9214	0
6531	447	1.5361	0.2591	0.2665	0.9215	0
6532	446.9	1.5352	0.2592	0.2664	0.9213	0
6533	446.8	1.5354	0.259	0.2665	0.9215	0
6534	446.7	1.5348	0.2591	0.2666	0.9216	0
6535	446.6	1.5365	0.2591	0.2667	0.9216	0
6536	446.5	1.5358	0.2591	0.2667	0.922	0
6537	446.4	1.5358	0.2592	0.2667	0.922	0
6538	446.3	1.5359	0.259	0.2667	0.9223	0
6539	446.2	1.5367	0.259	0.2667	0.9223	0
6540	446.1	1.5362	0.2594	0.2669	0.9226	0
6541	446	1.5369	0.2592	0.2668	0.9223	0
6542	445.9	1.5362	0.2592	0.2667	0.9226	0
6543	445.8	1.5361	0.2592	0.2665	0.9224	0
6544	445.7	1.537	0.2592	0.2668	0.9226	0
6545	445.6	1.537	0.2592	0.2666	0.9229	0
6546	445.5	1.5363	0.2593	0.267	0.9229	0
6547	445.4	1.5363	0.2594	0.2669	0.9229	0
6548	445.3	1.5364	0.2594	0.267	0.923	0
6549	445.2	1.5363	0.2592	0.2669	0.9237	0
6550	445.1	1.5356	0.2595	0.2672	0.9233	0
6551	445	1.5372	0.2595	0.2672	0.924	0
6552	444.9	1.538	0.2596	0.2674	0.9243	0
6553	444.8	1.5379	0.2595	0.2673	0.924	0
6554	444.7	1.5372	0.2595	0.2673	0.9237	0
6555	444.6	1.5372	0.2596	0.2674	0.9239	0
6556	444.5	1.5378	0.2594	0.2673	0.9242	0
6557	444.4	1.5378	0.2595	0.2675	0.9245	0
6558	444.3	1.5385	0.2596	0.2673	0.9245	0
6559	444.2	1.5385	0.2595	0.2672	0.9246	0
6560	444.1	1.5385	0.2597	0.2675	0.9249	0
6561	444	1.5392	0.2597	0.2675	0.9249	0
6562	443.9	1.5384	0.2598	0.2676	0.925	0
6563	443.8	1.5382	0.2599	0.2676	0.9249	0
6564	443.7	1.5397	0.2599	0.2676	0.925	0
6565	443.6	1.5389	0.2601	0.2677	0.9254	0

6566	443.5	1.5388	0.26	0.2679	0.9258	0
6567	443.4	1.5393	0.2599	0.2678	0.9256	0
6568	443.3	1.5391	0.26	0.2677	0.9258	0
6569	443.2	1.539	0.2601	0.2678	0.9257	0
6570	443.1	1.538	0.2599	0.2677	0.9256	0
6571	443	1.5394	0.2602	0.268	0.926	0
6572	442.9	1.5392	0.2602	0.2679	0.9262	0
6573	442.8	1.5391	0.2603	0.2679	0.9263	0
6574	442.7	1.5389	0.2602	0.2678	0.9265	0
6575	442.6	1.5394	0.2601	0.2679	0.9262	0
6576	442.5	1.5394	0.2605	0.268	0.9267	0
6577	442.4	1.5392	0.2604	0.2681	0.9267	0
6578	442.3	1.5389	0.2602	0.2679	0.9268	0
6579	442.2	1.5394	0.2601	0.268	0.9273	0
6580	442.1	1.5392	0.26	0.2681	0.9272	0
6581	442	1.5397	0.2602	0.2682	0.9275	0
6582	441.9	1.5395	0.2602	0.2681	0.9275	0
6583	441.8	1.5393	0.2601	0.268	0.9275	0
6584	441.7	1.5397	0.2603	0.2681	0.9274	0
6585	441.6	1.5403	0.2602	0.2681	0.9274	0
6586	441.5	1.5392	0.2603	0.2681	0.9276	0
6587	441.4	1.5405	0.2602	0.2682	0.9278	0
6588	441.3	1.5394	0.2603	0.2681	0.9277	0
6589	441.2	1.5399	0.2603	0.268	0.928	0
6590	441.1	1.5396	0.2602	0.2681	0.9277	0
6591	441	1.54	0.2602	0.2682	0.9279	0
6592	440.9	1.5397	0.2602	0.2681	0.9281	0
6593	440.8	1.5394	0.2602	0.268	0.9283	0
6594	440.7	1.5399	0.2601	0.268	0.9282	0
6595	440.6	1.5404	0.2605	0.268	0.9287	0
6596	440.5	1.54	0.2603	0.268	0.9287	0
6597	440.4	1.5411	0.2602	0.268	0.9283	0
6598	440.3	1.5401	0.2602	0.268	0.929	0
6599	440.2	1.5405	0.2603	0.2679	0.9288	0
6600	440.1	1.5402	0.26	0.2678	0.9287	0
6601	440	1.5405	0.2602	0.2678	0.9289	0
6602	439.9	1.5402	0.2599	0.2677	0.9289	0
6603	439.8	1.5399	0.26	0.2678	0.9289	0
6604	439.7	1.5402	0.2601	0.2677	0.9291	0
6605	439.6	1.5413	0.2599	0.2676	0.9295	0
6606	439.5	1.5402	0.2598	0.2677	0.9297	0
6607	439.4	1.5398	0.2598	0.2677	0.9291	0
6608	439.3	1.5402	0.2598	0.2676	0.9289	0

6609	439.2	1.5399	0.2598	0.2677	0.9298	0
6610	439.1	1.5401	0.2596	0.2676	0.9296	0
6611	439	1.5398	0.2596	0.2674	0.9295	0
6612	438.9	1.5409	0.2596	0.2673	0.9297	0
6613	438.8	1.5399	0.2596	0.2675	0.9299	0
6614	438.7	1.5403	0.2596	0.2673	0.9299	0
6615	438.6	1.5399	0.2595	0.2674	0.9296	0
6616	438.5	1.5395	0.2596	0.2673	0.9297	0
6617	438.4	1.5412	0.2594	0.2673	0.9298	0
6618	438.3	1.5393	0.2594	0.2672	0.9299	0
6619	438.2	1.5405	0.2596	0.2674	0.9302	0
6620	438.1	1.54	0.2594	0.2672	0.9303	0
6621	438	1.5398	0.2595	0.2675	0.9304	0
6622	437.9	1.5406	0.2592	0.2672	0.9305	0
6623	437.8	1.5394	0.2593	0.2671	0.9304	0
6624	437.7	1.5399	0.2593	0.2673	0.9307	0
6625	437.6	1.5401	0.2593	0.2672	0.931	0
6626	437.5	1.5405	0.2594	0.2673	0.9308	0
6627	437.4	1.5401	0.2595	0.2672	0.9307	0
6628	437.3	1.5403	0.2592	0.2671	0.9311	0
6629	437.2	1.5391	0.2592	0.2672	0.9312	0
6630	437.1	1.5396	0.2593	0.2674	0.9314	0
6631	437	1.5405	0.2593	0.2675	0.931	0
6632	436.9	1.5409	0.2593	0.2675	0.9317	0
6633	436.8	1.5404	0.2594	0.2673	0.9316	0
6634	436.7	1.54	0.2594	0.2672	0.9316	0
6635	436.6	1.5417	0.2595	0.2673	0.9317	0
6636	436.5	1.5407	0.2596	0.2673	0.9319	0
6637	436.4	1.5408	0.2594	0.2674	0.9321	0
6638	436.3	1.5403	0.2593	0.2674	0.9322	0
6639	436.2	1.5399	0.2593	0.2675	0.9326	0
6640	436.1	1.5402	0.2594	0.2675	0.9321	0
6641	436	1.5405	0.2596	0.2673	0.9324	0
6642	435.9	1.5408	0.2596	0.2675	0.9329	0
6643	435.8	1.5403	0.2596	0.2676	0.9331	0
6644	435.7	1.542	0.2598	0.2678	0.9329	0
6645	435.6	1.5423	0.2599	0.2676	0.9331	0
6646	435.5	1.541	0.2598	0.2676	0.9328	0
6647	435.4	1.542	0.2596	0.2679	0.9333	0
6648	435.3	1.5416	0.2598	0.2678	0.9332	0
6649	435.2	1.5418	0.2599	0.2678	0.9336	0
6650	435.1	1.5415	0.2601	0.2679	0.9336	0
6651	435	1.5423	0.2599	0.2679	0.9337	0

6652	434.9	1.5418	0.2601	0.2678	0.9336	0
6653	434.8	1.5421	0.2601	0.2678	0.9341	0
6654	434.7	1.5417	0.2602	0.2682	0.9342	0
6655	434.6	1.5427	0.2604	0.2681	0.9343	0
6656	434.5	1.543	0.2606	0.2683	0.9346	0
6657	434.4	1.5432	0.2606	0.2683	0.9348	0
6658	434.3	1.5428	0.2605	0.2685	0.9349	0
6659	434.2	1.5431	0.2608	0.2686	0.9348	0
6660	434.1	1.5432	0.2605	0.2685	0.935	0
6661	434	1.5441	0.2607	0.2686	0.9357	0
6662	433.9	1.5442	0.2605	0.2685	0.9356	0
6663	433.8	1.543	0.2607	0.2686	0.9353	0
6664	433.7	1.5442	0.261	0.269	0.9358	0
6665	433.6	1.5451	0.2611	0.2689	0.9361	0
6666	433.5	1.5444	0.261	0.269	0.9358	0
6667	433.4	1.5444	0.2609	0.2689	0.936	0
6668	433.3	1.5447	0.2609	0.2688	0.9363	0
6669	433.2	1.5442	0.261	0.2689	0.9364	0
6670	433.1	1.5451	0.2612	0.2691	0.9365	0
6671	433	1.5445	0.2611	0.269	0.9368	0
6672	432.9	1.5441	0.2612	0.2692	0.9366	0
6673	432.8	1.545	0.2613	0.2693	0.9368	0
6674	432.7	1.5451	0.2613	0.2692	0.9366	0
6675	432.6	1.5453	0.2613	0.2693	0.9371	0
6676	432.5	1.5461	0.2614	0.2694	0.9372	0
6677	432.4	1.5463	0.2614	0.2694	0.9374	0
6678	432.3	1.5457	0.2612	0.2694	0.9373	0
6679	432.2	1.545	0.2611	0.2692	0.9374	0
6680	432.1	1.5452	0.2612	0.2693	0.9378	0
6681	432	1.5453	0.2613	0.2691	0.9376	0
6682	431.9	1.5455	0.2614	0.2693	0.9379	0
6683	431.8	1.5457	0.2614	0.2693	0.938	0
6684	431.7	1.5457	0.2614	0.2693	0.938	0
6685	431.6	1.5465	0.2613	0.2692	0.9381	0
6686	431.5	1.545	0.2613	0.2691	0.9381	0
6687	431.4	1.546	0.2614	0.2693	0.9379	0
6688	431.3	1.5461	0.2615	0.2693	0.9382	0
6689	431.2	1.5447	0.2616	0.2691	0.9388	0
6690	431.1	1.5455	0.2613	0.2693	0.938	0
6691	431	1.5456	0.2614	0.2694	0.9385	0
6692	430.9	1.5464	0.2614	0.2693	0.9387	0
6693	430.8	1.545	0.2613	0.269	0.9387	0
6694	430.7	1.5465	0.2611	0.2692	0.9386	0

6695	430.6	1.5457	0.2611	0.269	0.9388	0
6696	430.5	1.5465	0.2612	0.2691	0.9388	0
6697	430.4	1.5466	0.2612	0.2691	0.939	0
6698	430.3	1.5451	0.2611	0.2689	0.9389	0
6699	430.2	1.5461	0.2612	0.269	0.9388	0
6700	430.1	1.5462	0.2614	0.2691	0.9394	0
6701	430	1.5468	0.2612	0.269	0.9395	0
6702	429.9	1.5475	0.261	0.2688	0.9395	0
6703	429.8	1.5454	0.2611	0.269	0.9393	0
6704	429.7	1.546	0.2609	0.2689	0.939	0
6705	429.6	1.5466	0.2607	0.2687	0.9393	0
6706	429.5	1.5467	0.2607	0.2688	0.9392	0
6707	429.4	1.5466	0.2605	0.2686	0.9396	0
6708	429.3	1.5454	0.2607	0.2687	0.94	0
6709	429.2	1.5454	0.2608	0.2688	0.9396	0
6710	429.1	1.5454	0.2606	0.2686	0.9401	0
6711	429	1.5459	0.2604	0.2685	0.94	0
6712	428.9	1.546	0.2607	0.2685	0.9401	0
6713	428.8	1.5446	0.2606	0.2684	0.9401	0
6714	428.7	1.5459	0.2606	0.2684	0.94	0
6715	428.6	1.545	0.2602	0.2681	0.9398	0
6716	428.5	1.5436	0.2603	0.2682	0.9401	0
6717	428.4	1.5451	0.2602	0.2684	0.9406	0
6718	428.3	1.5458	0.2601	0.2684	0.9406	0
6719	428.2	1.545	0.2599	0.2682	0.9404	0
6720	428.1	1.545	0.26	0.2681	0.9406	0
6721	428	1.5449	0.26	0.2679	0.9407	0
6722	427.9	1.5447	0.2597	0.2677	0.9405	0
6723	427.8	1.5441	0.2598	0.2679	0.9403	0
6724	427.7	1.5441	0.2599	0.2679	0.9406	0
6725	427.6	1.5454	0.2597	0.2678	0.9403	0
6726	427.5	1.544	0.2597	0.2679	0.9407	0
6727	427.4	1.5447	0.2597	0.2677	0.9407	0
6728	427.3	1.5453	0.2596	0.2677	0.9408	0
6729	427.2	1.546	0.2598	0.2677	0.9415	0
6730	427.1	1.5446	0.2596	0.2678	0.9409	0
6731	427	1.5445	0.2597	0.2679	0.9416	0
6732	426.9	1.5452	0.2597	0.2678	0.9415	0
6733	426.8	1.5444	0.2598	0.2677	0.9416	0
6734	426.7	1.5457	0.2597	0.2678	0.9415	0
6735	426.6	1.5449	0.2598	0.2677	0.9414	0
6736	426.5	1.5449	0.2596	0.2677	0.9416	0
6737	426.4	1.5457	0.2597	0.2679	0.942	0

6738	426.3	1.5456	0.2598	0.2678	0.9416	0
6739	426.2	1.5448	0.2597	0.2677	0.9422	0
6740	426.1	1.5461	0.2596	0.2677	0.9419	0
6741	426	1.5461	0.2597	0.2679	0.9423	0
6742	425.9	1.5459	0.2595	0.2677	0.9423	0
6743	425.8	1.5452	0.2597	0.2678	0.9426	0
6744	425.7	1.5458	0.2596	0.268	0.9425	0
6745	425.6	1.5458	0.2597	0.2681	0.9426	0
6746	425.5	1.5463	0.2597	0.2681	0.9423	0
6747	425.4	1.5463	0.2595	0.268	0.9431	0
6748	425.3	1.5463	0.2599	0.2684	0.9432	0
6749	425.2	1.5455	0.2599	0.2681	0.9433	0
6750	425.1	1.5462	0.2602	0.2682	0.9436	0
6751	425	1.5461	0.2601	0.2681	0.9434	0
6752	424.9	1.5467	0.26	0.2683	0.9439	0
6753	424.8	1.5467	0.26	0.2683	0.9437	0
6754	424.7	1.5466	0.2601	0.2683	0.944	0
6755	424.6	1.5473	0.2602	0.2684	0.9445	0
6756	424.5	1.5471	0.2602	0.2683	0.9445	0
6757	424.4	1.5471	0.2604	0.2685	0.9449	0
6758	424.3	1.5476	0.2605	0.2685	0.9447	0
6759	424.2	1.5476	0.2607	0.2687	0.9452	0
6760	424.1	1.5475	0.2604	0.2688	0.9453	0
6761	424	1.5481	0.2605	0.269	0.9451	0
6762	423.9	1.5487	0.2607	0.2689	0.9452	0
6763	423.8	1.5486	0.2606	0.269	0.9453	0
6764	423.7	1.5478	0.2608	0.269	0.9456	0
6765	423.6	1.5483	0.2607	0.2691	0.9457	0
6766	423.5	1.5496	0.2606	0.269	0.9463	0
6767	423.4	1.5481	0.2608	0.2691	0.9462	0
6768	423.3	1.5494	0.261	0.2694	0.9463	0
6769	423.2	1.5493	0.2609	0.2693	0.9463	0
6770	423.1	1.5483	0.2609	0.2692	0.9468	0
6771	423	1.5497	0.261	0.2694	0.9467	0
6772	422.9	1.5502	0.2611	0.2694	0.9471	0
6773	422.8	1.5493	0.2611	0.2695	0.9465	0
6774	422.7	1.5507	0.2615	0.2699	0.9472	0
6775	422.6	1.5505	0.2614	0.2696	0.9475	0
6776	422.5	1.551	0.2613	0.2698	0.9475	0
6777	422.4	1.5516	0.2615	0.2698	0.9477	0
6778	422.3	1.5499	0.2613	0.2699	0.9475	0
6779	422.2	1.5512	0.2614	0.2699	0.9476	0
6780	422.1	1.5509	0.2614	0.2698	0.9476	0

6781	422	1.5507	0.2614	0.2697	0.9477	0
6782	421.9	1.5504	0.2614	0.2698	0.9484	0
6783	421.8	1.551	0.2614	0.2699	0.948	0
6784	421.7	1.5507	0.2614	0.2699	0.9483	0
6785	421.6	1.5519	0.2612	0.2699	0.9482	0
6786	421.5	1.5509	0.2614	0.2699	0.948	0
6787	421.4	1.5508	0.2614	0.2697	0.9487	0
6788	421.3	1.552	0.2614	0.2699	0.9487	0
6789	421.2	1.5517	0.2615	0.2699	0.9484	0
6790	421.1	1.5515	0.2614	0.2702	0.9489	0
6791	421	1.5512	0.2614	0.2698	0.9491	0
6792	420.9	1.551	0.2614	0.2699	0.9493	0
6793	420.8	1.5522	0.2616	0.2698	0.9494	0
6794	420.7	1.5519	0.2616	0.2698	0.9493	0
6795	420.6	1.5517	0.2615	0.2698	0.9498	0
6796	420.5	1.5522	0.2614	0.2698	0.9497	0
6797	420.4	1.5518	0.2613	0.2696	0.95	0
6798	420.3	1.5516	0.2616	0.2699	0.9499	0
6799	420.2	1.552	0.2614	0.2698	0.9503	0
6800	420.1	1.5518	0.2616	0.2698	0.9503	0
6801	420	1.5522	0.2616	0.2698	0.9504	0
6802	419.9	1.5504	0.2615	0.2699	0.9502	0
6803	419.8	1.5516	0.2614	0.2699	0.9505	0
6804	419.7	1.5506	0.2615	0.2699	0.9506	0
6805	419.6	1.5516	0.2611	0.2697	0.9506	0
6806	419.5	1.5514	0.2612	0.2696	0.9504	0
6807	419.4	1.551	0.2612	0.2695	0.9503	0
6808	419.3	1.5516	0.2612	0.2696	0.9506	0
6809	419.2	1.5512	0.2611	0.2697	0.9506	0
6810	419.1	1.5508	0.2609	0.2695	0.9508	0
6811	419	1.5513	0.2609	0.2696	0.9509	0
6812	418.9	1.5509	0.261	0.2693	0.9509	0
6813	418.8	1.5506	0.2607	0.2693	0.9513	0
6814	418.7	1.5518	0.2608	0.2693	0.9509	0
6815	418.6	1.5522	0.2608	0.2692	0.9518	0
6816	418.5	1.5511	0.2607	0.2691	0.9514	0
6817	418.4	1.5514	0.2605	0.269	0.951	0
6818	418.3	1.5511	0.2604	0.269	0.9515	0
6819	418.2	1.5502	0.2605	0.2691	0.9516	0
6820	418.1	1.5513	0.2602	0.2691	0.9513	0
6821	418	1.5508	0.2601	0.2688	0.9514	0
6822	417.9	1.5506	0.2603	0.2688	0.9515	0
6823	417.8	1.5509	0.2601	0.2686	0.9516	0

6824	417.7	1.5513	0.2599	0.2685	0.9511	0
6825	417.6	1.551	0.2599	0.2684	0.9515	0
6826	417.5	1.5513	0.2598	0.2684	0.9519	0
6827	417.4	1.551	0.2599	0.2682	0.9519	0
6828	417.3	1.5506	0.2598	0.2685	0.9517	0
6829	417.2	1.5509	0.2596	0.2685	0.9519	0
6830	417.1	1.5507	0.2598	0.2685	0.9522	0
6831	417	1.5511	0.2597	0.2686	0.9522	0
6832	416.9	1.5508	0.2599	0.2686	0.9523	0
6833	416.8	1.5504	0.2597	0.2684	0.9525	0
6834	416.7	1.5508	0.2597	0.2683	0.9523	0
6835	416.6	1.5496	0.2598	0.2683	0.9521	0
6836	416.5	1.5508	0.2597	0.2682	0.9525	0
6837	416.4	1.5505	0.2598	0.2682	0.9526	0
6838	416.3	1.5509	0.2599	0.2684	0.9528	0
6839	416.2	1.5505	0.2597	0.2685	0.9528	0
6840	416.1	1.551	0.26	0.2684	0.9529	0
6841	416	1.5513	0.2597	0.2684	0.9532	0
6842	415.9	1.5501	0.2598	0.2684	0.9525	0
6843	415.8	1.5504	0.2599	0.2682	0.9527	0
6844	415.7	1.5501	0.2599	0.2681	0.953	0
6845	415.6	1.5505	0.26	0.2683	0.9536	0
6846	415.5	1.5508	0.2598	0.2682	0.9533	0
6847	415.4	1.5511	0.2599	0.2681	0.9536	0
6848	415.3	1.5514	0.2598	0.2683	0.9538	0
6849	415.2	1.551	0.2599	0.2683	0.954	0
6850	415.1	1.5507	0.2598	0.2683	0.954	0
6851	415	1.5502	0.2596	0.2685	0.9541	0
6852	414.9	1.5512	0.2596	0.2683	0.954	0
6853	414.8	1.5517	0.2598	0.2688	0.9544	0
6854	414.7	1.552	0.2597	0.2688	0.9543	0
6855	414.6	1.5516	0.26	0.2687	0.9547	0
6856	414.5	1.5511	0.2598	0.2689	0.9548	0
6857	414.4	1.5529	0.2599	0.2687	0.9549	0
6858	414.3	1.5525	0.2599	0.2686	0.9549	0
6859	414.2	1.5522	0.2601	0.2687	0.9552	0
6860	414.1	1.5526	0.2602	0.2691	0.9554	0
6861	414	1.5521	0.2604	0.2691	0.9557	0
6862	413.9	1.5524	0.2604	0.2691	0.9562	0
6863	413.8	1.5527	0.2605	0.2693	0.9561	0
6864	413.7	1.5532	0.2604	0.2694	0.9562	0
6865	413.6	1.5534	0.2607	0.2692	0.9563	0
6866	413.5	1.553	0.2609	0.2693	0.9564	0

6867	413.4	1.5541	0.2609	0.2694	0.9565	0
6868	413.3	1.5529	0.2612	0.2697	0.957	0
6869	413.2	1.5541	0.2612	0.2698	0.957	0
6870	413.1	1.5534	0.2611	0.2695	0.957	0
6871	413	1.5546	0.261	0.2696	0.9574	0
6872	412.9	1.554	0.261	0.2696	0.9575	0
6873	412.8	1.5545	0.2613	0.2698	0.9576	0
6874	412.7	1.5547	0.2612	0.2697	0.9577	0
6875	412.6	1.5541	0.2612	0.2697	0.9584	0
6876	412.5	1.5545	0.2613	0.2699	0.9578	0
6877	412.4	1.554	0.2613	0.27	0.9581	0
6878	412.3	1.5559	0.2614	0.27	0.9582	0
6879	412.2	1.5546	0.2614	0.2699	0.9587	0
6880	412.1	1.5556	0.2614	0.27	0.9587	0
6881	412	1.5544	0.2615	0.2702	0.9587	0
6882	411.9	1.5547	0.2618	0.2703	0.9591	0
6883	411.8	1.555	0.2615	0.2703	0.9593	0
6884	411.7	1.5561	0.2618	0.2704	0.9595	0
6885	411.6	1.5555	0.2616	0.2705	0.9594	0
6886	411.5	1.5558	0.2615	0.2702	0.9596	0
6887	411.4	1.5546	0.2615	0.2704	0.9597	0
6888	411.3	1.5556	0.2615	0.2702	0.9598	0
6889	411.2	1.5551	0.2616	0.2703	0.9597	0
6890	411.1	1.5552	0.2613	0.2703	0.9598	0
6891	411	1.5564	0.2614	0.2704	0.9604	0
6892	410.9	1.5559	0.2615	0.2705	0.9606	0
6893	410.8	1.5562	0.2616	0.2705	0.9604	0
6894	410.7	1.5565	0.2615	0.2706	0.9607	0
6895	410.6	1.5558	0.2615	0.2706	0.9612	0
6896	410.5	1.5552	0.2615	0.2704	0.9611	0
6897	410.4	1.5555	0.2614	0.2704	0.9612	0
6898	410.3	1.5566	0.2615	0.2706	0.9609	0
6899	410.2	1.556	0.2615	0.2706	0.9611	0
6900	410.1	1.5563	0.2617	0.2706	0.9612	0
6901	410	1.5565	0.2616	0.2706	0.9614	0
6902	409.9	1.5567	0.2615	0.2706	0.9617	0
6903	409.8	1.5561	0.2615	0.2706	0.9615	0
6904	409.7	1.5555	0.2618	0.2707	0.9615	0
6905	409.6	1.5559	0.2618	0.2705	0.9619	0
6906	409.5	1.5568	0.2616	0.2705	0.962	0
6907	409.4	1.5569	0.2615	0.2702	0.9619	0
6908	409.3	1.5571	0.2612	0.2702	0.9615	0
6909	409.2	1.5566	0.2612	0.2702	0.9622	0

6910	409.1	1.5576	0.2614	0.2703	0.9631	0
6911	409	1.5563	0.2613	0.2702	0.9625	0
6912	408.9	1.5573	0.2612	0.2701	0.9623	0
6913	408.8	1.5576	0.2612	0.2702	0.9626	0
6914	408.7	1.557	0.2613	0.2701	0.9627	0
6915	408.6	1.5572	0.2615	0.27	0.9627	0
6916	408.5	1.5565	0.2612	0.2698	0.9629	0
6917	408.4	1.5559	0.2611	0.2698	0.9629	0
6918	408.3	1.5568	0.2611	0.2697	0.9628	0
6919	408.2	1.5555	0.261	0.2698	0.9629	0
6920	408.1	1.5565	0.2609	0.27	0.9629	0
6921	408	1.5569	0.2609	0.27	0.9635	0
6922	407.9	1.5553	0.2608	0.2698	0.9634	0
6923	407.8	1.5555	0.2607	0.2697	0.9634	0
6924	407.7	1.5566	0.2607	0.2696	0.9635	0
6925	407.6	1.5569	0.2608	0.2696	0.9636	0
6926	407.5	1.5579	0.2607	0.2696	0.9636	0
6927	407.4	1.5564	0.2607	0.2697	0.9636	0
6928	407.3	1.5558	0.2606	0.2695	0.9638	0
6929	407.2	1.5552	0.2606	0.2694	0.9632	0
6930	407.1	1.5563	0.2606	0.2695	0.9637	0
6931	407	1.5567	0.2607	0.2696	0.9639	0
6932	406.9	1.5551	0.2604	0.2695	0.9638	0
6933	406.8	1.5562	0.2604	0.2694	0.9638	0
6934	406.7	1.5565	0.2605	0.2694	0.9639	0
6935	406.6	1.5568	0.2604	0.2695	0.964	0
6936	406.5	1.557	0.2605	0.2695	0.9647	0
6937	406.4	1.5572	0.2604	0.2693	0.9643	0
6938	406.3	1.5566	0.2603	0.2695	0.9643	0
6939	406.2	1.556	0.2604	0.2694	0.9644	0
6940	406.1	1.5572	0.2605	0.2694	0.9647	0
6941	406	1.5572	0.2604	0.2692	0.9643	0
6942	405.9	1.5559	0.2606	0.2694	0.9645	0
6943	405.8	1.556	0.2603	0.2691	0.9647	0
6944	405.7	1.5563	0.2603	0.2694	0.9648	0
6945	405.6	1.5565	0.2603	0.2693	0.965	0
6946	405.5	1.5577	0.2602	0.2691	0.9649	0
6947	405.4	1.5562	0.2602	0.2692	0.9656	0
6948	405.3	1.5582	0.2602	0.2693	0.9659	0
6949	405.2	1.5559	0.2604	0.2695	0.9657	0
6950	405.1	1.5569	0.2605	0.2695	0.9655	0
6951	405	1.5573	0.2606	0.2696	0.9659	0
6952	404.9	1.5574	0.2604	0.2692	0.966	0

6953	404.8	1.5584	0.2605	0.269	0.9659	0
6954	404.7	1.5569	0.2601	0.2697	0.9662	0
6955	404.6	1.5571	0.2605	0.2694	0.9657	0
6956	404.5	1.5582	0.2603	0.2694	0.9664	0
6957	404.4	1.5574	0.2601	0.2693	0.9661	0
6958	404.3	1.5576	0.2603	0.2694	0.9666	0
6959	404.2	1.5578	0.2604	0.2695	0.967	0
6960	404.1	1.558	0.2606	0.2695	0.967	0
6961	404	1.5573	0.2604	0.2696	0.967	0
6962	403.9	1.5574	0.2606	0.2697	0.9669	0
6963	403.8	1.5576	0.2606	0.2696	0.9671	0
6964	403.7	1.5587	0.2606	0.2698	0.9674	0
6965	403.6	1.5579	0.2607	0.2696	0.9672	0
6966	403.5	1.5582	0.2607	0.2697	0.9678	0
6967	403.4	1.5592	0.2609	0.2698	0.9677	0
6968	403.3	1.5586	0.2609	0.27	0.968	0
6969	403.2	1.5585	0.2607	0.2699	0.9682	0
6970	403.1	1.5578	0.2607	0.2699	0.9686	0
6971	403	1.5572	0.2609	0.2699	0.9687	0
6972	402.9	1.5582	0.261	0.27	0.9689	0
6973	402.8	1.5585	0.2611	0.2703	0.9689	0
6974	402.7	1.5594	0.261	0.2702	0.9694	0
6975	402.6	1.5587	0.261	0.2702	0.9694	0
6976	402.5	1.5587	0.261	0.2701	0.9692	0
6977	402.4	1.5581	0.2612	0.2704	0.9698	0
6978	402.3	1.5592	0.2612	0.2704	0.97	0
6979	402.2	1.5593	0.2612	0.2702	0.9701	0
6980	402.1	1.5604	0.2614	0.2704	0.9704	0
6981	402	1.5596	0.2615	0.2705	0.9705	0
6982	401.9	1.5588	0.2614	0.2704	0.9706	0
6983	401.8	1.5599	0.2614	0.2705	0.9701	0
6984	401.7	1.5592	0.2615	0.2707	0.9709	0
6985	401.6	1.5593	0.2614	0.2706	0.9715	0
6986	401.5	1.5603	0.2613	0.2709	0.9711	0
6987	401.4	1.5605	0.2613	0.2708	0.971	0
6988	401.3	1.5606	0.2613	0.2708	0.9715	0
6989	401.2	1.5607	0.2613	0.2707	0.9713	0
6990	401.1	1.56	0.2615	0.2709	0.9721	0
6991	401	1.56	0.2615	0.2708	0.9721	0
6992	400.9	1.56	0.2613	0.2707	0.9714	0
6993	400.8	1.5602	0.2614	0.2706	0.9711	0
6994	400.7	1.5612	0.2615	0.2706	0.9712	0
6995	400.6	1.5603	0.2614	0.2705	0.9714	0

6996	400.5	1.5586	0.2614	0.2706	0.9725	0
6997	400.4	1.5605	0.2613	0.2706	0.9719	0
6998	400.3	1.5607	0.2616	0.2706	0.9725	0
6999	400.2	1.5607	0.2613	0.2706	0.9723	0
7000	400.1	1.5598	0.2614	0.2706	0.9725	0
7001	400	1.5599	0.2613	0.2707	0.9726	0
7002	399.9	1.5601	0.2615	0.2707	0.9728	0
7003	399.8	1.5601	0.2613	0.2706	0.9729	0
7004	399.7	1.5611	0.2612	0.2707	0.9729	0
7005	399.6	1.5602	0.261	0.2704	0.9735	0
7006	399.5	1.5613	0.2612	0.2705	0.9734	0
7007	399.4	1.5606	0.2612	0.2705	0.9734	0
7008	399.3	1.5616	0.2613	0.2704	0.9727	0
7009	399.2	1.5616	0.2613	0.2704	0.973	0
7010	399.1	1.5609	0.2615	0.2707	0.9738	0
7011	399	1.5607	0.261	0.2706	0.9735	0
7012	398.9	1.5618	0.2609	0.2705	0.9739	0
7013	398.8	1.5599	0.2608	0.2704	0.9734	0
7014	398.7	1.5621	0.261	0.2706	0.9739	0
7015	398.6	1.5612	0.261	0.2705	0.9741	0
7016	398.5	1.5614	0.261	0.2705	0.9743	0
7017	398.4	1.5615	0.261	0.2704	0.9744	0
7018	398.3	1.5626	0.261	0.2705	0.9743	0
7019	398.2	1.5616	0.261	0.2705	0.9744	0
7020	398.1	1.5607	0.2609	0.2702	0.9746	0
7021	398	1.5609	0.261	0.2705	0.9747	0
7022	397.9	1.5619	0.2609	0.2703	0.9748	0
7023	397.8	1.5609	0.2607	0.2702	0.975	0
7024	397.7	1.5632	0.2609	0.2703	0.9753	0
7025	397.6	1.5612	0.2608	0.2702	0.9752	0
7026	397.5	1.5623	0.2607	0.2702	0.9756	0
7027	397.4	1.5613	0.2608	0.2702	0.9759	0
7028	397.3	1.5614	0.2609	0.2702	0.9752	0
7029	397.2	1.5636	0.261	0.2703	0.9755	0
7030	397.1	1.5617	0.261	0.2703	0.9753	0
7031	397	1.5606	0.2608	0.2701	0.9758	0
7032	396.9	1.5608	0.2608	0.2703	0.9761	0
7033	396.8	1.562	0.2608	0.2702	0.9765	0
7034	396.7	1.5611	0.2609	0.2702	0.9767	0
7035	396.6	1.5601	0.2609	0.2702	0.9765	0
7036	396.5	1.5611	0.2606	0.2702	0.9769	0
7037	396.4	1.5613	0.2605	0.2704	0.9768	0
7038	396.3	1.5614	0.2607	0.2704	0.9773	0

7039	396.2	1.5603	0.2604	0.27	0.9773	0
7040	396.1	1.5615	0.2605	0.2699	0.9769	0
7041	396	1.5617	0.2604	0.27	0.9774	0
7042	395.9	1.5618	0.2605	0.2701	0.9776	0
7043	395.8	1.563	0.2605	0.27	0.9778	0
7044	395.7	1.5631	0.2606	0.2701	0.9782	0
7045	395.6	1.5622	0.2604	0.27	0.9778	0
7046	395.5	1.5612	0.2606	0.2699	0.9779	0
7047	395.4	1.5623	0.2605	0.2699	0.9782	0
7048	395.3	1.5625	0.2606	0.2701	0.978	0
7049	395.2	1.5626	0.2605	0.2701	0.9781	0
7050	395.1	1.5629	0.2608	0.2702	0.9781	0
7051	395	1.5618	0.2606	0.2702	0.9787	0
7052	394.9	1.5628	0.2604	0.2701	0.9784	0
7053	394.8	1.562	0.2605	0.27	0.9787	0
7054	394.7	1.5632	0.2606	0.2701	0.9792	0
7055	394.6	1.5634	0.2605	0.2703	0.9794	0
7056	394.5	1.5636	0.2606	0.2702	0.9794	0
7057	394.4	1.5638	0.2607	0.2704	0.9797	0
7058	394.3	1.5627	0.2608	0.2704	0.9795	0
7059	394.2	1.5628	0.2607	0.2703	0.9799	0
7060	394.1	1.563	0.2608	0.2704	0.9798	0
7061	394	1.5641	0.2605	0.2703	0.9793	0
7062	393.9	1.5643	0.2608	0.2703	0.9798	0
7063	393.8	1.5645	0.2608	0.2704	0.98	0
7064	393.7	1.5646	0.2609	0.2705	0.9805	0
7065	393.6	1.5648	0.261	0.2707	0.9804	0
7066	393.5	1.5626	0.2608	0.2705	0.9805	0
7067	393.4	1.5638	0.2609	0.2705	0.9809	0
7068	393.3	1.5639	0.261	0.2706	0.9808	0
7069	393.2	1.5629	0.2608	0.2707	0.9807	0
7070	393.1	1.5641	0.2609	0.2706	0.9808	0
7071	393	1.5631	0.261	0.2708	0.9813	0
7072	392.9	1.5645	0.2611	0.2708	0.9816	0
7073	392.8	1.5644	0.261	0.2707	0.9819	0
7074	392.7	1.5657	0.261	0.2707	0.9814	0
7075	392.6	1.5646	0.2612	0.2708	0.9816	0
7076	392.5	1.5646	0.261	0.2708	0.9826	0
7077	392.4	1.5649	0.2611	0.271	0.982	0
7078	392.3	1.5649	0.261	0.2709	0.9823	0
7079	392.2	1.5637	0.2609	0.2707	0.982	0
7080	392.1	1.566	0.2608	0.2707	0.9824	0
7081	392	1.565	0.2609	0.2706	0.9823	0

7082	391.9	1.5652	0.2611	0.2708	0.9832	0
7083	391.8	1.5653	0.2612	0.2709	0.9827	0
7084	391.7	1.564	0.261	0.2705	0.9829	0
7085	391.6	1.5653	0.261	0.2706	0.9834	0
7086	391.5	1.5654	0.261	0.2708	0.9833	0
7087	391.4	1.5644	0.261	0.2707	0.9838	0
7088	391.3	1.5643	0.261	0.2708	0.9832	0
7089	391.2	1.5655	0.2608	0.2711	0.9829	0
7090	391.1	1.5666	0.2607	0.2708	0.9835	0
7091	391	1.5644	0.261	0.2708	0.9838	0
7092	390.9	1.5657	0.2609	0.2709	0.9833	0
7093	390.8	1.5658	0.2608	0.2709	0.9835	0
7094	390.7	1.5672	0.2611	0.271	0.9846	0
7095	390.6	1.566	0.2611	0.2709	0.9844	0
7096	390.5	1.5647	0.261	0.2706	0.984	0
7097	390.4	1.5658	0.2609	0.2707	0.9843	0
7098	390.3	1.5659	0.2608	0.2707	0.9841	0
7099	390.2	1.5659	0.2608	0.2708	0.9848	0
7100	390.1	1.5658	0.2607	0.2706	0.9848	0
7101	390	1.5659	0.2608	0.2706	0.9846	0
7102	389.9	1.5659	0.2607	0.2704	0.985	0
7103	389.8	1.566	0.2607	0.2705	0.9851	0
7104	389.7	1.5671	0.2608	0.2703	0.9851	0
7105	389.6	1.566	0.261	0.2706	0.985	0
7106	389.5	1.5672	0.261	0.2705	0.9857	0
7107	389.4	1.5661	0.261	0.2706	0.9865	0
7108	389.3	1.566	0.2609	0.2704	0.9855	0
7109	389.2	1.5658	0.2609	0.2702	0.9854	0
7110	389.1	1.5658	0.2604	0.2703	0.9855	0
7111	389	1.5659	0.2607	0.2705	0.986	0
7112	388.9	1.5672	0.2607	0.2701	0.986	0
7113	388.8	1.5672	0.2607	0.2704	0.986	0
7114	388.7	1.5671	0.2606	0.2703	0.9867	0
7115	388.6	1.566	0.2608	0.2703	0.9869	0
7116	388.5	1.5659	0.2605	0.2705	0.9866	0
7117	388.4	1.5659	0.2606	0.2706	0.9863	0
7118	388.3	1.5659	0.2606	0.2706	0.9867	0
7119	388.2	1.5659	0.2606	0.2703	0.9868	0
7120	388.1	1.5658	0.2605	0.27	0.9867	0
7121	388	1.5672	0.2605	0.2702	0.9873	0
7122	387.9	1.5645	0.2604	0.27	0.9872	0
7123	387.8	1.5671	0.2604	0.2701	0.9877	0
7124	387.7	1.5658	0.2605	0.27	0.9877	0

7125	387.6	1.5659	0.2606	0.2702	0.988	0
7126	387.5	1.5673	0.2604	0.2702	0.9877	0
7127	387.4	1.566	0.2606	0.2702	0.9882	0
7128	387.3	1.5673	0.2607	0.2703	0.9878	0
7129	387.2	1.5687	0.2608	0.2705	0.988	0
7130	387.1	1.5675	0.2609	0.2706	0.9886	0
7131	387	1.5676	0.2609	0.2705	0.9884	0
7132	386.9	1.5676	0.2608	0.2705	0.9888	0
7133	386.8	1.5663	0.261	0.2705	0.9889	0
7134	386.7	1.5662	0.2609	0.2704	0.9886	0
7135	386.6	1.5676	0.261	0.2704	0.9891	0
7136	386.5	1.5676	0.2608	0.2702	0.9888	0
7137	386.4	1.5691	0.261	0.2703	0.989	0
7138	386.3	1.5663	0.2608	0.2706	0.9901	0
7139	386.2	1.5678	0.2611	0.2706	0.9899	0
7140	386.1	1.5692	0.2609	0.2708	0.9897	0
7141	386	1.5694	0.2611	0.2707	0.9906	0
7142	385.9	1.5666	0.2611	0.2706	0.9904	0
7143	385.8	1.5681	0.2611	0.2707	0.9909	0
7144	385.7	1.5696	0.2611	0.2708	0.9907	0
7145	385.6	1.5697	0.2611	0.2708	0.9908	0
7146	385.5	1.5682	0.2612	0.2709	0.9909	0
7147	385.4	1.5683	0.2612	0.2707	0.991	0
7148	385.3	1.5697	0.2611	0.2707	0.9911	0
7149	385.2	1.5684	0.2614	0.271	0.9917	0
7150	385.1	1.5673	0.2615	0.2714	0.9921	0
7151	385	1.5702	0.2618	0.2714	0.9915	0
7152	384.9	1.5701	0.2615	0.271	0.9919	0
7153	384.8	1.5686	0.2613	0.2712	0.9911	0
7154	384.7	1.5688	0.2615	0.2714	0.9922	0
7155	384.6	1.5689	0.2616	0.2715	0.9924	0
7156	384.5	1.5705	0.2615	0.2714	0.9922	0
7157	384.4	1.5691	0.2616	0.2711	0.9915	0
7158	384.3	1.5723	0.2617	0.2715	0.9926	0
7159	384.2	1.5709	0.2616	0.2717	0.9932	0
7160	384.1	1.5711	0.2618	0.2712	0.9927	0
7161	384	1.5681	0.2617	0.2713	0.9932	0
7162	383.9	1.5698	0.2618	0.2715	0.9931	0
7163	383.8	1.5699	0.2618	0.2715	0.994	0
7164	383.7	1.5686	0.262	0.2716	0.9936	0
7165	383.6	1.5688	0.2621	0.2717	0.9942	0
7166	383.5	1.5703	0.2619	0.2719	0.9944	0
7167	383.4	1.5705	0.2621	0.2718	0.9943	0

7168	383.3	1.5707	0.2622	0.272	0.9945	0
7169	383.2	1.5708	0.2622	0.2719	0.9951	0
7170	383.1	1.5709	0.2621	0.2719	0.9945	0
7171	383	1.571	0.2622	0.2717	0.9951	0
7172	382.9	1.5712	0.2622	0.272	0.9954	0
7173	382.8	1.5714	0.262	0.2719	0.9945	0
7174	382.7	1.5717	0.2623	0.272	0.9957	0
7175	382.6	1.5735	0.2623	0.272	0.9956	0
7176	382.5	1.5735	0.2623	0.2718	0.9957	0
7177	382.4	1.5707	0.2621	0.2719	0.9961	0
7178	382.3	1.5709	0.2622	0.272	0.9956	0
7179	382.2	1.5712	0.2624	0.2719	0.9964	0
7180	382.1	1.5715	0.2624	0.272	0.9964	0
7181	382	1.5703	0.2624	0.2723	0.9961	0
7182	381.9	1.5704	0.2623	0.2719	0.9967	0
7183	381.8	1.5705	0.2621	0.2718	0.9961	0
7184	381.7	1.5727	0.2622	0.272	0.9963	0
7185	381.6	1.571	0.262	0.2718	0.9963	0
7186	381.5	1.5713	0.2621	0.2719	0.9968	0
7187	381.4	1.5717	0.2621	0.272	0.9968	0
7188	381.3	1.5722	0.2622	0.2722	0.9977	0
7189	381.2	1.5725	0.2622	0.272	0.9972	0
7190	381.1	1.5719	0.262	0.272	0.9971	0
7191	381	1.5722	0.2619	0.2719	0.9971	0
7192	380.9	1.5716	0.2618	0.2718	0.9974	0
7193	380.8	1.5718	0.2619	0.2718	0.9979	0
7194	380.7	1.5712	0.2618	0.2719	0.9976	0
7195	380.6	1.5724	0.2618	0.2721	0.9977	0
7196	380.5	1.5718	0.2617	0.2718	0.998	0
7197	380.4	1.572	0.2616	0.2719	0.9979	0
7198	380.3	1.5715	0.2617	0.2719	0.9983	0
7199	380.2	1.5726	0.2617	0.2718	0.9985	0
7200	380.1	1.572	0.2618	0.2719	0.9983	0
7201	380	1.5714	0.2618	0.2719	0.9983	0
7202	379.9	1.5725	0.2616	0.2717	0.9986	0
7203	379.8	1.571	0.2616	0.2718	0.9985	0
7204	379.7	1.5723	0.2618	0.2722	0.9991	0
7205	379.6	1.5716	0.2617	0.2719	0.9991	0
7206	379.5	1.5718	0.2616	0.2717	0.9993	0
7207	379.4	1.5712	0.2616	0.2716	0.9992	0
7208	379.3	1.5724	0.2616	0.2716	0.9991	0
7209	379.2	1.5716	0.2614	0.2714	0.9991	0
7210	379.1	1.5728	0.2614	0.2713	0.9995	0

7211	379	1.573	0.2612	0.2712	0.9994	0
7212	378.9	1.5724	0.2612	0.2712	0.9993	0
7213	378.8	1.5727	0.2613	0.2715	0.9997	0
7214	378.7	1.572	0.2613	0.2713	0.9998	0
7215	378.6	1.5731	0.2612	0.2712	0.9995	0
7216	378.5	1.5725	0.2612	0.2711	0.9996	0
7217	378.4	1.5718	0.261	0.2709	0.9996	0
7218	378.3	1.572	0.2612	0.2712	0.9998	0
7219	378.2	1.5723	0.2612	0.2708	1.0004	0
7220	378.1	1.5735	0.2611	0.271	1.0002	0
7221	378	1.572	0.2612	0.2712	1.0003	0
7222	377.9	1.573	0.261	0.271	1.0001	0
7223	377.8	1.5715	0.2609	0.271	1.0008	0
7224	377.7	1.5727	0.261	0.2709	1.001	0
7225	377.6	1.5721	0.2611	0.271	1.0014	0
7226	377.5	1.5733	0.2608	0.2709	1.0008	0
7227	377.4	1.5736	0.2609	0.2709	1.0012	0
7228	377.3	1.5729	0.2608	0.2709	1.0016	0
7229	377.2	1.5714	0.261	0.2711	1.0016	0
7230	377.1	1.5736	0.261	0.2712	1.0016	0
7231	377	1.572	0.2609	0.2713	1.0022	0
7232	376.9	1.5723	0.2608	0.2713	1.0022	0
7233	376.8	1.5715	0.2609	0.2708	1.0025	0
7234	376.7	1.5729	0.2612	0.271	1.0028	0
7235	376.6	1.5733	0.2612	0.2713	1.0023	0
7236	376.5	1.5735	0.261	0.2712	1.0027	0
7237	376.4	1.574	0.2611	0.2712	1.003	0
7238	376.3	1.5733	0.2611	0.2713	1.0033	0
7239	376.2	1.5737	0.261	0.2714	1.0033	0
7240	376.1	1.5732	0.2612	0.2716	1.0034	0
7241	376	1.5736	0.2613	0.2716	1.0037	0
7242	375.9	1.5731	0.2614	0.2715	1.0037	0
7243	375.8	1.5735	0.2614	0.2717	1.0043	0
7244	375.7	1.5729	0.2615	0.2717	1.004	0
7245	375.6	1.5732	0.2614	0.2718	1.0042	0
7246	375.5	1.5735	0.2612	0.2718	1.0039	0
7247	375.4	1.573	0.2615	0.2719	1.0049	0
7248	375.3	1.5745	0.2616	0.2719	1.0049	0
7249	375.2	1.5739	0.2617	0.2719	1.0047	0
7250	375.1	1.5754	0.2617	0.2718	1.005	0
7251	375	1.5759	0.262	0.272	1.0052	0
7252	374.9	1.5742	0.2618	0.2719	1.0057	0
7253	374.8	1.5749	0.262	0.2721	1.006	0

7254	374.7	1.5776	0.2622	0.2723	1.0065	0
7255	374.6	1.5748	0.2621	0.2722	1.0067	0
7256	374.5	1.5755	0.2623	0.2724	1.0064	0
7257	374.4	1.5749	0.262	0.2725	1.007	0
7258	374.3	1.5766	0.2623	0.2725	1.0067	0
7259	374.2	1.576	0.2622	0.2724	1.0071	0
7260	374.1	1.5755	0.2622	0.2729	1.0069	0
7261	374	1.5782	0.2623	0.2727	1.0077	0
7262	373.9	1.5756	0.2624	0.2729	1.007	0
7263	373.8	1.5772	0.2622	0.2726	1.0074	0
7264	373.7	1.5768	0.2623	0.2727	1.0082	0
7265	373.6	1.5774	0.2625	0.2728	1.0084	0
7266	373.5	1.577	0.2626	0.273	1.0084	0
7267	373.4	1.5766	0.2627	0.2731	1.009	0
7268	373.3	1.5772	0.2627	0.2732	1.0092	0
7269	373.2	1.5779	0.2627	0.2732	1.0089	0
7270	373.1	1.5774	0.2626	0.2731	1.0091	0
7271	373	1.577	0.2627	0.2731	1.0096	0
7272	372.9	1.5766	0.2628	0.2732	1.0094	0
7273	372.8	1.5773	0.2629	0.2732	1.0094	0
7274	372.7	1.577	0.263	0.2732	1.0097	0
7275	372.6	1.5766	0.2629	0.2731	1.0103	0
7276	372.5	1.5786	0.2632	0.2734	1.0102	0
7277	372.4	1.5782	0.2631	0.2734	1.0102	0
7278	372.3	1.5791	0.2629	0.2734	1.01	0
7279	372.2	1.5789	0.263	0.2735	1.0105	0
7280	372.1	1.5785	0.2631	0.2734	1.0106	0
7281	372	1.5783	0.2629	0.2735	1.011	0
7282	371.9	1.5781	0.2631	0.2734	1.0112	0
7283	371.8	1.5788	0.2629	0.2731	1.0106	0
7284	371.7	1.5774	0.2629	0.2731	1.0107	0
7285	371.6	1.5773	0.263	0.2734	1.0107	0
7286	371.5	1.5784	0.2631	0.2735	1.0111	0
7287	371.4	1.578	0.2629	0.2732	1.0111	0
7288	371.3	1.5779	0.2631	0.2734	1.0114	0
7289	371.2	1.5788	0.263	0.2732	1.0115	0
7290	371.1	1.5786	0.2629	0.2732	1.0118	0
7291	371	1.5796	0.2628	0.2731	1.012	0
7292	370.9	1.5782	0.2627	0.2731	1.0122	0
7293	370.8	1.5768	0.2627	0.273	1.0118	0
7294	370.7	1.5767	0.2626	0.273	1.0118	0
7295	370.6	1.5767	0.2629	0.273	1.0128	0
7296	370.5	1.5765	0.2626	0.2731	1.0121	0

7297	370.4	1.5764	0.2627	0.2729	1.0125	0
7298	370.3	1.5788	0.2627	0.273	1.0125	0
7299	370.2	1.5763	0.2624	0.2729	1.0129	0
7300	370.1	1.5786	0.2624	0.273	1.0129	0
7301	370	1.5785	0.2625	0.2729	1.0133	0
7302	369.9	1.5721	0.26	0.2707	1.011	0
7303	369.8	1.573	0.2595	0.2703	1.0107	0
7304	369.7	1.5735	0.2594	0.2706	1.0111	0
7305	369.6	1.5729	0.2596	0.2705	1.0113	0
7306	369.5	1.5731	0.2594	0.2705	1.0112	0
7307	369.4	1.5715	0.2592	0.2705	1.0116	0
7308	369.3	1.5727	0.2592	0.2703	1.0113	0
7309	369.2	1.5731	0.2595	0.2702	1.0113	0
7310	369.1	1.5724	0.2592	0.27	1.0116	0
7311	369	1.5726	0.2592	0.2701	1.0118	0
7312	368.9	1.5729	0.2592	0.2703	1.0117	0
7313	368.8	1.5732	0.2593	0.2703	1.0122	0
7314	368.7	1.5743	0.2592	0.2702	1.0121	0
7315	368.6	1.5737	0.2593	0.27	1.0123	0
7316	368.5	1.5728	0.2591	0.2698	1.0119	0
7317	368.4	1.5731	0.2592	0.2701	1.0121	0
7318	368.3	1.5732	0.2591	0.2701	1.0124	0
7319	368.2	1.5752	0.2592	0.2699	1.0133	0
7320	368.1	1.5744	0.2591	0.2697	1.0121	0
7321	368	1.5744	0.2589	0.2698	1.0127	0
7322	367.9	1.5745	0.2588	0.2696	1.013	0
7323	367.8	1.5738	0.2589	0.2696	1.013	0
7324	367.7	1.5747	0.2588	0.2695	1.013	0
7325	367.6	1.5731	0.2588	0.2694	1.013	0
7326	367.5	1.574	0.2587	0.2694	1.0132	0
7327	367.4	1.5738	0.2586	0.2691	1.0134	0
7328	367.3	1.5731	0.2587	0.2694	1.0134	0
7329	367.2	1.5734	0.2586	0.2695	1.0143	0
7330	367.1	1.5743	0.2586	0.2695	1.0143	0
7331	367	1.5732	0.2584	0.2694	1.0145	0
7332	366.9	1.5741	0.2585	0.2692	1.0146	0
7333	366.8	1.5724	0.2583	0.2692	1.0144	0
7334	366.7	1.5733	0.2585	0.2694	1.015	0
7335	366.6	1.5726	0.2586	0.2696	1.015	0
7336	366.5	1.5742	0.2586	0.2696	1.0154	0
7337	366.4	1.5732	0.2585	0.2695	1.0158	0
7338	366.3	1.574	0.2585	0.2695	1.0154	0
7339	366.2	1.573	0.2586	0.2696	1.0157	0

7340	366.1	1.5746	0.2587	0.2696	1.0155	0
7341	366	1.5744	0.2587	0.2696	1.0155	0
7342	365.9	1.5752	0.2587	0.2696	1.0155	0
7343	365.8	1.5744	0.2588	0.2698	1.0159	0
7344	365.7	1.5752	0.259	0.2699	1.0163	0
7345	365.6	1.5759	0.2589	0.2698	1.0165	0
7346	365.5	1.5747	0.2588	0.2698	1.0163	0
7347	365.4	1.5738	0.2589	0.2699	1.0171	0
7348	365.3	1.5753	0.2591	0.2698	1.0173	0
7349	365.2	1.5752	0.2591	0.27	1.0178	0
7350	365.1	1.5758	0.2594	0.2699	1.0184	0
7351	365	1.5748	0.2593	0.2701	1.0181	0
7352	364.9	1.5761	0.2591	0.27	1.0182	0
7353	364.8	1.5759	0.2592	0.27	1.0183	0
7354	364.7	1.5756	0.2591	0.27	1.0186	0
7355	364.6	1.5771	0.2595	0.2702	1.0191	0
7356	364.5	1.5768	0.2594	0.2702	1.0197	0
7357	364.4	1.5765	0.2593	0.2706	1.0192	0
7358	364.3	1.577	0.2594	0.2705	1.0198	0
7359	364.2	1.5769	0.2598	0.2706	1.0201	0
7360	364.1	1.5782	0.2599	0.2708	1.0202	0
7361	364	1.5777	0.2597	0.271	1.0205	0
7362	363.9	1.5789	0.2595	0.2709	1.0206	0
7363	363.8	1.5778	0.2598	0.271	1.0207	0
7364	363.7	1.5774	0.26	0.271	1.0213	0
7365	363.6	1.5786	0.2601	0.2714	1.0213	0
7366	363.5	1.5782	0.2602	0.2715	1.0217	0
7367	363.4	1.5794	0.2603	0.2714	1.0219	0
7368	363.3	1.5797	0.2602	0.2715	1.0225	0
7369	363.2	1.5801	0.2603	0.2714	1.0224	0
7370	363.1	1.5797	0.2603	0.2716	1.023	0
7371	363	1.5809	0.2605	0.2719	1.0236	0
7372	362.9	1.582	0.2604	0.2718	1.0234	0
7373	362.8	1.5807	0.2607	0.2719	1.0236	0
7374	362.7	1.5816	0.2604	0.2719	1.0237	0
7375	362.6	1.582	0.2607	0.2721	1.0243	0
7376	362.5	1.5821	0.2608	0.2721	1.024	0
7377	362.4	1.5824	0.2606	0.2722	1.0245	0
7378	362.3	1.5818	0.2605	0.2719	1.0244	0
7379	362.2	1.5828	0.2607	0.272	1.0242	0
7380	362.1	1.5813	0.2609	0.2718	1.0248	0
7381	362	1.5825	0.2608	0.272	1.0253	0
7382	361.9	1.581	0.2607	0.272	1.025	0

7383	361.8	1.582	0.2608	0.2722	1.0257	0
7384	361.7	1.5821	0.2607	0.2721	1.0258	0
7385	361.6	1.583	0.2609	0.2721	1.0261	0
7386	361.5	1.5824	0.2608	0.272	1.0265	0
7387	361.4	1.5817	0.2609	0.272	1.0261	0
7388	361.3	1.5818	0.2612	0.2719	1.0267	0
7389	361.2	1.5804	0.2611	0.272	1.0264	0
7390	361.1	1.5812	0.261	0.2721	1.0264	0
7391	361	1.5813	0.261	0.2721	1.0268	0
7392	360.9	1.5813	0.2608	0.2718	1.027	0
7393	360.8	1.5814	0.2611	0.2721	1.0269	0
7394	360.7	1.5814	0.2611	0.2719	1.0273	0
7395	360.6	1.5814	0.261	0.272	1.0275	0
7396	360.5	1.583	0.2611	0.272	1.0275	0
7397	360.4	1.5822	0.261	0.2719	1.0276	0
7398	360.3	1.5814	0.2611	0.272	1.0276	0
7399	360.2	1.583	0.261	0.272	1.0282	0
7400	360.1	1.5829	0.2609	0.2722	1.0281	0
7401	360	1.582	0.2608	0.2721	1.0283	0
7402	359.9	1.5826	0.2605	0.2718	1.0279	0
7403	359.8	1.5826	0.2606	0.2719	1.0283	0
7404	359.7	1.5817	0.2605	0.272	1.0284	0
7405	359.6	1.5816	0.2602	0.2717	1.0292	0
7406	359.5	1.5815	0.2604	0.2715	1.0289	0
7407	359.4	1.583	0.2604	0.2716	1.029	0
7408	359.3	1.5813	0.2604	0.2716	1.0293	0
7409	359.2	1.5827	0.2602	0.2715	1.029	0
7410	359.1	1.5819	0.2603	0.2713	1.0298	0
7411	359	1.581	0.2604	0.2714	1.0295	0
7412	358.9	1.5806	0.2601	0.2712	1.03	0
7413	358.8	1.5812	0.2599	0.2709	1.0298	0
7414	358.7	1.5819	0.26	0.271	1.0299	0
7415	358.6	1.5817	0.2598	0.271	1.0299	0
7416	358.5	1.5815	0.2599	0.2708	1.0306	0
7417	358.4	1.5804	0.2598	0.2706	1.0304	0
7418	358.3	1.5818	0.2596	0.2706	1.0301	0
7419	358.2	1.5816	0.2597	0.2708	1.0297	0
7420	358.1	1.5812	0.2594	0.2705	1.0305	0
7421	358	1.5819	0.2593	0.2706	1.0308	0
7422	357.9	1.5825	0.2593	0.2706	1.0308	0
7423	357.8	1.5823	0.2593	0.2706	1.0308	0
7424	357.7	1.582	0.2593	0.2702	1.0308	0
7425	357.6	1.5818	0.2592	0.2703	1.0313	0

7426	357.5	1.5815	0.2592	0.2703	1.0312	0
7427	357.4	1.5803	0.259	0.2701	1.0311	0
7428	357.3	1.5817	0.259	0.2698	1.0315	0
7429	357.2	1.5822	0.2586	0.27	1.0316	0
7430	357.1	1.5809	0.2588	0.27	1.0317	0
7431	357	1.5816	0.2588	0.2699	1.0315	0
7432	356.9	1.5804	0.2586	0.2699	1.0316	0
7433	356.8	1.5808	0.2585	0.2699	1.0316	0
7434	356.7	1.5813	0.2587	0.2699	1.0316	0
7435	356.6	1.581	0.2587	0.2698	1.0317	0
7436	356.5	1.5807	0.2588	0.2699	1.0326	0
7437	356.4	1.5803	0.2586	0.2697	1.0321	0
7438	356.3	1.5806	0.2585	0.2697	1.0324	0
7439	356.2	1.5803	0.2587	0.2699	1.0328	0
7440	356.1	1.5808	0.2589	0.27	1.0332	0
7441	356	1.5819	0.2586	0.2698	1.033	0
7442	355.9	1.5806	0.2587	0.2698	1.0328	0
7443	355.8	1.5818	0.2586	0.2696	1.0333	0
7444	355.7	1.5814	0.2587	0.2696	1.0331	0
7445	355.6	1.5801	0.2589	0.2697	1.0336	0
7446	355.5	1.5821	0.2588	0.27	1.0336	0
7447	355.4	1.5808	0.2587	0.27	1.0343	0
7448	355.3	1.5812	0.2589	0.27	1.0343	0
7449	355.2	1.5817	0.2591	0.2701	1.0344	0
7450	355.1	1.582	0.2591	0.2702	1.0344	0
7451	355	1.5814	0.259	0.2701	1.0342	0
7452	354.9	1.5816	0.2589	0.27	1.0348	0
7453	354.8	1.5821	0.2588	0.2701	1.0352	0
7454	354.7	1.5833	0.2589	0.2701	1.0354	0
7455	354.6	1.5835	0.2588	0.2703	1.0358	0
7456	354.5	1.5821	0.2592	0.2702	1.0364	0
7457	354.4	1.5834	0.2593	0.2705	1.0365	0
7458	354.3	1.5833	0.259	0.2703	1.0365	0
7459	354.2	1.5838	0.2593	0.2704	1.0371	0
7460	354.1	1.584	0.2593	0.2705	1.0372	0
7461	354	1.5826	0.2594	0.2706	1.0373	0
7462	353.9	1.5828	0.2594	0.2709	1.0375	0
7463	353.8	1.5829	0.2592	0.2707	1.0379	0
7464	353.7	1.584	0.2595	0.2707	1.0377	0
7465	353.6	1.5852	0.2597	0.2711	1.0384	0
7466	353.5	1.5854	0.2599	0.271	1.0387	0
7467	353.4	1.5846	0.2598	0.2713	1.0385	0
7468	353.3	1.5857	0.2597	0.2714	1.0391	0

7469	353.2	1.5859	0.2599	0.2715	1.0394	0
7470	353.1	1.586	0.2599	0.2715	1.0399	0
7471	353	1.5863	0.2602	0.2716	1.04	0
7472	352.9	1.5864	0.2601	0.2715	1.04	0
7473	352.8	1.5865	0.2601	0.2717	1.0395	0
7474	352.7	1.5868	0.2603	0.2719	1.0406	0
7475	352.6	1.5868	0.2604	0.2721	1.0403	0
7476	352.5	1.586	0.2604	0.2717	1.0407	0
7477	352.4	1.588	0.2603	0.2719	1.041	0
7478	352.3	1.5881	0.2604	0.2718	1.0415	0
7479	352.2	1.5882	0.2606	0.2721	1.0418	0
7480	352.1	1.5866	0.2608	0.2722	1.0418	0
7481	352	1.5876	0.2606	0.2722	1.0426	0
7482	351.9	1.5879	0.2608	0.2723	1.0435	0
7483	351.8	1.5898	0.2608	0.2723	1.0434	0
7484	351.7	1.5881	0.2608	0.2721	1.0434	0
7485	351.6	1.5891	0.2606	0.2722	1.0431	0
7486	351.5	1.5892	0.2609	0.2722	1.0436	0
7487	351.4	1.5886	0.2611	0.2724	1.044	0
7488	351.3	1.5886	0.2608	0.2723	1.0444	0
7489	351.2	1.5879	0.2609	0.2722	1.0445	0
7490	351.1	1.5881	0.2609	0.2721	1.0448	0
7491	351	1.5891	0.261	0.2721	1.045	0
7492	350.9	1.5884	0.261	0.2724	1.0456	0
7493	350.8	1.5893	0.2609	0.2723	1.0455	0
7494	350.7	1.5886	0.2611	0.2722	1.0456	0
7495	350.6	1.5897	0.2613	0.2725	1.0462	0
7496	350.5	1.5888	0.2612	0.2725	1.0453	0
7497	350.4	1.5898	0.2612	0.2723	1.0455	0
7498	350.3	1.5908	0.2611	0.2724	1.046	0
7499	350.2	1.5883	0.2613	0.2726	1.0463	0
7500	350.1	1.5893	0.2614	0.2724	1.0473	0
7501	350	1.5902	0.2611	0.2723	1.0472	0
7502	349.9	1.5902	0.2612	0.2724	1.0476	0
7503	349.8	1.5895	0.2613	0.2725	1.0474	0
7504	349.7	1.5903	0.2613	0.2722	1.0476	0
7505	349.6	1.5895	0.2614	0.2724	1.0477	0
7506	349.5	1.5897	0.2615	0.2725	1.048	0
7507	349.4	1.5897	0.2613	0.2728	1.0484	0
7508	349.3	1.5906	0.2613	0.2727	1.0486	0
7509	349.2	1.5906	0.2612	0.2728	1.0492	0
7510	349.1	1.5895	0.261	0.2726	1.0494	0
7511	349	1.5914	0.2611	0.2726	1.0494	0

7512	348.9	1.5923	0.2611	0.2725	1.0492	0
7513	348.8	1.5913	0.261	0.2724	1.0495	0
7514	348.7	1.5913	0.2612	0.2725	1.05	0
7515	348.6	1.5922	0.2611	0.2725	1.0498	0
7516	348.5	1.5902	0.261	0.2722	1.0498	0
7517	348.4	1.5901	0.2609	0.2722	1.0501	0
7518	348.3	1.591	0.2609	0.2723	1.0497	0
7519	348.2	1.5901	0.261	0.2724	1.0507	0
7520	348.1	1.5919	0.2611	0.2722	1.0507	0
7521	348	1.5899	0.2611	0.2722	1.0502	0
7522	347.9	1.5908	0.261	0.272	1.0503	0
7523	347.8	1.5898	0.2609	0.272	1.0516	0
7524	347.7	1.5907	0.2609	0.2722	1.0511	0
7525	347.6	1.5896	0.2608	0.2721	1.0505	0
7526	347.5	1.5904	0.2605	0.2719	1.0508	0
7527	347.4	1.5913	0.2606	0.2718	1.0512	0
7528	347.3	1.5923	0.2608	0.2718	1.051	0
7529	347.2	1.5911	0.2604	0.2717	1.0513	0
7530	347.1	1.5922	0.2609	0.272	1.0521	0
7531	347	1.5899	0.2606	0.2718	1.0517	0
7532	346.9	1.59	0.2606	0.2717	1.0516	0
7533	346.8	1.5898	0.2605	0.2719	1.0522	0
7534	346.7	1.5899	0.2607	0.2719	1.0527	0
7535	346.6	1.5898	0.2606	0.2718	1.0522	0
7536	346.5	1.5886	0.2603	0.2716	1.0519	0
7537	346.4	1.5896	0.2603	0.2715	1.0526	0
7538	346.3	1.5883	0.2602	0.2714	1.0525	0
7539	346.2	1.5894	0.2602	0.2715	1.0524	0
7540	346.1	1.5903	0.26	0.2714	1.0533	0
7541	346	1.5904	0.2604	0.2716	1.0539	0
7542	345.9	1.5913	0.2604	0.2715	1.0537	0
7543	345.8	1.5891	0.26	0.2715	1.054	0
7544	345.7	1.5912	0.2602	0.2718	1.0544	0
7545	345.6	1.592	0.2601	0.2717	1.0541	0
7546	345.5	1.591	0.2603	0.2716	1.0546	0
7547	345.4	1.5909	0.2602	0.2717	1.0541	0
7548	345.3	1.5899	0.2602	0.2719	1.0546	0
7549	345.2	1.5907	0.2602	0.2718	1.0545	0
7550	345.1	1.5906	0.2599	0.2717	1.0545	0
7551	345	1.5925	0.2598	0.2717	1.0552	0
7552	344.9	1.5912	0.26	0.2715	1.0551	0
7553	344.8	1.5913	0.2603	0.2718	1.0556	0
7554	344.7	1.5922	0.2605	0.2719	1.0566	0

7555	344.6	1.591	0.2605	0.2718	1.0569	0
7556	344.5	1.5928	0.2605	0.2718	1.0575	0
7557	344.4	1.5916	0.2605	0.2721	1.0572	0
7558	344.3	1.5903	0.2605	0.2718	1.0574	0
7559	344.2	1.5913	0.2607	0.2722	1.0581	0
7560	344.1	1.5932	0.2608	0.272	1.0581	0
7561	344	1.5909	0.2609	0.2722	1.0584	0
7562	343.9	1.5917	0.2607	0.272	1.0587	0
7563	343.8	1.5915	0.2608	0.2721	1.0583	0
7564	343.7	1.5925	0.261	0.2724	1.059	0
7565	343.6	1.5932	0.2607	0.2724	1.0595	0
7566	343.5	1.5932	0.2609	0.2723	1.0596	0
7567	343.4	1.5909	0.2609	0.2724	1.06	0
7568	343.3	1.5939	0.2611	0.2726	1.06	0
7569	343.2	1.5937	0.2613	0.2729	1.0603	0
7570	343.1	1.5947	0.2613	0.2729	1.0607	0
7571	343	1.5945	0.2612	0.2729	1.061	0
7572	342.9	1.5934	0.2616	0.2731	1.0618	0
7573	342.8	1.5943	0.2615	0.2731	1.0615	0
7574	342.7	1.5951	0.2616	0.273	1.0608	0
7575	342.6	1.596	0.2612	0.2728	1.062	0
7576	342.5	1.5938	0.2617	0.273	1.0629	0
7577	342.4	1.5959	0.2618	0.2732	1.062	0
7578	342.3	1.5957	0.2616	0.2733	1.0633	0
7579	342.2	1.5944	0.2618	0.2734	1.0633	0
7580	342.1	1.5951	0.2617	0.273	1.0631	0
7581	342	1.5962	0.2617	0.2732	1.0632	0
7582	341.9	1.596	0.2616	0.273	1.0633	0
7583	341.8	1.597	0.2619	0.2732	1.0643	0
7584	341.7	1.5979	0.2619	0.2732	1.0653	0
7585	341.6	1.5965	0.2619	0.2732	1.0649	0
7586	341.5	1.5975	0.2619	0.2733	1.065	0
7587	341.4	1.5974	0.2621	0.2734	1.0646	0
7588	341.3	1.5948	0.262	0.2734	1.0657	0
7589	341.2	1.5968	0.262	0.2732	1.0656	0
7590	341.1	1.5979	0.262	0.2733	1.066	0
7591	341	1.5978	0.2621	0.2735	1.0665	0
7592	340.9	1.5964	0.2623	0.2737	1.0664	0
7593	340.8	1.5961	0.2621	0.2731	1.0667	0
7594	340.7	1.5971	0.262	0.2733	1.0667	0
7595	340.6	1.5958	0.2621	0.2734	1.0672	0
7596	340.5	1.5968	0.2621	0.2738	1.0673	0
7597	340.4	1.5978	0.2621	0.2737	1.0672	0

7598	340.3	1.5976	0.2622	0.2735	1.0676	0
7599	340.2	1.5963	0.262	0.2735	1.0681	0
7600	340.1	1.5973	0.2621	0.2738	1.0689	0
7601	340	1.5971	0.2621	0.2735	1.0685	0
7602	339.9	1.3869	0.2533	0.2638	0.9526	0
7603	339.8	1.3863	0.2532	0.2639	0.9522	0
7604	339.7	1.386	0.2532	0.2639	0.9518	0
7605	339.6	1.3852	0.2531	0.264	0.952	0
7606	339.5	1.3848	0.2531	0.264	0.9519	0
7607	339.4	1.3855	0.253	0.2639	0.9515	0
7608	339.3	1.3847	0.253	0.2638	0.9517	0
7609	339.2	1.3831	0.2528	0.2637	0.9516	0
7610	339.1	1.3833	0.2528	0.2634	0.9518	0
7611	339	1.383	0.2529	0.2634	0.9512	0
7612	338.9	1.3821	0.2529	0.2636	0.951	0
7613	338.8	1.3818	0.2528	0.2637	0.9512	0
7614	338.7	1.3814	0.2529	0.2636	0.9507	0
7615	338.6	1.3806	0.2529	0.2637	0.9506	0
7616	338.5	1.3797	0.2527	0.2634	0.9503	0
7617	338.4	1.3795	0.2526	0.2635	0.9504	0
7618	338.3	1.3792	0.2527	0.2634	0.9502	0
7619	338.2	1.3789	0.2527	0.2634	0.9496	0
7620	338.1	1.3781	0.2527	0.2633	0.9499	0
7621	338	1.3773	0.2524	0.2633	0.9499	0
7622	337.9	1.3766	0.2526	0.2634	0.9495	0
7623	337.8	1.3756	0.2525	0.263	0.9489	0
7624	337.7	1.3764	0.2523	0.263	0.949	0
7625	337.6	1.3755	0.2521	0.2628	0.9488	0
7626	337.5	1.3742	0.2522	0.2628	0.9485	0
7627	337.4	1.3746	0.2522	0.2629	0.9485	0
7628	337.3	1.3738	0.2521	0.2628	0.9486	0
7629	337.2	1.373	0.2521	0.2628	0.9486	0
7630	337.1	1.3733	0.2521	0.2629	0.948	0
7631	337	1.372	0.2519	0.2629	0.9481	0
7632	336.9	1.3724	0.2519	0.2628	0.9478	0
7633	336.8	1.3717	0.2521	0.2627	0.9478	0
7634	336.7	1.3713	0.2519	0.2626	0.9474	0
7635	336.6	1.3706	0.2521	0.2625	0.9476	0
7636	336.5	1.3704	0.252	0.2626	0.9469	0
7637	336.4	1.3703	0.2519	0.2627	0.9475	0
7638	336.3	1.3699	0.2517	0.2627	0.9473	0
7639	336.2	1.3692	0.2517	0.2625	0.9467	0
7640	336.1	1.3684	0.2517	0.2624	0.9468	0

7641	336	1.3672	0.2516	0.2624	0.9464	0
7642	335.9	1.3681	0.2515	0.2625	0.9464	0
7643	335.8	1.3668	0.2516	0.2623	0.9465	0
7644	335.7	1.3659	0.2514	0.2621	0.9463	0
7645	335.6	1.3659	0.2514	0.2622	0.9461	0
7646	335.5	1.3659	0.2515	0.2622	0.946	0
7647	335.4	1.3652	0.2516	0.2623	0.946	0
7648	335.3	1.3645	0.2515	0.2623	0.946	0
7649	335.2	1.365	0.2516	0.2623	0.9463	0
7650	335.1	1.3648	0.2516	0.2621	0.9457	0
7651	335	1.3636	0.2514	0.262	0.9455	0
7652	334.9	1.3635	0.2515	0.2623	0.9456	0
7653	334.8	1.3635	0.2514	0.2625	0.9454	0
7654	334.7	1.3625	0.2511	0.262	0.9451	0
7655	334.6	1.362	0.2512	0.2623	0.9451	0
7656	334.5	1.3608	0.2511	0.2621	0.9449	0
7657	334.4	1.3612	0.2512	0.2621	0.9445	0
7658	334.3	1.3605	0.251	0.2621	0.945	0
7659	334.2	1.361	0.2511	0.262	0.9447	0
7660	334.1	1.3598	0.2511	0.262	0.9441	0
7661	334	1.3598	0.2513	0.2619	0.9441	0
7662	333.9	1.3591	0.2512	0.2618	0.9441	0
7663	333.8	1.3579	0.2511	0.262	0.944	0
7664	333.7	1.3585	0.2513	0.2621	0.9438	0
7665	333.6	1.358	0.2514	0.2622	0.9443	0
7666	333.5	1.3578	0.2513	0.2622	0.9436	0
7667	333.4	1.3562	0.2515	0.2621	0.9437	0
7668	333.3	1.356	0.2512	0.2619	0.9432	0
7669	333.2	1.3566	0.2512	0.2621	0.9432	0
7670	333.1	1.356	0.2514	0.2621	0.9436	0
7671	333	1.3555	0.2517	0.262	0.9434	0
7672	332.9	1.3549	0.2515	0.2621	0.9434	0
7673	332.8	1.3542	0.2512	0.2623	0.9432	0
7674	332.7	1.3542	0.2512	0.2622	0.9429	0
7675	332.6	1.3541	0.2512	0.262	0.9428	0
7676	332.5	1.353	0.2512	0.262	0.9426	0
7677	332.4	1.3536	0.2512	0.2622	0.9428	0
7678	332.3	1.3525	0.2514	0.2621	0.943	0
7679	332.2	1.3518	0.251	0.262	0.9427	0
7680	332.1	1.3519	0.2513	0.2622	0.9426	0
7681	332	1.3513	0.251	0.2621	0.9427	0
7682	331.9	1.3513	0.251	0.2618	0.9427	0
7683	331.8	1.3506	0.251	0.2617	0.9421	0

7684	331.7	1.35	0.2507	0.2619	0.9418	0
7685	331.6	1.3499	0.2509	0.2618	0.9417	0
7686	331.5	1.3488	0.2508	0.2617	0.9415	0
7687	331.4	1.3482	0.2508	0.2617	0.9415	0
7688	331.3	1.3482	0.2508	0.2616	0.9413	0
7689	331.2	1.3487	0.2507	0.2616	0.9412	0
7690	331.1	1.3476	0.2507	0.2615	0.9407	0
7691	331	1.347	0.2507	0.2616	0.9405	0
7692	330.9	1.3463	0.2505	0.2613	0.9404	0
7693	330.8	1.3457	0.2504	0.2612	0.9408	0
7694	330.7	1.3458	0.2505	0.2612	0.9404	0
7695	330.6	1.3454	0.2507	0.2614	0.9406	0
7696	330.5	1.3446	0.2502	0.2611	0.9399	0
7697	330.4	1.3446	0.25	0.2609	0.9399	0
7698	330.3	1.3439	0.2498	0.2607	0.94	0
7699	330.2	1.3439	0.2501	0.2606	0.94	0
7700	330.1	1.3433	0.2503	0.2608	0.9396	0
7701	330	1.3426	0.2501	0.2607	0.9392	0
7702	329.9	1.342	0.25	0.2607	0.939	0
7703	329.8	1.3415	0.25	0.2607	0.939	0
7704	329.7	1.3409	0.25	0.2607	0.9393	0
7705	329.6	1.3409	0.25	0.2606	0.9393	0
7706	329.5	1.341	0.2499	0.2606	0.9391	0
7707	329.4	1.3403	0.2495	0.2602	0.9388	0
7708	329.3	1.3402	0.2497	0.2601	0.9385	0
7709	329.2	1.339	0.2494	0.26	0.9377	0
7710	329.1	1.3397	0.2495	0.2601	0.9377	0
7711	329	1.339	0.2494	0.2599	0.9377	0
7712	328.9	1.337	0.2493	0.2597	0.9381	0
7713	328.8	1.3377	0.249	0.2598	0.9371	0
7714	328.7	1.3373	0.2492	0.26	0.9373	0
7715	328.6	1.3373	0.2491	0.2598	0.9371	0
7716	328.5	1.3366	0.249	0.2596	0.937	0
7717	328.4	1.3353	0.2492	0.2594	0.9368	0
7718	328.3	1.336	0.2491	0.2594	0.9368	0
7719	328.2	1.3348	0.2488	0.2595	0.9359	0
7720	328.1	1.3343	0.2488	0.2594	0.9362	0
7721	328	1.3337	0.2487	0.2593	0.9365	0
7722	327.9	1.333	0.2487	0.2593	0.936	0
7723	327.8	1.3331	0.2485	0.2592	0.936	0
7724	327.7	1.3317	0.2485	0.2591	0.9357	0
7725	327.6	1.3319	0.2484	0.259	0.9356	0
7726	327.5	1.3313	0.2486	0.2591	0.9354	0

7727	327.4	1.3302	0.2486	0.2593	0.9353	0
7728	327.3	1.3307	0.2482	0.2589	0.9353	0
7729	327.2	1.3302	0.2481	0.2587	0.9353	0
7730	327.1	1.3295	0.2482	0.2589	0.9348	0
7731	327	1.329	0.2481	0.2588	0.935	0
7732	326.9	1.3291	0.2481	0.2586	0.9348	0
7733	326.8	1.328	0.2482	0.2587	0.9347	0
7734	326.7	1.3267	0.248	0.2587	0.934	0
7735	326.6	1.3276	0.2482	0.2589	0.9342	0
7736	326.5	1.327	0.2481	0.2588	0.9343	0
7737	326.4	1.3265	0.2482	0.2589	0.934	0
7738	326.3	1.3253	0.248	0.2588	0.9341	0
7739	326.2	1.3261	0.2482	0.259	0.9337	0
7740	326.1	1.3255	0.2482	0.259	0.9338	0
7741	326	1.325	0.2481	0.2588	0.9334	0
7742	325.9	1.3244	0.248	0.2585	0.9332	0
7743	325.8	1.3233	0.248	0.2588	0.9332	0
7744	325.7	1.3241	0.2482	0.2589	0.9331	0
7745	325.6	1.3229	0.2483	0.2589	0.9327	0
7746	325.5	1.3223	0.2481	0.2587	0.9325	0
7747	325.4	1.3217	0.2481	0.2585	0.9326	0
7748	325.3	1.3218	0.248	0.2586	0.9325	0
7749	325.2	1.3215	0.2483	0.2586	0.9328	0
7750	325.1	1.3209	0.2482	0.2587	0.9321	0
7751	325	1.3211	0.2483	0.2588	0.9321	0
7752	324.9	1.3212	0.2482	0.2585	0.9323	0
7753	324.8	1.3214	0.2481	0.2588	0.9322	0
7754	324.7	1.3202	0.2483	0.2587	0.9321	0
7755	324.6	1.3204	0.2482	0.2587	0.932	0
7756	324.5	1.3184	0.2482	0.2585	0.9321	0
7757	324.4	1.3186	0.2481	0.2584	0.9312	0
7758	324.3	1.3181	0.2481	0.2588	0.9314	0
7759	324.2	1.3182	0.2481	0.2586	0.9313	0
7760	324.1	1.3177	0.248	0.2586	0.9307	0
7761	324	1.3173	0.248	0.2586	0.9309	0
7762	323.9	1.3175	0.2481	0.2586	0.9309	0
7763	323.8	1.3177	0.2482	0.2585	0.9312	0
7764	323.7	1.3173	0.2484	0.2586	0.9309	0
7765	323.6	1.3154	0.2484	0.2587	0.9303	0
7766	323.5	1.3156	0.2483	0.2585	0.9302	0
7767	323.4	1.3145	0.2483	0.2587	0.9306	0
7768	323.3	1.3141	0.2482	0.2588	0.93	0
7769	323.2	1.3135	0.2482	0.2587	0.9305	0

7770	323.1	1.313	0.2483	0.2587	0.9304	0
7771	323	1.3126	0.2482	0.2586	0.9299	0
7772	322.9	1.3122	0.2481	0.2586	0.9297	0
7773	322.8	1.3118	0.2481	0.2585	0.9297	0
7774	322.7	1.3113	0.2483	0.2585	0.9291	0
7775	322.6	1.3116	0.2484	0.2587	0.9289	0
7776	322.5	1.311	0.2483	0.2583	0.9287	0
7777	322.4	1.3113	0.248	0.2584	0.9285	0
7778	322.3	1.311	0.2481	0.2585	0.9287	0
7779	322.2	1.3092	0.2479	0.2585	0.9284	0
7780	322.1	1.3094	0.2478	0.2583	0.9281	0
7781	322	1.3083	0.2476	0.2581	0.9279	0
7782	321.9	1.3086	0.2476	0.258	0.9274	0
7783	321.8	1.3081	0.2476	0.2581	0.9274	0
7784	321.7	1.3072	0.2477	0.2581	0.9276	0
7785	321.6	1.3067	0.2477	0.2583	0.927	0
7786	321.5	1.3062	0.2474	0.2579	0.927	0
7787	321.4	1.305	0.2472	0.2578	0.9264	0
7788	321.3	1.3054	0.2473	0.2578	0.9266	0
7789	321.2	1.3049	0.2472	0.2576	0.926	0
7790	321.1	1.3046	0.247	0.2576	0.9261	0
7791	321	1.3036	0.2471	0.2577	0.926	0
7792	320.9	1.3031	0.2473	0.2574	0.9254	0
7793	320.8	1.3034	0.2472	0.2574	0.9255	0
7794	320.7	1.3024	0.2471	0.2576	0.9251	0
7795	320.6	1.3012	0.2469	0.2572	0.9251	0
7796	320.5	1.3029	0.2466	0.2569	0.9248	0
7797	320.4	1.3011	0.2467	0.2571	0.9247	0
7798	320.3	1.3013	0.2467	0.2569	0.9237	0
7799	320.2	1.2994	0.2466	0.2571	0.9238	0
7800	320.1	1.2998	0.2464	0.2568	0.9236	0
7801	320	1.2993	0.2464	0.2567	0.9236	

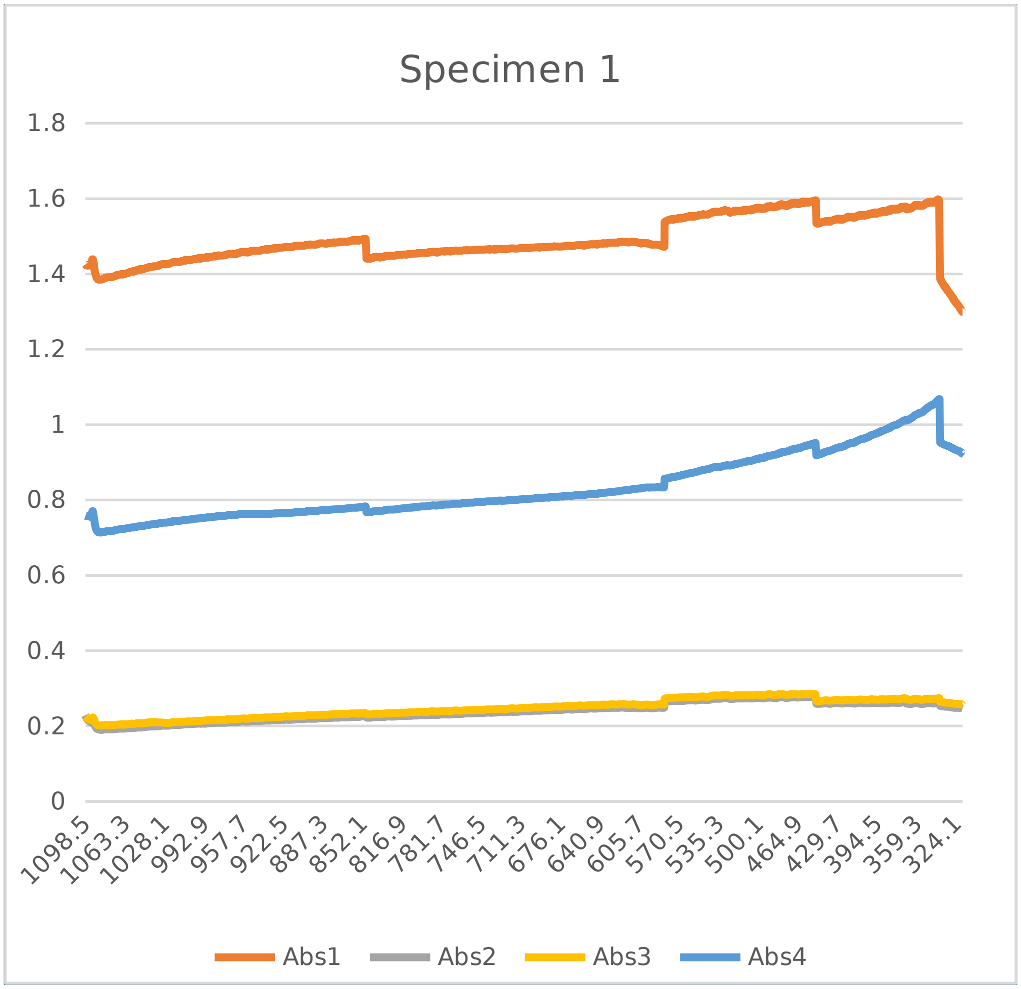

Specimen 1
1.8
1.6
1.4
1.2
1
0.8
0.6
0.4
0.2
0
1098.5
1063.3
1028.1
992.9
957.7
922.5
887.3
852.1
816.9
781.7
746.5
711.3
676.1
640.9
605.7
570.5
535.3
500.1
464.9
429.7
394.5
359.3
324.1
Abs1
Abs2
Abs3
Abs4
