# Supplementary material for: Coherent poly propagation materials with 3-dimensional photonic control over visible light
Source: PLoS One. 2019 Oct 17;14(10):e0223715. doi: 10.1371/journal.pone.0223715 (PMC6797174; doi:10.1371/journal.pone.0223715)
Supplement: S2 Table — Spreadsheet generated graph at bottom of table. (PDF) [file pone.0223715.s011.pdf]

Wavelength Scan Report - Specimen 2

Company	Complete Consulting Services LLC		
Operator	Dr. Michelle R. Stem	Test Time	2/11/2017 1:42
Document No.	WLS1702110001_1	Print Date	9/7/2019 3:59
Start wavelength	1100nm	Number of data	7801
End wavelength	320nm	K1	0.99
Unit		K0	0
Interval	0.1nm	K2	0
Calculate the Conc.	No	K3	0
Modification time	2/11/2017 1:42	Access time	2/11/2017 1:42
Remark	S13e027 - Specimen 2		

Scan Data

No.	Wavelength	Abs1	Abs2	Abs3	Abs4	Visible
1	1100	0.7947	0.4673	0.4307	0.4794	0
2	1099.9	0.7919	0.4672	0.4305	0.4785	0
3	1099.8	0.7895	0.4673	0.4304	0.4775	0
4	1099.7	0.7865	0.4672	0.4302	0.4768	0
5	1099.6	0.4711	0.4673	0.4301	0.4756	0
6	1099.5	0.4545	0.4674	0.4301	0.4745	0
7	1099.4	0.4431	0.4674	0.4296	0.4733	0
8	1099.3	0.4376	0.4674	0.4297	0.4729	0
9	1099.2	0.4295	0.4665	0.4295	0.4722	0
10	1099.1	0.4244	0.4662	0.4298	0.4716	0
11	1099	0.4195	0.4659	0.4297	0.4703	0
12	1098.9	0.4147	0.4654	0.4295	0.4695	0
13	1098.8	0.4112	0.4655	0.4296	0.4693	0
14	1098.7	0.4074	0.4653	0.4295	0.4683	0
15	1098.6	0.4046	0.4653	0.4295	0.468	0
16	1098.5	0.4014	0.4653	0.4294	0.467	0
17	1098.4	0.3984	0.4653	0.4298	0.4663	0
18	1098.3	0.395	0.4653	0.4296	0.4656	0
19	1098.2	0.3928	0.4651	0.4295	0.4649	0
20	1098.1	0.3904	0.4649	0.4296	0.4644	0
21	1098	0.3884	0.465	0.4295	0.4636	0
22	1097.9	0.386	0.4647	0.4295	0.4629	0
23	1097.8	0.3842	0.4649	0.4299	0.4625	0
24	1097.7	0.3819	0.4644	0.4298	0.4618	0
25	1097.6	0.3802	0.4639	0.4305	0.4615	0
26	1097.5	0.3779	0.4633	0.4302	0.4605	0
27	1097.4	0.3752	0.4624	0.43	0.4596	0
28	1097.3	0.3734	0.4623	0.43	0.4589	0
29	1097.2	0.3715	0.4618	0.43	0.4582	0

30	1097.1	0.3697	0.4611	0.4299	0.4574	0
31	1097	0.3683	0.4613	0.4302	0.457	0
32	1096.9	0.3668	0.4616	0.4304	0.4563	0
33	1096.8	0.3649	0.4613	0.4304	0.4553	0
34	1096.7	0.3646	0.4613	0.4304	0.4552	0
35	1096.6	0.3636	0.4611	0.4307	0.4542	0
36	1096.5	0.3623	0.4605	0.4307	0.4539	0
37	1096.4	0.3608	0.4599	0.4306	0.4534	0
38	1096.3	0.3597	0.46	0.4305	0.4529	0
39	1096.2	0.3587	0.4599	0.4304	0.4524	0
40	1096.1	0.3574	0.4593	0.4304	0.4519	0
41	1096	0.3564	0.4592	0.4311	0.4513	0
42	1095.9	0.3551	0.4588	0.4308	0.4507	0
43	1095.8	0.3545	0.4587	0.431	0.4498	0
44	1095.7	0.3534	0.4583	0.4309	0.4486	0
45	1095.6	0.3519	0.4577	0.4309	0.4477	0
46	1095.5	0.351	0.4573	0.4307	0.4468	0
47	1095.4	0.3501	0.4566	0.4305	0.4456	0
48	1095.3	0.3486	0.4559	0.4306	0.4446	0
49	1095.2	0.3476	0.4553	0.4307	0.4433	0
50	1095.1	0.3466	0.455	0.4311	0.4426	0
51	1095	0.3458	0.4546	0.4311	0.4424	0
52	1094.9	0.3451	0.4546	0.4314	0.442	0
53	1094.8	0.3448	0.4543	0.4317	0.4423	0
54	1094.7	0.3441	0.454	0.4314	0.4417	0
55	1094.6	0.3438	0.4539	0.4321	0.4421	0
56	1094.5	0.3438	0.4543	0.432	0.4422	0
57	1094.4	0.3441	0.4548	0.4324	0.4426	0
58	1094.3	0.3438	0.4551	0.4325	0.4426	0
59	1094.2	0.3445	0.4561	0.433	0.4434	0
60	1094.1	0.3449	0.457	0.4335	0.444	0
61	1094	0.3448	0.4572	0.4337	0.4445	0
62	1093.9	0.3451	0.458	0.4341	0.4452	0
63	1093.8	0.3453	0.4591	0.4342	0.4457	0
64	1093.7	0.3459	0.4598	0.4346	0.4459	0
65	1093.6	0.346	0.4598	0.4345	0.4461	0
66	1093.5	0.3457	0.4601	0.4346	0.446	0
67	1093.4	0.3458	0.4599	0.435	0.446	0
68	1093.3	0.3457	0.4599	0.4351	0.4459	0
69	1093.2	0.3453	0.4597	0.4351	0.4455	0
70	1093.1	0.3446	0.4596	0.4351	0.4449	0
71	1093	0.3444	0.4591	0.435	0.4443	0
72	1092.9	0.3436	0.4586	0.4351	0.4433	0

73	1092.8	0.3427	0.4574	0.4347	0.4418	0
74	1092.7	0.3414	0.4563	0.4343	0.4407	0
75	1092.6	0.3405	0.4554	0.4346	0.4392	0
76	1092.5	0.3393	0.4545	0.4344	0.438	0
77	1092.4	0.3381	0.453	0.4338	0.4362	0
78	1092.3	0.3371	0.4517	0.4339	0.4346	0
79	1092.2	0.3356	0.4502	0.4332	0.4327	0
80	1092.1	0.3345	0.4487	0.4331	0.4312	0
81	1092	0.3332	0.4472	0.4328	0.4293	0
82	1091.9	0.3321	0.4458	0.4326	0.4274	0
83	1091.8	0.3307	0.4444	0.4325	0.426	0
84	1091.7	0.3293	0.4428	0.4322	0.4243	0
85	1091.6	0.3278	0.4416	0.4319	0.4224	0
86	1091.5	0.3266	0.4404	0.4317	0.4213	0
87	1091.4	0.3258	0.4392	0.4318	0.4197	0
88	1091.3	0.3247	0.4377	0.4315	0.4185	0
89	1091.2	0.3235	0.4368	0.4314	0.4174	0
90	1091.1	0.3224	0.4358	0.4313	0.4161	0
91	1091	0.3212	0.4345	0.4309	0.4147	0
92	1090.9	0.3207	0.4337	0.4308	0.4139	0
93	1090.8	0.32	0.4331	0.4306	0.4129	0
94	1090.7	0.3195	0.4322	0.4308	0.4119	0
95	1090.6	0.3185	0.4312	0.4304	0.4107	0
96	1090.5	0.318	0.4306	0.4304	0.41	0
97	1090.4	0.3172	0.4298	0.4304	0.409	0
98	1090.3	0.3162	0.4291	0.4303	0.4081	0
99	1090.2	0.3162	0.4287	0.4305	0.4075	0
100	1090.1	0.3154	0.4277	0.4301	0.4068	0
101	1090	0.3152	0.4274	0.4303	0.4065	0
102	1089.9	0.3142	0.4268	0.43	0.4059	0
103	1089.8	0.3136	0.4264	0.4298	0.4052	0
104	1089.7	0.3132	0.4262	0.4299	0.4045	0
105	1089.6	0.3131	0.4259	0.4301	0.4041	0
106	1089.5	0.3127	0.4254	0.4298	0.4037	0
107	1089.4	0.3127	0.4251	0.43	0.4035	0
108	1089.3	0.3123	0.4248	0.4301	0.4032	0
109	1089.2	0.3117	0.4244	0.4299	0.4026	0
110	1089.1	0.3112	0.424	0.4299	0.4021	0
111	1089	0.3109	0.4237	0.4299	0.4017	0
112	1088.9	0.3106	0.4237	0.4297	0.4012	0
113	1088.8	0.3104	0.4233	0.43	0.4011	0
114	1088.7	0.3103	0.423	0.4296	0.4007	0
115	1088.6	0.31	0.4232	0.4298	0.4007	0

116	1088.5	0.3098	0.4232	0.4297	0.4002	0
117	1088.4	0.3098	0.4228	0.4299	0.4	0
118	1088.3	0.3093	0.4221	0.4297	0.3995	0
119	1088.2	0.3092	0.4219	0.4297	0.3994	0
120	1088.1	0.3095	0.4224	0.43	0.3995	0
121	1088	0.3091	0.4221	0.4301	0.3993	0
122	1087.9	0.3087	0.4221	0.4301	0.3993	0
123	1087.8	0.3086	0.422	0.43	0.3991	0
124	1087.7	0.3087	0.4219	0.43	0.3991	0
125	1087.6	0.3086	0.4221	0.4301	0.3989	0
126	1087.5	0.3084	0.4217	0.4301	0.3986	0
127	1087.4	0.3083	0.4215	0.4301	0.3988	0
128	1087.3	0.3083	0.4215	0.4304	0.3989	0
129	1087.2	0.3083	0.4219	0.4302	0.3989	0
130	1087.1	0.308	0.4215	0.4302	0.3984	0
131	1087	0.3079	0.4213	0.4302	0.3982	0
132	1086.9	0.3075	0.4213	0.4303	0.3982	0
133	1086.8	0.3077	0.4212	0.4304	0.3981	0
134	1086.7	0.3076	0.4209	0.4302	0.3979	0
135	1086.6	0.3073	0.4213	0.4304	0.398	0
136	1086.5	0.3071	0.4206	0.4302	0.3976	0
137	1086.4	0.307	0.4209	0.4305	0.3978	0
138	1086.3	0.3064	0.4203	0.4302	0.3973	0
139	1086.2	0.3066	0.4205	0.4302	0.3971	0
140	1086.1	0.3066	0.4201	0.4301	0.3967	0
141	1086	0.3066	0.4203	0.43	0.3969	0
142	1085.9	0.3064	0.4199	0.4304	0.3965	0
143	1085.8	0.3065	0.4201	0.4305	0.3964	0
144	1085.7	0.3059	0.4198	0.4302	0.3964	0
145	1085.6	0.3063	0.4199	0.4305	0.3966	0
146	1085.5	0.3064	0.4201	0.4304	0.3964	0
147	1085.4	0.3063	0.4205	0.4309	0.3964	0
148	1085.3	0.306	0.4198	0.4307	0.3963	0
149	1085.2	0.3061	0.4198	0.431	0.3963	0
150	1085.1	0.3059	0.4198	0.4311	0.3965	0
151	1085	0.3058	0.4201	0.4314	0.3961	0
152	1084.9	0.3058	0.4202	0.4313	0.3961	0
153	1084.8	0.3056	0.42	0.431	0.396	0
154	1084.7	0.3058	0.4204	0.431	0.396	0
155	1084.6	0.3059	0.4204	0.4315	0.396	0
156	1084.5	0.3058	0.4203	0.4316	0.396	0
157	1084.4	0.3059	0.4202	0.432	0.3959	0
158	1084.3	0.3058	0.4199	0.432	0.3957	0

159	1084.2	0.3059	0.4201	0.432	0.396	0
160	1084.1	0.3057	0.42	0.4322	0.3957	0
161	1084	0.3059	0.4196	0.4322	0.3956	0
162	1083.9	0.3062	0.4201	0.433	0.396	0
163	1083.8	0.306	0.4202	0.4328	0.3961	0
164	1083.7	0.3056	0.42	0.4325	0.3959	0
165	1083.6	0.3059	0.4202	0.4328	0.3961	0
166	1083.5	0.3058	0.4202	0.433	0.3959	0
167	1083.4	0.3055	0.4201	0.4327	0.3959	0
168	1083.3	0.3055	0.42	0.4331	0.3957	0
169	1083.2	0.3055	0.4198	0.4331	0.3954	0
170	1083.1	0.3055	0.4197	0.433	0.3956	0
171	1083	0.3054	0.4197	0.4332	0.3956	0
172	1082.9	0.3055	0.4197	0.4334	0.3956	0
173	1082.8	0.3052	0.4198	0.4336	0.3956	0
174	1082.7	0.3051	0.4193	0.4335	0.3958	0
175	1082.6	0.3054	0.4201	0.4338	0.3957	0
176	1082.5	0.3055	0.4202	0.434	0.3958	0
177	1082.4	0.3057	0.4202	0.434	0.3958	0
178	1082.3	0.3054	0.4202	0.434	0.3952	0
179	1082.2	0.3055	0.4201	0.4336	0.3951	0
180	1082.1	0.3053	0.4204	0.4341	0.3956	0
181	1082	0.3057	0.4204	0.4341	0.3956	0
182	1081.9	0.3057	0.4203	0.4343	0.3958	0
183	1081.8	0.3056	0.4201	0.4343	0.3958	0
184	1081.7	0.3056	0.4201	0.4344	0.3951	0
185	1081.6	0.3054	0.4201	0.4345	0.3949	0
186	1081.5	0.3055	0.4197	0.4345	0.3948	0
187	1081.4	0.3054	0.4199	0.4341	0.3948	0
188	1081.3	0.3055	0.4199	0.4343	0.3951	0
189	1081.2	0.3056	0.4199	0.4344	0.3949	0
190	1081.1	0.3053	0.4202	0.4346	0.3945	0
191	1081	0.3049	0.4199	0.4344	0.3947	0
192	1080.9	0.3053	0.4199	0.4346	0.3948	0
193	1080.8	0.3057	0.4201	0.4349	0.3952	0
194	1080.7	0.305	0.4196	0.4343	0.3945	0
195	1080.6	0.3054	0.4203	0.4349	0.3949	0
196	1080.5	0.3053	0.4199	0.435	0.3949	0
197	1080.4	0.3052	0.4197	0.4349	0.3946	0
198	1080.3	0.3054	0.4199	0.435	0.3946	0
199	1080.2	0.3054	0.4199	0.4353	0.3948	0
200	1080.1	0.3055	0.4199	0.4349	0.3945	0
201	1080	0.3051	0.4198	0.4348	0.3942	0

202	1079.9	0.3049	0.4201	0.4349	0.3938	0
203	1079.8	0.3049	0.4195	0.4345	0.3937	0
204	1079.7	0.3051	0.4198	0.4347	0.3939	0
205	1079.6	0.305	0.4196	0.4349	0.3936	0
206	1079.5	0.3052	0.4196	0.4351	0.394	0
207	1079.4	0.3057	0.4204	0.4359	0.3944	0
208	1079.3	0.3048	0.4198	0.4351	0.3938	0
209	1079.2	0.3047	0.4195	0.435	0.3933	0
210	1079.1	0.3047	0.4194	0.4351	0.393	0
211	1079	0.3048	0.4196	0.4348	0.3928	0
212	1078.9	0.3047	0.4194	0.435	0.3929	0
213	1078.8	0.3044	0.4191	0.4351	0.3925	0
214	1078.7	0.3049	0.4197	0.4355	0.3929	0
215	1078.6	0.3045	0.4196	0.4349	0.3922	0
216	1078.5	0.3042	0.4195	0.4345	0.3919	0
217	1078.4	0.3046	0.4192	0.4348	0.392	0
218	1078.3	0.3046	0.4193	0.4347	0.3917	0
219	1078.2	0.3042	0.4194	0.4348	0.3917	0
220	1078.1	0.3041	0.4189	0.4349	0.391	0
221	1078	0.304	0.419	0.4349	0.391	0
222	1077.9	0.3039	0.4189	0.435	0.391	0
223	1077.8	0.3041	0.4187	0.435	0.3909	0
224	1077.7	0.3046	0.4192	0.435	0.3908	0
225	1077.6	0.3042	0.4189	0.4353	0.3907	0
226	1077.5	0.3042	0.4189	0.4348	0.39	0
227	1077.4	0.3038	0.4186	0.4349	0.3896	0
228	1077.3	0.3041	0.4185	0.435	0.3897	0
229	1077.2	0.304	0.4189	0.435	0.3896	0
230	1077.1	0.3039	0.4188	0.435	0.3895	0
231	1077	0.304	0.4189	0.4354	0.3894	0
232	1076.9	0.304	0.4188	0.4355	0.3894	0
233	1076.8	0.304	0.4188	0.4352	0.3892	0
234	1076.7	0.3037	0.4186	0.4351	0.3888	0
235	1076.6	0.3039	0.4186	0.4355	0.3884	0
236	1076.5	0.304	0.4185	0.4354	0.3883	0
237	1076.4	0.3043	0.4187	0.4357	0.3886	0
238	1076.3	0.3038	0.4184	0.4353	0.3882	0
239	1076.2	0.3039	0.4183	0.4354	0.388	0
240	1076.1	0.3039	0.4182	0.4354	0.3875	0
241	1076	0.304	0.4183	0.4355	0.3876	0
242	1075.9	0.3039	0.4184	0.4354	0.3874	0
243	1075.8	0.3043	0.4188	0.4361	0.3879	0
244	1075.7	0.3042	0.419	0.4361	0.3877	0

245	1075.6	0.3042	0.4193	0.4361	0.3876	0
246	1075.5	0.304	0.4188	0.4362	0.3873	0
247	1075.4	0.3043	0.419	0.4364	0.3872	0
248	1075.3	0.3043	0.4188	0.436	0.3867	0
249	1075.2	0.3044	0.4193	0.4361	0.3866	0
250	1075.1	0.3044	0.4193	0.4362	0.3866	0
251	1075	0.3046	0.4195	0.4367	0.3865	0
252	1074.9	0.3044	0.4192	0.4366	0.3861	0
253	1074.8	0.3044	0.4194	0.4368	0.3858	0
254	1074.7	0.3046	0.4193	0.4369	0.3855	0
255	1074.6	0.3045	0.4193	0.4372	0.3853	0
256	1074.5	0.3044	0.4195	0.4369	0.3852	0
257	1074.4	0.3045	0.4196	0.4366	0.3849	0
258	1074.3	0.3045	0.4191	0.4368	0.3847	0
259	1074.2	0.3044	0.4195	0.4368	0.3846	0
260	1074.1	0.3046	0.4197	0.4374	0.3848	0
261	1074	0.3045	0.4199	0.4371	0.3847	0
262	1073.9	0.3048	0.4201	0.4378	0.3852	0
263	1073.8	0.3051	0.4197	0.4379	0.385	0
264	1073.7	0.3049	0.42	0.4379	0.3851	0
265	1073.6	0.305	0.4201	0.4381	0.3847	0
266	1073.5	0.3051	0.42	0.4379	0.3848	0
267	1073.4	0.305	0.4196	0.4378	0.3849	0
268	1073.3	0.3052	0.4199	0.4378	0.3849	0
269	1073.2	0.305	0.4203	0.4382	0.3853	0
270	1073.1	0.3052	0.4206	0.4384	0.3852	0
271	1073	0.3052	0.4202	0.4383	0.3849	0
272	1072.9	0.305	0.4201	0.4382	0.3845	0
273	1072.8	0.305	0.4198	0.4384	0.3843	0
274	1072.7	0.3054	0.4202	0.4387	0.3841	0
275	1072.6	0.3056	0.4206	0.4388	0.3845	0
276	1072.5	0.3051	0.4201	0.4389	0.3842	0
277	1072.4	0.3053	0.4203	0.439	0.384	0
278	1072.3	0.3055	0.4208	0.4389	0.3837	0
279	1072.2	0.3057	0.4202	0.4388	0.3837	0
280	1072.1	0.3056	0.4206	0.4392	0.384	0
281	1072	0.3056	0.4206	0.4393	0.3838	0
282	1071.9	0.3056	0.4206	0.4393	0.3835	0
283	1071.8	0.3058	0.4208	0.4396	0.3834	0
284	1071.7	0.3063	0.4208	0.4398	0.3833	0
285	1071.6	0.3061	0.421	0.4396	0.3832	0
286	1071.5	0.3054	0.421	0.4396	0.383	0
287	1071.4	0.3058	0.4211	0.44	0.383	0

288	1071.3	0.306	0.4211	0.4404	0.3829	0
289	1071.2	0.3058	0.421	0.4404	0.3827	0
290	1071.1	0.3059	0.4209	0.4401	0.3823	0
291	1071	0.3053	0.4208	0.4399	0.3822	0
292	1070.9	0.3057	0.421	0.4401	0.3823	0
293	1070.8	0.3059	0.4209	0.4402	0.3818	0
294	1070.7	0.3062	0.421	0.4404	0.3816	0
295	1070.6	0.3062	0.4212	0.4407	0.3815	0
296	1070.5	0.306	0.4214	0.4405	0.3812	0
297	1070.4	0.3058	0.4212	0.4403	0.3812	0
298	1070.3	0.3059	0.4214	0.44	0.381	0
299	1070.2	0.3062	0.4213	0.4403	0.3809	0
300	1070.1	0.3059	0.4211	0.4404	0.3807	0
301	1070	0.3061	0.4211	0.4406	0.3807	0
302	1069.9	0.306	0.4212	0.4408	0.3804	0
303	1069.8	0.3064	0.4215	0.4409	0.3804	0
304	1069.7	0.3064	0.4214	0.4406	0.3804	0
305	1069.6	0.3065	0.4211	0.4412	0.38	0
306	1069.5	0.3062	0.421	0.441	0.3797	0
307	1069.4	0.3062	0.4207	0.4409	0.3793	0
308	1069.3	0.3056	0.4205	0.4407	0.379	0
309	1069.2	0.3059	0.4207	0.4407	0.3793	0
310	1069.1	0.3061	0.4211	0.4409	0.3795	0
311	1069	0.3061	0.4211	0.441	0.3786	0
312	1068.9	0.3063	0.4213	0.4411	0.3789	0
313	1068.8	0.3062	0.4211	0.4409	0.3786	0
314	1068.7	0.306	0.4211	0.4407	0.3784	0
315	1068.6	0.3062	0.421	0.441	0.3781	0
316	1068.5	0.3059	0.4205	0.4407	0.3781	0
317	1068.4	0.3059	0.4206	0.4405	0.3779	0
318	1068.3	0.3062	0.4212	0.441	0.378	0
319	1068.2	0.3059	0.4209	0.4409	0.378	0
320	1068.1	0.3063	0.4212	0.4409	0.3778	0
321	1068	0.3061	0.4208	0.4408	0.3776	0
322	1067.9	0.306	0.4204	0.4408	0.3773	0
323	1067.8	0.3059	0.4204	0.4409	0.3768	0
324	1067.7	0.3056	0.4207	0.4411	0.377	0
325	1067.6	0.3056	0.421	0.4409	0.3769	0
326	1067.5	0.3062	0.421	0.4414	0.3767	0
327	1067.4	0.306	0.4209	0.4411	0.3769	0
328	1067.3	0.3061	0.4209	0.441	0.3765	0
329	1067.2	0.306	0.4208	0.4411	0.3761	0
330	1067.1	0.3061	0.4208	0.441	0.3756	0

331	1067	0.3057	0.4206	0.4405	0.3756	0
332	1066.9	0.3058	0.4203	0.4408	0.3754	0
333	1066.8	0.3057	0.4208	0.4409	0.375	0
334	1066.7	0.306	0.4212	0.4414	0.3755	0
335	1066.6	0.306	0.4212	0.4414	0.3753	0
336	1066.5	0.306	0.4209	0.4412	0.3751	0
337	1066.4	0.3058	0.4206	0.4414	0.3748	0
338	1066.3	0.3057	0.4203	0.4409	0.3746	0
339	1066.2	0.3058	0.4204	0.4411	0.3745	0
340	1066.1	0.3056	0.4203	0.4412	0.3746	0
341	1066	0.3055	0.4201	0.4409	0.3743	0
342	1065.9	0.3057	0.4203	0.4406	0.374	0
343	1065.8	0.3055	0.4204	0.4412	0.374	0
344	1065.7	0.3058	0.4205	0.4414	0.3739	0
345	1065.6	0.3061	0.4206	0.4417	0.3743	0
346	1065.5	0.3058	0.4207	0.4419	0.3744	0
347	1065.4	0.3063	0.4209	0.4418	0.3744	0
348	1065.3	0.3062	0.421	0.4417	0.3746	0
349	1065.2	0.3064	0.4211	0.4418	0.3742	0
350	1065.1	0.306	0.4206	0.4419	0.3739	0
351	1065	0.3061	0.4207	0.4415	0.3737	0
352	1064.9	0.3061	0.4208	0.442	0.3739	0
353	1064.8	0.3056	0.4206	0.4418	0.3737	0
354	1064.7	0.3057	0.4202	0.4418	0.3736	0
355	1064.6	0.3058	0.4203	0.4419	0.3735	0
356	1064.5	0.3054	0.4201	0.4419	0.3734	0
357	1064.4	0.3059	0.4202	0.4421	0.3737	0
358	1064.3	0.3059	0.4205	0.4422	0.3735	0
359	1064.2	0.3058	0.42	0.4423	0.3731	0
360	1064.1	0.3059	0.4202	0.4425	0.3729	0
361	1064	0.3058	0.4204	0.4423	0.3729	0
362	1063.9	0.3059	0.4202	0.442	0.3728	0
363	1063.8	0.3059	0.4203	0.4425	0.3732	0
364	1063.7	0.306	0.4204	0.4426	0.3728	0
365	1063.6	0.306	0.4205	0.4427	0.373	0
366	1063.5	0.3062	0.4202	0.443	0.3728	0
367	1063.4	0.3063	0.4206	0.4428	0.3727	0
368	1063.3	0.3065	0.4205	0.443	0.3724	0
369	1063.2	0.3066	0.4204	0.443	0.3723	0
370	1063.1	0.3067	0.4204	0.4431	0.3725	0
371	1063	0.3069	0.4204	0.4434	0.3726	0
372	1062.9	0.3068	0.421	0.4436	0.3728	0
373	1062.8	0.3066	0.4209	0.4435	0.3724	0

374	1062.7	0.3067	0.4207	0.4434	0.3724	0
375	1062.6	0.3068	0.4207	0.4434	0.3721	0
376	1062.5	0.307	0.4207	0.4435	0.3718	0
377	1062.4	0.307	0.4205	0.4437	0.3717	0
378	1062.3	0.3069	0.4206	0.4438	0.3718	0
379	1062.2	0.3071	0.421	0.4442	0.3719	0
380	1062.1	0.3072	0.421	0.4442	0.3719	0
381	1062	0.3074	0.421	0.4444	0.3718	0
382	1061.9	0.3071	0.4209	0.4441	0.3715	0
383	1061.8	0.3072	0.4208	0.4439	0.3715	0
384	1061.7	0.3073	0.421	0.4441	0.3712	0
385	1061.6	0.3071	0.4211	0.4446	0.3711	0
386	1061.5	0.3071	0.421	0.4443	0.3711	0
387	1061.4	0.3071	0.4209	0.4446	0.3708	0
388	1061.3	0.3071	0.4211	0.4445	0.3707	0
389	1061.2	0.3073	0.4209	0.4443	0.3705	0
390	1061.1	0.3071	0.421	0.4444	0.3708	0
391	1061	0.3073	0.4212	0.4448	0.3706	0
392	1060.9	0.307	0.4208	0.4443	0.3701	0
393	1060.8	0.3071	0.4214	0.4442	0.3702	0
394	1060.7	0.3068	0.4208	0.4443	0.3697	0
395	1060.6	0.3069	0.4213	0.4448	0.37	0
396	1060.5	0.3069	0.4207	0.445	0.3699	0
397	1060.4	0.3071	0.4212	0.445	0.3698	0
398	1060.3	0.3077	0.4214	0.4455	0.37	0
399	1060.2	0.3075	0.4212	0.4454	0.3696	0
400	1060.1	0.3076	0.4214	0.4456	0.3698	0
401	1060	0.3076	0.4213	0.4456	0.3694	0
402	1059.9	0.3074	0.4213	0.4452	0.3692	0
403	1059.8	0.3076	0.421	0.4454	0.3692	0
404	1059.7	0.3078	0.4215	0.4455	0.369	0
405	1059.6	0.3078	0.4217	0.4458	0.3689	0
406	1059.5	0.3077	0.4217	0.4458	0.3688	0
407	1059.4	0.3079	0.4217	0.4459	0.3686	0
408	1059.3	0.3077	0.4213	0.4461	0.3683	0
409	1059.2	0.3077	0.4214	0.4463	0.3683	0
410	1059.1	0.308	0.4214	0.4463	0.3682	0
411	1059	0.3079	0.4215	0.446	0.3679	0
412	1058.9	0.3076	0.4211	0.4461	0.3675	0
413	1058.8	0.3078	0.4212	0.4462	0.3669	0
414	1058.7	0.3083	0.422	0.4467	0.3673	0
415	1058.6	0.308	0.4214	0.4464	0.3667	0
416	1058.5	0.3081	0.4217	0.4461	0.3667	0

417	1058.4	0.3081	0.4219	0.4465	0.3665	0
418	1058.3	0.3081	0.4213	0.4468	0.3662	0
419	1058.2	0.3081	0.4217	0.4467	0.3662	0
420	1058.1	0.3082	0.4216	0.4464	0.3658	0
421	1058	0.3081	0.4215	0.4465	0.3653	0
422	1057.9	0.3082	0.4216	0.4468	0.3651	0
423	1057.8	0.3085	0.4218	0.4467	0.3651	0
424	1057.7	0.3083	0.4216	0.447	0.3648	0
425	1057.6	0.3085	0.4219	0.447	0.3643	0
426	1057.5	0.3082	0.4221	0.4468	0.3641	0
427	1057.4	0.3083	0.4219	0.4468	0.3637	0
428	1057.3	0.3083	0.4215	0.4471	0.3634	0
429	1057.2	0.3082	0.4215	0.4468	0.3629	0
430	1057.1	0.3081	0.4217	0.447	0.3625	0
431	1057	0.3082	0.4214	0.4473	0.3623	0
432	1056.9	0.3086	0.4217	0.4472	0.3619	0
433	1056.8	0.3086	0.4218	0.4477	0.3616	0
434	1056.7	0.3084	0.4217	0.4475	0.361	0
435	1056.6	0.3082	0.4219	0.4477	0.3606	0
436	1056.5	0.3083	0.422	0.4477	0.3603	0
437	1056.4	0.3085	0.4219	0.4477	0.3599	0
438	1056.3	0.3087	0.4217	0.4479	0.3594	0
439	1056.2	0.3091	0.4218	0.4481	0.3592	0
440	1056.1	0.3091	0.4217	0.448	0.3589	0
441	1056	0.3089	0.4219	0.4482	0.3585	0
442	1055.9	0.3089	0.422	0.448	0.3578	0
443	1055.8	0.309	0.422	0.4482	0.3576	0
444	1055.7	0.3087	0.422	0.4482	0.357	0
445	1055.6	0.3086	0.422	0.448	0.3565	0
446	1055.5	0.3092	0.4224	0.4487	0.3564	0
447	1055.4	0.3091	0.4222	0.4487	0.356	0
448	1055.3	0.3093	0.4224	0.4488	0.3555	0
449	1055.2	0.3094	0.4223	0.449	0.3555	0
450	1055.1	0.3094	0.4218	0.4488	0.3549	0
451	1055	0.3086	0.422	0.4492	0.3545	0
452	1054.9	0.3088	0.4219	0.4488	0.3541	0
453	1054.8	0.309	0.4221	0.4489	0.3538	0
454	1054.7	0.3093	0.4224	0.4492	0.3535	0
455	1054.6	0.3093	0.4224	0.4495	0.3532	0
456	1054.5	0.3089	0.4222	0.4493	0.353	0
457	1054.4	0.3094	0.4222	0.4494	0.3523	0
458	1054.3	0.3095	0.4223	0.4497	0.3519	0
459	1054.2	0.3093	0.4221	0.4494	0.3515	0

460	1054.1	0.3094	0.4224	0.4498	0.3517	0
461	1054	0.3095	0.4223	0.4496	0.3514	0
462	1053.9	0.3094	0.422	0.4497	0.3508	0
463	1053.8	0.3094	0.4221	0.45	0.3505	0
464	1053.7	0.31	0.4223	0.4503	0.3503	0
465	1053.6	0.3094	0.4222	0.4502	0.3505	0
466	1053.5	0.3097	0.4228	0.4502	0.3504	0
467	1053.4	0.3097	0.4227	0.4505	0.3504	0
468	1053.3	0.3098	0.4227	0.4506	0.3503	0
469	1053.2	0.31	0.423	0.4506	0.3505	0
470	1053.1	0.3102	0.4226	0.4506	0.3503	0
471	1053	0.3102	0.4229	0.4508	0.3502	0
472	1052.9	0.3102	0.4228	0.4509	0.3503	0
473	1052.8	0.3102	0.423	0.4507	0.3504	0
474	1052.7	0.3101	0.4231	0.4509	0.3501	0
475	1052.6	0.3103	0.4231	0.451	0.3501	0
476	1052.5	0.3102	0.423	0.4511	0.3499	0
477	1052.4	0.3104	0.4233	0.4516	0.3499	0
478	1052.3	0.3104	0.4232	0.4518	0.3496	0
479	1052.2	0.3104	0.4235	0.4518	0.3495	0
480	1052.1	0.3103	0.4235	0.4519	0.349	0
481	1052	0.3105	0.4232	0.4519	0.349	0
482	1051.9	0.3105	0.4234	0.4518	0.3488	0
483	1051.8	0.3102	0.4232	0.4517	0.3483	0
484	1051.7	0.3103	0.423	0.4518	0.348	0
485	1051.6	0.3103	0.4233	0.4521	0.348	0
486	1051.5	0.3103	0.4234	0.4519	0.3477	0
487	1051.4	0.3105	0.4236	0.4519	0.3478	0
488	1051.3	0.3105	0.4231	0.4521	0.3473	0
489	1051.2	0.3107	0.4233	0.452	0.3473	0
490	1051.1	0.3106	0.4232	0.4521	0.3472	0
491	1051	0.3106	0.4234	0.452	0.347	0
492	1050.9	0.3104	0.4234	0.4522	0.3469	0
493	1050.8	0.3107	0.4234	0.4524	0.3466	0
494	1050.7	0.3108	0.4238	0.4529	0.3464	0
495	1050.6	0.311	0.4238	0.4529	0.3462	0
496	1050.5	0.3105	0.4235	0.4526	0.3456	0
497	1050.4	0.3107	0.4235	0.4526	0.3455	0
498	1050.3	0.3108	0.4233	0.4524	0.3454	0
499	1050.2	0.3109	0.4234	0.4526	0.3452	0
500	1050.1	0.3112	0.4238	0.4533	0.3455	0
501	1050	0.3109	0.4234	0.4529	0.345	0
502	1049.9	0.3105	0.4231	0.4527	0.3444	0

503	1049.8	0.3106	0.4234	0.4531	0.3442	0
504	1049.7	0.3108	0.4236	0.4533	0.3444	0
505	1049.6	0.311	0.4235	0.4532	0.3439	0
506	1049.5	0.3109	0.4234	0.4528	0.3437	0
507	1049.4	0.311	0.4236	0.4532	0.3437	0
508	1049.3	0.3107	0.4235	0.453	0.3434	0
509	1049.2	0.311	0.4234	0.4533	0.3432	0
510	1049.1	0.311	0.4239	0.4535	0.3429	0
511	1049	0.3112	0.424	0.4537	0.3426	0
512	1048.9	0.3107	0.4237	0.4534	0.3423	0
513	1048.8	0.3112	0.4238	0.4535	0.342	0
514	1048.7	0.3109	0.4242	0.4541	0.3421	0
515	1048.6	0.3109	0.4239	0.4537	0.3416	0
516	1048.5	0.3112	0.4239	0.4538	0.3416	0
517	1048.4	0.3111	0.4238	0.4536	0.341	0
518	1048.3	0.311	0.4237	0.4538	0.341	0
519	1048.2	0.311	0.424	0.4541	0.3406	0
520	1048.1	0.3111	0.4239	0.454	0.3401	0
521	1048	0.311	0.4238	0.4542	0.3404	0
522	1047.9	0.3108	0.4236	0.4539	0.3402	0
523	1047.8	0.3112	0.4239	0.4541	0.3401	0
524	1047.7	0.3109	0.4241	0.4543	0.3397	0
525	1047.6	0.3112	0.4243	0.4545	0.3399	0
526	1047.5	0.3114	0.4244	0.4545	0.3396	0
527	1047.4	0.3116	0.4244	0.4549	0.3395	0
528	1047.3	0.3118	0.4246	0.455	0.3398	0
529	1047.2	0.3118	0.425	0.4554	0.3397	0
530	1047.1	0.3116	0.4246	0.4551	0.3391	0
531	1047	0.3111	0.4244	0.4548	0.3387	0
532	1046.9	0.3111	0.4246	0.4549	0.3382	0
533	1046.8	0.3118	0.4248	0.4554	0.3384	0
534	1046.7	0.3118	0.4248	0.4556	0.3383	0
535	1046.6	0.3118	0.4245	0.4553	0.3379	0
536	1046.5	0.3119	0.4248	0.4557	0.338	0
537	1046.4	0.3122	0.4249	0.456	0.338	0
538	1046.3	0.3122	0.4248	0.4564	0.3376	0
539	1046.2	0.3122	0.4251	0.456	0.3378	0
540	1046.1	0.3123	0.4248	0.456	0.3374	0
541	1046	0.312	0.4245	0.4554	0.337	0
542	1045.9	0.3118	0.4247	0.4555	0.3369	0
543	1045.8	0.3118	0.4251	0.4555	0.3367	0
544	1045.7	0.3123	0.4253	0.4556	0.3367	0
545	1045.6	0.312	0.4252	0.456	0.3368	0

546	1045.5	0.3122	0.4247	0.4563	0.3361	0
547	1045.4	0.3119	0.4252	0.4566	0.3361	0
548	1045.3	0.3125	0.4252	0.4567	0.3366	0
549	1045.2	0.3124	0.4254	0.4567	0.3361	0
550	1045.1	0.3124	0.4254	0.4568	0.3362	0
551	1045	0.3122	0.4252	0.4571	0.336	0
552	1044.9	0.3126	0.4254	0.4573	0.3362	0
553	1044.8	0.3122	0.4254	0.4574	0.3359	0
554	1044.7	0.3126	0.4253	0.4576	0.336	0
555	1044.6	0.313	0.4255	0.4575	0.3357	0
556	1044.5	0.3129	0.4256	0.4576	0.3357	0
557	1044.4	0.3129	0.4259	0.4578	0.336	0
558	1044.3	0.3126	0.4258	0.4577	0.3357	0
559	1044.2	0.3129	0.4258	0.4579	0.3356	0
560	1044.1	0.3136	0.4262	0.4582	0.3359	0
561	1044	0.3134	0.4261	0.458	0.3357	0
562	1043.9	0.3131	0.4263	0.4583	0.3357	0
563	1043.8	0.3132	0.4262	0.4583	0.3354	0
564	1043.7	0.3132	0.4266	0.4582	0.3356	0
565	1043.6	0.3132	0.4261	0.4583	0.3355	0
566	1043.5	0.3132	0.4264	0.4582	0.3353	0
567	1043.4	0.3134	0.4264	0.4587	0.3353	0
568	1043.3	0.3133	0.4263	0.4589	0.3353	0
569	1043.2	0.3134	0.4267	0.4589	0.3355	0
570	1043.1	0.3132	0.4266	0.4592	0.3352	0
571	1043	0.3134	0.4265	0.4592	0.3351	0
572	1042.9	0.3136	0.4267	0.4594	0.3356	0
573	1042.8	0.3137	0.4269	0.4595	0.3356	0
574	1042.7	0.3136	0.4269	0.4595	0.3352	0
575	1042.6	0.3138	0.4268	0.4598	0.3351	0
576	1042.5	0.3138	0.4271	0.4602	0.3353	0
577	1042.4	0.3137	0.4272	0.4604	0.3352	0
578	1042.3	0.3141	0.4275	0.4604	0.3351	0
579	1042.2	0.314	0.4271	0.4599	0.3349	0
580	1042.1	0.3139	0.4271	0.4601	0.3349	0
581	1042	0.3143	0.4272	0.4599	0.3348	0
582	1041.9	0.3142	0.4275	0.46	0.3346	0
583	1041.8	0.3138	0.427	0.46	0.3342	0
584	1041.7	0.314	0.427	0.4601	0.3344	0
585	1041.6	0.3142	0.4273	0.4605	0.3344	0
586	1041.5	0.3143	0.4273	0.4608	0.3345	0
587	1041.4	0.3142	0.4274	0.4607	0.3343	0
588	1041.3	0.3138	0.4272	0.4606	0.3339	0

589	1041.2	0.3143	0.4275	0.4607	0.334	0
590	1041.1	0.3142	0.4275	0.4606	0.3339	0
591	1041	0.314	0.4272	0.4608	0.3337	0
592	1040.9	0.3138	0.427	0.4606	0.3335	0
593	1040.8	0.314	0.4275	0.4608	0.3333	0
594	1040.7	0.314	0.4271	0.4612	0.3336	0
595	1040.6	0.3139	0.4275	0.4612	0.3331	0
596	1040.5	0.3141	0.4274	0.4614	0.3335	0
597	1040.4	0.3141	0.4271	0.4612	0.333	0
598	1040.3	0.3139	0.4273	0.4613	0.3331	0
599	1040.2	0.3139	0.4274	0.4612	0.3332	0
600	1040.1	0.314	0.4272	0.4612	0.3328	0
601	1040	0.3144	0.4274	0.4614	0.3328	0
602	1039.9	0.3139	0.4274	0.4611	0.3328	0
603	1039.8	0.3141	0.4272	0.4613	0.3328	0
604	1039.7	0.3139	0.427	0.4613	0.3327	0
605	1039.6	0.3139	0.427	0.4615	0.3325	0
606	1039.5	0.3139	0.4272	0.4616	0.3325	0
607	1039.4	0.3139	0.4271	0.4619	0.3324	0
608	1039.3	0.3138	0.427	0.4619	0.3325	0
609	1039.2	0.3138	0.4272	0.4621	0.3325	0
610	1039.1	0.3139	0.427	0.462	0.3323	0
611	1039	0.3137	0.4271	0.4618	0.332	0
612	1038.9	0.314	0.4272	0.4622	0.3323	0
613	1038.8	0.314	0.4272	0.4623	0.3324	0
614	1038.7	0.3142	0.4273	0.4623	0.3325	0
615	1038.6	0.3144	0.4275	0.4625	0.3326	0
616	1038.5	0.3139	0.4274	0.4626	0.3325	0
617	1038.4	0.3142	0.427	0.4627	0.3328	0
618	1038.3	0.3143	0.427	0.4631	0.3325	0
619	1038.2	0.3142	0.4271	0.4628	0.3324	0
620	1038.1	0.3143	0.4271	0.4626	0.3321	0
621	1038	0.314	0.4271	0.4625	0.3322	0
622	1037.9	0.3138	0.4269	0.4628	0.3322	0
623	1037.8	0.314	0.4271	0.4627	0.3317	0
624	1037.7	0.3139	0.4269	0.4628	0.3318	0
625	1037.6	0.314	0.4269	0.4629	0.3321	0
626	1037.5	0.3135	0.4265	0.4625	0.3317	0
627	1037.4	0.314	0.4267	0.4631	0.3319	0
628	1037.3	0.3142	0.4265	0.463	0.3318	0
629	1037.2	0.3139	0.4267	0.463	0.3316	0
630	1037.1	0.3138	0.4268	0.4631	0.3317	0
631	1037	0.3142	0.427	0.4632	0.3317	0

632	1036.9	0.3141	0.4271	0.4632	0.3318	0
633	1036.8	0.3143	0.4273	0.4634	0.332	0
634	1036.7	0.314	0.4273	0.4636	0.3317	0
635	1036.6	0.3146	0.4272	0.4641	0.3321	0
636	1036.5	0.3143	0.4276	0.4641	0.3321	0
637	1036.4	0.3141	0.4276	0.4641	0.3319	0
638	1036.3	0.3143	0.4273	0.4644	0.3319	0
639	1036.2	0.3146	0.4278	0.4645	0.3318	0
640	1036.1	0.3146	0.4277	0.4646	0.3319	0
641	1036	0.3148	0.428	0.465	0.3322	0
642	1035.9	0.315	0.4281	0.4652	0.332	0
643	1035.8	0.3149	0.4281	0.4653	0.332	0
644	1035.7	0.315	0.4279	0.4653	0.3321	0
645	1035.6	0.315	0.428	0.4657	0.3321	0
646	1035.5	0.315	0.4282	0.4654	0.3318	0
647	1035.4	0.3151	0.4281	0.4655	0.3319	0
648	1035.3	0.315	0.4286	0.4658	0.3318	0
649	1035.2	0.3152	0.4286	0.466	0.3319	0
650	1035.1	0.3152	0.4286	0.4661	0.3319	0
651	1035	0.3152	0.4284	0.4664	0.3319	0
652	1034.9	0.3151	0.4286	0.4665	0.3319	0
653	1034.8	0.3154	0.4287	0.4668	0.332	0
654	1034.7	0.3157	0.4291	0.4672	0.3324	0
655	1034.6	0.3159	0.4293	0.4671	0.3322	0
656	1034.5	0.3158	0.4291	0.4672	0.3323	0
657	1034.4	0.3158	0.4293	0.4675	0.3322	0
658	1034.3	0.3159	0.4292	0.4679	0.3326	0
659	1034.2	0.3159	0.4292	0.4679	0.3322	0
660	1034.1	0.3158	0.4297	0.4679	0.3324	0
661	1034	0.3161	0.4294	0.4679	0.3324	0
662	1033.9	0.3162	0.4296	0.4681	0.3323	0
663	1033.8	0.3163	0.4296	0.4683	0.3323	0
664	1033.7	0.3163	0.4299	0.4686	0.3326	0
665	1033.6	0.3162	0.4298	0.4685	0.3327	0
666	1033.5	0.3163	0.4299	0.4688	0.3325	0
667	1033.4	0.3165	0.4298	0.4688	0.3328	0
668	1033.3	0.3166	0.4298	0.4687	0.3323	0
669	1033.2	0.3162	0.4297	0.4688	0.3319	0
670	1033.1	0.3163	0.4301	0.469	0.3322	0
671	1033	0.3163	0.4299	0.4689	0.3321	0
672	1032.9	0.3165	0.4301	0.4694	0.3326	0
673	1032.8	0.3164	0.4305	0.4694	0.3322	0
674	1032.7	0.3164	0.4301	0.4693	0.3322	0

675	1032.6	0.3163	0.43	0.4697	0.3322	0
676	1032.5	0.3167	0.4301	0.4695	0.3324	0
677	1032.4	0.3167	0.4304	0.4699	0.3326	0
678	1032.3	0.3168	0.4305	0.47	0.3327	0
679	1032.2	0.3168	0.4304	0.4701	0.3323	0
680	1032.1	0.3166	0.4301	0.4702	0.3321	0
681	1032	0.3167	0.4299	0.4701	0.3325	0
682	1031.9	0.3171	0.4302	0.4704	0.3329	0
683	1031.8	0.317	0.4305	0.4705	0.3327	0
684	1031.7	0.317	0.4306	0.4707	0.3326	0
685	1031.6	0.3171	0.4307	0.4711	0.3326	0
686	1031.5	0.3174	0.4309	0.4711	0.3326	0
687	1031.4	0.3173	0.4308	0.4707	0.3325	0
688	1031.3	0.3172	0.4307	0.4708	0.3324	0
689	1031.2	0.3166	0.4308	0.4711	0.3324	0
690	1031.1	0.3171	0.431	0.4718	0.3327	0
691	1031	0.3171	0.4308	0.4715	0.3325	0
692	1030.9	0.317	0.4307	0.4713	0.3325	0
693	1030.8	0.3169	0.4307	0.4712	0.3322	0
694	1030.7	0.3169	0.4306	0.4713	0.3322	0
695	1030.6	0.3174	0.431	0.4718	0.3324	0
696	1030.5	0.3166	0.4305	0.4718	0.3319	0
697	1030.4	0.3172	0.4308	0.472	0.3323	0
698	1030.3	0.3172	0.4306	0.472	0.3321	0
699	1030.2	0.3173	0.4307	0.4724	0.3322	0
700	1030.1	0.3172	0.4307	0.472	0.3321	0
701	1030	0.3175	0.4308	0.4718	0.3321	0
702	1029.9	0.3172	0.4309	0.4717	0.332	0
703	1029.8	0.3172	0.4307	0.4718	0.332	0
704	1029.7	0.3169	0.4305	0.4722	0.3319	0
705	1029.6	0.3174	0.431	0.4724	0.3322	0
706	1029.5	0.3172	0.4308	0.4725	0.3318	0
707	1029.4	0.3168	0.4306	0.4722	0.3318	0
708	1029.3	0.3169	0.4306	0.4724	0.3318	0
709	1029.2	0.317	0.4305	0.4724	0.3319	0
710	1029.1	0.317	0.4305	0.4728	0.3318	0
711	1029	0.3172	0.4304	0.4725	0.3315	0
712	1028.9	0.3171	0.4306	0.4722	0.3317	0
713	1028.8	0.3166	0.4304	0.4723	0.3315	0
714	1028.7	0.3168	0.4306	0.4728	0.3317	0
715	1028.6	0.3168	0.4305	0.4726	0.3317	0
716	1028.5	0.3168	0.4303	0.4729	0.3316	0
717	1028.4	0.3167	0.4308	0.4728	0.3317	0

718	1028.3	0.317	0.4306	0.4729	0.3313	0
719	1028.2	0.317	0.4305	0.4731	0.3316	0
720	1028.1	0.317	0.4307	0.4733	0.3317	0
721	1028	0.3172	0.4308	0.4735	0.3318	0
722	1027.9	0.3167	0.4302	0.473	0.3313	0
723	1027.8	0.3168	0.4307	0.4732	0.3312	0
724	1027.7	0.3167	0.4308	0.4733	0.3313	0
725	1027.6	0.3168	0.4309	0.4734	0.3313	0
726	1027.5	0.3169	0.4307	0.4735	0.3312	0
727	1027.4	0.317	0.4307	0.4736	0.3314	0
728	1027.3	0.3166	0.4308	0.4735	0.3314	0
729	1027.2	0.3169	0.4304	0.4738	0.3312	0
730	1027.1	0.317	0.4306	0.4741	0.3313	0
731	1027	0.3168	0.4303	0.474	0.3309	0
732	1026.9	0.3168	0.4305	0.4737	0.331	0
733	1026.8	0.3168	0.4305	0.4738	0.331	0
734	1026.7	0.3172	0.4305	0.4739	0.3311	0
735	1026.6	0.317	0.4305	0.4742	0.331	0
736	1026.5	0.3167	0.4307	0.4743	0.3312	0
737	1026.4	0.317	0.4307	0.4744	0.3312	0
738	1026.3	0.3172	0.4313	0.4751	0.3311	0
739	1026.2	0.3172	0.4311	0.4751	0.3312	0
740	1026.1	0.3174	0.4307	0.4752	0.3311	0
741	1026	0.3173	0.4312	0.475	0.331	0
742	1025.9	0.3174	0.4314	0.4755	0.331	0
743	1025.8	0.3175	0.4313	0.4756	0.3307	0
744	1025.7	0.3175	0.4311	0.4758	0.3309	0
745	1025.6	0.3172	0.4313	0.4759	0.3309	0
746	1025.5	0.3174	0.4312	0.4756	0.3309	0
747	1025.4	0.3176	0.4314	0.4761	0.331	0
748	1025.3	0.3175	0.4315	0.4761	0.3309	0
749	1025.2	0.3178	0.4319	0.4764	0.331	0
750	1025.1	0.3179	0.4319	0.4767	0.3312	0
751	1025	0.3176	0.4317	0.4765	0.3311	0
752	1024.9	0.3177	0.4316	0.4765	0.3311	0
753	1024.8	0.318	0.4318	0.4769	0.331	0
754	1024.7	0.3182	0.4321	0.477	0.3315	0
755	1024.6	0.3183	0.4323	0.4775	0.3313	0
756	1024.5	0.3179	0.432	0.4776	0.3313	0
757	1024.4	0.318	0.4322	0.4777	0.3315	0
758	1024.3	0.3183	0.4325	0.4779	0.3314	0
759	1024.2	0.3184	0.4326	0.4781	0.3317	0
760	1024.1	0.3183	0.4326	0.4782	0.3317	0

761	1024	0.3184	0.433	0.4787	0.3318	0
762	1023.9	0.3184	0.4328	0.4786	0.3317	0
763	1023.8	0.3185	0.4329	0.4787	0.3317	0
764	1023.7	0.3184	0.433	0.4795	0.332	0
765	1023.6	0.3189	0.4334	0.4799	0.3322	0
766	1023.5	0.3189	0.4332	0.4794	0.3319	0
767	1023.4	0.3187	0.4332	0.4794	0.332	0
768	1023.3	0.3189	0.4334	0.4796	0.3322	0
769	1023.2	0.319	0.4334	0.4797	0.3322	0
770	1023.1	0.3193	0.4337	0.48	0.3326	0
771	1023	0.3192	0.4337	0.4802	0.3325	0
772	1022.9	0.3194	0.4337	0.4803	0.3323	0
773	1022.8	0.3193	0.4337	0.4804	0.3325	0
774	1022.7	0.3191	0.4337	0.4806	0.3324	0
775	1022.6	0.3194	0.4339	0.481	0.3324	0
776	1022.5	0.3198	0.434	0.481	0.3325	0
777	1022.4	0.3191	0.4339	0.481	0.3323	0
778	1022.3	0.3192	0.4342	0.4812	0.3326	0
779	1022.2	0.3196	0.4342	0.4813	0.3328	0
780	1022.1	0.3195	0.4342	0.4813	0.3326	0
781	1022	0.3197	0.4341	0.4815	0.3326	0
782	1021.9	0.3195	0.4339	0.4812	0.3327	0
783	1021.8	0.3198	0.4342	0.4815	0.3325	0
784	1021.7	0.3195	0.4339	0.4815	0.3322	0
785	1021.6	0.3194	0.434	0.4814	0.3324	0
786	1021.5	0.3193	0.4344	0.4821	0.3326	0
787	1021.4	0.3194	0.4341	0.4818	0.3324	0
788	1021.3	0.3196	0.4343	0.4816	0.3323	0
789	1021.2	0.3193	0.4341	0.4815	0.3322	0
790	1021.1	0.3189	0.4341	0.4818	0.3319	0
791	1021	0.3194	0.4342	0.4822	0.3327	0
792	1020.9	0.3197	0.4346	0.4824	0.3331	0
793	1020.8	0.3198	0.4348	0.4826	0.3329	0
794	1020.7	0.32	0.4347	0.4826	0.3327	0
795	1020.6	0.3202	0.4349	0.4826	0.3328	0
796	1020.5	0.3196	0.4345	0.4827	0.3326	0
797	1020.4	0.3197	0.4346	0.4829	0.3323	0
798	1020.3	0.3196	0.4344	0.4824	0.3324	0
799	1020.2	0.3199	0.4345	0.4831	0.3329	0
800	1020.1	0.3201	0.4345	0.4827	0.3328	0
801	1020	0.3199	0.4346	0.4831	0.3328	0
802	1019.9	0.3202	0.4349	0.4834	0.3334	0
803	1019.8	0.32	0.4347	0.4833	0.3327	0

804	1019.7	0.3197	0.4344	0.483	0.3323	0
805	1019.6	0.3201	0.4347	0.4835	0.3325	0
806	1019.5	0.3203	0.4347	0.4836	0.3324	0
807	1019.4	0.3204	0.4349	0.4836	0.3327	0
808	1019.3	0.3199	0.4351	0.4837	0.3328	0
809	1019.2	0.3199	0.4347	0.4836	0.3325	0
810	1019.1	0.3198	0.4347	0.4833	0.3325	0
811	1019	0.3199	0.435	0.4834	0.3324	0
812	1018.9	0.3194	0.4344	0.4832	0.3319	0
813	1018.8	0.3192	0.4345	0.4832	0.3321	0
814	1018.7	0.3193	0.4342	0.4833	0.332	0
815	1018.6	0.3195	0.4342	0.4834	0.3323	0
816	1018.5	0.3199	0.4343	0.4836	0.3321	0
817	1018.4	0.3198	0.4346	0.4839	0.3325	0
818	1018.3	0.3198	0.4347	0.4841	0.3322	0
819	1018.2	0.3194	0.4346	0.4838	0.3321	0
820	1018.1	0.3196	0.4345	0.4838	0.3319	0
821	1018	0.3201	0.4348	0.4843	0.3323	0
822	1017.9	0.3198	0.4349	0.4841	0.3323	0
823	1017.8	0.3194	0.4343	0.4839	0.3319	0
824	1017.7	0.3193	0.4342	0.4839	0.3318	0
825	1017.6	0.3194	0.4342	0.4838	0.3318	0
826	1017.5	0.3192	0.4341	0.4838	0.3317	0
827	1017.4	0.3188	0.4339	0.4837	0.3315	0
828	1017.3	0.3192	0.4341	0.4839	0.3314	0
829	1017.2	0.3194	0.434	0.4841	0.3317	0
830	1017.1	0.3195	0.4341	0.4843	0.3318	0
831	1017	0.3193	0.4341	0.4842	0.3315	0
832	1016.9	0.3195	0.4344	0.4842	0.3317	0
833	1016.8	0.3199	0.4343	0.4846	0.3318	0
834	1016.7	0.32	0.4343	0.4847	0.3318	0
835	1016.6	0.3199	0.4342	0.4849	0.3319	0
836	1016.5	0.3198	0.4346	0.4854	0.3322	0
837	1016.4	0.3197	0.4349	0.4854	0.332	0
838	1016.3	0.3199	0.4348	0.485	0.3318	0
839	1016.2	0.3199	0.4346	0.4853	0.332	0
840	1016.1	0.3201	0.4351	0.4858	0.332	0
841	1016	0.3199	0.4351	0.4857	0.3321	0
842	1015.9	0.3202	0.4349	0.4855	0.332	0
843	1015.8	0.3199	0.435	0.4859	0.3319	0
844	1015.7	0.32	0.4348	0.486	0.3321	0
845	1015.6	0.3201	0.4352	0.4862	0.3322	0
846	1015.5	0.3201	0.4351	0.4864	0.3322	0

847	1015.4	0.3201	0.4351	0.4862	0.3321	0
848	1015.3	0.3202	0.4351	0.4864	0.3319	0
849	1015.2	0.32	0.4349	0.4861	0.3319	0
850	1015.1	0.3199	0.435	0.4862	0.3319	0
851	1015	0.3201	0.435	0.4865	0.3319	0
852	1014.9	0.3199	0.4347	0.4862	0.3315	0
853	1014.8	0.3206	0.4356	0.4868	0.3321	0
854	1014.7	0.3201	0.4356	0.4868	0.3323	0
855	1014.6	0.3205	0.4356	0.4874	0.3322	0
856	1014.5	0.3206	0.4357	0.4873	0.3322	0
857	1014.4	0.3201	0.4357	0.4872	0.3323	0
858	1014.3	0.3204	0.4357	0.4873	0.3321	0
859	1014.2	0.3205	0.4357	0.4875	0.3324	0
860	1014.1	0.3208	0.4358	0.4878	0.3327	0
861	1014	0.3209	0.4358	0.4882	0.3324	0
862	1013.9	0.321	0.4359	0.488	0.3326	0
863	1013.8	0.3208	0.4358	0.488	0.3328	0
864	1013.7	0.3208	0.436	0.4882	0.3327	0
865	1013.6	0.3214	0.4364	0.4884	0.333	0
866	1013.5	0.3215	0.4367	0.4889	0.3332	0
867	1013.4	0.3214	0.4365	0.4887	0.3332	0
868	1013.3	0.3211	0.4365	0.4889	0.3331	0
869	1013.2	0.3214	0.4367	0.4891	0.3331	0
870	1013.1	0.3212	0.4367	0.4892	0.3331	0
871	1013	0.3212	0.4368	0.4892	0.3332	0
872	1012.9	0.3215	0.4369	0.4895	0.3333	0
873	1012.8	0.3215	0.437	0.4896	0.3333	0
874	1012.7	0.3214	0.4369	0.4894	0.3333	0
875	1012.6	0.3214	0.4367	0.4898	0.3334	0
876	1012.5	0.3214	0.4371	0.49	0.3335	0
877	1012.4	0.3217	0.4372	0.4901	0.3337	0
878	1012.3	0.3216	0.4373	0.49	0.3337	0
879	1012.2	0.3217	0.4375	0.4903	0.3338	0
880	1012.1	0.322	0.4376	0.4906	0.3339	0
881	1012	0.3218	0.437	0.4908	0.3337	0
882	1011.9	0.3221	0.4375	0.4909	0.3337	0
883	1011.8	0.3218	0.4372	0.4908	0.3335	0
884	1011.7	0.3217	0.4372	0.4906	0.3337	0
885	1011.6	0.3219	0.4376	0.4908	0.3337	0
886	1011.5	0.3219	0.4377	0.4911	0.3338	0
887	1011.4	0.322	0.4377	0.4908	0.3338	0
888	1011.3	0.3223	0.4376	0.4914	0.334	0
889	1011.2	0.3224	0.4377	0.4916	0.334	0

890	1011.1	0.3223	0.438	0.4915	0.3343	0
891	1011	0.3225	0.4382	0.4919	0.3344	0
892	1010.9	0.3225	0.4381	0.4919	0.3342	0
893	1010.8	0.3221	0.4378	0.4916	0.3342	0
894	1010.7	0.3226	0.4385	0.4921	0.3343	0
895	1010.6	0.3227	0.4382	0.4918	0.3343	0
896	1010.5	0.3226	0.4381	0.4919	0.3342	0
897	1010.4	0.3223	0.4378	0.4916	0.3341	0
898	1010.3	0.3227	0.438	0.4919	0.3343	0
899	1010.2	0.3226	0.4379	0.492	0.3341	0
900	1010.1	0.3224	0.438	0.4922	0.3342	0
901	1010	0.3226	0.4381	0.4924	0.3341	0
902	1009.9	0.3225	0.4385	0.4926	0.3344	0
903	1009.8	0.3227	0.4384	0.4925	0.3344	0
904	1009.7	0.3224	0.4381	0.4923	0.3338	0
905	1009.6	0.3226	0.4382	0.4923	0.3338	0
906	1009.5	0.3221	0.4378	0.4921	0.3338	0
907	1009.4	0.3222	0.4379	0.4924	0.3339	0
908	1009.3	0.3224	0.4381	0.4927	0.3339	0
909	1009.2	0.3225	0.4382	0.4929	0.3342	0
910	1009.1	0.3221	0.4381	0.4927	0.334	0
911	1009	0.3221	0.4383	0.4925	0.3337	0
912	1008.9	0.3224	0.4385	0.4927	0.3342	0
913	1008.8	0.322	0.4382	0.4927	0.3339	0
914	1008.7	0.322	0.438	0.4925	0.3336	0
915	1008.6	0.3221	0.4381	0.4921	0.3335	0
916	1008.5	0.322	0.4379	0.4922	0.3334	0
917	1008.4	0.3223	0.4385	0.4928	0.3338	0
918	1008.3	0.3221	0.4382	0.4925	0.3334	0
919	1008.2	0.3219	0.4381	0.4927	0.3335	0
920	1008.1	0.3223	0.4379	0.4927	0.3336	0
921	1008	0.3221	0.4379	0.4929	0.3337	0
922	1007.9	0.3225	0.4384	0.4929	0.334	0
923	1007.8	0.3225	0.4383	0.493	0.334	0
924	1007.7	0.3223	0.4384	0.4928	0.3337	0
925	1007.6	0.3223	0.4384	0.4933	0.3338	0
926	1007.5	0.3223	0.4384	0.4932	0.3335	0
927	1007.4	0.3222	0.438	0.4929	0.3336	0
928	1007.3	0.3225	0.4383	0.4929	0.3337	0
929	1007.2	0.3222	0.4383	0.4928	0.3336	0
930	1007.1	0.3224	0.4382	0.4928	0.3335	0
931	1007	0.3225	0.4381	0.4931	0.3333	0
932	1006.9	0.3224	0.4381	0.4931	0.3333	0

933	1006.8	0.3223	0.4383	0.4933	0.3334	0
934	1006.7	0.3225	0.4383	0.4935	0.3337	0
935	1006.6	0.3226	0.4384	0.4936	0.3336	0
936	1006.5	0.3226	0.4384	0.4936	0.3335	0
937	1006.4	0.3226	0.4385	0.4937	0.3334	0
938	1006.3	0.3226	0.4387	0.4939	0.3338	0
939	1006.2	0.3224	0.4384	0.4938	0.3335	0
940	1006.1	0.3224	0.4384	0.494	0.3335	0
941	1006	0.3223	0.4384	0.4936	0.3335	0
942	1005.9	0.3222	0.4384	0.4938	0.3333	0
943	1005.8	0.322	0.4384	0.4937	0.3332	0
944	1005.7	0.3223	0.4386	0.4936	0.3335	0
945	1005.6	0.3227	0.4385	0.494	0.3335	0
946	1005.5	0.3227	0.4387	0.4942	0.3335	0
947	1005.4	0.3229	0.4387	0.4944	0.3337	0
948	1005.3	0.3226	0.4386	0.4942	0.3333	0
949	1005.2	0.3224	0.4386	0.4944	0.3335	0
950	1005.1	0.3225	0.4387	0.4943	0.3338	0
951	1005	0.3227	0.439	0.4945	0.3338	0
952	1004.9	0.3224	0.4387	0.4943	0.3334	0
953	1004.8	0.3224	0.4385	0.4943	0.3331	0
954	1004.7	0.3227	0.4385	0.4947	0.3337	0
955	1004.6	0.3227	0.4383	0.4947	0.3334	0
956	1004.5	0.3229	0.439	0.4948	0.3336	0
957	1004.4	0.3226	0.4387	0.4946	0.3336	0
958	1004.3	0.3227	0.439	0.4949	0.3336	0
959	1004.2	0.3227	0.4391	0.4952	0.3337	0
960	1004.1	0.3229	0.4392	0.4952	0.3341	0
961	1004	0.323	0.4395	0.4951	0.3339	0
962	1003.9	0.3228	0.439	0.4951	0.3338	0
963	1003.8	0.323	0.4391	0.4952	0.3339	0
964	1003.7	0.3236	0.4391	0.4954	0.334	0
965	1003.6	0.3233	0.4393	0.4955	0.3339	0
966	1003.5	0.3234	0.4398	0.4956	0.334	0
967	1003.4	0.3234	0.4396	0.4957	0.334	0
968	1003.3	0.3234	0.4395	0.4958	0.3341	0
969	1003.2	0.3235	0.4397	0.4957	0.334	0
970	1003.1	0.3235	0.4397	0.496	0.334	0
971	1003	0.3233	0.4395	0.4957	0.3341	0
972	1002.9	0.3232	0.4396	0.4956	0.3338	0
973	1002.8	0.3234	0.4396	0.4959	0.334	0
974	1002.7	0.3233	0.4397	0.496	0.3339	0
975	1002.6	0.3236	0.4399	0.4965	0.3344	0

976	1002.5	0.3238	0.4402	0.4968	0.3348	0
977	1002.4	0.3237	0.44	0.4963	0.3343	0
978	1002.3	0.324	0.4401	0.4967	0.3344	0
979	1002.2	0.3238	0.4402	0.4966	0.3345	0
980	1002.1	0.324	0.4405	0.4967	0.3347	0
981	1002	0.3242	0.4401	0.497	0.3348	0
982	1001.9	0.3238	0.44	0.4969	0.3345	0
983	1001.8	0.3242	0.4401	0.4969	0.3346	0
984	1001.7	0.3237	0.44	0.4969	0.3344	0
985	1001.6	0.3241	0.4403	0.4973	0.3346	0
986	1001.5	0.3241	0.4403	0.4972	0.3346	0
987	1001.4	0.3244	0.4406	0.4974	0.335	0
988	1001.3	0.3244	0.4407	0.4976	0.3349	0
989	1001.2	0.3244	0.4408	0.4975	0.3349	0
990	1001.1	0.3242	0.4406	0.4975	0.3348	0
991	1001	0.324	0.4404	0.4974	0.3348	0
992	1000.9	0.3241	0.4406	0.4975	0.3349	0
993	1000.8	0.324	0.4406	0.4978	0.3349	0
994	1000.7	0.3241	0.4405	0.4976	0.3352	0
995	1000.6	0.3243	0.4404	0.4978	0.3351	0
996	1000.5	0.3242	0.4405	0.4976	0.335	0
997	1000.4	0.3243	0.4404	0.4977	0.3349	0
998	1000.3	0.3243	0.4405	0.4977	0.3349	0
999	1000.2	0.3242	0.4408	0.4979	0.3352	0
1000	1000.1	0.3246	0.4409	0.4978	0.3351	0
1001	1000	0.3242	0.4406	0.4978	0.335	0
1002	999.9	0.3246	0.4412	0.4978	0.3351	0
1003	999.8	0.3247	0.441	0.4978	0.3352	0
1004	999.7	0.3247	0.441	0.4979	0.3351	0
1005	999.6	0.3245	0.4407	0.4976	0.3349	0
1006	999.5	0.325	0.4412	0.4983	0.3355	0
1007	999.4	0.3248	0.4411	0.498	0.3353	0
1008	999.3	0.3247	0.441	0.4981	0.3351	0
1009	999.2	0.3248	0.4412	0.4981	0.3354	0
1010	999.1	0.3245	0.4409	0.4982	0.3352	0
1011	999	0.3246	0.4408	0.4982	0.3352	0
1012	998.9	0.3244	0.4408	0.4979	0.3351	0
1013	998.8	0.3248	0.4409	0.498	0.3352	0
1014	998.7	0.3246	0.441	0.4981	0.3352	0
1015	998.6	0.3248	0.4412	0.4982	0.3355	0
1016	998.5	0.3245	0.4408	0.4978	0.335	0
1017	998.4	0.3244	0.4408	0.498	0.3349	0
1018	998.3	0.3243	0.4411	0.4982	0.3349	0

1019	998.2	0.3246	0.4411	0.4984	0.3351	0
1020	998.1	0.3245	0.4408	0.498	0.3351	0
1021	998	0.3247	0.4409	0.498	0.3353	0
1022	997.9	0.3248	0.4411	0.4984	0.3353	0
1023	997.8	0.3246	0.4412	0.4982	0.3353	0
1024	997.7	0.3245	0.4412	0.4986	0.3352	0
1025	997.6	0.3245	0.4412	0.4983	0.3351	0
1026	997.5	0.3247	0.4411	0.4983	0.3351	0
1027	997.4	0.3246	0.4411	0.4982	0.3349	0
1028	997.3	0.3244	0.4412	0.498	0.335	0
1029	997.2	0.3247	0.4413	0.4984	0.3353	0
1030	997.1	0.3247	0.4416	0.4986	0.3353	0
1031	997	0.3246	0.4414	0.4982	0.3353	0
1032	996.9	0.3245	0.441	0.4984	0.3351	0
1033	996.8	0.3243	0.441	0.4981	0.335	0
1034	996.7	0.3247	0.4411	0.4981	0.335	0
1035	996.6	0.3251	0.4413	0.4987	0.3354	0
1036	996.5	0.3246	0.441	0.4984	0.3351	0
1037	996.4	0.3245	0.4411	0.4985	0.335	0
1038	996.3	0.3249	0.4412	0.4987	0.3352	0
1039	996.2	0.3249	0.4412	0.4985	0.3351	0
1040	996.1	0.325	0.4413	0.4988	0.3354	0
1041	996	0.3246	0.4413	0.4984	0.3352	0
1042	995.9	0.3246	0.4408	0.4984	0.3351	0
1043	995.8	0.3247	0.441	0.4984	0.3349	0
1044	995.7	0.3248	0.4412	0.4987	0.3349	0
1045	995.6	0.3252	0.4417	0.4988	0.3352	0
1046	995.5	0.3249	0.4415	0.4987	0.335	0
1047	995.4	0.3249	0.4416	0.4986	0.3351	0
1048	995.3	0.325	0.4415	0.4987	0.3351	0
1049	995.2	0.325	0.4411	0.4986	0.3351	0
1050	995.1	0.3252	0.4416	0.499	0.3353	0
1051	995	0.325	0.4415	0.4993	0.3351	0
1052	994.9	0.325	0.4416	0.4992	0.3351	0
1053	994.8	0.325	0.4416	0.4992	0.3351	0
1054	994.7	0.3251	0.4419	0.4995	0.3354	0
1055	994.6	0.3252	0.4419	0.4994	0.3356	0
1056	994.5	0.3252	0.4418	0.4992	0.3352	0
1057	994.4	0.3253	0.4421	0.4993	0.3351	0
1058	994.3	0.3254	0.4422	0.4995	0.3355	0
1059	994.2	0.3253	0.4422	0.4994	0.3354	0
1060	994.1	0.3254	0.4424	0.4996	0.3357	0
1061	994	0.3253	0.442	0.4994	0.3355	0

1062	993.9	0.3254	0.4424	0.4996	0.3354	0
1063	993.8	0.3251	0.4422	0.4993	0.3354	0
1064	993.7	0.325	0.4421	0.4995	0.3353	0
1065	993.6	0.3252	0.4423	0.4994	0.3355	0
1066	993.5	0.325	0.4423	0.4993	0.3352	0
1067	993.4	0.3252	0.4423	0.4993	0.3353	0
1068	993.3	0.3252	0.4423	0.4993	0.3352	0
1069	993.2	0.3253	0.4426	0.4997	0.3356	0
1070	993.1	0.3253	0.4425	0.4995	0.3353	0
1071	993	0.3256	0.4423	0.4997	0.3353	0
1072	992.9	0.3258	0.4426	0.5	0.3355	0
1073	992.8	0.3258	0.4424	0.5	0.3355	0
1074	992.7	0.3257	0.4424	0.5001	0.3353	0
1075	992.6	0.3255	0.4424	0.4999	0.3353	0
1076	992.5	0.3255	0.4423	0.4999	0.3351	0
1077	992.4	0.326	0.4428	0.5002	0.3359	0
1078	992.3	0.3259	0.443	0.5003	0.3359	0
1079	992.2	0.3258	0.4429	0.5002	0.3355	0
1080	992.1	0.3257	0.4427	0.5003	0.3356	0
1081	992	0.326	0.4427	0.5005	0.3357	0
1082	991.9	0.3261	0.4429	0.5003	0.3359	0
1083	991.8	0.3261	0.4429	0.5005	0.336	0
1084	991.7	0.3258	0.4428	0.5004	0.3358	0
1085	991.6	0.326	0.4432	0.5002	0.3359	0
1086	991.5	0.326	0.4432	0.5003	0.3359	0
1087	991.4	0.3255	0.4427	0.5003	0.3356	0
1088	991.3	0.3256	0.443	0.5003	0.3355	0
1089	991.2	0.3257	0.4429	0.5003	0.3353	0
1090	991.1	0.3256	0.4428	0.5003	0.3356	0
1091	991	0.3254	0.443	0.5003	0.3353	0
1092	990.9	0.3256	0.4436	0.5003	0.3357	0
1093	990.8	0.3257	0.4433	0.5002	0.3356	0
1094	990.7	0.3262	0.4433	0.5007	0.3359	0
1095	990.6	0.3261	0.4434	0.5007	0.3358	0
1096	990.5	0.3261	0.4433	0.5006	0.3358	0
1097	990.4	0.3257	0.4432	0.5004	0.3357	0
1098	990.3	0.3261	0.4435	0.5003	0.3357	0
1099	990.2	0.326	0.4432	0.5004	0.3357	0
1100	990.1	0.3261	0.4434	0.5006	0.3359	0
1101	990	0.3262	0.4434	0.5004	0.3356	0
1102	989.9	0.3262	0.4435	0.5006	0.336	0
1103	989.8	0.326	0.4432	0.5004	0.3354	0
1104	989.7	0.3261	0.4432	0.5003	0.3354	0

1105	989.6	0.3259	0.4434	0.5005	0.3355	0
1106	989.5	0.3258	0.4428	0.5001	0.3352	0
1107	989.4	0.3257	0.4432	0.5004	0.3352	0
1108	989.3	0.326	0.443	0.5005	0.3353	0
1109	989.2	0.3259	0.4432	0.5006	0.3354	0
1110	989.1	0.3257	0.4433	0.5003	0.335	0
1111	989	0.326	0.4432	0.5003	0.3352	0
1112	988.9	0.3259	0.4433	0.5005	0.3354	0
1113	988.8	0.3258	0.4434	0.5005	0.335	0
1114	988.7	0.3258	0.4432	0.5007	0.3351	0
1115	988.6	0.326	0.4433	0.5008	0.3354	0
1116	988.5	0.3264	0.4434	0.5009	0.3356	0
1117	988.4	0.326	0.4432	0.5006	0.3353	0
1118	988.3	0.3261	0.4431	0.5005	0.3351	0
1119	988.2	0.3262	0.4433	0.5006	0.3351	0
1120	988.1	0.326	0.4433	0.5004	0.3351	0
1121	988	0.3261	0.4433	0.5005	0.3353	0
1122	987.9	0.3263	0.4434	0.5005	0.3352	0
1123	987.8	0.326	0.4434	0.5003	0.3351	0
1124	987.7	0.3262	0.4434	0.5008	0.3351	0
1125	987.6	0.3264	0.4438	0.5008	0.3354	0
1126	987.5	0.3264	0.4435	0.5009	0.3354	0
1127	987.4	0.3262	0.4434	0.5006	0.3353	0
1128	987.3	0.3265	0.4436	0.5009	0.3353	0
1129	987.2	0.3264	0.4435	0.501	0.3354	0
1130	987.1	0.3262	0.4435	0.5007	0.3354	0
1131	987	0.3263	0.4435	0.501	0.3354	0
1132	986.9	0.3261	0.4435	0.5007	0.3351	0
1133	986.8	0.3263	0.4436	0.5009	0.3353	0
1134	986.7	0.3264	0.4438	0.501	0.3354	0
1135	986.6	0.3264	0.4438	0.5008	0.3354	0
1136	986.5	0.3263	0.4437	0.5008	0.3352	0
1137	986.4	0.3263	0.4439	0.5009	0.3352	0
1138	986.3	0.3264	0.4437	0.5008	0.3354	0
1139	986.2	0.3268	0.4444	0.5011	0.3358	0
1140	986.1	0.3267	0.444	0.5012	0.3356	0
1141	986	0.3264	0.4438	0.501	0.3352	0
1142	985.9	0.3265	0.4438	0.5009	0.3354	0
1143	985.8	0.3264	0.4438	0.5008	0.3353	0
1144	985.7	0.3263	0.4439	0.501	0.3353	0
1145	985.6	0.3264	0.4438	0.5012	0.3353	0
1146	985.5	0.3266	0.4441	0.5013	0.3356	0
1147	985.4	0.3268	0.4442	0.5013	0.3356	0

1148	985.3	0.3269	0.4443	0.5014	0.3354	0
1149	985.2	0.3266	0.4443	0.5015	0.3357	0
1150	985.1	0.3268	0.4444	0.5014	0.3356	0
1151	985	0.3267	0.4445	0.5012	0.3353	0
1152	984.9	0.327	0.4446	0.5015	0.3358	0
1153	984.8	0.3272	0.4449	0.5015	0.3359	0
1154	984.7	0.3271	0.4448	0.5017	0.3358	0
1155	984.6	0.3271	0.4447	0.5018	0.3358	0
1156	984.5	0.327	0.4445	0.5015	0.3358	0
1157	984.4	0.3272	0.4448	0.5017	0.3361	0
1158	984.3	0.3275	0.4451	0.502	0.3364	0
1159	984.2	0.3274	0.445	0.502	0.3361	0
1160	984.1	0.3272	0.4451	0.5019	0.336	0
1161	984	0.3277	0.4452	0.5018	0.3362	0
1162	983.9	0.3276	0.4453	0.5021	0.3362	0
1163	983.8	0.3277	0.4454	0.5022	0.3366	0
1164	983.7	0.3275	0.4453	0.5021	0.3363	0
1165	983.6	0.3274	0.4451	0.5023	0.3366	0
1166	983.5	0.3274	0.4454	0.5024	0.3361	0
1167	983.4	0.3274	0.4454	0.5023	0.3362	0
1168	983.3	0.3278	0.4455	0.5023	0.3363	0
1169	983.2	0.3281	0.4458	0.5027	0.3367	0
1170	983.1	0.3277	0.4453	0.5026	0.3366	0
1171	983	0.3275	0.445	0.502	0.3362	0
1172	982.9	0.3279	0.4454	0.5023	0.3364	0
1173	982.8	0.3277	0.4456	0.5024	0.3364	0
1174	982.7	0.3276	0.4454	0.5025	0.3362	0
1175	982.6	0.3278	0.4457	0.5027	0.3365	0
1176	982.5	0.3281	0.4458	0.5025	0.3366	0
1177	982.4	0.3278	0.446	0.5024	0.3366	0
1178	982.3	0.3277	0.4456	0.5024	0.3366	0
1179	982.2	0.3278	0.4457	0.5022	0.3364	0
1180	982.1	0.328	0.4457	0.5022	0.3365	0
1181	982	0.3277	0.4458	0.5026	0.3365	0
1182	981.9	0.3279	0.4459	0.5027	0.3368	0
1183	981.8	0.3278	0.4458	0.5025	0.3366	0
1184	981.7	0.3277	0.4456	0.5024	0.3366	0
1185	981.6	0.3277	0.4455	0.5022	0.3363	0
1186	981.5	0.328	0.4457	0.5026	0.3366	0
1187	981.4	0.3278	0.4458	0.5024	0.3365	0
1188	981.3	0.3276	0.4457	0.5023	0.3366	0
1189	981.2	0.3278	0.4455	0.5022	0.3366	0
1190	981.1	0.3278	0.4454	0.5024	0.3364	0

1191	981	0.3282	0.446	0.5027	0.3368	0
1192	980.9	0.3277	0.4456	0.5025	0.3366	0
1193	980.8	0.3275	0.4456	0.5022	0.3366	0
1194	980.7	0.3278	0.4456	0.5023	0.3366	0
1195	980.6	0.3278	0.4459	0.5023	0.3365	0
1196	980.5	0.3281	0.4457	0.5024	0.3365	0
1197	980.4	0.3278	0.4457	0.5025	0.3363	0
1198	980.3	0.3277	0.4456	0.5021	0.3363	0
1199	980.2	0.3281	0.4455	0.5023	0.3364	0
1200	980.1	0.3278	0.4455	0.502	0.3365	0
1201	980	0.3281	0.4455	0.5024	0.3365	0
1202	979.9	0.3278	0.4455	0.5022	0.3365	0
1203	979.8	0.3279	0.4456	0.5023	0.3367	0
1204	979.7	0.3277	0.4458	0.5022	0.3366	0
1205	979.6	0.328	0.4459	0.5023	0.3366	0
1206	979.5	0.3278	0.4456	0.502	0.3363	0
1207	979.4	0.3278	0.4458	0.5025	0.3365	0
1208	979.3	0.3276	0.4457	0.5024	0.3365	0
1209	979.2	0.3278	0.4458	0.5024	0.3361	0
1210	979.1	0.3277	0.4457	0.5021	0.3366	0
1211	979	0.3278	0.4457	0.502	0.3363	0
1212	978.9	0.3276	0.4455	0.502	0.3361	0
1213	978.8	0.3274	0.4452	0.5016	0.3361	0
1214	978.7	0.3274	0.4455	0.5017	0.3362	0
1215	978.6	0.3274	0.4453	0.5018	0.3362	0
1216	978.5	0.3278	0.4455	0.5019	0.3361	0
1217	978.4	0.3278	0.4455	0.502	0.3363	0
1218	978.3	0.3278	0.4452	0.5017	0.3361	0
1219	978.2	0.3276	0.4453	0.5016	0.3363	0
1220	978.1	0.3276	0.4453	0.5016	0.3362	0
1221	978	0.3276	0.4457	0.5017	0.3362	0
1222	977.9	0.3275	0.4452	0.5017	0.3362	0
1223	977.8	0.3274	0.445	0.5015	0.3361	0
1224	977.7	0.3276	0.4454	0.5017	0.3361	0
1225	977.6	0.3277	0.4452	0.5016	0.336	0
1226	977.5	0.3277	0.4453	0.5019	0.3364	0
1227	977.4	0.3278	0.4452	0.5021	0.3362	0
1228	977.3	0.3275	0.4451	0.5016	0.336	0
1229	977.2	0.3276	0.4453	0.5016	0.3363	0
1230	977.1	0.3275	0.4452	0.5017	0.336	0
1231	977	0.3275	0.4454	0.502	0.336	0
1232	976.9	0.3278	0.4457	0.5021	0.3362	0
1233	976.8	0.3275	0.4454	0.5016	0.336	0

1234	976.7	0.3279	0.4453	0.5017	0.336	0
1235	976.6	0.3276	0.4456	0.5018	0.336	0
1236	976.5	0.3275	0.4456	0.5018	0.3361	0
1237	976.4	0.3281	0.4459	0.5018	0.3364	0
1238	976.3	0.3279	0.4458	0.5021	0.3365	0
1239	976.2	0.3281	0.4458	0.502	0.3366	0
1240	976.1	0.3283	0.4458	0.5021	0.3366	0
1241	976	0.3281	0.4459	0.502	0.3365	0
1242	975.9	0.3281	0.4458	0.5018	0.3364	0
1243	975.8	0.3282	0.4459	0.502	0.3363	0
1244	975.7	0.3281	0.4461	0.502	0.3364	0
1245	975.6	0.328	0.4462	0.5021	0.3366	0
1246	975.5	0.3281	0.4461	0.5023	0.3367	0
1247	975.4	0.3285	0.4463	0.5026	0.337	0
1248	975.3	0.3283	0.4464	0.5027	0.3368	0
1249	975.2	0.3283	0.4462	0.5025	0.3368	0
1250	975.1	0.3283	0.4462	0.5023	0.3367	0
1251	975	0.3284	0.4463	0.5026	0.3367	0
1252	974.9	0.3286	0.4465	0.5024	0.3368	0
1253	974.8	0.3286	0.4464	0.5025	0.3371	0
1254	974.7	0.3283	0.4467	0.5025	0.3373	0
1255	974.6	0.3287	0.4468	0.5026	0.3371	0
1256	974.5	0.3289	0.4472	0.5031	0.3376	0
1257	974.4	0.3288	0.447	0.503	0.3374	0
1258	974.3	0.3285	0.4468	0.5031	0.3376	0
1259	974.2	0.329	0.4469	0.5032	0.3377	0
1260	974.1	0.3286	0.447	0.5031	0.3376	0
1261	974	0.3286	0.4471	0.5032	0.3376	0
1262	973.9	0.3288	0.4475	0.5031	0.3376	0
1263	973.8	0.3286	0.4473	0.503	0.3378	0
1264	973.7	0.3291	0.4478	0.5036	0.338	0
1265	973.6	0.3292	0.4479	0.5035	0.3379	0
1266	973.5	0.3294	0.4478	0.5035	0.3379	0
1267	973.4	0.3293	0.4478	0.5033	0.3378	0
1268	973.3	0.3296	0.4479	0.5038	0.3384	0
1269	973.2	0.3297	0.4481	0.5039	0.3383	0
1270	973.1	0.3298	0.4483	0.504	0.3387	0
1271	973	0.3298	0.4482	0.5038	0.3385	0
1272	972.9	0.3297	0.4482	0.5037	0.3383	0
1273	972.8	0.3296	0.4484	0.5038	0.3381	0
1274	972.7	0.3294	0.4481	0.5039	0.3382	0
1275	972.6	0.3296	0.448	0.5038	0.3382	0
1276	972.5	0.3297	0.4481	0.5038	0.3384	0

1277	972.4	0.3296	0.4481	0.5039	0.3384	0
1278	972.3	0.3297	0.4485	0.5041	0.3385	0
1279	972.2	0.3296	0.4483	0.5037	0.3384	0
1280	972.1	0.3298	0.4484	0.504	0.3387	0
1281	972	0.3296	0.4482	0.5039	0.3384	0
1282	971.9	0.3295	0.4483	0.5039	0.3383	0
1283	971.8	0.3295	0.448	0.5038	0.3383	0
1284	971.7	0.33	0.4482	0.5044	0.3385	0
1285	971.6	0.3299	0.4483	0.504	0.3386	0
1286	971.5	0.3298	0.4481	0.5039	0.3384	0
1287	971.4	0.33	0.4484	0.5042	0.3387	0
1288	971.3	0.3302	0.4484	0.504	0.3386	0
1289	971.2	0.3298	0.4484	0.5038	0.3386	0
1290	971.1	0.3301	0.4485	0.5042	0.3387	0
1291	971	0.3301	0.4487	0.5041	0.3387	0
1292	970.9	0.3299	0.4485	0.5038	0.3386	0
1293	970.8	0.3298	0.4485	0.5038	0.3385	0
1294	970.7	0.3295	0.4484	0.5037	0.3383	0
1295	970.6	0.3295	0.4485	0.5036	0.3383	0
1296	970.5	0.3294	0.4482	0.5036	0.3382	0
1297	970.4	0.3292	0.4483	0.5036	0.3382	0
1298	970.3	0.3298	0.4484	0.5036	0.3384	0
1299	970.2	0.3297	0.4485	0.5038	0.3384	0
1300	970.1	0.3299	0.4484	0.5034	0.3383	0
1301	970	0.3298	0.4486	0.5034	0.3385	0
1302	969.9	0.33	0.4486	0.5038	0.3387	0
1303	969.8	0.3301	0.4485	0.504	0.3385	0
1304	969.7	0.33	0.4486	0.504	0.3385	0
1305	969.6	0.3297	0.4486	0.5038	0.3384	0
1306	969.5	0.3296	0.4483	0.5033	0.3384	0
1307	969.4	0.3296	0.4482	0.5035	0.3384	0
1308	969.3	0.3297	0.4484	0.5038	0.3384	0
1309	969.2	0.3296	0.4484	0.5036	0.3383	0
1310	969.1	0.3296	0.4483	0.5034	0.3383	0
1311	969	0.3294	0.448	0.5031	0.3378	0
1312	968.9	0.3297	0.4483	0.5034	0.3382	0
1313	968.8	0.3293	0.4481	0.5032	0.3383	0
1314	968.7	0.3296	0.448	0.5034	0.3384	0
1315	968.6	0.3293	0.4479	0.5033	0.3383	0
1316	968.5	0.3292	0.448	0.5032	0.3383	0
1317	968.4	0.3294	0.4478	0.5033	0.3382	0
1318	968.3	0.3295	0.448	0.503	0.3382	0
1319	968.2	0.3296	0.448	0.503	0.3382	0

1320	968.1	0.3294	0.448	0.503	0.3383	0
1321	968	0.3293	0.4479	0.5031	0.3381	0
1322	967.9	0.3295	0.4482	0.503	0.338	0
1323	967.8	0.3293	0.4479	0.5026	0.3378	0
1324	967.7	0.3292	0.4479	0.5028	0.338	0
1325	967.6	0.3295	0.4481	0.5029	0.3382	0
1326	967.5	0.3293	0.4479	0.5026	0.3379	0
1327	967.4	0.3294	0.4479	0.5028	0.3377	0
1328	967.3	0.3294	0.4478	0.5028	0.3379	0
1329	967.2	0.3295	0.448	0.5029	0.3381	0
1330	967.1	0.3295	0.448	0.5027	0.338	0
1331	967	0.3294	0.4478	0.5026	0.338	0
1332	966.9	0.3292	0.448	0.5024	0.3378	0
1333	966.8	0.3295	0.4482	0.503	0.3379	0
1334	966.7	0.3297	0.4481	0.5031	0.3382	0
1335	966.6	0.3294	0.4479	0.5028	0.3379	0
1336	966.5	0.3296	0.448	0.503	0.338	0
1337	966.4	0.3292	0.4478	0.5028	0.3378	0
1338	966.3	0.3294	0.4477	0.5029	0.3377	0
1339	966.2	0.3295	0.4478	0.5028	0.3379	0
1340	966.1	0.3294	0.448	0.5032	0.3379	0
1341	966	0.3294	0.4481	0.5028	0.3377	0
1342	965.9	0.3294	0.4482	0.5028	0.3379	0
1343	965.8	0.3296	0.4482	0.5028	0.3381	0
1344	965.7	0.3297	0.4483	0.5031	0.3381	0
1345	965.6	0.3295	0.4482	0.5031	0.3379	0
1346	965.5	0.3296	0.448	0.5032	0.338	0
1347	965.4	0.3294	0.4481	0.5031	0.338	0
1348	965.3	0.3295	0.4479	0.5028	0.3377	0
1349	965.2	0.3296	0.4481	0.503	0.3381	0
1350	965.1	0.3297	0.4482	0.503	0.338	0
1351	965	0.3299	0.4482	0.5032	0.3383	0
1352	964.9	0.3296	0.4483	0.5032	0.3383	0
1353	964.8	0.3299	0.4487	0.5032	0.3382	0
1354	964.7	0.3298	0.4487	0.5034	0.3382	0
1355	964.6	0.33	0.4488	0.5035	0.3385	0
1356	964.5	0.3303	0.4492	0.5037	0.3387	0
1357	964.4	0.3303	0.4494	0.5039	0.3388	0
1358	964.3	0.33	0.4491	0.5037	0.3387	0
1359	964.2	0.3302	0.4493	0.5038	0.3389	0
1360	964.1	0.33	0.4494	0.5039	0.339	0
1361	964	0.3303	0.4495	0.5042	0.3392	0
1362	963.9	0.3306	0.4497	0.5042	0.3393	0

1363	963.8	0.3305	0.4498	0.5042	0.3393	0
1364	963.7	0.3306	0.4498	0.5043	0.3393	0
1365	963.6	0.3307	0.4499	0.5044	0.3394	0
1366	963.5	0.3309	0.4501	0.5043	0.3395	0
1367	963.4	0.331	0.4504	0.5047	0.3397	0
1368	963.3	0.3313	0.4506	0.505	0.3398	0
1369	963.2	0.3312	0.4502	0.505	0.3396	0
1370	963.1	0.3313	0.4504	0.5049	0.3399	0
1371	963	0.3315	0.4508	0.5052	0.34	0
1372	962.9	0.3314	0.4507	0.5052	0.3401	0
1373	962.8	0.3314	0.4506	0.5052	0.34	0
1374	962.7	0.3312	0.4504	0.5048	0.3398	0
1375	962.6	0.3313	0.4509	0.5051	0.3401	0
1376	962.5	0.3314	0.4507	0.5051	0.3402	0
1377	962.4	0.3313	0.4507	0.5051	0.3401	0
1378	962.3	0.3313	0.4508	0.5051	0.3401	0
1379	962.2	0.3314	0.4507	0.5053	0.34	0
1380	962.1	0.3317	0.4509	0.5052	0.3401	0
1381	962	0.3315	0.4508	0.5052	0.3401	0
1382	961.9	0.3316	0.4509	0.5053	0.3403	0
1383	961.8	0.3317	0.451	0.5053	0.3402	0
1384	961.7	0.3318	0.4512	0.5054	0.3403	0
1385	961.6	0.3315	0.4509	0.505	0.3403	0
1386	961.5	0.3315	0.4509	0.5052	0.3403	0
1387	961.4	0.3316	0.451	0.5054	0.3403	0
1388	961.3	0.3319	0.4515	0.5056	0.3405	0
1389	961.2	0.3317	0.4512	0.5054	0.3405	0
1390	961.1	0.3318	0.4513	0.5057	0.3406	0
1391	961	0.332	0.4515	0.5057	0.3405	0
1392	960.9	0.3317	0.4516	0.5055	0.3404	0
1393	960.8	0.3318	0.4517	0.5056	0.3405	0
1394	960.7	0.3321	0.4519	0.5058	0.3407	0
1395	960.6	0.3318	0.451	0.5053	0.3402	0
1396	960.5	0.3319	0.4512	0.5053	0.3405	0
1397	960.4	0.332	0.4512	0.5054	0.3405	0
1398	960.3	0.332	0.4515	0.5057	0.3406	0
1399	960.2	0.3319	0.4514	0.5058	0.3405	0
1400	960.1	0.3319	0.4512	0.5055	0.3405	0
1401	960	0.3319	0.4515	0.5054	0.3405	0
1402	959.9	0.3321	0.4514	0.5054	0.3405	0
1403	959.8	0.332	0.4516	0.5055	0.3407	0
1404	959.7	0.3319	0.4516	0.5055	0.3407	0
1405	959.6	0.332	0.4518	0.5057	0.3409	0

1406	959.5	0.3319	0.4515	0.5056	0.3405	0
1407	959.4	0.3318	0.4514	0.5055	0.3405	0
1408	959.3	0.3318	0.451	0.5052	0.3404	0
1409	959.2	0.3316	0.4511	0.505	0.3404	0
1410	959.1	0.3315	0.4511	0.5051	0.3402	0
1411	959	0.332	0.4513	0.5054	0.3405	0
1412	958.9	0.3318	0.4514	0.5053	0.3405	0
1413	958.8	0.3321	0.4515	0.5055	0.3406	0
1414	958.7	0.3319	0.4517	0.5051	0.3403	0
1415	958.6	0.3315	0.451	0.5048	0.34	0
1416	958.5	0.3317	0.451	0.5048	0.3402	0
1417	958.4	0.3315	0.4509	0.5046	0.3403	0
1418	958.3	0.3316	0.4511	0.5047	0.34	0
1419	958.2	0.3313	0.4509	0.5046	0.3397	0
1420	958.1	0.331	0.4509	0.5047	0.3399	0
1421	958	0.3316	0.4509	0.5051	0.3401	0
1422	957.9	0.3315	0.4508	0.5045	0.3399	0
1423	957.8	0.3313	0.4508	0.5043	0.3399	0
1424	957.7	0.3314	0.4509	0.5045	0.34	0
1425	957.6	0.3316	0.4509	0.5046	0.3401	0
1426	957.5	0.3317	0.4509	0.5045	0.34	0
1427	957.4	0.3314	0.4509	0.5047	0.34	0
1428	957.3	0.3315	0.4511	0.5046	0.34	0
1429	957.2	0.3315	0.451	0.5047	0.3402	0
1430	957.1	0.3313	0.4509	0.5044	0.3399	0
1431	957	0.3313	0.4507	0.5043	0.3399	0
1432	956.9	0.3311	0.4506	0.5042	0.3398	0
1433	956.8	0.3313	0.4506	0.5042	0.3398	0
1434	956.7	0.3311	0.4507	0.5041	0.3397	0
1435	956.6	0.3315	0.451	0.5042	0.3399	0
1436	956.5	0.3316	0.4509	0.5043	0.3398	0
1437	956.4	0.3312	0.4507	0.5041	0.3399	0
1438	956.3	0.3311	0.4507	0.5042	0.3396	0
1439	956.2	0.3313	0.4508	0.5041	0.3397	0
1440	956.1	0.3313	0.451	0.5042	0.3399	0
1441	956	0.3314	0.4509	0.5042	0.3401	0
1442	955.9	0.3317	0.4509	0.5043	0.3402	0
1443	955.8	0.3317	0.451	0.5043	0.34	0
1444	955.7	0.3315	0.451	0.5042	0.3398	0
1445	955.6	0.3313	0.4512	0.5042	0.3401	0
1446	955.5	0.3312	0.4507	0.504	0.3399	0
1447	955.4	0.3315	0.4511	0.5042	0.3401	0
1448	955.3	0.3315	0.451	0.5044	0.3401	0

1449	955.2	0.3315	0.4512	0.5046	0.3401	0
1450	955.1	0.3314	0.4515	0.5046	0.3403	0
1451	955	0.3315	0.4514	0.5046	0.3401	0
1452	954.9	0.3316	0.4513	0.5044	0.3402	0
1453	954.8	0.3316	0.4513	0.5041	0.3402	0
1454	954.7	0.3317	0.4513	0.5042	0.3403	0
1455	954.6	0.3314	0.4509	0.5041	0.3401	0
1456	954.5	0.3316	0.4511	0.5045	0.3401	0
1457	954.4	0.3318	0.4512	0.5046	0.3402	0
1458	954.3	0.3316	0.4515	0.5045	0.3403	0
1459	954.2	0.3322	0.4519	0.5045	0.3405	0
1460	954.1	0.3323	0.452	0.5045	0.3405	0
1461	954	0.3322	0.4519	0.5045	0.3406	0
1462	953.9	0.3323	0.4523	0.5047	0.3407	0
1463	953.8	0.3321	0.4521	0.5046	0.3406	0
1464	953.7	0.3323	0.4522	0.5045	0.3409	0
1465	953.6	0.3321	0.4522	0.5046	0.3409	0
1466	953.5	0.3325	0.4525	0.505	0.3411	0
1467	953.4	0.3328	0.4523	0.5051	0.3411	0
1468	953.3	0.3326	0.4524	0.505	0.3411	0
1469	953.2	0.3327	0.4528	0.5051	0.3413	0
1470	953.1	0.3328	0.4528	0.5055	0.3415	0
1471	953	0.3327	0.4528	0.5053	0.3413	0
1472	952.9	0.3326	0.4527	0.5051	0.3411	0
1473	952.8	0.3328	0.4529	0.5052	0.3413	0
1474	952.7	0.3329	0.4532	0.5052	0.3413	0
1475	952.6	0.333	0.4529	0.5054	0.3414	0
1476	952.5	0.3329	0.4531	0.5051	0.3414	0
1477	952.4	0.3332	0.4533	0.5055	0.3417	0
1478	952.3	0.3332	0.4535	0.5057	0.3416	0
1479	952.2	0.3333	0.4537	0.5056	0.3417	0
1480	952.1	0.3334	0.4536	0.5053	0.3416	0
1481	952	0.3335	0.4539	0.5057	0.3418	0
1482	951.9	0.3334	0.4539	0.5056	0.3419	0
1483	951.8	0.3334	0.4537	0.5057	0.3418	0
1484	951.7	0.3334	0.454	0.5056	0.3418	0
1485	951.6	0.3334	0.4541	0.5059	0.3422	0
1486	951.5	0.3335	0.4539	0.5059	0.3422	0
1487	951.4	0.3337	0.4541	0.5058	0.3421	0
1488	951.3	0.3334	0.454	0.5057	0.3421	0
1489	951.2	0.3334	0.4542	0.5057	0.3421	0
1490	951.1	0.3334	0.454	0.5058	0.342	0
1491	951	0.3336	0.4545	0.5058	0.3423	0

1492	950.9	0.3338	0.4544	0.5059	0.3424	0
1493	950.8	0.3339	0.4545	0.5058	0.3425	0
1494	950.7	0.3338	0.4542	0.5056	0.3422	0
1495	950.6	0.3339	0.4544	0.5056	0.3422	0
1496	950.5	0.3337	0.4543	0.5057	0.3422	0
1497	950.4	0.3339	0.4544	0.5058	0.3424	0
1498	950.3	0.3338	0.4543	0.5058	0.3423	0
1499	950.2	0.3336	0.4543	0.5057	0.3422	0
1500	950.1	0.3338	0.4544	0.5058	0.3423	0
1501	950	0.3339	0.4545	0.5057	0.3425	0
1502	949.9	0.3338	0.4547	0.5055	0.3425	0
1503	949.8	0.3337	0.4547	0.5055	0.3424	0
1504	949.7	0.3336	0.4545	0.5055	0.3422	0
1505	949.6	0.3338	0.4547	0.5055	0.3423	0
1506	949.5	0.3334	0.4541	0.5056	0.3423	0
1507	949.4	0.3338	0.4544	0.5053	0.3423	0
1508	949.3	0.3339	0.4544	0.506	0.3425	0
1509	949.2	0.3341	0.4548	0.5057	0.3426	0
1510	949.1	0.334	0.4546	0.5055	0.3427	0
1511	949	0.334	0.4545	0.5054	0.3425	0
1512	948.9	0.3338	0.4544	0.5053	0.3423	0
1513	948.8	0.3339	0.4543	0.5052	0.3423	0
1514	948.7	0.3336	0.4542	0.5053	0.3423	0
1515	948.6	0.3336	0.4543	0.5053	0.3422	0
1516	948.5	0.3337	0.4544	0.5052	0.3423	0
1517	948.4	0.3338	0.4544	0.5053	0.3423	0
1518	948.3	0.3336	0.4542	0.5051	0.3421	0
1519	948.2	0.3335	0.4542	0.5051	0.3421	0
1520	948.1	0.3337	0.4542	0.5047	0.3421	0
1521	948	0.3336	0.4541	0.5045	0.342	0
1522	947.9	0.3334	0.4542	0.5045	0.3421	0
1523	947.8	0.3335	0.454	0.5049	0.3421	0
1524	947.7	0.3337	0.4541	0.5049	0.3419	0
1525	947.6	0.3336	0.454	0.5046	0.3419	0
1526	947.5	0.3334	0.4541	0.5045	0.3418	0
1527	947.4	0.3334	0.4541	0.5043	0.3418	0
1528	947.3	0.3331	0.4539	0.5043	0.342	0
1529	947.2	0.3334	0.4541	0.5045	0.3417	0
1530	947.1	0.3331	0.4537	0.5043	0.3416	0
1531	947	0.3333	0.4541	0.5043	0.3419	0
1532	946.9	0.3331	0.4539	0.5043	0.342	0
1533	946.8	0.333	0.4537	0.5039	0.3415	0
1534	946.7	0.3333	0.454	0.5045	0.3417	0

1535	946.6	0.3335	0.454	0.5042	0.3418	0
1536	946.5	0.3335	0.4541	0.5043	0.3418	0
1537	946.4	0.3336	0.4538	0.5042	0.3417	0
1538	946.3	0.3336	0.454	0.5041	0.3417	0
1539	946.2	0.3334	0.4537	0.5039	0.3416	0
1540	946.1	0.3333	0.4537	0.504	0.3415	0
1541	946	0.3334	0.4538	0.504	0.3415	0
1542	945.9	0.3335	0.4539	0.5039	0.3416	0
1543	945.8	0.3333	0.4538	0.5042	0.3417	0
1544	945.7	0.3334	0.4537	0.5041	0.3418	0
1545	945.6	0.3334	0.4538	0.5039	0.3417	0
1546	945.5	0.3329	0.4536	0.5036	0.3414	0
1547	945.4	0.3334	0.4539	0.504	0.3417	0
1548	945.3	0.3334	0.4539	0.5039	0.3416	0
1549	945.2	0.3335	0.4542	0.504	0.3416	0
1550	945.1	0.3336	0.4538	0.5041	0.3418	0
1551	945	0.3336	0.4541	0.5041	0.3418	0
1552	944.9	0.3336	0.4542	0.504	0.3419	0
1553	944.8	0.3337	0.4544	0.5041	0.342	0
1554	944.7	0.3335	0.4542	0.504	0.342	0
1555	944.6	0.3336	0.4545	0.504	0.3421	0
1556	944.5	0.3335	0.4546	0.5042	0.3419	0
1557	944.4	0.3336	0.4547	0.5042	0.3421	0
1558	944.3	0.3338	0.4546	0.5044	0.3421	0
1559	944.2	0.3341	0.4545	0.5045	0.3422	0
1560	944.1	0.334	0.4546	0.5045	0.3422	0
1561	944	0.3341	0.4548	0.5045	0.3422	0
1562	943.9	0.3341	0.455	0.5044	0.3421	0
1563	943.8	0.3342	0.4548	0.5043	0.3422	0
1564	943.7	0.3343	0.455	0.5044	0.3424	0
1565	943.6	0.3344	0.4553	0.5048	0.3426	0
1566	943.5	0.3342	0.4553	0.5048	0.3426	0
1567	943.4	0.3342	0.4554	0.5046	0.3424	0
1568	943.3	0.3344	0.4554	0.5048	0.3424	0
1569	943.2	0.3343	0.4554	0.5048	0.3425	0
1570	943.1	0.3344	0.4552	0.5051	0.3427	0
1571	943	0.3347	0.4555	0.5048	0.3428	0
1572	942.9	0.3347	0.4558	0.5049	0.3428	0
1573	942.8	0.3345	0.4557	0.5047	0.3426	0
1574	942.7	0.3348	0.4557	0.5046	0.3426	0
1575	942.6	0.3349	0.4559	0.5048	0.3427	0
1576	942.5	0.3346	0.4558	0.5047	0.3427	0
1577	942.4	0.3349	0.4557	0.505	0.3429	0

1578	942.3	0.3348	0.4559	0.5048	0.343	0
1579	942.2	0.335	0.4559	0.5048	0.3431	0
1580	942.1	0.3349	0.456	0.5051	0.3431	0
1581	942	0.3351	0.4561	0.5053	0.3431	0
1582	941.9	0.3352	0.4561	0.5054	0.3432	0
1583	941.8	0.3351	0.4564	0.5053	0.343	0
1584	941.7	0.3351	0.4564	0.5053	0.3432	0
1585	941.6	0.335	0.4562	0.5049	0.3429	0
1586	941.5	0.3348	0.4564	0.5053	0.3431	0
1587	941.4	0.3351	0.4566	0.5052	0.3433	0
1588	941.3	0.3353	0.4568	0.5053	0.3433	0
1589	941.2	0.3347	0.4564	0.5053	0.3433	0
1590	941.1	0.3352	0.4563	0.5056	0.3432	0
1591	941	0.3351	0.4563	0.5055	0.3433	0
1592	940.9	0.3352	0.4564	0.5052	0.3432	0
1593	940.8	0.3353	0.4564	0.5052	0.3433	0
1594	940.7	0.3355	0.4565	0.5053	0.3433	0
1595	940.6	0.3355	0.4567	0.5053	0.3433	0
1596	940.5	0.3352	0.4567	0.5049	0.3432	0
1597	940.4	0.3352	0.4567	0.5052	0.3433	0
1598	940.3	0.3355	0.4569	0.5053	0.3434	0
1599	940.2	0.3357	0.4569	0.5054	0.3434	0
1600	940.1	0.3354	0.4569	0.5054	0.3433	0
1601	940	0.3353	0.457	0.5052	0.3433	0
1602	939.9	0.3354	0.457	0.5053	0.3433	0
1603	939.8	0.3352	0.457	0.5052	0.3433	0
1604	939.7	0.3355	0.4568	0.5054	0.3434	0
1605	939.6	0.3353	0.4568	0.5053	0.3433	0
1606	939.5	0.3351	0.4569	0.5053	0.3433	0
1607	939.4	0.3354	0.4569	0.5052	0.3433	0
1608	939.3	0.3355	0.457	0.5054	0.3434	0
1609	939.2	0.3354	0.4571	0.5052	0.3433	0
1610	939.1	0.3356	0.4572	0.5054	0.3435	0
1611	939	0.336	0.4576	0.5053	0.3438	0
1612	938.9	0.3357	0.4572	0.5052	0.3434	0
1613	938.8	0.3358	0.4573	0.5053	0.3434	0
1614	938.7	0.3357	0.4574	0.5055	0.3433	0
1615	938.6	0.3357	0.4569	0.5052	0.3432	0
1616	938.5	0.3354	0.4568	0.5053	0.3432	0
1617	938.4	0.3357	0.4573	0.5055	0.3434	0
1618	938.3	0.3357	0.4571	0.5054	0.3434	0
1619	938.2	0.3355	0.4571	0.5047	0.3432	0
1620	938.1	0.3354	0.4571	0.5049	0.3431	0

1621	938	0.3355	0.4571	0.505	0.3432	0
1622	937.9	0.3353	0.4569	0.5047	0.343	0
1623	937.8	0.3353	0.4569	0.5048	0.3429	0
1624	937.7	0.3355	0.4569	0.505	0.343	0
1625	937.6	0.3355	0.457	0.5048	0.3429	0
1626	937.5	0.3356	0.457	0.505	0.3429	0
1627	937.4	0.3356	0.4571	0.5049	0.3432	0
1628	937.3	0.3355	0.457	0.5047	0.3429	0
1629	937.2	0.3357	0.4568	0.5048	0.3429	0
1630	937.1	0.3355	0.4571	0.5049	0.343	0
1631	937	0.3355	0.4572	0.5047	0.3434	0
1632	936.9	0.3356	0.4574	0.5049	0.3432	0
1633	936.8	0.3354	0.4571	0.5049	0.3429	0
1634	936.7	0.3357	0.4572	0.5046	0.3431	0
1635	936.6	0.3355	0.4573	0.5048	0.3429	0
1636	936.5	0.3353	0.4573	0.5049	0.343	0
1637	936.4	0.3356	0.4571	0.5047	0.343	0
1638	936.3	0.3355	0.4571	0.5049	0.3429	0
1639	936.2	0.3356	0.4573	0.5049	0.3431	0
1640	936.1	0.3356	0.4573	0.5046	0.3428	0
1641	936	0.3354	0.4572	0.5046	0.3428	0
1642	935.9	0.3356	0.457	0.5048	0.3429	0
1643	935.8	0.3359	0.4577	0.5049	0.3431	0
1644	935.7	0.3357	0.4576	0.505	0.343	0
1645	935.6	0.336	0.4577	0.5049	0.343	0
1646	935.5	0.336	0.4575	0.5047	0.3431	0
1647	935.4	0.336	0.4576	0.505	0.3431	0
1648	935.3	0.3358	0.4577	0.5051	0.3431	0
1649	935.2	0.3358	0.4575	0.505	0.3432	0
1650	935.1	0.3357	0.4576	0.5048	0.3429	0
1651	935	0.3357	0.4577	0.5048	0.343	0
1652	934.9	0.3359	0.4576	0.5048	0.343	0
1653	934.8	0.3357	0.4578	0.5048	0.3428	0
1654	934.7	0.3357	0.4576	0.5047	0.3427	0
1655	934.6	0.3358	0.4577	0.5046	0.343	0
1656	934.5	0.3359	0.458	0.5048	0.3432	0
1657	934.4	0.3362	0.458	0.5049	0.3432	0
1658	934.3	0.3362	0.4583	0.5051	0.3433	0
1659	934.2	0.3362	0.4582	0.5053	0.3435	0
1660	934.1	0.3363	0.4581	0.5051	0.3434	0
1661	934	0.3364	0.4583	0.5052	0.3435	0
1662	933.9	0.3362	0.4582	0.5051	0.3433	0
1663	933.8	0.3361	0.4581	0.5052	0.3431	0

1664	933.7	0.3361	0.4583	0.5053	0.3432	0
1665	933.6	0.3363	0.4584	0.5053	0.3434	0
1666	933.5	0.3363	0.4586	0.5056	0.3435	0
1667	933.4	0.3364	0.4587	0.5055	0.3436	0
1668	933.3	0.3368	0.4588	0.5054	0.3434	0
1669	933.2	0.3365	0.4586	0.5052	0.3433	0
1670	933.1	0.3366	0.4589	0.5053	0.3435	0
1671	933	0.3367	0.4589	0.5052	0.3435	0
1672	932.9	0.3366	0.4586	0.5054	0.3435	0
1673	932.8	0.3367	0.4588	0.5056	0.3436	0
1674	932.7	0.3367	0.4589	0.5057	0.3435	0
1675	932.6	0.3367	0.459	0.5059	0.3439	0
1676	932.5	0.3367	0.4585	0.5056	0.3437	0
1677	932.4	0.3368	0.4589	0.5053	0.3435	0
1678	932.3	0.3367	0.4588	0.5053	0.3432	0
1679	932.2	0.3367	0.459	0.5057	0.3436	0
1680	932.1	0.3367	0.4588	0.5057	0.3435	0
1681	932	0.3367	0.4589	0.5059	0.3438	0
1682	931.9	0.3372	0.4595	0.5061	0.3439	0
1683	931.8	0.3369	0.4593	0.506	0.3436	0
1684	931.7	0.3367	0.4595	0.5059	0.3436	0
1685	931.6	0.3369	0.4594	0.5062	0.3437	0
1686	931.5	0.337	0.4594	0.5061	0.3438	0
1687	931.4	0.3373	0.4597	0.5062	0.3439	0
1688	931.3	0.337	0.4594	0.5059	0.3438	0
1689	931.2	0.3371	0.4596	0.5061	0.3439	0
1690	931.1	0.3371	0.4596	0.5061	0.3438	0
1691	931	0.337	0.4595	0.5059	0.3438	0
1692	930.9	0.3369	0.4594	0.5057	0.3437	0
1693	930.8	0.3371	0.4594	0.5058	0.3437	0
1694	930.7	0.337	0.4596	0.5059	0.3439	0
1695	930.6	0.3372	0.4596	0.5058	0.3439	0
1696	930.5	0.3369	0.4597	0.5059	0.3439	0
1697	930.4	0.3369	0.4596	0.5059	0.344	0
1698	930.3	0.3372	0.4596	0.5061	0.3441	0
1699	930.2	0.3371	0.4596	0.5058	0.3439	0
1700	930.1	0.3371	0.4598	0.5059	0.3438	0
1701	930	0.3371	0.4594	0.5059	0.3437	0
1702	929.9	0.337	0.4594	0.5059	0.3438	0
1703	929.8	0.3371	0.4594	0.5058	0.3436	0
1704	929.7	0.3371	0.4594	0.5057	0.3438	0
1705	929.6	0.3372	0.4596	0.5057	0.3437	0
1706	929.5	0.337	0.4591	0.5058	0.3436	0

1707	929.4	0.337	0.4593	0.5057	0.3436	0
1708	929.3	0.3372	0.4594	0.5057	0.3437	0
1709	929.2	0.337	0.4594	0.5057	0.3437	0
1710	929.1	0.3371	0.4594	0.5054	0.3436	0
1711	929	0.337	0.4594	0.5055	0.3435	0
1712	928.9	0.3369	0.4595	0.5057	0.3436	0
1713	928.8	0.3367	0.4594	0.5054	0.3434	0
1714	928.7	0.3369	0.4594	0.5054	0.3435	0
1715	928.6	0.3371	0.4594	0.5055	0.3435	0
1716	928.5	0.337	0.4595	0.5053	0.3437	0
1717	928.4	0.337	0.4594	0.5053	0.3433	0
1718	928.3	0.3368	0.459	0.5053	0.3433	0
1719	928.2	0.3366	0.4593	0.5053	0.3432	0
1720	928.1	0.3369	0.4594	0.5053	0.3431	0
1721	928	0.3369	0.4595	0.5051	0.3431	0
1722	927.9	0.3369	0.4593	0.5052	0.3432	0
1723	927.8	0.3372	0.4595	0.5055	0.3436	0
1724	927.7	0.337	0.4596	0.5055	0.3434	0
1725	927.6	0.3371	0.4591	0.5053	0.3431	0
1726	927.5	0.337	0.4594	0.5052	0.3432	0
1727	927.4	0.337	0.4595	0.5051	0.3429	0
1728	927.3	0.3372	0.4595	0.5051	0.3433	0
1729	927.2	0.3372	0.4597	0.5052	0.3433	0
1730	927.1	0.3373	0.4597	0.5056	0.3437	0
1731	927	0.3373	0.4596	0.5053	0.3436	0
1732	926.9	0.3371	0.4598	0.5055	0.3434	0
1733	926.8	0.3372	0.4598	0.5053	0.3437	0
1734	926.7	0.3372	0.46	0.5054	0.3436	0
1735	926.6	0.3373	0.4601	0.5056	0.3437	0
1736	926.5	0.3372	0.4601	0.5057	0.3437	0
1737	926.4	0.3372	0.4599	0.5055	0.3436	0
1738	926.3	0.3372	0.4602	0.5057	0.3437	0
1739	926.2	0.3375	0.4602	0.5057	0.3438	0
1740	926.1	0.3374	0.4601	0.5057	0.3435	0
1741	926	0.3375	0.4603	0.5057	0.3437	0
1742	925.9	0.3378	0.4603	0.5056	0.3437	0
1743	925.8	0.3376	0.4604	0.5059	0.3438	0
1744	925.7	0.3374	0.4606	0.5058	0.3437	0
1745	925.6	0.3376	0.4606	0.506	0.3438	0
1746	925.5	0.3375	0.4604	0.5059	0.3437	0
1747	925.4	0.3374	0.4605	0.5058	0.3437	0
1748	925.3	0.3378	0.4608	0.506	0.344	0
1749	925.2	0.3376	0.4605	0.5059	0.3439	0

1750	925.1	0.3377	0.4605	0.5056	0.3438	0
1751	925	0.3376	0.4606	0.5061	0.3438	0
1752	924.9	0.338	0.4609	0.5062	0.344	0
1753	924.8	0.3378	0.4608	0.5063	0.344	0
1754	924.7	0.3381	0.4611	0.5063	0.3443	0
1755	924.6	0.3382	0.4611	0.5066	0.3443	0
1756	924.5	0.3378	0.4612	0.5064	0.3438	0
1757	924.4	0.3381	0.461	0.5066	0.3443	0
1758	924.3	0.338	0.4612	0.5065	0.3443	0
1759	924.2	0.3381	0.4611	0.5067	0.3444	0
1760	924.1	0.3381	0.4613	0.5067	0.3443	0
1761	924	0.3381	0.4612	0.5067	0.3445	0
1762	923.9	0.3385	0.4616	0.5069	0.3446	0
1763	923.8	0.3384	0.4617	0.5068	0.3446	0
1764	923.7	0.3384	0.4616	0.5069	0.3446	0
1765	923.6	0.3385	0.4618	0.5068	0.3449	0
1766	923.5	0.3384	0.4618	0.5069	0.3448	0
1767	923.4	0.3385	0.4616	0.5067	0.3446	0
1768	923.3	0.3386	0.4616	0.507	0.3447	0
1769	923.2	0.3385	0.4616	0.507	0.3447	0
1770	923.1	0.3387	0.4616	0.5071	0.3446	0
1771	923	0.3385	0.4616	0.5067	0.3447	0
1772	922.9	0.3385	0.4617	0.5069	0.3448	0
1773	922.8	0.3388	0.4621	0.5072	0.3448	0
1774	922.7	0.3387	0.462	0.507	0.3446	0
1775	922.6	0.3388	0.4622	0.5071	0.3448	0
1776	922.5	0.3388	0.4621	0.5069	0.3448	0
1777	922.4	0.3389	0.4621	0.507	0.3448	0
1778	922.3	0.3384	0.4619	0.5068	0.3447	0
1779	922.2	0.3391	0.4624	0.5072	0.345	0
1780	922.1	0.3388	0.4624	0.507	0.3449	0
1781	922	0.3388	0.4622	0.5069	0.345	0
1782	921.9	0.3387	0.4622	0.5067	0.3448	0
1783	921.8	0.3391	0.4622	0.5071	0.345	0
1784	921.7	0.3389	0.4622	0.507	0.3448	0
1785	921.6	0.3385	0.4622	0.507	0.3446	0
1786	921.5	0.3387	0.4621	0.5069	0.3448	0
1787	921.4	0.3389	0.4624	0.507	0.3448	0
1788	921.3	0.3385	0.4622	0.5068	0.3448	0
1789	921.2	0.3388	0.4622	0.507	0.3449	0
1790	921.1	0.3387	0.4621	0.5067	0.3447	0
1791	921	0.3387	0.4623	0.5069	0.3447	0
1792	920.9	0.3388	0.4621	0.5068	0.3448	0

1793	920.8	0.3388	0.4622	0.5069	0.3449	0
1794	920.7	0.3389	0.4623	0.507	0.3451	0
1795	920.6	0.3387	0.4623	0.507	0.3446	0
1796	920.5	0.3385	0.4622	0.5068	0.3447	0
1797	920.4	0.3384	0.462	0.507	0.3445	0
1798	920.3	0.3384	0.462	0.5067	0.3445	0
1799	920.2	0.3384	0.462	0.5066	0.3446	0
1800	920.1	0.3387	0.462	0.507	0.3447	0
1801	920	0.3387	0.462	0.507	0.3447	0
1802	919.9	0.3386	0.4619	0.5066	0.3446	0
1803	919.8	0.3386	0.4619	0.5067	0.3445	0
1804	919.7	0.3385	0.4623	0.5068	0.3443	0
1805	919.6	0.3386	0.4621	0.5067	0.3445	0
1806	919.5	0.3389	0.4621	0.5069	0.3446	0
1807	919.4	0.3386	0.462	0.5066	0.3444	0
1808	919.3	0.3385	0.4623	0.5068	0.3445	0
1809	919.2	0.3387	0.4621	0.5067	0.3445	0
1810	919.1	0.3384	0.4619	0.5065	0.3442	0
1811	919	0.3383	0.4615	0.5064	0.3442	0
1812	918.9	0.3381	0.4616	0.5062	0.3442	0
1813	918.8	0.3383	0.4616	0.5061	0.3442	0
1814	918.7	0.3383	0.4616	0.5063	0.3444	0
1815	918.6	0.3385	0.4619	0.5064	0.3443	0
1816	918.5	0.3382	0.4616	0.5062	0.3442	0
1817	918.4	0.3384	0.4618	0.5063	0.3442	0
1818	918.3	0.3384	0.4616	0.5061	0.3441	0
1819	918.2	0.3384	0.4619	0.5062	0.3441	0
1820	918.1	0.3385	0.4619	0.5065	0.3441	0
1821	918	0.3383	0.4616	0.5061	0.3441	0
1822	917.9	0.3385	0.4618	0.5064	0.3441	0
1823	917.8	0.3384	0.462	0.5066	0.3444	0
1824	917.7	0.3385	0.4619	0.5061	0.3442	0
1825	917.6	0.3383	0.4618	0.5064	0.3442	0
1826	917.5	0.3386	0.462	0.5066	0.3444	0
1827	917.4	0.3385	0.4618	0.5064	0.3443	0
1828	917.3	0.3384	0.4618	0.5064	0.3444	0
1829	917.2	0.3387	0.4618	0.5064	0.3444	0
1830	917.1	0.3387	0.4619	0.5066	0.3444	0
1831	917	0.3387	0.4619	0.5063	0.3447	0
1832	916.9	0.3386	0.4616	0.5062	0.3441	0
1833	916.8	0.3383	0.4616	0.5063	0.3441	0
1834	916.7	0.3381	0.4616	0.5063	0.3441	0
1835	916.6	0.3384	0.4618	0.5062	0.3442	0

1836	916.5	0.3384	0.4618	0.5065	0.3439	0
1837	916.4	0.3385	0.462	0.5063	0.3441	0
1838	916.3	0.3386	0.4621	0.5064	0.3441	0
1839	916.2	0.3386	0.462	0.5064	0.344	0
1840	916.1	0.3387	0.4621	0.5064	0.3442	0
1841	916	0.3386	0.4621	0.5066	0.3443	0
1842	915.9	0.3388	0.4624	0.5068	0.3446	0
1843	915.8	0.339	0.4623	0.5068	0.3446	0
1844	915.7	0.3387	0.462	0.5067	0.3444	0
1845	915.6	0.3389	0.4624	0.5066	0.3446	0
1846	915.5	0.3391	0.4628	0.5067	0.3446	0
1847	915.4	0.3387	0.4624	0.5067	0.3446	0
1848	915.3	0.3389	0.4625	0.507	0.3448	0
1849	915.2	0.339	0.4625	0.5071	0.3449	0
1850	915.1	0.339	0.4627	0.507	0.3447	0
1851	915	0.3389	0.4626	0.507	0.3447	0
1852	914.9	0.3389	0.4628	0.5072	0.3447	0
1853	914.8	0.3392	0.463	0.5073	0.3449	0
1854	914.7	0.3393	0.4632	0.5072	0.3452	0
1855	914.6	0.3394	0.4631	0.5074	0.3449	0
1856	914.5	0.3395	0.4633	0.5075	0.3451	0
1857	914.4	0.3395	0.4632	0.5074	0.3451	0
1858	914.3	0.3398	0.4637	0.5076	0.3454	0
1859	914.2	0.3397	0.4635	0.5075	0.3454	0
1860	914.1	0.3396	0.4634	0.5076	0.3454	0
1861	914	0.3396	0.4638	0.5078	0.3454	0
1862	913.9	0.3397	0.4635	0.5077	0.3454	0
1863	913.8	0.3399	0.4639	0.5083	0.3456	0
1864	913.7	0.3398	0.4638	0.5081	0.3456	0
1865	913.6	0.3398	0.4641	0.5081	0.3456	0
1866	913.5	0.34	0.4642	0.5081	0.3461	0
1867	913.4	0.34	0.4641	0.508	0.3459	0
1868	913.3	0.3402	0.4641	0.5082	0.3458	0
1869	913.2	0.3401	0.4643	0.5081	0.3458	0
1870	913.1	0.34	0.4641	0.5078	0.3458	0
1871	913	0.3401	0.4643	0.508	0.3458	0
1872	912.9	0.3403	0.4643	0.508	0.3458	0
1873	912.8	0.3399	0.4642	0.5079	0.3457	0
1874	912.7	0.3402	0.4644	0.5082	0.346	0
1875	912.6	0.3401	0.4646	0.5083	0.3459	0
1876	912.5	0.3402	0.4647	0.5085	0.346	0
1877	912.4	0.3401	0.4643	0.5085	0.3459	0
1878	912.3	0.34	0.4644	0.5083	0.3458	0

1879	912.2	0.3404	0.4644	0.5084	0.346	0
1880	912.1	0.3401	0.4644	0.5083	0.3461	0
1881	912	0.3403	0.4645	0.5082	0.3458	0
1882	911.9	0.3403	0.4646	0.5086	0.3461	0
1883	911.8	0.3405	0.4648	0.5085	0.3462	0
1884	911.7	0.3403	0.4647	0.5083	0.3463	0
1885	911.6	0.3404	0.4648	0.5084	0.3462	0
1886	911.5	0.3402	0.4647	0.5083	0.3461	0
1887	911.4	0.3402	0.4646	0.5083	0.3459	0
1888	911.3	0.3405	0.4646	0.5084	0.346	0
1889	911.2	0.3402	0.4645	0.5081	0.3459	0
1890	911.1	0.3402	0.4646	0.5081	0.3461	0
1891	911	0.34	0.4646	0.5083	0.3458	0
1892	910.9	0.3404	0.4646	0.5083	0.346	0
1893	910.8	0.34	0.4645	0.5084	0.3458	0
1894	910.7	0.3401	0.4648	0.5083	0.3458	0
1895	910.6	0.3403	0.4646	0.5083	0.3459	0
1896	910.5	0.3403	0.4648	0.5083	0.3461	0
1897	910.4	0.3403	0.4647	0.5083	0.346	0
1898	910.3	0.3403	0.4646	0.5085	0.3459	0
1899	910.2	0.3402	0.4648	0.5083	0.346	0
1900	910.1	0.3404	0.4649	0.5084	0.3461	0
1901	910	0.3402	0.4647	0.5084	0.346	0
1902	909.9	0.3402	0.4646	0.5083	0.346	0
1903	909.8	0.3403	0.4646	0.5082	0.3459	0
1904	909.7	0.3402	0.4646	0.5082	0.3459	0
1905	909.6	0.3402	0.4644	0.5082	0.3458	0
1906	909.5	0.3401	0.4644	0.5082	0.3457	0
1907	909.4	0.34	0.4643	0.5082	0.3457	0
1908	909.3	0.3399	0.4642	0.508	0.3455	0
1909	909.2	0.34	0.4642	0.5083	0.3457	0
1910	909.1	0.3405	0.4647	0.5084	0.3461	0
1911	909	0.3401	0.4643	0.5082	0.3459	0
1912	908.9	0.3401	0.4644	0.5083	0.346	0
1913	908.8	0.3399	0.4641	0.5079	0.3454	0
1914	908.7	0.3399	0.4643	0.5078	0.3455	0
1915	908.6	0.3398	0.4641	0.5078	0.3455	0
1916	908.5	0.34	0.4643	0.5079	0.3457	0
1917	908.4	0.3399	0.4644	0.5077	0.3457	0
1918	908.3	0.3401	0.4643	0.5079	0.3456	0
1919	908.2	0.3401	0.4642	0.5077	0.3454	0
1920	908.1	0.3401	0.4642	0.5076	0.3455	0
1921	908	0.3401	0.4644	0.508	0.3455	0

1922	907.9	0.3398	0.4643	0.5077	0.3453	0
1923	907.8	0.3397	0.4641	0.5075	0.3455	0
1924	907.7	0.3398	0.4643	0.5078	0.3455	0
1925	907.6	0.3397	0.4642	0.5078	0.3455	0
1926	907.5	0.3396	0.4641	0.5078	0.3455	0
1927	907.4	0.3397	0.4643	0.5076	0.3453	0
1928	907.3	0.3399	0.4643	0.5077	0.3455	0
1929	907.2	0.3399	0.4642	0.5075	0.3454	0
1930	907.1	0.3399	0.4642	0.5077	0.3453	0
1931	907	0.3397	0.4638	0.5076	0.3453	0
1932	906.9	0.3395	0.4639	0.5075	0.3451	0
1933	906.8	0.3397	0.4642	0.5075	0.3451	0
1934	906.7	0.3398	0.4643	0.5076	0.3452	0
1935	906.6	0.3399	0.4644	0.508	0.3456	0
1936	906.5	0.3403	0.4646	0.5081	0.3458	0
1937	906.4	0.34	0.4646	0.508	0.3456	0
1938	906.3	0.34	0.4646	0.5078	0.3455	0
1939	906.2	0.3401	0.4644	0.5078	0.3455	0
1940	906.1	0.34	0.4645	0.5079	0.3455	0
1941	906	0.3401	0.4644	0.5079	0.3457	0
1942	905.9	0.3403	0.4645	0.5079	0.3457	0
1943	905.8	0.3405	0.4645	0.5081	0.3455	0
1944	905.7	0.3405	0.4648	0.5083	0.3459	0
1945	905.6	0.3402	0.4646	0.5079	0.3457	0
1946	905.5	0.3403	0.4648	0.508	0.3457	0
1947	905.4	0.3404	0.4648	0.5082	0.3458	0
1948	905.3	0.3403	0.4648	0.5078	0.3458	0
1949	905.2	0.3404	0.4652	0.5079	0.3459	0
1950	905.1	0.3408	0.4652	0.5081	0.3462	0
1951	905	0.3404	0.4652	0.508	0.3461	0
1952	904.9	0.3405	0.465	0.5082	0.346	0
1953	904.8	0.3406	0.465	0.5082	0.3459	0
1954	904.7	0.3406	0.4653	0.5082	0.3461	0
1955	904.6	0.3407	0.4653	0.5082	0.3463	0
1956	904.5	0.3409	0.4655	0.5085	0.3463	0
1957	904.4	0.3408	0.4655	0.5088	0.3465	0
1958	904.3	0.3408	0.4656	0.5088	0.3467	0
1959	904.2	0.341	0.4659	0.509	0.3466	0
1960	904.1	0.3412	0.4661	0.5091	0.3468	0
1961	904	0.3414	0.4665	0.5093	0.347	0
1962	903.9	0.3414	0.4662	0.5091	0.3468	0
1963	903.8	0.3415	0.4662	0.509	0.3469	0
1964	903.7	0.3416	0.4666	0.5093	0.3471	0

1965	903.6	0.3418	0.4666	0.5094	0.3472	0
1966	903.5	0.3417	0.4666	0.5095	0.3472	0
1967	903.4	0.3415	0.4664	0.5094	0.3469	0
1968	903.3	0.3418	0.4669	0.5095	0.3472	0
1969	903.2	0.3417	0.4669	0.5098	0.3474	0
1970	903.1	0.3419	0.467	0.5098	0.3474	0
1971	903	0.342	0.4671	0.5099	0.3476	0
1972	902.9	0.3421	0.4673	0.5102	0.3476	0
1973	902.8	0.3424	0.4673	0.5101	0.3477	0
1974	902.7	0.3422	0.4671	0.5098	0.3473	0
1975	902.6	0.3423	0.4673	0.5099	0.3476	0
1976	902.5	0.3423	0.4675	0.51	0.3478	0
1977	902.4	0.3422	0.4676	0.51	0.3478	0
1978	902.3	0.3423	0.4674	0.51	0.3478	0
1979	902.2	0.3424	0.4674	0.5101	0.3477	0
1980	902.1	0.3421	0.4672	0.5101	0.3477	0
1981	902	0.3423	0.4674	0.51	0.3478	0
1982	901.9	0.3424	0.4675	0.5102	0.3478	0
1983	901.8	0.3423	0.4675	0.51	0.3477	0
1984	901.7	0.3423	0.4675	0.5099	0.3479	0
1985	901.6	0.342	0.4673	0.5096	0.3474	0
1986	901.5	0.3423	0.4675	0.5098	0.3478	0
1987	901.4	0.3424	0.4675	0.5099	0.3478	0
1988	901.3	0.3423	0.4675	0.5097	0.348	0
1989	901.2	0.3425	0.4676	0.5099	0.3481	0
1990	901.1	0.3423	0.4677	0.5101	0.3481	0
1991	901	0.3424	0.4677	0.51	0.348	0
1992	900.9	0.3423	0.4676	0.5098	0.3479	0
1993	900.8	0.3425	0.4679	0.5102	0.3481	0
1994	900.7	0.3422	0.4676	0.5099	0.3477	0
1995	900.6	0.3423	0.4677	0.51	0.348	0
1996	900.5	0.3423	0.4678	0.5102	0.348	0
1997	900.4	0.3423	0.4676	0.5101	0.3479	0
1998	900.3	0.3423	0.4678	0.5101	0.3479	0
1999	900.2	0.3425	0.4675	0.51	0.348	0
2000	900.1	0.3424	0.4675	0.5099	0.348	0
2001	900	0.3423	0.4676	0.5098	0.3479	0
2002	899.9	0.3423	0.4679	0.5098	0.3479	0
2003	899.8	0.3423	0.4678	0.5098	0.3482	0
2004	899.7	0.3423	0.4678	0.51	0.3481	0
2005	899.6	0.3425	0.4678	0.5098	0.348	0
2006	899.5	0.3424	0.4677	0.5097	0.3481	0
2007	899.4	0.3424	0.4676	0.51	0.3481	0

2008	899.3	0.3422	0.4676	0.5099	0.3479	0
2009	899.2	0.3422	0.4673	0.5095	0.3477	0
2010	899.1	0.3422	0.4674	0.5095	0.3477	0
2011	899	0.3423	0.4674	0.5094	0.3476	0
2012	898.9	0.3424	0.4677	0.5097	0.3478	0
2013	898.8	0.3425	0.4676	0.5095	0.3478	0
2014	898.7	0.3424	0.4675	0.5096	0.3477	0
2015	898.6	0.3422	0.4675	0.5093	0.3474	0
2016	898.5	0.3421	0.4674	0.5095	0.3475	0
2017	898.4	0.3419	0.4672	0.5091	0.3474	0
2018	898.3	0.3422	0.4672	0.5093	0.3476	0
2019	898.2	0.3422	0.4676	0.5093	0.3478	0
2020	898.1	0.3422	0.4672	0.5093	0.3476	0
2021	898	0.3421	0.4671	0.509	0.3474	0
2022	897.9	0.3421	0.4669	0.5091	0.3474	0
2023	897.8	0.3422	0.4671	0.5092	0.3475	0
2024	897.7	0.3422	0.4672	0.5091	0.3475	0
2025	897.6	0.342	0.4671	0.5093	0.3477	0
2026	897.5	0.3421	0.4669	0.5091	0.3475	0
2027	897.4	0.3419	0.4671	0.5091	0.3474	0
2028	897.3	0.3422	0.4672	0.5092	0.3475	0
2029	897.2	0.3421	0.4671	0.5092	0.3476	0
2030	897.1	0.3421	0.4672	0.5088	0.3475	0
2031	897	0.3418	0.4668	0.5088	0.3472	0
2032	896.9	0.3419	0.4668	0.5089	0.3473	0
2033	896.8	0.3417	0.4667	0.5086	0.3473	0
2034	896.7	0.3419	0.4668	0.5087	0.3474	0
2035	896.6	0.3421	0.4671	0.5088	0.3475	0
2036	896.5	0.3419	0.4671	0.509	0.3476	0
2037	896.4	0.3418	0.4668	0.5087	0.3475	0
2038	896.3	0.3417	0.4668	0.5087	0.3473	0
2039	896.2	0.3418	0.4668	0.5088	0.3473	0
2040	896.1	0.3418	0.4668	0.5088	0.3474	0
2041	896	0.3418	0.4669	0.5088	0.3472	0
2042	895.9	0.3419	0.4671	0.5089	0.3474	0
2043	895.8	0.342	0.4671	0.5087	0.3475	0
2044	895.7	0.3423	0.4671	0.5089	0.3476	0
2045	895.6	0.3421	0.4669	0.5086	0.3475	0
2046	895.5	0.3422	0.4671	0.5086	0.3475	0
2047	895.4	0.3422	0.4672	0.5088	0.3476	0
2048	895.3	0.3422	0.4672	0.5089	0.3478	0
2049	895.2	0.3423	0.4676	0.5089	0.3479	0
2050	895.1	0.3423	0.4673	0.5089	0.3477	0

2051	895	0.3422	0.4676	0.5089	0.3477	0
2052	894.9	0.3421	0.4673	0.5087	0.3474	0
2053	894.8	0.342	0.4673	0.5088	0.3476	0
2054	894.7	0.342	0.4674	0.5089	0.3477	0
2055	894.6	0.3422	0.4674	0.5089	0.3479	0
2056	894.5	0.3423	0.4676	0.5091	0.348	0
2057	894.4	0.3422	0.4678	0.509	0.348	0
2058	894.3	0.3425	0.4679	0.5092	0.3481	0
2059	894.2	0.3426	0.4677	0.5091	0.3481	0
2060	894.1	0.3426	0.468	0.5091	0.3484	0
2061	894	0.3426	0.4678	0.5091	0.3483	0
2062	893.9	0.3426	0.468	0.5093	0.3482	0
2063	893.8	0.3429	0.4681	0.5094	0.3482	0
2064	893.7	0.3429	0.4682	0.5094	0.3482	0
2065	893.6	0.3428	0.4684	0.5096	0.3485	0
2066	893.5	0.3432	0.4688	0.51	0.3489	0
2067	893.4	0.343	0.4689	0.5099	0.3489	0
2068	893.3	0.3431	0.4687	0.5099	0.3487	0
2069	893.2	0.3432	0.4688	0.5098	0.3485	0
2070	893.1	0.3433	0.4687	0.5097	0.3487	0
2071	893	0.3432	0.4687	0.5099	0.3487	0
2072	892.9	0.3432	0.4689	0.51	0.3487	0
2073	892.8	0.3435	0.4693	0.5103	0.349	0
2074	892.7	0.3433	0.469	0.51	0.3488	0
2075	892.6	0.3432	0.4689	0.51	0.349	0
2076	892.5	0.3434	0.469	0.5101	0.3489	0
2077	892.4	0.3434	0.4691	0.5102	0.349	0
2078	892.3	0.3434	0.4691	0.5101	0.349	0
2079	892.2	0.3434	0.469	0.5103	0.3491	0
2080	892.1	0.3437	0.4694	0.5102	0.3492	0
2081	892	0.3436	0.4695	0.5104	0.3494	0
2082	891.9	0.3438	0.4697	0.5103	0.3493	0
2083	891.8	0.3438	0.4698	0.5104	0.3494	0
2084	891.7	0.3437	0.4696	0.5101	0.3494	0
2085	891.6	0.3436	0.4696	0.5102	0.3493	0
2086	891.5	0.3438	0.4698	0.5104	0.3495	0
2087	891.4	0.3438	0.4698	0.5103	0.3494	0
2088	891.3	0.3436	0.4697	0.5102	0.3494	0
2089	891.2	0.3436	0.4696	0.5101	0.3493	0
2090	891.1	0.3435	0.4696	0.5102	0.3495	0
2091	891	0.3438	0.4698	0.5105	0.3499	0
2092	890.9	0.3438	0.4698	0.5104	0.3497	0
2093	890.8	0.3436	0.4698	0.5102	0.3496	0

2094	890.7	0.3437	0.4699	0.5103	0.3496	0
2095	890.6	0.3437	0.47	0.5103	0.3496	0
2096	890.5	0.3436	0.4698	0.5102	0.3494	0
2097	890.4	0.3438	0.4696	0.5102	0.3493	0
2098	890.3	0.3438	0.4695	0.51	0.3493	0
2099	890.2	0.3437	0.4695	0.5104	0.3493	0
2100	890.1	0.3438	0.4699	0.5104	0.3497	0
2101	890	0.3442	0.47	0.5106	0.3499	0
2102	889.9	0.3438	0.4697	0.5103	0.3498	0
2103	889.8	0.3438	0.4697	0.5103	0.3497	0
2104	889.7	0.3439	0.47	0.5108	0.3498	0
2105	889.6	0.3441	0.4701	0.5108	0.35	0
2106	889.5	0.344	0.4703	0.5103	0.3499	0
2107	889.4	0.3439	0.4699	0.5101	0.3498	0
2108	889.3	0.3441	0.4701	0.5103	0.3498	0
2109	889.2	0.3441	0.47	0.5105	0.3498	0
2110	889.1	0.3438	0.47	0.5105	0.3497	0
2111	889	0.3441	0.47	0.5104	0.3499	0
2112	888.9	0.3441	0.4701	0.5106	0.3499	0
2113	888.8	0.3442	0.47	0.5104	0.3498	0
2114	888.7	0.3441	0.47	0.5103	0.3498	0
2115	888.6	0.3438	0.47	0.5103	0.3497	0
2116	888.5	0.3438	0.4699	0.5101	0.3496	0
2117	888.4	0.3439	0.47	0.5102	0.3496	0
2118	888.3	0.3438	0.4697	0.5101	0.3493	0
2119	888.2	0.3439	0.47	0.5103	0.3495	0
2120	888.1	0.344	0.4697	0.51	0.3495	0
2121	888	0.3438	0.4695	0.5099	0.3492	0
2122	887.9	0.3437	0.4697	0.5099	0.3494	0
2123	887.8	0.3437	0.4695	0.5097	0.3492	0
2124	887.7	0.344	0.4697	0.5099	0.3495	0
2125	887.6	0.3438	0.4695	0.51	0.3493	0
2126	887.5	0.3435	0.4693	0.5097	0.3492	0
2127	887.4	0.3436	0.4694	0.5098	0.3494	0
2128	887.3	0.3434	0.4695	0.5097	0.3492	0
2129	887.2	0.3436	0.4696	0.5095	0.3492	0
2130	887.1	0.3437	0.4696	0.5095	0.3491	0
2131	887	0.3437	0.4697	0.5096	0.3493	0
2132	886.9	0.3438	0.4698	0.5096	0.3494	0
2133	886.8	0.3437	0.4696	0.5095	0.3493	0
2134	886.7	0.3437	0.4694	0.5093	0.3491	0
2135	886.6	0.3438	0.4697	0.5095	0.3494	0
2136	886.5	0.3435	0.4694	0.5093	0.3493	0

2137	886.4	0.3433	0.4692	0.5094	0.3493	0
2138	886.3	0.3433	0.4692	0.5096	0.3492	0
2139	886.2	0.3433	0.4693	0.5095	0.3492	0
2140	886.1	0.3435	0.4695	0.5098	0.3492	0
2141	886	0.3437	0.4694	0.5096	0.3494	0
2142	885.9	0.3437	0.4695	0.5098	0.3494	0
2143	885.8	0.3437	0.4696	0.5097	0.3493	0
2144	885.7	0.3437	0.4698	0.5098	0.3495	0
2145	885.6	0.3438	0.4697	0.5095	0.3494	0
2146	885.5	0.3437	0.4695	0.5095	0.3494	0
2147	885.4	0.3436	0.4693	0.5096	0.3494	0
2148	885.3	0.3434	0.4697	0.5096	0.3493	0
2149	885.2	0.3436	0.4697	0.5095	0.3495	0
2150	885.1	0.3438	0.4694	0.5096	0.3493	0
2151	885	0.3438	0.4696	0.5095	0.3496	0
2152	884.9	0.3435	0.4693	0.5096	0.3492	0
2153	884.8	0.3436	0.4696	0.5097	0.3494	0
2154	884.7	0.3436	0.4696	0.5095	0.3495	0
2155	884.6	0.3439	0.4698	0.5097	0.3497	0
2156	884.5	0.3437	0.4697	0.5098	0.3496	0
2157	884.4	0.3442	0.4699	0.5101	0.3497	0
2158	884.3	0.3441	0.4699	0.5097	0.3495	0
2159	884.2	0.344	0.4702	0.5098	0.3497	0
2160	884.1	0.3438	0.47	0.5098	0.3496	0
2161	884	0.3438	0.4701	0.5097	0.3498	0
2162	883.9	0.3438	0.47	0.5096	0.3496	0
2163	883.8	0.3437	0.4698	0.5096	0.3496	0
2164	883.7	0.3441	0.4703	0.5099	0.35	0
2165	883.6	0.3444	0.4704	0.5103	0.3502	0
2166	883.5	0.3445	0.4705	0.5105	0.3503	0
2167	883.4	0.3443	0.4706	0.5102	0.3503	0
2168	883.3	0.3445	0.4706	0.5102	0.3502	0
2169	883.2	0.3445	0.4707	0.5102	0.3503	0
2170	883.1	0.3445	0.4709	0.5103	0.3504	0
2171	883	0.3444	0.4706	0.5101	0.3502	0
2172	882.9	0.3445	0.4705	0.5104	0.3501	0
2173	882.8	0.3448	0.4709	0.5106	0.3505	0
2174	882.7	0.3447	0.471	0.5105	0.3505	0
2175	882.6	0.3446	0.4709	0.5104	0.3504	0
2176	882.5	0.3445	0.4711	0.5103	0.3505	0
2177	882.4	0.3447	0.4708	0.5105	0.3504	0
2178	882.3	0.3446	0.471	0.5105	0.3506	0
2179	882.2	0.3449	0.4711	0.5106	0.3507	0

2180	882.1	0.3448	0.4713	0.5108	0.3507	0
2181	882	0.3447	0.4712	0.5106	0.3505	0
2182	881.9	0.3447	0.4712	0.5106	0.3507	0
2183	881.8	0.345	0.4715	0.5108	0.3509	0
2184	881.7	0.3449	0.4713	0.5108	0.3509	0
2185	881.6	0.3447	0.4712	0.5105	0.3509	0
2186	881.5	0.3447	0.4713	0.5107	0.3507	0
2187	881.4	0.3447	0.4711	0.5107	0.3507	0
2188	881.3	0.3448	0.4714	0.5104	0.3507	0
2189	881.2	0.345	0.4715	0.5107	0.3509	0
2190	881.1	0.3448	0.4715	0.5105	0.351	0
2191	881	0.345	0.4711	0.5106	0.3509	0
2192	880.9	0.3451	0.4713	0.511	0.3509	0
2193	880.8	0.345	0.4714	0.5109	0.351	0
2194	880.7	0.3452	0.4717	0.511	0.3511	0
2195	880.6	0.3453	0.4719	0.511	0.3512	0
2196	880.5	0.3451	0.4716	0.511	0.3512	0
2197	880.4	0.3451	0.4719	0.5111	0.3513	0
2198	880.3	0.3454	0.4719	0.511	0.3512	0
2199	880.2	0.3452	0.4721	0.511	0.3513	0
2200	880.1	0.3451	0.4718	0.511	0.3512	0
2201	880	0.3452	0.4718	0.511	0.3512	0
2202	879.9	0.3451	0.4719	0.5109	0.3511	0
2203	879.8	0.3456	0.4721	0.5113	0.3515	0
2204	879.7	0.3456	0.472	0.511	0.3515	0
2205	879.6	0.3451	0.4718	0.5112	0.3513	0
2206	879.5	0.3453	0.472	0.511	0.3514	0
2207	879.4	0.3451	0.472	0.511	0.3514	0
2208	879.3	0.3453	0.472	0.5112	0.3513	0
2209	879.2	0.3455	0.4722	0.511	0.3514	0
2210	879.1	0.3455	0.472	0.5111	0.3515	0
2211	879	0.3455	0.4722	0.5111	0.3514	0
2212	878.9	0.3453	0.4719	0.5111	0.3513	0
2213	878.8	0.3454	0.4718	0.5111	0.3513	0
2214	878.7	0.3452	0.4719	0.5111	0.3512	0
2215	878.6	0.3453	0.4722	0.5112	0.3514	0
2216	878.5	0.3455	0.4722	0.5113	0.3514	0
2217	878.4	0.3453	0.472	0.5111	0.3512	0
2218	878.3	0.3454	0.4721	0.5112	0.3512	0
2219	878.2	0.3453	0.4724	0.5112	0.3513	0
2220	878.1	0.3453	0.4723	0.511	0.3512	0
2221	878	0.3452	0.4721	0.5111	0.3512	0
2222	877.9	0.345	0.4719	0.5109	0.3511	0

2223	877.8	0.345	0.4717	0.5107	0.3512	0
2224	877.7	0.3451	0.4718	0.5109	0.3512	0
2225	877.6	0.3454	0.4719	0.5111	0.3513	0
2226	877.5	0.3455	0.4723	0.5113	0.3514	0
2227	877.4	0.3453	0.4721	0.5108	0.3511	0
2228	877.3	0.3452	0.4719	0.5109	0.3513	0
2229	877.2	0.345	0.4719	0.5108	0.3512	0
2230	877.1	0.3452	0.4718	0.5109	0.3511	0
2231	877	0.3454	0.4717	0.5109	0.3511	0
2232	876.9	0.3451	0.4715	0.5106	0.351	0
2233	876.8	0.3451	0.4718	0.5108	0.3512	0
2234	876.7	0.3453	0.4718	0.5109	0.3513	0
2235	876.6	0.3453	0.4721	0.5109	0.3516	0
2236	876.5	0.3454	0.4721	0.511	0.3513	0
2237	876.4	0.3452	0.4719	0.5109	0.3512	0
2238	876.3	0.3454	0.472	0.5108	0.351	0
2239	876.2	0.3454	0.4722	0.5109	0.3512	0
2240	876.1	0.3455	0.4723	0.511	0.3515	0
2241	876	0.3454	0.4721	0.5111	0.3515	0
2242	875.9	0.3453	0.472	0.5109	0.3512	0
2243	875.8	0.3454	0.472	0.511	0.3512	0
2244	875.7	0.3453	0.472	0.5108	0.3511	0
2245	875.6	0.3453	0.4719	0.5109	0.3513	0
2246	875.5	0.3454	0.4721	0.5108	0.3514	0
2247	875.4	0.3453	0.4722	0.5109	0.3512	0
2248	875.3	0.3453	0.4721	0.5108	0.3513	0
2249	875.2	0.3452	0.4722	0.5108	0.3514	0
2250	875.1	0.3453	0.4722	0.5109	0.3513	0
2251	875	0.3457	0.4721	0.511	0.3513	0
2252	874.9	0.3455	0.4722	0.5111	0.3514	0
2253	874.8	0.3456	0.4724	0.5111	0.3516	0
2254	874.7	0.3457	0.4725	0.5113	0.3517	0
2255	874.6	0.3456	0.4724	0.5112	0.3516	0
2256	874.5	0.3455	0.4724	0.5113	0.3516	0
2257	874.4	0.3453	0.4723	0.5112	0.3515	0
2258	874.3	0.3456	0.4725	0.5113	0.3517	0
2259	874.2	0.3458	0.4727	0.5113	0.3517	0
2260	874.1	0.3455	0.4727	0.5113	0.3516	0
2261	874	0.3457	0.4726	0.5113	0.3517	0
2262	873.9	0.3459	0.4728	0.5115	0.352	0
2263	873.8	0.346	0.4728	0.5117	0.3521	0
2264	873.7	0.3462	0.4731	0.5119	0.3523	0
2265	873.6	0.3459	0.4731	0.5118	0.3523	0

2266	873.5	0.3458	0.473	0.5115	0.3521	0
2267	873.4	0.3458	0.4733	0.5116	0.3522	0
2268	873.3	0.3458	0.4731	0.5114	0.3519	0
2269	873.2	0.346	0.4733	0.5115	0.3521	0
2270	873.1	0.3459	0.4729	0.5114	0.352	0
2271	873	0.3461	0.4734	0.5116	0.3521	0
2272	872.9	0.3463	0.4736	0.5119	0.3525	0
2273	872.8	0.3466	0.4736	0.5121	0.3524	0
2274	872.7	0.3463	0.4733	0.5118	0.3524	0
2275	872.6	0.3465	0.4736	0.5122	0.3526	0
2276	872.5	0.3463	0.4736	0.512	0.3526	0
2277	872.4	0.3464	0.4736	0.512	0.3525	0
2278	872.3	0.3464	0.4737	0.5121	0.3525	0
2279	872.2	0.3464	0.4736	0.512	0.3526	0
2280	872.1	0.3463	0.4732	0.5115	0.3525	0
2281	872	0.3465	0.4734	0.5118	0.3526	0
2282	871.9	0.3464	0.4736	0.5118	0.3526	0
2283	871.8	0.3465	0.4739	0.5121	0.3527	0
2284	871.7	0.3466	0.4741	0.5122	0.3527	0
2285	871.6	0.3465	0.4738	0.5119	0.3529	0
2286	871.5	0.3466	0.474	0.5124	0.3528	0
2287	871.4	0.3465	0.4739	0.5123	0.3527	0
2288	871.3	0.3468	0.474	0.5123	0.3529	0
2289	871.2	0.3466	0.4736	0.5123	0.3528	0
2290	871.1	0.3464	0.4733	0.5121	0.3526	0
2291	871	0.3464	0.4736	0.5121	0.3525	0
2292	870.9	0.3465	0.4737	0.5122	0.3525	0
2293	870.8	0.3467	0.4738	0.5121	0.3526	0
2294	870.7	0.3467	0.474	0.5122	0.3529	0
2295	870.6	0.3466	0.4738	0.5122	0.3528	0
2296	870.5	0.3467	0.474	0.5123	0.3529	0
2297	870.4	0.3464	0.474	0.5124	0.3529	0
2298	870.3	0.3468	0.474	0.5124	0.3528	0
2299	870.2	0.3466	0.474	0.5123	0.3529	0
2300	870.1	0.3465	0.474	0.5125	0.353	0
2301	870	0.3466	0.474	0.5124	0.3529	0
2302	869.9	0.3466	0.4741	0.5122	0.3528	0
2303	869.8	0.3466	0.4738	0.5123	0.3526	0
2304	869.7	0.3467	0.4741	0.5126	0.353	0
2305	869.6	0.3467	0.4739	0.5123	0.3528	0
2306	869.5	0.3466	0.4739	0.5123	0.3528	0
2307	869.4	0.3467	0.4738	0.5123	0.3527	0
2308	869.3	0.3466	0.4736	0.5122	0.3525	0

2309	869.2	0.3462	0.4736	0.512	0.3525	0
2310	869.1	0.3464	0.4736	0.5122	0.3527	0
2311	869	0.3465	0.4738	0.5121	0.3528	0
2312	868.9	0.3463	0.4739	0.5119	0.3527	0
2313	868.8	0.3464	0.4737	0.5119	0.3528	0
2314	868.7	0.3464	0.4739	0.5121	0.3527	0
2315	868.6	0.3465	0.4739	0.5121	0.3528	0
2316	868.5	0.3466	0.4738	0.5121	0.3527	0
2317	868.4	0.3466	0.4737	0.512	0.3527	0
2318	868.3	0.3465	0.4739	0.5122	0.3527	0
2319	868.2	0.3465	0.4738	0.5121	0.3527	0
2320	868.1	0.3465	0.4737	0.5119	0.3526	0
2321	868	0.3463	0.4736	0.512	0.3526	0
2322	867.9	0.3463	0.4736	0.5117	0.3525	0
2323	867.8	0.3461	0.4736	0.5118	0.3524	0
2324	867.7	0.3463	0.4736	0.5118	0.3526	0
2325	867.6	0.3466	0.4737	0.512	0.3525	0
2326	867.5	0.3462	0.4734	0.5117	0.3524	0
2327	867.4	0.3462	0.4735	0.5119	0.3527	0
2328	867.3	0.3465	0.4736	0.5117	0.3525	0
2329	867.2	0.3467	0.474	0.5121	0.3529	0
2330	867.1	0.3469	0.474	0.5125	0.3529	0
2331	867	0.3468	0.4738	0.5122	0.3528	0
2332	866.9	0.3466	0.4735	0.5121	0.3527	0
2333	866.8	0.3466	0.4737	0.5121	0.3528	0
2334	866.7	0.3465	0.4737	0.5121	0.3528	0
2335	866.6	0.3468	0.4739	0.5121	0.3529	0
2336	866.5	0.3466	0.474	0.5122	0.3527	0
2337	866.4	0.3465	0.4739	0.5122	0.3529	0
2338	866.3	0.3469	0.4743	0.5123	0.3531	0
2339	866.2	0.3469	0.4741	0.5122	0.353	0
2340	866.1	0.3468	0.4743	0.5121	0.353	0
2341	866	0.3467	0.4743	0.5123	0.353	0
2342	865.9	0.3469	0.4745	0.5125	0.353	0
2343	865.8	0.347	0.4743	0.5124	0.3531	0
2344	865.7	0.3469	0.4746	0.5125	0.3532	0
2345	865.6	0.3471	0.4746	0.5129	0.3533	0
2346	865.5	0.3471	0.4745	0.5129	0.3532	0
2347	865.4	0.3471	0.4748	0.5131	0.3535	0
2348	865.3	0.3472	0.4748	0.513	0.3536	0
2349	865.2	0.3473	0.475	0.5129	0.3536	0
2350	865.1	0.3472	0.4749	0.5129	0.3535	0
2351	865	0.3472	0.4749	0.5129	0.3537	0

2352	864.9	0.3471	0.4748	0.5129	0.3535	0
2353	864.8	0.3471	0.4749	0.513	0.3534	0
2354	864.7	0.3471	0.475	0.5131	0.3536	0
2355	864.6	0.3474	0.475	0.5132	0.3536	0
2356	864.5	0.3475	0.4752	0.5133	0.354	0
2357	864.4	0.3476	0.4754	0.5133	0.3539	0
2358	864.3	0.3475	0.4753	0.5134	0.354	0
2359	864.2	0.3476	0.4752	0.5135	0.3539	0
2360	864.1	0.3477	0.4752	0.5137	0.354	0
2361	864	0.3476	0.4754	0.5134	0.3539	0
2362	863.9	0.3475	0.4753	0.5133	0.3539	0
2363	863.8	0.3478	0.4755	0.5136	0.3539	0
2364	863.7	0.348	0.4757	0.5134	0.3541	0
2365	863.6	0.3477	0.4756	0.5134	0.354	0
2366	863.5	0.348	0.4758	0.5136	0.3541	0
2367	863.4	0.3479	0.4758	0.5135	0.3541	0
2368	863.3	0.3479	0.4759	0.5137	0.3541	0
2369	863.2	0.348	0.4759	0.5138	0.3543	0
2370	863.1	0.348	0.4759	0.5138	0.3544	0
2371	863	0.3479	0.4759	0.514	0.3543	0
2372	862.9	0.3479	0.4761	0.5139	0.3544	0
2373	862.8	0.348	0.4763	0.5139	0.3544	0
2374	862.7	0.3481	0.4762	0.5139	0.3546	0
2375	862.6	0.3479	0.4761	0.5139	0.3544	0
2376	862.5	0.3482	0.4762	0.5142	0.3546	0
2377	862.4	0.3481	0.4761	0.5142	0.3547	0
2378	862.3	0.3479	0.4759	0.5138	0.3545	0
2379	862.2	0.3482	0.4762	0.5141	0.3546	0
2380	862.1	0.3483	0.4762	0.514	0.3544	0
2381	862	0.3483	0.4763	0.5141	0.3547	0
2382	861.9	0.3481	0.4763	0.514	0.3546	0
2383	861.8	0.3481	0.4763	0.514	0.3547	0
2384	861.7	0.3482	0.4765	0.5142	0.3549	0
2385	861.6	0.3481	0.4765	0.5142	0.3549	0
2386	861.5	0.3482	0.4763	0.5142	0.3547	0
2387	861.4	0.3482	0.4762	0.5141	0.3545	0
2388	861.3	0.3482	0.4765	0.5139	0.3548	0
2389	861.2	0.3485	0.4764	0.514	0.3547	0
2390	861.1	0.3483	0.4763	0.5142	0.3548	0
2391	861	0.3483	0.4764	0.5143	0.3547	0
2392	860.9	0.3482	0.4763	0.5143	0.3546	0
2393	860.8	0.3483	0.4766	0.5142	0.3546	0
2394	860.7	0.3482	0.4762	0.5141	0.3547	0

2395	860.6	0.3484	0.4766	0.5142	0.3549	0
2396	860.5	0.3482	0.4764	0.514	0.3547	0
2397	860.4	0.3481	0.4764	0.5141	0.3547	0
2398	860.3	0.3481	0.4762	0.5142	0.3546	0
2399	860.2	0.3484	0.4765	0.5143	0.3549	0
2400	860.1	0.3485	0.4765	0.5142	0.355	0
2401	860	0.3483	0.4764	0.5141	0.3547	0
2402	859.9	0.3484	0.4765	0.5143	0.3549	0
2403	859.8	0.3483	0.4763	0.5141	0.355	0
2404	859.7	0.3484	0.4764	0.5142	0.3548	0
2405	859.6	0.3481	0.4765	0.5141	0.3547	0
2406	859.5	0.3482	0.4766	0.5142	0.3549	0
2407	859.4	0.3483	0.4766	0.5141	0.3549	0
2408	859.3	0.3483	0.4764	0.5143	0.3547	0
2409	859.2	0.3482	0.4766	0.514	0.3547	0
2410	859.1	0.348	0.4763	0.5139	0.3544	0
2411	859	0.3481	0.4763	0.5138	0.3544	0
2412	858.9	0.348	0.476	0.5138	0.3544	0
2413	858.8	0.3481	0.4761	0.5137	0.3544	0
2414	858.7	0.3481	0.4761	0.5138	0.3545	0
2415	858.6	0.348	0.4763	0.5139	0.3545	0
2416	858.5	0.348	0.4761	0.5139	0.3546	0
2417	858.4	0.3479	0.476	0.5139	0.3544	0
2418	858.3	0.3478	0.4759	0.5138	0.3544	0
2419	858.2	0.3478	0.4761	0.5137	0.3543	0
2420	858.1	0.3479	0.476	0.5136	0.3544	0
2421	858	0.3479	0.476	0.5134	0.3544	0
2422	857.9	0.3479	0.476	0.5137	0.3543	0
2423	857.8	0.3479	0.4758	0.5137	0.3544	0
2424	857.7	0.348	0.4759	0.5138	0.3543	0
2425	857.6	0.3478	0.4758	0.5138	0.3544	0
2426	857.5	0.3478	0.4757	0.5137	0.3543	0
2427	857.4	0.3478	0.4758	0.5137	0.3544	0
2428	857.3	0.3477	0.476	0.5136	0.3543	0
2429	857.2	0.3479	0.476	0.5137	0.3544	0
2430	857.1	0.3478	0.476	0.5135	0.3543	0
2431	857	0.3479	0.4762	0.5138	0.3544	0
2432	856.9	0.3481	0.4761	0.5138	0.3545	0
2433	856.8	0.348	0.4761	0.5137	0.3545	0
2434	856.7	0.3481	0.4762	0.5137	0.3547	0
2435	856.6	0.348	0.4762	0.5141	0.3545	0
2436	856.5	0.3481	0.4762	0.5141	0.3545	0
2437	856.4	0.3482	0.4763	0.514	0.3547	0

2438	856.3	0.3481	0.4764	0.5141	0.3545	0
2439	856.2	0.3482	0.4765	0.5142	0.3548	0
2440	856.1	0.3484	0.4764	0.5143	0.3546	0
2441	856	0.3484	0.4765	0.5142	0.3548	0
2442	855.9	0.3482	0.4764	0.5141	0.3549	0
2443	855.8	0.3482	0.4767	0.5142	0.3548	0
2444	855.7	0.3483	0.4769	0.5142	0.355	0
2445	855.6	0.3484	0.4771	0.5145	0.3551	0
2446	855.5	0.3485	0.4769	0.5143	0.355	0
2447	855.4	0.3485	0.4768	0.5145	0.3552	0
2448	855.3	0.3487	0.477	0.5147	0.3552	0
2449	855.2	0.3486	0.477	0.5147	0.3553	0
2450	855.1	0.3487	0.4773	0.5148	0.3553	0
2451	855	0.3487	0.4772	0.5148	0.3555	0
2452	854.9	0.3488	0.4773	0.5148	0.3555	0
2453	854.8	0.3488	0.4772	0.515	0.3556	0
2454	854.7	0.3487	0.4773	0.5147	0.3554	0
2455	854.6	0.349	0.4775	0.5149	0.3557	0
2456	854.5	0.3491	0.4776	0.515	0.3557	0
2457	854.4	0.3492	0.4778	0.5151	0.3557	0
2458	854.3	0.3493	0.4777	0.5152	0.3556	0
2459	854.2	0.3492	0.4776	0.5154	0.3559	0
2460	854.1	0.3492	0.4779	0.5154	0.356	0
2461	854	0.3492	0.4778	0.5153	0.3559	0
2462	853.9	0.3493	0.4779	0.5154	0.3559	0
2463	853.8	0.3492	0.4779	0.5154	0.356	0
2464	853.7	0.3492	0.4778	0.5154	0.356	0
2465	853.6	0.3492	0.478	0.5154	0.356	0
2466	853.5	0.3494	0.4783	0.5155	0.3562	0
2467	853.4	0.3493	0.4784	0.5155	0.3562	0
2468	853.3	0.3494	0.4784	0.5156	0.3561	0
2469	853.2	0.3497	0.4786	0.5156	0.3563	0
2470	853.1	0.3498	0.4785	0.5158	0.3564	0
2471	853	0.3497	0.4784	0.5158	0.3563	0
2472	852.9	0.3496	0.4786	0.5159	0.3563	0
2473	852.8	0.3496	0.4785	0.5156	0.3565	0
2474	852.7	0.35	0.4788	0.5162	0.3566	0
2475	852.6	0.3499	0.4787	0.5159	0.3566	0
2476	852.5	0.3499	0.4787	0.516	0.3566	0
2477	852.4	0.3497	0.4787	0.516	0.3565	0
2478	852.3	0.3498	0.4785	0.516	0.3567	0
2479	852.2	0.3499	0.4788	0.5161	0.3565	0
2480	852.1	0.3499	0.4788	0.5161	0.3566	0

2481	852	0.3501	0.4789	0.5163	0.3569	0
2482	851.9	0.35	0.4788	0.5164	0.3568	0
2483	851.8	0.3499	0.4786	0.516	0.3567	0
2484	851.7	0.3499	0.4787	0.5162	0.3567	0
2485	851.6	0.35	0.4788	0.5164	0.3567	0
2486	851.5	0.35	0.4787	0.5163	0.3567	0
2487	851.4	0.3499	0.4786	0.5159	0.3566	0
2488	851.3	0.35	0.4789	0.516	0.3567	0
2489	851.2	0.3499	0.4786	0.5161	0.3567	0
2490	851.1	0.3497	0.4786	0.5159	0.3566	0
2491	851	0.3498	0.4787	0.5158	0.3566	0
2492	850.9	0.3498	0.4788	0.5161	0.3567	0
2493	850.8	0.3498	0.4786	0.5157	0.3565	0
2494	850.7	0.3501	0.4789	0.5162	0.3566	0
2495	850.6	0.3497	0.4789	0.5162	0.3565	0
2496	850.5	0.3497	0.4789	0.516	0.3566	0
2497	850.4	0.3496	0.4786	0.5161	0.3563	0
2498	850.3	0.3497	0.4788	0.516	0.3564	0
2499	850.2	0.3499	0.4788	0.5161	0.3566	0
2500	850.1	0.3498	0.4786	0.5161	0.3564	0
2501	850	0.3497	0.4785	0.516	0.3564	0
2502	849.9	0.3447	0.4727	0.5126	0.3529	0
2503	849.8	0.3447	0.4725	0.5126	0.3529	0
2504	849.7	0.3448	0.4726	0.5125	0.353	0
2505	849.6	0.3449	0.4726	0.5125	0.3529	0
2506	849.5	0.345	0.4728	0.5127	0.3531	0
2507	849.4	0.3449	0.4725	0.5125	0.3529	0
2508	849.3	0.3447	0.4725	0.5125	0.3528	0
2509	849.2	0.3447	0.4723	0.5124	0.3527	0
2510	849.1	0.3446	0.4723	0.5123	0.3527	0
2511	849	0.3444	0.4721	0.5121	0.3523	0
2512	848.9	0.3444	0.4724	0.5123	0.3526	0
2513	848.8	0.3445	0.4722	0.5122	0.3527	0
2514	848.7	0.3444	0.4721	0.5121	0.3527	0
2515	848.6	0.3444	0.4721	0.5122	0.3525	0
2516	848.5	0.3444	0.4719	0.512	0.3523	0
2517	848.4	0.3442	0.4718	0.5119	0.3523	0
2518	848.3	0.3444	0.4721	0.512	0.3524	0
2519	848.2	0.3443	0.472	0.5122	0.3524	0
2520	848.1	0.3441	0.4718	0.5118	0.3523	0
2521	848	0.3445	0.472	0.512	0.3523	0
2522	847.9	0.3441	0.472	0.512	0.3522	0
2523	847.8	0.3441	0.4719	0.512	0.3522	0

2524	847.7	0.3441	0.4719	0.5117	0.3522	0
2525	847.6	0.344	0.4717	0.5117	0.3521	0
2526	847.5	0.3441	0.4717	0.5117	0.3522	0
2527	847.4	0.3441	0.4717	0.5119	0.352	0
2528	847.3	0.3441	0.4717	0.512	0.3522	0
2529	847.2	0.3443	0.4718	0.5118	0.3521	0
2530	847.1	0.3443	0.4718	0.5122	0.3522	0
2531	847	0.344	0.4717	0.5118	0.352	0
2532	846.9	0.3439	0.4718	0.5117	0.3522	0
2533	846.8	0.3439	0.4719	0.5116	0.3522	0
2534	846.7	0.3441	0.4717	0.5117	0.3521	0
2535	846.6	0.3442	0.4718	0.5121	0.3522	0
2536	846.5	0.3443	0.4719	0.5121	0.3523	0
2537	846.4	0.3444	0.4721	0.5121	0.3523	0
2538	846.3	0.3445	0.4721	0.5122	0.3526	0
2539	846.2	0.3445	0.4722	0.5124	0.3525	0
2540	846.1	0.3442	0.4719	0.5122	0.3524	0
2541	846	0.3443	0.4719	0.5121	0.3523	0
2542	845.9	0.3444	0.472	0.5122	0.3523	0
2543	845.8	0.3442	0.4719	0.5121	0.3524	0
2544	845.7	0.3443	0.4721	0.5119	0.3526	0
2545	845.6	0.3444	0.4722	0.5118	0.3524	0
2546	845.5	0.3447	0.4724	0.5123	0.3526	0
2547	845.4	0.3446	0.4723	0.5123	0.3525	0
2548	845.3	0.3448	0.4726	0.5124	0.3528	0
2549	845.2	0.3447	0.4725	0.5125	0.3528	0
2550	845.1	0.3448	0.4728	0.5127	0.353	0
2551	845	0.3448	0.4727	0.5126	0.3531	0
2552	844.9	0.3448	0.4728	0.5127	0.3531	0
2553	844.8	0.3449	0.473	0.5131	0.3531	0
2554	844.7	0.3452	0.4732	0.5131	0.3532	0
2555	844.6	0.3449	0.473	0.5128	0.3531	0
2556	844.5	0.345	0.4733	0.5132	0.3534	0
2557	844.4	0.3452	0.4733	0.5134	0.3536	0
2558	844.3	0.3453	0.4736	0.5135	0.3537	0
2559	844.2	0.3456	0.4736	0.5137	0.3539	0
2560	844.1	0.3455	0.4736	0.5138	0.3538	0
2561	844	0.3456	0.4737	0.5138	0.3538	0
2562	843.9	0.3456	0.4737	0.5139	0.3538	0
2563	843.8	0.3458	0.474	0.514	0.3541	0
2564	843.7	0.3459	0.474	0.514	0.354	0
2565	843.6	0.346	0.4743	0.5143	0.3543	0
2566	843.5	0.346	0.4743	0.5143	0.3543	0

2567	843.4	0.3459	0.4742	0.5142	0.3542	0
2568	843.3	0.346	0.4744	0.5143	0.3543	0
2569	843.2	0.3461	0.4749	0.5145	0.3544	0
2570	843.1	0.3461	0.4747	0.5146	0.3544	0
2571	843	0.3462	0.4745	0.5144	0.3544	0
2572	842.9	0.3463	0.4746	0.5144	0.3545	0
2573	842.8	0.3465	0.475	0.5147	0.3549	0
2574	842.7	0.3466	0.475	0.5147	0.3547	0
2575	842.6	0.3463	0.475	0.5147	0.3548	0
2576	842.5	0.3465	0.4751	0.5147	0.3549	0
2577	842.4	0.3463	0.475	0.5149	0.3548	0
2578	842.3	0.3465	0.4752	0.5149	0.3548	0
2579	842.2	0.3465	0.4753	0.515	0.3551	0
2580	842.1	0.3466	0.4753	0.5152	0.3547	0
2581	842	0.3465	0.4753	0.5149	0.355	0
2582	841.9	0.3464	0.4751	0.5151	0.3548	0
2583	841.8	0.3463	0.4752	0.5149	0.3549	0
2584	841.7	0.3466	0.4752	0.5152	0.3549	0
2585	841.6	0.3466	0.4753	0.5151	0.3549	0
2586	841.5	0.3464	0.4753	0.5152	0.3549	0
2587	841.4	0.3465	0.4753	0.515	0.3549	0
2588	841.3	0.3463	0.4752	0.5149	0.3547	0
2589	841.2	0.3467	0.4753	0.5151	0.355	0
2590	841.1	0.3464	0.4753	0.5152	0.355	0
2591	841	0.3464	0.4752	0.5151	0.355	0
2592	840.9	0.3466	0.4752	0.5153	0.3551	0
2593	840.8	0.3468	0.4755	0.5156	0.3553	0
2594	840.7	0.3466	0.4754	0.5154	0.3553	0
2595	840.6	0.3466	0.4752	0.5154	0.3551	0
2596	840.5	0.3466	0.4755	0.5154	0.3552	0
2597	840.4	0.3465	0.4754	0.5153	0.3551	0
2598	840.3	0.3465	0.4753	0.5154	0.3551	0
2599	840.2	0.3467	0.4754	0.5153	0.3552	0
2600	840.1	0.3467	0.4754	0.5155	0.3552	0
2601	840	0.3466	0.4755	0.5155	0.3551	0
2602	839.9	0.3466	0.4754	0.5155	0.3551	0
2603	839.8	0.3468	0.4756	0.5154	0.3551	0
2604	839.7	0.3467	0.4755	0.5155	0.3551	0
2605	839.6	0.3467	0.4754	0.5153	0.3551	0
2606	839.5	0.3465	0.4755	0.5152	0.3551	0
2607	839.4	0.3465	0.4754	0.5152	0.3551	0
2608	839.3	0.3465	0.4754	0.5152	0.355	0
2609	839.2	0.3465	0.4753	0.5151	0.3549	0

2610	839.1	0.3465	0.4752	0.5151	0.3549	0
2611	839	0.3465	0.4753	0.5151	0.3548	0
2612	838.9	0.3464	0.4751	0.515	0.3549	0
2613	838.8	0.3461	0.4749	0.5148	0.3547	0
2614	838.7	0.3462	0.475	0.515	0.3546	0
2615	838.6	0.346	0.4751	0.5149	0.3545	0
2616	838.5	0.3463	0.475	0.5151	0.3548	0
2617	838.4	0.346	0.4749	0.5148	0.3547	0
2618	838.3	0.3462	0.4749	0.5149	0.3547	0
2619	838.2	0.3463	0.4747	0.5149	0.3547	0
2620	838.1	0.3462	0.4749	0.5149	0.3546	0
2621	838	0.346	0.4752	0.5149	0.3545	0
2622	837.9	0.346	0.4748	0.5148	0.3545	0
2623	837.8	0.346	0.4748	0.5148	0.3545	0
2624	837.7	0.3461	0.4745	0.5146	0.3544	0
2625	837.6	0.346	0.4745	0.5147	0.3544	0
2626	837.5	0.346	0.4747	0.5147	0.3545	0
2627	837.4	0.3458	0.4745	0.5148	0.3543	0
2628	837.3	0.3458	0.4745	0.5147	0.3544	0
2629	837.2	0.3459	0.4744	0.5147	0.3543	0
2630	837.1	0.3459	0.4745	0.5147	0.3544	0
2631	837	0.3457	0.4744	0.5147	0.3543	0
2632	836.9	0.346	0.4745	0.5148	0.3543	0
2633	836.8	0.3459	0.4744	0.5146	0.3543	0
2634	836.7	0.3458	0.4742	0.5148	0.3541	0
2635	836.6	0.3461	0.4746	0.515	0.3545	0
2636	836.5	0.346	0.4746	0.5148	0.3543	0
2637	836.4	0.3461	0.4747	0.5149	0.3545	0
2638	836.3	0.3462	0.4747	0.5148	0.3544	0
2639	836.2	0.346	0.4746	0.5149	0.3542	0
2640	836.1	0.346	0.4747	0.5147	0.3543	0
2641	836	0.346	0.4747	0.5148	0.3544	0
2642	835.9	0.346	0.4746	0.5149	0.3542	0
2643	835.8	0.3459	0.4746	0.5149	0.3543	0
2644	835.7	0.346	0.4746	0.515	0.3544	0
2645	835.6	0.3462	0.4748	0.515	0.3545	0
2646	835.5	0.3463	0.4751	0.5153	0.3547	0
2647	835.4	0.3462	0.4748	0.5151	0.3546	0
2648	835.3	0.3464	0.4751	0.515	0.3547	0
2649	835.2	0.3463	0.4751	0.5151	0.3549	0
2650	835.1	0.3466	0.4751	0.5153	0.3547	0
2651	835	0.3464	0.475	0.5153	0.3548	0
2652	834.9	0.3464	0.4749	0.5154	0.355	0

2653	834.8	0.3465	0.4753	0.5156	0.355	0
2654	834.7	0.3465	0.4754	0.5157	0.3552	0
2655	834.6	0.3466	0.4756	0.5158	0.355	0
2656	834.5	0.3464	0.4755	0.5159	0.3551	0
2657	834.4	0.3464	0.4754	0.5157	0.3551	0
2658	834.3	0.3466	0.4753	0.5158	0.3551	0
2659	834.2	0.3466	0.4755	0.5161	0.3552	0
2660	834.1	0.3467	0.4758	0.516	0.3553	0
2661	834	0.3469	0.4758	0.5161	0.3555	0
2662	833.9	0.347	0.4759	0.516	0.3556	0
2663	833.8	0.3473	0.4762	0.5165	0.3558	0
2664	833.7	0.3468	0.4761	0.5163	0.3555	0
2665	833.6	0.3469	0.4762	0.5163	0.3557	0
2666	833.5	0.3473	0.4762	0.5164	0.3557	0
2667	833.4	0.3474	0.4765	0.5167	0.3558	0
2668	833.3	0.3475	0.4764	0.5168	0.356	0
2669	833.2	0.3475	0.4765	0.5169	0.356	0
2670	833.1	0.3476	0.4768	0.517	0.3561	0
2671	833	0.3475	0.4767	0.5172	0.3562	0
2672	832.9	0.3475	0.4768	0.517	0.3561	0
2673	832.8	0.3476	0.477	0.5174	0.3563	0
2674	832.7	0.3477	0.4772	0.5172	0.3563	0
2675	832.6	0.3478	0.4772	0.5175	0.3565	0
2676	832.5	0.348	0.4775	0.5174	0.3566	0
2677	832.4	0.348	0.4774	0.5176	0.3566	0
2678	832.3	0.3483	0.4777	0.5178	0.3568	0
2679	832.2	0.3482	0.4776	0.5176	0.3567	0
2680	832.1	0.3481	0.4775	0.5177	0.3567	0
2681	832	0.348	0.4778	0.5177	0.357	0
2682	831.9	0.3479	0.4776	0.5176	0.3567	0
2683	831.8	0.348	0.4776	0.5177	0.3568	0
2684	831.7	0.3481	0.4776	0.5179	0.3569	0
2685	831.6	0.348	0.4775	0.5179	0.3569	0
2686	831.5	0.3482	0.4775	0.5179	0.3568	0
2687	831.4	0.3481	0.4775	0.5179	0.3568	0
2688	831.3	0.3485	0.4779	0.5183	0.3572	0
2689	831.2	0.3486	0.478	0.5184	0.3572	0
2690	831.1	0.3484	0.4778	0.518	0.3571	0
2691	831	0.3484	0.478	0.5183	0.3572	0
2692	830.9	0.3483	0.478	0.5182	0.3571	0
2693	830.8	0.3484	0.4779	0.518	0.3569	0
2694	830.7	0.3481	0.4779	0.5179	0.3569	0
2695	830.6	0.3484	0.4779	0.5182	0.3571	0

2696	830.5	0.3485	0.478	0.5185	0.3573	0
2697	830.4	0.3487	0.4783	0.5186	0.3576	0
2698	830.3	0.3487	0.4783	0.5184	0.3574	0
2699	830.2	0.3484	0.478	0.5182	0.3572	0
2700	830.1	0.3487	0.4783	0.5187	0.3574	0
2701	830	0.3486	0.4781	0.5184	0.3573	0
2702	829.9	0.3486	0.4781	0.5184	0.3573	0
2703	829.8	0.3486	0.4781	0.5184	0.3571	0
2704	829.7	0.3484	0.4782	0.5183	0.3571	0
2705	829.6	0.3485	0.4781	0.5185	0.3572	0
2706	829.5	0.3485	0.4781	0.5184	0.3572	0
2707	829.4	0.3485	0.4783	0.5183	0.3572	0
2708	829.3	0.3483	0.478	0.5183	0.3572	0
2709	829.2	0.3486	0.4784	0.5186	0.3572	0
2710	829.1	0.3487	0.4781	0.5185	0.3571	0
2711	829	0.3485	0.478	0.5185	0.3572	0
2712	828.9	0.3485	0.478	0.5183	0.3572	0
2713	828.8	0.3485	0.4781	0.5186	0.3573	0
2714	828.7	0.3485	0.4781	0.5183	0.3572	0
2715	828.6	0.3483	0.4781	0.5185	0.3571	0
2716	828.5	0.348	0.4777	0.5181	0.3568	0
2717	828.4	0.3482	0.4781	0.5182	0.357	0
2718	828.3	0.3483	0.4781	0.5184	0.357	0
2719	828.2	0.3482	0.4779	0.5183	0.3569	0
2720	828.1	0.3483	0.4777	0.5185	0.3569	0
2721	828	0.3483	0.4776	0.5183	0.3569	0
2722	827.9	0.3479	0.4776	0.518	0.3566	0
2723	827.8	0.3478	0.4775	0.5182	0.3567	0
2724	827.7	0.3479	0.4775	0.5182	0.3569	0
2725	827.6	0.3479	0.4776	0.5181	0.3568	0
2726	827.5	0.3479	0.4775	0.518	0.3567	0
2727	827.4	0.3481	0.4776	0.5183	0.3569	0
2728	827.3	0.3481	0.4774	0.5181	0.3568	0
2729	827.2	0.3481	0.4776	0.5182	0.3568	0
2730	827.1	0.3481	0.4775	0.5181	0.3566	0
2731	827	0.3481	0.4776	0.5183	0.3568	0
2732	826.9	0.348	0.4775	0.5183	0.3567	0
2733	826.8	0.3479	0.4777	0.5181	0.3566	0
2734	826.7	0.3481	0.4778	0.5181	0.3567	0
2735	826.6	0.3481	0.4776	0.5181	0.3568	0
2736	826.5	0.3479	0.4774	0.518	0.3566	0
2737	826.4	0.3478	0.4774	0.5181	0.3565	0
2738	826.3	0.3479	0.4774	0.5182	0.3566	0

2739	826.2	0.3481	0.4775	0.5183	0.3568	0
2740	826.1	0.348	0.4774	0.5183	0.3566	0
2741	826	0.3481	0.4775	0.5183	0.3567	0
2742	825.9	0.348	0.4775	0.5182	0.3568	0
2743	825.8	0.3479	0.4775	0.5182	0.3568	0
2744	825.7	0.3479	0.4774	0.5182	0.3567	0
2745	825.6	0.3481	0.4776	0.5183	0.3567	0
2746	825.5	0.348	0.4775	0.5182	0.3566	0
2747	825.4	0.3482	0.4775	0.5186	0.3569	0
2748	825.3	0.3483	0.4778	0.5186	0.3569	0
2749	825.2	0.3484	0.478	0.5187	0.357	0
2750	825.1	0.3486	0.478	0.5188	0.3572	0
2751	825	0.3485	0.4782	0.5189	0.3575	0
2752	824.9	0.3482	0.478	0.5187	0.3571	0
2753	824.8	0.3481	0.4781	0.5189	0.357	0
2754	824.7	0.3484	0.4782	0.5188	0.3573	0
2755	824.6	0.3485	0.4782	0.5188	0.3575	0
2756	824.5	0.3484	0.4782	0.5189	0.3572	0
2757	824.4	0.3488	0.4786	0.5193	0.3575	0
2758	824.3	0.3486	0.4785	0.5191	0.3573	0
2759	824.2	0.3486	0.4784	0.5193	0.3572	0
2760	824.1	0.3488	0.4786	0.5193	0.3577	0
2761	824	0.3488	0.4786	0.5196	0.3576	0
2762	823.9	0.349	0.4788	0.5197	0.3578	0
2763	823.8	0.3489	0.4788	0.5195	0.3577	0
2764	823.7	0.349	0.4791	0.5195	0.3579	0
2765	823.6	0.349	0.479	0.5195	0.3577	0
2766	823.5	0.3491	0.4791	0.5197	0.3579	0
2767	823.4	0.3492	0.4791	0.5198	0.3581	0
2768	823.3	0.349	0.4791	0.5198	0.3579	0
2769	823.2	0.3493	0.479	0.5199	0.3579	0
2770	823.1	0.3492	0.4794	0.5198	0.358	0
2771	823	0.3494	0.4796	0.5197	0.3581	0
2772	822.9	0.3496	0.4796	0.5202	0.3582	0
2773	822.8	0.3495	0.4796	0.5202	0.3582	0
2774	822.7	0.3495	0.4796	0.5202	0.3582	0
2775	822.6	0.3494	0.4796	0.5205	0.3584	0
2776	822.5	0.3494	0.4795	0.5203	0.3583	0
2777	822.4	0.3496	0.4797	0.5205	0.3586	0
2778	822.3	0.3497	0.4797	0.5206	0.3587	0
2779	822.2	0.3496	0.4799	0.5206	0.3586	0
2780	822.1	0.3495	0.4799	0.5203	0.3585	0
2781	822	0.3498	0.4801	0.5207	0.3588	0

2782	821.9	0.3499	0.4801	0.5208	0.3588	0
2783	821.8	0.3496	0.4797	0.5205	0.3585	0
2784	821.7	0.3495	0.4797	0.5205	0.3585	0
2785	821.6	0.3496	0.4798	0.5206	0.3587	0
2786	821.5	0.3496	0.4798	0.5207	0.3586	0
2787	821.4	0.3498	0.4801	0.5209	0.3589	0
2788	821.3	0.3496	0.4799	0.5206	0.3587	0
2789	821.2	0.3497	0.48	0.5208	0.3588	0
2790	821.1	0.3498	0.48	0.5209	0.3587	0
2791	821	0.35	0.4803	0.5211	0.3588	0
2792	820.9	0.3499	0.4803	0.5212	0.3588	0
2793	820.8	0.3499	0.4801	0.5211	0.3588	0
2794	820.7	0.35	0.4802	0.5212	0.3588	0
2795	820.6	0.3496	0.4799	0.5208	0.3587	0
2796	820.5	0.3496	0.48	0.5208	0.3587	0
2797	820.4	0.3497	0.48	0.5211	0.3586	0
2798	820.3	0.3499	0.4803	0.5213	0.3589	0
2799	820.2	0.3499	0.4802	0.5212	0.3589	0
2800	820.1	0.3501	0.4805	0.5214	0.3591	0
2801	820	0.3501	0.4806	0.5214	0.3592	0
2802	819.9	0.3498	0.4803	0.5211	0.3591	0
2803	819.8	0.3498	0.4803	0.521	0.359	0
2804	819.7	0.35	0.4803	0.5213	0.359	0
2805	819.6	0.3499	0.4803	0.5213	0.359	0
2806	819.5	0.35	0.4804	0.5214	0.359	0
2807	819.4	0.35	0.4805	0.5215	0.3591	0
2808	819.3	0.35	0.4805	0.5214	0.3592	0
2809	819.2	0.3501	0.4806	0.5216	0.3593	0
2810	819.1	0.3502	0.4808	0.5217	0.3593	0
2811	819	0.35	0.4807	0.5215	0.3591	0
2812	818.9	0.35	0.4807	0.5216	0.3592	0
2813	818.8	0.3501	0.4806	0.5213	0.3591	0
2814	818.7	0.3502	0.4807	0.5215	0.3592	0
2815	818.6	0.3501	0.4805	0.5214	0.3591	0
2816	818.5	0.3501	0.4806	0.5215	0.3592	0
2817	818.4	0.3501	0.4804	0.5216	0.3592	0
2818	818.3	0.35	0.4804	0.5214	0.3591	0
2819	818.2	0.3501	0.4804	0.5215	0.3592	0
2820	818.1	0.3501	0.4804	0.5213	0.3591	0
2821	818	0.35	0.4803	0.5213	0.359	0
2822	817.9	0.3499	0.4803	0.5213	0.359	0
2823	817.8	0.3497	0.4803	0.5212	0.3589	0
2824	817.7	0.3497	0.4803	0.5212	0.359	0

2825	817.6	0.35	0.4804	0.5214	0.359	0
2826	817.5	0.3501	0.4805	0.5214	0.359	0
2827	817.4	0.3499	0.4805	0.5214	0.3589	0
2828	817.3	0.3501	0.4805	0.5215	0.359	0
2829	817.2	0.3501	0.4805	0.5216	0.3589	0
2830	817.1	0.3499	0.4804	0.5216	0.3589	0
2831	817	0.3501	0.4803	0.5215	0.3589	0
2832	816.9	0.3501	0.4802	0.5214	0.3589	0
2833	816.8	0.3499	0.4803	0.5214	0.3589	0
2834	816.7	0.3498	0.4803	0.5213	0.3588	0
2835	816.6	0.3497	0.4803	0.5212	0.3587	0
2836	816.5	0.3499	0.4804	0.5214	0.359	0
2837	816.4	0.3498	0.4804	0.5213	0.3589	0
2838	816.3	0.3499	0.4806	0.5214	0.359	0
2839	816.2	0.3499	0.4805	0.5213	0.3589	0
2840	816.1	0.35	0.4806	0.5214	0.359	0
2841	816	0.3501	0.4807	0.5218	0.3592	0
2842	815.9	0.3502	0.4807	0.5217	0.3592	0
2843	815.8	0.3502	0.4811	0.5218	0.3594	0
2844	815.7	0.3502	0.481	0.5218	0.3593	0
2845	815.6	0.35	0.4806	0.5217	0.3592	0
2846	815.5	0.3501	0.4808	0.5218	0.3592	0
2847	815.4	0.35	0.4809	0.5218	0.3593	0
2848	815.3	0.3499	0.4809	0.5217	0.3592	0
2849	815.2	0.35	0.4809	0.5217	0.359	0
2850	815.1	0.3502	0.4809	0.5216	0.3592	0
2851	815	0.3503	0.4809	0.5219	0.3593	0
2852	814.9	0.3505	0.481	0.5221	0.3594	0
2853	814.8	0.3504	0.4811	0.522	0.3593	0
2854	814.7	0.3504	0.4812	0.5221	0.3596	0
2855	814.6	0.3504	0.481	0.522	0.3596	0
2856	814.5	0.3503	0.4811	0.5221	0.3595	0
2857	814.4	0.3503	0.4812	0.522	0.3595	0
2858	814.3	0.3505	0.4812	0.522	0.3595	0
2859	814.2	0.3507	0.4815	0.5222	0.3596	0
2860	814.1	0.3505	0.4816	0.5222	0.3597	0
2861	814	0.3503	0.4812	0.5221	0.3596	0
2862	813.9	0.3505	0.4814	0.5222	0.3597	0
2863	813.8	0.3505	0.4815	0.5222	0.3598	0
2864	813.7	0.3505	0.4815	0.5224	0.3598	0
2865	813.6	0.3506	0.4815	0.5224	0.36	0
2866	813.5	0.3505	0.4815	0.5223	0.3598	0
2867	813.4	0.3509	0.4818	0.5226	0.3602	0

2868	813.3	0.3507	0.4818	0.5227	0.36	0
2869	813.2	0.3506	0.4819	0.5226	0.36	0
2870	813.1	0.3506	0.4818	0.5226	0.3599	0
2871	813	0.3509	0.4819	0.5227	0.3601	0
2872	812.9	0.351	0.4819	0.5228	0.3601	0
2873	812.8	0.3509	0.482	0.5227	0.3602	0
2874	812.7	0.3509	0.4819	0.5227	0.3601	0
2875	812.6	0.3511	0.482	0.5229	0.3603	0
2876	812.5	0.3511	0.482	0.5227	0.3603	0
2877	812.4	0.351	0.482	0.5228	0.3603	0
2878	812.3	0.3513	0.4822	0.5229	0.3605	0
2879	812.2	0.3512	0.4823	0.5232	0.3606	0
2880	812.1	0.3512	0.4821	0.523	0.3605	0
2881	812	0.3513	0.4823	0.5232	0.3606	0
2882	811.9	0.3512	0.4823	0.523	0.3606	0
2883	811.8	0.3514	0.4825	0.5231	0.3606	0
2884	811.7	0.3512	0.4825	0.5231	0.3608	0
2885	811.6	0.3512	0.4822	0.5231	0.3605	0
2886	811.5	0.3513	0.4826	0.5232	0.3609	0
2887	811.4	0.3514	0.4825	0.5233	0.3608	0
2888	811.3	0.3514	0.4825	0.5232	0.3607	0
2889	811.2	0.3512	0.4825	0.5232	0.3604	0
2890	811.1	0.3512	0.4826	0.523	0.3604	0
2891	811	0.3513	0.4826	0.5231	0.3606	0
2892	810.9	0.3512	0.4826	0.5231	0.3605	0
2893	810.8	0.3512	0.4825	0.5232	0.3606	0
2894	810.7	0.3515	0.4826	0.5233	0.3608	0
2895	810.6	0.3514	0.4824	0.5231	0.3607	0
2896	810.5	0.3515	0.4826	0.5235	0.361	0
2897	810.4	0.3514	0.4827	0.5233	0.3608	0
2898	810.3	0.3514	0.4828	0.5232	0.3609	0
2899	810.2	0.3515	0.4829	0.5233	0.3609	0
2900	810.1	0.3515	0.4828	0.5232	0.3608	0
2901	810	0.3513	0.4826	0.5231	0.3606	0
2902	809.9	0.3513	0.4828	0.5233	0.3605	0
2903	809.8	0.3513	0.4827	0.5233	0.3604	0
2904	809.7	0.3514	0.4827	0.5235	0.3607	0
2905	809.6	0.3514	0.4826	0.5233	0.3608	0
2906	809.5	0.3515	0.4826	0.5234	0.3608	0
2907	809.4	0.3514	0.4826	0.523	0.3607	0
2908	809.3	0.3513	0.4825	0.5231	0.3604	0
2909	809.2	0.3512	0.4825	0.5233	0.3605	0
2910	809.1	0.3514	0.4826	0.5232	0.3608	0

2911	809	0.3511	0.4824	0.523	0.3606	0
2912	808.9	0.3512	0.4823	0.5229	0.3606	0
2913	808.8	0.3513	0.4825	0.523	0.3606	0
2914	808.7	0.3513	0.4825	0.5232	0.3607	0
2915	808.6	0.3513	0.4825	0.5232	0.3607	0
2916	808.5	0.3514	0.4825	0.5232	0.3607	0
2917	808.4	0.3512	0.4825	0.5231	0.3606	0
2918	808.3	0.3512	0.4825	0.523	0.3607	0
2919	808.2	0.3513	0.4826	0.5231	0.3606	0
2920	808.1	0.3512	0.4826	0.523	0.3607	0
2921	808	0.3512	0.4826	0.5231	0.3606	0
2922	807.9	0.3514	0.4825	0.5231	0.3606	0
2923	807.8	0.3513	0.4825	0.5229	0.3605	0
2924	807.7	0.3512	0.4826	0.5231	0.3605	0
2925	807.6	0.3513	0.4827	0.523	0.3605	0
2926	807.5	0.3512	0.4825	0.5231	0.3605	0
2927	807.4	0.3516	0.4828	0.5232	0.3609	0
2928	807.3	0.3517	0.4829	0.5233	0.361	0
2929	807.2	0.3515	0.4827	0.5231	0.3608	0
2930	807.1	0.3513	0.4827	0.5231	0.3607	0
2931	807	0.3514	0.4827	0.5233	0.3607	0
2932	806.9	0.3515	0.4827	0.5234	0.3608	0
2933	806.8	0.3516	0.4827	0.5231	0.3609	0
2934	806.7	0.3516	0.4829	0.5232	0.3609	0
2935	806.6	0.3515	0.483	0.5233	0.3608	0
2936	806.5	0.3515	0.4827	0.5232	0.3609	0
2937	806.4	0.3516	0.4828	0.5231	0.3609	0
2938	806.3	0.3513	0.4829	0.5232	0.3609	0
2939	806.2	0.3516	0.4832	0.5235	0.3611	0
2940	806.1	0.3518	0.4832	0.5235	0.3613	0
2941	806	0.3519	0.4832	0.5234	0.3613	0
2942	805.9	0.3519	0.4833	0.5236	0.3613	0
2943	805.8	0.3519	0.4832	0.5236	0.3613	0
2944	805.7	0.3521	0.4832	0.5236	0.3614	0
2945	805.6	0.3521	0.4834	0.5237	0.3614	0
2946	805.5	0.352	0.4833	0.5236	0.3613	0
2947	805.4	0.3519	0.4833	0.5235	0.3612	0
2948	805.3	0.3519	0.4834	0.5236	0.3612	0
2949	805.2	0.3519	0.4835	0.5238	0.3613	0
2950	805.1	0.3522	0.4837	0.5239	0.3615	0
2951	805	0.352	0.4836	0.5239	0.3613	0
2952	804.9	0.3521	0.4837	0.5239	0.3614	0
2953	804.8	0.3523	0.4839	0.5241	0.3616	0

2954	804.7	0.3523	0.484	0.5241	0.3618	0
2955	804.6	0.3523	0.4839	0.5239	0.3616	0
2956	804.5	0.352	0.4836	0.5237	0.3614	0
2957	804.4	0.3519	0.4836	0.5237	0.3614	0
2958	804.3	0.3523	0.4838	0.5239	0.3618	0
2959	804.2	0.3524	0.4839	0.5242	0.3618	0
2960	804.1	0.3526	0.4842	0.5243	0.3622	0
2961	804	0.3527	0.4843	0.5242	0.362	0
2962	803.9	0.3527	0.4842	0.5244	0.3621	0
2963	803.8	0.3525	0.4842	0.5244	0.3621	0
2964	803.7	0.3528	0.4845	0.5244	0.3623	0
2965	803.6	0.3527	0.4845	0.5244	0.3622	0
2966	803.5	0.3528	0.4846	0.5244	0.3623	0
2967	803.4	0.3528	0.4847	0.5245	0.3622	0
2968	803.3	0.3529	0.4848	0.5246	0.3623	0
2969	803.2	0.353	0.4848	0.5248	0.3624	0
2970	803.1	0.353	0.485	0.5247	0.3626	0
2971	803	0.353	0.4849	0.5248	0.3627	0
2972	802.9	0.3529	0.4849	0.5247	0.3626	0
2973	802.8	0.3529	0.4848	0.5246	0.3626	0
2974	802.7	0.3531	0.4848	0.5248	0.3625	0
2975	802.6	0.353	0.4848	0.5249	0.3625	0
2976	802.5	0.353	0.4848	0.5248	0.3626	0
2977	802.4	0.3531	0.4849	0.5249	0.3627	0
2978	802.3	0.353	0.4849	0.5248	0.3628	0
2979	802.2	0.3529	0.4848	0.5247	0.3626	0
2980	802.1	0.353	0.4849	0.5249	0.3627	0
2981	802	0.353	0.485	0.5249	0.3627	0
2982	801.9	0.3532	0.4851	0.525	0.3629	0
2983	801.8	0.3535	0.4853	0.5252	0.363	0
2984	801.7	0.3533	0.4853	0.5251	0.3629	0
2985	801.6	0.3532	0.4853	0.5252	0.3629	0
2986	801.5	0.3533	0.4852	0.5252	0.3628	0
2987	801.4	0.3533	0.4853	0.5253	0.3629	0
2988	801.3	0.3533	0.4854	0.5252	0.3629	0
2989	801.2	0.3532	0.4853	0.5249	0.3628	0
2990	801.1	0.3532	0.4852	0.5252	0.3628	0
2991	801	0.3533	0.4854	0.5251	0.3628	0
2992	800.9	0.3533	0.4853	0.5252	0.3629	0
2993	800.8	0.3534	0.4854	0.5253	0.363	0
2994	800.7	0.3534	0.4854	0.5252	0.363	0
2995	800.6	0.3534	0.4852	0.5252	0.3629	0
2996	800.5	0.3532	0.4852	0.5252	0.3628	0

2997	800.4	0.3534	0.4851	0.5252	0.3628	0
2998	800.3	0.3533	0.4852	0.525	0.3629	0
2999	800.2	0.3532	0.4853	0.5251	0.3628	0
3000	800.1	0.3533	0.4852	0.525	0.3626	0
3001	800	0.3533	0.4853	0.5251	0.3628	0
3002	799.9	0.3532	0.4852	0.5251	0.3628	0
3003	799.8	0.3533	0.4853	0.5253	0.3628	0
3004	799.7	0.3531	0.4852	0.5251	0.3627	0
3005	799.6	0.3531	0.485	0.5249	0.3627	0
3006	799.5	0.3531	0.4851	0.525	0.3628	0
3007	799.4	0.3531	0.4853	0.5252	0.3631	0
3008	799.3	0.3533	0.4853	0.5253	0.363	0
3009	799.2	0.3532	0.4852	0.525	0.3629	0
3010	799.1	0.3531	0.4852	0.525	0.3628	0
3011	799	0.3532	0.4852	0.5248	0.3628	0
3012	798.9	0.3531	0.485	0.5247	0.3626	0
3013	798.8	0.3531	0.485	0.5246	0.3626	0
3014	798.7	0.353	0.485	0.5246	0.3625	0
3015	798.6	0.353	0.4848	0.5246	0.3626	0
3016	798.5	0.3528	0.4847	0.5246	0.3624	0
3017	798.4	0.353	0.4848	0.5247	0.3625	0
3018	798.3	0.3529	0.4849	0.5247	0.3625	0
3019	798.2	0.353	0.4851	0.5249	0.3625	0
3020	798.1	0.3529	0.485	0.5247	0.3625	0
3021	798	0.3528	0.4848	0.5247	0.3625	0
3022	797.9	0.3529	0.4848	0.5247	0.3626	0
3023	797.8	0.3529	0.4847	0.5247	0.3625	0
3024	797.7	0.3528	0.4846	0.5246	0.3627	0
3025	797.6	0.3527	0.4847	0.5246	0.3626	0
3026	797.5	0.3531	0.4849	0.5247	0.3626	0
3027	797.4	0.353	0.4849	0.5246	0.3624	0
3028	797.3	0.353	0.4847	0.5247	0.3625	0
3029	797.2	0.3529	0.4847	0.5247	0.3624	0
3030	797.1	0.3528	0.4848	0.5249	0.3624	0
3031	797	0.3527	0.4847	0.5245	0.3623	0
3032	796.9	0.3528	0.4846	0.5246	0.3624	0
3033	796.8	0.3529	0.4848	0.5247	0.3625	0
3034	796.7	0.3529	0.4849	0.5247	0.3625	0
3035	796.6	0.3527	0.4847	0.5245	0.3624	0
3036	796.5	0.3527	0.4849	0.5246	0.3626	0
3037	796.4	0.3527	0.4849	0.5246	0.3625	0
3038	796.3	0.3528	0.485	0.5245	0.3625	0
3039	796.2	0.3531	0.4851	0.5248	0.3628	0

3040	796.1	0.3531	0.4854	0.525	0.3628	0
3041	796	0.3531	0.4854	0.525	0.3629	0
3042	795.9	0.3532	0.4853	0.525	0.3629	0
3043	795.8	0.3532	0.4852	0.525	0.3628	0
3044	795.7	0.3533	0.4854	0.5252	0.363	0
3045	795.6	0.3535	0.4855	0.5254	0.3632	0
3046	795.5	0.3534	0.4855	0.5252	0.3632	0
3047	795.4	0.3535	0.4856	0.5254	0.3632	0
3048	795.3	0.3536	0.4859	0.5252	0.3632	0
3049	795.2	0.3535	0.4859	0.5254	0.3633	0
3050	795.1	0.3536	0.4858	0.5256	0.3633	0
3051	795	0.3537	0.4861	0.5259	0.3635	0
3052	794.9	0.3538	0.4861	0.5259	0.3636	0
3053	794.8	0.354	0.4861	0.526	0.3637	0
3054	794.7	0.3539	0.4862	0.5259	0.3637	0
3055	794.6	0.354	0.4863	0.526	0.3637	0
3056	794.5	0.354	0.4863	0.526	0.3639	0
3057	794.4	0.3541	0.4865	0.5261	0.3639	0
3058	794.3	0.3541	0.4866	0.5262	0.3641	0
3059	794.2	0.3543	0.4866	0.5263	0.3641	0
3060	794.1	0.3545	0.4867	0.5262	0.3641	0
3061	794	0.3546	0.4871	0.5264	0.3642	0
3062	793.9	0.3545	0.4872	0.5265	0.3644	0
3063	793.8	0.3547	0.4873	0.5267	0.3644	0
3064	793.7	0.3546	0.4872	0.5268	0.3647	0
3065	793.6	0.3548	0.4874	0.5269	0.3647	0
3066	793.5	0.3547	0.4874	0.5268	0.3646	0
3067	793.4	0.3547	0.4873	0.5267	0.3647	0
3068	793.3	0.3547	0.4874	0.5267	0.3645	0
3069	793.2	0.3548	0.4877	0.5268	0.3647	0
3070	793.1	0.3551	0.4877	0.527	0.3648	0
3071	793	0.3551	0.4877	0.5271	0.365	0
3072	792.9	0.355	0.4875	0.5269	0.3648	0
3073	792.8	0.3548	0.4874	0.5268	0.3648	0
3074	792.7	0.3548	0.4876	0.527	0.3647	0
3075	792.6	0.3548	0.4875	0.5272	0.3649	0
3076	792.5	0.3548	0.4875	0.5271	0.3649	0
3077	792.4	0.355	0.4876	0.5274	0.365	0
3078	792.3	0.3552	0.4877	0.5274	0.365	0
3079	792.2	0.3549	0.4877	0.5271	0.3648	0
3080	792.1	0.3549	0.4878	0.5272	0.3649	0
3081	792	0.3549	0.4879	0.5272	0.365	0
3082	791.9	0.3551	0.488	0.5273	0.365	0

3083	791.8	0.3553	0.488	0.5273	0.3651	0
3084	791.7	0.3552	0.4879	0.5272	0.3651	0
3085	791.6	0.3552	0.4882	0.5274	0.3653	0
3086	791.5	0.3551	0.4881	0.5275	0.3651	0
3087	791.4	0.3553	0.4882	0.5275	0.3652	0
3088	791.3	0.3553	0.4883	0.5275	0.3653	0
3089	791.2	0.3552	0.488	0.5273	0.365	0
3090	791.1	0.3551	0.4881	0.5274	0.3652	0
3091	791	0.3552	0.488	0.5274	0.3653	0
3092	790.9	0.3553	0.4883	0.5275	0.3652	0
3093	790.8	0.3552	0.4881	0.5274	0.3651	0
3094	790.7	0.3553	0.488	0.5274	0.365	0
3095	790.6	0.355	0.488	0.5274	0.3649	0
3096	790.5	0.3551	0.4878	0.5273	0.3648	0
3097	790.4	0.3551	0.4877	0.5274	0.3649	0
3098	790.3	0.3549	0.4877	0.5272	0.3647	0
3099	790.2	0.355	0.4877	0.5271	0.3649	0
3100	790.1	0.355	0.4878	0.5272	0.3649	0
3101	790	0.3548	0.4878	0.5271	0.3649	0
3102	789.9	0.3547	0.4878	0.5271	0.3648	0
3103	789.8	0.3548	0.4878	0.5273	0.3648	0
3104	789.7	0.355	0.4878	0.5274	0.3649	0
3105	789.6	0.3548	0.4879	0.527	0.3647	0
3106	789.5	0.3548	0.4878	0.527	0.3647	0
3107	789.4	0.3549	0.4878	0.5271	0.3648	0
3108	789.3	0.3547	0.4874	0.527	0.3647	0
3109	789.2	0.3548	0.4876	0.527	0.3648	0
3110	789.1	0.3548	0.4875	0.527	0.3646	0
3111	789	0.3547	0.4875	0.5269	0.3647	0
3112	788.9	0.3546	0.4875	0.5269	0.3645	0
3113	788.8	0.3546	0.4876	0.5268	0.3645	0
3114	788.7	0.3546	0.4875	0.5269	0.3644	0
3115	788.6	0.3545	0.4871	0.5269	0.3646	0
3116	788.5	0.3543	0.4872	0.5268	0.3643	0
3117	788.4	0.3545	0.4871	0.5266	0.3644	0
3118	788.3	0.3544	0.4871	0.5266	0.3643	0
3119	788.2	0.3544	0.4873	0.5268	0.3645	0
3120	788.1	0.3545	0.4873	0.5268	0.3645	0
3121	788	0.3543	0.4871	0.5268	0.3641	0
3122	787.9	0.3542	0.487	0.5265	0.3642	0
3123	787.8	0.3544	0.487	0.5265	0.3642	0
3124	787.7	0.3545	0.4871	0.5268	0.3645	0
3125	787.6	0.3545	0.487	0.5266	0.3643	0

3126	787.5	0.3542	0.4869	0.5264	0.3643	0
3127	787.4	0.3541	0.4867	0.5265	0.3641	0
3128	787.3	0.3542	0.4867	0.5264	0.364	0
3129	787.2	0.3541	0.4866	0.5264	0.3639	0
3130	787.1	0.3543	0.487	0.5267	0.3643	0
3131	787	0.3544	0.487	0.5267	0.3641	0
3132	786.9	0.3544	0.487	0.5266	0.364	0
3133	786.8	0.3542	0.4869	0.5265	0.3639	0
3134	786.7	0.3542	0.4869	0.5265	0.3639	0
3135	786.6	0.3541	0.487	0.5265	0.3641	0
3136	786.5	0.3542	0.4869	0.5266	0.364	0
3137	786.4	0.3542	0.4867	0.5266	0.364	0
3138	786.3	0.3544	0.4871	0.5266	0.364	0
3139	786.2	0.3546	0.4872	0.5269	0.3644	0
3140	786.1	0.3545	0.4872	0.5269	0.3642	0
3141	786	0.3543	0.4871	0.5269	0.3644	0
3142	785.9	0.3543	0.487	0.5268	0.3642	0
3143	785.8	0.3542	0.4871	0.5269	0.3641	0
3144	785.7	0.3545	0.4874	0.5271	0.3644	0
3145	785.6	0.3544	0.4872	0.5269	0.3643	0
3146	785.5	0.3543	0.4873	0.527	0.3643	0
3147	785.4	0.3544	0.4873	0.5271	0.3644	0
3148	785.3	0.3546	0.4878	0.5272	0.3646	0
3149	785.2	0.3546	0.4877	0.5272	0.3644	0
3150	785.1	0.3548	0.4876	0.5272	0.3646	0
3151	785	0.3549	0.4876	0.5276	0.3647	0
3152	784.9	0.3549	0.4878	0.5276	0.3649	0
3153	784.8	0.3551	0.488	0.5276	0.365	0
3154	784.7	0.3552	0.4882	0.5278	0.3652	0
3155	784.6	0.3553	0.4883	0.5279	0.3652	0
3156	784.5	0.3553	0.4884	0.5278	0.3653	0
3157	784.4	0.3554	0.4885	0.5278	0.3654	0
3158	784.3	0.3553	0.4884	0.5279	0.3654	0
3159	784.2	0.3554	0.4884	0.528	0.3655	0
3160	784.1	0.3557	0.4884	0.5281	0.3658	0
3161	784	0.3557	0.4888	0.5284	0.3659	0
3162	783.9	0.3559	0.4893	0.5284	0.3662	0
3163	783.8	0.3559	0.4891	0.5285	0.366	0
3164	783.7	0.3557	0.489	0.5285	0.366	0
3165	783.6	0.356	0.4892	0.5288	0.3663	0
3166	783.5	0.3563	0.4896	0.529	0.3664	0
3167	783.4	0.3563	0.4897	0.5289	0.3663	0
3168	783.3	0.3564	0.4897	0.5289	0.3663	0

3169	783.2	0.3562	0.4899	0.5291	0.3663	0
3170	783.1	0.3561	0.4897	0.5293	0.3664	0
3171	783	0.3565	0.4899	0.5293	0.3668	0
3172	782.9	0.3566	0.49	0.5292	0.3667	0
3173	782.8	0.3566	0.4899	0.5293	0.3668	0
3174	782.7	0.3566	0.4901	0.5293	0.3669	0
3175	782.6	0.3567	0.4902	0.5293	0.367	0
3176	782.5	0.3566	0.4901	0.5293	0.3667	0
3177	782.4	0.3569	0.4904	0.5296	0.3672	0
3178	782.3	0.357	0.4905	0.5297	0.3672	0
3179	782.2	0.357	0.4906	0.5297	0.3673	0
3180	782.1	0.3569	0.4903	0.5297	0.3671	0
3181	782	0.3567	0.4902	0.5297	0.367	0
3182	781.9	0.3568	0.4903	0.5296	0.3671	0
3183	781.8	0.3568	0.4902	0.5297	0.3671	0
3184	781.7	0.3567	0.4902	0.5295	0.367	0
3185	781.6	0.3569	0.4906	0.5297	0.3673	0
3186	781.5	0.3569	0.4906	0.5297	0.3672	0
3187	781.4	0.3568	0.4907	0.5298	0.367	0
3188	781.3	0.357	0.4907	0.5298	0.3671	0
3189	781.2	0.357	0.4907	0.53	0.3672	0
3190	781.1	0.357	0.4907	0.53	0.367	0
3191	781	0.3571	0.4906	0.5298	0.3672	0
3192	780.9	0.3571	0.4905	0.5297	0.3672	0
3193	780.8	0.3571	0.4907	0.53	0.3674	0
3194	780.7	0.3569	0.4906	0.5299	0.3671	0
3195	780.6	0.357	0.4908	0.5299	0.3672	0
3196	780.5	0.3569	0.4909	0.5297	0.3673	0
3197	780.4	0.357	0.4911	0.53	0.3675	0
3198	780.3	0.3571	0.4912	0.5301	0.3674	0
3199	780.2	0.357	0.4909	0.5301	0.3673	0
3200	780.1	0.3572	0.4909	0.5303	0.3674	0
3201	780	0.357	0.4907	0.5301	0.3674	0
3202	779.9	0.357	0.4907	0.5301	0.3674	0
3203	779.8	0.3571	0.4907	0.5302	0.3674	0
3204	779.7	0.3569	0.4906	0.5302	0.3673	0
3205	779.6	0.3571	0.4908	0.5301	0.3674	0
3206	779.5	0.3572	0.4908	0.53	0.3673	0
3207	779.4	0.357	0.4906	0.5298	0.3673	0
3208	779.3	0.357	0.4906	0.5299	0.3673	0
3209	779.2	0.357	0.4904	0.5299	0.3672	0
3210	779.1	0.357	0.4905	0.5298	0.3672	0
3211	779	0.3568	0.4905	0.5299	0.3672	0

3212	778.9	0.3566	0.4905	0.5299	0.3672	0
3213	778.8	0.3568	0.4904	0.5296	0.367	0
3214	778.7	0.3568	0.4904	0.5297	0.367	0
3215	778.6	0.3566	0.4902	0.5295	0.367	0
3216	778.5	0.3566	0.4903	0.5297	0.3668	0
3217	778.4	0.3566	0.4902	0.5298	0.3666	0
3218	778.3	0.3565	0.4901	0.5298	0.3669	0
3219	778.2	0.3565	0.4899	0.5296	0.3667	0
3220	778.1	0.3565	0.4899	0.5297	0.3667	0
3221	778	0.3564	0.4895	0.5294	0.3665	0
3222	777.9	0.3564	0.4896	0.5295	0.3665	0
3223	777.8	0.3564	0.4896	0.5294	0.3666	0
3224	777.7	0.3563	0.4896	0.5293	0.3666	0
3225	777.6	0.3563	0.4897	0.5293	0.3665	0
3226	777.5	0.3564	0.4897	0.5293	0.3666	0
3227	777.4	0.3565	0.4896	0.5293	0.3664	0
3228	777.3	0.356	0.4892	0.5291	0.3664	0
3229	777.2	0.356	0.4892	0.529	0.3662	0
3230	777.1	0.356	0.4893	0.529	0.3662	0
3231	777	0.356	0.4894	0.5292	0.3662	0
3232	776.9	0.356	0.4894	0.5293	0.3661	0
3233	776.8	0.3562	0.4894	0.5295	0.3662	0
3234	776.7	0.3563	0.4893	0.5291	0.3663	0
3235	776.6	0.3562	0.4895	0.5293	0.3663	0
3236	776.5	0.3562	0.4894	0.5289	0.3661	0
3237	776.4	0.3559	0.4894	0.5289	0.366	0
3238	776.3	0.3561	0.4896	0.529	0.3661	0
3239	776.2	0.3563	0.4897	0.5294	0.3664	0
3240	776.1	0.3562	0.4897	0.5293	0.3663	0
3241	776	0.3564	0.4899	0.5295	0.3664	0
3242	775.9	0.3562	0.4895	0.5294	0.3665	0
3243	775.8	0.3562	0.4898	0.5294	0.3664	0
3244	775.7	0.3562	0.4897	0.5293	0.3665	0
3245	775.6	0.3563	0.4898	0.5294	0.3666	0
3246	775.5	0.3562	0.4897	0.5294	0.3666	0
3247	775.4	0.3563	0.49	0.5297	0.3667	0
3248	775.3	0.3566	0.4903	0.5298	0.3669	0
3249	775.2	0.3566	0.4903	0.5297	0.3669	0
3250	775.1	0.3565	0.4902	0.5298	0.3669	0
3251	775	0.3566	0.4901	0.5299	0.3668	0
3252	774.9	0.3565	0.4899	0.5296	0.3667	0
3253	774.8	0.3566	0.4902	0.5298	0.3668	0
3254	774.7	0.3566	0.4903	0.5297	0.3668	0

3255	774.6	0.3568	0.4904	0.53	0.3671	0
3256	774.5	0.3568	0.4906	0.5302	0.3672	0
3257	774.4	0.357	0.4907	0.5301	0.3673	0
3258	774.3	0.3569	0.4905	0.5301	0.367	0
3259	774.2	0.357	0.4909	0.5304	0.3674	0
3260	774.1	0.357	0.4907	0.5303	0.3674	0
3261	774	0.3571	0.4909	0.5305	0.3675	0
3262	773.9	0.3571	0.4908	0.5306	0.3676	0
3263	773.8	0.3572	0.491	0.5306	0.3676	0
3264	773.7	0.3573	0.4911	0.5306	0.3678	0
3265	773.6	0.3574	0.4913	0.531	0.368	0
3266	773.5	0.3573	0.4912	0.5307	0.3679	0
3267	773.4	0.3575	0.4914	0.5308	0.368	0
3268	773.3	0.3575	0.4915	0.5309	0.368	0
3269	773.2	0.3576	0.4917	0.531	0.3681	0
3270	773.1	0.3579	0.4918	0.5313	0.3684	0
3271	773	0.3576	0.4917	0.5313	0.3682	0
3272	772.9	0.3578	0.4919	0.5315	0.3684	0
3273	772.8	0.358	0.4921	0.5316	0.3686	0
3274	772.7	0.358	0.4921	0.5317	0.3687	0
3275	772.6	0.3582	0.4922	0.5318	0.3687	0
3276	772.5	0.3582	0.4921	0.5318	0.3689	0
3277	772.4	0.3582	0.4925	0.532	0.3689	0
3278	772.3	0.3583	0.4924	0.5318	0.3687	0
3279	772.2	0.3582	0.4925	0.5318	0.3689	0
3280	772.1	0.3584	0.4927	0.532	0.369	0
3281	772	0.3584	0.4927	0.5319	0.3688	0
3282	771.9	0.3583	0.4928	0.5322	0.369	0
3283	771.8	0.3584	0.4929	0.5322	0.3691	0
3284	771.7	0.3584	0.4927	0.5322	0.3692	0
3285	771.6	0.3584	0.4928	0.532	0.3693	0
3286	771.5	0.3586	0.493	0.5323	0.3692	0
3287	771.4	0.3587	0.4929	0.5323	0.3693	0
3288	771.3	0.3587	0.4931	0.5325	0.3695	0
3289	771.2	0.3588	0.4932	0.5326	0.3695	0
3290	771.1	0.3591	0.4934	0.5326	0.3695	0
3291	771	0.3587	0.4932	0.5324	0.3693	0
3292	770.9	0.3589	0.4933	0.5325	0.3693	0
3293	770.8	0.3587	0.4931	0.5324	0.3693	0
3294	770.7	0.3589	0.4934	0.5326	0.3695	0
3295	770.6	0.3588	0.4932	0.5325	0.3694	0
3296	770.5	0.3586	0.4933	0.5325	0.3693	0
3297	770.4	0.3588	0.4935	0.5327	0.3695	0

3298	770.3	0.3589	0.4935	0.5327	0.3695	0
3299	770.2	0.3587	0.4932	0.5326	0.3694	0
3300	770.1	0.3588	0.4935	0.5326	0.3695	0
3301	770	0.3588	0.4934	0.5327	0.3694	0
3302	769.9	0.3589	0.4934	0.5327	0.3695	0
3303	769.8	0.3586	0.4932	0.5326	0.3695	0
3304	769.7	0.3588	0.4932	0.5326	0.3694	0
3305	769.6	0.3588	0.4932	0.5325	0.3693	0
3306	769.5	0.3589	0.4932	0.5326	0.3694	0
3307	769.4	0.3587	0.493	0.5324	0.3692	0
3308	769.3	0.3587	0.4928	0.5325	0.3693	0
3309	769.2	0.3591	0.4933	0.5326	0.3694	0
3310	769.1	0.359	0.4934	0.5327	0.3697	0
3311	769	0.3588	0.4931	0.5327	0.3695	0
3312	768.9	0.3586	0.4931	0.5326	0.3692	0
3313	768.8	0.3587	0.4932	0.5325	0.3694	0
3314	768.7	0.3585	0.4931	0.5324	0.3693	0
3315	768.6	0.3585	0.4931	0.5324	0.369	0
3316	768.5	0.3583	0.4929	0.5323	0.3691	0
3317	768.4	0.3586	0.4929	0.5323	0.3692	0
3318	768.3	0.3586	0.4929	0.5325	0.369	0
3319	768.2	0.3585	0.4926	0.5322	0.369	0
3320	768.1	0.3583	0.4926	0.5325	0.369	0
3321	768	0.3585	0.4927	0.5323	0.3691	0
3322	767.9	0.3583	0.4927	0.5323	0.3689	0
3323	767.8	0.3584	0.4927	0.5322	0.3689	0
3324	767.7	0.3584	0.4927	0.5323	0.3692	0
3325	767.6	0.3585	0.4927	0.5323	0.3692	0
3326	767.5	0.3584	0.4925	0.5321	0.3691	0
3327	767.4	0.3585	0.4927	0.5324	0.369	0
3328	767.3	0.3581	0.4927	0.5323	0.369	0
3329	767.2	0.3581	0.4926	0.5324	0.3687	0
3330	767.1	0.3581	0.4925	0.5322	0.3688	0
3331	767	0.3582	0.4926	0.5322	0.3688	0
3332	766.9	0.3581	0.4924	0.5322	0.3687	0
3333	766.8	0.3583	0.4925	0.5322	0.3687	0
3334	766.7	0.3581	0.4923	0.5322	0.3686	0
3335	766.6	0.3581	0.4923	0.532	0.3686	0
3336	766.5	0.358	0.4923	0.532	0.3685	0
3337	766.4	0.358	0.4923	0.5318	0.3686	0
3338	766.3	0.3579	0.4924	0.5321	0.3686	0
3339	766.2	0.358	0.4922	0.5321	0.3687	0
3340	766.1	0.358	0.4922	0.532	0.3687	0

3341	766	0.3578	0.4921	0.532	0.3684	0
3342	765.9	0.3579	0.4924	0.532	0.3685	0
3343	765.8	0.358	0.4924	0.532	0.3685	0
3344	765.7	0.3582	0.4926	0.5324	0.3687	0
3345	765.6	0.3582	0.4926	0.5325	0.3688	0
3346	765.5	0.3582	0.4925	0.5324	0.3687	0
3347	765.4	0.3584	0.4925	0.5323	0.3687	0
3348	765.3	0.3585	0.4925	0.5325	0.3689	0
3349	765.2	0.3581	0.4927	0.5322	0.3689	0
3350	765.1	0.358	0.4927	0.5324	0.3688	0
3351	765	0.3582	0.4926	0.5324	0.3687	0
3352	764.9	0.3582	0.4927	0.5324	0.3688	0
3353	764.8	0.3584	0.4927	0.5328	0.369	0
3354	764.7	0.3584	0.4929	0.5329	0.369	0
3355	764.6	0.3585	0.4928	0.5329	0.369	0
3356	764.5	0.3585	0.4926	0.5325	0.3688	0
3357	764.4	0.3586	0.4929	0.5329	0.369	0
3358	764.3	0.3587	0.4929	0.5329	0.3693	0
3359	764.2	0.3587	0.4932	0.533	0.3693	0
3360	764.1	0.359	0.4932	0.5331	0.3694	0
3361	764	0.3591	0.4935	0.5333	0.3696	0
3362	763.9	0.3588	0.4935	0.5332	0.3695	0
3363	763.8	0.359	0.4936	0.5335	0.3696	0
3364	763.7	0.3591	0.4937	0.5335	0.3695	0
3365	763.6	0.3592	0.4938	0.5336	0.3696	0
3366	763.5	0.359	0.4938	0.5337	0.3697	0
3367	763.4	0.3592	0.4939	0.5337	0.3699	0
3368	763.3	0.3593	0.4941	0.5338	0.37	0
3369	763.2	0.3594	0.494	0.5338	0.37	0
3370	763.1	0.3594	0.494	0.5339	0.37	0
3371	763	0.3594	0.494	0.5337	0.37	0
3372	762.9	0.3594	0.494	0.534	0.3701	0
3373	762.8	0.3597	0.4942	0.5341	0.3703	0
3374	762.7	0.3596	0.4943	0.534	0.3702	0
3375	762.6	0.3598	0.4945	0.5342	0.3704	0
3376	762.5	0.3597	0.4943	0.5343	0.3704	0
3377	762.4	0.3596	0.4943	0.5342	0.3702	0
3378	762.3	0.3598	0.4946	0.5346	0.3705	0
3379	762.2	0.3597	0.4946	0.5345	0.3704	0
3380	762.1	0.3598	0.4947	0.5345	0.3705	0
3381	762	0.3599	0.4947	0.5346	0.3706	0
3382	761.9	0.3599	0.4947	0.5346	0.3706	0
3383	761.8	0.3599	0.4945	0.5345	0.3706	0

3384	761.7	0.3597	0.4944	0.5344	0.3705	0
3385	761.6	0.3599	0.4944	0.5345	0.3705	0
3386	761.5	0.3599	0.495	0.5346	0.3708	0
3387	761.4	0.36	0.4948	0.5346	0.3709	0
3388	761.3	0.3601	0.495	0.5346	0.3707	0
3389	761.2	0.36	0.4951	0.5347	0.3707	0
3390	761.1	0.36	0.495	0.5347	0.3709	0
3391	761	0.3601	0.4949	0.5349	0.3709	0
3392	760.9	0.3601	0.4948	0.5349	0.3708	0
3393	760.8	0.3601	0.4948	0.5349	0.3707	0
3394	760.7	0.3599	0.4949	0.5349	0.3707	0
3395	760.6	0.36	0.4949	0.5349	0.3707	0
3396	760.5	0.36	0.495	0.535	0.3707	0
3397	760.4	0.3599	0.495	0.5351	0.3707	0
3398	760.3	0.3601	0.495	0.535	0.3709	0
3399	760.2	0.3599	0.495	0.5348	0.371	0
3400	760.1	0.3599	0.4951	0.5348	0.371	0
3401	760	0.36	0.4951	0.5352	0.371	0
3402	759.9	0.36	0.495	0.5349	0.3708	0
3403	759.8	0.36	0.4951	0.5352	0.3708	0
3404	759.7	0.3602	0.4953	0.5354	0.3711	0
3405	759.6	0.3601	0.4951	0.5351	0.371	0
3406	759.5	0.3602	0.4953	0.5353	0.3709	0
3407	759.4	0.36	0.4952	0.5354	0.3709	0
3408	759.3	0.3603	0.495	0.5352	0.3709	0
3409	759.2	0.36	0.4949	0.5352	0.3709	0
3410	759.1	0.3601	0.495	0.5353	0.3708	0
3411	759	0.3602	0.4953	0.5354	0.3712	0
3412	758.9	0.36	0.495	0.5354	0.3708	0
3413	758.8	0.3599	0.4952	0.5351	0.3708	0
3414	758.7	0.3599	0.495	0.535	0.3708	0
3415	758.6	0.3599	0.4951	0.5353	0.3709	0
3416	758.5	0.3602	0.4951	0.5354	0.371	0
3417	758.4	0.3601	0.495	0.5353	0.3708	0
3418	758.3	0.36	0.495	0.5352	0.3709	0
3419	758.2	0.3599	0.495	0.5351	0.3709	0
3420	758.1	0.3598	0.4949	0.5352	0.3708	0
3421	758	0.36	0.495	0.5354	0.3708	0
3422	757.9	0.3599	0.4948	0.5352	0.3707	0
3423	757.8	0.3601	0.495	0.5354	0.3709	0
3424	757.7	0.3601	0.4949	0.5352	0.3708	0
3425	757.6	0.3599	0.4949	0.5354	0.3709	0
3426	757.5	0.3599	0.4949	0.5354	0.3708	0

3427	757.4	0.36	0.4951	0.5356	0.3708	0
3428	757.3	0.3599	0.4949	0.5356	0.3707	0
3429	757.2	0.3599	0.4947	0.5353	0.3704	0
3430	757.1	0.36	0.4948	0.5355	0.3706	0
3431	757	0.3599	0.4948	0.5355	0.3705	0
3432	756.9	0.3599	0.4948	0.5357	0.3708	0
3433	756.8	0.3601	0.495	0.536	0.3709	0
3434	756.7	0.3601	0.495	0.536	0.371	0
3435	756.6	0.3599	0.4949	0.5356	0.371	0
3436	756.5	0.36	0.4949	0.5358	0.3708	0
3437	756.4	0.3599	0.495	0.5356	0.371	0
3438	756.3	0.3601	0.4951	0.5358	0.371	0
3439	756.2	0.36	0.4951	0.5357	0.3709	0
3440	756.1	0.36	0.4952	0.5357	0.371	0
3441	756	0.36	0.4952	0.5358	0.371	0
3442	755.9	0.36	0.495	0.5358	0.371	0
3443	755.8	0.3601	0.4951	0.5359	0.371	0
3444	755.7	0.3603	0.4953	0.5359	0.371	0
3445	755.6	0.3603	0.4954	0.5361	0.3713	0
3446	755.5	0.3603	0.4955	0.5361	0.3713	0
3447	755.4	0.3603	0.4955	0.5362	0.3712	0
3448	755.3	0.3603	0.4955	0.5362	0.3713	0
3449	755.2	0.3602	0.4953	0.536	0.3711	0
3450	755.1	0.3602	0.4953	0.5361	0.3712	0
3451	755	0.3603	0.4954	0.5364	0.3713	0
3452	754.9	0.3605	0.4955	0.5362	0.3711	0
3453	754.8	0.3605	0.4956	0.5364	0.3713	0
3454	754.7	0.3605	0.4955	0.5365	0.3713	0
3455	754.6	0.3604	0.4957	0.5365	0.3712	0
3456	754.5	0.3604	0.4955	0.5366	0.3713	0
3457	754.4	0.3606	0.4957	0.5366	0.3714	0
3458	754.3	0.3606	0.4958	0.5368	0.3716	0
3459	754.2	0.3606	0.496	0.5366	0.3715	0
3460	754.1	0.3609	0.496	0.5368	0.3719	0
3461	754	0.3608	0.4962	0.5368	0.3719	0
3462	753.9	0.3609	0.4963	0.537	0.3719	0
3463	753.8	0.361	0.496	0.537	0.3719	0
3464	753.7	0.3609	0.4962	0.5373	0.3719	0
3465	753.6	0.3609	0.4962	0.537	0.3717	0
3466	753.5	0.3607	0.4961	0.5369	0.3718	0
3467	753.4	0.3611	0.4965	0.5373	0.3722	0
3468	753.3	0.361	0.4965	0.5374	0.3723	0
3469	753.2	0.3609	0.4964	0.5373	0.372	0

3470	753.1	0.3611	0.4964	0.5373	0.3721	0
3471	753	0.361	0.4964	0.5375	0.3721	0
3472	752.9	0.3611	0.4964	0.5374	0.3723	0
3473	752.8	0.3612	0.4965	0.5376	0.3724	0
3474	752.7	0.361	0.4964	0.5375	0.3721	0
3475	752.6	0.3611	0.4966	0.5376	0.3722	0
3476	752.5	0.3612	0.4966	0.5375	0.3723	0
3477	752.4	0.3614	0.4969	0.5379	0.3727	0
3478	752.3	0.3612	0.4967	0.5379	0.3726	0
3479	752.2	0.3613	0.4967	0.5378	0.3724	0
3480	752.1	0.3612	0.4968	0.5378	0.3722	0
3481	752	0.3613	0.4969	0.538	0.3722	0
3482	751.9	0.3613	0.4969	0.5379	0.3723	0
3483	751.8	0.3614	0.4969	0.5379	0.3725	0
3484	751.7	0.3612	0.497	0.5381	0.3723	0
3485	751.6	0.3615	0.4971	0.5382	0.3725	0
3486	751.5	0.3614	0.4971	0.5382	0.3725	0
3487	751.4	0.3613	0.4969	0.5382	0.3726	0
3488	751.3	0.3615	0.4968	0.5381	0.3725	0
3489	751.2	0.3616	0.497	0.5383	0.3726	0
3490	751.1	0.3616	0.4968	0.5383	0.3727	0
3491	751	0.3615	0.497	0.5383	0.3726	0
3492	750.9	0.3615	0.4971	0.5385	0.3725	0
3493	750.8	0.3618	0.4971	0.5386	0.3728	0
3494	750.7	0.3615	0.497	0.5385	0.3727	0
3495	750.6	0.3615	0.4971	0.5384	0.3726	0
3496	750.5	0.3616	0.497	0.5385	0.3726	0
3497	750.4	0.3616	0.4971	0.5386	0.3725	0
3498	750.3	0.3614	0.4968	0.5383	0.3723	0
3499	750.2	0.3613	0.4969	0.5383	0.3723	0
3500	750.1	0.3617	0.4969	0.5384	0.3723	0
3501	750	0.3616	0.4968	0.5385	0.3724	0
3502	749.9	0.3614	0.497	0.5386	0.3725	0
3503	749.8	0.3614	0.4969	0.5383	0.3724	0
3504	749.7	0.3613	0.4968	0.5385	0.3723	0
3505	749.6	0.3612	0.4971	0.5385	0.3724	0
3506	749.5	0.3614	0.4974	0.5388	0.3726	0
3507	749.4	0.3614	0.497	0.5385	0.3725	0
3508	749.3	0.3614	0.497	0.5386	0.3725	0
3509	749.2	0.3613	0.4972	0.5387	0.3725	0
3510	749.1	0.3614	0.4972	0.5385	0.3725	0
3511	749	0.3614	0.4973	0.5387	0.3726	0
3512	748.9	0.3614	0.497	0.5388	0.3725	0

3513	748.8	0.3614	0.4971	0.5388	0.3726	0
3514	748.7	0.3613	0.497	0.5389	0.3724	0
3515	748.6	0.3613	0.497	0.5389	0.3726	0
3516	748.5	0.3616	0.4971	0.5391	0.3726	0
3517	748.4	0.3615	0.497	0.5391	0.3726	0
3518	748.3	0.3615	0.4969	0.539	0.3725	0
3519	748.2	0.3615	0.4969	0.5389	0.3726	0
3520	748.1	0.3614	0.4969	0.5391	0.3725	0
3521	748	0.3614	0.4969	0.539	0.3724	0
3522	747.9	0.3614	0.4971	0.5391	0.3724	0
3523	747.8	0.3616	0.4971	0.5392	0.3728	0
3524	747.7	0.3614	0.497	0.5394	0.3726	0
3525	747.6	0.3616	0.4972	0.5394	0.3725	0
3526	747.5	0.3617	0.4971	0.5392	0.3726	0
3527	747.4	0.3615	0.4974	0.5394	0.3726	0
3528	747.3	0.3616	0.4974	0.5394	0.3726	0
3529	747.2	0.3616	0.4973	0.5391	0.3726	0
3530	747.1	0.3614	0.4969	0.5391	0.3725	0
3531	747	0.3616	0.497	0.5395	0.3727	0
3532	746.9	0.3615	0.4972	0.5394	0.3728	0
3533	746.8	0.3617	0.4973	0.5396	0.3728	0
3534	746.7	0.3619	0.4973	0.5395	0.373	0
3535	746.6	0.3616	0.4972	0.5397	0.3728	0
3536	746.5	0.3617	0.4973	0.5396	0.3728	0
3537	746.4	0.3617	0.4975	0.5398	0.373	0
3538	746.3	0.3618	0.4972	0.5398	0.3729	0
3539	746.2	0.3615	0.4974	0.5398	0.3729	0
3540	746.1	0.3615	0.4977	0.5399	0.3729	0
3541	746	0.3617	0.4976	0.5401	0.3731	0
3542	745.9	0.3618	0.4974	0.54	0.373	0
3543	745.8	0.3619	0.4977	0.5403	0.373	0
3544	745.7	0.3619	0.4978	0.5402	0.3732	0
3545	745.6	0.3619	0.498	0.5405	0.3732	0
3546	745.5	0.3621	0.4981	0.5408	0.3733	0
3547	745.4	0.3621	0.498	0.5406	0.3733	0
3548	745.3	0.3623	0.498	0.5405	0.3734	0
3549	745.2	0.3622	0.4979	0.5407	0.3734	0
3550	745.1	0.3624	0.4981	0.5408	0.3736	0
3551	745	0.3621	0.4979	0.5407	0.3735	0
3552	744.9	0.3621	0.4979	0.5409	0.3735	0
3553	744.8	0.3625	0.4982	0.5411	0.3736	0
3554	744.7	0.3625	0.498	0.5409	0.3736	0
3555	744.6	0.3625	0.4982	0.5411	0.3736	0

3556	744.5	0.3622	0.4982	0.5409	0.3736	0
3557	744.4	0.3625	0.4984	0.5413	0.3738	0
3558	744.3	0.3625	0.4985	0.5414	0.3738	0
3559	744.2	0.3626	0.4985	0.5416	0.374	0
3560	744.1	0.3627	0.4985	0.5416	0.374	0
3561	744	0.3628	0.4987	0.5416	0.374	0
3562	743.9	0.3627	0.4988	0.5418	0.3741	0
3563	743.8	0.3628	0.499	0.5417	0.3743	0
3564	743.7	0.3627	0.4989	0.5417	0.3741	0
3565	743.6	0.363	0.499	0.5419	0.3742	0
3566	743.5	0.3629	0.499	0.5418	0.3742	0
3567	743.4	0.363	0.4989	0.542	0.3743	0
3568	743.3	0.363	0.499	0.5421	0.3742	0
3569	743.2	0.3631	0.4992	0.5421	0.3743	0
3570	743.1	0.363	0.4994	0.5422	0.3743	0
3571	743	0.3632	0.4996	0.5425	0.3744	0
3572	742.9	0.3632	0.4995	0.5426	0.3745	0
3573	742.8	0.3632	0.4994	0.5426	0.3745	0
3574	742.7	0.3633	0.4995	0.5424	0.3746	0
3575	742.6	0.3634	0.4994	0.5426	0.3747	0
3576	742.5	0.3633	0.4993	0.5425	0.3746	0
3577	742.4	0.3632	0.4993	0.5425	0.3747	0
3578	742.3	0.3633	0.4995	0.5427	0.3745	0
3579	742.2	0.3633	0.4995	0.5426	0.3746	0
3580	742.1	0.3633	0.4997	0.5429	0.3748	0
3581	742	0.3632	0.4996	0.5429	0.3747	0
3582	741.9	0.3632	0.4996	0.5429	0.3746	0
3583	741.8	0.3632	0.4995	0.5429	0.3747	0
3584	741.7	0.3634	0.4997	0.5429	0.3747	0
3585	741.6	0.3633	0.4997	0.5432	0.3747	0
3586	741.5	0.3635	0.4998	0.5432	0.3748	0
3587	741.4	0.3632	0.4995	0.5431	0.3748	0
3588	741.3	0.3633	0.4996	0.543	0.3747	0
3589	741.2	0.3632	0.4995	0.543	0.3746	0
3590	741.1	0.3631	0.4994	0.543	0.3746	0
3591	741	0.3633	0.4995	0.5433	0.3748	0
3592	740.9	0.3632	0.4995	0.5433	0.3747	0
3593	740.8	0.363	0.4992	0.5429	0.3744	0
3594	740.7	0.3631	0.4993	0.543	0.3744	0
3595	740.6	0.3631	0.4995	0.543	0.3744	0
3596	740.5	0.3631	0.4995	0.543	0.3746	0
3597	740.4	0.3632	0.4995	0.5432	0.3746	0
3598	740.3	0.3632	0.4994	0.5431	0.3746	0

3599	740.2	0.3631	0.4992	0.5432	0.3744	0
3600	740.1	0.363	0.4993	0.5429	0.3744	0
3601	740	0.3631	0.4993	0.5432	0.3744	0
3602	739.9	0.3631	0.4993	0.5431	0.3744	0
3603	739.8	0.3632	0.4991	0.5431	0.3745	0
3604	739.7	0.363	0.4995	0.5433	0.3745	0
3605	739.6	0.363	0.4994	0.5433	0.3745	0
3606	739.5	0.363	0.4992	0.5433	0.3744	0
3607	739.4	0.3628	0.4991	0.5431	0.3743	0
3608	739.3	0.3628	0.4992	0.5433	0.3742	0
3609	739.2	0.3629	0.4991	0.5432	0.3741	0
3610	739.1	0.3627	0.4991	0.5432	0.3741	0
3611	739	0.3627	0.4991	0.5433	0.3741	0
3612	738.9	0.3628	0.4991	0.5433	0.3742	0
3613	738.8	0.3628	0.4991	0.5432	0.3741	0
3614	738.7	0.3628	0.499	0.5433	0.3742	0
3615	738.6	0.3628	0.499	0.5433	0.3742	0
3616	738.5	0.3626	0.4988	0.5432	0.374	0
3617	738.4	0.3627	0.4989	0.5433	0.3741	0
3618	738.3	0.3623	0.4986	0.5431	0.3738	0
3619	738.2	0.3624	0.4988	0.5432	0.3737	0
3620	738.1	0.3624	0.4987	0.5433	0.3737	0
3621	738	0.3625	0.4984	0.5433	0.3738	0
3622	737.9	0.3624	0.4986	0.5435	0.3739	0
3623	737.8	0.3625	0.4987	0.5435	0.374	0
3624	737.7	0.3624	0.4984	0.5434	0.3739	0
3625	737.6	0.3627	0.4985	0.5435	0.374	0
3626	737.5	0.3625	0.4987	0.5437	0.3739	0
3627	737.4	0.3626	0.4986	0.5438	0.3738	0
3628	737.3	0.3624	0.4984	0.5435	0.3736	0
3629	737.2	0.3624	0.4985	0.5436	0.3738	0
3630	737.1	0.3626	0.4986	0.5437	0.374	0
3631	737	0.3624	0.4987	0.5438	0.3739	0
3632	736.9	0.3624	0.4987	0.5439	0.3738	0
3633	736.8	0.3627	0.4989	0.544	0.3741	0
3634	736.7	0.3629	0.499	0.5441	0.374	0
3635	736.6	0.3627	0.499	0.5439	0.374	0
3636	736.5	0.3629	0.4992	0.5442	0.3741	0
3637	736.4	0.3628	0.4989	0.5441	0.3741	0
3638	736.3	0.3629	0.4991	0.5444	0.3742	0
3639	736.2	0.3628	0.4992	0.5443	0.3742	0
3640	736.1	0.3628	0.4991	0.5443	0.3741	0
3641	736	0.3631	0.4992	0.5446	0.3744	0

3642	735.9	0.3631	0.4991	0.5446	0.3743	0
3643	735.8	0.3633	0.4995	0.5446	0.3744	0
3644	735.7	0.3633	0.4995	0.5448	0.3746	0
3645	735.6	0.3632	0.4996	0.5449	0.3745	0
3646	735.5	0.3633	0.4996	0.5452	0.3746	0
3647	735.4	0.3634	0.4997	0.5452	0.3747	0
3648	735.3	0.3633	0.4997	0.5451	0.3747	0
3649	735.2	0.3636	0.4998	0.5453	0.375	0
3650	735.1	0.3634	0.4999	0.5453	0.3748	0
3651	735	0.3635	0.4999	0.5455	0.3749	0
3652	734.9	0.3636	0.5001	0.5457	0.3751	0
3653	734.8	0.3637	0.5002	0.5457	0.375	0
3654	734.7	0.3639	0.5004	0.5458	0.3753	0
3655	734.6	0.3639	0.5004	0.546	0.3753	0
3656	734.5	0.364	0.5006	0.5462	0.3755	0
3657	734.4	0.3641	0.5007	0.5465	0.3757	0
3658	734.3	0.3645	0.5009	0.5467	0.3761	0
3659	734.2	0.3642	0.5007	0.5465	0.3758	0
3660	734.1	0.3643	0.5007	0.5466	0.3758	0
3661	734	0.3643	0.5008	0.5466	0.3759	0
3662	733.9	0.3644	0.501	0.5467	0.376	0
3663	733.8	0.3644	0.5007	0.5467	0.3761	0
3664	733.7	0.3646	0.501	0.5469	0.3762	0
3665	733.6	0.3647	0.5014	0.5472	0.3765	0
3666	733.5	0.3646	0.5015	0.5471	0.3763	0
3667	733.4	0.3646	0.5015	0.5471	0.3764	0
3668	733.3	0.3649	0.5016	0.5474	0.3764	0
3669	733.2	0.3647	0.5016	0.5474	0.3764	0
3670	733.1	0.3648	0.5017	0.5474	0.3766	0
3671	733	0.365	0.5017	0.5475	0.3767	0
3672	732.9	0.3647	0.5018	0.5479	0.3766	0
3673	732.8	0.3647	0.5017	0.5475	0.3765	0
3674	732.7	0.3649	0.5018	0.5477	0.3766	0
3675	732.6	0.3649	0.5019	0.5479	0.3767	0
3676	732.5	0.3653	0.5021	0.548	0.3769	0
3677	732.4	0.3652	0.5023	0.5481	0.3769	0
3678	732.3	0.3651	0.502	0.5481	0.3768	0
3679	732.2	0.3651	0.5019	0.548	0.3768	0
3680	732.1	0.365	0.5018	0.5481	0.3768	0
3681	732	0.365	0.5019	0.548	0.3767	0
3682	731.9	0.365	0.5018	0.5482	0.3767	0
3683	731.8	0.365	0.5018	0.5481	0.3768	0
3684	731.7	0.365	0.5019	0.5483	0.3767	0

3685	731.6	0.3649	0.502	0.5481	0.3768	0
3686	731.5	0.3651	0.5021	0.5483	0.3769	0
3687	731.4	0.3652	0.5023	0.5484	0.377	0
3688	731.3	0.3651	0.5021	0.5484	0.3768	0
3689	731.2	0.3652	0.5021	0.5484	0.3768	0
3690	731.1	0.3651	0.502	0.5484	0.3769	0
3691	731	0.3651	0.5021	0.5485	0.377	0
3692	730.9	0.365	0.5021	0.5484	0.377	0
3693	730.8	0.3648	0.5018	0.5482	0.3767	0
3694	730.7	0.3649	0.502	0.5485	0.3767	0
3695	730.6	0.3651	0.502	0.5488	0.3771	0
3696	730.5	0.365	0.5021	0.5487	0.3769	0
3697	730.4	0.365	0.5023	0.5486	0.3769	0
3698	730.3	0.3651	0.502	0.5488	0.3768	0
3699	730.2	0.365	0.502	0.5486	0.3766	0
3700	730.1	0.365	0.502	0.5486	0.3766	0
3701	730	0.3651	0.5021	0.5487	0.3768	0
3702	729.9	0.3651	0.502	0.5486	0.3767	0
3703	729.8	0.3648	0.5016	0.5486	0.3764	0
3704	729.7	0.3647	0.5016	0.5484	0.3766	0
3705	729.6	0.3647	0.5017	0.5485	0.3766	0
3706	729.5	0.3648	0.5017	0.5486	0.3767	0
3707	729.4	0.3647	0.5017	0.5485	0.3767	0
3708	729.3	0.3648	0.5016	0.5486	0.3767	0
3709	729.2	0.3648	0.5015	0.5486	0.3765	0
3710	729.1	0.3649	0.5014	0.5486	0.3764	0
3711	729	0.3644	0.5011	0.5483	0.376	0
3712	728.9	0.3646	0.5012	0.5484	0.3763	0
3713	728.8	0.3649	0.5015	0.5487	0.3763	0
3714	728.7	0.3647	0.5013	0.5488	0.3764	0
3715	728.6	0.3646	0.5014	0.5487	0.3764	0
3716	728.5	0.3646	0.5014	0.5485	0.3759	0
3717	728.4	0.3645	0.5011	0.5484	0.3759	0
3718	728.3	0.3645	0.5011	0.5485	0.3761	0
3719	728.2	0.3644	0.5011	0.5486	0.376	0
3720	728.1	0.3644	0.5011	0.5484	0.376	0
3721	728	0.3644	0.5012	0.5485	0.376	0
3722	727.9	0.3643	0.5009	0.5486	0.376	0
3723	727.8	0.3642	0.501	0.5485	0.3758	0
3724	727.7	0.3643	0.501	0.5485	0.3757	0
3725	727.6	0.3644	0.5011	0.5486	0.3758	0
3726	727.5	0.3641	0.5009	0.5485	0.3758	0
3727	727.4	0.364	0.5008	0.5487	0.3757	0

3728	727.3	0.3641	0.501	0.5487	0.3758	0
3729	727.2	0.364	0.5007	0.5485	0.3757	0
3730	727.1	0.364	0.5006	0.5486	0.3757	0
3731	727	0.3641	0.5007	0.5486	0.3758	0
3732	726.9	0.3641	0.5009	0.5486	0.3757	0
3733	726.8	0.3642	0.5006	0.5486	0.3757	0
3734	726.7	0.3641	0.5008	0.549	0.3757	0
3735	726.6	0.3641	0.5008	0.5488	0.3759	0
3736	726.5	0.3641	0.5009	0.5488	0.3759	0
3737	726.4	0.3641	0.5008	0.5488	0.3758	0
3738	726.3	0.3639	0.501	0.5489	0.3756	0
3739	726.2	0.3641	0.5008	0.549	0.3756	0
3740	726.1	0.3642	0.5008	0.549	0.3758	0
3741	726	0.364	0.5008	0.549	0.3758	0
3742	725.9	0.3643	0.5008	0.5492	0.3757	0
3743	725.8	0.3641	0.5008	0.549	0.3756	0
3744	725.7	0.3643	0.5011	0.5493	0.376	0
3745	725.6	0.3643	0.5011	0.5493	0.3759	0
3746	725.5	0.3643	0.5011	0.5493	0.3759	0
3747	725.4	0.3645	0.5013	0.5494	0.376	0
3748	725.3	0.3646	0.5015	0.5496	0.3761	0
3749	725.2	0.3646	0.5015	0.5498	0.3764	0
3750	725.1	0.3646	0.5017	0.5501	0.3763	0
3751	725	0.3646	0.5017	0.55	0.3764	0
3752	724.9	0.3647	0.5016	0.5503	0.3763	0
3753	724.8	0.3649	0.5017	0.5503	0.3764	0
3754	724.7	0.3652	0.502	0.5505	0.3767	0
3755	724.6	0.3651	0.5021	0.5505	0.3767	0
3756	724.5	0.3653	0.5022	0.5505	0.3767	0
3757	724.4	0.3653	0.5023	0.5506	0.3767	0
3758	724.3	0.3654	0.5024	0.5509	0.377	0
3759	724.2	0.3656	0.5027	0.5513	0.3772	0
3760	724.1	0.3658	0.5028	0.5513	0.3773	0
3761	724	0.3657	0.5027	0.5512	0.3773	0
3762	723.9	0.366	0.5031	0.5516	0.3775	0
3763	723.8	0.3661	0.5033	0.5517	0.3778	0
3764	723.7	0.3659	0.5033	0.5516	0.3777	0
3765	723.6	0.3657	0.5032	0.5518	0.3777	0
3766	723.5	0.3661	0.5033	0.552	0.3779	0
3767	723.4	0.3662	0.5033	0.5521	0.378	0
3768	723.3	0.3661	0.5032	0.552	0.378	0
3769	723.2	0.3661	0.5034	0.5521	0.3781	0
3770	723.1	0.3662	0.5035	0.5523	0.3784	0

3771	723	0.3663	0.5037	0.5524	0.3782	0
3772	722.9	0.3666	0.504	0.5526	0.3787	0
3773	722.8	0.3666	0.5038	0.5526	0.3786	0
3774	722.7	0.3665	0.5039	0.5527	0.3785	0
3775	722.6	0.3667	0.5042	0.5528	0.3786	0
3776	722.5	0.3665	0.5041	0.553	0.3788	0
3777	722.4	0.3667	0.5042	0.5532	0.3789	0
3778	722.3	0.3669	0.5044	0.5533	0.379	0
3779	722.2	0.3668	0.5041	0.5531	0.379	0
3780	722.1	0.3668	0.5042	0.553	0.3789	0
3781	722	0.3669	0.5042	0.5533	0.3788	0
3782	721.9	0.3667	0.504	0.5532	0.3788	0
3783	721.8	0.3668	0.5041	0.5533	0.3787	0
3784	721.7	0.367	0.5042	0.5535	0.3788	0
3785	721.6	0.3668	0.5045	0.5534	0.3788	0
3786	721.5	0.367	0.5045	0.5536	0.3789	0
3787	721.4	0.3668	0.5044	0.5533	0.3789	0
3788	721.3	0.367	0.5043	0.5532	0.3788	0
3789	721.2	0.3669	0.5045	0.5535	0.3789	0
3790	721.1	0.3667	0.5043	0.5536	0.3788	0
3791	721	0.3669	0.5044	0.5537	0.3791	0
3792	720.9	0.3672	0.5048	0.5539	0.3793	0
3793	720.8	0.367	0.5048	0.554	0.3791	0
3794	720.7	0.3669	0.5046	0.5538	0.3791	0
3795	720.6	0.367	0.5045	0.5539	0.3791	0
3796	720.5	0.3669	0.5047	0.554	0.3791	0
3797	720.4	0.3668	0.5047	0.5539	0.3789	0
3798	720.3	0.3669	0.5048	0.554	0.379	0
3799	720.2	0.367	0.5047	0.5541	0.3791	0
3800	720.1	0.3673	0.5047	0.5542	0.3792	0
3801	720	0.3672	0.5046	0.554	0.379	0
3802	719.9	0.3673	0.5047	0.5542	0.3791	0
3803	719.8	0.3672	0.5047	0.5543	0.3792	0
3804	719.7	0.3672	0.5045	0.5543	0.3792	0
3805	719.6	0.3672	0.5045	0.5543	0.3791	0
3806	719.5	0.3671	0.5046	0.5542	0.379	0
3807	719.4	0.3669	0.5042	0.5542	0.3788	0
3808	719.3	0.3667	0.5043	0.554	0.3786	0
3809	719.2	0.3669	0.5044	0.5541	0.3788	0
3810	719.1	0.3669	0.5045	0.5544	0.3789	0
3811	719	0.3667	0.5041	0.554	0.3786	0
3812	718.9	0.3668	0.5041	0.5542	0.3786	0
3813	718.8	0.3668	0.5042	0.554	0.3786	0

3814	718.7	0.3667	0.5041	0.5541	0.3786	0
3815	718.6	0.3665	0.504	0.554	0.3784	0
3816	718.5	0.3665	0.5041	0.5541	0.3784	0
3817	718.4	0.3664	0.5038	0.5538	0.3783	0
3818	718.3	0.3663	0.5036	0.5539	0.3781	0
3819	718.2	0.3663	0.5036	0.5538	0.3783	0
3820	718.1	0.3663	0.5036	0.554	0.3784	0
3821	718	0.3664	0.5038	0.5541	0.3785	0
3822	717.9	0.3663	0.5037	0.5541	0.3784	0
3823	717.8	0.3663	0.5037	0.5538	0.3783	0
3824	717.7	0.3662	0.5036	0.5541	0.378	0
3825	717.6	0.3662	0.5036	0.5541	0.378	0
3826	717.5	0.366	0.5035	0.5538	0.3778	0
3827	717.4	0.366	0.5035	0.5539	0.3778	0
3828	717.3	0.3662	0.5037	0.5537	0.378	0
3829	717.2	0.3659	0.5035	0.5539	0.378	0
3830	717.1	0.3661	0.5034	0.554	0.378	0
3831	717	0.3661	0.5034	0.5541	0.378	0
3832	716.9	0.366	0.5035	0.554	0.3779	0
3833	716.8	0.366	0.5035	0.5541	0.378	0
3834	716.7	0.3663	0.5038	0.5544	0.3781	0
3835	716.6	0.3662	0.5037	0.554	0.3781	0
3836	716.5	0.3662	0.5035	0.5542	0.378	0
3837	716.4	0.3661	0.5035	0.5543	0.378	0
3838	716.3	0.3663	0.5035	0.5543	0.378	0
3839	716.2	0.3661	0.5037	0.5544	0.3781	0
3840	716.1	0.3661	0.5037	0.5544	0.378	0
3841	716	0.3661	0.5035	0.5544	0.378	0
3842	715.9	0.3661	0.5033	0.5544	0.378	0
3843	715.8	0.3661	0.5035	0.5546	0.3782	0
3844	715.7	0.3662	0.5036	0.5545	0.3781	0
3845	715.6	0.3661	0.5036	0.5546	0.378	0
3846	715.5	0.3662	0.5038	0.5547	0.3783	0
3847	715.4	0.3664	0.5038	0.5548	0.3782	0
3848	715.3	0.3666	0.504	0.5549	0.3784	0
3849	715.2	0.3666	0.5039	0.5549	0.3783	0
3850	715.1	0.3664	0.5039	0.5549	0.3784	0
3851	715	0.3662	0.5038	0.5548	0.3783	0
3852	714.9	0.3665	0.504	0.5551	0.3785	0
3853	714.8	0.3666	0.5041	0.5552	0.3785	0
3854	714.7	0.3667	0.5042	0.5554	0.3787	0
3855	714.6	0.3665	0.504	0.5552	0.3785	0
3856	714.5	0.3666	0.5041	0.5555	0.3786	0

3857	714.4	0.367	0.5044	0.5558	0.3789	0
3858	714.3	0.367	0.5045	0.5557	0.3789	0
3859	714.2	0.3671	0.5045	0.5559	0.3789	0
3860	714.1	0.367	0.5046	0.5559	0.3788	0
3861	714	0.3672	0.5048	0.5561	0.379	0
3862	713.9	0.3671	0.5047	0.5561	0.3791	0
3863	713.8	0.3673	0.5048	0.5563	0.3793	0
3864	713.7	0.3674	0.5049	0.5563	0.3793	0
3865	713.6	0.3671	0.5048	0.5564	0.3795	0
3866	713.5	0.3673	0.5051	0.5564	0.3795	0
3867	713.4	0.3675	0.5053	0.5569	0.3798	0
3868	713.3	0.3676	0.5052	0.5569	0.3798	0
3869	713.2	0.3677	0.5053	0.557	0.3799	0
3870	713.1	0.3676	0.5054	0.5572	0.3798	0
3871	713	0.3679	0.5056	0.5572	0.38	0
3872	712.9	0.368	0.5058	0.5575	0.3799	0
3873	712.8	0.3681	0.5057	0.5575	0.3802	0
3874	712.7	0.368	0.5059	0.5575	0.3801	0
3875	712.6	0.3683	0.5061	0.5577	0.3803	0
3876	712.5	0.3682	0.5063	0.5579	0.3804	0
3877	712.4	0.3681	0.5063	0.5579	0.3805	0
3878	712.3	0.3683	0.5062	0.558	0.3806	0
3879	712.2	0.3683	0.5064	0.5582	0.3806	0
3880	712.1	0.3684	0.5064	0.5584	0.3806	0
3881	712	0.3686	0.5065	0.5583	0.3807	0
3882	711.9	0.3686	0.5067	0.5584	0.3808	0
3883	711.8	0.3686	0.5069	0.5585	0.3808	0
3884	711.7	0.3685	0.5066	0.5585	0.3808	0
3885	711.6	0.3685	0.5067	0.5586	0.381	0
3886	711.5	0.3686	0.5068	0.5589	0.3811	0
3887	711.4	0.3688	0.5069	0.5589	0.3809	0
3888	711.3	0.3689	0.5069	0.5589	0.3812	0
3889	711.2	0.3688	0.507	0.5589	0.3812	0
3890	711.1	0.369	0.5072	0.5592	0.3813	0
3891	711	0.3688	0.507	0.5591	0.3811	0
3892	710.9	0.3688	0.5071	0.559	0.3812	0
3893	710.8	0.3688	0.5071	0.5589	0.3812	0
3894	710.7	0.3689	0.5072	0.5591	0.3813	0
3895	710.6	0.3691	0.5074	0.5592	0.3813	0
3896	710.5	0.3692	0.5073	0.5594	0.3813	0
3897	710.4	0.3688	0.5069	0.5592	0.3813	0
3898	710.3	0.3687	0.507	0.5592	0.381	0
3899	710.2	0.369	0.5071	0.5593	0.3812	0

3900	710.1	0.369	0.5071	0.5596	0.3812	0
3901	710	0.3688	0.5073	0.5598	0.3813	0
3902	709.9	0.3689	0.5074	0.5595	0.3813	0
3903	709.8	0.3689	0.5073	0.5594	0.3812	0
3904	709.7	0.3688	0.5072	0.5594	0.3811	0
3905	709.6	0.369	0.5071	0.5597	0.3814	0
3906	709.5	0.369	0.5072	0.5595	0.3812	0
3907	709.4	0.3691	0.5074	0.5598	0.3813	0
3908	709.3	0.3692	0.5075	0.5598	0.3815	0
3909	709.2	0.3689	0.5074	0.5596	0.3812	0
3910	709.1	0.369	0.5073	0.5598	0.3813	0
3911	709	0.3689	0.5071	0.5597	0.3811	0
3912	708.9	0.3688	0.507	0.5596	0.3812	0
3913	708.8	0.3688	0.5069	0.5598	0.3812	0
3914	708.7	0.369	0.507	0.5598	0.3814	0
3915	708.6	0.3688	0.5071	0.5601	0.3813	0
3916	708.5	0.3686	0.5066	0.5598	0.3811	0
3917	708.4	0.3688	0.5065	0.5597	0.3809	0
3918	708.3	0.3687	0.5068	0.5598	0.3811	0
3919	708.2	0.3687	0.5068	0.5599	0.381	0
3920	708.1	0.3685	0.5065	0.5598	0.3808	0
3921	708	0.3685	0.5065	0.5595	0.3808	0
3922	707.9	0.3686	0.5066	0.5596	0.3808	0
3923	707.8	0.3683	0.5063	0.5594	0.3806	0
3924	707.7	0.3684	0.5065	0.5595	0.3807	0
3925	707.6	0.3685	0.5065	0.5595	0.3806	0
3926	707.5	0.3683	0.5066	0.5596	0.3805	0
3927	707.4	0.3684	0.5063	0.5597	0.3806	0
3928	707.3	0.3684	0.5062	0.5596	0.3805	0
3929	707.2	0.3683	0.5061	0.5597	0.3805	0
3930	707.1	0.3682	0.5061	0.5594	0.3803	0
3931	707	0.3681	0.506	0.5595	0.3802	0
3932	706.9	0.3681	0.506	0.5595	0.3804	0
3933	706.8	0.368	0.5061	0.5595	0.3804	0
3934	706.7	0.3681	0.5062	0.5595	0.3803	0
3935	706.6	0.368	0.506	0.5596	0.3803	0
3936	706.5	0.368	0.5061	0.5597	0.3805	0
3937	706.4	0.3682	0.5064	0.5598	0.3803	0
3938	706.3	0.3682	0.5061	0.5598	0.3803	0
3939	706.2	0.368	0.506	0.5597	0.3803	0
3940	706.1	0.3683	0.5062	0.5599	0.3805	0
3941	706	0.3682	0.5062	0.56	0.3807	0
3942	705.9	0.3681	0.506	0.5599	0.3806	0

3943	705.8	0.3684	0.5064	0.5602	0.3807	0
3944	705.7	0.3681	0.5061	0.56	0.3805	0
3945	705.6	0.3683	0.5062	0.5603	0.3807	0
3946	705.5	0.3681	0.5061	0.5603	0.3806	0
3947	705.4	0.3682	0.5061	0.5602	0.3805	0
3948	705.3	0.3683	0.5062	0.5602	0.3807	0
3949	705.2	0.3686	0.5065	0.5604	0.3806	0
3950	705.1	0.3685	0.5064	0.5604	0.3805	0
3951	705	0.3685	0.5064	0.5605	0.3805	0
3952	704.9	0.3685	0.5065	0.5605	0.3808	0
3953	704.8	0.3686	0.5067	0.5609	0.3808	0
3954	704.7	0.3688	0.5067	0.5608	0.3809	0
3955	704.6	0.3686	0.5067	0.5608	0.3809	0
3956	704.5	0.3685	0.5066	0.5607	0.3808	0
3957	704.4	0.3686	0.5067	0.5608	0.381	0
3958	704.3	0.3688	0.5067	0.5609	0.3809	0
3959	704.2	0.3686	0.5066	0.561	0.381	0
3960	704.1	0.3688	0.5068	0.5612	0.3812	0
3961	704	0.369	0.5071	0.5614	0.3813	0
3962	703.9	0.3688	0.5071	0.5612	0.3811	0
3963	703.8	0.369	0.5072	0.5616	0.3814	0
3964	703.7	0.369	0.5073	0.5616	0.3814	0
3965	703.6	0.3693	0.5076	0.562	0.3816	0
3966	703.5	0.3692	0.5075	0.5619	0.3815	0
3967	703.4	0.3691	0.5075	0.5618	0.3816	0
3968	703.3	0.3693	0.5077	0.5619	0.3818	0
3969	703.2	0.3694	0.5079	0.562	0.3819	0
3970	703.1	0.3693	0.508	0.5622	0.382	0
3971	703	0.3694	0.508	0.5619	0.3818	0
3972	702.9	0.3695	0.5079	0.5621	0.382	0
3973	702.8	0.3696	0.508	0.5625	0.3821	0
3974	702.7	0.3698	0.5082	0.5626	0.382	0
3975	702.6	0.3699	0.5083	0.5628	0.3821	0
3976	702.5	0.37	0.5083	0.5629	0.3822	0
3977	702.4	0.37	0.5081	0.5629	0.3822	0
3978	702.3	0.37	0.5082	0.5629	0.382	0
3979	702.2	0.3698	0.5083	0.5628	0.382	0
3980	702.1	0.37	0.5085	0.5629	0.3821	0
3981	702	0.3699	0.5084	0.563	0.3821	0
3982	701.9	0.3699	0.5084	0.5631	0.3822	0
3983	701.8	0.3701	0.5086	0.5633	0.3823	0
3984	701.7	0.3701	0.5086	0.5632	0.3824	0
3985	701.6	0.3697	0.5088	0.5634	0.3825	0

3986	701.5	0.3698	0.5087	0.5633	0.3825	0
3987	701.4	0.3699	0.5087	0.5635	0.3825	0
3988	701.3	0.3702	0.5088	0.5636	0.3828	0
3989	701.2	0.3698	0.5088	0.5635	0.3825	0
3990	701.1	0.37	0.5086	0.5636	0.3825	0
3991	701	0.3701	0.5085	0.5637	0.3825	0
3992	700.9	0.3703	0.5089	0.5638	0.3826	0
3993	700.8	0.3702	0.509	0.5638	0.3826	0
3994	700.7	0.3702	0.509	0.5638	0.3828	0
3995	700.6	0.3702	0.5088	0.5637	0.3826	0
3996	700.5	0.3704	0.5089	0.564	0.3826	0
3997	700.4	0.3705	0.509	0.5641	0.3828	0
3998	700.3	0.3703	0.5092	0.5642	0.3829	0
3999	700.2	0.3704	0.5091	0.5641	0.3827	0
4000	700.1	0.3705	0.5093	0.5642	0.3829	0
4001	700	0.3706	0.5093	0.5643	0.383	0
4002	699.9	0.3706	0.509	0.5642	0.3829	0
4003	699.8	0.3704	0.509	0.5643	0.3828	0
4004	699.7	0.3705	0.5089	0.5644	0.3828	0
4005	699.6	0.3704	0.5089	0.5644	0.3829	0
4006	699.5	0.3705	0.5092	0.5644	0.383	0
4007	699.4	0.3706	0.5091	0.5644	0.383	0
4008	699.3	0.3706	0.5092	0.5644	0.3829	0
4009	699.2	0.3703	0.509	0.5645	0.3829	0
4010	699.1	0.3705	0.5093	0.5645	0.3831	0
4011	699	0.3708	0.5094	0.5646	0.3832	0
4012	698.9	0.3706	0.5091	0.5644	0.3829	0
4013	698.8	0.3704	0.5088	0.5645	0.3828	0
4014	698.7	0.3701	0.5089	0.5644	0.3826	0
4015	698.6	0.3703	0.5093	0.5645	0.3829	0
4016	698.5	0.3704	0.5092	0.5645	0.383	0
4017	698.4	0.3703	0.5089	0.5645	0.3828	0
4018	698.3	0.3705	0.509	0.5644	0.383	0
4019	698.2	0.3704	0.5088	0.5645	0.3829	0
4020	698.1	0.3703	0.5087	0.5645	0.3828	0
4021	698	0.3702	0.5089	0.5646	0.3828	0
4022	697.9	0.3704	0.5087	0.5647	0.3829	0
4023	697.8	0.3703	0.5089	0.5646	0.3827	0
4024	697.7	0.3703	0.5089	0.5645	0.3826	0
4025	697.6	0.3702	0.5089	0.5645	0.3825	0
4026	697.5	0.3702	0.5088	0.5647	0.3824	0
4027	697.4	0.3702	0.5089	0.5644	0.3826	0
4028	697.3	0.3703	0.5091	0.5648	0.3828	0

4029	697.2	0.3702	0.5088	0.5647	0.3826	0
4030	697.1	0.3701	0.5086	0.5648	0.3827	0
4031	697	0.37	0.5086	0.5648	0.3825	0
4032	696.9	0.37	0.5084	0.5646	0.3825	0
4033	696.8	0.3702	0.5086	0.5646	0.3825	0
4034	696.7	0.37	0.5087	0.5646	0.3823	0
4035	696.6	0.3702	0.5089	0.5648	0.3825	0
4036	696.5	0.37	0.5088	0.5647	0.3824	0
4037	696.4	0.37	0.5086	0.5647	0.3825	0
4038	696.3	0.37	0.5085	0.5647	0.3825	0
4039	696.2	0.3701	0.5087	0.5649	0.3825	0
4040	696.1	0.3699	0.5086	0.5649	0.3825	0
4041	696	0.37	0.5086	0.5648	0.3825	0
4042	695.9	0.3702	0.5087	0.565	0.3826	0
4043	695.8	0.3702	0.5087	0.5649	0.3826	0
4044	695.7	0.3702	0.5088	0.5648	0.3825	0
4045	695.6	0.3704	0.5089	0.5652	0.3826	0
4046	695.5	0.3703	0.5089	0.565	0.3826	0
4047	695.4	0.3702	0.5091	0.565	0.3827	0
4048	695.3	0.3704	0.509	0.5652	0.3826	0
4049	695.2	0.3702	0.5088	0.5651	0.3827	0
4050	695.1	0.3704	0.5088	0.5652	0.3827	0
4051	695	0.3705	0.509	0.5653	0.3828	0
4052	694.9	0.3702	0.5088	0.5655	0.3828	0
4053	694.8	0.3705	0.509	0.5656	0.3828	0
4054	694.7	0.3705	0.5093	0.5656	0.383	0
4055	694.6	0.3706	0.5092	0.5656	0.3831	0
4056	694.5	0.3707	0.5094	0.5657	0.3831	0
4057	694.4	0.3707	0.5095	0.5655	0.3829	0
4058	694.3	0.3707	0.5096	0.5658	0.3833	0
4059	694.2	0.3707	0.5097	0.5659	0.3834	0
4060	694.1	0.3709	0.5098	0.566	0.3834	0
4061	694	0.371	0.5097	0.5661	0.3835	0
4062	693.9	0.371	0.5097	0.566	0.3835	0
4063	693.8	0.371	0.5097	0.566	0.3834	0
4064	693.7	0.3711	0.5098	0.5662	0.3835	0
4065	693.6	0.3712	0.51	0.5664	0.3837	0
4066	693.5	0.3713	0.51	0.5665	0.3838	0
4067	693.4	0.3713	0.5101	0.5667	0.3838	0
4068	693.3	0.3714	0.5104	0.5666	0.384	0
4069	693.2	0.3714	0.5104	0.5667	0.3842	0
4070	693.1	0.3715	0.5104	0.5669	0.3841	0
4071	693	0.3715	0.5104	0.5669	0.3841	0

4072	692.9	0.3715	0.5104	0.5669	0.3841	0
4073	692.8	0.3714	0.5101	0.5668	0.384	0
4074	692.7	0.3718	0.5105	0.5671	0.3843	0
4075	692.6	0.3721	0.5106	0.5673	0.3846	0
4076	692.5	0.3718	0.5106	0.5673	0.3844	0
4077	692.4	0.3717	0.5107	0.5673	0.3843	0
4078	692.3	0.3718	0.5106	0.5672	0.3844	0
4079	692.2	0.3717	0.5105	0.5672	0.3844	0
4080	692.1	0.3717	0.5107	0.5672	0.3845	0
4081	692	0.3718	0.5108	0.5674	0.3844	0
4082	691.9	0.3719	0.5109	0.5673	0.3845	0
4083	691.8	0.3718	0.511	0.5674	0.3845	0
4084	691.7	0.3719	0.511	0.5676	0.3846	0
4085	691.6	0.372	0.511	0.5677	0.3846	0
4086	691.5	0.3719	0.5109	0.5676	0.3846	0
4087	691.4	0.3721	0.511	0.5675	0.3846	0
4088	691.3	0.3718	0.5109	0.5674	0.3847	0
4089	691.2	0.372	0.5107	0.5676	0.3846	0
4090	691.1	0.3719	0.5109	0.5676	0.3848	0
4091	691	0.3718	0.511	0.5676	0.3847	0
4092	690.9	0.3719	0.511	0.5676	0.3848	0
4093	690.8	0.3719	0.5109	0.5678	0.3845	0
4094	690.7	0.3718	0.5112	0.5677	0.3846	0
4095	690.6	0.3715	0.5108	0.5676	0.3845	0
4096	690.5	0.3715	0.511	0.5676	0.3844	0
4097	690.4	0.3717	0.5111	0.5679	0.3845	0
4098	690.3	0.3717	0.5109	0.5677	0.3846	0
4099	690.2	0.3719	0.5109	0.568	0.3847	0
4100	690.1	0.3719	0.511	0.5677	0.3847	0
4101	690	0.3718	0.5107	0.5678	0.3846	0
4102	689.9	0.3718	0.5107	0.5675	0.3844	0
4103	689.8	0.3718	0.5108	0.5675	0.3845	0
4104	689.7	0.372	0.5109	0.5677	0.3845	0
4105	689.6	0.3721	0.511	0.5677	0.3846	0
4106	689.5	0.372	0.5109	0.5678	0.3846	0
4107	689.4	0.3719	0.511	0.5678	0.3846	0
4108	689.3	0.3716	0.5109	0.5676	0.3844	0
4109	689.2	0.3717	0.5108	0.5674	0.3843	0
4110	689.1	0.372	0.5111	0.5678	0.3845	0
4111	689	0.372	0.511	0.5677	0.3843	0
4112	688.9	0.3719	0.511	0.5678	0.3844	0
4113	688.8	0.372	0.511	0.5675	0.3844	0
4114	688.7	0.3719	0.5108	0.5677	0.3843	0

4115	688.6	0.372	0.5109	0.5679	0.3846	0
4116	688.5	0.3718	0.5108	0.5677	0.3845	0
4117	688.4	0.3717	0.5109	0.5677	0.3845	0
4118	688.3	0.3718	0.5107	0.5678	0.3844	0
4119	688.2	0.3717	0.5106	0.5678	0.3845	0
4120	688.1	0.3717	0.5109	0.5678	0.3846	0
4121	688	0.3717	0.5108	0.5677	0.3843	0
4122	687.9	0.3716	0.5108	0.5678	0.3843	0
4123	687.8	0.3717	0.5107	0.5678	0.3844	0
4124	687.7	0.3716	0.5109	0.5678	0.3843	0
4125	687.6	0.3719	0.511	0.568	0.3846	0
4126	687.5	0.3718	0.511	0.5681	0.3847	0
4127	687.4	0.3718	0.5111	0.568	0.3847	0
4128	687.3	0.3719	0.5112	0.5679	0.3849	0
4129	687.2	0.372	0.5112	0.5683	0.3848	0
4130	687.1	0.3719	0.5113	0.5682	0.3848	0
4131	687	0.3719	0.5113	0.5681	0.3847	0
4132	686.9	0.372	0.5113	0.568	0.3848	0
4133	686.8	0.3719	0.5114	0.5681	0.3847	0
4134	686.7	0.372	0.5114	0.5681	0.385	0
4135	686.6	0.372	0.5114	0.5682	0.3847	0
4136	686.5	0.372	0.5114	0.5682	0.3848	0
4137	686.4	0.372	0.5116	0.5683	0.3848	0
4138	686.3	0.372	0.5112	0.5682	0.3848	0
4139	686.2	0.3721	0.5114	0.5684	0.3848	0
4140	686.1	0.3722	0.5115	0.5686	0.3849	0
4141	686	0.3722	0.5114	0.5687	0.385	0
4142	685.9	0.3723	0.5115	0.5685	0.3849	0
4143	685.8	0.3723	0.5115	0.5685	0.3851	0
4144	685.7	0.3722	0.5115	0.5686	0.385	0
4145	685.6	0.3723	0.5116	0.5688	0.3851	0
4146	685.5	0.3724	0.5115	0.5686	0.3851	0
4147	685.4	0.3724	0.5116	0.5687	0.3852	0
4148	685.3	0.3723	0.5116	0.5687	0.3852	0
4149	685.2	0.3723	0.5117	0.5687	0.3851	0
4150	685.1	0.3724	0.5119	0.5689	0.3854	0
4151	685	0.3726	0.5121	0.5689	0.3855	0
4152	684.9	0.3726	0.5121	0.5689	0.3855	0
4153	684.8	0.3727	0.5124	0.5689	0.3855	0
4154	684.7	0.3728	0.5123	0.569	0.3857	0
4155	684.6	0.3729	0.5124	0.5692	0.386	0
4156	684.5	0.3729	0.5126	0.5691	0.3858	0
4157	684.4	0.3731	0.5125	0.5692	0.3858	0

4158	684.3	0.373	0.5126	0.5693	0.3859	0
4159	684.2	0.3728	0.5125	0.5692	0.3858	0
4160	684.1	0.3732	0.5128	0.5695	0.3859	0
4161	684	0.3731	0.5128	0.5694	0.386	0
4162	683.9	0.3731	0.5127	0.5694	0.3861	0
4163	683.8	0.3732	0.5128	0.5695	0.3861	0
4164	683.7	0.3733	0.5131	0.5698	0.3861	0
4165	683.6	0.3735	0.5134	0.5699	0.3863	0
4166	683.5	0.3737	0.5134	0.5699	0.3864	0
4167	683.4	0.3735	0.5132	0.5699	0.3864	0
4168	683.3	0.3737	0.5134	0.5699	0.3866	0
4169	683.2	0.3737	0.5134	0.57	0.3866	0
4170	683.1	0.3736	0.5132	0.5699	0.3867	0
4171	683	0.3736	0.5133	0.5699	0.3867	0
4172	682.9	0.3733	0.5131	0.5697	0.3864	0
4173	682.8	0.3733	0.5129	0.5695	0.3864	0
4174	682.7	0.3737	0.5132	0.5699	0.3866	0
4175	682.6	0.3738	0.5132	0.5699	0.3866	0
4176	682.5	0.3738	0.5134	0.5699	0.3866	0
4177	682.4	0.3738	0.5132	0.57	0.3866	0
4178	682.3	0.3739	0.5137	0.5701	0.3868	0
4179	682.2	0.3738	0.5136	0.5701	0.3868	0
4180	682.1	0.3737	0.5134	0.5701	0.3867	0
4181	682	0.3736	0.5135	0.5699	0.3868	0
4182	681.9	0.3737	0.5136	0.57	0.3868	0
4183	681.8	0.3739	0.5136	0.5701	0.3869	0
4184	681.7	0.3739	0.5136	0.5702	0.3869	0
4185	681.6	0.3739	0.5136	0.5702	0.3868	0
4186	681.5	0.3738	0.5138	0.5701	0.3867	0
4187	681.4	0.374	0.5135	0.57	0.3867	0
4188	681.3	0.374	0.5136	0.5702	0.3868	0
4189	681.2	0.3739	0.5138	0.5701	0.3869	0
4190	681.1	0.374	0.5139	0.5702	0.3868	0
4191	681	0.3739	0.514	0.5702	0.3868	0
4192	680.9	0.3739	0.5135	0.57	0.3867	0
4193	680.8	0.3738	0.5134	0.5702	0.3868	0
4194	680.7	0.3739	0.5135	0.5701	0.3866	0
4195	680.6	0.374	0.5135	0.57	0.3869	0
4196	680.5	0.3739	0.5135	0.5698	0.3868	0
4197	680.4	0.3738	0.5134	0.57	0.3868	0
4198	680.3	0.3739	0.5133	0.5698	0.3868	0
4199	680.2	0.3739	0.5133	0.5699	0.3866	0
4200	680.1	0.3738	0.5133	0.5699	0.3866	0

4201	680	0.3738	0.5133	0.5698	0.3866	0
4202	679.9	0.3737	0.5132	0.5697	0.3864	0
4203	679.8	0.3737	0.5131	0.5697	0.3865	0
4204	679.7	0.3736	0.5133	0.5698	0.3865	0
4205	679.6	0.3736	0.5133	0.5696	0.3864	0
4206	679.5	0.3735	0.513	0.5695	0.3863	0
4207	679.4	0.3736	0.5131	0.5696	0.3864	0
4208	679.3	0.3735	0.5132	0.5696	0.3865	0
4209	679.2	0.3735	0.5132	0.5696	0.3863	0
4210	679.1	0.3733	0.513	0.5694	0.3862	0
4211	679	0.3735	0.513	0.5694	0.3862	0
4212	678.9	0.3734	0.5131	0.5695	0.3864	0
4213	678.8	0.3733	0.5129	0.5692	0.3862	0
4214	678.7	0.3734	0.513	0.5693	0.3863	0
4215	678.6	0.3735	0.5131	0.5693	0.3861	0
4216	678.5	0.3733	0.5128	0.5691	0.386	0
4217	678.4	0.3733	0.5127	0.569	0.3858	0
4218	678.3	0.3732	0.5126	0.569	0.3859	0
4219	678.2	0.3732	0.5125	0.569	0.3855	0
4220	678.1	0.3731	0.5127	0.569	0.3856	0
4221	678	0.3732	0.5128	0.569	0.3857	0
4222	677.9	0.3734	0.5129	0.5691	0.3857	0
4223	677.8	0.3731	0.5127	0.569	0.3858	0
4224	677.7	0.373	0.5126	0.569	0.3857	0
4225	677.6	0.373	0.5127	0.569	0.3857	0
4226	677.5	0.3732	0.5128	0.569	0.386	0
4227	677.4	0.3731	0.5127	0.5689	0.3858	0
4228	677.3	0.373	0.5125	0.569	0.3857	0
4229	677.2	0.3732	0.5128	0.569	0.3862	0
4230	677.1	0.3732	0.5127	0.5691	0.3858	0
4231	677	0.3732	0.5129	0.5691	0.3859	0
4232	676.9	0.373	0.5126	0.5689	0.3858	0
4233	676.8	0.3734	0.513	0.5689	0.3862	0
4234	676.7	0.3737	0.5132	0.569	0.3863	0
4235	676.6	0.3733	0.5131	0.5691	0.3863	0
4236	676.5	0.3733	0.5129	0.5689	0.3861	0
4237	676.4	0.3735	0.5131	0.5693	0.3861	0
4238	676.3	0.3734	0.5132	0.5691	0.3862	0
4239	676.2	0.3737	0.5133	0.5692	0.3863	0
4240	676.1	0.3738	0.5134	0.5693	0.3864	0
4241	676	0.3737	0.5135	0.5693	0.3865	0
4242	675.9	0.3737	0.5137	0.5693	0.3866	0
4243	675.8	0.3737	0.5136	0.5695	0.3867	0

4244	675.7	0.3738	0.5138	0.5696	0.3869	0
4245	675.6	0.3738	0.5139	0.5695	0.387	0
4246	675.5	0.3738	0.5137	0.5695	0.3869	0
4247	675.4	0.374	0.5137	0.5697	0.3871	0
4248	675.3	0.374	0.5139	0.5697	0.3871	0
4249	675.2	0.3743	0.5143	0.57	0.3873	0
4250	675.1	0.3745	0.5146	0.57	0.3875	0
4251	675	0.3743	0.5143	0.57	0.3874	0
4252	674.9	0.3745	0.5145	0.57	0.3873	0
4253	674.8	0.3746	0.5146	0.5699	0.3874	0
4254	674.7	0.3746	0.5144	0.5701	0.3876	0
4255	674.6	0.3748	0.5147	0.5702	0.3879	0
4256	674.5	0.375	0.515	0.5703	0.3878	0
4257	674.4	0.3748	0.5149	0.5704	0.3877	0
4258	674.3	0.3748	0.515	0.5705	0.388	0
4259	674.2	0.3749	0.5152	0.5705	0.3878	0
4260	674.1	0.3752	0.5154	0.5706	0.3882	0
4261	674	0.3754	0.5156	0.5708	0.3884	0
4262	673.9	0.3754	0.5157	0.5709	0.3885	0
4263	673.8	0.3753	0.5157	0.5709	0.3884	0
4264	673.7	0.3753	0.5157	0.5708	0.3884	0
4265	673.6	0.3754	0.5155	0.5707	0.3885	0
4266	673.5	0.3755	0.5156	0.5708	0.3886	0
4267	673.4	0.3754	0.5156	0.5708	0.3887	0
4268	673.3	0.3754	0.5158	0.5709	0.3887	0
4269	673.2	0.3756	0.5162	0.571	0.389	0
4270	673.1	0.3756	0.5161	0.5709	0.3889	0
4271	673	0.3756	0.516	0.571	0.3887	0
4272	672.9	0.3757	0.5162	0.5711	0.3889	0
4273	672.8	0.3756	0.5162	0.571	0.3887	0
4274	672.7	0.3755	0.516	0.571	0.3886	0
4275	672.6	0.3755	0.5159	0.5708	0.3887	0
4276	672.5	0.3757	0.5161	0.5711	0.3886	0
4277	672.4	0.3756	0.5159	0.571	0.3886	0
4278	672.3	0.3757	0.5161	0.5708	0.3885	0
4279	672.2	0.3758	0.5161	0.571	0.3887	0
4280	672.1	0.3757	0.5161	0.5708	0.3886	0
4281	672	0.376	0.5161	0.5712	0.3889	0
4282	671.9	0.3759	0.5162	0.5711	0.389	0
4283	671.8	0.3759	0.5162	0.5709	0.389	0
4284	671.7	0.376	0.5162	0.571	0.3891	0
4285	671.6	0.3759	0.5162	0.571	0.3892	0
4286	671.5	0.3758	0.5162	0.571	0.3891	0

4287	671.4	0.3758	0.5162	0.571	0.3891	0
4288	671.3	0.3759	0.5162	0.5707	0.3891	0
4289	671.2	0.3761	0.5164	0.5711	0.3891	0
4290	671.1	0.3761	0.5164	0.5712	0.3892	0
4291	671	0.3759	0.5162	0.5708	0.3891	0
4292	670.9	0.376	0.5163	0.5709	0.3891	0
4293	670.8	0.376	0.5161	0.5709	0.3891	0
4294	670.7	0.3758	0.5162	0.5708	0.3889	0
4295	670.6	0.3758	0.5161	0.5706	0.3888	0
4296	670.5	0.3761	0.5163	0.5707	0.389	0
4297	670.4	0.3759	0.5162	0.5706	0.3889	0
4298	670.3	0.3757	0.5162	0.5706	0.3888	0
4299	670.2	0.3757	0.5158	0.5705	0.3887	0
4300	670.1	0.3756	0.5159	0.5706	0.3888	0
4301	670	0.3757	0.5159	0.5707	0.3889	0
4302	669.9	0.3756	0.5159	0.5705	0.3887	0
4303	669.8	0.3757	0.5159	0.5705	0.3887	0
4304	669.7	0.3755	0.516	0.5705	0.3887	0
4305	669.6	0.3755	0.5158	0.5703	0.3887	0
4306	669.5	0.3754	0.5156	0.5701	0.3886	0
4307	669.4	0.3756	0.5158	0.5702	0.3887	0
4308	669.3	0.3755	0.5156	0.57	0.3884	0
4309	669.2	0.3757	0.5159	0.5703	0.3886	0
4310	669.1	0.3755	0.5159	0.5702	0.3885	0
4311	669	0.3754	0.5158	0.5703	0.3885	0
4312	668.9	0.3752	0.5156	0.5701	0.3883	0
4313	668.8	0.3755	0.5156	0.57	0.3883	0
4314	668.7	0.3752	0.5155	0.5697	0.3881	0
4315	668.6	0.375	0.5154	0.5699	0.3882	0
4316	668.5	0.375	0.5153	0.5696	0.3882	0
4317	668.4	0.3751	0.5152	0.5696	0.3882	0
4318	668.3	0.375	0.5153	0.5695	0.3881	0
4319	668.2	0.3749	0.5152	0.5692	0.3881	0
4320	668.1	0.375	0.515	0.5692	0.3879	0
4321	668	0.3748	0.5149	0.569	0.3877	0
4322	667.9	0.3748	0.5149	0.5691	0.3878	0
4323	667.8	0.3748	0.5149	0.5691	0.3877	0
4324	667.7	0.3749	0.5147	0.5691	0.3876	0
4325	667.6	0.3747	0.5147	0.569	0.3875	0
4326	667.5	0.3745	0.5148	0.5689	0.3877	0
4327	667.4	0.3745	0.5147	0.5688	0.3875	0
4328	667.3	0.3745	0.5147	0.5687	0.3873	0
4329	667.2	0.3746	0.5149	0.5689	0.3876	0

4330	667.1	0.3745	0.5148	0.5688	0.3875	0
4331	667	0.3748	0.5149	0.569	0.3876	0
4332	666.9	0.3747	0.5149	0.5688	0.3875	0
4333	666.8	0.3747	0.5147	0.5688	0.3875	0
4334	666.7	0.3747	0.5147	0.5688	0.3875	0
4335	666.6	0.3747	0.5147	0.5688	0.3875	0
4336	666.5	0.3748	0.5148	0.5686	0.3876	0
4337	666.4	0.3747	0.5148	0.5688	0.3877	0
4338	666.3	0.3749	0.515	0.5689	0.3877	0
4339	666.2	0.3748	0.5149	0.569	0.3878	0
4340	666.1	0.375	0.5151	0.5689	0.3878	0
4341	666	0.375	0.5152	0.5688	0.388	0
4342	665.9	0.3749	0.5152	0.5687	0.3877	0
4343	665.8	0.3747	0.5151	0.5687	0.3877	0
4344	665.7	0.375	0.5152	0.5687	0.3879	0
4345	665.6	0.375	0.5153	0.5686	0.3879	0
4346	665.5	0.3749	0.5153	0.5687	0.3879	0
4347	665.4	0.375	0.5153	0.5688	0.3879	0
4348	665.3	0.3751	0.5154	0.569	0.3879	0
4349	665.2	0.3752	0.5154	0.569	0.3881	0
4350	665.1	0.3753	0.5156	0.5691	0.3882	0
4351	665	0.3753	0.5155	0.5691	0.3881	0
4352	664.9	0.3753	0.5156	0.569	0.3882	0
4353	664.8	0.3756	0.5159	0.5693	0.3884	0
4354	664.7	0.3756	0.5159	0.5694	0.3886	0
4355	664.6	0.3757	0.516	0.5695	0.3888	0
4356	664.5	0.3758	0.5159	0.5692	0.3888	0
4357	664.4	0.376	0.5163	0.5694	0.3889	0
4358	664.3	0.3759	0.5165	0.5696	0.389	0
4359	664.2	0.3761	0.5168	0.5696	0.3894	0
4360	664.1	0.3761	0.5166	0.5696	0.3893	0
4361	664	0.3762	0.5166	0.5696	0.3895	0
4362	663.9	0.3766	0.5168	0.5699	0.3894	0
4363	663.8	0.3766	0.5169	0.5697	0.3895	0
4364	663.7	0.3768	0.5171	0.5699	0.3897	0
4365	663.6	0.3766	0.5174	0.57	0.3899	0
4366	663.5	0.3769	0.5175	0.57	0.3899	0
4367	663.4	0.377	0.5176	0.5703	0.3901	0
4368	663.3	0.3772	0.5178	0.5703	0.3902	0
4369	663.2	0.3772	0.518	0.5705	0.3903	0
4370	663.1	0.3773	0.5179	0.5704	0.3903	0
4371	663	0.3773	0.5181	0.5704	0.3905	0
4372	662.9	0.3774	0.5182	0.5707	0.3905	0

4373	662.8	0.3774	0.5182	0.5703	0.3906	0
4374	662.7	0.3774	0.5182	0.5706	0.3905	0
4375	662.6	0.3774	0.5182	0.5706	0.3906	0
4376	662.5	0.3775	0.5183	0.5704	0.3906	0
4377	662.4	0.3775	0.5184	0.5706	0.3907	0
4378	662.3	0.3776	0.5186	0.5707	0.3909	0
4379	662.2	0.3777	0.5185	0.5707	0.3908	0
4380	662.1	0.3776	0.5185	0.5706	0.3909	0
4381	662	0.3776	0.5185	0.5706	0.3908	0
4382	661.9	0.3778	0.5185	0.5705	0.3909	0
4383	661.8	0.3777	0.5184	0.5706	0.3911	0
4384	661.7	0.3778	0.5186	0.5707	0.3912	0
4385	661.6	0.3779	0.5186	0.5707	0.3912	0
4386	661.5	0.3778	0.5187	0.5707	0.3913	0
4387	661.4	0.3779	0.5188	0.5708	0.3912	0
4388	661.3	0.3779	0.5186	0.5708	0.3913	0
4389	661.2	0.3776	0.5187	0.5706	0.3914	0
4390	661.1	0.3778	0.5187	0.5707	0.3913	0
4391	661	0.3778	0.5187	0.5703	0.3913	0
4392	660.9	0.3777	0.5186	0.5704	0.3912	0
4393	660.8	0.3777	0.5187	0.5704	0.3913	0
4394	660.7	0.3779	0.5188	0.5704	0.3912	0
4395	660.6	0.3779	0.519	0.5705	0.3911	0
4396	660.5	0.3778	0.519	0.5703	0.391	0
4397	660.4	0.3777	0.519	0.5705	0.391	0
4398	660.3	0.3778	0.5187	0.5706	0.3914	0
4399	660.2	0.378	0.5189	0.5705	0.3914	0
4400	660.1	0.3779	0.5188	0.5706	0.3912	0
4401	660	0.3778	0.5191	0.5704	0.3915	0
4402	659.9	0.3777	0.5189	0.5702	0.3912	0
4403	659.8	0.3776	0.5186	0.5701	0.3912	0
4404	659.7	0.3777	0.5188	0.5702	0.3912	0
4405	659.6	0.3777	0.5187	0.5702	0.3911	0
4406	659.5	0.3779	0.5189	0.57	0.391	0
4407	659.4	0.3776	0.5187	0.5699	0.391	0
4408	659.3	0.3777	0.5189	0.5701	0.3911	0
4409	659.2	0.3777	0.5186	0.5701	0.3911	0
4410	659.1	0.3775	0.5186	0.5699	0.3909	0
4411	659	0.3773	0.5182	0.5697	0.3907	0
4412	658.9	0.3772	0.5182	0.5697	0.3906	0
4413	658.8	0.3774	0.5183	0.5696	0.3906	0
4414	658.7	0.3775	0.5187	0.5696	0.3907	0
4415	658.6	0.3772	0.5184	0.5695	0.3905	0

4416	658.5	0.3772	0.518	0.5694	0.3902	0
4417	658.4	0.3772	0.5181	0.5695	0.3904	0
4418	658.3	0.3773	0.5179	0.5697	0.3903	0
4419	658.2	0.3771	0.5177	0.5692	0.3904	0
4420	658.1	0.3771	0.5178	0.5691	0.3903	0
4421	658	0.377	0.5177	0.5692	0.3902	0
4422	657.9	0.3771	0.5177	0.5693	0.3902	0
4423	657.8	0.377	0.5178	0.569	0.3901	0
4424	657.7	0.3768	0.5178	0.569	0.3902	0
4425	657.6	0.377	0.5178	0.5689	0.3901	0
4426	657.5	0.3768	0.5176	0.5689	0.3901	0
4427	657.4	0.3768	0.5175	0.5687	0.3901	0
4428	657.3	0.3769	0.5176	0.5688	0.3899	0
4429	657.2	0.3768	0.5174	0.5686	0.3896	0
4430	657.1	0.3768	0.5175	0.5686	0.3899	0
4431	657	0.377	0.5175	0.5687	0.3897	0
4432	656.9	0.3767	0.5174	0.5686	0.3897	0
4433	656.8	0.3767	0.5174	0.5684	0.3896	0
4434	656.7	0.3766	0.5175	0.5683	0.3896	0
4435	656.6	0.3767	0.5174	0.5683	0.3897	0
4436	656.5	0.3767	0.5175	0.5686	0.3898	0
4437	656.4	0.3767	0.5176	0.5685	0.3897	0
4438	656.3	0.3766	0.5176	0.5685	0.3896	0
4439	656.2	0.3765	0.5173	0.5683	0.3895	0
4440	656.1	0.3767	0.5176	0.5684	0.3898	0
4441	656	0.3769	0.5177	0.5682	0.3898	0
4442	655.9	0.3769	0.5176	0.5683	0.3896	0
4443	655.8	0.3771	0.5179	0.5686	0.39	0
4444	655.7	0.3768	0.5178	0.5686	0.39	0
4445	655.6	0.3769	0.518	0.5686	0.39	0
4446	655.5	0.3768	0.5176	0.5683	0.39	0
4447	655.4	0.3768	0.5178	0.5684	0.3898	0
4448	655.3	0.3768	0.5176	0.5683	0.3897	0
4449	655.2	0.3769	0.5178	0.5683	0.39	0
4450	655.1	0.3768	0.5179	0.5684	0.3901	0
4451	655	0.3768	0.5181	0.5685	0.3902	0
4452	654.9	0.377	0.5181	0.5685	0.3903	0
4453	654.8	0.377	0.5179	0.5684	0.3903	0
4454	654.7	0.3771	0.5181	0.5686	0.3903	0
4455	654.6	0.377	0.5181	0.5686	0.3903	0
4456	654.5	0.377	0.5182	0.5686	0.3902	0
4457	654.4	0.3771	0.5182	0.5686	0.3904	0
4458	654.3	0.3772	0.5182	0.5685	0.3904	0

4459	654.2	0.3774	0.5182	0.5686	0.3906	0
4460	654.1	0.3774	0.5184	0.5686	0.3908	0
4461	654	0.3775	0.5185	0.5687	0.3907	0
4462	653.9	0.3777	0.5186	0.5689	0.3907	0
4463	653.8	0.3778	0.5187	0.5688	0.391	0
4464	653.7	0.378	0.5188	0.5691	0.3913	0
4465	653.6	0.3781	0.5187	0.5689	0.3913	0
4466	653.5	0.378	0.5191	0.5691	0.3913	0
4467	653.4	0.378	0.5191	0.5691	0.3913	0
4468	653.3	0.3782	0.5194	0.5693	0.3914	0
4469	653.2	0.3784	0.5195	0.5692	0.3917	0
4470	653.1	0.3783	0.5197	0.5694	0.392	0
4471	653	0.3785	0.5198	0.5694	0.3922	0
4472	652.9	0.3785	0.5198	0.5694	0.3922	0
4473	652.8	0.3786	0.5199	0.5695	0.392	0
4474	652.7	0.3787	0.5201	0.5697	0.3922	0
4475	652.6	0.3789	0.5203	0.5698	0.3925	0
4476	652.5	0.379	0.5205	0.57	0.3924	0
4477	652.4	0.3791	0.5206	0.5699	0.3926	0
4478	652.3	0.379	0.5206	0.5699	0.3926	0
4479	652.2	0.3791	0.5206	0.57	0.3927	0
4480	652.1	0.3791	0.5207	0.57	0.3927	0
4481	652	0.3793	0.521	0.5702	0.3927	0
4482	651.9	0.3793	0.5211	0.5701	0.3929	0
4483	651.8	0.3792	0.521	0.5699	0.3927	0
4484	651.7	0.3792	0.5208	0.5701	0.3926	0
4485	651.6	0.3791	0.5209	0.5696	0.3926	0
4486	651.5	0.3795	0.5211	0.5701	0.3929	0
4487	651.4	0.3794	0.5212	0.5702	0.3928	0
4488	651.3	0.3793	0.521	0.57	0.3927	0
4489	651.2	0.3795	0.5212	0.5702	0.393	0
4490	651.1	0.3796	0.521	0.5701	0.3929	0
4491	651	0.3797	0.5211	0.5703	0.3932	0
4492	650.9	0.3797	0.5213	0.5704	0.3931	0
4493	650.8	0.3796	0.5213	0.5703	0.393	0
4494	650.7	0.3797	0.5212	0.57	0.393	0
4495	650.6	0.3795	0.5213	0.5699	0.393	0
4496	650.5	0.3797	0.5211	0.5699	0.3931	0
4497	650.4	0.3797	0.5213	0.5701	0.3931	0
4498	650.3	0.3797	0.5212	0.5699	0.3932	0
4499	650.2	0.3797	0.5212	0.57	0.3932	0
4500	650.1	0.3795	0.5211	0.5697	0.393	0
4501	650	0.3799	0.5213	0.5698	0.3932	0

4502	649.9	0.3798	0.5212	0.5701	0.3931	0
4503	649.8	0.3799	0.5215	0.5701	0.3932	0
4504	649.7	0.3799	0.5215	0.57	0.3933	0
4505	649.6	0.3796	0.5213	0.5696	0.3932	0
4506	649.5	0.3796	0.5214	0.5698	0.393	0
4507	649.4	0.3797	0.5212	0.5698	0.3931	0
4508	649.3	0.3796	0.521	0.5698	0.3932	0
4509	649.2	0.3797	0.5212	0.5697	0.3931	0
4510	649.1	0.3797	0.5212	0.5698	0.3932	0
4511	649	0.3797	0.5213	0.57	0.3932	0
4512	648.9	0.3796	0.5209	0.5697	0.393	0
4513	648.8	0.3795	0.5209	0.5697	0.3932	0
4514	648.7	0.3795	0.5209	0.5697	0.3931	0
4515	648.6	0.3794	0.5211	0.5695	0.3929	0
4516	648.5	0.3793	0.521	0.5693	0.3927	0
4517	648.4	0.3791	0.521	0.5696	0.3927	0
4518	648.3	0.3793	0.5211	0.5696	0.3927	0
4519	648.2	0.3793	0.5208	0.5694	0.3928	0
4520	648.1	0.3792	0.5206	0.5693	0.3925	0
4521	648	0.3792	0.5207	0.5691	0.3927	0
4522	647.9	0.3794	0.5206	0.5692	0.3926	0
4523	647.8	0.3792	0.5206	0.5691	0.3927	0
4524	647.7	0.3789	0.5205	0.5691	0.3928	0
4525	647.6	0.379	0.5205	0.569	0.3927	0
4526	647.5	0.379	0.5205	0.5691	0.3926	0
4527	647.4	0.3786	0.5203	0.5687	0.3923	0
4528	647.3	0.3787	0.5203	0.5688	0.3925	0
4529	647.2	0.3787	0.5204	0.5688	0.3923	0
4530	647.1	0.379	0.5204	0.5689	0.3923	0
4531	647	0.3786	0.5201	0.5686	0.392	0
4532	646.9	0.3788	0.5203	0.5686	0.3923	0
4533	646.8	0.3788	0.5203	0.5689	0.3923	0
4534	646.7	0.3788	0.5202	0.5688	0.3924	0
4535	646.6	0.3787	0.5201	0.5687	0.3924	0
4536	646.5	0.3789	0.5205	0.5687	0.3921	0
4537	646.4	0.3787	0.5205	0.5687	0.392	0
4538	646.3	0.3788	0.5204	0.5687	0.3922	0
4539	646.2	0.3788	0.5203	0.5688	0.3921	0
4540	646.1	0.3788	0.5203	0.5688	0.3922	0
4541	646	0.3787	0.5201	0.5685	0.392	0
4542	645.9	0.3787	0.5203	0.5686	0.392	0
4543	645.8	0.3788	0.5204	0.5684	0.3921	0
4544	645.7	0.3788	0.5206	0.5684	0.3919	0

4545	645.6	0.3788	0.5202	0.5685	0.392	0
4546	645.5	0.3786	0.5201	0.5683	0.3919	0
4547	645.4	0.3788	0.5203	0.5685	0.392	0
4548	645.3	0.3789	0.5204	0.5687	0.3923	0
4549	645.2	0.379	0.5205	0.5685	0.3921	0
4550	645.1	0.379	0.5205	0.5685	0.3922	0
4551	645	0.3791	0.5206	0.5684	0.3922	0
4552	644.9	0.3793	0.5207	0.5688	0.3923	0
4553	644.8	0.379	0.5207	0.5686	0.3923	0
4554	644.7	0.3791	0.5208	0.5686	0.3923	0
4555	644.6	0.3791	0.5204	0.5683	0.3921	0
4556	644.5	0.3792	0.5208	0.5686	0.3923	0
4557	644.4	0.3791	0.5209	0.5686	0.3923	0
4558	644.3	0.3792	0.5211	0.5687	0.3924	0
4559	644.2	0.3794	0.5211	0.5687	0.3925	0
4560	644.1	0.3794	0.521	0.5686	0.3923	0
4561	644	0.3794	0.5209	0.5687	0.3924	0
4562	643.9	0.3794	0.5211	0.5689	0.3926	0
4563	643.8	0.3795	0.5213	0.5692	0.3928	0
4564	643.7	0.3797	0.5215	0.5692	0.3929	0
4565	643.6	0.3798	0.5216	0.5692	0.3929	0
4566	643.5	0.3796	0.5215	0.569	0.3929	0
4567	643.4	0.3795	0.5216	0.5689	0.393	0
4568	643.3	0.3796	0.5217	0.5691	0.3932	0
4569	643.2	0.3798	0.5218	0.5692	0.3932	0
4570	643.1	0.3798	0.5219	0.5692	0.3932	0
4571	643	0.38	0.5219	0.5692	0.3933	0
4572	642.9	0.3803	0.5219	0.5694	0.3935	0
4573	642.8	0.3802	0.5219	0.5694	0.3935	0
4574	642.7	0.3804	0.5221	0.5695	0.3937	0
4575	642.6	0.3803	0.5219	0.5692	0.3935	0
4576	642.5	0.3802	0.5217	0.5692	0.3934	0
4577	642.4	0.3802	0.5219	0.5695	0.3936	0
4578	642.3	0.3803	0.5223	0.5696	0.3939	0
4579	642.2	0.3803	0.5223	0.5695	0.394	0
4580	642.1	0.3803	0.5223	0.5695	0.394	0
4581	642	0.3803	0.5223	0.5694	0.394	0
4582	641.9	0.3803	0.5227	0.5695	0.394	0
4583	641.8	0.3804	0.5227	0.5696	0.394	0
4584	641.7	0.3808	0.5228	0.5699	0.3942	0
4585	641.6	0.3806	0.5225	0.5696	0.3942	0
4586	641.5	0.3806	0.5227	0.5696	0.3941	0
4587	641.4	0.3808	0.5229	0.57	0.3942	0

4588	641.3	0.3808	0.5229	0.5698	0.3943	0
4589	641.2	0.3808	0.5229	0.57	0.3944	0
4590	641.1	0.3808	0.5229	0.5698	0.3945	0
4591	641	0.3807	0.523	0.5699	0.3943	0
4592	640.9	0.3807	0.5226	0.57	0.3942	0
4593	640.8	0.3807	0.5229	0.5695	0.3943	0
4594	640.7	0.3809	0.5231	0.5697	0.3944	0
4595	640.6	0.381	0.5228	0.5698	0.3945	0
4596	640.5	0.3812	0.523	0.5701	0.3946	0
4597	640.4	0.3811	0.523	0.57	0.3945	0
4598	640.3	0.3809	0.523	0.5701	0.3946	0
4599	640.2	0.3808	0.523	0.5699	0.3945	0
4600	640.1	0.3808	0.5231	0.5699	0.3945	0
4601	640	0.381	0.5234	0.5699	0.3947	0
4602	639.9	0.3809	0.5233	0.5699	0.3947	0
4603	639.8	0.3811	0.5234	0.5701	0.3946	0
4604	639.7	0.3808	0.5232	0.5699	0.3946	0
4605	639.6	0.3807	0.5234	0.5698	0.3947	0
4606	639.5	0.3808	0.5234	0.5699	0.3948	0
4607	639.4	0.3809	0.5233	0.57	0.3947	0
4608	639.3	0.3809	0.5235	0.5701	0.3948	0
4609	639.2	0.3809	0.5233	0.5701	0.3949	0
4610	639.1	0.3812	0.5234	0.5702	0.395	0
4611	639	0.381	0.5234	0.5699	0.3947	0
4612	638.9	0.381	0.5234	0.57	0.3947	0
4613	638.8	0.3812	0.5232	0.5698	0.3949	0
4614	638.7	0.381	0.5232	0.5699	0.3946	0
4615	638.6	0.3812	0.5234	0.5701	0.3947	0
4616	638.5	0.3812	0.5237	0.5701	0.3949	0
4617	638.4	0.3811	0.5237	0.57	0.3949	0
4618	638.3	0.381	0.5235	0.5699	0.3947	0
4619	638.2	0.381	0.5233	0.5699	0.3947	0
4620	638.1	0.3811	0.5232	0.5699	0.3947	0
4621	638	0.381	0.523	0.57	0.3945	0
4622	637.9	0.3809	0.5231	0.5699	0.3946	0
4623	637.8	0.381	0.5233	0.5699	0.3944	0
4624	637.7	0.3811	0.523	0.5699	0.3944	0
4625	637.6	0.3807	0.523	0.5698	0.3943	0
4626	637.5	0.3809	0.5231	0.5696	0.3942	0
4627	637.4	0.3809	0.523	0.5697	0.3943	0
4628	637.3	0.3808	0.5232	0.57	0.3945	0
4629	637.2	0.3809	0.5232	0.5701	0.3944	0
4630	637.1	0.3808	0.523	0.5697	0.3944	0

4631	637	0.3808	0.5229	0.5697	0.3942	0
4632	636.9	0.3807	0.5228	0.5696	0.3943	0
4633	636.8	0.3809	0.523	0.5697	0.3945	0
4634	636.7	0.3809	0.523	0.5697	0.3945	0
4635	636.6	0.3808	0.5229	0.5695	0.3945	0
4636	636.5	0.3805	0.5228	0.5695	0.3943	0
4637	636.4	0.3806	0.5228	0.5694	0.3943	0
4638	636.3	0.3808	0.5229	0.5697	0.3943	0
4639	636.2	0.3807	0.5231	0.5696	0.3941	0
4640	636.1	0.3808	0.5229	0.5695	0.3941	0
4641	636	0.3809	0.5229	0.5696	0.3942	0
4642	635.9	0.3809	0.5228	0.5697	0.3943	0
4643	635.8	0.3809	0.5231	0.5696	0.3944	0
4644	635.7	0.3808	0.5232	0.5697	0.3943	0
4645	635.6	0.3807	0.523	0.5694	0.3944	0
4646	635.5	0.3806	0.5229	0.5696	0.3944	0
4647	635.4	0.3809	0.5233	0.5696	0.3942	0
4648	635.3	0.3808	0.523	0.5695	0.3944	0
4649	635.2	0.3808	0.523	0.5695	0.3944	0
4650	635.1	0.3808	0.5231	0.5697	0.3945	0
4651	635	0.3808	0.5232	0.5695	0.3943	0
4652	634.9	0.3808	0.5231	0.5697	0.3943	0
4653	634.8	0.3809	0.5232	0.5697	0.3945	0
4654	634.7	0.381	0.5234	0.5697	0.3943	0
4655	634.6	0.3812	0.5234	0.5699	0.3949	0
4656	634.5	0.3812	0.5232	0.5697	0.3945	0
4657	634.4	0.3812	0.5233	0.5697	0.3945	0
4658	634.3	0.381	0.5234	0.5699	0.3945	0
4659	634.2	0.3811	0.5234	0.5698	0.3945	0
4660	634.1	0.3811	0.5235	0.5699	0.3945	0
4661	634	0.3811	0.5236	0.57	0.3948	0
4662	633.9	0.3811	0.5236	0.57	0.3947	0
4663	633.8	0.3813	0.5237	0.5703	0.3949	0
4664	633.7	0.381	0.5236	0.5701	0.3948	0
4665	633.6	0.3812	0.5237	0.5701	0.3949	0
4666	633.5	0.3814	0.5238	0.5703	0.3952	0
4667	633.4	0.3814	0.524	0.5703	0.395	0
4668	633.3	0.3813	0.5241	0.5702	0.3952	0
4669	633.2	0.3814	0.5239	0.5703	0.3952	0
4670	633.1	0.3815	0.5244	0.5705	0.395	0
4671	633	0.3815	0.5241	0.5707	0.395	0
4672	632.9	0.3815	0.524	0.5705	0.3951	0
4673	632.8	0.3815	0.524	0.5706	0.3951	0

4674	632.7	0.3817	0.5241	0.5704	0.3951	0
4675	632.6	0.3817	0.5242	0.5706	0.3952	0
4676	632.5	0.3818	0.5244	0.5706	0.3954	0
4677	632.4	0.3819	0.5247	0.5709	0.3955	0
4678	632.3	0.3819	0.5244	0.5706	0.3953	0
4679	632.2	0.3819	0.5244	0.5705	0.3954	0
4680	632.1	0.382	0.5242	0.5708	0.3953	0
4681	632	0.3818	0.5244	0.5708	0.3955	0
4682	631.9	0.3818	0.5245	0.5707	0.3954	0
4683	631.8	0.3819	0.5246	0.5708	0.3957	0
4684	631.7	0.3818	0.5246	0.5708	0.3958	0
4685	631.6	0.3819	0.5247	0.5707	0.3955	0
4686	631.5	0.3817	0.5246	0.5706	0.3954	0
4687	631.4	0.3817	0.5244	0.5708	0.3954	0
4688	631.3	0.3817	0.5243	0.5708	0.3953	0
4689	631.2	0.3817	0.5246	0.5707	0.3953	0
4690	631.1	0.3819	0.5246	0.571	0.3956	0
4691	631	0.3817	0.5246	0.571	0.3955	0
4692	630.9	0.3816	0.5246	0.5706	0.3955	0
4693	630.8	0.3818	0.5245	0.5707	0.3955	0
4694	630.7	0.382	0.5246	0.5707	0.3955	0
4695	630.6	0.3818	0.5244	0.5707	0.3956	0
4696	630.5	0.3818	0.5242	0.5705	0.3956	0
4697	630.4	0.3818	0.5242	0.5704	0.3955	0
4698	630.3	0.3817	0.524	0.5703	0.3953	0
4699	630.2	0.3817	0.5242	0.5703	0.3953	0
4700	630.1	0.3817	0.5244	0.5704	0.3954	0
4701	630	0.3817	0.5244	0.5704	0.3955	0
4702	629.9	0.3816	0.5246	0.5706	0.3955	0
4703	629.8	0.3815	0.5244	0.5705	0.3954	0
4704	629.7	0.3817	0.5243	0.5705	0.3954	0
4705	629.6	0.3819	0.5244	0.5708	0.3955	0
4706	629.5	0.3817	0.5244	0.5707	0.3954	0
4707	629.4	0.3817	0.5244	0.5706	0.3954	0
4708	629.3	0.3817	0.5245	0.5706	0.3955	0
4709	629.2	0.3818	0.5245	0.5706	0.3955	0
4710	629.1	0.3817	0.5243	0.5705	0.3956	0
4711	629	0.3815	0.5241	0.5704	0.3954	0
4712	628.9	0.3817	0.5242	0.5703	0.3954	0
4713	628.8	0.3817	0.5241	0.5703	0.3953	0
4714	628.7	0.3816	0.5241	0.5705	0.3954	0
4715	628.6	0.3814	0.5241	0.5702	0.3951	0
4716	628.5	0.3816	0.5244	0.5705	0.3953	0

4717	628.4	0.3816	0.5241	0.5704	0.3953	0
4718	628.3	0.3818	0.5245	0.5706	0.3955	0
4719	628.2	0.3814	0.5243	0.5705	0.3952	0
4720	628.1	0.3815	0.5245	0.5705	0.3954	0
4721	628	0.3818	0.5244	0.5704	0.3954	0
4722	627.9	0.3816	0.5242	0.5704	0.3953	0
4723	627.8	0.3814	0.5243	0.5704	0.3952	0
4724	627.7	0.3814	0.5242	0.5705	0.3955	0
4725	627.6	0.3816	0.5244	0.5707	0.3955	0
4726	627.5	0.3815	0.5247	0.5707	0.3955	0
4727	627.4	0.3816	0.5246	0.5708	0.3954	0
4728	627.3	0.3816	0.5245	0.5706	0.3954	0
4729	627.2	0.3817	0.5245	0.5707	0.3953	0
4730	627.1	0.3817	0.5245	0.5706	0.3955	0
4731	627	0.3817	0.5245	0.5704	0.3956	0
4732	626.9	0.3817	0.5245	0.5705	0.3956	0
4733	626.8	0.3817	0.5246	0.5708	0.3956	0
4734	626.7	0.3816	0.5244	0.5705	0.3955	0
4735	626.6	0.3819	0.5245	0.5708	0.3958	0
4736	626.5	0.3819	0.5245	0.5708	0.3957	0
4737	626.4	0.3818	0.5248	0.5707	0.3955	0
4738	626.3	0.3817	0.5248	0.5708	0.3956	0
4739	626.2	0.382	0.5247	0.5712	0.3957	0
4740	626.1	0.3817	0.5246	0.571	0.3957	0
4741	626	0.3819	0.5247	0.5712	0.3956	0
4742	625.9	0.3818	0.5243	0.5711	0.3954	0
4743	625.8	0.3819	0.5245	0.5709	0.3955	0
4744	625.7	0.382	0.5247	0.571	0.3955	0
4745	625.6	0.3821	0.5248	0.5709	0.3956	0
4746	625.5	0.382	0.5248	0.5711	0.3958	0
4747	625.4	0.382	0.5249	0.5714	0.3959	0
4748	625.3	0.3822	0.5246	0.5712	0.3959	0
4749	625.2	0.3821	0.5249	0.5714	0.396	0
4750	625.1	0.3821	0.525	0.5714	0.3959	0
4751	625	0.3821	0.5251	0.5716	0.3963	0
4752	624.9	0.382	0.5251	0.5715	0.3961	0
4753	624.8	0.3822	0.5254	0.5715	0.396	0
4754	624.7	0.3823	0.5255	0.5715	0.3962	0
4755	624.6	0.3823	0.5256	0.5717	0.3963	0
4756	624.5	0.3824	0.5257	0.5717	0.3964	0
4757	624.4	0.3824	0.5255	0.5718	0.3964	0
4758	624.3	0.3826	0.5253	0.5717	0.3964	0
4759	624.2	0.3825	0.5255	0.5718	0.3964	0

4760	624.1	0.3826	0.5256	0.5722	0.3967	0
4761	624	0.3827	0.5258	0.5721	0.3968	0
4762	623.9	0.3826	0.5258	0.5719	0.3968	0
4763	623.8	0.3827	0.5258	0.572	0.3969	0
4764	623.7	0.3827	0.5258	0.5721	0.3968	0
4765	623.6	0.3829	0.5258	0.5721	0.3968	0
4766	623.5	0.3826	0.5259	0.5723	0.3966	0
4767	623.4	0.3825	0.5259	0.5722	0.3966	0
4768	623.3	0.3829	0.5258	0.5719	0.3967	0
4769	623.2	0.3828	0.526	0.5721	0.3968	0
4770	623.1	0.3831	0.5261	0.5722	0.3968	0
4771	623	0.3825	0.5261	0.5721	0.3967	0
4772	622.9	0.3826	0.526	0.5721	0.3968	0
4773	622.8	0.3827	0.5261	0.5724	0.3967	0
4774	622.7	0.3827	0.5259	0.5723	0.3968	0
4775	622.6	0.3828	0.526	0.5722	0.3969	0
4776	622.5	0.3827	0.526	0.5722	0.3965	0
4777	622.4	0.3828	0.5263	0.5723	0.3967	0
4778	622.3	0.3828	0.526	0.5724	0.397	0
4779	622.2	0.3827	0.5261	0.5722	0.3969	0
4780	622.1	0.3829	0.5263	0.5726	0.3969	0
4781	622	0.3827	0.5261	0.5724	0.3968	0
4782	621.9	0.3828	0.5263	0.5726	0.3969	0
4783	621.8	0.3827	0.5259	0.5726	0.3969	0
4784	621.7	0.3828	0.5259	0.5722	0.3968	0
4785	621.6	0.3827	0.5258	0.5723	0.3967	0
4786	621.5	0.3826	0.5261	0.5725	0.3969	0
4787	621.4	0.3827	0.526	0.5725	0.3969	0
4788	621.3	0.3827	0.5262	0.5724	0.3968	0
4789	621.2	0.3826	0.526	0.5722	0.3964	0
4790	621.1	0.3828	0.5261	0.5723	0.3968	0
4791	621	0.3826	0.5259	0.5722	0.3966	0
4792	620.9	0.3824	0.5259	0.5724	0.3967	0
4793	620.8	0.3825	0.5257	0.5723	0.3965	0
4794	620.7	0.3824	0.5258	0.5723	0.3966	0
4795	620.6	0.3825	0.5258	0.5722	0.3968	0
4796	620.5	0.3824	0.5255	0.5721	0.3963	0
4797	620.4	0.3819	0.5254	0.5721	0.3962	0
4798	620.3	0.3822	0.5255	0.5721	0.3964	0
4799	620.2	0.3823	0.5257	0.5722	0.3964	0
4800	620.1	0.3823	0.5258	0.5723	0.3965	0
4801	620	0.3823	0.5256	0.5722	0.3966	0
4802	619.9	0.3823	0.5253	0.5722	0.3964	0

4803	619.8	0.3818	0.5251	0.5719	0.3962	0
4804	619.7	0.3818	0.5249	0.572	0.3962	0
4805	619.6	0.3818	0.5249	0.5721	0.3962	0
4806	619.5	0.3818	0.5251	0.5719	0.3961	0
4807	619.4	0.3821	0.5249	0.572	0.3962	0
4808	619.3	0.3817	0.5249	0.5719	0.3961	0
4809	619.2	0.3818	0.5251	0.5718	0.3961	0
4810	619.1	0.3816	0.5247	0.5719	0.3959	0
4811	619	0.3814	0.5247	0.5717	0.3957	0
4812	618.9	0.3815	0.5247	0.5718	0.396	0
4813	618.8	0.3812	0.5244	0.5716	0.3955	0
4814	618.7	0.3811	0.5243	0.5713	0.3952	0
4815	618.6	0.3812	0.5244	0.5715	0.3954	0
4816	618.5	0.3811	0.5245	0.5714	0.3953	0
4817	618.4	0.381	0.5242	0.5715	0.3951	0
4818	618.3	0.381	0.5241	0.5715	0.3953	0
4819	618.2	0.381	0.5242	0.5714	0.3952	0
4820	618.1	0.3809	0.5242	0.5714	0.3951	0
4821	618	0.3809	0.5241	0.5712	0.395	0
4822	617.9	0.3807	0.5238	0.5712	0.395	0
4823	617.8	0.3808	0.5238	0.5713	0.3948	0
4824	617.7	0.3809	0.524	0.5713	0.3949	0
4825	617.6	0.3808	0.5238	0.5714	0.3948	0
4826	617.5	0.3809	0.5238	0.5714	0.3947	0
4827	617.4	0.3809	0.5238	0.5716	0.3948	0
4828	617.3	0.3808	0.5238	0.5714	0.3947	0
4829	617.2	0.381	0.5241	0.5715	0.3949	0
4830	617.1	0.3807	0.524	0.5716	0.3947	0
4831	617	0.3806	0.5237	0.5714	0.3948	0
4832	616.9	0.3806	0.5238	0.5714	0.3949	0
4833	616.8	0.3808	0.524	0.5716	0.3949	0
4834	616.7	0.3809	0.524	0.5715	0.3951	0
4835	616.6	0.3809	0.5241	0.5717	0.3952	0
4836	616.5	0.3811	0.524	0.5714	0.3951	0
4837	616.4	0.3809	0.5239	0.5715	0.3951	0
4838	616.3	0.3809	0.524	0.5714	0.3951	0
4839	616.2	0.3813	0.5243	0.5718	0.3951	0
4840	616.1	0.3813	0.5243	0.5719	0.3953	0
4841	616	0.3812	0.5241	0.572	0.3953	0
4842	615.9	0.3814	0.5242	0.572	0.3951	0
4843	615.8	0.3811	0.5242	0.572	0.3952	0
4844	615.7	0.381	0.5241	0.5719	0.3952	0
4845	615.6	0.3812	0.5243	0.5721	0.3954	0

4846	615.5	0.3813	0.5242	0.572	0.3952	0
4847	615.4	0.3813	0.5243	0.5724	0.3954	0
4848	615.3	0.3814	0.5244	0.5722	0.3955	0
4849	615.2	0.3812	0.5246	0.5721	0.3957	0
4850	615.1	0.3812	0.5245	0.5722	0.3954	0
4851	615	0.3813	0.5247	0.5723	0.3954	0
4852	614.9	0.3814	0.5249	0.5724	0.3956	0
4853	614.8	0.3816	0.5249	0.5726	0.3956	0
4854	614.7	0.3819	0.525	0.5729	0.3959	0
4855	614.6	0.3818	0.525	0.5728	0.3958	0
4856	614.5	0.3817	0.525	0.573	0.3959	0
4857	614.4	0.3819	0.5251	0.5731	0.3961	0
4858	614.3	0.3819	0.5251	0.573	0.3961	0
4859	614.2	0.3818	0.5254	0.5731	0.3962	0
4860	614.1	0.3817	0.5252	0.5731	0.3961	0
4861	614	0.3817	0.5252	0.5732	0.3964	0
4862	613.9	0.3817	0.5254	0.5731	0.3964	0
4863	613.8	0.3816	0.5254	0.5732	0.3961	0
4864	613.7	0.3819	0.5255	0.5733	0.3962	0
4865	613.6	0.3819	0.5257	0.5733	0.3964	0
4866	613.5	0.382	0.5257	0.5733	0.3965	0
4867	613.4	0.3821	0.526	0.5739	0.3966	0
4868	613.3	0.3821	0.5259	0.5736	0.3967	0
4869	613.2	0.3821	0.5257	0.5736	0.3968	0
4870	613.1	0.3823	0.526	0.5738	0.3969	0
4871	613	0.3821	0.5256	0.5739	0.3969	0
4872	612.9	0.3822	0.5257	0.5738	0.3967	0
4873	612.8	0.3824	0.5256	0.5738	0.3967	0
4874	612.7	0.3821	0.5258	0.574	0.3967	0
4875	612.6	0.3823	0.5259	0.5739	0.3968	0
4876	612.5	0.3821	0.5257	0.574	0.3967	0
4877	612.4	0.3825	0.5259	0.5744	0.3968	0
4878	612.3	0.3825	0.526	0.5741	0.3971	0
4879	612.2	0.3824	0.5258	0.5741	0.3968	0
4880	612.1	0.3824	0.526	0.5741	0.3968	0
4881	612	0.3823	0.526	0.574	0.3969	0
4882	611.9	0.3826	0.5259	0.5742	0.3969	0
4883	611.8	0.3825	0.526	0.5743	0.397	0
4884	611.7	0.3822	0.5259	0.5741	0.3969	0
4885	611.6	0.3822	0.5256	0.5739	0.3967	0
4886	611.5	0.3822	0.5254	0.5738	0.3963	0
4887	611.4	0.3825	0.5256	0.574	0.3968	0
4888	611.3	0.3823	0.5255	0.5741	0.3969	0

4889	611.2	0.3822	0.5255	0.5739	0.3968	0
4890	611.1	0.3821	0.5254	0.5739	0.3964	0
4891	611	0.382	0.5253	0.5738	0.3964	0
4892	610.9	0.3822	0.5254	0.5738	0.3965	0
4893	610.8	0.382	0.5253	0.5738	0.3964	0
4894	610.7	0.3819	0.5252	0.5738	0.3963	0
4895	610.6	0.3819	0.5251	0.5738	0.3963	0
4896	610.5	0.3819	0.525	0.574	0.3962	0
4897	610.4	0.3819	0.5252	0.574	0.3964	0
4898	610.3	0.3814	0.525	0.5738	0.3963	0
4899	610.2	0.3817	0.5253	0.5738	0.3963	0
4900	610.1	0.3817	0.5254	0.5739	0.3962	0
4901	610	0.3816	0.5252	0.5739	0.3962	0
4902	609.9	0.3815	0.5249	0.5738	0.3958	0
4903	609.8	0.3815	0.5249	0.5738	0.396	0
4904	609.7	0.3813	0.5247	0.5738	0.3959	0
4905	609.6	0.3812	0.5245	0.5737	0.3958	0
4906	609.5	0.3811	0.5245	0.5737	0.3957	0
4907	609.4	0.381	0.5245	0.5732	0.3954	0
4908	609.3	0.3807	0.5243	0.5732	0.3952	0
4909	609.2	0.3808	0.5241	0.5732	0.3952	0
4910	609.1	0.3809	0.524	0.5735	0.3953	0
4911	609	0.3808	0.524	0.5735	0.3949	0
4912	608.9	0.3807	0.524	0.5733	0.3951	0
4913	608.8	0.3807	0.524	0.573	0.395	0
4914	608.7	0.3807	0.5239	0.5732	0.395	0
4915	608.6	0.3807	0.5236	0.5732	0.3949	0
4916	608.5	0.3805	0.5234	0.5731	0.3945	0
4917	608.4	0.3803	0.5233	0.573	0.3947	0
4918	608.3	0.38	0.5234	0.5729	0.3946	0
4919	608.2	0.3801	0.5232	0.5729	0.3945	0
4920	608.1	0.3801	0.523	0.5728	0.3945	0
4921	608	0.3799	0.5229	0.5726	0.3945	0
4922	607.9	0.3797	0.5227	0.5723	0.3941	0
4923	607.8	0.3796	0.5224	0.5724	0.3938	0
4924	607.7	0.3798	0.5227	0.5727	0.3941	0
4925	607.6	0.3798	0.5225	0.5726	0.3939	0
4926	607.5	0.3797	0.5223	0.5724	0.394	0
4927	607.4	0.3796	0.5224	0.5724	0.394	0
4928	607.3	0.3797	0.5224	0.5725	0.3939	0
4929	607.2	0.3798	0.5224	0.5726	0.394	0
4930	607.1	0.3795	0.5222	0.5725	0.3938	0
4931	607	0.3792	0.5221	0.5723	0.3937	0

4932	606.9	0.3791	0.5223	0.5723	0.3935	0
4933	606.8	0.3792	0.5223	0.5724	0.3937	0
4934	606.7	0.379	0.5223	0.5722	0.3935	0
4935	606.6	0.3791	0.5222	0.5725	0.3938	0
4936	606.5	0.3791	0.5222	0.5725	0.3936	0
4937	606.4	0.3793	0.5221	0.5724	0.3935	0
4938	606.3	0.3792	0.5221	0.5724	0.3935	0
4939	606.2	0.3794	0.5222	0.5726	0.3938	0
4940	606.1	0.3792	0.5221	0.5725	0.3936	0
4941	606	0.3791	0.5222	0.5723	0.3933	0
4942	605.9	0.3792	0.5223	0.5724	0.3935	0
4943	605.8	0.3792	0.5221	0.5725	0.3934	0
4944	605.7	0.3789	0.522	0.5726	0.3934	0
4945	605.6	0.3791	0.5219	0.5727	0.3936	0
4946	605.5	0.3789	0.522	0.5725	0.3935	0
4947	605.4	0.379	0.5221	0.5727	0.3933	0
4948	605.3	0.379	0.5219	0.5728	0.3935	0
4949	605.2	0.3791	0.522	0.5729	0.3934	0
4950	605.1	0.379	0.5218	0.5729	0.3935	0
4951	605	0.3792	0.5219	0.5731	0.3938	0
4952	604.9	0.3791	0.5221	0.573	0.3937	0
4953	604.8	0.3793	0.5222	0.5732	0.3937	0
4954	604.7	0.3793	0.5225	0.5731	0.3937	0
4955	604.6	0.3797	0.5226	0.5732	0.394	0
4956	604.5	0.3798	0.5226	0.5733	0.3942	0
4957	604.4	0.3798	0.5227	0.5737	0.3943	0
4958	604.3	0.3796	0.5225	0.5734	0.3943	0
4959	604.2	0.3798	0.5226	0.5736	0.3942	0
4960	604.1	0.3797	0.5226	0.5737	0.3942	0
4961	604	0.3798	0.5226	0.5737	0.3942	0
4962	603.9	0.3801	0.5229	0.5737	0.3942	0
4963	603.8	0.38	0.523	0.5738	0.3944	0
4964	603.7	0.38	0.5231	0.574	0.3944	0
4965	603.6	0.38	0.5232	0.5739	0.3946	0
4966	603.5	0.3804	0.5231	0.5742	0.3947	0
4967	603.4	0.3804	0.5233	0.5744	0.3949	0
4968	603.3	0.3802	0.5234	0.5744	0.3949	0
4969	603.2	0.3807	0.5236	0.5746	0.3952	0
4970	603.1	0.3809	0.5237	0.5747	0.3954	0
4971	603	0.3807	0.5237	0.5746	0.3953	0
4972	602.9	0.3805	0.5236	0.5744	0.3951	0
4973	602.8	0.3807	0.5239	0.5747	0.3953	0
4974	602.7	0.3806	0.5241	0.5747	0.3953	0

4975	602.6	0.3807	0.5241	0.575	0.3954	0
4976	602.5	0.3808	0.5239	0.5749	0.3955	0
4977	602.4	0.3807	0.5237	0.575	0.3955	0
4978	602.3	0.3806	0.5234	0.5749	0.3953	0
4979	602.2	0.3806	0.5236	0.5749	0.3952	0
4980	602.1	0.3807	0.5237	0.5751	0.3954	0
4981	602	0.3808	0.5239	0.5751	0.3955	0
4982	601.9	0.3808	0.5243	0.5753	0.3954	0
4983	601.8	0.3809	0.5239	0.5753	0.3952	0
4984	601.7	0.3809	0.524	0.5753	0.3955	0
4985	601.6	0.3811	0.5241	0.5754	0.3956	0
4986	601.5	0.3809	0.5237	0.5752	0.3954	0
4987	601.4	0.3809	0.5238	0.5753	0.3954	0
4988	601.3	0.3809	0.5239	0.5754	0.3954	0
4989	601.2	0.3809	0.5238	0.5754	0.3955	0
4990	601.1	0.381	0.5239	0.5755	0.3956	0
4991	601	0.3808	0.5238	0.5754	0.3956	0
4992	600.9	0.3811	0.5237	0.5756	0.3957	0
4993	600.8	0.381	0.5241	0.5756	0.3958	0
4994	600.7	0.3807	0.5237	0.5754	0.3958	0
4995	600.6	0.3807	0.5238	0.5754	0.3957	0
4996	600.5	0.3808	0.5237	0.5756	0.3956	0
4997	600.4	0.3808	0.5238	0.5756	0.3956	0
4998	600.3	0.3807	0.5235	0.5754	0.3953	0
4999	600.2	0.3809	0.5238	0.5757	0.3955	0
5000	600.1	0.3808	0.5237	0.5757	0.3956	0
5001	600	0.3807	0.5235	0.5755	0.3956	0
5002	599.9	0.3807	0.5233	0.5755	0.3953	0
5003	599.8	0.3806	0.5235	0.5756	0.3956	0
5004	599.7	0.3803	0.5233	0.5754	0.3953	0
5005	599.6	0.3803	0.5233	0.5753	0.3952	0
5006	599.5	0.38	0.5232	0.5751	0.3951	0
5007	599.4	0.3802	0.5234	0.5751	0.3952	0
5008	599.3	0.3802	0.5234	0.5754	0.3951	0
5009	599.2	0.3802	0.5232	0.5754	0.3952	0
5010	599.1	0.3801	0.523	0.5754	0.3949	0
5011	599	0.3798	0.5228	0.5752	0.3947	0
5012	598.9	0.38	0.5229	0.5752	0.3947	0
5013	598.8	0.3798	0.5227	0.575	0.3945	0
5014	598.7	0.3798	0.5227	0.5753	0.3945	0
5015	598.6	0.3799	0.5226	0.5752	0.3946	0
5016	598.5	0.3798	0.5226	0.5751	0.3944	0
5017	598.4	0.3797	0.5224	0.5751	0.3943	0

5018	598.3	0.3795	0.5222	0.575	0.3942	0
5019	598.2	0.3796	0.5223	0.5751	0.394	0
5020	598.1	0.3796	0.5221	0.5751	0.3943	0
5021	598	0.3792	0.522	0.5746	0.3938	0
5022	597.9	0.3793	0.5218	0.5747	0.3936	0
5023	597.8	0.3791	0.5217	0.5746	0.3936	0
5024	597.7	0.379	0.5216	0.5746	0.3937	0
5025	597.6	0.379	0.5217	0.5746	0.3936	0
5026	597.5	0.3788	0.5216	0.5745	0.3937	0
5027	597.4	0.3788	0.5214	0.5746	0.3935	0
5028	597.3	0.3788	0.5213	0.5745	0.3934	0
5029	597.2	0.3788	0.521	0.5744	0.3932	0
5030	597.1	0.3788	0.5208	0.5745	0.3932	0
5031	597	0.3786	0.521	0.5744	0.3931	0
5032	596.9	0.3786	0.5211	0.5745	0.3931	0
5033	596.8	0.3786	0.521	0.5744	0.3932	0
5034	596.7	0.3784	0.521	0.5744	0.3929	0
5035	596.6	0.3786	0.5213	0.5747	0.3929	0
5036	596.5	0.3786	0.5211	0.5744	0.3929	0
5037	596.4	0.3785	0.5209	0.5745	0.3928	0
5038	596.3	0.3784	0.5209	0.5745	0.3928	0
5039	596.2	0.3785	0.5209	0.5744	0.3931	0
5040	596.1	0.3785	0.521	0.5746	0.3932	0
5041	596	0.3784	0.5209	0.5747	0.3934	0
5042	595.9	0.3783	0.5209	0.5748	0.3932	0
5043	595.8	0.3785	0.5209	0.5749	0.3932	0
5044	595.7	0.3784	0.5207	0.5747	0.3931	0
5045	595.6	0.3785	0.5207	0.5749	0.3934	0
5046	595.5	0.3785	0.5206	0.5747	0.3932	0
5047	595.4	0.3785	0.5207	0.5748	0.3931	0
5048	595.3	0.3787	0.5212	0.575	0.3931	0
5049	595.2	0.3788	0.521	0.575	0.3931	0
5050	595.1	0.3787	0.5209	0.575	0.3931	0
5051	595	0.3788	0.5209	0.5749	0.3933	0
5052	594.9	0.3787	0.5209	0.5751	0.3933	0
5053	594.8	0.3787	0.5211	0.5751	0.3933	0
5054	594.7	0.3789	0.5212	0.5753	0.3934	0
5055	594.6	0.3788	0.5212	0.5756	0.3936	0
5056	594.5	0.3787	0.521	0.5755	0.3933	0
5057	594.4	0.3788	0.521	0.5756	0.3932	0
5058	594.3	0.3791	0.5212	0.576	0.3936	0
5059	594.2	0.379	0.5211	0.5759	0.3935	0
5060	594.1	0.3792	0.5214	0.5762	0.3936	0

5061	594	0.3792	0.5215	0.576	0.3937	0
5062	593.9	0.3794	0.5217	0.5764	0.394	0
5063	593.8	0.3793	0.5218	0.5762	0.3941	0
5064	593.7	0.3794	0.5219	0.5765	0.3944	0
5065	593.6	0.3796	0.5219	0.5766	0.3942	0
5066	593.5	0.3797	0.5218	0.5767	0.3942	0
5067	593.4	0.3797	0.5222	0.5764	0.3944	0
5068	593.3	0.3798	0.5222	0.5766	0.3945	0
5069	593.2	0.3801	0.5222	0.577	0.3946	0
5070	593.1	0.3803	0.5227	0.577	0.3948	0
5071	593	0.3802	0.5228	0.5769	0.3945	0
5072	592.9	0.3801	0.5227	0.5772	0.3947	0
5073	592.8	0.3801	0.5227	0.5772	0.3947	0
5074	592.7	0.3805	0.523	0.5772	0.3948	0
5075	592.6	0.3804	0.5233	0.5777	0.3951	0
5076	592.5	0.3804	0.5232	0.5775	0.3953	0
5077	592.4	0.3806	0.5231	0.5776	0.3954	0
5078	592.3	0.3807	0.5233	0.5776	0.3953	0
5079	592.2	0.3805	0.5232	0.5776	0.3952	0
5080	592.1	0.3806	0.5231	0.5776	0.3951	0
5081	592	0.3808	0.5234	0.5778	0.395	0
5082	591.9	0.3807	0.5235	0.5779	0.3952	0
5083	591.8	0.3808	0.5236	0.5782	0.3953	0
5084	591.7	0.3809	0.5237	0.5781	0.3953	0
5085	591.6	0.3808	0.5236	0.5784	0.3954	0
5086	591.5	0.3807	0.5233	0.5782	0.3954	0
5087	591.4	0.381	0.5236	0.5787	0.3957	0
5088	591.3	0.3811	0.5236	0.5783	0.3958	0
5089	591.2	0.3808	0.5235	0.5784	0.3954	0
5090	591.1	0.381	0.5234	0.5785	0.3956	0
5091	591	0.3812	0.524	0.5788	0.3959	0
5092	590.9	0.381	0.524	0.5788	0.3957	0
5093	590.8	0.3811	0.5238	0.5788	0.3957	0
5094	590.7	0.3811	0.5238	0.5789	0.3958	0
5095	590.6	0.3811	0.5238	0.579	0.396	0
5096	590.5	0.3812	0.524	0.5792	0.3958	0
5097	590.4	0.3813	0.5241	0.5792	0.3958	0
5098	590.3	0.3813	0.5239	0.5791	0.3959	0
5099	590.2	0.3814	0.5239	0.5792	0.3961	0
5100	590.1	0.3811	0.524	0.5793	0.3962	0
5101	590	0.3811	0.524	0.5791	0.3958	0
5102	589.9	0.3812	0.524	0.5792	0.3958	0
5103	589.8	0.3813	0.5239	0.5794	0.3959	0

5104	589.7	0.3813	0.5238	0.5794	0.3958	0
5105	589.6	0.3812	0.5236	0.5793	0.3958	0
5106	589.5	0.3816	0.524	0.5797	0.3962	0
5107	589.4	0.3813	0.5239	0.5793	0.396	0
5108	589.3	0.3813	0.5236	0.5795	0.3958	0
5109	589.2	0.3813	0.5236	0.5796	0.3959	0
5110	589.1	0.3814	0.5237	0.5799	0.3959	0
5111	589	0.3811	0.5237	0.5797	0.3958	0
5112	588.9	0.3813	0.5239	0.5798	0.3959	0
5113	588.8	0.3811	0.5236	0.5796	0.3958	0
5114	588.7	0.3811	0.5235	0.5797	0.3957	0
5115	588.6	0.3811	0.5237	0.5799	0.396	0
5116	588.5	0.3812	0.5236	0.5799	0.3959	0
5117	588.4	0.3806	0.5233	0.5796	0.3955	0
5118	588.3	0.3809	0.5231	0.5797	0.3955	0
5119	588.2	0.381	0.5236	0.5797	0.3954	0
5120	588.1	0.3809	0.5235	0.5797	0.3957	0
5121	588	0.3808	0.5232	0.5794	0.3953	0
5122	587.9	0.3805	0.5232	0.5797	0.3952	0
5123	587.8	0.3805	0.5232	0.5796	0.3952	0
5124	587.7	0.3804	0.5232	0.5796	0.3952	0
5125	587.6	0.3806	0.5231	0.5798	0.3952	0
5126	587.5	0.3806	0.5232	0.5799	0.3954	0
5127	587.4	0.3804	0.5232	0.5798	0.3951	0
5128	587.3	0.3804	0.523	0.5798	0.3951	0
5129	587.2	0.3804	0.523	0.5796	0.3953	0
5130	587.1	0.3803	0.523	0.5795	0.3951	0
5131	587	0.3803	0.5228	0.5798	0.3951	0
5132	586.9	0.3804	0.5229	0.5797	0.395	0
5133	586.8	0.3802	0.523	0.5798	0.395	0
5134	586.7	0.3802	0.523	0.5798	0.3951	0
5135	586.6	0.3804	0.5231	0.5799	0.3951	0
5136	586.5	0.3803	0.5231	0.58	0.3951	0
5137	586.4	0.38	0.5227	0.5801	0.3949	0
5138	586.3	0.3801	0.5225	0.5798	0.3949	0
5139	586.2	0.3803	0.5227	0.58	0.3951	0
5140	586.1	0.3804	0.5229	0.5803	0.3954	0
5141	586	0.3801	0.5228	0.58	0.3953	0
5142	585.9	0.3801	0.5226	0.58	0.3953	0
5143	585.8	0.3804	0.5224	0.5801	0.3953	0
5144	585.7	0.3804	0.5227	0.5802	0.3954	0
5145	585.6	0.3801	0.5227	0.5799	0.395	0
5146	585.5	0.3804	0.5231	0.5803	0.395	0

5147	585.4	0.3804	0.5228	0.5802	0.3949	0
5148	585.3	0.3803	0.5227	0.5803	0.3948	0
5149	585.2	0.3803	0.523	0.5805	0.3951	0
5150	585.1	0.3803	0.5231	0.5808	0.3952	0
5151	585	0.3808	0.5232	0.5809	0.3952	0
5152	584.9	0.3987	0.5464	0.6023	0.4147	0
5153	584.8	0.3988	0.5467	0.6024	0.4146	0
5154	584.7	0.3989	0.5465	0.6026	0.4147	0
5155	584.6	0.3988	0.5468	0.6027	0.4148	0
5156	584.5	0.399	0.5467	0.6027	0.4148	0
5157	584.4	0.3993	0.5469	0.6032	0.4152	0
5158	584.3	0.3991	0.5468	0.6032	0.4152	0
5159	584.2	0.3993	0.5469	0.6034	0.4153	0
5160	584.1	0.3993	0.5468	0.6032	0.4155	0
5161	584	0.3994	0.5472	0.6034	0.4155	0
5162	583.9	0.3994	0.5475	0.6038	0.4158	0
5163	583.8	0.3997	0.5476	0.6037	0.4159	0
5164	583.7	0.3998	0.5475	0.604	0.4159	0
5165	583.6	0.3998	0.548	0.6041	0.416	0
5166	583.5	0.3999	0.5482	0.6045	0.416	0
5167	583.4	0.4	0.548	0.6042	0.4162	0
5168	583.3	0.4001	0.5484	0.6046	0.4162	0
5169	583.2	0.4001	0.5485	0.6046	0.4164	0
5170	583.1	0.4003	0.5488	0.6049	0.4166	0
5171	583	0.4005	0.5489	0.6049	0.4165	0
5172	582.9	0.4005	0.549	0.6052	0.4168	0
5173	582.8	0.4004	0.5491	0.6053	0.4167	0
5174	582.7	0.4007	0.5493	0.6058	0.417	0
5175	582.6	0.4007	0.5493	0.6058	0.4171	0
5176	582.5	0.4011	0.5496	0.6058	0.4171	0
5177	582.4	0.4011	0.5498	0.606	0.417	0
5178	582.3	0.4011	0.5496	0.6061	0.4171	0
5179	582.2	0.4011	0.5496	0.6064	0.4172	0
5180	582.1	0.401	0.5497	0.6067	0.4172	0
5181	582	0.4012	0.5501	0.6066	0.4173	0
5182	581.9	0.4012	0.5502	0.6068	0.4172	0
5183	581.8	0.4011	0.5502	0.6068	0.4175	0
5184	581.7	0.4011	0.5503	0.6071	0.4176	0
5185	581.6	0.4013	0.5504	0.6076	0.4178	0
5186	581.5	0.4014	0.5502	0.6075	0.4177	0
5187	581.4	0.4014	0.5504	0.6073	0.4178	0
5188	581.3	0.4015	0.5505	0.6076	0.418	0
5189	581.2	0.4018	0.5505	0.6078	0.418	0

5190	581.1	0.4018	0.5503	0.6076	0.4178	0
5191	581	0.4017	0.5504	0.6078	0.4177	0
5192	580.9	0.4019	0.5509	0.6078	0.418	0
5193	580.8	0.4017	0.551	0.608	0.4177	0
5194	580.7	0.402	0.551	0.6081	0.4177	0
5195	580.6	0.4019	0.5507	0.6082	0.4179	0
5196	580.5	0.4018	0.5511	0.6085	0.4178	0
5197	580.4	0.4019	0.5513	0.6082	0.418	0
5198	580.3	0.4019	0.5509	0.6083	0.4177	0
5199	580.2	0.4018	0.5509	0.6084	0.4182	0
5200	580.1	0.4017	0.5508	0.6087	0.4182	0
5201	580	0.4018	0.5511	0.6089	0.4181	0
5202	579.9	0.4015	0.5509	0.6085	0.418	0
5203	579.8	0.4017	0.5509	0.6085	0.418	0
5204	579.7	0.4018	0.5508	0.6087	0.418	0
5205	579.6	0.402	0.551	0.6089	0.4181	0
5206	579.5	0.4021	0.5514	0.6091	0.4183	0
5207	579.4	0.402	0.5512	0.6091	0.4183	0
5208	579.3	0.402	0.5511	0.6092	0.4178	0
5209	579.2	0.4022	0.5511	0.6094	0.4182	0
5210	579.1	0.4022	0.5511	0.6094	0.4182	0
5211	579	0.4021	0.5511	0.6094	0.4179	0
5212	578.9	0.4019	0.551	0.6095	0.4178	0
5213	578.8	0.4018	0.551	0.6093	0.418	0
5214	578.7	0.402	0.551	0.6095	0.4178	0
5215	578.6	0.402	0.551	0.6095	0.4177	0
5216	578.5	0.4022	0.5511	0.6098	0.418	0
5217	578.4	0.4019	0.5511	0.6098	0.418	0
5218	578.3	0.4019	0.551	0.6099	0.418	0
5219	578.2	0.4017	0.5509	0.6097	0.418	0
5220	578.1	0.4019	0.5511	0.6099	0.4181	0
5221	578	0.4018	0.5512	0.6099	0.4181	0
5222	577.9	0.4018	0.5509	0.61	0.4179	0
5223	577.8	0.4018	0.5509	0.6102	0.4181	0
5224	577.7	0.4019	0.551	0.6102	0.418	0
5225	577.6	0.4017	0.551	0.6101	0.4178	0
5226	577.5	0.4016	0.5512	0.6104	0.4178	0
5227	577.4	0.4019	0.5512	0.6105	0.4179	0
5228	577.3	0.4019	0.5514	0.6102	0.4178	0
5229	577.2	0.402	0.5512	0.6105	0.418	0
5230	577.1	0.4021	0.5511	0.6104	0.4181	0
5231	577	0.4021	0.5511	0.6106	0.4181	0
5232	576.9	0.402	0.551	0.6106	0.418	0

5233	576.8	0.4022	0.5512	0.6107	0.4181	0
5234	576.7	0.402	0.5512	0.6108	0.4181	0
5235	576.6	0.4017	0.551	0.6108	0.418	0
5236	576.5	0.4019	0.551	0.6109	0.4181	0
5237	576.4	0.4016	0.5512	0.6109	0.4181	0
5238	576.3	0.4018	0.5511	0.6112	0.418	0
5239	576.2	0.4019	0.5513	0.6113	0.4183	0
5240	576.1	0.4019	0.5512	0.6115	0.418	0
5241	576	0.4018	0.5511	0.6113	0.4182	0
5242	575.9	0.4018	0.551	0.6113	0.4181	0
5243	575.8	0.4018	0.5509	0.6116	0.4181	0
5244	575.7	0.4019	0.5511	0.6115	0.418	0
5245	575.6	0.4018	0.5511	0.6113	0.4182	0
5246	575.5	0.4017	0.5514	0.6118	0.4183	0
5247	575.4	0.4017	0.5514	0.6118	0.4182	0
5248	575.3	0.4019	0.5515	0.612	0.4184	0
5249	575.2	0.4017	0.5517	0.612	0.4183	0
5250	575.1	0.4021	0.5515	0.612	0.4183	0
5251	575	0.4021	0.5517	0.6121	0.4183	0
5252	574.9	0.4022	0.5516	0.6121	0.4182	0
5253	574.8	0.4022	0.5519	0.6121	0.4184	0
5254	574.7	0.4024	0.5519	0.6123	0.4185	0
5255	574.6	0.4022	0.5517	0.6123	0.4187	0
5256	574.5	0.4024	0.552	0.6125	0.4183	0
5257	574.4	0.402	0.5515	0.6126	0.418	0
5258	574.3	0.4021	0.5516	0.6127	0.4182	0
5259	574.2	0.4024	0.552	0.6131	0.4187	0
5260	574.1	0.4024	0.5523	0.6134	0.4187	0
5261	574	0.4026	0.5523	0.6135	0.4187	0
5262	573.9	0.4025	0.5523	0.6133	0.4186	0
5263	573.8	0.4025	0.5524	0.6133	0.4185	0
5264	573.7	0.4024	0.5524	0.6137	0.4187	0
5265	573.6	0.4022	0.5524	0.6136	0.4188	0
5266	573.5	0.4022	0.5524	0.6137	0.4187	0
5267	573.4	0.4026	0.5524	0.614	0.4188	0
5268	573.3	0.4027	0.5525	0.6144	0.4189	0
5269	573.2	0.4027	0.5527	0.6141	0.4191	0
5270	573.1	0.4028	0.5528	0.6142	0.4192	0
5271	573	0.4031	0.5527	0.6144	0.4192	0
5272	572.9	0.4031	0.5528	0.6145	0.4189	0
5273	572.8	0.403	0.553	0.6147	0.419	0
5274	572.7	0.4032	0.5528	0.6149	0.4195	0
5275	572.6	0.4032	0.5531	0.615	0.4195	0

5276	572.5	0.4033	0.5532	0.6152	0.4196	0
5277	572.4	0.4032	0.5534	0.6152	0.4194	0
5278	572.3	0.4031	0.5531	0.6153	0.4195	0
5279	572.2	0.4032	0.5531	0.6153	0.4194	0
5280	572.1	0.4031	0.5532	0.6153	0.4196	0
5281	572	0.4034	0.5532	0.6156	0.4197	0
5282	571.9	0.4032	0.5533	0.6157	0.4196	0
5283	571.8	0.4034	0.553	0.6157	0.4195	0
5284	571.7	0.4032	0.553	0.6158	0.4194	0
5285	571.6	0.4034	0.5532	0.6158	0.4193	0
5286	571.5	0.4036	0.5531	0.6162	0.4195	0
5287	571.4	0.4035	0.5532	0.6159	0.4195	0
5288	571.3	0.4036	0.5533	0.6164	0.4198	0
5289	571.2	0.4036	0.5538	0.6165	0.4197	0
5290	571.1	0.4034	0.5533	0.6163	0.4197	0
5291	571	0.4032	0.5533	0.6166	0.4199	0
5292	570.9	0.4034	0.5534	0.6168	0.4197	0
5293	570.8	0.4033	0.5533	0.6167	0.42	0
5294	570.7	0.4035	0.5536	0.6168	0.4198	0
5295	570.6	0.4035	0.5535	0.6168	0.4198	0
5296	570.5	0.4034	0.5533	0.6168	0.4199	0
5297	570.4	0.4033	0.5533	0.6169	0.4196	0
5298	570.3	0.4035	0.5536	0.6175	0.4199	0
5299	570.2	0.4035	0.5536	0.6175	0.42	0
5300	570.1	0.4035	0.5532	0.6172	0.4198	0
5301	570	0.4035	0.5533	0.6173	0.4198	0
5302	569.9	0.4036	0.5533	0.6173	0.4195	0
5303	569.8	0.4035	0.5536	0.6177	0.4195	0
5304	569.7	0.4035	0.5534	0.6175	0.4194	0
5305	569.6	0.4033	0.5535	0.6175	0.4196	0
5306	569.5	0.4033	0.5533	0.6177	0.4195	0
5307	569.4	0.4031	0.5534	0.6177	0.4195	0
5308	569.3	0.4032	0.5535	0.6179	0.4195	0
5309	569.2	0.4032	0.5532	0.6178	0.4196	0
5310	569.1	0.4032	0.5533	0.6177	0.4195	0
5311	569	0.403	0.553	0.6181	0.4194	0
5312	568.9	0.4031	0.5534	0.6182	0.4196	0
5313	568.8	0.403	0.5534	0.6181	0.4195	0
5314	568.7	0.4031	0.5536	0.6184	0.4196	0
5315	568.6	0.4029	0.5532	0.6181	0.4192	0
5316	568.5	0.4029	0.5533	0.6181	0.4196	0
5317	568.4	0.4028	0.5532	0.6182	0.4193	0
5318	568.3	0.4029	0.5532	0.6185	0.4196	0

5319	568.2	0.4031	0.5532	0.6185	0.4195	0
5320	568.1	0.403	0.553	0.6187	0.4195	0
5321	568	0.4031	0.5531	0.6186	0.4197	0
5322	567.9	0.4031	0.5532	0.619	0.4197	0
5323	567.8	0.403	0.5532	0.619	0.4196	0
5324	567.7	0.4033	0.5532	0.6191	0.4195	0
5325	567.6	0.4031	0.5533	0.6193	0.4195	0
5326	567.5	0.4032	0.5531	0.6196	0.4194	0
5327	567.4	0.4033	0.5531	0.6194	0.4195	0
5328	567.3	0.403	0.5532	0.6194	0.4195	0
5329	567.2	0.4032	0.5532	0.6196	0.4195	0
5330	567.1	0.4031	0.5533	0.6198	0.4197	0
5331	567	0.4033	0.5531	0.6201	0.4196	0
5332	566.9	0.4036	0.5532	0.6204	0.4198	0
5333	566.8	0.4035	0.5534	0.6204	0.4198	0
5334	566.7	0.4036	0.5536	0.6204	0.4201	0
5335	566.6	0.4037	0.5537	0.6203	0.4201	0
5336	566.5	0.4033	0.5535	0.6204	0.42	0
5337	566.4	0.4034	0.5535	0.6205	0.4199	0
5338	566.3	0.4036	0.5538	0.6209	0.4199	0
5339	566.2	0.4035	0.5541	0.6208	0.4199	0
5340	566.1	0.4035	0.5539	0.6208	0.4198	0
5341	566	0.4034	0.5537	0.621	0.42	0
5342	565.9	0.4035	0.554	0.6212	0.4202	0
5343	565.8	0.4037	0.554	0.6213	0.4203	0
5344	565.7	0.4039	0.5543	0.6217	0.4205	0
5345	565.6	0.4039	0.5542	0.6215	0.4203	0
5346	565.5	0.404	0.5542	0.6216	0.4204	0
5347	565.4	0.4038	0.5542	0.6216	0.4205	0
5348	565.3	0.4041	0.5542	0.6217	0.4206	0
5349	565.2	0.4043	0.5544	0.6221	0.4207	0
5350	565.1	0.4042	0.5545	0.6222	0.4207	0
5351	565	0.4041	0.5545	0.6222	0.4208	0
5352	564.9	0.404	0.5545	0.6222	0.4207	0
5353	564.8	0.4042	0.5547	0.6225	0.4208	0
5354	564.7	0.4044	0.5549	0.6227	0.4211	0
5355	564.6	0.4045	0.555	0.6231	0.4212	0
5356	564.5	0.4042	0.5547	0.623	0.4209	0
5357	564.4	0.4044	0.5551	0.6233	0.421	0
5358	564.3	0.4047	0.5553	0.6235	0.4211	0
5359	564.2	0.405	0.5554	0.6239	0.4212	0
5360	564.1	0.4048	0.5552	0.6237	0.4212	0
5361	564	0.4046	0.5554	0.6238	0.4213	0

5362	563.9	0.405	0.5557	0.6241	0.4216	0
5363	563.8	0.4051	0.556	0.624	0.4214	0
5364	563.7	0.4051	0.5556	0.6244	0.4214	0
5365	563.6	0.4052	0.5556	0.6243	0.4215	0
5366	563.5	0.4053	0.5556	0.6245	0.4214	0
5367	563.4	0.4053	0.5558	0.6248	0.4216	0
5368	563.3	0.4049	0.5556	0.6247	0.4215	0
5369	563.2	0.4049	0.5558	0.6248	0.4215	0
5370	563.1	0.4051	0.5559	0.6248	0.4216	0
5371	563	0.4053	0.5562	0.6251	0.4219	0
5372	562.9	0.4052	0.5562	0.6253	0.4221	0
5373	562.8	0.4052	0.5563	0.6249	0.4217	0
5374	562.7	0.4052	0.556	0.6254	0.4218	0
5375	562.6	0.4051	0.5559	0.6254	0.4219	0
5376	562.5	0.4051	0.5559	0.6254	0.4217	0
5377	562.4	0.4051	0.556	0.6257	0.4216	0
5378	562.3	0.4052	0.556	0.6259	0.4218	0
5379	562.2	0.4051	0.5558	0.6259	0.4217	0
5380	562.1	0.4052	0.5558	0.626	0.4218	0
5381	562	0.4053	0.5559	0.6261	0.4217	0
5382	561.9	0.4052	0.5561	0.6263	0.4217	0
5383	561.8	0.4049	0.556	0.6263	0.4216	0
5384	561.7	0.405	0.5558	0.6263	0.4216	0
5385	561.6	0.4049	0.5559	0.6264	0.4216	0
5386	561.5	0.4051	0.5559	0.6265	0.4215	0
5387	561.4	0.4052	0.5562	0.6268	0.4215	0
5388	561.3	0.4051	0.5561	0.6268	0.4217	0
5389	561.2	0.4051	0.5561	0.627	0.4217	0
5390	561.1	0.405	0.5561	0.6269	0.4218	0
5391	561	0.4049	0.5561	0.6268	0.4216	0
5392	560.9	0.4049	0.5557	0.6268	0.4214	0
5393	560.8	0.4051	0.5556	0.6268	0.4215	0
5394	560.7	0.4048	0.5558	0.6272	0.4214	0
5395	560.6	0.4049	0.5555	0.6269	0.4215	0
5396	560.5	0.4048	0.5558	0.6272	0.4216	0
5397	560.4	0.4047	0.5554	0.6274	0.4215	0
5398	560.3	0.4048	0.5556	0.6271	0.4215	0
5399	560.2	0.4048	0.5556	0.6271	0.4216	0
5400	560.1	0.4048	0.5555	0.6275	0.4215	0
5401	560	0.4047	0.5557	0.6274	0.4216	0
5402	559.9	0.4046	0.5556	0.6275	0.4214	0
5403	559.8	0.4045	0.5555	0.6274	0.4213	0
5404	559.7	0.4048	0.5558	0.6278	0.4216	0

5405	559.6	0.4049	0.5557	0.6279	0.4215	0
5406	559.5	0.4043	0.5554	0.6277	0.4211	0
5407	559.4	0.4043	0.5555	0.6278	0.4213	0
5408	559.3	0.4043	0.5555	0.628	0.4212	0
5409	559.2	0.4042	0.5554	0.6279	0.4211	0
5410	559.1	0.4042	0.5554	0.6278	0.4209	0
5411	559	0.4043	0.555	0.6281	0.4209	0
5412	558.9	0.4045	0.5549	0.628	0.4209	0
5413	558.8	0.4043	0.5549	0.6282	0.4209	0
5414	558.7	0.404	0.5549	0.6282	0.4209	0
5415	558.6	0.4039	0.5551	0.6283	0.4209	0
5416	558.5	0.4042	0.5549	0.6284	0.4208	0
5417	558.4	0.4041	0.555	0.6285	0.4209	0
5418	558.3	0.4038	0.5548	0.6284	0.4208	0
5419	558.2	0.4039	0.5547	0.6284	0.4208	0
5420	558.1	0.4039	0.5547	0.6286	0.4207	0
5421	558	0.4036	0.5546	0.6284	0.4206	0
5422	557.9	0.4039	0.5544	0.6285	0.4205	0
5423	557.8	0.404	0.5548	0.6286	0.4205	0
5424	557.7	0.404	0.5549	0.6288	0.4208	0
5425	557.6	0.4042	0.5548	0.6291	0.4207	0
5426	557.5	0.404	0.5545	0.6289	0.4207	0
5427	557.4	0.4038	0.5547	0.6293	0.4206	0
5428	557.3	0.4038	0.5545	0.6293	0.4204	0
5429	557.2	0.4039	0.5547	0.6296	0.4202	0
5430	557.1	0.4039	0.5548	0.6295	0.4203	0
5431	557	0.4038	0.555	0.6301	0.4206	0
5432	556.9	0.404	0.555	0.6297	0.4205	0
5433	556.8	0.404	0.555	0.6299	0.4205	0
5434	556.7	0.4042	0.5549	0.63	0.4205	0
5435	556.6	0.4041	0.555	0.6302	0.4204	0
5436	556.5	0.4039	0.555	0.6302	0.4205	0
5437	556.4	0.4043	0.5552	0.6307	0.4206	0
5438	556.3	0.404	0.5552	0.6303	0.4208	0
5439	556.2	0.4043	0.5551	0.6307	0.4209	0
5440	556.1	0.4041	0.5555	0.631	0.421	0
5441	556	0.4041	0.5556	0.6309	0.421	0
5442	555.9	0.4042	0.5556	0.631	0.4211	0
5443	555.8	0.4044	0.5554	0.6311	0.4213	0
5444	555.7	0.4048	0.5555	0.6316	0.4215	0
5445	555.6	0.4048	0.5554	0.6319	0.4213	0
5446	555.5	0.4047	0.5559	0.6319	0.4214	0
5447	555.4	0.4047	0.556	0.632	0.4217	0

5448	555.3	0.4049	0.5559	0.6322	0.4219	0
5449	555.2	0.4049	0.5557	0.6323	0.422	0
5450	555.1	0.405	0.556	0.6324	0.4219	0
5451	555	0.4052	0.5562	0.6322	0.4219	0
5452	554.9	0.4053	0.5564	0.6329	0.4221	0
5453	554.8	0.4055	0.5566	0.6332	0.4222	0
5454	554.7	0.4053	0.5566	0.6331	0.4223	0
5455	554.6	0.4052	0.5567	0.6332	0.4222	0
5456	554.5	0.4054	0.5569	0.6334	0.4222	0
5457	554.4	0.4057	0.5571	0.6338	0.4226	0
5458	554.3	0.4056	0.5572	0.634	0.4229	0
5459	554.2	0.4056	0.5569	0.634	0.4227	0
5460	554.1	0.406	0.5572	0.6344	0.4227	0
5461	554	0.4061	0.5572	0.6346	0.4226	0
5462	553.9	0.4059	0.5575	0.6346	0.4226	0
5463	553.8	0.4062	0.5577	0.6349	0.4228	0
5464	553.7	0.4062	0.5573	0.635	0.4229	0
5465	553.6	0.4061	0.5577	0.6352	0.4228	0
5466	553.5	0.4061	0.5578	0.6352	0.4228	0
5467	553.4	0.4063	0.5578	0.6356	0.4231	0
5468	553.3	0.4063	0.5578	0.6357	0.4233	0
5469	553.2	0.4064	0.5581	0.6361	0.4232	0
5470	553.1	0.4067	0.5581	0.6362	0.4234	0
5471	553	0.4069	0.5582	0.6363	0.4237	0
5472	552.9	0.4069	0.5581	0.6366	0.4234	0
5473	552.8	0.4069	0.558	0.6365	0.4238	0
5474	552.7	0.4067	0.5581	0.6364	0.4237	0
5475	552.6	0.4068	0.5581	0.6366	0.4238	0
5476	552.5	0.4069	0.5579	0.6367	0.424	0
5477	552.4	0.4068	0.5581	0.6368	0.424	0
5478	552.3	0.4068	0.5581	0.6369	0.4238	0
5479	552.2	0.4066	0.5583	0.637	0.4238	0
5480	552.1	0.4065	0.5582	0.6372	0.4237	0
5481	552	0.4067	0.558	0.6372	0.4238	0
5482	551.9	0.4066	0.5583	0.6375	0.4238	0
5483	551.8	0.4069	0.5586	0.6376	0.4239	0
5484	551.7	0.4069	0.5584	0.6376	0.4238	0
5485	551.6	0.4066	0.5581	0.6378	0.424	0
5486	551.5	0.4065	0.5582	0.6377	0.4238	0
5487	551.4	0.4065	0.5582	0.6379	0.4238	0
5488	551.3	0.4067	0.5583	0.6381	0.4239	0
5489	551.2	0.4067	0.5583	0.6383	0.4239	0
5490	551.1	0.4068	0.5582	0.6382	0.4237	0

5491	551	0.4066	0.5582	0.6384	0.4237	0
5492	550.9	0.4068	0.5584	0.6387	0.4239	0
5493	550.8	0.407	0.5582	0.6388	0.4237	0
5494	550.7	0.4069	0.5583	0.6385	0.4236	0
5495	550.6	0.4072	0.5584	0.6387	0.4237	0
5496	550.5	0.4067	0.5584	0.6387	0.4236	0
5497	550.4	0.4067	0.5583	0.6387	0.4236	0
5498	550.3	0.4067	0.558	0.6389	0.4235	0
5499	550.2	0.4065	0.558	0.6387	0.4236	0
5500	550.1	0.4063	0.5582	0.639	0.4235	0
5501	550	0.4063	0.558	0.6391	0.4233	0
5502	549.9	0.4066	0.5583	0.6392	0.4235	0
5503	549.8	0.4064	0.5579	0.6394	0.4235	0
5504	549.7	0.4064	0.5581	0.6393	0.4235	0
5505	549.6	0.4062	0.5579	0.6393	0.4232	0
5506	549.5	0.4059	0.5579	0.6393	0.423	0
5507	549.4	0.406	0.5575	0.6395	0.4228	0
5508	549.3	0.4059	0.5574	0.6395	0.423	0
5509	549.2	0.4058	0.5575	0.6394	0.4228	0
5510	549.1	0.4061	0.5577	0.6397	0.4229	0
5511	549	0.406	0.5577	0.6397	0.4229	0
5512	548.9	0.406	0.5576	0.6397	0.4228	0
5513	548.8	0.4056	0.5576	0.6397	0.4228	0
5514	548.7	0.4058	0.5574	0.6397	0.4228	0
5515	548.6	0.4055	0.5572	0.6399	0.4224	0
5516	548.5	0.4055	0.557	0.6396	0.4221	0
5517	548.4	0.4054	0.5567	0.6397	0.4223	0
5518	548.3	0.4055	0.5567	0.6398	0.4222	0
5519	548.2	0.4054	0.5569	0.64	0.4222	0
5520	548.1	0.4051	0.5567	0.6398	0.4222	0
5521	548	0.4053	0.5565	0.6398	0.4224	0
5522	547.9	0.4052	0.5565	0.6399	0.4222	0
5523	547.8	0.4052	0.5566	0.6401	0.422	0
5524	547.7	0.4052	0.5568	0.6402	0.4218	0
5525	547.6	0.4053	0.5567	0.6404	0.4221	0
5526	547.5	0.4054	0.5567	0.6406	0.4221	0
5527	547.4	0.4054	0.5566	0.6404	0.422	0
5528	547.3	0.4053	0.5567	0.6407	0.4219	0
5529	547.2	0.405	0.5568	0.6409	0.4221	0
5530	547.1	0.4051	0.5567	0.6411	0.4219	0
5531	547	0.4052	0.5568	0.6411	0.422	0
5532	546.9	0.4052	0.5566	0.6411	0.4219	0
5533	546.8	0.4054	0.5567	0.6415	0.4219	0

5534	546.7	0.4055	0.5568	0.6416	0.4221	0
5535	546.6	0.4053	0.5569	0.6417	0.422	0
5536	546.5	0.4054	0.5567	0.6418	0.4221	0
5537	546.4	0.4054	0.5567	0.6418	0.4222	0
5538	546.3	0.4053	0.5568	0.6418	0.4221	0
5539	546.2	0.4056	0.5569	0.642	0.4223	0
5540	546.1	0.4055	0.5567	0.6422	0.4223	0
5541	546	0.4056	0.557	0.6425	0.4223	0
5542	545.9	0.4057	0.5572	0.6427	0.4222	0
5543	545.8	0.4056	0.5574	0.6428	0.4223	0
5544	545.7	0.4058	0.5573	0.6428	0.4223	0
5545	545.6	0.4057	0.5576	0.6434	0.4224	0
5546	545.5	0.4057	0.5573	0.6433	0.4225	0
5547	545.4	0.4057	0.5571	0.6434	0.4222	0
5548	545.3	0.4058	0.5576	0.6437	0.4226	0
5549	545.2	0.4062	0.5577	0.644	0.423	0
5550	545.1	0.4063	0.5579	0.6444	0.423	0
5551	545	0.4062	0.5578	0.6443	0.4231	0
5552	544.9	0.4062	0.5579	0.6446	0.4233	0
5553	544.8	0.4064	0.5582	0.6447	0.4231	0
5554	544.7	0.4065	0.5585	0.645	0.4232	0
5555	544.6	0.4067	0.5588	0.6454	0.4235	0
5556	544.5	0.4067	0.5588	0.6457	0.4237	0
5557	544.4	0.407	0.5588	0.646	0.4239	0
5558	544.3	0.407	0.5587	0.6459	0.4239	0
5559	544.2	0.4072	0.5588	0.6459	0.4238	0
5560	544.1	0.4075	0.5593	0.6466	0.4241	0
5561	544	0.4074	0.5597	0.6469	0.4243	0
5562	543.9	0.4076	0.5594	0.6471	0.4247	0
5563	543.8	0.4078	0.5596	0.6473	0.4245	0
5564	543.7	0.4079	0.5599	0.6475	0.4248	0
5565	543.6	0.4081	0.5601	0.648	0.4251	0
5566	543.5	0.4081	0.5604	0.6483	0.4256	0
5567	543.4	0.4081	0.5604	0.6482	0.4253	0
5568	543.3	0.4084	0.5607	0.6485	0.4255	0
5569	543.2	0.4083	0.5607	0.6486	0.4257	0
5570	543.1	0.4087	0.561	0.6489	0.4258	0
5571	543	0.4089	0.5613	0.6498	0.4263	0
5572	542.9	0.4091	0.5614	0.6497	0.4264	0
5573	542.8	0.4088	0.5614	0.6498	0.4264	0
5574	542.7	0.4088	0.5613	0.6499	0.4263	0
5575	542.6	0.409	0.5615	0.6501	0.4264	0
5576	542.5	0.4091	0.5615	0.6502	0.4264	0

5577	542.4	0.4095	0.5618	0.6506	0.4266	0
5578	542.3	0.4094	0.5619	0.6506	0.4268	0
5579	542.2	0.4095	0.5618	0.6507	0.4267	0
5580	542.1	0.4097	0.5618	0.6506	0.4267	0
5581	542	0.4098	0.5618	0.6512	0.4268	0
5582	541.9	0.4097	0.5619	0.6509	0.4267	0
5583	541.8	0.4097	0.5622	0.6513	0.4268	0
5584	541.7	0.4097	0.5623	0.6515	0.4269	0
5585	541.6	0.4099	0.5623	0.6516	0.4273	0
5586	541.5	0.4098	0.5624	0.6518	0.427	0
5587	541.4	0.4096	0.5624	0.6518	0.4272	0
5588	541.3	0.4097	0.5622	0.6519	0.4271	0
5589	541.2	0.4097	0.5626	0.6522	0.4269	0
5590	541.1	0.4097	0.5627	0.6522	0.4271	0
5591	541	0.41	0.5629	0.6527	0.4275	0
5592	540.9	0.4101	0.5633	0.6527	0.4273	0
5593	540.8	0.4101	0.5633	0.6528	0.4272	0
5594	540.7	0.4099	0.5632	0.6527	0.4271	0
5595	540.6	0.4102	0.5633	0.6532	0.4273	0
5596	540.5	0.4102	0.5631	0.6534	0.4274	0
5597	540.4	0.4104	0.5631	0.6536	0.4273	0
5598	540.3	0.4104	0.5632	0.6535	0.427	0
5599	540.2	0.4103	0.5629	0.6535	0.4271	0
5600	540.1	0.4108	0.5632	0.6539	0.4274	0
5601	540	0.4105	0.563	0.6538	0.4275	0
5602	539.9	0.4104	0.5632	0.6541	0.4276	0
5603	539.8	0.4103	0.5631	0.6541	0.4273	0
5604	539.7	0.4103	0.5629	0.6541	0.4273	0
5605	539.6	0.4101	0.563	0.6543	0.4274	0
5606	539.5	0.4104	0.5633	0.6541	0.4276	0
5607	539.4	0.4105	0.5634	0.6546	0.4274	0
5608	539.3	0.4104	0.5635	0.6548	0.4273	0
5609	539.2	0.4104	0.5635	0.6546	0.4275	0
5610	539.1	0.4104	0.5633	0.6546	0.4275	0
5611	539	0.4104	0.5635	0.6548	0.4271	0
5612	538.9	0.4105	0.5634	0.6549	0.427	0
5613	538.8	0.4104	0.5632	0.6548	0.427	0
5614	538.7	0.4102	0.5629	0.655	0.4268	0
5615	538.6	0.41	0.5629	0.655	0.4267	0
5616	538.5	0.4098	0.5629	0.6551	0.4268	0
5617	538.4	0.4101	0.563	0.6552	0.427	0
5618	538.3	0.41	0.5627	0.655	0.4267	0
5619	538.2	0.4098	0.5629	0.6553	0.4268	0

5620	538.1	0.4096	0.5627	0.6551	0.4265	0
5621	538	0.4098	0.5624	0.6556	0.4267	0
5622	537.9	0.41	0.5626	0.6556	0.4268	0
5623	537.8	0.4102	0.5625	0.6554	0.4265	0
5624	537.7	0.4099	0.5627	0.6556	0.4267	0
5625	537.6	0.4099	0.5627	0.6556	0.4268	0
5626	537.5	0.4095	0.5626	0.6557	0.4267	0
5627	537.4	0.4096	0.5624	0.6557	0.4265	0
5628	537.3	0.4096	0.5627	0.6559	0.4264	0
5629	537.2	0.4094	0.5625	0.6559	0.4265	0
5630	537.1	0.4094	0.5627	0.656	0.4266	0
5631	537	0.4094	0.5628	0.6561	0.4265	0
5632	536.9	0.4092	0.5626	0.6562	0.4262	0
5633	536.8	0.4095	0.5624	0.6563	0.4264	0
5634	536.7	0.4096	0.562	0.656	0.4264	0
5635	536.6	0.4094	0.5621	0.6565	0.4264	0
5636	536.5	0.4095	0.5621	0.6563	0.4265	0
5637	536.4	0.4094	0.5623	0.6566	0.4263	0
5638	536.3	0.4096	0.5624	0.6569	0.4263	0
5639	536.2	0.4097	0.5624	0.6568	0.4265	0
5640	536.1	0.4097	0.5624	0.6566	0.4266	0
5641	536	0.4094	0.5622	0.6567	0.4264	0
5642	535.9	0.4096	0.5622	0.6569	0.4263	0
5643	535.8	0.4096	0.5622	0.6573	0.4262	0
5644	535.7	0.4096	0.5625	0.6573	0.4265	0
5645	535.6	0.4097	0.5627	0.6576	0.4266	0
5646	535.5	0.4097	0.5626	0.6579	0.4268	0
5647	535.4	0.4097	0.5626	0.6579	0.4267	0
5648	535.3	0.4099	0.5628	0.6577	0.4267	0
5649	535.2	0.4098	0.5629	0.6579	0.4268	0
5650	535.1	0.4098	0.5628	0.658	0.4267	0
5651	535	0.4099	0.5629	0.6581	0.4269	0
5652	534.9	0.4101	0.5628	0.6582	0.4267	0
5653	534.8	0.4103	0.5629	0.6587	0.4269	0
5654	534.7	0.4101	0.5629	0.6587	0.4272	0
5655	534.6	0.4102	0.563	0.659	0.4272	0
5656	534.5	0.4102	0.5629	0.659	0.4272	0
5657	534.4	0.4105	0.5632	0.6592	0.4274	0
5658	534.3	0.4105	0.5633	0.6591	0.4274	0
5659	534.2	0.4106	0.5632	0.6594	0.4274	0
5660	534.1	0.4106	0.5634	0.6596	0.4277	0
5661	534	0.4106	0.5637	0.66	0.4276	0
5662	533.9	0.4107	0.5636	0.6602	0.4276	0

5663	533.8	0.4109	0.5638	0.6602	0.4274	0
5664	533.7	0.411	0.5638	0.6604	0.4276	0
5665	533.6	0.4108	0.5641	0.6605	0.4279	0
5666	533.5	0.411	0.564	0.6607	0.4278	0
5667	533.4	0.4113	0.5645	0.661	0.4282	0
5668	533.3	0.4116	0.5648	0.6612	0.4282	0
5669	533.2	0.4115	0.5645	0.6617	0.4283	0
5670	533.1	0.4116	0.5644	0.6617	0.4285	0
5671	533	0.4117	0.5648	0.662	0.4289	0
5672	532.9	0.4114	0.5648	0.6619	0.4288	0
5673	532.8	0.4117	0.5651	0.6622	0.4289	0
5674	532.7	0.412	0.5651	0.6622	0.4289	0
5675	532.6	0.4118	0.5652	0.6622	0.4289	0
5676	532.5	0.412	0.5652	0.6625	0.4288	0
5677	532.4	0.4121	0.5655	0.6627	0.4291	0
5678	532.3	0.4121	0.5653	0.6629	0.4293	0
5679	532.2	0.4122	0.5654	0.6633	0.4292	0
5680	532.1	0.412	0.5656	0.6631	0.4291	0
5681	532	0.4123	0.5657	0.6631	0.4294	0
5682	531.9	0.4121	0.5658	0.6634	0.4294	0
5683	531.8	0.4122	0.5661	0.6636	0.4295	0
5684	531.7	0.4121	0.566	0.6633	0.4295	0
5685	531.6	0.4123	0.5657	0.6637	0.4292	0
5686	531.5	0.4122	0.5657	0.6636	0.429	0
5687	531.4	0.4123	0.5656	0.6639	0.4293	0
5688	531.3	0.4126	0.5654	0.6637	0.4296	0
5689	531.2	0.4126	0.5656	0.6638	0.4297	0
5690	531.1	0.4127	0.5659	0.6642	0.4298	0
5691	531	0.4125	0.5658	0.6646	0.4296	0
5692	530.9	0.4123	0.5659	0.6646	0.4297	0
5693	530.8	0.4121	0.5659	0.6647	0.4294	0
5694	530.7	0.4124	0.5658	0.6646	0.4295	0
5695	530.6	0.4126	0.5657	0.6647	0.4295	0
5696	530.5	0.4124	0.5658	0.6649	0.4296	0
5697	530.4	0.4128	0.5659	0.6648	0.4297	0
5698	530.3	0.4129	0.5662	0.6653	0.43	0
5699	530.2	0.4127	0.5659	0.6655	0.4296	0
5700	530.1	0.4125	0.5658	0.6654	0.4297	0
5701	530	0.4126	0.5659	0.6652	0.4295	0
5702	529.9	0.4128	0.5661	0.6657	0.4297	0
5703	529.8	0.4127	0.5663	0.6659	0.4297	0
5704	529.7	0.4125	0.5663	0.6657	0.4296	0
5705	529.6	0.4126	0.566	0.6659	0.4292	0

5706	529.5	0.4125	0.5656	0.6657	0.4297	0
5707	529.4	0.4125	0.5658	0.6658	0.4297	0
5708	529.3	0.4123	0.5659	0.6658	0.4297	0
5709	529.2	0.4123	0.5657	0.6661	0.4294	0
5710	529.1	0.4122	0.5655	0.6663	0.4294	0
5711	529	0.412	0.5656	0.6661	0.4293	0
5712	528.9	0.4118	0.5652	0.6659	0.4289	0
5713	528.8	0.4119	0.5656	0.6661	0.4287	0
5714	528.7	0.412	0.5657	0.6661	0.4287	0
5715	528.6	0.4117	0.5656	0.6661	0.4287	0
5716	528.5	0.4117	0.5652	0.6658	0.4284	0
5717	528.4	0.4115	0.5648	0.6659	0.4283	0
5718	528.3	0.4114	0.5649	0.666	0.4283	0
5719	528.2	0.4111	0.5645	0.6656	0.4282	0
5720	528.1	0.4111	0.5645	0.6656	0.428	0
5721	528	0.411	0.5644	0.6656	0.4279	0
5722	527.9	0.4109	0.5641	0.6659	0.4278	0
5723	527.8	0.4108	0.5638	0.6658	0.4275	0
5724	527.7	0.4107	0.5638	0.6657	0.4276	0
5725	527.6	0.4106	0.5637	0.6657	0.4276	0
5726	527.5	0.4105	0.5636	0.6657	0.4274	0
5727	527.4	0.4105	0.5635	0.6659	0.4273	0
5728	527.3	0.4104	0.5638	0.6658	0.4274	0
5729	527.2	0.41	0.5636	0.6658	0.4271	0
5730	527.1	0.41	0.563	0.6657	0.4272	0
5731	527	0.4101	0.563	0.6659	0.4269	0
5732	526.9	0.41	0.5629	0.6657	0.4269	0
5733	526.8	0.4097	0.5629	0.6655	0.4266	0
5734	526.7	0.4096	0.5627	0.6657	0.4267	0
5735	526.6	0.4096	0.5626	0.666	0.4267	0
5736	526.5	0.4094	0.5628	0.666	0.4266	0
5737	526.4	0.4094	0.5625	0.6659	0.4266	0
5738	526.3	0.4094	0.5626	0.6662	0.4265	0
5739	526.2	0.4095	0.5627	0.6663	0.4267	0
5740	526.1	0.4096	0.5626	0.6664	0.4266	0
5741	526	0.4094	0.5622	0.6663	0.4264	0
5742	525.9	0.4096	0.5621	0.6665	0.4264	0
5743	525.8	0.4094	0.5622	0.6668	0.4264	0
5744	525.7	0.4096	0.5623	0.6667	0.4267	0
5745	525.6	0.4096	0.5623	0.6667	0.4266	0
5746	525.5	0.4098	0.5625	0.6667	0.4266	0
5747	525.4	0.4097	0.5628	0.667	0.4266	0
5748	525.3	0.4098	0.5626	0.6671	0.4266	0

5749	525.2	0.4097	0.5626	0.6674	0.4269	0
5750	525.1	0.4096	0.5626	0.6671	0.4267	0
5751	525	0.4095	0.5626	0.6674	0.4268	0
5752	524.9	0.4095	0.5629	0.6676	0.4269	0
5753	524.8	0.4097	0.5629	0.6677	0.4266	0
5754	524.7	0.4096	0.5629	0.6677	0.4267	0
5755	524.6	0.4097	0.5627	0.6677	0.4267	0
5756	524.5	0.4097	0.5627	0.6682	0.4267	0
5757	524.4	0.4097	0.5627	0.6682	0.4266	0
5758	524.3	0.4101	0.5627	0.6682	0.4268	0
5759	524.2	0.4101	0.5628	0.6686	0.4268	0
5760	524.1	0.4101	0.5632	0.6686	0.4271	0
5761	524	0.4101	0.5633	0.6688	0.427	0
5762	523.9	0.41	0.5632	0.6691	0.427	0
5763	523.8	0.4102	0.5635	0.6693	0.4271	0
5764	523.7	0.4104	0.5635	0.6693	0.4273	0
5765	523.6	0.4105	0.5635	0.6695	0.4273	0
5766	523.5	0.4104	0.5636	0.6697	0.4275	0
5767	523.4	0.4104	0.5638	0.6701	0.4276	0
5768	523.3	0.4104	0.5639	0.6703	0.4277	0
5769	523.2	0.4105	0.5638	0.6704	0.4277	0
5770	523.1	0.4104	0.564	0.6706	0.4277	0
5771	523	0.4108	0.5641	0.6708	0.4279	0
5772	522.9	0.4105	0.5641	0.6708	0.4279	0
5773	522.8	0.4109	0.5642	0.6708	0.428	0
5774	522.7	0.4111	0.5642	0.6712	0.4282	0
5775	522.6	0.4109	0.5641	0.6711	0.428	0
5776	522.5	0.4109	0.5642	0.6714	0.4282	0
5777	522.4	0.4109	0.5639	0.6713	0.4282	0
5778	522.3	0.4112	0.5644	0.6717	0.4282	0
5779	522.2	0.4113	0.5644	0.6718	0.4282	0
5780	522.1	0.4112	0.5646	0.6722	0.4281	0
5781	522	0.4111	0.5645	0.672	0.4283	0
5782	521.9	0.4107	0.5644	0.6721	0.4281	0
5783	521.8	0.4109	0.5646	0.6723	0.4281	0
5784	521.7	0.4113	0.5648	0.6725	0.4285	0
5785	521.6	0.411	0.5649	0.6727	0.4283	0
5786	521.5	0.411	0.5648	0.6728	0.4282	0
5787	521.4	0.4112	0.5646	0.673	0.4283	0
5788	521.3	0.4113	0.5646	0.673	0.4284	0
5789	521.2	0.4112	0.5649	0.6731	0.4283	0
5790	521.1	0.4116	0.5649	0.6736	0.4287	0
5791	521	0.4113	0.5646	0.6735	0.4287	0

5792	520.9	0.4115	0.5647	0.6736	0.4287	0
5793	520.8	0.4116	0.5649	0.6735	0.4288	0
5794	520.7	0.4112	0.5649	0.6737	0.4286	0
5795	520.6	0.4114	0.5653	0.6736	0.4288	0
5796	520.5	0.4114	0.5651	0.6739	0.4287	0
5797	520.4	0.4113	0.565	0.6741	0.4288	0
5798	520.3	0.4112	0.565	0.6732	0.4288	0
5799	520.2	0.4112	0.565	0.6741	0.4287	0
5800	520.1	0.4116	0.5652	0.6744	0.4287	0
5801	520	0.4115	0.5651	0.6743	0.4287	0
5802	519.9	0.4114	0.5651	0.6744	0.4288	0
5803	519.8	0.4115	0.5651	0.6749	0.4289	0
5804	519.7	0.4115	0.565	0.675	0.429	0
5805	519.6	0.4114	0.565	0.6753	0.4291	0
5806	519.5	0.4116	0.5651	0.6756	0.4291	0
5807	519.4	0.4112	0.5651	0.6755	0.4288	0
5808	519.3	0.4114	0.5653	0.6757	0.429	0
5809	519.2	0.4114	0.5652	0.6755	0.4288	0
5810	519.1	0.4115	0.5652	0.6756	0.4291	0
5811	519	0.4115	0.5653	0.6755	0.4288	0
5812	518.9	0.4113	0.5655	0.6757	0.4289	0
5813	518.8	0.4116	0.5657	0.6756	0.429	0
5814	518.7	0.4113	0.5656	0.6759	0.429	0
5815	518.6	0.4115	0.5653	0.6759	0.4291	0
5816	518.5	0.4112	0.5652	0.6758	0.4287	0
5817	518.4	0.411	0.5651	0.6759	0.4287	0
5818	518.3	0.4112	0.5651	0.6763	0.4285	0
5819	518.2	0.4111	0.5652	0.6767	0.4287	0
5820	518.1	0.4112	0.5652	0.6765	0.4286	0
5821	518	0.4112	0.5651	0.6765	0.4284	0
5822	517.9	0.4113	0.565	0.6767	0.4284	0
5823	517.8	0.4114	0.5652	0.6772	0.4283	0
5824	517.7	0.4113	0.5653	0.677	0.4283	0
5825	517.6	0.4114	0.5651	0.6769	0.4283	0
5826	517.5	0.4111	0.5651	0.677	0.4285	0
5827	517.4	0.4112	0.5649	0.6771	0.4284	0
5828	517.3	0.4111	0.5651	0.6772	0.4285	0
5829	517.2	0.4112	0.5652	0.6771	0.4285	0
5830	517.1	0.4112	0.5653	0.6774	0.4288	0
5831	517	0.4111	0.5652	0.6773	0.4285	0
5832	516.9	0.4111	0.5651	0.6775	0.4285	0
5833	516.8	0.4111	0.5651	0.6775	0.4288	0
5834	516.7	0.4112	0.5652	0.6776	0.4287	0

5835	516.6	0.4111	0.5649	0.6775	0.4287	0
5836	516.5	0.4111	0.565	0.6777	0.4283	0
5837	516.4	0.4111	0.5648	0.6779	0.4282	0
5838	516.3	0.4111	0.5648	0.6778	0.4284	0
5839	516.2	0.4113	0.565	0.6783	0.4286	0
5840	516.1	0.4114	0.5649	0.6783	0.4284	0
5841	516	0.4112	0.5646	0.6782	0.4282	0
5842	515.9	0.411	0.5649	0.6781	0.4284	0
5843	515.8	0.4109	0.5649	0.6778	0.4284	0
5844	515.7	0.4111	0.5649	0.6782	0.4284	0
5845	515.6	0.4112	0.5652	0.6787	0.4286	0
5846	515.5	0.4111	0.565	0.6787	0.4289	0
5847	515.4	0.4112	0.565	0.6789	0.4285	0
5848	515.3	0.4111	0.5647	0.6788	0.4285	0
5849	515.2	0.4112	0.5651	0.6791	0.4287	0
5850	515.1	0.4112	0.565	0.6794	0.4288	0
5851	515	0.4114	0.5649	0.6793	0.4287	0
5852	514.9	0.4113	0.5651	0.6795	0.4284	0
5853	514.8	0.4112	0.5651	0.6799	0.4286	0
5854	514.7	0.4115	0.5651	0.6798	0.4287	0
5855	514.6	0.4115	0.5651	0.6802	0.4285	0
5856	514.5	0.4113	0.5655	0.6799	0.4285	0
5857	514.4	0.4114	0.5654	0.6801	0.4287	0
5858	514.3	0.4115	0.5652	0.6805	0.4287	0
5859	514.2	0.4118	0.5654	0.6807	0.429	0
5860	514.1	0.4118	0.5654	0.6807	0.429	0
5861	514	0.4121	0.5654	0.6807	0.429	0
5862	513.9	0.4119	0.5657	0.681	0.4291	0
5863	513.8	0.4116	0.5655	0.6813	0.4293	0
5864	513.7	0.4116	0.5656	0.6817	0.4292	0
5865	513.6	0.4118	0.5656	0.6812	0.4293	0
5866	513.5	0.4119	0.5661	0.6817	0.4293	0
5867	513.4	0.4122	0.5659	0.6821	0.4296	0
5868	513.3	0.4123	0.566	0.6821	0.4296	0
5869	513.2	0.4122	0.5662	0.6824	0.4297	0
5870	513.1	0.4123	0.5661	0.6823	0.4296	0
5871	513	0.412	0.566	0.6825	0.4298	0
5872	512.9	0.4121	0.5662	0.6826	0.4297	0
5873	512.8	0.4122	0.5663	0.6829	0.4296	0
5874	512.7	0.4122	0.5662	0.6828	0.4295	0
5875	512.6	0.4124	0.5664	0.6834	0.4296	0
5876	512.5	0.4123	0.5663	0.683	0.4296	0
5877	512.4	0.4123	0.5664	0.683	0.4296	0

5878	512.3	0.4123	0.5663	0.6829	0.4296	0
5879	512.2	0.4124	0.5665	0.683	0.4295	0
5880	512.1	0.4124	0.5665	0.6832	0.4297	0
5881	512	0.4122	0.5666	0.6835	0.4297	0
5882	511.9	0.4121	0.5664	0.6832	0.4294	0
5883	511.8	0.4122	0.5664	0.6835	0.4294	0
5884	511.7	0.4124	0.5666	0.6839	0.4297	0
5885	511.6	0.4123	0.5665	0.6836	0.4298	0
5886	511.5	0.4123	0.5666	0.6841	0.4299	0
5887	511.4	0.4122	0.5668	0.684	0.4295	0
5888	511.3	0.4121	0.5667	0.684	0.4297	0
5889	511.2	0.4124	0.5667	0.6844	0.43	0
5890	511.1	0.4122	0.5666	0.6847	0.4297	0
5891	511	0.4122	0.5666	0.6844	0.4298	0
5892	510.9	0.4121	0.5667	0.6846	0.4299	0
5893	510.8	0.4121	0.5666	0.6843	0.4295	0
5894	510.7	0.4121	0.5666	0.6844	0.4297	0
5895	510.6	0.4123	0.5667	0.6846	0.4298	0
5896	510.5	0.4124	0.5665	0.6847	0.4298	0
5897	510.4	0.4122	0.5666	0.6847	0.4298	0
5898	510.3	0.4121	0.5663	0.685	0.4299	0
5899	510.2	0.4124	0.5668	0.6851	0.4299	0
5900	510.1	0.4123	0.5667	0.685	0.4298	0
5901	510	0.4122	0.5666	0.6852	0.4297	0
5902	509.9	0.412	0.5663	0.685	0.4296	0
5903	509.8	0.4119	0.5665	0.6853	0.4292	0
5904	509.7	0.4122	0.5667	0.685	0.4296	0
5905	509.6	0.4122	0.5668	0.6854	0.4298	0
5906	509.5	0.4123	0.5669	0.6856	0.4298	0
5907	509.4	0.4121	0.5666	0.6858	0.4293	0
5908	509.3	0.4121	0.5665	0.6857	0.4296	0
5909	509.2	0.412	0.5664	0.6859	0.4296	0
5910	509.1	0.412	0.5661	0.6858	0.4294	0
5911	509	0.4117	0.5663	0.6861	0.4296	0
5912	508.9	0.4117	0.5662	0.6863	0.4294	0
5913	508.8	0.4118	0.5665	0.686	0.4294	0
5914	508.7	0.4119	0.5664	0.6864	0.4292	0
5915	508.6	0.4116	0.5661	0.6859	0.4291	0
5916	508.5	0.4119	0.5661	0.6863	0.4292	0
5917	508.4	0.4119	0.5662	0.6867	0.4291	0
5918	508.3	0.4121	0.5663	0.6867	0.4295	0
5919	508.2	0.4121	0.5665	0.6868	0.4294	0
5920	508.1	0.412	0.5662	0.6868	0.4297	0

5921	508	0.4121	0.5661	0.687	0.4295	0
5922	507.9	0.4122	0.5663	0.687	0.4294	0
5923	507.8	0.4121	0.5664	0.6872	0.4294	0
5924	507.7	0.4122	0.5666	0.6874	0.4291	0
5925	507.6	0.4122	0.5667	0.6877	0.4293	0
5926	507.5	0.4123	0.5668	0.6876	0.4295	0
5927	507.4	0.4122	0.5666	0.6879	0.4297	0
5928	507.3	0.4121	0.5666	0.688	0.4294	0
5929	507.2	0.412	0.5666	0.688	0.4294	0
5930	507.1	0.4121	0.5664	0.6881	0.4294	0
5931	507	0.4121	0.5664	0.6881	0.4297	0
5932	506.9	0.412	0.5665	0.6879	0.4295	0
5933	506.8	0.4123	0.5666	0.6885	0.4296	0
5934	506.7	0.4122	0.5666	0.6885	0.4295	0
5935	506.6	0.4122	0.5667	0.6888	0.4297	0
5936	506.5	0.4121	0.5667	0.6887	0.4298	0
5937	506.4	0.4121	0.5666	0.6886	0.4298	0
5938	506.3	0.4122	0.5667	0.6892	0.4299	0
5939	506.2	0.4124	0.5669	0.6894	0.4299	0
5940	506.1	0.4125	0.5668	0.689	0.4298	0
5941	506	0.4126	0.5673	0.6894	0.4303	0
5942	505.9	0.4121	0.5671	0.6898	0.4299	0
5943	505.8	0.4123	0.5673	0.6898	0.43	0
5944	505.7	0.4124	0.5672	0.69	0.4302	0
5945	505.6	0.4126	0.5671	0.6903	0.4303	0
5946	505.5	0.4124	0.567	0.6902	0.4304	0
5947	505.4	0.4126	0.5669	0.6906	0.4303	0
5948	505.3	0.4126	0.5672	0.6905	0.4305	0
5949	505.2	0.4129	0.5675	0.6906	0.4306	0
5950	505.1	0.4129	0.5677	0.691	0.4306	0
5951	505	0.4128	0.5674	0.6908	0.4307	0
5952	504.9	0.4129	0.5676	0.6914	0.4305	0
5953	504.8	0.413	0.5679	0.6915	0.4308	0
5954	504.7	0.413	0.568	0.6915	0.4306	0
5955	504.6	0.413	0.568	0.6918	0.4307	0
5956	504.5	0.413	0.5679	0.6917	0.4311	0
5957	504.4	0.4133	0.5678	0.692	0.4312	0
5958	504.3	0.413	0.5682	0.692	0.431	0
5959	504.2	0.4132	0.5684	0.6923	0.4313	0
5960	504.1	0.4133	0.5682	0.6922	0.4313	0
5961	504	0.4136	0.5687	0.6927	0.4317	0
5962	503.9	0.4135	0.5686	0.6928	0.4314	0
5963	503.8	0.4136	0.5685	0.6929	0.4316	0

5964	503.7	0.4136	0.5686	0.6933	0.4315	0
5965	503.6	0.4139	0.569	0.6937	0.4319	0
5966	503.5	0.414	0.5686	0.6936	0.4317	0
5967	503.4	0.414	0.5687	0.6939	0.4315	0
5968	503.3	0.414	0.5686	0.6935	0.4316	0
5969	503.2	0.4136	0.5686	0.6938	0.4312	0
5970	503.1	0.4136	0.5689	0.6941	0.4315	0
5971	503	0.4137	0.5692	0.6941	0.4316	0
5972	502.9	0.4136	0.5693	0.694	0.4317	0
5973	502.8	0.4139	0.5693	0.6942	0.4318	0
5974	502.7	0.4139	0.5692	0.695	0.4319	0
5975	502.6	0.414	0.5695	0.6947	0.4318	0
5976	502.5	0.414	0.5693	0.6945	0.4318	0
5977	502.4	0.4142	0.5696	0.6947	0.4319	0
5978	502.3	0.414	0.5697	0.6949	0.4321	0
5979	502.2	0.4139	0.5697	0.6948	0.4319	0
5980	502.1	0.414	0.5694	0.6948	0.432	0
5981	502	0.4142	0.5694	0.6953	0.432	0
5982	501.9	0.4139	0.5694	0.6953	0.4321	0
5983	501.8	0.4142	0.5694	0.695	0.4323	0
5984	501.7	0.4143	0.5695	0.6957	0.4322	0
5985	501.6	0.4139	0.5693	0.6951	0.4318	0
5986	501.5	0.4139	0.569	0.6953	0.4319	0
5987	501.4	0.414	0.569	0.6957	0.4318	0
5988	501.3	0.4137	0.569	0.6958	0.4322	0
5989	501.2	0.4138	0.569	0.6955	0.4323	0
5990	501.1	0.4138	0.5692	0.6956	0.4319	0
5991	501	0.4139	0.5691	0.6957	0.4319	0
5992	500.9	0.4138	0.5695	0.6962	0.432	0
5993	500.8	0.4138	0.5693	0.6959	0.432	0
5994	500.7	0.4136	0.5691	0.6959	0.4318	0
5995	500.6	0.4135	0.5692	0.6962	0.4316	0
5996	500.5	0.4137	0.5692	0.6964	0.4318	0
5997	500.4	0.4134	0.5687	0.6962	0.4316	0
5998	500.3	0.4136	0.5691	0.6966	0.4317	0
5999	500.2	0.4137	0.5688	0.6965	0.4318	0
6000	500.1	0.4133	0.5688	0.6965	0.4316	0
6001	500	0.4133	0.5686	0.6967	0.4318	0
6002	499.9	0.4132	0.5688	0.6966	0.4317	0
6003	499.8	0.413	0.5687	0.6963	0.4313	0
6004	499.7	0.4133	0.5688	0.6965	0.4314	0
6005	499.6	0.4132	0.5685	0.6963	0.4313	0
6006	499.5	0.4131	0.5683	0.6962	0.4313	0

6007	499.4	0.4132	0.5683	0.6966	0.4312	0
6008	499.3	0.4131	0.5682	0.6966	0.4314	0
6009	499.2	0.413	0.5681	0.6966	0.4311	0
6010	499.1	0.413	0.5684	0.6968	0.4312	0
6011	499	0.4128	0.5681	0.6965	0.431	0
6012	498.9	0.4128	0.568	0.6966	0.4308	0
6013	498.8	0.4128	0.5681	0.6969	0.4307	0
6014	498.7	0.4128	0.5679	0.6971	0.4305	0
6015	498.6	0.4129	0.5678	0.6972	0.4307	0
6016	498.5	0.4128	0.568	0.6969	0.4306	0
6017	498.4	0.4128	0.5681	0.6971	0.4307	0
6018	498.3	0.4128	0.5681	0.6971	0.4308	0
6019	498.2	0.4126	0.5678	0.6973	0.4307	0
6020	498.1	0.4128	0.568	0.6975	0.4305	0
6021	498	0.4127	0.5679	0.6974	0.4303	0
6022	497.9	0.4129	0.5682	0.6975	0.4304	0
6023	497.8	0.4125	0.5678	0.6979	0.4304	0
6024	497.7	0.4127	0.5681	0.6979	0.4304	0
6025	497.6	0.4125	0.5677	0.6977	0.4304	0
6026	497.5	0.4123	0.5675	0.6977	0.4303	0
6027	497.4	0.4124	0.568	0.6977	0.4305	0
6028	497.3	0.4123	0.5677	0.698	0.4307	0
6029	497.2	0.4124	0.5678	0.6981	0.4305	0
6030	497.1	0.4125	0.5679	0.6982	0.4307	0
6031	497	0.4125	0.5677	0.6981	0.4307	0
6032	496.9	0.4125	0.5679	0.6982	0.4308	0
6033	496.8	0.4126	0.5681	0.6983	0.4308	0
6034	496.7	0.4126	0.568	0.6982	0.4309	0
6035	496.6	0.4128	0.5681	0.6987	0.4312	0
6036	496.5	0.4127	0.568	0.6984	0.4306	0
6037	496.4	0.4126	0.5683	0.6986	0.4308	0
6038	496.3	0.4129	0.5685	0.6992	0.431	0
6039	496.2	0.4129	0.5682	0.6993	0.431	0
6040	496.1	0.413	0.5682	0.699	0.4306	0
6041	496	0.4128	0.5682	0.6995	0.4309	0
6042	495.9	0.4127	0.5683	0.6997	0.431	0
6043	495.8	0.4129	0.5682	0.6997	0.4308	0
6044	495.7	0.413	0.5683	0.7	0.431	0
6045	495.6	0.4135	0.5687	0.7005	0.4313	0
6046	495.5	0.4135	0.5691	0.7002	0.4314	0
6047	495.4	0.4135	0.5692	0.7005	0.4312	0
6048	495.3	0.4136	0.5695	0.7011	0.4316	0
6049	495.2	0.4137	0.5695	0.7013	0.4316	0

6050	495.1	0.4137	0.5691	0.7015	0.4317	0
6051	495	0.4139	0.5694	0.7014	0.4318	0
6052	494.9	0.4139	0.5695	0.7017	0.4318	0
6053	494.8	0.414	0.5697	0.7018	0.4321	0
6054	494.7	0.4142	0.5697	0.7022	0.4325	0
6055	494.6	0.4142	0.5701	0.7026	0.4326	0
6056	494.5	0.4144	0.5701	0.7025	0.4325	0
6057	494.4	0.4142	0.5702	0.7024	0.4327	0
6058	494.3	0.4144	0.5703	0.7028	0.4327	0
6059	494.2	0.4145	0.5706	0.7031	0.4327	0
6060	494.1	0.4147	0.5705	0.7031	0.4329	0
6061	494	0.4146	0.5707	0.7035	0.4331	0
6062	493.9	0.4146	0.5709	0.7037	0.4334	0
6063	493.8	0.4149	0.571	0.704	0.4334	0
6064	493.7	0.415	0.5714	0.7038	0.4334	0
6065	493.6	0.4151	0.5713	0.7041	0.4335	0
6066	493.5	0.4152	0.5715	0.7046	0.4335	0
6067	493.4	0.4153	0.5716	0.7048	0.4334	0
6068	493.3	0.4153	0.5718	0.7047	0.4337	0
6069	493.2	0.4154	0.5719	0.7048	0.4339	0
6070	493.1	0.4156	0.5717	0.7049	0.4338	0
6071	493	0.4156	0.5715	0.7049	0.4337	0
6072	492.9	0.4153	0.5717	0.7052	0.4339	0
6073	492.8	0.4156	0.572	0.7055	0.4342	0
6074	492.7	0.4158	0.5718	0.7059	0.434	0
6075	492.6	0.4156	0.5721	0.7064	0.4342	0
6076	492.5	0.4156	0.572	0.706	0.4341	0
6077	492.4	0.4157	0.5718	0.7063	0.434	0
6078	492.3	0.4157	0.5721	0.7057	0.4342	0
6079	492.2	0.4156	0.5719	0.7062	0.4342	0
6080	492.1	0.4158	0.5722	0.7063	0.4343	0
6081	492	0.4158	0.5722	0.7062	0.4342	0
6082	491.9	0.4159	0.5724	0.7065	0.4344	0
6083	491.8	0.4158	0.5724	0.7064	0.4345	0
6084	491.7	0.4157	0.5723	0.7064	0.4342	0
6085	491.6	0.4155	0.5723	0.7066	0.4344	0
6086	491.5	0.4154	0.5726	0.7064	0.4344	0
6087	491.4	0.4154	0.5723	0.707	0.4343	0
6088	491.3	0.4156	0.5722	0.7073	0.4342	0
6089	491.2	0.4156	0.5721	0.7069	0.434	0
6090	491.1	0.4156	0.572	0.7069	0.4341	0
6091	491	0.4155	0.5721	0.7069	0.434	0
6092	490.9	0.4156	0.5719	0.7072	0.4342	0

6093	490.8	0.4155	0.5721	0.7075	0.4342	0
6094	490.7	0.4157	0.5722	0.7073	0.4342	0
6095	490.6	0.4157	0.572	0.7072	0.4343	0
6096	490.5	0.4154	0.5721	0.7072	0.4341	0
6097	490.4	0.4156	0.572	0.7074	0.4339	0
6098	490.3	0.4155	0.572	0.7072	0.4339	0
6099	490.2	0.4153	0.5723	0.7074	0.434	0
6100	490.1	0.4152	0.5722	0.7074	0.4337	0
6101	490	0.4153	0.5723	0.7073	0.4337	0
6102	489.9	0.415	0.5718	0.7072	0.4333	0
6103	489.8	0.4153	0.5719	0.7075	0.4334	0
6104	489.7	0.4152	0.5716	0.708	0.4337	0
6105	489.6	0.4154	0.5716	0.7079	0.4337	0
6106	489.5	0.4149	0.5713	0.7074	0.4331	0
6107	489.4	0.4151	0.5712	0.7073	0.4333	0
6108	489.3	0.415	0.5713	0.7072	0.4328	0
6109	489.2	0.4149	0.5713	0.7075	0.4327	0
6110	489.1	0.4147	0.5712	0.7078	0.4331	0
6111	489	0.4142	0.5713	0.7074	0.4329	0
6112	488.9	0.4144	0.5713	0.7075	0.4328	0
6113	488.8	0.4142	0.5712	0.7077	0.4326	0
6114	488.7	0.4144	0.5712	0.7078	0.4327	0
6115	488.6	0.4143	0.5706	0.7078	0.4324	0
6116	488.5	0.4141	0.5703	0.7075	0.4321	0
6117	488.4	0.4141	0.5704	0.7078	0.4322	0
6118	488.3	0.4143	0.5703	0.7078	0.432	0
6119	488.2	0.4145	0.5705	0.708	0.4322	0
6120	488.1	0.4138	0.57	0.7083	0.4324	0
6121	488	0.4136	0.5699	0.7078	0.4319	0
6122	487.9	0.4138	0.5697	0.7074	0.4318	0
6123	487.8	0.4134	0.5699	0.7075	0.4316	0
6124	487.7	0.4134	0.5699	0.7075	0.4317	0
6125	487.6	0.4133	0.57	0.7076	0.4316	0
6126	487.5	0.4134	0.5697	0.7079	0.4313	0
6127	487.4	0.4135	0.5696	0.7077	0.4314	0
6128	487.3	0.4135	0.5699	0.7082	0.4314	0
6129	487.2	0.4134	0.5696	0.7078	0.4313	0
6130	487.1	0.4134	0.5697	0.7078	0.4314	0
6131	487	0.4136	0.5697	0.7082	0.4314	0
6132	486.9	0.4133	0.5699	0.708	0.4315	0
6133	486.8	0.4135	0.5701	0.7078	0.4314	0
6134	486.7	0.4138	0.5698	0.7084	0.4316	0
6135	486.6	0.4135	0.57	0.7083	0.4318	0

6136	486.5	0.4137	0.57	0.7085	0.4316	0
6137	486.4	0.4139	0.5701	0.7082	0.432	0
6138	486.3	0.4133	0.5697	0.7087	0.4316	0
6139	486.2	0.4135	0.5701	0.7089	0.4318	0
6140	486.1	0.4136	0.5698	0.709	0.4315	0
6141	486	0.4138	0.5698	0.7094	0.4321	0
6142	485.9	0.4136	0.5702	0.7093	0.4319	0
6143	485.8	0.4138	0.5703	0.7094	0.432	0
6144	485.7	0.4141	0.5705	0.7098	0.4321	0
6145	485.6	0.414	0.5704	0.7095	0.4322	0
6146	485.5	0.4139	0.5706	0.7099	0.4325	0
6147	485.4	0.4142	0.5707	0.7102	0.4327	0
6148	485.3	0.414	0.5708	0.7101	0.4325	0
6149	485.2	0.4141	0.5709	0.7105	0.4322	0
6150	485.1	0.4143	0.571	0.7107	0.4325	0
6151	485	0.4143	0.5711	0.7106	0.4326	0
6152	484.9	0.4143	0.571	0.7109	0.433	0
6153	484.8	0.4145	0.5715	0.7114	0.4329	0
6154	484.7	0.4148	0.5712	0.7113	0.433	0
6155	484.6	0.4152	0.5716	0.7112	0.433	0
6156	484.5	0.4152	0.5715	0.7115	0.4332	0
6157	484.4	0.4152	0.5716	0.7117	0.4332	0
6158	484.3	0.4151	0.572	0.7116	0.4334	0
6159	484.2	0.4153	0.5723	0.7122	0.4335	0
6160	484.1	0.4156	0.5727	0.7126	0.4338	0
6161	484	0.4156	0.5725	0.7128	0.4339	0
6162	483.9	0.4154	0.5727	0.7131	0.434	0
6163	483.8	0.4154	0.573	0.7133	0.4341	0
6164	483.7	0.4158	0.5733	0.7135	0.4342	0
6165	483.6	0.4158	0.5736	0.7136	0.4343	0
6166	483.5	0.4159	0.5736	0.7137	0.4343	0
6167	483.4	0.4161	0.5736	0.7141	0.4345	0
6168	483.3	0.4161	0.5739	0.7145	0.4345	0
6169	483.2	0.416	0.574	0.7149	0.4347	0
6170	483.1	0.416	0.5739	0.7148	0.4347	0
6171	483	0.4166	0.5741	0.7151	0.435	0
6172	482.9	0.4165	0.5738	0.715	0.4348	0
6173	482.8	0.4164	0.574	0.7152	0.4348	0
6174	482.7	0.4165	0.5742	0.7155	0.4352	0
6175	482.6	0.4166	0.574	0.7153	0.435	0
6176	482.5	0.4169	0.5742	0.7155	0.4351	0
6177	482.4	0.417	0.5741	0.7159	0.4353	0
6178	482.3	0.4169	0.5742	0.7158	0.4353	0

6179	482.2	0.4172	0.5745	0.7162	0.4353	0
6180	482.1	0.4171	0.574	0.7164	0.4352	0
6181	482	0.417	0.5742	0.7161	0.4352	0
6182	481.9	0.417	0.5745	0.7162	0.4354	0
6183	481.8	0.417	0.5744	0.7163	0.4354	0
6184	481.7	0.417	0.5745	0.7163	0.4355	0
6185	481.6	0.417	0.5745	0.7165	0.4352	0
6186	481.5	0.4171	0.5742	0.7169	0.4353	0
6187	481.4	0.4171	0.5745	0.7169	0.4352	0
6188	481.3	0.4169	0.5741	0.7168	0.4353	0
6189	481.2	0.417	0.5749	0.7171	0.4356	0
6190	481.1	0.4171	0.5747	0.7172	0.4353	0
6191	481	0.417	0.5746	0.7172	0.4354	0
6192	480.9	0.4169	0.5744	0.7172	0.4354	0
6193	480.8	0.4168	0.5747	0.7178	0.4354	0
6194	480.7	0.4169	0.5748	0.7173	0.4355	0
6195	480.6	0.4169	0.5745	0.7173	0.4355	0
6196	480.5	0.4168	0.5745	0.7171	0.4355	0
6197	480.4	0.4169	0.5748	0.7174	0.4359	0
6198	480.3	0.4172	0.5748	0.7172	0.4359	0
6199	480.2	0.417	0.5747	0.7178	0.4359	0
6200	480.1	0.417	0.5748	0.7179	0.4357	0
6201	480	0.417	0.5747	0.7177	0.4354	0
6202	479.9	0.4169	0.5744	0.7178	0.4357	0
6203	479.8	0.4172	0.5746	0.7179	0.4357	0
6204	479.7	0.4168	0.5748	0.7177	0.4358	0
6205	479.6	0.4168	0.5739	0.718	0.4354	0
6206	479.5	0.417	0.5744	0.7182	0.4354	0
6207	479.4	0.4165	0.5741	0.718	0.4353	0
6208	479.3	0.4166	0.5742	0.7181	0.4354	0
6209	479.2	0.4166	0.5738	0.7181	0.4354	0
6210	479.1	0.4165	0.5742	0.7181	0.4353	0
6211	479	0.4162	0.574	0.7178	0.4351	0
6212	478.9	0.4167	0.5741	0.7183	0.4354	0
6213	478.8	0.4166	0.5738	0.7181	0.435	0
6214	478.7	0.4165	0.5739	0.718	0.4349	0
6215	478.6	0.4166	0.5738	0.7181	0.4352	0
6216	478.5	0.4163	0.5736	0.7179	0.4349	0
6217	478.4	0.4162	0.5733	0.7178	0.4344	0
6218	478.3	0.4161	0.5733	0.718	0.4346	0
6219	478.2	0.416	0.5736	0.7179	0.4346	0
6220	478.1	0.4159	0.5735	0.718	0.4344	0
6221	478	0.4157	0.5731	0.7177	0.4342	0

6222	477.9	0.4157	0.5736	0.7179	0.4343	0
6223	477.8	0.4157	0.5729	0.7178	0.4343	0
6224	477.7	0.4157	0.5728	0.718	0.4344	0
6225	477.6	0.4157	0.5731	0.718	0.4341	0
6226	477.5	0.4156	0.573	0.7179	0.4344	0
6227	477.4	0.4156	0.5729	0.718	0.4339	0
6228	477.3	0.4156	0.5733	0.7179	0.4341	0
6229	477.2	0.4153	0.573	0.7177	0.4339	0
6230	477.1	0.4154	0.5726	0.7177	0.4337	0
6231	477	0.4153	0.5727	0.7178	0.4337	0
6232	476.9	0.4153	0.5727	0.718	0.4338	0
6233	476.8	0.4154	0.5725	0.7178	0.4339	0
6234	476.7	0.4152	0.5727	0.7179	0.434	0
6235	476.6	0.4152	0.5725	0.7182	0.4337	0
6236	476.5	0.4153	0.5726	0.7182	0.4338	0
6237	476.4	0.4153	0.5726	0.7181	0.4339	0
6238	476.3	0.4154	0.5725	0.7183	0.4339	0
6239	476.2	0.4153	0.5727	0.718	0.4338	0
6240	476.1	0.4155	0.5728	0.7182	0.434	0
6241	476	0.4152	0.5728	0.7187	0.4339	0
6242	475.9	0.4154	0.5727	0.7186	0.434	0
6243	475.8	0.4157	0.5727	0.7189	0.4342	0
6244	475.7	0.4156	0.5725	0.7187	0.4341	0
6245	475.6	0.4157	0.573	0.7185	0.4341	0
6246	475.5	0.4155	0.5728	0.7188	0.4339	0
6247	475.4	0.4153	0.5731	0.7187	0.4338	0
6248	475.3	0.4156	0.5729	0.719	0.434	0
6249	475.2	0.4158	0.5728	0.7188	0.434	0
6250	475.1	0.4158	0.5731	0.7192	0.4345	0
6251	475	0.4157	0.5733	0.7195	0.4343	0
6252	474.9	0.4158	0.5733	0.7194	0.4343	0
6253	474.8	0.4158	0.5732	0.7202	0.4344	0
6254	474.7	0.4162	0.5733	0.7201	0.4346	0
6255	474.6	0.4161	0.5737	0.7199	0.4347	0
6256	474.5	0.416	0.5738	0.7202	0.4345	0
6257	474.4	0.416	0.5739	0.7203	0.4346	0
6258	474.3	0.4161	0.574	0.7209	0.4349	0
6259	474.2	0.416	0.5736	0.7208	0.4349	0
6260	474.1	0.4163	0.5738	0.7209	0.435	0
6261	474	0.4164	0.5738	0.7212	0.4352	0
6262	473.9	0.4169	0.5744	0.7212	0.4353	0
6263	473.8	0.4168	0.5745	0.7215	0.4356	0
6264	473.7	0.4171	0.5746	0.7219	0.4357	0

6265	473.6	0.4171	0.5749	0.7224	0.4358	0
6266	473.5	0.4172	0.5752	0.722	0.4362	0
6267	473.4	0.4172	0.5748	0.722	0.436	0
6268	473.3	0.4174	0.575	0.7221	0.4362	0
6269	473.2	0.4174	0.5756	0.7226	0.4364	0
6270	473.1	0.4172	0.5755	0.7226	0.4366	0
6271	473	0.4175	0.5755	0.723	0.4364	0
6272	472.9	0.4173	0.5755	0.7231	0.4362	0
6273	472.8	0.4175	0.5755	0.7233	0.4363	0
6274	472.7	0.4174	0.5754	0.7234	0.4363	0
6275	472.6	0.4177	0.5755	0.7233	0.4368	0
6276	472.5	0.4178	0.5761	0.7237	0.4369	0
6277	472.4	0.4179	0.576	0.724	0.4366	0
6278	472.3	0.4179	0.576	0.7245	0.4368	0
6279	472.2	0.4181	0.576	0.7245	0.4368	0
6280	472.1	0.4183	0.5761	0.7247	0.4371	0
6281	472	0.4185	0.5761	0.7248	0.437	0
6282	471.9	0.4185	0.5764	0.725	0.437	0
6283	471.8	0.4185	0.5765	0.725	0.4371	0
6284	471.7	0.4184	0.5766	0.7251	0.4373	0
6285	471.6	0.4184	0.5767	0.725	0.4373	0
6286	471.5	0.4186	0.5772	0.7257	0.4374	0
6287	471.4	0.4186	0.5768	0.7254	0.4375	0
6288	471.3	0.4185	0.5768	0.7256	0.4375	0
6289	471.2	0.4184	0.5768	0.7256	0.4373	0
6290	471.1	0.4184	0.5767	0.7253	0.4372	0
6291	471	0.4182	0.577	0.7256	0.4374	0
6292	470.9	0.418	0.5768	0.7254	0.4373	0
6293	470.8	0.418	0.5767	0.7255	0.4372	0
6294	470.7	0.4183	0.5766	0.7256	0.4372	0
6295	470.6	0.4184	0.5766	0.7258	0.4373	0
6296	470.5	0.4183	0.577	0.7263	0.4374	0
6297	470.4	0.4186	0.5769	0.7262	0.4374	0
6298	470.3	0.4188	0.5766	0.7264	0.4374	0
6299	470.2	0.4186	0.5771	0.7264	0.4374	0
6300	470.1	0.4188	0.5771	0.726	0.4376	0
6301	470	0.4188	0.5774	0.7265	0.438	0
6302	469.9	0.4189	0.5773	0.7268	0.4377	0
6303	469.8	0.4185	0.5771	0.7265	0.4378	0
6304	469.7	0.4187	0.5771	0.7268	0.4375	0
6305	469.6	0.4186	0.577	0.7268	0.4375	0
6306	469.5	0.4187	0.5768	0.7265	0.4374	0
6307	469.4	0.4185	0.5768	0.7262	0.4373	0

6308	469.3	0.4184	0.5768	0.7263	0.4375	0
6309	469.2	0.4184	0.5766	0.7267	0.4373	0
6310	469.1	0.4186	0.5768	0.7265	0.4374	0
6311	469	0.4184	0.5766	0.7265	0.4373	0
6312	468.9	0.4183	0.5767	0.7267	0.4375	0
6313	468.8	0.4184	0.5766	0.7267	0.4373	0
6314	468.7	0.4182	0.5766	0.7267	0.4377	0
6315	468.6	0.4184	0.5767	0.7262	0.4369	0
6316	468.5	0.4184	0.5766	0.7263	0.4368	0
6317	468.4	0.4183	0.5762	0.7265	0.437	0
6318	468.3	0.4182	0.5761	0.7264	0.4368	0
6319	468.2	0.4179	0.576	0.7263	0.4364	0
6320	468.1	0.418	0.576	0.7264	0.4365	0
6321	468	0.4179	0.5762	0.7268	0.4364	0
6322	467.9	0.418	0.576	0.7265	0.4363	0
6323	467.8	0.4179	0.5757	0.7263	0.4363	0
6324	467.7	0.4179	0.5759	0.7265	0.4366	0
6325	467.6	0.4177	0.5758	0.7266	0.4362	0
6326	467.5	0.4177	0.5761	0.7266	0.4364	0
6327	467.4	0.4176	0.5757	0.7269	0.4363	0
6328	467.3	0.4176	0.5757	0.7266	0.4362	0
6329	467.2	0.4175	0.5757	0.726	0.4362	0
6330	467.1	0.4173	0.5756	0.7267	0.436	0
6331	467	0.4176	0.5759	0.7265	0.4363	0
6332	466.9	0.4172	0.5753	0.7267	0.436	0
6333	466.8	0.417	0.5755	0.7263	0.4362	0
6334	466.7	0.4171	0.5755	0.7262	0.4361	0
6335	466.6	0.4174	0.5752	0.7266	0.4362	0
6336	466.5	0.4176	0.5754	0.7267	0.4363	0
6337	466.4	0.4173	0.5754	0.7266	0.4363	0
6338	466.3	0.4172	0.5752	0.7265	0.4364	0
6339	466.2	0.4176	0.5753	0.7269	0.4362	0
6340	466.1	0.4174	0.5756	0.727	0.4362	0
6341	466	0.4174	0.5755	0.7271	0.4362	0
6342	465.9	0.4175	0.5757	0.7269	0.4362	0
6343	465.8	0.4174	0.5758	0.7271	0.4365	0
6344	465.7	0.4174	0.5759	0.7271	0.4365	0
6345	465.6	0.4176	0.5759	0.7269	0.4365	0
6346	465.5	0.4172	0.5755	0.7271	0.4362	0
6347	465.4	0.4175	0.5755	0.7273	0.4359	0
6348	465.3	0.4175	0.5755	0.7277	0.4359	0
6349	465.2	0.4173	0.5755	0.7273	0.4363	0
6350	465.1	0.4175	0.5758	0.7273	0.4364	0

6351	465	0.4174	0.5759	0.7278	0.4362	0
6352	464.9	0.4174	0.5762	0.7277	0.4363	0
6353	464.8	0.4174	0.576	0.7274	0.4366	0
6354	464.7	0.4175	0.5756	0.728	0.4363	0
6355	464.6	0.4174	0.5758	0.7279	0.4366	0
6356	464.5	0.4176	0.5761	0.728	0.4367	0
6357	464.4	0.4176	0.5766	0.7281	0.4368	0
6358	464.3	0.4176	0.5767	0.7282	0.4365	0
6359	464.2	0.4177	0.5765	0.7284	0.4368	0
6360	464.1	0.418	0.5766	0.7281	0.437	0
6361	464	0.4182	0.5769	0.7281	0.4369	0
6362	463.9	0.4183	0.5769	0.7286	0.4369	0
6363	463.8	0.4181	0.5768	0.7289	0.4371	0
6364	463.7	0.4183	0.5765	0.7288	0.4372	0
6365	463.6	0.4183	0.5765	0.7292	0.437	0
6366	463.5	0.4184	0.5768	0.7289	0.437	0
6367	463.4	0.4185	0.5769	0.7295	0.4372	0
6368	463.3	0.4185	0.577	0.7299	0.4375	0
6369	463.2	0.4187	0.5773	0.7293	0.4375	0
6370	463.1	0.419	0.5773	0.7301	0.4377	0
6371	463	0.4187	0.577	0.73	0.4376	0
6372	462.9	0.4188	0.5772	0.7297	0.4376	0
6373	462.8	0.4186	0.5773	0.7299	0.4374	0
6374	462.7	0.4186	0.5772	0.7304	0.4375	0
6375	462.6	0.4187	0.5773	0.7306	0.4378	0
6376	462.5	0.419	0.5779	0.7305	0.4382	0
6377	462.4	0.4187	0.5778	0.7303	0.4381	0
6378	462.3	0.4189	0.5774	0.7301	0.4379	0
6379	462.2	0.419	0.5778	0.7303	0.4379	0
6380	462.1	0.4189	0.5776	0.7307	0.4376	0
6381	462	0.4191	0.5775	0.7308	0.4377	0
6382	461.9	0.4192	0.5776	0.7309	0.4378	0
6383	461.8	0.4193	0.5778	0.7307	0.4379	0
6384	461.7	0.4194	0.5777	0.731	0.4382	0
6385	461.6	0.4193	0.5776	0.7312	0.438	0
6386	461.5	0.4195	0.5777	0.7314	0.438	0
6387	461.4	0.4193	0.5778	0.7311	0.438	0
6388	461.3	0.4196	0.578	0.7314	0.438	0
6389	461.2	0.4194	0.5781	0.731	0.4379	0
6390	461.1	0.4196	0.5779	0.7314	0.438	0
6391	461	0.4195	0.5779	0.7315	0.4378	0
6392	460.9	0.4196	0.5779	0.7317	0.4381	0
6393	460.8	0.4193	0.578	0.7315	0.4381	0

6394	460.7	0.4194	0.5781	0.7317	0.4377	0
6395	460.6	0.4196	0.5783	0.7319	0.4381	0
6396	460.5	0.4196	0.5783	0.732	0.4381	0
6397	460.4	0.4195	0.5778	0.7322	0.438	0
6398	460.3	0.4194	0.578	0.732	0.4384	0
6399	460.2	0.4193	0.5784	0.7326	0.4383	0
6400	460.1	0.4195	0.5783	0.7323	0.4385	0
6401	460	0.4195	0.5783	0.7325	0.4387	0
6402	459.9	0.4196	0.5783	0.7325	0.4387	0
6403	459.8	0.4196	0.5785	0.7331	0.4389	0
6404	459.7	0.4195	0.5783	0.7331	0.4382	0
6405	459.6	0.4193	0.5783	0.7327	0.4384	0
6406	459.5	0.4195	0.5783	0.7327	0.4386	0
6407	459.4	0.4195	0.5784	0.7327	0.4385	0
6408	459.3	0.4195	0.5784	0.7332	0.4387	0
6409	459.2	0.4198	0.5784	0.7333	0.4385	0
6410	459.1	0.42	0.5785	0.7333	0.4386	0
6411	459	0.4195	0.5786	0.7332	0.4384	0
6412	458.9	0.4195	0.5787	0.7335	0.4382	0
6413	458.8	0.4195	0.5786	0.7334	0.4384	0
6414	458.7	0.4195	0.5784	0.7331	0.4385	0
6415	458.6	0.4192	0.578	0.7332	0.4384	0
6416	458.5	0.4195	0.5782	0.733	0.4384	0
6417	458.4	0.4193	0.5783	0.7332	0.4384	0
6418	458.3	0.4198	0.5786	0.7332	0.4385	0
6419	458.2	0.4195	0.5782	0.7335	0.4386	0
6420	458.1	0.4194	0.578	0.7334	0.4385	0
6421	458	0.4192	0.5783	0.7337	0.4384	0
6422	457.9	0.4193	0.5783	0.7337	0.4384	0
6423	457.8	0.4191	0.5781	0.7338	0.4383	0
6424	457.7	0.4191	0.5781	0.7335	0.4384	0
6425	457.6	0.4191	0.5782	0.7336	0.4382	0
6426	457.5	0.4191	0.5781	0.7334	0.4382	0
6427	457.4	0.4194	0.5782	0.7333	0.4379	0
6428	457.3	0.4193	0.5781	0.7336	0.4382	0
6429	457.2	0.4194	0.5782	0.7339	0.4382	0
6430	457.1	0.4194	0.5783	0.7337	0.4384	0
6431	457	0.4194	0.578	0.7338	0.4383	0
6432	456.9	0.4194	0.5779	0.734	0.4382	0
6433	456.8	0.4191	0.5779	0.7339	0.4382	0
6434	456.7	0.4193	0.578	0.734	0.4382	0
6435	456.6	0.4192	0.5782	0.734	0.4382	0
6436	456.5	0.4193	0.5782	0.7337	0.438	0

6437	456.4	0.4193	0.5782	0.7342	0.4382	0
6438	456.3	0.4193	0.5782	0.7342	0.4384	0
6439	456.2	0.4196	0.5783	0.7343	0.4383	0
6440	456.1	0.4193	0.5783	0.7344	0.4381	0
6441	456	0.4193	0.5784	0.7347	0.4385	0
6442	455.9	0.4192	0.5786	0.7343	0.4387	0
6443	455.8	0.4192	0.5785	0.7344	0.4386	0
6444	455.7	0.4192	0.5783	0.7344	0.4385	0
6445	455.6	0.4191	0.578	0.7346	0.4384	0
6446	455.5	0.4192	0.5782	0.7347	0.4384	0
6447	455.4	0.4191	0.578	0.7345	0.4385	0
6448	455.3	0.4195	0.5783	0.7346	0.4385	0
6449	455.2	0.4196	0.5785	0.7346	0.4385	0
6450	455.1	0.4197	0.5784	0.7352	0.4385	0
6451	455	0.4195	0.5784	0.7352	0.4388	0
6452	454.9	0.4194	0.5783	0.7353	0.4387	0
6453	454.8	0.4196	0.5785	0.7356	0.4387	0
6454	454.7	0.4197	0.5786	0.7351	0.4389	0
6455	454.6	0.4198	0.5788	0.7352	0.4389	0
6456	454.5	0.4197	0.5786	0.7352	0.439	0
6457	454.4	0.4197	0.5787	0.7357	0.4387	0
6458	454.3	0.4196	0.5788	0.735	0.439	0
6459	454.2	0.4199	0.5788	0.7355	0.439	0
6460	454.1	0.4199	0.5788	0.7355	0.4389	0
6461	454	0.4197	0.5789	0.7355	0.4391	0
6462	453.9	0.4199	0.579	0.7356	0.4391	0
6463	453.8	0.42	0.5792	0.7356	0.4394	0
6464	453.7	0.4199	0.5791	0.7359	0.4395	0
6465	453.6	0.4201	0.5792	0.7363	0.4398	0
6466	453.5	0.4201	0.5793	0.7361	0.4396	0
6467	453.4	0.42	0.5794	0.7365	0.4396	0
6468	453.3	0.42	0.5794	0.7364	0.4396	0
6469	453.2	0.4201	0.5793	0.7363	0.4395	0
6470	453.1	0.4203	0.5797	0.7367	0.44	0
6471	453	0.4203	0.5797	0.737	0.4399	0
6472	452.9	0.4203	0.5797	0.7369	0.4396	0
6473	452.8	0.4205	0.58	0.737	0.4399	0
6474	452.7	0.4205	0.58	0.7367	0.4398	0
6475	452.6	0.4206	0.5799	0.7371	0.4397	0
6476	452.5	0.4206	0.5801	0.7369	0.4396	0
6477	452.4	0.4206	0.5802	0.737	0.44	0
6478	452.3	0.4206	0.5798	0.7371	0.4399	0
6479	452.2	0.4206	0.5798	0.737	0.4396	0

6480	452.1	0.4204	0.58	0.7369	0.4402	0
6481	452	0.4203	0.5797	0.7371	0.4399	0
6482	451.9	0.4205	0.58	0.7369	0.4397	0
6483	451.8	0.4205	0.5805	0.737	0.4399	0
6484	451.7	0.4204	0.5804	0.7371	0.4396	0
6485	451.6	0.4203	0.5805	0.7374	0.4396	0
6486	451.5	0.4203	0.5803	0.7373	0.4395	0
6487	451.4	0.4203	0.5803	0.7372	0.4394	0
6488	451.3	0.4202	0.5804	0.7373	0.4394	0
6489	451.2	0.4203	0.5798	0.737	0.4393	0
6490	451.1	0.4202	0.5797	0.7373	0.4394	0
6491	451	0.4203	0.5796	0.7372	0.4396	0
6492	450.9	0.4204	0.5801	0.7375	0.4396	0
6493	450.8	0.4202	0.5797	0.7377	0.4393	0
6494	450.7	0.4202	0.5797	0.7368	0.4392	0
6495	450.6	0.4205	0.5799	0.7372	0.4393	0
6496	450.5	0.4204	0.5798	0.7373	0.4394	0
6497	450.4	0.4204	0.5798	0.7375	0.4395	0
6498	450.3	0.4204	0.5798	0.7376	0.4394	0
6499	450.2	0.4204	0.5797	0.7377	0.4396	0
6500	450.1	0.4203	0.5793	0.7371	0.4392	0
6501	450	0.4204	0.5795	0.7375	0.4391	0
6502	449.9	0.3965	0.5472	0.6953	0.4132	0
6503	449.8	0.3966	0.5472	0.6955	0.4132	0
6504	449.7	0.3965	0.5471	0.6957	0.4133	0
6505	449.6	0.3963	0.547	0.6956	0.4132	0
6506	449.5	0.3961	0.5471	0.6954	0.4133	0
6507	449.4	0.3963	0.5469	0.6957	0.4133	0
6508	449.3	0.3961	0.5472	0.6958	0.4133	0
6509	449.2	0.3962	0.547	0.6956	0.413	0
6510	449.1	0.3963	0.5471	0.6957	0.4132	0
6511	449	0.3962	0.5467	0.6955	0.4131	0
6512	448.9	0.3963	0.5469	0.6955	0.4133	0
6513	448.8	0.3964	0.5467	0.6954	0.4132	0
6514	448.7	0.3962	0.547	0.6955	0.413	0
6515	448.6	0.3962	0.547	0.6952	0.4129	0
6516	448.5	0.3958	0.547	0.6953	0.4125	0
6517	448.4	0.3961	0.5468	0.6954	0.4129	0
6518	448.3	0.3958	0.5468	0.6953	0.4128	0
6519	448.2	0.396	0.5466	0.6956	0.413	0
6520	448.1	0.396	0.5467	0.6953	0.4131	0
6521	448	0.3958	0.5465	0.6953	0.4127	0
6522	447.9	0.3959	0.5465	0.6953	0.4128	0

6523	447.8	0.3961	0.5469	0.6956	0.4129	0
6524	447.7	0.3961	0.5468	0.6953	0.4127	0
6525	447.6	0.396	0.5466	0.6955	0.4129	0
6526	447.5	0.3959	0.5468	0.6958	0.4125	0
6527	447.4	0.396	0.5468	0.6957	0.4126	0
6528	447.3	0.3958	0.5469	0.6956	0.4126	0
6529	447.2	0.3959	0.5469	0.6957	0.4127	0
6530	447.1	0.396	0.5466	0.6957	0.413	0
6531	447	0.3961	0.5469	0.6959	0.4129	0
6532	446.9	0.396	0.5466	0.6958	0.4129	0
6533	446.8	0.396	0.5469	0.6959	0.4131	0
6534	446.7	0.3962	0.547	0.6958	0.4132	0
6535	446.6	0.396	0.5471	0.6959	0.4128	0
6536	446.5	0.3962	0.5471	0.6963	0.4128	0
6537	446.4	0.3963	0.547	0.6963	0.413	0
6538	446.3	0.3965	0.547	0.696	0.4132	0
6539	446.2	0.3962	0.5469	0.6962	0.413	0
6540	446.1	0.3961	0.5474	0.6963	0.413	0
6541	446	0.3963	0.5477	0.6964	0.413	0
6542	445.9	0.3962	0.5477	0.6968	0.4129	0
6543	445.8	0.3963	0.5474	0.6964	0.4128	0
6544	445.7	0.3964	0.5473	0.6968	0.4134	0
6545	445.6	0.3966	0.5476	0.6972	0.4133	0
6546	445.5	0.3966	0.5476	0.6969	0.4135	0
6547	445.4	0.3963	0.5475	0.6966	0.4137	0
6548	445.3	0.3965	0.5474	0.6968	0.4137	0
6549	445.2	0.3968	0.5478	0.697	0.4138	0
6550	445.1	0.3968	0.5478	0.697	0.4137	0
6551	445	0.3969	0.5481	0.6971	0.4136	0
6552	444.9	0.3971	0.5482	0.697	0.4136	0
6553	444.8	0.3969	0.5483	0.6972	0.4139	0
6554	444.7	0.3969	0.5484	0.6976	0.414	0
6555	444.6	0.397	0.5483	0.6978	0.414	0
6556	444.5	0.397	0.5482	0.6978	0.414	0
6557	444.4	0.3973	0.5485	0.6976	0.4142	0
6558	444.3	0.3972	0.5484	0.6978	0.4143	0
6559	444.2	0.3973	0.5486	0.6978	0.4144	0
6560	444.1	0.3972	0.5488	0.698	0.4143	0
6561	444	0.3974	0.5483	0.6979	0.4144	0
6562	443.9	0.3974	0.5486	0.6979	0.4145	0
6563	443.8	0.3976	0.5488	0.6982	0.4145	0
6564	443.7	0.3976	0.5489	0.6984	0.4145	0
6565	443.6	0.3978	0.5489	0.6988	0.4145	0

6566	443.5	0.3977	0.549	0.6984	0.4145	0
6567	443.4	0.3978	0.5489	0.6982	0.4147	0
6568	443.3	0.3979	0.5492	0.6984	0.4149	0
6569	443.2	0.3978	0.5492	0.6987	0.4149	0
6570	443.1	0.3978	0.5494	0.6987	0.4148	0
6571	443	0.398	0.5495	0.6988	0.415	0
6572	442.9	0.3981	0.5496	0.6987	0.4153	0
6573	442.8	0.3981	0.5497	0.6991	0.4153	0
6574	442.7	0.3983	0.5495	0.6993	0.4154	0
6575	442.6	0.3981	0.5493	0.6991	0.4153	0
6576	442.5	0.398	0.5497	0.6993	0.4152	0
6577	442.4	0.398	0.5494	0.6992	0.4152	0
6578	442.3	0.398	0.5494	0.6994	0.4153	0
6579	442.2	0.3981	0.5494	0.6994	0.4153	0
6580	442.1	0.3981	0.5496	0.6992	0.4151	0
6581	442	0.398	0.5496	0.6992	0.4152	0
6582	441.9	0.3981	0.5494	0.6994	0.4152	0
6583	441.8	0.3979	0.5493	0.6995	0.4151	0
6584	441.7	0.398	0.5494	0.6994	0.4153	0
6585	441.6	0.3978	0.5494	0.6993	0.4152	0
6586	441.5	0.3979	0.5492	0.6995	0.4151	0
6587	441.4	0.398	0.5494	0.6998	0.415	0
6588	441.3	0.3981	0.5493	0.6998	0.4149	0
6589	441.2	0.3981	0.5495	0.6995	0.4149	0
6590	441.1	0.398	0.5494	0.6998	0.415	0
6591	441	0.3981	0.5494	0.6995	0.4152	0
6592	440.9	0.398	0.5494	0.6995	0.4152	0
6593	440.8	0.3977	0.5494	0.6997	0.415	0
6594	440.7	0.3978	0.5494	0.6995	0.415	0
6595	440.6	0.3977	0.5492	0.6994	0.4149	0
6596	440.5	0.3978	0.5494	0.6996	0.4149	0
6597	440.4	0.3978	0.5492	0.6997	0.4147	0
6598	440.3	0.3976	0.5492	0.6998	0.4147	0
6599	440.2	0.3976	0.5494	0.6999	0.4149	0
6600	440.1	0.3976	0.5493	0.6997	0.4148	0
6601	440	0.3976	0.5494	0.6999	0.4147	0
6602	439.9	0.3976	0.5493	0.6999	0.4146	0
6603	439.8	0.3978	0.5493	0.6998	0.4147	0
6604	439.7	0.3977	0.5492	0.6997	0.4145	0
6605	439.6	0.3976	0.549	0.6998	0.4144	0
6606	439.5	0.3976	0.5489	0.6995	0.4143	0
6607	439.4	0.3975	0.549	0.6996	0.4142	0
6608	439.3	0.3977	0.5491	0.6996	0.4141	0

6609	439.2	0.3975	0.5486	0.6995	0.414	0
6610	439.1	0.3977	0.5486	0.6995	0.4139	0
6611	439	0.3977	0.5483	0.6994	0.4142	0
6612	438.9	0.3975	0.5487	0.6993	0.4142	0
6613	438.8	0.3973	0.5487	0.6992	0.4142	0
6614	438.7	0.3973	0.5485	0.6992	0.4143	0
6615	438.6	0.3971	0.5484	0.6992	0.4137	0
6616	438.5	0.3972	0.5484	0.6991	0.4138	0
6617	438.4	0.3971	0.5484	0.6991	0.4139	0
6618	438.3	0.397	0.5484	0.6989	0.4141	0
6619	438.2	0.3971	0.5483	0.6991	0.4144	0
6620	438.1	0.397	0.5482	0.6989	0.414	0
6621	438	0.3969	0.548	0.6989	0.4138	0
6622	437.9	0.3969	0.5483	0.6994	0.4138	0
6623	437.8	0.3969	0.5482	0.6987	0.4136	0
6624	437.7	0.3971	0.5483	0.6987	0.4137	0
6625	437.6	0.3967	0.5481	0.6988	0.4137	0
6626	437.5	0.3968	0.5483	0.6991	0.4136	0
6627	437.4	0.3968	0.5482	0.6992	0.4134	0
6628	437.3	0.3966	0.5481	0.699	0.4133	0
6629	437.2	0.3969	0.548	0.6994	0.4134	0
6630	437.1	0.3967	0.5479	0.6994	0.4135	0
6631	437	0.3967	0.5483	0.6997	0.4136	0
6632	436.9	0.3967	0.5484	0.6995	0.4135	0
6633	436.8	0.3967	0.5486	0.6995	0.4137	0
6634	436.7	0.3967	0.5486	0.6994	0.4138	0
6635	436.6	0.3969	0.5489	0.6995	0.4138	0
6636	436.5	0.3968	0.5483	0.6997	0.4138	0
6637	436.4	0.3967	0.5485	0.6995	0.4138	0
6638	436.3	0.3969	0.5484	0.6995	0.4139	0
6639	436.2	0.3969	0.5484	0.6995	0.4139	0
6640	436.1	0.3969	0.5486	0.6997	0.4141	0
6641	436	0.3971	0.5488	0.7001	0.4144	0
6642	435.9	0.397	0.5487	0.7	0.414	0
6643	435.8	0.397	0.5487	0.7002	0.4143	0
6644	435.7	0.3971	0.5488	0.7007	0.4145	0
6645	435.6	0.3974	0.5488	0.7007	0.4142	0
6646	435.5	0.3976	0.5491	0.7009	0.4144	0
6647	435.4	0.3976	0.5491	0.7003	0.4144	0
6648	435.3	0.3975	0.5493	0.7004	0.4147	0
6649	435.2	0.3975	0.5494	0.7005	0.4145	0
6650	435.1	0.3977	0.5496	0.7012	0.4148	0
6651	435	0.3978	0.5498	0.7009	0.415	0

6652	434.9	0.3976	0.5497	0.701	0.415	0
6653	434.8	0.3974	0.5494	0.7009	0.4149	0
6654	434.7	0.3976	0.5498	0.7012	0.415	0
6655	434.6	0.3978	0.55	0.7017	0.415	0
6656	434.5	0.3977	0.5499	0.7018	0.4152	0
6657	434.4	0.3979	0.5502	0.7014	0.4153	0
6658	434.3	0.398	0.5501	0.7019	0.415	0
6659	434.2	0.3981	0.5504	0.702	0.4154	0
6660	434.1	0.3982	0.5505	0.7026	0.4156	0
6661	434	0.3983	0.5507	0.7023	0.4155	0
6662	433.9	0.3981	0.5509	0.7027	0.4157	0
6663	433.8	0.3983	0.5509	0.7025	0.4157	0
6664	433.7	0.3982	0.5509	0.7026	0.4158	0
6665	433.6	0.3985	0.5511	0.7033	0.4161	0
6666	433.5	0.3988	0.5513	0.703	0.416	0
6667	433.4	0.399	0.5515	0.7031	0.4163	0
6668	433.3	0.399	0.5516	0.7033	0.4162	0
6669	433.2	0.3992	0.5515	0.7032	0.4165	0
6670	433.1	0.3993	0.5516	0.7038	0.4166	0
6671	433	0.3994	0.5519	0.7034	0.4165	0
6672	432.9	0.3993	0.5517	0.7035	0.4164	0
6673	432.8	0.3994	0.5518	0.7038	0.4163	0
6674	432.7	0.3996	0.5518	0.7039	0.4165	0
6675	432.6	0.3992	0.552	0.7036	0.4166	0
6676	432.5	0.3995	0.5521	0.704	0.4167	0
6677	432.4	0.3995	0.5521	0.7036	0.4165	0
6678	432.3	0.3994	0.552	0.704	0.4165	0
6679	432.2	0.3992	0.552	0.7036	0.4166	0
6680	432.1	0.3995	0.5518	0.7043	0.4167	0
6681	432	0.3997	0.5522	0.7042	0.4168	0
6682	431.9	0.3996	0.5521	0.704	0.4167	0
6683	431.8	0.3997	0.552	0.704	0.4166	0
6684	431.7	0.3994	0.5521	0.7042	0.4166	0
6685	431.6	0.3996	0.5518	0.7037	0.4168	0
6686	431.5	0.3996	0.552	0.704	0.4167	0
6687	431.4	0.3999	0.5521	0.7043	0.4168	0
6688	431.3	0.3999	0.5521	0.7042	0.4167	0
6689	431.2	0.3998	0.5521	0.7042	0.4168	0
6690	431.1	0.3997	0.5521	0.704	0.4167	0
6691	431	0.3996	0.5521	0.7043	0.4168	0
6692	430.9	0.3997	0.5519	0.7042	0.4167	0
6693	430.8	0.3994	0.5519	0.7043	0.4166	0
6694	430.7	0.3994	0.5519	0.7043	0.4166	0

6695	430.6	0.3994	0.5519	0.7045	0.4166	0
6696	430.5	0.3996	0.552	0.7047	0.4168	0
6697	430.4	0.3997	0.5518	0.7048	0.4167	0
6698	430.3	0.3993	0.5516	0.7044	0.4166	0
6699	430.2	0.3994	0.5522	0.7046	0.4167	0
6700	430.1	0.3993	0.552	0.7048	0.4166	0
6701	430	0.3994	0.5518	0.7043	0.4164	0
6702	429.9	0.3991	0.5516	0.7044	0.4163	0
6703	429.8	0.399	0.5515	0.7043	0.4164	0
6704	429.7	0.399	0.5514	0.7043	0.4163	0
6705	429.6	0.399	0.5517	0.7042	0.4163	0
6706	429.5	0.3989	0.5515	0.7037	0.4161	0
6707	429.4	0.3989	0.5514	0.7038	0.416	0
6708	429.3	0.3991	0.5516	0.704	0.4163	0
6709	429.2	0.399	0.5516	0.7036	0.416	0
6710	429.1	0.399	0.5513	0.7036	0.4156	0
6711	429	0.3987	0.5515	0.7034	0.4157	0
6712	428.9	0.3987	0.5513	0.7036	0.4156	0
6713	428.8	0.3985	0.5512	0.7038	0.4154	0
6714	428.7	0.3983	0.5509	0.7036	0.4153	0
6715	428.6	0.3981	0.5511	0.7038	0.4155	0
6716	428.5	0.3979	0.5507	0.7034	0.4152	0
6717	428.4	0.3979	0.5507	0.7033	0.4153	0
6718	428.3	0.3981	0.5509	0.7033	0.4152	0
6719	428.2	0.3981	0.5505	0.7032	0.4153	0
6720	428.1	0.3979	0.5504	0.703	0.4148	0
6721	428	0.3981	0.5503	0.703	0.415	0
6722	427.9	0.3982	0.5505	0.703	0.4148	0
6723	427.8	0.3979	0.5504	0.703	0.4147	0
6724	427.7	0.3976	0.5501	0.7027	0.4147	0
6725	427.6	0.3978	0.5501	0.7026	0.4147	0
6726	427.5	0.3977	0.5498	0.7029	0.4148	0
6727	427.4	0.3978	0.55	0.7027	0.4147	0
6728	427.3	0.3979	0.5503	0.703	0.415	0
6729	427.2	0.3976	0.55	0.7031	0.4146	0
6730	427.1	0.3977	0.55	0.703	0.4147	0
6731	427	0.3974	0.55	0.7031	0.4148	0
6732	426.9	0.3974	0.5499	0.7029	0.4146	0
6733	426.8	0.3975	0.5499	0.7029	0.415	0
6734	426.7	0.3975	0.5499	0.7029	0.4148	0
6735	426.6	0.3976	0.5502	0.7032	0.4148	0
6736	426.5	0.3975	0.5502	0.703	0.4148	0
6737	426.4	0.3977	0.5501	0.7031	0.4151	0

6738	426.3	0.3976	0.5502	0.7034	0.4147	0
6739	426.2	0.3977	0.5501	0.7033	0.4147	0
6740	426.1	0.3979	0.5503	0.7033	0.4149	0
6741	426	0.3978	0.5506	0.7036	0.4147	0
6742	425.9	0.3979	0.5503	0.7037	0.415	0
6743	425.8	0.3979	0.5504	0.7036	0.4151	0
6744	425.7	0.3977	0.5503	0.7037	0.4151	0
6745	425.6	0.3978	0.5502	0.7038	0.4152	0
6746	425.5	0.3979	0.5503	0.7043	0.4153	0
6747	425.4	0.3979	0.5507	0.7042	0.4152	0
6748	425.3	0.3978	0.5506	0.7044	0.4152	0
6749	425.2	0.3978	0.5509	0.7043	0.4153	0
6750	425.1	0.398	0.5507	0.7044	0.4153	0
6751	425	0.3982	0.5507	0.7047	0.4156	0
6752	424.9	0.3982	0.5509	0.705	0.4158	0
6753	424.8	0.3983	0.551	0.705	0.4159	0
6754	424.7	0.3983	0.5509	0.7049	0.4158	0
6755	424.6	0.3984	0.5511	0.7051	0.4161	0
6756	424.5	0.3986	0.5514	0.7052	0.4161	0
6757	424.4	0.3986	0.5516	0.7056	0.4162	0
6758	424.3	0.3989	0.5516	0.7055	0.4163	0
6759	424.2	0.399	0.5519	0.7056	0.4163	0
6760	424.1	0.3991	0.5519	0.7056	0.4163	0
6761	424	0.3992	0.5525	0.7063	0.4167	0
6762	423.9	0.3991	0.5523	0.7063	0.4169	0
6763	423.8	0.3992	0.5526	0.7063	0.4171	0
6764	423.7	0.3995	0.5527	0.7064	0.4171	0
6765	423.6	0.3998	0.5526	0.7071	0.4174	0
6766	423.5	0.3996	0.5528	0.7065	0.4174	0
6767	423.4	0.3997	0.5529	0.7069	0.4171	0
6768	423.3	0.3997	0.553	0.7067	0.4173	0
6769	423.2	0.3999	0.5532	0.7075	0.4177	0
6770	423.1	0.4001	0.5533	0.7076	0.4174	0
6771	423	0.4	0.5534	0.7076	0.4177	0
6772	422.9	0.4001	0.5536	0.7075	0.4179	0
6773	422.8	0.4001	0.5535	0.7078	0.4179	0
6774	422.7	0.4003	0.5534	0.7076	0.4175	0
6775	422.6	0.4004	0.5535	0.7075	0.4177	0
6776	422.5	0.4006	0.5536	0.7076	0.4179	0
6777	422.4	0.4005	0.5536	0.7077	0.418	0
6778	422.3	0.4008	0.5539	0.708	0.4181	0
6779	422.2	0.4007	0.554	0.7082	0.4182	0
6780	422.1	0.4009	0.5538	0.708	0.4183	0

6781	422	0.4007	0.5537	0.7082	0.4182	0
6782	421.9	0.4007	0.5538	0.7082	0.4183	0
6783	421.8	0.4007	0.5538	0.7081	0.4182	0
6784	421.7	0.4007	0.554	0.7084	0.4181	0
6785	421.6	0.4009	0.5541	0.7087	0.418	0
6786	421.5	0.4008	0.554	0.7085	0.4183	0
6787	421.4	0.4007	0.5541	0.7085	0.4181	0
6788	421.3	0.4009	0.5543	0.7088	0.4183	0
6789	421.2	0.4009	0.5542	0.7091	0.4183	0
6790	421.1	0.401	0.5544	0.7088	0.4185	0
6791	421	0.4007	0.5538	0.7084	0.4182	0
6792	420.9	0.4005	0.554	0.7082	0.4183	0
6793	420.8	0.4005	0.5541	0.7087	0.4184	0
6794	420.7	0.4007	0.5539	0.7089	0.4184	0
6795	420.6	0.4006	0.5538	0.7088	0.4183	0
6796	420.5	0.4007	0.5537	0.7086	0.4181	0
6797	420.4	0.4008	0.5537	0.7087	0.418	0
6798	420.3	0.4007	0.5538	0.7089	0.4181	0
6799	420.2	0.4007	0.554	0.7089	0.4181	0
6800	420.1	0.4008	0.5541	0.7091	0.4183	0
6801	420	0.4006	0.5539	0.7094	0.4184	0
6802	419.9	0.4008	0.5538	0.7091	0.4183	0
6803	419.8	0.4005	0.5543	0.7087	0.418	0
6804	419.7	0.4005	0.5541	0.7085	0.4182	0
6805	419.6	0.4006	0.5541	0.7088	0.4183	0
6806	419.5	0.4006	0.554	0.7088	0.418	0
6807	419.4	0.4004	0.5536	0.709	0.4177	0
6808	419.3	0.4002	0.5535	0.7085	0.4176	0
6809	419.2	0.4002	0.5534	0.709	0.4177	0
6810	419.1	0.4002	0.5534	0.7084	0.4176	0
6811	419	0.4002	0.5537	0.7082	0.4174	0
6812	418.9	0.4001	0.5533	0.7083	0.4175	0
6813	418.8	0.3999	0.5533	0.708	0.4174	0
6814	418.7	0.3998	0.553	0.7082	0.4174	0
6815	418.6	0.3996	0.5528	0.7082	0.4172	0
6816	418.5	0.3994	0.5528	0.708	0.4171	0
6817	418.4	0.3993	0.5523	0.7082	0.4169	0
6818	418.3	0.3993	0.5522	0.7079	0.4169	0
6819	418.2	0.3992	0.5523	0.7078	0.4166	0
6820	418.1	0.399	0.5522	0.7073	0.4165	0
6821	418	0.3989	0.5525	0.7076	0.4166	0
6822	417.9	0.399	0.5524	0.7074	0.4162	0
6823	417.8	0.3989	0.5524	0.7074	0.4162	0

6824	417.7	0.3989	0.5522	0.7071	0.416	0
6825	417.6	0.3989	0.5521	0.7072	0.4161	0
6826	417.5	0.3988	0.5522	0.7072	0.4158	0
6827	417.4	0.3988	0.5517	0.7074	0.4157	0
6828	417.3	0.3987	0.5518	0.707	0.4157	0
6829	417.2	0.3989	0.5518	0.7074	0.416	0
6830	417.1	0.3989	0.552	0.707	0.4159	0
6831	417	0.3986	0.552	0.7072	0.4157	0
6832	416.9	0.3985	0.5518	0.707	0.4159	0
6833	416.8	0.3983	0.5515	0.7066	0.4158	0
6834	416.7	0.3983	0.5515	0.7068	0.4155	0
6835	416.6	0.3986	0.5515	0.7072	0.4158	0
6836	416.5	0.3986	0.5515	0.7069	0.4154	0
6837	416.4	0.3987	0.5516	0.7069	0.4153	0
6838	416.3	0.3986	0.5512	0.7069	0.4156	0
6839	416.2	0.3983	0.5511	0.7069	0.4154	0
6840	416.1	0.3981	0.5512	0.707	0.4153	0
6841	416	0.3983	0.5513	0.707	0.4154	0
6842	415.9	0.3985	0.5514	0.7071	0.4156	0
6843	415.8	0.3984	0.5516	0.7072	0.4155	0
6844	415.7	0.3988	0.5512	0.7075	0.4157	0
6845	415.6	0.3988	0.5516	0.7074	0.4156	0
6846	415.5	0.3987	0.5515	0.7071	0.4159	0
6847	415.4	0.3987	0.5515	0.7073	0.416	0
6848	415.3	0.3984	0.5517	0.7076	0.4158	0
6849	415.2	0.3985	0.5517	0.7075	0.4159	0
6850	415.1	0.3985	0.5516	0.7074	0.4157	0
6851	415	0.3986	0.5521	0.7073	0.4159	0
6852	414.9	0.3987	0.552	0.7078	0.4158	0
6853	414.8	0.3986	0.5521	0.7077	0.4161	0
6854	414.7	0.399	0.5523	0.708	0.4164	0
6855	414.6	0.3991	0.5524	0.7085	0.4166	0
6856	414.5	0.3989	0.5523	0.7085	0.4165	0
6857	414.4	0.3991	0.5522	0.7082	0.4166	0
6858	414.3	0.399	0.5522	0.7088	0.4167	0
6859	414.2	0.3994	0.5529	0.7091	0.4167	0
6860	414.1	0.3995	0.5527	0.7089	0.4168	0
6861	414	0.3994	0.5527	0.7092	0.4168	0
6862	413.9	0.3992	0.5529	0.7089	0.4171	0
6863	413.8	0.3997	0.5527	0.7089	0.4171	0
6864	413.7	0.3997	0.5529	0.7092	0.4171	0
6865	413.6	0.4001	0.5535	0.7096	0.4175	0
6866	413.5	0.4001	0.5536	0.7101	0.4171	0

6867	413.4	0.4001	0.5538	0.71	0.4175	0
6868	413.3	0.4001	0.554	0.71	0.4174	0
6869	413.2	0.4003	0.5542	0.7106	0.4178	0
6870	413.1	0.4005	0.5541	0.7106	0.4179	0
6871	413	0.4004	0.5542	0.7106	0.418	0
6872	412.9	0.4003	0.5541	0.7108	0.418	0
6873	412.8	0.4006	0.5542	0.7111	0.4181	0
6874	412.7	0.4008	0.5541	0.7111	0.4182	0
6875	412.6	0.4008	0.5541	0.7112	0.4179	0
6876	412.5	0.4007	0.5548	0.7114	0.4181	0
6877	412.4	0.4008	0.5547	0.7114	0.4181	0
6878	412.3	0.4009	0.555	0.7116	0.418	0
6879	412.2	0.4009	0.5549	0.7116	0.4181	0
6880	412.1	0.4009	0.5549	0.7116	0.4183	0
6881	412	0.401	0.5546	0.7117	0.4184	0
6882	411.9	0.4012	0.5549	0.7121	0.4185	0
6883	411.8	0.4012	0.5549	0.7118	0.4185	0
6884	411.7	0.4012	0.5551	0.7119	0.4185	0
6885	411.6	0.4013	0.5552	0.7118	0.4184	0
6886	411.5	0.4012	0.555	0.7119	0.4185	0
6887	411.4	0.4012	0.5551	0.7121	0.4188	0
6888	411.3	0.4012	0.5552	0.7124	0.4185	0
6889	411.2	0.4012	0.555	0.7123	0.4186	0
6890	411.1	0.4014	0.5553	0.7125	0.4187	0
6891	411	0.4015	0.5556	0.7123	0.4187	0
6892	410.9	0.4013	0.5557	0.7121	0.4187	0
6893	410.8	0.4014	0.5552	0.7124	0.4188	0
6894	410.7	0.4015	0.5554	0.7125	0.419	0
6895	410.6	0.4015	0.5556	0.7126	0.4189	0
6896	410.5	0.4015	0.5555	0.7128	0.4191	0
6897	410.4	0.4015	0.5555	0.7128	0.4191	0
6898	410.3	0.4017	0.5557	0.7126	0.4194	0
6899	410.2	0.4013	0.5554	0.7128	0.4193	0
6900	410.1	0.4012	0.5554	0.7124	0.419	0
6901	410	0.4014	0.5554	0.7129	0.4192	0
6902	409.9	0.4015	0.5555	0.7128	0.4191	0
6903	409.8	0.4016	0.5552	0.7127	0.4192	0
6904	409.7	0.4014	0.5554	0.713	0.4192	0
6905	409.6	0.4013	0.5551	0.7129	0.4193	0
6906	409.5	0.4015	0.5553	0.713	0.4193	0
6907	409.4	0.4016	0.5555	0.7129	0.419	0
6908	409.3	0.4014	0.5554	0.713	0.419	0
6909	409.2	0.4015	0.5556	0.7129	0.4188	0

6910	409.1	0.4013	0.5555	0.713	0.4187	0
6911	409	0.4015	0.5556	0.7132	0.4188	0
6912	408.9	0.4011	0.5551	0.7129	0.4186	0
6913	408.8	0.4012	0.5553	0.713	0.4189	0
6914	408.7	0.4009	0.5549	0.7127	0.4187	0
6915	408.6	0.401	0.555	0.7131	0.4189	0
6916	408.5	0.4009	0.5551	0.7128	0.419	0
6917	408.4	0.4003	0.5548	0.7129	0.4189	0
6918	408.3	0.4007	0.5547	0.7128	0.4186	0
6919	408.2	0.4006	0.5549	0.7125	0.4185	0
6920	408.1	0.4006	0.5549	0.7125	0.4185	0
6921	408	0.4002	0.5548	0.7121	0.4182	0
6922	407.9	0.4002	0.5547	0.7116	0.4179	0
6923	407.8	0.4005	0.5545	0.7122	0.4181	0
6924	407.7	0.4004	0.5546	0.7121	0.4179	0
6925	407.6	0.4004	0.5544	0.7121	0.4179	0
6926	407.5	0.4004	0.5543	0.7122	0.4177	0
6927	407.4	0.3999	0.5543	0.7121	0.4174	0
6928	407.3	0.4	0.5542	0.7123	0.4178	0
6929	407.2	0.4	0.554	0.7123	0.4175	0
6930	407.1	0.4	0.5541	0.7127	0.4173	0
6931	407	0.3998	0.5541	0.712	0.4174	0
6932	406.9	0.3998	0.5542	0.7116	0.4178	0
6933	406.8	0.4	0.5544	0.7116	0.4178	0
6934	406.7	0.4002	0.5541	0.7118	0.4175	0
6935	406.6	0.4002	0.5539	0.7118	0.4175	0
6936	406.5	0.4001	0.5537	0.7118	0.4171	0
6937	406.4	0.4002	0.5536	0.7116	0.4172	0
6938	406.3	0.3999	0.5537	0.7112	0.417	0
6939	406.2	0.4001	0.5537	0.7119	0.417	0
6940	406.1	0.4003	0.554	0.7118	0.4172	0
6941	406	0.4	0.5536	0.7115	0.4171	0
6942	405.9	0.3998	0.5537	0.7113	0.4172	0
6943	405.8	0.3999	0.5539	0.7115	0.4173	0
6944	405.7	0.3997	0.5538	0.7115	0.4175	0
6945	405.6	0.3999	0.5537	0.7119	0.4173	0
6946	405.5	0.3996	0.5535	0.712	0.4175	0
6947	405.4	0.3994	0.5536	0.7118	0.4174	0
6948	405.3	0.3998	0.5538	0.7121	0.4173	0
6949	405.2	0.3999	0.5537	0.7122	0.4174	0
6950	405.1	0.4	0.5536	0.7116	0.4176	0
6951	405	0.3999	0.5535	0.7118	0.4174	0
6952	404.9	0.4001	0.5538	0.7116	0.4171	0

6953	404.8	0.3999	0.5537	0.712	0.4172	0
6954	404.7	0.4001	0.5541	0.7123	0.4173	0
6955	404.6	0.4001	0.5541	0.7122	0.4172	0
6956	404.5	0.4	0.5542	0.7123	0.4173	0
6957	404.4	0.4001	0.5544	0.7123	0.4175	0
6958	404.3	0.4003	0.5546	0.7124	0.4175	0
6959	404.2	0.4005	0.5548	0.7128	0.418	0
6960	404.1	0.4004	0.5549	0.713	0.4181	0
6961	404	0.4006	0.555	0.713	0.4179	0
6962	403.9	0.4005	0.5549	0.713	0.4177	0
6963	403.8	0.4003	0.5547	0.713	0.4182	0
6964	403.7	0.4006	0.5549	0.7134	0.4183	0
6965	403.6	0.4008	0.5549	0.7135	0.4182	0
6966	403.5	0.401	0.5548	0.7137	0.4181	0
6967	403.4	0.4008	0.5552	0.714	0.4185	0
6968	403.3	0.4006	0.555	0.7137	0.4186	0
6969	403.2	0.4005	0.5551	0.714	0.4186	0
6970	403.1	0.4009	0.5554	0.7142	0.4187	0
6971	403	0.4013	0.5559	0.7144	0.4189	0
6972	402.9	0.4013	0.5556	0.7147	0.419	0
6973	402.8	0.4015	0.5557	0.7148	0.4192	0
6974	402.7	0.4013	0.5556	0.7147	0.4192	0
6975	402.6	0.4015	0.5559	0.7146	0.4194	0
6976	402.5	0.4016	0.5558	0.7148	0.4194	0
6977	402.4	0.4015	0.5557	0.7154	0.4195	0
6978	402.3	0.4016	0.5558	0.7149	0.4192	0
6979	402.2	0.4012	0.5558	0.7144	0.4194	0
6980	402.1	0.4013	0.556	0.7146	0.4193	0
6981	402	0.4013	0.5562	0.7146	0.4193	0
6982	401.9	0.4014	0.5561	0.7152	0.4191	0
6983	401.8	0.4013	0.5564	0.7151	0.4191	0
6984	401.7	0.4013	0.5561	0.7153	0.4191	0
6985	401.6	0.4017	0.5558	0.7148	0.419	0
6986	401.5	0.4018	0.5559	0.7152	0.4192	0
6987	401.4	0.4019	0.5564	0.7155	0.4195	0
6988	401.3	0.4019	0.5562	0.7157	0.4195	0
6989	401.2	0.4019	0.5566	0.7157	0.4195	0
6990	401.1	0.4019	0.5564	0.7153	0.4194	0
6991	401	0.4018	0.5566	0.716	0.4195	0
6992	400.9	0.4018	0.5564	0.7158	0.4195	0
6993	400.8	0.4018	0.5565	0.7156	0.4195	0
6994	400.7	0.4018	0.5564	0.7157	0.4197	0
6995	400.6	0.4018	0.5566	0.7157	0.42	0

6996	400.5	0.4019	0.5563	0.7156	0.4199	0
6997	400.4	0.4018	0.5562	0.7155	0.4193	0
6998	400.3	0.4017	0.5565	0.716	0.4197	0
6999	400.2	0.402	0.5569	0.7159	0.4198	0
7000	400.1	0.4018	0.5562	0.716	0.4196	0
7001	400	0.4021	0.5563	0.7159	0.42	0
7002	399.9	0.402	0.5565	0.716	0.42	0
7003	399.8	0.4019	0.5563	0.7158	0.4199	0
7004	399.7	0.4019	0.5565	0.716	0.4198	0
7005	399.6	0.4021	0.5564	0.7163	0.4197	0
7006	399.5	0.4019	0.5567	0.7157	0.4197	0
7007	399.4	0.4018	0.5565	0.7159	0.4194	0
7008	399.3	0.4019	0.5569	0.7161	0.4197	0
7009	399.2	0.402	0.557	0.7163	0.4197	0
7010	399.1	0.4018	0.5568	0.7161	0.4198	0
7011	399	0.4017	0.5564	0.7161	0.4196	0
7012	398.9	0.402	0.5563	0.7165	0.4195	0
7013	398.8	0.4019	0.5564	0.7162	0.4194	0
7014	398.7	0.402	0.5566	0.7165	0.4195	0
7015	398.6	0.4018	0.5564	0.7165	0.4195	0
7016	398.5	0.402	0.5564	0.7166	0.4197	0
7017	398.4	0.4019	0.5564	0.7163	0.4196	0
7018	398.3	0.4017	0.5563	0.7164	0.4194	0
7019	398.2	0.4017	0.5562	0.7168	0.4193	0
7020	398.1	0.4016	0.5561	0.716	0.4195	0
7021	398	0.4013	0.556	0.716	0.4192	0
7022	397.9	0.4013	0.5559	0.716	0.4191	0
7023	397.8	0.4016	0.5559	0.7161	0.4193	0
7024	397.7	0.4018	0.5561	0.7165	0.4192	0
7025	397.6	0.4018	0.5559	0.716	0.4193	0
7026	397.5	0.4018	0.5562	0.7158	0.4193	0
7027	397.4	0.4014	0.5558	0.7157	0.4192	0
7028	397.3	0.4016	0.5558	0.7158	0.4187	0
7029	397.2	0.4017	0.5561	0.7159	0.4191	0
7030	397.1	0.4016	0.5561	0.7169	0.4191	0
7031	397	0.4013	0.556	0.7167	0.419	0
7032	396.9	0.4012	0.5558	0.7166	0.4189	0
7033	396.8	0.4012	0.5559	0.7165	0.4188	0
7034	396.7	0.401	0.5562	0.7163	0.4189	0
7035	396.6	0.4009	0.5562	0.7162	0.4189	0
7036	396.5	0.4012	0.5563	0.7164	0.4189	0
7037	396.4	0.4011	0.5563	0.7163	0.4189	0
7038	396.3	0.4014	0.5565	0.7165	0.4191	0

7039	396.2	0.4009	0.5561	0.7162	0.4186	0
7040	396.1	0.4011	0.5563	0.7161	0.4188	0
7041	396	0.4013	0.5566	0.7167	0.4186	0
7042	395.9	0.4012	0.5562	0.7163	0.419	0
7043	395.8	0.4014	0.5566	0.7163	0.4189	0
7044	395.7	0.401	0.5562	0.7162	0.4187	0
7045	395.6	0.4014	0.5562	0.7161	0.4187	0
7046	395.5	0.4014	0.5562	0.716	0.4189	0
7047	395.4	0.4012	0.5565	0.716	0.4186	0
7048	395.3	0.4012	0.5565	0.7162	0.4187	0
7049	395.2	0.4011	0.5564	0.7163	0.4186	0
7050	395.1	0.4013	0.5564	0.7165	0.4186	0
7051	395	0.4013	0.5565	0.7169	0.4189	0
7052	394.9	0.401	0.5563	0.7171	0.4187	0
7053	394.8	0.401	0.556	0.7167	0.4189	0
7054	394.7	0.4011	0.5562	0.717	0.4189	0
7055	394.6	0.4012	0.5568	0.7166	0.4189	0
7056	394.5	0.401	0.5565	0.7169	0.419	0
7057	394.4	0.4011	0.5567	0.717	0.4187	0
7058	394.3	0.4012	0.5567	0.7171	0.4191	0
7059	394.2	0.4012	0.5565	0.7172	0.4194	0
7060	394.1	0.4013	0.5566	0.7173	0.4191	0
7061	394	0.4014	0.5566	0.7171	0.4191	0
7062	393.9	0.4014	0.5569	0.7176	0.4191	0
7063	393.8	0.4015	0.5567	0.7175	0.4192	0
7064	393.7	0.4014	0.5568	0.7175	0.419	0
7065	393.6	0.4018	0.557	0.7173	0.419	0
7066	393.5	0.4018	0.5574	0.718	0.4193	0
7067	393.4	0.4021	0.5575	0.7174	0.4196	0
7068	393.3	0.402	0.5574	0.7181	0.4196	0
7069	393.2	0.4019	0.5574	0.7175	0.4196	0
7070	393.1	0.4019	0.5574	0.7179	0.4196	0
7071	393	0.4019	0.5576	0.7183	0.4197	0
7072	392.9	0.4022	0.5579	0.7181	0.4198	0
7073	392.8	0.4022	0.558	0.718	0.42	0
7074	392.7	0.4021	0.558	0.718	0.42	0
7075	392.6	0.4023	0.5577	0.7182	0.4201	0
7076	392.5	0.402	0.5578	0.7183	0.4201	0
7077	392.4	0.4022	0.5579	0.7185	0.4199	0
7078	392.3	0.4022	0.5577	0.7186	0.4199	0
7079	392.2	0.4025	0.5582	0.7186	0.4204	0
7080	392.1	0.4022	0.5578	0.7186	0.4201	0
7081	392	0.4024	0.558	0.7185	0.4201	0

7082	391.9	0.4022	0.5578	0.7187	0.4204	0
7083	391.8	0.4021	0.5576	0.7185	0.4202	0
7084	391.7	0.4022	0.5579	0.7186	0.4202	0
7085	391.6	0.4024	0.558	0.7185	0.4203	0
7086	391.5	0.4025	0.5577	0.7188	0.4204	0
7087	391.4	0.4027	0.558	0.7187	0.4202	0
7088	391.3	0.4026	0.5582	0.719	0.4203	0
7089	391.2	0.4027	0.5582	0.7188	0.4203	0
7090	391.1	0.4023	0.5578	0.7184	0.4202	0
7091	391	0.4024	0.5582	0.719	0.4203	0
7092	390.9	0.402	0.5578	0.7191	0.4201	0
7093	390.8	0.4022	0.5577	0.719	0.4199	0
7094	390.7	0.4021	0.5575	0.7189	0.4199	0
7095	390.6	0.4022	0.558	0.7189	0.4202	0
7096	390.5	0.4024	0.5579	0.719	0.4203	0
7097	390.4	0.4023	0.5578	0.719	0.4202	0
7098	390.3	0.4024	0.558	0.7188	0.42	0
7099	390.2	0.4025	0.5581	0.7191	0.4202	0
7100	390.1	0.4022	0.5581	0.7195	0.4202	0
7101	390	0.4021	0.5582	0.7192	0.4202	0
7102	389.9	0.4019	0.5576	0.7189	0.4201	0
7103	389.8	0.402	0.5582	0.7192	0.4204	0
7104	389.7	0.4021	0.5582	0.7187	0.4203	0
7105	389.6	0.4018	0.558	0.7188	0.42	0
7106	389.5	0.4019	0.5579	0.7191	0.4198	0
7107	389.4	0.4018	0.5581	0.7191	0.4198	0
7108	389.3	0.402	0.5581	0.7188	0.42	0
7109	389.2	0.4021	0.5583	0.719	0.4197	0
7110	389.1	0.4022	0.5579	0.7187	0.4202	0
7111	389	0.4019	0.5578	0.7194	0.4199	0
7112	388.9	0.402	0.5575	0.7195	0.4196	0
7113	388.8	0.4019	0.5578	0.719	0.4198	0
7114	388.7	0.4019	0.5577	0.7191	0.4195	0
7115	388.6	0.4021	0.558	0.7191	0.4195	0
7116	388.5	0.4021	0.5578	0.7191	0.4196	0
7117	388.4	0.402	0.558	0.719	0.4197	0
7118	388.3	0.402	0.5576	0.7189	0.4195	0
7119	388.2	0.402	0.5573	0.7187	0.4193	0
7120	388.1	0.4018	0.557	0.7188	0.4195	0
7121	388	0.402	0.5572	0.7188	0.4195	0
7122	387.9	0.402	0.5574	0.7192	0.4195	0
7123	387.8	0.4021	0.5576	0.7194	0.4193	0
7124	387.7	0.4018	0.5575	0.719	0.4195	0

7125	387.6	0.4018	0.5576	0.7192	0.4196	0
7126	387.5	0.402	0.5574	0.7192	0.4198	0
7127	387.4	0.4023	0.5577	0.7197	0.4201	0
7128	387.3	0.4021	0.5575	0.7194	0.4197	0
7129	387.2	0.4019	0.5578	0.7192	0.4197	0
7130	387.1	0.4021	0.5578	0.7195	0.4195	0
7131	387	0.4023	0.5578	0.7194	0.4198	0
7132	386.9	0.4023	0.5578	0.7192	0.4197	0
7133	386.8	0.4024	0.5578	0.7188	0.4196	0
7134	386.7	0.402	0.5581	0.7193	0.4195	0
7135	386.6	0.402	0.5583	0.7198	0.4198	0
7136	386.5	0.4023	0.5583	0.7201	0.42	0
7137	386.4	0.4022	0.5582	0.7192	0.4199	0
7138	386.3	0.4021	0.5582	0.7193	0.4199	0
7139	386.2	0.4023	0.5584	0.72	0.42	0
7140	386.1	0.4025	0.5582	0.7196	0.4202	0
7141	386	0.4024	0.5583	0.72	0.42	0
7142	385.9	0.4024	0.5584	0.7202	0.4203	0
7143	385.8	0.4023	0.5584	0.7203	0.4203	0
7144	385.7	0.4028	0.5585	0.7203	0.4204	0
7145	385.6	0.4027	0.5585	0.7208	0.4206	0
7146	385.5	0.4028	0.5585	0.7202	0.4206	0
7147	385.4	0.4026	0.5584	0.7205	0.4206	0
7148	385.3	0.4026	0.5585	0.7204	0.4207	0
7149	385.2	0.4029	0.559	0.7208	0.4208	0
7150	385.1	0.403	0.5588	0.7207	0.4209	0
7151	385	0.4028	0.5588	0.7213	0.4208	0
7152	384.9	0.4029	0.559	0.721	0.4213	0
7153	384.8	0.4029	0.5589	0.7212	0.4211	0
7154	384.7	0.4032	0.5592	0.7214	0.4209	0
7155	384.6	0.4033	0.5594	0.7211	0.4211	0
7156	384.5	0.4035	0.5598	0.7217	0.4213	0
7157	384.4	0.4035	0.5599	0.722	0.4212	0
7158	384.3	0.4034	0.5597	0.722	0.4213	0
7159	384.2	0.4035	0.5599	0.7223	0.4214	0
7160	384.1	0.4037	0.56	0.7222	0.4218	0
7161	384	0.4038	0.5599	0.7224	0.4215	0
7162	383.9	0.4039	0.5598	0.7223	0.4216	0
7163	383.8	0.4038	0.5599	0.7226	0.4219	0
7164	383.7	0.4037	0.5601	0.7219	0.4216	0
7165	383.6	0.4042	0.5604	0.7229	0.4221	0
7166	383.5	0.404	0.5604	0.7228	0.4218	0
7167	383.4	0.4041	0.5603	0.7227	0.4217	0

7168	383.3	0.4042	0.5606	0.7229	0.4219	0
7169	383.2	0.4044	0.5607	0.7225	0.4221	0
7170	383.1	0.404	0.5602	0.7224	0.4219	0
7171	383	0.4039	0.5601	0.7228	0.422	0
7172	382.9	0.404	0.5607	0.7236	0.4223	0
7173	382.8	0.4043	0.5608	0.7239	0.4224	0
7174	382.7	0.4041	0.5606	0.7231	0.4224	0
7175	382.6	0.4042	0.5605	0.7232	0.4225	0
7176	382.5	0.4043	0.5605	0.7231	0.4225	0
7177	382.4	0.4042	0.5605	0.7237	0.4225	0
7178	382.3	0.4043	0.5608	0.7236	0.4224	0
7179	382.2	0.4045	0.5609	0.7233	0.422	0
7180	382.1	0.4043	0.561	0.7239	0.4221	0
7181	382	0.4045	0.5612	0.7238	0.4224	0
7182	381.9	0.4043	0.561	0.7236	0.4223	0
7183	381.8	0.4044	0.5612	0.7237	0.4223	0
7184	381.7	0.4044	0.5607	0.7234	0.4224	0
7185	381.6	0.4044	0.5608	0.7237	0.4223	0
7186	381.5	0.4044	0.5607	0.7238	0.4226	0
7187	381.4	0.4043	0.5609	0.7239	0.4225	0
7188	381.3	0.4046	0.5606	0.724	0.4222	0
7189	381.2	0.4047	0.5604	0.7236	0.4228	0
7190	381.1	0.4042	0.5607	0.7237	0.4227	0
7191	381	0.4043	0.5607	0.7235	0.4226	0
7192	380.9	0.4041	0.5604	0.7236	0.4224	0
7193	380.8	0.404	0.5604	0.7232	0.4223	0
7194	380.7	0.404	0.5606	0.7233	0.422	0
7195	380.6	0.404	0.5605	0.7231	0.4217	0
7196	380.5	0.404	0.5606	0.7235	0.4218	0
7197	380.4	0.404	0.5604	0.7237	0.4219	0
7198	380.3	0.4041	0.5602	0.7236	0.4218	0
7199	380.2	0.404	0.5605	0.7239	0.4218	0
7200	380.1	0.4039	0.5603	0.7237	0.4218	0
7201	380	0.404	0.5603	0.7236	0.4215	0
7202	379.9	0.404	0.5604	0.7236	0.4216	0
7203	379.8	0.4038	0.56	0.7236	0.4216	0
7204	379.7	0.4038	0.5603	0.7235	0.4216	0
7205	379.6	0.4038	0.5604	0.7231	0.4217	0
7206	379.5	0.4037	0.5602	0.7233	0.4218	0
7207	379.4	0.4037	0.56	0.7233	0.4216	0
7208	379.3	0.4036	0.5599	0.7234	0.4216	0
7209	379.2	0.4036	0.5599	0.7234	0.4214	0
7210	379.1	0.4038	0.5601	0.7235	0.4216	0

7211	379	0.4039	0.5598	0.7233	0.4213	0
7212	378.9	0.4039	0.5598	0.7232	0.4212	0
7213	378.8	0.4037	0.5597	0.7232	0.4213	0
7214	378.7	0.4036	0.5598	0.7227	0.4212	0
7215	378.6	0.4034	0.5598	0.7232	0.4212	0
7216	378.5	0.4032	0.5595	0.7231	0.4211	0
7217	378.4	0.4034	0.5595	0.7232	0.4207	0
7218	378.3	0.4032	0.5596	0.7233	0.4209	0
7219	378.2	0.4034	0.5597	0.723	0.4208	0
7220	378.1	0.4033	0.5596	0.7227	0.4208	0
7221	378	0.4035	0.5596	0.7227	0.4209	0
7222	377.9	0.4032	0.5598	0.7229	0.4211	0
7223	377.8	0.4029	0.5596	0.7228	0.4207	0
7224	377.7	0.4031	0.5597	0.7227	0.4206	0
7225	377.6	0.4032	0.56	0.7232	0.4206	0
7226	377.5	0.4031	0.56	0.7228	0.4206	0
7227	377.4	0.4031	0.56	0.723	0.4207	0
7228	377.3	0.4032	0.5599	0.7229	0.4204	0
7229	377.2	0.4033	0.5597	0.7232	0.4207	0
7230	377.1	0.4032	0.5595	0.7232	0.4209	0
7231	377	0.4033	0.5597	0.7235	0.4209	0
7232	376.9	0.4034	0.5601	0.7236	0.4208	0
7233	376.8	0.4034	0.5598	0.7237	0.4207	0
7234	376.7	0.4036	0.5598	0.7237	0.4212	0
7235	376.6	0.4037	0.5598	0.7236	0.4212	0
7236	376.5	0.4035	0.5596	0.724	0.421	0
7237	376.4	0.4036	0.5597	0.7238	0.4212	0
7238	376.3	0.4035	0.5597	0.7238	0.4216	0
7239	376.2	0.4035	0.5598	0.7245	0.4215	0
7240	376.1	0.4038	0.5602	0.7243	0.4216	0
7241	376	0.4039	0.5601	0.7246	0.4218	0
7242	375.9	0.4039	0.5607	0.7247	0.4219	0
7243	375.8	0.4041	0.5604	0.7249	0.4219	0
7244	375.7	0.4045	0.5607	0.7249	0.4221	0
7245	375.6	0.4048	0.5608	0.7247	0.4226	0
7246	375.5	0.4049	0.5609	0.7249	0.4226	0
7247	375.4	0.4045	0.561	0.7251	0.4225	0
7248	375.3	0.4044	0.561	0.7253	0.4225	0
7249	375.2	0.4043	0.561	0.7259	0.4226	0
7250	375.1	0.4045	0.5613	0.7255	0.4227	0
7251	375	0.4045	0.5615	0.7251	0.4228	0
7252	374.9	0.4045	0.5616	0.7254	0.4225	0
7253	374.8	0.4047	0.562	0.7258	0.4227	0

7254	374.7	0.405	0.562	0.7258	0.423	0
7255	374.6	0.405	0.5621	0.7259	0.4231	0
7256	374.5	0.4052	0.5623	0.726	0.4234	0
7257	374.4	0.4053	0.5625	0.7263	0.4234	0
7258	374.3	0.4056	0.5624	0.7264	0.4235	0
7259	374.2	0.4056	0.5624	0.7266	0.4233	0
7260	374.1	0.4057	0.5626	0.7269	0.4234	0
7261	374	0.4055	0.5629	0.7272	0.424	0
7262	373.9	0.4059	0.5628	0.7272	0.4238	0
7263	373.8	0.4057	0.5631	0.7271	0.4236	0
7264	373.7	0.4056	0.5631	0.7276	0.4239	0
7265	373.6	0.4058	0.5629	0.727	0.4239	0
7266	373.5	0.4064	0.5633	0.7275	0.4242	0
7267	373.4	0.4061	0.5634	0.7276	0.4242	0
7268	373.3	0.4065	0.5637	0.7275	0.4244	0
7269	373.2	0.4065	0.5638	0.7278	0.4244	0
7270	373.1	0.4067	0.5637	0.7276	0.4246	0
7271	373	0.4065	0.5637	0.7278	0.4245	0
7272	372.9	0.4063	0.5635	0.7281	0.4244	0
7273	372.8	0.4064	0.5637	0.7281	0.4244	0
7274	372.7	0.4067	0.5637	0.7278	0.4244	0
7275	372.6	0.4067	0.5635	0.7281	0.4244	0
7276	372.5	0.4066	0.564	0.728	0.4246	0
7277	372.4	0.4065	0.5643	0.7283	0.4247	0
7278	372.3	0.4065	0.5644	0.7283	0.4246	0
7279	372.2	0.4065	0.5638	0.7287	0.4245	0
7280	372.1	0.4067	0.564	0.7286	0.4246	0
7281	372	0.4066	0.5638	0.7282	0.4245	0
7282	371.9	0.4069	0.5642	0.7287	0.4248	0
7283	371.8	0.4068	0.5643	0.7284	0.4249	0
7284	371.7	0.4068	0.5643	0.7283	0.4249	0
7285	371.6	0.4068	0.5645	0.7282	0.4248	0
7286	371.5	0.4068	0.5642	0.7281	0.4246	0
7287	371.4	0.4067	0.5642	0.7282	0.4249	0
7288	371.3	0.4064	0.5641	0.7281	0.4249	0
7289	371.2	0.4064	0.5638	0.7283	0.4245	0
7290	371.1	0.4067	0.5638	0.7283	0.4245	0
7291	371	0.4065	0.564	0.7287	0.4245	0
7292	370.9	0.4065	0.5633	0.7288	0.4246	0
7293	370.8	0.4065	0.5634	0.7286	0.4245	0
7294	370.7	0.4064	0.5637	0.7285	0.4241	0
7295	370.6	0.4065	0.564	0.7283	0.4242	0
7296	370.5	0.4063	0.5641	0.7281	0.424	0

7297	370.4	0.4062	0.564	0.7282	0.4241	0
7298	370.3	0.4061	0.5636	0.7277	0.4236	0
7299	370.2	0.406	0.5634	0.7277	0.4235	0
7300	370.1	0.406	0.5636	0.7277	0.4237	0
7301	370	0.4062	0.5636	0.7282	0.4238	0
7302	369.9	0.4047	0.5625	0.7282	0.4222	0
7303	369.8	0.4047	0.5624	0.7284	0.4222	0
7304	369.7	0.4046	0.5621	0.7279	0.4222	0
7305	369.6	0.4048	0.5623	0.7279	0.422	0
7306	369.5	0.4046	0.5622	0.7277	0.4221	0
7307	369.4	0.4045	0.5621	0.7277	0.422	0
7308	369.3	0.4045	0.5621	0.7275	0.422	0
7309	369.2	0.4043	0.562	0.7275	0.4221	0
7310	369.1	0.4045	0.5617	0.7271	0.4218	0
7311	369	0.4042	0.5616	0.7268	0.4218	0
7312	368.9	0.404	0.5617	0.7272	0.4214	0
7313	368.8	0.4038	0.5614	0.727	0.4215	0
7314	368.7	0.4039	0.5616	0.7267	0.4214	0
7315	368.6	0.4041	0.5615	0.7269	0.4215	0
7316	368.5	0.4038	0.5613	0.7268	0.4214	0
7317	368.4	0.4036	0.5613	0.7266	0.4211	0
7318	368.3	0.4035	0.5614	0.7264	0.4212	0
7319	368.2	0.4034	0.5613	0.7266	0.421	0
7320	368.1	0.4036	0.5612	0.7262	0.4214	0
7321	368	0.4035	0.5608	0.7263	0.4209	0
7322	367.9	0.4034	0.5611	0.7262	0.4213	0
7323	367.8	0.4032	0.561	0.7263	0.4211	0
7324	367.7	0.4031	0.5606	0.7262	0.4209	0
7325	367.6	0.4031	0.5609	0.7261	0.421	0
7326	367.5	0.4031	0.561	0.7262	0.4208	0
7327	367.4	0.4032	0.5607	0.7262	0.4207	0
7328	367.3	0.4033	0.5604	0.7262	0.4209	0
7329	367.2	0.403	0.5604	0.726	0.4208	0
7330	367.1	0.4034	0.5608	0.7262	0.421	0
7331	367	0.403	0.5609	0.7258	0.4207	0
7332	366.9	0.4029	0.5604	0.7263	0.4205	0
7333	366.8	0.4027	0.5607	0.7259	0.4205	0
7334	366.7	0.4031	0.5609	0.7261	0.4205	0
7335	366.6	0.403	0.561	0.7261	0.4206	0
7336	366.5	0.4028	0.5608	0.7262	0.4207	0
7337	366.4	0.4028	0.5606	0.7265	0.4209	0
7338	366.3	0.403	0.5607	0.7263	0.4209	0
7339	366.2	0.403	0.5612	0.7264	0.421	0

7340	366.1	0.4031	0.5609	0.7261	0.4209	0
7341	366	0.4028	0.5607	0.7264	0.4207	0
7342	365.9	0.4029	0.5611	0.7264	0.4209	0
7343	365.8	0.4032	0.5612	0.7265	0.4211	0
7344	365.7	0.4033	0.5612	0.7264	0.4213	0
7345	365.6	0.4035	0.5612	0.7264	0.4214	0
7346	365.5	0.4036	0.5613	0.7264	0.4212	0
7347	365.4	0.4037	0.5613	0.7271	0.4212	0
7348	365.3	0.4037	0.5611	0.7274	0.4212	0
7349	365.2	0.4034	0.5611	0.7273	0.4213	0
7350	365.1	0.4036	0.5611	0.7276	0.4216	0
7351	365	0.4037	0.5616	0.7275	0.4219	0
7352	364.9	0.4036	0.5615	0.7279	0.4215	0
7353	364.8	0.4037	0.5615	0.728	0.4212	0
7354	364.7	0.4038	0.5617	0.728	0.4216	0
7355	364.6	0.4039	0.5619	0.7284	0.4217	0
7356	364.5	0.4041	0.562	0.7288	0.4218	0
7357	364.4	0.4043	0.5621	0.7285	0.4223	0
7358	364.3	0.4045	0.5619	0.7286	0.4222	0
7359	364.2	0.4047	0.5624	0.7292	0.4223	0
7360	364.1	0.4044	0.5625	0.7293	0.4225	0
7361	364	0.4046	0.5629	0.7294	0.4227	0
7362	363.9	0.4047	0.5631	0.7289	0.4225	0
7363	363.8	0.4049	0.5636	0.7297	0.4228	0
7364	363.7	0.4049	0.5637	0.7297	0.423	0
7365	363.6	0.4052	0.5638	0.7299	0.4233	0
7366	363.5	0.4055	0.5634	0.7299	0.4234	0
7367	363.4	0.4053	0.5634	0.7301	0.4235	0
7368	363.3	0.4053	0.5634	0.7307	0.4236	0
7369	363.2	0.4053	0.5637	0.7305	0.4235	0
7370	363.1	0.4054	0.5638	0.731	0.4236	0
7371	363	0.4053	0.564	0.731	0.4235	0
7372	362.9	0.4053	0.5642	0.731	0.4236	0
7373	362.8	0.4056	0.5641	0.731	0.4236	0
7374	362.7	0.4057	0.5642	0.7314	0.4238	0
7375	362.6	0.4059	0.5644	0.7314	0.424	0
7376	362.5	0.4058	0.5645	0.7317	0.424	0
7377	362.4	0.4061	0.5646	0.7324	0.4239	0
7378	362.3	0.4059	0.5647	0.7317	0.4238	0
7379	362.2	0.4061	0.5648	0.7315	0.4239	0
7380	362.1	0.4062	0.5644	0.7315	0.4242	0
7381	362	0.4061	0.5644	0.7319	0.4241	0
7382	361.9	0.4061	0.5648	0.7321	0.4244	0

7383	361.8	0.4062	0.5647	0.7318	0.4243	0
7384	361.7	0.4062	0.5645	0.7324	0.4244	0
7385	361.6	0.4058	0.5644	0.7322	0.4243	0
7386	361.5	0.406	0.5647	0.7322	0.4245	0
7387	361.4	0.406	0.5647	0.7321	0.4244	0
7388	361.3	0.4061	0.5648	0.7321	0.4244	0
7389	361.2	0.406	0.5646	0.7319	0.424	0
7390	361.1	0.4062	0.5646	0.7322	0.4245	0
7391	361	0.4061	0.5647	0.7319	0.4242	0
7392	360.9	0.4059	0.5646	0.7322	0.4242	0
7393	360.8	0.4059	0.5646	0.7324	0.4241	0
7394	360.7	0.4059	0.5645	0.7318	0.4241	0
7395	360.6	0.406	0.5646	0.7321	0.4243	0
7396	360.5	0.4061	0.5649	0.7323	0.4245	0
7397	360.4	0.406	0.5649	0.7325	0.4246	0
7398	360.3	0.4064	0.565	0.7324	0.4247	0
7399	360.2	0.4063	0.5644	0.7328	0.4245	0
7400	360.1	0.4062	0.5646	0.7321	0.4245	0
7401	360	0.406	0.5647	0.7323	0.4244	0
7402	359.9	0.4062	0.5648	0.7327	0.4244	0
7403	359.8	0.4059	0.5645	0.7322	0.4241	0
7404	359.7	0.4057	0.5647	0.7323	0.4242	0
7405	359.6	0.4057	0.5646	0.7323	0.4242	0
7406	359.5	0.4055	0.5644	0.7322	0.4239	0
7407	359.4	0.4052	0.5646	0.7318	0.4237	0
7408	359.3	0.4056	0.5644	0.7321	0.4239	0
7409	359.2	0.4055	0.5644	0.7321	0.4239	0
7410	359.1	0.4054	0.5643	0.7319	0.4236	0
7411	359	0.4051	0.564	0.7316	0.4233	0
7412	358.9	0.4051	0.5641	0.7312	0.4233	0
7413	358.8	0.4054	0.564	0.7315	0.4238	0
7414	358.7	0.4048	0.5636	0.7316	0.4235	0
7415	358.6	0.4051	0.5634	0.7314	0.4232	0
7416	358.5	0.405	0.5635	0.7311	0.423	0
7417	358.4	0.4048	0.563	0.7308	0.4227	0
7418	358.3	0.4045	0.5631	0.7309	0.4226	0
7419	358.2	0.4043	0.5628	0.7307	0.4229	0
7420	358.1	0.4043	0.5628	0.7304	0.4225	0
7421	358	0.4044	0.5627	0.7303	0.4224	0
7422	357.9	0.4044	0.5623	0.7309	0.4225	0
7423	357.8	0.4043	0.5626	0.7306	0.4226	0
7424	357.7	0.4042	0.5625	0.7302	0.4223	0
7425	357.6	0.404	0.5622	0.7302	0.4222	0

7426	357.5	0.404	0.5622	0.7298	0.4221	0
7427	357.4	0.4039	0.5622	0.7302	0.4221	0
7428	357.3	0.4039	0.5621	0.73	0.4218	0
7429	357.2	0.4037	0.5621	0.7299	0.4218	0
7430	357.1	0.4037	0.562	0.73	0.4219	0
7431	357	0.4037	0.5617	0.7297	0.4216	0
7432	356.9	0.404	0.5622	0.7303	0.4217	0
7433	356.8	0.4038	0.5623	0.7304	0.4217	0
7434	356.7	0.4037	0.5618	0.7304	0.4216	0
7435	356.6	0.4036	0.562	0.7303	0.4217	0
7436	356.5	0.4034	0.5616	0.7303	0.4216	0
7437	356.4	0.4035	0.5618	0.7303	0.4215	0
7438	356.3	0.4034	0.5617	0.7306	0.4214	0
7439	356.2	0.4032	0.5618	0.7299	0.4215	0
7440	356.1	0.4031	0.5615	0.73	0.4213	0
7441	356	0.4035	0.5615	0.7304	0.4214	0
7442	355.9	0.4037	0.5618	0.7306	0.4217	0
7443	355.8	0.4033	0.5618	0.7309	0.4219	0
7444	355.7	0.4032	0.5621	0.7305	0.4218	0
7445	355.6	0.4034	0.562	0.7313	0.4216	0
7446	355.5	0.4037	0.5616	0.7311	0.4214	0
7447	355.4	0.4037	0.5618	0.7308	0.4216	0
7448	355.3	0.4036	0.562	0.7312	0.4213	0
7449	355.2	0.4036	0.5623	0.7313	0.4217	0
7450	355.1	0.4039	0.5625	0.7314	0.4218	0
7451	355	0.4036	0.5626	0.7314	0.4216	0
7452	354.9	0.4037	0.5623	0.7312	0.4215	0
7453	354.8	0.4039	0.5625	0.7312	0.4218	0
7454	354.7	0.4039	0.5624	0.7318	0.422	0
7455	354.6	0.4041	0.5625	0.7319	0.4219	0
7456	354.5	0.4043	0.563	0.7319	0.4221	0
7457	354.4	0.4045	0.5635	0.732	0.4222	0
7458	354.3	0.4041	0.5635	0.7318	0.422	0
7459	354.2	0.4045	0.5635	0.7324	0.422	0
7460	354.1	0.4044	0.5635	0.7328	0.4221	0
7461	354	0.4044	0.5637	0.7325	0.4224	0
7462	353.9	0.4047	0.5636	0.733	0.4227	0
7463	353.8	0.4049	0.5638	0.7334	0.4226	0
7464	353.7	0.405	0.564	0.7337	0.4228	0
7465	353.6	0.4052	0.5643	0.7339	0.4234	0
7466	353.5	0.4051	0.5647	0.7334	0.4233	0
7467	353.4	0.405	0.5646	0.7336	0.4229	0
7468	353.3	0.4054	0.5647	0.7341	0.4233	0

7469	353.2	0.4055	0.5647	0.7341	0.4236	0
7470	353.1	0.4055	0.5648	0.7345	0.4236	0
7471	353	0.4062	0.5652	0.7343	0.4239	0
7472	352.9	0.4061	0.5651	0.7346	0.4241	0
7473	352.8	0.4062	0.5652	0.7351	0.424	0
7474	352.7	0.4065	0.5656	0.735	0.4245	0
7475	352.6	0.4062	0.5654	0.735	0.4245	0
7476	352.5	0.4061	0.5652	0.7354	0.4244	0
7477	352.4	0.4061	0.5655	0.7356	0.4245	0
7478	352.3	0.4062	0.5653	0.7353	0.4246	0
7479	352.2	0.4063	0.5655	0.7364	0.4242	0
7480	352.1	0.4064	0.5657	0.736	0.4243	0
7481	352	0.4064	0.5661	0.7361	0.4248	0
7482	351.9	0.4065	0.566	0.7363	0.4246	0
7483	351.8	0.4064	0.5659	0.7368	0.425	0
7484	351.7	0.4067	0.5668	0.7368	0.425	0
7485	351.6	0.407	0.5664	0.7368	0.4251	0
7486	351.5	0.4071	0.5662	0.7371	0.4251	0
7487	351.4	0.407	0.5665	0.7365	0.4251	0
7488	351.3	0.4068	0.5667	0.7368	0.4254	0
7489	351.2	0.4073	0.5666	0.7375	0.4255	0
7490	351.1	0.4074	0.5666	0.7374	0.4256	0
7491	351	0.4075	0.5667	0.7372	0.4257	0
7492	350.9	0.4074	0.567	0.737	0.4255	0
7493	350.8	0.4074	0.5669	0.7375	0.4256	0
7494	350.7	0.4078	0.5664	0.7379	0.4259	0
7495	350.6	0.4075	0.5665	0.7384	0.4257	0
7496	350.5	0.4074	0.5669	0.7379	0.4258	0
7497	350.4	0.4076	0.567	0.7377	0.4257	0
7498	350.3	0.4075	0.567	0.7378	0.4255	0
7499	350.2	0.4075	0.567	0.7383	0.4258	0
7500	350.1	0.4074	0.567	0.7383	0.4257	0
7501	350	0.4074	0.5669	0.7378	0.4254	0
7502	349.9	0.4073	0.5672	0.7379	0.4254	0
7503	349.8	0.4073	0.5674	0.7377	0.4253	0
7504	349.7	0.4075	0.5674	0.7382	0.4254	0
7505	349.6	0.4077	0.5674	0.7384	0.4257	0
7506	349.5	0.4076	0.5674	0.7385	0.4257	0
7507	349.4	0.4076	0.5677	0.7388	0.4254	0
7508	349.3	0.4075	0.5673	0.7384	0.4252	0
7509	349.2	0.4074	0.5671	0.7385	0.4257	0
7510	349.1	0.4073	0.5674	0.7383	0.4252	0
7511	349	0.4072	0.5674	0.7389	0.4252	0

7512	348.9	0.4071	0.5671	0.7384	0.4253	0
7513	348.8	0.4072	0.5674	0.7383	0.4253	0
7514	348.7	0.4072	0.567	0.7386	0.4254	0
7515	348.6	0.4074	0.5673	0.739	0.4253	0
7516	348.5	0.4072	0.5674	0.7387	0.4251	0
7517	348.4	0.4074	0.5672	0.7391	0.4249	0
7518	348.3	0.4072	0.5668	0.739	0.4252	0
7519	348.2	0.4075	0.5672	0.7391	0.4249	0
7520	348.1	0.4075	0.5671	0.739	0.425	0
7521	348	0.4074	0.5672	0.739	0.4249	0
7522	347.9	0.4073	0.5667	0.7388	0.4251	0
7523	347.8	0.4071	0.5669	0.739	0.425	0
7524	347.7	0.4068	0.5668	0.7388	0.425	0
7525	347.6	0.4069	0.5665	0.7386	0.4249	0
7526	347.5	0.4069	0.5668	0.7385	0.4248	0
7527	347.4	0.4068	0.5664	0.7387	0.4245	0
7528	347.3	0.4069	0.5665	0.7382	0.4248	0
7529	347.2	0.4068	0.5666	0.7386	0.4243	0
7530	347.1	0.4066	0.5663	0.7383	0.4246	0
7531	347	0.4063	0.5661	0.7382	0.4241	0
7532	346.9	0.4066	0.5664	0.7385	0.4241	0
7533	346.8	0.4066	0.5666	0.7387	0.4243	0
7534	346.7	0.4069	0.5663	0.739	0.4242	0
7535	346.6	0.4067	0.5665	0.7387	0.4243	0
7536	346.5	0.4065	0.5662	0.7387	0.4244	0
7537	346.4	0.4066	0.5664	0.7387	0.424	0
7538	346.3	0.4065	0.566	0.7389	0.424	0
7539	346.2	0.4067	0.566	0.7392	0.4238	0
7540	346.1	0.4065	0.5661	0.7393	0.424	0
7541	346	0.4065	0.566	0.7393	0.4241	0
7542	345.9	0.4064	0.5657	0.7389	0.4238	0
7543	345.8	0.4061	0.5658	0.7392	0.4239	0
7544	345.7	0.4064	0.5659	0.739	0.4241	0
7545	345.6	0.4064	0.566	0.739	0.424	0
7546	345.5	0.4064	0.5658	0.7393	0.4238	0
7547	345.4	0.4063	0.5662	0.7394	0.4242	0
7548	345.3	0.4064	0.5664	0.7391	0.4241	0
7549	345.2	0.4066	0.5664	0.7394	0.4245	0
7550	345.1	0.4059	0.5662	0.7399	0.4243	0
7551	345	0.4065	0.5665	0.7398	0.4245	0
7552	344.9	0.4064	0.5665	0.74	0.4243	0
7553	344.8	0.4065	0.5663	0.7399	0.4243	0
7554	344.7	0.4066	0.5666	0.7399	0.4246	0

7555	344.6	0.4067	0.5667	0.7405	0.4243	0
7556	344.5	0.4067	0.5667	0.7403	0.4246	0
7557	344.4	0.4071	0.5671	0.7402	0.4245	0
7558	344.3	0.4068	0.5668	0.7403	0.4245	0
7559	344.2	0.4071	0.5674	0.741	0.4249	0
7560	344.1	0.4073	0.567	0.741	0.4249	0
7561	344	0.4073	0.5672	0.7412	0.4249	0
7562	343.9	0.4075	0.5674	0.7415	0.4248	0
7563	343.8	0.4074	0.5678	0.7413	0.4251	0
7564	343.7	0.4075	0.5676	0.7422	0.4252	0
7565	343.6	0.4075	0.5677	0.7418	0.4251	0
7566	343.5	0.4079	0.5677	0.742	0.4252	0
7567	343.4	0.4077	0.5677	0.7424	0.4254	0
7568	343.3	0.4079	0.5678	0.7421	0.4256	0
7569	343.2	0.408	0.5682	0.7426	0.4254	0
7570	343.1	0.4082	0.5684	0.7425	0.4258	0
7571	343	0.4083	0.5684	0.7428	0.4258	0
7572	342.9	0.4084	0.5683	0.743	0.4261	0
7573	342.8	0.4086	0.5683	0.743	0.4263	0
7574	342.7	0.4083	0.5685	0.7432	0.4262	0
7575	342.6	0.4089	0.5686	0.743	0.4262	0
7576	342.5	0.4086	0.5686	0.7436	0.4265	0
7577	342.4	0.409	0.5688	0.744	0.4267	0
7578	342.3	0.4086	0.569	0.7441	0.4268	0
7579	342.2	0.4087	0.5692	0.744	0.4264	0
7580	342.1	0.4088	0.569	0.744	0.4268	0
7581	342	0.4087	0.5693	0.7441	0.4266	0
7582	341.9	0.4092	0.5697	0.744	0.4268	0
7583	341.8	0.4089	0.5695	0.7441	0.4268	0
7584	341.7	0.4091	0.5697	0.7445	0.4267	0
7585	341.6	0.409	0.5696	0.7442	0.4268	0
7586	341.5	0.4089	0.5699	0.7447	0.4267	0
7587	341.4	0.4091	0.5699	0.7453	0.4271	0
7588	341.3	0.4092	0.5702	0.7454	0.4273	0
7589	341.2	0.4091	0.5701	0.7454	0.427	0
7590	341.1	0.409	0.5703	0.7453	0.4273	0
7591	341	0.409	0.5701	0.7455	0.4272	0
7592	340.9	0.4091	0.57	0.7454	0.4271	0
7593	340.8	0.4091	0.5703	0.7457	0.4272	0
7594	340.7	0.4093	0.5699	0.7461	0.4269	0
7595	340.6	0.4094	0.5698	0.746	0.4269	0
7596	340.5	0.4095	0.57	0.7466	0.4269	0
7597	340.4	0.4095	0.5698	0.7462	0.427	0

7598	340.3	0.4094	0.5702	0.7458	0.4269	0
7599	340.2	0.4096	0.5702	0.7462	0.4272	0
7600	340.1	0.4093	0.5697	0.7462	0.427	0
7601	340	0.4096	0.57	0.7464	0.4277	0
7602	339.9	0.3913	0.5354	0.6832	0.4082	0
7603	339.8	0.3912	0.5355	0.6831	0.4082	0
7604	339.7	0.3911	0.5352	0.6833	0.4083	0
7605	339.6	0.3913	0.535	0.6832	0.4083	0
7606	339.5	0.3913	0.5354	0.683	0.4083	0
7607	339.4	0.3912	0.5351	0.6828	0.4081	0
7608	339.3	0.3912	0.5352	0.6833	0.4081	0
7609	339.2	0.391	0.5349	0.6828	0.408	0
7610	339.1	0.3911	0.5346	0.6832	0.4081	0
7611	339	0.3909	0.5347	0.6828	0.4078	0
7612	338.9	0.3909	0.5343	0.6823	0.4079	0
7613	338.8	0.3907	0.5341	0.6828	0.4077	0
7614	338.7	0.3909	0.5344	0.6823	0.4078	0
7615	338.6	0.3906	0.534	0.6821	0.4075	0
7616	338.5	0.3907	0.5338	0.6815	0.4076	0
7617	338.4	0.3907	0.5335	0.6821	0.4074	0
7618	338.3	0.3905	0.5337	0.6816	0.4075	0
7619	338.2	0.3903	0.5333	0.6812	0.4073	0
7620	338.1	0.3902	0.5331	0.6812	0.4073	0
7621	338	0.3901	0.533	0.6815	0.4071	0
7622	337.9	0.3899	0.5328	0.6806	0.4069	0
7623	337.8	0.3899	0.5331	0.6809	0.407	0
7624	337.7	0.3897	0.5328	0.6804	0.4069	0
7625	337.6	0.3897	0.5327	0.6804	0.4067	0
7626	337.5	0.3894	0.5324	0.6806	0.4067	0
7627	337.4	0.3898	0.5326	0.6802	0.407	0
7628	337.3	0.3897	0.5322	0.6797	0.407	0
7629	337.2	0.3896	0.5322	0.6803	0.4067	0
7630	337.1	0.3895	0.5321	0.6798	0.4066	0
7631	337	0.3895	0.532	0.6799	0.4063	0
7632	336.9	0.3895	0.5321	0.6799	0.4064	0
7633	336.8	0.3896	0.532	0.6802	0.4061	0
7634	336.7	0.3895	0.5321	0.6798	0.4062	0
7635	336.6	0.3888	0.5319	0.6797	0.4062	0
7636	336.5	0.3889	0.5316	0.6796	0.4061	0
7637	336.4	0.3892	0.5315	0.6794	0.4059	0
7638	336.3	0.3889	0.5316	0.6795	0.406	0
7639	336.2	0.3885	0.5314	0.6791	0.4062	0
7640	336.1	0.3884	0.5312	0.6792	0.4061	0

7641	336	0.3885	0.5312	0.679	0.406	0
7642	335.9	0.3886	0.5314	0.6789	0.4061	0
7643	335.8	0.3885	0.5309	0.6784	0.4058	0
7644	335.7	0.3884	0.5308	0.6785	0.4056	0
7645	335.6	0.3884	0.5307	0.679	0.4057	0
7646	335.5	0.3883	0.5305	0.6782	0.4055	0
7647	335.4	0.3886	0.5305	0.6784	0.4054	0
7648	335.3	0.3886	0.5304	0.6782	0.4056	0
7649	335.2	0.3887	0.5309	0.6785	0.4055	0
7650	335.1	0.3888	0.5307	0.678	0.4056	0
7651	335	0.3885	0.5307	0.6778	0.4055	0
7652	334.9	0.3884	0.5304	0.6777	0.4051	0
7653	334.8	0.3881	0.5302	0.6778	0.4052	0
7654	334.7	0.3881	0.5299	0.6775	0.4051	0
7655	334.6	0.3883	0.5296	0.6778	0.4054	0
7656	334.5	0.3882	0.5295	0.678	0.4052	0
7657	334.4	0.388	0.5295	0.6773	0.4049	0
7658	334.3	0.388	0.5295	0.677	0.4048	0
7659	334.2	0.3882	0.5295	0.6774	0.4049	0
7660	334.1	0.3883	0.5294	0.677	0.4048	0
7661	334	0.3882	0.5295	0.677	0.4048	0
7662	333.9	0.3881	0.5294	0.6772	0.4047	0
7663	333.8	0.3879	0.5291	0.6771	0.4045	0
7664	333.7	0.3877	0.5291	0.6772	0.4047	0
7665	333.6	0.3877	0.5292	0.6769	0.4046	0
7666	333.5	0.388	0.5293	0.6768	0.4047	0
7667	333.4	0.3881	0.5292	0.6764	0.4048	0
7668	333.3	0.3881	0.5291	0.6767	0.4046	0
7669	333.2	0.388	0.5288	0.6769	0.4044	0
7670	333.1	0.3883	0.529	0.6764	0.4049	0
7671	333	0.3879	0.5292	0.6766	0.4045	0
7672	332.9	0.3877	0.529	0.6766	0.4045	0
7673	332.8	0.3878	0.5288	0.6765	0.4044	0
7674	332.7	0.3878	0.5287	0.6765	0.4047	0
7675	332.6	0.3878	0.5288	0.6765	0.4046	0
7676	332.5	0.3875	0.5285	0.6764	0.4043	0
7677	332.4	0.3874	0.5285	0.6758	0.4044	0
7678	332.3	0.3874	0.5283	0.6761	0.4044	0
7679	332.2	0.3875	0.5284	0.6762	0.4043	0
7680	332.1	0.3877	0.5282	0.6761	0.4044	0
7681	332	0.3875	0.5279	0.6765	0.4044	0
7682	331.9	0.3876	0.5278	0.6758	0.4043	0
7683	331.8	0.3874	0.5278	0.6753	0.4044	0

7684	331.7	0.3876	0.5279	0.6756	0.404	0
7685	331.6	0.3872	0.5279	0.6752	0.4038	0
7686	331.5	0.3869	0.5277	0.6753	0.4038	0
7687	331.4	0.3872	0.5276	0.6751	0.4041	0
7688	331.3	0.3867	0.5272	0.6749	0.4039	0
7689	331.2	0.3866	0.5272	0.6748	0.4037	0
7690	331.1	0.3866	0.5266	0.6749	0.4036	0
7691	331	0.3863	0.5264	0.675	0.4035	0
7692	330.9	0.3864	0.5265	0.6746	0.4034	0
7693	330.8	0.3863	0.5266	0.6746	0.4035	0
7694	330.7	0.3865	0.5261	0.6738	0.4029	0
7695	330.6	0.3864	0.5263	0.6739	0.4029	0
7696	330.5	0.3862	0.526	0.6741	0.4031	0
7697	330.4	0.3863	0.5258	0.6737	0.4029	0
7698	330.3	0.3862	0.5257	0.6738	0.4025	0
7699	330.2	0.3865	0.5259	0.674	0.4028	0
7700	330.1	0.3862	0.5255	0.6738	0.4027	0
7701	330	0.3859	0.5251	0.6734	0.4026	0
7702	329.9	0.3857	0.5252	0.6731	0.4023	0
7703	329.8	0.3857	0.5249	0.6731	0.4019	0
7704	329.7	0.3859	0.5248	0.6732	0.402	0
7705	329.6	0.3858	0.525	0.673	0.4022	0
7706	329.5	0.3854	0.5245	0.6726	0.4019	0
7707	329.4	0.3848	0.5244	0.6722	0.4017	0
7708	329.3	0.385	0.5241	0.6724	0.4017	0
7709	329.2	0.385	0.524	0.6721	0.4016	0
7710	329.1	0.3847	0.524	0.6718	0.4014	0
7711	329	0.3848	0.5236	0.672	0.4011	0
7712	328.9	0.3848	0.5236	0.6716	0.4011	0
7713	328.8	0.3845	0.5235	0.6713	0.4009	0
7714	328.7	0.3845	0.5236	0.6715	0.4009	0
7715	328.6	0.3842	0.5231	0.6715	0.4009	0
7716	328.5	0.3842	0.5231	0.6712	0.4008	0
7717	328.4	0.3842	0.5231	0.6708	0.4008	0
7718	328.3	0.3841	0.5226	0.6709	0.4004	0
7719	328.2	0.3841	0.5228	0.6704	0.4004	0
7720	328.1	0.3841	0.5225	0.6706	0.4006	0
7721	328	0.3842	0.5228	0.6705	0.4003	0
7722	327.9	0.3841	0.5227	0.6705	0.4001	0
7723	327.8	0.3838	0.5222	0.6703	0.4002	0
7724	327.7	0.3838	0.522	0.6704	0.4002	0
7725	327.6	0.3836	0.5217	0.6701	0.4	0
7726	327.5	0.3835	0.5214	0.6695	0.3998	0

7727	327.4	0.3833	0.5214	0.67	0.3996	0
7728	327.3	0.3831	0.5214	0.6695	0.3997	0
7729	327.2	0.3833	0.5214	0.6696	0.3998	0
7730	327.1	0.3832	0.5214	0.6696	0.3997	0
7731	327	0.3832	0.5215	0.6693	0.3996	0
7732	326.9	0.383	0.5209	0.669	0.3994	0
7733	326.8	0.3828	0.5208	0.6693	0.3994	0
7734	326.7	0.383	0.5209	0.6694	0.3995	0
7735	326.6	0.3829	0.5208	0.6691	0.3994	0
7736	326.5	0.3826	0.5206	0.6687	0.3993	0
7737	326.4	0.3827	0.5205	0.6692	0.3992	0
7738	326.3	0.3828	0.5204	0.6686	0.3995	0
7739	326.2	0.3826	0.52	0.6689	0.3995	0
7740	326.1	0.3827	0.5201	0.6683	0.3996	0
7741	326	0.3829	0.5204	0.6686	0.3996	0
7742	325.9	0.3828	0.5207	0.6681	0.3994	0
7743	325.8	0.3825	0.5204	0.6681	0.399	0
7744	325.7	0.3825	0.5201	0.668	0.3988	0
7745	325.6	0.3823	0.5198	0.6679	0.3991	0
7746	325.5	0.3825	0.5199	0.6676	0.3993	0
7747	325.4	0.3822	0.5197	0.6677	0.3993	0
7748	325.3	0.3821	0.5196	0.6679	0.399	0
7749	325.2	0.3822	0.5195	0.6678	0.3989	0
7750	325.1	0.3824	0.5195	0.6679	0.399	0
7751	325	0.382	0.5195	0.6677	0.3987	0
7752	324.9	0.3822	0.5194	0.6676	0.3987	0
7753	324.8	0.3822	0.5196	0.6678	0.3988	0
7754	324.7	0.3823	0.5196	0.6676	0.3987	0
7755	324.6	0.3824	0.5194	0.6676	0.3986	0
7756	324.5	0.3822	0.5194	0.6675	0.3987	0
7757	324.4	0.3818	0.519	0.6677	0.3986	0
7758	324.3	0.3822	0.5193	0.6675	0.3987	0
7759	324.2	0.382	0.5187	0.6668	0.3985	0
7760	324.1	0.3821	0.5189	0.6669	0.3985	0
7761	324	0.3822	0.5189	0.6668	0.3987	0
7762	323.9	0.382	0.519	0.6668	0.3986	0
7763	323.8	0.3821	0.5189	0.6667	0.3987	0
7764	323.7	0.3821	0.5186	0.6667	0.3986	0
7765	323.6	0.3819	0.5185	0.6668	0.3986	0
7766	323.5	0.3821	0.5189	0.6667	0.3988	0
7767	323.4	0.3818	0.5184	0.6668	0.3984	0
7768	323.3	0.382	0.5184	0.6664	0.3986	0
7769	323.2	0.382	0.5183	0.6663	0.3985	0

7770	323.1	0.3817	0.5181	0.6662	0.3985	0
7771	323	0.3816	0.5182	0.6661	0.3985	0
7772	322.9	0.3817	0.5181	0.6661	0.3985	0
7773	322.8	0.3814	0.518	0.666	0.3982	0
7774	322.7	0.3814	0.5177	0.6656	0.3983	0
7775	322.6	0.3816	0.518	0.6658	0.3983	0
7776	322.5	0.3811	0.5177	0.6652	0.398	0
7777	322.4	0.3814	0.5177	0.6651	0.3983	0
7778	322.3	0.3812	0.5174	0.6651	0.3981	0
7779	322.2	0.381	0.5172	0.6652	0.3979	0
7780	322.1	0.3811	0.5172	0.6647	0.3977	0
7781	322	0.381	0.5168	0.6649	0.3977	0
7782	321.9	0.3809	0.517	0.6651	0.3976	0
7783	321.8	0.3805	0.5165	0.6648	0.3972	0
7784	321.7	0.3805	0.5163	0.6647	0.3976	0
7785	321.6	0.3804	0.5162	0.6646	0.3971	0
7786	321.5	0.3803	0.5164	0.6642	0.3971	0
7787	321.4	0.3799	0.5161	0.6639	0.3969	0
7788	321.3	0.3802	0.5157	0.6636	0.3969	0
7789	321.2	0.3803	0.5157	0.6636	0.3967	0
7790	321.1	0.3799	0.5154	0.6629	0.3964	0
7791	321	0.3798	0.5152	0.6628	0.3964	0
7792	320.9	0.3798	0.5151	0.6626	0.3962	0
7793	320.8	0.3798	0.5148	0.6623	0.3961	0
7794	320.7	0.3796	0.5146	0.6621	0.3959	0
7795	320.6	0.3796	0.5149	0.662	0.396	0
7796	320.5	0.3795	0.5146	0.6617	0.3958	0
7797	320.4	0.3795	0.514	0.6613	0.3955	0
7798	320.3	0.3792	0.5136	0.6613	0.3954	0
7799	320.2	0.3791	0.5137	0.6612	0.3954	0
7800	320.1	0.3791	0.5133	0.6612	0.3953	0
7801	320	0.3791	0.5132	0.6607	0.3952	

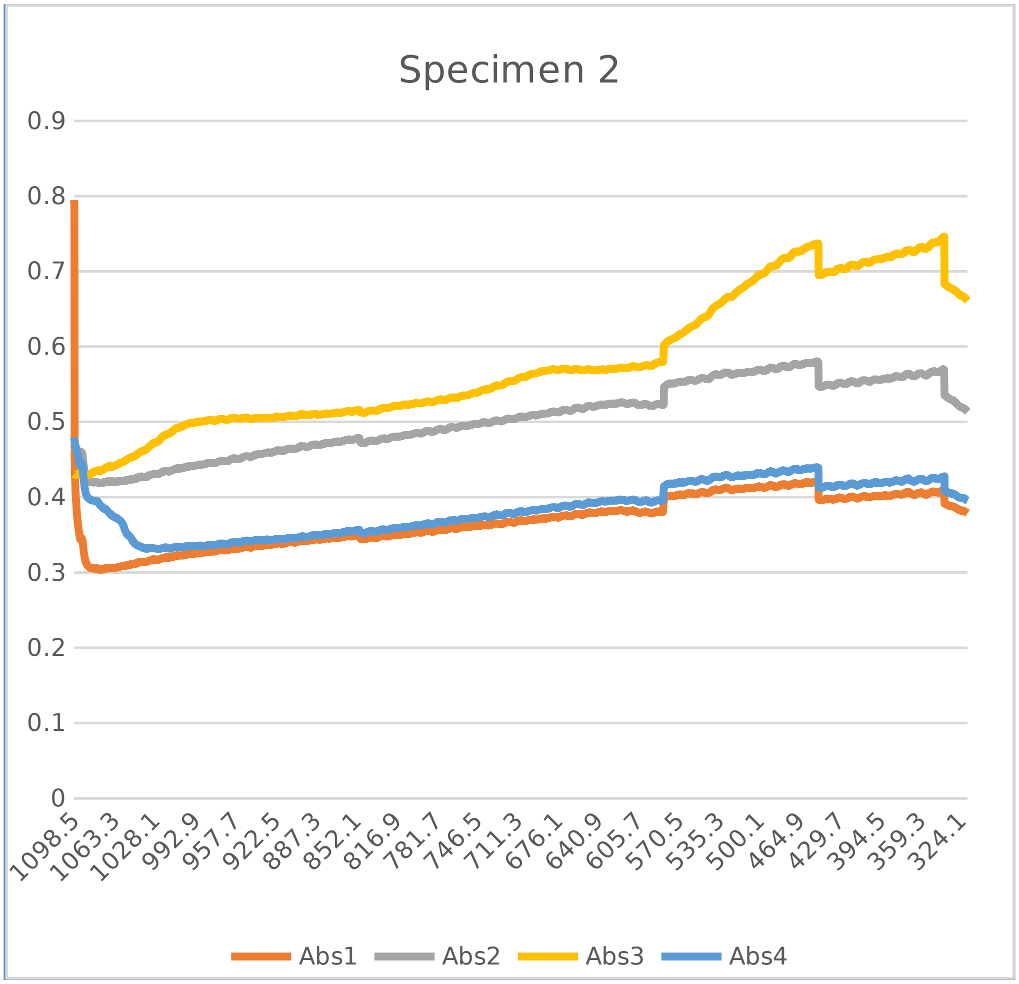
Specimen 2
0.9
0.8
0.7
0.6
0.5
0.4
0.3
0.2
0.1
0
1098.5
1063.3
1028.1
992.9
957.7
922.5
887.3
852.1
816.9
781.7
746.5
711.3
676.1
640.9
605.7
570.5
535.3
500.1
464.9
429.7
394.5
359.3
324.1
Abs1
Abs2
Abs3
Abs4
