# Supplementary material for: Coherent poly propagation materials with 3-dimensional photonic control over visible light
Source: PLoS One. 2019 Oct 17;14(10):e0223715. doi: 10.1371/journal.pone.0223715 (PMC6797174; doi:10.1371/journal.pone.0223715)
Supplement: S3 Table — Spreadsheet generated graph at bottom of table. (PDF) [file pone.0223715.s012.pdf]

Wavelength Scan Report - Specimen 3

Company	Complete Consulting Services LLC		
Operator	Dr. Michelle R. Stem	Test Time	2/10/2017 1:22
Document No.	WLS1702100001_1	Print Date	9/7/2019 4:28
Start wavelength	1100nm	Number of data	7801
End wavelength	320nm	K1	0.99
Unit		K0	0
Interval	0.1nm	K2	0
Calculate the Conc.	No	K3	0
Modification time	2/10/2017 1:22	Access time	2/10/2017 1:22
Remark	S14a030 - Specimen 3		

Scan Data

No.	Wavelength	Abs1	Abs2	Abs3	Abs4	Visible
1	1100	0.1917	0.3612	0.8951	0.0206	0
2	1099.9	0.1916	0.3603	0.8959	0.0203	0
3	1099.8	0.1916	0.359	0.8963	0.0199	0
4	1099.7	0.1914	0.3579	0.8965	0.0198	0
5	1099.6	0.1914	0.3568	0.8966	0.0197	0
6	1099.5	0.1913	0.3558	0.8971	0.0196	0
7	1099.4	0.1912	0.3547	0.897	0.0193	0
8	1099.3	0.1913	0.3542	0.8972	0.0192	0
9	1099.2	0.1911	0.3534	0.8983	0.0191	0
10	1099.1	0.1915	0.3521	0.8986	0.0193	0
11	1099	0.1909	0.3504	0.8983	0.0189	0
12	1098.9	0.1911	0.3486	0.8988	0.0188	0
13	1098.8	0.1911	0.3471	0.8996	0.0189	0
14	1098.7	0.1911	0.3457	0.8997	0.0187	0
15	1098.6	0.1913	0.3446	0.8997	0.0189	0
16	1098.5	0.1913	0.3441	0.9002	0.0188	0
17	1098.4	0.1911	0.3432	0.9006	0.0183	0
18	1098.3	0.1914	0.3422	0.9012	0.0184	0
19	1098.2	0.1915	0.3413	0.9011	0.0183	0
20	1098.1	0.1914	0.3408	0.9015	0.0184	0
21	1098	0.1915	0.3401	0.902	0.0182	0
22	1097.9	0.1914	0.3397	0.9026	0.0181	0
23	1097.8	0.1915	0.3398	0.9027	0.0182	0
24	1097.7	0.1915	0.3395	0.9037	0.0181	0
25	1097.6	0.1914	0.3395	0.9035	0.018	0
26	1097.5	0.1918	0.3395	0.9037	0.0182	0
27	1097.4	0.192	0.34	0.9046	0.0185	0
28	1097.3	0.1921	0.3394	0.9052	0.0182	0
29	1097.2	0.1918	0.3393	0.9051	0.018	0

30	1097.1	0.1918	0.3391	0.9054	0.0178	0
31	1097	0.1921	0.3395	0.9059	0.018	0
32	1096.9	0.1915	0.3394	0.9059	0.0178	0
33	1096.8	0.1913	0.339	0.9062	0.0174	0
34	1096.7	0.1915	0.3392	0.9059	0.0176	0
35	1096.6	0.1916	0.339	0.9061	0.0175	0
36	1096.5	0.1917	0.339	0.9063	0.0176	0
37	1096.4	0.1917	0.3385	0.9065	0.0177	0
38	1096.3	0.1917	0.3374	0.9063	0.0175	0
39	1096.2	0.1916	0.3366	0.9057	0.0172	0
40	1096.1	0.192	0.3364	0.9064	0.0177	0
41	1096	0.192	0.336	0.9068	0.0178	0
42	1095.9	0.1923	0.3355	0.9068	0.0179	0
43	1095.8	0.1927	0.3353	0.9076	0.0183	0
44	1095.7	0.1929	0.3351	0.9078	0.0183	0
45	1095.6	0.1931	0.335	0.9086	0.0185	0
46	1095.5	0.1933	0.3347	0.9086	0.0183	0
47	1095.4	0.1937	0.3347	0.9095	0.0186	0
48	1095.3	0.1934	0.3343	0.9097	0.0184	0
49	1095.2	0.1936	0.3341	0.9104	0.0187	0
50	1095.1	0.1939	0.3342	0.911	0.0186	0
51	1095	0.1943	0.3342	0.9123	0.0188	0
52	1094.9	0.1935	0.3338	0.9121	0.0181	0
53	1094.8	0.1934	0.3339	0.9124	0.018	0
54	1094.7	0.1932	0.3332	0.9123	0.0179	0
55	1094.6	0.1929	0.3331	0.9126	0.0177	0
56	1094.5	0.1926	0.3329	0.9128	0.0174	0
57	1094.4	0.1925	0.3332	0.9124	0.0173	0
58	1094.3	0.1922	0.3332	0.9125	0.0174	0
59	1094.2	0.1921	0.3331	0.9123	0.0174	0
60	1094.1	0.1915	0.3329	0.9115	0.0172	0
61	1094	0.1915	0.3331	0.9114	0.0171	0
62	1093.9	0.1914	0.3332	0.9105	0.0171	0
63	1093.8	0.1915	0.3334	0.9099	0.0172	0
64	1093.7	0.1912	0.3335	0.9099	0.0174	0
65	1093.6	0.1916	0.3338	0.9093	0.0177	0
66	1093.5	0.1917	0.3339	0.9093	0.0179	0
67	1093.4	0.1914	0.334	0.9095	0.018	0
68	1093.3	0.1919	0.3343	0.9091	0.0182	0
69	1093.2	0.1923	0.3342	0.9089	0.0185	0
70	1093.1	0.1928	0.3344	0.9098	0.019	0
71	1093	0.193	0.3344	0.9097	0.019	0
72	1092.9	0.1935	0.3346	0.9106	0.0192	0

73	1092.8	0.1938	0.3345	0.911	0.0193	0
74	1092.7	0.1944	0.3347	0.912	0.0195	0
75	1092.6	0.1953	0.3351	0.913	0.0199	0
76	1092.5	0.196	0.3353	0.914	0.0203	0
77	1092.4	0.1964	0.3354	0.9152	0.0205	0
78	1092.3	0.1969	0.3353	0.916	0.0206	0
79	1092.2	0.1971	0.3354	0.9172	0.0206	0
80	1092.1	0.1979	0.3355	0.9188	0.021	0
81	1092	0.1983	0.3351	0.9197	0.0209	0
82	1091.9	0.1987	0.3351	0.921	0.021	0
83	1091.8	0.1993	0.3351	0.9223	0.0212	0
84	1091.7	0.1999	0.3344	0.9235	0.0215	0
85	1091.6	0.2003	0.3335	0.9251	0.0216	0
86	1091.5	0.2009	0.3324	0.9262	0.0221	0
87	1091.4	0.2015	0.3317	0.9273	0.0222	0
88	1091.3	0.2016	0.331	0.928	0.0221	0
89	1091.2	0.2023	0.3302	0.9285	0.0223	0
90	1091.1	0.2028	0.3296	0.9301	0.0224	0
91	1091	0.2028	0.3284	0.931	0.0223	0
92	1090.9	0.203	0.3276	0.9313	0.0223	0
93	1090.8	0.2032	0.3266	0.9323	0.0221	0
94	1090.7	0.2033	0.3254	0.9332	0.0221	0
95	1090.6	0.2037	0.3248	0.9338	0.0223	0
96	1090.5	0.2039	0.3239	0.9344	0.0224	0
97	1090.4	0.2045	0.3233	0.9344	0.0224	0
98	1090.3	0.2046	0.3225	0.9356	0.0224	0
99	1090.2	0.2049	0.3214	0.9367	0.0224	0
100	1090.1	0.2049	0.3205	0.9367	0.0224	0
101	1090	0.2052	0.3193	0.9371	0.0225	0
102	1089.9	0.2051	0.3184	0.9376	0.0226	0
103	1089.8	0.2054	0.3173	0.9378	0.0226	0
104	1089.7	0.2054	0.3161	0.9388	0.0226	0
105	1089.6	0.2056	0.315	0.9385	0.0224	0
106	1089.5	0.2055	0.3142	0.9395	0.0226	0
107	1089.4	0.2059	0.3132	0.9399	0.0228	0
108	1089.3	0.2056	0.3122	0.9392	0.0222	0
109	1089.2	0.2059	0.3114	0.9398	0.0225	0
110	1089.1	0.2061	0.3105	0.94	0.0226	0
111	1089	0.2063	0.3101	0.9413	0.0226	0
112	1088.9	0.2061	0.31	0.9416	0.0224	0
113	1088.8	0.2062	0.3095	0.9412	0.0224	0
114	1088.7	0.2062	0.3084	0.9413	0.0223	0
115	1088.6	0.2063	0.3074	0.9416	0.0224	0

116	1088.5	0.2066	0.3068	0.9419	0.0227	0
117	1088.4	0.2067	0.3052	0.9419	0.0227	0
118	1088.3	0.2067	0.304	0.9423	0.0225	0
119	1088.2	0.2066	0.3031	0.9419	0.0223	0
120	1088.1	0.2066	0.3023	0.9419	0.0222	0
121	1088	0.2067	0.3014	0.9412	0.0221	0
122	1087.9	0.2068	0.3008	0.9425	0.0221	0
123	1087.8	0.2068	0.3004	0.9424	0.0222	0
124	1087.7	0.2069	0.2998	0.9426	0.0225	0
125	1087.6	0.2071	0.2993	0.9424	0.0224	0
126	1087.5	0.2068	0.2978	0.9431	0.0223	0
127	1087.4	0.2069	0.2969	0.9432	0.0223	0
128	1087.3	0.207	0.2959	0.9431	0.0223	0
129	1087.2	0.207	0.2955	0.9437	0.0224	0
130	1087.1	0.2071	0.2944	0.9431	0.0224	0
131	1087	0.207	0.2935	0.9437	0.0223	0
132	1086.9	0.2068	0.2922	0.9434	0.0222	0
133	1086.8	0.207	0.2916	0.9434	0.0225	0
134	1086.7	0.2071	0.2913	0.944	0.0224	0
135	1086.6	0.2071	0.2904	0.9444	0.0223	0
136	1086.5	0.2071	0.2901	0.9442	0.0224	0
137	1086.4	0.2076	0.2897	0.945	0.0227	0
138	1086.3	0.2073	0.2888	0.9446	0.0225	0
139	1086.2	0.2077	0.2879	0.9447	0.0227	0
140	1086.1	0.2076	0.287	0.9447	0.0227	0
141	1086	0.2076	0.2863	0.9451	0.0226	0
142	1085.9	0.208	0.2854	0.9456	0.0229	0
143	1085.8	0.2078	0.2845	0.9455	0.0228	0
144	1085.7	0.2081	0.2843	0.9454	0.0228	0
145	1085.6	0.208	0.2838	0.9457	0.0229	0
146	1085.5	0.2078	0.2835	0.9456	0.0226	0
147	1085.4	0.2082	0.2833	0.9459	0.0227	0
148	1085.3	0.2083	0.2831	0.9459	0.023	0
149	1085.2	0.2082	0.2829	0.9461	0.0232	0
150	1085.1	0.2085	0.2828	0.9462	0.0233	0
151	1085	0.2086	0.2823	0.9466	0.0235	0
152	1084.9	0.2089	0.281	0.9467	0.0235	0
153	1084.8	0.2088	0.2801	0.9466	0.0235	0
154	1084.7	0.2089	0.2801	0.9468	0.0234	0
155	1084.6	0.2087	0.2794	0.9464	0.0233	0
156	1084.5	0.2091	0.2796	0.9474	0.0237	0
157	1084.4	0.2092	0.2793	0.9474	0.0237	0
158	1084.3	0.2089	0.2786	0.9471	0.0236	0

159	1084.2	0.2091	0.2782	0.9474	0.0236	0
160	1084.1	0.209	0.2774	0.9475	0.0237	0
161	1084	0.2092	0.2769	0.9472	0.0238	0
162	1083.9	0.2096	0.2769	0.948	0.0243	0
163	1083.8	0.2096	0.2766	0.9479	0.0244	0
164	1083.7	0.2098	0.276	0.9479	0.0245	0
165	1083.6	0.2096	0.2756	0.9472	0.0242	0
166	1083.5	0.2097	0.2752	0.9486	0.0244	0
167	1083.4	0.2095	0.2744	0.9481	0.0244	0
168	1083.3	0.2096	0.274	0.948	0.0245	0
169	1083.2	0.2102	0.2732	0.9484	0.0248	0
170	1083.1	0.2103	0.2725	0.9486	0.0248	0
171	1083	0.2104	0.2717	0.9487	0.0248	0
172	1082.9	0.21	0.2709	0.9487	0.0248	0
173	1082.8	0.2104	0.27	0.949	0.0252	0
174	1082.7	0.2103	0.2691	0.9492	0.025	0
175	1082.6	0.2105	0.2686	0.9491	0.025	0
176	1082.5	0.2103	0.2681	0.9494	0.0249	0
177	1082.4	0.2106	0.2683	0.9491	0.0252	0
178	1082.3	0.2107	0.268	0.949	0.0254	0
179	1082.2	0.211	0.2682	0.9493	0.0254	0
180	1082.1	0.2109	0.268	0.9496	0.0253	0
181	1082	0.2107	0.2682	0.9497	0.0253	0
182	1081.9	0.2109	0.2683	0.95	0.0254	0
183	1081.8	0.2108	0.2682	0.9497	0.0254	0
184	1081.7	0.2108	0.2678	0.9495	0.0252	0
185	1081.6	0.2109	0.2678	0.9506	0.0254	0
186	1081.5	0.2104	0.2671	0.9503	0.025	0
187	1081.4	0.2107	0.2672	0.9501	0.0251	0
188	1081.3	0.2109	0.2673	0.95	0.0251	0
189	1081.2	0.2108	0.2674	0.9514	0.0252	0
190	1081.1	0.2108	0.2674	0.9511	0.0252	0
191	1081	0.2108	0.2672	0.9509	0.0254	0
192	1080.9	0.2113	0.2675	0.9511	0.0258	0
193	1080.8	0.211	0.2672	0.9509	0.0254	0
194	1080.7	0.2108	0.267	0.9506	0.0252	0
195	1080.6	0.2116	0.2674	0.9516	0.0258	0
196	1080.5	0.2116	0.2675	0.9512	0.0261	0
197	1080.4	0.2116	0.2671	0.9516	0.026	0
198	1080.3	0.2114	0.267	0.9516	0.0258	0
199	1080.2	0.2119	0.2669	0.9522	0.026	0
200	1080.1	0.2116	0.2668	0.9519	0.026	0
201	1080	0.2117	0.2666	0.9523	0.026	0

202	1079.9	0.2117	0.2665	0.9524	0.026	0
203	1079.8	0.2119	0.2661	0.9521	0.026	0
204	1079.7	0.2118	0.2657	0.9528	0.0259	0
205	1079.6	0.2114	0.2646	0.9522	0.0258	0
206	1079.5	0.2115	0.2634	0.9521	0.0259	0
207	1079.4	0.2114	0.2624	0.9521	0.0258	0
208	1079.3	0.2115	0.2613	0.9527	0.0257	0
209	1079.2	0.2116	0.2603	0.9527	0.0257	0
210	1079.1	0.2116	0.2597	0.9525	0.0258	0
211	1079	0.2119	0.2591	0.9531	0.0259	0
212	1078.9	0.2117	0.2575	0.9533	0.0256	0
213	1078.8	0.2121	0.2572	0.9538	0.0259	0
214	1078.7	0.2123	0.2564	0.9532	0.026	0
215	1078.6	0.2123	0.2553	0.9533	0.026	0
216	1078.5	0.2119	0.2544	0.9537	0.0261	0
217	1078.4	0.2121	0.2537	0.9537	0.026	0
218	1078.3	0.212	0.2529	0.9537	0.0259	0
219	1078.2	0.2124	0.2522	0.9536	0.0261	0
220	1078.1	0.212	0.2511	0.9545	0.0261	0
221	1078	0.2124	0.2506	0.9542	0.0261	0
222	1077.9	0.2123	0.2499	0.9532	0.0259	0
223	1077.8	0.2121	0.2494	0.9544	0.026	0
224	1077.7	0.2122	0.2495	0.9544	0.0261	0
225	1077.6	0.2122	0.249	0.9546	0.0259	0
226	1077.5	0.2123	0.2489	0.9549	0.0258	0
227	1077.4	0.212	0.2486	0.954	0.0258	0
228	1077.3	0.2122	0.2479	0.9547	0.0258	0
229	1077.2	0.2125	0.2477	0.9548	0.0261	0
230	1077.1	0.2122	0.2468	0.9545	0.0258	0
231	1077	0.2125	0.2461	0.9553	0.0261	0
232	1076.9	0.2123	0.2453	0.955	0.026	0
233	1076.8	0.2126	0.2444	0.9547	0.0263	0
234	1076.7	0.2129	0.2438	0.9555	0.0264	0
235	1076.6	0.2124	0.2425	0.9551	0.0258	0
236	1076.5	0.2127	0.242	0.9557	0.0261	0
237	1076.4	0.2127	0.2413	0.9556	0.0263	0
238	1076.3	0.2127	0.2408	0.9557	0.0264	0
239	1076.2	0.2127	0.2402	0.956	0.0265	0
240	1076.1	0.2127	0.2391	0.9556	0.0261	0
241	1076	0.2128	0.2385	0.9555	0.0264	0
242	1075.9	0.2125	0.238	0.9558	0.0261	0
243	1075.8	0.2124	0.2368	0.9558	0.026	0
244	1075.7	0.2126	0.236	0.9555	0.0261	0

245	1075.6	0.2129	0.2349	0.9559	0.0263	0
246	1075.5	0.2129	0.2335	0.956	0.0263	0
247	1075.4	0.2128	0.2323	0.9554	0.0265	0
248	1075.3	0.213	0.2323	0.9565	0.0266	0
249	1075.2	0.2133	0.2317	0.9561	0.0268	0
250	1075.1	0.213	0.23	0.9562	0.0264	0
251	1075	0.2133	0.2292	0.9571	0.0266	0
252	1074.9	0.2133	0.2285	0.9562	0.0267	0
253	1074.8	0.2136	0.2279	0.9568	0.0269	0
254	1074.7	0.2138	0.2271	0.9566	0.0271	0
255	1074.6	0.2135	0.2261	0.9569	0.0271	0
256	1074.5	0.2137	0.2241	0.9569	0.0272	0
257	1074.4	0.2133	0.2209	0.9569	0.0269	0
258	1074.3	0.2134	0.2169	0.9568	0.027	0
259	1074.2	0.2133	0.2126	0.9575	0.0271	0
260	1074.1	0.2136	0.2108	0.9569	0.0272	0
261	1074	0.2137	0.2085	0.9571	0.0273	0
262	1073.9	0.214	0.2066	0.9583	0.0276	0
263	1073.8	0.2138	0.2044	0.9582	0.0274	0
264	1073.7	0.2141	0.2029	0.9581	0.0278	0
265	1073.6	0.2139	0.2007	0.958	0.0278	0
266	1073.5	0.2142	0.1982	0.9581	0.0281	0
267	1073.4	0.2144	0.1944	0.9582	0.0282	0
268	1073.3	0.2142	0.1897	0.9585	0.028	0
269	1073.2	0.2139	0.1856	0.9584	0.0281	0
270	1073.1	0.2141	0.1833	0.9588	0.0281	0
271	1073	0.2141	0.1826	0.9584	0.0282	0
272	1072.9	0.2146	0.1821	0.9588	0.0286	0
273	1072.8	0.2142	0.1798	0.9585	0.0282	0
274	1072.7	0.2145	0.1793	0.9587	0.0284	0
275	1072.6	0.2146	0.1785	0.9586	0.0286	0
276	1072.5	0.2151	0.1748	0.9594	0.0288	0
277	1072.4	0.2148	0.1733	0.9595	0.0288	0
278	1072.3	0.215	0.1728	0.9591	0.0288	0
279	1072.2	0.2149	0.1726	0.9589	0.0289	0
280	1072.1	0.2148	0.1725	0.9595	0.0289	0
281	1072	0.2148	0.1723	0.96	0.029	0
282	1071.9	0.2147	0.1721	0.9594	0.029	0
283	1071.8	0.2152	0.1723	0.9591	0.029	0
284	1071.7	0.2153	0.1723	0.9596	0.0294	0
285	1071.6	0.2152	0.1725	0.9597	0.0293	0
286	1071.5	0.2154	0.1726	0.9601	0.0294	0
287	1071.4	0.2152	0.1723	0.9592	0.0293	0

288	1071.3	0.2152	0.1717	0.9599	0.0292	0
289	1071.2	0.2153	0.1713	0.9599	0.0294	0
290	1071.1	0.2155	0.1677	0.9606	0.0294	0
291	1071	0.2155	0.1666	0.9607	0.0294	0
292	1070.9	0.2153	0.1664	0.9603	0.0292	0
293	1070.8	0.2153	0.1665	0.9599	0.0293	0
294	1070.7	0.2154	0.1664	0.9599	0.0294	0
295	1070.6	0.2156	0.1665	0.9605	0.0297	0
296	1070.5	0.2158	0.1666	0.9606	0.03	0
297	1070.4	0.2159	0.1664	0.9607	0.0299	0
298	1070.3	0.2161	0.1666	0.961	0.0299	0
299	1070.2	0.2158	0.166	0.9603	0.0297	0
300	1070.1	0.2161	0.1657	0.9606	0.0299	0
301	1070	0.2158	0.1651	0.9608	0.0296	0
302	1069.9	0.2163	0.1653	0.9622	0.03	0
303	1069.8	0.2161	0.1653	0.9615	0.03	0
304	1069.7	0.2166	0.1656	0.962	0.0304	0
305	1069.6	0.216	0.1653	0.9624	0.0301	0
306	1069.5	0.2158	0.1652	0.9613	0.0299	0
307	1069.4	0.2162	0.1652	0.9614	0.0301	0
308	1069.3	0.2161	0.1652	0.9614	0.0301	0
309	1069.2	0.2161	0.165	0.9616	0.03	0
310	1069.1	0.216	0.1652	0.9616	0.03	0
311	1069	0.2159	0.1649	0.962	0.0298	0
312	1068.9	0.2158	0.1647	0.9614	0.0299	0
313	1068.8	0.2157	0.165	0.9618	0.0299	0
314	1068.7	0.216	0.1654	0.9624	0.03	0
315	1068.6	0.2162	0.1654	0.9619	0.0302	0
316	1068.5	0.2166	0.1656	0.9626	0.0305	0
317	1068.4	0.2165	0.1655	0.9625	0.0304	0
318	1068.3	0.2165	0.1657	0.9625	0.0303	0
319	1068.2	0.2164	0.1654	0.9626	0.0302	0
320	1068.1	0.2166	0.1653	0.9631	0.0304	0
321	1068	0.2165	0.165	0.9629	0.0303	0
322	1067.9	0.2164	0.1649	0.9628	0.0302	0
323	1067.8	0.2165	0.1647	0.9623	0.03	0
324	1067.7	0.2163	0.1646	0.9632	0.0299	0
325	1067.6	0.2163	0.1648	0.9632	0.0301	0
326	1067.5	0.2163	0.1647	0.9634	0.0302	0
327	1067.4	0.2167	0.1648	0.9628	0.0302	0
328	1067.3	0.2169	0.1651	0.9639	0.0305	0
329	1067.2	0.2168	0.1651	0.9632	0.0303	0
330	1067.1	0.2168	0.1646	0.9636	0.0304	0

331	1067	0.2169	0.1645	0.9639	0.0305	0
332	1066.9	0.2164	0.1641	0.9638	0.0301	0
333	1066.8	0.2162	0.1639	0.9635	0.0299	0
334	1066.7	0.2161	0.1638	0.9637	0.0299	0
335	1066.6	0.2166	0.1641	0.9641	0.0301	0
336	1066.5	0.2167	0.1643	0.9643	0.0301	0
337	1066.4	0.217	0.1645	0.9646	0.0305	0
338	1066.3	0.2169	0.1643	0.9643	0.0302	0
339	1066.2	0.2166	0.1642	0.9644	0.0301	0
340	1066.1	0.217	0.1644	0.9647	0.0304	0
341	1066	0.2168	0.1645	0.9645	0.0303	0
342	1065.9	0.2168	0.1646	0.9647	0.0302	0
343	1065.8	0.2168	0.1642	0.9645	0.0301	0
344	1065.7	0.2171	0.1643	0.965	0.0302	0
345	1065.6	0.2171	0.1645	0.9649	0.0304	0
346	1065.5	0.217	0.1645	0.9654	0.0304	0
347	1065.4	0.217	0.1645	0.9649	0.0302	0
348	1065.3	0.2173	0.1648	0.9657	0.0306	0
349	1065.2	0.2172	0.1648	0.9653	0.0304	0
350	1065.1	0.2172	0.1651	0.9657	0.0306	0
351	1065	0.2173	0.1648	0.9654	0.0304	0
352	1064.9	0.217	0.1649	0.9662	0.0304	0
353	1064.8	0.2171	0.1647	0.9657	0.0301	0
354	1064.7	0.2173	0.165	0.966	0.0306	0
355	1064.6	0.2174	0.1649	0.9663	0.0306	0
356	1064.5	0.2175	0.1652	0.966	0.0309	0
357	1064.4	0.2174	0.1651	0.9665	0.0308	0
358	1064.3	0.2175	0.1651	0.9662	0.0307	0
359	1064.2	0.2177	0.1653	0.9669	0.0307	0
360	1064.1	0.2175	0.1654	0.9668	0.0308	0
361	1064	0.2177	0.1656	0.9671	0.0309	0
362	1063.9	0.2176	0.1656	0.9672	0.0308	0
363	1063.8	0.2178	0.1658	0.9673	0.0311	0
364	1063.7	0.2182	0.1661	0.9676	0.0313	0
365	1063.6	0.2177	0.1653	0.967	0.0308	0
366	1063.5	0.2179	0.1656	0.967	0.0311	0
367	1063.4	0.2182	0.1659	0.9675	0.0313	0
368	1063.3	0.2179	0.166	0.9671	0.0312	0
369	1063.2	0.218	0.1659	0.9676	0.031	0
370	1063.1	0.2184	0.1661	0.9678	0.0312	0
371	1063	0.2183	0.1663	0.9679	0.0313	0
372	1062.9	0.2181	0.1658	0.9677	0.0311	0
373	1062.8	0.218	0.1658	0.9679	0.0312	0

374	1062.7	0.2181	0.166	0.9679	0.0312	0
375	1062.6	0.218	0.1661	0.9682	0.0313	0
376	1062.5	0.2181	0.1663	0.9676	0.0314	0
377	1062.4	0.218	0.1659	0.9675	0.0311	0
378	1062.3	0.2182	0.1664	0.9681	0.0314	0
379	1062.2	0.2183	0.1665	0.968	0.0313	0
380	1062.1	0.2183	0.1664	0.9683	0.0315	0
381	1062	0.2181	0.1663	0.968	0.0315	0
382	1061.9	0.2185	0.1669	0.9687	0.0318	0
383	1061.8	0.2186	0.1671	0.9691	0.032	0
384	1061.7	0.2188	0.167	0.9688	0.0318	0
385	1061.6	0.2188	0.1669	0.9687	0.0319	0
386	1061.5	0.2186	0.1666	0.9686	0.0317	0
387	1061.4	0.2184	0.1665	0.9688	0.0318	0
388	1061.3	0.2187	0.1669	0.969	0.032	0
389	1061.2	0.2188	0.1667	0.9693	0.0317	0
390	1061.1	0.2186	0.1669	0.9698	0.0318	0
391	1061	0.2189	0.1672	0.9699	0.0321	0
392	1060.9	0.2189	0.1672	0.97	0.0322	0
393	1060.8	0.219	0.1674	0.9703	0.0324	0
394	1060.7	0.219	0.1673	0.9698	0.0321	0
395	1060.6	0.2191	0.1673	0.9698	0.0322	0
396	1060.5	0.2191	0.1676	0.9697	0.0324	0
397	1060.4	0.2193	0.1679	0.9703	0.0325	0
398	1060.3	0.2196	0.1676	0.9702	0.0326	0
399	1060.2	0.2194	0.1676	0.9706	0.0326	0
400	1060.1	0.2194	0.1677	0.9706	0.0326	0
401	1060	0.22	0.1682	0.9709	0.0329	0
402	1059.9	0.2196	0.168	0.9709	0.0327	0
403	1059.8	0.2194	0.1682	0.9707	0.0328	0
404	1059.7	0.2193	0.168	0.9704	0.0327	0
405	1059.6	0.22	0.1684	0.9702	0.033	0
406	1059.5	0.2199	0.1682	0.9705	0.0329	0
407	1059.4	0.2202	0.1687	0.9713	0.0334	0
408	1059.3	0.2203	0.1688	0.9715	0.0334	0
409	1059.2	0.2199	0.1685	0.9715	0.0334	0
410	1059.1	0.2197	0.1682	0.9711	0.0329	0
411	1059	0.2198	0.1687	0.9714	0.0332	0
412	1058.9	0.2199	0.1686	0.971	0.0332	0
413	1058.8	0.2201	0.1689	0.9719	0.0335	0
414	1058.7	0.2201	0.1689	0.972	0.0335	0
415	1058.6	0.2201	0.1688	0.9718	0.0335	0
416	1058.5	0.2206	0.1691	0.9719	0.0337	0

417	1058.4	0.2201	0.1686	0.972	0.0334	0
418	1058.3	0.2201	0.1687	0.9718	0.0332	0
419	1058.2	0.2206	0.1691	0.9718	0.0337	0
420	1058.1	0.2206	0.1691	0.9719	0.0337	0
421	1058	0.2204	0.169	0.9729	0.0337	0
422	1057.9	0.2203	0.1688	0.9722	0.0334	0
423	1057.8	0.2205	0.1693	0.9725	0.0339	0
424	1057.7	0.2206	0.1694	0.9726	0.0341	0
425	1057.6	0.2206	0.169	0.972	0.0338	0
426	1057.5	0.2205	0.1693	0.9732	0.034	0
427	1057.4	0.2205	0.1692	0.9729	0.034	0
428	1057.3	0.2208	0.1693	0.9734	0.0342	0
429	1057.2	0.2211	0.1698	0.9728	0.0342	0
430	1057.1	0.221	0.1697	0.9729	0.0343	0
431	1057	0.2209	0.1696	0.973	0.0342	0
432	1056.9	0.2215	0.1699	0.974	0.0343	0
433	1056.8	0.2214	0.1699	0.9736	0.0343	0
434	1056.7	0.2214	0.17	0.9737	0.0344	0
435	1056.6	0.2214	0.17	0.9741	0.0346	0
436	1056.5	0.2214	0.17	0.9743	0.0348	0
437	1056.4	0.2214	0.1701	0.975	0.0348	0
438	1056.3	0.2216	0.1704	0.9749	0.035	0
439	1056.2	0.2218	0.1706	0.9749	0.035	0
440	1056.1	0.2219	0.1707	0.9748	0.0351	0
441	1056	0.2218	0.1708	0.9754	0.0352	0
442	1055.9	0.222	0.1708	0.9749	0.0352	0
443	1055.8	0.2219	0.1707	0.9753	0.0353	0
444	1055.7	0.2219	0.1708	0.9755	0.0352	0
445	1055.6	0.2217	0.1706	0.975	0.0352	0
446	1055.5	0.2216	0.1707	0.975	0.0353	0
447	1055.4	0.2219	0.171	0.975	0.0355	0
448	1055.3	0.2224	0.1714	0.976	0.0358	0
449	1055.2	0.2224	0.1711	0.9758	0.0358	0
450	1055.1	0.2223	0.1715	0.9757	0.0359	0
451	1055	0.2225	0.1716	0.976	0.0358	0
452	1054.9	0.2224	0.1716	0.9764	0.0359	0
453	1054.8	0.2223	0.1716	0.9761	0.0359	0
454	1054.7	0.2221	0.1716	0.9757	0.0359	0
455	1054.6	0.2224	0.1717	0.9765	0.0361	0
456	1054.5	0.2228	0.1718	0.9765	0.0362	0
457	1054.4	0.223	0.172	0.9772	0.0363	0
458	1054.3	0.2231	0.1721	0.9768	0.0364	0
459	1054.2	0.2229	0.1724	0.9771	0.0364	0

460	1054.1	0.223	0.1725	0.9772	0.0366	0
461	1054	0.223	0.1724	0.9767	0.0364	0
462	1053.9	0.2232	0.1722	0.977	0.0367	0
463	1053.8	0.2233	0.1724	0.9773	0.0369	0
464	1053.7	0.2233	0.1727	0.9776	0.037	0
465	1053.6	0.223	0.1725	0.9775	0.0368	0
466	1053.5	0.2233	0.1728	0.9778	0.037	0
467	1053.4	0.2233	0.1727	0.9777	0.0369	0
468	1053.3	0.2236	0.1732	0.9779	0.0371	0
469	1053.2	0.2232	0.173	0.9782	0.037	0
470	1053.1	0.2231	0.173	0.9783	0.037	0
471	1053	0.2235	0.1728	0.9785	0.0371	0
472	1052.9	0.2235	0.1729	0.978	0.037	0
473	1052.8	0.2233	0.173	0.9778	0.0369	0
474	1052.7	0.2237	0.1733	0.9783	0.0373	0
475	1052.6	0.2236	0.1733	0.9781	0.0373	0
476	1052.5	0.2237	0.1735	0.9782	0.0376	0
477	1052.4	0.224	0.1736	0.9784	0.0377	0
478	1052.3	0.224	0.1734	0.9781	0.0376	0
479	1052.2	0.2241	0.1736	0.9787	0.0378	0
480	1052.1	0.224	0.1734	0.9783	0.0376	0
481	1052	0.2239	0.1735	0.9782	0.0377	0
482	1051.9	0.2241	0.1739	0.9781	0.0377	0
483	1051.8	0.2239	0.1735	0.9786	0.0377	0
484	1051.7	0.224	0.1736	0.9791	0.0377	0
485	1051.6	0.2237	0.1736	0.9779	0.0376	0
486	1051.5	0.2239	0.174	0.9794	0.0379	0
487	1051.4	0.224	0.1737	0.9792	0.0379	0
488	1051.3	0.2244	0.174	0.9794	0.0382	0
489	1051.2	0.2242	0.1739	0.9798	0.0382	0
490	1051.1	0.2245	0.174	0.9796	0.0382	0
491	1051	0.2245	0.1741	0.98	0.0382	0
492	1050.9	0.2243	0.1743	0.9796	0.0383	0
493	1050.8	0.2246	0.1744	0.9794	0.0384	0
494	1050.7	0.2246	0.1747	0.9793	0.0383	0
495	1050.6	0.2246	0.1747	0.9801	0.0385	0
496	1050.5	0.2247	0.1745	0.98	0.0384	0
497	1050.4	0.2247	0.1747	0.9799	0.0384	0
498	1050.3	0.225	0.175	0.9799	0.0385	0
499	1050.2	0.225	0.1749	0.9799	0.0384	0
500	1050.1	0.2251	0.1749	0.9805	0.0385	0
501	1050	0.2251	0.175	0.9806	0.0386	0
502	1049.9	0.2251	0.1751	0.9805	0.0386	0

503	1049.8	0.2248	0.1746	0.9798	0.0384	0
504	1049.7	0.2249	0.1748	0.9805	0.0385	0
505	1049.6	0.2251	0.1752	0.9809	0.0386	0
506	1049.5	0.225	0.1752	0.9808	0.0385	0
507	1049.4	0.2253	0.1751	0.9812	0.0386	0
508	1049.3	0.225	0.1752	0.9807	0.0385	0
509	1049.2	0.2253	0.1754	0.9817	0.0386	0
510	1049.1	0.2251	0.1755	0.9812	0.0387	0
511	1049	0.2252	0.1755	0.9816	0.0388	0
512	1048.9	0.2254	0.1754	0.9818	0.0387	0
513	1048.8	0.2255	0.1755	0.9819	0.0389	0
514	1048.7	0.2256	0.1757	0.9818	0.0389	0
515	1048.6	0.2253	0.1755	0.9819	0.0387	0
516	1048.5	0.2254	0.1756	0.9821	0.0388	0
517	1048.4	0.2255	0.1757	0.9816	0.0388	0
518	1048.3	0.2252	0.1757	0.9819	0.0387	0
519	1048.2	0.2256	0.176	0.9822	0.0391	0
520	1048.1	0.2256	0.1759	0.9828	0.039	0
521	1048	0.2259	0.1761	0.9827	0.0392	0
522	1047.9	0.2258	0.1762	0.983	0.0393	0
523	1047.8	0.2258	0.1758	0.9833	0.0391	0
524	1047.7	0.2259	0.1761	0.9827	0.0392	0
525	1047.6	0.2261	0.1765	0.9828	0.0397	0
526	1047.5	0.226	0.1762	0.9826	0.0395	0
527	1047.4	0.2259	0.1764	0.9824	0.0396	0
528	1047.3	0.2261	0.1763	0.9823	0.0393	0
529	1047.2	0.226	0.1762	0.9823	0.0393	0
530	1047.1	0.2262	0.1765	0.9834	0.0395	0
531	1047	0.2263	0.1765	0.9835	0.0397	0
532	1046.9	0.2263	0.1766	0.9837	0.0397	0
533	1046.8	0.2261	0.1763	0.9838	0.0394	0
534	1046.7	0.2261	0.1768	0.984	0.0396	0
535	1046.6	0.2262	0.1768	0.9841	0.0399	0
536	1046.5	0.2265	0.1772	0.9838	0.0403	0
537	1046.4	0.2265	0.1771	0.9838	0.04	0
538	1046.3	0.2263	0.1771	0.9839	0.0402	0
539	1046.2	0.226	0.1768	0.9837	0.0399	0
540	1046.1	0.2264	0.1772	0.984	0.0402	0
541	1046	0.2266	0.177	0.9837	0.04	0
542	1045.9	0.2266	0.1771	0.9841	0.0401	0
543	1045.8	0.2265	0.1769	0.9841	0.04	0
544	1045.7	0.2271	0.1773	0.9847	0.0403	0
545	1045.6	0.2266	0.1771	0.984	0.0402	0

546	1045.5	0.2268	0.1775	0.9844	0.0404	0
547	1045.4	0.2266	0.1775	0.9839	0.0404	0
548	1045.3	0.2266	0.1771	0.9839	0.0401	0
549	1045.2	0.2269	0.1775	0.9846	0.0403	0
550	1045.1	0.2271	0.1776	0.9848	0.0405	0
551	1045	0.2269	0.1776	0.9845	0.0405	0
552	1044.9	0.2272	0.1775	0.9847	0.0405	0
553	1044.8	0.2272	0.1778	0.9852	0.0408	0
554	1044.7	0.2271	0.1774	0.9848	0.0405	0
555	1044.6	0.227	0.1774	0.985	0.0409	0
556	1044.5	0.2277	0.1775	0.9858	0.0411	0
557	1044.4	0.2275	0.1778	0.9849	0.0411	0
558	1044.3	0.2274	0.1773	0.9858	0.0411	0
559	1044.2	0.2277	0.1776	0.986	0.0417	0
560	1044.1	0.2277	0.1774	0.9858	0.0417	0
561	1044	0.2275	0.1773	0.9858	0.0415	0
562	1043.9	0.2275	0.1775	0.9861	0.0416	0
563	1043.8	0.2278	0.1773	0.9858	0.0416	0
564	1043.7	0.2279	0.1773	0.9859	0.0416	0
565	1043.6	0.2278	0.1774	0.9862	0.0418	0
566	1043.5	0.2279	0.1775	0.9862	0.0418	0
567	1043.4	0.2281	0.1778	0.9861	0.0418	0
568	1043.3	0.2281	0.1778	0.9869	0.0418	0
569	1043.2	0.2282	0.1778	0.9866	0.0419	0
570	1043.1	0.2281	0.1778	0.9863	0.0419	0
571	1043	0.2283	0.178	0.9864	0.0421	0
572	1042.9	0.2284	0.1782	0.9872	0.0421	0
573	1042.8	0.2281	0.1782	0.9863	0.042	0
574	1042.7	0.2284	0.1781	0.9864	0.0421	0
575	1042.6	0.2283	0.1781	0.9867	0.0421	0
576	1042.5	0.2283	0.1783	0.9867	0.0425	0
577	1042.4	0.2283	0.1783	0.9867	0.0424	0
578	1042.3	0.2283	0.1782	0.9869	0.0424	0
579	1042.2	0.2285	0.1783	0.9868	0.0424	0
580	1042.1	0.2286	0.1783	0.9875	0.0425	0
581	1042	0.2288	0.1787	0.9873	0.0425	0
582	1041.9	0.2287	0.1784	0.9869	0.0425	0
583	1041.8	0.2284	0.1785	0.9876	0.0424	0
584	1041.7	0.2285	0.1786	0.9878	0.0426	0
585	1041.6	0.2288	0.1789	0.9881	0.0426	0
586	1041.5	0.2291	0.1791	0.9878	0.0429	0
587	1041.4	0.2291	0.179	0.9877	0.0429	0
588	1041.3	0.229	0.179	0.988	0.0428	0

589	1041.2	0.2287	0.1788	0.9883	0.0427	0
590	1041.1	0.2289	0.1788	0.9875	0.0429	0
591	1041	0.2288	0.1789	0.988	0.0429	0
592	1040.9	0.229	0.1791	0.9882	0.0429	0
593	1040.8	0.2291	0.1794	0.9885	0.043	0
594	1040.7	0.2294	0.1794	0.9886	0.0432	0
595	1040.6	0.2291	0.1792	0.9884	0.0431	0
596	1040.5	0.2288	0.1789	0.9881	0.0426	0
597	1040.4	0.2292	0.1795	0.9889	0.0431	0
598	1040.3	0.2292	0.1795	0.9893	0.0432	0
599	1040.2	0.2291	0.1793	0.989	0.0432	0
600	1040.1	0.2293	0.1797	0.9893	0.0433	0
601	1040	0.2294	0.1795	0.9898	0.0433	0
602	1039.9	0.2295	0.1793	0.989	0.0432	0
603	1039.8	0.2295	0.1795	0.9895	0.0433	0
604	1039.7	0.2294	0.1794	0.9893	0.0433	0
605	1039.6	0.2296	0.1797	0.9895	0.0436	0
606	1039.5	0.2299	0.1796	0.9894	0.0434	0
607	1039.4	0.2296	0.1798	0.9895	0.0434	0
608	1039.3	0.2296	0.1798	0.9902	0.0435	0
609	1039.2	0.2298	0.1799	0.9898	0.0435	0
610	1039.1	0.2297	0.1798	0.9895	0.0434	0
611	1039	0.2295	0.1796	0.9897	0.0433	0
612	1038.9	0.2295	0.1793	0.9903	0.0431	0
613	1038.8	0.2293	0.1794	0.9897	0.043	0
614	1038.7	0.2295	0.1798	0.9896	0.0433	0
615	1038.6	0.2295	0.1798	0.9898	0.0435	0
616	1038.5	0.2299	0.1799	0.9902	0.0434	0
617	1038.4	0.2297	0.18	0.9904	0.0433	0
618	1038.3	0.2298	0.1799	0.9905	0.0433	0
619	1038.2	0.2297	0.18	0.9905	0.0433	0
620	1038.1	0.2297	0.1801	0.9898	0.0433	0
621	1038	0.2302	0.1805	0.9903	0.0436	0
622	1037.9	0.2298	0.18	0.9897	0.0433	0
623	1037.8	0.23	0.1804	0.9903	0.0436	0
624	1037.7	0.23	0.1804	0.9908	0.0435	0
625	1037.6	0.23	0.1804	0.9904	0.0435	0
626	1037.5	0.2301	0.1805	0.9903	0.0436	0
627	1037.4	0.23	0.1803	0.9901	0.0435	0
628	1037.3	0.2301	0.1805	0.991	0.0436	0
629	1037.2	0.23	0.1804	0.9911	0.0436	0
630	1037.1	0.23	0.1805	0.9906	0.0436	0
631	1037	0.2303	0.1807	0.9911	0.044	0

632	1036.9	0.2302	0.1806	0.9914	0.0439	0
633	1036.8	0.2305	0.1807	0.9915	0.0441	0
634	1036.7	0.2304	0.1807	0.9908	0.044	0
635	1036.6	0.2305	0.1809	0.9917	0.044	0
636	1036.5	0.2304	0.1808	0.9914	0.044	0
637	1036.4	0.2304	0.181	0.991	0.0442	0
638	1036.3	0.2307	0.181	0.9916	0.0442	0
639	1036.2	0.2306	0.181	0.9917	0.0442	0
640	1036.1	0.2306	0.181	0.9916	0.0442	0
641	1036	0.2307	0.1813	0.9918	0.0445	0
642	1035.9	0.2309	0.1813	0.9919	0.0447	0
643	1035.8	0.2309	0.1813	0.9923	0.0445	0
644	1035.7	0.2311	0.1815	0.9919	0.0447	0
645	1035.6	0.2312	0.1817	0.9925	0.045	0
646	1035.5	0.2309	0.1817	0.992	0.0449	0
647	1035.4	0.231	0.1819	0.9921	0.0451	0
648	1035.3	0.2309	0.1819	0.9924	0.045	0
649	1035.2	0.2312	0.1822	0.9931	0.0452	0
650	1035.1	0.2314	0.1822	0.9931	0.0453	0
651	1035	0.2317	0.1824	0.9929	0.0455	0
652	1034.9	0.2312	0.1821	0.9921	0.0451	0
653	1034.8	0.2314	0.1824	0.9926	0.0455	0
654	1034.7	0.2314	0.1823	0.9925	0.0455	0
655	1034.6	0.2312	0.1826	0.9925	0.0455	0
656	1034.5	0.2316	0.1827	0.9927	0.0457	0
657	1034.4	0.2315	0.1827	0.9933	0.0458	0
658	1034.3	0.2316	0.1827	0.9927	0.0458	0
659	1034.2	0.2319	0.1827	0.9932	0.0456	0
660	1034.1	0.2317	0.1827	0.9926	0.0457	0
661	1034	0.2319	0.1831	0.9925	0.046	0
662	1033.9	0.2319	0.1832	0.9932	0.0461	0
663	1033.8	0.2321	0.1833	0.9934	0.0461	0
664	1033.7	0.232	0.1835	0.9933	0.0463	0
665	1033.6	0.2318	0.1835	0.9929	0.0464	0
666	1033.5	0.232	0.1836	0.9935	0.0465	0
667	1033.4	0.232	0.1834	0.9929	0.0464	0
668	1033.3	0.2323	0.1837	0.9938	0.0468	0
669	1033.2	0.2324	0.1839	0.994	0.0469	0
670	1033.1	0.2322	0.1839	0.9939	0.0468	0
671	1033	0.2325	0.184	0.9941	0.047	0
672	1032.9	0.2327	0.1841	0.9938	0.0472	0
673	1032.8	0.2325	0.1841	0.9939	0.0473	0
674	1032.7	0.2326	0.1841	0.9942	0.0472	0

675	1032.6	0.2327	0.1843	0.9944	0.0473	0
676	1032.5	0.2329	0.1847	0.9947	0.0475	0
677	1032.4	0.2324	0.1844	0.994	0.0472	0
678	1032.3	0.2327	0.1845	0.9937	0.0472	0
679	1032.2	0.2329	0.1845	0.9949	0.0476	0
680	1032.1	0.233	0.1845	0.9944	0.0476	0
681	1032	0.2329	0.1847	0.994	0.0476	0
682	1031.9	0.2328	0.1846	0.9944	0.0474	0
683	1031.8	0.2328	0.1845	0.9943	0.0475	0
684	1031.7	0.2329	0.1846	0.9945	0.0476	0
685	1031.6	0.233	0.1846	0.9943	0.0475	0
686	1031.5	0.2333	0.1848	0.9946	0.0478	0
687	1031.4	0.2335	0.1849	0.9947	0.048	0
688	1031.3	0.2334	0.1849	0.9946	0.048	0
689	1031.2	0.233	0.1849	0.9948	0.0477	0
690	1031.1	0.2329	0.1847	0.9945	0.0478	0
691	1031	0.233	0.1848	0.9944	0.0477	0
692	1030.9	0.2335	0.1851	0.9948	0.048	0
693	1030.8	0.2335	0.1852	0.9949	0.0481	0
694	1030.7	0.2333	0.1851	0.995	0.048	0
695	1030.6	0.2332	0.1851	0.9955	0.048	0
696	1030.5	0.2334	0.1854	0.996	0.0482	0
697	1030.4	0.2334	0.1853	0.9953	0.0479	0
698	1030.3	0.2335	0.1855	0.9956	0.0481	0
699	1030.2	0.2335	0.1855	0.9953	0.0483	0
700	1030.1	0.2336	0.1853	0.9952	0.048	0
701	1030	0.2335	0.1853	0.9952	0.0479	0
702	1029.9	0.2336	0.1854	0.9954	0.0481	0
703	1029.8	0.2336	0.1854	0.9955	0.0481	0
704	1029.7	0.2334	0.1855	0.9958	0.0481	0
705	1029.6	0.2335	0.1852	0.9956	0.048	0
706	1029.5	0.2334	0.1852	0.9959	0.048	0
707	1029.4	0.2334	0.1851	0.9958	0.0478	0
708	1029.3	0.2334	0.1854	0.9951	0.0479	0
709	1029.2	0.2337	0.1856	0.9965	0.0482	0
710	1029.1	0.2337	0.1856	0.996	0.0483	0
711	1029	0.2338	0.1857	0.9963	0.0484	0
712	1028.9	0.234	0.1857	0.9964	0.0483	0
713	1028.8	0.2336	0.1854	0.9964	0.0481	0
714	1028.7	0.2338	0.1853	0.9968	0.048	0
715	1028.6	0.2336	0.1856	0.9962	0.0481	0
716	1028.5	0.2334	0.1852	0.9966	0.0477	0
717	1028.4	0.2336	0.1854	0.9966	0.048	0

718	1028.3	0.2336	0.1853	0.9963	0.0478	0
719	1028.2	0.234	0.1857	0.9962	0.0481	0
720	1028.1	0.2338	0.1857	0.997	0.0482	0
721	1028	0.2338	0.1857	0.9967	0.0483	0
722	1027.9	0.234	0.1858	0.997	0.0482	0
723	1027.8	0.2341	0.1858	0.9972	0.0482	0
724	1027.7	0.234	0.1858	0.9971	0.0481	0
725	1027.6	0.2342	0.186	0.9968	0.0485	0
726	1027.5	0.234	0.1857	0.9967	0.0485	0
727	1027.4	0.2337	0.1855	0.9965	0.0481	0
728	1027.3	0.234	0.1856	0.9972	0.048	0
729	1027.2	0.2341	0.1857	0.9975	0.0482	0
730	1027.1	0.2344	0.186	0.9981	0.0485	0
731	1027	0.2342	0.186	0.9977	0.0485	0
732	1026.9	0.2342	0.1859	0.997	0.0483	0
733	1026.8	0.2341	0.1858	0.9975	0.0484	0
734	1026.7	0.234	0.186	0.9971	0.0483	0
735	1026.6	0.234	0.186	0.9973	0.0483	0
736	1026.5	0.2344	0.1861	0.9974	0.0487	0
737	1026.4	0.2344	0.1862	0.9978	0.0488	0
738	1026.3	0.2342	0.1861	0.9973	0.0487	0
739	1026.2	0.2342	0.1863	0.9972	0.0488	0
740	1026.1	0.2344	0.1862	0.9977	0.0489	0
741	1026	0.2342	0.1863	0.9978	0.0489	0
742	1025.9	0.2344	0.1865	0.9976	0.0488	0
743	1025.8	0.2348	0.1864	0.9978	0.049	0
744	1025.7	0.2349	0.1869	0.9976	0.0494	0
745	1025.6	0.2349	0.1869	0.998	0.0493	0
746	1025.5	0.2352	0.187	0.9979	0.0495	0
747	1025.4	0.2349	0.1868	0.9983	0.0494	0
748	1025.3	0.2348	0.1869	0.998	0.0494	0
749	1025.2	0.2347	0.1869	0.9984	0.0495	0
750	1025.1	0.235	0.1869	0.998	0.0495	0
751	1025	0.2349	0.1872	0.9982	0.0496	0
752	1024.9	0.2353	0.1874	0.9989	0.0498	0
753	1024.8	0.2353	0.1876	0.9989	0.05	0
754	1024.7	0.2352	0.1877	0.9986	0.0501	0
755	1024.6	0.2353	0.1875	0.9987	0.0501	0
756	1024.5	0.2352	0.1876	0.9988	0.0501	0
757	1024.4	0.2351	0.1874	0.9987	0.0499	0
758	1024.3	0.2353	0.1877	0.9991	0.0502	0
759	1024.2	0.2356	0.1879	0.9987	0.0503	0
760	1024.1	0.2358	0.1883	0.9989	0.0505	0

761	1024	0.2359	0.1886	0.9992	0.0509	0
762	1023.9	0.236	0.1886	0.9988	0.051	0
763	1023.8	0.236	0.1887	0.9994	0.051	0
764	1023.7	0.2359	0.1888	0.9996	0.0511	0
765	1023.6	0.2358	0.1885	0.9995	0.0509	0
766	1023.5	0.2356	0.1885	0.9986	0.0508	0
767	1023.4	0.236	0.1887	0.9987	0.0509	0
768	1023.3	0.2361	0.1888	0.9994	0.0513	0
769	1023.2	0.236	0.1888	0.9996	0.0513	0
770	1023.1	0.2357	0.1886	0.9988	0.0513	0
771	1023	0.2357	0.1887	0.9988	0.0514	0
772	1022.9	0.2361	0.1893	0.999	0.0516	0
773	1022.8	0.2364	0.1894	0.9996	0.0517	0
774	1022.7	0.2363	0.1892	0.9993	0.0516	0
775	1022.6	0.236	0.1891	0.999	0.0516	0
776	1022.5	0.2359	0.1889	0.9995	0.0514	0
777	1022.4	0.236	0.1892	0.9994	0.0514	0
778	1022.3	0.2363	0.1893	0.9999	0.0517	0
779	1022.2	0.2363	0.1893	0.9995	0.0518	0
780	1022.1	0.2365	0.1894	0.9996	0.0519	0
781	1022	0.2366	0.1895	0.9997	0.0518	0
782	1021.9	0.2365	0.1895	1	0.0519	0
783	1021.8	0.2364	0.1895	1.0004	0.0519	0
784	1021.7	0.2366	0.1897	0.9996	0.052	0
785	1021.6	0.2368	0.1901	1.0006	0.0523	0
786	1021.5	0.2367	0.1901	0.9996	0.0525	0
787	1021.4	0.2368	0.1901	1.0003	0.0526	0
788	1021.3	0.2369	0.1902	1.0005	0.0527	0
789	1021.2	0.2371	0.1902	1.0005	0.0527	0
790	1021.1	0.2367	0.19	1	0.0525	0
791	1021	0.237	0.1899	1	0.0525	0
792	1020.9	0.237	0.1902	1.0003	0.0527	0
793	1020.8	0.2371	0.1902	1.0004	0.0527	0
794	1020.7	0.2372	0.1905	1.0002	0.0528	0
795	1020.6	0.2372	0.1903	1.0003	0.0528	0
796	1020.5	0.2373	0.1905	1.0008	0.053	0
797	1020.4	0.2374	0.1907	1.0006	0.0531	0
798	1020.3	0.2371	0.1905	1.0001	0.0528	0
799	1020.2	0.2372	0.1906	1.0006	0.0531	0
800	1020.1	0.2371	0.1907	1.0008	0.0532	0
801	1020	0.2375	0.1909	1.0012	0.0531	0
802	1019.9	0.2372	0.1907	1.0013	0.0528	0
803	1019.8	0.2374	0.1908	1.0012	0.053	0

804	1019.7	0.2372	0.1907	1.0011	0.053	0
805	1019.6	0.2372	0.1905	1.0011	0.053	0
806	1019.5	0.2374	0.1908	1.0015	0.053	0
807	1019.4	0.2376	0.1907	1.0015	0.053	0
808	1019.3	0.2375	0.1909	1.001	0.0532	0
809	1019.2	0.2377	0.1904	1.0013	0.0529	0
810	1019.1	0.2376	0.1907	1.0014	0.0529	0
811	1019	0.2378	0.191	1.0017	0.0532	0
812	1018.9	0.2377	0.1907	1.0017	0.0532	0
813	1018.8	0.2378	0.1909	1.0014	0.0534	0
814	1018.7	0.2375	0.1908	1.0011	0.053	0
815	1018.6	0.2379	0.1909	1.0017	0.0531	0
816	1018.5	0.2377	0.1906	1.0016	0.0531	0
817	1018.4	0.2378	0.1906	1.0016	0.0531	0
818	1018.3	0.238	0.1907	1.0018	0.0533	0
819	1018.2	0.238	0.1908	1.0022	0.0533	0
820	1018.1	0.2381	0.1913	1.002	0.0535	0
821	1018	0.2379	0.1912	1.0024	0.0533	0
822	1017.9	0.238	0.1913	1.0025	0.0531	0
823	1017.8	0.2379	0.1912	1.002	0.0531	0
824	1017.7	0.238	0.191	1.0023	0.0532	0
825	1017.6	0.2379	0.1913	1.0026	0.0532	0
826	1017.5	0.2383	0.1915	1.0028	0.0536	0
827	1017.4	0.2384	0.1916	1.0032	0.0536	0
828	1017.3	0.2381	0.1912	1.0022	0.0533	0
829	1017.2	0.2385	0.1916	1.0028	0.0537	0
830	1017.1	0.2383	0.1914	1.0029	0.0536	0
831	1017	0.2382	0.1914	1.003	0.0534	0
832	1016.9	0.2383	0.1915	1.003	0.0534	0
833	1016.8	0.2384	0.1916	1.0029	0.0535	0
834	1016.7	0.2383	0.1915	1.0032	0.0535	0
835	1016.6	0.2385	0.1917	1.0034	0.0537	0
836	1016.5	0.2385	0.1918	1.0037	0.0538	0
837	1016.4	0.2378	0.1912	1.0032	0.0533	0
838	1016.3	0.238	0.1914	1.0033	0.0534	0
839	1016.2	0.2383	0.1917	1.0039	0.0534	0
840	1016.1	0.2385	0.1919	1.0043	0.0537	0
841	1016	0.2384	0.1918	1.004	0.0538	0
842	1015.9	0.2385	0.1917	1.0037	0.0539	0
843	1015.8	0.2388	0.192	1.0042	0.054	0
844	1015.7	0.2387	0.192	1.0042	0.0539	0
845	1015.6	0.2388	0.1922	1.0046	0.0542	0
846	1015.5	0.2385	0.192	1.0045	0.054	0

847	1015.4	0.2384	0.192	1.004	0.054	0
848	1015.3	0.2387	0.192	1.004	0.0542	0
849	1015.2	0.2388	0.1923	1.0039	0.0544	0
850	1015.1	0.2388	0.1922	1.0037	0.0544	0
851	1015	0.2389	0.1924	1.004	0.0543	0
852	1014.9	0.2391	0.1925	1.0047	0.0543	0
853	1014.8	0.2394	0.193	1.0045	0.0546	0
854	1014.7	0.2394	0.1931	1.0046	0.0547	0
855	1014.6	0.2391	0.1924	1.0045	0.0545	0
856	1014.5	0.239	0.1925	1.0047	0.0545	0
857	1014.4	0.2389	0.1926	1.0047	0.0545	0
858	1014.3	0.2389	0.1926	1.0049	0.0545	0
859	1014.2	0.2393	0.1928	1.005	0.0549	0
860	1014.1	0.2391	0.193	1.005	0.0549	0
861	1014	0.2393	0.1931	1.0046	0.0549	0
862	1013.9	0.2392	0.1934	1.0055	0.0551	0
863	1013.8	0.2392	0.1932	1.0045	0.0551	0
864	1013.7	0.2394	0.1932	1.005	0.0551	0
865	1013.6	0.2394	0.1933	1.0047	0.0553	0
866	1013.5	0.2395	0.1933	1.0046	0.0553	0
867	1013.4	0.2396	0.1933	1.0054	0.0553	0
868	1013.3	0.2395	0.1935	1.0055	0.0554	0
869	1013.2	0.2398	0.1938	1.0052	0.0557	0
870	1013.1	0.2399	0.194	1.0056	0.056	0
871	1013	0.2402	0.1942	1.0058	0.0561	0
872	1012.9	0.2402	0.1942	1.0063	0.0561	0
873	1012.8	0.2402	0.1942	1.0053	0.0561	0
874	1012.7	0.2403	0.1943	1.0057	0.0563	0
875	1012.6	0.2403	0.1944	1.0055	0.0564	0
876	1012.5	0.2403	0.1943	1.0053	0.0564	0
877	1012.4	0.2404	0.1944	1.0054	0.0563	0
878	1012.3	0.2404	0.1944	1.0058	0.0564	0
879	1012.2	0.2404	0.1947	1.0054	0.0566	0
880	1012.1	0.2406	0.1946	1.0054	0.0567	0
881	1012	0.2406	0.1949	1.0059	0.057	0
882	1011.9	0.2407	0.1949	1.0063	0.0569	0
883	1011.8	0.2408	0.1949	1.0065	0.0569	0
884	1011.7	0.2406	0.1947	1.0064	0.0568	0
885	1011.6	0.2409	0.195	1.0057	0.057	0
886	1011.5	0.241	0.1951	1.0062	0.057	0
887	1011.4	0.2408	0.1952	1.0065	0.0571	0
888	1011.3	0.2407	0.1951	1.0061	0.0571	0
889	1011.2	0.2408	0.1952	1.006	0.0571	0

890	1011.1	0.241	0.1953	1.0066	0.0571	0
891	1011	0.2408	0.1953	1.0062	0.0572	0
892	1010.9	0.2411	0.1954	1.0063	0.0572	0
893	1010.8	0.241	0.1954	1.0065	0.0574	0
894	1010.7	0.2408	0.1952	1.0067	0.0572	0
895	1010.6	0.2409	0.1952	1.006	0.0572	0
896	1010.5	0.2407	0.1952	1.0059	0.0569	0
897	1010.4	0.241	0.1953	1.0061	0.0572	0
898	1010.3	0.241	0.1954	1.0067	0.0574	0
899	1010.2	0.2413	0.1957	1.0069	0.0576	0
900	1010.1	0.2413	0.1957	1.0066	0.0577	0
901	1010	0.2414	0.1958	1.0066	0.0577	0
902	1009.9	0.2413	0.1959	1.0066	0.0578	0
903	1009.8	0.2411	0.1956	1.0063	0.0574	0
904	1009.7	0.241	0.1955	1.0062	0.0573	0
905	1009.6	0.2414	0.1958	1.0067	0.0575	0
906	1009.5	0.2414	0.1956	1.0065	0.0578	0
907	1009.4	0.2417	0.1962	1.0071	0.058	0
908	1009.3	0.2415	0.196	1.0067	0.0577	0
909	1009.2	0.2416	0.1961	1.0072	0.0579	0
910	1009.1	0.2417	0.1961	1.0068	0.0579	0
911	1009	0.2415	0.1959	1.0068	0.0577	0
912	1008.9	0.2415	0.1962	1.0073	0.0578	0
913	1008.8	0.2417	0.1959	1.0073	0.0578	0
914	1008.7	0.2417	0.196	1.0075	0.0578	0
915	1008.6	0.2415	0.1959	1.0076	0.0578	0
916	1008.5	0.2418	0.1962	1.0075	0.058	0
917	1008.4	0.2417	0.1961	1.0075	0.0579	0
918	1008.3	0.2415	0.1961	1.0076	0.0578	0
919	1008.2	0.2415	0.196	1.0075	0.0579	0
920	1008.1	0.2417	0.196	1.0077	0.0581	0
921	1008	0.2416	0.196	1.008	0.0578	0
922	1007.9	0.2417	0.1962	1.0079	0.058	0
923	1007.8	0.2419	0.196	1.0078	0.0579	0
924	1007.7	0.242	0.1963	1.0081	0.058	0
925	1007.6	0.2416	0.1962	1.0078	0.0579	0
926	1007.5	0.2417	0.1961	1.0077	0.0578	0
927	1007.4	0.2416	0.1961	1.0083	0.0578	0
928	1007.3	0.2417	0.1961	1.0084	0.0578	0
929	1007.2	0.2417	0.1959	1.0077	0.0577	0
930	1007.1	0.242	0.1963	1.0084	0.058	0
931	1007	0.2421	0.1964	1.0077	0.0581	0
932	1006.9	0.2421	0.1963	1.0081	0.0579	0

933	1006.8	0.2417	0.1962	1.0081	0.0578	0
934	1006.7	0.2419	0.196	1.008	0.0579	0
935	1006.6	0.2422	0.1963	1.0086	0.0582	0
936	1006.5	0.2419	0.1962	1.0081	0.058	0
937	1006.4	0.242	0.1965	1.008	0.0581	0
938	1006.3	0.2421	0.1964	1.0078	0.058	0
939	1006.2	0.2421	0.1963	1.0082	0.058	0
940	1006.1	0.242	0.1965	1.0081	0.0582	0
941	1006	0.2421	0.1964	1.0086	0.0583	0
942	1005.9	0.2426	0.1967	1.0091	0.0584	0
943	1005.8	0.2424	0.1967	1.0086	0.0584	0
944	1005.7	0.2426	0.1968	1.0093	0.0584	0
945	1005.6	0.2426	0.1968	1.0094	0.0584	0
946	1005.5	0.2424	0.1969	1.0091	0.0584	0
947	1005.4	0.2425	0.1968	1.0088	0.0583	0
948	1005.3	0.2424	0.1969	1.0091	0.0585	0
949	1005.2	0.2424	0.1968	1.0093	0.0586	0
950	1005.1	0.2426	0.197	1.0094	0.0588	0
951	1005	0.2427	0.197	1.0091	0.0587	0
952	1004.9	0.2426	0.1972	1.0097	0.0587	0
953	1004.8	0.2424	0.1969	1.009	0.0584	0
954	1004.7	0.2427	0.1972	1.0092	0.0587	0
955	1004.6	0.2427	0.1975	1.0093	0.059	0
956	1004.5	0.2428	0.1974	1.0092	0.0589	0
957	1004.4	0.2425	0.1972	1.0087	0.059	0
958	1004.3	0.2427	0.1974	1.0093	0.0589	0
959	1004.2	0.2427	0.1973	1.0093	0.059	0
960	1004.1	0.2428	0.1973	1.01	0.059	0
961	1004	0.243	0.1977	1.0097	0.0595	0
962	1003.9	0.2431	0.1977	1.0095	0.0595	0
963	1003.8	0.2431	0.1975	1.0098	0.0593	0
964	1003.7	0.2431	0.1978	1.0099	0.0594	0
965	1003.6	0.2431	0.1978	1.0102	0.0596	0
966	1003.5	0.2432	0.1979	1.01	0.0596	0
967	1003.4	0.243	0.198	1.0098	0.0596	0
968	1003.3	0.2432	0.1978	1.0094	0.0597	0
969	1003.2	0.2431	0.198	1.0101	0.0597	0
970	1003.1	0.2436	0.1985	1.0102	0.0603	0
971	1003	0.2435	0.1982	1.0108	0.0603	0
972	1002.9	0.2434	0.1982	1.0097	0.06	0
973	1002.8	0.2435	0.1982	1.0105	0.0601	0
974	1002.7	0.2434	0.1983	1.0103	0.0601	0
975	1002.6	0.2434	0.1983	1.0102	0.0601	0

976	1002.5	0.2437	0.1987	1.0099	0.0603	0
977	1002.4	0.244	0.1988	1.0106	0.0605	0
978	1002.3	0.2438	0.1989	1.0101	0.0605	0
979	1002.2	0.2439	0.1989	1.0112	0.0606	0
980	1002.1	0.2439	0.1988	1.0106	0.0606	0
981	1002	0.2438	0.1989	1.0106	0.0606	0
982	1001.9	0.2439	0.1989	1.0109	0.0608	0
983	1001.8	0.244	0.199	1.0109	0.0607	0
984	1001.7	0.244	0.1991	1.0111	0.0607	0
985	1001.6	0.2442	0.1993	1.0108	0.0609	0
986	1001.5	0.2444	0.1996	1.0115	0.061	0
987	1001.4	0.2444	0.1995	1.0108	0.0612	0
988	1001.3	0.2446	0.1996	1.0117	0.0613	0
989	1001.2	0.2446	0.1998	1.0114	0.0614	0
990	1001.1	0.2443	0.1996	1.011	0.0612	0
991	1001	0.2445	0.1997	1.0109	0.0612	0
992	1000.9	0.2444	0.1996	1.0113	0.0613	0
993	1000.8	0.2443	0.1995	1.0113	0.0614	0
994	1000.7	0.2446	0.1996	1.011	0.0616	0
995	1000.6	0.2446	0.1998	1.0117	0.0617	0
996	1000.5	0.2446	0.1997	1.0112	0.0615	0
997	1000.4	0.2448	0.1999	1.0115	0.0617	0
998	1000.3	0.2448	0.2001	1.0117	0.0618	0
999	1000.2	0.2446	0.2	1.0112	0.0617	0
1000	1000.1	0.2447	0.2003	1.0115	0.0619	0
1001	1000	0.2449	0.2004	1.0118	0.0621	0
1002	999.9	0.2448	0.2001	1.0118	0.0616	0
1003	999.8	0.2448	0.2	1.0114	0.0619	0
1004	999.7	0.2449	0.2	1.0118	0.0618	0
1005	999.6	0.2448	0.2002	1.0111	0.0618	0
1006	999.5	0.2447	0.2001	1.0112	0.0616	0
1007	999.4	0.2451	0.2002	1.0121	0.0619	0
1008	999.3	0.2447	0.2002	1.0124	0.0618	0
1009	999.2	0.2449	0.2003	1.0121	0.062	0
1010	999.1	0.2452	0.2005	1.0122	0.0622	0
1011	999	0.2454	0.201	1.0125	0.0623	0
1012	998.9	0.2453	0.201	1.0123	0.0623	0
1013	998.8	0.2452	0.2006	1.0119	0.062	0
1014	998.7	0.2456	0.201	1.0123	0.0626	0
1015	998.6	0.2453	0.2008	1.012	0.0624	0
1016	998.5	0.2452	0.2007	1.0117	0.0624	0
1017	998.4	0.2452	0.2006	1.0123	0.0624	0
1018	998.3	0.2454	0.201	1.0127	0.0626	0

1019	998.2	0.2453	0.201	1.0127	0.0624	0
1020	998.1	0.2452	0.2007	1.0115	0.0623	0
1021	998	0.2454	0.2007	1.0116	0.0622	0
1022	997.9	0.2454	0.2009	1.0122	0.0626	0
1023	997.8	0.2453	0.2011	1.0134	0.0627	0
1024	997.7	0.2456	0.2012	1.0132	0.0629	0
1025	997.6	0.2454	0.201	1.013	0.0627	0
1026	997.5	0.2454	0.2007	1.0132	0.0624	0
1027	997.4	0.2458	0.201	1.0128	0.0627	0
1028	997.3	0.2456	0.2012	1.0131	0.0627	0
1029	997.2	0.2458	0.2012	1.0131	0.0628	0
1030	997.1	0.2458	0.201	1.0131	0.0628	0
1031	997	0.246	0.2014	1.0139	0.063	0
1032	996.9	0.2461	0.2015	1.0131	0.0631	0
1033	996.8	0.2457	0.2013	1.0131	0.0629	0
1034	996.7	0.2456	0.2012	1.0131	0.0626	0
1035	996.6	0.2456	0.2012	1.0128	0.0628	0
1036	996.5	0.2457	0.2012	1.0129	0.0627	0
1037	996.4	0.2457	0.2011	1.0128	0.0627	0
1038	996.3	0.2458	0.2014	1.0134	0.063	0
1039	996.2	0.2459	0.2016	1.0138	0.0628	0
1040	996.1	0.2458	0.2015	1.0134	0.0629	0
1041	996	0.2461	0.2016	1.0134	0.063	0
1042	995.9	0.2457	0.2014	1.013	0.063	0
1043	995.8	0.2458	0.2014	1.0135	0.0629	0
1044	995.7	0.2461	0.2015	1.0138	0.0632	0
1045	995.6	0.2461	0.2016	1.0133	0.0632	0
1046	995.5	0.2464	0.202	1.014	0.0634	0
1047	995.4	0.2461	0.2019	1.014	0.0634	0
1048	995.3	0.2462	0.2019	1.0136	0.0636	0
1049	995.2	0.2462	0.202	1.014	0.0636	0
1050	995.1	0.2463	0.202	1.0131	0.0635	0
1051	995	0.2466	0.2021	1.0139	0.0638	0
1052	994.9	0.2466	0.2021	1.0141	0.0636	0
1053	994.8	0.2463	0.202	1.0142	0.0636	0
1054	994.7	0.2464	0.202	1.0141	0.0636	0
1055	994.6	0.2465	0.2022	1.0139	0.0637	0
1056	994.5	0.2463	0.202	1.0141	0.0635	0
1057	994.4	0.2463	0.202	1.0138	0.0636	0
1058	994.3	0.2463	0.2021	1.014	0.0637	0
1059	994.2	0.2465	0.2022	1.0143	0.0637	0
1060	994.1	0.2464	0.2022	1.014	0.0637	0
1061	994	0.2464	0.2023	1.0143	0.0638	0

1062	993.9	0.2465	0.2026	1.0144	0.0641	0
1063	993.8	0.2468	0.2028	1.0141	0.0642	0
1064	993.7	0.2469	0.2026	1.0142	0.0642	0
1065	993.6	0.2469	0.2027	1.0142	0.0642	0
1066	993.5	0.2467	0.2028	1.0143	0.0642	0
1067	993.4	0.2469	0.2028	1.0141	0.0643	0
1068	993.3	0.2471	0.203	1.0147	0.0645	0
1069	993.2	0.2473	0.2032	1.0146	0.0647	0
1070	993.1	0.2472	0.2032	1.0148	0.0648	0
1071	993	0.2472	0.2031	1.0146	0.0647	0
1072	992.9	0.2473	0.2031	1.0145	0.0646	0
1073	992.8	0.2472	0.2031	1.0142	0.0647	0
1074	992.7	0.2473	0.2032	1.0145	0.0648	0
1075	992.6	0.2474	0.2033	1.0144	0.065	0
1076	992.5	0.2477	0.2037	1.0151	0.0653	0
1077	992.4	0.2477	0.2036	1.0151	0.0652	0
1078	992.3	0.2473	0.2035	1.015	0.065	0
1079	992.2	0.2471	0.2033	1.0146	0.0649	0
1080	992.1	0.2473	0.2035	1.0151	0.065	0
1081	992	0.2478	0.2039	1.0149	0.0653	0
1082	991.9	0.2476	0.2037	1.0153	0.0652	0
1083	991.8	0.2478	0.204	1.0154	0.0654	0
1084	991.7	0.248	0.2042	1.0158	0.0655	0
1085	991.6	0.2477	0.2039	1.0153	0.0654	0
1086	991.5	0.2479	0.204	1.0148	0.0653	0
1087	991.4	0.2477	0.2039	1.015	0.0654	0
1088	991.3	0.2478	0.2038	1.0154	0.0654	0
1089	991.2	0.2481	0.2041	1.0152	0.0655	0
1090	991.1	0.2478	0.2039	1.0154	0.0654	0
1091	991	0.248	0.2041	1.016	0.0657	0
1092	990.9	0.2485	0.2046	1.016	0.0661	0
1093	990.8	0.2484	0.2044	1.0163	0.0659	0
1094	990.7	0.2483	0.2045	1.0166	0.0659	0
1095	990.6	0.2482	0.2042	1.0163	0.0657	0
1096	990.5	0.2481	0.2044	1.0163	0.0658	0
1097	990.4	0.2481	0.2043	1.0162	0.0657	0
1098	990.3	0.2484	0.2043	1.0164	0.0659	0
1099	990.2	0.2484	0.2045	1.0164	0.0661	0
1100	990.1	0.2483	0.2044	1.0163	0.066	0
1101	990	0.2484	0.2045	1.0165	0.0661	0
1102	989.9	0.2486	0.2048	1.0164	0.0662	0
1103	989.8	0.2487	0.2046	1.0164	0.0663	0
1104	989.7	0.2484	0.2048	1.0165	0.0661	0

1105	989.6	0.2486	0.2046	1.0167	0.0661	0
1106	989.5	0.2488	0.2047	1.0167	0.0661	0
1107	989.4	0.2487	0.2046	1.017	0.0662	0
1108	989.3	0.2489	0.2047	1.0168	0.0662	0
1109	989.2	0.2488	0.205	1.0166	0.0664	0
1110	989.1	0.2488	0.205	1.0168	0.0663	0
1111	989	0.2488	0.2049	1.0168	0.0663	0
1112	988.9	0.249	0.2051	1.0166	0.0666	0
1113	988.8	0.2491	0.2051	1.017	0.0667	0
1114	988.7	0.2492	0.2052	1.0174	0.0666	0
1115	988.6	0.2492	0.2053	1.017	0.0667	0
1116	988.5	0.2492	0.2051	1.0173	0.0667	0
1117	988.4	0.2489	0.2052	1.0174	0.0667	0
1118	988.3	0.2491	0.2053	1.0171	0.0668	0
1119	988.2	0.2492	0.2056	1.0177	0.0669	0
1120	988.1	0.2494	0.2056	1.017	0.067	0
1121	988	0.2494	0.2054	1.017	0.0668	0
1122	987.9	0.2492	0.205	1.0168	0.0666	0
1123	987.8	0.2495	0.2055	1.0173	0.0671	0
1124	987.7	0.2493	0.2057	1.0178	0.0671	0
1125	987.6	0.2493	0.2057	1.018	0.0672	0
1126	987.5	0.2493	0.2056	1.0172	0.067	0
1127	987.4	0.2496	0.2058	1.0178	0.0674	0
1128	987.3	0.2495	0.206	1.0183	0.0675	0
1129	987.2	0.2496	0.2059	1.0178	0.0673	0
1130	987.1	0.2495	0.2059	1.018	0.0673	0
1131	987	0.2498	0.2061	1.0181	0.0673	0
1132	986.9	0.2498	0.2059	1.0181	0.0674	0
1133	986.8	0.2498	0.206	1.0185	0.0676	0
1134	986.7	0.2497	0.2059	1.018	0.0674	0
1135	986.6	0.2497	0.2061	1.0181	0.0674	0
1136	986.5	0.2499	0.2063	1.0183	0.0676	0
1137	986.4	0.2499	0.2063	1.0183	0.0676	0
1138	986.3	0.25	0.2063	1.0188	0.0677	0
1139	986.2	0.2501	0.2064	1.0184	0.068	0
1140	986.1	0.2503	0.2065	1.0184	0.0682	0
1141	986	0.2502	0.2063	1.0184	0.068	0
1142	985.9	0.25	0.2064	1.0193	0.0679	0
1143	985.8	0.2502	0.2066	1.0188	0.0681	0
1144	985.7	0.2504	0.2067	1.0192	0.0683	0
1145	985.6	0.2505	0.2069	1.0186	0.0682	0
1146	985.5	0.2506	0.2071	1.0184	0.0685	0
1147	985.4	0.2504	0.207	1.0183	0.0684	0

1148	985.3	0.2504	0.2069	1.0188	0.0685	0
1149	985.2	0.2503	0.2069	1.0186	0.0685	0
1150	985.1	0.2506	0.2072	1.0184	0.0686	0
1151	985	0.2507	0.2073	1.0188	0.0687	0
1152	984.9	0.2509	0.2074	1.0188	0.0688	0
1153	984.8	0.2508	0.2073	1.0194	0.069	0
1154	984.7	0.2505	0.2072	1.0194	0.0688	0
1155	984.6	0.2508	0.2075	1.0196	0.0691	0
1156	984.5	0.251	0.2077	1.0198	0.0691	0
1157	984.4	0.2512	0.2078	1.0197	0.0693	0
1158	984.3	0.2513	0.2078	1.0196	0.0693	0
1159	984.2	0.2513	0.2079	1.019	0.0695	0
1160	984.1	0.2513	0.2079	1.0196	0.0696	0
1161	984	0.2511	0.2078	1.0195	0.0695	0
1162	983.9	0.2513	0.2078	1.02	0.0695	0
1163	983.8	0.2514	0.2079	1.0192	0.0695	0
1164	983.7	0.2515	0.2079	1.0192	0.0696	0
1165	983.6	0.2516	0.2083	1.0191	0.0699	0
1166	983.5	0.2516	0.2083	1.0194	0.07	0
1167	983.4	0.2517	0.2086	1.0195	0.0702	0
1168	983.3	0.2517	0.2086	1.0197	0.0701	0
1169	983.2	0.2518	0.2086	1.0197	0.0701	0
1170	983.1	0.2518	0.2084	1.0199	0.0701	0
1171	983	0.2516	0.2085	1.0199	0.07	0
1172	982.9	0.2517	0.2086	1.0199	0.0702	0
1173	982.8	0.2521	0.2089	1.0203	0.0706	0
1174	982.7	0.2519	0.2085	1.0204	0.0702	0
1175	982.6	0.2519	0.2086	1.0203	0.0703	0
1176	982.5	0.2519	0.2087	1.0201	0.0705	0
1177	982.4	0.2518	0.2088	1.02	0.0705	0
1178	982.3	0.252	0.2091	1.0201	0.0706	0
1179	982.2	0.252	0.2091	1.0208	0.0706	0
1180	982.1	0.2521	0.2091	1.0205	0.0706	0
1181	982	0.2525	0.2091	1.0212	0.0708	0
1182	981.9	0.2523	0.2092	1.0204	0.0709	0
1183	981.8	0.2521	0.2093	1.0203	0.0708	0
1184	981.7	0.2521	0.2091	1.0209	0.0707	0
1185	981.6	0.2523	0.2092	1.0208	0.0708	0
1186	981.5	0.2525	0.2092	1.0209	0.071	0
1187	981.4	0.2524	0.2094	1.0205	0.0711	0
1188	981.3	0.2524	0.2092	1.0207	0.0709	0
1189	981.2	0.2522	0.2093	1.0206	0.0709	0
1190	981.1	0.2522	0.209	1.0209	0.0708	0

1191	981	0.2523	0.2093	1.0204	0.071	0
1192	980.9	0.2527	0.2096	1.0207	0.071	0
1193	980.8	0.2528	0.2096	1.0211	0.0712	0
1194	980.7	0.2528	0.2097	1.0216	0.0712	0
1195	980.6	0.2529	0.2098	1.0212	0.0713	0
1196	980.5	0.2528	0.2097	1.0216	0.0712	0
1197	980.4	0.2528	0.2096	1.0214	0.0712	0
1198	980.3	0.2528	0.2096	1.0217	0.0711	0
1199	980.2	0.2529	0.2096	1.0214	0.0712	0
1200	980.1	0.2527	0.2096	1.0216	0.0711	0
1201	980	0.2527	0.2096	1.0213	0.0712	0
1202	979.9	0.2529	0.2098	1.0215	0.0713	0
1203	979.8	0.2527	0.2099	1.0213	0.0714	0
1204	979.7	0.2528	0.2095	1.0213	0.0714	0
1205	979.6	0.2528	0.2096	1.0217	0.0713	0
1206	979.5	0.2529	0.2095	1.0218	0.0712	0
1207	979.4	0.2527	0.2095	1.0221	0.0712	0
1208	979.3	0.253	0.2099	1.0223	0.0714	0
1209	979.2	0.2529	0.2096	1.0219	0.0711	0
1210	979.1	0.2529	0.2098	1.0219	0.0713	0
1211	979	0.2528	0.2098	1.0218	0.0713	0
1212	978.9	0.2532	0.2099	1.0222	0.0714	0
1213	978.8	0.2532	0.2102	1.0224	0.0717	0
1214	978.7	0.2531	0.2101	1.0223	0.0716	0
1215	978.6	0.2531	0.2097	1.022	0.0714	0
1216	978.5	0.2532	0.2098	1.0224	0.0716	0
1217	978.4	0.2532	0.21	1.0227	0.0715	0
1218	978.3	0.253	0.2098	1.0219	0.0714	0
1219	978.2	0.2533	0.2101	1.0223	0.0716	0
1220	978.1	0.2531	0.21	1.0222	0.0714	0
1221	978	0.2533	0.2102	1.0225	0.0715	0
1222	977.9	0.2537	0.2104	1.0225	0.0718	0
1223	977.8	0.2535	0.2102	1.0229	0.0718	0
1224	977.7	0.2536	0.2106	1.0226	0.072	0
1225	977.6	0.2536	0.2106	1.0226	0.072	0
1226	977.5	0.2536	0.2102	1.0227	0.0718	0
1227	977.4	0.2536	0.2103	1.0226	0.0718	0
1228	977.3	0.2538	0.2101	1.0226	0.0718	0
1229	977.2	0.2539	0.2104	1.0232	0.0721	0
1230	977.1	0.2538	0.2102	1.0233	0.0719	0
1231	977	0.2538	0.2104	1.0232	0.072	0
1232	976.9	0.2539	0.2105	1.0232	0.0722	0
1233	976.8	0.2538	0.2107	1.0233	0.0723	0

1234	976.7	0.2537	0.2107	1.023	0.0724	0
1235	976.6	0.2539	0.2107	1.024	0.0724	0
1236	976.5	0.2541	0.2108	1.0232	0.0723	0
1237	976.4	0.2543	0.2109	1.0233	0.0725	0
1238	976.3	0.254	0.2108	1.0232	0.0725	0
1239	976.2	0.2543	0.2111	1.0233	0.0726	0
1240	976.1	0.2546	0.2113	1.023	0.0729	0
1241	976	0.2541	0.2111	1.0233	0.0728	0
1242	975.9	0.2542	0.2111	1.0234	0.0728	0
1243	975.8	0.2541	0.211	1.023	0.0729	0
1244	975.7	0.2541	0.2111	1.0233	0.0729	0
1245	975.6	0.2541	0.2114	1.0237	0.0729	0
1246	975.5	0.2544	0.2114	1.0235	0.073	0
1247	975.4	0.2546	0.2115	1.0237	0.0731	0
1248	975.3	0.2544	0.2115	1.0235	0.0731	0
1249	975.2	0.2545	0.2114	1.0231	0.0731	0
1250	975.1	0.2548	0.2114	1.0237	0.0733	0
1251	975	0.2548	0.2117	1.0236	0.0735	0
1252	974.9	0.2547	0.2118	1.0235	0.0734	0
1253	974.8	0.2548	0.2119	1.0233	0.0733	0
1254	974.7	0.255	0.212	1.0237	0.0734	0
1255	974.6	0.255	0.212	1.0244	0.0736	0
1256	974.5	0.2548	0.2117	1.0235	0.0736	0
1257	974.4	0.255	0.212	1.0238	0.0736	0
1258	974.3	0.2552	0.2123	1.0239	0.0738	0
1259	974.2	0.2552	0.2122	1.024	0.0739	0
1260	974.1	0.2553	0.2122	1.0234	0.074	0
1261	974	0.255	0.2123	1.0236	0.074	0
1262	973.9	0.2551	0.2124	1.0237	0.0741	0
1263	973.8	0.2556	0.2129	1.0239	0.0743	0
1264	973.7	0.2556	0.2129	1.0237	0.0743	0
1265	973.6	0.2554	0.2128	1.0236	0.0743	0
1266	973.5	0.2554	0.2127	1.024	0.0745	0
1267	973.4	0.2554	0.213	1.0241	0.0745	0
1268	973.3	0.2555	0.2129	1.0238	0.0746	0
1269	973.2	0.2555	0.2129	1.024	0.0744	0
1270	973.1	0.2556	0.2133	1.0236	0.0748	0
1271	973	0.2559	0.2134	1.0245	0.0749	0
1272	972.9	0.256	0.2135	1.0245	0.0749	0
1273	972.8	0.2558	0.2134	1.0242	0.0748	0
1274	972.7	0.2557	0.2133	1.0236	0.0747	0
1275	972.6	0.2556	0.2133	1.0239	0.0749	0
1276	972.5	0.256	0.2134	1.0241	0.0752	0

1277	972.4	0.2558	0.2132	1.0236	0.0749	0
1278	972.3	0.256	0.2136	1.0245	0.0752	0
1279	972.2	0.2563	0.2137	1.0246	0.0754	0
1280	972.1	0.2562	0.2137	1.0246	0.0754	0
1281	972	0.256	0.2133	1.025	0.0752	0
1282	971.9	0.2558	0.2133	1.0246	0.0752	0
1283	971.8	0.2559	0.2136	1.0247	0.0753	0
1284	971.7	0.2562	0.2138	1.0241	0.0753	0
1285	971.6	0.2563	0.214	1.0241	0.0756	0
1286	971.5	0.2562	0.2139	1.0246	0.0754	0
1287	971.4	0.2562	0.2141	1.0244	0.0755	0
1288	971.3	0.2563	0.2139	1.0244	0.0755	0
1289	971.2	0.2563	0.214	1.0246	0.0755	0
1290	971.1	0.2564	0.2143	1.0248	0.0758	0
1291	971	0.2566	0.2142	1.025	0.0758	0
1292	970.9	0.2566	0.2142	1.0251	0.0758	0
1293	970.8	0.2566	0.2142	1.0253	0.0758	0
1294	970.7	0.2567	0.2143	1.0249	0.0756	0
1295	970.6	0.2566	0.2141	1.0243	0.0756	0
1296	970.5	0.2569	0.2145	1.0252	0.0759	0
1297	970.4	0.257	0.2147	1.0251	0.0761	0
1298	970.3	0.2569	0.2147	1.0253	0.0761	0
1299	970.2	0.2568	0.2145	1.025	0.0759	0
1300	970.1	0.2566	0.2144	1.0252	0.0759	0
1301	970	0.2567	0.2144	1.0251	0.076	0
1302	969.9	0.2567	0.2146	1.0252	0.0759	0
1303	969.8	0.2566	0.2144	1.0248	0.0757	0
1304	969.7	0.2568	0.2145	1.0245	0.0761	0
1305	969.6	0.2568	0.2146	1.0251	0.076	0
1306	969.5	0.257	0.2145	1.0255	0.0759	0
1307	969.4	0.2568	0.2143	1.0252	0.0758	0
1308	969.3	0.2567	0.2144	1.0254	0.0758	0
1309	969.2	0.2566	0.2144	1.0248	0.0758	0
1310	969.1	0.2567	0.2145	1.0255	0.076	0
1311	969	0.2569	0.2146	1.0256	0.0762	0
1312	968.9	0.2567	0.2144	1.0257	0.0761	0
1313	968.8	0.2567	0.2145	1.0253	0.0758	0
1314	968.7	0.2571	0.2147	1.0253	0.0762	0
1315	968.6	0.2571	0.2149	1.026	0.0762	0
1316	968.5	0.257	0.2144	1.0253	0.076	0
1317	968.4	0.257	0.2144	1.0261	0.0761	0
1318	968.3	0.257	0.2144	1.0257	0.0759	0
1319	968.2	0.2569	0.2144	1.0253	0.0759	0

1320	968.1	0.2569	0.2144	1.0262	0.0759	0
1321	968	0.2572	0.2145	1.0267	0.076	0
1322	967.9	0.2572	0.2146	1.0261	0.076	0
1323	967.8	0.2573	0.2148	1.026	0.076	0
1324	967.7	0.2571	0.2144	1.0262	0.0759	0
1325	967.6	0.2571	0.2145	1.0258	0.0759	0
1326	967.5	0.2571	0.2146	1.0263	0.0758	0
1327	967.4	0.2571	0.2145	1.026	0.0758	0
1328	967.3	0.2572	0.2142	1.0261	0.0759	0
1329	967.2	0.2574	0.2148	1.0261	0.0761	0
1330	967.1	0.2574	0.2147	1.0267	0.076	0
1331	967	0.2571	0.2144	1.0264	0.076	0
1332	966.9	0.2573	0.2144	1.0259	0.0759	0
1333	966.8	0.2574	0.2145	1.0261	0.0761	0
1334	966.7	0.2575	0.2148	1.0267	0.0763	0
1335	966.6	0.2576	0.215	1.0265	0.0766	0
1336	966.5	0.2575	0.2147	1.0268	0.0765	0
1337	966.4	0.2573	0.2148	1.0266	0.0762	0
1338	966.3	0.2575	0.2149	1.027	0.0763	0
1339	966.2	0.2575	0.215	1.0266	0.0762	0
1340	966.1	0.2576	0.2151	1.0273	0.0765	0
1341	966	0.2576	0.2151	1.0267	0.0767	0
1342	965.9	0.2578	0.2151	1.0266	0.0767	0
1343	965.8	0.2577	0.2152	1.0267	0.0768	0
1344	965.7	0.2578	0.2151	1.0265	0.0766	0
1345	965.6	0.2578	0.2152	1.027	0.0767	0
1346	965.5	0.2577	0.2152	1.0271	0.0768	0
1347	965.4	0.2579	0.2151	1.0268	0.0769	0
1348	965.3	0.2579	0.2153	1.0267	0.0769	0
1349	965.2	0.2579	0.2153	1.0268	0.077	0
1350	965.1	0.2578	0.2156	1.0273	0.0771	0
1351	965	0.2578	0.2156	1.0272	0.0772	0
1352	964.9	0.258	0.2157	1.0272	0.0772	0
1353	964.8	0.2582	0.2157	1.0269	0.0774	0
1354	964.7	0.2583	0.2159	1.0267	0.0775	0
1355	964.6	0.2581	0.2158	1.0267	0.0773	0
1356	964.5	0.2581	0.2162	1.0273	0.0775	0
1357	964.4	0.2583	0.2161	1.0273	0.0776	0
1358	964.3	0.2584	0.2161	1.0267	0.0776	0
1359	964.2	0.2582	0.2159	1.0277	0.0778	0
1360	964.1	0.2584	0.2162	1.0277	0.0778	0
1361	964	0.2583	0.2162	1.0274	0.078	0
1362	963.9	0.2586	0.2165	1.0272	0.0782	0

1363	963.8	0.2587	0.2168	1.0274	0.0785	0
1364	963.7	0.2584	0.2166	1.0271	0.0783	0
1365	963.6	0.2584	0.2166	1.0266	0.0782	0
1366	963.5	0.2589	0.217	1.0275	0.0786	0
1367	963.4	0.259	0.217	1.0279	0.0786	0
1368	963.3	0.259	0.217	1.0274	0.0787	0
1369	963.2	0.2588	0.2169	1.0271	0.0786	0
1370	963.1	0.2588	0.2172	1.0277	0.0787	0
1371	963	0.2588	0.2171	1.0273	0.0788	0
1372	962.9	0.259	0.2172	1.0275	0.0788	0
1373	962.8	0.259	0.2172	1.027	0.0788	0
1374	962.7	0.2589	0.2172	1.0273	0.0787	0
1375	962.6	0.2593	0.2177	1.0272	0.0793	0
1376	962.5	0.2589	0.2171	1.027	0.079	0
1377	962.4	0.259	0.2172	1.0276	0.0789	0
1378	962.3	0.259	0.2173	1.0272	0.079	0
1379	962.2	0.2589	0.2173	1.0274	0.0789	0
1380	962.1	0.259	0.2173	1.0277	0.0791	0
1381	962	0.259	0.217	1.0276	0.079	0
1382	961.9	0.2591	0.2171	1.0269	0.0791	0
1383	961.8	0.259	0.2172	1.0274	0.0791	0
1384	961.7	0.2591	0.2175	1.0271	0.0794	0
1385	961.6	0.2591	0.2174	1.0272	0.0791	0
1386	961.5	0.2592	0.2174	1.0276	0.0791	0
1387	961.4	0.259	0.2174	1.0271	0.0792	0
1388	961.3	0.259	0.2174	1.0271	0.0792	0
1389	961.2	0.2592	0.2174	1.0272	0.0793	0
1390	961.1	0.2592	0.2174	1.0272	0.0793	0
1391	961	0.259	0.2175	1.0275	0.0791	0
1392	960.9	0.2589	0.2174	1.0272	0.0792	0
1393	960.8	0.2586	0.2173	1.0272	0.079	0
1394	960.7	0.2591	0.2175	1.0276	0.0792	0
1395	960.6	0.259	0.2176	1.0273	0.0793	0
1396	960.5	0.2591	0.2176	1.0274	0.0793	0
1397	960.4	0.2587	0.2173	1.0268	0.0792	0
1398	960.3	0.2591	0.2175	1.0276	0.0794	0
1399	960.2	0.2591	0.2175	1.0277	0.0793	0
1400	960.1	0.2591	0.2175	1.027	0.0794	0
1401	960	0.259	0.2176	1.027	0.0794	0
1402	959.9	0.2589	0.2175	1.0268	0.0792	0
1403	959.8	0.2589	0.2175	1.0272	0.0792	0
1404	959.7	0.2588	0.2173	1.0271	0.0791	0
1405	959.6	0.2591	0.2175	1.0275	0.0793	0

1406	959.5	0.2592	0.2176	1.0274	0.0793	0
1407	959.4	0.2591	0.2175	1.0273	0.0793	0
1408	959.3	0.2589	0.2173	1.0272	0.0791	0
1409	959.2	0.2591	0.2174	1.0274	0.0791	0
1410	959.1	0.2589	0.2173	1.0272	0.079	0
1411	959	0.2587	0.2171	1.0268	0.0789	0
1412	958.9	0.2586	0.2171	1.0269	0.0788	0
1413	958.8	0.2584	0.2167	1.027	0.0785	0
1414	958.7	0.2583	0.2168	1.0269	0.0785	0
1415	958.6	0.2584	0.2168	1.0271	0.0785	0
1416	958.5	0.2582	0.2164	1.0261	0.0783	0
1417	958.4	0.2583	0.2167	1.0267	0.0783	0
1418	958.3	0.2582	0.2164	1.0263	0.0781	0
1419	958.2	0.2583	0.2164	1.0274	0.0783	0
1420	958.1	0.2583	0.2165	1.0273	0.0783	0
1421	958	0.2583	0.2166	1.0268	0.0783	0
1422	957.9	0.2581	0.2166	1.027	0.0781	0
1423	957.8	0.2582	0.2166	1.027	0.078	0
1424	957.7	0.2582	0.2164	1.0268	0.078	0
1425	957.6	0.2581	0.2163	1.027	0.078	0
1426	957.5	0.2577	0.216	1.0273	0.0778	0
1427	957.4	0.2577	0.2161	1.027	0.0777	0
1428	957.3	0.2577	0.2159	1.0264	0.0778	0
1429	957.2	0.2578	0.2161	1.0264	0.0779	0
1430	957.1	0.2575	0.2159	1.0265	0.0776	0
1431	957	0.2576	0.2159	1.026	0.0775	0
1432	956.9	0.2576	0.216	1.0258	0.0776	0
1433	956.8	0.2576	0.216	1.0269	0.0777	0
1434	956.7	0.2573	0.2159	1.0263	0.0776	0
1435	956.6	0.2573	0.2158	1.0263	0.0773	0
1436	956.5	0.2577	0.2159	1.0264	0.0775	0
1437	956.4	0.2574	0.2158	1.0262	0.0774	0
1438	956.3	0.2576	0.2157	1.0264	0.0774	0
1439	956.2	0.2574	0.2156	1.0263	0.0775	0
1440	956.1	0.2573	0.2156	1.0268	0.0775	0
1441	956	0.2575	0.2157	1.0261	0.0775	0
1442	955.9	0.2574	0.2155	1.0255	0.0773	0
1443	955.8	0.2572	0.2156	1.0258	0.0772	0
1444	955.7	0.2573	0.2158	1.0259	0.0775	0
1445	955.6	0.2572	0.2155	1.0253	0.0773	0
1446	955.5	0.2571	0.2154	1.0252	0.077	0
1447	955.4	0.2571	0.2153	1.0249	0.077	0
1448	955.3	0.2569	0.2155	1.0258	0.0771	0

1449	955.2	0.2569	0.2155	1.0263	0.0771	0
1450	955.1	0.2568	0.2153	1.0261	0.077	0
1451	955	0.2569	0.2154	1.0255	0.077	0
1452	954.9	0.2567	0.2152	1.0251	0.0769	0
1453	954.8	0.2569	0.2154	1.0252	0.0771	0
1454	954.7	0.257	0.2155	1.0253	0.0772	0
1455	954.6	0.2568	0.2154	1.026	0.0772	0
1456	954.5	0.2568	0.2153	1.0259	0.0772	0
1457	954.4	0.2568	0.2153	1.0254	0.0771	0
1458	954.3	0.2565	0.2152	1.0255	0.077	0
1459	954.2	0.2564	0.2152	1.0248	0.0771	0
1460	954.1	0.2563	0.2152	1.0245	0.0771	0
1461	954	0.2566	0.2155	1.0247	0.0772	0
1462	953.9	0.2565	0.2154	1.0244	0.077	0
1463	953.8	0.2564	0.2153	1.0246	0.077	0
1464	953.7	0.2564	0.2153	1.0247	0.0771	0
1465	953.6	0.2562	0.2153	1.0239	0.077	0
1466	953.5	0.2563	0.2153	1.0242	0.0768	0
1467	953.4	0.2563	0.2152	1.0243	0.0768	0
1468	953.3	0.2563	0.2153	1.0248	0.077	0
1469	953.2	0.2564	0.2154	1.0238	0.0771	0
1470	953.1	0.2563	0.2153	1.0249	0.0773	0
1471	953	0.2564	0.2154	1.0241	0.0772	0
1472	952.9	0.2565	0.2155	1.0242	0.0774	0
1473	952.8	0.2564	0.2155	1.024	0.0774	0
1474	952.7	0.2565	0.2156	1.0242	0.0774	0
1475	952.6	0.2563	0.2152	1.0238	0.0772	0
1476	952.5	0.2564	0.2154	1.0239	0.0774	0
1477	952.4	0.256	0.2153	1.0233	0.0771	0
1478	952.3	0.2563	0.2155	1.0231	0.0772	0
1479	952.2	0.2561	0.2155	1.023	0.0771	0
1480	952.1	0.2562	0.2155	1.0231	0.0772	0
1481	952	0.2559	0.2153	1.0228	0.0771	0
1482	951.9	0.2558	0.2151	1.0232	0.0773	0
1483	951.8	0.2559	0.2152	1.0228	0.0771	0
1484	951.7	0.2559	0.2152	1.023	0.0772	0
1485	951.6	0.2558	0.215	1.023	0.0771	0
1486	951.5	0.2559	0.2151	1.0237	0.077	0
1487	951.4	0.2558	0.215	1.0238	0.077	0
1488	951.3	0.256	0.2154	1.023	0.0772	0
1489	951.2	0.2557	0.2152	1.0226	0.0771	0
1490	951.1	0.2559	0.2155	1.0232	0.0772	0
1491	951	0.256	0.2152	1.0231	0.0773	0

1492	950.9	0.2557	0.2152	1.0229	0.0771	0
1493	950.8	0.2556	0.2152	1.0228	0.077	0
1494	950.7	0.2557	0.2152	1.0236	0.0769	0
1495	950.6	0.2557	0.2151	1.0224	0.077	0
1496	950.5	0.2558	0.2152	1.0233	0.0771	0
1497	950.4	0.2558	0.2152	1.0229	0.077	0
1498	950.3	0.2557	0.2151	1.0231	0.0769	0
1499	950.2	0.2559	0.2153	1.0223	0.0771	0
1500	950.1	0.256	0.2154	1.0226	0.0774	0
1501	950	0.2557	0.2154	1.0221	0.0771	0
1502	949.9	0.2556	0.2151	1.0224	0.077	0
1503	949.8	0.2556	0.2151	1.0221	0.0771	0
1504	949.7	0.2557	0.215	1.0222	0.077	0
1505	949.6	0.2558	0.2151	1.0224	0.077	0
1506	949.5	0.2556	0.2151	1.022	0.0768	0
1507	949.4	0.2557	0.215	1.0224	0.077	0
1508	949.3	0.2554	0.2147	1.0224	0.0768	0
1509	949.2	0.2555	0.2149	1.0224	0.0768	0
1510	949.1	0.2554	0.215	1.0226	0.0769	0
1511	949	0.2553	0.2148	1.022	0.0766	0
1512	948.9	0.255	0.2147	1.0226	0.0766	0
1513	948.8	0.2552	0.2145	1.0224	0.0765	0
1514	948.7	0.2552	0.2147	1.0226	0.0766	0
1515	948.6	0.2552	0.2148	1.0224	0.0766	0
1516	948.5	0.2553	0.2146	1.0218	0.0763	0
1517	948.4	0.2553	0.2145	1.0229	0.0764	0
1518	948.3	0.2554	0.2146	1.0225	0.0763	0
1519	948.2	0.2554	0.2146	1.0231	0.0765	0
1520	948.1	0.2554	0.2145	1.0226	0.0764	0
1521	948	0.2554	0.2147	1.0225	0.0764	0
1522	947.9	0.255	0.2144	1.0222	0.076	0
1523	947.8	0.2551	0.2145	1.0223	0.076	0
1524	947.7	0.2551	0.2144	1.0219	0.0761	0
1525	947.6	0.255	0.2145	1.022	0.0762	0
1526	947.5	0.2551	0.2145	1.0224	0.0761	0
1527	947.4	0.2549	0.2145	1.022	0.076	0
1528	947.3	0.2551	0.2144	1.0221	0.0761	0
1529	947.2	0.255	0.2146	1.0217	0.0762	0
1530	947.1	0.2552	0.2144	1.0225	0.0762	0
1531	947	0.2549	0.2143	1.0221	0.0759	0
1532	946.9	0.2546	0.2141	1.0223	0.0757	0
1533	946.8	0.2546	0.2139	1.0215	0.0758	0
1534	946.7	0.2549	0.2142	1.0218	0.0758	0

1535	946.6	0.2547	0.2139	1.0217	0.0757	0
1536	946.5	0.2546	0.2139	1.0216	0.0757	0
1537	946.4	0.2547	0.2139	1.0215	0.0756	0
1538	946.3	0.2547	0.2138	1.0217	0.0756	0
1539	946.2	0.2547	0.2142	1.0222	0.0757	0
1540	946.1	0.2546	0.2141	1.0219	0.0758	0
1541	946	0.2546	0.214	1.0217	0.0758	0
1542	945.9	0.2545	0.2141	1.0216	0.0757	0
1543	945.8	0.2546	0.2139	1.0213	0.0757	0
1544	945.7	0.2548	0.214	1.0216	0.0758	0
1545	945.6	0.2547	0.2142	1.0223	0.0757	0
1546	945.5	0.2544	0.2139	1.0217	0.0755	0
1547	945.4	0.2544	0.2139	1.0211	0.0756	0
1548	945.3	0.2544	0.214	1.0218	0.0756	0
1549	945.2	0.2543	0.2139	1.0218	0.0756	0
1550	945.1	0.2542	0.2137	1.0216	0.0755	0
1551	945	0.2542	0.2138	1.0214	0.0756	0
1552	944.9	0.2541	0.2137	1.0216	0.0755	0
1553	944.8	0.2542	0.2136	1.0215	0.0757	0
1554	944.7	0.2545	0.2139	1.0222	0.0759	0
1555	944.6	0.2547	0.2141	1.0223	0.0759	0
1556	944.5	0.2546	0.2138	1.0212	0.0758	0
1557	944.4	0.2542	0.2141	1.0214	0.0758	0
1558	944.3	0.2542	0.2138	1.0217	0.0757	0
1559	944.2	0.2545	0.2141	1.0212	0.0758	0
1560	944.1	0.2544	0.2139	1.0213	0.0757	0
1561	944	0.2545	0.2139	1.0214	0.0758	0
1562	943.9	0.2543	0.2137	1.0213	0.0757	0
1563	943.8	0.2543	0.2138	1.0215	0.0758	0
1564	943.7	0.2544	0.2139	1.0212	0.0759	0
1565	943.6	0.2543	0.214	1.0212	0.0759	0
1566	943.5	0.2542	0.214	1.0209	0.0758	0
1567	943.4	0.2542	0.2139	1.0213	0.0759	0
1568	943.3	0.2544	0.2142	1.0213	0.0761	0
1569	943.2	0.2545	0.2142	1.0212	0.0761	0
1570	943.1	0.2545	0.214	1.0208	0.0761	0
1571	943	0.254	0.2135	1.0199	0.0757	0
1572	942.9	0.2541	0.2137	1.0204	0.0758	0
1573	942.8	0.2543	0.2139	1.0204	0.0759	0
1574	942.7	0.2541	0.2137	1.0208	0.0759	0
1575	942.6	0.2543	0.214	1.0208	0.076	0
1576	942.5	0.2543	0.2141	1.0204	0.0762	0
1577	942.4	0.2545	0.2142	1.0211	0.0763	0

1578	942.3	0.2544	0.2143	1.0208	0.0763	0
1579	942.2	0.2542	0.214	1.0207	0.0761	0
1580	942.1	0.2545	0.2143	1.0209	0.0763	0
1581	942	0.2541	0.2142	1.0214	0.0762	0
1582	941.9	0.2544	0.2144	1.0214	0.0763	0
1583	941.8	0.2543	0.2143	1.0207	0.0764	0
1584	941.7	0.2544	0.2144	1.0205	0.0765	0
1585	941.6	0.2542	0.2142	1.0208	0.0764	0
1586	941.5	0.2541	0.2142	1.0202	0.0764	0
1587	941.4	0.2541	0.2142	1.0203	0.0763	0
1588	941.3	0.2542	0.2142	1.0205	0.0765	0
1589	941.2	0.2539	0.214	1.0203	0.0761	0
1590	941.1	0.2542	0.2142	1.0205	0.0764	0
1591	941	0.254	0.2141	1.0202	0.0761	0
1592	940.9	0.2539	0.2142	1.0203	0.0762	0
1593	940.8	0.254	0.2139	1.0203	0.0762	0
1594	940.7	0.2537	0.2137	1.0201	0.0758	0
1595	940.6	0.2539	0.2139	1.0197	0.0762	0
1596	940.5	0.2541	0.2139	1.0201	0.0762	0
1597	940.4	0.2539	0.214	1.0198	0.0761	0
1598	940.3	0.254	0.214	1.0197	0.0763	0
1599	940.2	0.2539	0.2141	1.0196	0.0764	0
1600	940.1	0.2541	0.2144	1.0199	0.0765	0
1601	940	0.254	0.2144	1.0195	0.0767	0
1602	939.9	0.2539	0.2143	1.0199	0.0767	0
1603	939.8	0.2542	0.2142	1.0198	0.0765	0
1604	939.7	0.2543	0.2143	1.0196	0.0765	0
1605	939.6	0.2542	0.2144	1.0203	0.0767	0
1606	939.5	0.2542	0.2141	1.0195	0.0766	0
1607	939.4	0.254	0.2143	1.0196	0.0766	0
1608	939.3	0.2542	0.2143	1.02	0.0766	0
1609	939.2	0.254	0.2141	1.0201	0.0764	0
1610	939.1	0.2539	0.2142	1.0198	0.0765	0
1611	939	0.2544	0.2145	1.0203	0.0767	0
1612	938.9	0.2543	0.2143	1.0199	0.0765	0
1613	938.8	0.2545	0.2145	1.0202	0.0767	0
1614	938.7	0.2543	0.2145	1.02	0.0767	0
1615	938.6	0.254	0.2142	1.0195	0.0764	0
1616	938.5	0.2538	0.214	1.0195	0.0764	0
1617	938.4	0.254	0.2142	1.0198	0.0766	0
1618	938.3	0.2541	0.2144	1.0198	0.0765	0
1619	938.2	0.254	0.2143	1.0197	0.0766	0
1620	938.1	0.2541	0.2145	1.0199	0.0766	0

1621	938	0.2541	0.2145	1.02	0.0766	0
1622	937.9	0.2542	0.2142	1.0197	0.0767	0
1623	937.8	0.2541	0.2142	1.0194	0.0765	0
1624	937.7	0.2543	0.2142	1.0202	0.0768	0
1625	937.6	0.254	0.2143	1.0202	0.0767	0
1626	937.5	0.2539	0.2144	1.0198	0.0767	0
1627	937.4	0.2542	0.2146	1.0204	0.0768	0
1628	937.3	0.2538	0.2142	1.0193	0.0766	0
1629	937.2	0.254	0.2142	1.0199	0.0766	0
1630	937.1	0.2541	0.214	1.0193	0.0767	0
1631	937	0.254	0.2139	1.0198	0.0765	0
1632	936.9	0.2538	0.2139	1.0195	0.0764	0
1633	936.8	0.254	0.2141	1.0202	0.0765	0
1634	936.7	0.2539	0.214	1.0203	0.0765	0
1635	936.6	0.2542	0.2145	1.0201	0.077	0
1636	936.5	0.2539	0.2145	1.0206	0.0769	0
1637	936.4	0.254	0.2144	1.0202	0.077	0
1638	936.3	0.2544	0.2145	1.0203	0.0771	0
1639	936.2	0.2543	0.2144	1.0199	0.0769	0
1640	936.1	0.254	0.2145	1.02	0.0768	0
1641	936	0.254	0.2145	1.0204	0.0769	0
1642	935.9	0.2538	0.2142	1.0204	0.0767	0
1643	935.8	0.2541	0.2143	1.0199	0.0767	0
1644	935.7	0.254	0.2141	1.0196	0.0766	0
1645	935.6	0.2542	0.2142	1.02	0.0767	0
1646	935.5	0.2541	0.2144	1.0201	0.0768	0
1647	935.4	0.254	0.2143	1.0204	0.0768	0
1648	935.3	0.2541	0.2143	1.0198	0.0768	0
1649	935.2	0.254	0.2142	1.0199	0.0767	0
1650	935.1	0.2539	0.2142	1.0205	0.0767	0
1651	935	0.254	0.2143	1.02	0.0768	0
1652	934.9	0.2538	0.2144	1.0198	0.0768	0
1653	934.8	0.2541	0.2145	1.0199	0.0769	0
1654	934.7	0.2539	0.2144	1.0201	0.0769	0
1655	934.6	0.2539	0.2145	1.0201	0.077	0
1656	934.5	0.2538	0.2145	1.0197	0.0769	0
1657	934.4	0.2538	0.2144	1.0198	0.0769	0
1658	934.3	0.2539	0.2144	1.0197	0.077	0
1659	934.2	0.2541	0.2145	1.0197	0.077	0
1660	934.1	0.2539	0.2144	1.0194	0.077	0
1661	934	0.2541	0.2147	1.0202	0.0772	0
1662	933.9	0.254	0.2147	1.0201	0.0772	0
1663	933.8	0.254	0.2145	1.0196	0.0769	0

1664	933.7	0.2538	0.2143	1.0193	0.0768	0
1665	933.6	0.2538	0.2144	1.0196	0.0769	0
1666	933.5	0.2538	0.2144	1.0194	0.0768	0
1667	933.4	0.2538	0.2144	1.0195	0.077	0
1668	933.3	0.2539	0.2144	1.0196	0.077	0
1669	933.2	0.2538	0.2144	1.0201	0.0769	0
1670	933.1	0.2539	0.2145	1.0202	0.0771	0
1671	933	0.2539	0.2144	1.0201	0.077	0
1672	932.9	0.2541	0.2146	1.0203	0.0771	0
1673	932.8	0.2539	0.2145	1.0202	0.0771	0
1674	932.7	0.254	0.2147	1.0198	0.0773	0
1675	932.6	0.2542	0.2147	1.0203	0.0772	0
1676	932.5	0.254	0.2146	1.0198	0.0772	0
1677	932.4	0.2543	0.2148	1.0202	0.0774	0
1678	932.3	0.2539	0.2147	1.0201	0.0772	0
1679	932.2	0.2536	0.2146	1.0195	0.0771	0
1680	932.1	0.2538	0.2144	1.0199	0.0772	0
1681	932	0.2538	0.2144	1.0194	0.0771	0
1682	931.9	0.2541	0.2146	1.0197	0.0773	0
1683	931.8	0.2538	0.2147	1.0198	0.0773	0
1684	931.7	0.2537	0.2144	1.0196	0.0772	0
1685	931.6	0.2538	0.2145	1.0197	0.0771	0
1686	931.5	0.2538	0.2146	1.0196	0.0771	0
1687	931.4	0.2538	0.2146	1.0193	0.0771	0
1688	931.3	0.2539	0.2145	1.0194	0.0771	0
1689	931.2	0.2539	0.2145	1.019	0.0771	0
1690	931.1	0.2538	0.2143	1.0185	0.077	0
1691	931	0.2537	0.2143	1.0188	0.0769	0
1692	930.9	0.2537	0.2144	1.0192	0.0772	0
1693	930.8	0.2535	0.2145	1.0193	0.077	0
1694	930.7	0.2538	0.2144	1.0193	0.0772	0
1695	930.6	0.2537	0.2145	1.019	0.0771	0
1696	930.5	0.2536	0.2144	1.0192	0.0771	0
1697	930.4	0.2537	0.2144	1.0197	0.0769	0
1698	930.3	0.2534	0.2141	1.0197	0.0767	0
1699	930.2	0.2535	0.2142	1.0189	0.0767	0
1700	930.1	0.2536	0.2142	1.0192	0.0767	0
1701	930	0.2537	0.2143	1.0191	0.0768	0
1702	929.9	0.2535	0.2143	1.0194	0.0766	0
1703	929.8	0.2533	0.2139	1.0189	0.0765	0
1704	929.7	0.2533	0.2139	1.0186	0.0766	0
1705	929.6	0.2535	0.214	1.019	0.0766	0
1706	929.5	0.2534	0.2138	1.0182	0.0765	0

1707	929.4	0.2531	0.2139	1.0186	0.0763	0
1708	929.3	0.2533	0.2141	1.0185	0.0764	0
1709	929.2	0.2532	0.214	1.019	0.0763	0
1710	929.1	0.2531	0.2139	1.0187	0.0762	0
1711	929	0.253	0.2136	1.0187	0.076	0
1712	928.9	0.2532	0.2138	1.018	0.0762	0
1713	928.8	0.2532	0.2139	1.0184	0.0762	0
1714	928.7	0.253	0.214	1.0193	0.0763	0
1715	928.6	0.253	0.2139	1.0187	0.0762	0
1716	928.5	0.2531	0.214	1.0177	0.0761	0
1717	928.4	0.2527	0.2135	1.0183	0.0758	0
1718	928.3	0.2526	0.2133	1.0184	0.0758	0
1719	928.2	0.2527	0.2134	1.0184	0.076	0
1720	928.1	0.2527	0.2136	1.018	0.0759	0
1721	928	0.253	0.2138	1.0185	0.0759	0
1722	927.9	0.2532	0.2138	1.0178	0.0761	0
1723	927.8	0.2529	0.2136	1.0177	0.076	0
1724	927.7	0.2529	0.2137	1.0177	0.0762	0
1725	927.6	0.2527	0.2135	1.0178	0.0758	0
1726	927.5	0.2526	0.2138	1.0175	0.0759	0
1727	927.4	0.2524	0.2133	1.0174	0.0757	0
1728	927.3	0.2526	0.2133	1.0174	0.0758	0
1729	927.2	0.2527	0.2135	1.0178	0.0759	0
1730	927.1	0.2527	0.2134	1.0178	0.0758	0
1731	927	0.2527	0.2135	1.0177	0.0758	0
1732	926.9	0.2525	0.2134	1.0173	0.0758	0
1733	926.8	0.2525	0.2134	1.0173	0.0756	0
1734	926.7	0.2527	0.2134	1.017	0.0758	0
1735	926.6	0.2528	0.2138	1.0178	0.076	0
1736	926.5	0.253	0.2139	1.0178	0.0762	0
1737	926.4	0.2531	0.2139	1.0181	0.0761	0
1738	926.3	0.2529	0.2139	1.0179	0.0761	0
1739	926.2	0.2529	0.2136	1.0181	0.0761	0
1740	926.1	0.2529	0.214	1.0177	0.0762	0
1741	926	0.2529	0.2139	1.0178	0.0763	0
1742	925.9	0.2531	0.2139	1.018	0.0763	0
1743	925.8	0.253	0.2139	1.0178	0.0764	0
1744	925.7	0.253	0.2138	1.0182	0.0764	0
1745	925.6	0.2528	0.2138	1.0181	0.0763	0
1746	925.5	0.2526	0.2137	1.0176	0.0761	0
1747	925.4	0.2528	0.2138	1.0172	0.0762	0
1748	925.3	0.2526	0.2137	1.0173	0.0761	0
1749	925.2	0.253	0.2138	1.0178	0.0763	0

1750	925.1	0.2529	0.2138	1.0169	0.0764	0
1751	925	0.2527	0.2138	1.0175	0.0762	0
1752	924.9	0.253	0.2139	1.0177	0.0765	0
1753	924.8	0.2531	0.2141	1.0179	0.0765	0
1754	924.7	0.2529	0.2139	1.0173	0.0764	0
1755	924.6	0.2529	0.214	1.0172	0.0767	0
1756	924.5	0.2531	0.2142	1.0178	0.0767	0
1757	924.4	0.253	0.2141	1.0174	0.0766	0
1758	924.3	0.2532	0.2142	1.0175	0.0767	0
1759	924.2	0.253	0.214	1.0178	0.0766	0
1760	924.1	0.253	0.2141	1.0176	0.0766	0
1761	924	0.253	0.214	1.0166	0.0766	0
1762	923.9	0.2531	0.2144	1.0173	0.077	0
1763	923.8	0.253	0.2141	1.0174	0.0766	0
1764	923.7	0.2529	0.2142	1.0172	0.0766	0
1765	923.6	0.2527	0.2141	1.0171	0.0765	0
1766	923.5	0.2528	0.214	1.0172	0.0766	0
1767	923.4	0.2529	0.2142	1.0171	0.0766	0
1768	923.3	0.2531	0.2142	1.0167	0.0767	0
1769	923.2	0.2529	0.214	1.0173	0.0766	0
1770	923.1	0.2527	0.214	1.0167	0.0766	0
1771	923	0.253	0.2141	1.0176	0.0767	0
1772	922.9	0.2531	0.2142	1.0172	0.0769	0
1773	922.8	0.2529	0.2144	1.0175	0.0769	0
1774	922.7	0.2531	0.2144	1.0168	0.077	0
1775	922.6	0.2529	0.2141	1.017	0.0768	0
1776	922.5	0.2528	0.214	1.0169	0.0765	0
1777	922.4	0.2528	0.214	1.017	0.0766	0
1778	922.3	0.253	0.2144	1.0171	0.0767	0
1779	922.2	0.2532	0.2143	1.0171	0.0768	0
1780	922.1	0.2532	0.2142	1.0171	0.0769	0
1781	922	0.2532	0.2145	1.017	0.077	0
1782	921.9	0.2531	0.2144	1.0172	0.0768	0
1783	921.8	0.2529	0.2142	1.0172	0.0768	0
1784	921.7	0.2529	0.2144	1.0174	0.0768	0
1785	921.6	0.2533	0.2144	1.0172	0.0769	0
1786	921.5	0.253	0.2142	1.0171	0.077	0
1787	921.4	0.2529	0.2143	1.0164	0.077	0
1788	921.3	0.2529	0.2144	1.0172	0.0768	0
1789	921.2	0.253	0.2143	1.0171	0.0769	0
1790	921.1	0.2531	0.2144	1.0175	0.077	0
1791	921	0.2531	0.2146	1.0178	0.077	0
1792	920.9	0.2531	0.2145	1.0176	0.0769	0

1793	920.8	0.253	0.2143	1.0174	0.0768	0
1794	920.7	0.2532	0.2145	1.0171	0.077	0
1795	920.6	0.2528	0.2141	1.018	0.0766	0
1796	920.5	0.2528	0.2142	1.0177	0.0767	0
1797	920.4	0.253	0.2143	1.0174	0.0768	0
1798	920.3	0.2528	0.2142	1.0178	0.0768	0
1799	920.2	0.2528	0.2142	1.0173	0.0767	0
1800	920.1	0.253	0.2144	1.0178	0.0768	0
1801	920	0.253	0.2144	1.0177	0.0769	0
1802	919.9	0.2529	0.2143	1.0175	0.0767	0
1803	919.8	0.2529	0.2142	1.0176	0.0766	0
1804	919.7	0.253	0.2142	1.0176	0.0767	0
1805	919.6	0.2528	0.2142	1.0176	0.0766	0
1806	919.5	0.2527	0.214	1.0182	0.0766	0
1807	919.4	0.2528	0.2141	1.0178	0.0765	0
1808	919.3	0.2528	0.2142	1.0182	0.0764	0
1809	919.2	0.2529	0.2139	1.0177	0.0765	0
1810	919.1	0.253	0.214	1.0179	0.0764	0
1811	919	0.2525	0.2139	1.0174	0.0764	0
1812	918.9	0.2527	0.2139	1.0172	0.0765	0
1813	918.8	0.2526	0.2139	1.0176	0.0764	0
1814	918.7	0.2527	0.2138	1.0173	0.0764	0
1815	918.6	0.2527	0.214	1.0175	0.0765	0
1816	918.5	0.2529	0.2139	1.0183	0.0765	0
1817	918.4	0.2533	0.2143	1.0183	0.0769	0
1818	918.3	0.2528	0.2139	1.018	0.0764	0
1819	918.2	0.2527	0.2138	1.0177	0.0764	0
1820	918.1	0.2527	0.2137	1.0175	0.0763	0
1821	918	0.2528	0.2139	1.0179	0.0765	0
1822	917.9	0.253	0.2138	1.0167	0.0766	0
1823	917.8	0.2527	0.2138	1.0177	0.0764	0
1824	917.7	0.2528	0.2139	1.0178	0.0764	0
1825	917.6	0.2527	0.214	1.0179	0.0764	0
1826	917.5	0.2528	0.2141	1.0178	0.0764	0
1827	917.4	0.2528	0.2141	1.0176	0.0767	0
1828	917.3	0.2526	0.2138	1.0172	0.0765	0
1829	917.2	0.2526	0.2139	1.0173	0.0764	0
1830	917.1	0.2527	0.2139	1.0181	0.0764	0
1831	917	0.2528	0.214	1.0174	0.0766	0
1832	916.9	0.2531	0.2139	1.0178	0.0767	0
1833	916.8	0.2528	0.2138	1.0177	0.0765	0
1834	916.7	0.2528	0.214	1.0178	0.0765	0
1835	916.6	0.2528	0.2141	1.0178	0.0767	0

1836	916.5	0.2531	0.2142	1.0187	0.0767	0
1837	916.4	0.253	0.214	1.0178	0.0765	0
1838	916.3	0.253	0.2141	1.0178	0.0766	0
1839	916.2	0.2531	0.2141	1.0181	0.0768	0
1840	916.1	0.253	0.2143	1.0179	0.077	0
1841	916	0.2535	0.2145	1.0179	0.0771	0
1842	915.9	0.2529	0.2141	1.0178	0.0768	0
1843	915.8	0.253	0.2143	1.0179	0.077	0
1844	915.7	0.2531	0.2145	1.018	0.077	0
1845	915.6	0.2532	0.2144	1.018	0.077	0
1846	915.5	0.2533	0.2144	1.0179	0.077	0
1847	915.4	0.2532	0.2143	1.018	0.0769	0
1848	915.3	0.2531	0.2144	1.0183	0.0771	0
1849	915.2	0.2531	0.2144	1.0176	0.0771	0
1850	915.1	0.2531	0.2145	1.0177	0.0773	0
1851	915	0.253	0.2146	1.0173	0.0772	0
1852	914.9	0.2532	0.2147	1.0184	0.0772	0
1853	914.8	0.2532	0.2149	1.0183	0.0774	0
1854	914.7	0.2532	0.2148	1.0176	0.0773	0
1855	914.6	0.2534	0.2148	1.0179	0.0775	0
1856	914.5	0.2533	0.2146	1.0177	0.0775	0
1857	914.4	0.2534	0.2149	1.018	0.0776	0
1858	914.3	0.2534	0.2149	1.0175	0.0777	0
1859	914.2	0.2536	0.2149	1.0174	0.0777	0
1860	914.1	0.2535	0.2149	1.0176	0.0776	0
1861	914	0.2531	0.2148	1.0176	0.0774	0
1862	913.9	0.2536	0.2149	1.018	0.0777	0
1863	913.8	0.2533	0.2148	1.0176	0.0778	0
1864	913.7	0.2534	0.2152	1.0176	0.078	0
1865	913.6	0.2536	0.2152	1.0176	0.0779	0
1866	913.5	0.2538	0.2153	1.018	0.0781	0
1867	913.4	0.2536	0.2153	1.0175	0.0782	0
1868	913.3	0.2536	0.2155	1.0179	0.0783	0
1869	913.2	0.2535	0.2154	1.0178	0.0782	0
1870	913.1	0.2535	0.2153	1.0175	0.0781	0
1871	913	0.2536	0.2155	1.0177	0.0782	0
1872	912.9	0.2538	0.2156	1.0175	0.0784	0
1873	912.8	0.2538	0.2156	1.0181	0.0784	0
1874	912.7	0.2537	0.2154	1.0172	0.0782	0
1875	912.6	0.2537	0.2153	1.0173	0.0782	0
1876	912.5	0.2537	0.2154	1.0179	0.0783	0
1877	912.4	0.2539	0.2155	1.0178	0.0784	0
1878	912.3	0.2537	0.2156	1.0178	0.0784	0

1879	912.2	0.2538	0.2157	1.0173	0.0785	0
1880	912.1	0.2539	0.2158	1.0175	0.0785	0
1881	912	0.2539	0.2158	1.0173	0.0785	0
1882	911.9	0.254	0.2156	1.0175	0.0784	0
1883	911.8	0.2537	0.2155	1.0179	0.0784	0
1884	911.7	0.254	0.2159	1.0178	0.0787	0
1885	911.6	0.2541	0.2158	1.0175	0.0785	0
1886	911.5	0.2542	0.2158	1.0181	0.0787	0
1887	911.4	0.2539	0.2156	1.0177	0.0784	0
1888	911.3	0.254	0.2158	1.0182	0.0786	0
1889	911.2	0.2538	0.2156	1.0175	0.0784	0
1890	911.1	0.254	0.2157	1.0178	0.0786	0
1891	911	0.2539	0.2157	1.0174	0.0787	0
1892	910.9	0.2542	0.2159	1.0176	0.0787	0
1893	910.8	0.254	0.2157	1.0175	0.0785	0
1894	910.7	0.254	0.2157	1.0174	0.0787	0
1895	910.6	0.2541	0.2157	1.0179	0.0786	0
1896	910.5	0.254	0.2157	1.0177	0.0787	0
1897	910.4	0.2539	0.2157	1.0174	0.0784	0
1898	910.3	0.2539	0.2157	1.0179	0.0784	0
1899	910.2	0.2539	0.2158	1.0174	0.0785	0
1900	910.1	0.254	0.2157	1.0177	0.0784	0
1901	910	0.2537	0.2154	1.0178	0.0783	0
1902	909.9	0.2536	0.2155	1.0175	0.0784	0
1903	909.8	0.2538	0.2155	1.0179	0.0784	0
1904	909.7	0.2536	0.2156	1.018	0.0785	0
1905	909.6	0.2537	0.2156	1.0178	0.0783	0
1906	909.5	0.2536	0.2154	1.018	0.0783	0
1907	909.4	0.2537	0.2156	1.0176	0.0784	0
1908	909.3	0.2538	0.2156	1.0183	0.0784	0
1909	909.2	0.2539	0.2158	1.0185	0.0785	0
1910	909.1	0.2541	0.2159	1.0188	0.0786	0
1911	909	0.2539	0.2157	1.0189	0.0785	0
1912	908.9	0.254	0.2157	1.0189	0.0785	0
1913	908.8	0.254	0.2157	1.0187	0.0785	0
1914	908.7	0.2541	0.2157	1.0184	0.0784	0
1915	908.6	0.2539	0.2156	1.0189	0.0783	0
1916	908.5	0.254	0.2156	1.0184	0.0783	0
1917	908.4	0.2541	0.2158	1.0188	0.0785	0
1918	908.3	0.2541	0.2156	1.0187	0.0783	0
1919	908.2	0.2542	0.2155	1.0188	0.0784	0
1920	908.1	0.2539	0.2156	1.019	0.0783	0
1921	908	0.2539	0.2156	1.0187	0.0783	0

1922	907.9	0.2541	0.2157	1.0193	0.0783	0
1923	907.8	0.2542	0.2156	1.0196	0.0785	0
1924	907.7	0.2541	0.2156	1.0185	0.0784	0
1925	907.6	0.2541	0.2155	1.0194	0.0783	0
1926	907.5	0.254	0.2154	1.0188	0.0782	0
1927	907.4	0.2537	0.2154	1.0185	0.0781	0
1928	907.3	0.2539	0.2157	1.0193	0.0783	0
1929	907.2	0.2539	0.2157	1.019	0.0782	0
1930	907.1	0.2539	0.2155	1.0192	0.0781	0
1931	907	0.254	0.2157	1.0194	0.0784	0
1932	906.9	0.254	0.2156	1.0192	0.0783	0
1933	906.8	0.2539	0.2156	1.0193	0.0783	0
1934	906.7	0.2541	0.2157	1.019	0.0784	0
1935	906.6	0.254	0.2158	1.0194	0.0784	0
1936	906.5	0.2541	0.2158	1.019	0.0785	0
1937	906.4	0.254	0.2158	1.0189	0.0784	0
1938	906.3	0.2541	0.2158	1.019	0.0783	0
1939	906.2	0.254	0.2158	1.0191	0.0784	0
1940	906.1	0.2538	0.2156	1.0188	0.0784	0
1941	906	0.2541	0.2156	1.0191	0.0786	0
1942	905.9	0.2542	0.2159	1.0193	0.0786	0
1943	905.8	0.2542	0.2159	1.0197	0.0786	0
1944	905.7	0.254	0.2158	1.0193	0.0786	0
1945	905.6	0.2538	0.2159	1.019	0.0785	0
1946	905.5	0.254	0.2158	1.0188	0.0784	0
1947	905.4	0.254	0.2158	1.0194	0.0785	0
1948	905.3	0.2544	0.2161	1.019	0.0789	0
1949	905.2	0.2544	0.2161	1.0185	0.0788	0
1950	905.1	0.2545	0.2162	1.019	0.079	0
1951	905	0.2544	0.2162	1.0192	0.079	0
1952	904.9	0.2545	0.2161	1.0195	0.0791	0
1953	904.8	0.2546	0.2161	1.0199	0.0792	0
1954	904.7	0.2546	0.2164	1.0187	0.0792	0
1955	904.6	0.2548	0.2164	1.0187	0.0794	0
1956	904.5	0.2549	0.2167	1.0192	0.0795	0
1957	904.4	0.2545	0.2165	1.0189	0.0794	0
1958	904.3	0.2546	0.2166	1.0189	0.0794	0
1959	904.2	0.2548	0.2169	1.019	0.0798	0
1960	904.1	0.2549	0.217	1.0192	0.0797	0
1961	904	0.2549	0.217	1.0192	0.0798	0
1962	903.9	0.2548	0.2168	1.0192	0.0797	0
1963	903.8	0.2547	0.2169	1.0191	0.0798	0
1964	903.7	0.2547	0.2168	1.0188	0.0798	0

1965	903.6	0.2549	0.217	1.0194	0.08	0
1966	903.5	0.2547	0.2168	1.0188	0.0799	0
1967	903.4	0.2549	0.217	1.019	0.0801	0
1968	903.3	0.255	0.2173	1.0186	0.0801	0
1969	903.2	0.2552	0.2173	1.0193	0.0802	0
1970	903.1	0.2554	0.2175	1.019	0.0805	0
1971	903	0.2552	0.2175	1.019	0.0804	0
1972	902.9	0.2552	0.2175	1.0192	0.0804	0
1973	902.8	0.2552	0.2178	1.019	0.0805	0
1974	902.7	0.2551	0.2175	1.0189	0.0804	0
1975	902.6	0.255	0.2175	1.0186	0.0804	0
1976	902.5	0.2553	0.2175	1.0186	0.0805	0
1977	902.4	0.2552	0.2175	1.0185	0.0805	0
1978	902.3	0.2552	0.2175	1.0184	0.0804	0
1979	902.2	0.2552	0.2176	1.0187	0.0804	0
1980	902.1	0.2552	0.2176	1.0188	0.0807	0
1981	902	0.255	0.2175	1.019	0.0806	0
1982	901.9	0.2555	0.2178	1.0188	0.0808	0
1983	901.8	0.2553	0.2177	1.0193	0.0806	0
1984	901.7	0.2554	0.2177	1.0185	0.0806	0
1985	901.6	0.2552	0.2177	1.0189	0.0807	0
1986	901.5	0.2555	0.2179	1.0187	0.0807	0
1987	901.4	0.2554	0.2178	1.0188	0.0808	0
1988	901.3	0.2554	0.2179	1.0181	0.0808	0
1989	901.2	0.2552	0.2176	1.0188	0.0807	0
1990	901.1	0.2554	0.2177	1.0193	0.0808	0
1991	901	0.2555	0.218	1.0194	0.081	0
1992	900.9	0.2553	0.2178	1.019	0.0808	0
1993	900.8	0.2554	0.2178	1.0186	0.0809	0
1994	900.7	0.2554	0.218	1.0186	0.0809	0
1995	900.6	0.2554	0.218	1.0186	0.0808	0
1996	900.5	0.2552	0.2178	1.0191	0.0808	0
1997	900.4	0.2555	0.2178	1.0197	0.0809	0
1998	900.3	0.2553	0.2178	1.0192	0.0809	0
1999	900.2	0.2554	0.218	1.0195	0.081	0
2000	900.1	0.2557	0.2181	1.0196	0.0812	0
2001	900	0.2555	0.2179	1.0194	0.081	0
2002	899.9	0.2555	0.2181	1.0191	0.0811	0
2003	899.8	0.2557	0.2182	1.0195	0.0811	0
2004	899.7	0.2557	0.2182	1.019	0.081	0
2005	899.6	0.2554	0.2181	1.0191	0.0809	0
2006	899.5	0.2557	0.2181	1.0192	0.0811	0
2007	899.4	0.2556	0.218	1.0192	0.081	0

2008	899.3	0.2557	0.218	1.0194	0.0811	0
2009	899.2	0.2556	0.218	1.0192	0.0809	0
2010	899.1	0.2556	0.218	1.0192	0.0811	0
2011	899	0.2558	0.218	1.0195	0.0811	0
2012	898.9	0.2556	0.2178	1.0199	0.0809	0
2013	898.8	0.2557	0.2179	1.0194	0.0809	0
2014	898.7	0.2555	0.2178	1.0191	0.0807	0
2015	898.6	0.2556	0.2178	1.0192	0.0808	0
2016	898.5	0.2552	0.2176	1.0191	0.0807	0
2017	898.4	0.2553	0.2175	1.019	0.0806	0
2018	898.3	0.2552	0.2177	1.0198	0.0806	0
2019	898.2	0.2555	0.2178	1.0196	0.0807	0
2020	898.1	0.2554	0.2177	1.0192	0.0808	0
2021	898	0.2553	0.2175	1.0198	0.0806	0
2022	897.9	0.2554	0.2178	1.0196	0.0807	0
2023	897.8	0.2555	0.2179	1.0195	0.0809	0
2024	897.7	0.2555	0.2179	1.0194	0.0808	0
2025	897.6	0.2553	0.2175	1.0192	0.0806	0
2026	897.5	0.2554	0.2176	1.02	0.0806	0
2027	897.4	0.2556	0.2178	1.0201	0.0808	0
2028	897.3	0.2556	0.2177	1.0204	0.0808	0
2029	897.2	0.2555	0.2178	1.0199	0.0808	0
2030	897.1	0.2552	0.2177	1.0197	0.0808	0
2031	897	0.2554	0.2176	1.0191	0.0805	0
2032	896.9	0.2557	0.218	1.0199	0.0808	0
2033	896.8	0.2554	0.2177	1.0194	0.0806	0
2034	896.7	0.2554	0.2175	1.0195	0.0805	0
2035	896.6	0.2554	0.2175	1.0196	0.0806	0
2036	896.5	0.2554	0.2176	1.0195	0.0805	0
2037	896.4	0.2556	0.2179	1.0195	0.0808	0
2038	896.3	0.2556	0.2177	1.0205	0.0807	0
2039	896.2	0.2553	0.2176	1.0202	0.0807	0
2040	896.1	0.2553	0.2177	1.0193	0.0806	0
2041	896	0.2554	0.2177	1.0198	0.0806	0
2042	895.9	0.2554	0.2179	1.0201	0.0807	0
2043	895.8	0.2554	0.2178	1.0195	0.0807	0
2044	895.7	0.2556	0.218	1.0201	0.0808	0
2045	895.6	0.2555	0.2178	1.0197	0.0807	0
2046	895.5	0.2555	0.2177	1.0196	0.0808	0
2047	895.4	0.2557	0.218	1.0195	0.0809	0
2048	895.3	0.2554	0.2179	1.0195	0.0807	0
2049	895.2	0.2554	0.2178	1.0193	0.0807	0
2050	895.1	0.2557	0.2181	1.0197	0.0811	0

2051	895	0.2557	0.2179	1.0199	0.081	0
2052	894.9	0.2556	0.2179	1.02	0.081	0
2053	894.8	0.2557	0.218	1.0204	0.081	0
2054	894.7	0.2557	0.2182	1.0204	0.0811	0
2055	894.6	0.2559	0.2183	1.0198	0.0812	0
2056	894.5	0.2558	0.218	1.0199	0.0812	0
2057	894.4	0.2559	0.218	1.0195	0.0812	0
2058	894.3	0.2558	0.2182	1.0197	0.0813	0
2059	894.2	0.256	0.2185	1.02	0.0814	0
2060	894.1	0.2558	0.2184	1.0194	0.0814	0
2061	894	0.256	0.2186	1.0198	0.0816	0
2062	893.9	0.256	0.2187	1.0199	0.0815	0
2063	893.8	0.2558	0.2187	1.0197	0.0815	0
2064	893.7	0.2558	0.2186	1.0196	0.0815	0
2065	893.6	0.256	0.2186	1.0197	0.0816	0
2066	893.5	0.2563	0.2189	1.0195	0.0819	0
2067	893.4	0.256	0.2187	1.0195	0.0817	0
2068	893.3	0.2561	0.2188	1.0194	0.0818	0
2069	893.2	0.2562	0.2189	1.0193	0.082	0
2070	893.1	0.2562	0.219	1.0195	0.0821	0
2071	893	0.2564	0.2191	1.02	0.0822	0
2072	892.9	0.2565	0.2191	1.0193	0.0823	0
2073	892.8	0.2562	0.219	1.0195	0.0821	0
2074	892.7	0.2561	0.219	1.0189	0.0821	0
2075	892.6	0.2565	0.2192	1.0196	0.0824	0
2076	892.5	0.2566	0.2193	1.0193	0.0825	0
2077	892.4	0.2566	0.2193	1.0196	0.0825	0
2078	892.3	0.2567	0.2194	1.019	0.0825	0
2079	892.2	0.2566	0.2193	1.0191	0.0826	0
2080	892.1	0.2567	0.2193	1.0195	0.0826	0
2081	892	0.2566	0.2194	1.0201	0.0827	0
2082	891.9	0.2567	0.2196	1.0198	0.0826	0
2083	891.8	0.2567	0.2195	1.019	0.0828	0
2084	891.7	0.2568	0.2197	1.0194	0.0828	0
2085	891.6	0.2567	0.2197	1.0197	0.0829	0
2086	891.5	0.2568	0.2196	1.0196	0.0829	0
2087	891.4	0.2567	0.2195	1.0195	0.0828	0
2088	891.3	0.2568	0.2195	1.0191	0.0827	0
2089	891.2	0.257	0.2198	1.0193	0.0831	0
2090	891.1	0.2569	0.2198	1.0194	0.083	0
2091	891	0.2571	0.2201	1.02	0.0832	0
2092	890.9	0.2572	0.22	1.0196	0.0833	0
2093	890.8	0.2571	0.22	1.0196	0.0832	0

2094	890.7	0.2572	0.22	1.0199	0.0833	0
2095	890.6	0.2571	0.22	1.0198	0.0832	0
2096	890.5	0.2567	0.2201	1.0194	0.0831	0
2097	890.4	0.2569	0.2201	1.02	0.0832	0
2098	890.3	0.2569	0.22	1.0196	0.0831	0
2099	890.2	0.257	0.2201	1.0197	0.0833	0
2100	890.1	0.2571	0.2201	1.0191	0.0831	0
2101	890	0.2571	0.2201	1.0193	0.0833	0
2102	889.9	0.2571	0.2201	1.0192	0.0833	0
2103	889.8	0.257	0.2199	1.0193	0.0831	0
2104	889.7	0.257	0.2198	1.0193	0.0831	0
2105	889.6	0.257	0.2197	1.0196	0.0831	0
2106	889.5	0.257	0.2201	1.0195	0.0833	0
2107	889.4	0.2572	0.2202	1.0196	0.0834	0
2108	889.3	0.2571	0.2201	1.0193	0.0831	0
2109	889.2	0.2572	0.2204	1.0206	0.0834	0
2110	889.1	0.2572	0.2202	1.0192	0.0834	0
2111	889	0.2573	0.2204	1.0196	0.0835	0
2112	888.9	0.2572	0.2203	1.0195	0.0834	0
2113	888.8	0.2572	0.2202	1.0188	0.0834	0
2114	888.7	0.2571	0.2202	1.0197	0.0834	0
2115	888.6	0.257	0.2201	1.0196	0.0833	0
2116	888.5	0.2571	0.22	1.0198	0.0831	0
2117	888.4	0.257	0.2201	1.0199	0.0833	0
2118	888.3	0.2573	0.2204	1.0197	0.0835	0
2119	888.2	0.257	0.22	1.0195	0.0831	0
2120	888.1	0.2569	0.2198	1.0196	0.0831	0
2121	888	0.2569	0.2198	1.0202	0.0831	0
2122	887.9	0.2571	0.2201	1.0201	0.0832	0
2123	887.8	0.2571	0.2201	1.0197	0.0832	0
2124	887.7	0.2575	0.2202	1.0203	0.0834	0
2125	887.6	0.2572	0.22	1.0203	0.0832	0
2126	887.5	0.2571	0.22	1.0199	0.0831	0
2127	887.4	0.2571	0.2199	1.0199	0.0832	0
2128	887.3	0.2569	0.2198	1.0201	0.0831	0
2129	887.2	0.2568	0.2197	1.0193	0.083	0
2130	887.1	0.2568	0.2198	1.0197	0.083	0
2131	887	0.2571	0.2199	1.0199	0.0831	0
2132	886.9	0.257	0.2198	1.02	0.0829	0
2133	886.8	0.2572	0.2201	1.0198	0.0832	0
2134	886.7	0.2573	0.2203	1.0199	0.0831	0
2135	886.6	0.2572	0.2201	1.0198	0.0832	0
2136	886.5	0.2571	0.22	1.0202	0.0832	0

2137	886.4	0.257	0.2198	1.0195	0.0829	0
2138	886.3	0.2572	0.2199	1.0199	0.083	0
2139	886.2	0.2571	0.22	1.0201	0.0831	0
2140	886.1	0.257	0.22	1.02	0.083	0
2141	886	0.2569	0.2198	1.0196	0.0829	0
2142	885.9	0.2569	0.2198	1.0198	0.0829	0
2143	885.8	0.2568	0.2198	1.0198	0.0827	0
2144	885.7	0.2568	0.2196	1.0194	0.0829	0
2145	885.6	0.257	0.2199	1.0196	0.0832	0
2146	885.5	0.2572	0.22	1.02	0.0833	0
2147	885.4	0.2571	0.22	1.0196	0.0833	0
2148	885.3	0.2571	0.22	1.0202	0.0832	0
2149	885.2	0.257	0.2199	1.0195	0.0831	0
2150	885.1	0.257	0.2199	1.0192	0.083	0
2151	885	0.2572	0.2201	1.0199	0.0833	0
2152	884.9	0.2572	0.2201	1.0198	0.0832	0
2153	884.8	0.2573	0.2202	1.02	0.0833	0
2154	884.7	0.2573	0.22	1.0198	0.0832	0
2155	884.6	0.2571	0.2199	1.0199	0.0832	0
2156	884.5	0.257	0.2201	1.0196	0.0833	0
2157	884.4	0.2573	0.2202	1.0191	0.0834	0
2158	884.3	0.2572	0.2201	1.0192	0.0833	0
2159	884.2	0.2571	0.2199	1.019	0.0831	0
2160	884.1	0.2574	0.2202	1.0195	0.0836	0
2161	884	0.2574	0.2204	1.0199	0.0835	0
2162	883.9	0.2575	0.2204	1.0196	0.0837	0
2163	883.8	0.2578	0.2205	1.0195	0.0836	0
2164	883.7	0.2576	0.2207	1.0201	0.0837	0
2165	883.6	0.2575	0.2207	1.0197	0.0838	0
2166	883.5	0.2574	0.2206	1.0196	0.0838	0
2167	883.4	0.2575	0.2207	1.0197	0.084	0
2168	883.3	0.2578	0.2209	1.0198	0.084	0
2169	883.2	0.2576	0.2208	1.0196	0.0841	0
2170	883.1	0.2578	0.221	1.0198	0.0842	0
2171	883	0.2579	0.221	1.02	0.0842	0
2172	882.9	0.2576	0.2209	1.0198	0.084	0
2173	882.8	0.2578	0.2211	1.0202	0.0844	0
2174	882.7	0.2581	0.2212	1.0204	0.0844	0
2175	882.6	0.2581	0.2212	1.0202	0.0844	0
2176	882.5	0.2581	0.2212	1.0196	0.0844	0
2177	882.4	0.258	0.2212	1.0197	0.0844	0
2178	882.3	0.2582	0.2214	1.0194	0.0845	0
2179	882.2	0.2583	0.2214	1.0197	0.0846	0

2180	882.1	0.2582	0.2213	1.0203	0.0846	0
2181	882	0.258	0.2212	1.02	0.0845	0
2182	881.9	0.2582	0.2214	1.0194	0.0846	0
2183	881.8	0.2583	0.2217	1.0198	0.0848	0
2184	881.7	0.2583	0.2217	1.0204	0.0848	0
2185	881.6	0.2582	0.2217	1.0197	0.0848	0
2186	881.5	0.2583	0.2217	1.0199	0.0849	0
2187	881.4	0.2582	0.2215	1.0195	0.0849	0
2188	881.3	0.2583	0.2216	1.0201	0.0848	0
2189	881.2	0.2583	0.2218	1.0198	0.0849	0
2190	881.1	0.2583	0.2218	1.0195	0.0847	0
2191	881	0.2585	0.2217	1.02	0.0849	0
2192	880.9	0.2584	0.2218	1.0198	0.0849	0
2193	880.8	0.2584	0.2217	1.0204	0.0849	0
2194	880.7	0.2582	0.2217	1.0193	0.0848	0
2195	880.6	0.2583	0.2217	1.0198	0.0849	0
2196	880.5	0.2582	0.2217	1.0197	0.0849	0
2197	880.4	0.2585	0.2219	1.0197	0.085	0
2198	880.3	0.2583	0.2216	1.0197	0.0849	0
2199	880.2	0.2582	0.2217	1.0195	0.0849	0
2200	880.1	0.2584	0.2218	1.0197	0.0851	0
2201	880	0.2582	0.2217	1.0195	0.085	0
2202	879.9	0.2585	0.222	1.0201	0.0853	0
2203	879.8	0.2583	0.222	1.0199	0.085	0
2204	879.7	0.2583	0.2219	1.0192	0.0852	0
2205	879.6	0.2585	0.2218	1.0197	0.0852	0
2206	879.5	0.2586	0.222	1.0198	0.0852	0
2207	879.4	0.2586	0.2218	1.02	0.0851	0
2208	879.3	0.2586	0.2218	1.0193	0.085	0
2209	879.2	0.2585	0.2219	1.0202	0.085	0
2210	879.1	0.2586	0.2221	1.02	0.0852	0
2211	879	0.2585	0.2221	1.0203	0.0853	0
2212	878.9	0.2586	0.222	1.0204	0.0853	0
2213	878.8	0.2584	0.2218	1.0198	0.0852	0
2214	878.7	0.2586	0.2221	1.0198	0.0853	0
2215	878.6	0.2585	0.2222	1.0197	0.0854	0
2216	878.5	0.2586	0.222	1.0198	0.0852	0
2217	878.4	0.2586	0.2219	1.02	0.0851	0
2218	878.3	0.2587	0.2221	1.0195	0.0851	0
2219	878.2	0.2586	0.2221	1.02	0.0853	0
2220	878.1	0.2587	0.222	1.02	0.0853	0
2221	878	0.2586	0.222	1.0196	0.0853	0
2222	877.9	0.2583	0.2217	1.0193	0.085	0

2223	877.8	0.2586	0.2219	1.0198	0.0852	0
2224	877.7	0.2585	0.222	1.0193	0.0852	0
2225	877.6	0.2585	0.222	1.0198	0.0851	0
2226	877.5	0.2587	0.222	1.0207	0.0851	0
2227	877.4	0.2584	0.2219	1.0207	0.0851	0
2228	877.3	0.2587	0.2223	1.0202	0.0853	0
2229	877.2	0.2587	0.2221	1.0201	0.0851	0
2230	877.1	0.2586	0.2219	1.0206	0.0849	0
2231	877	0.2587	0.222	1.0204	0.0852	0
2232	876.9	0.2588	0.222	1.02	0.0853	0
2233	876.8	0.2587	0.222	1.0205	0.0851	0
2234	876.7	0.2589	0.2222	1.0206	0.0855	0
2235	876.6	0.2587	0.2222	1.0199	0.0854	0
2236	876.5	0.2588	0.2222	1.02	0.0853	0
2237	876.4	0.2588	0.222	1.0197	0.0851	0
2238	876.3	0.2589	0.2222	1.0204	0.0854	0
2239	876.2	0.2588	0.2221	1.02	0.0854	0
2240	876.1	0.2588	0.222	1.0203	0.0854	0
2241	876	0.2587	0.222	1.0205	0.0853	0
2242	875.9	0.2584	0.222	1.0202	0.0853	0
2243	875.8	0.2586	0.222	1.0204	0.0852	0
2244	875.7	0.2589	0.2223	1.0205	0.0855	0
2245	875.6	0.2591	0.2224	1.0207	0.0857	0
2246	875.5	0.2589	0.2222	1.0202	0.0856	0
2247	875.4	0.2591	0.2224	1.0206	0.0857	0
2248	875.3	0.259	0.2224	1.0205	0.0856	0
2249	875.2	0.2589	0.2223	1.0199	0.0856	0
2250	875.1	0.2591	0.2225	1.0205	0.0858	0
2251	875	0.259	0.2224	1.0199	0.0858	0
2252	874.9	0.259	0.2225	1.0203	0.0857	0
2253	874.8	0.2591	0.2226	1.0205	0.0857	0
2254	874.7	0.2591	0.2225	1.0206	0.0858	0
2255	874.6	0.259	0.2227	1.0205	0.0859	0
2256	874.5	0.2591	0.2225	1.0199	0.0859	0
2257	874.4	0.2595	0.2229	1.0205	0.0861	0
2258	874.3	0.2593	0.2229	1.0202	0.0859	0
2259	874.2	0.2594	0.2228	1.0211	0.086	0
2260	874.1	0.2593	0.2227	1.0204	0.0861	0
2261	874	0.2591	0.2227	1.0202	0.0861	0
2262	873.9	0.2591	0.2229	1.0203	0.0861	0
2263	873.8	0.2592	0.2227	1.0203	0.0861	0
2264	873.7	0.2593	0.2228	1.0201	0.0862	0
2265	873.6	0.2593	0.2229	1.0208	0.0861	0

2266	873.5	0.2594	0.2229	1.0208	0.0864	0
2267	873.4	0.2595	0.2231	1.0205	0.0865	0
2268	873.3	0.2596	0.2232	1.0208	0.0865	0
2269	873.2	0.2596	0.2232	1.0204	0.0865	0
2270	873.1	0.2596	0.2231	1.0206	0.0864	0
2271	873	0.2594	0.2232	1.0204	0.0866	0
2272	872.9	0.2595	0.2232	1.0203	0.0867	0
2273	872.8	0.2598	0.2235	1.0209	0.0868	0
2274	872.7	0.2597	0.2234	1.0203	0.0866	0
2275	872.6	0.2597	0.2235	1.0206	0.0869	0
2276	872.5	0.2596	0.2234	1.0205	0.0868	0
2277	872.4	0.2596	0.2233	1.0206	0.0866	0
2278	872.3	0.26	0.2234	1.0209	0.0869	0
2279	872.2	0.2597	0.2234	1.021	0.0869	0
2280	872.1	0.2595	0.2234	1.0205	0.0868	0
2281	872	0.2596	0.2233	1.0209	0.0867	0
2282	871.9	0.2598	0.2235	1.0209	0.0868	0
2283	871.8	0.26	0.2236	1.0206	0.0869	0
2284	871.7	0.2597	0.2234	1.0205	0.0869	0
2285	871.6	0.2597	0.2235	1.0201	0.0868	0
2286	871.5	0.2596	0.2235	1.0198	0.0868	0
2287	871.4	0.2596	0.2234	1.0203	0.0868	0
2288	871.3	0.2599	0.2237	1.021	0.0871	0
2289	871.2	0.2599	0.2239	1.021	0.0871	0
2290	871.1	0.2597	0.2235	1.0202	0.0869	0
2291	871	0.2596	0.2235	1.0207	0.087	0
2292	870.9	0.2598	0.2236	1.0205	0.087	0
2293	870.8	0.2598	0.2236	1.0205	0.0869	0
2294	870.7	0.26	0.2237	1.0203	0.0871	0
2295	870.6	0.2599	0.2238	1.0209	0.087	0
2296	870.5	0.2598	0.2236	1.0202	0.0869	0
2297	870.4	0.2599	0.2236	1.0209	0.087	0
2298	870.3	0.26	0.2238	1.0209	0.0871	0
2299	870.2	0.2602	0.2239	1.0209	0.0872	0
2300	870.1	0.2603	0.2241	1.0207	0.0872	0
2301	870	0.2602	0.224	1.0206	0.0871	0
2302	869.9	0.2602	0.2241	1.021	0.0872	0
2303	869.8	0.2601	0.2238	1.021	0.0872	0
2304	869.7	0.2601	0.2238	1.0208	0.087	0
2305	869.6	0.26	0.2239	1.0208	0.0872	0
2306	869.5	0.2601	0.2239	1.0213	0.0873	0
2307	869.4	0.2601	0.224	1.0216	0.0872	0
2308	869.3	0.2601	0.2239	1.021	0.0873	0

2309	869.2	0.2599	0.2237	1.0205	0.0871	0
2310	869.1	0.2599	0.2238	1.0202	0.0872	0
2311	869	0.2598	0.2237	1.0205	0.087	0
2312	868.9	0.2599	0.2236	1.0204	0.0871	0
2313	868.8	0.2598	0.2237	1.021	0.0871	0
2314	868.7	0.2598	0.2236	1.0209	0.0871	0
2315	868.6	0.2601	0.2238	1.0208	0.0872	0
2316	868.5	0.2602	0.2239	1.0213	0.0872	0
2317	868.4	0.2603	0.2239	1.0209	0.0871	0
2318	868.3	0.2601	0.2239	1.0209	0.0871	0
2319	868.2	0.2601	0.2238	1.0211	0.0872	0
2320	868.1	0.2601	0.2241	1.0212	0.0874	0
2321	868	0.26	0.224	1.0208	0.0872	0
2322	867.9	0.2602	0.2238	1.0212	0.0873	0
2323	867.8	0.2603	0.2239	1.0218	0.0873	0
2324	867.7	0.2604	0.2241	1.0213	0.0873	0
2325	867.6	0.2603	0.2238	1.0214	0.0874	0
2326	867.5	0.2601	0.2239	1.0219	0.0872	0
2327	867.4	0.2603	0.2242	1.0215	0.0874	0
2328	867.3	0.2605	0.2243	1.0215	0.0876	0
2329	867.2	0.2607	0.2246	1.0214	0.0877	0
2330	867.1	0.2604	0.2243	1.0211	0.0875	0
2331	867	0.2604	0.2241	1.0213	0.0874	0
2332	866.9	0.2602	0.224	1.0208	0.0873	0
2333	866.8	0.2602	0.224	1.0202	0.0872	0
2334	866.7	0.2603	0.2243	1.0212	0.0875	0
2335	866.6	0.2603	0.2241	1.0213	0.0873	0
2336	866.5	0.2603	0.2241	1.0213	0.0875	0
2337	866.4	0.2605	0.2242	1.0209	0.0877	0
2338	866.3	0.2603	0.2241	1.021	0.0875	0
2339	866.2	0.2603	0.2242	1.0209	0.0875	0
2340	866.1	0.2603	0.2242	1.021	0.0876	0
2341	866	0.2605	0.2243	1.0209	0.0877	0
2342	865.9	0.2606	0.2245	1.0214	0.0877	0
2343	865.8	0.2608	0.2246	1.0215	0.0879	0
2344	865.7	0.2607	0.2245	1.0212	0.0878	0
2345	865.6	0.2608	0.2247	1.0214	0.0878	0
2346	865.5	0.2606	0.2246	1.0208	0.0879	0
2347	865.4	0.2605	0.2245	1.0212	0.0878	0
2348	865.3	0.2607	0.2248	1.0217	0.0879	0
2349	865.2	0.261	0.2251	1.0217	0.0883	0
2350	865.1	0.2609	0.2248	1.0217	0.0882	0
2351	865	0.2608	0.2246	1.0208	0.0881	0

2352	864.9	0.2607	0.2246	1.021	0.088	0
2353	864.8	0.261	0.2249	1.0212	0.0882	0
2354	864.7	0.261	0.2248	1.0216	0.0882	0
2355	864.6	0.2611	0.225	1.0215	0.0883	0
2356	864.5	0.2612	0.2252	1.0215	0.0886	0
2357	864.4	0.2609	0.2251	1.0213	0.0884	0
2358	864.3	0.261	0.2251	1.0213	0.0885	0
2359	864.2	0.2611	0.2252	1.0218	0.0885	0
2360	864.1	0.2611	0.2251	1.021	0.0885	0
2361	864	0.2611	0.2251	1.0209	0.0885	0
2362	863.9	0.261	0.2253	1.0214	0.0887	0
2363	863.8	0.261	0.2253	1.0212	0.0887	0
2364	863.7	0.2609	0.2252	1.0209	0.0886	0
2365	863.6	0.2612	0.2255	1.0216	0.0889	0
2366	863.5	0.2614	0.2255	1.0212	0.089	0
2367	863.4	0.2613	0.2254	1.0212	0.0889	0
2368	863.3	0.2613	0.2254	1.021	0.0888	0
2369	863.2	0.2613	0.2254	1.0215	0.0889	0
2370	863.1	0.2612	0.2255	1.0211	0.0889	0
2371	863	0.2615	0.2256	1.0212	0.089	0
2372	862.9	0.2614	0.2255	1.0206	0.0891	0
2373	862.8	0.2612	0.2253	1.0213	0.0889	0
2374	862.7	0.2614	0.2256	1.0214	0.089	0
2375	862.6	0.2614	0.2258	1.0207	0.0892	0
2376	862.5	0.2616	0.2257	1.0206	0.0891	0
2377	862.4	0.2617	0.2259	1.0213	0.0893	0
2378	862.3	0.2617	0.2259	1.021	0.0893	0
2379	862.2	0.2617	0.2258	1.0208	0.0892	0
2380	862.1	0.2615	0.2258	1.0212	0.0893	0
2381	862	0.2616	0.2259	1.0209	0.0892	0
2382	861.9	0.2616	0.2259	1.0211	0.0893	0
2383	861.8	0.2616	0.2258	1.021	0.0892	0
2384	861.7	0.2617	0.2257	1.0213	0.0893	0
2385	861.6	0.2618	0.226	1.021	0.0894	0
2386	861.5	0.2617	0.226	1.0214	0.0895	0
2387	861.4	0.2617	0.226	1.0208	0.0895	0
2388	861.3	0.2616	0.226	1.0215	0.0894	0
2389	861.2	0.2617	0.226	1.0214	0.0893	0
2390	861.1	0.2618	0.2258	1.021	0.0892	0
2391	861	0.2619	0.2258	1.0215	0.0893	0
2392	860.9	0.2618	0.2259	1.0213	0.0895	0
2393	860.8	0.2618	0.2259	1.0213	0.0894	0
2394	860.7	0.2619	0.2263	1.0211	0.0895	0

2395	860.6	0.2619	0.2261	1.0212	0.0897	0
2396	860.5	0.2619	0.2262	1.0213	0.0897	0
2397	860.4	0.2618	0.226	1.0209	0.0894	0
2398	860.3	0.2617	0.2261	1.0211	0.0894	0
2399	860.2	0.2616	0.2258	1.0209	0.0894	0
2400	860.1	0.2615	0.2257	1.0208	0.0892	0
2401	860	0.2617	0.2259	1.0213	0.0894	0
2402	859.9	0.2618	0.2261	1.0218	0.0895	0
2403	859.8	0.2618	0.2261	1.0213	0.0897	0
2404	859.7	0.262	0.226	1.0215	0.0896	0
2405	859.6	0.262	0.2262	1.0218	0.0896	0
2406	859.5	0.2616	0.2258	1.0212	0.0893	0
2407	859.4	0.2616	0.226	1.0218	0.0894	0
2408	859.3	0.2617	0.2261	1.0214	0.0895	0
2409	859.2	0.2619	0.226	1.0216	0.0895	0
2410	859.1	0.262	0.2258	1.021	0.0894	0
2411	859	0.262	0.2259	1.0214	0.0895	0
2412	858.9	0.2618	0.2263	1.0222	0.0895	0
2413	858.8	0.2619	0.2261	1.0216	0.0895	0
2414	858.7	0.2618	0.2261	1.0219	0.0894	0
2415	858.6	0.262	0.226	1.0219	0.0895	0
2416	858.5	0.2618	0.226	1.0219	0.0893	0
2417	858.4	0.2619	0.226	1.0215	0.0893	0
2418	858.3	0.262	0.226	1.0222	0.0894	0
2419	858.2	0.2617	0.226	1.0216	0.0895	0
2420	858.1	0.2617	0.226	1.0213	0.0894	0
2421	858	0.2619	0.226	1.0219	0.0895	0
2422	857.9	0.2619	0.2259	1.0226	0.0893	0
2423	857.8	0.2621	0.2261	1.0229	0.0895	0
2424	857.7	0.2621	0.2264	1.0221	0.0895	0
2425	857.6	0.2622	0.2264	1.0223	0.0897	0
2426	857.5	0.2621	0.2262	1.0222	0.0896	0
2427	857.4	0.2619	0.2261	1.0221	0.0895	0
2428	857.3	0.2617	0.226	1.0217	0.0894	0
2429	857.2	0.2619	0.2261	1.0219	0.0894	0
2430	857.1	0.2621	0.2261	1.0223	0.0896	0
2431	857	0.2618	0.2261	1.0217	0.0895	0
2432	856.9	0.2619	0.2262	1.0222	0.0897	0
2433	856.8	0.262	0.2262	1.022	0.0896	0
2434	856.7	0.2622	0.2265	1.0221	0.0898	0
2435	856.6	0.2622	0.2264	1.0219	0.0899	0
2436	856.5	0.2622	0.2264	1.0219	0.0898	0
2437	856.4	0.2621	0.2263	1.0223	0.0898	0

2438	856.3	0.2622	0.2262	1.0218	0.0898	0
2439	856.2	0.2624	0.2264	1.0221	0.0899	0
2440	856.1	0.2624	0.2266	1.022	0.0901	0
2441	856	0.2623	0.2265	1.0223	0.09	0
2442	855.9	0.2623	0.2267	1.0221	0.09	0
2443	855.8	0.2626	0.2269	1.0226	0.0903	0
2444	855.7	0.2626	0.227	1.0217	0.0903	0
2445	855.6	0.2625	0.2269	1.022	0.0903	0
2446	855.5	0.2627	0.2269	1.0219	0.0904	0
2447	855.4	0.2626	0.2269	1.0222	0.0904	0
2448	855.3	0.2625	0.2269	1.0217	0.0903	0
2449	855.2	0.2625	0.2269	1.0211	0.0903	0
2450	855.1	0.2627	0.2272	1.0222	0.0905	0
2451	855	0.2626	0.2271	1.0217	0.0905	0
2452	854.9	0.2627	0.2272	1.0217	0.0905	0
2453	854.8	0.2628	0.2271	1.0223	0.0907	0
2454	854.7	0.2627	0.2271	1.0218	0.0907	0
2455	854.6	0.2627	0.2271	1.0223	0.0907	0
2456	854.5	0.263	0.2274	1.0222	0.091	0
2457	854.4	0.2629	0.2276	1.0222	0.091	0
2458	854.3	0.2629	0.2276	1.0218	0.0911	0
2459	854.2	0.2627	0.2275	1.0219	0.0909	0
2460	854.1	0.263	0.2276	1.0221	0.0912	0
2461	854	0.263	0.2276	1.0218	0.0913	0
2462	853.9	0.263	0.2275	1.0218	0.0911	0
2463	853.8	0.2631	0.2277	1.022	0.0912	0
2464	853.7	0.2631	0.2279	1.0216	0.0913	0
2465	853.6	0.2632	0.228	1.0217	0.0913	0
2466	853.5	0.2634	0.228	1.0214	0.0915	0
2467	853.4	0.2635	0.2281	1.0222	0.0916	0
2468	853.3	0.2633	0.228	1.022	0.0915	0
2469	853.2	0.2633	0.2282	1.0222	0.0915	0
2470	853.1	0.2634	0.2282	1.0215	0.0917	0
2471	853	0.2633	0.2282	1.0209	0.0915	0
2472	852.9	0.2634	0.2281	1.021	0.0917	0
2473	852.8	0.2636	0.2282	1.0218	0.0916	0
2474	852.7	0.2635	0.2282	1.0214	0.0918	0
2475	852.6	0.2637	0.2284	1.0222	0.0919	0
2476	852.5	0.2636	0.2282	1.0218	0.0917	0
2477	852.4	0.2634	0.2281	1.022	0.0916	0
2478	852.3	0.2635	0.2281	1.0219	0.0918	0
2479	852.2	0.2635	0.2281	1.022	0.0917	0
2480	852.1	0.2635	0.2282	1.0223	0.0917	0

2481	852	0.2637	0.2281	1.0217	0.0918	0
2482	851.9	0.2636	0.2283	1.0221	0.0918	0
2483	851.8	0.2635	0.2282	1.022	0.0917	0
2484	851.7	0.2635	0.2283	1.022	0.0918	0
2485	851.6	0.2636	0.2282	1.0219	0.0917	0
2486	851.5	0.2634	0.2281	1.0224	0.0918	0
2487	851.4	0.2634	0.2283	1.022	0.0919	0
2488	851.3	0.2637	0.2283	1.0229	0.092	0
2489	851.2	0.2636	0.2282	1.0217	0.0917	0
2490	851.1	0.2635	0.2283	1.0218	0.0918	0
2491	851	0.2637	0.2284	1.0218	0.092	0
2492	850.9	0.2636	0.2284	1.0219	0.0919	0
2493	850.8	0.2637	0.2285	1.022	0.0919	0
2494	850.7	0.2633	0.2282	1.0217	0.0918	0
2495	850.6	0.2637	0.2284	1.0215	0.0919	0
2496	850.5	0.2638	0.2285	1.0218	0.0921	0
2497	850.4	0.2638	0.2286	1.0219	0.092	0
2498	850.3	0.2637	0.2286	1.0222	0.092	0
2499	850.2	0.2637	0.2284	1.0229	0.092	0
2500	850.1	0.2636	0.2284	1.0228	0.0919	0
2501	850	0.2636	0.2283	1.0223	0.0918	0
2502	849.9	0.2583	0.2294	1.0388	0.098	0
2503	849.8	0.2584	0.2293	1.0391	0.0981	0
2504	849.7	0.2584	0.2292	1.0395	0.0981	0
2505	849.6	0.2584	0.2295	1.0392	0.098	0
2506	849.5	0.2583	0.2294	1.0397	0.0979	0
2507	849.4	0.2584	0.2293	1.0393	0.0979	0
2508	849.3	0.2585	0.2294	1.0399	0.0981	0
2509	849.2	0.2583	0.2293	1.0393	0.098	0
2510	849.1	0.2584	0.2293	1.0397	0.098	0
2511	849	0.2585	0.2293	1.0402	0.0981	0
2512	848.9	0.2584	0.2294	1.0399	0.0981	0
2513	848.8	0.2583	0.2291	1.0399	0.0979	0
2514	848.7	0.2584	0.2292	1.0404	0.098	0
2515	848.6	0.2584	0.2294	1.0403	0.0981	0
2516	848.5	0.2583	0.2294	1.0398	0.098	0
2517	848.4	0.2584	0.2294	1.0401	0.098	0
2518	848.3	0.2581	0.2292	1.0397	0.0978	0
2519	848.2	0.2583	0.2291	1.0397	0.0979	0
2520	848.1	0.2584	0.2293	1.04	0.0979	0
2521	848	0.2585	0.2295	1.0395	0.0981	0
2522	847.9	0.2586	0.2294	1.0398	0.0982	0
2523	847.8	0.2586	0.2293	1.0407	0.0981	0

2524	847.7	0.2583	0.2292	1.0407	0.0979	0
2525	847.6	0.2582	0.2292	1.0394	0.0978	0
2526	847.5	0.2584	0.2293	1.0399	0.0979	0
2527	847.4	0.2584	0.2293	1.0398	0.098	0
2528	847.3	0.2585	0.2295	1.0399	0.098	0
2529	847.2	0.2584	0.2294	1.0402	0.0979	0
2530	847.1	0.2585	0.2292	1.0399	0.098	0
2531	847	0.2586	0.2293	1.04	0.098	0
2532	846.9	0.2586	0.2294	1.0397	0.098	0
2533	846.8	0.2586	0.2296	1.0397	0.0981	0
2534	846.7	0.2587	0.2296	1.0397	0.0982	0
2535	846.6	0.2586	0.2298	1.0403	0.0982	0
2536	846.5	0.2586	0.2296	1.0402	0.0982	0
2537	846.4	0.2586	0.2294	1.0403	0.0982	0
2538	846.3	0.2587	0.2296	1.0403	0.0984	0
2539	846.2	0.2587	0.2295	1.0402	0.0983	0
2540	846.1	0.2586	0.2295	1.0402	0.0984	0
2541	846	0.2586	0.2296	1.0401	0.0983	0
2542	845.9	0.2586	0.2297	1.0401	0.0983	0
2543	845.8	0.2587	0.2298	1.0403	0.0983	0
2544	845.7	0.2589	0.2298	1.0401	0.0984	0
2545	845.6	0.259	0.2298	1.04	0.0985	0
2546	845.5	0.259	0.2298	1.04	0.0985	0
2547	845.4	0.259	0.2297	1.0408	0.0985	0
2548	845.3	0.259	0.23	1.0398	0.0986	0
2549	845.2	0.2592	0.2301	1.0404	0.0988	0
2550	845.1	0.259	0.2299	1.04	0.0987	0
2551	845	0.2589	0.2299	1.0398	0.0987	0
2552	844.9	0.2589	0.2299	1.04	0.0988	0
2553	844.8	0.2591	0.23	1.0399	0.0989	0
2554	844.7	0.259	0.2298	1.0398	0.0988	0
2555	844.6	0.2593	0.2301	1.04	0.099	0
2556	844.5	0.2592	0.2303	1.0397	0.0991	0
2557	844.4	0.2593	0.2305	1.0399	0.0993	0
2558	844.3	0.2594	0.2305	1.0398	0.0993	0
2559	844.2	0.2595	0.2305	1.0403	0.0993	0
2560	844.1	0.2598	0.2307	1.0396	0.0996	0
2561	844	0.2596	0.2307	1.0398	0.0996	0
2562	843.9	0.2597	0.2308	1.0401	0.0996	0
2563	843.8	0.2595	0.2307	1.0402	0.0995	0
2564	843.7	0.2597	0.2309	1.04	0.0997	0
2565	843.6	0.2596	0.2308	1.0397	0.0997	0
2566	843.5	0.2596	0.2307	1.0398	0.0999	0

2567	843.4	0.2598	0.2309	1.0397	0.0998	0
2568	843.3	0.2599	0.2311	1.0397	0.1	0
2569	843.2	0.2599	0.2312	1.0398	0.1001	0
2570	843.1	0.2598	0.2312	1.0397	0.1001	0
2571	843	0.2601	0.2314	1.0394	0.1002	0
2572	842.9	0.26	0.2313	1.0396	0.1003	0
2573	842.8	0.26	0.2312	1.0394	0.1002	0
2574	842.7	0.2601	0.2315	1.0393	0.1004	0
2575	842.6	0.2605	0.2316	1.0395	0.1005	0
2576	842.5	0.2601	0.2312	1.0397	0.1003	0
2577	842.4	0.2601	0.2314	1.0397	0.1004	0
2578	842.3	0.2601	0.2314	1.0393	0.1002	0
2579	842.2	0.26	0.2312	1.0395	0.1001	0
2580	842.1	0.2602	0.2314	1.0399	0.1003	0
2581	842	0.2601	0.2314	1.0402	0.1003	0
2582	841.9	0.2601	0.2313	1.0398	0.1004	0
2583	841.8	0.2602	0.2314	1.0395	0.1004	0
2584	841.7	0.2603	0.2316	1.0395	0.1004	0
2585	841.6	0.2603	0.2315	1.0397	0.1005	0
2586	841.5	0.2602	0.2316	1.04	0.1005	0
2587	841.4	0.2602	0.2314	1.04	0.1005	0
2588	841.3	0.2602	0.2315	1.0396	0.1005	0
2589	841.2	0.2603	0.2316	1.04	0.1005	0
2590	841.1	0.2603	0.2317	1.04	0.1006	0
2591	841	0.2603	0.2317	1.0395	0.1006	0
2592	840.9	0.2603	0.2316	1.0393	0.1005	0
2593	840.8	0.2603	0.2317	1.0403	0.1005	0
2594	840.7	0.2604	0.2318	1.04	0.1007	0
2595	840.6	0.2606	0.2317	1.0394	0.1007	0
2596	840.5	0.2603	0.2318	1.0395	0.1006	0
2597	840.4	0.2602	0.2316	1.0391	0.1005	0
2598	840.3	0.2605	0.2315	1.0397	0.1007	0
2599	840.2	0.2605	0.2318	1.0395	0.1008	0
2600	840.1	0.2606	0.2321	1.0401	0.101	0
2601	840	0.2606	0.2321	1.0401	0.1009	0
2602	839.9	0.2605	0.2318	1.0395	0.1008	0
2603	839.8	0.2605	0.2319	1.0393	0.1007	0
2604	839.7	0.2605	0.232	1.0401	0.1007	0
2605	839.6	0.2607	0.232	1.0394	0.1008	0
2606	839.5	0.2604	0.232	1.0395	0.1007	0
2607	839.4	0.2606	0.2319	1.0399	0.1008	0
2608	839.3	0.2607	0.2318	1.04	0.1009	0
2609	839.2	0.2609	0.2321	1.0397	0.1008	0

2610	839.1	0.2606	0.2319	1.0403	0.1008	0
2611	839	0.2608	0.2319	1.0406	0.1007	0
2612	838.9	0.2606	0.2321	1.0405	0.1007	0
2613	838.8	0.2607	0.2319	1.0402	0.1007	0
2614	838.7	0.2607	0.2319	1.0401	0.1008	0
2615	838.6	0.2608	0.2319	1.04	0.1006	0
2616	838.5	0.2607	0.2319	1.0399	0.1007	0
2617	838.4	0.2608	0.232	1.0405	0.1008	0
2618	838.3	0.2607	0.2321	1.0402	0.1008	0
2619	838.2	0.2605	0.2318	1.0401	0.1006	0
2620	838.1	0.2606	0.2318	1.0404	0.1006	0
2621	838	0.2604	0.2317	1.0401	0.1005	0
2622	837.9	0.2604	0.2316	1.0408	0.1003	0
2623	837.8	0.2606	0.2316	1.0403	0.1005	0
2624	837.7	0.2604	0.2318	1.0403	0.1004	0
2625	837.6	0.2606	0.2318	1.0404	0.1005	0
2626	837.5	0.2606	0.2318	1.0401	0.1005	0
2627	837.4	0.2608	0.2318	1.04	0.1007	0
2628	837.3	0.2609	0.2319	1.0403	0.1008	0
2629	837.2	0.261	0.232	1.0405	0.1008	0
2630	837.1	0.2608	0.2318	1.0401	0.1006	0
2631	837	0.2606	0.2318	1.0404	0.1005	0
2632	836.9	0.2604	0.2317	1.0409	0.1004	0
2633	836.8	0.2603	0.2318	1.0407	0.1005	0
2634	836.7	0.2605	0.2317	1.0409	0.1005	0
2635	836.6	0.2605	0.2319	1.0405	0.1008	0
2636	836.5	0.2608	0.232	1.0412	0.1008	0
2637	836.4	0.2609	0.2321	1.0408	0.1008	0
2638	836.3	0.2609	0.2321	1.0402	0.1008	0
2639	836.2	0.2609	0.232	1.0405	0.101	0
2640	836.1	0.2608	0.2319	1.0406	0.1008	0
2641	836	0.2609	0.232	1.0412	0.1008	0
2642	835.9	0.2606	0.2318	1.0404	0.1007	0
2643	835.8	0.2608	0.2318	1.0402	0.1008	0
2644	835.7	0.2608	0.232	1.0402	0.1008	0
2645	835.6	0.2609	0.2322	1.0402	0.1009	0
2646	835.5	0.2609	0.2322	1.0403	0.1009	0
2647	835.4	0.2613	0.2324	1.0405	0.1011	0
2648	835.3	0.2608	0.2321	1.04	0.1009	0
2649	835.2	0.2609	0.2321	1.0403	0.1009	0
2650	835.1	0.2607	0.2321	1.0399	0.101	0
2651	835	0.261	0.2322	1.0403	0.1011	0
2652	834.9	0.2609	0.2323	1.0402	0.1012	0

2653	834.8	0.261	0.2324	1.0408	0.1011	0
2654	834.7	0.2611	0.2323	1.0401	0.1011	0
2655	834.6	0.261	0.2324	1.0402	0.1012	0
2656	834.5	0.2611	0.2325	1.0411	0.1013	0
2657	834.4	0.2611	0.2324	1.0403	0.1013	0
2658	834.3	0.2611	0.2325	1.0407	0.1014	0
2659	834.2	0.2611	0.2325	1.0415	0.1013	0
2660	834.1	0.2613	0.2326	1.0401	0.1016	0
2661	834	0.2615	0.2328	1.0407	0.1018	0
2662	833.9	0.2616	0.2329	1.0406	0.1018	0
2663	833.8	0.2613	0.2327	1.0404	0.1017	0
2664	833.7	0.2614	0.2327	1.0401	0.1018	0
2665	833.6	0.2616	0.2329	1.0401	0.1018	0
2666	833.5	0.2616	0.2328	1.0396	0.1017	0
2667	833.4	0.2613	0.2328	1.0401	0.1018	0
2668	833.3	0.2615	0.233	1.04	0.102	0
2669	833.2	0.2617	0.2333	1.0405	0.1022	0
2670	833.1	0.2616	0.2332	1.04	0.1021	0
2671	833	0.2618	0.2334	1.0399	0.1024	0
2672	832.9	0.2619	0.2333	1.0399	0.1023	0
2673	832.8	0.2619	0.2335	1.04	0.1024	0
2674	832.7	0.2619	0.2333	1.0394	0.1024	0
2675	832.6	0.2619	0.2334	1.0396	0.1024	0
2676	832.5	0.262	0.2334	1.0397	0.1025	0
2677	832.4	0.2621	0.2337	1.04	0.1026	0
2678	832.3	0.262	0.2337	1.0403	0.1027	0
2679	832.2	0.2622	0.2336	1.0399	0.1026	0
2680	832.1	0.2623	0.2338	1.04	0.1028	0
2681	832	0.262	0.2335	1.0395	0.1027	0
2682	831.9	0.262	0.2336	1.0397	0.1029	0
2683	831.8	0.2621	0.2338	1.0396	0.1027	0
2684	831.7	0.2622	0.2339	1.0396	0.103	0
2685	831.6	0.2622	0.2339	1.0399	0.1029	0
2686	831.5	0.2623	0.2338	1.0401	0.1029	0
2687	831.4	0.2625	0.2339	1.0399	0.1031	0
2688	831.3	0.2624	0.2341	1.0394	0.1031	0
2689	831.2	0.2624	0.2339	1.0397	0.103	0
2690	831.1	0.2624	0.2339	1.0398	0.103	0
2691	831	0.2624	0.2339	1.04	0.103	0
2692	830.9	0.2624	0.2338	1.0401	0.1029	0
2693	830.8	0.2625	0.2339	1.0402	0.1031	0
2694	830.7	0.2624	0.234	1.0404	0.1032	0
2695	830.6	0.2622	0.2339	1.0395	0.1029	0

2696	830.5	0.2623	0.234	1.0393	0.1031	0
2697	830.4	0.2623	0.2341	1.0399	0.1031	0
2698	830.3	0.2622	0.234	1.04	0.103	0
2699	830.2	0.2624	0.234	1.0397	0.1031	0
2700	830.1	0.2624	0.2339	1.0397	0.1031	0
2701	830	0.2625	0.2342	1.0399	0.1033	0
2702	829.9	0.2626	0.2342	1.0397	0.1033	0
2703	829.8	0.2626	0.2342	1.04	0.1033	0
2704	829.7	0.2625	0.2341	1.0398	0.1032	0
2705	829.6	0.2627	0.2342	1.0393	0.1033	0
2706	829.5	0.2625	0.2342	1.0396	0.1032	0
2707	829.4	0.2625	0.2343	1.0397	0.1033	0
2708	829.3	0.2625	0.2342	1.0403	0.1033	0
2709	829.2	0.2626	0.2342	1.0401	0.1033	0
2710	829.1	0.2625	0.2343	1.0403	0.1034	0
2711	829	0.2625	0.2342	1.0398	0.1032	0
2712	828.9	0.2624	0.2342	1.0401	0.1032	0
2713	828.8	0.2626	0.2343	1.0403	0.1032	0
2714	828.7	0.2625	0.2343	1.0402	0.1034	0
2715	828.6	0.2626	0.2341	1.0398	0.1033	0
2716	828.5	0.2624	0.2339	1.0406	0.1031	0
2717	828.4	0.2626	0.234	1.0401	0.1031	0
2718	828.3	0.2626	0.2342	1.0403	0.1032	0
2719	828.2	0.2623	0.234	1.0397	0.1029	0
2720	828.1	0.2625	0.2342	1.0403	0.1031	0
2721	828	0.2624	0.2339	1.0405	0.103	0
2722	827.9	0.2624	0.234	1.0404	0.103	0
2723	827.8	0.2625	0.2342	1.0406	0.1032	0
2724	827.7	0.2624	0.2341	1.0405	0.103	0
2725	827.6	0.2623	0.2339	1.0402	0.103	0
2726	827.5	0.2624	0.2339	1.0404	0.1031	0
2727	827.4	0.2626	0.2339	1.0409	0.1032	0
2728	827.3	0.2626	0.234	1.0402	0.1031	0
2729	827.2	0.2626	0.234	1.0401	0.1033	0
2730	827.1	0.2624	0.2339	1.0408	0.1031	0
2731	827	0.2623	0.2338	1.0403	0.1029	0
2732	826.9	0.2624	0.2338	1.0403	0.103	0
2733	826.8	0.2624	0.2338	1.0407	0.103	0
2734	826.7	0.2625	0.234	1.041	0.1031	0
2735	826.6	0.2627	0.2341	1.0404	0.1031	0
2736	826.5	0.2626	0.234	1.0407	0.1032	0
2737	826.4	0.2626	0.234	1.0404	0.1032	0
2738	826.3	0.2627	0.2342	1.0405	0.1033	0

2739	826.2	0.2627	0.2341	1.0404	0.1033	0
2740	826.1	0.2626	0.234	1.0406	0.1032	0
2741	826	0.2627	0.2342	1.0407	0.1033	0
2742	825.9	0.2626	0.2342	1.0408	0.1032	0
2743	825.8	0.2626	0.2341	1.0407	0.1032	0
2744	825.7	0.2626	0.2343	1.0403	0.1033	0
2745	825.6	0.2627	0.2344	1.0405	0.1035	0
2746	825.5	0.2628	0.2344	1.0405	0.1035	0
2747	825.4	0.2628	0.2344	1.0404	0.1036	0
2748	825.3	0.2627	0.2345	1.0405	0.1035	0
2749	825.2	0.2628	0.2344	1.0405	0.1034	0
2750	825.1	0.2626	0.2342	1.0409	0.1034	0
2751	825	0.2629	0.2345	1.0409	0.1035	0
2752	824.9	0.2628	0.2346	1.0407	0.1035	0
2753	824.8	0.2628	0.2346	1.0399	0.1036	0
2754	824.7	0.2628	0.2343	1.0399	0.1035	0
2755	824.6	0.263	0.2346	1.0401	0.1038	0
2756	824.5	0.2631	0.2349	1.0406	0.1039	0
2757	824.4	0.263	0.2346	1.0411	0.1038	0
2758	824.3	0.2631	0.2348	1.0405	0.1038	0
2759	824.2	0.2629	0.2347	1.0404	0.1038	0
2760	824.1	0.263	0.2348	1.0404	0.1038	0
2761	824	0.2632	0.235	1.0405	0.104	0
2762	823.9	0.2633	0.2351	1.0399	0.1041	0
2763	823.8	0.2631	0.235	1.0402	0.104	0
2764	823.7	0.2632	0.2349	1.0404	0.1041	0
2765	823.6	0.2631	0.235	1.04	0.1041	0
2766	823.5	0.2633	0.2352	1.0405	0.1044	0
2767	823.4	0.2635	0.2354	1.0406	0.1045	0
2768	823.3	0.2635	0.2354	1.0399	0.1045	0
2769	823.2	0.2634	0.2354	1.0407	0.1045	0
2770	823.1	0.2635	0.2354	1.0404	0.1045	0
2771	823	0.2635	0.2353	1.0404	0.1045	0
2772	822.9	0.2637	0.2354	1.0406	0.1045	0
2773	822.8	0.2637	0.2355	1.0401	0.1045	0
2774	822.7	0.2636	0.2355	1.0405	0.1046	0
2775	822.6	0.2637	0.2356	1.0408	0.1047	0
2776	822.5	0.2636	0.2354	1.0406	0.1047	0
2777	822.4	0.2637	0.2356	1.0403	0.1048	0
2778	822.3	0.2638	0.2357	1.0407	0.1047	0
2779	822.2	0.2637	0.2356	1.0398	0.1046	0
2780	822.1	0.2637	0.2355	1.0396	0.1047	0
2781	822	0.2639	0.2356	1.0402	0.1048	0

2782	821.9	0.264	0.2356	1.04	0.105	0
2783	821.8	0.2641	0.2358	1.0407	0.105	0
2784	821.7	0.264	0.2357	1.0407	0.105	0
2785	821.6	0.2641	0.2358	1.0404	0.105	0
2786	821.5	0.2639	0.2359	1.0397	0.1051	0
2787	821.4	0.2638	0.2359	1.0403	0.1051	0
2788	821.3	0.2639	0.2359	1.0397	0.1051	0
2789	821.2	0.2641	0.236	1.04	0.1053	0
2790	821.1	0.2642	0.2359	1.0402	0.1053	0
2791	821	0.2642	0.236	1.0403	0.1053	0
2792	820.9	0.2642	0.2357	1.0402	0.1052	0
2793	820.8	0.2641	0.236	1.0397	0.1053	0
2794	820.7	0.2642	0.2361	1.0405	0.1053	0
2795	820.6	0.2639	0.236	1.0394	0.1051	0
2796	820.5	0.264	0.2359	1.04	0.1052	0
2797	820.4	0.2639	0.2359	1.0396	0.1051	0
2798	820.3	0.264	0.2359	1.0399	0.1051	0
2799	820.2	0.264	0.236	1.0401	0.1052	0
2800	820.1	0.2641	0.2361	1.0406	0.1052	0
2801	820	0.2641	0.2361	1.0401	0.1054	0
2802	819.9	0.2641	0.2361	1.0403	0.1054	0
2803	819.8	0.2641	0.2361	1.04	0.1053	0
2804	819.7	0.2644	0.2363	1.0403	0.1055	0
2805	819.6	0.2642	0.2363	1.0407	0.1056	0
2806	819.5	0.2644	0.2364	1.0398	0.1056	0
2807	819.4	0.2642	0.2363	1.0406	0.1055	0
2808	819.3	0.2643	0.2364	1.0406	0.1055	0
2809	819.2	0.2642	0.2363	1.0397	0.1055	0
2810	819.1	0.2643	0.2362	1.04	0.1054	0
2811	819	0.2643	0.2362	1.0405	0.1054	0
2812	818.9	0.2646	0.2364	1.0401	0.1055	0
2813	818.8	0.2644	0.2362	1.0406	0.1055	0
2814	818.7	0.2645	0.2364	1.0401	0.1057	0
2815	818.6	0.2644	0.2364	1.0405	0.1055	0
2816	818.5	0.2642	0.2363	1.0408	0.1055	0
2817	818.4	0.2645	0.2365	1.0404	0.1056	0
2818	818.3	0.2645	0.2363	1.0407	0.1055	0
2819	818.2	0.2645	0.2364	1.0404	0.1055	0
2820	818.1	0.2646	0.2365	1.0399	0.1055	0
2821	818	0.2644	0.2364	1.04	0.1055	0
2822	817.9	0.2645	0.2365	1.0405	0.1056	0
2823	817.8	0.2645	0.2365	1.0407	0.1056	0
2824	817.7	0.2645	0.2364	1.0404	0.1056	0

2825	817.6	0.2645	0.2364	1.0403	0.1056	0
2826	817.5	0.2644	0.2363	1.0403	0.1055	0
2827	817.4	0.2644	0.2363	1.0404	0.1056	0
2828	817.3	0.2646	0.2367	1.0405	0.1057	0
2829	817.2	0.2645	0.2365	1.041	0.1057	0
2830	817.1	0.2646	0.2366	1.041	0.1057	0
2831	817	0.2649	0.2367	1.0407	0.1059	0
2832	816.9	0.2645	0.2365	1.0401	0.1056	0
2833	816.8	0.2645	0.2366	1.041	0.1056	0
2834	816.7	0.2645	0.2365	1.0404	0.1057	0
2835	816.6	0.2645	0.2366	1.0403	0.1057	0
2836	816.5	0.2646	0.2366	1.0406	0.1057	0
2837	816.4	0.2649	0.2367	1.0407	0.1058	0
2838	816.3	0.2648	0.2367	1.0405	0.1059	0
2839	816.2	0.2647	0.2366	1.0411	0.1059	0
2840	816.1	0.2648	0.2367	1.0413	0.1059	0
2841	816	0.2648	0.2367	1.0407	0.1059	0
2842	815.9	0.2647	0.2366	1.0406	0.1058	0
2843	815.8	0.2646	0.2366	1.0404	0.1059	0
2844	815.7	0.2648	0.2367	1.041	0.106	0
2845	815.6	0.2648	0.2368	1.0413	0.106	0
2846	815.5	0.2648	0.2369	1.041	0.1061	0
2847	815.4	0.2647	0.2367	1.0413	0.106	0
2848	815.3	0.2647	0.2368	1.0407	0.1061	0
2849	815.2	0.2648	0.2368	1.0413	0.1061	0
2850	815.1	0.2648	0.2367	1.0408	0.106	0
2851	815	0.265	0.2369	1.0411	0.1061	0
2852	814.9	0.2651	0.2369	1.0414	0.1062	0
2853	814.8	0.2649	0.2366	1.0407	0.1059	0
2854	814.7	0.2651	0.2369	1.041	0.1064	0
2855	814.6	0.2651	0.2369	1.0412	0.1062	0
2856	814.5	0.2653	0.237	1.0409	0.1063	0
2857	814.4	0.2652	0.2371	1.0409	0.1064	0
2858	814.3	0.2651	0.237	1.0411	0.1063	0
2859	814.2	0.2651	0.237	1.0411	0.1063	0
2860	814.1	0.2653	0.2374	1.0412	0.1065	0
2861	814	0.2656	0.2377	1.0414	0.1067	0
2862	813.9	0.2655	0.2374	1.0409	0.1067	0
2863	813.8	0.2653	0.2373	1.0408	0.1067	0
2864	813.7	0.2654	0.2375	1.041	0.1067	0
2865	813.6	0.2654	0.2374	1.0407	0.1068	0
2866	813.5	0.2656	0.2375	1.0407	0.1068	0
2867	813.4	0.2653	0.2373	1.0406	0.1066	0

2868	813.3	0.2654	0.2374	1.0406	0.1067	0
2869	813.2	0.2654	0.2376	1.0407	0.1068	0
2870	813.1	0.2656	0.2377	1.0408	0.1071	0
2871	813	0.2658	0.2378	1.041	0.1072	0
2872	812.9	0.2658	0.2376	1.0408	0.1072	0
2873	812.8	0.2658	0.2377	1.0408	0.1072	0
2874	812.7	0.2657	0.2378	1.0406	0.1072	0
2875	812.6	0.2656	0.2377	1.041	0.1073	0
2876	812.5	0.2656	0.2376	1.041	0.1072	0
2877	812.4	0.2658	0.2378	1.0411	0.1073	0
2878	812.3	0.2657	0.2378	1.0416	0.1072	0
2879	812.2	0.2657	0.2377	1.0411	0.1073	0
2880	812.1	0.2658	0.2378	1.0409	0.1073	0
2881	812	0.2658	0.238	1.0408	0.1073	0
2882	811.9	0.2659	0.238	1.0407	0.1073	0
2883	811.8	0.2659	0.2379	1.0405	0.1074	0
2884	811.7	0.2659	0.2378	1.0405	0.1073	0
2885	811.6	0.2659	0.2377	1.0403	0.1073	0
2886	811.5	0.2658	0.2378	1.0405	0.1073	0
2887	811.4	0.2659	0.2381	1.0408	0.1075	0
2888	811.3	0.2658	0.238	1.0404	0.1074	0
2889	811.2	0.2661	0.2382	1.0409	0.1076	0
2890	811.1	0.2659	0.2381	1.0412	0.1076	0
2891	811	0.2661	0.2382	1.0408	0.1076	0
2892	810.9	0.2662	0.2383	1.0412	0.1076	0
2893	810.8	0.2661	0.2384	1.041	0.1076	0
2894	810.7	0.266	0.2383	1.041	0.1076	0
2895	810.6	0.2661	0.2382	1.0408	0.1078	0
2896	810.5	0.2661	0.2382	1.0408	0.1078	0
2897	810.4	0.2663	0.2383	1.0409	0.1078	0
2898	810.3	0.2661	0.2382	1.0404	0.1076	0
2899	810.2	0.2663	0.2385	1.041	0.1079	0
2900	810.1	0.2664	0.2385	1.0411	0.1079	0
2901	810	0.2663	0.2384	1.0409	0.1078	0
2902	809.9	0.2661	0.2383	1.0404	0.1076	0
2903	809.8	0.2662	0.2385	1.0406	0.1077	0
2904	809.7	0.2661	0.2381	1.041	0.1077	0
2905	809.6	0.2663	0.2382	1.0409	0.1077	0
2906	809.5	0.2663	0.2383	1.0412	0.1078	0
2907	809.4	0.2664	0.2387	1.0415	0.1079	0
2908	809.3	0.2663	0.2385	1.0417	0.1078	0
2909	809.2	0.2663	0.2383	1.0413	0.1077	0
2910	809.1	0.2664	0.2383	1.0411	0.1079	0

2911	809	0.2663	0.2384	1.0412	0.1079	0
2912	808.9	0.2664	0.2384	1.041	0.1079	0
2913	808.8	0.2665	0.2385	1.041	0.108	0
2914	808.7	0.2665	0.2385	1.0411	0.1079	0
2915	808.6	0.2665	0.2385	1.0416	0.1079	0
2916	808.5	0.2663	0.2384	1.0414	0.1078	0
2917	808.4	0.2664	0.2385	1.0413	0.1077	0
2918	808.3	0.2662	0.2386	1.0413	0.1078	0
2919	808.2	0.2665	0.2387	1.042	0.1079	0
2920	808.1	0.2664	0.2387	1.0415	0.1079	0
2921	808	0.2664	0.2385	1.0416	0.1079	0
2922	807.9	0.2664	0.2386	1.0415	0.1079	0
2923	807.8	0.2666	0.2386	1.0411	0.108	0
2924	807.7	0.2665	0.2388	1.0419	0.1081	0
2925	807.6	0.2668	0.2388	1.0416	0.1083	0
2926	807.5	0.2668	0.2387	1.0421	0.1083	0
2927	807.4	0.2668	0.239	1.0421	0.1083	0
2928	807.3	0.2666	0.2388	1.0421	0.1082	0
2929	807.2	0.2667	0.2388	1.0419	0.1082	0
2930	807.1	0.2666	0.2387	1.0414	0.1081	0
2931	807	0.2666	0.2388	1.0411	0.108	0
2932	806.9	0.2666	0.2388	1.0413	0.1082	0
2933	806.8	0.2667	0.2387	1.0414	0.1082	0
2934	806.7	0.2667	0.2388	1.0418	0.1085	0
2935	806.6	0.267	0.2389	1.0419	0.1085	0
2936	806.5	0.267	0.239	1.0419	0.1084	0
2937	806.4	0.2667	0.2389	1.042	0.1083	0
2938	806.3	0.2668	0.239	1.0417	0.1084	0
2939	806.2	0.2669	0.239	1.0416	0.1086	0
2940	806.1	0.2671	0.2393	1.0416	0.1085	0
2941	806	0.2669	0.2391	1.0413	0.1085	0
2942	805.9	0.2671	0.2392	1.0414	0.1086	0
2943	805.8	0.2671	0.2393	1.0411	0.1086	0
2944	805.7	0.2672	0.2393	1.0412	0.1086	0
2945	805.6	0.2671	0.2394	1.0419	0.1087	0
2946	805.5	0.2671	0.2394	1.0408	0.1087	0
2947	805.4	0.2671	0.2394	1.0413	0.1087	0
2948	805.3	0.2672	0.2394	1.0411	0.1087	0
2949	805.2	0.2672	0.2395	1.0411	0.1088	0
2950	805.1	0.2674	0.2396	1.0414	0.109	0
2951	805	0.2673	0.2395	1.0416	0.109	0
2952	804.9	0.2673	0.2395	1.0417	0.109	0
2953	804.8	0.2672	0.2394	1.0413	0.1089	0

2954	804.7	0.2673	0.2395	1.0415	0.109	0
2955	804.6	0.2676	0.2396	1.0422	0.1092	0
2956	804.5	0.2676	0.2396	1.0423	0.1092	0
2957	804.4	0.2675	0.2395	1.0415	0.1092	0
2958	804.3	0.2674	0.2396	1.0417	0.1091	0
2959	804.2	0.2676	0.2398	1.0417	0.1094	0
2960	804.1	0.2676	0.24	1.0417	0.1093	0
2961	804	0.2677	0.2401	1.0418	0.1096	0
2962	803.9	0.2675	0.2399	1.0412	0.1093	0
2963	803.8	0.2676	0.2399	1.0411	0.1094	0
2964	803.7	0.2676	0.24	1.0415	0.1096	0
2965	803.6	0.2678	0.2402	1.0417	0.1098	0
2966	803.5	0.2679	0.2402	1.0417	0.1098	0
2967	803.4	0.2678	0.2404	1.0413	0.1099	0
2968	803.3	0.2677	0.2403	1.0411	0.1098	0
2969	803.2	0.2678	0.2403	1.041	0.1098	0
2970	803.1	0.2679	0.2401	1.0412	0.1097	0
2971	803	0.2677	0.2401	1.0413	0.1097	0
2972	802.9	0.2678	0.24	1.0413	0.1096	0
2973	802.8	0.2679	0.2403	1.0408	0.1098	0
2974	802.7	0.2681	0.2404	1.0413	0.1101	0
2975	802.6	0.2679	0.2404	1.0411	0.1099	0
2976	802.5	0.2679	0.2404	1.041	0.1099	0
2977	802.4	0.2678	0.2405	1.0407	0.1098	0
2978	802.3	0.2682	0.2407	1.0404	0.1101	0
2979	802.2	0.2681	0.2405	1.0414	0.1102	0
2980	802.1	0.2679	0.2403	1.041	0.11	0
2981	802	0.2681	0.2403	1.0411	0.1101	0
2982	801.9	0.2679	0.2402	1.0409	0.1099	0
2983	801.8	0.2679	0.2402	1.0407	0.11	0
2984	801.7	0.268	0.2402	1.0407	0.1099	0
2985	801.6	0.2682	0.2404	1.0414	0.1102	0
2986	801.5	0.2681	0.2405	1.0411	0.1102	0
2987	801.4	0.2681	0.2406	1.0411	0.1101	0
2988	801.3	0.2682	0.2406	1.0409	0.1101	0
2989	801.2	0.2685	0.2407	1.041	0.1102	0
2990	801.1	0.2686	0.2407	1.0413	0.1103	0
2991	801	0.2685	0.2408	1.0407	0.1103	0
2992	800.9	0.2682	0.2408	1.0418	0.1103	0
2993	800.8	0.2683	0.2408	1.0419	0.1102	0
2994	800.7	0.2685	0.2409	1.0417	0.1103	0
2995	800.6	0.2683	0.2408	1.0411	0.1102	0
2996	800.5	0.2682	0.2408	1.0418	0.1103	0

2997	800.4	0.2685	0.2409	1.042	0.1103	0
2998	800.3	0.2685	0.2408	1.0417	0.1104	0
2999	800.2	0.2684	0.241	1.0414	0.1106	0
3000	800.1	0.2684	0.2408	1.0412	0.1105	0
3001	800	0.2686	0.241	1.0413	0.1106	0
3002	799.9	0.2686	0.241	1.0418	0.1107	0
3003	799.8	0.2689	0.241	1.0417	0.1106	0
3004	799.7	0.2689	0.2412	1.0415	0.1108	0
3005	799.6	0.2687	0.2412	1.0412	0.1107	0
3006	799.5	0.2686	0.2409	1.0411	0.1107	0
3007	799.4	0.2688	0.241	1.0418	0.1107	0
3008	799.3	0.2687	0.2411	1.0421	0.1107	0
3009	799.2	0.2687	0.241	1.0421	0.1107	0
3010	799.1	0.2686	0.241	1.0423	0.1106	0
3011	799	0.2687	0.241	1.0422	0.1105	0
3012	798.9	0.2686	0.2409	1.0423	0.1107	0
3013	798.8	0.2686	0.241	1.0425	0.1107	0
3014	798.7	0.2686	0.2409	1.0426	0.1105	0
3015	798.6	0.2687	0.241	1.0425	0.1107	0
3016	798.5	0.2686	0.2409	1.0427	0.1105	0
3017	798.4	0.2687	0.2409	1.0423	0.1106	0
3018	798.3	0.2684	0.2406	1.0426	0.1103	0
3019	798.2	0.2685	0.2407	1.0425	0.1105	0
3020	798.1	0.2685	0.2408	1.0423	0.1105	0
3021	798	0.2688	0.241	1.0423	0.1107	0
3022	797.9	0.2688	0.2408	1.0421	0.1106	0
3023	797.8	0.2687	0.2409	1.0417	0.1106	0
3024	797.7	0.2688	0.241	1.0422	0.1107	0
3025	797.6	0.2688	0.2412	1.0426	0.1107	0
3026	797.5	0.2688	0.2412	1.0426	0.1107	0
3027	797.4	0.2689	0.241	1.0426	0.1106	0
3028	797.3	0.2689	0.2411	1.0422	0.1106	0
3029	797.2	0.2688	0.2411	1.0425	0.1107	0
3030	797.1	0.269	0.2413	1.043	0.1108	0
3031	797	0.269	0.2412	1.043	0.1108	0
3032	796.9	0.2688	0.2411	1.0426	0.1106	0
3033	796.8	0.269	0.2411	1.0436	0.1107	0
3034	796.7	0.269	0.2412	1.0426	0.1107	0
3035	796.6	0.2691	0.2414	1.0427	0.1109	0
3036	796.5	0.2692	0.2414	1.043	0.1109	0
3037	796.4	0.2691	0.2413	1.0427	0.111	0
3038	796.3	0.2692	0.2414	1.0426	0.111	0
3039	796.2	0.2692	0.2415	1.0425	0.1111	0

3040	796.1	0.2691	0.2415	1.0426	0.111	0
3041	796	0.2692	0.2414	1.0422	0.1109	0
3042	795.9	0.2693	0.2416	1.0428	0.111	0
3043	795.8	0.2692	0.2416	1.0428	0.1111	0
3044	795.7	0.2691	0.2414	1.042	0.1111	0
3045	795.6	0.2693	0.2416	1.0426	0.1114	0
3046	795.5	0.2693	0.2418	1.0425	0.1114	0
3047	795.4	0.2696	0.2419	1.0425	0.1114	0
3048	795.3	0.2694	0.2417	1.0424	0.1114	0
3049	795.2	0.2696	0.2419	1.0433	0.1116	0
3050	795.1	0.2697	0.242	1.0426	0.1117	0
3051	795	0.2697	0.2419	1.043	0.1117	0
3052	794.9	0.2696	0.2419	1.0423	0.1116	0
3053	794.8	0.2696	0.2421	1.0423	0.1117	0
3054	794.7	0.2696	0.2422	1.042	0.1118	0
3055	794.6	0.2696	0.2421	1.0424	0.1119	0
3056	794.5	0.2697	0.2423	1.0427	0.112	0
3057	794.4	0.2696	0.2423	1.0428	0.1121	0
3058	794.3	0.2698	0.2423	1.0427	0.112	0
3059	794.2	0.27	0.2424	1.0423	0.1123	0
3060	794.1	0.2699	0.2425	1.0422	0.1122	0
3061	794	0.27	0.2427	1.0426	0.1124	0
3062	793.9	0.2701	0.2428	1.0423	0.1126	0
3063	793.8	0.2701	0.2429	1.0421	0.1125	0
3064	793.7	0.27	0.2428	1.0419	0.1125	0
3065	793.6	0.27	0.2428	1.0422	0.1124	0
3066	793.5	0.2702	0.243	1.0423	0.1126	0
3067	793.4	0.2702	0.2429	1.0418	0.1126	0
3068	793.3	0.2703	0.243	1.0427	0.1128	0
3069	793.2	0.2701	0.2427	1.0422	0.1125	0
3070	793.1	0.2704	0.2428	1.0428	0.1128	0
3071	793	0.2705	0.2432	1.0427	0.1128	0
3072	792.9	0.2704	0.2431	1.0429	0.1127	0
3073	792.8	0.2706	0.2432	1.0419	0.1128	0
3074	792.7	0.2707	0.2431	1.043	0.1131	0
3075	792.6	0.2707	0.2435	1.0427	0.1132	0
3076	792.5	0.2707	0.2432	1.0427	0.1131	0
3077	792.4	0.2706	0.2432	1.0429	0.113	0
3078	792.3	0.2705	0.2434	1.042	0.1132	0
3079	792.2	0.2707	0.2434	1.0419	0.1133	0
3080	792.1	0.2707	0.2434	1.0424	0.1132	0
3081	792	0.2706	0.2433	1.0412	0.1131	0
3082	791.9	0.2706	0.2433	1.0419	0.1131	0

3083	791.8	0.2706	0.2434	1.042	0.1133	0
3084	791.7	0.2708	0.2434	1.0421	0.1133	0
3085	791.6	0.271	0.2435	1.0422	0.1134	0
3086	791.5	0.2708	0.2435	1.042	0.1133	0
3087	791.4	0.2709	0.2435	1.0424	0.1134	0
3088	791.3	0.2707	0.2434	1.042	0.1134	0
3089	791.2	0.2708	0.2436	1.0422	0.1134	0
3090	791.1	0.2708	0.2437	1.0426	0.1136	0
3091	791	0.2708	0.2436	1.0423	0.1134	0
3092	790.9	0.2708	0.2434	1.0422	0.1132	0
3093	790.8	0.2708	0.2434	1.0425	0.1133	0
3094	790.7	0.2709	0.2435	1.0425	0.1134	0
3095	790.6	0.271	0.2438	1.0424	0.1136	0
3096	790.5	0.271	0.2438	1.0426	0.1136	0
3097	790.4	0.2711	0.2437	1.0428	0.1134	0
3098	790.3	0.2712	0.2439	1.0427	0.1136	0
3099	790.2	0.2712	0.2437	1.0428	0.1134	0
3100	790.1	0.271	0.2438	1.0429	0.1135	0
3101	790	0.2712	0.2439	1.0433	0.1136	0
3102	789.9	0.2712	0.2438	1.0428	0.1135	0
3103	789.8	0.271	0.2437	1.0425	0.1135	0
3104	789.7	0.2709	0.2436	1.0426	0.1134	0
3105	789.6	0.2709	0.2436	1.0427	0.1133	0
3106	789.5	0.2712	0.2437	1.0427	0.1135	0
3107	789.4	0.2713	0.2437	1.0432	0.1136	0
3108	789.3	0.2712	0.2437	1.0432	0.1135	0
3109	789.2	0.2711	0.2436	1.0427	0.1135	0
3110	789.1	0.2711	0.2436	1.043	0.1133	0
3111	789	0.271	0.2436	1.0426	0.1133	0
3112	788.9	0.2713	0.2438	1.0429	0.1135	0
3113	788.8	0.2713	0.2438	1.0433	0.1137	0
3114	788.7	0.2712	0.2438	1.0434	0.1136	0
3115	788.6	0.2709	0.2436	1.043	0.1132	0
3116	788.5	0.2711	0.2436	1.0433	0.1134	0
3117	788.4	0.2711	0.2437	1.0433	0.1135	0
3118	788.3	0.271	0.2436	1.0434	0.1134	0
3119	788.2	0.271	0.2437	1.0435	0.1134	0
3120	788.1	0.2712	0.244	1.0432	0.1135	0
3121	788	0.2712	0.2438	1.0438	0.1135	0
3122	787.9	0.2713	0.2439	1.0437	0.1135	0
3123	787.8	0.271	0.2437	1.0433	0.1133	0
3124	787.7	0.2709	0.2436	1.0438	0.1134	0
3125	787.6	0.2711	0.2436	1.0433	0.1133	0

3126	787.5	0.2711	0.2437	1.0435	0.1133	0
3127	787.4	0.2714	0.2439	1.0439	0.1136	0
3128	787.3	0.2713	0.2439	1.0441	0.1136	0
3129	787.2	0.2713	0.244	1.044	0.1137	0
3130	787.1	0.2713	0.2438	1.0434	0.1135	0
3131	787	0.2714	0.2439	1.0439	0.1136	0
3132	786.9	0.2716	0.244	1.0438	0.1137	0
3133	786.8	0.2714	0.2441	1.0443	0.1137	0
3134	786.7	0.2714	0.2438	1.0439	0.1136	0
3135	786.6	0.2713	0.2438	1.0438	0.1137	0
3136	786.5	0.2714	0.2438	1.0437	0.1137	0
3137	786.4	0.2713	0.2439	1.0437	0.1135	0
3138	786.3	0.2714	0.2439	1.0438	0.1137	0
3139	786.2	0.2712	0.2437	1.0439	0.1136	0
3140	786.1	0.2714	0.2438	1.044	0.1137	0
3141	786	0.2715	0.2441	1.0442	0.1138	0
3142	785.9	0.2715	0.2441	1.0437	0.1138	0
3143	785.8	0.2713	0.244	1.0439	0.1137	0
3144	785.7	0.2716	0.2442	1.0442	0.1139	0
3145	785.6	0.2716	0.2442	1.0439	0.1139	0
3146	785.5	0.2716	0.2441	1.0445	0.1139	0
3147	785.4	0.2719	0.2443	1.0445	0.1142	0
3148	785.3	0.2718	0.2443	1.0443	0.1141	0
3149	785.2	0.2719	0.2444	1.0445	0.1142	0
3150	785.1	0.272	0.2444	1.0439	0.1143	0
3151	785	0.272	0.2445	1.0438	0.1144	0
3152	784.9	0.2721	0.2447	1.044	0.1144	0
3153	784.8	0.2721	0.2448	1.0443	0.1146	0
3154	784.7	0.2721	0.2447	1.0446	0.1145	0
3155	784.6	0.2723	0.2448	1.0445	0.1147	0
3156	784.5	0.2723	0.2449	1.0447	0.1147	0
3157	784.4	0.2723	0.2449	1.0441	0.1148	0
3158	784.3	0.2722	0.245	1.0443	0.1149	0
3159	784.2	0.2724	0.245	1.044	0.115	0
3160	784.1	0.2724	0.2451	1.0431	0.1151	0
3161	784	0.2724	0.245	1.0432	0.1151	0
3162	783.9	0.2724	0.2451	1.0432	0.1151	0
3163	783.8	0.2724	0.2452	1.0433	0.1152	0
3164	783.7	0.2725	0.2453	1.0432	0.1153	0
3165	783.6	0.2726	0.2454	1.0435	0.1153	0
3166	783.5	0.2727	0.2454	1.0438	0.1154	0
3167	783.4	0.2727	0.2456	1.0439	0.1155	0
3168	783.3	0.2726	0.2456	1.0432	0.1153	0

3169	783.2	0.2729	0.2456	1.0431	0.1156	0
3170	783.1	0.2728	0.2456	1.043	0.1156	0
3171	783	0.273	0.2458	1.0436	0.1157	0
3172	782.9	0.2731	0.2457	1.0436	0.1158	0
3173	782.8	0.2732	0.2458	1.0435	0.1158	0
3174	782.7	0.2729	0.2457	1.0435	0.1158	0
3175	782.6	0.273	0.2457	1.0431	0.1159	0
3176	782.5	0.2731	0.2459	1.0433	0.116	0
3177	782.4	0.2733	0.246	1.0427	0.1161	0
3178	782.3	0.2732	0.2459	1.0435	0.1161	0
3179	782.2	0.2732	0.2461	1.0432	0.1162	0
3180	782.1	0.2733	0.246	1.0432	0.1162	0
3181	782	0.2733	0.246	1.0437	0.1162	0
3182	781.9	0.2734	0.2463	1.044	0.1164	0
3183	781.8	0.2735	0.2463	1.0432	0.1164	0
3184	781.7	0.2734	0.2463	1.0431	0.1164	0
3185	781.6	0.2733	0.2462	1.0437	0.1163	0
3186	781.5	0.2735	0.2462	1.0441	0.1164	0
3187	781.4	0.2733	0.2463	1.0438	0.1166	0
3188	781.3	0.2737	0.2465	1.0438	0.1166	0
3189	781.2	0.2733	0.2463	1.0437	0.1164	0
3190	781.1	0.2733	0.2463	1.0436	0.1165	0
3191	781	0.2733	0.2461	1.0433	0.1163	0
3192	780.9	0.2736	0.2465	1.0433	0.1166	0
3193	780.8	0.2736	0.2464	1.043	0.1166	0
3194	780.7	0.2736	0.2465	1.0437	0.1166	0
3195	780.6	0.274	0.2466	1.0434	0.1166	0
3196	780.5	0.2738	0.2468	1.0438	0.1167	0
3197	780.4	0.2738	0.2467	1.0437	0.1167	0
3198	780.3	0.2735	0.2467	1.0435	0.1166	0
3199	780.2	0.2737	0.2466	1.0437	0.1167	0
3200	780.1	0.2738	0.2466	1.0434	0.1167	0
3201	780	0.2737	0.2468	1.0434	0.1167	0
3202	779.9	0.2736	0.2467	1.0433	0.1165	0
3203	779.8	0.2738	0.2465	1.0437	0.1166	0
3204	779.7	0.2737	0.2464	1.0433	0.1167	0
3205	779.6	0.2735	0.2465	1.0434	0.1166	0
3206	779.5	0.2738	0.2466	1.0442	0.1167	0
3207	779.4	0.2738	0.2466	1.0445	0.1167	0
3208	779.3	0.2738	0.2466	1.0438	0.1168	0
3209	779.2	0.2739	0.2466	1.0445	0.1168	0
3210	779.1	0.2738	0.2466	1.0441	0.1168	0
3211	779	0.2738	0.2466	1.0443	0.1166	0

3212	778.9	0.274	0.2469	1.0443	0.1169	0
3213	778.8	0.2741	0.247	1.0447	0.1169	0
3214	778.7	0.2739	0.2467	1.0443	0.1167	0
3215	778.6	0.2739	0.2465	1.0444	0.1165	0
3216	778.5	0.2738	0.2465	1.0446	0.1166	0
3217	778.4	0.2737	0.2465	1.044	0.1165	0
3218	778.3	0.2736	0.2462	1.044	0.1163	0
3219	778.2	0.2738	0.2464	1.0445	0.1164	0
3220	778.1	0.2739	0.2464	1.0443	0.1165	0
3221	778	0.2737	0.2464	1.0442	0.1165	0
3222	777.9	0.2737	0.2464	1.0444	0.1166	0
3223	777.8	0.2737	0.2464	1.0442	0.1165	0
3224	777.7	0.2737	0.2464	1.0441	0.1164	0
3225	777.6	0.2738	0.2465	1.0442	0.1166	0
3226	777.5	0.274	0.2466	1.045	0.1166	0
3227	777.4	0.2739	0.2465	1.0455	0.1165	0
3228	777.3	0.2738	0.2465	1.0448	0.1166	0
3229	777.2	0.2739	0.2467	1.0449	0.1166	0
3230	777.1	0.274	0.2468	1.0448	0.1167	0
3231	777	0.274	0.2467	1.0445	0.1166	0
3232	776.9	0.2739	0.2466	1.0446	0.1165	0
3233	776.8	0.274	0.2467	1.0449	0.1167	0
3234	776.7	0.2739	0.2467	1.045	0.1166	0
3235	776.6	0.2739	0.2467	1.0448	0.1166	0
3236	776.5	0.2739	0.2467	1.0448	0.1166	0
3237	776.4	0.2739	0.2467	1.0451	0.1166	0
3238	776.3	0.2739	0.2467	1.0449	0.1167	0
3239	776.2	0.274	0.2466	1.0453	0.1166	0
3240	776.1	0.2739	0.2465	1.045	0.1165	0
3241	776	0.2739	0.2467	1.0449	0.1166	0
3242	775.9	0.2739	0.2469	1.0439	0.1166	0
3243	775.8	0.2739	0.2466	1.045	0.1165	0
3244	775.7	0.2739	0.2467	1.0449	0.1166	0
3245	775.6	0.2739	0.2467	1.045	0.1166	0
3246	775.5	0.2742	0.2468	1.0453	0.117	0
3247	775.4	0.2743	0.247	1.0456	0.1171	0
3248	775.3	0.2744	0.2471	1.0457	0.117	0
3249	775.2	0.2742	0.2471	1.0451	0.1171	0
3250	775.1	0.2743	0.2471	1.0457	0.1173	0
3251	775	0.2745	0.2473	1.0457	0.1172	0
3252	774.9	0.2747	0.2475	1.0451	0.1174	0
3253	774.8	0.2745	0.2475	1.0454	0.1174	0
3254	774.7	0.2746	0.2475	1.0455	0.1174	0

3255	774.6	0.2748	0.2476	1.0449	0.1176	0
3256	774.5	0.2746	0.2475	1.0452	0.1175	0
3257	774.4	0.2749	0.2475	1.0452	0.1176	0
3258	774.3	0.2747	0.2474	1.0447	0.1175	0
3259	774.2	0.2747	0.2474	1.0445	0.1175	0
3260	774.1	0.2749	0.2476	1.0449	0.1176	0
3261	774	0.2748	0.2476	1.0444	0.1177	0
3262	773.9	0.2752	0.2478	1.0446	0.118	0
3263	773.8	0.275	0.2478	1.045	0.1178	0
3264	773.7	0.2753	0.2481	1.0452	0.1181	0
3265	773.6	0.2754	0.2483	1.0456	0.1183	0
3266	773.5	0.2752	0.248	1.0455	0.1182	0
3267	773.4	0.2751	0.2481	1.0454	0.1182	0
3268	773.3	0.2753	0.2482	1.045	0.1183	0
3269	773.2	0.2754	0.2482	1.0445	0.1183	0
3270	773.1	0.2756	0.2484	1.0451	0.1186	0
3271	773	0.2754	0.2484	1.0451	0.1186	0
3272	772.9	0.2755	0.2485	1.0449	0.1188	0
3273	772.8	0.2756	0.2486	1.045	0.1188	0
3274	772.7	0.2757	0.2485	1.0451	0.1187	0
3275	772.6	0.2756	0.2486	1.0449	0.1187	0
3276	772.5	0.2754	0.2485	1.0449	0.1187	0
3277	772.4	0.2756	0.2486	1.045	0.1187	0
3278	772.3	0.2758	0.2488	1.0447	0.119	0
3279	772.2	0.2758	0.2488	1.0447	0.119	0
3280	772.1	0.2757	0.249	1.045	0.119	0
3281	772	0.2758	0.2488	1.0446	0.1189	0
3282	771.9	0.2759	0.249	1.0446	0.1191	0
3283	771.8	0.2761	0.2491	1.0453	0.1193	0
3284	771.7	0.276	0.2493	1.045	0.1193	0
3285	771.6	0.2761	0.249	1.0454	0.1193	0
3286	771.5	0.2761	0.2491	1.0451	0.1194	0
3287	771.4	0.2761	0.2491	1.0451	0.1194	0
3288	771.3	0.2761	0.2493	1.045	0.1195	0
3289	771.2	0.2763	0.2494	1.0449	0.1196	0
3290	771.1	0.2762	0.2493	1.0452	0.1196	0
3291	771	0.2763	0.2493	1.045	0.1197	0
3292	770.9	0.2761	0.2493	1.0443	0.1195	0
3293	770.8	0.2761	0.2491	1.0444	0.1193	0
3294	770.7	0.2762	0.2494	1.0447	0.1196	0
3295	770.6	0.2764	0.2495	1.0448	0.1197	0
3296	770.5	0.2764	0.2494	1.0444	0.1197	0
3297	770.4	0.2764	0.2495	1.0448	0.1198	0

3298	770.3	0.2764	0.2495	1.0448	0.1197	0
3299	770.2	0.2764	0.2496	1.0446	0.1198	0
3300	770.1	0.2765	0.2495	1.0445	0.1198	0
3301	770	0.2763	0.2496	1.0452	0.1197	0
3302	769.9	0.2765	0.2496	1.0446	0.1199	0
3303	769.8	0.2763	0.2495	1.0447	0.1197	0
3304	769.7	0.2767	0.2498	1.0446	0.12	0
3305	769.6	0.2766	0.2497	1.045	0.1199	0
3306	769.5	0.2766	0.2497	1.0449	0.12	0
3307	769.4	0.2766	0.2496	1.0449	0.1199	0
3308	769.3	0.2765	0.2497	1.0445	0.12	0
3309	769.2	0.2767	0.2497	1.0448	0.1199	0
3310	769.1	0.2767	0.2496	1.045	0.1199	0
3311	769	0.2767	0.2496	1.0447	0.1199	0
3312	768.9	0.2764	0.2494	1.0452	0.1197	0
3313	768.8	0.2767	0.2497	1.0456	0.12	0
3314	768.7	0.2766	0.2498	1.0451	0.12	0
3315	768.6	0.2766	0.2498	1.0454	0.1198	0
3316	768.5	0.2767	0.2498	1.0455	0.1199	0
3317	768.4	0.2766	0.2497	1.0454	0.1198	0
3318	768.3	0.2766	0.2497	1.0452	0.1198	0
3319	768.2	0.2766	0.2496	1.0452	0.1198	0
3320	768.1	0.2767	0.2496	1.0458	0.1199	0
3321	768	0.2767	0.2496	1.0452	0.1199	0
3322	767.9	0.2765	0.2495	1.0455	0.1197	0
3323	767.8	0.2767	0.2496	1.0462	0.1197	0
3324	767.7	0.2769	0.2498	1.0464	0.1201	0
3325	767.6	0.2768	0.2496	1.0459	0.1199	0
3326	767.5	0.2769	0.2496	1.0461	0.12	0
3327	767.4	0.2769	0.2499	1.046	0.1201	0
3328	767.3	0.2769	0.2499	1.0462	0.1199	0
3329	767.2	0.2768	0.2498	1.0458	0.1199	0
3330	767.1	0.2767	0.2499	1.0459	0.1199	0
3331	767	0.2768	0.2499	1.0464	0.12	0
3332	766.9	0.2769	0.2499	1.0463	0.1201	0
3333	766.8	0.2768	0.2496	1.0454	0.12	0
3334	766.7	0.2767	0.2497	1.0457	0.12	0
3335	766.6	0.2767	0.2497	1.0462	0.12	0
3336	766.5	0.2766	0.2497	1.0464	0.1198	0
3337	766.4	0.2767	0.2496	1.046	0.1199	0
3338	766.3	0.2768	0.2497	1.0464	0.1199	0
3339	766.2	0.2769	0.2499	1.0462	0.12	0
3340	766.1	0.2768	0.2499	1.0469	0.12	0

3341	766	0.2769	0.2499	1.0459	0.1201	0
3342	765.9	0.2771	0.2501	1.0466	0.1203	0
3343	765.8	0.2772	0.2501	1.0463	0.1204	0
3344	765.7	0.277	0.2501	1.0466	0.1202	0
3345	765.6	0.2769	0.25	1.0465	0.1201	0
3346	765.5	0.2769	0.2498	1.0464	0.1201	0
3347	765.4	0.277	0.2501	1.0461	0.1203	0
3348	765.3	0.2772	0.2501	1.0463	0.1203	0
3349	765.2	0.2773	0.2502	1.0465	0.1203	0
3350	765.1	0.2773	0.2502	1.0462	0.1202	0
3351	765	0.2775	0.2503	1.0458	0.1203	0
3352	764.9	0.2773	0.2502	1.0461	0.1204	0
3353	764.8	0.2772	0.2502	1.047	0.1204	0
3354	764.7	0.2773	0.2502	1.046	0.1204	0
3355	764.6	0.2771	0.2503	1.0461	0.1204	0
3356	764.5	0.2774	0.2502	1.0465	0.1205	0
3357	764.4	0.2775	0.2503	1.0468	0.1207	0
3358	764.3	0.2774	0.2504	1.0466	0.1207	0
3359	764.2	0.2774	0.2504	1.046	0.1207	0
3360	764.1	0.2774	0.2504	1.0461	0.1207	0
3361	764	0.2775	0.2504	1.0457	0.1207	0
3362	763.9	0.2776	0.2505	1.0461	0.1208	0
3363	763.8	0.2776	0.2505	1.0463	0.1209	0
3364	763.7	0.2777	0.2506	1.0462	0.121	0
3365	763.6	0.2778	0.2507	1.046	0.1211	0
3366	763.5	0.278	0.2508	1.0464	0.1211	0
3367	763.4	0.2779	0.2507	1.0465	0.1211	0
3368	763.3	0.2777	0.2507	1.0456	0.121	0
3369	763.2	0.2778	0.2508	1.0458	0.1211	0
3370	763.1	0.278	0.2509	1.0462	0.1213	0
3371	763	0.278	0.251	1.046	0.1214	0
3372	762.9	0.2781	0.2511	1.0454	0.1215	0
3373	762.8	0.2782	0.2512	1.0461	0.1215	0
3374	762.7	0.278	0.2513	1.046	0.1215	0
3375	762.6	0.2781	0.2514	1.0456	0.1216	0
3376	762.5	0.2782	0.2516	1.0461	0.1217	0
3377	762.4	0.2784	0.2517	1.0459	0.1218	0
3378	762.3	0.2784	0.2515	1.0462	0.1218	0
3379	762.2	0.2783	0.2516	1.0462	0.1218	0
3380	762.1	0.2784	0.2516	1.0459	0.1219	0
3381	762	0.2785	0.2517	1.0463	0.122	0
3382	761.9	0.2784	0.2515	1.046	0.1219	0
3383	761.8	0.2785	0.2517	1.0467	0.1221	0

3384	761.7	0.2787	0.2519	1.0467	0.1221	0
3385	761.6	0.2786	0.2518	1.0464	0.1222	0
3386	761.5	0.2787	0.252	1.0457	0.1222	0
3387	761.4	0.2788	0.252	1.0458	0.1223	0
3388	761.3	0.2791	0.2521	1.0462	0.1225	0
3389	761.2	0.2789	0.252	1.0461	0.1224	0
3390	761.1	0.2788	0.2519	1.0461	0.1224	0
3391	761	0.2785	0.252	1.0458	0.1223	0
3392	760.9	0.2788	0.252	1.0462	0.1224	0
3393	760.8	0.2787	0.252	1.0459	0.1223	0
3394	760.7	0.279	0.2522	1.0458	0.1225	0
3395	760.6	0.279	0.2522	1.046	0.1225	0
3396	760.5	0.279	0.2522	1.0465	0.1226	0
3397	760.4	0.2787	0.2521	1.0462	0.1224	0
3398	760.3	0.279	0.2521	1.0463	0.1224	0
3399	760.2	0.279	0.2521	1.0468	0.1226	0
3400	760.1	0.279	0.2521	1.0464	0.1226	0
3401	760	0.2791	0.2524	1.047	0.1226	0
3402	759.9	0.2791	0.2522	1.0469	0.1226	0
3403	759.8	0.2791	0.2522	1.0463	0.1226	0
3404	759.7	0.2791	0.2523	1.0466	0.1226	0
3405	759.6	0.2791	0.2524	1.0464	0.1226	0
3406	759.5	0.279	0.2523	1.0465	0.1226	0
3407	759.4	0.2791	0.2523	1.0467	0.1227	0
3408	759.3	0.2792	0.2525	1.0465	0.1227	0
3409	759.2	0.2792	0.2524	1.0468	0.1227	0
3410	759.1	0.2794	0.2526	1.0474	0.1228	0
3411	759	0.2792	0.2525	1.0468	0.1227	0
3412	758.9	0.2793	0.2526	1.0468	0.1227	0
3413	758.8	0.2791	0.2523	1.0465	0.1226	0
3414	758.7	0.2791	0.2522	1.0465	0.1226	0
3415	758.6	0.2791	0.2524	1.0466	0.1227	0
3416	758.5	0.2794	0.2524	1.0467	0.1228	0
3417	758.4	0.2793	0.2526	1.0465	0.1229	0
3418	758.3	0.2794	0.2525	1.0469	0.123	0
3419	758.2	0.2794	0.2525	1.0465	0.1228	0
3420	758.1	0.2794	0.2525	1.047	0.1228	0
3421	758	0.2794	0.2527	1.0472	0.123	0
3422	757.9	0.2794	0.2527	1.0473	0.1229	0
3423	757.8	0.2795	0.2526	1.0473	0.123	0
3424	757.7	0.2794	0.2525	1.0474	0.123	0
3425	757.6	0.2795	0.2526	1.0476	0.123	0
3426	757.5	0.2796	0.2527	1.0469	0.1231	0

3427	757.4	0.2796	0.2528	1.047	0.1232	0
3428	757.3	0.2797	0.2527	1.0471	0.1232	0
3429	757.2	0.2795	0.2527	1.0472	0.1231	0
3430	757.1	0.2795	0.2528	1.0474	0.1232	0
3431	757	0.2795	0.2527	1.0476	0.123	0
3432	756.9	0.2797	0.2529	1.0477	0.1232	0
3433	756.8	0.2798	0.253	1.048	0.1233	0
3434	756.7	0.2798	0.2529	1.0483	0.1234	0
3435	756.6	0.28	0.2529	1.0478	0.1233	0
3436	756.5	0.2798	0.2529	1.0477	0.1234	0
3437	756.4	0.2798	0.2528	1.0474	0.1233	0
3438	756.3	0.2799	0.253	1.0478	0.1234	0
3439	756.2	0.2797	0.2529	1.0474	0.1232	0
3440	756.1	0.2797	0.253	1.0474	0.1233	0
3441	756	0.2799	0.2529	1.0476	0.1233	0
3442	755.9	0.2801	0.2531	1.0479	0.1235	0
3443	755.8	0.2803	0.2532	1.047	0.1236	0
3444	755.7	0.2799	0.2531	1.0477	0.1235	0
3445	755.6	0.2799	0.2531	1.0479	0.1234	0
3446	755.5	0.2799	0.253	1.0481	0.1236	0
3447	755.4	0.2799	0.2533	1.0481	0.1236	0
3448	755.3	0.2801	0.2533	1.0481	0.1237	0
3449	755.2	0.2801	0.2533	1.0481	0.1239	0
3450	755.1	0.2799	0.2531	1.0479	0.1236	0
3451	755	0.2801	0.2533	1.0476	0.1237	0
3452	754.9	0.2802	0.2532	1.0475	0.1238	0
3453	754.8	0.2804	0.2535	1.0481	0.1239	0
3454	754.7	0.2805	0.2536	1.0478	0.124	0
3455	754.6	0.2804	0.2536	1.0481	0.124	0
3456	754.5	0.2803	0.2536	1.0474	0.124	0
3457	754.4	0.2804	0.2536	1.0475	0.124	0
3458	754.3	0.2804	0.2537	1.0473	0.1239	0
3459	754.2	0.2804	0.2537	1.0474	0.1241	0
3460	754.1	0.2805	0.2538	1.0478	0.124	0
3461	754	0.2804	0.2536	1.0478	0.1241	0
3462	753.9	0.2807	0.254	1.0476	0.1243	0
3463	753.8	0.2807	0.254	1.0477	0.1243	0
3464	753.7	0.2806	0.254	1.0473	0.1243	0
3465	753.6	0.2807	0.2541	1.0473	0.1244	0
3466	753.5	0.2806	0.2541	1.0477	0.1244	0
3467	753.4	0.2806	0.254	1.0477	0.1245	0
3468	753.3	0.2808	0.2541	1.0479	0.1246	0
3469	753.2	0.2808	0.2543	1.0475	0.1247	0

3470	753.1	0.281	0.2544	1.0482	0.1249	0
3471	753	0.2811	0.2544	1.0476	0.1248	0
3472	752.9	0.2812	0.2545	1.0477	0.1249	0
3473	752.8	0.2812	0.2545	1.0471	0.1249	0
3474	752.7	0.2812	0.2546	1.0477	0.1251	0
3475	752.6	0.2813	0.2546	1.0477	0.1251	0
3476	752.5	0.2813	0.2545	1.0477	0.125	0
3477	752.4	0.2812	0.2544	1.0482	0.1249	0
3478	752.3	0.2813	0.2546	1.0487	0.1249	0
3479	752.2	0.2814	0.2546	1.048	0.125	0
3480	752.1	0.2814	0.2546	1.0475	0.125	0
3481	752	0.2813	0.2546	1.0479	0.1251	0
3482	751.9	0.2814	0.2548	1.048	0.1253	0
3483	751.8	0.2815	0.2549	1.0482	0.1252	0
3484	751.7	0.2815	0.2548	1.048	0.1252	0
3485	751.6	0.2816	0.2548	1.0486	0.1252	0
3486	751.5	0.2815	0.255	1.0482	0.1252	0
3487	751.4	0.2816	0.255	1.0478	0.1254	0
3488	751.3	0.2815	0.255	1.0478	0.1253	0
3489	751.2	0.2818	0.2551	1.0482	0.1254	0
3490	751.1	0.2818	0.2551	1.0483	0.1254	0
3491	751	0.2819	0.2549	1.048	0.1255	0
3492	750.9	0.2819	0.2548	1.0484	0.1255	0
3493	750.8	0.2817	0.2548	1.0483	0.1255	0
3494	750.7	0.2818	0.2551	1.0484	0.1256	0
3495	750.6	0.2816	0.2549	1.0477	0.1254	0
3496	750.5	0.2817	0.2549	1.0485	0.1254	0
3497	750.4	0.2817	0.255	1.0484	0.1254	0
3498	750.3	0.2818	0.2552	1.0484	0.1256	0
3499	750.2	0.2818	0.2552	1.0489	0.1255	0
3500	750.1	0.2818	0.2553	1.0489	0.1256	0
3501	750	0.2819	0.2553	1.0484	0.1256	0
3502	749.9	0.2819	0.2552	1.0483	0.1256	0
3503	749.8	0.2819	0.2553	1.0488	0.1256	0
3504	749.7	0.282	0.2554	1.0488	0.1258	0
3505	749.6	0.2819	0.2553	1.0482	0.1257	0
3506	749.5	0.2821	0.2553	1.0486	0.1257	0
3507	749.4	0.282	0.2553	1.0486	0.1258	0
3508	749.3	0.282	0.2554	1.049	0.1258	0
3509	749.2	0.2822	0.2553	1.0488	0.1259	0
3510	749.1	0.2823	0.2556	1.0491	0.1261	0
3511	749	0.2821	0.2554	1.0487	0.1257	0
3512	748.9	0.282	0.2554	1.049	0.1257	0

3513	748.8	0.2822	0.2556	1.0494	0.126	0
3514	748.7	0.2824	0.2557	1.0491	0.1261	0
3515	748.6	0.2824	0.2559	1.0494	0.1262	0
3516	748.5	0.2822	0.2557	1.0489	0.1261	0
3517	748.4	0.2824	0.2558	1.0487	0.1261	0
3518	748.3	0.2824	0.2558	1.0485	0.1261	0
3519	748.2	0.2824	0.2556	1.049	0.1261	0
3520	748.1	0.2825	0.2555	1.0485	0.1262	0
3521	748	0.2826	0.2557	1.0488	0.1262	0
3522	747.9	0.2825	0.2559	1.0494	0.1262	0
3523	747.8	0.2826	0.2559	1.0493	0.1263	0
3524	747.7	0.2828	0.2561	1.0491	0.1263	0
3525	747.6	0.2828	0.2563	1.0491	0.1265	0
3526	747.5	0.2827	0.2562	1.0494	0.1264	0
3527	747.4	0.2828	0.2561	1.0494	0.1265	0
3528	747.3	0.2829	0.2562	1.0495	0.1264	0
3529	747.2	0.2827	0.2561	1.0493	0.1266	0
3530	747.1	0.2828	0.2561	1.0489	0.1265	0
3531	747	0.2829	0.2561	1.0488	0.1266	0
3532	746.9	0.283	0.2562	1.0492	0.1266	0
3533	746.8	0.2829	0.2562	1.0496	0.1266	0
3534	746.7	0.2831	0.2564	1.0499	0.1267	0
3535	746.6	0.283	0.2562	1.0491	0.1268	0
3536	746.5	0.2829	0.2563	1.049	0.1268	0
3537	746.4	0.2828	0.2564	1.0494	0.1268	0
3538	746.3	0.283	0.2563	1.0492	0.1268	0
3539	746.2	0.283	0.2563	1.0492	0.1268	0
3540	746.1	0.283	0.2564	1.0492	0.1268	0
3541	746	0.2831	0.2565	1.0492	0.1269	0
3542	745.9	0.2833	0.2566	1.0492	0.1269	0
3543	745.8	0.2833	0.2566	1.0485	0.127	0
3544	745.7	0.2834	0.2568	1.0492	0.1271	0
3545	745.6	0.2834	0.2568	1.0499	0.1272	0
3546	745.5	0.2834	0.2567	1.0493	0.1273	0
3547	745.4	0.2834	0.2567	1.0493	0.1273	0
3548	745.3	0.2834	0.2568	1.0495	0.1273	0
3549	745.2	0.2834	0.2569	1.0496	0.1273	0
3550	745.1	0.2835	0.2571	1.0497	0.1275	0
3551	745	0.2834	0.2569	1.0498	0.1273	0
3552	744.9	0.2835	0.2568	1.0496	0.1273	0
3553	744.8	0.2835	0.257	1.0493	0.1274	0
3554	744.7	0.2837	0.2572	1.0498	0.1276	0
3555	744.6	0.2836	0.2571	1.0498	0.1276	0

3556	744.5	0.2837	0.2572	1.0494	0.1275	0
3557	744.4	0.2838	0.2573	1.0497	0.1277	0
3558	744.3	0.2839	0.2572	1.0496	0.1277	0
3559	744.2	0.2838	0.2572	1.0495	0.1278	0
3560	744.1	0.2841	0.2575	1.0497	0.1279	0
3561	744	0.2839	0.2574	1.0495	0.128	0
3562	743.9	0.2841	0.2575	1.0498	0.1282	0
3563	743.8	0.2842	0.2576	1.0494	0.1281	0
3564	743.7	0.2841	0.2575	1.05	0.128	0
3565	743.6	0.2843	0.2575	1.0499	0.1282	0
3566	743.5	0.2843	0.2578	1.0494	0.1285	0
3567	743.4	0.2844	0.2579	1.0489	0.1283	0
3568	743.3	0.2844	0.2579	1.0493	0.1283	0
3569	743.2	0.2844	0.2579	1.0494	0.1284	0
3570	743.1	0.2846	0.2579	1.0491	0.1284	0
3571	743	0.2845	0.2579	1.0493	0.1285	0
3572	742.9	0.2843	0.2578	1.0495	0.1285	0
3573	742.8	0.2847	0.2581	1.0492	0.1286	0
3574	742.7	0.2847	0.2581	1.0487	0.1287	0
3575	742.6	0.2845	0.258	1.0491	0.1286	0
3576	742.5	0.2848	0.2582	1.0497	0.1288	0
3577	742.4	0.2846	0.2581	1.0498	0.1286	0
3578	742.3	0.2845	0.258	1.0498	0.1286	0
3579	742.2	0.2843	0.258	1.0498	0.1285	0
3580	742.1	0.2846	0.2582	1.0494	0.1288	0
3581	742	0.2848	0.258	1.0495	0.1287	0
3582	741.9	0.2848	0.2581	1.0498	0.1287	0
3583	741.8	0.2847	0.2582	1.05	0.1287	0
3584	741.7	0.2847	0.2582	1.0494	0.1288	0
3585	741.6	0.2847	0.2583	1.0499	0.1287	0
3586	741.5	0.285	0.2584	1.0496	0.1288	0
3587	741.4	0.2848	0.2583	1.0495	0.1287	0
3588	741.3	0.2847	0.2583	1.0495	0.1287	0
3589	741.2	0.285	0.2585	1.0497	0.129	0
3590	741.1	0.285	0.2584	1.0496	0.1289	0
3591	741	0.2851	0.2585	1.0495	0.1289	0
3592	740.9	0.285	0.2585	1.05	0.1289	0
3593	740.8	0.2851	0.2586	1.0502	0.129	0
3594	740.7	0.2851	0.2584	1.0502	0.1289	0
3595	740.6	0.2852	0.2585	1.0497	0.1291	0
3596	740.5	0.2851	0.2585	1.0501	0.1291	0
3597	740.4	0.2853	0.2586	1.0504	0.1291	0
3598	740.3	0.2856	0.2588	1.0504	0.1294	0

3599	740.2	0.2855	0.2587	1.0505	0.1292	0
3600	740.1	0.2852	0.2588	1.0499	0.1291	0
3601	740	0.2852	0.2586	1.0496	0.129	0
3602	739.9	0.2852	0.2587	1.05	0.129	0
3603	739.8	0.2852	0.2585	1.0502	0.1289	0
3604	739.7	0.2855	0.2588	1.0505	0.1291	0
3605	739.6	0.2857	0.2589	1.0508	0.1293	0
3606	739.5	0.2856	0.2588	1.0506	0.1293	0
3607	739.4	0.2854	0.2587	1.051	0.1291	0
3608	739.3	0.2856	0.2588	1.0508	0.1295	0
3609	739.2	0.2856	0.2589	1.0505	0.1294	0
3610	739.1	0.2857	0.2589	1.0504	0.1292	0
3611	739	0.2855	0.2588	1.0506	0.1293	0
3612	738.9	0.2855	0.2588	1.0505	0.1292	0
3613	738.8	0.2855	0.2589	1.0506	0.1291	0
3614	738.7	0.2856	0.2586	1.051	0.1291	0
3615	738.6	0.2855	0.2587	1.0505	0.1291	0
3616	738.5	0.2855	0.2587	1.0504	0.1292	0
3617	738.4	0.2855	0.2588	1.051	0.1292	0
3618	738.3	0.2854	0.2587	1.0507	0.1292	0
3619	738.2	0.2857	0.2588	1.0515	0.1292	0
3620	738.1	0.2856	0.2588	1.0511	0.1293	0
3621	738	0.2856	0.2588	1.0508	0.1292	0
3622	737.9	0.2857	0.2589	1.0508	0.1292	0
3623	737.8	0.2855	0.2588	1.0511	0.1291	0
3624	737.7	0.2857	0.259	1.0516	0.1293	0
3625	737.6	0.2855	0.259	1.0511	0.1292	0
3626	737.5	0.2858	0.259	1.0511	0.1293	0
3627	737.4	0.2857	0.2589	1.0511	0.1294	0
3628	737.3	0.286	0.2591	1.0518	0.1295	0
3629	737.2	0.2862	0.2592	1.0515	0.1297	0
3630	737.1	0.286	0.2591	1.0516	0.1296	0
3631	737	0.2858	0.2589	1.0513	0.1294	0
3632	736.9	0.2859	0.2591	1.0516	0.1295	0
3633	736.8	0.2862	0.2592	1.0513	0.1296	0
3634	736.7	0.2859	0.259	1.0512	0.1294	0
3635	736.6	0.2861	0.2591	1.0516	0.1296	0
3636	736.5	0.2861	0.2592	1.051	0.1297	0
3637	736.4	0.2861	0.2592	1.0519	0.1298	0
3638	736.3	0.2862	0.2594	1.0516	0.1298	0
3639	736.2	0.2862	0.2593	1.0514	0.1298	0
3640	736.1	0.2864	0.2594	1.0517	0.1299	0
3641	736	0.2863	0.2596	1.0517	0.13	0

3642	735.9	0.2864	0.2596	1.0513	0.1301	0
3643	735.8	0.2864	0.2596	1.0512	0.13	0
3644	735.7	0.2865	0.2597	1.0509	0.13	0
3645	735.6	0.2864	0.2595	1.0515	0.13	0
3646	735.5	0.2866	0.2596	1.0516	0.1302	0
3647	735.4	0.2865	0.2598	1.0511	0.1302	0
3648	735.3	0.2866	0.26	1.0507	0.1304	0
3649	735.2	0.2869	0.2601	1.0516	0.1306	0
3650	735.1	0.2869	0.2602	1.0515	0.1306	0
3651	735	0.287	0.2603	1.0516	0.1307	0
3652	734.9	0.2869	0.2602	1.0513	0.1306	0
3653	734.8	0.2869	0.2601	1.0512	0.1305	0
3654	734.7	0.2869	0.2602	1.051	0.1306	0
3655	734.6	0.2871	0.2603	1.051	0.1308	0
3656	734.5	0.287	0.2603	1.0504	0.1307	0
3657	734.4	0.287	0.2604	1.0508	0.1308	0
3658	734.3	0.2873	0.2606	1.0513	0.1311	0
3659	734.2	0.2875	0.2608	1.0508	0.1313	0
3660	734.1	0.2875	0.2608	1.0505	0.1312	0
3661	734	0.2876	0.2607	1.0515	0.1315	0
3662	733.9	0.2876	0.2608	1.0509	0.1314	0
3663	733.8	0.2875	0.2609	1.0509	0.1314	0
3664	733.7	0.2876	0.2609	1.0509	0.1315	0
3665	733.6	0.2877	0.2611	1.0509	0.1317	0
3666	733.5	0.2876	0.261	1.0506	0.1316	0
3667	733.4	0.2877	0.2611	1.0512	0.1316	0
3668	733.3	0.2877	0.2612	1.0505	0.1317	0
3669	733.2	0.288	0.2613	1.0508	0.1319	0
3670	733.1	0.2881	0.2615	1.0512	0.1321	0
3671	733	0.288	0.2614	1.0506	0.1319	0
3672	732.9	0.288	0.2614	1.051	0.132	0
3673	732.8	0.2879	0.2614	1.0509	0.1321	0
3674	732.7	0.2881	0.2613	1.051	0.132	0
3675	732.6	0.288	0.2613	1.0506	0.1319	0
3676	732.5	0.2881	0.2613	1.0505	0.132	0
3677	732.4	0.2882	0.2617	1.0513	0.1322	0
3678	732.3	0.2882	0.2616	1.0509	0.1321	0
3679	732.2	0.2883	0.2617	1.051	0.1322	0
3680	732.1	0.2883	0.2617	1.0511	0.1321	0
3681	732	0.2882	0.2617	1.0509	0.1322	0
3682	731.9	0.2882	0.2616	1.0507	0.1322	0
3683	731.8	0.2885	0.2618	1.0514	0.1323	0
3684	731.7	0.2885	0.2619	1.0512	0.1324	0

3685	731.6	0.2885	0.2618	1.0512	0.1325	0
3686	731.5	0.2884	0.2618	1.051	0.1323	0
3687	731.4	0.2884	0.2618	1.051	0.1325	0
3688	731.3	0.2885	0.2617	1.051	0.1323	0
3689	731.2	0.2886	0.2619	1.0509	0.1325	0
3690	731.1	0.2885	0.2619	1.0512	0.1324	0
3691	731	0.2888	0.2621	1.0512	0.1326	0
3692	730.9	0.2887	0.2619	1.0515	0.1325	0
3693	730.8	0.2887	0.262	1.051	0.1325	0
3694	730.7	0.2886	0.262	1.0513	0.1325	0
3695	730.6	0.2888	0.2621	1.0512	0.1326	0
3696	730.5	0.2888	0.2621	1.0515	0.1326	0
3697	730.4	0.2888	0.262	1.0515	0.1325	0
3698	730.3	0.2889	0.2622	1.0516	0.1329	0
3699	730.2	0.2888	0.2622	1.0515	0.1327	0
3700	730.1	0.289	0.2622	1.0513	0.1327	0
3701	730	0.289	0.2621	1.0509	0.1327	0
3702	729.9	0.2891	0.2622	1.0516	0.1328	0
3703	729.8	0.289	0.2621	1.0511	0.1326	0
3704	729.7	0.2889	0.2623	1.0519	0.1327	0
3705	729.6	0.2888	0.2623	1.0517	0.1327	0
3706	729.5	0.289	0.2623	1.0518	0.1327	0
3707	729.4	0.2889	0.2622	1.0518	0.1325	0
3708	729.3	0.2889	0.2622	1.0519	0.1326	0
3709	729.2	0.2891	0.2623	1.0521	0.1328	0
3710	729.1	0.2892	0.2624	1.0518	0.1327	0
3711	729	0.2892	0.2623	1.052	0.1326	0
3712	728.9	0.2892	0.2624	1.0524	0.1327	0
3713	728.8	0.2891	0.2625	1.0526	0.1328	0
3714	728.7	0.2894	0.2626	1.052	0.1328	0
3715	728.6	0.2893	0.2624	1.0518	0.1328	0
3716	728.5	0.2894	0.2626	1.0521	0.1329	0
3717	728.4	0.2893	0.2626	1.0525	0.1327	0
3718	728.3	0.2893	0.2625	1.0522	0.1328	0
3719	728.2	0.2892	0.2622	1.0526	0.1327	0
3720	728.1	0.2894	0.2623	1.0525	0.1327	0
3721	728	0.2891	0.2622	1.0527	0.1325	0
3722	727.9	0.2895	0.2624	1.0531	0.1328	0
3723	727.8	0.2893	0.2624	1.053	0.1327	0
3724	727.7	0.2893	0.2626	1.0525	0.1327	0
3725	727.6	0.2893	0.2623	1.0526	0.1327	0
3726	727.5	0.2893	0.2624	1.0531	0.1327	0
3727	727.4	0.2894	0.2624	1.0529	0.1328	0

3728	727.3	0.2895	0.2624	1.0526	0.1328	0
3729	727.2	0.2894	0.2625	1.0527	0.1328	0
3730	727.1	0.2894	0.2625	1.0531	0.1328	0
3731	727	0.2895	0.2626	1.0532	0.1329	0
3732	726.9	0.2898	0.2627	1.053	0.133	0
3733	726.8	0.2897	0.2628	1.0531	0.133	0
3734	726.7	0.2896	0.2627	1.0525	0.1329	0
3735	726.6	0.2895	0.2626	1.0534	0.1329	0
3736	726.5	0.2897	0.2628	1.0536	0.1329	0
3737	726.4	0.2898	0.2629	1.0534	0.1331	0
3738	726.3	0.2898	0.2628	1.0533	0.1331	0
3739	726.2	0.2897	0.2627	1.0531	0.1331	0
3740	726.1	0.29	0.2631	1.0531	0.1332	0
3741	726	0.2902	0.263	1.0533	0.1332	0
3742	725.9	0.2899	0.2629	1.0531	0.1332	0
3743	725.8	0.2899	0.263	1.053	0.1333	0
3744	725.7	0.2899	0.2629	1.0531	0.1333	0
3745	725.6	0.2902	0.2632	1.0534	0.1336	0
3746	725.5	0.2902	0.2632	1.0533	0.1335	0
3747	725.4	0.2903	0.2635	1.0534	0.1336	0
3748	725.3	0.2905	0.2635	1.0533	0.1338	0
3749	725.2	0.2905	0.2636	1.0527	0.1338	0
3750	725.1	0.2907	0.2636	1.0535	0.134	0
3751	725	0.2906	0.2636	1.0537	0.1341	0
3752	724.9	0.2906	0.2637	1.0532	0.134	0
3753	724.8	0.2907	0.2636	1.0536	0.1341	0
3754	724.7	0.2908	0.2637	1.0532	0.1342	0
3755	724.6	0.2908	0.2638	1.0529	0.1343	0
3756	724.5	0.291	0.2639	1.0534	0.1343	0
3757	724.4	0.2909	0.264	1.0531	0.1344	0
3758	724.3	0.2909	0.264	1.0536	0.1344	0
3759	724.2	0.2909	0.2639	1.053	0.1345	0
3760	724.1	0.2912	0.2642	1.0529	0.1347	0
3761	724	0.2911	0.2641	1.0527	0.1348	0
3762	723.9	0.2913	0.2643	1.0532	0.1348	0
3763	723.8	0.2914	0.2645	1.0534	0.135	0
3764	723.7	0.2914	0.2645	1.0534	0.135	0
3765	723.6	0.2914	0.2646	1.053	0.1351	0
3766	723.5	0.2915	0.2646	1.0525	0.1352	0
3767	723.4	0.2917	0.2647	1.0527	0.1352	0
3768	723.3	0.2918	0.2649	1.053	0.1353	0
3769	723.2	0.2919	0.2651	1.0525	0.1355	0
3770	723.1	0.292	0.2651	1.0524	0.1355	0

3771	723	0.292	0.2652	1.0526	0.1356	0
3772	722.9	0.292	0.2651	1.0537	0.1356	0
3773	722.8	0.2921	0.2653	1.0533	0.1356	0
3774	722.7	0.2922	0.2655	1.0532	0.1359	0
3775	722.6	0.2922	0.2654	1.0529	0.1359	0
3776	722.5	0.2923	0.2655	1.0526	0.136	0
3777	722.4	0.2923	0.2656	1.053	0.136	0
3778	722.3	0.2924	0.2656	1.0532	0.136	0
3779	722.2	0.2924	0.2657	1.0533	0.1361	0
3780	722.1	0.2927	0.2658	1.0532	0.1362	0
3781	722	0.2926	0.2659	1.0531	0.1362	0
3782	721.9	0.2925	0.2657	1.0531	0.1362	0
3783	721.8	0.2924	0.2658	1.0532	0.1362	0
3784	721.7	0.2926	0.2658	1.0532	0.1363	0
3785	721.6	0.2926	0.2658	1.0532	0.1363	0
3786	721.5	0.2926	0.2658	1.0534	0.1363	0
3787	721.4	0.2926	0.2658	1.0528	0.1362	0
3788	721.3	0.2929	0.2658	1.0531	0.1364	0
3789	721.2	0.2931	0.2661	1.0535	0.1366	0
3790	721.1	0.2931	0.2661	1.0535	0.1366	0
3791	721	0.2929	0.2659	1.0527	0.1364	0
3792	720.9	0.2933	0.2662	1.0526	0.1368	0
3793	720.8	0.2932	0.2661	1.0535	0.1367	0
3794	720.7	0.2933	0.2663	1.0528	0.1368	0
3795	720.6	0.2932	0.2662	1.0531	0.1367	0
3796	720.5	0.2931	0.2663	1.053	0.1368	0
3797	720.4	0.2933	0.2665	1.0536	0.1368	0
3798	720.3	0.2936	0.2666	1.0536	0.137	0
3799	720.2	0.2935	0.2666	1.0534	0.1369	0
3800	720.1	0.2934	0.2665	1.0532	0.1369	0
3801	720	0.2934	0.2664	1.0536	0.1369	0
3802	719.9	0.2935	0.2665	1.0532	0.1369	0
3803	719.8	0.2936	0.2666	1.0538	0.1369	0
3804	719.7	0.2937	0.2666	1.0539	0.137	0
3805	719.6	0.2937	0.2668	1.0536	0.137	0
3806	719.5	0.2938	0.2667	1.0534	0.1371	0
3807	719.4	0.2937	0.2667	1.0532	0.1371	0
3808	719.3	0.2939	0.2667	1.0539	0.1372	0
3809	719.2	0.294	0.2666	1.0541	0.1371	0
3810	719.1	0.2936	0.2666	1.054	0.137	0
3811	719	0.2939	0.2667	1.0536	0.1371	0
3812	718.9	0.2939	0.2666	1.0544	0.137	0
3813	718.8	0.2939	0.2667	1.0545	0.1371	0

3814	718.7	0.2938	0.2667	1.0539	0.1371	0
3815	718.6	0.2939	0.2667	1.0541	0.137	0
3816	718.5	0.2938	0.2666	1.0541	0.1371	0
3817	718.4	0.2938	0.2667	1.0539	0.137	0
3818	718.3	0.2939	0.2666	1.0547	0.1371	0
3819	718.2	0.2939	0.2667	1.0541	0.1371	0
3820	718.1	0.2939	0.2667	1.0542	0.1371	0
3821	718	0.2941	0.2668	1.0541	0.1372	0
3822	717.9	0.294	0.2666	1.0548	0.1372	0
3823	717.8	0.2942	0.2668	1.0548	0.1372	0
3824	717.7	0.2939	0.2668	1.0549	0.1371	0
3825	717.6	0.2942	0.267	1.0552	0.1373	0
3826	717.5	0.2945	0.267	1.055	0.1373	0
3827	717.4	0.2942	0.2668	1.0551	0.1371	0
3828	717.3	0.2942	0.2668	1.055	0.1372	0
3829	717.2	0.2945	0.267	1.055	0.1373	0
3830	717.1	0.2944	0.267	1.0549	0.1374	0
3831	717	0.2945	0.2671	1.0554	0.1374	0
3832	716.9	0.2945	0.2671	1.0552	0.1375	0
3833	716.8	0.2947	0.2672	1.0557	0.1375	0
3834	716.7	0.2949	0.2672	1.0551	0.1375	0
3835	716.6	0.295	0.2673	1.0555	0.1376	0
3836	716.5	0.2947	0.2672	1.0552	0.1374	0
3837	716.4	0.2948	0.2672	1.0551	0.1374	0
3838	716.3	0.2946	0.2673	1.0553	0.1374	0
3839	716.2	0.2948	0.2674	1.0552	0.1377	0
3840	716.1	0.2949	0.2674	1.0548	0.1377	0
3841	716	0.2951	0.2674	1.0551	0.1377	0
3842	715.9	0.295	0.2674	1.0554	0.1377	0
3843	715.8	0.2951	0.2677	1.0547	0.1378	0
3844	715.7	0.2951	0.2676	1.0544	0.1377	0
3845	715.6	0.2953	0.2678	1.0548	0.138	0
3846	715.5	0.2951	0.2676	1.055	0.1379	0
3847	715.4	0.2953	0.2678	1.0555	0.1379	0
3848	715.3	0.2953	0.2679	1.0557	0.138	0
3849	715.2	0.2955	0.2678	1.0553	0.1382	0
3850	715.1	0.2956	0.268	1.056	0.1383	0
3851	715	0.2957	0.2681	1.0553	0.1384	0
3852	714.9	0.2957	0.2682	1.0556	0.1384	0
3853	714.8	0.2957	0.2682	1.0557	0.1383	0
3854	714.7	0.296	0.2685	1.0562	0.1386	0
3855	714.6	0.2959	0.2684	1.0555	0.1386	0
3856	714.5	0.2962	0.2685	1.0551	0.1387	0

3857	714.4	0.2962	0.2685	1.0558	0.1388	0
3858	714.3	0.2961	0.2685	1.0553	0.1388	0
3859	714.2	0.2961	0.2686	1.0562	0.1388	0
3860	714.1	0.2962	0.2688	1.0554	0.139	0
3861	714	0.2965	0.2689	1.0553	0.1392	0
3862	713.9	0.2966	0.2689	1.0557	0.1392	0
3863	713.8	0.2967	0.2688	1.0549	0.1391	0
3864	713.7	0.2967	0.269	1.0556	0.1393	0
3865	713.6	0.2967	0.2691	1.0558	0.1394	0
3866	713.5	0.297	0.2693	1.0561	0.1396	0
3867	713.4	0.2971	0.2694	1.0556	0.1396	0
3868	713.3	0.2971	0.2694	1.0559	0.1396	0
3869	713.2	0.2973	0.2694	1.0555	0.1398	0
3870	713.1	0.2973	0.2695	1.0554	0.1398	0
3871	713	0.2975	0.2696	1.055	0.14	0
3872	712.9	0.2975	0.2697	1.0551	0.1401	0
3873	712.8	0.2978	0.2699	1.0554	0.1402	0
3874	712.7	0.2979	0.2699	1.0556	0.1402	0
3875	712.6	0.2978	0.27	1.0554	0.1403	0
3876	712.5	0.2978	0.27	1.0551	0.1404	0
3877	712.4	0.2982	0.2703	1.0555	0.1406	0
3878	712.3	0.2982	0.2702	1.0554	0.1406	0
3879	712.2	0.2983	0.2703	1.0546	0.1406	0
3880	712.1	0.2982	0.2703	1.055	0.1406	0
3881	712	0.2983	0.2703	1.0543	0.1407	0
3882	711.9	0.2985	0.2704	1.0547	0.1409	0
3883	711.8	0.2987	0.2706	1.0551	0.141	0
3884	711.7	0.2987	0.2706	1.0553	0.1409	0
3885	711.6	0.2988	0.2707	1.0556	0.141	0
3886	711.5	0.2988	0.2708	1.0555	0.1411	0
3887	711.4	0.2989	0.2708	1.0554	0.1412	0
3888	711.3	0.2991	0.2709	1.0548	0.1413	0
3889	711.2	0.2991	0.271	1.0554	0.1412	0
3890	711.1	0.2992	0.271	1.0553	0.1413	0
3891	711	0.2993	0.2711	1.0552	0.1415	0
3892	710.9	0.2995	0.2711	1.0552	0.1414	0
3893	710.8	0.2994	0.2712	1.0555	0.1415	0
3894	710.7	0.2995	0.2713	1.0552	0.1416	0
3895	710.6	0.2997	0.2715	1.0557	0.1416	0
3896	710.5	0.2994	0.2712	1.0549	0.1415	0
3897	710.4	0.2994	0.2714	1.0557	0.1415	0
3898	710.3	0.2997	0.2716	1.0564	0.1417	0
3899	710.2	0.3001	0.2717	1.0559	0.1419	0

3900	710.1	0.2999	0.2716	1.0556	0.1418	0
3901	710	0.3002	0.2719	1.0558	0.1421	0
3902	709.9	0.3002	0.2717	1.0562	0.1419	0
3903	709.8	0.3003	0.272	1.0565	0.142	0
3904	709.7	0.3004	0.2718	1.0556	0.1421	0
3905	709.6	0.3004	0.2719	1.056	0.142	0
3906	709.5	0.3005	0.2721	1.0558	0.142	0
3907	709.4	0.3004	0.2719	1.0558	0.142	0
3908	709.3	0.3005	0.272	1.0559	0.1422	0
3909	709.2	0.3006	0.2721	1.0564	0.1421	0
3910	709.1	0.3009	0.2724	1.0573	0.1424	0
3911	709	0.3009	0.2723	1.0573	0.1423	0
3912	708.9	0.3008	0.2723	1.0556	0.1422	0
3913	708.8	0.3008	0.2722	1.0567	0.1422	0
3914	708.7	0.3009	0.2722	1.0565	0.1422	0
3915	708.6	0.301	0.2723	1.057	0.1423	0
3916	708.5	0.3011	0.2725	1.0571	0.1425	0
3917	708.4	0.3012	0.2725	1.0573	0.1425	0
3918	708.3	0.3012	0.2726	1.0574	0.1425	0
3919	708.2	0.3012	0.2725	1.0579	0.1423	0
3920	708.1	0.3014	0.2726	1.058	0.1425	0
3921	708	0.3015	0.2726	1.0573	0.1425	0
3922	707.9	0.3016	0.2727	1.0574	0.1425	0
3923	707.8	0.3017	0.2728	1.0574	0.1426	0
3924	707.7	0.3018	0.2728	1.0573	0.1425	0
3925	707.6	0.3018	0.2727	1.058	0.1425	0
3926	707.5	0.3018	0.2725	1.0582	0.1424	0
3927	707.4	0.3021	0.2727	1.0578	0.1427	0
3928	707.3	0.302	0.2729	1.0579	0.1426	0
3929	707.2	0.3019	0.2729	1.0583	0.1426	0
3930	707.1	0.3019	0.273	1.0577	0.1426	0
3931	707	0.3021	0.273	1.0582	0.1426	0
3932	706.9	0.3023	0.273	1.058	0.1427	0
3933	706.8	0.3024	0.273	1.0576	0.1427	0
3934	706.7	0.3025	0.2733	1.0578	0.1427	0
3935	706.6	0.3025	0.2732	1.0583	0.1427	0
3936	706.5	0.3024	0.2731	1.0584	0.1426	0
3937	706.4	0.3026	0.2731	1.059	0.1427	0
3938	706.3	0.3026	0.2732	1.0582	0.1426	0
3939	706.2	0.3027	0.2732	1.0582	0.1426	0
3940	706.1	0.3028	0.2735	1.0586	0.1428	0
3941	706	0.303	0.2734	1.0586	0.1429	0
3942	705.9	0.3029	0.2734	1.0589	0.1428	0

3943	705.8	0.3028	0.2734	1.0585	0.1427	0
3944	705.7	0.3029	0.2735	1.0584	0.1426	0
3945	705.6	0.3032	0.2735	1.0584	0.1429	0
3946	705.5	0.3034	0.2738	1.0584	0.1431	0
3947	705.4	0.3035	0.2737	1.0582	0.1431	0
3948	705.3	0.3034	0.2737	1.0585	0.143	0
3949	705.2	0.3037	0.2738	1.0586	0.1431	0
3950	705.1	0.3037	0.274	1.0582	0.1431	0
3951	705	0.3039	0.2741	1.0584	0.1434	0
3952	704.9	0.304	0.2742	1.0589	0.1434	0
3953	704.8	0.3042	0.2743	1.0589	0.1435	0
3954	704.7	0.3043	0.2745	1.0589	0.1436	0
3955	704.6	0.3043	0.2746	1.0589	0.1436	0
3956	704.5	0.3046	0.2746	1.0596	0.1437	0
3957	704.4	0.3046	0.2746	1.059	0.1437	0
3958	704.3	0.3046	0.2747	1.0587	0.1436	0
3959	704.2	0.3048	0.2747	1.059	0.1439	0
3960	704.1	0.3051	0.2749	1.0588	0.1439	0
3961	704	0.3051	0.2751	1.0586	0.144	0
3962	703.9	0.3052	0.2752	1.0589	0.1442	0
3963	703.8	0.3055	0.2753	1.0585	0.1443	0
3964	703.7	0.3055	0.2753	1.0584	0.1441	0
3965	703.6	0.3057	0.2755	1.0591	0.1444	0
3966	703.5	0.3058	0.2755	1.0589	0.1444	0
3967	703.4	0.3056	0.2756	1.0591	0.1444	0
3968	703.3	0.306	0.2759	1.0586	0.1447	0
3969	703.2	0.3062	0.2759	1.0582	0.1448	0
3970	703.1	0.3062	0.2759	1.0592	0.1446	0
3971	703	0.3063	0.2759	1.0589	0.1447	0
3972	702.9	0.3065	0.276	1.0591	0.1449	0
3973	702.8	0.3067	0.2762	1.0592	0.145	0
3974	702.7	0.3068	0.2764	1.0591	0.1451	0
3975	702.6	0.307	0.2765	1.0589	0.1452	0
3976	702.5	0.307	0.2764	1.0585	0.1451	0
3977	702.4	0.3071	0.2766	1.0583	0.1452	0
3978	702.3	0.3072	0.2768	1.059	0.1453	0
3979	702.2	0.3073	0.2768	1.0592	0.1454	0
3980	702.1	0.3075	0.2769	1.0591	0.1456	0
3981	702	0.3074	0.277	1.059	0.1454	0
3982	701.9	0.3075	0.2769	1.0593	0.1455	0
3983	701.8	0.3076	0.2769	1.0595	0.1455	0
3984	701.7	0.3077	0.2771	1.0591	0.1457	0
3985	701.6	0.308	0.2773	1.0594	0.1458	0

3986	701.5	0.3081	0.2774	1.059	0.1458	0
3987	701.4	0.3084	0.2775	1.0598	0.146	0
3988	701.3	0.3083	0.2778	1.0593	0.146	0
3989	701.2	0.3084	0.2778	1.0597	0.1459	0
3990	701.1	0.3084	0.2778	1.0598	0.146	0
3991	701	0.3086	0.2779	1.0596	0.1461	0
3992	700.9	0.3087	0.2781	1.06	0.1462	0
3993	700.8	0.3088	0.2782	1.0599	0.1463	0
3994	700.7	0.309	0.2783	1.0595	0.1464	0
3995	700.6	0.3091	0.2782	1.0595	0.1463	0
3996	700.5	0.3089	0.2782	1.0597	0.1463	0
3997	700.4	0.309	0.2783	1.0599	0.1463	0
3998	700.3	0.3092	0.2784	1.0597	0.1465	0
3999	700.2	0.3094	0.2788	1.0601	0.1466	0
4000	700.1	0.3095	0.2787	1.06	0.1466	0
4001	700	0.3095	0.2788	1.0599	0.1467	0
4002	699.9	0.3095	0.2787	1.0601	0.1465	0
4003	699.8	0.3097	0.2789	1.0596	0.1468	0
4004	699.7	0.3099	0.2791	1.0595	0.1469	0
4005	699.6	0.3101	0.2791	1.0605	0.1471	0
4006	699.5	0.3103	0.2793	1.06	0.147	0
4007	699.4	0.3103	0.2794	1.0601	0.1472	0
4008	699.3	0.3103	0.2794	1.0602	0.1472	0
4009	699.2	0.3105	0.2796	1.0605	0.1472	0
4010	699.1	0.3105	0.2796	1.0608	0.1472	0
4011	699	0.3104	0.2795	1.0604	0.1471	0
4012	698.9	0.3104	0.2795	1.0601	0.1471	0
4013	698.8	0.3104	0.2796	1.0603	0.147	0
4014	698.7	0.3107	0.2799	1.0607	0.1472	0
4015	698.6	0.3108	0.2801	1.0605	0.1473	0
4016	698.5	0.3109	0.2801	1.0608	0.1473	0
4017	698.4	0.311	0.2802	1.0608	0.1474	0
4018	698.3	0.311	0.2802	1.0614	0.1475	0
4019	698.2	0.3111	0.2803	1.0613	0.1475	0
4020	698.1	0.3112	0.2803	1.061	0.1476	0
4021	698	0.3114	0.2805	1.0613	0.1477	0
4022	697.9	0.3113	0.2806	1.0612	0.1476	0
4023	697.8	0.3114	0.2807	1.0609	0.1476	0
4024	697.7	0.3115	0.2808	1.0618	0.1478	0
4025	697.6	0.3115	0.2808	1.0618	0.1477	0
4026	697.5	0.3116	0.2808	1.0618	0.1477	0
4027	697.4	0.3116	0.2809	1.062	0.1477	0
4028	697.3	0.3118	0.281	1.0619	0.1479	0

4029	697.2	0.3122	0.2813	1.0621	0.148	0
4030	697.1	0.3121	0.2815	1.062	0.148	0
4031	697	0.3118	0.2814	1.0621	0.148	0
4032	696.9	0.3119	0.2814	1.0623	0.148	0
4033	696.8	0.3122	0.2814	1.0623	0.148	0
4034	696.7	0.3123	0.2814	1.0621	0.1481	0
4035	696.6	0.3122	0.2816	1.0623	0.148	0
4036	696.5	0.3123	0.2816	1.0621	0.148	0
4037	696.4	0.3124	0.2817	1.0621	0.1481	0
4038	696.3	0.3126	0.2817	1.0622	0.1482	0
4039	696.2	0.3126	0.2818	1.0625	0.1483	0
4040	696.1	0.3126	0.282	1.0624	0.1483	0
4041	696	0.3127	0.2821	1.0625	0.1484	0
4042	695.9	0.3128	0.282	1.0631	0.1483	0
4043	695.8	0.3129	0.2823	1.0634	0.1485	0
4044	695.7	0.313	0.2825	1.0627	0.1486	0
4045	695.6	0.3131	0.2825	1.0628	0.1487	0
4046	695.5	0.3131	0.2827	1.0631	0.1488	0
4047	695.4	0.3133	0.2828	1.0628	0.1489	0
4048	695.3	0.3131	0.2828	1.0627	0.1487	0
4049	695.2	0.3133	0.2828	1.0622	0.1488	0
4050	695.1	0.3135	0.2831	1.0627	0.149	0
4051	695	0.3136	0.2832	1.0633	0.1492	0
4052	694.9	0.3136	0.2832	1.0628	0.1491	0
4053	694.8	0.3137	0.2835	1.0629	0.1493	0
4054	694.7	0.3137	0.2835	1.063	0.1491	0
4055	694.6	0.3138	0.2834	1.0629	0.1491	0
4056	694.5	0.3141	0.2838	1.0631	0.1494	0
4057	694.4	0.3141	0.284	1.0635	0.1495	0
4058	694.3	0.3141	0.2838	1.063	0.1494	0
4059	694.2	0.3143	0.284	1.0629	0.1496	0
4060	694.1	0.3144	0.2842	1.0632	0.1497	0
4061	694	0.3144	0.2843	1.0628	0.1498	0
4062	693.9	0.3146	0.2844	1.063	0.1497	0
4063	693.8	0.3146	0.2844	1.0637	0.1499	0
4064	693.7	0.3148	0.2845	1.0636	0.1501	0
4065	693.6	0.3149	0.2849	1.0634	0.1502	0
4066	693.5	0.3149	0.285	1.0628	0.1502	0
4067	693.4	0.3152	0.2852	1.0632	0.1504	0
4068	693.3	0.315	0.2851	1.0633	0.1503	0
4069	693.2	0.3151	0.2852	1.0635	0.1504	0
4070	693.1	0.3152	0.2853	1.0635	0.1505	0
4071	693	0.3153	0.2854	1.0638	0.1505	0

4072	692.9	0.3152	0.2855	1.0633	0.1505	0
4073	692.8	0.3155	0.2857	1.0631	0.1506	0
4074	692.7	0.3156	0.2859	1.063	0.1508	0
4075	692.6	0.3158	0.2859	1.0632	0.1509	0
4076	692.5	0.3159	0.2862	1.0637	0.1511	0
4077	692.4	0.3161	0.2862	1.0638	0.1512	0
4078	692.3	0.3161	0.2864	1.0639	0.1511	0
4079	692.2	0.3161	0.2864	1.0635	0.1513	0
4080	692.1	0.316	0.2864	1.0636	0.1512	0
4081	692	0.3161	0.2864	1.0636	0.1512	0
4082	691.9	0.3162	0.2864	1.0634	0.1512	0
4083	691.8	0.3163	0.2865	1.063	0.1513	0
4084	691.7	0.3165	0.2867	1.0635	0.1515	0
4085	691.6	0.3167	0.2869	1.0639	0.1517	0
4086	691.5	0.3167	0.2869	1.0639	0.1515	0
4087	691.4	0.3168	0.287	1.0635	0.1515	0
4088	691.3	0.3168	0.2872	1.0636	0.1517	0
4089	691.2	0.3167	0.2872	1.0641	0.1517	0
4090	691.1	0.3169	0.2873	1.0637	0.1518	0
4091	691	0.317	0.2874	1.0644	0.1519	0
4092	690.9	0.3171	0.2876	1.0641	0.1519	0
4093	690.8	0.317	0.2875	1.0639	0.1519	0
4094	690.7	0.3172	0.2877	1.0644	0.152	0
4095	690.6	0.3173	0.2879	1.0636	0.1523	0
4096	690.5	0.3175	0.2879	1.0644	0.1523	0
4097	690.4	0.3176	0.2881	1.0644	0.1524	0
4098	690.3	0.3177	0.2881	1.0648	0.1524	0
4099	690.2	0.3176	0.2882	1.0645	0.1525	0
4100	690.1	0.3179	0.2884	1.0643	0.1526	0
4101	690	0.3179	0.2885	1.0654	0.1526	0
4102	689.9	0.318	0.2886	1.0651	0.1526	0
4103	689.8	0.3184	0.2888	1.065	0.1528	0
4104	689.7	0.3185	0.2889	1.0655	0.153	0
4105	689.6	0.3184	0.289	1.0649	0.1529	0
4106	689.5	0.3182	0.2889	1.0655	0.1529	0
4107	689.4	0.3183	0.2889	1.066	0.1529	0
4108	689.3	0.3184	0.289	1.0654	0.1529	0
4109	689.2	0.3185	0.2891	1.0656	0.1529	0
4110	689.1	0.3186	0.2891	1.0654	0.153	0
4111	689	0.3185	0.2891	1.0649	0.153	0
4112	688.9	0.3187	0.2893	1.066	0.1532	0
4113	688.8	0.3189	0.2895	1.0654	0.1532	0
4114	688.7	0.3189	0.2896	1.0656	0.1533	0

4115	688.6	0.3188	0.2896	1.0657	0.1533	0
4116	688.5	0.319	0.2896	1.0657	0.1534	0
4117	688.4	0.319	0.2897	1.0658	0.1535	0
4118	688.3	0.3191	0.2897	1.0665	0.1536	0
4119	688.2	0.3193	0.2899	1.0663	0.1537	0
4120	688.1	0.3194	0.29	1.0662	0.1537	0
4121	688	0.3194	0.2901	1.0665	0.1537	0
4122	687.9	0.3195	0.2903	1.0663	0.1538	0
4123	687.8	0.3197	0.2904	1.0663	0.1539	0
4124	687.7	0.3196	0.2904	1.0659	0.154	0
4125	687.6	0.3196	0.2905	1.0662	0.1541	0
4126	687.5	0.3197	0.2906	1.0664	0.1542	0
4127	687.4	0.32	0.2907	1.0661	0.1542	0
4128	687.3	0.3202	0.2907	1.067	0.1544	0
4129	687.2	0.3201	0.2908	1.0662	0.1544	0
4130	687.1	0.3201	0.2907	1.0663	0.1543	0
4131	687	0.3202	0.2909	1.0671	0.1543	0
4132	686.9	0.3203	0.291	1.0675	0.1544	0
4133	686.8	0.3204	0.2911	1.0674	0.1545	0
4134	686.7	0.3205	0.2913	1.0673	0.1545	0
4135	686.6	0.3206	0.2913	1.0671	0.1547	0
4136	686.5	0.3208	0.2915	1.0669	0.1549	0
4137	686.4	0.3209	0.2916	1.0681	0.155	0
4138	686.3	0.3209	0.2917	1.0673	0.1551	0
4139	686.2	0.321	0.2918	1.0671	0.1551	0
4140	686.1	0.3211	0.2918	1.0678	0.1552	0
4141	686	0.3212	0.2919	1.0678	0.1553	0
4142	685.9	0.3214	0.292	1.0674	0.1554	0
4143	685.8	0.3214	0.292	1.0673	0.1555	0
4144	685.7	0.3213	0.2921	1.0675	0.1555	0
4145	685.6	0.3215	0.2923	1.0684	0.1558	0
4146	685.5	0.3217	0.2923	1.0684	0.1557	0
4147	685.4	0.3218	0.2925	1.068	0.1558	0
4148	685.3	0.3219	0.2925	1.0679	0.1559	0
4149	685.2	0.322	0.2927	1.0678	0.1561	0
4150	685.1	0.322	0.2928	1.0675	0.156	0
4151	685	0.322	0.2927	1.0674	0.156	0
4152	684.9	0.322	0.2929	1.0676	0.1561	0
4153	684.8	0.3223	0.2929	1.0674	0.1561	0
4154	684.7	0.3225	0.293	1.0677	0.1565	0
4155	684.6	0.3227	0.2932	1.0682	0.1566	0
4156	684.5	0.3227	0.2934	1.0682	0.1566	0
4157	684.4	0.3228	0.2934	1.0679	0.1566	0

4158	684.3	0.3228	0.2932	1.0679	0.1566	0
4159	684.2	0.323	0.2934	1.068	0.1567	0
4160	684.1	0.3229	0.2934	1.0687	0.1567	0
4161	684	0.3231	0.2936	1.0681	0.157	0
4162	683.9	0.3231	0.2937	1.0683	0.157	0
4163	683.8	0.3233	0.2938	1.0682	0.1571	0
4164	683.7	0.3234	0.2939	1.0688	0.1572	0
4165	683.6	0.3236	0.294	1.0684	0.1574	0
4166	683.5	0.3236	0.2941	1.0685	0.1573	0
4167	683.4	0.3236	0.2941	1.0683	0.1574	0
4168	683.3	0.3236	0.2941	1.0687	0.1576	0
4169	683.2	0.3239	0.2944	1.0683	0.1576	0
4170	683.1	0.3239	0.2944	1.0684	0.1577	0
4171	683	0.3242	0.2946	1.0687	0.158	0
4172	682.9	0.3241	0.2948	1.0688	0.158	0
4173	682.8	0.3243	0.2947	1.0684	0.158	0
4174	682.7	0.3243	0.2947	1.0683	0.158	0
4175	682.6	0.3245	0.2948	1.0688	0.1582	0
4176	682.5	0.3245	0.2949	1.0691	0.1584	0
4177	682.4	0.3247	0.295	1.0691	0.1585	0
4178	682.3	0.3247	0.2952	1.0692	0.1585	0
4179	682.2	0.3247	0.2951	1.0691	0.1584	0
4180	682.1	0.3248	0.295	1.0694	0.1586	0
4181	682	0.3249	0.2953	1.0696	0.1587	0
4182	681.9	0.3251	0.2954	1.0697	0.1587	0
4183	681.8	0.325	0.2953	1.069	0.1586	0
4184	681.7	0.3251	0.2953	1.0694	0.1588	0
4185	681.6	0.3254	0.2957	1.0699	0.1589	0
4186	681.5	0.3252	0.2955	1.0702	0.1589	0
4187	681.4	0.3254	0.2956	1.0703	0.159	0
4188	681.3	0.3255	0.2955	1.0701	0.159	0
4189	681.2	0.3256	0.2958	1.0705	0.1591	0
4190	681.1	0.3256	0.2957	1.0701	0.1591	0
4191	681	0.3257	0.2959	1.0701	0.1593	0
4192	680.9	0.3258	0.2959	1.0699	0.1593	0
4193	680.8	0.3259	0.296	1.0708	0.1594	0
4194	680.7	0.3258	0.296	1.0704	0.1594	0
4195	680.6	0.3258	0.2959	1.07	0.1593	0
4196	680.5	0.3259	0.2959	1.0704	0.1594	0
4197	680.4	0.3263	0.2965	1.071	0.1597	0
4198	680.3	0.326	0.2963	1.0702	0.1595	0
4199	680.2	0.3261	0.2962	1.0704	0.1596	0
4200	680.1	0.3263	0.2965	1.0711	0.1597	0

4201	680	0.3262	0.2964	1.0706	0.1597	0
4202	679.9	0.3263	0.2964	1.0707	0.1596	0
4203	679.8	0.3262	0.2964	1.0713	0.1597	0
4204	679.7	0.3263	0.2966	1.0714	0.1597	0
4205	679.6	0.3266	0.2967	1.0716	0.16	0
4206	679.5	0.3268	0.2968	1.0718	0.1602	0
4207	679.4	0.3267	0.2968	1.0719	0.16	0
4208	679.3	0.3267	0.2966	1.072	0.1601	0
4209	679.2	0.3269	0.2968	1.0716	0.1602	0
4210	679.1	0.3269	0.2969	1.0718	0.1601	0
4211	679	0.3268	0.2969	1.0722	0.1601	0
4212	678.9	0.3268	0.297	1.0723	0.1602	0
4213	678.8	0.3269	0.297	1.0717	0.1603	0
4214	678.7	0.3269	0.2972	1.072	0.1604	0
4215	678.6	0.3271	0.2971	1.0724	0.1604	0
4216	678.5	0.3271	0.2972	1.0726	0.1604	0
4217	678.4	0.3271	0.2971	1.0731	0.1604	0
4218	678.3	0.3272	0.2973	1.073	0.1607	0
4219	678.2	0.3273	0.2974	1.0733	0.1607	0
4220	678.1	0.3274	0.2973	1.0733	0.1607	0
4221	678	0.3272	0.2973	1.0726	0.1608	0
4222	677.9	0.3272	0.2975	1.0729	0.1608	0
4223	677.8	0.3274	0.2977	1.0737	0.1608	0
4224	677.7	0.3275	0.2978	1.0736	0.161	0
4225	677.6	0.3275	0.2978	1.0732	0.161	0
4226	677.5	0.3277	0.2977	1.0733	0.161	0
4227	677.4	0.3278	0.2978	1.0736	0.1611	0
4228	677.3	0.3278	0.2979	1.0742	0.1612	0
4229	677.2	0.3278	0.298	1.0741	0.1612	0
4230	677.1	0.3278	0.2982	1.0739	0.1613	0
4231	677	0.3278	0.2982	1.0745	0.1614	0
4232	676.9	0.328	0.2984	1.0745	0.1615	0
4233	676.8	0.328	0.2984	1.0737	0.1616	0
4234	676.7	0.3281	0.2986	1.0741	0.1617	0
4235	676.6	0.3284	0.2985	1.0741	0.1619	0
4236	676.5	0.3287	0.2988	1.0746	0.1622	0
4237	676.4	0.3286	0.2989	1.0744	0.1622	0
4238	676.3	0.3286	0.2989	1.0749	0.1623	0
4239	676.2	0.3285	0.2989	1.0741	0.1623	0
4240	676.1	0.3285	0.2989	1.0744	0.1623	0
4241	676	0.3287	0.299	1.0746	0.1624	0
4242	675.9	0.3288	0.2991	1.0748	0.1625	0
4243	675.8	0.3289	0.2994	1.0748	0.1626	0

4244	675.7	0.3288	0.2994	1.0751	0.1627	0
4245	675.6	0.3289	0.2994	1.0752	0.1626	0
4246	675.5	0.3291	0.2995	1.0753	0.1628	0
4247	675.4	0.3291	0.2996	1.0753	0.163	0
4248	675.3	0.3293	0.2997	1.0747	0.163	0
4249	675.2	0.3295	0.2999	1.0748	0.1633	0
4250	675.1	0.3295	0.3	1.0743	0.1635	0
4251	675	0.3297	0.3003	1.0748	0.1637	0
4252	674.9	0.3296	0.3001	1.0747	0.1635	0
4253	674.8	0.3296	0.3004	1.0751	0.1639	0
4254	674.7	0.3296	0.3002	1.0753	0.1639	0
4255	674.6	0.3296	0.3003	1.0752	0.1639	0
4256	674.5	0.3299	0.3004	1.0756	0.1641	0
4257	674.4	0.3298	0.3005	1.075	0.164	0
4258	674.3	0.3299	0.3006	1.075	0.1642	0
4259	674.2	0.33	0.3009	1.075	0.1644	0
4260	674.1	0.33	0.3009	1.0751	0.1645	0
4261	674	0.3302	0.301	1.0747	0.1646	0
4262	673.9	0.3302	0.3011	1.0748	0.1647	0
4263	673.8	0.3302	0.3012	1.0751	0.1649	0
4264	673.7	0.3304	0.3014	1.0753	0.1649	0
4265	673.6	0.3306	0.3015	1.0757	0.1651	0
4266	673.5	0.3306	0.3015	1.0756	0.1651	0
4267	673.4	0.3306	0.3017	1.0756	0.1653	0
4268	673.3	0.3305	0.3018	1.0749	0.1654	0
4269	673.2	0.3308	0.3018	1.0756	0.1655	0
4270	673.1	0.3307	0.3018	1.0762	0.1655	0
4271	673	0.331	0.3018	1.0761	0.1656	0
4272	672.9	0.331	0.3021	1.0759	0.1657	0
4273	672.8	0.3311	0.3022	1.0764	0.1659	0
4274	672.7	0.3311	0.3022	1.0761	0.1658	0
4275	672.6	0.3312	0.3023	1.0762	0.166	0
4276	672.5	0.3311	0.3024	1.0763	0.1661	0
4277	672.4	0.3313	0.3025	1.0761	0.1663	0
4278	672.3	0.3316	0.3026	1.0765	0.1665	0
4279	672.2	0.3315	0.3026	1.0769	0.1664	0
4280	672.1	0.3315	0.3026	1.0765	0.1665	0
4281	672	0.3315	0.3027	1.0769	0.1665	0
4282	671.9	0.3316	0.303	1.0766	0.1666	0
4283	671.8	0.3316	0.3029	1.0766	0.1668	0
4284	671.7	0.3315	0.3029	1.0765	0.1667	0
4285	671.6	0.3316	0.3029	1.0767	0.1667	0
4286	671.5	0.3318	0.3032	1.0775	0.167	0

4287	671.4	0.3317	0.3031	1.077	0.167	0
4288	671.3	0.3316	0.3029	1.0771	0.167	0
4289	671.2	0.3319	0.3032	1.0774	0.1671	0
4290	671.1	0.3319	0.3032	1.0769	0.1672	0
4291	671	0.332	0.3033	1.0773	0.1672	0
4292	670.9	0.332	0.3034	1.0776	0.1673	0
4293	670.8	0.3319	0.3035	1.0773	0.1674	0
4294	670.7	0.332	0.3035	1.0775	0.1675	0
4295	670.6	0.3321	0.3035	1.0784	0.1676	0
4296	670.5	0.3323	0.3038	1.078	0.1676	0
4297	670.4	0.3324	0.3038	1.0781	0.1677	0
4298	670.3	0.3324	0.3038	1.0781	0.1677	0
4299	670.2	0.3324	0.3038	1.0782	0.1677	0
4300	670.1	0.3325	0.3039	1.078	0.1678	0
4301	670	0.3324	0.3039	1.0783	0.1678	0
4302	669.9	0.3322	0.3038	1.0782	0.1677	0
4303	669.8	0.3325	0.304	1.0784	0.1679	0
4304	669.7	0.3324	0.304	1.078	0.1678	0
4305	669.6	0.3325	0.3041	1.0785	0.1678	0
4306	669.5	0.3327	0.3042	1.0789	0.168	0
4307	669.4	0.3326	0.3041	1.0789	0.168	0
4308	669.3	0.3327	0.304	1.079	0.168	0
4309	669.2	0.3328	0.3041	1.0787	0.168	0
4310	669.1	0.3329	0.3043	1.0792	0.1681	0
4311	669	0.3327	0.3042	1.0788	0.1679	0
4312	668.9	0.333	0.3044	1.0793	0.1683	0
4313	668.8	0.333	0.3044	1.0799	0.1682	0
4314	668.7	0.3329	0.3044	1.0804	0.1683	0
4315	668.6	0.3331	0.3047	1.0804	0.1684	0
4316	668.5	0.3331	0.3046	1.0804	0.1683	0
4317	668.4	0.3332	0.3047	1.0803	0.1684	0
4318	668.3	0.3332	0.3046	1.0802	0.1683	0
4319	668.2	0.3334	0.3047	1.0808	0.1685	0
4320	668.1	0.3334	0.3048	1.0808	0.1686	0
4321	668	0.3334	0.3048	1.0807	0.1686	0
4322	667.9	0.3334	0.3049	1.0806	0.1686	0
4323	667.8	0.3336	0.305	1.0811	0.1689	0
4324	667.7	0.3337	0.305	1.0813	0.1689	0
4325	667.6	0.3336	0.3051	1.0814	0.1689	0
4326	667.5	0.3336	0.3051	1.0807	0.1689	0
4327	667.4	0.3339	0.3051	1.0812	0.169	0
4328	667.3	0.3341	0.3053	1.0817	0.1692	0
4329	667.2	0.3341	0.3053	1.0819	0.1691	0

4330	667.1	0.334	0.3053	1.082	0.1693	0
4331	667	0.3341	0.3056	1.0818	0.1694	0
4332	666.9	0.3342	0.3056	1.0819	0.1693	0
4333	666.8	0.3345	0.3059	1.0824	0.1696	0
4334	666.7	0.3345	0.3058	1.0822	0.1695	0
4335	666.6	0.3345	0.3057	1.0822	0.1696	0
4336	666.5	0.3346	0.3058	1.0831	0.1696	0
4337	666.4	0.3347	0.3059	1.0824	0.1697	0
4338	666.3	0.3346	0.306	1.0822	0.1697	0
4339	666.2	0.3348	0.3061	1.0825	0.1699	0
4340	666.1	0.3347	0.3061	1.0827	0.1699	0
4341	666	0.335	0.3064	1.0823	0.17	0
4342	665.9	0.3352	0.3064	1.0825	0.1703	0
4343	665.8	0.3352	0.3066	1.0835	0.1703	0
4344	665.7	0.3352	0.3066	1.0833	0.1703	0
4345	665.6	0.3352	0.3067	1.0833	0.1705	0
4346	665.5	0.3353	0.3068	1.0837	0.1707	0
4347	665.4	0.3355	0.3072	1.0837	0.1708	0
4348	665.3	0.3354	0.307	1.0836	0.1708	0
4349	665.2	0.3355	0.3072	1.0832	0.171	0
4350	665.1	0.3355	0.3072	1.0841	0.1711	0
4351	665	0.3357	0.3074	1.084	0.1713	0
4352	664.9	0.3359	0.3075	1.0839	0.1714	0
4353	664.8	0.3362	0.3077	1.0836	0.1717	0
4354	664.7	0.3361	0.3078	1.0841	0.1719	0
4355	664.6	0.3363	0.308	1.0838	0.1719	0
4356	664.5	0.3362	0.308	1.0839	0.1719	0
4357	664.4	0.3363	0.3081	1.0846	0.1722	0
4358	664.3	0.3364	0.3082	1.0846	0.1723	0
4359	664.2	0.3367	0.3083	1.0841	0.1724	0
4360	664.1	0.3367	0.3084	1.0845	0.1725	0
4361	664	0.3369	0.3087	1.0839	0.1727	0
4362	663.9	0.3369	0.3087	1.0842	0.1726	0
4363	663.8	0.337	0.309	1.0849	0.1729	0
4364	663.7	0.337	0.3091	1.0842	0.1731	0
4365	663.6	0.3372	0.3093	1.084	0.1734	0
4366	663.5	0.3373	0.3094	1.0833	0.1734	0
4367	663.4	0.3375	0.3095	1.0847	0.1736	0
4368	663.3	0.3377	0.3098	1.0846	0.174	0
4369	663.2	0.3377	0.3098	1.0854	0.1738	0
4370	663.1	0.3376	0.3099	1.0842	0.1739	0
4371	663	0.3375	0.31	1.0846	0.174	0
4372	662.9	0.3377	0.3101	1.0855	0.1742	0

4373	662.8	0.3377	0.3102	1.0846	0.1742	0
4374	662.7	0.3379	0.3103	1.0846	0.1743	0
4375	662.6	0.3378	0.3104	1.085	0.1745	0
4376	662.5	0.3379	0.3106	1.085	0.1746	0
4377	662.4	0.3381	0.3106	1.0848	0.1747	0
4378	662.3	0.3383	0.3109	1.085	0.1749	0
4379	662.2	0.3383	0.3108	1.0851	0.1749	0
4380	662.1	0.3385	0.3109	1.0857	0.1751	0
4381	662	0.3385	0.3111	1.0857	0.1752	0
4382	661.9	0.3385	0.3111	1.0855	0.1753	0
4383	661.8	0.3387	0.3113	1.0857	0.1754	0
4384	661.7	0.3387	0.3114	1.0856	0.1755	0
4385	661.6	0.3388	0.3115	1.086	0.1755	0
4386	661.5	0.3389	0.3115	1.0859	0.1756	0
4387	661.4	0.3388	0.3115	1.0863	0.1755	0
4388	661.3	0.3389	0.3116	1.0858	0.1757	0
4389	661.2	0.3389	0.3116	1.0866	0.1758	0
4390	661.1	0.3392	0.3119	1.0865	0.176	0
4391	661	0.3393	0.3121	1.0865	0.1761	0
4392	660.9	0.3393	0.3122	1.0866	0.1762	0
4393	660.8	0.3394	0.3123	1.0872	0.1763	0
4394	660.7	0.3395	0.3122	1.0878	0.1764	0
4395	660.6	0.3396	0.3125	1.0875	0.1765	0
4396	660.5	0.3395	0.3124	1.087	0.1765	0
4397	660.4	0.3397	0.3126	1.0867	0.1766	0
4398	660.3	0.3398	0.3127	1.0871	0.1768	0
4399	660.2	0.3396	0.3128	1.0872	0.1768	0
4400	660.1	0.3397	0.3128	1.0876	0.1769	0
4401	660	0.3399	0.3131	1.0877	0.177	0
4402	659.9	0.34	0.3131	1.0876	0.1771	0
4403	659.8	0.3403	0.3134	1.0879	0.1773	0
4404	659.7	0.3402	0.3135	1.0884	0.1774	0
4405	659.6	0.3403	0.3134	1.0882	0.1773	0
4406	659.5	0.3404	0.3135	1.0882	0.1774	0
4407	659.4	0.3404	0.3134	1.0891	0.1775	0
4408	659.3	0.3407	0.3137	1.0888	0.1777	0
4409	659.2	0.3407	0.3137	1.089	0.1777	0
4410	659.1	0.3407	0.3139	1.0895	0.1777	0
4411	659	0.3406	0.3138	1.0898	0.1776	0
4412	658.9	0.3406	0.3139	1.0902	0.1777	0
4413	658.8	0.3407	0.314	1.0896	0.1778	0
4414	658.7	0.3408	0.3141	1.0899	0.1779	0
4415	658.6	0.3409	0.3142	1.0899	0.1779	0

4416	658.5	0.3411	0.3143	1.0901	0.178	0
4417	658.4	0.341	0.3141	1.0903	0.1781	0
4418	658.3	0.341	0.3142	1.0908	0.1781	0
4419	658.2	0.3411	0.3145	1.0906	0.1782	0
4420	658.1	0.3414	0.3146	1.0909	0.1783	0
4421	658	0.3414	0.3146	1.0909	0.1783	0
4422	657.9	0.3415	0.3149	1.0916	0.1785	0
4423	657.8	0.3415	0.3149	1.092	0.1785	0
4424	657.7	0.3415	0.3149	1.0918	0.1787	0
4425	657.6	0.3416	0.3152	1.0918	0.1789	0
4426	657.5	0.3418	0.3152	1.0915	0.1789	0
4427	657.4	0.3418	0.3153	1.092	0.1789	0
4428	657.3	0.3418	0.3154	1.0919	0.179	0
4429	657.2	0.3419	0.3154	1.0924	0.179	0
4430	657.1	0.3417	0.3153	1.0917	0.1789	0
4431	657	0.3419	0.3155	1.0918	0.1792	0
4432	656.9	0.3418	0.3154	1.0928	0.1791	0
4433	656.8	0.3417	0.3155	1.0925	0.179	0
4434	656.7	0.3421	0.3157	1.093	0.1794	0
4435	656.6	0.3419	0.3158	1.0929	0.1794	0
4436	656.5	0.3422	0.316	1.0934	0.1796	0
4437	656.4	0.3422	0.316	1.0937	0.1796	0
4438	656.3	0.3425	0.3162	1.094	0.1797	0
4439	656.2	0.3426	0.3166	1.0934	0.18	0
4440	656.1	0.3425	0.3165	1.0938	0.18	0
4441	656	0.3425	0.3164	1.0938	0.18	0
4442	655.9	0.3426	0.3166	1.0942	0.1802	0
4443	655.8	0.3428	0.3169	1.0939	0.1803	0
4444	655.7	0.3428	0.3169	1.0947	0.1804	0
4445	655.6	0.3431	0.3172	1.0938	0.1806	0
4446	655.5	0.3432	0.3173	1.0951	0.1806	0
4447	655.4	0.3433	0.3176	1.0953	0.1808	0
4448	655.3	0.3433	0.3175	1.0948	0.1809	0
4449	655.2	0.3434	0.3177	1.0948	0.1811	0
4450	655.1	0.3436	0.3181	1.0951	0.1812	0
4451	655	0.3437	0.318	1.0945	0.1814	0
4452	654.9	0.3438	0.3182	1.0948	0.1817	0
4453	654.8	0.344	0.3184	1.0953	0.1818	0
4454	654.7	0.3442	0.3186	1.0956	0.1819	0
4455	654.6	0.3441	0.319	1.0958	0.1821	0
4456	654.5	0.3442	0.3188	1.096	0.1821	0
4457	654.4	0.3445	0.3191	1.0955	0.1825	0
4458	654.3	0.3446	0.3194	1.0964	0.1826	0

4459	654.2	0.3446	0.3196	1.0963	0.1829	0
4460	654.1	0.345	0.3198	1.0969	0.1831	0
4461	654	0.345	0.3199	1.0967	0.1832	0
4462	653.9	0.3453	0.3202	1.0967	0.1834	0
4463	653.8	0.3454	0.3204	1.0968	0.1836	0
4464	653.7	0.3455	0.3205	1.0967	0.1837	0
4465	653.6	0.3456	0.3209	1.097	0.184	0
4466	653.5	0.3457	0.321	1.0977	0.1841	0
4467	653.4	0.346	0.3212	1.0977	0.1843	0
4468	653.3	0.3462	0.3215	1.0973	0.1843	0
4469	653.2	0.3463	0.3216	1.0969	0.1845	0
4470	653.1	0.3465	0.3218	1.0972	0.1848	0
4471	653	0.3466	0.3221	1.0974	0.1851	0
4472	652.9	0.3469	0.3222	1.0975	0.1851	0
4473	652.8	0.3469	0.3224	1.0977	0.1853	0
4474	652.7	0.3469	0.3226	1.0984	0.1856	0
4475	652.6	0.3472	0.3229	1.0977	0.1858	0
4476	652.5	0.3472	0.323	1.0983	0.186	0
4477	652.4	0.3474	0.3232	1.0978	0.1861	0
4478	652.3	0.3475	0.3233	1.0977	0.1861	0
4479	652.2	0.3478	0.3236	1.0985	0.1865	0
4480	652.1	0.348	0.3239	1.0988	0.1867	0
4481	652	0.348	0.3238	1.0988	0.1867	0
4482	651.9	0.348	0.3241	1.0991	0.1869	0
4483	651.8	0.3481	0.324	1.099	0.1869	0
4484	651.7	0.3485	0.3244	1.0992	0.1873	0
4485	651.6	0.3485	0.3245	1.0991	0.1873	0
4486	651.5	0.3487	0.3249	1.0996	0.1875	0
4487	651.4	0.3489	0.325	1.1001	0.1877	0
4488	651.3	0.3488	0.3251	1.0995	0.1877	0
4489	651.2	0.3492	0.3254	1.1003	0.1879	0
4490	651.1	0.3493	0.3256	1.0999	0.1882	0
4491	651	0.3495	0.3257	1.1006	0.1883	0
4492	650.9	0.3495	0.3257	1.1007	0.1883	0
4493	650.8	0.3496	0.326	1.1006	0.1884	0
4494	650.7	0.3495	0.3258	1.1004	0.1885	0
4495	650.6	0.3496	0.3261	1.1008	0.1887	0
4496	650.5	0.3499	0.3263	1.1008	0.1889	0
4497	650.4	0.3501	0.3267	1.1015	0.1891	0
4498	650.3	0.3504	0.3269	1.1017	0.1893	0
4499	650.2	0.3507	0.3271	1.1023	0.1894	0
4500	650.1	0.3504	0.327	1.1024	0.1893	0
4501	650	0.3505	0.3272	1.1019	0.1895	0

4502	649.9	0.3507	0.3275	1.1024	0.1897	0
4503	649.8	0.3507	0.3275	1.102	0.1898	0
4504	649.7	0.3508	0.3274	1.1018	0.1897	0
4505	649.6	0.3512	0.3278	1.1022	0.19	0
4506	649.5	0.3512	0.3278	1.1027	0.1901	0
4507	649.4	0.3514	0.328	1.1026	0.1902	0
4508	649.3	0.3515	0.328	1.1028	0.1904	0
4509	649.2	0.3517	0.3282	1.1036	0.1906	0
4510	649.1	0.3519	0.3284	1.1034	0.1907	0
4511	649	0.3519	0.3284	1.1035	0.1909	0
4512	648.9	0.352	0.3285	1.1034	0.1909	0
4513	648.8	0.3522	0.3286	1.104	0.191	0
4514	648.7	0.3522	0.3287	1.1044	0.1911	0
4515	648.6	0.3524	0.3291	1.1051	0.1913	0
4516	648.5	0.3525	0.3291	1.1048	0.1914	0
4517	648.4	0.3525	0.3292	1.1051	0.1916	0
4518	648.3	0.3525	0.3291	1.1053	0.1916	0
4519	648.2	0.3529	0.3295	1.1054	0.1918	0
4520	648.1	0.3529	0.3295	1.1058	0.1919	0
4521	648	0.3531	0.3296	1.1055	0.1921	0
4522	647.9	0.3532	0.3298	1.1054	0.1922	0
4523	647.8	0.3534	0.3299	1.1061	0.1923	0
4524	647.7	0.3536	0.3302	1.1061	0.1925	0
4525	647.6	0.3536	0.3301	1.1069	0.1926	0
4526	647.5	0.3537	0.3301	1.1075	0.1926	0
4527	647.4	0.3538	0.3303	1.1071	0.1926	0
4528	647.3	0.3541	0.3306	1.107	0.193	0
4529	647.2	0.354	0.3308	1.1079	0.1931	0
4530	647.1	0.3542	0.3309	1.1083	0.1932	0
4531	647	0.3543	0.3308	1.107	0.1932	0
4532	646.9	0.3545	0.331	1.1079	0.1933	0
4533	646.8	0.3547	0.3311	1.108	0.1934	0
4534	646.7	0.3549	0.3312	1.1084	0.1934	0
4535	646.6	0.355	0.3314	1.108	0.1936	0
4536	646.5	0.3548	0.3313	1.1085	0.1935	0
4537	646.4	0.355	0.3316	1.1095	0.1937	0
4538	646.3	0.3551	0.3318	1.1095	0.1939	0
4539	646.2	0.3555	0.332	1.1094	0.194	0
4540	646.1	0.3556	0.3321	1.1091	0.1942	0
4541	646	0.3558	0.3322	1.1092	0.1942	0
4542	645.9	0.3558	0.3323	1.1097	0.1942	0
4543	645.8	0.356	0.3325	1.1096	0.1944	0
4544	645.7	0.3563	0.3327	1.1094	0.1946	0

4545	645.6	0.3563	0.3328	1.1101	0.1947	0
4546	645.5	0.3564	0.3329	1.1103	0.1948	0
4547	645.4	0.3566	0.3332	1.1106	0.195	0
4548	645.3	0.3567	0.3333	1.1107	0.1951	0
4549	645.2	0.3569	0.3333	1.1109	0.1951	0
4550	645.1	0.3571	0.3336	1.1108	0.1954	0
4551	645	0.3572	0.3336	1.1106	0.1953	0
4552	644.9	0.3573	0.3339	1.111	0.1955	0
4553	644.8	0.3576	0.3342	1.111	0.1957	0
4554	644.7	0.3576	0.3341	1.1111	0.1957	0
4555	644.6	0.358	0.3343	1.1112	0.1959	0
4556	644.5	0.358	0.3344	1.1114	0.196	0
4557	644.4	0.3582	0.3345	1.1118	0.1962	0
4558	644.3	0.3582	0.3345	1.1118	0.1962	0
4559	644.2	0.3584	0.3346	1.1117	0.1963	0
4560	644.1	0.3586	0.3347	1.1116	0.1965	0
4561	644	0.3587	0.3348	1.112	0.1965	0
4562	643.9	0.3589	0.3349	1.1122	0.1967	0
4563	643.8	0.3593	0.3352	1.112	0.197	0
4564	643.7	0.3596	0.3355	1.1125	0.1971	0
4565	643.6	0.3597	0.3355	1.1128	0.1972	0
4566	643.5	0.3598	0.3357	1.1129	0.1973	0
4567	643.4	0.3601	0.3358	1.1127	0.1974	0
4568	643.3	0.3601	0.3359	1.1123	0.1976	0
4569	643.2	0.3603	0.3358	1.1123	0.1976	0
4570	643.1	0.3605	0.3361	1.1125	0.1978	0
4571	643	0.3608	0.3364	1.1139	0.1981	0
4572	642.9	0.3611	0.3364	1.1137	0.1981	0
4573	642.8	0.3612	0.3366	1.1138	0.1983	0
4574	642.7	0.3612	0.3366	1.1136	0.1984	0
4575	642.6	0.3614	0.3368	1.1136	0.1984	0
4576	642.5	0.3616	0.3369	1.1139	0.1985	0
4577	642.4	0.3618	0.337	1.1144	0.1988	0
4578	642.3	0.3621	0.3373	1.1144	0.199	0
4579	642.2	0.3622	0.3372	1.1143	0.199	0
4580	642.1	0.3624	0.3374	1.1141	0.1992	0
4581	642	0.3625	0.3374	1.1141	0.1992	0
4582	641.9	0.3628	0.3376	1.1144	0.1993	0
4583	641.8	0.3629	0.3378	1.1144	0.1993	0
4584	641.7	0.3631	0.3379	1.1147	0.1994	0
4585	641.6	0.3633	0.3383	1.1151	0.1996	0
4586	641.5	0.3635	0.3384	1.115	0.1998	0
4587	641.4	0.3637	0.3385	1.1148	0.1998	0

4588	641.3	0.3639	0.3386	1.1156	0.1998	0
4589	641.2	0.364	0.3386	1.1152	0.1999	0
4590	641.1	0.3642	0.339	1.1159	0.2001	0
4591	641	0.3645	0.339	1.116	0.2001	0
4592	640.9	0.3645	0.3392	1.1164	0.2003	0
4593	640.8	0.3648	0.3395	1.1161	0.2003	0
4594	640.7	0.3649	0.3397	1.1163	0.2006	0
4595	640.6	0.3651	0.3395	1.1164	0.2005	0
4596	640.5	0.3653	0.3397	1.1167	0.2006	0
4597	640.4	0.3655	0.34	1.1165	0.2008	0
4598	640.3	0.3657	0.3401	1.117	0.2008	0
4599	640.2	0.3657	0.3403	1.1167	0.2009	0
4600	640.1	0.3661	0.3405	1.1172	0.201	0
4601	640	0.3664	0.3407	1.1173	0.2012	0
4602	639.9	0.3664	0.3408	1.1174	0.2012	0
4603	639.8	0.3665	0.341	1.1181	0.2013	0
4604	639.7	0.3667	0.3411	1.1173	0.2014	0
4605	639.6	0.3669	0.3411	1.1177	0.2014	0
4606	639.5	0.3669	0.3413	1.1173	0.2016	0
4607	639.4	0.3672	0.3415	1.1182	0.2017	0
4608	639.3	0.3674	0.3415	1.1182	0.2018	0
4609	639.2	0.3674	0.3417	1.1186	0.2018	0
4610	639.1	0.3677	0.342	1.119	0.2019	0
4611	639	0.3679	0.3421	1.1183	0.202	0
4612	638.9	0.3679	0.3422	1.1194	0.2021	0
4613	638.8	0.368	0.3421	1.119	0.202	0
4614	638.7	0.3683	0.3425	1.1194	0.2022	0
4615	638.6	0.3684	0.3426	1.1194	0.2023	0
4616	638.5	0.3686	0.343	1.1196	0.2026	0
4617	638.4	0.3688	0.3431	1.1203	0.2027	0
4618	638.3	0.3691	0.3433	1.12	0.2027	0
4619	638.2	0.3692	0.3435	1.1205	0.2027	0
4620	638.1	0.3693	0.3435	1.1203	0.2028	0
4621	638	0.3696	0.3438	1.1208	0.2029	0
4622	637.9	0.3698	0.3438	1.121	0.203	0
4623	637.8	0.3701	0.3441	1.1213	0.2033	0
4624	637.7	0.3701	0.3441	1.121	0.2032	0
4625	637.6	0.3702	0.3441	1.1217	0.2032	0
4626	637.5	0.3703	0.3443	1.1212	0.2033	0
4627	637.4	0.3705	0.3447	1.1226	0.2035	0
4628	637.3	0.3708	0.3451	1.1227	0.2037	0
4629	637.2	0.371	0.345	1.1223	0.2037	0
4630	637.1	0.3712	0.3452	1.1222	0.2038	0

4631	637	0.3712	0.3453	1.1224	0.2038	0
4632	636.9	0.3714	0.3454	1.1227	0.2039	0
4633	636.8	0.3713	0.3454	1.1232	0.2039	0
4634	636.7	0.3715	0.3456	1.1235	0.204	0
4635	636.6	0.3717	0.3458	1.1239	0.2041	0
4636	636.5	0.372	0.3461	1.124	0.2044	0
4637	636.4	0.3723	0.3464	1.1244	0.2045	0
4638	636.3	0.3724	0.3466	1.1246	0.2046	0
4639	636.2	0.3727	0.3467	1.1249	0.2047	0
4640	636.1	0.3728	0.3468	1.1244	0.2046	0
4641	636	0.3728	0.3467	1.1245	0.2047	0
4642	635.9	0.373	0.3468	1.1247	0.2045	0
4643	635.8	0.3732	0.347	1.1258	0.2049	0
4644	635.7	0.3733	0.3474	1.1257	0.205	0
4645	635.6	0.3735	0.3476	1.1258	0.2052	0
4646	635.5	0.3736	0.3477	1.1256	0.2054	0
4647	635.4	0.3736	0.3477	1.1251	0.2054	0
4648	635.3	0.3739	0.348	1.1256	0.2054	0
4649	635.2	0.3742	0.3483	1.126	0.2057	0
4650	635.1	0.3743	0.3483	1.1265	0.2057	0
4651	635	0.3745	0.3484	1.1262	0.2058	0
4652	634.9	0.3746	0.3487	1.1266	0.206	0
4653	634.8	0.3748	0.349	1.1268	0.206	0
4654	634.7	0.3748	0.349	1.1268	0.2061	0
4655	634.6	0.3752	0.3493	1.127	0.2065	0
4656	634.5	0.3753	0.3493	1.1268	0.2065	0
4657	634.4	0.3755	0.3496	1.1276	0.2066	0
4658	634.3	0.3759	0.35	1.1276	0.207	0
4659	634.2	0.376	0.3501	1.1275	0.2069	0
4660	634.1	0.3761	0.3504	1.1284	0.2071	0
4661	634	0.3763	0.3505	1.1287	0.2072	0
4662	633.9	0.3764	0.3508	1.1284	0.2075	0
4663	633.8	0.3766	0.3509	1.1283	0.2074	0
4664	633.7	0.3766	0.351	1.1287	0.2076	0
4665	633.6	0.3769	0.3512	1.1288	0.2076	0
4666	633.5	0.377	0.3513	1.1287	0.2078	0
4667	633.4	0.3772	0.3517	1.1286	0.208	0
4668	633.3	0.3774	0.3517	1.1288	0.2082	0
4669	633.2	0.3776	0.3521	1.1294	0.2085	0
4670	633.1	0.3776	0.3524	1.1295	0.2085	0
4671	633	0.3777	0.3522	1.1291	0.2086	0
4672	632.9	0.378	0.3526	1.13	0.2088	0
4673	632.8	0.3782	0.3527	1.1302	0.2088	0

4674	632.7	0.3783	0.353	1.1301	0.209	0
4675	632.6	0.3784	0.3531	1.1307	0.2092	0
4676	632.5	0.3786	0.3533	1.1301	0.2092	0
4677	632.4	0.3788	0.3535	1.13	0.2095	0
4678	632.3	0.379	0.3539	1.1309	0.2097	0
4679	632.2	0.3792	0.3539	1.1308	0.21	0
4680	632.1	0.3794	0.3543	1.1319	0.2102	0
4681	632	0.3797	0.3544	1.1315	0.2103	0
4682	631.9	0.3796	0.3545	1.1316	0.2101	0
4683	631.8	0.38	0.3547	1.1321	0.2103	0
4684	631.7	0.3799	0.355	1.1316	0.2105	0
4685	631.6	0.3801	0.3549	1.1324	0.2105	0
4686	631.5	0.3801	0.3552	1.1325	0.2106	0
4687	631.4	0.3802	0.3555	1.1325	0.2108	0
4688	631.3	0.3804	0.3556	1.1332	0.2109	0
4689	631.2	0.3804	0.3556	1.1328	0.211	0
4690	631.1	0.3804	0.3558	1.133	0.2111	0
4691	631	0.3807	0.3559	1.1336	0.2113	0
4692	630.9	0.3809	0.3562	1.1337	0.2114	0
4693	630.8	0.3811	0.3563	1.1336	0.2115	0
4694	630.7	0.3813	0.3566	1.1335	0.2116	0
4695	630.6	0.3813	0.3569	1.1343	0.2119	0
4696	630.5	0.3816	0.357	1.1349	0.2121	0
4697	630.4	0.3816	0.357	1.1346	0.2121	0
4698	630.3	0.3818	0.3571	1.1349	0.2123	0
4699	630.2	0.3818	0.3574	1.1357	0.2124	0
4700	630.1	0.3821	0.3577	1.1357	0.2127	0
4701	630	0.3822	0.3577	1.1355	0.2128	0
4702	629.9	0.3824	0.3579	1.136	0.2128	0
4703	629.8	0.3826	0.358	1.1361	0.2129	0
4704	629.7	0.3826	0.3582	1.1358	0.213	0
4705	629.6	0.3829	0.3583	1.1359	0.2133	0
4706	629.5	0.3831	0.3586	1.1365	0.2135	0
4707	629.4	0.3833	0.3588	1.1371	0.2136	0
4708	629.3	0.3833	0.3588	1.1376	0.2136	0
4709	629.2	0.3835	0.3591	1.1374	0.2138	0
4710	629.1	0.3835	0.3594	1.1382	0.214	0
4711	629	0.3837	0.3596	1.1383	0.2141	0
4712	628.9	0.3836	0.3597	1.1382	0.2142	0
4713	628.8	0.384	0.36	1.1383	0.2144	0
4714	628.7	0.3842	0.3599	1.1389	0.2144	0
4715	628.6	0.3843	0.3603	1.1392	0.2146	0
4716	628.5	0.3842	0.3605	1.1394	0.2145	0

4717	628.4	0.3846	0.3607	1.1399	0.2148	0
4718	628.3	0.3845	0.3607	1.1404	0.2149	0
4719	628.2	0.3848	0.3609	1.1401	0.2151	0
4720	628.1	0.3851	0.361	1.1408	0.2153	0
4721	628	0.3851	0.3612	1.1403	0.2155	0
4722	627.9	0.3853	0.3615	1.1409	0.2156	0
4723	627.8	0.3855	0.3617	1.1411	0.2157	0
4724	627.7	0.3858	0.3619	1.1413	0.216	0
4725	627.6	0.3858	0.3621	1.1417	0.2161	0
4726	627.5	0.3859	0.3622	1.1422	0.2164	0
4727	627.4	0.3858	0.3621	1.142	0.2163	0
4728	627.3	0.386	0.3625	1.1424	0.2165	0
4729	627.2	0.3862	0.3625	1.1422	0.2167	0
4730	627.1	0.3864	0.3627	1.1426	0.2168	0
4731	627	0.3866	0.3629	1.1427	0.2171	0
4732	626.9	0.3868	0.3631	1.1434	0.2172	0
4733	626.8	0.3871	0.3633	1.143	0.2173	0
4734	626.7	0.3872	0.3634	1.1438	0.2175	0
4735	626.6	0.3872	0.3636	1.1438	0.2175	0
4736	626.5	0.3874	0.3638	1.1438	0.2177	0
4737	626.4	0.3875	0.364	1.1446	0.2178	0
4738	626.3	0.3877	0.3642	1.1447	0.218	0
4739	626.2	0.3878	0.3641	1.1449	0.218	0
4740	626.1	0.388	0.3645	1.145	0.2183	0
4741	626	0.3879	0.3644	1.1459	0.2184	0
4742	625.9	0.3882	0.3646	1.1455	0.2185	0
4743	625.8	0.3882	0.3646	1.1452	0.2186	0
4744	625.7	0.3884	0.3649	1.1457	0.2188	0
4745	625.6	0.3888	0.3653	1.1465	0.2191	0
4746	625.5	0.3888	0.3653	1.1466	0.2192	0
4747	625.4	0.3889	0.3654	1.1467	0.2194	0
4748	625.3	0.389	0.3655	1.1476	0.2195	0
4749	625.2	0.389	0.3658	1.1471	0.2196	0
4750	625.1	0.3892	0.3659	1.1471	0.2199	0
4751	625	0.3895	0.3661	1.1478	0.22	0
4752	624.9	0.3896	0.3662	1.1471	0.2201	0
4753	624.8	0.3898	0.3664	1.148	0.2205	0
4754	624.7	0.3901	0.3666	1.1483	0.2207	0
4755	624.6	0.3901	0.3666	1.1483	0.2208	0
4756	624.5	0.3902	0.3668	1.148	0.2209	0
4757	624.4	0.3905	0.3668	1.1489	0.2211	0
4758	624.3	0.3906	0.3671	1.1487	0.2212	0
4759	624.2	0.3904	0.3671	1.1492	0.2212	0

4760	624.1	0.3907	0.3673	1.1497	0.2215	0
4761	624	0.3909	0.3675	1.1497	0.2217	0
4762	623.9	0.391	0.3677	1.1497	0.2218	0
4763	623.8	0.3912	0.3678	1.15	0.222	0
4764	623.7	0.3914	0.368	1.1501	0.2222	0
4765	623.6	0.3916	0.368	1.1509	0.2224	0
4766	623.5	0.3917	0.3683	1.1505	0.2226	0
4767	623.4	0.3917	0.3684	1.1513	0.2228	0
4768	623.3	0.392	0.3683	1.1515	0.223	0
4769	623.2	0.3919	0.3687	1.1521	0.2232	0
4770	623.1	0.392	0.3687	1.152	0.2233	0
4771	623	0.3923	0.3689	1.1517	0.2234	0
4772	622.9	0.3924	0.3691	1.1516	0.2237	0
4773	622.8	0.3925	0.3692	1.1522	0.2238	0
4774	622.7	0.3928	0.3694	1.1528	0.224	0
4775	622.6	0.3927	0.3695	1.1526	0.2239	0
4776	622.5	0.3928	0.3695	1.1531	0.2242	0
4777	622.4	0.3929	0.3695	1.1539	0.2243	0
4778	622.3	0.3929	0.3695	1.1538	0.2244	0
4779	622.2	0.3933	0.3699	1.1544	0.2247	0
4780	622.1	0.3934	0.3699	1.1549	0.2248	0
4781	622	0.3935	0.3701	1.1542	0.2249	0
4782	621.9	0.3936	0.3702	1.1553	0.2252	0
4783	621.8	0.3937	0.3702	1.1556	0.2254	0
4784	621.7	0.3939	0.3704	1.1557	0.2255	0
4785	621.6	0.3942	0.3706	1.1562	0.2258	0
4786	621.5	0.3942	0.3704	1.1565	0.2258	0
4787	621.4	0.3942	0.3704	1.1566	0.2259	0
4788	621.3	0.3943	0.3706	1.1565	0.2262	0
4789	621.2	0.3945	0.3708	1.1571	0.2263	0
4790	621.1	0.3945	0.3708	1.1573	0.2263	0
4791	621	0.3945	0.3708	1.1571	0.2264	0
4792	620.9	0.3947	0.371	1.1575	0.2267	0
4793	620.8	0.395	0.3711	1.1575	0.2268	0
4794	620.7	0.3949	0.3711	1.1585	0.227	0
4795	620.6	0.395	0.3712	1.1592	0.2273	0
4796	620.5	0.3949	0.3712	1.1596	0.2274	0
4797	620.4	0.395	0.3712	1.1599	0.2274	0
4798	620.3	0.3951	0.3713	1.1604	0.2277	0
4799	620.2	0.3951	0.3713	1.1601	0.2278	0
4800	620.1	0.3953	0.3715	1.1608	0.228	0
4801	620	0.3953	0.3714	1.1613	0.228	0
4802	619.9	0.3955	0.3716	1.1621	0.2282	0

4803	619.8	0.3956	0.3718	1.1618	0.2284	0
4804	619.7	0.3955	0.3719	1.1624	0.2286	0
4805	619.6	0.3956	0.3719	1.1624	0.2287	0
4806	619.5	0.3957	0.3717	1.1621	0.2288	0
4807	619.4	0.3959	0.3719	1.1636	0.2291	0
4808	619.3	0.396	0.3719	1.1641	0.2292	0
4809	619.2	0.3961	0.372	1.1648	0.2294	0
4810	619.1	0.3964	0.3722	1.1651	0.2298	0
4811	619	0.3963	0.3722	1.1656	0.23	0
4812	618.9	0.3964	0.3722	1.1656	0.23	0
4813	618.8	0.3967	0.3724	1.1662	0.2303	0
4814	618.7	0.3968	0.3725	1.1666	0.2305	0
4815	618.6	0.3967	0.3723	1.1666	0.2304	0
4816	618.5	0.397	0.3725	1.1676	0.2308	0
4817	618.4	0.3969	0.3726	1.1681	0.2309	0
4818	618.3	0.397	0.3726	1.1683	0.2311	0
4819	618.2	0.397	0.3728	1.1682	0.2314	0
4820	618.1	0.3972	0.3728	1.1688	0.2315	0
4821	618	0.3972	0.3728	1.1689	0.2317	0
4822	617.9	0.3971	0.3729	1.1698	0.232	0
4823	617.8	0.3973	0.3732	1.1699	0.2322	0
4824	617.7	0.3973	0.373	1.1708	0.2324	0
4825	617.6	0.3975	0.3731	1.1704	0.2325	0
4826	617.5	0.3976	0.3731	1.1712	0.2329	0
4827	617.4	0.3976	0.3732	1.1712	0.233	0
4828	617.3	0.3976	0.3732	1.1721	0.2332	0
4829	617.2	0.3978	0.3733	1.1729	0.2334	0
4830	617.1	0.3981	0.3737	1.1731	0.2339	0
4831	617	0.3982	0.3736	1.1739	0.234	0
4832	616.9	0.3983	0.3737	1.1738	0.2343	0
4833	616.8	0.3983	0.3736	1.174	0.2345	0
4834	616.7	0.3983	0.3736	1.174	0.2345	0
4835	616.6	0.3986	0.3736	1.1744	0.2348	0
4836	616.5	0.3986	0.3738	1.1751	0.2352	0
4837	616.4	0.3987	0.3738	1.1754	0.2354	0
4838	616.3	0.3986	0.3738	1.1755	0.2355	0
4839	616.2	0.3986	0.374	1.1759	0.2358	0
4840	616.1	0.3989	0.3741	1.1767	0.2361	0
4841	616	0.3989	0.374	1.1762	0.2363	0
4842	615.9	0.3992	0.3742	1.1761	0.2366	0
4843	615.8	0.3993	0.3744	1.1774	0.2369	0
4844	615.7	0.3992	0.3743	1.1763	0.2371	0
4845	615.6	0.3994	0.3745	1.1777	0.2374	0

4846	615.5	0.3992	0.3745	1.1775	0.2375	0
4847	615.4	0.3993	0.3745	1.178	0.2377	0
4848	615.3	0.3996	0.3747	1.1782	0.2381	0
4849	615.2	0.3997	0.3747	1.1783	0.2384	0
4850	615.1	0.3997	0.3746	1.178	0.2386	0
4851	615	0.3997	0.3746	1.1783	0.2387	0
4852	614.9	0.3997	0.3747	1.18	0.2391	0
4853	614.8	0.3999	0.375	1.1797	0.2396	0
4854	614.7	0.4	0.375	1.1797	0.2398	0
4855	614.6	0.4	0.3749	1.1797	0.24	0
4856	614.5	0.4002	0.3751	1.18	0.2401	0
4857	614.4	0.4002	0.3751	1.1801	0.2404	0
4858	614.3	0.4002	0.3751	1.1806	0.2405	0
4859	614.2	0.4004	0.3754	1.1807	0.241	0
4860	614.1	0.4005	0.3754	1.1812	0.2413	0
4861	614	0.4006	0.3755	1.1811	0.2416	0
4862	613.9	0.4006	0.3754	1.1807	0.2417	0
4863	613.8	0.4008	0.3754	1.1813	0.2421	0
4864	613.7	0.4009	0.3756	1.1812	0.2425	0
4865	613.6	0.4009	0.3756	1.1817	0.2428	0
4866	613.5	0.4012	0.3759	1.1823	0.2433	0
4867	613.4	0.4012	0.376	1.1822	0.2434	0
4868	613.3	0.4011	0.3759	1.1823	0.2436	0
4869	613.2	0.4013	0.3761	1.1821	0.2439	0
4870	613.1	0.4013	0.3759	1.1825	0.244	0
4871	613	0.4014	0.3761	1.1824	0.2444	0
4872	612.9	0.4014	0.376	1.1825	0.2447	0
4873	612.8	0.4015	0.3761	1.1831	0.2449	0
4874	612.7	0.4015	0.3762	1.1837	0.2451	0
4875	612.6	0.4017	0.3763	1.184	0.2453	0
4876	612.5	0.4016	0.3762	1.1831	0.2457	0
4877	612.4	0.4017	0.3763	1.1833	0.2458	0
4878	612.3	0.4017	0.3763	1.1836	0.246	0
4879	612.2	0.4017	0.3764	1.1844	0.2463	0
4880	612.1	0.4018	0.3762	1.1841	0.2466	0
4881	612	0.4019	0.3763	1.1842	0.2469	0
4882	611.9	0.4019	0.3761	1.1847	0.247	0
4883	611.8	0.402	0.3764	1.1846	0.2472	0
4884	611.7	0.4019	0.3764	1.1843	0.2473	0
4885	611.6	0.402	0.3765	1.185	0.2476	0
4886	611.5	0.4018	0.3762	1.1858	0.2478	0
4887	611.4	0.4019	0.3763	1.1856	0.2479	0
4888	611.3	0.4019	0.3764	1.1858	0.2481	0

4889	611.2	0.4021	0.3765	1.1853	0.2484	0
4890	611.1	0.402	0.3764	1.1855	0.2486	0
4891	611	0.4022	0.3766	1.1852	0.2488	0
4892	610.9	0.4022	0.3765	1.1855	0.249	0
4893	610.8	0.4022	0.3764	1.1856	0.2491	0
4894	610.7	0.4022	0.3764	1.1859	0.2493	0
4895	610.6	0.4021	0.3764	1.1865	0.2496	0
4896	610.5	0.4023	0.3764	1.1866	0.2498	0
4897	610.4	0.4022	0.3765	1.1867	0.2501	0
4898	610.3	0.4024	0.3764	1.1868	0.2502	0
4899	610.2	0.4023	0.3764	1.1869	0.2504	0
4900	610.1	0.4025	0.3766	1.1865	0.2506	0
4901	610	0.4024	0.3764	1.1866	0.2508	0
4902	609.9	0.4024	0.3763	1.1875	0.2509	0
4903	609.8	0.4026	0.3764	1.1872	0.2511	0
4904	609.7	0.4026	0.3765	1.1876	0.2514	0
4905	609.6	0.4027	0.3765	1.1879	0.2516	0
4906	609.5	0.4027	0.3764	1.1883	0.2516	0
4907	609.4	0.4029	0.3766	1.1887	0.252	0
4908	609.3	0.4029	0.3765	1.1888	0.2521	0
4909	609.2	0.4027	0.3765	1.1884	0.2523	0
4910	609.1	0.4027	0.3763	1.1886	0.2523	0
4911	609	0.4024	0.3763	1.1888	0.2525	0
4912	608.9	0.4025	0.3765	1.189	0.2526	0
4913	608.8	0.4026	0.3764	1.1889	0.2527	0
4914	608.7	0.4027	0.3766	1.1896	0.2531	0
4915	608.6	0.4029	0.3766	1.1899	0.2534	0
4916	608.5	0.4027	0.3764	1.1894	0.2535	0
4917	608.4	0.4027	0.3763	1.1893	0.2536	0
4918	608.3	0.4026	0.3761	1.1895	0.2535	0
4919	608.2	0.4028	0.3762	1.1904	0.2538	0
4920	608.1	0.403	0.3763	1.1898	0.2542	0
4921	608	0.4029	0.3763	1.1905	0.2543	0
4922	607.9	0.4031	0.3763	1.1912	0.2545	0
4923	607.8	0.403	0.3763	1.1909	0.2545	0
4924	607.7	0.4028	0.3762	1.1908	0.2546	0
4925	607.6	0.403	0.3763	1.1915	0.2549	0
4926	607.5	0.4029	0.3762	1.1903	0.2551	0
4927	607.4	0.403	0.3763	1.1908	0.2553	0
4928	607.3	0.4031	0.3763	1.1911	0.2555	0
4929	607.2	0.4029	0.3762	1.1915	0.2555	0
4930	607.1	0.403	0.3762	1.1915	0.2558	0
4931	607	0.4031	0.3761	1.1913	0.256	0

4932	606.9	0.4032	0.3763	1.1909	0.2562	0
4933	606.8	0.4031	0.3764	1.1914	0.2566	0
4934	606.7	0.4031	0.3762	1.1917	0.2567	0
4935	606.6	0.4031	0.3762	1.1909	0.2568	0
4936	606.5	0.4029	0.376	1.1919	0.2571	0
4937	606.4	0.4031	0.3761	1.192	0.2572	0
4938	606.3	0.4031	0.3762	1.1917	0.2575	0
4939	606.2	0.4032	0.3762	1.1917	0.2576	0
4940	606.1	0.4033	0.3763	1.1917	0.2578	0
4941	606	0.4033	0.3766	1.1921	0.258	0
4942	605.9	0.4035	0.3765	1.1924	0.2583	0
4943	605.8	0.4034	0.3763	1.1916	0.2584	0
4944	605.7	0.4034	0.3763	1.1922	0.2586	0
4945	605.6	0.4034	0.3764	1.1928	0.2589	0
4946	605.5	0.4035	0.3766	1.1922	0.259	0
4947	605.4	0.4034	0.3762	1.1923	0.259	0
4948	605.3	0.4033	0.3762	1.1916	0.2592	0
4949	605.2	0.4034	0.3762	1.1919	0.2594	0
4950	605.1	0.4035	0.3764	1.1916	0.2597	0
4951	605	0.4036	0.3763	1.1921	0.26	0
4952	604.9	0.4037	0.3765	1.1915	0.2602	0
4953	604.8	0.4035	0.3766	1.1921	0.2602	0
4954	604.7	0.4037	0.3768	1.1919	0.2606	0
4955	604.6	0.4037	0.3767	1.1919	0.2608	0
4956	604.5	0.4038	0.3768	1.1914	0.261	0
4957	604.4	0.4038	0.3766	1.1919	0.2611	0
4958	604.3	0.4039	0.3767	1.1916	0.2613	0
4959	604.2	0.4039	0.3766	1.1909	0.2615	0
4960	604.1	0.4041	0.3769	1.1917	0.2619	0
4961	604	0.4039	0.3769	1.1915	0.2621	0
4962	603.9	0.4042	0.3772	1.1911	0.2624	0
4963	603.8	0.404	0.3769	1.1911	0.2626	0
4964	603.7	0.4041	0.3771	1.1917	0.2629	0
4965	603.6	0.4043	0.3772	1.1912	0.2631	0
4966	603.5	0.4042	0.377	1.1913	0.2631	0
4967	603.4	0.4044	0.3772	1.1911	0.2634	0
4968	603.3	0.4043	0.3771	1.1914	0.2636	0
4969	603.2	0.4043	0.3771	1.1915	0.2636	0
4970	603.1	0.4043	0.3772	1.1908	0.2638	0
4971	603	0.4045	0.3772	1.1905	0.264	0
4972	602.9	0.4045	0.3773	1.1908	0.2642	0
4973	602.8	0.4046	0.3775	1.1908	0.2645	0
4974	602.7	0.4048	0.3777	1.1907	0.2646	0

4975	602.6	0.4044	0.3773	1.1909	0.2648	0
4976	602.5	0.4045	0.3773	1.1903	0.2648	0
4977	602.4	0.4046	0.3774	1.1903	0.2649	0
4978	602.3	0.4046	0.3774	1.1909	0.2651	0
4979	602.2	0.4048	0.3776	1.1907	0.2654	0
4980	602.1	0.4049	0.3778	1.191	0.2657	0
4981	602	0.405	0.3777	1.1912	0.2658	0
4982	601.9	0.4048	0.3774	1.1916	0.2658	0
4983	601.8	0.405	0.3776	1.1914	0.2661	0
4984	601.7	0.4048	0.3776	1.1907	0.2662	0
4985	601.6	0.4049	0.3776	1.1912	0.2662	0
4986	601.5	0.4048	0.3777	1.1906	0.2663	0
4987	601.4	0.4049	0.3774	1.191	0.2664	0
4988	601.3	0.4049	0.3776	1.1906	0.2666	0
4989	601.2	0.4049	0.3775	1.1907	0.2668	0
4990	601.1	0.405	0.3775	1.1903	0.2668	0
4991	601	0.4049	0.3774	1.191	0.2668	0
4992	600.9	0.4051	0.3776	1.1911	0.2672	0
4993	600.8	0.4051	0.3776	1.191	0.2673	0
4994	600.7	0.4051	0.3776	1.1917	0.2674	0
4995	600.6	0.4052	0.3776	1.1915	0.2676	0
4996	600.5	0.4053	0.3776	1.192	0.2677	0
4997	600.4	0.4054	0.3778	1.1916	0.268	0
4998	600.3	0.4052	0.3776	1.1924	0.2679	0
4999	600.2	0.4052	0.3777	1.192	0.2681	0
5000	600.1	0.4052	0.3776	1.192	0.268	0
5001	600	0.4052	0.3776	1.1918	0.2681	0
5002	599.9	0.4053	0.3776	1.1915	0.2683	0
5003	599.8	0.4054	0.3776	1.1916	0.2685	0
5004	599.7	0.4054	0.3775	1.192	0.2686	0
5005	599.6	0.4056	0.3775	1.1919	0.2686	0
5006	599.5	0.4054	0.3775	1.1916	0.2687	0
5007	599.4	0.4055	0.3775	1.1913	0.2688	0
5008	599.3	0.4052	0.3774	1.1922	0.2688	0
5009	599.2	0.4054	0.3774	1.192	0.269	0
5010	599.1	0.4055	0.3773	1.1921	0.269	0
5011	599	0.4054	0.3772	1.1929	0.2691	0
5012	598.9	0.4055	0.3774	1.1928	0.2692	0
5013	598.8	0.4054	0.3774	1.1928	0.2694	0
5014	598.7	0.4056	0.3773	1.1925	0.2695	0
5015	598.6	0.4055	0.3774	1.1933	0.2693	0
5016	598.5	0.4056	0.3772	1.1937	0.2693	0
5017	598.4	0.4055	0.3771	1.1939	0.2694	0

5018	598.3	0.4056	0.377	1.1936	0.2694	0
5019	598.2	0.4055	0.3772	1.1937	0.2696	0
5020	598.1	0.4056	0.3771	1.1939	0.2696	0
5021	598	0.4057	0.3772	1.1942	0.2698	0
5022	597.9	0.4055	0.3771	1.1941	0.2697	0
5023	597.8	0.4055	0.3771	1.1942	0.2698	0
5024	597.7	0.4055	0.3772	1.1936	0.2699	0
5025	597.6	0.4057	0.3772	1.1942	0.27	0
5026	597.5	0.4055	0.3771	1.1947	0.27	0
5027	597.4	0.4055	0.377	1.194	0.27	0
5028	597.3	0.4057	0.377	1.1943	0.2701	0
5029	597.2	0.4057	0.377	1.1942	0.2702	0
5030	597.1	0.4056	0.377	1.1944	0.2703	0
5031	597	0.4056	0.3769	1.1947	0.2704	0
5032	596.9	0.4057	0.3769	1.195	0.2705	0
5033	596.8	0.4059	0.377	1.1946	0.2706	0
5034	596.7	0.4059	0.3769	1.1952	0.2706	0
5035	596.6	0.406	0.377	1.1947	0.2706	0
5036	596.5	0.4059	0.3768	1.1953	0.2708	0
5037	596.4	0.4062	0.377	1.1961	0.271	0
5038	596.3	0.4061	0.3769	1.1952	0.2709	0
5039	596.2	0.4061	0.3768	1.1946	0.2709	0
5040	596.1	0.4062	0.3772	1.1947	0.2711	0
5041	596	0.4062	0.3771	1.1956	0.2709	0
5042	595.9	0.4062	0.3771	1.1953	0.2712	0
5043	595.8	0.4061	0.3768	1.1954	0.2711	0
5044	595.7	0.4063	0.377	1.1957	0.2712	0
5045	595.6	0.4062	0.3768	1.1952	0.2713	0
5046	595.5	0.4063	0.377	1.1951	0.2713	0
5047	595.4	0.4065	0.3772	1.1954	0.2717	0
5048	595.3	0.4065	0.3771	1.1949	0.2717	0
5049	595.2	0.4067	0.3772	1.1954	0.2718	0
5050	595.1	0.4066	0.3773	1.195	0.272	0
5051	595	0.4066	0.3772	1.1961	0.2719	0
5052	594.9	0.4068	0.3773	1.1957	0.272	0
5053	594.8	0.4067	0.3772	1.1956	0.2719	0
5054	594.7	0.4069	0.3776	1.1954	0.2722	0
5055	594.6	0.4072	0.3775	1.1964	0.2725	0
5056	594.5	0.4071	0.3774	1.1959	0.2725	0
5057	594.4	0.407	0.3775	1.1962	0.2726	0
5058	594.3	0.407	0.3775	1.1959	0.2726	0
5059	594.2	0.4069	0.3774	1.1952	0.2727	0
5060	594.1	0.4069	0.3774	1.1952	0.2727	0

5061	594	0.4072	0.3775	1.1957	0.2729	0
5062	593.9	0.4073	0.3776	1.1957	0.273	0
5063	593.8	0.4073	0.3778	1.1951	0.2731	0
5064	593.7	0.4072	0.3778	1.1951	0.2731	0
5065	593.6	0.4074	0.378	1.1953	0.2732	0
5066	593.5	0.4075	0.378	1.1953	0.2734	0
5067	593.4	0.4077	0.3781	1.1953	0.2735	0
5068	593.3	0.4076	0.378	1.1951	0.2735	0
5069	593.2	0.4076	0.3779	1.1954	0.2735	0
5070	593.1	0.4079	0.3782	1.1953	0.2737	0
5071	593	0.4078	0.3782	1.1946	0.2736	0
5072	592.9	0.4077	0.3781	1.1954	0.2739	0
5073	592.8	0.4076	0.3781	1.1951	0.2739	0
5074	592.7	0.408	0.3784	1.1952	0.2741	0
5075	592.6	0.408	0.3784	1.195	0.2742	0
5076	592.5	0.4079	0.3784	1.1951	0.2741	0
5077	592.4	0.4079	0.3786	1.1949	0.2742	0
5078	592.3	0.4078	0.3784	1.1953	0.2742	0
5079	592.2	0.408	0.3785	1.195	0.2744	0
5080	592.1	0.408	0.3785	1.1949	0.2744	0
5081	592	0.408	0.3785	1.1951	0.2744	0
5082	591.9	0.4083	0.3786	1.1947	0.2745	0
5083	591.8	0.4085	0.3789	1.1954	0.2748	0
5084	591.7	0.4084	0.3788	1.1949	0.2748	0
5085	591.6	0.4085	0.3787	1.1949	0.2749	0
5086	591.5	0.4085	0.3788	1.1945	0.2748	0
5087	591.4	0.4085	0.3788	1.195	0.2749	0
5088	591.3	0.4087	0.3789	1.1949	0.2751	0
5089	591.2	0.4085	0.3788	1.1952	0.2748	0
5090	591.1	0.4085	0.379	1.1953	0.275	0
5091	591	0.4087	0.3791	1.1952	0.2752	0
5092	590.9	0.4087	0.379	1.1953	0.2751	0
5093	590.8	0.4087	0.3791	1.1948	0.2753	0
5094	590.7	0.4085	0.379	1.1957	0.2753	0
5095	590.6	0.4087	0.379	1.1955	0.2754	0
5096	590.5	0.4088	0.3791	1.1952	0.2754	0
5097	590.4	0.4089	0.3791	1.1944	0.2755	0
5098	590.3	0.409	0.3792	1.1947	0.2756	0
5099	590.2	0.409	0.3793	1.1959	0.2756	0
5100	590.1	0.409	0.3793	1.1946	0.2756	0
5101	590	0.4089	0.3793	1.1944	0.2757	0
5102	589.9	0.4089	0.3795	1.195	0.2756	0
5103	589.8	0.4089	0.3793	1.1949	0.2756	0

5104	589.7	0.409	0.3793	1.1946	0.2757	0
5105	589.6	0.4092	0.3795	1.1953	0.2759	0
5106	589.5	0.4093	0.3796	1.1951	0.2759	0
5107	589.4	0.4092	0.3794	1.1948	0.2758	0
5108	589.3	0.4093	0.3796	1.1952	0.276	0
5109	589.2	0.4091	0.3794	1.1957	0.2761	0
5110	589.1	0.4092	0.3795	1.1962	0.276	0
5111	589	0.4093	0.3794	1.1961	0.276	0
5112	588.9	0.4091	0.3795	1.1953	0.2758	0
5113	588.8	0.4092	0.3796	1.1952	0.2758	0
5114	588.7	0.4092	0.3795	1.1956	0.2759	0
5115	588.6	0.4092	0.3795	1.1961	0.2758	0
5116	588.5	0.4091	0.3795	1.1965	0.2759	0
5117	588.4	0.4091	0.3795	1.1969	0.2758	0
5118	588.3	0.4091	0.3796	1.1958	0.2757	0
5119	588.2	0.4091	0.3796	1.1965	0.2757	0
5120	588.1	0.4091	0.3795	1.1971	0.2757	0
5121	588	0.4091	0.3797	1.1966	0.2758	0
5122	587.9	0.4092	0.3798	1.197	0.2759	0
5123	587.8	0.4092	0.3797	1.1969	0.2758	0
5124	587.7	0.4093	0.3797	1.1979	0.2759	0
5125	587.6	0.4093	0.3797	1.198	0.2759	0
5126	587.5	0.4092	0.3798	1.1983	0.2757	0
5127	587.4	0.4091	0.3796	1.1981	0.2758	0
5128	587.3	0.409	0.3797	1.1979	0.2758	0
5129	587.2	0.4091	0.3799	1.1974	0.2758	0
5130	587.1	0.4093	0.3797	1.1971	0.2759	0
5131	587	0.4093	0.3798	1.1979	0.2757	0
5132	586.9	0.4094	0.3799	1.1974	0.2758	0
5133	586.8	0.4094	0.3798	1.1981	0.2759	0
5134	586.7	0.4091	0.3798	1.1982	0.2757	0
5135	586.6	0.4091	0.3798	1.1981	0.2758	0
5136	586.5	0.4092	0.3798	1.1986	0.2759	0
5137	586.4	0.4093	0.3799	1.1983	0.2758	0
5138	586.3	0.4094	0.3801	1.199	0.2759	0
5139	586.2	0.4093	0.38	1.199	0.2759	0
5140	586.1	0.4091	0.3798	1.1983	0.2758	0
5141	586	0.4093	0.3799	1.1985	0.2758	0
5142	585.9	0.4093	0.3799	1.1983	0.2758	0
5143	585.8	0.4094	0.3801	1.1988	0.276	0
5144	585.7	0.4093	0.3802	1.198	0.2758	0
5145	585.6	0.4095	0.3802	1.1986	0.2759	0
5146	585.5	0.4093	0.3801	1.1985	0.2759	0

5147	585.4	0.4094	0.3803	1.1993	0.2759	0
5148	585.3	0.4093	0.3802	1.1992	0.2759	0
5149	585.2	0.4094	0.3804	1.1995	0.2759	0
5150	585.1	0.4095	0.3805	1.1996	0.2761	0
5151	585	0.4094	0.3804	1.1995	0.2759	0
5152	584.9	0.4207	0.385	1.1889	0.2785	0
5153	584.8	0.4206	0.3849	1.1892	0.2785	0
5154	584.7	0.4206	0.385	1.1884	0.2785	0
5155	584.6	0.4207	0.3852	1.1885	0.2785	0
5156	584.5	0.4208	0.3854	1.1889	0.2785	0
5157	584.4	0.4209	0.3853	1.1887	0.2785	0
5158	584.3	0.421	0.3854	1.1896	0.2786	0
5159	584.2	0.4208	0.3852	1.1882	0.2785	0
5160	584.1	0.4209	0.3853	1.1887	0.2786	0
5161	584	0.4209	0.3855	1.189	0.2785	0
5162	583.9	0.4209	0.3855	1.1882	0.2785	0
5163	583.8	0.421	0.3859	1.1892	0.2786	0
5164	583.7	0.4212	0.386	1.1898	0.2787	0
5165	583.6	0.4213	0.3858	1.1892	0.2786	0
5166	583.5	0.4211	0.3858	1.1894	0.2787	0
5167	583.4	0.4212	0.386	1.1898	0.2787	0
5168	583.3	0.4211	0.3861	1.1898	0.2787	0
5169	583.2	0.421	0.3859	1.1894	0.2787	0
5170	583.1	0.4211	0.3859	1.19	0.2786	0
5171	583	0.4211	0.3862	1.1911	0.2788	0
5172	582.9	0.4213	0.3864	1.1907	0.2788	0
5173	582.8	0.4213	0.3863	1.1912	0.2788	0
5174	582.7	0.4213	0.3863	1.1912	0.2786	0
5175	582.6	0.421	0.3861	1.1912	0.2785	0
5176	582.5	0.4209	0.3861	1.1918	0.2786	0
5177	582.4	0.4214	0.3864	1.1924	0.2786	0
5178	582.3	0.4213	0.3864	1.1916	0.2787	0
5179	582.2	0.4215	0.3865	1.1925	0.2786	0
5180	582.1	0.4213	0.3865	1.193	0.2787	0
5181	582	0.4212	0.3864	1.1928	0.2785	0
5182	581.9	0.4213	0.3865	1.1931	0.2785	0
5183	581.8	0.4214	0.3866	1.1939	0.2786	0
5184	581.7	0.4213	0.3867	1.1935	0.2786	0
5185	581.6	0.4212	0.3867	1.1934	0.2785	0
5186	581.5	0.4212	0.3867	1.1946	0.2785	0
5187	581.4	0.4213	0.387	1.1951	0.2786	0
5188	581.3	0.4217	0.3871	1.1953	0.2788	0
5189	581.2	0.4215	0.3872	1.195	0.2787	0

5190	581.1	0.4215	0.3873	1.1946	0.2788	0
5191	581	0.4215	0.3873	1.1948	0.2787	0
5192	580.9	0.4216	0.3873	1.1952	0.2789	0
5193	580.8	0.4216	0.3873	1.1948	0.279	0
5194	580.7	0.4217	0.3874	1.1951	0.2789	0
5195	580.6	0.4218	0.3876	1.195	0.279	0
5196	580.5	0.4217	0.3875	1.1948	0.279	0
5197	580.4	0.4217	0.3876	1.1951	0.279	0
5198	580.3	0.4218	0.3876	1.1949	0.279	0
5199	580.2	0.4217	0.3877	1.1951	0.2791	0
5200	580.1	0.422	0.388	1.1957	0.2791	0
5201	580	0.4219	0.3879	1.195	0.279	0
5202	579.9	0.422	0.3879	1.1956	0.2791	0
5203	579.8	0.4218	0.3879	1.1956	0.2789	0
5204	579.7	0.422	0.3879	1.1958	0.279	0
5205	579.6	0.422	0.3881	1.1959	0.2791	0
5206	579.5	0.422	0.3882	1.1952	0.2791	0
5207	579.4	0.4221	0.3882	1.1961	0.2792	0
5208	579.3	0.4219	0.388	1.1964	0.279	0
5209	579.2	0.4221	0.3882	1.1965	0.279	0
5210	579.1	0.4222	0.3883	1.1971	0.2792	0
5211	579	0.4224	0.3885	1.1969	0.2792	0
5212	578.9	0.422	0.3882	1.1971	0.279	0
5213	578.8	0.422	0.3883	1.1968	0.279	0
5214	578.7	0.4223	0.3884	1.1966	0.2792	0
5215	578.6	0.4222	0.3884	1.1969	0.279	0
5216	578.5	0.4224	0.3885	1.1974	0.279	0
5217	578.4	0.4223	0.3884	1.1974	0.2791	0
5218	578.3	0.4222	0.3884	1.1978	0.2792	0
5219	578.2	0.4224	0.3886	1.1982	0.2792	0
5220	578.1	0.4224	0.3887	1.1978	0.2792	0
5221	578	0.4223	0.3886	1.1981	0.2791	0
5222	577.9	0.4223	0.3888	1.1981	0.2792	0
5223	577.8	0.4223	0.3888	1.1985	0.2792	0
5224	577.7	0.4225	0.389	1.1989	0.2793	0
5225	577.6	0.4223	0.389	1.1988	0.2793	0
5226	577.5	0.4224	0.3891	1.1989	0.2794	0
5227	577.4	0.4226	0.3893	1.1989	0.2794	0
5228	577.3	0.4226	0.3894	1.1994	0.2795	0
5229	577.2	0.4224	0.3893	1.1992	0.2794	0
5230	577.1	0.4226	0.3893	1.1988	0.2794	0
5231	577	0.4225	0.3893	1.199	0.2794	0
5232	576.9	0.4225	0.3894	1.1997	0.2795	0

5233	576.8	0.4227	0.3894	1.1998	0.2795	0
5234	576.7	0.4228	0.3895	1.1998	0.2796	0
5235	576.6	0.4228	0.3895	1.2	0.2795	0
5236	576.5	0.4227	0.3897	1.1999	0.2796	0
5237	576.4	0.4228	0.3896	1.1998	0.2795	0
5238	576.3	0.4227	0.3896	1.2004	0.2794	0
5239	576.2	0.4227	0.3896	1.2004	0.2795	0
5240	576.1	0.4225	0.3896	1.2003	0.2795	0
5241	576	0.4226	0.3895	1.2002	0.2795	0
5242	575.9	0.4227	0.3896	1.2002	0.2796	0
5243	575.8	0.4229	0.3899	1.2003	0.2796	0
5244	575.7	0.4229	0.39	1.2009	0.2797	0
5245	575.6	0.4228	0.39	1.2012	0.2797	0
5246	575.5	0.4228	0.39	1.2008	0.2797	0
5247	575.4	0.4228	0.3901	1.2014	0.2797	0
5248	575.3	0.4227	0.39	1.1999	0.2796	0
5249	575.2	0.4228	0.39	1.2005	0.2796	0
5250	575.1	0.423	0.3902	1.2012	0.2798	0
5251	575	0.4228	0.39	1.2017	0.2798	0
5252	574.9	0.4228	0.3902	1.2017	0.2798	0
5253	574.8	0.4228	0.3903	1.2012	0.2798	0
5254	574.7	0.4229	0.3904	1.2015	0.28	0
5255	574.6	0.4227	0.3905	1.2014	0.2798	0
5256	574.5	0.423	0.3903	1.2007	0.2799	0
5257	574.4	0.4232	0.3908	1.2017	0.28	0
5258	574.3	0.4232	0.3908	1.2021	0.2801	0
5259	574.2	0.4233	0.391	1.2017	0.2802	0
5260	574.1	0.4232	0.3909	1.2012	0.2802	0
5261	574	0.4232	0.3908	1.2021	0.2802	0
5262	573.9	0.4232	0.3908	1.2014	0.2802	0
5263	573.8	0.4232	0.391	1.202	0.2802	0
5264	573.7	0.4232	0.3911	1.2013	0.2803	0
5265	573.6	0.4235	0.3913	1.202	0.2804	0
5266	573.5	0.4234	0.3914	1.2015	0.2805	0
5267	573.4	0.4235	0.3913	1.2027	0.2804	0
5268	573.3	0.4236	0.3916	1.2018	0.2806	0
5269	573.2	0.4236	0.3914	1.2017	0.2806	0
5270	573.1	0.4237	0.3916	1.2017	0.2806	0
5271	573	0.4235	0.3916	1.2016	0.2806	0
5272	572.9	0.4235	0.3918	1.2022	0.2807	0
5273	572.8	0.4236	0.3917	1.2017	0.2807	0
5274	572.7	0.4237	0.3919	1.2028	0.2807	0
5275	572.6	0.4236	0.3917	1.2022	0.2807	0

5276	572.5	0.4237	0.3918	1.2019	0.2808	0
5277	572.4	0.4233	0.3916	1.2025	0.2804	0
5278	572.3	0.4235	0.3919	1.202	0.2808	0
5279	572.2	0.4238	0.392	1.2024	0.2809	0
5280	572.1	0.4239	0.3921	1.2018	0.2808	0
5281	572	0.4239	0.3923	1.2023	0.281	0
5282	571.9	0.4238	0.3923	1.2025	0.281	0
5283	571.8	0.4239	0.3925	1.2031	0.281	0
5284	571.7	0.424	0.3924	1.2027	0.281	0
5285	571.6	0.424	0.3926	1.2027	0.2811	0
5286	571.5	0.4239	0.3924	1.2031	0.281	0
5287	571.4	0.4239	0.3925	1.2027	0.2811	0
5288	571.3	0.424	0.3927	1.2033	0.2811	0
5289	571.2	0.424	0.3924	1.2032	0.2811	0
5290	571.1	0.4241	0.3926	1.2035	0.281	0
5291	571	0.4243	0.3928	1.2032	0.2813	0
5292	570.9	0.4244	0.3929	1.2037	0.2812	0
5293	570.8	0.4244	0.3929	1.2037	0.2813	0
5294	570.7	0.4244	0.393	1.2037	0.2814	0
5295	570.6	0.4244	0.393	1.2029	0.2813	0
5296	570.5	0.4242	0.393	1.2034	0.2813	0
5297	570.4	0.4245	0.3931	1.2033	0.2814	0
5298	570.3	0.4244	0.3932	1.2037	0.2815	0
5299	570.2	0.4244	0.3931	1.2032	0.2815	0
5300	570.1	0.4243	0.3933	1.2032	0.2815	0
5301	570	0.4245	0.3933	1.2044	0.2815	0
5302	569.9	0.4247	0.3936	1.2051	0.2817	0
5303	569.8	0.4246	0.3936	1.2043	0.2817	0
5304	569.7	0.4247	0.3937	1.204	0.2818	0
5305	569.6	0.4247	0.3936	1.2045	0.2816	0
5306	569.5	0.4247	0.3937	1.2048	0.2818	0
5307	569.4	0.4248	0.3939	1.2052	0.2818	0
5308	569.3	0.4249	0.3937	1.205	0.2819	0
5309	569.2	0.4249	0.3939	1.2043	0.2819	0
5310	569.1	0.4249	0.394	1.2046	0.282	0
5311	569	0.4249	0.394	1.2054	0.2819	0
5312	568.9	0.425	0.3941	1.2058	0.282	0
5313	568.8	0.425	0.3939	1.2062	0.2817	0
5314	568.7	0.425	0.3941	1.206	0.282	0
5315	568.6	0.425	0.3943	1.2053	0.282	0
5316	568.5	0.4248	0.3941	1.2057	0.2819	0
5317	568.4	0.4249	0.3943	1.2061	0.282	0
5318	568.3	0.4251	0.3945	1.2059	0.2823	0

5319	568.2	0.4252	0.3944	1.2063	0.2822	0
5320	568.1	0.4252	0.3944	1.2056	0.2821	0
5321	568	0.4253	0.3946	1.2065	0.2823	0
5322	567.9	0.4254	0.3948	1.2063	0.2824	0
5323	567.8	0.4255	0.3948	1.2059	0.2823	0
5324	567.7	0.4256	0.3949	1.2059	0.2824	0
5325	567.6	0.4255	0.3949	1.2066	0.2824	0
5326	567.5	0.4255	0.3951	1.2059	0.2824	0
5327	567.4	0.4255	0.3951	1.2067	0.2824	0
5328	567.3	0.4255	0.3951	1.2065	0.2824	0
5329	567.2	0.4257	0.3951	1.2061	0.2825	0
5330	567.1	0.4258	0.3951	1.2063	0.2825	0
5331	567	0.4257	0.3954	1.2062	0.2826	0
5332	566.9	0.4257	0.3954	1.2066	0.2828	0
5333	566.8	0.4258	0.3956	1.2065	0.2827	0
5334	566.7	0.4261	0.3958	1.2065	0.2828	0
5335	566.6	0.426	0.3958	1.2071	0.283	0
5336	566.5	0.4261	0.3958	1.2069	0.2828	0
5337	566.4	0.4261	0.396	1.2069	0.283	0
5338	566.3	0.4263	0.3959	1.2072	0.283	0
5339	566.2	0.4262	0.3959	1.2074	0.283	0
5340	566.1	0.4261	0.396	1.2069	0.2831	0
5341	566	0.4261	0.3961	1.2069	0.2833	0
5342	565.9	0.4261	0.3961	1.2068	0.2832	0
5343	565.8	0.4262	0.3962	1.2074	0.2833	0
5344	565.7	0.4264	0.3964	1.2074	0.2835	0
5345	565.6	0.4263	0.3965	1.2076	0.2835	0
5346	565.5	0.4265	0.3966	1.2072	0.2835	0
5347	565.4	0.4266	0.3967	1.2072	0.2835	0
5348	565.3	0.4266	0.3969	1.2072	0.2838	0
5349	565.2	0.4267	0.3969	1.2075	0.2838	0
5350	565.1	0.4266	0.3967	1.207	0.2839	0
5351	565	0.4268	0.397	1.207	0.2839	0
5352	564.9	0.4267	0.3969	1.2076	0.2839	0
5353	564.8	0.427	0.3971	1.2073	0.2841	0
5354	564.7	0.427	0.3973	1.2072	0.2841	0
5355	564.6	0.427	0.3975	1.2075	0.2842	0
5356	564.5	0.427	0.3975	1.2076	0.2841	0
5357	564.4	0.4272	0.3975	1.2076	0.2842	0
5358	564.3	0.4272	0.3975	1.2072	0.2843	0
5359	564.2	0.4271	0.3975	1.2073	0.2842	0
5360	564.1	0.4272	0.3978	1.2074	0.2845	0
5361	564	0.4273	0.3979	1.2077	0.2845	0

5362	563.9	0.4276	0.3981	1.2084	0.2848	0
5363	563.8	0.4276	0.3982	1.2076	0.2847	0
5364	563.7	0.4274	0.398	1.2081	0.2847	0
5365	563.6	0.4274	0.398	1.2064	0.2847	0
5366	563.5	0.4275	0.398	1.2066	0.2847	0
5367	563.4	0.4274	0.3982	1.2074	0.2847	0
5368	563.3	0.4276	0.3983	1.2071	0.2849	0
5369	563.2	0.4278	0.3984	1.2068	0.285	0
5370	563.1	0.4276	0.3984	1.2072	0.2849	0
5371	563	0.4278	0.3986	1.208	0.2853	0
5372	562.9	0.4278	0.3985	1.2078	0.2852	0
5373	562.8	0.4279	0.3987	1.2071	0.2853	0
5374	562.7	0.4278	0.3986	1.2073	0.2853	0
5375	562.6	0.428	0.3988	1.2073	0.2854	0
5376	562.5	0.4279	0.3988	1.2076	0.2854	0
5377	562.4	0.428	0.3989	1.2076	0.2855	0
5378	562.3	0.428	0.3991	1.2084	0.2855	0
5379	562.2	0.4283	0.3992	1.2084	0.2857	0
5380	562.1	0.4283	0.3994	1.208	0.2857	0
5381	562	0.4284	0.3994	1.2082	0.2858	0
5382	561.9	0.4284	0.3993	1.2083	0.2857	0
5383	561.8	0.4284	0.3995	1.2082	0.2859	0
5384	561.7	0.4284	0.3996	1.2081	0.286	0
5385	561.6	0.4286	0.3996	1.2083	0.2859	0
5386	561.5	0.4286	0.3996	1.2084	0.2859	0
5387	561.4	0.4286	0.3995	1.208	0.2861	0
5388	561.3	0.4284	0.3998	1.2087	0.286	0
5389	561.2	0.4284	0.3996	1.2085	0.2859	0
5390	561.1	0.4287	0.3997	1.2087	0.2861	0
5391	561	0.4287	0.3999	1.2089	0.2862	0
5392	560.9	0.4288	0.3999	1.2094	0.2863	0
5393	560.8	0.4288	0.4001	1.2088	0.2865	0
5394	560.7	0.429	0.4003	1.2096	0.2866	0
5395	560.6	0.4289	0.4001	1.2093	0.2866	0
5396	560.5	0.4289	0.4001	1.2088	0.2865	0
5397	560.4	0.4291	0.4003	1.2094	0.2865	0
5398	560.3	0.4291	0.4002	1.2098	0.2864	0
5399	560.2	0.4291	0.4002	1.2098	0.2866	0
5400	560.1	0.4289	0.4003	1.2099	0.2866	0
5401	560	0.429	0.4004	1.2098	0.2868	0
5402	559.9	0.4291	0.4005	1.2103	0.2869	0
5403	559.8	0.4293	0.4004	1.2102	0.2869	0
5404	559.7	0.4294	0.4008	1.2099	0.2871	0

5405	559.6	0.4292	0.4005	1.2099	0.2869	0
5406	559.5	0.4293	0.4005	1.2103	0.2869	0
5407	559.4	0.4293	0.4006	1.2102	0.287	0
5408	559.3	0.4295	0.4007	1.2105	0.2871	0
5409	559.2	0.4294	0.4007	1.2103	0.2872	0
5410	559.1	0.4293	0.4006	1.2103	0.2871	0
5411	559	0.4295	0.4008	1.2104	0.2872	0
5412	558.9	0.4295	0.4009	1.2104	0.2873	0
5413	558.8	0.4299	0.4011	1.2108	0.2875	0
5414	558.7	0.4297	0.4012	1.2107	0.2874	0
5415	558.6	0.4298	0.4012	1.2109	0.2876	0
5416	558.5	0.4297	0.4012	1.2111	0.2875	0
5417	558.4	0.4298	0.4012	1.2111	0.2876	0
5418	558.3	0.4299	0.4013	1.2113	0.2876	0
5419	558.2	0.43	0.4016	1.2122	0.2879	0
5420	558.1	0.4301	0.4018	1.2115	0.288	0
5421	558	0.4302	0.4017	1.2119	0.288	0
5422	557.9	0.43	0.4017	1.2114	0.2879	0
5423	557.8	0.4301	0.4016	1.2119	0.2879	0
5424	557.7	0.4302	0.4018	1.2124	0.288	0
5425	557.6	0.4303	0.402	1.2126	0.2883	0
5426	557.5	0.4303	0.4019	1.2122	0.2883	0
5427	557.4	0.4302	0.4019	1.2126	0.2884	0
5428	557.3	0.4305	0.4019	1.2137	0.2885	0
5429	557.2	0.4303	0.402	1.2138	0.2885	0
5430	557.1	0.4304	0.4021	1.2126	0.2886	0
5431	557	0.4304	0.4021	1.2125	0.2887	0
5432	556.9	0.4307	0.4023	1.2122	0.2888	0
5433	556.8	0.4307	0.4024	1.2126	0.2889	0
5434	556.7	0.4307	0.4027	1.2137	0.2891	0
5435	556.6	0.4309	0.4027	1.2128	0.2891	0
5436	556.5	0.4309	0.4027	1.2127	0.2891	0
5437	556.4	0.4311	0.4029	1.2134	0.2894	0
5438	556.3	0.4308	0.4028	1.2134	0.2893	0
5439	556.2	0.4309	0.4031	1.2129	0.2896	0
5440	556.1	0.4311	0.4029	1.213	0.2896	0
5441	556	0.4312	0.4031	1.2137	0.2896	0
5442	555.9	0.4315	0.4035	1.2135	0.2899	0
5443	555.8	0.4314	0.4036	1.2142	0.2899	0
5444	555.7	0.4316	0.4037	1.2136	0.2902	0
5445	555.6	0.4317	0.4037	1.2132	0.2903	0
5446	555.5	0.4317	0.4038	1.2139	0.2904	0
5447	555.4	0.4318	0.4039	1.2135	0.2907	0

5448	555.3	0.432	0.404	1.2129	0.2907	0
5449	555.2	0.432	0.404	1.2133	0.2908	0
5450	555.1	0.4319	0.4041	1.2128	0.2907	0
5451	555	0.4319	0.4041	1.2126	0.2908	0
5452	554.9	0.4321	0.4043	1.2134	0.2912	0
5453	554.8	0.4323	0.4045	1.2142	0.2913	0
5454	554.7	0.4322	0.4047	1.2137	0.2914	0
5455	554.6	0.4323	0.4046	1.2134	0.2912	0
5456	554.5	0.4323	0.4047	1.2127	0.2914	0
5457	554.4	0.4326	0.4048	1.2133	0.2917	0
5458	554.3	0.4327	0.4052	1.2132	0.2919	0
5459	554.2	0.4325	0.4054	1.2135	0.2919	0
5460	554.1	0.4327	0.4053	1.2136	0.292	0
5461	554	0.4328	0.4055	1.2131	0.2921	0
5462	553.9	0.4327	0.4055	1.2129	0.2921	0
5463	553.8	0.4327	0.4055	1.2133	0.2923	0
5464	553.7	0.433	0.4058	1.2135	0.2925	0
5465	553.6	0.4331	0.4057	1.2128	0.2927	0
5466	553.5	0.4332	0.406	1.2131	0.2928	0
5467	553.4	0.4332	0.406	1.2129	0.2929	0
5468	553.3	0.4333	0.4061	1.2132	0.293	0
5469	553.2	0.4334	0.4061	1.2128	0.2932	0
5470	553.1	0.4335	0.4063	1.2131	0.2933	0
5471	553	0.4335	0.4063	1.2134	0.2933	0
5472	552.9	0.4335	0.4066	1.214	0.2935	0
5473	552.8	0.4337	0.4066	1.2135	0.2935	0
5474	552.7	0.434	0.4067	1.2141	0.2936	0
5475	552.6	0.4339	0.4069	1.2141	0.2938	0
5476	552.5	0.434	0.4069	1.2136	0.294	0
5477	552.4	0.434	0.4068	1.2145	0.294	0
5478	552.3	0.4341	0.407	1.2142	0.2939	0
5479	552.2	0.434	0.407	1.2136	0.2941	0
5480	552.1	0.4343	0.4072	1.214	0.2943	0
5481	552	0.4343	0.4073	1.2148	0.2944	0
5482	551.9	0.4344	0.4072	1.2144	0.2944	0
5483	551.8	0.4344	0.4072	1.2143	0.2945	0
5484	551.7	0.4345	0.4075	1.2149	0.2946	0
5485	551.6	0.4344	0.4073	1.2144	0.2947	0
5486	551.5	0.4347	0.4075	1.2139	0.2948	0
5487	551.4	0.4345	0.4075	1.2139	0.2947	0
5488	551.3	0.4346	0.4077	1.2137	0.2948	0
5489	551.2	0.4348	0.4078	1.2144	0.295	0
5490	551.1	0.4351	0.4079	1.2147	0.2952	0

5491	551	0.4351	0.4081	1.2146	0.2953	0
5492	550.9	0.4351	0.4082	1.2146	0.2955	0
5493	550.8	0.4351	0.408	1.2148	0.2953	0
5494	550.7	0.4352	0.4082	1.2144	0.2955	0
5495	550.6	0.4353	0.4081	1.2148	0.2955	0
5496	550.5	0.4354	0.408	1.2149	0.2955	0
5497	550.4	0.4355	0.4082	1.2151	0.2957	0
5498	550.3	0.4356	0.4084	1.2155	0.2958	0
5499	550.2	0.4356	0.4084	1.2155	0.2958	0
5500	550.1	0.4356	0.4085	1.2154	0.296	0
5501	550	0.4358	0.4085	1.2151	0.296	0
5502	549.9	0.4358	0.4086	1.215	0.296	0
5503	549.8	0.4359	0.4089	1.2149	0.2963	0
5504	549.7	0.4361	0.4089	1.216	0.2963	0
5505	549.6	0.4362	0.4091	1.2154	0.2965	0
5506	549.5	0.4362	0.409	1.2159	0.2965	0
5507	549.4	0.4362	0.409	1.2162	0.2966	0
5508	549.3	0.4363	0.4091	1.2165	0.2966	0
5509	549.2	0.4363	0.4093	1.2163	0.2968	0
5510	549.1	0.4365	0.4092	1.2168	0.2968	0
5511	549	0.4366	0.4094	1.2163	0.2969	0
5512	548.9	0.4367	0.4096	1.2171	0.2971	0
5513	548.8	0.4369	0.4096	1.2174	0.2972	0
5514	548.7	0.437	0.4097	1.2169	0.2972	0
5515	548.6	0.437	0.4098	1.2172	0.2972	0
5516	548.5	0.4369	0.4096	1.2164	0.2972	0
5517	548.4	0.4369	0.4099	1.2169	0.2974	0
5518	548.3	0.4372	0.41	1.2174	0.2975	0
5519	548.2	0.4373	0.4101	1.2171	0.2976	0
5520	548.1	0.4374	0.4103	1.2177	0.2977	0
5521	548	0.4376	0.4103	1.2178	0.2979	0
5522	547.9	0.4375	0.4104	1.2175	0.2979	0
5523	547.8	0.4376	0.4106	1.2174	0.2981	0
5524	547.7	0.4377	0.4105	1.2175	0.2979	0
5525	547.6	0.4378	0.4106	1.2176	0.298	0
5526	547.5	0.4379	0.4107	1.2177	0.298	0
5527	547.4	0.4381	0.4107	1.2176	0.2982	0
5528	547.3	0.438	0.4109	1.218	0.2984	0
5529	547.2	0.4382	0.4109	1.2181	0.2984	0
5530	547.1	0.4382	0.411	1.2182	0.2984	0
5531	547	0.4384	0.4111	1.2186	0.2986	0
5532	546.9	0.4387	0.4113	1.2184	0.2987	0
5533	546.8	0.4386	0.4113	1.218	0.2989	0

5534	546.7	0.4386	0.4115	1.2173	0.2991	0
5535	546.6	0.4389	0.4116	1.2184	0.2991	0
5536	546.5	0.4389	0.4116	1.2181	0.2992	0
5537	546.4	0.4391	0.4117	1.2184	0.2993	0
5538	546.3	0.439	0.4116	1.2183	0.2993	0
5539	546.2	0.4393	0.4119	1.2177	0.2996	0
5540	546.1	0.4396	0.4122	1.2187	0.2997	0
5541	546	0.4396	0.4123	1.2186	0.2998	0
5542	545.9	0.4398	0.4124	1.2186	0.2999	0
5543	545.8	0.4398	0.4125	1.2186	0.3001	0
5544	545.7	0.44	0.4128	1.2189	0.3003	0
5545	545.6	0.4403	0.4129	1.218	0.3005	0
5546	545.5	0.4405	0.4132	1.2183	0.3006	0
5547	545.4	0.4405	0.413	1.2183	0.3006	0
5548	545.3	0.4407	0.4133	1.2182	0.3009	0
5549	545.2	0.4408	0.4135	1.2181	0.301	0
5550	545.1	0.4408	0.4135	1.2182	0.301	0
5551	545	0.4409	0.4135	1.2182	0.301	0
5552	544.9	0.4411	0.4136	1.2178	0.3012	0
5553	544.8	0.4412	0.4137	1.2182	0.3013	0
5554	544.7	0.4416	0.4142	1.2176	0.3017	0
5555	544.6	0.4415	0.4143	1.2177	0.3019	0
5556	544.5	0.4416	0.4144	1.218	0.3018	0
5557	544.4	0.4417	0.4146	1.2179	0.3021	0
5558	544.3	0.442	0.4149	1.2177	0.3022	0
5559	544.2	0.4421	0.4149	1.2176	0.3026	0
5560	544.1	0.4425	0.4153	1.2167	0.3029	0
5561	544	0.4426	0.4155	1.2173	0.3029	0
5562	543.9	0.4427	0.4155	1.2172	0.3029	0
5563	543.8	0.4429	0.4155	1.2164	0.3031	0
5564	543.7	0.4428	0.4157	1.2173	0.3031	0
5565	543.6	0.443	0.4159	1.217	0.3033	0
5566	543.5	0.4432	0.4162	1.2168	0.3035	0
5567	543.4	0.4434	0.4164	1.2163	0.3038	0
5568	543.3	0.4435	0.4164	1.2164	0.3038	0
5569	543.2	0.4435	0.4165	1.2161	0.3039	0
5570	543.1	0.4437	0.4167	1.2152	0.304	0
5571	543	0.4437	0.4167	1.2154	0.3041	0
5572	542.9	0.4443	0.4171	1.2164	0.3044	0
5573	542.8	0.4444	0.4171	1.2166	0.3046	0
5574	542.7	0.4444	0.4172	1.2164	0.3046	0
5575	542.6	0.4446	0.4175	1.216	0.3048	0
5576	542.5	0.4445	0.4177	1.2164	0.3048	0

5577	542.4	0.4447	0.4178	1.2155	0.305	0
5578	542.3	0.445	0.4179	1.2155	0.3052	0
5579	542.2	0.445	0.4181	1.2156	0.3052	0
5580	542.1	0.4452	0.4182	1.2154	0.3053	0
5581	542	0.4453	0.4182	1.2166	0.3054	0
5582	541.9	0.4453	0.4184	1.2159	0.3055	0
5583	541.8	0.4455	0.4186	1.2161	0.3058	0
5584	541.7	0.4458	0.4189	1.2166	0.3059	0
5585	541.6	0.446	0.419	1.2153	0.3061	0
5586	541.5	0.4464	0.4191	1.2155	0.3063	0
5587	541.4	0.4463	0.4192	1.2158	0.3063	0
5588	541.3	0.4464	0.4192	1.2156	0.3064	0
5589	541.2	0.4465	0.4193	1.2154	0.3065	0
5590	541.1	0.4466	0.4194	1.2151	0.3066	0
5591	541	0.4466	0.4196	1.2157	0.3067	0
5592	540.9	0.447	0.4199	1.2154	0.307	0
5593	540.8	0.4469	0.42	1.2151	0.3069	0
5594	540.7	0.4473	0.4202	1.2161	0.3073	0
5595	540.6	0.4474	0.4203	1.2161	0.3072	0
5596	540.5	0.4475	0.4203	1.2149	0.3073	0
5597	540.4	0.4475	0.4205	1.2147	0.3075	0
5598	540.3	0.4477	0.4207	1.2153	0.3076	0
5599	540.2	0.4478	0.4208	1.2156	0.3077	0
5600	540.1	0.448	0.4208	1.216	0.3078	0
5601	540	0.4479	0.4208	1.2159	0.3078	0
5602	539.9	0.4481	0.4211	1.2162	0.3078	0
5603	539.8	0.4483	0.4211	1.2156	0.3079	0
5604	539.7	0.4485	0.4215	1.2161	0.3082	0
5605	539.6	0.4485	0.4216	1.2161	0.3082	0
5606	539.5	0.4486	0.4215	1.2157	0.3083	0
5607	539.4	0.4488	0.4218	1.2163	0.3085	0
5608	539.3	0.449	0.422	1.2158	0.3085	0
5609	539.2	0.4491	0.4221	1.2159	0.3088	0
5610	539.1	0.4492	0.4221	1.2158	0.3087	0
5611	539	0.4493	0.4224	1.2164	0.3088	0
5612	538.9	0.4494	0.4224	1.2171	0.3089	0
5613	538.8	0.4496	0.4226	1.2168	0.3091	0
5614	538.7	0.4495	0.4225	1.2164	0.3091	0
5615	538.6	0.4499	0.4228	1.2175	0.3093	0
5616	538.5	0.4498	0.4227	1.2168	0.3093	0
5617	538.4	0.4498	0.4227	1.217	0.3094	0
5618	538.3	0.45	0.4231	1.2165	0.3095	0
5619	538.2	0.4502	0.4232	1.2175	0.3097	0

5620	538.1	0.4503	0.4233	1.2178	0.3098	0
5621	538	0.4503	0.4232	1.218	0.3096	0
5622	537.9	0.4504	0.4235	1.2178	0.3098	0
5623	537.8	0.4508	0.4236	1.2176	0.31	0
5624	537.7	0.4508	0.4236	1.2179	0.3102	0
5625	537.6	0.4507	0.4237	1.2175	0.31	0
5626	537.5	0.451	0.4237	1.2186	0.3102	0
5627	537.4	0.4514	0.4241	1.2185	0.3106	0
5628	537.3	0.4513	0.4243	1.2172	0.3105	0
5629	537.2	0.4516	0.4244	1.2182	0.3107	0
5630	537.1	0.4516	0.4246	1.2185	0.3107	0
5631	537	0.4517	0.4245	1.2182	0.3108	0
5632	536.9	0.4518	0.4246	1.2185	0.3108	0
5633	536.8	0.4518	0.4247	1.2191	0.3109	0
5634	536.7	0.4522	0.4249	1.2189	0.3112	0
5635	536.6	0.4522	0.4251	1.2189	0.3113	0
5636	536.5	0.4522	0.4252	1.2192	0.3113	0
5637	536.4	0.4524	0.4253	1.2186	0.3114	0
5638	536.3	0.4525	0.4254	1.2186	0.3115	0
5639	536.2	0.4525	0.4255	1.2198	0.3117	0
5640	536.1	0.4527	0.4257	1.2193	0.3119	0
5641	536	0.4528	0.4258	1.2193	0.3119	0
5642	535.9	0.4532	0.426	1.2201	0.3123	0
5643	535.8	0.4533	0.426	1.2194	0.3122	0
5644	535.7	0.4533	0.4261	1.2193	0.3124	0
5645	535.6	0.4536	0.4262	1.2187	0.3124	0
5646	535.5	0.4534	0.4264	1.219	0.3125	0
5647	535.4	0.4537	0.4268	1.2195	0.3129	0
5648	535.3	0.454	0.427	1.2196	0.3133	0
5649	535.2	0.4539	0.427	1.2191	0.3131	0
5650	535.1	0.4541	0.4271	1.2187	0.3132	0
5651	535	0.4543	0.4275	1.2192	0.3135	0
5652	534.9	0.4543	0.4274	1.2197	0.3134	0
5653	534.8	0.4545	0.4277	1.2198	0.3137	0
5654	534.7	0.4547	0.4278	1.2201	0.3138	0
5655	534.6	0.4548	0.428	1.2197	0.314	0
5656	534.5	0.4549	0.4281	1.2197	0.3141	0
5657	534.4	0.455	0.4284	1.2194	0.3142	0
5658	534.3	0.4551	0.4285	1.2191	0.3144	0
5659	534.2	0.4554	0.4287	1.2195	0.3146	0
5660	534.1	0.4556	0.429	1.2189	0.3149	0
5661	534	0.4557	0.4291	1.2192	0.3151	0
5662	533.9	0.4559	0.4293	1.22	0.3152	0

5663	533.8	0.4559	0.4294	1.2194	0.3151	0
5664	533.7	0.4561	0.4294	1.2191	0.3153	0
5665	533.6	0.4564	0.4296	1.2189	0.3156	0
5666	533.5	0.4563	0.4299	1.2189	0.3158	0
5667	533.4	0.4565	0.4301	1.2193	0.3158	0
5668	533.3	0.4569	0.4305	1.2188	0.3161	0
5669	533.2	0.4568	0.4304	1.2186	0.3161	0
5670	533.1	0.4573	0.4307	1.2181	0.3164	0
5671	533	0.4574	0.431	1.2193	0.3167	0
5672	532.9	0.4571	0.4307	1.2191	0.3166	0
5673	532.8	0.4571	0.4307	1.219	0.3166	0
5674	532.7	0.4575	0.4309	1.2191	0.3169	0
5675	532.6	0.4579	0.4313	1.2191	0.3174	0
5676	532.5	0.458	0.4315	1.219	0.3174	0
5677	532.4	0.4582	0.4316	1.2192	0.3176	0
5678	532.3	0.4583	0.4319	1.2186	0.3178	0
5679	532.2	0.4583	0.4319	1.2189	0.3178	0
5680	532.1	0.4585	0.432	1.2187	0.318	0
5681	532	0.4584	0.4321	1.2187	0.318	0
5682	531.9	0.4585	0.4321	1.2187	0.3182	0
5683	531.8	0.4588	0.4325	1.2193	0.3184	0
5684	531.7	0.4589	0.4327	1.2195	0.3186	0
5685	531.6	0.4591	0.4329	1.2185	0.3188	0
5686	531.5	0.4592	0.4331	1.2193	0.3189	0
5687	531.4	0.4593	0.4332	1.2197	0.3188	0
5688	531.3	0.4593	0.4332	1.2191	0.3191	0
5689	531.2	0.4595	0.4336	1.2198	0.3193	0
5690	531.1	0.4597	0.4336	1.2196	0.3195	0
5691	531	0.4598	0.4338	1.219	0.3196	0
5692	530.9	0.4598	0.4338	1.2187	0.3198	0
5693	530.8	0.4598	0.4337	1.2202	0.3196	0
5694	530.7	0.46	0.434	1.2196	0.3199	0
5695	530.6	0.4604	0.4342	1.2206	0.3201	0
5696	530.5	0.4605	0.4345	1.2193	0.3203	0
5697	530.4	0.4605	0.4347	1.2196	0.3205	0
5698	530.3	0.4607	0.4347	1.2198	0.3208	0
5699	530.2	0.4609	0.4349	1.2199	0.321	0
5700	530.1	0.461	0.4349	1.2196	0.3209	0
5701	530	0.461	0.4351	1.2204	0.3209	0
5702	529.9	0.4611	0.4352	1.2198	0.3211	0
5703	529.8	0.4613	0.4355	1.2201	0.3213	0
5704	529.7	0.4614	0.4355	1.2206	0.3214	0
5705	529.6	0.4616	0.4356	1.2204	0.3216	0

5706	529.5	0.4619	0.4358	1.2203	0.3217	0
5707	529.4	0.4619	0.4359	1.2215	0.3219	0
5708	529.3	0.4619	0.4358	1.2205	0.322	0
5709	529.2	0.4619	0.436	1.2202	0.3221	0
5710	529.1	0.4621	0.4362	1.2205	0.3221	0
5711	529	0.4622	0.4363	1.221	0.3224	0
5712	528.9	0.4625	0.4365	1.2213	0.3226	0
5713	528.8	0.4624	0.4364	1.2214	0.3224	0
5714	528.7	0.4625	0.4365	1.221	0.3227	0
5715	528.6	0.4624	0.4366	1.2213	0.3228	0
5716	528.5	0.4627	0.4367	1.2216	0.3229	0
5717	528.4	0.4627	0.4369	1.2224	0.3229	0
5718	528.3	0.4629	0.4371	1.2225	0.3232	0
5719	528.2	0.463	0.4372	1.2226	0.3232	0
5720	528.1	0.463	0.4371	1.2226	0.3232	0
5721	528	0.463	0.4374	1.2231	0.3233	0
5722	527.9	0.4631	0.4375	1.2238	0.3235	0
5723	527.8	0.4632	0.4374	1.224	0.3236	0
5724	527.7	0.4634	0.4377	1.2243	0.3237	0
5725	527.6	0.4633	0.4376	1.2242	0.3236	0
5726	527.5	0.4634	0.4377	1.2257	0.3236	0
5727	527.4	0.4637	0.4379	1.2254	0.324	0
5728	527.3	0.4637	0.4379	1.2246	0.3241	0
5729	527.2	0.4636	0.4379	1.2257	0.3241	0
5730	527.1	0.4638	0.4382	1.2262	0.3241	0
5731	527	0.464	0.4385	1.2264	0.3245	0
5732	526.9	0.464	0.4385	1.2273	0.3245	0
5733	526.8	0.4643	0.4387	1.2272	0.3246	0
5734	526.7	0.4641	0.4387	1.2273	0.3247	0
5735	526.6	0.4642	0.4389	1.228	0.3249	0
5736	526.5	0.4644	0.439	1.2278	0.3249	0
5737	526.4	0.4644	0.4391	1.228	0.3249	0
5738	526.3	0.4645	0.4391	1.2278	0.3251	0
5739	526.2	0.4648	0.4393	1.2284	0.3253	0
5740	526.1	0.4647	0.4393	1.2278	0.3253	0
5741	526	0.4649	0.4392	1.2288	0.3254	0
5742	525.9	0.4649	0.4394	1.2287	0.3256	0
5743	525.8	0.4651	0.4396	1.2293	0.3258	0
5744	525.7	0.4652	0.4398	1.2295	0.3257	0
5745	525.6	0.4653	0.4397	1.2284	0.3258	0
5746	525.5	0.4656	0.4401	1.2292	0.3262	0
5747	525.4	0.4655	0.4401	1.2297	0.3261	0
5748	525.3	0.4656	0.4402	1.2291	0.3264	0

5749	525.2	0.4657	0.4404	1.2293	0.3265	0
5750	525.1	0.466	0.4405	1.2283	0.3266	0
5751	525	0.4661	0.4406	1.2293	0.3269	0
5752	524.9	0.4663	0.4409	1.2298	0.3269	0
5753	524.8	0.4665	0.441	1.2296	0.3272	0
5754	524.7	0.4667	0.4411	1.2306	0.3273	0
5755	524.6	0.4667	0.4414	1.2293	0.3276	0
5756	524.5	0.4671	0.4418	1.2299	0.3278	0
5757	524.4	0.4671	0.4418	1.2296	0.328	0
5758	524.3	0.467	0.4418	1.2296	0.3281	0
5759	524.2	0.4673	0.4419	1.2298	0.3282	0
5760	524.1	0.4675	0.4421	1.2303	0.3284	0
5761	524	0.4677	0.4424	1.2303	0.3287	0
5762	523.9	0.4679	0.4427	1.2296	0.3288	0
5763	523.8	0.4682	0.4427	1.2294	0.3291	0
5764	523.7	0.4682	0.443	1.2303	0.3294	0
5765	523.6	0.4683	0.443	1.23	0.3294	0
5766	523.5	0.4682	0.4432	1.2301	0.3295	0
5767	523.4	0.4686	0.4434	1.2302	0.3296	0
5768	523.3	0.4686	0.4435	1.23	0.3299	0
5769	523.2	0.4688	0.4436	1.23	0.3301	0
5770	523.1	0.469	0.4439	1.2306	0.3304	0
5771	523	0.4692	0.4439	1.2311	0.3305	0
5772	522.9	0.4693	0.4442	1.2304	0.3306	0
5773	522.8	0.4695	0.4443	1.2305	0.3308	0
5774	522.7	0.4697	0.4444	1.231	0.3308	0
5775	522.6	0.4698	0.4446	1.2315	0.3311	0
5776	522.5	0.4701	0.4449	1.2309	0.3313	0
5777	522.4	0.4701	0.4448	1.2305	0.3313	0
5778	522.3	0.4702	0.445	1.2311	0.3315	0
5779	522.2	0.4703	0.4452	1.2307	0.3316	0
5780	522.1	0.4704	0.4452	1.2312	0.3318	0
5781	522	0.4704	0.4455	1.2312	0.332	0
5782	521.9	0.4707	0.4457	1.2314	0.3322	0
5783	521.8	0.471	0.4459	1.2311	0.3324	0
5784	521.7	0.471	0.446	1.2316	0.3325	0
5785	521.6	0.4711	0.4462	1.2308	0.3326	0
5786	521.5	0.4713	0.4463	1.2309	0.3327	0
5787	521.4	0.4715	0.4467	1.2315	0.333	0
5788	521.3	0.4716	0.4468	1.2311	0.3332	0
5789	521.2	0.4717	0.4469	1.232	0.3335	0
5790	521.1	0.472	0.4472	1.2322	0.3337	0
5791	521	0.472	0.4472	1.2326	0.3337	0

5792	520.9	0.4722	0.4473	1.2322	0.3338	0
5793	520.8	0.4724	0.4474	1.2327	0.334	0
5794	520.7	0.4727	0.4477	1.2321	0.3344	0
5795	520.6	0.4726	0.4479	1.232	0.3344	0
5796	520.5	0.4729	0.4481	1.2323	0.3349	0
5797	520.4	0.4732	0.4483	1.2323	0.3349	0
5798	520.3	0.4732	0.4485	1.2324	0.3351	0
5799	520.2	0.4733	0.4486	1.2319	0.3351	0
5800	520.1	0.4735	0.4488	1.2333	0.3354	0
5801	520	0.4738	0.4491	1.2331	0.3356	0
5802	519.9	0.4739	0.4491	1.2335	0.3358	0
5803	519.8	0.474	0.4492	1.2335	0.3359	0
5804	519.7	0.4741	0.4495	1.2341	0.3362	0
5805	519.6	0.4744	0.4498	1.2342	0.3364	0
5806	519.5	0.4745	0.4499	1.2346	0.3365	0
5807	519.4	0.4747	0.45	1.2339	0.3366	0
5808	519.3	0.4748	0.4501	1.2344	0.337	0
5809	519.2	0.4751	0.4504	1.2344	0.3371	0
5810	519.1	0.4749	0.4505	1.2352	0.337	0
5811	519	0.4752	0.4505	1.2354	0.3373	0
5812	518.9	0.4755	0.4507	1.2346	0.3375	0
5813	518.8	0.4757	0.4509	1.2347	0.3377	0
5814	518.7	0.4759	0.4512	1.2349	0.338	0
5815	518.6	0.4759	0.4513	1.2344	0.3381	0
5816	518.5	0.4762	0.4515	1.2354	0.3382	0
5817	518.4	0.4759	0.4516	1.2357	0.3382	0
5818	518.3	0.4763	0.4518	1.2354	0.3384	0
5819	518.2	0.4764	0.4519	1.2355	0.3387	0
5820	518.1	0.4768	0.4521	1.2357	0.339	0
5821	518	0.4769	0.4522	1.2366	0.339	0
5822	517.9	0.477	0.4523	1.2367	0.3394	0
5823	517.8	0.4772	0.4526	1.2368	0.3396	0
5824	517.7	0.4773	0.4526	1.2363	0.3395	0
5825	517.6	0.4773	0.4525	1.2366	0.3396	0
5826	517.5	0.4775	0.4527	1.2364	0.3398	0
5827	517.4	0.4776	0.4527	1.2364	0.34	0
5828	517.3	0.478	0.4529	1.2369	0.3401	0
5829	517.2	0.4781	0.4533	1.2372	0.3404	0
5830	517.1	0.4784	0.4535	1.2374	0.3408	0
5831	517	0.4783	0.4535	1.2374	0.3407	0
5832	516.9	0.4786	0.4537	1.2379	0.3408	0
5833	516.8	0.4786	0.4537	1.2379	0.341	0
5834	516.7	0.4789	0.4537	1.2384	0.3412	0

5835	516.6	0.4788	0.4536	1.2384	0.3413	0
5836	516.5	0.4793	0.4537	1.2385	0.3415	0
5837	516.4	0.4794	0.4539	1.2396	0.3416	0
5838	516.3	0.4796	0.4539	1.2387	0.3418	0
5839	516.2	0.4797	0.4542	1.239	0.3422	0
5840	516.1	0.4799	0.4546	1.2392	0.3425	0
5841	516	0.48	0.4543	1.2392	0.3425	0
5842	515.9	0.4802	0.4544	1.2401	0.3426	0
5843	515.8	0.4802	0.4546	1.2389	0.3427	0
5844	515.7	0.4803	0.4547	1.2394	0.3428	0
5845	515.6	0.4804	0.455	1.2396	0.3431	0
5846	515.5	0.4807	0.4548	1.24	0.3432	0
5847	515.4	0.4807	0.4551	1.2396	0.3433	0
5848	515.3	0.481	0.4555	1.2398	0.3436	0
5849	515.2	0.4813	0.4555	1.2395	0.3438	0
5850	515.1	0.4814	0.4555	1.2395	0.3439	0
5851	515	0.4817	0.4559	1.2407	0.3442	0
5852	514.9	0.4816	0.456	1.2398	0.3443	0
5853	514.8	0.4819	0.4561	1.2409	0.3447	0
5854	514.7	0.4819	0.4563	1.2401	0.3448	0
5855	514.6	0.4824	0.4565	1.2408	0.3451	0
5856	514.5	0.4822	0.4565	1.2403	0.3451	0
5857	514.4	0.4824	0.4566	1.2408	0.3453	0
5858	514.3	0.4826	0.4568	1.2405	0.3456	0
5859	514.2	0.4825	0.4568	1.2401	0.3455	0
5860	514.1	0.4829	0.457	1.2407	0.3458	0
5861	514	0.4831	0.4572	1.2413	0.346	0
5862	513.9	0.4832	0.4572	1.2419	0.3461	0
5863	513.8	0.4834	0.4574	1.2415	0.3464	0
5864	513.7	0.4834	0.4577	1.2425	0.3466	0
5865	513.6	0.4836	0.4577	1.2413	0.3467	0
5866	513.5	0.4839	0.4579	1.2414	0.3469	0
5867	513.4	0.4841	0.4582	1.2416	0.3471	0
5868	513.3	0.4841	0.4581	1.2421	0.3472	0
5869	513.2	0.4844	0.4585	1.2413	0.3475	0
5870	513.1	0.4845	0.4584	1.2419	0.3476	0
5871	513	0.4847	0.4587	1.2421	0.3479	0
5872	512.9	0.4849	0.4588	1.2421	0.348	0
5873	512.8	0.485	0.459	1.2422	0.3481	0
5874	512.7	0.4853	0.4592	1.2415	0.3483	0
5875	512.6	0.4854	0.4594	1.243	0.3486	0
5876	512.5	0.4857	0.4596	1.2427	0.3487	0
5877	512.4	0.4856	0.4597	1.2432	0.3489	0

5878	512.3	0.4857	0.46	1.2429	0.3491	0
5879	512.2	0.4858	0.46	1.2433	0.3491	0
5880	512.1	0.4861	0.4603	1.2431	0.3494	0
5881	512	0.4866	0.4604	1.2432	0.3497	0
5882	511.9	0.4865	0.4605	1.2438	0.3499	0
5883	511.8	0.4866	0.4607	1.2438	0.35	0
5884	511.7	0.4869	0.4608	1.2435	0.3502	0
5885	511.6	0.4871	0.461	1.2436	0.3504	0
5886	511.5	0.4872	0.4611	1.2433	0.3505	0
5887	511.4	0.4875	0.4614	1.244	0.351	0
5888	511.3	0.4878	0.4614	1.2449	0.351	0
5889	511.2	0.4879	0.4614	1.2444	0.3511	0
5890	511.1	0.4879	0.4614	1.2445	0.3512	0
5891	511	0.488	0.4616	1.2442	0.3514	0
5892	510.9	0.4882	0.4618	1.2452	0.3518	0
5893	510.8	0.4883	0.4619	1.2448	0.3519	0
5894	510.7	0.4886	0.4619	1.2453	0.3519	0
5895	510.6	0.4888	0.4622	1.246	0.3522	0
5896	510.5	0.4889	0.4624	1.2456	0.3522	0
5897	510.4	0.4889	0.4626	1.2457	0.3525	0
5898	510.3	0.4891	0.4625	1.2462	0.3527	0
5899	510.2	0.4892	0.4628	1.2454	0.3529	0
5900	510.1	0.4895	0.4631	1.2464	0.3531	0
5901	510	0.4897	0.4632	1.2465	0.3532	0
5902	509.9	0.4898	0.4635	1.2471	0.3535	0
5903	509.8	0.49	0.4636	1.2475	0.3535	0
5904	509.7	0.4902	0.4637	1.2463	0.3539	0
5905	509.6	0.4903	0.4638	1.2468	0.354	0
5906	509.5	0.4906	0.4641	1.2474	0.3542	0
5907	509.4	0.4907	0.4642	1.2474	0.3543	0
5908	509.3	0.4908	0.4642	1.2478	0.3545	0
5909	509.2	0.4911	0.4645	1.2491	0.3548	0
5910	509.1	0.4914	0.4649	1.2478	0.3551	0
5911	509	0.4913	0.4648	1.2487	0.3551	0
5912	508.9	0.4916	0.4652	1.2493	0.3554	0
5913	508.8	0.4916	0.4653	1.2498	0.3556	0
5914	508.7	0.4919	0.4654	1.249	0.3557	0
5915	508.6	0.4921	0.4656	1.2496	0.356	0
5916	508.5	0.4924	0.4658	1.2492	0.3562	0
5917	508.4	0.4925	0.466	1.2494	0.3565	0
5918	508.3	0.4926	0.4661	1.2503	0.3564	0
5919	508.2	0.4929	0.4661	1.2504	0.3566	0
5920	508.1	0.4931	0.4664	1.25	0.3568	0

5921	508	0.4933	0.4668	1.2508	0.3571	0
5922	507.9	0.4934	0.4668	1.2508	0.3571	0
5923	507.8	0.4934	0.467	1.2503	0.3572	0
5924	507.7	0.4937	0.467	1.251	0.3575	0
5925	507.6	0.4941	0.4675	1.2513	0.3578	0
5926	507.5	0.4942	0.4677	1.2513	0.3581	0
5927	507.4	0.4946	0.468	1.2515	0.3583	0
5928	507.3	0.4946	0.468	1.2511	0.3585	0
5929	507.2	0.4945	0.4681	1.2516	0.3586	0
5930	507.1	0.495	0.4684	1.2508	0.3589	0
5931	507	0.4952	0.4686	1.2515	0.3591	0
5932	506.9	0.4955	0.4689	1.2517	0.3594	0
5933	506.8	0.4955	0.4691	1.2518	0.3595	0
5934	506.7	0.4957	0.4693	1.2514	0.3597	0
5935	506.6	0.496	0.4694	1.2515	0.3598	0
5936	506.5	0.4962	0.4696	1.2526	0.36	0
5937	506.4	0.4962	0.4698	1.2532	0.3603	0
5938	506.3	0.4965	0.4698	1.2528	0.3605	0
5939	506.2	0.4968	0.4704	1.2528	0.3609	0
5940	506.1	0.4971	0.4705	1.2528	0.3611	0
5941	506	0.497	0.4704	1.2528	0.3611	0
5942	505.9	0.4973	0.4706	1.2535	0.3614	0
5943	505.8	0.4974	0.471	1.2528	0.3616	0
5944	505.7	0.4974	0.4713	1.2538	0.3619	0
5945	505.6	0.4979	0.4716	1.2536	0.3621	0
5946	505.5	0.498	0.4718	1.2532	0.3623	0
5947	505.4	0.4981	0.4718	1.2528	0.3623	0
5948	505.3	0.4984	0.472	1.2544	0.3626	0
5949	505.2	0.4986	0.4723	1.2552	0.3629	0
5950	505.1	0.4987	0.4722	1.2532	0.363	0
5951	505	0.4987	0.4726	1.2534	0.3632	0
5952	504.9	0.4993	0.4729	1.2536	0.3635	0
5953	504.8	0.4992	0.4731	1.2537	0.3637	0
5954	504.7	0.4995	0.4735	1.2549	0.3639	0
5955	504.6	0.4996	0.4732	1.2544	0.364	0
5956	504.5	0.5	0.4737	1.2548	0.3645	0
5957	504.4	0.5001	0.4739	1.2549	0.3646	0
5958	504.3	0.5003	0.4741	1.255	0.3647	0
5959	504.2	0.5005	0.4744	1.2557	0.365	0
5960	504.1	0.5007	0.4745	1.2554	0.3652	0
5961	504	0.5009	0.4746	1.256	0.3653	0
5962	503.9	0.5013	0.4749	1.2557	0.3657	0
5963	503.8	0.5011	0.4749	1.2548	0.3659	0

5964	503.7	0.5016	0.4752	1.2551	0.3662	0
5965	503.6	0.5017	0.4756	1.2557	0.3663	0
5966	503.5	0.5018	0.476	1.2554	0.3665	0
5967	503.4	0.5021	0.476	1.2556	0.3667	0
5968	503.3	0.5022	0.476	1.2557	0.3669	0
5969	503.2	0.5024	0.4764	1.2559	0.3671	0
5970	503.1	0.5025	0.4764	1.257	0.3671	0
5971	503	0.5027	0.4766	1.2571	0.3673	0
5972	502.9	0.503	0.4769	1.2568	0.3676	0
5973	502.8	0.5033	0.4772	1.2565	0.3677	0
5974	502.7	0.5035	0.4773	1.2573	0.3682	0
5975	502.6	0.5035	0.4773	1.2563	0.3682	0
5976	502.5	0.5039	0.4778	1.258	0.3683	0
5977	502.4	0.5041	0.478	1.2574	0.3687	0
5978	502.3	0.5043	0.4781	1.2575	0.3689	0
5979	502.2	0.5044	0.4785	1.2581	0.3691	0
5980	502.1	0.5046	0.4785	1.2588	0.3692	0
5981	502	0.5049	0.4787	1.2586	0.3694	0
5982	501.9	0.505	0.4789	1.2582	0.3696	0
5983	501.8	0.5054	0.4791	1.2579	0.37	0
5984	501.7	0.5052	0.4791	1.259	0.3699	0
5985	501.6	0.5055	0.4792	1.2587	0.37	0
5986	501.5	0.5057	0.4794	1.2587	0.3702	0
5987	501.4	0.5057	0.4797	1.2584	0.3704	0
5988	501.3	0.5061	0.4801	1.2592	0.3706	0
5989	501.2	0.5063	0.4802	1.2595	0.3709	0
5990	501.1	0.5065	0.4803	1.2591	0.3712	0
5991	501	0.5066	0.4804	1.2598	0.3713	0
5992	500.9	0.5068	0.4807	1.2594	0.3714	0
5993	500.8	0.507	0.4809	1.2601	0.3716	0
5994	500.7	0.5073	0.481	1.2608	0.3718	0
5995	500.6	0.5073	0.4811	1.261	0.3719	0
5996	500.5	0.5076	0.4814	1.2605	0.3721	0
5997	500.4	0.5077	0.4814	1.2608	0.3724	0
5998	500.3	0.508	0.4818	1.2615	0.3726	0
5999	500.2	0.5083	0.4821	1.2618	0.3728	0
6000	500.1	0.5084	0.4822	1.2619	0.373	0
6001	500	0.5087	0.4824	1.2621	0.3732	0
6002	499.9	0.5085	0.4824	1.2626	0.3732	0
6003	499.8	0.5089	0.4828	1.2629	0.3735	0
6004	499.7	0.509	0.4827	1.2624	0.3736	0
6005	499.6	0.5093	0.4831	1.2637	0.3737	0
6006	499.5	0.5095	0.4832	1.2638	0.374	0

6007	499.4	0.5096	0.4835	1.264	0.3742	0
6008	499.3	0.5099	0.4836	1.2642	0.3744	0
6009	499.2	0.51	0.4838	1.2643	0.3745	0
6010	499.1	0.5103	0.4842	1.2639	0.3747	0
6011	499	0.5104	0.4841	1.264	0.3749	0
6012	498.9	0.5107	0.4841	1.2652	0.375	0
6013	498.8	0.511	0.4846	1.2654	0.3752	0
6014	498.7	0.511	0.4848	1.265	0.3754	0
6015	498.6	0.5114	0.485	1.2652	0.3757	0
6016	498.5	0.5116	0.4852	1.2665	0.3758	0
6017	498.4	0.5118	0.4853	1.2661	0.376	0
6018	498.3	0.512	0.4854	1.2673	0.3761	0
6019	498.2	0.5121	0.4859	1.2679	0.3762	0
6020	498.1	0.5121	0.4859	1.2675	0.3765	0
6021	498	0.5125	0.4861	1.2671	0.3767	0
6022	497.9	0.5127	0.4865	1.2674	0.377	0
6023	497.8	0.513	0.4868	1.2681	0.3773	0
6024	497.7	0.5131	0.4871	1.2682	0.3775	0
6025	497.6	0.5137	0.4872	1.2684	0.3776	0
6026	497.5	0.5138	0.4874	1.2686	0.3778	0
6027	497.4	0.5138	0.4877	1.2693	0.3782	0
6028	497.3	0.514	0.4879	1.2689	0.3782	0
6029	497.2	0.5142	0.4881	1.2685	0.3784	0
6030	497.1	0.5144	0.4883	1.2697	0.3786	0
6031	497	0.5149	0.4885	1.2689	0.3791	0
6032	496.9	0.5149	0.4886	1.2695	0.3791	0
6033	496.8	0.515	0.4887	1.2702	0.3792	0
6034	496.7	0.5154	0.4892	1.2699	0.3796	0
6035	496.6	0.5153	0.4893	1.2706	0.3798	0
6036	496.5	0.5157	0.4894	1.2701	0.3799	0
6037	496.4	0.516	0.4897	1.2698	0.3801	0
6038	496.3	0.5165	0.4901	1.2701	0.3807	0
6039	496.2	0.5165	0.4903	1.2708	0.3807	0
6040	496.1	0.5166	0.4904	1.2709	0.3808	0
6041	496	0.517	0.4908	1.2711	0.3811	0
6042	495.9	0.5172	0.4909	1.2708	0.3813	0
6043	495.8	0.5175	0.4911	1.2705	0.3817	0
6044	495.7	0.5176	0.4915	1.2712	0.3818	0
6045	495.6	0.5182	0.4918	1.271	0.382	0
6046	495.5	0.5183	0.4919	1.2717	0.3824	0
6047	495.4	0.5185	0.4921	1.2719	0.3826	0
6048	495.3	0.5186	0.4925	1.2715	0.3828	0
6049	495.2	0.5189	0.4927	1.2711	0.383	0

6050	495.1	0.519	0.4929	1.2724	0.3832	0
6051	495	0.5191	0.4929	1.272	0.3834	0
6052	494.9	0.5193	0.4931	1.2716	0.3835	0
6053	494.8	0.5196	0.4933	1.2728	0.3837	0
6054	494.7	0.5199	0.4936	1.2731	0.3841	0
6055	494.6	0.5202	0.4939	1.2718	0.3844	0
6056	494.5	0.5205	0.4943	1.272	0.3847	0
6057	494.4	0.5206	0.4946	1.2726	0.3848	0
6058	494.3	0.5208	0.4945	1.2727	0.385	0
6059	494.2	0.5213	0.4949	1.2724	0.3853	0
6060	494.1	0.5215	0.4949	1.2721	0.3855	0
6061	494	0.5216	0.4955	1.2729	0.3858	0
6062	493.9	0.5218	0.4957	1.2725	0.386	0
6063	493.8	0.522	0.4958	1.2721	0.3861	0
6064	493.7	0.5222	0.4962	1.2713	0.3865	0
6065	493.6	0.5225	0.4963	1.2725	0.3866	0
6066	493.5	0.5227	0.4965	1.2722	0.387	0
6067	493.4	0.5233	0.4971	1.2737	0.3874	0
6068	493.3	0.5234	0.4971	1.2733	0.3874	0
6069	493.2	0.5237	0.4972	1.2735	0.3877	0
6070	493.1	0.5238	0.4975	1.2731	0.3878	0
6071	493	0.524	0.4978	1.2733	0.3881	0
6072	492.9	0.5243	0.4981	1.2741	0.3883	0
6073	492.8	0.5244	0.4981	1.2748	0.3885	0
6074	492.7	0.5247	0.4985	1.275	0.3887	0
6075	492.6	0.5249	0.4987	1.2748	0.3891	0
6076	492.5	0.5252	0.4991	1.2756	0.3893	0
6077	492.4	0.5252	0.4992	1.2757	0.3894	0
6078	492.3	0.5258	0.4992	1.2759	0.3896	0
6079	492.2	0.5259	0.4995	1.2756	0.3898	0
6080	492.1	0.5261	0.4997	1.2758	0.3899	0
6081	492	0.5263	0.5001	1.2755	0.3902	0
6082	491.9	0.5264	0.5002	1.2752	0.3904	0
6083	491.8	0.5269	0.5006	1.2755	0.3907	0
6084	491.7	0.5269	0.5005	1.2762	0.3908	0
6085	491.6	0.5271	0.5009	1.2764	0.3911	0
6086	491.5	0.5273	0.5011	1.2773	0.3912	0
6087	491.4	0.5276	0.5014	1.2764	0.3916	0
6088	491.3	0.5277	0.5015	1.2772	0.3916	0
6089	491.2	0.5282	0.5018	1.2775	0.3921	0
6090	491.1	0.5285	0.5019	1.2772	0.3922	0
6091	491	0.5285	0.5024	1.278	0.3924	0
6092	490.9	0.5288	0.5025	1.2776	0.3926	0

6093	490.8	0.5291	0.5028	1.279	0.3928	0
6094	490.7	0.5293	0.5028	1.2791	0.3928	0
6095	490.6	0.5295	0.5031	1.2788	0.3931	0
6096	490.5	0.5296	0.5033	1.2796	0.3932	0
6097	490.4	0.5301	0.5037	1.2799	0.3933	0
6098	490.3	0.5302	0.5037	1.2802	0.3938	0
6099	490.2	0.5302	0.5039	1.2803	0.3938	0
6100	490.1	0.5305	0.5041	1.2799	0.394	0
6101	490	0.531	0.5042	1.2813	0.3942	0
6102	489.9	0.5311	0.5046	1.2798	0.3945	0
6103	489.8	0.5312	0.5045	1.2801	0.3947	0
6104	489.7	0.5314	0.5048	1.2813	0.3947	0
6105	489.6	0.5316	0.505	1.2822	0.3951	0
6106	489.5	0.5318	0.5052	1.2819	0.3953	0
6107	489.4	0.5321	0.5056	1.2815	0.3954	0
6108	489.3	0.5323	0.5055	1.2817	0.3955	0
6109	489.2	0.5324	0.5056	1.282	0.3956	0
6110	489.1	0.5328	0.5062	1.2829	0.396	0
6111	489	0.5329	0.5063	1.2826	0.3962	0
6112	488.9	0.5333	0.5066	1.2835	0.3965	0
6113	488.8	0.5334	0.5066	1.283	0.3964	0
6114	488.7	0.5337	0.5069	1.2839	0.3968	0
6115	488.6	0.5339	0.5069	1.2847	0.3969	0
6116	488.5	0.534	0.5072	1.2854	0.3969	0
6117	488.4	0.5343	0.5074	1.2845	0.397	0
6118	488.3	0.5345	0.5078	1.286	0.3973	0
6119	488.2	0.5347	0.5076	1.2861	0.3975	0
6120	488.1	0.5348	0.5079	1.2856	0.3977	0
6121	488	0.5351	0.5081	1.2866	0.3978	0
6122	487.9	0.5353	0.5084	1.2868	0.3981	0
6123	487.8	0.5355	0.5086	1.2864	0.3983	0
6124	487.7	0.5358	0.5087	1.2872	0.3985	0
6125	487.6	0.5361	0.509	1.2875	0.3988	0
6126	487.5	0.5361	0.5092	1.2877	0.3988	0
6127	487.4	0.5365	0.5096	1.2875	0.3991	0
6128	487.3	0.5368	0.5099	1.2889	0.3995	0
6129	487.2	0.537	0.5101	1.2892	0.3996	0
6130	487.1	0.5375	0.5104	1.2888	0.3998	0
6131	487	0.5374	0.5103	1.2889	0.4001	0
6132	486.9	0.5377	0.5108	1.2892	0.4002	0
6133	486.8	0.538	0.5109	1.2888	0.4005	0
6134	486.7	0.5381	0.5109	1.2888	0.4005	0
6135	486.6	0.5384	0.5114	1.2892	0.4008	0

6136	486.5	0.5386	0.5115	1.29	0.401	0
6137	486.4	0.539	0.5116	1.2904	0.4012	0
6138	486.3	0.5391	0.5121	1.2912	0.4015	0
6139	486.2	0.5392	0.5122	1.2914	0.4016	0
6140	486.1	0.5399	0.5126	1.2911	0.402	0
6141	486	0.5399	0.5128	1.2913	0.4023	0
6142	485.9	0.5402	0.513	1.291	0.4025	0
6143	485.8	0.5406	0.5133	1.2912	0.4027	0
6144	485.7	0.5409	0.5134	1.2907	0.4028	0
6145	485.6	0.5412	0.5137	1.2917	0.4032	0
6146	485.5	0.5413	0.514	1.2925	0.4033	0
6147	485.4	0.5416	0.5142	1.2928	0.4035	0
6148	485.3	0.5421	0.5146	1.2936	0.4038	0
6149	485.2	0.5422	0.5151	1.2927	0.4042	0
6150	485.1	0.5425	0.515	1.2935	0.4045	0
6151	485	0.5428	0.5153	1.2932	0.4046	0
6152	484.9	0.5431	0.5155	1.294	0.4048	0
6153	484.8	0.5432	0.5157	1.2936	0.405	0
6154	484.7	0.5437	0.5161	1.2933	0.4055	0
6155	484.6	0.544	0.5166	1.2924	0.4059	0
6156	484.5	0.5442	0.5169	1.2932	0.406	0
6157	484.4	0.5447	0.5172	1.2936	0.4063	0
6158	484.3	0.545	0.5174	1.2939	0.4066	0
6159	484.2	0.5451	0.5177	1.2935	0.4068	0
6160	484.1	0.5452	0.5178	1.2944	0.4071	0
6161	484	0.5457	0.5182	1.2941	0.4076	0
6162	483.9	0.5459	0.5183	1.2937	0.4077	0
6163	483.8	0.5461	0.5185	1.2939	0.4079	0
6164	483.7	0.5466	0.5191	1.2949	0.4081	0
6165	483.6	0.5465	0.5192	1.2938	0.4085	0
6166	483.5	0.5468	0.5193	1.294	0.4085	0
6167	483.4	0.5471	0.5195	1.2949	0.409	0
6168	483.3	0.5476	0.5199	1.2946	0.4092	0
6169	483.2	0.5479	0.5201	1.2949	0.4095	0
6170	483.1	0.5481	0.5207	1.2952	0.4099	0
6171	483	0.5484	0.521	1.2956	0.4101	0
6172	482.9	0.5487	0.5212	1.2959	0.4104	0
6173	482.8	0.5489	0.5213	1.2953	0.4104	0
6174	482.7	0.5489	0.5216	1.2962	0.4107	0
6175	482.6	0.5494	0.522	1.2952	0.411	0
6176	482.5	0.5496	0.5221	1.2961	0.4111	0
6177	482.4	0.5501	0.5224	1.2971	0.4113	0
6178	482.3	0.5502	0.5226	1.2966	0.4116	0

6179	482.2	0.5504	0.5227	1.2969	0.4116	0
6180	482.1	0.5509	0.5231	1.2967	0.4122	0
6181	482	0.551	0.5234	1.2962	0.4122	0
6182	481.9	0.5513	0.5236	1.2971	0.4124	0
6183	481.8	0.5517	0.5238	1.2974	0.4128	0
6184	481.7	0.5519	0.5241	1.2976	0.413	0
6185	481.6	0.552	0.5242	1.2979	0.4131	0
6186	481.5	0.5524	0.5246	1.2983	0.4134	0
6187	481.4	0.5526	0.5248	1.2991	0.4136	0
6188	481.3	0.5528	0.5252	1.2981	0.4138	0
6189	481.2	0.553	0.5255	1.2983	0.4139	0
6190	481.1	0.5531	0.5256	1.2986	0.4141	0
6191	481	0.5536	0.5257	1.3003	0.4145	0
6192	480.9	0.5538	0.5259	1.3006	0.4147	0
6193	480.8	0.5543	0.5264	1.3003	0.4151	0
6194	480.7	0.5547	0.5265	1.3006	0.4153	0
6195	480.6	0.5548	0.5267	1.3009	0.4155	0
6196	480.5	0.5549	0.527	1.3011	0.4158	0
6197	480.4	0.5551	0.527	1.3006	0.4158	0
6198	480.3	0.5556	0.5275	1.3016	0.4161	0
6199	480.2	0.5555	0.5276	1.301	0.4161	0
6200	480.1	0.5558	0.5279	1.3015	0.4165	0
6201	480	0.5562	0.5283	1.3018	0.4168	0
6202	479.9	0.5565	0.5285	1.3027	0.4171	0
6203	479.8	0.5568	0.5288	1.303	0.4173	0
6204	479.7	0.5571	0.5289	1.3033	0.4173	0
6205	479.6	0.5572	0.5291	1.3036	0.4178	0
6206	479.5	0.5577	0.5295	1.3031	0.4177	0
6207	479.4	0.5577	0.5296	1.3034	0.418	0
6208	479.3	0.5582	0.5299	1.3046	0.4185	0
6209	479.2	0.5584	0.5301	1.3048	0.4186	0
6210	479.1	0.5584	0.5302	1.3049	0.4186	0
6211	479	0.5588	0.5307	1.3047	0.4191	0
6212	478.9	0.5589	0.5308	1.3049	0.4192	0
6213	478.8	0.5593	0.531	1.3053	0.4194	0
6214	478.7	0.5595	0.5312	1.3055	0.4195	0
6215	478.6	0.5598	0.5316	1.3073	0.4198	0
6216	478.5	0.56	0.5316	1.3054	0.4198	0
6217	478.4	0.5603	0.5317	1.3078	0.4201	0
6218	478.3	0.5607	0.532	1.3081	0.4204	0
6219	478.2	0.5609	0.5322	1.3084	0.4206	0
6220	478.1	0.5612	0.5327	1.3096	0.4209	0
6221	478	0.5611	0.5329	1.3097	0.4208	0

6222	477.9	0.5615	0.533	1.3094	0.4212	0
6223	477.8	0.5618	0.533	1.3096	0.4211	0
6224	477.7	0.562	0.5334	1.3092	0.4214	0
6225	477.6	0.5623	0.5335	1.3088	0.4217	0
6226	477.5	0.5624	0.5337	1.3097	0.4217	0
6227	477.4	0.5628	0.5342	1.3109	0.4221	0
6228	477.3	0.563	0.5345	1.3106	0.4224	0
6229	477.2	0.5634	0.5345	1.3115	0.4224	0
6230	477.1	0.5635	0.5347	1.3111	0.4228	0
6231	477	0.5636	0.535	1.3114	0.4228	0
6232	476.9	0.5638	0.5351	1.3124	0.4231	0
6233	476.8	0.5642	0.5352	1.3128	0.4232	0
6234	476.7	0.5646	0.5357	1.3131	0.4235	0
6235	476.6	0.5649	0.5357	1.312	0.4238	0
6236	476.5	0.5647	0.5358	1.3122	0.4238	0
6237	476.4	0.5649	0.536	1.3131	0.4239	0
6238	476.3	0.5653	0.5365	1.3129	0.4242	0
6239	476.2	0.5656	0.5366	1.3131	0.4244	0
6240	476.1	0.5658	0.5367	1.3134	0.4245	0
6241	476	0.5662	0.5371	1.3145	0.4247	0
6242	475.9	0.5664	0.5376	1.3142	0.4253	0
6243	475.8	0.5668	0.5378	1.3146	0.4255	0
6244	475.7	0.5672	0.538	1.3142	0.4257	0
6245	475.6	0.5677	0.5384	1.3146	0.426	0
6246	475.5	0.5676	0.5386	1.3156	0.4263	0
6247	475.4	0.568	0.5389	1.3159	0.4265	0
6248	475.3	0.5681	0.539	1.3154	0.4265	0
6249	475.2	0.5685	0.5395	1.3157	0.427	0
6250	475.1	0.5688	0.5395	1.3162	0.4272	0
6251	475	0.5691	0.5399	1.3173	0.4277	0
6252	474.9	0.5695	0.5399	1.3168	0.4277	0
6253	474.8	0.5698	0.5403	1.3171	0.428	0
6254	474.7	0.5701	0.5408	1.3167	0.4282	0
6255	474.6	0.5701	0.5409	1.3175	0.4285	0
6256	474.5	0.5706	0.5412	1.3165	0.4287	0
6257	474.4	0.5708	0.5416	1.3183	0.4288	0
6258	474.3	0.571	0.5419	1.3179	0.4294	0
6259	474.2	0.5715	0.5421	1.3175	0.4297	0
6260	474.1	0.5716	0.5424	1.317	0.4298	0
6261	474	0.5722	0.5426	1.3181	0.43	0
6262	473.9	0.5724	0.543	1.3176	0.4305	0
6263	473.8	0.5725	0.5432	1.3179	0.4306	0
6264	473.7	0.573	0.5434	1.3182	0.4308	0

6265	473.6	0.5731	0.5436	1.3177	0.4311	0
6266	473.5	0.5733	0.5441	1.3195	0.4315	0
6267	473.4	0.5736	0.5443	1.3183	0.4316	0
6268	473.3	0.5738	0.5444	1.3193	0.4318	0
6269	473.2	0.5745	0.5449	1.3198	0.4324	0
6270	473.1	0.5748	0.5452	1.3193	0.4325	0
6271	473	0.5751	0.5456	1.3204	0.4329	0
6272	472.9	0.5754	0.5458	1.32	0.4329	0
6273	472.8	0.5757	0.5461	1.3194	0.4332	0
6274	472.7	0.5759	0.5461	1.3205	0.4335	0
6275	472.6	0.5762	0.5467	1.3193	0.4338	0
6276	472.5	0.5766	0.5469	1.3197	0.4341	0
6277	472.4	0.5771	0.5475	1.3207	0.4343	0
6278	472.3	0.5771	0.5475	1.321	0.4346	0
6279	472.2	0.5774	0.5479	1.3206	0.435	0
6280	472.1	0.5778	0.548	1.3224	0.4354	0
6281	472	0.578	0.5483	1.3219	0.4356	0
6282	471.9	0.5783	0.5485	1.3207	0.4358	0
6283	471.8	0.5788	0.5491	1.3219	0.436	0
6284	471.7	0.579	0.5493	1.3229	0.4362	0
6285	471.6	0.5794	0.5497	1.3217	0.4366	0
6286	471.5	0.5796	0.5498	1.3219	0.4369	0
6287	471.4	0.5797	0.5501	1.3222	0.4369	0
6288	471.3	0.5802	0.5505	1.3219	0.4374	0
6289	471.2	0.5806	0.5507	1.3237	0.4375	0
6290	471.1	0.5807	0.5509	1.3231	0.4379	0
6291	471	0.581	0.5511	1.3249	0.438	0
6292	470.9	0.5813	0.5514	1.3237	0.4383	0
6293	470.8	0.5817	0.552	1.3242	0.4387	0
6294	470.7	0.5821	0.5522	1.3245	0.4391	0
6295	470.6	0.5822	0.5524	1.3255	0.4393	0
6296	470.5	0.5824	0.5524	1.325	0.4393	0
6297	470.4	0.5828	0.5526	1.3253	0.4396	0
6298	470.3	0.5833	0.5534	1.3257	0.4401	0
6299	470.2	0.5837	0.5535	1.3252	0.4403	0
6300	470.1	0.5837	0.5538	1.3262	0.4404	0
6301	470	0.5838	0.5541	1.3266	0.4406	0
6302	469.9	0.5844	0.5542	1.327	0.441	0
6303	469.8	0.5847	0.5545	1.3266	0.4413	0
6304	469.7	0.585	0.5547	1.3276	0.4415	0
6305	469.6	0.5851	0.5552	1.3278	0.4417	0
6306	469.5	0.5856	0.5555	1.3282	0.4421	0
6307	469.4	0.5855	0.5555	1.3275	0.4422	0

6308	469.3	0.5861	0.556	1.3288	0.4425	0
6309	469.2	0.5861	0.5564	1.3274	0.4425	0
6310	469.1	0.5866	0.5564	1.3293	0.4427	0
6311	469	0.5869	0.5566	1.3288	0.4431	0
6312	468.9	0.5874	0.5569	1.3291	0.4434	0
6313	468.8	0.5874	0.5572	1.3303	0.4435	0
6314	468.7	0.5877	0.5574	1.329	0.4437	0
6315	468.6	0.588	0.5576	1.3292	0.444	0
6316	468.5	0.5881	0.5579	1.3303	0.444	0
6317	468.4	0.5883	0.558	1.3306	0.4442	0
6318	468.3	0.5885	0.5584	1.3308	0.4443	0
6319	468.2	0.5889	0.5586	1.3304	0.4448	0
6320	468.1	0.5894	0.5591	1.3316	0.445	0
6321	468	0.5896	0.5591	1.332	0.4455	0
6322	467.9	0.5899	0.5595	1.3315	0.4456	0
6323	467.8	0.5902	0.5597	1.3334	0.4458	0
6324	467.7	0.5906	0.5599	1.333	0.4462	0
6325	467.6	0.5906	0.5602	1.3339	0.446	0
6326	467.5	0.5911	0.5605	1.3335	0.4463	0
6327	467.4	0.5912	0.5607	1.3338	0.4466	0
6328	467.3	0.5915	0.5609	1.3342	0.4467	0
6329	467.2	0.5919	0.5612	1.3346	0.4472	0
6330	467.1	0.5921	0.5614	1.3358	0.4474	0
6331	467	0.5924	0.5619	1.3352	0.4475	0
6332	466.9	0.5925	0.562	1.3348	0.4478	0
6333	466.8	0.5928	0.5624	1.3351	0.4479	0
6334	466.7	0.5929	0.5626	1.3354	0.4482	0
6335	466.6	0.5935	0.563	1.3368	0.4488	0
6336	466.5	0.5938	0.5631	1.3362	0.4487	0
6337	466.4	0.5941	0.5631	1.3373	0.449	0
6338	466.3	0.5943	0.5635	1.3377	0.4492	0
6339	466.2	0.5943	0.5638	1.338	0.4493	0
6340	466.1	0.595	0.564	1.3393	0.4496	0
6341	466	0.595	0.5642	1.3371	0.4499	0
6342	465.9	0.5954	0.5645	1.3392	0.4502	0
6343	465.8	0.5957	0.5647	1.3379	0.4504	0
6344	465.7	0.5958	0.565	1.339	0.4505	0
6345	465.6	0.5962	0.5655	1.3387	0.4509	0
6346	465.5	0.5967	0.5656	1.3382	0.4513	0
6347	465.4	0.5966	0.5658	1.3401	0.4514	0
6348	465.3	0.597	0.5659	1.3404	0.4516	0
6349	465.2	0.5975	0.5661	1.34	0.452	0
6350	465.1	0.5975	0.5665	1.3402	0.4521	0

6351	465	0.598	0.5668	1.3406	0.4524	0
6352	464.9	0.5983	0.5672	1.3402	0.4525	0
6353	464.8	0.5984	0.5674	1.3405	0.4529	0
6354	464.7	0.5988	0.5677	1.3399	0.4531	0
6355	464.6	0.599	0.5679	1.3411	0.4533	0
6356	464.5	0.5993	0.5681	1.3414	0.4535	0
6357	464.4	0.6	0.5686	1.3402	0.454	0
6358	464.3	0.5998	0.569	1.3421	0.4542	0
6359	464.2	0.6002	0.569	1.3416	0.4544	0
6360	464.1	0.6008	0.5697	1.3421	0.4549	0
6361	464	0.601	0.5698	1.3433	0.455	0
6362	463.9	0.6015	0.5702	1.3427	0.4554	0
6363	463.8	0.6015	0.5705	1.3429	0.4554	0
6364	463.7	0.6021	0.5708	1.3441	0.4559	0
6365	463.6	0.6023	0.571	1.3444	0.4561	0
6366	463.5	0.6025	0.5713	1.3439	0.4564	0
6367	463.4	0.6028	0.5717	1.3425	0.4567	0
6368	463.3	0.6031	0.5716	1.3435	0.4569	0
6369	463.2	0.6034	0.5723	1.3439	0.4572	0
6370	463.1	0.6038	0.5724	1.3452	0.4577	0
6371	463	0.604	0.5728	1.3437	0.4579	0
6372	462.9	0.6042	0.5731	1.3439	0.458	0
6373	462.8	0.6047	0.5731	1.3451	0.4585	0
6374	462.7	0.6051	0.5737	1.3453	0.4587	0
6375	462.6	0.6056	0.5741	1.3458	0.4591	0
6376	462.5	0.6059	0.5744	1.3451	0.4592	0
6377	462.4	0.6059	0.5747	1.3454	0.4595	0
6378	462.3	0.6064	0.575	1.3467	0.4598	0
6379	462.2	0.6066	0.5751	1.345	0.46	0
6380	462.1	0.6069	0.5756	1.3463	0.4604	0
6381	462	0.6072	0.5758	1.3457	0.4606	0
6382	461.9	0.6078	0.576	1.3478	0.4609	0
6383	461.8	0.6081	0.5764	1.3472	0.4613	0
6384	461.7	0.6083	0.5766	1.3474	0.4617	0
6385	461.6	0.6086	0.577	1.3467	0.4616	0
6386	461.5	0.6087	0.5772	1.3468	0.462	0
6387	461.4	0.609	0.5777	1.3472	0.4621	0
6388	461.3	0.6095	0.578	1.3484	0.4627	0
6389	461.2	0.6098	0.5782	1.3477	0.4628	0
6390	461.1	0.6104	0.5785	1.3489	0.4631	0
6391	461	0.6103	0.5789	1.3482	0.4633	0
6392	460.9	0.6109	0.5791	1.3504	0.4636	0
6393	460.8	0.6112	0.5796	1.3507	0.464	0

6394	460.7	0.6112	0.5797	1.3499	0.4643	0
6395	460.6	0.6117	0.5803	1.3502	0.4645	0
6396	460.5	0.6121	0.5805	1.3497	0.4649	0
6397	460.4	0.6126	0.5806	1.349	0.4651	0
6398	460.3	0.6127	0.5809	1.351	0.4654	0
6399	460.2	0.613	0.5811	1.3503	0.4657	0
6400	460.1	0.6135	0.5817	1.3507	0.4661	0
6401	460	0.6139	0.5821	1.352	0.4665	0
6402	459.9	0.6142	0.5821	1.3512	0.4665	0
6403	459.8	0.6144	0.5826	1.3513	0.4666	0
6404	459.7	0.6147	0.5828	1.3516	0.4671	0
6405	459.6	0.615	0.5829	1.3528	0.4674	0
6406	459.5	0.6158	0.5836	1.3532	0.4678	0
6407	459.4	0.6159	0.5841	1.3516	0.4682	0
6408	459.3	0.6162	0.5843	1.3538	0.4684	0
6409	459.2	0.6166	0.5848	1.3541	0.4687	0
6410	459.1	0.6168	0.5848	1.3553	0.4693	0
6411	459	0.6173	0.5854	1.3546	0.4693	0
6412	458.9	0.6176	0.5858	1.3559	0.4697	0
6413	458.8	0.618	0.5861	1.3562	0.4699	0
6414	458.7	0.6183	0.5863	1.3545	0.4703	0
6415	458.6	0.6186	0.5864	1.3566	0.4705	0
6416	458.5	0.6189	0.5869	1.3559	0.4707	0
6417	458.4	0.6195	0.5869	1.3571	0.4708	0
6418	458.3	0.6197	0.5873	1.3563	0.4712	0
6419	458.2	0.6201	0.5876	1.3586	0.4715	0
6420	458.1	0.6203	0.5878	1.358	0.4719	0
6421	458	0.6208	0.5883	1.3573	0.4721	0
6422	457.9	0.6212	0.5885	1.359	0.4726	0
6423	457.8	0.6213	0.5887	1.3587	0.4727	0
6424	457.7	0.6216	0.5889	1.3592	0.4728	0
6425	457.6	0.6221	0.5892	1.3591	0.4732	0
6426	457.5	0.622	0.5896	1.3601	0.4734	0
6427	457.4	0.6225	0.59	1.3607	0.4739	0
6428	457.3	0.623	0.5904	1.3612	0.4741	0
6429	457.2	0.6231	0.5906	1.361	0.4745	0
6430	457.1	0.6234	0.5908	1.3614	0.4744	0
6431	457	0.6239	0.591	1.3614	0.4748	0
6432	456.9	0.624	0.5913	1.3612	0.475	0
6433	456.8	0.6243	0.5917	1.363	0.4754	0
6434	456.7	0.6248	0.5919	1.3635	0.4757	0
6435	456.6	0.6251	0.5924	1.3628	0.4761	0
6436	456.5	0.6255	0.5926	1.3639	0.4763	0

6437	456.4	0.6258	0.5927	1.3644	0.4764	0
6438	456.3	0.6261	0.5931	1.3648	0.4767	0
6439	456.2	0.6264	0.5934	1.3641	0.477	0
6440	456.1	0.6268	0.5939	1.3647	0.4773	0
6441	456	0.6271	0.5941	1.3646	0.4775	0
6442	455.9	0.6275	0.5944	1.3652	0.478	0
6443	455.8	0.6278	0.5946	1.3656	0.4781	0
6444	455.7	0.6281	0.595	1.3655	0.4785	0
6445	455.6	0.6284	0.5951	1.3654	0.4789	0
6446	455.5	0.6286	0.5957	1.366	0.4791	0
6447	455.4	0.629	0.5958	1.3659	0.4793	0
6448	455.3	0.6294	0.5963	1.3664	0.4797	0
6449	455.2	0.6296	0.5966	1.367	0.4801	0
6450	455.1	0.63	0.597	1.3676	0.4804	0
6451	455	0.6304	0.5972	1.3668	0.4805	0
6452	454.9	0.6308	0.5973	1.368	0.4808	0
6453	454.8	0.6311	0.5977	1.3672	0.4811	0
6454	454.7	0.6313	0.5981	1.3684	0.4814	0
6455	454.6	0.6315	0.5983	1.3689	0.4817	0
6456	454.5	0.632	0.5987	1.3701	0.4819	0
6457	454.4	0.632	0.5988	1.37	0.4822	0
6458	454.3	0.6327	0.5993	1.37	0.4824	0
6459	454.2	0.6328	0.5996	1.3706	0.483	0
6460	454.1	0.6333	0.6001	1.3711	0.4832	0
6461	454	0.6337	0.6003	1.371	0.4833	0
6462	453.9	0.6339	0.6005	1.3708	0.4836	0
6463	453.8	0.6342	0.6007	1.3707	0.484	0
6464	453.7	0.6344	0.6012	1.3705	0.4842	0
6465	453.6	0.6347	0.6012	1.3698	0.4844	0
6466	453.5	0.635	0.6018	1.3704	0.4847	0
6467	453.4	0.6355	0.6019	1.3723	0.485	0
6468	453.3	0.6356	0.6022	1.3722	0.4854	0
6469	453.2	0.6361	0.6026	1.3723	0.4857	0
6470	453.1	0.6363	0.6031	1.3728	0.4861	0
6471	453	0.6368	0.6032	1.3733	0.4862	0
6472	452.9	0.637	0.6037	1.3732	0.4865	0
6473	452.8	0.6374	0.6041	1.3738	0.487	0
6474	452.7	0.6379	0.6043	1.373	0.4871	0
6475	452.6	0.6381	0.6045	1.3729	0.4875	0
6476	452.5	0.6384	0.6049	1.3743	0.4881	0
6477	452.4	0.6389	0.6053	1.3741	0.488	0
6478	452.3	0.6392	0.6054	1.3753	0.4885	0
6479	452.2	0.6395	0.6059	1.3753	0.4887	0

6480	452.1	0.6399	0.6062	1.3758	0.489	0
6481	452	0.64	0.6066	1.3757	0.4892	0
6482	451.9	0.6405	0.6066	1.3756	0.4896	0
6483	451.8	0.6409	0.6071	1.3762	0.4898	0
6484	451.7	0.6412	0.6075	1.376	0.4902	0
6485	451.6	0.6413	0.6077	1.3757	0.4903	0
6486	451.5	0.6416	0.608	1.3764	0.4909	0
6487	451.4	0.6421	0.6085	1.3777	0.4912	0
6488	451.3	0.6425	0.6088	1.3782	0.4914	0
6489	451.2	0.6428	0.6086	1.3786	0.4914	0
6490	451.1	0.6431	0.609	1.3778	0.4919	0
6491	451	0.6434	0.6095	1.3784	0.4922	0
6492	450.9	0.644	0.6098	1.3791	0.4925	0
6493	450.8	0.6444	0.6102	1.3789	0.4929	0
6494	450.7	0.6446	0.6103	1.3794	0.4932	0
6495	450.6	0.6448	0.6107	1.3792	0.4934	0
6496	450.5	0.6454	0.6111	1.3805	0.4937	0
6497	450.4	0.6456	0.6111	1.3818	0.494	0
6498	450.3	0.646	0.6117	1.3823	0.4943	0
6499	450.2	0.6463	0.612	1.3828	0.4947	0
6500	450.1	0.6465	0.6124	1.3813	0.4951	0
6501	450	0.647	0.6127	1.3818	0.4953	0
6502	449.9	0.637	0.6095	1.409	0.4933	0
6503	449.8	0.6374	0.6098	1.4089	0.4937	0
6504	449.7	0.6376	0.61	1.4104	0.4936	0
6505	449.6	0.6383	0.6106	1.4106	0.4944	0
6506	449.5	0.6385	0.6108	1.4104	0.4945	0
6507	449.4	0.6386	0.611	1.4108	0.4948	0
6508	449.3	0.6393	0.6116	1.412	0.4951	0
6509	449.2	0.6396	0.6118	1.4131	0.4955	0
6510	449.1	0.6401	0.6122	1.4124	0.496	0
6511	449	0.6404	0.6126	1.4115	0.4962	0
6512	448.9	0.6408	0.6129	1.4126	0.4966	0
6513	448.8	0.6413	0.6132	1.4124	0.4967	0
6514	448.7	0.6417	0.6135	1.4134	0.497	0
6515	448.6	0.6419	0.6139	1.4139	0.4974	0
6516	448.5	0.642	0.6142	1.4143	0.4979	0
6517	448.4	0.6426	0.6144	1.4148	0.4981	0
6518	448.3	0.643	0.615	1.4158	0.4984	0
6519	448.2	0.6436	0.6155	1.415	0.4988	0
6520	448.1	0.6438	0.6155	1.416	0.499	0
6521	448	0.6441	0.6159	1.4163	0.4993	0
6522	447.9	0.6444	0.616	1.4166	0.4996	0

6523	447.8	0.6448	0.6166	1.4177	0.5	0
6524	447.7	0.6451	0.6171	1.4163	0.5005	0
6525	447.6	0.6455	0.6173	1.4184	0.5008	0
6526	447.5	0.646	0.6178	1.4188	0.5011	0
6527	447.4	0.6461	0.618	1.4185	0.5014	0
6528	447.3	0.6465	0.6183	1.4188	0.5017	0
6529	447.2	0.6471	0.6187	1.4204	0.502	0
6530	447.1	0.6476	0.619	1.4201	0.5024	0
6531	447	0.6478	0.6192	1.4192	0.5027	0
6532	446.9	0.6483	0.6196	1.4208	0.5032	0
6533	446.8	0.6487	0.62	1.4217	0.5033	0
6534	446.7	0.6491	0.6203	1.4213	0.5038	0
6535	446.6	0.6495	0.6207	1.4217	0.5042	0
6536	446.5	0.6499	0.6212	1.4233	0.5046	0
6537	446.4	0.6501	0.6214	1.4218	0.5048	0
6538	446.3	0.6508	0.6217	1.4227	0.5053	0
6539	446.2	0.6509	0.622	1.4235	0.5056	0
6540	446.1	0.6512	0.6226	1.4231	0.506	0
6541	446	0.6517	0.6228	1.4227	0.5061	0
6542	445.9	0.6519	0.6231	1.4241	0.5064	0
6543	445.8	0.6527	0.6235	1.4251	0.5069	0
6544	445.7	0.6528	0.6237	1.4246	0.5072	0
6545	445.6	0.6532	0.6241	1.4255	0.5076	0
6546	445.5	0.6534	0.6245	1.4246	0.508	0
6547	445.4	0.6539	0.6248	1.4247	0.5083	0
6548	445.3	0.6543	0.6251	1.4249	0.5087	0
6549	445.2	0.6547	0.6254	1.4262	0.5089	0
6550	445.1	0.6551	0.6258	1.4252	0.5092	0
6551	445	0.6552	0.6262	1.4259	0.5096	0
6552	444.9	0.6557	0.6266	1.4268	0.51	0
6553	444.8	0.6561	0.627	1.427	0.5104	0
6554	444.7	0.6565	0.6275	1.426	0.5107	0
6555	444.6	0.657	0.6276	1.4268	0.5111	0
6556	444.5	0.6573	0.628	1.4281	0.5115	0
6557	444.4	0.6578	0.6285	1.4283	0.5118	0
6558	444.3	0.6579	0.6287	1.4284	0.5122	0
6559	444.2	0.6584	0.629	1.4278	0.5124	0
6560	444.1	0.6588	0.6295	1.428	0.513	0
6561	444	0.6592	0.6298	1.4282	0.5134	0
6562	443.9	0.6595	0.6301	1.4288	0.5136	0
6563	443.8	0.6598	0.6305	1.4283	0.5141	0
6564	443.7	0.6602	0.6311	1.429	0.5144	0
6565	443.6	0.6605	0.6313	1.4296	0.5147	0

6566	443.5	0.6609	0.6316	1.4291	0.515	0
6567	443.4	0.6614	0.632	1.4303	0.5155	0
6568	443.3	0.6617	0.632	1.4309	0.5157	0
6569	443.2	0.662	0.6324	1.4314	0.516	0
6570	443.1	0.6624	0.6328	1.4314	0.5163	0
6571	443	0.6626	0.633	1.4308	0.5167	0
6572	442.9	0.663	0.6334	1.4308	0.5171	0
6573	442.8	0.6637	0.6339	1.4322	0.5175	0
6574	442.7	0.664	0.6344	1.4329	0.5179	0
6575	442.6	0.6641	0.6347	1.4328	0.5183	0
6576	442.5	0.6645	0.6351	1.4322	0.5186	0
6577	442.4	0.6649	0.6354	1.4332	0.5187	0
6578	442.3	0.6654	0.6357	1.4338	0.5191	0
6579	442.2	0.6659	0.6358	1.4338	0.5196	0
6580	442.1	0.6665	0.6363	1.4343	0.52	0
6581	442	0.6666	0.6366	1.4343	0.5202	0
6582	441.9	0.6669	0.637	1.4347	0.5204	0
6583	441.8	0.6673	0.6372	1.4352	0.5208	0
6584	441.7	0.6676	0.6376	1.4352	0.5212	0
6585	441.6	0.6679	0.6377	1.4351	0.5215	0
6586	441.5	0.6681	0.638	1.436	0.5218	0
6587	441.4	0.6686	0.6384	1.436	0.5221	0
6588	441.3	0.6688	0.6388	1.4376	0.5225	0
6589	441.2	0.669	0.6391	1.4362	0.5227	0
6590	441.1	0.6695	0.6394	1.4379	0.5231	0
6591	441	0.6699	0.6396	1.4384	0.5234	0
6592	440.9	0.6702	0.6401	1.4382	0.5237	0
6593	440.8	0.6707	0.6404	1.4391	0.5239	0
6594	440.7	0.671	0.6407	1.4396	0.5244	0
6595	440.6	0.6714	0.6412	1.4401	0.5247	0
6596	440.5	0.6717	0.6413	1.4405	0.525	0
6597	440.4	0.672	0.6418	1.4415	0.5253	0
6598	440.3	0.6721	0.6422	1.4401	0.5257	0
6599	440.2	0.6727	0.6426	1.4418	0.5259	0
6600	440.1	0.6733	0.6429	1.4417	0.5263	0
6601	440	0.6733	0.6431	1.442	0.5266	0
6602	439.9	0.6737	0.6433	1.4429	0.5269	0
6603	439.8	0.6741	0.6436	1.4422	0.5273	0
6604	439.7	0.6745	0.644	1.4432	0.5276	0
6605	439.6	0.6745	0.6441	1.4435	0.5279	0
6606	439.5	0.6749	0.6447	1.4444	0.5283	0
6607	439.4	0.6756	0.645	1.4466	0.5288	0
6608	439.3	0.6758	0.6452	1.4469	0.5287	0

6609	439.2	0.676	0.6456	1.4461	0.5292	0
6610	439.1	0.6766	0.6459	1.4471	0.5296	0
6611	439	0.6769	0.6462	1.4474	0.5299	0
6612	438.9	0.6772	0.6466	1.4472	0.5303	0
6613	438.8	0.6776	0.6469	1.4481	0.5306	0
6614	438.7	0.6779	0.6472	1.4484	0.531	0
6615	438.6	0.6787	0.6477	1.4489	0.5314	0
6616	438.5	0.679	0.6483	1.4487	0.5318	0
6617	438.4	0.6792	0.6486	1.4496	0.5321	0
6618	438.3	0.6798	0.6489	1.4505	0.5327	0
6619	438.2	0.6801	0.6492	1.4514	0.5328	0
6620	438.1	0.6804	0.6495	1.4523	0.5332	0
6621	438	0.6809	0.6498	1.4532	0.5335	0
6622	437.9	0.6813	0.6503	1.453	0.534	0
6623	437.8	0.6814	0.6506	1.4521	0.5343	0
6624	437.7	0.682	0.651	1.4536	0.5347	0
6625	437.6	0.6822	0.6512	1.4532	0.535	0
6626	437.5	0.6827	0.6516	1.4547	0.5351	0
6627	437.4	0.6831	0.6519	1.4537	0.5353	0
6628	437.3	0.6833	0.6524	1.4541	0.5358	0
6629	437.2	0.6838	0.6527	1.4555	0.5364	0
6630	437.1	0.6841	0.653	1.4558	0.5366	0
6631	437	0.6846	0.6532	1.4555	0.5369	0
6632	436.9	0.6847	0.6537	1.4557	0.5372	0
6633	436.8	0.6852	0.654	1.4566	0.5377	0
6634	436.7	0.6855	0.6544	1.4575	0.5382	0
6635	436.6	0.6861	0.655	1.4579	0.5387	0
6636	436.5	0.6866	0.6553	1.457	0.5392	0
6637	436.4	0.6871	0.6556	1.4579	0.5396	0
6638	436.3	0.6872	0.6559	1.458	0.5399	0
6639	436.2	0.6878	0.6565	1.4595	0.5404	0
6640	436.1	0.6882	0.657	1.4593	0.5407	0
6641	436	0.6885	0.6573	1.4589	0.5411	0
6642	435.9	0.689	0.6576	1.4605	0.5417	0
6643	435.8	0.6894	0.6581	1.4602	0.5421	0
6644	435.7	0.6897	0.6585	1.4597	0.5425	0
6645	435.6	0.6899	0.6587	1.4605	0.5429	0
6646	435.5	0.6905	0.6591	1.4601	0.543	0
6647	435.4	0.6914	0.6595	1.4606	0.5437	0
6648	435.3	0.6915	0.6598	1.4607	0.544	0
6649	435.2	0.692	0.6603	1.4622	0.5444	0
6650	435.1	0.6924	0.6607	1.4626	0.5449	0
6651	435	0.6927	0.6613	1.4628	0.5454	0

6652	434.9	0.693	0.6617	1.4623	0.5456	0
6653	434.8	0.6933	0.662	1.4637	0.5461	0
6654	434.7	0.6937	0.6625	1.464	0.5466	0
6655	434.6	0.6943	0.6629	1.4637	0.547	0
6656	434.5	0.6948	0.6635	1.464	0.5475	0
6657	434.4	0.6953	0.6637	1.463	0.5477	0
6658	434.3	0.6956	0.6641	1.4643	0.5481	0
6659	434.2	0.6959	0.6645	1.4646	0.5487	0
6660	434.1	0.6963	0.6649	1.4655	0.5491	0
6661	434	0.6967	0.6654	1.4663	0.5496	0
6662	433.9	0.6973	0.6658	1.4653	0.55	0
6663	433.8	0.6975	0.6662	1.4654	0.5502	0
6664	433.7	0.6979	0.6666	1.4663	0.5507	0
6665	433.6	0.6984	0.667	1.4671	0.5511	0
6666	433.5	0.6988	0.6673	1.4667	0.5515	0
6667	433.4	0.6992	0.6678	1.4669	0.5521	0
6668	433.3	0.6997	0.668	1.4682	0.5524	0
6669	433.2	0.6998	0.6683	1.4665	0.5528	0
6670	433.1	0.7003	0.6689	1.468	0.5533	0
6671	433	0.7006	0.6692	1.4681	0.5536	0
6672	432.9	0.7013	0.6698	1.4684	0.554	0
6673	432.8	0.7016	0.67	1.4685	0.5544	0
6674	432.7	0.702	0.6703	1.4692	0.5548	0
6675	432.6	0.7021	0.6706	1.4698	0.5551	0
6676	432.5	0.7026	0.671	1.4699	0.5554	0
6677	432.4	0.7029	0.6715	1.4707	0.5558	0
6678	432.3	0.7035	0.6718	1.4715	0.5563	0
6679	432.2	0.7039	0.6723	1.4711	0.5568	0
6680	432.1	0.7043	0.6726	1.4706	0.557	0
6681	432	0.7045	0.673	1.4706	0.5573	0
6682	431.9	0.705	0.6737	1.4721	0.5578	0
6683	431.8	0.7055	0.6739	1.4728	0.5583	0
6684	431.7	0.7058	0.6742	1.4735	0.5587	0
6685	431.6	0.7062	0.6745	1.4742	0.559	0
6686	431.5	0.7066	0.6748	1.4731	0.5594	0
6687	431.4	0.7069	0.6752	1.4732	0.5599	0
6688	431.3	0.7074	0.6754	1.4745	0.5602	0
6689	431.2	0.7076	0.6758	1.4745	0.5604	0
6690	431.1	0.708	0.6762	1.4764	0.5609	0
6691	431	0.7083	0.6765	1.4759	0.5612	0
6692	430.9	0.7088	0.6769	1.4765	0.5616	0
6693	430.8	0.7092	0.6775	1.4773	0.562	0
6694	430.7	0.7095	0.6779	1.4774	0.5625	0

6695	430.6	0.7098	0.6783	1.4768	0.5628	0
6696	430.5	0.7103	0.6785	1.4786	0.5631	0
6697	430.4	0.7107	0.6787	1.4786	0.5634	0
6698	430.3	0.7112	0.6793	1.4794	0.5639	0
6699	430.2	0.7113	0.6795	1.4799	0.5642	0
6700	430.1	0.7115	0.6796	1.4798	0.5644	0
6701	430	0.7118	0.6801	1.4793	0.5649	0
6702	429.9	0.7124	0.6804	1.4799	0.5652	0
6703	429.8	0.7127	0.6806	1.4804	0.5655	0
6704	429.7	0.713	0.6809	1.4816	0.5657	0
6705	429.6	0.7133	0.6813	1.4816	0.566	0
6706	429.5	0.7137	0.6818	1.4829	0.5665	0
6707	429.4	0.7141	0.6821	1.4836	0.5669	0
6708	429.3	0.7142	0.6824	1.484	0.5671	0
6709	429.2	0.7145	0.6827	1.4847	0.5674	0
6710	429.1	0.7149	0.6829	1.4858	0.5677	0
6711	429	0.7153	0.6834	1.4853	0.5683	0
6712	428.9	0.7157	0.6839	1.4859	0.5687	0
6713	428.8	0.7161	0.6843	1.4859	0.5689	0
6714	428.7	0.7166	0.6846	1.4865	0.5693	0
6715	428.6	0.7169	0.6848	1.4876	0.5697	0
6716	428.5	0.7172	0.6853	1.4876	0.57	0
6717	428.4	0.7175	0.6855	1.4875	0.5704	0
6718	428.3	0.7179	0.686	1.4895	0.5709	0
6719	428.2	0.7183	0.6864	1.4906	0.5712	0
6720	428.1	0.7186	0.6867	1.4899	0.5714	0
6721	428	0.719	0.6871	1.4899	0.5719	0
6722	427.9	0.7194	0.6871	1.491	0.5722	0
6723	427.8	0.7198	0.6878	1.4917	0.5725	0
6724	427.7	0.72	0.6883	1.4917	0.5729	0
6725	427.6	0.7204	0.6884	1.4921	0.5732	0
6726	427.5	0.7209	0.6887	1.4922	0.5736	0
6727	427.4	0.7213	0.6891	1.4933	0.574	0
6728	427.3	0.7217	0.6895	1.4945	0.5744	0
6729	427.2	0.7221	0.6898	1.4943	0.5746	0
6730	427.1	0.7223	0.69	1.4936	0.575	0
6731	427	0.7231	0.6905	1.4955	0.5755	0
6732	426.9	0.7232	0.691	1.4948	0.5759	0
6733	426.8	0.7234	0.6914	1.4967	0.5764	0
6734	426.7	0.7238	0.6918	1.4965	0.5768	0
6735	426.6	0.7245	0.6924	1.4973	0.5773	0
6736	426.5	0.7248	0.6927	1.4972	0.5777	0
6737	426.4	0.7253	0.6929	1.4983	0.5779	0

6738	426.3	0.7258	0.6935	1.499	0.5787	0
6739	426.2	0.7263	0.694	1.4996	0.5791	0
6740	426.1	0.7267	0.6944	1.4995	0.5795	0
6741	426	0.7269	0.6948	1.5013	0.5801	0
6742	425.9	0.7275	0.6951	1.5007	0.5806	0
6743	425.8	0.7283	0.6957	1.5007	0.5811	0
6744	425.7	0.7284	0.6961	1.5012	0.5815	0
6745	425.6	0.7289	0.6966	1.5024	0.582	0
6746	425.5	0.7292	0.6969	1.503	0.5826	0
6747	425.4	0.7298	0.6973	1.5017	0.583	0
6748	425.3	0.7301	0.698	1.5028	0.5834	0
6749	425.2	0.7304	0.6983	1.504	0.5838	0
6750	425.1	0.7312	0.6988	1.504	0.5844	0
6751	425	0.7318	0.6992	1.504	0.5849	0
6752	424.9	0.7318	0.6996	1.5043	0.5851	0
6753	424.8	0.7322	0.6999	1.5037	0.5857	0
6754	424.7	0.7328	0.7006	1.5055	0.5863	0
6755	424.6	0.7333	0.7011	1.5048	0.5868	0
6756	424.5	0.7337	0.7015	1.506	0.5872	0
6757	424.4	0.7341	0.7018	1.5051	0.5876	0
6758	424.3	0.735	0.7025	1.506	0.5885	0
6759	424.2	0.7354	0.7028	1.5063	0.5889	0
6760	424.1	0.7357	0.7035	1.5076	0.5893	0
6761	424	0.7362	0.7038	1.5062	0.59	0
6762	423.9	0.7364	0.704	1.5073	0.5903	0
6763	423.8	0.7368	0.7047	1.5073	0.5908	0
6764	423.7	0.7372	0.7053	1.5077	0.5913	0
6765	423.6	0.7377	0.7058	1.5082	0.5918	0
6766	423.5	0.7382	0.7061	1.5081	0.5924	0
6767	423.4	0.7386	0.7065	1.5086	0.5926	0
6768	423.3	0.7392	0.707	1.5091	0.5933	0
6769	423.2	0.7397	0.7074	1.5077	0.5937	0
6770	423.1	0.74	0.7079	1.5088	0.5942	0
6771	423	0.7407	0.7084	1.5092	0.5946	0
6772	422.9	0.741	0.7087	1.5104	0.5952	0
6773	422.8	0.7416	0.7095	1.5097	0.5959	0
6774	422.7	0.7421	0.7098	1.5121	0.5963	0
6775	422.6	0.7424	0.7102	1.5101	0.5967	0
6776	422.5	0.7429	0.7108	1.5118	0.5973	0
6777	422.4	0.7433	0.7112	1.5116	0.5975	0
6778	422.3	0.7438	0.7116	1.5127	0.598	0
6779	422.2	0.7443	0.7121	1.5132	0.5985	0
6780	422.1	0.7447	0.7124	1.5136	0.599	0

6781	422	0.7453	0.7129	1.5142	0.5997	0
6782	421.9	0.7456	0.7134	1.5145	0.6	0
6783	421.8	0.746	0.7137	1.5149	0.6005	0
6784	421.7	0.7463	0.7142	1.5147	0.6009	0
6785	421.6	0.7469	0.7143	1.5144	0.6013	0
6786	421.5	0.7471	0.7149	1.5154	0.6017	0
6787	421.4	0.7475	0.7155	1.5173	0.6024	0
6788	421.3	0.7481	0.716	1.5171	0.6028	0
6789	421.2	0.7484	0.7164	1.5175	0.6031	0
6790	421.1	0.7487	0.7166	1.5185	0.6035	0
6791	421	0.7493	0.7171	1.5183	0.6041	0
6792	420.9	0.7497	0.7175	1.5186	0.6045	0
6793	420.8	0.7501	0.718	1.5185	0.6049	0
6794	420.7	0.7507	0.7185	1.5196	0.6055	0
6795	420.6	0.7511	0.719	1.5208	0.6061	0
6796	420.5	0.7516	0.7193	1.5199	0.6064	0
6797	420.4	0.7518	0.7196	1.5208	0.6068	0
6798	420.3	0.7523	0.72	1.5219	0.6073	0
6799	420.2	0.7527	0.7204	1.523	0.6078	0
6800	420.1	0.7529	0.721	1.5233	0.6083	0
6801	420	0.7534	0.7212	1.5231	0.6088	0
6802	419.9	0.7541	0.7218	1.5236	0.6093	0
6803	419.8	0.7547	0.7221	1.524	0.6097	0
6804	419.7	0.7547	0.7224	1.5249	0.61	0
6805	419.6	0.755	0.7229	1.5251	0.6103	0
6806	419.5	0.7554	0.7233	1.5248	0.6107	0
6807	419.4	0.7559	0.7235	1.5266	0.6113	0
6808	419.3	0.7565	0.7241	1.5271	0.6117	0
6809	419.2	0.7567	0.7244	1.5273	0.6121	0
6810	419.1	0.757	0.7248	1.5277	0.6125	0
6811	419	0.7578	0.7254	1.5294	0.6129	0
6812	418.9	0.7581	0.7259	1.5279	0.6135	0
6813	418.8	0.7583	0.7263	1.5282	0.6138	0
6814	418.7	0.7589	0.7268	1.53	0.6144	0
6815	418.6	0.7593	0.7271	1.5311	0.6146	0
6816	418.5	0.7598	0.7271	1.5314	0.6151	0
6817	418.4	0.7601	0.7277	1.5325	0.6155	0
6818	418.3	0.7604	0.728	1.5335	0.616	0
6819	418.2	0.7609	0.7284	1.5325	0.6164	0
6820	418.1	0.7613	0.7287	1.5335	0.6168	0
6821	418	0.7617	0.7293	1.5339	0.617	0
6822	417.9	0.7621	0.7297	1.5351	0.6175	0
6823	417.8	0.7624	0.73	1.5361	0.6178	0

6824	417.7	0.7625	0.7302	1.5364	0.6182	0
6825	417.6	0.7632	0.7306	1.5382	0.6187	0
6826	417.5	0.7636	0.7309	1.5358	0.6191	0
6827	417.4	0.7639	0.7314	1.5383	0.6195	0
6828	417.3	0.7644	0.7319	1.5387	0.6199	0
6829	417.2	0.7649	0.7323	1.5397	0.6203	0
6830	417.1	0.765	0.7328	1.5401	0.6209	0
6831	417	0.7654	0.733	1.5396	0.6211	0
6832	416.9	0.7658	0.7334	1.54	0.6216	0
6833	416.8	0.7661	0.7338	1.541	0.622	0
6834	416.7	0.7667	0.7343	1.5407	0.6225	0
6835	416.6	0.7671	0.7347	1.5426	0.6232	0
6836	416.5	0.7673	0.7352	1.5428	0.6235	0
6837	416.4	0.768	0.7354	1.5431	0.6237	0
6838	416.3	0.7684	0.736	1.5436	0.6243	0
6839	416.2	0.7689	0.7364	1.5431	0.6247	0
6840	416.1	0.7693	0.7368	1.5458	0.6254	0
6841	416	0.77	0.7376	1.5447	0.6258	0
6842	415.9	0.7703	0.7378	1.5457	0.6262	0
6843	415.8	0.7706	0.7383	1.546	0.6267	0
6844	415.7	0.7713	0.7387	1.5463	0.6273	0
6845	415.6	0.7716	0.7392	1.5482	0.6277	0
6846	415.5	0.772	0.7396	1.5485	0.6282	0
6847	415.4	0.7726	0.74	1.5482	0.6287	0
6848	415.3	0.7727	0.7405	1.547	0.6293	0
6849	415.2	0.7735	0.7412	1.5489	0.6298	0
6850	415.1	0.7739	0.7415	1.5484	0.6302	0
6851	415	0.7745	0.742	1.5488	0.6308	0
6852	414.9	0.7748	0.7422	1.5491	0.6312	0
6853	414.8	0.7754	0.7429	1.5517	0.6319	0
6854	414.7	0.776	0.7435	1.5506	0.6325	0
6855	414.6	0.7765	0.7441	1.551	0.6332	0
6856	414.5	0.7768	0.7445	1.5513	0.6337	0
6857	414.4	0.7775	0.7452	1.5516	0.634	0
6858	414.3	0.7781	0.7455	1.5519	0.6347	0
6859	414.2	0.7788	0.746	1.5514	0.6353	0
6860	414.1	0.7791	0.7466	1.5533	0.6358	0
6861	414	0.7794	0.747	1.5536	0.6363	0
6862	413.9	0.7799	0.7474	1.5531	0.6369	0
6863	413.8	0.7805	0.748	1.5534	0.6372	0
6864	413.7	0.7812	0.7486	1.5538	0.6381	0
6865	413.6	0.7815	0.7494	1.555	0.6389	0
6866	413.5	0.782	0.7499	1.5544	0.6395	0

6867	413.4	0.7826	0.7504	1.5548	0.6399	0
6868	413.3	0.7831	0.7507	1.555	0.6404	0
6869	413.2	0.7836	0.7513	1.5561	0.6411	0
6870	413.1	0.784	0.7519	1.5565	0.6418	0
6871	413	0.7847	0.7524	1.5567	0.6424	0
6872	412.9	0.7854	0.753	1.5571	0.6429	0
6873	412.8	0.7855	0.7534	1.5573	0.6433	0
6874	412.7	0.7863	0.7543	1.5569	0.6439	0
6875	412.6	0.7868	0.7546	1.5587	0.6448	0
6876	412.5	0.7873	0.755	1.559	0.6452	0
6877	412.4	0.7877	0.7556	1.5584	0.6456	0
6878	412.3	0.7883	0.7562	1.5588	0.6462	0
6879	412.2	0.7886	0.7565	1.5583	0.6468	0
6880	412.1	0.789	0.7572	1.5586	0.6474	0
6881	412	0.7898	0.7576	1.5587	0.6477	0
6882	411.9	0.7901	0.758	1.5599	0.6485	0
6883	411.8	0.7909	0.7587	1.5619	0.6491	0
6884	411.7	0.7913	0.7591	1.5629	0.6496	0
6885	411.6	0.792	0.7596	1.5616	0.6502	0
6886	411.5	0.7923	0.76	1.5618	0.6507	0
6887	411.4	0.7929	0.7607	1.5614	0.6514	0
6888	411.3	0.7932	0.7611	1.5633	0.6518	0
6889	411.2	0.7938	0.7619	1.5644	0.6524	0
6890	411.1	0.7945	0.7624	1.5656	0.6531	0
6891	411	0.7951	0.7628	1.565	0.6537	0
6892	410.9	0.7957	0.7634	1.5653	0.6544	0
6893	410.8	0.796	0.7637	1.5654	0.6547	0
6894	410.7	0.7963	0.7642	1.5673	0.6553	0
6895	410.6	0.7969	0.7648	1.5651	0.6559	0
6896	410.5	0.7977	0.7655	1.5671	0.6564	0
6897	410.4	0.7981	0.7659	1.569	0.6569	0
6898	410.3	0.7984	0.7662	1.5692	0.6575	0
6899	410.2	0.799	0.7668	1.5695	0.658	0
6900	410.1	0.7997	0.7675	1.5705	0.6586	0
6901	410	0.8	0.768	1.57	0.6591	0
6902	409.9	0.8008	0.7686	1.5704	0.6596	0
6903	409.8	0.8009	0.7689	1.5722	0.6602	0
6904	409.7	0.8016	0.7696	1.5715	0.6609	0
6905	409.6	0.8021	0.7702	1.5711	0.6613	0
6906	409.5	0.8023	0.7706	1.5721	0.662	0
6907	409.4	0.8028	0.7711	1.5731	0.6625	0
6908	409.3	0.8034	0.7717	1.5735	0.6633	0
6909	409.2	0.804	0.7723	1.5737	0.6637	0

6910	409.1	0.8045	0.7723	1.5748	0.6642	0
6911	409	0.805	0.773	1.5759	0.6649	0
6912	408.9	0.8053	0.7736	1.5762	0.6654	0
6913	408.8	0.806	0.774	1.5772	0.6658	0
6914	408.7	0.8067	0.7746	1.5775	0.6665	0
6915	408.6	0.8072	0.7755	1.5787	0.6672	0
6916	408.5	0.8076	0.7756	1.5781	0.6675	0
6917	408.4	0.8081	0.7761	1.58	0.6681	0
6918	408.3	0.8084	0.7766	1.5803	0.6686	0
6919	408.2	0.8095	0.777	1.5823	0.6693	0
6920	408.1	0.8096	0.7776	1.5817	0.6697	0
6921	408	0.8099	0.778	1.5827	0.6701	0
6922	407.9	0.8103	0.7785	1.5837	0.6706	0
6923	407.8	0.8112	0.7793	1.5851	0.6714	0
6924	407.7	0.8115	0.7795	1.5836	0.6719	0
6925	407.6	0.8125	0.7801	1.5857	0.6724	0
6926	407.5	0.8125	0.7805	1.5849	0.6728	0
6927	407.4	0.8129	0.7812	1.587	0.6735	0
6928	407.3	0.8136	0.7815	1.5882	0.6739	0
6929	407.2	0.8142	0.7823	1.5877	0.6745	0
6930	407.1	0.8146	0.7827	1.5881	0.675	0
6931	407	0.815	0.7832	1.5892	0.6755	0
6932	406.9	0.8155	0.7834	1.5895	0.6762	0
6933	406.8	0.8162	0.7838	1.5897	0.6764	0
6934	406.7	0.8167	0.7846	1.5902	0.6773	0
6935	406.6	0.817	0.7851	1.5914	0.6776	0
6936	406.5	0.8174	0.7854	1.5916	0.6781	0
6937	406.4	0.818	0.7858	1.591	0.6788	0
6938	406.3	0.8185	0.7863	1.5923	0.6793	0
6939	406.2	0.8189	0.7871	1.5926	0.6796	0
6940	406.1	0.8195	0.7873	1.5937	0.6803	0
6941	406	0.8198	0.7878	1.594	0.6807	0
6942	405.9	0.8204	0.7883	1.5954	0.6812	0
6943	405.8	0.8208	0.789	1.5966	0.6818	0
6944	405.7	0.8214	0.7895	1.5979	0.6822	0
6945	405.6	0.822	0.7899	1.5953	0.6829	0
6946	405.5	0.8223	0.7904	1.5976	0.6833	0
6947	405.4	0.8231	0.7909	1.5979	0.6842	0
6948	405.3	0.8235	0.7914	1.5982	0.6848	0
6949	405.2	0.8238	0.7918	1.5975	0.6852	0
6950	405.1	0.8245	0.7926	1.5987	0.6856	0
6951	405	0.8251	0.7932	1.5981	0.6863	0
6952	404.9	0.8258	0.7938	1.5985	0.6872	0

6953	404.8	0.8261	0.7942	1.6007	0.6876	0
6954	404.7	0.8269	0.7949	1.6	0.6882	0
6955	404.6	0.8272	0.7954	1.5992	0.6888	0
6956	404.5	0.8278	0.7959	1.6014	0.6893	0
6957	404.4	0.828	0.7962	1.6026	0.6898	0
6958	404.3	0.8289	0.7968	1.6018	0.6905	0
6959	404.2	0.8294	0.7975	1.6003	0.6913	0
6960	404.1	0.8302	0.7982	1.6024	0.6918	0
6961	404	0.8307	0.7987	1.6028	0.6927	0
6962	403.9	0.8313	0.7992	1.603	0.6931	0
6963	403.8	0.8319	0.8001	1.6034	0.6939	0
6964	403.7	0.8322	0.8007	1.6046	0.6945	0
6965	403.6	0.8329	0.8013	1.6049	0.6952	0
6966	403.5	0.8337	0.8021	1.6052	0.6958	0
6967	403.4	0.8342	0.8024	1.6064	0.6963	0
6968	403.3	0.8352	0.8033	1.6048	0.6972	0
6969	403.2	0.8355	0.8038	1.6061	0.6979	0
6970	403.1	0.836	0.8042	1.6062	0.6984	0
6971	403	0.8363	0.8048	1.6054	0.6989	0
6972	402.9	0.8371	0.8055	1.6077	0.6996	0
6973	402.8	0.8378	0.806	1.6069	0.7003	0
6974	402.7	0.8382	0.8066	1.6082	0.7009	0
6975	402.6	0.839	0.8072	1.6084	0.7016	0
6976	402.5	0.8395	0.808	1.6098	0.7025	0
6977	402.4	0.8402	0.8084	1.6089	0.7032	0
6978	402.3	0.8407	0.8091	1.6112	0.7038	0
6979	402.2	0.8411	0.8098	1.6115	0.7046	0
6980	402.1	0.8421	0.8106	1.6119	0.7053	0
6981	402	0.8429	0.8112	1.6121	0.7058	0
6982	401.9	0.8434	0.8119	1.6113	0.7064	0
6983	401.8	0.8438	0.8125	1.6125	0.7071	0
6984	401.7	0.8446	0.813	1.6128	0.708	0
6985	401.6	0.8449	0.8138	1.613	0.7086	0
6986	401.5	0.8458	0.8142	1.6121	0.7091	0
6987	401.4	0.8464	0.8149	1.6144	0.7098	0
6988	401.3	0.847	0.8154	1.6157	0.7105	0
6989	401.2	0.8474	0.8162	1.6171	0.7113	0
6990	401.1	0.8482	0.8168	1.6174	0.712	0
6991	401	0.8488	0.8176	1.6176	0.7126	0
6992	400.9	0.8492	0.8181	1.6167	0.7131	0
6993	400.8	0.8502	0.8186	1.6181	0.7138	0
6994	400.7	0.8508	0.8196	1.6195	0.7145	0
6995	400.6	0.8511	0.82	1.6196	0.7152	0

6996	400.5	0.8519	0.8205	1.6198	0.7159	0
6997	400.4	0.8524	0.821	1.62	0.7165	0
6998	400.3	0.8529	0.8217	1.6213	0.7171	0
6999	400.2	0.8536	0.8223	1.6214	0.7176	0
7000	400.1	0.8544	0.823	1.6217	0.7183	0
7001	400	0.855	0.8237	1.622	0.7188	0
7002	399.9	0.8553	0.8244	1.6212	0.7199	0
7003	399.8	0.8562	0.825	1.6237	0.7207	0
7004	399.7	0.8569	0.8256	1.6227	0.7213	0
7005	399.6	0.8575	0.8264	1.6242	0.7222	0
7006	399.5	0.8581	0.8271	1.6255	0.7227	0
7007	399.4	0.8589	0.8276	1.6257	0.7232	0
7008	399.3	0.8594	0.8286	1.626	0.7242	0
7009	399.2	0.8599	0.8289	1.6273	0.7248	0
7010	399.1	0.8605	0.8294	1.6275	0.7251	0
7011	399	0.8613	0.8303	1.6278	0.7258	0
7012	398.9	0.862	0.8309	1.6291	0.7265	0
7013	398.8	0.8627	0.8315	1.6294	0.7273	0
7014	398.7	0.8631	0.8324	1.631	0.728	0
7015	398.6	0.8638	0.833	1.6322	0.7286	0
7016	398.5	0.8644	0.8336	1.6325	0.7293	0
7017	398.4	0.865	0.8339	1.6315	0.7299	0
7018	398.3	0.8657	0.8348	1.6331	0.7307	0
7019	398.2	0.8662	0.8353	1.6321	0.7314	0
7020	398.1	0.8669	0.8359	1.6323	0.7319	0
7021	398	0.8672	0.8368	1.6326	0.7328	0
7022	397.9	0.868	0.8375	1.6354	0.7333	0
7023	397.8	0.8687	0.838	1.6355	0.7339	0
7024	397.7	0.8696	0.8387	1.6359	0.7347	0
7025	397.6	0.8703	0.8393	1.6361	0.7353	0
7026	397.5	0.8707	0.8398	1.6376	0.7358	0
7027	397.4	0.8712	0.8404	1.6402	0.7367	0
7028	397.3	0.872	0.8411	1.6393	0.7372	0
7029	397.2	0.8726	0.8418	1.6397	0.7381	0
7030	397.1	0.8736	0.8427	1.64	0.7388	0
7031	397	0.8744	0.8433	1.6391	0.7394	0
7032	396.9	0.8746	0.8436	1.6393	0.7401	0
7033	396.8	0.8753	0.8445	1.642	0.7408	0
7034	396.7	0.8757	0.8448	1.6435	0.7411	0
7035	396.6	0.8767	0.8455	1.6425	0.7419	0
7036	396.5	0.8771	0.8461	1.6453	0.7425	0
7037	396.4	0.8777	0.847	1.6445	0.7434	0
7038	396.3	0.8783	0.8479	1.6448	0.7441	0

7039	396.2	0.879	0.8483	1.645	0.7446	0
7040	396.1	0.8792	0.8488	1.6452	0.7453	0
7041	396	0.88	0.8493	1.6468	0.746	0
7042	395.9	0.8806	0.85	1.6474	0.7467	0
7043	395.8	0.8814	0.8509	1.649	0.7475	0
7044	395.7	0.8818	0.8518	1.6479	0.748	0
7045	395.6	0.8826	0.8522	1.6484	0.7488	0
7046	395.5	0.8833	0.8527	1.65	0.7495	0
7047	395.4	0.8839	0.8533	1.649	0.7501	0
7048	395.3	0.8845	0.8541	1.6506	0.7505	0
7049	395.2	0.8853	0.8551	1.6498	0.7515	0
7050	395.1	0.8857	0.8553	1.6513	0.752	0
7051	395	0.8862	0.8565	1.6531	0.7526	0
7052	394.9	0.8868	0.857	1.652	0.7532	0
7053	394.8	0.8876	0.8577	1.6511	0.7539	0
7054	394.7	0.8882	0.8579	1.6514	0.7544	0
7055	394.6	0.8889	0.8588	1.6545	0.7554	0
7056	394.5	0.8896	0.8594	1.6549	0.7562	0
7057	394.4	0.8901	0.86	1.6551	0.7566	0
7058	394.3	0.8909	0.8608	1.6556	0.7574	0
7059	394.2	0.8917	0.8611	1.656	0.7581	0
7060	394.1	0.8921	0.8621	1.655	0.7589	0
7061	394	0.8927	0.8625	1.6553	0.7593	0
7062	393.9	0.8935	0.8634	1.6544	0.76	0
7063	393.8	0.8941	0.8637	1.6575	0.7607	0
7064	393.7	0.8946	0.8646	1.6564	0.7614	0
7065	393.6	0.8954	0.8651	1.6567	0.7622	0
7066	393.5	0.8958	0.866	1.6572	0.7627	0
7067	393.4	0.8969	0.8668	1.6592	0.7639	0
7068	393.3	0.8974	0.8676	1.6594	0.7643	0
7069	393.2	0.8978	0.8681	1.6597	0.7652	0
7070	393.1	0.8989	0.8684	1.66	0.7659	0
7071	393	0.8994	0.8692	1.6602	0.7663	0
7072	392.9	0.8997	0.8702	1.6607	0.767	0
7073	392.8	0.9006	0.8709	1.6611	0.7676	0
7074	392.7	0.9013	0.8717	1.6614	0.7683	0
7075	392.6	0.902	0.8723	1.6618	0.7693	0
7076	392.5	0.9027	0.8731	1.6608	0.7699	0
7077	392.4	0.9033	0.8736	1.664	0.7706	0
7078	392.3	0.9042	0.8743	1.6643	0.7713	0
7079	392.2	0.9046	0.8752	1.6647	0.772	0
7080	392.1	0.9055	0.8759	1.665	0.7725	0
7081	392	0.9062	0.8765	1.6668	0.7735	0

7082	391.9	0.9068	0.877	1.6657	0.774	0
7083	391.8	0.9075	0.8778	1.666	0.7748	0
7084	391.7	0.9085	0.8786	1.6708	0.7754	0
7085	391.6	0.9092	0.8792	1.6683	0.7762	0
7086	391.5	0.9098	0.8799	1.67	0.7767	0
7087	391.4	0.9102	0.8807	1.6689	0.7776	0
7088	391.3	0.9108	0.8815	1.6692	0.778	0
7089	391.2	0.9115	0.8823	1.6696	0.7788	0
7090	391.1	0.9126	0.8828	1.6701	0.7797	0
7091	391	0.9131	0.8836	1.6719	0.7801	0
7092	390.9	0.9139	0.8845	1.6694	0.7814	0
7093	390.8	0.9142	0.8855	1.6711	0.7817	0
7094	390.7	0.915	0.8857	1.6744	0.7828	0
7095	390.6	0.916	0.8866	1.6748	0.7834	0
7096	390.5	0.9166	0.8875	1.6753	0.7843	0
7097	390.4	0.9175	0.8883	1.6772	0.7847	0
7098	390.3	0.9182	0.8889	1.676	0.7855	0
7099	390.2	0.9188	0.8896	1.6763	0.7866	0
7100	390.1	0.9196	0.8905	1.6782	0.7871	0
7101	390	0.9205	0.8913	1.677	0.7876	0
7102	389.9	0.9209	0.8918	1.6804	0.7886	0
7103	389.8	0.9218	0.8928	1.6791	0.7891	0
7104	389.7	0.9225	0.8934	1.6794	0.7899	0
7105	389.6	0.9232	0.8943	1.6798	0.7907	0
7106	389.5	0.924	0.8952	1.6787	0.7914	0
7107	389.4	0.9246	0.8959	1.6837	0.7923	0
7108	389.3	0.9257	0.8966	1.6825	0.7932	0
7109	389.2	0.9268	0.8978	1.683	0.7939	0
7110	389.1	0.9273	0.8985	1.6834	0.795	0
7111	389	0.9279	0.899	1.682	0.7958	0
7112	388.9	0.9289	0.8999	1.6856	0.7964	0
7113	388.8	0.9294	0.9008	1.686	0.7973	0
7114	388.7	0.9303	0.9018	1.6882	0.798	0
7115	388.6	0.931	0.9023	1.6852	0.7984	0
7116	388.5	0.9318	0.903	1.6873	0.7991	0
7117	388.4	0.9322	0.9042	1.6877	0.8002	0
7118	388.3	0.9333	0.9048	1.688	0.8006	0
7119	388.2	0.9337	0.9051	1.6901	0.8015	0
7120	388.1	0.9346	0.9062	1.6905	0.8022	0
7121	388	0.9357	0.9068	1.6892	0.8031	0
7122	387.9	0.9365	0.9081	1.6914	0.8041	0
7123	387.8	0.937	0.9088	1.6918	0.8046	0
7124	387.7	0.9379	0.9095	1.6905	0.8055	0

7125	387.6	0.9387	0.9102	1.6927	0.8064	0
7126	387.5	0.9392	0.9111	1.693	0.8069	0
7127	387.4	0.9401	0.9122	1.6936	0.808	0
7128	387.3	0.9406	0.9126	1.694	0.8087	0
7129	387.2	0.9415	0.9133	1.6927	0.8095	0
7130	387.1	0.9426	0.9146	1.6951	0.8106	0
7131	387	0.9434	0.9153	1.6973	0.8113	0
7132	386.9	0.944	0.916	1.6942	0.8119	0
7133	386.8	0.945	0.9169	1.6948	0.8128	0
7134	386.7	0.9455	0.9175	1.6969	0.8135	0
7135	386.6	0.946	0.9188	1.6973	0.8142	0
7136	386.5	0.947	0.9194	1.6997	0.8151	0
7137	386.4	0.9478	0.9203	1.6985	0.816	0
7138	386.3	0.9486	0.9207	1.6971	0.8168	0
7139	386.2	0.9496	0.9222	1.6976	0.8179	0
7140	386.1	0.9503	0.9228	1.6981	0.8186	0
7141	386	0.9505	0.9235	1.6986	0.8193	0
7142	385.9	0.9519	0.9247	1.701	0.82	0
7143	385.8	0.9525	0.9253	1.7015	0.8209	0
7144	385.7	0.9533	0.9262	1.7003	0.8218	0
7145	385.6	0.9544	0.9268	1.7009	0.8228	0
7146	385.5	0.9544	0.9274	1.703	0.8231	0
7147	385.4	0.9552	0.9284	1.7037	0.8242	0
7148	385.3	0.9567	0.9294	1.7024	0.825	0
7149	385.2	0.9574	0.9303	1.7049	0.8261	0
7150	385.1	0.9578	0.9315	1.7035	0.8266	0
7151	385	0.9585	0.9315	1.7061	0.8275	0
7152	384.9	0.9603	0.9328	1.7067	0.8282	0
7153	384.8	0.9604	0.9341	1.7053	0.8292	0
7154	384.7	0.9608	0.9351	1.704	0.8303	0
7155	384.6	0.962	0.9358	1.7086	0.8311	0
7156	384.5	0.9632	0.9366	1.7074	0.8322	0
7157	384.4	0.9635	0.937	1.7098	0.8327	0
7158	384.3	0.9643	0.9381	1.7085	0.8335	0
7159	384.2	0.9653	0.9389	1.711	0.8344	0
7160	384.1	0.9659	0.9397	1.7077	0.8351	0
7161	384	0.9667	0.9411	1.7083	0.8358	0
7162	383.9	0.9679	0.9415	1.709	0.8367	0
7163	383.8	0.9685	0.9426	1.7098	0.8376	0
7164	383.7	0.969	0.9434	1.7105	0.8387	0
7165	383.6	0.9699	0.9445	1.7091	0.8393	0
7166	383.5	0.9704	0.9453	1.7119	0.8407	0
7167	383.4	0.972	0.946	1.7105	0.841	0

7168	383.3	0.9725	0.9464	1.7112	0.8418	0
7169	383.2	0.973	0.9475	1.7119	0.8424	0
7170	383.1	0.9739	0.9483	1.7147	0.8436	0
7171	383	0.9749	0.9491	1.7133	0.8442	0
7172	382.9	0.9754	0.9506	1.714	0.8448	0
7173	382.8	0.9763	0.9506	1.7147	0.8454	0
7174	382.7	0.9773	0.9515	1.7113	0.8467	0
7175	382.6	0.978	0.9528	1.7164	0.8474	0
7176	382.5	0.9788	0.9535	1.7149	0.8485	0
7177	382.4	0.9791	0.9541	1.7157	0.8487	0
7178	382.3	0.9799	0.9551	1.7145	0.8495	0
7179	382.2	0.9813	0.9564	1.7175	0.8508	0
7180	382.1	0.9816	0.9566	1.7205	0.8515	0
7181	382	0.9825	0.9574	1.7191	0.8525	0
7182	381.9	0.9831	0.9587	1.7199	0.8526	0
7183	381.8	0.9838	0.9592	1.7185	0.8539	0
7184	381.7	0.9846	0.9598	1.7194	0.8541	0
7185	381.6	0.9854	0.9602	1.7204	0.8557	0
7186	381.5	0.9865	0.9612	1.7169	0.8564	0
7187	381.4	0.9872	0.9618	1.7222	0.8572	0
7188	381.3	0.9881	0.9633	1.7232	0.8578	0
7189	381.2	0.9893	0.9639	1.7219	0.8584	0
7190	381.1	0.9896	0.965	1.7228	0.8595	0
7191	381	0.9904	0.9664	1.7261	0.8598	0
7192	380.9	0.9911	0.9667	1.7247	0.8609	0
7193	380.8	0.9925	0.9676	1.7296	0.862	0
7194	380.7	0.9927	0.9684	1.7293	0.8627	0
7195	380.6	0.9937	0.9695	1.7279	0.8634	0
7196	380.5	0.9944	0.9703	1.7289	0.8641	0
7197	380.4	0.995	0.9709	1.7286	0.865	0
7198	380.3	0.996	0.9718	1.7308	0.866	0
7199	380.2	0.9967	0.9726	1.7329	0.8668	0
7200	380.1	0.9974	0.9736	1.7328	0.8674	0
7201	380	0.998	0.9743	1.7324	0.8681	0
7202	379.9	0.999	0.9752	1.7333	0.869	0
7203	379.8	0.9996	0.9761	1.7343	0.8699	0
7204	379.7	1.0009	0.977	1.7342	0.8708	0
7205	379.6	1.0015	0.9777	1.7352	0.8717	0
7206	379.5	1.0027	0.9788	1.7363	0.8723	0
7207	379.4	1.0033	0.9799	1.7373	0.8735	0
7208	379.3	1.0039	0.9804	1.7383	0.8742	0
7209	379.2	1.0051	0.9812	1.7393	0.8753	0
7210	379.1	1.0057	0.982	1.7417	0.8759	0

7211	379	1.0063	0.9831	1.7401	0.8769	0
7212	378.9	1.0074	0.9838	1.7399	0.8779	0
7213	378.8	1.0077	0.9847	1.7421	0.8784	0
7214	378.7	1.0091	0.986	1.7446	0.8797	0
7215	378.6	1.0101	0.9864	1.7431	0.8805	0
7216	378.5	1.0106	0.9877	1.7428	0.8814	0
7217	378.4	1.012	0.9885	1.7452	0.8824	0
7218	378.3	1.0126	0.9892	1.7463	0.8832	0
7219	378.2	1.0136	0.9905	1.7475	0.8841	0
7220	378.1	1.0145	0.9911	1.7473	0.8849	0
7221	378	1.0153	0.992	1.7483	0.8858	0
7222	377.9	1.0162	0.9932	1.7455	0.887	0
7223	377.8	1.0171	0.9942	1.7493	0.8876	0
7224	377.7	1.0183	0.9951	1.7505	0.8887	0
7225	377.6	1.019	0.9961	1.7502	0.8895	0
7226	377.5	1.0199	0.9969	1.75	0.8905	0
7227	377.4	1.0207	0.998	1.7512	0.8913	0
7228	377.3	1.0216	0.9991	1.7523	0.8921	0
7229	377.2	1.0227	1	1.7522	0.8935	0
7230	377.1	1.0234	1.0006	1.7534	0.8942	0
7231	377	1.0242	1.0018	1.7533	0.8952	0
7232	376.9	1.0256	1.0028	1.7532	0.8963	0
7233	376.8	1.0267	1.0038	1.7544	0.8971	0
7234	376.7	1.0272	1.0047	1.7556	0.8982	0
7235	376.6	1.0284	1.006	1.7556	0.8991	0
7236	376.5	1.029	1.0067	1.754	0.9001	0
7237	376.4	1.0299	1.0075	1.7568	0.9012	0
7238	376.3	1.0307	1.0085	1.7566	0.9018	0
7239	376.2	1.0317	1.0101	1.7595	0.9027	0
7240	376.1	1.0325	1.0106	1.7578	0.904	0
7241	376	1.0333	1.0118	1.7579	0.905	0
7242	375.9	1.0343	1.0124	1.7593	0.9058	0
7243	375.8	1.0353	1.0135	1.7578	0.9068	0
7244	375.7	1.0358	1.0147	1.7606	0.9073	0
7245	375.6	1.0368	1.0156	1.7575	0.9086	0
7246	375.5	1.0381	1.0167	1.7589	0.9093	0
7247	375.4	1.0392	1.0174	1.7589	0.9108	0
7248	375.3	1.0402	1.0181	1.7589	0.9116	0
7249	375.2	1.041	1.0196	1.7604	0.9127	0
7250	375.1	1.0418	1.0206	1.7619	0.9137	0
7251	375	1.0432	1.0215	1.7635	0.9146	0
7252	374.9	1.0436	1.0224	1.7618	0.9154	0
7253	374.8	1.0443	1.0239	1.762	0.9166	0

7254	374.7	1.0461	1.0255	1.762	0.9176	0
7255	374.6	1.0466	1.0259	1.7619	0.9186	0
7256	374.5	1.0476	1.0265	1.7618	0.919	0
7257	374.4	1.0485	1.0278	1.7618	0.9201	0
7258	374.3	1.0491	1.0289	1.7634	0.9213	0
7259	374.2	1.05	1.0294	1.7619	0.9223	0
7260	374.1	1.0513	1.0306	1.7636	0.9231	0
7261	374	1.0524	1.0321	1.7638	0.9245	0
7262	373.9	1.0533	1.033	1.7638	0.9252	0
7263	373.8	1.0547	1.0346	1.764	0.9266	0
7264	373.7	1.0554	1.0351	1.7657	0.9279	0
7265	373.6	1.0564	1.0354	1.7674	0.9287	0
7266	373.5	1.0574	1.0366	1.7675	0.9298	0
7267	373.4	1.0584	1.0377	1.7675	0.9304	0
7268	373.3	1.0589	1.0388	1.7661	0.9316	0
7269	373.2	1.0596	1.0397	1.7662	0.9322	0
7270	373.1	1.0612	1.0411	1.7681	0.9335	0
7271	373	1.062	1.0422	1.7684	0.9345	0
7272	372.9	1.0636	1.0433	1.7703	0.9356	0
7273	372.8	1.064	1.0446	1.7704	0.9367	0
7274	372.7	1.0649	1.045	1.7707	0.9375	0
7275	372.6	1.0657	1.0461	1.7726	0.9385	0
7276	372.5	1.0666	1.0472	1.7693	0.9392	0
7277	372.4	1.0677	1.0482	1.7697	0.9402	0
7278	372.3	1.0687	1.0491	1.7701	0.9417	0
7279	372.2	1.0698	1.0507	1.7722	0.9427	0
7280	372.1	1.0707	1.0512	1.7742	0.9432	0
7281	372	1.0717	1.0521	1.7728	0.9446	0
7282	371.9	1.0723	1.053	1.7731	0.9453	0
7283	371.8	1.0734	1.054	1.7752	0.946	0
7284	371.7	1.0745	1.0553	1.7756	0.947	0
7285	371.6	1.0753	1.056	1.776	0.9483	0
7286	371.5	1.076	1.0569	1.7764	0.9487	0
7287	371.4	1.0772	1.058	1.7769	0.9501	0
7288	371.3	1.0779	1.0587	1.7773	0.9508	0
7289	371.2	1.0783	1.0597	1.7758	0.9518	0
7290	371.1	1.0796	1.0601	1.7782	0.9527	0
7291	371	1.0809	1.0617	1.7806	0.9536	0
7292	370.9	1.0815	1.0626	1.7794	0.9546	0
7293	370.8	1.0827	1.0636	1.7816	0.9551	0
7294	370.7	1.0832	1.0644	1.7784	0.9563	0
7295	370.6	1.0838	1.0653	1.7808	0.9572	0
7296	370.5	1.0856	1.0667	1.7854	0.9586	0

7297	370.4	1.0866	1.0675	1.784	0.9593	0
7298	370.3	1.087	1.0683	1.7883	0.9598	0
7299	370.2	1.0882	1.0693	1.7852	0.9615	0
7300	370.1	1.0889	1.0703	1.7859	0.962	0
7301	370	1.0894	1.0711	1.7865	0.9627	0
7302	369.9	1.0887	1.0706	1.7926	0.9632	0
7303	369.8	1.0898	1.0712	1.7923	0.9635	0
7304	369.7	1.0909	1.072	1.7937	0.9646	0
7305	369.6	1.0914	1.0732	1.7963	0.9655	0
7306	369.5	1.0923	1.0738	1.7975	0.9667	0
7307	369.4	1.0932	1.075	1.7986	0.9672	0
7308	369.3	1.0943	1.0755	1.7998	0.9681	0
7309	369.2	1.0952	1.0769	1.7994	0.9693	0
7310	369.1	1.096	1.0775	1.8005	0.97	0
7311	369	1.0971	1.0789	1.8002	0.9711	0
7312	368.9	1.0975	1.0793	1.7996	0.9714	0
7313	368.8	1.0984	1.0808	1.8009	0.9726	0
7314	368.7	1.1002	1.0817	1.8021	0.9738	0
7315	368.6	1.1006	1.0824	1.8045	0.975	0
7316	368.5	1.1014	1.0835	1.8042	0.9757	0
7317	368.4	1.1026	1.0842	1.8051	0.9766	0
7318	368.3	1.1038	1.0859	1.8063	0.9778	0
7319	368.2	1.1044	1.0865	1.8057	0.9787	0
7320	368.1	1.106	1.0875	1.8081	0.9792	0
7321	368	1.1069	1.0884	1.8091	0.9806	0
7322	367.9	1.1076	1.0897	1.81	0.9813	0
7323	367.8	1.1087	1.0905	1.8097	0.9824	0
7324	367.7	1.1097	1.0915	1.8122	0.9835	0
7325	367.6	1.1107	1.0923	1.8129	0.9846	0
7326	367.5	1.1117	1.0935	1.8125	0.9856	0
7327	367.4	1.1125	1.0946	1.8132	0.9864	0
7328	367.3	1.1138	1.0956	1.8142	0.9874	0
7329	367.2	1.1148	1.0968	1.8138	0.989	0
7330	367.1	1.1163	1.0981	1.8147	0.9898	0
7331	367	1.117	1.0988	1.8154	0.9907	0
7332	366.9	1.1176	1.0997	1.8149	0.9918	0
7333	366.8	1.1189	1.1012	1.817	0.9927	0
7334	366.7	1.1204	1.1027	1.8179	0.9941	0
7335	366.6	1.1213	1.1033	1.8174	0.9952	0
7336	366.5	1.122	1.1043	1.8195	0.9964	0
7337	366.4	1.1234	1.1057	1.8233	0.9976	0
7338	366.3	1.1245	1.1067	1.8226	0.9986	0
7339	366.2	1.1259	1.1075	1.819	0.9994	0

7340	366.1	1.1265	1.109	1.8226	1.0005	0
7341	366	1.1276	1.1101	1.8219	1.0017	0
7342	365.9	1.1289	1.1114	1.8241	1.0026	0
7343	365.8	1.1299	1.1124	1.8248	1.0042	0
7344	365.7	1.1312	1.1134	1.8226	1.0052	0
7345	365.6	1.132	1.1144	1.826	1.0066	0
7346	365.5	1.1332	1.1162	1.8267	1.0074	0
7347	365.4	1.1341	1.117	1.826	1.0089	0
7348	365.3	1.1356	1.1186	1.8253	1.01	0
7349	365.2	1.1365	1.1197	1.8258	1.0108	0
7350	365.1	1.1377	1.1206	1.8265	1.0121	0
7351	365	1.1389	1.1218	1.83	1.0132	0
7352	364.9	1.1398	1.123	1.8292	1.0145	0
7353	364.8	1.1408	1.1242	1.8313	1.0158	0
7354	364.7	1.1425	1.1256	1.8289	1.0168	0
7355	364.6	1.1437	1.1271	1.8295	1.018	0
7356	364.5	1.1448	1.1281	1.8285	1.0196	0
7357	364.4	1.1451	1.1296	1.8306	1.0204	0
7358	364.3	1.147	1.1303	1.8296	1.0216	0
7359	364.2	1.1483	1.1316	1.8317	1.0234	0
7360	364.1	1.1489	1.1331	1.832	1.0244	0
7361	364	1.1504	1.1342	1.834	1.0256	0
7362	363.9	1.1514	1.1352	1.8315	1.0269	0
7363	363.8	1.1532	1.1369	1.8321	1.0279	0
7364	363.7	1.1541	1.1378	1.831	1.0294	0
7365	363.6	1.1554	1.1391	1.8314	1.0304	0
7366	363.5	1.156	1.1402	1.8331	1.0316	0
7367	363.4	1.1571	1.1413	1.8351	1.0328	0
7368	363.3	1.1585	1.1421	1.8339	1.0342	0
7369	363.2	1.1596	1.1435	1.8343	1.0353	0
7370	363.1	1.1609	1.145	1.8361	1.0366	0
7371	363	1.1621	1.1465	1.8335	1.0378	0
7372	362.9	1.1632	1.1481	1.8354	1.0392	0
7373	362.8	1.1645	1.1488	1.8355	1.04	0
7374	362.7	1.1654	1.1503	1.8358	1.0412	0
7375	362.6	1.1669	1.1509	1.8376	1.0427	0
7376	362.5	1.1681	1.1526	1.8363	1.0438	0
7377	362.4	1.1687	1.1535	1.8352	1.045	0
7378	362.3	1.1702	1.1547	1.8369	1.0463	0
7379	362.2	1.1716	1.1566	1.8385	1.0474	0
7380	362.1	1.1727	1.1571	1.8387	1.0484	0
7381	362	1.1738	1.1585	1.8389	1.0493	0
7382	361.9	1.1746	1.1598	1.8405	1.0506	0

7383	361.8	1.1763	1.1612	1.8406	1.0517	0
7384	361.7	1.1773	1.1619	1.8407	1.053	0
7385	361.6	1.1781	1.1627	1.8409	1.0539	0
7386	361.5	1.1795	1.1643	1.841	1.0552	0
7387	361.4	1.1813	1.166	1.8426	1.0566	0
7388	361.3	1.1817	1.1667	1.8427	1.0578	0
7389	361.2	1.1829	1.1683	1.8442	1.059	0
7390	361.1	1.1845	1.1688	1.8444	1.0605	0
7391	361	1.1854	1.1706	1.8444	1.0616	0
7392	360.9	1.1865	1.1713	1.8445	1.0627	0
7393	360.8	1.1877	1.1729	1.8474	1.0634	0
7394	360.7	1.1884	1.1738	1.8459	1.0651	0
7395	360.6	1.1902	1.1756	1.8459	1.066	0
7396	360.5	1.1913	1.1767	1.8504	1.0676	0
7397	360.4	1.1923	1.1776	1.8489	1.069	0
7398	360.3	1.1932	1.1788	1.8503	1.0697	0
7399	360.2	1.1949	1.1802	1.849	1.0711	0
7400	360.1	1.1957	1.1813	1.8517	1.0724	0
7401	360	1.197	1.1825	1.8532	1.0736	0
7402	359.9	1.198	1.1837	1.8516	1.0745	0
7403	359.8	1.199	1.1853	1.8517	1.076	0
7404	359.7	1.2005	1.1865	1.8516	1.0774	0
7405	359.6	1.2014	1.1877	1.856	1.0783	0
7406	359.5	1.2026	1.1889	1.8558	1.0794	0
7407	359.4	1.2038	1.1904	1.8587	1.0811	0
7408	359.3	1.2051	1.191	1.8617	1.0818	0
7409	359.2	1.2064	1.1922	1.8586	1.0832	0
7410	359.1	1.2072	1.193	1.86	1.0846	0
7411	359	1.2087	1.1947	1.8612	1.0853	0
7412	358.9	1.2091	1.1955	1.8625	1.0861	0
7413	358.8	1.2107	1.1969	1.8623	1.0878	0
7414	358.7	1.212	1.1979	1.8638	1.089	0
7415	358.6	1.2133	1.1989	1.862	1.0902	0
7416	358.5	1.2145	1.2003	1.8648	1.091	0
7417	358.4	1.215	1.2014	1.8646	1.0921	0
7418	358.3	1.2165	1.2019	1.8675	1.0932	0
7419	358.2	1.2173	1.2034	1.8657	1.0943	0
7420	358.1	1.2181	1.2044	1.8654	1.0951	0
7421	358	1.2193	1.2056	1.87	1.0965	0
7422	357.9	1.2208	1.2067	1.8697	1.0976	0
7423	357.8	1.2221	1.2083	1.8726	1.099	0
7424	357.7	1.2227	1.2086	1.8738	1.0999	0
7425	357.6	1.2242	1.2099	1.8735	1.1009	0

7426	357.5	1.2253	1.2113	1.8716	1.1019	0
7427	357.4	1.2264	1.2124	1.8761	1.103	0
7428	357.3	1.2272	1.2136	1.8743	1.1041	0
7429	357.2	1.2287	1.2146	1.8755	1.1054	0
7430	357.1	1.2298	1.2156	1.8751	1.1069	0
7431	357	1.231	1.2172	1.8798	1.1081	0
7432	356.9	1.2321	1.2182	1.8762	1.1093	0
7433	356.8	1.234	1.2197	1.8775	1.1104	0
7434	356.7	1.2348	1.2205	1.8788	1.1118	0
7435	356.6	1.2359	1.2222	1.8833	1.1131	0
7436	356.5	1.2369	1.2232	1.8812	1.1139	0
7437	356.4	1.2388	1.2247	1.8809	1.1156	0
7438	356.3	1.2399	1.2257	1.8805	1.1166	0
7439	356.2	1.241	1.2268	1.8818	1.1176	0
7440	356.1	1.2423	1.228	1.8828	1.119	0
7441	356	1.2431	1.2295	1.8842	1.1198	0
7442	355.9	1.2449	1.2313	1.8854	1.1214	0
7443	355.8	1.2459	1.2322	1.8849	1.1226	0
7444	355.7	1.2478	1.2333	1.8861	1.1239	0
7445	355.6	1.2492	1.2346	1.8873	1.1254	0
7446	355.5	1.2499	1.236	1.8868	1.1267	0
7447	355.4	1.2513	1.2374	1.8864	1.1279	0
7448	355.3	1.2526	1.2389	1.8877	1.1294	0
7449	355.2	1.2539	1.2402	1.8888	1.1308	0
7450	355.1	1.2545	1.2412	1.8883	1.132	0
7451	355	1.2562	1.2428	1.8844	1.1334	0
7452	354.9	1.2577	1.2442	1.8888	1.1344	0
7453	354.8	1.2582	1.2455	1.8898	1.1358	0
7454	354.7	1.2601	1.2466	1.8911	1.1375	0
7455	354.6	1.2613	1.2477	1.889	1.1389	0
7456	354.5	1.2627	1.2498	1.8901	1.1401	0
7457	354.4	1.2642	1.2508	1.8896	1.1416	0
7458	354.3	1.2661	1.2527	1.8925	1.143	0
7459	354.2	1.2667	1.2536	1.8952	1.1444	0
7460	354.1	1.268	1.2553	1.8946	1.1459	0
7461	354	1.2691	1.2563	1.894	1.1469	0
7462	353.9	1.2706	1.2577	1.8952	1.1488	0
7463	353.8	1.2719	1.2594	1.8928	1.1508	0
7464	353.7	1.2741	1.2611	1.8921	1.1514	0
7465	353.6	1.2747	1.262	1.8949	1.1529	0
7466	353.5	1.2762	1.2635	1.8944	1.1543	0
7467	353.4	1.278	1.2656	1.8988	1.1561	0
7468	353.3	1.279	1.267	1.8964	1.1573	0

7469	353.2	1.2808	1.2683	1.8939	1.1588	0
7470	353.1	1.2822	1.2701	1.8967	1.1603	0
7471	353	1.2831	1.2709	1.8976	1.1614	0
7472	352.9	1.284	1.2727	1.8969	1.1633	0
7473	352.8	1.2864	1.2741	1.8963	1.1646	0
7474	352.7	1.2879	1.2755	1.8938	1.1662	0
7475	352.6	1.289	1.277	1.8967	1.1678	0
7476	352.5	1.29	1.2788	1.8978	1.1688	0
7477	352.4	1.2914	1.2797	1.8987	1.1706	0
7478	352.3	1.2929	1.2812	1.9017	1.172	0
7479	352.2	1.2939	1.283	1.8973	1.1739	0
7480	352.1	1.2962	1.284	1.8965	1.1748	0
7481	352	1.2973	1.2863	1.903	1.1764	0
7482	351.9	1.2988	1.2877	1.9004	1.178	0
7483	351.8	1.3004	1.2884	1.8998	1.1795	0
7484	351.7	1.302	1.2899	1.8991	1.1812	0
7485	351.6	1.3026	1.2922	1.9001	1.1828	0
7486	351.5	1.3041	1.2933	1.8994	1.1838	0
7487	351.4	1.3061	1.2947	1.9004	1.1854	0
7488	351.3	1.3078	1.2968	1.8998	1.1866	0
7489	351.2	1.3084	1.2978	1.9008	1.1886	0
7490	351.1	1.3099	1.2997	1.9036	1.1895	0
7491	351	1.3115	1.3004	1.9047	1.192	0
7492	350.9	1.313	1.3023	1.9039	1.1929	0
7493	350.8	1.3145	1.3038	1.9031	1.1939	0
7494	350.7	1.3163	1.3049	1.9061	1.1957	0
7495	350.6	1.3175	1.307	1.9072	1.1972	0
7496	350.5	1.3186	1.3086	1.9083	1.199	0
7497	350.4	1.3211	1.3101	1.9093	1.2003	0
7498	350.3	1.3223	1.3117	1.9048	1.2017	0
7499	350.2	1.3233	1.3131	1.9039	1.203	0
7500	350.1	1.325	1.3148	1.9087	1.2045	0
7501	350	1.3261	1.3153	1.9097	1.2055	0
7502	349.9	1.3276	1.3178	1.9069	1.208	0
7503	349.8	1.3289	1.3185	1.9119	1.2095	0
7504	349.7	1.3305	1.3205	1.9129	1.2108	0
7505	349.6	1.3321	1.3221	1.912	1.2119	0
7506	349.5	1.3333	1.3237	1.9111	1.2133	0
7507	349.4	1.3359	1.3248	1.9102	1.2147	0
7508	349.3	1.3371	1.327	1.9113	1.2166	0
7509	349.2	1.3381	1.3279	1.9141	1.2178	0
7510	349.1	1.3393	1.3295	1.9132	1.2193	0
7511	349	1.3405	1.3312	1.9162	1.2212	0

7512	348.9	1.3421	1.3328	1.9172	1.223	0
7513	348.8	1.3442	1.3343	1.9181	1.2239	0
7514	348.7	1.3448	1.3353	1.9191	1.2253	0
7515	348.6	1.3469	1.3363	1.918	1.2267	0
7516	348.5	1.3481	1.338	1.917	1.2281	0
7517	348.4	1.3492	1.3396	1.918	1.2299	0
7518	348.3	1.351	1.3408	1.9191	1.2319	0
7519	348.2	1.3526	1.3424	1.92	1.2325	0
7520	348.1	1.3538	1.344	1.919	1.234	0
7521	348	1.3555	1.3451	1.9199	1.2362	0
7522	347.9	1.3571	1.3467	1.9209	1.2368	0
7523	347.8	1.3574	1.3485	1.922	1.2389	0
7524	347.7	1.3592	1.3497	1.921	1.2405	0
7525	347.6	1.3609	1.3514	1.9241	1.2415	0
7526	347.5	1.3632	1.3525	1.9271	1.2434	0
7527	347.4	1.364	1.3538	1.9262	1.2447	0
7528	347.3	1.3663	1.3561	1.9293	1.2462	0
7529	347.2	1.3665	1.3573	1.9282	1.2478	0
7530	347.1	1.3683	1.3585	1.9293	1.249	0
7531	347	1.3696	1.3609	1.9304	1.2506	0
7532	346.9	1.3716	1.3623	1.9294	1.2523	0
7533	346.8	1.3722	1.3634	1.9325	1.2533	0
7534	346.7	1.3748	1.3648	1.9337	1.2551	0
7535	346.6	1.3761	1.366	1.9305	1.2559	0
7536	346.5	1.3767	1.3677	1.9357	1.2573	0
7537	346.4	1.3781	1.3685	1.9326	1.2591	0
7538	346.3	1.3799	1.3697	1.9379	1.2597	0
7539	346.2	1.3808	1.3711	1.9392	1.2611	0
7540	346.1	1.3829	1.3731	1.936	1.2634	0
7541	346	1.3842	1.3744	1.937	1.2645	0
7542	345.9	1.3843	1.3756	1.9336	1.2658	0
7543	345.8	1.3866	1.3779	1.9395	1.2678	0
7544	345.7	1.3879	1.3786	1.9383	1.2695	0
7545	345.6	1.39	1.3806	1.9395	1.2708	0
7546	345.5	1.3913	1.3812	1.9405	1.2715	0
7547	345.4	1.3926	1.3825	1.9393	1.2732	0
7548	345.3	1.3949	1.3847	1.9383	1.2747	0
7549	345.2	1.3958	1.3861	1.9372	1.276	0
7550	345.1	1.3972	1.3874	1.9406	1.2778	0
7551	345	1.3988	1.3904	1.9419	1.2799	0
7552	344.9	1.4001	1.3916	1.9406	1.2815	0
7553	344.8	1.4024	1.3926	1.9419	1.282	0
7554	344.7	1.4038	1.3951	1.9405	1.2842	0

7555	344.6	1.4047	1.396	1.9394	1.2862	0
7556	344.5	1.4069	1.3982	1.9429	1.2876	0
7557	344.4	1.4085	1.3997	1.9417	1.289	0
7558	344.3	1.4092	1.4011	1.9404	1.2909	0
7559	344.2	1.4113	1.4031	1.9414	1.2921	0
7560	344.1	1.4144	1.404	1.9426	1.2931	0
7561	344	1.4152	1.4054	1.939	1.295	0
7562	343.9	1.416	1.4069	1.9401	1.2969	0
7563	343.8	1.4182	1.4083	1.9411	1.2983	0
7564	343.7	1.4205	1.4106	1.9446	1.3008	0
7565	343.6	1.4216	1.4123	1.9435	1.3019	0
7566	343.5	1.4225	1.4139	1.9446	1.3039	0
7567	343.4	1.4239	1.416	1.9432	1.3058	0
7568	343.3	1.4256	1.4176	1.9468	1.3067	0
7569	343.2	1.428	1.4192	1.9455	1.3094	0
7570	343.1	1.4288	1.4214	1.949	1.3102	0
7571	343	1.4313	1.4224	1.9477	1.3118	0
7572	342.9	1.4323	1.4241	1.9465	1.314	0
7573	342.8	1.4348	1.4257	1.9427	1.3156	0
7574	342.7	1.4359	1.4283	1.9439	1.3173	0
7575	342.6	1.4367	1.4298	1.9424	1.3187	0
7576	342.5	1.4394	1.4316	1.9437	1.3217	0
7577	342.4	1.4404	1.4327	1.9474	1.3234	0
7578	342.3	1.4431	1.4345	1.9437	1.3245	0
7579	342.2	1.4442	1.4371	1.9424	1.3262	0
7580	342.1	1.4468	1.4373	1.9486	1.3285	0
7581	342	1.4471	1.4399	1.9473	1.3297	0
7582	341.9	1.4489	1.4433	1.9484	1.3307	0
7583	341.8	1.4508	1.4427	1.9471	1.3337	0
7584	341.7	1.4528	1.4455	1.9509	1.3349	0
7585	341.6	1.454	1.4474	1.947	1.3373	0
7586	341.5	1.4559	1.4493	1.9483	1.3385	0
7587	341.4	1.457	1.4512	1.9495	1.3403	0
7588	341.3	1.459	1.4523	1.9507	1.3415	0
7589	341.2	1.4628	1.4544	1.9494	1.3441	0
7590	341.1	1.4631	1.4571	1.948	1.3452	0
7591	341	1.4642	1.4582	1.9518	1.3478	0
7592	340.9	1.4663	1.4594	1.9531	1.3497	0
7593	340.8	1.4684	1.4615	1.9544	1.3516	0
7594	340.7	1.4706	1.4636	1.953	1.353	0
7595	340.6	1.4719	1.4658	1.9571	1.3551	0
7596	340.5	1.4733	1.4671	1.9531	1.3571	0
7597	340.4	1.4755	1.4683	1.9517	1.3585	0

7598	340.3	1.4768	1.4714	1.953	1.3605	0
7599	340.2	1.4789	1.4735	1.957	1.3633	0
7600	340.1	1.4802	1.4756	1.9555	1.3625	0
7601	340	1.4825	1.4779	1.9541	1.366	0
7602	339.9	1.0075	1.0285	1.7355	0.954	0
7603	339.8	1.0076	1.0283	1.7355	0.9538	0
7604	339.7	1.007	1.028	1.7353	0.9541	0
7605	339.6	1.0068	1.0276	1.7371	0.9539	0
7606	339.5	1.0068	1.0272	1.7359	0.9538	0
7607	339.4	1.0061	1.0273	1.7355	0.9534	0
7608	339.3	1.0058	1.0266	1.7341	0.9533	0
7609	339.2	1.0051	1.0268	1.735	0.9529	0
7610	339.1	1.0049	1.0262	1.7347	0.9529	0
7611	339	1.0045	1.0261	1.7335	0.9528	0
7612	338.9	1.0044	1.0255	1.7332	0.9524	0
7613	338.8	1.0041	1.0255	1.7318	0.9523	0
7614	338.7	1.0035	1.0252	1.7315	0.952	0
7615	338.6	1.0031	1.0251	1.7302	0.9517	0
7616	338.5	1.0032	1.0241	1.73	0.9515	0
7617	338.4	1.0024	1.0245	1.7287	0.9514	0
7618	338.3	1.002	1.0241	1.7284	0.951	0
7619	338.2	1.0024	1.0237	1.7306	0.9511	0
7620	338.1	1.0017	1.0237	1.7292	0.951	0
7621	338	1.0011	1.0229	1.7266	0.9504	0
7622	337.9	1.0006	1.0229	1.7289	0.9506	0
7623	337.8	1.0005	1.0229	1.7287	0.9506	0
7624	337.7	1.0001	1.0226	1.7262	0.9504	0
7625	337.6	0.9998	1.0223	1.726	0.9496	0
7626	337.5	0.9996	1.0215	1.7271	0.9497	0
7627	337.4	0.999	1.0214	1.7258	0.9494	0
7628	337.3	0.999	1.0207	1.7244	0.9494	0
7629	337.2	0.9986	1.0207	1.7232	0.9496	0
7630	337.1	0.9984	1.0205	1.7254	0.949	0
7631	337	0.9983	1.02	1.723	0.9486	0
7632	336.9	0.9973	1.0195	1.724	0.9488	0
7633	336.8	0.9969	1.0194	1.7215	0.9483	0
7634	336.7	0.9967	1.0192	1.7225	0.9482	0
7635	336.6	0.9966	1.0187	1.7212	0.9481	0
7636	336.5	0.9963	1.0187	1.7211	0.9476	0
7637	336.4	0.9958	1.0183	1.7199	0.9474	0
7638	336.3	0.9957	1.018	1.7199	0.9474	0
7639	336.2	0.9949	1.0174	1.7185	0.9469	0
7640	336.1	0.9946	1.0169	1.7183	0.9467	0

7641	336	0.9946	1.0167	1.7196	0.9461	0
7642	335.9	0.9943	1.0165	1.7184	0.9459	0
7643	335.8	0.9937	1.0164	1.7171	0.9459	0
7644	335.7	0.9932	1.0162	1.7169	0.946	0
7645	335.6	0.993	1.0156	1.7168	0.9452	0
7646	335.5	0.9929	1.0155	1.7169	0.945	0
7647	335.4	0.9921	1.0155	1.7144	0.945	0
7648	335.3	0.992	1.015	1.7156	0.9448	0
7649	335.2	0.9914	1.0142	1.7156	0.9443	0
7650	335.1	0.9908	1.0143	1.7146	0.9445	0
7651	335	0.9907	1.014	1.7132	0.944	0
7652	334.9	0.9902	1.0136	1.712	0.9441	0
7653	334.8	0.9893	1.0133	1.712	0.9434	0
7654	334.7	0.9896	1.0131	1.7121	0.9432	0
7655	334.6	0.989	1.0126	1.7108	0.9429	0
7656	334.5	0.9888	1.0121	1.7108	0.9425	0
7657	334.4	0.9882	1.012	1.7083	0.9425	0
7658	334.3	0.9876	1.0115	1.7082	0.9425	0
7659	334.2	0.9875	1.0118	1.7085	0.9422	0
7660	334.1	0.9871	1.0111	1.7086	0.9416	0
7661	334	0.9865	1.0111	1.7073	0.9415	0
7662	333.9	0.9862	1.0108	1.7087	0.9414	0
7663	333.8	0.9865	1.0105	1.7061	0.941	0
7664	333.7	0.9858	1.01	1.7062	0.9405	0
7665	333.6	0.9851	1.0096	1.705	0.9403	0
7666	333.5	0.9845	1.0088	1.705	0.9399	0
7667	333.4	0.984	1.0086	1.705	0.9396	0
7668	333.3	0.9834	1.0085	1.7052	0.9393	0
7669	333.2	0.9833	1.0082	1.7026	0.9395	0
7670	333.1	0.9832	1.0077	1.7028	0.9389	0
7671	333	0.9827	1.0067	1.7029	0.9386	0
7672	332.9	0.9819	1.007	1.7016	0.9381	0
7673	332.8	0.9815	1.0066	1.7004	0.9379	0
7674	332.7	0.9814	1.0066	1.6994	0.9379	0
7675	332.6	0.9808	1.0058	1.6994	0.9373	0
7676	332.5	0.9804	1.0052	1.7008	0.9368	0
7677	332.4	0.9802	1.0045	1.6983	0.9372	0
7678	332.3	0.9793	1.0046	1.6984	0.9364	0
7679	332.2	0.9794	1.0043	1.6986	0.9362	0
7680	332.1	0.9791	1.004	1.6988	0.9361	0
7681	332	0.9785	1.0032	1.6975	0.9354	0
7682	331.9	0.9781	1.0029	1.6964	0.9352	0
7683	331.8	0.9776	1.0024	1.6951	0.9343	0

7684	331.7	0.9771	1.0019	1.6938	0.9342	0
7685	331.6	0.977	1.0019	1.694	0.9338	0
7686	331.5	0.9768	1.001	1.6942	0.9335	0
7687	331.4	0.9762	1.0004	1.6928	0.9331	0
7688	331.3	0.9753	1.0003	1.6916	0.9326	0
7689	331.2	0.9748	1.0004	1.6904	0.9323	0
7690	331.1	0.9748	0.9999	1.6918	0.9319	0
7691	331	0.9744	0.9991	1.688	0.9322	0
7692	330.9	0.9737	0.9989	1.6894	0.9318	0
7693	330.8	0.9735	0.9982	1.6895	0.9313	0
7694	330.7	0.973	0.9983	1.6895	0.9307	0
7695	330.6	0.9724	0.9974	1.6897	0.9305	0
7696	330.5	0.972	0.9971	1.6886	0.9303	0
7697	330.4	0.9714	0.9968	1.6875	0.9301	0
7698	330.3	0.9708	0.9963	1.6848	0.93	0
7699	330.2	0.9707	0.9962	1.6864	0.9291	0
7700	330.1	0.9701	0.9957	1.6837	0.9289	0
7701	330	0.9698	0.9949	1.6837	0.9285	0
7702	329.9	0.9689	0.9943	1.6838	0.928	0
7703	329.8	0.9684	0.9941	1.6839	0.9272	0
7704	329.7	0.9684	0.9936	1.6829	0.9274	0
7705	329.6	0.9676	0.9929	1.6815	0.9267	0
7706	329.5	0.9671	0.9924	1.6803	0.9259	0
7707	329.4	0.966	0.9914	1.6803	0.9255	0
7708	329.3	0.966	0.9911	1.6792	0.9254	0
7709	329.2	0.9654	0.9905	1.6805	0.9247	0
7710	329.1	0.9647	0.9899	1.678	0.9245	0
7711	329	0.9639	0.9899	1.6782	0.9243	0
7712	328.9	0.9639	0.9898	1.6783	0.9238	0
7713	328.8	0.9638	0.9891	1.6798	0.9235	0
7714	328.7	0.9629	0.9883	1.6757	0.9229	0
7715	328.6	0.9622	0.9879	1.6758	0.9226	0
7716	328.5	0.9624	0.9873	1.676	0.9219	0
7717	328.4	0.9614	0.9869	1.6748	0.9217	0
7718	328.3	0.9608	0.9866	1.6751	0.9213	0
7719	328.2	0.9603	0.9859	1.6751	0.9207	0
7720	328.1	0.9599	0.9855	1.6753	0.9204	0
7721	328	0.9593	0.9849	1.6728	0.92	0
7722	327.9	0.9592	0.9849	1.6717	0.9196	0
7723	327.8	0.9585	0.9843	1.6718	0.9196	0
7724	327.7	0.9581	0.9833	1.6706	0.9191	0
7725	327.6	0.9571	0.9829	1.6693	0.9184	0
7726	327.5	0.957	0.9823	1.6695	0.9179	0

7727	327.4	0.9562	0.9816	1.6682	0.9174	0
7728	327.3	0.9554	0.9811	1.6682	0.9167	0
7729	327.2	0.9551	0.9805	1.6699	0.9163	0
7730	327.1	0.9548	0.9797	1.666	0.9159	0
7731	327	0.9535	0.9793	1.6675	0.9154	0
7732	326.9	0.9534	0.9789	1.6677	0.9152	0
7733	326.8	0.9524	0.9779	1.665	0.9144	0
7734	326.7	0.9518	0.9776	1.6667	0.9138	0
7735	326.6	0.9513	0.9772	1.664	0.9135	0
7736	326.5	0.951	0.9764	1.6615	0.9129	0
7737	326.4	0.9503	0.9757	1.6616	0.9121	0
7738	326.3	0.9499	0.9751	1.6632	0.9117	0
7739	326.2	0.9488	0.9749	1.6593	0.9113	0
7740	326.1	0.949	0.9745	1.6596	0.9111	0
7741	326	0.9478	0.974	1.6584	0.9105	0
7742	325.9	0.9474	0.9733	1.6572	0.9095	0
7743	325.8	0.9471	0.9727	1.6588	0.9096	0
7744	325.7	0.9461	0.9724	1.6562	0.9086	0
7745	325.6	0.9454	0.9717	1.6578	0.9081	0
7746	325.5	0.9449	0.9709	1.6567	0.908	0
7747	325.4	0.9443	0.9703	1.6555	0.9076	0
7748	325.3	0.9436	0.97	1.6543	0.9064	0
7749	325.2	0.9432	0.9688	1.6531	0.9065	0
7750	325.1	0.9422	0.9681	1.6518	0.906	0
7751	325	0.9419	0.9675	1.6507	0.9053	0
7752	324.9	0.9408	0.9668	1.6507	0.9045	0
7753	324.8	0.9405	0.9659	1.6496	0.9039	0
7754	324.7	0.9398	0.9656	1.6498	0.9033	0
7755	324.6	0.939	0.9645	1.6473	0.9033	0
7756	324.5	0.9382	0.9643	1.6462	0.9024	0
7757	324.4	0.9375	0.9639	1.6477	0.9017	0
7758	324.3	0.9368	0.9632	1.6465	0.901	0
7759	324.2	0.9362	0.9624	1.6454	0.9001	0
7760	324.1	0.9354	0.9619	1.6443	0.8998	0
7761	324	0.935	0.9609	1.6444	0.8988	0
7762	323.9	0.9345	0.9605	1.642	0.8985	0
7763	323.8	0.9335	0.9595	1.6421	0.8978	0
7764	323.7	0.9328	0.9591	1.6422	0.8969	0
7765	323.6	0.9318	0.9581	1.641	0.8962	0
7766	323.5	0.9307	0.9573	1.6384	0.8959	0
7767	323.4	0.9301	0.9568	1.6402	0.895	0
7768	323.3	0.9299	0.9566	1.6391	0.8944	0
7769	323.2	0.9295	0.9554	1.6379	0.8943	0

7770	323.1	0.9284	0.9546	1.6353	0.8931	0
7771	323	0.9281	0.9544	1.6371	0.8928	0
7772	322.9	0.927	0.9536	1.6346	0.8922	0
7773	322.8	0.9268	0.9531	1.6335	0.8917	0
7774	322.7	0.9261	0.9524	1.6337	0.8907	0
7775	322.6	0.925	0.9516	1.634	0.8906	0
7776	322.5	0.9247	0.9505	1.6329	0.8895	0
7777	322.4	0.9236	0.9503	1.6303	0.8889	0
7778	322.3	0.9232	0.9496	1.6293	0.8887	0
7779	322.2	0.9223	0.9491	1.6296	0.8881	0
7780	322.1	0.9215	0.9482	1.6285	0.8872	0
7781	322	0.9206	0.9471	1.6288	0.8868	0
7782	321.9	0.9204	0.9467	1.6292	0.8861	0
7783	321.8	0.9192	0.946	1.6265	0.8851	0
7784	321.7	0.9193	0.9453	1.6255	0.8846	0
7785	321.6	0.9178	0.9441	1.6258	0.884	0
7786	321.5	0.9174	0.944	1.6247	0.8835	0
7787	321.4	0.9164	0.943	1.625	0.8827	0
7788	321.3	0.9159	0.942	1.6224	0.882	0
7789	321.2	0.915	0.9417	1.6227	0.8815	0
7790	321.1	0.914	0.941	1.6217	0.8805	0
7791	321	0.9139	0.9398	1.6205	0.8801	0
7792	320.9	0.9134	0.9399	1.6179	0.8792	0
7793	320.8	0.9121	0.9386	1.6196	0.8785	0
7794	320.7	0.9112	0.9381	1.6184	0.8776	0
7795	320.6	0.9108	0.937	1.6188	0.8771	0
7796	320.5	0.9099	0.9362	1.6192	0.8763	0
7797	320.4	0.9094	0.936	1.615	0.8759	0
7798	320.3	0.9086	0.9352	1.617	0.8748	0
7799	320.2	0.9082	0.9345	1.6157	0.8746	0
7800	320.1	0.9073	0.9334	1.6145	0.8737	0
7801	320	0.9066	0.933	1.615	0.873	

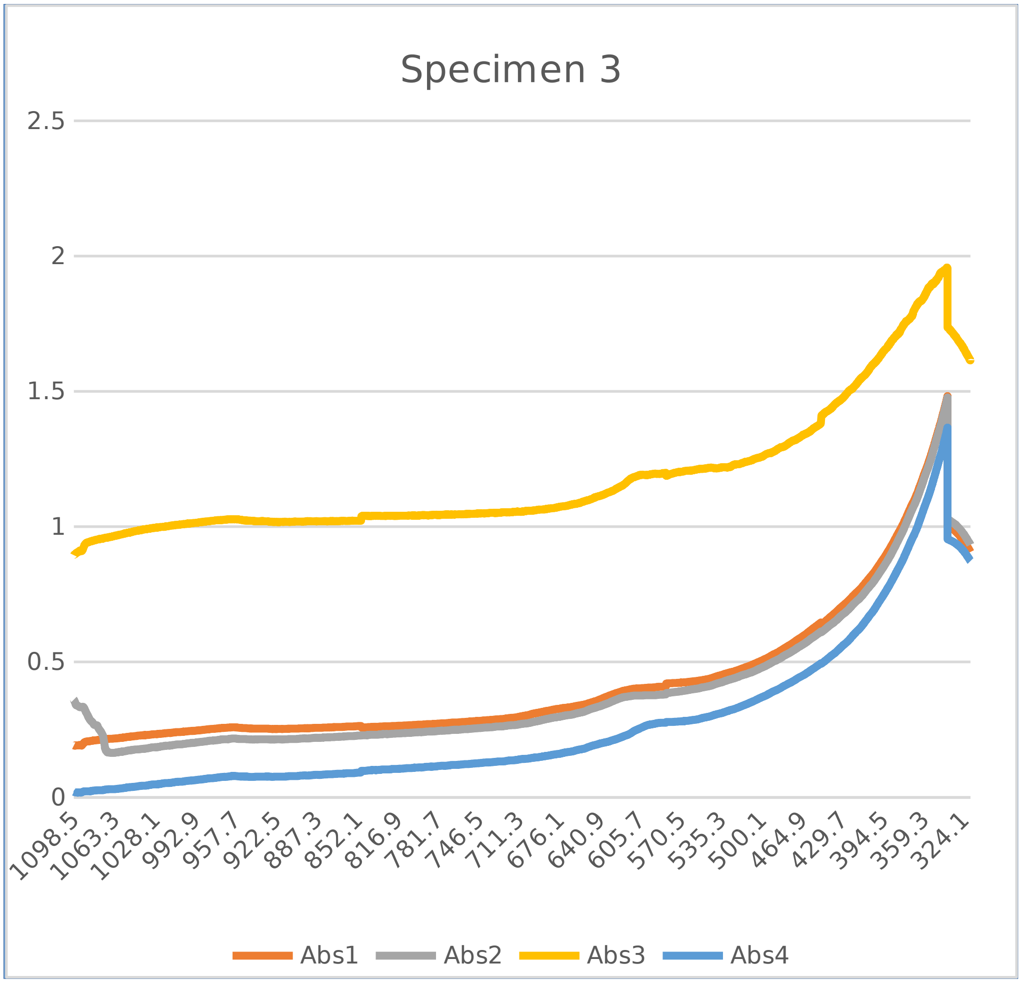

Specimen 3
2.5
2
1.5
1
0.5
0
1098.5
1063.3
1028.1
992.9
957.7
922.5
887.3
852.1
816.9
781.7
746.5
711.3
676.1
640.9
605.7
570.5
535.3
500.1
464.9
429.7
394.5
359.3
324.1
Abs1
Abs2
Abs3
Abs4
