# Supplementary material for: Coherent poly propagation materials with 3-dimensional photonic control over visible light
Source: PLoS One. 2019 Oct 17;14(10):e0223715. doi: 10.1371/journal.pone.0223715 (PMC6797174; doi:10.1371/journal.pone.0223715)
Supplement: S5 Table — Spreadsheet generated graph at bottom of table. (PDF) [file pone.0223715.s014.pdf]

Wavelength Scan Report - Specimen 5

Company	Complete Consulting Services LLC		
Operator	Dr. Michelle R. Stem	Test Time	11/8/2017 0:33
Document No.	WLS1711080001_1	Print Date	9/6/2019 20:15
Start wavelength	1100nm	Number of data	7801
End wavelength	320nm	K1	0.99
Unit		K0	0
Interval	0.1nm	K2	0
Calculate the Conc.	No	K3	0
Modification time	11/8/2017 0:33	Access time	11/8/2017 0:33
Remark	S16a068 - Specimen 5		

Scan Data

No.	Wavelength	Abs1	Abs2	Abs3	Abs4	Visible
1	1100	0.4013	0.7469	1.5575	1.147	0
2	1099.9	0.4014	0.7486	1.5635	1.147	0
3	1099.8	0.4014	0.7505	1.5686	1.1463	0
4	1099.7	0.4021	0.7521	1.5734	1.1465	0
5	1099.6	0.4029	0.7537	1.5727	1.1467	0
6	1099.5	0.4086	0.7557	1.5698	1.1467	0
7	1099.4	0.4119	0.7576	1.566	1.1471	0
8	1099.3	0.4145	0.7603	1.5649	1.1471	0
9	1099.2	0.4163	0.7618	1.5678	1.1468	0
10	1099.1	0.4204	0.7642	1.5673	1.147	0
11	1099	0.4253	0.766	1.5739	1.147	0
12	1098.9	0.449	0.7684	1.5834	1.1476	0
13	1098.8	0.4557	0.7703	1.6035	1.1474	0
14	1098.7	0.4556	0.7721	1.6156	1.1472	0
15	1098.6	0.4556	0.7747	1.62	1.1475	0
16	1098.5	0.4551	0.7771	1.621	1.1475	0
17	1098.4	0.4544	0.7795	1.6188	1.1474	0
18	1098.3	0.4542	0.7829	1.6206	1.1478	0
19	1098.2	0.4541	0.7874	1.6154	1.1477	0
20	1098.1	0.4538	0.7919	1.6045	1.1481	0
21	1098	0.4536	0.7965	1.5895	1.1482	0
22	1097.9	0.4532	0.8014	1.5772	1.1497	0
23	1097.8	0.4532	0.8061	1.5715	1.15	0
24	1097.7	0.4529	0.8112	1.5651	1.151	0
25	1097.6	0.4535	0.8173	1.546	1.1516	0
26	1097.5	0.4536	0.8234	1.5134	1.1523	0
27	1097.4	0.4537	0.8287	1.3794	1.1517	0
28	1097.3	0.4533	0.8349	0.6831	1.1521	0
29	1097.2	0.453	0.8408	0.6842	1.1524	0

30	1097.1	0.4526	0.846	0.6853	1.1525	0
31	1097	0.4525	0.851	0.6865	1.1523	0
32	1096.9	0.4522	0.8547	0.6881	1.1523	0
33	1096.8	0.4516	0.8588	0.6899	1.153	0
34	1096.7	0.4508	0.8624	0.6919	1.1538	0
35	1096.6	0.4502	0.8662	0.6933	1.1538	0
36	1096.5	0.4492	0.8694	0.6954	1.1545	0
37	1096.4	0.4486	0.873	0.6968	1.1551	0
38	1096.3	0.448	0.8755	0.6982	1.1552	0
39	1096.2	0.4476	0.8779	0.6995	1.1557	0
40	1096.1	0.4474	0.8806	0.7013	1.1561	0
41	1096	0.4476	0.8826	0.7021	1.1568	0
42	1095.9	0.4485	0.8846	0.7034	1.157	0
43	1095.8	0.4497	0.8872	0.7041	1.1573	0
44	1095.7	0.4502	0.8894	0.7044	1.1577	0
45	1095.6	0.4509	0.8919	0.7046	1.1573	0
46	1095.5	0.4526	0.8952	0.7047	1.1574	0
47	1095.4	0.4533	0.8976	0.705	1.1574	0
48	1095.3	0.4541	0.8996	0.7048	1.157	0
49	1095.2	0.4547	0.9009	0.7049	1.1566	0
50	1095.1	0.4552	0.9026	0.7053	1.1559	0
51	1095	0.4553	0.9027	0.7058	1.155	0
52	1094.9	0.4555	0.9029	0.7071	1.1543	0
53	1094.8	0.4552	0.9024	0.7083	1.1538	0
54	1094.7	0.4542	0.9008	0.7094	1.1526	0
55	1094.6	0.4529	0.8992	0.7108	1.1521	0
56	1094.5	0.4517	0.897	0.7126	1.1513	0
57	1094.4	0.45	0.8945	0.714	1.1508	0
58	1094.3	0.4486	0.8918	0.715	1.1506	0
59	1094.2	0.4468	0.8888	0.7159	1.1502	0
60	1094.1	0.4456	0.8864	0.7181	1.1502	0
61	1094	0.4439	0.8839	0.7188	1.1499	0
62	1093.9	0.4426	0.8821	0.7202	1.1502	0
63	1093.8	0.4413	0.8808	0.7214	1.1505	0
64	1093.7	0.4403	0.8799	0.7225	1.1508	0
65	1093.6	0.4403	0.8799	0.7229	1.1514	0
66	1093.5	0.44	0.8803	0.7228	1.1519	0
67	1093.4	0.4405	0.8813	0.7235	1.1526	0
68	1093.3	0.4409	0.8827	0.7238	1.1534	0
69	1093.2	0.442	0.8846	0.7239	1.1542	0
70	1093.1	0.4434	0.8873	0.7233	1.1549	0
71	1093	0.445	0.8905	0.7218	1.1556	0
72	1092.9	0.4474	0.8944	0.7202	1.1564	0

73	1092.8	0.4493	0.8986	0.7188	1.1571	0
74	1092.7	0.4517	0.9032	0.7171	1.1577	0
75	1092.6	0.4547	0.9082	0.7153	1.1584	0
76	1092.5	0.4576	0.9133	0.7131	1.1589	0
77	1092.4	0.4606	0.9186	0.7113	1.1603	0
78	1092.3	0.4632	0.9248	0.7095	1.1608	0
79	1092.2	0.4666	0.9303	0.7074	1.161	0
80	1092.1	0.47	0.9359	0.7056	1.1619	0
81	1092	0.473	0.9419	0.7039	1.1626	0
82	1091.9	0.4763	0.9477	0.7024	1.163	0
83	1091.8	0.4794	0.9534	0.7	1.1633	0
84	1091.7	0.4822	0.9591	0.6978	1.1639	0
85	1091.6	0.4853	0.9647	0.6957	1.1644	0
86	1091.5	0.4886	0.97	0.6937	1.1649	0
87	1091.4	0.4913	0.975	0.6918	1.1656	0
88	1091.3	0.4935	0.9804	0.6908	1.1655	0
89	1091.2	0.4961	0.985	0.6896	1.1662	0
90	1091.1	0.4987	0.9896	0.6881	1.167	0
91	1091	0.5006	0.994	0.6867	1.1665	0
92	1090.9	0.5028	0.9982	0.685	1.1668	0
93	1090.8	0.5044	1.0023	0.681	1.1668	0
94	1090.7	0.5064	1.0062	0.6774	1.1675	0
95	1090.6	0.5079	1.0098	0.6736	1.168	0
96	1090.5	0.5094	1.0131	0.6698	1.1682	0
97	1090.4	0.5113	1.016	0.6668	1.1679	0
98	1090.3	0.5128	1.0191	0.6654	1.1685	0
99	1090.2	0.514	1.0211	0.6644	1.1685	0
100	1090.1	0.5149	1.0244	0.6641	1.1693	0
101	1090	0.5159	1.0263	0.6631	1.1693	0
102	1089.9	0.5172	1.0283	0.6632	1.1694	0
103	1089.8	0.518	1.0301	0.6628	1.1694	0
104	1089.7	0.5192	1.0324	0.6629	1.1701	0
105	1089.6	0.5199	1.0341	0.6621	1.1703	0
106	1089.5	0.5205	1.0356	0.662	1.1701	0
107	1089.4	0.5216	1.0369	0.6613	1.1706	0
108	1089.3	0.522	1.0382	0.6612	1.1707	0
109	1089.2	0.5227	1.0404	0.6613	1.1705	0
110	1089.1	0.5235	1.0418	0.6608	1.1704	0
111	1089	0.524	1.0428	0.6608	1.1707	0
112	1088.9	0.5241	1.044	0.6605	1.1706	0
113	1088.8	0.5251	1.0452	0.6605	1.1709	0
114	1088.7	0.5252	1.0466	0.6604	1.1712	0
115	1088.6	0.5254	1.0473	0.6599	1.1712	0

116	1088.5	0.5259	1.0495	0.6602	1.1712	0
117	1088.4	0.5264	1.0511	0.6601	1.1712	0
118	1088.3	0.5266	1.0536	0.6602	1.1714	0
119	1088.2	0.5269	1.055	0.6602	1.1712	0
120	1088.1	0.5271	1.0566	0.6605	1.172	0
121	1088	0.5272	1.0574	0.6601	1.172	0
122	1087.9	0.5275	1.0582	0.6599	1.1718	0
123	1087.8	0.527	1.0598	0.66	1.1713	0
124	1087.7	0.5277	1.0625	0.6602	1.1719	0
125	1087.6	0.5277	1.0633	0.6601	1.1718	0
126	1087.5	0.5278	1.0643	0.6601	1.1722	0
127	1087.4	0.5277	1.0655	0.6601	1.1721	0
128	1087.3	0.5283	1.0657	0.6602	1.1724	0
129	1087.2	0.5285	1.0682	0.6602	1.1727	0
130	1087.1	0.5283	1.0688	0.6605	1.1725	0
131	1087	0.528	1.069	0.6601	1.1723	0
132	1086.9	0.5284	1.0694	0.6604	1.1721	0
133	1086.8	0.5286	1.0705	0.6603	1.1726	0
134	1086.7	0.5291	1.0718	0.6606	1.1729	0
135	1086.6	0.5288	1.0723	0.6605	1.1731	0
136	1086.5	0.5287	1.0735	0.6607	1.1728	0
137	1086.4	0.5295	1.0734	0.6605	1.1728	0
138	1086.3	0.5297	1.0748	0.6602	1.1729	0
139	1086.2	0.5298	1.0769	0.6604	1.1728	0
140	1086.1	0.5298	1.0773	0.6603	1.1729	0
141	1086	0.5299	1.0781	0.6605	1.1733	0
142	1085.9	0.5304	1.0794	0.6607	1.1732	0
143	1085.8	0.5305	1.0802	0.6607	1.1732	0
144	1085.7	0.5306	1.0804	0.6604	1.1735	0
145	1085.6	0.5304	1.0819	0.6605	1.1736	0
146	1085.5	0.5309	1.0828	0.6608	1.1735	0
147	1085.4	0.5311	1.083	0.6608	1.1737	0
148	1085.3	0.5309	1.0844	0.6608	1.1736	0
149	1085.2	0.5317	1.0856	0.6612	1.1735	0
150	1085.1	0.5318	1.0859	0.6613	1.1736	0
151	1085	0.5315	1.0871	0.6614	1.1738	0
152	1084.9	0.5316	1.0874	0.6615	1.1737	0
153	1084.8	0.5317	1.0883	0.6619	1.1739	0
154	1084.7	0.5319	1.0891	0.6623	1.1744	0
155	1084.6	0.5317	1.0898	0.6621	1.1742	0
156	1084.5	0.5318	1.0907	0.6624	1.1739	0
157	1084.4	0.5318	1.0902	0.6623	1.1739	0
158	1084.3	0.5321	1.0908	0.6623	1.1739	0

159	1084.2	0.5318	1.0911	0.6624	1.1731	0
160	1084.1	0.5323	1.0907	0.6627	1.174	0
161	1084	0.532	1.092	0.6626	1.1738	0
162	1083.9	0.5319	1.0919	0.6631	1.1744	0
163	1083.8	0.5321	1.0926	0.663	1.1741	0
164	1083.7	0.5326	1.0935	0.6636	1.1744	0
165	1083.6	0.532	1.0946	0.6633	1.1739	0
166	1083.5	0.5321	1.0951	0.6632	1.1738	0
167	1083.4	0.5322	1.0958	0.6633	1.1742	0
168	1083.3	0.5321	1.0967	0.6639	1.174	0
169	1083.2	0.5322	1.0976	0.6641	1.1739	0
170	1083.1	0.532	1.0978	0.6641	1.1742	0
171	1083	0.5321	1.0989	0.6641	1.1739	0
172	1082.9	0.5319	1.1	0.6645	1.1738	0
173	1082.8	0.5318	1.1009	0.6646	1.1739	0
174	1082.7	0.532	1.1016	0.6646	1.1738	0
175	1082.6	0.5321	1.1019	0.6643	1.1743	0
176	1082.5	0.5323	1.1026	0.6645	1.174	0
177	1082.4	0.5319	1.1032	0.6648	1.1742	0
178	1082.3	0.5317	1.1038	0.6649	1.1739	0
179	1082.2	0.5317	1.1042	0.665	1.1742	0
180	1082.1	0.5323	1.1051	0.6652	1.1741	0
181	1082	0.5325	1.1056	0.6655	1.1739	0
182	1081.9	0.5322	1.1058	0.6653	1.1742	0
183	1081.8	0.5325	1.1064	0.6652	1.1744	0
184	1081.7	0.5327	1.1069	0.6657	1.1741	0
185	1081.6	0.5327	1.107	0.6659	1.174	0
186	1081.5	0.5327	1.1076	0.6659	1.1744	0
187	1081.4	0.5325	1.1081	0.6658	1.1743	0
188	1081.3	0.5325	1.1083	0.666	1.1744	0
189	1081.2	0.5326	1.1086	0.6659	1.1742	0
190	1081.1	0.5329	1.1092	0.6661	1.1745	0
191	1081	0.5329	1.1093	0.6662	1.1747	0
192	1080.9	0.5331	1.1094	0.6663	1.1746	0
193	1080.8	0.5327	1.1104	0.6663	1.1743	0
194	1080.7	0.5331	1.1106	0.666	1.175	0
195	1080.6	0.5329	1.1109	0.6663	1.1748	0
196	1080.5	0.5329	1.1117	0.6667	1.1753	0
197	1080.4	0.5329	1.1118	0.6664	1.1751	0
198	1080.3	0.5327	1.1124	0.6666	1.1752	0
199	1080.2	0.5329	1.1123	0.6664	1.1754	0
200	1080.1	0.5332	1.1132	0.6666	1.1754	0
201	1080	0.5331	1.1134	0.6665	1.1754	0

202	1079.9	0.5333	1.1135	0.6666	1.1753	0
203	1079.8	0.5329	1.114	0.6665	1.1755	0
204	1079.7	0.5332	1.1146	0.6662	1.1755	0
205	1079.6	0.533	1.1143	0.6665	1.1747	0
206	1079.5	0.5333	1.1154	0.6661	1.1752	0
207	1079.4	0.5338	1.116	0.666	1.1752	0
208	1079.3	0.534	1.1175	0.6664	1.1757	0
209	1079.2	0.534	1.1177	0.666	1.1756	0
210	1079.1	0.5342	1.1181	0.6659	1.1756	0
211	1079	0.5343	1.1182	0.666	1.1755	0
212	1078.9	0.5342	1.1182	0.6661	1.1754	0
213	1078.8	0.5339	1.1186	0.6659	1.1753	0
214	1078.7	0.5341	1.1194	0.6655	1.1755	0
215	1078.6	0.5345	1.1199	0.6658	1.1759	0
216	1078.5	0.5345	1.1204	0.6658	1.1753	0
217	1078.4	0.5345	1.1211	0.6655	1.1758	0
218	1078.3	0.5347	1.121	0.6648	1.1757	0
219	1078.2	0.5347	1.1215	0.6644	1.1756	0
220	1078.1	0.5349	1.1214	0.6639	1.1758	0
221	1078	0.5345	1.1214	0.6636	1.1759	0
222	1077.9	0.5352	1.122	0.6636	1.1756	0
223	1077.8	0.5351	1.1222	0.6634	1.176	0
224	1077.7	0.5349	1.1224	0.6628	1.176	0
225	1077.6	0.5349	1.1231	0.6625	1.1764	0
226	1077.5	0.5353	1.1233	0.662	1.176	0
227	1077.4	0.5357	1.1232	0.6616	1.176	0
228	1077.3	0.5354	1.1237	0.6607	1.1763	0
229	1077.2	0.5354	1.1232	0.6595	1.1759	0
230	1077.1	0.5356	1.1247	0.6587	1.1763	0
231	1077	0.5355	1.1243	0.6581	1.176	0
232	1076.9	0.5354	1.1245	0.6575	1.1765	0
233	1076.8	0.5356	1.1252	0.6572	1.1768	0
234	1076.7	0.5358	1.1255	0.6568	1.1764	0
235	1076.6	0.5358	1.1254	0.6564	1.1764	0
236	1076.5	0.536	1.1259	0.6563	1.1766	0
237	1076.4	0.5354	1.1256	0.6559	1.1764	0
238	1076.3	0.5353	1.1263	0.6558	1.1759	0
239	1076.2	0.5356	1.1262	0.6556	1.1765	0
240	1076.1	0.5358	1.1273	0.6556	1.1765	0
241	1076	0.5355	1.1275	0.6554	1.1764	0
242	1075.9	0.5361	1.127	0.6555	1.1768	0
243	1075.8	0.5358	1.1273	0.6553	1.1762	0
244	1075.7	0.536	1.1275	0.6552	1.1765	0

245	1075.6	0.5358	1.128	0.6554	1.177	0
246	1075.5	0.5358	1.1278	0.6552	1.177	0
247	1075.4	0.5363	1.1282	0.655	1.1769	0
248	1075.3	0.5362	1.1281	0.6549	1.1771	0
249	1075.2	0.536	1.1286	0.6546	1.1768	0
250	1075.1	0.5364	1.1294	0.6548	1.1771	0
251	1075	0.5362	1.1283	0.6546	1.177	0
252	1074.9	0.5362	1.1278	0.6548	1.1771	0
253	1074.8	0.5357	1.129	0.6546	1.177	0
254	1074.7	0.536	1.1288	0.6546	1.1772	0
255	1074.6	0.5363	1.1294	0.6546	1.1769	0
256	1074.5	0.536	1.129	0.6546	1.1768	0
257	1074.4	0.5363	1.1289	0.6545	1.1769	0
258	1074.3	0.5366	1.1294	0.6547	1.1774	0
259	1074.2	0.5368	1.129	0.6549	1.1773	0
260	1074.1	0.536	1.1288	0.6543	1.1769	0
261	1074	0.5362	1.129	0.6544	1.1772	0
262	1073.9	0.5362	1.1291	0.6546	1.1769	0
263	1073.8	0.5361	1.1292	0.6543	1.1768	0
264	1073.7	0.5364	1.1292	0.6546	1.177	0
265	1073.6	0.5367	1.129	0.6548	1.1767	0
266	1073.5	0.5367	1.1293	0.6549	1.1773	0
267	1073.4	0.5364	1.1286	0.6548	1.1768	0
268	1073.3	0.5362	1.1292	0.6549	1.1769	0
269	1073.2	0.5364	1.1296	0.6551	1.1771	0
270	1073.1	0.5364	1.1289	0.6552	1.1773	0
271	1073	0.5365	1.1295	0.6553	1.1774	0
272	1072.9	0.5362	1.1295	0.6553	1.1772	0
273	1072.8	0.5364	1.1294	0.6552	1.1773	0
274	1072.7	0.5365	1.1292	0.655	1.1769	0
275	1072.6	0.5364	1.1291	0.6549	1.1768	0
276	1072.5	0.5361	1.1294	0.6552	1.1773	0
277	1072.4	0.5362	1.1297	0.6552	1.1772	0
278	1072.3	0.5363	1.1291	0.6551	1.1772	0
279	1072.2	0.5365	1.129	0.6552	1.1772	0
280	1072.1	0.5363	1.13	0.6551	1.1774	0
281	1072	0.5365	1.13	0.6554	1.1775	0
282	1071.9	0.5365	1.1301	0.6555	1.1777	0
283	1071.8	0.5367	1.13	0.6554	1.1776	0
284	1071.7	0.5365	1.1304	0.6554	1.1774	0
285	1071.6	0.5366	1.1302	0.6552	1.1773	0
286	1071.5	0.5365	1.1307	0.6551	1.1772	0
287	1071.4	0.5367	1.131	0.6552	1.1774	0

288	1071.3	0.5368	1.1312	0.6554	1.1774	0
289	1071.2	0.5367	1.1303	0.6553	1.1772	0
290	1071.1	0.5369	1.1309	0.6553	1.1777	0
291	1071	0.537	1.1314	0.6553	1.1778	0
292	1070.9	0.5367	1.1314	0.6554	1.1776	0
293	1070.8	0.5367	1.1311	0.6551	1.1773	0
294	1070.7	0.5373	1.1311	0.6553	1.1775	0
295	1070.6	0.5371	1.1313	0.6553	1.1777	0
296	1070.5	0.537	1.1321	0.6555	1.1775	0
297	1070.4	0.5368	1.1321	0.6552	1.1777	0
298	1070.3	0.537	1.1324	0.6555	1.1781	0
299	1070.2	0.5373	1.132	0.655	1.1775	0
300	1070.1	0.5373	1.1323	0.6552	1.1777	0
301	1070	0.5369	1.1321	0.6552	1.1778	0
302	1069.9	0.5372	1.1327	0.6551	1.1777	0
303	1069.8	0.5375	1.1323	0.6553	1.1778	0
304	1069.7	0.5374	1.1328	0.6551	1.1777	0
305	1069.6	0.5379	1.1326	0.6552	1.1778	0
306	1069.5	0.5375	1.1331	0.6552	1.1774	0
307	1069.4	0.5374	1.1328	0.655	1.1781	0
308	1069.3	0.5375	1.133	0.6548	1.1778	0
309	1069.2	0.5375	1.1329	0.6548	1.178	0
310	1069.1	0.538	1.1336	0.6548	1.1784	0
311	1069	0.5378	1.1335	0.6543	1.1779	0
312	1068.9	0.538	1.1341	0.6546	1.1787	0
313	1068.8	0.5381	1.1343	0.6543	1.1785	0
314	1068.7	0.5385	1.1343	0.6542	1.1782	0
315	1068.6	0.5381	1.1343	0.6539	1.1783	0
316	1068.5	0.538	1.1348	0.6537	1.1785	0
317	1068.4	0.538	1.1344	0.6535	1.1782	0
318	1068.3	0.5383	1.1344	0.6535	1.1783	0
319	1068.2	0.5382	1.1347	0.6535	1.1783	0
320	1068.1	0.538	1.1352	0.6532	1.1786	0
321	1068	0.5386	1.1353	0.6532	1.1787	0
322	1067.9	0.5383	1.1352	0.6529	1.1783	0
323	1067.8	0.5385	1.1346	0.6529	1.1782	0
324	1067.7	0.5387	1.1352	0.6527	1.1786	0
325	1067.6	0.5386	1.1357	0.6532	1.1789	0
326	1067.5	0.5384	1.1358	0.6528	1.1786	0
327	1067.4	0.5384	1.1364	0.6528	1.1789	0
328	1067.3	0.5387	1.1365	0.6527	1.1787	0
329	1067.2	0.5392	1.1369	0.653	1.1792	0
330	1067.1	0.5397	1.1368	0.6533	1.1794	0

331	1067	0.539	1.1371	0.6528	1.1791	0
332	1066.9	0.5393	1.1376	0.6529	1.1789	0
333	1066.8	0.5393	1.1373	0.6527	1.1788	0
334	1066.7	0.5389	1.1369	0.6526	1.1792	0
335	1066.6	0.5388	1.1374	0.6525	1.179	0
336	1066.5	0.5387	1.1375	0.6524	1.1788	0
337	1066.4	0.539	1.1375	0.6524	1.179	0
338	1066.3	0.5394	1.1374	0.6527	1.1792	0
339	1066.2	0.5392	1.1374	0.6524	1.1792	0
340	1066.1	0.5395	1.1379	0.6528	1.179	0
341	1066	0.5393	1.1374	0.6525	1.1792	0
342	1065.9	0.5394	1.138	0.6526	1.1798	0
343	1065.8	0.5389	1.1377	0.6524	1.1791	0
344	1065.7	0.5392	1.1382	0.6525	1.1788	0
345	1065.6	0.5392	1.1375	0.6523	1.1787	0
346	1065.5	0.5393	1.1383	0.6526	1.1792	0
347	1065.4	0.5391	1.1382	0.6527	1.1783	0
348	1065.3	0.5393	1.1381	0.6526	1.1785	0
349	1065.2	0.5394	1.1391	0.6528	1.1789	0
350	1065.1	0.5393	1.1387	0.6528	1.1786	0
351	1065	0.5397	1.1392	0.6528	1.1788	0
352	1064.9	0.5394	1.1388	0.6525	1.1789	0
353	1064.8	0.5391	1.1387	0.6526	1.1784	0
354	1064.7	0.5393	1.1388	0.6525	1.1787	0
355	1064.6	0.5392	1.1382	0.6527	1.1786	0
356	1064.5	0.539	1.1389	0.6526	1.1783	0
357	1064.4	0.5392	1.1382	0.6528	1.1785	0
358	1064.3	0.5389	1.1382	0.6525	1.178	0
359	1064.2	0.5391	1.1384	0.6528	1.1781	0
360	1064.1	0.539	1.1387	0.6526	1.1781	0
361	1064	0.5392	1.1385	0.6528	1.1784	0
362	1063.9	0.5388	1.1388	0.653	1.178	0
363	1063.8	0.5391	1.1395	0.6532	1.1783	0
364	1063.7	0.5387	1.1392	0.6532	1.1779	0
365	1063.6	0.539	1.1396	0.6531	1.1786	0
366	1063.5	0.5392	1.1387	0.653	1.1778	0
367	1063.4	0.5387	1.1386	0.653	1.1779	0
368	1063.3	0.539	1.1396	0.6532	1.1782	0
369	1063.2	0.5382	1.1389	0.6531	1.1778	0
370	1063.1	0.5388	1.139	0.653	1.1781	0
371	1063	0.5388	1.1387	0.6532	1.1776	0
372	1062.9	0.5385	1.1387	0.6531	1.1772	0
373	1062.8	0.5386	1.139	0.6532	1.1778	0

374	1062.7	0.5388	1.1393	0.6533	1.178	0
375	1062.6	0.5385	1.1388	0.6529	1.177	0
376	1062.5	0.5386	1.1391	0.653	1.1772	0
377	1062.4	0.5385	1.139	0.6531	1.1771	0
378	1062.3	0.5382	1.1391	0.6534	1.1777	0
379	1062.2	0.5376	1.1389	0.6531	1.177	0
380	1062.1	0.5388	1.1397	0.6534	1.1775	0
381	1062	0.5387	1.1402	0.6533	1.1779	0
382	1061.9	0.5389	1.1388	0.6535	1.1775	0
383	1061.8	0.5389	1.1396	0.6537	1.1776	0
384	1061.7	0.5391	1.1396	0.6537	1.1773	0
385	1061.6	0.5385	1.1393	0.6535	1.1766	0
386	1061.5	0.5388	1.139	0.6534	1.1774	0
387	1061.4	0.5382	1.1394	0.6537	1.1771	0
388	1061.3	0.5387	1.1401	0.654	1.1771	0
389	1061.2	0.5391	1.1397	0.6538	1.1774	0
390	1061.1	0.5388	1.1398	0.6538	1.1769	0
391	1061	0.5388	1.14	0.654	1.1773	0
392	1060.9	0.5383	1.1401	0.6536	1.1776	0
393	1060.8	0.539	1.1393	0.6539	1.177	0
394	1060.7	0.5392	1.1401	0.6542	1.1774	0
395	1060.6	0.539	1.1402	0.6542	1.1774	0
396	1060.5	0.5387	1.1402	0.6542	1.1776	0
397	1060.4	0.5388	1.1402	0.6543	1.1775	0
398	1060.3	0.539	1.1402	0.6545	1.1777	0
399	1060.2	0.5396	1.1404	0.6546	1.1779	0
400	1060.1	0.5392	1.1407	0.6547	1.1772	0
401	1060	0.5392	1.1403	0.6546	1.1776	0
402	1059.9	0.5396	1.1404	0.6547	1.1776	0
403	1059.8	0.5397	1.1401	0.6549	1.1773	0
404	1059.7	0.5395	1.1406	0.6551	1.1776	0
405	1059.6	0.5397	1.1413	0.6548	1.1779	0
406	1059.5	0.54	1.1413	0.6552	1.1775	0
407	1059.4	0.5397	1.1409	0.6554	1.1779	0
408	1059.3	0.5397	1.1404	0.6555	1.1783	0
409	1059.2	0.54	1.1409	0.6556	1.1783	0
410	1059.1	0.5401	1.1409	0.6556	1.1781	0
411	1059	0.5404	1.1408	0.6556	1.1782	0
412	1058.9	0.5405	1.1416	0.6556	1.1785	0
413	1058.8	0.5406	1.1417	0.6558	1.1784	0
414	1058.7	0.5406	1.1413	0.6557	1.178	0
415	1058.6	0.5409	1.1418	0.656	1.1787	0
416	1058.5	0.5407	1.1421	0.656	1.1789	0

417	1058.4	0.5409	1.1422	0.6561	1.1789	0
418	1058.3	0.5409	1.1422	0.656	1.1785	0
419	1058.2	0.5408	1.1423	0.6561	1.1789	0
420	1058.1	0.5413	1.1423	0.6559	1.1788	0
421	1058	0.5413	1.1427	0.656	1.1789	0
422	1057.9	0.5415	1.1426	0.6564	1.1789	0
423	1057.8	0.5414	1.1431	0.6564	1.1791	0
424	1057.7	0.5412	1.143	0.6567	1.1789	0
425	1057.6	0.5418	1.1432	0.6569	1.1796	0
426	1057.5	0.5418	1.1433	0.6567	1.1793	0
427	1057.4	0.5422	1.1435	0.6569	1.1798	0
428	1057.3	0.5427	1.1438	0.6569	1.1797	0
429	1057.2	0.5425	1.1434	0.6572	1.18	0
430	1057.1	0.5427	1.1443	0.6571	1.1804	0
431	1057	0.5427	1.1441	0.6572	1.1801	0
432	1056.9	0.5428	1.1443	0.6573	1.1802	0
433	1056.8	0.5432	1.1438	0.6574	1.1804	0
434	1056.7	0.543	1.1443	0.6576	1.1803	0
435	1056.6	0.5431	1.1446	0.6573	1.1803	0
436	1056.5	0.5436	1.1451	0.6579	1.1805	0
437	1056.4	0.5439	1.1452	0.6581	1.1811	0
438	1056.3	0.5437	1.1449	0.6578	1.1811	0
439	1056.2	0.5438	1.1451	0.6578	1.1808	0
440	1056.1	0.5438	1.1454	0.6581	1.181	0
441	1056	0.5437	1.1451	0.6581	1.1808	0
442	1055.9	0.5437	1.1453	0.6583	1.1813	0
443	1055.8	0.5437	1.1452	0.6583	1.1809	0
444	1055.7	0.5442	1.1452	0.6585	1.1813	0
445	1055.6	0.5441	1.1456	0.6585	1.1811	0
446	1055.5	0.5445	1.1462	0.6587	1.1816	0
447	1055.4	0.5445	1.1462	0.6586	1.1816	0
448	1055.3	0.5448	1.1465	0.659	1.1814	0
449	1055.2	0.5447	1.1464	0.6589	1.1819	0
450	1055.1	0.5445	1.1458	0.6589	1.1817	0
451	1055	0.5447	1.1462	0.6593	1.1818	0
452	1054.9	0.5448	1.1465	0.6595	1.1818	0
453	1054.8	0.545	1.1462	0.6595	1.1822	0
454	1054.7	0.5449	1.146	0.6594	1.1819	0
455	1054.6	0.5448	1.1462	0.6596	1.1821	0
456	1054.5	0.5449	1.1468	0.6598	1.1818	0
457	1054.4	0.5451	1.1468	0.66	1.182	0
458	1054.3	0.5451	1.1471	0.6601	1.1822	0
459	1054.2	0.5452	1.1461	0.66	1.1821	0

460	1054.1	0.5455	1.1463	0.6602	1.1822	0
461	1054	0.5455	1.1466	0.6603	1.1823	0
462	1053.9	0.5456	1.1468	0.6605	1.1824	0
463	1053.8	0.5457	1.1466	0.6607	1.1821	0
464	1053.7	0.5456	1.1468	0.6607	1.1825	0
465	1053.6	0.5455	1.1471	0.6609	1.1823	0
466	1053.5	0.5458	1.147	0.6611	1.1828	0
467	1053.4	0.5452	1.1464	0.661	1.1826	0
468	1053.3	0.5456	1.1468	0.6608	1.1827	0
469	1053.2	0.5456	1.147	0.6611	1.1829	0
470	1053.1	0.5458	1.1468	0.6613	1.1831	0
471	1053	0.5466	1.1476	0.6615	1.1835	0
472	1052.9	0.5464	1.1469	0.6614	1.1832	0
473	1052.8	0.5465	1.1472	0.6615	1.183	0
474	1052.7	0.546	1.1471	0.6615	1.1826	0
475	1052.6	0.546	1.1473	0.6615	1.1827	0
476	1052.5	0.5465	1.1476	0.6618	1.183	0
477	1052.4	0.5466	1.1478	0.6619	1.1834	0
478	1052.3	0.5465	1.1473	0.6619	1.183	0
479	1052.2	0.5466	1.1479	0.6621	1.1831	0
480	1052.1	0.5469	1.1481	0.6622	1.1832	0
481	1052	0.547	1.1476	0.662	1.1836	0
482	1051.9	0.547	1.1487	0.6622	1.1832	0
483	1051.8	0.5474	1.1489	0.6625	1.183	0
484	1051.7	0.5474	1.1488	0.6627	1.1837	0
485	1051.6	0.5471	1.1484	0.6626	1.1832	0
486	1051.5	0.5473	1.1485	0.6628	1.1837	0
487	1051.4	0.5476	1.148	0.6628	1.184	0
488	1051.3	0.5475	1.1483	0.6629	1.1839	0
489	1051.2	0.5476	1.1484	0.6627	1.1836	0
490	1051.1	0.5477	1.1482	0.6629	1.1841	0
491	1051	0.5477	1.1489	0.6631	1.1843	0
492	1050.9	0.548	1.1486	0.6634	1.1841	0
493	1050.8	0.548	1.1488	0.6631	1.1841	0
494	1050.7	0.5475	1.1483	0.663	1.184	0
495	1050.6	0.5476	1.1488	0.6631	1.1837	0
496	1050.5	0.5481	1.149	0.663	1.1838	0
497	1050.4	0.5482	1.1492	0.6632	1.1843	0
498	1050.3	0.5484	1.1498	0.6634	1.1846	0
499	1050.2	0.5486	1.1496	0.6636	1.1845	0
500	1050.1	0.5485	1.1495	0.6637	1.1841	0
501	1050	0.5488	1.1497	0.6637	1.1843	0
502	1049.9	0.5489	1.1494	0.6637	1.1843	0

503	1049.8	0.5491	1.1499	0.6638	1.1849	0
504	1049.7	0.5489	1.1495	0.6639	1.1848	0
505	1049.6	0.5493	1.1499	0.664	1.1849	0
506	1049.5	0.549	1.15	0.6636	1.185	0
507	1049.4	0.5494	1.1503	0.6639	1.1854	0
508	1049.3	0.5495	1.1503	0.664	1.1847	0
509	1049.2	0.5492	1.1495	0.6637	1.1848	0
510	1049.1	0.5491	1.1501	0.6638	1.185	0
511	1049	0.5494	1.1505	0.664	1.185	0
512	1048.9	0.5496	1.1508	0.664	1.1855	0
513	1048.8	0.5493	1.1508	0.6639	1.1854	0
514	1048.7	0.5495	1.1507	0.664	1.185	0
515	1048.6	0.5502	1.1511	0.664	1.1854	0
516	1048.5	0.5501	1.1509	0.6642	1.1853	0
517	1048.4	0.5505	1.1507	0.6641	1.1853	0
518	1048.3	0.5503	1.1505	0.6639	1.1855	0
519	1048.2	0.5499	1.1511	0.6648	1.1855	0
520	1048.1	0.5497	1.151	0.6645	1.1859	0
521	1048	0.5497	1.1507	0.6646	1.1857	0
522	1047.9	0.5503	1.1508	0.6648	1.1857	0
523	1047.8	0.5508	1.1508	0.6649	1.1855	0
524	1047.7	0.5508	1.1512	0.665	1.1856	0
525	1047.6	0.5506	1.1514	0.6653	1.1855	0
526	1047.5	0.5509	1.1517	0.6652	1.1853	0
527	1047.4	0.5507	1.1517	0.6651	1.1856	0
528	1047.3	0.55	1.1524	0.6654	1.1859	0
529	1047.2	0.5502	1.1517	0.6651	1.1856	0
530	1047.1	0.5511	1.1518	0.6652	1.1859	0
531	1047	0.5511	1.152	0.6653	1.1856	0
532	1046.9	0.5509	1.1519	0.6655	1.1858	0
533	1046.8	0.5507	1.1518	0.6655	1.1859	0
534	1046.7	0.5513	1.1519	0.6656	1.1862	0
535	1046.6	0.551	1.1514	0.6657	1.1856	0
536	1046.5	0.5512	1.1516	0.6661	1.1866	0
537	1046.4	0.5514	1.1519	0.666	1.186	0
538	1046.3	0.5518	1.1521	0.6663	1.1863	0
539	1046.2	0.5518	1.1523	0.6662	1.1857	0
540	1046.1	0.5524	1.1521	0.6664	1.1864	0
541	1046	0.5515	1.152	0.6666	1.1868	0
542	1045.9	0.5521	1.1523	0.6667	1.1872	0
543	1045.8	0.5519	1.1515	0.6666	1.1867	0
544	1045.7	0.5519	1.1526	0.6668	1.1861	0
545	1045.6	0.5524	1.1522	0.6669	1.1869	0

546	1045.5	0.5525	1.1521	0.667	1.1863	0
547	1045.4	0.5522	1.1519	0.6671	1.1867	0
548	1045.3	0.5522	1.1516	0.667	1.1868	0
549	1045.2	0.5525	1.1521	0.6673	1.1866	0
550	1045.1	0.5525	1.1526	0.6678	1.1868	0
551	1045	0.5522	1.1518	0.6673	1.1868	0
552	1044.9	0.5526	1.1523	0.6676	1.1864	0
553	1044.8	0.5525	1.1516	0.6677	1.1865	0
554	1044.7	0.5522	1.1522	0.6677	1.1868	0
555	1044.6	0.5527	1.152	0.6679	1.1869	0
556	1044.5	0.553	1.1522	0.668	1.1869	0
557	1044.4	0.5524	1.1519	0.6681	1.1867	0
558	1044.3	0.5526	1.1523	0.6681	1.1873	0
559	1044.2	0.5529	1.1526	0.6685	1.1869	0
560	1044.1	0.553	1.1519	0.6687	1.1874	0
561	1044	0.5534	1.152	0.6686	1.1873	0
562	1043.9	0.5532	1.1515	0.6686	1.1873	0
563	1043.8	0.5532	1.1519	0.6688	1.1872	0
564	1043.7	0.5531	1.1518	0.6687	1.1871	0
565	1043.6	0.5531	1.1514	0.6689	1.1874	0
566	1043.5	0.5534	1.1518	0.6693	1.1872	0
567	1043.4	0.5536	1.1517	0.6691	1.1876	0
568	1043.3	0.554	1.1517	0.6693	1.1878	0
569	1043.2	0.5541	1.1521	0.6695	1.1874	0
570	1043.1	0.5534	1.1515	0.6692	1.187	0
571	1043	0.5532	1.151	0.6692	1.1869	0
572	1042.9	0.5537	1.1517	0.6696	1.1871	0
573	1042.8	0.5538	1.1518	0.6696	1.1874	0
574	1042.7	0.554	1.152	0.6697	1.188	0
575	1042.6	0.5541	1.1521	0.6699	1.188	0
576	1042.5	0.5541	1.1519	0.6699	1.1875	0
577	1042.4	0.5541	1.1521	0.6699	1.1871	0
578	1042.3	0.5543	1.1519	0.6702	1.1873	0
579	1042.2	0.5542	1.1522	0.6701	1.1871	0
580	1042.1	0.5543	1.1525	0.6702	1.1876	0
581	1042	0.5543	1.1525	0.6704	1.1882	0
582	1041.9	0.5546	1.1522	0.6703	1.188	0
583	1041.8	0.5547	1.1524	0.6703	1.1879	0
584	1041.7	0.5548	1.1529	0.6704	1.188	0
585	1041.6	0.5548	1.1525	0.6703	1.1881	0
586	1041.5	0.555	1.1525	0.6703	1.1885	0
587	1041.4	0.5553	1.1527	0.6705	1.1878	0
588	1041.3	0.5554	1.1529	0.6706	1.1882	0

589	1041.2	0.5554	1.1528	0.6707	1.1887	0
590	1041.1	0.5556	1.1528	0.6708	1.1886	0
591	1041	0.5554	1.1532	0.6709	1.1882	0
592	1040.9	0.5557	1.1529	0.6708	1.1884	0
593	1040.8	0.5557	1.1525	0.6708	1.1884	0
594	1040.7	0.5556	1.1532	0.6708	1.1882	0
595	1040.6	0.556	1.1536	0.6708	1.1888	0
596	1040.5	0.5562	1.1535	0.6707	1.1885	0
597	1040.4	0.5561	1.1532	0.6709	1.1887	0
598	1040.3	0.5557	1.1533	0.6706	1.1886	0
599	1040.2	0.5563	1.1538	0.6709	1.1885	0
600	1040.1	0.5567	1.1537	0.6711	1.1891	0
601	1040	0.5564	1.1535	0.6709	1.189	0
602	1039.9	0.5566	1.1541	0.6708	1.1887	0
603	1039.8	0.5564	1.1539	0.6706	1.1888	0
604	1039.7	0.5566	1.1541	0.6709	1.1892	0
605	1039.6	0.557	1.1537	0.6709	1.1891	0
606	1039.5	0.557	1.1542	0.6707	1.189	0
607	1039.4	0.5573	1.1547	0.6711	1.1894	0
608	1039.3	0.5572	1.1548	0.6708	1.1895	0
609	1039.2	0.5571	1.1547	0.6709	1.1894	0
610	1039.1	0.5575	1.1549	0.6709	1.1902	0
611	1039	0.5575	1.1549	0.6708	1.1899	0
612	1038.9	0.5578	1.1555	0.671	1.1895	0
613	1038.8	0.5578	1.1556	0.6709	1.1892	0
614	1038.7	0.5571	1.1556	0.6711	1.1898	0
615	1038.6	0.5578	1.1556	0.6713	1.1898	0
616	1038.5	0.5579	1.1556	0.6712	1.19	0
617	1038.4	0.5577	1.1558	0.671	1.1904	0
618	1038.3	0.5582	1.1559	0.671	1.1905	0
619	1038.2	0.558	1.1556	0.6711	1.1899	0
620	1038.1	0.5581	1.1559	0.6712	1.1904	0
621	1038	0.5586	1.1557	0.6714	1.1911	0
622	1037.9	0.5585	1.1556	0.6715	1.1904	0
623	1037.8	0.5586	1.156	0.6712	1.1906	0
624	1037.7	0.5584	1.1564	0.6714	1.1907	0
625	1037.6	0.5587	1.1566	0.6715	1.1905	0
626	1037.5	0.5588	1.1565	0.6717	1.1912	0
627	1037.4	0.5589	1.1566	0.672	1.1908	0
628	1037.3	0.559	1.1562	0.6718	1.1909	0
629	1037.2	0.5593	1.1561	0.6721	1.1913	0
630	1037.1	0.5593	1.1562	0.6722	1.191	0
631	1037	0.5589	1.1559	0.6719	1.1912	0

632	1036.9	0.559	1.1565	0.672	1.191	0
633	1036.8	0.559	1.1566	0.6724	1.1909	0
634	1036.7	0.5595	1.1566	0.6723	1.1912	0
635	1036.6	0.5591	1.1562	0.6725	1.1911	0
636	1036.5	0.5585	1.1559	0.6724	1.1912	0
637	1036.4	0.559	1.1559	0.6728	1.1911	0
638	1036.3	0.5593	1.1563	0.6726	1.1905	0
639	1036.2	0.5592	1.156	0.673	1.1915	0
640	1036.1	0.559	1.1561	0.6731	1.1912	0
641	1036	0.5591	1.1563	0.6731	1.1915	0
642	1035.9	0.5593	1.1561	0.6733	1.1912	0
643	1035.8	0.5595	1.1555	0.6735	1.191	0
644	1035.7	0.5593	1.1555	0.6737	1.1913	0
645	1035.6	0.5593	1.1562	0.6737	1.1912	0
646	1035.5	0.5591	1.1552	0.6738	1.191	0
647	1035.4	0.5596	1.1552	0.6739	1.1912	0
648	1035.3	0.5596	1.1551	0.6741	1.1915	0
649	1035.2	0.5597	1.1549	0.674	1.1915	0
650	1035.1	0.5593	1.1552	0.674	1.1914	0
651	1035	0.5598	1.1562	0.6746	1.1915	0
652	1034.9	0.5595	1.1552	0.6745	1.1915	0
653	1034.8	0.5596	1.1553	0.6747	1.1911	0
654	1034.7	0.5599	1.1556	0.6748	1.1916	0
655	1034.6	0.5594	1.1547	0.6749	1.1912	0
656	1034.5	0.5595	1.1547	0.6747	1.191	0
657	1034.4	0.56	1.1551	0.6753	1.1915	0
658	1034.3	0.5595	1.155	0.6752	1.191	0
659	1034.2	0.5598	1.1547	0.6753	1.191	0
660	1034.1	0.5596	1.1551	0.6755	1.1908	0
661	1034	0.5596	1.1543	0.6755	1.1916	0
662	1033.9	0.5594	1.1545	0.6757	1.1914	0
663	1033.8	0.5598	1.1552	0.6756	1.1915	0
664	1033.7	0.5597	1.1545	0.6758	1.1909	0
665	1033.6	0.5597	1.154	0.6759	1.1914	0
666	1033.5	0.56	1.1544	0.6761	1.1915	0
667	1033.4	0.5599	1.1542	0.6762	1.1914	0
668	1033.3	0.5599	1.1544	0.6763	1.1914	0
669	1033.2	0.5594	1.155	0.6763	1.1916	0
670	1033.1	0.5598	1.1552	0.6764	1.1918	0
671	1033	0.5599	1.1551	0.6765	1.1913	0
672	1032.9	0.5607	1.1552	0.6769	1.1917	0
673	1032.8	0.56	1.1545	0.6766	1.1915	0
674	1032.7	0.5603	1.1548	0.6767	1.1914	0

675	1032.6	0.5601	1.1548	0.6769	1.1916	0
676	1032.5	0.5605	1.155	0.677	1.1917	0
677	1032.4	0.5602	1.1546	0.6769	1.1916	0
678	1032.3	0.5603	1.1551	0.6772	1.1915	0
679	1032.2	0.5601	1.1551	0.6772	1.192	0
680	1032.1	0.5606	1.1553	0.6773	1.1919	0
681	1032	0.5602	1.1553	0.6771	1.1918	0
682	1031.9	0.5608	1.1551	0.6772	1.1917	0
683	1031.8	0.5604	1.155	0.6775	1.1922	0
684	1031.7	0.5607	1.1549	0.6774	1.1919	0
685	1031.6	0.5604	1.1549	0.6773	1.1921	0
686	1031.5	0.5605	1.1546	0.6773	1.192	0
687	1031.4	0.5606	1.155	0.6774	1.1921	0
688	1031.3	0.5605	1.1548	0.6775	1.1923	0
689	1031.2	0.5604	1.1547	0.6777	1.1925	0
690	1031.1	0.5604	1.1548	0.6776	1.1922	0
691	1031	0.5609	1.155	0.6776	1.1924	0
692	1030.9	0.5604	1.1551	0.6777	1.1924	0
693	1030.8	0.5609	1.1557	0.678	1.1925	0
694	1030.7	0.5608	1.1552	0.6778	1.1924	0
695	1030.6	0.5612	1.1561	0.678	1.1932	0
696	1030.5	0.5609	1.1558	0.6779	1.1929	0
697	1030.4	0.5612	1.1557	0.6779	1.193	0
698	1030.3	0.5608	1.1556	0.6776	1.193	0
699	1030.2	0.5612	1.1557	0.6777	1.1929	0
700	1030.1	0.5611	1.1563	0.6776	1.1929	0
701	1030	0.5611	1.1561	0.6777	1.1934	0
702	1029.9	0.5612	1.156	0.6777	1.1932	0
703	1029.8	0.5611	1.1565	0.6779	1.1934	0
704	1029.7	0.5616	1.1565	0.6777	1.1937	0
705	1029.6	0.5613	1.1566	0.6779	1.1937	0
706	1029.5	0.5616	1.1563	0.6778	1.1937	0
707	1029.4	0.5619	1.1567	0.6776	1.1937	0
708	1029.3	0.5616	1.1567	0.6778	1.1938	0
709	1029.2	0.5614	1.1566	0.6775	1.1938	0
710	1029.1	0.562	1.1573	0.6779	1.1936	0
711	1029	0.5616	1.1573	0.6778	1.1935	0
712	1028.9	0.562	1.1573	0.6779	1.1937	0
713	1028.8	0.5619	1.1573	0.6779	1.194	0
714	1028.7	0.5617	1.1574	0.6777	1.1944	0
715	1028.6	0.562	1.1578	0.6779	1.1946	0
716	1028.5	0.5619	1.1578	0.6777	1.1943	0
717	1028.4	0.5619	1.1576	0.6777	1.1946	0

718	1028.3	0.5619	1.1577	0.6779	1.1943	0
719	1028.2	0.5624	1.1578	0.6776	1.1943	0
720	1028.1	0.562	1.1578	0.6775	1.1943	0
721	1028	0.5616	1.1582	0.678	1.1946	0
722	1027.9	0.562	1.1578	0.678	1.1946	0
723	1027.8	0.5616	1.158	0.6779	1.1946	0
724	1027.7	0.5616	1.1584	0.6779	1.1947	0
725	1027.6	0.5617	1.1586	0.6777	1.1945	0
726	1027.5	0.5614	1.1581	0.6777	1.1943	0
727	1027.4	0.5617	1.1585	0.6778	1.1952	0
728	1027.3	0.5615	1.1589	0.678	1.1947	0
729	1027.2	0.5613	1.1585	0.6778	1.1945	0
730	1027.1	0.5616	1.1591	0.6781	1.195	0
731	1027	0.5617	1.1584	0.6781	1.1951	0
732	1026.9	0.5618	1.1585	0.6781	1.195	0
733	1026.8	0.5617	1.1588	0.6781	1.1951	0
734	1026.7	0.5616	1.1592	0.6781	1.1954	0
735	1026.6	0.5617	1.1594	0.6782	1.1954	0
736	1026.5	0.5615	1.1588	0.678	1.1956	0
737	1026.4	0.5613	1.1586	0.6782	1.1951	0
738	1026.3	0.5618	1.1588	0.678	1.1952	0
739	1026.2	0.5618	1.1593	0.6787	1.1959	0
740	1026.1	0.5618	1.1589	0.6786	1.1956	0
741	1026	0.5618	1.1592	0.6788	1.1956	0
742	1025.9	0.5615	1.1587	0.6788	1.1958	0
743	1025.8	0.5606	1.1589	0.6792	1.1956	0
744	1025.7	0.5613	1.159	0.679	1.1956	0
745	1025.6	0.5611	1.1586	0.6789	1.1954	0
746	1025.5	0.5611	1.1588	0.6789	1.1953	0
747	1025.4	0.5611	1.1585	0.6795	1.1956	0
748	1025.3	0.5613	1.1584	0.6793	1.1954	0
749	1025.2	0.5614	1.1583	0.6796	1.1958	0
750	1025.1	0.5614	1.158	0.6796	1.1956	0
751	1025	0.5614	1.158	0.6797	1.1957	0
752	1024.9	0.561	1.1581	0.6797	1.1953	0
753	1024.8	0.5609	1.1577	0.6799	1.1955	0
754	1024.7	0.5613	1.1581	0.6803	1.1956	0
755	1024.6	0.5609	1.1576	0.6805	1.1956	0
756	1024.5	0.5612	1.1575	0.6807	1.1958	0
757	1024.4	0.5613	1.1575	0.6806	1.1963	0
758	1024.3	0.5611	1.1573	0.6808	1.1959	0
759	1024.2	0.5611	1.1576	0.6811	1.1963	0
760	1024.1	0.5607	1.157	0.6813	1.1958	0

761	1024	0.561	1.1572	0.6814	1.1959	0
762	1023.9	0.5607	1.1571	0.6817	1.1962	0
763	1023.8	0.561	1.1567	0.6815	1.1961	0
764	1023.7	0.5608	1.1568	0.6819	1.1961	0
765	1023.6	0.5609	1.1569	0.6821	1.196	0
766	1023.5	0.5611	1.1571	0.6823	1.1961	0
767	1023.4	0.5605	1.1573	0.6823	1.1958	0
768	1023.3	0.5607	1.157	0.6823	1.196	0
769	1023.2	0.5607	1.1566	0.6823	1.1959	0
770	1023.1	0.5604	1.1566	0.6824	1.1958	0
771	1023	0.5607	1.1566	0.6828	1.196	0
772	1022.9	0.5606	1.1563	0.6827	1.1958	0
773	1022.8	0.5604	1.1562	0.6826	1.1958	0
774	1022.7	0.5605	1.1563	0.683	1.1958	0
775	1022.6	0.5611	1.1563	0.6831	1.1958	0
776	1022.5	0.5606	1.1565	0.6829	1.1951	0
777	1022.4	0.5607	1.1572	0.6832	1.1958	0
778	1022.3	0.5606	1.1566	0.6831	1.1958	0
779	1022.2	0.5605	1.1565	0.6833	1.1959	0
780	1022.1	0.5607	1.1563	0.6833	1.1959	0
781	1022	0.5608	1.1561	0.6833	1.1957	0
782	1021.9	0.5609	1.1562	0.6835	1.1963	0
783	1021.8	0.561	1.1562	0.6838	1.1962	0
784	1021.7	0.5608	1.1564	0.6838	1.1962	0
785	1021.6	0.5609	1.1566	0.6837	1.1959	0
786	1021.5	0.5604	1.1562	0.6838	1.1959	0
787	1021.4	0.5604	1.1566	0.6837	1.1961	0
788	1021.3	0.5609	1.1566	0.6838	1.1965	0
789	1021.2	0.5605	1.1561	0.6836	1.196	0
790	1021.1	0.561	1.1564	0.6838	1.1963	0
791	1021	0.5608	1.1565	0.6839	1.1967	0
792	1020.9	0.561	1.157	0.6839	1.1966	0
793	1020.8	0.5606	1.1562	0.6838	1.1968	0
794	1020.7	0.5606	1.1568	0.684	1.1964	0
795	1020.6	0.5607	1.1569	0.6839	1.1969	0
796	1020.5	0.5606	1.1565	0.6839	1.1966	0
797	1020.4	0.5609	1.1562	0.6839	1.1967	0
798	1020.3	0.5604	1.1569	0.684	1.1966	0
799	1020.2	0.5608	1.1569	0.6842	1.1968	0
800	1020.1	0.5611	1.1571	0.6842	1.1971	0
801	1020	0.5611	1.1575	0.6842	1.1972	0
802	1019.9	0.5613	1.1572	0.6841	1.1973	0
803	1019.8	0.5615	1.1574	0.6844	1.1975	0

804	1019.7	0.5612	1.1572	0.6844	1.1968	0
805	1019.6	0.5617	1.1574	0.6841	1.1972	0
806	1019.5	0.5611	1.1575	0.684	1.1973	0
807	1019.4	0.5615	1.1575	0.6843	1.1978	0
808	1019.3	0.5612	1.1578	0.6841	1.1976	0
809	1019.2	0.5615	1.1572	0.6842	1.1978	0
810	1019.1	0.5612	1.1581	0.6842	1.1979	0
811	1019	0.5616	1.1583	0.6843	1.1983	0
812	1018.9	0.5612	1.1575	0.6839	1.1975	0
813	1018.8	0.5614	1.1575	0.6839	1.1977	0
814	1018.7	0.5615	1.1579	0.684	1.1979	0
815	1018.6	0.5614	1.1581	0.6838	1.1984	0
816	1018.5	0.5618	1.1585	0.6839	1.1982	0
817	1018.4	0.5618	1.1584	0.684	1.1981	0
818	1018.3	0.5619	1.1587	0.684	1.1984	0
819	1018.2	0.5622	1.1593	0.6842	1.1985	0
820	1018.1	0.562	1.1582	0.6838	1.1986	0
821	1018	0.5618	1.1587	0.6838	1.1992	0
822	1017.9	0.5622	1.1584	0.6839	1.1989	0
823	1017.8	0.5624	1.1587	0.684	1.1997	0
824	1017.7	0.5622	1.1592	0.6841	1.1999	0
825	1017.6	0.5622	1.1597	0.6842	1.1999	0
826	1017.5	0.5623	1.1596	0.684	1.1995	0
827	1017.4	0.5621	1.1595	0.684	1.1997	0
828	1017.3	0.5622	1.1591	0.6839	1.1993	0
829	1017.2	0.5623	1.1592	0.6841	1.1996	0
830	1017.1	0.5622	1.1596	0.6839	1.1997	0
831	1017	0.5623	1.1592	0.6842	1.2003	0
832	1016.9	0.5624	1.1596	0.6839	1.2008	0
833	1016.8	0.5626	1.16	0.6841	1.2014	0
834	1016.7	0.5624	1.1594	0.6841	1.2038	0
835	1016.6	0.5622	1.1592	0.6839	1.2047	0
836	1016.5	0.5624	1.1591	0.684	1.2045	0
837	1016.4	0.5624	1.1594	0.6841	1.2054	0
838	1016.3	0.5623	1.1589	0.6839	1.2051	0
839	1016.2	0.5621	1.1593	0.6843	1.2055	0
840	1016.1	0.5619	1.1595	0.6844	1.2062	0
841	1016	0.5625	1.1595	0.6844	1.206	0
842	1015.9	0.5625	1.1592	0.6846	1.2059	0
843	1015.8	0.5617	1.1597	0.6843	1.2061	0
844	1015.7	0.5623	1.1591	0.6842	1.2058	0
845	1015.6	0.5624	1.159	0.6846	1.2053	0
846	1015.5	0.5624	1.1596	0.6847	1.2056	0

847	1015.4	0.5624	1.1591	0.6848	1.2056	0
848	1015.3	0.5622	1.1588	0.6847	1.2058	0
849	1015.2	0.5621	1.1591	0.6848	1.2061	0
850	1015.1	0.5624	1.1592	0.6849	1.2059	0
851	1015	0.5622	1.1585	0.6849	1.2062	0
852	1014.9	0.5623	1.1591	0.6852	1.2055	0
853	1014.8	0.5624	1.1591	0.685	1.2062	0
854	1014.7	0.562	1.1594	0.6853	1.206	0
855	1014.6	0.5622	1.1593	0.6855	1.2061	0
856	1014.5	0.5622	1.1585	0.6856	1.206	0
857	1014.4	0.5623	1.1593	0.6859	1.206	0
858	1014.3	0.5624	1.159	0.686	1.206	0
859	1014.2	0.5625	1.1587	0.6862	1.2061	0
860	1014.1	0.5622	1.159	0.6862	1.2064	0
861	1014	0.5623	1.1587	0.6861	1.2063	0
862	1013.9	0.5625	1.1584	0.6865	1.207	0
863	1013.8	0.5622	1.158	0.6864	1.2065	0
864	1013.7	0.5623	1.1584	0.6866	1.2072	0
865	1013.6	0.5623	1.1584	0.6867	1.2075	0
866	1013.5	0.562	1.1579	0.6868	1.2085	0
867	1013.4	0.5621	1.1578	0.6871	1.2098	0
868	1013.3	0.5622	1.1578	0.6871	1.2106	0
869	1013.2	0.5618	1.1577	0.6871	1.2113	0
870	1013.1	0.5618	1.1573	0.6871	1.2138	0
871	1013	0.5621	1.157	0.6871	1.2146	0
872	1012.9	0.5622	1.1576	0.6874	1.2146	0
873	1012.8	0.562	1.157	0.6874	1.2148	0
874	1012.7	0.5621	1.1567	0.6875	1.216	0
875	1012.6	0.5623	1.1569	0.6878	1.2206	0
876	1012.5	0.5623	1.1573	0.6881	1.2214	0
877	1012.4	0.5624	1.1571	0.6882	1.2217	0
878	1012.3	0.5623	1.1572	0.6881	1.222	0
879	1012.2	0.5621	1.1571	0.6883	1.2226	0
880	1012.1	0.5621	1.1566	0.6882	1.2223	0
881	1012	0.562	1.1574	0.6885	1.2246	0
882	1011.9	0.5618	1.1568	0.6886	1.2264	0
883	1011.8	0.5621	1.1572	0.6887	1.2311	0
884	1011.7	0.5622	1.1573	0.6887	1.2316	0
885	1011.6	0.562	1.157	0.6884	1.2317	0
886	1011.5	0.5618	1.1568	0.6889	1.2323	0
887	1011.4	0.5624	1.1565	0.6888	1.2322	0
888	1011.3	0.562	1.1567	0.6886	1.2326	0
889	1011.2	0.5621	1.1564	0.6888	1.2332	0

890	1011.1	0.562	1.1562	0.6892	1.2334	0
891	1011	0.5621	1.1569	0.689	1.2342	0
892	1010.9	0.5616	1.1568	0.689	1.2355	0
893	1010.8	0.5617	1.157	0.6891	1.2356	0
894	1010.7	0.5622	1.1571	0.6893	1.2354	0
895	1010.6	0.562	1.1567	0.6893	1.2358	0
896	1010.5	0.5619	1.1569	0.6892	1.2354	0
897	1010.4	0.5622	1.1571	0.6893	1.2359	0
898	1010.3	0.5621	1.157	0.6891	1.2357	0
899	1010.2	0.5621	1.1567	0.6891	1.2359	0
900	1010.1	0.5621	1.1569	0.6891	1.2361	0
901	1010	0.5623	1.1571	0.6892	1.2366	0
902	1009.9	0.5623	1.1575	0.6894	1.2374	0
903	1009.8	0.5621	1.157	0.6892	1.2379	0
904	1009.7	0.5624	1.1567	0.6895	1.2392	0
905	1009.6	0.5625	1.1567	0.6892	1.243	0
906	1009.5	0.5626	1.1578	0.6895	1.2534	0
907	1009.4	0.5624	1.157	0.6895	1.2979	0
908	1009.3	0.5629	1.1572	0.6895	1.3013	0
909	1009.2	0.5628	1.1571	0.6891	1.3022	0
910	1009.1	0.5629	1.1575	0.6894	1.3044	0
911	1009	0.5632	1.1575	0.6895	1.3093	0
912	1008.9	0.563	1.1581	0.6893	1.3101	0
913	1008.8	0.5631	1.1572	0.6894	1.3105	0
914	1008.7	0.5632	1.1581	0.6893	1.311	0
915	1008.6	0.5631	1.1583	0.6894	1.3119	0
916	1008.5	0.5631	1.1582	0.6893	1.3117	0
917	1008.4	0.5633	1.1586	0.6892	1.3122	0
918	1008.3	0.5633	1.1582	0.6893	1.312	0
919	1008.2	0.5635	1.1589	0.6893	1.3125	0
920	1008.1	0.5632	1.1581	0.6897	1.3132	0
921	1008	0.5634	1.159	0.6894	1.3132	0
922	1007.9	0.5637	1.1593	0.6896	1.3135	0
923	1007.8	0.5638	1.1586	0.6896	1.3134	0
924	1007.7	0.564	1.1582	0.6896	1.3145	0
925	1007.6	0.5639	1.1586	0.6893	1.3141	0
926	1007.5	0.5635	1.1583	0.6891	1.3144	0
927	1007.4	0.5635	1.1589	0.6893	1.3147	0
928	1007.3	0.5634	1.1585	0.6892	1.3144	0
929	1007.2	0.5635	1.1583	0.6892	1.3148	0
930	1007.1	0.5639	1.159	0.6892	1.3147	0
931	1007	0.564	1.1593	0.6891	1.3151	0
932	1006.9	0.5635	1.1591	0.6893	1.3157	0

933	1006.8	0.5636	1.1589	0.6893	1.3158	0
934	1006.7	0.5639	1.159	0.6894	1.3155	0
935	1006.6	0.5637	1.1595	0.6898	1.3157	0
936	1006.5	0.5639	1.1592	0.6893	1.3153	0
937	1006.4	0.564	1.1587	0.6894	1.3158	0
938	1006.3	0.5634	1.159	0.6893	1.3162	0
939	1006.2	0.5634	1.1587	0.6894	1.3161	0
940	1006.1	0.5636	1.1593	0.6897	1.3152	0
941	1006	0.564	1.1595	0.6892	1.3152	0
942	1005.9	0.564	1.1588	0.6896	1.3157	0
943	1005.8	0.5637	1.1589	0.6892	1.3155	0
944	1005.7	0.5637	1.1593	0.6894	1.3157	0
945	1005.6	0.564	1.1591	0.6896	1.3156	0
946	1005.5	0.5641	1.159	0.6895	1.3165	0
947	1005.4	0.5642	1.1588	0.6895	1.3164	0
948	1005.3	0.5642	1.1591	0.6897	1.3163	0
949	1005.2	0.5644	1.1589	0.6898	1.317	0
950	1005.1	0.5641	1.1592	0.6898	1.3168	0
951	1005	0.5642	1.159	0.6899	1.3171	0
952	1004.9	0.5637	1.1591	0.6903	1.3167	0
953	1004.8	0.5638	1.1589	0.6901	1.3173	0
954	1004.7	0.564	1.1591	0.6905	1.3169	0
955	1004.6	0.5644	1.1588	0.6903	1.3163	0
956	1004.5	0.564	1.1586	0.6905	1.3165	0
957	1004.4	0.5644	1.1586	0.6906	1.3155	0
958	1004.3	0.5642	1.1588	0.6906	1.3163	0
959	1004.2	0.5644	1.1589	0.6908	1.3167	0
960	1004.1	0.5642	1.1582	0.6908	1.3153	0
961	1004	0.5644	1.1582	0.691	1.3156	0
962	1003.9	0.5645	1.1585	0.6911	1.3155	0
963	1003.8	0.5642	1.1582	0.6909	1.3157	0
964	1003.7	0.5643	1.1584	0.6912	1.3159	0
965	1003.6	0.5641	1.1582	0.6911	1.3159	0
966	1003.5	0.5642	1.1583	0.6913	1.3163	0
967	1003.4	0.564	1.1585	0.6913	1.3158	0
968	1003.3	0.564	1.1583	0.6916	1.3161	0
969	1003.2	0.5643	1.1584	0.6917	1.3157	0
970	1003.1	0.5643	1.1584	0.6916	1.3154	0
971	1003	0.5642	1.1588	0.6918	1.3155	0
972	1002.9	0.5641	1.1582	0.6916	1.3157	0
973	1002.8	0.5642	1.1582	0.692	1.3154	0
974	1002.7	0.5644	1.1578	0.6919	1.3155	0
975	1002.6	0.5641	1.1581	0.6919	1.3153	0

976	1002.5	0.5639	1.158	0.692	1.3161	0
977	1002.4	0.5642	1.1583	0.6922	1.3151	0
978	1002.3	0.5645	1.1584	0.6923	1.3156	0
979	1002.2	0.5642	1.1577	0.6923	1.3163	0
980	1002.1	0.5643	1.1581	0.6923	1.3161	0
981	1002	0.5643	1.1582	0.6924	1.3156	0
982	1001.9	0.5642	1.1577	0.6924	1.3155	0
983	1001.8	0.5643	1.1577	0.6928	1.3156	0
984	1001.7	0.5643	1.1575	0.6928	1.316	0
985	1001.6	0.5647	1.157	0.6927	1.3165	0
986	1001.5	0.5647	1.1579	0.6928	1.3163	0
987	1001.4	0.5647	1.1578	0.6928	1.3158	0
988	1001.3	0.5642	1.1573	0.6927	1.316	0
989	1001.2	0.5647	1.1578	0.693	1.3159	0
990	1001.1	0.5648	1.1578	0.6931	1.3165	0
991	1001	0.5648	1.1581	0.6932	1.3163	0
992	1000.9	0.5647	1.1582	0.6931	1.3153	0
993	1000.8	0.5642	1.1578	0.6931	1.3148	0
994	1000.7	0.5647	1.1576	0.6932	1.316	0
995	1000.6	0.5642	1.1578	0.693	1.3163	0
996	1000.5	0.5646	1.1576	0.6929	1.3164	0
997	1000.4	0.5639	1.1571	0.693	1.3159	0
998	1000.3	0.5643	1.1574	0.6932	1.3159	0
999	1000.2	0.5645	1.1579	0.6933	1.3155	0
1000	1000.1	0.5649	1.1581	0.6934	1.316	0
1001	1000	0.5647	1.1579	0.6935	1.3154	0
1002	999.9	0.5646	1.1585	0.6935	1.3159	0
1003	999.8	0.5649	1.1576	0.6937	1.3154	0
1004	999.7	0.5642	1.1577	0.6937	1.3155	0
1005	999.6	0.5646	1.1578	0.6937	1.3163	0
1006	999.5	0.5644	1.1584	0.6935	1.316	0
1007	999.4	0.5647	1.1584	0.6936	1.316	0
1008	999.3	0.5645	1.1582	0.6937	1.3154	0
1009	999.2	0.5646	1.1575	0.6936	1.3153	0
1010	999.1	0.5646	1.1577	0.6939	1.3164	0
1011	999	0.5647	1.1583	0.6938	1.3164	0
1012	998.9	0.5645	1.1579	0.6937	1.3164	0
1013	998.8	0.5646	1.1581	0.6937	1.3166	0
1014	998.7	0.5648	1.1588	0.6936	1.3163	0
1015	998.6	0.5648	1.158	0.6938	1.3154	0
1016	998.5	0.5652	1.1585	0.6939	1.316	0
1017	998.4	0.5649	1.1579	0.6938	1.3167	0
1018	998.3	0.5652	1.158	0.6936	1.3166	0

1019	998.2	0.5649	1.1579	0.6936	1.3168	0
1020	998.1	0.5652	1.158	0.6938	1.3172	0
1021	998	0.5649	1.1582	0.6937	1.3174	0
1022	997.9	0.5651	1.1586	0.6939	1.3162	0
1023	997.8	0.5649	1.1581	0.6936	1.3175	0
1024	997.7	0.5652	1.1582	0.6938	1.3188	0
1025	997.6	0.565	1.1582	0.6938	1.3175	0
1026	997.5	0.5651	1.158	0.6938	1.3179	0
1027	997.4	0.5652	1.1583	0.6936	1.3179	0
1028	997.3	0.565	1.1585	0.6936	1.3178	0
1029	997.2	0.565	1.1583	0.6936	1.3178	0
1030	997.1	0.5652	1.1585	0.6938	1.318	0
1031	997	0.5654	1.1587	0.6937	1.3183	0
1032	996.9	0.5652	1.1581	0.6937	1.3178	0
1033	996.8	0.5653	1.1585	0.694	1.318	0
1034	996.7	0.5652	1.1586	0.694	1.3183	0
1035	996.6	0.5654	1.158	0.6939	1.3187	0
1036	996.5	0.5656	1.1586	0.694	1.3178	0
1037	996.4	0.5656	1.1591	0.6939	1.3182	0
1038	996.3	0.5653	1.1588	0.694	1.3181	0
1039	996.2	0.5658	1.1589	0.6939	1.3185	0
1040	996.1	0.5657	1.1588	0.694	1.3186	0
1041	996	0.5658	1.1591	0.694	1.3183	0
1042	995.9	0.5657	1.1587	0.6941	1.3184	0
1043	995.8	0.5658	1.159	0.6944	1.3187	0
1044	995.7	0.5656	1.159	0.6941	1.3197	0
1045	995.6	0.5656	1.1588	0.6942	1.3194	0
1046	995.5	0.5655	1.1589	0.694	1.3198	0
1047	995.4	0.5657	1.1585	0.6944	1.3198	0
1048	995.3	0.5656	1.1586	0.6943	1.3202	0
1049	995.2	0.5658	1.1585	0.6946	1.3214	0
1050	995.1	0.5658	1.1589	0.6945	1.3222	0
1051	995	0.5658	1.1591	0.6946	1.3223	0
1052	994.9	0.5655	1.1589	0.6947	1.323	0
1053	994.8	0.5658	1.1586	0.6948	1.3245	0
1054	994.7	0.5656	1.159	0.6947	1.324	0
1055	994.6	0.566	1.1593	0.6948	1.3242	0
1056	994.5	0.5659	1.1581	0.6945	1.3244	0
1057	994.4	0.566	1.1585	0.6948	1.3241	0
1058	994.3	0.5657	1.1583	0.6949	1.3245	0
1059	994.2	0.5659	1.1589	0.6951	1.3248	0
1060	994.1	0.5655	1.1585	0.6949	1.324	0
1061	994	0.5659	1.1583	0.695	1.3239	0

1062	993.9	0.5656	1.1578	0.6949	1.3239	0
1063	993.8	0.5657	1.1579	0.6951	1.3237	0
1064	993.7	0.5657	1.1584	0.6951	1.3236	0
1065	993.6	0.5661	1.1583	0.6954	1.3238	0
1066	993.5	0.5656	1.1578	0.6954	1.3243	0
1067	993.4	0.5657	1.1583	0.6957	1.3232	0
1068	993.3	0.5657	1.1585	0.6958	1.3234	0
1069	993.2	0.5659	1.1583	0.696	1.3239	0
1070	993.1	0.5658	1.1583	0.6958	1.3229	0
1071	993	0.5657	1.1584	0.6959	1.3234	0
1072	992.9	0.5661	1.1583	0.696	1.3232	0
1073	992.8	0.5655	1.1583	0.696	1.3234	0
1074	992.7	0.566	1.1581	0.6962	1.3231	0
1075	992.6	0.5659	1.1583	0.6962	1.3234	0
1076	992.5	0.566	1.1584	0.6963	1.3231	0
1077	992.4	0.5659	1.1583	0.6963	1.3235	0
1078	992.3	0.5658	1.1579	0.6962	1.3226	0
1079	992.2	0.5662	1.1579	0.6966	1.323	0
1080	992.1	0.5663	1.1579	0.6966	1.3231	0
1081	992	0.5657	1.1584	0.6963	1.3222	0
1082	991.9	0.5654	1.1578	0.6965	1.322	0
1083	991.8	0.5657	1.158	0.6968	1.3218	0
1084	991.7	0.5657	1.1583	0.6965	1.3226	0
1085	991.6	0.566	1.1584	0.6968	1.3229	0
1086	991.5	0.5658	1.1578	0.6968	1.3231	0
1087	991.4	0.5659	1.1587	0.6968	1.3229	0
1088	991.3	0.5659	1.1591	0.6966	1.3226	0
1089	991.2	0.5656	1.1587	0.697	1.3229	0
1090	991.1	0.566	1.158	0.6966	1.3222	0
1091	991	0.5659	1.1583	0.6967	1.3226	0
1092	990.9	0.566	1.1584	0.6968	1.3224	0
1093	990.8	0.5663	1.1586	0.6969	1.3229	0
1094	990.7	0.5664	1.1581	0.6968	1.3228	0
1095	990.6	0.5663	1.1587	0.697	1.3227	0
1096	990.5	0.5666	1.1584	0.6968	1.3231	0
1097	990.4	0.5665	1.1589	0.6968	1.3229	0
1098	990.3	0.5664	1.1583	0.6968	1.3227	0
1099	990.2	0.5665	1.1587	0.6968	1.3231	0
1100	990.1	0.5667	1.1587	0.6971	1.3232	0
1101	990	0.5662	1.1588	0.6969	1.3239	0
1102	989.9	0.5665	1.1584	0.6968	1.3233	0
1103	989.8	0.5663	1.1582	0.6971	1.3236	0
1104	989.7	0.5668	1.1586	0.697	1.3232	0

1105	989.6	0.5667	1.1586	0.6969	1.3232	0
1106	989.5	0.5666	1.1586	0.6971	1.3237	0
1107	989.4	0.5666	1.1586	0.6969	1.3237	0
1108	989.3	0.5668	1.1594	0.697	1.3235	0
1109	989.2	0.567	1.1588	0.6971	1.3229	0
1110	989.1	0.5669	1.1594	0.697	1.3234	0
1111	989	0.5673	1.1589	0.6971	1.3231	0
1112	988.9	0.5665	1.159	0.6972	1.3228	0
1113	988.8	0.5667	1.1592	0.6974	1.3233	0
1114	988.7	0.5668	1.1592	0.6971	1.3234	0
1115	988.6	0.5665	1.159	0.6971	1.3234	0
1116	988.5	0.567	1.1593	0.6973	1.3239	0
1117	988.4	0.5669	1.1589	0.697	1.3228	0
1118	988.3	0.5668	1.159	0.6973	1.3235	0
1119	988.2	0.5668	1.159	0.6974	1.3232	0
1120	988.1	0.567	1.1589	0.6974	1.3236	0
1121	988	0.5668	1.1592	0.6975	1.324	0
1122	987.9	0.5667	1.159	0.6972	1.3237	0
1123	987.8	0.5666	1.1591	0.6973	1.3228	0
1124	987.7	0.5668	1.1587	0.6973	1.3226	0
1125	987.6	0.5669	1.1589	0.6977	1.3236	0
1126	987.5	0.5668	1.159	0.6975	1.3231	0
1127	987.4	0.5669	1.1591	0.6975	1.3219	0
1128	987.3	0.567	1.1588	0.6978	1.3237	0
1129	987.2	0.567	1.1585	0.6975	1.323	0
1130	987.1	0.5671	1.1591	0.6977	1.3221	0
1131	987	0.5669	1.1587	0.6978	1.3219	0
1132	986.9	0.5672	1.159	0.698	1.3228	0
1133	986.8	0.5671	1.1589	0.6982	1.3229	0
1134	986.7	0.5669	1.1582	0.6981	1.3223	0
1135	986.6	0.567	1.1585	0.6983	1.3226	0
1136	986.5	0.5669	1.1584	0.6981	1.3222	0
1137	986.4	0.567	1.1584	0.6982	1.322	0
1138	986.3	0.5672	1.1586	0.6981	1.3211	0
1139	986.2	0.567	1.1592	0.6982	1.3216	0
1140	986.1	0.5672	1.1589	0.6983	1.3223	0
1141	986	0.5674	1.1589	0.6986	1.3219	0
1142	985.9	0.5672	1.1589	0.6985	1.3226	0
1143	985.8	0.5669	1.1585	0.6983	1.3216	0
1144	985.7	0.5666	1.159	0.6986	1.322	0
1145	985.6	0.5667	1.1591	0.6985	1.3218	0
1146	985.5	0.5666	1.1588	0.6987	1.3216	0
1147	985.4	0.5668	1.1584	0.6986	1.3214	0

1148	985.3	0.567	1.1585	0.699	1.3217	0
1149	985.2	0.5672	1.1589	0.699	1.3212	0
1150	985.1	0.567	1.1586	0.6992	1.3213	0
1151	985	0.5668	1.1582	0.6992	1.322	0
1152	984.9	0.5671	1.1585	0.6991	1.3215	0
1153	984.8	0.5667	1.1585	0.6993	1.3213	0
1154	984.7	0.5671	1.1583	0.6994	1.3207	0
1155	984.6	0.5671	1.1582	0.6994	1.3212	0
1156	984.5	0.567	1.1578	0.6995	1.3212	0
1157	984.4	0.5666	1.1577	0.6996	1.3214	0
1158	984.3	0.5668	1.1584	0.6998	1.3216	0
1159	984.2	0.5672	1.1588	0.6999	1.3209	0
1160	984.1	0.5672	1.158	0.7003	1.322	0
1161	984	0.567	1.1585	0.7001	1.3206	0
1162	983.9	0.5672	1.158	0.7001	1.3211	0
1163	983.8	0.5674	1.1584	0.7002	1.3205	0
1164	983.7	0.5672	1.1579	0.7005	1.3208	0
1165	983.6	0.567	1.158	0.7003	1.3207	0
1166	983.5	0.5668	1.1574	0.7001	1.3199	0
1167	983.4	0.5672	1.158	0.7005	1.3203	0
1168	983.3	0.5674	1.1579	0.7007	1.3207	0
1169	983.2	0.5675	1.1585	0.7008	1.3206	0
1170	983.1	0.5675	1.1579	0.7007	1.3199	0
1171	983	0.5673	1.1579	0.7006	1.3203	0
1172	982.9	0.5676	1.1581	0.7007	1.3198	0
1173	982.8	0.5677	1.1581	0.7009	1.3199	0
1174	982.7	0.5675	1.1579	0.7008	1.3202	0
1175	982.6	0.567	1.1574	0.7008	1.3202	0
1176	982.5	0.5674	1.1578	0.7009	1.3203	0
1177	982.4	0.5675	1.158	0.7008	1.3203	0
1178	982.3	0.5678	1.1579	0.7008	1.3204	0
1179	982.2	0.5675	1.158	0.7008	1.3192	0
1180	982.1	0.5675	1.1583	0.7008	1.3203	0
1181	982	0.5677	1.1582	0.701	1.3198	0
1182	981.9	0.5676	1.1582	0.7009	1.3207	0
1183	981.8	0.5676	1.1579	0.701	1.3206	0
1184	981.7	0.5679	1.158	0.7009	1.3204	0
1185	981.6	0.5676	1.158	0.7009	1.3206	0
1186	981.5	0.5677	1.1583	0.7009	1.3207	0
1187	981.4	0.5677	1.1584	0.7009	1.3203	0
1188	981.3	0.5678	1.1586	0.7011	1.3202	0
1189	981.2	0.5678	1.1584	0.701	1.3199	0
1190	981.1	0.5681	1.159	0.701	1.3209	0

1191	981	0.5679	1.1591	0.701	1.3202	0
1192	980.9	0.5681	1.159	0.7011	1.3213	0
1193	980.8	0.5681	1.159	0.701	1.3206	0
1194	980.7	0.5679	1.1589	0.701	1.3206	0
1195	980.6	0.568	1.1593	0.7009	1.321	0
1196	980.5	0.5682	1.1594	0.7009	1.3211	0
1197	980.4	0.5683	1.1591	0.701	1.3212	0
1198	980.3	0.5677	1.1595	0.7007	1.3213	0
1199	980.2	0.5682	1.159	0.701	1.3215	0
1200	980.1	0.5684	1.159	0.7006	1.3216	0
1201	980	0.5683	1.1594	0.7007	1.3214	0
1202	979.9	0.5682	1.1601	0.7008	1.3209	0
1203	979.8	0.5686	1.1599	0.701	1.3224	0
1204	979.7	0.5687	1.1594	0.7009	1.3223	0
1205	979.6	0.5686	1.1593	0.7007	1.3219	0
1206	979.5	0.5687	1.1595	0.7005	1.3222	0
1207	979.4	0.5687	1.16	0.7007	1.3218	0
1208	979.3	0.5686	1.1602	0.7006	1.3228	0
1209	979.2	0.5687	1.1605	0.7005	1.3219	0
1210	979.1	0.5688	1.16	0.7005	1.3226	0
1211	979	0.5692	1.1605	0.7004	1.3224	0
1212	978.9	0.5688	1.1603	0.7002	1.3225	0
1213	978.8	0.5693	1.1606	0.7003	1.3231	0
1214	978.7	0.5691	1.1604	0.7001	1.3239	0
1215	978.6	0.5689	1.1606	0.7002	1.3235	0
1216	978.5	0.5691	1.1605	0.7001	1.3233	0
1217	978.4	0.5693	1.1609	0.7	1.3239	0
1218	978.3	0.5693	1.1607	0.7	1.3239	0
1219	978.2	0.5694	1.1612	0.6998	1.3236	0
1220	978.1	0.5696	1.1613	0.7002	1.3246	0
1221	978	0.5694	1.1613	0.7003	1.3243	0
1222	977.9	0.5694	1.1611	0.7002	1.324	0
1223	977.8	0.5695	1.1612	0.7001	1.3247	0
1224	977.7	0.5698	1.1613	0.7	1.3243	0
1225	977.6	0.5694	1.1615	0.7001	1.3239	0
1226	977.5	0.5693	1.1612	0.7	1.3239	0
1227	977.4	0.5697	1.1616	0.7001	1.3244	0
1228	977.3	0.5694	1.1613	0.7002	1.3248	0
1229	977.2	0.5689	1.1613	0.7002	1.3245	0
1230	977.1	0.5698	1.161	0.7003	1.324	0
1231	977	0.5698	1.1614	0.7003	1.3237	0
1232	976.9	0.5693	1.1612	0.7004	1.3243	0
1233	976.8	0.57	1.161	0.7007	1.3243	0

1234	976.7	0.5698	1.1611	0.7007	1.3237	0
1235	976.6	0.5698	1.1614	0.7008	1.3237	0
1236	976.5	0.5693	1.1613	0.7007	1.3236	0
1237	976.4	0.5694	1.1611	0.7011	1.3228	0
1238	976.3	0.5694	1.1607	0.7009	1.3235	0
1239	976.2	0.5696	1.1609	0.7013	1.3231	0
1240	976.1	0.5694	1.1607	0.7011	1.3231	0
1241	976	0.5695	1.1613	0.7013	1.3233	0
1242	975.9	0.5693	1.1614	0.7016	1.3221	0
1243	975.8	0.5695	1.1612	0.7015	1.3227	0
1244	975.7	0.5692	1.1607	0.7016	1.3219	0
1245	975.6	0.5692	1.1609	0.7017	1.3224	0
1246	975.5	0.5694	1.1611	0.702	1.322	0
1247	975.4	0.5693	1.1605	0.7019	1.3217	0
1248	975.3	0.5694	1.1606	0.7021	1.3222	0
1249	975.2	0.5691	1.1607	0.7021	1.3216	0
1250	975.1	0.5692	1.1604	0.702	1.3216	0
1251	975	0.5696	1.1598	0.7023	1.3216	0
1252	974.9	0.5695	1.16	0.7026	1.3214	0
1253	974.8	0.569	1.1604	0.7026	1.3205	0
1254	974.7	0.5692	1.1597	0.7027	1.3203	0
1255	974.6	0.5687	1.1594	0.7028	1.3207	0
1256	974.5	0.569	1.1596	0.7026	1.3198	0
1257	974.4	0.5697	1.1598	0.7031	1.3198	0
1258	974.3	0.5693	1.1594	0.7033	1.3196	0
1259	974.2	0.5692	1.1602	0.7032	1.3194	0
1260	974.1	0.5693	1.1601	0.7035	1.3195	0
1261	974	0.5693	1.1597	0.7035	1.3189	0
1262	973.9	0.5692	1.1596	0.7038	1.3194	0
1263	973.8	0.5693	1.1596	0.7038	1.3192	0
1264	973.7	0.569	1.1593	0.704	1.3197	0
1265	973.6	0.5688	1.1591	0.704	1.3188	0
1266	973.5	0.5689	1.1594	0.7041	1.3173	0
1267	973.4	0.5692	1.1591	0.7042	1.318	0
1268	973.3	0.569	1.1587	0.7043	1.3191	0
1269	973.2	0.5692	1.1584	0.7044	1.3186	0
1270	973.1	0.5688	1.1587	0.7047	1.3184	0
1271	973	0.5688	1.1584	0.7046	1.3178	0
1272	972.9	0.569	1.1589	0.7046	1.3185	0
1273	972.8	0.569	1.1586	0.7045	1.3178	0
1274	972.7	0.5692	1.1586	0.7045	1.3185	0
1275	972.6	0.5691	1.1582	0.7047	1.3174	0
1276	972.5	0.5691	1.1584	0.7047	1.3174	0

1277	972.4	0.5693	1.1585	0.7047	1.3178	0
1278	972.3	0.5692	1.1584	0.7045	1.3178	0
1279	972.2	0.5693	1.1586	0.7048	1.3179	0
1280	972.1	0.5692	1.1581	0.7049	1.3173	0
1281	972	0.5693	1.1588	0.7049	1.3178	0
1282	971.9	0.5693	1.1589	0.7051	1.3176	0
1283	971.8	0.5693	1.1584	0.7052	1.3172	0
1284	971.7	0.5696	1.1582	0.7051	1.317	0
1285	971.6	0.5692	1.1584	0.7051	1.3172	0
1286	971.5	0.5696	1.1585	0.7051	1.317	0
1287	971.4	0.5694	1.1588	0.7051	1.3177	0
1288	971.3	0.5696	1.1588	0.7053	1.3184	0
1289	971.2	0.5697	1.159	0.7055	1.3189	0
1290	971.1	0.5696	1.1585	0.7055	1.3191	0
1291	971	0.5694	1.1591	0.7053	1.3175	0
1292	970.9	0.5693	1.1584	0.7053	1.3178	0
1293	970.8	0.5693	1.1591	0.7052	1.3178	0
1294	970.7	0.5696	1.1588	0.7052	1.3179	0
1295	970.6	0.5692	1.1585	0.7049	1.3181	0
1296	970.5	0.5699	1.1594	0.7055	1.3185	0
1297	970.4	0.5696	1.1594	0.705	1.3182	0
1298	970.3	0.5696	1.1594	0.705	1.3176	0
1299	970.2	0.5699	1.1596	0.7049	1.3179	0
1300	970.1	0.5697	1.1594	0.7051	1.3184	0
1301	970	0.5698	1.1596	0.7049	1.3182	0
1302	969.9	0.5693	1.1594	0.705	1.3191	0
1303	969.8	0.5696	1.1599	0.7052	1.3188	0
1304	969.7	0.5701	1.1596	0.7051	1.319	0
1305	969.6	0.5702	1.1601	0.7048	1.3192	0
1306	969.5	0.5708	1.1602	0.7049	1.3193	0
1307	969.4	0.5704	1.1605	0.7048	1.3191	0
1308	969.3	0.5702	1.1598	0.7046	1.3194	0
1309	969.2	0.5702	1.1596	0.7045	1.3198	0
1310	969.1	0.5705	1.1604	0.7045	1.3194	0
1311	969	0.5708	1.1606	0.7049	1.3199	0
1312	968.9	0.5706	1.1606	0.7045	1.3206	0
1313	968.8	0.5705	1.1606	0.7043	1.3203	0
1314	968.7	0.571	1.1609	0.7046	1.3203	0
1315	968.6	0.5707	1.1607	0.7044	1.3211	0
1316	968.5	0.5706	1.1604	0.7044	1.3213	0
1317	968.4	0.5708	1.1606	0.7043	1.3218	0
1318	968.3	0.5707	1.1609	0.7041	1.3214	0
1319	968.2	0.5703	1.1609	0.704	1.3208	0

1320	968.1	0.5708	1.1609	0.7038	1.3214	0
1321	968	0.5711	1.1612	0.704	1.3215	0
1322	967.9	0.5712	1.1617	0.704	1.3216	0
1323	967.8	0.5711	1.1615	0.7041	1.3216	0
1324	967.7	0.5712	1.1613	0.7039	1.321	0
1325	967.6	0.5716	1.1614	0.7039	1.3215	0
1326	967.5	0.5713	1.1611	0.7039	1.322	0
1327	967.4	0.5713	1.1613	0.7039	1.3214	0
1328	967.3	0.5712	1.1615	0.7038	1.3221	0
1329	967.2	0.5718	1.1619	0.7041	1.3224	0
1330	967.1	0.5715	1.1621	0.7041	1.3224	0
1331	967	0.5718	1.1616	0.7039	1.3218	0
1332	966.9	0.5718	1.1616	0.7039	1.3221	0
1333	966.8	0.5716	1.1612	0.7039	1.3224	0
1334	966.7	0.5711	1.1619	0.7039	1.3231	0
1335	966.6	0.5712	1.1618	0.7036	1.3221	0
1336	966.5	0.572	1.162	0.7042	1.3222	0
1337	966.4	0.572	1.1622	0.7042	1.3222	0
1338	966.3	0.5717	1.1616	0.704	1.3217	0
1339	966.2	0.5718	1.1614	0.7041	1.3219	0
1340	966.1	0.5717	1.1612	0.7041	1.3211	0
1341	966	0.5717	1.1617	0.7041	1.3212	0
1342	965.9	0.5716	1.1616	0.7042	1.3219	0
1343	965.8	0.5719	1.1619	0.7042	1.322	0
1344	965.7	0.5718	1.162	0.7044	1.3212	0
1345	965.6	0.5718	1.1621	0.7044	1.3209	0
1346	965.5	0.5715	1.1617	0.7046	1.3203	0
1347	965.4	0.5718	1.1617	0.7047	1.3215	0
1348	965.3	0.5713	1.1612	0.7047	1.3211	0
1349	965.2	0.5718	1.1617	0.7051	1.3208	0
1350	965.1	0.5717	1.1611	0.705	1.3208	0
1351	965	0.5719	1.1619	0.7052	1.3206	0
1352	964.9	0.5717	1.1614	0.7053	1.3208	0
1353	964.8	0.5719	1.1609	0.7054	1.3205	0
1354	964.7	0.5717	1.1608	0.7056	1.3197	0
1355	964.6	0.5716	1.1613	0.7059	1.3196	0
1356	964.5	0.5715	1.1613	0.7061	1.3191	0
1357	964.4	0.5714	1.1603	0.706	1.3191	0
1358	964.3	0.5713	1.1604	0.706	1.3185	0
1359	964.2	0.5712	1.1605	0.7063	1.319	0
1360	964.1	0.5716	1.1606	0.7063	1.3186	0
1361	964	0.5715	1.1606	0.7066	1.3185	0
1362	963.9	0.5715	1.1599	0.7067	1.3187	0

1363	963.8	0.5711	1.1596	0.707	1.318	0
1364	963.7	0.571	1.16	0.707	1.3172	0
1365	963.6	0.5711	1.16	0.7071	1.3172	0
1366	963.5	0.5709	1.1593	0.7072	1.3175	0
1367	963.4	0.5709	1.1592	0.7071	1.317	0
1368	963.3	0.5713	1.1598	0.7076	1.317	0
1369	963.2	0.5715	1.1597	0.7078	1.3162	0
1370	963.1	0.5713	1.1594	0.7077	1.3159	0
1371	963	0.5706	1.1597	0.7077	1.3159	0
1372	962.9	0.5704	1.1592	0.7077	1.3156	0
1373	962.8	0.5704	1.1593	0.7077	1.3154	0
1374	962.7	0.571	1.159	0.7079	1.3161	0
1375	962.6	0.5706	1.1589	0.7076	1.3153	0
1376	962.5	0.5707	1.1589	0.7081	1.3156	0
1377	962.4	0.5706	1.1588	0.708	1.3152	0
1378	962.3	0.5711	1.1585	0.7081	1.3156	0
1379	962.2	0.5711	1.1588	0.7081	1.3154	0
1380	962.1	0.5711	1.1588	0.7082	1.3157	0
1381	962	0.5709	1.1588	0.7081	1.3154	0
1382	961.9	0.5708	1.1589	0.7081	1.3151	0
1383	961.8	0.5708	1.1587	0.7083	1.3149	0
1384	961.7	0.5712	1.1588	0.7084	1.3152	0
1385	961.6	0.5711	1.1585	0.7085	1.3148	0
1386	961.5	0.571	1.1587	0.7084	1.3151	0
1387	961.4	0.5714	1.1584	0.7087	1.3156	0
1388	961.3	0.5712	1.1586	0.7085	1.3146	0
1389	961.2	0.5712	1.1585	0.7087	1.3157	0
1390	961.1	0.5713	1.1583	0.7087	1.3132	0
1391	961	0.5711	1.1582	0.7087	1.3147	0
1392	960.9	0.5711	1.1586	0.7088	1.3148	0
1393	960.8	0.5711	1.1585	0.7086	1.3147	0
1394	960.7	0.5707	1.1585	0.7086	1.3141	0
1395	960.6	0.5708	1.1583	0.7088	1.3144	0
1396	960.5	0.5712	1.1585	0.7088	1.3144	0
1397	960.4	0.5713	1.1586	0.7089	1.3148	0
1398	960.3	0.5711	1.159	0.7087	1.3148	0
1399	960.2	0.5711	1.1586	0.7088	1.3151	0
1400	960.1	0.5709	1.1586	0.7088	1.3142	0
1401	960	0.5709	1.1587	0.7086	1.3141	0
1402	959.9	0.5707	1.1588	0.7087	1.3148	0
1403	959.8	0.5711	1.1596	0.7088	1.3147	0
1404	959.7	0.5712	1.1586	0.7088	1.3148	0
1405	959.6	0.5709	1.159	0.7086	1.3145	0

1406	959.5	0.571	1.1587	0.7086	1.3138	0
1407	959.4	0.5713	1.1591	0.7084	1.315	0
1408	959.3	0.5711	1.158	0.7085	1.315	0
1409	959.2	0.5707	1.1593	0.7085	1.3156	0
1410	959.1	0.5714	1.1589	0.7086	1.3153	0
1411	959	0.5713	1.1595	0.7083	1.3162	0
1412	958.9	0.5714	1.1589	0.7082	1.3164	0
1413	958.8	0.5717	1.159	0.7084	1.3164	0
1414	958.7	0.5712	1.1591	0.7081	1.3157	0
1415	958.6	0.571	1.1587	0.708	1.3161	0
1416	958.5	0.5714	1.1588	0.708	1.3167	0
1417	958.4	0.5716	1.1596	0.7081	1.3162	0
1418	958.3	0.5716	1.1592	0.7081	1.3168	0
1419	958.2	0.5714	1.1595	0.7079	1.3161	0
1420	958.1	0.5714	1.1599	0.7078	1.3164	0
1421	958	0.572	1.1599	0.708	1.3166	0
1422	957.9	0.5714	1.1598	0.7077	1.3172	0
1423	957.8	0.5714	1.1602	0.7077	1.3169	0
1424	957.7	0.5714	1.1598	0.7075	1.3172	0
1425	957.6	0.5715	1.1599	0.7076	1.3183	0
1426	957.5	0.5716	1.1593	0.7075	1.3174	0
1427	957.4	0.5717	1.1595	0.7077	1.3174	0
1428	957.3	0.5715	1.1595	0.7075	1.3177	0
1429	957.2	0.5712	1.1592	0.7074	1.3177	0
1430	957.1	0.5713	1.1592	0.7073	1.3176	0
1431	957	0.5714	1.1592	0.7074	1.3167	0
1432	956.9	0.5715	1.1601	0.7078	1.3172	0
1433	956.8	0.5716	1.1601	0.7077	1.3178	0
1434	956.7	0.5715	1.1598	0.7077	1.3169	0
1435	956.6	0.5715	1.1596	0.7074	1.3173	0
1436	956.5	0.5715	1.16	0.7077	1.3178	0
1437	956.4	0.5717	1.1599	0.7075	1.3168	0
1438	956.3	0.5715	1.1593	0.7076	1.3171	0
1439	956.2	0.5716	1.1596	0.7077	1.3172	0
1440	956.1	0.5714	1.1598	0.7078	1.3178	0
1441	956	0.5713	1.1595	0.7078	1.3174	0
1442	955.9	0.5711	1.1592	0.7078	1.3167	0
1443	955.8	0.5715	1.1592	0.7078	1.3173	0
1444	955.7	0.571	1.1593	0.7078	1.3171	0
1445	955.6	0.5709	1.1595	0.7082	1.3172	0
1446	955.5	0.571	1.1595	0.7079	1.316	0
1447	955.4	0.5712	1.159	0.7083	1.3159	0
1448	955.3	0.5708	1.1587	0.708	1.3159	0

1449	955.2	0.571	1.1589	0.7081	1.3159	0
1450	955.1	0.5709	1.1587	0.7081	1.3155	0
1451	955	0.5711	1.1586	0.7083	1.316	0
1452	954.9	0.5703	1.1588	0.7083	1.3153	0
1453	954.8	0.5704	1.1579	0.7084	1.3153	0
1454	954.7	0.5705	1.1577	0.7082	1.3153	0
1455	954.6	0.5703	1.1575	0.7084	1.315	0
1456	954.5	0.5705	1.1576	0.7088	1.3156	0
1457	954.4	0.5706	1.1574	0.7088	1.3147	0
1458	954.3	0.5704	1.1581	0.7088	1.3139	0
1459	954.2	0.5704	1.1576	0.7091	1.3136	0
1460	954.1	0.5704	1.1575	0.7092	1.3135	0
1461	954	0.5703	1.1577	0.7095	1.3134	0
1462	953.9	0.5701	1.1572	0.7094	1.3133	0
1463	953.8	0.5699	1.1569	0.7096	1.3131	0
1464	953.7	0.5698	1.1569	0.7095	1.313	0
1465	953.6	0.57	1.157	0.7098	1.3125	0
1466	953.5	0.5698	1.1563	0.7097	1.3122	0
1467	953.4	0.5699	1.1565	0.7099	1.3121	0
1468	953.3	0.5695	1.1564	0.7101	1.3119	0
1469	953.2	0.5693	1.1562	0.7103	1.3121	0
1470	953.1	0.5695	1.1565	0.7105	1.3119	0
1471	953	0.5694	1.1563	0.7106	1.3116	0
1472	952.9	0.5693	1.1559	0.7105	1.311	0
1473	952.8	0.5692	1.1555	0.7106	1.3104	0
1474	952.7	0.5693	1.1555	0.7105	1.3095	0
1475	952.6	0.5695	1.1556	0.7108	1.3099	0
1476	952.5	0.5693	1.1553	0.7107	1.3098	0
1477	952.4	0.569	1.155	0.7108	1.3096	0
1478	952.3	0.569	1.1551	0.711	1.3095	0
1479	952.2	0.5691	1.1548	0.711	1.3094	0
1480	952.1	0.5688	1.1546	0.7111	1.309	0
1481	952	0.569	1.1548	0.7111	1.3091	0
1482	951.9	0.5687	1.1546	0.7111	1.3091	0
1483	951.8	0.5687	1.1545	0.7113	1.3088	0
1484	951.7	0.5689	1.1547	0.7112	1.3083	0
1485	951.6	0.5686	1.1549	0.7112	1.3086	0
1486	951.5	0.5683	1.1549	0.7112	1.309	0
1487	951.4	0.5686	1.155	0.7116	1.3091	0
1488	951.3	0.5689	1.1545	0.7115	1.3085	0
1489	951.2	0.5689	1.1542	0.7116	1.308	0
1490	951.1	0.5686	1.154	0.7114	1.3086	0
1491	951	0.5689	1.1544	0.7118	1.3088	0

1492	950.9	0.568	1.1541	0.7118	1.309	0
1493	950.8	0.5682	1.1542	0.7117	1.3088	0
1494	950.7	0.5685	1.1542	0.712	1.3085	0
1495	950.6	0.5686	1.1541	0.712	1.308	0
1496	950.5	0.5684	1.1538	0.7121	1.3073	0
1497	950.4	0.5687	1.1536	0.7119	1.308	0
1498	950.3	0.5687	1.1539	0.7121	1.3083	0
1499	950.2	0.5689	1.154	0.7123	1.3075	0
1500	950.1	0.569	1.1538	0.7124	1.3072	0
1501	950	0.5689	1.1537	0.7122	1.3077	0
1502	949.9	0.5689	1.1535	0.7122	1.3081	0
1503	949.8	0.5689	1.1539	0.7122	1.3072	0
1504	949.7	0.5688	1.1537	0.7119	1.307	0
1505	949.6	0.5689	1.1533	0.712	1.308	0
1506	949.5	0.5689	1.1535	0.7119	1.3082	0
1507	949.4	0.5687	1.1534	0.7119	1.3083	0
1508	949.3	0.5689	1.1536	0.7118	1.3072	0
1509	949.2	0.5689	1.1538	0.7118	1.3081	0
1510	949.1	0.569	1.154	0.7119	1.3078	0
1511	949	0.569	1.1539	0.7118	1.3089	0
1512	948.9	0.569	1.1542	0.7117	1.3093	0
1513	948.8	0.5693	1.1548	0.712	1.3088	0
1514	948.7	0.569	1.1544	0.7116	1.309	0
1515	948.6	0.5685	1.1544	0.7115	1.3087	0
1516	948.5	0.5686	1.1542	0.7114	1.3088	0
1517	948.4	0.5692	1.1548	0.7116	1.3087	0
1518	948.3	0.5692	1.1543	0.7116	1.3092	0
1519	948.2	0.5692	1.1544	0.7114	1.3087	0
1520	948.1	0.5693	1.1545	0.7112	1.3087	0
1521	948	0.5688	1.1542	0.7112	1.3086	0
1522	947.9	0.5693	1.1543	0.7113	1.3086	0
1523	947.8	0.5687	1.1545	0.7114	1.3092	0
1524	947.7	0.5689	1.1547	0.711	1.3096	0
1525	947.6	0.5689	1.1552	0.7111	1.3103	0
1526	947.5	0.569	1.1554	0.711	1.3103	0
1527	947.4	0.5692	1.1547	0.7111	1.3094	0
1528	947.3	0.5694	1.1547	0.711	1.3094	0
1529	947.2	0.5693	1.1557	0.7111	1.3097	0
1530	947.1	0.5694	1.1553	0.711	1.3102	0
1531	947	0.5694	1.1555	0.7109	1.3102	0
1532	946.9	0.5696	1.1553	0.7109	1.3111	0
1533	946.8	0.5692	1.1553	0.711	1.3105	0
1534	946.7	0.569	1.1553	0.7111	1.3112	0

1535	946.6	0.5692	1.1549	0.7108	1.3097	0
1536	946.5	0.5696	1.1551	0.7109	1.3106	0
1537	946.4	0.5695	1.1549	0.7109	1.31	0
1538	946.3	0.5695	1.1548	0.7109	1.3102	0
1539	946.2	0.5696	1.1554	0.7108	1.3105	0
1540	946.1	0.5696	1.1551	0.7108	1.31	0
1541	946	0.5692	1.1545	0.7107	1.3104	0
1542	945.9	0.5692	1.155	0.7108	1.3105	0
1543	945.8	0.5691	1.1552	0.7108	1.3105	0
1544	945.7	0.5693	1.1551	0.7108	1.3101	0
1545	945.6	0.5691	1.1554	0.7109	1.3108	0
1546	945.5	0.5692	1.1551	0.7111	1.3106	0
1547	945.4	0.5696	1.1551	0.7111	1.3103	0
1548	945.3	0.5696	1.155	0.711	1.3099	0
1549	945.2	0.5698	1.1553	0.711	1.3103	0
1550	945.1	0.5697	1.1554	0.711	1.3099	0
1551	945	0.5695	1.1548	0.711	1.3102	0
1552	944.9	0.5697	1.155	0.7112	1.3089	0
1553	944.8	0.5696	1.1549	0.7112	1.3088	0
1554	944.7	0.5693	1.1547	0.7113	1.3091	0
1555	944.6	0.5695	1.1546	0.7116	1.3089	0
1556	944.5	0.5693	1.1547	0.7117	1.3093	0
1557	944.4	0.5694	1.1546	0.7117	1.3086	0
1558	944.3	0.5692	1.1544	0.7119	1.3091	0
1559	944.2	0.5692	1.1541	0.712	1.3085	0
1560	944.1	0.5692	1.1546	0.7121	1.3089	0
1561	944	0.5692	1.1538	0.7119	1.309	0
1562	943.9	0.5692	1.1537	0.712	1.3091	0
1563	943.8	0.569	1.1535	0.7123	1.3085	0
1564	943.7	0.5691	1.1533	0.7123	1.3076	0
1565	943.6	0.569	1.1537	0.7126	1.3081	0
1566	943.5	0.5688	1.1539	0.7125	1.308	0
1567	943.4	0.5688	1.1534	0.7124	1.3074	0
1568	943.3	0.5689	1.1531	0.7127	1.3075	0
1569	943.2	0.569	1.1533	0.7126	1.3069	0
1570	943.1	0.5688	1.153	0.7125	1.3068	0
1571	943	0.5686	1.1535	0.7126	1.3069	0
1572	942.9	0.5689	1.1528	0.7128	1.3058	0
1573	942.8	0.5686	1.1525	0.7129	1.306	0
1574	942.7	0.5687	1.1528	0.7129	1.3061	0
1575	942.6	0.5685	1.1522	0.7129	1.3058	0
1576	942.5	0.5687	1.1527	0.7131	1.3063	0
1577	942.4	0.5686	1.1526	0.7132	1.3058	0

1578	942.3	0.5683	1.1524	0.7133	1.3061	0
1579	942.2	0.5684	1.1525	0.7133	1.3058	0
1580	942.1	0.5684	1.1524	0.7134	1.3055	0
1581	942	0.5684	1.1524	0.7131	1.3058	0
1582	941.9	0.5682	1.1523	0.7133	1.3053	0
1583	941.8	0.568	1.1521	0.7133	1.3049	0
1584	941.7	0.5682	1.1525	0.7133	1.3046	0
1585	941.6	0.5682	1.152	0.7131	1.3051	0
1586	941.5	0.5681	1.152	0.7135	1.3053	0
1587	941.4	0.568	1.1518	0.7132	1.3048	0
1588	941.3	0.568	1.1516	0.7134	1.3047	0
1589	941.2	0.568	1.1519	0.7136	1.3049	0
1590	941.1	0.5683	1.1519	0.7136	1.3049	0
1591	941	0.5683	1.1518	0.7137	1.3046	0
1592	940.9	0.568	1.152	0.7135	1.3052	0
1593	940.8	0.5679	1.1518	0.7137	1.3052	0
1594	940.7	0.5683	1.1515	0.7137	1.3047	0
1595	940.6	0.5682	1.1513	0.7139	1.3045	0
1596	940.5	0.5679	1.1514	0.7138	1.3043	0
1597	940.4	0.5682	1.1515	0.714	1.3042	0
1598	940.3	0.5678	1.1516	0.714	1.3036	0
1599	940.2	0.5679	1.1512	0.714	1.3041	0
1600	940.1	0.5679	1.1505	0.714	1.3031	0
1601	940	0.5676	1.1511	0.7141	1.3033	0
1602	939.9	0.5675	1.1511	0.7141	1.3042	0
1603	939.8	0.5674	1.1509	0.7141	1.3039	0
1604	939.7	0.5675	1.1513	0.7141	1.3033	0
1605	939.6	0.5678	1.1516	0.7142	1.3033	0
1606	939.5	0.5678	1.1513	0.7141	1.3035	0
1607	939.4	0.5677	1.1512	0.7145	1.3034	0
1608	939.3	0.5676	1.1509	0.7142	1.3027	0
1609	939.2	0.5677	1.1508	0.7142	1.3032	0
1610	939.1	0.5677	1.1509	0.7144	1.3027	0
1611	939	0.5677	1.1511	0.7143	1.3026	0
1612	938.9	0.5679	1.1513	0.7145	1.3029	0
1613	938.8	0.5679	1.1514	0.7143	1.3037	0
1614	938.7	0.5677	1.1511	0.7144	1.3037	0
1615	938.6	0.5679	1.1513	0.7144	1.3031	0
1616	938.5	0.5679	1.1511	0.7142	1.3038	0
1617	938.4	0.5681	1.1514	0.7144	1.3032	0
1618	938.3	0.5683	1.1507	0.7144	1.3037	0
1619	938.2	0.5682	1.151	0.7144	1.3033	0
1620	938.1	0.5679	1.1509	0.7144	1.3039	0

1621	938	0.5678	1.1512	0.7144	1.3029	0
1622	937.9	0.5679	1.1511	0.7142	1.303	0
1623	937.8	0.5682	1.1514	0.7144	1.304	0
1624	937.7	0.5682	1.1515	0.7145	1.3035	0
1625	937.6	0.5681	1.1513	0.7142	1.3033	0
1626	937.5	0.5681	1.1517	0.7143	1.3033	0
1627	937.4	0.5682	1.1517	0.7142	1.3036	0
1628	937.3	0.5679	1.1512	0.7143	1.3035	0
1629	937.2	0.568	1.1512	0.7143	1.3044	0
1630	937.1	0.5677	1.151	0.7143	1.3042	0
1631	937	0.5679	1.1511	0.7139	1.3041	0
1632	936.9	0.5678	1.1512	0.714	1.3045	0
1633	936.8	0.568	1.1517	0.7144	1.3036	0
1634	936.7	0.5682	1.1513	0.7142	1.304	0
1635	936.6	0.5679	1.1513	0.7142	1.3038	0
1636	936.5	0.5678	1.1512	0.7142	1.3028	0
1637	936.4	0.5679	1.1513	0.7144	1.3029	0
1638	936.3	0.5678	1.1512	0.7144	1.3033	0
1639	936.2	0.5678	1.151	0.7141	1.3035	0
1640	936.1	0.5677	1.1509	0.7145	1.3039	0
1641	936	0.5679	1.1514	0.7145	1.3036	0
1642	935.9	0.5679	1.1508	0.7147	1.3025	0
1643	935.8	0.5678	1.1511	0.7149	1.3031	0
1644	935.7	0.5678	1.1515	0.7148	1.3034	0
1645	935.6	0.5677	1.1507	0.7145	1.3032	0
1646	935.5	0.5679	1.151	0.7148	1.303	0
1647	935.4	0.5679	1.1511	0.7147	1.3023	0
1648	935.3	0.568	1.1514	0.7149	1.3021	0
1649	935.2	0.5679	1.1506	0.7148	1.3025	0
1650	935.1	0.568	1.1509	0.7149	1.3021	0
1651	935	0.5678	1.1509	0.715	1.3021	0
1652	934.9	0.5681	1.1507	0.7151	1.3023	0
1653	934.8	0.5677	1.1506	0.7153	1.3019	0
1654	934.7	0.5678	1.1506	0.7153	1.3018	0
1655	934.6	0.5676	1.1502	0.7152	1.302	0
1656	934.5	0.5677	1.1504	0.7153	1.302	0
1657	934.4	0.5676	1.1504	0.7153	1.3011	0
1658	934.3	0.5676	1.1501	0.7155	1.3027	0
1659	934.2	0.5675	1.1498	0.7153	1.3017	0
1660	934.1	0.5671	1.1496	0.7154	1.3009	0
1661	934	0.5675	1.1502	0.7157	1.3002	0
1662	933.9	0.5677	1.1498	0.7159	1.3016	0
1663	933.8	0.5677	1.1498	0.7159	1.301	0

1664	933.7	0.5673	1.1498	0.7157	1.3002	0
1665	933.6	0.5673	1.1499	0.7158	1.3003	0
1666	933.5	0.5672	1.149	0.7158	1.3003	0
1667	933.4	0.5675	1.1495	0.7157	1.2998	0
1668	933.3	0.5673	1.1494	0.7161	1.2999	0
1669	933.2	0.5673	1.1489	0.7161	1.3008	0
1670	933.1	0.567	1.1491	0.7161	1.3008	0
1671	933	0.5672	1.1493	0.7161	1.3005	0
1672	932.9	0.567	1.1488	0.7162	1.2999	0
1673	932.8	0.5672	1.1489	0.7161	1.2988	0
1674	932.7	0.5667	1.149	0.7161	1.2991	0
1675	932.6	0.5665	1.1492	0.7163	1.2995	0
1676	932.5	0.5669	1.1493	0.7165	1.2998	0
1677	932.4	0.5672	1.149	0.7164	1.2994	0
1678	932.3	0.5671	1.1488	0.7164	1.2988	0
1679	932.2	0.5672	1.1488	0.7164	1.2988	0
1680	932.1	0.567	1.1491	0.7164	1.299	0
1681	932	0.5668	1.1487	0.7165	1.2993	0
1682	931.9	0.5669	1.1483	0.7164	1.2993	0
1683	931.8	0.5666	1.1491	0.7168	1.2987	0
1684	931.7	0.5671	1.1491	0.7165	1.299	0
1685	931.6	0.5664	1.1485	0.7166	1.299	0
1686	931.5	0.5669	1.1485	0.7165	1.299	0
1687	931.4	0.5669	1.1488	0.7164	1.2989	0
1688	931.3	0.5672	1.1487	0.7165	1.2987	0
1689	931.2	0.567	1.1493	0.7166	1.299	0
1690	931.1	0.5669	1.1488	0.7165	1.2988	0
1691	931	0.5667	1.1487	0.7164	1.299	0
1692	930.9	0.5669	1.1488	0.7166	1.2989	0
1693	930.8	0.5671	1.1482	0.7165	1.2977	0
1694	930.7	0.5668	1.149	0.7166	1.2989	0
1695	930.6	0.5669	1.1488	0.7166	1.2989	0
1696	930.5	0.5669	1.1487	0.7166	1.2988	0
1697	930.4	0.5669	1.149	0.7166	1.2992	0
1698	930.3	0.5665	1.1485	0.7166	1.2991	0
1699	930.2	0.5668	1.1485	0.7166	1.2985	0
1700	930.1	0.5667	1.1484	0.7163	1.299	0
1701	930	0.5665	1.1491	0.7164	1.2988	0
1702	929.9	0.5664	1.1488	0.7164	1.2991	0
1703	929.8	0.5667	1.1488	0.7166	1.2989	0
1704	929.7	0.5665	1.1487	0.7164	1.2982	0
1705	929.6	0.5666	1.1489	0.7165	1.2991	0
1706	929.5	0.567	1.1485	0.7165	1.2994	0

1707	929.4	0.5671	1.1486	0.7167	1.2989	0
1708	929.3	0.5672	1.1489	0.7166	1.2983	0
1709	929.2	0.567	1.1485	0.7165	1.2988	0
1710	929.1	0.5671	1.1486	0.7164	1.2993	0
1711	929	0.567	1.1481	0.7163	1.2989	0
1712	928.9	0.5668	1.1484	0.7164	1.2995	0
1713	928.8	0.567	1.1489	0.7165	1.2991	0
1714	928.7	0.5671	1.1483	0.7164	1.2988	0
1715	928.6	0.5669	1.1484	0.7165	1.299	0
1716	928.5	0.5669	1.1484	0.7165	1.2987	0
1717	928.4	0.5672	1.1479	0.7165	1.2986	0
1718	928.3	0.567	1.1486	0.7166	1.2986	0
1719	928.2	0.5671	1.1483	0.7166	1.2983	0
1720	928.1	0.5669	1.1485	0.7164	1.2975	0
1721	928	0.5668	1.1484	0.7169	1.2978	0
1722	927.9	0.5669	1.1481	0.7166	1.298	0
1723	927.8	0.5668	1.1477	0.7168	1.2986	0
1724	927.7	0.5669	1.1479	0.7169	1.2986	0
1725	927.6	0.5668	1.1481	0.7169	1.2977	0
1726	927.5	0.5667	1.148	0.717	1.2984	0
1727	927.4	0.5668	1.1484	0.717	1.2981	0
1728	927.3	0.5667	1.1482	0.7171	1.2979	0
1729	927.2	0.5669	1.1483	0.7172	1.2983	0
1730	927.1	0.5669	1.1476	0.7171	1.298	0
1731	927	0.5667	1.1472	0.717	1.2977	0
1732	926.9	0.5667	1.1474	0.717	1.2979	0
1733	926.8	0.5663	1.1474	0.7169	1.2966	0
1734	926.7	0.5667	1.1477	0.7174	1.2968	0
1735	926.6	0.5664	1.1477	0.7174	1.2971	0
1736	926.5	0.5667	1.1479	0.7174	1.2966	0
1737	926.4	0.5669	1.1471	0.7175	1.2969	0
1738	926.3	0.5666	1.1471	0.7176	1.2969	0
1739	926.2	0.5663	1.1472	0.7176	1.2968	0
1740	926.1	0.5665	1.1473	0.7177	1.2966	0
1741	926	0.5665	1.1473	0.7178	1.2959	0
1742	925.9	0.5666	1.1476	0.718	1.2964	0
1743	925.8	0.5665	1.1469	0.7177	1.2969	0
1744	925.7	0.5663	1.1472	0.7181	1.2967	0
1745	925.6	0.5664	1.1472	0.718	1.2961	0
1746	925.5	0.5666	1.1469	0.7181	1.2959	0
1747	925.4	0.5666	1.1471	0.7182	1.2959	0
1748	925.3	0.5663	1.1472	0.7183	1.2958	0
1749	925.2	0.5664	1.1468	0.7182	1.2952	0

1750	925.1	0.5668	1.1465	0.7184	1.2954	0
1751	925	0.5665	1.1465	0.7186	1.2959	0
1752	924.9	0.5665	1.1465	0.7185	1.2954	0
1753	924.8	0.5666	1.1472	0.7187	1.2951	0
1754	924.7	0.5663	1.1465	0.7187	1.2947	0
1755	924.6	0.5664	1.1462	0.7186	1.2952	0
1756	924.5	0.5665	1.1459	0.7189	1.2954	0
1757	924.4	0.5663	1.1462	0.719	1.2937	0
1758	924.3	0.5665	1.1458	0.7189	1.2944	0
1759	924.2	0.5662	1.1459	0.7191	1.2936	0
1760	924.1	0.5664	1.1456	0.7191	1.295	0
1761	924	0.5664	1.146	0.7193	1.2943	0
1762	923.9	0.5664	1.1454	0.7194	1.2941	0
1763	923.8	0.5662	1.1461	0.7192	1.2939	0
1764	923.7	0.5662	1.1455	0.7192	1.2935	0
1765	923.6	0.5662	1.1454	0.7197	1.2931	0
1766	923.5	0.5663	1.1457	0.7195	1.293	0
1767	923.4	0.5663	1.1453	0.7195	1.2936	0
1768	923.3	0.566	1.1451	0.7194	1.2932	0
1769	923.2	0.566	1.1457	0.7196	1.293	0
1770	923.1	0.5663	1.1456	0.7198	1.2931	0
1771	923	0.5663	1.1459	0.7196	1.2936	0
1772	922.9	0.5664	1.1459	0.72	1.2928	0
1773	922.8	0.5664	1.1458	0.7199	1.2934	0
1774	922.7	0.5662	1.1453	0.7201	1.2934	0
1775	922.6	0.5665	1.1462	0.7198	1.2923	0
1776	922.5	0.5668	1.1457	0.72	1.2936	0
1777	922.4	0.5666	1.1457	0.7199	1.2936	0
1778	922.3	0.5663	1.1458	0.7199	1.2935	0
1779	922.2	0.5663	1.1455	0.72	1.2932	0
1780	922.1	0.5664	1.1456	0.7199	1.293	0
1781	922	0.5664	1.1457	0.7201	1.2937	0
1782	921.9	0.5665	1.1459	0.7199	1.2932	0
1783	921.8	0.5665	1.1455	0.7198	1.2935	0
1784	921.7	0.5659	1.1453	0.7198	1.293	0
1785	921.6	0.5659	1.146	0.7196	1.2931	0
1786	921.5	0.5661	1.1458	0.7198	1.2929	0
1787	921.4	0.5665	1.1462	0.7198	1.2931	0
1788	921.3	0.5666	1.1462	0.7198	1.293	0
1789	921.2	0.5667	1.1458	0.7197	1.2935	0
1790	921.1	0.5666	1.1462	0.7196	1.2934	0
1791	921	0.5665	1.1459	0.7196	1.2942	0
1792	920.9	0.5667	1.1465	0.7199	1.2946	0

1793	920.8	0.5666	1.146	0.7198	1.2944	0
1794	920.7	0.5666	1.1465	0.7195	1.2931	0
1795	920.6	0.5664	1.1457	0.7195	1.2929	0
1796	920.5	0.5667	1.146	0.7196	1.2928	0
1797	920.4	0.5667	1.1463	0.7194	1.2936	0
1798	920.3	0.5666	1.146	0.7195	1.2941	0
1799	920.2	0.5666	1.1465	0.7194	1.2945	0
1800	920.1	0.5667	1.1463	0.7194	1.2939	0
1801	920	0.5665	1.1463	0.7193	1.2943	0
1802	919.9	0.5667	1.1464	0.7193	1.294	0
1803	919.8	0.5664	1.1465	0.719	1.2941	0
1804	919.7	0.5669	1.1466	0.7191	1.295	0
1805	919.6	0.5665	1.1467	0.7192	1.2946	0
1806	919.5	0.5665	1.1472	0.7193	1.2956	0
1807	919.4	0.5668	1.1471	0.7193	1.2952	0
1808	919.3	0.5669	1.1473	0.7189	1.295	0
1809	919.2	0.5666	1.1476	0.7189	1.2952	0
1810	919.1	0.5667	1.1472	0.7188	1.2952	0
1811	919	0.567	1.1468	0.7187	1.2941	0
1812	918.9	0.567	1.1467	0.7184	1.2949	0
1813	918.8	0.5669	1.1471	0.7188	1.2955	0
1814	918.7	0.5673	1.1477	0.7185	1.2952	0
1815	918.6	0.5673	1.1473	0.7187	1.2959	0
1816	918.5	0.5675	1.1477	0.7188	1.2957	0
1817	918.4	0.5674	1.1478	0.719	1.296	0
1818	918.3	0.5669	1.1474	0.7186	1.2957	0
1819	918.2	0.5667	1.1477	0.7184	1.2962	0
1820	918.1	0.5671	1.1478	0.7186	1.2955	0
1821	918	0.5673	1.148	0.7184	1.2964	0
1822	917.9	0.5675	1.1479	0.7185	1.2965	0
1823	917.8	0.5675	1.1482	0.7185	1.2964	0
1824	917.7	0.5672	1.1483	0.7186	1.2969	0
1825	917.6	0.5674	1.1479	0.7187	1.2972	0
1826	917.5	0.5676	1.1481	0.7187	1.2971	0
1827	917.4	0.5674	1.148	0.7187	1.2969	0
1828	917.3	0.5672	1.148	0.7186	1.2958	0
1829	917.2	0.5673	1.148	0.7187	1.2968	0
1830	917.1	0.5672	1.1476	0.7189	1.2964	0
1831	917	0.5674	1.1478	0.7186	1.2966	0
1832	916.9	0.5674	1.1476	0.7188	1.296	0
1833	916.8	0.5671	1.1478	0.7189	1.2962	0
1834	916.7	0.567	1.1476	0.7191	1.2958	0
1835	916.6	0.5674	1.1476	0.7193	1.2956	0

1836	916.5	0.5672	1.1475	0.7193	1.2955	0
1837	916.4	0.5673	1.1477	0.7193	1.2955	0
1838	916.3	0.567	1.1472	0.7193	1.2945	0
1839	916.2	0.5671	1.1468	0.7193	1.2949	0
1840	916.1	0.5672	1.147	0.7194	1.2951	0
1841	916	0.5672	1.1472	0.7195	1.2948	0
1842	915.9	0.5674	1.1474	0.7199	1.2944	0
1843	915.8	0.5672	1.1465	0.7196	1.2946	0
1844	915.7	0.5674	1.1473	0.7198	1.2948	0
1845	915.6	0.5672	1.1463	0.7201	1.2935	0
1846	915.5	0.5672	1.1461	0.7202	1.2935	0
1847	915.4	0.5672	1.1464	0.7203	1.2936	0
1848	915.3	0.5674	1.1465	0.7205	1.2934	0
1849	915.2	0.5671	1.1461	0.7204	1.2932	0
1850	915.1	0.5673	1.1464	0.7207	1.2939	0
1851	915	0.567	1.1464	0.7205	1.2933	0
1852	914.9	0.567	1.1459	0.7208	1.2927	0
1853	914.8	0.5667	1.1461	0.7208	1.2923	0
1854	914.7	0.567	1.1464	0.7211	1.2926	0
1855	914.6	0.5666	1.1458	0.721	1.2921	0
1856	914.5	0.567	1.1458	0.721	1.2915	0
1857	914.4	0.5668	1.1453	0.7211	1.2909	0
1858	914.3	0.5667	1.1452	0.7213	1.2915	0
1859	914.2	0.5668	1.1451	0.7214	1.2913	0
1860	914.1	0.5668	1.1452	0.7214	1.2913	0
1861	914	0.5667	1.1454	0.7214	1.2913	0
1862	913.9	0.5665	1.1459	0.7216	1.2907	0
1863	913.8	0.5665	1.1448	0.7217	1.2901	0
1864	913.7	0.5665	1.1445	0.7219	1.2899	0
1865	913.6	0.5667	1.1443	0.7221	1.2896	0
1866	913.5	0.5667	1.1451	0.7219	1.2905	0
1867	913.4	0.5666	1.1448	0.722	1.2895	0
1868	913.3	0.5668	1.1451	0.7221	1.2899	0
1869	913.2	0.5667	1.1452	0.7222	1.2901	0
1870	913.1	0.5666	1.1447	0.7222	1.2898	0
1871	913	0.5665	1.1444	0.7223	1.2896	0
1872	912.9	0.5664	1.1446	0.7226	1.2893	0
1873	912.8	0.5662	1.145	0.7226	1.2895	0
1874	912.7	0.5662	1.145	0.7226	1.2893	0
1875	912.6	0.5664	1.144	0.7226	1.2891	0
1876	912.5	0.5666	1.1449	0.7228	1.2897	0
1877	912.4	0.5667	1.1442	0.7226	1.2893	0
1878	912.3	0.5664	1.1445	0.7226	1.2898	0

1879	912.2	0.5666	1.1448	0.7228	1.2897	0
1880	912.1	0.5662	1.1447	0.7227	1.2892	0
1881	912	0.5663	1.1443	0.7227	1.289	0
1882	911.9	0.5661	1.1447	0.7229	1.2892	0
1883	911.8	0.5661	1.1448	0.7229	1.2891	0
1884	911.7	0.566	1.1445	0.7225	1.2893	0
1885	911.6	0.5662	1.1449	0.7226	1.2893	0
1886	911.5	0.5667	1.1449	0.7226	1.2887	0
1887	911.4	0.5663	1.1446	0.7227	1.2898	0
1888	911.3	0.5667	1.1445	0.7227	1.2882	0
1889	911.2	0.5667	1.1441	0.7228	1.2883	0
1890	911.1	0.5666	1.1447	0.7227	1.2891	0
1891	911	0.5666	1.1443	0.7226	1.2887	0
1892	910.9	0.567	1.1451	0.7226	1.2892	0
1893	910.8	0.5665	1.1448	0.7226	1.2893	0
1894	910.7	0.566	1.1446	0.7228	1.2894	0
1895	910.6	0.567	1.1449	0.7227	1.2895	0
1896	910.5	0.5668	1.1449	0.7226	1.2904	0
1897	910.4	0.567	1.1455	0.7227	1.2901	0
1898	910.3	0.5667	1.1453	0.7224	1.29	0
1899	910.2	0.5666	1.1449	0.7225	1.2899	0
1900	910.1	0.567	1.1447	0.7224	1.2906	0
1901	910	0.5673	1.1449	0.7224	1.2906	0
1902	909.9	0.567	1.1451	0.7223	1.2901	0
1903	909.8	0.5672	1.1447	0.7224	1.2902	0
1904	909.7	0.5671	1.1451	0.7223	1.2902	0
1905	909.6	0.5665	1.145	0.7223	1.2904	0
1906	909.5	0.5669	1.1452	0.7223	1.2907	0
1907	909.4	0.5668	1.1458	0.7224	1.2909	0
1908	909.3	0.5671	1.1455	0.7222	1.2905	0
1909	909.2	0.5672	1.1459	0.7223	1.2902	0
1910	909.1	0.5671	1.1461	0.7222	1.2909	0
1911	909	0.5674	1.1457	0.7219	1.2907	0
1912	908.9	0.5675	1.146	0.7219	1.2907	0
1913	908.8	0.5672	1.1457	0.722	1.2912	0
1914	908.7	0.5673	1.1459	0.7218	1.2914	0
1915	908.6	0.5674	1.1461	0.7219	1.2913	0
1916	908.5	0.5677	1.1459	0.7218	1.2907	0
1917	908.4	0.5676	1.1459	0.7216	1.2912	0
1918	908.3	0.5673	1.1462	0.7215	1.2913	0
1919	908.2	0.5678	1.1463	0.7217	1.2926	0
1920	908.1	0.5677	1.1468	0.7216	1.2927	0
1921	908	0.5675	1.1469	0.7214	1.2923	0

1922	907.9	0.5678	1.1467	0.7216	1.2923	0
1923	907.8	0.5677	1.1468	0.7217	1.292	0
1924	907.7	0.5681	1.1467	0.7214	1.2924	0
1925	907.6	0.5681	1.1472	0.7214	1.2925	0
1926	907.5	0.568	1.1468	0.7215	1.2923	0
1927	907.4	0.5676	1.1469	0.7216	1.2926	0
1928	907.3	0.568	1.1467	0.7215	1.2918	0
1929	907.2	0.5678	1.1471	0.7215	1.2923	0
1930	907.1	0.5679	1.147	0.7215	1.2928	0
1931	907	0.5681	1.1472	0.7214	1.2928	0
1932	906.9	0.5683	1.147	0.7217	1.2928	0
1933	906.8	0.5683	1.1474	0.7216	1.2927	0
1934	906.7	0.5683	1.147	0.7216	1.2923	0
1935	906.6	0.5681	1.1472	0.7215	1.2925	0
1936	906.5	0.5683	1.1471	0.7215	1.2928	0
1937	906.4	0.5682	1.1471	0.7216	1.2928	0
1938	906.3	0.568	1.1468	0.7214	1.2921	0
1939	906.2	0.568	1.1468	0.7216	1.2923	0
1940	906.1	0.5681	1.1473	0.7215	1.2923	0
1941	906	0.5682	1.1472	0.7217	1.2918	0
1942	905.9	0.5681	1.1473	0.7217	1.2923	0
1943	905.8	0.5683	1.1469	0.7217	1.2911	0
1944	905.7	0.5682	1.1466	0.7218	1.2925	0
1945	905.6	0.5681	1.1468	0.7218	1.2919	0
1946	905.5	0.5681	1.1467	0.722	1.292	0
1947	905.4	0.5679	1.1467	0.7221	1.2917	0
1948	905.3	0.5682	1.1467	0.7223	1.2914	0
1949	905.2	0.5681	1.1468	0.7225	1.2912	0
1950	905.1	0.5679	1.1464	0.7224	1.2901	0
1951	905	0.5682	1.1467	0.7227	1.2911	0
1952	904.9	0.5681	1.146	0.7226	1.2901	0
1953	904.8	0.568	1.1463	0.7227	1.2899	0
1954	904.7	0.568	1.1456	0.7228	1.2899	0
1955	904.6	0.568	1.1461	0.723	1.289	0
1956	904.5	0.568	1.1456	0.7231	1.2896	0
1957	904.4	0.5678	1.1462	0.7233	1.2891	0
1958	904.3	0.5675	1.1453	0.7235	1.2889	0
1959	904.2	0.5678	1.1449	0.7237	1.289	0
1960	904.1	0.568	1.145	0.7239	1.288	0
1961	904	0.5679	1.1448	0.7242	1.2889	0
1962	903.9	0.568	1.1451	0.7243	1.2878	0
1963	903.8	0.5677	1.1448	0.7243	1.2874	0
1964	903.7	0.568	1.1449	0.7244	1.2875	0

1965	903.6	0.5675	1.1445	0.7247	1.287	0
1966	903.5	0.5677	1.1444	0.7249	1.2872	0
1967	903.4	0.5677	1.1443	0.725	1.2867	0
1968	903.3	0.5676	1.1441	0.7249	1.2865	0
1969	903.2	0.5673	1.1438	0.7249	1.2855	0
1970	903.1	0.5671	1.1439	0.7254	1.2856	0
1971	903	0.5676	1.1443	0.7254	1.2871	0
1972	902.9	0.5673	1.1441	0.7252	1.2863	0
1973	902.8	0.5675	1.1438	0.7254	1.2864	0
1974	902.7	0.5673	1.1434	0.7252	1.285	0
1975	902.6	0.5673	1.1438	0.7256	1.2852	0
1976	902.5	0.5673	1.1435	0.7256	1.2848	0
1977	902.4	0.5674	1.1441	0.7256	1.2851	0
1978	902.3	0.5674	1.1435	0.7256	1.2855	0
1979	902.2	0.5673	1.1438	0.7256	1.2848	0
1980	902.1	0.5672	1.1436	0.7259	1.2847	0
1981	902	0.5675	1.1432	0.726	1.2853	0
1982	901.9	0.5677	1.1435	0.7259	1.2849	0
1983	901.8	0.5674	1.1438	0.7257	1.2854	0
1984	901.7	0.5678	1.1439	0.726	1.2843	0
1985	901.6	0.5673	1.1434	0.726	1.2851	0
1986	901.5	0.5677	1.1436	0.726	1.2856	0
1987	901.4	0.5675	1.1436	0.7261	1.2843	0
1988	901.3	0.5677	1.1432	0.7261	1.2849	0
1989	901.2	0.5677	1.1431	0.7261	1.2842	0
1990	901.1	0.5675	1.1435	0.726	1.2848	0
1991	901	0.5675	1.1433	0.7261	1.285	0
1992	900.9	0.5679	1.1436	0.7263	1.2851	0
1993	900.8	0.568	1.1438	0.7264	1.2852	0
1994	900.7	0.5675	1.1439	0.7262	1.2844	0
1995	900.6	0.5679	1.1441	0.7263	1.2844	0
1996	900.5	0.568	1.144	0.7263	1.2843	0
1997	900.4	0.5678	1.1434	0.7262	1.2845	0
1998	900.3	0.5675	1.1432	0.7263	1.2847	0
1999	900.2	0.5679	1.1434	0.7264	1.2842	0
2000	900.1	0.5678	1.1436	0.7265	1.285	0
2001	900	0.5677	1.1435	0.7262	1.2853	0
2002	899.9	0.5678	1.1438	0.7263	1.2846	0
2003	899.8	0.5679	1.1436	0.7261	1.284	0
2004	899.7	0.5682	1.1437	0.7261	1.2845	0
2005	899.6	0.5681	1.1437	0.7259	1.2852	0
2006	899.5	0.5681	1.1438	0.726	1.2854	0
2007	899.4	0.5681	1.1446	0.7258	1.2849	0

2008	899.3	0.5683	1.1442	0.7257	1.2842	0
2009	899.2	0.5681	1.1442	0.7256	1.2846	0
2010	899.1	0.5684	1.1446	0.7257	1.2852	0
2011	899	0.5686	1.1447	0.7257	1.2859	0
2012	898.9	0.5685	1.1446	0.7257	1.2859	0
2013	898.8	0.5681	1.1443	0.7255	1.2857	0
2014	898.7	0.5683	1.1447	0.7255	1.2854	0
2015	898.6	0.5683	1.1452	0.7256	1.2864	0
2016	898.5	0.5684	1.1455	0.7256	1.2858	0
2017	898.4	0.5685	1.1449	0.7254	1.286	0
2018	898.3	0.5688	1.1448	0.7254	1.2854	0
2019	898.2	0.5686	1.1454	0.7253	1.2863	0
2020	898.1	0.5686	1.145	0.7251	1.2855	0
2021	898	0.5691	1.1454	0.7253	1.2864	0
2022	897.9	0.569	1.1453	0.7254	1.2862	0
2023	897.8	0.569	1.1456	0.7251	1.2867	0
2024	897.7	0.5687	1.1456	0.7253	1.2868	0
2025	897.6	0.5687	1.1454	0.725	1.2866	0
2026	897.5	0.5689	1.1458	0.7248	1.2872	0
2027	897.4	0.5689	1.1461	0.7248	1.2865	0
2028	897.3	0.569	1.1457	0.725	1.2876	0
2029	897.2	0.5691	1.1459	0.7249	1.2879	0
2030	897.1	0.5694	1.1462	0.725	1.2875	0
2031	897	0.5694	1.1457	0.7248	1.2871	0
2032	896.9	0.5689	1.1455	0.7248	1.2878	0
2033	896.8	0.5693	1.1456	0.725	1.2882	0
2034	896.7	0.5692	1.1455	0.7248	1.2879	0
2035	896.6	0.5694	1.1461	0.7248	1.2886	0
2036	896.5	0.5692	1.1464	0.7249	1.2873	0
2037	896.4	0.569	1.146	0.7251	1.2871	0
2038	896.3	0.5693	1.1458	0.7249	1.288	0
2039	896.2	0.5693	1.1462	0.7251	1.2876	0
2040	896.1	0.5693	1.1459	0.725	1.2873	0
2041	896	0.5697	1.1463	0.7252	1.2872	0
2042	895.9	0.5696	1.146	0.7249	1.2876	0
2043	895.8	0.5695	1.1459	0.725	1.2875	0
2044	895.7	0.5694	1.146	0.7251	1.2881	0
2045	895.6	0.5695	1.1455	0.7251	1.288	0
2046	895.5	0.5696	1.1459	0.7251	1.2874	0
2047	895.4	0.5695	1.1462	0.7252	1.287	0
2048	895.3	0.5692	1.1456	0.7251	1.2871	0
2049	895.2	0.5693	1.1459	0.7251	1.2881	0
2050	895.1	0.5692	1.146	0.7252	1.2885	0

2051	895	0.5695	1.1465	0.7255	1.288	0
2052	894.9	0.5695	1.1461	0.7253	1.2875	0
2053	894.8	0.5697	1.146	0.7255	1.2874	0
2054	894.7	0.5694	1.1456	0.7253	1.2869	0
2055	894.6	0.5694	1.1455	0.7253	1.286	0
2056	894.5	0.5694	1.1456	0.7254	1.2858	0
2057	894.4	0.5694	1.1457	0.7257	1.2858	0
2058	894.3	0.5694	1.1456	0.7259	1.2863	0
2059	894.2	0.5694	1.145	0.7259	1.2854	0
2060	894.1	0.5695	1.1446	0.7259	1.2856	0
2061	894	0.5693	1.1453	0.7262	1.286	0
2062	893.9	0.5695	1.1449	0.7261	1.2854	0
2063	893.8	0.5694	1.1449	0.7265	1.2859	0
2064	893.7	0.5694	1.1449	0.7266	1.2858	0
2065	893.6	0.5696	1.1451	0.7267	1.285	0
2066	893.5	0.5692	1.1453	0.7268	1.285	0
2067	893.4	0.5692	1.1443	0.7268	1.2845	0
2068	893.3	0.5693	1.1446	0.727	1.2843	0
2069	893.2	0.5692	1.1441	0.7268	1.2837	0
2070	893.1	0.5697	1.1447	0.7275	1.2835	0
2071	893	0.5696	1.1446	0.7275	1.2835	0
2072	892.9	0.5696	1.1444	0.7276	1.2832	0
2073	892.8	0.5695	1.1444	0.7277	1.2834	0
2074	892.7	0.5696	1.1444	0.7277	1.282	0
2075	892.6	0.5698	1.1444	0.7278	1.2829	0
2076	892.5	0.5698	1.1442	0.7279	1.2831	0
2077	892.4	0.5694	1.1443	0.7279	1.283	0
2078	892.3	0.5693	1.1441	0.7277	1.2826	0
2079	892.2	0.5693	1.1437	0.7277	1.2821	0
2080	892.1	0.5697	1.1437	0.728	1.2823	0
2081	892	0.5695	1.1436	0.7281	1.2822	0
2082	891.9	0.5698	1.1441	0.7284	1.2814	0
2083	891.8	0.5699	1.1442	0.7283	1.2817	0
2084	891.7	0.5697	1.1441	0.7284	1.282	0
2085	891.6	0.5695	1.1442	0.7284	1.2818	0
2086	891.5	0.5696	1.1438	0.7283	1.2819	0
2087	891.4	0.5695	1.144	0.7284	1.282	0
2088	891.3	0.5694	1.1436	0.7285	1.2808	0
2089	891.2	0.5693	1.1437	0.7286	1.2813	0
2090	891.1	0.5698	1.1435	0.7288	1.2825	0
2091	891	0.5697	1.1434	0.7286	1.2815	0
2092	890.9	0.57	1.143	0.7286	1.2818	0
2093	890.8	0.5698	1.1438	0.7287	1.2809	0

2094	890.7	0.5698	1.1437	0.729	1.2819	0
2095	890.6	0.5698	1.1437	0.7289	1.2802	0
2096	890.5	0.5694	1.1437	0.7289	1.2808	0
2097	890.4	0.5696	1.1434	0.7289	1.2807	0
2098	890.3	0.5696	1.1433	0.7288	1.2811	0
2099	890.2	0.5696	1.1435	0.7289	1.2815	0
2100	890.1	0.5699	1.1432	0.7289	1.2815	0
2101	890	0.5698	1.1439	0.7289	1.2809	0
2102	889.9	0.57	1.1439	0.7289	1.2818	0
2103	889.8	0.5699	1.1438	0.7289	1.2814	0
2104	889.7	0.57	1.1433	0.7289	1.2813	0
2105	889.6	0.5702	1.1439	0.7288	1.2811	0
2106	889.5	0.5699	1.1439	0.729	1.2811	0
2107	889.4	0.5697	1.1441	0.7289	1.2813	0
2108	889.3	0.5698	1.1437	0.7289	1.2818	0
2109	889.2	0.57	1.144	0.729	1.2818	0
2110	889.1	0.5703	1.1441	0.7286	1.2818	0
2111	889	0.5701	1.1441	0.7287	1.2816	0
2112	888.9	0.5701	1.1449	0.7288	1.2817	0
2113	888.8	0.5703	1.1448	0.7286	1.2817	0
2114	888.7	0.5699	1.1449	0.7284	1.2809	0
2115	888.6	0.5705	1.1446	0.7283	1.2823	0
2116	888.5	0.5704	1.1447	0.7284	1.2819	0
2117	888.4	0.5705	1.1448	0.7285	1.2827	0
2118	888.3	0.5708	1.1449	0.7284	1.2831	0
2119	888.2	0.5708	1.145	0.7283	1.2828	0
2120	888.1	0.5709	1.1449	0.7283	1.2829	0
2121	888	0.5708	1.1452	0.7281	1.2828	0
2122	887.9	0.5708	1.1453	0.7283	1.2827	0
2123	887.8	0.5708	1.1454	0.7281	1.2835	0
2124	887.7	0.5706	1.1453	0.7277	1.2826	0
2125	887.6	0.5708	1.1454	0.7279	1.2824	0
2126	887.5	0.5711	1.1455	0.728	1.2829	0
2127	887.4	0.571	1.1454	0.7279	1.2831	0
2128	887.3	0.5712	1.1456	0.7278	1.2834	0
2129	887.2	0.571	1.1454	0.7276	1.2831	0
2130	887.1	0.5712	1.1458	0.7277	1.2837	0
2131	887	0.5712	1.146	0.7277	1.2843	0
2132	886.9	0.5713	1.1454	0.7278	1.2833	0
2133	886.8	0.5712	1.1458	0.7277	1.2834	0
2134	886.7	0.5715	1.1464	0.7277	1.2843	0
2135	886.6	0.5713	1.1464	0.7275	1.2841	0
2136	886.5	0.5715	1.1465	0.7276	1.2846	0

2137	886.4	0.5714	1.1463	0.7275	1.2844	0
2138	886.3	0.5714	1.1461	0.7276	1.2841	0
2139	886.2	0.5716	1.147	0.7276	1.2841	0
2140	886.1	0.5714	1.1463	0.7273	1.2836	0
2141	886	0.5717	1.1464	0.7275	1.2846	0
2142	885.9	0.5716	1.1465	0.7275	1.2846	0
2143	885.8	0.5715	1.1462	0.7274	1.2851	0
2144	885.7	0.5715	1.1464	0.7273	1.285	0
2145	885.6	0.5716	1.146	0.7271	1.2839	0
2146	885.5	0.5717	1.1463	0.7275	1.2846	0
2147	885.4	0.5714	1.1463	0.7275	1.2843	0
2148	885.3	0.5715	1.1463	0.7276	1.2843	0
2149	885.2	0.572	1.1464	0.7276	1.2848	0
2150	885.1	0.5722	1.1463	0.728	1.284	0
2151	885	0.5722	1.1464	0.7278	1.2848	0
2152	884.9	0.572	1.1463	0.7277	1.2844	0
2153	884.8	0.5719	1.1468	0.728	1.285	0
2154	884.7	0.5718	1.1466	0.728	1.284	0
2155	884.6	0.5717	1.1461	0.7277	1.2845	0
2156	884.5	0.5719	1.1461	0.7279	1.284	0
2157	884.4	0.5722	1.1467	0.7282	1.2834	0
2158	884.3	0.5724	1.1462	0.7283	1.2822	0
2159	884.2	0.5721	1.146	0.7281	1.2835	0
2160	884.1	0.5721	1.1459	0.7282	1.2831	0
2161	884	0.5719	1.1455	0.7281	1.2839	0
2162	883.9	0.5716	1.1456	0.7285	1.2829	0
2163	883.8	0.5719	1.1463	0.7286	1.2827	0
2164	883.7	0.572	1.1459	0.7286	1.2826	0
2165	883.6	0.5719	1.146	0.7288	1.2823	0
2166	883.5	0.5722	1.1455	0.7289	1.2829	0
2167	883.4	0.5719	1.1459	0.729	1.2825	0
2168	883.3	0.5722	1.1459	0.7291	1.2816	0
2169	883.2	0.5726	1.1461	0.7291	1.2822	0
2170	883.1	0.5724	1.1461	0.7291	1.2817	0
2171	883	0.572	1.1456	0.7291	1.2817	0
2172	882.9	0.5723	1.1456	0.7293	1.2821	0
2173	882.8	0.5721	1.1454	0.7294	1.2814	0
2174	882.7	0.5723	1.1456	0.7293	1.282	0
2175	882.6	0.5719	1.1455	0.7292	1.2818	0
2176	882.5	0.5722	1.146	0.7297	1.2824	0
2177	882.4	0.5721	1.1455	0.7295	1.2812	0
2178	882.3	0.5721	1.1455	0.7295	1.2818	0
2179	882.2	0.572	1.1448	0.7295	1.2813	0

2180	882.1	0.572	1.1456	0.7295	1.2806	0
2181	882	0.5724	1.1454	0.7294	1.2812	0
2182	881.9	0.5721	1.1454	0.7295	1.2804	0
2183	881.8	0.572	1.1452	0.7296	1.2801	0
2184	881.7	0.5721	1.1449	0.7296	1.2805	0
2185	881.6	0.5721	1.1455	0.7297	1.2805	0
2186	881.5	0.5721	1.145	0.7299	1.2804	0
2187	881.4	0.572	1.1454	0.7297	1.2807	0
2188	881.3	0.572	1.1452	0.7299	1.2816	0
2189	881.2	0.5723	1.1457	0.7302	1.2808	0
2190	881.1	0.5722	1.1451	0.7301	1.2795	0
2191	881	0.5724	1.1455	0.7301	1.2803	0
2192	880.9	0.5722	1.1456	0.73	1.2796	0
2193	880.8	0.5727	1.1456	0.7302	1.2799	0
2194	880.7	0.5727	1.1454	0.7303	1.2804	0
2195	880.6	0.5724	1.1452	0.7301	1.2803	0
2196	880.5	0.5726	1.1453	0.7301	1.2802	0
2197	880.4	0.5725	1.1458	0.7303	1.2803	0
2198	880.3	0.5727	1.1456	0.7302	1.2794	0
2199	880.2	0.5725	1.1458	0.7302	1.2797	0
2200	880.1	0.5723	1.1455	0.7303	1.2796	0
2201	880	0.5721	1.1454	0.7301	1.2795	0
2202	879.9	0.5723	1.1455	0.73	1.28	0
2203	879.8	0.5723	1.1454	0.7301	1.2799	0
2204	879.7	0.5724	1.1458	0.7305	1.2797	0
2205	879.6	0.5725	1.1451	0.7303	1.2801	0
2206	879.5	0.5726	1.1457	0.7305	1.2795	0
2207	879.4	0.5725	1.1456	0.7302	1.2794	0
2208	879.3	0.5727	1.1457	0.7301	1.2792	0
2209	879.2	0.5728	1.1455	0.7305	1.2802	0
2210	879.1	0.5727	1.1451	0.7301	1.28	0
2211	879	0.5729	1.1458	0.7306	1.2799	0
2212	878.9	0.5728	1.1458	0.7306	1.2798	0
2213	878.8	0.5728	1.1458	0.7306	1.2797	0
2214	878.7	0.5728	1.146	0.7303	1.2806	0
2215	878.6	0.5727	1.146	0.7304	1.2807	0
2216	878.5	0.5729	1.1463	0.7304	1.2802	0
2217	878.4	0.5734	1.146	0.7304	1.2807	0
2218	878.3	0.5737	1.1455	0.7304	1.2807	0
2219	878.2	0.5734	1.1456	0.7303	1.2803	0
2220	878.1	0.5731	1.1461	0.7303	1.2806	0
2221	878	0.5731	1.1463	0.7303	1.2807	0
2222	877.9	0.5734	1.1463	0.7303	1.281	0

2223	877.8	0.5731	1.1462	0.7303	1.2809	0
2224	877.7	0.5734	1.1465	0.7301	1.2808	0
2225	877.6	0.5734	1.1467	0.7301	1.281	0
2226	877.5	0.5737	1.1469	0.7303	1.2813	0
2227	877.4	0.5732	1.1464	0.73	1.2811	0
2228	877.3	0.5735	1.1469	0.7301	1.2814	0
2229	877.2	0.5736	1.1467	0.7303	1.2815	0
2230	877.1	0.5739	1.1467	0.7298	1.28	0
2231	877	0.5738	1.1463	0.7302	1.2809	0
2232	876.9	0.5737	1.1468	0.7301	1.2808	0
2233	876.8	0.5733	1.1467	0.7302	1.281	0
2234	876.7	0.5735	1.1467	0.7302	1.2801	0
2235	876.6	0.5735	1.1468	0.7302	1.2814	0
2236	876.5	0.5739	1.1467	0.7303	1.2814	0
2237	876.4	0.5738	1.1465	0.7302	1.281	0
2238	876.3	0.5738	1.1464	0.7302	1.2813	0
2239	876.2	0.5739	1.1466	0.7301	1.2807	0
2240	876.1	0.574	1.1468	0.7304	1.2813	0
2241	876	0.5739	1.1466	0.7301	1.2808	0
2242	875.9	0.5737	1.1471	0.7305	1.2811	0
2243	875.8	0.5742	1.1472	0.7303	1.281	0
2244	875.7	0.5741	1.1466	0.7303	1.281	0
2245	875.6	0.574	1.1465	0.7304	1.2809	0
2246	875.5	0.5739	1.1468	0.7304	1.2811	0
2247	875.4	0.5738	1.1464	0.7301	1.2806	0
2248	875.3	0.5735	1.1465	0.7301	1.2805	0
2249	875.2	0.5738	1.1467	0.7302	1.2814	0
2250	875.1	0.5741	1.1468	0.7304	1.2815	0
2251	875	0.5744	1.1469	0.7307	1.2803	0
2252	874.9	0.5743	1.1467	0.7305	1.2808	0
2253	874.8	0.5738	1.1465	0.7306	1.2805	0
2254	874.7	0.5739	1.1466	0.7307	1.2798	0
2255	874.6	0.5741	1.1469	0.7307	1.2795	0
2256	874.5	0.5737	1.147	0.7308	1.2797	0
2257	874.4	0.5738	1.1471	0.7308	1.2801	0
2258	874.3	0.5741	1.1471	0.7311	1.2792	0
2259	874.2	0.5741	1.1467	0.7312	1.2796	0
2260	874.1	0.5743	1.1465	0.7312	1.2794	0
2261	874	0.5742	1.1468	0.7313	1.2794	0
2262	873.9	0.5739	1.1469	0.7312	1.2796	0
2263	873.8	0.5741	1.1465	0.7313	1.2793	0
2264	873.7	0.574	1.1464	0.7313	1.2786	0
2265	873.6	0.5742	1.1471	0.7315	1.2793	0

2266	873.5	0.574	1.1463	0.7313	1.2787	0
2267	873.4	0.5741	1.1462	0.7314	1.2784	0
2268	873.3	0.5744	1.1461	0.7314	1.2784	0
2269	873.2	0.574	1.146	0.7314	1.2786	0
2270	873.1	0.5741	1.147	0.7314	1.2787	0
2271	873	0.5738	1.1461	0.7314	1.2782	0
2272	872.9	0.574	1.1465	0.7316	1.2785	0
2273	872.8	0.574	1.1461	0.7316	1.2784	0
2274	872.7	0.574	1.1459	0.7315	1.2779	0
2275	872.6	0.5739	1.146	0.7316	1.2777	0
2276	872.5	0.5747	1.1465	0.7318	1.2776	0
2277	872.4	0.5747	1.1462	0.7318	1.2784	0
2278	872.3	0.5745	1.1463	0.7318	1.2787	0
2279	872.2	0.5746	1.1465	0.7318	1.2781	0
2280	872.1	0.5743	1.1469	0.7321	1.278	0
2281	872	0.5743	1.1465	0.7319	1.2782	0
2282	871.9	0.5744	1.146	0.7319	1.2787	0
2283	871.8	0.5744	1.1462	0.7318	1.2784	0
2284	871.7	0.5745	1.1464	0.732	1.2778	0
2285	871.6	0.5743	1.1458	0.732	1.2773	0
2286	871.5	0.5745	1.1462	0.7319	1.2773	0
2287	871.4	0.5745	1.1464	0.732	1.2778	0
2288	871.3	0.5745	1.1464	0.7322	1.2773	0
2289	871.2	0.5746	1.1463	0.7321	1.2781	0
2290	871.1	0.5747	1.1467	0.7319	1.2783	0
2291	871	0.5746	1.1466	0.732	1.2782	0
2292	870.9	0.5745	1.1461	0.732	1.2779	0
2293	870.8	0.5745	1.1474	0.732	1.2775	0
2294	870.7	0.5747	1.1469	0.7319	1.2783	0
2295	870.6	0.5744	1.1467	0.7318	1.2785	0
2296	870.5	0.5747	1.1468	0.7318	1.2784	0
2297	870.4	0.5744	1.1467	0.7318	1.2783	0
2298	870.3	0.5748	1.147	0.7319	1.2789	0
2299	870.2	0.5747	1.1473	0.7319	1.2788	0
2300	870.1	0.575	1.1472	0.7318	1.2793	0
2301	870	0.5752	1.1472	0.7318	1.2796	0
2302	869.9	0.5748	1.1472	0.7315	1.2788	0
2303	869.8	0.575	1.1474	0.7318	1.2787	0
2304	869.7	0.5752	1.1471	0.7315	1.279	0
2305	869.6	0.5752	1.1475	0.7316	1.2789	0
2306	869.5	0.5748	1.1471	0.7315	1.2787	0
2307	869.4	0.5752	1.1478	0.7316	1.2794	0
2308	869.3	0.5753	1.1481	0.7318	1.2798	0

2309	869.2	0.5753	1.1479	0.7316	1.279	0
2310	869.1	0.5758	1.1486	0.7315	1.2799	0
2311	869	0.5758	1.1478	0.7316	1.2804	0
2312	868.9	0.5756	1.1479	0.7317	1.2793	0
2313	868.8	0.5756	1.1478	0.7317	1.2802	0
2314	868.7	0.5758	1.1479	0.7316	1.2791	0
2315	868.6	0.5755	1.1478	0.7315	1.2797	0
2316	868.5	0.5757	1.148	0.7315	1.2796	0
2317	868.4	0.576	1.1479	0.7315	1.2796	0
2318	868.3	0.5756	1.1482	0.7316	1.2793	0
2319	868.2	0.5759	1.148	0.7318	1.2799	0
2320	868.1	0.5758	1.1482	0.7316	1.2797	0
2321	868	0.5757	1.1485	0.7317	1.2792	0
2322	867.9	0.576	1.1476	0.7317	1.2794	0
2323	867.8	0.5757	1.1479	0.7317	1.279	0
2324	867.7	0.5755	1.148	0.7317	1.2792	0
2325	867.6	0.576	1.1484	0.7319	1.2796	0
2326	867.5	0.5759	1.1483	0.7318	1.2789	0
2327	867.4	0.5761	1.148	0.7318	1.2785	0
2328	867.3	0.5764	1.1485	0.732	1.2783	0
2329	867.2	0.5761	1.1477	0.7319	1.2788	0
2330	867.1	0.5757	1.1481	0.732	1.2793	0
2331	867	0.5762	1.1482	0.732	1.2797	0
2332	866.9	0.576	1.1482	0.7318	1.2789	0
2333	866.8	0.576	1.1481	0.732	1.279	0
2334	866.7	0.5762	1.1479	0.7323	1.2785	0
2335	866.6	0.576	1.148	0.7321	1.2796	0
2336	866.5	0.5762	1.1478	0.7319	1.2793	0
2337	866.4	0.5761	1.1487	0.7322	1.2785	0
2338	866.3	0.5762	1.1483	0.7323	1.2779	0
2339	866.2	0.5758	1.1483	0.7324	1.2779	0
2340	866.1	0.5758	1.1481	0.7323	1.278	0
2341	866	0.5757	1.1479	0.7323	1.278	0
2342	865.9	0.5757	1.1479	0.7324	1.2783	0
2343	865.8	0.5759	1.148	0.7326	1.2781	0
2344	865.7	0.5759	1.1481	0.7325	1.2777	0
2345	865.6	0.5759	1.1481	0.7325	1.277	0
2346	865.5	0.5761	1.1479	0.7327	1.2777	0
2347	865.4	0.5761	1.1476	0.7328	1.2773	0
2348	865.3	0.576	1.1476	0.7329	1.2782	0
2349	865.2	0.576	1.1476	0.733	1.277	0
2350	865.1	0.5759	1.1479	0.7331	1.2772	0
2351	865	0.5761	1.148	0.7332	1.277	0

2352	864.9	0.5763	1.1476	0.7333	1.2774	0
2353	864.8	0.5762	1.1476	0.7335	1.2759	0
2354	864.7	0.5761	1.1473	0.7334	1.2764	0
2355	864.6	0.5761	1.1472	0.7334	1.2763	0
2356	864.5	0.5763	1.1473	0.7336	1.2764	0
2357	864.4	0.5764	1.1476	0.7338	1.2777	0
2358	864.3	0.5761	1.1476	0.7338	1.2758	0
2359	864.2	0.5761	1.1471	0.7339	1.2749	0
2360	864.1	0.5763	1.1472	0.7339	1.2753	0
2361	864	0.576	1.1471	0.734	1.2757	0
2362	863.9	0.5762	1.1471	0.7339	1.2751	0
2363	863.8	0.5761	1.1472	0.7343	1.2756	0
2364	863.7	0.5763	1.1474	0.7341	1.2755	0
2365	863.6	0.576	1.1473	0.7339	1.2754	0
2366	863.5	0.5762	1.1475	0.7343	1.2753	0
2367	863.4	0.5762	1.1472	0.7344	1.275	0
2368	863.3	0.5756	1.1469	0.7343	1.2747	0
2369	863.2	0.5759	1.1471	0.7344	1.2745	0
2370	863.1	0.5761	1.1469	0.7345	1.2752	0
2371	863	0.5761	1.147	0.7343	1.2745	0
2372	862.9	0.5761	1.1469	0.7344	1.275	0
2373	862.8	0.5759	1.1467	0.7344	1.2747	0
2374	862.7	0.5763	1.1467	0.7346	1.2744	0
2375	862.6	0.5761	1.1473	0.7345	1.274	0
2376	862.5	0.5764	1.147	0.7346	1.2742	0
2377	862.4	0.5761	1.1469	0.7345	1.2739	0
2378	862.3	0.5761	1.1468	0.7346	1.2748	0
2379	862.2	0.5761	1.1468	0.7345	1.2744	0
2380	862.1	0.5763	1.1468	0.7345	1.2735	0
2381	862	0.5763	1.147	0.7346	1.2743	0
2382	861.9	0.5763	1.147	0.7346	1.2737	0
2383	861.8	0.5761	1.1466	0.7344	1.2739	0
2384	861.7	0.5764	1.1471	0.7346	1.2742	0
2385	861.6	0.5765	1.1471	0.7346	1.2745	0
2386	861.5	0.5766	1.1474	0.7347	1.2743	0
2387	861.4	0.5765	1.1473	0.7348	1.2745	0
2388	861.3	0.5766	1.1476	0.7348	1.2742	0
2389	861.2	0.5769	1.1473	0.7347	1.2748	0
2390	861.1	0.5769	1.1474	0.7347	1.2747	0
2391	861	0.5767	1.1477	0.7346	1.2742	0
2392	860.9	0.5767	1.1475	0.7347	1.2748	0
2393	860.8	0.5767	1.1476	0.7345	1.2744	0
2394	860.7	0.5769	1.1474	0.7345	1.2753	0

2395	860.6	0.577	1.1484	0.7345	1.2752	0
2396	860.5	0.5767	1.1481	0.7344	1.2751	0
2397	860.4	0.577	1.1482	0.7345	1.2752	0
2398	860.3	0.577	1.1484	0.7346	1.2752	0
2399	860.2	0.5771	1.1487	0.7346	1.2752	0
2400	860.1	0.5771	1.1485	0.7344	1.2751	0
2401	860	0.5771	1.1483	0.7343	1.2751	0
2402	859.9	0.5774	1.1485	0.7343	1.2764	0
2403	859.8	0.5772	1.1485	0.7341	1.2761	0
2404	859.7	0.5773	1.1486	0.7341	1.2759	0
2405	859.6	0.5772	1.1487	0.734	1.2761	0
2406	859.5	0.5776	1.1492	0.7338	1.275	0
2407	859.4	0.5778	1.1489	0.7341	1.2776	0
2408	859.3	0.578	1.1491	0.7339	1.2772	0
2409	859.2	0.5777	1.1487	0.7339	1.2768	0
2410	859.1	0.5779	1.1498	0.7338	1.2774	0
2411	859	0.5776	1.1496	0.7337	1.2769	0
2412	858.9	0.5777	1.1498	0.7338	1.2775	0
2413	858.8	0.5779	1.1498	0.7338	1.278	0
2414	858.7	0.578	1.1493	0.7336	1.278	0
2415	858.6	0.5782	1.1499	0.7336	1.2773	0
2416	858.5	0.5783	1.15	0.7335	1.2778	0
2417	858.4	0.578	1.1499	0.7334	1.2774	0
2418	858.3	0.578	1.15	0.7332	1.2779	0
2419	858.2	0.578	1.1502	0.7334	1.2776	0
2420	858.1	0.5782	1.1503	0.7332	1.2777	0
2421	858	0.5784	1.1504	0.7334	1.2778	0
2422	857.9	0.5784	1.1504	0.7333	1.2792	0
2423	857.8	0.5784	1.1505	0.7335	1.278	0
2424	857.7	0.5784	1.1504	0.7332	1.2791	0
2425	857.6	0.5786	1.1507	0.7332	1.2788	0
2426	857.5	0.5784	1.1508	0.7331	1.2788	0
2427	857.4	0.5783	1.1505	0.733	1.2787	0
2428	857.3	0.5782	1.1501	0.7332	1.2778	0
2429	857.2	0.5784	1.1503	0.7331	1.2781	0
2430	857.1	0.5784	1.1504	0.7332	1.2777	0
2431	857	0.5786	1.1504	0.7331	1.2777	0
2432	856.9	0.5785	1.1504	0.7331	1.278	0
2433	856.8	0.5788	1.1502	0.7335	1.278	0
2434	856.7	0.5789	1.15	0.7338	1.2775	0
2435	856.6	0.5785	1.1508	0.7338	1.2775	0
2436	856.5	0.5786	1.1508	0.7336	1.2779	0
2437	856.4	0.5786	1.1502	0.7339	1.2771	0

2438	856.3	0.5784	1.1501	0.7341	1.2771	0
2439	856.2	0.5785	1.15	0.7341	1.2768	0
2440	856.1	0.5787	1.1503	0.734	1.2765	0
2441	856	0.5785	1.1503	0.7342	1.2762	0
2442	855.9	0.5784	1.1499	0.7342	1.2763	0
2443	855.8	0.5783	1.1499	0.7345	1.2769	0
2444	855.7	0.5785	1.1504	0.7344	1.2766	0
2445	855.6	0.5784	1.1495	0.7346	1.2764	0
2446	855.5	0.5782	1.1498	0.7347	1.2762	0
2447	855.4	0.5778	1.1496	0.7346	1.2759	0
2448	855.3	0.5783	1.1493	0.7349	1.2755	0
2449	855.2	0.5785	1.1493	0.7349	1.2752	0
2450	855.1	0.5783	1.1491	0.7349	1.2754	0
2451	855	0.5782	1.1495	0.7353	1.2744	0
2452	854.9	0.5786	1.1491	0.735	1.2757	0
2453	854.8	0.5786	1.1493	0.7353	1.2742	0
2454	854.7	0.5789	1.1491	0.7354	1.2745	0
2455	854.6	0.5783	1.1491	0.7353	1.2745	0
2456	854.5	0.5785	1.1488	0.7358	1.2737	0
2457	854.4	0.5784	1.1488	0.7356	1.2737	0
2458	854.3	0.5785	1.1493	0.7359	1.2746	0
2459	854.2	0.578	1.1488	0.7361	1.2737	0
2460	854.1	0.578	1.1487	0.736	1.2733	0
2461	854	0.5782	1.1486	0.7362	1.2725	0
2462	853.9	0.5783	1.1488	0.7363	1.273	0
2463	853.8	0.5784	1.1478	0.7364	1.2721	0
2464	853.7	0.5785	1.1483	0.7364	1.2725	0
2465	853.6	0.5786	1.1483	0.7366	1.272	0
2466	853.5	0.5784	1.1485	0.7365	1.2721	0
2467	853.4	0.5787	1.1485	0.7367	1.2722	0
2468	853.3	0.5785	1.1484	0.7368	1.2718	0
2469	853.2	0.5787	1.1482	0.7368	1.2727	0
2470	853.1	0.5785	1.1482	0.7369	1.2722	0
2471	853	0.5783	1.1479	0.7368	1.2712	0
2472	852.9	0.5783	1.1486	0.737	1.2718	0
2473	852.8	0.5781	1.1481	0.7371	1.2718	0
2474	852.7	0.5781	1.1481	0.7369	1.2714	0
2475	852.6	0.5784	1.1481	0.737	1.2712	0
2476	852.5	0.5788	1.1483	0.7372	1.2714	0
2477	852.4	0.5787	1.1479	0.7372	1.2717	0
2478	852.3	0.5786	1.1478	0.7372	1.2711	0
2479	852.2	0.5787	1.1485	0.7372	1.2716	0
2480	852.1	0.5786	1.1485	0.7375	1.2717	0

2481	852	0.5786	1.1481	0.7374	1.2711	0
2482	851.9	0.579	1.1485	0.7376	1.2705	0
2483	851.8	0.5786	1.1481	0.7374	1.2702	0
2484	851.7	0.5789	1.1482	0.7374	1.2715	0
2485	851.6	0.5786	1.1479	0.7376	1.2711	0
2486	851.5	0.5787	1.1479	0.7376	1.2715	0
2487	851.4	0.5786	1.1487	0.7377	1.2715	0
2488	851.3	0.5789	1.1486	0.7378	1.2722	0
2489	851.2	0.5789	1.1487	0.7375	1.2718	0
2490	851.1	0.5792	1.1481	0.7374	1.272	0
2491	851	0.579	1.149	0.7375	1.2717	0
2492	850.9	0.5788	1.1485	0.7374	1.2714	0
2493	850.8	0.5793	1.1487	0.7374	1.2719	0
2494	850.7	0.5793	1.1485	0.7373	1.2717	0
2495	850.6	0.5793	1.1494	0.7374	1.2726	0
2496	850.5	0.5794	1.149	0.7375	1.2729	0
2497	850.4	0.5794	1.1493	0.7373	1.2725	0
2498	850.3	0.5793	1.1493	0.7371	1.2722	0
2499	850.2	0.5792	1.1496	0.7373	1.2722	0
2500	850.1	0.5793	1.1498	0.7373	1.2727	0
2501	850	0.579	1.1492	0.7368	1.2722	0
2502	849.9	0.5531	1.1305	0.7311	1.2664	0
2503	849.8	0.5532	1.1305	0.7311	1.2666	0
2504	849.7	0.5531	1.1306	0.731	1.2668	0
2505	849.6	0.5532	1.1303	0.7307	1.2664	0
2506	849.5	0.553	1.1305	0.7309	1.267	0
2507	849.4	0.5532	1.1307	0.7309	1.2667	0
2508	849.3	0.5536	1.1308	0.7311	1.2674	0
2509	849.2	0.5537	1.1307	0.7307	1.2667	0
2510	849.1	0.5535	1.1311	0.7306	1.2673	0
2511	849	0.5534	1.1313	0.7304	1.2681	0
2512	848.9	0.5538	1.1311	0.7304	1.2673	0
2513	848.8	0.5539	1.1315	0.7304	1.267	0
2514	848.7	0.5541	1.1312	0.7306	1.2685	0
2515	848.6	0.5541	1.1318	0.7302	1.268	0
2516	848.5	0.554	1.1317	0.7302	1.2685	0
2517	848.4	0.5537	1.1314	0.7301	1.2687	0
2518	848.3	0.5539	1.1319	0.7302	1.2691	0
2519	848.2	0.554	1.1318	0.73	1.2687	0
2520	848.1	0.554	1.1321	0.73	1.269	0
2521	848	0.5539	1.132	0.7301	1.2684	0
2522	847.9	0.5541	1.1322	0.73	1.2687	0
2523	847.8	0.554	1.1323	0.73	1.2691	0

2524	847.7	0.5544	1.133	0.7298	1.2692	0
2525	847.6	0.5544	1.1325	0.7297	1.2691	0
2526	847.5	0.5541	1.1326	0.7298	1.2689	0
2527	847.4	0.5542	1.1325	0.7297	1.2694	0
2528	847.3	0.5542	1.1329	0.7297	1.2691	0
2529	847.2	0.5543	1.1329	0.7298	1.2697	0
2530	847.1	0.5542	1.133	0.7296	1.2685	0
2531	847	0.5543	1.1328	0.7295	1.2693	0
2532	846.9	0.5546	1.1329	0.7296	1.2691	0
2533	846.8	0.5544	1.1331	0.7296	1.2699	0
2534	846.7	0.5545	1.133	0.7298	1.2695	0
2535	846.6	0.5546	1.133	0.7298	1.2695	0
2536	846.5	0.555	1.1332	0.7299	1.2691	0
2537	846.4	0.5545	1.1327	0.7296	1.2689	0
2538	846.3	0.5549	1.1332	0.7297	1.2687	0
2539	846.2	0.5551	1.1334	0.73	1.2699	0
2540	846.1	0.5547	1.1338	0.7302	1.2694	0
2541	846	0.555	1.1338	0.7301	1.2694	0
2542	845.9	0.5548	1.133	0.73	1.2692	0
2543	845.8	0.5548	1.1329	0.7299	1.2692	0
2544	845.7	0.5548	1.1334	0.7303	1.269	0
2545	845.6	0.5548	1.1336	0.7303	1.2691	0
2546	845.5	0.5548	1.1327	0.7305	1.2692	0
2547	845.4	0.5548	1.1327	0.7306	1.2691	0
2548	845.3	0.5551	1.1326	0.7308	1.2675	0
2549	845.2	0.555	1.1331	0.731	1.2687	0
2550	845.1	0.555	1.1327	0.731	1.2679	0
2551	845	0.555	1.1327	0.7309	1.2677	0
2552	844.9	0.5547	1.1323	0.7311	1.2657	0
2553	844.8	0.5547	1.1323	0.7312	1.267	0
2554	844.7	0.5548	1.1322	0.7314	1.2669	0
2555	844.6	0.5547	1.1323	0.7315	1.2658	0
2556	844.5	0.5549	1.1321	0.7315	1.2656	0
2557	844.4	0.5548	1.1319	0.7322	1.2661	0
2558	844.3	0.5549	1.132	0.7321	1.2654	0
2559	844.2	0.5548	1.1318	0.7325	1.2647	0
2560	844.1	0.5545	1.1311	0.7324	1.2652	0
2561	844	0.5544	1.1319	0.7325	1.2637	0
2562	843.9	0.5548	1.1317	0.7327	1.2642	0
2563	843.8	0.555	1.1318	0.7329	1.2643	0
2564	843.7	0.5547	1.1314	0.733	1.2646	0
2565	843.6	0.5546	1.1312	0.7329	1.264	0
2566	843.5	0.5547	1.1311	0.733	1.2633	0

2567	843.4	0.5547	1.1308	0.7332	1.2637	0
2568	843.3	0.5546	1.1311	0.7335	1.2641	0
2569	843.2	0.5546	1.131	0.7337	1.2634	0
2570	843.1	0.5548	1.1311	0.7336	1.2631	0
2571	843	0.5551	1.1309	0.7336	1.2632	0
2572	842.9	0.555	1.131	0.7338	1.2628	0
2573	842.8	0.555	1.1311	0.7337	1.2626	0
2574	842.7	0.5547	1.1311	0.7338	1.2621	0
2575	842.6	0.555	1.1312	0.7339	1.2618	0
2576	842.5	0.5548	1.1308	0.7338	1.2622	0
2577	842.4	0.555	1.1305	0.7341	1.2617	0
2578	842.3	0.5551	1.131	0.7341	1.2618	0
2579	842.2	0.555	1.1312	0.734	1.2621	0
2580	842.1	0.555	1.1312	0.7341	1.2618	0
2581	842	0.555	1.1313	0.7343	1.2616	0
2582	841.9	0.5552	1.1312	0.7343	1.2617	0
2583	841.8	0.5551	1.1317	0.7344	1.2616	0
2584	841.7	0.5552	1.1314	0.7343	1.2622	0
2585	841.6	0.5552	1.1314	0.7345	1.2617	0
2586	841.5	0.5549	1.1305	0.7343	1.2612	0
2587	841.4	0.5551	1.1311	0.7342	1.2613	0
2588	841.3	0.5549	1.131	0.7343	1.2617	0
2589	841.2	0.555	1.1306	0.7345	1.2608	0
2590	841.1	0.555	1.1311	0.7346	1.261	0
2591	841	0.5555	1.1315	0.7347	1.2609	0
2592	840.9	0.5554	1.1312	0.7347	1.2611	0
2593	840.8	0.5553	1.1315	0.7345	1.2614	0
2594	840.7	0.5552	1.1314	0.7348	1.2613	0
2595	840.6	0.5554	1.1319	0.7346	1.2611	0
2596	840.5	0.5555	1.1317	0.7345	1.2616	0
2597	840.4	0.5556	1.1317	0.7345	1.2614	0
2598	840.3	0.5556	1.132	0.7344	1.2616	0
2599	840.2	0.5556	1.1318	0.7346	1.2615	0
2600	840.1	0.5551	1.132	0.7345	1.261	0
2601	840	0.5554	1.1313	0.7344	1.2619	0
2602	839.9	0.5557	1.1319	0.7344	1.2624	0
2603	839.8	0.5556	1.1325	0.7342	1.2614	0
2604	839.7	0.556	1.1324	0.7343	1.2615	0
2605	839.6	0.5557	1.1324	0.7343	1.2623	0
2606	839.5	0.5559	1.1328	0.7342	1.2622	0
2607	839.4	0.5562	1.1325	0.7341	1.2624	0
2608	839.3	0.556	1.1326	0.734	1.262	0
2609	839.2	0.5562	1.1331	0.734	1.2629	0

2610	839.1	0.5561	1.1329	0.7339	1.2629	0
2611	839	0.5566	1.1333	0.7341	1.2633	0
2612	838.9	0.5565	1.133	0.7338	1.2636	0
2613	838.8	0.5566	1.1333	0.7336	1.264	0
2614	838.7	0.5567	1.1333	0.7336	1.2631	0
2615	838.6	0.5565	1.1334	0.7335	1.2634	0
2616	838.5	0.5566	1.1336	0.7333	1.2641	0
2617	838.4	0.5566	1.1338	0.7331	1.2642	0
2618	838.3	0.5567	1.1343	0.7334	1.2648	0
2619	838.2	0.5569	1.134	0.7332	1.2645	0
2620	838.1	0.5571	1.1341	0.7332	1.2656	0
2621	838	0.5572	1.134	0.7331	1.2653	0
2622	837.9	0.557	1.1339	0.7328	1.2649	0
2623	837.8	0.5571	1.1347	0.733	1.2648	0
2624	837.7	0.5572	1.1344	0.7331	1.2647	0
2625	837.6	0.5572	1.1346	0.733	1.2655	0
2626	837.5	0.5573	1.135	0.7329	1.2654	0
2627	837.4	0.5573	1.1346	0.7328	1.2654	0
2628	837.3	0.5574	1.1352	0.7328	1.2657	0
2629	837.2	0.5574	1.1353	0.7325	1.2656	0
2630	837.1	0.5575	1.1354	0.7324	1.2657	0
2631	837	0.5576	1.1353	0.7325	1.2666	0
2632	836.9	0.5575	1.1343	0.7327	1.2662	0
2633	836.8	0.5575	1.1353	0.7328	1.266	0
2634	836.7	0.5576	1.1361	0.7326	1.266	0
2635	836.6	0.5578	1.1355	0.7326	1.2658	0
2636	836.5	0.5578	1.1352	0.7326	1.2664	0
2637	836.4	0.5577	1.1357	0.7327	1.2662	0
2638	836.3	0.5576	1.1358	0.7326	1.2663	0
2639	836.2	0.5578	1.1355	0.7324	1.2665	0
2640	836.1	0.5578	1.1359	0.7324	1.2662	0
2641	836	0.5578	1.1356	0.7325	1.2658	0
2642	835.9	0.5579	1.1362	0.7326	1.2657	0
2643	835.8	0.5578	1.1357	0.7327	1.266	0
2644	835.7	0.5581	1.1358	0.7329	1.2664	0
2645	835.6	0.5585	1.1359	0.7328	1.2668	0
2646	835.5	0.5582	1.1354	0.733	1.2667	0
2647	835.4	0.5584	1.1358	0.7329	1.2658	0
2648	835.3	0.5583	1.1356	0.7331	1.2663	0
2649	835.2	0.5581	1.1358	0.7329	1.2663	0
2650	835.1	0.5581	1.1358	0.7331	1.2659	0
2651	835	0.5583	1.135	0.7333	1.2655	0
2652	834.9	0.5583	1.1355	0.7334	1.2652	0

2653	834.8	0.5582	1.136	0.7336	1.2655	0
2654	834.7	0.5583	1.1358	0.7334	1.2647	0
2655	834.6	0.5583	1.1354	0.7335	1.2657	0
2656	834.5	0.5584	1.1356	0.7339	1.2648	0
2657	834.4	0.5585	1.1357	0.734	1.265	0
2658	834.3	0.5585	1.1354	0.734	1.2643	0
2659	834.2	0.5585	1.1355	0.7341	1.2644	0
2660	834.1	0.5585	1.1352	0.734	1.2636	0
2661	834	0.5585	1.1355	0.7341	1.2646	0
2662	833.9	0.5582	1.1351	0.7341	1.2641	0
2663	833.8	0.5583	1.1352	0.7345	1.2638	0
2664	833.7	0.5583	1.1346	0.7345	1.2636	0
2665	833.6	0.5584	1.1348	0.7347	1.263	0
2666	833.5	0.5584	1.1348	0.7349	1.2626	0
2667	833.4	0.5585	1.135	0.7351	1.2628	0
2668	833.3	0.5583	1.1345	0.7351	1.2628	0
2669	833.2	0.5584	1.1353	0.7352	1.2617	0
2670	833.1	0.5584	1.1356	0.7354	1.2619	0
2671	833	0.5583	1.1347	0.7355	1.2619	0
2672	832.9	0.5583	1.1345	0.7356	1.2621	0
2673	832.8	0.5581	1.1344	0.7356	1.2609	0
2674	832.7	0.5581	1.1345	0.7357	1.2613	0
2675	832.6	0.5583	1.1346	0.7357	1.2611	0
2676	832.5	0.558	1.1347	0.7359	1.2615	0
2677	832.4	0.5582	1.1343	0.7359	1.2615	0
2678	832.3	0.5585	1.1347	0.7361	1.2613	0
2679	832.2	0.5585	1.1345	0.7362	1.26	0
2680	832.1	0.5587	1.1347	0.7362	1.261	0
2681	832	0.5586	1.1342	0.7363	1.2609	0
2682	831.9	0.5584	1.1341	0.7365	1.2605	0
2683	831.8	0.5587	1.1347	0.7364	1.2616	0
2684	831.7	0.5587	1.1344	0.7365	1.2599	0
2685	831.6	0.5589	1.1348	0.7368	1.2598	0
2686	831.5	0.5586	1.1344	0.7367	1.2604	0
2687	831.4	0.5588	1.1343	0.7367	1.2597	0
2688	831.3	0.559	1.1348	0.7368	1.2596	0
2689	831.2	0.5589	1.1345	0.7366	1.2599	0
2690	831.1	0.5588	1.1347	0.7365	1.2588	0
2691	831	0.5591	1.1347	0.7367	1.2593	0
2692	830.9	0.5591	1.1341	0.7367	1.2596	0
2693	830.8	0.559	1.1346	0.7368	1.2594	0
2694	830.7	0.5588	1.1344	0.7368	1.2599	0
2695	830.6	0.5589	1.1342	0.7369	1.2599	0

2696	830.5	0.5592	1.1343	0.737	1.2597	0
2697	830.4	0.5588	1.1346	0.7369	1.2592	0
2698	830.3	0.5589	1.1344	0.7369	1.2594	0
2699	830.2	0.5591	1.1345	0.7369	1.2596	0
2700	830.1	0.559	1.1349	0.737	1.2594	0
2701	830	0.5592	1.1348	0.7371	1.2592	0
2702	829.9	0.5592	1.1349	0.737	1.2592	0
2703	829.8	0.5587	1.135	0.7371	1.2601	0
2704	829.7	0.5592	1.1354	0.7371	1.2603	0
2705	829.6	0.5593	1.135	0.737	1.2595	0
2706	829.5	0.5592	1.1348	0.737	1.2602	0
2707	829.4	0.5594	1.135	0.7369	1.2597	0
2708	829.3	0.5595	1.1349	0.7369	1.2598	0
2709	829.2	0.5593	1.1351	0.737	1.2599	0
2710	829.1	0.5594	1.1357	0.7371	1.2603	0
2711	829	0.56	1.1356	0.7369	1.2604	0
2712	828.9	0.5596	1.1356	0.7369	1.2606	0
2713	828.8	0.5599	1.1358	0.7368	1.2602	0
2714	828.7	0.5601	1.1357	0.7368	1.2602	0
2715	828.6	0.5603	1.1361	0.7367	1.2611	0
2716	828.5	0.5599	1.136	0.7366	1.2609	0
2717	828.4	0.5601	1.136	0.7366	1.2609	0
2718	828.3	0.56	1.1362	0.7365	1.2613	0
2719	828.2	0.5603	1.1364	0.7364	1.2611	0
2720	828.1	0.5602	1.1368	0.7363	1.2615	0
2721	828	0.5603	1.1365	0.7361	1.2617	0
2722	827.9	0.5604	1.136	0.7362	1.2618	0
2723	827.8	0.5604	1.1363	0.7364	1.2617	0
2724	827.7	0.5603	1.1366	0.7364	1.2612	0
2725	827.6	0.5607	1.1367	0.7362	1.2628	0
2726	827.5	0.5606	1.1364	0.7361	1.2629	0
2727	827.4	0.5605	1.1368	0.7361	1.2625	0
2728	827.3	0.5608	1.137	0.7363	1.2624	0
2729	827.2	0.5611	1.1375	0.7362	1.2626	0
2730	827.1	0.5612	1.1372	0.7361	1.2627	0
2731	827	0.561	1.1371	0.7362	1.2625	0
2732	826.9	0.5609	1.1378	0.7362	1.2616	0
2733	826.8	0.561	1.1377	0.7361	1.263	0
2734	826.7	0.5607	1.1374	0.7361	1.2623	0
2735	826.6	0.561	1.1369	0.7361	1.2627	0
2736	826.5	0.5612	1.1375	0.7359	1.2622	0
2737	826.4	0.5613	1.1377	0.7359	1.2623	0
2738	826.3	0.5616	1.1377	0.736	1.2628	0

2739	826.2	0.5612	1.1379	0.736	1.2633	0
2740	826.1	0.5614	1.1378	0.736	1.2636	0
2741	826	0.5615	1.138	0.7361	1.2636	0
2742	825.9	0.5613	1.138	0.736	1.2631	0
2743	825.8	0.5614	1.138	0.7359	1.2634	0
2744	825.7	0.5612	1.1384	0.736	1.2632	0
2745	825.6	0.5612	1.1384	0.7359	1.2636	0
2746	825.5	0.5614	1.1381	0.7359	1.2637	0
2747	825.4	0.5615	1.1377	0.736	1.2638	0
2748	825.3	0.5615	1.1382	0.736	1.2631	0
2749	825.2	0.5617	1.1389	0.736	1.2633	0
2750	825.1	0.5618	1.1383	0.736	1.2626	0
2751	825	0.5617	1.1384	0.7361	1.2622	0
2752	824.9	0.5617	1.138	0.7364	1.2622	0
2753	824.8	0.5617	1.1383	0.7364	1.263	0
2754	824.7	0.5616	1.1379	0.7363	1.2629	0
2755	824.6	0.562	1.1383	0.7367	1.2629	0
2756	824.5	0.5619	1.138	0.7365	1.2626	0
2757	824.4	0.562	1.1382	0.7364	1.2628	0
2758	824.3	0.5618	1.138	0.7365	1.2627	0
2759	824.2	0.5618	1.138	0.7367	1.2618	0
2760	824.1	0.5619	1.1379	0.7367	1.2622	0
2761	824	0.562	1.1381	0.7368	1.2616	0
2762	823.9	0.5622	1.1383	0.7369	1.2616	0
2763	823.8	0.562	1.1385	0.737	1.2615	0
2764	823.7	0.5621	1.1383	0.7371	1.2616	0
2765	823.6	0.5622	1.1382	0.7371	1.2625	0
2766	823.5	0.5624	1.1382	0.7372	1.2622	0
2767	823.4	0.5621	1.1381	0.7373	1.2613	0
2768	823.3	0.5621	1.138	0.7374	1.2612	0
2769	823.2	0.5626	1.1375	0.7374	1.2614	0
2770	823.1	0.5624	1.1378	0.7376	1.2616	0
2771	823	0.5622	1.1377	0.7376	1.2616	0
2772	822.9	0.5624	1.1381	0.7374	1.261	0
2773	822.8	0.5626	1.1379	0.7379	1.2618	0
2774	822.7	0.5625	1.1384	0.7379	1.2606	0
2775	822.6	0.5625	1.1386	0.7378	1.261	0
2776	822.5	0.5623	1.1381	0.7379	1.2609	0
2777	822.4	0.5625	1.138	0.7381	1.2606	0
2778	822.3	0.5625	1.1379	0.7381	1.261	0
2779	822.2	0.5629	1.1384	0.7382	1.2613	0
2780	822.1	0.5627	1.1381	0.7382	1.2609	0
2781	822	0.5625	1.1384	0.7383	1.26	0

2782	821.9	0.5626	1.138	0.7384	1.2606	0
2783	821.8	0.5625	1.1377	0.7384	1.2602	0
2784	821.7	0.5627	1.1383	0.7384	1.2607	0
2785	821.6	0.5627	1.1382	0.7385	1.26	0
2786	821.5	0.5629	1.1383	0.7387	1.2603	0
2787	821.4	0.563	1.1382	0.7388	1.2606	0
2788	821.3	0.563	1.1383	0.7388	1.2596	0
2789	821.2	0.5629	1.1384	0.7389	1.2603	0
2790	821.1	0.5631	1.1384	0.7388	1.2596	0
2791	821	0.5628	1.1385	0.7386	1.2596	0
2792	820.9	0.5629	1.1383	0.7388	1.26	0
2793	820.8	0.5631	1.1384	0.7388	1.2597	0
2794	820.7	0.563	1.1386	0.7388	1.2598	0
2795	820.6	0.563	1.1388	0.7387	1.26	0
2796	820.5	0.5632	1.1385	0.7388	1.2604	0
2797	820.4	0.5633	1.1382	0.7389	1.2591	0
2798	820.3	0.5635	1.1386	0.739	1.2601	0
2799	820.2	0.5632	1.1382	0.7388	1.2599	0
2800	820.1	0.5634	1.1384	0.7391	1.26	0
2801	820	0.5635	1.1384	0.7391	1.2594	0
2802	819.9	0.5635	1.1384	0.7392	1.2599	0
2803	819.8	0.5638	1.1385	0.7394	1.2598	0
2804	819.7	0.5636	1.1386	0.7392	1.2595	0
2805	819.6	0.5636	1.1387	0.7393	1.2592	0
2806	819.5	0.5638	1.1387	0.7392	1.2594	0
2807	819.4	0.5637	1.1394	0.7393	1.2595	0
2808	819.3	0.564	1.1388	0.7394	1.2589	0
2809	819.2	0.5637	1.1386	0.7395	1.2598	0
2810	819.1	0.5635	1.1388	0.7392	1.2606	0
2811	819	0.5634	1.1388	0.7393	1.26	0
2812	818.9	0.5638	1.1393	0.7392	1.2606	0
2813	818.8	0.5642	1.1394	0.7395	1.2599	0
2814	818.7	0.5641	1.1394	0.7394	1.2592	0
2815	818.6	0.5644	1.1395	0.7393	1.2596	0
2816	818.5	0.5643	1.1394	0.739	1.2586	0
2817	818.4	0.5644	1.1396	0.7393	1.2598	0
2818	818.3	0.5644	1.1393	0.7392	1.2597	0
2819	818.2	0.5643	1.1396	0.7391	1.26	0
2820	818.1	0.5647	1.1395	0.7392	1.2602	0
2821	818	0.5644	1.1393	0.7391	1.2606	0
2822	817.9	0.5648	1.1394	0.7392	1.2609	0
2823	817.8	0.5645	1.1397	0.7393	1.2605	0
2824	817.7	0.5647	1.14	0.7392	1.2604	0

2825	817.6	0.5647	1.1396	0.7392	1.2605	0
2826	817.5	0.5647	1.14	0.7391	1.2607	0
2827	817.4	0.5651	1.1408	0.7393	1.261	0
2828	817.3	0.5648	1.1405	0.7392	1.26	0
2829	817.2	0.5649	1.1402	0.7393	1.2611	0
2830	817.1	0.5649	1.1407	0.7392	1.2608	0
2831	817	0.5651	1.1404	0.7393	1.2609	0
2832	816.9	0.5651	1.1402	0.7392	1.2618	0
2833	816.8	0.5654	1.1404	0.739	1.261	0
2834	816.7	0.5651	1.1403	0.7393	1.2613	0
2835	816.6	0.5655	1.1407	0.7393	1.261	0
2836	816.5	0.5654	1.1408	0.7392	1.2613	0
2837	816.4	0.5653	1.1404	0.7391	1.2609	0
2838	816.3	0.5654	1.1404	0.7392	1.2603	0
2839	816.2	0.5655	1.1406	0.7392	1.2604	0
2840	816.1	0.5653	1.1403	0.7392	1.2605	0
2841	816	0.5655	1.1408	0.7392	1.2612	0
2842	815.9	0.5655	1.1407	0.7392	1.2606	0
2843	815.8	0.5656	1.1409	0.7396	1.2601	0
2844	815.7	0.5657	1.1411	0.7393	1.26	0
2845	815.6	0.5656	1.1412	0.7395	1.2594	0
2846	815.5	0.5656	1.1412	0.7394	1.2605	0
2847	815.4	0.5657	1.141	0.7394	1.261	0
2848	815.3	0.5658	1.1415	0.7397	1.2611	0
2849	815.2	0.5657	1.1413	0.7395	1.261	0
2850	815.1	0.5654	1.141	0.7395	1.2607	0
2851	815	0.5655	1.141	0.7396	1.2605	0
2852	814.9	0.5661	1.1414	0.7397	1.2604	0
2853	814.8	0.5663	1.1412	0.7399	1.261	0
2854	814.7	0.5662	1.1409	0.7397	1.2601	0
2855	814.6	0.5661	1.1412	0.7399	1.26	0
2856	814.5	0.5661	1.141	0.7399	1.2602	0
2857	814.4	0.5663	1.1412	0.74	1.2611	0
2858	814.3	0.5662	1.1414	0.7402	1.2608	0
2859	814.2	0.5661	1.1408	0.7401	1.2602	0
2860	814.1	0.5662	1.1415	0.7401	1.2603	0
2861	814	0.5662	1.1412	0.7402	1.2603	0
2862	813.9	0.5663	1.1409	0.7403	1.2597	0
2863	813.8	0.5663	1.1411	0.7405	1.2595	0
2864	813.7	0.5663	1.141	0.7405	1.2597	0
2865	813.6	0.5662	1.1409	0.7405	1.2598	0
2866	813.5	0.5666	1.1419	0.7406	1.2595	0
2867	813.4	0.5665	1.1415	0.7408	1.2587	0

2868	813.3	0.5668	1.1409	0.7407	1.2604	0
2869	813.2	0.5667	1.1411	0.7409	1.2595	0
2870	813.1	0.5666	1.141	0.7408	1.2601	0
2871	813	0.5666	1.1412	0.7408	1.2596	0
2872	812.9	0.5665	1.1421	0.7409	1.2599	0
2873	812.8	0.5667	1.1416	0.7407	1.2594	0
2874	812.7	0.5667	1.1415	0.7408	1.2591	0
2875	812.6	0.5669	1.1413	0.7408	1.2597	0
2876	812.5	0.5666	1.141	0.7407	1.259	0
2877	812.4	0.5668	1.1411	0.7408	1.2592	0
2878	812.3	0.5669	1.1414	0.741	1.259	0
2879	812.2	0.5671	1.1416	0.7409	1.2597	0
2880	812.1	0.567	1.1418	0.741	1.2584	0
2881	812	0.5668	1.1414	0.741	1.2594	0
2882	811.9	0.5671	1.1413	0.7411	1.2596	0
2883	811.8	0.5671	1.1409	0.7411	1.2596	0
2884	811.7	0.5668	1.1411	0.741	1.2597	0
2885	811.6	0.5673	1.1417	0.7413	1.26	0
2886	811.5	0.5673	1.1412	0.7413	1.2601	0
2887	811.4	0.5674	1.1414	0.7412	1.26	0
2888	811.3	0.5674	1.1417	0.7412	1.2592	0
2889	811.2	0.5676	1.1414	0.7414	1.2596	0
2890	811.1	0.5673	1.1422	0.7415	1.2599	0
2891	811	0.5676	1.1418	0.7414	1.2599	0
2892	810.9	0.5676	1.1416	0.7413	1.2595	0
2893	810.8	0.5674	1.1421	0.7414	1.2606	0
2894	810.7	0.5678	1.1421	0.7416	1.2598	0
2895	810.6	0.5677	1.1422	0.7413	1.26	0
2896	810.5	0.5677	1.1422	0.7411	1.2597	0
2897	810.4	0.5679	1.1418	0.7411	1.2598	0
2898	810.3	0.568	1.1417	0.7412	1.2593	0
2899	810.2	0.568	1.1424	0.7411	1.2597	0
2900	810.1	0.5682	1.1422	0.7412	1.2607	0
2901	810	0.5682	1.1425	0.7412	1.2608	0
2902	809.9	0.5679	1.1423	0.7412	1.2602	0
2903	809.8	0.568	1.1425	0.7413	1.2601	0
2904	809.7	0.5681	1.1427	0.7414	1.2605	0
2905	809.6	0.5681	1.143	0.7414	1.2605	0
2906	809.5	0.5684	1.1431	0.7415	1.2607	0
2907	809.4	0.5686	1.1428	0.7414	1.2606	0
2908	809.3	0.5684	1.143	0.7415	1.2605	0
2909	809.2	0.5685	1.1427	0.7412	1.2603	0
2910	809.1	0.5686	1.1431	0.7414	1.2613	0

2911	809	0.5688	1.1432	0.7414	1.2611	0
2912	808.9	0.5688	1.1436	0.7412	1.2605	0
2913	808.8	0.5689	1.1437	0.7413	1.2609	0
2914	808.7	0.5689	1.1436	0.7414	1.2611	0
2915	808.6	0.5689	1.1432	0.7412	1.2606	0
2916	808.5	0.5692	1.1436	0.7414	1.2606	0
2917	808.4	0.5691	1.1427	0.7413	1.2597	0
2918	808.3	0.5691	1.143	0.7411	1.2601	0
2919	808.2	0.569	1.143	0.7412	1.2603	0
2920	808.1	0.5692	1.1431	0.7411	1.26	0
2921	808	0.5691	1.1433	0.7413	1.2605	0
2922	807.9	0.5691	1.1435	0.7415	1.2604	0
2923	807.8	0.5691	1.1437	0.7415	1.2605	0
2924	807.7	0.5695	1.1434	0.7416	1.2603	0
2925	807.6	0.5694	1.1436	0.7416	1.2607	0
2926	807.5	0.5697	1.1434	0.7416	1.2605	0
2927	807.4	0.5697	1.1431	0.7416	1.2611	0
2928	807.3	0.5693	1.1432	0.7418	1.2605	0
2929	807.2	0.5697	1.1431	0.7417	1.2602	0
2930	807.1	0.5697	1.1431	0.7417	1.2606	0
2931	807	0.5694	1.143	0.7417	1.261	0
2932	806.9	0.5697	1.1433	0.7418	1.2609	0
2933	806.8	0.5697	1.1431	0.742	1.26	0
2934	806.7	0.5699	1.1432	0.742	1.2605	0
2935	806.6	0.5699	1.1434	0.7421	1.2599	0
2936	806.5	0.5698	1.1436	0.7421	1.2598	0
2937	806.4	0.57	1.1435	0.7425	1.2596	0
2938	806.3	0.5702	1.1433	0.7422	1.2605	0
2939	806.2	0.5698	1.1436	0.7421	1.2604	0
2940	806.1	0.57	1.1433	0.7423	1.2609	0
2941	806	0.5701	1.1439	0.7425	1.2599	0
2942	805.9	0.5704	1.1436	0.7425	1.2603	0
2943	805.8	0.5704	1.1427	0.7427	1.2599	0
2944	805.7	0.5703	1.1432	0.7426	1.2593	0
2945	805.6	0.5703	1.1436	0.7425	1.2597	0
2946	805.5	0.5705	1.1437	0.7427	1.2595	0
2947	805.4	0.5703	1.1431	0.7428	1.2594	0
2948	805.3	0.5701	1.1434	0.743	1.2599	0
2949	805.2	0.5705	1.1436	0.7431	1.2596	0
2950	805.1	0.5705	1.1432	0.7431	1.2593	0
2951	805	0.5704	1.1432	0.743	1.2585	0
2952	804.9	0.5707	1.143	0.7431	1.2587	0
2953	804.8	0.5705	1.1431	0.7433	1.2585	0

2954	804.7	0.5707	1.1433	0.7432	1.2587	0
2955	804.6	0.5708	1.1429	0.7435	1.2573	0
2956	804.5	0.5709	1.1428	0.7435	1.2589	0
2957	804.4	0.5708	1.1428	0.7436	1.2593	0
2958	804.3	0.5707	1.1426	0.7435	1.2583	0
2959	804.2	0.5709	1.143	0.7437	1.2581	0
2960	804.1	0.5708	1.1428	0.7436	1.2582	0
2961	804	0.5707	1.1427	0.7438	1.2585	0
2962	803.9	0.5708	1.1427	0.7439	1.2573	0
2963	803.8	0.5706	1.143	0.7441	1.2573	0
2964	803.7	0.5709	1.1428	0.744	1.2565	0
2965	803.6	0.5709	1.1428	0.7442	1.2567	0
2966	803.5	0.5712	1.1426	0.744	1.2568	0
2967	803.4	0.5709	1.143	0.7441	1.2568	0
2968	803.3	0.5711	1.1429	0.7445	1.2552	0
2969	803.2	0.5713	1.1432	0.7445	1.2554	0
2970	803.1	0.571	1.1428	0.7445	1.2567	0
2971	803	0.5712	1.1431	0.7443	1.2567	0
2972	802.9	0.5715	1.1429	0.7444	1.2571	0
2973	802.8	0.5716	1.1433	0.7446	1.258	0
2974	802.7	0.5713	1.1433	0.7446	1.2568	0
2975	802.6	0.5715	1.1435	0.7446	1.2558	0
2976	802.5	0.5718	1.1433	0.7446	1.2572	0
2977	802.4	0.572	1.143	0.7448	1.2569	0
2978	802.3	0.5718	1.143	0.7447	1.2571	0
2979	802.2	0.5717	1.1428	0.7447	1.2569	0
2980	802.1	0.5718	1.1432	0.7449	1.2557	0
2981	802	0.5716	1.1431	0.7448	1.2567	0
2982	801.9	0.5718	1.1434	0.7446	1.2559	0
2983	801.8	0.572	1.1431	0.7448	1.2566	0
2984	801.7	0.5721	1.1433	0.7448	1.2572	0
2985	801.6	0.5722	1.1432	0.7445	1.2571	0
2986	801.5	0.5722	1.1436	0.7446	1.2569	0
2987	801.4	0.5722	1.1431	0.7448	1.257	0
2988	801.3	0.572	1.1433	0.7443	1.2575	0
2989	801.2	0.5723	1.1432	0.7445	1.2573	0
2990	801.1	0.5725	1.1435	0.7446	1.2572	0
2991	801	0.5727	1.144	0.7445	1.2581	0
2992	800.9	0.5724	1.1438	0.7444	1.2575	0
2993	800.8	0.5727	1.1436	0.7445	1.2586	0
2994	800.7	0.5727	1.144	0.7446	1.2579	0
2995	800.6	0.5727	1.1434	0.7443	1.2577	0
2996	800.5	0.5728	1.1441	0.7444	1.2581	0

2997	800.4	0.573	1.1438	0.7446	1.2587	0
2998	800.3	0.5731	1.1442	0.7446	1.2589	0
2999	800.2	0.5731	1.1445	0.7445	1.2587	0
3000	800.1	0.5731	1.1443	0.7442	1.2592	0
3001	800	0.5732	1.1443	0.7443	1.2585	0
3002	799.9	0.5733	1.1442	0.7441	1.2588	0
3003	799.8	0.5735	1.1448	0.7442	1.2593	0
3004	799.7	0.5735	1.1449	0.744	1.2589	0
3005	799.6	0.5733	1.1445	0.7439	1.2596	0
3006	799.5	0.5736	1.145	0.744	1.2594	0
3007	799.4	0.5735	1.1449	0.744	1.2591	0
3008	799.3	0.5736	1.1452	0.744	1.2596	0
3009	799.2	0.5738	1.1451	0.744	1.2596	0
3010	799.1	0.5738	1.1448	0.744	1.2602	0
3011	799	0.5738	1.145	0.7439	1.2592	0
3012	798.9	0.5739	1.1451	0.7438	1.2601	0
3013	798.8	0.5737	1.1454	0.7439	1.2601	0
3014	798.7	0.5741	1.1456	0.7439	1.2607	0
3015	798.6	0.5743	1.1457	0.7436	1.2604	0
3016	798.5	0.5743	1.1455	0.7438	1.261	0
3017	798.4	0.5743	1.1461	0.7434	1.2603	0
3018	798.3	0.5745	1.1463	0.7437	1.261	0
3019	798.2	0.5747	1.1459	0.7438	1.2614	0
3020	798.1	0.5745	1.1464	0.7437	1.2613	0
3021	798	0.5745	1.1466	0.7436	1.2613	0
3022	797.9	0.575	1.1466	0.7436	1.2615	0
3023	797.8	0.5751	1.1464	0.7437	1.2613	0
3024	797.7	0.5752	1.1463	0.7435	1.2614	0
3025	797.6	0.5749	1.1467	0.7436	1.2613	0
3026	797.5	0.5749	1.1467	0.7439	1.2611	0
3027	797.4	0.5752	1.1472	0.7439	1.2609	0
3028	797.3	0.575	1.147	0.744	1.2611	0
3029	797.2	0.5755	1.147	0.7441	1.261	0
3030	797.1	0.5754	1.1467	0.7438	1.2605	0
3031	797	0.5753	1.1466	0.7435	1.2614	0
3032	796.9	0.5753	1.1462	0.744	1.2601	0
3033	796.8	0.5755	1.1461	0.744	1.2605	0
3034	796.7	0.5755	1.1466	0.7442	1.2606	0
3035	796.6	0.5756	1.1466	0.7441	1.2608	0
3036	796.5	0.5756	1.1467	0.7444	1.2601	0
3037	796.4	0.5756	1.1464	0.7445	1.2602	0
3038	796.3	0.5754	1.1464	0.7448	1.2593	0
3039	796.2	0.5757	1.1465	0.7449	1.2588	0

3040	796.1	0.5754	1.1462	0.7448	1.2586	0
3041	796	0.5756	1.1459	0.7447	1.2593	0
3042	795.9	0.5756	1.1458	0.745	1.2596	0
3043	795.8	0.5757	1.1459	0.7452	1.2587	0
3044	795.7	0.5757	1.1458	0.7453	1.2583	0
3045	795.6	0.5755	1.1458	0.7453	1.2582	0
3046	795.5	0.5756	1.1459	0.7452	1.2586	0
3047	795.4	0.5758	1.1457	0.7453	1.2588	0
3048	795.3	0.5756	1.1456	0.7456	1.2578	0
3049	795.2	0.5757	1.1458	0.7456	1.2584	0
3050	795.1	0.5758	1.1463	0.7458	1.2582	0
3051	795	0.576	1.1456	0.7458	1.2576	0
3052	794.9	0.5759	1.1456	0.7458	1.2575	0
3053	794.8	0.5757	1.1458	0.746	1.2565	0
3054	794.7	0.576	1.1452	0.7461	1.2563	0
3055	794.6	0.576	1.1451	0.7462	1.2566	0
3056	794.5	0.576	1.1455	0.7462	1.2566	0
3057	794.4	0.576	1.1458	0.7467	1.256	0
3058	794.3	0.5761	1.1454	0.7465	1.2559	0
3059	794.2	0.5761	1.1453	0.7468	1.2556	0
3060	794.1	0.5763	1.1452	0.7468	1.2553	0
3061	794	0.5761	1.1452	0.747	1.2558	0
3062	793.9	0.5762	1.1456	0.7472	1.256	0
3063	793.8	0.5762	1.1449	0.7471	1.2556	0
3064	793.7	0.5764	1.1453	0.7473	1.2544	0
3065	793.6	0.5766	1.1455	0.7475	1.2552	0
3066	793.5	0.5766	1.1454	0.7473	1.2551	0
3067	793.4	0.5763	1.1455	0.7474	1.2547	0
3068	793.3	0.5766	1.1453	0.7474	1.2539	0
3069	793.2	0.5768	1.1453	0.7478	1.254	0
3070	793.1	0.5768	1.1451	0.7477	1.2545	0
3071	793	0.5767	1.1454	0.7479	1.2545	0
3072	792.9	0.5769	1.1452	0.7479	1.254	0
3073	792.8	0.577	1.1451	0.748	1.2542	0
3074	792.7	0.5767	1.1453	0.7478	1.2538	0
3075	792.6	0.5767	1.145	0.7477	1.2544	0
3076	792.5	0.5772	1.1455	0.748	1.2548	0
3077	792.4	0.5772	1.1451	0.7481	1.2541	0
3078	792.3	0.5771	1.1448	0.748	1.2541	0
3079	792.2	0.5772	1.1455	0.7481	1.2553	0
3080	792.1	0.577	1.1451	0.7478	1.2547	0
3081	792	0.5771	1.1456	0.748	1.2542	0
3082	791.9	0.5769	1.1455	0.748	1.2548	0

3083	791.8	0.5771	1.1452	0.7478	1.2542	0
3084	791.7	0.5772	1.1454	0.748	1.2546	0
3085	791.6	0.5774	1.1454	0.7481	1.2547	0
3086	791.5	0.5774	1.1456	0.748	1.2542	0
3087	791.4	0.5777	1.1456	0.7483	1.2542	0
3088	791.3	0.5776	1.1459	0.7481	1.2539	0
3089	791.2	0.5775	1.146	0.7482	1.2546	0
3090	791.1	0.5775	1.1457	0.748	1.2542	0
3091	791	0.5778	1.1463	0.7482	1.254	0
3092	790.9	0.5781	1.146	0.7483	1.2542	0
3093	790.8	0.5777	1.146	0.7482	1.2549	0
3094	790.7	0.5781	1.1465	0.748	1.2546	0
3095	790.6	0.5781	1.1464	0.7483	1.2551	0
3096	790.5	0.5782	1.1462	0.748	1.2543	0
3097	790.4	0.5785	1.1464	0.7481	1.2544	0
3098	790.3	0.5783	1.1467	0.7481	1.2542	0
3099	790.2	0.5783	1.1468	0.7478	1.2549	0
3100	790.1	0.5784	1.1466	0.7477	1.2539	0
3101	790	0.5784	1.1466	0.7477	1.2547	0
3102	789.9	0.5786	1.1473	0.7479	1.2553	0
3103	789.8	0.5782	1.1472	0.7478	1.2556	0
3104	789.7	0.5785	1.1471	0.7475	1.2556	0
3105	789.6	0.5788	1.1477	0.7475	1.2554	0
3106	789.5	0.5787	1.1476	0.7475	1.2562	0
3107	789.4	0.5791	1.1473	0.7474	1.2567	0
3108	789.3	0.579	1.1472	0.7475	1.2562	0
3109	789.2	0.579	1.1482	0.7474	1.2559	0
3110	789.1	0.5791	1.1478	0.7474	1.2563	0
3111	789	0.5796	1.1478	0.7472	1.2558	0
3112	788.9	0.5795	1.1487	0.7473	1.2569	0
3113	788.8	0.5794	1.1484	0.7472	1.2574	0
3114	788.7	0.5796	1.1483	0.7471	1.2569	0
3115	788.6	0.5798	1.1489	0.7468	1.2574	0
3116	788.5	0.5798	1.1484	0.7471	1.2577	0
3117	788.4	0.5796	1.1487	0.747	1.2573	0
3118	788.3	0.5798	1.1484	0.7469	1.257	0
3119	788.2	0.5799	1.1488	0.7471	1.2582	0
3120	788.1	0.5802	1.1494	0.7468	1.2577	0
3121	788	0.5801	1.1494	0.7468	1.2582	0
3122	787.9	0.5801	1.1494	0.7467	1.2584	0
3123	787.8	0.5798	1.1493	0.7466	1.2581	0
3124	787.7	0.5798	1.1494	0.7466	1.2588	0
3125	787.6	0.5802	1.1498	0.7465	1.2589	0

3126	787.5	0.5801	1.1498	0.7465	1.2581	0
3127	787.4	0.5805	1.15	0.7466	1.2582	0
3128	787.3	0.5804	1.1503	0.7464	1.259	0
3129	787.2	0.5805	1.1503	0.7467	1.2586	0
3130	787.1	0.5806	1.1502	0.7466	1.258	0
3131	787	0.5808	1.1503	0.7466	1.2585	0
3132	786.9	0.5806	1.1507	0.7464	1.2592	0
3133	786.8	0.5806	1.1503	0.7464	1.2591	0
3134	786.7	0.5808	1.1505	0.7465	1.2593	0
3135	786.6	0.5809	1.1504	0.7464	1.2591	0
3136	786.5	0.5809	1.15	0.7466	1.2591	0
3137	786.4	0.581	1.1503	0.7466	1.2585	0
3138	786.3	0.5808	1.1503	0.7466	1.2591	0
3139	786.2	0.5814	1.1503	0.7469	1.2588	0
3140	786.1	0.5815	1.1505	0.7468	1.2594	0
3141	786	0.5813	1.1506	0.7468	1.2588	0
3142	785.9	0.5815	1.1504	0.747	1.2591	0
3143	785.8	0.5817	1.1506	0.747	1.2587	0
3144	785.7	0.5813	1.1502	0.7471	1.2579	0
3145	785.6	0.5816	1.1501	0.7473	1.2578	0
3146	785.5	0.5813	1.1504	0.7471	1.2582	0
3147	785.4	0.5814	1.1503	0.7473	1.2577	0
3148	785.3	0.5814	1.1505	0.7475	1.2576	0
3149	785.2	0.5813	1.1505	0.7476	1.2574	0
3150	785.1	0.5814	1.1505	0.7476	1.257	0
3151	785	0.5816	1.1505	0.748	1.2569	0
3152	784.9	0.5819	1.1503	0.7482	1.2565	0
3153	784.8	0.5818	1.1501	0.7482	1.256	0
3154	784.7	0.5818	1.1506	0.7484	1.2561	0
3155	784.6	0.5817	1.1502	0.7489	1.256	0
3156	784.5	0.5816	1.1497	0.7489	1.2558	0
3157	784.4	0.5818	1.1497	0.749	1.2553	0
3158	784.3	0.5817	1.1498	0.749	1.2544	0
3159	784.2	0.5816	1.1498	0.7493	1.2544	0
3160	784.1	0.5817	1.1495	0.7493	1.2548	0
3161	784	0.5816	1.1492	0.7494	1.253	0
3162	783.9	0.5816	1.1491	0.7495	1.2527	0
3163	783.8	0.5817	1.1492	0.7497	1.2531	0
3164	783.7	0.5815	1.1491	0.7498	1.2542	0
3165	783.6	0.5818	1.1495	0.7498	1.2534	0
3166	783.5	0.5817	1.1491	0.7502	1.2526	0
3167	783.4	0.5815	1.1493	0.75	1.2528	0
3168	783.3	0.5819	1.149	0.7503	1.2519	0

3169	783.2	0.5819	1.149	0.7505	1.2521	0
3170	783.1	0.5821	1.1487	0.7505	1.2512	0
3171	783	0.5821	1.1491	0.7508	1.2507	0
3172	782.9	0.5821	1.1491	0.7508	1.2518	0
3173	782.8	0.582	1.1488	0.7509	1.2527	0
3174	782.7	0.5821	1.1485	0.7508	1.2514	0
3175	782.6	0.5823	1.1489	0.751	1.2521	0
3176	782.5	0.5823	1.1492	0.7511	1.2521	0
3177	782.4	0.5823	1.1487	0.7511	1.2517	0
3178	782.3	0.5825	1.1486	0.7511	1.2524	0
3179	782.2	0.5826	1.1493	0.7513	1.2517	0
3180	782.1	0.5825	1.1495	0.7512	1.2515	0
3181	782	0.5827	1.1494	0.7512	1.2515	0
3182	781.9	0.5827	1.1494	0.7513	1.2518	0
3183	781.8	0.5827	1.1493	0.7513	1.2517	0
3184	781.7	0.5827	1.1495	0.7515	1.2512	0
3185	781.6	0.5829	1.1495	0.7515	1.2514	0
3186	781.5	0.5829	1.1492	0.7514	1.2508	0
3187	781.4	0.5827	1.1495	0.7514	1.2509	0
3188	781.3	0.5828	1.1496	0.7515	1.2513	0
3189	781.2	0.5829	1.1497	0.7515	1.2517	0
3190	781.1	0.5831	1.1496	0.7515	1.251	0
3191	781	0.5832	1.1498	0.7516	1.2517	0
3192	780.9	0.5832	1.1494	0.7516	1.2518	0
3193	780.8	0.5832	1.1496	0.7517	1.2512	0
3194	780.7	0.5832	1.1501	0.7517	1.2513	0
3195	780.6	0.5834	1.15	0.7518	1.2515	0
3196	780.5	0.5834	1.15	0.7515	1.2517	0
3197	780.4	0.5835	1.15	0.7516	1.2506	0
3198	780.3	0.5833	1.1496	0.7516	1.2507	0
3199	780.2	0.5834	1.1502	0.7518	1.2514	0
3200	780.1	0.5836	1.1505	0.7517	1.2515	0
3201	780	0.5837	1.1507	0.7518	1.2515	0
3202	779.9	0.5839	1.1506	0.7517	1.2524	0
3203	779.8	0.5838	1.1506	0.7517	1.2521	0
3204	779.7	0.5839	1.1507	0.7516	1.2521	0
3205	779.6	0.584	1.1512	0.7514	1.2516	0
3206	779.5	0.584	1.1516	0.7513	1.2521	0
3207	779.4	0.5841	1.1512	0.7511	1.2522	0
3208	779.3	0.5847	1.1509	0.7511	1.2535	0
3209	779.2	0.5843	1.1517	0.7512	1.2535	0
3210	779.1	0.5842	1.1515	0.7511	1.2539	0
3211	779	0.5845	1.1518	0.7511	1.2537	0

3212	778.9	0.5846	1.1515	0.7508	1.2537	0
3213	778.8	0.5847	1.1523	0.751	1.2538	0
3214	778.7	0.5846	1.1524	0.7507	1.2541	0
3215	778.6	0.5849	1.1522	0.7506	1.2542	0
3216	778.5	0.5848	1.1526	0.7505	1.2548	0
3217	778.4	0.5853	1.153	0.7504	1.2548	0
3218	778.3	0.5855	1.153	0.7505	1.2551	0
3219	778.2	0.5856	1.1532	0.7503	1.2558	0
3220	778.1	0.5852	1.1538	0.7502	1.2548	0
3221	778	0.5854	1.1535	0.7501	1.2557	0
3222	777.9	0.5854	1.1538	0.7501	1.2558	0
3223	777.8	0.5857	1.1543	0.7502	1.2559	0
3224	777.7	0.5855	1.1538	0.75	1.2562	0
3225	777.6	0.5857	1.1539	0.75	1.2565	0
3226	777.5	0.5858	1.1541	0.7499	1.2558	0
3227	777.4	0.5858	1.154	0.7498	1.2561	0
3228	777.3	0.5862	1.1542	0.7497	1.2571	0
3229	777.2	0.5861	1.1543	0.7497	1.2571	0
3230	777.1	0.586	1.1547	0.7495	1.2567	0
3231	777	0.586	1.1546	0.7497	1.2568	0
3232	776.9	0.5862	1.1549	0.7498	1.2572	0
3233	776.8	0.5861	1.1544	0.7498	1.2566	0
3234	776.7	0.5864	1.1552	0.7499	1.2573	0
3235	776.6	0.5867	1.1551	0.7499	1.258	0
3236	776.5	0.5867	1.1553	0.7499	1.258	0
3237	776.4	0.5868	1.1552	0.7498	1.2574	0
3238	776.3	0.5866	1.1553	0.7497	1.2575	0
3239	776.2	0.5865	1.1555	0.7498	1.2579	0
3240	776.1	0.5869	1.1549	0.7498	1.2567	0
3241	776	0.5871	1.1554	0.7498	1.2577	0
3242	775.9	0.5871	1.1554	0.7499	1.2573	0
3243	775.8	0.587	1.1555	0.7499	1.2574	0
3244	775.7	0.5871	1.1554	0.7499	1.2576	0
3245	775.6	0.5874	1.1556	0.75	1.2571	0
3246	775.5	0.587	1.1555	0.7498	1.257	0
3247	775.4	0.5873	1.1558	0.7502	1.2568	0
3248	775.3	0.5872	1.1556	0.7503	1.2568	0
3249	775.2	0.5873	1.1562	0.7504	1.2561	0
3250	775.1	0.5874	1.1557	0.7504	1.2568	0
3251	775	0.5874	1.1561	0.7503	1.2566	0
3252	774.9	0.5875	1.1557	0.7503	1.2573	0
3253	774.8	0.5874	1.1556	0.7506	1.2559	0
3254	774.7	0.5874	1.1557	0.7507	1.2568	0

3255	774.6	0.5875	1.1561	0.7509	1.2564	0
3256	774.5	0.588	1.1558	0.7508	1.2564	0
3257	774.4	0.5879	1.1559	0.7511	1.2562	0
3258	774.3	0.5878	1.1558	0.7513	1.2556	0
3259	774.2	0.5879	1.1559	0.7513	1.2552	0
3260	774.1	0.5879	1.1563	0.7513	1.2556	0
3261	774	0.5877	1.1554	0.7514	1.2547	0
3262	773.9	0.5878	1.1557	0.7515	1.2548	0
3263	773.8	0.588	1.1553	0.7518	1.2547	0
3264	773.7	0.5878	1.1553	0.7519	1.2546	0
3265	773.6	0.588	1.1547	0.752	1.2536	0
3266	773.5	0.5877	1.1552	0.7519	1.254	0
3267	773.4	0.5878	1.1557	0.752	1.2536	0
3268	773.3	0.5883	1.1554	0.7523	1.2536	0
3269	773.2	0.5884	1.156	0.7526	1.2535	0
3270	773.1	0.5884	1.1558	0.753	1.2532	0
3271	773	0.5884	1.1555	0.7528	1.2537	0
3272	772.9	0.5882	1.1551	0.7528	1.2537	0
3273	772.8	0.5884	1.1548	0.753	1.2528	0
3274	772.7	0.5882	1.1552	0.7532	1.2525	0
3275	772.6	0.5882	1.1552	0.7533	1.2524	0
3276	772.5	0.5884	1.155	0.7534	1.2531	0
3277	772.4	0.5882	1.1548	0.7534	1.2526	0
3278	772.3	0.5883	1.1551	0.7534	1.2523	0
3279	772.2	0.5886	1.1553	0.7534	1.2522	0
3280	772.1	0.5886	1.1552	0.7535	1.2522	0
3281	772	0.5889	1.1555	0.7536	1.2518	0
3282	771.9	0.5886	1.1553	0.7539	1.2521	0
3283	771.8	0.5886	1.1552	0.7538	1.2522	0
3284	771.7	0.5888	1.1554	0.7537	1.2524	0
3285	771.6	0.5887	1.1554	0.7539	1.2527	0
3286	771.5	0.5889	1.1554	0.7539	1.2515	0
3287	771.4	0.5888	1.155	0.7539	1.251	0
3288	771.3	0.589	1.1554	0.7539	1.2504	0
3289	771.2	0.5887	1.1552	0.7537	1.2511	0
3290	771.1	0.589	1.1555	0.754	1.2515	0
3291	771	0.589	1.1552	0.754	1.2515	0
3292	770.9	0.5892	1.1551	0.7541	1.2511	0
3293	770.8	0.5894	1.1554	0.7541	1.251	0
3294	770.7	0.5892	1.1553	0.7541	1.2506	0
3295	770.6	0.5895	1.1555	0.7543	1.2502	0
3296	770.5	0.5895	1.1559	0.7542	1.2502	0
3297	770.4	0.5894	1.156	0.7543	1.2507	0

3298	770.3	0.5896	1.1563	0.7542	1.2504	0
3299	770.2	0.5898	1.1561	0.7543	1.251	0
3300	770.1	0.5898	1.1558	0.754	1.251	0
3301	770	0.5902	1.1554	0.7541	1.2515	0
3302	769.9	0.59	1.1569	0.7542	1.2517	0
3303	769.8	0.5901	1.1563	0.7542	1.2519	0
3304	769.7	0.59	1.1566	0.7541	1.252	0
3305	769.6	0.5901	1.1567	0.7541	1.2512	0
3306	769.5	0.59	1.1573	0.7543	1.2514	0
3307	769.4	0.5905	1.1571	0.7543	1.2515	0
3308	769.3	0.5903	1.1572	0.754	1.2512	0
3309	769.2	0.5904	1.1573	0.754	1.2523	0
3310	769.1	0.5904	1.1574	0.754	1.2525	0
3311	769	0.5906	1.1573	0.754	1.2527	0
3312	768.9	0.5908	1.1577	0.754	1.2521	0
3313	768.8	0.591	1.1578	0.7539	1.2528	0
3314	768.7	0.5911	1.1574	0.7538	1.2538	0
3315	768.6	0.5913	1.1581	0.7537	1.2536	0
3316	768.5	0.5913	1.1583	0.7536	1.2532	0
3317	768.4	0.5915	1.1586	0.7536	1.2534	0
3318	768.3	0.5913	1.1586	0.7537	1.2533	0
3319	768.2	0.5914	1.1592	0.7537	1.2539	0
3320	768.1	0.5917	1.1589	0.7536	1.254	0
3321	768	0.5918	1.159	0.7535	1.2543	0
3322	767.9	0.5918	1.1591	0.7534	1.255	0
3323	767.8	0.5918	1.1591	0.7535	1.2553	0
3324	767.7	0.5918	1.1594	0.7533	1.2551	0
3325	767.6	0.5922	1.1593	0.7532	1.2552	0
3326	767.5	0.592	1.1596	0.7533	1.2557	0
3327	767.4	0.5921	1.1594	0.7532	1.255	0
3328	767.3	0.5922	1.16	0.7533	1.2555	0
3329	767.2	0.5924	1.1598	0.7531	1.2556	0
3330	767.1	0.5925	1.1597	0.7534	1.2547	0
3331	767	0.5922	1.1599	0.7531	1.2563	0
3332	766.9	0.5923	1.1602	0.7531	1.256	0
3333	766.8	0.5927	1.1603	0.753	1.256	0
3334	766.7	0.5928	1.1602	0.7531	1.256	0
3335	766.6	0.5929	1.1602	0.7534	1.2557	0
3336	766.5	0.5928	1.1608	0.7533	1.2556	0
3337	766.4	0.5931	1.1605	0.7532	1.2552	0
3338	766.3	0.5929	1.1608	0.7532	1.2562	0
3339	766.2	0.593	1.1604	0.7531	1.2562	0
3340	766.1	0.5933	1.1607	0.7531	1.2566	0

3341	766	0.5933	1.161	0.7531	1.2566	0
3342	765.9	0.5933	1.1608	0.7532	1.2564	0
3343	765.8	0.5937	1.1607	0.7532	1.2565	0
3344	765.7	0.5934	1.1614	0.7533	1.2567	0
3345	765.6	0.5936	1.1615	0.7534	1.2562	0
3346	765.5	0.5934	1.1614	0.7533	1.2561	0
3347	765.4	0.5931	1.1613	0.7532	1.2557	0
3348	765.3	0.5934	1.1614	0.7529	1.2558	0
3349	765.2	0.5935	1.1617	0.7531	1.2569	0
3350	765.1	0.5938	1.1615	0.7532	1.2569	0
3351	765	0.5938	1.1617	0.7533	1.2566	0
3352	764.9	0.5935	1.1611	0.7534	1.2563	0
3353	764.8	0.5937	1.1614	0.7534	1.256	0
3354	764.7	0.5939	1.1613	0.7535	1.2558	0
3355	764.6	0.5941	1.1614	0.7537	1.2563	0
3356	764.5	0.594	1.1613	0.7536	1.2563	0
3357	764.4	0.5942	1.1618	0.7537	1.2565	0
3358	764.3	0.5942	1.1619	0.7539	1.2564	0
3359	764.2	0.5941	1.1617	0.7539	1.2553	0
3360	764.1	0.5941	1.1618	0.7538	1.2548	0
3361	764	0.5944	1.1619	0.7541	1.255	0
3362	763.9	0.5945	1.1616	0.7541	1.2548	0
3363	763.8	0.5944	1.1616	0.7544	1.255	0
3364	763.7	0.5944	1.162	0.7543	1.2549	0
3365	763.6	0.5942	1.1617	0.7545	1.2549	0
3366	763.5	0.5942	1.1615	0.7542	1.2546	0
3367	763.4	0.5944	1.1617	0.7543	1.2542	0
3368	763.3	0.5944	1.162	0.7546	1.2535	0
3369	763.2	0.5948	1.1618	0.7548	1.2542	0
3370	763.1	0.5949	1.1618	0.7548	1.2541	0
3371	763	0.5946	1.1617	0.7547	1.254	0
3372	762.9	0.5948	1.162	0.7549	1.2543	0
3373	762.8	0.5946	1.1619	0.7551	1.2542	0
3374	762.7	0.595	1.1619	0.7551	1.2536	0
3375	762.6	0.595	1.1622	0.7552	1.2536	0
3376	762.5	0.595	1.1624	0.7553	1.2528	0
3377	762.4	0.5953	1.1619	0.7551	1.2538	0
3378	762.3	0.5953	1.1622	0.7551	1.2539	0
3379	762.2	0.5952	1.1624	0.7552	1.2539	0
3380	762.1	0.5949	1.1622	0.7555	1.2529	0
3381	762	0.5953	1.1625	0.7553	1.2536	0
3382	761.9	0.5954	1.1625	0.7555	1.2534	0
3383	761.8	0.5953	1.1624	0.7556	1.2536	0

3384	761.7	0.5956	1.163	0.7554	1.2534	0
3385	761.6	0.5959	1.1628	0.7558	1.2536	0
3386	761.5	0.5957	1.1628	0.7556	1.2532	0
3387	761.4	0.5959	1.163	0.7557	1.2534	0
3388	761.3	0.5958	1.1626	0.7556	1.2533	0
3389	761.2	0.5958	1.1631	0.7558	1.2533	0
3390	761.1	0.5957	1.1627	0.7556	1.2527	0
3391	761	0.5961	1.1635	0.7558	1.2533	0
3392	760.9	0.5961	1.1633	0.7557	1.2532	0
3393	760.8	0.5962	1.1634	0.7559	1.2536	0
3394	760.7	0.5963	1.1635	0.756	1.2536	0
3395	760.6	0.5966	1.1634	0.756	1.2532	0
3396	760.5	0.5965	1.1635	0.7557	1.2531	0
3397	760.4	0.5965	1.1634	0.7559	1.2531	0
3398	760.3	0.5962	1.1634	0.756	1.2529	0
3399	760.2	0.5963	1.1636	0.756	1.2521	0
3400	760.1	0.5965	1.1638	0.756	1.2525	0
3401	760	0.5966	1.1642	0.7558	1.2533	0
3402	759.9	0.5966	1.1637	0.7558	1.2535	0
3403	759.8	0.597	1.1642	0.7559	1.2534	0
3404	759.7	0.5969	1.1641	0.7559	1.2535	0
3405	759.6	0.5971	1.1645	0.7561	1.2529	0
3406	759.5	0.597	1.1649	0.7562	1.2532	0
3407	759.4	0.597	1.1651	0.7562	1.2541	0
3408	759.3	0.5972	1.1647	0.756	1.2535	0
3409	759.2	0.5973	1.1647	0.7561	1.2534	0
3410	759.1	0.5975	1.1652	0.7562	1.2534	0
3411	759	0.5977	1.1646	0.756	1.2535	0
3412	758.9	0.5978	1.1648	0.7561	1.2539	0
3413	758.8	0.5977	1.1651	0.756	1.2532	0
3414	758.7	0.5977	1.1655	0.7561	1.2533	0
3415	758.6	0.5978	1.1653	0.7561	1.2539	0
3416	758.5	0.5979	1.1656	0.756	1.2539	0
3417	758.4	0.598	1.1652	0.7559	1.254	0
3418	758.3	0.5982	1.1653	0.7561	1.2543	0
3419	758.2	0.5981	1.1658	0.7561	1.2529	0
3420	758.1	0.5982	1.166	0.7561	1.2547	0
3421	758	0.5982	1.1661	0.756	1.2546	0
3422	757.9	0.5985	1.1662	0.756	1.2548	0
3423	757.8	0.5983	1.1658	0.756	1.2547	0
3424	757.7	0.5984	1.1662	0.7559	1.256	0
3425	757.6	0.5987	1.1665	0.756	1.2555	0
3426	757.5	0.5988	1.1662	0.7559	1.2543	0

3427	757.4	0.5986	1.1663	0.7559	1.2551	0
3428	757.3	0.5988	1.1665	0.7561	1.2545	0
3429	757.2	0.5987	1.1666	0.7561	1.2543	0
3430	757.1	0.5989	1.167	0.7561	1.2551	0
3431	757	0.5992	1.1669	0.7561	1.2548	0
3432	756.9	0.5988	1.1671	0.7559	1.2545	0
3433	756.8	0.5989	1.1676	0.7561	1.2548	0
3434	756.7	0.5989	1.1673	0.756	1.2549	0
3435	756.6	0.5991	1.1674	0.7561	1.255	0
3436	756.5	0.5989	1.1676	0.756	1.2551	0
3437	756.4	0.5992	1.1672	0.7561	1.2547	0
3438	756.3	0.5993	1.1674	0.7563	1.2547	0
3439	756.2	0.5991	1.1674	0.7561	1.2543	0
3440	756.1	0.5993	1.1678	0.7563	1.2541	0
3441	756	0.5995	1.168	0.7561	1.254	0
3442	755.9	0.5995	1.1682	0.7562	1.2546	0
3443	755.8	0.5995	1.1678	0.7564	1.2546	0
3444	755.7	0.5998	1.1681	0.7564	1.2542	0
3445	755.6	0.6001	1.1685	0.7565	1.2549	0
3446	755.5	0.6	1.1685	0.7567	1.2551	0
3447	755.4	0.5999	1.1684	0.7566	1.2548	0
3448	755.3	0.6002	1.1684	0.7567	1.2542	0
3449	755.2	0.6	1.1685	0.7567	1.2551	0
3450	755.1	0.6001	1.1685	0.7567	1.2548	0
3451	755	0.6001	1.1687	0.7565	1.2549	0
3452	754.9	0.6004	1.169	0.7569	1.2544	0
3453	754.8	0.6007	1.1688	0.757	1.2546	0
3454	754.7	0.6003	1.1689	0.7569	1.2548	0
3455	754.6	0.6006	1.1689	0.757	1.2556	0
3456	754.5	0.6007	1.1691	0.7573	1.2552	0
3457	754.4	0.6009	1.1692	0.7573	1.2541	0
3458	754.3	0.6009	1.1689	0.7572	1.2539	0
3459	754.2	0.6007	1.1691	0.7572	1.2541	0
3460	754.1	0.601	1.1694	0.7573	1.2545	0
3461	754	0.6008	1.1693	0.7571	1.2535	0
3462	753.9	0.6009	1.1693	0.7572	1.2541	0
3463	753.8	0.6006	1.1689	0.7574	1.2532	0
3464	753.7	0.6009	1.169	0.7574	1.2537	0
3465	753.6	0.6013	1.1695	0.7575	1.2537	0
3466	753.5	0.601	1.1694	0.7576	1.2537	0
3467	753.4	0.6012	1.1692	0.7576	1.2532	0
3468	753.3	0.6013	1.1692	0.7578	1.2542	0
3469	753.2	0.6009	1.1693	0.7579	1.2531	0

3470	753.1	0.6013	1.1695	0.7578	1.2531	0
3471	753	0.6015	1.1696	0.7581	1.2533	0
3472	752.9	0.6013	1.1693	0.7582	1.253	0
3473	752.8	0.6016	1.1696	0.758	1.253	0
3474	752.7	0.6017	1.17	0.7583	1.253	0
3475	752.6	0.6017	1.1697	0.7581	1.2524	0
3476	752.5	0.6019	1.1695	0.758	1.2525	0
3477	752.4	0.6018	1.1702	0.7581	1.2528	0
3478	752.3	0.6018	1.1699	0.7581	1.253	0
3479	752.2	0.6023	1.1701	0.7581	1.2529	0
3480	752.1	0.6021	1.1701	0.7582	1.2529	0
3481	752	0.6024	1.1702	0.7583	1.2539	0
3482	751.9	0.6023	1.1703	0.7581	1.2538	0
3483	751.8	0.6022	1.1704	0.7583	1.2536	0
3484	751.7	0.6025	1.1705	0.7583	1.253	0
3485	751.6	0.6025	1.1707	0.7581	1.2535	0
3486	751.5	0.6027	1.1711	0.7582	1.254	0
3487	751.4	0.6029	1.1708	0.758	1.2525	0
3488	751.3	0.6029	1.1706	0.7582	1.2526	0
3489	751.2	0.6028	1.1705	0.7584	1.2534	0
3490	751.1	0.603	1.1711	0.7587	1.2538	0
3491	751	0.6028	1.1713	0.7584	1.2547	0
3492	750.9	0.6033	1.1712	0.7583	1.2538	0
3493	750.8	0.6032	1.1714	0.7585	1.2538	0
3494	750.7	0.6033	1.1713	0.7583	1.2538	0
3495	750.6	0.6033	1.1716	0.7583	1.2541	0
3496	750.5	0.6034	1.1721	0.7582	1.2542	0
3497	750.4	0.6034	1.1717	0.7582	1.2541	0
3498	750.3	0.6037	1.1719	0.7582	1.2541	0
3499	750.2	0.6035	1.1719	0.7583	1.2536	0
3500	750.1	0.6036	1.1725	0.7582	1.254	0
3501	750	0.6038	1.1722	0.7583	1.2539	0
3502	749.9	0.6038	1.1726	0.7581	1.2539	0
3503	749.8	0.6039	1.1722	0.7581	1.2542	0
3504	749.7	0.6042	1.1726	0.7582	1.2544	0
3505	749.6	0.6043	1.1727	0.7582	1.2551	0
3506	749.5	0.6045	1.1728	0.7581	1.2555	0
3507	749.4	0.6044	1.1732	0.7579	1.255	0
3508	749.3	0.6043	1.1732	0.7583	1.255	0
3509	749.2	0.6044	1.1732	0.7581	1.2546	0
3510	749.1	0.6046	1.1733	0.758	1.2555	0
3511	749	0.6048	1.1739	0.7583	1.2555	0
3512	748.9	0.6048	1.1737	0.7582	1.2557	0

3513	748.8	0.605	1.1737	0.7583	1.2557	0
3514	748.7	0.6048	1.1739	0.7582	1.2559	0
3515	748.6	0.6052	1.1742	0.7583	1.2558	0
3516	748.5	0.6049	1.1741	0.7583	1.2558	0
3517	748.4	0.6051	1.1741	0.7583	1.2561	0
3518	748.3	0.6051	1.1739	0.7584	1.2559	0
3519	748.2	0.6051	1.1741	0.7583	1.2546	0
3520	748.1	0.605	1.1741	0.7583	1.2554	0
3521	748	0.6051	1.1742	0.7583	1.2548	0
3522	747.9	0.6049	1.1737	0.7583	1.2543	0
3523	747.8	0.605	1.1741	0.7582	1.2539	0
3524	747.7	0.6054	1.1746	0.7586	1.2549	0
3525	747.6	0.6056	1.1743	0.7584	1.2546	0
3526	747.5	0.6058	1.1745	0.7584	1.2539	0
3527	747.4	0.6059	1.1746	0.7584	1.2546	0
3528	747.3	0.6058	1.1743	0.7587	1.255	0
3529	747.2	0.6059	1.1743	0.7585	1.2546	0
3530	747.1	0.6062	1.1748	0.7586	1.2545	0
3531	747	0.6063	1.175	0.7587	1.2554	0
3532	746.9	0.6062	1.175	0.7588	1.2553	0
3533	746.8	0.6065	1.1753	0.7589	1.2542	0
3534	746.7	0.6069	1.1752	0.7592	1.2549	0
3535	746.6	0.6068	1.175	0.7591	1.2548	0
3536	746.5	0.6067	1.1754	0.759	1.2553	0
3537	746.4	0.6068	1.1749	0.7591	1.2549	0
3538	746.3	0.6069	1.1752	0.7592	1.2548	0
3539	746.2	0.6067	1.1752	0.7592	1.2544	0
3540	746.1	0.6067	1.1748	0.7591	1.2548	0
3541	746	0.6071	1.1751	0.7591	1.2541	0
3542	745.9	0.6072	1.175	0.7593	1.255	0
3543	745.8	0.607	1.1753	0.7594	1.2544	0
3544	745.7	0.6071	1.1755	0.7595	1.2549	0
3545	745.6	0.6073	1.1755	0.7595	1.2548	0
3546	745.5	0.6073	1.1753	0.7597	1.2535	0
3547	745.4	0.6073	1.1757	0.7597	1.2531	0
3548	745.3	0.6073	1.1749	0.7596	1.2532	0
3549	745.2	0.6076	1.1752	0.7599	1.254	0
3550	745.1	0.6075	1.1751	0.7599	1.2531	0
3551	745	0.6078	1.1755	0.76	1.2531	0
3552	744.9	0.6078	1.1755	0.76	1.2529	0
3553	744.8	0.6077	1.1758	0.7603	1.2528	0
3554	744.7	0.6075	1.176	0.7604	1.2527	0
3555	744.6	0.6075	1.1755	0.7603	1.2526	0

3556	744.5	0.6078	1.1756	0.7603	1.2531	0
3557	744.4	0.6079	1.1757	0.7603	1.2521	0
3558	744.3	0.6081	1.1759	0.7606	1.2517	0
3559	744.2	0.608	1.1757	0.7605	1.2525	0
3560	744.1	0.6081	1.1756	0.7608	1.2522	0
3561	744	0.6081	1.1759	0.761	1.2518	0
3562	743.9	0.6082	1.1758	0.7609	1.252	0
3563	743.8	0.6083	1.1763	0.7611	1.2515	0
3564	743.7	0.6086	1.1765	0.7611	1.2517	0
3565	743.6	0.6086	1.1762	0.761	1.2507	0
3566	743.5	0.6086	1.1761	0.7613	1.2514	0
3567	743.4	0.6089	1.1765	0.7614	1.2512	0
3568	743.3	0.609	1.1764	0.7614	1.2515	0
3569	743.2	0.6087	1.1764	0.7613	1.2511	0
3570	743.1	0.6088	1.1763	0.7614	1.2511	0
3571	743	0.6091	1.1763	0.7614	1.2521	0
3572	742.9	0.6092	1.1765	0.7613	1.2513	0
3573	742.8	0.6089	1.1766	0.7614	1.2517	0
3574	742.7	0.6091	1.1767	0.7613	1.2521	0
3575	742.6	0.6091	1.1769	0.7616	1.2515	0
3576	742.5	0.6093	1.177	0.7615	1.2517	0
3577	742.4	0.6095	1.1771	0.7617	1.2523	0
3578	742.3	0.6095	1.177	0.7615	1.2518	0
3579	742.2	0.6097	1.1772	0.7614	1.2516	0
3580	742.1	0.6097	1.1771	0.7614	1.2524	0
3581	742	0.6099	1.1774	0.7616	1.2519	0
3582	741.9	0.6099	1.1775	0.7616	1.2523	0
3583	741.8	0.6099	1.178	0.7614	1.252	0
3584	741.7	0.6101	1.1777	0.7613	1.2529	0
3585	741.6	0.6101	1.1781	0.7615	1.2524	0
3586	741.5	0.6101	1.1777	0.7614	1.2525	0
3587	741.4	0.61	1.178	0.7614	1.2527	0
3588	741.3	0.6103	1.1782	0.7614	1.2529	0
3589	741.2	0.6105	1.1785	0.7613	1.2524	0
3590	741.1	0.6105	1.1784	0.7615	1.2527	0
3591	741	0.6107	1.1786	0.7612	1.2531	0
3592	740.9	0.6104	1.1785	0.7613	1.2527	0
3593	740.8	0.6108	1.1787	0.7612	1.254	0
3594	740.7	0.6108	1.1794	0.7614	1.2542	0
3595	740.6	0.6108	1.1792	0.7614	1.2545	0
3596	740.5	0.611	1.1787	0.7611	1.2535	0
3597	740.4	0.6108	1.179	0.7611	1.2539	0
3598	740.3	0.611	1.179	0.761	1.254	0

3599	740.2	0.6114	1.1794	0.7611	1.254	0
3600	740.1	0.6113	1.1796	0.7611	1.2544	0
3601	740	0.6114	1.1797	0.7609	1.2541	0
3602	739.9	0.6116	1.18	0.761	1.2541	0
3603	739.8	0.6119	1.1799	0.761	1.2534	0
3604	739.7	0.6119	1.18	0.7607	1.2543	0
3605	739.6	0.612	1.1805	0.7608	1.2552	0
3606	739.5	0.6122	1.1803	0.7608	1.2549	0
3607	739.4	0.6119	1.1803	0.7608	1.255	0
3608	739.3	0.6123	1.1813	0.7609	1.2557	0
3609	739.2	0.6125	1.1812	0.7607	1.2552	0
3610	739.1	0.6126	1.181	0.7608	1.2554	0
3611	739	0.6126	1.1813	0.7605	1.2558	0
3612	738.9	0.6127	1.1819	0.7607	1.2558	0
3613	738.8	0.6128	1.1819	0.7605	1.2558	0
3614	738.7	0.6128	1.1819	0.7607	1.2558	0
3615	738.6	0.6129	1.1822	0.7603	1.2559	0
3616	738.5	0.6131	1.182	0.7604	1.2559	0
3617	738.4	0.6133	1.1823	0.7606	1.2562	0
3618	738.3	0.6135	1.1826	0.7603	1.2566	0
3619	738.2	0.6133	1.1832	0.7605	1.2565	0
3620	738.1	0.6136	1.1832	0.7604	1.257	0
3621	738	0.6135	1.1828	0.7604	1.2573	0
3622	737.9	0.6138	1.1827	0.7605	1.2567	0
3623	737.8	0.6137	1.1832	0.7604	1.2567	0
3624	737.7	0.6139	1.183	0.7604	1.2564	0
3625	737.6	0.6138	1.1835	0.7604	1.2567	0
3626	737.5	0.6142	1.1837	0.7606	1.2566	0
3627	737.4	0.6141	1.1839	0.7605	1.257	0
3628	737.3	0.6142	1.1839	0.7608	1.2568	0
3629	737.2	0.6139	1.1838	0.7606	1.2569	0
3630	737.1	0.6141	1.1836	0.7607	1.2574	0
3631	737	0.6144	1.1841	0.7607	1.2568	0
3632	736.9	0.6143	1.1841	0.7607	1.2558	0
3633	736.8	0.6144	1.1841	0.7608	1.2564	0
3634	736.7	0.6146	1.1842	0.7608	1.2562	0
3635	736.6	0.6146	1.1841	0.7609	1.2571	0
3636	736.5	0.6148	1.1839	0.761	1.2555	0
3637	736.4	0.6145	1.184	0.7611	1.2554	0
3638	736.3	0.6148	1.1843	0.7613	1.2556	0
3639	736.2	0.6148	1.1841	0.7612	1.2563	0
3640	736.1	0.6149	1.1842	0.7614	1.2555	0
3641	736	0.6148	1.1839	0.7617	1.2543	0

3642	735.9	0.6149	1.1845	0.7617	1.2554	0
3643	735.8	0.615	1.184	0.7619	1.2553	0
3644	735.7	0.6153	1.1839	0.7619	1.2541	0
3645	735.6	0.6153	1.1843	0.7621	1.2553	0
3646	735.5	0.6149	1.1844	0.7622	1.2546	0
3647	735.4	0.6152	1.1845	0.7623	1.2539	0
3648	735.3	0.6153	1.1846	0.7624	1.2537	0
3649	735.2	0.6153	1.1846	0.7624	1.2537	0
3650	735.1	0.6153	1.1845	0.7626	1.2541	0
3651	735	0.6155	1.1843	0.7625	1.2535	0
3652	734.9	0.6153	1.1845	0.7628	1.253	0
3653	734.8	0.6153	1.1845	0.7631	1.2532	0
3654	734.7	0.6155	1.1848	0.7631	1.2527	0
3655	734.6	0.616	1.1844	0.7634	1.2524	0
3656	734.5	0.616	1.1847	0.7633	1.2523	0
3657	734.4	0.616	1.1848	0.7633	1.2523	0
3658	734.3	0.6162	1.1845	0.7635	1.2527	0
3659	734.2	0.616	1.1848	0.7639	1.252	0
3660	734.1	0.616	1.1849	0.7637	1.2516	0
3661	734	0.6162	1.1851	0.764	1.2513	0
3662	733.9	0.616	1.1856	0.764	1.2518	0
3663	733.8	0.616	1.1851	0.764	1.2506	0
3664	733.7	0.6163	1.1852	0.7643	1.2508	0
3665	733.6	0.6164	1.1846	0.7641	1.2507	0
3666	733.5	0.6165	1.1854	0.7644	1.2506	0
3667	733.4	0.6165	1.1849	0.7643	1.2511	0
3668	733.3	0.6164	1.185	0.7644	1.2508	0
3669	733.2	0.6166	1.185	0.7645	1.2505	0
3670	733.1	0.6166	1.1853	0.7646	1.2496	0
3671	733	0.6168	1.1856	0.7646	1.2504	0
3672	732.9	0.6167	1.186	0.7645	1.251	0
3673	732.8	0.6169	1.186	0.7644	1.2512	0
3674	732.7	0.6169	1.1855	0.7646	1.2505	0
3675	732.6	0.617	1.1858	0.765	1.2512	0
3676	732.5	0.617	1.1863	0.7647	1.251	0
3677	732.4	0.6173	1.186	0.7647	1.2503	0
3678	732.3	0.6173	1.1863	0.7649	1.2501	0
3679	732.2	0.6174	1.1867	0.7648	1.2505	0
3680	732.1	0.6175	1.1867	0.7647	1.2507	0
3681	732	0.6176	1.1869	0.7647	1.2505	0
3682	731.9	0.6178	1.1873	0.765	1.2506	0
3683	731.8	0.6178	1.1872	0.7649	1.2514	0
3684	731.7	0.6182	1.1869	0.7648	1.2504	0

3685	731.6	0.6183	1.1871	0.765	1.2513	0
3686	731.5	0.618	1.1873	0.7649	1.2517	0
3687	731.4	0.6182	1.1875	0.7649	1.2515	0
3688	731.3	0.6178	1.1875	0.7648	1.2521	0
3689	731.2	0.6185	1.1877	0.765	1.2519	0
3690	731.1	0.6183	1.1878	0.7651	1.2521	0
3691	731	0.6184	1.1878	0.7649	1.2526	0
3692	730.9	0.6187	1.1884	0.7645	1.2519	0
3693	730.8	0.619	1.1884	0.7648	1.2525	0
3694	730.7	0.6187	1.1884	0.7648	1.2518	0
3695	730.6	0.6186	1.1885	0.7646	1.2518	0
3696	730.5	0.6188	1.1889	0.7644	1.2516	0
3697	730.4	0.6193	1.1889	0.7645	1.2524	0
3698	730.3	0.6194	1.1894	0.7646	1.252	0
3699	730.2	0.6194	1.1896	0.7646	1.2524	0
3700	730.1	0.6196	1.1899	0.7643	1.2532	0
3701	730	0.6196	1.1902	0.7643	1.2532	0
3702	729.9	0.6197	1.1904	0.7645	1.2538	0
3703	729.8	0.62	1.1903	0.7644	1.2535	0
3704	729.7	0.6201	1.1902	0.7642	1.2538	0
3705	729.6	0.6199	1.1909	0.764	1.2543	0
3706	729.5	0.6202	1.1907	0.7639	1.254	0
3707	729.4	0.6202	1.1903	0.7639	1.2542	0
3708	729.3	0.6206	1.1912	0.7639	1.2541	0
3709	729.2	0.6205	1.1913	0.7637	1.2546	0
3710	729.1	0.6207	1.1914	0.7638	1.2547	0
3711	729	0.621	1.1917	0.7637	1.2547	0
3712	728.9	0.6211	1.1918	0.7637	1.2553	0
3713	728.8	0.6211	1.1921	0.7636	1.2555	0
3714	728.7	0.6211	1.1922	0.7635	1.2555	0
3715	728.6	0.621	1.1923	0.7634	1.2557	0
3716	728.5	0.6213	1.1927	0.7634	1.2558	0
3717	728.4	0.6216	1.1929	0.7635	1.2567	0
3718	728.3	0.6214	1.1929	0.7635	1.2561	0
3719	728.2	0.6215	1.1935	0.7633	1.256	0
3720	728.1	0.6218	1.1932	0.7633	1.2572	0
3721	728	0.6217	1.1934	0.7634	1.2567	0
3722	727.9	0.622	1.1934	0.7634	1.2569	0
3723	727.8	0.6222	1.1936	0.7633	1.2571	0
3724	727.7	0.6222	1.194	0.7635	1.257	0
3725	727.6	0.6224	1.1939	0.7634	1.2571	0
3726	727.5	0.6223	1.1939	0.7634	1.2573	0
3727	727.4	0.6225	1.1946	0.7634	1.2575	0

3728	727.3	0.6224	1.1945	0.7631	1.2573	0
3729	727.2	0.6229	1.195	0.7631	1.2579	0
3730	727.1	0.6229	1.1947	0.7632	1.2581	0
3731	727	0.6231	1.1949	0.7633	1.2583	0
3732	726.9	0.6231	1.1951	0.7632	1.2575	0
3733	726.8	0.6229	1.1949	0.7631	1.2573	0
3734	726.7	0.6232	1.1952	0.7633	1.2575	0
3735	726.6	0.6232	1.1955	0.7635	1.2583	0
3736	726.5	0.6237	1.1956	0.7635	1.2584	0
3737	726.4	0.6234	1.1955	0.7632	1.2577	0
3738	726.3	0.6237	1.1958	0.7632	1.2582	0
3739	726.2	0.6236	1.1959	0.7632	1.2577	0
3740	726.1	0.6237	1.1955	0.7632	1.2577	0
3741	726	0.6237	1.1955	0.7636	1.2575	0
3742	725.9	0.6242	1.1958	0.7636	1.2578	0
3743	725.8	0.624	1.1959	0.7637	1.2578	0
3744	725.7	0.624	1.1958	0.7636	1.2575	0
3745	725.6	0.6243	1.1958	0.7638	1.2573	0
3746	725.5	0.6243	1.1957	0.764	1.2572	0
3747	725.4	0.6242	1.1954	0.764	1.2571	0
3748	725.3	0.6242	1.1956	0.764	1.2565	0
3749	725.2	0.6243	1.1956	0.7641	1.2566	0
3750	725.1	0.6244	1.1954	0.7643	1.2563	0
3751	725	0.6245	1.1956	0.7646	1.2563	0
3752	724.9	0.6246	1.1958	0.7646	1.2559	0
3753	724.8	0.6248	1.1956	0.7651	1.2557	0
3754	724.7	0.6249	1.1958	0.7651	1.2554	0
3755	724.6	0.6247	1.1956	0.765	1.2557	0
3756	724.5	0.6251	1.1956	0.7653	1.2549	0
3757	724.4	0.6246	1.1953	0.7654	1.2546	0
3758	724.3	0.6249	1.1954	0.7655	1.2543	0
3759	724.2	0.6248	1.1955	0.7657	1.2541	0
3760	724.1	0.6249	1.1949	0.7658	1.2541	0
3761	724	0.6254	1.195	0.7663	1.2536	0
3762	723.9	0.625	1.1952	0.7662	1.2536	0
3763	723.8	0.6252	1.1952	0.7664	1.2542	0
3764	723.7	0.6253	1.1953	0.7665	1.2526	0
3765	723.6	0.6255	1.195	0.7666	1.2525	0
3766	723.5	0.6257	1.1949	0.7668	1.2527	0
3767	723.4	0.6253	1.1949	0.7671	1.2523	0
3768	723.3	0.6255	1.195	0.7668	1.2512	0
3769	723.2	0.6256	1.1946	0.7671	1.2518	0
3770	723.1	0.6256	1.195	0.7671	1.2519	0

3771	723	0.6258	1.1949	0.7674	1.2521	0
3772	722.9	0.6258	1.1951	0.7674	1.2514	0
3773	722.8	0.6261	1.1949	0.7674	1.2513	0
3774	722.7	0.626	1.1954	0.7676	1.2509	0
3775	722.6	0.6263	1.1955	0.7676	1.2518	0
3776	722.5	0.626	1.1951	0.7677	1.2514	0
3777	722.4	0.6262	1.1954	0.7678	1.2512	0
3778	722.3	0.6265	1.1952	0.7678	1.2512	0
3779	722.2	0.6263	1.1957	0.7676	1.2512	0
3780	722.1	0.6266	1.1956	0.7678	1.2517	0
3781	722	0.6266	1.1954	0.7676	1.2512	0
3782	721.9	0.6266	1.1956	0.7677	1.251	0
3783	721.8	0.6271	1.1961	0.7677	1.2507	0
3784	721.7	0.6269	1.1957	0.7679	1.2503	0
3785	721.6	0.6269	1.1959	0.7679	1.2505	0
3786	721.5	0.6269	1.1956	0.7682	1.2496	0
3787	721.4	0.6272	1.1966	0.768	1.2504	0
3788	721.3	0.6273	1.1961	0.7681	1.2511	0
3789	721.2	0.6274	1.1959	0.7682	1.2512	0
3790	721.1	0.6277	1.1957	0.7684	1.2515	0
3791	721	0.6273	1.1963	0.7681	1.251	0
3792	720.9	0.6274	1.1962	0.768	1.2509	0
3793	720.8	0.6276	1.1962	0.768	1.2512	0
3794	720.7	0.6279	1.1961	0.7681	1.2504	0
3795	720.6	0.6279	1.1961	0.7679	1.2505	0
3796	720.5	0.6282	1.1964	0.7684	1.2503	0
3797	720.4	0.6282	1.1966	0.7683	1.2509	0
3798	720.3	0.6282	1.1963	0.7681	1.2511	0
3799	720.2	0.6281	1.1965	0.768	1.2513	0
3800	720.1	0.6283	1.1968	0.7682	1.2511	0
3801	720	0.6285	1.1967	0.768	1.2521	0
3802	719.9	0.6285	1.1971	0.7679	1.2526	0
3803	719.8	0.6287	1.1972	0.7679	1.2526	0
3804	719.7	0.6288	1.1971	0.7679	1.2526	0
3805	719.6	0.629	1.1973	0.7681	1.253	0
3806	719.5	0.6292	1.1975	0.768	1.2522	0
3807	719.4	0.6294	1.1978	0.7677	1.2531	0
3808	719.3	0.6294	1.1979	0.7678	1.2533	0
3809	719.2	0.6295	1.198	0.7675	1.2534	0
3810	719.1	0.63	1.1986	0.7677	1.2539	0
3811	719	0.6298	1.1987	0.7675	1.2543	0
3812	718.9	0.6298	1.1986	0.7675	1.2537	0
3813	718.8	0.6301	1.1991	0.7674	1.2546	0

3814	718.7	0.6302	1.1992	0.7673	1.254	0
3815	718.6	0.6302	1.199	0.7671	1.2549	0
3816	718.5	0.6302	1.1992	0.7671	1.2552	0
3817	718.4	0.6305	1.1998	0.767	1.2543	0
3818	718.3	0.6307	1.1999	0.7671	1.2543	0
3819	718.2	0.6307	1.1995	0.767	1.2553	0
3820	718.1	0.6308	1.1999	0.7669	1.2562	0
3821	718	0.6311	1.2003	0.767	1.2557	0
3822	717.9	0.6312	1.2003	0.7667	1.2547	0
3823	717.8	0.6313	1.2006	0.7668	1.2565	0
3824	717.7	0.6313	1.2007	0.7668	1.2568	0
3825	717.6	0.6311	1.2007	0.7665	1.2572	0
3826	717.5	0.6315	1.2008	0.7666	1.2568	0
3827	717.4	0.6318	1.2011	0.7665	1.2571	0
3828	717.3	0.6318	1.2013	0.7665	1.2569	0
3829	717.2	0.6317	1.2015	0.7662	1.2576	0
3830	717.1	0.6321	1.2017	0.7664	1.2577	0
3831	717	0.632	1.2015	0.7663	1.258	0
3832	716.9	0.6323	1.2015	0.7663	1.2582	0
3833	716.8	0.6324	1.2018	0.7665	1.2578	0
3834	716.7	0.6322	1.202	0.7664	1.258	0
3835	716.6	0.6323	1.2015	0.7664	1.2581	0
3836	716.5	0.6326	1.2018	0.7664	1.2576	0
3837	716.4	0.6328	1.202	0.7664	1.2575	0
3838	716.3	0.6325	1.202	0.7663	1.2579	0
3839	716.2	0.6327	1.2019	0.7666	1.2579	0
3840	716.1	0.6328	1.2022	0.7665	1.2581	0
3841	716	0.633	1.2026	0.7664	1.259	0
3842	715.9	0.6331	1.2031	0.7666	1.2583	0
3843	715.8	0.6334	1.2026	0.7664	1.2582	0
3844	715.7	0.6336	1.2027	0.7666	1.2579	0
3845	715.6	0.6335	1.2026	0.7664	1.2578	0
3846	715.5	0.6334	1.2028	0.7665	1.2579	0
3847	715.4	0.6334	1.2026	0.7666	1.258	0
3848	715.3	0.6338	1.2026	0.7667	1.2578	0
3849	715.2	0.6343	1.2029	0.7669	1.2583	0
3850	715.1	0.6345	1.2029	0.7671	1.2586	0
3851	715	0.6346	1.2028	0.7671	1.2584	0
3852	714.9	0.6344	1.2029	0.767	1.2581	0
3853	714.8	0.6343	1.2032	0.7673	1.2581	0
3854	714.7	0.6344	1.2031	0.767	1.2585	0
3855	714.6	0.6348	1.2029	0.7672	1.2575	0
3856	714.5	0.6346	1.2028	0.7673	1.2575	0

3857	714.4	0.6345	1.2031	0.7674	1.2576	0
3858	714.3	0.6348	1.203	0.7674	1.2574	0
3859	714.2	0.6349	1.2028	0.7676	1.2571	0
3860	714.1	0.6348	1.203	0.7676	1.2561	0
3861	714	0.6352	1.2031	0.7678	1.2564	0
3862	713.9	0.635	1.2026	0.7681	1.2558	0
3863	713.8	0.6352	1.203	0.7681	1.2558	0
3864	713.7	0.6352	1.2029	0.7683	1.2559	0
3865	713.6	0.6352	1.2032	0.7685	1.2557	0
3866	713.5	0.6355	1.2026	0.7686	1.2558	0
3867	713.4	0.6358	1.2031	0.7689	1.2557	0
3868	713.3	0.6355	1.2029	0.7689	1.2548	0
3869	713.2	0.6358	1.2032	0.7691	1.2544	0
3870	713.1	0.6358	1.2031	0.7693	1.2546	0
3871	713	0.6358	1.2029	0.7692	1.2544	0
3872	712.9	0.636	1.2031	0.7693	1.2546	0
3873	712.8	0.6361	1.2033	0.7697	1.254	0
3874	712.7	0.6361	1.2031	0.7696	1.2543	0
3875	712.6	0.6361	1.2031	0.7696	1.2531	0
3876	712.5	0.6362	1.2032	0.7699	1.254	0
3877	712.4	0.6361	1.2032	0.7698	1.2533	0
3878	712.3	0.6364	1.2031	0.7702	1.2531	0
3879	712.2	0.6362	1.2031	0.7701	1.2532	0
3880	712.1	0.6364	1.2036	0.7702	1.2532	0
3881	712	0.6366	1.2037	0.7702	1.2528	0
3882	711.9	0.6367	1.2037	0.7701	1.2531	0
3883	711.8	0.6367	1.2032	0.7702	1.2522	0
3884	711.7	0.6368	1.2033	0.7701	1.2531	0
3885	711.6	0.6369	1.2035	0.7704	1.2522	0
3886	711.5	0.637	1.2037	0.7704	1.2516	0
3887	711.4	0.6371	1.2031	0.7704	1.2525	0
3888	711.3	0.6373	1.2034	0.7705	1.2522	0
3889	711.2	0.6373	1.2033	0.7704	1.2524	0
3890	711.1	0.6373	1.2037	0.7707	1.2516	0
3891	711	0.6374	1.2041	0.7705	1.252	0
3892	710.9	0.6377	1.204	0.7708	1.2523	0
3893	710.8	0.6378	1.204	0.7709	1.2524	0
3894	710.7	0.6379	1.2041	0.7708	1.2526	0
3895	710.6	0.638	1.2039	0.7707	1.2521	0
3896	710.5	0.6382	1.2042	0.7711	1.2523	0
3897	710.4	0.6384	1.2043	0.7711	1.2532	0
3898	710.3	0.6384	1.2044	0.7711	1.2522	0
3899	710.2	0.6384	1.2044	0.771	1.2515	0

3900	710.1	0.6386	1.2044	0.7711	1.2518	0
3901	710	0.6388	1.2047	0.7711	1.2522	0
3902	709.9	0.6389	1.205	0.7711	1.252	0
3903	709.8	0.6391	1.2049	0.771	1.2523	0
3904	709.7	0.639	1.2049	0.7711	1.2521	0
3905	709.6	0.6391	1.205	0.771	1.2529	0
3906	709.5	0.6394	1.2053	0.7711	1.2527	0
3907	709.4	0.6395	1.2057	0.7711	1.2525	0
3908	709.3	0.6394	1.2057	0.7711	1.2527	0
3909	709.2	0.6396	1.2059	0.771	1.2536	0
3910	709.1	0.64	1.2061	0.7711	1.2536	0
3911	709	0.6403	1.2064	0.7708	1.2541	0
3912	708.9	0.6403	1.2065	0.7709	1.2542	0
3913	708.8	0.6404	1.2067	0.7709	1.2535	0
3914	708.7	0.6405	1.2067	0.7707	1.2539	0
3915	708.6	0.6403	1.2063	0.7705	1.2533	0
3916	708.5	0.6406	1.2069	0.7706	1.2548	0
3917	708.4	0.6409	1.2074	0.7706	1.2548	0
3918	708.3	0.6408	1.2072	0.7708	1.2539	0
3919	708.2	0.641	1.2076	0.7706	1.2561	0
3920	708.1	0.6413	1.2077	0.7704	1.2556	0
3921	708	0.6415	1.2078	0.7705	1.2555	0
3922	707.9	0.6415	1.2077	0.7704	1.2548	0
3923	707.8	0.6418	1.2083	0.7707	1.2547	0
3924	707.7	0.6416	1.2081	0.7702	1.2547	0
3925	707.6	0.6421	1.2085	0.7703	1.2556	0
3926	707.5	0.6419	1.2086	0.7701	1.2559	0
3927	707.4	0.6422	1.2089	0.77	1.2572	0
3928	707.3	0.6424	1.2088	0.7701	1.2576	0
3929	707.2	0.6424	1.2092	0.77	1.2565	0
3930	707.1	0.6425	1.2091	0.7699	1.2566	0
3931	707	0.6426	1.2095	0.7698	1.2578	0
3932	706.9	0.6427	1.2096	0.7697	1.257	0
3933	706.8	0.6428	1.2097	0.7698	1.2573	0
3934	706.7	0.6428	1.2101	0.77	1.2579	0
3935	706.6	0.6431	1.2103	0.7701	1.2577	0
3936	706.5	0.6433	1.2103	0.7698	1.2574	0
3937	706.4	0.6435	1.2103	0.7698	1.2586	0
3938	706.3	0.6435	1.2106	0.7698	1.2575	0
3939	706.2	0.6436	1.2104	0.77	1.257	0
3940	706.1	0.6437	1.2104	0.7697	1.2578	0
3941	706	0.644	1.2106	0.7699	1.2584	0
3942	705.9	0.6441	1.2112	0.7699	1.2583	0

3943	705.8	0.644	1.2108	0.7698	1.2585	0
3944	705.7	0.6441	1.2113	0.7698	1.2582	0
3945	705.6	0.6444	1.2117	0.7698	1.258	0
3946	705.5	0.6446	1.2114	0.7698	1.2586	0
3947	705.4	0.6447	1.2115	0.77	1.2582	0
3948	705.3	0.6445	1.2117	0.77	1.2582	0
3949	705.2	0.6447	1.2118	0.7699	1.258	0
3950	705.1	0.6449	1.2116	0.77	1.2579	0
3951	705	0.6451	1.2117	0.77	1.2586	0
3952	704.9	0.645	1.2117	0.7702	1.2582	0
3953	704.8	0.6451	1.2119	0.7703	1.2584	0
3954	704.7	0.6451	1.2121	0.7703	1.2581	0
3955	704.6	0.6454	1.2119	0.7705	1.2583	0
3956	704.5	0.6453	1.2119	0.7705	1.2578	0
3957	704.4	0.6455	1.2119	0.7709	1.2583	0
3958	704.3	0.6454	1.2117	0.7708	1.2575	0
3959	704.2	0.6454	1.2118	0.7706	1.2577	0
3960	704.1	0.6455	1.2121	0.7709	1.258	0
3961	704	0.6458	1.212	0.7708	1.2575	0
3962	703.9	0.6462	1.2121	0.7711	1.2578	0
3963	703.8	0.646	1.2121	0.7711	1.2565	0
3964	703.7	0.6461	1.212	0.7713	1.2568	0
3965	703.6	0.6464	1.2121	0.7715	1.2567	0
3966	703.5	0.6464	1.2127	0.7717	1.2565	0
3967	703.4	0.6464	1.2124	0.7715	1.2569	0
3968	703.3	0.6465	1.2124	0.7715	1.2561	0
3969	703.2	0.6463	1.2128	0.7717	1.2562	0
3970	703.1	0.6465	1.2123	0.7716	1.2558	0
3971	703	0.6469	1.2126	0.7719	1.2556	0
3972	702.9	0.647	1.2128	0.7718	1.2559	0
3973	702.8	0.6471	1.2126	0.7721	1.2552	0
3974	702.7	0.6474	1.2127	0.7723	1.256	0
3975	702.6	0.6471	1.2124	0.7719	1.2561	0
3976	702.5	0.6473	1.213	0.7721	1.2562	0
3977	702.4	0.6476	1.2129	0.7724	1.2559	0
3978	702.3	0.6476	1.2129	0.7725	1.2554	0
3979	702.2	0.6479	1.213	0.7726	1.2549	0
3980	702.1	0.6478	1.213	0.7724	1.255	0
3981	702	0.6478	1.2128	0.7723	1.255	0
3982	701.9	0.6481	1.2133	0.7725	1.2554	0
3983	701.8	0.6481	1.2136	0.7723	1.2558	0
3984	701.7	0.6483	1.2132	0.7724	1.2545	0
3985	701.6	0.6485	1.2137	0.7726	1.2545	0

3986	701.5	0.6485	1.2136	0.7727	1.2553	0
3987	701.4	0.6485	1.2138	0.7726	1.2549	0
3988	701.3	0.6488	1.2136	0.7726	1.2539	0
3989	701.2	0.6487	1.2138	0.7731	1.2544	0
3990	701.1	0.649	1.2137	0.7731	1.2548	0
3991	701	0.6494	1.2145	0.7729	1.2556	0
3992	700.9	0.6493	1.2141	0.7728	1.2548	0
3993	700.8	0.6494	1.2144	0.773	1.2544	0
3994	700.7	0.6496	1.2139	0.7733	1.255	0
3995	700.6	0.6497	1.2144	0.7733	1.2542	0
3996	700.5	0.6496	1.2145	0.7732	1.2551	0
3997	700.4	0.6498	1.2145	0.7732	1.2553	0
3998	700.3	0.6497	1.2145	0.7734	1.2547	0
3999	700.2	0.6499	1.2144	0.7733	1.2544	0
4000	700.1	0.6502	1.2147	0.7736	1.2545	0
4001	700	0.6504	1.2153	0.7736	1.2546	0
4002	699.9	0.6503	1.215	0.7737	1.2552	0
4003	699.8	0.6507	1.2149	0.7739	1.2546	0
4004	699.7	0.6506	1.2152	0.7735	1.2547	0
4005	699.6	0.6508	1.2154	0.7735	1.2553	0
4006	699.5	0.6511	1.2154	0.7733	1.2548	0
4007	699.4	0.6511	1.2156	0.7734	1.2555	0
4008	699.3	0.651	1.2153	0.7735	1.2543	0
4009	699.2	0.6515	1.2156	0.7737	1.2543	0
4010	699.1	0.6515	1.2156	0.7738	1.2553	0
4011	699	0.6517	1.2156	0.7737	1.2554	0
4012	698.9	0.6518	1.2163	0.7736	1.2558	0
4013	698.8	0.6521	1.2163	0.7736	1.2558	0
4014	698.7	0.6519	1.2164	0.7735	1.2558	0
4015	698.6	0.6524	1.2161	0.7735	1.2561	0
4016	698.5	0.6524	1.2166	0.7735	1.2568	0
4017	698.4	0.6524	1.217	0.7734	1.2552	0
4018	698.3	0.6528	1.217	0.7734	1.2557	0
4019	698.2	0.6529	1.217	0.7736	1.2566	0
4020	698.1	0.6532	1.2176	0.7734	1.2562	0
4021	698	0.6528	1.2173	0.7735	1.2562	0
4022	697.9	0.6532	1.2179	0.7736	1.2563	0
4023	697.8	0.6533	1.2178	0.7735	1.2565	0
4024	697.7	0.6536	1.2179	0.7737	1.2572	0
4025	697.6	0.6537	1.2177	0.7734	1.2572	0
4026	697.5	0.6538	1.2183	0.7736	1.2565	0
4027	697.4	0.6536	1.218	0.7737	1.257	0
4028	697.3	0.654	1.2184	0.7734	1.2561	0

4029	697.2	0.654	1.2185	0.7735	1.2563	0
4030	697.1	0.6542	1.2189	0.7734	1.2565	0
4031	697	0.6544	1.2187	0.7734	1.2573	0
4032	696.9	0.654	1.2187	0.7734	1.2569	0
4033	696.8	0.6542	1.2191	0.7734	1.2577	0
4034	696.7	0.6545	1.2187	0.7734	1.2569	0
4035	696.6	0.6545	1.2193	0.7737	1.2572	0
4036	696.5	0.6548	1.2192	0.7735	1.2573	0
4037	696.4	0.6551	1.2192	0.7735	1.2568	0
4038	696.3	0.6549	1.2194	0.7734	1.257	0
4039	696.2	0.6551	1.2193	0.7737	1.2567	0
4040	696.1	0.6553	1.2197	0.7737	1.2572	0
4041	696	0.6554	1.22	0.7736	1.2575	0
4042	695.9	0.6555	1.2197	0.7735	1.2563	0
4043	695.8	0.6556	1.2201	0.7738	1.256	0
4044	695.7	0.6557	1.22	0.7741	1.2566	0
4045	695.6	0.6559	1.2197	0.7739	1.2575	0
4046	695.5	0.6559	1.2204	0.7741	1.2572	0
4047	695.4	0.6557	1.2198	0.774	1.2572	0
4048	695.3	0.6561	1.2202	0.7741	1.2565	0
4049	695.2	0.6561	1.2204	0.7741	1.2571	0
4050	695.1	0.6563	1.2204	0.7741	1.2569	0
4051	695	0.6564	1.2202	0.7742	1.2568	0
4052	694.9	0.6564	1.2205	0.7742	1.2555	0
4053	694.8	0.6568	1.221	0.7746	1.2563	0
4054	694.7	0.6567	1.2208	0.7744	1.2559	0
4055	694.6	0.6571	1.2206	0.7742	1.2561	0
4056	694.5	0.657	1.2207	0.7744	1.2568	0
4057	694.4	0.6573	1.2211	0.7744	1.2557	0
4058	694.3	0.6576	1.2209	0.7747	1.2557	0
4059	694.2	0.6571	1.221	0.7747	1.2559	0
4060	694.1	0.6572	1.2211	0.775	1.2559	0
4061	694	0.6576	1.2213	0.775	1.256	0
4062	693.9	0.6578	1.2209	0.7749	1.2558	0
4063	693.8	0.6578	1.2211	0.7748	1.2559	0
4064	693.7	0.6581	1.2211	0.775	1.255	0
4065	693.6	0.6579	1.2218	0.775	1.2555	0
4066	693.5	0.6581	1.2218	0.775	1.2553	0
4067	693.4	0.6582	1.2214	0.7754	1.2561	0
4068	693.3	0.6583	1.2217	0.7754	1.2548	0
4069	693.2	0.6586	1.2218	0.7755	1.2559	0
4070	693.1	0.6586	1.2221	0.7754	1.2548	0
4071	693	0.6588	1.2222	0.7753	1.2556	0

4072	692.9	0.6591	1.222	0.7756	1.2549	0
4073	692.8	0.6589	1.2221	0.7755	1.2545	0
4074	692.7	0.6592	1.2223	0.7757	1.2553	0
4075	692.6	0.6593	1.2227	0.7756	1.2554	0
4076	692.5	0.6592	1.2221	0.7756	1.255	0
4077	692.4	0.6594	1.2227	0.7757	1.2545	0
4078	692.3	0.6594	1.2228	0.7757	1.2549	0
4079	692.2	0.6598	1.2229	0.776	1.2563	0
4080	692.1	0.66	1.2232	0.7759	1.256	0
4081	692	0.6602	1.2229	0.776	1.257	0
4082	691.9	0.6601	1.2231	0.7758	1.2559	0
4083	691.8	0.6602	1.2232	0.7757	1.2559	0
4084	691.7	0.6603	1.2233	0.776	1.2562	0
4085	691.6	0.6603	1.2233	0.776	1.256	0
4086	691.5	0.6605	1.2236	0.776	1.2554	0
4087	691.4	0.6606	1.2236	0.7759	1.256	0
4088	691.3	0.6607	1.2239	0.776	1.255	0
4089	691.2	0.661	1.2233	0.7759	1.2559	0
4090	691.1	0.6608	1.2239	0.7758	1.2549	0
4091	691	0.6611	1.2243	0.7761	1.2551	0
4092	690.9	0.6615	1.2241	0.7763	1.2557	0
4093	690.8	0.6613	1.2243	0.7761	1.2565	0
4094	690.7	0.6618	1.2248	0.7761	1.2563	0
4095	690.6	0.6617	1.2247	0.7761	1.2567	0
4096	690.5	0.6619	1.2251	0.7761	1.2574	0
4097	690.4	0.6622	1.2251	0.7763	1.257	0
4098	690.3	0.6622	1.225	0.7762	1.2568	0
4099	690.2	0.6625	1.2253	0.7761	1.2569	0
4100	690.1	0.6623	1.226	0.7761	1.257	0
4101	690	0.6628	1.2262	0.7761	1.2573	0
4102	689.9	0.6629	1.2259	0.776	1.2572	0
4103	689.8	0.663	1.2262	0.7761	1.2573	0
4104	689.7	0.6631	1.2261	0.7759	1.2572	0
4105	689.6	0.6633	1.2264	0.7758	1.2576	0
4106	689.5	0.6636	1.2268	0.7758	1.2577	0
4107	689.4	0.6638	1.227	0.7757	1.2579	0
4108	689.3	0.6637	1.227	0.7757	1.2583	0
4109	689.2	0.6637	1.227	0.7757	1.2589	0
4110	689.1	0.6641	1.2271	0.7757	1.2581	0
4111	689	0.6641	1.2277	0.7757	1.2581	0
4112	688.9	0.6642	1.2279	0.7759	1.2576	0
4113	688.8	0.6641	1.2279	0.7763	1.2584	0
4114	688.7	0.6647	1.2281	0.7762	1.2581	0

4115	688.6	0.6645	1.2281	0.7758	1.2586	0
4116	688.5	0.665	1.2283	0.776	1.2589	0
4117	688.4	0.6651	1.2282	0.776	1.2586	0
4118	688.3	0.6652	1.2284	0.776	1.2582	0
4119	688.2	0.6655	1.2283	0.776	1.2585	0
4120	688.1	0.6654	1.2287	0.7757	1.2582	0
4121	688	0.6655	1.2287	0.776	1.258	0
4122	687.9	0.6655	1.2287	0.7763	1.2582	0
4123	687.8	0.6657	1.229	0.7759	1.2583	0
4124	687.7	0.6657	1.2294	0.7762	1.258	0
4125	687.6	0.6657	1.2297	0.7761	1.2582	0
4126	687.5	0.6659	1.2298	0.7761	1.2588	0
4127	687.4	0.6661	1.2296	0.7761	1.2577	0
4128	687.3	0.6664	1.2295	0.7763	1.258	0
4129	687.2	0.6666	1.2298	0.7765	1.2591	0
4130	687.1	0.6667	1.2296	0.7761	1.259	0
4131	687	0.6668	1.2299	0.7765	1.2588	0
4132	686.9	0.6669	1.2308	0.7764	1.2586	0
4133	686.8	0.6673	1.2308	0.7765	1.2579	0
4134	686.7	0.6674	1.2308	0.7765	1.2579	0
4135	686.6	0.6672	1.2308	0.7766	1.2582	0
4136	686.5	0.6674	1.2306	0.7766	1.2577	0
4137	686.4	0.6675	1.2311	0.7767	1.2579	0
4138	686.3	0.6677	1.2314	0.7768	1.2582	0
4139	686.2	0.6677	1.2316	0.7768	1.2582	0
4140	686.1	0.6678	1.2316	0.777	1.2578	0
4141	686	0.6679	1.232	0.7771	1.2576	0
4142	685.9	0.6682	1.232	0.7773	1.2571	0
4143	685.8	0.6684	1.2315	0.7774	1.2569	0
4144	685.7	0.6685	1.2317	0.7773	1.2583	0
4145	685.6	0.6685	1.2322	0.7774	1.2574	0
4146	685.5	0.6688	1.2321	0.7775	1.2584	0
4147	685.4	0.6687	1.232	0.7776	1.2572	0
4148	685.3	0.669	1.2322	0.7778	1.2567	0
4149	685.2	0.6693	1.2323	0.7777	1.2571	0
4150	685.1	0.6691	1.2328	0.7779	1.2567	0
4151	685	0.6689	1.233	0.7778	1.2566	0
4152	684.9	0.6692	1.233	0.7781	1.2573	0
4153	684.8	0.6692	1.2328	0.778	1.2566	0
4154	684.7	0.6693	1.2329	0.7779	1.2555	0
4155	684.6	0.6698	1.2333	0.7781	1.2551	0
4156	684.5	0.6698	1.2332	0.7782	1.2558	0
4157	684.4	0.67	1.2337	0.7782	1.2556	0

4158	684.3	0.6695	1.2334	0.7783	1.2553	0
4159	684.2	0.6698	1.234	0.7784	1.256	0
4160	684.1	0.67	1.2341	0.7782	1.2545	0
4161	684	0.67	1.2339	0.7784	1.2549	0
4162	683.9	0.6698	1.2337	0.7787	1.2542	0
4163	683.8	0.6704	1.2338	0.7789	1.2548	0
4164	683.7	0.6703	1.2344	0.7789	1.255	0
4165	683.6	0.6703	1.2345	0.7787	1.2547	0
4166	683.5	0.6707	1.2347	0.7789	1.2547	0
4167	683.4	0.6708	1.2346	0.7789	1.2549	0
4168	683.3	0.6709	1.2354	0.7791	1.2551	0
4169	683.2	0.6709	1.2352	0.7792	1.2547	0
4170	683.1	0.6715	1.2358	0.7793	1.2547	0
4171	683	0.6712	1.236	0.7793	1.2546	0
4172	682.9	0.6716	1.2365	0.7793	1.2553	0
4173	682.8	0.6716	1.2366	0.7791	1.2549	0
4174	682.7	0.6717	1.2366	0.7794	1.2547	0
4175	682.6	0.6717	1.2366	0.7794	1.255	0
4176	682.5	0.6719	1.2365	0.7795	1.255	0
4177	682.4	0.6721	1.2368	0.7795	1.2545	0
4178	682.3	0.6724	1.2371	0.7793	1.255	0
4179	682.2	0.6724	1.2374	0.7796	1.2556	0
4180	682.1	0.6727	1.2379	0.7793	1.2549	0
4181	682	0.6725	1.2388	0.7791	1.2554	0
4182	681.9	0.6727	1.2385	0.7794	1.2556	0
4183	681.8	0.6729	1.2385	0.7791	1.2561	0
4184	681.7	0.6729	1.239	0.7794	1.2557	0
4185	681.6	0.6733	1.2397	0.7798	1.2565	0
4186	681.5	0.673	1.2397	0.7795	1.2554	0
4187	681.4	0.6731	1.2398	0.7792	1.2555	0
4188	681.3	0.6737	1.2404	0.7795	1.255	0
4189	681.2	0.6739	1.2406	0.7793	1.2557	0
4190	681.1	0.6738	1.2406	0.7793	1.2562	0
4191	681	0.6738	1.2412	0.779	1.256	0
4192	680.9	0.6739	1.2413	0.7789	1.2559	0
4193	680.8	0.6742	1.2417	0.779	1.2571	0
4194	680.7	0.6744	1.242	0.7792	1.2564	0
4195	680.6	0.6746	1.2421	0.7793	1.2565	0
4196	680.5	0.6746	1.2424	0.779	1.2571	0
4197	680.4	0.6749	1.2428	0.7789	1.2571	0
4198	680.3	0.6752	1.2434	0.7791	1.2577	0
4199	680.2	0.6751	1.2437	0.7789	1.257	0
4200	680.1	0.6755	1.2439	0.7789	1.2577	0

4201	680	0.6754	1.2437	0.7787	1.2577	0
4202	679.9	0.6754	1.2441	0.7789	1.2583	0
4203	679.8	0.6758	1.2444	0.7789	1.2586	0
4204	679.7	0.676	1.245	0.779	1.2584	0
4205	679.6	0.6761	1.2457	0.7788	1.2585	0
4206	679.5	0.6765	1.2455	0.7787	1.2584	0
4207	679.4	0.6767	1.2458	0.7784	1.259	0
4208	679.3	0.6768	1.2463	0.7784	1.2592	0
4209	679.2	0.677	1.2466	0.7784	1.2601	0
4210	679.1	0.6771	1.2472	0.7784	1.2607	0
4211	679	0.6773	1.2473	0.7782	1.2604	0
4212	678.9	0.6776	1.2474	0.7784	1.2608	0
4213	678.8	0.6775	1.2481	0.7783	1.2605	0
4214	678.7	0.6777	1.2478	0.7781	1.2603	0
4215	678.6	0.6778	1.2484	0.7782	1.2613	0
4216	678.5	0.6779	1.2485	0.7784	1.2609	0
4217	678.4	0.6783	1.2487	0.7785	1.2613	0
4218	678.3	0.6783	1.2493	0.7784	1.2609	0
4219	678.2	0.6787	1.25	0.7782	1.2617	0
4220	678.1	0.6786	1.2496	0.7779	1.2602	0
4221	678	0.6787	1.2496	0.7781	1.2624	0
4222	677.9	0.6787	1.2498	0.7781	1.2627	0
4223	677.8	0.6791	1.2504	0.7782	1.2633	0
4224	677.7	0.6795	1.2504	0.7783	1.2631	0
4225	677.6	0.6796	1.2507	0.7782	1.2632	0
4226	677.5	0.6794	1.2514	0.7778	1.2624	0
4227	677.4	0.6796	1.2514	0.778	1.2623	0
4228	677.3	0.6797	1.2517	0.7781	1.2626	0
4229	677.2	0.6797	1.2517	0.778	1.263	0
4230	677.1	0.6801	1.2519	0.7779	1.2618	0
4231	677	0.6802	1.2525	0.7781	1.2621	0
4232	676.9	0.6803	1.2524	0.7784	1.2615	0
4233	676.8	0.6803	1.2526	0.7782	1.2625	0
4234	676.7	0.6804	1.253	0.7784	1.2616	0
4235	676.6	0.6806	1.2532	0.7782	1.2617	0
4236	676.5	0.6808	1.2535	0.7786	1.2624	0
4237	676.4	0.6809	1.2536	0.7786	1.2615	0
4238	676.3	0.681	1.2539	0.7788	1.2616	0
4239	676.2	0.681	1.2535	0.7791	1.2614	0
4240	676.1	0.6811	1.2541	0.7792	1.2603	0
4241	676	0.6814	1.2537	0.7791	1.2607	0
4242	675.9	0.6813	1.2546	0.7794	1.2602	0
4243	675.8	0.6815	1.2546	0.7794	1.2604	0

4244	675.7	0.6817	1.2546	0.7797	1.2604	0
4245	675.6	0.6818	1.255	0.7797	1.2596	0
4246	675.5	0.6817	1.2554	0.7795	1.2596	0
4247	675.4	0.6817	1.2551	0.7798	1.2599	0
4248	675.3	0.6814	1.2555	0.78	1.2596	0
4249	675.2	0.6822	1.2555	0.7802	1.2589	0
4250	675.1	0.6822	1.2556	0.7801	1.2587	0
4251	675	0.6823	1.2558	0.7805	1.2579	0
4252	674.9	0.6825	1.2562	0.7807	1.2581	0
4253	674.8	0.6825	1.2568	0.7809	1.2579	0
4254	674.7	0.6827	1.2561	0.7806	1.258	0
4255	674.6	0.6828	1.2564	0.7808	1.2579	0
4256	674.5	0.683	1.2569	0.7813	1.2584	0
4257	674.4	0.683	1.2574	0.7813	1.2572	0
4258	674.3	0.683	1.2576	0.7816	1.2567	0
4259	674.2	0.683	1.2582	0.7818	1.2563	0
4260	674.1	0.6834	1.258	0.7815	1.2566	0
4261	674	0.6835	1.2579	0.7817	1.2564	0
4262	673.9	0.6834	1.2582	0.7817	1.2562	0
4263	673.8	0.6836	1.2585	0.7819	1.2562	0
4264	673.7	0.6839	1.2584	0.7819	1.2561	0
4265	673.6	0.6839	1.2587	0.7822	1.2564	0
4266	673.5	0.6839	1.2592	0.7823	1.2557	0
4267	673.4	0.6841	1.2596	0.7822	1.2563	0
4268	673.3	0.6841	1.2593	0.7822	1.2562	0
4269	673.2	0.6846	1.2596	0.7823	1.2562	0
4270	673.1	0.6844	1.2598	0.7824	1.2556	0
4271	673	0.6849	1.2601	0.7829	1.2551	0
4272	672.9	0.6848	1.2603	0.7827	1.2551	0
4273	672.8	0.6848	1.2605	0.7827	1.2554	0
4274	672.7	0.685	1.2604	0.7826	1.2554	0
4275	672.6	0.685	1.2609	0.7825	1.2551	0
4276	672.5	0.685	1.2609	0.7826	1.2546	0
4277	672.4	0.6855	1.2612	0.7826	1.2557	0
4278	672.3	0.6854	1.2612	0.7828	1.2549	0
4279	672.2	0.6856	1.2618	0.783	1.2552	0
4280	672.1	0.6858	1.2618	0.7827	1.2553	0
4281	672	0.6858	1.2616	0.7829	1.2557	0
4282	671.9	0.6858	1.2621	0.7828	1.2549	0
4283	671.8	0.686	1.2623	0.7827	1.2551	0
4284	671.7	0.686	1.2623	0.783	1.2554	0
4285	671.6	0.6866	1.2626	0.783	1.2552	0
4286	671.5	0.6864	1.2628	0.7829	1.2571	0

4287	671.4	0.6865	1.2626	0.7828	1.2559	0
4288	671.3	0.6869	1.2631	0.7828	1.2556	0
4289	671.2	0.6873	1.2634	0.783	1.257	0
4290	671.1	0.687	1.2638	0.7829	1.2568	0
4291	671	0.6873	1.2638	0.7827	1.257	0
4292	670.9	0.6874	1.2643	0.7827	1.2564	0
4293	670.8	0.6879	1.2644	0.7828	1.2571	0
4294	670.7	0.6878	1.2646	0.7827	1.2569	0
4295	670.6	0.6878	1.2645	0.7827	1.2577	0
4296	670.5	0.6878	1.2649	0.7823	1.2572	0
4297	670.4	0.6879	1.2651	0.782	1.2574	0
4298	670.3	0.6883	1.2653	0.7824	1.2574	0
4299	670.2	0.6885	1.2658	0.7822	1.2573	0
4300	670.1	0.6886	1.266	0.7822	1.258	0
4301	670	0.6886	1.2662	0.7822	1.2583	0
4302	669.9	0.6889	1.2661	0.7821	1.2592	0
4303	669.8	0.6889	1.2663	0.782	1.259	0
4304	669.7	0.689	1.2668	0.782	1.2588	0
4305	669.6	0.6895	1.267	0.7817	1.2597	0
4306	669.5	0.6897	1.2671	0.7817	1.2598	0
4307	669.4	0.69	1.2673	0.7815	1.2595	0
4308	669.3	0.6902	1.2673	0.7813	1.2597	0
4309	669.2	0.6902	1.2675	0.7811	1.2598	0
4310	669.1	0.6904	1.2674	0.7814	1.2602	0
4311	669	0.6906	1.2681	0.7813	1.2607	0
4312	668.9	0.6905	1.2683	0.7811	1.2616	0
4313	668.8	0.6909	1.2682	0.7808	1.2612	0
4314	668.7	0.691	1.2685	0.7807	1.2617	0
4315	668.6	0.6913	1.2687	0.7808	1.2628	0
4316	668.5	0.6915	1.269	0.7808	1.262	0
4317	668.4	0.6919	1.269	0.7808	1.2628	0
4318	668.3	0.6918	1.2687	0.7807	1.2628	0
4319	668.2	0.6919	1.2689	0.7806	1.2628	0
4320	668.1	0.6921	1.2692	0.7804	1.2633	0
4321	668	0.6922	1.2691	0.7805	1.2634	0
4322	667.9	0.6924	1.2691	0.7806	1.2635	0
4323	667.8	0.6926	1.2692	0.7807	1.2635	0
4324	667.7	0.6927	1.2692	0.7804	1.264	0
4325	667.6	0.6929	1.2687	0.7802	1.2639	0
4326	667.5	0.6928	1.2693	0.78	1.2632	0
4327	667.4	0.6929	1.2689	0.7803	1.2637	0
4328	667.3	0.6932	1.2695	0.7803	1.2642	0
4329	667.2	0.6934	1.2693	0.78	1.2651	0

4330	667.1	0.6936	1.2696	0.7802	1.2636	0
4331	667	0.6936	1.2694	0.7802	1.2659	0
4332	666.9	0.6939	1.2694	0.7804	1.2648	0
4333	666.8	0.694	1.2693	0.7802	1.2653	0
4334	666.7	0.6943	1.2694	0.7803	1.2641	0
4335	666.6	0.6942	1.2694	0.7803	1.2648	0
4336	666.5	0.6946	1.2694	0.7803	1.2652	0
4337	666.4	0.6947	1.2692	0.7804	1.2646	0
4338	666.3	0.695	1.2692	0.7805	1.264	0
4339	666.2	0.6952	1.269	0.7806	1.2646	0
4340	666.1	0.6954	1.2691	0.7806	1.2647	0
4341	666	0.6955	1.2691	0.7807	1.264	0
4342	665.9	0.6955	1.2685	0.7807	1.265	0
4343	665.8	0.6957	1.2691	0.781	1.2649	0
4344	665.7	0.6957	1.269	0.7809	1.2642	0
4345	665.6	0.6959	1.2687	0.7811	1.2638	0
4346	665.5	0.6957	1.2685	0.781	1.2637	0
4347	665.4	0.696	1.2683	0.7813	1.2637	0
4348	665.3	0.696	1.268	0.7814	1.2628	0
4349	665.2	0.6962	1.2678	0.7815	1.2643	0
4350	665.1	0.6966	1.2678	0.7817	1.2626	0
4351	665	0.6967	1.2675	0.7817	1.2613	0
4352	664.9	0.6967	1.2677	0.782	1.2627	0
4353	664.8	0.6968	1.2671	0.7821	1.2625	0
4354	664.7	0.6967	1.2671	0.7822	1.2616	0
4355	664.6	0.697	1.2671	0.7824	1.2619	0
4356	664.5	0.6968	1.2672	0.7824	1.2622	0
4357	664.4	0.6968	1.2666	0.7826	1.2614	0
4358	664.3	0.6973	1.2667	0.783	1.2603	0
4359	664.2	0.6974	1.2663	0.7831	1.261	0
4360	664.1	0.6974	1.266	0.783	1.2608	0
4361	664	0.6975	1.2661	0.7833	1.2598	0
4362	663.9	0.6975	1.2664	0.7837	1.2589	0
4363	663.8	0.6977	1.2659	0.7836	1.2593	0
4364	663.7	0.698	1.2659	0.7842	1.2589	0
4365	663.6	0.698	1.2654	0.7844	1.2586	0
4366	663.5	0.6981	1.2653	0.7843	1.2585	0
4367	663.4	0.6979	1.2647	0.784	1.2575	0
4368	663.3	0.6981	1.2649	0.7842	1.2577	0
4369	663.2	0.6986	1.2651	0.7845	1.2584	0
4370	663.1	0.6984	1.2648	0.7847	1.2574	0
4371	663	0.6985	1.2646	0.7848	1.258	0
4372	662.9	0.6988	1.2656	0.7849	1.2578	0

4373	662.8	0.6987	1.2649	0.7848	1.257	0
4374	662.7	0.6992	1.2647	0.785	1.2569	0
4375	662.6	0.6992	1.2647	0.785	1.2566	0
4376	662.5	0.6994	1.2649	0.7851	1.2573	0
4377	662.4	0.6992	1.2647	0.785	1.256	0
4378	662.3	0.6995	1.2649	0.785	1.2575	0
4379	662.2	0.6994	1.2645	0.7848	1.258	0
4380	662.1	0.6997	1.2647	0.7853	1.2575	0
4381	662	0.7	1.2649	0.7858	1.2566	0
4382	661.9	0.7001	1.2645	0.7852	1.2573	0
4383	661.8	0.7005	1.2643	0.7854	1.2576	0
4384	661.7	0.7005	1.2647	0.7856	1.2571	0
4385	661.6	0.7006	1.2651	0.7855	1.2574	0
4386	661.5	0.7005	1.2649	0.7857	1.2575	0
4387	661.4	0.7004	1.2646	0.7856	1.258	0
4388	661.3	0.701	1.2641	0.7856	1.258	0
4389	661.2	0.7013	1.265	0.7859	1.2577	0
4390	661.1	0.7014	1.2644	0.7859	1.2581	0
4391	661	0.7018	1.2653	0.7858	1.2577	0
4392	660.9	0.7018	1.265	0.7857	1.2578	0
4393	660.8	0.7018	1.2649	0.7858	1.2573	0
4394	660.7	0.7019	1.2654	0.7858	1.258	0
4395	660.6	0.7023	1.2653	0.7859	1.2583	0
4396	660.5	0.7026	1.2654	0.786	1.2584	0
4397	660.4	0.7027	1.2658	0.7861	1.2592	0
4398	660.3	0.7026	1.2659	0.7862	1.2588	0
4399	660.2	0.7029	1.2655	0.7856	1.2587	0
4400	660.1	0.7029	1.2656	0.7859	1.2591	0
4401	660	0.7033	1.2657	0.7862	1.2587	0
4402	659.9	0.7033	1.2658	0.7858	1.259	0
4403	659.8	0.7036	1.2662	0.7857	1.2594	0
4404	659.7	0.7033	1.2661	0.7857	1.2591	0
4405	659.6	0.7035	1.266	0.7856	1.2594	0
4406	659.5	0.7037	1.2662	0.7854	1.2604	0
4407	659.4	0.704	1.2662	0.7854	1.2611	0
4408	659.3	0.7043	1.2663	0.7852	1.2608	0
4409	659.2	0.7045	1.2672	0.7851	1.2614	0
4410	659.1	0.7047	1.2674	0.7854	1.261	0
4411	659	0.7049	1.2677	0.785	1.2611	0
4412	658.9	0.7051	1.2674	0.7848	1.2614	0
4413	658.8	0.7056	1.2676	0.7852	1.2616	0
4414	658.7	0.7057	1.2681	0.7851	1.2619	0
4415	658.6	0.7057	1.2683	0.7849	1.2623	0

4416	658.5	0.7059	1.2684	0.7846	1.2633	0
4417	658.4	0.7058	1.2685	0.7844	1.2623	0
4418	658.3	0.7064	1.2685	0.7844	1.2631	0
4419	658.2	0.7064	1.2689	0.7843	1.2633	0
4420	658.1	0.7063	1.2687	0.7841	1.2641	0
4421	658	0.7065	1.2689	0.784	1.2647	0
4422	657.9	0.7067	1.2691	0.7837	1.2647	0
4423	657.8	0.7068	1.2693	0.7843	1.2647	0
4424	657.7	0.7071	1.2693	0.784	1.2653	0
4425	657.6	0.7073	1.2702	0.784	1.2659	0
4426	657.5	0.7076	1.2703	0.7837	1.2665	0
4427	657.4	0.7074	1.2704	0.7839	1.2664	0
4428	657.3	0.7077	1.2704	0.7838	1.2669	0
4429	657.2	0.7078	1.2704	0.7836	1.2673	0
4430	657.1	0.7077	1.2709	0.7836	1.2671	0
4431	657	0.7082	1.2711	0.7836	1.2671	0
4432	656.9	0.7087	1.2714	0.7838	1.2667	0
4433	656.8	0.7086	1.2715	0.7837	1.2675	0
4434	656.7	0.7091	1.2715	0.7837	1.2675	0
4435	656.6	0.7091	1.2715	0.7836	1.2675	0
4436	656.5	0.7094	1.2715	0.7834	1.2668	0
4437	656.4	0.7095	1.2719	0.7837	1.2672	0
4438	656.3	0.7095	1.2718	0.7836	1.2678	0
4439	656.2	0.7098	1.2726	0.7837	1.2679	0
4440	656.1	0.7099	1.2729	0.7837	1.2686	0
4441	656	0.71	1.2724	0.7838	1.269	0
4442	655.9	0.7103	1.2729	0.7839	1.2682	0
4443	655.8	0.7104	1.2723	0.7836	1.2681	0
4444	655.7	0.7108	1.2724	0.7835	1.2685	0
4445	655.6	0.7107	1.2728	0.7839	1.2686	0
4446	655.5	0.7108	1.2726	0.784	1.2681	0
4447	655.4	0.7113	1.2732	0.7839	1.2676	0
4448	655.3	0.7113	1.2734	0.7841	1.268	0
4449	655.2	0.7114	1.2737	0.7842	1.2683	0
4450	655.1	0.7112	1.2739	0.7842	1.2678	0
4451	655	0.7118	1.2735	0.7842	1.2678	0
4452	654.9	0.7118	1.2736	0.7843	1.2677	0
4453	654.8	0.7118	1.2738	0.7845	1.2675	0
4454	654.7	0.7118	1.2738	0.7844	1.2682	0
4455	654.6	0.712	1.2742	0.7844	1.269	0
4456	654.5	0.7124	1.2741	0.7847	1.2673	0
4457	654.4	0.7123	1.2742	0.7847	1.2672	0
4458	654.3	0.7125	1.274	0.7849	1.267	0

4459	654.2	0.7127	1.2741	0.7851	1.2676	0
4460	654.1	0.7131	1.2745	0.7851	1.2671	0
4461	654	0.7131	1.2745	0.7854	1.2671	0
4462	653.9	0.7134	1.2747	0.7856	1.2673	0
4463	653.8	0.7136	1.2747	0.7858	1.2675	0
4464	653.7	0.7136	1.2748	0.7858	1.2672	0
4465	653.6	0.7138	1.2746	0.7857	1.2665	0
4466	653.5	0.7139	1.275	0.7859	1.2658	0
4467	653.4	0.7141	1.2749	0.7859	1.2662	0
4468	653.3	0.7142	1.2753	0.7863	1.2654	0
4469	653.2	0.7146	1.2751	0.7862	1.2655	0
4470	653.1	0.7144	1.2753	0.7862	1.2657	0
4471	653	0.7146	1.2752	0.7865	1.2663	0
4472	652.9	0.7147	1.2748	0.7865	1.2648	0
4473	652.8	0.7148	1.2747	0.7866	1.2655	0
4474	652.7	0.7148	1.2754	0.7869	1.2651	0
4475	652.6	0.7152	1.2752	0.7868	1.2649	0
4476	652.5	0.7155	1.2757	0.7872	1.2641	0
4477	652.4	0.7154	1.2758	0.787	1.2637	0
4478	652.3	0.7158	1.2759	0.7872	1.2647	0
4479	652.2	0.7157	1.2757	0.7873	1.2639	0
4480	652.1	0.716	1.2761	0.7874	1.264	0
4481	652	0.7159	1.2761	0.7877	1.2647	0
4482	651.9	0.7164	1.2763	0.7874	1.264	0
4483	651.8	0.7162	1.2766	0.7875	1.264	0
4484	651.7	0.7162	1.2764	0.7877	1.2637	0
4485	651.6	0.7166	1.2763	0.7876	1.2634	0
4486	651.5	0.7167	1.2767	0.7879	1.2629	0
4487	651.4	0.717	1.2767	0.788	1.2636	0
4488	651.3	0.7171	1.2768	0.7877	1.2634	0
4489	651.2	0.7173	1.2771	0.7878	1.2634	0
4490	651.1	0.7173	1.2775	0.7882	1.2632	0
4491	651	0.7176	1.2772	0.7883	1.2633	0
4492	650.9	0.7176	1.2774	0.7882	1.2638	0
4493	650.8	0.7177	1.2778	0.788	1.2631	0
4494	650.7	0.7178	1.2778	0.7884	1.2639	0
4495	650.6	0.7182	1.2782	0.7886	1.2639	0
4496	650.5	0.7182	1.2782	0.7883	1.2637	0
4497	650.4	0.7183	1.2785	0.7884	1.264	0
4498	650.3	0.7187	1.2783	0.7885	1.2636	0
4499	650.2	0.7189	1.2784	0.7884	1.2637	0
4500	650.1	0.7192	1.2787	0.7884	1.2646	0
4501	650	0.7193	1.279	0.7881	1.2631	0

4502	649.9	0.7197	1.2791	0.7882	1.2634	0
4503	649.8	0.72	1.2794	0.7884	1.2636	0
4504	649.7	0.7201	1.2793	0.7886	1.2639	0
4505	649.6	0.7203	1.28	0.7884	1.2639	0
4506	649.5	0.7203	1.28	0.7882	1.2641	0
4507	649.4	0.7208	1.2801	0.7881	1.2646	0
4508	649.3	0.7207	1.28	0.7882	1.2648	0
4509	649.2	0.7208	1.2803	0.7886	1.2654	0
4510	649.1	0.7211	1.2808	0.7882	1.2649	0
4511	649	0.7213	1.2816	0.788	1.2646	0
4512	648.9	0.7213	1.2816	0.7885	1.2651	0
4513	648.8	0.7218	1.2822	0.7886	1.2654	0
4514	648.7	0.7216	1.2815	0.7883	1.2665	0
4515	648.6	0.7219	1.2821	0.7881	1.2662	0
4516	648.5	0.7223	1.2825	0.7876	1.2661	0
4517	648.4	0.7224	1.2826	0.7879	1.2671	0
4518	648.3	0.7227	1.2829	0.7879	1.2665	0
4519	648.2	0.7229	1.2833	0.7881	1.2671	0
4520	648.1	0.7229	1.2835	0.7879	1.268	0
4521	648	0.7233	1.284	0.7877	1.2683	0
4522	647.9	0.7235	1.2839	0.7878	1.2673	0
4523	647.8	0.7236	1.2841	0.7877	1.2679	0
4524	647.7	0.7239	1.2842	0.7877	1.2674	0
4525	647.6	0.7242	1.2845	0.7876	1.2681	0
4526	647.5	0.7242	1.2848	0.7875	1.2686	0
4527	647.4	0.7244	1.285	0.7873	1.269	0
4528	647.3	0.7249	1.2853	0.7874	1.2685	0
4529	647.2	0.7249	1.2855	0.7873	1.269	0
4530	647.1	0.7248	1.2858	0.787	1.2696	0
4531	647	0.7252	1.2858	0.7873	1.2702	0
4532	646.9	0.7254	1.2858	0.7872	1.2709	0
4533	646.8	0.7254	1.2862	0.787	1.2709	0
4534	646.7	0.7255	1.2863	0.7872	1.2694	0
4535	646.6	0.7258	1.2861	0.787	1.2705	0
4536	646.5	0.7262	1.2868	0.7874	1.2706	0
4537	646.4	0.7262	1.2867	0.787	1.2706	0
4538	646.3	0.7264	1.2873	0.7871	1.2703	0
4539	646.2	0.7267	1.2878	0.7871	1.2702	0
4540	646.1	0.7267	1.2874	0.7868	1.2705	0
4541	646	0.727	1.288	0.7869	1.2706	0
4542	645.9	0.7271	1.2881	0.7869	1.2714	0
4543	645.8	0.7274	1.288	0.7871	1.2706	0
4544	645.7	0.7277	1.2881	0.7873	1.2716	0

4545	645.6	0.7279	1.2886	0.7871	1.2712	0
4546	645.5	0.7281	1.2883	0.7871	1.2718	0
4547	645.4	0.7282	1.2885	0.7872	1.2718	0
4548	645.3	0.728	1.2885	0.7873	1.2707	0
4549	645.2	0.7283	1.2885	0.7873	1.2715	0
4550	645.1	0.7285	1.2888	0.7872	1.271	0
4551	645	0.7287	1.2889	0.7872	1.2715	0
4552	644.9	0.7289	1.2898	0.7872	1.2708	0
4553	644.8	0.7289	1.2897	0.7874	1.2703	0
4554	644.7	0.7293	1.29	0.7874	1.2713	0
4555	644.6	0.7295	1.2897	0.7875	1.271	0
4556	644.5	0.7296	1.2897	0.7875	1.271	0
4557	644.4	0.7296	1.2901	0.7875	1.2712	0
4558	644.3	0.73	1.2906	0.7879	1.2705	0
4559	644.2	0.7298	1.2905	0.7879	1.2702	0
4560	644.1	0.73	1.2906	0.7878	1.2703	0
4561	644	0.7303	1.2905	0.7878	1.2698	0
4562	643.9	0.7306	1.2912	0.7879	1.2696	0
4563	643.8	0.7308	1.2909	0.7881	1.2707	0
4564	643.7	0.7304	1.2912	0.7883	1.2703	0
4565	643.6	0.7307	1.2911	0.7883	1.2705	0
4566	643.5	0.7311	1.291	0.7884	1.2701	0
4567	643.4	0.7311	1.2911	0.7883	1.2705	0
4568	643.3	0.7311	1.2913	0.7885	1.2702	0
4569	643.2	0.7315	1.2918	0.7887	1.27	0
4570	643.1	0.7318	1.2919	0.7885	1.2708	0
4571	643	0.7321	1.292	0.7888	1.2709	0
4572	642.9	0.7322	1.2924	0.7891	1.2704	0
4573	642.8	0.7324	1.2922	0.7891	1.27	0
4574	642.7	0.7326	1.2921	0.7893	1.2695	0
4575	642.6	0.7327	1.2923	0.7893	1.2699	0
4576	642.5	0.7329	1.2921	0.7895	1.2695	0
4577	642.4	0.733	1.2932	0.7894	1.269	0
4578	642.3	0.7332	1.2928	0.7893	1.2693	0
4579	642.2	0.7335	1.2935	0.7894	1.2708	0
4580	642.1	0.7334	1.2931	0.7895	1.2703	0
4581	642	0.7339	1.2933	0.7893	1.2704	0
4582	641.9	0.734	1.2935	0.7894	1.2703	0
4583	641.8	0.7341	1.2937	0.7897	1.2704	0
4584	641.7	0.7342	1.2937	0.7896	1.2707	0
4585	641.6	0.7346	1.2943	0.7896	1.2695	0
4586	641.5	0.7349	1.2944	0.7898	1.2711	0
4587	641.4	0.7349	1.2948	0.7899	1.2702	0

4588	641.3	0.7353	1.2949	0.79	1.2697	0
4589	641.2	0.7354	1.2948	0.7901	1.2707	0
4590	641.1	0.7353	1.2949	0.7899	1.271	0
4591	641	0.7355	1.2954	0.7899	1.2701	0
4592	640.9	0.7359	1.2954	0.7901	1.2697	0
4593	640.8	0.7358	1.2953	0.7901	1.2699	0
4594	640.7	0.7361	1.2957	0.79	1.2703	0
4595	640.6	0.7366	1.2959	0.7901	1.27	0
4596	640.5	0.7363	1.2961	0.7899	1.27	0
4597	640.4	0.737	1.2961	0.7902	1.2708	0
4598	640.3	0.737	1.2967	0.7904	1.2711	0
4599	640.2	0.7374	1.297	0.7903	1.2702	0
4600	640.1	0.7374	1.2973	0.7905	1.271	0
4601	640	0.7374	1.2969	0.7904	1.2696	0
4602	639.9	0.7379	1.2972	0.7905	1.2707	0
4603	639.8	0.7379	1.2974	0.7906	1.2709	0
4604	639.7	0.7382	1.2977	0.7909	1.2705	0
4605	639.6	0.7382	1.2978	0.791	1.2706	0
4606	639.5	0.7383	1.2984	0.7906	1.2712	0
4607	639.4	0.7386	1.2981	0.7904	1.2707	0
4608	639.3	0.7388	1.2986	0.7908	1.271	0
4609	639.2	0.7391	1.2988	0.7908	1.2716	0
4610	639.1	0.7394	1.2989	0.7906	1.2713	0
4611	639	0.7395	1.299	0.7906	1.2714	0
4612	638.9	0.7399	1.2997	0.7909	1.2718	0
4613	638.8	0.7401	1.2997	0.7909	1.2721	0
4614	638.7	0.7403	1.3	0.7907	1.2718	0
4615	638.6	0.7406	1.3002	0.7908	1.2732	0
4616	638.5	0.7405	1.3003	0.7907	1.2735	0
4617	638.4	0.741	1.3007	0.7904	1.2732	0
4618	638.3	0.741	1.301	0.7905	1.2729	0
4619	638.2	0.7412	1.3014	0.7905	1.2729	0
4620	638.1	0.7414	1.3012	0.7902	1.2732	0
4621	638	0.7417	1.3016	0.7906	1.2737	0
4622	637.9	0.7418	1.3019	0.7906	1.2747	0
4623	637.8	0.7421	1.3022	0.7904	1.2741	0
4624	637.7	0.7425	1.3024	0.7906	1.2749	0
4625	637.6	0.7426	1.3026	0.7907	1.2751	0
4626	637.5	0.7427	1.3027	0.7905	1.275	0
4627	637.4	0.7427	1.3028	0.7905	1.2745	0
4628	637.3	0.7432	1.303	0.7905	1.2758	0
4629	637.2	0.7434	1.3038	0.7907	1.2754	0
4630	637.1	0.7436	1.3035	0.7906	1.2767	0

4631	637	0.7438	1.3038	0.7905	1.2759	0
4632	636.9	0.744	1.3037	0.7903	1.2756	0
4633	636.8	0.7441	1.304	0.7905	1.2756	0
4634	636.7	0.7443	1.3044	0.7905	1.2752	0
4635	636.6	0.7445	1.3043	0.7906	1.2758	0
4636	636.5	0.7447	1.3052	0.7907	1.276	0
4637	636.4	0.7448	1.305	0.7905	1.276	0
4638	636.3	0.745	1.3051	0.7906	1.2757	0
4639	636.2	0.7453	1.3054	0.7905	1.2766	0
4640	636.1	0.7456	1.3053	0.7904	1.2766	0
4641	636	0.7454	1.3056	0.7904	1.2766	0
4642	635.9	0.7459	1.3056	0.7905	1.2769	0
4643	635.8	0.7462	1.306	0.7906	1.277	0
4644	635.7	0.7462	1.3062	0.7905	1.2763	0
4645	635.6	0.7463	1.3065	0.7904	1.2764	0
4646	635.5	0.7465	1.3067	0.7905	1.2771	0
4647	635.4	0.7469	1.3068	0.7908	1.2765	0
4648	635.3	0.7471	1.3072	0.7908	1.2769	0
4649	635.2	0.7474	1.3073	0.7911	1.2762	0
4650	635.1	0.7473	1.3071	0.7907	1.2768	0
4651	635	0.7476	1.3074	0.7909	1.2765	0
4652	634.9	0.7477	1.3078	0.7909	1.2765	0
4653	634.8	0.7476	1.3078	0.791	1.2758	0
4654	634.7	0.7482	1.3082	0.7909	1.2767	0
4655	634.6	0.7482	1.3084	0.7913	1.2773	0
4656	634.5	0.7488	1.309	0.7914	1.277	0
4657	634.4	0.7487	1.3085	0.7913	1.2774	0
4658	634.3	0.7487	1.3088	0.7912	1.2768	0
4659	634.2	0.7491	1.3088	0.7911	1.2777	0
4660	634.1	0.749	1.3086	0.7915	1.2771	0
4661	634	0.7494	1.3089	0.7918	1.2776	0
4662	633.9	0.7496	1.3096	0.7916	1.2763	0
4663	633.8	0.7498	1.3092	0.7917	1.2762	0
4664	633.7	0.7501	1.3094	0.7919	1.2768	0
4665	633.6	0.7499	1.3098	0.792	1.2766	0
4666	633.5	0.7502	1.31	0.7918	1.2761	0
4667	633.4	0.7504	1.3099	0.7918	1.2765	0
4668	633.3	0.7507	1.3097	0.7923	1.2764	0
4669	633.2	0.751	1.3102	0.7922	1.276	0
4670	633.1	0.7509	1.3104	0.7923	1.2753	0
4671	633	0.7509	1.3108	0.7922	1.2765	0
4672	632.9	0.7512	1.3112	0.7924	1.2769	0
4673	632.8	0.7513	1.3111	0.7925	1.2768	0

4674	632.7	0.752	1.3114	0.7925	1.2767	0
4675	632.6	0.7518	1.3118	0.7925	1.2762	0
4676	632.5	0.7521	1.3116	0.7925	1.2763	0
4677	632.4	0.7523	1.3118	0.7924	1.2769	0
4678	632.3	0.7527	1.312	0.7926	1.2763	0
4679	632.2	0.7527	1.3122	0.7928	1.2765	0
4680	632.1	0.7529	1.3125	0.7925	1.277	0
4681	632	0.7531	1.3129	0.7927	1.2774	0
4682	631.9	0.7536	1.313	0.7927	1.2775	0
4683	631.8	0.7537	1.3132	0.7926	1.2775	0
4684	631.7	0.7536	1.3129	0.7926	1.2769	0
4685	631.6	0.7541	1.3132	0.7927	1.2771	0
4686	631.5	0.7542	1.3137	0.7924	1.2772	0
4687	631.4	0.7544	1.314	0.793	1.2779	0
4688	631.3	0.7546	1.3143	0.7928	1.2771	0
4689	631.2	0.7548	1.314	0.7927	1.2767	0
4690	631.1	0.755	1.3149	0.7926	1.2769	0
4691	631	0.7552	1.315	0.7928	1.278	0
4692	630.9	0.7554	1.3154	0.7927	1.2776	0
4693	630.8	0.7558	1.3154	0.7928	1.2785	0
4694	630.7	0.7559	1.3156	0.7928	1.2776	0
4695	630.6	0.7561	1.3158	0.7926	1.2774	0
4696	630.5	0.7563	1.3161	0.7928	1.2778	0
4697	630.4	0.7565	1.3165	0.7928	1.2783	0
4698	630.3	0.7569	1.3165	0.7926	1.2783	0
4699	630.2	0.7572	1.3166	0.7929	1.2791	0
4700	630.1	0.7571	1.317	0.7925	1.2792	0
4701	630	0.7574	1.3174	0.7926	1.2792	0
4702	629.9	0.7575	1.3174	0.7926	1.2794	0
4703	629.8	0.7577	1.3179	0.7926	1.2786	0
4704	629.7	0.7579	1.3182	0.7927	1.2792	0
4705	629.6	0.7585	1.3187	0.7926	1.2788	0
4706	629.5	0.7588	1.3185	0.7928	1.2792	0
4707	629.4	0.7589	1.3189	0.7925	1.2796	0
4708	629.3	0.7592	1.3192	0.7926	1.2801	0
4709	629.2	0.7595	1.3191	0.7926	1.2803	0
4710	629.1	0.7597	1.3201	0.7926	1.2799	0
4711	629	0.7598	1.3201	0.7928	1.2797	0
4712	628.9	0.76	1.3204	0.7927	1.2794	0
4713	628.8	0.7601	1.3203	0.7924	1.2795	0
4714	628.7	0.7606	1.3209	0.7926	1.2799	0
4715	628.6	0.7606	1.321	0.7925	1.2794	0
4716	628.5	0.761	1.3213	0.7925	1.28	0

4717	628.4	0.7612	1.3218	0.7924	1.2801	0
4718	628.3	0.7613	1.3217	0.7926	1.2801	0
4719	628.2	0.7615	1.3223	0.793	1.2809	0
4720	628.1	0.7617	1.3229	0.7928	1.2796	0
4721	628	0.7619	1.3224	0.7926	1.2793	0
4722	627.9	0.7623	1.3231	0.793	1.2804	0
4723	627.8	0.7623	1.3233	0.7929	1.2806	0
4724	627.7	0.7626	1.3238	0.7928	1.2806	0
4725	627.6	0.7627	1.3238	0.7929	1.2807	0
4726	627.5	0.7631	1.324	0.7928	1.2813	0
4727	627.4	0.7636	1.3243	0.7932	1.281	0
4728	627.3	0.7636	1.3245	0.7929	1.28	0
4729	627.2	0.7639	1.3249	0.7931	1.2805	0
4730	627.1	0.7639	1.3246	0.7932	1.2803	0
4731	627	0.7644	1.3249	0.7929	1.2806	0
4732	626.9	0.7644	1.325	0.7928	1.2805	0
4733	626.8	0.7649	1.3253	0.793	1.2808	0
4734	626.7	0.765	1.3258	0.793	1.2806	0
4735	626.6	0.7651	1.3256	0.7931	1.2798	0
4736	626.5	0.7654	1.326	0.7933	1.28	0
4737	626.4	0.7654	1.3262	0.7928	1.28	0
4738	626.3	0.7656	1.3262	0.7931	1.2801	0
4739	626.2	0.7658	1.3265	0.7933	1.2807	0
4740	626.1	0.7659	1.3271	0.7933	1.2801	0
4741	626	0.7659	1.3264	0.7931	1.2797	0
4742	625.9	0.7663	1.3269	0.7933	1.2798	0
4743	625.8	0.7665	1.3272	0.7935	1.2794	0
4744	625.7	0.7667	1.3275	0.7936	1.2799	0
4745	625.6	0.7672	1.3275	0.7935	1.2787	0
4746	625.5	0.767	1.3278	0.7938	1.2782	0
4747	625.4	0.767	1.3278	0.7939	1.2784	0
4748	625.3	0.7676	1.3287	0.7938	1.2793	0
4749	625.2	0.7676	1.3281	0.794	1.2788	0
4750	625.1	0.7679	1.3286	0.7939	1.2789	0
4751	625	0.7682	1.3289	0.7941	1.2787	0
4752	624.9	0.7683	1.3289	0.794	1.2788	0
4753	624.8	0.7686	1.3288	0.7941	1.2793	0
4754	624.7	0.7687	1.3293	0.7944	1.2784	0
4755	624.6	0.769	1.3298	0.7945	1.278	0
4756	624.5	0.7693	1.3301	0.7946	1.2777	0
4757	624.4	0.7693	1.3303	0.7944	1.2775	0
4758	624.3	0.7695	1.3304	0.7947	1.2766	0
4759	624.2	0.7698	1.3305	0.7946	1.2775	0

4760	624.1	0.77	1.3305	0.7947	1.2776	0
4761	624	0.7703	1.3312	0.7947	1.2784	0
4762	623.9	0.7706	1.3313	0.7947	1.2771	0
4763	623.8	0.7707	1.3313	0.7949	1.2775	0
4764	623.7	0.771	1.3318	0.7952	1.277	0
4765	623.6	0.771	1.3321	0.7954	1.2773	0
4766	623.5	0.771	1.3325	0.7953	1.2767	0
4767	623.4	0.7715	1.3325	0.7955	1.277	0
4768	623.3	0.7714	1.3325	0.7953	1.2763	0
4769	623.2	0.7715	1.3328	0.7952	1.2768	0
4770	623.1	0.7721	1.333	0.7952	1.2751	0
4771	623	0.7724	1.3332	0.7951	1.2765	0
4772	622.9	0.7723	1.3335	0.795	1.2763	0
4773	622.8	0.7727	1.334	0.7952	1.2766	0
4774	622.7	0.7728	1.3347	0.7952	1.276	0
4775	622.6	0.7732	1.334	0.7954	1.2763	0
4776	622.5	0.7736	1.3349	0.795	1.2763	0
4777	622.4	0.7737	1.3349	0.7951	1.2763	0
4778	622.3	0.7741	1.3353	0.7953	1.2765	0
4779	622.2	0.7742	1.3351	0.7953	1.2763	0
4780	622.1	0.7742	1.3356	0.7954	1.2764	0
4781	622	0.7746	1.3359	0.7956	1.2771	0
4782	621.9	0.7747	1.3363	0.7955	1.2777	0
4783	621.8	0.775	1.3363	0.7955	1.2767	0
4784	621.7	0.7754	1.3365	0.7953	1.2751	0
4785	621.6	0.7753	1.3367	0.7956	1.2762	0
4786	621.5	0.7754	1.3376	0.7954	1.2767	0
4787	621.4	0.776	1.3377	0.7956	1.2763	0
4788	621.3	0.776	1.3378	0.7952	1.2776	0
4789	621.2	0.7763	1.338	0.7954	1.2764	0
4790	621.1	0.7767	1.3386	0.7954	1.2773	0
4791	621	0.7768	1.3385	0.7954	1.2772	0
4792	620.9	0.7772	1.3389	0.7953	1.2769	0
4793	620.8	0.7772	1.3391	0.7952	1.2774	0
4794	620.7	0.7775	1.3398	0.7952	1.2775	0
4795	620.6	0.7773	1.3402	0.7948	1.2784	0
4796	620.5	0.7776	1.3402	0.7948	1.2784	0
4797	620.4	0.7778	1.3406	0.7947	1.2784	0
4798	620.3	0.7783	1.3407	0.7948	1.2785	0
4799	620.2	0.7786	1.3414	0.7949	1.2796	0
4800	620.1	0.779	1.3419	0.7947	1.2797	0
4801	620	0.7793	1.3424	0.7948	1.2797	0
4802	619.9	0.7797	1.3428	0.7949	1.2796	0

4803	619.8	0.7797	1.3431	0.7947	1.28	0
4804	619.7	0.78	1.3426	0.7945	1.2798	0
4805	619.6	0.7801	1.3433	0.7945	1.2792	0
4806	619.5	0.7808	1.3439	0.7945	1.2804	0
4807	619.4	0.781	1.344	0.7949	1.2803	0
4808	619.3	0.7809	1.3444	0.7946	1.2807	0
4809	619.2	0.7811	1.3444	0.7943	1.2809	0
4810	619.1	0.7816	1.3446	0.7943	1.281	0
4811	619	0.7819	1.3453	0.7942	1.2812	0
4812	618.9	0.782	1.346	0.7944	1.2814	0
4813	618.8	0.7822	1.3464	0.7939	1.2817	0
4814	618.7	0.7827	1.3466	0.7942	1.281	0
4815	618.6	0.7829	1.3468	0.7942	1.2813	0
4816	618.5	0.7832	1.347	0.794	1.2816	0
4817	618.4	0.7835	1.3473	0.794	1.2821	0
4818	618.3	0.7837	1.3477	0.794	1.2822	0
4819	618.2	0.784	1.3482	0.7939	1.2829	0
4820	618.1	0.7843	1.3484	0.7941	1.2827	0
4821	618	0.7844	1.3486	0.7937	1.2835	0
4822	617.9	0.7848	1.3491	0.7939	1.283	0
4823	617.8	0.785	1.3494	0.794	1.2837	0
4824	617.7	0.7854	1.3501	0.794	1.2835	0
4825	617.6	0.7857	1.3498	0.7937	1.2841	0
4826	617.5	0.7858	1.3497	0.7937	1.2835	0
4827	617.4	0.786	1.3502	0.7935	1.2844	0
4828	617.3	0.7863	1.3506	0.7937	1.2851	0
4829	617.2	0.7866	1.3508	0.7937	1.2845	0
4830	617.1	0.7869	1.3516	0.7937	1.2842	0
4831	617	0.787	1.3519	0.7934	1.2842	0
4832	616.9	0.7872	1.3517	0.7938	1.2843	0
4833	616.8	0.7877	1.3521	0.7941	1.2841	0
4834	616.7	0.788	1.3525	0.794	1.2841	0
4835	616.6	0.7882	1.3527	0.794	1.2833	0
4836	616.5	0.7883	1.3529	0.794	1.2841	0
4837	616.4	0.7886	1.353	0.7939	1.2834	0
4838	616.3	0.7888	1.3536	0.7942	1.2834	0
4839	616.2	0.789	1.3533	0.7944	1.2842	0
4840	616.1	0.7893	1.3535	0.7942	1.2833	0
4841	616	0.7892	1.3541	0.7941	1.2823	0
4842	615.9	0.7897	1.3537	0.7942	1.2832	0
4843	615.8	0.7899	1.3542	0.7944	1.2827	0
4844	615.7	0.7903	1.3547	0.7948	1.2828	0
4845	615.6	0.7903	1.3544	0.7946	1.2815	0

4846	615.5	0.7906	1.3546	0.7947	1.2808	0
4847	615.4	0.7908	1.3549	0.7945	1.2812	0
4848	615.3	0.7912	1.3554	0.7949	1.2822	0
4849	615.2	0.7914	1.3554	0.7948	1.2819	0
4850	615.1	0.7916	1.3558	0.7949	1.281	0
4851	615	0.792	1.3563	0.795	1.2804	0
4852	614.9	0.7923	1.3563	0.7949	1.2801	0
4853	614.8	0.7924	1.3562	0.7954	1.2797	0
4854	614.7	0.7926	1.3567	0.7958	1.2811	0
4855	614.6	0.7927	1.3569	0.7956	1.2805	0
4856	614.5	0.7929	1.3576	0.7955	1.2811	0
4857	614.4	0.7934	1.3568	0.7958	1.2805	0
4858	614.3	0.7935	1.3575	0.7959	1.28	0
4859	614.2	0.7939	1.3578	0.7958	1.2804	0
4860	614.1	0.7939	1.3577	0.7961	1.2802	0
4861	614	0.7944	1.358	0.7962	1.2797	0
4862	613.9	0.7944	1.3586	0.7963	1.2794	0
4863	613.8	0.7947	1.3586	0.796	1.2797	0
4864	613.7	0.7945	1.359	0.7964	1.2804	0
4865	613.6	0.7952	1.3587	0.7965	1.2794	0
4866	613.5	0.7955	1.3593	0.7964	1.2795	0
4867	613.4	0.7955	1.3594	0.7965	1.2789	0
4868	613.3	0.7959	1.3594	0.7966	1.2795	0
4869	613.2	0.7963	1.3601	0.7969	1.278	0
4870	613.1	0.7965	1.3604	0.7971	1.2793	0
4871	613	0.7965	1.3603	0.797	1.279	0
4872	612.9	0.7967	1.3609	0.797	1.2785	0
4873	612.8	0.7971	1.3607	0.7966	1.2786	0
4874	612.7	0.7971	1.3603	0.7966	1.2781	0
4875	612.6	0.7971	1.3612	0.7967	1.2789	0
4876	612.5	0.7975	1.3619	0.7963	1.2789	0
4877	612.4	0.7981	1.3618	0.7969	1.279	0
4878	612.3	0.7982	1.3626	0.7969	1.2791	0
4879	612.2	0.7985	1.3629	0.7971	1.279	0
4880	612.1	0.799	1.3632	0.797	1.2793	0
4881	612	0.7991	1.3634	0.7968	1.2797	0
4882	611.9	0.7994	1.3638	0.7971	1.2794	0
4883	611.8	0.7996	1.3638	0.7968	1.2806	0
4884	611.7	0.7999	1.3639	0.7969	1.2799	0
4885	611.6	0.8001	1.3647	0.797	1.2799	0
4886	611.5	0.8002	1.3648	0.7968	1.2803	0
4887	611.4	0.8007	1.3648	0.7968	1.28	0
4888	611.3	0.8009	1.3651	0.7968	1.2806	0

4889	611.2	0.801	1.3654	0.7965	1.2804	0
4890	611.1	0.8012	1.3661	0.7965	1.2803	0
4891	611	0.8016	1.3667	0.7968	1.28	0
4892	610.9	0.802	1.3666	0.7966	1.2813	0
4893	610.8	0.8022	1.3669	0.7965	1.281	0
4894	610.7	0.8024	1.3677	0.7966	1.2816	0
4895	610.6	0.8026	1.368	0.7966	1.2807	0
4896	610.5	0.8033	1.3678	0.7965	1.2823	0
4897	610.4	0.8036	1.3687	0.7967	1.2819	0
4898	610.3	0.8038	1.369	0.7966	1.2819	0
4899	610.2	0.8039	1.3694	0.7966	1.2827	0
4900	610.1	0.8039	1.3692	0.7963	1.2826	0
4901	610	0.8045	1.3697	0.7962	1.2826	0
4902	609.9	0.805	1.3701	0.7961	1.2842	0
4903	609.8	0.8048	1.3706	0.7958	1.2842	0
4904	609.7	0.805	1.3709	0.7957	1.2839	0
4905	609.6	0.8052	1.3715	0.7956	1.2842	0
4906	609.5	0.8059	1.3716	0.7958	1.2837	0
4907	609.4	0.8062	1.3721	0.7957	1.2843	0
4908	609.3	0.8065	1.3727	0.7954	1.2842	0
4909	609.2	0.8067	1.3724	0.7953	1.2855	0
4910	609.1	0.8071	1.3733	0.7952	1.2862	0
4911	609	0.8076	1.3739	0.795	1.2856	0
4912	608.9	0.8076	1.3738	0.795	1.2864	0
4913	608.8	0.8081	1.3746	0.795	1.2867	0
4914	608.7	0.8082	1.3749	0.7949	1.2871	0
4915	608.6	0.8085	1.3754	0.7947	1.2884	0
4916	608.5	0.8091	1.3753	0.7947	1.2882	0
4917	608.4	0.8093	1.3763	0.7948	1.288	0
4918	608.3	0.8098	1.3767	0.7946	1.2885	0
4919	608.2	0.8098	1.3772	0.7943	1.2886	0
4920	608.1	0.8102	1.3774	0.7941	1.2886	0
4921	608	0.8104	1.378	0.794	1.2895	0
4922	607.9	0.8107	1.3789	0.7939	1.2887	0
4923	607.8	0.8112	1.3789	0.7939	1.2889	0
4924	607.7	0.8117	1.379	0.794	1.2907	0
4925	607.6	0.8116	1.3796	0.7938	1.29	0
4926	607.5	0.812	1.3799	0.7935	1.2906	0
4927	607.4	0.8124	1.3803	0.7937	1.2907	0
4928	607.3	0.8125	1.3812	0.7936	1.2914	0
4929	607.2	0.8132	1.3815	0.7934	1.2907	0
4930	607.1	0.8135	1.3811	0.7933	1.2909	0
4931	607	0.8138	1.3819	0.7933	1.2912	0

4932	606.9	0.8142	1.3826	0.7935	1.2917	0
4933	606.8	0.8145	1.3828	0.7933	1.291	0
4934	606.7	0.8147	1.383	0.7936	1.2921	0
4935	606.6	0.8149	1.3837	0.7933	1.2916	0
4936	606.5	0.8152	1.384	0.7934	1.2917	0
4937	606.4	0.8155	1.3844	0.7937	1.2922	0
4938	606.3	0.8157	1.3845	0.7936	1.292	0
4939	606.2	0.816	1.3851	0.7933	1.2925	0
4940	606.1	0.8165	1.3859	0.7935	1.2927	0
4941	606	0.8168	1.3865	0.7933	1.2929	0
4942	605.9	0.8172	1.3868	0.7936	1.2917	0
4943	605.8	0.8175	1.387	0.7936	1.2919	0
4944	605.7	0.8178	1.387	0.7936	1.2922	0
4945	605.6	0.8181	1.3873	0.7938	1.2922	0
4946	605.5	0.8186	1.3884	0.7937	1.2926	0
4947	605.4	0.8187	1.3888	0.7941	1.2923	0
4948	605.3	0.8192	1.389	0.794	1.2917	0
4949	605.2	0.819	1.3895	0.7939	1.2912	0
4950	605.1	0.8195	1.3902	0.7938	1.2927	0
4951	605	0.8198	1.3899	0.794	1.2914	0
4952	604.9	0.8199	1.3902	0.7942	1.2914	0
4953	604.8	0.8201	1.3908	0.7943	1.2912	0
4954	604.7	0.8205	1.3912	0.7946	1.2919	0
4955	604.6	0.8209	1.3917	0.7946	1.2914	0
4956	604.5	0.8208	1.3917	0.7947	1.2909	0
4957	604.4	0.8214	1.3923	0.7945	1.2903	0
4958	604.3	0.8215	1.3928	0.795	1.291	0
4959	604.2	0.822	1.3932	0.795	1.2905	0
4960	604.1	0.8221	1.3939	0.7951	1.2903	0
4961	604	0.8226	1.3941	0.795	1.2901	0
4962	603.9	0.8228	1.3951	0.7956	1.2896	0
4963	603.8	0.8233	1.395	0.7956	1.2896	0
4964	603.7	0.8236	1.3955	0.7961	1.2898	0
4965	603.6	0.8238	1.3966	0.7961	1.2883	0
4966	603.5	0.824	1.3964	0.7961	1.2887	0
4967	603.4	0.8244	1.3971	0.7959	1.2881	0
4968	603.3	0.8245	1.3977	0.7959	1.2891	0
4969	603.2	0.8249	1.3983	0.7961	1.2885	0
4970	603.1	0.8252	1.3987	0.7965	1.2883	0
4971	603	0.8256	1.3994	0.7964	1.2885	0
4972	602.9	0.8258	1.3997	0.7966	1.2885	0
4973	602.8	0.8261	1.4004	0.7963	1.2881	0
4974	602.7	0.8264	1.401	0.7964	1.2884	0

4975	602.6	0.8269	1.4013	0.7964	1.2879	0
4976	602.5	0.827	1.402	0.7964	1.2882	0
4977	602.4	0.8274	1.4026	0.7967	1.2885	0
4978	602.3	0.828	1.4034	0.7966	1.2884	0
4979	602.2	0.8282	1.4034	0.7966	1.2883	0
4980	602.1	0.8283	1.4048	0.7968	1.2882	0
4981	602	0.8289	1.4056	0.797	1.2885	0
4982	601.9	0.8291	1.4055	0.7969	1.2886	0
4983	601.8	0.8295	1.406	0.7968	1.2891	0
4984	601.7	0.8298	1.4062	0.7969	1.2874	0
4985	601.6	0.83	1.4075	0.7968	1.2883	0
4986	601.5	0.8303	1.4079	0.7965	1.2885	0
4987	601.4	0.8305	1.4084	0.7968	1.2883	0
4988	601.3	0.8309	1.4089	0.7969	1.2887	0
4989	601.2	0.8315	1.4094	0.797	1.2888	0
4990	601.1	0.8319	1.4099	0.7972	1.2886	0
4991	601	0.8319	1.4105	0.797	1.2883	0
4992	600.9	0.8323	1.4113	0.7971	1.2891	0
4993	600.8	0.8327	1.4117	0.7969	1.2896	0
4994	600.7	0.8332	1.4121	0.797	1.29	0
4995	600.6	0.8331	1.413	0.7971	1.2893	0
4996	600.5	0.8336	1.4137	0.797	1.2902	0
4997	600.4	0.8341	1.4141	0.7968	1.2901	0
4998	600.3	0.8344	1.4148	0.7968	1.289	0
4999	600.2	0.8345	1.4153	0.7969	1.2901	0
5000	600.1	0.8347	1.4155	0.7968	1.2896	0
5001	600	0.8352	1.4162	0.7965	1.2902	0
5002	599.9	0.8351	1.4174	0.7961	1.2908	0
5003	599.8	0.8357	1.4175	0.7966	1.2901	0
5004	599.7	0.8363	1.4187	0.7965	1.2915	0
5005	599.6	0.8365	1.419	0.7961	1.2912	0
5006	599.5	0.8372	1.4197	0.7963	1.2913	0
5007	599.4	0.8375	1.4207	0.7964	1.2915	0
5008	599.3	0.8381	1.4212	0.796	1.2916	0
5009	599.2	0.8384	1.4222	0.7959	1.2925	0
5010	599.1	0.8386	1.4225	0.796	1.2917	0
5011	599	0.8389	1.4234	0.7958	1.2926	0
5012	598.9	0.8395	1.4238	0.7956	1.2925	0
5013	598.8	0.84	1.4242	0.7956	1.2932	0
5014	598.7	0.8403	1.4245	0.7957	1.2939	0
5015	598.6	0.8403	1.426	0.7954	1.2945	0
5016	598.5	0.8408	1.4265	0.7949	1.2949	0
5017	598.4	0.8411	1.4273	0.7948	1.2952	0

5018	598.3	0.8419	1.4279	0.795	1.2962	0
5019	598.2	0.8421	1.4288	0.7949	1.2959	0
5020	598.1	0.8422	1.4294	0.7948	1.297	0
5021	598	0.8425	1.4298	0.7949	1.2964	0
5022	597.9	0.8432	1.4311	0.7947	1.2974	0
5023	597.8	0.8435	1.4316	0.7944	1.2968	0
5024	597.7	0.8438	1.4324	0.7947	1.2978	0
5025	597.6	0.8441	1.4322	0.7941	1.2979	0
5026	597.5	0.8444	1.4332	0.7943	1.298	0
5027	597.4	0.8448	1.4338	0.7942	1.2979	0
5028	597.3	0.8452	1.4351	0.7942	1.2986	0
5029	597.2	0.8454	1.4349	0.7939	1.2986	0
5030	597.1	0.8457	1.4356	0.7936	1.2991	0
5031	597	0.8463	1.436	0.7938	1.2998	0
5032	596.9	0.8467	1.4365	0.7939	1.2993	0
5033	596.8	0.847	1.4374	0.7937	1.3001	0
5034	596.7	0.8475	1.4378	0.7939	1.2997	0
5035	596.6	0.8479	1.4385	0.7936	1.3004	0
5036	596.5	0.848	1.4386	0.7936	1.2999	0
5037	596.4	0.8488	1.4396	0.7941	1.3002	0
5038	596.3	0.8486	1.4399	0.7937	1.2997	0
5039	596.2	0.8492	1.4402	0.7933	1.3001	0
5040	596.1	0.8493	1.4404	0.7934	1.3011	0
5041	596	0.8498	1.4406	0.7934	1.3004	0
5042	595.9	0.8501	1.4412	0.7934	1.3002	0
5043	595.8	0.8505	1.4412	0.7937	1.3006	0
5044	595.7	0.851	1.4414	0.7939	1.3011	0
5045	595.6	0.8513	1.442	0.7938	1.3013	0
5046	595.5	0.8518	1.4422	0.7936	1.3009	0
5047	595.4	0.852	1.4421	0.7935	1.3	0
5048	595.3	0.8524	1.4418	0.7935	1.2997	0
5049	595.2	0.8527	1.4424	0.7936	1.2994	0
5050	595.1	0.8529	1.442	0.7937	1.2999	0
5051	595	0.8535	1.4422	0.794	1.2998	0
5052	594.9	0.8539	1.442	0.7941	1.3006	0
5053	594.8	0.8545	1.442	0.7943	1.2997	0
5054	594.7	0.8544	1.4421	0.7941	1.3002	0
5055	594.6	0.8547	1.4412	0.7942	1.2992	0
5056	594.5	0.8555	1.4411	0.7943	1.299	0
5057	594.4	0.8556	1.4411	0.7946	1.299	0
5058	594.3	0.8562	1.4408	0.7947	1.2987	0
5059	594.2	0.8566	1.4403	0.7946	1.299	0
5060	594.1	0.857	1.44	0.7949	1.2984	0

5061	594	0.8574	1.4395	0.7948	1.2985	0
5062	593.9	0.8575	1.4397	0.795	1.299	0
5063	593.8	0.8579	1.4388	0.7949	1.2987	0
5064	593.7	0.8582	1.4382	0.795	1.2982	0
5065	593.6	0.8583	1.4383	0.7948	1.2977	0
5066	593.5	0.8588	1.4381	0.7951	1.2972	0
5067	593.4	0.8593	1.4377	0.7954	1.2971	0
5068	593.3	0.8602	1.4365	0.7954	1.2979	0
5069	593.2	0.8602	1.4363	0.7955	1.2977	0
5070	593.1	0.8602	1.4361	0.7953	1.2972	0
5071	593	0.8608	1.4356	0.7958	1.2977	0
5072	592.9	0.8614	1.4354	0.7956	1.296	0
5073	592.8	0.8617	1.4349	0.7959	1.2968	0
5074	592.7	0.8623	1.4347	0.796	1.2974	0
5075	592.6	0.8626	1.4342	0.7958	1.2967	0
5076	592.5	0.8629	1.4337	0.7959	1.2968	0
5077	592.4	0.8635	1.4332	0.7959	1.2961	0
5078	592.3	0.8638	1.4334	0.7961	1.2962	0
5079	592.2	0.8643	1.4331	0.7964	1.2964	0
5080	592.1	0.8647	1.4329	0.7966	1.2959	0
5081	592	0.865	1.432	0.7964	1.2949	0
5082	591.9	0.8653	1.4314	0.7962	1.2967	0
5083	591.8	0.8655	1.4309	0.7964	1.296	0
5084	591.7	0.866	1.4308	0.7959	1.2972	0
5085	591.6	0.8667	1.4311	0.7964	1.2953	0
5086	591.5	0.8673	1.4306	0.7963	1.2955	0
5087	591.4	0.8677	1.4299	0.7966	1.2962	0
5088	591.3	0.8678	1.4305	0.7967	1.296	0
5089	591.2	0.8685	1.4299	0.7968	1.2958	0
5090	591.1	0.869	1.4302	0.7966	1.2957	0
5091	591	0.8695	1.4294	0.7965	1.2964	0
5092	590.9	0.8701	1.4297	0.7965	1.2955	0
5093	590.8	0.8704	1.4289	0.7968	1.2959	0
5094	590.7	0.8708	1.4288	0.7969	1.2953	0
5095	590.6	0.8716	1.429	0.7968	1.2962	0
5096	590.5	0.8719	1.4284	0.7969	1.2957	0
5097	590.4	0.8725	1.4287	0.7972	1.2959	0
5098	590.3	0.873	1.4289	0.7967	1.2951	0
5099	590.2	0.8737	1.4284	0.7967	1.2958	0
5100	590.1	0.8739	1.4284	0.7968	1.2958	0
5101	590	0.8741	1.4278	0.7967	1.2963	0
5102	589.9	0.8747	1.4284	0.7967	1.2959	0
5103	589.8	0.8754	1.4284	0.7967	1.2961	0

5104	589.7	0.8758	1.4281	0.7968	1.2973	0
5105	589.6	0.8766	1.4284	0.7968	1.2972	0
5106	589.5	0.8767	1.4283	0.7967	1.2971	0
5107	589.4	0.8771	1.4286	0.7964	1.2973	0
5108	589.3	0.8781	1.4284	0.7963	1.2976	0
5109	589.2	0.8786	1.4287	0.7962	1.2973	0
5110	589.1	0.8793	1.4284	0.7963	1.2973	0
5111	589	0.8795	1.4292	0.7961	1.2976	0
5112	588.9	0.88	1.4292	0.7963	1.2976	0
5113	588.8	0.8804	1.4287	0.7964	1.298	0
5114	588.7	0.881	1.4295	0.796	1.2981	0
5115	588.6	0.8816	1.4295	0.7958	1.2977	0
5116	588.5	0.8821	1.4297	0.7957	1.2993	0
5117	588.4	0.8828	1.4298	0.7958	1.2997	0
5118	588.3	0.8832	1.4298	0.7957	1.2988	0
5119	588.2	0.8839	1.4299	0.7954	1.2995	0
5120	588.1	0.8845	1.4303	0.7953	1.2997	0
5121	588	0.8848	1.4304	0.7953	1.2995	0
5122	587.9	0.8855	1.4305	0.7954	1.3002	0
5123	587.8	0.8861	1.431	0.7953	1.3008	0
5124	587.7	0.8868	1.4311	0.7955	1.3001	0
5125	587.6	0.8876	1.4313	0.7954	1.3012	0
5126	587.5	0.8879	1.4318	0.7953	1.3009	0
5127	587.4	0.8882	1.4323	0.7952	1.3016	0
5128	587.3	0.8888	1.4316	0.7953	1.3017	0
5129	587.2	0.8897	1.4323	0.7952	1.3019	0
5130	587.1	0.8898	1.4324	0.7949	1.3017	0
5131	587	0.8905	1.4338	0.7951	1.3019	0
5132	586.9	0.891	1.4331	0.795	1.3038	0
5133	586.8	0.8914	1.4334	0.7951	1.3029	0
5134	586.7	0.8922	1.4337	0.7951	1.3028	0
5135	586.6	0.8924	1.4339	0.7949	1.3027	0
5136	586.5	0.8931	1.4341	0.7948	1.3036	0
5137	586.4	0.8933	1.4343	0.795	1.3037	0
5138	586.3	0.8941	1.4345	0.7949	1.3033	0
5139	586.2	0.8948	1.4357	0.795	1.3037	0
5140	586.1	0.8954	1.4351	0.795	1.3039	0
5141	586	0.8956	1.4356	0.7946	1.3037	0
5142	585.9	0.896	1.4355	0.7945	1.3036	0
5143	585.8	0.8966	1.4363	0.7947	1.304	0
5144	585.7	0.8972	1.4358	0.7949	1.3034	0
5145	585.6	0.8982	1.436	0.795	1.3045	0
5146	585.5	0.8985	1.4367	0.795	1.3039	0

5147	585.4	0.8991	1.4366	0.7948	1.3031	0
5148	585.3	0.8994	1.4372	0.795	1.303	0
5149	585.2	0.9001	1.4375	0.795	1.303	0
5150	585.1	0.9007	1.4375	0.7952	1.3034	0
5151	585	0.901	1.4377	0.7951	1.3029	0
5152	584.9	0.9056	1.419	0.8349	1.2991	0
5153	584.8	0.9061	1.4192	0.835	1.2986	0
5154	584.7	0.9068	1.4195	0.8353	1.2983	0
5155	584.6	0.9077	1.4204	0.8352	1.2974	0
5156	584.5	0.9082	1.4206	0.8353	1.298	0
5157	584.4	0.9084	1.4209	0.8354	1.2981	0
5158	584.3	0.909	1.4212	0.8356	1.2987	0
5159	584.2	0.9096	1.4208	0.8357	1.2978	0
5160	584.1	0.9102	1.4212	0.8358	1.2986	0
5161	584	0.9105	1.422	0.8356	1.2971	0
5162	583.9	0.9112	1.4224	0.8358	1.2968	0
5163	583.8	0.9119	1.4223	0.8361	1.2972	0
5164	583.7	0.9125	1.4227	0.836	1.2969	0
5165	583.6	0.9134	1.4232	0.8361	1.2963	0
5166	583.5	0.9133	1.4232	0.836	1.2963	0
5167	583.4	0.9141	1.4238	0.8363	1.2957	0
5168	583.3	0.9147	1.4246	0.8363	1.2974	0
5169	583.2	0.9152	1.4241	0.8364	1.297	0
5170	583.1	0.916	1.4251	0.8366	1.2959	0
5171	583	0.9165	1.4254	0.8364	1.2962	0
5172	582.9	0.9171	1.4258	0.8363	1.2956	0
5173	582.8	0.9178	1.4264	0.8365	1.2966	0
5174	582.7	0.9184	1.4263	0.8364	1.2963	0
5175	582.6	0.9191	1.4276	0.8362	1.296	0
5176	582.5	0.92	1.4272	0.837	1.2964	0
5177	582.4	0.9201	1.4286	0.8372	1.2956	0
5178	582.3	0.9204	1.428	0.8369	1.2969	0
5179	582.2	0.9215	1.429	0.8368	1.2959	0
5180	582.1	0.9217	1.4286	0.8366	1.2963	0
5181	582	0.9222	1.4303	0.8368	1.2964	0
5182	581.9	0.9231	1.4295	0.8369	1.2967	0
5183	581.8	0.9239	1.43	0.8372	1.2968	0
5184	581.7	0.9242	1.4298	0.8371	1.2965	0
5185	581.6	0.9251	1.4309	0.8372	1.2966	0
5186	581.5	0.9256	1.4308	0.837	1.2973	0
5187	581.4	0.9262	1.432	0.8373	1.2971	0
5188	581.3	0.9271	1.4319	0.8377	1.2969	0
5189	581.2	0.9272	1.4316	0.8377	1.2965	0

5190	581.1	0.9282	1.4315	0.8377	1.2976	0
5191	581	0.9288	1.4319	0.8376	1.2973	0
5192	580.9	0.9295	1.4328	0.8374	1.2968	0
5193	580.8	0.9304	1.4333	0.8375	1.2969	0
5194	580.7	0.9311	1.4329	0.8376	1.2974	0
5195	580.6	0.9315	1.4332	0.8378	1.2974	0
5196	580.5	0.9323	1.4336	0.8378	1.298	0
5197	580.4	0.9332	1.4339	0.8379	1.2975	0
5198	580.3	0.9338	1.4348	0.838	1.2975	0
5199	580.2	0.9344	1.4352	0.8381	1.2981	0
5200	580.1	0.935	1.4348	0.8383	1.297	0
5201	580	0.9352	1.4356	0.8381	1.2983	0
5202	579.9	0.936	1.4351	0.8381	1.2981	0
5203	579.8	0.937	1.436	0.8384	1.2987	0
5204	579.7	0.9377	1.4374	0.8384	1.2971	0
5205	579.6	0.9385	1.437	0.8381	1.2979	0
5206	579.5	0.9395	1.4379	0.8382	1.2975	0
5207	579.4	0.9398	1.4381	0.8384	1.2983	0
5208	579.3	0.9402	1.4382	0.8383	1.2981	0
5209	579.2	0.9409	1.4383	0.8384	1.2979	0
5210	579.1	0.9413	1.4384	0.8383	1.2987	0
5211	579	0.9427	1.438	0.8388	1.2986	0
5212	578.9	0.9433	1.4394	0.8389	1.2972	0
5213	578.8	0.9437	1.4398	0.8388	1.2977	0
5214	578.7	0.9446	1.4399	0.8386	1.298	0
5215	578.6	0.9453	1.4412	0.8388	1.2991	0
5216	578.5	0.9461	1.4407	0.8384	1.2981	0
5217	578.4	0.9466	1.4408	0.8388	1.2988	0
5218	578.3	0.9472	1.4416	0.8386	1.2987	0
5219	578.2	0.948	1.4418	0.8386	1.2995	0
5220	578.1	0.9486	1.4432	0.8386	1.2998	0
5221	578	0.9494	1.4431	0.8388	1.2995	0
5222	577.9	0.9501	1.4432	0.8387	1.2998	0
5223	577.8	0.9505	1.4446	0.8386	1.3006	0
5224	577.7	0.9511	1.444	0.8389	1.2994	0
5225	577.6	0.952	1.4447	0.8388	1.3005	0
5226	577.5	0.9525	1.4448	0.8386	1.3004	0
5227	577.4	0.9532	1.4449	0.8387	1.3011	0
5228	577.3	0.9537	1.4456	0.8388	1.3	0
5229	577.2	0.9548	1.4463	0.8386	1.3007	0
5230	577.1	0.9551	1.4464	0.8388	1.3014	0
5231	577	0.9559	1.4463	0.8386	1.3011	0
5232	576.9	0.9561	1.4476	0.8384	1.3013	0

5233	576.8	0.9568	1.4476	0.8384	1.3006		0
5234	576.7	0.9575	1.4477	0.8385	1.3018		0
5235	576.6	0.9583	1.4489	0.8388	1.3015		0
5236	576.5	0.959	1.4485	0.8388	1.3018		0
5237	576.4	0.9593	1.4491	0.8388	1.302		0
5238	576.3	0.96	1.4491	0.8388	1.3008		0
5239	576.2	0.9608	1.4492	0.8389	1.3011		0
5240	576.1	0.9616	1.4498	0.8385	1.3022		0
5241	576	0.9619	1.4505	0.8387	1.3024		0
5242	575.9	0.9626	1.4506	0.8388	1.3018		0
5243	575.8	0.9629	1.4517	0.8387	1.3019		0
5244	575.7	0.9639	1.4517	0.8387	1.3017		0
5245	575.6	0.964	1.4517	0.839	1.3023		0
5246	575.5	0.9646	1.4523	0.8391	1.302		0
5247	575.4	0.9654	1.4524	0.8392	1.3031		0
5248	575.3	0.9658	1.4535	0.8394	1.3033		0
5249	575.2	0.9661	1.4529	0.8392	1.3029		0
5250	575.1	0.9669	1.4535	0.8393	1.3018		0
5251	575	0.9679	1.4537	0.8392	1.3022		0
5252	574.9	0.9682	1.4538	0.8399	1.302		0
5253	574.8	0.9685	1.4542	0.8393	1.3021		0
5254	574.7	0.9692	1.4548	0.8395	1.3022		0
5255	574.6	0.9698	1.4554	0.8394	1.3015		0
5256	574.5	0.9703	1.4555	0.8397	1.3026		0
5257	574.4	0.9712	1.4555	0.8397	1.3029		0
5258	574.3	0.9718	1.4555	0.8402	1.3022		0
5259	574.2	0.9723	1.4574	0.8399	1.3033		0
5260	574.1	0.9728	1.4574	0.8401	1.3022		0
5261	574	0.9733	1.4574	0.8405	1.3028		0
5262	573.9	0.9736	1.458	0.8402	1.3021		0
5263	573.8	0.9742	1.4579	0.84	1.3022		0
5264	573.7	0.9749	1.4586	0.8406	1.3017		0
5265	573.6	0.9753	1.4592	0.8407	1.301		0
5266	573.5	0.9761	1.4586	0.8407	1.3003		0
5267	573.4	0.9767	1.4598	0.8408	1.3022		0
5268	573.3	0.9769	1.4604	0.8406	1.3015		0
5269	573.2	0.9774	1.4599	0.841	1.3014		0
5270	573.1	0.9783	1.46	0.8413	1.3013		0
5271	573	0.9786	1.4605	0.8409	1.3014		0
5272	572.9	0.9792	1.4617	0.8409	1.3015		0
5273	572.8	0.9796	1.4617	0.8412	1.3021		0
5274	572.7	0.9803	1.4616	0.8409	1.3014		0
5275	572.6	0.9809	1.4622	0.8411	1.302		0

5276	572.5	0.9812	1.4621	0.8413	1.3029	0
5277	572.4	0.9818	1.4628	0.8416	1.3016	0
5278	572.3	0.9826	1.4635	0.8413	1.3022	0
5279	572.2	0.9828	1.4641	0.8415	1.302	0
5280	572.1	0.9836	1.4641	0.8414	1.3018	0
5281	572	0.9841	1.4652	0.8415	1.3019	0
5282	571.9	0.9845	1.4658	0.8412	1.302	0
5283	571.8	0.9848	1.4657	0.8416	1.3025	0
5284	571.7	0.9856	1.4663	0.8415	1.3027	0
5285	571.6	0.9862	1.4669	0.8416	1.3025	0
5286	571.5	0.987	1.4669	0.842	1.3032	0
5287	571.4	0.9876	1.4675	0.8418	1.3029	0
5288	571.3	0.9877	1.4682	0.8422	1.3028	0
5289	571.2	0.9884	1.4681	0.842	1.3029	0
5290	571.1	0.9889	1.4688	0.8418	1.3039	0
5291	571	0.9894	1.4692	0.842	1.304	0
5292	570.9	0.9899	1.4698	0.8415	1.305	0
5293	570.8	0.9904	1.4697	0.8415	1.3047	0
5294	570.7	0.9908	1.4709	0.8415	1.304	0
5295	570.6	0.9916	1.4715	0.8415	1.3034	0
5296	570.5	0.9921	1.472	0.8416	1.3055	0
5297	570.4	0.9923	1.472	0.8415	1.3053	0
5298	570.3	0.9931	1.4732	0.8416	1.3052	0
5299	570.2	0.9937	1.4732	0.8416	1.3037	0
5300	570.1	0.9942	1.4737	0.8415	1.305	0
5301	570	0.9946	1.4749	0.8418	1.3064	0
5302	569.9	0.9952	1.4749	0.8416	1.3058	0
5303	569.8	0.9959	1.4756	0.8418	1.3057	0
5304	569.7	0.9962	1.4755	0.8419	1.3062	0
5305	569.6	0.9967	1.476	0.8416	1.3063	0
5306	569.5	0.9973	1.4766	0.8418	1.3065	0
5307	569.4	0.9975	1.4772	0.8415	1.3067	0
5308	569.3	0.9983	1.4772	0.8417	1.3081	0
5309	569.2	0.9991	1.4784	0.8416	1.3071	0
5310	569.1	0.9993	1.4784	0.8417	1.3077	0
5311	569	0.9999	1.4796	0.8417	1.3071	0
5312	568.9	1.0002	1.4801	0.8417	1.308	0
5313	568.8	1.0006	1.4795	0.842	1.3074	0
5314	568.7	1.0012	1.4807	0.8414	1.3084	0
5315	568.6	1.0017	1.4812	0.8416	1.3077	0
5316	568.5	1.0021	1.4818	0.8415	1.3095	0
5317	568.4	1.0029	1.4818	0.8416	1.3081	0
5318	568.3	1.0029	1.483	0.8419	1.3083	0

5319	568.2	1.0036	1.4836	0.8417	1.3081	0
5320	568.1	1.0041	1.4842	0.8414	1.3094	0
5321	568	1.0045	1.4842	0.842	1.3089	0
5322	567.9	1.0049	1.4848	0.8421	1.3087	0
5323	567.8	1.0053	1.4853	0.8418	1.3088	0
5324	567.7	1.006	1.4859	0.8417	1.3078	0
5325	567.6	1.0061	1.4858	0.8418	1.3091	0
5326	567.5	1.0064	1.4857	0.8421	1.3096	0
5327	567.4	1.0068	1.4869	0.842	1.3082	0
5328	567.3	1.0074	1.4874	0.842	1.3084	0
5329	567.2	1.0077	1.4886	0.8419	1.3081	0
5330	567.1	1.0081	1.4885	0.8419	1.3087	0
5331	567	1.0087	1.4892	0.8421	1.3082	0
5332	566.9	1.009	1.4897	0.8424	1.3079	0
5333	566.8	1.0094	1.4903	0.8422	1.3089	0
5334	566.7	1.0101	1.4903	0.8425	1.3084	0
5335	566.6	1.0104	1.4909	0.8426	1.3081	0
5336	566.5	1.0108	1.4919	0.8425	1.3089	0
5337	566.4	1.0111	1.4914	0.8426	1.3077	0
5338	566.3	1.0118	1.4926	0.843	1.3076	0
5339	566.2	1.0119	1.4924	0.8428	1.3071	0
5340	566.1	1.0126	1.4925	0.8428	1.3086	0
5341	566	1.013	1.4942	0.8434	1.3084	0
5342	565.9	1.0132	1.4937	0.8434	1.3086	0
5343	565.8	1.0133	1.4941	0.843	1.3087	0
5344	565.7	1.0139	1.4946	0.8431	1.3092	0
5345	565.6	1.0141	1.4952	0.8435	1.3082	0
5346	565.5	1.0148	1.4964	0.8434	1.3073	0
5347	565.4	1.0151	1.4963	0.8434	1.3059	0
5348	565.3	1.0157	1.4969	0.8434	1.3077	0
5349	565.2	1.016	1.4968	0.8437	1.3085	0
5350	565.1	1.0164	1.4974	0.844	1.3068	0
5351	565	1.0167	1.4974	0.8442	1.307	0
5352	564.9	1.0171	1.4974	0.8439	1.3079	0
5353	564.8	1.0176	1.499	0.8441	1.3073	0
5354	564.7	1.0179	1.4995	0.8438	1.3063	0
5355	564.6	1.0184	1.4996	0.8443	1.3062	0
5356	564.5	1.0186	1.5006	0.8439	1.3066	0
5357	564.4	1.019	1.5	0.8445	1.3053	0
5358	564.3	1.0196	1.5006	0.8444	1.306	0
5359	564.2	1.0198	1.5011	0.8445	1.3052	0
5360	564.1	1.0204	1.5022	0.8448	1.3051	0
5361	564	1.0209	1.5021	0.8448	1.3048	0

5362	563.9	1.0213	1.5021	0.8448	1.3047	0
5363	563.8	1.0216	1.5038	0.845	1.3048	0
5364	563.7	1.0218	1.5038	0.8452	1.3057	0
5365	563.6	1.0222	1.5042	0.8451	1.3058	0
5366	563.5	1.0228	1.5048	0.8452	1.3052	0
5367	563.4	1.0227	1.5041	0.8454	1.3057	0
5368	563.3	1.0232	1.5052	0.8455	1.304	0
5369	563.2	1.0237	1.5057	0.8451	1.3049	0
5370	563.1	1.0245	1.5069	0.8454	1.3059	0
5371	563	1.0248	1.5062	0.8453	1.3056	0
5372	562.9	1.0255	1.5073	0.8457	1.3058	0
5373	562.8	1.0255	1.5077	0.8455	1.3047	0
5374	562.7	1.026	1.5082	0.8459	1.3063	0
5375	562.6	1.0264	1.5087	0.8456	1.3061	0
5376	562.5	1.0264	1.5098	0.8455	1.3054	0
5377	562.4	1.0273	1.5097	0.8453	1.3059	0
5378	562.3	1.0276	1.5102	0.8456	1.3075	0
5379	562.2	1.0281	1.51	0.8456	1.3065	0
5380	562.1	1.0283	1.5111	0.8456	1.3063	0
5381	562	1.029	1.5116	0.8452	1.3056	0
5382	561.9	1.0293	1.5115	0.8452	1.3068	0
5383	561.8	1.0299	1.5125	0.8454	1.3058	0
5384	561.7	1.0303	1.513	0.8456	1.3067	0
5385	561.6	1.0306	1.5135	0.8454	1.3065	0
5386	561.5	1.031	1.514	0.8454	1.3069	0
5387	561.4	1.0317	1.5157	0.8454	1.3071	0
5388	561.3	1.0322	1.515	0.8455	1.3075	0
5389	561.2	1.0327	1.5167	0.8457	1.3074	0
5390	561.1	1.0333	1.5165	0.8455	1.3074	0
5391	561	1.0335	1.5175	0.8455	1.3071	0
5392	560.9	1.0337	1.5184	0.8455	1.3075	0
5393	560.8	1.0343	1.5184	0.8452	1.3076	0
5394	560.7	1.0349	1.5189	0.8452	1.3088	0
5395	560.6	1.0356	1.5188	0.8453	1.3093	0
5396	560.5	1.0357	1.5198	0.8453	1.3091	0
5397	560.4	1.0365	1.5209	0.8455	1.3095	0
5398	560.3	1.037	1.5213	0.8455	1.3096	0
5399	560.2	1.0372	1.5223	0.8451	1.31	0
5400	560.1	1.0378	1.5222	0.8451	1.309	0
5401	560	1.0381	1.5232	0.8453	1.3109	0
5402	559.9	1.0386	1.5237	0.845	1.3103	0
5403	559.8	1.0391	1.5242	0.8451	1.3098	0
5404	559.7	1.0399	1.5247	0.8449	1.3095	0

5405	559.6	1.0406	1.5257	0.8446	1.3107	0
5406	559.5	1.0408	1.5261	0.8446	1.3107	0
5407	559.4	1.0417	1.526	0.8448	1.3098	0
5408	559.3	1.0421	1.5277	0.8447	1.3109	0
5409	559.2	1.0429	1.5282	0.8447	1.3118	0
5410	559.1	1.0433	1.5292	0.8443	1.3112	0
5411	559	1.0437	1.529	0.8445	1.3112	0
5412	558.9	1.0443	1.5302	0.8446	1.3115	0
5413	558.8	1.0452	1.5308	0.8448	1.3127	0
5414	558.7	1.0457	1.5318	0.8444	1.3121	0
5415	558.6	1.0462	1.5316	0.8444	1.3128	0
5416	558.5	1.0464	1.532	0.8443	1.3129	0
5417	558.4	1.0473	1.5331	0.8444	1.3137	0
5418	558.3	1.0479	1.5341	0.8444	1.3138	0
5419	558.2	1.0488	1.5345	0.8441	1.3131	0
5420	558.1	1.0495	1.535	0.8438	1.3133	0
5421	558	1.05	1.5361	0.8442	1.3141	0
5422	557.9	1.0507	1.5366	0.8442	1.3139	0
5423	557.8	1.051	1.5369	0.8438	1.3146	0
5424	557.7	1.0516	1.5374	0.8447	1.3144	0
5425	557.6	1.052	1.5384	0.8446	1.3133	0
5426	557.5	1.0528	1.539	0.8442	1.3132	0
5427	557.4	1.0532	1.5399	0.8442	1.3136	0
5428	557.3	1.0543	1.5404	0.8447	1.3148	0
5429	557.2	1.0547	1.5415	0.8441	1.3152	0
5430	557.1	1.0556	1.5414	0.8444	1.3154	0
5431	557	1.0561	1.5425	0.8446	1.3148	0
5432	556.9	1.0567	1.5417	0.8448	1.3146	0
5433	556.8	1.0578	1.5434	0.8444	1.3154	0
5434	556.7	1.0583	1.5439	0.8445	1.3148	0
5435	556.6	1.0585	1.5443	0.8445	1.3141	0
5436	556.5	1.0596	1.5448	0.8445	1.315	0
5437	556.4	1.0601	1.5463	0.8445	1.315	0
5438	556.3	1.0607	1.5457	0.8447	1.3142	0
5439	556.2	1.0611	1.5467	0.8448	1.3139	0
5440	556.1	1.0625	1.5478	0.8451	1.3147	0
5441	556	1.0629	1.5481	0.8455	1.313	0
5442	555.9	1.0635	1.548	0.8454	1.3127	0
5443	555.8	1.0641	1.549	0.8457	1.3127	0
5444	555.7	1.065	1.5495	0.8457	1.3133	0
5445	555.6	1.0655	1.5504	0.8457	1.3125	0
5446	555.5	1.0661	1.5508	0.8461	1.3126	0
5447	555.4	1.0669	1.552	0.8463	1.3121	0

5448	555.3	1.0677	1.5518	0.8466	1.3122	0
5449	555.2	1.0685	1.5528	0.8465	1.3105	0
5450	555.1	1.069	1.5537	0.8464	1.3108	0
5451	555	1.0698	1.5529	0.8465	1.3105	0
5452	554.9	1.0703	1.5546	0.8467	1.3103	0
5453	554.8	1.0714	1.5557	0.8468	1.3108	0
5454	554.7	1.072	1.5559	0.8466	1.3104	0
5455	554.6	1.0725	1.557	0.8472	1.3098	0
5456	554.5	1.0737	1.5568	0.8471	1.3092	0
5457	554.4	1.0745	1.5578	0.8473	1.3093	0
5458	554.3	1.0754	1.5587	0.8474	1.3089	0
5459	554.2	1.0759	1.559	0.8474	1.3085	0
5460	554.1	1.0768	1.5601	0.848	1.3083	0
5461	554	1.0774	1.5604	0.8477	1.3086	0
5462	553.9	1.0785	1.5614	0.8482	1.3087	0
5463	553.8	1.079	1.5624	0.8486	1.308	0
5464	553.7	1.08	1.5633	0.8486	1.3077	0
5465	553.6	1.0809	1.5631	0.8489	1.3081	0
5466	553.5	1.0817	1.564	0.8489	1.3084	0
5467	553.4	1.0824	1.5644	0.8488	1.3084	0
5468	553.3	1.0836	1.5659	0.8492	1.3074	0
5469	553.2	1.0844	1.5656	0.8489	1.307	0
5470	553.1	1.0856	1.5665	0.8492	1.3077	0
5471	553	1.0864	1.5668	0.8492	1.3073	0
5472	552.9	1.0867	1.5677	0.8487	1.3066	0
5473	552.8	1.0876	1.5679	0.8488	1.3068	0
5474	552.7	1.0883	1.5688	0.8488	1.3071	0
5475	552.6	1.0893	1.5703	0.849	1.3084	0
5476	552.5	1.0902	1.5712	0.8492	1.3073	0
5477	552.4	1.0913	1.5721	0.8493	1.3079	0
5478	552.3	1.0922	1.573	0.8494	1.3068	0
5479	552.2	1.0931	1.5726	0.8494	1.3064	0
5480	552.1	1.0943	1.5747	0.8494	1.307	0
5481	552	1.095	1.575	0.8492	1.3059	0
5482	551.9	1.0957	1.5757	0.8493	1.3067	0
5483	551.8	1.0966	1.5766	0.8493	1.3067	0
5484	551.7	1.0976	1.5768	0.8499	1.3079	0
5485	551.6	1.0986	1.5782	0.8496	1.308	0
5486	551.5	1.0991	1.579	0.8494	1.3078	0
5487	551.4	1.1005	1.5798	0.8493	1.3074	0
5488	551.3	1.1013	1.5795	0.8492	1.3073	0
5489	551.2	1.1023	1.5809	0.8494	1.3078	0
5490	551.1	1.1028	1.5816	0.8496	1.3076	0

5491	551	1.1038	1.5824	0.8496	1.3081	0
5492	550.9	1.1051	1.5833	0.8495	1.308	0
5493	550.8	1.1062	1.5841	0.85	1.3089	0
5494	550.7	1.107	1.5854	0.8498	1.3079	0
5495	550.6	1.1077	1.5867	0.8497	1.308	0
5496	550.5	1.1088	1.5869	0.8496	1.3082	0
5497	550.4	1.1096	1.5889	0.8495	1.3086	0
5498	550.3	1.111	1.5883	0.8495	1.308	0
5499	550.2	1.1118	1.5897	0.8497	1.3085	0
5500	550.1	1.1129	1.5904	0.8496	1.3096	0
5501	550	1.1137	1.591	0.849	1.3098	0
5502	549.9	1.1148	1.5924	0.849	1.3103	0
5503	549.8	1.1158	1.5931	0.8488	1.3104	0
5504	549.7	1.1167	1.5951	0.849	1.3104	0
5505	549.6	1.1175	1.5959	0.8492	1.3106	0
5506	549.5	1.1185	1.5965	0.8489	1.3109	0
5507	549.4	1.1195	1.5966	0.8491	1.3113	0
5508	549.3	1.1202	1.5991	0.8489	1.3115	0
5509	549.2	1.1213	1.5991	0.849	1.3112	0
5510	549.1	1.1223	1.6003	0.849	1.3114	0
5511	549	1.1232	1.6016	0.8482	1.3111	0
5512	548.9	1.1238	1.6022	0.8487	1.3118	0
5513	548.8	1.1251	1.6035	0.8485	1.3127	0
5514	548.7	1.1262	1.6042	0.8482	1.3128	0
5515	548.6	1.1269	1.6047	0.8482	1.3136	0
5516	548.5	1.1283	1.6061	0.8483	1.3133	0
5517	548.4	1.1293	1.6073	0.8482	1.3133	0
5518	548.3	1.13	1.6087	0.8485	1.3133	0
5519	548.2	1.1312	1.6092	0.8482	1.3135	0
5520	548.1	1.1323	1.6098	0.8483	1.3138	0
5521	548	1.1329	1.6118	0.8484	1.3141	0
5522	547.9	1.1341	1.613	0.8482	1.3156	0
5523	547.8	1.1347	1.6136	0.8481	1.3159	0
5524	547.7	1.1357	1.6141	0.8482	1.3151	0
5525	547.6	1.1368	1.6154	0.848	1.3158	0
5526	547.5	1.1379	1.616	0.8484	1.3163	0
5527	547.4	1.1391	1.6191	0.8479	1.3161	0
5528	547.3	1.14	1.6196	0.8482	1.3159	0
5529	547.2	1.1403	1.6202	0.848	1.3168	0
5530	547.1	1.1417	1.6213	0.8478	1.3145	0
5531	547	1.1424	1.6218	0.8482	1.316	0
5532	546.9	1.1433	1.6237	0.8485	1.3168	0
5533	546.8	1.1445	1.6242	0.8489	1.317	0

5534	546.7	1.1452	1.6253	0.8483	1.3161	0
5535	546.6	1.1464	1.6272	0.8483	1.3159	0
5536	546.5	1.1472	1.6283	0.8484	1.3152	0
5537	546.4	1.1483	1.6296	0.8486	1.3161	0
5538	546.3	1.149	1.6307	0.849	1.3156	0
5539	546.2	1.1498	1.6318	0.849	1.3149	0
5540	546.1	1.1509	1.6329	0.8488	1.316	0
5541	546	1.1517	1.6342	0.8491	1.3148	0
5542	545.9	1.1523	1.6358	0.8489	1.3153	0
5543	545.8	1.1535	1.637	0.849	1.3154	0
5544	545.7	1.1542	1.6389	0.8493	1.3148	0
5545	545.6	1.1555	1.64	0.8493	1.3154	0
5546	545.5	1.1563	1.6411	0.8493	1.3151	0
5547	545.4	1.1571	1.6422	0.8495	1.3144	0
5548	545.3	1.1577	1.6432	0.8499	1.315	0
5549	545.2	1.1582	1.6442	0.8499	1.315	0
5550	545.1	1.1596	1.6469	0.8498	1.315	0
5551	545	1.1607	1.6473	0.8499	1.315	0
5552	544.9	1.1615	1.6491	0.8505	1.3153	0
5553	544.8	1.1625	1.6502	0.8506	1.3142	0
5554	544.7	1.1629	1.6513	0.8512	1.3148	0
5555	544.6	1.1638	1.6522	0.8509	1.3136	0
5556	544.5	1.1648	1.6548	0.8508	1.3138	0
5557	544.4	1.1654	1.6551	0.8514	1.3114	0
5558	544.3	1.1662	1.6577	0.852	1.3127	0
5559	544.2	1.1666	1.6579	0.8522	1.3118	0
5560	544.1	1.1676	1.6598	0.8524	1.3117	0
5561	544	1.1682	1.6616	0.8523	1.3109	0
5562	543.9	1.1692	1.6625	0.8526	1.312	0
5563	543.8	1.1695	1.6643	0.8527	1.3118	0
5564	543.7	1.1704	1.6661	0.853	1.3099	0
5565	543.6	1.1713	1.6678	0.8531	1.3092	0
5566	543.5	1.1715	1.6688	0.8535	1.3097	0
5567	543.4	1.1727	1.6699	0.8537	1.3088	0
5568	543.3	1.1731	1.6717	0.8541	1.3082	0
5569	543.2	1.1742	1.6742	0.8539	1.3079	0
5570	543.1	1.1746	1.6752	0.854	1.308	0
5571	543	1.1756	1.677	0.8543	1.3081	0
5572	542.9	1.1762	1.6796	0.8545	1.3071	0
5573	542.8	1.1771	1.6804	0.8543	1.3076	0
5574	542.7	1.1782	1.6824	0.8545	1.3075	0
5575	542.6	1.1783	1.6841	0.8548	1.3054	0
5576	542.5	1.1791	1.6851	0.8549	1.3072	0

5577	542.4	1.1798	1.687	0.8552	1.3073	0
5578	542.3	1.1804	1.6896	0.8553	1.3076	0
5579	542.2	1.1812	1.6905	0.8552	1.3068	0
5580	542.1	1.1822	1.6934	0.8556	1.3056	0
5581	542	1.1831	1.6953	0.8554	1.307	0
5582	541.9	1.1836	1.6953	0.8554	1.3076	0
5583	541.8	1.1842	1.698	0.8556	1.3068	0
5584	541.7	1.1856	1.6999	0.8559	1.3068	0
5585	541.6	1.1856	1.7017	0.8559	1.3064	0
5586	541.5	1.1861	1.7035	0.8561	1.307	0
5587	541.4	1.1867	1.7054	0.8563	1.3066	0
5588	541.3	1.1879	1.7074	0.8564	1.3059	0
5589	541.2	1.1887	1.7084	0.856	1.3054	0
5590	541.1	1.189	1.7102	0.8561	1.3057	0
5591	541	1.1895	1.713	0.8561	1.306	0
5592	540.9	1.1904	1.7141	0.856	1.3055	0
5593	540.8	1.1915	1.716	0.8564	1.3058	0
5594	540.7	1.1921	1.718	0.8564	1.305	0
5595	540.6	1.1928	1.7199	0.8567	1.3055	0
5596	540.5	1.1935	1.7228	0.8567	1.3048	0
5597	540.4	1.1942	1.7238	0.8566	1.3057	0
5598	540.3	1.1947	1.7257	0.857	1.3066	0
5599	540.2	1.1956	1.7278	0.8571	1.3061	0
5600	540.1	1.1965	1.7289	0.8568	1.3061	0
5601	540	1.1973	1.731	0.8573	1.3067	0
5602	539.9	1.1982	1.7349	0.8572	1.3062	0
5603	539.8	1.1983	1.7359	0.8567	1.3066	0
5604	539.7	1.1993	1.7371	0.8572	1.3066	0
5605	539.6	1.2003	1.7401	0.8569	1.3071	0
5606	539.5	1.2009	1.7422	0.8571	1.307	0
5607	539.4	1.2014	1.7433	0.8567	1.3079	0
5608	539.3	1.2023	1.7465	0.857	1.3078	0
5609	539.2	1.2028	1.7476	0.8573	1.3083	0
5610	539.1	1.204	1.7488	0.8569	1.3071	0
5611	539	1.2046	1.7521	0.8565	1.3089	0
5612	538.9	1.2051	1.7532	0.8562	1.3083	0
5613	538.8	1.2063	1.7544	0.8567	1.3093	0
5614	538.7	1.2067	1.7588	0.8566	1.3092	0
5615	538.6	1.2075	1.759	0.8564	1.3098	0
5616	538.5	1.2083	1.7622	0.8562	1.3092	0
5617	538.4	1.2091	1.7625	0.856	1.3107	0
5618	538.3	1.21	1.7659	0.8561	1.311	0
5619	538.2	1.2106	1.7683	0.8564	1.3113	0

5620	538.1	1.2116	1.7697	0.8563	1.3123	0
5621	538	1.2124	1.7719	0.8561	1.3109	0
5622	537.9	1.2131	1.7733	0.856	1.3105	0
5623	537.8	1.2133	1.7746	0.8556	1.3122	0
5624	537.7	1.2143	1.777	0.8566	1.3128	0
5625	537.6	1.2152	1.7795	0.8564	1.3123	0
5626	537.5	1.2162	1.782	0.8561	1.313	0
5627	537.4	1.2169	1.7834	0.8557	1.3128	0
5628	537.3	1.2178	1.786	0.8559	1.3139	0
5629	537.2	1.2185	1.7874	0.8557	1.3134	0
5630	537.1	1.219	1.7901	0.8555	1.313	0
5631	537	1.2204	1.7926	0.8556	1.3139	0
5632	536.9	1.2211	1.794	0.8556	1.3133	0
5633	536.8	1.2218	1.7967	0.8555	1.3136	0
5634	536.7	1.2224	1.7981	0.8558	1.3138	0
5635	536.6	1.2234	1.8008	0.8555	1.3137	0
5636	536.5	1.2249	1.8034	0.8555	1.3135	0
5637	536.4	1.2254	1.8039	0.8557	1.3143	0
5638	536.3	1.2268	1.8066	0.8558	1.3141	0
5639	536.2	1.2272	1.8095	0.8559	1.3136	0
5640	536.1	1.2282	1.811	0.8558	1.3139	0
5641	536	1.229	1.8126	0.8557	1.3146	0
5642	535.9	1.23	1.8155	0.8562	1.3144	0
5643	535.8	1.2313	1.8196	0.8561	1.3138	0
5644	535.7	1.2323	1.8199	0.8558	1.3144	0
5645	535.6	1.2331	1.8216	0.8559	1.3139	0
5646	535.5	1.2344	1.8245	0.856	1.3145	0
5647	535.4	1.2351	1.8274	0.8562	1.3155	0
5648	535.3	1.2361	1.829	0.8561	1.3153	0
5649	535.2	1.238	1.8296	0.8563	1.3153	0
5650	535.1	1.2388	1.8327	0.8561	1.3153	0
5651	535	1.2397	1.8356	0.8559	1.3153	0
5652	534.9	1.2408	1.8374	0.8558	1.3156	0
5653	534.8	1.2419	1.8405	0.8559	1.3142	0
5654	534.7	1.2431	1.8423	0.8562	1.3149	0
5655	534.6	1.2449	1.8455	0.8562	1.3143	0
5656	534.5	1.246	1.8459	0.8562	1.3129	0
5657	534.4	1.2467	1.8492	0.8565	1.3135	0
5658	534.3	1.2483	1.8525	0.8565	1.3134	0
5659	534.2	1.2491	1.8515	0.857	1.3128	0
5660	534.1	1.2503	1.8564	0.8568	1.3131	0
5661	534	1.2519	1.8583	0.857	1.313	0
5662	533.9	1.2535	1.8603	0.8577	1.3133	0

5663	533.8	1.2549	1.8622	0.8571	1.3126	0
5664	533.7	1.2561	1.8656	0.8575	1.3116	0
5665	533.6	1.2574	1.8692	0.8578	1.3119	0
5666	533.5	1.259	1.8697	0.8574	1.3117	0
5667	533.4	1.2607	1.8719	0.8579	1.3117	0
5668	533.3	1.2623	1.8739	0.8576	1.3123	0
5669	533.2	1.2636	1.8745	0.8581	1.3126	0
5670	533.1	1.266	1.8781	0.8578	1.312	0
5671	533	1.2669	1.8818	0.8576	1.3118	0
5672	532.9	1.2687	1.8825	0.8579	1.3113	0
5673	532.8	1.2709	1.8848	0.8584	1.3104	0
5674	532.7	1.2724	1.8872	0.8581	1.3113	0
5675	532.6	1.2738	1.8895	0.8584	1.3117	0
5676	532.5	1.2758	1.8916	0.858	1.311	0
5677	532.4	1.2777	1.8939	0.8583	1.3109	0
5678	532.3	1.2792	1.898	0.8585	1.3104	0
5679	532.2	1.2814	1.8987	0.8587	1.3103	0
5680	532.1	1.2828	1.8993	0.859	1.3096	0
5681	532	1.2856	1.9035	0.8589	1.3101	0
5682	531.9	1.2878	1.9042	0.8588	1.3108	0
5683	531.8	1.2893	1.9066	0.8592	1.3102	0
5684	531.7	1.2913	1.9074	0.8587	1.3106	0
5685	531.6	1.2935	1.9101	0.8587	1.3107	0
5686	531.5	1.2954	1.9126	0.8589	1.3097	0
5687	531.4	1.2974	1.9134	0.8587	1.3109	0
5688	531.3	1.2996	1.9161	0.859	1.3109	0
5689	531.2	1.3021	1.9187	0.8594	1.3113	0
5690	531.1	1.3049	1.9212	0.8593	1.3102	0
5691	531	1.3067	1.9222	0.8594	1.3111	0
5692	530.9	1.3097	1.923	0.8595	1.3106	0
5693	530.8	1.3118	1.9257	0.8591	1.3091	0
5694	530.7	1.314	1.9266	0.8591	1.31	0
5695	530.6	1.3168	1.9276	0.8595	1.31	0
5696	530.5	1.3191	1.9323	0.8596	1.3101	0
5697	530.4	1.3228	1.9314	0.8594	1.3109	0
5698	530.3	1.3261	1.9343	0.8594	1.3123	0
5699	530.2	1.3285	1.9353	0.8598	1.3114	0
5700	530.1	1.3317	1.9381	0.8597	1.3117	0
5701	530	1.3342	1.9372	0.8596	1.3113	0
5702	529.9	1.3379	1.9419	0.8592	1.3116	0
5703	529.8	1.341	1.9431	0.859	1.3108	0
5704	529.7	1.3444	1.944	0.8593	1.3121	0
5705	529.6	1.3475	1.947	0.8589	1.313	0

5706	529.5	1.351	1.946	0.8592	1.3129	0
5707	529.4	1.3541	1.9511	0.8592	1.3133	0
5708	529.3	1.3583	1.9502	0.8592	1.3133	0
5709	529.2	1.3615	1.9512	0.8593	1.3133	0
5710	529.1	1.3653	1.9523	0.8597	1.3133	0
5711	529	1.3695	1.9534	0.8592	1.3137	0
5712	528.9	1.3728	1.9564	0.8591	1.3146	0
5713	528.8	1.3764	1.9557	0.8586	1.3148	0
5714	528.7	1.3798	1.9569	0.858	1.3153	0
5715	528.6	1.3845	1.9602	0.858	1.3154	0
5716	528.5	1.3883	1.9617	0.8582	1.3162	0
5717	528.4	1.3926	1.9629	0.8577	1.3168	0
5718	528.3	1.3959	1.9644	0.8574	1.3171	0
5719	528.2	1.4004	1.9659	0.8572	1.3193	0
5720	528.1	1.4052	1.9675	0.857	1.3188	0
5721	528	1.4092	1.969	0.8567	1.3181	0
5722	527.9	1.4133	1.9705	0.8567	1.3184	0
5723	527.8	1.4177	1.97	0.856	1.3199	0
5724	527.7	1.422	1.9695	0.8559	1.3194	0
5725	527.6	1.4265	1.9733	0.8556	1.3199	0
5726	527.5	1.431	1.9729	0.8551	1.3204	0
5727	527.4	1.4358	1.977	0.8553	1.3206	0
5728	527.3	1.4405	1.9744	0.8551	1.3217	0
5729	527.2	1.4455	1.9786	0.8547	1.322	0
5730	527.1	1.451	1.9805	0.8547	1.3221	0
5731	527	1.4554	1.9803	0.8547	1.3219	0
5732	526.9	1.4611	1.98	0.8544	1.3226	0
5733	526.8	1.4664	1.982	0.8541	1.3239	0
5734	526.7	1.471	1.9841	0.8541	1.3232	0
5735	526.6	1.477	1.9838	0.8541	1.3249	0
5736	526.5	1.4817	1.9881	0.8539	1.3237	0
5737	526.4	1.488	1.9857	0.8539	1.3236	0
5738	526.3	1.4929	1.9901	0.8541	1.3244	0
5739	526.2	1.4987	1.9878	0.854	1.3248	0
5740	526.1	1.5052	1.9854	0.8538	1.3232	0
5741	526	1.5104	1.9897	0.854	1.3245	0
5742	525.9	1.5163	1.9896	0.8542	1.3258	0
5743	525.8	1.524	1.9895	0.8541	1.3252	0
5744	525.7	1.53	1.9916	0.8542	1.3255	0
5745	525.6	1.537	1.9913	0.8541	1.3253	0
5746	525.5	1.5432	1.9933	0.8543	1.326	0
5747	525.4	1.5505	1.9908	0.8545	1.3253	0
5748	525.3	1.5569	1.9952	0.8542	1.3255	0

5749	525.2	1.5636	1.9928	0.8548	1.3264	0
5750	525.1	1.5712	1.9972	0.8546	1.3257	0
5751	525	1.5781	1.9971	0.8548	1.3265	0
5752	524.9	1.5868	1.9967	0.8546	1.3262	0
5753	524.8	1.5948	1.9988	0.8549	1.3265	0
5754	524.7	1.602	1.9985	0.8547	1.3262	0
5755	524.6	1.6094	2.0007	0.8548	1.3269	0
5756	524.5	1.6188	2.0004	0.8549	1.3262	0
5757	524.4	1.6276	2.0003	0.855	1.3266	0
5758	524.3	1.6355	2.0025	0.8555	1.3264	0
5759	524.2	1.6456	2.0022	0.8552	1.3271	0
5760	524.1	1.6525	2.0018	0.8555	1.3267	0
5761	524	1.662	2.004	0.8553	1.327	0
5762	523.9	1.6706	2.0038	0.8558	1.3259	0
5763	523.8	1.6815	2.0034	0.8554	1.327	0
5764	523.7	1.6894	2.0057	0.856	1.3274	0
5765	523.6	1.6996	2.0078	0.8563	1.327	0
5766	523.5	1.7089	2.0052	0.8563	1.3273	0
5767	523.4	1.7197	2.0072	0.8561	1.327	0
5768	523.3	1.7282	2.0094	0.8562	1.3262	0
5769	523.2	1.7395	2.0116	0.8565	1.3265	0
5770	523.1	1.7485	2.0113	0.8565	1.3272	0
5771	523	1.759	2.0086	0.8564	1.328	0
5772	522.9	1.7698	2.0107	0.857	1.3272	0
5773	522.8	1.7795	2.0154	0.8573	1.3269	0
5774	522.7	1.7922	2.015	0.8567	1.328	0
5775	522.6	1.8009	2.0148	0.8568	1.3273	0
5776	522.5	1.8113	2.0145	0.8572	1.328	0
5777	522.4	1.8235	2.0166	0.8567	1.3282	0
5778	522.3	1.8345	2.0163	0.8571	1.3279	0
5779	522.2	1.8441	2.016	0.8571	1.3276	0
5780	522.1	1.8573	2.0182	0.8572	1.3263	0
5781	522	1.8693	2.0205	0.8573	1.3286	0
5782	521.9	1.8815	2.02	0.8577	1.3292	0
5783	521.8	1.8904	2.0197	0.8574	1.3283	0
5784	521.7	1.9033	2.0194	0.8574	1.3285	0
5785	521.6	1.9166	2.024	0.8575	1.3281	0
5786	521.5	1.9265	2.0239	0.8575	1.328	0
5787	521.4	1.9384	2.0235	0.8575	1.3291	0
5788	521.3	1.9487	2.0257	0.8575	1.3288	0
5789	521.2	1.9613	2.0254	0.8577	1.3284	0
5790	521.1	1.9744	2.025	0.8575	1.3286	0
5791	521	1.9856	2.0298	0.8575	1.3283	0

5792	520.9	1.9946	2.0294	0.8574	1.3289	0
5793	520.8	2.009	2.0291	0.8577	1.3286	0
5794	520.7	2.0185	2.0288	0.8577	1.3288	0
5795	520.6	2.0284	2.0284	0.8574	1.329	0
5796	520.5	2.0413	2.0333	0.8574	1.3287	0
5797	520.4	2.0518	2.0331	0.8578	1.3299	0
5798	520.3	2.0625	2.0327	0.8578	1.3296	0
5799	520.2	2.0677	2.0349	0.858	1.3313	0
5800	520.1	2.0847	2.0372	0.8576	1.3309	0
5801	520	2.0934	2.0396	0.8577	1.3311	0
5802	519.9	2.1052	2.0446	0.858	1.3307	0
5803	519.8	2.1144	2.0415	0.8578	1.3325	0
5804	519.7	2.1204	2.0413	0.8577	1.3312	0
5805	519.6	2.1364	2.0462	0.8577	1.3302	0
5806	519.5	2.1462	2.0459	0.8576	1.333	0
5807	519.4	2.1563	2.0456	0.8577	1.3311	0
5808	519.3	2.1665	2.0479	0.8582	1.3302	0
5809	519.2	2.177	2.0502	0.8583	1.3308	0
5810	519.1	2.1842	2.05	0.858	1.3311	0
5811	519	2.1952	2.0524	0.8577	1.3318	0
5812	518.9	2.2065	2.0519	0.8574	1.3319	0
5813	518.8	2.2141	2.0544	0.8576	1.3316	0
5814	518.7	2.2179	2.057	0.8581	1.3324	0
5815	518.6	2.2298	2.0593	0.858	1.3314	0
5816	518.5	2.2337	2.059	0.8585	1.3317	0
5817	518.4	2.2462	2.0615	0.8577	1.3323	0
5818	518.3	2.2545	2.0696	0.858	1.3319	0
5819	518.2	2.2677	2.0664	0.858	1.3332	0
5820	518.1	2.2719	2.0689	0.858	1.3339	0
5821	518	2.2808	2.0685	0.8575	1.334	0
5822	517.9	2.29	2.074	0.8575	1.3336	0
5823	517.8	2.2945	2.0737	0.8588	1.3333	0
5824	517.7	2.3041	2.0763	0.858	1.3346	0
5825	517.6	2.3138	2.0818	0.8576	1.3347	0
5826	517.5	2.3186	2.0844	0.8574	1.3338	0
5827	517.4	2.3184	2.0873	0.8579	1.3342	0
5828	517.3	2.3285	2.09	0.8579	1.3338	0
5829	517.2	2.3281	2.0896	0.8578	1.3324	0
5830	517.1	2.3385	2.0985	0.8575	1.3352	0
5831	517	2.3547	2.0951	0.8576	1.3354	0
5832	516.9	2.3545	2.1011	0.8579	1.3346	0
5833	516.8	2.3655	2.1007	0.8579	1.3354	0
5834	516.7	2.3653	2.1036	0.8582	1.334	0

5835	516.6	2.3766	2.1064	0.8585	1.3358	0
5836	516.5	2.3762	2.1092	0.8581	1.336	0
5837	516.4	2.382	2.109	0.8582	1.3346	0
5838	516.3	2.3876	2.1151	0.858	1.3364	0
5839	516.2	2.3935	2.1213	0.8582	1.3355	0
5840	516.1	2.3994	2.1242	0.8578	1.3356	0
5841	516	2.4054	2.1273	0.8585	1.3353	0
5842	515.9	2.4051	2.1338	0.8584	1.335	0
5843	515.8	2.4177	2.13	0.8585	1.3352	0
5844	515.7	2.4173	2.1364	0.8579	1.3343	0
5845	515.6	2.4237	2.1432	0.8582	1.3351	0
5846	515.5	2.43	2.1464	0.8579	1.3364	0
5847	515.4	2.4296	2.1459	0.8581	1.3349	0
5848	515.3	2.4292	2.149	0.8583	1.3345	0
5849	515.2	2.4359	2.156	0.8586	1.3349	0
5850	515.1	2.4567	2.1629	0.8584	1.3355	0
5851	515	2.4492	2.1663	0.8583	1.3357	0
5852	514.9	2.4561	2.1698	0.8586	1.3355	0
5853	514.8	2.4557	2.1731	0.8584	1.3346	0
5854	514.7	2.4627	2.1843	0.8586	1.3359	0
5855	514.6	2.4624	2.184	0.859	1.334	0
5856	514.5	2.477	2.1915	0.8591	1.3337	0
5857	514.4	2.469	2.1951	0.8583	1.3343	0
5858	514.3	2.4763	2.1947	0.8587	1.3345	0
5859	514.2	2.4761	2.1985	0.8594	1.3343	0
5860	514.1	2.4757	2.2063	0.859	1.3334	0
5861	514	2.4831	2.2142	0.8591	1.3331	0
5862	513.9	2.4905	2.2181	0.8591	1.3338	0
5863	513.8	2.4903	2.222	0.8596	1.3341	0
5864	513.7	2.4978	2.2259	0.8597	1.3326	0
5865	513.6	2.4976	2.2343	0.8594	1.3329	0
5866	513.5	2.5053	2.2383	0.8596	1.3319	0
5867	513.4	2.505	2.2425	0.8598	1.3321	0
5868	513.3	2.5047	2.2467	0.8601	1.3335	0
5869	513.2	2.5211	2.251	0.86	1.3344	0
5870	513.1	2.5207	2.2598	0.86	1.3334	0
5871	513	2.5206	2.2643	0.86	1.3327	0
5872	512.9	2.5201	2.2687	0.8601	1.3334	0
5873	512.8	2.5198	2.278	0.8601	1.3337	0
5874	512.7	2.5281	2.2777	0.8599	1.3334	0
5875	512.6	2.5191	2.2871	0.8602	1.3329	0
5876	512.5	2.5188	2.297	0.8603	1.3332	0
5877	512.4	2.5271	2.2966	0.8602	1.3329	0

5878	512.3	2.5355	2.3065	0.86	1.3319	0
5879	512.2	2.5264	2.3115	0.86	1.3327	0
5880	512.1	2.5348	2.3165	0.8599	1.3335	0
5881	512	2.5255	2.3268	0.8597	1.3323	0
5882	511.9	2.5252	2.3321	0.86	1.3327	0
5883	511.8	2.5249	2.3373	0.8602	1.3329	0
5884	511.7	2.5245	2.3427	0.8601	1.3331	0
5885	511.6	2.533	2.348	0.8601	1.3333	0
5886	511.5	2.5325	2.3536	0.8603	1.3335	0
5887	511.4	2.5236	2.3533	0.8602	1.3332	0
5888	511.3	2.5232	2.3648	0.8606	1.3334	0
5889	511.2	2.5141	2.3704	0.8603	1.3335	0
5890	511.1	2.5137	2.3762	0.8598	1.3331	0
5891	511	2.5134	2.3884	0.86	1.3333	0
5892	510.9	2.513	2.3946	0.8598	1.3336	0
5893	510.8	2.5042	2.3942	0.8598	1.3349	0
5894	510.7	2.4957	2.407	0.8601	1.3334	0
5895	510.6	2.5036	2.4134	0.8601	1.3342	0
5896	510.5	2.4949	2.4198	0.8599	1.3355	0
5897	510.4	2.511	2.4193	0.8599	1.3338	0
5898	510.3	2.494	2.4331	0.8599	1.3336	0
5899	510.2	2.4777	2.4399	0.8597	1.3355	0
5900	510.1	2.4853	2.4468	0.8602	1.3352	0
5901	510	2.4769	2.4465	0.8597	1.3347	0
5902	509.9	2.4766	2.4389	0.8601	1.3351	0
5903	509.8	2.4684	2.4608	0.8602	1.3347	0
5904	509.7	2.4681	2.4681	0.8599	1.3344	0
5905	509.6	2.4676	2.4755	0.8597	1.3356	0
5906	509.5	2.452	2.4672	0.8595	1.3357	0
5907	509.4	2.4442	2.4747	0.8597	1.3359	0
5908	509.3	2.4365	2.4904	0.8599	1.3373	0
5909	509.2	2.436	2.4983	0.8599	1.3357	0
5910	509.1	2.4286	2.4896	0.86	1.3371	0
5911	509	2.4142	2.506	0.8598	1.3373	0
5912	508.9	2.4138	2.5141	0.859	1.3369	0
5913	508.8	2.4066	2.5226	0.8597	1.3366	0
5914	508.7	2.3994	2.5311	0.8596	1.3367	0
5915	508.6	2.3991	2.5308	0.8592	1.3381	0
5916	508.5	2.3856	2.5396	0.8593	1.3378	0
5917	508.4	2.3852	2.53	0.8595	1.3362	0
5918	508.3	2.3657	2.5388	0.8594	1.3375	0
5919	508.2	2.3654	2.5384	0.8596	1.3377	0
5920	508.1	2.359	2.5474	0.8596	1.3375	0

5921	508	2.3524	2.547	0.8593	1.3376	0
5922	507.9	2.3461	2.5467	0.8598	1.3385	0
5923	507.8	2.34	2.556	0.8596	1.3376	0
5924	507.7	2.328	2.5556	0.8595	1.3367	0
5925	507.6	2.3219	2.575	0.8597	1.3368	0
5926	507.5	2.3161	2.5646	0.8596	1.3371	0
5927	507.4	2.3101	2.5743	0.86	1.3367	0
5928	507.3	2.2989	2.5842	0.86	1.3364	0
5929	507.2	2.2879	2.5839	0.8601	1.3361	0
5930	507.1	2.2824	2.5835	0.8604	1.3369	0
5931	507	2.277	2.583	0.8602	1.3383	0
5932	506.9	2.2614	2.5931	0.8599	1.3366	0
5933	506.8	2.2561	2.5928	0.8603	1.3363	0
5934	506.7	2.251	2.6031	0.8606	1.336	0
5935	506.6	2.246	2.5817	0.8602	1.3363	0
5936	506.5	2.2314	2.5918	0.8602	1.3365	0
5937	506.4	2.2221	2.5916	0.8607	1.3374	0
5938	506.3	2.2171	2.5911	0.8603	1.3358	0
5939	506.2	2.2123	2.5907	0.8607	1.3366	0
5940	506.1	2.2032	2.5904	0.8608	1.3358	0
5941	506	2.19	2.6008	0.8604	1.336	0
5942	505.9	2.1814	2.6005	0.8609	1.3357	0
5943	505.8	2.181	2.6112	0.8612	1.3341	0
5944	505.7	2.1725	2.5998	0.861	1.3338	0
5945	505.6	2.1681	2.5995	0.8615	1.3347	0
5946	505.5	2.1559	2.5991	0.8614	1.3344	0
5947	505.4	2.1517	2.5988	0.8615	1.3346	0
5948	505.3	2.136	2.6094	0.8616	1.3336	0
5949	505.2	2.1283	2.5981	0.8617	1.3339	0
5950	505.1	2.1208	2.6089	0.8619	1.3337	0
5951	505	2.1168	2.6085	0.8623	1.3333	0
5952	504.9	2.1093	2.5972	0.8619	1.3329	0
5953	504.8	2.1019	2.6077	0.8619	1.3319	0
5954	504.7	2.0947	2.6075	0.8623	1.3322	0
5955	504.6	2.0841	2.6183	0.8618	1.3318	0
5956	504.5	2.0838	2.6066	0.8623	1.3314	0
5957	504.4	2.0703	2.6064	0.8628	1.3318	0
5958	504.3	2.0636	2.6061	0.8627	1.332	0
5959	504.2	2.0601	2.6057	0.8633	1.3311	0
5960	504.1	2.0503	2.6054	0.8632	1.3295	0
5961	504	2.047	2.6164	0.8636	1.3287	0
5962	503.9	2.0375	2.6048	0.8634	1.3301	0
5963	503.8	2.0312	2.6045	0.8637	1.3316	0

5964	503.7	2.0249	2.6041	0.864	1.3307	0
5965	503.6	2.0187	2.5928	0.8644	1.3297	0
5966	503.5	2.0097	2.5923	0.8638	1.3299	0
5967	503.4	2.0067	2.5814	0.8642	1.3291	0
5968	503.3	2.0036	2.6028	0.8641	1.3288	0
5969	503.2	1.9948	2.6024	0.8641	1.3284	0
5970	503.1	1.989	2.5911	0.8641	1.3286	0
5971	503	1.9808	2.6019	0.8643	1.329	0
5972	502.9	1.975	2.5904	0.8645	1.328	0
5973	502.8	1.9694	2.5901	0.8645	1.3276	0
5974	502.7	1.964	2.5897	0.8645	1.3291	0
5975	502.6	1.9584	2.5786	0.8643	1.3287	0
5976	502.5	1.953	2.5782	0.8641	1.3283	0
5977	502.4	1.9477	2.5779	0.864	1.3286	0
5978	502.3	1.9449	2.5884	0.8644	1.3282	0
5979	502.2	1.9372	2.588	0.8645	1.3279	0
5980	502.1	1.9297	2.5877	0.8648	1.3282	0
5981	502	1.9199	2.5874	0.8643	1.3273	0
5982	501.9	1.9197	2.5872	0.8647	1.3294	0
5983	501.8	1.9147	2.5761	0.8647	1.3291	0
5984	501.7	1.9074	2.5864	0.8643	1.3293	0
5985	501.6	1.9048	2.5753	0.8643	1.3284	0
5986	501.5	1.8978	2.575	0.8645	1.3293	0
5987	501.4	1.8952	2.5746	0.8648	1.3283	0
5988	501.3	1.8881	2.5741	0.8641	1.3284	0
5989	501.2	1.8835	2.5847	0.8643	1.3282	0
5990	501.1	1.879	2.5844	0.8642	1.3291	0
5991	501	1.8744	2.584	0.8643	1.3288	0
5992	500.9	1.8677	2.5837	0.8646	1.3302	0
5993	500.8	1.8654	2.5835	0.8645	1.33	0
5994	500.7	1.8588	2.5941	0.8644	1.3303	0
5995	500.6	1.8563	2.5936	0.8646	1.3298	0
5996	500.5	1.8499	2.5715	0.8649	1.3301	0
5997	500.4	1.8475	2.5819	0.8642	1.329	0
5998	500.3	1.8392	2.5815	0.8639	1.3299	0
5999	500.2	1.837	2.5812	0.8636	1.329	0
6000	500.1	1.8308	2.5702	0.8637	1.3299	0
6001	500	1.8266	2.5698	0.8641	1.3314	0
6002	499.9	1.8223	2.58	0.8635	1.3303	0
6003	499.8	1.8164	2.5799	0.8637	1.3319	0
6004	499.7	1.8161	2.5688	0.8636	1.3322	0
6005	499.6	1.8082	2.5683	0.8635	1.3305	0
6006	499.5	1.8042	2.5787	0.8638	1.332	0

6007	499.4	1.8038	2.5572	0.8634	1.3335	0
6008	499.3	1.7962	2.5779	0.8629	1.3324	0
6009	499.2	1.7905	2.5776	0.863	1.3333	0
6010	499.1	1.7883	2.5772	0.8631	1.333	0
6011	499	1.7827	2.5661	0.8635	1.3332	0
6012	498.9	1.7789	2.5766	0.8634	1.3335	0
6013	498.8	1.7768	2.5654	0.8635	1.3344	0
6014	498.7	1.773	2.5758	0.8635	1.3346	0
6015	498.6	1.7675	2.5646	0.8629	1.3342	0
6016	498.5	1.7654	2.575	0.8627	1.3338	0
6017	498.4	1.7586	2.5642	0.8626	1.3349	0
6018	498.3	1.7565	2.5743	0.8628	1.3338	0
6019	498.2	1.7544	2.5632	0.863	1.3353	0
6020	498.1	1.7491	2.5627	0.8628	1.3348	0
6021	498	1.7455	2.5842	0.8626	1.3351	0
6022	497.9	1.7419	2.5839	0.8625	1.3373	0
6023	497.8	1.7384	2.5835	0.8626	1.3357	0
6024	497.7	1.7347	2.583	0.8625	1.3364	0
6025	497.6	1.7314	2.5717	0.8627	1.3375	0
6026	497.5	1.7278	2.5714	0.8625	1.337	0
6027	497.4	1.7243	2.5709	0.8625	1.3367	0
6028	497.3	1.7193	2.5928	0.8623	1.3356	0
6029	497.2	1.7159	2.5812	0.863	1.3366	0
6030	497.1	1.714	2.5809	0.8633	1.3362	0
6031	497	1.7105	2.5694	0.8627	1.3364	0
6032	496.9	1.7071	2.5799	0.8626	1.3359	0
6033	496.8	1.7038	2.5909	0.8626	1.3356	0
6034	496.7	1.699	2.5682	0.8626	1.3352	0
6035	496.6	1.6973	2.5902	0.863	1.3356	0
6036	496.5	1.6954	2.6014	0.8634	1.3359	0
6037	496.4	1.6921	2.5894	0.8632	1.3367	0
6038	496.3	1.6889	2.5891	0.8631	1.337	0
6039	496.2	1.6871	2.5887	0.8632	1.3354	0
6040	496.1	1.6825	2.5885	0.864	1.3351	0
6041	496	1.6792	2.588	0.8635	1.334	0
6042	495.9	1.6763	2.5879	0.8642	1.3338	0
6043	495.8	1.676	2.5875	0.8639	1.3342	0
6044	495.7	1.6727	2.5986	0.8643	1.3343	0
6045	495.6	1.6696	2.5984	0.8647	1.3334	0
6046	495.5	1.6665	2.5981	0.8648	1.3337	0
6047	495.4	1.6621	2.5977	0.8648	1.3334	0
6048	495.3	1.6617	2.6092	0.8653	1.333	0
6049	495.2	1.6586	2.5969	0.8652	1.3326	0

6050	495.1	1.6555	2.6084	0.865	1.3322	0
6051	495	1.6538	2.608	0.8648	1.3325	0
6052	494.9	1.652	2.6198	0.8653	1.3295	0
6053	494.8	1.6478	2.6196	0.8659	1.3305	0
6054	494.7	1.6449	2.6071	0.8661	1.3303	0
6055	494.6	1.6445	2.6066	0.8659	1.3305	0
6056	494.5	1.6415	2.6312	0.8666	1.3307	0
6057	494.4	1.6411	2.6308	0.8665	1.3298	0
6058	494.3	1.6369	2.6304	0.8666	1.3307	0
6059	494.2	1.639	2.6174	0.8663	1.3296	0
6060	494.1	1.6335	2.6297	0.8673	1.3312	0
6061	494	1.632	2.6295	0.8675	1.329	0
6062	493.9	1.6303	2.6165	0.8672	1.3293	0
6063	493.8	1.6275	2.6162	0.8673	1.329	0
6064	493.7	1.6258	2.6284	0.8676	1.3299	0
6065	493.6	1.6241	2.6409	0.8678	1.3295	0
6066	493.5	1.6226	2.654	0.8684	1.3286	0
6067	493.4	1.6234	2.6402	0.8682	1.3275	0
6068	493.3	1.6206	2.6269	0.868	1.3292	0
6069	493.2	1.6191	2.6396	0.8686	1.3289	0
6070	493.1	1.6162	2.6527	0.8682	1.3279	0
6071	493	1.6157	2.6521	0.8691	1.3267	0
6072	492.9	1.6155	2.6519	0.8688	1.3271	0
6073	492.8	1.6113	2.6653	0.8688	1.328	0
6074	492.7	1.6122	2.679	0.8682	1.3276	0
6075	492.6	1.6095	2.6788	0.8694	1.3267	0
6076	492.5	1.6079	2.6643	0.8692	1.327	0
6077	492.4	1.6087	2.6782	0.869	1.3279	0
6078	492.3	1.6071	2.6778	0.8688	1.3281	0
6079	492.2	1.6067	2.6921	0.8692	1.3278	0
6080	492.1	1.604	2.6772	0.8693	1.3275	0
6081	492	1.6036	2.7066	0.8694	1.3277	0
6082	491.9	1.6032	2.691	0.8692	1.328	0
6083	491.8	1.6017	2.6908	0.8694	1.3277	0
6084	491.7	1.6	2.7055	0.8692	1.3279	0
6085	491.6	1.5997	2.6899	0.8696	1.3283	0
6086	491.5	1.5994	2.6897	0.8692	1.3266	0
6087	491.4	1.599	2.7047	0.8693	1.3276	0
6088	491.3	1.5974	2.7042	0.8694	1.3272	0
6089	491.2	1.5971	2.7038	0.8695	1.3282	0
6090	491.1	1.598	2.7192	0.8698	1.3278	0
6091	491	1.5977	2.719	0.8698	1.3282	0
6092	490.9	1.5961	2.7185	0.8694	1.3291	0

6093	490.8	1.5957	2.7347	0.8695	1.3294	0
6094	490.7	1.5942	2.7342	0.869	1.3291	0
6095	490.6	1.5949	2.7338	0.8689	1.3299	0
6096	490.5	1.5946	2.7335	0.8693	1.3309	0
6097	490.4	1.5942	2.7167	0.8686	1.3311	0
6098	490.3	1.5928	2.7165	0.8691	1.3303	0
6099	490.2	1.5923	2.7324	0.8691	1.3318	0
6100	490.1	1.5944	2.7491	0.8694	1.3321	0
6101	490	1.5952	2.7317	0.8692	1.331	0
6102	489.9	1.5934	2.7481	0.8687	1.3305	0
6103	489.8	1.5931	2.7307	0.8688	1.3301	0
6104	489.7	1.5928	2.7474	0.8683	1.3319	0
6105	489.6	1.5947	2.73	0.8683	1.3327	0
6106	489.5	1.5945	2.7469	0.8685	1.3325	0
6107	489.4	1.5929	2.7464	0.8684	1.3335	0
6108	489.3	1.5937	2.7459	0.8681	1.3337	0
6109	489.2	1.5947	2.7457	0.8679	1.3341	0
6110	489.1	1.5944	2.7632	0.8681	1.3352	0
6111	489	1.5952	2.745	0.8681	1.3347	0
6112	488.9	1.5947	2.7445	0.8679	1.335	0
6113	488.8	1.5956	2.7442	0.8682	1.336	0
6114	488.7	1.5966	2.7617	0.8677	1.3357	0
6115	488.6	1.5962	2.7435	0.8674	1.3367	0
6116	488.5	1.5957	2.7607	0.8673	1.3362	0
6117	488.4	1.5965	2.7605	0.8675	1.3371	0
6118	488.3	1.5975	2.7423	0.8666	1.3375	0
6119	488.2	1.5983	2.7418	0.8668	1.3364	0
6120	488.1	1.5991	2.7416	0.8671	1.3373	0
6121	488	1.5987	2.7411	0.8672	1.337	0
6122	487.9	1.5984	2.7585	0.8668	1.338	0
6123	487.8	1.6006	2.7582	0.8671	1.3371	0
6124	487.7	1.6002	2.7402	0.8666	1.338	0
6125	487.6	1.601	2.7397	0.8664	1.3389	0
6126	487.5	1.6017	2.7222	0.8659	1.3391	0
6127	487.4	1.6014	2.7387	0.8662	1.3408	0
6128	487.3	1.6035	2.7383	0.8665	1.3397	0
6129	487.2	1.6018	2.738	0.8663	1.34	0
6130	487.1	1.6015	2.7552	0.8658	1.3396	0
6131	487	1.6051	2.7203	0.8662	1.3394	0
6132	486.9	1.6045	2.7545	0.8659	1.3395	0
6133	486.8	1.6069	2.7031	0.8662	1.3393	0
6134	486.7	1.6077	2.7192	0.8662	1.341	0
6135	486.6	1.6075	2.719	0.8669	1.3408	0

6136	486.5	1.6083	2.7354	0.8669	1.3403	0
6137	486.4	1.6107	2.7181	0.8671	1.34	0
6138	486.3	1.6115	2.7347	0.8662	1.3403	0
6139	486.2	1.6112	2.7174	0.8664	1.34	0
6140	486.1	1.6109	2.7169	0.8665	1.3396	0
6141	486	1.6131	2.7167	0.867	1.3406	0
6142	485.9	1.6127	2.7162	0.8669	1.3409	0
6143	485.8	1.6149	2.6994	0.8669	1.3384	0
6144	485.7	1.6147	2.6992	0.8677	1.3396	0
6145	485.6	1.6183	2.683	0.8675	1.3406	0
6146	485.5	1.6179	2.6826	0.8673	1.3402	0
6147	485.4	1.6176	2.6981	0.8677	1.3406	0
6148	485.3	1.6198	2.6819	0.8675	1.3401	0
6149	485.2	1.6222	2.6815	0.8679	1.3398	0
6150	485.1	1.6231	2.6659	0.8677	1.3394	0
6151	485	1.6227	2.6653	0.8675	1.3382	0
6152	484.9	1.625	2.6651	0.8683	1.3386	0
6153	484.8	1.6247	2.6647	0.8683	1.3376	0
6154	484.7	1.6243	2.6643	0.8685	1.3385	0
6155	484.6	1.6281	2.6494	0.8685	1.3389	0
6156	484.5	1.6291	2.649	0.8688	1.3379	0
6157	484.4	1.6302	2.6343	0.869	1.3375	0
6158	484.3	1.6313	2.6203	0.8694	1.3365	0
6159	484.2	1.6308	2.6336	0.8695	1.3361	0
6160	484.1	1.6333	2.6196	0.8702	1.3358	0
6161	484	1.6344	2.6192	0.87	1.3355	0
6162	483.9	1.6354	2.5924	0.8699	1.3365	0
6163	483.8	1.6379	2.605	0.8705	1.3355	0
6164	483.7	1.6375	2.5918	0.8706	1.3343	0
6165	483.6	1.64	2.5914	0.8708	1.3354	0
6166	483.5	1.641	2.5909	0.8706	1.3343	0
6167	483.4	1.6435	2.5658	0.8712	1.3346	0
6168	483.3	1.6447	2.5656	0.8714	1.3337	0
6169	483.2	1.6458	2.5533	0.8713	1.3326	0
6170	483.1	1.6469	2.5528	0.8714	1.3343	0
6171	483	1.648	2.5525	0.8718	1.3332	0
6172	482.9	1.6504	2.5291	0.872	1.3342	0
6173	482.8	1.6501	2.5402	0.8717	1.3339	0
6174	482.7	1.6542	2.5176	0.8713	1.3328	0
6175	482.6	1.6553	2.5171	0.8719	1.3338	0
6176	482.5	1.6564	2.5167	0.8715	1.3334	0
6177	482.4	1.659	2.5056	0.8719	1.3331	0
6178	482.3	1.6603	2.5054	0.8724	1.3329	0

6179	482.2	1.6612	2.4943	0.8721	1.333	0
6180	482.1	1.664	2.494	0.8723	1.3326	0
6181	482	1.6651	2.4936	0.8724	1.3337	0
6182	481.9	1.6664	2.4731	0.8723	1.3327	0
6183	481.8	1.6674	2.4726	0.8726	1.3329	0
6184	481.7	1.6702	2.4724	0.8727	1.3355	0
6185	481.6	1.6714	2.4622	0.8729	1.3337	0
6186	481.5	1.6725	2.4716	0.8722	1.3325	0
6187	481.4	1.6754	2.4426	0.8725	1.333	0
6188	481.3	1.6766	2.4516	0.8721	1.3341	0
6189	481.2	1.6777	2.4511	0.8726	1.3328	0
6190	481.1	1.6821	2.4414	0.8729	1.3339	0
6191	481	1.6833	2.4409	0.873	1.3334	0
6192	480.9	1.6862	2.4314	0.8727	1.3346	0
6193	480.8	1.6874	2.4222	0.8725	1.3327	0
6194	480.7	1.6887	2.413	0.8729	1.3331	0
6195	480.6	1.6899	2.3954	0.873	1.3334	0
6196	480.5	1.6928	2.3951	0.8731	1.3315	0
6197	480.4	1.6956	2.4031	0.8728	1.3332	0
6198	480.3	1.697	2.3943	0.8725	1.3336	0
6199	480.2	1.7017	2.3939	0.8726	1.3325	0
6200	480.1	1.703	2.3852	0.8725	1.3335	0
6201	480	1.706	2.3848	0.8721	1.3331	0
6202	479.9	1.7073	2.3762	0.8726	1.3327	0
6203	479.8	1.7103	2.3679	0.8723	1.3331	0
6204	479.7	1.7134	2.3675	0.8726	1.3341	0
6205	479.6	1.7131	2.3752	0.8725	1.3345	0
6206	479.5	1.716	2.3587	0.8722	1.3353	0
6207	479.4	1.7173	2.3583	0.872	1.3349	0
6208	479.3	1.7243	2.358	0.872	1.3369	0
6209	479.2	1.7257	2.35	0.8724	1.3365	0
6210	479.1	1.7289	2.3496	0.8724	1.3361	0
6211	479	1.7285	2.3417	0.8724	1.3365	0
6212	478.9	1.7318	2.3339	0.8719	1.3375	0
6213	478.8	1.7351	2.3408	0.8721	1.3379	0
6214	478.7	1.7383	2.3257	0.8718	1.3366	0
6215	478.6	1.7379	2.3325	0.8716	1.3369	0
6216	478.5	1.745	2.3248	0.8715	1.338	0
6217	478.4	1.7485	2.3245	0.8719	1.337	0
6218	478.3	1.7499	2.3239	0.8712	1.3402	0
6219	478.2	1.7535	2.3236	0.8713	1.3383	0
6220	478.1	1.7571	2.3161	0.8716	1.3388	0
6221	478	1.7565	2.3228	0.8715	1.339	0

6222	477.9	1.7622	2.3153	0.8718	1.3395	0
6223	477.8	1.7658	2.3079	0.8714	1.3391	0
6224	477.7	1.7672	2.3073	0.871	1.3408	0
6225	477.6	1.771	2.307	0.8707	1.3412	0
6226	477.5	1.7746	2.3065	0.8705	1.34	0
6227	477.4	1.7806	2.3063	0.8705	1.3405	0
6228	477.3	1.7802	2.3059	0.8709	1.3401	0
6229	477.2	1.7862	2.3055	0.8711	1.3413	0
6230	477.1	1.7879	2.2982	0.8712	1.3417	0
6231	477	1.7938	2.2909	0.8704	1.3412	0
6232	476.9	1.7957	2.2974	0.8704	1.3416	0
6233	476.8	1.7974	2.2901	0.8707	1.3427	0
6234	476.7	1.8014	2.2829	0.87	1.3423	0
6235	476.6	1.8077	2.2894	0.8705	1.3412	0
6236	476.5	1.8096	2.2822	0.8706	1.3415	0
6237	476.4	1.8159	2.2817	0.8707	1.3419	0
6238	476.3	1.818	2.2883	0.8707	1.3409	0
6239	476.2	1.8222	2.2879	0.8705	1.342	0
6240	476.1	1.8265	2.2808	0.8704	1.3424	0
6241	476	1.8284	2.2803	0.8703	1.3435	0
6242	475.9	1.8351	2.2732	0.8708	1.343	0
6243	475.8	1.842	2.2729	0.8707	1.3434	0
6244	475.7	1.8466	2.266	0.8707	1.3431	0
6245	475.6	1.8511	2.2721	0.8707	1.3419	0
6246	475.5	1.8531	2.2716	0.8707	1.3421	0
6247	475.4	1.8578	2.2712	0.8709	1.3418	0
6248	475.3	1.8625	2.2708	0.8711	1.3422	0
6249	475.2	1.8673	2.2771	0.8707	1.3426	0
6250	475.1	1.8722	2.2636	0.8714	1.3415	0
6251	475	1.8797	2.2696	0.8712	1.3426	0
6252	474.9	1.8847	2.2627	0.8713	1.3429	0
6253	474.8	1.8896	2.2623	0.8716	1.3417	0
6254	474.7	1.892	2.2619	0.872	1.3405	0
6255	474.6	1.8972	2.2615	0.8714	1.3409	0
6256	474.5	1.9025	2.2611	0.8716	1.3429	0
6257	474.4	1.9049	2.2607	0.8719	1.3417	0
6258	474.3	1.9131	2.2538	0.8719	1.3404	0
6259	474.2	1.9215	2.2598	0.8726	1.34	0
6260	474.1	1.9272	2.2595	0.8723	1.3405	0
6261	474	1.9299	2.2592	0.8731	1.3394	0
6262	473.9	1.9355	2.2587	0.8728	1.3397	0
6263	473.8	1.9444	2.2647	0.8732	1.3392	0
6264	473.7	1.9471	2.2577	0.8732	1.3395	0

6265	473.6	1.9562	2.2508	0.8727	1.339	0
6266	473.5	1.959	2.2503	0.8733	1.3378	0
6267	473.4	1.9686	2.2564	0.8733	1.3382	0
6268	473.3	1.9782	2.2559	0.8731	1.3377	0
6269	473.2	1.9812	2.2555	0.8734	1.3381	0
6270	473.1	1.9842	2.255	0.8737	1.3368	0
6271	473	1.9943	2.2482	0.8741	1.3364	0
6272	472.9	1.9977	2.248	0.8744	1.3362	0
6273	472.8	2.0081	2.2538	0.8742	1.3372	0
6274	472.7	2.0113	2.247	0.8742	1.3375	0
6275	472.6	2.0183	2.2529	0.874	1.3362	0
6276	472.5	2.0178	2.2524	0.8741	1.3365	0
6277	472.4	2.0287	2.2454	0.8746	1.3352	0
6278	472.3	2.0401	2.245	0.8746	1.3364	0
6279	472.2	2.0435	2.2509	0.8745	1.3358	0
6280	472.1	2.0514	2.2506	0.8747	1.3355	0
6281	472	2.0592	2.2502	0.8748	1.3359	0
6282	471.9	2.0629	2.2497	0.8744	1.3362	0
6283	471.8	2.071	2.2558	0.8753	1.3365	0
6284	471.7	2.0791	2.2423	0.8753	1.336	0
6285	471.6	2.0832	2.2483	0.8757	1.3356	0
6286	471.5	2.0917	2.2545	0.8754	1.3352	0
6287	471.4	2.1006	2.2541	0.8761	1.3373	0
6288	471.3	2.1046	2.247	0.8755	1.3351	0
6289	471.2	2.1135	2.2596	0.8753	1.3353	0
6290	471.1	2.1227	2.246	0.8756	1.3357	0
6291	471	2.1271	2.252	0.8757	1.3352	0
6292	470.9	2.1316	2.245	0.8752	1.3363	0
6293	470.8	2.1413	2.2447	0.8759	1.3359	0
6294	470.7	2.1459	2.2441	0.876	1.3354	0
6295	470.6	2.1612	2.2502	0.8757	1.3349	0
6296	470.5	2.1608	2.2497	0.8759	1.3369	0
6297	470.4	2.1602	2.2491	0.8761	1.3363	0
6298	470.3	2.1816	2.2421	0.8758	1.3357	0
6299	470.2	2.1811	2.2547	0.8759	1.3344	0
6300	470.1	2.1807	2.2411	0.8763	1.3348	0
6301	470	2.1916	2.2537	0.8757	1.335	0
6302	469.9	2.2029	2.2467	0.8756	1.3355	0
6303	469.8	2.2083	2.2527	0.8756	1.3349	0
6304	469.7	2.214	2.2523	0.876	1.3344	0
6305	469.6	2.2195	2.245	0.8757	1.3346	0
6306	469.5	2.2253	2.2513	0.8758	1.335	0
6307	469.4	2.2311	2.2507	0.8758	1.3361	0

6308	469.3	2.2371	2.2503	0.8759	1.3356	0
6309	469.2	2.2497	2.2633	0.8766	1.3359	0
6310	469.1	2.2559	2.2492	0.8757	1.3354	0
6311	469	2.2624	2.2488	0.8756	1.3366	0
6312	468.9	2.2688	2.255	0.8759	1.3369	0
6313	468.8	2.2683	2.2545	0.8754	1.338	0
6314	468.7	2.282	2.254	0.8754	1.3375	0
6315	468.6	2.2743	2.2534	0.8755	1.337	0
6316	468.5	2.2882	2.2529	0.8758	1.3397	0
6317	468.4	2.3027	2.2524	0.8756	1.3384	0
6318	468.3	2.3022	2.2519	0.8751	1.3387	0
6319	468.2	2.2941	2.2582	0.8751	1.339	0
6320	468.1	2.3089	2.2577	0.875	1.3394	0
6321	468	2.3084	2.2504	0.875	1.3398	0
6322	467.9	2.3236	2.2635	0.875	1.34	0
6323	467.8	2.3231	2.2631	0.8751	1.3395	0
6324	467.7	2.3308	2.2557	0.8749	1.3407	0
6325	467.6	2.3303	2.2552	0.8747	1.3394	0
6326	467.5	2.338	2.2615	0.8741	1.3405	0
6327	467.4	2.346	2.2542	0.8746	1.34	0
6328	467.3	2.3454	2.2537	0.8752	1.3404	0
6329	467.2	2.3624	2.2673	0.8748	1.34	0
6330	467.1	2.3531	2.2596	0.8748	1.3411	0
6331	467	2.3527	2.2592	0.8741	1.3415	0
6332	466.9	2.3611	2.2588	0.8746	1.3411	0
6333	466.8	2.3787	2.2654	0.8753	1.3415	0
6334	466.7	2.3691	2.265	0.8742	1.3427	0
6335	466.6	2.3686	2.2645	0.8749	1.3431	0
6336	466.5	2.3772	2.264	0.8744	1.3417	0
6337	466.4	2.3768	2.2708	0.8742	1.3422	0
6338	466.3	2.3763	2.2703	0.8743	1.3425	0
6339	466.2	2.3946	2.2625	0.8746	1.342	0
6340	466.1	2.3942	2.2692	0.8745	1.3432	0
6341	466	2.3936	2.2687	0.8745	1.3418	0
6342	465.9	2.403	2.2611	0.8753	1.3414	0
6343	465.8	2.3927	2.2751	0.8754	1.3426	0
6344	465.7	2.4019	2.2672	0.8751	1.3412	0
6345	465.6	2.4014	2.2667	0.8746	1.3416	0
6346	465.5	2.4011	2.2663	0.8748	1.3421	0
6347	465.4	2.4004	2.2731	0.8749	1.3424	0
6348	465.3	2.4099	2.2725	0.8752	1.341	0
6349	465.2	2.4094	2.2721	0.8753	1.3422	0
6350	465.1	2.3988	2.2788	0.8752	1.3407	0

6351	465	2.4185	2.2709	0.8754	1.3402	0
6352	464.9	2.4078	2.2779	0.8757	1.3406	0
6353	464.8	2.4281	2.2699	0.8756	1.3401	0
6354	464.7	2.4171	2.277	0.8758	1.3406	0
6355	464.6	2.427	2.2689	0.8757	1.3408	0
6356	464.5	2.4264	2.2758	0.8758	1.3403	0
6357	464.4	2.4261	2.2755	0.876	1.3417	0
6358	464.3	2.4255	2.2749	0.8763	1.3402	0
6359	464.2	2.4357	2.2743	0.876	1.3387	0
6360	464.1	2.4245	2.2813	0.8762	1.34	0
6361	464	2.4346	2.2808	0.8768	1.3385	0
6362	463.9	2.434	2.2803	0.8765	1.3389	0
6363	463.8	2.4227	2.2796	0.8768	1.3391	0
6364	463.7	2.4222	2.2791	0.8769	1.3386	0
6365	463.6	2.4324	2.2785	0.8776	1.3389	0
6366	463.5	2.4429	2.2858	0.8774	1.3393	0
6367	463.4	2.4312	2.2851	0.8771	1.3387	0
6368	463.3	2.4418	2.2925	0.8775	1.3391	0
6369	463.2	2.4303	2.2765	0.8773	1.3394	0
6370	463.1	2.4297	2.2914	0.8773	1.338	0
6371	463	2.4514	2.2829	0.8776	1.3392	0
6372	462.9	2.4394	2.2823	0.8776	1.3385	0
6373	462.8	2.4501	2.2975	0.8779	1.3379	0
6374	462.7	2.4383	2.2812	0.8783	1.3383	0
6375	462.6	2.4489	2.2963	0.8782	1.3376	0
6376	462.5	2.4483	2.2958	0.878	1.3379	0
6377	462.4	2.4477	2.2872	0.8774	1.3391	0
6378	462.3	2.4248	2.2945	0.878	1.3394	0
6379	462.2	2.4465	2.2939	0.878	1.3379	0
6380	462.1	2.4574	2.2933	0.8776	1.3381	0
6381	462	2.4453	2.3009	0.878	1.3385	0
6382	461.9	2.4447	2.3002	0.8784	1.3396	0
6383	461.8	2.4441	2.2996	0.878	1.3399	0
6384	461.7	2.4436	2.2909	0.8778	1.3394	0
6385	461.6	2.4546	2.3067	0.8778	1.3388	0
6386	461.5	2.4421	2.2977	0.8783	1.3371	0
6387	461.4	2.4415	2.3053	0.8783	1.3383	0
6388	461.3	2.4525	2.3046	0.8786	1.3376	0
6389	461.2	2.4637	2.304	0.8786	1.3379	0
6390	461.1	2.4396	2.3118	0.8782	1.3372	0
6391	461	2.439	2.3111	0.8785	1.3375	0
6392	460.9	2.4617	2.3104	0.8778	1.3395	0
6393	460.8	2.4492	2.3098	0.8778	1.3398	0

6394	460.7	2.4604	2.3265	0.8784	1.3391	0
6395	460.6	2.4598	2.3171	0.8784	1.3385	0
6396	460.5	2.459	2.3163	0.8783	1.3386	0
6397	460.4	2.4465	2.3245	0.8783	1.3389	0
6398	460.3	2.4575	2.3236	0.878	1.338	0
6399	460.2	2.4571	2.3231	0.8785	1.3384	0
6400	460.1	2.4443	2.3135	0.8787	1.3386	0
6401	460	2.4437	2.3398	0.8793	1.3389	0
6402	459.9	2.4548	2.3298	0.8788	1.339	0
6403	459.8	2.4541	2.3291	0.8781	1.3374	0
6404	459.7	2.4535	2.3376	0.8784	1.3385	0
6405	459.6	2.465	2.337	0.879	1.3379	0
6406	459.5	2.4642	2.3362	0.8792	1.338	0
6407	459.4	2.4514	2.3355	0.8786	1.3392	0
6408	459.3	2.4627	2.3536	0.8781	1.3384	0
6409	459.2	2.4622	2.353	0.8788	1.3397	0
6410	459.1	2.4492	2.3522	0.879	1.3399	0
6411	459	2.4733	2.3515	0.879	1.3402	0
6412	458.9	2.4602	2.351	0.8787	1.3405	0
6413	458.8	2.4719	2.3502	0.8785	1.3397	0
6414	458.7	2.4585	2.3591	0.879	1.3398	0
6415	458.6	2.4704	2.3584	0.8794	1.3392	0
6416	458.5	2.4825	2.3576	0.8792	1.3383	0
6417	458.4	2.4689	2.3669	0.8789	1.3386	0
6418	458.3	2.4811	2.3661	0.8787	1.3378	0
6419	458.2	2.4564	2.3625	0.879	1.3396	0
6420	458.1	2.4708	2.3677	0.8792	1.3405	0
6421	458	2.4701	2.367	0.8786	1.3409	0
6422	457.9	2.4693	2.3662	0.8787	1.3413	0
6423	457.8	2.4609	2.3655	0.8789	1.34	0
6424	457.7	2.4679	2.3648	0.879	1.341	0
6425	457.6	2.475	2.3641	0.8795	1.3403	0
6426	457.5	2.4664	2.3757	0.8789	1.3395	0
6427	457.4	2.4656	2.3749	0.8789	1.341	0
6428	457.3	2.4726	2.3742	0.8788	1.3409	0
6429	457.2	2.4641	2.3798	0.8793	1.3413	0
6430	457.1	2.4557	2.379	0.8795	1.3417	0
6431	457	2.4704	2.3913	0.8792	1.3416	0
6432	456.9	2.4697	2.3905	0.8792	1.342	0
6433	456.8	2.4612	2.3965	0.879	1.3419	0
6434	456.7	2.4683	2.3957	0.8788	1.3412	0
6435	456.6	2.4597	2.4016	0.8788	1.3421	0
6436	456.5	2.4748	2.4011	0.8793	1.3415	0

6437	456.4	2.4584	2.4073	0.8794	1.3415	0
6438	456.3	2.4655	2.4065	0.8799	1.3425	0
6439	456.2	2.4647	2.4127	0.8796	1.3436	0
6440	456.1	2.4639	2.419	0.8794	1.3428	0
6441	456	2.4712	2.4183	0.8791	1.3427	0
6442	455.9	2.4623	2.4174	0.8795	1.343	0
6443	455.8	2.4697	2.4239	0.8797	1.3423	0
6444	455.7	2.4689	2.4305	0.8795	1.341	0
6445	455.6	2.4683	2.4448	0.8797	1.3415	0
6446	455.5	2.4675	2.4365	0.88	1.342	0
6447	455.4	2.4667	2.4432	0.88	1.3418	0
6448	455.3	2.466	2.4425	0.8798	1.3416	0
6449	455.2	2.4652	2.4418	0.88	1.3415	0
6450	455.1	2.4725	2.4486	0.88	1.3413	0
6451	455	2.4717	2.4478	0.8796	1.3411	0
6452	454.9	2.4712	2.4552	0.88	1.34	0
6453	454.8	2.4704	2.4623	0.8802	1.3404	0
6454	454.7	2.4697	2.4616	0.8805	1.3403	0
6455	454.6	2.469	2.4609	0.8811	1.3402	0
6456	454.5	2.4683	2.4683	0.8808	1.3425	0
6457	454.4	2.4676	2.4676	0.8805	1.3412	0
6458	454.3	2.4751	2.4751	0.8808	1.3417	0
6459	454.2	2.4743	2.4743	0.8809	1.3416	0
6460	454.1	2.482	2.482	0.8812	1.3401	0
6461	454	2.4812	2.4897	0.881	1.3393	0
6462	453.9	2.4804	2.4891	0.881	1.3392	0
6463	453.8	2.4796	2.497	0.8814	1.3396	0
6464	453.7	2.4789	2.5051	0.8817	1.3383	0
6465	453.6	2.4782	2.5137	0.8812	1.3394	0
6466	453.5	2.486	2.5129	0.8813	1.3405	0
6467	453.4	2.4852	2.5122	0.8814	1.3391	0
6468	453.3	2.4844	2.5206	0.8813	1.3389	0
6469	453.2	2.4837	2.52	0.8813	1.3389	0
6470	453.1	2.4831	2.5193	0.8817	1.3388	0
6471	453	2.4823	2.5378	0.8817	1.3386	0
6472	452.9	2.4903	2.537	0.8816	1.3385	0
6473	452.8	2.4895	2.5462	0.8814	1.3389	0
6474	452.7	2.4887	2.5455	0.8815	1.34	0
6475	452.6	2.5058	2.5547	0.8817	1.3391	0
6476	452.5	2.4959	2.5645	0.8817	1.3403	0
6477	452.4	2.4951	2.5635	0.8819	1.3388	0
6478	452.3	2.4944	2.5629	0.8817	1.3381	0
6479	452.2	2.4935	2.5727	0.8816	1.3397	0

6480	452.1	2.502	2.583	0.8818	1.3409	0
6481	452	2.501	2.5711	0.8818	1.34	0
6482	451.9	2.5005	2.5928	0.8818	1.3394	0
6483	451.8	2.5089	2.5919	0.8826	1.3398	0
6484	451.7	2.5176	2.6026	0.8821	1.3396	0
6485	451.6	2.5072	2.6017	0.8824	1.34	0
6486	451.5	2.516	2.5896	0.8817	1.34	0
6487	451.4	2.5056	2.6119	0.8814	1.3396	0
6488	451.3	2.524	2.5993	0.8817	1.3394	0
6489	451.2	2.523	2.5984	0.8817	1.3405	0
6490	451.1	2.5223	2.6094	0.8814	1.3404	0
6491	451	2.5117	2.6209	0.8812	1.3409	0
6492	450.9	2.5306	2.62	0.8812	1.3399	0
6493	450.8	2.5197	2.6447	0.8813	1.3411	0
6494	450.7	2.519	2.631	0.8817	1.3397	0
6495	450.6	2.5283	2.6302	0.882	1.3408	0
6496	450.5	2.5271	2.6291	0.8816	1.341	0
6497	450.4	2.5366	2.6413	0.8813	1.341	0
6498	450.3	2.5255	2.6404	0.8811	1.34	0
6499	450.2	2.5349	2.6396	0.8812	1.3412	0
6500	450.1	2.5339	2.6657	0.8809	1.3409	0
6501	450	2.533	2.6647	0.8813	1.3433	0
6502	449.9	2.5792	2.6857	0.825	1.371	0
6503	449.8	2.5804	2.6868	0.825	1.371	0
6504	449.7	2.5724	2.688	0.8249	1.3711	0
6505	449.6	2.5827	2.7014	0.8248	1.3727	0
6506	449.5	2.5839	2.7025	0.8248	1.3721	0
6507	449.4	2.5847	2.716	0.8246	1.3731	0
6508	449.3	2.5859	2.7172	0.8246	1.3719	0
6509	449.2	2.5967	2.7185	0.8248	1.3725	0
6510	449.1	2.5882	2.7194	0.8254	1.3724	0
6511	449	2.5894	2.708	0.8246	1.373	0
6512	448.9	2.6	2.7347	0.8246	1.373	0
6513	448.8	2.611	2.7104	0.825	1.373	0
6514	448.7	2.6022	2.724	0.8247	1.3723	0
6515	448.6	2.6033	2.738	0.8248	1.3734	0
6516	448.5	2.6043	2.7525	0.8248	1.3728	0
6517	448.4	2.6055	2.7272	0.8248	1.3728	0
6518	448.3	2.6064	2.7411	0.825	1.3738	0
6519	448.2	2.5979	2.7555	0.8249	1.3737	0
6520	448.1	2.6183	2.7565	0.8243	1.3736	0
6521	448	2.6194	2.7713	0.8244	1.3741	0
6522	447.9	2.6107	2.7587	0.8249	1.3735	0

6523	447.8	2.6212	2.7597	0.825	1.3738	0
6524	447.7	2.6323	2.7607	0.8252	1.3748	0
6525	447.6	2.6232	2.7755	0.8247	1.3741	0
6526	447.5	2.6341	2.7762	0.8251	1.3745	0
6527	447.4	2.6253	2.7773	0.8255	1.3744	0
6528	447.3	2.6364	2.7783	0.8253	1.3749	0
6529	447.2	2.6373	2.7794	0.8254	1.3747	0
6530	447.1	2.6282	2.7802	0.8252	1.3751	0
6531	447	2.6289	2.7673	0.8249	1.3737	0
6532	446.9	2.64	2.782	0.8255	1.3753	0
6533	446.8	2.6407	2.7828	0.8254	1.3722	0
6534	446.7	2.6319	2.7701	0.8253	1.3716	0
6535	446.6	2.6326	2.7849	0.8256	1.3742	0
6536	446.5	2.6538	2.8	0.8261	1.373	0
6537	446.4	2.6445	2.8008	0.8265	1.3749	0
6538	446.3	2.6455	2.7737	0.826	1.3736	0
6539	446.2	2.6461	2.7881	0.8265	1.3738	0
6540	446.1	2.6469	2.8033	0.8264	1.3735	0
6541	446	2.6478	2.8187	0.8264	1.3733	0
6542	445.9	2.6486	2.777	0.826	1.3742	0
6543	445.8	2.6496	2.806	0.8262	1.3746	0
6544	445.7	2.6503	2.8213	0.8265	1.3732	0
6545	445.6	2.6509	2.8074	0.8267	1.3739	0
6546	445.5	2.6519	2.8083	0.8267	1.372	0
6547	445.4	2.6525	2.8088	0.8265	1.3738	0
6548	445.3	2.6637	2.8097	0.8263	1.3726	0
6549	445.2	2.6442	2.8105	0.8267	1.3732	0
6550	445.1	2.655	2.8114	0.8272	1.373	0
6551	445	2.6659	2.8122	0.8271	1.3728	0
6552	444.9	2.6467	2.8277	0.8269	1.3721	0
6553	444.8	2.6676	2.8283	0.8268	1.3717	0
6554	444.7	2.658	2.8145	0.8273	1.3719	0
6555	444.6	2.669	2.8297	0.827	1.371	0
6556	444.5	2.6594	2.8306	0.8273	1.3707	0
6557	444.4	2.6704	2.8312	0.8275	1.3709	0
6558	444.3	2.6609	2.8471	0.8275	1.3711	0
6559	444.2	2.6718	2.8327	0.8274	1.3713	0
6560	444.1	2.6724	2.8333	0.8277	1.371	0
6561	444	2.6631	2.8342	0.828	1.3712	0
6562	443.9	2.6739	2.8348	0.828	1.3708	0
6563	443.8	2.6745	2.8353	0.8279	1.3694	0
6564	443.7	2.6651	2.8671	0.8284	1.3702	0
6565	443.6	2.6657	2.8368	0.8286	1.3698	0

6566	443.5	2.6664	2.8526	0.8279	1.3719	0
6567	443.4	2.667	2.869	0.8281	1.3695	0
6568	443.3	2.6678	2.8386	0.8286	1.3697	0
6569	443.2	2.6682	2.8703	0.8284	1.3697	0
6570	443.1	2.6688	2.8398	0.8287	1.3692	0
6571	443	2.6694	2.8557	0.8288	1.3699	0
6572	442.9	2.67	2.8564	0.8287	1.3694	0
6573	442.8	2.6807	2.8567	0.8287	1.3695	0
6574	442.7	2.671	2.8573	0.8289	1.3684	0
6575	442.6	2.6819	2.8739	0.829	1.3691	0
6576	442.5	2.6722	2.8586	0.8289	1.3702	0
6577	442.4	2.6726	2.8748	0.8285	1.3707	0
6578	442.3	2.6834	2.8752	0.8288	1.3696	0
6579	442.2	2.6838	2.8758	0.829	1.3701	0
6580	442.1	2.6741	2.8604	0.8289	1.3691	0
6581	442	2.6745	2.8768	0.8286	1.3685	0
6582	441.9	2.6751	2.8771	0.8288	1.3695	0
6583	441.8	2.6755	2.862	0.8288	1.3695	0
6584	441.7	2.6761	2.8781	0.8288	1.3705	0
6585	441.6	2.6765	2.8788	0.8287	1.3705	0
6586	441.5	2.677	2.8633	0.8289	1.3695	0
6587	441.4	2.6776	2.8798	0.829	1.3705	0
6588	441.3	2.678	2.8801	0.8288	1.3695	0
6589	441.2	2.6784	2.8969	0.8288	1.3694	0
6590	441.1	2.679	2.8652	0.8288	1.3699	0
6591	441	2.6694	2.8979	0.8288	1.3703	0
6592	440.9	2.66	2.8661	0.8289	1.3697	0
6593	440.8	2.6702	2.8824	0.8289	1.3712	0
6594	440.7	2.6807	2.8989	0.8289	1.3706	0
6595	440.6	2.671	2.8996	0.8287	1.3715	0
6596	440.5	2.6815	2.9	0.8286	1.3704	0
6597	440.4	2.6718	2.9176	0.8285	1.3724	0
6598	440.3	2.6724	2.901	0.8287	1.3704	0
6599	440.2	2.6828	2.9014	0.8289	1.3712	0
6600	440.1	2.6832	2.8851	0.8285	1.3726	0
6601	440	2.6834	2.902	0.828	1.373	0
6602	439.9	2.6741	2.8861	0.8285	1.3725	0
6603	439.8	2.6743	2.8864	0.8282	1.3729	0
6604	439.7	2.6747	2.9031	0.8281	1.3731	0
6605	439.6	2.6751	2.8871	0.828	1.3731	0
6606	439.5	2.6755	2.9038	0.8277	1.3734	0
6607	439.4	2.6659	2.8877	0.828	1.3747	0
6608	439.3	2.6761	2.9215	0.8281	1.3746	0

6609	439.2	2.6765	2.9052	0.8278	1.375	0
6610	439.1	2.677	2.9052	0.8278	1.3753	0
6611	439	2.6772	2.9055	0.8279	1.3747	0
6612	438.9	2.668	2.9062	0.8276	1.3756	0
6613	438.8	2.678	2.8901	0.8277	1.3765	0
6614	438.7	2.6782	2.8904	0.8274	1.3763	0
6615	438.6	2.6786	2.9241	0.8272	1.3766	0
6616	438.5	2.6692	2.9073	0.8273	1.377	0
6617	438.4	2.6696	2.8914	0.8277	1.3774	0
6618	438.3	2.6796	2.908	0.8272	1.378	0
6619	438.2	2.6605	2.8921	0.8269	1.3779	0
6620	438.1	2.6704	2.9087	0.8269	1.3782	0
6621	438	2.6805	2.909	0.827	1.379	0
6622	437.9	2.6809	2.9094	0.827	1.3789	0
6623	437.8	2.6811	2.9097	0.8269	1.3796	0
6624	437.7	2.6815	2.927	0.827	1.3814	0
6625	437.6	2.6718	2.9101	0.827	1.3797	0
6626	437.5	2.6819	2.9104	0.8269	1.38	0
6627	437.4	2.6824	2.9108	0.8269	1.3798	0
6628	437.3	2.6826	2.9112	0.8269	1.3796	0
6629	437.2	2.683	2.8952	0.827	1.3799	0
6630	437.1	2.6735	2.9115	0.8269	1.3807	0
6631	437	2.6739	2.8955	0.827	1.381	0
6632	436.9	2.6838	2.8959	0.827	1.3814	0
6633	436.8	2.6743	2.9126	0.8272	1.3806	0
6634	436.7	2.6844	2.9129	0.8271	1.3815	0
6635	436.6	2.6847	2.9133	0.8271	1.3797	0
6636	436.5	2.6751	2.9133	0.8271	1.379	0
6637	436.4	2.6853	2.8972	0.8272	1.3788	0
6638	436.3	2.6757	2.914	0.8277	1.3811	0
6639	436.2	2.6959	2.9314	0.8279	1.38	0
6640	436.1	2.6763	2.9147	0.8278	1.3782	0
6641	436	2.6861	2.9318	0.8275	1.3802	0
6642	435.9	2.6866	2.9322	0.8282	1.3786	0
6643	435.8	2.6868	2.8989	0.8283	1.3784	0
6644	435.7	2.6872	2.9158	0.8281	1.3788	0
6645	435.6	2.6776	2.9158	0.8284	1.3771	0
6646	435.5	2.6876	2.9161	0.8285	1.3788	0
6647	435.4	2.6782	2.9165	0.8289	1.3781	0
6648	435.3	2.688	2.9	0.8292	1.3777	0
6649	435.2	2.6882	2.9169	0.8291	1.3785	0
6650	435.1	2.6885	2.9169	0.8286	1.3777	0
6651	435	2.6889	2.9172	0.8289	1.3771	0

6652	434.9	2.6889	2.9176	0.829	1.3767	0
6653	434.8	2.6994	2.9179	0.8295	1.3766	0
6654	434.7	2.6996	2.9179	0.8299	1.3759	0
6655	434.6	2.6998	2.9183	0.8301	1.3747	0
6656	434.5	2.7001	2.9183	0.83	1.3749	0
6657	434.4	2.7003	2.9024	0.8299	1.3751	0
6658	434.3	2.7003	2.9024	0.83	1.3748	0
6659	434.2	2.6906	2.9027	0.8304	1.3746	0
6660	434.1	2.7009	2.9194	0.8306	1.3749	0
6661	434	2.6912	2.9367	0.8311	1.3746	0
6662	433.9	2.6914	2.937	0.8309	1.3739	0
6663	433.8	2.7016	2.9034	0.8312	1.3745	0
6664	433.7	2.7018	2.9038	0.8313	1.3733	0
6665	433.6	2.702	2.9041	0.8316	1.3735	0
6666	433.5	2.702	2.8884	0.8312	1.3732	0
6667	433.4	2.7124	2.9208	0.8311	1.3738	0
6668	433.3	2.7025	2.9045	0.8315	1.3744	0
6669	433.2	2.7027	2.9212	0.8322	1.3732	0
6670	433.1	2.7027	2.9048	0.8318	1.3739	0
6671	433	2.7029	2.9052	0.8319	1.3731	0
6672	432.9	2.7031	2.9385	0.8319	1.3728	0
6673	432.8	2.7031	2.9215	0.8319	1.3729	0
6674	432.7	2.6934	2.9055	0.8319	1.3725	0
6675	432.6	2.7036	2.9219	0.8323	1.3727	0
6676	432.5	2.714	2.9059	0.8322	1.373	0
6677	432.4	2.714	2.9223	0.8327	1.373	0
6678	432.3	2.7142	2.9062	0.8325	1.3728	0
6679	432.2	2.7042	2.9226	0.8323	1.3719	0
6680	432.1	2.7042	2.8904	0.8325	1.373	0
6681	432	2.7147	2.9066	0.8324	1.3726	0
6682	431.9	2.7044	2.9066	0.8322	1.3722	0
6683	431.8	2.7047	2.9066	0.8325	1.3719	0
6684	431.7	2.7047	2.923	0.8325	1.3719	0
6685	431.6	2.7149	2.9069	0.8328	1.3711	0
6686	431.5	2.7256	2.9069	0.8328	1.3726	0
6687	431.4	2.7151	2.9069	0.8324	1.3722	0
6688	431.3	2.7049	2.9069	0.833	1.3723	0
6689	431.2	2.7153	2.9237	0.8327	1.373	0
6690	431.1	2.7053	2.9073	0.8328	1.373	0
6691	431	2.7153	2.9237	0.8324	1.3734	0
6692	430.9	2.6953	2.9073	0.8324	1.3722	0
6693	430.8	2.7156	2.9076	0.8326	1.3727	0
6694	430.7	2.7156	2.9241	0.8327	1.3728	0

6695	430.6	2.7055	2.9076	0.8327	1.3742	0
6696	430.5	2.7158	2.8918	0.8323	1.3737	0
6697	430.4	2.7158	2.8918	0.8323	1.3743	0
6698	430.3	2.7158	2.8918	0.8323	1.3753	0
6699	430.2	2.7158	2.9076	0.8321	1.3738	0
6700	430.1	2.7158	2.9076	0.8323	1.3743	0
6701	430	2.7058	2.9241	0.8321	1.3749	0
6702	429.9	2.7158	2.9076	0.832	1.3748	0
6703	429.8	2.7158	2.9241	0.8317	1.3749	0
6704	429.7	2.7263	2.8918	0.8317	1.3757	0
6705	429.6	2.7058	2.8921	0.8313	1.3767	0
6706	429.5	2.7055	2.8918	0.8315	1.3753	0
6707	429.4	2.7265	2.8768	0.8319	1.3775	0
6708	429.3	2.7058	2.9076	0.8315	1.3782	0
6709	429.2	2.7158	2.8921	0.8313	1.3777	0
6710	429.1	2.7055	2.8765	0.8312	1.3781	0
6711	429	2.7158	2.9076	0.8311	1.3781	0
6712	428.9	2.7055	2.9076	0.8309	1.3775	0
6713	428.8	2.7158	2.9076	0.8309	1.3781	0
6714	428.7	2.7156	2.9076	0.8306	1.3798	0
6715	428.6	2.7055	2.9076	0.8307	1.3804	0
6716	428.5	2.7055	2.9076	0.8302	1.3784	0
6717	428.4	2.7156	2.9237	0.8307	1.3798	0
6718	428.3	2.7055	2.9076	0.8304	1.3799	0
6719	428.2	2.7053	2.9073	0.8304	1.3807	0
6720	428.1	2.7053	2.9073	0.8306	1.3821	0
6721	428	2.7051	2.8914	0.8305	1.3811	0
6722	427.9	2.7051	2.8914	0.8301	1.3825	0
6723	427.8	2.7051	2.8761	0.8298	1.3839	0
6724	427.7	2.7051	2.9073	0.8293	1.3838	0
6725	427.6	2.6951	2.9069	0.83	1.3837	0
6726	427.5	2.7051	2.8761	0.8297	1.3838	0
6727	427.4	2.6949	2.8758	0.8297	1.3836	0
6728	427.3	2.7049	2.8911	0.8299	1.3837	0
6729	427.2	2.6949	2.8758	0.8297	1.3849	0
6730	427.1	2.6946	2.9069	0.8295	1.3839	0
6731	427	2.6949	2.8911	0.8297	1.384	0
6732	426.9	2.6849	2.9066	0.8294	1.3853	0
6733	426.8	2.7047	2.8755	0.8296	1.3843	0
6734	426.7	2.7044	2.8908	0.8293	1.3847	0
6735	426.6	2.6946	2.8755	0.8293	1.3842	0
6736	426.5	2.6944	2.9066	0.8296	1.3851	0
6737	426.4	2.6844	2.8908	0.8298	1.3845	0

6738	426.3	2.6844	2.8904	0.8295	1.3848	0
6739	426.2	2.6844	2.8752	0.8296	1.3862	0
6740	426.1	2.694	2.8904	0.8296	1.3857	0
6741	426	2.6844	2.8904	0.8295	1.3858	0
6742	425.9	2.6842	2.9062	0.8296	1.3842	0
6743	425.8	2.6747	2.8904	0.8295	1.3861	0
6744	425.7	2.6842	2.8901	0.8298	1.3855	0
6745	425.6	2.6745	2.9059	0.8302	1.3854	0
6746	425.5	2.6745	2.8748	0.83	1.3849	0
6747	425.4	2.6649	2.8748	0.8303	1.3833	0
6748	425.3	2.6649	2.8745	0.8301	1.3847	0
6749	425.2	2.6647	2.8897	0.8304	1.3841	0
6750	425.1	2.6647	2.8745	0.8303	1.3835	0
6751	425	2.6645	2.8894	0.8305	1.3844	0
6752	424.9	2.6647	2.8745	0.8307	1.3835	0
6753	424.8	2.6645	2.8894	0.8308	1.3833	0
6754	424.7	2.6554	2.8742	0.8308	1.3839	0
6755	424.6	2.6552	2.8742	0.8311	1.3822	0
6756	424.5	2.655	2.8891	0.8311	1.3831	0
6757	424.4	2.655	2.8891	0.8315	1.3815	0
6758	424.3	2.6548	2.8891	0.8315	1.3828	0
6759	424.2	2.6639	2.8891	0.8316	1.3814	0
6760	424.1	2.6546	2.8732	0.8319	1.3806	0
6761	424	2.6544	2.9045	0.832	1.3825	0
6762	423.9	2.6544	2.8884	0.8322	1.3815	0
6763	423.8	2.6455	2.8884	0.8326	1.3805	0
6764	423.7	2.6455	2.8884	0.8332	1.3795	0
6765	423.6	2.6451	2.8881	0.8331	1.3806	0
6766	423.5	2.6451	2.8582	0.8336	1.3791	0
6767	423.4	2.6362	2.8579	0.8334	1.378	0
6768	423.3	2.636	2.8726	0.8334	1.3793	0
6769	423.2	2.6358	2.8877	0.8339	1.3787	0
6770	423.1	2.6356	2.8722	0.8335	1.379	0
6771	423	2.6356	2.8874	0.8336	1.3793	0
6772	422.9	2.6354	2.8719	0.8337	1.3787	0
6773	422.8	2.6351	2.8716	0.8338	1.3784	0
6774	422.7	2.6264	2.8716	0.8336	1.3783	0
6775	422.6	2.6264	2.8716	0.8338	1.3796	0
6776	422.5	2.6262	2.8713	0.8338	1.3789	0
6777	422.4	2.626	2.8864	0.8342	1.3788	0
6778	422.3	2.6258	2.871	0.8345	1.3791	0
6779	422.2	2.6171	2.8861	0.8347	1.3794	0
6780	422.1	2.6167	2.8857	0.8343	1.3776	0

6781	422	2.6251	2.8703	0.8345	1.3775	0
6782	421.9	2.6164	2.87	0.8343	1.3777	0
6783	421.8	2.6162	2.8851	0.8348	1.3765	0
6784	421.7	2.616	2.9007	0.8349	1.3773	0
6785	421.6	2.6158	2.8847	0.8349	1.3771	0
6786	421.5	2.6155	2.8844	0.835	1.3778	0
6787	421.4	2.6153	2.8542	0.8349	1.3775	0
6788	421.3	2.6151	2.8687	0.8347	1.3773	0
6789	421.2	2.6064	2.8684	0.8348	1.3785	0
6790	421.1	2.6062	2.8392	0.8349	1.3792	0
6791	421	2.6142	2.8677	0.835	1.3775	0
6792	420.9	2.6057	2.8831	0.8351	1.3787	0
6793	420.8	2.6139	2.8827	0.835	1.3771	0
6794	420.7	2.6054	2.8983	0.8352	1.3793	0
6795	420.6	2.605	2.8821	0.8353	1.3779	0
6796	420.5	2.6048	2.8817	0.8352	1.3787	0
6797	420.4	2.6045	2.8665	0.8349	1.3793	0
6798	420.3	2.6041	2.8661	0.8352	1.38	0
6799	420.2	2.5957	2.8807	0.8347	1.3781	0
6800	420.1	2.6035	2.8807	0.8347	1.3803	0
6801	420	2.6033	2.8962	0.835	1.3796	0
6802	419.9	2.5948	2.8649	0.8349	1.3802	0
6803	419.8	2.5945	2.8798	0.8346	1.3804	0
6804	419.7	2.6022	2.8952	0.8349	1.3805	0
6805	419.6	2.6019	2.8791	0.8351	1.3807	0
6806	419.5	2.5935	2.8788	0.8342	1.3809	0
6807	419.4	2.6098	2.8784	0.8347	1.3803	0
6808	419.3	2.601	2.863	0.8343	1.3818	0
6809	419.2	2.5926	2.8778	0.8342	1.382	0
6810	419.1	2.5923	2.8931	0.834	1.3822	0
6811	419	2.5919	2.8771	0.8339	1.3817	0
6812	418.9	2.5916	2.8768	0.8339	1.3834	0
6813	418.8	2.5911	2.8765	0.8339	1.383	0
6814	418.7	2.5909	2.8608	0.8336	1.3842	0
6815	418.6	2.5986	2.8758	0.8333	1.3858	0
6816	418.5	2.5983	2.8755	0.8328	1.385	0
6817	418.4	2.5979	2.8748	0.8333	1.3851	0
6818	418.3	2.5896	2.8748	0.8329	1.3859	0
6819	418.2	2.5971	2.8742	0.8328	1.3864	0
6820	418.1	2.5967	2.8897	0.8327	1.3861	0
6821	418	2.5965	2.8735	0.8325	1.3878	0
6822	417.9	2.5962	2.8891	0.8323	1.3885	0
6823	417.8	2.5959	2.8729	0.8328	1.3881	0

6824	417.7	2.5955	2.8726	0.8328	1.3884	0
6825	417.6	2.587	2.857	0.8323	1.3885	0
6826	417.5	2.5948	2.8877	0.8324	1.3891	0
6827	417.4	2.5943	2.8716	0.8327	1.3893	0
6828	417.3	2.6024	2.8871	0.8322	1.3894	0
6829	417.2	2.6105	2.8867	0.8319	1.3891	0
6830	417.1	2.5933	2.8706	0.8319	1.3908	0
6831	417	2.5931	2.8861	0.8317	1.3894	0
6832	416.9	2.6094	2.8857	0.8319	1.3906	0
6833	416.8	2.6005	2.8851	0.8321	1.3897	0
6834	416.7	2.5919	2.8847	0.8322	1.3904	0
6835	416.6	2.5998	2.8844	0.8321	1.39	0
6836	416.5	2.5995	2.8841	0.8317	1.3912	0
6837	416.4	2.5991	2.8837	0.8315	1.3913	0
6838	416.3	2.6071	2.8834	0.8316	1.3914	0
6839	416.2	2.5984	2.8831	0.8322	1.3911	0
6840	416.1	2.6062	2.8668	0.8317	1.3922	0
6841	416	2.6059	2.8821	0.8322	1.3918	0
6842	415.9	2.6142	2.8983	0.832	1.3914	0
6843	415.8	2.6052	2.8814	0.8318	1.3906	0
6844	415.7	2.6048	2.8976	0.8321	1.3902	0
6845	415.6	2.613	2.8969	0.832	1.3913	0
6846	415.5	2.6041	2.9136	0.8319	1.391	0
6847	415.4	2.6036	2.8962	0.8324	1.391	0
6848	415.3	2.6117	2.8794	0.8326	1.3901	0
6849	415.2	2.6115	2.9126	0.8323	1.3898	0
6850	415.1	2.611	2.9122	0.8326	1.3904	0
6851	415	2.6107	2.8945	0.8325	1.3916	0
6852	414.9	2.6103	2.8941	0.8328	1.3901	0
6853	414.8	2.6099	2.8938	0.8327	1.3919	0
6854	414.7	2.6273	2.8935	0.8331	1.3899	0
6855	414.6	2.6267	2.9101	0.833	1.3911	0
6856	414.5	2.6354	2.9097	0.8331	1.3901	0
6857	414.4	2.626	2.9094	0.8331	1.3902	0
6858	414.3	2.6165	2.9087	0.8329	1.3892	0
6859	414.2	2.6253	2.9083	0.8331	1.39	0
6860	414.1	2.6249	2.908	0.8337	1.3896	0
6861	414	2.6243	2.9252	0.8335	1.3891	0
6862	413.9	2.633	2.9248	0.8338	1.3876	0
6863	413.8	2.6325	2.9066	0.834	1.3881	0
6864	413.7	2.6413	2.9237	0.8341	1.3892	0
6865	413.6	2.6409	2.9234	0.8343	1.3883	0
6866	413.5	2.6405	2.9416	0.8345	1.3878	0

6867	413.4	2.6496	2.9226	0.8346	1.3884	0
6868	413.3	2.6396	2.9223	0.8347	1.3869	0
6869	413.2	2.6486	2.9215	0.8349	1.3869	0
6870	413.1	2.648	2.9034	0.8348	1.3859	0
6871	413	2.6381	2.9205	0.835	1.387	0
6872	412.9	2.6472	2.9385	0.8354	1.386	0
6873	412.8	2.6467	2.9382	0.8355	1.3865	0
6874	412.7	2.6558	2.919	0.8353	1.3866	0
6875	412.6	2.6554	2.937	0.8359	1.3867	0
6876	412.5	2.655	2.9183	0.8356	1.3862	0
6877	412.4	2.6447	2.9363	0.8354	1.3863	0
6878	412.3	2.6639	2.9355	0.8356	1.3857	0
6879	412.2	2.6635	2.9547	0.8358	1.3859	0
6880	412.1	2.6733	2.9348	0.8357	1.3853	0
6881	412	2.6728	2.9344	0.836	1.3848	0
6882	411.9	2.672	2.9531	0.836	1.3842	0
6883	411.8	2.6716	2.9329	0.8362	1.3858	0
6884	411.7	2.6712	2.9519	0.8359	1.3864	0
6885	411.6	2.6811	2.9516	0.8357	1.3849	0
6886	411.5	2.6702	2.9318	0.8362	1.3854	0
6887	411.4	2.6696	2.9504	0.8364	1.3866	0
6888	411.3	2.6904	2.9307	0.8363	1.386	0
6889	411.2	2.679	2.9492	0.8364	1.3866	0
6890	411.1	2.6893	2.9488	0.8362	1.3855	0
6891	411	2.6887	2.9686	0.8361	1.3859	0
6892	410.9	2.688	2.9889	0.8366	1.3848	0
6893	410.8	2.6985	2.9674	0.8366	1.3853	0
6894	410.7	2.6979	2.9465	0.8369	1.3854	0
6895	410.6	2.6863	2.9662	0.8371	1.3853	0
6896	410.5	2.708	2.9658	0.8368	1.3842	0
6897	410.4	2.6964	2.945	0.837	1.3848	0
6898	410.3	2.6957	2.9646	0.8367	1.3848	0
6899	410.2	2.7064	2.9642	0.8371	1.3848	0
6900	410.1	2.6946	2.9634	0.8371	1.3848	0
6901	410	2.7053	2.963	0.8368	1.3857	0
6902	409.9	2.7047	2.9834	0.8369	1.3862	0
6903	409.8	2.7156	2.9614	0.8364	1.3872	0
6904	409.7	2.7149	2.961	0.8363	1.386	0
6905	409.6	2.7029	2.9602	0.8371	1.3877	0
6906	409.5	2.7138	2.9809	0.837	1.3865	0
6907	409.4	2.7131	2.959	0.8367	1.3871	0
6908	409.3	2.7245	2.9796	0.8368	1.3869	0
6909	409.2	2.7003	2.9792	0.8368	1.388	0

6910	409.1	2.7111	2.9784	0.8363	1.389	0
6911	409	2.7347	2.9776	0.836	1.3883	0
6912	408.9	2.7219	2.9772	0.8362	1.3872	0
6913	408.8	2.7335	2.9763	0.836	1.3882	0
6914	408.7	2.7328	2.9547	0.8361	1.3893	0
6915	408.6	2.7324	2.9543	0.8362	1.3887	0
6916	408.5	2.7317	2.997	0.8362	1.3892	0
6917	408.4	2.731	2.9739	0.836	1.3897	0
6918	408.3	2.743	2.9735	0.8361	1.3908	0
6919	408.2	2.7423	2.9516	0.8361	1.3907	0
6920	408.1	2.7547	2.9944	0.8362	1.3919	0
6921	408	2.7542	2.9718	0.8361	1.3913	0
6922	407.9	2.7535	2.971	0.8362	1.3924	0
6923	407.8	2.7399	2.9706	0.8358	1.3918	0
6924	407.7	2.7525	2.9923	0.8359	1.3913	0
6925	407.6	2.7518	2.9914	0.8358	1.394	0
6926	407.5	2.751	2.9686	0.8352	1.3921	0
6927	407.4	2.7637	3.0137	0.8357	1.392	0
6928	407.3	2.763	2.967	0.8352	1.3937	0
6929	407.2	2.7762	2.9889	0.835	1.3937	0
6930	407.1	2.762	2.9662	0.8354	1.3938	0
6931	407	2.7612	2.9654	0.8352	1.3937	0
6932	406.9	2.7886	3.0106	0.8353	1.3953	0
6933	406.8	2.76	2.9863	0.8351	1.393	0
6934	406.7	2.7595	2.9859	0.8354	1.393	0
6935	406.6	2.7726	2.9851	0.8348	1.3941	0
6936	406.5	2.7719	3.0079	0.8349	1.3941	0
6937	406.4	2.7713	2.9838	0.8354	1.3947	0
6938	406.3	2.7706	2.961	0.835	1.3951	0
6939	406.2	2.7701	2.9825	0.8352	1.3952	0
6940	406.1	2.7836	2.9821	0.8354	1.3963	0
6941	406	2.7552	2.9817	0.8357	1.3958	0
6942	405.9	2.7825	3.0044	0.8351	1.3952	0
6943	405.8	2.7967	3.0039	0.835	1.3957	0
6944	405.7	2.7959	3.0031	0.8352	1.3963	0
6945	405.6	2.7953	3.0026	0.8353	1.3962	0
6946	405.5	2.7799	3.0017	0.8357	1.3972	0
6947	405.4	2.794	2.9776	0.8352	1.3954	0
6948	405.3	2.8245	3.0004	0.8355	1.3949	0
6949	405.2	2.808	3	0.8353	1.3965	0
6950	405.1	2.7775	2.9991	0.8359	1.396	0
6951	405	2.7913	2.9987	0.8353	1.397	0
6952	404.9	2.7908	3.0227	0.8358	1.3945	0

6953	404.8	2.8052	2.9974	0.836	1.394	0
6954	404.7	2.8044	3.0214	0.8363	1.3937	0
6955	404.6	2.7739	2.9957	0.8365	1.395	0
6956	404.5	2.8033	2.9952	0.8363	1.3943	0
6957	404.4	2.8184	2.9944	0.8362	1.3931	0
6958	404.3	2.7867	2.994	0.8362	1.3941	0
6959	404.2	2.817	2.9931	0.8366	1.3934	0
6960	404.1	2.8005	3.0173	0.8363	1.3939	0
6961	404	2.8156	3.0164	0.8368	1.3932	0
6962	403.9	2.815	3.0159	0.8367	1.3926	0
6963	403.8	2.7986	3.015	0.8371	1.3938	0
6964	403.7	2.8136	3.0146	0.8371	1.3944	0
6965	403.6	2.813	3.0141	0.8374	1.3926	0
6966	403.5	2.8122	3.0132	0.8371	1.3924	0
6967	403.4	2.8116	3.0123	0.8373	1.3923	0
6968	403.3	2.8271	3.0119	0.8374	1.3928	0
6969	403.2	2.8102	3.011	0.8375	1.3927	0
6970	403.1	2.8094	3.0106	0.8373	1.3926	0
6971	403	2.8251	3.0097	0.8375	1.3925	0
6972	402.9	2.8245	3.0088	0.8378	1.393	0
6973	402.8	2.8407	2.9834	0.838	1.393	0
6974	402.7	2.8579	3.0339	0.838	1.3923	0
6975	402.6	2.8392	3.007	0.838	1.3928	0
6976	402.5	2.8216	3.0325	0.8377	1.392	0
6977	402.4	2.8207	3.0052	0.8382	1.392	0
6978	402.3	2.8371	3.0044	0.8383	1.3918	0
6979	402.2	2.8365	3.0039	0.8385	1.3924	0
6980	402.1	2.8533	3.0297	0.8382	1.3928	0
6981	402	2.8348	3.0287	0.8379	1.3926	0
6982	401.9	2.8517	3.0278	0.838	1.393	0
6983	401.8	2.8333	3.055	0.8382	1.3916	0
6984	401.7	2.8502	3.0259	0.8383	1.3914	0
6985	401.6	2.8492	2.9991	0.8384	1.3925	0
6986	401.5	2.8483	2.9983	0.8385	1.3922	0
6987	401.4	2.8297	2.9974	0.8386	1.3927	0
6988	401.3	2.8844	3.0227	0.8384	1.3912	0
6989	401.2	2.8642	3.0218	0.8383	1.3935	0
6990	401.1	2.8636	3.0209	0.8385	1.3927	0
6991	401	2.8626	3.0205	0.8384	1.3938	0
6992	400.9	2.8435	3.0477	0.8387	1.393	0
6993	400.8	2.8611	3.0467	0.8389	1.3948	0
6994	400.7	2.8416	3.0177	0.8386	1.3938	0
6995	400.6	2.8407	3.0448	0.8386	1.393	0

6996	400.5	2.8778	3.0164	0.8385	1.3923	0
6997	400.4	2.8576	3.0155	0.8383	1.3933	0
6998	400.3	2.8567	3.0424	0.8385	1.3937	0
6999	400.2	2.8557	3.0137	0.8385	1.3941	0
7000	400.1	2.8365	3.0123	0.8386	1.3945	0
7001	400	2.8732	3.0395	0.8385	1.3949	0
7002	399.9	2.8722	3.0386	0.8384	1.3946	0
7003	399.8	2.8523	3.0101	0.8386	1.3938	0
7004	399.7	2.8706	3.0372	0.8382	1.3949	0
7005	399.6	2.8505	3.0079	0.8384	1.394	0
7006	399.5	2.869	3.0353	0.8386	1.3958	0
7007	399.4	2.8681	3.0343	0.8382	1.3962	0
7008	399.3	2.8671	3.0635	0.8378	1.3959	0
7009	399.2	2.8661	3.0625	0.8381	1.3944	0
7010	399.1	2.8652	3.0615	0.8384	1.3961	0
7011	399	2.8642	3.0926	0.838	1.3971	0
7012	398.9	2.8441	3.0297	0.838	1.3969	0
7013	398.8	2.8626	3.059	0.8385	1.3981	0
7014	398.7	2.8817	3.0278	0.838	1.397	0
7015	398.6	2.8604	3.057	0.8386	1.3961	0
7016	398.5	2.8595	3.056	0.8379	1.3979	0
7017	398.4	2.8589	3.055	0.8381	1.3977	0
7018	398.3	2.8778	3.0862	0.839	1.3988	0
7019	398.2	2.8567	3.0531	0.8385	1.4006	0
7020	398.1	2.8761	3.0521	0.8382	1.3989	0
7021	398	2.8752	3.0511	0.8382	1.3986	0
7022	397.9	2.8955	3.0205	0.8381	1.3999	0
7023	397.8	2.8732	3.0491	0.8383	1.3996	0
7024	397.7	2.8722	3.0804	0.8384	1.3992	0
7025	397.6	2.8713	3.0472	0.838	1.399	0
7026	397.5	2.8703	3.0783	0.8378	1.4001	0
7027	397.4	2.8693	3.0453	0.8381	1.4013	0
7028	397.3	2.8897	3.0768	0.838	1.3996	0
7029	397.2	2.8887	3.0434	0.8381	1.4001	0
7030	397.1	2.8877	3.1096	0.8384	1.4021	0
7031	397	2.8867	3.0737	0.8382	1.4018	0
7032	396.9	2.908	3.0731	0.838	1.4001	0
7033	396.8	2.8851	3.1068	0.8381	1.4007	0
7034	396.7	2.8841	3.0711	0.838	1.4003	0
7035	396.6	2.8831	3.0701	0.8382	1.4001	0
7036	396.5	2.8821	3.0691	0.8381	1.402	0
7037	396.4	2.9034	3.068	0.8385	1.4011	0
7038	396.3	2.8801	3.1018	0.8393	1.4015	0

7039	396.2	2.8794	3.0343	0.8383	1.4022	0
7040	396.1	2.8781	3.0655	0.8384	1.4018	0
7041	396	2.8996	3.0645	0.8385	1.4009	0
7042	395.9	2.8765	3.098	0.8385	1.4014	0
7043	395.8	2.9212	3.0975	0.8388	1.402	0
7044	395.7	2.8969	3.1343	0.8387	1.4025	0
7045	395.6	2.8959	3.0605	0.8388	1.4023	0
7046	395.5	2.8952	3.0947	0.8389	1.4015	0
7047	395.4	2.8938	3.0937	0.8389	1.4011	0
7048	395.3	2.9165	3.0926	0.8387	1.4002	0
7049	395.2	2.8921	3.1296	0.8383	1.4014	0
7050	395.1	2.869	3.056	0.8389	1.4013	0
7051	395	2.8901	3.0899	0.839	1.4011	0
7052	394.9	2.8891	3.0888	0.8393	1.4	0
7053	394.8	2.8884	3.0531	0.8395	1.4006	0
7054	394.7	2.9108	3.0521	0.8392	1.4011	0
7055	394.6	2.9097	3.1238	0.8392	1.4008	0
7056	394.5	2.909	3.1226	0.8395	1.4022	0
7057	394.4	2.8844	3.0841	0.8395	1.3997	0
7058	394.3	2.9069	3.0482	0.8392	1.4009	0
7059	394.2	2.9059	3.082	0.8395	1.4009	0
7060	394.1	2.8814	3.0809	0.8394	1.3998	0
7061	394	2.9038	3.0799	0.8394	1.4002	0
7062	393.9	2.8794	3.0788	0.8394	1.4	0
7063	393.8	2.9266	3.0778	0.8399	1.4005	0
7064	393.7	2.8775	3.1146	0.8404	1.3995	0
7065	393.6	2.9	3.1135	0.8402	1.3993	0
7066	393.5	2.8989	3.1129	0.84	1.399	0
7067	393.4	2.9226	3.0742	0.8401	1.4003	0
7068	393.3	2.8969	3.1524	0.8404	1.4001	0
7069	393.2	2.8959	3.1096	0.8402	1.399	0
7070	393.1	2.9197	3.109	0.8407	1.399	0
7071	393	2.8938	3.1079	0.8405	1.3993	0
7072	392.9	2.9176	3.1068	0.8405	1.3998	0
7073	392.8	2.8918	3.1057	0.8406	1.3988	0
7074	392.7	2.8908	3.1046	0.8407	1.3994	0
7075	392.6	2.9144	3.1035	0.8409	1.3998	0
7076	392.5	2.8887	3.1024	0.8405	1.4004	0
7077	392.4	2.9389	3.1013	0.8406	1.3978	0
7078	392.3	2.9115	3.1421	0.8408	1.3999	0
7079	392.2	2.9101	3.1409	0.8405	1.3995	0
7080	392.1	2.909	3.098	0.8401	1.4	0
7081	392	2.9344	3.0969	0.8412	1.399	0

7082	391.9	2.9069	3.1373	0.8399	1.3987	0
7083	391.8	2.9322	3.1367	0.84	1.3993	0
7084	391.7	2.9314	3.0942	0.8399	1.3999	0
7085	391.6	2.9038	3.1343	0.8401	1.4004	0
7086	391.5	2.9027	3.1331	0.8402	1.4008	0
7087	391.4	2.9017	3.1319	0.84	1.4013	0
7088	391.3	2.9547	3.1308	0.8402	1.4009	0
7089	391.2	2.8996	3.1302	0.8399	1.4016	0
7090	391.1	2.8983	3.0872	0.8402	1.4011	0
7091	391	2.9516	3.0482	0.84	1.4008	0
7092	390.9	2.9223	3.1267	0.8402	1.4005	0
7093	390.8	2.9492	3.1249	0.8401	1.4001	0
7094	390.7	2.9201	3.1244	0.8398	1.4008	0
7095	390.6	2.9469	3.1232	0.8403	1.4022	0
7096	390.5	2.9176	3.1221	0.8403	1.4017	0
7097	390.4	2.9446	3.1209	0.8404	1.4006	0
7098	390.3	2.9154	3.1198	0.8399	1.4019	0
7099	390.2	2.9423	3.0768	0.8403	1.4024	0
7100	390.1	2.9129	3.163	0.8407	1.4028	0
7101	390	2.9119	3.1618	0.8404	1.4016	0
7102	389.9	2.9385	3.1146	0.8402	1.4036	0
7103	389.8	2.9674	3.1135	0.84	1.4017	0
7104	389.7	2.9363	3.1124	0.84	1.4022	0
7105	389.6	2.9348	3.1113	0.8403	1.4035	0
7106	389.5	2.9337	3.1555	0.8399	1.4046	0
7107	389.4	2.9045	3.1543	0.8401	1.4043	0
7108	389.3	2.9031	3.153	0.8401	1.4029	0
7109	389.2	2.9296	3.1057	0.84	1.4042	0
7110	389.1	2.9285	3.1046	0.84	1.4039	0
7111	389	2.927	3.1035	0.8401	1.4043	0
7112	388.9	2.9559	3.1475	0.8397	1.4047	0
7113	388.8	2.9547	3.1007	0.8401	1.4052	0
7114	388.7	2.9531	3.1451	0.8397	1.4056	0
7115	388.6	2.9519	3.098	0.8401	1.4052	0
7116	388.5	2.983	3.1427	0.8398	1.4066	0
7117	388.4	2.9194	3.1415	0.8397	1.4061	0
7118	388.3	2.9477	3.1911	0.8396	1.4064	0
7119	388.2	2.9788	3.1385	0.8398	1.4062	0
7120	388.1	2.9454	3.0915	0.8399	1.4067	0
7121	388	2.9763	3.1361	0.8397	1.4053	0
7122	387.9	2.9423	3.0888	0.8397	1.4056	0
7123	387.8	2.9735	3.1331	0.8397	1.4071	0
7124	387.7	2.9401	3.1319	0.8398	1.4075	0

7125	387.6	2.9385	3.1308	0.8395	1.4071	0
7126	387.5	2.9374	3.1805	0.8393	1.4076	0
7127	387.4	2.9059	3.1278	0.84	1.4072	0
7128	387.3	2.9344	3.2358	0.8392	1.4068	0
7129	387.2	2.9654	3.1249	0.8396	1.4073	0
7130	387.1	2.9642	3.1238	0.8394	1.4068	0
7131	387	2.9626	3.1226	0.8399	1.4092	0
7132	386.9	2.9292	3.172	0.8399	1.4087	0
7133	386.8	2.9598	3.1707	0.8401	1.4074	0
7134	386.7	2.9935	3.118	0.8403	1.406	0
7135	386.6	2.9574	3.1169	0.8395	1.4075	0
7136	386.5	2.9905	3.1158	0.8402	1.4062	0
7137	386.4	2.9547	3.1656	0.8407	1.4068	0
7138	386.3	2.988	3.1129	0.8409	1.4073	0
7139	386.2	2.9867	3.1118	0.8411	1.4069	0
7140	386.1	2.9504	3.1612	0.8405	1.4065	0
7141	386	2.9169	3.1599	0.8404	1.407	0
7142	385.9	2.9825	3.1073	0.8405	1.4065	0
7143	385.8	2.9813	3.1574	0.841	1.4062	0
7144	385.7	2.945	3.1046	0.8408	1.4049	0
7145	385.6	2.9784	3.1035	0.841	1.4055	0
7146	385.5	2.9772	3.153	0.8413	1.4042	0
7147	385.4	2.9759	3.2097	0.8409	1.4056	0
7148	385.3	2.9743	3.1506	0.8409	1.4043	0
7149	385.2	2.9382	3.1494	0.8408	1.4039	0
7150	385.1	2.9718	3.2062	0.8412	1.4055	0
7151	385	3.0083	3.1463	0.8414	1.4051	0
7152	384.9	2.969	3.0937	0.8416	1.4057	0
7153	384.8	3.0052	3.1439	0.8419	1.4043	0
7154	384.7	3.0039	3.1421	0.8421	1.4049	0
7155	384.6	3.0026	3.1409	0.8421	1.4046	0
7156	384.5	2.9634	3.1397	0.8421	1.4042	0
7157	384.4	3	3.1385	0.8424	1.4038	0
7158	384.3	2.961	3.1367	0.8419	1.4035	0
7159	384.2	2.9594	3.1355	0.8419	1.4041	0
7160	384.1	2.9961	3.1343	0.842	1.4048	0
7161	384	2.9944	3.1904	0.8422	1.4034	0
7162	383.9	2.9931	3.1898	0.8423	1.4041	0
7163	383.8	2.9918	3.1884	0.8423	1.4028	0
7164	383.7	3.032	3.1864	0.8429	1.4034	0
7165	383.6	2.9512	3.1273	0.8432	1.4011	0
7166	383.5	3.0292	3.1838	0.8428	1.4028	0
7167	383.4	2.9863	3.1824	0.8431	1.4036	0

7168	383.3	3.0264	3.1811	0.8434	1.4032	0
7169	383.2	2.9838	3.1221	0.8433	1.4029	0
7170	383.1	2.9825	3.1209	0.8431	1.4026	0
7171	383	3.0223	3.1772	0.8434	1.4012	0
7172	382.9	2.9796	3.2426	0.8434	1.4019	0
7173	382.8	3.0195	3.1746	0.8436	1.4038	0
7174	382.7	2.9772	3.1733	0.8438	1.4035	0
7175	382.6	2.9755	3.1141	0.8437	1.4041	0
7176	382.5	2.9743	3.1707	0.8436	1.4028	0
7177	382.4	2.9727	3.1113	0.844	1.4025	0
7178	382.3	3.0128	3.1096	0.8434	1.4042	0
7179	382.2	3.0114	3.1662	0.8429	1.404	0
7180	382.1	3.0101	3.1649	0.8437	1.4048	0
7181	382	2.9674	3.2306	0.8433	1.4035	0
7182	381.9	3.0074	3.1624	0.8432	1.4041	0
7183	381.8	3.0516	3.1612	0.8434	1.4039	0
7184	381.7	3.0044	3.2262	0.8435	1.4047	0
7185	381.6	3.0035	3.158	0.8438	1.4046	0
7186	381.5	3.0477	3.1568	0.8437	1.4066	0
7187	381.4	3.0004	3.2226	0.8438	1.4051	0
7188	381.3	3.0448	3.2211	0.8438	1.407	0
7189	381.2	3.0429	3.1518	0.8439	1.4061	0
7190	381.1	3.0182	3.1831	0.8435	1.4058	0
7191	381	3.0168	3.2161	0.8438	1.4061	0
7192	380.9	3.0155	3.1798	0.8436	1.4059	0
7193	380.8	3.0376	3.1785	0.8442	1.4079	0
7194	380.7	3.0357	3.2118	0.844	1.4098	0
7195	380.6	3.011	3.2487	0.8439	1.409	0
7196	380.5	3.0329	3.1746	0.8437	1.4081	0
7197	380.4	3.0315	3.2076	0.8437	1.409	0
7198	380.3	3.0301	3.1713	0.8431	1.4092	0
7199	380.2	3.0287	3.1701	0.8433	1.409	0
7200	380.1	3.0783	3.2034	0.843	1.4087	0
7201	380	3.0506	3.1675	0.8431	1.4108	0
7202	379.9	3.0491	3.1656	0.8429	1.4103	0
7203	379.8	3.0227	3.1643	0.843	1.4102	0
7204	379.7	3.0218	3.163	0.8428	1.4106	0
7205	379.6	3.0448	3.1965	0.8431	1.4121	0
7206	379.5	3.0434	3.1945	0.8428	1.4117	0
7207	379.4	3.0173	3.1931	0.8427	1.411	0
7208	379.3	3.0405	3.2299	0.843	1.4112	0
7209	379.2	3.0391	3.1904	0.8429	1.4128	0
7210	379.1	3.0376	3.2262	0.8429	1.4131	0

7211	379	3.0625	3.2248	0.8427	1.4133	0
7212	378.9	3.0343	3.1858	0.8427	1.413	0
7213	378.8	3.0872	3.1844	0.8426	1.4128	0
7214	378.7	3.058	3.2204	0.8429	1.4126	0
7215	378.6	3.056	3.219	0.8423	1.4127	0
7216	378.5	3.0545	3.2175	0.8425	1.4143	0
7217	378.4	3.0535	3.1785	0.8424	1.4143	0
7218	378.3	3.0516	3.2147	0.8425	1.4158	0
7219	378.2	3.0501	3.2132	0.8425	1.4136	0
7220	378.1	3.0768	3.1733	0.8421	1.4145	0
7221	378	3.0752	3.2097	0.8428	1.4149	0
7222	377.9	3.1035	3.2083	0.8425	1.4151	0
7223	377.8	3.0721	3.1688	0.8427	1.4168	0
7224	377.7	3.0424	3.2048	0.8423	1.4159	0
7225	377.6	3.0685	3.2449	0.8426	1.4162	0
7226	377.5	3.0391	3.202	0.8425	1.4152	0
7227	377.4	3.0655	3.2418	0.8422	1.4161	0
7228	377.3	3.064	3.1986	0.8425	1.4164	0
7229	377.2	3.0921	3.2381	0.8428	1.4162	0
7230	377.1	3.0605	3.1952	0.8429	1.4152	0
7231	377	3.059	3.2351	0.8432	1.4148	0
7232	376.9	3.1198	3.1918	0.8432	1.4146	0
7233	376.8	3.0857	3.1904	0.8436	1.4163	0
7234	376.7	3.0841	3.2306	0.8436	1.4169	0
7235	376.6	3.0526	3.1878	0.8438	1.4166	0
7236	376.5	3.0511	3.2733	0.8435	1.4164	0
7237	376.4	3.1118	3.1844	0.8436	1.4162	0
7238	376.3	3.0783	3.224	0.8438	1.416	0
7239	376.2	3.1085	3.1811	0.8443	1.4165	0
7240	376.1	3.0752	3.1798	0.8434	1.4156	0
7241	376	3.0737	3.2197	0.8439	1.4154	0
7242	375.9	3.104	3.2636	0.8443	1.4145	0
7243	375.8	3.0701	3.2161	0.8444	1.4136	0
7244	375.7	3.1007	3.2147	0.8441	1.4162	0
7245	375.6	3.0991	3.2588	0.844	1.4146	0
7246	375.5	3.0975	3.2118	0.8447	1.413	0
7247	375.4	3.0958	3.2557	0.8449	1.4134	0
7248	375.3	3.129	3.2083	0.8451	1.4132	0
7249	375.2	3.0931	3.2069	0.8452	1.4131	0
7250	375.1	3.059	3.2055	0.8457	1.4136	0
7251	375	3.0899	3.2034	0.8449	1.4112	0
7252	374.9	3.0883	3.248	0.8453	1.4125	0
7253	374.8	3.1215	3.2464	0.8456	1.4123	0

7254	374.7	3.0851	3.2449	0.8455	1.4121	0
7255	374.6	3.0835	3.1972	0.8464	1.4112	0
7256	374.5	3.082	3.1959	0.8465	1.4112	0
7257	374.4	3.1146	3.2396	0.8462	1.4116	0
7258	374.3	3.0783	3.2381	0.8459	1.4114	0
7259	374.2	3.0768	3.2882	0.8465	1.4098	0
7260	374.1	3.1101	3.1891	0.8473	1.4111	0
7261	374	3.109	3.2848	0.8469	1.4098	0
7262	373.9	3.1451	3.2321	0.8466	1.4081	0
7263	373.8	3.1057	3.2815	0.8469	1.4081	0
7264	373.7	3.1421	3.2807	0.8467	1.4096	0
7265	373.6	3.1403	3.279	0.8476	1.4096	0
7266	373.5	3.1391	3.2774	0.8478	1.4103	0
7267	373.4	3.1373	3.2248	0.8475	1.4081	0
7268	373.3	3.1361	3.2233	0.847	1.4086	0
7269	373.2	3.1343	3.2218	0.8472	1.4087	0
7270	373.1	3.1331	3.2716	0.8474	1.4094	0
7271	373	3.1314	3.2182	0.8483	1.4086	0
7272	372.9	3.1713	3.2168	0.8482	1.4094	0
7273	372.8	3.1701	3.3251	0.8478	1.4094	0
7274	372.7	3.1273	3.3233	0.8478	1.408	0
7275	372.6	3.1669	3.2636	0.8482	1.411	0
7276	372.5	3.0862	3.3206	0.8481	1.4089	0
7277	372.4	3.1226	3.2097	0.8481	1.4082	0
7278	372.3	3.1624	3.2596	0.8485	1.4098	0
7279	372.2	3.2069	3.2581	0.8491	1.4108	0
7280	372.1	3.1599	3.2565	0.8486	1.4092	0
7281	372	3.1586	3.2041	0.8477	1.411	0
7282	371.9	3.1568	3.2541	0.8484	1.4111	0
7283	371.8	3.1555	3.3107	0.8484	1.408	0
7284	371.7	3.1543	3.251	0.8486	1.4106	0
7285	371.6	3.153	3.308	0.8484	1.4094	0
7286	371.5	3.1972	3.1972	0.848	1.4103	0
7287	371.4	3.15	3.3054	0.8486	1.4097	0
7288	371.3	3.1945	3.1945	0.8485	1.4099	0
7289	371.2	3.1475	3.3019	0.8489	1.4101	0
7290	371.1	3.1463	3.2426	0.8487	1.4105	0
7291	371	3.1445	3.2993	0.8482	1.4098	0
7292	370.9	3.1891	3.2984	0.8478	1.4101	0
7293	370.8	3.2388	3.2388	0.848	1.411	0
7294	370.7	3.1409	3.2373	0.8483	1.4098	0
7295	370.6	3.1391	3.3615	0.8476	1.4125	0
7296	370.5	3.1838	3.2924	0.8476	1.4128	0

7297	370.4	3.1361	3.2916	0.8476	1.4121	0
7298	370.3	3.1805	3.2899	0.8479	1.4132	0
7299	370.2	3.1798	3.3556	0.8475	1.4119	0
7300	370.1	3.1778	3.3546	0.8475	1.4122	0
7301	370	3.1772	3.2277	0.8474	1.4134	0
7302	369.9	3.1778	3.3028	0.8469	1.4254	0
7303	369.8	3.1791	3.3045	0.8468	1.4254	0
7304	369.7	3.1805	3.2596	0.8462	1.4261	0
7305	369.6	3.2197	3.3072	0.8462	1.426	0
7306	369.5	3.2211	3.308	0.8467	1.4265	0
7307	369.4	3.2218	3.2636	0.8464	1.4258	0
7308	369.3	3.1506	3.2644	0.8462	1.4282	0
7309	369.2	3.1518	3.266	0.8457	1.4268	0
7310	369.1	3.153	3.2668	0.8458	1.4285	0
7311	369	3.1884	3.2676	0.8459	1.4278	0
7312	368.9	3.1898	3.2692	0.8458	1.4289	0
7313	368.8	3.1911	3.2284	0.8453	1.4294	0
7314	368.7	3.1918	3.317	0.8456	1.431	0
7315	368.6	3.2306	3.2725	0.8454	1.4313	0
7316	368.5	3.2321	3.2733	0.8454	1.4306	0
7317	368.4	3.1952	3.2741	0.8455	1.4329	0
7318	368.3	3.1959	3.2749	0.8451	1.432	0
7319	368.2	3.1972	3.2765	0.845	1.4324	0
7320	368.1	3.1979	3.2358	0.845	1.4321	0
7321	368	3.1993	3.2782	0.8452	1.4324	0
7322	367.9	3.2	3.279	0.8448	1.4333	0
7323	367.8	3.2007	3.3261	0.8447	1.4336	0
7324	367.7	3.2013	3.2807	0.8446	1.4339	0
7325	367.6	3.1681	3.2823	0.8451	1.4343	0
7326	367.5	3.2034	3.2832	0.8448	1.434	0
7327	367.4	3.2426	3.2426	0.8444	1.435	0
7328	367.3	3.2055	3.2434	0.8445	1.4352	0
7329	367.2	3.2441	3.2857	0.8443	1.4335	0
7330	367.1	3.2069	3.2449	0.8447	1.435	0
7331	367	3.2076	3.2874	0.8445	1.4345	0
7332	366.9	3.2464	3.2464	0.8446	1.4348	0
7333	366.8	3.2097	3.289	0.8443	1.435	0
7334	366.7	3.2104	3.2899	0.8451	1.4358	0
7335	366.6	3.2111	3.2487	0.8447	1.4359	0
7336	366.5	3.2118	3.2907	0.8446	1.4361	0
7337	366.4	3.2125	3.2916	0.8442	1.435	0
7338	366.3	3.251	3.2924	0.8449	1.4345	0
7339	366.2	3.2518	3.2518	0.8451	1.4351	0

7340	366.1	3.2147	3.3391	0.8447	1.4352	0
7341	366	3.2154	3.2941	0.8452	1.4354	0
7342	365.9	3.2541	3.295	0.8453	1.4349	0
7343	365.8	3.2541	3.2958	0.8453	1.4355	0
7344	365.7	3.2175	3.2967	0.8452	1.4356	0
7345	365.6	3.2182	3.2976	0.8451	1.4362	0
7346	365.5	3.2565	3.3439	0.8452	1.4356	0
7347	365.4	3.2573	3.2984	0.8459	1.4346	0
7348	365.3	3.2197	3.2993	0.846	1.4353	0
7349	365.2	3.2581	3.2993	0.8456	1.4346	0
7350	365.1	3.2588	3.3002	0.8461	1.434	0
7351	365	3.2218	3.301	0.8463	1.4335	0
7352	364.9	3.2604	3.2604	0.8466	1.4347	0
7353	364.8	3.2226	3.3019	0.8471	1.4346	0
7354	364.7	3.2233	3.3028	0.8473	1.4335	0
7355	364.6	3.224	3.3487	0.8474	1.4346	0
7356	364.5	3.2248	3.3497	0.8476	1.4333	0
7357	364.4	3.2628	3.3497	0.8475	1.4326	0
7358	364.3	3.2636	3.3045	0.8476	1.432	0
7359	364.2	3.2636	3.3054	0.8481	1.4312	0
7360	364.1	3.2644	3.3054	0.8483	1.4318	0
7361	364	3.2644	3.3516	0.848	1.4304	0
7362	363.9	3.3063	3.3526	0.849	1.4303	0
7363	363.8	3.3072	3.4034	0.8486	1.43	0
7364	363.7	3.266	3.3072	0.8488	1.4294	0
7365	363.6	3.266	3.308	0.849	1.4308	0
7366	363.5	3.2291	3.3536	0.8487	1.4295	0
7367	363.4	3.2291	3.308	0.849	1.4276	0
7368	363.3	3.2676	3.3546	0.8492	1.4308	0
7369	363.2	3.3089	3.3089	0.85	1.4295	0
7370	363.1	3.2684	3.3556	0.8497	1.4292	0
7371	363	3.3098	3.3556	0.8501	1.4289	0
7372	362.9	3.2684	3.3556	0.8498	1.4287	0
7373	362.8	3.2692	3.3107	0.8502	1.429	0
7374	362.7	3.3107	3.3565	0.8503	1.4292	0
7375	362.6	3.3107	3.3107	0.85	1.4288	0
7376	362.5	3.3107	3.3107	0.85	1.4297	0
7377	362.4	3.27	3.3116	0.8506	1.4299	0
7378	362.3	3.3116	3.3116	0.8503	1.4284	0
7379	362.2	3.3116	3.3116	0.8505	1.4303	0
7380	362.1	3.3116	3.3116	0.8507	1.4305	0
7381	362	3.3125	3.3125	0.8504	1.4306	0
7382	361.9	3.3125	3.2708	0.8512	1.4303	0

7383	361.8	3.2708	3.3125	0.8502	1.4303	0
7384	361.7	3.3125	3.3585	0.8505	1.4299	0
7385	361.6	3.3125	3.3585	0.8505	1.4295	0
7386	361.5	3.3125	3.3125	0.8508	1.4302	0
7387	361.4	3.2716	3.3585	0.8509	1.4292	0
7388	361.3	3.3125	3.3585	0.8511	1.4298	0
7389	361.2	3.3134	3.2716	0.851	1.4304	0
7390	361.1	3.3585	3.3585	0.8512	1.4294	0
7391	361	3.3585	3.3585	0.8509	1.4288	0
7392	360.9	3.3585	3.3585	0.851	1.43	0
7393	360.8	3.3585	3.3585	0.8511	1.4306	0
7394	360.7	3.3134	3.3585	0.8511	1.4295	0
7395	360.6	3.3134	3.3585	0.8511	1.4301	0
7396	360.5	3.3134	3.4101	0.8512	1.4317	0
7397	360.4	3.3585	3.3585	0.8511	1.4317	0
7398	360.3	3.3134	3.3134	0.8511	1.433	0
7399	360.2	3.3585	3.3585	0.8509	1.4324	0
7400	360.1	3.3134	3.3585	0.8511	1.4305	0
7401	360	3.3585	3.3134	0.8514	1.4321	0
7402	359.9	3.3585	3.3125	0.8509	1.4327	0
7403	359.8	3.3585	3.3585	0.8512	1.4314	0
7404	359.7	3.4101	3.4101	0.8512	1.4324	0
7405	359.6	3.3585	3.3585	0.851	1.4335	0
7406	359.5	3.3125	3.3585	0.8505	1.4346	0
7407	359.4	3.3585	3.3585	0.8505	1.4334	0
7408	359.3	3.4089	3.3585	0.8504	1.4327	0
7409	359.2	3.3575	3.3575	0.8503	1.4336	0
7410	359.1	3.4089	3.4089	0.8506	1.4352	0
7411	359	3.3575	3.3575	0.8508	1.435	0
7412	358.9	3.4089	3.3575	0.8508	1.4354	0
7413	358.8	3.3575	3.4089	0.8508	1.4359	0
7414	358.7	3.4078	3.4078	0.8498	1.4356	0
7415	358.6	3.4078	3.3565	0.8494	1.4377	0
7416	358.5	3.3565	3.4078	0.8496	1.438	0
7417	358.4	3.4078	3.4078	0.8496	1.439	0
7418	358.3	3.3556	3.3556	0.8492	1.4369	0
7419	358.2	3.4067	3.3098	0.8495	1.4391	0
7420	358.1	3.4067	3.3556	0.8496	1.4401	0
7421	358	3.4067	3.3556	0.8495	1.4416	0
7422	357.9	3.4647	3.3556	0.849	1.4408	0
7423	357.8	3.3546	3.3546	0.849	1.4417	0
7424	357.7	3.3546	3.4056	0.8485	1.4413	0
7425	357.6	3.3546	3.4056	0.8484	1.4417	0

7426	357.5	3.4056	3.3536	0.8483	1.4426	0
7427	357.4	3.3536	3.3536	0.8484	1.4423	0
7428	357.3	3.4045	3.4622	0.8485	1.4432	0
7429	357.2	3.4045	3.3526	0.8482	1.4434	0
7430	357.1	3.3526	3.4034	0.8482	1.4443	0
7431	357	3.4622	3.4622	0.8482	1.4421	0
7432	356.9	3.4034	3.3516	0.8479	1.4436	0
7433	356.8	3.4034	3.4034	0.8479	1.4445	0
7434	356.7	3.4023	3.4023	0.848	1.4447	0
7435	356.6	3.4597	3.3507	0.8481	1.4449	0
7436	356.5	3.4597	3.4012	0.8482	1.4451	0
7437	356.4	3.3507	3.3507	0.848	1.4466	0
7438	356.3	3.4012	3.4012	0.8482	1.4456	0
7439	356.2	3.3497	3.4001	0.8482	1.4445	0
7440	356.1	3.4001	3.4584	0.8483	1.4454	0
7441	356	3.4001	3.4001	0.8481	1.4462	0
7442	355.9	3.399	3.399	0.8476	1.4464	0
7443	355.8	3.4572	3.4572	0.8482	1.4454	0
7444	355.7	3.4559	3.399	0.8483	1.4455	0
7445	355.6	3.3979	3.3979	0.8487	1.4456	0
7446	355.5	3.5229	3.3979	0.8481	1.4452	0
7447	355.4	3.4547	3.3969	0.8484	1.4454	0
7448	355.3	3.4547	3.4547	0.8485	1.4454	0
7449	355.2	3.3958	3.3958	0.8483	1.4449	0
7450	355.1	3.3958	3.4535	0.8488	1.4449	0
7451	355	3.4535	3.52	0.8492	1.4463	0
7452	354.9	3.4522	3.3947	0.849	1.4459	0
7453	354.8	3.4522	3.3936	0.8491	1.4453	0
7454	354.7	3.3936	3.4522	0.8496	1.4455	0
7455	354.6	3.451	3.451	0.8493	1.4444	0
7456	354.5	3.3925	3.451	0.8494	1.4444	0
7457	354.4	3.4498	3.4498	0.85	1.4445	0
7458	354.3	3.4498	3.4498	0.8503	1.4434	0
7459	354.2	3.4486	3.3904	0.8503	1.4428	0
7460	354.1	3.4486	3.4486	0.8504	1.4441	0
7461	354	3.4473	3.3893	0.8501	1.4441	0
7462	353.9	3.4473	3.3893	0.8506	1.4435	0
7463	353.8	3.5129	3.3883	0.8506	1.4427	0
7464	353.7	3.4461	3.4461	0.8511	1.4433	0
7465	353.6	3.4449	3.3872	0.8515	1.4434	0
7466	353.5	3.5114	3.3862	0.8517	1.442	0
7467	353.4	3.4437	3.4437	0.8521	1.4414	0
7468	353.3	3.4437	3.3851	0.8528	1.442	0

7469	353.2	3.51	3.4425	0.8528	1.4401	0
7470	353.1	3.4413	3.384	0.8527	1.4412	0
7471	353	3.5086	3.4413	0.8527	1.4393	0
7472	352.9	3.4401	3.4401	0.8526	1.4391	0
7473	352.8	3.5072	3.4401	0.8527	1.4402	0
7474	352.7	3.5058	3.4389	0.8534	1.4403	0
7475	352.6	3.5058	3.3799	0.8538	1.4402	0
7476	352.5	3.5045	3.5045	0.854	1.4419	0
7477	352.4	3.4365	3.4365	0.8538	1.4406	0
7478	352.3	3.5031	3.4365	0.8533	1.4417	0
7479	352.2	3.5017	3.4353	0.8538	1.4404	0
7480	352.1	3.5017	3.4342	0.8539	1.4415	0
7481	352	3.5003	3.3757	0.8538	1.4408	0
7482	351.9	3.3747	3.433	0.8537	1.4419	0
7483	351.8	3.4989	3.433	0.8541	1.4399	0
7484	351.7	3.4989	3.4318	0.8541	1.4404	0
7485	351.6	3.4976	3.4306	0.8543	1.4409	0
7486	351.5	3.4976	3.4306	0.8546	1.4403	0
7487	351.4	3.5751	3.4295	0.8547	1.4383	0
7488	351.3	3.4962	3.4283	0.8547	1.4414	0
7489	351.2	3.4283	3.4949	0.8549	1.4405	0
7490	351.1	3.5735	3.4935	0.855	1.4416	0
7491	351	3.4935	3.4935	0.8548	1.4396	0
7492	350.9	3.4921	3.426	0.8551	1.4395	0
7493	350.8	3.5719	3.4921	0.855	1.4402	0
7494	350.7	3.5702	3.4237	0.8552	1.44	0
7495	350.6	3.4895	3.4895	0.8551	1.4409	0
7496	350.5	3.4895	3.4895	0.8552	1.4408	0
7497	350.4	3.4881	3.4881	0.855	1.4406	0
7498	350.3	3.567	3.4214	0.8554	1.4412	0
7499	350.2	3.4868	3.4202	0.8546	1.4409	0
7500	350.1	3.5654	3.4191	0.8555	1.4427	0
7501	350	3.5638	3.4179	0.8554	1.4406	0
7502	349.9	3.5638	3.4841	0.8557	1.4411	0
7503	349.8	3.6596	3.4168	0.8557	1.4407	0
7504	349.7	3.6596	3.4157	0.8559	1.4398	0
7505	349.6	3.4815	3.4145	0.8556	1.4416	0
7506	349.5	3.5591	3.4802	0.8557	1.4406	0
7507	349.4	3.5591	3.4123	0.8556	1.4417	0
7508	349.3	3.4789	3.4123	0.8551	1.442	0
7509	349.2	3.5575	3.4112	0.8556	1.443	0
7510	349.1	3.556	3.4763	0.8555	1.442	0
7511	349	3.6517	3.4089	0.8553	1.4424	0

7512	348.9	3.5544	3.475	0.8553	1.4427	0
7513	348.8	3.5528	3.4737	0.8551	1.4444	0
7514	348.7	3.5513	3.4056	0.8545	1.444	0
7515	348.6	3.5513	3.4045	0.8548	1.445	0
7516	348.5	3.6459	3.4034	0.8549	1.4447	0
7517	348.4	3.6459	3.4023	0.855	1.4463	0
7518	348.3	3.5482	3.4012	0.8544	1.4458	0
7519	348.2	3.644	3.4001	0.8547	1.447	0
7520	348.1	3.5452	3.399	0.8548	1.4467	0
7521	348	3.6421	3.3979	0.8545	1.4469	0
7522	347.9	3.6402	3.3969	0.8547	1.4486	0
7523	347.8	3.6402	3.4634	0.8544	1.4484	0
7524	347.7	3.5406	3.3947	0.8547	1.4465	0
7525	347.6	3.5406	3.4609	0.8551	1.449	0
7526	347.5	3.762	3.3936	0.8548	1.448	0
7527	347.4	3.6345	3.4584	0.854	1.4482	0
7528	347.3	3.6345	3.4584	0.8544	1.4479	0
7529	347.2	3.5361	3.4572	0.8542	1.4489	0
7530	347.1	3.6326	3.4559	0.8537	1.4493	0
7531	347	3.5331	3.4547	0.8544	1.4503	0
7532	346.9	3.6289	3.5331	0.8537	1.45	0
7533	346.8	3.6289	3.4522	0.8542	1.4512	0
7534	346.7	3.6271	3.451	0.8542	1.4508	0
7535	346.6	3.5302	3.451	0.8541	1.4513	0
7536	346.5	3.5287	3.4498	0.854	1.451	0
7537	346.4	3.6253	3.4486	0.8543	1.452	0
7538	346.3	3.5258	3.4473	0.8538	1.4516	0
7539	346.2	3.5258	3.5258	0.8537	1.4519	0
7540	346.1	3.6216	3.4449	0.8538	1.4531	0
7541	346	3.6198	3.3768	0.8542	1.4513	0
7542	345.9	3.6198	3.4425	0.8542	1.4531	0
7543	345.8	3.618	3.4413	0.8547	1.4534	0
7544	345.7	3.6162	3.52	0.8544	1.4523	0
7545	345.6	3.5186	3.4389	0.8543	1.4519	0
7546	345.5	3.6144	3.5171	0.8544	1.4522	0
7547	345.4	3.6126	3.4365	0.8542	1.4532	0
7548	345.3	3.7375	3.5157	0.8541	1.4514	0
7549	345.2	3.5143	3.5143	0.8542	1.4517	0
7550	345.1	3.7352	3.5129	0.8544	1.4521	0
7551	345	3.6091	3.5114	0.8543	1.4524	0
7552	344.9	3.7328	3.51	0.8549	1.4512	0
7553	344.8	3.7305	3.4306	0.8547	1.4523	0
7554	344.7	3.5086	3.4283	0.8548	1.4519	0

7555	344.6	3.6038	3.5072	0.8546	1.4515	0
7556	344.5	3.7282	3.5058	0.8547	1.4518	0
7557	344.4	3.6021	3.5045	0.8553	1.4506	0
7558	344.3	3.7258	3.5031	0.8558	1.4516	0
7559	344.2	3.7235	3.4225	0.8561	1.4514	0
7560	344.1	3.5986	3.5003	0.8557	1.4517	0
7561	344	3.8962	3.5003	0.8556	1.4489	0
7562	343.9	3.8962	3.4191	0.8561	1.4507	0
7563	343.8	3.719	3.4179	0.8561	1.4511	0
7564	343.7	3.5935	3.4168	0.8558	1.4498	0
7565	343.6	3.8928	3.4157	0.8563	1.4495	0
7566	343.5	3.5901	3.4145	0.8565	1.4489	0
7567	343.4	3.7144	3.4921	0.8564	1.4486	0
7568	343.3	3.7122	3.5884	0.8567	1.4488	0
7569	343.2	3.7122	3.4895	0.8568	1.4493	0
7570	343.1	3.71	3.4089	0.8571	1.4487	0
7571	343	3.8861	3.4868	0.8572	1.4498	0
7572	342.9	3.7077	3.4855	0.8571	1.4494	0
7573	342.8	3.7055	3.5817	0.857	1.4481	0
7574	342.7	3.8827	3.58	0.8571	1.4484	0
7575	342.6	3.7033	3.4034	0.8573	1.448	0
7576	342.5	3.7033	3.4815	0.8582	1.4485	0
7577	342.4	3.5768	3.4802	0.8578	1.4504	0
7578	342.3	3.5751	3.4789	0.8579	1.4499	0
7579	342.2	3.8761	3.4776	0.8577	1.4478	0
7580	342.1	3.8729	3.3298	0.8578	1.4497	0
7581	342	3.6968	3.475	0.8574	1.4499	0
7582	341.9	3.5702	3.4737	0.8579	1.4496	0
7583	341.8	3.6946	3.4724	0.8579	1.4499	0
7584	341.7	3.8697	3.4711	0.8584	1.4495	0
7585	341.6	3.6904	3.4698	0.8586	1.4506	0
7586	341.5	3.6904	3.4685	0.859	1.4484	0
7587	341.4	3.5638	3.466	0.8588	1.4495	0
7588	341.3	3.5622	3.4647	0.8586	1.4498	0
7589	341.2	3.6861	3.4634	0.8583	1.4501	0
7590	341.1	3.5591	3.5591	0.8584	1.4487	0
7591	341	3.6819	3.4609	0.859	1.4499	0
7592	340.9	3.6819	3.556	0.8598	1.4511	0
7593	340.8	3.6799	3.4584	0.8592	1.4505	0
7594	340.7	3.8539	3.4572	0.8593	1.4518	0
7595	340.6	3.8539	3.5528	0.8594	1.4503	0
7596	340.5	3.6757	3.4547	0.8593	1.4507	0
7597	340.4	3.6737	3.4522	0.8592	1.4501	0

7598	340.3	3.6737	3.451	0.8589	1.4522	0
7599	340.2	3.8477	3.4498	0.8593	1.4508	0
7600	340.1	3.6696	3.5452	0.859	1.4519	0
7601	340	3.6696	3.4473	0.8594	1.4506	0
7602	339.9	1.3106	2.0156	0.8088	1.3778	0
7603	339.8	1.3095	2.0141	0.8086	1.3773	0
7604	339.7	1.3081	2.0127	0.8084	1.3769	0
7605	339.6	1.3068	2.0135	0.8085	1.3766	0
7606	339.5	1.3054	2.01	0.8086	1.3777	0
7607	339.4	1.3045	2.0107	0.8081	1.3768	0
7608	339.3	1.303	2.0094	0.8079	1.3769	0
7609	339.2	1.3023	2.006	0.8079	1.3772	0
7610	339.1	1.3007	2.0044	0.8078	1.3756	0
7611	339	1.2989	2.0052	0.8076	1.3758	0
7612	338.9	1.2983	2.0016	0.8074	1.3759	0
7613	338.8	1.2973	2.0023	0.8072	1.376	0
7614	338.7	1.2964	1.9988	0.8071	1.3756	0
7615	338.6	1.2945	1.9973	0.8066	1.3757	0
7616	338.5	1.2931	1.9959	0.8062	1.3748	0
7617	338.4	1.2921	1.9945	0.8061	1.3755	0
7618	338.3	1.2908	1.9975	0.8062	1.3758	0
7619	338.2	1.2898	1.994	0.8056	1.3749	0
7620	338.1	1.2875	1.9904	0.8053	1.3745	0
7621	338	1.2869	1.9867	0.8052	1.3746	0
7622	337.9	1.2865	1.9898	0.805	1.3738	0
7623	337.8	1.2846	1.9862	0.8046	1.3734	0
7624	337.7	1.2838	1.9871	0.8051	1.3733	0
7625	337.6	1.283	1.9837	0.8043	1.3741	0
7626	337.5	1.282	1.9844	0.8041	1.3732	0
7627	337.4	1.2807	1.9853	0.8039	1.3725	0
7628	337.3	1.2796	1.9816	0.804	1.372	0
7629	337.2	1.2784	1.9783	0.8038	1.3719	0
7630	337.1	1.277	1.9768	0.8032	1.371	0
7631	337	1.2761	1.9777	0.8033	1.3708	0
7632	336.9	1.2752	1.9763	0.8033	1.371	0
7633	336.8	1.2744	1.9751	0.8029	1.3709	0
7634	336.7	1.2736	1.9739	0.8029	1.3713	0
7635	336.6	1.2726	1.9725	0.8025	1.3699	0
7636	336.5	1.2709	1.9711	0.8024	1.3707	0
7637	336.4	1.2705	1.9678	0.8025	1.3701	0
7638	336.3	1.2682	1.9685	0.8021	1.3691	0
7639	336.2	1.2679	1.9673	0.8019	1.3685	0
7640	336.1	1.2671	1.9661	0.8021	1.3695	0

7641	336	1.2658	1.9648	0.8018	1.3682	0
7642	335.9	1.2645	1.9635	0.8015	1.3686	0
7643	335.8	1.2636	1.9622	0.8012	1.3683	0
7644	335.7	1.2625	1.961	0.8011	1.3688	0
7645	335.6	1.2615	1.9596	0.8008	1.368	0
7646	335.5	1.2608	1.9585	0.8007	1.3663	0
7647	335.4	1.2595	1.9571	0.8008	1.3655	0
7648	335.3	1.2587	1.9559	0.8007	1.3671	0
7649	335.2	1.2571	1.9526	0.8008	1.3665	0
7650	335.1	1.2568	1.9536	0.8008	1.3653	0
7651	335	1.2551	1.9523	0.8005	1.3652	0
7652	334.9	1.2547	1.9511	0.8003	1.3639	0
7653	334.8	1.2531	1.9542	0.8002	1.3639	0
7654	334.7	1.2524	1.9488	0.8002	1.3639	0
7655	334.6	1.2516	1.9475	0.8006	1.3648	0
7656	334.5	1.2503	1.9462	0.7996	1.3636	0
7657	334.4	1.2492	1.9451	0.7996	1.3624	0
7658	334.3	1.249	1.944	0.7996	1.3631	0
7659	334.2	1.2481	1.9427	0.7995	1.3618	0
7660	334.1	1.2459	1.9414	0.7993	1.3605	0
7661	334	1.2448	1.9402	0.7989	1.3616	0
7662	333.9	1.2444	1.939	0.799	1.361	0
7663	333.8	1.2432	1.9378	0.7992	1.3597	0
7664	333.7	1.2424	1.9388	0.7992	1.3585	0
7665	333.6	1.2413	1.9333	0.799	1.3579	0
7666	333.5	1.2401	1.9342	0.7986	1.3579	0
7667	333.4	1.2394	1.9331	0.7988	1.3579	0
7668	333.3	1.2386	1.9341	0.7987	1.3579	0
7669	333.2	1.2379	1.9329	0.7985	1.3573	0
7670	333.1	1.2362	1.9295	0.798	1.3572	0
7671	333	1.2354	1.9261	0.7977	1.3565	0
7672	332.9	1.2342	1.9293	0.7982	1.3554	0
7673	332.8	1.2331	1.9259	0.7982	1.3542	0
7674	332.7	1.2328	1.9226	0.7982	1.3548	0
7675	332.6	1.2313	1.9259	0.7977	1.3555	0
7676	332.5	1.2309	1.9224	0.7974	1.3542	0
7677	332.4	1.2298	1.9213	0.7975	1.3537	0
7678	332.3	1.2291	1.9201	0.7978	1.3549	0
7679	332.2	1.2275	1.919	0.7974	1.3531	0
7680	332.1	1.2262	1.9199	0.7968	1.3543	0
7681	332	1.226	1.9166	0.7971	1.3532	0
7682	331.9	1.2253	1.9134	0.7969	1.3538	0
7683	331.8	1.2232	1.9121	0.7967	1.3514	0

7684	331.7	1.2225	1.9132	0.7966	1.3521	0
7685	331.6	1.2214	1.912	0.7963	1.3527	0
7686	331.5	1.2202	1.9108	0.7961	1.3515	0
7687	331.4	1.22	1.9098	0.7958	1.3529	0
7688	331.3	1.2193	1.9086	0.7959	1.3505	0
7689	331.2	1.2182	1.9053	0.7952	1.3506	0
7690	331.1	1.2169	1.9063	0.7948	1.3506	0
7691	331	1.2158	1.903	0.7952	1.3512	0
7692	330.9	1.2147	1.9018	0.7947	1.3501	0
7693	330.8	1.214	1.9007	0.7945	1.3508	0
7694	330.7	1.2132	1.9016	0.7944	1.3496	0
7695	330.6	1.212	1.8983	0.7942	1.349	0
7696	330.5	1.2112	1.897	0.794	1.3496	0
7697	330.4	1.2101	1.8981	0.7938	1.3497	0
7698	330.3	1.209	1.8969	0.7936	1.3485	0
7699	330.2	1.2087	1.8958	0.7933	1.3492	0
7700	330.1	1.207	1.8945	0.7933	1.3473	0
7701	330	1.2063	1.8933	0.7931	1.3468	0
7702	329.9	1.2051	1.8922	0.7934	1.3481	0
7703	329.8	1.2043	1.891	0.7933	1.3475	0
7704	329.7	1.2027	1.8898	0.7929	1.3476	0
7705	329.6	1.2019	1.8885	0.7924	1.3475	0
7706	329.5	1.2015	1.8873	0.7923	1.3476	0
7707	329.4	1.2	1.8841	0.7923	1.3478	0
7708	329.3	1.1996	1.8849	0.7925	1.3471	0
7709	329.2	1.198	1.8859	0.7921	1.3466	0
7710	329.1	1.197	1.8845	0.7914	1.347	0
7711	329	1.1958	1.8832	0.7911	1.3457	0
7712	328.9	1.1941	1.8799	0.7908	1.3471	0
7713	328.8	1.1938	1.8765	0.7909	1.3459	0
7714	328.7	1.1926	1.8774	0.7907	1.3453	0
7715	328.6	1.1919	1.8741	0.7903	1.3461	0
7716	328.5	1.1906	1.875	0.7902	1.3448	0
7717	328.4	1.1894	1.8738	0.7895	1.3442	0
7718	328.3	1.1886	1.8725	0.7896	1.3443	0
7719	328.2	1.1878	1.8713	0.7894	1.345	0
7720	328.1	1.1865	1.87	0.7892	1.3437	0
7721	328	1.1853	1.8688	0.7889	1.3431	0
7722	327.9	1.184	1.8675	0.789	1.3431	0
7723	327.8	1.1829	1.8685	0.7884	1.3433	0
7724	327.7	1.182	1.8651	0.7884	1.342	0
7725	327.6	1.1809	1.864	0.7888	1.3415	0
7726	327.5	1.1802	1.8649	0.7881	1.341	0

7727	327.4	1.1785	1.8616	0.7882	1.3405	0
7728	327.3	1.1777	1.8603	0.7878	1.3398	0
7729	327.2	1.1769	1.8591	0.7877	1.3386	0
7730	327.1	1.1756	1.8578	0.7874	1.3393	0
7731	327	1.1754	1.8588	0.7872	1.3388	0
7732	326.9	1.1737	1.8575	0.7872	1.3382	0
7733	326.8	1.1726	1.8565	0.7872	1.3377	0
7734	326.7	1.1713	1.853	0.787	1.3378	0
7735	326.6	1.171	1.8539	0.7867	1.3365	0
7736	326.5	1.1693	1.8506	0.7867	1.3367	0
7737	326.4	1.1681	1.8494	0.7862	1.3368	0
7738	326.3	1.1674	1.8503	0.786	1.3356	0
7739	326.2	1.1662	1.8492	0.7861	1.3358	0
7740	326.1	1.165	1.848	0.7862	1.3366	0
7741	326	1.1647	1.8446	0.7856	1.3354	0
7742	325.9	1.1634	1.8433	0.7854	1.3341	0
7743	325.8	1.1622	1.84	0.7852	1.3336	0
7744	325.7	1.161	1.8387	0.7855	1.333	0
7745	325.6	1.1602	1.8418	0.7852	1.3325	0
7746	325.5	1.1595	1.8385	0.7859	1.3333	0
7747	325.4	1.1582	1.8372	0.7851	1.3314	0
7748	325.3	1.1566	1.836	0.7852	1.3309	0
7749	325.2	1.1567	1.8348	0.785	1.3303	0
7750	325.1	1.155	1.8335	0.7851	1.3297	0
7751	325	1.1544	1.8324	0.7851	1.3279	0
7752	324.9	1.1532	1.8312	0.7845	1.3288	0
7753	324.8	1.1519	1.83	0.7849	1.3276	0
7754	324.7	1.1502	1.8266	0.785	1.327	0
7755	324.6	1.15	1.8276	0.7848	1.3272	0
7756	324.5	1.1493	1.8243	0.7847	1.3275	0
7757	324.4	1.1481	1.8253	0.7841	1.3263	0
7758	324.3	1.1473	1.824	0.784	1.3257	0
7759	324.2	1.1461	1.8228	0.784	1.3245	0
7760	324.1	1.1446	1.8239	0.7835	1.3234	0
7761	324	1.1434	1.8205	0.7835	1.3243	0
7762	323.9	1.1422	1.8194	0.7843	1.3231	0
7763	323.8	1.1416	1.8204	0.7834	1.3227	0
7764	323.7	1.1403	1.8192	0.7833	1.3221	0
7765	323.6	1.1396	1.8158	0.7832	1.3217	0
7766	323.5	1.138	1.8168	0.7835	1.3205	0
7767	323.4	1.1368	1.8157	0.7835	1.3201	0
7768	323.3	1.1361	1.8124	0.7832	1.3203	0
7769	323.2	1.1346	1.8092	0.783	1.3193	0

7770	323.1	1.1341	1.8103	0.783	1.3183	0
7771	323	1.1329	1.807	0.7828	1.3192	0
7772	322.9	1.1322	1.808	0.7823	1.3187	0
7773	322.8	1.1311	1.8047	0.7824	1.3176	0
7774	322.7	1.13	1.8014	0.7825	1.3179	0
7775	322.6	1.1289	1.8047	0.782	1.3168	0
7776	322.5	1.1276	1.8055	0.782	1.3155	0
7777	322.4	1.1265	1.8001	0.7813	1.3151	0
7778	322.3	1.1253	1.7989	0.7816	1.3161	0
7779	322.2	1.1245	1.7999	0.7809	1.3148	0
7780	322.1	1.1237	1.799	0.7814	1.3147	0
7781	322	1.1229	1.7978	0.7811	1.3142	0
7782	321.9	1.1217	1.7966	0.7804	1.3151	0
7783	321.8	1.121	1.7933	0.7801	1.3154	0
7784	321.7	1.1201	1.7944	0.7801	1.3152	0
7785	321.6	1.1195	1.7934	0.7801	1.3134	0
7786	321.5	1.1184	1.7923	0.7801	1.3138	0
7787	321.4	1.1167	1.7889	0.7795	1.3132	0
7788	321.3	1.1161	1.79	0.7793	1.3129	0
7789	321.2	1.1154	1.7889	0.7786	1.3118	0
7790	321.1	1.1142	1.7834	0.7785	1.3113	0
7791	321	1.1128	1.7845	0.7785	1.311	0
7792	320.9	1.1121	1.7834	0.7783	1.3099	0
7793	320.8	1.111	1.7823	0.7782	1.3103	0
7794	320.7	1.1108	1.7812	0.778	1.3106	0
7795	320.6	1.1097	1.7801	0.7779	1.3102	0
7796	320.5	1.1081	1.779	0.7783	1.3098	0
7797	320.4	1.1078	1.7756	0.7771	1.3101	0
7798	320.3	1.1068	1.7789	0.7778	1.3098	0
7799	320.2	1.106	1.7755	0.7772	1.3108	0
7800	320.1	1.1048	1.7743	0.7767	1.3089	0
7801	320	1.1033	1.7732	0.7758	1.3077	

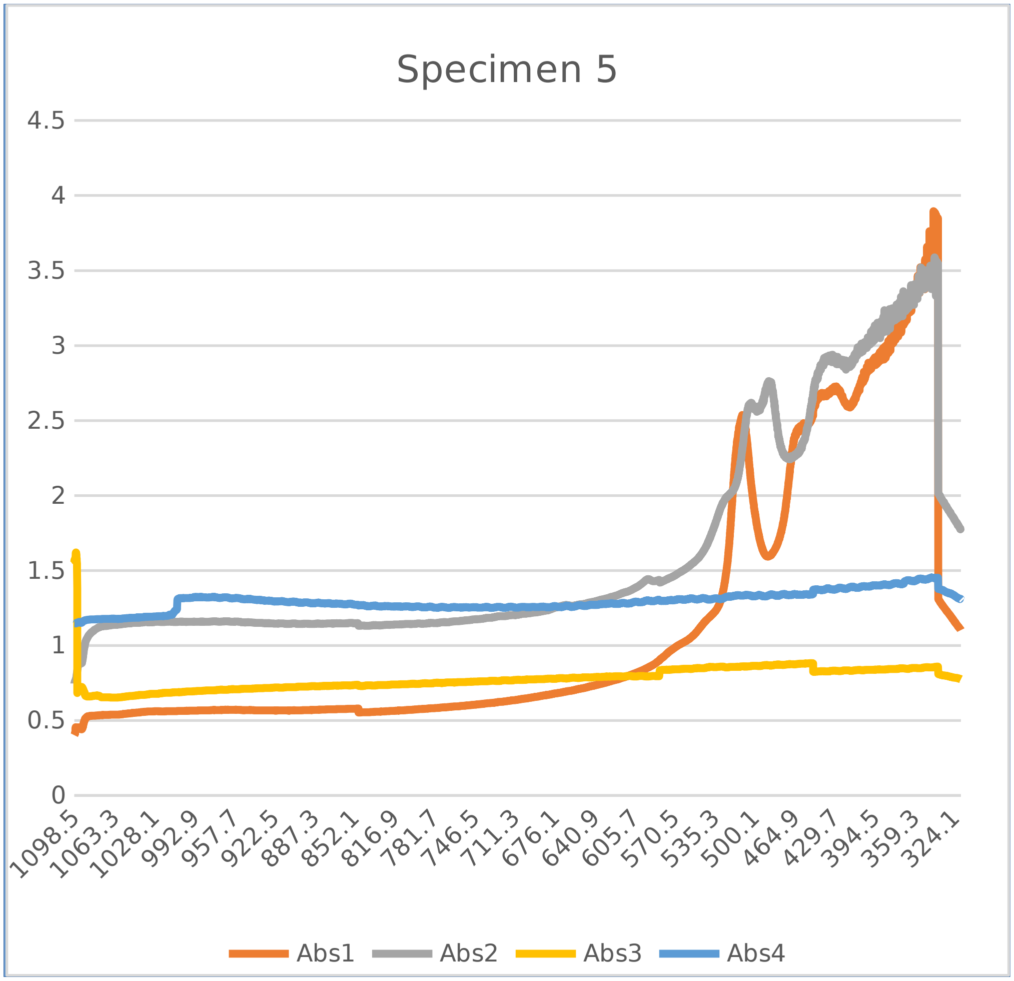

Specimen 5
4.5
4
3.5
3
2.5
2
1.5
1
0.5
0
1098.5
1063.3
1028.1
992.9
957.7
922.5
887.3
852.1
816.9
781.7
746.5
711.3
676.1
640.9
605.7
570.5
535.3
500.1
464.9
429.7
394.5
359.3
324.1
Abs1
Abs2
Abs3
Abs4
