# Supplementary material for: Coherent poly propagation materials with 3-dimensional photonic control over visible light
Source: PLoS One. 2019 Oct 17;14(10):e0223715. doi: 10.1371/journal.pone.0223715 (PMC6797174; doi:10.1371/journal.pone.0223715)
Supplement: S6 Table — Spreadsheet generated graph at bottom of table. (PDF) [file pone.0223715.s015.pdf]

Wavelength Scan Report - Specimen 6

Company	Complete Consulting Services LLC		
Operator	Dr. Michelle R. Stem	Test Time	11/8/2017 0:33
Document No.	WLS1711080001_2	Print Date	9/6/2019 18:34
Start wavelength	1100nm	Number of data	7801
End wavelength	320nm	K1	0.99
Unit		K0	0
Interval	0.1nm	K2	0
Calculate the Conc.	No	K3	0
Modification time	11/8/2017 0:33	Access time	11/8/2017 0:33
Remark	S01b072 - Specimen 6		

Scan Data

No.	Wavelength	Abs1	Abs2	Abs3	Abs4	Visible
1	1100	0.5411	0.6588	0.4553	1.2796	0
2	1099.9	0.5417	0.6584	0.4501	1.2759	0
3	1099.8	0.5417	0.6578	0.4459	1.2654	0
4	1099.7	0.5421	0.6574	0.4415	1.2434	0
5	1099.6	0.542	0.6564	0.4386	1.2256	0
6	1099.5	0.5414	0.6594	0.4357	1.2107	0
7	1099.4	0.5402	0.662	0.4319	0.91	0
8	1099.3	0.5399	0.6662	0.4281	0.7995	0
9	1099.2	0.5393	0.6674	0.425	0.7988	0
10	1099.1	0.5398	0.6682	0.4223	0.7988	0
11	1099	0.5397	0.6685	0.4195	0.7985	0
12	1098.9	0.5401	0.6693	0.4171	0.7987	0
13	1098.8	0.54	0.6695	0.4145	0.7986	0
14	1098.7	0.5398	0.6699	0.4122	0.7992	0
15	1098.6	0.5401	0.6703	0.4099	0.7994	0
16	1098.5	0.5402	0.6706	0.408	0.7996	0
17	1098.4	0.5402	0.6709	0.406	0.7999	0
18	1098.3	0.5402	0.6712	0.4041	0.8	0
19	1098.2	0.5405	0.6717	0.4022	0.8007	0
20	1098.1	0.5404	0.6723	0.4006	0.8004	0
21	1098	0.5404	0.6727	0.3987	0.801	0
22	1097.9	0.5407	0.6733	0.3971	0.8013	0
23	1097.8	0.5403	0.6741	0.3954	0.8014	0
24	1097.7	0.5396	0.6743	0.3946	0.8012	0
25	1097.6	0.5388	0.6751	0.3941	0.8016	0
26	1097.5	0.5383	0.6756	0.3931	0.8017	0
27	1097.4	0.5375	0.6759	0.3916	0.8018	0
28	1097.3	0.5364	0.6761	0.3905	0.802	0
29	1097.2	0.5347	0.6763	0.3895	0.8017	0

30	1097.1	0.5334	0.6764	0.3884	0.8023	0
31	1097	0.5333	0.6767	0.3874	0.8028	0
32	1096.9	0.5328	0.6767	0.386	0.8029	0
33	1096.8	0.5327	0.6769	0.3852	0.8037	0
34	1096.7	0.5325	0.6768	0.3841	0.8039	0
35	1096.6	0.5323	0.6769	0.383	0.8045	0
36	1096.5	0.5326	0.6773	0.3823	0.8051	0
37	1096.4	0.5328	0.6781	0.3813	0.8057	0
38	1096.3	0.5334	0.6787	0.3802	0.8058	0
39	1096.2	0.534	0.6797	0.379	0.8062	0
40	1096.1	0.5352	0.6807	0.3786	0.8059	0
41	1096	0.5363	0.6819	0.3771	0.806	0
42	1095.9	0.5375	0.6833	0.3761	0.8058	0
43	1095.8	0.5384	0.6843	0.3748	0.8055	0
44	1095.7	0.5398	0.6855	0.3736	0.8048	0
45	1095.6	0.541	0.6866	0.3726	0.8042	0
46	1095.5	0.5423	0.6879	0.3718	0.8041	0
47	1095.4	0.5429	0.6886	0.3703	0.8031	0
48	1095.3	0.5433	0.6887	0.3692	0.803	0
49	1095.2	0.5434	0.6887	0.3681	0.8027	0
50	1095.1	0.5432	0.6889	0.3671	0.8028	0
51	1095	0.5426	0.6883	0.3666	0.8033	0
52	1094.9	0.542	0.6875	0.366	0.8036	0
53	1094.8	0.5409	0.6867	0.3655	0.8052	0
54	1094.7	0.5395	0.6859	0.365	0.8062	0
55	1094.6	0.5382	0.6851	0.3649	0.8071	0
56	1094.5	0.5369	0.6843	0.3649	0.8088	0
57	1094.4	0.5357	0.6836	0.3654	0.8112	0
58	1094.3	0.5345	0.6829	0.365	0.8132	0
59	1094.2	0.5336	0.6826	0.365	0.8152	0
60	1094.1	0.5328	0.6824	0.365	0.817	0
61	1094	0.5321	0.6823	0.3648	0.8183	0
62	1093.9	0.5317	0.6827	0.3646	0.8198	0
63	1093.8	0.5316	0.6833	0.3647	0.8207	0
64	1093.7	0.532	0.684	0.3639	0.8217	0
65	1093.6	0.533	0.685	0.3633	0.8222	0
66	1093.5	0.5342	0.6864	0.363	0.8222	0
67	1093.4	0.5355	0.6878	0.3623	0.8222	0
68	1093.3	0.5374	0.6897	0.3613	0.8219	0
69	1093.2	0.5396	0.6912	0.3602	0.8209	0
70	1093.1	0.5419	0.6931	0.3591	0.8197	0
71	1093	0.5443	0.6948	0.3573	0.8186	0
72	1092.9	0.5469	0.6969	0.3558	0.8168	0

73	1092.8	0.55	0.6986	0.354	0.8151	0
74	1092.7	0.5526	0.7007	0.3523	0.8129	0
75	1092.6	0.5559	0.7029	0.351	0.8112	0
76	1092.5	0.5585	0.7049	0.3492	0.8087	0
77	1092.4	0.5616	0.7071	0.3475	0.8066	0
78	1092.3	0.5642	0.7089	0.3459	0.8041	0
79	1092.2	0.5672	0.7106	0.3436	0.8018	0
80	1092.1	0.5701	0.7124	0.3422	0.7996	0
81	1092	0.5728	0.7142	0.3404	0.7975	0
82	1091.9	0.5757	0.7162	0.3385	0.795	0
83	1091.8	0.5783	0.7174	0.3366	0.7931	0
84	1091.7	0.5808	0.7191	0.3351	0.7908	0
85	1091.6	0.583	0.7203	0.3334	0.7889	0
86	1091.5	0.5854	0.7219	0.3319	0.7867	0
87	1091.4	0.5876	0.7231	0.3303	0.7852	0
88	1091.3	0.5894	0.724	0.3286	0.7833	0
89	1091.2	0.5911	0.7251	0.3271	0.7818	0
90	1091.1	0.5929	0.7262	0.3256	0.7804	0
91	1091	0.5944	0.727	0.3243	0.779	0
92	1090.9	0.5956	0.7279	0.3229	0.7778	0
93	1090.8	0.5969	0.7283	0.3216	0.7763	0
94	1090.7	0.5979	0.7291	0.3205	0.7754	0
95	1090.6	0.5994	0.7298	0.3195	0.7746	0
96	1090.5	0.6006	0.7304	0.3182	0.7736	0
97	1090.4	0.6014	0.7309	0.3176	0.7729	0
98	1090.3	0.6025	0.7313	0.3168	0.7722	0
99	1090.2	0.6032	0.7319	0.3159	0.7718	0
100	1090.1	0.6041	0.7325	0.3152	0.771	0
101	1090	0.6045	0.7327	0.3141	0.7703	0
102	1089.9	0.6053	0.7331	0.3134	0.7697	0
103	1089.8	0.6058	0.7333	0.3125	0.7693	0
104	1089.7	0.607	0.734	0.3127	0.7692	0
105	1089.6	0.607	0.7342	0.3123	0.7685	0
106	1089.5	0.6076	0.7343	0.3119	0.7682	0
107	1089.4	0.608	0.7349	0.3113	0.7678	0
108	1089.3	0.6086	0.7352	0.3108	0.7677	0
109	1089.2	0.6088	0.7352	0.3105	0.7672	0
110	1089.1	0.6091	0.7353	0.3102	0.7668	0
111	1089	0.6093	0.7358	0.31	0.7665	0
112	1088.9	0.6096	0.7358	0.3093	0.7664	0
113	1088.8	0.61	0.7359	0.3092	0.7663	0
114	1088.7	0.6102	0.736	0.3088	0.7662	0
115	1088.6	0.61	0.736	0.3083	0.766	0

116	1088.5	0.6106	0.7362	0.3079	0.7659	0
117	1088.4	0.6109	0.7362	0.3079	0.7658	0
118	1088.3	0.6112	0.7362	0.3075	0.766	0
119	1088.2	0.6112	0.7364	0.3073	0.766	0
120	1088.1	0.6114	0.7365	0.3073	0.7658	0
121	1088	0.6113	0.7363	0.3071	0.7658	0
122	1087.9	0.6113	0.7364	0.3067	0.7659	0
123	1087.8	0.6114	0.7364	0.3064	0.7656	0
124	1087.7	0.6115	0.7369	0.3065	0.766	0
125	1087.6	0.6117	0.7371	0.3064	0.7663	0
126	1087.5	0.6117	0.737	0.3061	0.7664	0
127	1087.4	0.612	0.7372	0.306	0.7664	0
128	1087.3	0.6124	0.7374	0.3057	0.7667	0
129	1087.2	0.6125	0.7376	0.3055	0.7668	0
130	1087.1	0.6125	0.7375	0.3054	0.767	0
131	1087	0.6126	0.7376	0.3047	0.7667	0
132	1086.9	0.6128	0.7377	0.3048	0.7669	0
133	1086.8	0.6129	0.7381	0.3045	0.7675	0
134	1086.7	0.6132	0.7383	0.3046	0.7675	0
135	1086.6	0.6131	0.7382	0.3042	0.7674	0
136	1086.5	0.6137	0.7386	0.3041	0.7677	0
137	1086.4	0.6137	0.7386	0.3036	0.7674	0
138	1086.3	0.6138	0.7386	0.3034	0.7672	0
139	1086.2	0.6139	0.7388	0.3033	0.767	0
140	1086.1	0.6139	0.7389	0.3029	0.7671	0
141	1086	0.6139	0.7391	0.3028	0.7672	0
142	1085.9	0.6142	0.7393	0.3028	0.7677	0
143	1085.8	0.6144	0.7394	0.3026	0.7677	0
144	1085.7	0.6146	0.7395	0.3024	0.7676	0
145	1085.6	0.6148	0.7395	0.3025	0.7677	0
146	1085.5	0.6149	0.7399	0.3022	0.7678	0
147	1085.4	0.6153	0.7401	0.3021	0.7682	0
148	1085.3	0.6151	0.7401	0.3019	0.7679	0
149	1085.2	0.6154	0.7403	0.3021	0.7682	0
150	1085.1	0.6156	0.7405	0.3016	0.7685	0
151	1085	0.6154	0.7405	0.3013	0.7684	0
152	1084.9	0.6158	0.7408	0.3012	0.7685	0
153	1084.8	0.6159	0.7409	0.3019	0.7689	0
154	1084.7	0.6165	0.7415	0.3022	0.7695	0
155	1084.6	0.6164	0.7413	0.3016	0.7693	0
156	1084.5	0.6164	0.7416	0.3019	0.7695	0
157	1084.4	0.6165	0.7418	0.3017	0.7696	0
158	1084.3	0.6167	0.7418	0.3018	0.7697	0

159	1084.2	0.6168	0.7418	0.3017	0.7698	0
160	1084.1	0.6168	0.7421	0.3019	0.7701	0
161	1084	0.6166	0.7418	0.3017	0.7701	0
162	1083.9	0.617	0.7422	0.3018	0.7702	0
163	1083.8	0.6171	0.7422	0.3016	0.7704	0
164	1083.7	0.6175	0.7428	0.3019	0.7711	0
165	1083.6	0.6171	0.7424	0.3014	0.7707	0
166	1083.5	0.6171	0.7426	0.3014	0.7709	0
167	1083.4	0.6172	0.7426	0.3014	0.7708	0
168	1083.3	0.6174	0.7427	0.3013	0.771	0
169	1083.2	0.6174	0.743	0.3015	0.7712	0
170	1083.1	0.6176	0.7432	0.3012	0.7713	0
171	1083	0.6176	0.7432	0.3011	0.7713	0
172	1082.9	0.6177	0.7432	0.3011	0.7715	0
173	1082.8	0.6176	0.7433	0.3013	0.7718	0
174	1082.7	0.6178	0.7434	0.3011	0.7719	0
175	1082.6	0.6179	0.7436	0.301	0.772	0
176	1082.5	0.6179	0.7435	0.301	0.772	0
177	1082.4	0.6178	0.7434	0.3009	0.7722	0
178	1082.3	0.6178	0.7437	0.3009	0.7721	0
179	1082.2	0.618	0.7439	0.301	0.7722	0
180	1082.1	0.6183	0.744	0.301	0.7724	0
181	1082	0.6182	0.7441	0.3012	0.7727	0
182	1081.9	0.6183	0.744	0.3005	0.7728	0
183	1081.8	0.6185	0.7442	0.3008	0.7728	0
184	1081.7	0.6185	0.7443	0.301	0.773	0
185	1081.6	0.6184	0.7444	0.3006	0.773	0
186	1081.5	0.6186	0.7445	0.3007	0.773	0
187	1081.4	0.6185	0.7445	0.3006	0.7731	0
188	1081.3	0.6186	0.7446	0.3006	0.773	0
189	1081.2	0.6183	0.7447	0.3005	0.7733	0
190	1081.1	0.6182	0.7447	0.3004	0.7735	0
191	1081	0.6184	0.7448	0.3005	0.7735	0
192	1080.9	0.6184	0.7452	0.3006	0.7735	0
193	1080.8	0.618	0.7448	0.3005	0.7734	0
194	1080.7	0.6173	0.745	0.3003	0.7736	0
195	1080.6	0.6169	0.7451	0.3006	0.7736	0
196	1080.5	0.6167	0.7455	0.3008	0.7739	0
197	1080.4	0.6163	0.7452	0.3007	0.7738	0
198	1080.3	0.6163	0.7452	0.3005	0.7739	0
199	1080.2	0.6159	0.7454	0.3004	0.7738	0
200	1080.1	0.6162	0.7457	0.3006	0.7742	0
201	1080	0.6159	0.7456	0.3008	0.7742	0

202	1079.9	0.6159	0.7455	0.3005	0.7742	0
203	1079.8	0.6157	0.7457	0.3005	0.7742	0
204	1079.7	0.6158	0.7457	0.3005	0.7742	0
205	1079.6	0.6158	0.7456	0.3002	0.774	0
206	1079.5	0.6158	0.7454	0.3	0.7739	0
207	1079.4	0.6158	0.7456	0.3	0.7743	0
208	1079.3	0.6159	0.7459	0.3001	0.7745	0
209	1079.2	0.616	0.746	0.3002	0.7744	0
210	1079.1	0.616	0.746	0.3002	0.7743	0
211	1079	0.6161	0.7462	0.3003	0.7745	0
212	1078.9	0.6161	0.7461	0.3003	0.7745	0
213	1078.8	0.6159	0.746	0.3002	0.7742	0
214	1078.7	0.6159	0.7459	0.2999	0.774	0
215	1078.6	0.6164	0.7462	0.3002	0.7742	0
216	1078.5	0.6163	0.7463	0.3003	0.7745	0
217	1078.4	0.6164	0.7466	0.3004	0.7744	0
218	1078.3	0.6163	0.7462	0.3003	0.7742	0
219	1078.2	0.6166	0.7465	0.2999	0.7745	0
220	1078.1	0.6163	0.7465	0.2997	0.7743	0
221	1078	0.6163	0.7464	0.2999	0.774	0
222	1077.9	0.6164	0.7463	0.2999	0.7742	0
223	1077.8	0.6162	0.7465	0.2998	0.7742	0
224	1077.7	0.6163	0.7467	0.2998	0.7742	0
225	1077.6	0.6166	0.7466	0.3001	0.7744	0
226	1077.5	0.6165	0.7467	0.2998	0.7744	0
227	1077.4	0.6163	0.7468	0.3001	0.7745	0
228	1077.3	0.6167	0.7471	0.3	0.7747	0
229	1077.2	0.6166	0.7467	0.2995	0.7745	0
230	1077.1	0.6166	0.7469	0.2997	0.7746	0
231	1077	0.6164	0.7466	0.2993	0.7741	0
232	1076.9	0.6168	0.7468	0.2993	0.7744	0
233	1076.8	0.6171	0.7471	0.2995	0.7747	0
234	1076.7	0.6172	0.7471	0.2997	0.7749	0
235	1076.6	0.6176	0.747	0.2993	0.7747	0
236	1076.5	0.6181	0.7471	0.2997	0.7749	0
237	1076.4	0.618	0.747	0.2992	0.7746	0
238	1076.3	0.6183	0.7469	0.2995	0.7747	0
239	1076.2	0.6183	0.7472	0.2993	0.7748	0
240	1076.1	0.6187	0.7477	0.2996	0.7752	0
241	1076	0.6186	0.7474	0.2995	0.7751	0
242	1075.9	0.6187	0.7477	0.2994	0.7752	0
243	1075.8	0.6188	0.7475	0.2996	0.7752	0
244	1075.7	0.6192	0.7478	0.2993	0.7752	0

245	1075.6	0.6195	0.7483	0.2998	0.7756	0
246	1075.5	0.6196	0.748	0.2997	0.7754	0
247	1075.4	0.6196	0.748	0.2994	0.7753	0
248	1075.3	0.6196	0.7479	0.2996	0.7756	0
249	1075.2	0.6196	0.748	0.2992	0.7755	0
250	1075.1	0.62	0.7485	0.2993	0.7759	0
251	1075	0.6199	0.7483	0.2991	0.7758	0
252	1074.9	0.6202	0.7483	0.2994	0.776	0
253	1074.8	0.6206	0.7483	0.2995	0.7758	0
254	1074.7	0.6208	0.7485	0.2992	0.7761	0
255	1074.6	0.6211	0.7488	0.2992	0.7763	0
256	1074.5	0.6212	0.7489	0.2991	0.7764	0
257	1074.4	0.6211	0.7487	0.2992	0.7764	0
258	1074.3	0.6215	0.7492	0.2996	0.7768	0
259	1074.2	0.6218	0.7494	0.2998	0.7771	0
260	1074.1	0.6216	0.7492	0.2994	0.7769	0
261	1074	0.622	0.7495	0.2997	0.7773	0
262	1073.9	0.6221	0.7497	0.2998	0.7774	0
263	1073.8	0.6224	0.7494	0.2995	0.7774	0
264	1073.7	0.6226	0.7503	0.2999	0.7775	0
265	1073.6	0.6227	0.7502	0.3	0.7778	0
266	1073.5	0.6231	0.7501	0.3002	0.778	0
267	1073.4	0.623	0.7503	0.3	0.7779	0
268	1073.3	0.6234	0.7505	0.3001	0.7783	0
269	1073.2	0.6237	0.7506	0.3	0.7782	0
270	1073.1	0.6239	0.7507	0.3003	0.7787	0
271	1073	0.6243	0.751	0.3005	0.7786	0
272	1072.9	0.6244	0.751	0.3005	0.7787	0
273	1072.8	0.6246	0.751	0.3003	0.7788	0
274	1072.7	0.6248	0.7512	0.3	0.7791	0
275	1072.6	0.6248	0.7509	0.3	0.7788	0
276	1072.5	0.6252	0.7512	0.3002	0.7792	0
277	1072.4	0.6254	0.7513	0.3003	0.7793	0
278	1072.3	0.6255	0.7513	0.3002	0.7794	0
279	1072.2	0.6259	0.7515	0.3003	0.7795	0
280	1072.1	0.6262	0.7515	0.3004	0.7796	0
281	1072	0.6266	0.7519	0.3005	0.7797	0
282	1071.9	0.627	0.752	0.3007	0.7801	0
283	1071.8	0.6272	0.7521	0.3005	0.7801	0
284	1071.7	0.6273	0.752	0.3004	0.7803	0
285	1071.6	0.6272	0.7521	0.3003	0.7801	0
286	1071.5	0.6273	0.7522	0.3004	0.7803	0
287	1071.4	0.6275	0.7525	0.3003	0.7805	0

288	1071.3	0.6277	0.7525	0.3004	0.7805	0
289	1071.2	0.6277	0.7523	0.3004	0.7802	0
290	1071.1	0.6279	0.7525	0.3005	0.7804	0
291	1071	0.628	0.7526	0.3005	0.7808	0
292	1070.9	0.6281	0.7528	0.3006	0.7806	0
293	1070.8	0.6281	0.7527	0.3005	0.7805	0
294	1070.7	0.6284	0.7529	0.3006	0.7809	0
295	1070.6	0.6286	0.7532	0.3005	0.781	0
296	1070.5	0.6287	0.7533	0.3005	0.7811	0
297	1070.4	0.6288	0.7532	0.3003	0.7812	0
298	1070.3	0.6291	0.7536	0.3006	0.7817	0
299	1070.2	0.6291	0.7534	0.3005	0.7815	0
300	1070.1	0.6292	0.7534	0.3007	0.7816	0
301	1070	0.6294	0.7533	0.3007	0.7813	0
302	1069.9	0.6295	0.7534	0.3007	0.7815	0
303	1069.8	0.6299	0.7535	0.3006	0.7817	0
304	1069.7	0.6301	0.7538	0.3009	0.7819	0
305	1069.6	0.6305	0.7538	0.3007	0.7817	0
306	1069.5	0.6308	0.7539	0.3004	0.7817	0
307	1069.4	0.6312	0.7538	0.3004	0.7817	0
308	1069.3	0.6352	0.7537	0.3004	0.7815	0
309	1069.2	0.6368	0.7539	0.3006	0.7816	0
310	1069.1	0.6383	0.7541	0.3007	0.7818	0
311	1069	0.6404	0.754	0.3007	0.7818	0
312	1068.9	0.6439	0.7545	0.3011	0.7821	0
313	1068.8	0.6461	0.7544	0.301	0.7819	0
314	1068.7	0.6481	0.7543	0.3007	0.782	0
315	1068.6	0.65	0.7543	0.3007	0.7818	0
316	1068.5	0.6763	0.7543	0.3006	0.7818	0
317	1068.4	0.6784	0.7543	0.3005	0.7818	0
318	1068.3	0.6796	0.7546	0.3007	0.7819	0
319	1068.2	0.6803	0.7547	0.3005	0.7819	0
320	1068.1	0.6811	0.7546	0.3005	0.7818	0
321	1068	0.6821	0.7546	0.3005	0.7818	0
322	1067.9	0.6829	0.7545	0.3004	0.7819	0
323	1067.8	0.6841	0.7546	0.3003	0.7815	0
324	1067.7	0.7216	0.7547	0.3005	0.7818	0
325	1067.6	0.7287	0.7552	0.3007	0.782	0
326	1067.5	0.736	0.7553	0.3004	0.7818	0
327	1067.4	0.8167	0.7553	0.3005	0.782	0
328	1067.3	0.8169	0.7552	0.3004	0.7818	0
329	1067.2	0.8172	0.7553	0.3005	0.7818	0
330	1067.1	0.8175	0.7556	0.3008	0.7823	0

331	1067	0.8177	0.7556	0.3007	0.782	0
332	1066.9	0.8176	0.7555	0.3004	0.7819	0
333	1066.8	0.8177	0.7554	0.3006	0.7821	0
334	1066.7	0.8178	0.7555	0.3004	0.782	0
335	1066.6	0.8178	0.7556	0.3006	0.782	0
336	1066.5	0.8178	0.7556	0.3003	0.7818	0
337	1066.4	0.8179	0.7556	0.3004	0.7816	0
338	1066.3	0.8181	0.756	0.3007	0.7821	0
339	1066.2	0.8179	0.7558	0.3005	0.782	0
340	1066.1	0.8183	0.7559	0.3003	0.782	0
341	1066	0.818	0.7558	0.3002	0.7818	0
342	1065.9	0.8186	0.7561	0.3004	0.7819	0
343	1065.8	0.8183	0.7559	0.3004	0.782	0
344	1065.7	0.8185	0.7561	0.3003	0.7821	0
345	1065.6	0.8185	0.7562	0.3003	0.7821	0
346	1065.5	0.8189	0.7564	0.3005	0.7824	0
347	1065.4	0.8191	0.7561	0.3005	0.7821	0
348	1065.3	0.8189	0.7565	0.3004	0.7823	0
349	1065.2	0.8189	0.7566	0.3006	0.7825	0
350	1065.1	0.8187	0.7564	0.3004	0.7825	0
351	1065	0.8191	0.7567	0.3005	0.7825	0
352	1064.9	0.8189	0.7565	0.3	0.7825	0
353	1064.8	0.8187	0.7565	0.3001	0.7823	0
354	1064.7	0.8189	0.7567	0.3001	0.7823	0
355	1064.6	0.8187	0.7566	0.3	0.7823	0
356	1064.5	0.8186	0.7567	0.3003	0.7821	0
357	1064.4	0.8188	0.7568	0.3003	0.7823	0
358	1064.3	0.8188	0.7566	0.3001	0.7824	0
359	1064.2	0.8189	0.7568	0.3001	0.7825	0
360	1064.1	0.819	0.7568	0.3002	0.7824	0
361	1064	0.8193	0.7571	0.3004	0.7828	0
362	1063.9	0.8188	0.7572	0.3	0.7825	0
363	1063.8	0.8191	0.7572	0.3004	0.7828	0
364	1063.7	0.819	0.7572	0.3001	0.7827	0
365	1063.6	0.8187	0.757	0.2998	0.7826	0
366	1063.5	0.8194	0.7574	0.3	0.7827	0
367	1063.4	0.8192	0.7572	0.3001	0.7826	0
368	1063.3	0.8193	0.7575	0.3005	0.7828	0
369	1063.2	0.8186	0.7572	0.3001	0.7827	0
370	1063.1	0.8192	0.7575	0.3002	0.783	0
371	1063	0.8193	0.7576	0.3	0.7829	0
372	1062.9	0.819	0.7572	0.2999	0.7827	0
373	1062.8	0.8191	0.7576	0.3001	0.783	0

374	1062.7	0.8191	0.7577	0.3	0.7829	0
375	1062.6	0.8189	0.7574	0.2999	0.7828	0
376	1062.5	0.8191	0.7576	0.3	0.7828	0
377	1062.4	0.819	0.7575	0.2997	0.7826	0
378	1062.3	0.8191	0.7576	0.3001	0.7829	0
379	1062.2	0.8189	0.7575	0.2997	0.783	0
380	1062.1	0.8194	0.758	0.3002	0.7832	0
381	1062	0.8193	0.7579	0.3001	0.7831	0
382	1061.9	0.819	0.7578	0.3	0.7831	0
383	1061.8	0.8191	0.7581	0.3003	0.7834	0
384	1061.7	0.8195	0.7582	0.3002	0.7836	0
385	1061.6	0.8191	0.7581	0.2997	0.7833	0
386	1061.5	0.8191	0.7579	0.2997	0.7832	0
387	1061.4	0.8193	0.7582	0.3	0.7835	0
388	1061.3	0.8196	0.7583	0.3	0.7837	0
389	1061.2	0.8194	0.7585	0.3002	0.7838	0
390	1061.1	0.8195	0.7583	0.3002	0.7839	0
391	1061	0.8198	0.7585	0.3003	0.784	0
392	1060.9	0.8196	0.7585	0.2999	0.7839	0
393	1060.8	0.8193	0.7585	0.3001	0.784	0
394	1060.7	0.8199	0.7586	0.3003	0.7842	0
395	1060.6	0.8201	0.7591	0.3001	0.7844	0
396	1060.5	0.8198	0.759	0.3003	0.7844	0
397	1060.4	0.8197	0.7591	0.3003	0.7846	0
398	1060.3	0.8198	0.7591	0.3004	0.7844	0
399	1060.2	0.8202	0.7594	0.3006	0.7849	0
400	1060.1	0.8201	0.7594	0.3006	0.7848	0
401	1060	0.8202	0.7594	0.3003	0.785	0
402	1059.9	0.8204	0.7597	0.3006	0.7849	0
403	1059.8	0.8203	0.7597	0.3007	0.7853	0
404	1059.7	0.8205	0.7598	0.3009	0.7853	0
405	1059.6	0.8205	0.76	0.3009	0.7856	0
406	1059.5	0.8209	0.7601	0.301	0.7856	0
407	1059.4	0.8208	0.7599	0.3009	0.7857	0
408	1059.3	0.8209	0.7602	0.3011	0.786	0
409	1059.2	0.8209	0.7605	0.3013	0.786	0
410	1059.1	0.8213	0.7607	0.3014	0.786	0
411	1059	0.8213	0.7607	0.3015	0.7859	0
412	1058.9	0.8212	0.7606	0.3014	0.7861	0
413	1058.8	0.8213	0.761	0.3016	0.7862	0
414	1058.7	0.8214	0.761	0.3018	0.7864	0
415	1058.6	0.8219	0.7612	0.302	0.7866	0
416	1058.5	0.8217	0.7612	0.3018	0.7866	0

417	1058.4	0.8218	0.7616	0.3019	0.7866	0
418	1058.3	0.8219	0.7611	0.3019	0.7866	0
419	1058.2	0.8217	0.7614	0.302	0.7866	0
420	1058.1	0.8218	0.7615	0.3021	0.7869	0
421	1058	0.8221	0.7615	0.302	0.7869	0
422	1057.9	0.8221	0.7618	0.3025	0.7871	0
423	1057.8	0.8224	0.7619	0.3024	0.7873	0
424	1057.7	0.8224	0.762	0.3024	0.7873	0
425	1057.6	0.8229	0.7625	0.3028	0.7875	0
426	1057.5	0.8228	0.7625	0.3028	0.7877	0
427	1057.4	0.8229	0.7625	0.3028	0.7878	0
428	1057.3	0.8229	0.7628	0.303	0.7879	0
429	1057.2	0.8232	0.7629	0.3031	0.7881	0
430	1057.1	0.8233	0.763	0.3029	0.7881	0
431	1057	0.8234	0.7633	0.3032	0.7884	0
432	1056.9	0.8235	0.7631	0.3033	0.7883	0
433	1056.8	0.8236	0.7634	0.3035	0.7886	0
434	1056.7	0.8238	0.7633	0.3037	0.7887	0
435	1056.6	0.8237	0.7637	0.3035	0.7886	0
436	1056.5	0.8237	0.7638	0.3036	0.7889	0
437	1056.4	0.8242	0.7641	0.3039	0.7892	0
438	1056.3	0.8245	0.764	0.3039	0.7891	0
439	1056.2	0.824	0.764	0.304	0.7889	0
440	1056.1	0.8242	0.7643	0.3041	0.7893	0
441	1056	0.8245	0.7644	0.3042	0.7892	0
442	1055.9	0.8245	0.7645	0.3041	0.7895	0
443	1055.8	0.8245	0.7647	0.3043	0.7898	0
444	1055.7	0.8246	0.7648	0.3044	0.7897	0
445	1055.6	0.825	0.765	0.3047	0.7898	0
446	1055.5	0.8251	0.7653	0.3048	0.7903	0
447	1055.4	0.8257	0.7654	0.305	0.7904	0
448	1055.3	0.8259	0.7655	0.3051	0.7906	0
449	1055.2	0.8253	0.7656	0.3049	0.7903	0
450	1055.1	0.8254	0.7656	0.3048	0.7906	0
451	1055	0.8253	0.7657	0.3049	0.7906	0
452	1054.9	0.8256	0.7658	0.305	0.7908	0
453	1054.8	0.8259	0.7661	0.3052	0.7911	0
454	1054.7	0.8258	0.7663	0.3053	0.7912	0
455	1054.6	0.8258	0.7662	0.3054	0.7912	0
456	1054.5	0.826	0.7665	0.3056	0.7915	0
457	1054.4	0.8265	0.7665	0.3055	0.7917	0
458	1054.3	0.8264	0.7669	0.3058	0.7918	0
459	1054.2	0.8264	0.7667	0.3054	0.7916	0

460	1054.1	0.8263	0.7666	0.3057	0.7918	0
461	1054	0.8266	0.7671	0.306	0.7919	0
462	1053.9	0.8264	0.7675	0.306	0.7922	0
463	1053.8	0.827	0.7675	0.3062	0.7924	0
464	1053.7	0.827	0.7675	0.306	0.7926	0
465	1053.6	0.8269	0.7677	0.306	0.7925	0
466	1053.5	0.8271	0.7679	0.3064	0.7928	0
467	1053.4	0.8274	0.7681	0.3064	0.7929	0
468	1053.3	0.8273	0.7681	0.3064	0.7928	0
469	1053.2	0.8275	0.7682	0.3064	0.7934	0
470	1053.1	0.8272	0.7685	0.3068	0.7935	0
471	1053	0.8276	0.7687	0.3069	0.7935	0
472	1052.9	0.8277	0.7685	0.3069	0.7931	0
473	1052.8	0.8276	0.7686	0.3067	0.7933	0
474	1052.7	0.8276	0.7686	0.3067	0.7934	0
475	1052.6	0.8276	0.7688	0.3068	0.7935	0
476	1052.5	0.8276	0.7689	0.3068	0.7936	0
477	1052.4	0.8276	0.769	0.3069	0.7937	0
478	1052.3	0.8279	0.7692	0.3066	0.794	0
479	1052.2	0.828	0.7693	0.3066	0.7938	0
480	1052.1	0.828	0.7694	0.3067	0.7938	0
481	1052	0.8283	0.7695	0.3068	0.7941	0
482	1051.9	0.8283	0.7697	0.3071	0.7944	0
483	1051.8	0.8284	0.7698	0.3072	0.7945	0
484	1051.7	0.8285	0.7697	0.3072	0.7943	0
485	1051.6	0.8283	0.7696	0.307	0.7941	0
486	1051.5	0.8287	0.7702	0.3073	0.7948	0
487	1051.4	0.8288	0.7701	0.3073	0.7949	0
488	1051.3	0.8285	0.7702	0.3072	0.795	0
489	1051.2	0.8289	0.7702	0.3072	0.795	0
490	1051.1	0.829	0.7704	0.3073	0.7951	0
491	1051	0.8291	0.7708	0.3074	0.7951	0
492	1050.9	0.8292	0.7708	0.3077	0.7954	0
493	1050.8	0.8292	0.7707	0.3077	0.7952	0
494	1050.7	0.8293	0.7706	0.3073	0.7952	0
495	1050.6	0.8292	0.7706	0.3074	0.7953	0
496	1050.5	0.8291	0.7707	0.3075	0.7953	0
497	1050.4	0.8295	0.7709	0.3076	0.7955	0
498	1050.3	0.8296	0.7712	0.3076	0.7956	0
499	1050.2	0.8297	0.7714	0.308	0.796	0
500	1050.1	0.8297	0.7711	0.3075	0.7958	0
501	1050	0.83	0.7714	0.3079	0.7958	0
502	1049.9	0.8299	0.7714	0.3076	0.7959	0

503	1049.8	0.83	0.7716	0.3079	0.7961	0
504	1049.7	0.8299	0.7717	0.3078	0.7961	0
505	1049.6	0.8301	0.772	0.3078	0.7962	0
506	1049.5	0.83	0.7718	0.3077	0.7961	0
507	1049.4	0.8302	0.772	0.3079	0.7962	0
508	1049.3	0.83	0.7719	0.3078	0.7961	0
509	1049.2	0.8301	0.7719	0.3078	0.7959	0
510	1049.1	0.8302	0.7718	0.3075	0.7959	0
511	1049	0.8301	0.7719	0.3077	0.7963	0
512	1048.9	0.8303	0.7723	0.3079	0.7965	0
513	1048.8	0.8304	0.7721	0.3079	0.7964	0
514	1048.7	0.8308	0.7724	0.3077	0.7968	0
515	1048.6	0.8308	0.7724	0.3082	0.7967	0
516	1048.5	0.8308	0.7728	0.3084	0.7968	0
517	1048.4	0.8307	0.7728	0.3083	0.7967	0
518	1048.3	0.8308	0.7727	0.3083	0.7969	0
519	1048.2	0.831	0.773	0.3083	0.7971	0
520	1048.1	0.8311	0.7729	0.3085	0.7972	0
521	1048	0.831	0.7731	0.3084	0.7973	0
522	1047.9	0.8312	0.7733	0.3087	0.7973	0
523	1047.8	0.8316	0.7736	0.3087	0.7977	0
524	1047.7	0.8315	0.7737	0.309	0.7977	0
525	1047.6	0.8316	0.7737	0.3089	0.7978	0
526	1047.5	0.8317	0.7737	0.3088	0.7978	0
527	1047.4	0.8316	0.7736	0.3088	0.7977	0
528	1047.3	0.8318	0.7739	0.3089	0.7979	0
529	1047.2	0.8318	0.7737	0.3087	0.7977	0
530	1047.1	0.8318	0.7738	0.3087	0.798	0
531	1047	0.8318	0.7742	0.3092	0.7981	0
532	1046.9	0.8319	0.7741	0.3089	0.7982	0
533	1046.8	0.8319	0.7741	0.309	0.7983	0
534	1046.7	0.8319	0.7743	0.3091	0.7984	0
535	1046.6	0.8322	0.7745	0.3092	0.7984	0
536	1046.5	0.8325	0.7745	0.3093	0.7988	0
537	1046.4	0.8325	0.7749	0.3095	0.7989	0
538	1046.3	0.8325	0.7748	0.3097	0.7991	0
539	1046.2	0.8325	0.775	0.3096	0.7991	0
540	1046.1	0.8328	0.7755	0.31	0.7997	0
541	1046	0.8328	0.7752	0.3097	0.7993	0
542	1045.9	0.8329	0.7755	0.3098	0.7996	0
543	1045.8	0.8327	0.7756	0.3096	0.7998	0
544	1045.7	0.833	0.7756	0.3096	0.7998	0
545	1045.6	0.833	0.7757	0.3097	0.8	0

546	1045.5	0.833	0.7759	0.3097	0.8	0
547	1045.4	0.8333	0.7762	0.3098	0.8003	0
548	1045.3	0.8331	0.7761	0.3101	0.8003	0
549	1045.2	0.8333	0.7765	0.3101	0.8004	0
550	1045.1	0.8337	0.7769	0.3103	0.8007	0
551	1045	0.8334	0.7765	0.31	0.8005	0
552	1044.9	0.8334	0.7765	0.3099	0.8006	0
553	1044.8	0.8337	0.7767	0.3101	0.8008	0
554	1044.7	0.8338	0.7768	0.3101	0.8008	0
555	1044.6	0.8339	0.7768	0.3102	0.801	0
556	1044.5	0.8339	0.7772	0.3105	0.8013	0
557	1044.4	0.8338	0.7771	0.3103	0.8009	0
558	1044.3	0.8335	0.7772	0.3104	0.8012	0
559	1044.2	0.8339	0.7776	0.3106	0.8016	0
560	1044.1	0.8341	0.7776	0.3106	0.8017	0
561	1044	0.8344	0.7777	0.3107	0.8018	0
562	1043.9	0.8345	0.7779	0.3107	0.8018	0
563	1043.8	0.8346	0.7779	0.3108	0.802	0
564	1043.7	0.8345	0.7781	0.311	0.8021	0
565	1043.6	0.8345	0.7781	0.3109	0.8022	0
566	1043.5	0.8344	0.7784	0.3111	0.8025	0
567	1043.4	0.8346	0.7785	0.3111	0.8023	0
568	1043.3	0.8346	0.7787	0.3115	0.8025	0
569	1043.2	0.8348	0.7787	0.3112	0.8026	0
570	1043.1	0.8349	0.7787	0.3111	0.8027	0
571	1043	0.8345	0.7786	0.3111	0.8027	0
572	1042.9	0.835	0.7787	0.3114	0.803	0
573	1042.8	0.835	0.779	0.3114	0.8029	0
574	1042.7	0.8352	0.7789	0.3114	0.803	0
575	1042.6	0.8351	0.7791	0.3113	0.8032	0
576	1042.5	0.8354	0.7793	0.3113	0.8034	0
577	1042.4	0.8351	0.7792	0.3115	0.8033	0
578	1042.3	0.8354	0.7794	0.3117	0.8036	0
579	1042.2	0.8355	0.7795	0.3117	0.8034	0
580	1042.1	0.8353	0.7798	0.3119	0.8036	0
581	1042	0.8354	0.7796	0.3119	0.8037	0
582	1041.9	0.8358	0.7797	0.312	0.8036	0
583	1041.8	0.8356	0.7797	0.3121	0.8037	0
584	1041.7	0.8359	0.78	0.3123	0.804	0
585	1041.6	0.8358	0.7801	0.312	0.804	0
586	1041.5	0.836	0.7801	0.3122	0.804	0
587	1041.4	0.8363	0.7804	0.3124	0.8041	0
588	1041.3	0.8363	0.7802	0.3124	0.8043	0

589	1041.2	0.8364	0.7805	0.3126	0.8045	0
590	1041.1	0.8362	0.7807	0.3124	0.8045	0
591	1041	0.8365	0.7805	0.3125	0.8046	0
592	1040.9	0.8364	0.7806	0.3127	0.8044	0
593	1040.8	0.8364	0.7806	0.3126	0.8045	0
594	1040.7	0.8365	0.7809	0.3126	0.8047	0
595	1040.6	0.8368	0.7808	0.3127	0.8045	0
596	1040.5	0.8366	0.7808	0.3127	0.8045	0
597	1040.4	0.8369	0.7809	0.3129	0.8047	0
598	1040.3	0.8366	0.7808	0.3127	0.8047	0
599	1040.2	0.837	0.7812	0.3127	0.8048	0
600	1040.1	0.8371	0.7814	0.3129	0.805	0
601	1040	0.8371	0.7811	0.3128	0.8048	0
602	1039.9	0.8368	0.7811	0.3127	0.8044	0
603	1039.8	0.8368	0.781	0.3127	0.8045	0
604	1039.7	0.8371	0.7811	0.3129	0.8046	0
605	1039.6	0.8371	0.7813	0.313	0.8047	0
606	1039.5	0.8372	0.7812	0.3129	0.8043	0
607	1039.4	0.8374	0.7816	0.3129	0.805	0
608	1039.3	0.8373	0.7816	0.3132	0.8049	0
609	1039.2	0.8375	0.7816	0.313	0.8047	0
610	1039.1	0.8376	0.7817	0.3131	0.805	0
611	1039	0.8377	0.7816	0.3131	0.805	0
612	1038.9	0.8377	0.782	0.313	0.8051	0
613	1038.8	0.8379	0.7817	0.313	0.8051	0
614	1038.7	0.838	0.7819	0.3133	0.8049	0
615	1038.6	0.8381	0.7821	0.3137	0.8053	0
616	1038.5	0.8379	0.7819	0.3135	0.8049	0
617	1038.4	0.8378	0.7819	0.3135	0.805	0
618	1038.3	0.8383	0.7822	0.3138	0.8053	0
619	1038.2	0.8379	0.7821	0.3136	0.805	0
620	1038.1	0.8382	0.7824	0.3137	0.8052	0
621	1038	0.8383	0.7825	0.3137	0.8055	0
622	1037.9	0.8386	0.7825	0.3137	0.8053	0
623	1037.8	0.8383	0.7825	0.3138	0.8052	0
624	1037.7	0.8387	0.7829	0.3141	0.8056	0
625	1037.6	0.8388	0.7828	0.3141	0.8057	0
626	1037.5	0.8389	0.7831	0.3141	0.8058	0
627	1037.4	0.8391	0.7831	0.3142	0.8059	0
628	1037.3	0.8388	0.7831	0.3142	0.8061	0
629	1037.2	0.8393	0.7834	0.3144	0.8062	0
630	1037.1	0.8391	0.7836	0.3145	0.8062	0
631	1037	0.8393	0.7837	0.3144	0.8062	0

632	1036.9	0.8392	0.7836	0.3148	0.8065	0
633	1036.8	0.8391	0.7838	0.3145	0.8065	0
634	1036.7	0.8391	0.7838	0.3145	0.8066	0
635	1036.6	0.8395	0.7838	0.3145	0.8068	0
636	1036.5	0.8393	0.784	0.3145	0.8066	0
637	1036.4	0.8394	0.7841	0.3146	0.8069	0
638	1036.3	0.8394	0.784	0.3148	0.8067	0
639	1036.2	0.8399	0.7842	0.315	0.8074	0
640	1036.1	0.8397	0.7844	0.3151	0.8074	0
641	1036	0.8397	0.7845	0.3152	0.8073	0
642	1035.9	0.8398	0.7846	0.3153	0.8077	0
643	1035.8	0.84	0.785	0.3155	0.8079	0
644	1035.7	0.84	0.785	0.3153	0.8079	0
645	1035.6	0.8403	0.7849	0.3152	0.808	0
646	1035.5	0.8402	0.7852	0.3152	0.8083	0
647	1035.4	0.8401	0.7851	0.3155	0.8083	0
648	1035.3	0.8402	0.7853	0.3158	0.8084	0
649	1035.2	0.8404	0.7853	0.3158	0.8087	0
650	1035.1	0.8403	0.7855	0.3156	0.8087	0
651	1035	0.8408	0.7859	0.3161	0.809	0
652	1034.9	0.8407	0.7858	0.3161	0.809	0
653	1034.8	0.8408	0.7861	0.316	0.8091	0
654	1034.7	0.8407	0.7861	0.316	0.8094	0
655	1034.6	0.8407	0.7859	0.3161	0.8094	0
656	1034.5	0.8406	0.7862	0.316	0.8094	0
657	1034.4	0.8414	0.7868	0.3166	0.8099	0
658	1034.3	0.841	0.7865	0.3163	0.8098	0
659	1034.2	0.8411	0.7867	0.3163	0.8099	0
660	1034.1	0.8408	0.7867	0.3163	0.81	0
661	1034	0.841	0.7869	0.3162	0.8101	0
662	1033.9	0.8412	0.7869	0.3164	0.8101	0
663	1033.8	0.8411	0.7872	0.3165	0.8103	0
664	1033.7	0.8412	0.7872	0.3165	0.8103	0
665	1033.6	0.8413	0.7872	0.3165	0.8105	0
666	1033.5	0.8414	0.7874	0.3169	0.8106	0
667	1033.4	0.8417	0.7876	0.3171	0.8109	0
668	1033.3	0.8414	0.7874	0.317	0.8109	0
669	1033.2	0.8414	0.7875	0.3169	0.8111	0
670	1033.1	0.8417	0.7878	0.3174	0.8112	0
671	1033	0.8417	0.7878	0.3171	0.8113	0
672	1032.9	0.8421	0.7882	0.3174	0.8113	0
673	1032.8	0.842	0.7879	0.3172	0.8113	0
674	1032.7	0.8418	0.7883	0.3173	0.8114	0

675	1032.6	0.8421	0.7884	0.3174	0.8116	0
676	1032.5	0.8423	0.7885	0.3173	0.8117	0
677	1032.4	0.8424	0.7885	0.3176	0.8117	0
678	1032.3	0.8424	0.7886	0.3177	0.8117	0
679	1032.2	0.8424	0.7887	0.3175	0.8119	0
680	1032.1	0.8425	0.7888	0.3176	0.812	0
681	1032	0.8425	0.7888	0.3177	0.8119	0
682	1031.9	0.8427	0.7889	0.3177	0.8121	0
683	1031.8	0.8425	0.7891	0.3177	0.8121	0
684	1031.7	0.8429	0.7889	0.3179	0.8123	0
685	1031.6	0.8427	0.7889	0.3179	0.8122	0
686	1031.5	0.8426	0.7892	0.3179	0.8123	0
687	1031.4	0.8428	0.7893	0.318	0.8124	0
688	1031.3	0.8427	0.7891	0.318	0.8123	0
689	1031.2	0.8429	0.7894	0.3181	0.8127	0
690	1031.1	0.8429	0.7895	0.318	0.8125	0
691	1031	0.8434	0.7896	0.3183	0.8127	0
692	1030.9	0.8433	0.7896	0.3183	0.8126	0
693	1030.8	0.8436	0.79	0.3186	0.8129	0
694	1030.7	0.8436	0.7897	0.3183	0.8128	0
695	1030.6	0.8436	0.79	0.3186	0.8129	0
696	1030.5	0.8436	0.7899	0.3187	0.8128	0
697	1030.4	0.8436	0.7899	0.3186	0.8129	0
698	1030.3	0.8434	0.7897	0.3186	0.8128	0
699	1030.2	0.8437	0.7901	0.3187	0.8129	0
700	1030.1	0.8434	0.7899	0.3185	0.8127	0
701	1030	0.8432	0.79	0.3187	0.8128	0
702	1029.9	0.8436	0.7901	0.3186	0.8128	0
703	1029.8	0.8437	0.7902	0.3186	0.8127	0
704	1029.7	0.8436	0.7902	0.3186	0.8128	0
705	1029.6	0.8439	0.7901	0.3186	0.8127	0
706	1029.5	0.8438	0.7901	0.3187	0.8128	0
707	1029.4	0.8438	0.7902	0.3187	0.8129	0
708	1029.3	0.8439	0.7902	0.3186	0.8129	0
709	1029.2	0.8439	0.7904	0.3187	0.8129	0
710	1029.1	0.8441	0.7905	0.3186	0.8131	0
711	1029	0.8442	0.7904	0.3188	0.8128	0
712	1028.9	0.8441	0.7905	0.3188	0.813	0
713	1028.8	0.8443	0.7907	0.3188	0.8132	0
714	1028.7	0.8443	0.7906	0.3188	0.813	0
715	1028.6	0.8443	0.7908	0.3187	0.813	0
716	1028.5	0.8443	0.7908	0.3187	0.813	0
717	1028.4	0.8446	0.7909	0.319	0.8131	0

718	1028.3	0.8445	0.7907	0.3188	0.813	0
719	1028.2	0.8443	0.7906	0.3186	0.8128	0
720	1028.1	0.8444	0.7907	0.3187	0.8129	0
721	1028	0.8447	0.7911	0.3188	0.8132	0
722	1027.9	0.8447	0.791	0.3191	0.8133	0
723	1027.8	0.8449	0.791	0.3192	0.8132	0
724	1027.7	0.8448	0.7912	0.319	0.8133	0
725	1027.6	0.8446	0.7912	0.319	0.8133	0
726	1027.5	0.8447	0.7909	0.3189	0.8131	0
727	1027.4	0.8446	0.7911	0.319	0.8131	0
728	1027.3	0.845	0.7913	0.3189	0.8132	0
729	1027.2	0.845	0.7913	0.3189	0.8132	0
730	1027.1	0.8452	0.7914	0.3192	0.8134	0
731	1027	0.8454	0.7917	0.3192	0.8134	0
732	1026.9	0.8452	0.7917	0.3193	0.8135	0
733	1026.8	0.8452	0.7918	0.3195	0.8137	0
734	1026.7	0.8455	0.7917	0.3194	0.8137	0
735	1026.6	0.8453	0.792	0.3195	0.8137	0
736	1026.5	0.8449	0.7917	0.3194	0.8134	0
737	1026.4	0.8453	0.7916	0.3193	0.8136	0
738	1026.3	0.8456	0.792	0.3194	0.8138	0
739	1026.2	0.8458	0.7924	0.3199	0.8141	0
740	1026.1	0.8458	0.7925	0.3198	0.814	0
741	1026	0.8463	0.7926	0.32	0.8142	0
742	1025.9	0.846	0.7927	0.32	0.8143	0
743	1025.8	0.8459	0.7927	0.32	0.8144	0
744	1025.7	0.846	0.7926	0.32	0.8145	0
745	1025.6	0.846	0.7928	0.3197	0.8145	0
746	1025.5	0.8458	0.7927	0.3197	0.8143	0
747	1025.4	0.8461	0.7929	0.32	0.8148	0
748	1025.3	0.846	0.7929	0.3201	0.8148	0
749	1025.2	0.8464	0.7932	0.3204	0.8152	0
750	1025.1	0.8461	0.7932	0.3204	0.8151	0
751	1025	0.8462	0.7935	0.3205	0.8152	0
752	1024.9	0.846	0.7935	0.3203	0.8153	0
753	1024.8	0.8464	0.7935	0.3205	0.8156	0
754	1024.7	0.8464	0.7937	0.3208	0.8158	0
755	1024.6	0.8467	0.7939	0.3208	0.8158	0
756	1024.5	0.8467	0.7941	0.321	0.8161	0
757	1024.4	0.8468	0.7942	0.321	0.8163	0
758	1024.3	0.8469	0.7943	0.321	0.8164	0
759	1024.2	0.8469	0.7945	0.3212	0.8168	0
760	1024.1	0.847	0.7948	0.3214	0.8168	0

761	1024	0.8471	0.795	0.3215	0.8171	0
762	1023.9	0.8473	0.795	0.3214	0.8172	0
763	1023.8	0.8472	0.7951	0.3215	0.8172	0
764	1023.7	0.8474	0.7951	0.3216	0.8174	0
765	1023.6	0.8475	0.7953	0.3219	0.8176	0
766	1023.5	0.8474	0.7954	0.3219	0.8178	0
767	1023.4	0.8475	0.7956	0.3219	0.8178	0
768	1023.3	0.8474	0.7956	0.3218	0.8178	0
769	1023.2	0.8473	0.7955	0.3218	0.8177	0
770	1023.1	0.8473	0.7957	0.3217	0.8178	0
771	1023	0.8477	0.796	0.3221	0.8182	0
772	1022.9	0.8476	0.7959	0.3221	0.8182	0
773	1022.8	0.8479	0.796	0.3218	0.8185	0
774	1022.7	0.8483	0.7963	0.3224	0.8188	0
775	1022.6	0.8481	0.7964	0.3223	0.8188	0
776	1022.5	0.8481	0.7963	0.3222	0.8184	0
777	1022.4	0.8478	0.7965	0.3223	0.8189	0
778	1022.3	0.8482	0.7966	0.3224	0.8189	0
779	1022.2	0.8482	0.7964	0.3226	0.819	0
780	1022.1	0.8481	0.7965	0.3226	0.8191	0
781	1022	0.8483	0.7964	0.3225	0.8191	0
782	1021.9	0.8485	0.7967	0.3225	0.8189	0
783	1021.8	0.8486	0.7969	0.3228	0.8194	0
784	1021.7	0.8486	0.797	0.3229	0.8193	0
785	1021.6	0.8485	0.7971	0.3226	0.8192	0
786	1021.5	0.8484	0.7972	0.3227	0.8192	0
787	1021.4	0.8485	0.7969	0.3228	0.8195	0
788	1021.3	0.8485	0.7972	0.3228	0.8195	0
789	1021.2	0.8484	0.7969	0.3227	0.8192	0
790	1021.1	0.8488	0.7974	0.3229	0.8195	0
791	1021	0.8489	0.7977	0.3229	0.8198	0
792	1020.9	0.8488	0.7974	0.323	0.8196	0
793	1020.8	0.8489	0.7974	0.3229	0.8197	0
794	1020.7	0.8487	0.7976	0.3231	0.8199	0
795	1020.6	0.8489	0.7977	0.323	0.8199	0
796	1020.5	0.8489	0.7976	0.323	0.8199	0
797	1020.4	0.849	0.7977	0.3232	0.8201	0
798	1020.3	0.8491	0.7978	0.3232	0.82	0
799	1020.2	0.8495	0.7979	0.3232	0.8202	0
800	1020.1	0.8492	0.7982	0.3233	0.8202	0
801	1020	0.8495	0.7983	0.3233	0.8202	0
802	1019.9	0.8496	0.7982	0.3234	0.8202	0
803	1019.8	0.8498	0.7984	0.3237	0.8205	0

804	1019.7	0.8495	0.7984	0.3235	0.8202	0
805	1019.6	0.8496	0.7984	0.3235	0.8202	0
806	1019.5	0.8496	0.7982	0.3231	0.8199	0
807	1019.4	0.8502	0.7984	0.3233	0.8203	0
808	1019.3	0.8497	0.7983	0.3235	0.8203	0
809	1019.2	0.8497	0.7983	0.3233	0.82	0
810	1019.1	0.8501	0.7985	0.3236	0.8201	0
811	1019	0.85	0.7984	0.3236	0.8201	0
812	1018.9	0.85	0.7983	0.3235	0.8199	0
813	1018.8	0.8497	0.7984	0.3233	0.8199	0
814	1018.7	0.8498	0.7984	0.3233	0.8199	0
815	1018.6	0.8499	0.7984	0.3234	0.8201	0
816	1018.5	0.8499	0.7986	0.3234	0.8202	0
817	1018.4	0.8502	0.7986	0.3235	0.8202	0
818	1018.3	0.8501	0.7987	0.3235	0.8204	0
819	1018.2	0.8503	0.7988	0.3237	0.8204	0
820	1018.1	0.8504	0.7986	0.3234	0.8202	0
821	1018	0.8504	0.7987	0.3236	0.8202	0
822	1017.9	0.8505	0.7986	0.3235	0.8201	0
823	1017.8	0.8506	0.7988	0.3236	0.8204	0
824	1017.7	0.8506	0.7988	0.3236	0.8202	0
825	1017.6	0.8507	0.7988	0.3238	0.8201	0
826	1017.5	0.8508	0.7988	0.3236	0.8201	0
827	1017.4	0.8505	0.7989	0.3236	0.8201	0
828	1017.3	0.8504	0.7987	0.3233	0.82	0
829	1017.2	0.8505	0.799	0.3234	0.8201	0
830	1017.1	0.8506	0.7989	0.3237	0.8199	0
831	1017	0.8507	0.7991	0.3237	0.8202	0
832	1016.9	0.8507	0.7991	0.3239	0.8203	0
833	1016.8	0.8509	0.799	0.3237	0.8203	0
834	1016.7	0.8508	0.7991	0.3235	0.8203	0
835	1016.6	0.8505	0.7989	0.3236	0.8201	0
836	1016.5	0.8508	0.799	0.3238	0.8203	0
837	1016.4	0.8508	0.7993	0.3235	0.8204	0
838	1016.3	0.8511	0.7994	0.3237	0.8204	0
839	1016.2	0.8513	0.7997	0.3241	0.8205	0
840	1016.1	0.851	0.7995	0.324	0.8204	0
841	1016	0.8509	0.7995	0.3239	0.8209	0
842	1015.9	0.8511	0.7995	0.3237	0.8206	0
843	1015.8	0.8511	0.7997	0.3236	0.8207	0
844	1015.7	0.8511	0.7996	0.3238	0.8207	0
845	1015.6	0.8515	0.7999	0.324	0.8209	0
846	1015.5	0.8513	0.8001	0.3241	0.8211	0

847	1015.4	0.8514	0.7998	0.3239	0.8208	0
848	1015.3	0.8514	0.7999	0.3239	0.8208	0
849	1015.2	0.8516	0.8001	0.3238	0.821	0
850	1015.1	0.8515	0.8003	0.324	0.8211	0
851	1015	0.8516	0.8001	0.3241	0.8211	0
852	1014.9	0.8518	0.8005	0.3243	0.8212	0
853	1014.8	0.8517	0.8004	0.3242	0.8215	0
854	1014.7	0.8519	0.8006	0.3244	0.8215	0
855	1014.6	0.8519	0.8009	0.324	0.8216	0
856	1014.5	0.852	0.8007	0.3243	0.8216	0
857	1014.4	0.8521	0.801	0.3245	0.8219	0
858	1014.3	0.8521	0.801	0.3247	0.822	0
859	1014.2	0.8521	0.801	0.3247	0.8223	0
860	1014.1	0.8525	0.8013	0.3248	0.8222	0
861	1014	0.8524	0.8013	0.3248	0.8225	0
862	1013.9	0.8523	0.8017	0.325	0.8227	0
863	1013.8	0.8526	0.8017	0.325	0.8224	0
864	1013.7	0.8525	0.8019	0.3251	0.8229	0
865	1013.6	0.8526	0.802	0.3249	0.823	0
866	1013.5	0.8525	0.8019	0.3252	0.8229	0
867	1013.4	0.8528	0.8022	0.3252	0.8232	0
868	1013.3	0.8528	0.8023	0.3253	0.8233	0
869	1013.2	0.8526	0.8021	0.3253	0.8235	0
870	1013.1	0.8524	0.8022	0.3252	0.8235	0
871	1013	0.8525	0.8024	0.3254	0.8235	0
872	1012.9	0.8528	0.8024	0.3253	0.8237	0
873	1012.8	0.8526	0.8025	0.3252	0.8239	0
874	1012.7	0.8528	0.8025	0.3253	0.8239	0
875	1012.6	0.8527	0.8027	0.3254	0.8241	0
876	1012.5	0.8532	0.8029	0.3258	0.8243	0
877	1012.4	0.8531	0.8027	0.3256	0.8244	0
878	1012.3	0.8533	0.803	0.3256	0.8244	0
879	1012.2	0.8533	0.803	0.3257	0.8246	0
880	1012.1	0.8534	0.803	0.326	0.8246	0
881	1012	0.8534	0.8034	0.3261	0.8248	0
882	1011.9	0.8534	0.8034	0.3259	0.8247	0
883	1011.8	0.8537	0.8035	0.3262	0.8251	0
884	1011.7	0.8537	0.8035	0.3261	0.8249	0
885	1011.6	0.8534	0.8037	0.326	0.8246	0
886	1011.5	0.8534	0.8037	0.3262	0.8248	0
887	1011.4	0.8537	0.8036	0.3262	0.8249	0
888	1011.3	0.8537	0.8039	0.3261	0.8252	0
889	1011.2	0.8535	0.8038	0.3261	0.8253	0

890	1011.1	0.8538	0.804	0.3265	0.8253	0
891	1011	0.8537	0.8039	0.3263	0.8252	0
892	1010.9	0.8539	0.804	0.3264	0.8252	0
893	1010.8	0.854	0.8043	0.3267	0.8254	0
894	1010.7	0.8543	0.8044	0.3266	0.8258	0
895	1010.6	0.8543	0.8043	0.3266	0.8255	0
896	1010.5	0.8541	0.8044	0.3265	0.8256	0
897	1010.4	0.8541	0.8045	0.3265	0.8256	0
898	1010.3	0.8542	0.8045	0.3265	0.8256	0
899	1010.2	0.8542	0.8046	0.3265	0.8257	0
900	1010.1	0.8544	0.8044	0.3265	0.8256	0
901	1010	0.8541	0.8046	0.3266	0.8257	0
902	1009.9	0.8542	0.8047	0.3266	0.8258	0
903	1009.8	0.8543	0.8047	0.3264	0.826	0
904	1009.7	0.8545	0.8048	0.3267	0.8258	0
905	1009.6	0.8544	0.8049	0.3267	0.8258	0
906	1009.5	0.8546	0.8049	0.3267	0.8258	0
907	1009.4	0.8545	0.8047	0.3266	0.8257	0
908	1009.3	0.8547	0.8047	0.3267	0.8258	0
909	1009.2	0.8547	0.8051	0.3265	0.8257	0
910	1009.1	0.8544	0.8049	0.3265	0.8259	0
911	1009	0.8548	0.8049	0.3267	0.826	0
912	1008.9	0.8548	0.8052	0.3266	0.8259	0
913	1008.8	0.8548	0.805	0.3266	0.8257	0
914	1008.7	0.8549	0.8051	0.3266	0.8258	0
915	1008.6	0.855	0.805	0.3267	0.826	0
916	1008.5	0.855	0.805	0.3267	0.8257	0
917	1008.4	0.8553	0.8051	0.3268	0.8259	0
918	1008.3	0.8551	0.8054	0.3266	0.8258	0
919	1008.2	0.8551	0.8052	0.3267	0.826	0
920	1008.1	0.8555	0.8053	0.3269	0.8259	0
921	1008	0.8555	0.8056	0.3268	0.826	0
922	1007.9	0.8555	0.8055	0.3271	0.826	0
923	1007.8	0.8556	0.8057	0.3271	0.8261	0
924	1007.7	0.8555	0.8056	0.3271	0.826	0
925	1007.6	0.8553	0.8055	0.3268	0.8261	0
926	1007.5	0.8553	0.8053	0.3267	0.8256	0
927	1007.4	0.8556	0.8056	0.3269	0.8259	0
928	1007.3	0.8554	0.8055	0.3269	0.8259	0
929	1007.2	0.8553	0.8054	0.3267	0.8253	0
930	1007.1	0.8556	0.8054	0.3267	0.8259	0
931	1007	0.8555	0.8056	0.3267	0.826	0
932	1006.9	0.8556	0.8059	0.3268	0.8259	0

933	1006.8	0.8556	0.8059	0.3267	0.8258	0
934	1006.7	0.8558	0.806	0.3268	0.8259	0
935	1006.6	0.856	0.8062	0.3269	0.8262	0
936	1006.5	0.8557	0.806	0.3267	0.826	0
937	1006.4	0.856	0.8059	0.3265	0.8261	0
938	1006.3	0.8558	0.806	0.3263	0.8262	0
939	1006.2	0.8559	0.8059	0.3265	0.8262	0
940	1006.1	0.8559	0.8059	0.3262	0.8262	0
941	1006	0.8559	0.8059	0.3261	0.8259	0
942	1005.9	0.856	0.8059	0.3262	0.8259	0
943	1005.8	0.8559	0.8059	0.3262	0.826	0
944	1005.7	0.8562	0.8059	0.3261	0.8261	0
945	1005.6	0.8562	0.8063	0.3258	0.8261	0
946	1005.5	0.8562	0.8062	0.3259	0.8262	0
947	1005.4	0.856	0.8065	0.326	0.8263	0
948	1005.3	0.8563	0.8065	0.326	0.8265	0
949	1005.2	0.8562	0.8063	0.3258	0.8265	0
950	1005.1	0.8563	0.8064	0.3258	0.8265	0
951	1005	0.8562	0.8065	0.3259	0.8264	0
952	1004.9	0.8566	0.8068	0.3263	0.8269	0
953	1004.8	0.8566	0.807	0.3263	0.827	0
954	1004.7	0.8565	0.8071	0.3263	0.8271	0
955	1004.6	0.8567	0.8071	0.3263	0.827	0
956	1004.5	0.8568	0.8072	0.3261	0.8272	0
957	1004.4	0.8567	0.8074	0.3262	0.8275	0
958	1004.3	0.8569	0.8074	0.3263	0.8275	0
959	1004.2	0.8572	0.8074	0.3264	0.8276	0
960	1004.1	0.8569	0.8074	0.3262	0.8275	0
961	1004	0.857	0.8075	0.326	0.8277	0
962	1003.9	0.8573	0.8079	0.3261	0.8278	0
963	1003.8	0.857	0.808	0.3262	0.8278	0
964	1003.7	0.857	0.8077	0.3263	0.8279	0
965	1003.6	0.8568	0.8078	0.3262	0.8278	0
966	1003.5	0.857	0.8081	0.3263	0.828	0
967	1003.4	0.8574	0.8082	0.3263	0.828	0
968	1003.3	0.8574	0.8082	0.3263	0.8282	0
969	1003.2	0.8576	0.8083	0.3264	0.8284	0
970	1003.1	0.8577	0.8083	0.3261	0.8282	0
971	1003	0.8572	0.8083	0.3261	0.8283	0
972	1002.9	0.8573	0.8085	0.326	0.8284	0
973	1002.8	0.8576	0.8088	0.3264	0.8285	0
974	1002.7	0.8575	0.8085	0.3264	0.8285	0
975	1002.6	0.8576	0.8084	0.3263	0.8286	0

976	1002.5	0.8576	0.8088	0.3263	0.8287	0
977	1002.4	0.8579	0.8088	0.3264	0.8288	0
978	1002.3	0.8579	0.809	0.3264	0.8289	0
979	1002.2	0.8576	0.809	0.3264	0.8288	0
980	1002.1	0.8576	0.8091	0.3264	0.829	0
981	1002	0.8577	0.8089	0.3263	0.8289	0
982	1001.9	0.8578	0.8091	0.3264	0.8291	0
983	1001.8	0.8582	0.8095	0.3266	0.8293	0
984	1001.7	0.8582	0.8094	0.3266	0.8294	0
985	1001.6	0.8584	0.8095	0.3264	0.8293	0
986	1001.5	0.8584	0.8096	0.3263	0.8293	0
987	1001.4	0.8583	0.8096	0.3265	0.8297	0
988	1001.3	0.8585	0.8097	0.3266	0.8295	0
989	1001.2	0.8585	0.8098	0.3267	0.8297	0
990	1001.1	0.8586	0.81	0.3266	0.83	0
991	1001	0.8586	0.8098	0.3269	0.8299	0
992	1000.9	0.8587	0.8101	0.3267	0.8299	0
993	1000.8	0.8585	0.81	0.3268	0.8299	0
994	1000.7	0.8588	0.8102	0.3267	0.83	0
995	1000.6	0.8586	0.81	0.3265	0.8299	0
996	1000.5	0.8588	0.8099	0.3267	0.8296	0
997	1000.4	0.8586	0.8101	0.3269	0.8301	0
998	1000.3	0.8587	0.8102	0.3268	0.8302	0
999	1000.2	0.8592	0.8105	0.3271	0.8302	0
1000	1000.1	0.859	0.8107	0.327	0.8302	0
1001	1000	0.8592	0.8105	0.3271	0.83	0
1002	999.9	0.8591	0.8107	0.3271	0.8303	0
1003	999.8	0.8592	0.8109	0.3271	0.8304	0
1004	999.7	0.8591	0.8107	0.327	0.8305	0
1005	999.6	0.8595	0.8109	0.3271	0.8305	0
1006	999.5	0.8593	0.8108	0.3269	0.8305	0
1007	999.4	0.8594	0.8109	0.3273	0.8306	0
1008	999.3	0.8593	0.8113	0.327	0.8306	0
1009	999.2	0.8593	0.8113	0.3272	0.8306	0
1010	999.1	0.8595	0.8114	0.3272	0.8307	0
1011	999	0.8594	0.8113	0.327	0.8306	0
1012	998.9	0.8592	0.8112	0.327	0.8306	0
1013	998.8	0.8592	0.811	0.3269	0.8306	0
1014	998.7	0.8592	0.8113	0.327	0.8306	0
1015	998.6	0.8595	0.8115	0.327	0.8305	0
1016	998.5	0.8595	0.8115	0.3273	0.8308	0
1017	998.4	0.8597	0.8117	0.3273	0.8306	0
1018	998.3	0.8595	0.8115	0.3269	0.8303	0

1019	998.2	0.8598	0.8116	0.3271	0.8306	0
1020	998.1	0.8598	0.8115	0.3271	0.8305	0
1021	998	0.8598	0.8118	0.3273	0.8309	0
1022	997.9	0.8597	0.8116	0.3271	0.8307	0
1023	997.8	0.8597	0.8119	0.3272	0.8307	0
1024	997.7	0.8599	0.8121	0.3274	0.8309	0
1025	997.6	0.8601	0.812	0.3273	0.8309	0
1026	997.5	0.8598	0.8121	0.3274	0.8306	0
1027	997.4	0.8599	0.8121	0.3271	0.8306	0
1028	997.3	0.8601	0.8121	0.3272	0.8305	0
1029	997.2	0.8602	0.8122	0.3271	0.8309	0
1030	997.1	0.8604	0.8123	0.3274	0.8309	0
1031	997	0.8602	0.8121	0.327	0.8306	0
1032	996.9	0.86	0.8124	0.3273	0.8308	0
1033	996.8	0.8604	0.8127	0.3273	0.8307	0
1034	996.7	0.8606	0.8129	0.3276	0.8309	0
1035	996.6	0.8606	0.8128	0.3275	0.8311	0
1036	996.5	0.8605	0.8127	0.3276	0.8311	0
1037	996.4	0.8606	0.8131	0.3276	0.831	0
1038	996.3	0.8607	0.8132	0.3276	0.8312	0
1039	996.2	0.8606	0.8132	0.3276	0.831	0
1040	996.1	0.8608	0.8134	0.3276	0.8313	0
1041	996	0.8606	0.8134	0.3275	0.8312	0
1042	995.9	0.8608	0.8135	0.3276	0.8312	0
1043	995.8	0.8609	0.8137	0.3278	0.8315	0
1044	995.7	0.8608	0.8136	0.3275	0.8311	0
1045	995.6	0.861	0.8135	0.3276	0.8312	0
1046	995.5	0.8607	0.8138	0.3275	0.8313	0
1047	995.4	0.8611	0.8141	0.3278	0.8314	0
1048	995.3	0.8611	0.8141	0.3276	0.8314	0
1049	995.2	0.8613	0.8143	0.3277	0.8316	0
1050	995.1	0.8613	0.8143	0.3278	0.8315	0
1051	995	0.8616	0.8146	0.3279	0.8316	0
1052	994.9	0.8612	0.8143	0.3276	0.8316	0
1053	994.8	0.8616	0.8144	0.3276	0.8318	0
1054	994.7	0.8614	0.8148	0.3277	0.8319	0
1055	994.6	0.8616	0.8147	0.328	0.832	0
1056	994.5	0.8613	0.8145	0.3276	0.8318	0
1057	994.4	0.8618	0.815	0.3282	0.8321	0
1058	994.3	0.8615	0.8149	0.3281	0.8319	0
1059	994.2	0.8618	0.8153	0.3283	0.8322	0
1060	994.1	0.8616	0.8147	0.3279	0.8321	0
1061	994	0.8618	0.8149	0.328	0.8322	0

1062	993.9	0.8618	0.8152	0.3279	0.8322	0
1063	993.8	0.8616	0.8152	0.328	0.8323	0
1064	993.7	0.8618	0.8154	0.3279	0.8323	0
1065	993.6	0.862	0.8156	0.3281	0.8325	0
1066	993.5	0.8623	0.8155	0.3281	0.8327	0
1067	993.4	0.8621	0.8156	0.3283	0.8328	0
1068	993.3	0.8621	0.8157	0.3283	0.833	0
1069	993.2	0.8622	0.8161	0.3284	0.833	0
1070	993.1	0.8625	0.8162	0.3284	0.8329	0
1071	993	0.8622	0.8162	0.3284	0.8331	0
1072	992.9	0.8625	0.8162	0.3285	0.8332	0
1073	992.8	0.8626	0.8163	0.3286	0.8332	0
1074	992.7	0.8625	0.8163	0.3287	0.8333	0
1075	992.6	0.863	0.8165	0.3287	0.8334	0
1076	992.5	0.8628	0.8167	0.3289	0.8333	0
1077	992.4	0.8627	0.8168	0.3286	0.8333	0
1078	992.3	0.8628	0.8166	0.3287	0.8334	0
1079	992.2	0.8632	0.8171	0.3293	0.8338	0
1080	992.1	0.8631	0.8172	0.3291	0.8339	0
1081	992	0.863	0.8169	0.3287	0.8336	0
1082	991.9	0.863	0.817	0.3289	0.8335	0
1083	991.8	0.8629	0.8172	0.3288	0.8338	0
1084	991.7	0.8629	0.8171	0.3289	0.8336	0
1085	991.6	0.8632	0.8175	0.329	0.8338	0
1086	991.5	0.8628	0.8174	0.3288	0.8337	0
1087	991.4	0.8631	0.8175	0.3289	0.8338	0
1088	991.3	0.8632	0.8173	0.3288	0.8337	0
1089	991.2	0.8635	0.8175	0.3291	0.834	0
1090	991.1	0.8632	0.8174	0.3289	0.8339	0
1091	991	0.8635	0.8175	0.3291	0.8339	0
1092	990.9	0.8635	0.8177	0.329	0.8339	0
1093	990.8	0.8633	0.8176	0.329	0.8339	0
1094	990.7	0.8634	0.8176	0.3287	0.8339	0
1095	990.6	0.8635	0.8179	0.329	0.834	0
1096	990.5	0.8636	0.818	0.3291	0.834	0
1097	990.4	0.8638	0.8181	0.3291	0.8341	0
1098	990.3	0.8636	0.8181	0.3291	0.8341	0
1099	990.2	0.8639	0.8184	0.3291	0.8341	0
1100	990.1	0.8639	0.818	0.329	0.8339	0
1101	990	0.864	0.8182	0.3293	0.834	0
1102	989.9	0.8639	0.818	0.329	0.8341	0
1103	989.8	0.8639	0.8179	0.3289	0.8341	0
1104	989.7	0.8641	0.8184	0.329	0.8342	0

1105	989.6	0.8641	0.8184	0.3291	0.834	0
1106	989.5	0.8643	0.8186	0.3291	0.8342	0
1107	989.4	0.8643	0.8183	0.3291	0.8342	0
1108	989.3	0.8641	0.8185	0.3289	0.8344	0
1109	989.2	0.8644	0.8188	0.3292	0.8342	0
1110	989.1	0.8645	0.8186	0.329	0.8343	0
1111	989	0.8645	0.8186	0.3292	0.8343	0
1112	988.9	0.8646	0.8188	0.3293	0.8345	0
1113	988.8	0.8646	0.8189	0.3293	0.8343	0
1114	988.7	0.8647	0.8191	0.3294	0.8344	0
1115	988.6	0.8647	0.8187	0.3293	0.8343	0
1116	988.5	0.8647	0.8191	0.3293	0.8345	0
1117	988.4	0.8647	0.8188	0.3291	0.8342	0
1118	988.3	0.8649	0.8191	0.3295	0.8346	0
1119	988.2	0.8649	0.819	0.3295	0.8346	0
1120	988.1	0.8649	0.8195	0.3295	0.8346	0
1121	988	0.8649	0.8193	0.3296	0.8345	0
1122	987.9	0.865	0.8197	0.3294	0.8347	0
1123	987.8	0.865	0.8193	0.3294	0.8345	0
1124	987.7	0.8652	0.8193	0.3294	0.8346	0
1125	987.6	0.8654	0.8199	0.3299	0.8348	0
1126	987.5	0.8653	0.82	0.3297	0.8349	0
1127	987.4	0.8651	0.8197	0.3296	0.8347	0
1128	987.3	0.8654	0.8199	0.3296	0.835	0
1129	987.2	0.8652	0.8197	0.3295	0.8349	0
1130	987.1	0.8655	0.8197	0.3294	0.8351	0
1131	987	0.8654	0.8199	0.3296	0.8351	0
1132	986.9	0.8657	0.82	0.3298	0.8353	0
1133	986.8	0.8656	0.8201	0.3299	0.8353	0
1134	986.7	0.8654	0.8202	0.3298	0.8353	0
1135	986.6	0.8658	0.8204	0.3301	0.8356	0
1136	986.5	0.8658	0.8203	0.3298	0.8353	0
1137	986.4	0.8658	0.8207	0.3298	0.8356	0
1138	986.3	0.8659	0.8202	0.3299	0.8355	0
1139	986.2	0.866	0.8208	0.3301	0.8354	0
1140	986.1	0.866	0.8206	0.3301	0.8355	0
1141	986	0.8663	0.8208	0.3302	0.8358	0
1142	985.9	0.866	0.8208	0.3302	0.836	0
1143	985.8	0.8662	0.8209	0.33	0.8358	0
1144	985.7	0.8661	0.821	0.33	0.8359	0
1145	985.6	0.8663	0.8208	0.3301	0.836	0
1146	985.5	0.8663	0.8211	0.3302	0.836	0
1147	985.4	0.8663	0.8211	0.3301	0.836	0

1148	985.3	0.8665	0.8214	0.3301	0.8364	0
1149	985.2	0.8664	0.8215	0.3302	0.8363	0
1150	985.1	0.8667	0.8218	0.3303	0.8366	0
1151	985	0.8668	0.8216	0.3304	0.8366	0
1152	984.9	0.8668	0.8216	0.3305	0.8365	0
1153	984.8	0.8668	0.8218	0.3308	0.8368	0
1154	984.7	0.8668	0.8221	0.3307	0.8367	0
1155	984.6	0.867	0.8222	0.3307	0.8368	0
1156	984.5	0.867	0.8221	0.3306	0.8367	0
1157	984.4	0.8671	0.8222	0.3306	0.837	0
1158	984.3	0.8671	0.8224	0.331	0.8372	0
1159	984.2	0.8675	0.8226	0.3311	0.8375	0
1160	984.1	0.8676	0.8229	0.3312	0.8377	0
1161	984	0.8672	0.8224	0.331	0.8373	0
1162	983.9	0.8675	0.8227	0.3311	0.8374	0
1163	983.8	0.8674	0.8226	0.331	0.8374	0
1164	983.7	0.8677	0.8229	0.3311	0.8379	0
1165	983.6	0.8676	0.823	0.331	0.8377	0
1166	983.5	0.8675	0.8226	0.3309	0.8376	0
1167	983.4	0.8677	0.8232	0.3312	0.8377	0
1168	983.3	0.8679	0.8232	0.3312	0.8377	0
1169	983.2	0.8679	0.8232	0.3311	0.838	0
1170	983.1	0.868	0.8234	0.3313	0.838	0
1171	983	0.868	0.8233	0.3312	0.8379	0
1172	982.9	0.868	0.8237	0.3313	0.8383	0
1173	982.8	0.8682	0.8236	0.3312	0.8381	0
1174	982.7	0.8681	0.8235	0.3311	0.8381	0
1175	982.6	0.8681	0.8233	0.331	0.8378	0
1176	982.5	0.8681	0.8236	0.331	0.838	0
1177	982.4	0.8681	0.8237	0.3311	0.838	0
1178	982.3	0.8681	0.8237	0.3312	0.8381	0
1179	982.2	0.8685	0.8238	0.3311	0.838	0
1180	982.1	0.8683	0.8239	0.3312	0.838	0
1181	982	0.8686	0.8238	0.3312	0.8382	0
1182	981.9	0.8685	0.824	0.3311	0.8382	0
1183	981.8	0.8684	0.8238	0.3311	0.8383	0
1184	981.7	0.8688	0.824	0.3312	0.8382	0
1185	981.6	0.8688	0.8242	0.3315	0.8383	0
1186	981.5	0.8689	0.8244	0.3315	0.8384	0
1187	981.4	0.8685	0.8242	0.3313	0.8384	0
1188	981.3	0.8688	0.8244	0.3315	0.8385	0
1189	981.2	0.8688	0.8245	0.3315	0.8384	0
1190	981.1	0.869	0.8246	0.3316	0.8384	0

1191	981	0.8691	0.8246	0.3314	0.8383	0
1192	980.9	0.8692	0.8247	0.3316	0.8385	0
1193	980.8	0.8692	0.8247	0.3316	0.8384	0
1194	980.7	0.8691	0.8245	0.3315	0.8383	0
1195	980.6	0.8691	0.8247	0.3315	0.8385	0
1196	980.5	0.8691	0.8249	0.3314	0.8385	0
1197	980.4	0.8694	0.8249	0.3316	0.8384	0
1198	980.3	0.8693	0.8248	0.3314	0.8382	0
1199	980.2	0.8694	0.8248	0.3315	0.8383	0
1200	980.1	0.8692	0.8246	0.3313	0.8381	0
1201	980	0.8694	0.8248	0.3314	0.8382	0
1202	979.9	0.8695	0.825	0.3313	0.8383	0
1203	979.8	0.8697	0.8252	0.3317	0.8386	0
1204	979.7	0.8697	0.8252	0.3315	0.8383	0
1205	979.6	0.8697	0.825	0.3314	0.8382	0
1206	979.5	0.8697	0.8251	0.3315	0.8382	0
1207	979.4	0.8701	0.8252	0.3316	0.8384	0
1208	979.3	0.8702	0.8252	0.3315	0.8383	0
1209	979.2	0.8703	0.8255	0.3314	0.8383	0
1210	979.1	0.8699	0.825	0.3313	0.838	0
1211	979	0.8703	0.8252	0.3313	0.838	0
1212	978.9	0.8696	0.825	0.3312	0.8377	0
1213	978.8	0.87	0.8255	0.3314	0.8381	0
1214	978.7	0.8699	0.8251	0.3313	0.838	0
1215	978.6	0.87	0.8252	0.3312	0.8377	0
1216	978.5	0.87	0.8253	0.3311	0.8378	0
1217	978.4	0.87	0.8252	0.331	0.8376	0
1218	978.3	0.8702	0.8252	0.3311	0.8377	0
1219	978.2	0.8702	0.8254	0.331	0.8376	0
1220	978.1	0.8704	0.8258	0.3313	0.838	0
1221	978	0.8707	0.8257	0.3313	0.8379	0
1222	977.9	0.8706	0.8258	0.3313	0.8378	0
1223	977.8	0.8707	0.8258	0.3312	0.8379	0
1224	977.7	0.8703	0.8257	0.3311	0.8379	0
1225	977.6	0.8705	0.8256	0.331	0.8378	0
1226	977.5	0.8707	0.8256	0.3311	0.8376	0
1227	977.4	0.8707	0.8256	0.3312	0.8378	0
1228	977.3	0.8708	0.8259	0.3312	0.838	0
1229	977.2	0.8709	0.8259	0.3311	0.8379	0
1230	977.1	0.8707	0.8261	0.3313	0.8381	0
1231	977	0.8707	0.826	0.3312	0.8381	0
1232	976.9	0.8706	0.8262	0.3312	0.8379	0
1233	976.8	0.8709	0.8262	0.3314	0.8381	0

1234	976.7	0.8712	0.8262	0.3311	0.8381	0
1235	976.6	0.871	0.8263	0.3314	0.8381	0
1236	976.5	0.8711	0.8262	0.3315	0.8383	0
1237	976.4	0.8713	0.8266	0.3317	0.8386	0
1238	976.3	0.8712	0.8266	0.3318	0.8386	0
1239	976.2	0.8716	0.8268	0.3318	0.839	0
1240	976.1	0.8715	0.8269	0.3316	0.8389	0
1241	976	0.8714	0.8271	0.3316	0.8389	0
1242	975.9	0.8717	0.8269	0.3317	0.8393	0
1243	975.8	0.8715	0.827	0.332	0.8392	0
1244	975.7	0.8715	0.8271	0.3319	0.8392	0
1245	975.6	0.8716	0.8273	0.332	0.8395	0
1246	975.5	0.8718	0.8275	0.3321	0.8396	0
1247	975.4	0.8717	0.8275	0.3321	0.8396	0
1248	975.3	0.8718	0.8277	0.3322	0.8399	0
1249	975.2	0.8717	0.8277	0.3322	0.8397	0
1250	975.1	0.8719	0.8275	0.3322	0.8397	0
1251	975	0.8718	0.8281	0.3322	0.8401	0
1252	974.9	0.8721	0.828	0.3324	0.8401	0
1253	974.8	0.872	0.828	0.3324	0.8402	0
1254	974.7	0.8722	0.8281	0.3325	0.8402	0
1255	974.6	0.872	0.8283	0.3326	0.8403	0
1256	974.5	0.872	0.8281	0.3326	0.8405	0
1257	974.4	0.8725	0.8285	0.3328	0.841	0
1258	974.3	0.8723	0.8285	0.3328	0.8409	0
1259	974.2	0.8723	0.8285	0.3329	0.8411	0
1260	974.1	0.8724	0.8289	0.3329	0.8411	0
1261	974	0.8725	0.8289	0.333	0.8411	0
1262	973.9	0.8725	0.8289	0.3331	0.8415	0
1263	973.8	0.8727	0.8291	0.3331	0.8417	0
1264	973.7	0.8725	0.8292	0.3332	0.8417	0
1265	973.6	0.8725	0.8292	0.3332	0.8416	0
1266	973.5	0.8726	0.8295	0.3331	0.8418	0
1267	973.4	0.8728	0.8294	0.3333	0.8421	0
1268	973.3	0.8728	0.8296	0.3332	0.8421	0
1269	973.2	0.8728	0.8297	0.3333	0.842	0
1270	973.1	0.8728	0.8298	0.3334	0.8422	0
1271	973	0.8729	0.8297	0.3333	0.8424	0
1272	972.9	0.8729	0.8298	0.3334	0.8422	0
1273	972.8	0.8727	0.8297	0.3332	0.8423	0
1274	972.7	0.8728	0.8298	0.3333	0.8422	0
1275	972.6	0.8731	0.83	0.3335	0.8425	0
1276	972.5	0.8731	0.83	0.3333	0.8425	0

1277	972.4	0.8731	0.83	0.3334	0.8426	0
1278	972.3	0.8731	0.83	0.3334	0.8423	0
1279	972.2	0.8732	0.8303	0.3335	0.8424	0
1280	972.1	0.8734	0.8301	0.3333	0.8424	0
1281	972	0.8736	0.8303	0.3335	0.8426	0
1282	971.9	0.8732	0.8304	0.3336	0.8427	0
1283	971.8	0.8735	0.8303	0.3336	0.8425	0
1284	971.7	0.8734	0.8306	0.3335	0.8427	0
1285	971.6	0.8734	0.8304	0.3334	0.8427	0
1286	971.5	0.8734	0.8305	0.3334	0.8429	0
1287	971.4	0.8736	0.8306	0.3336	0.8428	0
1288	971.3	0.8738	0.8306	0.3336	0.8429	0
1289	971.2	0.8739	0.8308	0.3337	0.843	0
1290	971.1	0.8737	0.8307	0.3337	0.8431	0
1291	971	0.8737	0.8306	0.3337	0.8429	0
1292	970.9	0.8739	0.8308	0.3336	0.8429	0
1293	970.8	0.8735	0.8308	0.3336	0.8428	0
1294	970.7	0.8739	0.831	0.3336	0.8429	0
1295	970.6	0.8737	0.831	0.3336	0.8428	0
1296	970.5	0.8743	0.8311	0.334	0.8429	0
1297	970.4	0.874	0.8308	0.3336	0.8427	0
1298	970.3	0.8739	0.8309	0.3336	0.8428	0
1299	970.2	0.8739	0.8309	0.3335	0.8429	0
1300	970.1	0.8743	0.8308	0.3337	0.8428	0
1301	970	0.874	0.8311	0.3337	0.8428	0
1302	969.9	0.8741	0.8309	0.3334	0.8426	0
1303	969.8	0.8745	0.8313	0.3338	0.8428	0
1304	969.7	0.8743	0.8313	0.3338	0.8428	0
1305	969.6	0.8744	0.8312	0.3336	0.8427	0
1306	969.5	0.8744	0.8312	0.3337	0.8427	0
1307	969.4	0.8745	0.8313	0.3339	0.8426	0
1308	969.3	0.8741	0.831	0.3334	0.8424	0
1309	969.2	0.8741	0.8311	0.3333	0.8425	0
1310	969.1	0.8743	0.8309	0.3335	0.8423	0
1311	969	0.8746	0.8313	0.3335	0.8425	0
1312	968.9	0.8746	0.8311	0.3333	0.8423	0
1313	968.8	0.8745	0.8311	0.3333	0.8423	0
1314	968.7	0.8746	0.8314	0.3334	0.8425	0
1315	968.6	0.8748	0.8312	0.3334	0.8423	0
1316	968.5	0.8747	0.8312	0.3333	0.8423	0
1317	968.4	0.8748	0.8312	0.3331	0.8421	0
1318	968.3	0.8745	0.8312	0.3333	0.8419	0
1319	968.2	0.8747	0.8311	0.333	0.8421	0

1320	968.1	0.8746	0.8313	0.3332	0.842	0
1321	968	0.8747	0.8313	0.3333	0.8421	0
1322	967.9	0.8749	0.8315	0.3332	0.8421	0
1323	967.8	0.8751	0.8316	0.3332	0.8421	0
1324	967.7	0.8748	0.8311	0.333	0.8418	0
1325	967.6	0.875	0.8314	0.3332	0.8419	0
1326	967.5	0.875	0.8312	0.3328	0.8417	0
1327	967.4	0.875	0.8313	0.333	0.8418	0
1328	967.3	0.8749	0.8313	0.3329	0.8418	0
1329	967.2	0.8754	0.8318	0.3332	0.8421	0
1330	967.1	0.8754	0.8317	0.3333	0.842	0
1331	967	0.8752	0.8318	0.333	0.8419	0
1332	966.9	0.8751	0.8317	0.3332	0.8418	0
1333	966.8	0.8753	0.8315	0.3329	0.8416	0
1334	966.7	0.8751	0.8317	0.3331	0.8419	0
1335	966.6	0.8751	0.8313	0.3331	0.8416	0
1336	966.5	0.8754	0.8322	0.3335	0.842	0
1337	966.4	0.8756	0.8322	0.3334	0.842	0
1338	966.3	0.8754	0.832	0.3331	0.8418	0
1339	966.2	0.8755	0.8321	0.3334	0.8421	0
1340	966.1	0.8754	0.8319	0.3332	0.842	0
1341	966	0.8755	0.8322	0.3332	0.8422	0
1342	965.9	0.8756	0.8321	0.3333	0.8422	0
1343	965.8	0.8756	0.8323	0.3334	0.8425	0
1344	965.7	0.8756	0.8323	0.3335	0.8424	0
1345	965.6	0.8759	0.8324	0.3335	0.8425	0
1346	965.5	0.8756	0.8325	0.3334	0.8425	0
1347	965.4	0.8758	0.8326	0.3335	0.8427	0
1348	965.3	0.8756	0.8326	0.3334	0.8427	0
1349	965.2	0.8759	0.8327	0.3337	0.843	0
1350	965.1	0.8759	0.8327	0.3339	0.843	0
1351	965	0.8761	0.8328	0.334	0.843	0
1352	964.9	0.8761	0.833	0.3339	0.8433	0
1353	964.8	0.8759	0.833	0.3338	0.8432	0
1354	964.7	0.8759	0.8333	0.3342	0.8436	0
1355	964.6	0.8761	0.8333	0.3342	0.8437	0
1356	964.5	0.8761	0.8336	0.3344	0.844	0
1357	964.4	0.8761	0.8335	0.3342	0.8439	0
1358	964.3	0.8761	0.8335	0.3341	0.844	0
1359	964.2	0.8761	0.8336	0.3344	0.8442	0
1360	964.1	0.8765	0.8338	0.3344	0.8446	0
1361	964	0.8765	0.8338	0.3347	0.8447	0
1362	963.9	0.8764	0.8338	0.3346	0.8447	0

1363	963.8	0.8763	0.834	0.3347	0.8448	0
1364	963.7	0.8765	0.834	0.3348	0.8449	0
1365	963.6	0.8764	0.8343	0.3349	0.845	0
1366	963.5	0.8764	0.8344	0.3348	0.8451	0
1367	963.4	0.8765	0.8342	0.3347	0.8452	0
1368	963.3	0.8766	0.8345	0.3348	0.8456	0
1369	963.2	0.8768	0.8347	0.3351	0.8458	0
1370	963.1	0.8765	0.8342	0.3351	0.8455	0
1371	963	0.8764	0.8345	0.335	0.8456	0
1372	962.9	0.8763	0.8347	0.335	0.8454	0
1373	962.8	0.8766	0.8345	0.3351	0.8455	0
1374	962.7	0.8766	0.8347	0.335	0.8458	0
1375	962.6	0.8764	0.8344	0.3349	0.8456	0
1376	962.5	0.8765	0.8349	0.3351	0.846	0
1377	962.4	0.8765	0.8346	0.3349	0.8461	0
1378	962.3	0.8766	0.8348	0.335	0.846	0
1379	962.2	0.8766	0.8348	0.335	0.8462	0
1380	962.1	0.8764	0.8349	0.3352	0.8462	0
1381	962	0.8764	0.8348	0.3352	0.8461	0
1382	961.9	0.8764	0.8348	0.3354	0.8461	0
1383	961.8	0.8767	0.835	0.3354	0.8463	0
1384	961.7	0.8768	0.835	0.3357	0.8466	0
1385	961.6	0.8767	0.8352	0.3357	0.8466	0
1386	961.5	0.8767	0.8351	0.3355	0.8466	0
1387	961.4	0.8766	0.8351	0.3357	0.8467	0
1388	961.3	0.8767	0.835	0.3357	0.8465	0
1389	961.2	0.8767	0.8353	0.3359	0.8469	0
1390	961.1	0.8768	0.8351	0.3358	0.8467	0
1391	961	0.8768	0.835	0.3358	0.8468	0
1392	960.9	0.8766	0.835	0.3358	0.8469	0
1393	960.8	0.8766	0.835	0.3357	0.8468	0
1394	960.7	0.8765	0.835	0.3358	0.8469	0
1395	960.6	0.8765	0.8348	0.3357	0.8466	0
1396	960.5	0.8764	0.8348	0.3357	0.8469	0
1397	960.4	0.8766	0.835	0.3357	0.8468	0
1398	960.3	0.8766	0.8349	0.3356	0.8467	0
1399	960.2	0.8765	0.8347	0.3358	0.8468	0
1400	960.1	0.8765	0.8349	0.3356	0.8469	0
1401	960	0.8763	0.8347	0.3355	0.8467	0
1402	959.9	0.8763	0.8347	0.3357	0.8469	0
1403	959.8	0.8765	0.8347	0.3359	0.847	0
1404	959.7	0.8762	0.8346	0.3358	0.8468	0
1405	959.6	0.8763	0.8347	0.3356	0.8468	0

1406	959.5	0.8763	0.8345	0.3357	0.8468	0
1407	959.4	0.8761	0.8346	0.3356	0.8467	0
1408	959.3	0.8761	0.8346	0.3353	0.8465	0
1409	959.2	0.8761	0.8343	0.3354	0.8467	0
1410	959.1	0.8761	0.8343	0.3355	0.8466	0
1411	959	0.8763	0.8344	0.3356	0.8465	0
1412	958.9	0.8761	0.8343	0.3352	0.8464	0
1413	958.8	0.8761	0.8341	0.3353	0.8464	0
1414	958.7	0.8758	0.8341	0.3355	0.8465	0
1415	958.6	0.8758	0.8338	0.3353	0.8462	0
1416	958.5	0.8757	0.8337	0.3353	0.846	0
1417	958.4	0.8757	0.8338	0.3355	0.8461	0
1418	958.3	0.8754	0.8337	0.3353	0.8461	0
1419	958.2	0.8756	0.8337	0.3353	0.846	0
1420	958.1	0.8756	0.8335	0.3353	0.846	0
1421	958	0.8759	0.8336	0.3356	0.8461	0
1422	957.9	0.8755	0.8332	0.3352	0.846	0
1423	957.8	0.8754	0.8333	0.3353	0.8459	0
1424	957.7	0.8754	0.8332	0.3354	0.8457	0
1425	957.6	0.8752	0.833	0.3351	0.8457	0
1426	957.5	0.8751	0.8331	0.3351	0.8456	0
1427	957.4	0.8752	0.8328	0.3351	0.8458	0
1428	957.3	0.8753	0.833	0.3348	0.8455	0
1429	957.2	0.8748	0.8329	0.3351	0.8457	0
1430	957.1	0.8747	0.8326	0.3349	0.8456	0
1431	957	0.8745	0.8325	0.3351	0.8456	0
1432	956.9	0.8748	0.8327	0.3353	0.8458	0
1433	956.8	0.8748	0.8328	0.3352	0.8457	0
1434	956.7	0.8747	0.8326	0.3351	0.8457	0
1435	956.6	0.8744	0.8322	0.335	0.8455	0
1436	956.5	0.8746	0.8324	0.3352	0.8457	0
1437	956.4	0.8744	0.8323	0.3352	0.8457	0
1438	956.3	0.8743	0.8321	0.3352	0.8457	0
1439	956.2	0.8744	0.8321	0.3354	0.8459	0
1440	956.1	0.8743	0.8322	0.3353	0.8458	0
1441	956	0.8739	0.8319	0.3351	0.8458	0
1442	955.9	0.8739	0.8318	0.3351	0.8459	0
1443	955.8	0.8743	0.8322	0.3353	0.8462	0
1444	955.7	0.8739	0.8319	0.3353	0.8461	0
1445	955.6	0.874	0.8322	0.3355	0.8462	0
1446	955.5	0.874	0.8316	0.3354	0.846	0
1447	955.4	0.8739	0.8319	0.3355	0.846	0
1448	955.3	0.8737	0.8316	0.3354	0.846	0

1449	955.2	0.8736	0.8314	0.3352	0.846	0
1450	955.1	0.8734	0.8314	0.3353	0.8463	0
1451	955	0.8735	0.8313	0.3354	0.8464	0
1452	954.9	0.873	0.8312	0.3354	0.8462	0
1453	954.8	0.873	0.8312	0.3353	0.8464	0
1454	954.7	0.8728	0.8312	0.3351	0.8463	0
1455	954.6	0.8728	0.831	0.3352	0.8464	0
1456	954.5	0.8731	0.8314	0.3355	0.8468	0
1457	954.4	0.8728	0.8312	0.3355	0.8467	0
1458	954.3	0.8731	0.8311	0.3355	0.8467	0
1459	954.2	0.8727	0.8311	0.3356	0.8469	0
1460	954.1	0.8729	0.8312	0.3358	0.8469	0
1461	954	0.8727	0.8314	0.3359	0.8473	0
1462	953.9	0.8727	0.8311	0.3358	0.8472	0
1463	953.8	0.8727	0.8312	0.3358	0.8474	0
1464	953.7	0.8727	0.8311	0.336	0.8475	0
1465	953.6	0.8725	0.8313	0.3358	0.8476	0
1466	953.5	0.8723	0.8312	0.336	0.8478	0
1467	953.4	0.8722	0.8312	0.3361	0.8479	0
1468	953.3	0.8722	0.8313	0.3362	0.8479	0
1469	953.2	0.8722	0.8312	0.3364	0.8482	0
1470	953.1	0.8723	0.8313	0.3366	0.8482	0
1471	953	0.8721	0.8313	0.3364	0.8484	0
1472	952.9	0.8721	0.8313	0.3365	0.8484	0
1473	952.8	0.8718	0.8309	0.3364	0.8485	0
1474	952.7	0.8719	0.8308	0.3366	0.8486	0
1475	952.6	0.8718	0.8314	0.3366	0.8487	0
1476	952.5	0.8716	0.8311	0.3363	0.8486	0
1477	952.4	0.8716	0.831	0.3365	0.8487	0
1478	952.3	0.8719	0.8312	0.3367	0.8489	0
1479	952.2	0.8717	0.8309	0.3367	0.8488	0
1480	952.1	0.8714	0.8308	0.3364	0.8489	0
1481	952	0.8714	0.8308	0.3366	0.8488	0
1482	951.9	0.8714	0.8306	0.3365	0.849	0
1483	951.8	0.8714	0.8307	0.3367	0.849	0
1484	951.7	0.8714	0.8308	0.3367	0.8491	0
1485	951.6	0.8711	0.8306	0.3366	0.849	0
1486	951.5	0.8712	0.8307	0.3367	0.8492	0
1487	951.4	0.8713	0.8309	0.3368	0.8493	0
1488	951.3	0.8711	0.8307	0.3368	0.8493	0
1489	951.2	0.8714	0.8307	0.3368	0.8495	0
1490	951.1	0.8711	0.8305	0.3368	0.8494	0
1491	951	0.8712	0.8308	0.3369	0.8496	0

1492	950.9	0.8709	0.8306	0.3367	0.8495	0
1493	950.8	0.8708	0.8304	0.3368	0.8495	0
1494	950.7	0.8708	0.8306	0.3369	0.8496	0
1495	950.6	0.8706	0.8306	0.3371	0.8497	0
1496	950.5	0.8708	0.8305	0.337	0.8497	0
1497	950.4	0.8706	0.8304	0.337	0.8498	0
1498	950.3	0.8704	0.8307	0.3372	0.8499	0
1499	950.2	0.8711	0.8308	0.3372	0.85	0
1500	950.1	0.8709	0.8307	0.3373	0.85	0
1501	950	0.8707	0.8307	0.3372	0.85	0
1502	949.9	0.8706	0.8305	0.3371	0.85	0
1503	949.8	0.8706	0.8303	0.3371	0.8498	0
1504	949.7	0.8704	0.8301	0.3369	0.8496	0
1505	949.6	0.8704	0.8302	0.3372	0.8498	0
1506	949.5	0.8703	0.8301	0.3371	0.8497	0
1507	949.4	0.8704	0.8304	0.3372	0.8498	0
1508	949.3	0.8703	0.83	0.3369	0.8496	0
1509	949.2	0.8702	0.8301	0.3371	0.8496	0
1510	949.1	0.8702	0.8299	0.337	0.8497	0
1511	949	0.8703	0.8298	0.3368	0.8496	0
1512	948.9	0.8704	0.8299	0.337	0.8495	0
1513	948.8	0.8703	0.8299	0.3372	0.8497	0
1514	948.7	0.8701	0.8297	0.3369	0.8496	0
1515	948.6	0.8699	0.8295	0.3367	0.8492	0
1516	948.5	0.8699	0.8294	0.3368	0.8492	0
1517	948.4	0.8701	0.8298	0.337	0.8494	0
1518	948.3	0.8701	0.83	0.3369	0.8494	0
1519	948.2	0.8701	0.8296	0.3369	0.8492	0
1520	948.1	0.8698	0.8292	0.3366	0.8491	0
1521	948	0.87	0.8293	0.3366	0.8491	0
1522	947.9	0.87	0.8293	0.3366	0.8492	0
1523	947.8	0.8701	0.8294	0.3366	0.8492	0
1524	947.7	0.8697	0.8292	0.3364	0.8488	0
1525	947.6	0.8697	0.8292	0.3364	0.8489	0
1526	947.5	0.8696	0.829	0.3365	0.8489	0
1527	947.4	0.8696	0.829	0.3365	0.8488	0
1528	947.3	0.8697	0.8291	0.3364	0.849	0
1529	947.2	0.8697	0.8292	0.3365	0.8489	0
1530	947.1	0.8693	0.8291	0.3366	0.8487	0
1531	947	0.8695	0.8291	0.3365	0.8486	0
1532	946.9	0.8695	0.8288	0.3364	0.8487	0
1533	946.8	0.8693	0.8289	0.3364	0.8489	0
1534	946.7	0.8697	0.829	0.3367	0.8488	0

1535	946.6	0.8692	0.8285	0.3364	0.8486	0
1536	946.5	0.8694	0.8288	0.3367	0.8487	0
1537	946.4	0.8694	0.8286	0.3364	0.8487	0
1538	946.3	0.8694	0.8288	0.3364	0.8486	0
1539	946.2	0.8689	0.8286	0.3363	0.8485	0
1540	946.1	0.8693	0.8287	0.3365	0.8486	0
1541	946	0.8687	0.8284	0.3363	0.8486	0
1542	945.9	0.8694	0.8285	0.3364	0.8487	0
1543	945.8	0.869	0.8285	0.3365	0.8487	0
1544	945.7	0.8691	0.8284	0.3363	0.8486	0
1545	945.6	0.8691	0.8285	0.3363	0.8488	0
1546	945.5	0.8693	0.8285	0.3365	0.8489	0
1547	945.4	0.8693	0.8284	0.3365	0.8489	0
1548	945.3	0.869	0.8283	0.3365	0.8488	0
1549	945.2	0.8691	0.8285	0.3366	0.8488	0
1550	945.1	0.8689	0.8283	0.3365	0.8489	0
1551	945	0.8689	0.8284	0.3365	0.849	0
1552	944.9	0.869	0.8286	0.3368	0.8492	0
1553	944.8	0.8688	0.8285	0.3366	0.8492	0
1554	944.7	0.8689	0.8284	0.3366	0.8492	0
1555	944.6	0.8688	0.8287	0.3367	0.8492	0
1556	944.5	0.8689	0.8286	0.3369	0.8493	0
1557	944.4	0.8687	0.8284	0.3367	0.8492	0
1558	944.3	0.8691	0.8287	0.3368	0.8496	0
1559	944.2	0.8688	0.8287	0.337	0.8496	0
1560	944.1	0.869	0.8288	0.337	0.8499	0
1561	944	0.8689	0.8286	0.3368	0.8496	0
1562	943.9	0.8687	0.8288	0.337	0.8498	0
1563	943.8	0.8688	0.8288	0.3369	0.8499	0
1564	943.7	0.8686	0.8287	0.337	0.8499	0
1565	943.6	0.8687	0.8287	0.3372	0.8501	0
1566	943.5	0.8688	0.8286	0.337	0.8501	0
1567	943.4	0.8689	0.8288	0.3372	0.8504	0
1568	943.3	0.8689	0.8288	0.3371	0.8505	0
1569	943.2	0.8687	0.8289	0.3373	0.8505	0
1570	943.1	0.8687	0.8286	0.3372	0.8503	0
1571	943	0.8687	0.8286	0.3371	0.8504	0
1572	942.9	0.8687	0.8289	0.3372	0.8505	0
1573	942.8	0.8685	0.8286	0.3372	0.8504	0
1574	942.7	0.8684	0.8288	0.3372	0.8507	0
1575	942.6	0.8685	0.8287	0.3374	0.8505	0
1576	942.5	0.8685	0.8287	0.3376	0.8507	0
1577	942.4	0.8686	0.8288	0.3375	0.8507	0

1578	942.3	0.8686	0.8288	0.3374	0.8506	0
1579	942.2	0.8683	0.8289	0.3376	0.8509	0
1580	942.1	0.8682	0.8286	0.3375	0.8508	0
1581	942	0.868	0.8286	0.3374	0.8507	0
1582	941.9	0.8683	0.8288	0.3375	0.8507	0
1583	941.8	0.868	0.8284	0.3374	0.8508	0
1584	941.7	0.8682	0.8286	0.3376	0.851	0
1585	941.6	0.8681	0.8285	0.3376	0.8509	0
1586	941.5	0.8682	0.8285	0.3376	0.8511	0
1587	941.4	0.868	0.8283	0.3374	0.8509	0
1588	941.3	0.868	0.8285	0.3375	0.8509	0
1589	941.2	0.8681	0.8287	0.3374	0.8511	0
1590	941.1	0.8679	0.8286	0.3376	0.8511	0
1591	941	0.868	0.8284	0.3374	0.8512	0
1592	940.9	0.8679	0.8286	0.3376	0.8513	0
1593	940.8	0.8676	0.8285	0.3376	0.8512	0
1594	940.7	0.8678	0.8285	0.3377	0.8514	0
1595	940.6	0.8678	0.8284	0.3377	0.8514	0
1596	940.5	0.8676	0.8284	0.3374	0.8513	0
1597	940.4	0.868	0.8285	0.3377	0.8515	0
1598	940.3	0.8678	0.8285	0.3378	0.8515	0
1599	940.2	0.8676	0.8286	0.3374	0.8513	0
1600	940.1	0.8674	0.8282	0.3374	0.8513	0
1601	940	0.8677	0.8283	0.3376	0.8514	0
1602	939.9	0.8677	0.8282	0.3376	0.8514	0
1603	939.8	0.8675	0.8284	0.3377	0.8515	0
1604	939.7	0.8676	0.8282	0.3375	0.8517	0
1605	939.6	0.8675	0.8282	0.3377	0.8517	0
1606	939.5	0.8675	0.8281	0.3377	0.8516	0
1607	939.4	0.8677	0.8285	0.3378	0.8518	0
1608	939.3	0.8673	0.8282	0.3376	0.8515	0
1609	939.2	0.8674	0.8283	0.3375	0.8518	0
1610	939.1	0.8676	0.8283	0.3378	0.8518	0
1611	939	0.8674	0.8282	0.3378	0.8518	0
1612	938.9	0.8672	0.8283	0.3378	0.8519	0
1613	938.8	0.8672	0.8279	0.3378	0.8517	0
1614	938.7	0.867	0.8282	0.3378	0.8518	0
1615	938.6	0.8675	0.8282	0.3379	0.852	0
1616	938.5	0.8672	0.8279	0.3377	0.8519	0
1617	938.4	0.8673	0.8282	0.3379	0.852	0
1618	938.3	0.8672	0.8279	0.3378	0.8519	0
1619	938.2	0.8671	0.8279	0.3378	0.8518	0
1620	938.1	0.8669	0.8279	0.3378	0.8518	0

1621	938	0.8673	0.828	0.3378	0.8519	0
1622	937.9	0.8672	0.8279	0.3377	0.8519	0
1623	937.8	0.8672	0.8279	0.3377	0.852	0
1624	937.7	0.8673	0.8282	0.338	0.8522	0
1625	937.6	0.8673	0.8279	0.3378	0.8521	0
1626	937.5	0.8671	0.828	0.3377	0.852	0
1627	937.4	0.8669	0.8279	0.338	0.8521	0
1628	937.3	0.867	0.828	0.338	0.8521	0
1629	937.2	0.8671	0.8279	0.3378	0.8521	0
1630	937.1	0.8669	0.8278	0.3377	0.8518	0
1631	937	0.8667	0.8277	0.3376	0.8519	0
1632	936.9	0.8668	0.8276	0.3377	0.8518	0
1633	936.8	0.8668	0.8278	0.338	0.852	0
1634	936.7	0.8671	0.8279	0.3379	0.852	0
1635	936.6	0.867	0.8276	0.3378	0.8518	0
1636	936.5	0.8667	0.8275	0.3378	0.8518	0
1637	936.4	0.8667	0.8278	0.3379	0.852	0
1638	936.3	0.8669	0.8278	0.3379	0.8519	0
1639	936.2	0.8668	0.8277	0.3377	0.852	0
1640	936.1	0.8668	0.8277	0.3378	0.852	0
1641	936	0.8669	0.8276	0.3378	0.8522	0
1642	935.9	0.867	0.8277	0.3379	0.8521	0
1643	935.8	0.867	0.8277	0.3379	0.8523	0
1644	935.7	0.8671	0.8277	0.338	0.8521	0
1645	935.6	0.8667	0.8276	0.3378	0.8522	0
1646	935.5	0.8667	0.828	0.3379	0.8523	0
1647	935.4	0.8666	0.8276	0.338	0.8523	0
1648	935.3	0.8669	0.8277	0.3381	0.8525	0
1649	935.2	0.8669	0.8279	0.3381	0.8526	0
1650	935.1	0.8669	0.8279	0.3381	0.8526	0
1651	935	0.8668	0.8279	0.3381	0.8528	0
1652	934.9	0.8666	0.828	0.3381	0.8526	0
1653	934.8	0.867	0.8281	0.3383	0.8529	0
1654	934.7	0.867	0.8279	0.338	0.8531	0
1655	934.6	0.8667	0.8277	0.3382	0.8529	0
1656	934.5	0.8666	0.8278	0.3383	0.8528	0
1657	934.4	0.8669	0.8278	0.3381	0.8529	0
1658	934.3	0.8667	0.8279	0.3382	0.8531	0
1659	934.2	0.8667	0.8278	0.3383	0.8532	0
1660	934.1	0.8664	0.8278	0.3381	0.8529	0
1661	934	0.8665	0.8279	0.3383	0.8531	0
1662	933.9	0.8671	0.828	0.3387	0.8533	0
1663	933.8	0.8669	0.828	0.3385	0.8533	0

1664	933.7	0.8666	0.8278	0.3385	0.8531	0
1665	933.6	0.8667	0.8279	0.3386	0.8531	0
1666	933.5	0.8666	0.828	0.3384	0.8533	0
1667	933.4	0.8666	0.8278	0.3385	0.8533	0
1668	933.3	0.8666	0.8281	0.3386	0.8534	0
1669	933.2	0.8666	0.8279	0.3386	0.8537	0
1670	933.1	0.8662	0.8278	0.3387	0.8533	0
1671	933	0.8663	0.8279	0.3386	0.8536	0
1672	932.9	0.8664	0.8277	0.3384	0.8534	0
1673	932.8	0.8665	0.8279	0.3386	0.8535	0
1674	932.7	0.8664	0.8279	0.3386	0.8534	0
1675	932.6	0.8664	0.8279	0.3387	0.8536	0
1676	932.5	0.8666	0.8282	0.339	0.8538	0
1677	932.4	0.8665	0.828	0.3386	0.8538	0
1678	932.3	0.8663	0.8281	0.3386	0.8538	0
1679	932.2	0.8661	0.8281	0.3388	0.8537	0
1680	932.1	0.8661	0.8277	0.3386	0.8539	0
1681	932	0.8663	0.8279	0.3386	0.8537	0
1682	931.9	0.8663	0.8281	0.3387	0.8539	0
1683	931.8	0.8662	0.828	0.3386	0.854	0
1684	931.7	0.8663	0.8278	0.3386	0.8539	0
1685	931.6	0.8661	0.8279	0.3387	0.8538	0
1686	931.5	0.8662	0.8279	0.3387	0.8539	0
1687	931.4	0.8662	0.8278	0.3386	0.8538	0
1688	931.3	0.8663	0.828	0.3387	0.8541	0
1689	931.2	0.866	0.8277	0.3389	0.8541	0
1690	931.1	0.8661	0.8277	0.3387	0.8538	0
1691	931	0.866	0.8277	0.3385	0.8539	0
1692	930.9	0.8663	0.8277	0.3387	0.8539	0
1693	930.8	0.866	0.8279	0.3387	0.8539	0
1694	930.7	0.866	0.8276	0.3386	0.8539	0
1695	930.6	0.866	0.8277	0.3387	0.854	0
1696	930.5	0.866	0.8275	0.3387	0.8538	0
1697	930.4	0.8657	0.8277	0.3389	0.854	0
1698	930.3	0.866	0.8275	0.3387	0.8538	0
1699	930.2	0.8659	0.8275	0.3385	0.8539	0
1700	930.1	0.8658	0.8274	0.3387	0.8537	0
1701	930	0.8658	0.8274	0.3388	0.8539	0
1702	929.9	0.8659	0.8276	0.3386	0.8538	0
1703	929.8	0.8656	0.8276	0.3387	0.8539	0
1704	929.7	0.8655	0.8276	0.3386	0.8538	0
1705	929.6	0.8657	0.8272	0.3386	0.8539	0
1706	929.5	0.8655	0.8274	0.3386	0.8539	0

1707	929.4	0.8658	0.8275	0.3389	0.8539	0
1708	929.3	0.8659	0.8275	0.3389	0.8539	0
1709	929.2	0.8658	0.8274	0.3386	0.8539	0
1710	929.1	0.8659	0.8271	0.3385	0.8538	0
1711	929	0.8656	0.8273	0.3386	0.8536	0
1712	928.9	0.8656	0.8273	0.3385	0.8538	0
1713	928.8	0.8657	0.8273	0.3387	0.854	0
1714	928.7	0.8656	0.8274	0.3387	0.8539	0
1715	928.6	0.8655	0.8273	0.3386	0.8539	0
1716	928.5	0.8658	0.8273	0.3386	0.8538	0
1717	928.4	0.8656	0.8273	0.3387	0.8539	0
1718	928.3	0.8657	0.8272	0.3388	0.8541	0
1719	928.2	0.8655	0.8271	0.3386	0.8541	0
1720	928.1	0.8654	0.827	0.3385	0.8537	0
1721	928	0.8658	0.8273	0.3388	0.8541	0
1722	927.9	0.8656	0.8274	0.3386	0.854	0
1723	927.8	0.8658	0.8272	0.3385	0.854	0
1724	927.7	0.8656	0.8273	0.3387	0.8544	0
1725	927.6	0.8656	0.8272	0.3387	0.8541	0
1726	927.5	0.8658	0.8274	0.3388	0.8543	0
1727	927.4	0.8657	0.8273	0.3388	0.8544	0
1728	927.3	0.8655	0.8274	0.3386	0.8544	0
1729	927.2	0.8656	0.8274	0.3388	0.8545	0
1730	927.1	0.8655	0.8272	0.3386	0.8544	0
1731	927	0.8654	0.8272	0.3387	0.8544	0
1732	926.9	0.8654	0.8273	0.3387	0.8542	0
1733	926.8	0.8651	0.827	0.3384	0.8545	0
1734	926.7	0.8653	0.8272	0.3388	0.8548	0
1735	926.6	0.8656	0.8274	0.3388	0.8549	0
1736	926.5	0.8655	0.8273	0.3386	0.8549	0
1737	926.4	0.8657	0.8275	0.3389	0.8549	0
1738	926.3	0.8655	0.8275	0.3391	0.8549	0
1739	926.2	0.8655	0.8273	0.3389	0.8549	0
1740	926.1	0.8654	0.8275	0.3389	0.855	0
1741	926	0.8655	0.8273	0.3387	0.8551	0
1742	925.9	0.8656	0.8275	0.3393	0.8553	0
1743	925.8	0.8653	0.8276	0.339	0.8552	0
1744	925.7	0.8657	0.8279	0.339	0.8553	0
1745	925.6	0.8657	0.8277	0.3391	0.8553	0
1746	925.5	0.8653	0.8279	0.3392	0.8554	0
1747	925.4	0.8654	0.8278	0.3393	0.8555	0
1748	925.3	0.8655	0.8279	0.3392	0.8555	0
1749	925.2	0.8657	0.8277	0.339	0.8556	0

1750	925.1	0.8657	0.828	0.3393	0.8558	0
1751	925	0.8654	0.828	0.3393	0.8556	0
1752	924.9	0.8655	0.828	0.3394	0.8558	0
1753	924.8	0.8658	0.8282	0.3394	0.856	0
1754	924.7	0.8656	0.8278	0.3394	0.8561	0
1755	924.6	0.8656	0.8278	0.3392	0.856	0
1756	924.5	0.8656	0.8281	0.3394	0.8559	0
1757	924.4	0.8656	0.8281	0.3394	0.856	0
1758	924.3	0.8656	0.8279	0.3394	0.8562	0
1759	924.2	0.8656	0.828	0.3396	0.8562	0
1760	924.1	0.8656	0.828	0.3396	0.8562	0
1761	924	0.8657	0.8282	0.3397	0.8565	0
1762	923.9	0.8657	0.8283	0.3398	0.8565	0
1763	923.8	0.8655	0.8281	0.3397	0.8566	0
1764	923.7	0.8655	0.8279	0.3395	0.8564	0
1765	923.6	0.8657	0.8283	0.3397	0.8568	0
1766	923.5	0.8657	0.828	0.3397	0.8567	0
1767	923.4	0.8658	0.8285	0.3397	0.8567	0
1768	923.3	0.8655	0.828	0.3397	0.8565	0
1769	923.2	0.8655	0.8283	0.3395	0.8569	0
1770	923.1	0.8657	0.8286	0.3396	0.857	0
1771	923	0.8656	0.8283	0.3397	0.857	0
1772	922.9	0.8656	0.8285	0.3397	0.857	0
1773	922.8	0.8654	0.8284	0.3399	0.857	0
1774	922.7	0.8657	0.8284	0.3398	0.8571	0
1775	922.6	0.8654	0.8284	0.3398	0.8568	0
1776	922.5	0.8657	0.8283	0.3399	0.8573	0
1777	922.4	0.8657	0.8285	0.3399	0.8571	0
1778	922.3	0.8656	0.8285	0.34	0.8572	0
1779	922.2	0.8657	0.8286	0.3398	0.8571	0
1780	922.1	0.8655	0.8285	0.3399	0.8569	0
1781	922	0.8657	0.8285	0.34	0.8571	0
1782	921.9	0.8655	0.8284	0.3399	0.8569	0
1783	921.8	0.8656	0.8283	0.3399	0.857	0
1784	921.7	0.8654	0.8285	0.3397	0.857	0
1785	921.6	0.8655	0.8285	0.3398	0.857	0
1786	921.5	0.8654	0.8283	0.3397	0.8568	0
1787	921.4	0.8656	0.8284	0.34	0.8571	0
1788	921.3	0.8656	0.8283	0.3399	0.857	0
1789	921.2	0.8655	0.8284	0.3398	0.857	0
1790	921.1	0.8655	0.8284	0.3396	0.857	0
1791	921	0.8653	0.8285	0.3395	0.857	0
1792	920.9	0.8656	0.8284	0.3396	0.857	0

1793	920.8	0.8655	0.8283	0.3395	0.8569	0
1794	920.7	0.8655	0.8281	0.3394	0.8567	0
1795	920.6	0.8652	0.8281	0.3392	0.8567	0
1796	920.5	0.8655	0.8281	0.3394	0.8569	0
1797	920.4	0.8654	0.828	0.3394	0.8566	0
1798	920.3	0.8654	0.8282	0.3393	0.8569	0
1799	920.2	0.8654	0.828	0.3393	0.8567	0
1800	920.1	0.8653	0.8281	0.3392	0.8566	0
1801	920	0.8652	0.8281	0.3392	0.8565	0
1802	919.9	0.8653	0.828	0.3391	0.8567	0
1803	919.8	0.8652	0.8277	0.3392	0.8565	0
1804	919.7	0.8654	0.828	0.3392	0.8566	0
1805	919.6	0.8654	0.8277	0.3391	0.8565	0
1806	919.5	0.8654	0.8279	0.3392	0.8565	0
1807	919.4	0.8652	0.8279	0.3391	0.8565	0
1808	919.3	0.8651	0.8279	0.3391	0.8563	0
1809	919.2	0.8651	0.8279	0.339	0.8561	0
1810	919.1	0.8652	0.8277	0.3389	0.8562	0
1811	919	0.8652	0.828	0.3389	0.8561	0
1812	918.9	0.8651	0.8276	0.3389	0.8561	0
1813	918.8	0.8652	0.8277	0.339	0.8559	0
1814	918.7	0.8653	0.8277	0.3389	0.8559	0
1815	918.6	0.8651	0.8275	0.3388	0.8561	0
1816	918.5	0.8653	0.8277	0.3391	0.8562	0
1817	918.4	0.8654	0.8278	0.3391	0.8561	0
1818	918.3	0.8652	0.8278	0.3389	0.8559	0
1819	918.2	0.8652	0.8277	0.3386	0.8557	0
1820	918.1	0.8653	0.8276	0.3386	0.8558	0
1821	918	0.8652	0.8278	0.3388	0.8557	0
1822	917.9	0.8651	0.8274	0.3387	0.8557	0
1823	917.8	0.8651	0.8276	0.3387	0.8559	0
1824	917.7	0.8653	0.8278	0.3389	0.8559	0
1825	917.6	0.8656	0.8276	0.3389	0.8559	0
1826	917.5	0.8654	0.8277	0.3389	0.8558	0
1827	917.4	0.8654	0.8278	0.3389	0.8561	0
1828	917.3	0.8651	0.8278	0.3388	0.8559	0
1829	917.2	0.8654	0.8277	0.339	0.8562	0
1830	917.1	0.8651	0.8279	0.3389	0.8561	0
1831	917	0.8651	0.8277	0.3388	0.8561	0
1832	916.9	0.8653	0.8276	0.3389	0.8561	0
1833	916.8	0.865	0.8277	0.3387	0.856	0
1834	916.7	0.8653	0.828	0.3388	0.8563	0
1835	916.6	0.8655	0.8285	0.339	0.8567	0

1836	916.5	0.8653	0.828	0.3389	0.8565	0
1837	916.4	0.8655	0.8283	0.3391	0.8565	0
1838	916.3	0.8654	0.828	0.339	0.8566	0
1839	916.2	0.8656	0.8281	0.3392	0.8567	0
1840	916.1	0.8654	0.8282	0.339	0.8568	0
1841	916	0.8652	0.8281	0.3389	0.8567	0
1842	915.9	0.8657	0.8286	0.3393	0.8569	0
1843	915.8	0.8654	0.8284	0.3393	0.8569	0
1844	915.7	0.8655	0.8287	0.3395	0.8573	0
1845	915.6	0.8656	0.8285	0.3394	0.8571	0
1846	915.5	0.8654	0.8286	0.3393	0.8572	0
1847	915.4	0.8658	0.8285	0.3396	0.8575	0
1848	915.3	0.8656	0.829	0.3397	0.8577	0
1849	915.2	0.8656	0.8287	0.3395	0.8577	0
1850	915.1	0.8655	0.8287	0.3394	0.8579	0
1851	915	0.8656	0.8289	0.3396	0.8577	0
1852	914.9	0.8656	0.8288	0.3394	0.858	0
1853	914.8	0.8656	0.829	0.3396	0.8576	0
1854	914.7	0.8657	0.8291	0.3399	0.8582	0
1855	914.6	0.8655	0.8291	0.3397	0.8581	0
1856	914.5	0.8657	0.8292	0.3398	0.858	0
1857	914.4	0.8656	0.8291	0.3398	0.8584	0
1858	914.3	0.8657	0.8291	0.3399	0.8584	0
1859	914.2	0.8658	0.8293	0.3399	0.8586	0
1860	914.1	0.8656	0.8291	0.34	0.8586	0
1861	914	0.8657	0.8294	0.34	0.8586	0
1862	913.9	0.8659	0.8295	0.34	0.8589	0
1863	913.8	0.8659	0.8296	0.3402	0.859	0
1864	913.7	0.8657	0.8296	0.3402	0.8591	0
1865	913.6	0.8657	0.8296	0.3402	0.8594	0
1866	913.5	0.8659	0.8296	0.3402	0.8593	0
1867	913.4	0.8657	0.8297	0.3403	0.8591	0
1868	913.3	0.8659	0.8298	0.3404	0.8594	0
1869	913.2	0.8659	0.8295	0.3403	0.8593	0
1870	913.1	0.8658	0.83	0.3403	0.8593	0
1871	913	0.866	0.8299	0.3405	0.8595	0
1872	912.9	0.866	0.8299	0.3407	0.8595	0
1873	912.8	0.8661	0.83	0.3405	0.8596	0
1874	912.7	0.8661	0.8302	0.3405	0.8597	0
1875	912.6	0.8658	0.8299	0.3405	0.8596	0
1876	912.5	0.8662	0.8299	0.3406	0.8597	0
1877	912.4	0.8659	0.8299	0.3406	0.8597	0
1878	912.3	0.8658	0.8299	0.3404	0.8595	0

1879	912.2	0.8661	0.8299	0.3407	0.8599	0
1880	912.1	0.8659	0.8298	0.3406	0.8596	0
1881	912	0.866	0.83	0.3407	0.8597	0
1882	911.9	0.8661	0.8305	0.3406	0.8596	0
1883	911.8	0.8661	0.83	0.3406	0.8597	0
1884	911.7	0.8659	0.8299	0.3406	0.8597	0
1885	911.6	0.866	0.8302	0.3405	0.8596	0
1886	911.5	0.8661	0.8301	0.3407	0.8597	0
1887	911.4	0.8661	0.83	0.3406	0.8598	0
1888	911.3	0.8663	0.8304	0.3407	0.86	0
1889	911.2	0.8661	0.8303	0.3406	0.86	0
1890	911.1	0.8662	0.8303	0.3407	0.8599	0
1891	911	0.8658	0.8298	0.3404	0.8597	0
1892	910.9	0.866	0.83	0.3405	0.8598	0
1893	910.8	0.8661	0.83	0.3405	0.8597	0
1894	910.7	0.8661	0.8302	0.3405	0.8599	0
1895	910.6	0.8661	0.83	0.3404	0.8596	0
1896	910.5	0.8663	0.8302	0.3404	0.8598	0
1897	910.4	0.8664	0.83	0.3405	0.8598	0
1898	910.3	0.8662	0.8304	0.3404	0.8596	0
1899	910.2	0.8661	0.8302	0.3405	0.8595	0
1900	910.1	0.866	0.83	0.3404	0.8595	0
1901	910	0.8663	0.8303	0.3405	0.8596	0
1902	909.9	0.8659	0.8302	0.3401	0.8593	0
1903	909.8	0.8661	0.8302	0.3404	0.8595	0
1904	909.7	0.8659	0.8301	0.3403	0.8593	0
1905	909.6	0.8661	0.83	0.3404	0.8593	0
1906	909.5	0.8661	0.8299	0.3402	0.8592	0
1907	909.4	0.866	0.8301	0.3403	0.8593	0
1908	909.3	0.866	0.83	0.3402	0.8591	0
1909	909.2	0.8662	0.8301	0.34	0.8592	0
1910	909.1	0.8661	0.8299	0.3401	0.859	0
1911	909	0.8661	0.8298	0.3399	0.8588	0
1912	908.9	0.8662	0.8301	0.3402	0.8591	0
1913	908.8	0.8661	0.8299	0.3401	0.8589	0
1914	908.7	0.866	0.8299	0.3399	0.8589	0
1915	908.6	0.8661	0.8298	0.3401	0.8589	0
1916	908.5	0.8663	0.83	0.3401	0.8588	0
1917	908.4	0.8662	0.83	0.3401	0.8587	0
1918	908.3	0.866	0.8298	0.3397	0.8586	0
1919	908.2	0.8661	0.83	0.3399	0.8589	0
1920	908.1	0.8662	0.8299	0.3399	0.8588	0
1921	908	0.8662	0.8299	0.3399	0.8587	0

1922	907.9	0.8662	0.83	0.3399	0.8588	0
1923	907.8	0.8663	0.83	0.3402	0.8587	0
1924	907.7	0.8663	0.8296	0.34	0.8587	0
1925	907.6	0.8663	0.8301	0.34	0.8587	0
1926	907.5	0.8662	0.8301	0.34	0.8586	0
1927	907.4	0.8664	0.83	0.3396	0.8587	0
1928	907.3	0.8663	0.8298	0.3397	0.8587	0
1929	907.2	0.8661	0.8298	0.3398	0.8583	0
1930	907.1	0.8662	0.8299	0.3398	0.8587	0
1931	907	0.8665	0.83	0.3397	0.8585	0
1932	906.9	0.8663	0.83	0.3399	0.8586	0
1933	906.8	0.8665	0.83	0.3399	0.8585	0
1934	906.7	0.8663	0.8298	0.3399	0.8585	0
1935	906.6	0.8663	0.83	0.34	0.8585	0
1936	906.5	0.8663	0.8299	0.3396	0.8588	0
1937	906.4	0.8666	0.8298	0.3399	0.8586	0
1938	906.3	0.8664	0.83	0.3398	0.8587	0
1939	906.2	0.8663	0.83	0.3398	0.8588	0
1940	906.1	0.8663	0.83	0.3399	0.8588	0
1941	906	0.8665	0.8301	0.34	0.8587	0
1942	905.9	0.8664	0.8301	0.34	0.8588	0
1943	905.8	0.8665	0.8302	0.34	0.8588	0
1944	905.7	0.8666	0.8303	0.3399	0.8588	0
1945	905.6	0.8665	0.8302	0.34	0.8589	0
1946	905.5	0.8666	0.83	0.3402	0.8593	0
1947	905.4	0.8666	0.8304	0.3401	0.8592	0
1948	905.3	0.8666	0.8307	0.3403	0.8594	0
1949	905.2	0.8666	0.8306	0.3404	0.8594	0
1950	905.1	0.8666	0.8305	0.34	0.8593	0
1951	905	0.8666	0.8307	0.3403	0.8597	0
1952	904.9	0.8666	0.8307	0.3403	0.8597	0
1953	904.8	0.8667	0.831	0.3403	0.8597	0
1954	904.7	0.8666	0.8307	0.3404	0.8599	0
1955	904.6	0.8669	0.8311	0.3404	0.8599	0
1956	904.5	0.8667	0.831	0.3405	0.8603	0
1957	904.4	0.8668	0.8313	0.3407	0.8605	0
1958	904.3	0.8665	0.8311	0.3407	0.8608	0
1959	904.2	0.8668	0.8316	0.3409	0.861	0
1960	904.1	0.8667	0.8314	0.3409	0.8609	0
1961	904	0.8671	0.8317	0.3411	0.8614	0
1962	903.9	0.8671	0.8318	0.3412	0.8615	0
1963	903.8	0.8669	0.8317	0.3412	0.8615	0
1964	903.7	0.8671	0.832	0.3412	0.8616	0

1965	903.6	0.8669	0.8317	0.3413	0.8618	0
1966	903.5	0.8671	0.8323	0.3414	0.8617	0
1967	903.4	0.867	0.8319	0.3414	0.8618	0
1968	903.3	0.867	0.8321	0.3411	0.8621	0
1969	903.2	0.8672	0.8321	0.3414	0.862	0
1970	903.1	0.867	0.8323	0.3415	0.8622	0
1971	903	0.8672	0.8322	0.3415	0.8623	0
1972	902.9	0.867	0.8324	0.3415	0.8623	0
1973	902.8	0.867	0.8324	0.3415	0.8623	0
1974	902.7	0.8671	0.8322	0.3413	0.8623	0
1975	902.6	0.8671	0.8323	0.3415	0.8626	0
1976	902.5	0.8672	0.8323	0.3415	0.8626	0
1977	902.4	0.8671	0.8327	0.3416	0.8626	0
1978	902.3	0.8672	0.8324	0.3415	0.8626	0
1979	902.2	0.8672	0.8325	0.3415	0.8627	0
1980	902.1	0.8672	0.8325	0.3416	0.8627	0
1981	902	0.8674	0.8327	0.3418	0.863	0
1982	901.9	0.8676	0.8328	0.3417	0.863	0
1983	901.8	0.8675	0.8328	0.3417	0.863	0
1984	901.7	0.8678	0.8328	0.3418	0.863	0
1985	901.6	0.8671	0.833	0.3419	0.863	0
1986	901.5	0.8675	0.833	0.3418	0.8632	0
1987	901.4	0.8674	0.8329	0.3417	0.8631	0
1988	901.3	0.8677	0.8329	0.3419	0.863	0
1989	901.2	0.8673	0.833	0.3418	0.863	0
1990	901.1	0.8675	0.8329	0.3418	0.8631	0
1991	901	0.8676	0.8332	0.3419	0.8633	0
1992	900.9	0.8676	0.8332	0.3421	0.8634	0
1993	900.8	0.8676	0.8333	0.3422	0.8633	0
1994	900.7	0.8679	0.833	0.3421	0.8632	0
1995	900.6	0.8679	0.833	0.342	0.8635	0
1996	900.5	0.8676	0.8331	0.3421	0.8635	0
1997	900.4	0.8677	0.8334	0.342	0.8633	0
1998	900.3	0.8678	0.8335	0.342	0.8633	0
1999	900.2	0.8679	0.8335	0.342	0.8636	0
2000	900.1	0.8679	0.8333	0.3421	0.8636	0
2001	900	0.8676	0.8331	0.3418	0.8633	0
2002	899.9	0.8676	0.833	0.3417	0.8633	0
2003	899.8	0.8676	0.8334	0.3419	0.8633	0
2004	899.7	0.8677	0.8332	0.3419	0.8634	0
2005	899.6	0.8677	0.8333	0.3417	0.8633	0
2006	899.5	0.8677	0.8333	0.3417	0.863	0
2007	899.4	0.8678	0.8332	0.3417	0.863	0

2008	899.3	0.8676	0.833	0.3416	0.8629	0
2009	899.2	0.8676	0.833	0.3415	0.8628	0
2010	899.1	0.8677	0.8329	0.3416	0.8626	0
2011	899	0.8676	0.8331	0.3416	0.8627	0
2012	898.9	0.8676	0.833	0.3416	0.8627	0
2013	898.8	0.8676	0.8329	0.3415	0.8627	0
2014	898.7	0.8677	0.8329	0.3416	0.8625	0
2015	898.6	0.8675	0.8329	0.3416	0.8626	0
2016	898.5	0.8679	0.8329	0.3415	0.8624	0
2017	898.4	0.8677	0.833	0.3416	0.8627	0
2018	898.3	0.8679	0.8329	0.3414	0.8625	0
2019	898.2	0.8677	0.8327	0.3414	0.8623	0
2020	898.1	0.8675	0.8329	0.3412	0.8623	0
2021	898	0.8677	0.8328	0.3413	0.8623	0
2022	897.9	0.8679	0.8328	0.3414	0.8623	0
2023	897.8	0.8678	0.8329	0.3414	0.8624	0
2024	897.7	0.8678	0.8328	0.3413	0.8624	0
2025	897.6	0.8677	0.8327	0.3412	0.862	0
2026	897.5	0.8678	0.8326	0.3413	0.862	0
2027	897.4	0.8677	0.8329	0.3413	0.8619	0
2028	897.3	0.8678	0.8327	0.3411	0.8621	0
2029	897.2	0.8679	0.8328	0.3412	0.8622	0
2030	897.1	0.8679	0.8329	0.3412	0.862	0
2031	897	0.868	0.8328	0.341	0.862	0
2032	896.9	0.8676	0.8327	0.3409	0.8619	0
2033	896.8	0.868	0.8331	0.3412	0.8622	0
2034	896.7	0.8677	0.8326	0.341	0.8617	0
2035	896.6	0.8679	0.8329	0.3412	0.8619	0
2036	896.5	0.8677	0.8329	0.341	0.862	0
2037	896.4	0.8683	0.833	0.3413	0.8621	0
2038	896.3	0.8681	0.833	0.3413	0.8622	0
2039	896.2	0.8679	0.8332	0.3411	0.862	0
2040	896.1	0.8677	0.8329	0.341	0.8618	0
2041	896	0.8681	0.8333	0.3414	0.8621	0
2042	895.9	0.8678	0.8332	0.3412	0.862	0
2043	895.8	0.8681	0.833	0.3412	0.8621	0
2044	895.7	0.868	0.8331	0.3412	0.8622	0
2045	895.6	0.8682	0.833	0.3412	0.8621	0
2046	895.5	0.868	0.833	0.3413	0.8622	0
2047	895.4	0.868	0.8331	0.3412	0.8622	0
2048	895.3	0.868	0.833	0.3411	0.862	0
2049	895.2	0.868	0.8333	0.3413	0.8622	0
2050	895.1	0.8682	0.8335	0.3415	0.8624	0

2051	895	0.8683	0.8337	0.3414	0.8627	0
2052	894.9	0.8682	0.8334	0.3414	0.8625	0
2053	894.8	0.8683	0.8333	0.3415	0.8624	0
2054	894.7	0.868	0.8333	0.3413	0.8622	0
2055	894.6	0.8682	0.8334	0.3414	0.8624	0
2056	894.5	0.8683	0.8336	0.3415	0.8627	0
2057	894.4	0.8682	0.8335	0.3415	0.8628	0
2058	894.3	0.8684	0.8336	0.3415	0.863	0
2059	894.2	0.8682	0.8337	0.3416	0.863	0
2060	894.1	0.8682	0.8337	0.3416	0.863	0
2061	894	0.8683	0.8338	0.3417	0.8631	0
2062	893.9	0.8682	0.8338	0.3418	0.8634	0
2063	893.8	0.8684	0.8342	0.342	0.8637	0
2064	893.7	0.8685	0.8342	0.342	0.864	0
2065	893.6	0.8686	0.8343	0.3421	0.8639	0
2066	893.5	0.8686	0.8345	0.3421	0.8638	0
2067	893.4	0.8684	0.8345	0.342	0.8638	0
2068	893.3	0.8683	0.8346	0.3422	0.864	0
2069	893.2	0.8681	0.8343	0.3419	0.8637	0
2070	893.1	0.8687	0.8349	0.342	0.8643	0
2071	893	0.8688	0.835	0.3423	0.8645	0
2072	892.9	0.8688	0.8348	0.3424	0.8646	0
2073	892.8	0.8689	0.8352	0.3423	0.8647	0
2074	892.7	0.869	0.8353	0.3425	0.8648	0
2075	892.6	0.8689	0.8355	0.3426	0.8651	0
2076	892.5	0.8689	0.8353	0.3426	0.865	0
2077	892.4	0.8687	0.8353	0.3426	0.8649	0
2078	892.3	0.8687	0.8352	0.3425	0.8649	0
2079	892.2	0.8684	0.8351	0.3424	0.8648	0
2080	892.1	0.8687	0.8352	0.3424	0.865	0
2081	892	0.8685	0.8352	0.3425	0.8652	0
2082	891.9	0.8689	0.8354	0.3427	0.8654	0
2083	891.8	0.8691	0.8356	0.3427	0.8654	0
2084	891.7	0.8689	0.8355	0.3427	0.8654	0
2085	891.6	0.8688	0.8354	0.3428	0.8653	0
2086	891.5	0.8687	0.8353	0.3426	0.8651	0
2087	891.4	0.8689	0.8356	0.3428	0.8656	0
2088	891.3	0.8687	0.8357	0.3428	0.8657	0
2089	891.2	0.8688	0.8357	0.3428	0.8656	0
2090	891.1	0.8693	0.836	0.3429	0.8659	0
2091	891	0.8691	0.8356	0.3426	0.8657	0
2092	890.9	0.869	0.836	0.3428	0.8659	0
2093	890.8	0.8691	0.8359	0.3428	0.8657	0

2094	890.7	0.8693	0.8361	0.3431	0.8661	0
2095	890.6	0.8693	0.836	0.3429	0.8661	0
2096	890.5	0.8693	0.8359	0.3428	0.866	0
2097	890.4	0.8693	0.836	0.3431	0.8661	0
2098	890.3	0.8693	0.8359	0.3429	0.8659	0
2099	890.2	0.8693	0.8363	0.3429	0.866	0
2100	890.1	0.8691	0.836	0.3427	0.8658	0
2101	890	0.8695	0.836	0.3427	0.8659	0
2102	889.9	0.8694	0.836	0.3431	0.8659	0
2103	889.8	0.8695	0.8361	0.343	0.8659	0
2104	889.7	0.8695	0.8362	0.3428	0.8661	0
2105	889.6	0.8693	0.8357	0.3428	0.8658	0
2106	889.5	0.8694	0.8359	0.3428	0.866	0
2107	889.4	0.8696	0.836	0.3428	0.866	0
2108	889.3	0.8696	0.8361	0.3429	0.8659	0
2109	889.2	0.8697	0.8361	0.343	0.8659	0
2110	889.1	0.8695	0.8362	0.3428	0.8656	0
2111	889	0.8694	0.8358	0.3425	0.8655	0
2112	888.9	0.8698	0.8362	0.3427	0.8658	0
2113	888.8	0.8696	0.8362	0.3429	0.866	0
2114	888.7	0.8698	0.8362	0.3427	0.8657	0
2115	888.6	0.8694	0.836	0.3427	0.8656	0
2116	888.5	0.8697	0.8362	0.3426	0.8656	0
2117	888.4	0.8697	0.8361	0.3427	0.8655	0
2118	888.3	0.8696	0.8363	0.3426	0.8656	0
2119	888.2	0.8696	0.836	0.3428	0.8655	0
2120	888.1	0.8696	0.8361	0.3426	0.8654	0
2121	888	0.8694	0.8359	0.3424	0.8654	0
2122	887.9	0.8694	0.8361	0.3425	0.8653	0
2123	887.8	0.8695	0.8359	0.3425	0.8653	0
2124	887.7	0.8693	0.8359	0.3424	0.8651	0
2125	887.6	0.8699	0.8359	0.3425	0.865	0
2126	887.5	0.8695	0.836	0.3426	0.8651	0
2127	887.4	0.8695	0.8359	0.3425	0.865	0
2128	887.3	0.8696	0.836	0.3425	0.865	0
2129	887.2	0.8696	0.8359	0.3423	0.865	0
2130	887.1	0.8698	0.8361	0.3423	0.8649	0
2131	887	0.8698	0.8361	0.3425	0.8649	0
2132	886.9	0.8698	0.8362	0.3423	0.8649	0
2133	886.8	0.8697	0.8358	0.3423	0.8649	0
2134	886.7	0.8696	0.8359	0.3423	0.8648	0
2135	886.6	0.8696	0.8359	0.3423	0.8647	0
2136	886.5	0.8696	0.8363	0.3423	0.8648	0

2137	886.4	0.8695	0.8359	0.3421	0.8647	0
2138	886.3	0.8696	0.8361	0.3421	0.8646	0
2139	886.2	0.8696	0.836	0.3423	0.8647	0
2140	886.1	0.8697	0.8358	0.3421	0.8647	0
2141	886	0.8696	0.8359	0.3419	0.8646	0
2142	885.9	0.8694	0.8358	0.3421	0.8645	0
2143	885.8	0.8696	0.8359	0.3422	0.8646	0
2144	885.7	0.8696	0.8359	0.342	0.8646	0
2145	885.6	0.8694	0.8357	0.342	0.8644	0
2146	885.5	0.8697	0.836	0.3421	0.8646	0
2147	885.4	0.8696	0.8361	0.3422	0.8647	0
2148	885.3	0.8695	0.8362	0.3422	0.8646	0
2149	885.2	0.8696	0.836	0.3421	0.8647	0
2150	885.1	0.8698	0.8363	0.3422	0.8649	0
2151	885	0.8699	0.8365	0.3424	0.865	0
2152	884.9	0.8697	0.8364	0.3423	0.8649	0
2153	884.8	0.8699	0.8364	0.3424	0.865	0
2154	884.7	0.8697	0.8363	0.3422	0.865	0
2155	884.6	0.8698	0.8363	0.3423	0.8648	0
2156	884.5	0.8699	0.8364	0.3423	0.8648	0
2157	884.4	0.8701	0.8365	0.3425	0.8653	0
2158	884.3	0.8699	0.8367	0.3425	0.8653	0
2159	884.2	0.8699	0.8365	0.3422	0.8652	0
2160	884.1	0.8699	0.8365	0.3423	0.8652	0
2161	884	0.8698	0.8366	0.3421	0.8653	0
2162	883.9	0.8702	0.8367	0.3425	0.8656	0
2163	883.8	0.8703	0.8369	0.3427	0.8658	0
2164	883.7	0.8702	0.8369	0.3427	0.8657	0
2165	883.6	0.8705	0.8371	0.3427	0.8659	0
2166	883.5	0.8704	0.8372	0.3427	0.8661	0
2167	883.4	0.8704	0.8372	0.3429	0.8661	0
2168	883.3	0.8703	0.8373	0.3428	0.866	0
2169	883.2	0.8704	0.8374	0.343	0.8663	0
2170	883.1	0.8702	0.8373	0.3429	0.8662	0
2171	883	0.8702	0.8375	0.3428	0.8662	0
2172	882.9	0.8703	0.8376	0.3431	0.8665	0
2173	882.8	0.8705	0.8373	0.3431	0.8663	0
2174	882.7	0.8704	0.8376	0.3431	0.8667	0
2175	882.6	0.8704	0.8376	0.3428	0.8663	0
2176	882.5	0.8707	0.8378	0.3431	0.8669	0
2177	882.4	0.8705	0.8377	0.3431	0.8666	0
2178	882.3	0.8707	0.8376	0.3431	0.8669	0
2179	882.2	0.8707	0.8375	0.3428	0.8665	0

2180	882.1	0.8704	0.8376	0.3428	0.8666	0
2181	882	0.8703	0.8377	0.3428	0.8667	0
2182	881.9	0.8703	0.8377	0.3428	0.8667	0
2183	881.8	0.8704	0.8373	0.3428	0.8668	0
2184	881.7	0.8706	0.8378	0.343	0.8672	0
2185	881.6	0.8707	0.8377	0.3428	0.867	0
2186	881.5	0.8707	0.838	0.3432	0.8671	0
2187	881.4	0.8706	0.8382	0.3431	0.867	0
2188	881.3	0.8708	0.838	0.3432	0.8671	0
2189	881.2	0.8709	0.8384	0.3433	0.8675	0
2190	881.1	0.871	0.8384	0.3433	0.8672	0
2191	881	0.871	0.8381	0.3434	0.8673	0
2192	880.9	0.8707	0.8386	0.3434	0.8673	0
2193	880.8	0.8708	0.8383	0.3433	0.8674	0
2194	880.7	0.8709	0.8385	0.3434	0.8676	0
2195	880.6	0.8708	0.8383	0.3434	0.8674	0
2196	880.5	0.8708	0.8383	0.3433	0.8673	0
2197	880.4	0.8707	0.8383	0.3434	0.8674	0
2198	880.3	0.8708	0.8385	0.3434	0.8675	0
2199	880.2	0.8711	0.8385	0.3434	0.8677	0
2200	880.1	0.8709	0.8385	0.3434	0.8674	0
2201	880	0.8708	0.8385	0.3432	0.8675	0
2202	879.9	0.871	0.8384	0.3433	0.8672	0
2203	879.8	0.8709	0.8384	0.3432	0.8674	0
2204	879.7	0.8712	0.8386	0.3436	0.8677	0
2205	879.6	0.871	0.8387	0.3434	0.8676	0
2206	879.5	0.8711	0.8388	0.3434	0.8675	0
2207	879.4	0.8708	0.8386	0.3433	0.8676	0
2208	879.3	0.8708	0.8384	0.3432	0.8677	0
2209	879.2	0.871	0.8387	0.3433	0.868	0
2210	879.1	0.8709	0.8386	0.3431	0.8677	0
2211	879	0.8712	0.8388	0.3436	0.868	0
2212	878.9	0.8713	0.8387	0.3434	0.8677	0
2213	878.8	0.8714	0.8388	0.3435	0.8678	0
2214	878.7	0.871	0.8387	0.3432	0.8677	0
2215	878.6	0.8713	0.8389	0.3433	0.8675	0
2216	878.5	0.8712	0.839	0.3434	0.8674	0
2217	878.4	0.8712	0.8389	0.3433	0.8675	0
2218	878.3	0.871	0.8386	0.3434	0.8678	0
2219	878.2	0.8712	0.8386	0.3434	0.8677	0
2220	878.1	0.8714	0.839	0.3435	0.8678	0
2221	878	0.8714	0.8388	0.3434	0.8677	0
2222	877.9	0.8714	0.8388	0.3434	0.868	0

2223	877.8	0.8714	0.8386	0.3433	0.8678	0
2224	877.7	0.8712	0.8388	0.3433	0.8678	0
2225	877.6	0.8715	0.839	0.3433	0.8679	0
2226	877.5	0.8714	0.8388	0.3434	0.8678	0
2227	877.4	0.8713	0.8387	0.3432	0.8676	0
2228	877.3	0.8713	0.839	0.3434	0.8676	0
2229	877.2	0.8711	0.8388	0.3434	0.8675	0
2230	877.1	0.8715	0.8388	0.343	0.8676	0
2231	877	0.8715	0.8391	0.3432	0.8677	0
2232	876.9	0.8715	0.839	0.3433	0.8675	0
2233	876.8	0.8712	0.839	0.3432	0.8674	0
2234	876.7	0.8714	0.839	0.3433	0.8676	0
2235	876.6	0.8715	0.839	0.3432	0.8674	0
2236	876.5	0.8713	0.839	0.3432	0.8675	0
2237	876.4	0.8716	0.8391	0.3433	0.8673	0
2238	876.3	0.8714	0.8391	0.3433	0.8675	0
2239	876.2	0.8717	0.8391	0.3432	0.8676	0
2240	876.1	0.8715	0.8394	0.3433	0.8677	0
2241	876	0.8714	0.8394	0.3432	0.8676	0
2242	875.9	0.8717	0.8394	0.3433	0.8675	0
2243	875.8	0.8714	0.8393	0.3433	0.8677	0
2244	875.7	0.8717	0.8394	0.3434	0.8677	0
2245	875.6	0.8716	0.8396	0.3433	0.8678	0
2246	875.5	0.8716	0.8392	0.3431	0.8678	0
2247	875.4	0.8718	0.8391	0.343	0.8676	0
2248	875.3	0.8714	0.8392	0.3429	0.8675	0
2249	875.2	0.8717	0.8391	0.3431	0.8677	0
2250	875.1	0.8717	0.8395	0.3432	0.8678	0
2251	875	0.8717	0.8395	0.3431	0.868	0
2252	874.9	0.872	0.8396	0.3432	0.868	0
2253	874.8	0.8718	0.8397	0.3433	0.8681	0
2254	874.7	0.8718	0.8394	0.3431	0.8681	0
2255	874.6	0.8719	0.8396	0.3434	0.8681	0
2256	874.5	0.8717	0.8397	0.3432	0.8681	0
2257	874.4	0.8718	0.8399	0.3434	0.8682	0
2258	874.3	0.8721	0.84	0.3434	0.8687	0
2259	874.2	0.872	0.84	0.3436	0.8686	0
2260	874.1	0.8722	0.8401	0.3434	0.8685	0
2261	874	0.8722	0.8399	0.3435	0.8689	0
2262	873.9	0.8719	0.8399	0.3434	0.8687	0
2263	873.8	0.872	0.8401	0.3436	0.8689	0
2264	873.7	0.8722	0.84	0.3435	0.8686	0
2265	873.6	0.872	0.8402	0.3435	0.8689	0

2266	873.5	0.8718	0.8401	0.3433	0.8685	0
2267	873.4	0.872	0.8402	0.3435	0.8687	0
2268	873.3	0.872	0.8401	0.3434	0.8689	0
2269	873.2	0.8718	0.8403	0.3433	0.8688	0
2270	873.1	0.8719	0.8405	0.3436	0.8689	0
2271	873	0.8721	0.8403	0.3435	0.8689	0
2272	872.9	0.8724	0.8403	0.3435	0.8689	0
2273	872.8	0.872	0.8403	0.3435	0.8692	0
2274	872.7	0.8726	0.8404	0.3436	0.8692	0
2275	872.6	0.8721	0.8404	0.3435	0.8691	0
2276	872.5	0.8724	0.8407	0.3437	0.8695	0
2277	872.4	0.8721	0.8405	0.3437	0.8691	0
2278	872.3	0.8724	0.8407	0.3437	0.8693	0
2279	872.2	0.8723	0.8408	0.3438	0.8694	0
2280	872.1	0.8725	0.8407	0.3436	0.8693	0
2281	872	0.8724	0.8409	0.3439	0.8694	0
2282	871.9	0.8724	0.8405	0.3437	0.8693	0
2283	871.8	0.8725	0.8408	0.3438	0.8695	0
2284	871.7	0.8728	0.8409	0.344	0.8695	0
2285	871.6	0.8727	0.8409	0.3438	0.8693	0
2286	871.5	0.8725	0.841	0.3439	0.8694	0
2287	871.4	0.8725	0.8409	0.3438	0.8693	0
2288	871.3	0.8729	0.8411	0.3441	0.8696	0
2289	871.2	0.8727	0.8409	0.3439	0.8695	0
2290	871.1	0.8726	0.8411	0.3439	0.8697	0
2291	871	0.8725	0.8409	0.344	0.8695	0
2292	870.9	0.8724	0.841	0.3441	0.8695	0
2293	870.8	0.8725	0.8412	0.344	0.8696	0
2294	870.7	0.8726	0.8412	0.3442	0.8695	0
2295	870.6	0.8725	0.8409	0.3438	0.8693	0
2296	870.5	0.8726	0.8409	0.3438	0.8695	0
2297	870.4	0.8727	0.8411	0.3438	0.8694	0
2298	870.3	0.8727	0.8412	0.3439	0.8695	0
2299	870.2	0.8728	0.8412	0.3438	0.8694	0
2300	870.1	0.8727	0.8411	0.3437	0.8693	0
2301	870	0.873	0.8411	0.344	0.8694	0
2302	869.9	0.873	0.8411	0.3436	0.8693	0
2303	869.8	0.8729	0.8412	0.3437	0.8695	0
2304	869.7	0.873	0.841	0.3435	0.8692	0
2305	869.6	0.873	0.8413	0.3437	0.8693	0
2306	869.5	0.8728	0.8411	0.3434	0.8691	0
2307	869.4	0.8729	0.8413	0.3437	0.8693	0
2308	869.3	0.8731	0.8415	0.3439	0.8693	0

2309	869.2	0.8731	0.8414	0.3437	0.8695	0
2310	869.1	0.873	0.8415	0.3438	0.8691	0
2311	869	0.8732	0.8414	0.3438	0.8692	0
2312	868.9	0.8732	0.8413	0.3437	0.8691	0
2313	868.8	0.8731	0.8412	0.3436	0.869	0
2314	868.7	0.8731	0.8414	0.3438	0.8691	0
2315	868.6	0.873	0.8411	0.3436	0.869	0
2316	868.5	0.8732	0.8412	0.3437	0.8687	0
2317	868.4	0.873	0.8414	0.3436	0.869	0
2318	868.3	0.8733	0.8414	0.3436	0.869	0
2319	868.2	0.8734	0.8415	0.3437	0.8693	0
2320	868.1	0.8733	0.8414	0.3435	0.8689	0
2321	868	0.8733	0.8415	0.3438	0.8693	0
2322	867.9	0.8733	0.8415	0.3438	0.8694	0
2323	867.8	0.8734	0.8414	0.3437	0.8694	0
2324	867.7	0.8732	0.8415	0.3435	0.8692	0
2325	867.6	0.8733	0.8419	0.3436	0.8694	0
2326	867.5	0.8732	0.8416	0.3436	0.8695	0
2327	867.4	0.8735	0.8416	0.3436	0.8694	0
2328	867.3	0.8734	0.842	0.3437	0.8696	0
2329	867.2	0.8733	0.8418	0.3438	0.8695	0
2330	867.1	0.8733	0.8418	0.3436	0.8695	0
2331	867	0.8734	0.8418	0.344	0.8696	0
2332	866.9	0.8735	0.8418	0.3436	0.8694	0
2333	866.8	0.8737	0.8419	0.3437	0.8698	0
2334	866.7	0.8737	0.8423	0.344	0.8697	0
2335	866.6	0.8736	0.8419	0.344	0.8698	0
2336	866.5	0.8735	0.8417	0.3436	0.8697	0
2337	866.4	0.8735	0.842	0.3438	0.8697	0
2338	866.3	0.8735	0.8422	0.3437	0.8697	0
2339	866.2	0.8735	0.8422	0.3439	0.87	0
2340	866.1	0.8736	0.8421	0.3438	0.8699	0
2341	866	0.8735	0.8423	0.3438	0.8698	0
2342	865.9	0.8736	0.8422	0.3437	0.8699	0
2343	865.8	0.8736	0.8424	0.3439	0.8702	0
2344	865.7	0.8737	0.8423	0.3439	0.87	0
2345	865.6	0.8737	0.8424	0.344	0.8703	0
2346	865.5	0.8737	0.8423	0.344	0.8703	0
2347	865.4	0.8736	0.8423	0.344	0.8703	0
2348	865.3	0.8737	0.8426	0.344	0.8706	0
2349	865.2	0.8736	0.8427	0.3441	0.8707	0
2350	865.1	0.8738	0.8426	0.344	0.8706	0
2351	865	0.8736	0.8428	0.344	0.8706	0

2352	864.9	0.8739	0.8429	0.3442	0.8708	0
2353	864.8	0.874	0.8428	0.3441	0.8708	0
2354	864.7	0.8739	0.8427	0.3441	0.8707	0
2355	864.6	0.8739	0.843	0.344	0.8708	0
2356	864.5	0.8738	0.8431	0.3441	0.871	0
2357	864.4	0.874	0.8433	0.3442	0.8713	0
2358	864.3	0.8741	0.8431	0.3443	0.8714	0
2359	864.2	0.8742	0.8433	0.3443	0.8712	0
2360	864.1	0.8742	0.8435	0.3444	0.8715	0
2361	864	0.874	0.8432	0.3443	0.8714	0
2362	863.9	0.8741	0.8434	0.3442	0.8715	0
2363	863.8	0.8742	0.8437	0.3446	0.8716	0
2364	863.7	0.8743	0.8437	0.3445	0.8717	0
2365	863.6	0.8744	0.8435	0.3443	0.8717	0
2366	863.5	0.8743	0.8438	0.3445	0.8721	0
2367	863.4	0.8743	0.844	0.3448	0.872	0
2368	863.3	0.8743	0.8436	0.3442	0.8719	0
2369	863.2	0.8744	0.8438	0.3445	0.8719	0
2370	863.1	0.8745	0.8438	0.3446	0.8721	0
2371	863	0.8743	0.8439	0.3444	0.8721	0
2372	862.9	0.8744	0.8438	0.3446	0.872	0
2373	862.8	0.8743	0.8436	0.3446	0.8719	0
2374	862.7	0.8744	0.8439	0.3447	0.8723	0
2375	862.6	0.8746	0.8438	0.3444	0.8722	0
2376	862.5	0.8746	0.8443	0.3449	0.8723	0
2377	862.4	0.8743	0.8439	0.3445	0.8721	0
2378	862.3	0.8746	0.8442	0.3447	0.8722	0
2379	862.2	0.8744	0.844	0.3446	0.8721	0
2380	862.1	0.8744	0.844	0.3444	0.8722	0
2381	862	0.8745	0.8441	0.3445	0.8722	0
2382	861.9	0.8746	0.844	0.3446	0.8722	0
2383	861.8	0.8744	0.8443	0.3445	0.8719	0
2384	861.7	0.8747	0.844	0.3445	0.8721	0
2385	861.6	0.8745	0.8444	0.3447	0.872	0
2386	861.5	0.8745	0.8445	0.3447	0.8723	0
2387	861.4	0.8747	0.8441	0.3448	0.872	0
2388	861.3	0.8745	0.8444	0.3447	0.8724	0
2389	861.2	0.8747	0.8446	0.3447	0.8722	0
2390	861.1	0.8749	0.8444	0.3446	0.8721	0
2391	861	0.8747	0.8443	0.3446	0.872	0
2392	860.9	0.875	0.8444	0.3447	0.8719	0
2393	860.8	0.8748	0.8442	0.3445	0.8718	0
2394	860.7	0.8748	0.8444	0.3448	0.8718	0

2395	860.6	0.8748	0.8444	0.3446	0.8718	0
2396	860.5	0.875	0.8444	0.3446	0.8719	0
2397	860.4	0.8749	0.8446	0.3447	0.8721	0
2398	860.3	0.8751	0.8447	0.3448	0.8721	0
2399	860.2	0.8752	0.8446	0.345	0.8722	0
2400	860.1	0.8752	0.8445	0.3446	0.8721	0
2401	860	0.8748	0.8443	0.3446	0.8718	0
2402	859.9	0.8751	0.8443	0.3446	0.8717	0
2403	859.8	0.8749	0.8444	0.3445	0.8717	0
2404	859.7	0.8753	0.8445	0.3444	0.8716	0
2405	859.6	0.8752	0.8444	0.3443	0.8715	0
2406	859.5	0.875	0.8445	0.3444	0.8715	0
2407	859.4	0.8751	0.8443	0.3445	0.8714	0
2408	859.3	0.8752	0.8447	0.3445	0.8714	0
2409	859.2	0.8752	0.8445	0.3444	0.8714	0
2410	859.1	0.8751	0.8445	0.3444	0.8714	0
2411	859	0.8751	0.8444	0.3442	0.8712	0
2412	858.9	0.8752	0.8444	0.3443	0.8712	0
2413	858.8	0.8753	0.8446	0.3442	0.8712	0
2414	858.7	0.8752	0.8445	0.3442	0.8715	0
2415	858.6	0.8753	0.8444	0.3441	0.8712	0
2416	858.5	0.8753	0.8443	0.3442	0.8712	0
2417	858.4	0.8753	0.8443	0.344	0.871	0
2418	858.3	0.8751	0.8441	0.3439	0.8709	0
2419	858.2	0.8752	0.8442	0.344	0.8707	0
2420	858.1	0.8752	0.8443	0.3439	0.8707	0
2421	858	0.8752	0.8443	0.3439	0.8708	0
2422	857.9	0.875	0.8443	0.3439	0.8707	0
2423	857.8	0.8755	0.8444	0.3439	0.8709	0
2424	857.7	0.8755	0.8444	0.3437	0.8707	0
2425	857.6	0.8754	0.8447	0.3439	0.8707	0
2426	857.5	0.8754	0.8445	0.3439	0.8705	0
2427	857.4	0.8754	0.8444	0.3438	0.8708	0
2428	857.3	0.8755	0.8448	0.3439	0.8709	0
2429	857.2	0.8754	0.8444	0.3439	0.8706	0
2430	857.1	0.8754	0.8446	0.3437	0.8707	0
2431	857	0.8753	0.8446	0.3438	0.8709	0
2432	856.9	0.8753	0.8446	0.3438	0.871	0
2433	856.8	0.8755	0.8448	0.3439	0.8714	0
2434	856.7	0.8759	0.8452	0.3442	0.8714	0
2435	856.6	0.8759	0.8451	0.3443	0.8716	0
2436	856.5	0.8755	0.845	0.3441	0.8712	0
2437	856.4	0.8759	0.845	0.3442	0.8716	0

2438	856.3	0.8757	0.8452	0.3443	0.8718	0
2439	856.2	0.8755	0.8452	0.3442	0.8716	0
2440	856.1	0.8757	0.8452	0.344	0.8718	0
2441	856	0.876	0.8454	0.3442	0.8721	0
2442	855.9	0.8758	0.8454	0.3444	0.8719	0
2443	855.8	0.8757	0.8454	0.3444	0.8721	0
2444	855.7	0.876	0.8455	0.3444	0.8723	0
2445	855.6	0.8758	0.8456	0.3445	0.8722	0
2446	855.5	0.8759	0.8455	0.3443	0.8723	0
2447	855.4	0.8756	0.8454	0.3441	0.8722	0
2448	855.3	0.876	0.8458	0.3444	0.8724	0
2449	855.2	0.8759	0.8458	0.3445	0.8724	0
2450	855.1	0.876	0.8458	0.3444	0.8725	0
2451	855	0.8761	0.846	0.3446	0.8727	0
2452	854.9	0.8763	0.8461	0.3448	0.8727	0
2453	854.8	0.876	0.8461	0.3447	0.8729	0
2454	854.7	0.8764	0.8461	0.3447	0.8731	0
2455	854.6	0.8762	0.8463	0.3448	0.8732	0
2456	854.5	0.8763	0.8464	0.3449	0.8734	0
2457	854.4	0.8764	0.8464	0.3449	0.8735	0
2458	854.3	0.8764	0.8465	0.345	0.8739	0
2459	854.2	0.8764	0.8467	0.3452	0.8736	0
2460	854.1	0.8764	0.8465	0.3451	0.8739	0
2461	854	0.8765	0.8467	0.3452	0.874	0
2462	853.9	0.8763	0.8469	0.3451	0.8738	0
2463	853.8	0.8762	0.8468	0.3451	0.8738	0
2464	853.7	0.8763	0.8471	0.345	0.8739	0
2465	853.6	0.8766	0.847	0.3452	0.8743	0
2466	853.5	0.8767	0.8472	0.3453	0.8743	0
2467	853.4	0.8769	0.8475	0.3455	0.8747	0
2468	853.3	0.8765	0.8472	0.3453	0.8745	0
2469	853.2	0.8768	0.8473	0.3454	0.8745	0
2470	853.1	0.8767	0.8473	0.3454	0.8744	0
2471	853	0.8765	0.8473	0.3452	0.8744	0
2472	852.9	0.8767	0.8476	0.3454	0.8747	0
2473	852.8	0.8765	0.8475	0.3454	0.8747	0
2474	852.7	0.8765	0.8474	0.3452	0.8744	0
2475	852.6	0.8766	0.8472	0.3455	0.8748	0
2476	852.5	0.8768	0.8476	0.3456	0.8747	0
2477	852.4	0.8768	0.8476	0.3456	0.875	0
2478	852.3	0.8769	0.8477	0.3455	0.875	0
2479	852.2	0.8769	0.8477	0.3456	0.8749	0
2480	852.1	0.8769	0.8478	0.3456	0.8749	0

2481	852	0.8768	0.8477	0.3456	0.875	0
2482	851.9	0.8769	0.8479	0.3456	0.8751	0
2483	851.8	0.8769	0.848	0.3456	0.875	0
2484	851.7	0.8771	0.8479	0.3457	0.8751	0
2485	851.6	0.8771	0.848	0.3455	0.875	0
2486	851.5	0.877	0.848	0.3457	0.8753	0
2487	851.4	0.8772	0.8482	0.3458	0.8752	0
2488	851.3	0.8772	0.8481	0.3459	0.875	0
2489	851.2	0.8773	0.8483	0.3458	0.8751	0
2490	851.1	0.8773	0.8481	0.3458	0.8749	0
2491	851	0.8773	0.8481	0.3457	0.8752	0
2492	850.9	0.8771	0.8483	0.3457	0.8751	0
2493	850.8	0.8772	0.8484	0.3457	0.875	0
2494	850.7	0.8773	0.8484	0.3456	0.8751	0
2495	850.6	0.8773	0.8482	0.3456	0.8751	0
2496	850.5	0.8773	0.8484	0.3456	0.8752	0
2497	850.4	0.8774	0.8482	0.3453	0.8749	0
2498	850.3	0.8771	0.8482	0.3455	0.8748	0
2499	850.2	0.8774	0.8483	0.3453	0.875	0
2500	850.1	0.8775	0.8484	0.3453	0.8749	0
2501	850	0.8771	0.8479	0.345	0.8745	0
2502	849.9	0.8874	0.8657	0.338	0.8636	0
2503	849.8	0.8873	0.8656	0.3381	0.8636	0
2504	849.7	0.8872	0.8657	0.3379	0.8633	0
2505	849.6	0.8873	0.8654	0.3378	0.8631	0
2506	849.5	0.8871	0.8655	0.3379	0.8633	0
2507	849.4	0.8872	0.8656	0.3378	0.8629	0
2508	849.3	0.8876	0.8658	0.338	0.8632	0
2509	849.2	0.8873	0.8656	0.3379	0.8629	0
2510	849.1	0.8873	0.8658	0.3377	0.8631	0
2511	849	0.8873	0.8656	0.3376	0.8628	0
2512	848.9	0.8875	0.8656	0.3378	0.8629	0
2513	848.8	0.8873	0.8657	0.3377	0.8629	0
2514	848.7	0.8876	0.8657	0.3379	0.8626	0
2515	848.6	0.8874	0.8656	0.3376	0.8627	0
2516	848.5	0.8875	0.8654	0.3376	0.8625	0
2517	848.4	0.8875	0.8652	0.3375	0.8623	0
2518	848.3	0.8877	0.8656	0.3375	0.8626	0
2519	848.2	0.8875	0.8655	0.3374	0.8624	0
2520	848.1	0.8875	0.8656	0.3375	0.8623	0
2521	848	0.8875	0.8655	0.3374	0.862	0
2522	847.9	0.8876	0.8656	0.3374	0.8625	0
2523	847.8	0.8876	0.8655	0.3375	0.8622	0

2524	847.7	0.8877	0.8654	0.3375	0.862	0
2525	847.6	0.8875	0.8655	0.3374	0.8621	0
2526	847.5	0.8875	0.8655	0.3374	0.8622	0
2527	847.4	0.8874	0.8655	0.3373	0.862	0
2528	847.3	0.8875	0.8655	0.3373	0.8622	0
2529	847.2	0.8875	0.8657	0.3373	0.8623	0
2530	847.1	0.8874	0.8655	0.3372	0.862	0
2531	847	0.8875	0.8655	0.3372	0.8619	0
2532	846.9	0.8875	0.8657	0.3373	0.8621	0
2533	846.8	0.8877	0.8657	0.3372	0.8623	0
2534	846.7	0.8876	0.8658	0.3372	0.8622	0
2535	846.6	0.8876	0.8657	0.3374	0.8623	0
2536	846.5	0.8878	0.8659	0.3375	0.8625	0
2537	846.4	0.8877	0.8656	0.3372	0.8622	0
2538	846.3	0.888	0.8659	0.3372	0.8621	0
2539	846.2	0.888	0.866	0.3375	0.8625	0
2540	846.1	0.8881	0.866	0.3374	0.8624	0
2541	846	0.8879	0.866	0.3376	0.8624	0
2542	845.9	0.8879	0.8659	0.3374	0.8626	0
2543	845.8	0.8879	0.8661	0.3375	0.8626	0
2544	845.7	0.8881	0.8665	0.3374	0.8627	0
2545	845.6	0.8882	0.8663	0.3376	0.8628	0
2546	845.5	0.8881	0.8665	0.3377	0.8629	0
2547	845.4	0.8883	0.8665	0.3378	0.8631	0
2548	845.3	0.8883	0.8665	0.3378	0.8631	0
2549	845.2	0.8884	0.8669	0.338	0.8635	0
2550	845.1	0.8884	0.8668	0.3379	0.8635	0
2551	845	0.8885	0.8671	0.3379	0.8634	0
2552	844.9	0.8881	0.8669	0.3379	0.8634	0
2553	844.8	0.8883	0.867	0.3379	0.864	0
2554	844.7	0.8885	0.8671	0.3381	0.8639	0
2555	844.6	0.8885	0.8674	0.3378	0.8639	0
2556	844.5	0.8886	0.8675	0.3381	0.8641	0
2557	844.4	0.8887	0.8673	0.3382	0.8641	0
2558	844.3	0.8887	0.8674	0.3382	0.8644	0
2559	844.2	0.8888	0.8678	0.3384	0.8648	0
2560	844.1	0.8887	0.8676	0.3384	0.8645	0
2561	844	0.8887	0.868	0.3384	0.8648	0
2562	843.9	0.8889	0.8681	0.3387	0.8651	0
2563	843.8	0.889	0.8681	0.3386	0.8651	0
2564	843.7	0.8889	0.8682	0.3387	0.8653	0
2565	843.6	0.8889	0.8681	0.3387	0.8652	0
2566	843.5	0.8889	0.8682	0.3388	0.8655	0

2567	843.4	0.8888	0.8684	0.3385	0.8657	0
2568	843.3	0.8892	0.8686	0.3387	0.8657	0
2569	843.2	0.8891	0.8689	0.3389	0.8658	0
2570	843.1	0.8891	0.8689	0.3388	0.8659	0
2571	843	0.8891	0.8686	0.3389	0.866	0
2572	842.9	0.8893	0.8686	0.339	0.866	0
2573	842.8	0.8894	0.8688	0.339	0.8661	0
2574	842.7	0.8893	0.8685	0.339	0.8662	0
2575	842.6	0.8894	0.8686	0.339	0.8663	0
2576	842.5	0.8894	0.8689	0.3391	0.8664	0
2577	842.4	0.8893	0.869	0.3391	0.8664	0
2578	842.3	0.8895	0.869	0.3392	0.8664	0
2579	842.2	0.8898	0.8691	0.3391	0.8664	0
2580	842.1	0.8894	0.8691	0.3392	0.8666	0
2581	842	0.8895	0.8692	0.3392	0.8665	0
2582	841.9	0.8896	0.8693	0.3393	0.8664	0
2583	841.8	0.8896	0.8694	0.3394	0.8666	0
2584	841.7	0.8898	0.8695	0.3393	0.8668	0
2585	841.6	0.8898	0.8693	0.3393	0.8667	0
2586	841.5	0.8897	0.8692	0.3392	0.8667	0
2587	841.4	0.8897	0.8694	0.3392	0.8666	0
2588	841.3	0.8893	0.8693	0.339	0.8665	0
2589	841.2	0.8897	0.8692	0.339	0.8666	0
2590	841.1	0.8897	0.8696	0.3392	0.8668	0
2591	841	0.8899	0.8696	0.3394	0.867	0
2592	840.9	0.8897	0.8695	0.3393	0.8667	0
2593	840.8	0.8899	0.8697	0.3393	0.8668	0
2594	840.7	0.8899	0.8697	0.3393	0.8671	0
2595	840.6	0.89	0.8699	0.3395	0.867	0
2596	840.5	0.8901	0.8701	0.3393	0.867	0
2597	840.4	0.89	0.87	0.3394	0.8669	0
2598	840.3	0.8898	0.8699	0.3392	0.8669	0
2599	840.2	0.8901	0.8699	0.3393	0.8669	0
2600	840.1	0.89	0.8699	0.3395	0.867	0
2601	840	0.89	0.8697	0.3392	0.8666	0
2602	839.9	0.89	0.8695	0.3392	0.8666	0
2603	839.8	0.89	0.8698	0.3391	0.8667	0
2604	839.7	0.8901	0.8699	0.3391	0.8669	0
2605	839.6	0.89	0.8698	0.3392	0.8665	0
2606	839.5	0.8899	0.8696	0.3391	0.8666	0
2607	839.4	0.8902	0.87	0.3391	0.8666	0
2608	839.3	0.8901	0.8697	0.3391	0.8665	0
2609	839.2	0.8902	0.8696	0.3391	0.8662	0

2610	839.1	0.8902	0.8698	0.339	0.8664	0
2611	839	0.8904	0.8699	0.3392	0.8666	0
2612	838.9	0.8901	0.8699	0.3389	0.8661	0
2613	838.8	0.8903	0.8696	0.3389	0.8663	0
2614	838.7	0.8903	0.8699	0.3388	0.8661	0
2615	838.6	0.8901	0.8699	0.3389	0.8658	0
2616	838.5	0.8903	0.8698	0.3388	0.8658	0
2617	838.4	0.8903	0.8697	0.3388	0.8657	0
2618	838.3	0.8902	0.8698	0.3388	0.8658	0
2619	838.2	0.8904	0.8698	0.3388	0.8657	0
2620	838.1	0.8902	0.8695	0.3389	0.8657	0
2621	838	0.8901	0.8697	0.3386	0.8656	0
2622	837.9	0.8902	0.8694	0.3384	0.8654	0
2623	837.8	0.8903	0.8696	0.3387	0.8655	0
2624	837.7	0.8903	0.8696	0.3386	0.8654	0
2625	837.6	0.8906	0.8697	0.3386	0.8655	0
2626	837.5	0.8905	0.8696	0.3386	0.8653	0
2627	837.4	0.8904	0.8695	0.3385	0.8653	0
2628	837.3	0.8903	0.8696	0.3384	0.8653	0
2629	837.2	0.8902	0.8698	0.3384	0.8652	0
2630	837.1	0.8903	0.8696	0.3383	0.8652	0
2631	837	0.8903	0.8696	0.3384	0.8653	0
2632	836.9	0.8906	0.8698	0.3384	0.8653	0
2633	836.8	0.8905	0.8697	0.3386	0.8653	0
2634	836.7	0.8906	0.8697	0.3384	0.8651	0
2635	836.6	0.8904	0.8697	0.3383	0.865	0
2636	836.5	0.8903	0.8698	0.3384	0.865	0
2637	836.4	0.8904	0.8699	0.3383	0.8649	0
2638	836.3	0.8903	0.8697	0.3381	0.8652	0
2639	836.2	0.8905	0.8698	0.3382	0.865	0
2640	836.1	0.8904	0.8698	0.3383	0.8648	0
2641	836	0.8906	0.8697	0.3383	0.8651	0
2642	835.9	0.8907	0.87	0.3384	0.8652	0
2643	835.8	0.8906	0.8701	0.3383	0.8653	0
2644	835.7	0.8907	0.8701	0.3384	0.8652	0
2645	835.6	0.8909	0.8702	0.3383	0.8653	0
2646	835.5	0.8907	0.8705	0.3384	0.8654	0
2647	835.4	0.8907	0.8702	0.3384	0.8653	0
2648	835.3	0.8911	0.8705	0.3386	0.8656	0
2649	835.2	0.8907	0.8703	0.3384	0.8655	0
2650	835.1	0.8909	0.8704	0.3386	0.8657	0
2651	835	0.8908	0.8703	0.3386	0.8656	0
2652	834.9	0.8909	0.8707	0.3388	0.8659	0

2653	834.8	0.891	0.8707	0.3387	0.866	0
2654	834.7	0.8909	0.8708	0.3386	0.8658	0
2655	834.6	0.8911	0.8705	0.3387	0.8661	0
2656	834.5	0.8912	0.871	0.3388	0.8661	0
2657	834.4	0.8912	0.8713	0.339	0.8663	0
2658	834.3	0.8912	0.8711	0.3391	0.8665	0
2659	834.2	0.8915	0.8713	0.339	0.8667	0
2660	834.1	0.8911	0.8711	0.3389	0.8665	0
2661	834	0.8913	0.8711	0.3391	0.8667	0
2662	833.9	0.8913	0.8713	0.3391	0.8667	0
2663	833.8	0.8913	0.8712	0.3392	0.867	0
2664	833.7	0.8914	0.8715	0.3391	0.867	0
2665	833.6	0.8914	0.8715	0.3391	0.8671	0
2666	833.5	0.8914	0.8718	0.3392	0.8672	0
2667	833.4	0.8915	0.8716	0.3394	0.8673	0
2668	833.3	0.8914	0.8716	0.3392	0.8676	0
2669	833.2	0.8916	0.872	0.3393	0.8678	0
2670	833.1	0.8917	0.872	0.3395	0.8679	0
2671	833	0.8917	0.872	0.3395	0.8678	0
2672	832.9	0.8917	0.8721	0.3397	0.8678	0
2673	832.8	0.8919	0.8721	0.3397	0.8678	0
2674	832.7	0.8918	0.8722	0.3397	0.8681	0
2675	832.6	0.8916	0.8722	0.3397	0.8682	0
2676	832.5	0.8918	0.8722	0.3397	0.8682	0
2677	832.4	0.892	0.8722	0.3397	0.8682	0
2678	832.3	0.8921	0.8726	0.3399	0.8684	0
2679	832.2	0.8921	0.8726	0.3399	0.8686	0
2680	832.1	0.8923	0.8727	0.3398	0.8688	0
2681	832	0.892	0.8726	0.34	0.8685	0
2682	831.9	0.892	0.8726	0.3401	0.8687	0
2683	831.8	0.8921	0.8728	0.3401	0.8689	0
2684	831.7	0.8922	0.873	0.3401	0.869	0
2685	831.6	0.8924	0.873	0.3402	0.869	0
2686	831.5	0.8923	0.873	0.3401	0.869	0
2687	831.4	0.8923	0.8729	0.3401	0.869	0
2688	831.3	0.8922	0.8732	0.3402	0.8692	0
2689	831.2	0.8922	0.8731	0.3402	0.8692	0
2690	831.1	0.8924	0.8732	0.3402	0.8691	0
2691	831	0.8923	0.8733	0.3402	0.8691	0
2692	830.9	0.8922	0.8731	0.3402	0.869	0
2693	830.8	0.8922	0.8729	0.3401	0.8688	0
2694	830.7	0.8926	0.8733	0.3403	0.8693	0
2695	830.6	0.8924	0.8733	0.3402	0.8693	0

2696	830.5	0.8924	0.8731	0.3404	0.8692	0
2697	830.4	0.8921	0.8732	0.3401	0.8692	0
2698	830.3	0.8924	0.8733	0.3403	0.8693	0
2699	830.2	0.8925	0.8734	0.3403	0.8695	0
2700	830.1	0.8923	0.8734	0.3403	0.8695	0
2701	830	0.8928	0.8733	0.3402	0.8694	0
2702	829.9	0.8926	0.8735	0.3402	0.8694	0
2703	829.8	0.8925	0.8734	0.3403	0.8693	0
2704	829.7	0.8927	0.8737	0.3403	0.8696	0
2705	829.6	0.8925	0.8737	0.3402	0.8694	0
2706	829.5	0.8926	0.8735	0.3403	0.8693	0
2707	829.4	0.8926	0.8737	0.3403	0.8693	0
2708	829.3	0.8925	0.8735	0.3402	0.8695	0
2709	829.2	0.8927	0.8737	0.3401	0.8692	0
2710	829.1	0.8927	0.8737	0.3403	0.8693	0
2711	829	0.8928	0.8738	0.3402	0.8693	0
2712	828.9	0.8927	0.8737	0.3403	0.8693	0
2713	828.8	0.8928	0.8737	0.3402	0.8692	0
2714	828.7	0.8928	0.8738	0.3402	0.8691	0
2715	828.6	0.8927	0.8736	0.34	0.8691	0
2716	828.5	0.8928	0.8736	0.34	0.869	0
2717	828.4	0.8929	0.8737	0.3399	0.869	0
2718	828.3	0.8929	0.8736	0.34	0.8691	0
2719	828.2	0.893	0.8737	0.3401	0.869	0
2720	828.1	0.8927	0.8738	0.34	0.8691	0
2721	828	0.8928	0.8736	0.3398	0.8688	0
2722	827.9	0.8927	0.8735	0.3399	0.8689	0
2723	827.8	0.8929	0.8737	0.3399	0.8688	0
2724	827.7	0.8929	0.8737	0.3399	0.8688	0
2725	827.6	0.8928	0.8738	0.3399	0.8688	0
2726	827.5	0.893	0.8735	0.3399	0.8685	0
2727	827.4	0.893	0.8738	0.3398	0.8687	0
2728	827.3	0.893	0.8738	0.3399	0.8686	0
2729	827.2	0.8929	0.8736	0.3397	0.8685	0
2730	827.1	0.8929	0.8735	0.3397	0.8685	0
2731	827	0.893	0.8739	0.3398	0.8686	0
2732	826.9	0.893	0.8737	0.3397	0.8688	0
2733	826.8	0.8931	0.8737	0.3397	0.8686	0
2734	826.7	0.8931	0.8737	0.3396	0.869	0
2735	826.6	0.8929	0.8737	0.3396	0.8686	0
2736	826.5	0.8933	0.874	0.3396	0.8685	0
2737	826.4	0.8932	0.8737	0.3397	0.8686	0
2738	826.3	0.8933	0.8738	0.3399	0.8687	0

2739	826.2	0.8931	0.8739	0.3397	0.8691	0
2740	826.1	0.8932	0.8739	0.3397	0.8686	0
2741	826	0.8933	0.8741	0.3397	0.8687	0
2742	825.9	0.8931	0.8738	0.3397	0.8685	0
2743	825.8	0.893	0.8741	0.3396	0.8684	0
2744	825.7	0.8932	0.8739	0.3397	0.8686	0
2745	825.6	0.8931	0.874	0.3396	0.8685	0
2746	825.5	0.8931	0.8739	0.3395	0.8686	0
2747	825.4	0.8935	0.8741	0.3397	0.8686	0
2748	825.3	0.8932	0.8742	0.3397	0.8685	0
2749	825.2	0.8934	0.8741	0.3398	0.8685	0
2750	825.1	0.8931	0.8741	0.3397	0.8686	0
2751	825	0.8933	0.8741	0.3397	0.8686	0
2752	824.9	0.8932	0.8741	0.3396	0.8687	0
2753	824.8	0.8934	0.8744	0.3398	0.8689	0
2754	824.7	0.8937	0.8744	0.3399	0.8689	0
2755	824.6	0.8937	0.8745	0.3399	0.8692	0
2756	824.5	0.8935	0.8744	0.3398	0.8689	0
2757	824.4	0.8937	0.8747	0.3398	0.869	0
2758	824.3	0.8938	0.8745	0.3398	0.8692	0
2759	824.2	0.8936	0.8748	0.3401	0.8693	0
2760	824.1	0.8936	0.8749	0.34	0.8693	0
2761	824	0.8938	0.8749	0.3401	0.8694	0
2762	823.9	0.894	0.8749	0.3401	0.8695	0
2763	823.8	0.8937	0.8751	0.3402	0.8696	0
2764	823.7	0.8938	0.8752	0.3403	0.8695	0
2765	823.6	0.8939	0.8753	0.3403	0.8696	0
2766	823.5	0.8939	0.8753	0.3404	0.8699	0
2767	823.4	0.894	0.8753	0.3404	0.8698	0
2768	823.3	0.894	0.8753	0.3404	0.8698	0
2769	823.2	0.8942	0.8755	0.3405	0.8698	0
2770	823.1	0.8944	0.8757	0.3404	0.8702	0
2771	823	0.8942	0.8755	0.3404	0.8701	0
2772	822.9	0.8942	0.8754	0.3405	0.8702	0
2773	822.8	0.8944	0.8758	0.3405	0.8702	0
2774	822.7	0.8943	0.8758	0.3406	0.8703	0
2775	822.6	0.8941	0.8758	0.3406	0.8703	0
2776	822.5	0.8943	0.8761	0.3405	0.8704	0
2777	822.4	0.8945	0.8761	0.3407	0.8704	0
2778	822.3	0.8944	0.8761	0.3407	0.8704	0
2779	822.2	0.8945	0.8765	0.3408	0.8705	0
2780	822.1	0.8945	0.8764	0.3408	0.8706	0
2781	822	0.8947	0.8763	0.3408	0.8705	0

2782	821.9	0.8947	0.8762	0.3407	0.8707	0
2783	821.8	0.8946	0.8762	0.3407	0.8707	0
2784	821.7	0.8947	0.8764	0.3408	0.8709	0
2785	821.6	0.8947	0.8763	0.3407	0.8709	0
2786	821.5	0.8949	0.8767	0.3409	0.8709	0
2787	821.4	0.895	0.8767	0.3411	0.8714	0
2788	821.3	0.8949	0.8767	0.341	0.8711	0
2789	821.2	0.8949	0.8767	0.3409	0.871	0
2790	821.1	0.895	0.8768	0.341	0.871	0
2791	821	0.8948	0.8768	0.3409	0.871	0
2792	820.9	0.8948	0.8767	0.3409	0.8713	0
2793	820.8	0.895	0.8769	0.341	0.8712	0
2794	820.7	0.895	0.8771	0.341	0.8711	0
2795	820.6	0.895	0.877	0.341	0.8711	0
2796	820.5	0.8951	0.8769	0.3411	0.8714	0
2797	820.4	0.8951	0.8773	0.3411	0.8713	0
2798	820.3	0.8953	0.8772	0.3412	0.8714	0
2799	820.2	0.8952	0.8771	0.3412	0.8713	0
2800	820.1	0.8953	0.8771	0.3411	0.8714	0
2801	820	0.8952	0.8772	0.341	0.8715	0
2802	819.9	0.8953	0.8775	0.3413	0.8717	0
2803	819.8	0.8953	0.8775	0.3413	0.8719	0
2804	819.7	0.8951	0.8773	0.3412	0.8716	0
2805	819.6	0.8954	0.8774	0.3412	0.8717	0
2806	819.5	0.8956	0.8774	0.3412	0.8717	0
2807	819.4	0.8954	0.8776	0.3412	0.8717	0
2808	819.3	0.8954	0.8775	0.3413	0.8717	0
2809	819.2	0.8955	0.8778	0.3414	0.8719	0
2810	819.1	0.8953	0.8776	0.3411	0.8715	0
2811	819	0.8956	0.8776	0.3412	0.8717	0
2812	818.9	0.8957	0.8778	0.3413	0.8717	0
2813	818.8	0.8956	0.878	0.3413	0.8721	0
2814	818.7	0.8956	0.8779	0.3412	0.8718	0
2815	818.6	0.8958	0.8779	0.3412	0.8718	0
2816	818.5	0.8956	0.878	0.3412	0.8717	0
2817	818.4	0.8957	0.878	0.3412	0.8718	0
2818	818.3	0.8956	0.878	0.3412	0.8718	0
2819	818.2	0.8956	0.878	0.3413	0.8719	0
2820	818.1	0.8957	0.8779	0.3411	0.8718	0
2821	818	0.8956	0.8777	0.3411	0.8715	0
2822	817.9	0.8958	0.8779	0.3411	0.8718	0
2823	817.8	0.896	0.8779	0.3412	0.8715	0
2824	817.7	0.896	0.878	0.3412	0.8717	0

2825	817.6	0.8958	0.878	0.3413	0.8717	0
2826	817.5	0.896	0.8779	0.3412	0.8719	0
2827	817.4	0.8961	0.8785	0.3414	0.872	0
2828	817.3	0.8958	0.8784	0.3411	0.872	0
2829	817.2	0.8959	0.8782	0.3413	0.8719	0
2830	817.1	0.896	0.8783	0.3411	0.8717	0
2831	817	0.896	0.8783	0.3411	0.8716	0
2832	816.9	0.896	0.8785	0.3411	0.8717	0
2833	816.8	0.896	0.8784	0.3412	0.8719	0
2834	816.7	0.8962	0.8784	0.3412	0.8719	0
2835	816.6	0.8961	0.8785	0.3413	0.8718	0
2836	816.5	0.8962	0.8784	0.3412	0.8718	0
2837	816.4	0.8961	0.8783	0.3411	0.8719	0
2838	816.3	0.8962	0.8786	0.3411	0.8719	0
2839	816.2	0.8962	0.8782	0.3412	0.8718	0
2840	816.1	0.8963	0.8785	0.3412	0.8719	0
2841	816	0.8963	0.8786	0.3412	0.872	0
2842	815.9	0.8962	0.8786	0.3411	0.8719	0
2843	815.8	0.8964	0.8788	0.3414	0.8723	0
2844	815.7	0.8963	0.8791	0.3414	0.8722	0
2845	815.6	0.8965	0.879	0.3413	0.8721	0
2846	815.5	0.8964	0.8788	0.3412	0.8721	0
2847	815.4	0.8966	0.879	0.3414	0.8723	0
2848	815.3	0.8965	0.8789	0.3414	0.8724	0
2849	815.2	0.8966	0.879	0.3414	0.8724	0
2850	815.1	0.8966	0.8787	0.3412	0.8721	0
2851	815	0.8965	0.8791	0.3414	0.8723	0
2852	814.9	0.8967	0.8795	0.3416	0.8727	0
2853	814.8	0.8968	0.8793	0.3415	0.8726	0
2854	814.7	0.8968	0.8793	0.3413	0.8728	0
2855	814.6	0.8968	0.8794	0.3414	0.8726	0
2856	814.5	0.8968	0.8793	0.3415	0.8725	0
2857	814.4	0.8968	0.8794	0.3416	0.8727	0
2858	814.3	0.8968	0.8795	0.3416	0.8727	0
2859	814.2	0.8968	0.8796	0.3416	0.8728	0
2860	814.1	0.8968	0.8795	0.3416	0.8729	0
2861	814	0.8969	0.8798	0.3416	0.8728	0
2862	813.9	0.8972	0.8796	0.3418	0.8729	0
2863	813.8	0.8971	0.8801	0.3419	0.8731	0
2864	813.7	0.897	0.8801	0.3418	0.8731	0
2865	813.6	0.897	0.88	0.3419	0.8732	0
2866	813.5	0.897	0.8801	0.3419	0.8731	0
2867	813.4	0.8972	0.8801	0.3418	0.873	0

2868	813.3	0.8974	0.8801	0.342	0.8733	0
2869	813.2	0.8972	0.8804	0.342	0.8735	0
2870	813.1	0.8973	0.8806	0.342	0.8733	0
2871	813	0.8975	0.8803	0.342	0.8733	0
2872	812.9	0.8977	0.8804	0.3421	0.8734	0
2873	812.8	0.8973	0.8806	0.3421	0.8734	0
2874	812.7	0.8977	0.8804	0.342	0.8737	0
2875	812.6	0.8976	0.8805	0.3419	0.8737	0
2876	812.5	0.8974	0.8803	0.3419	0.8733	0
2877	812.4	0.8974	0.8804	0.3419	0.8734	0
2878	812.3	0.8976	0.8805	0.342	0.8736	0
2879	812.2	0.8977	0.8803	0.342	0.8737	0
2880	812.1	0.8977	0.8807	0.3422	0.8738	0
2881	812	0.8974	0.8807	0.3419	0.8735	0
2882	811.9	0.8977	0.8809	0.342	0.8735	0
2883	811.8	0.8977	0.8808	0.3421	0.8738	0
2884	811.7	0.8977	0.8808	0.3419	0.874	0
2885	811.6	0.8981	0.8811	0.3421	0.8738	0
2886	811.5	0.8979	0.8811	0.3421	0.8738	0
2887	811.4	0.8979	0.8811	0.3421	0.8739	0
2888	811.3	0.8979	0.8812	0.3422	0.8738	0
2889	811.2	0.8979	0.8812	0.3422	0.8738	0
2890	811.1	0.898	0.8812	0.3423	0.8741	0
2891	811	0.8981	0.8812	0.3421	0.8737	0
2892	810.9	0.8979	0.8811	0.3421	0.874	0
2893	810.8	0.8983	0.8814	0.3421	0.874	0
2894	810.7	0.8983	0.8814	0.3421	0.8741	0
2895	810.6	0.8981	0.8813	0.342	0.8738	0
2896	810.5	0.8981	0.8812	0.3421	0.8738	0
2897	810.4	0.8982	0.8814	0.342	0.8738	0
2898	810.3	0.8983	0.8813	0.3421	0.8738	0
2899	810.2	0.8984	0.8814	0.3421	0.8738	0
2900	810.1	0.8984	0.8818	0.3422	0.8739	0
2901	810	0.8983	0.8816	0.3421	0.8738	0
2902	809.9	0.8985	0.8814	0.342	0.8738	0
2903	809.8	0.8984	0.8817	0.3421	0.8737	0
2904	809.7	0.8985	0.8817	0.3421	0.8739	0
2905	809.6	0.8985	0.8818	0.3422	0.8738	0
2906	809.5	0.8986	0.8818	0.3423	0.8743	0
2907	809.4	0.8985	0.8816	0.3421	0.874	0
2908	809.3	0.8987	0.8818	0.3422	0.8738	0
2909	809.2	0.8984	0.8817	0.3421	0.8739	0
2910	809.1	0.8987	0.8818	0.3424	0.874	0

2911	809	0.899	0.8818	0.3423	0.8743	0
2912	808.9	0.899	0.8821	0.3423	0.8743	0
2913	808.8	0.8989	0.882	0.3425	0.8741	0
2914	808.7	0.8987	0.8822	0.3424	0.8739	0
2915	808.6	0.8988	0.8819	0.3422	0.8739	0
2916	808.5	0.8989	0.8821	0.3424	0.8741	0
2917	808.4	0.8988	0.882	0.3422	0.874	0
2918	808.3	0.899	0.8821	0.3422	0.8739	0
2919	808.2	0.8987	0.882	0.3423	0.874	0
2920	808.1	0.8989	0.882	0.3425	0.8742	0
2921	808	0.8989	0.8825	0.3424	0.8741	0
2922	807.9	0.899	0.8824	0.3424	0.8743	0
2923	807.8	0.899	0.8823	0.3423	0.8741	0
2924	807.7	0.8991	0.8825	0.3423	0.8742	0
2925	807.6	0.8991	0.8824	0.3424	0.8743	0
2926	807.5	0.8994	0.8826	0.3425	0.8744	0
2927	807.4	0.8993	0.8827	0.3424	0.8742	0
2928	807.3	0.8994	0.8826	0.3424	0.8744	0
2929	807.2	0.8992	0.8825	0.3424	0.8745	0
2930	807.1	0.8992	0.8827	0.3425	0.8745	0
2931	807	0.8992	0.8827	0.3424	0.8745	0
2932	806.9	0.8994	0.883	0.3427	0.8748	0
2933	806.8	0.8994	0.883	0.3427	0.8747	0
2934	806.7	0.8995	0.883	0.3426	0.8747	0
2935	806.6	0.8995	0.8831	0.3425	0.8748	0
2936	806.5	0.8993	0.8833	0.3424	0.8748	0
2937	806.4	0.8997	0.8834	0.3427	0.875	0
2938	806.3	0.8998	0.8833	0.3426	0.875	0
2939	806.2	0.8997	0.8832	0.3427	0.875	0
2940	806.1	0.8997	0.8833	0.3428	0.875	0
2941	806	0.8999	0.8834	0.3429	0.8751	0
2942	805.9	0.8995	0.8835	0.3428	0.8753	0
2943	805.8	0.8998	0.8833	0.3429	0.8754	0
2944	805.7	0.8997	0.8836	0.3429	0.8753	0
2945	805.6	0.9	0.8837	0.3429	0.8756	0
2946	805.5	0.9001	0.8838	0.343	0.8756	0
2947	805.4	0.9001	0.8839	0.343	0.8756	0
2948	805.3	0.9002	0.884	0.3431	0.8759	0
2949	805.2	0.9003	0.8839	0.3432	0.8758	0
2950	805.1	0.9002	0.8843	0.3432	0.876	0
2951	805	0.9003	0.8842	0.343	0.8758	0
2952	804.9	0.9005	0.8847	0.3434	0.8761	0
2953	804.8	0.9002	0.8842	0.3433	0.8764	0

2954	804.7	0.9004	0.8844	0.3434	0.8762	0
2955	804.6	0.9003	0.8846	0.3433	0.8764	0
2956	804.5	0.9005	0.8844	0.3434	0.8762	0
2957	804.4	0.9005	0.8846	0.3434	0.8762	0
2958	804.3	0.9005	0.8848	0.3435	0.8765	0
2959	804.2	0.9006	0.8847	0.3434	0.8766	0
2960	804.1	0.9005	0.8847	0.3436	0.8767	0
2961	804	0.9007	0.885	0.3436	0.8767	0
2962	803.9	0.9007	0.885	0.3435	0.877	0
2963	803.8	0.9009	0.8849	0.3437	0.8769	0
2964	803.7	0.9007	0.8853	0.3437	0.8771	0
2965	803.6	0.9009	0.8851	0.3436	0.8772	0
2966	803.5	0.9008	0.885	0.3437	0.8769	0
2967	803.4	0.901	0.8852	0.3437	0.877	0
2968	803.3	0.9013	0.8852	0.3438	0.8774	0
2969	803.2	0.9012	0.8852	0.3439	0.8772	0
2970	803.1	0.901	0.8853	0.3438	0.8774	0
2971	803	0.9013	0.8856	0.3439	0.8772	0
2972	802.9	0.9012	0.8854	0.344	0.8775	0
2973	802.8	0.9017	0.8857	0.3441	0.8775	0
2974	802.7	0.9014	0.8858	0.3439	0.8774	0
2975	802.6	0.9013	0.8857	0.3441	0.8775	0
2976	802.5	0.9015	0.8858	0.344	0.8778	0
2977	802.4	0.9016	0.8859	0.3442	0.8775	0
2978	802.3	0.9018	0.8859	0.3441	0.8776	0
2979	802.2	0.9012	0.8856	0.3441	0.8775	0
2980	802.1	0.9015	0.8862	0.3441	0.8774	0
2981	802	0.9015	0.8859	0.344	0.8776	0
2982	801.9	0.9014	0.886	0.3439	0.8774	0
2983	801.8	0.9016	0.886	0.3439	0.8776	0
2984	801.7	0.9016	0.8862	0.344	0.8776	0
2985	801.6	0.9016	0.8861	0.3439	0.8775	0
2986	801.5	0.9018	0.8861	0.3441	0.8775	0
2987	801.4	0.9018	0.8861	0.3441	0.8776	0
2988	801.3	0.9016	0.8862	0.344	0.8776	0
2989	801.2	0.9018	0.886	0.3441	0.8777	0
2990	801.1	0.9019	0.8862	0.344	0.8776	0
2991	801	0.9018	0.8862	0.344	0.8775	0
2992	800.9	0.9019	0.8863	0.344	0.8774	0
2993	800.8	0.9021	0.8863	0.344	0.8773	0
2994	800.7	0.9019	0.8864	0.344	0.8777	0
2995	800.6	0.9018	0.8863	0.344	0.8774	0
2996	800.5	0.9018	0.886	0.3438	0.8772	0

2997	800.4	0.9021	0.8865	0.3441	0.8774	0
2998	800.3	0.9021	0.8866	0.3441	0.8775	0
2999	800.2	0.9021	0.8868	0.344	0.8775	0
3000	800.1	0.9019	0.8865	0.3439	0.8772	0
3001	800	0.9021	0.8866	0.3439	0.8774	0
3002	799.9	0.9022	0.8864	0.3438	0.8772	0
3003	799.8	0.9021	0.8866	0.3438	0.8773	0
3004	799.7	0.9025	0.8864	0.3436	0.8773	0
3005	799.6	0.9022	0.8863	0.3436	0.8771	0
3006	799.5	0.9021	0.8865	0.3436	0.877	0
3007	799.4	0.9021	0.8864	0.3436	0.877	0
3008	799.3	0.9022	0.8864	0.3438	0.877	0
3009	799.2	0.9022	0.8866	0.3436	0.8769	0
3010	799.1	0.9023	0.8865	0.3435	0.877	0
3011	799	0.9022	0.8864	0.3435	0.877	0
3012	798.9	0.9025	0.887	0.3435	0.8768	0
3013	798.8	0.9022	0.8868	0.3436	0.8768	0
3014	798.7	0.9024	0.8867	0.3436	0.8769	0
3015	798.6	0.9025	0.8866	0.3436	0.8765	0
3016	798.5	0.9024	0.8865	0.3436	0.8771	0
3017	798.4	0.9027	0.8864	0.3436	0.8769	0
3018	798.3	0.9027	0.8866	0.3436	0.8769	0
3019	798.2	0.9026	0.8869	0.3437	0.8767	0
3020	798.1	0.9028	0.8867	0.3435	0.8766	0
3021	798	0.9027	0.8868	0.3436	0.8766	0
3022	797.9	0.903	0.887	0.3437	0.8771	0
3023	797.8	0.903	0.8871	0.3438	0.8769	0
3024	797.7	0.9029	0.8871	0.3437	0.8769	0
3025	797.6	0.9028	0.887	0.3435	0.8767	0
3026	797.5	0.9029	0.8871	0.3437	0.8767	0
3027	797.4	0.9029	0.8873	0.3438	0.877	0
3028	797.3	0.9029	0.8871	0.3436	0.8769	0
3029	797.2	0.9033	0.8872	0.3438	0.8767	0
3030	797.1	0.9032	0.8871	0.3436	0.877	0
3031	797	0.9031	0.8871	0.3435	0.8768	0
3032	796.9	0.903	0.8873	0.3437	0.877	0
3033	796.8	0.9033	0.8873	0.3436	0.8772	0
3034	796.7	0.9033	0.8877	0.3438	0.8773	0
3035	796.6	0.9034	0.8877	0.3439	0.8772	0
3036	796.5	0.9035	0.8878	0.3438	0.8774	0
3037	796.4	0.9036	0.888	0.3441	0.8776	0
3038	796.3	0.9036	0.888	0.3442	0.8777	0
3039	796.2	0.9037	0.888	0.3442	0.8778	0

3040	796.1	0.9034	0.8881	0.344	0.8776	0
3041	796	0.9035	0.8881	0.3442	0.8779	0
3042	795.9	0.9034	0.888	0.3442	0.878	0
3043	795.8	0.9037	0.8883	0.3443	0.8784	0
3044	795.7	0.9039	0.8887	0.3443	0.8782	0
3045	795.6	0.9039	0.8883	0.3444	0.8783	0
3046	795.5	0.904	0.8884	0.3443	0.8784	0
3047	795.4	0.9039	0.8884	0.3445	0.8784	0
3048	795.3	0.9041	0.8886	0.3446	0.8783	0
3049	795.2	0.9041	0.8887	0.3445	0.8785	0
3050	795.1	0.904	0.8891	0.3448	0.8789	0
3051	795	0.9039	0.8887	0.3446	0.8787	0
3052	794.9	0.9041	0.8889	0.3447	0.879	0
3053	794.8	0.9042	0.889	0.3448	0.8788	0
3054	794.7	0.9042	0.8893	0.3449	0.8791	0
3055	794.6	0.9044	0.8891	0.345	0.8793	0
3056	794.5	0.9045	0.8893	0.345	0.8793	0
3057	794.4	0.9046	0.8893	0.3452	0.8796	0
3058	794.3	0.9045	0.8895	0.3451	0.8796	0
3059	794.2	0.9045	0.8897	0.3453	0.8799	0
3060	794.1	0.9045	0.8897	0.3452	0.8798	0
3061	794	0.9047	0.8896	0.3454	0.88	0
3062	793.9	0.9048	0.89	0.3454	0.8799	0
3063	793.8	0.9049	0.8902	0.3455	0.8801	0
3064	793.7	0.9048	0.8901	0.3455	0.8803	0
3065	793.6	0.905	0.8902	0.3456	0.8804	0
3066	793.5	0.905	0.8901	0.3455	0.8803	0
3067	793.4	0.9052	0.8907	0.3457	0.8807	0
3068	793.3	0.9052	0.8903	0.3457	0.8807	0
3069	793.2	0.9054	0.8902	0.3458	0.8809	0
3070	793.1	0.9054	0.8905	0.3457	0.881	0
3071	793	0.9055	0.8907	0.3459	0.881	0
3072	792.9	0.9055	0.8909	0.3459	0.881	0
3073	792.8	0.9055	0.8908	0.346	0.881	0
3074	792.7	0.9055	0.8906	0.3458	0.8808	0
3075	792.6	0.9054	0.8908	0.3459	0.8808	0
3076	792.5	0.9057	0.891	0.3461	0.8811	0
3077	792.4	0.9058	0.891	0.346	0.8811	0
3078	792.3	0.9056	0.8908	0.3459	0.881	0
3079	792.2	0.9056	0.8912	0.3461	0.8812	0
3080	792.1	0.9055	0.8908	0.3458	0.8813	0
3081	792	0.9058	0.891	0.3458	0.8813	0
3082	791.9	0.9059	0.891	0.3459	0.8811	0

3083	791.8	0.9057	0.8911	0.3458	0.8811	0
3084	791.7	0.9058	0.891	0.3458	0.8811	0
3085	791.6	0.9058	0.8912	0.3459	0.8813	0
3086	791.5	0.9058	0.8912	0.3459	0.8813	0
3087	791.4	0.906	0.8914	0.346	0.8813	0
3088	791.3	0.9059	0.8912	0.3462	0.8816	0
3089	791.2	0.9062	0.8916	0.346	0.8815	0
3090	791.1	0.9061	0.8915	0.3459	0.8814	0
3091	791	0.9063	0.8915	0.3459	0.8812	0
3092	790.9	0.906	0.8914	0.3461	0.8813	0
3093	790.8	0.9063	0.8913	0.3458	0.8812	0
3094	790.7	0.9063	0.8915	0.346	0.8814	0
3095	790.6	0.9062	0.8918	0.3461	0.8814	0
3096	790.5	0.9061	0.8916	0.346	0.8812	0
3097	790.4	0.9063	0.8918	0.3462	0.8813	0
3098	790.3	0.9063	0.8915	0.346	0.8813	0
3099	790.2	0.9063	0.8914	0.3459	0.8812	0
3100	790.1	0.9064	0.8915	0.3458	0.8812	0
3101	790	0.9062	0.8916	0.3457	0.8809	0
3102	789.9	0.9066	0.8919	0.3459	0.881	0
3103	789.8	0.9067	0.8916	0.3459	0.881	0
3104	789.7	0.9065	0.8915	0.3457	0.8809	0
3105	789.6	0.9066	0.8916	0.3457	0.8809	0
3106	789.5	0.9067	0.8917	0.3458	0.8808	0
3107	789.4	0.9067	0.8913	0.3456	0.8808	0
3108	789.3	0.9067	0.8918	0.3456	0.8807	0
3109	789.2	0.9066	0.8915	0.3457	0.8806	0
3110	789.1	0.9067	0.8914	0.3456	0.8807	0
3111	789	0.9068	0.8916	0.3456	0.8806	0
3112	788.9	0.907	0.8915	0.3457	0.8805	0
3113	788.8	0.9067	0.8916	0.3454	0.8804	0
3114	788.7	0.907	0.8916	0.3455	0.8803	0
3115	788.6	0.907	0.8916	0.3455	0.8803	0
3116	788.5	0.9069	0.8919	0.3455	0.8803	0
3117	788.4	0.9071	0.8917	0.3454	0.8803	0
3118	788.3	0.907	0.8916	0.3453	0.8802	0
3119	788.2	0.9069	0.8919	0.3453	0.8803	0
3120	788.1	0.907	0.8919	0.3454	0.8802	0
3121	788	0.9072	0.892	0.3455	0.8802	0
3122	787.9	0.9069	0.8917	0.3452	0.8799	0
3123	787.8	0.9075	0.8916	0.3452	0.8803	0
3124	787.7	0.9072	0.8919	0.3453	0.8801	0
3125	787.6	0.9069	0.8918	0.3452	0.88	0

3126	787.5	0.9072	0.8917	0.3451	0.8798	0
3127	787.4	0.9073	0.8919	0.3452	0.8799	0
3128	787.3	0.9074	0.8917	0.3451	0.88	0
3129	787.2	0.9076	0.8918	0.3452	0.88	0
3130	787.1	0.9076	0.8918	0.3451	0.8797	0
3131	787	0.9075	0.892	0.3452	0.8799	0
3132	786.9	0.9075	0.8919	0.3448	0.8798	0
3133	786.8	0.9073	0.8919	0.345	0.8801	0
3134	786.7	0.9077	0.8921	0.3452	0.8799	0
3135	786.6	0.9076	0.892	0.3452	0.8799	0
3136	786.5	0.9075	0.892	0.3451	0.8799	0
3137	786.4	0.9077	0.8924	0.3452	0.8801	0
3138	786.3	0.9078	0.8923	0.3453	0.88	0
3139	786.2	0.9081	0.8927	0.3455	0.8803	0
3140	786.1	0.908	0.8928	0.3455	0.8803	0
3141	786	0.9082	0.8925	0.3454	0.8802	0
3142	785.9	0.908	0.8927	0.3454	0.8802	0
3143	785.8	0.9082	0.8928	0.3454	0.8805	0
3144	785.7	0.9081	0.8927	0.3454	0.8804	0
3145	785.6	0.9082	0.8929	0.3455	0.8806	0
3146	785.5	0.9079	0.8926	0.3454	0.8806	0
3147	785.4	0.9082	0.8931	0.3455	0.8808	0
3148	785.3	0.9085	0.8929	0.3457	0.8808	0
3149	785.2	0.9083	0.8931	0.3458	0.8808	0
3150	785.1	0.9084	0.8932	0.3458	0.8812	0
3151	785	0.9086	0.8935	0.3461	0.8814	0
3152	784.9	0.9088	0.8936	0.3461	0.8816	0
3153	784.8	0.9086	0.8937	0.3462	0.8816	0
3154	784.7	0.9087	0.8936	0.3462	0.8817	0
3155	784.6	0.909	0.894	0.3464	0.882	0
3156	784.5	0.9089	0.8939	0.3465	0.8822	0
3157	784.4	0.9088	0.8941	0.3465	0.8822	0
3158	784.3	0.909	0.8942	0.3466	0.8823	0
3159	784.2	0.9089	0.8945	0.3467	0.8825	0
3160	784.1	0.9091	0.8944	0.3469	0.8827	0
3161	784	0.9089	0.8946	0.3468	0.8827	0
3162	783.9	0.9091	0.8944	0.3467	0.8828	0
3163	783.8	0.9091	0.8948	0.347	0.8832	0
3164	783.7	0.9093	0.8947	0.3469	0.8833	0
3165	783.6	0.9094	0.8949	0.3471	0.8833	0
3166	783.5	0.9096	0.8951	0.3472	0.8837	0
3167	783.4	0.9094	0.8952	0.3473	0.8836	0
3168	783.3	0.9096	0.8952	0.3473	0.8836	0

3169	783.2	0.9099	0.8954	0.3475	0.884	0
3170	783.1	0.91	0.8955	0.3474	0.8839	0
3171	783	0.9099	0.8958	0.3476	0.8843	0
3172	782.9	0.9099	0.8957	0.3475	0.8841	0
3173	782.8	0.91	0.8958	0.3475	0.8842	0
3174	782.7	0.9102	0.8958	0.3476	0.8841	0
3175	782.6	0.91	0.8957	0.3476	0.8846	0
3176	782.5	0.9103	0.8955	0.3478	0.8845	0
3177	782.4	0.9101	0.896	0.3477	0.8843	0
3178	782.3	0.9102	0.896	0.3477	0.8846	0
3179	782.2	0.9102	0.8958	0.3478	0.8846	0
3180	782.1	0.9105	0.896	0.3479	0.8845	0
3181	782	0.9104	0.8962	0.3479	0.8846	0
3182	781.9	0.9106	0.8965	0.348	0.8849	0
3183	781.8	0.9105	0.8962	0.3477	0.8847	0
3184	781.7	0.9106	0.8965	0.3481	0.8848	0
3185	781.6	0.9105	0.8964	0.3479	0.8849	0
3186	781.5	0.9104	0.8962	0.348	0.8848	0
3187	781.4	0.9107	0.8965	0.348	0.8851	0
3188	781.3	0.9107	0.8964	0.348	0.8852	0
3189	781.2	0.9104	0.8965	0.3481	0.885	0
3190	781.1	0.9107	0.8966	0.348	0.885	0
3191	781	0.9109	0.8968	0.3481	0.8852	0
3192	780.9	0.9109	0.8968	0.3482	0.8851	0
3193	780.8	0.9109	0.8968	0.3481	0.8851	0
3194	780.7	0.911	0.897	0.3481	0.8851	0
3195	780.6	0.911	0.8969	0.3479	0.8851	0
3196	780.5	0.911	0.897	0.3481	0.8853	0
3197	780.4	0.9111	0.897	0.348	0.8851	0
3198	780.3	0.9109	0.8968	0.3481	0.8853	0
3199	780.2	0.9114	0.8972	0.348	0.8856	0
3200	780.1	0.9112	0.897	0.3481	0.8852	0
3201	780	0.9112	0.8971	0.348	0.8849	0
3202	779.9	0.9114	0.897	0.3483	0.8853	0
3203	779.8	0.9112	0.897	0.348	0.8849	0
3204	779.7	0.9113	0.8973	0.348	0.8851	0
3205	779.6	0.9115	0.8971	0.3479	0.8849	0
3206	779.5	0.9114	0.8972	0.3479	0.8847	0
3207	779.4	0.9114	0.8971	0.3478	0.8849	0
3208	779.3	0.9115	0.897	0.3479	0.885	0
3209	779.2	0.9118	0.8972	0.3479	0.8847	0
3210	779.1	0.9117	0.8971	0.3477	0.8847	0
3211	779	0.9116	0.8974	0.3478	0.885	0

3212	778.9	0.9117	0.8971	0.3476	0.8845	0
3213	778.8	0.9115	0.8971	0.3476	0.8845	0
3214	778.7	0.9116	0.8971	0.3475	0.8844	0
3215	778.6	0.9117	0.897	0.3475	0.8842	0
3216	778.5	0.9118	0.8973	0.3475	0.8842	0
3217	778.4	0.9119	0.8973	0.3476	0.8843	0
3218	778.3	0.9119	0.8973	0.3474	0.8844	0
3219	778.2	0.9119	0.8973	0.3474	0.8841	0
3220	778.1	0.9118	0.897	0.3473	0.884	0
3221	778	0.9119	0.897	0.3471	0.884	0
3222	777.9	0.9119	0.8969	0.3473	0.8839	0
3223	777.8	0.9119	0.8971	0.3473	0.8838	0
3224	777.7	0.912	0.8971	0.3472	0.8836	0
3225	777.6	0.912	0.897	0.3471	0.8834	0
3226	777.5	0.9121	0.8972	0.3472	0.8837	0
3227	777.4	0.912	0.8969	0.3471	0.8838	0
3228	777.3	0.9121	0.8972	0.3471	0.8837	0
3229	777.2	0.9122	0.8972	0.3471	0.8837	0
3230	777.1	0.9122	0.8971	0.347	0.8834	0
3231	777	0.9125	0.8972	0.3472	0.8835	0
3232	776.9	0.9124	0.8973	0.3471	0.8836	0
3233	776.8	0.9125	0.8971	0.347	0.8838	0
3234	776.7	0.9125	0.8972	0.347	0.8835	0
3235	776.6	0.9126	0.8974	0.347	0.8836	0
3236	776.5	0.9125	0.8974	0.3472	0.8835	0
3237	776.4	0.9125	0.8976	0.347	0.8837	0
3238	776.3	0.9125	0.8975	0.347	0.8838	0
3239	776.2	0.9125	0.8976	0.347	0.8838	0
3240	776.1	0.9125	0.8976	0.3471	0.8835	0
3241	776	0.9129	0.8977	0.3473	0.8837	0
3242	775.9	0.9127	0.8979	0.3472	0.8837	0
3243	775.8	0.9129	0.8978	0.3472	0.8837	0
3244	775.7	0.9131	0.8979	0.3473	0.8838	0
3245	775.6	0.913	0.8978	0.3473	0.8841	0
3246	775.5	0.9131	0.8977	0.3472	0.8839	0
3247	775.4	0.9132	0.8983	0.3475	0.8841	0
3248	775.3	0.9133	0.8982	0.3474	0.8842	0
3249	775.2	0.9133	0.8984	0.3475	0.8841	0
3250	775.1	0.9132	0.8982	0.3474	0.884	0
3251	775	0.9134	0.898	0.3474	0.8842	0
3252	774.9	0.9133	0.8984	0.3475	0.8842	0
3253	774.8	0.9134	0.8988	0.3476	0.8845	0
3254	774.7	0.9137	0.899	0.3477	0.8847	0

3255	774.6	0.914	0.899	0.3477	0.8847	0
3256	774.5	0.9136	0.899	0.3478	0.8847	0
3257	774.4	0.9138	0.8991	0.348	0.8848	0
3258	774.3	0.9137	0.8991	0.3479	0.8849	0
3259	774.2	0.914	0.899	0.3478	0.8852	0
3260	774.1	0.9141	0.8992	0.348	0.8853	0
3261	774	0.914	0.8994	0.3481	0.8853	0
3262	773.9	0.914	0.8993	0.3483	0.8853	0
3263	773.8	0.9144	0.8996	0.3483	0.8855	0
3264	773.7	0.9142	0.8998	0.3483	0.8856	0
3265	773.6	0.9142	0.8998	0.3484	0.8858	0
3266	773.5	0.9143	0.8999	0.3483	0.8859	0
3267	773.4	0.9145	0.9	0.3484	0.886	0
3268	773.3	0.9146	0.9002	0.3487	0.8863	0
3269	773.2	0.9148	0.9003	0.3488	0.8866	0
3270	773.1	0.9148	0.9004	0.349	0.8869	0
3271	773	0.9148	0.9005	0.349	0.8868	0
3272	772.9	0.9148	0.9005	0.349	0.8868	0
3273	772.8	0.915	0.9006	0.3489	0.887	0
3274	772.7	0.9148	0.9006	0.349	0.887	0
3275	772.6	0.9149	0.9008	0.3491	0.8871	0
3276	772.5	0.9151	0.901	0.3492	0.8871	0
3277	772.4	0.915	0.9008	0.3492	0.8871	0
3278	772.3	0.9153	0.901	0.3491	0.8874	0
3279	772.2	0.9152	0.9012	0.3493	0.8874	0
3280	772.1	0.9154	0.9012	0.3492	0.8876	0
3281	772	0.9155	0.9011	0.3494	0.8875	0
3282	771.9	0.9157	0.9014	0.3495	0.8876	0
3283	771.8	0.9156	0.9014	0.3496	0.8876	0
3284	771.7	0.9157	0.9015	0.3495	0.8875	0
3285	771.6	0.9156	0.9016	0.3494	0.8876	0
3286	771.5	0.9156	0.9016	0.3494	0.8878	0
3287	771.4	0.9158	0.9017	0.3495	0.8877	0
3288	771.3	0.9157	0.9018	0.3495	0.8878	0
3289	771.2	0.9157	0.9018	0.3494	0.8878	0
3290	771.1	0.9158	0.9018	0.3494	0.8878	0
3291	771	0.9159	0.9016	0.3494	0.8877	0
3292	770.9	0.9159	0.9019	0.3496	0.8882	0
3293	770.8	0.9161	0.9019	0.3494	0.888	0
3294	770.7	0.916	0.9018	0.3495	0.8882	0
3295	770.6	0.9161	0.9022	0.3496	0.8882	0
3296	770.5	0.9162	0.9022	0.3496	0.8881	0
3297	770.4	0.9164	0.902	0.3495	0.8882	0

3298	770.3	0.9163	0.9023	0.3497	0.8883	0
3299	770.2	0.9165	0.9024	0.3497	0.8882	0
3300	770.1	0.9165	0.9023	0.3496	0.8881	0
3301	770	0.9164	0.9025	0.3495	0.8882	0
3302	769.9	0.9165	0.9025	0.3495	0.8881	0
3303	769.8	0.9165	0.9026	0.3495	0.8882	0
3304	769.7	0.9166	0.9026	0.3497	0.888	0
3305	769.6	0.9167	0.9025	0.3497	0.8882	0
3306	769.5	0.9169	0.9026	0.3496	0.8882	0
3307	769.4	0.9168	0.9026	0.3496	0.8882	0
3308	769.3	0.9166	0.9025	0.3494	0.888	0
3309	769.2	0.9168	0.9026	0.3495	0.8882	0
3310	769.1	0.9169	0.9026	0.3495	0.8881	0
3311	769	0.9169	0.9025	0.3493	0.888	0
3312	768.9	0.917	0.9028	0.3495	0.8879	0
3313	768.8	0.917	0.9029	0.3495	0.8879	0
3314	768.7	0.917	0.9027	0.3494	0.8878	0
3315	768.6	0.9173	0.903	0.3493	0.8878	0
3316	768.5	0.917	0.9028	0.3493	0.8877	0
3317	768.4	0.9174	0.9026	0.3494	0.8879	0
3318	768.3	0.9173	0.9027	0.3494	0.8877	0
3319	768.2	0.9175	0.9031	0.3495	0.888	0
3320	768.1	0.9174	0.9028	0.3494	0.8876	0
3321	768	0.9174	0.9029	0.3493	0.8876	0
3322	767.9	0.9174	0.9029	0.3494	0.8874	0
3323	767.8	0.9174	0.903	0.3493	0.8875	0
3324	767.7	0.9176	0.9029	0.3494	0.8876	0
3325	767.6	0.9175	0.9028	0.3492	0.8875	0
3326	767.5	0.9178	0.9031	0.3493	0.8875	0
3327	767.4	0.9177	0.9029	0.3492	0.8875	0
3328	767.3	0.9178	0.9032	0.3492	0.8876	0
3329	767.2	0.9179	0.903	0.3492	0.8873	0
3330	767.1	0.918	0.9032	0.3493	0.8872	0
3331	767	0.9178	0.9031	0.3491	0.8872	0
3332	766.9	0.9179	0.9031	0.349	0.8872	0
3333	766.8	0.9179	0.9034	0.3491	0.8873	0
3334	766.7	0.9179	0.9034	0.3491	0.8872	0
3335	766.6	0.9181	0.9035	0.3491	0.8874	0
3336	766.5	0.9182	0.9035	0.349	0.8872	0
3337	766.4	0.9183	0.9035	0.3492	0.8874	0
3338	766.3	0.9183	0.9037	0.3492	0.8872	0
3339	766.2	0.9184	0.9036	0.3492	0.8874	0
3340	766.1	0.9183	0.9035	0.3491	0.8874	0

3341	766	0.9184	0.9035	0.3492	0.8873	0
3342	765.9	0.9184	0.9035	0.3492	0.8873	0
3343	765.8	0.9184	0.9036	0.3492	0.8875	0
3344	765.7	0.9185	0.9036	0.3492	0.8874	0
3345	765.6	0.9186	0.9039	0.3492	0.8874	0
3346	765.5	0.9187	0.9038	0.3491	0.8872	0
3347	765.4	0.9185	0.9036	0.3491	0.8873	0
3348	765.3	0.9186	0.9039	0.3491	0.8872	0
3349	765.2	0.9186	0.9041	0.3491	0.8874	0
3350	765.1	0.9187	0.9038	0.3492	0.8878	0
3351	765	0.9187	0.9042	0.3492	0.8879	0
3352	764.9	0.9187	0.9041	0.3491	0.8876	0
3353	764.8	0.9193	0.9043	0.3493	0.8879	0
3354	764.7	0.9191	0.9043	0.3494	0.8877	0
3355	764.6	0.9189	0.9046	0.3493	0.888	0
3356	764.5	0.9193	0.9044	0.3494	0.8878	0
3357	764.4	0.9192	0.9045	0.3495	0.888	0
3358	764.3	0.9191	0.9047	0.3495	0.888	0
3359	764.2	0.9193	0.9048	0.3496	0.8881	0
3360	764.1	0.9195	0.9049	0.3496	0.8881	0
3361	764	0.9196	0.9049	0.3497	0.8884	0
3362	763.9	0.9198	0.9054	0.3498	0.8885	0
3363	763.8	0.9197	0.905	0.3498	0.8888	0
3364	763.7	0.9198	0.9051	0.3498	0.8884	0
3365	763.6	0.9197	0.9054	0.3499	0.8883	0
3366	763.5	0.9199	0.905	0.3498	0.8885	0
3367	763.4	0.9199	0.9053	0.3499	0.8885	0
3368	763.3	0.92	0.9057	0.35	0.8888	0
3369	763.2	0.9201	0.9058	0.3501	0.8889	0
3370	763.1	0.9203	0.9057	0.3501	0.889	0
3371	763	0.9202	0.9058	0.35	0.889	0
3372	762.9	0.9201	0.9059	0.3502	0.889	0
3373	762.8	0.9203	0.9061	0.3502	0.8892	0
3374	762.7	0.9203	0.9062	0.3503	0.8891	0
3375	762.6	0.9208	0.9061	0.3502	0.8894	0
3376	762.5	0.9204	0.9062	0.3503	0.8894	0
3377	762.4	0.9207	0.9062	0.3503	0.8895	0
3378	762.3	0.9207	0.9065	0.3503	0.8897	0
3379	762.2	0.9206	0.9064	0.3504	0.8892	0
3380	762.1	0.921	0.9065	0.3505	0.8895	0
3381	762	0.9209	0.9065	0.3505	0.8895	0
3382	761.9	0.921	0.9066	0.3504	0.8896	0
3383	761.8	0.9209	0.9069	0.3505	0.8897	0

3384	761.7	0.9211	0.9068	0.3505	0.8896	0
3385	761.6	0.9211	0.9068	0.3504	0.8897	0
3386	761.5	0.9212	0.9069	0.3505	0.8898	0
3387	761.4	0.9213	0.9072	0.3506	0.89	0
3388	761.3	0.9212	0.9073	0.3505	0.8898	0
3389	761.2	0.9214	0.9075	0.3506	0.89	0
3390	761.1	0.9214	0.9071	0.3505	0.89	0
3391	761	0.9215	0.9074	0.3505	0.89	0
3392	760.9	0.9215	0.9073	0.3506	0.8902	0
3393	760.8	0.9215	0.9074	0.3505	0.89	0
3394	760.7	0.9218	0.9076	0.3507	0.89	0
3395	760.6	0.922	0.9077	0.3507	0.8901	0
3396	760.5	0.9219	0.9078	0.3506	0.8899	0
3397	760.4	0.922	0.9077	0.3507	0.89	0
3398	760.3	0.9219	0.9076	0.3506	0.8904	0
3399	760.2	0.9221	0.9079	0.3509	0.8904	0
3400	760.1	0.922	0.9079	0.3508	0.8903	0
3401	760	0.9222	0.9077	0.3507	0.8901	0
3402	759.9	0.9223	0.908	0.3508	0.8901	0
3403	759.8	0.9224	0.9079	0.3509	0.8903	0
3404	759.7	0.9223	0.9083	0.3509	0.8904	0
3405	759.6	0.9224	0.9081	0.3507	0.8902	0
3406	759.5	0.9227	0.9084	0.3508	0.8903	0
3407	759.4	0.9225	0.9081	0.3508	0.8905	0
3408	759.3	0.9226	0.908	0.3508	0.8904	0
3409	759.2	0.9227	0.9086	0.3511	0.8905	0
3410	759.1	0.9228	0.9089	0.3511	0.8907	0
3411	759	0.9228	0.9086	0.3508	0.8905	0
3412	758.9	0.9229	0.9086	0.3509	0.8906	0
3413	758.8	0.9231	0.9087	0.3508	0.8905	0
3414	758.7	0.9228	0.9088	0.3509	0.8906	0
3415	758.6	0.9232	0.9087	0.351	0.8907	0
3416	758.5	0.9233	0.9088	0.3509	0.8906	0
3417	758.4	0.9232	0.909	0.3509	0.8905	0
3418	758.3	0.9232	0.9089	0.3509	0.8906	0
3419	758.2	0.9229	0.909	0.3508	0.8904	0
3420	758.1	0.9234	0.909	0.3507	0.8905	0
3421	758	0.9234	0.9091	0.3507	0.8903	0
3422	757.9	0.9234	0.9093	0.3509	0.8905	0
3423	757.8	0.9235	0.909	0.3508	0.8905	0
3424	757.7	0.9236	0.9093	0.3507	0.8906	0
3425	757.6	0.9237	0.9093	0.351	0.8906	0
3426	757.5	0.9239	0.9093	0.3507	0.8906	0

3427	757.4	0.9237	0.9093	0.3507	0.8906	0
3428	757.3	0.924	0.9097	0.351	0.8908	0
3429	757.2	0.9238	0.9095	0.3509	0.8905	0
3430	757.1	0.9238	0.9096	0.3507	0.8906	0
3431	757	0.9241	0.9099	0.3509	0.8906	0
3432	756.9	0.924	0.9097	0.3507	0.8906	0
3433	756.8	0.9242	0.9098	0.3507	0.8907	0
3434	756.7	0.9241	0.9096	0.3508	0.8906	0
3435	756.6	0.9243	0.9102	0.3509	0.8907	0
3436	756.5	0.9242	0.91	0.3509	0.8909	0
3437	756.4	0.9244	0.9101	0.3509	0.8909	0
3438	756.3	0.9244	0.9104	0.351	0.8908	0
3439	756.2	0.9243	0.9099	0.3509	0.8909	0
3440	756.1	0.9245	0.9102	0.351	0.8908	0
3441	756	0.9244	0.9104	0.351	0.8908	0
3442	755.9	0.9245	0.9104	0.3511	0.8909	0
3443	755.8	0.9247	0.9107	0.3511	0.891	0
3444	755.7	0.9248	0.9105	0.3511	0.8913	0
3445	755.6	0.9248	0.9109	0.3512	0.8914	0
3446	755.5	0.9249	0.9109	0.3513	0.8913	0
3447	755.4	0.9248	0.911	0.3511	0.8912	0
3448	755.3	0.9251	0.911	0.3512	0.8915	0
3449	755.2	0.9252	0.9114	0.3513	0.8913	0
3450	755.1	0.9252	0.9113	0.3512	0.8912	0
3451	755	0.9253	0.9113	0.3512	0.8911	0
3452	754.9	0.9253	0.9113	0.3513	0.8916	0
3453	754.8	0.9256	0.9116	0.3513	0.8916	0
3454	754.7	0.9254	0.9116	0.3514	0.8915	0
3455	754.6	0.9259	0.9118	0.3513	0.8914	0
3456	754.5	0.9258	0.9118	0.3515	0.8918	0
3457	754.4	0.9258	0.912	0.3515	0.8916	0
3458	754.3	0.9259	0.9118	0.3514	0.8917	0
3459	754.2	0.9257	0.9121	0.3516	0.8919	0
3460	754.1	0.926	0.9122	0.3516	0.8919	0
3461	754	0.926	0.9124	0.3515	0.8918	0
3462	753.9	0.926	0.9126	0.3516	0.892	0
3463	753.8	0.926	0.9123	0.3517	0.892	0
3464	753.7	0.9261	0.9124	0.3516	0.892	0
3465	753.6	0.9261	0.9127	0.3516	0.8921	0
3466	753.5	0.9262	0.9129	0.3516	0.8921	0
3467	753.4	0.9262	0.9129	0.3517	0.8921	0
3468	753.3	0.9263	0.9129	0.3517	0.8922	0
3469	753.2	0.9263	0.9127	0.3517	0.8923	0

3470	753.1	0.9264	0.913	0.3518	0.8923	0
3471	753	0.9266	0.913	0.3518	0.8925	0
3472	752.9	0.9268	0.913	0.3518	0.8926	0
3473	752.8	0.9267	0.9131	0.3519	0.8926	0
3474	752.7	0.9267	0.9134	0.352	0.8925	0
3475	752.6	0.9268	0.9133	0.3518	0.8923	0
3476	752.5	0.9268	0.9135	0.3517	0.8924	0
3477	752.4	0.9269	0.9134	0.3519	0.8925	0
3478	752.3	0.927	0.9137	0.3519	0.8927	0
3479	752.2	0.9273	0.9135	0.3521	0.893	0
3480	752.1	0.9272	0.9138	0.3518	0.8928	0
3481	752	0.9273	0.9136	0.3519	0.8929	0
3482	751.9	0.9274	0.9139	0.3518	0.8929	0
3483	751.8	0.9277	0.914	0.3521	0.8929	0
3484	751.7	0.9274	0.9142	0.3521	0.8928	0
3485	751.6	0.9276	0.914	0.3522	0.8928	0
3486	751.5	0.9277	0.9143	0.3521	0.8928	0
3487	751.4	0.9276	0.9143	0.3519	0.8928	0
3488	751.3	0.9278	0.9143	0.352	0.893	0
3489	751.2	0.9279	0.9143	0.3521	0.8929	0
3490	751.1	0.9278	0.9145	0.3522	0.893	0
3491	751	0.9279	0.9142	0.3521	0.8928	0
3492	750.9	0.928	0.9144	0.3522	0.8931	0
3493	750.8	0.928	0.9147	0.3522	0.8932	0
3494	750.7	0.928	0.9146	0.3521	0.893	0
3495	750.6	0.9282	0.9146	0.3521	0.8928	0
3496	750.5	0.9283	0.9145	0.352	0.8928	0
3497	750.4	0.9282	0.9148	0.352	0.8928	0
3498	750.3	0.9285	0.9149	0.352	0.8928	0
3499	750.2	0.9285	0.9148	0.352	0.8927	0
3500	750.1	0.9285	0.9149	0.3521	0.8928	0
3501	750	0.9287	0.9151	0.352	0.893	0
3502	749.9	0.9286	0.9151	0.3519	0.8929	0
3503	749.8	0.9289	0.9152	0.352	0.8931	0
3504	749.7	0.929	0.9152	0.352	0.8929	0
3505	749.6	0.9291	0.9152	0.352	0.893	0
3506	749.5	0.9292	0.9154	0.3521	0.893	0
3507	749.4	0.9292	0.9155	0.3522	0.8929	0
3508	749.3	0.9293	0.9154	0.3522	0.8931	0
3509	749.2	0.9292	0.9152	0.3521	0.8929	0
3510	749.1	0.9293	0.9155	0.352	0.893	0
3511	749	0.9295	0.9157	0.3519	0.8931	0
3512	748.9	0.9296	0.9157	0.3519	0.8931	0

3513	748.8	0.9296	0.9158	0.352	0.8933	0
3514	748.7	0.9294	0.9158	0.352	0.893	0
3515	748.6	0.9299	0.9159	0.3521	0.8933	0
3516	748.5	0.9296	0.9156	0.352	0.8931	0
3517	748.4	0.9298	0.9159	0.352	0.8931	0
3518	748.3	0.9299	0.916	0.352	0.8931	0
3519	748.2	0.9299	0.916	0.3519	0.8931	0
3520	748.1	0.9299	0.916	0.3521	0.8931	0
3521	748	0.9299	0.9162	0.352	0.8931	0
3522	747.9	0.9301	0.9161	0.3521	0.893	0
3523	747.8	0.9302	0.9164	0.3521	0.8933	0
3524	747.7	0.9303	0.9164	0.3522	0.8934	0
3525	747.6	0.9305	0.9163	0.3523	0.8931	0
3526	747.5	0.9303	0.9162	0.3521	0.8932	0
3527	747.4	0.9304	0.9165	0.3521	0.8931	0
3528	747.3	0.9305	0.9165	0.3522	0.8932	0
3529	747.2	0.9305	0.9167	0.3523	0.8935	0
3530	747.1	0.9304	0.9168	0.3523	0.8933	0
3531	747	0.9307	0.917	0.3524	0.8936	0
3532	746.9	0.931	0.9171	0.3523	0.8934	0
3533	746.8	0.9309	0.9172	0.3524	0.8937	0
3534	746.7	0.9311	0.9174	0.3526	0.8938	0
3535	746.6	0.9312	0.9174	0.3526	0.8938	0
3536	746.5	0.9311	0.9173	0.3524	0.8937	0
3537	746.4	0.931	0.917	0.3525	0.8938	0
3538	746.3	0.9312	0.9175	0.3525	0.894	0
3539	746.2	0.9312	0.9175	0.3524	0.8939	0
3540	746.1	0.9314	0.9173	0.3525	0.8939	0
3541	746	0.9314	0.9176	0.3526	0.8937	0
3542	745.9	0.9314	0.9178	0.3528	0.8942	0
3543	745.8	0.9318	0.918	0.3526	0.8942	0
3544	745.7	0.9318	0.9179	0.3528	0.8944	0
3545	745.6	0.9317	0.9181	0.3527	0.8942	0
3546	745.5	0.9319	0.9184	0.3529	0.8944	0
3547	745.4	0.9319	0.9184	0.3529	0.8944	0
3548	745.3	0.9321	0.9185	0.3529	0.8945	0
3549	745.2	0.9322	0.9183	0.353	0.8946	0
3550	745.1	0.9321	0.9186	0.353	0.8947	0
3551	745	0.9324	0.9187	0.3531	0.8949	0
3552	744.9	0.9325	0.9187	0.3532	0.8948	0
3553	744.8	0.9325	0.9189	0.3532	0.895	0
3554	744.7	0.9323	0.919	0.3532	0.8951	0
3555	744.6	0.9324	0.9189	0.3532	0.8949	0

3556	744.5	0.9325	0.9189	0.3534	0.8951	0
3557	744.4	0.9326	0.9191	0.3533	0.8951	0
3558	744.3	0.9328	0.919	0.3533	0.8953	0
3559	744.2	0.9331	0.9193	0.3534	0.8955	0
3560	744.1	0.9329	0.9196	0.3535	0.8953	0
3561	744	0.9332	0.9195	0.3535	0.8955	0
3562	743.9	0.9332	0.9198	0.3536	0.8956	0
3563	743.8	0.9335	0.92	0.3538	0.8958	0
3564	743.7	0.9335	0.92	0.3536	0.8958	0
3565	743.6	0.9336	0.9199	0.3537	0.8958	0
3566	743.5	0.9337	0.9201	0.3537	0.8959	0
3567	743.4	0.9337	0.9202	0.3537	0.8961	0
3568	743.3	0.9338	0.92	0.3538	0.8961	0
3569	743.2	0.9337	0.9202	0.3537	0.8961	0
3570	743.1	0.934	0.9203	0.3538	0.896	0
3571	743	0.9338	0.9204	0.3538	0.896	0
3572	742.9	0.934	0.9205	0.3539	0.8961	0
3573	742.8	0.9343	0.9207	0.354	0.8963	0
3574	742.7	0.9342	0.9206	0.3538	0.8962	0
3575	742.6	0.9344	0.9206	0.3539	0.8962	0
3576	742.5	0.9343	0.9207	0.3538	0.8961	0
3577	742.4	0.9345	0.9209	0.354	0.8963	0
3578	742.3	0.9343	0.9207	0.3539	0.8963	0
3579	742.2	0.9345	0.9209	0.3539	0.8964	0
3580	742.1	0.9346	0.9209	0.3538	0.8962	0
3581	742	0.9347	0.921	0.3539	0.8962	0
3582	741.9	0.9348	0.9211	0.3539	0.8962	0
3583	741.8	0.9347	0.921	0.3538	0.8963	0
3584	741.7	0.9348	0.9212	0.3539	0.8964	0
3585	741.6	0.935	0.9214	0.354	0.8966	0
3586	741.5	0.9353	0.9213	0.354	0.8963	0
3587	741.4	0.9351	0.9211	0.3538	0.8961	0
3588	741.3	0.9352	0.9215	0.354	0.8961	0
3589	741.2	0.9354	0.9214	0.354	0.8964	0
3590	741.1	0.9354	0.9216	0.3539	0.8965	0
3591	741	0.9354	0.9214	0.3539	0.8962	0
3592	740.9	0.9354	0.9215	0.3537	0.8961	0
3593	740.8	0.9355	0.9216	0.3539	0.8962	0
3594	740.7	0.9358	0.9217	0.3539	0.8962	0
3595	740.6	0.9358	0.9216	0.3538	0.8962	0
3596	740.5	0.9356	0.9215	0.3537	0.896	0
3597	740.4	0.9356	0.9216	0.3535	0.8958	0
3598	740.3	0.9356	0.9217	0.3537	0.8958	0

3599	740.2	0.9359	0.9217	0.3537	0.8958	0
3600	740.1	0.9357	0.9217	0.3536	0.8958	0
3601	740	0.936	0.922	0.3537	0.8959	0
3602	739.9	0.936	0.9217	0.3536	0.8958	0
3603	739.8	0.9361	0.9219	0.3539	0.8961	0
3604	739.7	0.9362	0.9222	0.3536	0.8958	0
3605	739.6	0.9363	0.922	0.3537	0.8957	0
3606	739.5	0.9362	0.922	0.3536	0.8957	0
3607	739.4	0.9362	0.9223	0.3536	0.8958	0
3608	739.3	0.9365	0.9222	0.3537	0.8961	0
3609	739.2	0.9365	0.9222	0.3536	0.8958	0
3610	739.1	0.9366	0.9221	0.3535	0.8956	0
3611	739	0.9366	0.9224	0.3534	0.8955	0
3612	738.9	0.9369	0.9224	0.3536	0.8956	0
3613	738.8	0.9368	0.9223	0.3534	0.8956	0
3614	738.7	0.9368	0.9225	0.3534	0.8955	0
3615	738.6	0.9367	0.9224	0.3531	0.8953	0
3616	738.5	0.9369	0.9227	0.3533	0.8954	0
3617	738.4	0.9372	0.9228	0.3531	0.8955	0
3618	738.3	0.9371	0.9227	0.3534	0.8956	0
3619	738.2	0.9374	0.9226	0.3534	0.8955	0
3620	738.1	0.9375	0.9226	0.3535	0.8955	0
3621	738	0.9373	0.9227	0.3532	0.8953	0
3622	737.9	0.9373	0.9228	0.3533	0.8954	0
3623	737.8	0.9374	0.9229	0.3532	0.8955	0
3624	737.7	0.9375	0.9228	0.3532	0.8956	0
3625	737.6	0.9374	0.923	0.3532	0.8955	0
3626	737.5	0.938	0.9232	0.3534	0.8956	0
3627	737.4	0.9379	0.9235	0.3534	0.8956	0
3628	737.3	0.9381	0.9234	0.3536	0.8959	0
3629	737.2	0.938	0.9232	0.3533	0.8956	0
3630	737.1	0.938	0.9232	0.3534	0.8956	0
3631	737	0.9381	0.9235	0.3536	0.8958	0
3632	736.9	0.9382	0.9236	0.3536	0.8958	0
3633	736.8	0.9381	0.9235	0.3536	0.8959	0
3634	736.7	0.9383	0.9237	0.3536	0.8962	0
3635	736.6	0.9385	0.9239	0.3537	0.896	0
3636	736.5	0.9383	0.924	0.3538	0.8961	0
3637	736.4	0.9386	0.9239	0.3537	0.8966	0
3638	736.3	0.9387	0.9242	0.3538	0.8965	0
3639	736.2	0.9389	0.9245	0.3538	0.8964	0
3640	736.1	0.9388	0.9242	0.354	0.8966	0
3641	736	0.9391	0.9245	0.3541	0.8968	0

3642	735.9	0.9392	0.9246	0.3542	0.8969	0
3643	735.8	0.9393	0.9249	0.3542	0.8972	0
3644	735.7	0.9392	0.925	0.3543	0.8971	0
3645	735.6	0.9393	0.9249	0.3542	0.897	0
3646	735.5	0.9394	0.9251	0.3543	0.8971	0
3647	735.4	0.9395	0.9252	0.3543	0.8973	0
3648	735.3	0.9396	0.9254	0.3544	0.8975	0
3649	735.2	0.9397	0.9253	0.3545	0.8976	0
3650	735.1	0.9399	0.9255	0.3544	0.8976	0
3651	735	0.9398	0.9255	0.3546	0.8979	0
3652	734.9	0.9401	0.9256	0.3547	0.8977	0
3653	734.8	0.9401	0.9257	0.3547	0.898	0
3654	734.7	0.9405	0.926	0.3548	0.8982	0
3655	734.6	0.9405	0.9259	0.3548	0.8984	0
3656	734.5	0.9403	0.9261	0.3548	0.8985	0
3657	734.4	0.9404	0.9262	0.3549	0.8984	0
3658	734.3	0.9406	0.9265	0.3549	0.8985	0
3659	734.2	0.9408	0.9267	0.3552	0.8991	0
3660	734.1	0.9408	0.9268	0.3551	0.8986	0
3661	734	0.941	0.9269	0.3553	0.899	0
3662	733.9	0.941	0.927	0.3552	0.8988	0
3663	733.8	0.9411	0.9268	0.3552	0.8989	0
3664	733.7	0.9414	0.9273	0.3553	0.8992	0
3665	733.6	0.9412	0.927	0.3554	0.8992	0
3666	733.5	0.9415	0.9274	0.3556	0.8995	0
3667	733.4	0.9417	0.9273	0.3553	0.8992	0
3668	733.3	0.9415	0.9272	0.3555	0.8992	0
3669	733.2	0.9416	0.9273	0.3554	0.8996	0
3670	733.1	0.9416	0.9273	0.3556	0.8995	0
3671	733	0.9416	0.9279	0.3555	0.8995	0
3672	732.9	0.942	0.9276	0.3556	0.8995	0
3673	732.8	0.942	0.9277	0.3555	0.8996	0
3674	732.7	0.942	0.9276	0.3554	0.8996	0
3675	732.6	0.9419	0.9279	0.3555	0.8995	0
3676	732.5	0.9421	0.928	0.3557	0.8996	0
3677	732.4	0.9421	0.9279	0.3555	0.8994	0
3678	732.3	0.9423	0.9278	0.3557	0.8996	0
3679	732.2	0.9423	0.9281	0.3556	0.8998	0
3680	732.1	0.9426	0.9281	0.3557	0.8998	0
3681	732	0.9425	0.9284	0.3557	0.8996	0
3682	731.9	0.9429	0.9284	0.3556	0.8999	0
3683	731.8	0.9427	0.9282	0.3558	0.8999	0
3684	731.7	0.9428	0.9284	0.3557	0.8999	0

3685	731.6	0.943	0.9287	0.3558	0.8999	0
3686	731.5	0.943	0.9284	0.3557	0.8999	0
3687	731.4	0.9432	0.9288	0.3557	0.9	0
3688	731.3	0.9431	0.9285	0.3557	0.8999	0
3689	731.2	0.9433	0.9286	0.3557	0.8999	0
3690	731.1	0.9433	0.9288	0.3557	0.9	0
3691	731	0.9433	0.9287	0.3556	0.8998	0
3692	730.9	0.9434	0.9288	0.3555	0.8998	0
3693	730.8	0.9436	0.9291	0.3555	0.8998	0
3694	730.7	0.9433	0.9291	0.3555	0.8996	0
3695	730.6	0.9438	0.9289	0.3555	0.8996	0
3696	730.5	0.9436	0.9289	0.3556	0.8997	0
3697	730.4	0.9437	0.9291	0.3554	0.8994	0
3698	730.3	0.944	0.9291	0.3555	0.8996	0
3699	730.2	0.9441	0.929	0.3555	0.8996	0
3700	730.1	0.944	0.9291	0.3556	0.8996	0
3701	730	0.944	0.9292	0.3555	0.8995	0
3702	729.9	0.9442	0.9293	0.3555	0.8997	0
3703	729.8	0.9443	0.9292	0.3554	0.8993	0
3704	729.7	0.9442	0.9294	0.3554	0.8994	0
3705	729.6	0.9443	0.9292	0.3553	0.8993	0
3706	729.5	0.9444	0.9294	0.3554	0.8991	0
3707	729.4	0.9444	0.9295	0.3552	0.8991	0
3708	729.3	0.9446	0.9297	0.3552	0.899	0
3709	729.2	0.9444	0.9295	0.3551	0.899	0
3710	729.1	0.9446	0.9295	0.355	0.8991	0
3711	729	0.9446	0.9296	0.3551	0.899	0
3712	728.9	0.9448	0.9296	0.3552	0.8989	0
3713	728.8	0.9449	0.9295	0.3551	0.8988	0
3714	728.7	0.9449	0.9298	0.3551	0.8989	0
3715	728.6	0.945	0.9297	0.355	0.8989	0
3716	728.5	0.9453	0.9298	0.3551	0.8987	0
3717	728.4	0.9452	0.9298	0.3551	0.8987	0
3718	728.3	0.9454	0.93	0.3551	0.8988	0
3719	728.2	0.9454	0.9301	0.355	0.8985	0
3720	728.1	0.9455	0.93	0.3551	0.8987	0
3721	728	0.9457	0.93	0.3551	0.8988	0
3722	727.9	0.9456	0.9302	0.355	0.8985	0
3723	727.8	0.9457	0.9304	0.355	0.8984	0
3724	727.7	0.9461	0.9303	0.355	0.8987	0
3725	727.6	0.9461	0.9303	0.3549	0.8985	0
3726	727.5	0.9461	0.9304	0.355	0.8986	0
3727	727.4	0.9465	0.9306	0.3551	0.8986	0

3728	727.3	0.9462	0.9306	0.3548	0.8983	0
3729	727.2	0.9463	0.9305	0.355	0.8986	0
3730	727.1	0.9463	0.9305	0.3549	0.8985	0
3731	727	0.9464	0.9308	0.3549	0.8987	0
3732	726.9	0.9465	0.9311	0.355	0.8985	0
3733	726.8	0.9465	0.931	0.3549	0.8985	0
3734	726.7	0.9468	0.931	0.355	0.8985	0
3735	726.6	0.9468	0.9311	0.355	0.8986	0
3736	726.5	0.947	0.9313	0.3551	0.8985	0
3737	726.4	0.9472	0.9313	0.355	0.8987	0
3738	726.3	0.9471	0.9314	0.3551	0.8988	0
3739	726.2	0.9471	0.9315	0.3551	0.8988	0
3740	726.1	0.9473	0.9314	0.355	0.8986	0
3741	726	0.9473	0.9315	0.3551	0.8989	0
3742	725.9	0.9476	0.9317	0.3553	0.8989	0
3743	725.8	0.9476	0.9317	0.3552	0.8989	0
3744	725.7	0.9475	0.9319	0.3553	0.8992	0
3745	725.6	0.9478	0.932	0.3554	0.8992	0
3746	725.5	0.9479	0.9321	0.3554	0.8993	0
3747	725.4	0.948	0.9321	0.3554	0.8993	0
3748	725.3	0.948	0.9326	0.3555	0.8993	0
3749	725.2	0.948	0.9324	0.3556	0.8995	0
3750	725.1	0.9481	0.9324	0.3555	0.8996	0
3751	725	0.9484	0.9328	0.3556	0.8999	0
3752	724.9	0.9486	0.933	0.3559	0.8998	0
3753	724.8	0.9489	0.9331	0.3558	0.9002	0
3754	724.7	0.9489	0.9331	0.3559	0.9003	0
3755	724.6	0.9488	0.9333	0.3559	0.9005	0
3756	724.5	0.9491	0.9338	0.3562	0.9009	0
3757	724.4	0.9491	0.9335	0.3561	0.9009	0
3758	724.3	0.9491	0.9338	0.3562	0.901	0
3759	724.2	0.9491	0.9338	0.3563	0.9011	0
3760	724.1	0.9494	0.934	0.3563	0.9013	0
3761	724	0.9495	0.9341	0.3565	0.9014	0
3762	723.9	0.9496	0.9344	0.3566	0.9012	0
3763	723.8	0.9497	0.9344	0.3568	0.9017	0
3764	723.7	0.9499	0.9345	0.3567	0.902	0
3765	723.6	0.95	0.9345	0.3567	0.9019	0
3766	723.5	0.9503	0.9351	0.3569	0.9022	0
3767	723.4	0.9504	0.9351	0.3568	0.9022	0
3768	723.3	0.9502	0.935	0.3568	0.9022	0
3769	723.2	0.9503	0.9349	0.357	0.9022	0
3770	723.1	0.9505	0.935	0.357	0.9025	0

3771	723	0.9507	0.9354	0.3572	0.9028	0
3772	722.9	0.9507	0.9356	0.3572	0.9025	0
3773	722.8	0.9505	0.9357	0.3571	0.9026	0
3774	722.7	0.9509	0.9357	0.3574	0.9029	0
3775	722.6	0.9512	0.9358	0.3573	0.9029	0
3776	722.5	0.951	0.936	0.3572	0.903	0
3777	722.4	0.9512	0.936	0.3573	0.9027	0
3778	722.3	0.9513	0.9359	0.3574	0.9029	0
3779	722.2	0.9513	0.9363	0.3573	0.9029	0
3780	722.1	0.9517	0.9363	0.3575	0.9032	0
3781	722	0.9517	0.9364	0.3574	0.9034	0
3782	721.9	0.9517	0.9364	0.3575	0.9032	0
3783	721.8	0.9518	0.9366	0.3575	0.9035	0
3784	721.7	0.9519	0.9366	0.3574	0.9033	0
3785	721.6	0.9521	0.9367	0.3576	0.9035	0
3786	721.5	0.9519	0.9367	0.3574	0.9033	0
3787	721.4	0.952	0.9369	0.3575	0.9033	0
3788	721.3	0.9522	0.9371	0.3576	0.9032	0
3789	721.2	0.9521	0.9372	0.3576	0.9033	0
3790	721.1	0.9526	0.9373	0.3578	0.9036	0
3791	721	0.9524	0.9373	0.3576	0.9034	0
3792	720.9	0.9526	0.9371	0.3576	0.9035	0
3793	720.8	0.9526	0.9372	0.3575	0.9034	0
3794	720.7	0.9527	0.9376	0.3577	0.9035	0
3795	720.6	0.9529	0.9375	0.3576	0.9034	0
3796	720.5	0.953	0.9379	0.3578	0.9036	0
3797	720.4	0.953	0.9378	0.3576	0.9035	0
3798	720.3	0.9533	0.9378	0.3575	0.9035	0
3799	720.2	0.9532	0.9376	0.3576	0.9037	0
3800	720.1	0.9533	0.9379	0.3576	0.9035	0
3801	720	0.9535	0.9378	0.3576	0.9035	0
3802	719.9	0.9537	0.938	0.3574	0.9036	0
3803	719.8	0.9534	0.9381	0.3575	0.9036	0
3804	719.7	0.9536	0.9381	0.3575	0.9035	0
3805	719.6	0.9538	0.9382	0.3576	0.9035	0
3806	719.5	0.9538	0.9384	0.3575	0.9034	0
3807	719.4	0.9541	0.9383	0.3575	0.9032	0
3808	719.3	0.9542	0.9385	0.3575	0.9033	0
3809	719.2	0.954	0.9383	0.3574	0.9032	0
3810	719.1	0.9545	0.9385	0.3574	0.9031	0
3811	719	0.9544	0.9387	0.3573	0.903	0
3812	718.9	0.9544	0.9385	0.3574	0.9029	0
3813	718.8	0.9545	0.9386	0.3573	0.9029	0

3814	718.7	0.9548	0.9386	0.3574	0.9029	0
3815	718.6	0.9547	0.9389	0.3572	0.903	0
3816	718.5	0.9548	0.9386	0.3571	0.9028	0
3817	718.4	0.9548	0.9387	0.3571	0.9028	0
3818	718.3	0.9548	0.9389	0.3572	0.9024	0
3819	718.2	0.955	0.9388	0.357	0.9023	0
3820	718.1	0.9551	0.9393	0.357	0.9024	0
3821	718	0.9552	0.9389	0.3571	0.9026	0
3822	717.9	0.9551	0.939	0.3569	0.9023	0
3823	717.8	0.9552	0.9391	0.357	0.9025	0
3824	717.7	0.9554	0.9393	0.357	0.9023	0
3825	717.6	0.9557	0.9391	0.3569	0.9023	0
3826	717.5	0.9555	0.9391	0.3568	0.9024	0
3827	717.4	0.9556	0.9392	0.3569	0.9023	0
3828	717.3	0.9555	0.9395	0.357	0.9023	0
3829	717.2	0.9555	0.9394	0.3566	0.902	0
3830	717.1	0.9559	0.9397	0.3567	0.9022	0
3831	717	0.9558	0.9393	0.3566	0.9019	0
3832	716.9	0.956	0.9396	0.3566	0.902	0
3833	716.8	0.9561	0.9395	0.3566	0.9023	0
3834	716.7	0.9561	0.9398	0.3566	0.9021	0
3835	716.6	0.9563	0.9398	0.3566	0.9019	0
3836	716.5	0.9561	0.9399	0.3567	0.902	0
3837	716.4	0.9564	0.9399	0.3566	0.9021	0
3838	716.3	0.9565	0.9399	0.3565	0.9021	0
3839	716.2	0.9567	0.9401	0.3566	0.9022	0
3840	716.1	0.9566	0.9403	0.3565	0.902	0
3841	716	0.9568	0.9401	0.3566	0.9021	0
3842	715.9	0.9569	0.94	0.3566	0.9022	0
3843	715.8	0.9571	0.9405	0.3567	0.9024	0
3844	715.7	0.9571	0.9404	0.3566	0.9021	0
3845	715.6	0.9573	0.9408	0.3569	0.9025	0
3846	715.5	0.9573	0.9407	0.3567	0.9021	0
3847	715.4	0.9573	0.941	0.3569	0.9024	0
3848	715.3	0.9576	0.9408	0.3569	0.9024	0
3849	715.2	0.9578	0.9411	0.357	0.9026	0
3850	715.1	0.9579	0.9414	0.3572	0.903	0
3851	715	0.958	0.9416	0.3571	0.9027	0
3852	714.9	0.9582	0.9415	0.3571	0.9029	0
3853	714.8	0.9583	0.9417	0.3572	0.9029	0
3854	714.7	0.9583	0.9417	0.357	0.9028	0
3855	714.6	0.9583	0.9417	0.3571	0.9029	0
3856	714.5	0.9584	0.942	0.3573	0.903	0

3857	714.4	0.9586	0.942	0.3573	0.9033	0
3858	714.3	0.9588	0.9421	0.3574	0.9035	0
3859	714.2	0.9589	0.9424	0.3573	0.9036	0
3860	714.1	0.9587	0.9426	0.3574	0.9034	0
3861	714	0.959	0.9427	0.3574	0.9033	0
3862	713.9	0.9594	0.9429	0.3576	0.9036	0
3863	713.8	0.9592	0.9431	0.3576	0.9039	0
3864	713.7	0.9595	0.9433	0.3577	0.9043	0
3865	713.6	0.9595	0.9433	0.3579	0.9043	0
3866	713.5	0.9595	0.9433	0.3578	0.9043	0
3867	713.4	0.9599	0.9438	0.3581	0.9047	0
3868	713.3	0.9601	0.9438	0.3581	0.9045	0
3869	713.2	0.9602	0.9439	0.3581	0.9047	0
3870	713.1	0.9602	0.9442	0.3583	0.9047	0
3871	713	0.9603	0.9442	0.3583	0.9049	0
3872	712.9	0.9605	0.9443	0.3584	0.9051	0
3873	712.8	0.9605	0.9442	0.3584	0.9052	0
3874	712.7	0.9607	0.9447	0.3584	0.9053	0
3875	712.6	0.9605	0.9445	0.3584	0.9052	0
3876	712.5	0.961	0.945	0.3587	0.9053	0
3877	712.4	0.961	0.9451	0.3586	0.9055	0
3878	712.3	0.9613	0.9452	0.3588	0.9057	0
3879	712.2	0.9611	0.9454	0.3587	0.9057	0
3880	712.1	0.9615	0.9454	0.3587	0.9058	0
3881	712	0.9614	0.9454	0.3588	0.9057	0
3882	711.9	0.9613	0.9455	0.3588	0.9057	0
3883	711.8	0.9616	0.9457	0.3588	0.9059	0
3884	711.7	0.9617	0.9457	0.3588	0.9059	0
3885	711.6	0.9619	0.946	0.3589	0.906	0
3886	711.5	0.9621	0.9459	0.3591	0.9061	0
3887	711.4	0.962	0.946	0.3588	0.9061	0
3888	711.3	0.9622	0.9463	0.359	0.9061	0
3889	711.2	0.9621	0.9461	0.3588	0.9061	0
3890	711.1	0.9623	0.9464	0.359	0.9062	0
3891	711	0.9624	0.9465	0.3591	0.9064	0
3892	710.9	0.9625	0.9467	0.3591	0.9067	0
3893	710.8	0.9626	0.9467	0.3591	0.9067	0
3894	710.7	0.9628	0.947	0.3593	0.9068	0
3895	710.6	0.9629	0.9471	0.3592	0.9065	0
3896	710.5	0.9629	0.9473	0.3591	0.9066	0
3897	710.4	0.9631	0.9473	0.3592	0.9067	0
3898	710.3	0.9633	0.9474	0.3593	0.9066	0
3899	710.2	0.9633	0.9472	0.3593	0.9066	0

3900	710.1	0.9634	0.9476	0.3594	0.9067	0
3901	710	0.9639	0.9478	0.3592	0.9069	0
3902	709.9	0.9636	0.9477	0.3593	0.9067	0
3903	709.8	0.9641	0.9479	0.3593	0.9067	0
3904	709.7	0.9639	0.9481	0.3593	0.9067	0
3905	709.6	0.9641	0.9479	0.3592	0.9066	0
3906	709.5	0.9642	0.9482	0.3592	0.9067	0
3907	709.4	0.9644	0.9481	0.3593	0.9068	0
3908	709.3	0.9642	0.9483	0.3593	0.9067	0
3909	709.2	0.9644	0.9483	0.3592	0.9066	0
3910	709.1	0.9646	0.9485	0.3593	0.9066	0
3911	709	0.9647	0.9486	0.3592	0.9068	0
3912	708.9	0.9646	0.9486	0.3594	0.9066	0
3913	708.8	0.9646	0.9486	0.3592	0.9067	0
3914	708.7	0.965	0.949	0.3592	0.9065	0
3915	708.6	0.9652	0.9486	0.3591	0.9065	0
3916	708.5	0.9648	0.9486	0.359	0.9065	0
3917	708.4	0.9652	0.9489	0.3592	0.9064	0
3918	708.3	0.9652	0.949	0.359	0.9063	0
3919	708.2	0.9653	0.949	0.3591	0.9064	0
3920	708.1	0.9656	0.9493	0.359	0.9065	0
3921	708	0.9655	0.9492	0.3591	0.9063	0
3922	707.9	0.9659	0.9493	0.359	0.9064	0
3923	707.8	0.9659	0.9495	0.3589	0.9063	0
3924	707.7	0.9657	0.9493	0.359	0.9063	0
3925	707.6	0.9658	0.9495	0.3588	0.9064	0
3926	707.5	0.966	0.9497	0.3589	0.9062	0
3927	707.4	0.9662	0.9496	0.3587	0.9062	0
3928	707.3	0.9661	0.9497	0.3586	0.9061	0
3929	707.2	0.9663	0.95	0.3587	0.9061	0
3930	707.1	0.9664	0.9499	0.3587	0.906	0
3931	707	0.9666	0.9498	0.3587	0.9062	0
3932	706.9	0.9666	0.9497	0.3587	0.9058	0
3933	706.8	0.9666	0.9503	0.3587	0.906	0
3934	706.7	0.9667	0.9501	0.3586	0.906	0
3935	706.6	0.9669	0.95	0.3586	0.9061	0
3936	706.5	0.9671	0.9502	0.3587	0.9059	0
3937	706.4	0.967	0.9503	0.3587	0.9058	0
3938	706.3	0.967	0.9505	0.3586	0.906	0
3939	706.2	0.9675	0.9508	0.3588	0.906	0
3940	706.1	0.9671	0.9508	0.3586	0.9058	0
3941	706	0.9674	0.9509	0.3587	0.906	0
3942	705.9	0.9677	0.951	0.3587	0.9058	0

3943	705.8	0.9675	0.951	0.3586	0.9059	0
3944	705.7	0.9679	0.951	0.3587	0.9061	0
3945	705.6	0.968	0.9511	0.3587	0.9061	0
3946	705.5	0.968	0.9514	0.3588	0.906	0
3947	705.4	0.9682	0.9516	0.3589	0.9061	0
3948	705.3	0.9683	0.9517	0.3588	0.9061	0
3949	705.2	0.9685	0.9519	0.3588	0.906	0
3950	705.1	0.9685	0.9518	0.3588	0.9063	0
3951	705	0.9686	0.9519	0.3589	0.9064	0
3952	704.9	0.9688	0.9521	0.359	0.9064	0
3953	704.8	0.9686	0.9522	0.359	0.9063	0
3954	704.7	0.9688	0.9523	0.3589	0.9065	0
3955	704.6	0.9691	0.9524	0.359	0.9066	0
3956	704.5	0.9691	0.9525	0.3589	0.9066	0
3957	704.4	0.9692	0.9525	0.359	0.9067	0
3958	704.3	0.9694	0.9529	0.3591	0.907	0
3959	704.2	0.9696	0.9533	0.359	0.9069	0
3960	704.1	0.9698	0.9533	0.3592	0.907	0
3961	704	0.9697	0.9533	0.3593	0.907	0
3962	703.9	0.9701	0.9535	0.3594	0.9073	0
3963	703.8	0.9699	0.9537	0.3595	0.9073	0
3964	703.7	0.9702	0.9537	0.3595	0.9072	0
3965	703.6	0.9702	0.9541	0.3594	0.9074	0
3966	703.5	0.9703	0.9541	0.3595	0.9077	0
3967	703.4	0.9703	0.9542	0.3596	0.9076	0
3968	703.3	0.9706	0.9543	0.3596	0.9074	0
3969	703.2	0.9707	0.9545	0.3597	0.9077	0
3970	703.1	0.9707	0.9545	0.3596	0.9078	0
3971	703	0.971	0.9546	0.3598	0.9079	0
3972	702.9	0.9713	0.9549	0.3597	0.9083	0
3973	702.8	0.9712	0.955	0.3598	0.9081	0
3974	702.7	0.9715	0.9552	0.36	0.9083	0
3975	702.6	0.9714	0.9552	0.3598	0.9082	0
3976	702.5	0.9716	0.9555	0.3598	0.9081	0
3977	702.4	0.9716	0.9555	0.36	0.9083	0
3978	702.3	0.9719	0.9557	0.3602	0.9086	0
3979	702.2	0.9721	0.956	0.3603	0.909	0
3980	702.1	0.9721	0.956	0.3603	0.9088	0
3981	702	0.9722	0.956	0.36	0.9087	0
3982	701.9	0.9724	0.956	0.36	0.9087	0
3983	701.8	0.9723	0.9562	0.3601	0.909	0
3984	701.7	0.9725	0.9564	0.3601	0.9089	0
3985	701.6	0.9727	0.9567	0.3604	0.9089	0

3986	701.5	0.9729	0.9567	0.3601	0.9089	0
3987	701.4	0.9729	0.9569	0.3601	0.9089	0
3988	701.3	0.973	0.9571	0.3603	0.9091	0
3989	701.2	0.9732	0.9572	0.3604	0.909	0
3990	701.1	0.9733	0.9573	0.3604	0.9094	0
3991	701	0.9736	0.9575	0.3606	0.9095	0
3992	700.9	0.9736	0.9575	0.3605	0.9094	0
3993	700.8	0.9738	0.9577	0.3607	0.9095	0
3994	700.7	0.9738	0.958	0.3605	0.9094	0
3995	700.6	0.9737	0.9577	0.3605	0.9095	0
3996	700.5	0.974	0.9577	0.3604	0.9094	0
3997	700.4	0.9742	0.9581	0.3604	0.9094	0
3998	700.3	0.9742	0.9582	0.3606	0.9097	0
3999	700.2	0.9745	0.9584	0.3606	0.9097	0
4000	700.1	0.9746	0.9587	0.3608	0.9099	0
4001	700	0.9746	0.9587	0.3608	0.9096	0
4002	699.9	0.9748	0.9589	0.3609	0.9099	0
4003	699.8	0.9752	0.9592	0.3608	0.91	0
4004	699.7	0.975	0.9591	0.3608	0.9099	0
4005	699.6	0.9753	0.9593	0.3607	0.9098	0
4006	699.5	0.9752	0.9592	0.3607	0.9102	0
4007	699.4	0.9754	0.9594	0.3607	0.91	0
4008	699.3	0.9755	0.9597	0.3608	0.9097	0
4009	699.2	0.9757	0.9598	0.361	0.9101	0
4010	699.1	0.9759	0.9601	0.361	0.9104	0
4011	699	0.9758	0.9597	0.361	0.9103	0
4012	698.9	0.9761	0.9601	0.3609	0.9101	0
4013	698.8	0.9764	0.9602	0.3609	0.9102	0
4014	698.7	0.9763	0.9601	0.3608	0.9101	0
4015	698.6	0.9763	0.9605	0.3608	0.91	0
4016	698.5	0.9764	0.9603	0.3609	0.91	0
4017	698.4	0.9765	0.9606	0.3609	0.9099	0
4018	698.3	0.9766	0.9606	0.3608	0.9097	0
4019	698.2	0.9769	0.9607	0.3609	0.91	0
4020	698.1	0.9769	0.9609	0.3608	0.91	0
4021	698	0.9768	0.961	0.3607	0.91	0
4022	697.9	0.977	0.961	0.3607	0.9099	0
4023	697.8	0.9774	0.9612	0.3607	0.91	0
4024	697.7	0.9775	0.9614	0.3609	0.9099	0
4025	697.6	0.9775	0.9615	0.3609	0.91	0
4026	697.5	0.9778	0.9617	0.3609	0.9103	0
4027	697.4	0.9778	0.9616	0.3608	0.9104	0
4028	697.3	0.978	0.9617	0.361	0.9103	0

4029	697.2	0.9781	0.9617	0.3609	0.91	0
4030	697.1	0.9781	0.9618	0.3607	0.9102	0
4031	697	0.9782	0.962	0.3608	0.9102	0
4032	696.9	0.9785	0.9621	0.3608	0.9104	0
4033	696.8	0.9784	0.9622	0.3608	0.9103	0
4034	696.7	0.9784	0.9625	0.3607	0.9103	0
4035	696.6	0.9786	0.9622	0.3608	0.9103	0
4036	696.5	0.9786	0.9626	0.3608	0.91	0
4037	696.4	0.9786	0.9626	0.3607	0.9102	0
4038	696.3	0.979	0.9628	0.3608	0.9101	0
4039	696.2	0.9792	0.963	0.3609	0.9101	0
4040	696.1	0.9794	0.963	0.361	0.9104	0
4041	696	0.9793	0.9629	0.3609	0.9102	0
4042	695.9	0.9796	0.9634	0.3611	0.9106	0
4043	695.8	0.9797	0.9635	0.361	0.9104	0
4044	695.7	0.9799	0.9634	0.3609	0.9105	0
4045	695.6	0.9797	0.9634	0.3609	0.9104	0
4046	695.5	0.9799	0.9637	0.361	0.9109	0
4047	695.4	0.98	0.964	0.3611	0.9109	0
4048	695.3	0.9803	0.964	0.3611	0.9107	0
4049	695.2	0.9804	0.9642	0.3611	0.9108	0
4050	695.1	0.9804	0.9642	0.3611	0.911	0
4051	695	0.9805	0.9645	0.3612	0.9108	0
4052	694.9	0.9808	0.9646	0.3612	0.9108	0
4053	694.8	0.981	0.9649	0.3613	0.9111	0
4054	694.7	0.981	0.9651	0.3614	0.911	0
4055	694.6	0.9811	0.9652	0.3613	0.9114	0
4056	694.5	0.9813	0.9652	0.3613	0.9114	0
4057	694.4	0.9813	0.9652	0.3614	0.9114	0
4058	694.3	0.9816	0.9656	0.3616	0.9115	0
4059	694.2	0.9815	0.9658	0.3616	0.9117	0
4060	694.1	0.9817	0.966	0.3615	0.9117	0
4061	694	0.9818	0.9659	0.3615	0.9119	0
4062	693.9	0.9819	0.9661	0.3615	0.9117	0
4063	693.8	0.9819	0.9661	0.3615	0.9119	0
4064	693.7	0.9822	0.9663	0.3617	0.9118	0
4065	693.6	0.9823	0.9666	0.3616	0.9119	0
4066	693.5	0.9823	0.9667	0.3618	0.9119	0
4067	693.4	0.9826	0.9668	0.3617	0.9122	0
4068	693.3	0.9827	0.967	0.3618	0.912	0
4069	693.2	0.9828	0.9675	0.3619	0.9122	0
4070	693.1	0.983	0.9672	0.3618	0.9122	0
4071	693	0.9831	0.9671	0.3619	0.9124	0

4072	692.9	0.9832	0.9675	0.3619	0.9125	0
4073	692.8	0.9832	0.9676	0.3619	0.9125	0
4074	692.7	0.9835	0.9678	0.362	0.9124	0
4075	692.6	0.9836	0.9677	0.3619	0.9123	0
4076	692.5	0.9837	0.968	0.3619	0.9125	0
4077	692.4	0.9838	0.9681	0.362	0.9128	0
4078	692.3	0.9835	0.9683	0.362	0.9126	0
4079	692.2	0.9842	0.9684	0.3622	0.9127	0
4080	692.1	0.9843	0.9686	0.3622	0.913	0
4081	692	0.9845	0.9689	0.3622	0.913	0
4082	691.9	0.9847	0.9689	0.3621	0.9128	0
4083	691.8	0.9845	0.969	0.3622	0.9128	0
4084	691.7	0.9848	0.9693	0.3623	0.9128	0
4085	691.6	0.985	0.9692	0.3621	0.9126	0
4086	691.5	0.9848	0.969	0.3621	0.9129	0
4087	691.4	0.985	0.9694	0.362	0.9128	0
4088	691.3	0.985	0.9696	0.3621	0.9129	0
4089	691.2	0.9852	0.9696	0.362	0.9129	0
4090	691.1	0.9855	0.9697	0.3621	0.9127	0
4091	691	0.9855	0.9698	0.3621	0.9129	0
4092	690.9	0.9857	0.9703	0.3621	0.9129	0
4093	690.8	0.9859	0.9705	0.3624	0.9129	0
4094	690.7	0.9859	0.9706	0.3622	0.913	0
4095	690.6	0.9861	0.9704	0.3622	0.9127	0
4096	690.5	0.9863	0.9708	0.3622	0.9128	0
4097	690.4	0.9863	0.9708	0.3622	0.9127	0
4098	690.3	0.9867	0.971	0.3623	0.9129	0
4099	690.2	0.9866	0.971	0.3622	0.9132	0
4100	690.1	0.9868	0.9712	0.3622	0.9131	0
4101	690	0.9871	0.9713	0.3623	0.913	0
4102	689.9	0.9869	0.9716	0.3623	0.913	0
4103	689.8	0.9873	0.9717	0.3624	0.913	0
4104	689.7	0.9873	0.9719	0.3623	0.9129	0
4105	689.6	0.9873	0.972	0.3622	0.913	0
4106	689.5	0.9875	0.9723	0.3622	0.9129	0
4107	689.4	0.9879	0.9723	0.3623	0.9131	0
4108	689.3	0.9878	0.9724	0.3621	0.9133	0
4109	689.2	0.988	0.9724	0.3621	0.9129	0
4110	689.1	0.9881	0.9726	0.3621	0.9132	0
4111	689	0.9881	0.9727	0.3623	0.9132	0
4112	688.9	0.9885	0.9726	0.3622	0.913	0
4113	688.8	0.9885	0.9727	0.3623	0.9129	0
4114	688.7	0.9887	0.9731	0.3623	0.9132	0

4115	688.6	0.9886	0.973	0.3621	0.913	0
4116	688.5	0.989	0.9733	0.3624	0.9132	0
4117	688.4	0.989	0.9734	0.3623	0.9133	0
4118	688.3	0.9892	0.9736	0.3623	0.9131	0
4119	688.2	0.9891	0.9735	0.3622	0.9131	0
4120	688.1	0.9893	0.9737	0.3621	0.913	0
4121	688	0.9892	0.9738	0.3622	0.9134	0
4122	687.9	0.9896	0.974	0.3623	0.9134	0
4123	687.8	0.9898	0.9743	0.3623	0.9134	0
4124	687.7	0.9898	0.9744	0.3623	0.9132	0
4125	687.6	0.9899	0.9746	0.3625	0.9135	0
4126	687.5	0.99	0.9746	0.3624	0.9133	0
4127	687.4	0.9903	0.9748	0.3625	0.9135	0
4128	687.3	0.9905	0.9753	0.3627	0.9137	0
4129	687.2	0.9906	0.9753	0.3626	0.9136	0
4130	687.1	0.9905	0.9753	0.3625	0.9137	0
4131	687	0.991	0.9756	0.3626	0.9138	0
4132	686.9	0.9909	0.976	0.3626	0.9139	0
4133	686.8	0.9912	0.9759	0.3628	0.9138	0
4134	686.7	0.9913	0.9762	0.3629	0.9139	0
4135	686.6	0.9913	0.9764	0.3627	0.9141	0
4136	686.5	0.9916	0.9764	0.3628	0.9139	0
4137	686.4	0.9918	0.9766	0.3628	0.9142	0
4138	686.3	0.9918	0.9769	0.363	0.9143	0
4139	686.2	0.992	0.977	0.3629	0.9141	0
4140	686.1	0.9922	0.9771	0.3628	0.9139	0
4141	686	0.9921	0.9772	0.363	0.9143	0
4142	685.9	0.9925	0.9772	0.363	0.9146	0
4143	685.8	0.9924	0.9777	0.3629	0.9147	0
4144	685.7	0.9925	0.9778	0.363	0.9145	0
4145	685.6	0.9928	0.9779	0.3629	0.9147	0
4146	685.5	0.9929	0.9781	0.3631	0.9147	0
4147	685.4	0.993	0.9782	0.3632	0.9147	0
4148	685.3	0.9931	0.9783	0.3632	0.915	0
4149	685.2	0.9932	0.9786	0.3635	0.9153	0
4150	685.1	0.9934	0.9789	0.3635	0.9153	0
4151	685	0.9936	0.9791	0.3635	0.9153	0
4152	684.9	0.9936	0.9791	0.3634	0.9156	0
4153	684.8	0.9935	0.9792	0.3633	0.9152	0
4154	684.7	0.9941	0.9796	0.3635	0.9155	0
4155	684.6	0.9943	0.9798	0.3636	0.9156	0
4156	684.5	0.9941	0.9796	0.3635	0.9157	0
4157	684.4	0.9942	0.98	0.3636	0.9158	0

4158	684.3	0.9945	0.98	0.3636	0.9159	0
4159	684.2	0.9949	0.9804	0.3638	0.9158	0
4160	684.1	0.9947	0.9807	0.3637	0.9159	0
4161	684	0.9948	0.9807	0.3638	0.9161	0
4162	683.9	0.9949	0.9809	0.3637	0.916	0
4163	683.8	0.9949	0.981	0.3638	0.9163	0
4164	683.7	0.9953	0.9812	0.3638	0.9163	0
4165	683.6	0.9954	0.9813	0.3639	0.9164	0
4166	683.5	0.9953	0.9817	0.3638	0.9164	0
4167	683.4	0.9954	0.9818	0.3639	0.9163	0
4168	683.3	0.9955	0.9819	0.3641	0.9166	0
4169	683.2	0.9959	0.9822	0.364	0.9167	0
4170	683.1	0.9961	0.9823	0.3641	0.9167	0
4171	683	0.9962	0.9825	0.3643	0.9168	0
4172	682.9	0.9964	0.9827	0.3642	0.9169	0
4173	682.8	0.9962	0.9828	0.3641	0.9167	0
4174	682.7	0.9962	0.9828	0.364	0.9167	0
4175	682.6	0.9964	0.983	0.3642	0.9166	0
4176	682.5	0.9966	0.9832	0.3641	0.9168	0
4177	682.4	0.997	0.9834	0.3642	0.917	0
4178	682.3	0.9973	0.9836	0.3642	0.9167	0
4179	682.2	0.9971	0.9837	0.3643	0.917	0
4180	682.1	0.9973	0.9838	0.3642	0.9168	0
4181	682	0.9974	0.9839	0.3641	0.9169	0
4182	681.9	0.9973	0.984	0.3641	0.9167	0
4183	681.8	0.9975	0.9844	0.3642	0.9169	0
4184	681.7	0.9977	0.9844	0.3642	0.9169	0
4185	681.6	0.9977	0.9846	0.3643	0.9168	0
4186	681.5	0.998	0.9846	0.3642	0.9168	0
4187	681.4	0.9976	0.9846	0.364	0.9168	0
4188	681.3	0.998	0.9851	0.3641	0.9169	0
4189	681.2	0.9984	0.9852	0.3641	0.9168	0
4190	681.1	0.9983	0.9852	0.3641	0.9168	0
4191	681	0.9983	0.9853	0.3641	0.9171	0
4192	680.9	0.9984	0.9855	0.364	0.9165	0
4193	680.8	0.9987	0.9854	0.3641	0.9169	0
4194	680.7	0.9988	0.9859	0.3642	0.9166	0
4195	680.6	0.999	0.9859	0.3641	0.9166	0
4196	680.5	0.9991	0.986	0.364	0.9166	0
4197	680.4	0.9993	0.9863	0.3641	0.9168	0
4198	680.3	0.9995	0.9866	0.364	0.9168	0
4199	680.2	0.9996	0.9865	0.3639	0.9167	0
4200	680.1	0.9997	0.9869	0.3641	0.9168	0

4201	680	0.9998	0.9867	0.3641	0.9166	0
4202	679.9	0.9998	0.9871	0.3641	0.9166	0
4203	679.8	1.0001	0.9873	0.364	0.9165	0
4204	679.7	1.0001	0.9873	0.3641	0.9167	0
4205	679.6	1.0003	0.9876	0.364	0.9165	0
4206	679.5	1.0006	0.9876	0.3639	0.9163	0
4207	679.4	1.0006	0.9878	0.3639	0.9165	0
4208	679.3	1.0006	0.9882	0.364	0.9165	0
4209	679.2	1.0009	0.988	0.3638	0.9161	0
4210	679.1	1.0008	0.9884	0.3639	0.9162	0
4211	679	1.0009	0.9885	0.3639	0.9163	0
4212	678.9	1.001	0.9886	0.3639	0.9162	0
4213	678.8	1.0013	0.9885	0.3638	0.9163	0
4214	678.7	1.0013	0.9885	0.3637	0.916	0
4215	678.6	1.0015	0.989	0.3637	0.916	0
4216	678.5	1.0015	0.9892	0.3638	0.9161	0
4217	678.4	1.0015	0.9895	0.3638	0.916	0
4218	678.3	1.0017	0.9895	0.3637	0.916	0
4219	678.2	1.0021	0.9899	0.3641	0.9164	0
4220	678.1	1.002	0.9899	0.3639	0.916	0
4221	678	1.0022	0.9901	0.3638	0.9162	0
4222	677.9	1.0023	0.9901	0.3637	0.9157	0
4223	677.8	1.0025	0.9903	0.3637	0.9161	0
4224	677.7	1.0028	0.9907	0.3638	0.9163	0
4225	677.6	1.0029	0.9904	0.3638	0.916	0
4226	677.5	1.0025	0.9904	0.3637	0.9161	0
4227	677.4	1.003	0.991	0.3639	0.9162	0
4228	677.3	1.0028	0.991	0.3638	0.9161	0
4229	677.2	1.0031	0.9912	0.3638	0.9161	0
4230	677.1	1.0033	0.9916	0.3639	0.9164	0
4231	677	1.0034	0.9918	0.3639	0.9164	0
4232	676.9	1.0035	0.9919	0.3638	0.9163	0
4233	676.8	1.0036	0.9923	0.3638	0.9162	0
4234	676.7	1.0036	0.9924	0.3639	0.9163	0
4235	676.6	1.0038	0.9928	0.3639	0.9162	0
4236	676.5	1.0041	0.9927	0.3642	0.9166	0
4237	676.4	1.0042	0.9929	0.364	0.9167	0
4238	676.3	1.0044	0.993	0.3642	0.917	0
4239	676.2	1.0045	0.9934	0.3644	0.917	0
4240	676.1	1.0047	0.9936	0.3643	0.9171	0
4241	676	1.0046	0.9935	0.3643	0.917	0
4242	675.9	1.0048	0.9943	0.3646	0.9173	0
4243	675.8	1.005	0.9943	0.3645	0.9175	0

4244	675.7	1.0052	0.9946	0.3646	0.9175	0
4245	675.6	1.0054	0.9948	0.3647	0.9176	0
4246	675.5	1.0054	0.995	0.3648	0.9177	0
4247	675.4	1.0056	0.9952	0.3647	0.9177	0
4248	675.3	1.005	0.9953	0.3648	0.918	0
4249	675.2	1.006	0.9957	0.365	0.918	0
4250	675.1	1.0057	0.9957	0.365	0.9183	0
4251	675	1.0059	0.9962	0.3652	0.9183	0
4252	674.9	1.0065	0.9967	0.3651	0.9185	0
4253	674.8	1.0065	0.9968	0.3654	0.9189	0
4254	674.7	1.0066	0.997	0.3653	0.9187	0
4255	674.6	1.0067	0.9971	0.3654	0.9188	0
4256	674.5	1.0069	0.9977	0.3656	0.919	0
4257	674.4	1.0071	0.9976	0.3656	0.919	0
4258	674.3	1.0072	0.998	0.3657	0.9192	0
4259	674.2	1.007	0.9982	0.3657	0.9197	0
4260	674.1	1.0074	0.9983	0.3659	0.9197	0
4261	674	1.0076	0.9985	0.3657	0.9199	0
4262	673.9	1.0077	0.9988	0.3659	0.9195	0
4263	673.8	1.0079	0.999	0.3659	0.9195	0
4264	673.7	1.008	0.9991	0.366	0.9199	0
4265	673.6	1.008	0.9995	0.366	0.9198	0
4266	673.5	1.0084	0.9998	0.3661	0.9203	0
4267	673.4	1.0082	0.9999	0.3661	0.92	0
4268	673.3	1.0086	1.0003	0.3663	0.9203	0
4269	673.2	1.0086	1.0004	0.3662	0.9202	0
4270	673.1	1.0085	1.0006	0.3663	0.9202	0
4271	673	1.0089	1.0009	0.3664	0.9202	0
4272	672.9	1.009	1.001	0.3664	0.9202	0
4273	672.8	1.0092	1.0011	0.3663	0.9205	0
4274	672.7	1.0093	1.0012	0.3662	0.9204	0
4275	672.6	1.0095	1.0016	0.3664	0.9204	0
4276	672.5	1.0096	1.0018	0.3662	0.9204	0
4277	672.4	1.0098	1.0019	0.3665	0.9204	0
4278	672.3	1.0098	1.002	0.3663	0.9204	0
4279	672.2	1.01	1.0025	0.3666	0.9204	0
4280	672.1	1.0102	1.0028	0.3665	0.9208	0
4281	672	1.0102	1.003	0.3665	0.9208	0
4282	671.9	1.01	1.0028	0.3665	0.9209	0
4283	671.8	1.0104	1.0032	0.3664	0.9208	0
4284	671.7	1.0105	1.0033	0.3666	0.9207	0
4285	671.6	1.0108	1.0035	0.3665	0.9206	0
4286	671.5	1.0108	1.0037	0.3665	0.9206	0

4287	671.4	1.0108	1.0041	0.3665	0.9207	0
4288	671.3	1.0109	1.0043	0.3666	0.9207	0
4289	671.2	1.0111	1.0045	0.3665	0.9208	0
4290	671.1	1.0112	1.0045	0.3665	0.9211	0
4291	671	1.0112	1.0047	0.3666	0.9209	0
4292	670.9	1.0116	1.0048	0.3665	0.9206	0
4293	670.8	1.0116	1.0051	0.3664	0.9205	0
4294	670.7	1.0119	1.0051	0.3664	0.9204	0
4295	670.6	1.0118	1.0054	0.3663	0.9206	0
4296	670.5	1.0118	1.0053	0.3662	0.9202	0
4297	670.4	1.0118	1.0057	0.3661	0.9204	0
4298	670.3	1.0121	1.006	0.3663	0.9204	0
4299	670.2	1.0124	1.0061	0.3664	0.9206	0
4300	670.1	1.0125	1.0059	0.3662	0.9202	0
4301	670	1.0127	1.0063	0.3662	0.9201	0
4302	669.9	1.013	1.0066	0.3662	0.9201	0
4303	669.8	1.0127	1.0066	0.3661	0.92	0
4304	669.7	1.0128	1.0069	0.3662	0.9198	0
4305	669.6	1.0132	1.0072	0.366	0.9199	0
4306	669.5	1.0131	1.0073	0.366	0.92	0
4307	669.4	1.0133	1.0071	0.3659	0.9197	0
4308	669.3	1.0133	1.0075	0.3659	0.9196	0
4309	669.2	1.0136	1.0077	0.3658	0.9193	0
4310	669.1	1.0137	1.0078	0.3657	0.9194	0
4311	669	1.0137	1.008	0.3657	0.9193	0
4312	668.9	1.0138	1.0083	0.3656	0.9195	0
4313	668.8	1.0139	1.0082	0.3657	0.9193	0
4314	668.7	1.0139	1.0085	0.3656	0.9193	0
4315	668.6	1.0142	1.0087	0.3656	0.9194	0
4316	668.5	1.0144	1.0088	0.3656	0.9192	0
4317	668.4	1.0145	1.0091	0.3657	0.9194	0
4318	668.3	1.0146	1.0095	0.3655	0.9191	0
4319	668.2	1.0149	1.0095	0.3654	0.9189	0
4320	668.1	1.0148	1.0096	0.3655	0.9189	0
4321	668	1.0148	1.0099	0.3652	0.9188	0
4322	667.9	1.015	1.0103	0.3655	0.919	0
4323	667.8	1.0154	1.0103	0.3655	0.9189	0
4324	667.7	1.0155	1.0105	0.3655	0.9189	0
4325	667.6	1.0156	1.0106	0.3655	0.9189	0
4326	667.5	1.0157	1.0108	0.3653	0.9187	0
4327	667.4	1.0156	1.011	0.3652	0.9187	0
4328	667.3	1.0158	1.0113	0.3652	0.9188	0
4329	667.2	1.0158	1.0115	0.3653	0.9188	0

4330	667.1	1.016	1.0116	0.3652	0.9186	0
4331	667	1.0161	1.0117	0.3653	0.9187	0
4332	666.9	1.0163	1.0121	0.3654	0.919	0
4333	666.8	1.0166	1.0124	0.3654	0.9187	0
4334	666.7	1.0167	1.0126	0.3654	0.9187	0
4335	666.6	1.0168	1.0126	0.3653	0.9187	0
4336	666.5	1.0171	1.013	0.3654	0.9191	0
4337	666.4	1.0172	1.0132	0.3654	0.9189	0
4338	666.3	1.0176	1.0138	0.3656	0.9192	0
4339	666.2	1.0178	1.0141	0.3655	0.9192	0
4340	666.1	1.0177	1.0143	0.3655	0.9192	0
4341	666	1.018	1.0145	0.3657	0.9191	0
4342	665.9	1.018	1.0147	0.3656	0.919	0
4343	665.8	1.0183	1.0149	0.3656	0.9193	0
4344	665.7	1.0183	1.0153	0.3658	0.9196	0
4345	665.6	1.0186	1.0155	0.366	0.9196	0
4346	665.5	1.0186	1.0157	0.3661	0.9192	0
4347	665.4	1.0189	1.0162	0.3661	0.9198	0
4348	665.3	1.0191	1.0164	0.3661	0.9201	0
4349	665.2	1.0194	1.0168	0.3663	0.9202	0
4350	665.1	1.0194	1.017	0.3662	0.9199	0
4351	665	1.0195	1.0173	0.3663	0.9204	0
4352	664.9	1.0199	1.0175	0.3664	0.9203	0
4353	664.8	1.0198	1.0179	0.3664	0.9203	0
4354	664.7	1.0201	1.0181	0.3664	0.9209	0
4355	664.6	1.0205	1.0186	0.3666	0.9208	0
4356	664.5	1.0205	1.0186	0.3665	0.9209	0
4357	664.4	1.0205	1.019	0.3665	0.9209	0
4358	664.3	1.0206	1.0193	0.3668	0.9214	0
4359	664.2	1.0211	1.0196	0.367	0.9215	0
4360	664.1	1.0211	1.02	0.3669	0.9216	0
4361	664	1.0214	1.0202	0.3671	0.9218	0
4362	663.9	1.0216	1.0206	0.3671	0.9221	0
4363	663.8	1.0219	1.021	0.3673	0.9223	0
4364	663.7	1.0221	1.0214	0.3675	0.9226	0
4365	663.6	1.0222	1.0217	0.3675	0.9224	0
4366	663.5	1.0222	1.0219	0.3676	0.9223	0
4367	663.4	1.0223	1.0221	0.3677	0.9225	0
4368	663.3	1.0225	1.0225	0.3677	0.9226	0
4369	663.2	1.0227	1.023	0.3678	0.9232	0
4370	663.1	1.023	1.023	0.3679	0.9231	0
4371	663	1.0234	1.0235	0.368	0.9233	0
4372	662.9	1.0232	1.0236	0.368	0.9232	0

4373	662.8	1.0234	1.0241	0.3679	0.9232	0
4374	662.7	1.0238	1.0246	0.3682	0.9235	0
4375	662.6	1.0239	1.0246	0.3683	0.9234	0
4376	662.5	1.0241	1.0251	0.3683	0.9238	0
4377	662.4	1.0242	1.0255	0.3683	0.9237	0
4378	662.3	1.0244	1.0254	0.3683	0.9236	0
4379	662.2	1.0245	1.0258	0.3682	0.9236	0
4380	662.1	1.0246	1.0257	0.3682	0.9242	0
4381	662	1.025	1.0263	0.3684	0.924	0
4382	661.9	1.0248	1.0265	0.3683	0.9239	0
4383	661.8	1.0252	1.0266	0.3684	0.9239	0
4384	661.7	1.0253	1.0271	0.3683	0.9238	0
4385	661.6	1.0256	1.0274	0.3686	0.9239	0
4386	661.5	1.0256	1.0277	0.3686	0.9238	0
4387	661.4	1.0259	1.0281	0.3686	0.9239	0
4388	661.3	1.0262	1.0283	0.3685	0.924	0
4389	661.2	1.0264	1.0287	0.3687	0.9242	0
4390	661.1	1.0265	1.0288	0.3686	0.9242	0
4391	661	1.0264	1.0291	0.3687	0.9246	0
4392	660.9	1.0268	1.0294	0.3687	0.9242	0
4393	660.8	1.027	1.0296	0.3687	0.9242	0
4394	660.7	1.027	1.0299	0.3687	0.924	0
4395	660.6	1.0273	1.0303	0.3689	0.924	0
4396	660.5	1.0275	1.0306	0.3688	0.9245	0
4397	660.4	1.0278	1.0309	0.3688	0.9242	0
4398	660.3	1.0279	1.0313	0.3687	0.9244	0
4399	660.2	1.0278	1.0314	0.3685	0.9244	0
4400	660.1	1.028	1.0316	0.3686	0.9243	0
4401	660	1.0282	1.0321	0.3688	0.9246	0
4402	659.9	1.0285	1.0323	0.3686	0.9242	0
4403	659.8	1.0287	1.0327	0.3686	0.9244	0
4404	659.7	1.0287	1.0327	0.3687	0.9242	0
4405	659.6	1.0289	1.0329	0.3685	0.9238	0
4406	659.5	1.0289	1.0333	0.3684	0.9242	0
4407	659.4	1.0292	1.0337	0.3686	0.9241	0
4408	659.3	1.0295	1.0339	0.3685	0.9239	0
4409	659.2	1.0295	1.034	0.3685	0.9238	0
4410	659.1	1.0299	1.0344	0.3686	0.9239	0
4411	659	1.0298	1.0345	0.3683	0.924	0
4412	658.9	1.03	1.0348	0.3683	0.9235	0
4413	658.8	1.0302	1.0353	0.3685	0.9241	0
4414	658.7	1.0302	1.0356	0.3682	0.9236	0
4415	658.6	1.0306	1.0358	0.3683	0.9236	0

4416	658.5	1.0306	1.0358	0.3682	0.9237	0
4417	658.4	1.0308	1.036	0.3681	0.9235	0
4418	658.3	1.031	1.0362	0.368	0.9236	0
4419	658.2	1.0313	1.0366	0.368	0.9233	0
4420	658.1	1.0314	1.0366	0.3678	0.9233	0
4421	658	1.0314	1.0369	0.3677	0.9231	0
4422	657.9	1.0315	1.0375	0.3679	0.923	0
4423	657.8	1.0317	1.0378	0.3678	0.9232	0
4424	657.7	1.0317	1.038	0.3678	0.9228	0
4425	657.6	1.032	1.0382	0.3678	0.923	0
4426	657.5	1.0319	1.0386	0.3676	0.9229	0
4427	657.4	1.0322	1.0388	0.3678	0.9228	0
4428	657.3	1.0326	1.0389	0.3677	0.9227	0
4429	657.2	1.033	1.0393	0.3677	0.9226	0
4430	657.1	1.0328	1.0397	0.3678	0.9229	0
4431	657	1.0332	1.04	0.3678	0.9232	0
4432	656.9	1.0332	1.0404	0.3678	0.9228	0
4433	656.8	1.0333	1.0407	0.3677	0.9228	0
4434	656.7	1.0336	1.041	0.3678	0.9228	0
4435	656.6	1.0337	1.041	0.3676	0.9228	0
4436	656.5	1.0339	1.0416	0.3676	0.9227	0
4437	656.4	1.0339	1.0417	0.3677	0.9228	0
4438	656.3	1.034	1.042	0.3676	0.9228	0
4439	656.2	1.0344	1.0424	0.3677	0.9228	0
4440	656.1	1.0348	1.0428	0.3679	0.9229	0
4441	656	1.0346	1.0434	0.3678	0.9228	0
4442	655.9	1.0349	1.0438	0.3679	0.923	0
4443	655.8	1.0351	1.0438	0.3678	0.9229	0
4444	655.7	1.0354	1.0442	0.3677	0.923	0
4445	655.6	1.0354	1.0444	0.3676	0.9232	0
4446	655.5	1.0356	1.0453	0.3677	0.9229	0
4447	655.4	1.0359	1.0455	0.3679	0.9234	0
4448	655.3	1.0361	1.0459	0.3679	0.9234	0
4449	655.2	1.0361	1.0463	0.3679	0.9232	0
4450	655.1	1.0368	1.0464	0.368	0.9234	0
4451	655	1.0364	1.0468	0.368	0.9234	0
4452	654.9	1.037	1.0473	0.368	0.9237	0
4453	654.8	1.0373	1.0479	0.3681	0.9239	0
4454	654.7	1.0374	1.0482	0.3681	0.9239	0
4455	654.6	1.0375	1.0487	0.3683	0.9243	0
4456	654.5	1.0379	1.0491	0.3684	0.9243	0
4457	654.4	1.038	1.0493	0.3682	0.9242	0
4458	654.3	1.038	1.0498	0.3683	0.924	0

4459	654.2	1.0384	1.0505	0.3684	0.9244	0
4460	654.1	1.0388	1.0511	0.3687	0.9247	0
4461	654	1.0388	1.0512	0.3687	0.9248	0
4462	653.9	1.0391	1.0519	0.3688	0.9249	0
4463	653.8	1.0391	1.0522	0.3689	0.9251	0
4464	653.7	1.0395	1.0528	0.369	0.9252	0
4465	653.6	1.0397	1.0531	0.369	0.9251	0
4466	653.5	1.0399	1.0536	0.3692	0.9252	0
4467	653.4	1.04	1.0538	0.3691	0.9255	0
4468	653.3	1.0404	1.0543	0.3693	0.9257	0
4469	653.2	1.0404	1.0549	0.3693	0.926	0
4470	653.1	1.0406	1.0552	0.3692	0.9259	0
4471	653	1.041	1.0557	0.3694	0.9262	0
4472	652.9	1.0411	1.0562	0.3694	0.9261	0
4473	652.8	1.0413	1.0564	0.3695	0.9263	0
4474	652.7	1.0417	1.057	0.3695	0.9265	0
4475	652.6	1.0418	1.0573	0.3695	0.9263	0
4476	652.5	1.0419	1.058	0.3695	0.9265	0
4477	652.4	1.0422	1.0584	0.3696	0.9266	0
4478	652.3	1.0425	1.0587	0.3696	0.9266	0
4479	652.2	1.0425	1.0591	0.3697	0.9269	0
4480	652.1	1.0426	1.0595	0.3698	0.9271	0
4481	652	1.0428	1.0602	0.3698	0.9272	0
4482	651.9	1.0431	1.0604	0.3698	0.9269	0
4483	651.8	1.0434	1.0607	0.3698	0.9271	0
4484	651.7	1.0437	1.0611	0.3698	0.9273	0
4485	651.6	1.0438	1.0617	0.3701	0.9273	0
4486	651.5	1.0441	1.0621	0.3702	0.9274	0
4487	651.4	1.0442	1.0627	0.3702	0.9275	0
4488	651.3	1.0443	1.0631	0.3702	0.9275	0
4489	651.2	1.0446	1.0635	0.3702	0.9275	0
4490	651.1	1.0448	1.064	0.3702	0.9278	0
4491	651	1.0452	1.0647	0.3705	0.9278	0
4492	650.9	1.0453	1.0648	0.3705	0.928	0
4493	650.8	1.0455	1.0651	0.3704	0.9281	0
4494	650.7	1.0456	1.0658	0.3705	0.9283	0
4495	650.6	1.046	1.0667	0.3706	0.9281	0
4496	650.5	1.0461	1.0668	0.3705	0.9281	0
4497	650.4	1.0465	1.0673	0.3705	0.9284	0
4498	650.3	1.0468	1.0678	0.3705	0.9282	0
4499	650.2	1.0467	1.0681	0.3705	0.9285	0
4500	650.1	1.047	1.0685	0.3706	0.9285	0
4501	650	1.0474	1.0688	0.3705	0.9282	0

4502	649.9	1.0473	1.0693	0.3704	0.9283	0
4503	649.8	1.0476	1.0699	0.3705	0.9282	0
4504	649.7	1.0478	1.0704	0.3705	0.9283	0
4505	649.6	1.0479	1.0706	0.3705	0.9282	0
4506	649.5	1.0482	1.0709	0.3705	0.9285	0
4507	649.4	1.0483	1.0716	0.3705	0.9283	0
4508	649.3	1.0484	1.072	0.3706	0.9282	0
4509	649.2	1.0487	1.0724	0.3707	0.9286	0
4510	649.1	1.0492	1.0728	0.3704	0.9282	0
4511	649	1.0494	1.0733	0.3705	0.9285	0
4512	648.9	1.0494	1.0739	0.3704	0.9284	0
4513	648.8	1.0498	1.0741	0.3704	0.9283	0
4514	648.7	1.0499	1.0747	0.3703	0.9284	0
4515	648.6	1.0501	1.075	0.3705	0.9284	0
4516	648.5	1.0503	1.0753	0.3702	0.9282	0
4517	648.4	1.0505	1.0758	0.3702	0.9283	0
4518	648.3	1.0504	1.0763	0.3703	0.9283	0
4519	648.2	1.051	1.0769	0.3703	0.9281	0
4520	648.1	1.0511	1.0771	0.3702	0.9281	0
4521	648	1.0512	1.0776	0.3702	0.9281	0
4522	647.9	1.0513	1.0781	0.3702	0.9283	0
4523	647.8	1.0515	1.0784	0.3701	0.9281	0
4524	647.7	1.0518	1.0788	0.3701	0.928	0
4525	647.6	1.0519	1.0792	0.3699	0.9278	0
4526	647.5	1.052	1.0795	0.3701	0.9277	0
4527	647.4	1.0523	1.0802	0.3702	0.928	0
4528	647.3	1.0527	1.0806	0.3701	0.9279	0
4529	647.2	1.0527	1.081	0.3703	0.9278	0
4530	647.1	1.053	1.0815	0.3699	0.9277	0
4531	647	1.0532	1.0821	0.37	0.928	0
4532	646.9	1.0534	1.0825	0.37	0.9276	0
4533	646.8	1.0534	1.0827	0.3699	0.9279	0
4534	646.7	1.0536	1.0832	0.3698	0.9279	0
4535	646.6	1.0539	1.0837	0.3698	0.9275	0
4536	646.5	1.0541	1.0842	0.3701	0.9277	0
4537	646.4	1.0542	1.0847	0.37	0.9278	0
4538	646.3	1.0542	1.0851	0.37	0.928	0
4539	646.2	1.0544	1.0856	0.3699	0.9278	0
4540	646.1	1.0547	1.0858	0.3699	0.9274	0
4541	646	1.0549	1.0864	0.37	0.9276	0
4542	645.9	1.0551	1.0868	0.3698	0.9276	0
4543	645.8	1.0556	1.0873	0.3699	0.9279	0
4544	645.7	1.0554	1.0879	0.37	0.9278	0

4545	645.6	1.0558	1.0883	0.3699	0.9276	0
4546	645.5	1.056	1.0891	0.3698	0.9279	0
4547	645.4	1.0564	1.0896	0.3699	0.9279	0
4548	645.3	1.0563	1.0896	0.3699	0.9279	0
4549	645.2	1.0566	1.0904	0.37	0.9282	0
4550	645.1	1.0571	1.0909	0.37	0.9281	0
4551	645	1.057	1.0912	0.37	0.928	0
4552	644.9	1.0573	1.092	0.37	0.928	0
4553	644.8	1.0574	1.0925	0.3701	0.9281	0
4554	644.7	1.0577	1.0928	0.3702	0.9282	0
4555	644.6	1.058	1.0936	0.3702	0.9285	0
4556	644.5	1.058	1.0942	0.3702	0.9286	0
4557	644.4	1.0586	1.0949	0.3702	0.9286	0
4558	644.3	1.0587	1.0954	0.3703	0.9288	0
4559	644.2	1.059	1.0959	0.3704	0.9289	0
4560	644.1	1.0591	1.0964	0.3704	0.9286	0
4561	644	1.0591	1.097	0.3704	0.9288	0
4562	643.9	1.0597	1.0976	0.3705	0.9291	0
4563	643.8	1.0599	1.0984	0.3706	0.9293	0
4564	643.7	1.0598	1.0986	0.3707	0.9293	0
4565	643.6	1.0601	1.0993	0.3708	0.9292	0
4566	643.5	1.0602	1.0997	0.3707	0.9292	0
4567	643.4	1.0608	1.1006	0.3707	0.9293	0
4568	643.3	1.0606	1.1011	0.3706	0.9293	0
4569	643.2	1.061	1.1017	0.3709	0.9295	0
4570	643.1	1.0612	1.1023	0.3708	0.9296	0
4571	643	1.0615	1.103	0.3709	0.9297	0
4572	642.9	1.0619	1.1038	0.371	0.9299	0
4573	642.8	1.0622	1.1042	0.371	0.93	0
4574	642.7	1.0623	1.1048	0.371	0.9301	0
4575	642.6	1.0623	1.1054	0.3711	0.9301	0
4576	642.5	1.0628	1.1061	0.3712	0.9303	0
4577	642.4	1.0628	1.1068	0.3711	0.9306	0
4578	642.3	1.0632	1.1074	0.3712	0.9305	0
4579	642.2	1.0635	1.1079	0.3712	0.9306	0
4580	642.1	1.0636	1.1084	0.3711	0.9304	0
4581	642	1.0638	1.1092	0.3712	0.9304	0
4582	641.9	1.0642	1.1099	0.3712	0.9304	0
4583	641.8	1.0642	1.1107	0.3712	0.9307	0
4584	641.7	1.0646	1.1114	0.3712	0.9307	0
4585	641.6	1.0649	1.1118	0.3713	0.9309	0
4586	641.5	1.065	1.1126	0.3715	0.9311	0
4587	641.4	1.0655	1.1132	0.3717	0.9312	0

4588	641.3	1.0655	1.1139	0.3716	0.9312	0
4589	641.2	1.066	1.1145	0.3716	0.9313	0
4590	641.1	1.066	1.1153	0.3715	0.9317	0
4591	641	1.0664	1.1158	0.3716	0.9312	0
4592	640.9	1.0667	1.1164	0.3716	0.9308	0
4593	640.8	1.067	1.1172	0.3717	0.9311	0
4594	640.7	1.0672	1.1178	0.3715	0.9314	0
4595	640.6	1.0674	1.1187	0.3716	0.9312	0
4596	640.5	1.0677	1.1191	0.3717	0.9311	0
4597	640.4	1.0679	1.1198	0.3718	0.9314	0
4598	640.3	1.0681	1.1208	0.3717	0.9313	0
4599	640.2	1.0685	1.1213	0.3718	0.9315	0
4600	640.1	1.0686	1.1219	0.3718	0.9315	0
4601	640	1.0689	1.1223	0.3717	0.9314	0
4602	639.9	1.0693	1.1234	0.3718	0.932	0
4603	639.8	1.0695	1.124	0.3719	0.9317	0
4604	639.7	1.0697	1.1249	0.372	0.932	0
4605	639.6	1.07	1.1252	0.3718	0.9321	0
4606	639.5	1.0704	1.1258	0.3718	0.9319	0
4607	639.4	1.0703	1.1266	0.3719	0.9319	0
4608	639.3	1.0707	1.1275	0.3721	0.9321	0
4609	639.2	1.0712	1.128	0.372	0.932	0
4610	639.1	1.0713	1.1289	0.3719	0.9322	0
4611	639	1.0717	1.1293	0.372	0.9321	0
4612	638.9	1.0719	1.13	0.372	0.9322	0
4613	638.8	1.0722	1.1308	0.3722	0.9321	0
4614	638.7	1.0722	1.1315	0.372	0.9322	0
4615	638.6	1.073	1.1323	0.3722	0.9322	0
4616	638.5	1.073	1.133	0.3721	0.9322	0
4617	638.4	1.0734	1.1336	0.3719	0.9323	0
4618	638.3	1.0736	1.1339	0.3718	0.9321	0
4619	638.2	1.0738	1.1348	0.3719	0.9323	0
4620	638.1	1.0737	1.1352	0.3718	0.9319	0
4621	638	1.0742	1.1361	0.372	0.9323	0
4622	637.9	1.0745	1.1367	0.3719	0.9319	0
4623	637.8	1.0748	1.1376	0.3719	0.932	0
4624	637.7	1.0752	1.1381	0.372	0.932	0
4625	637.6	1.0755	1.1391	0.3719	0.9323	0
4626	637.5	1.0755	1.1395	0.3718	0.9321	0
4627	637.4	1.0758	1.1402	0.3718	0.932	0
4628	637.3	1.0761	1.1408	0.3717	0.9319	0
4629	637.2	1.0764	1.1416	0.3719	0.9319	0
4630	637.1	1.0767	1.1424	0.3719	0.9322	0

4631	637	1.0768	1.143	0.3718	0.9322	0
4632	636.9	1.0771	1.1434	0.3718	0.9319	0
4633	636.8	1.0774	1.1443	0.3718	0.932	0
4634	636.7	1.0776	1.1448	0.3718	0.932	0
4635	636.6	1.078	1.1458	0.3717	0.9321	0
4636	636.5	1.0782	1.1463	0.3717	0.932	0
4637	636.4	1.0784	1.147	0.3718	0.9322	0
4638	636.3	1.0785	1.1476	0.3717	0.932	0
4639	636.2	1.0793	1.1484	0.3718	0.9324	0
4640	636.1	1.0795	1.1489	0.3718	0.9325	0
4641	636	1.0797	1.1494	0.3715	0.9324	0
4642	635.9	1.0799	1.1499	0.3717	0.9321	0
4643	635.8	1.0804	1.151	0.3717	0.9323	0
4644	635.7	1.0807	1.1513	0.3718	0.9322	0
4645	635.6	1.081	1.1523	0.3717	0.9323	0
4646	635.5	1.0808	1.1526	0.3718	0.9324	0
4647	635.4	1.0815	1.1534	0.3719	0.9326	0
4648	635.3	1.0817	1.1539	0.3719	0.9325	0
4649	635.2	1.082	1.1547	0.372	0.9327	0
4650	635.1	1.0822	1.1553	0.3719	0.9324	0
4651	635	1.0827	1.1559	0.3719	0.9327	0
4652	634.9	1.0828	1.1566	0.3721	0.9327	0
4653	634.8	1.0831	1.1572	0.3719	0.9329	0
4654	634.7	1.0836	1.1579	0.372	0.9328	0
4655	634.6	1.0837	1.1586	0.372	0.9327	0
4656	634.5	1.0841	1.1593	0.3721	0.9332	0
4657	634.4	1.0843	1.1601	0.3721	0.9333	0
4658	634.3	1.0846	1.1605	0.3721	0.933	0
4659	634.2	1.0849	1.1611	0.3721	0.9333	0
4660	634.1	1.0852	1.1617	0.3723	0.9333	0
4661	634	1.0855	1.1624	0.3721	0.9333	0
4662	633.9	1.0858	1.1627	0.3724	0.9336	0
4663	633.8	1.0861	1.1636	0.3725	0.9336	0
4664	633.7	1.0866	1.1643	0.3724	0.9337	0
4665	633.6	1.0869	1.165	0.3725	0.9338	0
4666	633.5	1.0871	1.1659	0.3725	0.9337	0
4667	633.4	1.0873	1.166	0.3725	0.9339	0
4668	633.3	1.0877	1.1668	0.3726	0.9338	0
4669	633.2	1.0881	1.1676	0.3727	0.9343	0
4670	633.1	1.0882	1.1682	0.3725	0.9342	0
4671	633	1.0886	1.1689	0.3726	0.9341	0
4672	632.9	1.0891	1.1693	0.3727	0.9341	0
4673	632.8	1.0892	1.1701	0.3728	0.9342	0

4674	632.7	1.0894	1.1707	0.3728	0.9344	0
4675	632.6	1.09	1.1714	0.3728	0.9344	0
4676	632.5	1.0902	1.1723	0.3728	0.9345	0
4677	632.4	1.0906	1.1725	0.3728	0.9346	0
4678	632.3	1.0908	1.1734	0.3727	0.9345	0
4679	632.2	1.0913	1.1741	0.3727	0.9347	0
4680	632.1	1.0916	1.1744	0.3728	0.9345	0
4681	632	1.0918	1.1753	0.3728	0.9344	0
4682	631.9	1.0923	1.176	0.3728	0.9345	0
4683	631.8	1.0924	1.1768	0.3729	0.9349	0
4684	631.7	1.0927	1.1773	0.3728	0.9348	0
4685	631.6	1.0934	1.1779	0.3728	0.9347	0
4686	631.5	1.0934	1.1783	0.3728	0.9344	0
4687	631.4	1.0938	1.1791	0.3727	0.9347	0
4688	631.3	1.0943	1.1797	0.3729	0.935	0
4689	631.2	1.0945	1.1804	0.3729	0.935	0
4690	631.1	1.0946	1.1809	0.3728	0.9346	0
4691	631	1.0953	1.1817	0.3728	0.9345	0
4692	630.9	1.0955	1.1823	0.3728	0.9347	0
4693	630.8	1.096	1.183	0.3728	0.9349	0
4694	630.7	1.0962	1.1836	0.3728	0.9351	0
4695	630.6	1.0963	1.1843	0.3728	0.9346	0
4696	630.5	1.0968	1.185	0.3727	0.9348	0
4697	630.4	1.0972	1.1859	0.3728	0.9348	0
4698	630.3	1.0975	1.1863	0.3728	0.9348	0
4699	630.2	1.0979	1.1872	0.3729	0.9348	0
4700	630.1	1.0984	1.1878	0.3727	0.9347	0
4701	630	1.0986	1.1884	0.3727	0.9345	0
4702	629.9	1.0988	1.1889	0.3726	0.9347	0
4703	629.8	1.0993	1.1899	0.3728	0.9347	0
4704	629.7	1.0999	1.1907	0.3729	0.9347	0
4705	629.6	1.1002	1.1915	0.3728	0.9349	0
4706	629.5	1.1006	1.1922	0.3728	0.9351	0
4707	629.4	1.1008	1.1928	0.3728	0.9351	0
4708	629.3	1.1013	1.1934	0.373	0.9345	0
4709	629.2	1.1014	1.1941	0.3726	0.9348	0
4710	629.1	1.1021	1.1949	0.3729	0.935	0
4711	629	1.1022	1.1956	0.3727	0.9351	0
4712	628.9	1.1027	1.1962	0.3728	0.9351	0
4713	628.8	1.103	1.1971	0.3729	0.935	0
4714	628.7	1.1036	1.1974	0.3728	0.9352	0
4715	628.6	1.104	1.1981	0.3726	0.9349	0
4716	628.5	1.1042	1.1992	0.3727	0.9352	0

4717	628.4	1.1045	1.1997	0.3726	0.9351	0
4718	628.3	1.1047	1.2005	0.3726	0.9349	0
4719	628.2	1.1053	1.2013	0.3729	0.9351	0
4720	628.1	1.1056	1.2018	0.3728	0.9353	0
4721	628	1.106	1.2024	0.3726	0.9348	0
4722	627.9	1.1062	1.2034	0.3728	0.9352	0
4723	627.8	1.1068	1.204	0.3728	0.9353	0
4724	627.7	1.1072	1.2049	0.3728	0.9353	0
4725	627.6	1.1075	1.2055	0.3729	0.9357	0
4726	627.5	1.1081	1.2063	0.3728	0.9354	0
4727	627.4	1.1085	1.2069	0.3729	0.9354	0
4728	627.3	1.1089	1.208	0.3729	0.9356	0
4729	627.2	1.1091	1.2083	0.3729	0.9358	0
4730	627.1	1.1096	1.2095	0.3729	0.9354	0
4731	627	1.11	1.21	0.3728	0.9356	0
4732	626.9	1.1101	1.2109	0.3727	0.9356	0
4733	626.8	1.1106	1.2115	0.3729	0.9356	0
4734	626.7	1.1111	1.2122	0.373	0.9356	0
4735	626.6	1.1113	1.2127	0.373	0.9357	0
4736	626.5	1.1116	1.2134	0.3728	0.9355	0
4737	626.4	1.112	1.214	0.3728	0.9357	0
4738	626.3	1.1123	1.215	0.3729	0.9359	0
4739	626.2	1.1126	1.2158	0.3729	0.9361	0
4740	626.1	1.113	1.2163	0.3729	0.9363	0
4741	626	1.1137	1.2171	0.3727	0.936	0
4742	625.9	1.1138	1.2179	0.3729	0.9361	0
4743	625.8	1.1146	1.2185	0.3728	0.9362	0
4744	625.7	1.1147	1.2195	0.373	0.9363	0
4745	625.6	1.1151	1.2203	0.3729	0.9362	0
4746	625.5	1.1157	1.2209	0.373	0.9364	0
4747	625.4	1.1159	1.2215	0.3729	0.9366	0
4748	625.3	1.1163	1.2225	0.373	0.9367	0
4749	625.2	1.1169	1.2232	0.3732	0.9367	0
4750	625.1	1.1173	1.2238	0.3733	0.9372	0
4751	625	1.1176	1.225	0.3733	0.9368	0
4752	624.9	1.118	1.2254	0.3733	0.937	0
4753	624.8	1.1186	1.2263	0.3733	0.9372	0
4754	624.7	1.1192	1.2271	0.3735	0.9374	0
4755	624.6	1.1197	1.2278	0.3735	0.9374	0
4756	624.5	1.12	1.2286	0.3736	0.9375	0
4757	624.4	1.1201	1.2291	0.3736	0.9372	0
4758	624.3	1.1206	1.23	0.3735	0.9373	0
4759	624.2	1.1214	1.2309	0.3737	0.9375	0

4760	624.1	1.1215	1.2319	0.3738	0.9375	0
4761	624	1.122	1.2325	0.3737	0.9377	0
4762	623.9	1.1224	1.2332	0.3738	0.9379	0
4763	623.8	1.1229	1.2337	0.3737	0.9377	0
4764	623.7	1.1233	1.2349	0.3738	0.9379	0
4765	623.6	1.1239	1.2356	0.3739	0.9381	0
4766	623.5	1.1242	1.2362	0.3738	0.9378	0
4767	623.4	1.1243	1.2371	0.3738	0.9382	0
4768	623.3	1.1249	1.2377	0.3738	0.938	0
4769	623.2	1.1252	1.2384	0.3736	0.9379	0
4770	623.1	1.1257	1.2391	0.3739	0.938	0
4771	623	1.1262	1.2401	0.3738	0.9382	0
4772	622.9	1.1265	1.2407	0.3737	0.9381	0
4773	622.8	1.1271	1.2417	0.3739	0.9383	0
4774	622.7	1.1271	1.2423	0.3738	0.9388	0
4775	622.6	1.1277	1.2429	0.3738	0.9385	0
4776	622.5	1.1281	1.2439	0.3739	0.9387	0
4777	622.4	1.1289	1.2447	0.3739	0.9386	0
4778	622.3	1.1295	1.2453	0.3739	0.9388	0
4779	622.2	1.1294	1.246	0.3738	0.9385	0
4780	622.1	1.1298	1.2465	0.3736	0.9384	0
4781	622	1.1303	1.2474	0.3739	0.9389	0
4782	621.9	1.1304	1.2481	0.3739	0.9385	0
4783	621.8	1.131	1.2489	0.3739	0.9384	0
4784	621.7	1.1316	1.2497	0.3736	0.9386	0
4785	621.6	1.1321	1.2506	0.3737	0.9387	0
4786	621.5	1.1322	1.2512	0.3736	0.9386	0
4787	621.4	1.1332	1.2521	0.374	0.9385	0
4788	621.3	1.1333	1.2526	0.3737	0.9386	0
4789	621.2	1.1335	1.2531	0.3736	0.9383	0
4790	621.1	1.1342	1.2542	0.3737	0.9383	0
4791	621	1.1346	1.2551	0.3736	0.9383	0
4792	620.9	1.1347	1.2557	0.3736	0.9386	0
4793	620.8	1.1355	1.2565	0.3735	0.9385	0
4794	620.7	1.136	1.2575	0.3737	0.9384	0
4795	620.6	1.1361	1.2578	0.3735	0.9384	0
4796	620.5	1.1366	1.2588	0.3734	0.9379	0
4797	620.4	1.1373	1.2596	0.3732	0.9382	0
4798	620.3	1.1379	1.2606	0.3734	0.9382	0
4799	620.2	1.1383	1.2613	0.3735	0.9381	0
4800	620.1	1.1386	1.2621	0.3734	0.9381	0
4801	620	1.1395	1.2629	0.3733	0.9379	0
4802	619.9	1.1398	1.2637	0.3733	0.9382	0

4803	619.8	1.1402	1.2648	0.3733	0.9385	0
4804	619.7	1.1406	1.2655	0.3732	0.9386	0
4805	619.6	1.141	1.2661	0.3732	0.938	0
4806	619.5	1.1415	1.2669	0.3732	0.9379	0
4807	619.4	1.1421	1.268	0.3732	0.9382	0
4808	619.3	1.1425	1.2689	0.373	0.9381	0
4809	619.2	1.1429	1.2693	0.3731	0.9378	0
4810	619.1	1.1434	1.2706	0.373	0.938	0
4811	619	1.1439	1.2715	0.373	0.9378	0
4812	618.9	1.1446	1.2721	0.3728	0.9379	0
4813	618.8	1.1449	1.2729	0.3728	0.938	0
4814	618.7	1.1456	1.2741	0.3727	0.9379	0
4815	618.6	1.1459	1.2748	0.3726	0.9378	0
4816	618.5	1.1462	1.2755	0.3726	0.9379	0
4817	618.4	1.1469	1.2766	0.3728	0.9382	0
4818	618.3	1.1473	1.2773	0.3726	0.9378	0
4819	618.2	1.1482	1.2784	0.3725	0.9377	0
4820	618.1	1.1488	1.2792	0.3726	0.9378	0
4821	618	1.149	1.28	0.3724	0.9378	0
4822	617.9	1.1497	1.2808	0.3724	0.938	0
4823	617.8	1.1501	1.2817	0.3722	0.9378	0
4824	617.7	1.1507	1.2829	0.3725	0.9377	0
4825	617.6	1.1511	1.2837	0.3724	0.9376	0
4826	617.5	1.1516	1.2848	0.3724	0.9375	0
4827	617.4	1.1521	1.2852	0.3723	0.9375	0
4828	617.3	1.1528	1.2861	0.3723	0.9374	0
4829	617.2	1.1535	1.2871	0.3722	0.9375	0
4830	617.1	1.1536	1.2878	0.372	0.9373	0
4831	617	1.1542	1.2886	0.3722	0.9378	0
4832	616.9	1.1549	1.2897	0.3722	0.9373	0
4833	616.8	1.1555	1.2907	0.3723	0.9376	0
4834	616.7	1.156	1.2916	0.3724	0.9377	0
4835	616.6	1.1567	1.2922	0.3725	0.9376	0
4836	616.5	1.1573	1.2928	0.3724	0.9376	0
4837	616.4	1.1575	1.294	0.3723	0.938	0
4838	616.3	1.1582	1.2947	0.3724	0.9377	0
4839	616.2	1.1586	1.2954	0.3725	0.9378	0
4840	616.1	1.1592	1.2963	0.3723	0.9381	0
4841	616	1.1595	1.2971	0.3723	0.938	0
4842	615.9	1.1604	1.2978	0.3723	0.9378	0
4843	615.8	1.1611	1.2987	0.3725	0.9383	0
4844	615.7	1.1617	1.2996	0.3726	0.9383	0
4845	615.6	1.162	1.3005	0.3725	0.9382	0

4846	615.5	1.1624	1.3011	0.3724	0.9385	0
4847	615.4	1.163	1.302	0.3726	0.9385	0
4848	615.3	1.1638	1.3026	0.3727	0.9387	0
4849	615.2	1.1644	1.3044	0.3728	0.9389	0
4850	615.1	1.1651	1.3044	0.3727	0.9388	0
4851	615	1.1656	1.3055	0.3727	0.9389	0
4852	614.9	1.1663	1.3061	0.3727	0.9389	0
4853	614.8	1.1668	1.3067	0.3729	0.9388	0
4854	614.7	1.1673	1.3075	0.373	0.9393	0
4855	614.6	1.1678	1.3081	0.3729	0.9395	0
4856	614.5	1.1685	1.309	0.373	0.9393	0
4857	614.4	1.1691	1.3098	0.3729	0.9392	0
4858	614.3	1.17	1.3104	0.3731	0.9398	0
4859	614.2	1.1705	1.3113	0.3732	0.9396	0
4860	614.1	1.1711	1.3119	0.3733	0.9396	0
4861	614	1.1717	1.3124	0.3733	0.9399	0
4862	613.9	1.1723	1.3131	0.3733	0.9396	0
4863	613.8	1.1731	1.3138	0.3732	0.94	0
4864	613.7	1.1736	1.3145	0.3733	0.9401	0
4865	613.6	1.1742	1.3151	0.3733	0.9403	0
4866	613.5	1.1748	1.3157	0.3733	0.9406	0
4867	613.4	1.1756	1.3162	0.3733	0.9403	0
4868	613.3	1.1759	1.3169	0.3734	0.9404	0
4869	613.2	1.1766	1.3175	0.3734	0.9409	0
4870	613.1	1.1771	1.3178	0.3734	0.9406	0
4871	613	1.1779	1.3184	0.3734	0.9409	0
4872	612.9	1.1784	1.3189	0.3733	0.9407	0
4873	612.8	1.1791	1.3196	0.3733	0.9404	0
4874	612.7	1.1796	1.3201	0.3733	0.9403	0
4875	612.6	1.1801	1.3207	0.3732	0.9412	0
4876	612.5	1.1806	1.321	0.373	0.9407	0
4877	612.4	1.1815	1.3219	0.3733	0.9406	0
4878	612.3	1.1821	1.3222	0.3733	0.9405	0
4879	612.2	1.1826	1.3227	0.3733	0.9409	0
4880	612.1	1.1837	1.3232	0.3734	0.9411	0
4881	612	1.184	1.3237	0.3732	0.9406	0
4882	611.9	1.1844	1.3242	0.3732	0.9402	0
4883	611.8	1.1848	1.3244	0.373	0.9409	0
4884	611.7	1.1858	1.3247	0.3732	0.9404	0
4885	611.6	1.1864	1.3253	0.3731	0.9404	0
4886	611.5	1.1871	1.3254	0.373	0.9404	0
4887	611.4	1.1873	1.3261	0.3731	0.9407	0
4888	611.3	1.1882	1.3263	0.373	0.9405	0

4889	611.2	1.1887	1.3266	0.3728	0.9403	0
4890	611.1	1.1893	1.3271	0.3729	0.9405	0
4891	611	1.1899	1.3274	0.3728	0.9403	0
4892	610.9	1.1907	1.3277	0.3727	0.9402	0
4893	610.8	1.1913	1.328	0.3728	0.9402	0
4894	610.7	1.1918	1.3283	0.3727	0.9403	0
4895	610.6	1.1923	1.3285	0.3727	0.9401	0
4896	610.5	1.1932	1.3292	0.3727	0.9401	0
4897	610.4	1.1936	1.3294	0.3726	0.9402	0
4898	610.3	1.1941	1.3294	0.3726	0.9402	0
4899	610.2	1.1947	1.3299	0.3724	0.9399	0
4900	610.1	1.1953	1.3304	0.3724	0.94	0
4901	610	1.1961	1.3306	0.3725	0.9399	0
4902	609.9	1.197	1.3311	0.3724	0.9398	0
4903	609.8	1.1971	1.3314	0.3722	0.9395	0
4904	609.7	1.198	1.3312	0.3723	0.9395	0
4905	609.6	1.1987	1.3314	0.3721	0.9395	0
4906	609.5	1.1989	1.3321	0.3722	0.9395	0
4907	609.4	1.1999	1.3321	0.372	0.9394	0
4908	609.3	1.2004	1.3324	0.3719	0.9392	0
4909	609.2	1.2012	1.3326	0.3719	0.939	0
4910	609.1	1.2016	1.3328	0.3717	0.9392	0
4911	609	1.202	1.3333	0.3717	0.9389	0
4912	608.9	1.2027	1.3336	0.3716	0.939	0
4913	608.8	1.2035	1.3339	0.3715	0.9387	0
4914	608.7	1.204	1.334	0.3715	0.9383	0
4915	608.6	1.2048	1.3342	0.3715	0.9382	0
4916	608.5	1.2054	1.3348	0.3714	0.9386	0
4917	608.4	1.2059	1.3345	0.3714	0.9383	0
4918	608.3	1.2068	1.3349	0.3713	0.9383	0
4919	608.2	1.2071	1.335	0.3711	0.938	0
4920	608.1	1.2081	1.3355	0.3709	0.9379	0
4921	608	1.2088	1.3356	0.371	0.9379	0
4922	607.9	1.2093	1.336	0.371	0.9381	0
4923	607.8	1.2103	1.336	0.3709	0.9379	0
4924	607.7	1.211	1.3361	0.3708	0.9379	0
4925	607.6	1.2114	1.3367	0.3707	0.9379	0
4926	607.5	1.2119	1.3364	0.3707	0.9376	0
4927	607.4	1.2125	1.337	0.3706	0.9376	0
4928	607.3	1.2134	1.3376	0.3706	0.9374	0
4929	607.2	1.214	1.3373	0.3705	0.9373	0
4930	607.1	1.2149	1.3375	0.3704	0.9373	0
4931	607	1.2154	1.3376	0.3704	0.9374	0

4932	606.9	1.216	1.3379	0.3705	0.9373	0
4933	606.8	1.2171	1.3381	0.3705	0.9376	0
4934	606.7	1.2174	1.3383	0.3703	0.9373	0
4935	606.6	1.2181	1.3386	0.3702	0.9375	0
4936	606.5	1.2189	1.3385	0.3702	0.9374	0
4937	606.4	1.2197	1.339	0.3704	0.9374	0
4938	606.3	1.2205	1.3393	0.3704	0.9379	0
4939	606.2	1.2212	1.3394	0.3704	0.9376	0
4940	606.1	1.222	1.3396	0.3704	0.9375	0
4941	606	1.2229	1.34	0.3705	0.9376	0
4942	605.9	1.2235	1.34	0.3703	0.9377	0
4943	605.8	1.2241	1.3404	0.3702	0.9379	0
4944	605.7	1.2249	1.3404	0.3703	0.9375	0
4945	605.6	1.2254	1.3406	0.3704	0.9375	0
4946	605.5	1.2264	1.3411	0.3705	0.9379	0
4947	605.4	1.2269	1.341	0.3703	0.9376	0
4948	605.3	1.2277	1.3412	0.3703	0.9379	0
4949	605.2	1.2286	1.3415	0.3703	0.9379	0
4950	605.1	1.2295	1.3415	0.3706	0.9378	0
4951	605	1.2301	1.3417	0.3705	0.9379	0
4952	604.9	1.2306	1.3419	0.3704	0.9382	0
4953	604.8	1.2316	1.3424	0.3704	0.9385	0
4954	604.7	1.2323	1.3423	0.3705	0.9382	0
4955	604.6	1.2329	1.3427	0.3705	0.9385	0
4956	604.5	1.2339	1.3428	0.3706	0.939	0
4957	604.4	1.2349	1.3429	0.3705	0.9388	0
4958	604.3	1.2351	1.3435	0.3708	0.9388	0
4959	604.2	1.2362	1.3435	0.3709	0.939	0
4960	604.1	1.2369	1.344	0.3709	0.9391	0
4961	604	1.2377	1.3441	0.371	0.9392	0
4962	603.9	1.2387	1.3443	0.3713	0.9394	0
4963	603.8	1.239	1.3444	0.3711	0.9395	0
4964	603.7	1.2398	1.3449	0.3713	0.9399	0
4965	603.6	1.2405	1.3453	0.3712	0.9395	0
4966	603.5	1.2414	1.3456	0.3712	0.9399	0
4967	603.4	1.2421	1.3454	0.3712	0.9399	0
4968	603.3	1.243	1.3459	0.3712	0.94	0
4969	603.2	1.2438	1.3457	0.3714	0.9401	0
4970	603.1	1.2447	1.3463	0.3714	0.9403	0
4971	603	1.245	1.3468	0.3714	0.9403	0
4972	602.9	1.2461	1.3471	0.3714	0.9404	0
4973	602.8	1.2466	1.3469	0.3714	0.9403	0
4974	602.7	1.2476	1.3472	0.3714	0.9404	0

4975	602.6	1.2483	1.3475	0.3714	0.9406	0
4976	602.5	1.2487	1.3479	0.3713	0.9404	0
4977	602.4	1.2496	1.3484	0.3714	0.9406	0
4978	602.3	1.2503	1.3486	0.3715	0.9409	0
4979	602.2	1.2513	1.3488	0.3716	0.9409	0
4980	602.1	1.2521	1.3494	0.3716	0.9412	0
4981	602	1.2525	1.3496	0.3717	0.9407	0
4982	601.9	1.2532	1.3497	0.3716	0.9409	0
4983	601.8	1.2541	1.3501	0.3715	0.9409	0
4984	601.7	1.2546	1.3506	0.3715	0.9411	0
4985	601.6	1.2554	1.3508	0.3716	0.9409	0
4986	601.5	1.2561	1.3508	0.3716	0.9408	0
4987	601.4	1.257	1.3514	0.3714	0.9409	0
4988	601.3	1.2575	1.3517	0.3716	0.9412	0
4989	601.2	1.2585	1.3521	0.3715	0.9414	0
4990	601.1	1.2592	1.3525	0.3717	0.9413	0
4991	601	1.2599	1.353	0.3716	0.9413	0
4992	600.9	1.2608	1.3533	0.3717	0.9411	0
4993	600.8	1.2615	1.3535	0.3716	0.9411	0
4994	600.7	1.2622	1.3543	0.3717	0.9414	0
4995	600.6	1.2626	1.3543	0.3717	0.9414	0
4996	600.5	1.2636	1.3548	0.3716	0.9411	0
4997	600.4	1.2643	1.355	0.3715	0.9413	0
4998	600.3	1.2654	1.3555	0.3715	0.9411	0
4999	600.2	1.266	1.3562	0.3717	0.9411	0
5000	600.1	1.2667	1.3565	0.3714	0.9409	0
5001	600	1.2674	1.357	0.3715	0.941	0
5002	599.9	1.2681	1.3569	0.3713	0.9412	0
5003	599.8	1.2691	1.3577	0.3715	0.9409	0
5004	599.7	1.2699	1.3582	0.3713	0.9409	0
5005	599.6	1.2707	1.3586	0.3712	0.9408	0
5006	599.5	1.2715	1.3593	0.3712	0.9405	0
5007	599.4	1.272	1.3596	0.3713	0.9407	0
5008	599.3	1.2725	1.3602	0.3711	0.9405	0
5009	599.2	1.2736	1.3606	0.371	0.9406	0
5010	599.1	1.2744	1.3608	0.371	0.9405	0
5011	599	1.275	1.3612	0.3709	0.9403	0
5012	598.9	1.2759	1.3623	0.3709	0.9401	0
5013	598.8	1.2764	1.3625	0.3709	0.94	0
5014	598.7	1.2772	1.3631	0.3708	0.94	0
5015	598.6	1.278	1.3635	0.3706	0.9396	0
5016	598.5	1.2783	1.3636	0.3706	0.9395	0
5017	598.4	1.2788	1.364	0.3702	0.9393	0

5018	598.3	1.2799	1.3654	0.3705	0.9395	0
5019	598.2	1.2803	1.3655	0.3704	0.9393	0
5020	598.1	1.2811	1.3659	0.3702	0.9391	0
5021	598	1.2818	1.3661	0.3703	0.9395	0
5022	597.9	1.2824	1.3666	0.3702	0.9392	0
5023	597.8	1.2834	1.3672	0.3702	0.939	0
5024	597.7	1.2839	1.3677	0.3702	0.9388	0
5025	597.6	1.2846	1.3682	0.3701	0.9391	0
5026	597.5	1.2855	1.369	0.3702	0.939	0
5027	597.4	1.2861	1.3697	0.3701	0.9392	0
5028	597.3	1.2872	1.3701	0.3699	0.9392	0
5029	597.2	1.2874	1.3709	0.3699	0.9388	0
5030	597.1	1.2883	1.3713	0.3699	0.9389	0
5031	597	1.2892	1.3718	0.3698	0.9392	0
5032	596.9	1.2898	1.3727	0.3698	0.9389	0
5033	596.8	1.2908	1.3733	0.3698	0.9387	0
5034	596.7	1.2917	1.3741	0.3698	0.9386	0
5035	596.6	1.2921	1.3748	0.3698	0.9384	0
5036	596.5	1.2926	1.375	0.3696	0.9388	0
5037	596.4	1.2938	1.3761	0.3699	0.9386	0
5038	596.3	1.2946	1.3765	0.3699	0.9387	0
5039	596.2	1.2952	1.3769	0.3699	0.9388	0
5040	596.1	1.2962	1.378	0.3697	0.9386	0
5041	596	1.2969	1.3783	0.3697	0.9387	0
5042	595.9	1.2976	1.3792	0.3698	0.9386	0
5043	595.8	1.2982	1.38	0.3698	0.9387	0
5044	595.7	1.299	1.3805	0.3698	0.9389	0
5045	595.6	1.3001	1.3815	0.3698	0.9389	0
5046	595.5	1.3009	1.3824	0.3698	0.9389	0
5047	595.4	1.3017	1.383	0.3699	0.9387	0
5048	595.3	1.3023	1.3835	0.3698	0.9387	0
5049	595.2	1.3032	1.3845	0.3698	0.939	0
5050	595.1	1.3035	1.3851	0.3698	0.939	0
5051	595	1.3049	1.3863	0.37	0.939	0
5052	594.9	1.3056	1.3866	0.3701	0.9391	0
5053	594.8	1.3065	1.3876	0.3701	0.9393	0
5054	594.7	1.3078	1.3882	0.3701	0.9392	0
5055	594.6	1.3083	1.3894	0.3701	0.9396	0
5056	594.5	1.3094	1.3903	0.3702	0.9396	0
5057	594.4	1.3098	1.3908	0.3702	0.9394	0
5058	594.3	1.3108	1.3922	0.3704	0.9399	0
5059	594.2	1.3121	1.3931	0.3706	0.9399	0
5060	594.1	1.3126	1.3937	0.3704	0.9401	0

5061	594	1.3135	1.3946	0.3706	0.9401	0
5062	593.9	1.3143	1.3956	0.3707	0.9403	0
5063	593.8	1.3151	1.3965	0.3707	0.9403	0
5064	593.7	1.3162	1.3971	0.3707	0.9408	0
5065	593.6	1.317	1.398	0.3706	0.9404	0
5066	593.5	1.3176	1.3987	0.3707	0.9404	0
5067	593.4	1.3186	1.3998	0.3709	0.9404	0
5068	593.3	1.3192	1.4005	0.3711	0.9408	0
5069	593.2	1.3206	1.4015	0.3711	0.9408	0
5070	593.1	1.321	1.4028	0.371	0.9408	0
5071	593	1.3219	1.4032	0.3711	0.9409	0
5072	592.9	1.3225	1.4042	0.3712	0.9409	0
5073	592.8	1.3237	1.405	0.3714	0.941	0
5074	592.7	1.3244	1.406	0.3712	0.9413	0
5075	592.6	1.3254	1.4071	0.3713	0.9412	0
5076	592.5	1.3261	1.4079	0.3712	0.9418	0
5077	592.4	1.3271	1.4087	0.3713	0.9413	0
5078	592.3	1.3275	1.4098	0.3713	0.9412	0
5079	592.2	1.3284	1.4111	0.3714	0.9415	0
5080	592.1	1.3296	1.4115	0.3714	0.9417	0
5081	592	1.3301	1.4126	0.3715	0.9415	0
5082	591.9	1.3313	1.413	0.3713	0.9417	0
5083	591.8	1.332	1.4142	0.3713	0.9415	0
5084	591.7	1.3326	1.4151	0.3713	0.9417	0
5085	591.6	1.3335	1.416	0.3716	0.9421	0
5086	591.5	1.3346	1.4166	0.3716	0.942	0
5087	591.4	1.3352	1.4176	0.3717	0.9421	0
5088	591.3	1.336	1.4184	0.3718	0.942	0
5089	591.2	1.3364	1.4192	0.3716	0.942	0
5090	591.1	1.3377	1.4206	0.3717	0.9423	0
5091	591	1.3385	1.4212	0.3718	0.9424	0
5092	590.9	1.3394	1.4225	0.3718	0.9426	0
5093	590.8	1.34	1.4234	0.3719	0.9426	0
5094	590.7	1.3412	1.4241	0.372	0.9426	0
5095	590.6	1.3421	1.4254	0.3721	0.9424	0
5096	590.5	1.3425	1.4262	0.3721	0.9423	0
5097	590.4	1.3438	1.4272	0.3721	0.9424	0
5098	590.3	1.3443	1.4278	0.3721	0.9424	0
5099	590.2	1.3455	1.4284	0.3721	0.9425	0
5100	590.1	1.3462	1.4295	0.3722	0.9425	0
5101	590	1.3467	1.4304	0.372	0.9425	0
5102	589.9	1.3476	1.431	0.3721	0.9425	0
5103	589.8	1.3486	1.4321	0.372	0.9422	0

5104	589.7	1.3495	1.4338	0.3721	0.9425	0
5105	589.6	1.3504	1.4344	0.372	0.9426	0
5106	589.5	1.3511	1.4355	0.372	0.9426	0
5107	589.4	1.3521	1.4358	0.372	0.9425	0
5108	589.3	1.3527	1.4368	0.3719	0.9423	0
5109	589.2	1.3531	1.4379	0.3719	0.9425	0
5110	589.1	1.3546	1.4388	0.372	0.9423	0
5111	589	1.3554	1.4396	0.372	0.9421	0
5112	588.9	1.3565	1.4408	0.3721	0.9421	0
5113	588.8	1.3568	1.4415	0.3719	0.9423	0
5114	588.7	1.3573	1.4424	0.3719	0.9422	0
5115	588.6	1.3588	1.4428	0.3718	0.9422	0
5116	588.5	1.3595	1.4443	0.3717	0.9417	0
5117	588.4	1.36	1.4448	0.3718	0.9414	0
5118	588.3	1.3612	1.4461	0.3717	0.9421	0
5119	588.2	1.3618	1.447	0.3718	0.9421	0
5120	588.1	1.3626	1.4483	0.3717	0.9415	0
5121	588	1.3632	1.4484	0.3717	0.9414	0
5122	587.9	1.3645	1.4503	0.3718	0.9418	0
5123	587.8	1.3651	1.4504	0.3717	0.9417	0
5124	587.7	1.3661	1.451	0.3717	0.9418	0
5125	587.6	1.3668	1.4525	0.3716	0.9415	0
5126	587.5	1.3678	1.4531	0.3716	0.9419	0
5127	587.4	1.3684	1.4541	0.3717	0.9418	0
5128	587.3	1.3691	1.4547	0.3718	0.942	0
5129	587.2	1.3702	1.4562	0.3718	0.9417	0
5130	587.1	1.3708	1.4568	0.3716	0.9415	0
5131	587	1.3712	1.4571	0.3717	0.9415	0
5132	586.9	1.3722	1.4577	0.3716	0.9411	0
5133	586.8	1.3731	1.4589	0.3717	0.9416	0
5134	586.7	1.3739	1.4597	0.3718	0.9414	0
5135	586.6	1.375	1.4605	0.3717	0.9416	0
5136	586.5	1.3755	1.4612	0.3719	0.9414	0
5137	586.4	1.3762	1.4623	0.3718	0.9413	0
5138	586.3	1.377	1.4631	0.3718	0.9413	0
5139	586.2	1.3783	1.4636	0.3718	0.9414	0
5140	586.1	1.3787	1.4647	0.3719	0.9415	0
5141	586	1.3794	1.4658	0.3716	0.9412	0
5142	585.9	1.3803	1.4658	0.3716	0.9415	0
5143	585.8	1.3813	1.4672	0.3717	0.9417	0
5144	585.7	1.3818	1.4685	0.3718	0.9418	0
5145	585.6	1.383	1.4693	0.3718	0.9412	0
5146	585.5	1.3839	1.4701	0.3719	0.9414	0

5147	585.4	1.384	1.47	0.3718	0.9417	0
5148	585.3	1.3852	1.4717	0.3718	0.9415	0
5149	585.2	1.3854	1.4721	0.3719	0.9419	0
5150	585.1	1.3864	1.4731	0.3719	0.942	0
5151	585	1.3876	1.4739	0.372	0.9419	0
5152	584.9	1.379	1.4754	0.3891	0.9906	0
5153	584.8	1.3791	1.4772	0.3892	0.9911	0
5154	584.7	1.3805	1.4776	0.3893	0.9917	0
5155	584.6	1.3813	1.4786	0.3892	0.991	0
5156	584.5	1.3815	1.479	0.3894	0.9913	0
5157	584.4	1.3823	1.4801	0.3896	0.9917	0
5158	584.3	1.3837	1.4812	0.3896	0.9918	0
5159	584.2	1.3852	1.4816	0.3897	0.9919	0
5160	584.1	1.3855	1.4829	0.3898	0.9919	0
5161	584	1.3862	1.4838	0.3897	0.9919	0
5162	583.9	1.3871	1.4843	0.3898	0.9918	0
5163	583.8	1.3882	1.4857	0.3901	0.9927	0
5164	583.7	1.3892	1.487	0.3903	0.9925	0
5165	583.6	1.3903	1.4869	0.3903	0.9924	0
5166	583.5	1.3902	1.4876	0.3904	0.9925	0
5167	583.4	1.3914	1.4876	0.3903	0.9932	0
5168	583.3	1.3922	1.49	0.3904	0.9934	0
5169	583.2	1.3929	1.4902	0.3903	0.9933	0
5170	583.1	1.3945	1.4913	0.3905	0.9931	0
5171	583	1.3955	1.4924	0.3906	0.9935	0
5172	582.9	1.3971	1.4935	0.3906	0.9934	0
5173	582.8	1.397	1.4933	0.3907	0.9934	0
5174	582.7	1.3988	1.4947	0.3908	0.9938	0
5175	582.6	1.3989	1.4946	0.391	0.994	0
5176	582.5	1.4004	1.4962	0.3911	0.9939	0
5177	582.4	1.4011	1.4962	0.3911	0.9941	0
5178	582.3	1.4019	1.4977	0.3911	0.9942	0
5179	582.2	1.4029	1.4979	0.3912	0.9942	0
5180	582.1	1.4038	1.4996	0.3911	0.9941	0
5181	582	1.4048	1.4999	0.3913	0.9944	0
5182	581.9	1.4066	1.5003	0.3912	0.9947	0
5183	581.8	1.4065	1.5006	0.3914	0.9948	0
5184	581.7	1.4083	1.5026	0.3915	0.9945	0
5185	581.6	1.4087	1.5029	0.3913	0.9947	0
5186	581.5	1.4105	1.5033	0.3914	0.9949	0
5187	581.4	1.4105	1.5037	0.3915	0.9951	0
5188	581.3	1.4124	1.505	0.3917	0.9957	0
5189	581.2	1.4127	1.5051	0.3914	0.995	0

5190	581.1	1.4139	1.5063	0.3914	0.9947	0
5191	581	1.4149	1.5073	0.3914	0.9953	0
5192	580.9	1.4158	1.509	0.3916	0.995	0
5193	580.8	1.4169	1.5094	0.3917	0.9954	0
5194	580.7	1.4172	1.5095	0.3917	0.9949	0
5195	580.6	1.4188	1.5098	0.3918	0.9959	0
5196	580.5	1.4192	1.5115	0.3917	0.9962	0
5197	580.4	1.4201	1.5124	0.3918	0.996	0
5198	580.3	1.421	1.5118	0.392	0.996	0
5199	580.2	1.4227	1.5129	0.3921	0.9961	0
5200	580.1	1.4235	1.5137	0.3919	0.996	0
5201	580	1.4243	1.5145	0.392	0.9961	0
5202	579.9	1.4251	1.5153	0.392	0.9964	0
5203	579.8	1.4267	1.517	0.3921	0.9965	0
5204	579.7	1.4274	1.5178	0.392	0.9966	0
5205	579.6	1.4282	1.517	0.3919	0.9964	0
5206	579.5	1.4298	1.518	0.3921	0.9967	0
5207	579.4	1.4312	1.5195	0.3921	0.9959	0
5208	579.3	1.4313	1.5202	0.3922	0.9961	0
5209	579.2	1.4327	1.5202	0.392	0.9964	0
5210	579.1	1.4334	1.521	0.3921	0.9968	0
5211	579	1.4336	1.5218	0.3921	0.9967	0
5212	578.9	1.4351	1.5219	0.392	0.9966	0
5213	578.8	1.4367	1.5236	0.3923	0.9964	0
5214	578.7	1.438	1.5251	0.3922	0.9972	0
5215	578.6	1.4387	1.5243	0.3921	0.9972	0
5216	578.5	1.4401	1.525	0.3921	0.9971	0
5217	578.4	1.4408	1.5265	0.3923	0.9969	0
5218	578.3	1.4416	1.5273	0.3922	0.9972	0
5219	578.2	1.4436	1.5281	0.3923	0.9973	0
5220	578.1	1.445	1.5289	0.3923	0.9972	0
5221	578	1.445	1.5287	0.3922	0.9969	0
5222	577.9	1.4457	1.5294	0.3921	0.9972	0
5223	577.8	1.4478	1.5302	0.3923	0.9971	0
5224	577.7	1.4484	1.5308	0.392	0.997	0
5225	577.6	1.4497	1.5308	0.3921	0.9966	0
5226	577.5	1.4504	1.5315	0.392	0.9973	0
5227	577.4	1.4518	1.5322	0.3922	0.9974	0
5228	577.3	1.4531	1.5329	0.392	0.9974	0
5229	577.2	1.4538	1.5343	0.3921	0.9972	0
5230	577.1	1.4551	1.5343	0.3921	0.9973	0
5231	577	1.4569	1.5349	0.392	0.9972	0
5232	576.9	1.4576	1.5355	0.392	0.997	0

5233	576.8	1.4576	1.5362	0.3922	0.9976	0
5234	576.7	1.4596	1.5368	0.3922	0.9977	0
5235	576.6	1.4608	1.5375	0.3921	0.9974	0
5236	576.5	1.4622	1.5382	0.3921	0.9979	0
5237	576.4	1.4628	1.5389	0.3924	0.9975	0
5238	576.3	1.464	1.5394	0.3922	0.9972	0
5239	576.2	1.4654	1.5394	0.3922	0.9983	0
5240	576.1	1.4673	1.54	0.3924	0.9975	0
5241	576	1.4674	1.5407	0.3922	0.9977	0
5242	575.9	1.4687	1.5414	0.3924	0.9978	0
5243	575.8	1.4704	1.5426	0.3922	0.9978	0
5244	575.7	1.4699	1.5418	0.3922	0.9981	0
5245	575.6	1.4724	1.5432	0.3923	0.9976	0
5246	575.5	1.473	1.5438	0.3921	0.9978	0
5247	575.4	1.4749	1.5437	0.3922	0.998	0
5248	575.3	1.4748	1.5442	0.3923	0.9981	0
5249	575.2	1.476	1.5448	0.3922	0.998	0
5250	575.1	1.4773	1.5462	0.3924	0.998	0
5251	575	1.4793	1.5462	0.3924	0.9986	0
5252	574.9	1.4801	1.5469	0.3926	0.9987	0
5253	574.8	1.4812	1.5473	0.3925	0.9987	0
5254	574.7	1.4817	1.5479	0.3924	0.999	0
5255	574.6	1.4829	1.5477	0.3923	0.9988	0
5256	574.5	1.4843	1.5492	0.3923	0.9986	0
5257	574.4	1.4856	1.5499	0.3927	0.9993	0
5258	574.3	1.4868	1.5497	0.3926	0.9992	0
5259	574.2	1.4876	1.5504	0.3928	0.9995	0
5260	574.1	1.4894	1.5502	0.3926	0.9992	0
5261	574	1.4901	1.5516	0.3929	0.9995	0
5262	573.9	1.4913	1.5529	0.3929	0.9998	0
5263	573.8	1.4925	1.5527	0.3928	0.9993	0
5264	573.7	1.4938	1.5534	0.3929	0.9998	0
5265	573.6	1.4951	1.554	0.393	0.9998	0
5266	573.5	1.4969	1.5538	0.3931	0.9999	0
5267	573.4	1.497	1.5545	0.3932	0.9999	0
5268	573.3	1.4988	1.555	0.3932	0.9996	0
5269	573.2	1.5002	1.5565	0.3934	1	0
5270	573.1	1.5016	1.5565	0.3934	1.0004	0
5271	573	1.5034	1.557	0.3933	1.0005	0
5272	572.9	1.5033	1.5575	0.3934	1.0007	0
5273	572.8	1.5052	1.5574	0.3934	1.0004	0
5274	572.7	1.5071	1.5579	0.3934	1.0008	0
5275	572.6	1.5077	1.5585	0.3934	1.0007	0

5276	572.5	1.5089	1.559	0.3934	1.0006	0
5277	572.4	1.5103	1.5598	0.3936	1.0012	0
5278	572.3	1.5122	1.5596	0.3936	1.0009	0
5279	572.2	1.5128	1.5602	0.3936	1.0007	0
5280	572.1	1.5134	1.5608	0.3935	1.0011	0
5281	572	1.5153	1.5614	0.3935	1.0009	0
5282	571.9	1.5159	1.5619	0.3935	1.0008	0
5283	571.8	1.517	1.5624	0.3935	1.0009	0
5284	571.7	1.519	1.563	0.3933	1.0011	0
5285	571.6	1.5203	1.5629	0.3936	1.0013	0
5286	571.5	1.5223	1.5635	0.3936	1.0011	0
5287	571.4	1.5229	1.5649	0.3937	1.0011	0
5288	571.3	1.5249	1.5648	0.3936	1.0015	0
5289	571.2	1.5254	1.5654	0.3937	1.0017	0
5290	571.1	1.5267	1.566	0.3936	1.0019	0
5291	571	1.5272	1.5657	0.3937	1.0014	0
5292	570.9	1.5291	1.567	0.3937	1.0011	0
5293	570.8	1.5304	1.5668	0.3936	1.0012	0
5294	570.7	1.5316	1.5673	0.3936	1.0014	0
5295	570.6	1.5322	1.5672	0.3935	1.0011	0
5296	570.5	1.5341	1.5684	0.3933	1.0011	0
5297	570.4	1.5347	1.5698	0.3935	1.0015	0
5298	570.3	1.5367	1.5689	0.3934	1.0015	0
5299	570.2	1.5379	1.5695	0.3934	1.0014	0
5300	570.1	1.5399	1.5693	0.3935	1.001	0
5301	570	1.5404	1.5713	0.3937	1.0014	0
5302	569.9	1.5417	1.5712	0.3936	1.0013	0
5303	569.8	1.5438	1.5719	0.3936	1.0014	0
5304	569.7	1.5457	1.5724	0.3935	1.0014	0
5305	569.6	1.5463	1.5729	0.3933	1.0015	0
5306	569.5	1.5475	1.5735	0.3934	1.0017	0
5307	569.4	1.5488	1.5741	0.3933	1.0012	0
5308	569.3	1.5508	1.574	0.3935	1.0017	0
5309	569.2	1.5507	1.5753	0.3936	1.0015	0
5310	569.1	1.552	1.5752	0.3935	1.0013	0
5311	569	1.554	1.5758	0.3936	1.0013	0
5312	568.9	1.5553	1.577	0.3937	1.0012	0
5313	568.8	1.5558	1.5769	0.3936	1.0012	0
5314	568.7	1.5572	1.5775	0.3935	1.0014	0
5315	568.6	1.5591	1.5787	0.3937	1.0013	0
5316	568.5	1.5597	1.5779	0.3936	1.0017	0
5317	568.4	1.561	1.5792	0.3934	1.0019	0
5318	568.3	1.563	1.5797	0.3936	1.0021	0

5319	568.2	1.5637	1.5804	0.3938	1.0022	0
5320	568.1	1.5656	1.5817	0.3938	1.0019	0
5321	568	1.5663	1.5816	0.3938	1.0018	0
5322	567.9	1.5676	1.5822	0.3939	1.0016	0
5323	567.8	1.5688	1.5819	0.3939	1.0017	0
5324	567.7	1.5701	1.5832	0.3937	1.0019	0
5325	567.6	1.5713	1.583	0.3939	1.0022	0
5326	567.5	1.5725	1.5842	0.3937	1.0021	0
5327	567.4	1.5738	1.5848	0.3938	1.0023	0
5328	567.3	1.5751	1.5854	0.3937	1.0023	0
5329	567.2	1.5764	1.5859	0.3939	1.0028	0
5330	567.1	1.5777	1.5857	0.394	1.0026	0
5331	567	1.5797	1.5871	0.3938	1.0025	0
5332	566.9	1.5795	1.5884	0.3936	1.0022	0
5333	566.8	1.5809	1.5882	0.3939	1.0028	0
5334	566.7	1.583	1.5889	0.3941	1.0027	0
5335	566.6	1.5843	1.5894	0.394	1.0027	0
5336	566.5	1.5847	1.5906	0.3938	1.0025	0
5337	566.4	1.5861	1.5905	0.3943	1.0026	0
5338	566.3	1.5882	1.5919	0.3942	1.0029	0
5339	566.2	1.5885	1.5915	0.394	1.003	0
5340	566.1	1.59	1.5929	0.3942	1.0033	0
5341	566	1.5913	1.5935	0.3942	1.0033	0
5342	565.9	1.5927	1.5941	0.3944	1.0034	0
5343	565.8	1.5938	1.5946	0.3942	1.0032	0
5344	565.7	1.595	1.595	0.3941	1.0035	0
5345	565.6	1.5964	1.5964	0.3943	1.0035	0
5346	565.5	1.5978	1.5963	0.3946	1.0038	0
5347	565.4	1.599	1.5975	0.3943	1.0037	0
5348	565.3	1.6004	1.5981	0.3945	1.004	0
5349	565.2	1.6009	1.5994	0.3945	1.0041	0
5350	565.1	1.6022	1.5992	0.3945	1.0041	0
5351	565	1.6035	1.6005	0.3944	1.0037	0
5352	564.9	1.6048	1.6011	0.3947	1.0046	0
5353	564.8	1.6068	1.6015	0.3946	1.0045	0
5354	564.7	1.6073	1.6028	0.3946	1.0039	0
5355	564.6	1.6087	1.6035	0.3947	1.0048	0
5356	564.5	1.6099	1.6039	0.3948	1.0046	0
5357	564.4	1.612	1.6037	0.3949	1.0053	0
5358	564.3	1.6126	1.6059	0.395	1.0047	0
5359	564.2	1.6138	1.6063	0.395	1.005	0
5360	564.1	1.6144	1.6068	0.395	1.005	0
5361	564	1.6157	1.6081	0.3951	1.0051	0

5362	563.9	1.617	1.608	0.3952	1.0051	0
5363	563.8	1.6183	1.6092	0.3951	1.005	0
5364	563.7	1.6188	1.6098	0.3952	1.0053	0
5365	563.6	1.6208	1.6102	0.3953	1.0053	0
5366	563.5	1.6221	1.6115	0.3952	1.0057	0
5367	563.4	1.6226	1.612	0.3953	1.0058	0
5368	563.3	1.6247	1.614	0.3952	1.0059	0
5369	563.2	1.626	1.6145	0.3952	1.0059	0
5370	563.1	1.6274	1.6151	0.3953	1.006	0
5371	563	1.6286	1.6148	0.3954	1.0062	0
5372	562.9	1.6291	1.6161	0.3954	1.0062	0
5373	562.8	1.6311	1.6173	0.3951	1.0064	0
5374	562.7	1.6323	1.6177	0.3952	1.0061	0
5375	562.6	1.6329	1.6183	0.3954	1.0062	0
5376	562.5	1.6349	1.6195	0.3955	1.0062	0
5377	562.4	1.6346	1.6192	0.3954	1.0062	0
5378	562.3	1.6367	1.6197	0.3952	1.0056	0
5379	562.2	1.6379	1.6209	0.3954	1.0058	0
5380	562.1	1.6384	1.6222	0.3953	1.0063	0
5381	562	1.6396	1.6226	0.3952	1.0059	0
5382	561.9	1.6401	1.6239	0.3953	1.0056	0
5383	561.8	1.6422	1.6243	0.3951	1.0057	0
5384	561.7	1.6435	1.6248	0.3954	1.0059	0
5385	561.6	1.6448	1.6269	0.3954	1.0058	0
5386	561.5	1.646	1.6273	0.3953	1.0061	0
5387	561.4	1.6473	1.6263	0.3953	1.0059	0
5388	561.3	1.6486	1.6283	0.3953	1.006	0
5389	561.2	1.6499	1.6296	0.3952	1.0058	0
5390	561.1	1.6495	1.6308	0.3953	1.0058	0
5391	561	1.6515	1.6304	0.395	1.0059	0
5392	560.9	1.6535	1.6316	0.395	1.0057	0
5393	560.8	1.6549	1.6329	0.395	1.0055	0
5394	560.7	1.6553	1.6326	0.3951	1.006	0
5395	560.6	1.6567	1.6338	0.395	1.0058	0
5396	560.5	1.6579	1.6358	0.3949	1.0059	0
5397	560.4	1.6592	1.6363	0.3949	1.0056	0
5398	560.3	1.6596	1.6375	0.395	1.0059	0
5399	560.2	1.6608	1.6386	0.3949	1.0055	0
5400	560.1	1.6621	1.6391	0.3947	1.0058	0
5401	560	1.6633	1.6403	0.3947	1.0059	0
5402	559.9	1.6646	1.6416	0.3946	1.0055	0
5403	559.8	1.6668	1.6421	0.3948	1.0053	0
5404	559.7	1.6681	1.6441	0.3948	1.0059	0

5405	559.6	1.6694	1.6454	0.3947	1.0053	0
5406	559.5	1.6697	1.645	0.3945	1.0052	0
5407	559.4	1.6711	1.6463	0.3945	1.0055	0
5408	559.3	1.6724	1.6475	0.3947	1.0053	0
5409	559.2	1.6745	1.6488	0.3945	1.0054	0
5410	559.1	1.6758	1.65	0.3945	1.0052	0
5411	559	1.677	1.6504	0.3944	1.0054	0
5412	558.9	1.6777	1.6518	0.3945	1.0056	0
5413	558.8	1.6799	1.6516	0.3945	1.0054	0
5414	558.7	1.6803	1.6528	0.3945	1.0054	0
5415	558.6	1.6815	1.6547	0.3945	1.0052	0
5416	558.5	1.6827	1.6551	0.3943	1.0049	0
5417	558.4	1.6849	1.6564	0.3943	1.0052	0
5418	558.3	1.6853	1.6576	0.3943	1.0049	0
5419	558.2	1.6875	1.6581	0.3942	1.005	0
5420	558.1	1.6888	1.6594	0.3942	1.0054	0
5421	558	1.6885	1.6599	0.3943	1.0053	0
5422	557.9	1.6907	1.6627	0.3944	1.0048	0
5423	557.8	1.6918	1.6631	0.3941	1.0046	0
5424	557.7	1.6932	1.6635	0.394	1.0049	0
5425	557.6	1.6953	1.6647	0.394	1.0047	0
5426	557.5	1.6959	1.6661	0.3943	1.005	0
5427	557.4	1.6962	1.6673	0.3941	1.0048	0
5428	557.3	1.6976	1.6686	0.3942	1.0048	0
5429	557.2	1.6988	1.669	0.3943	1.0052	0
5430	557.1	1.7003	1.6704	0.3943	1.0049	0
5431	557	1.7016	1.6724	0.3943	1.005	0
5432	556.9	1.7029	1.6729	0.3944	1.0053	0
5433	556.8	1.7042	1.6733	0.3944	1.0054	0
5434	556.7	1.7056	1.6747	0.3946	1.0056	0
5435	556.6	1.7068	1.6766	0.3943	1.0058	0
5436	556.5	1.7074	1.6788	0.3946	1.006	0
5437	556.4	1.7094	1.6791	0.3945	1.0058	0
5438	556.3	1.7118	1.6805	0.3947	1.006	0
5439	556.2	1.713	1.6826	0.3947	1.0063	0
5440	556.1	1.7135	1.6838	0.3946	1.0063	0
5441	556	1.7148	1.685	0.3946	1.006	0
5442	555.9	1.7169	1.6854	0.3948	1.0065	0
5443	555.8	1.7173	1.6875	0.3948	1.0064	0
5444	555.7	1.7196	1.688	0.395	1.0065	0
5445	555.6	1.7208	1.69	0.3949	1.0065	0
5446	555.5	1.7212	1.692	0.3948	1.0069	0
5447	555.4	1.7218	1.6926	0.3952	1.0071	0

5448	555.3	1.7249	1.6938	0.3952	1.0074	0
5449	555.2	1.7253	1.6951	0.3953	1.0076	0
5450	555.1	1.7256	1.6962	0.3951	1.0077	0
5451	555	1.7269	1.6983	0.3952	1.0081	0
5452	554.9	1.73	1.6996	0.3954	1.0083	0
5453	554.8	1.7306	1.701	0.3954	1.0083	0
5454	554.7	1.7317	1.702	0.3954	1.0078	0
5455	554.6	1.7331	1.7034	0.3956	1.0085	0
5456	554.5	1.7353	1.7046	0.3957	1.0085	0
5457	554.4	1.7357	1.7067	0.3958	1.009	0
5458	554.3	1.7378	1.7078	0.3957	1.0089	0
5459	554.2	1.7381	1.7099	0.3956	1.0087	0
5460	554.1	1.7405	1.7104	0.3957	1.0087	0
5461	554	1.7407	1.7123	0.3958	1.0087	0
5462	553.9	1.7421	1.7128	0.3959	1.0091	0
5463	553.8	1.7443	1.714	0.3959	1.0096	0
5464	553.7	1.7455	1.7169	0.396	1.0097	0
5465	553.6	1.7468	1.7181	0.3962	1.0096	0
5466	553.5	1.7481	1.7194	0.3963	1.0097	0
5467	553.4	1.7484	1.7214	0.3961	1.0097	0
5468	553.3	1.7507	1.7218	0.3963	1.0101	0
5469	553.2	1.7518	1.7238	0.396	1.0105	0
5470	553.1	1.754	1.725	0.3962	1.0097	0
5471	553	1.7553	1.7279	0.3963	1.01	0
5472	552.9	1.7565	1.7282	0.3962	1.0101	0
5473	552.8	1.7567	1.7293	0.3962	1.0098	0
5474	552.7	1.7589	1.7323	0.3964	1.0101	0
5475	552.6	1.7601	1.7325	0.3962	1.0102	0
5476	552.5	1.7623	1.7337	0.3963	1.0106	0
5477	552.4	1.7625	1.7358	0.3962	1.0106	0
5478	552.3	1.7638	1.7378	0.3965	1.0103	0
5479	552.2	1.7659	1.7399	0.3964	1.0104	0
5480	552.1	1.769	1.7419	0.3962	1.0106	0
5481	552	1.7703	1.7431	0.3964	1.0109	0
5482	551.9	1.7704	1.7441	0.3963	1.0104	0
5483	551.8	1.7717	1.7454	0.3965	1.011	0
5484	551.7	1.7729	1.7474	0.3964	1.0109	0
5485	551.6	1.7739	1.7484	0.3962	1.0108	0
5486	551.5	1.7751	1.7495	0.3961	1.0103	0
5487	551.4	1.7763	1.7507	0.3963	1.0106	0
5488	551.3	1.7775	1.7528	0.3963	1.0106	0
5489	551.2	1.7797	1.7529	0.3964	1.0106	0
5490	551.1	1.7807	1.7549	0.3962	1.0107	0

5491	551	1.7829	1.7579	0.3964	1.0109	0
5492	550.9	1.7831	1.7591	0.3964	1.0108	0
5493	550.8	1.7854	1.7602	0.3965	1.0107	0
5494	550.7	1.7864	1.7621	0.3964	1.0106	0
5495	550.6	1.7885	1.7631	0.3961	1.0105	0
5496	550.5	1.7897	1.7652	0.3962	1.0105	0
5497	550.4	1.7917	1.7662	0.3963	1.0104	0
5498	550.3	1.7918	1.7682	0.3961	1.0104	0
5499	550.2	1.794	1.7693	0.3961	1.0105	0
5500	550.1	1.7961	1.7713	0.3961	1.0107	0
5501	550	1.796	1.7732	0.3959	1.011	0
5502	549.9	1.7982	1.7752	0.3958	1.0109	0
5503	549.8	1.7983	1.7763	0.3957	1.0108	0
5504	549.7	1.8014	1.7783	0.3957	1.0104	0
5505	549.6	1.8016	1.7794	0.3957	1.0104	0
5506	549.5	1.8036	1.7814	0.3957	1.0103	0
5507	549.4	1.8047	1.7824	0.3958	1.0108	0
5508	549.3	1.8067	1.7843	0.3956	1.0103	0
5509	549.2	1.8077	1.7863	0.3956	1.0102	0
5510	549.1	1.8087	1.7882	0.3955	1.01	0
5511	549	1.8098	1.7892	0.3954	1.0101	0
5512	548.9	1.8108	1.7912	0.3953	1.0099	0
5513	548.8	1.8129	1.7922	0.3952	1.0097	0
5514	548.7	1.815	1.7932	0.3952	1.0099	0
5515	548.6	1.816	1.7962	0.395	1.0098	0
5516	548.5	1.8171	1.7972	0.395	1.0098	0
5517	548.4	1.8192	1.7982	0.3951	1.0096	0
5518	548.3	1.8192	1.8002	0.3951	1.0101	0
5519	548.2	1.8202	1.8012	0.395	1.0101	0
5520	548.1	1.8233	1.8042	0.3949	1.0101	0
5521	548	1.8233	1.8062	0.3949	1.0099	0
5522	547.9	1.8264	1.8082	0.3951	1.0101	0
5523	547.8	1.8263	1.8081	0.395	1.0097	0
5524	547.7	1.8273	1.8112	0.3951	1.0097	0
5525	547.6	1.8295	1.8122	0.395	1.01	0
5526	547.5	1.8304	1.8142	0.395	1.0099	0
5527	547.4	1.8324	1.8161	0.395	1.01	0
5528	547.3	1.8334	1.8181	0.3951	1.0095	0
5529	547.2	1.8344	1.8201	0.3951	1.0097	0
5530	547.1	1.8364	1.8209	0.395	1.0098	0
5531	547	1.8373	1.8229	0.3949	1.0097	0
5532	546.9	1.8382	1.8249	0.3949	1.0101	0
5533	546.8	1.8392	1.8258	0.3949	1.0099	0

5534	546.7	1.8412	1.8289	0.395	1.0105	0
5535	546.6	1.8422	1.8298	0.395	1.0103	0
5536	546.5	1.8441	1.8328	0.3949	1.0103	0
5537	546.4	1.844	1.8327	0.3952	1.0105	0
5538	546.3	1.8461	1.8347	0.3953	1.0105	0
5539	546.2	1.848	1.8366	0.3952	1.0103	0
5540	546.1	1.8489	1.8386	0.3951	1.0103	0
5541	546	1.8488	1.8407	0.3953	1.0105	0
5542	545.9	1.8506	1.8425	0.3951	1.0107	0
5543	545.8	1.8516	1.8446	0.3954	1.0107	0
5544	545.7	1.8524	1.8466	0.3953	1.011	0
5545	545.6	1.8544	1.8474	0.3953	1.011	0
5546	545.5	1.8565	1.8506	0.3956	1.0114	0
5547	545.4	1.8573	1.8526	0.3956	1.0115	0
5548	545.3	1.8581	1.8533	0.3955	1.0113	0
5549	545.2	1.8601	1.8565	0.3955	1.0114	0
5550	545.1	1.8609	1.8573	0.3957	1.0117	0
5551	545	1.8618	1.8594	0.3958	1.0121	0
5552	544.9	1.8638	1.8626	0.396	1.0123	0
5553	544.8	1.8634	1.8622	0.396	1.0125	0
5554	544.7	1.8667	1.8655	0.3961	1.0126	0
5555	544.6	1.8674	1.8674	0.3962	1.0128	0
5556	544.5	1.8694	1.8682	0.3964	1.0128	0
5557	544.4	1.869	1.869	0.3965	1.013	0
5558	544.3	1.8698	1.8723	0.3967	1.0135	0
5559	544.2	1.8718	1.873	0.3967	1.0135	0
5560	544.1	1.8725	1.8763	0.3968	1.0137	0
5561	544	1.8733	1.8797	0.3969	1.0144	0
5562	543.9	1.8752	1.879	0.3968	1.0142	0
5563	543.8	1.876	1.881	0.3972	1.0141	0
5564	543.7	1.8779	1.8831	0.3973	1.0151	0
5565	543.6	1.8786	1.885	0.3973	1.0146	0
5566	543.5	1.8793	1.887	0.3974	1.0151	0
5567	543.4	1.88	1.8891	0.3974	1.0151	0
5568	543.3	1.882	1.8911	0.3977	1.0157	0
5569	543.2	1.8814	1.8918	0.3976	1.0158	0
5570	543.1	1.8833	1.8938	0.3978	1.0159	0
5571	543	1.8841	1.8973	0.3979	1.0156	0
5572	542.9	1.886	1.8979	0.3978	1.0157	0
5573	542.8	1.8852	1.8984	0.3977	1.0159	0
5574	542.7	1.8873	1.902	0.398	1.0163	0
5575	542.6	1.8892	1.9027	0.3979	1.0163	0
5576	542.5	1.8899	1.9061	0.3981	1.0169	0

5577	542.4	1.8933	1.9069	0.3982	1.0166	0
5578	542.3	1.8926	1.9089	0.3983	1.0164	0
5579	542.2	1.8945	1.911	0.3982	1.0164	0
5580	542.1	1.8953	1.9104	0.3984	1.0171	0
5581	542	1.8973	1.914	0.3985	1.017	0
5582	541.9	1.8965	1.9174	0.3984	1.017	0
5583	541.8	1.8985	1.9181	0.3985	1.0168	0
5584	541.7	1.8992	1.9203	0.3986	1.0174	0
5585	541.6	1.8997	1.9223	0.3984	1.0175	0
5586	541.5	1.9017	1.923	0.3984	1.0174	0
5587	541.4	1.9024	1.9238	0.3986	1.0172	0
5588	541.3	1.9031	1.926	0.3989	1.0178	0
5589	541.2	1.905	1.9281	0.3989	1.0177	0
5590	541.1	1.9056	1.9288	0.3989	1.0178	0
5591	541	1.9048	1.9295	0.399	1.0177	0
5592	540.9	1.9083	1.9347	0.3991	1.018	0
5593	540.8	1.9103	1.9354	0.3991	1.0181	0
5594	540.7	1.9096	1.9362	0.3992	1.0185	0
5595	540.6	1.9115	1.9399	0.3992	1.0178	0
5596	540.5	1.9108	1.9407	0.3993	1.0185	0
5597	540.4	1.9142	1.9429	0.3993	1.0183	0
5598	540.3	1.9133	1.942	0.3993	1.0185	0
5599	540.2	1.914	1.9458	0.3995	1.0185	0
5600	540.1	1.9176	1.9467	0.3996	1.0188	0
5601	540	1.9183	1.9474	0.3994	1.0185	0
5602	539.9	1.9189	1.9513	0.3994	1.0186	0
5603	539.8	1.9194	1.9519	0.3994	1.0185	0
5604	539.7	1.9201	1.9528	0.3997	1.0186	0
5605	539.6	1.9222	1.9551	0.3994	1.0183	0
5606	539.5	1.9229	1.9559	0.3995	1.0188	0
5607	539.4	1.9249	1.9582	0.3995	1.0188	0
5608	539.3	1.9256	1.9573	0.3995	1.0183	0
5609	539.2	1.9247	1.9597	0.3995	1.0187	0
5610	539.1	1.9269	1.9622	0.3996	1.0188	0
5611	539	1.9276	1.9647	0.3996	1.0188	0
5612	538.9	1.9283	1.9655	0.3995	1.0186	0
5613	538.8	1.9289	1.9663	0.3996	1.0187	0
5614	538.7	1.9296	1.9688	0.3994	1.0184	0
5615	538.6	1.9303	1.9696	0.3994	1.0183	0
5616	538.5	1.9325	1.9704	0.3993	1.0185	0
5617	538.4	1.9349	1.9731	0.3994	1.0185	0
5618	538.3	1.934	1.974	0.3995	1.0184	0
5619	538.2	1.9363	1.9749	0.3994	1.0185	0

5620	538.1	1.9371	1.9758	0.3994	1.0188	0
5621	538	1.9377	1.9783	0.3994	1.0182	0
5622	537.9	1.9385	1.9792	0.3993	1.0185	0
5623	537.8	1.9391	1.9818	0.3993	1.0185	0
5624	537.7	1.9399	1.9809	0.3994	1.0178	0
5625	537.6	1.9389	1.9836	0.3993	1.0181	0
5626	537.5	1.943	1.9864	0.3994	1.0186	0
5627	537.4	1.9437	1.9855	0.3993	1.0181	0
5628	537.3	1.9445	1.9883	0.3992	1.0183	0
5629	537.2	1.9437	1.9892	0.3992	1.0185	0
5630	537.1	1.9462	1.9903	0.3993	1.0186	0
5631	537	1.9451	1.9911	0.3993	1.0182	0
5632	536.9	1.9475	1.9957	0.3992	1.0182	0
5633	536.8	1.9467	1.9949	0.3994	1.0183	0
5634	536.7	1.9491	1.9958	0.3991	1.0181	0
5635	536.6	1.9499	1.9968	0.3993	1.0178	0
5636	536.5	1.9505	1.9977	0.3991	1.0174	0
5637	536.4	1.9515	1.9988	0.3993	1.018	0
5638	536.3	1.9522	1.9997	0.3992	1.0181	0
5639	536.2	1.9531	2.0027	0.3995	1.018	0
5640	536.1	1.9539	2.0018	0.3994	1.0183	0
5641	536	1.9548	2.0048	0.3995	1.0182	0
5642	535.9	1.9555	2.0038	0.3994	1.0184	0
5643	535.8	1.9581	2.0048	0.3994	1.0186	0
5644	535.7	1.957	2.0078	0.3994	1.0182	0
5645	535.6	1.9597	2.0089	0.3995	1.0184	0
5646	535.5	1.9605	2.0118	0.3995	1.0184	0
5647	535.4	1.9595	2.0109	0.3996	1.0186	0
5648	535.3	1.962	2.0118	0.3995	1.0188	0
5649	535.2	1.9631	2.0151	0.3996	1.0188	0
5650	535.1	1.964	2.0142	0.3998	1.0188	0
5651	535	1.9665	2.0171	0.3997	1.0184	0
5652	534.9	1.9655	2.0183	0.3999	1.0191	0
5653	534.8	1.9664	2.0173	0.3997	1.0192	0
5654	534.7	1.9655	2.0205	0.3999	1.0191	0
5655	534.6	1.9681	2.0216	0.4	1.0187	0
5656	534.5	1.9671	2.0227	0.4	1.02	0
5657	534.4	1.9679	2.0238	0.3997	1.0196	0
5658	534.3	1.9708	2.0229	0.3995	1.0195	0
5659	534.2	1.9698	2.0261	0.3999	1.0191	0
5660	534.1	1.9727	2.0231	0.4	1.0195	0
5661	534	1.9755	2.0264	0.4	1.0196	0
5662	533.9	1.9746	2.0276	0.4001	1.0197	0

5663	533.8	1.9754	2.0287	0.4001	1.0196	0
5664	533.7	1.9763	2.0277	0.4	1.0193	0
5665	533.6	1.9773	2.0311	0.4003	1.0194	0
5666	533.5	1.9782	2.0301	0.4001	1.0196	0
5667	533.4	1.9793	2.0337	0.4003	1.0198	0
5668	533.3	1.9801	2.0303	0.4002	1.0196	0
5669	533.2	1.9812	2.0339	0.4003	1.0202	0
5670	533.1	1.9802	2.035	0.4004	1.0198	0
5671	533	1.9811	2.0363	0.4004	1.0201	0
5672	532.9	1.9802	2.0354	0.4005	1.0203	0
5673	532.8	1.9833	2.039	0.4005	1.02	0
5674	532.7	1.9845	2.0382	0.4008	1.0205	0
5675	532.6	1.9856	2.0373	0.4007	1.0204	0
5676	532.5	1.9865	2.043	0.4008	1.0208	0
5677	532.4	1.9855	2.0398	0.4007	1.021	0
5678	532.3	1.9867	2.0411	0.4008	1.021	0
5679	532.2	1.9877	2.0448	0.4007	1.0209	0
5680	532.1	1.9887	2.0414	0.4008	1.0209	0
5681	532	1.992	2.0429	0.4011	1.0211	0
5682	531.9	1.9889	2.0442	0.4009	1.0203	0
5683	531.8	1.9921	2.0433	0.4009	1.0209	0
5684	531.7	1.9911	2.0446	0.401	1.0211	0
5685	531.6	1.9925	2.0462	0.4013	1.0212	0
5686	531.5	1.9935	2.0452	0.401	1.0209	0
5687	531.4	1.9947	2.0466	0.4008	1.0217	0
5688	531.3	1.996	2.0482	0.4008	1.0213	0
5689	531.2	1.995	2.0497	0.4009	1.0215	0
5690	531.1	1.9961	2.051	0.4009	1.0222	0
5691	531	1.9952	2.0526	0.4009	1.0216	0
5692	530.9	1.9964	2.0492	0.4009	1.0213	0
5693	530.8	1.9954	2.0506	0.401	1.021	0
5694	530.7	1.9966	2.0522	0.4009	1.0212	0
5695	530.6	2.0002	2.0538	0.4011	1.0213	0
5696	530.5	1.9993	2.0529	0.4012	1.0213	0
5697	530.4	1.9983	2.057	0.401	1.0215	0
5698	530.3	1.9996	2.0535	0.4012	1.022	0
5699	530.2	2.001	2.0577	0.4013	1.0221	0
5700	530.1	2.0021	2.0541	0.4013	1.022	0
5701	530	2.0035	2.0583	0.4014	1.0218	0
5702	529.9	2.0047	2.0572	0.4011	1.0214	0
5703	529.8	2.0038	2.0564	0.4013	1.0215	0
5704	529.7	2.0074	2.0579	0.4013	1.0212	0
5705	529.6	2.0042	2.057	0.4013	1.0213	0

5706	529.5	2.0055	2.0586	0.4011	1.0212	0
5707	529.4	2.0068	2.0576	0.4012	1.0212	0
5708	529.3	2.0105	2.0593	0.4013	1.0214	0
5709	529.2	2.0072	2.0584	0.4013	1.0215	0
5710	529.1	2.0086	2.0626	0.4011	1.0213	0
5711	529	2.0076	2.0616	0.4008	1.021	0
5712	528.9	2.0089	2.0659	0.4008	1.0204	0
5713	528.8	2.0105	2.0625	0.401	1.0207	0
5714	528.7	2.0096	2.0643	0.4007	1.02	0
5715	528.6	2.0111	2.0634	0.4007	1.0208	0
5716	528.5	2.0128	2.0628	0.4007	1.0204	0
5717	528.4	2.012	2.0646	0.4004	1.0203	0
5718	528.3	2.0161	2.064	0.4006	1.0199	0
5719	528.2	2.013	2.0633	0.4005	1.0195	0
5720	528.1	2.0149	2.0628	0.4003	1.0195	0
5721	528	2.0142	2.0648	0.4	1.0188	0
5722	527.9	2.016	2.0668	0.3999	1.0186	0
5723	527.8	2.0131	2.0691	0.4	1.0186	0
5724	527.7	2.015	2.0632	0.3998	1.0184	0
5725	527.6	2.0169	2.0654	0.3996	1.0184	0
5726	527.5	2.0165	2.0676	0.3995	1.0179	0
5727	527.4	2.0186	2.0674	0.3994	1.0181	0
5728	527.3	2.0182	2.0697	0.3991	1.0175	0
5729	527.2	2.0205	2.0668	0.3991	1.0178	0
5730	527.1	2.0201	2.0664	0.3991	1.0174	0
5731	527	2.0175	2.0689	0.3988	1.0172	0
5732	526.9	2.022	2.0659	0.3989	1.0172	0
5733	526.8	2.0195	2.0685	0.399	1.017	0
5734	526.7	2.0216	2.0682	0.3989	1.017	0
5735	526.6	2.019	2.0707	0.3986	1.017	0
5736	526.5	2.0237	2.0705	0.3985	1.0168	0
5737	526.4	2.0235	2.0731	0.3985	1.0164	0
5738	526.3	2.0209	2.0702	0.3985	1.0165	0
5739	526.2	2.0233	2.0729	0.3987	1.0169	0
5740	526.1	2.0207	2.0727	0.3985	1.0168	0
5741	526	2.0255	2.0726	0.3985	1.0169	0
5742	525.9	2.0253	2.0724	0.3985	1.0164	0
5743	525.8	2.0252	2.0724	0.3985	1.0171	0
5744	525.7	2.025	2.0721	0.3986	1.0166	0
5745	525.6	2.0247	2.0746	0.3985	1.0166	0
5746	525.5	2.0269	2.0716	0.3984	1.0166	0
5747	525.4	2.0268	2.0714	0.3987	1.0171	0
5748	525.3	2.0265	2.0711	0.3985	1.0168	0

5749	525.2	2.0289	2.0738	0.3986	1.017	0
5750	525.1	2.0261	2.0763	0.3987	1.017	0
5751	525	2.0311	2.0735	0.3986	1.0176	0
5752	524.9	2.0257	2.0759	0.3986	1.0173	0
5753	524.8	2.028	2.0757	0.3986	1.0176	0
5754	524.7	2.0302	2.0782	0.3986	1.0177	0
5755	524.6	2.0299	2.0779	0.3985	1.0175	0
5756	524.5	2.0271	2.0805	0.3986	1.0175	0
5757	524.4	2.0296	2.0748	0.3989	1.0176	0
5758	524.3	2.032	2.0746	0.399	1.018	0
5759	524.2	2.0317	2.0771	0.3991	1.0179	0
5760	524.1	2.0312	2.0739	0.399	1.018	0
5761	524	2.0311	2.0794	0.3991	1.0185	0
5762	523.9	2.0334	2.0763	0.3992	1.0184	0
5763	523.8	2.0331	2.0788	0.3991	1.0185	0
5764	523.7	2.033	2.0816	0.3994	1.0189	0
5765	523.6	2.0326	2.0784	0.3993	1.0188	0
5766	523.5	2.0349	2.081	0.3994	1.019	0
5767	523.4	2.0346	2.0807	0.3994	1.0187	0
5768	523.3	2.0369	2.0775	0.3995	1.0187	0
5769	523.2	2.0367	2.0801	0.3995	1.0189	0
5770	523.1	2.0364	2.0798	0.3996	1.0192	0
5771	523	2.0362	2.0796	0.3995	1.0194	0
5772	522.9	2.0384	2.0822	0.3996	1.0196	0
5773	522.8	2.0356	2.0791	0.3999	1.0196	0
5774	522.7	2.0378	2.0815	0.3998	1.0195	0
5775	522.6	2.0376	2.0813	0.3998	1.02	0
5776	522.5	2.0373	2.081	0.3998	1.0197	0
5777	522.4	2.037	2.0836	0.3998	1.0196	0
5778	522.3	2.0366	2.0832	0.4	1.0195	0
5779	522.2	2.0389	2.0858	0.3999	1.0197	0
5780	522.1	2.0386	2.0885	0.3997	1.0197	0
5781	522	2.041	2.0824	0.4	1.0194	0
5782	521.9	2.0406	2.0849	0.3998	1.0195	0
5783	521.8	2.0402	2.0845	0.4001	1.0197	0
5784	521.7	2.0426	2.0842	0.4	1.0204	0
5785	521.6	2.0395	2.0839	0.3999	1.0202	0
5786	521.5	2.0394	2.0866	0.4003	1.0201	0
5787	521.4	2.0416	2.0892	0.4002	1.0202	0
5788	521.3	2.044	2.0859	0.4	1.0201	0
5789	521.2	2.0436	2.0856	0.4	1.0203	0
5790	521.1	2.046	2.0852	0.3999	1.0199	0
5791	521	2.0456	2.0878	0.3999	1.0204	0

5792	520.9	2.0425	2.0874	0.3995	1.0204	0
5793	520.8	2.0476	2.0871	0.3997	1.0197	0
5794	520.7	2.0446	2.0897	0.3997	1.0201	0
5795	520.6	2.0442	2.0894	0.3997	1.02	0
5796	520.5	2.0466	2.0891	0.3997	1.02	0
5797	520.4	2.0463	2.0918	0.4	1.0204	0
5798	520.3	2.046	2.0885	0.3999	1.0201	0
5799	520.2	2.0483	2.091	0.3999	1.0204	0
5800	520.1	2.0479	2.0877	0.3997	1.0203	0
5801	520	2.0476	2.0904	0.3996	1.0203	0
5802	519.9	2.05	2.093	0.3996	1.0202	0
5803	519.8	2.0496	2.0957	0.3999	1.0201	0
5804	519.7	2.0467	2.0924	0.4	1.0203	0
5805	519.6	2.049	2.089	0.3998	1.0202	0
5806	519.5	2.0513	2.0946	0.3997	1.0206	0
5807	519.4	2.0483	2.0943	0.3998	1.0205	0
5808	519.3	2.0506	2.097	0.3997	1.0201	0
5809	519.2	2.0502	2.0935	0.3997	1.0202	0
5810	519.1	2.05	2.0963	0.3999	1.021	0
5811	519	2.0524	2.0929	0.3999	1.0206	0
5812	518.9	2.0492	2.0956	0.3997	1.0202	0
5813	518.8	2.0489	2.0983	0.3999	1.0204	0
5814	518.7	2.0541	2.0981	0.4	1.0202	0
5815	518.6	2.0509	2.0945	0.3999	1.0205	0
5816	518.5	2.0534	2.0973	0.3999	1.0205	0
5817	518.4	2.053	2.1	0.3997	1.0203	0
5818	518.3	2.0554	2.0965	0.3997	1.0204	0
5819	518.2	2.0551	2.0993	0.3997	1.0203	0
5820	518.1	2.0519	2.102	0.3996	1.02	0
5821	518	2.0514	2.0984	0.3996	1.0208	0
5822	517.9	2.0511	2.0981	0.3995	1.0202	0
5823	517.8	2.0564	2.1009	0.3997	1.0204	0
5824	517.7	2.0561	2.1006	0.3997	1.0204	0
5825	517.6	2.0557	2.1033	0.3995	1.0205	0
5826	517.5	2.0553	2.0998	0.3996	1.0201	0
5827	517.4	2.0579	2.0996	0.3998	1.0207	0
5828	517.3	2.0576	2.0993	0.3998	1.0209	0
5829	517.2	2.0572	2.0989	0.3997	1.0205	0
5830	517.1	2.054	2.1017	0.3997	1.0206	0
5831	517	2.0593	2.1045	0.3997	1.0206	0
5832	516.9	2.0591	2.1042	0.3999	1.0206	0
5833	516.8	2.0559	2.1039	0.3998	1.021	0
5834	516.7	2.0585	2.1036	0.3999	1.021	0

5835	516.6	2.061	2.1033	0.3998	1.0206	0
5836	516.5	2.0606	2.1029	0.3996	1.0211	0
5837	516.4	2.0603	2.1057	0.3998	1.0213	0
5838	516.3	2.0571	2.1054	0.3998	1.0209	0
5839	516.2	2.0625	2.105	0.3998	1.0208	0
5840	516.1	2.0621	2.1046	0.3997	1.0207	0
5841	516	2.0617	2.1075	0.3997	1.0214	0
5842	515.9	2.0615	2.1072	0.3999	1.0217	0
5843	515.8	2.064	2.1101	0.3998	1.0218	0
5844	515.7	2.0607	2.1097	0.3998	1.0212	0
5845	515.6	2.0634	2.1095	0.3999	1.0209	0
5846	515.5	2.0631	2.1091	0.3999	1.0206	0
5847	515.4	2.0626	2.1119	0.3998	1.0209	0
5848	515.3	2.0651	2.1083	0.3998	1.0211	0
5849	515.2	2.062	2.1081	0.4002	1.0209	0
5850	515.1	2.0616	2.1109	0.3999	1.0215	0
5851	515	2.0642	2.1106	0.3999	1.0215	0
5852	514.9	2.064	2.1137	0.4	1.0218	0
5853	514.8	2.0665	2.1133	0.4	1.0214	0
5854	514.7	2.0662	2.1129	0.3998	1.0216	0
5855	514.6	2.063	2.1127	0.4001	1.0213	0
5856	514.5	2.0686	2.1157	0.4	1.0216	0
5857	514.4	2.0681	2.1186	0.4001	1.0225	0
5858	514.3	2.0678	2.1149	0.4	1.0216	0
5859	514.2	2.0706	2.1114	0.4001	1.0219	0
5860	514.1	2.0702	2.1143	0.4002	1.0221	0
5861	514	2.0729	2.1174	0.4002	1.0223	0
5862	513.9	2.0696	2.1203	0.4001	1.0222	0
5863	513.8	2.0723	2.1167	0.4002	1.0225	0
5864	513.7	2.0719	2.1163	0.4001	1.0226	0
5865	513.6	2.0716	2.116	0.4002	1.022	0
5866	513.5	2.0742	2.119	0.4002	1.0224	0
5867	513.4	2.0708	2.1186	0.4002	1.0221	0
5868	513.3	2.0736	2.1218	0.4002	1.0224	0
5869	513.2	2.0733	2.1181	0.4005	1.0227	0
5870	513.1	2.0729	2.1245	0.4004	1.0226	0
5871	513	2.0727	2.1209	0.4006	1.0223	0
5872	512.9	2.0754	2.1239	0.4004	1.023	0
5873	512.8	2.0752	2.1236	0.4005	1.0233	0
5874	512.7	2.0779	2.1267	0.4007	1.0233	0
5875	512.6	2.0774	2.1263	0.4006	1.0225	0
5876	512.5	2.0772	2.1226	0.4006	1.0225	0
5877	512.4	2.0769	2.1257	0.4006	1.0238	0

5878	512.3	2.0795	2.1253	0.4005	1.0229	0
5879	512.2	2.0792	2.1249	0.4005	1.0228	0
5880	512.1	2.082	2.1246	0.4006	1.0231	0
5881	512	2.0814	2.1275	0.4004	1.023	0
5882	511.9	2.0812	2.1273	0.4006	1.0231	0
5883	511.8	2.0777	2.127	0.4006	1.023	0
5884	511.7	2.0836	2.1301	0.4005	1.0226	0
5885	511.6	2.0801	2.1297	0.4004	1.0231	0
5886	511.5	2.0829	2.1294	0.4004	1.023	0
5887	511.4	2.0795	2.1291	0.4006	1.0233	0
5888	511.3	2.0823	2.1252	0.4005	1.0232	0
5889	511.2	2.0818	2.1248	0.4005	1.023	0
5890	511.1	2.0878	2.1279	0.4004	1.0227	0
5891	511	2.0874	2.1275	0.4003	1.0231	0
5892	510.9	2.0839	2.1307	0.4004	1.0234	0
5893	510.8	2.0835	2.1303	0.4003	1.0232	0
5894	510.7	2.0832	2.13	0.4005	1.0232	0
5895	510.6	2.086	2.1332	0.4002	1.0229	0
5896	510.5	2.0856	2.1328	0.4002	1.0224	0
5897	510.4	2.0883	2.1323	0.4	1.0225	0
5898	510.3	2.088	2.1356	0.4004	1.0233	0
5899	510.2	2.0877	2.1316	0.3999	1.023	0
5900	510.1	2.0842	2.135	0.4001	1.0227	0
5901	510	2.0869	2.1381	0.4001	1.0228	0
5902	509.9	2.09	2.1379	0.4001	1.0223	0
5903	509.8	2.0928	2.1338	0.3999	1.023	0
5904	509.7	2.0892	2.1372	0.4	1.0224	0
5905	509.6	2.0888	2.1367	0.4001	1.0225	0
5906	509.5	2.0948	2.1362	0.3997	1.0228	0
5907	509.4	2.0944	2.1358	0.4	1.0222	0
5908	509.3	2.094	2.1391	0.3999	1.0218	0
5909	509.2	2.0903	2.1386	0.3998	1.0225	0
5910	509.1	2.0933	2.142	0.3998	1.0222	0
5911	509	2.0929	2.1343	0.3998	1.022	0
5912	508.9	2.0926	2.1413	0.3997	1.0223	0
5913	508.8	2.0956	2.1447	0.3996	1.0225	0
5914	508.7	2.0984	2.1404	0.3999	1.0223	0
5915	508.6	2.0947	2.1438	0.3997	1.0222	0
5916	508.5	2.0977	2.1435	0.3996	1.0225	0
5917	508.4	2.0973	2.1431	0.3997	1.0229	0
5918	508.3	2.1003	2.1389	0.3997	1.0228	0
5919	508.2	2.0999	2.1423	0.3994	1.0218	0
5920	508.1	2.0996	2.1421	0.3997	1.0219	0

5921	508	2.1025	2.149	0.3996	1.0225	0
5922	507.9	2.1023	2.145	0.3995	1.0225	0
5923	507.8	2.0987	2.141	0.3997	1.0228	0
5924	507.7	2.105	2.1481	0.3997	1.023	0
5925	507.6	2.1046	2.1477	0.3998	1.0226	0
5926	507.5	2.1077	2.1474	0.3997	1.0226	0
5927	507.4	2.1073	2.147	0.3996	1.0227	0
5928	507.3	2.107	2.1467	0.3996	1.0224	0
5929	507.2	2.1033	2.1502	0.3998	1.0226	0
5930	507.1	2.1098	2.1537	0.3997	1.0229	0
5931	507	2.106	2.1494	0.3996	1.0228	0
5932	506.9	2.109	2.1528	0.3994	1.0229	0
5933	506.8	2.1086	2.1486	0.3997	1.0225	0
5934	506.7	2.1118	2.1522	0.3999	1.0231	0
5935	506.6	2.1081	2.1558	0.4	1.0231	0
5936	506.5	2.1147	2.1553	0.4	1.0233	0
5937	506.4	2.1109	2.1551	0.4001	1.0233	0
5938	506.3	2.114	2.1547	0.4001	1.0235	0
5939	506.2	2.1101	2.1582	0.4	1.0234	0
5940	506.1	2.1098	2.1618	0.4001	1.0234	0
5941	506	2.1129	2.1575	0.4002	1.0236	0
5942	505.9	2.1162	2.1573	0.4003	1.0242	0
5943	505.8	2.1123	2.1608	0.4002	1.0238	0
5944	505.7	2.1155	2.1565	0.4003	1.0243	0
5945	505.6	2.1151	2.1601	0.4005	1.024	0
5946	505.5	2.1184	2.1598	0.4007	1.0237	0
5947	505.4	2.1145	2.1595	0.4005	1.0242	0
5948	505.3	2.1177	2.1591	0.4009	1.0247	0
5949	505.2	2.121	2.1588	0.4008	1.0247	0
5950	505.1	2.1208	2.1625	0.401	1.0248	0
5951	505	2.124	2.1581	0.4008	1.0243	0
5952	504.9	2.12	2.1618	0.401	1.0254	0
5953	504.8	2.1196	2.1613	0.4009	1.025	0
5954	504.7	2.123	2.1611	0.4009	1.0256	0
5955	504.6	2.1225	2.1606	0.4007	1.0252	0
5956	504.5	2.1259	2.1643	0.401	1.0254	0
5957	504.4	2.1219	2.164	0.4011	1.0251	0
5958	504.3	2.1252	2.1637	0.4012	1.0254	0
5959	504.2	2.1249	2.1634	0.4012	1.0256	0
5960	504.1	2.1282	2.167	0.4012	1.0261	0
5961	504	2.1243	2.1668	0.4015	1.0259	0
5962	503.9	2.124	2.1706	0.4016	1.0262	0
5963	503.8	2.1274	2.1662	0.4015	1.0265	0

5964	503.7	2.127	2.1618	0.4017	1.0267	0
5965	503.6	2.1304	2.1654	0.4016	1.0266	0
5966	503.5	2.1262	2.165	0.4017	1.0265	0
5967	503.4	2.1298	2.1648	0.4017	1.0266	0
5968	503.3	2.1294	2.1645	0.4017	1.0269	0
5969	503.2	2.1328	2.1682	0.4015	1.0268	0
5970	503.1	2.1324	2.1637	0.4016	1.0264	0
5971	503	2.1322	2.1676	0.4017	1.0268	0
5972	502.9	2.1356	2.1713	0.4018	1.0266	0
5973	502.8	2.1352	2.1709	0.4018	1.0271	0
5974	502.7	2.135	2.1665	0.4018	1.0275	0
5975	502.6	2.1345	2.1702	0.4018	1.0271	0
5976	502.5	2.1341	2.174	0.4016	1.0269	0
5977	502.4	2.1338	2.1695	0.4017	1.0267	0
5978	502.3	2.1335	2.1692	0.4016	1.0275	0
5979	502.2	2.1369	2.1688	0.4017	1.0272	0
5980	502.1	2.1367	2.1727	0.4018	1.0272	0
5981	502	2.1402	2.1683	0.4017	1.0275	0
5982	501.9	2.14	2.1722	0.4019	1.0275	0
5983	501.8	2.1396	2.1676	0.4017	1.0277	0
5984	501.7	2.1431	2.1714	0.4018	1.0271	0
5985	501.6	2.1428	2.1711	0.402	1.0274	0
5986	501.5	2.1464	2.1708	0.4016	1.027	0
5987	501.4	2.1421	2.1704	0.4017	1.0273	0
5988	501.3	2.1456	2.1741	0.4017	1.0268	0
5989	501.2	2.1453	2.1697	0.4017	1.0272	0
5990	501.1	2.145	2.1736	0.4016	1.0275	0
5991	501	2.1487	2.1733	0.4017	1.0275	0
5992	500.9	2.1483	2.173	0.4016	1.0271	0
5993	500.8	2.1481	2.1812	0.4016	1.0271	0
5994	500.7	2.1478	2.1766	0.4016	1.0274	0
5995	500.6	2.1513	2.1719	0.4015	1.0273	0
5996	500.5	2.147	2.1758	0.4014	1.0269	0
5997	500.4	2.1505	2.1754	0.4014	1.0268	0
5998	500.3	2.1502	2.1708	0.4014	1.0271	0
5999	500.2	2.1539	2.1789	0.4013	1.0267	0
6000	500.1	2.1536	2.1787	0.4012	1.027	0
6001	500	2.1492	2.1783	0.4011	1.0267	0
6002	499.9	2.1487	2.1735	0.4011	1.0265	0
6003	499.8	2.1526	2.1733	0.4011	1.0266	0
6004	499.7	2.1563	2.1773	0.4012	1.0266	0
6005	499.6	2.1517	2.1768	0.4009	1.0264	0
6006	499.5	2.1555	2.1808	0.4008	1.0261	0

6007	499.4	2.1592	2.1761	0.4007	1.0266	0
6008	499.3	2.1547	2.1799	0.4006	1.0264	0
6009	499.2	2.1585	2.1753	0.4006	1.0258	0
6010	499.1	2.1581	2.1793	0.4005	1.0261	0
6011	499	2.1577	2.1789	0.4004	1.026	0
6012	498.9	2.1615	2.1742	0.4005	1.026	0
6013	498.8	2.157	2.1826	0.4005	1.0259	0
6014	498.7	2.1608	2.1778	0.4004	1.0258	0
6015	498.6	2.1646	2.1774	0.4006	1.0258	0
6016	498.5	2.16	2.177	0.4003	1.0253	0
6017	498.4	2.1641	2.1812	0.4005	1.0252	0
6018	498.3	2.1635	2.1764	0.4004	1.0257	0
6019	498.2	2.1674	2.1804	0.4003	1.0252	0
6020	498.1	2.1627	2.1799	0.4001	1.0254	0
6021	498	2.1624	2.1796	0.4	1.0251	0
6022	497.9	2.1663	2.1792	0.4	1.0253	0
6023	497.8	2.1702	2.1833	0.4002	1.0256	0
6024	497.7	2.1697	2.1828	0.3999	1.0257	0
6025	497.6	2.1695	2.1826	0.4002	1.0255	0
6026	497.5	2.169	2.1821	0.4001	1.0257	0
6027	497.4	2.173	2.1862	0.4	1.0259	0
6028	497.3	2.1726	2.1814	0.4001	1.0258	0
6029	497.2	2.1679	2.181	0.4001	1.0252	0
6030	497.1	2.1719	2.1851	0.4002	1.0251	0
6031	497	2.1758	2.1847	0.4	1.0253	0
6032	496.9	2.171	2.1798	0.4001	1.0252	0
6033	496.8	2.1751	2.184	0.4001	1.0255	0
6034	496.7	2.1746	2.1835	0.4001	1.0257	0
6035	496.6	2.1744	2.1788	0.4003	1.0261	0
6036	496.5	2.1829	2.1829	0.4003	1.0263	0
6037	496.4	2.178	2.187	0.4002	1.0262	0
6038	496.3	2.1777	2.1822	0.4004	1.0262	0
6039	496.2	2.1818	2.1863	0.4003	1.0261	0
6040	496.1	2.186	2.1815	0.4004	1.0262	0
6041	496	2.1855	2.1766	0.4003	1.0263	0
6042	495.9	2.1854	2.1809	0.4008	1.0265	0
6043	495.8	2.1896	2.185	0.4009	1.0268	0
6044	495.7	2.1891	2.1801	0.4009	1.0273	0
6045	495.6	2.1888	2.1843	0.4009	1.0273	0
6046	495.5	2.1885	2.184	0.4012	1.0279	0
6047	495.4	2.1882	2.1882	0.401	1.0279	0
6048	495.3	2.1878	2.1878	0.4012	1.0284	0
6049	495.2	2.1874	2.1829	0.4014	1.0284	0

6050	495.1	2.187	2.1824	0.4013	1.0283	0
6051	495	2.1912	2.1866	0.4015	1.0282	0
6052	494.9	2.1908	2.1908	0.4016	1.0285	0
6053	494.8	2.1952	2.1814	0.4015	1.0285	0
6054	494.7	2.1996	2.1811	0.4019	1.0289	0
6055	494.6	2.1945	2.1807	0.4018	1.0291	0
6056	494.5	2.1941	2.1849	0.4017	1.0291	0
6057	494.4	2.1985	2.1846	0.4018	1.0294	0
6058	494.3	2.1981	2.1888	0.4021	1.0294	0
6059	494.2	2.2024	2.1884	0.4022	1.0295	0
6060	494.1	2.2021	2.1834	0.4022	1.0298	0
6061	494	2.2066	2.1832	0.4022	1.0296	0
6062	493.9	2.2015	2.1828	0.4024	1.0299	0
6063	493.8	2.2012	2.1871	0.4028	1.0299	0
6064	493.7	2.2008	2.1914	0.4027	1.0305	0
6065	493.6	2.2004	2.1817	0.4028	1.0307	0
6066	493.5	2.2049	2.186	0.4027	1.0311	0
6067	493.4	2.2045	2.1856	0.4027	1.0313	0
6068	493.3	2.2042	2.1807	0.4028	1.0307	0
6069	493.2	2.2088	2.1851	0.4031	1.0311	0
6070	493.1	2.2036	2.1894	0.4031	1.0314	0
6071	493	2.2079	2.1888	0.403	1.0312	0
6072	492.9	2.2077	2.1886	0.4032	1.0319	0
6073	492.8	2.2122	2.1882	0.4029	1.0318	0
6074	492.7	2.2118	2.1831	0.403	1.0317	0
6075	492.6	2.2165	2.1829	0.4032	1.0322	0
6076	492.5	2.2112	2.1872	0.4033	1.0322	0
6077	492.4	2.2157	2.1868	0.4035	1.0321	0
6078	492.3	2.2153	2.1817	0.4031	1.0313	0
6079	492.2	2.215	2.186	0.4031	1.0319	0
6080	492.1	2.2197	2.1858	0.4033	1.032	0
6081	492	2.2193	2.1854	0.4033	1.0326	0
6082	491.9	2.2139	2.1849	0.4033	1.0325	0
6083	491.8	2.2187	2.1894	0.4034	1.0323	0
6084	491.7	2.2182	2.1937	0.4034	1.0325	0
6085	491.6	2.223	2.1886	0.4034	1.0322	0
6086	491.5	2.2175	2.1836	0.4035	1.0321	0
6087	491.4	2.2172	2.1879	0.4034	1.0325	0
6088	491.3	2.227	2.1923	0.4032	1.0321	0
6089	491.2	2.2267	2.1919	0.4033	1.032	0
6090	491.1	2.2264	2.1868	0.4033	1.032	0
6091	491	2.2261	2.1913	0.4034	1.0324	0
6092	490.9	2.2257	2.1862	0.4034	1.0327	0

6093	490.8	2.2253	2.1858	0.4033	1.0327	0
6094	490.7	2.225	2.1902	0.4031	1.033	0
6095	490.6	2.2297	2.185	0.4029	1.0325	0
6096	490.5	2.2346	2.1847	0.4031	1.0322	0
6097	490.4	2.229	2.189	0.4031	1.0318	0
6098	490.3	2.2288	2.1936	0.4034	1.0323	0
6099	490.2	2.2336	2.1884	0.403	1.0321	0
6100	490.1	2.228	2.1928	0.4028	1.0318	0
6101	490	2.2381	2.1876	0.403	1.0323	0
6102	489.9	2.2323	2.1871	0.4026	1.0315	0
6103	489.8	2.2426	2.1819	0.4027	1.0318	0
6104	489.7	2.2317	2.1864	0.4027	1.0315	0
6105	489.6	2.2365	2.1859	0.4024	1.031	0
6106	489.5	2.2416	2.1905	0.4024	1.0315	0
6107	489.4	2.2359	2.1901	0.4023	1.0314	0
6108	489.3	2.2409	2.1849	0.4022	1.031	0
6109	489.2	2.2406	2.1894	0.4024	1.0314	0
6110	489.1	2.2403	2.1892	0.4022	1.0315	0
6111	489	2.2453	2.1887	0.4024	1.0317	0
6112	488.9	2.2449	2.1883	0.4022	1.0309	0
6113	488.8	2.2501	2.188	0.4022	1.0306	0
6114	488.7	2.2442	2.1925	0.402	1.0306	0
6115	488.6	2.2439	2.1873	0.4022	1.0306	0
6116	488.5	2.249	2.1868	0.4018	1.0304	0
6117	488.4	2.2484	2.1912	0.4019	1.031	0
6118	488.3	2.2483	2.1861	0.4021	1.0304	0
6119	488.2	2.2477	2.1904	0.4018	1.0303	0
6120	488.1	2.253	2.19	0.4018	1.0302	0
6121	488	2.247	2.1897	0.4017	1.0298	0
6122	487.9	2.2466	2.1893	0.4015	1.0301	0
6123	487.8	2.252	2.194	0.4017	1.0302	0
6124	487.7	2.2515	2.1886	0.4016	1.0295	0
6125	487.6	2.251	2.1882	0.4015	1.0297	0
6126	487.5	2.2506	2.1876	0.401	1.0295	0
6127	487.4	2.2559	2.1873	0.4011	1.0295	0
6128	487.3	2.2554	2.1869	0.401	1.0294	0
6129	487.2	2.2551	2.1914	0.401	1.0297	0
6130	487.1	2.2547	2.196	0.401	1.0297	0
6131	487	2.2545	2.1908	0.4011	1.0298	0
6132	486.9	2.2539	2.1902	0.4012	1.0296	0
6133	486.8	2.2537	2.19	0.4012	1.0297	0
6134	486.7	2.259	2.1946	0.4011	1.0299	0
6135	486.6	2.2646	2.1894	0.4013	1.0301	0

6136	486.5	2.2641	2.1938	0.4011	1.0295	0
6137	486.4	2.2581	2.1886	0.4013	1.0297	0
6138	486.3	2.2576	2.1931	0.4012	1.0296	0
6139	486.2	2.2574	2.1879	0.4012	1.03	0
6140	486.1	2.2628	2.1925	0.4012	1.0296	0
6141	486	2.2624	2.1872	0.4012	1.0299	0
6142	485.9	2.262	2.1917	0.4012	1.0298	0
6143	485.8	2.2615	2.1863	0.4012	1.0304	0
6144	485.7	2.2614	2.191	0.4013	1.0302	0
6145	485.6	2.261	2.1907	0.4015	1.0305	0
6146	485.5	2.2606	2.1953	0.4014	1.0308	0
6147	485.4	2.2662	2.195	0.4018	1.0305	0
6148	485.3	2.2716	2.1945	0.4016	1.0307	0
6149	485.2	2.2654	2.1942	0.4017	1.0304	0
6150	485.1	2.2709	2.1938	0.4019	1.031	0
6151	485	2.2645	2.1933	0.4017	1.032	0
6152	484.9	2.2762	2.1929	0.4017	1.0312	0
6153	484.8	2.2698	2.1977	0.4019	1.0316	0
6154	484.7	2.2634	2.1922	0.4019	1.0319	0
6155	484.6	2.2691	2.1919	0.402	1.0316	0
6156	484.5	2.2687	2.1966	0.4021	1.0312	0
6157	484.4	2.2744	2.1963	0.4021	1.0316	0
6158	484.3	2.2681	2.196	0.4025	1.0323	0
6159	484.2	2.2738	2.1956	0.4024	1.0323	0
6160	484.1	2.2796	2.1952	0.4027	1.0327	0
6161	484	2.2731	2.1949	0.4026	1.033	0
6162	483.9	2.2788	2.1945	0.4029	1.0333	0
6163	483.8	2.2724	2.1891	0.403	1.0337	0
6164	483.7	2.2781	2.1989	0.4031	1.0333	0
6165	483.6	2.2778	2.1935	0.4031	1.0341	0
6166	483.5	2.2836	2.193	0.403	1.0336	0
6167	483.4	2.277	2.1927	0.4033	1.0336	0
6168	483.3	2.2767	2.1924	0.4035	1.0337	0
6169	483.2	2.2764	2.2024	0.4035	1.034	0
6170	483.1	2.2885	2.202	0.4035	1.034	0
6171	483	2.2756	2.2016	0.4036	1.0343	0
6172	482.9	2.2814	2.196	0.4033	1.0346	0
6173	482.8	2.2874	2.2009	0.4034	1.0346	0
6174	482.7	2.2807	2.2005	0.4036	1.0346	0
6175	482.6	2.2803	2.1949	0.4037	1.0346	0
6176	482.5	2.2863	2.1997	0.4037	1.0349	0
6177	482.4	2.2859	2.1941	0.404	1.0349	0
6178	482.3	2.2793	2.1991	0.404	1.0358	0

6179	482.2	2.2788	2.1985	0.4039	1.0352	0
6180	482.1	2.2847	2.1982	0.4041	1.0352	0
6181	482	2.2844	2.1978	0.4039	1.0359	0
6182	481.9	2.2841	2.2028	0.404	1.0353	0
6183	481.8	2.2901	2.197	0.404	1.0358	0
6184	481.7	2.2832	2.202	0.404	1.0359	0
6185	481.6	2.2829	2.1963	0.404	1.0359	0
6186	481.5	2.2825	2.2012	0.4039	1.0358	0
6187	481.4	2.2887	2.1957	0.4042	1.0359	0
6188	481.3	2.2818	2.2059	0.4043	1.0363	0
6189	481.2	2.2878	2.2054	0.4041	1.0361	0
6190	481.1	2.2874	2.1997	0.4044	1.0357	0
6191	481	2.2869	2.2045	0.4042	1.0364	0
6192	480.9	2.2932	2.2096	0.4042	1.0361	0
6193	480.8	2.2928	2.2038	0.4043	1.036	0
6194	480.7	2.2924	2.2034	0.4042	1.0364	0
6195	480.6	2.2854	2.1977	0.404	1.0363	0
6196	480.5	2.2916	2.208	0.4041	1.0367	0
6197	480.4	2.2911	2.2075	0.4039	1.0361	0
6198	480.3	2.2974	2.2018	0.4043	1.0358	0
6199	480.2	2.2838	2.2123	0.4042	1.0366	0
6200	480.1	2.29	2.2064	0.4042	1.0373	0
6201	480	2.2895	2.206	0.4042	1.0365	0
6202	479.9	2.2891	2.2056	0.4041	1.0364	0
6203	479.8	2.2888	2.2052	0.4042	1.0364	0
6204	479.7	2.295	2.1994	0.4041	1.036	0
6205	479.6	2.2946	2.2045	0.404	1.0368	0
6206	479.5	2.2941	2.2093	0.4037	1.0358	0
6207	479.4	2.2936	2.2034	0.4037	1.0357	0
6208	479.3	2.2934	2.2142	0.4039	1.0362	0
6209	479.2	2.2929	2.2082	0.4038	1.0362	0
6210	479.1	2.2993	2.2078	0.4039	1.0358	0
6211	479	2.2922	2.213	0.4038	1.0358	0
6212	478.9	2.2917	2.2126	0.4037	1.0358	0
6213	478.8	2.2847	2.2123	0.4038	1.0358	0
6214	478.7	2.2977	2.2117	0.4034	1.0364	0
6215	478.6	2.2905	2.2113	0.4033	1.0348	0
6216	478.5	2.3036	2.2108	0.4032	1.0344	0
6217	478.4	2.2965	2.2106	0.4033	1.0352	0
6218	478.3	2.2959	2.21	0.4032	1.0351	0
6219	478.2	2.3024	2.2152	0.403	1.035	0
6220	478.1	2.3022	2.215	0.4031	1.0352	0
6221	478	2.2947	2.2144	0.403	1.0346	0

6222	477.9	2.3014	2.2086	0.4029	1.0347	0
6223	477.8	2.2941	2.2137	0.403	1.0347	0
6224	477.7	2.2935	2.2189	0.4028	1.0345	0
6225	477.6	2.3	2.2128	0.4027	1.0349	0
6226	477.5	2.2996	2.2124	0.4029	1.0341	0
6227	477.4	2.2993	2.2122	0.4027	1.0346	0
6228	477.3	2.299	2.2118	0.4028	1.0346	0
6229	477.2	2.2986	2.2114	0.4028	1.0342	0
6230	477.1	2.2982	2.2167	0.4029	1.0342	0
6231	477	2.2977	2.2162	0.4029	1.0345	0
6232	476.9	2.3043	2.2159	0.403	1.0345	0
6233	476.8	2.2969	2.2212	0.4028	1.0344	0
6234	476.7	2.3035	2.215	0.4027	1.0352	0
6235	476.6	2.2962	2.2147	0.4026	1.0344	0
6236	476.5	2.3028	2.2143	0.4026	1.0344	0
6237	476.4	2.3022	2.2139	0.4027	1.0344	0
6238	476.3	2.2951	2.2136	0.4027	1.0345	0
6239	476.2	2.2946	2.219	0.4028	1.0348	0
6240	476.1	2.3083	2.2186	0.4028	1.0345	0
6241	476	2.3009	2.2182	0.4028	1.0348	0
6242	475.9	2.3074	2.2177	0.4026	1.0347	0
6243	475.8	2.3	2.2173	0.4027	1.0344	0
6244	475.7	2.2997	2.2229	0.4028	1.0348	0
6245	475.6	2.2992	2.2224	0.4027	1.0351	0
6246	475.5	2.2987	2.216	0.4027	1.0346	0
6247	475.4	2.3054	2.2215	0.4027	1.0354	0
6248	475.3	2.298	2.2153	0.403	1.035	0
6249	475.2	2.3047	2.2208	0.4028	1.0354	0
6250	475.1	2.3043	2.2146	0.4031	1.0355	0
6251	475	2.311	2.22	0.4033	1.0357	0
6252	474.9	2.3035	2.2195	0.4031	1.0353	0
6253	474.8	2.3029	2.2191	0.4031	1.0356	0
6254	474.7	2.3098	2.2246	0.4031	1.0356	0
6255	474.6	2.3022	2.2242	0.403	1.036	0
6256	474.5	2.3019	2.2299	0.4032	1.0364	0
6257	474.4	2.3086	2.2234	0.4032	1.0364	0
6258	474.3	2.3081	2.2229	0.403	1.0359	0
6259	474.2	2.3005	2.2226	0.4034	1.0363	0
6260	474.1	2.3002	2.2163	0.4034	1.0359	0
6261	474	2.3071	2.2279	0.4037	1.0368	0
6262	473.9	2.2994	2.2275	0.4038	1.0363	0
6263	473.8	2.3061	2.221	0.4038	1.0366	0
6264	473.7	2.3057	2.2265	0.4038	1.0366	0

6265	473.6	2.3051	2.22	0.4038	1.0368	0
6266	473.5	2.2976	2.2316	0.404	1.0371	0
6267	473.4	2.2971	2.2252	0.4039	1.0376	0
6268	473.3	2.3039	2.2308	0.404	1.0371	0
6269	473.2	2.3034	2.2242	0.404	1.037	0
6270	473.1	2.3029	2.2177	0.4039	1.0373	0
6271	473	2.3025	2.2294	0.4039	1.0377	0
6272	472.9	2.3096	2.2292	0.4041	1.0379	0
6273	472.8	2.3091	2.2349	0.4044	1.0385	0
6274	472.7	2.3086	2.2282	0.4044	1.038	0
6275	472.6	2.3081	2.2339	0.4044	1.0384	0
6276	472.5	2.3076	2.2272	0.4042	1.0386	0
6277	472.4	2.3071	2.2391	0.4043	1.0381	0
6278	472.3	2.3067	2.2325	0.4044	1.0394	0
6279	472.2	2.2988	2.2319	0.4041	1.0387	0
6280	472.1	2.3058	2.2316	0.4045	1.0389	0
6281	472	2.3054	2.2311	0.4044	1.0384	0
6282	471.9	2.305	2.2307	0.4045	1.0387	0
6283	471.8	2.3045	2.2303	0.4047	1.0387	0
6284	471.7	2.3039	2.2359	0.4044	1.0386	0
6285	471.6	2.2962	2.2355	0.4047	1.0381	0
6286	471.5	2.3031	2.2289	0.4049	1.039	0
6287	471.4	2.3029	2.2349	0.405	1.0391	0
6288	471.3	2.3096	2.2343	0.4048	1.0393	0
6289	471.2	2.3017	2.2465	0.4048	1.0387	0
6290	471.1	2.3087	2.2333	0.4048	1.0395	0
6291	471	2.3082	2.2328	0.405	1.0394	0
6292	470.9	2.3003	2.2323	0.4048	1.0393	0
6293	470.8	2.2998	2.2319	0.4049	1.0397	0
6294	470.7	2.3068	2.2377	0.4052	1.04	0
6295	470.6	2.2989	2.2372	0.4053	1.0403	0
6296	470.5	2.3058	2.2368	0.4051	1.0399	0
6297	470.4	2.3128	2.2361	0.4052	1.0401	0
6298	470.3	2.3047	2.2421	0.4052	1.0399	0
6299	470.2	2.3042	2.2415	0.4055	1.0399	0
6300	470.1	2.3037	2.2411	0.4053	1.0398	0
6301	470	2.3032	2.2406	0.4051	1.0401	0
6302	469.9	2.3029	2.2402	0.4051	1.0401	0
6303	469.8	2.3022	2.2396	0.4053	1.0403	0
6304	469.7	2.317	2.2391	0.4052	1.0399	0
6305	469.6	2.3012	2.245	0.405	1.0397	0
6306	469.5	2.3008	2.2446	0.405	1.0397	0
6307	469.4	2.3078	2.2441	0.405	1.0404	0

6308	469.3	2.3072	2.2436	0.405	1.0403	0
6309	469.2	2.3068	2.2431	0.4051	1.0398	0
6310	469.1	2.3063	2.2492	0.405	1.0397	0
6311	469	2.3058	2.2421	0.4052	1.0397	0
6312	468.9	2.2977	2.2483	0.4047	1.0408	0
6313	468.8	2.3048	2.2477	0.4049	1.0403	0
6314	468.7	2.3043	2.2472	0.4046	1.0398	0
6315	468.6	2.3037	2.2467	0.4047	1.0392	0
6316	468.5	2.3109	2.2529	0.4048	1.04	0
6317	468.4	2.3027	2.2457	0.4046	1.0394	0
6318	468.3	2.3022	2.2519	0.4044	1.0398	0
6319	468.2	2.3016	2.2447	0.4043	1.0397	0
6320	468.1	2.3012	2.2509	0.4046	1.0392	0
6321	468	2.3084	2.2504	0.4046	1.04	0
6322	467.9	2.3001	2.2498	0.4045	1.0398	0
6323	467.8	2.2996	2.2493	0.4045	1.0393	0
6324	467.7	2.3147	2.2489	0.4042	1.0397	0
6325	467.6	2.3142	2.2484	0.4045	1.0396	0
6326	467.5	2.3057	2.2546	0.4042	1.039	0
6327	467.4	2.3132	2.2542	0.4047	1.039	0
6328	467.3	2.3049	2.2537	0.404	1.0389	0
6329	467.2	2.3044	2.2533	0.4044	1.0394	0
6330	467.1	2.3038	2.2527	0.4043	1.0384	0
6331	467	2.3113	2.2523	0.4043	1.0384	0
6332	466.9	2.3188	2.2659	0.4045	1.0389	0
6333	466.8	2.3104	2.2654	0.4044	1.0388	0
6334	466.7	2.31	2.2579	0.4043	1.0392	0
6335	466.6	2.3174	2.2574	0.4043	1.0391	0
6336	466.5	2.3089	2.264	0.4043	1.0386	0
6337	466.4	2.3085	2.2565	0.4044	1.039	0
6338	466.3	2.308	2.2422	0.4047	1.0394	0
6339	466.2	2.3074	2.2625	0.4047	1.0396	0
6340	466.1	2.307	2.2549	0.4046	1.0388	0
6341	466	2.3064	2.2545	0.4044	1.0391	0
6342	465.9	2.3061	2.2611	0.4045	1.0391	0
6343	465.8	2.3135	2.2606	0.4045	1.039	0
6344	465.7	2.3129	2.26	0.4045	1.0384	0
6345	465.6	2.2966	2.2667	0.4044	1.0392	0
6346	465.5	2.3042	2.2592	0.4047	1.0393	0
6347	465.4	2.3036	2.2658	0.4047	1.0396	0
6348	465.3	2.303	2.2653	0.4044	1.0395	0
6349	465.2	2.3025	2.2647	0.4044	1.0403	0
6350	465.1	2.3099	2.2641	0.4043	1.0392	0

6351	465	2.3015	2.2709	0.4044	1.0396	0
6352	464.9	2.3089	2.2704	0.4046	1.0391	0
6353	464.8	2.3084	2.2626	0.4045	1.0399	0
6354	464.7	2.3161	2.2623	0.4048	1.0404	0
6355	464.6	2.2994	2.2616	0.4047	1.0398	0
6356	464.5	2.3069	2.2611	0.4048	1.0397	0
6357	464.4	2.3065	2.2755	0.4051	1.0411	0
6358	464.3	2.3059	2.2602	0.4051	1.0409	0
6359	464.2	2.3135	2.2669	0.405	1.0403	0
6360	464.1	2.3049	2.2664	0.4053	1.0412	0
6361	464	2.3124	2.2658	0.4052	1.0414	0
6362	463.9	2.2958	2.2727	0.4054	1.0409	0
6363	463.8	2.3112	2.2646	0.4051	1.0407	0
6364	463.7	2.2946	2.2716	0.4053	1.0407	0
6365	463.6	2.3021	2.271	0.4052	1.0406	0
6366	463.5	2.3016	2.2781	0.4054	1.0414	0
6367	463.4	2.3009	2.2699	0.4054	1.0407	0
6368	463.3	2.3086	2.2695	0.4056	1.0407	0
6369	463.2	2.308	2.2765	0.4054	1.0406	0
6370	463.1	2.3074	2.2759	0.4056	1.0409	0
6371	463	2.3069	2.2752	0.4056	1.0421	0
6372	462.9	2.2981	2.2746	0.4055	1.0424	0
6373	462.8	2.3139	2.274	0.4054	1.0418	0
6374	462.7	2.297	2.2734	0.4057	1.0417	0
6375	462.6	2.3043	2.2728	0.4054	1.0419	0
6376	462.5	2.3038	2.2799	0.4057	1.0414	0
6377	462.4	2.3032	2.2793	0.4054	1.0416	0
6378	462.3	2.3109	2.271	0.4055	1.0415	0
6379	462.2	2.3102	2.2704	0.4054	1.0418	0
6380	462.1	2.3014	2.2774	0.4053	1.0416	0
6381	462	2.3009	2.2692	0.4053	1.0419	0
6382	461.9	2.3085	2.2842	0.4055	1.0418	0
6383	461.8	2.2996	2.2756	0.4054	1.042	0
6384	461.7	2.3073	2.2752	0.4058	1.042	0
6385	461.6	2.3067	2.2823	0.4058	1.0423	0
6386	461.5	2.306	2.2816	0.4057	1.0424	0
6387	461.4	2.3053	2.2731	0.4057	1.0422	0
6388	461.3	2.3217	2.2725	0.4058	1.042	0
6389	461.2	2.304	2.2797	0.4057	1.0423	0
6390	461.1	2.3118	2.2712	0.4056	1.0412	0
6391	461	2.3028	2.2784	0.4054	1.0424	0
6392	460.9	2.3021	2.2777	0.4056	1.0426	0
6393	460.8	2.3098	2.277	0.4056	1.0419	0

6394	460.7	2.3008	2.2924	0.4057	1.0422	0
6395	460.6	2.3086	2.2918	0.4057	1.0425	0
6396	460.5	2.3163	2.2829	0.4056	1.0422	0
6397	460.4	2.2987	2.2823	0.4057	1.0425	0
6398	460.3	2.3148	2.2814	0.4054	1.0421	0
6399	460.2	2.3143	2.289	0.4058	1.0425	0
6400	460.1	2.3135	2.2802	0.4056	1.0427	0
6401	460	2.3043	2.2876	0.4057	1.0429	0
6402	459.9	2.2951	2.2951	0.4058	1.0421	0
6403	459.8	2.3029	2.2861	0.4056	1.0419	0
6404	459.7	2.3022	2.2937	0.4057	1.0426	0
6405	459.6	2.3188	2.293	0.4057	1.0424	0
6406	459.5	2.3093	2.2923	0.4055	1.0431	0
6407	459.4	2.3174	2.2916	0.4057	1.0424	0
6408	459.3	2.3079	2.2908	0.4055	1.043	0
6409	459.2	2.3072	2.2819	0.4056	1.0419	0
6410	459.1	2.3065	2.2979	0.4056	1.0426	0
6411	459	2.3058	2.2888	0.4057	1.0429	0
6412	458.9	2.314	2.2966	0.4058	1.0423	0
6413	458.8	2.3044	2.2791	0.4058	1.0424	0
6414	458.7	2.3124	2.3036	0.4057	1.0426	0
6415	458.6	2.3207	2.2859	0.4058	1.0429	0
6416	458.5	2.3021	2.2935	0.4057	1.0425	0
6417	458.4	2.3102	2.2929	0.4058	1.0428	0
6418	458.3	2.3094	2.3006	0.4056	1.0424	0
6419	458.2	2.306	2.2955	0.4057	1.0426	0
6420	458.1	2.3052	2.2947	0.4057	1.0429	0
6421	458	2.3098	2.2992	0.4056	1.043	0
6422	457.9	2.309	2.2984	0.4055	1.0435	0
6423	457.8	2.3137	2.3029	0.4056	1.0433	0
6424	457.7	2.3129	2.297	0.4058	1.0431	0
6425	457.6	2.3069	2.3016	0.4057	1.043	0
6426	457.5	2.317	2.3008	0.4055	1.0431	0
6427	457.4	2.3107	2.2999	0.4055	1.0429	0
6428	457.3	2.3045	2.2992	0.4056	1.0428	0
6429	457.2	2.3092	2.2985	0.4058	1.0426	0
6430	457.1	2.3139	2.303	0.4056	1.043	0
6431	457	2.3078	2.2971	0.4055	1.0435	0
6432	456.9	2.307	2.3016	0.4057	1.0436	0
6433	456.8	2.3064	2.3009	0.4059	1.0436	0
6434	456.7	2.311	2.3056	0.4057	1.0434	0
6435	456.6	2.3102	2.3047	0.4057	1.0435	0
6436	456.5	2.3096	2.3042	0.4057	1.0438	0

6437	456.4	2.3144	2.3035	0.4059	1.0437	0
6438	456.3	2.3137	2.3028	0.4059	1.0442	0
6439	456.2	2.313	2.3075	0.4059	1.0434	0
6440	456.1	2.3066	2.3066	0.4059	1.0432	0
6441	456	2.306	2.306	0.4058	1.0435	0
6442	455.9	2.305	2.305	0.4058	1.0431	0
6443	455.8	2.3156	2.3044	0.4059	1.044	0
6444	455.7	2.3093	2.3093	0.4058	1.0442	0
6445	455.6	2.3086	2.303	0.4059	1.0441	0
6446	455.5	2.3079	2.3023	0.4059	1.044	0
6447	455.4	2.307	2.307	0.4059	1.0441	0
6448	455.3	2.3118	2.3063	0.4058	1.0436	0
6449	455.2	2.3169	2.3111	0.4061	1.0442	0
6450	455.1	2.316	2.3103	0.406	1.0439	0
6451	455	2.3095	2.3095	0.406	1.0441	0
6452	454.9	2.309	2.3034	0.4061	1.0441	0
6453	454.8	2.3082	2.3026	0.4063	1.0446	0
6454	454.7	2.3132	2.3075	0.4061	1.0441	0
6455	454.6	2.3125	2.3068	0.4062	1.0444	0
6456	454.5	2.3117	2.306	0.4063	1.0445	0
6457	454.4	2.311	2.3054	0.4062	1.0448	0
6458	454.3	2.3103	2.3103	0.4064	1.0447	0
6459	454.2	2.3095	2.3095	0.4065	1.0452	0
6460	454.1	2.3087	2.3145	0.4064	1.0453	0
6461	454	2.3136	2.3136	0.4066	1.0448	0
6462	453.9	2.313	2.3016	0.4067	1.045	0
6463	453.8	2.3121	2.3121	0.4065	1.0451	0
6464	453.7	2.3114	2.3172	0.4066	1.045	0
6465	453.6	2.3107	2.3107	0.4066	1.0452	0
6466	453.5	2.3158	2.3158	0.4065	1.0451	0
6467	453.4	2.315	2.3092	0.4068	1.045	0
6468	453.3	2.32	2.3083	0.4065	1.0457	0
6469	453.2	2.3135	2.3135	0.4067	1.046	0
6470	453.1	2.3128	2.3128	0.407	1.0463	0
6471	453	2.3062	2.312	0.4069	1.0458	0
6472	452.9	2.3112	2.3171	0.4069	1.046	0
6473	452.8	2.3104	2.3104	0.4069	1.0455	0
6474	452.7	2.3155	2.3155	0.407	1.046	0
6475	452.6	2.3146	2.3146	0.4069	1.046	0
6476	452.5	2.3139	2.3079	0.4069	1.0462	0
6477	452.4	2.313	2.313	0.4066	1.0457	0
6478	452.3	2.3184	2.3124	0.4069	1.046	0
6479	452.2	2.3175	2.3115	0.4065	1.0457	0

6480	452.1	2.3168	2.3168	0.4067	1.0457	0
6481	452	2.3158	2.3098	0.4064	1.0457	0
6482	451.9	2.3213	2.3213	0.4066	1.0457	0
6483	451.8	2.3143	2.3205	0.4067	1.0456	0
6484	451.7	2.3196	2.3135	0.4066	1.0456	0
6485	451.6	2.3187	2.3126	0.4066	1.0458	0
6486	451.5	2.318	2.318	0.4065	1.0461	0
6487	451.4	2.3231	2.317	0.4063	1.0457	0
6488	451.3	2.3223	2.3101	0.4067	1.0462	0
6489	451.2	2.3215	2.3092	0.4065	1.0456	0
6490	451.1	2.3145	2.3145	0.4063	1.0456	0
6491	451	2.3198	2.3137	0.4063	1.0457	0
6492	450.9	2.3251	2.3127	0.4061	1.0451	0
6493	450.8	2.3244	2.3119	0.4061	1.0456	0
6494	450.7	2.3174	2.3174	0.4062	1.0459	0
6495	450.6	2.3166	2.3104	0.4062	1.0457	0
6496	450.5	2.3218	2.3218	0.406	1.0453	0
6497	450.4	2.321	2.3148	0.4062	1.0453	0
6498	450.3	2.3201	2.3138	0.4062	1.045	0
6499	450.2	2.3193	2.3193	0.4061	1.0455	0
6500	450.1	2.3246	2.312	0.4058	1.0459	0
6501	450	2.3237	2.311	0.4059	1.0453	0
6502	449.9	2.3336	2.3229	0.3857	0.98	0
6503	449.8	2.3347	2.3188	0.3856	0.9807	0
6504	449.7	2.3306	2.3201	0.3857	0.9807	0
6505	449.6	2.3264	2.3212	0.3857	0.9806	0
6506	449.5	2.3328	2.3171	0.3855	0.9806	0
6507	449.4	2.3338	2.3232	0.3852	0.9804	0
6508	449.3	2.3349	2.3193	0.3854	0.9801	0
6509	449.2	2.3308	2.3256	0.3853	0.9801	0
6510	449.1	2.3319	2.3216	0.3853	0.9807	0
6511	449	2.333	2.3176	0.3854	0.981	0
6512	448.9	2.3341	2.3238	0.3855	0.9804	0
6513	448.8	2.3301	2.325	0.3855	0.9805	0
6514	448.7	2.3363	2.326	0.3853	0.9803	0
6515	448.6	2.3374	2.322	0.3854	0.9803	0
6516	448.5	2.3386	2.3181	0.3855	0.9805	0
6517	448.4	2.3396	2.3242	0.3855	0.9806	0
6518	448.3	2.3407	2.3252	0.3856	0.9803	0
6519	448.2	2.3365	2.3262	0.3855	0.9808	0
6520	448.1	2.3427	2.3222	0.3853	0.9803	0
6521	448	2.3437	2.3183	0.3852	0.9804	0
6522	447.9	2.3396	2.3243	0.3854	0.9808	0

6523	447.8	2.3405	2.3252	0.3853	0.9808	0
6524	447.7	2.3467	2.3262	0.3853	0.9809	0
6525	447.6	2.3425	2.3272	0.3852	0.9805	0
6526	447.5	2.3434	2.3232	0.3853	0.9806	0
6527	447.4	2.3445	2.3292	0.3858	0.9809	0
6528	447.3	2.3508	2.3253	0.3856	0.9813	0
6529	447.2	2.3464	2.3312	0.3856	0.9816	0
6530	447.1	2.3422	2.3272	0.3856	0.9811	0
6531	447	2.3481	2.323	0.3855	0.981	0
6532	446.9	2.3492	2.3289	0.3855	0.9812	0
6533	446.8	2.35	2.3298	0.3857	0.9813	0
6534	446.7	2.3458	2.3308	0.3859	0.981	0
6535	446.6	2.3518	2.3367	0.3857	0.9815	0
6536	446.5	2.3528	2.3277	0.3858	0.9816	0
6537	446.4	2.3537	2.3285	0.3856	0.9819	0
6538	446.3	2.3546	2.3295	0.386	0.9818	0
6539	446.2	2.3553	2.3253	0.3857	0.9818	0
6540	446.1	2.351	2.3262	0.3856	0.9815	0
6541	446	2.3569	2.3319	0.3856	0.982	0
6542	445.9	2.3527	2.3279	0.3857	0.9819	0
6543	445.8	2.3587	2.3337	0.3859	0.9822	0
6544	445.7	2.3493	2.3248	0.3859	0.9819	0
6545	445.6	2.3551	2.3254	0.3858	0.9821	0
6546	445.5	2.3559	2.3262	0.3859	0.9823	0
6547	445.4	2.3566	2.3318	0.3858	0.9824	0
6548	445.3	2.3575	2.3279	0.3857	0.9824	0
6549	445.2	2.3581	2.3333	0.3857	0.9824	0
6550	445.1	2.3539	2.3341	0.3859	0.9826	0
6551	445	2.3598	2.3302	0.3861	0.9828	0
6552	444.9	2.3556	2.3311	0.3862	0.9834	0
6553	444.8	2.3614	2.3318	0.3861	0.9831	0
6554	444.7	2.3621	2.3325	0.386	0.9836	0
6555	444.6	2.3628	2.3331	0.3862	0.9836	0
6556	444.5	2.3634	2.3291	0.386	0.9839	0
6557	444.4	2.3591	2.3298	0.3863	0.9836	0
6558	444.3	2.3649	2.3306	0.3863	0.9836	0
6559	444.2	2.3656	2.336	0.3864	0.9839	0
6560	444.1	2.3613	2.332	0.3863	0.9838	0
6561	444	2.3671	2.3374	0.3864	0.9841	0
6562	443.9	2.3676	2.3333	0.3864	0.9843	0
6563	443.8	2.3633	2.334	0.3865	0.9847	0
6564	443.7	2.364	2.33	0.3868	0.984	0
6565	443.6	2.3699	2.3262	0.3868	0.9848	0

6566	443.5	2.3652	2.3312	0.387	0.9844	0
6567	443.4	2.3659	2.3366	0.3867	0.9848	0
6568	443.3	2.3666	2.3326	0.3871	0.985	0
6569	443.2	2.3722	2.3331	0.3868	0.985	0
6570	443.1	2.3727	2.3384	0.3867	0.9851	0
6571	443	2.3735	2.3343	0.3868	0.9854	0
6572	442.9	2.374	2.3396	0.3867	0.9851	0
6573	442.8	2.3745	2.3355	0.3869	0.9853	0
6574	442.7	2.375	2.3359	0.3867	0.9853	0
6575	442.6	2.3808	2.3366	0.3869	0.9854	0
6576	442.5	2.3763	2.3372	0.387	0.9852	0
6577	442.4	2.3768	2.3377	0.387	0.9854	0
6578	442.3	2.3772	2.3335	0.387	0.9854	0
6579	442.2	2.3777	2.3386	0.3867	0.9855	0
6580	442.1	2.3782	2.3391	0.3868	0.9854	0
6581	442	2.3837	2.3396	0.3867	0.9856	0
6582	441.9	2.3741	2.3401	0.3865	0.9853	0
6583	441.8	2.3797	2.3406	0.3865	0.9856	0
6584	441.7	2.3802	2.3364	0.3866	0.9853	0
6585	441.6	2.3755	2.3415	0.3866	0.9853	0
6586	441.5	2.381	2.342	0.3865	0.9856	0
6587	441.4	2.3867	2.3378	0.3867	0.9857	0
6588	441.3	2.3821	2.343	0.3866	0.9856	0
6589	441.2	2.3876	2.3388	0.3866	0.9856	0
6590	441.1	2.3881	2.3439	0.3866	0.9854	0
6591	441	2.3833	2.3443	0.3865	0.9854	0
6592	440.9	2.3942	2.3401	0.3866	0.9852	0
6593	440.8	2.3894	2.3406	0.3863	0.9855	0
6594	440.7	2.3898	2.3409	0.3864	0.9853	0
6595	440.6	2.3902	2.3413	0.3865	0.9855	0
6596	440.5	2.3906	2.3372	0.3864	0.9855	0
6597	440.4	2.391	2.3423	0.3867	0.9854	0
6598	440.3	2.3916	2.3474	0.3867	0.9852	0
6599	440.2	2.3971	2.3478	0.3864	0.9852	0
6600	440.1	2.3923	2.3481	0.3862	0.9852	0
6601	440	2.3927	2.3438	0.3862	0.9851	0
6602	439.9	2.3932	2.3489	0.3863	0.9854	0
6603	439.8	2.3988	2.3447	0.3863	0.9852	0
6604	439.7	2.3938	2.345	0.386	0.9848	0
6605	439.6	2.3995	2.3454	0.3861	0.9849	0
6606	439.5	2.3998	2.3457	0.386	0.9846	0
6607	439.4	2.4001	2.3415	0.3858	0.9848	0
6608	439.3	2.3952	2.3373	0.3859	0.9847	0

6609	439.2	2.4009	2.3469	0.386	0.9847	0
6610	439.1	2.4012	2.3518	0.386	0.9845	0
6611	439	2.4015	2.3476	0.3861	0.9846	0
6612	438.9	2.402	2.3479	0.386	0.9848	0
6613	438.8	2.4077	2.3483	0.3859	0.9848	0
6614	438.7	2.4026	2.344	0.3857	0.9843	0
6615	438.6	2.4083	2.3536	0.3856	0.9846	0
6616	438.5	2.4086	2.354	0.3855	0.9846	0
6617	438.4	2.409	2.3544	0.3858	0.9842	0
6618	438.3	2.4092	2.3546	0.3855	0.9842	0
6619	438.2	2.4203	2.3549	0.3854	0.9841	0
6620	438.1	2.4098	2.3506	0.3853	0.9844	0
6621	438	2.4102	2.3509	0.3854	0.9841	0
6622	437.9	2.4159	2.3559	0.3853	0.9835	0
6623	437.8	2.4107	2.3514	0.3852	0.9835	0
6624	437.7	2.4163	2.3517	0.3853	0.9842	0
6625	437.6	2.4167	2.3566	0.3853	0.9839	0
6626	437.5	2.4169	2.3523	0.3851	0.9836	0
6627	437.4	2.412	2.3572	0.3852	0.9837	0
6628	437.3	2.4284	2.3576	0.3853	0.9836	0
6629	437.2	2.4179	2.3579	0.3853	0.9838	0
6630	437.1	2.4235	2.3535	0.3851	0.9838	0
6631	437	2.4239	2.3632	0.3853	0.984	0
6632	436.9	2.4242	2.3635	0.3854	0.9843	0
6633	436.8	2.4299	2.3544	0.3854	0.9841	0
6634	436.7	2.4249	2.3549	0.3854	0.9838	0
6635	436.6	2.425	2.3551	0.3855	0.984	0
6636	436.5	2.4253	2.3599	0.3854	0.9844	0
6637	436.4	2.4311	2.3602	0.3854	0.9839	0
6638	436.3	2.4313	2.3652	0.3854	0.9846	0
6639	436.2	2.4318	2.3656	0.3856	0.9846	0
6640	436.1	2.4375	2.3657	0.3855	0.9845	0
6641	436	2.4265	2.3564	0.3854	0.9848	0
6642	435.9	2.4324	2.3615	0.3857	0.9848	0
6643	435.8	2.4382	2.3618	0.3856	0.9847	0
6644	435.7	2.4385	2.3621	0.3859	0.985	0
6645	435.6	2.4388	2.367	0.3857	0.9857	0
6646	435.5	2.439	2.3673	0.386	0.9853	0
6647	435.4	2.4449	2.3629	0.3861	0.9858	0
6648	435.3	2.445	2.3677	0.386	0.9855	0
6649	435.2	2.4453	2.3727	0.3861	0.9858	0
6650	435.1	2.4511	2.3682	0.3862	0.9856	0
6651	435	2.4515	2.3686	0.3864	0.9864	0

6652	434.9	2.4459	2.3687	0.3861	0.9864	0
6653	434.8	2.452	2.369	0.3865	0.987	0
6654	434.7	2.4465	2.3692	0.3865	0.9872	0
6655	434.6	2.4467	2.3742	0.3862	0.9869	0
6656	434.5	2.4526	2.3744	0.3867	0.9869	0
6657	434.4	2.4528	2.3747	0.3868	0.9871	0
6658	434.3	2.453	2.3748	0.3866	0.9875	0
6659	434.2	2.459	2.3703	0.3865	0.9873	0
6660	434.1	2.4536	2.3754	0.3869	0.9881	0
6661	434	2.4537	2.3803	0.3869	0.988	0
6662	433.9	2.4598	2.3806	0.3869	0.9877	0
6663	433.8	2.4542	2.3807	0.387	0.9882	0
6664	433.7	2.4602	2.381	0.3872	0.9887	0
6665	433.6	2.4603	2.3812	0.3872	0.9885	0
6666	433.5	2.4664	2.3765	0.3872	0.988	0
6667	433.4	2.4665	2.3814	0.3871	0.9886	0
6668	433.3	2.4666	2.3816	0.3871	0.9887	0
6669	433.2	2.4669	2.3818	0.3874	0.9889	0
6670	433.1	2.4729	2.382	0.3872	0.9887	0
6671	433	2.4731	2.387	0.3874	0.9889	0
6672	432.9	2.4672	2.3823	0.3873	0.9888	0
6673	432.8	2.4734	2.3825	0.3874	0.9895	0
6674	432.7	2.4734	2.3874	0.3875	0.9891	0
6675	432.6	2.4678	2.3925	0.3875	0.9897	0
6676	432.5	2.4739	2.3879	0.3875	0.9895	0
6677	432.4	2.4739	2.383	0.3875	0.9894	0
6678	432.3	2.4742	2.3931	0.3876	0.9902	0
6679	432.2	2.4803	2.3882	0.3875	0.9897	0
6680	432.1	2.4804	2.3933	0.3877	0.99	0
6681	432	2.4804	2.3933	0.3877	0.9899	0
6682	431.9	2.4866	2.3934	0.3877	0.9894	0
6683	431.8	2.4807	2.3985	0.3876	0.9897	0
6684	431.7	2.4868	2.3936	0.3877	0.99	0
6685	431.6	2.4869	2.3937	0.3878	0.9897	0
6686	431.5	2.487	2.3938	0.3878	0.9908	0
6687	431.4	2.487	2.3938	0.3877	0.9894	0
6688	431.3	2.4872	2.3989	0.3876	0.9897	0
6689	431.2	2.4874	2.3942	0.3879	0.99	0
6690	431.1	2.4936	2.3991	0.3877	0.9902	0
6691	431	2.4873	2.4041	0.3876	0.9901	0
6692	430.9	2.4936	2.3992	0.3877	0.9899	0
6693	430.8	2.5	2.3944	0.3876	0.9899	0
6694	430.7	2.4938	2.4044	0.3878	0.99	0

6695	430.6	2.5	2.3994	0.3876	0.99	0
6696	430.5	2.4939	2.4044	0.3875	0.9897	0
6697	430.4	2.5002	2.3996	0.3876	0.9898	0
6698	430.3	2.5002	2.4045	0.3875	0.9898	0
6699	430.2	2.4939	2.4045	0.3876	0.9895	0
6700	430.1	2.5002	2.3996	0.3875	0.9896	0
6701	430	2.5003	2.4047	0.3874	0.9897	0
6702	429.9	2.5065	2.4096	0.3873	0.9896	0
6703	429.8	2.5067	2.4046	0.3872	0.9891	0
6704	429.7	2.5065	2.4096	0.387	0.989	0
6705	429.6	2.5065	2.4097	0.387	0.9891	0
6706	429.5	2.5065	2.4096	0.3868	0.9888	0
6707	429.4	2.5133	2.4099	0.387	0.9891	0
6708	429.3	2.5196	2.4149	0.3868	0.9889	0
6709	429.2	2.5131	2.4097	0.3868	0.9885	0
6710	429.1	2.5194	2.4096	0.3867	0.9884	0
6711	429	2.5196	2.4148	0.3866	0.989	0
6712	428.9	2.5129	2.4147	0.3865	0.9887	0
6713	428.8	2.513	2.4148	0.3866	0.9882	0
6714	428.7	2.5193	2.4145	0.3862	0.9882	0
6715	428.6	2.5194	2.4147	0.3864	0.9879	0
6716	428.5	2.5129	2.4147	0.3863	0.9881	0
6717	428.4	2.5193	2.4198	0.3863	0.9878	0
6718	428.3	2.5259	2.4199	0.3864	0.9883	0
6719	428.2	2.5193	2.425	0.3862	0.9882	0
6720	428.1	2.5191	2.4249	0.386	0.9879	0
6721	428	2.5258	2.4195	0.3857	0.9878	0
6722	427.9	2.5258	2.4195	0.3859	0.988	0
6723	427.8	2.5256	2.4248	0.3859	0.9877	0
6724	427.7	2.5256	2.4247	0.3858	0.9875	0
6725	427.6	2.5322	2.4246	0.3857	0.9872	0
6726	427.5	2.5256	2.43	0.3858	0.9877	0
6727	427.4	2.5321	2.4245	0.3856	0.9879	0
6728	427.3	2.5391	2.4246	0.3857	0.9876	0
6729	427.2	2.5321	2.4297	0.3855	0.987	0
6730	427.1	2.5388	2.4351	0.3856	0.9871	0
6731	427	2.539	2.4298	0.3856	0.9874	0
6732	426.9	2.5388	2.4296	0.3856	0.9871	0
6733	426.8	2.5387	2.4296	0.3855	0.9876	0
6734	426.7	2.5318	2.4349	0.3854	0.9873	0
6735	426.6	2.5456	2.4349	0.3854	0.9874	0
6736	426.5	2.5525	2.4347	0.3853	0.9874	0
6737	426.4	2.5384	2.4346	0.3853	0.9871	0

6738	426.3	2.5453	2.4455	0.3853	0.987	0
6739	426.2	2.5452	2.4345	0.3854	0.9873	0
6740	426.1	2.5522	2.4345	0.3854	0.9869	0
6741	426	2.5596	2.44	0.3854	0.9874	0
6742	425.9	2.5521	2.4454	0.3854	0.9874	0
6743	425.8	2.5521	2.4344	0.3856	0.9872	0
6744	425.7	2.5592	2.4453	0.3854	0.9875	0
6745	425.6	2.5591	2.4451	0.3855	0.9879	0
6746	425.5	2.5519	2.445	0.3855	0.9881	0
6747	425.4	2.5518	2.4506	0.3855	0.9878	0
6748	425.3	2.5589	2.4505	0.3855	0.9885	0
6749	425.2	2.5661	2.4562	0.3855	0.9882	0
6750	425.1	2.5586	2.4559	0.3857	0.9883	0
6751	425	2.5659	2.4559	0.3858	0.9882	0
6752	424.9	2.5733	2.4503	0.3859	0.9883	0
6753	424.8	2.5732	2.4501	0.3861	0.9885	0
6754	424.7	2.5658	2.4616	0.3861	0.9891	0
6755	424.6	2.573	2.4614	0.3861	0.9888	0
6756	424.5	2.5728	2.4613	0.3861	0.9886	0
6757	424.4	2.5728	2.4554	0.3862	0.9889	0
6758	424.3	2.5727	2.4553	0.3861	0.9896	0
6759	424.2	2.5802	2.461	0.3865	0.9895	0
6760	424.1	2.5799	2.4607	0.3862	0.9898	0
6761	424	2.5724	2.4665	0.3865	0.9898	0
6762	423.9	2.5875	2.4665	0.3867	0.99	0
6763	423.8	2.5799	2.4665	0.3869	0.9903	0
6764	423.7	2.5874	2.4724	0.387	0.9907	0
6765	423.6	2.5872	2.472	0.3869	0.9908	0
6766	423.5	2.595	2.4661	0.387	0.9907	0
6767	423.4	2.5869	2.4719	0.3872	0.9913	0
6768	423.3	2.5869	2.4777	0.3871	0.9912	0
6769	423.2	2.5867	2.4776	0.3871	0.991	0
6770	423.1	2.5943	2.4774	0.3871	0.9915	0
6771	423	2.5941	2.4833	0.3872	0.9917	0
6772	422.9	2.594	2.4831	0.3874	0.9919	0
6773	422.8	2.5938	2.4768	0.3873	0.9917	0
6774	422.7	2.6015	2.4766	0.3875	0.9918	0
6775	422.6	2.6015	2.4827	0.3875	0.9916	0
6776	422.5	2.5933	2.4885	0.3875	0.9918	0
6777	422.4	2.6092	2.4823	0.3874	0.9917	0
6778	422.3	2.601	2.4821	0.3877	0.9915	0
6779	422.2	2.6007	2.488	0.3878	0.9921	0
6780	422.1	2.6085	2.4877	0.3877	0.9921	0

6781	422	2.6084	2.4938	0.3877	0.9926	0
6782	421.9	2.6082	2.4873	0.3876	0.9923	0
6783	421.8	2.6078	2.487	0.3876	0.9925	0
6784	421.7	2.6077	2.4995	0.3878	0.9926	0
6785	421.6	2.6075	2.4994	0.3878	0.9928	0
6786	421.5	2.6073	2.4927	0.3878	0.9929	0
6787	421.4	2.6153	2.4924	0.3879	0.9928	0
6788	421.3	2.6068	2.4921	0.3878	0.9928	0
6789	421.2	2.6148	2.4983	0.3879	0.9931	0
6790	421.1	2.6146	2.4981	0.388	0.9927	0
6791	421	2.6142	2.4977	0.3877	0.9924	0
6792	420.9	2.614	2.504	0.3879	0.9928	0
6793	420.8	2.6223	2.5038	0.3879	0.9928	0
6794	420.7	2.6137	2.51	0.3878	0.9936	0
6795	420.6	2.6218	2.5098	0.3877	0.993	0
6796	420.5	2.613	2.5029	0.3877	0.9926	0
6797	420.4	2.6212	2.5092	0.3878	0.9927	0
6798	420.3	2.6209	2.5089	0.3876	0.9927	0
6799	420.2	2.6291	2.5085	0.3875	0.9928	0
6800	420.1	2.6202	2.5017	0.3875	0.9925	0
6801	420	2.6373	2.5213	0.3875	0.9926	0
6802	419.9	2.637	2.5143	0.3877	0.9927	0
6803	419.8	2.6278	2.5207	0.3874	0.9926	0
6804	419.7	2.6364	2.5136	0.3876	0.9924	0
6805	419.6	2.636	2.52	0.3874	0.9921	0
6806	419.5	2.6356	2.5197	0.3873	0.992	0
6807	419.4	2.6356	2.5196	0.3875	0.9927	0
6808	419.3	2.6264	2.5191	0.3873	0.9924	0
6809	419.2	2.6438	2.5256	0.3872	0.9921	0
6810	419.1	2.6434	2.5253	0.3872	0.9918	0
6811	419	2.6521	2.5249	0.387	0.992	0
6812	418.9	2.6426	2.5315	0.387	0.9919	0
6813	418.8	2.6515	2.5312	0.3868	0.9915	0
6814	418.7	2.6511	2.5308	0.3867	0.9912	0
6815	418.6	2.6417	2.5376	0.3865	0.9916	0
6816	418.5	2.6503	2.5302	0.3865	0.9911	0
6817	418.4	2.6499	2.5439	0.3865	0.9911	0
6818	418.3	2.6498	2.5366	0.3865	0.9913	0
6819	418.2	2.6494	2.5361	0.3862	0.991	0
6820	418.1	2.649	2.5429	0.3863	0.9904	0
6821	418	2.6488	2.5355	0.3863	0.9904	0
6822	417.9	2.6484	2.5423	0.3863	0.9905	0
6823	417.8	2.648	2.5418	0.3862	0.9902	0

6824	417.7	2.657	2.5417	0.3862	0.9902	0
6825	417.6	2.6474	2.5487	0.386	0.9907	0
6826	417.5	2.6659	2.5409	0.3861	0.9907	0
6827	417.4	2.656	2.5478	0.386	0.9903	0
6828	417.3	2.6651	2.5549	0.386	0.99	0
6829	417.2	2.6647	2.5471	0.3859	0.9905	0
6830	417.1	2.6643	2.5542	0.3859	0.9901	0
6831	417	2.6641	2.5539	0.386	0.9904	0
6832	416.9	2.6637	2.5535	0.3859	0.9904	0
6833	416.8	2.6633	2.5683	0.3859	0.9902	0
6834	416.7	2.6728	2.5604	0.386	0.9903	0
6835	416.6	2.6625	2.56	0.3859	0.9901	0
6836	416.5	2.6527	2.5596	0.3858	0.9906	0
6837	416.4	2.6815	2.5592	0.3858	0.9904	0
6838	416.3	2.6811	2.5588	0.3857	0.9905	0
6839	416.2	2.6708	2.574	0.3858	0.9903	0
6840	416.1	2.6704	2.5658	0.3857	0.9902	0
6841	416	2.6801	2.5653	0.3858	0.9898	0
6842	415.9	2.6696	2.5727	0.3856	0.9901	0
6843	415.8	2.6794	2.5725	0.3858	0.9908	0
6844	415.7	2.679	2.5642	0.3858	0.9904	0
6845	415.6	2.6887	2.5795	0.3858	0.991	0
6846	415.5	2.6885	2.5792	0.3859	0.9907	0
6847	415.4	2.6878	2.5707	0.3857	0.9908	0
6848	415.3	2.6874	2.5784	0.3858	0.9912	0
6849	415.2	2.6872	2.5862	0.3858	0.9909	0
6850	415.1	2.6972	2.5776	0.3858	0.9911	0
6851	415	2.6863	2.5854	0.3861	0.9907	0
6852	414.9	2.6859	2.585	0.3862	0.991	0
6853	414.8	2.6962	2.5764	0.3863	0.9914	0
6854	414.7	2.6957	2.5842	0.3861	0.9916	0
6855	414.6	2.706	2.5839	0.3863	0.9917	0
6856	414.5	2.6949	2.5834	0.3862	0.9916	0
6857	414.4	2.7051	2.5829	0.3861	0.9919	0
6858	414.3	2.694	2.5907	0.3862	0.992	0
6859	414.2	2.7044	2.5988	0.3862	0.9915	0
6860	414.1	2.704	2.5984	0.3866	0.9922	0
6861	414	2.7033	2.5896	0.3864	0.9923	0
6862	413.9	2.7031	2.5976	0.3864	0.9925	0
6863	413.8	2.7135	2.5885	0.3866	0.9926	0
6864	413.7	2.702	2.6052	0.3865	0.9926	0
6865	413.6	2.7126	2.5962	0.3863	0.9924	0
6866	413.5	2.7011	2.6043	0.3868	0.993	0

6867	413.4	2.7118	2.5952	0.3867	0.9929	0
6868	413.3	2.7113	2.6033	0.3868	0.9931	0
6869	413.2	2.7106	2.6115	0.3869	0.9934	0
6870	413.1	2.7102	2.611	0.3869	0.9929	0
6871	413	2.721	2.6196	0.3868	0.9929	0
6872	412.9	2.7206	2.6014	0.387	0.9935	0
6873	412.8	2.7201	2.6096	0.3869	0.9934	0
6874	412.7	2.7194	2.618	0.3869	0.994	0
6875	412.6	2.7192	2.6176	0.3872	0.994	0
6876	412.5	2.7187	2.6173	0.3874	0.994	0
6877	412.4	2.7069	2.6258	0.3872	0.9941	0
6878	412.3	2.7062	2.6253	0.3872	0.9944	0
6879	412.2	2.706	2.6249	0.3874	0.9936	0
6880	412.1	2.7167	2.6243	0.3875	0.9937	0
6881	412	2.7162	2.6332	0.3876	0.9943	0
6882	411.9	2.7272	2.6326	0.3873	0.9941	0
6883	411.8	2.7265	2.6227	0.3874	0.994	0
6884	411.7	2.7147	2.6317	0.3873	0.9938	0
6885	411.6	2.7142	2.6407	0.3875	0.9942	0
6886	411.5	2.7251	2.6402	0.3876	0.9945	0
6887	411.4	2.7247	2.6302	0.3875	0.9949	0
6888	411.3	2.7361	2.649	0.3875	0.9944	0
6889	411.2	2.7235	2.6484	0.3877	0.9949	0
6890	411.1	2.7231	2.6381	0.3877	0.9949	0
6891	411	2.7342	2.6472	0.3875	0.9946	0
6892	410.9	2.7338	2.6467	0.3875	0.9952	0
6893	410.8	2.7333	2.6461	0.3876	0.9948	0
6894	410.7	2.7326	2.6455	0.3876	0.9947	0
6895	410.6	2.7321	2.6353	0.3878	0.9946	0
6896	410.5	2.7438	2.6444	0.3879	0.9949	0
6897	410.4	2.7312	2.6538	0.3878	0.9951	0
6898	410.3	2.7305	2.6533	0.3879	0.9952	0
6899	410.2	2.7421	2.6529	0.3879	0.9953	0
6900	410.1	2.7542	2.6523	0.3879	0.9952	0
6901	410	2.7409	2.6515	0.3878	0.9954	0
6902	409.9	2.7404	2.6611	0.3877	0.9952	0
6903	409.8	2.7397	2.6605	0.3875	0.9948	0
6904	409.7	2.7515	2.6598	0.3876	0.9952	0
6905	409.6	2.7385	2.6594	0.3878	0.995	0
6906	409.5	2.7378	2.6588	0.3876	0.9948	0
6907	409.4	2.7373	2.6686	0.3877	0.9951	0
6908	409.3	2.7366	2.6574	0.3876	0.9946	0
6909	409.2	2.7615	2.678	0.3875	0.9947	0

6910	409.1	2.7354	2.6774	0.3874	0.9947	0
6911	409	2.7347	2.6876	0.3874	0.995	0
6912	408.9	2.7467	2.6761	0.3873	0.9947	0
6913	408.8	2.7462	2.6755	0.3873	0.9945	0
6914	408.7	2.7455	2.6749	0.3873	0.9943	0
6915	408.6	2.7711	2.6853	0.3873	0.9944	0
6916	408.5	2.7442	2.6737	0.3872	0.9949	0
6917	408.4	2.7565	2.684	0.3872	0.9947	0
6918	408.3	2.743	2.6834	0.3872	0.9944	0
6919	408.2	2.7688	2.6828	0.3871	0.9948	0
6920	408.1	2.7547	2.6936	0.3871	0.9943	0
6921	408	2.7675	2.6817	0.3871	0.9946	0
6922	407.9	2.7535	2.67	0.3871	0.9947	0
6923	407.8	2.7662	2.7033	0.3869	0.9948	0
6924	407.7	2.7525	2.6912	0.387	0.9942	0
6925	407.6	2.765	2.6904	0.3868	0.9942	0
6926	407.5	2.7642	2.6897	0.3867	0.9944	0
6927	407.4	2.7503	2.6891	0.3868	0.994	0
6928	407.3	2.7496	2.6998	0.3866	0.9935	0
6929	407.2	2.7625	2.6878	0.3868	0.9942	0
6930	407.1	2.762	2.7109	0.3868	0.9936	0
6931	407	2.7749	2.7102	0.3865	0.9946	0
6932	406.9	2.7744	2.7093	0.3866	0.9939	0
6933	406.8	2.76	2.697	0.3866	0.9945	0
6934	406.7	2.7595	2.7082	0.3867	0.9936	0
6935	406.6	2.7726	2.7075	0.3866	0.9934	0
6936	406.5	2.7582	2.6951	0.3867	0.994	0
6937	406.4	2.7713	2.7064	0.3868	0.9942	0
6938	406.3	2.7706	2.7058	0.3867	0.9949	0
6939	406.2	2.7701	2.7051	0.3867	0.9939	0
6940	406.1	2.7557	2.7044	0.3866	0.9943	0
6941	406	2.783	2.7162	0.3866	0.9938	0
6942	405.9	2.7825	2.7156	0.3867	0.9944	0
6943	405.8	2.782	2.7149	0.3866	0.9945	0
6944	405.7	2.7812	2.7142	0.3868	0.9945	0
6945	405.6	2.7662	2.7263	0.3867	0.9946	0
6946	405.5	2.7799	2.7256	0.3867	0.9943	0
6947	405.4	2.7791	2.7122	0.3865	0.9946	0
6948	405.3	2.7786	2.7242	0.3867	0.9943	0
6949	405.2	2.7637	2.7235	0.387	0.9943	0
6950	405.1	2.7921	2.7231	0.3867	0.9945	0
6951	405	2.7768	2.7352	0.3868	0.9949	0
6952	404.9	2.7908	2.7345	0.387	0.9949	0

6953	404.8	2.7902	2.734	0.387	0.9953	0
6954	404.7	2.7891	2.7464	0.3869	0.9952	0
6955	404.6	2.7886	2.7326	0.3868	0.9951	0
6956	404.5	2.7881	2.7452	0.387	0.9956	0
6957	404.4	2.7726	2.7312	0.3871	0.9957	0
6958	404.3	2.8019	2.7305	0.3871	0.9954	0
6959	404.2	2.8011	2.7433	0.3872	0.995	0
6960	404.1	2.8005	2.7423	0.387	0.9954	0
6961	404	2.7846	2.7418	0.3871	0.9958	0
6962	403.9	2.7991	2.7411	0.3871	0.9961	0
6963	403.8	2.7986	2.7404	0.3873	0.9962	0
6964	403.7	2.7978	2.7537	0.3874	0.996	0
6965	403.6	2.7972	2.753	0.3877	0.996	0
6966	403.5	2.8122	2.7523	0.3877	0.9967	0
6967	403.4	2.7959	2.7515	0.3875	0.9957	0
6968	403.3	2.7951	2.7508	0.3875	0.9965	0
6969	403.2	2.7943	2.7645	0.3875	0.9962	0
6970	403.1	2.8094	2.7637	0.3875	0.9965	0
6971	403	2.8088	2.763	0.3875	0.9966	0
6972	402.9	2.808	2.7481	0.3876	0.9968	0
6973	402.8	2.8074	2.7617	0.3876	0.9972	0
6974	402.7	2.823	2.7757	0.3875	0.997	0
6975	402.6	2.806	2.7902	0.3878	0.997	0
6976	402.5	2.8052	2.7742	0.3877	0.9974	0
6977	402.4	2.8044	2.7734	0.3877	0.9973	0
6978	402.3	2.8035	2.7726	0.3878	0.9967	0
6979	402.2	2.803	2.7719	0.3877	0.997	0
6980	402.1	2.8184	2.7565	0.3877	0.9973	0
6981	402	2.8179	2.7703	0.3876	0.9969	0
6982	401.9	2.817	2.7846	0.3877	0.9976	0
6983	401.8	2.8333	2.7838	0.3877	0.9973	0
6984	401.7	2.7989	2.783	0.3875	0.9972	0
6985	401.6	2.8145	2.7823	0.3876	0.997	0
6986	401.5	2.8136	2.7972	0.3876	0.9971	0
6987	401.4	2.8128	2.7964	0.3876	0.9971	0
6988	401.3	2.8119	2.7956	0.3875	0.9972	0
6989	401.2	2.828	2.7945	0.3876	0.9974	0
6990	401.1	2.8102	2.8102	0.3874	0.9971	0
6991	401	2.8094	2.8094	0.3874	0.9975	0
6992	400.9	2.8085	2.7765	0.3874	0.9975	0
6993	400.8	2.808	2.808	0.3876	0.9978	0
6994	400.7	2.8239	2.7905	0.3874	0.9973	0
6995	400.6	2.823	2.806	0.3876	0.9975	0

6996	400.5	2.8052	2.7889	0.3878	0.9971	0
6997	400.4	2.8213	2.8044	0.3878	0.9972	0
6998	400.3	2.8204	2.8035	0.3876	0.9979	0
6999	400.2	2.8374	2.8196	0.3876	0.9972	0
7000	400.1	2.8365	2.8187	0.3877	0.9971	0
7001	400	2.8179	2.8179	0.3877	0.9978	0
7002	399.9	2.8345	2.8345	0.3874	0.9973	0
7003	399.8	2.8162	2.8162	0.3875	0.9974	0
7004	399.7	2.833	2.8153	0.3874	0.9975	0
7005	399.6	2.8321	2.8142	0.3874	0.9971	0
7006	399.5	2.8312	2.8133	0.3876	0.9973	0
7007	399.4	2.8303	2.8125	0.3875	0.9974	0
7008	399.3	2.8292	2.8116	0.3875	0.997	0
7009	399.2	2.8283	2.8108	0.3875	0.9969	0
7010	399.1	2.8459	2.8274	0.3876	0.9973	0
7011	399	2.845	2.8265	0.3873	0.9972	0
7012	398.9	2.8441	2.8256	0.3874	0.9976	0
7013	398.8	2.8431	2.8431	0.3875	0.9978	0
7014	398.7	2.8422	2.8236	0.3873	0.9976	0
7015	398.6	2.8413	2.8049	0.3873	0.997	0
7016	398.5	2.8219	2.8041	0.3873	0.9968	0
7017	398.4	2.8589	2.8395	0.3873	0.9973	0
7018	398.3	2.8576	2.8199	0.3871	0.9974	0
7019	398.2	2.8567	2.819	0.3873	0.9978	0
7020	398.1	2.8365	2.8365	0.387	0.9971	0
7021	398	2.817	2.8356	0.387	0.9973	0
7022	397.9	2.8348	2.8162	0.3871	0.9969	0
7023	397.8	2.8339	2.8153	0.3871	0.9979	0
7024	397.7	2.852	2.8327	0.3871	0.9971	0
7025	397.6	2.8511	2.8318	0.3872	0.9965	0
7026	397.5	2.8502	2.8502	0.387	0.9969	0
7027	397.4	2.83	2.8492	0.387	0.9977	0
7028	397.3	2.8483	2.8483	0.387	0.9975	0
7029	397.2	2.8471	2.8674	0.3867	0.9971	0
7030	397.1	2.8465	2.8465	0.3869	0.9974	0
7031	397	2.8453	2.8453	0.387	0.9975	0
7032	396.9	2.8444	2.8645	0.387	0.9976	0
7033	396.8	2.8435	2.8435	0.3871	0.9976	0
7034	396.7	2.8626	2.8626	0.3871	0.9974	0
7035	396.6	2.8617	2.8416	0.3872	0.9973	0
7036	396.5	2.8608	2.8407	0.387	0.9978	0
7037	396.4	2.8398	2.8398	0.3871	0.998	0
7038	396.3	2.8386	2.8589	0.387	0.997	0

7039	396.2	2.8582	2.8582	0.3871	0.9977	0
7040	396.1	2.857	2.8781	0.3868	0.998	0
7041	396	2.856	2.8775	0.3869	0.9974	0
7042	395.9	2.8551	2.8551	0.387	0.9973	0
7043	395.8	2.8755	2.8979	0.3871	0.9978	0
7044	395.7	2.8533	2.8745	0.3872	0.9983	0
7045	395.6	2.8321	2.8735	0.387	0.9982	0
7046	395.5	2.8729	2.8517	0.3874	0.9983	0
7047	395.4	2.8505	2.8938	0.3872	0.9987	0
7048	395.3	2.8496	2.8931	0.3872	0.998	0
7049	395.2	2.8486	2.8486	0.387	0.9979	0
7050	395.1	2.8477	2.8911	0.3871	0.9982	0
7051	395	2.8681	2.8681	0.3875	0.9987	0
7052	394.9	2.8456	2.8668	0.3872	0.9994	0
7053	394.8	2.8661	2.8661	0.3872	0.9986	0
7054	394.7	2.8649	2.8649	0.3873	0.9984	0
7055	394.6	2.8639	2.8861	0.3873	0.9988	0
7056	394.5	2.8633	2.8854	0.3875	0.9986	0
7057	394.4	2.862	2.8844	0.3877	0.9985	0
7058	394.3	2.8611	2.8611	0.3874	0.9986	0
7059	394.2	2.8824	2.8824	0.3875	0.9993	0
7060	394.1	2.838	2.9048	0.3875	0.9991	0
7061	394	2.8582	2.8804	0.3875	0.9992	0
7062	393.9	2.8794	2.8794	0.3873	0.9995	0
7063	393.8	2.856	2.902	0.3876	0.9988	0
7064	393.7	2.8551	2.901	0.3877	0.9996	0
7065	393.6	2.8542	2.9	0.3876	1.0001	0
7066	393.5	2.8529	2.8989	0.3877	0.9997	0
7067	393.4	2.8745	2.8745	0.3878	0.9996	0
7068	393.3	2.8969	2.8735	0.3879	0.9999	0
7069	393.2	2.8502	2.8722	0.388	0.9997	0
7070	393.1	2.8716	2.9197	0.3881	0.9998	0
7071	393	2.8703	2.8938	0.3879	1.0002	0
7072	392.9	2.8693	2.8928	0.3879	1.0003	0
7073	392.8	2.8684	2.8684	0.3878	1.0006	0
7074	392.7	2.8674	2.8908	0.388	1.0002	0
7075	392.6	2.8661	2.9144	0.3879	1.0003	0
7076	392.5	2.8887	2.9133	0.3879	0.9999	0
7077	392.4	2.8419	2.8877	0.388	1.0012	0
7078	392.3	2.863	2.9115	0.3877	1	0
7079	392.2	2.862	2.9101	0.3878	1.0002	0
7080	392.1	2.8844	2.909	0.3877	0.9997	0
7081	392	2.8598	2.908	0.3877	0.9996	0

7082	391.9	2.8821	2.9069	0.3876	1.0004	0
7083	391.8	2.8811	2.9059	0.3875	0.9997	0
7084	391.7	2.8567	2.9048	0.3876	1.0003	0
7085	391.6	2.8791	2.9303	0.3876	1.0008	0
7086	391.5	2.8545	2.9027	0.3873	0.9999	0
7087	391.4	2.8768	2.8768	0.3875	0.9998	0
7088	391.3	2.852	2.9003	0.3873	0.9996	0
7089	391.2	2.8745	2.9539	0.3875	1.0003	0
7090	391.1	2.8983	2.9245	0.3873	1.0006	0
7091	391	2.8722	2.9234	0.3872	1.0001	0
7092	390.9	2.8713	2.8959	0.3873	1	0
7093	390.8	2.87	2.8948	0.3873	0.9997	0
7094	390.7	2.8938	2.9201	0.3873	0.9997	0
7095	390.6	2.8677	2.8928	0.3873	0.9996	0
7096	390.5	2.8665	2.9176	0.3873	0.9996	0
7097	390.4	2.8904	2.9165	0.3873	0.9995	0
7098	390.3	2.8891	2.8891	0.3872	1	0
7099	390.2	2.8633	2.9144	0.3874	0.9998	0
7100	390.1	2.862	2.9412	0.3872	0.9996	0
7101	390	2.8854	2.9119	0.3872	0.9996	0
7102	389.9	2.8841	2.9385	0.3868	0.9996	0
7103	389.8	2.8582	2.9374	0.3867	0.9995	0
7104	389.7	2.8817	2.908	0.387	0.9997	0
7105	389.6	2.9069	2.9069	0.3872	0.9994	0
7106	389.5	2.8791	2.9337	0.3869	0.9997	0
7107	389.4	2.8781	2.9326	0.3869	0.9996	0
7108	389.3	2.8765	2.961	0.387	0.9992	0
7109	389.2	2.8755	2.9296	0.3869	0.9993	0
7110	389.1	2.8742	2.9003	0.3868	0.9997	0
7111	389	2.8993	2.8993	0.3868	0.9998	0
7112	388.9	2.8716	2.9559	0.3868	0.9995	0
7113	388.8	2.8965	2.9245	0.3869	0.9993	0
7114	388.7	2.8687	2.9234	0.3869	0.9993	0
7115	388.6	2.8938	2.9519	0.3868	0.9994	0
7116	388.5	2.8928	2.8928	0.3867	0.9988	0
7117	388.4	2.8914	2.9194	0.3868	0.9999	0
7118	388.3	2.8636	2.9179	0.3865	0.9995	0
7119	388.2	2.8887	2.9469	0.3867	0.9998	0
7120	388.1	2.8874	2.9154	0.3866	0.9992	0
7121	388	2.8861	2.9439	0.3868	0.9995	0
7122	387.9	2.8844	2.9126	0.3865	0.9991	0
7123	387.8	2.8834	2.9412	0.3863	0.9993	0
7124	387.7	2.8821	2.9401	0.3863	0.9993	0

7125	387.6	2.9087	2.9706	0.3865	0.9995	0
7126	387.5	2.9073	2.9374	0.3866	0.9999	0
7127	387.4	2.8778	2.9359	0.3865	0.9992	0
7128	387.3	2.9045	2.9344	0.3867	0.999	0
7129	387.2	2.8752	2.9333	0.3866	0.9995	0
7130	387.1	2.8474	2.9318	0.3866	0.9999	0
7131	387	2.9307	2.9626	0.3866	0.9993	0
7132	386.9	2.9292	2.9292	0.3866	0.9997	0
7133	386.8	2.9278	2.9278	0.3867	0.9994	0
7134	386.7	2.8965	2.9263	0.3868	0.9995	0
7135	386.6	2.9252	2.9252	0.3867	0.9993	0
7136	386.5	2.8658	2.9559	0.3868	1.0002	0
7137	386.4	2.8645	2.9893	0.3868	1	0
7138	386.3	2.8911	2.9212	0.387	1.0002	0
7139	386.2	2.8897	2.9867	0.387	0.9999	0
7140	386.1	2.9504	2.9183	0.3869	1.0004	0
7141	386	2.8871	3.0218	0.3867	1.0001	0
7142	385.9	2.8854	2.9477	0.3868	1.0002	0
7143	385.8	2.9144	2.9813	0.387	0.9993	0
7144	385.7	2.8831	2.9801	0.3872	1.001	0
7145	385.6	2.9115	2.9439	0.387	1.0008	0
7146	385.5	2.9104	2.9772	0.3873	1.0006	0
7147	385.4	2.9412	2.9759	0.3871	1.0007	0
7148	385.3	2.9076	2.9397	0.3873	1.0017	0
7149	385.2	2.9382	3.011	0.3874	1.0007	0
7150	385.1	2.9048	2.9718	0.3874	1.0014	0
7151	385	2.9034	2.9702	0.3873	1.0011	0
7152	384.9	2.934	2.969	0.3873	1.0017	0
7153	384.8	2.9329	2.9678	0.3873	1.0019	0
7154	384.7	2.8993	3.0039	0.3873	1.0013	0
7155	384.6	2.8979	3.0026	0.3875	1.0023	0
7156	384.5	2.9634	2.9634	0.3875	1.0024	0
7157	384.4	2.9274	2.9622	0.3877	1.0023	0
7158	384.3	2.8938	2.9987	0.3879	1.0022	0
7159	384.2	2.9248	2.9974	0.3878	1.002	0
7160	384.1	2.9234	2.9234	0.388	1.0023	0
7161	384	2.8897	2.9944	0.3881	1.0028	0
7162	383.9	2.9205	2.9931	0.3879	1.0031	0
7163	383.8	2.8871	2.9918	0.3881	1.0034	0
7164	383.7	2.9179	2.9905	0.388	1.0028	0
7165	383.6	2.9512	2.9512	0.3881	1.0027	0
7166	383.5	2.9151	2.9876	0.3881	1.0035	0
7167	383.4	2.914	2.9863	0.3882	1.0038	0

7168	383.3	2.9126	3.0264	0.3883	1.0036	0
7169	383.2	2.9112	2.9838	0.3884	1.004	0
7170	383.1	2.9446	2.9446	0.3884	1.0047	0
7171	383	2.9083	2.9431	0.3883	1.0032	0
7172	382.9	2.9069	2.9796	0.3884	1.0035	0
7173	382.8	2.9404	2.9404	0.3885	1.0044	0
7174	382.7	2.9393	2.9772	0.3889	1.0039	0
7175	382.6	2.9378	2.9755	0.3885	1.0041	0
7176	382.5	2.9363	2.9743	0.3887	1.0045	0
7177	382.4	2.9003	2.9727	0.3886	1.0044	0
7178	382.3	2.8989	2.9714	0.3885	1.0046	0
7179	382.2	2.8976	2.9702	0.3884	1.0041	0
7180	382.1	2.8962	3.0101	0.3887	1.0045	0
7181	382	2.9296	2.9674	0.3885	1.0054	0
7182	381.9	2.9281	2.9658	0.3885	1.0047	0
7183	381.8	2.8921	3.0061	0.3886	1.0047	0
7184	381.7	2.9255	3.0044	0.3885	1.0054	0
7185	381.6	2.8894	3.0035	0.3886	1.0047	0
7186	381.5	2.923	2.9606	0.3887	1.0051	0
7187	381.4	2.8867	2.9215	0.3888	1.0045	0
7188	381.3	2.9201	2.9991	0.3888	1.0035	0
7189	381.2	2.9367	3.0195	0.3887	1.0049	0
7190	381.1	2.9165	2.9957	0.3887	1.005	0
7191	381	2.9337	3.0168	0.3886	1.0049	0
7192	380.9	2.9322	2.9931	0.3886	1.0045	0
7193	380.8	2.9311	2.9918	0.3885	1.0045	0
7194	380.7	2.9112	3.0123	0.3886	1.0047	0
7195	380.6	2.9281	2.9889	0.3886	1.005	0
7196	380.5	2.9083	3.0329	0.3884	1.0044	0
7197	380.4	2.9252	3.0083	0.3886	1.0044	0
7198	380.3	2.9431	3.0066	0.3884	1.0044	0
7199	380.2	2.9223	3.0052	0.3884	1.0041	0
7200	380.1	2.9401	3.0039	0.3884	1.0043	0
7201	380	2.9389	3.0026	0.3883	1.004	0
7202	379.9	2.937	3.0009	0.3882	1.0038	0
7203	379.8	2.9359	2.9996	0.3884	1.0041	0
7204	379.7	2.9151	3.0218	0.3882	1.0046	0
7205	379.6	2.9329	3.02	0.3881	1.0045	0
7206	379.5	2.9314	3.0434	0.3879	1.0041	0
7207	379.4	2.93	3.0173	0.3881	1.0041	0
7208	379.3	2.9285	3.0155	0.388	1.004	0
7209	379.2	2.9473	3.0141	0.3881	1.004	0
7210	379.1	2.9255	3.0128	0.3878	1.0034	0

7211	379	2.9442	3.011	0.3877	1.0033	0
7212	378.9	2.9427	2.9863	0.3879	1.0034	0
7213	378.8	2.9412	3.0595	0.3879	1.0034	0
7214	378.7	2.9397	3.0066	0.388	1.0038	0
7215	378.6	2.9179	3.0297	0.3879	1.0034	0
7216	378.5	2.9367	3.0035	0.3876	1.0035	0
7217	378.4	2.9566	3.0022	0.3879	1.0035	0
7218	378.3	2.9551	3.0004	0.3877	1.0033	0
7219	378.2	2.9322	3.0237	0.3877	1.0029	0
7220	378.1	2.9303	2.9974	0.3877	1.0033	0
7221	378	2.95	3.0209	0.3877	1.0035	0
7222	377.9	2.9485	3.0191	0.3877	1.0031	0
7223	377.8	2.9469	3.0438	0.3875	1.003	0
7224	377.7	2.9245	3.0159	0.3878	1.0032	0
7225	377.6	2.9662	3.0146	0.3875	1.0033	0
7226	377.5	2.9208	3.0391	0.3875	1.0034	0
7227	377.4	2.9404	3.011	0.3877	1.0035	0
7228	377.3	2.9389	3.0092	0.3875	1.0033	0
7229	377.2	2.9374	3.0079	0.3875	1.0038	0
7230	377.1	2.9355	3.0325	0.3875	1.0039	0
7231	377	2.934	3.0311	0.3875	1.0038	0
7232	376.9	2.9547	3.0031	0.3875	1.0038	0
7233	376.8	2.9531	3.0278	0.3879	1.0038	0
7234	376.7	2.9296	3.0264	0.3878	1.0041	0
7235	376.6	2.95	3.0246	0.3876	1.0043	0
7236	376.5	2.9485	3.0232	0.3879	1.0043	0
7237	376.4	2.9469	3.0496	0.3879	1.0041	0
7238	376.3	2.9454	2.994	0.3881	1.0041	0
7239	376.2	2.9439	3.0186	0.3881	1.0044	0
7240	376.1	2.9423	3.0168	0.3883	1.0044	0
7241	376	2.9408	3.0155	0.3884	1.0047	0
7242	375.9	2.9626	3.0137	0.3881	1.0047	0
7243	375.8	2.9378	3.0123	0.388	1.0047	0
7244	375.7	2.9594	3.0106	0.3882	1.005	0
7245	375.6	2.9344	3.0092	0.3883	1.0051	0
7246	375.5	2.9562	3.0074	0.3886	1.0059	0
7247	375.4	2.9547	3.0061	0.3884	1.0062	0
7248	375.3	2.9531	3.0044	0.3885	1.0053	0
7249	375.2	2.9516	3.0306	0.3887	1.0066	0
7250	375.1	2.95	3.0292	0.3887	1.0067	0
7251	375	2.9248	2.9996	0.3886	1.006	0
7252	374.9	2.9469	3.0259	0.3889	1.0068	0
7253	374.8	2.9454	3.0545	0.3892	1.0068	0

7254	374.7	2.9201	3.0227	0.3891	1.0066	0
7255	374.6	2.9187	3.0214	0.3889	1.0066	0
7256	374.5	2.9404	3.0195	0.3891	1.0069	0
7257	374.4	2.9389	3.0482	0.389	1.0068	0
7258	374.3	2.9622	2.9884	0.3891	1.0069	0
7259	374.2	2.9606	3.0448	0.3891	1.0073	0
7260	374.1	2.9851	3.0132	0.3891	1.0075	0
7261	374	2.9574	3.0119	0.3895	1.007	0
7262	373.9	2.9559	3.0101	0.3897	1.0079	0
7263	373.8	2.9543	3.0088	0.3899	1.0078	0
7264	373.7	2.9527	3.0372	0.3898	1.0078	0
7265	373.6	2.9516	3.0357	0.3898	1.0084	0
7266	373.5	2.95	3.0343	0.3896	1.0084	0
7267	373.4	2.9485	3.0026	0.39	1.0087	0
7268	373.3	2.9469	3.0013	0.3898	1.0093	0
7269	373.2	2.9718	3.0297	0.3899	1.0088	0
7270	373.1	2.9439	3.0605	0.3899	1.0091	0
7271	373	2.9686	3.0269	0.3899	1.009	0
7272	372.9	2.9408	3.025	0.3897	1.009	0
7273	372.8	2.9393	3.056	0.3898	1.0091	0
7274	372.7	2.9642	3.0223	0.3902	1.0088	0
7275	372.6	2.963	3.0209	0.3901	1.0088	0
7276	372.5	2.9614	3.0195	0.3901	1.009	0
7277	372.4	2.9337	3.0182	0.3902	1.0091	0
7278	372.3	2.9586	3.0164	0.3903	1.0094	0
7279	372.2	2.957	3.0472	0.3904	1.0096	0
7280	372.1	2.9555	3.0458	0.3903	1.0093	0
7281	372	2.9543	3.0123	0.3902	1.0101	0
7282	371.9	2.9527	2.9809	0.3904	1.0093	0
7283	371.8	2.9512	3.0414	0.3902	1.0096	0
7284	371.7	2.95	3.0079	0.3902	1.0099	0
7285	371.6	2.9767	2.9767	0.3906	1.0099	0
7286	371.5	2.9473	3.0052	0.3905	1.0097	0
7287	371.4	2.9462	3.0362	0.3906	1.0099	0
7288	371.3	2.9446	3.0348	0.3907	1.0098	0
7289	371.2	2.9431	3.0334	0.3905	1.01	0
7290	371.1	2.9698	3.032	0.3905	1.01	0
7291	371	2.9404	2.9987	0.3904	1.0098	0
7292	370.9	2.9674	3.0292	0.3904	1.0098	0
7293	370.8	2.9378	2.9957	0.3902	1.0099	0
7294	370.7	2.9646	2.9944	0.3904	1.0093	0
7295	370.6	2.963	2.963	0.3903	1.0095	0
7296	370.5	2.9337	3.0241	0.3904	1.0098	0

7297	370.4	2.9322	3.0223	0.3902	1.0096	0
7298	370.3	2.9307	2.9889	0.3901	1.0092	0
7299	370.2	2.9578	2.9876	0.3901	1.0092	0
7300	370.1	2.9863	3.0531	0.3901	1.0088	0
7301	370	2.9551	3.0173	0.3903	1.0089	0
7302	369.9	2.8771	2.9352	0.3906	1.0086	0
7303	369.8	2.8784	2.9363	0.3904	1.0089	0
7304	369.7	2.8798	2.9176	0.3903	1.009	0
7305	369.6	2.8993	2.9389	0.3902	1.0089	0
7306	369.5	2.9003	2.9197	0.3901	1.0084	0
7307	369.4	2.9017	2.9412	0.3898	1.0084	0
7308	369.3	2.8844	2.9223	0.3897	1.0083	0
7309	369.2	2.8854	2.9435	0.3896	1.008	0
7310	369.1	2.8864	2.9245	0.3896	1.0078	0
7311	369	2.8877	2.9255	0.3896	1.0078	0
7312	368.9	2.871	2.9266	0.3896	1.0075	0
7313	368.8	2.8901	2.9278	0.3894	1.0075	0
7314	368.7	2.8908	2.9488	0.3891	1.0075	0
7315	368.6	2.8742	2.9296	0.389	1.0075	0
7316	368.5	2.8931	2.9307	0.389	1.0072	0
7317	368.4	2.9126	2.9519	0.389	1.0074	0
7318	368.3	2.8771	2.9531	0.3889	1.0078	0
7319	368.2	2.9144	2.9751	0.3886	1.0079	0
7320	368.1	2.8969	2.9348	0.3886	1.0076	0
7321	368	2.9165	2.9355	0.3887	1.0072	0
7322	367.9	2.8989	2.9566	0.3886	1.007	0
7323	367.8	2.8996	2.9578	0.3885	1.0067	0
7324	367.7	2.9007	2.9385	0.3884	1.0072	0
7325	367.6	2.8841	2.9397	0.3885	1.0073	0
7326	367.5	2.9027	2.9404	0.3886	1.0077	0
7327	367.4	2.9034	2.9614	0.3887	1.0073	0
7328	367.3	2.9045	2.9423	0.3888	1.007	0
7329	367.2	2.9052	2.9431	0.3885	1.0073	0
7330	367.1	2.9245	2.9439	0.3885	1.0068	0
7331	367	2.9069	2.9446	0.3885	1.0073	0
7332	366.9	2.9076	2.9658	0.3885	1.0071	0
7333	366.8	2.9083	2.9462	0.3885	1.0074	0
7334	366.7	2.9094	2.9674	0.3882	1.0071	0
7335	366.6	2.9285	2.9477	0.3883	1.0069	0
7336	366.5	2.8931	2.9686	0.3883	1.0074	0
7337	366.4	2.9115	2.9492	0.3883	1.0075	0
7338	366.3	2.9307	2.95	0.3884	1.0074	0
7339	366.2	2.9314	2.971	0.3882	1.0077	0

7340	366.1	2.9136	2.9714	0.3881	1.008	0
7341	366	2.9329	2.9722	0.3881	1.0078	0
7342	365.9	2.9151	2.9527	0.3882	1.009	0
7343	365.8	2.9158	2.9735	0.3884	1.0081	0
7344	365.7	2.8986	2.9743	0.3882	1.0083	0
7345	365.6	2.9355	2.9751	0.3884	1.008	0
7346	365.5	2.9176	2.9555	0.3883	1.0082	0
7347	365.4	2.9183	2.9562	0.3885	1.0081	0
7348	365.3	2.9374	2.9983	0.3884	1.0089	0
7349	365.2	2.9194	2.9776	0.3883	1.0083	0
7350	365.1	2.9385	2.9578	0.3884	1.0089	0
7351	365	2.9208	2.9586	0.3887	1.0092	0
7352	364.9	2.9212	3.0004	0.3887	1.0089	0
7353	364.8	2.9404	2.9598	0.3887	1.0092	0
7354	364.7	2.9226	3.0017	0.3889	1.0091	0
7355	364.6	2.923	2.9809	0.3889	1.0101	0
7356	364.5	2.9234	2.9813	0.389	1.0099	0
7357	364.4	2.9241	2.9821	0.3891	1.0106	0
7358	364.3	2.9245	3.0035	0.3892	1.0109	0
7359	364.2	2.9248	3.0039	0.389	1.0111	0
7360	364.1	2.9439	3.0048	0.3893	1.0108	0
7361	364	2.9259	2.9638	0.3894	1.0109	0
7362	363.9	2.945	2.9642	0.3894	1.0112	0
7363	363.8	2.909	3.0057	0.3895	1.0117	0
7364	363.7	2.927	2.965	0.3896	1.012	0
7365	363.6	2.9274	2.9855	0.3897	1.0119	0
7366	363.5	2.9462	2.9859	0.3897	1.0124	0
7367	363.4	2.9104	2.9863	0.3897	1.0117	0
7368	363.3	2.9285	2.9867	0.3897	1.0119	0
7369	363.2	2.9289	3.0083	0.3899	1.0125	0
7370	363.1	2.9292	3.0083	0.3897	1.0126	0
7371	363	2.9481	3.0088	0.3898	1.0124	0
7372	362.9	2.9485	2.988	0.3899	1.0125	0
7373	362.8	2.9303	2.988	0.3899	1.0128	0
7374	362.7	2.9682	2.9884	0.39	1.0132	0
7375	362.6	2.9492	2.9884	0.3899	1.013	0
7376	362.5	2.9492	2.9889	0.3899	1.0129	0
7377	362.4	2.9133	2.9893	0.39	1.0133	0
7378	362.3	2.9314	2.9893	0.39	1.0135	0
7379	362.2	2.95	2.9897	0.39	1.0135	0
7380	362.1	2.9318	3.011	0.3901	1.0131	0
7381	362	2.9318	2.9897	0.3901	1.0138	0
7382	361.9	2.9504	3.0114	0.3901	1.0132	0

7383	361.8	2.9504	2.9901	0.3898	1.013	0
7384	361.7	2.9702	2.9702	0.39	1.0138	0
7385	361.6	2.9508	2.9905	0.39	1.0142	0
7386	361.5	2.9512	2.9905	0.3898	1.0136	0
7387	361.4	2.9512	2.9706	0.3898	1.0138	0
7388	361.3	2.9512	2.9905	0.3897	1.0136	0
7389	361.2	2.9326	2.9905	0.3897	1.0134	0
7390	361.1	2.9706	2.991	0.3899	1.0142	0
7391	361	2.9512	2.991	0.3898	1.0137	0
7392	360.9	2.9516	2.991	0.3897	1.014	0
7393	360.8	2.9516	3.0123	0.39	1.0141	0
7394	360.7	2.9516	2.991	0.3899	1.0141	0
7395	360.6	2.9516	2.9706	0.3899	1.0141	0
7396	360.5	2.9516	2.991	0.3898	1.014	0
7397	360.4	2.9512	3.0119	0.3896	1.0142	0
7398	360.3	2.9516	2.991	0.3898	1.0137	0
7399	360.2	2.9516	2.991	0.39	1.0139	0
7400	360.1	2.9706	2.991	0.3898	1.014	0
7401	360	2.9512	3.0119	0.3898	1.0145	0
7402	359.9	2.9512	3.0119	0.3897	1.014	0
7403	359.8	2.9512	3.0119	0.3895	1.0144	0
7404	359.7	2.9702	3.0339	0.3893	1.0142	0
7405	359.6	2.9508	3.0114	0.3895	1.0137	0
7406	359.5	2.9326	2.9905	0.3895	1.014	0
7407	359.4	2.9702	3.0114	0.3895	1.0132	0
7408	359.3	2.9508	2.9901	0.3893	1.0135	0
7409	359.2	2.9698	3.011	0.3893	1.0137	0
7410	359.1	2.9694	2.9897	0.3892	1.0131	0
7411	359	2.9694	2.9897	0.3891	1.0132	0
7412	358.9	2.95	3.0106	0.3891	1.0135	0
7413	358.8	2.969	3.0329	0.389	1.0134	0
7414	358.7	2.969	3.0101	0.3888	1.0127	0
7415	358.6	2.9686	2.9889	0.3887	1.0131	0
7416	358.5	2.9492	3.0097	0.3886	1.0128	0
7417	358.4	2.9682	2.9884	0.3886	1.0126	0
7418	358.3	2.9678	3.0315	0.3882	1.0124	0
7419	358.2	2.9485	3.0092	0.3886	1.0126	0
7420	358.1	2.9481	3.0311	0.3884	1.0119	0
7421	358	2.9876	3.0088	0.388	1.0124	0
7422	357.9	2.967	3.0083	0.3881	1.0121	0
7423	357.8	2.9666	3.0083	0.3882	1.0118	0
7424	357.7	2.9867	3.0301	0.3878	1.0121	0
7425	357.6	2.9662	3.0074	0.3878	1.0123	0

7426	357.5	2.9658	3.0297	0.3877	1.0123	0
7427	357.4	2.9462	3.007	0.3878	1.012	0
7428	357.3	2.9654	3.0066	0.388	1.0117	0
7429	357.2	2.9458	3.0061	0.3878	1.0115	0
7430	357.1	2.9646	3.0061	0.3876	1.0112	0
7431	357	2.945	3.0278	0.3875	1.0118	0
7432	356.9	2.9638	3.0052	0.3877	1.0117	0
7433	356.8	2.9638	3.0052	0.3877	1.0116	0
7434	356.7	2.963	3.0269	0.3876	1.0116	0
7435	356.6	2.9435	3.0044	0.3875	1.0119	0
7436	356.5	2.9626	3.0039	0.3875	1.0119	0
7437	356.4	2.9622	3.0259	0.3874	1.0118	0
7438	356.3	2.9618	3.0255	0.3875	1.0121	0
7439	356.2	2.9813	3.025	0.3873	1.0118	0
7440	356.1	2.9416	3.0246	0.3872	1.0115	0
7441	356	2.9412	3.0241	0.3874	1.012	0
7442	355.9	2.9602	3.0237	0.3873	1.012	0
7443	355.8	2.9598	3.0009	0.3875	1.0116	0
7444	355.7	2.9594	3.0462	0.3874	1.0116	0
7445	355.6	2.9393	3.0223	0.3873	1.012	0
7446	355.5	2.9582	3.0218	0.3874	1.0121	0
7447	355.4	2.978	3.0214	0.3876	1.0123	0
7448	355.3	2.9987	3.0443	0.3874	1.0119	0
7449	355.2	2.9772	3.0205	0.3875	1.0121	0
7450	355.1	2.9562	3.0434	0.3874	1.0124	0
7451	355	2.9759	3.0195	0.3874	1.0126	0
7452	354.9	2.9555	2.9965	0.3875	1.0124	0
7453	354.8	2.9355	3.0182	0.3875	1.0125	0
7454	354.7	2.9957	3.0182	0.3878	1.0132	0
7455	354.6	2.9743	3.041	0.3878	1.0135	0
7456	354.5	2.9948	3.0168	0.3879	1.014	0
7457	354.4	2.9731	2.994	0.3882	1.0139	0
7458	354.3	2.9523	3.0159	0.3881	1.014	0
7459	354.2	2.9718	3.0386	0.3882	1.0141	0
7460	354.1	2.9714	3.0381	0.3883	1.0143	0
7461	354	2.9706	3.0376	0.3883	1.0144	0
7462	353.9	2.9702	3.0372	0.3886	1.0147	0
7463	353.8	2.9694	3.0362	0.3886	1.0146	0
7464	353.7	2.9686	3.0123	0.3888	1.0147	0
7465	353.6	2.9682	3.0114	0.3886	1.015	0
7466	353.5	2.9674	2.9884	0.3887	1.0152	0
7467	353.4	2.9666	3.0101	0.3887	1.0155	0
7468	353.3	2.9662	3.0097	0.3889	1.016	0

7469	353.2	2.9867	3.0088	0.3891	1.0156	0
7470	353.1	2.9859	2.9859	0.3888	1.016	0
7471	353	2.9642	3.0074	0.3891	1.0158	0
7472	352.9	2.9634	3.0301	0.3892	1.0164	0
7473	352.8	2.9626	3.0061	0.3892	1.0161	0
7474	352.7	2.9618	2.983	0.3896	1.0165	0
7475	352.6	3.0048	3.0048	0.3896	1.0167	0
7476	352.5	2.9606	3.0039	0.3896	1.0169	0
7477	352.4	2.9809	2.9809	0.3897	1.0167	0
7478	352.3	2.959	3.0259	0.3896	1.0165	0
7479	352.2	2.9796	3.0017	0.3898	1.0168	0
7480	352.1	2.9574	3.0009	0.39	1.0173	0
7481	352	2.978	3.0004	0.3898	1.0172	0
7482	351.9	2.9559	3.0232	0.3899	1.0175	0
7483	351.8	2.9555	2.9987	0.3897	1.0179	0
7484	351.7	2.9755	2.9978	0.3899	1.0183	0
7485	351.6	2.9747	2.997	0.3898	1.0182	0
7486	351.5	2.9743	3.02	0.3902	1.018	0
7487	351.4	2.9735	2.9957	0.3901	1.0179	0
7488	351.3	2.9727	2.9952	0.3901	1.0176	0
7489	351.2	2.9508	2.9944	0.39	1.0182	0
7490	351.1	2.95	3.0168	0.3901	1.0183	0
7491	351	2.9706	2.9927	0.39	1.0191	0
7492	350.9	2.9485	2.9918	0.3903	1.0186	0
7493	350.8	2.969	3.015	0.3905	1.0189	0
7494	350.7	2.9905	3.0141	0.3903	1.0189	0
7495	350.6	2.9462	3.0376	0.3903	1.0186	0
7496	350.5	2.9666	3.0123	0.3901	1.019	0
7497	350.4	2.9442	3.0114	0.3904	1.0187	0
7498	350.3	2.9439	3.0357	0.3904	1.0185	0
7499	350.2	2.9863	3.0348	0.3903	1.0193	0
7500	350.1	2.9634	3.0339	0.3903	1.0187	0
7501	350	2.9626	3.0083	0.3904	1.0199	0
7502	349.9	2.9614	2.9838	0.3906	1.0196	0
7503	349.8	2.9393	3.0311	0.3904	1.0193	0
7504	349.7	2.9821	3.0301	0.3902	1.0186	0
7505	349.6	2.9809	3.0044	0.3904	1.0185	0
7506	349.5	2.9578	3.0035	0.3903	1.019	0
7507	349.4	2.9792	3.0026	0.3901	1.0193	0
7508	349.3	2.978	3.0264	0.39	1.0181	0
7509	349.2	2.9551	3.0004	0.3902	1.0191	0
7510	349.1	2.9763	2.9996	0.3901	1.0194	0
7511	349	2.9527	2.9987	0.3901	1.0189	0

7512	348.9	2.9743	3.0223	0.3901	1.0184	0
7513	348.8	2.9508	2.9965	0.3901	1.019	0
7514	348.7	2.9722	2.9957	0.3901	1.0187	0
7515	348.6	2.971	2.9944	0.3898	1.0188	0
7516	348.5	2.9477	2.9935	0.39	1.0179	0
7517	348.4	2.9465	3.0173	0.3898	1.018	0
7518	348.3	2.9454	2.9678	0.3897	1.0184	0
7519	348.2	2.9234	2.9905	0.3898	1.0185	0
7520	348.1	2.9658	2.9893	0.3898	1.0188	0
7521	348	2.9646	2.988	0.3899	1.0187	0
7522	347.9	2.9201	3.0119	0.3898	1.0184	0
7523	347.8	2.9626	2.9863	0.3897	1.0187	0
7524	347.7	2.9614	2.9851	0.3896	1.0178	0
7525	347.6	2.9606	3.0088	0.3895	1.0184	0
7526	347.5	2.9374	3.0079	0.3898	1.0182	0
7527	347.4	2.9582	3.0066	0.3896	1.0183	0
7528	347.3	2.9574	2.9809	0.3896	1.0178	0
7529	347.2	2.9796	3.0044	0.3892	1.0174	0
7530	347.1	2.9551	2.9788	0.3892	1.018	0
7531	347	2.9539	2.9776	0.3893	1.0174	0
7532	346.9	2.9531	3.0013	0.3893	1.0177	0
7533	346.8	2.9755	2.9755	0.3893	1.0183	0
7534	346.7	2.9743	2.9743	0.3894	1.0177	0
7535	346.6	2.95	2.9735	0.3895	1.0184	0
7536	346.5	2.9722	2.9722	0.3898	1.0187	0
7537	346.4	2.971	2.971	0.3896	1.0183	0
7538	346.3	2.9465	2.9702	0.3894	1.018	0
7539	346.2	2.9454	2.969	0.3894	1.018	0
7540	346.1	2.9446	2.9927	0.3895	1.0186	0
7541	346	2.967	3.0182	0.3895	1.018	0
7542	345.9	2.9658	2.9905	0.3894	1.018	0
7543	345.8	2.9412	2.9893	0.3894	1.0181	0
7544	345.7	2.9634	2.9884	0.3892	1.0179	0
7545	345.6	2.9622	2.9872	0.3895	1.0178	0
7546	345.5	2.9374	2.961	0.3893	1.0185	0
7547	345.4	2.9363	2.9846	0.3895	1.0184	0
7548	345.3	2.959	2.959	0.3899	1.0188	0
7549	345.2	2.934	2.9825	0.3894	1.0186	0
7550	345.1	2.9329	2.9813	0.3894	1.0192	0
7551	345	2.9801	3.0066	0.3894	1.0191	0
7552	344.9	2.9543	3.0334	0.3894	1.0185	0
7553	344.8	2.9531	2.9776	0.3896	1.0187	0
7554	344.7	2.9516	3.0031	0.3896	1.0195	0

7555	344.6	2.927	2.9755	0.3897	1.0194	0
7556	344.5	2.9492	3.0004	0.3898	1.0196	0
7557	344.4	2.9481	2.9991	0.3897	1.0199	0
7558	344.3	2.9469	2.9978	0.39	1.0197	0
7559	344.2	2.9458	2.9706	0.3902	1.0195	0
7560	344.1	2.9446	2.9957	0.3901	1.02	0
7561	344	2.9435	2.9435	0.3902	1.0197	0
7562	343.9	2.942	2.9931	0.3904	1.0198	0
7563	343.8	2.9408	2.9918	0.3905	1.0203	0
7564	343.7	2.9646	2.9905	0.3902	1.0205	0
7565	343.6	2.9634	2.9897	0.3906	1.0212	0
7566	343.5	2.9618	2.9884	0.3904	1.0201	0
7567	343.4	2.961	2.9872	0.3904	1.0201	0
7568	343.3	2.9594	2.9594	0.3905	1.0211	0
7569	343.2	2.9586	2.9586	0.3906	1.0213	0
7570	343.1	2.9322	3.0414	0.3906	1.0214	0
7571	343	2.9559	2.9821	0.3906	1.021	0
7572	342.9	2.9296	2.9296	0.3907	1.0225	0
7573	342.8	2.9531	3.0074	0.3905	1.0215	0
7574	342.7	2.927	2.9519	0.3908	1.0222	0
7575	342.6	2.9508	2.9772	0.3908	1.0216	0
7576	342.5	2.9248	2.9759	0.391	1.022	0
7577	342.4	2.9485	2.9485	0.3909	1.0225	0
7578	342.3	2.9223	2.9735	0.3912	1.0224	0
7579	342.2	2.9458	2.9722	0.3912	1.0221	0
7580	342.1	2.9442	2.9706	0.3912	1.0229	0
7581	342	2.9431	2.9694	0.391	1.0229	0
7582	341.9	2.942	2.942	0.3912	1.0224	0
7583	341.8	2.9404	2.9404	0.3911	1.0229	0
7584	341.7	2.9393	2.9658	0.3916	1.0236	0
7585	341.6	2.9378	2.9378	0.3916	1.0231	0
7586	341.5	2.9363	2.991	0.3915	1.0233	0
7587	341.4	2.9101	2.9614	0.3913	1.0237	0
7588	341.3	2.909	2.9602	0.3914	1.024	0
7589	341.2	2.9322	2.9867	0.3914	1.0241	0
7590	341.1	2.9062	2.9574	0.3914	1.0234	0
7591	341	2.9559	2.9838	0.3916	1.0236	0
7592	340.9	2.9034	2.9825	0.3915	1.0239	0
7593	340.8	2.9266	2.9531	0.3915	1.0234	0
7594	340.7	2.9519	2.9519	0.3915	1.0237	0
7595	340.6	2.9241	2.9504	0.3914	1.0235	0
7596	340.5	2.8979	3.007	0.3913	1.0234	0
7597	340.4	2.9477	2.9477	0.3913	1.0236	0

7598	340.3	2.9197	2.9743	0.3914	1.0232	0
7599	340.2	2.9183	2.9727	0.3915	1.0237	0
7600	340.1	2.8921	3.0013	0.3916	1.0236	0
7601	340	2.942	2.942	0.3917	1.0235	0
7602	339.9	1.9823	2.0492	0.3716	0.9529	0
7603	339.8	1.9789	2.0431	0.3715	0.9525	0
7604	339.7	1.9814	2.0464	0.3716	0.9528	0
7605	339.6	1.9781	2.0427	0.3715	0.9523	0
7606	339.5	1.9767	2.0413	0.3714	0.9522	0
7607	339.4	1.9753	2.0399	0.3714	0.9521	0
7608	339.3	1.9739	2.0385	0.3712	0.9517	0
7609	339.2	1.9727	2.035	0.3714	0.9517	0
7610	339.1	1.9692	2.0357	0.371	0.9516	0
7611	339	1.9698	2.0344	0.371	0.9511	0
7612	338.9	1.9663	2.033	0.371	0.9507	0
7613	338.8	1.9689	2.0315	0.3708	0.9504	0
7614	338.7	1.9655	2.0301	0.3707	0.9506	0
7615	338.6	1.964	2.0286	0.3706	0.95	0
7616	338.5	1.9606	2.0296	0.3705	0.9496	0
7617	338.4	1.9612	2.0282	0.3703	0.9494	0
7618	338.3	1.9619	2.0246	0.3702	0.9489	0
7619	338.2	1.9605	2.0208	0.3705	0.949	0
7620	338.1	1.9571	2.0217	0.3701	0.9484	0
7621	338	1.9577	2.0203	0.3699	0.9487	0
7622	337.9	1.9564	2.0213	0.3701	0.9482	0
7623	337.8	1.9509	2.0175	0.3699	0.9477	0
7624	337.7	1.9517	2.014	0.3696	0.9481	0
7625	337.6	1.9484	2.0174	0.3697	0.9476	0
7626	337.5	1.949	2.0136	0.3696	0.947	0
7627	337.4	1.9517	2.0147	0.3697	0.9471	0
7628	337.3	1.9482	2.0108	0.3695	0.9468	0
7629	337.2	1.945	2.0096	0.3695	0.9464	0
7630	337.1	1.9456	2.0082	0.3694	0.9464	0
7631	337	1.9402	2.0045	0.3692	0.9463	0
7632	336.9	1.9429	2.0078	0.3691	0.9459	0
7633	336.8	1.9377	2.0043	0.3692	0.9459	0
7634	336.7	1.9384	2.0031	0.3692	0.9459	0
7635	336.6	1.9391	2.0017	0.3691	0.9455	0
7636	336.5	1.9357	1.9981	0.369	0.9454	0
7637	336.4	1.9345	1.9991	0.3691	0.9451	0
7638	336.3	1.9371	2	0.3687	0.9446	0
7639	336.2	1.9339	1.9965	0.3687	0.9441	0
7640	336.1	1.9306	1.9929	0.3687	0.9447	0

7641	336	1.9314	1.994	0.3686	0.9442	0
7642	335.9	1.9281	1.9904	0.3686	0.9446	0
7643	335.8	1.9267	1.9937	0.3685	0.9437	0
7644	335.7	1.9255	1.9925	0.3685	0.9435	0
7645	335.6	1.9282	1.9888	0.3684	0.9434	0
7646	335.5	1.9251	1.9854	0.3687	0.9436	0
7647	335.4	1.9237	1.9863	0.3684	0.9428	0
7648	335.3	1.9225	1.9851	0.3684	0.9424	0
7649	335.2	1.9193	1.984	0.3686	0.9428	0
7650	335.1	1.9222	1.9828	0.3682	0.9431	0
7651	335	1.9189	1.9815	0.3683	0.9426	0
7652	334.9	1.9156	1.9802	0.3681	0.9422	0
7653	334.8	1.9145	1.9791	0.3682	0.9423	0
7654	334.7	1.9133	1.9779	0.3682	0.9421	0
7655	334.6	1.9141	1.9767	0.3682	0.9419	0
7656	334.5	1.9108	1.9754	0.3681	0.9419	0
7657	334.4	1.9117	1.972	0.3682	0.9414	0
7658	334.3	1.9086	1.9732	0.3684	0.9418	0
7659	334.2	1.9093	1.9719	0.3682	0.9421	0
7660	334.1	1.908	1.9683	0.3682	0.9416	0
7661	334	1.9068	1.9694	0.3681	0.9411	0
7662	333.9	1.9056	1.9682	0.3681	0.9412	0
7663	333.8	1.9024	1.967	0.3682	0.9408	0
7664	333.7	1.9011	1.9658	0.3682	0.9407	0
7665	333.6	1.902	1.9623	0.3678	0.9407	0
7666	333.5	1.9008	1.9634	0.3679	0.9401	0
7667	333.4	1.8997	1.9623	0.368	0.9406	0
7668	333.3	1.9005	1.9611	0.3678	0.9402	0
7669	333.2	1.8973	1.9599	0.3678	0.9399	0
7670	333.1	1.894	1.961	0.3675	0.9396	0
7671	333	1.8948	1.9551	0.3674	0.939	0
7672	332.9	1.8937	1.954	0.3674	0.939	0
7673	332.8	1.8905	1.9551	0.3675	0.9394	0
7674	332.7	1.8894	1.9517	0.3673	0.9385	0
7675	332.6	1.8883	1.9529	0.3676	0.9385	0
7676	332.5	1.889	1.9539	0.3674	0.9383	0
7677	332.4	1.8879	1.9505	0.3674	0.9386	0
7678	332.3	1.8867	1.947	0.3673	0.9381	0
7679	332.2	1.8856	1.9505	0.3673	0.9378	0
7680	332.1	1.8843	1.9469	0.3669	0.9377	0
7681	332	1.8832	1.9458	0.367	0.9375	0
7682	331.9	1.8821	1.9447	0.3668	0.9376	0
7683	331.8	1.8809	1.9435	0.3667	0.9375	0

7684	331.7	1.8798	1.9424	0.3666	0.9373	0
7685	331.6	1.8786	1.9436	0.3666	0.9368	0
7686	331.5	1.8774	1.9377	0.3666	0.9363	0
7687	331.4	1.8784	1.9413	0.3664	0.936	0
7688	331.3	1.8752	1.9355	0.3665	0.9362	0
7689	331.2	1.8741	1.9367	0.3663	0.9358	0
7690	331.1	1.8728	1.9354	0.3661	0.9354	0
7691	331	1.8717	1.9366	0.3658	0.9352	0
7692	330.9	1.8706	1.9332	0.366	0.9348	0
7693	330.8	1.8715	1.9321	0.3659	0.9351	0
7694	330.7	1.8682	1.9309	0.3658	0.9346	0
7695	330.6	1.867	1.9296	0.3656	0.9343	0
7696	330.5	1.8658	1.9307	0.3653	0.9335	0
7697	330.4	1.8647	1.9273	0.3654	0.9334	0
7698	330.3	1.8635	1.9238	0.3653	0.9334	0
7699	330.2	1.8624	1.925	0.3656	0.9334	0
7700	330.1	1.8611	1.9237	0.3651	0.9329	0
7701	330	1.862	1.9225	0.3651	0.9324	0
7702	329.9	1.8567	1.9214	0.3651	0.9324	0
7703	329.8	1.8596	1.9178	0.3651	0.9321	0
7704	329.7	1.8564	1.919	0.3649	0.9324	0
7705	329.6	1.8571	1.92	0.3647	0.9318	0
7706	329.5	1.8558	1.9164	0.3645	0.9311	0
7707	329.4	1.8548	1.9154	0.3648	0.931	0
7708	329.3	1.8515	1.9141	0.3646	0.9309	0
7709	329.2	1.8503	1.9129	0.3645	0.931	0
7710	329.1	1.8489	1.9115	0.3641	0.93	0
7711	329	1.8456	1.9102	0.364	0.9304	0
7712	328.9	1.8465	1.9091	0.3639	0.9297	0
7713	328.8	1.8473	1.9079	0.3639	0.9298	0
7714	328.7	1.844	1.9089	0.3636	0.93	0
7715	328.6	1.8429	1.9055	0.3636	0.9296	0
7716	328.5	1.8436	1.9042	0.3635	0.9288	0
7717	328.4	1.8403	1.9006	0.3635	0.9285	0
7718	328.3	1.8391	1.9017	0.3635	0.9285	0
7719	328.2	1.8379	1.8982	0.3633	0.9281	0
7720	328.1	1.8366	1.8992	0.363	0.929	0
7721	328	1.8354	1.8956	0.363	0.928	0
7722	327.9	1.8341	1.8967	0.363	0.9277	0
7723	327.8	1.8329	1.8932	0.363	0.9278	0
7724	327.7	1.8316	1.8919	0.363	0.9273	0
7725	327.6	1.8326	1.8932	0.3628	0.927	0
7726	327.5	1.8293	1.8943	0.3629	0.927	0

7727	327.4	1.8302	1.8908	0.3628	0.9266	0
7728	327.3	1.8268	1.8895	0.3627	0.9265	0
7729	327.2	1.8277	1.8883	0.3627	0.9269	0
7730	327.1	1.8244	1.887	0.3627	0.9261	0
7731	327	1.8232	1.8859	0.3625	0.9262	0
7732	326.9	1.8199	1.8846	0.3621	0.9257	0
7733	326.8	1.8209	1.8835	0.3624	0.9254	0
7734	326.7	1.8176	1.8822	0.3623	0.9249	0
7735	326.6	1.8183	1.8809	0.3622	0.9249	0
7736	326.5	1.8171	1.8774	0.3621	0.9247	0
7737	326.4	1.8159	1.8785	0.3621	0.9248	0
7738	326.3	1.8168	1.875	0.3619	0.9247	0
7739	326.2	1.8115	1.8762	0.3619	0.9243	0
7740	326.1	1.8124	1.8727	0.3617	0.9241	0
7741	326	1.8112	1.8738	0.3617	0.924	0
7742	325.9	1.8119	1.8725	0.3617	0.9237	0
7743	325.8	1.8087	1.869	0.3618	0.9241	0
7744	325.7	1.8095	1.8701	0.3616	0.9239	0
7745	325.6	1.8062	1.8689	0.3615	0.9229	0
7746	325.5	1.8091	1.8676	0.3614	0.9233	0
7747	325.4	1.8038	1.8687	0.3615	0.9231	0
7748	325.3	1.8006	1.8629	0.3617	0.9227	0
7749	325.2	1.8013	1.8616	0.3614	0.9223	0
7750	325.1	1.8001	1.8604	0.3612	0.9223	0
7751	325	1.799	1.8616	0.3613	0.9223	0
7752	324.9	1.7958	1.8627	0.3613	0.9225	0
7753	324.8	1.7966	1.8569	0.3612	0.9218	0
7754	324.7	1.7953	1.8556	0.3612	0.9219	0
7755	324.6	1.7941	1.8568	0.3612	0.9215	0
7756	324.5	1.793	1.8533	0.3612	0.9215	0
7757	324.4	1.7899	1.8522	0.3612	0.9214	0
7758	324.3	1.7906	1.8556	0.3611	0.9215	0
7759	324.2	1.7874	1.852	0.3609	0.9208	0
7760	324.1	1.7883	1.8509	0.3611	0.9206	0
7761	324	1.7871	1.8497	0.3607	0.9204	0
7762	323.9	1.786	1.8486	0.3607	0.9207	0
7763	323.8	1.7848	1.8498	0.3608	0.9209	0
7764	323.7	1.7835	1.8438	0.3605	0.9202	0
7765	323.6	1.7844	1.8427	0.3606	0.9198	0
7766	323.5	1.7792	1.8415	0.3607	0.9198	0
7767	323.4	1.7801	1.8381	0.3607	0.9198	0
7768	323.3	1.779	1.8393	0.3604	0.9194	0
7769	323.2	1.7759	1.8405	0.3605	0.9195	0

7770	323.1	1.7769	1.8349	0.3607	0.9194	0
7771	323	1.7777	1.8383	0.3604	0.9193	0
7772	322.9	1.7726	1.8326	0.3605	0.9187	0
7773	322.8	1.7714	1.836	0.3605	0.9187	0
7774	322.7	1.7723	1.8349	0.3603	0.9184	0
7775	322.6	1.7712	1.8315	0.3603	0.9187	0
7776	322.5	1.7699	1.8302	0.3601	0.918	0
7777	322.4	1.7688	1.8314	0.3602	0.9177	0
7778	322.3	1.7677	1.828	0.3601	0.918	0
7779	322.2	1.7664	1.829	0.3601	0.9176	0
7780	322.1	1.7656	1.8259	0.36	0.9174	0
7781	322	1.7643	1.8269	0.3598	0.9172	0
7782	321.9	1.7632	1.8258	0.3596	0.916	0
7783	321.8	1.762	1.8246	0.3596	0.916	0
7784	321.7	1.759	1.8213	0.3595	0.9162	0
7785	321.6	1.756	1.8226	0.3593	0.9155	0
7786	321.5	1.7569	1.8192	0.3593	0.9155	0
7787	321.4	1.7576	1.8156	0.3592	0.9148	0
7788	321.3	1.7566	1.8192	0.359	0.9155	0
7789	321.2	1.7555	1.8181	0.3588	0.9144	0
7790	321.1	1.7543	1.8169	0.3587	0.9144	0
7791	321	1.7513	1.8136	0.3586	0.9146	0
7792	320.9	1.7522	1.8125	0.3588	0.9143	0
7793	320.8	1.749	1.8091	0.3586	0.9132	0
7794	320.7	1.7479	1.8126	0.3584	0.9133	0
7795	320.6	1.7488	1.8091	0.3583	0.913	0
7796	320.5	1.7457	1.8103	0.3583	0.9131	0
7797	320.4	1.7465	1.8068	0.3582	0.9127	0
7798	320.3	1.7435	1.8081	0.3583	0.9129	0
7799	320.2	1.7442	1.8045	0.358	0.9125	0
7800	320.1	1.7431	1.8057	0.3577	0.9117	0
7801	320	1.7399	1.8022	0.3576	0.9114	

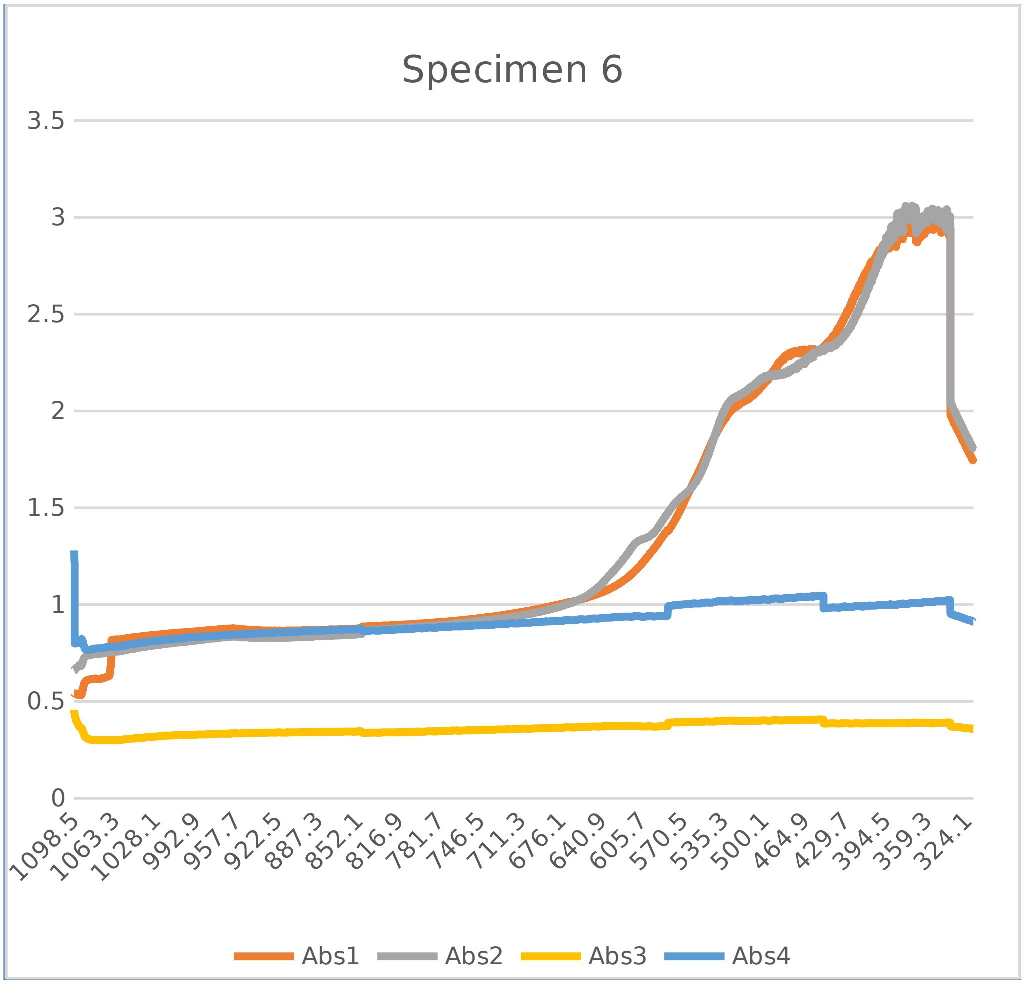

Specimen 6
3.5
3
2.5
2
1.5
1
0.5
0
1098.5
1063.3
1028.1
992.9
957.7
922.5
887.3
852.1
816.9
781.7
746.5
711.3
676.1
640.9
605.7
570.5
535.3
500.1
464.9
429.7
394.5
359.3
324.1
Abs1
Abs2
Abs3
Abs4
