# Supplementary material for: Coherent poly propagation materials with 3-dimensional photonic control over visible light
Source: PLoS One. 2019 Oct 17;14(10):e0223715. doi: 10.1371/journal.pone.0223715 (PMC6797174; doi:10.1371/journal.pone.0223715)
Supplement: S7 Table — Spreadsheet generated graph at bottom of table. (PDF) [file pone.0223715.s016.pdf]

Wavelength Scan Report - Specimen 7

Company	Complete Consulting Services LLC		
Operator	Dr. Michelle R. Stem	Test Time	11/8/2017 0:33
Document No.	WLS1711080001_3	Print Date	9/6/2019 19:48
Start wavelength	1100nm	Number of data	7801
End wavelength	320nm	K1	0.99
Unit		K0	0
Interval	0.1nm	K2	0
Calculate the Conc.	No	K3	0
Modification time	11/8/2017 0:33	Access time	11/8/2017 0:33
Remark	S06a082 - Specimen 7		

Scan Data

No.	Wavelength	Abs1	Abs2	Abs3	Abs4	Visible
1	1100	0.4682	0.3961	0.5433	1.3689	0
2	1099.9	0.4679	0.4006	0.5413	1.3694	0
3	1099.8	0.4661	0.4052	0.5395	1.3697	0
4	1099.7	0.4599	0.4099	0.5381	1.3695	0
5	1099.6	0.7972	0.4151	0.5371	1.3703	0
6	1099.5	0.7945	0.4199	0.5365	1.3708	0
7	1099.4	0.7917	0.4245	0.5356	1.3707	0
8	1099.3	0.79	0.4292	0.5349	1.3711	0
9	1099.2	0.7895	0.435	0.5333	1.3708	0
10	1099.1	0.7895	0.4418	0.5329	1.3714	0
11	1099	0.7896	0.4495	0.5318	1.3714	0
12	1098.9	0.7895	0.4608	0.5315	1.372	0
13	1098.8	0.7891	0.4731	0.5304	1.3718	0
14	1098.7	0.789	0.4817	0.5298	1.3719	0
15	1098.6	0.7891	0.4904	0.5291	1.3725	0
16	1098.5	0.7888	0.4999	0.5286	1.3721	0
17	1098.4	0.789	0.5105	0.5281	1.3729	0
18	1098.3	0.7889	0.5213	0.5273	1.3734	0
19	1098.2	0.7891	0.5349	0.5267	1.3737	0
20	1098.1	0.789	0.5462	0.5259	1.3742	0
21	1098	0.7889	0.5554	0.5252	1.3752	0
22	1097.9	0.7886	0.5672	0.524	1.3756	0
23	1097.8	0.7886	0.5817	0.5231	1.376	0
24	1097.7	0.7881	0.5936	0.5218	1.3771	0
25	1097.6	0.7878	0.6065	0.5211	1.3771	0
26	1097.5	0.7876	0.619	0.5202	1.3784	0
27	1097.4	0.7875	0.631	0.5194	1.3793	0
28	1097.3	0.7874	0.6428	0.5185	1.3804	0
29	1097.2	0.7872	0.6478	0.5177	1.3805	0

30	1097.1	0.7875	0.6539	0.5172	1.3817	0
31	1097	0.7875	0.6629	0.5168	1.3832	0
32	1096.9	0.7875	0.6772	0.516	1.3843	0
33	1096.8	0.7877	0.6826	0.5162	1.3849	0
34	1096.7	0.7881	0.6831	0.5159	1.3854	0
35	1096.6	0.7881	0.6833	0.5155	1.3866	0
36	1096.5	0.7887	0.684	0.515	1.3882	0
37	1096.4	0.7888	0.6845	0.5146	1.39	0
38	1096.3	0.7892	0.6854	0.5142	1.3914	0
39	1096.2	0.7888	0.6858	0.5135	1.3927	0
40	1096.1	0.7886	0.6865	0.5128	1.3939	0
41	1096	0.7885	0.6875	0.5123	1.394	0
42	1095.9	0.7879	0.6886	0.5114	1.3959	0
43	1095.8	0.7876	0.6899	0.5106	1.3979	0
44	1095.7	0.7866	0.6907	0.5094	1.3984	0
45	1095.6	0.7857	0.6916	0.5083	1.3994	0
46	1095.5	0.7854	0.6926	0.5072	1.4006	0
47	1095.4	0.7844	0.6932	0.5062	1.4004	0
48	1095.3	0.784	0.6941	0.5056	1.4011	0
49	1095.2	0.7835	0.6944	0.5049	1.4008	0
50	1095.1	0.7834	0.6947	0.5042	1.401	0
51	1095	0.7837	0.6946	0.5037	1.4015	0
52	1094.9	0.7842	0.6946	0.5033	1.4006	0
53	1094.8	0.7847	0.694	0.5031	1.4002	0
54	1094.7	0.7854	0.6933	0.5029	1.3992	0
55	1094.6	0.7865	0.6927	0.5032	1.3987	0
56	1094.5	0.7882	0.6925	0.5042	1.3982	0
57	1094.4	0.7895	0.6921	0.5048	1.3976	0
58	1094.3	0.7909	0.6914	0.5054	1.3974	0
59	1094.2	0.7922	0.6906	0.5059	1.397	0
60	1094.1	0.7934	0.6904	0.5064	1.3971	0
61	1094	0.7948	0.6902	0.5069	1.3973	0
62	1093.9	0.7959	0.6903	0.5076	1.3975	0
63	1093.8	0.7966	0.6904	0.5078	1.3977	0
64	1093.7	0.7972	0.6909	0.5082	1.3985	0
65	1093.6	0.7976	0.6915	0.5087	1.4	0
66	1093.5	0.7974	0.6921	0.5085	1.4009	0
67	1093.4	0.7972	0.6931	0.5082	1.4018	0
68	1093.3	0.7969	0.6943	0.508	1.4035	0
69	1093.2	0.796	0.6955	0.5071	1.4048	0
70	1093.1	0.7949	0.6969	0.5062	1.406	0
71	1093	0.7936	0.6983	0.5048	1.4074	0
72	1092.9	0.7923	0.7002	0.5039	1.4092	0

73	1092.8	0.7907	0.7016	0.5023	1.4105	0
74	1092.7	0.7889	0.7033	0.5007	1.4116	0
75	1092.6	0.7868	0.7051	0.4992	1.4132	0
76	1092.5	0.7847	0.7065	0.4973	1.4146	0
77	1092.4	0.7832	0.7083	0.4959	1.416	0
78	1092.3	0.7809	0.7099	0.4933	1.4168	0
79	1092.2	0.7792	0.7117	0.491	1.4178	0
80	1092.1	0.777	0.7136	0.4893	1.4199	0
81	1092	0.7749	0.7148	0.4868	1.4205	0
82	1091.9	0.773	0.7163	0.4847	1.4211	0
83	1091.8	0.7711	0.7179	0.4829	1.4224	0
84	1091.7	0.7692	0.7193	0.4802	1.4238	0
85	1091.6	0.7675	0.7207	0.4775	1.4243	0
86	1091.5	0.7655	0.7217	0.4753	1.4254	0
87	1091.4	0.7641	0.7228	0.4732	1.4257	0
88	1091.3	0.7628	0.7242	0.4714	1.4263	0
89	1091.2	0.7612	0.7251	0.4698	1.4272	0
90	1091.1	0.7602	0.7262	0.4683	1.4281	0
91	1091	0.7588	0.7268	0.466	1.4289	0
92	1090.9	0.758	0.7279	0.4644	1.429	0
93	1090.8	0.7565	0.7286	0.4625	1.4293	0
94	1090.7	0.7556	0.7291	0.4606	1.4294	0
95	1090.6	0.7546	0.7298	0.4595	1.4296	0
96	1090.5	0.7538	0.7303	0.4582	1.4299	0
97	1090.4	0.7531	0.7309	0.4575	1.4304	0
98	1090.3	0.7526	0.7317	0.4566	1.4307	0
99	1090.2	0.7519	0.7321	0.4557	1.4313	0
100	1090.1	0.7514	0.7326	0.4543	1.4315	0
101	1090	0.7505	0.7324	0.4525	1.4313	0
102	1089.9	0.7501	0.7329	0.4518	1.4313	0
103	1089.8	0.7495	0.7329	0.4505	1.4314	0
104	1089.7	0.7492	0.7336	0.4498	1.4314	0
105	1089.6	0.7489	0.734	0.4489	1.4318	0
106	1089.5	0.7487	0.7345	0.4481	1.4319	0
107	1089.4	0.7483	0.7347	0.4475	1.432	0
108	1089.3	0.7479	0.7349	0.4468	1.4314	0
109	1089.2	0.7473	0.7351	0.4459	1.4323	0
110	1089.1	0.7472	0.7353	0.4451	1.4324	0
111	1089	0.7469	0.7354	0.4444	1.4316	0
112	1088.9	0.7467	0.7356	0.4437	1.4323	0
113	1088.8	0.7465	0.7358	0.4433	1.4326	0
114	1088.7	0.7462	0.7358	0.4422	1.4321	0
115	1088.6	0.7461	0.736	0.4417	1.4324	0

116	1088.5	0.746	0.7364	0.4411	1.4323	0
117	1088.4	0.7457	0.7363	0.4397	1.432	0
118	1088.3	0.7457	0.7365	0.4389	1.432	0
119	1088.2	0.7457	0.7363	0.4382	1.4322	0
120	1088.1	0.7456	0.7365	0.4377	1.4322	0
121	1088	0.7455	0.7364	0.4369	1.4321	0
122	1087.9	0.7456	0.7366	0.4366	1.4317	0
123	1087.8	0.7455	0.7369	0.4363	1.4325	0
124	1087.7	0.7454	0.7368	0.4359	1.4323	0
125	1087.6	0.7452	0.7367	0.4355	1.4322	0
126	1087.5	0.7451	0.7369	0.4347	1.4327	0
127	1087.4	0.7453	0.7369	0.4341	1.4324	0
128	1087.3	0.7451	0.7372	0.4335	1.4319	0
129	1087.2	0.7456	0.7374	0.4327	1.433	0
130	1087.1	0.7453	0.7375	0.4321	1.4329	0
131	1087	0.7452	0.7376	0.4311	1.4324	0
132	1086.9	0.7452	0.7374	0.4306	1.4328	0
133	1086.8	0.7452	0.7377	0.43	1.4332	0
134	1086.7	0.7452	0.7377	0.4299	1.4328	0
135	1086.6	0.745	0.7378	0.4292	1.4333	0
136	1086.5	0.7451	0.7379	0.4283	1.4334	0
137	1086.4	0.7449	0.7381	0.4274	1.4338	0
138	1086.3	0.7451	0.7384	0.4269	1.4344	0
139	1086.2	0.7452	0.7384	0.4262	1.434	0
140	1086.1	0.7453	0.7387	0.4253	1.4349	0
141	1086	0.7453	0.7388	0.4247	1.4351	0
142	1085.9	0.7451	0.7389	0.424	1.4346	0
143	1085.8	0.7451	0.7387	0.4231	1.4345	0
144	1085.7	0.745	0.7386	0.4222	1.4349	0
145	1085.6	0.7451	0.7388	0.4216	1.4355	0
146	1085.5	0.7448	0.7391	0.4209	1.4355	0
147	1085.4	0.7448	0.7391	0.4206	1.4358	0
148	1085.3	0.7451	0.7394	0.4199	1.4364	0
149	1085.2	0.745	0.7392	0.4192	1.4368	0
150	1085.1	0.7448	0.7392	0.4186	1.4364	0
151	1085	0.7452	0.7396	0.4184	1.4363	0
152	1084.9	0.7452	0.7395	0.4178	1.4368	0
153	1084.8	0.7454	0.7397	0.4172	1.4376	0
154	1084.7	0.7454	0.7397	0.4164	1.4372	0
155	1084.6	0.7453	0.7397	0.4155	1.4377	0
156	1084.5	0.7453	0.7401	0.415	1.4384	0
157	1084.4	0.7455	0.7402	0.4145	1.4386	0
158	1084.3	0.7456	0.7401	0.414	1.4393	0

159	1084.2	0.7457	0.7401	0.4132	1.439	0
160	1084.1	0.7461	0.7405	0.4128	1.4398	0
161	1084	0.7461	0.7403	0.4123	1.4399	0
162	1083.9	0.7462	0.7406	0.4121	1.4402	0
163	1083.8	0.7463	0.7406	0.4115	1.4404	0
164	1083.7	0.7462	0.7405	0.4109	1.4411	0
165	1083.6	0.7464	0.7406	0.41	1.4412	0
166	1083.5	0.7467	0.7412	0.4096	1.4415	0
167	1083.4	0.7465	0.7409	0.4088	1.4415	0
168	1083.3	0.7468	0.7412	0.4082	1.4418	0
169	1083.2	0.7468	0.7412	0.4075	1.4424	0
170	1083.1	0.7469	0.7413	0.4068	1.4426	0
171	1083	0.7469	0.7413	0.406	1.4422	0
172	1082.9	0.7469	0.7412	0.4054	1.4427	0
173	1082.8	0.7469	0.7411	0.4046	1.4433	0
174	1082.7	0.7469	0.7414	0.4038	1.4434	0
175	1082.6	0.7469	0.7414	0.4028	1.4434	0
176	1082.5	0.7469	0.7413	0.402	1.443	0
177	1082.4	0.7473	0.7416	0.4015	1.4429	0
178	1082.3	0.7471	0.7414	0.4008	1.4424	0
179	1082.2	0.7474	0.742	0.4006	1.4432	0
180	1082.1	0.7473	0.7416	0.3999	1.4436	0
181	1082	0.7475	0.7417	0.3993	1.4437	0
182	1081.9	0.7473	0.7416	0.3985	1.4433	0
183	1081.8	0.7475	0.7417	0.3981	1.4442	0
184	1081.7	0.7476	0.742	0.3977	1.4439	0
185	1081.6	0.7477	0.7421	0.3975	1.4446	0
186	1081.5	0.7475	0.742	0.3968	1.4441	0
187	1081.4	0.7477	0.7423	0.3963	1.444	0
188	1081.3	0.748	0.7423	0.3959	1.445	0
189	1081.2	0.7479	0.7424	0.3954	1.445	0
190	1081.1	0.7478	0.7424	0.3946	1.4451	0
191	1081	0.7478	0.7425	0.3937	1.4448	0
192	1080.9	0.748	0.7424	0.393	1.4457	0
193	1080.8	0.7477	0.7424	0.392	1.4448	0
194	1080.7	0.7478	0.7425	0.3911	1.4449	0
195	1080.6	0.748	0.7427	0.3904	1.4451	0
196	1080.5	0.7479	0.743	0.3899	1.4451	0
197	1080.4	0.7481	0.7427	0.3892	1.4453	0
198	1080.3	0.7482	0.7431	0.3885	1.4454	0
199	1080.2	0.7481	0.7431	0.3878	1.445	0
200	1080.1	0.7482	0.7431	0.3874	1.4456	0
201	1080	0.748	0.7428	0.3865	1.4451	0

202	1079.9	0.7483	0.7432	0.386	1.4454	0
203	1079.8	0.7481	0.7432	0.3854	1.4457	0
204	1079.7	0.7484	0.7432	0.3849	1.446	0
205	1079.6	0.7485	0.7436	0.3844	1.4461	0
206	1079.5	0.7482	0.7433	0.3837	1.4455	0
207	1079.4	0.7481	0.7431	0.3829	1.4455	0
208	1079.3	0.748	0.7433	0.3823	1.445	0
209	1079.2	0.748	0.7433	0.3821	1.4455	0
210	1079.1	0.7479	0.7433	0.3813	1.4447	0
211	1079	0.7479	0.7432	0.3804	1.4451	0
212	1078.9	0.7482	0.7433	0.3801	1.4452	0
213	1078.8	0.7482	0.7433	0.3798	1.4452	0
214	1078.7	0.748	0.7436	0.3792	1.4457	0
215	1078.6	0.7481	0.7434	0.3785	1.4449	0
216	1078.5	0.7478	0.7435	0.3779	1.4451	0
217	1078.4	0.748	0.7437	0.3775	1.4449	0
218	1078.3	0.7481	0.7437	0.377	1.445	0
219	1078.2	0.7482	0.7438	0.3764	1.4447	0
220	1078.1	0.748	0.7439	0.3758	1.4451	0
221	1078	0.7478	0.7436	0.3748	1.4449	0
222	1077.9	0.748	0.7439	0.3743	1.4451	0
223	1077.8	0.7479	0.7439	0.3734	1.4451	0
224	1077.7	0.748	0.7442	0.3729	1.4445	0
225	1077.6	0.7481	0.7443	0.3724	1.4446	0
226	1077.5	0.7479	0.7441	0.3718	1.4449	0
227	1077.4	0.748	0.7443	0.3711	1.4448	0
228	1077.3	0.7479	0.7442	0.3703	1.445	0
229	1077.2	0.7481	0.7443	0.3699	1.4448	0
230	1077.1	0.7479	0.7444	0.3692	1.4448	0
231	1077	0.7481	0.7445	0.3688	1.4454	0
232	1076.9	0.7482	0.7446	0.3685	1.4448	0
233	1076.8	0.7481	0.7445	0.368	1.4449	0
234	1076.7	0.7483	0.7447	0.3676	1.4448	0
235	1076.6	0.7484	0.7448	0.3669	1.4449	0
236	1076.5	0.7482	0.7449	0.3663	1.445	0
237	1076.4	0.7481	0.7446	0.3657	1.445	0
238	1076.3	0.7481	0.7445	0.3651	1.4447	0
239	1076.2	0.7481	0.7447	0.3648	1.445	0
240	1076.1	0.7481	0.7445	0.3643	1.4454	0
241	1076	0.7482	0.7446	0.3639	1.4452	0
242	1075.9	0.7484	0.7449	0.3636	1.4455	0
243	1075.8	0.7485	0.7451	0.3631	1.4457	0
244	1075.7	0.7484	0.7449	0.363	1.4462	0

245	1075.6	0.7485	0.745	0.3627	1.4457	0
246	1075.5	0.7486	0.7449	0.3622	1.4455	0
247	1075.4	0.7483	0.7449	0.3619	1.4459	0
248	1075.3	0.7486	0.7453	0.3619	1.4456	0
249	1075.2	0.7486	0.7452	0.3614	1.4457	0
250	1075.1	0.7485	0.7452	0.3608	1.4466	0
251	1075	0.7487	0.7454	0.3609	1.4462	0
252	1074.9	0.749	0.7454	0.3606	1.4465	0
253	1074.8	0.7491	0.7456	0.3605	1.4468	0
254	1074.7	0.7489	0.7455	0.3602	1.4466	0
255	1074.6	0.7489	0.7455	0.3598	1.4467	0
256	1074.5	0.7491	0.7454	0.3596	1.4475	0
257	1074.4	0.7492	0.7458	0.3595	1.4475	0
258	1074.3	0.7493	0.7459	0.3593	1.4477	0
259	1074.2	0.7495	0.7461	0.3592	1.4477	0
260	1074.1	0.7496	0.7462	0.3592	1.4484	0
261	1074	0.7495	0.746	0.3591	1.4481	0
262	1073.9	0.7495	0.746	0.3588	1.4483	0
263	1073.8	0.7501	0.7462	0.3591	1.4486	0
264	1073.7	0.7502	0.7465	0.3589	1.449	0
265	1073.6	0.7503	0.7467	0.3589	1.4497	0
266	1073.5	0.7502	0.7466	0.3586	1.4495	0
267	1073.4	0.7505	0.7468	0.3587	1.4503	0
268	1073.3	0.7505	0.7468	0.3585	1.4501	0
269	1073.2	0.7505	0.7467	0.3587	1.4501	0
270	1073.1	0.7505	0.7468	0.3585	1.451	0
271	1073	0.7508	0.7468	0.3587	1.4511	0
272	1072.9	0.751	0.7469	0.3587	1.4511	0
273	1072.8	0.7509	0.7469	0.3587	1.451	0
274	1072.7	0.7508	0.7471	0.3585	1.4511	0
275	1072.6	0.751	0.747	0.3582	1.4514	0
276	1072.5	0.7512	0.7472	0.3582	1.4516	0
277	1072.4	0.7514	0.7475	0.3581	1.4516	0
278	1072.3	0.7514	0.7476	0.3581	1.4519	0
279	1072.2	0.7515	0.7475	0.358	1.452	0
280	1072.1	0.7516	0.7476	0.3578	1.4527	0
281	1072	0.7517	0.7476	0.358	1.4529	0
282	1071.9	0.7519	0.748	0.3578	1.4528	0
283	1071.8	0.7518	0.7477	0.3575	1.453	0
284	1071.7	0.7519	0.748	0.3573	1.4525	0
285	1071.6	0.7519	0.748	0.3571	1.4533	0
286	1071.5	0.752	0.7479	0.357	1.4532	0
287	1071.4	0.752	0.748	0.3567	1.453	0

288	1071.3	0.7521	0.748	0.3564	1.4532	0
289	1071.2	0.7524	0.7484	0.3563	1.4536	0
290	1071.1	0.7523	0.7482	0.356	1.4538	0
291	1071	0.7524	0.7482	0.3558	1.4535	0
292	1070.9	0.7524	0.7485	0.3556	1.454	0
293	1070.8	0.7522	0.7481	0.3552	1.4544	0
294	1070.7	0.7524	0.7484	0.3549	1.4544	0
295	1070.6	0.7525	0.7487	0.3551	1.4544	0
296	1070.5	0.7525	0.7488	0.3549	1.4543	0
297	1070.4	0.7523	0.7486	0.3543	1.4539	0
298	1070.3	0.7524	0.7486	0.3542	1.454	0
299	1070.2	0.7527	0.749	0.354	1.4545	0
300	1070.1	0.7527	0.7489	0.3534	1.4549	0
301	1070	0.7527	0.7489	0.3531	1.4547	0
302	1069.9	0.7528	0.7491	0.353	1.4546	0
303	1069.8	0.7527	0.7491	0.3526	1.4542	0
304	1069.7	0.7528	0.7491	0.3524	1.4545	0
305	1069.6	0.7527	0.7488	0.352	1.4544	0
306	1069.5	0.7528	0.7494	0.352	1.4548	0
307	1069.4	0.7527	0.7493	0.3515	1.4546	0
308	1069.3	0.7529	0.7493	0.3511	1.4551	0
309	1069.2	0.7528	0.7492	0.3507	1.4544	0
310	1069.1	0.7529	0.7493	0.3506	1.4543	0
311	1069	0.7529	0.7493	0.3502	1.4544	0
312	1068.9	0.753	0.7494	0.3502	1.4546	0
313	1068.8	0.7529	0.7495	0.3499	1.4547	0
314	1068.7	0.7531	0.7497	0.3497	1.4549	0
315	1068.6	0.7531	0.7498	0.3497	1.4547	0
316	1068.5	0.7529	0.7498	0.3495	1.4541	0
317	1068.4	0.753	0.7496	0.3494	1.4542	0
318	1068.3	0.753	0.7497	0.3493	1.4539	0
319	1068.2	0.7527	0.7495	0.3488	1.4539	0
320	1068.1	0.7529	0.7497	0.3488	1.4547	0
321	1068	0.753	0.7497	0.3486	1.4542	0
322	1067.9	0.753	0.7498	0.3483	1.4544	0
323	1067.8	0.753	0.7499	0.348	1.4541	0
324	1067.7	0.7528	0.7496	0.3475	1.4548	0
325	1067.6	0.7529	0.7499	0.3476	1.4545	0
326	1067.5	0.7528	0.7499	0.3473	1.4542	0
327	1067.4	0.7529	0.7497	0.3471	1.4543	0
328	1067.3	0.7526	0.7497	0.3469	1.4548	0
329	1067.2	0.7527	0.7498	0.3468	1.4543	0
330	1067.1	0.7527	0.7496	0.3465	1.4541	0

331	1067	0.7529	0.7499	0.3465	1.454	0
332	1066.9	0.7529	0.7499	0.3464	1.4538	0
333	1066.8	0.753	0.7499	0.3464	1.4539	0
334	1066.7	0.7532	0.7501	0.3464	1.4546	0
335	1066.6	0.753	0.75	0.3461	1.4542	0
336	1066.5	0.7531	0.7504	0.3461	1.4541	0
337	1066.4	0.7531	0.7501	0.346	1.4542	0
338	1066.3	0.7529	0.7502	0.3459	1.4543	0
339	1066.2	0.7531	0.7503	0.346	1.4543	0
340	1066.1	0.753	0.7502	0.3456	1.4539	0
341	1066	0.7531	0.7505	0.3456	1.4542	0
342	1065.9	0.7532	0.7504	0.3457	1.4549	0
343	1065.8	0.7533	0.7505	0.3456	1.4545	0
344	1065.7	0.7532	0.7506	0.345	1.4542	0
345	1065.6	0.7536	0.7509	0.3451	1.4541	0
346	1065.5	0.7535	0.7507	0.3452	1.4543	0
347	1065.4	0.7532	0.7504	0.3451	1.4544	0
348	1065.3	0.7536	0.7509	0.345	1.4542	0
349	1065.2	0.7536	0.7508	0.3449	1.4544	0
350	1065.1	0.7536	0.7507	0.3449	1.4553	0
351	1065	0.7535	0.751	0.3448	1.4549	0
352	1064.9	0.7536	0.751	0.3447	1.4546	0
353	1064.8	0.7537	0.7509	0.3445	1.4545	0
354	1064.7	0.7537	0.751	0.3443	1.4546	0
355	1064.6	0.7539	0.751	0.3443	1.455	0
356	1064.5	0.7539	0.7511	0.3443	1.4553	0
357	1064.4	0.7539	0.7513	0.3439	1.4551	0
358	1064.3	0.7543	0.7513	0.3443	1.4558	0
359	1064.2	0.7543	0.7515	0.3441	1.4556	0
360	1064.1	0.7541	0.7513	0.3438	1.4557	0
361	1064	0.7543	0.7514	0.3438	1.4562	0
362	1063.9	0.7542	0.7514	0.3434	1.4559	0
363	1063.8	0.7545	0.7511	0.3435	1.456	0
364	1063.7	0.7541	0.751	0.3431	1.4556	0
365	1063.6	0.7542	0.7512	0.3432	1.456	0
366	1063.5	0.7543	0.7514	0.3431	1.4559	0
367	1063.4	0.7545	0.7515	0.3431	1.4558	0
368	1063.3	0.7545	0.7517	0.3427	1.4563	0
369	1063.2	0.7548	0.7516	0.343	1.4569	0
370	1063.1	0.7546	0.7513	0.3428	1.4567	0
371	1063	0.7547	0.7516	0.3428	1.4566	0
372	1062.9	0.7549	0.7516	0.343	1.4572	0
373	1062.8	0.7552	0.7517	0.3428	1.4573	0

374	1062.7	0.755	0.7518	0.3427	1.4577	0
375	1062.6	0.7553	0.7521	0.3429	1.4578	0
376	1062.5	0.7552	0.7519	0.3427	1.4582	0
377	1062.4	0.7552	0.7521	0.3425	1.4582	0
378	1062.3	0.7552	0.7519	0.3422	1.4569	0
379	1062.2	0.7554	0.7519	0.3421	1.458	0
380	1062.1	0.7551	0.7518	0.3419	1.4577	0
381	1062	0.755	0.7515	0.3417	1.4582	0
382	1061.9	0.7554	0.7518	0.3419	1.459	0
383	1061.8	0.7556	0.7524	0.3421	1.4586	0
384	1061.7	0.7554	0.7522	0.3418	1.4583	0
385	1061.6	0.7556	0.7522	0.3418	1.4585	0
386	1061.5	0.7556	0.7523	0.3416	1.459	0
387	1061.4	0.7555	0.7526	0.3412	1.4589	0
388	1061.3	0.7559	0.7525	0.3415	1.4591	0
389	1061.2	0.7556	0.7526	0.3414	1.4596	0
390	1061.1	0.7557	0.7526	0.3414	1.4596	0
391	1061	0.7558	0.7528	0.3411	1.4595	0
392	1060.9	0.7555	0.7528	0.3406	1.4593	0
393	1060.8	0.7559	0.7528	0.3408	1.4589	0
394	1060.7	0.756	0.7528	0.3407	1.4596	0
395	1060.6	0.756	0.753	0.3407	1.4595	0
396	1060.5	0.756	0.7527	0.3405	1.4588	0
397	1060.4	0.7563	0.7529	0.3402	1.4594	0
398	1060.3	0.7564	0.7532	0.3402	1.4601	0
399	1060.2	0.7564	0.7534	0.3403	1.4603	0
400	1060.1	0.7565	0.7533	0.34	1.4608	0
401	1060	0.7565	0.7535	0.3401	1.4602	0
402	1059.9	0.7564	0.7534	0.3396	1.4601	0
403	1059.8	0.7564	0.7534	0.3396	1.4606	0
404	1059.7	0.7564	0.7536	0.3394	1.4605	0
405	1059.6	0.7568	0.7537	0.3398	1.4607	0
406	1059.5	0.7566	0.7539	0.3395	1.4606	0
407	1059.4	0.7567	0.7538	0.3391	1.4605	0
408	1059.3	0.7566	0.754	0.3391	1.461	0
409	1059.2	0.757	0.754	0.3389	1.4611	0
410	1059.1	0.757	0.7541	0.339	1.461	0
411	1059	0.757	0.7541	0.3387	1.461	0
412	1058.9	0.7572	0.7541	0.3386	1.4615	0
413	1058.8	0.7571	0.7544	0.3386	1.4604	0
414	1058.7	0.7573	0.7546	0.3387	1.4616	0
415	1058.6	0.7573	0.7545	0.3389	1.4615	0
416	1058.5	0.7574	0.7549	0.3389	1.4616	0

417	1058.4	0.7574	0.7548	0.3387	1.4617	0
418	1058.3	0.7574	0.7549	0.3389	1.4622	0
419	1058.2	0.7576	0.7547	0.3389	1.4616	0
420	1058.1	0.7572	0.7549	0.3386	1.4618	0
421	1058	0.7575	0.7549	0.3386	1.4617	0
422	1057.9	0.7577	0.7551	0.3389	1.4623	0
423	1057.8	0.7576	0.7551	0.3387	1.4626	0
424	1057.7	0.7576	0.7553	0.3391	1.4625	0
425	1057.6	0.7578	0.7554	0.3389	1.4623	0
426	1057.5	0.7578	0.7554	0.3389	1.4623	0
427	1057.4	0.7578	0.7556	0.3389	1.4627	0
428	1057.3	0.7578	0.7555	0.339	1.4626	0
429	1057.2	0.7578	0.7559	0.339	1.4631	0
430	1057.1	0.7582	0.7558	0.3392	1.4629	0
431	1057	0.7579	0.7557	0.3388	1.4627	0
432	1056.9	0.7581	0.7559	0.3388	1.4622	0
433	1056.8	0.7582	0.7562	0.3388	1.4627	0
434	1056.7	0.7584	0.7562	0.3388	1.463	0
435	1056.6	0.7584	0.7561	0.3389	1.4635	0
436	1056.5	0.7585	0.7563	0.3388	1.4628	0
437	1056.4	0.7588	0.7568	0.3389	1.4639	0
438	1056.3	0.7589	0.7567	0.3389	1.4632	0
439	1056.2	0.7586	0.7567	0.3387	1.4633	0
440	1056.1	0.7587	0.7567	0.3388	1.4632	0
441	1056	0.7588	0.7567	0.3389	1.4631	0
442	1055.9	0.7592	0.7571	0.3393	1.4638	0
443	1055.8	0.759	0.7571	0.3391	1.4637	0
444	1055.7	0.759	0.7572	0.339	1.4638	0
445	1055.6	0.7589	0.7571	0.339	1.4641	0
446	1055.5	0.7592	0.7572	0.3391	1.4647	0
447	1055.4	0.7594	0.7575	0.3393	1.4641	0
448	1055.3	0.7593	0.7573	0.339	1.4643	0
449	1055.2	0.7594	0.7577	0.3389	1.4648	0
450	1055.1	0.7593	0.7574	0.3389	1.4645	0
451	1055	0.7595	0.7576	0.3391	1.4644	0
452	1054.9	0.7596	0.7577	0.3391	1.4654	0
453	1054.8	0.7599	0.7579	0.3395	1.4655	0
454	1054.7	0.7599	0.7578	0.3394	1.4652	0
455	1054.6	0.7599	0.7579	0.3392	1.4655	0
456	1054.5	0.7597	0.7579	0.3393	1.4653	0
457	1054.4	0.7603	0.7581	0.3393	1.4663	0
458	1054.3	0.7603	0.7584	0.3394	1.4663	0
459	1054.2	0.7604	0.7584	0.3397	1.4668	0

460	1054.1	0.7604	0.7586	0.3397	1.4666	0
461	1054	0.7604	0.7585	0.3396	1.4665	0
462	1053.9	0.7604	0.7584	0.3391	1.4661	0
463	1053.8	0.7606	0.7586	0.3397	1.4666	0
464	1053.7	0.7608	0.7589	0.3398	1.4667	0
465	1053.6	0.7609	0.7589	0.3398	1.4671	0
466	1053.5	0.7608	0.7588	0.3395	1.4674	0
467	1053.4	0.7608	0.7591	0.3393	1.4677	0
468	1053.3	0.7608	0.7588	0.3395	1.467	0
469	1053.2	0.761	0.7589	0.3393	1.4679	0
470	1053.1	0.7613	0.7591	0.3395	1.4684	0
471	1053	0.7614	0.7593	0.3394	1.4677	0
472	1052.9	0.7615	0.7595	0.3395	1.4687	0
473	1052.8	0.7615	0.7595	0.3394	1.468	0
474	1052.7	0.7615	0.7596	0.3394	1.468	0
475	1052.6	0.7615	0.7596	0.3395	1.4687	0
476	1052.5	0.7615	0.7595	0.3393	1.4684	0
477	1052.4	0.7616	0.7595	0.3395	1.4688	0
478	1052.3	0.7617	0.7596	0.3395	1.4687	0
479	1052.2	0.7619	0.7598	0.3396	1.4687	0
480	1052.1	0.7618	0.7598	0.3394	1.4688	0
481	1052	0.762	0.76	0.3395	1.4698	0
482	1051.9	0.7618	0.76	0.3394	1.4691	0
483	1051.8	0.762	0.76	0.3395	1.469	0
484	1051.7	0.762	0.7601	0.3395	1.4694	0
485	1051.6	0.7623	0.7604	0.3396	1.4694	0
486	1051.5	0.7623	0.7603	0.3395	1.4692	0
487	1051.4	0.7622	0.7605	0.3394	1.4696	0
488	1051.3	0.7624	0.7607	0.3396	1.47	0
489	1051.2	0.7626	0.7608	0.3395	1.4703	0
490	1051.1	0.7625	0.7608	0.3393	1.4701	0
491	1051	0.7623	0.7606	0.3393	1.4699	0
492	1050.9	0.7626	0.7607	0.3393	1.4699	0
493	1050.8	0.7628	0.7609	0.3394	1.4702	0
494	1050.7	0.7625	0.7609	0.3393	1.4703	0
495	1050.6	0.7627	0.7608	0.3393	1.4702	0
496	1050.5	0.7627	0.7609	0.3392	1.47	0
497	1050.4	0.7628	0.7614	0.3395	1.471	0
498	1050.3	0.7629	0.7613	0.3395	1.4704	0
499	1050.2	0.7628	0.7612	0.3393	1.4705	0
500	1050.1	0.7629	0.7613	0.3396	1.4705	0
501	1050	0.7628	0.7612	0.3394	1.4701	0
502	1049.9	0.7629	0.7614	0.3397	1.4711	0

503	1049.8	0.763	0.7614	0.3396	1.4701	0
504	1049.7	0.7629	0.7614	0.3395	1.4707	0
505	1049.6	0.763	0.7617	0.3395	1.4702	0
506	1049.5	0.7631	0.7617	0.3397	1.4705	0
507	1049.4	0.7631	0.7615	0.3396	1.4709	0
508	1049.3	0.763	0.7615	0.3393	1.4714	0
509	1049.2	0.7632	0.7618	0.3396	1.4709	0
510	1049.1	0.7632	0.7617	0.3396	1.4712	0
511	1049	0.7633	0.762	0.3397	1.4711	0
512	1048.9	0.7635	0.7621	0.3396	1.471	0
513	1048.8	0.7635	0.762	0.3397	1.4713	0
514	1048.7	0.7637	0.7625	0.3401	1.4714	0
515	1048.6	0.7636	0.7625	0.34	1.4712	0
516	1048.5	0.7637	0.7624	0.3401	1.4714	0
517	1048.4	0.7638	0.7625	0.3401	1.4713	0
518	1048.3	0.7636	0.7625	0.34	1.471	0
519	1048.2	0.7638	0.7625	0.34	1.4713	0
520	1048.1	0.7638	0.7625	0.3401	1.4721	0
521	1048	0.764	0.7626	0.3403	1.4721	0
522	1047.9	0.764	0.7628	0.3404	1.4723	0
523	1047.8	0.7642	0.7629	0.3404	1.4723	0
524	1047.7	0.7641	0.7629	0.3406	1.472	0
525	1047.6	0.764	0.7628	0.3404	1.4721	0
526	1047.5	0.764	0.7629	0.3403	1.4724	0
527	1047.4	0.7645	0.7633	0.3407	1.4726	0
528	1047.3	0.7646	0.7633	0.3409	1.4729	0
529	1047.2	0.7647	0.7636	0.3408	1.4727	0
530	1047.1	0.7645	0.7633	0.3408	1.4733	0
531	1047	0.7651	0.7639	0.3412	1.474	0
532	1046.9	0.7648	0.7637	0.341	1.4736	0
533	1046.8	0.765	0.7639	0.3412	1.474	0
534	1046.7	0.7649	0.7638	0.3413	1.4747	0
535	1046.6	0.7651	0.7638	0.341	1.474	0
536	1046.5	0.7649	0.764	0.341	1.4739	0
537	1046.4	0.7653	0.764	0.3412	1.4739	0
538	1046.3	0.7652	0.764	0.3413	1.4741	0
539	1046.2	0.7653	0.764	0.3415	1.4748	0
540	1046.1	0.7653	0.7641	0.3414	1.4746	0
541	1046	0.7656	0.7645	0.3416	1.4747	0
542	1045.9	0.7658	0.7644	0.3416	1.4747	0
543	1045.8	0.7654	0.7642	0.3413	1.4746	0
544	1045.7	0.7656	0.7644	0.3416	1.4754	0
545	1045.6	0.766	0.7645	0.3418	1.4758	0

546	1045.5	0.7659	0.7646	0.3419	1.4756	0
547	1045.4	0.7661	0.7646	0.3419	1.476	0
548	1045.3	0.7662	0.7648	0.342	1.4759	0
549	1045.2	0.7663	0.7649	0.3421	1.4757	0
550	1045.1	0.7665	0.7652	0.3423	1.4765	0
551	1045	0.7665	0.765	0.3423	1.476	0
552	1044.9	0.7665	0.7651	0.3424	1.4767	0
553	1044.8	0.7664	0.765	0.3423	1.4764	0
554	1044.7	0.7664	0.7651	0.3424	1.4768	0
555	1044.6	0.7666	0.7651	0.3425	1.4771	0
556	1044.5	0.7668	0.765	0.3425	1.4774	0
557	1044.4	0.7668	0.7652	0.3425	1.4772	0
558	1044.3	0.7669	0.7656	0.3427	1.478	0
559	1044.2	0.7672	0.7657	0.3428	1.4781	0
560	1044.1	0.7673	0.7658	0.3425	1.4782	0
561	1044	0.7674	0.7661	0.3427	1.4781	0
562	1043.9	0.7676	0.7657	0.3428	1.4781	0
563	1043.8	0.7677	0.766	0.3428	1.4779	0
564	1043.7	0.7677	0.7661	0.3428	1.4786	0
565	1043.6	0.7677	0.7661	0.3425	1.4787	0
566	1043.5	0.768	0.7663	0.3427	1.4792	0
567	1043.4	0.7677	0.7664	0.3425	1.4792	0
568	1043.3	0.7678	0.7663	0.3426	1.479	0
569	1043.2	0.7683	0.7666	0.3426	1.4795	0
570	1043.1	0.7683	0.7666	0.3425	1.4797	0
571	1043	0.7683	0.7669	0.3424	1.4795	0
572	1042.9	0.7682	0.7666	0.3424	1.4797	0
573	1042.8	0.7685	0.7669	0.3422	1.4795	0
574	1042.7	0.7685	0.7671	0.3423	1.4799	0
575	1042.6	0.7686	0.767	0.3423	1.4801	0
576	1042.5	0.7687	0.767	0.3423	1.4806	0
577	1042.4	0.7687	0.7672	0.3423	1.4801	0
578	1042.3	0.7686	0.7671	0.3423	1.4802	0
579	1042.2	0.7687	0.7672	0.3423	1.4803	0
580	1042.1	0.769	0.7676	0.3424	1.4808	0
581	1042	0.7689	0.7675	0.3424	1.4804	0
582	1041.9	0.769	0.7674	0.3423	1.4811	0
583	1041.8	0.7688	0.7674	0.3421	1.4806	0
584	1041.7	0.7687	0.7676	0.3421	1.48	0
585	1041.6	0.7691	0.768	0.3424	1.4809	0
586	1041.5	0.769	0.7675	0.3423	1.4809	0
587	1041.4	0.7692	0.7679	0.3424	1.4808	0
588	1041.3	0.7691	0.7676	0.3421	1.4807	0

589	1041.2	0.7693	0.768	0.3421	1.4811	0
590	1041.1	0.7692	0.7679	0.342	1.4811	0
591	1041	0.7695	0.7682	0.3422	1.4808	0
592	1040.9	0.7695	0.7682	0.3421	1.4809	0
593	1040.8	0.7692	0.768	0.3415	1.4811	0
594	1040.7	0.7692	0.7679	0.3414	1.4809	0
595	1040.6	0.7696	0.7682	0.3415	1.4812	0
596	1040.5	0.7693	0.7682	0.3411	1.4809	0
597	1040.4	0.7695	0.7684	0.341	1.4816	0
598	1040.3	0.7696	0.7682	0.3405	1.4814	0
599	1040.2	0.7695	0.7684	0.34	1.4811	0
600	1040.1	0.7697	0.7685	0.3399	1.4813	0
601	1040	0.7696	0.7684	0.3396	1.4809	0
602	1039.9	0.7696	0.7685	0.3394	1.4807	0
603	1039.8	0.7698	0.7687	0.3395	1.481	0
604	1039.7	0.7696	0.7687	0.3391	1.4809	0
605	1039.6	0.7697	0.7687	0.3389	1.481	0
606	1039.5	0.7696	0.7687	0.3391	1.4809	0
607	1039.4	0.7697	0.7689	0.3389	1.481	0
608	1039.3	0.77	0.7691	0.3389	1.4814	0
609	1039.2	0.7699	0.7692	0.3389	1.4815	0
610	1039.1	0.7698	0.7689	0.3388	1.481	0
611	1039	0.7698	0.7691	0.3386	1.4807	0
612	1038.9	0.7697	0.7691	0.3386	1.4809	0
613	1038.8	0.7698	0.769	0.3385	1.4806	0
614	1038.7	0.7699	0.7694	0.3385	1.4812	0
615	1038.6	0.77	0.7695	0.3384	1.4806	0
616	1038.5	0.7699	0.7696	0.3384	1.4816	0
617	1038.4	0.7697	0.7692	0.3379	1.4807	0
618	1038.3	0.7699	0.7693	0.3378	1.4809	0
619	1038.2	0.7698	0.7694	0.3376	1.481	0
620	1038.1	0.7699	0.7696	0.3374	1.4804	0
621	1038	0.77	0.7694	0.3374	1.4817	0
622	1037.9	0.7702	0.7698	0.3372	1.4812	0
623	1037.8	0.7701	0.7698	0.3369	1.4809	0
624	1037.7	0.7701	0.7698	0.3365	1.481	0
625	1037.6	0.7702	0.7699	0.3365	1.4812	0
626	1037.5	0.7703	0.7697	0.3364	1.481	0
627	1037.4	0.7705	0.7701	0.3366	1.4814	0
628	1037.3	0.7705	0.7701	0.3362	1.4813	0
629	1037.2	0.7704	0.7701	0.3362	1.4815	0
630	1037.1	0.7706	0.77	0.3361	1.4816	0
631	1037	0.7707	0.7701	0.336	1.4815	0

632	1036.9	0.7709	0.7705	0.336	1.4818	0
633	1036.8	0.771	0.7705	0.3359	1.4829	0
634	1036.7	0.771	0.7705	0.3356	1.4826	0
635	1036.6	0.7709	0.7704	0.3354	1.4823	0
636	1036.5	0.771	0.7707	0.3352	1.4829	0
637	1036.4	0.7714	0.7706	0.3351	1.4832	0
638	1036.3	0.7716	0.7709	0.3353	1.4835	0
639	1036.2	0.7717	0.7711	0.3354	1.4838	0
640	1036.1	0.7718	0.7711	0.3351	1.4835	0
641	1036	0.7719	0.7713	0.335	1.4842	0
642	1035.9	0.7722	0.7713	0.335	1.4841	0
643	1035.8	0.7721	0.7712	0.3349	1.4841	0
644	1035.7	0.7722	0.7713	0.3349	1.4851	0
645	1035.6	0.7725	0.7714	0.335	1.4852	0
646	1035.5	0.7723	0.7714	0.3349	1.485	0
647	1035.4	0.7724	0.7714	0.3347	1.4851	0
648	1035.3	0.7725	0.7715	0.3348	1.4857	0
649	1035.2	0.7728	0.7718	0.3348	1.4855	0
650	1035.1	0.7728	0.7717	0.335	1.4859	0
651	1035	0.7729	0.7715	0.3352	1.486	0
652	1034.9	0.7728	0.7719	0.3348	1.4862	0
653	1034.8	0.7734	0.7724	0.335	1.487	0
654	1034.7	0.7733	0.7722	0.3351	1.4867	0
655	1034.6	0.7736	0.7723	0.3352	1.4871	0
656	1034.5	0.7735	0.7721	0.3349	1.487	0
657	1034.4	0.7737	0.7725	0.3353	1.4878	0
658	1034.3	0.774	0.7726	0.3354	1.4881	0
659	1034.2	0.7738	0.7723	0.3351	1.4876	0
660	1034.1	0.7739	0.7724	0.3351	1.4884	0
661	1034	0.7741	0.7726	0.335	1.4886	0
662	1033.9	0.7741	0.7724	0.335	1.4887	0
663	1033.8	0.7741	0.7726	0.3348	1.4889	0
664	1033.7	0.7745	0.7729	0.3351	1.4888	0
665	1033.6	0.7745	0.7729	0.3351	1.4885	0
666	1033.5	0.7746	0.7728	0.335	1.4887	0
667	1033.4	0.7747	0.7729	0.3349	1.4895	0
668	1033.3	0.7747	0.7729	0.3347	1.4899	0
669	1033.2	0.7747	0.7729	0.3348	1.4897	0
670	1033.1	0.7746	0.773	0.3347	1.4897	0
671	1033	0.7748	0.7731	0.3347	1.4899	0
672	1032.9	0.775	0.7732	0.3348	1.4904	0
673	1032.8	0.7751	0.773	0.3348	1.4905	0
674	1032.7	0.7751	0.7731	0.3349	1.4898	0

675	1032.6	0.7749	0.7732	0.3345	1.4901	0
676	1032.5	0.7751	0.7734	0.3345	1.4903	0
677	1032.4	0.7751	0.7735	0.3344	1.4903	0
678	1032.3	0.7752	0.7735	0.3343	1.4904	0
679	1032.2	0.7754	0.7737	0.3343	1.4905	0
680	1032.1	0.7755	0.7738	0.3343	1.4903	0
681	1032	0.7755	0.7737	0.334	1.4906	0
682	1031.9	0.7753	0.7734	0.3336	1.4909	0
683	1031.8	0.7755	0.7738	0.3335	1.49	0
684	1031.7	0.7757	0.7739	0.3335	1.4904	0
685	1031.6	0.7758	0.7743	0.3336	1.4907	0
686	1031.5	0.776	0.7741	0.3337	1.4915	0
687	1031.4	0.7759	0.7742	0.3336	1.4911	0
688	1031.3	0.776	0.7744	0.3335	1.4912	0
689	1031.2	0.776	0.7741	0.3334	1.4911	0
690	1031.1	0.776	0.7743	0.3331	1.4912	0
691	1031	0.776	0.7744	0.3329	1.4911	0
692	1030.9	0.7761	0.7743	0.3329	1.4911	0
693	1030.8	0.7763	0.7745	0.3329	1.4913	0
694	1030.7	0.7762	0.7746	0.3329	1.4913	0
695	1030.6	0.7762	0.7746	0.3328	1.4917	0
696	1030.5	0.7762	0.7745	0.3327	1.4916	0
697	1030.4	0.7761	0.7745	0.3325	1.4912	0
698	1030.3	0.7764	0.7748	0.3327	1.4914	0
699	1030.2	0.7763	0.7747	0.3326	1.4914	0
700	1030.1	0.7761	0.7746	0.3323	1.4912	0
701	1030	0.7764	0.7747	0.3324	1.4917	0
702	1029.9	0.7763	0.7748	0.3324	1.4909	0
703	1029.8	0.7762	0.7748	0.3322	1.4908	0
704	1029.7	0.7763	0.775	0.3321	1.4912	0
705	1029.6	0.7764	0.775	0.3322	1.4912	0
706	1029.5	0.7764	0.7752	0.3323	1.4909	0
707	1029.4	0.7761	0.7751	0.3321	1.4907	0
708	1029.3	0.7764	0.7751	0.3321	1.4912	0
709	1029.2	0.7764	0.7753	0.3322	1.4906	0
710	1029.1	0.7765	0.7755	0.3321	1.4908	0
711	1029	0.7766	0.7753	0.3322	1.4912	0
712	1028.9	0.7766	0.7753	0.3321	1.4905	0
713	1028.8	0.7766	0.7753	0.3321	1.4908	0
714	1028.7	0.7764	0.7753	0.3319	1.4906	0
715	1028.6	0.7765	0.7752	0.3318	1.4902	0
716	1028.5	0.7764	0.7754	0.3318	1.4903	0
717	1028.4	0.7765	0.7754	0.3316	1.4902	0

718	1028.3	0.7766	0.7755	0.3315	1.4908	0
719	1028.2	0.7765	0.7755	0.3314	1.4905	0
720	1028.1	0.7765	0.7754	0.3315	1.4904	0
721	1028	0.7766	0.7755	0.3314	1.4905	0
722	1027.9	0.7767	0.7756	0.3314	1.4905	0
723	1027.8	0.7766	0.7757	0.3315	1.4909	0
724	1027.7	0.7765	0.7757	0.3314	1.4905	0
725	1027.6	0.7765	0.7757	0.331	1.4904	0
726	1027.5	0.7765	0.7756	0.3311	1.4903	0
727	1027.4	0.7766	0.7757	0.3311	1.4909	0
728	1027.3	0.7768	0.776	0.3313	1.491	0
729	1027.2	0.7769	0.7758	0.3308	1.4909	0
730	1027.1	0.7769	0.7761	0.3307	1.4908	0
731	1027	0.777	0.7761	0.3308	1.491	0
732	1026.9	0.777	0.7763	0.3308	1.4911	0
733	1026.8	0.7771	0.7763	0.3307	1.4906	0
734	1026.7	0.7771	0.7763	0.3306	1.4904	0
735	1026.6	0.7769	0.7761	0.3303	1.4909	0
736	1026.5	0.7771	0.7763	0.3303	1.491	0
737	1026.4	0.7772	0.7763	0.3304	1.4915	0
738	1026.3	0.7773	0.7765	0.3306	1.4916	0
739	1026.2	0.7773	0.7765	0.3302	1.4912	0
740	1026.1	0.7775	0.7765	0.3303	1.4915	0
741	1026	0.7773	0.7763	0.3301	1.4916	0
742	1025.9	0.7778	0.7768	0.3303	1.4922	0
743	1025.8	0.7777	0.7769	0.3301	1.4922	0
744	1025.7	0.778	0.7769	0.3301	1.4923	0
745	1025.6	0.778	0.7769	0.3299	1.4927	0
746	1025.5	0.778	0.7771	0.3298	1.4928	0
747	1025.4	0.7783	0.7772	0.3298	1.493	0
748	1025.3	0.7784	0.7771	0.3295	1.4933	0
749	1025.2	0.7786	0.7775	0.3299	1.4933	0
750	1025.1	0.7786	0.7774	0.3299	1.4941	0
751	1025	0.7789	0.7776	0.3298	1.4942	0
752	1024.9	0.779	0.7776	0.3299	1.4943	0
753	1024.8	0.7791	0.7776	0.33	1.4944	0
754	1024.7	0.7792	0.7778	0.3301	1.4948	0
755	1024.6	0.7793	0.7778	0.33	1.4952	0
756	1024.5	0.7794	0.7778	0.3298	1.4952	0
757	1024.4	0.7795	0.7779	0.3301	1.4953	0
758	1024.3	0.7797	0.7781	0.3301	1.4951	0
759	1024.2	0.78	0.7782	0.3303	1.4961	0
760	1024.1	0.78	0.7781	0.33	1.4959	0

761	1024	0.7801	0.7783	0.3302	1.4964	0
762	1023.9	0.7802	0.7783	0.3303	1.4966	0
763	1023.8	0.7804	0.7783	0.3302	1.4973	0
764	1023.7	0.7804	0.7784	0.3301	1.4973	0
765	1023.6	0.7807	0.7786	0.3305	1.4975	0
766	1023.5	0.7808	0.7786	0.3306	1.4976	0
767	1023.4	0.7808	0.7786	0.3306	1.4978	0
768	1023.3	0.7811	0.7788	0.3306	1.4984	0
769	1023.2	0.7814	0.7792	0.3308	1.4983	0
770	1023.1	0.7813	0.7791	0.3306	1.4986	0
771	1023	0.7813	0.7788	0.3306	1.4988	0
772	1022.9	0.7815	0.779	0.3305	1.4991	0
773	1022.8	0.7816	0.7793	0.3307	1.4993	0
774	1022.7	0.7817	0.7793	0.3305	1.4997	0
775	1022.6	0.782	0.7796	0.3305	1.4998	0
776	1022.5	0.7818	0.7796	0.3303	1.4997	0
777	1022.4	0.7816	0.7791	0.3297	1.499	0
778	1022.3	0.7816	0.7791	0.3296	1.4993	0
779	1022.2	0.7817	0.7792	0.3295	1.4997	0
780	1022.1	0.7818	0.7794	0.3294	1.4994	0
781	1022	0.782	0.7795	0.3293	1.5	0
782	1021.9	0.7822	0.7796	0.3294	1.5	0
783	1021.8	0.7825	0.7798	0.3295	1.5003	0
784	1021.7	0.7825	0.78	0.3295	1.5006	0
785	1021.6	0.7823	0.7799	0.3292	1.4999	0
786	1021.5	0.7827	0.7802	0.3295	1.5004	0
787	1021.4	0.7827	0.7802	0.3295	1.5	0
788	1021.3	0.7828	0.7801	0.3297	1.5003	0
789	1021.2	0.7827	0.7803	0.3295	1.5006	0
790	1021.1	0.7828	0.7803	0.3296	1.5003	0
791	1021	0.783	0.7804	0.3297	1.5001	0
792	1020.9	0.7832	0.7804	0.3296	1.5005	0
793	1020.8	0.7832	0.7805	0.3296	1.5013	0
794	1020.7	0.7832	0.7806	0.3297	1.5011	0
795	1020.6	0.7833	0.7807	0.3296	1.5008	0
796	1020.5	0.7833	0.7807	0.3297	1.5015	0
797	1020.4	0.7834	0.781	0.3297	1.5013	0
798	1020.3	0.7833	0.7809	0.3296	1.5009	0
799	1020.2	0.7835	0.7807	0.3296	1.5012	0
800	1020.1	0.7834	0.7808	0.3297	1.5009	0
801	1020	0.7834	0.781	0.3298	1.5012	0
802	1019.9	0.7832	0.781	0.3296	1.5008	0
803	1019.8	0.7836	0.781	0.3299	1.5017	0

804	1019.7	0.7837	0.7812	0.3299	1.5015	0
805	1019.6	0.7837	0.7812	0.3299	1.5005	0
806	1019.5	0.7836	0.7813	0.3299	1.5013	0
807	1019.4	0.7837	0.7813	0.3298	1.5009	0
808	1019.3	0.7838	0.7811	0.3298	1.5012	0
809	1019.2	0.7839	0.7813	0.33	1.501	0
810	1019.1	0.7837	0.7814	0.3298	1.501	0
811	1019	0.7836	0.7813	0.3297	1.5006	0
812	1018.9	0.7835	0.7813	0.3298	1.5003	0
813	1018.8	0.7836	0.7812	0.3296	1.501	0
814	1018.7	0.7838	0.7815	0.3297	1.5008	0
815	1018.6	0.7837	0.7816	0.3297	1.5009	0
816	1018.5	0.7837	0.7815	0.3297	1.5006	0
817	1018.4	0.7836	0.7814	0.3297	1.5002	0
818	1018.3	0.7838	0.7818	0.3296	1.5005	0
819	1018.2	0.7835	0.7817	0.3296	1.5001	0
820	1018.1	0.7839	0.7819	0.3297	1.5004	0
821	1018	0.7837	0.7818	0.3298	1.5005	0
822	1017.9	0.7837	0.7818	0.3298	1.5001	0
823	1017.8	0.7837	0.7818	0.3296	1.5008	0
824	1017.7	0.7837	0.7818	0.3296	1.4999	0
825	1017.6	0.784	0.782	0.3299	1.5004	0
826	1017.5	0.7839	0.7818	0.3297	1.5004	0
827	1017.4	0.7839	0.7822	0.3299	1.5002	0
828	1017.3	0.7842	0.782	0.33	1.5011	0
829	1017.2	0.784	0.7821	0.3298	1.5006	0
830	1017.1	0.7838	0.782	0.3298	1.5002	0
831	1017	0.7841	0.7822	0.3297	1.5004	0
832	1016.9	0.7839	0.7821	0.3297	1.5006	0
833	1016.8	0.7841	0.7824	0.3299	1.5004	0
834	1016.7	0.7839	0.7822	0.3296	1.5004	0
835	1016.6	0.7839	0.7823	0.3297	1.5007	0
836	1016.5	0.7839	0.782	0.3297	1.5008	0
837	1016.4	0.7841	0.7822	0.3297	1.5006	0
838	1016.3	0.7843	0.7823	0.3298	1.5015	0
839	1016.2	0.7844	0.7825	0.3299	1.5012	0
840	1016.1	0.7845	0.7825	0.3298	1.5008	0
841	1016	0.7842	0.7825	0.3299	1.501	0
842	1015.9	0.7845	0.7827	0.33	1.5014	0
843	1015.8	0.7845	0.7826	0.33	1.5016	0
844	1015.7	0.7848	0.7826	0.3303	1.5009	0
845	1015.6	0.7847	0.7827	0.3303	1.5017	0
846	1015.5	0.7847	0.7827	0.3302	1.5014	0

847	1015.4	0.785	0.7829	0.3303	1.5017	0
848	1015.3	0.785	0.7829	0.3302	1.502	0
849	1015.2	0.7848	0.7829	0.3303	1.5016	0
850	1015.1	0.7852	0.7832	0.3305	1.502	0
851	1015	0.7851	0.7832	0.3305	1.5023	0
852	1014.9	0.7855	0.7835	0.3306	1.5028	0
853	1014.8	0.7855	0.7832	0.3305	1.5029	0
854	1014.7	0.7854	0.7832	0.3307	1.5031	0
855	1014.6	0.7857	0.7836	0.3308	1.5024	0
856	1014.5	0.786	0.7834	0.3308	1.5033	0
857	1014.4	0.7859	0.7835	0.331	1.503	0
858	1014.3	0.7861	0.7836	0.3312	1.5034	0
859	1014.2	0.7863	0.784	0.3314	1.5044	0
860	1014.1	0.7864	0.784	0.3314	1.5036	0
861	1014	0.7865	0.784	0.3314	1.5043	0
862	1013.9	0.7866	0.7838	0.3315	1.5047	0
863	1013.8	0.7867	0.7842	0.3318	1.5045	0
864	1013.7	0.7869	0.7842	0.3318	1.5048	0
865	1013.6	0.787	0.7843	0.3319	1.5051	0
866	1013.5	0.7873	0.7843	0.332	1.5049	0
867	1013.4	0.7874	0.7847	0.3323	1.5054	0
868	1013.3	0.7876	0.7847	0.3324	1.5062	0
869	1013.2	0.7875	0.7846	0.3324	1.5063	0
870	1013.1	0.7877	0.7847	0.3325	1.5062	0
871	1013	0.7879	0.785	0.3326	1.5066	0
872	1012.9	0.788	0.7852	0.3328	1.5063	0
873	1012.8	0.788	0.7848	0.3326	1.5064	0
874	1012.7	0.7881	0.7847	0.3325	1.5071	0
875	1012.6	0.7882	0.7848	0.3326	1.5074	0
876	1012.5	0.7881	0.7849	0.3327	1.5077	0
877	1012.4	0.7883	0.7852	0.3328	1.5081	0
878	1012.3	0.7884	0.7852	0.333	1.5078	0
879	1012.2	0.7885	0.7852	0.3331	1.5075	0
880	1012.1	0.7886	0.7853	0.3331	1.5084	0
881	1012	0.7888	0.7854	0.3333	1.5081	0
882	1011.9	0.7888	0.7852	0.3333	1.5082	0
883	1011.8	0.7887	0.7853	0.3332	1.5079	0
884	1011.7	0.789	0.7856	0.3334	1.5083	0
885	1011.6	0.7891	0.7856	0.3334	1.5084	0
886	1011.5	0.7893	0.7858	0.3335	1.5088	0
887	1011.4	0.7891	0.7858	0.3335	1.5083	0
888	1011.3	0.7892	0.7856	0.3335	1.5091	0
889	1011.2	0.7892	0.7857	0.3335	1.5088	0

890	1011.1	0.7893	0.7855	0.3334	1.5084	0
891	1011	0.7894	0.7858	0.3335	1.5092	0
892	1010.9	0.7892	0.7857	0.3335	1.5093	0
893	1010.8	0.7894	0.7859	0.3334	1.5101	0
894	1010.7	0.7894	0.7859	0.3335	1.5097	0
895	1010.6	0.7898	0.786	0.3337	1.5095	0
896	1010.5	0.7898	0.786	0.3337	1.5097	0
897	1010.4	0.7896	0.786	0.3335	1.5098	0
898	1010.3	0.7899	0.7863	0.3336	1.5106	0
899	1010.2	0.7898	0.7864	0.3339	1.5103	0
900	1010.1	0.7901	0.7866	0.3339	1.5101	0
901	1010	0.79	0.7863	0.3339	1.5101	0
902	1009.9	0.79	0.7864	0.3339	1.5103	0
903	1009.8	0.79	0.7864	0.3337	1.5109	0
904	1009.7	0.7901	0.7864	0.3338	1.5106	0
905	1009.6	0.79	0.7865	0.3338	1.5102	0
906	1009.5	0.7902	0.7864	0.3338	1.5104	0
907	1009.4	0.7901	0.7865	0.3339	1.5111	0
908	1009.3	0.7902	0.7866	0.3339	1.5097	0
909	1009.2	0.7904	0.7869	0.3341	1.51	0
910	1009.1	0.7901	0.7867	0.3337	1.5099	0
911	1009	0.7905	0.787	0.334	1.5104	0
912	1008.9	0.7904	0.7869	0.334	1.5098	0
913	1008.8	0.7905	0.7869	0.334	1.5105	0
914	1008.7	0.7907	0.787	0.334	1.5102	0
915	1008.6	0.7905	0.7873	0.3341	1.5104	0
916	1008.5	0.7903	0.7867	0.334	1.5098	0
917	1008.4	0.7905	0.7871	0.3341	1.51	0
918	1008.3	0.7904	0.787	0.3339	1.5105	0
919	1008.2	0.7904	0.7871	0.3338	1.5096	0
920	1008.1	0.7903	0.7868	0.3339	1.5096	0
921	1008	0.7903	0.7873	0.3339	1.5098	0
922	1007.9	0.7905	0.7872	0.3339	1.51	0
923	1007.8	0.7904	0.7871	0.3339	1.5096	0
924	1007.7	0.7904	0.7871	0.3338	1.5097	0
925	1007.6	0.7906	0.7874	0.3341	1.5099	0
926	1007.5	0.7905	0.7872	0.3339	1.5094	0
927	1007.4	0.7906	0.7876	0.3339	1.5096	0
928	1007.3	0.7907	0.7873	0.3338	1.5096	0
929	1007.2	0.7908	0.7874	0.3339	1.5099	0
930	1007.1	0.7907	0.7874	0.3338	1.5094	0
931	1007	0.7908	0.7877	0.3338	1.509	0
932	1006.9	0.7909	0.7877	0.334	1.5099	0

933	1006.8	0.7909	0.7877	0.3339	1.51	0
934	1006.7	0.7909	0.7876	0.3338	1.51	0
935	1006.6	0.7909	0.7876	0.3338	1.5096	0
936	1006.5	0.7909	0.7874	0.3337	1.5102	0
937	1006.4	0.7909	0.7876	0.3339	1.5094	0
938	1006.3	0.7909	0.7878	0.3339	1.5101	0
939	1006.2	0.791	0.7877	0.3341	1.5098	0
940	1006.1	0.7912	0.7878	0.3341	1.51	0
941	1006	0.7911	0.7881	0.3342	1.5102	0
942	1005.9	0.7914	0.7879	0.334	1.5104	0
943	1005.8	0.7915	0.7882	0.3342	1.5106	0
944	1005.7	0.7914	0.7879	0.3341	1.5101	0
945	1005.6	0.7913	0.7878	0.3341	1.5107	0
946	1005.5	0.7914	0.7883	0.3339	1.5109	0
947	1005.4	0.7917	0.7884	0.3343	1.5107	0
948	1005.3	0.7918	0.7884	0.3344	1.5115	0
949	1005.2	0.7918	0.7885	0.3343	1.511	0
950	1005.1	0.7918	0.7881	0.3343	1.511	0
951	1005	0.7919	0.7885	0.3345	1.5109	0
952	1004.9	0.7922	0.7885	0.3346	1.5117	0
953	1004.8	0.7921	0.7886	0.3346	1.5112	0
954	1004.7	0.7926	0.789	0.3349	1.5117	0
955	1004.6	0.7923	0.7886	0.3349	1.5112	0
956	1004.5	0.7926	0.7888	0.335	1.512	0
957	1004.4	0.7925	0.7888	0.335	1.5122	0
958	1004.3	0.7927	0.789	0.3351	1.5124	0
959	1004.2	0.7928	0.7891	0.3351	1.5125	0
960	1004.1	0.7929	0.7891	0.3352	1.5122	0
961	1004	0.7932	0.7893	0.3353	1.5131	0
962	1003.9	0.7932	0.7892	0.3355	1.5128	0
963	1003.8	0.7937	0.7897	0.3358	1.5132	0
964	1003.7	0.7937	0.7896	0.3356	1.5138	0
965	1003.6	0.7934	0.7896	0.3356	1.5133	0
966	1003.5	0.7937	0.7895	0.3358	1.5135	0
967	1003.4	0.7938	0.7895	0.3358	1.5137	0
968	1003.3	0.794	0.7896	0.336	1.5145	0
969	1003.2	0.7939	0.7896	0.3358	1.5141	0
970	1003.1	0.7939	0.7896	0.3358	1.5148	0
971	1003	0.7941	0.7899	0.336	1.5145	0
972	1002.9	0.7941	0.7899	0.336	1.5147	0
973	1002.8	0.7942	0.79	0.336	1.5149	0
974	1002.7	0.7943	0.79	0.3361	1.5151	0
975	1002.6	0.7944	0.7899	0.3361	1.5146	0

976	1002.5	0.7945	0.7901	0.3362	1.5148	0
977	1002.4	0.7945	0.7903	0.3363	1.515	0
978	1002.3	0.7946	0.7903	0.3362	1.5157	0
979	1002.2	0.7946	0.7902	0.3363	1.5158	0
980	1002.1	0.7947	0.7902	0.3363	1.516	0
981	1002	0.7946	0.7902	0.336	1.5154	0
982	1001.9	0.7947	0.7902	0.3363	1.5158	0
983	1001.8	0.795	0.7904	0.3365	1.516	0
984	1001.7	0.795	0.7905	0.3365	1.5162	0
985	1001.6	0.7951	0.7904	0.3365	1.5163	0
986	1001.5	0.7951	0.7903	0.3365	1.5159	0
987	1001.4	0.7952	0.7906	0.3366	1.5162	0
988	1001.3	0.7954	0.7905	0.3367	1.5163	0
989	1001.2	0.7955	0.7908	0.3367	1.516	0
990	1001.1	0.7956	0.7908	0.3367	1.5162	0
991	1001	0.7955	0.7908	0.3367	1.5168	0
992	1000.9	0.7959	0.7909	0.3367	1.517	0
993	1000.8	0.7957	0.791	0.3369	1.5166	0
994	1000.7	0.796	0.7909	0.3369	1.5172	0
995	1000.6	0.796	0.7912	0.337	1.5175	0
996	1000.5	0.7961	0.7911	0.3371	1.5171	0
997	1000.4	0.7961	0.7913	0.3373	1.5174	0
998	1000.3	0.7963	0.7916	0.3373	1.5176	0
999	1000.2	0.7961	0.7913	0.3371	1.5175	0
1000	1000.1	0.7962	0.7913	0.3372	1.5183	0
1001	1000	0.7962	0.7914	0.3371	1.5178	0
1002	999.9	0.7965	0.7915	0.3373	1.5179	0
1003	999.8	0.7968	0.7917	0.3375	1.5183	0
1004	999.7	0.7966	0.7917	0.3374	1.5178	0
1005	999.6	0.7968	0.7919	0.3376	1.5186	0
1006	999.5	0.7969	0.7918	0.3376	1.5187	0
1007	999.4	0.7968	0.7918	0.3377	1.5182	0
1008	999.3	0.7968	0.7917	0.3374	1.5183	0
1009	999.2	0.7968	0.7918	0.3376	1.5184	0
1010	999.1	0.797	0.792	0.3377	1.5187	0
1011	999	0.797	0.792	0.3376	1.5187	0
1012	998.9	0.797	0.7921	0.3375	1.5182	0
1013	998.8	0.7969	0.792	0.3376	1.5188	0
1014	998.7	0.7972	0.7922	0.3377	1.5191	0
1015	998.6	0.797	0.792	0.3376	1.5179	0
1016	998.5	0.7971	0.7922	0.3376	1.5181	0
1017	998.4	0.7974	0.7923	0.3376	1.5188	0
1018	998.3	0.7973	0.7922	0.3376	1.5189	0

1019	998.2	0.7972	0.7923	0.3377	1.5184	0
1020	998.1	0.7972	0.7923	0.3374	1.519	0
1021	998	0.7973	0.7923	0.3374	1.5185	0
1022	997.9	0.7973	0.7923	0.3374	1.5186	0
1023	997.8	0.7975	0.7925	0.3375	1.5188	0
1024	997.7	0.7975	0.7925	0.3376	1.5183	0
1025	997.6	0.7976	0.7925	0.3376	1.5191	0
1026	997.5	0.7975	0.7926	0.3376	1.5191	0
1027	997.4	0.7977	0.7927	0.3377	1.5192	0
1028	997.3	0.7978	0.7927	0.3379	1.5195	0
1029	997.2	0.7977	0.7926	0.3378	1.519	0
1030	997.1	0.798	0.7928	0.3377	1.5197	0
1031	997	0.7976	0.7926	0.3376	1.519	0
1032	996.9	0.7979	0.7927	0.3377	1.5191	0
1033	996.8	0.7978	0.7928	0.3376	1.5187	0
1034	996.7	0.798	0.7928	0.3377	1.5194	0
1035	996.6	0.7981	0.793	0.3378	1.5201	0
1036	996.5	0.7982	0.7933	0.3377	1.5192	0
1037	996.4	0.7981	0.7932	0.3379	1.5198	0
1038	996.3	0.7982	0.7931	0.3378	1.5193	0
1039	996.2	0.7981	0.7931	0.3377	1.5199	0
1040	996.1	0.7982	0.7932	0.3376	1.5193	0
1041	996	0.7984	0.7933	0.3377	1.5201	0
1042	995.9	0.7985	0.7934	0.3379	1.5198	0
1043	995.8	0.7985	0.7932	0.3378	1.5198	0
1044	995.7	0.7988	0.7935	0.338	1.5196	0
1045	995.6	0.7989	0.7938	0.3382	1.5204	0
1046	995.5	0.799	0.7937	0.3382	1.5205	0
1047	995.4	0.7989	0.7937	0.3381	1.5199	0
1048	995.3	0.7991	0.7935	0.3381	1.5211	0
1049	995.2	0.7989	0.7938	0.3383	1.5207	0
1050	995.1	0.7991	0.7937	0.3383	1.5203	0
1051	995	0.7992	0.7939	0.3382	1.5204	0
1052	994.9	0.7991	0.7939	0.3383	1.5205	0
1053	994.8	0.7994	0.7942	0.3383	1.5217	0
1054	994.7	0.7996	0.794	0.3385	1.5209	0
1055	994.6	0.7995	0.7941	0.3384	1.521	0
1056	994.5	0.7994	0.7942	0.3384	1.521	0
1057	994.4	0.7994	0.7939	0.3384	1.5216	0
1058	994.3	0.7999	0.7944	0.3385	1.5214	0
1059	994.2	0.7999	0.7942	0.3386	1.5215	0
1060	994.1	0.8	0.7943	0.3386	1.5217	0
1061	994	0.8002	0.7947	0.3388	1.5219	0

1062	993.9	0.8003	0.7946	0.3387	1.5225	0
1063	993.8	0.8001	0.7945	0.3388	1.5226	0
1064	993.7	0.8005	0.7945	0.3391	1.5229	0
1065	993.6	0.8004	0.7947	0.3389	1.5224	0
1066	993.5	0.8005	0.7949	0.3391	1.5231	0
1067	993.4	0.8009	0.7951	0.3394	1.5224	0
1068	993.3	0.8008	0.795	0.3392	1.5229	0
1069	993.2	0.801	0.7951	0.3392	1.5236	0
1070	993.1	0.8012	0.795	0.3393	1.5233	0
1071	993	0.8013	0.7952	0.3393	1.5233	0
1072	992.9	0.8012	0.7953	0.3393	1.5238	0
1073	992.8	0.8013	0.7953	0.3393	1.5241	0
1074	992.7	0.8013	0.7954	0.3396	1.5243	0
1075	992.6	0.8016	0.7955	0.3395	1.5238	0
1076	992.5	0.8016	0.7953	0.3394	1.5238	0
1077	992.4	0.8016	0.7955	0.3395	1.5244	0
1078	992.3	0.8018	0.7956	0.3398	1.5247	0
1079	992.2	0.8016	0.7956	0.3395	1.5247	0
1080	992.1	0.8017	0.7953	0.3396	1.5242	0
1081	992	0.8019	0.7958	0.3396	1.5243	0
1082	991.9	0.8019	0.7958	0.3399	1.5245	0
1083	991.8	0.8022	0.7961	0.3398	1.5253	0
1084	991.7	0.8022	0.7961	0.34	1.5248	0
1085	991.6	0.8024	0.7964	0.34	1.5245	0
1086	991.5	0.8021	0.7961	0.3398	1.5248	0
1087	991.4	0.8024	0.7963	0.34	1.5257	0
1088	991.3	0.8023	0.7961	0.3399	1.5251	0
1089	991.2	0.8026	0.7963	0.34	1.5248	0
1090	991.1	0.8025	0.796	0.3399	1.5259	0
1091	991	0.8027	0.7964	0.3401	1.5249	0
1092	990.9	0.8026	0.7964	0.3401	1.5256	0
1093	990.8	0.8028	0.7966	0.3401	1.5257	0
1094	990.7	0.803	0.7966	0.3402	1.5253	0
1095	990.6	0.8029	0.7966	0.3401	1.5259	0
1096	990.5	0.8031	0.7967	0.3403	1.5261	0
1097	990.4	0.8031	0.7965	0.3401	1.526	0
1098	990.3	0.8031	0.7969	0.3401	1.5256	0
1099	990.2	0.803	0.7968	0.34	1.5255	0
1100	990.1	0.8031	0.797	0.34	1.5251	0
1101	990	0.8032	0.7972	0.3402	1.5254	0
1102	989.9	0.8032	0.7969	0.3401	1.5258	0
1103	989.8	0.8033	0.7969	0.3401	1.5259	0
1104	989.7	0.8033	0.797	0.3402	1.526	0

1105	989.6	0.8033	0.797	0.3401	1.5254	0
1106	989.5	0.8034	0.7972	0.3402	1.5255	0
1107	989.4	0.8035	0.7972	0.3404	1.5267	0
1108	989.3	0.8039	0.7972	0.3403	1.5264	0
1109	989.2	0.8036	0.7972	0.3402	1.5263	0
1110	989.1	0.8037	0.7973	0.3402	1.5265	0
1111	989	0.8038	0.7974	0.3402	1.5265	0
1112	988.9	0.8039	0.7975	0.3403	1.5266	0
1113	988.8	0.8039	0.7974	0.3402	1.5266	0
1114	988.7	0.8041	0.7978	0.3404	1.5264	0
1115	988.6	0.804	0.7976	0.3403	1.5264	0
1116	988.5	0.8042	0.7977	0.3404	1.527	0
1117	988.4	0.8042	0.7978	0.3404	1.5265	0
1118	988.3	0.8044	0.7979	0.3405	1.5266	0
1119	988.2	0.8044	0.7978	0.3405	1.5273	0
1120	988.1	0.8044	0.7978	0.3405	1.5273	0
1121	988	0.8045	0.798	0.3407	1.527	0
1122	987.9	0.8047	0.798	0.3405	1.5275	0
1123	987.8	0.8045	0.798	0.3405	1.5276	0
1124	987.7	0.8046	0.7982	0.3406	1.5272	0
1125	987.6	0.8049	0.7982	0.3405	1.5272	0
1126	987.5	0.805	0.7982	0.3406	1.5278	0
1127	987.4	0.8049	0.7981	0.3407	1.5273	0
1128	987.3	0.8049	0.7986	0.3407	1.528	0
1129	987.2	0.8052	0.7983	0.3407	1.5282	0
1130	987.1	0.8051	0.7985	0.3406	1.5282	0
1131	987	0.8054	0.7988	0.3409	1.529	0
1132	986.9	0.8054	0.7988	0.3408	1.5284	0
1133	986.8	0.8055	0.7988	0.341	1.5287	0
1134	986.7	0.8056	0.7989	0.3408	1.5292	0
1135	986.6	0.8057	0.799	0.341	1.5288	0
1136	986.5	0.8058	0.799	0.3409	1.5289	0
1137	986.4	0.8058	0.799	0.3409	1.53	0
1138	986.3	0.806	0.799	0.3411	1.529	0
1139	986.2	0.8063	0.7989	0.3412	1.5294	0
1140	986.1	0.8063	0.7992	0.3413	1.5294	0
1141	986	0.8064	0.7993	0.3413	1.5295	0
1142	985.9	0.8065	0.7995	0.3415	1.5298	0
1143	985.8	0.8064	0.7994	0.3414	1.5302	0
1144	985.7	0.8065	0.7991	0.3414	1.5302	0
1145	985.6	0.8067	0.7994	0.3417	1.531	0
1146	985.5	0.8069	0.7997	0.3418	1.5306	0
1147	985.4	0.807	0.7997	0.3417	1.5312	0

1148	985.3	0.8072	0.7998	0.3418	1.5314	0
1149	985.2	0.8072	0.7998	0.3418	1.5321	0
1150	985.1	0.8074	0.7999	0.3419	1.5316	0
1151	985	0.8072	0.8001	0.3417	1.5316	0
1152	984.9	0.8075	0.8	0.3419	1.5312	0
1153	984.8	0.8078	0.8	0.3421	1.532	0
1154	984.7	0.8076	0.7999	0.3421	1.532	0
1155	984.6	0.8078	0.8001	0.3421	1.5321	0
1156	984.5	0.8077	0.8	0.3422	1.5321	0
1157	984.4	0.8081	0.8005	0.3424	1.5323	0
1158	984.3	0.8082	0.8004	0.3423	1.5332	0
1159	984.2	0.8084	0.8008	0.3425	1.5327	0
1160	984.1	0.8084	0.8007	0.3425	1.5328	0
1161	984	0.8083	0.8007	0.3426	1.5339	0
1162	983.9	0.8086	0.8006	0.3426	1.534	0
1163	983.8	0.8088	0.8008	0.3428	1.5337	0
1164	983.7	0.8088	0.8008	0.3428	1.5342	0
1165	983.6	0.8088	0.801	0.3428	1.5333	0
1166	983.5	0.809	0.801	0.3428	1.5339	0
1167	983.4	0.809	0.8011	0.3428	1.5333	0
1168	983.3	0.8093	0.8011	0.343	1.5346	0
1169	983.2	0.8095	0.8012	0.343	1.5342	0
1170	983.1	0.8095	0.8014	0.3432	1.5343	0
1171	983	0.8096	0.8014	0.3433	1.5345	0
1172	982.9	0.8095	0.8013	0.3432	1.5343	0
1173	982.8	0.8095	0.8016	0.343	1.5348	0
1174	982.7	0.8097	0.8015	0.3431	1.5346	0
1175	982.6	0.8096	0.8014	0.3428	1.5348	0
1176	982.5	0.8098	0.8014	0.3431	1.5351	0
1177	982.4	0.8097	0.8015	0.343	1.5352	0
1178	982.3	0.8101	0.8021	0.3433	1.5359	0
1179	982.2	0.8101	0.802	0.3433	1.5356	0
1180	982.1	0.8101	0.802	0.3432	1.5355	0
1181	982	0.8104	0.802	0.3432	1.5356	0
1182	981.9	0.8102	0.8022	0.3435	1.5357	0
1183	981.8	0.8104	0.8023	0.3433	1.5352	0
1184	981.7	0.8103	0.8021	0.3433	1.5357	0
1185	981.6	0.8106	0.8021	0.3433	1.5358	0
1186	981.5	0.8106	0.8025	0.3435	1.5359	0
1187	981.4	0.8106	0.8023	0.3432	1.5359	0
1188	981.3	0.8108	0.8024	0.3434	1.5355	0
1189	981.2	0.8109	0.8024	0.3435	1.5361	0
1190	981.1	0.811	0.8027	0.3436	1.5357	0

1191	981	0.8108	0.8024	0.3434	1.5356	0
1192	980.9	0.811	0.8025	0.3435	1.5362	0
1193	980.8	0.8111	0.8025	0.3435	1.5361	0
1194	980.7	0.8106	0.8025	0.3432	1.5354	0
1195	980.6	0.811	0.8028	0.3434	1.5357	0
1196	980.5	0.811	0.8028	0.3435	1.5357	0
1197	980.4	0.8113	0.8028	0.3434	1.5358	0
1198	980.3	0.811	0.803	0.3434	1.5357	0
1199	980.2	0.8113	0.8028	0.3434	1.5364	0
1200	980.1	0.8109	0.8028	0.3431	1.5356	0
1201	980	0.8112	0.803	0.3434	1.5353	0
1202	979.9	0.8114	0.8031	0.3434	1.536	0
1203	979.8	0.8114	0.8031	0.3433	1.5359	0
1204	979.7	0.8112	0.803	0.3432	1.5359	0
1205	979.6	0.8115	0.8032	0.3432	1.5353	0
1206	979.5	0.8112	0.8031	0.3431	1.5359	0
1207	979.4	0.8112	0.8032	0.3431	1.5353	0
1208	979.3	0.8113	0.8031	0.3431	1.5359	0
1209	979.2	0.8113	0.8033	0.3431	1.5354	0
1210	979.1	0.8114	0.8035	0.3431	1.5355	0
1211	979	0.8116	0.8033	0.343	1.5355	0
1212	978.9	0.8113	0.8033	0.343	1.5354	0
1213	978.8	0.8117	0.8036	0.3431	1.5355	0
1214	978.7	0.8115	0.8035	0.343	1.5359	0
1215	978.6	0.8115	0.8037	0.3429	1.5349	0
1216	978.5	0.8114	0.8034	0.3428	1.5359	0
1217	978.4	0.8114	0.8036	0.3428	1.5355	0
1218	978.3	0.8116	0.8037	0.3429	1.535	0
1219	978.2	0.8116	0.8036	0.3428	1.5349	0
1220	978.1	0.8116	0.8038	0.343	1.5361	0
1221	978	0.8118	0.8037	0.3428	1.5356	0
1222	977.9	0.8117	0.8038	0.3428	1.5361	0
1223	977.8	0.8118	0.804	0.3429	1.5356	0
1224	977.7	0.8119	0.8039	0.3428	1.5356	0
1225	977.6	0.812	0.804	0.343	1.5358	0
1226	977.5	0.8123	0.8042	0.343	1.5354	0
1227	977.4	0.8121	0.8041	0.343	1.5364	0
1228	977.3	0.812	0.804	0.343	1.5359	0
1229	977.2	0.812	0.8042	0.343	1.5359	0
1230	977.1	0.8122	0.8041	0.3429	1.5354	0
1231	977	0.8122	0.8043	0.343	1.5366	0
1232	976.9	0.8124	0.8045	0.343	1.5361	0
1233	976.8	0.8125	0.8045	0.3431	1.5363	0

1234	976.7	0.8128	0.8045	0.3431	1.5358	0
1235	976.6	0.8125	0.8043	0.3431	1.5363	0
1236	976.5	0.8126	0.8045	0.3432	1.5369	0
1237	976.4	0.8129	0.8044	0.3433	1.5372	0
1238	976.3	0.8132	0.8048	0.3434	1.5374	0
1239	976.2	0.8131	0.8047	0.3433	1.5373	0
1240	976.1	0.813	0.8046	0.3433	1.5379	0
1241	976	0.8133	0.8047	0.3435	1.538	0
1242	975.9	0.8134	0.8049	0.3437	1.5383	0
1243	975.8	0.8135	0.8049	0.3437	1.5383	0
1244	975.7	0.8136	0.8049	0.3438	1.5384	0
1245	975.6	0.8137	0.805	0.344	1.5392	0
1246	975.5	0.814	0.8052	0.3442	1.5393	0
1247	975.4	0.8141	0.8052	0.3442	1.5393	0
1248	975.3	0.8142	0.8053	0.344	1.5394	0
1249	975.2	0.8142	0.8056	0.3444	1.5396	0
1250	975.1	0.8143	0.8055	0.3444	1.5402	0
1251	975	0.8144	0.8054	0.3443	1.5397	0
1252	974.9	0.8144	0.8056	0.3446	1.5399	0
1253	974.8	0.8147	0.8057	0.3446	1.5406	0
1254	974.7	0.815	0.8058	0.3449	1.5408	0
1255	974.6	0.8149	0.8058	0.3447	1.5413	0
1256	974.5	0.8151	0.8059	0.345	1.5415	0
1257	974.4	0.8154	0.8057	0.345	1.5417	0
1258	974.3	0.8154	0.8061	0.3452	1.5413	0
1259	974.2	0.8155	0.8061	0.3452	1.5419	0
1260	974.1	0.8156	0.8059	0.3452	1.5419	0
1261	974	0.8156	0.8061	0.3452	1.542	0
1262	973.9	0.8159	0.8064	0.3453	1.5422	0
1263	973.8	0.8159	0.8062	0.3455	1.5429	0
1264	973.7	0.816	0.8064	0.3456	1.5425	0
1265	973.6	0.8162	0.8064	0.3456	1.5431	0
1266	973.5	0.8163	0.8065	0.3458	1.5433	0
1267	973.4	0.8166	0.8068	0.3458	1.5435	0
1268	973.3	0.8165	0.8065	0.3459	1.5434	0
1269	973.2	0.8168	0.8067	0.346	1.5442	0
1270	973.1	0.8168	0.8067	0.346	1.5436	0
1271	973	0.8169	0.807	0.3462	1.5443	0
1272	972.9	0.817	0.807	0.3461	1.5444	0
1273	972.8	0.817	0.8068	0.3462	1.5444	0
1274	972.7	0.8169	0.8068	0.3462	1.5444	0
1275	972.6	0.8171	0.807	0.346	1.5439	0
1276	972.5	0.8169	0.8072	0.3461	1.544	0

1277	972.4	0.8173	0.807	0.3463	1.5447	0
1278	972.3	0.8176	0.8075	0.3465	1.5444	0
1279	972.2	0.8176	0.8073	0.3465	1.5449	0
1280	972.1	0.8174	0.8072	0.3464	1.5442	0
1281	972	0.8177	0.8073	0.3464	1.545	0
1282	971.9	0.8177	0.8074	0.3465	1.545	0
1283	971.8	0.8178	0.8074	0.3465	1.5451	0
1284	971.7	0.8178	0.8076	0.3468	1.5451	0
1285	971.6	0.8181	0.8077	0.3467	1.5453	0
1286	971.5	0.818	0.8076	0.3467	1.5452	0
1287	971.4	0.8181	0.8078	0.3467	1.5458	0
1288	971.3	0.8181	0.8078	0.3468	1.5453	0
1289	971.2	0.8182	0.8079	0.3467	1.5453	0
1290	971.1	0.8183	0.8079	0.3467	1.5459	0
1291	971	0.8184	0.8078	0.3468	1.5449	0
1292	970.9	0.8183	0.8077	0.3465	1.5453	0
1293	970.8	0.8184	0.8079	0.347	1.545	0
1294	970.7	0.8187	0.808	0.347	1.5456	0
1295	970.6	0.8185	0.8083	0.3468	1.546	0
1296	970.5	0.8185	0.808	0.3468	1.5454	0
1297	970.4	0.8186	0.8081	0.3466	1.5454	0
1298	970.3	0.8188	0.8083	0.347	1.5456	0
1299	970.2	0.8187	0.8084	0.3466	1.5455	0
1300	970.1	0.8188	0.8084	0.3468	1.5462	0
1301	970	0.8187	0.8085	0.3466	1.5455	0
1302	969.9	0.8186	0.8084	0.3465	1.5455	0
1303	969.8	0.8186	0.8084	0.3466	1.545	0
1304	969.7	0.8187	0.8084	0.3466	1.5456	0
1305	969.6	0.8185	0.8083	0.3463	1.5453	0
1306	969.5	0.8186	0.8084	0.3464	1.5454	0
1307	969.4	0.8185	0.8084	0.3463	1.5448	0
1308	969.3	0.8187	0.8086	0.3462	1.5448	0
1309	969.2	0.8187	0.8083	0.3464	1.5459	0
1310	969.1	0.819	0.8086	0.3464	1.5449	0
1311	969	0.8189	0.8089	0.3466	1.5456	0
1312	968.9	0.8189	0.8088	0.3465	1.5449	0
1313	968.8	0.8188	0.8084	0.3463	1.5453	0
1314	968.7	0.8188	0.8086	0.3463	1.5459	0
1315	968.6	0.8188	0.8087	0.3462	1.5458	0
1316	968.5	0.8187	0.8088	0.3461	1.5447	0
1317	968.4	0.819	0.8089	0.3462	1.5447	0
1318	968.3	0.819	0.8089	0.3461	1.5442	0
1319	968.2	0.8192	0.809	0.3462	1.5455	0

1320	968.1	0.8189	0.8087	0.3459	1.5446	0
1321	968	0.819	0.809	0.3461	1.5452	0
1322	967.9	0.819	0.8089	0.346	1.5447	0
1323	967.8	0.819	0.8089	0.3459	1.5446	0
1324	967.7	0.819	0.809	0.346	1.5446	0
1325	967.6	0.819	0.809	0.3457	1.5446	0
1326	967.5	0.819	0.8088	0.3457	1.5451	0
1327	967.4	0.819	0.809	0.3458	1.5446	0
1328	967.3	0.819	0.8092	0.3457	1.5446	0
1329	967.2	0.8193	0.8091	0.3457	1.5446	0
1330	967.1	0.8191	0.8091	0.3458	1.5445	0
1331	967	0.8191	0.8092	0.3458	1.5446	0
1332	966.9	0.8191	0.8092	0.346	1.5452	0
1333	966.8	0.8194	0.8093	0.346	1.5447	0
1334	966.7	0.8193	0.8095	0.3459	1.5447	0
1335	966.6	0.8195	0.8098	0.346	1.5449	0
1336	966.5	0.8194	0.8095	0.346	1.5448	0
1337	966.4	0.8195	0.8095	0.3461	1.5448	0
1338	966.3	0.8195	0.8095	0.3459	1.5453	0
1339	966.2	0.8197	0.8095	0.3461	1.5454	0
1340	966.1	0.82	0.8097	0.3463	1.5445	0
1341	966	0.8198	0.8097	0.3463	1.5455	0
1342	965.9	0.8201	0.8099	0.3464	1.5452	0
1343	965.8	0.8201	0.8098	0.3464	1.5457	0
1344	965.7	0.82	0.8098	0.3463	1.5457	0
1345	965.6	0.8204	0.8101	0.3465	1.5472	0
1346	965.5	0.8205	0.81	0.3464	1.5461	0
1347	965.4	0.8205	0.8101	0.3467	1.5467	0
1348	965.3	0.8206	0.8101	0.3468	1.5462	0
1349	965.2	0.8209	0.8104	0.3468	1.5469	0
1350	965.1	0.821	0.8106	0.347	1.5466	0
1351	965	0.8211	0.8105	0.3471	1.5472	0
1352	964.9	0.8212	0.8106	0.3472	1.5479	0
1353	964.8	0.8212	0.8104	0.3471	1.5479	0
1354	964.7	0.8215	0.8103	0.3472	1.5474	0
1355	964.6	0.8217	0.8109	0.3477	1.5488	0
1356	964.5	0.8218	0.8108	0.3475	1.5484	0
1357	964.4	0.822	0.811	0.3478	1.5486	0
1358	964.3	0.8218	0.8109	0.3478	1.549	0
1359	964.2	0.8219	0.8107	0.3477	1.5491	0
1360	964.1	0.8223	0.8108	0.3479	1.5492	0
1361	964	0.822	0.8109	0.3478	1.5492	0
1362	963.9	0.8221	0.811	0.348	1.5499	0

1363	963.8	0.8224	0.8111	0.3481	1.5495	0
1364	963.7	0.8224	0.8112	0.3483	1.5502	0
1365	963.6	0.8225	0.8112	0.3481	1.5502	0
1366	963.5	0.8228	0.8111	0.3483	1.5504	0
1367	963.4	0.823	0.8111	0.3486	1.5511	0
1368	963.3	0.8229	0.8112	0.3486	1.5512	0
1369	963.2	0.8231	0.8113	0.3487	1.5513	0
1370	963.1	0.8233	0.8113	0.3487	1.5513	0
1371	963	0.823	0.8113	0.3486	1.5513	0
1372	962.9	0.8232	0.8116	0.3488	1.5516	0
1373	962.8	0.8234	0.8114	0.3488	1.5515	0
1374	962.7	0.8233	0.8115	0.349	1.5522	0
1375	962.6	0.8234	0.8115	0.349	1.5516	0
1376	962.5	0.8234	0.8117	0.3493	1.5524	0
1377	962.4	0.8235	0.8116	0.3491	1.5518	0
1378	962.3	0.8236	0.8115	0.3491	1.5514	0
1379	962.2	0.8235	0.8117	0.3493	1.552	0
1380	962.1	0.8236	0.8117	0.3494	1.5525	0
1381	962	0.8235	0.8114	0.3492	1.5519	0
1382	961.9	0.8236	0.8117	0.3493	1.5525	0
1383	961.8	0.8237	0.8115	0.3493	1.552	0
1384	961.7	0.8236	0.8115	0.3492	1.552	0
1385	961.6	0.8236	0.8114	0.3493	1.552	0
1386	961.5	0.8238	0.8117	0.3496	1.5529	0
1387	961.4	0.8239	0.8117	0.3495	1.5523	0
1388	961.3	0.8238	0.812	0.3496	1.5524	0
1389	961.2	0.8237	0.8116	0.3494	1.5529	0
1390	961.1	0.8238	0.8118	0.3494	1.5524	0
1391	961	0.8239	0.8118	0.3496	1.5525	0
1392	960.9	0.8238	0.8118	0.3497	1.553	0
1393	960.8	0.824	0.8114	0.3496	1.5524	0
1394	960.7	0.8238	0.8117	0.3495	1.553	0
1395	960.6	0.8239	0.8117	0.3498	1.5527	0
1396	960.5	0.824	0.8116	0.3498	1.5532	0
1397	960.4	0.8238	0.8118	0.3497	1.5527	0
1398	960.3	0.8238	0.8115	0.3497	1.5526	0
1399	960.2	0.8239	0.8117	0.3497	1.5527	0
1400	960.1	0.8238	0.8115	0.3499	1.5527	0
1401	960	0.8238	0.8115	0.3498	1.5526	0
1402	959.9	0.8237	0.8113	0.3496	1.5521	0
1403	959.8	0.8236	0.8114	0.3497	1.552	0
1404	959.7	0.8235	0.8113	0.3496	1.552	0
1405	959.6	0.8234	0.8114	0.3494	1.553	0

1406	959.5	0.8234	0.811	0.3493	1.5519	0
1407	959.4	0.8233	0.8112	0.3494	1.5519	0
1408	959.3	0.8232	0.8109	0.3493	1.5513	0
1409	959.2	0.8231	0.8111	0.3494	1.5513	0
1410	959.1	0.823	0.8109	0.3491	1.5511	0
1411	959	0.8234	0.8113	0.3497	1.5515	0
1412	958.9	0.8232	0.8112	0.3495	1.5509	0
1413	958.8	0.8228	0.8107	0.3491	1.5504	0
1414	958.7	0.823	0.8109	0.3491	1.55	0
1415	958.6	0.8227	0.8106	0.3489	1.5504	0
1416	958.5	0.8227	0.8107	0.349	1.5504	0
1417	958.4	0.8224	0.8106	0.349	1.5503	0
1418	958.3	0.8224	0.8106	0.349	1.5499	0
1419	958.2	0.8221	0.8103	0.3486	1.5496	0
1420	958.1	0.822	0.8102	0.3488	1.549	0
1421	958	0.8221	0.8103	0.3487	1.549	0
1422	957.9	0.8221	0.8104	0.3488	1.5498	0
1423	957.8	0.8219	0.8102	0.3487	1.5491	0
1424	957.7	0.8218	0.8102	0.3487	1.5491	0
1425	957.6	0.8216	0.81	0.3486	1.5489	0
1426	957.5	0.8216	0.81	0.3484	1.5495	0
1427	957.4	0.8216	0.8101	0.3487	1.5491	0
1428	957.3	0.8216	0.81	0.3487	1.5486	0
1429	957.2	0.8215	0.81	0.3486	1.5485	0
1430	957.1	0.8215	0.8099	0.3486	1.548	0
1431	957	0.8215	0.8099	0.3488	1.5487	0
1432	956.9	0.8212	0.8097	0.3484	1.5479	0
1433	956.8	0.8214	0.8099	0.3488	1.5482	0
1434	956.7	0.8212	0.8097	0.3487	1.5481	0
1435	956.6	0.821	0.8095	0.3486	1.548	0
1436	956.5	0.8208	0.8093	0.3484	1.5468	0
1437	956.4	0.8212	0.8095	0.3488	1.5481	0
1438	956.3	0.8212	0.8091	0.3487	1.5481	0
1439	956.2	0.8209	0.8091	0.3486	1.5475	0
1440	956.1	0.8209	0.8093	0.3486	1.5475	0
1441	956	0.8208	0.8091	0.3484	1.5474	0
1442	955.9	0.8208	0.8091	0.3485	1.5474	0
1443	955.8	0.8207	0.8088	0.3484	1.5469	0
1444	955.7	0.8207	0.8087	0.3487	1.5469	0
1445	955.6	0.8205	0.8087	0.3485	1.547	0
1446	955.5	0.8204	0.8085	0.3485	1.5469	0
1447	955.4	0.8204	0.8087	0.3487	1.5466	0
1448	955.3	0.8204	0.8085	0.3488	1.5476	0

1449	955.2	0.8204	0.8086	0.3488	1.5478	0
1450	955.1	0.8204	0.8085	0.3489	1.5473	0
1451	955	0.8202	0.8084	0.3489	1.5473	0
1452	954.9	0.8203	0.8083	0.3489	1.5468	0
1453	954.8	0.8201	0.8081	0.3489	1.5468	0
1454	954.7	0.8201	0.8082	0.3489	1.5463	0
1455	954.6	0.82	0.8081	0.3491	1.5475	0
1456	954.5	0.8203	0.808	0.3491	1.5471	0
1457	954.4	0.8203	0.808	0.3492	1.5477	0
1458	954.3	0.8202	0.808	0.3492	1.5472	0
1459	954.2	0.8203	0.8078	0.3495	1.5479	0
1460	954.1	0.8205	0.8081	0.3499	1.5483	0
1461	954	0.8204	0.808	0.3498	1.5477	0
1462	953.9	0.8205	0.8078	0.35	1.5484	0
1463	953.8	0.8205	0.8076	0.3498	1.5473	0
1464	953.7	0.8204	0.8078	0.35	1.548	0
1465	953.6	0.8203	0.8076	0.35	1.5485	0
1466	953.5	0.8206	0.8077	0.3502	1.5487	0
1467	953.4	0.8204	0.8074	0.3501	1.5483	0
1468	953.3	0.8204	0.8075	0.3504	1.5485	0
1469	953.2	0.8204	0.8074	0.3504	1.549	0
1470	953.1	0.8203	0.8074	0.3504	1.5491	0
1471	953	0.8202	0.8073	0.3503	1.548	0
1472	952.9	0.8204	0.8071	0.3504	1.5491	0
1473	952.8	0.8202	0.8071	0.3503	1.5492	0
1474	952.7	0.8202	0.8071	0.3502	1.5486	0
1475	952.6	0.8201	0.8068	0.3505	1.5486	0
1476	952.5	0.8203	0.8071	0.3506	1.5488	0
1477	952.4	0.8201	0.8071	0.3506	1.5488	0
1478	952.3	0.8201	0.8067	0.3506	1.5483	0
1479	952.2	0.8203	0.8066	0.3507	1.5489	0
1480	952.1	0.8202	0.8067	0.3507	1.5494	0
1481	952	0.8202	0.8068	0.3506	1.5489	0
1482	951.9	0.8202	0.8069	0.3508	1.549	0
1483	951.8	0.82	0.8067	0.3509	1.549	0
1484	951.7	0.82	0.8066	0.3509	1.5485	0
1485	951.6	0.8202	0.807	0.3511	1.5493	0
1486	951.5	0.8201	0.8066	0.3512	1.5487	0
1487	951.4	0.82	0.8066	0.3511	1.5487	0
1488	951.3	0.82	0.8066	0.3511	1.5488	0
1489	951.2	0.8201	0.8064	0.3512	1.5488	0
1490	951.1	0.82	0.8061	0.3513	1.5479	0
1491	951	0.8201	0.8063	0.3513	1.5489	0

1492	950.9	0.8199	0.8063	0.3513	1.5484	0
1493	950.8	0.8199	0.8061	0.3512	1.549	0
1494	950.7	0.8199	0.8063	0.3513	1.5485	0
1495	950.6	0.8199	0.806	0.3512	1.5485	0
1496	950.5	0.8199	0.8063	0.3513	1.5491	0
1497	950.4	0.8199	0.8059	0.3512	1.5485	0
1498	950.3	0.8199	0.8058	0.3513	1.5491	0
1499	950.2	0.8198	0.8057	0.3513	1.5484	0
1500	950.1	0.82	0.806	0.3515	1.548	0
1501	950	0.8199	0.8059	0.3515	1.548	0
1502	949.9	0.8197	0.8058	0.3513	1.5479	0
1503	949.8	0.8198	0.8058	0.3513	1.5484	0
1504	949.7	0.8197	0.8059	0.3515	1.5485	0
1505	949.6	0.82	0.8061	0.3517	1.5486	0
1506	949.5	0.8198	0.8059	0.3516	1.548	0
1507	949.4	0.8196	0.8057	0.3515	1.5478	0
1508	949.3	0.8195	0.8056	0.3513	1.5476	0
1509	949.2	0.8195	0.8057	0.3514	1.5473	0
1510	949.1	0.8195	0.8056	0.3513	1.5472	0
1511	949	0.8194	0.8054	0.3512	1.5477	0
1512	948.9	0.8193	0.8056	0.3512	1.5476	0
1513	948.8	0.8192	0.8053	0.3512	1.547	0
1514	948.7	0.8191	0.8054	0.3512	1.5476	0
1515	948.6	0.8189	0.8053	0.3511	1.5469	0
1516	948.5	0.819	0.8053	0.3511	1.5464	0
1517	948.4	0.8187	0.8051	0.3508	1.5468	0
1518	948.3	0.8189	0.8053	0.351	1.5463	0
1519	948.2	0.8188	0.805	0.3509	1.5462	0
1520	948.1	0.8184	0.8047	0.3507	1.5455	0
1521	948	0.8184	0.8048	0.3505	1.546	0
1522	947.9	0.8186	0.8048	0.3507	1.5461	0
1523	947.8	0.8186	0.8049	0.3507	1.5461	0
1524	947.7	0.8185	0.8047	0.3508	1.5455	0
1525	947.6	0.8185	0.8048	0.3506	1.5455	0
1526	947.5	0.8183	0.8048	0.3506	1.5449	0
1527	947.4	0.8184	0.8048	0.3507	1.5455	0
1528	947.3	0.8184	0.8048	0.3507	1.5455	0
1529	947.2	0.8183	0.8047	0.3506	1.5449	0
1530	947.1	0.8181	0.8045	0.3505	1.5453	0
1531	947	0.8181	0.8043	0.3503	1.5452	0
1532	946.9	0.8179	0.8046	0.3505	1.5447	0
1533	946.8	0.818	0.8043	0.3504	1.5447	0
1534	946.7	0.8181	0.8045	0.3505	1.5443	0

1535	946.6	0.8178	0.8045	0.3504	1.5447	0
1536	946.5	0.8181	0.8043	0.3503	1.5442	0
1537	946.4	0.8181	0.8043	0.3504	1.5442	0
1538	946.3	0.8178	0.804	0.3502	1.5441	0
1539	946.2	0.8176	0.8042	0.3502	1.5445	0
1540	946.1	0.8178	0.8041	0.3502	1.544	0
1541	946	0.8178	0.8045	0.3502	1.5441	0
1542	945.9	0.8177	0.8043	0.3504	1.5436	0
1543	945.8	0.8179	0.8042	0.3505	1.5442	0
1544	945.7	0.8179	0.8042	0.3504	1.5441	0
1545	945.6	0.8178	0.804	0.3504	1.5446	0
1546	945.5	0.8177	0.8042	0.3502	1.5445	0
1547	945.4	0.8175	0.804	0.3501	1.5439	0
1548	945.3	0.8176	0.8039	0.3504	1.5439	0
1549	945.2	0.8176	0.8041	0.3505	1.5445	0
1550	945.1	0.8177	0.8043	0.3505	1.544	0
1551	945	0.8177	0.8041	0.3506	1.5441	0
1552	944.9	0.8177	0.8041	0.3505	1.5441	0
1553	944.8	0.8179	0.8041	0.3507	1.5442	0
1554	944.7	0.8177	0.804	0.3506	1.5441	0
1555	944.6	0.818	0.8041	0.3507	1.5451	0
1556	944.5	0.8179	0.8037	0.3507	1.5446	0
1557	944.4	0.8179	0.8039	0.3509	1.5442	0
1558	944.3	0.8181	0.804	0.3508	1.5453	0
1559	944.2	0.8181	0.8041	0.3509	1.5449	0
1560	944.1	0.8182	0.8041	0.351	1.5449	0
1561	944	0.818	0.8039	0.351	1.5449	0
1562	943.9	0.8183	0.8042	0.3513	1.5451	0
1563	943.8	0.8182	0.804	0.3512	1.5449	0
1564	943.7	0.8182	0.804	0.3512	1.5454	0
1565	943.6	0.8183	0.8041	0.3515	1.5456	0
1566	943.5	0.8182	0.804	0.3514	1.5456	0
1567	943.4	0.8183	0.8041	0.3513	1.5461	0
1568	943.3	0.8183	0.804	0.3515	1.5456	0
1569	943.2	0.8185	0.8042	0.3515	1.5457	0
1570	943.1	0.8183	0.8041	0.3516	1.5457	0
1571	943	0.8184	0.804	0.3516	1.5457	0
1572	942.9	0.8184	0.8039	0.3516	1.5457	0
1573	942.8	0.8185	0.8039	0.3517	1.5458	0
1574	942.7	0.8185	0.8041	0.3517	1.5458	0
1575	942.6	0.8182	0.8038	0.3518	1.5463	0
1576	942.5	0.8184	0.804	0.3517	1.5458	0
1577	942.4	0.8184	0.8039	0.3518	1.5459	0

1578	942.3	0.8185	0.8039	0.3518	1.546	0
1579	942.2	0.8185	0.8038	0.3519	1.546	0
1580	942.1	0.8185	0.8039	0.3518	1.5461	0
1581	942	0.8184	0.8037	0.3518	1.5461	0
1582	941.9	0.8183	0.8037	0.3521	1.5462	0
1583	941.8	0.8184	0.8037	0.3519	1.5468	0
1584	941.7	0.8185	0.8037	0.352	1.5458	0
1585	941.6	0.8185	0.8035	0.3521	1.5459	0
1586	941.5	0.8187	0.8038	0.3521	1.546	0
1587	941.4	0.8184	0.8037	0.352	1.5465	0
1588	941.3	0.8186	0.8035	0.3521	1.546	0
1589	941.2	0.8186	0.8035	0.3522	1.5462	0
1590	941.1	0.8187	0.8038	0.3522	1.5457	0
1591	941	0.8185	0.8036	0.3521	1.5457	0
1592	940.9	0.8185	0.8035	0.3522	1.5462	0
1593	940.8	0.8188	0.8036	0.3523	1.5464	0
1594	940.7	0.8187	0.8035	0.3524	1.5465	0
1595	940.6	0.8186	0.8037	0.3523	1.5465	0
1596	940.5	0.8187	0.8035	0.3526	1.5464	0
1597	940.4	0.8184	0.8033	0.3522	1.5458	0
1598	940.3	0.8187	0.8032	0.3522	1.5458	0
1599	940.2	0.8187	0.8036	0.3526	1.5466	0
1600	940.1	0.8188	0.8034	0.3525	1.5461	0
1601	940	0.8189	0.8035	0.3525	1.5461	0
1602	939.9	0.8188	0.8035	0.3526	1.5461	0
1603	939.8	0.8188	0.8033	0.3526	1.5461	0
1604	939.7	0.8189	0.8034	0.3526	1.5466	0
1605	939.6	0.8187	0.8031	0.3524	1.5465	0
1606	939.5	0.8188	0.8035	0.3528	1.5467	0
1607	939.4	0.8187	0.8035	0.3526	1.5465	0
1608	939.3	0.8187	0.8032	0.3526	1.5465	0
1609	939.2	0.8189	0.8034	0.3527	1.5466	0
1610	939.1	0.8188	0.8032	0.3526	1.5459	0
1611	939	0.8186	0.8031	0.3525	1.5457	0
1612	938.9	0.8187	0.8031	0.3525	1.5458	0
1613	938.8	0.8186	0.8032	0.3527	1.5462	0
1614	938.7	0.8187	0.8033	0.3526	1.5462	0
1615	938.6	0.8184	0.8031	0.3525	1.546	0
1616	938.5	0.8185	0.8031	0.3525	1.546	0
1617	938.4	0.8185	0.803	0.3526	1.5465	0
1618	938.3	0.8187	0.8033	0.3528	1.546	0
1619	938.2	0.8188	0.8032	0.3527	1.5466	0
1620	938.1	0.8187	0.8032	0.3526	1.5459	0

1621	938	0.8187	0.803	0.3526	1.5463	0
1622	937.9	0.8188	0.8031	0.3527	1.5457	0
1623	937.8	0.8187	0.8033	0.3528	1.5463	0
1624	937.7	0.8186	0.8033	0.3528	1.5456	0
1625	937.6	0.8186	0.8031	0.3526	1.546	0
1626	937.5	0.8187	0.8032	0.3526	1.5455	0
1627	937.4	0.8186	0.803	0.3526	1.5459	0
1628	937.3	0.8187	0.8033	0.3527	1.5455	0
1629	937.2	0.8188	0.8032	0.3528	1.546	0
1630	937.1	0.8186	0.8033	0.3527	1.5459	0
1631	937	0.8187	0.8033	0.3526	1.5459	0
1632	936.9	0.8185	0.8032	0.3526	1.5458	0
1633	936.8	0.8188	0.8033	0.3529	1.546	0
1634	936.7	0.8189	0.8034	0.3528	1.5459	0
1635	936.6	0.8186	0.8031	0.3526	1.5458	0
1636	936.5	0.8186	0.8034	0.3528	1.5459	0
1637	936.4	0.8187	0.8033	0.3528	1.5464	0
1638	936.3	0.8186	0.8031	0.3527	1.5458	0
1639	936.2	0.8186	0.8032	0.3527	1.5458	0
1640	936.1	0.8184	0.8028	0.3525	1.5456	0
1641	936	0.8183	0.8031	0.3526	1.5462	0
1642	935.9	0.8184	0.803	0.3527	1.5457	0
1643	935.8	0.8184	0.803	0.3525	1.5451	0
1644	935.7	0.8185	0.8029	0.3526	1.5462	0
1645	935.6	0.8187	0.8032	0.353	1.5458	0
1646	935.5	0.8186	0.8029	0.3529	1.5461	0
1647	935.4	0.8187	0.803	0.353	1.5457	0
1648	935.3	0.8185	0.8029	0.353	1.5457	0
1649	935.2	0.8186	0.8029	0.353	1.5462	0
1650	935.1	0.8187	0.803	0.353	1.5462	0
1651	935	0.8188	0.8031	0.3531	1.5461	0
1652	934.9	0.8188	0.8031	0.3531	1.5462	0
1653	934.8	0.8189	0.8032	0.3532	1.5462	0
1654	934.7	0.8189	0.8031	0.3533	1.5463	0
1655	934.6	0.8188	0.8031	0.3532	1.5467	0
1656	934.5	0.819	0.8031	0.3533	1.5467	0
1657	934.4	0.8188	0.8029	0.3533	1.5472	0
1658	934.3	0.819	0.8031	0.3534	1.5467	0
1659	934.2	0.8188	0.8031	0.3534	1.5466	0
1660	934.1	0.8189	0.803	0.3534	1.5466	0
1661	934	0.8191	0.8032	0.3535	1.5467	0
1662	933.9	0.8191	0.8033	0.3535	1.5473	0
1663	933.8	0.8189	0.8031	0.3535	1.5466	0

1664	933.7	0.8191	0.8031	0.3535	1.5468	0
1665	933.6	0.819	0.8031	0.3536	1.5463	0
1666	933.5	0.8193	0.8033	0.3536	1.5469	0
1667	933.4	0.8192	0.8032	0.3537	1.5475	0
1668	933.3	0.8192	0.803	0.3538	1.547	0
1669	933.2	0.8193	0.8032	0.3538	1.5476	0
1670	933.1	0.8194	0.8034	0.3538	1.5477	0
1671	933	0.8194	0.8034	0.354	1.5472	0
1672	932.9	0.8194	0.8031	0.3539	1.5476	0
1673	932.8	0.8194	0.8032	0.3538	1.5472	0
1674	932.7	0.8194	0.8032	0.3538	1.5472	0
1675	932.6	0.8193	0.8031	0.3539	1.5478	0
1676	932.5	0.8193	0.8031	0.3539	1.5473	0
1677	932.4	0.8195	0.8032	0.354	1.5474	0
1678	932.3	0.8195	0.8029	0.354	1.5484	0
1679	932.2	0.8194	0.8028	0.3539	1.5483	0
1680	932.1	0.8196	0.8029	0.3539	1.5474	0
1681	932	0.8195	0.8029	0.354	1.5474	0
1682	931.9	0.8194	0.8028	0.354	1.5474	0
1683	931.8	0.8195	0.8029	0.3542	1.5469	0
1684	931.7	0.8195	0.803	0.354	1.5479	0
1685	931.6	0.8196	0.8033	0.3542	1.548	0
1686	931.5	0.8194	0.803	0.3541	1.5473	0
1687	931.4	0.8196	0.8032	0.3542	1.5473	0
1688	931.3	0.8196	0.8029	0.3542	1.5473	0
1689	931.2	0.8194	0.8027	0.354	1.5472	0
1690	931.1	0.8192	0.8029	0.3539	1.5471	0
1691	931	0.8192	0.8027	0.3539	1.5471	0
1692	930.9	0.8195	0.8029	0.3541	1.5472	0
1693	930.8	0.8194	0.8028	0.3541	1.5473	0
1694	930.7	0.8194	0.8027	0.3539	1.5467	0
1695	930.6	0.8193	0.8028	0.3538	1.5471	0
1696	930.5	0.8193	0.8026	0.3539	1.5466	0
1697	930.4	0.8192	0.8026	0.3538	1.5471	0
1698	930.3	0.8194	0.8027	0.3537	1.5467	0
1699	930.2	0.8192	0.8025	0.3538	1.5466	0
1700	930.1	0.8191	0.8026	0.3537	1.5465	0
1701	930	0.8191	0.8026	0.3538	1.5465	0
1702	929.9	0.8192	0.8026	0.3538	1.5465	0
1703	929.8	0.8192	0.8027	0.3539	1.5467	0
1704	929.7	0.8193	0.8026	0.3539	1.5466	0
1705	929.6	0.8193	0.8027	0.3539	1.5466	0
1706	929.5	0.8193	0.8028	0.3538	1.5472	0

1707	929.4	0.8192	0.8025	0.3537	1.5459	0
1708	929.3	0.819	0.8023	0.3536	1.5463	0
1709	929.2	0.8191	0.8024	0.3537	1.5464	0
1710	929.1	0.8191	0.8024	0.3537	1.5459	0
1711	929	0.8193	0.8025	0.3538	1.546	0
1712	928.9	0.8192	0.8024	0.3537	1.546	0
1713	928.8	0.8192	0.8025	0.3539	1.5459	0
1714	928.7	0.8193	0.8025	0.3539	1.546	0
1715	928.6	0.8192	0.8024	0.3538	1.5464	0
1716	928.5	0.8194	0.8024	0.3539	1.546	0
1717	928.4	0.8195	0.8026	0.3539	1.5466	0
1718	928.3	0.8192	0.8024	0.3539	1.5463	0
1719	928.2	0.8195	0.8023	0.354	1.5465	0
1720	928.1	0.8193	0.8025	0.3539	1.5464	0
1721	928	0.8192	0.8025	0.3538	1.5458	0
1722	927.9	0.8194	0.8024	0.3539	1.5459	0
1723	927.8	0.8193	0.8025	0.354	1.547	0
1724	927.7	0.8194	0.8024	0.354	1.5464	0
1725	927.6	0.8193	0.8023	0.3539	1.5463	0
1726	927.5	0.8195	0.8024	0.3541	1.5464	0
1727	927.4	0.8196	0.8027	0.3541	1.5465	0
1728	927.3	0.8195	0.8022	0.3541	1.5463	0
1729	927.2	0.8196	0.8022	0.3541	1.5463	0
1730	927.1	0.8197	0.8025	0.3543	1.5469	0
1731	927	0.8195	0.8023	0.354	1.5463	0
1732	926.9	0.8197	0.8024	0.3542	1.5464	0
1733	926.8	0.8198	0.8023	0.3543	1.5469	0
1734	926.7	0.8196	0.8021	0.3542	1.5468	0
1735	926.6	0.8196	0.8023	0.3542	1.5469	0
1736	926.5	0.8197	0.8022	0.3542	1.5474	0
1737	926.4	0.8198	0.8022	0.3544	1.5469	0
1738	926.3	0.8201	0.8025	0.3546	1.5471	0
1739	926.2	0.82	0.8026	0.3547	1.547	0
1740	926.1	0.8199	0.8025	0.3545	1.5474	0
1741	926	0.82	0.8026	0.3546	1.5476	0
1742	925.9	0.8201	0.8026	0.3548	1.5481	0
1743	925.8	0.8201	0.8025	0.3548	1.5476	0
1744	925.7	0.8201	0.8025	0.3547	1.547	0
1745	925.6	0.8203	0.8026	0.3549	1.5476	0
1746	925.5	0.8206	0.8026	0.3551	1.5478	0
1747	925.4	0.8204	0.8025	0.355	1.5482	0
1748	925.3	0.8204	0.8026	0.3551	1.5482	0
1749	925.2	0.8203	0.8025	0.3549	1.5481	0

1750	925.1	0.8202	0.8025	0.355	1.5481	0
1751	925	0.8206	0.8025	0.355	1.5481	0
1752	924.9	0.8205	0.8026	0.3552	1.5482	0
1753	924.8	0.8207	0.8027	0.3554	1.5489	0
1754	924.7	0.8206	0.8028	0.3553	1.5493	0
1755	924.6	0.8208	0.8029	0.3555	1.5484	0
1756	924.5	0.8207	0.8027	0.3553	1.5493	0
1757	924.4	0.8206	0.8026	0.3553	1.5492	0
1758	924.3	0.8208	0.8028	0.3555	1.5494	0
1759	924.2	0.8208	0.8027	0.3555	1.5493	0
1760	924.1	0.8208	0.8027	0.3555	1.5493	0
1761	924	0.821	0.8031	0.3558	1.55	0
1762	923.9	0.8211	0.8029	0.3558	1.5494	0
1763	923.8	0.8209	0.8027	0.3555	1.5492	0
1764	923.7	0.821	0.8027	0.3556	1.5497	0
1765	923.6	0.8212	0.8027	0.3556	1.5492	0
1766	923.5	0.8211	0.8028	0.3557	1.5498	0
1767	923.4	0.821	0.8028	0.3557	1.5497	0
1768	923.3	0.8211	0.8027	0.3557	1.5498	0
1769	923.2	0.8211	0.8029	0.3558	1.5498	0
1770	923.1	0.8214	0.803	0.3559	1.5498	0
1771	923	0.8213	0.803	0.356	1.5498	0
1772	922.9	0.8213	0.8029	0.3559	1.5498	0
1773	922.8	0.8213	0.8028	0.356	1.5504	0
1774	922.7	0.8211	0.8028	0.3558	1.5496	0
1775	922.6	0.8211	0.8029	0.3558	1.5502	0
1776	922.5	0.8213	0.8027	0.3558	1.5501	0
1777	922.4	0.8212	0.8029	0.3559	1.5496	0
1778	922.3	0.8214	0.8029	0.356	1.5496	0
1779	922.2	0.8215	0.803	0.3561	1.5502	0
1780	922.1	0.8215	0.803	0.3562	1.5496	0
1781	922	0.8215	0.8029	0.356	1.5495	0
1782	921.9	0.8214	0.8029	0.3559	1.5494	0
1783	921.8	0.8213	0.8028	0.356	1.5499	0
1784	921.7	0.8215	0.8029	0.356	1.5493	0
1785	921.6	0.8214	0.8028	0.356	1.5493	0
1786	921.5	0.8213	0.8029	0.3561	1.5498	0
1787	921.4	0.8217	0.8031	0.3563	1.55	0
1788	921.3	0.8215	0.803	0.356	1.5497	0
1789	921.2	0.8214	0.8028	0.3558	1.5495	0
1790	921.1	0.8214	0.8029	0.3559	1.5495	0
1791	921	0.8214	0.8029	0.3559	1.5495	0
1792	920.9	0.8216	0.803	0.3561	1.549	0

1793	920.8	0.8214	0.8027	0.3559	1.5488	0
1794	920.7	0.8212	0.8027	0.3557	1.5492	0
1795	920.6	0.8214	0.8031	0.3558	1.5493	0
1796	920.5	0.8214	0.8029	0.3558	1.5487	0
1797	920.4	0.8213	0.8027	0.3559	1.5486	0
1798	920.3	0.8211	0.8029	0.3558	1.549	0
1799	920.2	0.8212	0.8029	0.3557	1.5489	0
1800	920.1	0.8212	0.8029	0.3556	1.5488	0
1801	920	0.821	0.803	0.3557	1.5487	0
1802	919.9	0.8211	0.8029	0.3556	1.5493	0
1803	919.8	0.8213	0.8029	0.3557	1.5482	0
1804	919.7	0.8213	0.8032	0.3557	1.5487	0
1805	919.6	0.8213	0.8032	0.3556	1.5481	0
1806	919.5	0.8213	0.8031	0.3557	1.5486	0
1807	919.4	0.8214	0.8031	0.3557	1.5491	0
1808	919.3	0.8212	0.8029	0.3555	1.5484	0
1809	919.2	0.8213	0.803	0.3556	1.5478	0
1810	919.1	0.8211	0.8029	0.3555	1.5476	0
1811	919	0.8212	0.8032	0.3556	1.5482	0
1812	918.9	0.8212	0.803	0.3556	1.5481	0
1813	918.8	0.8211	0.803	0.3554	1.548	0
1814	918.7	0.8213	0.803	0.3554	1.5474	0
1815	918.6	0.8211	0.8029	0.3553	1.5477	0
1816	918.5	0.821	0.803	0.3552	1.5472	0
1817	918.4	0.8211	0.8028	0.3553	1.5476	0
1818	918.3	0.821	0.8028	0.355	1.5474	0
1819	918.2	0.8208	0.8029	0.3551	1.5473	0
1820	918.1	0.8209	0.8028	0.3549	1.5473	0
1821	918	0.8209	0.8028	0.3549	1.5472	0
1822	917.9	0.8209	0.8026	0.355	1.5471	0
1823	917.8	0.821	0.803	0.3552	1.5472	0
1824	917.7	0.821	0.803	0.3551	1.5471	0
1825	917.6	0.821	0.8029	0.355	1.5476	0
1826	917.5	0.8211	0.803	0.355	1.5471	0
1827	917.4	0.821	0.8031	0.3552	1.5476	0
1828	917.3	0.8214	0.8033	0.3554	1.5478	0
1829	917.2	0.8211	0.8032	0.3552	1.5475	0
1830	917.1	0.8214	0.803	0.3552	1.5475	0
1831	917	0.8213	0.8032	0.3551	1.5474	0
1832	916.9	0.8215	0.803	0.3552	1.548	0
1833	916.8	0.8214	0.8031	0.3554	1.5485	0
1834	916.7	0.8215	0.803	0.3553	1.5474	0
1835	916.6	0.8216	0.8029	0.3553	1.5479	0

1836	916.5	0.8216	0.8029	0.3552	1.5478	0
1837	916.4	0.8216	0.8031	0.3554	1.5479	0
1838	916.3	0.8217	0.8032	0.3554	1.5484	0
1839	916.2	0.8219	0.8031	0.3555	1.548	0
1840	916.1	0.8218	0.8032	0.3555	1.5485	0
1841	916	0.822	0.8035	0.3557	1.5492	0
1842	915.9	0.8221	0.8035	0.356	1.5487	0
1843	915.8	0.8222	0.8034	0.356	1.5497	0
1844	915.7	0.8223	0.8033	0.3559	1.5492	0
1845	915.6	0.8221	0.8033	0.3561	1.5491	0
1846	915.5	0.8223	0.8035	0.3561	1.5491	0
1847	915.4	0.8224	0.8035	0.3561	1.5492	0
1848	915.3	0.8224	0.8036	0.3564	1.5498	0
1849	915.2	0.8227	0.8035	0.3564	1.5497	0
1850	915.1	0.8226	0.8037	0.3565	1.5497	0
1851	915	0.8227	0.8037	0.3565	1.5503	0
1852	914.9	0.8229	0.8036	0.3566	1.5498	0
1853	914.8	0.8228	0.8038	0.3567	1.5504	0
1854	914.7	0.823	0.8039	0.3567	1.5499	0
1855	914.6	0.8229	0.8038	0.3567	1.5509	0
1856	914.5	0.8232	0.8039	0.3568	1.5504	0
1857	914.4	0.8234	0.8041	0.3571	1.5517	0
1858	914.3	0.8233	0.804	0.3571	1.551	0
1859	914.2	0.8233	0.804	0.357	1.5515	0
1860	914.1	0.8236	0.8041	0.3572	1.5516	0
1861	914	0.8236	0.8041	0.3573	1.5516	0
1862	913.9	0.8237	0.8042	0.3574	1.5516	0
1863	913.8	0.8238	0.8041	0.3574	1.552	0
1864	913.7	0.8239	0.8043	0.3574	1.5517	0
1865	913.6	0.824	0.8042	0.3575	1.5511	0
1866	913.5	0.8239	0.8041	0.3575	1.5521	0
1867	913.4	0.8241	0.8042	0.3577	1.5527	0
1868	913.3	0.8243	0.8045	0.3578	1.5523	0
1869	913.2	0.8243	0.8045	0.3579	1.5527	0
1870	913.1	0.8242	0.8044	0.3578	1.5527	0
1871	913	0.8242	0.8044	0.3577	1.552	0
1872	912.9	0.8242	0.8044	0.3578	1.5519	0
1873	912.8	0.8241	0.8044	0.3577	1.5535	0
1874	912.7	0.8242	0.8045	0.3577	1.5535	0
1875	912.6	0.8244	0.8046	0.3579	1.5536	0
1876	912.5	0.8245	0.8046	0.358	1.5525	0
1877	912.4	0.8244	0.8046	0.3578	1.5529	0
1878	912.3	0.8245	0.8046	0.3579	1.5535	0

1879	912.2	0.8246	0.8047	0.3581	1.5535	0
1880	912.1	0.8245	0.8046	0.358	1.5534	0
1881	912	0.8245	0.8045	0.3581	1.5522	0
1882	911.9	0.8246	0.8047	0.358	1.5527	0
1883	911.8	0.8246	0.8047	0.3579	1.5526	0
1884	911.7	0.8247	0.8047	0.3582	1.5533	0
1885	911.6	0.8248	0.8049	0.3582	1.5533	0
1886	911.5	0.8248	0.8049	0.3583	1.5539	0
1887	911.4	0.8248	0.8047	0.3582	1.5537	0
1888	911.3	0.8248	0.8048	0.3583	1.5532	0
1889	911.2	0.8249	0.8048	0.3582	1.5536	0
1890	911.1	0.825	0.8048	0.358	1.5535	0
1891	911	0.8249	0.8047	0.3581	1.5534	0
1892	910.9	0.8248	0.8045	0.3579	1.5532	0
1893	910.8	0.8248	0.8047	0.3578	1.5531	0
1894	910.7	0.8247	0.8047	0.3578	1.5531	0
1895	910.6	0.8247	0.8048	0.3579	1.553	0
1896	910.5	0.8249	0.8048	0.3579	1.553	0
1897	910.4	0.8249	0.8048	0.358	1.553	0
1898	910.3	0.8251	0.8049	0.3581	1.5535	0
1899	910.2	0.8249	0.8047	0.3579	1.5523	0
1900	910.1	0.8248	0.805	0.3578	1.5527	0
1901	910	0.8249	0.805	0.3579	1.5533	0
1902	909.9	0.8249	0.8049	0.3579	1.5521	0
1903	909.8	0.825	0.805	0.3579	1.5531	0
1904	909.7	0.8247	0.8048	0.3578	1.5529	0
1905	909.6	0.8247	0.8049	0.3576	1.5522	0
1906	909.5	0.8248	0.805	0.3576	1.5528	0
1907	909.4	0.8249	0.8048	0.3576	1.5527	0
1908	909.3	0.825	0.8051	0.3576	1.5527	0
1909	909.2	0.8249	0.805	0.3576	1.553	0
1910	909.1	0.8248	0.8048	0.3577	1.5513	0
1911	909	0.8248	0.8047	0.3576	1.5518	0
1912	908.9	0.825	0.8049	0.3577	1.5524	0
1913	908.8	0.825	0.8051	0.3576	1.5517	0
1914	908.7	0.8249	0.8052	0.3578	1.5523	0
1915	908.6	0.8246	0.8051	0.3574	1.5514	0
1916	908.5	0.8247	0.8051	0.3574	1.5519	0
1917	908.4	0.8249	0.805	0.3573	1.5518	0
1918	908.3	0.8248	0.8051	0.3574	1.5518	0
1919	908.2	0.8248	0.805	0.3573	1.5516	0
1920	908.1	0.8249	0.8051	0.3574	1.5516	0
1921	908	0.8248	0.805	0.3572	1.5515	0

1922	907.9	0.8249	0.8053	0.3573	1.5514	0
1923	907.8	0.8248	0.8051	0.3573	1.5514	0
1924	907.7	0.8244	0.8047	0.357	1.551	0
1925	907.6	0.8247	0.805	0.3571	1.551	0
1926	907.5	0.8248	0.8051	0.357	1.551	0
1927	907.4	0.8249	0.8051	0.3571	1.551	0
1928	907.3	0.8248	0.8051	0.357	1.5513	0
1929	907.2	0.8247	0.8051	0.3571	1.5508	0
1930	907.1	0.8248	0.8049	0.3571	1.5512	0
1931	907	0.8248	0.8052	0.3571	1.5517	0
1932	906.9	0.8249	0.8052	0.3571	1.5511	0
1933	906.8	0.8249	0.8051	0.3571	1.5511	0
1934	906.7	0.8249	0.805	0.357	1.5509	0
1935	906.6	0.8247	0.8051	0.357	1.5514	0
1936	906.5	0.8251	0.8054	0.3571	1.551	0
1937	906.4	0.825	0.8051	0.3571	1.5514	0
1938	906.3	0.8249	0.8052	0.3569	1.5507	0
1939	906.2	0.8251	0.8053	0.3573	1.552	0
1940	906.1	0.8255	0.8056	0.3574	1.5521	0
1941	906	0.8252	0.8055	0.3574	1.552	0
1942	905.9	0.8253	0.8054	0.3573	1.5519	0
1943	905.8	0.8253	0.8053	0.3574	1.5519	0
1944	905.7	0.8255	0.8055	0.3574	1.5524	0
1945	905.6	0.8256	0.8054	0.3574	1.5524	0
1946	905.5	0.8257	0.8055	0.3575	1.5524	0
1947	905.4	0.8257	0.8056	0.3577	1.5525	0
1948	905.3	0.8256	0.8053	0.3573	1.5522	0
1949	905.2	0.826	0.8055	0.3576	1.5523	0
1950	905.1	0.8261	0.8057	0.3579	1.553	0
1951	905	0.826	0.8055	0.3578	1.5529	0
1952	904.9	0.8262	0.8059	0.358	1.5536	0
1953	904.8	0.8262	0.8058	0.358	1.553	0
1954	904.7	0.8264	0.8059	0.3582	1.5536	0
1955	904.6	0.8266	0.8061	0.3583	1.5542	0
1956	904.5	0.8266	0.8059	0.3583	1.5541	0
1957	904.4	0.8266	0.806	0.3585	1.5547	0
1958	904.3	0.8268	0.806	0.3586	1.5542	0
1959	904.2	0.8268	0.806	0.3587	1.5548	0
1960	904.1	0.8272	0.8061	0.3588	1.5554	0
1961	904	0.8274	0.8064	0.3591	1.5545	0
1962	903.9	0.8275	0.8065	0.3592	1.5551	0
1963	903.8	0.8277	0.8066	0.3594	1.5551	0
1964	903.7	0.8276	0.8064	0.3594	1.5556	0

1965	903.6	0.8277	0.8064	0.3594	1.5561	0
1966	903.5	0.828	0.8069	0.3596	1.5562	0
1967	903.4	0.828	0.8068	0.3596	1.5562	0
1968	903.3	0.828	0.8066	0.3596	1.5561	0
1969	903.2	0.8282	0.8066	0.3598	1.5562	0
1970	903.1	0.8283	0.8069	0.3599	1.5562	0
1971	903	0.8284	0.8068	0.36	1.5568	0
1972	902.9	0.8284	0.807	0.36	1.5568	0
1973	902.8	0.8285	0.807	0.3599	1.5567	0
1974	902.7	0.8284	0.8068	0.3601	1.5571	0
1975	902.6	0.8284	0.8068	0.36	1.557	0
1976	902.5	0.8285	0.8069	0.3604	1.5571	0
1977	902.4	0.8286	0.807	0.3602	1.5576	0
1978	902.3	0.8287	0.8071	0.3602	1.5576	0
1979	902.2	0.8288	0.8069	0.3602	1.558	0
1980	902.1	0.8288	0.807	0.3601	1.5574	0
1981	902	0.8288	0.807	0.3602	1.5579	0
1982	901.9	0.8288	0.8072	0.3603	1.5579	0
1983	901.8	0.8289	0.807	0.3602	1.5572	0
1984	901.7	0.8291	0.8071	0.3604	1.5579	0
1985	901.6	0.8293	0.8073	0.3606	1.558	0
1986	901.5	0.8292	0.8073	0.3605	1.5579	0
1987	901.4	0.8291	0.8075	0.3605	1.5584	0
1988	901.3	0.8291	0.8072	0.3603	1.5587	0
1989	901.2	0.8292	0.8072	0.3604	1.5575	0
1990	901.1	0.8294	0.8073	0.3604	1.5587	0
1991	901	0.8291	0.807	0.3603	1.5579	0
1992	900.9	0.8292	0.8074	0.3603	1.5585	0
1993	900.8	0.8292	0.8074	0.3604	1.5584	0
1994	900.7	0.8293	0.8074	0.3606	1.5585	0
1995	900.6	0.8294	0.8073	0.3606	1.559	0
1996	900.5	0.8296	0.8075	0.3606	1.5589	0
1997	900.4	0.8297	0.8076	0.3605	1.5594	0
1998	900.3	0.8296	0.8074	0.3605	1.5581	0
1999	900.2	0.8295	0.8074	0.3606	1.5586	0
2000	900.1	0.8297	0.8076	0.3607	1.5592	0
2001	900	0.8295	0.8074	0.3607	1.5579	0
2002	899.9	0.8298	0.8075	0.3607	1.5585	0
2003	899.8	0.8296	0.8077	0.3609	1.5584	0
2004	899.7	0.8297	0.8076	0.3607	1.5584	0
2005	899.6	0.8297	0.8076	0.3605	1.5582	0
2006	899.5	0.8295	0.8077	0.3604	1.5591	0
2007	899.4	0.8296	0.8077	0.3604	1.5585	0

2008	899.3	0.8298	0.8079	0.3607	1.5586	0
2009	899.2	0.8297	0.8079	0.3605	1.5578	0
2010	899.1	0.8296	0.8078	0.3604	1.5577	0
2011	899	0.8296	0.8077	0.3603	1.5587	0
2012	898.9	0.8296	0.8076	0.3603	1.558	0
2013	898.8	0.8296	0.8076	0.3602	1.5573	0
2014	898.7	0.8293	0.8075	0.3601	1.5578	0
2015	898.6	0.8295	0.8078	0.3601	1.5577	0
2016	898.5	0.8298	0.8077	0.3601	1.5582	0
2017	898.4	0.8296	0.8077	0.36	1.5575	0
2018	898.3	0.8297	0.8076	0.36	1.5574	0
2019	898.2	0.8294	0.8076	0.3599	1.5573	0
2020	898.1	0.8294	0.8075	0.3598	1.5577	0
2021	898	0.8293	0.8077	0.3597	1.557	0
2022	897.9	0.8293	0.8075	0.3596	1.5574	0
2023	897.8	0.8294	0.8076	0.3598	1.5569	0
2024	897.7	0.8295	0.8077	0.3596	1.5568	0
2025	897.6	0.8294	0.8078	0.3596	1.5567	0
2026	897.5	0.8294	0.8077	0.3597	1.5572	0
2027	897.4	0.8295	0.8077	0.3597	1.5564	0
2028	897.3	0.8295	0.8077	0.3597	1.5569	0
2029	897.2	0.8295	0.8079	0.3597	1.5564	0
2030	897.1	0.8292	0.8077	0.3596	1.5562	0
2031	897	0.8293	0.8079	0.3596	1.5567	0
2032	896.9	0.8296	0.8077	0.3595	1.5573	0
2033	896.8	0.8297	0.8081	0.3597	1.5567	0
2034	896.7	0.8294	0.8077	0.3596	1.5571	0
2035	896.6	0.8294	0.8079	0.3595	1.557	0
2036	896.5	0.8296	0.808	0.3596	1.5569	0
2037	896.4	0.8297	0.8079	0.3597	1.5563	0
2038	896.3	0.8298	0.808	0.3597	1.5564	0
2039	896.2	0.8298	0.8081	0.3598	1.5574	0
2040	896.1	0.8299	0.8083	0.3598	1.5563	0
2041	896	0.8299	0.8081	0.3597	1.5573	0
2042	895.9	0.8298	0.8082	0.3598	1.5573	0
2043	895.8	0.8298	0.8081	0.3597	1.5566	0
2044	895.7	0.8298	0.8082	0.3597	1.5565	0
2045	895.6	0.8296	0.808	0.3597	1.5569	0
2046	895.5	0.8299	0.8081	0.3598	1.5575	0
2047	895.4	0.8302	0.8081	0.3597	1.5564	0
2048	895.3	0.83	0.8083	0.3599	1.5569	0
2049	895.2	0.8302	0.8083	0.3598	1.5569	0
2050	895.1	0.8305	0.8085	0.36	1.5575	0

2051	895	0.8306	0.8084	0.3601	1.5574	0
2052	894.9	0.8304	0.8083	0.36	1.5574	0
2053	894.8	0.8304	0.8083	0.3601	1.5584	0
2054	894.7	0.8306	0.8085	0.3601	1.5578	0
2055	894.6	0.8309	0.8085	0.3602	1.5584	0
2056	894.5	0.8308	0.8086	0.3603	1.5578	0
2057	894.4	0.8309	0.8086	0.3603	1.5584	0
2058	894.3	0.831	0.8087	0.3605	1.5584	0
2059	894.2	0.831	0.8086	0.3605	1.5584	0
2060	894.1	0.8312	0.8089	0.3607	1.5584	0
2061	894	0.8313	0.8088	0.3608	1.5589	0
2062	893.9	0.8311	0.8087	0.3607	1.5593	0
2063	893.8	0.831	0.8086	0.3607	1.5593	0
2064	893.7	0.8315	0.809	0.3609	1.5594	0
2065	893.6	0.8316	0.8089	0.3608	1.5588	0
2066	893.5	0.8317	0.8089	0.3611	1.56	0
2067	893.4	0.8318	0.8091	0.3612	1.5595	0
2068	893.3	0.8318	0.8092	0.3612	1.5594	0
2069	893.2	0.832	0.809	0.3613	1.5605	0
2070	893.1	0.8319	0.8091	0.3613	1.5604	0
2071	893	0.8319	0.8092	0.3613	1.5598	0
2072	892.9	0.8321	0.8092	0.3615	1.5605	0
2073	892.8	0.8323	0.8094	0.3616	1.5599	0
2074	892.7	0.8324	0.8093	0.3615	1.5603	0
2075	892.6	0.8324	0.8093	0.3618	1.561	0
2076	892.5	0.8326	0.8095	0.3618	1.5611	0
2077	892.4	0.8326	0.8095	0.362	1.561	0
2078	892.3	0.8328	0.8098	0.3621	1.5616	0
2079	892.2	0.8329	0.8097	0.3622	1.5617	0
2080	892.1	0.8329	0.8095	0.3621	1.5616	0
2081	892	0.8329	0.8096	0.3621	1.562	0
2082	891.9	0.8329	0.8095	0.362	1.5619	0
2083	891.8	0.8331	0.8096	0.3622	1.5626	0
2084	891.7	0.8331	0.8097	0.3622	1.5619	0
2085	891.6	0.833	0.8098	0.3621	1.5618	0
2086	891.5	0.8331	0.8096	0.3622	1.5622	0
2087	891.4	0.8332	0.8098	0.3622	1.5617	0
2088	891.3	0.8334	0.8099	0.3622	1.5617	0
2089	891.2	0.8333	0.8098	0.3621	1.5627	0
2090	891.1	0.8337	0.8102	0.3626	1.5624	0
2091	891	0.8337	0.81	0.3626	1.5623	0
2092	890.9	0.8336	0.8101	0.3624	1.5627	0
2093	890.8	0.8337	0.8101	0.3624	1.5627	0

2094	890.7	0.8337	0.8101	0.3626	1.5633	0
2095	890.6	0.8339	0.8104	0.3626	1.5626	0
2096	890.5	0.8337	0.8103	0.3627	1.5625	0
2097	890.4	0.8339	0.8103	0.3626	1.5636	0
2098	890.3	0.8339	0.8102	0.3628	1.5636	0
2099	890.2	0.8341	0.8102	0.3625	1.5634	0
2100	890.1	0.834	0.8104	0.3629	1.5636	0
2101	890	0.8341	0.8104	0.3628	1.5633	0
2102	889.9	0.8341	0.8103	0.3627	1.5627	0
2103	889.8	0.834	0.8103	0.3627	1.5631	0
2104	889.7	0.834	0.8105	0.3626	1.563	0
2105	889.6	0.834	0.8105	0.3625	1.5629	0
2106	889.5	0.8342	0.8103	0.3626	1.5629	0
2107	889.4	0.8341	0.8105	0.3626	1.5628	0
2108	889.3	0.8343	0.8106	0.3627	1.5634	0
2109	889.2	0.8342	0.8106	0.3626	1.5621	0
2110	889.1	0.8341	0.8105	0.3627	1.5631	0
2111	889	0.8342	0.8105	0.3625	1.563	0
2112	888.9	0.834	0.8103	0.3625	1.5628	0
2113	888.8	0.8341	0.8104	0.3624	1.5622	0
2114	888.7	0.8342	0.8103	0.3625	1.5626	0
2115	888.6	0.8341	0.8106	0.3625	1.562	0
2116	888.5	0.8342	0.8106	0.3624	1.5625	0
2117	888.4	0.834	0.8106	0.3623	1.5629	0
2118	888.3	0.8341	0.8106	0.3623	1.5622	0
2119	888.2	0.834	0.8106	0.3622	1.5621	0
2120	888.1	0.8339	0.8106	0.3622	1.562	0
2121	888	0.8341	0.8106	0.3622	1.5619	0
2122	887.9	0.8342	0.8107	0.3622	1.5618	0
2123	887.8	0.834	0.8106	0.3622	1.5616	0
2124	887.7	0.8339	0.8105	0.3621	1.561	0
2125	887.6	0.834	0.8105	0.362	1.5614	0
2126	887.5	0.8339	0.8106	0.3618	1.5617	0
2127	887.4	0.8341	0.8106	0.362	1.5624	0
2128	887.3	0.8339	0.8106	0.3619	1.5617	0
2129	887.2	0.8339	0.8105	0.3618	1.561	0
2130	887.1	0.8339	0.8104	0.3617	1.5614	0
2131	887	0.8342	0.8105	0.3619	1.5609	0
2132	886.9	0.8341	0.8107	0.362	1.5614	0
2133	886.8	0.8339	0.8105	0.3617	1.5618	0
2134	886.7	0.8342	0.8107	0.3618	1.5612	0
2135	886.6	0.8339	0.8106	0.3618	1.5616	0
2136	886.5	0.834	0.8109	0.3617	1.5616	0

2137	886.4	0.834	0.8107	0.3617	1.5614	0
2138	886.3	0.8342	0.8109	0.362	1.561	0
2139	886.2	0.8341	0.8107	0.3618	1.5614	0
2140	886.1	0.8341	0.8107	0.3618	1.5618	0
2141	886	0.8341	0.8108	0.3618	1.5612	0
2142	885.9	0.8343	0.8108	0.3618	1.5617	0
2143	885.8	0.8343	0.8109	0.362	1.5611	0
2144	885.7	0.8344	0.8109	0.362	1.5622	0
2145	885.6	0.8344	0.8108	0.3618	1.5615	0
2146	885.5	0.8345	0.811	0.362	1.5614	0
2147	885.4	0.8346	0.811	0.362	1.5613	0
2148	885.3	0.8342	0.8109	0.3619	1.5612	0
2149	885.2	0.8345	0.8111	0.3619	1.5617	0
2150	885.1	0.8346	0.811	0.3619	1.5611	0
2151	885	0.8346	0.8109	0.3618	1.5617	0
2152	884.9	0.8348	0.8109	0.362	1.5611	0
2153	884.8	0.8347	0.8112	0.362	1.5616	0
2154	884.7	0.8348	0.8113	0.362	1.5622	0
2155	884.6	0.8349	0.8112	0.3621	1.5621	0
2156	884.5	0.8348	0.8113	0.3621	1.5621	0
2157	884.4	0.835	0.8112	0.3622	1.5621	0
2158	884.3	0.8353	0.8115	0.3623	1.5616	0
2159	884.2	0.8352	0.8115	0.3621	1.5631	0
2160	884.1	0.8352	0.8115	0.3623	1.5632	0
2161	884	0.8354	0.8117	0.3624	1.5632	0
2162	883.9	0.8355	0.8118	0.3626	1.5633	0
2163	883.8	0.8356	0.8118	0.3626	1.5626	0
2164	883.7	0.8356	0.8117	0.3625	1.5625	0
2165	883.6	0.8355	0.8115	0.3625	1.5641	0
2166	883.5	0.8356	0.8119	0.3626	1.563	0
2167	883.4	0.8359	0.8118	0.3629	1.563	0
2168	883.3	0.8362	0.812	0.3631	1.5637	0
2169	883.2	0.8362	0.812	0.363	1.5636	0
2170	883.1	0.8361	0.8118	0.363	1.564	0
2171	883	0.8362	0.8119	0.3629	1.5634	0
2172	882.9	0.8361	0.8119	0.363	1.5639	0
2173	882.8	0.8363	0.8122	0.363	1.5639	0
2174	882.7	0.8362	0.8119	0.3629	1.5643	0
2175	882.6	0.8364	0.8122	0.3631	1.565	0
2176	882.5	0.8367	0.8123	0.3632	1.5645	0
2177	882.4	0.8365	0.8123	0.3632	1.565	0
2178	882.3	0.8367	0.8125	0.3634	1.5651	0
2179	882.2	0.8366	0.8123	0.3633	1.5649	0

2180	882.1	0.8367	0.8124	0.3633	1.5648	0
2181	882	0.8367	0.8123	0.3632	1.5653	0
2182	881.9	0.8369	0.8123	0.3633	1.5653	0
2183	881.8	0.8368	0.8123	0.3634	1.5658	0
2184	881.7	0.8369	0.8124	0.3634	1.5657	0
2185	881.6	0.8373	0.8127	0.3637	1.5653	0
2186	881.5	0.8372	0.8125	0.3637	1.5657	0
2187	881.4	0.8375	0.8126	0.3639	1.5652	0
2188	881.3	0.8373	0.8127	0.3637	1.5657	0
2189	881.2	0.8372	0.8125	0.3637	1.5662	0
2190	881.1	0.8375	0.8125	0.3637	1.5661	0
2191	881	0.8376	0.8129	0.364	1.5656	0
2192	880.9	0.8376	0.8129	0.3639	1.5655	0
2193	880.8	0.8379	0.8129	0.3641	1.5662	0
2194	880.7	0.8374	0.8128	0.3638	1.5664	0
2195	880.6	0.8376	0.8132	0.364	1.5659	0
2196	880.5	0.8376	0.8129	0.3639	1.5657	0
2197	880.4	0.8376	0.813	0.364	1.5657	0
2198	880.3	0.8378	0.8129	0.3639	1.5662	0
2199	880.2	0.8379	0.813	0.364	1.5662	0
2200	880.1	0.8379	0.813	0.364	1.566	0
2201	880	0.838	0.8132	0.364	1.5667	0
2202	879.9	0.8381	0.8131	0.3641	1.5661	0
2203	879.8	0.8379	0.8132	0.364	1.5665	0
2204	879.7	0.8381	0.8132	0.364	1.5664	0
2205	879.6	0.8382	0.8132	0.3642	1.5671	0
2206	879.5	0.8382	0.8132	0.3641	1.5669	0
2207	879.4	0.8382	0.813	0.3641	1.5662	0
2208	879.3	0.8382	0.8133	0.3641	1.5668	0
2209	879.2	0.8382	0.8133	0.3641	1.5668	0
2210	879.1	0.8381	0.8132	0.3642	1.5666	0
2211	879	0.8385	0.8134	0.364	1.5667	0
2212	878.9	0.8384	0.8134	0.3641	1.5666	0
2213	878.8	0.8384	0.8136	0.3642	1.5665	0
2214	878.7	0.8385	0.8137	0.3643	1.566	0
2215	878.6	0.8384	0.8135	0.3641	1.5663	0
2216	878.5	0.8384	0.8134	0.364	1.5667	0
2217	878.4	0.8384	0.8135	0.364	1.5666	0
2218	878.3	0.8385	0.8135	0.3639	1.5665	0
2219	878.2	0.8384	0.8134	0.364	1.5664	0
2220	878.1	0.8386	0.8135	0.364	1.5657	0
2221	878	0.8385	0.8135	0.3639	1.5662	0
2222	877.9	0.8386	0.8135	0.3639	1.5668	0

2223	877.8	0.8386	0.8135	0.364	1.5661	0
2224	877.7	0.8385	0.8136	0.3639	1.566	0
2225	877.6	0.8384	0.8133	0.3637	1.5658	0
2226	877.5	0.8383	0.8133	0.3637	1.5662	0
2227	877.4	0.8384	0.8136	0.3638	1.5657	0
2228	877.3	0.8386	0.8135	0.3639	1.5663	0
2229	877.2	0.8385	0.8133	0.3638	1.5655	0
2230	877.1	0.8386	0.8139	0.3639	1.5662	0
2231	877	0.8388	0.8138	0.364	1.5661	0
2232	876.9	0.8388	0.8138	0.364	1.5662	0
2233	876.8	0.8391	0.8137	0.3641	1.5673	0
2234	876.7	0.839	0.8139	0.3639	1.5665	0
2235	876.6	0.8389	0.8139	0.3642	1.566	0
2236	876.5	0.8389	0.8139	0.3639	1.5664	0
2237	876.4	0.8391	0.8138	0.3642	1.5659	0
2238	876.3	0.8391	0.814	0.3641	1.5664	0
2239	876.2	0.8392	0.8141	0.3642	1.5664	0
2240	876.1	0.8391	0.8142	0.3642	1.5663	0
2241	876	0.8389	0.8137	0.364	1.566	0
2242	875.9	0.8394	0.8141	0.3642	1.5662	0
2243	875.8	0.8394	0.814	0.3642	1.5667	0
2244	875.7	0.8393	0.8141	0.3642	1.5666	0
2245	875.6	0.8393	0.8141	0.3643	1.5671	0
2246	875.5	0.8395	0.8142	0.3644	1.5671	0
2247	875.4	0.8395	0.8141	0.3643	1.567	0
2248	875.3	0.8395	0.8141	0.3642	1.5669	0
2249	875.2	0.8394	0.8143	0.3642	1.5669	0
2250	875.1	0.8397	0.8145	0.3644	1.5669	0
2251	875	0.8398	0.8143	0.3643	1.5668	0
2252	874.9	0.8398	0.8145	0.3644	1.5675	0
2253	874.8	0.8397	0.8142	0.3645	1.5673	0
2254	874.7	0.8399	0.8145	0.3644	1.5673	0
2255	874.6	0.8399	0.8144	0.3645	1.5672	0
2256	874.5	0.84	0.8143	0.3643	1.567	0
2257	874.4	0.8399	0.8144	0.3644	1.5677	0
2258	874.3	0.84	0.8143	0.3645	1.5676	0
2259	874.2	0.8401	0.8145	0.3645	1.5676	0
2260	874.1	0.8401	0.8145	0.3646	1.5682	0
2261	874	0.8402	0.8146	0.3646	1.5682	0
2262	873.9	0.8403	0.8145	0.3646	1.5681	0
2263	873.8	0.8404	0.8145	0.3648	1.5681	0
2264	873.7	0.8406	0.8147	0.3648	1.5687	0
2265	873.6	0.8404	0.8146	0.3648	1.5686	0

2266	873.5	0.8406	0.8144	0.365	1.5686	0
2267	873.4	0.8408	0.8148	0.365	1.5687	0
2268	873.3	0.8409	0.8149	0.3651	1.5687	0
2269	873.2	0.8407	0.8148	0.365	1.569	0
2270	873.1	0.8409	0.815	0.3652	1.5697	0
2271	873	0.8408	0.8148	0.365	1.5695	0
2272	872.9	0.8412	0.815	0.3651	1.5696	0
2273	872.8	0.8412	0.8153	0.3653	1.5696	0
2274	872.7	0.8412	0.8151	0.3652	1.5695	0
2275	872.6	0.841	0.815	0.3652	1.57	0
2276	872.5	0.8411	0.815	0.3652	1.5693	0
2277	872.4	0.8411	0.8152	0.3653	1.5699	0
2278	872.3	0.8414	0.8155	0.3654	1.5694	0
2279	872.2	0.8413	0.8151	0.3653	1.5698	0
2280	872.1	0.8413	0.8152	0.3654	1.5698	0
2281	872	0.8412	0.8153	0.3653	1.5691	0
2282	871.9	0.8412	0.8152	0.3653	1.569	0
2283	871.8	0.8415	0.8154	0.3653	1.5696	0
2284	871.7	0.8415	0.8154	0.3654	1.5695	0
2285	871.6	0.8418	0.8157	0.3655	1.5703	0
2286	871.5	0.8417	0.8156	0.3655	1.5695	0
2287	871.4	0.8418	0.8154	0.3654	1.5694	0
2288	871.3	0.8418	0.8154	0.3654	1.5705	0
2289	871.2	0.8417	0.8157	0.3655	1.5699	0
2290	871.1	0.8417	0.8155	0.3654	1.5698	0
2291	871	0.8417	0.8153	0.3652	1.5696	0
2292	870.9	0.8417	0.8156	0.3653	1.5702	0
2293	870.8	0.8421	0.8155	0.3654	1.5702	0
2294	870.7	0.8418	0.8154	0.3653	1.5694	0
2295	870.6	0.8416	0.8155	0.3652	1.5698	0
2296	870.5	0.8419	0.8156	0.3652	1.5693	0
2297	870.4	0.8419	0.8157	0.3652	1.5698	0
2298	870.3	0.8419	0.8156	0.3652	1.5697	0
2299	870.2	0.8418	0.8156	0.3651	1.569	0
2300	870.1	0.8419	0.8156	0.3653	1.5697	0
2301	870	0.8418	0.8155	0.365	1.5694	0
2302	869.9	0.8419	0.8159	0.3652	1.5695	0
2303	869.8	0.842	0.8159	0.3654	1.5694	0
2304	869.7	0.842	0.8157	0.3652	1.5699	0
2305	869.6	0.8419	0.8158	0.3651	1.5698	0
2306	869.5	0.8421	0.816	0.3651	1.5691	0
2307	869.4	0.8422	0.8159	0.3651	1.5702	0
2308	869.3	0.8423	0.8159	0.3651	1.5697	0

2309	869.2	0.8424	0.8161	0.3652	1.5696	0
2310	869.1	0.8424	0.8161	0.3651	1.5701	0
2311	869	0.8421	0.816	0.365	1.5693	0
2312	868.9	0.8421	0.8161	0.365	1.5699	0
2313	868.8	0.8422	0.8161	0.365	1.5697	0
2314	868.7	0.8423	0.8159	0.365	1.5697	0
2315	868.6	0.8423	0.816	0.3649	1.569	0
2316	868.5	0.8424	0.8161	0.3652	1.5696	0
2317	868.4	0.8423	0.8162	0.365	1.5695	0
2318	868.3	0.8425	0.8161	0.3651	1.5689	0
2319	868.2	0.8425	0.8162	0.3651	1.5701	0
2320	868.1	0.8429	0.8164	0.3652	1.5702	0
2321	868	0.8427	0.8164	0.3654	1.5696	0
2322	867.9	0.8427	0.8165	0.3654	1.5701	0
2323	867.8	0.8428	0.8163	0.3653	1.5707	0
2324	867.7	0.8429	0.8164	0.3654	1.5706	0
2325	867.6	0.8429	0.8164	0.3652	1.5698	0
2326	867.5	0.843	0.8165	0.3653	1.5704	0
2327	867.4	0.8428	0.8165	0.3652	1.5703	0
2328	867.3	0.8432	0.8168	0.3655	1.5705	0
2329	867.2	0.8431	0.8164	0.3653	1.5708	0
2330	867.1	0.8431	0.8165	0.3654	1.5702	0
2331	867	0.8432	0.8166	0.3654	1.5708	0
2332	866.9	0.8431	0.8164	0.3654	1.5701	0
2333	866.8	0.8433	0.8167	0.3654	1.5701	0
2334	866.7	0.8432	0.8167	0.3655	1.5706	0
2335	866.6	0.8434	0.8168	0.3655	1.5707	0
2336	866.5	0.8435	0.8169	0.3655	1.5707	0
2337	866.4	0.8436	0.8169	0.3656	1.5712	0
2338	866.3	0.8437	0.817	0.3656	1.5712	0
2339	866.2	0.8438	0.817	0.3657	1.5707	0
2340	866.1	0.8438	0.817	0.3658	1.5712	0
2341	866	0.8438	0.8168	0.3657	1.5705	0
2342	865.9	0.8437	0.8169	0.3656	1.5717	0
2343	865.8	0.844	0.8171	0.3658	1.5718	0
2344	865.7	0.8442	0.8172	0.366	1.5712	0
2345	865.6	0.8442	0.8171	0.366	1.5717	0
2346	865.5	0.8444	0.8174	0.3662	1.5718	0
2347	865.4	0.8445	0.8175	0.3662	1.5718	0
2348	865.3	0.8444	0.8172	0.3661	1.5722	0
2349	865.2	0.8445	0.8174	0.3662	1.5723	0
2350	865.1	0.8446	0.8174	0.3663	1.5724	0
2351	865	0.8445	0.8174	0.3664	1.5729	0

2352	864.9	0.8447	0.8175	0.3663	1.5722	0
2353	864.8	0.8446	0.8175	0.3664	1.5722	0
2354	864.7	0.8448	0.8173	0.3664	1.5728	0
2355	864.6	0.845	0.8178	0.3666	1.5723	0
2356	864.5	0.8451	0.8178	0.3668	1.5731	0
2357	864.4	0.845	0.8176	0.3666	1.5734	0
2358	864.3	0.845	0.8176	0.3666	1.5734	0
2359	864.2	0.8452	0.8176	0.3667	1.5735	0
2360	864.1	0.8453	0.8176	0.3667	1.5734	0
2361	864	0.8454	0.8179	0.367	1.5734	0
2362	863.9	0.8453	0.8181	0.3669	1.5734	0
2363	863.8	0.8456	0.8179	0.367	1.5734	0
2364	863.7	0.8457	0.8181	0.3671	1.5741	0
2365	863.6	0.8456	0.8179	0.3671	1.574	0
2366	863.5	0.8457	0.8178	0.3671	1.5745	0
2367	863.4	0.846	0.8183	0.3673	1.5747	0
2368	863.3	0.8461	0.8183	0.3674	1.5747	0
2369	863.2	0.846	0.8184	0.3673	1.5747	0
2370	863.1	0.846	0.8181	0.3672	1.5739	0
2371	863	0.8459	0.8183	0.3673	1.5745	0
2372	862.9	0.8459	0.8181	0.3673	1.5738	0
2373	862.8	0.8461	0.8185	0.3672	1.5744	0
2374	862.7	0.846	0.8184	0.3674	1.575	0
2375	862.6	0.8461	0.8182	0.3674	1.5744	0
2376	862.5	0.8462	0.8183	0.3673	1.5744	0
2377	862.4	0.8462	0.8183	0.3674	1.575	0
2378	862.3	0.8465	0.8185	0.3676	1.5756	0
2379	862.2	0.8465	0.8186	0.3675	1.575	0
2380	862.1	0.8467	0.8188	0.3675	1.575	0
2381	862	0.8466	0.8187	0.3675	1.5749	0
2382	861.9	0.8466	0.8187	0.3676	1.5755	0
2383	861.8	0.8467	0.8188	0.3676	1.5755	0
2384	861.7	0.8467	0.8188	0.3675	1.5754	0
2385	861.6	0.8467	0.8187	0.3675	1.5759	0
2386	861.5	0.8467	0.8187	0.3676	1.5752	0
2387	861.4	0.8466	0.8187	0.3675	1.5752	0
2388	861.3	0.8468	0.8191	0.3674	1.5752	0
2389	861.2	0.8467	0.8188	0.3674	1.5757	0
2390	861.1	0.8467	0.8189	0.3674	1.575	0
2391	861	0.8468	0.8188	0.3672	1.5749	0
2392	860.9	0.8466	0.8187	0.3672	1.5748	0
2393	860.8	0.8469	0.819	0.3674	1.5755	0
2394	860.7	0.8469	0.8189	0.3673	1.5748	0

2395	860.6	0.8468	0.8191	0.3673	1.5748	0
2396	860.5	0.8468	0.8192	0.3674	1.5754	0
2397	860.4	0.8468	0.8188	0.3672	1.5752	0
2398	860.3	0.8468	0.819	0.3672	1.5751	0
2399	860.2	0.8471	0.819	0.3674	1.5753	0
2400	860.1	0.8471	0.8193	0.3673	1.5752	0
2401	860	0.8469	0.8192	0.3673	1.5751	0
2402	859.9	0.8469	0.8191	0.367	1.5749	0
2403	859.8	0.8469	0.8192	0.3671	1.5749	0
2404	859.7	0.847	0.8193	0.3671	1.5755	0
2405	859.6	0.8469	0.8191	0.367	1.5747	0
2406	859.5	0.8468	0.819	0.3669	1.5746	0
2407	859.4	0.8468	0.8192	0.367	1.574	0
2408	859.3	0.8469	0.8195	0.3671	1.5752	0
2409	859.2	0.847	0.8197	0.367	1.5745	0
2410	859.1	0.8467	0.8194	0.3668	1.5743	0
2411	859	0.8469	0.8194	0.3669	1.5743	0
2412	858.9	0.847	0.8194	0.3669	1.5742	0
2413	858.8	0.8467	0.8193	0.3667	1.5747	0
2414	858.7	0.847	0.8193	0.3666	1.574	0
2415	858.6	0.8469	0.8195	0.3668	1.5735	0
2416	858.5	0.8471	0.8193	0.3666	1.5739	0
2417	858.4	0.847	0.8195	0.3668	1.574	0
2418	858.3	0.847	0.8195	0.3667	1.5739	0
2419	858.2	0.8469	0.8194	0.3666	1.5738	0
2420	858.1	0.847	0.8195	0.3664	1.5737	0
2421	858	0.847	0.8196	0.3666	1.5737	0
2422	857.9	0.8472	0.8195	0.3665	1.5737	0
2423	857.8	0.8472	0.8196	0.3666	1.5743	0
2424	857.7	0.8473	0.8197	0.3666	1.5736	0
2425	857.6	0.8472	0.8196	0.3665	1.5735	0
2426	857.5	0.8473	0.8196	0.3665	1.5735	0
2427	857.4	0.8474	0.8197	0.3666	1.5742	0
2428	857.3	0.8472	0.8198	0.3665	1.5747	0
2429	857.2	0.8476	0.8199	0.3665	1.5741	0
2430	857.1	0.8475	0.8199	0.3666	1.5741	0
2431	857	0.8475	0.8198	0.3665	1.5741	0
2432	856.9	0.8477	0.8199	0.3667	1.5742	0
2433	856.8	0.8479	0.8199	0.3666	1.5747	0
2434	856.7	0.8479	0.8199	0.3667	1.5747	0
2435	856.6	0.848	0.8201	0.3668	1.5747	0
2436	856.5	0.8482	0.8203	0.367	1.575	0
2437	856.4	0.8483	0.8201	0.3672	1.575	0

2438	856.3	0.8483	0.8203	0.3673	1.5756	0
2439	856.2	0.8484	0.8206	0.3673	1.5758	0
2440	856.1	0.8486	0.8205	0.3675	1.5758	0
2441	856	0.8487	0.8204	0.3674	1.5757	0
2442	855.9	0.8487	0.8206	0.3675	1.5763	0
2443	855.8	0.8487	0.8203	0.3674	1.5762	0
2444	855.7	0.8489	0.8206	0.3676	1.5764	0
2445	855.6	0.8488	0.8205	0.3675	1.5763	0
2446	855.5	0.8489	0.8208	0.3677	1.5764	0
2447	855.4	0.8491	0.8207	0.3678	1.577	0
2448	855.3	0.8492	0.8208	0.3679	1.5765	0
2449	855.2	0.8493	0.8207	0.3678	1.5764	0
2450	855.1	0.8494	0.8208	0.368	1.5772	0
2451	855	0.8495	0.8208	0.3679	1.5766	0
2452	854.9	0.8497	0.8208	0.368	1.5772	0
2453	854.8	0.8498	0.8211	0.3682	1.5774	0
2454	854.7	0.8498	0.8211	0.3682	1.5779	0
2455	854.6	0.8498	0.821	0.3682	1.5779	0
2456	854.5	0.8498	0.8208	0.3681	1.5772	0
2457	854.4	0.8501	0.8211	0.3685	1.5787	0
2458	854.3	0.8502	0.8214	0.3686	1.5788	0
2459	854.2	0.8503	0.8214	0.3685	1.5782	0
2460	854.1	0.8504	0.8215	0.3688	1.5795	0
2461	854	0.8505	0.8215	0.3688	1.5789	0
2462	853.9	0.8506	0.8215	0.369	1.579	0
2463	853.8	0.8507	0.8215	0.3691	1.579	0
2464	853.7	0.8508	0.8216	0.3693	1.5797	0
2465	853.6	0.851	0.8218	0.3692	1.5797	0
2466	853.5	0.8511	0.8219	0.3693	1.5797	0
2467	853.4	0.851	0.8218	0.3691	1.5791	0
2468	853.3	0.8509	0.8218	0.3693	1.5797	0
2469	853.2	0.8512	0.8219	0.3694	1.5811	0
2470	853.1	0.8512	0.822	0.3694	1.5805	0
2471	853	0.8513	0.8222	0.3694	1.5799	0
2472	852.9	0.8513	0.822	0.3695	1.5798	0
2473	852.8	0.8513	0.822	0.3695	1.5805	0
2474	852.7	0.8514	0.8221	0.3694	1.5804	0
2475	852.6	0.8517	0.8224	0.3696	1.5807	0
2476	852.5	0.8517	0.8222	0.3694	1.5799	0
2477	852.4	0.8518	0.8222	0.3697	1.5806	0
2478	852.3	0.8521	0.8226	0.3698	1.5814	0
2479	852.2	0.8519	0.8223	0.3697	1.58	0
2480	852.1	0.852	0.8225	0.3698	1.5807	0

2481	852	0.852	0.8224	0.3698	1.5801	0
2482	851.9	0.8521	0.8225	0.3699	1.5807	0
2483	851.8	0.8522	0.8224	0.3698	1.5807	0
2484	851.7	0.8521	0.8225	0.3698	1.5807	0
2485	851.6	0.8524	0.8227	0.37	1.5808	0
2486	851.5	0.8521	0.8227	0.3698	1.5813	0
2487	851.4	0.8524	0.8228	0.3698	1.5807	0
2488	851.3	0.8525	0.8229	0.37	1.5814	0
2489	851.2	0.8524	0.8228	0.3699	1.5807	0
2490	851.1	0.8524	0.8226	0.3698	1.5813	0
2491	851	0.8522	0.8227	0.3696	1.5811	0
2492	850.9	0.8523	0.8228	0.3696	1.581	0
2493	850.8	0.8523	0.8228	0.3697	1.5811	0
2494	850.7	0.8524	0.8228	0.3697	1.5811	0
2495	850.6	0.8525	0.8229	0.3696	1.5805	0
2496	850.5	0.8525	0.8229	0.3696	1.581	0
2497	850.4	0.8525	0.823	0.3696	1.5804	0
2498	850.3	0.8525	0.8231	0.3697	1.5811	0
2499	850.2	0.8525	0.8231	0.3696	1.5805	0
2500	850.1	0.8524	0.823	0.3695	1.5803	0
2501	850	0.8524	0.8228	0.3693	1.5802	0
2502	849.9	0.869	0.833	0.368	1.6046	0
2503	849.8	0.869	0.833	0.368	1.604	0
2504	849.7	0.869	0.833	0.3679	1.6044	0
2505	849.6	0.8688	0.833	0.3677	1.6037	0
2506	849.5	0.869	0.8331	0.3677	1.6038	0
2507	849.4	0.8691	0.833	0.3677	1.6043	0
2508	849.3	0.8689	0.833	0.3676	1.6031	0
2509	849.2	0.8691	0.8331	0.3676	1.6031	0
2510	849.1	0.869	0.8328	0.3673	1.6035	0
2511	849	0.8689	0.8331	0.3675	1.6029	0
2512	848.9	0.8691	0.8332	0.3676	1.6036	0
2513	848.8	0.8689	0.8332	0.3674	1.6029	0
2514	848.7	0.8691	0.8334	0.3675	1.6037	0
2515	848.6	0.869	0.8332	0.3674	1.6024	0
2516	848.5	0.8689	0.8332	0.3673	1.6028	0
2517	848.4	0.8689	0.8332	0.3674	1.6028	0
2518	848.3	0.8691	0.8333	0.3673	1.6028	0
2519	848.2	0.8691	0.8334	0.3673	1.6028	0
2520	848.1	0.869	0.8334	0.3672	1.6028	0
2521	848	0.8691	0.8335	0.3672	1.6028	0
2522	847.9	0.8692	0.8335	0.3672	1.6028	0
2523	847.8	0.8691	0.8334	0.367	1.6027	0

2524	847.7	0.8692	0.8338	0.3672	1.6021	0
2525	847.6	0.869	0.8334	0.367	1.6026	0
2526	847.5	0.869	0.8336	0.367	1.6026	0
2527	847.4	0.8692	0.8334	0.3669	1.6026	0
2528	847.3	0.869	0.8334	0.3669	1.6025	0
2529	847.2	0.869	0.8333	0.3667	1.603	0
2530	847.1	0.8691	0.8335	0.3668	1.6025	0
2531	847	0.869	0.8333	0.3668	1.6019	0
2532	846.9	0.8691	0.8334	0.3667	1.6018	0
2533	846.8	0.8691	0.8335	0.3667	1.6024	0
2534	846.7	0.8691	0.8335	0.3667	1.6024	0
2535	846.6	0.8693	0.8336	0.3668	1.6025	0
2536	846.5	0.8693	0.8337	0.3669	1.6031	0
2537	846.4	0.8694	0.8339	0.3668	1.6026	0
2538	846.3	0.8695	0.8337	0.3669	1.6026	0
2539	846.2	0.8693	0.8336	0.3667	1.6025	0
2540	846.1	0.8695	0.8337	0.3667	1.6026	0
2541	846	0.8697	0.834	0.367	1.6033	0
2542	845.9	0.8698	0.8342	0.3671	1.6035	0
2543	845.8	0.87	0.8344	0.3672	1.6041	0
2544	845.7	0.87	0.8342	0.3672	1.6036	0
2545	845.6	0.87	0.8341	0.3672	1.6036	0
2546	845.5	0.8701	0.8343	0.3674	1.6043	0
2547	845.4	0.8701	0.8342	0.3674	1.6043	0
2548	845.3	0.8706	0.8345	0.3676	1.6046	0
2549	845.2	0.8706	0.8346	0.3677	1.604	0
2550	845.1	0.8707	0.8346	0.3678	1.6041	0
2551	845	0.8707	0.8346	0.3678	1.6047	0
2552	844.9	0.871	0.8351	0.3681	1.605	0
2553	844.8	0.8711	0.8349	0.3683	1.605	0
2554	844.7	0.8713	0.835	0.3683	1.6051	0
2555	844.6	0.8713	0.8349	0.3681	1.6056	0
2556	844.5	0.8714	0.835	0.3684	1.6059	0
2557	844.4	0.8715	0.8352	0.3686	1.606	0
2558	844.3	0.8717	0.8354	0.3686	1.6061	0
2559	844.2	0.8719	0.8352	0.3688	1.6068	0
2560	844.1	0.872	0.8354	0.3689	1.6069	0
2561	844	0.8721	0.8355	0.369	1.607	0
2562	843.9	0.8723	0.8357	0.3691	1.6066	0
2563	843.8	0.8723	0.8356	0.3693	1.6078	0
2564	843.7	0.8725	0.8354	0.3693	1.6073	0
2565	843.6	0.8725	0.8358	0.3695	1.6074	0
2566	843.5	0.8727	0.8358	0.3694	1.6081	0

2567	843.4	0.8727	0.8358	0.3694	1.6082	0
2568	843.3	0.873	0.8359	0.3696	1.6083	0
2569	843.2	0.8729	0.8359	0.3697	1.6083	0
2570	843.1	0.8732	0.8361	0.3699	1.6085	0
2571	843	0.8732	0.8362	0.3701	1.6086	0
2572	842.9	0.8734	0.8362	0.3701	1.6087	0
2573	842.8	0.8733	0.8363	0.37	1.6092	0
2574	842.7	0.8736	0.8365	0.3702	1.6093	0
2575	842.6	0.8737	0.8362	0.3703	1.6095	0
2576	842.5	0.8736	0.8363	0.3703	1.6101	0
2577	842.4	0.8737	0.8363	0.3702	1.6095	0
2578	842.3	0.8738	0.8366	0.3703	1.6096	0
2579	842.2	0.8738	0.8365	0.3702	1.6096	0
2580	842.1	0.8738	0.8365	0.3703	1.6102	0
2581	842	0.8738	0.8365	0.3704	1.6097	0
2582	841.9	0.8739	0.8365	0.3703	1.6097	0
2583	841.8	0.874	0.8365	0.3702	1.6097	0
2584	841.7	0.874	0.8366	0.3703	1.6104	0
2585	841.6	0.8737	0.8366	0.3702	1.6097	0
2586	841.5	0.8742	0.8371	0.3706	1.6095	0
2587	841.4	0.8744	0.837	0.3706	1.6108	0
2588	841.3	0.8744	0.837	0.3706	1.6096	0
2589	841.2	0.8745	0.8369	0.3705	1.6101	0
2590	841.1	0.8743	0.8375	0.3705	1.6102	0
2591	841	0.8744	0.8373	0.3704	1.6102	0
2592	840.9	0.8745	0.8369	0.3703	1.6101	0
2593	840.8	0.8745	0.837	0.3706	1.6103	0
2594	840.7	0.8745	0.8372	0.3706	1.6104	0
2595	840.6	0.8749	0.8373	0.3707	1.6118	0
2596	840.5	0.8747	0.8372	0.3706	1.6098	0
2597	840.4	0.8748	0.8372	0.3706	1.6104	0
2598	840.3	0.8748	0.8372	0.3704	1.6111	0
2599	840.2	0.875	0.8375	0.3706	1.6106	0
2600	840.1	0.8749	0.8376	0.3706	1.6101	0
2601	840	0.875	0.8376	0.3706	1.6101	0
2602	839.9	0.875	0.8376	0.3706	1.6101	0
2603	839.8	0.8749	0.8375	0.3705	1.61	0
2604	839.7	0.8748	0.8374	0.3705	1.6094	0
2605	839.6	0.8749	0.8375	0.3705	1.6095	0
2606	839.5	0.8749	0.8375	0.3704	1.6101	0
2607	839.4	0.8749	0.8376	0.3703	1.6101	0
2608	839.3	0.8749	0.8376	0.3703	1.6101	0
2609	839.2	0.8749	0.8378	0.3702	1.6101	0

2610	839.1	0.8749	0.8377	0.3702	1.6101	0
2611	839	0.8749	0.8376	0.3701	1.6095	0
2612	838.9	0.8752	0.8377	0.3701	1.6101	0
2613	838.8	0.8749	0.8377	0.37	1.6089	0
2614	838.7	0.8748	0.8377	0.37	1.6089	0
2615	838.6	0.8746	0.8375	0.3698	1.6093	0
2616	838.5	0.8746	0.8375	0.3698	1.6088	0
2617	838.4	0.8746	0.8377	0.3696	1.6093	0
2618	838.3	0.8747	0.8378	0.3698	1.6089	0
2619	838.2	0.8747	0.8378	0.3697	1.6089	0
2620	838.1	0.8747	0.838	0.3697	1.609	0
2621	838	0.8749	0.838	0.3698	1.6097	0
2622	837.9	0.8748	0.8378	0.3696	1.6083	0
2623	837.8	0.8748	0.8379	0.3695	1.609	0
2624	837.7	0.8751	0.8381	0.3696	1.6092	0
2625	837.6	0.8748	0.8379	0.3696	1.6091	0
2626	837.5	0.8747	0.838	0.3695	1.6079	0
2627	837.4	0.8749	0.8381	0.3694	1.6085	0
2628	837.3	0.8749	0.8381	0.3696	1.6086	0
2629	837.2	0.875	0.838	0.3694	1.6085	0
2630	837.1	0.875	0.8382	0.3695	1.6093	0
2631	837	0.875	0.8381	0.3693	1.6087	0
2632	836.9	0.8751	0.8384	0.3694	1.6088	0
2633	836.8	0.8752	0.8383	0.3695	1.6094	0
2634	836.7	0.8751	0.8383	0.3694	1.6083	0
2635	836.6	0.8752	0.8384	0.3693	1.6082	0
2636	836.5	0.875	0.8384	0.3693	1.6089	0
2637	836.4	0.8752	0.8384	0.3694	1.6084	0
2638	836.3	0.8754	0.8383	0.3695	1.6085	0
2639	836.2	0.875	0.8384	0.3693	1.6085	0
2640	836.1	0.8753	0.8384	0.3695	1.6086	0
2641	836	0.8753	0.8384	0.3693	1.6092	0
2642	835.9	0.8751	0.8384	0.3694	1.6086	0
2643	835.8	0.8753	0.8386	0.3694	1.6087	0
2644	835.7	0.8756	0.8386	0.3695	1.6089	0
2645	835.6	0.8756	0.8387	0.3695	1.609	0
2646	835.5	0.8757	0.8388	0.3695	1.6091	0
2647	835.4	0.8759	0.8391	0.3696	1.6093	0
2648	835.3	0.8758	0.8387	0.3695	1.6092	0
2649	835.2	0.876	0.8388	0.3696	1.6099	0
2650	835.1	0.876	0.8388	0.3696	1.6094	0
2651	835	0.8759	0.8391	0.3698	1.6096	0
2652	834.9	0.8761	0.839	0.3698	1.6109	0

2653	834.8	0.8764	0.8393	0.3701	1.61	0
2654	834.7	0.8764	0.8393	0.37	1.6106	0
2655	834.6	0.8764	0.8393	0.3701	1.6107	0
2656	834.5	0.8765	0.8392	0.37	1.6096	0
2657	834.4	0.8767	0.8395	0.3702	1.6104	0
2658	834.3	0.877	0.8398	0.3704	1.6106	0
2659	834.2	0.8769	0.8397	0.3705	1.6107	0
2660	834.1	0.877	0.8397	0.3703	1.6114	0
2661	834	0.877	0.8396	0.3704	1.6108	0
2662	833.9	0.8771	0.8397	0.3708	1.6112	0
2663	833.8	0.8773	0.8397	0.3707	1.6112	0
2664	833.7	0.8773	0.8399	0.3708	1.6119	0
2665	833.6	0.8774	0.8398	0.3709	1.612	0
2666	833.5	0.8775	0.8397	0.371	1.6116	0
2667	833.4	0.8776	0.8401	0.371	1.6123	0
2668	833.3	0.8778	0.8401	0.3713	1.6125	0
2669	833.2	0.8778	0.8399	0.3712	1.612	0
2670	833.1	0.8779	0.8401	0.3714	1.6127	0
2671	833	0.8779	0.8401	0.3713	1.6121	0
2672	832.9	0.8781	0.8402	0.3714	1.613	0
2673	832.8	0.878	0.8403	0.3716	1.6124	0
2674	832.7	0.8783	0.8403	0.3717	1.6132	0
2675	832.6	0.8783	0.8403	0.3716	1.6134	0
2676	832.5	0.8782	0.8403	0.3717	1.6134	0
2677	832.4	0.8785	0.8406	0.3718	1.6136	0
2678	832.3	0.8785	0.8406	0.372	1.6138	0
2679	832.2	0.8784	0.8405	0.3718	1.6137	0
2680	832.1	0.8786	0.8404	0.3718	1.6132	0
2681	832	0.8787	0.8405	0.3719	1.6133	0
2682	831.9	0.8787	0.8408	0.3719	1.6135	0
2683	831.8	0.8788	0.8407	0.372	1.6148	0
2684	831.7	0.8791	0.841	0.3723	1.6138	0
2685	831.6	0.8791	0.841	0.3723	1.6146	0
2686	831.5	0.8791	0.8409	0.3723	1.614	0
2687	831.4	0.8792	0.8408	0.3723	1.6142	0
2688	831.3	0.8794	0.841	0.3725	1.6149	0
2689	831.2	0.8792	0.8412	0.3726	1.6138	0
2690	831.1	0.8793	0.8409	0.3724	1.6139	0
2691	831	0.8795	0.841	0.3726	1.6146	0
2692	830.9	0.8796	0.841	0.3726	1.6141	0
2693	830.8	0.8795	0.8412	0.3725	1.6154	0
2694	830.7	0.8796	0.8412	0.3727	1.615	0
2695	830.6	0.8796	0.8411	0.3727	1.615	0

2696	830.5	0.8797	0.8412	0.3725	1.6145	0
2697	830.4	0.8797	0.8413	0.3727	1.614	0
2698	830.3	0.8797	0.8413	0.3728	1.6153	0
2699	830.2	0.8797	0.8413	0.3728	1.6148	0
2700	830.1	0.8799	0.8413	0.3727	1.6155	0
2701	830	0.8797	0.8413	0.3728	1.6149	0
2702	829.9	0.8798	0.8413	0.3726	1.6149	0
2703	829.8	0.88	0.8414	0.3728	1.6151	0
2704	829.7	0.8801	0.8414	0.3728	1.6153	0
2705	829.6	0.88	0.8416	0.3726	1.6147	0
2706	829.5	0.88	0.8416	0.3726	1.6148	0
2707	829.4	0.8799	0.8418	0.3726	1.6154	0
2708	829.3	0.8801	0.8416	0.3726	1.615	0
2709	829.2	0.8801	0.8415	0.3725	1.615	0
2710	829.1	0.8799	0.8414	0.3723	1.6149	0
2711	829	0.8799	0.8414	0.3724	1.6145	0
2712	828.9	0.88	0.8416	0.3724	1.6151	0
2713	828.8	0.88	0.8417	0.3723	1.6153	0
2714	828.7	0.8801	0.8416	0.3723	1.6147	0
2715	828.6	0.8801	0.8416	0.3723	1.6148	0
2716	828.5	0.8799	0.8414	0.3722	1.6142	0
2717	828.4	0.88	0.8416	0.3722	1.6143	0
2718	828.3	0.8802	0.8415	0.3723	1.6145	0
2719	828.2	0.8801	0.8415	0.3721	1.6151	0
2720	828.1	0.8802	0.8415	0.3721	1.6146	0
2721	828	0.8803	0.8418	0.3722	1.6147	0
2722	827.9	0.8801	0.8417	0.3721	1.6141	0
2723	827.8	0.8801	0.8417	0.372	1.6147	0
2724	827.7	0.88	0.8415	0.3719	1.6142	0
2725	827.6	0.8802	0.8418	0.3719	1.6137	0
2726	827.5	0.8799	0.8416	0.3718	1.6143	0
2727	827.4	0.88	0.8417	0.372	1.6139	0
2728	827.3	0.8802	0.8418	0.3721	1.6147	0
2729	827.2	0.8802	0.8417	0.372	1.6141	0
2730	827.1	0.8802	0.8417	0.3719	1.6141	0
2731	827	0.8801	0.8418	0.3718	1.6142	0
2732	826.9	0.8802	0.8418	0.3719	1.6143	0
2733	826.8	0.8802	0.8418	0.3717	1.6136	0
2734	826.7	0.8804	0.8418	0.3719	1.6145	0
2735	826.6	0.8803	0.8418	0.3718	1.6146	0
2736	826.5	0.8803	0.8418	0.3718	1.614	0
2737	826.4	0.8803	0.8418	0.3719	1.6148	0
2738	826.3	0.8806	0.842	0.372	1.6144	0

2739	826.2	0.8804	0.8422	0.3719	1.6138	0
2740	826.1	0.8805	0.842	0.3718	1.6144	0
2741	826	0.8807	0.8422	0.372	1.6147	0
2742	825.9	0.8805	0.8421	0.3719	1.6136	0
2743	825.8	0.8808	0.8424	0.3721	1.615	0
2744	825.7	0.8807	0.8423	0.3719	1.6144	0
2745	825.6	0.8808	0.8425	0.372	1.6152	0
2746	825.5	0.8808	0.8424	0.3719	1.614	0
2747	825.4	0.8806	0.8423	0.3718	1.6146	0
2748	825.3	0.8808	0.8423	0.3719	1.6148	0
2749	825.2	0.881	0.8424	0.3719	1.6143	0
2750	825.1	0.8811	0.8427	0.372	1.6146	0
2751	825	0.881	0.8426	0.372	1.6146	0
2752	824.9	0.8811	0.8426	0.3722	1.6148	0
2753	824.8	0.8813	0.8427	0.3722	1.615	0
2754	824.7	0.8814	0.8425	0.3722	1.6151	0
2755	824.6	0.8815	0.8425	0.3722	1.6158	0
2756	824.5	0.8814	0.8425	0.3723	1.6153	0
2757	824.4	0.8814	0.8426	0.3724	1.6155	0
2758	824.3	0.8816	0.8427	0.3724	1.6156	0
2759	824.2	0.8818	0.8429	0.3726	1.6152	0
2760	824.1	0.8817	0.843	0.3726	1.6154	0
2761	824	0.8818	0.8428	0.3724	1.616	0
2762	823.9	0.882	0.843	0.3726	1.6168	0
2763	823.8	0.882	0.8429	0.3727	1.6158	0
2764	823.7	0.8821	0.843	0.3727	1.6165	0
2765	823.6	0.8821	0.843	0.3727	1.616	0
2766	823.5	0.8822	0.8431	0.3728	1.6162	0
2767	823.4	0.8823	0.8431	0.3728	1.6158	0
2768	823.3	0.8825	0.8435	0.3729	1.6166	0
2769	823.2	0.8826	0.8431	0.3729	1.6167	0
2770	823.1	0.8825	0.843	0.373	1.6168	0
2771	823	0.8825	0.8435	0.373	1.6175	0
2772	822.9	0.8825	0.8432	0.3729	1.6163	0
2773	822.8	0.8827	0.8435	0.3729	1.6167	0
2774	822.7	0.8827	0.8434	0.3732	1.6174	0
2775	822.6	0.8828	0.8434	0.3732	1.617	0
2776	822.5	0.883	0.8433	0.3733	1.6177	0
2777	822.4	0.8831	0.8436	0.3734	1.6174	0
2778	822.3	0.8832	0.8436	0.3734	1.6169	0
2779	822.2	0.8833	0.8438	0.3736	1.6177	0
2780	822.1	0.8834	0.8435	0.3735	1.6179	0
2781	822	0.8833	0.8438	0.3736	1.6169	0

2782	821.9	0.8835	0.8438	0.3737	1.6182	0
2783	821.8	0.8834	0.8438	0.3736	1.6177	0
2784	821.7	0.8836	0.8436	0.3736	1.6184	0
2785	821.6	0.8838	0.844	0.3739	1.6176	0
2786	821.5	0.8838	0.844	0.3738	1.6183	0
2787	821.4	0.8839	0.844	0.374	1.6179	0
2788	821.3	0.884	0.844	0.3738	1.618	0
2789	821.2	0.884	0.8441	0.3738	1.6187	0
2790	821.1	0.8841	0.8437	0.3738	1.6187	0
2791	821	0.884	0.8439	0.3738	1.6177	0
2792	820.9	0.884	0.8439	0.3737	1.6184	0
2793	820.8	0.8843	0.8439	0.3739	1.6181	0
2794	820.7	0.8842	0.8443	0.374	1.6188	0
2795	820.6	0.8842	0.8441	0.374	1.6183	0
2796	820.5	0.884	0.8443	0.3739	1.6185	0
2797	820.4	0.8842	0.8442	0.3738	1.6185	0
2798	820.3	0.8842	0.844	0.374	1.6187	0
2799	820.2	0.8843	0.8443	0.3741	1.6195	0
2800	820.1	0.8845	0.8443	0.3741	1.6185	0
2801	820	0.8845	0.8443	0.3742	1.6192	0
2802	819.9	0.8848	0.8444	0.3742	1.6194	0
2803	819.8	0.8846	0.8445	0.3743	1.6195	0
2804	819.7	0.885	0.8446	0.3744	1.6186	0
2805	819.6	0.8849	0.8448	0.3743	1.6193	0
2806	819.5	0.885	0.8446	0.3744	1.6188	0
2807	819.4	0.8849	0.8445	0.3744	1.6189	0
2808	819.3	0.8851	0.8448	0.3745	1.6197	0
2809	819.2	0.8853	0.8447	0.3745	1.6199	0
2810	819.1	0.8851	0.8446	0.3744	1.6193	0
2811	819	0.8853	0.8447	0.3745	1.6196	0
2812	818.9	0.8852	0.8448	0.3745	1.6191	0
2813	818.8	0.8855	0.8449	0.3744	1.6193	0
2814	818.7	0.8855	0.8447	0.3746	1.62	0
2815	818.6	0.8854	0.845	0.3744	1.6195	0
2816	818.5	0.8854	0.8449	0.3743	1.6196	0
2817	818.4	0.8855	0.845	0.3744	1.6192	0
2818	818.3	0.8855	0.845	0.3744	1.6193	0
2819	818.2	0.8855	0.845	0.3743	1.6199	0
2820	818.1	0.8855	0.845	0.3742	1.6195	0
2821	818	0.8855	0.8451	0.3743	1.619	0
2822	817.9	0.8854	0.8448	0.3742	1.6197	0
2823	817.8	0.8853	0.845	0.3741	1.6191	0
2824	817.7	0.8857	0.8451	0.3743	1.6194	0

2825	817.6	0.8857	0.8452	0.3743	1.6195	0
2826	817.5	0.8857	0.8452	0.3742	1.6196	0
2827	817.4	0.8858	0.8451	0.3743	1.6192	0
2828	817.3	0.8858	0.8451	0.3742	1.6199	0
2829	817.2	0.8858	0.845	0.3742	1.6194	0
2830	817.1	0.8861	0.8453	0.3743	1.6202	0
2831	817	0.8858	0.8451	0.3743	1.6197	0
2832	816.9	0.8862	0.8454	0.3744	1.62	0
2833	816.8	0.8861	0.8453	0.3744	1.6194	0
2834	816.7	0.8862	0.8453	0.3744	1.6203	0
2835	816.6	0.8861	0.8453	0.3744	1.6197	0
2836	816.5	0.8862	0.8453	0.3744	1.6192	0
2837	816.4	0.8861	0.8456	0.3744	1.62	0
2838	816.3	0.8864	0.8456	0.3744	1.6202	0
2839	816.2	0.8863	0.8457	0.3746	1.6209	0
2840	816.1	0.8865	0.8456	0.3744	1.6199	0
2841	816	0.8864	0.8457	0.3745	1.6207	0
2842	815.9	0.8867	0.8459	0.3746	1.6197	0
2843	815.8	0.8865	0.8458	0.3745	1.621	0
2844	815.7	0.8867	0.8457	0.3746	1.6205	0
2845	815.6	0.8867	0.8457	0.3746	1.6207	0
2846	815.5	0.8866	0.8458	0.3745	1.6213	0
2847	815.4	0.8866	0.8457	0.3746	1.6209	0
2848	815.3	0.8869	0.8458	0.3746	1.6205	0
2849	815.2	0.8869	0.8459	0.3745	1.6206	0
2850	815.1	0.8868	0.8459	0.3744	1.6207	0
2851	815	0.8869	0.8461	0.3746	1.621	0
2852	814.9	0.8871	0.8461	0.3747	1.6211	0
2853	814.8	0.8874	0.8463	0.375	1.6214	0
2854	814.7	0.8874	0.8465	0.375	1.6216	0
2855	814.6	0.8875	0.8464	0.3751	1.6207	0
2856	814.5	0.8875	0.8465	0.375	1.6214	0
2857	814.4	0.8876	0.8463	0.3751	1.621	0
2858	814.3	0.8875	0.8462	0.375	1.6216	0
2859	814.2	0.8877	0.8464	0.375	1.6212	0
2860	814.1	0.8877	0.8463	0.3751	1.622	0
2861	814	0.8879	0.8464	0.3751	1.621	0
2862	813.9	0.8878	0.8463	0.3752	1.6217	0
2863	813.8	0.8879	0.8463	0.3752	1.6213	0
2864	813.7	0.8881	0.8466	0.3754	1.6215	0
2865	813.6	0.888	0.8465	0.3754	1.6222	0
2866	813.5	0.8881	0.8465	0.3753	1.6223	0
2867	813.4	0.8881	0.8465	0.3753	1.6231	0

2868	813.3	0.8883	0.8467	0.3756	1.6228	0
2869	813.2	0.8882	0.8467	0.3755	1.6223	0
2870	813.1	0.8883	0.8468	0.3756	1.6226	0
2871	813	0.8884	0.8469	0.3755	1.6215	0
2872	812.9	0.8883	0.8469	0.3756	1.6223	0
2873	812.8	0.8887	0.8469	0.3756	1.6219	0
2874	812.7	0.8885	0.8471	0.3757	1.6227	0
2875	812.6	0.8887	0.8473	0.3759	1.623	0
2876	812.5	0.8888	0.8472	0.3758	1.6225	0
2877	812.4	0.889	0.8473	0.3759	1.6221	0
2878	812.3	0.8891	0.8474	0.3761	1.6231	0
2879	812.2	0.889	0.8475	0.3761	1.6232	0
2880	812.1	0.8892	0.8474	0.3761	1.6233	0
2881	812	0.8892	0.8474	0.3761	1.6235	0
2882	811.9	0.889	0.8473	0.3759	1.6229	0
2883	811.8	0.8893	0.8474	0.376	1.6231	0
2884	811.7	0.8893	0.8476	0.3761	1.6227	0
2885	811.6	0.8893	0.8474	0.376	1.6234	0
2886	811.5	0.8896	0.8478	0.3763	1.6232	0
2887	811.4	0.8895	0.8476	0.3761	1.6244	0
2888	811.3	0.8896	0.8478	0.3762	1.6229	0
2889	811.2	0.8896	0.8478	0.3761	1.6241	0
2890	811.1	0.8896	0.8475	0.3762	1.6242	0
2891	811	0.8896	0.8478	0.376	1.6238	0
2892	810.9	0.89	0.8478	0.3762	1.6234	0
2893	810.8	0.8899	0.8478	0.3762	1.6236	0
2894	810.7	0.8896	0.8476	0.376	1.6236	0
2895	810.6	0.8897	0.8477	0.3759	1.6238	0
2896	810.5	0.8899	0.8478	0.376	1.6241	0
2897	810.4	0.89	0.848	0.3762	1.6231	0
2898	810.3	0.8901	0.848	0.3762	1.6239	0
2899	810.2	0.8901	0.848	0.3761	1.624	0
2900	810.1	0.8901	0.848	0.3763	1.6236	0
2901	810	0.8901	0.848	0.3762	1.6238	0
2902	809.9	0.8902	0.8481	0.3761	1.6234	0
2903	809.8	0.8903	0.8483	0.3761	1.6235	0
2904	809.7	0.8903	0.8482	0.3762	1.6243	0
2905	809.6	0.8902	0.8482	0.3761	1.6238	0
2906	809.5	0.8905	0.8483	0.3763	1.6241	0
2907	809.4	0.8904	0.8483	0.3761	1.6236	0
2908	809.3	0.8905	0.8484	0.3763	1.6239	0
2909	809.2	0.8907	0.8486	0.3763	1.6235	0
2910	809.1	0.8906	0.8483	0.3761	1.624	0

2911	809	0.8908	0.8486	0.3762	1.6238	0
2912	808.9	0.8907	0.8486	0.3763	1.6246	0
2913	808.8	0.8908	0.8486	0.3763	1.6241	0
2914	808.7	0.8907	0.8484	0.3763	1.6243	0
2915	808.6	0.8907	0.8485	0.3761	1.6238	0
2916	808.5	0.8909	0.8485	0.3762	1.624	0
2917	808.4	0.8911	0.8487	0.3763	1.6242	0
2918	808.3	0.8911	0.8487	0.3762	1.6243	0
2919	808.2	0.8909	0.8485	0.3763	1.625	0
2920	808.1	0.891	0.8488	0.3762	1.6246	0
2921	808	0.8912	0.8488	0.3763	1.6248	0
2922	807.9	0.891	0.8487	0.3762	1.6244	0
2923	807.8	0.8913	0.8489	0.3764	1.6247	0
2924	807.7	0.8913	0.8489	0.3764	1.6249	0
2925	807.6	0.8916	0.849	0.3764	1.6245	0
2926	807.5	0.8917	0.849	0.3763	1.6246	0
2927	807.4	0.8917	0.8491	0.3766	1.6249	0
2928	807.3	0.8918	0.849	0.3765	1.6249	0
2929	807.2	0.8918	0.849	0.3766	1.6251	0
2930	807.1	0.8918	0.8491	0.3766	1.6248	0
2931	807	0.892	0.8494	0.377	1.6252	0
2932	806.9	0.892	0.8494	0.3768	1.6253	0
2933	806.8	0.892	0.8495	0.3768	1.6256	0
2934	806.7	0.8922	0.8496	0.3769	1.6257	0
2935	806.6	0.8922	0.8496	0.3768	1.6264	0
2936	806.5	0.8924	0.8495	0.3769	1.6254	0
2937	806.4	0.8923	0.8495	0.3769	1.6251	0
2938	806.3	0.8923	0.8494	0.3769	1.6264	0
2939	806.2	0.8925	0.8495	0.377	1.626	0
2940	806.1	0.8927	0.8497	0.3771	1.6257	0
2941	806	0.8928	0.85	0.3773	1.6265	0
2942	805.9	0.8929	0.8498	0.3773	1.6261	0
2943	805.8	0.893	0.8501	0.3773	1.6269	0
2944	805.7	0.8929	0.8501	0.3773	1.6265	0
2945	805.6	0.8929	0.8499	0.3773	1.6266	0
2946	805.5	0.893	0.8501	0.3773	1.6275	0
2947	805.4	0.8932	0.85	0.3774	1.6276	0
2948	805.3	0.8934	0.85	0.3776	1.6272	0
2949	805.2	0.8935	0.8504	0.3777	1.6264	0
2950	805.1	0.8937	0.8502	0.3777	1.6271	0
2951	805	0.8934	0.8502	0.3777	1.6266	0
2952	804.9	0.8935	0.8502	0.3777	1.6268	0
2953	804.8	0.8938	0.8506	0.3778	1.6278	0

2954	804.7	0.8939	0.8505	0.3779	1.6279	0
2955	804.6	0.8938	0.8504	0.3779	1.6275	0
2956	804.5	0.8939	0.8506	0.3778	1.6271	0
2957	804.4	0.8941	0.8505	0.378	1.6273	0
2958	804.3	0.8943	0.8506	0.3782	1.6287	0
2959	804.2	0.894	0.8506	0.3781	1.6283	0
2960	804.1	0.8945	0.8508	0.3782	1.628	0
2961	804	0.8944	0.8508	0.3782	1.6281	0
2962	803.9	0.8945	0.8508	0.3782	1.6283	0
2963	803.8	0.8946	0.8509	0.3784	1.628	0
2964	803.7	0.8946	0.8511	0.3784	1.6282	0
2965	803.6	0.8949	0.8511	0.3785	1.6285	0
2966	803.5	0.895	0.8512	0.3787	1.6287	0
2967	803.4	0.8948	0.8509	0.3785	1.6287	0
2968	803.3	0.8951	0.8512	0.3788	1.6285	0
2969	803.2	0.8952	0.8511	0.3787	1.6292	0
2970	803.1	0.8956	0.8514	0.3789	1.629	0
2971	803	0.8954	0.8516	0.3788	1.6291	0
2972	802.9	0.8954	0.8512	0.3789	1.6292	0
2973	802.8	0.8954	0.8515	0.3787	1.6288	0
2974	802.7	0.8954	0.8513	0.3789	1.6296	0
2975	802.6	0.8956	0.8516	0.3788	1.6297	0
2976	802.5	0.8956	0.8515	0.3788	1.6293	0
2977	802.4	0.8956	0.8515	0.3789	1.6295	0
2978	802.3	0.8958	0.8517	0.3788	1.6296	0
2979	802.2	0.8958	0.8518	0.379	1.6299	0
2980	802.1	0.8957	0.8516	0.3789	1.6294	0
2981	802	0.8958	0.8515	0.379	1.6296	0
2982	801.9	0.8958	0.8518	0.3789	1.6292	0
2983	801.8	0.8958	0.8519	0.3789	1.6287	0
2984	801.7	0.896	0.8519	0.379	1.6302	0
2985	801.6	0.896	0.852	0.379	1.6297	0
2986	801.5	0.8961	0.852	0.379	1.6304	0
2987	801.4	0.8961	0.8521	0.3789	1.63	0
2988	801.3	0.8961	0.8521	0.3789	1.6296	0
2989	801.2	0.8963	0.852	0.3789	1.6298	0
2990	801.1	0.8962	0.852	0.3789	1.6299	0
2991	801	0.8963	0.8523	0.3789	1.6296	0
2992	800.9	0.8963	0.8522	0.3789	1.6297	0
2993	800.8	0.8963	0.8522	0.3788	1.6298	0
2994	800.7	0.8963	0.8521	0.3788	1.6293	0
2995	800.6	0.8964	0.8522	0.3788	1.6301	0
2996	800.5	0.8964	0.8523	0.3787	1.6297	0

2997	800.4	0.8965	0.8523	0.3789	1.6293	0
2998	800.3	0.8963	0.8522	0.3786	1.6299	0
2999	800.2	0.8965	0.8523	0.3787	1.629	0
3000	800.1	0.8965	0.8524	0.3787	1.6291	0
3001	800	0.8966	0.8523	0.3787	1.6294	0
3002	799.9	0.8967	0.8526	0.3788	1.6302	0
3003	799.8	0.8967	0.8525	0.3788	1.6297	0
3004	799.7	0.8967	0.8524	0.3787	1.6293	0
3005	799.6	0.8966	0.8525	0.3786	1.6294	0
3006	799.5	0.8966	0.8526	0.3785	1.6301	0
3007	799.4	0.8969	0.8527	0.3786	1.6304	0
3008	799.3	0.8969	0.8528	0.3787	1.6295	0
3009	799.2	0.8969	0.8527	0.3786	1.6296	0
3010	799.1	0.8969	0.8527	0.3784	1.6292	0
3011	799	0.8968	0.8529	0.3784	1.6294	0
3012	798.9	0.897	0.8529	0.3784	1.6295	0
3013	798.8	0.897	0.8528	0.3786	1.6292	0
3014	798.7	0.897	0.853	0.3785	1.6298	0
3015	798.6	0.8972	0.853	0.3785	1.6295	0
3016	798.5	0.8971	0.8531	0.3784	1.6291	0
3017	798.4	0.8972	0.8531	0.3784	1.6292	0
3018	798.3	0.897	0.8531	0.3783	1.63	0
3019	798.2	0.8972	0.853	0.3782	1.6295	0
3020	798.1	0.897	0.853	0.3782	1.629	0
3021	798	0.8972	0.853	0.3782	1.6293	0
3022	797.9	0.8975	0.8533	0.3783	1.6289	0
3023	797.8	0.8974	0.8533	0.3783	1.6296	0
3024	797.7	0.8975	0.8534	0.3784	1.6294	0
3025	797.6	0.8977	0.8532	0.3784	1.6295	0
3026	797.5	0.8977	0.8534	0.3784	1.6292	0
3027	797.4	0.8977	0.8536	0.3785	1.6305	0
3028	797.3	0.8977	0.8535	0.3783	1.6295	0
3029	797.2	0.8978	0.8535	0.3784	1.6291	0
3030	797.1	0.8976	0.8536	0.3785	1.6298	0
3031	797	0.898	0.8537	0.3785	1.6296	0
3032	796.9	0.898	0.8536	0.3785	1.6303	0
3033	796.8	0.898	0.8538	0.3784	1.6299	0
3034	796.7	0.8983	0.8536	0.3786	1.6296	0
3035	796.6	0.8983	0.854	0.3788	1.6299	0
3036	796.5	0.8985	0.854	0.3788	1.6313	0
3037	796.4	0.8987	0.8545	0.3789	1.631	0
3038	796.3	0.8985	0.8542	0.379	1.6306	0
3039	796.2	0.8988	0.8542	0.3791	1.6308	0

3040	796.1	0.8989	0.8545	0.3791	1.6316	0
3041	796	0.899	0.8545	0.3792	1.6314	0
3042	795.9	0.8991	0.8544	0.3791	1.6309	0
3043	795.8	0.8993	0.8547	0.3794	1.6318	0
3044	795.7	0.8995	0.8547	0.3795	1.6316	0
3045	795.6	0.8993	0.8545	0.3796	1.6317	0
3046	795.5	0.8994	0.8546	0.3795	1.6318	0
3047	795.4	0.8996	0.8547	0.3797	1.6321	0
3048	795.3	0.8996	0.8548	0.3798	1.6323	0
3049	795.2	0.8999	0.855	0.38	1.6327	0
3050	795.1	0.8998	0.8549	0.3798	1.6327	0
3051	795	0.8999	0.8551	0.3801	1.6331	0
3052	794.9	0.9001	0.8552	0.3803	1.6333	0
3053	794.8	0.9003	0.8551	0.3802	1.6329	0
3054	794.7	0.9003	0.8551	0.3803	1.6331	0
3055	794.6	0.9005	0.8551	0.3804	1.6329	0
3056	794.5	0.9008	0.8555	0.3806	1.6338	0
3057	794.4	0.9007	0.8554	0.3807	1.634	0
3058	794.3	0.9009	0.8553	0.3807	1.6336	0
3059	794.2	0.901	0.8556	0.3809	1.6344	0
3060	794.1	0.9009	0.8556	0.3808	1.6341	0
3061	794	0.9012	0.8558	0.3811	1.6343	0
3062	793.9	0.9011	0.8558	0.3812	1.6351	0
3063	793.8	0.9013	0.8558	0.3811	1.6348	0
3064	793.7	0.9014	0.8557	0.3811	1.6344	0
3065	793.6	0.9016	0.8559	0.3812	1.6351	0
3066	793.5	0.9015	0.8558	0.3811	1.6354	0
3067	793.4	0.9017	0.856	0.3814	1.6356	0
3068	793.3	0.902	0.8562	0.3816	1.6354	0
3069	793.2	0.9021	0.8564	0.3817	1.6356	0
3070	793.1	0.9021	0.8563	0.3817	1.6353	0
3071	793	0.9023	0.8563	0.3817	1.6355	0
3072	792.9	0.9024	0.8563	0.3817	1.6357	0
3073	792.8	0.9022	0.8562	0.3816	1.6352	0
3074	792.7	0.9022	0.8564	0.3816	1.636	0
3075	792.6	0.9023	0.8566	0.3819	1.6362	0
3076	792.5	0.9025	0.8565	0.3818	1.637	0
3077	792.4	0.9026	0.8567	0.3819	1.6366	0
3078	792.3	0.9026	0.8568	0.3819	1.6357	0
3079	792.2	0.9028	0.8568	0.382	1.6359	0
3080	792.1	0.9028	0.8568	0.382	1.6367	0
3081	792	0.9027	0.8568	0.382	1.6357	0
3082	791.9	0.903	0.8569	0.382	1.6359	0

3083	791.8	0.903	0.8569	0.382	1.6366	0
3084	791.7	0.9031	0.857	0.382	1.6363	0
3085	791.6	0.9033	0.8572	0.3821	1.6365	0
3086	791.5	0.9035	0.8571	0.3823	1.6367	0
3087	791.4	0.9032	0.8573	0.3822	1.6363	0
3088	791.3	0.9035	0.8572	0.3822	1.6365	0
3089	791.2	0.9036	0.8574	0.3821	1.6367	0
3090	791.1	0.9035	0.8573	0.3822	1.6373	0
3091	791	0.9034	0.8572	0.3821	1.637	0
3092	790.9	0.9035	0.8576	0.3821	1.6376	0
3093	790.8	0.9037	0.8575	0.3821	1.6367	0
3094	790.7	0.9037	0.8574	0.3822	1.637	0
3095	790.6	0.9039	0.8577	0.3822	1.6371	0
3096	790.5	0.904	0.8576	0.3822	1.6373	0
3097	790.4	0.9039	0.8576	0.3821	1.6363	0
3098	790.3	0.904	0.8575	0.3821	1.637	0
3099	790.2	0.9039	0.8577	0.3822	1.6367	0
3100	790.1	0.9039	0.8578	0.3819	1.6368	0
3101	790	0.9038	0.8579	0.382	1.6369	0
3102	789.9	0.9039	0.8575	0.3818	1.6369	0
3103	789.8	0.9039	0.8578	0.3817	1.6365	0
3104	789.7	0.9039	0.8578	0.3817	1.6367	0
3105	789.6	0.9039	0.858	0.3817	1.6363	0
3106	789.5	0.9037	0.8578	0.3815	1.6363	0
3107	789.4	0.9037	0.8577	0.3815	1.6364	0
3108	789.3	0.904	0.8578	0.3816	1.6361	0
3109	789.2	0.9038	0.8579	0.3816	1.6363	0
3110	789.1	0.9039	0.8579	0.3814	1.6359	0
3111	789	0.904	0.858	0.3814	1.6355	0
3112	788.9	0.9039	0.8581	0.3814	1.6367	0
3113	788.8	0.9041	0.8579	0.3813	1.6362	0
3114	788.7	0.9041	0.8581	0.3812	1.6359	0
3115	788.6	0.9039	0.8578	0.3812	1.6354	0
3116	788.5	0.9039	0.8581	0.3811	1.6356	0
3117	788.4	0.9041	0.8581	0.3811	1.6357	0
3118	788.3	0.9042	0.8583	0.3812	1.636	0
3119	788.2	0.9043	0.8582	0.3813	1.6362	0
3120	788.1	0.9045	0.8586	0.3812	1.6358	0
3121	788	0.9044	0.8586	0.3812	1.6354	0
3122	787.9	0.9043	0.8584	0.3811	1.6366	0
3123	787.8	0.9044	0.8585	0.3811	1.6357	0
3124	787.7	0.9045	0.8586	0.3811	1.6359	0
3125	787.6	0.9044	0.8584	0.381	1.6359	0

3126	787.5	0.9041	0.8583	0.3809	1.6355	0
3127	787.4	0.9045	0.8585	0.381	1.6357	0
3128	787.3	0.9045	0.8586	0.3809	1.6353	0
3129	787.2	0.9045	0.8586	0.3809	1.636	0
3130	787.1	0.9046	0.8585	0.3808	1.6362	0
3131	787	0.9046	0.8586	0.3809	1.6364	0
3132	786.9	0.9048	0.8588	0.3809	1.6356	0
3133	786.8	0.9047	0.8588	0.3808	1.6361	0
3134	786.7	0.905	0.8587	0.381	1.6359	0
3135	786.6	0.9049	0.8588	0.3809	1.6366	0
3136	786.5	0.905	0.8591	0.3811	1.6363	0
3137	786.4	0.9052	0.859	0.3811	1.6365	0
3138	786.3	0.9053	0.8589	0.381	1.6361	0
3139	786.2	0.9053	0.8593	0.381	1.6369	0
3140	786.1	0.9055	0.8592	0.3812	1.6372	0
3141	786	0.9053	0.8592	0.3812	1.6362	0
3142	785.9	0.9054	0.8591	0.3812	1.6364	0
3143	785.8	0.9058	0.8594	0.3814	1.6368	0
3144	785.7	0.906	0.8594	0.3816	1.6371	0
3145	785.6	0.9061	0.8596	0.3816	1.6367	0
3146	785.5	0.9061	0.8597	0.3817	1.638	0
3147	785.4	0.9063	0.8599	0.3817	1.6377	0
3148	785.3	0.9065	0.8599	0.382	1.6375	0
3149	785.2	0.9067	0.86	0.3821	1.6383	0
3150	785.1	0.9065	0.86	0.382	1.6379	0
3151	785	0.9067	0.86	0.3822	1.6387	0
3152	784.9	0.907	0.8603	0.3824	1.6385	0
3153	784.8	0.9071	0.8603	0.3824	1.6392	0
3154	784.7	0.9071	0.8604	0.3826	1.6379	0
3155	784.6	0.9073	0.8605	0.3827	1.6387	0
3156	784.5	0.9074	0.8606	0.3828	1.6389	0
3157	784.4	0.9077	0.8607	0.383	1.6392	0
3158	784.3	0.9079	0.8608	0.3832	1.64	0
3159	784.2	0.9079	0.8608	0.3833	1.6403	0
3160	784.1	0.9081	0.8609	0.3834	1.64	0
3161	784	0.9083	0.8611	0.3836	1.6408	0
3162	783.9	0.9083	0.8608	0.3835	1.641	0
3163	783.8	0.9086	0.8613	0.3839	1.6403	0
3164	783.7	0.9087	0.8613	0.3838	1.641	0
3165	783.6	0.9088	0.8612	0.384	1.6412	0
3166	783.5	0.9088	0.8615	0.3841	1.641	0
3167	783.4	0.9091	0.8615	0.3841	1.6417	0
3168	783.3	0.9091	0.8615	0.3843	1.642	0

3169	783.2	0.9092	0.8616	0.3845	1.6417	0
3170	783.1	0.9095	0.8618	0.3845	1.6425	0
3171	783	0.9095	0.8618	0.3845	1.6421	0
3172	782.9	0.9096	0.8618	0.3845	1.6423	0
3173	782.8	0.9096	0.8619	0.3845	1.6419	0
3174	782.7	0.9098	0.8619	0.3847	1.6416	0
3175	782.6	0.9099	0.8621	0.3848	1.6424	0
3176	782.5	0.9099	0.8621	0.3847	1.6421	0
3177	782.4	0.9098	0.8621	0.3847	1.6416	0
3178	782.3	0.9101	0.862	0.3848	1.6424	0
3179	782.2	0.9102	0.8621	0.3849	1.6427	0
3180	782.1	0.9102	0.8623	0.385	1.6429	0
3181	782	0.9102	0.8622	0.3849	1.6424	0
3182	781.9	0.9102	0.8624	0.385	1.6431	0
3183	781.8	0.9105	0.8624	0.385	1.6434	0
3184	781.7	0.9105	0.8625	0.3852	1.643	0
3185	781.6	0.9109	0.8625	0.3853	1.6428	0
3186	781.5	0.9109	0.8628	0.3853	1.6436	0
3187	781.4	0.9109	0.8627	0.3853	1.6437	0
3188	781.3	0.9111	0.8627	0.3853	1.6439	0
3189	781.2	0.9109	0.8626	0.3851	1.6433	0
3190	781.1	0.9112	0.863	0.3853	1.6438	0
3191	781	0.9112	0.863	0.3853	1.6439	0
3192	780.9	0.9112	0.863	0.3855	1.6441	0
3193	780.8	0.9112	0.8631	0.3854	1.6432	0
3194	780.7	0.9114	0.8631	0.3854	1.6444	0
3195	780.6	0.9113	0.8632	0.3854	1.644	0
3196	780.5	0.9114	0.8633	0.3854	1.6448	0
3197	780.4	0.9117	0.8634	0.3855	1.6434	0
3198	780.3	0.9115	0.8634	0.3854	1.6435	0
3199	780.2	0.9117	0.8632	0.3852	1.6441	0
3200	780.1	0.9117	0.8634	0.3854	1.6444	0
3201	780	0.9118	0.8633	0.3853	1.6439	0
3202	779.9	0.9121	0.8635	0.3854	1.6441	0
3203	779.8	0.912	0.8636	0.3854	1.6438	0
3204	779.7	0.9118	0.8634	0.3852	1.6444	0
3205	779.6	0.9117	0.8636	0.3851	1.644	0
3206	779.5	0.912	0.8638	0.3852	1.6442	0
3207	779.4	0.9118	0.8636	0.385	1.6442	0
3208	779.3	0.9118	0.8636	0.385	1.6438	0
3209	779.2	0.9119	0.8637	0.385	1.6435	0
3210	779.1	0.9119	0.8636	0.385	1.6441	0
3211	779	0.9119	0.8638	0.3849	1.6437	0

3212	778.9	0.9118	0.8636	0.3848	1.6438	0
3213	778.8	0.9121	0.8639	0.3848	1.6435	0
3214	778.7	0.912	0.8637	0.3847	1.6436	0
3215	778.6	0.912	0.8641	0.3848	1.6437	0
3216	778.5	0.9117	0.8638	0.3845	1.6431	0
3217	778.4	0.9118	0.864	0.3845	1.6433	0
3218	778.3	0.912	0.8641	0.3846	1.643	0
3219	778.2	0.9121	0.864	0.3845	1.6431	0
3220	778.1	0.912	0.8638	0.3843	1.6437	0
3221	778	0.912	0.864	0.3841	1.6432	0
3222	777.9	0.9121	0.8642	0.3843	1.644	0
3223	777.8	0.9123	0.8646	0.3845	1.6427	0
3224	777.7	0.9122	0.8643	0.3844	1.6428	0
3225	777.6	0.9124	0.8645	0.3842	1.643	0
3226	777.5	0.9122	0.8643	0.3843	1.643	0
3227	777.4	0.9123	0.8644	0.3842	1.6432	0
3228	777.3	0.9123	0.8643	0.384	1.6432	0
3229	777.2	0.9123	0.8644	0.3841	1.6428	0
3230	777.1	0.9124	0.8643	0.3841	1.643	0
3231	777	0.9124	0.8645	0.384	1.6432	0
3232	776.9	0.9124	0.8647	0.3841	1.6428	0
3233	776.8	0.9126	0.8647	0.384	1.6434	0
3234	776.7	0.9125	0.8644	0.384	1.6425	0
3235	776.6	0.9126	0.8645	0.3839	1.6426	0
3236	776.5	0.9125	0.8647	0.3841	1.6428	0
3237	776.4	0.9127	0.8648	0.3841	1.6431	0
3238	776.3	0.9126	0.8648	0.3839	1.6426	0
3239	776.2	0.9128	0.8649	0.3839	1.6428	0
3240	776.1	0.9126	0.8648	0.3838	1.6428	0
3241	776	0.9128	0.8649	0.3839	1.6426	0
3242	775.9	0.913	0.865	0.384	1.6433	0
3243	775.8	0.9132	0.8651	0.3841	1.643	0
3244	775.7	0.9132	0.8651	0.3841	1.6432	0
3245	775.6	0.9133	0.8652	0.3842	1.6434	0
3246	775.5	0.9134	0.8652	0.3842	1.6431	0
3247	775.4	0.9135	0.8651	0.3844	1.6438	0
3248	775.3	0.9137	0.8652	0.3844	1.6435	0
3249	775.2	0.9138	0.8654	0.3844	1.6432	0
3250	775.1	0.914	0.8656	0.3844	1.6439	0
3251	775	0.914	0.8656	0.3845	1.6442	0
3252	774.9	0.9141	0.8657	0.3847	1.6444	0
3253	774.8	0.9142	0.8659	0.3849	1.6442	0
3254	774.7	0.9143	0.8659	0.3848	1.6443	0

3255	774.6	0.9145	0.8661	0.3849	1.6451	0
3256	774.5	0.9146	0.8661	0.3849	1.6448	0
3257	774.4	0.9148	0.8663	0.3853	1.6456	0
3258	774.3	0.9149	0.8664	0.3854	1.6453	0
3259	774.2	0.9149	0.8662	0.3854	1.6455	0
3260	774.1	0.9152	0.8665	0.3855	1.6452	0
3261	774	0.9151	0.8664	0.3854	1.6459	0
3262	773.9	0.9153	0.8664	0.3856	1.6467	0
3263	773.8	0.9154	0.8668	0.3858	1.6464	0
3264	773.7	0.9156	0.8667	0.3859	1.6471	0
3265	773.6	0.9156	0.8667	0.3859	1.6468	0
3266	773.5	0.9158	0.8667	0.386	1.6464	0
3267	773.4	0.9159	0.8669	0.3861	1.6467	0
3268	773.3	0.9162	0.8672	0.3864	1.6472	0
3269	773.2	0.9165	0.8672	0.3866	1.6474	0
3270	773.1	0.9163	0.8672	0.3865	1.6475	0
3271	773	0.9167	0.8674	0.3866	1.6483	0
3272	772.9	0.9166	0.8673	0.3867	1.6485	0
3273	772.8	0.9168	0.8673	0.3868	1.6477	0
3274	772.7	0.9169	0.8677	0.387	1.648	0
3275	772.6	0.9169	0.8677	0.3869	1.6481	0
3276	772.5	0.9171	0.8677	0.3869	1.6482	0
3277	772.4	0.917	0.8677	0.387	1.6483	0
3278	772.3	0.9174	0.8678	0.3872	1.6492	0
3279	772.2	0.9174	0.8679	0.3872	1.6489	0
3280	772.1	0.9174	0.8679	0.3872	1.6485	0
3281	772	0.9175	0.8681	0.3874	1.6493	0
3282	771.9	0.9177	0.868	0.3873	1.6489	0
3283	771.8	0.9177	0.8681	0.3874	1.6491	0
3284	771.7	0.9178	0.8682	0.3876	1.6498	0
3285	771.6	0.9177	0.8682	0.3873	1.6488	0
3286	771.5	0.9177	0.8683	0.3875	1.6491	0
3287	771.4	0.9178	0.8682	0.3874	1.6492	0
3288	771.3	0.918	0.8685	0.3876	1.65	0
3289	771.2	0.9181	0.8686	0.3876	1.6507	0
3290	771.1	0.9182	0.8686	0.3877	1.6504	0
3291	771	0.9183	0.8688	0.3876	1.6499	0
3292	770.9	0.9184	0.8686	0.3878	1.6502	0
3293	770.8	0.9184	0.8689	0.3878	1.6499	0
3294	770.7	0.9187	0.8691	0.3881	1.6513	0
3295	770.6	0.9187	0.869	0.3879	1.6503	0
3296	770.5	0.9186	0.869	0.388	1.6499	0
3297	770.4	0.9186	0.8689	0.3878	1.65	0

3298	770.3	0.9188	0.8693	0.388	1.6507	0
3299	770.2	0.9191	0.8694	0.388	1.6505	0
3300	770.1	0.9192	0.8695	0.3881	1.6501	0
3301	770	0.9193	0.8695	0.3881	1.6503	0
3302	769.9	0.919	0.8693	0.388	1.6508	0
3303	769.8	0.9192	0.8695	0.388	1.6505	0
3304	769.7	0.9196	0.8698	0.3881	1.6508	0
3305	769.6	0.9194	0.8696	0.3881	1.6509	0
3306	769.5	0.9195	0.8696	0.3881	1.6505	0
3307	769.4	0.9194	0.8697	0.3881	1.6507	0
3308	769.3	0.9195	0.8698	0.388	1.6508	0
3309	769.2	0.9196	0.8699	0.3881	1.651	0
3310	769.1	0.9197	0.8698	0.3878	1.6504	0
3311	769	0.9196	0.8699	0.388	1.6507	0
3312	768.9	0.9198	0.8701	0.3879	1.6504	0
3313	768.8	0.9197	0.8702	0.3878	1.6511	0
3314	768.7	0.9198	0.8699	0.3877	1.65	0
3315	768.6	0.9196	0.8701	0.3876	1.6501	0
3316	768.5	0.9195	0.87	0.3875	1.65	0
3317	768.4	0.9198	0.87	0.3874	1.6502	0
3318	768.3	0.9196	0.8702	0.3874	1.6499	0
3319	768.2	0.9198	0.8701	0.3874	1.65	0
3320	768.1	0.9198	0.8703	0.3874	1.6502	0
3321	768	0.9199	0.8703	0.3875	1.6503	0
3322	767.9	0.92	0.8703	0.3874	1.6499	0
3323	767.8	0.9201	0.8703	0.3875	1.6502	0
3324	767.7	0.9202	0.8704	0.3874	1.6497	0
3325	767.6	0.92	0.8704	0.3872	1.6502	0
3326	767.5	0.92	0.8705	0.3872	1.6498	0
3327	767.4	0.9198	0.8704	0.3871	1.6499	0
3328	767.3	0.9201	0.8704	0.3873	1.6496	0
3329	767.2	0.9201	0.8705	0.3871	1.6497	0
3330	767.1	0.9202	0.8706	0.3871	1.6498	0
3331	767	0.9203	0.8707	0.3871	1.65	0
3332	766.9	0.9203	0.8707	0.3871	1.6502	0
3333	766.8	0.9203	0.8708	0.3871	1.6503	0
3334	766.7	0.9203	0.8709	0.3871	1.6504	0
3335	766.6	0.9205	0.8708	0.3871	1.6511	0
3336	766.5	0.9204	0.871	0.3871	1.6501	0
3337	766.4	0.9205	0.871	0.3871	1.6503	0
3338	766.3	0.9207	0.871	0.3871	1.6504	0
3339	766.2	0.9207	0.8712	0.3871	1.6505	0
3340	766.1	0.9208	0.8712	0.3872	1.6503	0

3341	766	0.9209	0.8714	0.3872	1.6503	0
3342	765.9	0.9209	0.8713	0.387	1.6505	0
3343	765.8	0.9212	0.8716	0.3874	1.6508	0
3344	765.7	0.921	0.8715	0.3872	1.6502	0
3345	765.6	0.9211	0.8714	0.3872	1.651	0
3346	765.5	0.9213	0.8715	0.3874	1.6506	0
3347	765.4	0.9214	0.8719	0.3875	1.6508	0
3348	765.3	0.9214	0.8716	0.3874	1.6513	0
3349	765.2	0.9214	0.8718	0.3874	1.651	0
3350	765.1	0.9213	0.8717	0.3874	1.6506	0
3351	765	0.9216	0.8718	0.3873	1.6513	0
3352	764.9	0.9218	0.872	0.3876	1.651	0
3353	764.8	0.9219	0.8721	0.3876	1.6522	0
3354	764.7	0.9219	0.872	0.3877	1.6513	0
3355	764.6	0.922	0.8722	0.3877	1.6515	0
3356	764.5	0.9222	0.8722	0.3878	1.6517	0
3357	764.4	0.9222	0.8724	0.3878	1.6519	0
3358	764.3	0.9223	0.8723	0.3879	1.6526	0
3359	764.2	0.9224	0.8727	0.388	1.6528	0
3360	764.1	0.9225	0.8725	0.3878	1.6528	0
3361	764	0.9224	0.8725	0.388	1.6525	0
3362	763.9	0.9227	0.8726	0.3881	1.6527	0
3363	763.8	0.9227	0.8726	0.3882	1.6529	0
3364	763.7	0.9229	0.8729	0.3882	1.6536	0
3365	763.6	0.9231	0.8728	0.3882	1.6527	0
3366	763.5	0.923	0.8729	0.3883	1.6529	0
3367	763.4	0.9232	0.8729	0.3885	1.6531	0
3368	763.3	0.9234	0.8729	0.3885	1.6532	0
3369	763.2	0.9232	0.873	0.3884	1.6533	0
3370	763.1	0.9234	0.873	0.3885	1.6536	0
3371	763	0.9236	0.8734	0.3887	1.6539	0
3372	762.9	0.9237	0.8735	0.3888	1.6541	0
3373	762.8	0.9236	0.8735	0.3887	1.6542	0
3374	762.7	0.9239	0.8737	0.3889	1.6545	0
3375	762.6	0.9241	0.8738	0.3891	1.6542	0
3376	762.5	0.9241	0.8737	0.389	1.6543	0
3377	762.4	0.9242	0.8737	0.3891	1.6544	0
3378	762.3	0.9243	0.8737	0.3891	1.6541	0
3379	762.2	0.9244	0.8739	0.389	1.6553	0
3380	762.1	0.9245	0.8739	0.3892	1.6549	0
3381	762	0.9247	0.8741	0.3893	1.6546	0
3382	761.9	0.9248	0.8741	0.3894	1.6554	0
3383	761.8	0.9249	0.8743	0.3895	1.6555	0

3384	761.7	0.925	0.8742	0.3895	1.6556	0
3385	761.6	0.9251	0.8744	0.3895	1.6558	0
3386	761.5	0.9251	0.8746	0.3895	1.655	0
3387	761.4	0.9252	0.8746	0.3894	1.6555	0
3388	761.3	0.9253	0.8747	0.3896	1.6557	0
3389	761.2	0.9254	0.8749	0.3897	1.6559	0
3390	761.1	0.9254	0.8748	0.3896	1.6555	0
3391	761	0.9254	0.8747	0.3895	1.6555	0
3392	760.9	0.9255	0.8749	0.3896	1.6557	0
3393	760.8	0.9256	0.875	0.3896	1.6554	0
3394	760.7	0.9259	0.8754	0.3897	1.6561	0
3395	760.6	0.9257	0.8752	0.3896	1.6562	0
3396	760.5	0.9259	0.8752	0.3898	1.6569	0
3397	760.4	0.9261	0.8753	0.3898	1.6567	0
3398	760.3	0.9262	0.8754	0.3899	1.6563	0
3399	760.2	0.9261	0.8752	0.3898	1.6564	0
3400	760.1	0.9263	0.8755	0.3898	1.6561	0
3401	760	0.9263	0.8755	0.3897	1.6567	0
3402	759.9	0.9263	0.8756	0.3898	1.6564	0
3403	759.8	0.9264	0.8757	0.39	1.6567	0
3404	759.7	0.9266	0.8758	0.3899	1.6563	0
3405	759.6	0.9266	0.8758	0.3898	1.657	0
3406	759.5	0.9266	0.8759	0.3899	1.6572	0
3407	759.4	0.9268	0.876	0.39	1.657	0
3408	759.3	0.9268	0.8761	0.39	1.6577	0
3409	759.2	0.9269	0.8763	0.3901	1.6575	0
3410	759.1	0.927	0.8762	0.3901	1.6576	0
3411	759	0.9269	0.8762	0.3899	1.6571	0
3412	758.9	0.927	0.8761	0.3899	1.6571	0
3413	758.8	0.9271	0.8762	0.3899	1.6573	0
3414	758.7	0.9271	0.8761	0.3896	1.6574	0
3415	758.6	0.9271	0.8763	0.3898	1.6575	0
3416	758.5	0.9272	0.8764	0.3899	1.6573	0
3417	758.4	0.9274	0.8765	0.3899	1.657	0
3418	758.3	0.9275	0.8766	0.3899	1.6566	0
3419	758.2	0.9275	0.8765	0.3899	1.6571	0
3420	758.1	0.9275	0.8768	0.3899	1.6568	0
3421	758	0.9277	0.8767	0.3898	1.6574	0
3422	757.9	0.9277	0.8768	0.3899	1.6576	0
3423	757.8	0.9276	0.8767	0.3898	1.6577	0
3424	757.7	0.9278	0.877	0.3899	1.6574	0
3425	757.6	0.928	0.877	0.39	1.6575	0
3426	757.5	0.9279	0.8772	0.39	1.6576	0

3427	757.4	0.9281	0.8771	0.39	1.6573	0
3428	757.3	0.9281	0.8771	0.3899	1.6578	0
3429	757.2	0.928	0.8773	0.3898	1.658	0
3430	757.1	0.9284	0.8774	0.3901	1.6578	0
3431	757	0.9283	0.8773	0.39	1.6583	0
3432	756.9	0.9283	0.8774	0.39	1.658	0
3433	756.8	0.9284	0.8775	0.39	1.6581	0
3434	756.7	0.9285	0.8776	0.3901	1.6587	0
3435	756.6	0.9286	0.8776	0.3901	1.6583	0
3436	756.5	0.9286	0.8778	0.3902	1.6585	0
3437	756.4	0.9288	0.878	0.3902	1.6582	0
3438	756.3	0.9289	0.8777	0.3901	1.6588	0
3439	756.2	0.929	0.8778	0.3902	1.6589	0
3440	756.1	0.9289	0.8777	0.39	1.6583	0
3441	756	0.9289	0.878	0.3901	1.6586	0
3442	755.9	0.9291	0.8779	0.3902	1.6588	0
3443	755.8	0.9292	0.8783	0.3903	1.659	0
3444	755.7	0.9293	0.8783	0.3902	1.6585	0
3445	755.6	0.9293	0.8782	0.3902	1.6597	0
3446	755.5	0.9295	0.8784	0.3904	1.6589	0
3447	755.4	0.9298	0.8784	0.3906	1.6592	0
3448	755.3	0.9297	0.8787	0.3907	1.6598	0
3449	755.2	0.9298	0.8787	0.3905	1.6594	0
3450	755.1	0.9296	0.8788	0.3905	1.6595	0
3451	755	0.9299	0.8788	0.3905	1.6596	0
3452	754.9	0.93	0.8787	0.3906	1.6592	0
3453	754.8	0.9302	0.8791	0.3908	1.6601	0
3454	754.7	0.9304	0.879	0.3908	1.6602	0
3455	754.6	0.9302	0.8791	0.3907	1.6602	0
3456	754.5	0.9303	0.8791	0.3908	1.6604	0
3457	754.4	0.9304	0.8792	0.3908	1.66	0
3458	754.3	0.9306	0.8792	0.3907	1.6606	0
3459	754.2	0.9306	0.8794	0.3908	1.6608	0
3460	754.1	0.9309	0.8794	0.391	1.6605	0
3461	754	0.9307	0.8796	0.3909	1.661	0
3462	753.9	0.9311	0.8799	0.3912	1.6608	0
3463	753.8	0.9311	0.8797	0.3913	1.6615	0
3464	753.7	0.9311	0.8798	0.3912	1.6616	0
3465	753.6	0.9314	0.8799	0.3914	1.6618	0
3466	753.5	0.9315	0.8799	0.3913	1.6614	0
3467	753.4	0.9316	0.8798	0.3914	1.6621	0
3468	753.3	0.9317	0.8802	0.3915	1.6623	0
3469	753.2	0.9318	0.8803	0.3916	1.6624	0

3470	753.1	0.9318	0.8803	0.3915	1.6619	0
3471	753	0.9319	0.8803	0.3915	1.662	0
3472	752.9	0.9321	0.8805	0.3917	1.6618	0
3473	752.8	0.9322	0.8805	0.3916	1.6618	0
3474	752.7	0.9323	0.8805	0.3917	1.6626	0
3475	752.6	0.9322	0.8807	0.3918	1.6622	0
3476	752.5	0.9324	0.8809	0.3919	1.6624	0
3477	752.4	0.9325	0.8808	0.392	1.6625	0
3478	752.3	0.9326	0.8809	0.3918	1.6626	0
3479	752.2	0.9325	0.8809	0.3917	1.6627	0
3480	752.1	0.9327	0.8812	0.392	1.6625	0
3481	752	0.9328	0.8812	0.3919	1.6626	0
3482	751.9	0.9329	0.8812	0.3919	1.6627	0
3483	751.8	0.933	0.8812	0.3919	1.6632	0
3484	751.7	0.9329	0.8813	0.3918	1.6634	0
3485	751.6	0.933	0.8815	0.3919	1.6629	0
3486	751.5	0.9331	0.8816	0.3918	1.6631	0
3487	751.4	0.9332	0.8815	0.3918	1.6631	0
3488	751.3	0.9333	0.8816	0.3919	1.6629	0
3489	751.2	0.9333	0.8816	0.3918	1.6629	0
3490	751.1	0.9334	0.8817	0.3918	1.6635	0
3491	751	0.9334	0.8816	0.3918	1.6636	0
3492	750.9	0.9336	0.8818	0.3919	1.6633	0
3493	750.8	0.9336	0.882	0.3919	1.6634	0
3494	750.7	0.9337	0.882	0.3919	1.6635	0
3495	750.6	0.9336	0.882	0.3919	1.6641	0
3496	750.5	0.934	0.8822	0.3919	1.6633	0
3497	750.4	0.9339	0.8823	0.3921	1.6634	0
3498	750.3	0.934	0.8823	0.3919	1.664	0
3499	750.2	0.9341	0.8824	0.3919	1.6636	0
3500	750.1	0.9342	0.8826	0.3919	1.6643	0
3501	750	0.9342	0.8827	0.3918	1.6643	0
3502	749.9	0.9344	0.8825	0.3919	1.664	0
3503	749.8	0.9344	0.8826	0.3918	1.6641	0
3504	749.7	0.9346	0.883	0.3921	1.6643	0
3505	749.6	0.9347	0.883	0.3921	1.6639	0
3506	749.5	0.9347	0.8831	0.392	1.6645	0
3507	749.4	0.9349	0.8832	0.3921	1.6647	0
3508	749.3	0.9348	0.8832	0.3921	1.6647	0
3509	749.2	0.9347	0.8832	0.3919	1.6643	0
3510	749.1	0.9348	0.8833	0.392	1.6639	0
3511	749	0.9349	0.8834	0.3921	1.6641	0
3512	748.9	0.935	0.8835	0.3919	1.6637	0

3513	748.8	0.9351	0.8838	0.392	1.6649	0
3514	748.7	0.9354	0.8836	0.3919	1.6645	0
3515	748.6	0.9352	0.8837	0.3918	1.6646	0
3516	748.5	0.9355	0.8839	0.3921	1.6647	0
3517	748.4	0.9355	0.8839	0.392	1.6643	0
3518	748.3	0.9355	0.8838	0.3921	1.6649	0
3519	748.2	0.9357	0.884	0.3921	1.665	0
3520	748.1	0.9357	0.8841	0.392	1.6646	0
3521	748	0.9357	0.8842	0.392	1.6643	0
3522	747.9	0.936	0.8846	0.3922	1.665	0
3523	747.8	0.9358	0.8842	0.3921	1.665	0
3524	747.7	0.9361	0.8846	0.3923	1.6647	0
3525	747.6	0.9361	0.8846	0.3923	1.6653	0
3526	747.5	0.9363	0.8847	0.3923	1.6654	0
3527	747.4	0.9364	0.8848	0.3921	1.6655	0
3528	747.3	0.9365	0.8849	0.3922	1.6652	0
3529	747.2	0.9364	0.885	0.3921	1.6661	0
3530	747.1	0.9366	0.885	0.3922	1.6659	0
3531	747	0.9368	0.8851	0.3923	1.6655	0
3532	746.9	0.9371	0.8853	0.3925	1.6658	0
3533	746.8	0.9372	0.8855	0.3927	1.6665	0
3534	746.7	0.9372	0.8856	0.3926	1.6666	0
3535	746.6	0.9374	0.8857	0.3925	1.6661	0
3536	746.5	0.9373	0.8856	0.3925	1.6666	0
3537	746.4	0.9376	0.8858	0.3926	1.6664	0
3538	746.3	0.9376	0.8861	0.3927	1.6665	0
3539	746.2	0.9377	0.8862	0.3929	1.6667	0
3540	746.1	0.9379	0.8863	0.3929	1.6674	0
3541	746	0.938	0.8863	0.3929	1.6675	0
3542	745.9	0.938	0.8862	0.393	1.6675	0
3543	745.8	0.9381	0.8864	0.393	1.6677	0
3544	745.7	0.9384	0.8867	0.3931	1.6679	0
3545	745.6	0.9383	0.8867	0.3932	1.6675	0
3546	745.5	0.9387	0.8868	0.3933	1.6681	0
3547	745.4	0.9386	0.8868	0.3932	1.6678	0
3548	745.3	0.9389	0.887	0.3935	1.6679	0
3549	745.2	0.939	0.887	0.3934	1.669	0
3550	745.1	0.9392	0.8875	0.3934	1.6682	0
3551	745	0.9392	0.8872	0.3936	1.6688	0
3552	744.9	0.9394	0.8876	0.3936	1.669	0
3553	744.8	0.9395	0.8875	0.3937	1.6686	0
3554	744.7	0.9397	0.8878	0.3937	1.6692	0
3555	744.6	0.9396	0.8878	0.3937	1.6688	0

3556	744.5	0.94	0.8881	0.3938	1.6695	0
3557	744.4	0.94	0.8882	0.394	1.6702	0
3558	744.3	0.9402	0.8883	0.394	1.6699	0
3559	744.2	0.9402	0.8885	0.3942	1.67	0
3560	744.1	0.9404	0.8885	0.3941	1.6696	0
3561	744	0.9406	0.8886	0.3943	1.6703	0
3562	743.9	0.9405	0.8887	0.3943	1.6693	0
3563	743.8	0.9408	0.8889	0.3945	1.6706	0
3564	743.7	0.9409	0.8889	0.3944	1.6706	0
3565	743.6	0.9411	0.889	0.3944	1.6702	0
3566	743.5	0.941	0.8891	0.3945	1.6703	0
3567	743.4	0.9412	0.8891	0.3946	1.6714	0
3568	743.3	0.9414	0.8893	0.3946	1.6706	0
3569	743.2	0.9413	0.8895	0.3947	1.6707	0
3570	743.1	0.9415	0.8895	0.3946	1.6713	0
3571	743	0.9417	0.8896	0.3946	1.6714	0
3572	742.9	0.9418	0.8898	0.3947	1.6705	0
3573	742.8	0.9418	0.8898	0.3947	1.6716	0
3574	742.7	0.9421	0.89	0.3947	1.6716	0
3575	742.6	0.9419	0.8901	0.3948	1.6713	0
3576	742.5	0.9421	0.8903	0.3948	1.6714	0
3577	742.4	0.9422	0.8903	0.3948	1.672	0
3578	742.3	0.9423	0.8902	0.3947	1.672	0
3579	742.2	0.9424	0.8907	0.3947	1.6717	0
3580	742.1	0.9424	0.8905	0.3948	1.6717	0
3581	742	0.9426	0.8906	0.3948	1.6718	0
3582	741.9	0.9426	0.8908	0.3947	1.6724	0
3583	741.8	0.9429	0.891	0.3948	1.672	0
3584	741.7	0.9429	0.8911	0.3948	1.6726	0
3585	741.6	0.943	0.8911	0.3949	1.6717	0
3586	741.5	0.9431	0.8911	0.3949	1.6724	0
3587	741.4	0.9433	0.8913	0.3948	1.6724	0
3588	741.3	0.9432	0.8913	0.3948	1.6725	0
3589	741.2	0.9435	0.8915	0.3949	1.6721	0
3590	741.1	0.9435	0.8914	0.3947	1.6721	0
3591	741	0.9434	0.8915	0.3946	1.6726	0
3592	740.9	0.9438	0.8917	0.3948	1.6723	0
3593	740.8	0.9438	0.8918	0.3947	1.6729	0
3594	740.7	0.9438	0.8919	0.3946	1.6729	0
3595	740.6	0.9439	0.8919	0.3946	1.672	0
3596	740.5	0.944	0.8921	0.3944	1.6721	0
3597	740.4	0.9439	0.8921	0.3945	1.6731	0
3598	740.3	0.9441	0.8922	0.3945	1.6727	0

3599	740.2	0.9442	0.8924	0.3946	1.6729	0
3600	740.1	0.9445	0.893	0.3946	1.673	0
3601	740	0.9444	0.8928	0.3946	1.6725	0
3602	739.9	0.9446	0.8929	0.3947	1.6727	0
3603	739.8	0.9447	0.893	0.3947	1.6728	0
3604	739.7	0.9448	0.8931	0.3948	1.6724	0
3605	739.6	0.9449	0.8932	0.3945	1.6734	0
3606	739.5	0.9448	0.8931	0.3944	1.6729	0
3607	739.4	0.9451	0.8932	0.3944	1.673	0
3608	739.3	0.9451	0.8935	0.3945	1.6726	0
3609	739.2	0.9453	0.8936	0.3945	1.6727	0
3610	739.1	0.9454	0.8937	0.3945	1.6732	0
3611	739	0.9456	0.8938	0.3945	1.6723	0
3612	738.9	0.9456	0.8937	0.3944	1.6724	0
3613	738.8	0.9455	0.8938	0.3943	1.6735	0
3614	738.7	0.9456	0.8939	0.3941	1.6729	0
3615	738.6	0.9458	0.8941	0.3943	1.6731	0
3616	738.5	0.9458	0.8942	0.3942	1.6736	0
3617	738.4	0.946	0.8943	0.3942	1.6732	0
3618	738.3	0.9461	0.8945	0.3942	1.6728	0
3619	738.2	0.9461	0.8946	0.3942	1.6734	0
3620	738.1	0.9463	0.8946	0.3941	1.6729	0
3621	738	0.9463	0.8947	0.3942	1.6735	0
3622	737.9	0.9464	0.8948	0.3942	1.6726	0
3623	737.8	0.9463	0.8948	0.394	1.6736	0
3624	737.7	0.9466	0.8949	0.3941	1.6738	0
3625	737.6	0.9468	0.895	0.3943	1.6734	0
3626	737.5	0.9469	0.895	0.3942	1.6735	0
3627	737.4	0.947	0.8953	0.3942	1.674	0
3628	737.3	0.9471	0.8953	0.3941	1.674	0
3629	737.2	0.9472	0.8956	0.3943	1.6743	0
3630	737.1	0.9474	0.8956	0.3943	1.6739	0
3631	737	0.9477	0.8958	0.3946	1.6741	0
3632	736.9	0.9476	0.8958	0.3946	1.6741	0
3633	736.8	0.9477	0.896	0.3945	1.6747	0
3634	736.7	0.9478	0.8961	0.3946	1.6748	0
3635	736.6	0.948	0.8963	0.3946	1.6744	0
3636	736.5	0.9481	0.8962	0.3948	1.675	0
3637	736.4	0.9484	0.8965	0.3949	1.6748	0
3638	736.3	0.9487	0.8965	0.3951	1.6754	0
3639	736.2	0.9487	0.8966	0.3951	1.6755	0
3640	736.1	0.9491	0.8968	0.3951	1.6761	0
3641	736	0.9492	0.8969	0.3952	1.6758	0

3642	735.9	0.9493	0.8969	0.3956	1.6764	0
3643	735.8	0.9495	0.897	0.3955	1.6765	0
3644	735.7	0.9496	0.8973	0.3955	1.6762	0
3645	735.6	0.9499	0.8975	0.3957	1.6764	0
3646	735.5	0.95	0.8975	0.3957	1.6764	0
3647	735.4	0.9501	0.8975	0.3957	1.6776	0
3648	735.3	0.9505	0.8978	0.3959	1.6772	0
3649	735.2	0.9506	0.8979	0.3961	1.6773	0
3650	735.1	0.9506	0.8981	0.3961	1.6773	0
3651	735	0.9508	0.8981	0.3962	1.6775	0
3652	734.9	0.951	0.898	0.3962	1.6781	0
3653	734.8	0.9512	0.8984	0.3963	1.6783	0
3654	734.7	0.9513	0.8983	0.3966	1.6784	0
3655	734.6	0.9515	0.8983	0.3967	1.6786	0
3656	734.5	0.9517	0.8985	0.3966	1.6791	0
3657	734.4	0.9519	0.8987	0.3968	1.6793	0
3658	734.3	0.9518	0.8987	0.3969	1.6789	0
3659	734.2	0.952	0.8988	0.3969	1.6795	0
3660	734.1	0.952	0.8988	0.3971	1.6801	0
3661	734	0.9525	0.899	0.3971	1.6797	0
3662	733.9	0.9525	0.8991	0.3972	1.6803	0
3663	733.8	0.9527	0.8992	0.3972	1.6804	0
3664	733.7	0.9529	0.8994	0.3974	1.6806	0
3665	733.6	0.9528	0.8995	0.3974	1.6806	0
3666	733.5	0.953	0.8994	0.3974	1.6807	0
3667	733.4	0.9533	0.8996	0.3976	1.6809	0
3668	733.3	0.9533	0.8997	0.3975	1.6814	0
3669	733.2	0.9535	0.8998	0.3975	1.681	0
3670	733.1	0.9534	0.8998	0.3976	1.6811	0
3671	733	0.9536	0.8997	0.3977	1.6816	0
3672	732.9	0.9537	0.8998	0.3975	1.6816	0
3673	732.8	0.9538	0.9002	0.3977	1.6812	0
3674	732.7	0.9539	0.9	0.3978	1.6819	0
3675	732.6	0.9539	0.9001	0.3977	1.682	0
3676	732.5	0.9542	0.9002	0.3978	1.6816	0
3677	732.4	0.9542	0.9002	0.3979	1.6821	0
3678	732.3	0.9545	0.9005	0.3979	1.6818	0
3679	732.2	0.9544	0.9007	0.3979	1.6818	0
3680	732.1	0.9546	0.9007	0.3979	1.6819	0
3681	732	0.9548	0.9007	0.3977	1.6829	0
3682	731.9	0.9547	0.9008	0.3979	1.682	0
3683	731.8	0.9548	0.9008	0.3978	1.6825	0
3684	731.7	0.9548	0.9009	0.3979	1.6821	0

3685	731.6	0.9551	0.9008	0.398	1.6832	0
3686	731.5	0.9552	0.9012	0.3981	1.6828	0
3687	731.4	0.9554	0.9012	0.3981	1.6825	0
3688	731.3	0.9553	0.9015	0.3979	1.6829	0
3689	731.2	0.9554	0.9013	0.3979	1.6824	0
3690	731.1	0.9553	0.9013	0.3979	1.683	0
3691	731	0.9554	0.9014	0.3979	1.684	0
3692	730.9	0.9555	0.9014	0.3978	1.683	0
3693	730.8	0.9555	0.9014	0.3978	1.683	0
3694	730.7	0.9557	0.9015	0.3978	1.6826	0
3695	730.6	0.9555	0.9014	0.3977	1.6836	0
3696	730.5	0.9556	0.9016	0.3978	1.6826	0
3697	730.4	0.9558	0.9016	0.3979	1.6832	0
3698	730.3	0.9558	0.9018	0.3977	1.6833	0
3699	730.2	0.9559	0.9018	0.3976	1.6839	0
3700	730.1	0.9559	0.9019	0.3978	1.6839	0
3701	730	0.9561	0.9019	0.3977	1.6829	0
3702	729.9	0.9561	0.902	0.3976	1.6835	0
3703	729.8	0.9562	0.9021	0.3976	1.6835	0
3704	729.7	0.9562	0.902	0.3974	1.6826	0
3705	729.6	0.9562	0.902	0.3973	1.6835	0
3706	729.5	0.9561	0.9022	0.3973	1.6835	0
3707	729.4	0.9562	0.902	0.3972	1.683	0
3708	729.3	0.9561	0.9021	0.3973	1.683	0
3709	729.2	0.9563	0.9021	0.3972	1.683	0
3710	729.1	0.9563	0.9024	0.3972	1.6828	0
3711	729	0.9564	0.9025	0.3973	1.6828	0
3712	728.9	0.9564	0.9024	0.3971	1.6837	0
3713	728.8	0.9565	0.9024	0.3972	1.6833	0
3714	728.7	0.9566	0.9025	0.3971	1.6834	0
3715	728.6	0.9568	0.9026	0.3971	1.6829	0
3716	728.5	0.9566	0.9026	0.3968	1.6823	0
3717	728.4	0.9566	0.9027	0.3968	1.6828	0
3718	728.3	0.9567	0.9027	0.3968	1.6834	0
3719	728.2	0.9568	0.9028	0.3968	1.6828	0
3720	728.1	0.9569	0.9028	0.3968	1.683	0
3721	728	0.9569	0.9027	0.3967	1.6829	0
3722	727.9	0.9571	0.9031	0.3969	1.6836	0
3723	727.8	0.957	0.9029	0.3968	1.6835	0
3724	727.7	0.9569	0.9027	0.3967	1.6835	0
3725	727.6	0.957	0.9029	0.3967	1.6831	0
3726	727.5	0.9571	0.9032	0.3967	1.6832	0
3727	727.4	0.9573	0.9034	0.3967	1.6832	0

3728	727.3	0.9574	0.9033	0.3968	1.6833	0
3729	727.2	0.9575	0.9034	0.3968	1.6839	0
3730	727.1	0.9575	0.9033	0.3966	1.6834	0
3731	727	0.9575	0.9032	0.3965	1.6843	0
3732	726.9	0.9576	0.9035	0.3967	1.6839	0
3733	726.8	0.9576	0.9034	0.3966	1.6839	0
3734	726.7	0.9578	0.9035	0.3968	1.6841	0
3735	726.6	0.9578	0.9037	0.3968	1.6841	0
3736	726.5	0.958	0.9035	0.3968	1.6842	0
3737	726.4	0.958	0.9036	0.3967	1.6837	0
3738	726.3	0.9581	0.9038	0.3967	1.6842	0
3739	726.2	0.9581	0.9038	0.3967	1.6842	0
3740	726.1	0.9583	0.9039	0.3967	1.6848	0
3741	726	0.9583	0.904	0.3969	1.6844	0
3742	725.9	0.9586	0.904	0.3971	1.6851	0
3743	725.8	0.9587	0.9041	0.3971	1.6846	0
3744	725.7	0.9588	0.9042	0.3972	1.6847	0
3745	725.6	0.959	0.9042	0.3972	1.6853	0
3746	725.5	0.959	0.9043	0.3973	1.6853	0
3747	725.4	0.9594	0.9045	0.3974	1.686	0
3748	725.3	0.9595	0.9045	0.3974	1.6855	0
3749	725.2	0.9594	0.9043	0.3974	1.6864	0
3750	725.1	0.9595	0.9046	0.3974	1.6861	0
3751	725	0.9597	0.9047	0.3977	1.6862	0
3752	724.9	0.9597	0.9049	0.3978	1.6873	0
3753	724.8	0.9601	0.9049	0.3981	1.687	0
3754	724.7	0.96	0.9049	0.3981	1.687	0
3755	724.6	0.9602	0.9051	0.3982	1.6871	0
3756	724.5	0.9605	0.9054	0.3986	1.6873	0
3757	724.4	0.9607	0.9052	0.3987	1.6879	0
3758	724.3	0.9608	0.9055	0.3987	1.688	0
3759	724.2	0.9611	0.9056	0.3988	1.6877	0
3760	724.1	0.9611	0.9056	0.3991	1.6887	0
3761	724	0.9613	0.9056	0.399	1.6893	0
3762	723.9	0.9613	0.9056	0.3992	1.6893	0
3763	723.8	0.9617	0.906	0.3995	1.6896	0
3764	723.7	0.9619	0.9058	0.3997	1.6901	0
3765	723.6	0.9619	0.9059	0.3996	1.6897	0
3766	723.5	0.9621	0.9061	0.3998	1.6898	0
3767	723.4	0.9624	0.9063	0.3999	1.6899	0
3768	723.3	0.9624	0.9064	0.4001	1.69	0
3769	723.2	0.9625	0.9064	0.4	1.69	0
3770	723.1	0.9626	0.9064	0.4001	1.6906	0

3771	723	0.9625	0.9064	0.4	1.6905	0
3772	722.9	0.9628	0.9066	0.4003	1.6907	0
3773	722.8	0.9629	0.9065	0.4003	1.6907	0
3774	722.7	0.963	0.9066	0.4005	1.6908	0
3775	722.6	0.9632	0.9069	0.4004	1.6914	0
3776	722.5	0.9634	0.9068	0.4006	1.6911	0
3777	722.4	0.9633	0.9069	0.4005	1.6915	0
3778	722.3	0.9635	0.9073	0.4006	1.6917	0
3779	722.2	0.9635	0.9071	0.4006	1.6916	0
3780	722.1	0.9636	0.9071	0.4006	1.6921	0
3781	722	0.9638	0.9074	0.4009	1.6918	0
3782	721.9	0.964	0.9074	0.4007	1.6918	0
3783	721.8	0.9639	0.9074	0.4006	1.6917	0
3784	721.7	0.964	0.9076	0.4009	1.6923	0
3785	721.6	0.9641	0.9077	0.4009	1.6924	0
3786	721.5	0.9643	0.9077	0.4009	1.6924	0
3787	721.4	0.9642	0.9075	0.4008	1.6923	0
3788	721.3	0.9643	0.9078	0.4009	1.6919	0
3789	721.2	0.9644	0.9076	0.4009	1.6924	0
3790	721.1	0.9646	0.908	0.401	1.6924	0
3791	721	0.9648	0.9081	0.401	1.6925	0
3792	720.9	0.9647	0.9081	0.401	1.6926	0
3793	720.8	0.9647	0.908	0.401	1.6925	0
3794	720.7	0.9649	0.9082	0.401	1.693	0
3795	720.6	0.965	0.9083	0.4011	1.6931	0
3796	720.5	0.9651	0.9083	0.4012	1.6931	0
3797	720.4	0.965	0.908	0.4012	1.6926	0
3798	720.3	0.9652	0.9084	0.4012	1.6927	0
3799	720.2	0.9653	0.9083	0.4011	1.6936	0
3800	720.1	0.9653	0.9086	0.401	1.6932	0
3801	720	0.9653	0.9085	0.401	1.6931	0
3802	719.9	0.9655	0.9087	0.4011	1.6926	0
3803	719.8	0.9655	0.9086	0.4011	1.6926	0
3804	719.7	0.9658	0.9087	0.4011	1.6931	0
3805	719.6	0.9657	0.9087	0.4009	1.6931	0
3806	719.5	0.9658	0.9088	0.4008	1.6931	0
3807	719.4	0.9657	0.909	0.4008	1.6931	0
3808	719.3	0.966	0.9091	0.4009	1.6928	0
3809	719.2	0.9658	0.9091	0.4008	1.6931	0
3810	719.1	0.9659	0.9091	0.4007	1.6926	0
3811	719	0.9658	0.909	0.4006	1.693	0
3812	718.9	0.966	0.9091	0.4007	1.6925	0
3813	718.8	0.9661	0.9093	0.4007	1.693	0

3814	718.7	0.966	0.9092	0.4005	1.6924	0
3815	718.6	0.9662	0.9093	0.4004	1.693	0
3816	718.5	0.9659	0.9093	0.4003	1.6929	0
3817	718.4	0.9661	0.9092	0.4003	1.6928	0
3818	718.3	0.9661	0.9093	0.4002	1.6928	0
3819	718.2	0.9662	0.9094	0.4003	1.6928	0
3820	718.1	0.9662	0.9094	0.4	1.6917	0
3821	718	0.9659	0.9094	0.3999	1.6926	0
3822	717.9	0.9662	0.9094	0.4	1.6932	0
3823	717.8	0.9663	0.9095	0.4	1.6927	0
3824	717.7	0.9665	0.9096	0.3999	1.6927	0
3825	717.6	0.9664	0.9097	0.4	1.6926	0
3826	717.5	0.9665	0.9096	0.3997	1.6921	0
3827	717.4	0.9665	0.9096	0.3998	1.6926	0
3828	717.3	0.9666	0.9096	0.3996	1.692	0
3829	717.2	0.9667	0.9099	0.3996	1.6921	0
3830	717.1	0.9666	0.9097	0.3996	1.6925	0
3831	717	0.9667	0.9098	0.3996	1.692	0
3832	716.9	0.9669	0.9103	0.3998	1.6926	0
3833	716.8	0.9668	0.91	0.3996	1.692	0
3834	716.7	0.9669	0.9101	0.3995	1.692	0
3835	716.6	0.967	0.9104	0.3997	1.6925	0
3836	716.5	0.9671	0.9103	0.3998	1.6925	0
3837	716.4	0.9671	0.9102	0.3997	1.693	0
3838	716.3	0.9671	0.9103	0.3997	1.6924	0
3839	716.2	0.9673	0.9103	0.3998	1.6925	0
3840	716.1	0.9674	0.9105	0.3999	1.693	0
3841	716	0.9677	0.9107	0.4	1.6931	0
3842	715.9	0.9678	0.9108	0.4001	1.6937	0
3843	715.8	0.9677	0.9107	0.3999	1.6931	0
3844	715.7	0.9676	0.9107	0.4	1.693	0
3845	715.6	0.9677	0.9108	0.3998	1.6934	0
3846	715.5	0.9678	0.9109	0.3999	1.6934	0
3847	715.4	0.9679	0.9108	0.4	1.6935	0
3848	715.3	0.968	0.9111	0.3998	1.6935	0
3849	715.2	0.9683	0.9113	0.4001	1.6936	0
3850	715.1	0.9684	0.9112	0.4001	1.694	0
3851	715	0.9684	0.9112	0.4002	1.6935	0
3852	714.9	0.9685	0.9112	0.4001	1.6934	0
3853	714.8	0.9686	0.9115	0.4003	1.694	0
3854	714.7	0.9687	0.9114	0.4003	1.694	0
3855	714.6	0.9691	0.9117	0.4005	1.6947	0
3856	714.5	0.9691	0.9119	0.4005	1.6952	0

3857	714.4	0.9692	0.9117	0.4006	1.6942	0
3858	714.3	0.9693	0.912	0.4007	1.6957	0
3859	714.2	0.9697	0.912	0.4009	1.6948	0
3860	714.1	0.9698	0.9121	0.4011	1.6953	0
3861	714	0.9699	0.9122	0.4011	1.6958	0
3862	713.9	0.9698	0.9121	0.4011	1.6958	0
3863	713.8	0.9701	0.9125	0.4013	1.6959	0
3864	713.7	0.9704	0.9127	0.4015	1.6965	0
3865	713.6	0.9704	0.9128	0.4016	1.696	0
3866	713.5	0.9705	0.9127	0.4018	1.6965	0
3867	713.4	0.9706	0.9129	0.4018	1.6966	0
3868	713.3	0.9707	0.913	0.4019	1.6971	0
3869	713.2	0.971	0.913	0.4021	1.697	0
3870	713.1	0.9711	0.9131	0.4021	1.6971	0
3871	713	0.9712	0.9132	0.4022	1.6971	0
3872	712.9	0.9713	0.9133	0.4023	1.6976	0
3873	712.8	0.9716	0.9136	0.4027	1.6978	0
3874	712.7	0.9717	0.9136	0.4027	1.6977	0
3875	712.6	0.972	0.9138	0.4027	1.6978	0
3876	712.5	0.9719	0.914	0.4027	1.6982	0
3877	712.4	0.9721	0.9139	0.4028	1.6982	0
3878	712.3	0.9722	0.9138	0.4029	1.6987	0
3879	712.2	0.9724	0.9141	0.403	1.6982	0
3880	712.1	0.9723	0.9142	0.4031	1.6987	0
3881	712	0.9726	0.9141	0.4031	1.6981	0
3882	711.9	0.9725	0.9142	0.4032	1.6992	0
3883	711.8	0.9727	0.9144	0.4033	1.6991	0
3884	711.7	0.9729	0.9145	0.4034	1.6992	0
3885	711.6	0.973	0.9147	0.4033	1.6991	0
3886	711.5	0.9729	0.9145	0.4033	1.699	0
3887	711.4	0.9732	0.9147	0.4033	1.6996	0
3888	711.3	0.9733	0.9147	0.4035	1.6996	0
3889	711.2	0.9734	0.9148	0.4034	1.7	0
3890	711.1	0.9737	0.9149	0.4036	1.6995	0
3891	711	0.9736	0.9151	0.4037	1.699	0
3892	710.9	0.9737	0.9153	0.4038	1.7	0
3893	710.8	0.9739	0.9152	0.404	1.6994	0
3894	710.7	0.974	0.9152	0.404	1.6999	0
3895	710.6	0.974	0.9153	0.404	1.7009	0
3896	710.5	0.9741	0.9155	0.4039	1.7004	0
3897	710.4	0.9743	0.9154	0.404	1.7004	0
3898	710.3	0.9745	0.9158	0.4041	1.7004	0
3899	710.2	0.9747	0.9161	0.4041	1.7004	0

3900	710.1	0.9746	0.9158	0.4038	1.7008	0
3901	710	0.9747	0.9159	0.4041	1.7008	0
3902	709.9	0.9748	0.916	0.404	1.7012	0
3903	709.8	0.9749	0.9163	0.4041	1.7006	0
3904	709.7	0.9749	0.9162	0.4039	1.7005	0
3905	709.6	0.9749	0.9162	0.404	1.7004	0
3906	709.5	0.975	0.9162	0.4041	1.7009	0
3907	709.4	0.9752	0.9164	0.404	1.7009	0
3908	709.3	0.9754	0.9165	0.404	1.7003	0
3909	709.2	0.9752	0.9164	0.4039	1.7007	0
3910	709.1	0.9754	0.9166	0.4041	1.7008	0
3911	709	0.9754	0.9168	0.404	1.7016	0
3912	708.9	0.9754	0.9167	0.4039	1.701	0
3913	708.8	0.9757	0.917	0.404	1.7016	0
3914	708.7	0.9756	0.9169	0.4038	1.7014	0
3915	708.6	0.9759	0.9171	0.4038	1.7014	0
3916	708.5	0.9759	0.9171	0.4037	1.7009	0
3917	708.4	0.9758	0.9171	0.4036	1.7012	0
3918	708.3	0.9759	0.9169	0.4036	1.7011	0
3919	708.2	0.976	0.9171	0.4037	1.701	0
3920	708.1	0.976	0.9174	0.4037	1.7015	0
3921	708	0.9762	0.9173	0.4034	1.7009	0
3922	707.9	0.9763	0.9175	0.4035	1.7013	0
3923	707.8	0.9762	0.9176	0.4035	1.7007	0
3924	707.7	0.9762	0.9176	0.4034	1.7011	0
3925	707.6	0.9764	0.9175	0.4033	1.7015	0
3926	707.5	0.9765	0.9178	0.4033	1.701	0
3927	707.4	0.9762	0.9179	0.4033	1.7013	0
3928	707.3	0.9764	0.9179	0.4033	1.7008	0
3929	707.2	0.9764	0.918	0.4031	1.7007	0
3930	707.1	0.9766	0.918	0.4032	1.7006	0
3931	707	0.9765	0.9182	0.4033	1.7016	0
3932	706.9	0.9766	0.9182	0.4032	1.7015	0
3933	706.8	0.9771	0.9182	0.4033	1.7014	0
3934	706.7	0.977	0.9187	0.4034	1.7015	0
3935	706.6	0.9771	0.9185	0.4032	1.7019	0
3936	706.5	0.977	0.9184	0.4031	1.7012	0
3937	706.4	0.9772	0.9186	0.4032	1.7011	0
3938	706.3	0.9774	0.9188	0.4034	1.7012	0
3939	706.2	0.9775	0.9191	0.4034	1.7016	0
3940	706.1	0.9773	0.9188	0.4031	1.7018	0
3941	706	0.9773	0.9188	0.4032	1.7017	0
3942	705.9	0.9774	0.9189	0.4032	1.7017	0

3943	705.8	0.9776	0.919	0.4032	1.701	0
3944	705.7	0.9776	0.919	0.4032	1.7015	0
3945	705.6	0.9776	0.9192	0.4031	1.7019	0
3946	705.5	0.9778	0.9196	0.4032	1.7019	0
3947	705.4	0.978	0.9196	0.4033	1.7023	0
3948	705.3	0.978	0.9195	0.4032	1.7027	0
3949	705.2	0.9784	0.9197	0.4034	1.7033	0
3950	705.1	0.9782	0.9196	0.4033	1.7026	0
3951	705	0.9783	0.9196	0.4032	1.7025	0
3952	704.9	0.9786	0.9199	0.4035	1.7025	0
3953	704.8	0.9786	0.9202	0.4038	1.7031	0
3954	704.7	0.9787	0.92	0.4037	1.7029	0
3955	704.6	0.979	0.9203	0.4039	1.7029	0
3956	704.5	0.9791	0.9203	0.4038	1.7027	0
3957	704.4	0.9791	0.9205	0.4037	1.7032	0
3958	704.3	0.9791	0.9203	0.4037	1.7036	0
3959	704.2	0.9794	0.9205	0.4039	1.7031	0
3960	704.1	0.9796	0.9208	0.4041	1.7041	0
3961	704	0.9798	0.921	0.4042	1.704	0
3962	703.9	0.9798	0.921	0.4043	1.704	0
3963	703.8	0.98	0.921	0.4043	1.7049	0
3964	703.7	0.98	0.9211	0.4043	1.7048	0
3965	703.6	0.9803	0.9213	0.4044	1.7048	0
3966	703.5	0.9806	0.9214	0.4045	1.7053	0
3967	703.4	0.9806	0.9214	0.4045	1.7046	0
3968	703.3	0.9807	0.9215	0.4047	1.705	0
3969	703.2	0.981	0.9216	0.4048	1.7055	0
3970	703.1	0.981	0.9218	0.4048	1.7054	0
3971	703	0.9812	0.9218	0.405	1.7049	0
3972	702.9	0.9814	0.922	0.405	1.7059	0
3973	702.8	0.9814	0.9221	0.4052	1.7052	0
3974	702.7	0.9814	0.9223	0.4051	1.7056	0
3975	702.6	0.9815	0.9223	0.4052	1.7061	0
3976	702.5	0.9815	0.9225	0.4052	1.7064	0
3977	702.4	0.9816	0.9225	0.4051	1.7063	0
3978	702.3	0.982	0.9228	0.4055	1.7064	0
3979	702.2	0.982	0.9226	0.4053	1.7062	0
3980	702.1	0.9822	0.9228	0.4053	1.7066	0
3981	702	0.9822	0.9229	0.4055	1.7071	0
3982	701.9	0.9824	0.9231	0.4054	1.7069	0
3983	701.8	0.9825	0.9235	0.4056	1.7074	0
3984	701.7	0.9827	0.9234	0.4057	1.7073	0
3985	701.6	0.9827	0.9233	0.4056	1.707	0

3986	701.5	0.9827	0.9235	0.4057	1.7075	0
3987	701.4	0.9829	0.9236	0.4057	1.7079	0
3988	701.3	0.9831	0.9237	0.4058	1.7073	0
3989	701.2	0.9832	0.9238	0.4059	1.7077	0
3990	701.1	0.9834	0.9242	0.4059	1.7083	0
3991	701	0.9833	0.924	0.406	1.7081	0
3992	700.9	0.9836	0.9242	0.406	1.7085	0
3993	700.8	0.9837	0.9242	0.4061	1.7083	0
3994	700.7	0.9839	0.9245	0.406	1.7083	0
3995	700.6	0.984	0.9246	0.4061	1.7082	0
3996	700.5	0.9841	0.9246	0.4061	1.7085	0
3997	700.4	0.9842	0.9246	0.406	1.7083	0
3998	700.3	0.9842	0.9247	0.4062	1.7087	0
3999	700.2	0.9845	0.9249	0.4061	1.7091	0
4000	700.1	0.9846	0.9249	0.4062	1.7095	0
4001	700	0.9846	0.925	0.4062	1.7084	0
4002	699.9	0.9847	0.9253	0.4064	1.7094	0
4003	699.8	0.9848	0.9253	0.4065	1.7092	0
4004	699.7	0.9849	0.9254	0.4064	1.7101	0
4005	699.6	0.985	0.9255	0.4062	1.7094	0
4006	699.5	0.9852	0.9256	0.4064	1.7099	0
4007	699.4	0.9854	0.9259	0.4065	1.7098	0
4008	699.3	0.9855	0.926	0.4066	1.7096	0
4009	699.2	0.9856	0.9259	0.4067	1.7101	0
4010	699.1	0.9858	0.9262	0.4065	1.7099	0
4011	699	0.9858	0.9263	0.4065	1.7098	0
4012	698.9	0.9857	0.9262	0.4064	1.7101	0
4013	698.8	0.986	0.9264	0.4065	1.71	0
4014	698.7	0.9863	0.9266	0.4065	1.7104	0
4015	698.6	0.9862	0.9268	0.4067	1.7108	0
4016	698.5	0.9862	0.9267	0.4066	1.7106	0
4017	698.4	0.9862	0.927	0.4064	1.7105	0
4018	698.3	0.9864	0.927	0.4066	1.7108	0
4019	698.2	0.9867	0.9272	0.4065	1.7107	0
4020	698.1	0.9866	0.9274	0.4065	1.71	0
4021	698	0.9867	0.9275	0.4065	1.7109	0
4022	697.9	0.9869	0.9275	0.4064	1.7107	0
4023	697.8	0.9872	0.9277	0.4066	1.7112	0
4024	697.7	0.9873	0.9277	0.4065	1.7109	0
4025	697.6	0.9873	0.9279	0.4066	1.7114	0
4026	697.5	0.9873	0.9278	0.4066	1.7107	0
4027	697.4	0.9875	0.9281	0.4066	1.7117	0
4028	697.3	0.9876	0.9281	0.4066	1.7115	0

4029	697.2	0.9876	0.9282	0.4065	1.7113	0
4030	697.1	0.9878	0.9285	0.4065	1.7118	0
4031	697	0.9879	0.9284	0.4066	1.7115	0
4032	696.9	0.9881	0.9285	0.4067	1.7114	0
4033	696.8	0.9882	0.9288	0.4069	1.7118	0
4034	696.7	0.9883	0.9289	0.4067	1.7116	0
4035	696.6	0.9884	0.9289	0.4067	1.712	0
4036	696.5	0.9883	0.9289	0.4067	1.7124	0
4037	696.4	0.9884	0.9291	0.4067	1.7122	0
4038	696.3	0.9886	0.9292	0.4067	1.712	0
4039	696.2	0.9886	0.9293	0.4066	1.7123	0
4040	696.1	0.9888	0.9293	0.4067	1.7122	0
4041	696	0.989	0.9295	0.4069	1.7127	0
4042	695.9	0.9892	0.9296	0.4069	1.7131	0
4043	695.8	0.9894	0.9299	0.407	1.713	0
4044	695.7	0.9895	0.9298	0.4071	1.7134	0
4045	695.6	0.9896	0.9299	0.4071	1.7132	0
4046	695.5	0.9896	0.9301	0.4073	1.7129	0
4047	695.4	0.9899	0.9302	0.4071	1.7135	0
4048	695.3	0.99	0.9302	0.4072	1.7127	0
4049	695.2	0.9901	0.9305	0.4073	1.7132	0
4050	695.1	0.9902	0.9306	0.4073	1.7141	0
4051	695	0.9903	0.9308	0.4074	1.7139	0
4052	694.9	0.9904	0.9307	0.4074	1.7137	0
4053	694.8	0.9905	0.9307	0.4075	1.7141	0
4054	694.7	0.9904	0.9307	0.4074	1.7139	0
4055	694.6	0.9905	0.9309	0.4075	1.7143	0
4056	694.5	0.9906	0.9311	0.4075	1.7147	0
4057	694.4	0.991	0.9313	0.4078	1.7152	0
4058	694.3	0.991	0.9314	0.4077	1.7144	0
4059	694.2	0.9914	0.9315	0.4077	1.7148	0
4060	694.1	0.9914	0.9317	0.4079	1.7153	0
4061	694	0.9916	0.9319	0.408	1.7158	0
4062	693.9	0.9916	0.932	0.4081	1.7156	0
4063	693.8	0.992	0.932	0.4082	1.7154	0
4064	693.7	0.9919	0.932	0.4081	1.7156	0
4065	693.6	0.9921	0.9324	0.4082	1.7161	0
4066	693.5	0.9921	0.9323	0.4082	1.7159	0
4067	693.4	0.9924	0.9327	0.4083	1.7164	0
4068	693.3	0.9924	0.9327	0.4085	1.7162	0
4069	693.2	0.9928	0.9329	0.4085	1.7161	0
4070	693.1	0.9928	0.9328	0.4085	1.7159	0
4071	693	0.9928	0.933	0.4086	1.7168	0

4072	692.9	0.9929	0.9331	0.4086	1.7172	0
4073	692.8	0.9932	0.9332	0.4087	1.7175	0
4074	692.7	0.9933	0.9336	0.4087	1.7168	0
4075	692.6	0.9934	0.9334	0.4087	1.7166	0
4076	692.5	0.9936	0.9337	0.4089	1.7166	0
4077	692.4	0.9933	0.9337	0.4085	1.7173	0
4078	692.3	0.9937	0.934	0.4088	1.7172	0
4079	692.2	0.9939	0.9341	0.4088	1.7181	0
4080	692.1	0.9939	0.9342	0.4087	1.7179	0
4081	692	0.9941	0.9342	0.4089	1.7176	0
4082	691.9	0.9941	0.9345	0.4089	1.7181	0
4083	691.8	0.9944	0.9347	0.4089	1.7184	0
4084	691.7	0.9944	0.9348	0.4089	1.7183	0
4085	691.6	0.9946	0.9349	0.409	1.7186	0
4086	691.5	0.9948	0.9348	0.4089	1.7183	0
4087	691.4	0.9949	0.9351	0.409	1.7182	0
4088	691.3	0.9951	0.9353	0.4091	1.7181	0
4089	691.2	0.9952	0.9352	0.409	1.7183	0
4090	691.1	0.9953	0.9357	0.409	1.7187	0
4091	691	0.9953	0.9355	0.409	1.719	0
4092	690.9	0.9955	0.9355	0.4092	1.7188	0
4093	690.8	0.9954	0.9358	0.4091	1.7191	0
4094	690.7	0.9957	0.936	0.4089	1.7189	0
4095	690.6	0.9957	0.9361	0.4091	1.7192	0
4096	690.5	0.996	0.9365	0.4093	1.7191	0
4097	690.4	0.9961	0.9363	0.4089	1.7188	0
4098	690.3	0.9962	0.9366	0.4091	1.7197	0
4099	690.2	0.9963	0.9368	0.409	1.7195	0
4100	690.1	0.9963	0.9368	0.4089	1.7186	0
4101	690	0.9964	0.937	0.409	1.719	0
4102	689.9	0.9966	0.9374	0.409	1.7199	0
4103	689.8	0.9967	0.9373	0.409	1.7196	0
4104	689.7	0.9968	0.9376	0.409	1.7194	0
4105	689.6	0.9968	0.9376	0.409	1.7192	0
4106	689.5	0.9969	0.9378	0.4089	1.72	0
4107	689.4	0.9974	0.9382	0.4091	1.7194	0
4108	689.3	0.9975	0.9384	0.409	1.7203	0
4109	689.2	0.9975	0.9383	0.409	1.72	0
4110	689.1	0.9976	0.9385	0.4091	1.7198	0
4111	689	0.9977	0.9386	0.4091	1.7196	0
4112	688.9	0.9979	0.9391	0.4092	1.7199	0
4113	688.8	0.9979	0.939	0.4089	1.7202	0
4114	688.7	0.998	0.9393	0.4089	1.7199	0

4115	688.6	0.9983	0.9395	0.4091	1.7209	0
4116	688.5	0.9983	0.9395	0.4089	1.72	0
4117	688.4	0.9987	0.9398	0.4092	1.7209	0
4118	688.3	0.9985	0.9399	0.409	1.7213	0
4119	688.2	0.9987	0.9399	0.409	1.7215	0
4120	688.1	0.9989	0.9399	0.409	1.7207	0
4121	688	0.999	0.9404	0.4092	1.7211	0
4122	687.9	0.9993	0.9406	0.4094	1.721	0
4123	687.8	0.9994	0.9408	0.4094	1.7219	0
4124	687.7	0.9995	0.9408	0.4093	1.7216	0
4125	687.6	0.9997	0.9411	0.4094	1.7214	0
4126	687.5	0.9997	0.9412	0.4093	1.7212	0
4127	687.4	1	0.9413	0.4095	1.7215	0
4128	687.3	1.0001	0.9417	0.4095	1.7225	0
4129	687.2	1.0004	0.9418	0.4096	1.7223	0
4130	687.1	1.0005	0.9421	0.4096	1.7221	0
4131	687	1.0004	0.9422	0.4097	1.7224	0
4132	686.9	1.0007	0.9424	0.4096	1.7227	0
4133	686.8	1.0008	0.9423	0.4096	1.7224	0
4134	686.7	1.0009	0.9425	0.4097	1.7228	0
4135	686.6	1.001	0.9426	0.4097	1.7225	0
4136	686.5	1.0012	0.9429	0.4097	1.7229	0
4137	686.4	1.0015	0.943	0.41	1.7227	0
4138	686.3	1.0017	0.9434	0.4101	1.7243	0
4139	686.2	1.0018	0.9435	0.4101	1.7241	0
4140	686.1	1.0018	0.9438	0.4101	1.7238	0
4141	686	1.002	0.9437	0.4102	1.7242	0
4142	685.9	1.0023	0.9439	0.4103	1.7246	0
4143	685.8	1.0026	0.9443	0.4103	1.7243	0
4144	685.7	1.0024	0.9442	0.4103	1.724	0
4145	685.6	1.0027	0.9446	0.4105	1.7244	0
4146	685.5	1.0029	0.9446	0.4103	1.7247	0
4147	685.4	1.0031	0.9449	0.4106	1.7245	0
4148	685.3	1.0034	0.9452	0.4108	1.725	0
4149	685.2	1.0033	0.945	0.4106	1.7252	0
4150	685.1	1.0036	0.9456	0.4109	1.7257	0
4151	685	1.004	0.9457	0.4111	1.7255	0
4152	684.9	1.0041	0.9461	0.4111	1.7265	0
4153	684.8	1.0043	0.946	0.4111	1.7268	0
4154	684.7	1.0044	0.9463	0.411	1.7266	0
4155	684.6	1.0048	0.9461	0.411	1.7263	0
4156	684.5	1.0049	0.9466	0.4111	1.7267	0
4157	684.4	1.0052	0.9465	0.4113	1.7264	0

4158	684.3	1.0053	0.9469	0.4115	1.7268	0
4159	684.2	1.0055	0.9471	0.4116	1.7278	0
4160	684.1	1.0056	0.9473	0.4115	1.7282	0
4161	684	1.0059	0.9473	0.4115	1.7273	0
4162	683.9	1.006	0.9475	0.4117	1.7271	0
4163	683.8	1.0063	0.9477	0.4118	1.728	0
4164	683.7	1.0063	0.9478	0.4119	1.7277	0
4165	683.6	1.0067	0.9481	0.4119	1.7276	0
4166	683.5	1.0069	0.9482	0.4121	1.7286	0
4167	683.4	1.0072	0.9485	0.4121	1.7284	0
4168	683.3	1.0073	0.9487	0.4121	1.7287	0
4169	683.2	1.0076	0.9488	0.4122	1.7291	0
4170	683.1	1.0076	0.9489	0.4122	1.7288	0
4171	683	1.008	0.9493	0.4121	1.7286	0
4172	682.9	1.0082	0.9493	0.4123	1.729	0
4173	682.8	1.0083	0.9494	0.4122	1.7293	0
4174	682.7	1.0084	0.9495	0.4121	1.7291	0
4175	682.6	1.0086	0.9497	0.4122	1.7293	0
4176	682.5	1.009	0.9499	0.4123	1.7299	0
4177	682.4	1.0092	0.9501	0.4121	1.7296	0
4178	682.3	1.0096	0.9504	0.4124	1.7295	0
4179	682.2	1.0097	0.9504	0.4122	1.7292	0
4180	682.1	1.0099	0.9507	0.4123	1.7295	0
4181	682	1.0101	0.9508	0.4125	1.7305	0
4182	681.9	1.0103	0.9508	0.4123	1.7302	0
4183	681.8	1.0107	0.9511	0.4123	1.7295	0
4184	681.7	1.0107	0.9511	0.4123	1.7303	0
4185	681.6	1.0108	0.9514	0.4124	1.7302	0
4186	681.5	1.011	0.9516	0.4123	1.7299	0
4187	681.4	1.0114	0.9518	0.4124	1.7297	0
4188	681.3	1.0116	0.952	0.4123	1.73	0
4189	681.2	1.0118	0.9521	0.4123	1.7303	0
4190	681.1	1.012	0.9522	0.4123	1.73	0
4191	681	1.0123	0.9525	0.4123	1.7311	0
4192	680.9	1.0127	0.9527	0.4123	1.7308	0
4193	680.8	1.0129	0.9526	0.4123	1.7312	0
4194	680.7	1.0131	0.9527	0.4124	1.7315	0
4195	680.6	1.0132	0.953	0.4122	1.7312	0
4196	680.5	1.0136	0.9531	0.4121	1.7316	0
4197	680.4	1.0139	0.9531	0.4121	1.7314	0
4198	680.3	1.0141	0.9532	0.412	1.731	0
4199	680.2	1.0142	0.9535	0.412	1.7321	0
4200	680.1	1.0147	0.9538	0.4122	1.7319	0

4201	680	1.0149	0.9537	0.412	1.7309	0
4202	679.9	1.0151	0.9541	0.4122	1.7314	0
4203	679.8	1.0155	0.9541	0.4122	1.731	0
4204	679.7	1.0157	0.9544	0.4121	1.7314	0
4205	679.6	1.0159	0.9543	0.4119	1.731	0
4206	679.5	1.0162	0.9546	0.4119	1.7314	0
4207	679.4	1.0165	0.9549	0.4119	1.7317	0
4208	679.3	1.0167	0.9548	0.4118	1.7314	0
4209	679.2	1.017	0.9552	0.4119	1.7318	0
4210	679.1	1.0171	0.9551	0.4118	1.7315	0
4211	679	1.0174	0.9554	0.4116	1.7318	0
4212	678.9	1.0177	0.9556	0.4116	1.7316	0
4213	678.8	1.018	0.9557	0.4116	1.7313	0
4214	678.7	1.0183	0.9559	0.4115	1.7316	0
4215	678.6	1.0185	0.9559	0.4117	1.732	0
4216	678.5	1.0188	0.9562	0.4115	1.7317	0
4217	678.4	1.019	0.9564	0.4114	1.732	0
4218	678.3	1.0194	0.9566	0.4115	1.7318	0
4219	678.2	1.0198	0.9569	0.4115	1.7316	0
4220	678.1	1.0201	0.9569	0.4114	1.732	0
4221	678	1.0205	0.9571	0.4115	1.7324	0
4222	677.9	1.021	0.9574	0.4116	1.7322	0
4223	677.8	1.0211	0.9575	0.4117	1.7326	0
4224	677.7	1.0214	0.9576	0.4116	1.7329	0
4225	677.6	1.0216	0.9579	0.4115	1.7333	0
4226	677.5	1.0218	0.9582	0.4115	1.7336	0
4227	677.4	1.0223	0.9584	0.4117	1.7335	0
4228	677.3	1.0226	0.9583	0.4116	1.7332	0
4229	677.2	1.023	0.9586	0.4118	1.733	0
4230	677.1	1.023	0.9586	0.4116	1.7339	0
4231	677	1.0233	0.9588	0.4117	1.733	0
4232	676.9	1.0238	0.959	0.4117	1.7334	0
4233	676.8	1.0241	0.9593	0.4119	1.7339	0
4234	676.7	1.0241	0.9597	0.4118	1.7335	0
4235	676.6	1.0247	0.9598	0.412	1.734	0
4236	676.5	1.0251	0.96	0.412	1.7345	0
4237	676.4	1.0255	0.9602	0.4122	1.7344	0
4238	676.3	1.0257	0.9604	0.4123	1.7347	0
4239	676.2	1.026	0.9606	0.4125	1.7352	0
4240	676.1	1.0262	0.9608	0.4125	1.7355	0
4241	676	1.0264	0.961	0.4125	1.736	0
4242	675.9	1.0268	0.9612	0.4127	1.7358	0
4243	675.8	1.0274	0.9613	0.4128	1.7357	0

4244	675.7	1.0275	0.9616	0.413	1.736	0
4245	675.6	1.028	0.9616	0.4131	1.7371	0
4246	675.5	1.0284	0.962	0.4131	1.7363	0
4247	675.4	1.0286	0.9621	0.4133	1.7374	0
4248	675.3	1.0287	0.9623	0.4135	1.7371	0
4249	675.2	1.029	0.9624	0.4134	1.7389	0
4250	675.1	1.0295	0.9625	0.4137	1.7373	0
4251	675	1.0297	0.9629	0.4136	1.7379	0
4252	674.9	1.0301	0.963	0.4138	1.7389	0
4253	674.8	1.0302	0.9631	0.4137	1.7386	0
4254	674.7	1.0307	0.9633	0.4141	1.7385	0
4255	674.6	1.0309	0.9635	0.4141	1.7389	0
4256	674.5	1.0315	0.9641	0.4143	1.7395	0
4257	674.4	1.0314	0.9639	0.4143	1.7392	0
4258	674.3	1.032	0.9644	0.4147	1.7399	0
4259	674.2	1.0323	0.9643	0.4146	1.7402	0
4260	674.1	1.0326	0.9648	0.4146	1.7394	0
4261	674	1.0329	0.9648	0.415	1.7405	0
4262	673.9	1.0334	0.965	0.415	1.7404	0
4263	673.8	1.0335	0.9652	0.4151	1.7409	0
4264	673.7	1.034	0.9654	0.4152	1.7413	0
4265	673.6	1.0342	0.9656	0.4152	1.7412	0
4266	673.5	1.0345	0.9657	0.4153	1.7416	0
4267	673.4	1.0347	0.9659	0.4156	1.7421	0
4268	673.3	1.0351	0.9657	0.4154	1.7418	0
4269	673.2	1.0351	0.966	0.4156	1.7423	0
4270	673.1	1.0355	0.9662	0.4155	1.7422	0
4271	673	1.0357	0.9663	0.4155	1.7425	0
4272	672.9	1.0359	0.9666	0.4156	1.743	0
4273	672.8	1.0363	0.9666	0.4156	1.7435	0
4274	672.7	1.0365	0.9668	0.4157	1.7427	0
4275	672.6	1.0368	0.9671	0.4157	1.7424	0
4276	672.5	1.0371	0.9672	0.4159	1.743	0
4277	672.4	1.0373	0.9672	0.4158	1.7435	0
4278	672.3	1.0375	0.9671	0.4158	1.7432	0
4279	672.2	1.0377	0.9676	0.4157	1.7437	0
4280	672.1	1.038	0.9677	0.4161	1.7436	0
4281	672	1.0382	0.9679	0.4161	1.7447	0
4282	671.9	1.0384	0.9679	0.416	1.7438	0
4283	671.8	1.0387	0.9683	0.4161	1.7444	0
4284	671.7	1.0389	0.9682	0.4161	1.7435	0
4285	671.6	1.0392	0.9683	0.416	1.7446	0
4286	671.5	1.0393	0.9686	0.4163	1.7439	0

4287	671.4	1.0397	0.9686	0.4159	1.7442	0
4288	671.3	1.0398	0.9689	0.416	1.7441	0
4289	671.2	1.04	0.9688	0.4159	1.7445	0
4290	671.1	1.0401	0.969	0.416	1.7451	0
4291	671	1.0404	0.969	0.416	1.7448	0
4292	670.9	1.0405	0.9692	0.4159	1.7453	0
4293	670.8	1.0407	0.9693	0.4159	1.7444	0
4294	670.7	1.0407	0.9694	0.4158	1.7442	0
4295	670.6	1.0411	0.9697	0.4158	1.7441	0
4296	670.5	1.0411	0.9696	0.4157	1.7445	0
4297	670.4	1.0413	0.9697	0.4156	1.7444	0
4298	670.3	1.0415	0.9699	0.4155	1.7454	0
4299	670.2	1.0418	0.9701	0.4157	1.7453	0
4300	670.1	1.042	0.9702	0.4157	1.7458	0
4301	670	1.0421	0.9701	0.4155	1.7462	0
4302	669.9	1.0423	0.9704	0.4155	1.7447	0
4303	669.8	1.0424	0.9704	0.4153	1.7458	0
4304	669.7	1.0427	0.9706	0.4154	1.7457	0
4305	669.6	1.0427	0.9705	0.4153	1.7448	0
4306	669.5	1.043	0.9706	0.4152	1.7446	0
4307	669.4	1.043	0.9706	0.415	1.745	0
4308	669.3	1.043	0.9708	0.415	1.7455	0
4309	669.2	1.0433	0.9708	0.4152	1.7461	0
4310	669.1	1.0435	0.971	0.4149	1.7451	0
4311	669	1.0437	0.9712	0.4149	1.745	0
4312	668.9	1.0438	0.9711	0.4146	1.7461	0
4313	668.8	1.044	0.971	0.4146	1.7459	0
4314	668.7	1.0442	0.9715	0.4146	1.7465	0
4315	668.6	1.0443	0.9715	0.4146	1.7456	0
4316	668.5	1.0446	0.9716	0.4147	1.7456	0
4317	668.4	1.0449	0.9716	0.4146	1.7453	0
4318	668.3	1.0449	0.9717	0.4145	1.7459	0
4319	668.2	1.0452	0.9719	0.4144	1.7464	0
4320	668.1	1.0453	0.972	0.4142	1.7448	0
4321	668	1.0456	0.9719	0.4141	1.7466	0
4322	667.9	1.0457	0.972	0.414	1.7457	0
4323	667.8	1.0459	0.9722	0.4142	1.7463	0
4324	667.7	1.046	0.9723	0.4142	1.7462	0
4325	667.6	1.0462	0.9721	0.414	1.746	0
4326	667.5	1.0467	0.9723	0.4141	1.7472	0
4327	667.4	1.0468	0.9724	0.4139	1.7463	0
4328	667.3	1.047	0.9726	0.4141	1.7469	0
4329	667.2	1.0472	0.9726	0.4143	1.7468	0

4330	667.1	1.0478	0.9727	0.4142	1.7467	0
4331	667	1.0479	0.9728	0.4143	1.7466	0
4332	666.9	1.0479	0.9728	0.4142	1.747	0
4333	666.8	1.0484	0.9732	0.4143	1.7476	0
4334	666.7	1.0485	0.973	0.4142	1.7475	0
4335	666.6	1.0486	0.9731	0.414	1.7473	0
4336	666.5	1.0491	0.9732	0.4144	1.7479	0
4337	666.4	1.0493	0.9734	0.4144	1.7472	0
4338	666.3	1.0495	0.9732	0.4143	1.7477	0
4339	666.2	1.0498	0.9736	0.4143	1.7469	0
4340	666.1	1.05	0.9736	0.4145	1.7475	0
4341	666	1.0502	0.9735	0.4146	1.748	0
4342	665.9	1.0505	0.9737	0.4144	1.7486	0
4343	665.8	1.0508	0.9739	0.4146	1.7491	0
4344	665.7	1.051	0.9738	0.4147	1.7484	0
4345	665.6	1.0513	0.974	0.4148	1.7489	0
4346	665.5	1.0515	0.9741	0.415	1.7489	0
4347	665.4	1.0518	0.9742	0.4151	1.7501	0
4348	665.3	1.0519	0.9743	0.4151	1.7494	0
4349	665.2	1.0524	0.9744	0.4153	1.75	0
4350	665.1	1.0525	0.9746	0.4154	1.7498	0
4351	665	1.053	0.9747	0.4155	1.7505	0
4352	664.9	1.0529	0.9745	0.4154	1.7516	0
4353	664.8	1.0535	0.9749	0.4158	1.7518	0
4354	664.7	1.0535	0.9749	0.4159	1.7517	0
4355	664.6	1.0536	0.9751	0.4162	1.7523	0
4356	664.5	1.0543	0.9751	0.4163	1.7524	0
4357	664.4	1.0546	0.9754	0.4167	1.7524	0
4358	664.3	1.0548	0.9753	0.4167	1.753	0
4359	664.2	1.0551	0.9758	0.417	1.753	0
4360	664.1	1.0553	0.9759	0.417	1.7529	0
4361	664	1.0554	0.9756	0.4171	1.7535	0
4362	663.9	1.0557	0.976	0.4173	1.7535	0
4363	663.8	1.0559	0.976	0.4174	1.7548	0
4364	663.7	1.0562	0.9761	0.4177	1.7548	0
4365	663.6	1.0564	0.9763	0.4176	1.7547	0
4366	663.5	1.0566	0.9763	0.4177	1.7553	0
4367	663.4	1.0569	0.9765	0.4177	1.756	0
4368	663.3	1.0568	0.9767	0.418	1.7553	0
4369	663.2	1.0572	0.9769	0.418	1.7552	0
4370	663.1	1.0574	0.9768	0.4183	1.7559	0
4371	663	1.0573	0.9769	0.4183	1.7558	0
4372	662.9	1.0577	0.9773	0.4184	1.7559	0

4373	662.8	1.058	0.9772	0.4184	1.7571	0
4374	662.7	1.0581	0.9773	0.4185	1.757	0
4375	662.6	1.0582	0.9775	0.4186	1.7563	0
4376	662.5	1.0584	0.9776	0.4188	1.757	0
4377	662.4	1.0587	0.9776	0.4189	1.757	0
4378	662.3	1.0586	0.9777	0.419	1.7568	0
4379	662.2	1.0589	0.9777	0.4189	1.7575	0
4380	662.1	1.0589	0.9778	0.4189	1.758	0
4381	662	1.0592	0.9779	0.4189	1.758	0
4382	661.9	1.0593	0.9782	0.4189	1.758	0
4383	661.8	1.0595	0.9781	0.4192	1.7587	0
4384	661.7	1.0597	0.9784	0.4193	1.7587	0
4385	661.6	1.0597	0.9784	0.4192	1.7592	0
4386	661.5	1.0599	0.9785	0.4194	1.7585	0
4387	661.4	1.06	0.9786	0.4191	1.7591	0
4388	661.3	1.06	0.9789	0.4192	1.7604	0
4389	661.2	1.0603	0.979	0.4193	1.7591	0
4390	661.1	1.0604	0.9788	0.4193	1.7596	0
4391	661	1.0605	0.9793	0.4194	1.7598	0
4392	660.9	1.0608	0.9791	0.4191	1.761	0
4393	660.8	1.0606	0.9792	0.4191	1.7601	0
4394	660.7	1.0608	0.9792	0.4193	1.7608	0
4395	660.6	1.0608	0.9794	0.4192	1.7615	0
4396	660.5	1.0611	0.9792	0.4193	1.7601	0
4397	660.4	1.061	0.9795	0.4193	1.7607	0
4398	660.3	1.0614	0.9796	0.4192	1.7606	0
4399	660.2	1.0615	0.9797	0.4193	1.7606	0
4400	660.1	1.0615	0.9796	0.4193	1.7618	0
4401	660	1.0616	0.9798	0.4192	1.7611	0
4402	659.9	1.0617	0.9799	0.4192	1.7611	0
4403	659.8	1.0617	0.98	0.4192	1.7624	0
4404	659.7	1.0618	0.9799	0.4192	1.7616	0
4405	659.6	1.062	0.9801	0.4192	1.7609	0
4406	659.5	1.062	0.9801	0.419	1.7615	0
4407	659.4	1.062	0.9803	0.4189	1.7614	0
4408	659.3	1.0621	0.9802	0.4189	1.7621	0
4409	659.2	1.0621	0.9803	0.4187	1.7613	0
4410	659.1	1.0623	0.9806	0.4187	1.7613	0
4411	659	1.0624	0.9805	0.4186	1.7613	0
4412	658.9	1.0624	0.9804	0.4186	1.7618	0
4413	658.8	1.0623	0.9804	0.4185	1.761	0
4414	658.7	1.0626	0.9806	0.4183	1.7624	0
4415	658.6	1.0626	0.9806	0.4182	1.7616	0

4416	658.5	1.0626	0.9806	0.4185	1.7622	0
4417	658.4	1.0627	0.981	0.4182	1.7623	0
4418	658.3	1.0627	0.9808	0.4181	1.7614	0
4419	658.2	1.0629	0.981	0.418	1.7622	0
4420	658.1	1.063	0.9812	0.4181	1.7621	0
4421	658	1.0632	0.9812	0.4182	1.7622	0
4422	657.9	1.0634	0.9812	0.4181	1.7621	0
4423	657.8	1.0635	0.9813	0.418	1.762	0
4424	657.7	1.0633	0.9813	0.4179	1.7627	0
4425	657.6	1.0637	0.9815	0.4179	1.7628	0
4426	657.5	1.0637	0.9815	0.4178	1.7634	0
4427	657.4	1.0637	0.9817	0.4179	1.7626	0
4428	657.3	1.0639	0.9815	0.4178	1.7633	0
4429	657.2	1.0638	0.9818	0.4177	1.7625	0
4430	657.1	1.0639	0.9819	0.4176	1.7625	0
4431	657	1.0641	0.9817	0.4176	1.7618	0
4432	656.9	1.0643	0.9821	0.4177	1.7631	0
4433	656.8	1.0644	0.9822	0.4178	1.7632	0
4434	656.7	1.0644	0.9821	0.4177	1.7637	0
4435	656.6	1.0644	0.9821	0.4177	1.7637	0
4436	656.5	1.0643	0.9821	0.4176	1.7637	0
4437	656.4	1.0646	0.9824	0.4177	1.763	0
4438	656.3	1.0646	0.9825	0.4177	1.7637	0
4439	656.2	1.0647	0.9825	0.4177	1.7644	0
4440	656.1	1.0648	0.9827	0.4176	1.7644	0
4441	656	1.0652	0.9827	0.4177	1.7638	0
4442	655.9	1.065	0.9827	0.4177	1.7644	0
4443	655.8	1.0651	0.983	0.4179	1.7652	0
4444	655.7	1.0652	0.9829	0.4178	1.7652	0
4445	655.6	1.0653	0.9829	0.4178	1.7651	0
4446	655.5	1.0655	0.9832	0.4178	1.7645	0
4447	655.4	1.0659	0.9835	0.4179	1.7653	0
4448	655.3	1.0659	0.9834	0.418	1.7653	0
4449	655.2	1.066	0.9836	0.4181	1.7667	0
4450	655.1	1.066	0.9837	0.4182	1.7661	0
4451	655	1.0663	0.984	0.4183	1.7661	0
4452	654.9	1.0664	0.9841	0.4184	1.7669	0
4453	654.8	1.0665	0.9842	0.4184	1.7663	0
4454	654.7	1.0666	0.9844	0.4185	1.767	0
4455	654.6	1.0668	0.9845	0.4186	1.7677	0
4456	654.5	1.0669	0.9845	0.4186	1.7677	0
4457	654.4	1.0671	0.9846	0.4188	1.7685	0
4458	654.3	1.0672	0.9849	0.4189	1.7679	0

4459	654.2	1.0673	0.9851	0.419	1.7687	0
4460	654.1	1.0674	0.9849	0.4191	1.7693	0
4461	654	1.0677	0.9855	0.4194	1.769	0
4462	653.9	1.0678	0.9854	0.4194	1.7689	0
4463	653.8	1.068	0.9855	0.4195	1.7697	0
4464	653.7	1.0682	0.9857	0.4195	1.7711	0
4465	653.6	1.0683	0.9858	0.4196	1.7705	0
4466	653.5	1.0685	0.9858	0.4197	1.7706	0
4467	653.4	1.0686	0.9861	0.4199	1.7714	0
4468	653.3	1.0688	0.9862	0.4203	1.7715	0
4469	653.2	1.069	0.9864	0.4204	1.7715	0
4470	653.1	1.0689	0.9864	0.4202	1.7723	0
4471	653	1.0693	0.9867	0.4204	1.7725	0
4472	652.9	1.0694	0.9869	0.4205	1.7725	0
4473	652.8	1.0694	0.9872	0.4207	1.7726	0
4474	652.7	1.0695	0.987	0.4207	1.7733	0
4475	652.6	1.0697	0.9873	0.4207	1.7727	0
4476	652.5	1.0697	0.9875	0.4208	1.7727	0
4477	652.4	1.0698	0.9876	0.4209	1.7735	0
4478	652.3	1.07	0.9878	0.421	1.7736	0
4479	652.2	1.07	0.988	0.4211	1.7737	0
4480	652.1	1.0703	0.988	0.4211	1.7737	0
4481	652	1.0705	0.9883	0.4214	1.7746	0
4482	651.9	1.0705	0.9884	0.4212	1.7739	0
4483	651.8	1.0708	0.9885	0.4213	1.7754	0
4484	651.7	1.0707	0.9887	0.4215	1.7747	0
4485	651.6	1.071	0.9889	0.4217	1.7756	0
4486	651.5	1.0711	0.989	0.4216	1.7743	0
4487	651.4	1.0712	0.989	0.4217	1.7758	0
4488	651.3	1.0714	0.9894	0.4219	1.7766	0
4489	651.2	1.0716	0.9892	0.4217	1.7759	0
4490	651.1	1.0717	0.9894	0.4218	1.776	0
4491	651	1.0717	0.9894	0.4219	1.7767	0
4492	650.9	1.0717	0.9897	0.4219	1.776	0
4493	650.8	1.0719	0.9897	0.422	1.776	0
4494	650.7	1.0722	0.9899	0.4219	1.7769	0
4495	650.6	1.0723	0.99	0.4219	1.777	0
4496	650.5	1.0724	0.9904	0.422	1.7771	0
4497	650.4	1.0727	0.9904	0.422	1.778	0
4498	650.3	1.0724	0.9904	0.422	1.7771	0
4499	650.2	1.0729	0.9907	0.4222	1.778	0
4500	650.1	1.073	0.9909	0.4222	1.7781	0
4501	650	1.0729	0.991	0.4221	1.7774	0

4502	649.9	1.0729	0.9911	0.4222	1.7781	0
4503	649.8	1.0731	0.991	0.4221	1.7782	0
4504	649.7	1.073	0.9912	0.4221	1.7782	0
4505	649.6	1.0732	0.9913	0.4222	1.7784	0
4506	649.5	1.0732	0.9915	0.422	1.7798	0
4507	649.4	1.0735	0.9916	0.4221	1.7792	0
4508	649.3	1.0734	0.9918	0.4221	1.7785	0
4509	649.2	1.0738	0.992	0.4221	1.7794	0
4510	649.1	1.0737	0.9921	0.422	1.7794	0
4511	649	1.0737	0.9922	0.422	1.7795	0
4512	648.9	1.0739	0.9925	0.4219	1.7803	0
4513	648.8	1.0738	0.9924	0.4218	1.7797	0
4514	648.7	1.074	0.9927	0.4219	1.7804	0
4515	648.6	1.0742	0.9929	0.4218	1.7798	0
4516	648.5	1.0741	0.9927	0.4217	1.7798	0
4517	648.4	1.0742	0.993	0.4217	1.7799	0
4518	648.3	1.0742	0.9931	0.4218	1.78	0
4519	648.2	1.0744	0.9933	0.422	1.7808	0
4520	648.1	1.0743	0.9931	0.4214	1.7807	0
4521	648	1.0742	0.9933	0.4215	1.78	0
4522	647.9	1.0745	0.9935	0.4215	1.781	0
4523	647.8	1.0744	0.9937	0.4218	1.7811	0
4524	647.7	1.0746	0.9937	0.4215	1.781	0
4525	647.6	1.0745	0.9939	0.4213	1.781	0
4526	647.5	1.0745	0.9941	0.4213	1.7811	0
4527	647.4	1.0748	0.9943	0.4213	1.7805	0
4528	647.3	1.0748	0.9943	0.4212	1.7812	0
4529	647.2	1.0746	0.9943	0.4211	1.7806	0
4530	647.1	1.0749	0.9946	0.4212	1.7815	0
4531	647	1.0747	0.9946	0.4211	1.7814	0
4532	646.9	1.0749	0.9946	0.4211	1.7815	0
4533	646.8	1.075	0.9948	0.4211	1.7817	0
4534	646.7	1.0747	0.995	0.421	1.7825	0
4535	646.6	1.0751	0.995	0.4212	1.7826	0
4536	646.5	1.0752	0.9952	0.4209	1.7819	0
4537	646.4	1.0753	0.9954	0.4212	1.7828	0
4538	646.3	1.0753	0.9957	0.4212	1.7829	0
4539	646.2	1.0755	0.9957	0.421	1.7829	0
4540	646.1	1.0758	0.9961	0.4212	1.7825	0
4541	646	1.0758	0.996	0.4212	1.7833	0
4542	645.9	1.0757	0.9961	0.4212	1.7833	0
4543	645.8	1.076	0.9965	0.4212	1.7835	0
4544	645.7	1.0759	0.9965	0.4212	1.7835	0

4545	645.6	1.0759	0.9964	0.4211	1.7829	0
4546	645.5	1.0762	0.9968	0.4213	1.7838	0
4547	645.4	1.0764	0.997	0.4213	1.784	0
4548	645.3	1.0765	0.9968	0.4211	1.7848	0
4549	645.2	1.0766	0.9971	0.4212	1.7849	0
4550	645.1	1.0767	0.9972	0.4213	1.7843	0
4551	645	1.0767	0.9974	0.4213	1.7844	0
4552	644.9	1.077	0.9977	0.4214	1.7853	0
4553	644.8	1.077	0.998	0.4217	1.7848	0
4554	644.7	1.0772	0.9981	0.4217	1.785	0
4555	644.6	1.0771	0.9982	0.4217	1.7866	0
4556	644.5	1.0773	0.9984	0.4219	1.7852	0
4557	644.4	1.0777	0.9986	0.4219	1.7862	0
4558	644.3	1.0779	0.9988	0.4222	1.7871	0
4559	644.2	1.0779	0.999	0.4221	1.7872	0
4560	644.1	1.078	0.999	0.4223	1.7873	0
4561	644	1.0782	0.9992	0.4224	1.7875	0
4562	643.9	1.0787	0.9995	0.4226	1.7886	0
4563	643.8	1.0784	0.9995	0.4225	1.7886	0
4564	643.7	1.0785	0.9996	0.4222	1.7886	0
4565	643.6	1.0788	1	0.4225	1.7896	0
4566	643.5	1.0789	0.9999	0.4225	1.789	0
4567	643.4	1.0791	1.0002	0.4227	1.7892	0
4568	643.3	1.0792	1.0003	0.4228	1.79	0
4569	643.2	1.0797	1.0006	0.423	1.7903	0
4570	643.1	1.08	1.0007	0.4232	1.7905	0
4571	643	1.0801	1.0009	0.4234	1.79	0
4572	642.9	1.08	1.001	0.4234	1.7901	0
4573	642.8	1.0805	1.0013	0.4235	1.791	0
4574	642.7	1.0805	1.0013	0.4236	1.7919	0
4575	642.6	1.0806	1.0013	0.4235	1.792	0
4576	642.5	1.0807	1.0018	0.4235	1.7915	0
4577	642.4	1.081	1.0018	0.4234	1.7916	0
4578	642.3	1.0811	1.0018	0.4233	1.7917	0
4579	642.2	1.0811	1.002	0.4235	1.7926	0
4580	642.1	1.0814	1.0022	0.4235	1.7921	0
4581	642	1.0816	1.0025	0.4235	1.7922	0
4582	641.9	1.0815	1.0026	0.4235	1.7932	0
4583	641.8	1.082	1.0027	0.4237	1.7926	0
4584	641.7	1.0823	1.0031	0.4238	1.7937	0
4585	641.6	1.0823	1.0031	0.4238	1.7938	0
4586	641.5	1.0821	1.0031	0.4238	1.7939	0
4587	641.4	1.0825	1.0033	0.4238	1.7941	0

4588	641.3	1.0827	1.0036	0.424	1.795	0
4589	641.2	1.0828	1.0037	0.424	1.7952	0
4590	641.1	1.0831	1.0038	0.4241	1.7954	0
4591	641	1.0831	1.0043	0.4241	1.7963	0
4592	640.9	1.0833	1.0043	0.4243	1.7958	0
4593	640.8	1.0836	1.0046	0.4244	1.7953	0
4594	640.7	1.0838	1.0047	0.4242	1.7968	0
4595	640.6	1.0841	1.0048	0.4243	1.7963	0
4596	640.5	1.084	1.005	0.4243	1.7966	0
4597	640.4	1.0842	1.0052	0.4243	1.7975	0
4598	640.3	1.0842	1.0054	0.4245	1.7963	0
4599	640.2	1.0847	1.0055	0.4247	1.7979	0
4600	640.1	1.0846	1.0059	0.4246	1.7973	0
4601	640	1.085	1.0057	0.4247	1.7983	0
4602	639.9	1.0848	1.006	0.4249	1.7977	0
4603	639.8	1.0852	1.006	0.4246	1.7979	0
4604	639.7	1.0852	1.0063	0.4247	1.7979	0
4605	639.6	1.0853	1.0067	0.4247	1.7981	0
4606	639.5	1.0855	1.0067	0.4247	1.7998	0
4607	639.4	1.0857	1.0067	0.4248	1.7992	0
4608	639.3	1.0859	1.0069	0.4247	1.8002	0
4609	639.2	1.086	1.0071	0.4247	1.7996	0
4610	639.1	1.0861	1.0074	0.4247	1.8005	0
4611	639	1.0861	1.0075	0.4249	1.8007	0
4612	638.9	1.0864	1.0079	0.4247	1.7994	0
4613	638.8	1.0865	1.0076	0.4247	1.7995	0
4614	638.7	1.0866	1.0077	0.4245	1.8003	0
4615	638.6	1.087	1.0082	0.4248	1.8006	0
4616	638.5	1.0869	1.0083	0.4246	1.8008	0
4617	638.4	1.087	1.0086	0.4247	1.8017	0
4618	638.3	1.0873	1.0085	0.4249	1.8012	0
4619	638.2	1.0875	1.0088	0.4247	1.8014	0
4620	638.1	1.0876	1.0089	0.4244	1.8015	0
4621	638	1.0876	1.0092	0.4247	1.8017	0
4622	637.9	1.0879	1.0094	0.4247	1.8019	0
4623	637.8	1.088	1.0098	0.4247	1.8021	0
4624	637.7	1.0881	1.0098	0.4247	1.803	0
4625	637.6	1.0882	1.0099	0.4246	1.8025	0
4626	637.5	1.0885	1.01	0.4247	1.8019	0
4627	637.4	1.0888	1.0105	0.4246	1.8029	0
4628	637.3	1.0887	1.0104	0.4249	1.803	0
4629	637.2	1.0887	1.0107	0.4247	1.8032	0
4630	637.1	1.089	1.0107	0.4247	1.8027	0

4631	637	1.0892	1.0107	0.4245	1.8035	0
4632	636.9	1.0892	1.0111	0.4246	1.8038	0
4633	636.8	1.0894	1.0113	0.4246	1.8047	0
4634	636.7	1.0895	1.0115	0.4246	1.8049	0
4635	636.6	1.0899	1.0116	0.4246	1.8044	0
4636	636.5	1.09	1.0118	0.4246	1.8038	0
4637	636.4	1.09	1.0119	0.4247	1.8047	0
4638	636.3	1.0903	1.0124	0.4248	1.805	0
4639	636.2	1.0904	1.0125	0.4249	1.8059	0
4640	636.1	1.0904	1.0125	0.4245	1.806	0
4641	636	1.0905	1.0127	0.4248	1.8062	0
4642	635.9	1.0908	1.0129	0.4248	1.8058	0
4643	635.8	1.0909	1.013	0.4248	1.8067	0
4644	635.7	1.0913	1.013	0.4249	1.8068	0
4645	635.6	1.0911	1.0135	0.425	1.8071	0
4646	635.5	1.0914	1.0135	0.4249	1.8072	0
4647	635.4	1.0918	1.0138	0.4249	1.8075	0
4648	635.3	1.0916	1.0138	0.4249	1.8076	0
4649	635.2	1.0918	1.0142	0.4251	1.808	0
4650	635.1	1.0922	1.0143	0.425	1.8089	0
4651	635	1.0922	1.0144	0.4251	1.8083	0
4652	634.9	1.0923	1.0145	0.4252	1.8078	0
4653	634.8	1.0926	1.0148	0.4251	1.8095	0
4654	634.7	1.0926	1.015	0.425	1.8088	0
4655	634.6	1.0927	1.015	0.4251	1.8097	0
4656	634.5	1.093	1.0154	0.4252	1.81	0
4657	634.4	1.0932	1.0155	0.4254	1.8102	0
4658	634.3	1.0933	1.0159	0.4253	1.8112	0
4659	634.2	1.0935	1.016	0.4255	1.8108	0
4660	634.1	1.0936	1.0162	0.4255	1.8109	0
4661	634	1.0941	1.0166	0.4257	1.8112	0
4662	633.9	1.094	1.0166	0.4257	1.8121	0
4663	633.8	1.0946	1.0171	0.4261	1.8117	0
4664	633.7	1.0945	1.0171	0.426	1.8133	0
4665	633.6	1.0945	1.0173	0.426	1.8121	0
4666	633.5	1.0947	1.0175	0.426	1.8123	0
4667	633.4	1.0951	1.0178	0.426	1.8133	0
4668	633.3	1.0954	1.0179	0.4264	1.8143	0
4669	633.2	1.0953	1.0181	0.4265	1.8145	0
4670	633.1	1.0956	1.0183	0.4265	1.8141	0
4671	633	1.0956	1.0184	0.4264	1.8149	0
4672	632.9	1.0959	1.0187	0.4264	1.8144	0
4673	632.8	1.0959	1.019	0.4265	1.8154	0

4674	632.7	1.0961	1.0194	0.4265	1.8156	0
4675	632.6	1.0963	1.0194	0.4265	1.8158	0
4676	632.5	1.0965	1.0198	0.4265	1.816	0
4677	632.4	1.0966	1.0197	0.4265	1.8155	0
4678	632.3	1.0967	1.0197	0.4265	1.8164	0
4679	632.2	1.0971	1.0203	0.4266	1.8168	0
4680	632.1	1.0972	1.0203	0.4265	1.8162	0
4681	632	1.0973	1.0204	0.4266	1.8178	0
4682	631.9	1.0973	1.0207	0.4264	1.8166	0
4683	631.8	1.0978	1.0212	0.4267	1.8177	0
4684	631.7	1.098	1.0213	0.4269	1.8187	0
4685	631.6	1.0979	1.0215	0.4268	1.8181	0
4686	631.5	1.0982	1.0216	0.4267	1.8175	0
4687	631.4	1.0983	1.0219	0.4268	1.8185	0
4688	631.3	1.0987	1.0222	0.4268	1.8188	0
4689	631.2	1.0987	1.0224	0.4268	1.819	0
4690	631.1	1.0991	1.0228	0.4267	1.8193	0
4691	631	1.0991	1.0228	0.4268	1.8209	0
4692	630.9	1.0994	1.023	0.4269	1.8197	0
4693	630.8	1.0995	1.0235	0.4269	1.8215	0
4694	630.7	1.0998	1.0234	0.4267	1.8201	0
4695	630.6	1.0997	1.0237	0.4267	1.8203	0
4696	630.5	1.0999	1.0238	0.4268	1.8205	0
4697	630.4	1.1002	1.0242	0.4268	1.8215	0
4698	630.3	1.1005	1.0244	0.4268	1.8219	0
4699	630.2	1.1005	1.0247	0.4268	1.822	0
4700	630.1	1.1009	1.0247	0.4268	1.8222	0
4701	630	1.1009	1.0252	0.4268	1.8225	0
4702	629.9	1.1011	1.0253	0.4269	1.8227	0
4703	629.8	1.1012	1.0257	0.427	1.823	0
4704	629.7	1.1014	1.0257	0.427	1.824	0
4705	629.6	1.1018	1.0262	0.4269	1.8242	0
4706	629.5	1.1018	1.0261	0.4267	1.8243	0
4707	629.4	1.1019	1.0266	0.4268	1.8246	0
4708	629.3	1.1023	1.0268	0.4267	1.8242	0
4709	629.2	1.1022	1.0272	0.427	1.8244	0
4710	629.1	1.1026	1.0274	0.4268	1.8261	0
4711	629	1.1025	1.0274	0.4268	1.8256	0
4712	628.9	1.1027	1.0278	0.4268	1.8251	0
4713	628.8	1.103	1.0279	0.4269	1.8261	0
4714	628.7	1.103	1.0283	0.4268	1.8263	0
4715	628.6	1.1034	1.0285	0.4266	1.8265	0
4716	628.5	1.1035	1.0287	0.4267	1.8267	0

4717	628.4	1.1037	1.029	0.4267	1.8262	0
4718	628.3	1.1039	1.0292	0.4267	1.8272	0
4719	628.2	1.1041	1.0296	0.4268	1.8274	0
4720	628.1	1.1042	1.0299	0.4268	1.8278	0
4721	628	1.1046	1.03	0.4267	1.828	0
4722	627.9	1.1048	1.0302	0.4268	1.8283	0
4723	627.8	1.1049	1.0306	0.427	1.8285	0
4724	627.7	1.1051	1.0308	0.4269	1.8294	0
4725	627.6	1.1052	1.0309	0.4269	1.8298	0
4726	627.5	1.1056	1.0313	0.427	1.8285	0
4727	627.4	1.1056	1.0314	0.427	1.8295	0
4728	627.3	1.1058	1.0318	0.4271	1.8305	0
4729	627.2	1.1059	1.0321	0.427	1.8307	0
4730	627.1	1.1062	1.0325	0.4272	1.8303	0
4731	627	1.106	1.0324	0.4271	1.8304	0
4732	626.9	1.1067	1.0329	0.4271	1.8315	0
4733	626.8	1.1067	1.0331	0.4272	1.8317	0
4734	626.7	1.107	1.0334	0.4273	1.8312	0
4735	626.6	1.1073	1.0338	0.4275	1.8315	0
4736	626.5	1.1074	1.0338	0.4273	1.8324	0
4737	626.4	1.1075	1.0342	0.4275	1.832	0
4738	626.3	1.1081	1.0344	0.4277	1.8324	0
4739	626.2	1.1082	1.0348	0.4279	1.8334	0
4740	626.1	1.1083	1.0349	0.4276	1.8336	0
4741	626	1.1086	1.0352	0.4277	1.8338	0
4742	625.9	1.1087	1.0357	0.4277	1.834	0
4743	625.8	1.1088	1.0356	0.4278	1.8342	0
4744	625.7	1.1091	1.036	0.4278	1.8345	0
4745	625.6	1.1093	1.0364	0.4281	1.8348	0
4746	625.5	1.1095	1.0366	0.428	1.8358	0
4747	625.4	1.1097	1.0368	0.4279	1.8352	0
4748	625.3	1.1101	1.0372	0.4282	1.8356	0
4749	625.2	1.1105	1.0374	0.4282	1.8366	0
4750	625.1	1.1107	1.0377	0.4284	1.8362	0
4751	625	1.111	1.0381	0.4283	1.8365	0
4752	624.9	1.1111	1.0383	0.4284	1.8382	0
4753	624.8	1.1115	1.0388	0.4286	1.837	0
4754	624.7	1.1115	1.0389	0.4285	1.8379	0
4755	624.6	1.1118	1.0392	0.4287	1.8382	0
4756	624.5	1.1121	1.0396	0.4286	1.8385	0
4757	624.4	1.1123	1.0399	0.429	1.8396	0
4758	624.3	1.1125	1.04	0.4288	1.839	0
4759	624.2	1.1126	1.0403	0.4288	1.8393	0

4760	624.1	1.1129	1.0406	0.429	1.8403	0
4761	624	1.1132	1.041	0.4289	1.8413	0
4762	623.9	1.1134	1.0413	0.429	1.8401	0
4763	623.8	1.1136	1.0417	0.4292	1.8404	0
4764	623.7	1.114	1.042	0.4292	1.8414	0
4765	623.6	1.1142	1.0422	0.4292	1.8409	0
4766	623.5	1.1145	1.0424	0.429	1.8419	0
4767	623.4	1.1148	1.0426	0.4294	1.8415	0
4768	623.3	1.1147	1.0428	0.4295	1.8417	0
4769	623.2	1.115	1.043	0.4293	1.8418	0
4770	623.1	1.1151	1.0434	0.4294	1.8421	0
4771	623	1.1154	1.0438	0.4295	1.8423	0
4772	622.9	1.1159	1.0443	0.4296	1.8442	0
4773	622.8	1.1159	1.0443	0.4297	1.8429	0
4774	622.7	1.1165	1.0449	0.4296	1.8432	0
4775	622.6	1.1164	1.0449	0.4295	1.8433	0
4776	622.5	1.1165	1.0453	0.4296	1.8435	0
4777	622.4	1.1168	1.0454	0.4295	1.8445	0
4778	622.3	1.1171	1.0458	0.4298	1.8433	0
4779	622.2	1.1174	1.0461	0.4296	1.8442	0
4780	622.1	1.1175	1.0463	0.4296	1.8453	0
4781	622	1.1177	1.0467	0.4297	1.8448	0
4782	621.9	1.1181	1.0471	0.4297	1.8458	0
4783	621.8	1.1182	1.0473	0.4298	1.8452	0
4784	621.7	1.1184	1.0475	0.4297	1.8455	0
4785	621.6	1.1189	1.0477	0.4299	1.8457	0
4786	621.5	1.1188	1.048	0.4299	1.8459	0
4787	621.4	1.119	1.0483	0.4297	1.8461	0
4788	621.3	1.1192	1.0485	0.4298	1.8463	0
4789	621.2	1.1196	1.0487	0.4298	1.8466	0
4790	621.1	1.1198	1.0492	0.43	1.8461	0
4791	621	1.1201	1.0493	0.4298	1.847	0
4792	620.9	1.1202	1.0499	0.43	1.8473	0
4793	620.8	1.1203	1.0498	0.4297	1.8475	0
4794	620.7	1.1208	1.0503	0.4298	1.8477	0
4795	620.6	1.1211	1.0506	0.4298	1.848	0
4796	620.5	1.1211	1.0508	0.4295	1.8481	0
4797	620.4	1.1215	1.0511	0.4296	1.8483	0
4798	620.3	1.1215	1.0514	0.4295	1.8485	0
4799	620.2	1.1218	1.0516	0.4295	1.8488	0
4800	620.1	1.122	1.0517	0.4293	1.8489	0
4801	620	1.1222	1.0518	0.4292	1.8484	0
4802	619.9	1.1227	1.0526	0.4296	1.8495	0

4803	619.8	1.1228	1.0527	0.4294	1.8496	0
4804	619.7	1.1227	1.0527	0.4292	1.8489	0
4805	619.6	1.123	1.053	0.4291	1.8492	0
4806	619.5	1.1231	1.0534	0.4291	1.8493	0
4807	619.4	1.1236	1.0535	0.429	1.8496	0
4808	619.3	1.1239	1.0541	0.4292	1.85	0
4809	619.2	1.1241	1.0543	0.4293	1.8502	0
4810	619.1	1.1243	1.0545	0.4291	1.8504	0
4811	619	1.1248	1.055	0.4289	1.8513	0
4812	618.9	1.1248	1.0552	0.429	1.8501	0
4813	618.8	1.1253	1.0555	0.4288	1.8511	0
4814	618.7	1.1254	1.0559	0.4287	1.8513	0
4815	618.6	1.1256	1.0558	0.4287	1.8514	0
4816	618.5	1.126	1.0564	0.4288	1.8518	0
4817	618.4	1.1263	1.0565	0.4287	1.8512	0
4818	618.3	1.1266	1.0571	0.4287	1.853	0
4819	618.2	1.1269	1.0574	0.429	1.8518	0
4820	618.1	1.1274	1.0575	0.4288	1.8528	0
4821	618	1.1275	1.0578	0.4287	1.8521	0
4822	617.9	1.1277	1.0581	0.4286	1.8538	0
4823	617.8	1.128	1.0585	0.4287	1.8533	0
4824	617.7	1.1283	1.0587	0.4287	1.8528	0
4825	617.6	1.1286	1.0589	0.4285	1.8537	0
4826	617.5	1.1289	1.0592	0.4288	1.854	0
4827	617.4	1.1292	1.0594	0.4287	1.8542	0
4828	617.3	1.1294	1.0599	0.4289	1.8545	0
4829	617.2	1.1299	1.0605	0.429	1.8547	0
4830	617.1	1.1299	1.0605	0.4286	1.8556	0
4831	617	1.1302	1.0608	0.4287	1.855	0
4832	616.9	1.1306	1.0611	0.4289	1.856	0
4833	616.8	1.1307	1.0613	0.4288	1.8561	0
4834	616.7	1.1311	1.0617	0.4288	1.8564	0
4835	616.6	1.1317	1.0619	0.4289	1.8566	0
4836	616.5	1.1319	1.0625	0.4289	1.8561	0
4837	616.4	1.132	1.0626	0.4289	1.857	0
4838	616.3	1.1324	1.0629	0.429	1.8572	0
4839	616.2	1.133	1.0634	0.4293	1.8584	0
4840	616.1	1.1331	1.0636	0.4293	1.8578	0
4841	616	1.1334	1.0637	0.4294	1.8587	0
4842	615.9	1.1337	1.064	0.4294	1.8582	0
4843	615.8	1.1342	1.0643	0.4296	1.8577	0
4844	615.7	1.1344	1.0649	0.4298	1.8587	0
4845	615.6	1.1349	1.0652	0.4297	1.8589	0

4846	615.5	1.1353	1.0654	0.43	1.8607	0
4847	615.4	1.1357	1.0659	0.43	1.8594	0
4848	615.3	1.1362	1.0662	0.4301	1.8604	0
4849	615.2	1.1365	1.0666	0.4303	1.8614	0
4850	615.1	1.1365	1.0667	0.4303	1.8601	0
4851	615	1.1371	1.0671	0.4303	1.8604	0
4852	614.9	1.1374	1.0674	0.4306	1.8606	0
4853	614.8	1.1378	1.0676	0.4307	1.8623	0
4854	614.7	1.1383	1.0683	0.4307	1.8619	0
4855	614.6	1.1386	1.0684	0.4307	1.862	0
4856	614.5	1.1389	1.0689	0.4309	1.863	0
4857	614.4	1.1394	1.0692	0.4312	1.8632	0
4858	614.3	1.1401	1.0695	0.4315	1.8635	0
4859	614.2	1.1403	1.0699	0.4314	1.8637	0
4860	614.1	1.1406	1.0702	0.4315	1.8639	0
4861	614	1.1409	1.0704	0.4316	1.864	0
4862	613.9	1.1413	1.0709	0.4318	1.8649	0
4863	613.8	1.1416	1.0711	0.4319	1.8651	0
4864	613.7	1.142	1.0716	0.4319	1.8645	0
4865	613.6	1.1423	1.0719	0.4318	1.8646	0
4866	613.5	1.1426	1.072	0.432	1.8656	0
4867	613.4	1.143	1.0724	0.432	1.865	0
4868	613.3	1.1434	1.073	0.4323	1.866	0
4869	613.2	1.1436	1.0732	0.4323	1.8668	0
4870	613.1	1.1443	1.0737	0.4324	1.8664	0
4871	613	1.1446	1.0739	0.4324	1.8665	0
4872	612.9	1.1446	1.074	0.4325	1.8672	0
4873	612.8	1.1451	1.0744	0.4325	1.8674	0
4874	612.7	1.1453	1.0748	0.4325	1.8668	0
4875	612.6	1.1457	1.0751	0.4326	1.8677	0
4876	612.5	1.1461	1.0755	0.4328	1.8686	0
4877	612.4	1.1463	1.0758	0.4328	1.868	0
4878	612.3	1.1468	1.0762	0.4329	1.8688	0
4879	612.2	1.1472	1.0765	0.433	1.8697	0
4880	612.1	1.1475	1.0767	0.433	1.8691	0
4881	612	1.1477	1.0769	0.4329	1.8692	0
4882	611.9	1.1479	1.0772	0.4332	1.8693	0
4883	611.8	1.1484	1.0777	0.4331	1.8702	0
4884	611.7	1.1484	1.0779	0.4327	1.8694	0
4885	611.6	1.1489	1.0781	0.4329	1.8696	0
4886	611.5	1.1493	1.0784	0.433	1.8696	0
4887	611.4	1.1498	1.0789	0.4331	1.8714	0
4888	611.3	1.15	1.0789	0.4331	1.8699	0

4889	611.2	1.1502	1.0795	0.4329	1.8707	0
4890	611.1	1.1507	1.0797	0.4332	1.8701	0
4891	611	1.1508	1.0802	0.433	1.8708	0
4892	610.9	1.1513	1.0802	0.4329	1.8708	0
4893	610.8	1.1515	1.0807	0.4328	1.8709	0
4894	610.7	1.1519	1.0808	0.4329	1.8711	0
4895	610.6	1.1522	1.0813	0.433	1.8727	0
4896	610.5	1.1526	1.0815	0.4328	1.8719	0
4897	610.4	1.1527	1.0819	0.4327	1.8719	0
4898	610.3	1.1532	1.0821	0.4329	1.8712	0
4899	610.2	1.1535	1.0824	0.4329	1.8727	0
4900	610.1	1.1538	1.0828	0.4329	1.8727	0
4901	610	1.1541	1.083	0.4329	1.8735	0
4902	609.9	1.1542	1.0832	0.4328	1.8726	0
4903	609.8	1.1546	1.0834	0.4327	1.8734	0
4904	609.7	1.1547	1.0837	0.4328	1.8726	0
4905	609.6	1.1552	1.084	0.4325	1.8734	0
4906	609.5	1.1556	1.0842	0.4326	1.8734	0
4907	609.4	1.1557	1.0845	0.4325	1.8733	0
4908	609.3	1.1561	1.0848	0.4324	1.8748	0
4909	609.2	1.1566	1.085	0.4325	1.8733	0
4910	609.1	1.1566	1.0852	0.4324	1.874	0
4911	609	1.1569	1.0858	0.4324	1.8732	0
4912	608.9	1.1573	1.0861	0.4323	1.8732	0
4913	608.8	1.1573	1.0861	0.4322	1.8738	0
4914	608.7	1.1579	1.0866	0.4322	1.8738	0
4915	608.6	1.1585	1.0868	0.4323	1.8738	0
4916	608.5	1.1587	1.0872	0.4323	1.8745	0
4917	608.4	1.1591	1.0875	0.4321	1.8744	0
4918	608.3	1.1592	1.0878	0.432	1.8751	0
4919	608.2	1.1595	1.0881	0.4321	1.8751	0
4920	608.1	1.16	1.0886	0.4323	1.8759	0
4921	608	1.1604	1.0887	0.4319	1.8749	0
4922	607.9	1.1608	1.0891	0.432	1.8763	0
4923	607.8	1.1612	1.0896	0.4321	1.8763	0
4924	607.7	1.1613	1.0897	0.4322	1.8754	0
4925	607.6	1.1614	1.0898	0.4319	1.8752	0
4926	607.5	1.162	1.0901	0.4319	1.8752	0
4927	607.4	1.1621	1.0906	0.4317	1.8758	0
4928	607.3	1.1626	1.091	0.4319	1.8757	0
4929	607.2	1.1631	1.0912	0.4318	1.8763	0
4930	607.1	1.1634	1.0915	0.4319	1.877	0
4931	607	1.1638	1.0917	0.4318	1.876	0

4932	606.9	1.1641	1.0921	0.432	1.8767	0
4933	606.8	1.1644	1.0922	0.432	1.8765	0
4934	606.7	1.1647	1.0926	0.4319	1.8771	0
4935	606.6	1.1653	1.0932	0.432	1.8762	0
4936	606.5	1.1657	1.0934	0.4322	1.8777	0
4937	606.4	1.166	1.0935	0.4322	1.8774	0
4938	606.3	1.1664	1.0938	0.4323	1.878	0
4939	606.2	1.1668	1.0945	0.4326	1.8779	0
4940	606.1	1.1673	1.0948	0.4326	1.8777	0
4941	606	1.1678	1.0949	0.4324	1.8782	0
4942	605.9	1.1679	1.0952	0.4326	1.8788	0
4943	605.8	1.1684	1.0956	0.4326	1.8785	0
4944	605.7	1.1689	1.0961	0.4329	1.8799	0
4945	605.6	1.1691	1.0961	0.4326	1.8803	0
4946	605.5	1.1695	1.0967	0.4328	1.8802	0
4947	605.4	1.1698	1.0967	0.4329	1.8798	0
4948	605.3	1.1705	1.0969	0.4332	1.8803	0
4949	605.2	1.1708	1.0972	0.4332	1.8809	0
4950	605.1	1.1714	1.0981	0.4336	1.8816	0
4951	605	1.1717	1.0983	0.4337	1.8812	0
4952	604.9	1.172	1.0984	0.4336	1.8808	0
4953	604.8	1.1723	1.0987	0.4339	1.8822	0
4954	604.7	1.173	1.0989	0.434	1.8819	0
4955	604.6	1.1733	1.0996	0.4342	1.8824	0
4956	604.5	1.1739	1.0998	0.4344	1.8829	0
4957	604.4	1.1741	1.1	0.4346	1.8826	0
4958	604.3	1.1746	1.1004	0.4347	1.8831	0
4959	604.2	1.1751	1.1007	0.4347	1.8843	0
4960	604.1	1.1757	1.1012	0.4352	1.8848	0
4961	604	1.1757	1.1014	0.4352	1.8844	0
4962	603.9	1.1763	1.1015	0.4354	1.8849	0
4963	603.8	1.1768	1.1021	0.4354	1.8853	0
4964	603.7	1.1772	1.1024	0.4357	1.8858	0
4965	603.6	1.1776	1.1025	0.4358	1.8861	0
4966	603.5	1.178	1.1028	0.4357	1.8865	0
4967	603.4	1.1786	1.1033	0.436	1.887	0
4968	603.3	1.1788	1.1037	0.4361	1.8866	0
4969	603.2	1.1796	1.1039	0.4363	1.8871	0
4970	603.1	1.1801	1.1043	0.4364	1.8866	0
4971	603	1.1805	1.1046	0.4365	1.887	0
4972	602.9	1.1807	1.1046	0.4365	1.888	0
4973	602.8	1.1813	1.1053	0.4367	1.8868	0
4974	602.7	1.1817	1.1052	0.4369	1.8887	0

4975	602.6	1.1822	1.106	0.4368	1.8891	0
4976	602.5	1.1823	1.1061	0.4369	1.8893	0
4977	602.4	1.1829	1.1062	0.4368	1.8888	0
4978	602.3	1.1831	1.1065	0.4368	1.889	0
4979	602.2	1.1837	1.1068	0.4367	1.8893	0
4980	602.1	1.184	1.107	0.4371	1.8896	0
4981	602	1.1843	1.1075	0.437	1.8898	0
4982	601.9	1.1849	1.1076	0.4371	1.8894	0
4983	601.8	1.1853	1.1079	0.4373	1.8905	0
4984	601.7	1.1855	1.1084	0.4374	1.8907	0
4985	601.6	1.1859	1.1082	0.4373	1.8924	0
4986	601.5	1.1863	1.1088	0.4372	1.8911	0
4987	601.4	1.1866	1.109	0.4374	1.8921	0
4988	601.3	1.1871	1.1095	0.4374	1.8923	0
4989	601.2	1.1873	1.1094	0.4372	1.8924	0
4990	601.1	1.1878	1.1097	0.4374	1.8919	0
4991	601	1.1882	1.1103	0.4372	1.8929	0
4992	600.9	1.189	1.1105	0.4377	1.8931	0
4993	600.8	1.189	1.1108	0.4376	1.8933	0
4994	600.7	1.1892	1.1107	0.4376	1.8933	0
4995	600.6	1.1897	1.1113	0.4375	1.8935	0
4996	600.5	1.1902	1.1114	0.4377	1.8937	0
4997	600.4	1.1907	1.1119	0.4376	1.8947	0
4998	600.3	1.1909	1.112	0.4379	1.8948	0
4999	600.2	1.1914	1.1122	0.4379	1.894	0
5000	600.1	1.1916	1.1125	0.4375	1.895	0
5001	600	1.1922	1.1127	0.4377	1.8951	0
5002	599.9	1.1928	1.1131	0.4376	1.8953	0
5003	599.8	1.193	1.1132	0.4373	1.8953	0
5004	599.7	1.1932	1.1135	0.4374	1.8946	0
5005	599.6	1.1935	1.1137	0.4373	1.8956	0
5006	599.5	1.1935	1.1139	0.4372	1.8963	0
5007	599.4	1.1942	1.1141	0.4372	1.8964	0
5008	599.3	1.1945	1.1144	0.4372	1.8958	0
5009	599.2	1.1949	1.1148	0.437	1.8967	0
5010	599.1	1.1949	1.1149	0.4368	1.8966	0
5011	599	1.1955	1.1153	0.4369	1.8976	0
5012	598.9	1.1956	1.1152	0.4369	1.8976	0
5013	598.8	1.196	1.1155	0.4368	1.8967	0
5014	598.7	1.1961	1.1158	0.4365	1.8976	0
5015	598.6	1.1968	1.116	0.4363	1.8977	0
5016	598.5	1.197	1.1162	0.4364	1.8986	0
5017	598.4	1.1974	1.1163	0.4363	1.8986	0

5018	598.3	1.1975	1.1167	0.4363	1.8987	0
5019	598.2	1.1978	1.1169	0.436	1.8978	0
5020	598.1	1.1983	1.1171	0.4359	1.8988	0
5021	598	1.1986	1.1172	0.4359	1.8988	0
5022	597.9	1.1988	1.1176	0.4359	1.8988	0
5023	597.8	1.1993	1.1178	0.4356	1.8997	0
5024	597.7	1.1995	1.118	0.4357	1.8997	0
5025	597.6	1.2	1.1181	0.4358	1.9008	0
5026	597.5	1.2002	1.1184	0.4356	1.9006	0
5027	597.4	1.2005	1.1185	0.4356	1.9006	0
5028	597.3	1.2007	1.1188	0.4354	1.8997	0
5029	597.2	1.2011	1.119	0.4354	1.9006	0
5030	597.1	1.2012	1.1194	0.4354	1.9006	0
5031	597	1.2017	1.1196	0.4353	1.9023	0
5032	596.9	1.2021	1.1197	0.4353	1.9014	0
5033	596.8	1.2024	1.12	0.4353	1.9014	0
5034	596.7	1.2027	1.1204	0.4352	1.9013	0
5035	596.6	1.2031	1.1207	0.4353	1.9032	0
5036	596.5	1.2035	1.1205	0.435	1.902	0
5037	596.4	1.2035	1.1208	0.435	1.9028	0
5038	596.3	1.204	1.1211	0.4352	1.9038	0
5039	596.2	1.2042	1.1216	0.4352	1.9038	0
5040	596.1	1.2047	1.1216	0.435	1.9047	0
5041	596	1.2051	1.1217	0.4351	1.9044	0
5042	595.9	1.2052	1.1221	0.4351	1.9043	0
5043	595.8	1.2059	1.1222	0.4353	1.9052	0
5044	595.7	1.2063	1.1227	0.435	1.9051	0
5045	595.6	1.2066	1.1228	0.435	1.9059	0
5046	595.5	1.2069	1.1232	0.4351	1.9057	0
5047	595.4	1.2072	1.1231	0.4351	1.9066	0
5048	595.3	1.208	1.1237	0.4353	1.9065	0
5049	595.2	1.2081	1.1239	0.4356	1.9074	0
5050	595.1	1.2084	1.1242	0.4354	1.9082	0
5051	595	1.2088	1.1243	0.4354	1.908	0
5052	594.9	1.2094	1.1244	0.4353	1.9078	0
5053	594.8	1.2097	1.1247	0.4356	1.9087	0
5054	594.7	1.2102	1.125	0.4357	1.9096	0
5055	594.6	1.2105	1.1254	0.4356	1.9103	0
5056	594.5	1.211	1.1257	0.4358	1.9101	0
5057	594.4	1.2113	1.126	0.4358	1.9099	0
5058	594.3	1.2119	1.126	0.4361	1.9128	0
5059	594.2	1.2123	1.1268	0.4363	1.9117	0
5060	594.1	1.2127	1.1266	0.4361	1.9124	0

5061	594	1.2129	1.1272	0.4363	1.9132	0
5062	593.9	1.2136	1.1274	0.4365	1.9121	0
5063	593.8	1.214	1.1277	0.4364	1.913	0
5064	593.7	1.2144	1.1278	0.4365	1.9128	0
5065	593.6	1.2144	1.1281	0.4367	1.9136	0
5066	593.5	1.2153	1.1285	0.4367	1.9145	0
5067	593.4	1.2156	1.1285	0.4366	1.9152	0
5068	593.3	1.2161	1.129	0.437	1.9153	0
5069	593.2	1.2166	1.1292	0.4369	1.916	0
5070	593.1	1.2167	1.1295	0.437	1.9159	0
5071	593	1.2173	1.1295	0.4369	1.9167	0
5072	592.9	1.2176	1.1299	0.437	1.9166	0
5073	592.8	1.2186	1.1305	0.4374	1.9188	0
5074	592.7	1.2182	1.1306	0.4372	1.9185	0
5075	592.6	1.2187	1.1308	0.4371	1.9173	0
5076	592.5	1.219	1.1309	0.4372	1.9193	0
5077	592.4	1.2194	1.1313	0.4373	1.9203	0
5078	592.3	1.2195	1.1317	0.4377	1.9191	0
5079	592.2	1.2203	1.1319	0.4376	1.9201	0
5080	592.1	1.2204	1.1323	0.4377	1.9211	0
5081	592	1.2209	1.1323	0.4375	1.9209	0
5082	591.9	1.2212	1.1325	0.4374	1.9219	0
5083	591.8	1.2218	1.1326	0.4376	1.9207	0
5084	591.7	1.2221	1.1332	0.4376	1.9217	0
5085	591.6	1.2223	1.1336	0.4376	1.9228	0
5086	591.5	1.2229	1.1335	0.4376	1.9216	0
5087	591.4	1.223	1.1338	0.4377	1.9238	0
5088	591.3	1.2236	1.1341	0.4377	1.9226	0
5089	591.2	1.2237	1.1343	0.4377	1.9236	0
5090	591.1	1.2243	1.1346	0.4376	1.9258	0
5091	591	1.225	1.1351	0.438	1.9235	0
5092	590.9	1.2252	1.1352	0.4382	1.9258	0
5093	590.8	1.2255	1.1358	0.4379	1.9245	0
5094	590.7	1.2262	1.1358	0.4378	1.9245	0
5095	590.6	1.2265	1.1361	0.4381	1.9255	0
5096	590.5	1.2266	1.1366	0.4382	1.9255	0
5097	590.4	1.2272	1.137	0.4381	1.9266	0
5098	590.3	1.2275	1.1371	0.4382	1.9277	0
5099	590.2	1.2277	1.1373	0.4382	1.9264	0
5100	590.1	1.2283	1.1374	0.4381	1.9263	0
5101	590	1.2284	1.1376	0.4382	1.9286	0
5102	589.9	1.2288	1.1381	0.4378	1.9287	0
5103	589.8	1.2292	1.1384	0.4378	1.9298	0

5104	589.7	1.2294	1.1385	0.438	1.9286	0
5105	589.6	1.23	1.1392	0.4379	1.9299	0
5106	589.5	1.2304	1.1392	0.4376	1.9298	0
5107	589.4	1.2306	1.1396	0.4375	1.9298	0
5108	589.3	1.2309	1.14	0.4377	1.9299	0
5109	589.2	1.2311	1.1404	0.4375	1.9299	0
5110	589.1	1.2316	1.1405	0.4373	1.9299	0
5111	589	1.2317	1.1405	0.4375	1.9299	0
5112	588.9	1.2324	1.1412	0.4375	1.93	0
5113	588.8	1.2326	1.1415	0.4376	1.9301	0
5114	588.7	1.2333	1.1416	0.4374	1.9313	0
5115	588.6	1.2333	1.142	0.4372	1.9313	0
5116	588.5	1.2338	1.1424	0.4372	1.9327	0
5117	588.4	1.2338	1.1426	0.437	1.9316	0
5118	588.3	1.2342	1.1429	0.437	1.9329	0
5119	588.2	1.2345	1.1431	0.4369	1.933	0
5120	588.1	1.2349	1.1434	0.4366	1.9319	0
5121	588	1.2354	1.1438	0.4367	1.9321	0
5122	587.9	1.2355	1.1443	0.4368	1.9335	0
5123	587.8	1.236	1.1445	0.4367	1.9325	0
5124	587.7	1.2364	1.1446	0.4366	1.9326	0
5125	587.6	1.2365	1.1447	0.4365	1.934	0
5126	587.5	1.2368	1.1452	0.4365	1.9317	0
5127	587.4	1.2372	1.1457	0.4364	1.9331	0
5128	587.3	1.2375	1.1461	0.4363	1.9346	0
5129	587.2	1.2379	1.1462	0.4362	1.9348	0
5130	587.1	1.2379	1.1464	0.4362	1.9337	0
5131	587	1.2385	1.147	0.4361	1.9354	0
5132	586.9	1.2389	1.1469	0.436	1.9356	0
5133	586.8	1.239	1.1474	0.4361	1.9358	0
5134	586.7	1.2393	1.1479	0.436	1.9362	0
5135	586.6	1.2399	1.1477	0.4359	1.9365	0
5136	586.5	1.24	1.1483	0.436	1.9368	0
5137	586.4	1.2407	1.1487	0.4361	1.9373	0
5138	586.3	1.2409	1.1489	0.4363	1.9362	0
5139	586.2	1.2412	1.1491	0.4361	1.9364	0
5140	586.1	1.2415	1.1494	0.436	1.9368	0
5141	586	1.2418	1.1497	0.4358	1.9357	0
5142	585.9	1.2422	1.15	0.436	1.9375	0
5143	585.8	1.2423	1.1504	0.436	1.9378	0
5144	585.7	1.2428	1.151	0.4362	1.9396	0
5145	585.6	1.2432	1.1514	0.4361	1.9373	0
5146	585.5	1.2434	1.1518	0.4361	1.9378	0

5147	585.4	1.2439	1.1522	0.4362	1.9396	0
5148	585.3	1.2447	1.1525	0.4362	1.9399	0
5149	585.2	1.245	1.1528	0.4361	1.9402	0
5150	585.1	1.2449	1.1529	0.4361	1.9392	0
5151	585	1.2453	1.1535	0.4362	1.9396	0
5152	584.9	1.241	1.152	0.4583	1.9585	0
5153	584.8	1.241	1.1523	0.4581	1.9581	0
5154	584.7	1.2414	1.1529	0.4581	1.9575	0
5155	584.6	1.2423	1.1535	0.4582	1.9594	0
5156	584.5	1.2425	1.154	0.4584	1.9615	0
5157	584.4	1.2429	1.1543	0.4586	1.961	0
5158	584.3	1.2434	1.1546	0.4588	1.9606	0
5159	584.2	1.2434	1.1549	0.459	1.9579	0
5160	584.1	1.2439	1.1552	0.4589	1.9576	0
5161	584	1.244	1.1559	0.459	1.9618	0
5162	583.9	1.2442	1.1561	0.459	1.9615	0
5163	583.8	1.2451	1.1564	0.4589	1.9611	0
5164	583.7	1.2451	1.1574	0.4591	1.9634	0
5165	583.6	1.2458	1.1576	0.4591	1.9632	0
5166	583.5	1.2457	1.1579	0.4593	1.9631	0
5167	583.4	1.2467	1.1587	0.4593	1.9632	0
5168	583.3	1.2467	1.1587	0.4596	1.9633	0
5169	583.2	1.2469	1.1596	0.4596	1.9635	0
5170	583.1	1.2475	1.1602	0.4595	1.9659	0
5171	583	1.2475	1.1606	0.4596	1.964	0
5172	582.9	1.248	1.1611	0.4598	1.9669	0
5173	582.8	1.2485	1.1612	0.4596	1.965	0
5174	582.7	1.249	1.1617	0.4597	1.9655	0
5175	582.6	1.2488	1.1617	0.4598	1.9639	0
5176	582.5	1.2495	1.1624	0.4597	1.9669	0
5177	582.4	1.25	1.1633	0.4599	1.9655	0
5178	582.3	1.2498	1.1633	0.4599	1.9663	0
5179	582.2	1.2507	1.1638	0.4598	1.9671	0
5180	582.1	1.2509	1.1649	0.4601	1.9682	0
5181	582	1.2515	1.1649	0.4601	1.9716	0
5182	581.9	1.2514	1.1657	0.4603	1.9682	0
5183	581.8	1.2521	1.1661	0.4604	1.9693	0
5184	581.7	1.2527	1.1672	0.4602	1.9703	0
5185	581.6	1.253	1.1672	0.4602	1.9715	0
5186	581.5	1.2532	1.1673	0.4601	1.9703	0
5187	581.4	1.2544	1.1677	0.4604	1.9715	0
5188	581.3	1.2542	1.1685	0.4605	1.9704	0
5189	581.2	1.2553	1.1689	0.4606	1.9737	0

5190	581.1	1.2559	1.1693	0.4604	1.9703	0
5191	581	1.2563	1.1702	0.4606	1.9716	0
5192	580.9	1.2572	1.1704	0.4608	1.9747	0
5193	580.8	1.2575	1.1707	0.4608	1.9713	0
5194	580.7	1.2584	1.1713	0.461	1.9723	0
5195	580.6	1.2589	1.1719	0.461	1.971	0
5196	580.5	1.2593	1.1724	0.4606	1.9718	0
5197	580.4	1.2599	1.1727	0.4608	1.9728	0
5198	580.3	1.2607	1.1735	0.4607	1.9736	0
5199	580.2	1.2616	1.1737	0.4609	1.9745	0
5200	580.1	1.2621	1.1743	0.4609	1.9755	0
5201	580	1.2629	1.1747	0.4607	1.974	0
5202	579.9	1.2633	1.1756	0.4607	1.9749	0
5203	579.8	1.2643	1.1762	0.461	1.9736	0
5204	579.7	1.2642	1.1766	0.4612	1.9766	0
5205	579.6	1.2655	1.1775	0.461	1.9753	0
5206	579.5	1.2658	1.1779	0.4607	1.9782	0
5207	579.4	1.2664	1.1785	0.461	1.977	0
5208	579.3	1.2671	1.1789	0.4609	1.9778	0
5209	579.2	1.2679	1.1797	0.4609	1.9742	0
5210	579.1	1.2686	1.18	0.4611	1.9771	0
5211	579	1.2693	1.1804	0.461	1.9777	0
5212	578.9	1.2701	1.1813	0.4608	1.9765	0
5213	578.8	1.271	1.1821	0.4606	1.9773	0
5214	578.7	1.2713	1.1822	0.461	1.9781	0
5215	578.6	1.2722	1.1837	0.461	1.9811	0
5216	578.5	1.273	1.1838	0.461	1.9797	0
5217	578.4	1.2736	1.1848	0.4608	1.9803	0
5218	578.3	1.2744	1.1849	0.4612	1.979	0
5219	578.2	1.2752	1.1857	0.4612	1.9798	0
5220	578.1	1.2755	1.1864	0.4611	1.9827	0
5221	578	1.277	1.1871	0.4611	1.9833	0
5222	577.9	1.2773	1.1875	0.4609	1.9819	0
5223	577.8	1.2785	1.1882	0.461	1.9827	0
5224	577.7	1.2788	1.1893	0.461	1.9834	0
5225	577.6	1.2799	1.19	0.461	1.9819	0
5226	577.5	1.281	1.191	0.4608	1.9825	0
5227	577.4	1.2817	1.1914	0.4609	1.9833	0
5228	577.3	1.2819	1.1923	0.4608	1.9839	0
5229	577.2	1.2831	1.1934	0.4609	1.9846	0
5230	577.1	1.2842	1.1938	0.461	1.9853	0
5231	577	1.2844	1.1947	0.461	1.9859	0
5232	576.9	1.2856	1.1951	0.4608	1.9867	0

5233	576.8	1.2866	1.196	0.4609	1.9851	0
5234	576.7	1.2871	1.1963	0.4608	1.9857	0
5235	576.6	1.2882	1.1976	0.4607	1.9885	0
5236	576.5	1.2889	1.1986	0.4607	1.987	0
5237	576.4	1.29	1.1993	0.4606	1.9876	0
5238	576.3	1.2907	1.2	0.4608	1.9884	0
5239	576.2	1.2912	1.2006	0.4609	1.9868	0
5240	576.1	1.2923	1.2018	0.4608	1.9874	0
5241	576	1.2934	1.2029	0.4607	1.9902	0
5242	575.9	1.294	1.2035	0.4609	1.9866	0
5243	575.8	1.2956	1.2046	0.4609	1.9873	0
5244	575.7	1.2962	1.2055	0.4611	1.99	0
5245	575.6	1.2972	1.2064	0.4608	1.9884	0
5246	575.5	1.2983	1.2071	0.4608	1.9913	0
5247	575.4	1.2993	1.2081	0.461	1.9919	0
5248	575.3	1.2999	1.209	0.461	1.9903	0
5249	575.2	1.3019	1.2103	0.4611	1.991	0
5250	575.1	1.3025	1.211	0.4613	1.9916	0
5251	575	1.304	1.2123	0.4612	1.9923	0
5252	574.9	1.3046	1.2133	0.4613	1.9929	0
5253	574.8	1.3058	1.2147	0.4614	1.9936	0
5254	574.7	1.3067	1.2156	0.4612	1.9941	0
5255	574.6	1.3078	1.2173	0.4614	1.9926	0
5256	574.5	1.3093	1.2183	0.4614	1.9933	0
5257	574.4	1.3105	1.2193	0.4617	1.9919	0
5258	574.3	1.3119	1.2206	0.4617	1.9945	0
5259	574.2	1.3135	1.2217	0.4619	1.9931	0
5260	574.1	1.3145	1.2226	0.4619	1.9937	0
5261	574	1.3156	1.2247	0.462	1.9944	0
5262	573.9	1.3171	1.226	0.4622	1.9928	0
5263	573.8	1.3181	1.2273	0.4623	1.9956	0
5264	573.7	1.3201	1.2287	0.4625	1.9962	0
5265	573.6	1.3211	1.23	0.4623	1.9968	0
5266	573.5	1.3226	1.2313	0.4625	1.9974	0
5267	573.4	1.324	1.2329	0.4626	1.9979	0
5268	573.3	1.3255	1.2346	0.4625	1.9965	0
5269	573.2	1.327	1.2359	0.4628	1.9949	0
5270	573.1	1.3281	1.2376	0.4626	1.9997	0
5271	573	1.3297	1.2395	0.4628	1.9984	0
5272	572.9	1.3317	1.2408	0.4628	2.0011	0
5273	572.8	1.3331	1.2421	0.4631	1.9974	0
5274	572.7	1.3346	1.2445	0.4629	2.0022	0
5275	572.6	1.336	1.2457	0.4627	2.0006	0

5276	572.5	1.3374	1.2474	0.4628	2.0012	0
5277	572.4	1.3395	1.2488	0.463	2.0039	0
5278	572.3	1.3409	1.2508	0.463	2.0024	0
5279	572.2	1.3429	1.2525	0.4631	2.001	0
5280	572.1	1.3444	1.255	0.4631	2.0036	0
5281	572	1.346	1.2571	0.4632	2.0022	0
5282	571.9	1.3484	1.2588	0.4633	2.0027	0
5283	571.8	1.3504	1.2605	0.4632	2.0033	0
5284	571.7	1.3523	1.2626	0.463	2.0018	0
5285	571.6	1.3534	1.2647	0.4629	2.0024	0
5286	571.5	1.3557	1.2667	0.463	2.005	0
5287	571.4	1.3578	1.2689	0.4632	2.0057	0
5288	571.3	1.3597	1.271	0.463	2.0062	0
5289	571.2	1.3617	1.2731	0.4631	2.0047	0
5290	571.1	1.3632	1.2748	0.4628	2.0052	0
5291	571	1.3656	1.2772	0.4628	2.0037	0
5292	570.9	1.3676	1.2797	0.463	2.0063	0
5293	570.8	1.37	1.2814	0.4628	2.0089	0
5294	570.7	1.372	1.2839	0.4627	2.0053	0
5295	570.6	1.3745	1.2861	0.4629	2.008	0
5296	570.5	1.3769	1.2885	0.4629	2.0085	0
5297	570.4	1.3791	1.2908	0.4627	2.0113	0
5298	570.3	1.3821	1.2934	0.4629	2.0098	0
5299	570.2	1.3842	1.296	0.463	2.0104	0
5300	570.1	1.3866	1.2976	0.4629	2.0108	0
5301	570	1.3891	1.2998	0.4629	2.0093	0
5302	569.9	1.3917	1.3028	0.4628	2.0099	0
5303	569.8	1.3947	1.3054	0.4626	2.0126	0
5304	569.7	1.3974	1.3085	0.4627	2.0111	0
5305	569.6	1.4	1.3102	0.4626	2.0117	0
5306	569.5	1.4026	1.3129	0.4627	2.0143	0
5307	569.4	1.4056	1.3159	0.4627	2.0128	0
5308	569.3	1.4083	1.3186	0.4628	2.0135	0
5309	569.2	1.411	1.3213	0.4628	2.0141	0
5310	569.1	1.4136	1.324	0.4628	2.0126	0
5311	569	1.4162	1.3266	0.4628	2.0152	0
5312	568.9	1.4194	1.3293	0.4626	2.0157	0
5313	568.8	1.422	1.332	0.4628	2.0163	0
5314	568.7	1.4247	1.3347	0.4627	2.0169	0
5315	568.6	1.4273	1.3369	0.4627	2.0153	0
5316	568.5	1.4306	1.3393	0.4628	2.016	0
5317	568.4	1.4332	1.3415	0.463	2.0165	0
5318	568.3	1.436	1.3447	0.4627	2.015	0

5319	568.2	1.4393	1.3475	0.4629	2.0177	0
5320	568.1	1.4421	1.3504	0.4632	2.0183	0
5321	568	1.4448	1.3526	0.4634	2.0168	0
5322	567.9	1.4481	1.3554	0.4633	2.0194	0
5323	567.8	1.4514	1.3581	0.4633	2.0179	0
5324	567.7	1.4541	1.3605	0.4633	2.0205	0
5325	567.6	1.4574	1.3628	0.4631	2.021	0
5326	567.5	1.4597	1.366	0.463	2.0195	0
5327	567.4	1.4625	1.3679	0.463	2.022	0
5328	567.3	1.4665	1.3713	0.4633	2.0206	0
5329	567.2	1.4693	1.3741	0.4634	2.0212	0
5330	567.1	1.4727	1.377	0.4633	2.0238	0
5331	567	1.4768	1.3794	0.4634	2.0244	0
5332	566.9	1.4792	1.3819	0.4634	2.025	0
5333	566.8	1.4839	1.3853	0.4636	2.0236	0
5334	566.7	1.4868	1.3878	0.4638	2.0241	0
5335	566.6	1.4902	1.3906	0.4635	2.0266	0
5336	566.5	1.4945	1.3941	0.4638	2.0252	0
5337	566.4	1.4986	1.3965	0.4639	2.0237	0
5338	566.3	1.5017	1.3991	0.4641	2.0264	0
5339	566.2	1.5052	1.4025	0.464	2.0269	0
5340	566.1	1.5089	1.4055	0.464	2.0254	0
5341	566	1.5131	1.4074	0.464	2.028	0
5342	565.9	1.5169	1.4106	0.4642	2.0307	0
5343	565.8	1.5206	1.413	0.4644	2.0291	0
5344	565.7	1.5243	1.4156	0.4643	2.0297	0
5345	565.6	1.5288	1.4187	0.4647	2.0304	0
5346	565.5	1.5332	1.4218	0.4646	2.0309	0
5347	565.4	1.537	1.4244	0.4646	2.0335	0
5348	565.3	1.5414	1.4273	0.4644	2.0299	0
5349	565.2	1.5446	1.4299	0.4644	2.0325	0
5350	565.1	1.5486	1.4331	0.4646	2.0311	0
5351	565	1.5531	1.4357	0.4648	2.0356	0
5352	564.9	1.5578	1.4389	0.4651	2.0342	0
5353	564.8	1.561	1.4415	0.4651	2.0327	0
5354	564.7	1.5657	1.4446	0.4651	2.0373	0
5355	564.6	1.5697	1.4472	0.4651	2.0358	0
5356	564.5	1.5744	1.4498	0.4654	2.0363	0
5357	564.4	1.5785	1.453	0.4656	2.0369	0
5358	564.3	1.5826	1.4557	0.4656	2.0394	0
5359	564.2	1.5867	1.4588	0.4654	2.0379	0
5360	564.1	1.5902	1.4611	0.4655	2.0385	0
5361	564	1.595	1.4648	0.4655	2.0389	0

5362	563.9	1.5992	1.4675	0.4657	2.0395	0
5363	563.8	1.6028	1.4702	0.466	2.0421	0
5364	563.7	1.607	1.4735	0.466	2.0426	0
5365	563.6	1.6114	1.4769	0.466	2.0411	0
5366	563.5	1.6156	1.4801	0.4662	2.0436	0
5367	563.4	1.6192	1.4823	0.4663	2.0442	0
5368	563.3	1.6229	1.4857	0.4664	2.0427	0
5369	563.2	1.6273	1.4885	0.4664	2.0433	0
5370	563.1	1.631	1.4913	0.4663	2.0438	0
5371	563	1.634	1.4948	0.4666	2.0423	0
5372	562.9	1.6393	1.4976	0.4664	2.0449	0
5373	562.8	1.643	1.5016	0.4666	2.0454	0
5374	562.7	1.6466	1.5038	0.4664	2.0458	0
5375	562.6	1.6495	1.5071	0.4663	2.0482	0
5376	562.5	1.6542	1.51	0.4666	2.0468	0
5377	562.4	1.6572	1.5135	0.4664	2.0473	0
5378	562.3	1.661	1.5164	0.4666	2.0477	0
5379	562.2	1.6639	1.5192	0.4665	2.0502	0
5380	562.1	1.6677	1.5227	0.4663	2.0507	0
5381	562	1.6717	1.5251	0.4666	2.0492	0
5382	561.9	1.6747	1.5286	0.4667	2.0496	0
5383	561.8	1.6777	1.5321	0.4665	2.0501	0
5384	561.7	1.6808	1.5351	0.4666	2.0526	0
5385	561.6	1.6856	1.5367	0.4665	2.051	0
5386	561.5	1.6888	1.5404	0.4664	2.0535	0
5387	561.4	1.691	1.544	0.4665	2.054	0
5388	561.3	1.6942	1.5477	0.4665	2.0545	0
5389	561.2	1.6965	1.5501	0.4664	2.053	0
5390	561.1	1.7015	1.5538	0.4662	2.0555	0
5391	561	1.7038	1.5563	0.4664	2.0539	0
5392	560.9	1.7068	1.5598	0.4661	2.0563	0
5393	560.8	1.71	1.5622	0.4661	2.0567	0
5394	560.7	1.7124	1.566	0.4663	2.0572	0
5395	560.6	1.7175	1.5691	0.4661	2.0556	0
5396	560.5	1.7189	1.5723	0.4662	2.0562	0
5397	560.4	1.7212	1.5754	0.4662	2.0566	0
5398	560.3	1.7246	1.5786	0.466	2.057	0
5399	560.2	1.7288	1.5824	0.4662	2.0595	0
5400	560.1	1.7302	1.5849	0.4661	2.06	0
5401	560	1.7336	1.5882	0.4659	2.0605	0
5402	559.9	1.737	1.5914	0.4659	2.0589	0
5403	559.8	1.7404	1.5954	0.4659	2.0615	0
5404	559.7	1.7438	1.5979	0.4661	2.0598	0

5405	559.6	1.7462	1.6012	0.4659	2.0603	0
5406	559.5	1.7487	1.6052	0.4657	2.0607	0
5407	559.4	1.7521	1.6078	0.4658	2.0653	0
5408	559.3	1.7567	1.612	0.4659	2.0638	0
5409	559.2	1.759	1.6145	0.4656	2.0621	0
5410	559.1	1.7626	1.6187	0.4657	2.0626	0
5411	559	1.7652	1.6214	0.4655	2.0651	0
5412	558.9	1.7687	1.6248	0.4656	2.0657	0
5413	558.8	1.7722	1.6282	0.4656	2.064	0
5414	558.7	1.7758	1.6309	0.4654	2.0685	0
5415	558.6	1.7784	1.6343	0.4655	2.069	0
5416	558.5	1.7809	1.6378	0.4654	2.0674	0
5417	558.4	1.7846	1.6406	0.465	2.0678	0
5418	558.3	1.7883	1.6434	0.4654	2.0683	0
5419	558.2	1.7908	1.6476	0.4653	2.0687	0
5420	558.1	1.7946	1.6512	0.4652	2.0692	0
5421	558	1.7971	1.6539	0.4652	2.0716	0
5422	557.9	1.7998	1.6568	0.4652	2.0722	0
5423	557.8	1.8036	1.6613	0.4655	2.0726	0
5424	557.7	1.8073	1.6633	0.4653	2.073	0
5425	557.6	1.8089	1.6677	0.4651	2.0735	0
5426	557.5	1.8115	1.6698	0.4653	2.0739	0
5427	557.4	1.8154	1.6735	0.4653	2.0764	0
5428	557.3	1.816	1.6773	0.4653	2.0749	0
5429	557.2	1.8198	1.6803	0.4654	2.0795	0
5430	557.1	1.8225	1.684	0.4654	2.0757	0
5431	557	1.8252	1.6869	0.4655	2.0761	0
5432	556.9	1.828	1.6908	0.4656	2.0787	0
5433	556.8	1.8319	1.6938	0.4653	2.0791	0
5434	556.7	1.8324	1.6968	0.4658	2.0796	0
5435	556.6	1.8364	1.6998	0.4658	2.08	0
5436	556.5	1.8367	1.7027	0.4657	2.0803	0
5437	556.4	1.8397	1.7059	0.4659	2.0809	0
5438	556.3	1.8412	1.7098	0.4659	2.0813	0
5439	556.2	1.8429	1.7121	0.4663	2.0818	0
5440	556.1	1.8468	1.7159	0.4658	2.0841	0
5441	556	1.8474	1.7191	0.4662	2.0847	0
5442	555.9	1.8503	1.7214	0.4666	2.0852	0
5443	555.8	1.8532	1.7255	0.4665	2.0836	0
5444	555.7	1.8537	1.7278	0.4668	2.0883	0
5445	555.6	1.8578	1.731	0.4669	2.0887	0
5446	555.5	1.858	1.734	0.4668	2.089	0
5447	555.4	1.8623	1.7373	0.4669	2.0895	0

5448	555.3	1.8639	1.7415	0.4673	2.0879	0
5449	555.2	1.8669	1.7438	0.4674	2.0904	0
5450	555.1	1.8686	1.7471	0.4675	2.0929	0
5451	555	1.8715	1.7494	0.4676	2.0933	0
5452	554.9	1.8732	1.7518	0.4676	2.0938	0
5453	554.8	1.8761	1.7551	0.4679	2.0942	0
5454	554.7	1.8791	1.7584	0.4681	2.0946	0
5455	554.6	1.8808	1.7607	0.468	2.0971	0
5456	554.5	1.8825	1.763	0.4683	2.0975	0
5457	554.4	1.8855	1.7664	0.4685	2.098	0
5458	554.3	1.8885	1.7698	0.4687	2.0984	0
5459	554.2	1.8914	1.7711	0.4686	2.0987	0
5460	554.1	1.8944	1.7744	0.4687	2.1012	0
5461	554	1.8975	1.7768	0.4688	2.1016	0
5462	553.9	1.8991	1.7802	0.469	2.104	0
5463	553.8	1.9035	1.7836	0.4691	2.1023	0
5464	553.7	1.9064	1.7849	0.4692	2.1025	0
5465	553.6	1.9094	1.7871	0.4694	2.1028	0
5466	553.5	1.9111	1.7906	0.4694	2.1053	0
5467	553.4	1.9142	1.792	0.4693	2.1056	0
5468	553.3	1.9173	1.7954	0.4694	2.1081	0
5469	553.2	1.9204	1.7979	0.4696	2.1063	0
5470	553.1	1.9249	1.8014	0.4697	2.1066	0
5471	553	1.9253	1.8018	0.4699	2.1091	0
5472	552.9	1.927	1.8042	0.47	2.1073	0
5473	552.8	1.9314	1.8065	0.4698	2.1097	0
5474	552.7	1.9346	1.8089	0.47	2.11	0
5475	552.6	1.9378	1.8114	0.4704	2.1125	0
5476	552.5	1.9409	1.8138	0.4702	2.1148	0
5477	552.4	1.944	1.8161	0.4701	2.1107	0
5478	552.3	1.9457	1.8175	0.4702	2.1132	0
5479	552.2	1.9488	1.8198	0.4701	2.1154	0
5480	552.1	1.9505	1.8223	0.4701	2.1179	0
5481	552	1.9523	1.8237	0.47	2.116	0
5482	551.9	1.9541	1.8251	0.4701	2.1184	0
5483	551.8	1.9589	1.8276	0.4702	2.1187	0
5484	551.7	1.9606	1.8289	0.4701	2.1168	0
5485	551.6	1.9622	1.8301	0.4702	2.1191	0
5486	551.5	1.967	1.8338	0.4703	2.1193	0
5487	551.4	1.9657	1.8339	0.4705	2.1173	0
5488	551.3	1.9705	1.8353	0.4705	2.1219	0
5489	551.2	1.9692	1.8389	0.4704	2.1221	0
5490	551.1	1.9738	1.8389	0.4705	2.1221	0

5491	551	1.9755	1.8402	0.4702	2.1244	0
5492	550.9	1.9756	1.8426	0.4703	2.1245	0
5493	550.8	1.9775	1.8441	0.4703	2.1249	0
5494	550.7	1.976	1.8464	0.4705	2.1271	0
5495	550.6	1.9793	1.8477	0.4703	2.1273	0
5496	550.5	1.9793	1.8501	0.47	2.1295	0
5497	550.4	1.9795	1.8514	0.47	2.1275	0
5498	550.3	1.9827	1.8527	0.4703	2.1298	0
5499	550.2	1.9844	1.8539	0.4702	2.1299	0
5500	550.1	1.9829	1.8552	0.4701	2.1322	0
5501	550	1.9845	1.8564	0.47	2.1322	0
5502	549.9	1.9862	1.8577	0.4701	2.1346	0
5503	549.8	1.9862	1.8589	0.47	2.1324	0
5504	549.7	1.988	1.8602	0.4699	2.1347	0
5505	549.6	1.988	1.8627	0.4697	2.1348	0
5506	549.5	1.988	1.8627	0.4698	2.1348	0
5507	549.4	1.9898	1.8653	0.4698	2.1372	0
5508	549.3	1.9882	1.8665	0.4696	2.1372	0
5509	549.2	1.9897	1.8675	0.4697	2.1394	0
5510	549.1	1.9913	1.8676	0.4696	2.1371	0
5511	549	1.9914	1.8688	0.4695	2.1394	0
5512	548.9	1.9913	1.8712	0.4696	2.1416	0
5513	548.8	1.9913	1.8712	0.4695	2.1393	0
5514	548.7	1.9912	1.8724	0.4696	2.1439	0
5515	548.6	1.9896	1.8723	0.4692	2.1415	0
5516	548.5	1.9911	1.8747	0.4693	2.1437	0
5517	548.4	1.9879	1.8759	0.4694	2.1437	0
5518	548.3	1.9877	1.8758	0.4691	2.1413	0
5519	548.2	1.9878	1.877	0.4693	2.1482	0
5520	548.1	1.9845	1.8781	0.4691	2.1458	0
5521	548	1.9843	1.878	0.4689	2.1502	0
5522	547.9	1.9843	1.8792	0.4693	2.1478	0
5523	547.8	1.9826	1.8804	0.4691	2.1478	0
5524	547.7	1.9809	1.8802	0.469	2.15	0
5525	547.6	1.9793	1.8814	0.4691	2.1499	0
5526	547.5	1.9775	1.8812	0.4692	2.1521	0
5527	547.4	1.9774	1.8812	0.4692	2.1497	0
5528	547.3	1.9757	1.8835	0.4693	2.1517	0
5529	547.2	1.9739	1.882	0.4692	2.1538	0
5530	547.1	1.9722	1.8831	0.469	2.1561	0
5531	547	1.9705	1.883	0.4691	2.156	0
5532	546.9	1.9703	1.884	0.4692	2.1581	0
5533	546.8	1.967	1.8838	0.4692	2.1578	0

5534	546.7	1.9653	1.8836	0.4694	2.1577	0
5535	546.6	1.9636	1.8859	0.4693	2.1598	0
5536	546.5	1.9634	1.8858	0.4696	2.1597	0
5537	546.4	1.9601	1.8855	0.4696	2.1618	0
5538	546.3	1.9569	1.8853	0.4698	2.1616	0
5539	546.2	1.9582	1.8863	0.4698	2.1638	0
5540	546.1	1.9564	1.8873	0.4698	2.1659	0
5541	546	1.9531	1.8858	0.4697	2.1656	0
5542	545.9	1.9528	1.8854	0.4699	2.1677	0
5543	545.8	1.9509	1.8851	0.4699	2.1699	0
5544	545.7	1.9491	1.8847	0.4699	2.1695	0
5545	545.6	1.9474	1.887	0.4701	2.1717	0
5546	545.5	1.947	1.8854	0.4704	2.1713	0
5547	545.4	1.9467	1.8863	0.4703	2.171	0
5548	545.3	1.945	1.8835	0.4709	2.1757	0
5549	545.2	1.9431	1.8857	0.4709	2.1778	0
5550	545.1	1.9441	1.8852	0.4707	2.1749	0
5551	545	1.9437	1.8836	0.4708	2.1795	0
5552	544.9	1.9404	1.8844	0.4711	2.1765	0
5553	544.8	1.9414	1.884	0.4713	2.1812	0
5554	544.7	1.9395	1.8835	0.4713	2.1807	0
5555	544.6	1.9406	1.8832	0.4716	2.1829	0
5556	544.5	1.9387	1.8827	0.4718	2.1825	0
5557	544.4	1.9383	1.8823	0.472	2.1846	0
5558	544.3	1.9365	1.8807	0.4722	2.1868	0
5559	544.2	1.9375	1.8802	0.4726	2.1864	0
5560	544.1	1.9355	1.8822	0.4726	2.1858	0
5561	544	1.9365	1.8805	0.4728	2.188	0
5562	543.9	1.9344	1.8799	0.4731	2.1926	0
5563	543.8	1.9326	1.8795	0.4735	2.1922	0
5564	543.7	1.9335	1.879	0.4737	2.1917	0
5565	543.6	1.9329	1.8784	0.4737	2.1964	0
5566	543.5	1.931	1.8779	0.474	2.1959	0
5567	543.4	1.9318	1.8773	0.4739	2.198	0
5568	543.3	1.9313	1.8755	0.4742	2.2002	0
5569	543.2	1.9307	1.8749	0.4743	2.2022	0
5570	543.1	1.9301	1.8756	0.4748	2.2043	0
5571	543	1.928	1.8737	0.4748	2.2038	0
5572	542.9	1.9302	1.8742	0.4747	2.2058	0
5573	542.8	1.9281	1.8723	0.4749	2.2052	0
5574	542.7	1.9288	1.8703	0.4752	2.2071	0
5575	542.6	1.9268	1.871	0.475	2.2066	0
5576	542.5	1.9262	1.8704	0.4752	2.2115	0

5577	542.4	1.9241	1.8697	0.4756	2.2108	0
5578	542.3	1.9262	1.869	0.4755	2.2157	0
5579	542.2	1.9241	1.867	0.4755	2.2123	0
5580	542.1	1.9234	1.8676	0.4758	2.2144	0
5581	542	1.9256	1.8669	0.4758	2.2137	0
5582	541.9	1.9234	1.8649	0.476	2.2187	0
5583	541.8	1.9242	1.8642	0.4759	2.218	0
5584	541.7	1.9235	1.8648	0.4762	2.2202	0
5585	541.6	1.9227	1.8653	0.4761	2.2223	0
5586	541.5	1.9234	1.8632	0.4761	2.2245	0
5587	541.4	1.9227	1.8638	0.4764	2.2237	0
5588	541.3	1.9234	1.8617	0.4765	2.2259	0
5589	541.2	1.9211	1.8596	0.4764	2.228	0
5590	541.1	1.9218	1.8589	0.4764	2.2332	0
5591	541	1.9211	1.8594	0.4766	2.2295	0
5592	540.9	1.9217	1.8585	0.4766	2.2346	0
5593	540.8	1.9224	1.8578	0.4765	2.2309	0
5594	540.7	1.9231	1.857	0.4769	2.2362	0
5595	540.6	1.9222	1.8561	0.4767	2.2384	0
5596	540.5	1.9229	1.8566	0.4768	2.2346	0
5597	540.4	1.922	1.8531	0.4769	2.2397	0
5598	540.3	1.9211	1.8548	0.4767	2.2483	0
5599	540.2	1.9218	1.854	0.4771	2.2412	0
5600	540.1	1.9225	1.8545	0.477	2.2466	0
5601	540	1.9216	1.8523	0.4773	2.249	0
5602	539.9	1.9221	1.8513	0.4771	2.2512	0
5603	539.8	1.9228	1.8518	0.4773	2.2503	0
5604	539.7	1.9234	1.8496	0.4771	2.2527	0
5605	539.6	1.9225	1.8512	0.4769	2.255	0
5606	539.5	1.9247	1.8478	0.477	2.2541	0
5607	539.4	1.9237	1.8494	0.4768	2.2598	0
5608	539.3	1.9229	1.8486	0.477	2.2623	0
5609	539.2	1.9251	1.8478	0.4771	2.2581	0
5610	539.1	1.9243	1.8469	0.477	2.2639	0
5611	539	1.9218	1.846	0.4769	2.263	0
5612	538.9	1.9241	1.8451	0.4769	2.2655	0
5613	538.8	1.9233	1.8456	0.4769	2.2682	0
5614	538.7	1.9222	1.8472	0.4768	2.2706	0
5615	538.6	1.9228	1.8449	0.4765	2.273	0
5616	538.5	1.9234	1.844	0.4764	2.2721	0
5617	538.4	1.9226	1.8432	0.4761	2.2713	0
5618	538.3	1.9233	1.8435	0.4766	2.2738	0
5619	538.2	1.9224	1.8427	0.4764	2.2765	0

5620	538.1	1.9231	1.8431	0.4764	2.2757	0
5621	538	1.9205	1.8421	0.4761	2.2782	0
5622	537.9	1.9227	1.8425	0.4762	2.2808	0
5623	537.8	1.9218	1.8416	0.4763	2.2835	0
5624	537.7	1.921	1.8421	0.4765	2.2863	0
5625	537.6	1.92	1.8411	0.4761	2.289	0
5626	537.5	1.9191	1.8402	0.4759	2.2881	0
5627	537.4	1.9181	1.8392	0.4759	2.2908	0
5628	537.3	1.9188	1.8396	0.4761	2.2937	0
5629	537.2	1.9149	1.8415	0.4763	2.2966	0
5630	537.1	1.917	1.8418	0.4759	2.2994	0
5631	537	1.9145	1.8409	0.476	2.3023	0
5632	536.9	1.9152	1.8387	0.4764	2.3015	0
5633	536.8	1.9143	1.8404	0.4763	2.3083	0
5634	536.7	1.9133	1.8407	0.4762	2.3112	0
5635	536.6	1.9108	1.8399	0.4761	2.3103	0
5636	536.5	1.9115	1.8403	0.4763	2.3135	0
5637	536.4	1.9106	1.8394	0.4765	2.3165	0
5638	536.3	1.9096	1.8398	0.4761	2.3196	0
5639	536.2	1.9103	1.8388	0.476	2.3228	0
5640	536.1	1.9077	1.8392	0.476	2.3177	0
5641	536	1.9069	1.8411	0.476	2.325	0
5642	535.9	1.9074	1.8413	0.4763	2.3239	0
5643	535.8	1.9049	1.8404	0.4765	2.323	0
5644	535.7	1.9055	1.8408	0.476	2.3304	0
5645	535.6	1.906	1.8425	0.476	2.3379	0
5646	535.5	1.905	1.8414	0.476	2.3368	0
5647	535.4	1.904	1.8419	0.4761	2.3402	0
5648	535.3	1.9015	1.8437	0.4763	2.3392	0
5649	535.2	1.9037	1.8427	0.4761	2.3471	0
5650	535.1	1.9027	1.8432	0.4762	2.3506	0
5651	535	1.9018	1.845	0.4763	2.3541	0
5652	534.9	1.9008	1.844	0.4766	2.3576	0
5653	534.8	1.9013	1.8457	0.4765	2.3565	0
5654	534.7	1.9005	1.8463	0.4768	2.3649	0
5655	534.6	1.8979	1.8481	0.4767	2.3687	0
5656	534.5	1.9001	1.8472	0.4769	2.3724	0
5657	534.4	1.9008	1.8491	0.4769	2.3715	0
5658	534.3	1.8997	1.8495	0.477	2.3753	0
5659	534.2	1.9005	1.8515	0.4769	2.3792	0
5660	534.1	1.8996	1.8507	0.4773	2.3833	0
5661	534	1.9003	1.8512	0.4776	2.3873	0
5662	533.9	1.8993	1.8531	0.4779	2.3914	0

5663	533.8	1.9	1.8536	0.4778	2.3954	0
5664	533.7	1.899	1.8526	0.4779	2.3945	0
5665	533.6	1.898	1.856	0.4779	2.4037	0
5666	533.5	1.8986	1.8565	0.4779	2.4029	0
5667	533.4	1.8992	1.8569	0.4776	2.4016	0
5668	533.3	1.8983	1.859	0.478	2.4114	0
5669	533.2	1.899	1.8596	0.4781	2.4214	0
5670	533.1	1.8964	1.8601	0.4785	2.4203	0
5671	533	1.8954	1.8606	0.4782	2.4249	0
5672	532.9	1.896	1.8611	0.4784	2.4352	0
5673	532.8	1.8967	1.8632	0.4787	2.44	0
5674	532.7	1.8956	1.8652	0.4785	2.4331	0
5675	532.6	1.8948	1.8644	0.4785	2.4438	0
5676	532.5	1.8939	1.8651	0.4787	2.443	0
5677	532.4	1.8946	1.8656	0.4787	2.442	0
5678	532.3	1.8935	1.8693	0.479	2.4589	0
5679	532.2	1.8926	1.8683	0.4788	2.4519	0
5680	532.1	1.8915	1.8689	0.479	2.4569	0
5681	532	1.8906	1.8711	0.4788	2.4622	0
5682	531.9	1.8896	1.8733	0.4789	2.4612	0
5683	531.8	1.8887	1.8723	0.4789	2.4665	0
5684	531.7	1.8862	1.8747	0.4789	2.4656	0
5685	531.6	1.8871	1.8756	0.4794	2.4712	0
5686	531.5	1.8844	1.8778	0.4794	2.4766	0
5687	531.4	1.8834	1.8785	0.4794	2.4821	0
5688	531.3	1.8825	1.8792	0.4793	2.4812	0
5689	531.2	1.8799	1.8815	0.4793	2.4803	0
5690	531.1	1.8806	1.8823	0.4794	2.4996	0
5691	531	1.878	1.883	0.4792	2.4919	0
5692	530.9	1.8773	1.8839	0.4795	2.4978	0
5693	530.8	1.8764	1.8881	0.4795	2.4901	0
5694	530.7	1.8755	1.8889	0.4798	2.5031	0
5695	530.6	1.8761	1.8879	0.4798	2.4951	0
5696	530.5	1.8718	1.8903	0.4797	2.5081	0
5697	530.4	1.8724	1.891	0.4794	2.5071	0
5698	530.3	1.8731	1.8917	0.4794	2.5133	0
5699	530.2	1.8689	1.8943	0.4797	2.5123	0
5700	530.1	1.8679	1.8968	0.4796	2.5113	0
5701	530	1.8669	1.8976	0.4795	2.5177	0
5702	529.9	1.8676	1.8984	0.4796	2.5167	0
5703	529.8	1.8651	1.9029	0.4796	2.5158	0
5704	529.7	1.8656	1.9036	0.4796	2.5075	0
5705	529.6	1.863	1.9043	0.4791	2.5211	0

5706	529.5	1.862	1.9071	0.4795	2.5278	0
5707	529.4	1.8611	1.9079	0.4794	2.5268	0
5708	529.3	1.8617	1.9088	0.4792	2.5334	0
5709	529.2	1.8608	1.9116	0.4794	2.5174	0
5710	529.1	1.8566	1.9144	0.4792	2.5396	0
5711	529	1.8572	1.9152	0.4791	2.5306	0
5712	528.9	1.8564	1.9181	0.4789	2.5376	0
5713	528.8	1.8553	1.919	0.4786	2.5366	0
5714	528.7	1.8546	1.9182	0.4786	2.5438	0
5715	528.6	1.852	1.9211	0.4783	2.5349	0
5716	528.5	1.8527	1.9241	0.478	2.542	0
5717	528.4	1.852	1.9273	0.478	2.5493	0
5718	528.3	1.8529	1.9285	0.4781	2.5405	0
5719	528.2	1.8506	1.9298	0.4777	2.5479	0
5720	528.1	1.85	1.9311	0.4775	2.5473	0
5721	528	1.8477	1.9325	0.4775	2.5467	0
5722	527.9	1.8489	1.934	0.4772	2.5462	0
5723	527.8	1.8483	1.9375	0.4771	2.5539	0
5724	527.7	1.8461	1.9389	0.4769	2.5533	0
5725	527.6	1.8472	1.9384	0.4767	2.5528	0
5726	527.5	1.8452	1.9401	0.4764	2.5525	0
5727	527.4	1.8465	1.9438	0.4764	2.5521	0
5728	527.3	1.846	1.9454	0.4762	2.5516	0
5729	527.2	1.8459	1.9453	0.4761	2.5685	0
5730	527.1	1.8439	1.947	0.4758	2.5596	0
5731	527	1.8437	1.9489	0.4759	2.5594	0
5732	526.9	1.8451	1.9507	0.4757	2.5677	0
5733	526.8	1.8433	1.9506	0.4759	2.5589	0
5734	526.7	1.8431	1.9546	0.4757	2.5588	0
5735	526.6	1.8429	1.9565	0.4755	2.5672	0
5736	526.5	1.841	1.9562	0.4755	2.5583	0
5737	526.4	1.8409	1.9583	0.4754	2.5667	0
5738	526.3	1.8425	1.9604	0.4755	2.5667	0
5739	526.2	1.8422	1.9601	0.4753	2.5664	0
5740	526.1	1.8404	1.9599	0.4752	2.5751	0
5741	526	1.8419	1.9598	0.4753	2.5662	0
5742	525.9	1.8403	1.9641	0.4758	2.5662	0
5743	525.8	1.8417	1.9639	0.4758	2.5748	0
5744	525.7	1.8398	1.9637	0.4757	2.5658	0
5745	525.6	1.8396	1.9656	0.4755	2.5743	0
5746	525.5	1.841	1.9654	0.4757	2.5653	0
5747	525.4	1.8393	1.9675	0.476	2.5829	0
5748	525.3	1.8392	1.9696	0.476	2.5738	0

5749	525.2	1.8389	1.9715	0.4759	2.5737	0
5750	525.1	1.8387	1.9713	0.4757	2.5824	0
5751	525	1.8385	1.9733	0.4759	2.5732	0
5752	524.9	1.8398	1.973	0.4757	2.5819	0
5753	524.8	1.8379	1.975	0.476	2.5815	0
5754	524.7	1.8377	1.9771	0.476	2.5724	0
5755	524.6	1.8359	1.9769	0.4762	2.5812	0
5756	524.5	1.8357	1.9767	0.4762	2.5901	0
5757	524.4	1.8371	1.9787	0.4764	2.5807	0
5758	524.3	1.8352	1.9807	0.4764	2.5989	0
5759	524.2	1.835	1.9804	0.4766	2.5802	0
5760	524.1	1.8364	1.9802	0.4767	2.5984	0
5761	524	1.8328	1.9822	0.4766	2.5797	0
5762	523.9	1.8342	1.9819	0.4769	2.5885	0
5763	523.8	1.8338	1.9839	0.477	2.5882	0
5764	523.7	1.8304	1.9814	0.4769	2.588	0
5765	523.6	1.8302	1.9858	0.4771	2.5879	0
5766	523.5	1.8299	1.9832	0.477	2.5784	0
5767	523.4	1.8296	1.9829	0.4771	2.5781	0
5768	523.3	1.8261	1.9848	0.4772	2.5869	0
5769	523.2	1.8291	1.987	0.4772	2.596	0
5770	523.1	1.8271	1.9866	0.477	2.5864	0
5771	523	1.8253	1.9864	0.4771	2.5955	0
5772	522.9	1.8249	1.986	0.4771	2.5952	0
5773	522.8	1.823	1.988	0.4773	2.5948	0
5774	522.7	1.8243	1.9878	0.477	2.5945	0
5775	522.6	1.8224	1.9897	0.4772	2.5941	0
5776	522.5	1.8237	1.9872	0.4776	2.6035	0
5777	522.4	1.8218	1.9892	0.4775	2.5936	0
5778	522.3	1.8215	1.9865	0.4777	2.6028	0
5779	522.2	1.8213	1.9887	0.4775	2.6026	0
5780	522.1	1.821	1.9908	0.4778	2.5928	0
5781	522	1.8207	1.9905	0.4775	2.6021	0
5782	521.9	1.8188	1.9901	0.4776	2.5921	0
5783	521.8	1.8185	1.9898	0.4778	2.6014	0
5784	521.7	1.818	1.9894	0.4775	2.601	0
5785	521.6	1.8177	1.9891	0.4776	2.5911	0
5786	521.5	1.8175	1.9912	0.4779	2.6005	0
5787	521.4	1.8187	1.9908	0.4777	2.6	0
5788	521.3	1.8167	1.9904	0.4776	2.5901	0
5789	521.2	1.818	1.9878	0.4776	2.5899	0
5790	521.1	1.8162	1.9947	0.4778	2.5991	0
5791	521	1.8176	1.9897	0.4779	2.5989	0

5792	520.9	1.8155	1.9892	0.4779	2.5984	0
5793	520.8	1.8168	1.9889	0.4779	2.5981	0
5794	520.7	1.818	1.9886	0.4782	2.5977	0
5795	520.6	1.8194	1.9906	0.478	2.5976	0
5796	520.5	1.8174	1.9903	0.4779	2.5972	0
5797	520.4	1.8186	1.99	0.4778	2.5967	0
5798	520.3	1.8183	1.9872	0.4779	2.6062	0
5799	520.2	1.818	1.9892	0.4777	2.5962	0
5800	520.1	1.8208	1.9889	0.4777	2.6055	0
5801	520	1.8173	1.9886	0.4779	2.5953	0
5802	519.9	1.8185	1.9905	0.478	2.595	0
5803	519.8	1.8182	1.9878	0.4779	2.5947	0
5804	519.7	1.8195	1.9876	0.478	2.5849	0
5805	519.6	1.8223	1.9871	0.4778	2.594	0
5806	519.5	1.8203	1.9891	0.4777	2.5936	0
5807	519.4	1.8201	1.9865	0.4781	2.5839	0
5808	519.3	1.8214	1.9862	0.4785	2.6028	0
5809	519.2	1.821	1.9882	0.4781	2.6024	0
5810	519.1	1.8222	1.9878	0.4776	2.6021	0
5811	519	1.8234	1.9873	0.4777	2.6015	0
5812	518.9	1.8216	1.9848	0.4782	2.6114	0
5813	518.8	1.8229	1.9868	0.4781	2.601	0
5814	518.7	1.8208	1.984	0.4779	2.6007	0
5815	518.6	1.8237	1.9884	0.4776	2.6103	0
5816	518.5	1.8235	1.9882	0.4778	2.5902	0
5817	518.4	1.8214	1.9854	0.4779	2.5996	0
5818	518.3	1.8227	1.9849	0.4779	2.5991	0
5819	518.2	1.8223	1.9846	0.4779	2.5989	0
5820	518.1	1.8219	1.9866	0.4775	2.5887	0
5821	518	1.8232	1.9838	0.4775	2.5882	0
5822	517.9	1.8213	1.9859	0.478	2.5977	0
5823	517.8	1.8243	1.9856	0.4781	2.5974	0
5824	517.7	1.8222	1.9852	0.4777	2.5872	0
5825	517.6	1.8219	1.9873	0.4776	2.5967	0
5826	517.5	1.8215	1.9869	0.4775	2.5964	0
5827	517.4	1.8211	1.9865	0.4776	2.5959	0
5828	517.3	1.8207	1.9837	0.4777	2.5955	0
5829	517.2	1.8203	1.9833	0.4776	2.5854	0
5830	517.1	1.8216	1.983	0.4776	2.5948	0
5831	517	1.8196	1.9826	0.4775	2.5945	0
5832	516.9	1.8193	1.9847	0.4774	2.5941	0
5833	516.8	1.8189	1.982	0.4775	2.5938	0
5834	516.7	1.8202	1.9816	0.4776	2.6035	0

5835	516.6	1.8216	1.9862	0.4776	2.5931	0
5836	516.5	1.8197	1.9859	0.4778	2.6029	0
5837	516.4	1.8193	1.9831	0.4777	2.5924	0
5838	516.3	1.8189	1.9827	0.4777	2.5824	0
5839	516.2	1.8204	1.9825	0.4777	2.6019	0
5840	516.1	1.8184	1.9798	0.4778	2.6015	0
5841	516	1.8164	1.9843	0.4777	2.5913	0
5842	515.9	1.8176	1.9839	0.4777	2.5909	0
5843	515.8	1.8173	1.9836	0.4777	2.6005	0
5844	515.7	1.8186	1.9832	0.4779	2.6002	0
5845	515.6	1.8183	1.9853	0.4777	2.5998	0
5846	515.5	1.818	1.9825	0.4777	2.5894	0
5847	515.4	1.8176	1.9846	0.4778	2.5991	0
5848	515.3	1.8173	1.9867	0.478	2.5988	0
5849	515.2	1.817	1.984	0.4779	2.5984	0
5850	515.1	1.8183	1.9812	0.4778	2.5882	0
5851	515	1.818	1.9833	0.4781	2.5977	0
5852	514.9	1.8176	1.9829	0.4778	2.5974	0
5853	514.8	1.8156	1.9851	0.4781	2.6073	0
5854	514.7	1.817	1.9823	0.478	2.5967	0
5855	514.6	1.8183	1.9819	0.4781	2.5964	0
5856	514.5	1.8163	1.9866	0.4782	2.5962	0
5857	514.4	1.8161	1.9838	0.4781	2.5959	0
5858	514.3	1.8157	1.9835	0.4781	2.5955	0
5859	514.2	1.8153	1.9831	0.4783	2.6054	0
5860	514.1	1.8166	1.9827	0.4785	2.5948	0
5861	514	1.8181	1.9825	0.4784	2.5945	0
5862	513.9	1.8178	1.9847	0.4784	2.5943	0
5863	513.8	1.8175	1.9819	0.4787	2.6041	0
5864	513.7	1.817	1.9839	0.4785	2.6036	0
5865	513.6	1.8167	1.9836	0.4786	2.5933	0
5866	513.5	1.8164	1.9833	0.4786	2.6031	0
5867	513.4	1.8162	1.9856	0.4787	2.6029	0
5868	513.3	1.8159	1.9828	0.4788	2.6026	0
5869	513.2	1.8156	1.9849	0.479	2.6128	0
5870	513.1	1.8151	1.9819	0.4788	2.6017	0
5871	513	1.8165	1.9842	0.4789	2.6119	0
5872	512.9	1.8145	1.9864	0.4791	2.6012	0
5873	512.8	1.8142	1.9836	0.4791	2.6114	0
5874	512.7	1.8155	1.9832	0.4789	2.611	0
5875	512.6	1.8153	1.983	0.479	2.6107	0
5876	512.5	1.8132	1.9851	0.4794	2.6105	0
5877	512.4	1.8128	1.9847	0.4791	2.6099	0

5878	512.3	1.8141	1.9843	0.4791	2.6096	0
5879	512.2	1.8138	1.9815	0.4791	2.6092	0
5880	512.1	1.8152	1.9862	0.4791	2.6089	0
5881	512	1.8148	1.9833	0.4789	2.6192	0
5882	511.9	1.8128	1.9856	0.479	2.6084	0
5883	511.8	1.8141	1.9825	0.4787	2.6078	0
5884	511.7	1.8137	1.9822	0.4787	2.6075	0
5885	511.6	1.8116	1.9843	0.4789	2.6178	0
5886	511.5	1.813	1.9815	0.4788	2.6068	0
5887	511.4	1.811	1.9838	0.4791	2.6064	0
5888	511.3	1.8124	1.9809	0.4789	2.6062	0
5889	511.2	1.812	1.9831	0.4788	2.6165	0
5890	511.1	1.8116	1.9851	0.4786	2.6054	0
5891	511	1.8113	1.9848	0.4788	2.605	0
5892	510.9	1.8109	1.982	0.4788	2.6155	0
5893	510.8	1.8122	1.9815	0.4787	2.6149	0
5894	510.7	1.8135	1.9836	0.4788	2.6038	0
5895	510.6	1.8114	1.9807	0.4785	2.6142	0
5896	510.5	1.8128	1.9829	0.4786	2.6249	0
5897	510.4	1.8124	1.9825	0.4784	2.6135	0
5898	510.3	1.8138	1.9822	0.4787	2.6131	0
5899	510.2	1.8117	1.9844	0.4784	2.6238	0
5900	510.1	1.8096	1.984	0.4783	2.6124	0
5901	510	1.811	1.9836	0.4783	2.6121	0
5902	509.9	1.8123	1.9833	0.4784	2.6227	0
5903	509.8	1.8119	1.9855	0.4783	2.6114	0
5904	509.7	1.8117	1.9826	0.4785	2.622	0
5905	509.6	1.8147	1.9822	0.4781	2.6216	0
5906	509.5	1.8126	1.9819	0.4786	2.6212	0
5907	509.4	1.8123	1.9841	0.4783	2.6209	0
5908	509.3	1.8136	1.9836	0.4783	2.6205	0
5909	509.2	1.8149	1.9859	0.478	2.6313	0
5910	509.1	1.8146	1.9829	0.4781	2.6198	0
5911	509	1.8159	1.9877	0.4782	2.6306	0
5912	508.9	1.8155	1.9847	0.4779	2.6189	0
5913	508.8	1.8135	1.9896	0.4782	2.6187	0
5914	508.7	1.8166	1.984	0.4781	2.6295	0
5915	508.6	1.8162	1.9836	0.4778	2.6178	0
5916	508.5	1.8176	1.9858	0.4776	2.6174	0
5917	508.4	1.8173	1.9855	0.4782	2.64	0
5918	508.3	1.8187	1.9877	0.478	2.6396	0
5919	508.2	1.8182	1.9873	0.4782	2.6392	0
5920	508.1	1.8161	1.987	0.4778	2.6388	0

5921	508	1.8192	1.9891	0.478	2.6269	0
5922	507.9	1.8188	1.9913	0.4777	2.6379	0
5923	507.8	1.8203	1.9884	0.4778	2.6377	0
5924	507.7	1.82	1.9908	0.4783	2.6375	0
5925	507.6	1.8214	1.9904	0.478	2.637	0
5926	507.5	1.8229	1.9901	0.478	2.6368	0
5927	507.4	1.8207	1.9897	0.4778	2.6364	0
5928	507.3	1.8223	1.9895	0.4782	2.6245	0
5929	507.2	1.8218	1.9943	0.4783	2.6356	0
5930	507.1	1.8215	1.9913	0.4779	2.6472	0
5931	507	1.8229	1.991	0.478	2.6469	0
5932	506.9	1.8226	1.9933	0.4782	2.6347	0
5933	506.8	1.8259	1.9956	0.4782	2.6461	0
5934	506.7	1.8236	1.9952	0.4781	2.6457	0
5935	506.6	1.8234	1.9949	0.4785	2.6455	0
5936	506.5	1.8248	1.9972	0.4781	2.6451	0
5937	506.4	1.8245	1.9942	0.4785	2.657	0
5938	506.3	1.8261	1.9967	0.4786	2.6445	0
5939	506.2	1.8238	1.9962	0.4785	2.6562	0
5940	506.1	1.8271	1.9986	0.4787	2.656	0
5941	506	1.8249	1.9983	0.4786	2.6556	0
5942	505.9	1.8263	1.9978	0.4785	2.6552	0
5943	505.8	1.8279	1.9976	0.4789	2.6426	0
5944	505.7	1.8276	1.9973	0.479	2.6546	0
5945	505.6	1.8272	1.9997	0.4793	2.6542	0
5946	505.5	1.8287	1.9993	0.479	2.6538	0
5947	505.4	1.8266	1.999	0.4791	2.679	0
5948	505.3	1.8299	2.0013	0.4792	2.6409	0
5949	505.2	1.8296	2.0011	0.4796	2.6287	0
5950	505.1	1.8293	2.0035	0.4796	2.6404	0
5951	505	1.8307	2.0031	0.4793	2.6521	0
5952	504.9	1.8285	2.0028	0.4796	2.6643	0
5953	504.8	1.8301	2.0025	0.4798	2.6515	0
5954	504.7	1.8297	2.0049	0.4797	2.6511	0
5955	504.6	1.8313	2.0017	0.4795	2.6507	0
5956	504.5	1.831	2.0015	0.48	2.6631	0
5957	504.4	1.8307	2.004	0.4801	2.6627	0
5958	504.3	1.8321	2.0063	0.4801	2.6753	0
5959	504.2	1.8319	2.0033	0.4801	2.6621	0
5960	504.1	1.8316	2.0058	0.4807	2.6617	0
5961	504	1.8312	2.0054	0.4802	2.6878	0
5962	503.9	1.8327	2.005	0.4802	2.6741	0
5963	503.8	1.8343	2.0048	0.4807	2.6737	0

5964	503.7	1.834	2.0045	0.4807	2.6735	0
5965	503.6	1.8337	2.0041	0.4809	2.6863	0
5966	503.5	1.8353	2.0066	0.4809	2.6728	0
5967	503.4	1.837	2.0064	0.4811	2.6996	0
5968	503.3	1.8365	2.0059	0.481	2.672	0
5969	503.2	1.8381	2.0056	0.4811	2.6588	0
5970	503.1	1.8377	2.0052	0.4811	2.6584	0
5971	503	1.8414	2.005	0.4811	2.6844	0
5972	502.9	1.841	2.0074	0.481	2.6708	0
5973	502.8	1.8406	2.0071	0.4811	2.6838	0
5974	502.7	1.8404	2.0068	0.4811	2.6836	0
5975	502.6	1.842	2.0065	0.481	2.6832	0
5976	502.5	1.8416	2.0061	0.4813	2.6828	0
5977	502.4	1.8432	2.0058	0.4811	2.6824	0
5978	502.3	1.8449	2.0055	0.4813	2.6959	0
5979	502.2	1.8446	2.008	0.4811	2.6819	0
5980	502.1	1.8442	2.0048	0.4812	2.6953	0
5981	502	1.8458	2.0073	0.4812	2.6949	0
5982	501.9	1.8475	2.0041	0.4813	2.6946	0
5983	501.8	1.8492	2.0067	0.481	2.6942	0
5984	501.7	1.8469	2.0064	0.4813	2.694	0
5985	501.6	1.8486	2.006	0.4811	2.708	0
5986	501.5	1.8522	2.0057	0.4809	2.6794	0
5987	501.4	1.8519	2.0053	0.4811	2.6792	0
5988	501.3	1.8515	2.0078	0.481	2.7069	0
5989	501.2	1.8553	2.0047	0.4812	2.7066	0
5990	501.1	1.8549	2.0072	0.4808	2.6919	0
5991	501	1.8566	2.0069	0.4808	2.7058	0
5992	500.9	1.8562	2.0036	0.4807	2.7055	0
5993	500.8	1.8599	2.0032	0.4807	2.7051	0
5994	500.7	1.8575	2.0057	0.4806	2.7047	0
5995	500.6	1.8592	2.0083	0.4807	2.7044	0
5996	500.5	1.863	2.005	0.4806	2.6897	0
5997	500.4	1.8627	2.0046	0.4805	2.7183	0
5998	500.3	1.8625	2.0045	0.4807	2.7033	0
5999	500.2	1.8641	2.004	0.4805	2.7176	0
6000	500.1	1.868	2.0007	0.4808	2.7027	0
6001	500	1.8676	2.0033	0.4803	2.7169	0
6002	499.9	1.8695	2.0001	0.4805	2.702	0
6003	499.8	1.8712	2.0026	0.48	2.7016	0
6004	499.7	1.8707	2.0021	0.48	2.731	0
6005	499.6	1.8703	2.0046	0.4797	2.7153	0
6006	499.5	1.8722	2.0014	0.4796	2.7462	0

6007	499.4	1.874	2.0011	0.4796	2.73	0
6008	499.3	1.878	2.0008	0.4796	2.7144	0
6009	499.2	1.8777	2.0004	0.4795	2.714	0
6010	499.1	1.8816	1.9971	0.4793	2.7289	0
6011	499	1.8791	1.9996	0.4792	2.7286	0
6012	498.9	1.8831	1.9993	0.4792	2.7131	0
6013	498.8	1.885	1.9989	0.4791	2.7279	0
6014	498.7	1.8868	2.0013	0.4791	2.7433	0
6015	498.6	1.8842	1.9981	0.4789	2.7118	0
6016	498.5	1.8883	1.9977	0.4789	2.7268	0
6017	498.4	1.8902	1.9974	0.479	2.7263	0
6018	498.3	1.8921	1.9971	0.4791	2.7582	0
6019	498.2	1.894	1.9967	0.4788	2.7414	0
6020	498.1	1.8959	1.9934	0.4786	2.7251	0
6021	498	1.8933	1.9959	0.4787	2.7406	0
6022	497.9	1.8975	1.9927	0.4791	2.7402	0
6023	497.8	1.8993	1.9922	0.4786	2.7562	0
6024	497.7	1.9013	1.9918	0.4789	2.7557	0
6025	497.6	1.9033	1.9944	0.4785	2.7555	0
6026	497.5	1.9029	1.9911	0.4785	2.7387	0
6027	497.4	1.9049	1.9907	0.4786	2.7547	0
6028	497.3	1.9022	1.9903	0.4786	2.7542	0
6029	497.2	1.9089	1.99	0.4783	2.7711	0
6030	497.1	1.9086	1.9926	0.4787	2.7373	0
6031	497	1.9106	1.9893	0.4787	2.7533	0
6032	496.9	1.915	1.9889	0.4788	2.7698	0
6033	496.8	1.9147	1.9886	0.4787	2.7696	0
6034	496.7	1.9144	1.9854	0.4789	2.7693	0
6035	496.6	1.9189	1.985	0.4785	2.7518	0
6036	496.5	1.9161	1.9904	0.4787	2.7685	0
6037	496.4	1.9231	1.9843	0.4787	2.7683	0
6038	496.3	1.9227	1.9868	0.479	2.7508	0
6039	496.2	1.9225	1.9865	0.4791	2.7675	0
6040	496.1	1.927	1.9861	0.4792	2.767	0
6041	496	1.9291	1.9828	0.4791	2.7844	0
6042	495.9	1.9314	1.9854	0.4795	2.7665	0
6043	495.8	1.931	1.9822	0.4797	2.7838	0
6044	495.7	1.9333	1.9847	0.4798	2.7836	0
6045	495.6	1.9328	1.9843	0.4796	2.783	0
6046	495.5	1.9376	1.981	0.48	2.8011	0
6047	495.4	1.9373	1.9836	0.4801	2.7647	0
6048	495.3	1.9395	1.9833	0.4801	2.782	0
6049	495.2	1.9418	1.9801	0.4805	2.7817	0

6050	495.1	1.9442	1.9798	0.4805	2.8	0
6051	495	1.9438	1.9794	0.4806	2.7632	0
6052	494.9	1.9487	1.979	0.4809	2.7807	0
6053	494.8	1.951	1.9815	0.4811	2.7804	0
6054	494.7	1.9506	1.9783	0.481	2.7983	0
6055	494.6	1.9529	1.9779	0.4813	2.7981	0
6056	494.5	1.958	1.9804	0.4815	2.7978	0
6057	494.4	1.9603	1.98	0.4812	2.7972	0
6058	494.3	1.9599	1.9796	0.4816	2.797	0
6059	494.2	1.9596	1.9765	0.4816	2.8159	0
6060	494.1	1.9648	1.9789	0.4817	2.7962	0
6061	494	1.9701	1.9758	0.4818	2.8153	0
6062	493.9	1.9697	1.9754	0.4822	2.8147	0
6063	493.8	1.975	1.9779	0.4822	2.8145	0
6064	493.7	1.9775	1.9747	0.4822	2.7948	0
6065	493.6	1.9772	1.9743	0.4823	2.8136	0
6066	493.5	1.9769	1.974	0.4826	2.8133	0
6067	493.4	1.9823	1.9765	0.4824	2.813	0
6068	493.3	1.982	1.9791	0.4826	2.833	0
6069	493.2	1.9875	1.9729	0.4827	2.7932	0
6070	493.1	1.9901	1.9755	0.4829	2.8119	0
6071	493	1.9927	1.9722	0.483	2.8116	0
6072	492.9	1.9923	1.9747	0.483	2.8114	0
6073	492.8	1.9919	1.9743	0.4828	2.8312	0
6074	492.7	2.0007	1.974	0.483	2.852	0
6075	492.6	1.9973	1.9736	0.4831	2.8303	0
6076	492.5	1.997	1.9733	0.4834	2.83	0
6077	492.4	2.0058	1.973	0.4832	2.8297	0
6078	492.3	2.0054	1.9725	0.4832	2.8091	0
6079	492.2	2.0112	1.9721	0.4827	2.8502	0
6080	492.1	2.0109	1.9718	0.4831	2.8286	0
6081	492	2.0138	1.9715	0.4831	2.8716	0
6082	491.9	2.0134	1.9712	0.4835	2.8492	0
6083	491.8	2.0163	1.9709	0.4833	2.8489	0
6084	491.7	2.0191	1.9705	0.483	2.8483	0
6085	491.6	2.022	1.9702	0.4833	2.8268	0
6086	491.5	2.0248	1.9696	0.4831	2.8265	0
6087	491.4	2.0278	1.9723	0.4831	2.8474	0
6088	491.3	2.0307	1.9719	0.4833	2.8471	0
6089	491.2	2.0336	1.9715	0.4835	2.8687	0
6090	491.1	2.0334	1.9713	0.4834	2.8687	0
6091	491	2.0364	1.968	0.4834	2.8459	0
6092	490.9	2.0393	1.9705	0.4832	2.8914	0

6093	490.8	2.0424	1.9673	0.483	2.8453	0
6094	490.7	2.0455	1.9697	0.4829	2.8447	0
6095	490.6	2.0485	1.9694	0.483	2.8668	0
6096	490.5	2.0481	1.966	0.4827	2.8441	0
6097	490.4	2.0512	1.9686	0.4824	2.8894	0
6098	490.3	2.0545	1.9683	0.483	2.8891	0
6099	490.2	2.0576	1.9708	0.4827	2.8887	0
6100	490.1	2.0608	1.9675	0.4823	2.8649	0
6101	490	2.0568	1.967	0.4823	2.8642	0
6102	489.9	2.0636	1.9667	0.4821	2.9122	0
6103	489.8	2.0632	1.9663	0.4821	2.8871	0
6104	489.7	2.0665	1.966	0.4823	2.9115	0
6105	489.6	2.0699	1.9686	0.4819	2.8864	0
6106	489.5	2.0732	1.9653	0.4821	2.8861	0
6107	489.4	2.0729	1.965	0.4819	2.9108	0
6108	489.3	2.0762	1.9646	0.4818	2.8854	0
6109	489.2	2.0795	1.9641	0.482	2.9097	0
6110	489.1	2.083	1.9638	0.4814	2.9355	0
6111	489	2.0864	1.9663	0.4815	2.8841	0
6112	488.9	2.0938	1.9631	0.4812	2.9087	0
6113	488.8	2.0973	1.9656	0.4812	2.8834	0
6114	488.7	2.097	1.9681	0.4813	2.8831	0
6115	488.6	2.1005	1.9648	0.4811	2.9076	0
6116	488.5	2.1041	1.9615	0.4808	2.9069	0
6117	488.4	2.1037	1.9611	0.4806	2.9066	0
6118	488.3	2.1075	1.9637	0.4809	2.9066	0
6119	488.2	2.1112	1.9604	0.4809	2.9062	0
6120	488.1	2.1108	1.96	0.4804	2.9055	0
6121	488	2.1227	1.9595	0.4804	2.9314	0
6122	487.9	2.1225	1.9622	0.4804	2.9048	0
6123	487.8	2.1221	1.9617	0.4805	2.9045	0
6124	487.7	2.1259	1.9584	0.4804	2.9041	0
6125	487.6	2.1297	1.9608	0.4799	2.9034	0
6126	487.5	2.1338	1.9606	0.4802	2.9876	0
6127	487.4	2.1377	1.9602	0.4801	2.9031	0
6128	487.3	2.1418	1.957	0.48	2.9027	0
6129	487.2	2.1415	1.9595	0.48	2.9024	0
6130	487.1	2.1499	1.959	0.4798	2.9017	0
6131	487	2.1452	1.9559	0.48	2.9278	0
6132	486.9	2.1537	1.9612	0.4799	2.9274	0
6133	486.8	2.158	1.955	0.48	2.9551	0
6134	486.7	2.1623	1.9576	0.4797	2.9266	0
6135	486.6	2.1665	1.9571	0.4797	2.9543	0

6136	486.5	2.171	1.9597	0.48	2.9259	0
6137	486.4	2.1706	1.9594	0.4801	2.9255	0
6138	486.3	2.1799	1.959	0.4799	2.9531	0
6139	486.2	2.1795	1.9557	0.4798	2.9248	0
6140	486.1	2.184	1.9553	0.4799	2.9523	0
6141	486	2.1887	1.955	0.4798	2.9241	0
6142	485.9	2.1934	1.9547	0.4801	2.9817	0
6143	485.8	2.1929	1.9571	0.4799	2.9512	0
6144	485.7	2.1977	1.9568	0.4803	2.9512	0
6145	485.6	2.2024	1.9535	0.4803	2.9226	0
6146	485.5	2.202	1.959	0.4802	2.9801	0
6147	485.4	2.2123	1.9588	0.4806	2.95	0
6148	485.3	2.2173	1.9555	0.4809	2.9496	0
6149	485.2	2.2223	1.9551	0.4806	2.9792	0
6150	485.1	2.2274	1.9547	0.4809	2.9488	0
6151	485	2.2215	1.9543	0.4809	2.9485	0
6152	484.9	2.2322	1.957	0.4812	2.978	0
6153	484.8	2.2375	1.9537	0.4813	2.978	0
6154	484.7	2.2427	1.9562	0.4815	2.9776	0
6155	484.6	2.2423	1.9588	0.4814	2.9772	0
6156	484.5	2.2478	1.9555	0.4817	2.9767	0
6157	484.4	2.2591	1.9551	0.4821	2.9763	0
6158	484.3	2.2527	1.9547	0.4821	2.9759	0
6159	484.2	2.2583	1.9573	0.482	2.9454	0
6160	484.1	2.2581	1.9541	0.4823	2.9751	0
6161	484	2.2695	1.9535	0.4822	2.9446	0
6162	483.9	2.2693	1.9562	0.483	2.9743	0
6163	483.8	2.2814	1.953	0.4829	3.0061	0
6164	483.7	2.281	1.9555	0.483	2.9739	0
6165	483.6	2.287	1.9581	0.4829	2.9735	0
6166	483.5	2.2867	1.9518	0.4833	2.9731	0
6167	483.4	2.2927	1.9544	0.483	2.9727	0
6168	483.3	2.2923	1.9541	0.4833	3.0044	0
6169	483.2	2.3118	1.9567	0.4836	2.9718	0
6170	483.1	2.3048	1.9563	0.4835	3.0039	0
6171	483	2.3111	1.953	0.4837	2.971	0
6172	482.9	2.3107	1.9555	0.4835	2.9706	0
6173	482.8	2.3243	1.9553	0.4839	3.0026	0
6174	482.7	2.3308	1.9548	0.4838	3.0372	0
6175	482.6	2.3305	1.9575	0.4841	3.0017	0
6176	482.5	2.33	1.954	0.4838	3.0013	0
6177	482.4	2.3442	1.9537	0.4842	2.969	0
6178	482.3	2.3437	1.9562	0.4839	3.0004	0

6179	482.2	2.3509	1.9559	0.4841	3.0004	0
6180	482.1	2.358	1.9555	0.4842	2.9678	0
6181	482	2.3653	1.9551	0.4837	2.9996	0
6182	481.9	2.3573	1.9579	0.4841	2.9991	0
6183	481.8	2.3725	1.9575	0.4843	2.9991	0
6184	481.7	2.3721	1.9571	0.4845	3.0334	0
6185	481.6	2.3716	1.9567	0.4844	2.9983	0
6186	481.5	2.3792	1.9563	0.4844	2.9978	0
6187	481.4	2.387	1.956	0.4843	2.9974	0
6188	481.3	2.3949	1.9556	0.4846	2.997	0
6189	481.2	2.3945	1.9551	0.4843	2.9642	0
6190	481.1	2.3942	1.9548	0.4842	2.9961	0
6191	481	2.3937	1.9544	0.4843	2.9957	0
6192	480.9	2.4103	1.9539	0.4842	2.9952	0
6193	480.8	2.4187	1.9566	0.4842	2.9948	0
6194	480.7	2.4095	1.9562	0.4841	2.9944	0
6195	480.6	2.4359	1.9526	0.4843	2.994	0
6196	480.5	2.4356	1.9554	0.4843	2.9935	0
6197	480.4	2.4351	1.958	0.4844	2.9931	0
6198	480.3	2.4443	1.9578	0.4846	3.0278	0
6199	480.2	2.4344	1.9573	0.4843	2.9927	0
6200	480.1	2.4527	1.9537	0.4842	3.0269	0
6201	480	2.4621	1.9563	0.4841	2.9594	0
6202	479.9	2.4619	1.9561	0.4844	2.9914	0
6203	479.8	2.4613	1.9525	0.484	3.0255	0
6204	479.7	2.471	1.9552	0.484	2.9905	0
6205	479.6	2.4706	1.9611	0.4843	3.025	0
6206	479.5	2.4804	1.9575	0.484	3.0246	0
6207	479.4	2.4799	1.9602	0.4843	2.9893	0
6208	479.3	2.49	1.9598	0.4839	3.0237	0
6209	479.2	2.4896	1.9594	0.4838	3.0232	0
6210	479.1	2.4893	1.9591	0.4836	2.988	0
6211	479	2.4996	1.9555	0.4836	2.9876	0
6212	478.9	2.4992	1.9583	0.4838	2.9551	0
6213	478.8	2.4987	1.9546	0.4837	2.9867	0
6214	478.7	2.5093	1.9605	0.4833	3.0214	0
6215	478.6	2.5089	1.957	0.4834	2.9859	0
6216	478.5	2.5085	1.9566	0.4833	3.0205	0
6217	478.4	2.5082	1.9562	0.4832	3.02	0
6218	478.3	2.519	1.9557	0.483	3.0195	0
6219	478.2	2.5186	1.9554	0.483	3.0191	0
6220	478.1	2.5299	1.9582	0.4831	3.0191	0
6221	478	2.5294	1.9578	0.483	3.056	0

6222	477.9	2.529	1.9573	0.483	3.0555	0
6223	477.8	2.517	1.96	0.4827	3.0177	0
6224	477.7	2.54	1.9565	0.4827	3.055	0
6225	477.6	2.5397	1.9593	0.4826	3.0545	0
6226	477.5	2.5514	1.9588	0.4826	3.0164	0
6227	477.4	2.551	1.9552	0.4825	2.9813	0
6228	477.3	2.5508	1.9581	0.4825	3.0155	0
6229	477.2	2.563	1.9577	0.4823	3.0155	0
6230	477.1	2.5626	1.9574	0.4826	3.0526	0
6231	477	2.5622	1.957	0.4825	3.0521	0
6232	476.9	2.5618	1.9596	0.4824	3.0516	0
6233	476.8	2.5743	1.9593	0.4822	3.0137	0
6234	476.7	2.561	1.9589	0.4822	3.0511	0
6235	476.6	2.5737	1.9618	0.4824	3.0128	0
6236	476.5	2.5733	1.9551	0.4824	3.0506	0
6237	476.4	2.5862	1.9579	0.4822	3.0501	0
6238	476.3	2.5724	1.9573	0.4823	3.0496	0
6239	476.2	2.5854	1.957	0.4822	3.0491	0
6240	476.1	2.5849	1.9597	0.4822	3.0487	0
6241	476	2.5711	1.956	0.4822	3.0482	0
6242	475.9	2.5979	1.9557	0.4822	2.9751	0
6243	475.8	2.5976	1.9586	0.4824	3.0477	0
6244	475.7	2.5972	1.9582	0.4823	3.0092	0
6245	475.6	2.611	1.9611	0.4825	3.0088	0
6246	475.5	2.6107	1.9574	0.4825	3.0462	0
6247	475.4	2.5959	1.957	0.4825	3.0458	0
6248	475.3	2.6099	1.9535	0.4827	3.0458	0
6249	475.2	2.6094	1.9595	0.4826	3.0453	0
6250	475.1	2.5948	1.9559	0.483	3.007	0
6251	475	2.6087	1.9587	0.4828	3.0443	0
6252	474.9	2.6084	1.9583	0.483	3.0857	0
6253	474.8	2.6225	1.9611	0.4831	3.0434	0
6254	474.7	2.6223	1.9641	0.4831	3.0057	0
6255	474.6	2.6218	1.9603	0.4831	3.0841	0
6256	474.5	2.6214	1.9599	0.4832	3.0424	0
6257	474.4	2.6211	1.9629	0.4833	3.0419	0
6258	474.3	2.6358	1.9591	0.4834	3.0039	0
6259	474.2	2.6353	1.9619	0.4833	3.041	0
6260	474.1	2.6198	1.9616	0.4837	3.041	0
6261	474	2.6192	1.9578	0.4836	3.04	0
6262	473.9	2.6499	1.964	0.4834	3.04	0
6263	473.8	2.6336	1.957	0.4836	3.0395	0
6264	473.7	2.6332	1.9631	0.4838	3.0391	0

6265	473.6	2.6486	1.9627	0.4843	3.0386	0
6266	473.5	2.6325	1.9656	0.4843	3.0381	0
6267	473.4	2.6478	1.9653	0.4846	3	0
6268	473.3	2.6315	1.9647	0.4844	2.9996	0
6269	473.2	2.6631	1.9642	0.4843	3.0367	0
6270	473.1	2.6463	1.9638	0.4846	2.9987	0
6271	473	2.6623	1.9633	0.4844	3.0357	0
6272	472.9	2.6455	1.9663	0.4845	3.0768	0
6273	472.8	2.6451	1.9659	0.4846	3.0762	0
6274	472.7	2.6609	1.9654	0.4848	3.0762	0
6275	472.6	2.6776	1.9684	0.485	3.0343	0
6276	472.5	2.6772	1.9645	0.4852	3.0339	0
6277	472.4	2.66	1.9676	0.4855	3.0334	0
6278	472.3	2.6763	1.9637	0.4851	3.0742	0
6279	472.2	2.659	1.97	0.4853	3.0737	0
6280	472.1	2.6755	1.9697	0.4855	3.032	0
6281	472	2.6749	1.9657	0.4854	3.0731	0
6282	471.9	2.6745	1.9686	0.4855	3.0726	0
6283	471.8	2.6916	1.9681	0.4856	3.0721	0
6284	471.7	2.6735	1.9711	0.4853	3.0716	0
6285	471.6	2.6908	1.9673	0.4858	3.0297	0
6286	471.5	2.6904	1.9703	0.4856	3.0292	0
6287	471.4	2.6722	1.9697	0.4855	3.0701	0
6288	471.3	2.6895	1.9728	0.4857	3.0283	0
6289	471.2	2.7073	1.9722	0.4853	3.0691	0
6290	471.1	2.7069	1.9718	0.4855	3.0273	0
6291	471	2.7064	1.9712	0.4855	3.068	0
6292	470.9	2.6876	1.9709	0.4856	3.0675	0
6293	470.8	2.7055	1.9703	0.4857	3.067	0
6294	470.7	2.7051	1.9769	0.4859	3.067	0
6295	470.6	2.6861	1.973	0.4861	3.0665	0
6296	470.5	2.7042	1.976	0.4859	3.066	0
6297	470.4	2.7036	1.9753	0.4855	3.065	0
6298	470.3	2.7031	1.9714	0.4858	3.065	0
6299	470.2	2.7219	1.9744	0.4862	3.1101	0
6300	470.1	2.7416	1.9739	0.4859	3.064	0
6301	470	2.721	1.977	0.486	3.0635	0
6302	469.9	2.7203	1.9765	0.486	3.063	0
6303	469.8	2.7007	1.9796	0.486	3.0209	0
6304	469.7	2.7194	1.979	0.4857	3.062	0
6305	469.6	2.719	1.9821	0.4859	3.0615	0
6306	469.5	2.7387	1.9781	0.4859	3.061	0
6307	469.4	2.738	1.981	0.486	3.0605	0

6308	469.3	2.7375	1.9806	0.4858	3.06	0
6309	469.2	2.7371	1.9765	0.4858	3.1051	0
6310	469.1	2.7577	1.9796	0.4861	3.1046	0
6311	469	2.7361	1.9829	0.4861	3.0585	0
6312	468.9	2.7567	1.9786	0.4857	3.058	0
6313	468.8	2.7562	1.9854	0.4862	3.057	0
6314	468.7	2.7345	1.9848	0.486	3.1024	0
6315	468.6	2.7552	1.9808	0.4858	3.015	0
6316	468.5	2.7547	1.9839	0.4855	3.1018	0
6317	468.4	2.7542	1.9834	0.4857	3.0555	0
6318	468.3	2.7537	1.9865	0.4851	3.0545	0
6319	468.2	2.7533	1.9861	0.4851	3.054	0
6320	468.1	2.7749	1.9855	0.4849	3.0535	0
6321	468	2.7981	1.985	0.4849	3.0991	0
6322	467.9	2.7739	1.9882	0.4849	3.0526	0
6323	467.8	2.7734	1.9877	0.4847	3.0521	0
6324	467.7	2.7731	1.9873	0.485	3.0975	0
6325	467.6	2.7501	1.9905	0.4848	3.0511	0
6326	467.5	2.7719	1.9899	0.4844	3.0964	0
6327	467.4	2.7713	1.9933	0.4846	3.0958	0
6328	467.3	2.7486	1.989	0.4844	3.0496	0
6329	467.2	2.7703	1.9923	0.4846	3.0079	0
6330	467.1	2.7934	1.988	0.4846	3.0942	0
6331	467	2.7471	1.9914	0.4845	3.0482	0
6332	466.9	2.7924	1.9908	0.4846	3.0477	0
6333	466.8	2.7685	1.9981	0.4844	3.0472	0
6334	466.7	2.768	1.9975	0.4844	3.0926	0
6335	466.6	2.7675	1.9971	0.4847	3.0462	0
6336	466.5	2.7905	1.9966	0.4845	3.0458	0
6337	466.4	2.7665	1.9961	0.4845	3.091	0
6338	466.3	2.7662	1.9919	0.4849	3.0448	0
6339	466.2	2.7891	1.9991	0.4845	3.0443	0
6340	466.1	2.8133	1.9986	0.485	3.0899	0
6341	466	2.813	1.9981	0.4844	3.0894	0
6342	465.9	2.7878	1.9977	0.4847	3.0888	0
6343	465.8	2.8119	2.0049	0.4845	3.0883	0
6344	465.7	2.7867	2.0005	0.4844	3.0878	0
6345	465.6	2.786	2.0039	0.4845	3.0414	0
6346	465.5	2.7857	1.9996	0.4844	3.1379	0
6347	465.4	2.8099	2.0031	0.485	3.0862	0
6348	465.3	2.8094	2.0025	0.4852	3.0857	0
6349	465.2	2.8091	2.0061	0.485	3.0395	0
6350	465.1	2.7602	2.0056	0.4849	3.0846	0

6351	465	2.808	2.0051	0.485	3.0841	0
6352	464.9	2.8336	2.0044	0.485	3.0376	0
6353	464.8	2.782	2.008	0.4851	3.083	0
6354	464.7	2.8063	2.0115	0.485	3.0825	0
6355	464.6	2.8321	2.0069	0.4852	3.0362	0
6356	464.5	2.757	2.0105	0.4853	3.0814	0
6357	464.4	2.8049	2.01	0.4857	3.0809	0
6358	464.3	2.8044	2.0053	0.4855	3.0804	0
6359	464.2	2.8035	2.0088	0.4855	3.0799	0
6360	464.1	2.8576	2.0125	0.4855	3.0339	0
6361	464	2.8292	2.0161	0.4855	3.1302	0
6362	463.9	2.8286	2.0156	0.4859	3.0783	0
6363	463.8	2.828	2.0108	0.486	3.032	0
6364	463.7	2.8554	2.0144	0.4859	3.0773	0
6365	463.6	2.8268	2.0181	0.4858	3.1278	0
6366	463.5	2.8542	2.0175	0.4863	3.0762	0
6367	463.4	2.8256	2.0169	0.4859	3.0757	0
6368	463.3	2.8251	2.0163	0.486	3.0747	0
6369	463.2	2.8248	2.0159	0.4858	3.0747	0
6370	463.1	2.852	2.0196	0.486	3.0283	0
6371	463	2.8514	2.019	0.4862	3.0731	0
6372	462.9	2.8508	2.0184	0.4862	3.1238	0
6373	462.8	2.8505	2.0179	0.4862	3.0721	0
6374	462.7	2.7953	2.0216	0.4863	3.0716	0
6375	462.6	2.8492	2.021	0.4863	3.1221	0
6376	462.5	2.8486	2.0248	0.4863	3.0706	0
6377	462.4	2.8202	2.0242	0.4864	3.0701	0
6378	462.3	2.8196	2.028	0.4867	3.0696	0
6379	462.2	2.8471	2.0275	0.4865	3.0691	0
6380	462.1	2.8462	2.0312	0.4862	3.068	0
6381	462	2.8176	2.035	0.4865	3.0675	0
6382	461.9	2.817	2.0299	0.4866	3.067	0
6383	461.8	2.8444	2.0337	0.4863	3.1175	0
6384	461.7	2.8159	2.0288	0.4865	3.0655	0
6385	461.6	2.8153	2.0282	0.4868	3.065	0
6386	461.5	2.8425	2.0318	0.4865	3.1152	0
6387	461.4	2.8719	2.0357	0.4864	3.064	0
6388	461.3	2.8413	2.0306	0.4864	3.063	0
6389	461.2	2.8706	2.0344	0.4866	3.0625	0
6390	461.1	2.8401	2.0384	0.4867	3.1129	0
6391	461	2.8392	2.0376	0.4863	3.061	0
6392	460.9	2.8105	2.0416	0.4863	3.1118	0
6393	460.8	2.8681	2.041	0.4865	3.1113	0

6394	460.7	2.8374	2.0498	0.4864	3.0595	0
6395	460.6	2.8365	2.0396	0.4864	3.0128	0
6396	460.5	2.808	2.0391	0.4865	3.109	0
6397	460.4	2.8353	2.0431	0.4864	3.1085	0
6398	460.3	2.8348	2.047	0.4864	3.0565	0
6399	460.2	2.8342	2.0464	0.4867	3.056	0
6400	460.1	2.8333	2.0409	0.4866	3.1643	0
6401	460	2.8327	2.0449	0.4865	3.1057	0
6402	459.9	2.862	2.0444	0.4867	3.1051	0
6403	459.8	2.8614	2.0532	0.4869	3.1046	0
6404	459.7	2.8604	2.0476	0.4867	3.0526	0
6405	459.6	2.83	2.0517	0.4867	3.0516	0
6406	459.5	2.8592	2.051	0.4865	3.1024	0
6407	459.4	2.8586	2.0551	0.4867	3.1593	0
6408	459.3	2.8576	2.0545	0.4866	3.1007	0
6409	459.2	2.8271	2.0537	0.4867	3.0487	0
6410	459.1	2.8564	2.0579	0.4866	3.1574	0
6411	459	2.8557	2.0623	0.4864	3.0985	0
6412	458.9	2.8548	2.0565	0.4865	3.1561	0
6413	458.8	2.8539	2.0606	0.4866	3.0458	0
6414	458.7	2.8401	2.0589	0.4863	3.1062	0
6415	458.6	2.8579	2.0613	0.4865	3.1057	0
6416	458.5	2.8386	2.0665	0.4865	3.1397	0
6417	458.4	2.8564	2.0657	0.4865	3.104	0
6418	458.3	2.8554	2.0649	0.4866	3.1035	0
6419	458.2	2.8548	2.0643	0.4863	3.1373	0
6420	458.1	2.8542	2.0697	0.4864	3.1367	0
6421	458	2.8348	2.0689	0.4865	3.1361	0
6422	457.9	2.8526	2.0682	0.4862	3.1002	0
6423	457.8	2.8517	2.0705	0.486	3.0996	0
6424	457.7	2.8703	2.0698	0.486	3.1337	0
6425	457.6	2.8318	2.0722	0.4861	3.1331	0
6426	457.5	2.8496	2.0745	0.486	3.1701	0
6427	457.4	2.8681	2.0737	0.4861	3.1314	0
6428	457.3	2.8874	2.0762	0.4861	3.0958	0
6429	457.2	2.8471	2.0754	0.4858	3.1675	0
6430	457.1	2.8462	2.0777	0.4856	3.1669	0
6431	457	2.8456	2.0802	0.4857	3.0937	0
6432	456.9	2.845	2.0796	0.4859	3.1273	0
6433	456.8	2.8259	2.0822	0.4856	3.1267	0
6434	456.7	2.8435	2.0814	0.4859	3.0915	0
6435	456.6	2.8425	2.0838	0.4859	3.0904	0
6436	456.5	2.8419	2.0865	0.4859	3.0899	0

6437	456.4	2.8604	2.0857	0.4858	3.1238	0
6438	456.3	2.8404	2.0882	0.4859	3.1232	0
6439	456.2	2.8213	2.0876	0.4863	3.1226	0
6440	456.1	2.8582	2.0868	0.4862	3.1215	0
6441	456	2.8383	2.0861	0.4862	3.1586	0
6442	455.9	2.8377	2.0887	0.4861	3.1203	0
6443	455.8	2.856	2.0914	0.486	3.1574	0
6444	455.7	2.8554	2.0907	0.4859	3.1568	0
6445	455.6	2.8548	2.09	0.486	3.118	0
6446	455.5	2.8348	2.0926	0.4862	3.1549	0
6447	455.4	2.8533	2.0988	0.4862	3.1163	0
6448	455.3	2.8333	2.0946	0.4859	3.1537	0
6449	455.2	2.8327	2.0975	0.4862	3.153	0
6450	455.1	2.8318	2.1001	0.4863	3.1524	0
6451	455	2.8309	2.0994	0.4861	3.1512	0
6452	454.9	2.8496	2.0987	0.4864	3.1506	0
6453	454.8	2.8489	2.1051	0.4863	3.1124	0
6454	454.7	2.8483	2.1043	0.4864	3.1494	0
6455	454.6	2.8477	2.1036	0.4864	3.1487	0
6456	454.5	2.8468	2.1027	0.4865	3.1475	0
6457	454.4	2.8265	2.1056	0.4866	3.1884	0
6458	454.3	2.8453	2.1085	0.4867	3.1463	0
6459	454.2	2.8444	2.1114	0.4865	3.1871	0
6460	454.1	2.8639	2.1106	0.4866	3.1864	0
6461	454	2.8428	2.1099	0.4869	3.1439	0
6462	453.9	2.823	2.1129	0.4871	3.1433	0
6463	453.8	2.8416	2.1158	0.4876	3.1838	0
6464	453.7	2.8407	2.115	0.4872	3.1831	0
6465	453.6	2.8401	2.1144	0.4871	3.1415	0
6466	453.5	2.8595	2.1136	0.4871	3.1403	0
6467	453.4	2.8383	2.1164	0.4871	3.1397	0
6468	453.3	2.8377	2.1195	0.4874	3.1391	0
6469	453.2	2.8371	2.1226	0.4875	3.1379	0
6470	453.1	2.8362	2.1218	0.4874	3.2248	0
6471	453	2.8353	2.121	0.4874	3.1778	0
6472	452.9	2.8348	2.1203	0.4872	3.098	0
6473	452.8	2.8339	2.1233	0.4875	3.0975	0
6474	452.7	2.8333	2.1263	0.4873	3.1759	0
6475	452.6	2.8324	2.1256	0.4874	3.1337	0
6476	452.5	2.8315	2.1287	0.4875	3.1739	0
6477	452.4	2.8114	2.1278	0.4873	3.0942	0
6478	452.3	2.8502	2.1271	0.4872	3.1308	0
6479	452.2	2.8292	2.1302	0.4867	3.2175	0

6480	452.1	2.8283	2.1334	0.4871	3.1707	0
6481	452	2.8477	2.1366	0.487	3.1701	0
6482	451.9	2.8268	2.1358	0.4871	3.1694	0
6483	451.8	2.8259	2.139	0.4868	3.214	0
6484	451.7	2.8254	2.1342	0.4867	3.2132	0
6485	451.6	2.8245	2.1375	0.4867	3.2125	0
6486	451.5	2.8438	2.1366	0.4866	3.1662	0
6487	451.4	2.8228	2.1439	0.4865	3.2111	0
6488	451.3	2.8219	2.1473	0.4865	3.1643	0
6489	451.2	2.8213	2.1424	0.4867	3.1637	0
6490	451.1	2.8404	2.1456	0.4863	3.1624	0
6491	451	2.8196	2.149	0.4863	3.1203	0
6492	450.9	2.8187	2.1482	0.4862	3.1612	0
6493	450.8	2.838	2.1558	0.4864	3.1186	0
6494	450.7	2.8371	2.1508	0.4865	3.2048	0
6495	450.6	2.8159	2.1541	0.486	3.2041	0
6496	450.5	2.815	2.1532	0.4859	3.2034	0
6497	450.4	2.8345	2.1522	0.4858	3.202	0
6498	450.3	2.8336	2.1601	0.4862	3.1555	0
6499	450.2	2.8327	2.1593	0.4857	3.1549	0
6500	450.1	2.8116	2.154	0.4857	3.2	0
6501	450	2.8309	2.1618	0.4856	3.1986	0
6502	449.9	2.8225	2.1692	0.454	3.1959	0
6503	449.8	2.8586	2.1742	0.4541	3.1972	0
6504	449.7	2.8245	2.1751	0.4539	3.1979	0
6505	449.6	2.8094	2.1725	0.4538	3.1993	0
6506	449.5	2.8438	2.1737	0.4541	3.2418	0
6507	449.4	2.8453	2.1786	0.4544	3.1637	0
6508	449.3	2.8292	2.176	0.454	3.1649	0
6509	449.2	2.8303	2.1809	0.4539	3.2457	0
6510	449.1	2.8315	2.182	0.4539	3.2464	0
6511	449	2.8496	2.1831	0.4537	3.2062	0
6512	448.9	2.8336	2.1841	0.4534	3.2487	0
6513	448.8	2.852	2.1854	0.4538	3.2083	0
6514	448.7	2.8359	2.1864	0.4538	3.2097	0
6515	448.6	2.8368	2.1912	0.4539	3.2104	0
6516	448.5	2.8216	2.1923	0.4538	3.1739	0
6517	448.4	2.8225	2.1933	0.4535	3.2125	0
6518	448.3	2.8236	2.1942	0.4531	3.2549	0
6519	448.2	2.8088	2.1991	0.4533	3.2147	0
6520	448.1	2.8256	2.204	0.4535	3.2154	0
6521	448	2.8428	2.2011	0.4534	3.2168	0
6522	447.9	2.8277	2.2061	0.4536	3.2175	0

6523	447.8	2.8289	2.2032	0.4537	3.2604	0
6524	447.7	2.8297	2.2041	0.4534	3.2197	0
6525	447.6	2.8306	2.2089	0.4534	3.262	0
6526	447.5	2.8315	2.2099	0.4536	3.2218	0
6527	447.4	2.8167	2.2108	0.4534	3.1844	0
6528	447.3	2.8176	2.2156	0.4533	3.2233	0
6529	447.2	2.8187	2.2127	0.4535	3.2248	0
6530	447.1	2.8353	2.2175	0.4534	3.2255	0
6531	447	2.8362	2.2185	0.4534	3.1884	0
6532	446.9	2.8374	2.2195	0.4537	3.2684	0
6533	446.8	2.8225	2.2203	0.4535	3.1904	0
6534	446.7	2.8392	2.2174	0.4536	3.2708	0
6535	446.6	2.8242	2.226	0.4534	3.2716	0
6536	446.5	2.8099	2.2269	0.4537	3.2306	0
6537	446.4	2.8259	2.2317	0.4538	3.2314	0
6538	446.3	2.8116	2.2287	0.454	3.2741	0
6539	446.2	2.8125	2.2336	0.4536	3.2336	0
6540	446.1	2.8136	2.2305	0.454	3.2757	0
6541	446	2.8142	2.2352	0.4538	3.1972	0
6542	445.9	2.815	2.236	0.4539	3.2358	0
6543	445.8	2.8011	2.2369	0.4539	3.1993	0
6544	445.7	2.8321	2.2378	0.4538	3.2381	0
6545	445.6	2.8176	2.2426	0.4543	3.2388	0
6546	445.5	2.8184	2.2434	0.4541	3.2013	0
6547	445.4	2.8193	2.2483	0.4541	3.2403	0
6548	445.3	2.8052	2.249	0.4543	3.2411	0
6549	445.2	2.8207	2.2498	0.4542	3.2418	0
6550	445.1	2.8216	2.2507	0.4542	3.2048	0
6551	445	2.8225	2.2514	0.4544	3.2434	0
6552	444.9	2.8085	2.2562	0.4545	3.2441	0
6553	444.8	2.8091	2.257	0.4542	3.2449	0
6554	444.7	2.8401	2.2619	0.4546	3.2457	0
6555	444.6	2.7967	2.2626	0.4551	3.2464	0
6556	444.5	2.8116	2.2633	0.4548	3.289	0
6557	444.4	2.8122	2.264	0.4547	3.248	0
6558	444.3	2.7986	2.2647	0.4549	3.2487	0
6559	444.2	2.8136	2.2696	0.4552	3.2495	0
6560	444.1	2.8145	2.2746	0.455	3.2503	0
6561	444	2.8008	2.2752	0.455	3.251	0
6562	443.9	2.8303	2.2716	0.455	3.2924	0
6563	443.8	2.8164	2.2765	0.4551	3.2518	0
6564	443.7	2.817	2.2772	0.455	3.2526	0
6565	443.6	2.8035	2.282	0.4551	3.2154	0

6566	443.5	2.8041	2.2868	0.455	3.2161	0
6567	443.4	2.8047	2.2832	0.4549	3.2549	0
6568	443.3	2.8196	2.2881	0.455	3.2175	0
6569	443.2	2.8202	2.2887	0.4548	3.2182	0
6570	443.1	2.8066	2.285	0.4546	3.2565	0
6571	443	2.8213	2.2942	0.4546	3.219	0
6572	442.9	2.8077	2.2905	0.4547	3.2581	0
6573	442.8	2.8225	2.2912	0.4548	3.2581	0
6574	442.7	2.8088	2.296	0.4548	3.2588	0
6575	442.6	2.8236	2.2965	0.4547	3.2596	0
6576	442.5	2.8102	2.3016	0.4548	3.2226	0
6577	442.4	2.8108	2.302	0.4548	3.2604	0
6578	442.3	2.8254	2.3025	0.4547	3.2612	0
6579	442.2	2.8116	2.3073	0.4545	3.3028	0
6580	442.1	2.7852	2.3036	0.4547	3.3036	0
6581	442	2.7989	2.3085	0.4546	3.2628	0
6582	441.9	2.8133	2.3089	0.4546	3.2628	0
6583	441.8	2.8	2.3094	0.4547	3.2636	0
6584	441.7	2.8005	2.31	0.4547	3.3054	0
6585	441.6	2.8008	2.3148	0.4547	3.3063	0
6586	441.5	2.8153	2.3153	0.4544	3.2652	0
6587	441.4	2.8156	2.3202	0.4542	3.3072	0
6588	441.3	2.8022	2.3162	0.4542	3.3072	0
6589	441.2	2.8167	2.3212	0.4541	3.2668	0
6590	441.1	2.8033	2.3216	0.4542	3.2668	0
6591	441	2.8173	2.3219	0.454	3.2676	0
6592	440.9	2.8179	2.3225	0.4542	3.2676	0
6593	440.8	2.791	2.3273	0.4541	3.2684	0
6594	440.7	2.7916	2.3233	0.4542	3.3098	0
6595	440.6	2.8052	2.3282	0.4539	3.3107	0
6596	440.5	2.8196	2.3332	0.4538	3.2692	0
6597	440.4	2.806	2.329	0.4536	3.27	0
6598	440.3	2.8204	2.3387	0.4539	3.2328	0
6599	440.2	2.8072	2.339	0.454	3.3125	0
6600	440.1	2.8213	2.3441	0.4537	3.3125	0
6601	440	2.8077	2.3445	0.4536	3.3134	0
6602	439.9	2.808	2.3402	0.4538	3.3134	0
6603	439.8	2.8365	2.3453	0.4539	3.3134	0
6604	439.7	2.8088	2.3456	0.4534	3.2725	0
6605	439.6	2.823	2.3507	0.4533	3.2733	0
6606	439.5	2.8097	2.3511	0.4532	3.3152	0
6607	439.4	2.8099	2.3513	0.453	3.3152	0
6608	439.3	2.8105	2.3566	0.4532	3.3161	0

6609	439.2	2.8108	2.3568	0.4529	3.3161	0
6610	439.1	2.8111	2.3621	0.4527	3.3161	0
6611	439	2.8254	2.3623	0.4527	3.317	0
6612	438.9	2.8119	2.3627	0.4527	3.2381	0
6613	438.8	2.8122	2.3678	0.4526	3.2757	0
6614	438.7	2.8125	2.3682	0.4525	3.3179	0
6615	438.6	2.8407	2.3685	0.4527	3.2765	0
6616	438.5	2.8268	2.3689	0.4524	3.3179	0
6617	438.4	2.8274	2.3693	0.4522	3.3188	0
6618	438.3	2.8139	2.3697	0.4522	3.3188	0
6619	438.2	2.828	2.3749	0.4521	3.2782	0
6620	438.1	2.8283	2.3801	0.452	3.2782	0
6621	438	2.8147	2.3853	0.4518	3.2782	0
6622	437.9	2.815	2.3857	0.4518	3.279	0
6623	437.8	2.8153	2.3861	0.4517	3.3206	0
6624	437.7	2.8159	2.3915	0.4519	3.2798	0
6625	437.6	2.8159	2.3866	0.4517	3.2798	0
6626	437.5	2.8162	2.3869	0.4517	3.2798	0
6627	437.4	2.8164	2.3922	0.4516	3.2807	0
6628	437.3	2.8306	2.3978	0.4519	3.2807	0
6629	437.2	2.8312	2.3931	0.4518	3.3224	0
6630	437.1	2.8173	2.3983	0.4516	3.2815	0
6631	437	2.8179	2.3987	0.4519	3.2815	0
6632	436.9	2.8182	2.4042	0.452	3.2815	0
6633	436.8	2.8184	2.4046	0.452	3.2823	0
6634	436.7	2.8324	2.4047	0.452	3.2823	0
6635	436.6	2.8327	2.4102	0.4519	3.2823	0
6636	436.5	2.819	2.4052	0.4518	3.3242	0
6637	436.4	2.8193	2.4107	0.452	3.2832	0
6638	436.3	2.8336	2.4163	0.4519	3.2457	0
6639	436.2	2.8199	2.4166	0.4521	3.2457	0
6640	436.1	2.8339	2.4115	0.452	3.284	0
6641	436	2.8342	2.4171	0.4521	3.3251	0
6642	435.9	2.8207	2.4174	0.4522	3.3261	0
6643	435.8	2.8348	2.4231	0.4525	3.3261	0
6644	435.7	2.8213	2.4233	0.4526	3.3261	0
6645	435.6	2.8353	2.4235	0.4527	3.2857	0
6646	435.5	2.8356	2.4292	0.4526	3.327	0
6647	435.4	2.8359	2.4352	0.4529	3.327	0
6648	435.3	2.8362	2.4244	0.4532	3.2857	0
6649	435.2	2.8225	2.43	0.4531	3.3279	0
6650	435.1	2.8228	2.4414	0.4534	3.3279	0
6651	435	2.8368	2.436	0.4532	3.2865	0

6652	434.9	2.8371	2.4419	0.4533	3.3288	0
6653	434.8	2.8371	2.4421	0.4533	3.3288	0
6654	434.7	2.8377	2.4425	0.4536	3.3288	0
6655	434.6	2.852	2.4484	0.4537	3.3288	0
6656	434.5	2.8523	2.4543	0.4538	3.3298	0
6657	434.4	2.8383	2.4488	0.4538	3.2882	0
6658	434.3	2.8526	2.4548	0.4541	3.2882	0
6659	434.2	2.8386	2.4551	0.4542	3.289	0
6660	434.1	2.8533	2.4495	0.4544	3.289	0
6661	434	2.8533	2.4613	0.4542	3.3768	0
6662	433.9	2.8256	2.4674	0.4546	3.3768	0
6663	433.8	2.8395	2.4676	0.4545	3.3307	0
6664	433.7	2.8539	2.4678	0.4546	3.2899	0
6665	433.6	2.8542	2.4679	0.4548	3.3316	0
6666	433.5	2.8401	2.4683	0.4551	3.3316	0
6667	433.4	2.8693	2.4744	0.4549	3.3316	0
6668	433.3	2.8548	2.4746	0.4549	3.2907	0
6669	433.2	2.8548	2.4808	0.455	3.3778	0
6670	433.1	2.8551	2.4811	0.4552	3.3325	0
6671	433	2.87	2.4811	0.4551	3.2907	0
6672	432.9	2.8413	2.4814	0.4553	3.3788	0
6673	432.8	2.8554	2.4876	0.455	3.3325	0
6674	432.7	2.8703	2.4877	0.4552	3.3325	0
6675	432.6	2.856	2.488	0.4555	3.2916	0
6676	432.5	2.871	2.5007	0.4556	3.3788	0
6677	432.4	2.871	2.4944	0.4557	3.3335	0
6678	432.3	2.8564	2.4947	0.4555	3.2924	0
6679	432.2	2.8567	2.4949	0.4555	3.3799	0
6680	432.1	2.8713	2.5013	0.4559	3.3335	0
6681	432	2.8867	2.5013	0.4556	3.3799	0
6682	431.9	2.8716	2.5014	0.4559	3.3344	0
6683	431.8	2.857	2.5016	0.456	3.3799	0
6684	431.7	2.857	2.5081	0.4559	3.3799	0
6685	431.6	2.8719	2.5082	0.4556	3.3344	0
6686	431.5	2.8719	2.5148	0.456	3.3344	0
6687	431.4	2.8874	2.515	0.4556	3.3799	0
6688	431.3	2.8719	2.515	0.4554	3.3799	0
6689	431.2	2.8722	2.515	0.4556	3.3344	0
6690	431.1	2.8874	2.5217	0.4557	3.3344	0
6691	431	2.8874	2.5217	0.4556	3.3344	0
6692	430.9	2.8877	2.5219	0.4556	3.3809	0
6693	430.8	2.8877	2.522	0.4556	3.3354	0
6694	430.7	2.8877	2.5222	0.4555	3.3809	0

6695	430.6	2.8881	2.529	0.4557	3.3354	0
6696	430.5	2.8729	2.5358	0.4557	3.3809	0
6697	430.4	2.8881	2.5358	0.4555	3.3809	0
6698	430.3	2.8881	2.5358	0.4555	3.3809	0
6699	430.2	2.9038	2.5358	0.4555	3.3809	0
6700	430.1	2.8881	2.536	0.4556	3.3809	0
6701	430	2.9038	2.5358	0.4551	3.3354	0
6702	429.9	2.8881	2.5429	0.4553	3.3809	0
6703	429.8	2.9041	2.543	0.4551	3.3809	0
6704	429.7	2.9205	2.5429	0.4551	3.3809	0
6705	429.6	2.8729	2.5501	0.455	3.3354	0
6706	429.5	2.9038	2.5429	0.455	3.3809	0
6707	429.4	2.9038	2.5499	0.4545	3.3354	0
6708	429.3	2.9201	2.5499	0.4545	3.3354	0
6709	429.2	2.9038	2.5571	0.4546	3.4318	0
6710	429.1	2.9201	2.5571	0.4545	3.3354	0
6711	429	2.9038	2.5643	0.454	3.3809	0
6712	428.9	2.9201	2.5643	0.4542	3.3354	0
6713	428.8	2.9201	2.5569	0.4543	3.3809	0
6714	428.7	2.9038	2.5642	0.4538	3.2557	0
6715	428.6	2.9201	2.5717	0.454	3.3809	0
6716	428.5	2.9038	2.5715	0.4537	3.3809	0
6717	428.4	2.9038	2.5715	0.4535	3.3809	0
6718	428.3	2.9201	2.5791	0.4536	3.3809	0
6719	428.2	2.9201	2.5791	0.4535	3.3809	0
6720	428.1	2.9034	2.5789	0.4535	3.3809	0
6721	428	2.9197	2.5865	0.4532	3.3809	0
6722	427.9	2.9197	2.5945	0.4534	3.3809	0
6723	427.8	2.9034	2.5865	0.4533	3.3809	0
6724	427.7	2.9367	2.5943	0.453	3.3344	0
6725	427.6	2.9367	2.5943	0.4529	3.3799	0
6726	427.5	2.9367	2.5943	0.4532	3.3799	0
6727	427.4	2.9197	2.6021	0.4529	3.4318	0
6728	427.3	2.9194	2.6021	0.4528	3.3344	0
6729	427.2	2.9363	2.6183	0.4525	3.3799	0
6730	427.1	2.9363	2.6021	0.4527	3.3799	0
6731	427	2.9363	2.6182	0.4528	3.3799	0
6732	426.9	2.9539	2.6182	0.4526	3.3799	0
6733	426.8	2.9363	2.6099	0.4527	3.3799	0
6734	426.7	2.9363	2.6265	0.4525	3.3799	0
6735	426.6	2.9363	2.6182	0.4526	3.3799	0
6736	426.5	2.9539	2.618	0.4525	3.3799	0
6737	426.4	2.9539	2.618	0.4527	3.3799	0

6738	426.3	2.9535	2.6264	0.4528	3.3799	0
6739	426.2	2.9535	2.6349	0.4526	3.3799	0
6740	426.1	2.9355	2.6347	0.4525	3.3799	0
6741	426	2.9535	2.6347	0.4527	3.3799	0
6742	425.9	2.9535	2.6347	0.4527	3.3799	0
6743	425.8	2.9535	2.6345	0.453	3.3788	0
6744	425.7	2.9531	2.6434	0.4529	3.3788	0
6745	425.6	2.9718	2.6432	0.4527	3.3335	0
6746	425.5	2.9718	2.6521	0.4529	3.3788	0
6747	425.4	2.9714	2.6611	0.4528	3.4306	0
6748	425.3	2.9531	2.6521	0.4532	3.4306	0
6749	425.2	2.991	2.6519	0.4532	3.3788	0
6750	425.1	2.9714	2.6609	0.4532	3.4295	0
6751	425	2.9714	2.6702	0.453	3.3788	0
6752	424.9	2.9905	2.6609	0.4535	3.3788	0
6753	424.8	2.971	2.6702	0.4536	3.4295	0
6754	424.7	2.971	2.6607	0.4537	3.3788	0
6755	424.6	3.0106	2.67	0.4539	3.4295	0
6756	424.5	2.9901	2.6698	0.454	3.3788	0
6757	424.4	3.0106	2.6794	0.454	3.4295	0
6758	424.3	2.9901	2.6891	0.4543	3.3778	0
6759	424.2	2.9901	2.6792	0.4543	3.4295	0
6760	424.1	3.0101	2.6889	0.4547	3.4295	0
6761	424	2.9897	2.6988	0.4544	3.4295	0
6762	423.9	2.9897	2.6887	0.4547	3.3778	0
6763	423.8	3.0097	2.6985	0.455	3.4295	0
6764	423.7	3.0097	2.6983	0.455	3.4283	0
6765	423.6	3.0306	2.7084	0.4551	3.4283	0
6766	423.5	3.0306	2.719	0.4551	3.3778	0
6767	423.4	3.0092	2.7187	0.4552	3.3778	0
6768	423.3	2.9889	2.708	0.4552	3.4283	0
6769	423.2	3.0088	2.7183	0.4555	3.3768	0
6770	423.1	3.0088	2.7289	0.4558	3.4283	0
6771	423	3.0088	2.7289	0.4558	3.3307	0
6772	422.9	3.0083	2.7286	0.4557	3.4271	0
6773	422.8	3.0297	2.7284	0.4559	3.3757	0
6774	422.7	3.0292	2.7392	0.4558	3.3757	0
6775	422.6	3.0516	2.7392	0.4562	3.3757	0
6776	422.5	3.0292	2.7279	0.4561	3.4271	0
6777	422.4	3.0287	2.7387	0.4564	3.4855	0
6778	422.3	3.0287	2.7498	0.4561	3.426	0
6779	422.2	3.0506	2.7496	0.456	3.426	0
6780	422.1	3.0283	2.7493	0.456	3.426	0

6781	422	3.0278	2.7493	0.4564	3.426	0
6782	421.9	3.0278	2.7607	0.4563	3.4841	0
6783	421.8	3.0278	2.7605	0.4562	3.426	0
6784	421.7	3.0273	2.7602	0.4566	3.3747	0
6785	421.6	3.0496	2.76	0.4564	3.3737	0
6786	421.5	3.0491	2.7719	0.4564	3.4248	0
6787	421.4	3.0491	2.7716	0.4564	3.4828	0
6788	421.3	3.0264	2.7836	0.4564	3.4248	0
6789	421.2	3.0487	2.7833	0.4565	3.4248	0
6790	421.1	3.0482	2.783	0.4567	3.3726	0
6791	421	3.0482	2.7828	0.4565	3.4237	0
6792	420.9	3.0477	2.7951	0.4564	3.3726	0
6793	420.8	3.0477	2.8077	0.4567	3.4237	0
6794	420.7	3.0472	2.7698	0.4567	3.4225	0
6795	420.6	3.0246	2.8072	0.4567	3.4225	0
6796	420.5	3.0467	2.8069	0.4564	3.3716	0
6797	420.4	3.0701	2.8066	0.4565	3.4225	0
6798	420.3	3.0462	2.8063	0.4567	3.4214	0
6799	420.2	3.0462	2.8196	0.4567	3.4214	0
6800	420.1	3.0458	2.8058	0.4567	3.4789	0
6801	420	3.0691	2.819	0.4566	3.4789	0
6802	419.9	3.0685	2.8187	0.4562	3.4789	0
6803	419.8	3.0931	2.8184	0.4564	3.4202	0
6804	419.7	3.0443	2.8318	0.4563	3.3686	0
6805	419.6	3.0675	2.8315	0.456	3.4776	0
6806	419.5	3.067	2.8312	0.456	3.3686	0
6807	419.4	3.067	2.8309	0.4559	3.4191	0
6808	419.3	3.0915	2.8303	0.4558	3.4191	0
6809	419.2	3.091	2.8441	0.4555	3.4179	0
6810	419.1	3.0904	2.8441	0.4557	3.4179	0
6811	419	3.0655	2.8586	0.4554	3.4179	0
6812	418.9	3.065	2.8579	0.4551	3.475	0
6813	418.8	3.065	2.8576	0.4552	3.475	0
6814	418.7	3.0645	2.8722	0.4547	3.3655	0
6815	418.6	3.0888	2.857	0.4549	3.4737	0
6816	418.5	3.1427	2.8716	0.4549	3.4737	0
6817	418.4	3.0635	2.856	0.4544	3.4737	0
6818	418.3	3.0878	2.871	0.4545	3.4157	0
6819	418.2	3.1141	2.8867	0.4545	3.5391	0
6820	418.1	3.0872	2.8861	0.454	3.4724	0
6821	418	3.0867	2.87	0.4538	3.4145	0
6822	417.9	3.0862	2.8854	0.4539	3.4711	0
6823	417.8	3.1124	2.9014	0.454	3.4711	0

6824	417.7	3.0857	2.9183	0.454	3.4711	0
6825	417.6	3.1118	2.901	0.454	3.6162	0
6826	417.5	3.1113	2.9003	0.4538	3.4123	0
6827	417.4	3.1107	2.9172	0.4539	3.4123	0
6828	417.3	3.1107	2.8996	0.4537	3.4698	0
6829	417.2	3.1385	2.9165	0.4537	3.4112	0
6830	417.1	3.1379	2.9337	0.4535	3.4685	0
6831	417	3.1096	2.9333	0.4538	3.4101	0
6832	416.9	3.1373	2.9329	0.4536	3.4101	0
6833	416.8	3.0825	2.9147	0.4537	3.4101	0
6834	416.7	3.1361	2.9508	0.4536	3.4672	0
6835	416.6	3.1079	2.9504	0.4536	3.4672	0
6836	416.5	3.1079	2.95	0.4536	3.4672	0
6837	416.4	3.1355	2.9311	0.4537	3.5331	0
6838	416.3	3.1068	2.9307	0.4536	3.5331	0
6839	416.2	3.1068	2.9488	0.4536	3.4078	0
6840	416.1	3.1343	2.9678	0.4535	3.5317	0
6841	416	3.1637	2.9481	0.4535	3.4647	0
6842	415.9	3.1337	2.967	0.4536	3.4067	0
6843	415.8	3.1331	2.9872	0.4536	3.5317	0
6844	415.7	3.1325	2.9666	0.4537	3.4634	0
6845	415.6	3.1325	2.9863	0.4536	3.4634	0
6846	415.5	3.1319	2.9654	0.4535	3.4634	0
6847	415.4	3.1314	3.0066	0.4536	3.4622	0
6848	415.3	3.1612	2.9851	0.4537	3.4622	0
6849	415.2	3.1308	3.0057	0.4539	3.4622	0
6850	415.1	3.1605	2.9842	0.454	3.4609	0
6851	415	3.1599	3.0048	0.4537	3.5272	0
6852	414.9	3.1593	3.0044	0.4538	3.5272	0
6853	414.8	3.1593	2.983	0.454	3.5272	0
6854	414.7	3.1586	3.0259	0.454	3.4597	0
6855	414.6	3.1284	3.0035	0.454	3.4597	0
6856	414.5	3.158	2.9817	0.454	3.4584	0
6857	414.4	3.1574	3.0026	0.4541	3.4001	0
6858	414.3	3.1574	3.0246	0.4544	3.4584	0
6859	414.2	3.1568	3.0241	0.4543	3.4572	0
6860	414.1	3.1884	3.0237	0.4543	3.399	0
6861	414	3.1261	3.0232	0.4546	3.4572	0
6862	413.9	3.1878	3.0462	0.4549	3.399	0
6863	413.8	3.1549	3	0.4551	3.4559	0
6864	413.7	3.1543	3.0453	0.4551	3.4559	0
6865	413.6	3.1543	3.0701	0.4552	3.5214	0
6866	413.5	3.1858	3.0443	0.4552	3.5214	0

6867	413.4	3.1851	3.0438	0.4551	3.5214	0
6868	413.3	3.1851	3.02	0.4555	3.4535	0
6869	413.2	3.1844	3.068	0.4554	3.4535	0
6870	413.1	3.219	3.0675	0.4556	3.52	0
6871	413	3.1512	3.0424	0.4555	3.52	0
6872	412.9	3.1831	3.0665	0.4555	3.4522	0
6873	412.8	3.2168	3.066	0.4557	3.5186	0
6874	412.7	3.2168	3.0921	0.456	3.451	0
6875	412.6	3.1818	3.0915	0.456	3.5969	0
6876	412.5	3.1811	3.091	0.4564	3.5171	0
6877	412.4	3.2154	3.0904	0.4562	3.5157	0
6878	412.3	3.1798	3.0635	0.456	3.4486	0
6879	412.2	3.214	3.063	0.4561	3.5157	0
6880	412.1	3.214	3.0888	0.456	3.5143	0
6881	412	3.2132	3.0883	0.4562	3.4473	0
6882	411.9	3.2503	3.0878	0.456	3.5143	0
6883	411.8	3.2503	3.1152	0.4558	3.4461	0
6884	411.7	3.2118	3.1146	0.4564	3.4461	0
6885	411.6	3.2111	3.1141	0.4564	3.5129	0
6886	411.5	3.2104	3.0857	0.4564	3.4449	0
6887	411.4	3.1752	3.1433	0.4567	3.5114	0
6888	411.3	3.2472	3.1427	0.4565	3.4437	0
6889	411.2	3.209	3.1421	0.4565	3.51	0
6890	411.1	3.2464	3.1415	0.4564	3.4425	0
6891	411	3.2457	3.1113	0.4564	3.5086	0
6892	410.9	3.2076	3.1403	0.4566	3.5086	0
6893	410.8	3.2857	3.1397	0.4565	3.5072	0
6894	410.7	3.2441	3.1096	0.4566	3.5072	0
6895	410.6	3.2434	3.1385	0.4566	3.4401	0
6896	410.5	3.2434	3.1385	0.4567	3.5058	0
6897	410.4	3.2426	3.1379	0.4564	3.4389	0
6898	410.3	3.2418	3.1694	0.4566	3.5045	0
6899	410.2	3.2411	3.1688	0.4562	3.3799	0
6900	410.1	3.2823	3.1681	0.4564	3.5045	0
6901	410	3.2815	3.202	0.4568	3.5031	0
6902	409.9	3.2807	3.202	0.4566	3.5031	0
6903	409.8	3.2013	3.1662	0.4568	3.4353	0
6904	409.7	3.2381	3.2007	0.4566	3.4342	0
6905	409.6	3.2373	3.2	0.4567	3.4342	0
6906	409.5	3.2373	3.1993	0.4567	3.5003	0
6907	409.4	3.2366	3.2366	0.4563	3.5003	0
6908	409.3	3.2358	3.1979	0.4564	3.4989	0
6909	409.2	3.2351	3.2351	0.4567	3.4989	0

6910	409.1	3.2757	3.1972	0.4565	3.4976	0
6911	409	3.2343	3.1965	0.4565	3.4306	0
6912	408.9	3.3206	3.2336	0.4563	3.4962	0
6913	408.8	3.2741	3.1952	0.4562	3.4962	0
6914	408.7	3.2321	3.2733	0.4561	3.4949	0
6915	408.6	3.2733	3.1938	0.4558	3.5735	0
6916	408.5	3.2725	3.2725	0.4559	3.4271	0
6917	408.4	3.2306	3.2306	0.4558	3.4935	0
6918	408.3	3.317	3.2299	0.4555	3.4921	0
6919	408.2	3.3161	3.2291	0.4559	3.5719	0
6920	408.1	3.3152	3.2284	0.4554	3.4921	0
6921	408	3.3152	3.2277	0.4552	3.4237	0
6922	407.9	3.3143	3.2684	0.4552	3.4908	0
6923	407.8	3.2676	3.2676	0.4553	3.5686	0
6924	407.7	3.3134	3.3134	0.4554	3.4895	0
6925	407.6	3.3125	3.2668	0.455	3.4881	0
6926	407.5	3.3116	3.266	0.4549	3.4881	0
6927	407.4	3.2652	3.2652	0.455	3.4868	0
6928	407.3	3.3107	3.2644	0.4553	3.4868	0
6929	407.2	3.3615	3.3098	0.4549	3.5654	0
6930	407.1	3.3089	3.2636	0.4549	3.5638	0
6931	407	3.3089	3.2211	0.4548	3.4179	0
6932	406.9	3.262	3.262	0.4548	3.4168	0
6933	406.8	3.3072	3.3072	0.4549	3.4828	0
6934	406.7	3.2612	3.3063	0.4548	3.5622	0
6935	406.6	3.3063	3.3063	0.4549	3.5607	0
6936	406.5	3.3565	3.3054	0.4548	3.4815	0
6937	406.4	3.3565	3.3565	0.4545	3.4815	0
6938	406.3	3.3045	3.3045	0.4546	3.5591	0
6939	406.2	3.3546	3.3036	0.4546	3.4802	0
6940	406.1	3.3028	3.3028	0.4546	3.4789	0
6941	406	3.3536	3.2565	0.4548	3.5575	0
6942	405.9	3.3526	3.2557	0.4547	3.4776	0
6943	405.8	3.4101	3.4101	0.4547	3.4776	0
6944	405.7	3.3516	3.3002	0.4548	3.556	0
6945	405.6	3.4089	3.3507	0.4546	3.4763	0
6946	405.5	3.3507	3.3507	0.4548	3.475	0
6947	405.4	3.3497	3.3497	0.455	3.5544	0
6948	405.3	3.3487	3.3487	0.4546	3.5528	0
6949	405.2	3.3487	3.3487	0.4549	3.5528	0
6950	405.1	3.2967	3.251	0.4549	3.5513	0
6951	405	3.4056	3.3468	0.4553	3.4724	0
6952	404.9	3.4045	3.4045	0.4554	3.4045	0

6953	404.8	3.4711	3.295	0.4554	3.4711	0
6954	404.7	3.3449	3.3449	0.4555	3.5498	0
6955	404.6	3.4023	3.3449	0.4554	3.5482	0
6956	404.5	3.3439	3.4023	0.4556	3.5482	0
6957	404.4	3.4012	3.4012	0.4556	3.5467	0
6958	404.3	3.4001	3.3429	0.4559	3.4672	0
6959	404.2	3.4672	3.4001	0.4558	3.4672	0
6960	404.1	3.399	3.341	0.4558	3.466	0
6961	404	3.3979	3.3979	0.4556	3.466	0
6962	403.9	3.3979	3.3401	0.4557	3.4647	0
6963	403.8	3.4634	3.4634	0.4558	3.4634	0
6964	403.7	3.3969	3.3969	0.4561	3.5421	0
6965	403.6	3.3958	3.3958	0.4559	3.5421	0
6966	403.5	3.3947	3.3372	0.456	3.5406	0
6967	403.4	3.3363	3.3363	0.4562	3.5406	0
6968	403.3	3.3936	3.3354	0.4562	3.4609	0
6969	403.2	3.3925	3.3925	0.4563	3.5391	0
6970	403.1	3.3925	3.3344	0.4564	3.5391	0
6971	403	3.4584	3.3915	0.4564	3.5376	0
6972	402.9	3.3904	3.3904	0.4566	3.6345	0
6973	402.8	3.4572	3.3904	0.4569	3.5361	0
6974	402.7	3.4559	3.3316	0.4565	3.5361	0
6975	402.6	3.4559	3.3883	0.4565	3.5346	0
6976	402.5	3.3883	3.4547	0.4566	3.5346	0
6977	402.4	3.4547	3.3872	0.4567	3.6308	0
6978	402.3	3.4535	3.4535	0.4569	3.4535	0
6979	402.2	3.4522	3.3862	0.4569	3.4522	0
6980	402.1	3.4522	3.4522	0.4568	3.6289	0
6981	402	3.451	3.451	0.4568	3.5302	0
6982	401.9	3.451	3.451	0.457	3.5302	0
6983	401.8	3.5287	3.383	0.457	3.6253	0
6984	401.7	3.4486	3.4486	0.457	3.6253	0
6985	401.6	3.4486	3.4486	0.4571	3.5272	0
6986	401.5	3.3799	3.4473	0.4565	3.6234	0
6987	401.4	3.4461	3.4461	0.457	3.4461	0
6988	401.3	3.4449	3.4449	0.457	3.4449	0
6989	401.2	3.5243	3.3778	0.4568	3.5243	0
6990	401.1	3.4437	3.4437	0.4567	3.4437	0
6991	401	3.4437	3.3757	0.4568	3.4437	0
6992	400.9	3.3747	3.5214	0.4565	3.5214	0
6993	400.8	3.3747	3.52	0.4567	3.4413	0
6994	400.7	3.3737	3.4401	0.4567	3.52	0
6995	400.6	3.4401	3.5186	0.4569	3.5186	0

6996	400.5	3.4389	3.3716	0.4568	3.5186	0
6997	400.4	3.4377	3.3716	0.4569	3.5171	0
6998	400.3	3.5157	3.5157	0.4567	3.5157	0
6999	400.2	3.5157	3.5157	0.4569	3.5157	0
7000	400.1	3.5143	3.4353	0.4569	3.5143	0
7001	400	3.5129	3.4342	0.4566	3.5129	0
7002	399.9	3.4342	3.5129	0.4566	3.5129	0
7003	399.8	3.433	3.5114	0.4567	3.5114	0
7004	399.7	3.4318	3.5114	0.4567	3.5114	0
7005	399.6	3.4306	3.4306	0.4565	3.51	0
7006	399.5	3.5086	3.4295	0.4566	3.6055	0
7007	399.4	3.5086	3.5086	0.4566	3.6055	0
7008	399.3	3.4283	3.5072	0.4567	3.6038	0
7009	399.2	3.6038	3.5058	0.4567	3.6038	0
7010	399.1	3.6021	3.5058	0.4565	3.6021	0
7011	399	3.6021	3.5045	0.4564	3.5045	0
7012	398.9	3.5031	3.5031	0.4564	3.5031	0
7013	398.8	3.5031	3.5031	0.4563	3.6003	0
7014	398.7	3.5017	3.5017	0.4563	3.5017	0
7015	398.6	3.4214	3.4214	0.4562	3.5986	0
7016	398.5	3.5003	3.5003	0.456	3.5003	0
7017	398.4	3.4989	3.4989	0.4561	3.4989	0
7018	398.3	3.4191	3.4976	0.4558	3.4976	0
7019	398.2	3.4976	3.5935	0.4559	3.5935	0
7020	398.1	3.5935	3.4962	0.456	3.5935	0
7021	398	3.5918	3.5918	0.456	3.5918	0
7022	397.9	3.4935	3.4935	0.4559	3.4935	0
7023	397.8	3.5901	3.4935	0.4559	3.4935	0
7024	397.7	3.4921	3.5901	0.4557	3.4921	0
7025	397.6	3.5884	3.4908	0.4559	3.4908	0
7026	397.5	3.5867	3.4112	0.4559	3.5867	0
7027	397.4	3.5867	3.4895	0.456	3.7122	0
7028	397.3	3.4881	3.4881	0.456	3.585	0
7029	397.2	3.4881	3.585	0.456	3.4881	0
7030	397.1	3.5834	3.5834	0.4557	3.5834	0
7031	397	3.5834	3.4855	0.4558	3.5834	0
7032	396.9	3.5817	3.4841	0.4559	3.4841	0
7033	396.8	3.7055	3.4841	0.4556	3.4045	0
7034	396.7	3.58	3.58	0.4554	3.58	0
7035	396.6	3.5784	3.5784	0.4554	3.4815	0
7036	396.5	3.5784	3.4012	0.4555	3.4815	0
7037	396.4	3.5768	3.4802	0.4552	3.4802	0
7038	396.3	3.4789	3.5751	0.4555	3.4789	0

7039	396.2	3.5751	3.5751	0.4558	3.5751	0
7040	396.1	3.5735	3.4776	0.456	3.3979	0
7041	396	3.699	3.4763	0.4556	3.5735	0
7042	395.9	3.6968	3.475	0.4557	3.5719	0
7043	395.8	3.4737	3.5719	0.4559	3.5719	0
7044	395.7	3.4737	3.4737	0.4562	3.5702	0
7045	395.6	3.4724	3.5686	0.4561	3.4724	0
7046	395.5	3.5686	3.4711	0.4559	3.5686	0
7047	395.4	3.567	3.4711	0.4562	3.567	0
7048	395.3	3.567	3.3904	0.4562	3.4698	0
7049	395.2	3.5654	3.5654	0.456	3.4685	0
7050	395.1	3.4672	3.5638	0.4563	3.5638	0
7051	395	3.5638	3.5638	0.4562	3.4672	0
7052	394.9	3.466	3.5622	0.4562	3.466	0
7053	394.8	3.5622	3.4647	0.4561	3.4647	0
7054	394.7	3.5607	3.6861	0.4565	3.5607	0
7055	394.6	3.5591	3.5591	0.4564	3.4634	0
7056	394.5	3.684	3.5591	0.4564	3.5591	0
7057	394.4	3.6819	3.5575	0.4562	3.5575	0
7058	394.3	3.5575	3.5575	0.4568	3.5575	0
7059	394.2	3.556	3.556	0.4567	3.556	0
7060	394.1	3.6799	3.5544	0.4568	3.4584	0
7061	394	3.6799	3.5544	0.4565	3.4572	0
7062	393.9	3.5528	3.5528	0.4571	3.5528	0
7063	393.8	3.5528	3.6778	0.4571	3.6778	0
7064	393.7	3.5513	3.5513	0.457	3.5513	0
7065	393.6	3.6757	3.5498	0.457	3.5498	0
7066	393.5	3.6737	3.5498	0.4569	3.5498	0
7067	393.4	3.6737	3.451	0.4571	3.5482	0
7068	393.3	3.8477	3.5467	0.4573	3.5467	0
7069	393.2	3.8477	3.5452	0.4573	3.6716	0
7070	393.1	3.5452	3.5452	0.4576	3.5452	0
7071	393	3.6696	3.5436	0.4574	3.4473	0
7072	392.9	3.6676	3.6676	0.4577	3.4461	0
7073	392.8	3.5421	3.5421	0.4575	3.5421	0
7074	392.7	3.6655	3.6655	0.4575	3.5406	0
7075	392.6	3.6655	3.4425	0.4578	3.4425	0
7076	392.5	3.6635	3.5391	0.4577	3.5391	0
7077	392.4	3.5376	3.6635	0.4578	3.5376	0
7078	392.3	3.8386	3.6615	0.458	3.5361	0
7079	392.2	3.6615	3.5361	0.4577	3.4389	0
7080	392.1	3.5346	3.5346	0.4574	3.5346	0
7081	392	3.5346	3.6596	0.4576	3.5346	0

7082	391.9	3.5331	3.5331	0.4578	3.5331	0
7083	391.8	3.6576	3.6576	0.4578	3.5317	0
7084	391.7	3.6556	3.6556	0.4576	3.6556	0
7085	391.6	3.8297	3.5302	0.4575	3.5302	0
7086	391.5	3.6536	3.6536	0.4575	3.4318	0
7087	391.4	3.6517	3.5272	0.4574	3.5272	0
7088	391.3	3.6517	3.4295	0.4573	3.6517	0
7089	391.2	3.6498	3.5258	0.4574	3.5258	0
7090	391.1	3.8239	3.5243	0.4573	3.5243	0
7091	391	3.8239	3.5229	0.4571	3.6478	0
7092	390.9	3.6459	3.5214	0.4572	3.5214	0
7093	390.8	3.6459	3.6459	0.4572	3.52	0
7094	390.7	3.52	3.644	0.4575	3.4225	0
7095	390.6	3.8182	3.5186	0.4572	3.5186	0
7096	390.5	3.6421	3.6421	0.4569	3.5171	0
7097	390.4	3.6421	3.5157	0.4572	3.5157	0
7098	390.3	3.8153	3.6402	0.4572	3.6402	0
7099	390.2	3.6383	3.6383	0.457	3.6383	0
7100	390.1	3.6383	3.5129	0.4571	3.5129	0
7101	390	3.8125	3.6364	0.4571	3.6364	0
7102	389.9	3.8125	3.6345	0.4568	3.51	0
7103	389.8	3.8097	3.5086	0.4567	3.5086	0
7104	389.7	3.8097	3.6326	0.4565	3.5072	0
7105	389.6	4.1079	3.8069	0.457	3.5058	0
7106	389.5	3.6308	3.5045	0.4569	3.5045	0
7107	389.4	3.6289	3.6289	0.4568	3.5045	0
7108	389.3	3.6271	3.6271	0.4567	3.4056	0
7109	389.2	3.6271	3.6271	0.4568	3.5017	0
7110	389.1	3.8013	3.8013	0.4569	3.5003	0
7111	389	3.7986	3.4989	0.4566	3.4989	0
7112	388.9	3.6216	3.4976	0.4567	3.6216	0
7113	388.8	4.0969	3.6216	0.4566	3.4962	0
7114	388.7	3.6198	3.6198	0.4565	3.4949	0
7115	388.6	4.0969	3.618	0.4564	3.4935	0
7116	388.5	3.7932	3.4921	0.4565	3.618	0
7117	388.4	3.7932	3.6162	0.4563	3.4908	0
7118	388.3	3.6144	3.7905	0.4565	3.4895	0
7119	388.2	3.7905	3.6126	0.4563	3.4881	0
7120	388.1	4.0915	3.6126	0.4564	3.4868	0
7121	388	3.7878	3.6108	0.4564	3.4855	0
7122	387.9	3.7852	3.4841	0.4564	3.4841	0
7123	387.8	3.7852	3.4828	0.4564	3.6073	0
7124	387.7	4.0862	3.7825	0.4565	3.4815	0

7125	387.6	3.7825	3.7825	0.4565	3.383	0
7126	387.5	3.7799	3.6038	0.4565	3.6038	0
7127	387.4	3.6021	3.6021	0.4565	3.4776	0
7128	387.3	3.7773	3.4763	0.4567	3.6003	0
7129	387.2	3.7773	3.6003	0.4566	3.475	0
7130	387.1	3.5986	3.7747	0.4569	3.4737	0
7131	387	3.7721	3.5969	0.4567	3.5969	0
7132	386.9	3.7721	3.5952	0.457	3.4711	0
7133	386.8	4.0706	3.5952	0.457	3.5952	0
7134	386.7	3.5935	3.5935	0.4571	3.4685	0
7135	386.6	4.0706	3.5918	0.4569	3.5918	0
7136	386.5	3.767	3.5901	0.4566	3.466	0
7137	386.4	3.7645	3.7645	0.457	3.4647	0
7138	386.3	3.7645	3.7645	0.4568	3.3665	0
7139	386.2	4.0655	3.5867	0.4573	3.4622	0
7140	386.1	3.762	3.585	0.4575	3.4597	0
7141	386	3.7595	3.5834	0.4572	3.4584	0
7142	385.9	3.7595	3.7595	0.4575	3.4572	0
7143	385.8	3.757	3.4559	0.4572	3.5817	0
7144	385.7	3.757	3.58	0.4575	3.58	0
7145	385.6	3.7545	3.5784	0.4577	3.4535	0
7146	385.5	3.7545	3.4522	0.4575	3.7545	0
7147	385.4	3.752	3.752	0.4575	3.451	0
7148	385.3	3.7496	3.4498	0.4576	3.4498	0
7149	385.2	3.7496	3.5735	0.4578	3.5735	0
7150	385.1	3.5719	3.7471	0.458	3.5719	0
7151	385	4.0458	3.5702	0.4581	3.4461	0
7152	384.9	4.0458	3.5686	0.458	3.4437	0
7153	384.8	4.0458	3.567	0.4581	3.567	0
7154	384.7	3.7423	4.0458	0.4581	3.4413	0
7155	384.6	3.7399	3.7399	0.4579	3.4401	0
7156	384.5	3.7399	3.5638	0.4583	3.7399	0
7157	384.4	3.7375	3.5622	0.4583	3.4377	0
7158	384.3	3.7375	3.5607	0.4585	3.5607	0
7159	384.2	3.7352	3.5591	0.4583	3.5591	0
7160	384.1	3.7352	3.5575	0.4583	3.5575	0
7161	384	4.0362	3.5575	0.4584	3.7328	0
7162	383.9	3.7305	3.7305	0.4588	3.556	0
7163	383.8	3.7305	3.7305	0.4588	3.7305	0
7164	383.7	4.0315	3.5528	0.4586	3.5528	0
7165	383.6	3.7282	3.5513	0.4591	3.426	0
7166	383.5	3.7258	3.5498	0.4589	3.5498	0
7167	383.4	3.7258	3.5482	0.4591	3.5482	0

7168	383.3	4.0223	3.7235	0.4592	3.4225	0
7169	383.2	3.7212	3.7212	0.4591	3.5467	0
7170	383.1	4.0223	3.5452	0.459	3.4191	0
7171	383	4.0223	3.5436	0.459	3.4179	0
7172	382.9	4.0177	3.4168	0.459	3.5421	0
7173	382.8	4.0177	3.7167	0.4591	3.5406	0
7174	382.7	3.7144	3.7144	0.4594	3.5391	0
7175	382.6	3.7144	3.5376	0.4595	3.5376	0
7176	382.5	3.7122	3.7122	0.4592	3.4112	0
7177	382.4	4.0132	3.7122	0.4593	3.5346	0
7178	382.3	3.71	3.71	0.4594	3.5346	0
7179	382.2	4.0088	3.7077	0.4595	3.5331	0
7180	382.1	4.0088	3.7077	0.4595	3.5317	0
7181	382	4.0088	3.7055	0.4593	3.4045	0
7182	381.9	4.0044	3.7033	0.4598	3.4034	0
7183	381.8	3.7033	3.7033	0.4595	3.7033	0
7184	381.7	3.7011	3.7011	0.4592	3.4012	0
7185	381.6	3.7011	3.5243	0.4593	3.5243	0
7186	381.5	4	3.699	0.4592	3.3979	0
7187	381.4	3.5214	3.5214	0.4592	3.6968	0
7188	381.3	3.9957	3.6946	0.4591	3.52	0
7189	381.2	3.9957	3.6946	0.4593	3.5969	0
7190	381.1	4.2924	3.6925	0.4592	3.5171	0
7191	381	4.2924	3.6904	0.4593	3.5157	0
7192	380.9	4.2924	3.6904	0.4588	3.5935	0
7193	380.8	4.2924	3.6882	0.459	3.5129	0
7194	380.7	3.9872	3.5901	0.4589	3.5901	0
7195	380.6	4.284	3.6861	0.4588	3.51	0
7196	380.5	4.284	3.684	0.459	3.5086	0
7197	380.4	4.284	3.6819	0.4587	3.5072	0
7198	380.3	4	3.6819	0.4589	3.5058	0
7199	380.2	3.983	3.6799	0.459	3.5045	0
7200	380.1	4	3.6778	0.4588	3.5817	0
7201	380	3.9788	3.6778	0.4586	3.5017	0
7202	379.9	4.2757	3.8013	0.4583	3.4989	0
7203	379.8	4.2757	3.7986	0.4586	3.5768	0
7204	379.7	3.9747	3.6737	0.4587	3.5751	0
7205	379.6	4.2757	3.7959	0.4583	3.6716	0
7206	379.5	3.9706	3.6696	0.4584	3.4935	0
7207	379.4	4.2676	3.7932	0.4584	3.5719	0
7208	379.3	4.2676	3.9666	0.4584	3.4908	0
7209	379.2	4	3.6655	0.4586	3.5686	0
7210	379.1	4	3.7878	0.4579	3.567	0

7211	379	4	3.9626	0.4581	3.4868	0
7212	378.9	4.2596	3.7852	0.4579	3.5638	0
7213	378.8	4.2596	3.7852	0.458	3.4828	0
7214	378.7	4	3.9586	0.4582	3.5607	0
7215	378.6	4.2596	3.7799	0.4579	3.6556	0
7216	378.5	4.2596	3.6556	0.4575	3.4789	0
7217	378.4	4.2518	3.7773	0.4576	3.4776	0
7218	378.3	4	3.7773	0.4576	3.475	0
7219	378.2	4.2518	3.6498	0.4577	3.4067	0
7220	378.1	4	3.6478	0.4577	3.4724	0
7221	378	4	3.9469	0.4576	3.4711	0
7222	377.9	4.2441	3.9469	0.4575	3.4698	0
7223	377.8	4.2441	3.7696	0.4576	3.5467	0
7224	377.7	4.2441	3.767	0.4575	3.5452	0
7225	377.6	4	3.7645	0.4576	3.5436	0
7226	377.5	4	3.7645	0.4577	3.4634	0
7227	377.4	4.2366	3.762	0.4579	3.5406	0
7228	377.3	4	3.9355	0.4581	3.4597	0
7229	377.2	4	3.7595	0.4579	3.4584	0
7230	377.1	4.2366	3.9318	0.4579	3.4559	0
7231	377	4.2366	3.757	0.4582	3.5346	0
7232	376.9	4	3.6289	0.458	3.4535	0
7233	376.8	4.2291	3.6271	0.4585	3.6271	0
7234	376.7	4.2291	3.752	0.4581	3.5287	0
7235	376.6	3.9245	3.7496	0.4582	3.5272	0
7236	376.5	4	3.9245	0.4583	3.5258	0
7237	376.4	4	3.7471	0.4584	3.3788	0
7238	376.3	4.2218	3.9208	0.4584	3.5229	0
7239	376.2	4.2218	3.618	0.4583	3.5214	0
7240	376.1	4.2218	3.6162	0.4585	3.52	0
7241	376	4.2147	3.9172	0.4585	3.4389	0
7242	375.9	4	3.9136	0.4588	3.4377	0
7243	375.8	4.2147	3.9136	0.4589	3.6126	0
7244	375.7	4	3.7352	0.4593	3.5143	0
7245	375.6	4.2147	3.7352	0.4591	3.5129	0
7246	375.5	4	3.7328	0.4591	3.51	0
7247	375.4	4	3.7305	0.4592	3.5086	0
7248	375.3	4.2076	3.9066	0.4595	3.6038	0
7249	375.2	4.2076	3.7282	0.4598	3.5058	0
7250	375.1	4.2007	3.9031	0.4594	3.5045	0
7251	375	4	3.7235	0.4596	3.5031	0
7252	374.9	4.2007	3.7235	0.4597	3.5017	0
7253	374.8	4	3.7212	0.46	3.4202	0

7254	374.7	4.1938	3.8962	0.4603	3.4976	0
7255	374.6	4.1938	3.8928	0.46	3.4962	0
7256	374.5	4	4.1938	0.4602	3.4157	0
7257	374.4	4.1938	3.8928	0.4604	3.4935	0
7258	374.3	4	3.7144	0.4607	3.4123	0
7259	374.2	4	3.8894	0.4605	3.4895	0
7260	374.1	4	3.71	0.4606	3.4881	0
7261	374	4	3.71	0.4609	3.4868	0
7262	373.9	4.1871	3.7077	0.4607	3.4855	0
7263	373.8	4	3.7055	0.4609	3.4841	0
7264	373.7	4	3.58	0.461	3.58	0
7265	373.6	4	3.8794	0.4611	3.5784	0
7266	373.5	4	3.7011	0.4611	3.4789	0
7267	373.4	4	3.699	0.4609	3.4776	0
7268	373.3	4	3.8729	0.4611	3.5735	0
7269	373.2	4	3.5719	0.4611	3.5719	0
7270	373.1	4	3.6946	0.4612	3.4737	0
7271	373	4	3.8697	0.4609	3.5686	0
7272	372.9	4	3.8697	0.4614	3.4698	0
7273	372.8	4	3.8665	0.4615	3.5654	0
7274	372.7	4	3.5638	0.4611	3.4672	0
7275	372.6	4	3.8633	0.4615	3.5622	0
7276	372.5	4	3.8633	0.4616	3.5607	0
7277	372.4	4.1612	3.8601	0.4614	3.5607	0
7278	372.3	4	3.684	0.4616	3.4609	0
7279	372.2	4	3.6819	0.4615	3.5575	0
7280	372.1	4	3.6799	0.4615	3.4584	0
7281	372	4	3.8539	0.4613	3.5544	0
7282	371.9	4	3.8539	0.4611	3.5528	0
7283	371.8	4	3.8539	0.4612	3.3757	0
7284	371.7	4	3.6757	0.4613	3.5498	0
7285	371.6	4	3.6737	0.4615	3.5482	0
7286	371.5	4	3.8477	0.4617	3.4498	0
7287	371.4	4	3.6716	0.4614	3.5452	0
7288	371.3	4	3.8447	0.4613	3.4473	0
7289	371.2	4	3.6676	0.4614	3.4461	0
7290	371.1	4	4.1427	0.4616	3.5421	0
7291	371	4	3.6655	0.4615	3.3645	0
7292	370.9	4	3.6635	0.4614	3.4425	0
7293	370.8	4	3.8386	0.4616	3.4401	0
7294	370.7	4	3.8386	0.4612	3.6615	0
7295	370.6	4	3.8356	0.461	3.3585	0
7296	370.5	4	3.8356	0.461	3.3575	0

7297	370.4	4	3.8327	0.461	3.4353	0
7298	370.3	4	3.6556	0.461	3.5302	0
7299	370.2	4.1308	3.8297	0.461	3.433	0
7300	370.1	4	4.1308	0.4611	3.5287	0
7301	370	4	3.8268	0.4607	3.5272	0
7302	369.9	4	3.9508	0.4593	3.4067	0
7303	369.8	4	3.6517	0.4594	3.475	0
7304	369.7	4	3.6517	0.4592	3.4089	0
7305	369.6	4	3.7773	0.4594	3.4776	0
7306	369.5	4	3.9547	0.4591	3.4123	0
7307	369.4	4	3.7799	0.4591	3.4134	0
7308	369.3	4	3.6576	0.4589	3.4815	0
7309	369.2	4	3.6576	0.4588	3.4828	0
7310	369.1	4	3.9586	0.4587	3.4168	0
7311	369	4	3.7852	0.4585	3.4841	0
7312	368.9	4	3.9626	0.4584	3.4191	0
7313	368.8	4	3.7878	0.4584	3.3615	0
7314	368.7	4	3.7878	0.4583	3.4881	0
7315	368.6	4	3.6655	0.4581	3.3134	0
7316	368.5	4	3.7905	0.458	3.3655	0
7317	368.4	4	3.6676	0.458	3.4248	0
7318	368.3	4	3.9706	0.4576	3.4248	0
7319	368.2	4	3.9706	0.4577	3.4935	0
7320	368.1	4	4.2757	0.4578	3.4271	0
7321	368	4	3.7959	0.4577	3.4283	0
7322	367.9	4	3.7959	0.4579	3.4295	0
7323	367.8	4	3.9747	0.4574	3.4306	0
7324	367.7	4	3.7986	0.4575	3.3726	0
7325	367.6	4	3.9747	0.4573	3.4318	0
7326	367.5	4	3.8013	0.4573	3.433	0
7327	367.4	4	3.8013	0.4576	3.3757	0
7328	367.3	4	3.8013	0.4574	3.4342	0
7329	367.2	4	3.8041	0.4573	3.4353	0
7330	367.1	4	3.8041	0.4572	3.4365	0
7331	367	4	3.6799	0.4571	3.4377	0
7332	366.9	4	3.983	0.4571	3.4377	0
7333	366.8	4	3.983	0.4573	3.4389	0
7334	366.7	4	3.8069	0.4574	3.382	0
7335	366.6	4	4.284	0.4574	3.4401	0
7336	366.5	4	3.8097	0.4571	3.383	0
7337	366.4	4	3.9872	0.4573	3.384	0
7338	366.3	4	3.9872	0.4575	3.4425	0
7339	366.2	4	4.2924	0.4574	3.4437	0

7340	366.1	4	3.9872	0.4574	3.4437	0
7341	366	4	3.9872	0.4571	3.3872	0
7342	365.9	4	3.9914	0.4575	3.4461	0
7343	365.8	4	3.8153	0.4575	3.3883	0
7344	365.7	4	4.2924	0.4577	3.4473	0
7345	365.6	4	3.8153	0.4573	3.4473	0
7346	365.5	4	3.6904	0.4577	3.4486	0
7347	365.4	4	3.8182	0.4579	3.3904	0
7348	365.3	4	3.9957	0.4578	3.3915	0
7349	365.2	4	3.6925	0.4579	3.4498	0
7350	365.1	4	3.8182	0.4579	3.5171	0
7351	365	4	3.6946	0.4579	3.451	0
7352	364.9	4	3.821	0.458	3.4522	0
7353	364.8	4	3.6946	0.4587	3.4522	0
7354	364.7	4	3.6968	0.4584	3.3947	0
7355	364.6	4	3.821	0.4586	3.3958	0
7356	364.5	4	3.821	0.4584	3.3958	0
7357	364.4	4	3.8239	0.4587	3.3969	0
7358	364.3	4	3.699	0.4589	3.4547	0
7359	364.2	4	4	0.4592	3.3979	0
7360	364.1	4	4	0.4593	3.3979	0
7361	364	4	4	0.4593	3.3478	0
7362	363.9	4	4	0.4593	3.5243	0
7363	363.8	4	3.8268	0.4599	3.399	0
7364	363.7	4	3.8268	0.4597	3.4584	0
7365	363.6	4	3.8268	0.4598	3.3487	0
7366	363.5	4	4.0044	0.46	3.4012	0
7367	363.4	4	3.8268	0.4603	3.4597	0
7368	363.3	4	4.0044	0.4604	3.3507	0
7369	363.2	4	3.8268	0.4605	3.3507	0
7370	363.1	4	3.8268	0.4605	3.4023	0
7371	363	4	3.8297	0.4605	3.4609	0
7372	362.9	4	3.8297	0.4605	3.4609	0
7373	362.8	4	3.8297	0.4609	3.4034	0
7374	362.7	4	3.7033	0.4608	3.4034	0
7375	362.6	4	3.8297	0.461	3.3526	0
7376	362.5	4	4.0044	0.461	3.3526	0
7377	362.4	4	4.0044	0.4607	3.4045	0
7378	362.3	4	3.8297	0.4611	3.4045	0
7379	362.2	4	3.7055	0.4608	3.4622	0
7380	362.1	4	4.0088	0.4608	3.4045	0
7381	362	4	4.0088	0.461	3.4045	0
7382	361.9	4	3.8297	0.4612	3.4056	0

7383	361.8	4	3.8327	0.4612	3.4056	0
7384	361.7	4	4.0088	0.4609	3.4056	0
7385	361.6	4	3.7055	0.4611	3.4634	0
7386	361.5	4	4.0088	0.4609	3.4056	0
7387	361.4	4	3.8327	0.4609	3.4056	0
7388	361.3	4	4.0088	0.4609	3.3546	0
7389	361.2	4	3.8327	0.461	3.4056	0
7390	361.1	4	4.0088	0.4612	3.3546	0
7391	361	4	3.8327	0.4614	3.3089	0
7392	360.9	4	3.8327	0.4615	3.4067	0
7393	360.8	4	4.0088	0.4612	3.4647	0
7394	360.7	4	3.8327	0.4611	3.4067	0
7395	360.6	4	3.6108	0.4613	3.4067	0
7396	360.5	4	3.7077	0.4611	3.4647	0
7397	360.4	4	3.8327	0.4614	3.4647	0
7398	360.3	4	3.8327	0.4612	3.4067	0
7399	360.2	4	4.0088	0.4615	3.4067	0
7400	360.1	4	4.0088	0.4613	3.4067	0
7401	360	4	3.8327	0.4612	3.4067	0
7402	359.9	4	3.8327	0.4613	3.4647	0
7403	359.8	4	3.8327	0.461	3.4067	0
7404	359.7	4	4.0088	0.4612	3.4647	0
7405	359.6	4	4.0088	0.461	3.4056	0
7406	359.5	4	3.8327	0.461	3.4634	0
7407	359.4	4	3.7077	0.4607	3.4056	0
7408	359.3	4	4.0088	0.4604	3.4056	0
7409	359.2	4	3.7055	0.4607	3.4056	0
7410	359.1	4	3.8327	0.4604	3.4056	0
7411	359	4	3.7055	0.4603	3.4634	0
7412	358.9	4	3.7055	0.4603	3.4634	0
7413	358.8	4	3.7055	0.4602	3.4622	0
7414	358.7	4	3.8297	0.4602	3.4622	0
7415	358.6	4	3.7055	0.4597	3.4045	0
7416	358.5	4	3.7055	0.4599	3.4045	0
7417	358.4	4	4.0044	0.4597	3.4622	0
7418	358.3	4	3.8297	0.4596	3.4622	0
7419	358.2	4	3.8297	0.4595	3.4622	0
7420	358.1	4	4.0044	0.4594	3.4609	0
7421	358	4	3.7033	0.4594	3.4609	0
7422	357.9	4	3.8297	0.4592	3.4609	0
7423	357.8	4	4.0044	0.4593	3.4609	0
7424	357.7	4	3.7033	0.4591	3.4609	0
7425	357.6	4	3.8268	0.4593	3.4597	0

7426	357.5	4	3.8268	0.459	3.4597	0
7427	357.4	4	3.8268	0.4588	3.4597	0
7428	357.3	4	3.8268	0.4589	3.4597	0
7429	357.2	4	3.8268	0.459	3.4012	0
7430	357.1	4	4.0044	0.4589	3.4001	0
7431	357	4	3.8268	0.4587	3.4001	0
7432	356.9	4	3.8268	0.4585	3.4001	0
7433	356.8	4	4	0.4585	3.4572	0
7434	356.7	4	3.7011	0.4585	3.399	0
7435	356.6	4	3.8239	0.4583	3.399	0
7436	356.5	4	3.6021	0.4584	3.3979	0
7437	356.4	4	3.8239	0.4584	3.3979	0
7438	356.3	4	3.8239	0.4583	3.4559	0
7439	356.2	4	3.699	0.4582	3.4559	0
7440	356.1	4	4	0.4581	3.3969	0
7441	356	4	3.6968	0.4581	3.4547	0
7442	355.9	4	3.6968	0.4581	3.4535	0
7443	355.8	4	3.821	0.4582	3.3958	0
7444	355.7	4	3.9957	0.4584	3.52	0
7445	355.6	4	3.821	0.4585	3.52	0
7446	355.5	4	3.821	0.4579	3.4522	0
7447	355.4	4	3.821	0.4585	3.4522	0
7448	355.3	4	3.8182	0.4586	3.451	0
7449	355.2	4	3.8182	0.4584	3.451	0
7450	355.1	4	3.8182	0.4587	3.5171	0
7451	355	4	3.9957	0.4586	3.4498	0
7452	354.9	4	3.8182	0.4587	3.4498	0
7453	354.8	4	3.8182	0.459	3.5157	0
7454	354.7	4	3.8153	0.459	3.4486	0
7455	354.6	4	3.8153	0.4592	3.4486	0
7456	354.5	4	3.9914	0.4595	3.4473	0
7457	354.4	4	3.8153	0.4592	3.3893	0
7458	354.3	4	3.8153	0.4596	3.5129	0
7459	354.2	4	3.9914	0.4596	3.4461	0
7460	354.1	4	3.6882	0.4597	3.4449	0
7461	354	4	3.9872	0.4597	3.3862	0
7462	353.9	4	3.8125	0.46	3.4437	0
7463	353.8	4	3.8125	0.4601	3.4437	0
7464	353.7	4	3.8097	0.4604	3.51	0
7465	353.6	4	3.8097	0.4603	3.4425	0
7466	353.5	4	3.8097	0.461	3.384	0
7467	353.4	4	3.8097	0.4609	3.4413	0
7468	353.3	4	3.983	0.4607	3.382	0

7469	353.2	4	3.8069	0.4611	3.4401	0
7470	353.1	4	3.8069	0.4612	3.3298	0
7471	353	4	3.8069	0.4614	3.4389	0
7472	352.9	4	3.8069	0.4611	3.3799	0
7473	352.8	4	3.983	0.4613	3.4365	0
7474	352.7	4	3.8041	0.4614	3.5031	0
7475	352.6	4	3.8041	0.4617	3.4353	0
7476	352.5	4	3.8013	0.4616	3.5017	0
7477	352.4	4	3.8013	0.4614	3.4342	0
7478	352.3	4	3.8013	0.462	3.433	0
7479	352.2	4	3.6757	0.4621	3.3747	0
7480	352.1	4	3.9747	0.4619	3.4318	0
7481	352	4	3.9747	0.4619	3.3726	0
7482	351.9	4	3.7986	0.4621	3.4976	0
7483	351.8	4	3.7986	0.4622	3.3716	0
7484	351.7	4	3.7959	0.4623	3.4283	0
7485	351.6	4	3.7959	0.4624	3.4283	0
7486	351.5	4	3.9706	0.4624	3.4271	0
7487	351.4	4	3.7959	0.4625	3.5735	0
7488	351.3	4	3.9706	0.4624	3.4921	0
7489	351.2	4	3.6676	0.4622	3.4921	0
7490	351.1	4	3.7932	0.4627	3.3665	0
7491	351	4	3.7905	0.4624	3.4237	0
7492	350.9	4	3.7905	0.4626	3.3645	0
7493	350.8	4	3.9666	0.4622	3.4214	0
7494	350.7	4	3.7878	0.4625	3.4881	0
7495	350.6	4	3.9626	0.4624	3.4202	0
7496	350.5	4	3.6635	0.4624	3.4191	0
7497	350.4	4	3.6615	0.4629	3.4191	0
7498	350.3	4	3.9626	0.4626	3.3595	0
7499	350.2	4	3.7852	0.4628	3.3595	0
7500	350.1	4	3.6596	0.4628	3.4168	0
7501	350	4	3.6576	0.4627	3.3575	0
7502	349.9	4	3.7825	0.4627	3.3565	0
7503	349.8	4	3.7825	0.4628	3.3556	0
7504	349.7	4	3.7799	0.4628	3.4123	0
7505	349.6	4	3.7799	0.4626	3.4789	0
7506	349.5	4	3.7799	0.4626	3.4776	0
7507	349.4	4	3.6536	0.4628	3.3516	0
7508	349.3	4	3.9547	0.4624	3.3002	0
7509	349.2	4	3.6517	0.4626	3.4078	0
7510	349.1	4	3.7747	0.4627	3.4067	0
7511	349	4	3.7747	0.4623	3.4056	0

7512	348.9	4	3.6478	0.4622	3.4056	0
7513	348.8	4	3.7721	0.4622	3.3458	0
7514	348.7	4	3.7721	0.4622	3.3449	0
7515	348.6	4	3.7696	0.4625	3.3439	0
7516	348.5	4	3.9469	0.4623	3.4012	0
7517	348.4	4	3.767	0.4622	3.4672	0
7518	348.3	4	3.767	0.4622	3.341	0
7519	348.2	4	3.767	0.4621	3.3979	0
7520	348.1	4	3.6402	0.4623	3.3969	0
7521	348	4	3.9393	0.4623	3.3382	0
7522	347.9	4	3.6383	0.4622	3.4622	0
7523	347.8	4	3.762	0.4622	3.3936	0
7524	347.7	4	3.7595	0.4621	3.3925	0
7525	347.6	4	3.7595	0.4619	3.3915	0
7526	347.5	4	3.9355	0.4618	3.3325	0
7527	347.4	4	3.757	0.4617	3.4572	0
7528	347.3	4	3.6326	0.4618	3.3307	0
7529	347.2	4	3.7545	0.4617	3.3872	0
7530	347.1	4	3.9318	0.4618	3.3862	0
7531	347	4	3.7545	0.4618	3.3851	0
7532	346.9	4	3.9281	0.4618	3.384	0
7533	346.8	4	3.9281	0.4617	3.4498	0
7534	346.7	4	3.9245	0.4616	3.4486	0
7535	346.6	4	3.9245	0.4615	3.3233	0
7536	346.5	4	3.9245	0.4615	3.4473	0
7537	346.4	4	3.6216	0.4612	3.3788	0
7538	346.3	4	4.2218	0.4615	3.4449	0
7539	346.2	4	3.9208	0.4614	3.3768	0
7540	346.1	4	3.7423	0.4618	3.3757	0
7541	346	4	3.7423	0.4613	3.3747	0
7542	345.9	4	3.7423	0.4613	3.3737	0
7543	345.8	4	3.9172	0.4614	3.3726	0
7544	345.7	4	3.9136	0.4615	3.3706	0
7545	345.6	4	3.7375	0.4617	3.3696	0
7546	345.5	4	3.7375	0.4615	3.4353	0
7547	345.4	4	3.9101	0.4616	3.3675	0
7548	345.3	4	3.7352	0.4612	3.433	0
7549	345.2	4	3.7328	0.4614	3.3655	0
7550	345.1	4	3.9066	0.4616	3.3645	0
7551	345	4	3.7305	0.4614	3.3635	0
7552	344.9	4	3.7305	0.4616	3.4283	0
7553	344.8	4	3.7282	0.4616	3.4271	0
7554	344.7	4	3.7282	0.4618	3.4271	0

7555	344.6	4	3.9031	0.4618	3.3585	0
7556	344.5	4	3.8996	0.4621	3.3575	0
7557	344.4	4	3.5986	0.4621	3.5017	0
7558	344.3	4	3.7235	0.4624	3.3546	0
7559	344.2	4	3.7212	0.4624	3.3536	0
7560	344.1	4	4.2007	0.4624	3.3526	0
7561	344	4	3.8962	0.4626	3.3507	0
7562	343.9	4	3.8928	0.4622	3.4168	0
7563	343.8	4	3.7167	0.4629	3.3487	0
7564	343.7	4	3.8928	0.463	3.3478	0
7565	343.6	4	3.8894	0.4628	3.2882	0
7566	343.5	4	3.7122	0.4628	3.3449	0
7567	343.4	4	4.1871	0.4633	3.3439	0
7568	343.3	4	3.8861	0.4633	3.4101	0
7569	343.2	4	3.8861	0.4633	3.342	0
7570	343.1	4	3.5834	0.4634	3.341	0
7571	343	4	3.7077	0.4635	3.3391	0
7572	342.9	4	3.7055	0.4637	3.3382	0
7573	342.8	4	3.8794	0.4639	3.4034	0
7574	342.7	4	3.7033	0.4636	3.3354	0
7575	342.6	4	3.8794	0.4638	3.4012	0
7576	342.5	4	3.8761	0.4639	3.3335	0
7577	342.4	4	3.8761	0.4639	3.3316	0
7578	342.3	4	4.1739	0.464	3.2725	0
7579	342.2	4	3.6968	0.4642	3.2716	0
7580	342.1	4	3.6968	0.4639	3.3279	0
7581	342	4	3.6946	0.464	3.327	0
7582	341.9	4	3.6925	0.4639	3.3251	0
7583	341.8	4	4.1675	0.4643	3.3242	0
7584	341.7	4	3.8665	0.4643	3.3224	0
7585	341.6	4	3.6882	0.4641	3.3883	0
7586	341.5	4	3.6882	0.4641	3.3197	0
7587	341.4	4	3.6861	0.4641	3.3851	0
7588	341.3	4	3.684	0.4641	3.384	0
7589	341.2	4	3.684	0.4643	3.3161	0
7590	341.1	4	3.6819	0.4646	3.3143	0
7591	341	4	3.6819	0.4642	3.3134	0
7592	340.9	4	3.6799	0.4644	3.4572	0
7593	340.8	4	3.6778	0.4645	3.3768	0
7594	340.7	4	3.6757	0.4644	3.3089	0
7595	340.6	4	3.6757	0.4644	3.4535	0
7596	340.5	4	3.8508	0.4645	3.3737	0
7597	340.4	4	3.6737	0.4643	3.3716	0

7598	340.3	4	3.8477	0.4645	3.3706	0
7599	340.2	4	3.6696	0.4643	3.3686	0
7600	340.1	4	3.6676	0.4645	3.3675	0
7601	340	4	3.6676	0.4651	3.3655	0
7602	339.9	2.2874	2.005	0.4428	2.5686	0
7603	339.8	2.2819	2.0036	0.4426	2.5519	0
7604	339.7	2.2845	1.9979	0.4425	2.5505	0
7605	339.6	2.2791	2.0008	0.4427	2.5567	0
7606	339.5	2.2776	1.995	0.4425	2.5476	0
7607	339.4	2.2722	1.9957	0.4421	2.5462	0
7608	339.3	2.2708	1.9943	0.442	2.5447	0
7609	339.2	2.2734	1.9929	0.4417	2.5508	0
7610	339.1	2.268	1.9916	0.442	2.542	0
7611	339	2.2706	1.9902	0.4417	2.5406	0
7612	338.9	2.2651	1.9845	0.4416	2.5467	0
7613	338.8	2.2638	1.9874	0.4419	2.5378	0
7614	338.7	2.2664	1.986	0.4417	2.5364	0
7615	338.6	2.265	1.9845	0.4413	2.5349	0
7616	338.5	2.2636	1.9831	0.4412	2.5336	0
7617	338.4	2.2621	1.9838	0.4409	2.5321	0
7618	338.3	2.2567	1.9803	0.4407	2.5384	0
7619	338.2	2.2554	1.9768	0.4406	2.5293	0
7620	338.1	2.2542	1.9757	0.4409	2.5358	0
7621	338	2.2486	1.9783	0.4406	2.5342	0
7622	337.9	2.2552	1.9747	0.4405	2.5252	0
7623	337.8	2.2539	1.9756	0.4404	2.5239	0
7624	337.7	2.2524	1.972	0.4403	2.53	0
7625	337.6	2.2471	1.9707	0.4404	2.5287	0
7626	337.5	2.2418	1.9673	0.4402	2.5274	0
7627	337.4	2.2445	1.9659	0.4398	2.5186	0
7628	337.3	2.2431	1.9667	0.4399	2.5246	0
7629	337.2	2.2418	1.9633	0.44	2.5084	0
7630	337.1	2.2484	1.966	0.4397	2.5219	0
7631	337	2.2391	1.9627	0.4398	2.5131	0
7632	336.9	2.2418	1.9614	0.4403	2.5119	0
7633	336.8	2.2366	1.9601	0.4397	2.5181	0
7634	336.7	2.2352	1.9567	0.4396	2.5167	0
7635	336.6	2.234	1.9575	0.4394	2.5079	0
7636	336.5	2.2326	1.9541	0.4393	2.5067	0
7637	336.4	2.2314	1.955	0.4395	2.5054	0
7638	336.3	2.2341	1.9537	0.4396	2.504	0
7639	336.2	2.2328	1.9544	0.4392	2.4953	0
7640	336.1	2.2275	1.951	0.4389	2.494	0

7641	336	2.2302	1.9497	0.4391	2.5078	0
7642	335.9	2.2248	1.9463	0.4389	2.4988	0
7643	335.8	2.2236	1.9472	0.4389	2.4976	0
7644	335.7	2.2265	1.946	0.439	2.504	0
7645	335.6	2.2252	1.9447	0.4387	2.5027	0
7646	335.5	2.2159	1.9434	0.4389	2.5014	0
7647	335.4	2.2185	1.9421	0.4386	2.5	0
7648	335.3	2.2174	1.9388	0.4386	2.4913	0
7649	335.2	2.2121	1.9375	0.4387	2.49	0
7650	335.1	2.2148	1.9342	0.4387	2.4888	0
7651	335	2.2135	1.935	0.4385	2.4876	0
7652	334.9	2.2083	1.9359	0.4385	2.4939	0
7653	334.8	2.2071	1.9346	0.4386	2.4927	0
7654	334.7	2.2138	1.9334	0.4384	2.4839	0
7655	334.6	2.2047	1.9301	0.4383	2.4827	0
7656	334.5	2.2034	1.9288	0.4383	2.4889	0
7657	334.4	2.2021	1.9296	0.4382	2.4876	0
7658	334.3	2.2009	1.9284	0.4385	2.4789	0
7659	334.2	2.2037	1.9273	0.4384	2.4777	0
7660	334.1	2.2025	1.9261	0.4383	2.484	0
7661	334	2.1974	1.9227	0.4383	2.4753	0
7662	333.9	2.1961	1.9237	0.4381	2.4816	0
7663	333.8	2.199	1.9205	0.4382	2.4806	0
7664	333.7	2.1898	1.9213	0.4381	2.487	0
7665	333.6	2.1967	1.9223	0.4384	2.4707	0
7666	333.5	2.1915	1.9169	0.4382	2.477	0
7667	333.4	2.1904	1.9158	0.4381	2.4684	0
7668	333.3	2.1891	1.9166	0.438	2.4671	0
7669	333.2	2.1882	1.9157	0.4383	2.4661	0
7670	333.1	2.1907	1.9122	0.4379	2.4722	0
7671	333	2.1857	1.9132	0.4382	2.4637	0
7672	332.9	2.1846	1.9121	0.4381	2.4702	0
7673	332.8	2.1834	1.9109	0.438	2.4614	0
7674	332.7	2.1822	1.9077	0.4384	2.4678	0
7675	332.6	2.1851	1.9086	0.4381	2.459	0
7676	332.5	2.1838	1.9052	0.438	2.4578	0
7677	332.4	2.1787	1.902	0.4378	2.4642	0
7678	332.3	2.1815	1.903	0.4377	2.4631	0
7679	332.2	2.1765	1.904	0.4378	2.4544	0
7680	332.1	2.1752	1.9027	0.4376	2.4607	0
7681	332	2.178	1.8995	0.4375	2.4595	0
7682	331.9	2.1769	1.9005	0.4377	2.4584	0
7683	331.8	2.1718	1.8972	0.4373	2.4498	0

7684	331.7	2.1706	1.8982	0.4371	2.4562	0
7685	331.6	2.1695	1.8971	0.4372	2.4476	0
7686	331.5	2.1684	1.898	0.437	2.454	0
7687	331.4	2.1634	1.8948	0.4371	2.4528	0
7688	331.3	2.166	1.8936	0.4367	2.4516	0
7689	331.2	2.1651	1.8926	0.4369	2.4506	0
7690	331.1	2.16	1.8893	0.4368	2.4418	0
7691	331	2.1627	1.8881	0.4364	2.4482	0
7692	330.9	2.1615	1.8911	0.4363	2.447	0
7693	330.8	2.1603	1.8879	0.4361	2.4459	0
7694	330.7	2.1553	1.8846	0.4363	2.4371	0
7695	330.6	2.158	1.8834	0.4361	2.4435	0
7696	330.5	2.1608	1.8844	0.436	2.4424	0
7697	330.4	2.1517	1.8811	0.4357	2.4488	0
7698	330.3	2.1544	1.8819	0.4357	2.4324	0
7699	330.2	2.1494	1.8787	0.4357	2.4312	0
7700	330.1	2.152	1.8795	0.4356	2.4375	0
7701	330	2.1469	1.8762	0.4355	2.4363	0
7702	329.9	2.1496	1.875	0.4354	2.4276	0
7703	329.8	2.1483	1.8737	0.4351	2.4338	0
7704	329.7	2.1431	1.8745	0.435	2.4325	0
7705	329.6	2.1419	1.8712	0.4348	2.4313	0
7706	329.5	2.1445	1.87	0.4347	2.43	0
7707	329.4	2.1394	1.8687	0.4345	2.4365	0
7708	329.3	2.1421	1.8654	0.4346	2.4201	0
7709	329.2	2.1368	1.8641	0.4343	2.4262	0
7710	329.1	2.1355	1.8649	0.4338	2.4175	0
7711	329	2.1383	1.8638	0.4343	2.4239	0
7712	328.9	2.1331	1.8624	0.434	2.4225	0
7713	328.8	2.1319	1.8613	0.4337	2.4291	0
7714	328.7	2.1307	1.86	0.4335	2.4201	0
7715	328.6	2.1334	1.8588	0.4336	2.419	0
7716	328.5	2.1321	1.8597	0.4334	2.4177	0
7717	328.4	2.1232	1.8585	0.4331	2.4089	0
7718	328.3	2.1258	1.8572	0.4332	2.4152	0
7719	328.2	2.1285	1.856	0.433	2.414	0
7720	328.1	2.1233	1.8527	0.433	2.4052	0
7721	328	2.126	1.8514	0.4326	2.404	0
7722	327.9	2.1207	1.8501	0.4326	2.4102	0
7723	327.8	2.1235	1.8489	0.4326	2.4014	0
7724	327.7	2.1183	1.8456	0.4324	2.4002	0
7725	327.6	2.1133	1.8465	0.4326	2.399	0
7726	327.5	2.1159	1.8495	0.4324	2.3978	0

7727	327.4	2.1147	1.8462	0.4324	2.4042	0
7728	327.3	2.1135	1.8449	0.4323	2.403	0
7729	327.2	2.1084	1.8395	0.4321	2.4094	0
7730	327.1	2.1072	1.8405	0.4321	2.4006	0
7731	327	2.1099	1.8393	0.4321	2.3918	0
7732	326.9	2.1086	1.8401	0.432	2.3905	0
7733	326.8	2.1036	1.8369	0.4323	2.4046	0
7734	326.7	2.1024	1.8356	0.4323	2.3958	0
7735	326.6	2.1051	1.8345	0.432	2.3946	0
7736	326.5	2.1039	1.8332	0.4319	2.3858	0
7737	326.4	2.1027	1.832	0.4319	2.3846	0
7738	326.3	2.1055	1.8288	0.4319	2.3834	0
7739	326.2	2.1003	1.8297	0.4318	2.3822	0
7740	326.1	2.0991	1.8306	0.4317	2.381	0
7741	326	2.098	1.8274	0.4317	2.3874	0
7742	325.9	2.0968	1.8241	0.4318	2.3713	0
7743	325.8	2.0918	1.825	0.4317	2.3776	0
7744	325.7	2.0944	1.8217	0.4316	2.3763	0
7745	325.6	2.0932	1.8225	0.4316	2.3751	0
7746	325.5	2.0881	1.8213	0.4317	2.3739	0
7747	325.4	2.083	1.818	0.4315	2.3726	0
7748	325.3	2.0895	1.8168	0.4314	2.3714	0
7749	325.2	2.0885	1.8157	0.4315	2.3704	0
7750	325.1	2.0833	1.8144	0.4314	2.3691	0
7751	325	2.086	1.8154	0.4314	2.3679	0
7752	324.9	2.0809	1.8121	0.4314	2.3667	0
7753	324.8	2.0836	1.813	0.4314	2.373	0
7754	324.7	2.0786	1.8119	0.4316	2.3644	0
7755	324.6	2.0814	1.8107	0.4316	2.3708	0
7756	324.5	2.0762	1.8074	0.4315	2.362	0
7757	324.4	2.079	1.8083	0.4315	2.3685	0
7758	324.3	2.074	1.8072	0.4316	2.3597	0
7759	324.2	2.0767	1.8039	0.4315	2.3585	0
7760	324.1	2.0755	1.8028	0.4314	2.3726	0
7761	324	2.0743	1.8016	0.4314	2.3638	0
7762	323.9	2.0692	1.7983	0.4313	2.3625	0
7763	323.8	2.0721	1.7994	0.4319	2.3616	0
7764	323.7	2.067	1.7982	0.4315	2.3528	0
7765	323.6	2.0697	1.799	0.4313	2.3591	0
7766	323.5	2.0685	1.7937	0.4312	2.3504	0
7767	323.4	2.0636	1.7927	0.4314	2.357	0
7768	323.3	2.0665	1.7937	0.4315	2.3483	0
7769	323.2	2.0613	1.7904	0.4312	2.3471	0

7770	323.1	2.064	1.7913	0.4314	2.3535	0
7771	323	2.0628	1.7922	0.4314	2.3448	0
7772	322.9	2.0578	1.7911	0.4311	2.3512	0
7773	322.8	2.0567	1.7899	0.4308	2.3576	0
7774	322.7	2.0555	1.7866	0.4308	2.3412	0
7775	322.6	2.0543	1.7875	0.4307	2.3401	0
7776	322.5	2.0493	1.7864	0.4304	2.3315	0
7777	322.4	2.052	1.7832	0.4306	2.3378	0
7778	322.3	2.0471	1.7821	0.4307	2.3368	0
7779	322.2	2.046	1.7789	0.4303	2.3431	0
7780	322.1	2.0566	1.7799	0.4299	2.3421	0
7781	322	2.0476	1.7788	0.43	2.341	0
7782	321.9	2.0465	1.7777	0.4303	2.3324	0
7783	321.8	2.0415	1.7723	0.4299	2.3311	0
7784	321.7	2.0482	1.7775	0.4301	2.33	0
7785	321.6	2.0432	1.7743	0.4298	2.3365	0
7786	321.5	2.0382	1.7753	0.4296	2.3354	0
7787	321.4	2.0448	1.7741	0.4296	2.3193	0
7788	321.3	2.0399	1.771	0.4295	2.3256	0
7789	321.2	2.0387	1.7699	0.4292	2.3245	0
7790	321.1	2.0338	1.7709	0.4293	2.3234	0
7791	321	2.0404	1.7656	0.4291	2.3149	0
7792	320.9	2.0356	1.7646	0.4292	2.3289	0
7793	320.8	2.0382	1.7655	0.4294	2.3126	0
7794	320.7	2.0292	1.7642	0.4286	2.3264	0
7795	320.6	2.0319	1.7631	0.4283	2.3178	0
7796	320.5	2.0308	1.7599	0.4283	2.3166	0
7797	320.4	2.0297	1.7609	0.4282	2.3155	0
7798	320.3	2.0285	1.7597	0.4282	2.3143	0
7799	320.2	2.0236	1.7565	0.4281	2.3133	0
7800	320.1	2.0263	1.7575	0.4285	2.2974	0
7801	320	2.0213	1.7585	0.4279	2.3036	

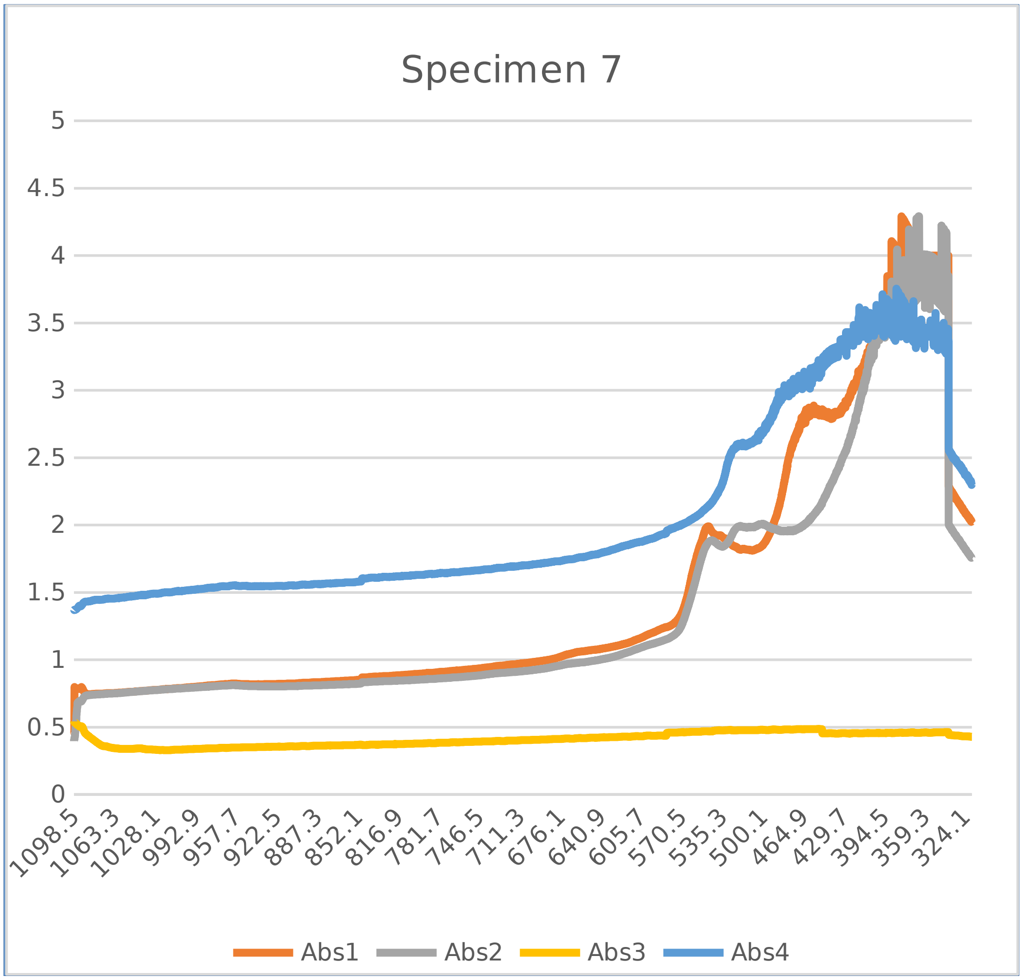
Specimen 7
5
4.5
4
3.5
3
2.5
2
1.5
1
0.5
0
1098.5
1063.3
1028.1
992.9
957.7
922.5
887.3
852.1
816.9
781.7
746.5
711.3
676.1
640.9
605.7
570.5
535.3
500.1
464.9
429.7
394.5
359.3
324.1
Abs1
Abs2
Abs3
Abs4
