# Supplementary material for: Coherent poly propagation materials with 3-dimensional photonic control over visible light
Source: PLoS One. 2019 Oct 17;14(10):e0223715. doi: 10.1371/journal.pone.0223715 (PMC6797174; doi:10.1371/journal.pone.0223715)
Supplement: S8 Table — Spreadsheet generated graph at bottom of table. (PDF) [file pone.0223715.s017.pdf]

Wavelength Scan Report - Specimen 8

Company	Complete Consulting Services LLC		
Operator	Dr. Michelle R. Stem	Test Time	2/13/2017 1:21
Document No.	WLS1702130001_2	Print Date	9/6/2019 21:07
Start wavelength	1100nm	Number of data	7801
End wavelength	320nm	K1	0.99
Unit		K0	0
Interval	0.1nm	K2	0
Calculate the Conc.	No	K3	0
Modification time	2/13/2017 1:21	Access time	2/13/2017 1:21
Remark	S13b038 - Specimen 8		

Scan Data

No.	Wavelength	Abs1	Abs2	Abs3	Abs4	Visible
1	1100	0.9184	1.2856	1.174	1.0029	0
2	1099.9	0.9195	1.2826	1.1762	1.0025	0
3	1099.8	0.9317	1.2813	1.1784	1.0023	0
4	1099.7	0.9365	1.2801	1.1799	1.0019	0
5	1099.6	0.9483	1.2784	1.181	1.0016	0
6	1099.5	0.9584	1.2768	1.181	1.0013	0
7	1099.4	0.9618	1.2763	1.1817	1.0013	0
8	1099.3	0.9641	1.2757	1.1843	1.0013	0
9	1099.2	0.9683	1.2754	1.1842	1.0018	0
10	1099.1	0.9743	1.2746	1.1845	1.0016	0
11	1099	0.9801	1.2742	1.1865	1.0019	0
12	1098.9	0.9841	1.2739	1.1864	1.0019	0
13	1098.8	0.9864	1.2737	1.1867	1.002	0
14	1098.7	0.9876	1.2734	1.1877	1.0016	0
15	1098.6	0.9886	1.2734	1.1888	1.0021	0
16	1098.5	0.9899	1.2734	1.1899	1.0023	0
17	1098.4	0.9906	1.273	1.1906	1.0025	0
18	1098.3	0.9913	1.2731	1.1915	1.0025	0
19	1098.2	0.9915	1.2719	1.1915	1.0028	0
20	1098.1	0.9917	1.2714	1.1921	1.0028	0
21	1098	0.9923	1.2711	1.1926	1.003	0
22	1097.9	0.9928	1.2706	1.1927	1.0033	0
23	1097.8	0.9929	1.2701	1.1933	1.0033	0
24	1097.7	0.9932	1.2695	1.1932	1.0031	0
25	1097.6	0.9937	1.2694	1.1941	1.0033	0
26	1097.5	0.9937	1.2695	1.1937	1.0033	0
27	1097.4	0.9937	1.2682	1.1933	1.003	0
28	1097.3	0.9938	1.2679	1.1932	1.0033	0
29	1097.2	0.9938	1.2671	1.1923	1.003	0

30	1097.1	0.9941	1.2661	1.1927	1.003	0
31	1097	0.9939	1.2658	1.1927	1.003	0
32	1096.9	0.9937	1.2652	1.1941	1.003	0
33	1096.8	0.9941	1.2645	1.1936	1.003	0
34	1096.7	0.9943	1.2646	1.1934	1.003	0
35	1096.6	0.9945	1.2646	1.1942	1.0037	0
36	1096.5	0.9949	1.2642	1.1943	1.0037	0
37	1096.4	0.9951	1.2635	1.1939	1.0039	0
38	1096.3	0.9953	1.2628	1.1937	1.0041	0
39	1096.2	0.9957	1.2626	1.1932	1.0043	0
40	1096.1	0.9959	1.2622	1.1924	1.0043	0
41	1096	0.9963	1.2619	1.1932	1.0047	0
42	1095.9	0.9968	1.2613	1.1921	1.005	0
43	1095.8	0.9971	1.261	1.1926	1.0055	0
44	1095.7	0.9972	1.2607	1.1926	1.0056	0
45	1095.6	0.9976	1.2606	1.192	1.0057	0
46	1095.5	0.9978	1.2607	1.1919	1.0058	0
47	1095.4	0.9982	1.2609	1.1915	1.0058	0
48	1095.3	0.9981	1.2612	1.1918	1.0059	0
49	1095.2	0.998	1.2616	1.1922	1.0056	0
50	1095.1	0.9979	1.2617	1.1919	1.0056	0
51	1095	0.9977	1.2616	1.1905	1.0055	0
52	1094.9	0.9974	1.2613	1.1907	1.0052	0
53	1094.8	0.9973	1.2615	1.1919	1.005	0
54	1094.7	0.9967	1.2612	1.1914	1.0048	0
55	1094.6	0.9962	1.2606	1.1911	1.0044	0
56	1094.5	0.9965	1.2607	1.1914	1.0046	0
57	1094.4	0.9966	1.2606	1.1919	1.0048	0
58	1094.3	0.9964	1.2599	1.1925	1.0046	0
59	1094.2	0.9964	1.2597	1.1917	1.0049	0
60	1094.1	0.9963	1.2593	1.1923	1.0049	0
61	1094	0.9972	1.2598	1.1916	1.0057	0
62	1093.9	0.9969	1.2593	1.1915	1.0056	0
63	1093.8	0.9973	1.2594	1.1912	1.0063	0
64	1093.7	0.9979	1.2593	1.1916	1.0066	0
65	1093.6	0.9982	1.2585	1.1915	1.0066	0
66	1093.5	0.9983	1.2587	1.1905	1.0069	0
67	1093.4	0.9989	1.2589	1.1906	1.0073	0
68	1093.3	0.9996	1.2587	1.1902	1.0078	0
69	1093.2	1.0004	1.2594	1.1902	1.0081	0
70	1093.1	1.0007	1.2591	1.1895	1.0085	0
71	1093	1.0013	1.2593	1.1896	1.0087	0
72	1092.9	1.0017	1.2595	1.1889	1.0087	0

73	1092.8	1.0023	1.2599	1.1891	1.0091	0
74	1092.7	1.0027	1.2599	1.1884	1.0092	0
75	1092.6	1.0032	1.26	1.1883	1.0093	0
76	1092.5	1.0034	1.2601	1.1882	1.0094	0
77	1092.4	1.0039	1.2607	1.1876	1.0096	0
78	1092.3	1.0043	1.2606	1.1873	1.0097	0
79	1092.2	1.0049	1.2605	1.1872	1.0096	0
80	1092.1	1.0052	1.2606	1.1876	1.0098	0
81	1092	1.0057	1.2613	1.1881	1.0098	0
82	1091.9	1.0059	1.2614	1.1873	1.0097	0
83	1091.8	1.0063	1.2621	1.1862	1.0097	0
84	1091.7	1.0063	1.2619	1.1861	1.0094	0
85	1091.6	1.0063	1.2617	1.1856	1.0095	0
86	1091.5	1.0066	1.2626	1.1865	1.0094	0
87	1091.4	1.0072	1.2626	1.1866	1.0094	0
88	1091.3	1.0074	1.263	1.187	1.0091	0
89	1091.2	1.0076	1.2634	1.1869	1.0097	0
90	1091.1	1.0074	1.2632	1.186	1.0092	0
91	1091	1.0077	1.2627	1.1855	1.0092	0
92	1090.9	1.0077	1.262	1.1864	1.009	0
93	1090.8	1.0079	1.2623	1.1855	1.0088	0
94	1090.7	1.0081	1.2613	1.1856	1.0087	0
95	1090.6	1.0082	1.2615	1.186	1.0087	0
96	1090.5	1.0084	1.2613	1.1866	1.0089	0
97	1090.4	1.0083	1.2611	1.1869	1.0085	0
98	1090.3	1.0084	1.2608	1.1867	1.0083	0
99	1090.2	1.0085	1.2609	1.1867	1.0086	0
100	1090.1	1.0089	1.2611	1.1859	1.0089	0
101	1090	1.009	1.2609	1.1868	1.009	0
102	1089.9	1.009	1.2608	1.187	1.0089	0
103	1089.8	1.0091	1.261	1.1863	1.0087	0
104	1089.7	1.0092	1.2608	1.1868	1.0089	0
105	1089.6	1.0093	1.2605	1.1869	1.0089	0
106	1089.5	1.0094	1.2605	1.1874	1.009	0
107	1089.4	1.0092	1.2606	1.1873	1.0086	0
108	1089.3	1.0092	1.2602	1.1873	1.0088	0
109	1089.2	1.0091	1.2603	1.1875	1.0087	0
110	1089.1	1.0092	1.2598	1.1871	1.0088	0
111	1089	1.0095	1.26	1.1876	1.0088	0
112	1088.9	1.0092	1.26	1.1879	1.0087	0
113	1088.8	1.0091	1.2596	1.1872	1.0084	0
114	1088.7	1.0092	1.2598	1.1879	1.0088	0
115	1088.6	1.0093	1.2598	1.1881	1.009	0

116	1088.5	1.0093	1.2599	1.1877	1.0091	0
117	1088.4	1.0092	1.2599	1.1878	1.0089	0
118	1088.3	1.0094	1.2598	1.1887	1.0092	0
119	1088.2	1.0095	1.2599	1.1889	1.0091	0
120	1088.1	1.0096	1.2597	1.1893	1.0089	0
121	1088	1.0095	1.26	1.1881	1.009	0
122	1087.9	1.0093	1.2595	1.1875	1.0088	0
123	1087.8	1.0095	1.26	1.1879	1.009	0
124	1087.7	1.0096	1.26	1.1883	1.0091	0
125	1087.6	1.0097	1.2598	1.1887	1.0092	0
126	1087.5	1.0095	1.2595	1.1881	1.0093	0
127	1087.4	1.0097	1.2596	1.1883	1.0089	0
128	1087.3	1.0099	1.2599	1.1882	1.0092	0
129	1087.2	1.0096	1.2594	1.1887	1.0089	0
130	1087.1	1.0099	1.2593	1.1889	1.0093	0
131	1087	1.0099	1.2595	1.1893	1.0094	0
132	1086.9	1.01	1.2597	1.1888	1.0092	0
133	1086.8	1.0101	1.259	1.1886	1.0094	0
134	1086.7	1.0102	1.2594	1.188	1.0095	0
135	1086.6	1.0105	1.2589	1.1883	1.0099	0
136	1086.5	1.0104	1.2596	1.188	1.0095	0
137	1086.4	1.0105	1.2589	1.1885	1.0096	0
138	1086.3	1.0108	1.2587	1.1887	1.0096	0
139	1086.2	1.0109	1.2589	1.1885	1.0098	0
140	1086.1	1.0106	1.2587	1.1877	1.0096	0
141	1086	1.0111	1.2585	1.1884	1.0097	0
142	1085.9	1.011	1.2591	1.1878	1.0098	0
143	1085.8	1.0112	1.2587	1.1876	1.0102	0
144	1085.7	1.0112	1.2586	1.1882	1.0102	0
145	1085.6	1.0111	1.2584	1.1874	1.0101	0
146	1085.5	1.0116	1.2582	1.1881	1.0105	0
147	1085.4	1.0116	1.2581	1.1872	1.0105	0
148	1085.3	1.0121	1.2584	1.1871	1.0107	0
149	1085.2	1.0122	1.2586	1.1869	1.0113	0
150	1085.1	1.0123	1.2584	1.1876	1.0111	0
151	1085	1.0123	1.258	1.1877	1.0111	0
152	1084.9	1.0123	1.2576	1.1868	1.0113	0
153	1084.8	1.0124	1.2572	1.1868	1.0116	0
154	1084.7	1.013	1.2575	1.1868	1.012	0
155	1084.6	1.0129	1.2575	1.1865	1.0119	0
156	1084.5	1.013	1.2569	1.1865	1.0119	0
157	1084.4	1.0134	1.2569	1.1853	1.0121	0
158	1084.3	1.0135	1.2571	1.1849	1.0125	0

159	1084.2	1.0137	1.2566	1.1858	1.0121	0
160	1084.1	1.0138	1.2565	1.1845	1.0124	0
161	1084	1.014	1.2564	1.1847	1.0127	0
162	1083.9	1.014	1.2566	1.1843	1.0127	0
163	1083.8	1.0141	1.2569	1.1836	1.0131	0
164	1083.7	1.0144	1.2571	1.184	1.0134	0
165	1083.6	1.0147	1.257	1.1835	1.0136	0
166	1083.5	1.0147	1.2567	1.1837	1.0137	0
167	1083.4	1.0148	1.2569	1.1833	1.0139	0
168	1083.3	1.0149	1.2567	1.183	1.0139	0
169	1083.2	1.015	1.2565	1.1824	1.0138	0
170	1083.1	1.0153	1.2563	1.1816	1.0139	0
171	1083	1.0154	1.256	1.1824	1.0139	0
172	1082.9	1.0156	1.2564	1.182	1.0142	0
173	1082.8	1.0157	1.2564	1.1824	1.0143	0
174	1082.7	1.0154	1.2561	1.1825	1.0143	0
175	1082.6	1.0158	1.2563	1.1826	1.0143	0
176	1082.5	1.0161	1.2563	1.183	1.0144	0
177	1082.4	1.0162	1.2562	1.1824	1.0146	0
178	1082.3	1.016	1.256	1.1824	1.0146	0
179	1082.2	1.0159	1.2561	1.182	1.0145	0
180	1082.1	1.0162	1.2556	1.1815	1.0145	0
181	1082	1.0159	1.2556	1.1817	1.0145	0
182	1081.9	1.0164	1.2555	1.1825	1.0152	0
183	1081.8	1.0163	1.2555	1.1826	1.015	0
184	1081.7	1.0165	1.2553	1.1819	1.0153	0
185	1081.6	1.0169	1.2559	1.182	1.0152	0
186	1081.5	1.0167	1.2559	1.1809	1.0152	0
187	1081.4	1.0169	1.2559	1.182	1.0153	0
188	1081.3	1.017	1.2556	1.1825	1.0154	0
189	1081.2	1.0175	1.2558	1.1822	1.0157	0
190	1081.1	1.0171	1.2557	1.1824	1.0156	0
191	1081	1.0172	1.2555	1.1817	1.0157	0
192	1080.9	1.0173	1.2552	1.1816	1.0154	0
193	1080.8	1.0175	1.2554	1.1822	1.0162	0
194	1080.7	1.0174	1.2551	1.1825	1.0157	0
195	1080.6	1.0174	1.2549	1.1816	1.0158	0
196	1080.5	1.0174	1.2549	1.1817	1.0159	0
197	1080.4	1.0174	1.2549	1.1822	1.0159	0
198	1080.3	1.0175	1.2548	1.1817	1.0157	0
199	1080.2	1.0177	1.2545	1.1815	1.0159	0
200	1080.1	1.0177	1.2542	1.1818	1.0159	0
201	1080	1.0177	1.2545	1.1811	1.016	0

202	1079.9	1.0178	1.2545	1.1811	1.0162	0
203	1079.8	1.0178	1.2548	1.1819	1.0163	0
204	1079.7	1.0177	1.2547	1.1815	1.0163	0
205	1079.6	1.018	1.2538	1.1815	1.0164	0
206	1079.5	1.0177	1.2538	1.1814	1.016	0
207	1079.4	1.0176	1.2537	1.182	1.0157	0
208	1079.3	1.0177	1.2539	1.1819	1.0159	0
209	1079.2	1.0178	1.2543	1.1828	1.0162	0
210	1079.1	1.0179	1.2549	1.183	1.0164	0
211	1079	1.0181	1.2541	1.1835	1.016	0
212	1078.9	1.0181	1.2542	1.1833	1.016	0
213	1078.8	1.0181	1.2542	1.1828	1.0162	0
214	1078.7	1.0178	1.2539	1.1826	1.0159	0
215	1078.6	1.0181	1.2544	1.183	1.0161	0
216	1078.5	1.0179	1.2541	1.1826	1.0159	0
217	1078.4	1.0174	1.2537	1.1822	1.0155	0
218	1078.3	1.0178	1.2537	1.1827	1.0155	0
219	1078.2	1.0178	1.2543	1.1831	1.0159	0
220	1078.1	1.0178	1.2539	1.1825	1.0161	0
221	1078	1.0179	1.2537	1.1832	1.0159	0
222	1077.9	1.0181	1.2541	1.184	1.0159	0
223	1077.8	1.0178	1.2545	1.1831	1.0158	0
224	1077.7	1.0181	1.2548	1.1835	1.0161	0
225	1077.6	1.0183	1.2544	1.1843	1.0162	0
226	1077.5	1.0175	1.2538	1.1829	1.0155	0
227	1077.4	1.0179	1.2539	1.1833	1.0159	0
228	1077.3	1.0181	1.2542	1.184	1.016	0
229	1077.2	1.0182	1.2541	1.1843	1.0161	0
230	1077.1	1.0182	1.2531	1.184	1.0158	0
231	1077	1.0182	1.253	1.1847	1.0162	0
232	1076.9	1.0181	1.2525	1.1844	1.016	0
233	1076.8	1.018	1.2522	1.1842	1.0156	0
234	1076.7	1.0182	1.2525	1.1844	1.0161	0
235	1076.6	1.0182	1.2518	1.1845	1.0158	0
236	1076.5	1.0182	1.2517	1.1844	1.016	0
237	1076.4	1.0182	1.2516	1.1842	1.0161	0
238	1076.3	1.0181	1.2512	1.1841	1.016	0
239	1076.2	1.0184	1.2512	1.1833	1.0161	0
240	1076.1	1.0183	1.2509	1.1834	1.0163	0
241	1076	1.0184	1.2509	1.1841	1.0164	0
242	1075.9	1.0187	1.2511	1.184	1.0166	0
243	1075.8	1.0185	1.2508	1.1832	1.0163	0
244	1075.7	1.0187	1.251	1.1837	1.0167	0

245	1075.6	1.0185	1.2507	1.1834	1.0164	0
246	1075.5	1.0189	1.2507	1.1834	1.0167	0
247	1075.4	1.0188	1.2506	1.1834	1.0166	0
248	1075.3	1.0189	1.2506	1.1825	1.0166	0
249	1075.2	1.0187	1.2506	1.1835	1.0165	0
250	1075.1	1.0189	1.2502	1.1823	1.0167	0
251	1075	1.0193	1.2502	1.1832	1.017	0
252	1074.9	1.0194	1.2502	1.1829	1.0171	0
253	1074.8	1.0192	1.2497	1.1834	1.0165	0
254	1074.7	1.0195	1.25	1.1832	1.0171	0
255	1074.6	1.0193	1.2497	1.1825	1.0172	0
256	1074.5	1.0194	1.2501	1.182	1.0172	0
257	1074.4	1.0195	1.2498	1.1823	1.0172	0
258	1074.3	1.0197	1.2493	1.1822	1.0172	0
259	1074.2	1.0199	1.2495	1.1816	1.0178	0
260	1074.1	1.0198	1.2498	1.182	1.0179	0
261	1074	1.0198	1.2497	1.1823	1.0179	0
262	1073.9	1.0202	1.2494	1.1819	1.0182	0
263	1073.8	1.02	1.2495	1.1821	1.0181	0
264	1073.7	1.0203	1.2495	1.1813	1.0183	0
265	1073.6	1.0203	1.249	1.1814	1.0182	0
266	1073.5	1.0206	1.2495	1.1813	1.0185	0
267	1073.4	1.0208	1.2496	1.1805	1.0187	0
268	1073.3	1.0208	1.2499	1.1807	1.0186	0
269	1073.2	1.0205	1.2494	1.1797	1.019	0
270	1073.1	1.0209	1.2499	1.1801	1.0191	0
271	1073	1.0211	1.2494	1.1801	1.019	0
272	1072.9	1.0213	1.2501	1.1799	1.0192	0
273	1072.8	1.0212	1.2498	1.1799	1.0193	0
274	1072.7	1.0216	1.2496	1.1798	1.0196	0
275	1072.6	1.0218	1.2498	1.1794	1.0196	0
276	1072.5	1.0217	1.2499	1.1794	1.0197	0
277	1072.4	1.022	1.2497	1.1797	1.0199	0
278	1072.3	1.022	1.2498	1.1789	1.0197	0
279	1072.2	1.0221	1.2498	1.1794	1.0201	0
280	1072.1	1.0222	1.2494	1.1784	1.0199	0
281	1072	1.0223	1.2493	1.1785	1.0203	0
282	1071.9	1.0223	1.2493	1.1795	1.0203	0
283	1071.8	1.0224	1.2495	1.1787	1.0201	0
284	1071.7	1.0226	1.2496	1.1773	1.0205	0
285	1071.6	1.0225	1.2493	1.1765	1.0206	0
286	1071.5	1.0227	1.2492	1.1778	1.0204	0
287	1071.4	1.023	1.2495	1.1777	1.0208	0

288	1071.3	1.023	1.2493	1.1777	1.021	0
289	1071.2	1.0228	1.2488	1.1772	1.0207	0
290	1071.1	1.0231	1.249	1.1774	1.0205	0
291	1071	1.023	1.2487	1.1773	1.0209	0
292	1070.9	1.0232	1.2485	1.1777	1.021	0
293	1070.8	1.0233	1.249	1.1767	1.021	0
294	1070.7	1.0233	1.2485	1.1775	1.0212	0
295	1070.6	1.0236	1.2485	1.1775	1.0213	0
296	1070.5	1.0236	1.2488	1.1773	1.0215	0
297	1070.4	1.0239	1.2488	1.1778	1.0218	0
298	1070.3	1.0237	1.249	1.1776	1.0217	0
299	1070.2	1.0235	1.2487	1.1765	1.0216	0
300	1070.1	1.0237	1.2483	1.1765	1.0215	0
301	1070	1.0238	1.2489	1.1768	1.0216	0
302	1069.9	1.0239	1.2494	1.1775	1.0216	0
303	1069.8	1.0237	1.2488	1.1764	1.0214	0
304	1069.7	1.0239	1.2488	1.1774	1.0218	0
305	1069.6	1.0239	1.2489	1.1757	1.0218	0
306	1069.5	1.024	1.2493	1.1765	1.0218	0
307	1069.4	1.0242	1.2491	1.1763	1.0219	0
308	1069.3	1.0242	1.249	1.1761	1.0219	0
309	1069.2	1.0241	1.2489	1.1762	1.0219	0
310	1069.1	1.0243	1.2493	1.1774	1.0219	0
311	1069	1.0244	1.2493	1.1773	1.0221	0
312	1068.9	1.0243	1.2491	1.1762	1.022	0
313	1068.8	1.0243	1.2489	1.176	1.0221	0
314	1068.7	1.0245	1.2493	1.1775	1.0223	0
315	1068.6	1.0248	1.2498	1.1768	1.0226	0
316	1068.5	1.025	1.2496	1.1761	1.0226	0
317	1068.4	1.0245	1.2492	1.1771	1.0223	0
318	1068.3	1.0245	1.249	1.1777	1.0219	0
319	1068.2	1.0242	1.2485	1.1775	1.0217	0
320	1068.1	1.0245	1.249	1.1781	1.0222	0
321	1068	1.0245	1.2491	1.1779	1.0222	0
322	1067.9	1.0244	1.2493	1.1782	1.0222	0
323	1067.8	1.0244	1.2492	1.1777	1.0222	0
324	1067.7	1.0246	1.2493	1.1781	1.022	0
325	1067.6	1.0245	1.2492	1.1771	1.0222	0
326	1067.5	1.0246	1.2491	1.1792	1.0221	0
327	1067.4	1.0246	1.2492	1.1787	1.0221	0
328	1067.3	1.0246	1.2489	1.1782	1.0221	0
329	1067.2	1.0245	1.2489	1.1778	1.022	0
330	1067.1	1.0245	1.2485	1.1769	1.0223	0

331	1067	1.0248	1.2487	1.178	1.0224	0
332	1066.9	1.0248	1.249	1.1779	1.0223	0
333	1066.8	1.0248	1.2488	1.1785	1.0223	0
334	1066.7	1.0248	1.2488	1.1776	1.0225	0
335	1066.6	1.0246	1.2488	1.1777	1.0222	0
336	1066.5	1.0246	1.2491	1.1777	1.0221	0
337	1066.4	1.0248	1.2491	1.178	1.0223	0
338	1066.3	1.0249	1.249	1.1775	1.0224	0
339	1066.2	1.0248	1.2493	1.1776	1.0223	0
340	1066.1	1.0245	1.2488	1.1783	1.0223	0
341	1066	1.0247	1.2489	1.1775	1.0225	0
342	1065.9	1.0247	1.2491	1.1778	1.0224	0
343	1065.8	1.0252	1.2497	1.1781	1.0224	0
344	1065.7	1.0248	1.2493	1.1789	1.0228	0
345	1065.6	1.0251	1.2493	1.1776	1.0223	0
346	1065.5	1.0251	1.2497	1.1785	1.0231	0
347	1065.4	1.0249	1.2491	1.1784	1.0227	0
348	1065.3	1.0251	1.2494	1.1792	1.0231	0
349	1065.2	1.0253	1.2492	1.1781	1.0226	0
350	1065.1	1.0252	1.2496	1.1776	1.0229	0
351	1065	1.0252	1.2493	1.1788	1.0231	0
352	1064.9	1.0253	1.249	1.1777	1.0231	0
353	1064.8	1.0254	1.2494	1.1779	1.0234	0
354	1064.7	1.026	1.2493	1.1777	1.0233	0
355	1064.6	1.0258	1.2495	1.178	1.0235	0
356	1064.5	1.0261	1.2498	1.1777	1.0239	0
357	1064.4	1.0263	1.2495	1.1779	1.0239	0
358	1064.3	1.0262	1.2498	1.1777	1.024	0
359	1064.2	1.0261	1.2491	1.1776	1.0239	0
360	1064.1	1.0263	1.2496	1.1776	1.0239	0
361	1064	1.0265	1.2497	1.1765	1.0241	0
362	1063.9	1.0263	1.2494	1.1764	1.0238	0
363	1063.8	1.0267	1.25	1.1774	1.0245	0
364	1063.7	1.0268	1.25	1.1764	1.0246	0
365	1063.6	1.0269	1.2499	1.1765	1.0245	0
366	1063.5	1.0271	1.2495	1.1769	1.0247	0
367	1063.4	1.0269	1.2497	1.1754	1.0243	0
368	1063.3	1.0272	1.2502	1.1753	1.0244	0
369	1063.2	1.0269	1.2497	1.1757	1.025	0
370	1063.1	1.0272	1.2502	1.1766	1.0248	0
371	1063	1.0276	1.2496	1.1769	1.0254	0
372	1062.9	1.0274	1.2498	1.1763	1.0253	0
373	1062.8	1.0275	1.2502	1.1765	1.0253	0

374	1062.7	1.0275	1.2502	1.1764	1.0255	0
375	1062.6	1.0279	1.2504	1.1752	1.0258	0
376	1062.5	1.0278	1.25	1.1757	1.0257	0
377	1062.4	1.0279	1.2503	1.1758	1.0262	0
378	1062.3	1.0277	1.2504	1.1754	1.0258	0
379	1062.2	1.0279	1.25	1.1756	1.026	0
380	1062.1	1.0278	1.2503	1.1751	1.0259	0
381	1062	1.0281	1.2507	1.1747	1.0258	0
382	1061.9	1.0278	1.2503	1.1742	1.0259	0
383	1061.8	1.0284	1.2505	1.1752	1.0261	0
384	1061.7	1.0285	1.2505	1.1748	1.0261	0
385	1061.6	1.0287	1.2506	1.1747	1.0264	0
386	1061.5	1.0287	1.2504	1.1745	1.0264	0
387	1061.4	1.0287	1.2505	1.1733	1.0267	0
388	1061.3	1.0288	1.2509	1.1743	1.0265	0
389	1061.2	1.0287	1.25	1.1741	1.0264	0
390	1061.1	1.029	1.2509	1.1741	1.0267	0
391	1061	1.0289	1.2509	1.1738	1.0268	0
392	1060.9	1.029	1.2503	1.1743	1.0269	0
393	1060.8	1.0294	1.2509	1.1744	1.0273	0
394	1060.7	1.0291	1.2512	1.1737	1.0271	0
395	1060.6	1.0293	1.2513	1.1737	1.0272	0
396	1060.5	1.0295	1.251	1.1733	1.0277	0
397	1060.4	1.0296	1.2515	1.1739	1.0274	0
398	1060.3	1.0293	1.2507	1.1742	1.0275	0
399	1060.2	1.0296	1.2507	1.1742	1.0278	0
400	1060.1	1.0297	1.2516	1.1734	1.0277	0
401	1060	1.0298	1.251	1.1736	1.028	0
402	1059.9	1.0304	1.2517	1.1736	1.0282	0
403	1059.8	1.0305	1.252	1.1742	1.0283	0
404	1059.7	1.0302	1.2515	1.1737	1.0282	0
405	1059.6	1.0305	1.2521	1.1733	1.0281	0
406	1059.5	1.0305	1.2516	1.1724	1.028	0
407	1059.4	1.0304	1.252	1.1726	1.028	0
408	1059.3	1.0305	1.2516	1.1727	1.0281	0
409	1059.2	1.0305	1.2516	1.1735	1.0285	0
410	1059.1	1.0306	1.2517	1.1737	1.0286	0
411	1059	1.0304	1.2515	1.1736	1.0284	0
412	1058.9	1.0308	1.2512	1.1725	1.0285	0
413	1058.8	1.031	1.2518	1.1731	1.0282	0
414	1058.7	1.031	1.2523	1.1729	1.029	0
415	1058.6	1.0307	1.2517	1.1726	1.0288	0
416	1058.5	1.031	1.2517	1.1724	1.0284	0

417	1058.4	1.0309	1.2525	1.1738	1.029	0
418	1058.3	1.0309	1.252	1.1732	1.0286	0
419	1058.2	1.0312	1.2527	1.1746	1.0289	0
420	1058.1	1.031	1.252	1.1736	1.0285	0
421	1058	1.0309	1.2522	1.1733	1.0286	0
422	1057.9	1.0313	1.253	1.1736	1.0286	0
423	1057.8	1.0309	1.2525	1.1728	1.0284	0
424	1057.7	1.0312	1.2529	1.173	1.0287	0
425	1057.6	1.0313	1.253	1.173	1.029	0
426	1057.5	1.0311	1.253	1.173	1.0286	0
427	1057.4	1.0311	1.2524	1.1732	1.0288	0
428	1057.3	1.0315	1.2529	1.1736	1.0294	0
429	1057.2	1.0314	1.253	1.173	1.0289	0
430	1057.1	1.0316	1.2533	1.1736	1.0293	0
431	1057	1.0313	1.2534	1.1733	1.0289	0
432	1056.9	1.0316	1.2538	1.1728	1.0297	0
433	1056.8	1.0317	1.2537	1.1734	1.0295	0
434	1056.7	1.0315	1.2535	1.1728	1.0296	0
435	1056.6	1.0319	1.2538	1.1735	1.0298	0
436	1056.5	1.032	1.2537	1.1735	1.0295	0
437	1056.4	1.0319	1.2546	1.1741	1.0298	0
438	1056.3	1.0318	1.254	1.1737	1.0299	0
439	1056.2	1.0323	1.2544	1.1735	1.0301	0
440	1056.1	1.0319	1.2543	1.1733	1.0301	0
441	1056	1.032	1.2542	1.1734	1.0296	0
442	1055.9	1.0323	1.2545	1.1722	1.0299	0
443	1055.8	1.0323	1.2543	1.1732	1.0299	0
444	1055.7	1.0324	1.2547	1.1727	1.0304	0
445	1055.6	1.0322	1.2545	1.1731	1.0301	0
446	1055.5	1.0322	1.2547	1.1736	1.0302	0
447	1055.4	1.0322	1.2548	1.1733	1.0302	0
448	1055.3	1.0327	1.2552	1.1733	1.0307	0
449	1055.2	1.0327	1.2558	1.1732	1.0307	0
450	1055.1	1.0327	1.2553	1.1722	1.0308	0
451	1055	1.0332	1.2563	1.173	1.031	0
452	1054.9	1.0331	1.2562	1.173	1.0311	0
453	1054.8	1.033	1.2554	1.1725	1.0308	0
454	1054.7	1.033	1.2554	1.1716	1.0308	0
455	1054.6	1.0331	1.2555	1.1713	1.0309	0
456	1054.5	1.0333	1.2558	1.1712	1.0311	0
457	1054.4	1.0334	1.2561	1.172	1.0312	0
458	1054.3	1.0337	1.2564	1.1718	1.0315	0
459	1054.2	1.0339	1.2569	1.1708	1.0318	0

460	1054.1	1.034	1.2564	1.1713	1.0318	0
461	1054	1.034	1.2566	1.1712	1.0318	0
462	1053.9	1.0341	1.2566	1.1718	1.0321	0
463	1053.8	1.0342	1.2566	1.1704	1.0321	0
464	1053.7	1.0343	1.2569	1.1714	1.0323	0
465	1053.6	1.0342	1.2567	1.1703	1.032	0
466	1053.5	1.0344	1.257	1.17	1.0324	0
467	1053.4	1.0344	1.2567	1.1707	1.0326	0
468	1053.3	1.0347	1.2573	1.1708	1.0327	0
469	1053.2	1.0349	1.2572	1.1706	1.0326	0
470	1053.1	1.0345	1.2569	1.169	1.0325	0
471	1053	1.0348	1.2572	1.1689	1.0329	0
472	1052.9	1.0346	1.2571	1.1697	1.0328	0
473	1052.8	1.0347	1.2569	1.1711	1.0326	0
474	1052.7	1.0351	1.2574	1.1706	1.0332	0
475	1052.6	1.0352	1.2573	1.1701	1.0331	0
476	1052.5	1.0355	1.2577	1.1704	1.0333	0
477	1052.4	1.0354	1.2575	1.1708	1.0335	0
478	1052.3	1.0355	1.2578	1.1692	1.0334	0
479	1052.2	1.0353	1.2576	1.1697	1.0332	0
480	1052.1	1.0354	1.2583	1.1687	1.0335	0
481	1052	1.0353	1.2579	1.1695	1.0335	0
482	1051.9	1.0358	1.2579	1.1697	1.0337	0
483	1051.8	1.0357	1.2579	1.1696	1.0338	0
484	1051.7	1.0356	1.2578	1.1696	1.0337	0
485	1051.6	1.036	1.2576	1.1696	1.0337	0
486	1051.5	1.0361	1.2582	1.1695	1.0339	0
487	1051.4	1.0359	1.2577	1.1687	1.034	0
488	1051.3	1.0361	1.2577	1.1694	1.0339	0
489	1051.2	1.0361	1.2578	1.1691	1.0337	0
490	1051.1	1.0358	1.2575	1.169	1.0339	0
491	1051	1.0357	1.2578	1.1682	1.034	0
492	1050.9	1.0358	1.2576	1.168	1.0339	0
493	1050.8	1.0362	1.2578	1.1685	1.0341	0
494	1050.7	1.0362	1.2578	1.1687	1.0343	0
495	1050.6	1.036	1.2578	1.1683	1.0338	0
496	1050.5	1.0365	1.2581	1.1687	1.0342	0
497	1050.4	1.0365	1.2583	1.169	1.0341	0
498	1050.3	1.0366	1.2584	1.1688	1.0344	0
499	1050.2	1.0365	1.2576	1.168	1.0343	0
500	1050.1	1.0362	1.2578	1.1685	1.0345	0
501	1050	1.0365	1.2584	1.1687	1.0343	0
502	1049.9	1.0366	1.2579	1.1684	1.0345	0

503	1049.8	1.0366	1.2582	1.1686	1.0346	0
504	1049.7	1.0368	1.2587	1.168	1.0346	0
505	1049.6	1.0369	1.2584	1.1695	1.0347	0
506	1049.5	1.0369	1.2582	1.1695	1.0347	0
507	1049.4	1.0367	1.2587	1.169	1.0347	0
508	1049.3	1.0367	1.2585	1.1688	1.0346	0
509	1049.2	1.0369	1.2586	1.1695	1.0349	0
510	1049.1	1.0373	1.2589	1.1694	1.0353	0
511	1049	1.0371	1.2589	1.1696	1.0351	0
512	1048.9	1.037	1.2587	1.1695	1.0352	0
513	1048.8	1.0371	1.2584	1.1694	1.0349	0
514	1048.7	1.0369	1.2591	1.1691	1.0347	0
515	1048.6	1.0372	1.2591	1.1686	1.0347	0
516	1048.5	1.0374	1.2594	1.1688	1.035	0
517	1048.4	1.0375	1.259	1.1691	1.035	0
518	1048.3	1.0376	1.2589	1.1694	1.0353	0
519	1048.2	1.0377	1.2587	1.1684	1.0354	0
520	1048.1	1.0374	1.2587	1.1676	1.0352	0
521	1048	1.0376	1.2589	1.1681	1.0352	0
522	1047.9	1.0378	1.2593	1.1684	1.0352	0
523	1047.8	1.0377	1.2589	1.169	1.0352	0
524	1047.7	1.0374	1.2588	1.1673	1.0351	0
525	1047.6	1.0378	1.2589	1.1689	1.0357	0
526	1047.5	1.0381	1.2586	1.1688	1.0356	0
527	1047.4	1.0381	1.2589	1.1698	1.0358	0
528	1047.3	1.0377	1.2583	1.1688	1.0358	0
529	1047.2	1.0381	1.258	1.1687	1.036	0
530	1047.1	1.0385	1.2585	1.1684	1.0363	0
531	1047	1.0384	1.2586	1.1681	1.0363	0
532	1046.9	1.0384	1.2584	1.1677	1.0363	0
533	1046.8	1.0384	1.2583	1.1682	1.0363	0
534	1046.7	1.0388	1.2586	1.1684	1.0365	0
535	1046.6	1.039	1.2585	1.1684	1.0369	0
536	1046.5	1.0386	1.258	1.1683	1.0365	0
537	1046.4	1.0386	1.2574	1.168	1.0365	0
538	1046.3	1.039	1.2572	1.1675	1.0369	0
539	1046.2	1.0392	1.2578	1.1681	1.0371	0
540	1046.1	1.0391	1.2576	1.1689	1.0369	0
541	1046	1.0393	1.2575	1.1684	1.037	0
542	1045.9	1.0394	1.2576	1.1677	1.037	0
543	1045.8	1.0397	1.2576	1.1679	1.0373	0
544	1045.7	1.0396	1.2574	1.168	1.0376	0
545	1045.6	1.0395	1.257	1.1672	1.0374	0

546	1045.5	1.0395	1.2573	1.1673	1.0373	0
547	1045.4	1.0397	1.2575	1.1676	1.0378	0
548	1045.3	1.0399	1.2575	1.1676	1.0377	0
549	1045.2	1.0399	1.2572	1.1666	1.0378	0
550	1045.1	1.0401	1.257	1.1674	1.0381	0
551	1045	1.0402	1.2575	1.1673	1.0382	0
552	1044.9	1.0405	1.2572	1.167	1.0383	0
553	1044.8	1.0402	1.2571	1.1666	1.0383	0
554	1044.7	1.0404	1.2574	1.166	1.0386	0
555	1044.6	1.0407	1.2573	1.1666	1.0383	0
556	1044.5	1.0406	1.257	1.1665	1.0385	0
557	1044.4	1.0408	1.2569	1.1661	1.0386	0
558	1044.3	1.0407	1.2574	1.1662	1.0385	0
559	1044.2	1.0409	1.2569	1.166	1.0389	0
560	1044.1	1.0407	1.2566	1.165	1.0387	0
561	1044	1.041	1.2568	1.1653	1.0389	0
562	1043.9	1.0409	1.257	1.1649	1.0386	0
563	1043.8	1.0412	1.2568	1.1649	1.0388	0
564	1043.7	1.0413	1.2566	1.1645	1.0389	0
565	1043.6	1.0412	1.2569	1.1652	1.0391	0
566	1043.5	1.0415	1.2565	1.1638	1.0391	0
567	1043.4	1.0415	1.2569	1.1651	1.0393	0
568	1043.3	1.042	1.2574	1.1644	1.0398	0
569	1043.2	1.0419	1.2571	1.1649	1.0397	0
570	1043.1	1.042	1.2572	1.1653	1.0397	0
571	1043	1.0417	1.2565	1.1644	1.0396	0
572	1042.9	1.0419	1.257	1.1641	1.0399	0
573	1042.8	1.042	1.2574	1.1639	1.0397	0
574	1042.7	1.0421	1.2572	1.1635	1.04	0
575	1042.6	1.0423	1.2575	1.1637	1.0402	0
576	1042.5	1.0424	1.2575	1.1632	1.0401	0
577	1042.4	1.0424	1.2573	1.1629	1.0402	0
578	1042.3	1.0425	1.2576	1.1639	1.0405	0
579	1042.2	1.0426	1.2575	1.1639	1.0404	0
580	1042.1	1.0423	1.2572	1.1634	1.0404	0
581	1042	1.0426	1.2571	1.1636	1.0405	0
582	1041.9	1.0429	1.2575	1.1639	1.0405	0
583	1041.8	1.0427	1.2575	1.1638	1.0404	0
584	1041.7	1.0429	1.2569	1.1634	1.0404	0
585	1041.6	1.0428	1.2575	1.1634	1.0407	0
586	1041.5	1.0429	1.2577	1.1634	1.0408	0
587	1041.4	1.0426	1.2571	1.1629	1.0408	0
588	1041.3	1.0429	1.2574	1.1632	1.0406	0

589	1041.2	1.0428	1.2573	1.1637	1.041	0
590	1041.1	1.0428	1.2576	1.1642	1.0408	0
591	1041	1.043	1.2572	1.1635	1.0407	0
592	1040.9	1.0432	1.2575	1.1634	1.0411	0
593	1040.8	1.0434	1.2578	1.1637	1.0411	0
594	1040.7	1.0437	1.2575	1.1636	1.0412	0
595	1040.6	1.0433	1.2581	1.1636	1.0413	0
596	1040.5	1.0435	1.2581	1.1637	1.0412	0
597	1040.4	1.0437	1.2577	1.1635	1.0416	0
598	1040.3	1.0434	1.2575	1.1632	1.0413	0
599	1040.2	1.0434	1.2577	1.1641	1.0415	0
600	1040.1	1.0435	1.2578	1.1646	1.0413	0
601	1040	1.0435	1.2582	1.1639	1.0412	0
602	1039.9	1.0437	1.2578	1.1636	1.0415	0
603	1039.8	1.0438	1.258	1.1637	1.0413	0
604	1039.7	1.0438	1.2578	1.1632	1.0412	0
605	1039.6	1.0435	1.258	1.1629	1.0413	0
606	1039.5	1.0437	1.2582	1.1644	1.0416	0
607	1039.4	1.0439	1.2586	1.1639	1.0417	0
608	1039.3	1.0439	1.2581	1.1641	1.0414	0
609	1039.2	1.0439	1.2583	1.1633	1.0416	0
610	1039.1	1.0438	1.2581	1.1632	1.0416	0
611	1039	1.0441	1.2582	1.1643	1.0418	0
612	1038.9	1.0439	1.2581	1.1645	1.0419	0
613	1038.8	1.0437	1.2583	1.1632	1.0417	0
614	1038.7	1.044	1.2584	1.1651	1.0416	0
615	1038.6	1.0436	1.2581	1.1655	1.0413	0
616	1038.5	1.0442	1.2585	1.1648	1.0417	0
617	1038.4	1.0441	1.2584	1.1648	1.0415	0
618	1038.3	1.0442	1.2582	1.1644	1.0417	0
619	1038.2	1.044	1.2583	1.1644	1.0417	0
620	1038.1	1.0443	1.2581	1.1646	1.0419	0
621	1038	1.0443	1.2584	1.1654	1.0417	0
622	1037.9	1.0439	1.2582	1.1644	1.0417	0
623	1037.8	1.0443	1.2582	1.1647	1.0418	0
624	1037.7	1.0443	1.2581	1.1649	1.0417	0
625	1037.6	1.0443	1.258	1.1647	1.0418	0
626	1037.5	1.0443	1.2583	1.1646	1.0419	0
627	1037.4	1.0446	1.2581	1.1646	1.042	0
628	1037.3	1.0445	1.2583	1.165	1.042	0
629	1037.2	1.0445	1.2584	1.1644	1.0418	0
630	1037.1	1.0445	1.2582	1.1655	1.0422	0
631	1037	1.0451	1.258	1.1653	1.0425	0

632	1036.9	1.0449	1.2579	1.1655	1.0425	0
633	1036.8	1.045	1.2578	1.1654	1.0427	0
634	1036.7	1.0455	1.2583	1.1652	1.0431	0
635	1036.6	1.0454	1.2582	1.1646	1.0428	0
636	1036.5	1.0456	1.2582	1.1653	1.0432	0
637	1036.4	1.0454	1.2581	1.1647	1.0427	0
638	1036.3	1.0454	1.2578	1.1634	1.043	0
639	1036.2	1.0456	1.2579	1.1646	1.043	0
640	1036.1	1.0458	1.2577	1.1636	1.0431	0
641	1036	1.0458	1.2579	1.1633	1.0433	0
642	1035.9	1.046	1.2584	1.1619	1.0432	0
643	1035.8	1.0462	1.2578	1.1619	1.0435	0
644	1035.7	1.046	1.258	1.1615	1.0436	0
645	1035.6	1.0461	1.2581	1.1622	1.0437	0
646	1035.5	1.0462	1.2572	1.1618	1.0438	0
647	1035.4	1.0462	1.2572	1.1619	1.0438	0
648	1035.3	1.0463	1.2571	1.1611	1.0438	0
649	1035.2	1.0466	1.257	1.1608	1.0441	0
650	1035.1	1.0464	1.2566	1.16	1.0439	0
651	1035	1.0466	1.2568	1.1598	1.0441	0
652	1034.9	1.0468	1.2563	1.1596	1.0443	0
653	1034.8	1.047	1.2567	1.1596	1.0446	0
654	1034.7	1.047	1.2566	1.1597	1.0449	0
655	1034.6	1.0475	1.2566	1.1597	1.0452	0
656	1034.5	1.0479	1.2568	1.1608	1.0455	0
657	1034.4	1.0477	1.2571	1.1602	1.0454	0
658	1034.3	1.0474	1.2569	1.1595	1.0452	0
659	1034.2	1.0475	1.2567	1.1584	1.0451	0
660	1034.1	1.0479	1.2567	1.1589	1.0454	0
661	1034	1.0479	1.2567	1.159	1.0451	0
662	1033.9	1.0481	1.2569	1.1601	1.0457	0
663	1033.8	1.0482	1.257	1.1587	1.0459	0
664	1033.7	1.0481	1.2567	1.1571	1.046	0
665	1033.6	1.0484	1.2571	1.1587	1.0463	0
666	1033.5	1.0483	1.2567	1.1586	1.0463	0
667	1033.4	1.0483	1.2568	1.1581	1.0461	0
668	1033.3	1.0486	1.2569	1.1586	1.0467	0
669	1033.2	1.0489	1.2571	1.1576	1.0466	0
670	1033.1	1.0489	1.2573	1.158	1.0465	0
671	1033	1.049	1.2572	1.1573	1.0467	0
672	1032.9	1.0491	1.2571	1.1574	1.0472	0
673	1032.8	1.0493	1.2568	1.1572	1.0468	0
674	1032.7	1.0493	1.2571	1.1571	1.0471	0

675	1032.6	1.0494	1.2572	1.1569	1.0472	0
676	1032.5	1.0492	1.257	1.1562	1.047	0
677	1032.4	1.0491	1.2569	1.1562	1.0471	0
678	1032.3	1.0492	1.2571	1.1581	1.0472	0
679	1032.2	1.0497	1.2571	1.1567	1.0476	0
680	1032.1	1.0496	1.2574	1.1583	1.0476	0
681	1032	1.0497	1.2571	1.1573	1.0475	0
682	1031.9	1.0497	1.2576	1.1568	1.0476	0
683	1031.8	1.0498	1.2575	1.1568	1.0479	0
684	1031.7	1.0499	1.2575	1.1572	1.0479	0
685	1031.6	1.05	1.2574	1.1574	1.0479	0
686	1031.5	1.0503	1.2576	1.1566	1.0483	0
687	1031.4	1.0504	1.2572	1.1566	1.0482	0
688	1031.3	1.0506	1.2574	1.1568	1.0484	0
689	1031.2	1.0505	1.2578	1.157	1.0481	0
690	1031.1	1.0504	1.2575	1.156	1.0485	0
691	1031	1.0503	1.2577	1.1571	1.0483	0
692	1030.9	1.0504	1.2577	1.1561	1.0482	0
693	1030.8	1.0506	1.2573	1.1564	1.0485	0
694	1030.7	1.0505	1.2572	1.1562	1.0481	0
695	1030.6	1.0506	1.2573	1.1563	1.0486	0
696	1030.5	1.0506	1.2573	1.156	1.0483	0
697	1030.4	1.0506	1.2577	1.1569	1.0485	0
698	1030.3	1.0503	1.2572	1.1564	1.0483	0
699	1030.2	1.0507	1.257	1.1562	1.0484	0
700	1030.1	1.0507	1.257	1.1561	1.0486	0
701	1030	1.0509	1.2574	1.1567	1.0487	0
702	1029.9	1.0509	1.2569	1.1567	1.0487	0
703	1029.8	1.0511	1.257	1.1574	1.0488	0
704	1029.7	1.0507	1.2568	1.1562	1.0483	0
705	1029.6	1.051	1.2573	1.1567	1.0486	0
706	1029.5	1.0509	1.2571	1.1564	1.0487	0
707	1029.4	1.0509	1.2571	1.1556	1.0489	0
708	1029.3	1.051	1.2572	1.1563	1.0488	0
709	1029.2	1.051	1.257	1.1569	1.049	0
710	1029.1	1.0509	1.2569	1.157	1.0489	0
711	1029	1.0507	1.2572	1.1575	1.0486	0
712	1028.9	1.0509	1.2566	1.1569	1.0484	0
713	1028.8	1.0507	1.2564	1.1575	1.0481	0
714	1028.7	1.051	1.2568	1.1587	1.0487	0
715	1028.6	1.051	1.2568	1.1587	1.0485	0
716	1028.5	1.0508	1.2566	1.1586	1.0485	0
717	1028.4	1.0512	1.2575	1.158	1.0488	0

718	1028.3	1.0509	1.2566	1.1583	1.0484	0
719	1028.2	1.0509	1.2569	1.1581	1.0486	0
720	1028.1	1.051	1.2575	1.1588	1.0485	0
721	1028	1.051	1.257	1.1576	1.0487	0
722	1027.9	1.051	1.2566	1.1582	1.0486	0
723	1027.8	1.0511	1.2563	1.1583	1.0488	0
724	1027.7	1.051	1.2568	1.1577	1.0487	0
725	1027.6	1.0513	1.2563	1.1584	1.0487	0
726	1027.5	1.0512	1.2569	1.1591	1.0492	0
727	1027.4	1.0512	1.2568	1.1586	1.049	0
728	1027.3	1.0512	1.2566	1.159	1.0487	0
729	1027.2	1.0513	1.2568	1.1587	1.0488	0
730	1027.1	1.0512	1.2562	1.1583	1.0488	0
731	1027	1.0515	1.2566	1.1589	1.049	0
732	1026.9	1.0512	1.2569	1.1587	1.0489	0
733	1026.8	1.0516	1.2568	1.1585	1.0491	0
734	1026.7	1.0516	1.2569	1.1586	1.0493	0
735	1026.6	1.0516	1.2566	1.1586	1.0491	0
736	1026.5	1.0517	1.2567	1.1583	1.0493	0
737	1026.4	1.0516	1.2564	1.1586	1.0492	0
738	1026.3	1.0518	1.2564	1.159	1.0491	0
739	1026.2	1.0519	1.2567	1.1585	1.0492	0
740	1026.1	1.0522	1.2571	1.1581	1.0494	0
741	1026	1.0522	1.2566	1.1588	1.0497	0
742	1025.9	1.0522	1.2569	1.1586	1.0496	0
743	1025.8	1.0526	1.2564	1.1581	1.05	0
744	1025.7	1.0524	1.2562	1.158	1.0499	0
745	1025.6	1.0526	1.2561	1.1575	1.0498	0
746	1025.5	1.0527	1.2561	1.1584	1.05	0
747	1025.4	1.0528	1.2561	1.1579	1.0502	0
748	1025.3	1.0531	1.2564	1.158	1.0506	0
749	1025.2	1.0532	1.2562	1.157	1.0509	0
750	1025.1	1.0536	1.2565	1.1575	1.051	0
751	1025	1.0536	1.256	1.1572	1.051	0
752	1024.9	1.0535	1.2558	1.1565	1.051	0
753	1024.8	1.0533	1.256	1.1559	1.051	0
754	1024.7	1.0535	1.256	1.156	1.0511	0
755	1024.6	1.0539	1.2559	1.156	1.0513	0
756	1024.5	1.0542	1.256	1.1565	1.0517	0
757	1024.4	1.0542	1.2562	1.1557	1.0519	0
758	1024.3	1.0545	1.2568	1.1566	1.052	0
759	1024.2	1.0543	1.2557	1.1555	1.0519	0
760	1024.1	1.0544	1.2558	1.1547	1.0521	0

761	1024	1.0544	1.2558	1.1547	1.0521	0
762	1023.9	1.0545	1.2556	1.1535	1.052	0
763	1023.8	1.0551	1.2564	1.1546	1.0526	0
764	1023.7	1.0553	1.2564	1.1535	1.0528	0
765	1023.6	1.0556	1.2565	1.1541	1.0531	0
766	1023.5	1.0553	1.2561	1.1541	1.053	0
767	1023.4	1.0556	1.2562	1.1534	1.0531	0
768	1023.3	1.0557	1.2561	1.1528	1.0531	0
769	1023.2	1.0557	1.2556	1.1526	1.0533	0
770	1023.1	1.0556	1.256	1.1524	1.0535	0
771	1023	1.0561	1.2561	1.1518	1.0538	0
772	1022.9	1.056	1.2556	1.1527	1.0536	0
773	1022.8	1.056	1.2558	1.1532	1.0536	0
774	1022.7	1.0558	1.2558	1.1515	1.0538	0
775	1022.6	1.0562	1.2556	1.1518	1.0538	0
776	1022.5	1.0565	1.2558	1.1518	1.0543	0
777	1022.4	1.0565	1.2558	1.1519	1.0542	0
778	1022.3	1.057	1.2564	1.1517	1.0547	0
779	1022.2	1.0567	1.2557	1.1515	1.0544	0
780	1022.1	1.0568	1.2556	1.1517	1.0546	0
781	1022	1.0567	1.2558	1.1512	1.0545	0
782	1021.9	1.0567	1.2557	1.1512	1.0547	0
783	1021.8	1.0569	1.256	1.1511	1.0549	0
784	1021.7	1.0569	1.2562	1.1518	1.0548	0
785	1021.6	1.057	1.2559	1.1516	1.0547	0
786	1021.5	1.0566	1.2557	1.151	1.0545	0
787	1021.4	1.0572	1.2557	1.1506	1.0549	0
788	1021.3	1.0571	1.2559	1.1509	1.0549	0
789	1021.2	1.0571	1.2563	1.151	1.055	0
790	1021.1	1.0575	1.2566	1.1503	1.0551	0
791	1021	1.0574	1.2564	1.1509	1.0551	0
792	1020.9	1.0576	1.2567	1.1511	1.0552	0
793	1020.8	1.0576	1.2561	1.1515	1.0554	0
794	1020.7	1.0575	1.2564	1.15	1.0552	0
795	1020.6	1.0576	1.2562	1.1499	1.0553	0
796	1020.5	1.0576	1.2563	1.1501	1.0551	0
797	1020.4	1.0577	1.2564	1.1499	1.0554	0
798	1020.3	1.0576	1.2563	1.1497	1.0553	0
799	1020.2	1.0577	1.2566	1.149	1.0553	0
800	1020.1	1.0579	1.2564	1.1506	1.0556	0
801	1020	1.0581	1.2567	1.1504	1.0556	0
802	1019.9	1.0581	1.2566	1.15	1.0555	0
803	1019.8	1.0579	1.2562	1.1507	1.0554	0

804	1019.7	1.0584	1.2564	1.1512	1.0556	0
805	1019.6	1.0579	1.2563	1.1506	1.0557	0
806	1019.5	1.0582	1.2571	1.1517	1.0559	0
807	1019.4	1.0578	1.2564	1.1518	1.0554	0
808	1019.3	1.0581	1.2567	1.151	1.0557	0
809	1019.2	1.0581	1.2567	1.15	1.0555	0
810	1019.1	1.0581	1.2562	1.1511	1.0558	0
811	1019	1.0583	1.2566	1.1512	1.0558	0
812	1018.9	1.0583	1.2565	1.1514	1.0556	0
813	1018.8	1.0582	1.2565	1.1516	1.0557	0
814	1018.7	1.0582	1.2566	1.1521	1.0557	0
815	1018.6	1.0581	1.2568	1.1521	1.0558	0
816	1018.5	1.0583	1.2566	1.1517	1.0559	0
817	1018.4	1.0581	1.2567	1.1529	1.0557	0
818	1018.3	1.0583	1.2566	1.1528	1.0558	0
819	1018.2	1.0583	1.2566	1.1518	1.056	0
820	1018.1	1.0585	1.2568	1.1525	1.056	0
821	1018	1.0582	1.2566	1.1527	1.0557	0
822	1017.9	1.0583	1.2564	1.1527	1.0559	0
823	1017.8	1.0586	1.2568	1.1525	1.0562	0
824	1017.7	1.0589	1.2571	1.1532	1.0563	0
825	1017.6	1.0585	1.2569	1.1531	1.0562	0
826	1017.5	1.0583	1.2569	1.1516	1.0561	0
827	1017.4	1.0583	1.2567	1.1522	1.0559	0
828	1017.3	1.0584	1.2569	1.152	1.056	0
829	1017.2	1.0587	1.2571	1.1525	1.0563	0
830	1017.1	1.0587	1.257	1.1522	1.0564	0
831	1017	1.0589	1.2571	1.1534	1.0565	0
832	1016.9	1.0588	1.2565	1.1531	1.0561	0
833	1016.8	1.0585	1.2559	1.1519	1.0558	0
834	1016.7	1.0587	1.2566	1.1515	1.0561	0
835	1016.6	1.0589	1.2565	1.1523	1.0563	0
836	1016.5	1.0586	1.2564	1.1519	1.0563	0
837	1016.4	1.0587	1.2563	1.1519	1.0563	0
838	1016.3	1.0588	1.2563	1.1508	1.0562	0
839	1016.2	1.059	1.2562	1.1522	1.0565	0
840	1016.1	1.0593	1.256	1.1519	1.0568	0
841	1016	1.0598	1.2568	1.1525	1.0574	0
842	1015.9	1.0598	1.2569	1.1541	1.0575	0
843	1015.8	1.0597	1.2562	1.1533	1.0571	0
844	1015.7	1.0596	1.2562	1.1526	1.0571	0
845	1015.6	1.0598	1.2567	1.1525	1.0571	0
846	1015.5	1.0596	1.256	1.1528	1.0572	0

847	1015.4	1.06	1.2563	1.1531	1.0573	0
848	1015.3	1.0597	1.2562	1.1525	1.0572	0
849	1015.2	1.06	1.256	1.1522	1.0575	0
850	1015.1	1.0603	1.256	1.1523	1.0576	0
851	1015	1.0602	1.2558	1.1524	1.0577	0
852	1014.9	1.0603	1.2558	1.1522	1.0576	0
853	1014.8	1.0603	1.2556	1.1513	1.058	0
854	1014.7	1.0603	1.2564	1.1521	1.0581	0
855	1014.6	1.061	1.2559	1.1522	1.0585	0
856	1014.5	1.0607	1.2555	1.1517	1.0581	0
857	1014.4	1.0606	1.2555	1.15	1.0581	0
858	1014.3	1.061	1.2559	1.1501	1.0586	0
859	1014.2	1.0615	1.2557	1.1514	1.0588	0
860	1014.1	1.0611	1.256	1.1508	1.0589	0
861	1014	1.0616	1.2562	1.1511	1.0593	0
862	1013.9	1.0613	1.2559	1.1507	1.059	0
863	1013.8	1.0615	1.2554	1.1509	1.059	0
864	1013.7	1.0619	1.2565	1.1505	1.0592	0
865	1013.6	1.0618	1.2556	1.1496	1.0593	0
866	1013.5	1.0617	1.2557	1.1495	1.0595	0
867	1013.4	1.0622	1.2558	1.1497	1.0595	0
868	1013.3	1.0622	1.256	1.1493	1.0597	0
869	1013.2	1.0625	1.256	1.1497	1.06	0
870	1013.1	1.0624	1.2557	1.1487	1.0601	0
871	1013	1.0624	1.2556	1.1483	1.06	0
872	1012.9	1.0627	1.2553	1.1483	1.0605	0
873	1012.8	1.063	1.2553	1.1486	1.0606	0
874	1012.7	1.0629	1.2559	1.1472	1.0607	0
875	1012.6	1.0633	1.2553	1.1485	1.0608	0
876	1012.5	1.0633	1.2555	1.1482	1.0608	0
877	1012.4	1.0634	1.2555	1.1481	1.0612	0
878	1012.3	1.0636	1.2556	1.1482	1.0614	0
879	1012.2	1.0636	1.2555	1.1477	1.0614	0
880	1012.1	1.0637	1.2552	1.1477	1.0614	0
881	1012	1.0636	1.255	1.1476	1.0614	0
882	1011.9	1.0636	1.2554	1.1483	1.0616	0
883	1011.8	1.0639	1.2551	1.1481	1.0619	0
884	1011.7	1.0639	1.255	1.1473	1.0617	0
885	1011.6	1.064	1.255	1.1478	1.0615	0
886	1011.5	1.0645	1.2546	1.1471	1.0618	0
887	1011.4	1.0646	1.2549	1.1467	1.0623	0
888	1011.3	1.0645	1.255	1.1466	1.0621	0
889	1011.2	1.0647	1.2552	1.1465	1.0622	0

890	1011.1	1.0644	1.2551	1.1469	1.062	0
891	1011	1.0646	1.2547	1.1471	1.0624	0
892	1010.9	1.0648	1.2554	1.147	1.0624	0
893	1010.8	1.0648	1.2552	1.1461	1.0624	0
894	1010.7	1.0647	1.2548	1.1463	1.0624	0
895	1010.6	1.0649	1.255	1.1461	1.0627	0
896	1010.5	1.065	1.2559	1.1458	1.0629	0
897	1010.4	1.0652	1.2558	1.1459	1.0628	0
898	1010.3	1.0649	1.2553	1.1459	1.063	0
899	1010.2	1.0652	1.2553	1.1462	1.0629	0
900	1010.1	1.0652	1.2555	1.1455	1.0632	0
901	1010	1.0653	1.2553	1.1458	1.0631	0
902	1009.9	1.0655	1.2554	1.1468	1.0635	0
903	1009.8	1.0653	1.2553	1.1457	1.063	0
904	1009.7	1.0653	1.2553	1.145	1.0633	0
905	1009.6	1.0655	1.2552	1.1457	1.0631	0
906	1009.5	1.0658	1.2556	1.1458	1.0634	0
907	1009.4	1.0656	1.2552	1.146	1.0632	0
908	1009.3	1.0655	1.2553	1.1461	1.0633	0
909	1009.2	1.0657	1.2549	1.146	1.0633	0
910	1009.1	1.0658	1.2552	1.1463	1.0631	0
911	1009	1.0661	1.2552	1.1457	1.0637	0
912	1008.9	1.0661	1.2554	1.1461	1.0637	0
913	1008.8	1.066	1.2552	1.1465	1.0636	0
914	1008.7	1.0662	1.2554	1.1472	1.0636	0
915	1008.6	1.0662	1.2554	1.1466	1.0636	0
916	1008.5	1.0662	1.2553	1.1469	1.0638	0
917	1008.4	1.066	1.2548	1.1462	1.0636	0
918	1008.3	1.0665	1.2556	1.1479	1.0639	0
919	1008.2	1.0663	1.255	1.1486	1.0635	0
920	1008.1	1.0662	1.2554	1.1463	1.0638	0
921	1008	1.0664	1.2553	1.1479	1.0638	0
922	1007.9	1.0664	1.2549	1.1484	1.0634	0
923	1007.8	1.0663	1.255	1.1471	1.0635	0
924	1007.7	1.0665	1.2552	1.1478	1.0639	0
925	1007.6	1.0664	1.255	1.1478	1.0637	0
926	1007.5	1.0664	1.255	1.1474	1.0638	0
927	1007.4	1.0666	1.2551	1.1473	1.0638	0
928	1007.3	1.0666	1.2553	1.1478	1.064	0
929	1007.2	1.0665	1.2551	1.1488	1.0641	0
930	1007.1	1.0664	1.2551	1.1485	1.064	0
931	1007	1.0666	1.2549	1.1477	1.064	0
932	1006.9	1.0669	1.2552	1.148	1.0643	0

933	1006.8	1.0668	1.255	1.1471	1.064	0
934	1006.7	1.0666	1.255	1.1471	1.0642	0
935	1006.6	1.0667	1.2554	1.1487	1.0641	0
936	1006.5	1.0667	1.2557	1.1484	1.0643	0
937	1006.4	1.0666	1.2554	1.1478	1.0644	0
938	1006.3	1.067	1.2556	1.148	1.0648	0
939	1006.2	1.067	1.2552	1.1477	1.0646	0
940	1006.1	1.067	1.2554	1.148	1.0646	0
941	1006	1.0669	1.2553	1.1478	1.0647	0
942	1005.9	1.0669	1.2556	1.1485	1.0643	0
943	1005.8	1.0673	1.2559	1.1481	1.0647	0
944	1005.7	1.0672	1.2557	1.1477	1.065	0
945	1005.6	1.0674	1.2556	1.1489	1.065	0
946	1005.5	1.0675	1.2557	1.1486	1.0649	0
947	1005.4	1.0675	1.2559	1.1485	1.0651	0
948	1005.3	1.0675	1.2558	1.1488	1.0651	0
949	1005.2	1.0676	1.2559	1.1479	1.0651	0
950	1005.1	1.0679	1.2555	1.1479	1.0653	0
951	1005	1.0679	1.2556	1.1482	1.0653	0
952	1004.9	1.0679	1.2553	1.1485	1.0659	0
953	1004.8	1.0678	1.2559	1.1483	1.0652	0
954	1004.7	1.0682	1.2556	1.1484	1.0658	0
955	1004.6	1.0683	1.2559	1.1484	1.0661	0
956	1004.5	1.0685	1.2558	1.1484	1.0661	0
957	1004.4	1.0686	1.2558	1.1474	1.0658	0
958	1004.3	1.0685	1.2558	1.1477	1.0662	0
959	1004.2	1.0689	1.2558	1.1473	1.0665	0
960	1004.1	1.0689	1.2555	1.1466	1.0665	0
961	1004	1.0691	1.256	1.1468	1.0665	0
962	1003.9	1.0689	1.256	1.1473	1.0668	0
963	1003.8	1.0693	1.2557	1.1467	1.0669	0
964	1003.7	1.0692	1.2558	1.1472	1.0668	0
965	1003.6	1.0692	1.2557	1.147	1.0672	0
966	1003.5	1.0696	1.2559	1.1467	1.0672	0
967	1003.4	1.0697	1.2561	1.1463	1.0677	0
968	1003.3	1.0696	1.2559	1.1461	1.0676	0
969	1003.2	1.0696	1.2562	1.1457	1.0675	0
970	1003.1	1.0699	1.2559	1.1448	1.0679	0
971	1003	1.0704	1.2564	1.1453	1.0682	0
972	1002.9	1.0699	1.2558	1.1447	1.0681	0
973	1002.8	1.0703	1.2565	1.1451	1.0681	0
974	1002.7	1.0699	1.256	1.1445	1.0682	0
975	1002.6	1.0704	1.2557	1.1446	1.0681	0

976	1002.5	1.0702	1.2558	1.1449	1.0682	0
977	1002.4	1.0707	1.2564	1.1464	1.0685	0
978	1002.3	1.0708	1.2565	1.1446	1.0686	0
979	1002.2	1.0708	1.2562	1.1439	1.0688	0
980	1002.1	1.0707	1.2562	1.144	1.0687	0
981	1002	1.0706	1.2561	1.1448	1.0688	0
982	1001.9	1.0709	1.2558	1.1444	1.0685	0
983	1001.8	1.0712	1.2558	1.1435	1.0692	0
984	1001.7	1.0713	1.2561	1.1445	1.0691	0
985	1001.6	1.0711	1.2562	1.1441	1.0689	0
986	1001.5	1.0711	1.256	1.1441	1.0691	0
987	1001.4	1.0709	1.2557	1.1443	1.0691	0
988	1001.3	1.0718	1.2558	1.1449	1.0695	0
989	1001.2	1.0717	1.2563	1.1446	1.0695	0
990	1001.1	1.0717	1.2562	1.1446	1.0697	0
991	1001	1.0722	1.2559	1.1428	1.0698	0
992	1000.9	1.0721	1.256	1.1438	1.0697	0
993	1000.8	1.0719	1.2563	1.1436	1.0699	0
994	1000.7	1.072	1.2558	1.1435	1.07	0
995	1000.6	1.072	1.2563	1.1441	1.0702	0
996	1000.5	1.0722	1.2557	1.1444	1.07	0
997	1000.4	1.0723	1.2562	1.1446	1.0705	0
998	1000.3	1.0725	1.2565	1.1437	1.0703	0
999	1000.2	1.0726	1.2563	1.1433	1.0704	0
1000	1000.1	1.0729	1.2565	1.1445	1.0711	0
1001	1000	1.0728	1.2561	1.1439	1.0708	0
1002	999.9	1.0728	1.2564	1.1433	1.0704	0
1003	999.8	1.073	1.2568	1.143	1.071	0
1004	999.7	1.0734	1.2567	1.1439	1.0714	0
1005	999.6	1.0733	1.2565	1.1435	1.0713	0
1006	999.5	1.073	1.2561	1.1427	1.0712	0
1007	999.4	1.0732	1.2566	1.1441	1.0712	0
1008	999.3	1.0731	1.2564	1.1442	1.0713	0
1009	999.2	1.0734	1.2565	1.1438	1.0715	0
1010	999.1	1.0734	1.2564	1.1435	1.0714	0
1011	999	1.0735	1.2565	1.1441	1.0717	0
1012	998.9	1.0737	1.2573	1.1445	1.0715	0
1013	998.8	1.0738	1.2564	1.1436	1.072	0
1014	998.7	1.0734	1.2568	1.1439	1.0718	0
1015	998.6	1.0733	1.2562	1.1429	1.0715	0
1016	998.5	1.0737	1.2563	1.1433	1.0719	0
1017	998.4	1.0738	1.2567	1.1434	1.0716	0
1018	998.3	1.0738	1.2572	1.1434	1.0718	0

1019	998.2	1.074	1.2567	1.1436	1.072	0
1020	998.1	1.0742	1.2571	1.1438	1.0722	0
1021	998	1.074	1.2568	1.1441	1.0722	0
1022	997.9	1.0738	1.2568	1.1443	1.0718	0
1023	997.8	1.0737	1.2566	1.144	1.0722	0
1024	997.7	1.0741	1.2567	1.1439	1.0721	0
1025	997.6	1.0741	1.2565	1.1441	1.0722	0
1026	997.5	1.0741	1.2564	1.1429	1.0719	0
1027	997.4	1.0744	1.2574	1.1438	1.0723	0
1028	997.3	1.0742	1.2566	1.144	1.0721	0
1029	997.2	1.0745	1.2574	1.1443	1.0725	0
1030	997.1	1.0749	1.2573	1.144	1.0725	0
1031	997	1.0745	1.2573	1.1428	1.0727	0
1032	996.9	1.0744	1.2574	1.1436	1.0726	0
1033	996.8	1.075	1.2576	1.1441	1.073	0
1034	996.7	1.075	1.2574	1.1451	1.073	0
1035	996.6	1.0751	1.2576	1.1444	1.0734	0
1036	996.5	1.0749	1.2569	1.1427	1.0731	0
1037	996.4	1.0752	1.2574	1.1433	1.0733	0
1038	996.3	1.0754	1.2576	1.1447	1.0734	0
1039	996.2	1.0754	1.2578	1.1442	1.0737	0
1040	996.1	1.0756	1.2577	1.143	1.0739	0
1041	996	1.0753	1.2573	1.1431	1.0738	0
1042	995.9	1.0758	1.2578	1.1443	1.074	0
1043	995.8	1.0757	1.258	1.1435	1.074	0
1044	995.7	1.0754	1.2575	1.1436	1.0738	0
1045	995.6	1.0758	1.2575	1.1434	1.0741	0
1046	995.5	1.0756	1.2577	1.1427	1.0742	0
1047	995.4	1.0759	1.2577	1.1434	1.0743	0
1048	995.3	1.076	1.2578	1.1438	1.0742	0
1049	995.2	1.076	1.2575	1.1435	1.074	0
1050	995.1	1.0758	1.2575	1.1433	1.0744	0
1051	995	1.0761	1.2581	1.1429	1.0745	0
1052	994.9	1.0763	1.2584	1.1427	1.075	0
1053	994.8	1.0764	1.2579	1.1432	1.0748	0
1054	994.7	1.0765	1.2579	1.1431	1.0751	0
1055	994.6	1.0767	1.2585	1.1422	1.0752	0
1056	994.5	1.077	1.259	1.1431	1.0756	0
1057	994.4	1.0769	1.2586	1.1423	1.0753	0
1058	994.3	1.077	1.2581	1.1429	1.0754	0
1059	994.2	1.0773	1.2578	1.1425	1.0757	0
1060	994.1	1.0773	1.2583	1.1429	1.0761	0
1061	994	1.0775	1.2587	1.1423	1.0759	0

1062	993.9	1.0776	1.2586	1.1425	1.0762	0
1063	993.8	1.0775	1.2582	1.1429	1.0759	0
1064	993.7	1.0777	1.2582	1.142	1.0763	0
1065	993.6	1.0773	1.2582	1.142	1.0764	0
1066	993.5	1.0775	1.2581	1.1429	1.0765	0
1067	993.4	1.078	1.2582	1.1423	1.0767	0
1068	993.3	1.0782	1.2574	1.1418	1.0768	0
1069	993.2	1.078	1.2574	1.1427	1.0768	0
1070	993.1	1.0784	1.2575	1.1408	1.0772	0
1071	993	1.0784	1.2581	1.1419	1.0774	0
1072	992.9	1.0785	1.2573	1.1418	1.0775	0
1073	992.8	1.0787	1.2581	1.1427	1.0775	0
1074	992.7	1.0789	1.2578	1.142	1.0779	0
1075	992.6	1.079	1.2572	1.142	1.0778	0
1076	992.5	1.079	1.2575	1.1412	1.0782	0
1077	992.4	1.0791	1.2576	1.1413	1.0781	0
1078	992.3	1.079	1.2578	1.141	1.0783	0
1079	992.2	1.0791	1.2576	1.1413	1.0781	0
1080	992.1	1.079	1.2576	1.1409	1.0784	0
1081	992	1.0792	1.2578	1.1408	1.0784	0
1082	991.9	1.0796	1.2581	1.1415	1.0788	0
1083	991.8	1.0796	1.258	1.141	1.0792	0
1084	991.7	1.0797	1.2578	1.1409	1.0791	0
1085	991.6	1.0798	1.2581	1.1403	1.0792	0
1086	991.5	1.0799	1.258	1.1404	1.0789	0
1087	991.4	1.08	1.2587	1.1412	1.0792	0
1088	991.3	1.0803	1.2588	1.1416	1.0793	0
1089	991.2	1.0802	1.2583	1.1403	1.0794	0
1090	991.1	1.0806	1.2585	1.1405	1.0798	0
1091	991	1.0804	1.2586	1.1396	1.0799	0
1092	990.9	1.0803	1.2584	1.1392	1.0795	0
1093	990.8	1.0806	1.2589	1.1409	1.0798	0
1094	990.7	1.0804	1.2585	1.1398	1.0796	0
1095	990.6	1.0805	1.2582	1.1401	1.0797	0
1096	990.5	1.0804	1.2588	1.1407	1.0798	0
1097	990.4	1.0807	1.2591	1.1399	1.0801	0
1098	990.3	1.0805	1.2585	1.1417	1.0801	0
1099	990.2	1.0809	1.2593	1.1414	1.0801	0
1100	990.1	1.0812	1.2593	1.1404	1.0806	0
1101	990	1.0812	1.259	1.142	1.0806	0
1102	989.9	1.0812	1.2592	1.1415	1.0808	0
1103	989.8	1.0812	1.2596	1.1413	1.0806	0
1104	989.7	1.0814	1.2591	1.1417	1.0808	0

1105	989.6	1.0812	1.2586	1.1408	1.081	0
1106	989.5	1.0817	1.2592	1.1411	1.0811	0
1107	989.4	1.0816	1.2591	1.1408	1.0812	0
1108	989.3	1.0818	1.2594	1.1414	1.0814	0
1109	989.2	1.0822	1.2599	1.141	1.0812	0
1110	989.1	1.0825	1.2602	1.1408	1.0819	0
1111	989	1.0818	1.2601	1.1405	1.0818	0
1112	988.9	1.0823	1.2599	1.1408	1.0819	0
1113	988.8	1.0821	1.2598	1.1406	1.0817	0
1114	988.7	1.0824	1.2602	1.1404	1.0822	0
1115	988.6	1.0826	1.2601	1.1413	1.0826	0
1116	988.5	1.0829	1.2602	1.1419	1.0827	0
1117	988.4	1.0827	1.2605	1.1419	1.0825	0
1118	988.3	1.0826	1.2598	1.1411	1.0826	0
1119	988.2	1.0829	1.2604	1.1412	1.0825	0
1120	988.1	1.0829	1.26	1.1418	1.0827	0
1121	988	1.0831	1.26	1.1416	1.0829	0
1122	987.9	1.083	1.26	1.1402	1.0828	0
1123	987.8	1.0832	1.2607	1.141	1.0828	0
1124	987.7	1.0832	1.2604	1.1401	1.0832	0
1125	987.6	1.0835	1.2605	1.1404	1.0833	0
1126	987.5	1.083	1.2605	1.1408	1.083	0
1127	987.4	1.0838	1.2603	1.1406	1.0834	0
1128	987.3	1.0835	1.2605	1.1412	1.0835	0
1129	987.2	1.0838	1.2611	1.1419	1.0838	0
1130	987.1	1.0839	1.2608	1.1406	1.0839	0
1131	987	1.084	1.261	1.1414	1.0838	0
1132	986.9	1.0841	1.2609	1.1398	1.0839	0
1133	986.8	1.0842	1.2606	1.1409	1.0844	0
1134	986.7	1.0842	1.2609	1.1403	1.084	0
1135	986.6	1.0842	1.2603	1.1396	1.0842	0
1136	986.5	1.0848	1.261	1.1402	1.0846	0
1137	986.4	1.0848	1.2612	1.1408	1.0848	0
1138	986.3	1.0849	1.2608	1.1407	1.0849	0
1139	986.2	1.0849	1.2618	1.1411	1.0851	0
1140	986.1	1.085	1.2612	1.1407	1.085	0
1141	986	1.0851	1.2615	1.1412	1.0853	0
1142	985.9	1.0853	1.2615	1.1391	1.0853	0
1143	985.8	1.0856	1.2616	1.1395	1.0854	0
1144	985.7	1.0856	1.2618	1.1387	1.0858	0
1145	985.6	1.0859	1.2616	1.14	1.0859	0
1146	985.5	1.0858	1.2615	1.1394	1.0858	0
1147	985.4	1.0858	1.2617	1.1379	1.0858	0

1148	985.3	1.086	1.2619	1.1399	1.0862	0
1149	985.2	1.0861	1.262	1.1402	1.0861	0
1150	985.1	1.0862	1.2618	1.1393	1.0866	0
1151	985	1.0861	1.2617	1.139	1.0863	0
1152	984.9	1.086	1.2618	1.1387	1.0866	0
1153	984.8	1.0867	1.2621	1.1392	1.0867	0
1154	984.7	1.0868	1.2624	1.1405	1.087	0
1155	984.6	1.0869	1.2623	1.1399	1.0871	0
1156	984.5	1.0871	1.2622	1.1387	1.0873	0
1157	984.4	1.0871	1.2623	1.1383	1.0871	0
1158	984.3	1.0873	1.2624	1.1391	1.0877	0
1159	984.2	1.0878	1.2627	1.1401	1.0878	0
1160	984.1	1.0873	1.262	1.1383	1.0877	0
1161	984	1.0876	1.2624	1.1388	1.0882	0
1162	983.9	1.0878	1.2623	1.1388	1.0882	0
1163	983.8	1.0879	1.2622	1.1387	1.0881	0
1164	983.7	1.0879	1.2621	1.138	1.0885	0
1165	983.6	1.0882	1.263	1.138	1.0886	0
1166	983.5	1.0885	1.2634	1.1382	1.0887	0
1167	983.4	1.0884	1.263	1.1387	1.0886	0
1168	983.3	1.0884	1.2636	1.1376	1.0886	0
1169	983.2	1.0887	1.2639	1.1377	1.0887	0
1170	983.1	1.0887	1.2635	1.1377	1.0891	0
1171	983	1.0891	1.264	1.1379	1.0895	0
1172	982.9	1.0891	1.2635	1.1383	1.0891	0
1173	982.8	1.0891	1.2636	1.1362	1.0891	0
1174	982.7	1.0893	1.2635	1.1371	1.0895	0
1175	982.6	1.0895	1.2639	1.137	1.0895	0
1176	982.5	1.0894	1.2636	1.1374	1.0894	0
1177	982.4	1.0895	1.2642	1.1378	1.0899	0
1178	982.3	1.0899	1.2638	1.1383	1.0899	0
1179	982.2	1.0897	1.2644	1.1383	1.0901	0
1180	982.1	1.0899	1.2641	1.1385	1.0899	0
1181	982	1.0896	1.2638	1.138	1.09	0
1182	981.9	1.09	1.2636	1.1377	1.0902	0
1183	981.8	1.0905	1.2642	1.1378	1.0907	0
1184	981.7	1.0902	1.2645	1.1378	1.0902	0
1185	981.6	1.09	1.2646	1.1378	1.0902	0
1186	981.5	1.0904	1.2644	1.1375	1.0902	0
1187	981.4	1.0904	1.2646	1.1372	1.0908	0
1188	981.3	1.0904	1.2649	1.1383	1.0904	0
1189	981.2	1.0902	1.2641	1.1379	1.0904	0
1190	981.1	1.0905	1.2647	1.1384	1.0909	0

1191	981	1.091	1.2648	1.1383	1.091	0
1192	980.9	1.0913	1.2655	1.1378	1.0909	0
1193	980.8	1.0907	1.265	1.1383	1.0909	0
1194	980.7	1.0909	1.2655	1.1377	1.0909	0
1195	980.6	1.091	1.265	1.1378	1.0912	0
1196	980.5	1.0912	1.265	1.1386	1.091	0
1197	980.4	1.0906	1.2657	1.1382	1.0912	0
1198	980.3	1.0912	1.2653	1.1381	1.0912	0
1199	980.2	1.0915	1.2656	1.1397	1.0915	0
1200	980.1	1.0918	1.2656	1.1386	1.0915	0
1201	980	1.0915	1.2656	1.1388	1.0915	0
1202	979.9	1.0917	1.2656	1.1384	1.0917	0
1203	979.8	1.0919	1.2659	1.1384	1.0917	0
1204	979.7	1.0917	1.2659	1.1395	1.0919	0
1205	979.6	1.0919	1.2659	1.1391	1.0917	0
1206	979.5	1.0916	1.2661	1.1392	1.0918	0
1207	979.4	1.0919	1.2662	1.1387	1.0915	0
1208	979.3	1.0918	1.2664	1.1392	1.0916	0
1209	979.2	1.092	1.2669	1.1398	1.0918	0
1210	979.1	1.0921	1.2668	1.1389	1.0917	0
1211	979	1.0919	1.2668	1.1395	1.0919	0
1212	978.9	1.0922	1.2666	1.1398	1.092	0
1213	978.8	1.0925	1.2669	1.1402	1.0919	0
1214	978.7	1.0926	1.2666	1.1405	1.092	0
1215	978.6	1.0922	1.267	1.1401	1.0918	0
1216	978.5	1.0925	1.2668	1.1409	1.0921	0
1217	978.4	1.0924	1.2667	1.1398	1.092	0
1218	978.3	1.0925	1.2669	1.1393	1.0919	0
1219	978.2	1.0927	1.2671	1.1402	1.0921	0
1220	978.1	1.0929	1.2675	1.1401	1.0927	0
1221	978	1.0926	1.2672	1.1401	1.0922	0
1222	977.9	1.0925	1.2671	1.1393	1.0919	0
1223	977.8	1.0928	1.2672	1.1396	1.0922	0
1224	977.7	1.0929	1.2673	1.1397	1.0925	0
1225	977.6	1.0933	1.2675	1.1399	1.0925	0
1226	977.5	1.0931	1.2675	1.1399	1.0925	0
1227	977.4	1.0934	1.2676	1.1402	1.0928	0
1228	977.3	1.0933	1.2675	1.1404	1.0925	0
1229	977.2	1.0933	1.2675	1.1408	1.0927	0
1230	977.1	1.0933	1.2672	1.1404	1.0927	0
1231	977	1.0931	1.2674	1.141	1.0929	0
1232	976.9	1.0935	1.2677	1.1399	1.0929	0
1233	976.8	1.0939	1.2676	1.1401	1.0931	0

1234	976.7	1.0941	1.2681	1.1405	1.0933	0
1235	976.6	1.0943	1.268	1.1403	1.0937	0
1236	976.5	1.0939	1.2684	1.1405	1.0935	0
1237	976.4	1.0941	1.2678	1.1401	1.0931	0
1238	976.3	1.0942	1.2679	1.1395	1.0936	0
1239	976.2	1.0941	1.268	1.139	1.0935	0
1240	976.1	1.0944	1.268	1.1388	1.0938	0
1241	976	1.0944	1.2678	1.1391	1.0936	0
1242	975.9	1.0945	1.2681	1.1393	1.0941	0
1243	975.8	1.0951	1.2683	1.1391	1.0943	0
1244	975.7	1.095	1.2681	1.1387	1.094	0
1245	975.6	1.0952	1.2678	1.1387	1.0944	0
1246	975.5	1.0953	1.268	1.1383	1.0947	0
1247	975.4	1.0953	1.2687	1.138	1.0947	0
1248	975.3	1.0957	1.268	1.1377	1.0949	0
1249	975.2	1.0958	1.2683	1.1379	1.0946	0
1250	975.1	1.096	1.2686	1.1374	1.0952	0
1251	975	1.0961	1.2686	1.1375	1.0951	0
1252	974.9	1.096	1.2687	1.1374	1.0956	0
1253	974.8	1.096	1.2683	1.1358	1.0954	0
1254	974.7	1.0963	1.2686	1.1359	1.0957	0
1255	974.6	1.0966	1.2682	1.1356	1.096	0
1256	974.5	1.0967	1.269	1.1359	1.0963	0
1257	974.4	1.0968	1.2689	1.1356	1.0962	0
1258	974.3	1.0969	1.2688	1.1349	1.0963	0
1259	974.2	1.0975	1.2688	1.1358	1.0967	0
1260	974.1	1.0976	1.2686	1.1357	1.0968	0
1261	974	1.0974	1.2684	1.136	1.097	0
1262	973.9	1.0973	1.2686	1.134	1.0969	0
1263	973.8	1.0976	1.2692	1.1345	1.097	0
1264	973.7	1.0978	1.2693	1.1342	1.097	0
1265	973.6	1.0978	1.2694	1.1338	1.0972	0
1266	973.5	1.0979	1.2695	1.1333	1.0973	0
1267	973.4	1.0981	1.2694	1.135	1.0977	0
1268	973.3	1.098	1.2698	1.134	1.0978	0
1269	973.2	1.0984	1.2696	1.1333	1.0978	0
1270	973.1	1.0985	1.2697	1.1338	1.0981	0
1271	973	1.0986	1.2699	1.1331	1.0982	0
1272	972.9	1.0983	1.2692	1.1339	1.0979	0
1273	972.8	1.0991	1.2696	1.1336	1.0983	0
1274	972.7	1.0989	1.2699	1.133	1.0983	0
1275	972.6	1.0991	1.2698	1.1327	1.0987	0
1276	972.5	1.0991	1.2699	1.133	1.0987	0

1277	972.4	1.099	1.2698	1.1322	1.0986	0
1278	972.3	1.0992	1.2696	1.1328	1.0988	0
1279	972.2	1.0991	1.2696	1.1328	1.0985	0
1280	972.1	1.099	1.2697	1.1329	1.0988	0
1281	972	1.0994	1.2702	1.1329	1.099	0
1282	971.9	1.1001	1.2705	1.1325	1.0993	0
1283	971.8	1.0999	1.2704	1.1331	1.0997	0
1284	971.7	1.1	1.2703	1.1337	1.0994	0
1285	971.6	1.1	1.2706	1.1333	1.0996	0
1286	971.5	1.1001	1.2705	1.1329	1.0995	0
1287	971.4	1.0998	1.2706	1.1339	1.0996	0
1288	971.3	1.1003	1.2703	1.1335	1.0997	0
1289	971.2	1.0999	1.2705	1.1338	1.0995	0
1290	971.1	1.1001	1.2705	1.1333	1.0993	0
1291	971	1.1001	1.2711	1.1329	1.0993	0
1292	970.9	1.1	1.2707	1.1329	1.0996	0
1293	970.8	1.1003	1.2711	1.1321	1.0996	0
1294	970.7	1.1005	1.2709	1.1331	1.1001	0
1295	970.6	1.1004	1.2711	1.133	1.1002	0
1296	970.5	1.1007	1.2709	1.1342	1.1001	0
1297	970.4	1.1008	1.2711	1.1339	1.1	0
1298	970.3	1.1008	1.271	1.1336	1.1	0
1299	970.2	1.1008	1.2713	1.1338	1.1002	0
1300	970.1	1.1009	1.2718	1.1342	1.1003	0
1301	970	1.101	1.2714	1.133	1.1004	0
1302	969.9	1.101	1.2719	1.133	1.1006	0
1303	969.8	1.1009	1.2717	1.1344	1.1005	0
1304	969.7	1.101	1.2719	1.1341	1.1006	0
1305	969.6	1.101	1.2714	1.1341	1.1004	0
1306	969.5	1.1012	1.2725	1.1341	1.1004	0
1307	969.4	1.1011	1.2722	1.1344	1.1003	0
1308	969.3	1.1013	1.2718	1.1337	1.1007	0
1309	969.2	1.1014	1.2722	1.134	1.1008	0
1310	969.1	1.1013	1.2721	1.1337	1.1005	0
1311	969	1.1013	1.2724	1.135	1.1005	0
1312	968.9	1.1013	1.272	1.1347	1.1003	0
1313	968.8	1.1013	1.272	1.1345	1.1003	0
1314	968.7	1.1014	1.2728	1.1349	1.1008	0
1315	968.6	1.1011	1.2724	1.1342	1.1007	0
1316	968.5	1.1014	1.2724	1.1352	1.1006	0
1317	968.4	1.1015	1.273	1.1348	1.1009	0
1318	968.3	1.1015	1.2727	1.1353	1.1009	0
1319	968.2	1.1019	1.2729	1.1356	1.1011	0

1320	968.1	1.1014	1.2729	1.1343	1.1008	0
1321	968	1.102	1.2729	1.135	1.1012	0
1322	967.9	1.1022	1.2734	1.1358	1.1014	0
1323	967.8	1.1017	1.2729	1.1355	1.1009	0
1324	967.7	1.1017	1.2734	1.1348	1.1011	0
1325	967.6	1.1022	1.273	1.1354	1.101	0
1326	967.5	1.1017	1.2729	1.1353	1.1009	0
1327	967.4	1.102	1.273	1.1354	1.101	0
1328	967.3	1.1018	1.2733	1.1365	1.101	0
1329	967.2	1.1017	1.2732	1.1364	1.1011	0
1330	967.1	1.1022	1.2736	1.1358	1.1012	0
1331	967	1.1019	1.2739	1.1356	1.1013	0
1332	966.9	1.1022	1.2742	1.1356	1.1012	0
1333	966.8	1.1022	1.2739	1.1367	1.1016	0
1334	966.7	1.1023	1.274	1.136	1.1015	0
1335	966.6	1.1027	1.2739	1.1363	1.1021	0
1336	966.5	1.1024	1.2741	1.1356	1.102	0
1337	966.4	1.1028	1.274	1.1362	1.102	0
1338	966.3	1.1028	1.274	1.1353	1.1022	0
1339	966.2	1.1025	1.2737	1.1357	1.1023	0
1340	966.1	1.1029	1.2744	1.1361	1.1025	0
1341	966	1.1032	1.2745	1.136	1.1026	0
1342	965.9	1.1032	1.2748	1.136	1.1026	0
1343	965.8	1.1037	1.2746	1.1355	1.1025	0
1344	965.7	1.1033	1.2746	1.1358	1.1029	0
1345	965.6	1.1035	1.2746	1.1361	1.1029	0
1346	965.5	1.1033	1.2746	1.1366	1.1027	0
1347	965.4	1.1037	1.2748	1.1357	1.1029	0
1348	965.3	1.1034	1.2744	1.1351	1.1028	0
1349	965.2	1.1039	1.2747	1.135	1.1029	0
1350	965.1	1.104	1.2751	1.1358	1.1034	0
1351	965	1.1042	1.2747	1.1357	1.1036	0
1352	964.9	1.1042	1.275	1.135	1.1036	0
1353	964.8	1.1044	1.2752	1.1344	1.1036	0
1354	964.7	1.1046	1.2751	1.134	1.104	0
1355	964.6	1.1051	1.2748	1.1341	1.1045	0
1356	964.5	1.1047	1.2753	1.1345	1.1045	0
1357	964.4	1.1052	1.2759	1.1336	1.1046	0
1358	964.3	1.1054	1.2757	1.1333	1.1052	0
1359	964.2	1.1054	1.2754	1.1328	1.1052	0
1360	964.1	1.1054	1.2755	1.1338	1.1052	0
1361	964	1.1052	1.2754	1.1324	1.1052	0
1362	963.9	1.1055	1.2752	1.1326	1.1051	0

1363	963.8	1.1059	1.2753	1.1321	1.1059	0
1364	963.7	1.106	1.2751	1.1324	1.1058	0
1365	963.6	1.1062	1.2762	1.1317	1.106	0
1366	963.5	1.1063	1.2757	1.1316	1.1061	0
1367	963.4	1.1064	1.2758	1.1317	1.1064	0
1368	963.3	1.1061	1.2755	1.131	1.1063	0
1369	963.2	1.1062	1.2753	1.1307	1.1062	0
1370	963.1	1.1065	1.2756	1.1307	1.1065	0
1371	963	1.1064	1.2756	1.1301	1.1064	0
1372	962.9	1.1066	1.2752	1.1303	1.1066	0
1373	962.8	1.1068	1.2751	1.1309	1.1068	0
1374	962.7	1.1069	1.2755	1.1308	1.1071	0
1375	962.6	1.1069	1.2754	1.1305	1.1071	0
1376	962.5	1.1069	1.2756	1.1307	1.1071	0
1377	962.4	1.1069	1.2755	1.1304	1.1069	0
1378	962.3	1.107	1.2756	1.1309	1.1072	0
1379	962.2	1.1073	1.2751	1.1304	1.1071	0
1380	962.1	1.1073	1.2751	1.1295	1.1073	0
1381	962	1.1076	1.2757	1.1302	1.1076	0
1382	961.9	1.1075	1.2757	1.1307	1.1077	0
1383	961.8	1.1075	1.2752	1.1295	1.1077	0
1384	961.7	1.1075	1.2746	1.1301	1.1079	0
1385	961.6	1.1075	1.274	1.1305	1.1079	0
1386	961.5	1.1075	1.2737	1.1301	1.1077	0
1387	961.4	1.1076	1.2729	1.1298	1.1078	0
1388	961.3	1.1077	1.2725	1.1293	1.1073	0
1389	961.2	1.1076	1.2723	1.1297	1.108	0
1390	961.1	1.1076	1.2719	1.1297	1.108	0
1391	961	1.1077	1.2712	1.1299	1.1079	0
1392	960.9	1.1081	1.2712	1.129	1.1081	0
1393	960.8	1.1079	1.2709	1.1299	1.1079	0
1394	960.7	1.1076	1.2711	1.1302	1.108	0
1395	960.6	1.1074	1.2705	1.1285	1.108	0
1396	960.5	1.1077	1.2709	1.1292	1.1079	0
1397	960.4	1.1078	1.2707	1.1289	1.108	0
1398	960.3	1.1079	1.2705	1.1288	1.1081	0
1399	960.2	1.108	1.2704	1.1294	1.108	0
1400	960.1	1.1078	1.2701	1.1291	1.108	0
1401	960	1.1077	1.2697	1.1294	1.1079	0
1402	959.9	1.1072	1.2694	1.1288	1.1076	0
1403	959.8	1.1072	1.2699	1.1291	1.1074	0
1404	959.7	1.1071	1.269	1.1293	1.1075	0
1405	959.6	1.1078	1.2695	1.1297	1.1078	0

1406	959.5	1.1072	1.2689	1.1298	1.1074	0
1407	959.4	1.1073	1.2692	1.1307	1.1075	0
1408	959.3	1.1074	1.2691	1.1294	1.1072	0
1409	959.2	1.1074	1.2687	1.1295	1.1076	0
1410	959.1	1.1074	1.269	1.13	1.1076	0
1411	959	1.1071	1.2688	1.1292	1.1075	0
1412	958.9	1.107	1.2685	1.1298	1.1072	0
1413	958.8	1.107	1.2684	1.1299	1.1072	0
1414	958.7	1.107	1.2682	1.1302	1.1074	0
1415	958.6	1.1069	1.2678	1.1298	1.1071	0
1416	958.5	1.1071	1.268	1.1303	1.1071	0
1417	958.4	1.1068	1.2679	1.13	1.107	0
1418	958.3	1.1067	1.2675	1.1307	1.1071	0
1419	958.2	1.1065	1.2679	1.1303	1.1067	0
1420	958.1	1.1064	1.2675	1.13	1.1066	0
1421	958	1.1063	1.267	1.1292	1.1063	0
1422	957.9	1.1064	1.2669	1.1297	1.1062	0
1423	957.8	1.1058	1.2675	1.1289	1.106	0
1424	957.7	1.1059	1.2668	1.1301	1.1061	0
1425	957.6	1.1059	1.2671	1.1301	1.1055	0
1426	957.5	1.1058	1.2672	1.1304	1.106	0
1427	957.4	1.1054	1.2664	1.13	1.1058	0
1428	957.3	1.1055	1.2668	1.1307	1.1057	0
1429	957.2	1.1052	1.2667	1.1302	1.1054	0
1430	957.1	1.1054	1.2664	1.1302	1.1054	0
1431	957	1.1052	1.2666	1.1308	1.1056	0
1432	956.9	1.1055	1.2668	1.1313	1.1053	0
1433	956.8	1.1051	1.2662	1.1301	1.1049	0
1434	956.7	1.105	1.2655	1.1304	1.105	0
1435	956.6	1.1049	1.2656	1.1299	1.1051	0
1436	956.5	1.1052	1.2666	1.1309	1.105	0
1437	956.4	1.105	1.2661	1.1311	1.105	0
1438	956.3	1.1049	1.2662	1.1312	1.1051	0
1439	956.2	1.1048	1.2657	1.1302	1.105	0
1440	956.1	1.1048	1.266	1.13	1.105	0
1441	956	1.1047	1.2652	1.1303	1.1047	0
1442	955.9	1.1045	1.2652	1.1301	1.1047	0
1443	955.8	1.1044	1.2652	1.1302	1.1044	0
1444	955.7	1.1043	1.2644	1.1291	1.1043	0
1445	955.6	1.1041	1.2646	1.1298	1.1041	0
1446	955.5	1.104	1.2643	1.129	1.1038	0
1447	955.4	1.104	1.2648	1.1293	1.104	0
1448	955.3	1.1041	1.2646	1.1284	1.1039	0

1449	955.2	1.1041	1.264	1.1282	1.1039	0
1450	955.1	1.1036	1.2635	1.1279	1.1036	0
1451	955	1.1036	1.2636	1.1287	1.1036	0
1452	954.9	1.1033	1.2632	1.1278	1.1035	0
1453	954.8	1.1031	1.2631	1.1277	1.1035	0
1454	954.7	1.1034	1.2633	1.1279	1.1034	0
1455	954.6	1.1034	1.2628	1.1274	1.1036	0
1456	954.5	1.1035	1.2622	1.1284	1.1031	0
1457	954.4	1.1033	1.2624	1.1277	1.1033	0
1458	954.3	1.1033	1.2626	1.1271	1.1035	0
1459	954.2	1.1032	1.2622	1.127	1.103	0
1460	954.1	1.1029	1.2619	1.1263	1.1029	0
1461	954	1.1032	1.2616	1.1266	1.103	0
1462	953.9	1.1027	1.2609	1.1262	1.1029	0
1463	953.8	1.1028	1.261	1.1263	1.1028	0
1464	953.7	1.1027	1.2608	1.1254	1.1029	0
1465	953.6	1.103	1.2611	1.125	1.103	0
1466	953.5	1.103	1.2612	1.1247	1.1027	0
1467	953.4	1.1028	1.2613	1.1252	1.1031	0
1468	953.3	1.1028	1.261	1.1252	1.1028	0
1469	953.2	1.1028	1.2612	1.1242	1.103	0
1470	953.1	1.1028	1.2601	1.1247	1.1026	0
1471	953	1.1027	1.2608	1.1233	1.1029	0
1472	952.9	1.1024	1.2605	1.1242	1.1028	0
1473	952.8	1.1024	1.26	1.1238	1.1028	0
1474	952.7	1.1025	1.2598	1.1239	1.1027	0
1475	952.6	1.1026	1.2596	1.1229	1.1026	0
1476	952.5	1.1022	1.2593	1.1232	1.1024	0
1477	952.4	1.1022	1.2594	1.1218	1.1028	0
1478	952.3	1.1022	1.259	1.1223	1.1024	0
1479	952.2	1.1024	1.2587	1.1225	1.1028	0
1480	952.1	1.1026	1.2586	1.1211	1.1026	0
1481	952	1.1021	1.2582	1.1216	1.1025	0
1482	951.9	1.1021	1.2585	1.1214	1.1027	0
1483	951.8	1.1024	1.2582	1.1227	1.1026	0
1484	951.7	1.1024	1.2582	1.1221	1.1024	0
1485	951.6	1.1024	1.2587	1.1223	1.1026	0
1486	951.5	1.102	1.2582	1.1211	1.102	0
1487	951.4	1.1022	1.2577	1.1219	1.102	0
1488	951.3	1.1019	1.2578	1.1214	1.1023	0
1489	951.2	1.102	1.2574	1.1207	1.102	0
1490	951.1	1.1018	1.2574	1.1221	1.1018	0
1491	951	1.102	1.2573	1.1214	1.1021	0

1492	950.9	1.1016	1.257	1.1208	1.1018	0
1493	950.8	1.1018	1.2569	1.1206	1.1018	0
1494	950.7	1.1017	1.257	1.1209	1.1021	0
1495	950.6	1.1015	1.2568	1.1218	1.1017	0
1496	950.5	1.1015	1.2566	1.1212	1.1017	0
1497	950.4	1.1016	1.2563	1.1211	1.102	0
1498	950.3	1.1018	1.2565	1.1214	1.1018	0
1499	950.2	1.1013	1.2568	1.1202	1.1017	0
1500	950.1	1.1015	1.2567	1.1192	1.1015	0
1501	950	1.1015	1.2565	1.1197	1.1015	0
1502	949.9	1.1016	1.2565	1.1208	1.1016	0
1503	949.8	1.1015	1.2564	1.1201	1.1016	0
1504	949.7	1.1014	1.2561	1.1202	1.1014	0
1505	949.6	1.1012	1.2561	1.1204	1.1012	0
1506	949.5	1.1012	1.2561	1.1202	1.101	0
1507	949.4	1.1011	1.2564	1.1217	1.1013	0
1508	949.3	1.1015	1.2559	1.1214	1.1015	0
1509	949.2	1.1011	1.2557	1.1218	1.1009	0
1510	949.1	1.1009	1.2554	1.1214	1.1009	0
1511	949	1.1009	1.2554	1.1209	1.1011	0
1512	948.9	1.101	1.2558	1.1212	1.1014	0
1513	948.8	1.101	1.2558	1.1211	1.101	0
1514	948.7	1.1009	1.2555	1.1206	1.1007	0
1515	948.6	1.1004	1.2553	1.1206	1.1006	0
1516	948.5	1.1004	1.2561	1.1216	1.1008	0
1517	948.4	1.1004	1.2555	1.1216	1.1006	0
1518	948.3	1.1006	1.2556	1.1219	1.1006	0
1519	948.2	1.1006	1.2556	1.1217	1.1004	0
1520	948.1	1.1005	1.2555	1.1213	1.1003	0
1521	948	1.1007	1.2559	1.1219	1.1005	0
1522	947.9	1.1002	1.2559	1.1219	1.1004	0
1523	947.8	1.1003	1.2554	1.1213	1.1001	0
1524	947.7	1.1002	1.2556	1.1216	1.1004	0
1525	947.6	1.1002	1.2557	1.1221	1.1002	0
1526	947.5	1.1001	1.2556	1.1228	1.1001	0
1527	947.4	1.1001	1.2553	1.1221	1.0999	0
1528	947.3	1.1	1.2552	1.1221	1.0997	0
1529	947.2	1.0999	1.2554	1.1216	1.0999	0
1530	947.1	1.0996	1.2557	1.1226	1.0999	0
1531	947	1.0996	1.2554	1.1222	1.0998	0
1532	946.9	1.0996	1.2554	1.1233	1.0996	0
1533	946.8	1.1	1.2556	1.1235	1.0998	0
1534	946.7	1.0999	1.2556	1.1226	1.0995	0

1535	946.6	1.0998	1.2559	1.1231	1.0998	0
1536	946.5	1.0995	1.2556	1.1221	1.0993	0
1537	946.4	1.0997	1.2559	1.123	1.0991	0
1538	946.3	1.0995	1.2559	1.1227	1.0993	0
1539	946.2	1.0995	1.2563	1.1224	1.0989	0
1540	946.1	1.0993	1.2562	1.1224	1.0991	0
1541	946	1.0995	1.2567	1.123	1.0993	0
1542	945.9	1.0995	1.2575	1.123	1.0993	0
1543	945.8	1.0994	1.2571	1.1219	1.099	0
1544	945.7	1.0995	1.2569	1.1219	1.0993	0
1545	945.6	1.0996	1.2573	1.1222	1.0994	0
1546	945.5	1.0992	1.2574	1.1222	1.0988	0
1547	945.4	1.0991	1.2579	1.1216	1.0991	0
1548	945.3	1.0989	1.2574	1.1217	1.0989	0
1549	945.2	1.099	1.2578	1.1221	1.0989	0
1550	945.1	1.0989	1.2581	1.1215	1.099	0
1551	945	1.0993	1.2579	1.1224	1.0989	0
1552	944.9	1.0991	1.2576	1.1217	1.0989	0
1553	944.8	1.099	1.2583	1.1217	1.099	0
1554	944.7	1.0993	1.2584	1.1214	1.0991	0
1555	944.6	1.0995	1.2582	1.1211	1.0991	0
1556	944.5	1.0994	1.2579	1.1212	1.0992	0
1557	944.4	1.0993	1.2582	1.1209	1.0995	0
1558	944.3	1.0992	1.2579	1.1204	1.0994	0
1559	944.2	1.0992	1.2581	1.1199	1.099	0
1560	944.1	1.0992	1.2586	1.1194	1.0988	0
1561	944	1.0995	1.2582	1.1192	1.0991	0
1562	943.9	1.0994	1.2584	1.1198	1.099	0
1563	943.8	1.0991	1.2583	1.1195	1.0989	0
1564	943.7	1.0993	1.2585	1.1193	1.0989	0
1565	943.6	1.0991	1.258	1.1192	1.0991	0
1566	943.5	1.0993	1.2583	1.1193	1.0991	0
1567	943.4	1.0991	1.258	1.1192	1.0989	0
1568	943.3	1.0992	1.2583	1.1199	1.0992	0
1569	943.2	1.0994	1.2585	1.1201	1.0992	0
1570	943.1	1.0994	1.2589	1.119	1.0992	0
1571	943	1.0993	1.2589	1.1198	1.0995	0
1572	942.9	1.0995	1.2588	1.1182	1.0995	0
1573	942.8	1.0995	1.2587	1.1183	1.0991	0
1574	942.7	1.0991	1.2589	1.118	1.0991	0
1575	942.6	1.0991	1.2587	1.1178	1.0989	0
1576	942.5	1.0992	1.2584	1.1183	1.099	0
1577	942.4	1.0993	1.2584	1.1182	1.0991	0

1578	942.3	1.0991	1.2584	1.1181	1.0989	0
1579	942.2	1.0991	1.2583	1.1177	1.0991	0
1580	942.1	1.0993	1.2588	1.118	1.0993	0
1581	942	1.0994	1.2585	1.1183	1.0994	0
1582	941.9	1.0994	1.2585	1.1175	1.0994	0
1583	941.8	1.0996	1.2588	1.1179	1.0992	0
1584	941.7	1.0994	1.2587	1.1173	1.0992	0
1585	941.6	1.0995	1.2586	1.1176	1.0995	0
1586	941.5	1.0997	1.2589	1.116	1.0991	0
1587	941.4	1.0997	1.2585	1.1162	1.0997	0
1588	941.3	1.0996	1.2587	1.1161	1.0993	0
1589	941.2	1.0993	1.2588	1.1161	1.0991	0
1590	941.1	1.0996	1.2584	1.1162	1.0994	0
1591	941	1.0997	1.259	1.1161	1.0993	0
1592	940.9	1.0997	1.259	1.117	1.0993	0
1593	940.8	1.0996	1.2591	1.1173	1.0996	0
1594	940.7	1.0997	1.2587	1.1168	1.0993	0
1595	940.6	1.0999	1.2586	1.1157	1.0995	0
1596	940.5	1.0996	1.2588	1.116	1.0992	0
1597	940.4	1.0994	1.2583	1.1165	1.0992	0
1598	940.3	1.0994	1.2586	1.1161	1.0992	0
1599	940.2	1.0994	1.2588	1.1163	1.0994	0
1600	940.1	1.0997	1.2587	1.1152	1.0996	0
1601	940	1.0997	1.2586	1.1156	1.0993	0
1602	939.9	1.0995	1.2586	1.1147	1.0993	0
1603	939.8	1.0996	1.2582	1.1152	1.0992	0
1604	939.7	1.0994	1.2588	1.1142	1.0992	0
1605	939.6	1.0996	1.259	1.1141	1.0996	0
1606	939.5	1.1	1.2589	1.1142	1.0994	0
1607	939.4	1.0996	1.2587	1.1143	1.0994	0
1608	939.3	1.0994	1.2587	1.1148	1.0994	0
1609	939.2	1.0996	1.2587	1.1154	1.0994	0
1610	939.1	1.1	1.2589	1.115	1.0996	0
1611	939	1.1001	1.2587	1.1158	1.0999	0
1612	938.9	1.0996	1.2587	1.1152	1.0994	0
1613	938.8	1.0997	1.2586	1.1152	1.0993	0
1614	938.7	1.0997	1.2589	1.1149	1.0993	0
1615	938.6	1.0996	1.2585	1.1152	1.0996	0
1616	938.5	1.0996	1.2582	1.1154	1.0994	0
1617	938.4	1.0996	1.2588	1.1156	1.0994	0
1618	938.3	1.0997	1.2587	1.1162	1.0993	0
1619	938.2	1.0995	1.2583	1.115	1.0992	0
1620	938.1	1.0996	1.2584	1.1149	1.0995	0

1621	938	1.0995	1.2585	1.1154	1.0993	0
1622	937.9	1.0993	1.2584	1.116	1.0993	0
1623	937.8	1.0995	1.2585	1.1153	1.0995	0
1624	937.7	1.0995	1.2583	1.1156	1.0993	0
1625	937.6	1.0995	1.2589	1.1148	1.0993	0
1626	937.5	1.0995	1.2589	1.1154	1.0993	0
1627	937.4	1.0994	1.259	1.1148	1.099	0
1628	937.3	1.0996	1.2594	1.1145	1.099	0
1629	937.2	1.0999	1.2595	1.116	1.0993	0
1630	937.1	1.0996	1.259	1.1152	1.0992	0
1631	937	1.0995	1.2598	1.1141	1.0993	0
1632	936.9	1.0992	1.2593	1.1149	1.0988	0
1633	936.8	1.0994	1.2593	1.1154	1.0994	0
1634	936.7	1.0999	1.2596	1.115	1.0993	0
1635	936.6	1.0993	1.2593	1.1141	1.0987	0
1636	936.5	1.0994	1.2592	1.1147	1.0992	0
1637	936.4	1.0994	1.2589	1.1149	1.0994	0
1638	936.3	1.0994	1.2591	1.1152	1.0992	0
1639	936.2	1.0998	1.2594	1.1145	1.0992	0
1640	936.1	1.0997	1.2589	1.1143	1.0993	0
1641	936	1.0997	1.2592	1.1141	1.0993	0
1642	935.9	1.0997	1.2595	1.1154	1.0993	0
1643	935.8	1.0994	1.259	1.1151	1.0992	0
1644	935.7	1.0993	1.2586	1.1146	1.0991	0
1645	935.6	1.0995	1.259	1.1148	1.0993	0
1646	935.5	1.0999	1.2584	1.1149	1.0995	0
1647	935.4	1.0997	1.2587	1.1155	1.0993	0
1648	935.3	1.0998	1.2586	1.1143	1.099	0
1649	935.2	1.0994	1.2578	1.1145	1.099	0
1650	935.1	1.0992	1.2581	1.1145	1.099	0
1651	935	1.0995	1.2582	1.1139	1.0993	0
1652	934.9	1.0997	1.2579	1.1144	1.0991	0
1653	934.8	1.0996	1.2577	1.1132	1.0992	0
1654	934.7	1.0996	1.2574	1.1141	1.0994	0
1655	934.6	1.0996	1.2573	1.1135	1.099	0
1656	934.5	1.0997	1.258	1.1124	1.0994	0
1657	934.4	1.1	1.2574	1.1137	1.0992	0
1658	934.3	1.0995	1.2573	1.1129	1.0993	0
1659	934.2	1.0999	1.2565	1.1132	1.0991	0
1660	934.1	1.0997	1.2568	1.1129	1.0993	0
1661	934	1.0995	1.2562	1.1122	1.0993	0
1662	933.9	1.0997	1.256	1.1134	1.0993	0
1663	933.8	1.0997	1.2563	1.1118	1.0994	0

1664	933.7	1.0997	1.256	1.1121	1.0993	0
1665	933.6	1.1002	1.2555	1.112	1.0994	0
1666	933.5	1.1001	1.2559	1.1128	1.0993	0
1667	933.4	1.1	1.2557	1.1123	1.0994	0
1668	933.3	1.0999	1.2551	1.111	1.0996	0
1669	933.2	1.1004	1.2549	1.1106	1.1002	0
1670	933.1	1.1002	1.2548	1.1116	1.0996	0
1671	933	1.1004	1.2547	1.113	1.0998	0
1672	932.9	1.1004	1.2545	1.1129	1.0998	0
1673	932.8	1.1003	1.2546	1.1121	1.0997	0
1674	932.7	1.1002	1.2541	1.112	1.1	0
1675	932.6	1.1001	1.2546	1.1112	1.1	0
1676	932.5	1.1001	1.2537	1.1105	1.0995	0
1677	932.4	1.1001	1.2537	1.1119	1.0999	0
1678	932.3	1.1001	1.2537	1.112	1.0999	0
1679	932.2	1.1003	1.2531	1.1112	1.0999	0
1680	932.1	1.1004	1.2533	1.1109	1.0998	0
1681	932	1.1001	1.2529	1.1102	1.0997	0
1682	931.9	1.1001	1.2529	1.1106	1.0998	0
1683	931.8	1.1003	1.2532	1.1104	1.0999	0
1684	931.7	1.1001	1.252	1.1111	1.0997	0
1685	931.6	1.1005	1.2522	1.1109	1.1001	0
1686	931.5	1.1005	1.2527	1.1099	1.1001	0
1687	931.4	1.1006	1.2524	1.1099	1.0999	0
1688	931.3	1.1004	1.2518	1.1097	1.1	0
1689	931.2	1.1005	1.2519	1.1101	1.1001	0
1690	931.1	1.1003	1.2516	1.1103	1.0999	0
1691	931	1.1003	1.2516	1.1095	1.0997	0
1692	930.9	1.1002	1.2508	1.109	1.0998	0
1693	930.8	1.1	1.2505	1.1104	1.0994	0
1694	930.7	1.0999	1.2509	1.1102	1.0995	0
1695	930.6	1.1	1.2508	1.1102	1.0994	0
1696	930.5	1.1003	1.2503	1.1091	1.0997	0
1697	930.4	1.1003	1.2506	1.1098	1.0999	0
1698	930.3	1.1001	1.2501	1.1108	1.0995	0
1699	930.2	1.0999	1.2494	1.1116	1.0995	0
1700	930.1	1.0998	1.2493	1.1105	1.0993	0
1701	930	1.1001	1.2496	1.1104	1.0993	0
1702	929.9	1.0999	1.2493	1.1105	1.0991	0
1703	929.8	1.0998	1.2492	1.1104	1.099	0
1704	929.7	1.0997	1.249	1.1111	1.0991	0
1705	929.6	1.0996	1.2487	1.1105	1.099	0
1706	929.5	1.1	1.2488	1.1107	1.0989	0

1707	929.4	1.1	1.2489	1.1103	1.0993	0
1708	929.3	1.1	1.2486	1.1106	1.0992	0
1709	929.2	1.0997	1.2483	1.11	1.0991	0
1710	929.1	1.0999	1.2483	1.1094	1.0991	0
1711	929	1.0998	1.2486	1.1104	1.099	0
1712	928.9	1.0997	1.2478	1.1092	1.0989	0
1713	928.8	1.1	1.2478	1.1096	1.099	0
1714	928.7	1.0998	1.2471	1.1086	1.099	0
1715	928.6	1.0999	1.2471	1.1091	1.0993	0
1716	928.5	1.0999	1.2474	1.1101	1.0993	0
1717	928.4	1.0994	1.2465	1.1102	1.099	0
1718	928.3	1.0995	1.247	1.1098	1.0991	0
1719	928.2	1.0996	1.2466	1.1107	1.0991	0
1720	928.1	1.0997	1.2471	1.1099	1.0991	0
1721	928	1.0999	1.2466	1.1105	1.0993	0
1722	927.9	1.1	1.2466	1.1097	1.0993	0
1723	927.8	1.0999	1.2464	1.11	1.0992	0
1724	927.7	1.1001	1.2466	1.1101	1.0995	0
1725	927.6	1.0997	1.2462	1.11	1.0991	0
1726	927.5	1.0998	1.2462	1.1104	1.0992	0
1727	927.4	1.1	1.246	1.1101	1.0994	0
1728	927.3	1.0996	1.246	1.1099	1.099	0
1729	927.2	1.0999	1.2459	1.1093	1.0993	0
1730	927.1	1.0996	1.2463	1.1096	1.0992	0
1731	927	1.0998	1.2462	1.1092	1.0994	0
1732	926.9	1.1001	1.246	1.1089	1.0995	0
1733	926.8	1.1002	1.2464	1.1084	1.0994	0
1734	926.7	1.1002	1.246	1.1093	1.0994	0
1735	926.6	1.1	1.2459	1.1088	1.0994	0
1736	926.5	1.1	1.246	1.1087	1.0994	0
1737	926.4	1.1003	1.2466	1.1092	1.0997	0
1738	926.3	1.1003	1.2457	1.1087	1.0999	0
1739	926.2	1.1004	1.2459	1.1089	1.0998	0
1740	926.1	1.1	1.246	1.1099	1.0996	0
1741	926	1.1003	1.2456	1.1096	1.0997	0
1742	925.9	1.1001	1.2456	1.1091	1.0993	0
1743	925.8	1.1	1.2457	1.1097	1.0996	0
1744	925.7	1.1004	1.2454	1.1079	1.0996	0
1745	925.6	1.1007	1.2462	1.1089	1.1001	0
1746	925.5	1.1005	1.2464	1.108	1.0997	0
1747	925.4	1.1005	1.2469	1.108	1.0999	0
1748	925.3	1.1004	1.246	1.1087	1.0999	0
1749	925.2	1.1008	1.2462	1.1081	1.1	0

1750	925.1	1.1005	1.2463	1.1078	1.1001	0
1751	925	1.1002	1.2455	1.1071	1.0998	0
1752	924.9	1.1003	1.2459	1.1078	1.1	0
1753	924.8	1.1002	1.2457	1.1069	1.0998	0
1754	924.7	1.1006	1.2455	1.1069	1.0998	0
1755	924.6	1.1005	1.246	1.1074	1.0999	0
1756	924.5	1.1009	1.246	1.1064	1.0999	0
1757	924.4	1.1004	1.2463	1.1057	1.1002	0
1758	924.3	1.1003	1.2461	1.1068	1.0999	0
1759	924.2	1.1005	1.2459	1.1058	1.0999	0
1760	924.1	1.1007	1.2459	1.106	1.1	0
1761	924	1.1005	1.2459	1.1056	1.1003	0
1762	923.9	1.1008	1.2462	1.1053	1.1004	0
1763	923.8	1.1005	1.2459	1.1054	1.1003	0
1764	923.7	1.101	1.246	1.1044	1.1002	0
1765	923.6	1.1011	1.2463	1.1054	1.1005	0
1766	923.5	1.1011	1.246	1.1047	1.1007	0
1767	923.4	1.1006	1.2458	1.1038	1.1004	0
1768	923.3	1.1007	1.2456	1.1047	1.1005	0
1769	923.2	1.1008	1.2452	1.1041	1.1006	0
1770	923.1	1.1011	1.2453	1.1041	1.1008	0
1771	923	1.101	1.2454	1.104	1.1009	0
1772	922.9	1.101	1.2456	1.1039	1.101	0
1773	922.8	1.1013	1.2459	1.1043	1.101	0
1774	922.7	1.1012	1.2455	1.1048	1.1008	0
1775	922.6	1.1011	1.2455	1.1034	1.1011	0
1776	922.5	1.1015	1.2457	1.105	1.1009	0
1777	922.4	1.1014	1.2457	1.1044	1.101	0
1778	922.3	1.1012	1.2458	1.1044	1.1008	0
1779	922.2	1.1012	1.2457	1.1045	1.101	0
1780	922.1	1.1011	1.2451	1.1043	1.1009	0
1781	922	1.1014	1.2458	1.1052	1.1012	0
1782	921.9	1.1014	1.2458	1.1049	1.101	0
1783	921.8	1.101	1.2453	1.1051	1.1012	0
1784	921.7	1.101	1.2455	1.1049	1.1008	0
1785	921.6	1.1013	1.2454	1.1055	1.1011	0
1786	921.5	1.1012	1.2455	1.1049	1.101	0
1787	921.4	1.101	1.2458	1.1044	1.101	0
1788	921.3	1.1013	1.2456	1.1042	1.1013	0
1789	921.2	1.1012	1.2458	1.1041	1.101	0
1790	921.1	1.1015	1.2456	1.1052	1.1011	0
1791	921	1.1014	1.2455	1.1057	1.1014	0
1792	920.9	1.1009	1.2455	1.1054	1.1007	0

1793	920.8	1.101	1.2454	1.1053	1.1008	0
1794	920.7	1.101	1.2457	1.105	1.1008	0
1795	920.6	1.1012	1.2457	1.1045	1.101	0
1796	920.5	1.101	1.2454	1.1049	1.101	0
1797	920.4	1.1012	1.2459	1.1053	1.1008	0
1798	920.3	1.1012	1.2455	1.1057	1.101	0
1799	920.2	1.1012	1.2458	1.1056	1.101	0
1800	920.1	1.1013	1.2456	1.1056	1.1011	0
1801	920	1.101	1.2453	1.1058	1.1008	0
1802	919.9	1.1008	1.2453	1.1064	1.101	0
1803	919.8	1.1009	1.2455	1.1062	1.1007	0
1804	919.7	1.101	1.2453	1.1061	1.1008	0
1805	919.6	1.1011	1.2452	1.1061	1.1009	0
1806	919.5	1.1013	1.2456	1.106	1.1011	0
1807	919.4	1.101	1.2454	1.1057	1.101	0
1808	919.3	1.101	1.2455	1.106	1.1008	0
1809	919.2	1.101	1.2452	1.1069	1.1008	0
1810	919.1	1.1008	1.2457	1.1055	1.1012	0
1811	919	1.1007	1.2456	1.1054	1.1011	0
1812	918.9	1.1006	1.2454	1.1068	1.1006	0
1813	918.8	1.1009	1.2454	1.1062	1.1009	0
1814	918.7	1.1005	1.2452	1.1062	1.1007	0
1815	918.6	1.1009	1.2455	1.1069	1.1007	0
1816	918.5	1.1007	1.2453	1.1066	1.1007	0
1817	918.4	1.1006	1.2449	1.1063	1.1004	0
1818	918.3	1.1006	1.2461	1.1062	1.1006	0
1819	918.2	1.1007	1.2456	1.1065	1.1009	0
1820	918.1	1.1004	1.2455	1.1062	1.1008	0
1821	918	1.1008	1.2457	1.1061	1.101	0
1822	917.9	1.1006	1.2458	1.1057	1.1006	0
1823	917.8	1.1006	1.2456	1.1072	1.1008	0
1824	917.7	1.101	1.2463	1.1066	1.1008	0
1825	917.6	1.101	1.2463	1.1067	1.101	0
1826	917.5	1.1009	1.2459	1.1068	1.1011	0
1827	917.4	1.1007	1.2457	1.1056	1.1007	0
1828	917.3	1.101	1.2461	1.1054	1.101	0
1829	917.2	1.101	1.2458	1.1059	1.1012	0
1830	917.1	1.1011	1.2462	1.1068	1.1011	0
1831	917	1.101	1.2461	1.1064	1.101	0
1832	916.9	1.1009	1.246	1.1064	1.1009	0
1833	916.8	1.1009	1.2463	1.1064	1.1013	0
1834	916.7	1.1012	1.2458	1.1067	1.1014	0
1835	916.6	1.1011	1.2457	1.1062	1.1013	0

1836	916.5	1.1009	1.2455	1.1064	1.1013	0
1837	916.4	1.1011	1.2455	1.1056	1.1014	0
1838	916.3	1.1014	1.2456	1.1055	1.1014	0
1839	916.2	1.1011	1.2458	1.1055	1.1017	0
1840	916.1	1.1013	1.2457	1.1048	1.1017	0
1841	916	1.1014	1.2462	1.1058	1.1016	0
1842	915.9	1.1014	1.2456	1.1044	1.1016	0
1843	915.8	1.1013	1.2458	1.1059	1.1019	0
1844	915.7	1.1016	1.2459	1.1047	1.102	0
1845	915.6	1.1017	1.2455	1.1051	1.1021	0
1846	915.5	1.1017	1.2458	1.1055	1.1023	0
1847	915.4	1.1017	1.2458	1.1043	1.1023	0
1848	915.3	1.1015	1.2453	1.1031	1.1019	0
1849	915.2	1.1018	1.2448	1.1037	1.1021	0
1850	915.1	1.1017	1.2448	1.1027	1.1023	0
1851	915	1.1021	1.2455	1.1027	1.1025	0
1852	914.9	1.1019	1.2452	1.1035	1.1027	0
1853	914.8	1.1022	1.2456	1.1042	1.1026	0
1854	914.7	1.1021	1.2451	1.1031	1.1029	0
1855	914.6	1.1023	1.2451	1.1035	1.1029	0
1856	914.5	1.1022	1.2451	1.1036	1.1028	0
1857	914.4	1.1025	1.2452	1.1025	1.1027	0
1858	914.3	1.1023	1.2449	1.1029	1.1029	0
1859	914.2	1.1025	1.2451	1.1023	1.1031	0
1860	914.1	1.1023	1.245	1.1023	1.1027	0
1861	914	1.1025	1.2455	1.1017	1.1031	0
1862	913.9	1.1023	1.2455	1.1023	1.1031	0
1863	913.8	1.1024	1.2451	1.1012	1.103	0
1864	913.7	1.1027	1.2452	1.1017	1.1033	0
1865	913.6	1.1029	1.2452	1.1005	1.1033	0
1866	913.5	1.1025	1.245	1.1014	1.1033	0
1867	913.4	1.103	1.2456	1.1016	1.1034	0
1868	913.3	1.1029	1.245	1.1012	1.1035	0
1869	913.2	1.103	1.2449	1.1015	1.1034	0
1870	913.1	1.1031	1.2452	1.1019	1.1035	0
1871	913	1.1029	1.2449	1.1013	1.1037	0
1872	912.9	1.1029	1.2449	1.1003	1.1037	0
1873	912.8	1.103	1.2454	1.1001	1.1038	0
1874	912.7	1.103	1.2451	1.1002	1.104	0
1875	912.6	1.1036	1.2456	1.101	1.1042	0
1876	912.5	1.1033	1.2455	1.1014	1.1041	0
1877	912.4	1.1035	1.2455	1.1002	1.1041	0
1878	912.3	1.1034	1.2454	1.1006	1.1042	0

1879	912.2	1.1035	1.2457	1.1011	1.1042	0
1880	912.1	1.1035	1.2452	1.1006	1.1043	0
1881	912	1.1035	1.2457	1.1007	1.1047	0
1882	911.9	1.1039	1.2457	1.0995	1.1046	0
1883	911.8	1.1038	1.245	1.1008	1.1046	0
1884	911.7	1.1039	1.2457	1.1005	1.1049	0
1885	911.6	1.1038	1.246	1.0999	1.1048	0
1886	911.5	1.1043	1.2458	1.1003	1.1047	0
1887	911.4	1.1042	1.246	1.1009	1.105	0
1888	911.3	1.104	1.2462	1.1004	1.105	0
1889	911.2	1.104	1.246	1.1011	1.105	0
1890	911.1	1.1042	1.2459	1.1004	1.1049	0
1891	911	1.1038	1.2459	1.1001	1.105	0
1892	910.9	1.1042	1.2461	1.0997	1.105	0
1893	910.8	1.1043	1.2462	1.0996	1.1049	0
1894	910.7	1.104	1.2459	1.0998	1.1048	0
1895	910.6	1.104	1.2462	1.1013	1.1048	0
1896	910.5	1.1043	1.2462	1.1007	1.1049	0
1897	910.4	1.1043	1.2466	1.1003	1.1049	0
1898	910.3	1.1043	1.2461	1.1003	1.1047	0
1899	910.2	1.1041	1.2464	1.1007	1.1051	0
1900	910.1	1.104	1.2461	1.1001	1.1044	0
1901	910	1.104	1.2458	1.1004	1.1048	0
1902	909.9	1.1043	1.2464	1.1007	1.1049	0
1903	909.8	1.1043	1.2467	1.1015	1.1051	0
1904	909.7	1.1045	1.2467	1.1001	1.1051	0
1905	909.6	1.1044	1.2464	1.0995	1.105	0
1906	909.5	1.1042	1.2465	1.0999	1.1048	0
1907	909.4	1.1041	1.2467	1.0996	1.1049	0
1908	909.3	1.1041	1.2461	1.1007	1.1051	0
1909	909.2	1.1043	1.2461	1.0999	1.1049	0
1910	909.1	1.1042	1.2465	1.1004	1.1052	0
1911	909	1.1042	1.2464	1.1007	1.105	0
1912	908.9	1.1043	1.2465	1.1003	1.1047	0
1913	908.8	1.1041	1.2465	1.1009	1.1049	0
1914	908.7	1.1042	1.2465	1.1014	1.1048	0
1915	908.6	1.1043	1.2461	1.1009	1.1047	0
1916	908.5	1.1042	1.2463	1.1019	1.1048	0
1917	908.4	1.1042	1.2465	1.1014	1.105	0
1918	908.3	1.1043	1.2462	1.1015	1.1051	0
1919	908.2	1.1041	1.2462	1.1011	1.1047	0
1920	908.1	1.1041	1.2463	1.1007	1.1045	0
1921	908	1.1038	1.2464	1.101	1.1048	0

1922	907.9	1.1043	1.2469	1.1013	1.1049	0
1923	907.8	1.1042	1.2466	1.1014	1.1049	0
1924	907.7	1.1041	1.2468	1.1019	1.1049	0
1925	907.6	1.1044	1.2468	1.1011	1.105	0
1926	907.5	1.104	1.2469	1.1007	1.1048	0
1927	907.4	1.1044	1.2466	1.1013	1.105	0
1928	907.3	1.1042	1.2466	1.1018	1.1048	0
1929	907.2	1.1042	1.2466	1.1022	1.105	0
1930	907.1	1.1042	1.2469	1.102	1.105	0
1931	907	1.1044	1.2467	1.102	1.1052	0
1932	906.9	1.1043	1.2468	1.1025	1.1053	0
1933	906.8	1.1046	1.2469	1.1024	1.1054	0
1934	906.7	1.1044	1.2469	1.1026	1.1054	0
1935	906.6	1.1049	1.2473	1.1033	1.1057	0
1936	906.5	1.1046	1.247	1.1014	1.1052	0
1937	906.4	1.1049	1.2474	1.1009	1.1055	0
1938	906.3	1.1047	1.2469	1.1015	1.1053	0
1939	906.2	1.1046	1.2468	1.1014	1.1056	0
1940	906.1	1.1046	1.247	1.1018	1.1056	0
1941	906	1.1048	1.2468	1.1008	1.1056	0
1942	905.9	1.105	1.2468	1.1013	1.1058	0
1943	905.8	1.1051	1.2469	1.1013	1.1059	0
1944	905.7	1.1047	1.2469	1.1009	1.1057	0
1945	905.6	1.1051	1.2477	1.1015	1.1059	0
1946	905.5	1.1052	1.2474	1.102	1.1062	0
1947	905.4	1.1054	1.2475	1.1012	1.1064	0
1948	905.3	1.1054	1.2477	1.1015	1.1062	0
1949	905.2	1.1054	1.247	1.1006	1.1062	0
1950	905.1	1.1054	1.2471	1.1012	1.1064	0
1951	905	1.1054	1.247	1.1008	1.1066	0
1952	904.9	1.1054	1.2471	1.1006	1.1066	0
1953	904.8	1.1059	1.2474	1.1005	1.1069	0
1954	904.7	1.106	1.2476	1.1002	1.1068	0
1955	904.6	1.1058	1.2473	1.0998	1.1068	0
1956	904.5	1.1065	1.2477	1.0997	1.1071	0
1957	904.4	1.1061	1.2469	1.0989	1.1069	0
1958	904.3	1.1062	1.2473	1.0987	1.1076	0
1959	904.2	1.1065	1.2471	1.0986	1.1075	0
1960	904.1	1.1066	1.2472	1.0976	1.1078	0
1961	904	1.1065	1.2477	1.0986	1.1077	0
1962	903.9	1.107	1.2473	1.098	1.1082	0
1963	903.8	1.1068	1.2475	1.0977	1.108	0
1964	903.7	1.1072	1.2472	1.0977	1.1082	0

1965	903.6	1.1068	1.2471	1.0978	1.1082	0
1966	903.5	1.1071	1.2475	1.0969	1.1085	0
1967	903.4	1.1071	1.2476	1.0964	1.1083	0
1968	903.3	1.1073	1.2481	1.0968	1.1087	0
1969	903.2	1.1074	1.2475	1.0971	1.1085	0
1970	903.1	1.1076	1.2474	1.0973	1.109	0
1971	903	1.1074	1.2472	1.0965	1.1086	0
1972	902.9	1.1076	1.2472	1.0961	1.109	0
1973	902.8	1.1077	1.2476	1.0962	1.1091	0
1974	902.7	1.108	1.2472	1.0965	1.109	0
1975	902.6	1.1077	1.2471	1.0964	1.1092	0
1976	902.5	1.1079	1.2473	1.0958	1.1091	0
1977	902.4	1.1078	1.247	1.0959	1.109	0
1978	902.3	1.1079	1.2474	1.0956	1.1091	0
1979	902.2	1.1079	1.2471	1.0962	1.1093	0
1980	902.1	1.1084	1.2476	1.0955	1.1092	0
1981	902	1.108	1.247	1.0951	1.1092	0
1982	901.9	1.1081	1.2477	1.095	1.1095	0
1983	901.8	1.1085	1.2476	1.0956	1.1097	0
1984	901.7	1.1084	1.2478	1.0953	1.11	0
1985	901.6	1.1081	1.2472	1.095	1.1095	0
1986	901.5	1.1086	1.2478	1.0955	1.1098	0
1987	901.4	1.1087	1.2478	1.0953	1.11	0
1988	901.3	1.1088	1.2481	1.0953	1.1102	0
1989	901.2	1.1086	1.2479	1.0945	1.11	0
1990	901.1	1.1085	1.2478	1.0952	1.1101	0
1991	901	1.1086	1.2475	1.0947	1.1098	0
1992	900.9	1.1084	1.2478	1.0955	1.1098	0
1993	900.8	1.1084	1.2479	1.0953	1.1096	0
1994	900.7	1.1088	1.2478	1.0949	1.11	0
1995	900.6	1.1085	1.2474	1.095	1.1099	0
1996	900.5	1.1089	1.2479	1.0952	1.1099	0
1997	900.4	1.1084	1.2474	1.0947	1.11	0
1998	900.3	1.1087	1.2476	1.0946	1.1099	0
1999	900.2	1.1087	1.2476	1.0938	1.1102	0
2000	900.1	1.1088	1.248	1.0947	1.1101	0
2001	900	1.1089	1.248	1.0938	1.1105	0
2002	899.9	1.1088	1.2478	1.0939	1.1103	0
2003	899.8	1.1088	1.248	1.0952	1.1102	0
2004	899.7	1.1089	1.2479	1.0946	1.1103	0
2005	899.6	1.1091	1.2482	1.0942	1.1106	0
2006	899.5	1.1089	1.2483	1.0944	1.1103	0
2007	899.4	1.109	1.2482	1.0943	1.1104	0

2008	899.3	1.109	1.2484	1.0944	1.1106	0
2009	899.2	1.1088	1.248	1.0951	1.1102	0
2010	899.1	1.1092	1.2482	1.0968	1.1106	0
2011	899	1.109	1.2485	1.0956	1.1104	0
2012	898.9	1.1091	1.2481	1.0954	1.1104	0
2013	898.8	1.1089	1.2484	1.0955	1.1105	0
2014	898.7	1.1089	1.2482	1.0954	1.1103	0
2015	898.6	1.1092	1.248	1.0946	1.1102	0
2016	898.5	1.1088	1.2479	1.0953	1.1105	0
2017	898.4	1.1093	1.2486	1.096	1.1108	0
2018	898.3	1.109	1.2485	1.0958	1.1104	0
2019	898.2	1.109	1.249	1.0966	1.1104	0
2020	898.1	1.1091	1.2489	1.0963	1.1103	0
2021	898	1.1085	1.2483	1.0955	1.1099	0
2022	897.9	1.1089	1.2487	1.096	1.1103	0
2023	897.8	1.109	1.2486	1.097	1.1104	0
2024	897.7	1.1087	1.2483	1.0961	1.1103	0
2025	897.6	1.1086	1.2488	1.0968	1.1102	0
2026	897.5	1.1087	1.2484	1.0969	1.1105	0
2027	897.4	1.1088	1.2486	1.0964	1.1103	0
2028	897.3	1.1091	1.2489	1.0973	1.1105	0
2029	897.2	1.1088	1.2489	1.0964	1.1107	0
2030	897.1	1.1086	1.2487	1.0968	1.1103	0
2031	897	1.1087	1.2488	1.0963	1.1105	0
2032	896.9	1.1084	1.2485	1.0966	1.1103	0
2033	896.8	1.1087	1.2486	1.0966	1.1105	0
2034	896.7	1.1089	1.2489	1.097	1.1105	0
2035	896.6	1.109	1.2489	1.0969	1.1106	0
2036	896.5	1.109	1.2488	1.097	1.1103	0
2037	896.4	1.1087	1.2487	1.0961	1.1103	0
2038	896.3	1.1088	1.2486	1.0966	1.1105	0
2039	896.2	1.1087	1.249	1.0957	1.1105	0
2040	896.1	1.109	1.249	1.0954	1.1105	0
2041	896	1.109	1.2492	1.0967	1.1104	0
2042	895.9	1.1091	1.2494	1.0965	1.1108	0
2043	895.8	1.1089	1.2491	1.0962	1.1107	0
2044	895.7	1.1091	1.249	1.0958	1.1107	0
2045	895.6	1.109	1.2489	1.095	1.1108	0
2046	895.5	1.1095	1.2488	1.095	1.1107	0
2047	895.4	1.1092	1.2487	1.0959	1.1106	0
2048	895.3	1.1093	1.2487	1.0957	1.1108	0
2049	895.2	1.1093	1.2489	1.0956	1.1107	0
2050	895.1	1.1093	1.2486	1.095	1.1109	0

2051	895	1.1095	1.2492	1.096	1.1111	0
2052	894.9	1.1094	1.2496	1.0963	1.1113	0
2053	894.8	1.1096	1.2493	1.0959	1.1114	0
2054	894.7	1.1096	1.249	1.0953	1.1116	0
2055	894.6	1.1096	1.2488	1.0965	1.1114	0
2056	894.5	1.1098	1.2488	1.0959	1.1116	0
2057	894.4	1.1097	1.249	1.0958	1.1114	0
2058	894.3	1.1099	1.2487	1.0948	1.1117	0
2059	894.2	1.1099	1.2487	1.0954	1.112	0
2060	894.1	1.1101	1.2487	1.095	1.112	0
2061	894	1.1099	1.249	1.095	1.112	0
2062	893.9	1.1099	1.2493	1.0952	1.1122	0
2063	893.8	1.1102	1.2494	1.0953	1.1123	0
2064	893.7	1.1105	1.2492	1.0947	1.1126	0
2065	893.6	1.1106	1.25	1.0941	1.1128	0
2066	893.5	1.1106	1.2493	1.0932	1.1129	0
2067	893.4	1.1107	1.2491	1.0935	1.1132	0
2068	893.3	1.1111	1.2491	1.0942	1.1131	0
2069	893.2	1.1108	1.249	1.0936	1.1133	0
2070	893.1	1.1109	1.2486	1.0938	1.1134	0
2071	893	1.111	1.2495	1.0935	1.1132	0
2072	892.9	1.1113	1.2493	1.0934	1.1135	0
2073	892.8	1.1111	1.2491	1.0928	1.1136	0
2074	892.7	1.1113	1.2495	1.0931	1.1136	0
2075	892.6	1.1116	1.2492	1.0921	1.1136	0
2076	892.5	1.1114	1.2496	1.093	1.1141	0
2077	892.4	1.1114	1.2496	1.0928	1.1139	0
2078	892.3	1.1119	1.249	1.093	1.1141	0
2079	892.2	1.1118	1.2493	1.0928	1.1141	0
2080	892.1	1.1116	1.2495	1.0921	1.1141	0
2081	892	1.112	1.2498	1.0912	1.1143	0
2082	891.9	1.112	1.2495	1.0917	1.1142	0
2083	891.8	1.1124	1.2495	1.0919	1.1145	0
2084	891.7	1.1121	1.2494	1.092	1.1144	0
2085	891.6	1.1121	1.2494	1.0916	1.1144	0
2086	891.5	1.1126	1.2498	1.0919	1.1149	0
2087	891.4	1.1121	1.25	1.0916	1.1148	0
2088	891.3	1.1122	1.2496	1.0915	1.1147	0
2089	891.2	1.1125	1.2501	1.0917	1.1148	0
2090	891.1	1.1122	1.2497	1.0913	1.1149	0
2091	891	1.1122	1.2495	1.0903	1.1147	0
2092	890.9	1.1125	1.2498	1.0908	1.1149	0
2093	890.8	1.1125	1.2496	1.091	1.1148	0

2094	890.7	1.1124	1.2495	1.0907	1.1149	0
2095	890.6	1.1124	1.25	1.091	1.1149	0
2096	890.5	1.1122	1.2499	1.0912	1.1151	0
2097	890.4	1.1127	1.2499	1.0906	1.1154	0
2098	890.3	1.1125	1.2499	1.0896	1.1152	0
2099	890.2	1.1127	1.2497	1.0904	1.1156	0
2100	890.1	1.1128	1.2504	1.0909	1.1155	0
2101	890	1.1126	1.2496	1.0903	1.1155	0
2102	889.9	1.1128	1.2501	1.0909	1.1155	0
2103	889.8	1.1129	1.2503	1.0916	1.1156	0
2104	889.7	1.1128	1.2502	1.0913	1.1157	0
2105	889.6	1.1127	1.2503	1.0912	1.1154	0
2106	889.5	1.1128	1.2503	1.0917	1.1157	0
2107	889.4	1.1129	1.2498	1.0911	1.1158	0
2108	889.3	1.1129	1.2502	1.0909	1.1156	0
2109	889.2	1.1128	1.2503	1.0924	1.1155	0
2110	889.1	1.1125	1.25	1.0908	1.1158	0
2111	889	1.1129	1.2503	1.0913	1.1156	0
2112	888.9	1.1131	1.2504	1.0917	1.1158	0
2113	888.8	1.1128	1.2504	1.091	1.1159	0
2114	888.7	1.1127	1.2503	1.0927	1.1156	0
2115	888.6	1.1126	1.25	1.0925	1.1157	0
2116	888.5	1.1128	1.2502	1.092	1.1157	0
2117	888.4	1.113	1.2503	1.0924	1.1157	0
2118	888.3	1.1128	1.2506	1.0918	1.1159	0
2119	888.2	1.1129	1.2505	1.0919	1.1158	0
2120	888.1	1.1129	1.2505	1.0915	1.1158	0
2121	888	1.1125	1.2504	1.0919	1.1154	0
2122	887.9	1.1125	1.2499	1.0923	1.1154	0
2123	887.8	1.1123	1.25	1.0921	1.1154	0
2124	887.7	1.1128	1.2504	1.0926	1.1158	0
2125	887.6	1.1129	1.2507	1.0926	1.1158	0
2126	887.5	1.1126	1.2507	1.0924	1.1159	0
2127	887.4	1.1128	1.2509	1.0928	1.1159	0
2128	887.3	1.1129	1.2508	1.0932	1.1158	0
2129	887.2	1.1126	1.2508	1.0933	1.1157	0
2130	887.1	1.1126	1.2506	1.0926	1.1156	0
2131	887	1.1126	1.2506	1.0923	1.1153	0
2132	886.9	1.1128	1.2506	1.0929	1.1155	0
2133	886.8	1.1127	1.2505	1.0928	1.1158	0
2134	886.7	1.1126	1.2507	1.0924	1.1158	0
2135	886.6	1.1128	1.2507	1.0926	1.116	0
2136	886.5	1.1126	1.2502	1.0924	1.116	0

2137	886.4	1.1129	1.2509	1.0932	1.1163	0
2138	886.3	1.1128	1.2507	1.0933	1.1159	0
2139	886.2	1.1127	1.2506	1.0928	1.116	0
2140	886.1	1.1127	1.2507	1.0932	1.1159	0
2141	886	1.1129	1.2513	1.0926	1.116	0
2142	885.9	1.113	1.2517	1.0937	1.1162	0
2143	885.8	1.1132	1.2514	1.0931	1.1163	0
2144	885.7	1.1127	1.2511	1.0928	1.1161	0
2145	885.6	1.1131	1.2516	1.0936	1.1165	0
2146	885.5	1.113	1.252	1.0932	1.1161	0
2147	885.4	1.113	1.2519	1.0929	1.1162	0
2148	885.3	1.113	1.2515	1.0928	1.1163	0
2149	885.2	1.1132	1.2518	1.0926	1.1163	0
2150	885.1	1.1135	1.2516	1.0928	1.1167	0
2151	885	1.1132	1.2521	1.0923	1.1165	0
2152	884.9	1.1133	1.252	1.092	1.1167	0
2153	884.8	1.1132	1.2519	1.0925	1.1166	0
2154	884.7	1.1136	1.2521	1.0927	1.1168	0
2155	884.6	1.1138	1.2523	1.0914	1.1171	0
2156	884.5	1.1137	1.2522	1.0923	1.117	0
2157	884.4	1.1143	1.2521	1.0929	1.117	0
2158	884.3	1.1137	1.2524	1.0922	1.1169	0
2159	884.2	1.1138	1.2519	1.0923	1.1174	0
2160	884.1	1.1138	1.2518	1.0924	1.1173	0
2161	884	1.114	1.2522	1.0928	1.1176	0
2162	883.9	1.1139	1.2523	1.0915	1.1172	0
2163	883.8	1.114	1.2517	1.0911	1.1174	0
2164	883.7	1.1142	1.252	1.0916	1.1174	0
2165	883.6	1.1139	1.2521	1.0913	1.1177	0
2166	883.5	1.1142	1.2522	1.0908	1.1176	0
2167	883.4	1.1144	1.2522	1.09	1.1182	0
2168	883.3	1.1146	1.2519	1.0894	1.118	0
2169	883.2	1.1144	1.2522	1.0892	1.1182	0
2170	883.1	1.1145	1.2523	1.0901	1.1181	0
2171	883	1.1147	1.252	1.0903	1.1187	0
2172	882.9	1.1145	1.2523	1.0897	1.118	0
2173	882.8	1.1147	1.2523	1.0903	1.1182	0
2174	882.7	1.1146	1.2522	1.0901	1.1183	0
2175	882.6	1.1148	1.252	1.0896	1.1184	0
2176	882.5	1.1152	1.2524	1.0903	1.1185	0
2177	882.4	1.1151	1.2524	1.0897	1.1187	0
2178	882.3	1.1147	1.2521	1.0893	1.1189	0
2179	882.2	1.115	1.2519	1.089	1.1185	0

2180	882.1	1.115	1.2523	1.0895	1.1185	0
2181	882	1.1152	1.2523	1.0891	1.1187	0
2182	881.9	1.1155	1.2527	1.0885	1.1189	0
2183	881.8	1.1151	1.2525	1.0893	1.1189	0
2184	881.7	1.115	1.2526	1.089	1.119	0
2185	881.6	1.1155	1.2524	1.0887	1.1193	0
2186	881.5	1.1155	1.2525	1.0887	1.1191	0
2187	881.4	1.1155	1.2525	1.0875	1.1193	0
2188	881.3	1.1155	1.2525	1.0883	1.1193	0
2189	881.2	1.1156	1.2526	1.0882	1.1196	0
2190	881.1	1.1155	1.2526	1.0873	1.1193	0
2191	881	1.1154	1.2528	1.0872	1.1197	0
2192	880.9	1.1158	1.2524	1.0877	1.1196	0
2193	880.8	1.1157	1.2529	1.0875	1.1201	0
2194	880.7	1.1161	1.2528	1.0874	1.1199	0
2195	880.6	1.1162	1.2527	1.0877	1.1198	0
2196	880.5	1.1159	1.2529	1.0868	1.1201	0
2197	880.4	1.1163	1.2532	1.0878	1.1199	0
2198	880.3	1.116	1.2534	1.0873	1.1198	0
2199	880.2	1.116	1.2528	1.0874	1.1203	0
2200	880.1	1.116	1.2529	1.0873	1.12	0
2201	880	1.116	1.2529	1.0869	1.1202	0
2202	879.9	1.1164	1.2534	1.0877	1.1204	0
2203	879.8	1.116	1.2527	1.0873	1.1201	0
2204	879.7	1.1162	1.2529	1.0872	1.12	0
2205	879.6	1.1161	1.2535	1.0866	1.1202	0
2206	879.5	1.116	1.253	1.0865	1.1202	0
2207	879.4	1.1161	1.2532	1.0866	1.1201	0
2208	879.3	1.116	1.2534	1.0865	1.1204	0
2209	879.2	1.1164	1.2533	1.0868	1.1206	0
2210	879.1	1.1166	1.2533	1.0866	1.1204	0
2211	879	1.1163	1.2535	1.0866	1.1206	0
2212	878.9	1.1164	1.2531	1.0868	1.1202	0
2213	878.8	1.1164	1.2536	1.086	1.1204	0
2214	878.7	1.1163	1.2535	1.0861	1.1205	0
2215	878.6	1.1165	1.2537	1.0863	1.1203	0
2216	878.5	1.1165	1.2537	1.0871	1.1206	0
2217	878.4	1.1162	1.2534	1.0874	1.1202	0
2218	878.3	1.1163	1.2534	1.0879	1.1205	0
2219	878.2	1.1164	1.2536	1.0876	1.1204	0
2220	878.1	1.1167	1.2537	1.0881	1.1208	0
2221	878	1.1165	1.2537	1.089	1.1209	0
2222	877.9	1.1164	1.2542	1.0876	1.1208	0

2223	877.8	1.1167	1.2538	1.087	1.1207	0
2224	877.7	1.1164	1.2543	1.0878	1.1211	0
2225	877.6	1.117	1.2544	1.0881	1.1212	0
2226	877.5	1.1168	1.2539	1.0883	1.1208	0
2227	877.4	1.1168	1.2543	1.0889	1.1208	0
2228	877.3	1.1168	1.2541	1.0882	1.1209	0
2229	877.2	1.1168	1.2538	1.0871	1.1206	0
2230	877.1	1.117	1.2547	1.0881	1.121	0
2231	877	1.1164	1.2537	1.0875	1.1205	0
2232	876.9	1.1167	1.2538	1.0876	1.1209	0
2233	876.8	1.1169	1.2544	1.0884	1.1209	0
2234	876.7	1.117	1.2537	1.0885	1.1209	0
2235	876.6	1.1168	1.2543	1.0885	1.1209	0
2236	876.5	1.1171	1.2547	1.0876	1.1214	0
2237	876.4	1.1168	1.2544	1.0889	1.1209	0
2238	876.3	1.1171	1.2542	1.088	1.1212	0
2239	876.2	1.1172	1.2545	1.0873	1.1212	0
2240	876.1	1.1173	1.2541	1.0875	1.1211	0
2241	876	1.1172	1.2544	1.0883	1.1213	0
2242	875.9	1.1173	1.2547	1.0885	1.1213	0
2243	875.8	1.1171	1.2549	1.0882	1.1214	0
2244	875.7	1.1172	1.2546	1.0883	1.1217	0
2245	875.6	1.1173	1.2545	1.088	1.1218	0
2246	875.5	1.1171	1.2546	1.0879	1.1216	0
2247	875.4	1.1169	1.2544	1.0876	1.1214	0
2248	875.3	1.1172	1.2545	1.0875	1.1215	0
2249	875.2	1.1174	1.2548	1.0873	1.1215	0
2250	875.1	1.1175	1.2547	1.0875	1.1218	0
2251	875	1.1174	1.2542	1.0874	1.1218	0
2252	874.9	1.1175	1.2546	1.0873	1.1218	0
2253	874.8	1.1175	1.2546	1.0877	1.122	0
2254	874.7	1.1175	1.2549	1.0875	1.122	0
2255	874.6	1.1176	1.2549	1.0874	1.1219	0
2256	874.5	1.1177	1.2549	1.0875	1.1222	0
2257	874.4	1.1176	1.2552	1.0872	1.1221	0
2258	874.3	1.1178	1.2551	1.087	1.1223	0
2259	874.2	1.1178	1.2548	1.0864	1.1227	0
2260	874.1	1.1179	1.255	1.0863	1.1224	0
2261	874	1.1182	1.2548	1.0869	1.1224	0
2262	873.9	1.1181	1.2555	1.0864	1.123	0
2263	873.8	1.1183	1.2555	1.0871	1.1228	0
2264	873.7	1.1183	1.2552	1.086	1.1228	0
2265	873.6	1.1184	1.255	1.0861	1.1231	0

2266	873.5	1.1181	1.255	1.0857	1.123	0
2267	873.4	1.1183	1.2556	1.0866	1.123	0
2268	873.3	1.1185	1.2549	1.0862	1.123	0
2269	873.2	1.1184	1.2551	1.0855	1.1233	0
2270	873.1	1.1183	1.2551	1.0863	1.1231	0
2271	873	1.1185	1.255	1.0866	1.123	0
2272	872.9	1.1184	1.2553	1.0846	1.1231	0
2273	872.8	1.1183	1.2554	1.0841	1.1233	0
2274	872.7	1.1185	1.2551	1.0854	1.1232	0
2275	872.6	1.1185	1.2551	1.0842	1.1235	0
2276	872.5	1.1186	1.2552	1.0855	1.1237	0
2277	872.4	1.1188	1.2555	1.0845	1.124	0
2278	872.3	1.1187	1.2554	1.085	1.1238	0
2279	872.2	1.1191	1.2553	1.0848	1.1243	0
2280	872.1	1.1187	1.2554	1.0854	1.1241	0
2281	872	1.1189	1.2551	1.0856	1.1239	0
2282	871.9	1.1194	1.2557	1.0858	1.1241	0
2283	871.8	1.1191	1.2551	1.0854	1.1243	0
2284	871.7	1.1188	1.2553	1.0855	1.1242	0
2285	871.6	1.1192	1.2562	1.0845	1.1244	0
2286	871.5	1.1192	1.2558	1.0854	1.1246	0
2287	871.4	1.1193	1.2557	1.0853	1.1242	0
2288	871.3	1.119	1.2556	1.0848	1.1241	0
2289	871.2	1.1192	1.2556	1.0854	1.1243	0
2290	871.1	1.119	1.2559	1.0858	1.1246	0
2291	871	1.1193	1.2563	1.0859	1.1245	0
2292	870.9	1.1191	1.2558	1.0853	1.1247	0
2293	870.8	1.1194	1.2557	1.0846	1.1246	0
2294	870.7	1.1197	1.2561	1.0853	1.1249	0
2295	870.6	1.1196	1.2556	1.086	1.125	0
2296	870.5	1.1193	1.2559	1.0859	1.1249	0
2297	870.4	1.1192	1.256	1.086	1.1248	0
2298	870.3	1.1194	1.2559	1.0848	1.1246	0
2299	870.2	1.1194	1.2559	1.0838	1.1248	0
2300	870.1	1.1192	1.2559	1.0858	1.1248	0
2301	870	1.1197	1.2557	1.0853	1.1253	0
2302	869.9	1.1196	1.2558	1.0843	1.1252	0
2303	869.8	1.1192	1.2555	1.0846	1.1248	0
2304	869.7	1.1195	1.2559	1.0849	1.1252	0
2305	869.6	1.1196	1.2562	1.0859	1.1252	0
2306	869.5	1.1194	1.2554	1.0854	1.1253	0
2307	869.4	1.1195	1.2556	1.085	1.1251	0
2308	869.3	1.1195	1.2562	1.0849	1.1254	0

2309	869.2	1.1197	1.2559	1.0848	1.1253	0
2310	869.1	1.1196	1.2561	1.0847	1.1254	0
2311	869	1.1192	1.2562	1.0845	1.1253	0
2312	868.9	1.1193	1.2561	1.0845	1.1252	0
2313	868.8	1.1199	1.2568	1.0848	1.1253	0
2314	868.7	1.1198	1.2561	1.0854	1.1256	0
2315	868.6	1.1197	1.2563	1.0854	1.1253	0
2316	868.5	1.1198	1.2559	1.086	1.1252	0
2317	868.4	1.1199	1.2566	1.0852	1.1255	0
2318	868.3	1.1201	1.2563	1.0857	1.1257	0
2319	868.2	1.1202	1.2562	1.0857	1.1257	0
2320	868.1	1.1203	1.2563	1.0847	1.1259	0
2321	868	1.12	1.2563	1.0855	1.1259	0
2322	867.9	1.1205	1.257	1.0861	1.1261	0
2323	867.8	1.1204	1.2569	1.0852	1.1262	0
2324	867.7	1.12	1.2563	1.0847	1.1259	0
2325	867.6	1.1205	1.2565	1.0855	1.1261	0
2326	867.5	1.1204	1.2568	1.085	1.1258	0
2327	867.4	1.1203	1.2567	1.0853	1.1261	0
2328	867.3	1.1203	1.2564	1.0849	1.1259	0
2329	867.2	1.1204	1.2569	1.0864	1.1263	0
2330	867.1	1.1206	1.2569	1.0856	1.1262	0
2331	867	1.1204	1.2567	1.0856	1.1265	0
2332	866.9	1.1205	1.2576	1.0862	1.1266	0
2333	866.8	1.1205	1.2571	1.0857	1.1266	0
2334	866.7	1.1206	1.2572	1.0862	1.1265	0
2335	866.6	1.121	1.2572	1.0857	1.1269	0
2336	866.5	1.1211	1.2576	1.086	1.1267	0
2337	866.4	1.1208	1.257	1.0855	1.1267	0
2338	866.3	1.1209	1.2571	1.0851	1.1268	0
2339	866.2	1.1206	1.257	1.0852	1.1269	0
2340	866.1	1.1209	1.2571	1.0851	1.1272	0
2341	866	1.1211	1.257	1.0854	1.1272	0
2342	865.9	1.1211	1.257	1.0856	1.1274	0
2343	865.8	1.1211	1.257	1.0846	1.1274	0
2344	865.7	1.1213	1.2571	1.0848	1.1274	0
2345	865.6	1.1213	1.2573	1.0848	1.1278	0
2346	865.5	1.1215	1.2573	1.0856	1.1276	0
2347	865.4	1.1215	1.2569	1.085	1.1278	0
2348	865.3	1.1215	1.2568	1.085	1.1277	0
2349	865.2	1.1214	1.2569	1.0843	1.1277	0
2350	865.1	1.1215	1.2573	1.0842	1.128	0
2351	865	1.1217	1.2567	1.0842	1.128	0

2352	864.9	1.1214	1.2569	1.0839	1.128	0
2353	864.8	1.1216	1.2566	1.0837	1.1279	0
2354	864.7	1.1219	1.257	1.084	1.128	0
2355	864.6	1.1219	1.2566	1.0839	1.1284	0
2356	864.5	1.1222	1.257	1.084	1.1285	0
2357	864.4	1.1218	1.2572	1.0839	1.1284	0
2358	864.3	1.1219	1.257	1.084	1.1289	0
2359	864.2	1.1224	1.2573	1.083	1.1287	0
2360	864.1	1.1224	1.257	1.0836	1.1287	0
2361	864	1.1221	1.2567	1.0829	1.1289	0
2362	863.9	1.1226	1.2576	1.0832	1.1289	0
2363	863.8	1.1224	1.257	1.0828	1.1292	0
2364	863.7	1.1227	1.2571	1.0831	1.1293	0
2365	863.6	1.1226	1.2574	1.0823	1.1294	0
2366	863.5	1.1227	1.2575	1.0814	1.1293	0
2367	863.4	1.1228	1.2574	1.0824	1.1292	0
2368	863.3	1.1229	1.2572	1.0831	1.1295	0
2369	863.2	1.1226	1.2574	1.0819	1.1301	0
2370	863.1	1.1231	1.258	1.0813	1.1299	0
2371	863	1.1233	1.2573	1.0812	1.13	0
2372	862.9	1.1231	1.2574	1.0813	1.1299	0
2373	862.8	1.123	1.2574	1.08	1.13	0
2374	862.7	1.1229	1.257	1.0809	1.1297	0
2375	862.6	1.123	1.2568	1.0818	1.13	0
2376	862.5	1.1235	1.257	1.0818	1.1303	0
2377	862.4	1.1236	1.2577	1.0821	1.1306	0
2378	862.3	1.1235	1.258	1.0819	1.1305	0
2379	862.2	1.1232	1.2574	1.0822	1.1305	0
2380	862.1	1.1234	1.2572	1.0814	1.1302	0
2381	862	1.1237	1.2577	1.0812	1.1307	0
2382	861.9	1.1237	1.2578	1.0817	1.1307	0
2383	861.8	1.1237	1.2577	1.0822	1.1305	0
2384	861.7	1.1234	1.258	1.0812	1.1305	0
2385	861.6	1.1236	1.2579	1.0815	1.1304	0
2386	861.5	1.1235	1.2578	1.0819	1.1308	0
2387	861.4	1.1235	1.2578	1.0816	1.1307	0
2388	861.3	1.1235	1.2581	1.0818	1.1308	0
2389	861.2	1.1233	1.2573	1.0812	1.1305	0
2390	861.1	1.1239	1.2581	1.0812	1.1309	0
2391	861	1.1242	1.2581	1.0807	1.1312	0
2392	860.9	1.1239	1.2575	1.082	1.1314	0
2393	860.8	1.1241	1.2578	1.0808	1.1312	0
2394	860.7	1.1243	1.258	1.0803	1.1313	0

2395	860.6	1.1237	1.2579	1.0799	1.1312	0
2396	860.5	1.1239	1.2578	1.0802	1.1314	0
2397	860.4	1.1239	1.2582	1.0814	1.1312	0
2398	860.3	1.1239	1.2577	1.0814	1.1312	0
2399	860.2	1.1242	1.2583	1.0808	1.1312	0
2400	860.1	1.1243	1.2587	1.0811	1.1315	0
2401	860	1.1241	1.2582	1.0803	1.1313	0
2402	859.9	1.1242	1.2583	1.0814	1.1314	0
2403	859.8	1.124	1.2586	1.0808	1.1317	0
2404	859.7	1.1243	1.2582	1.0817	1.1315	0
2405	859.6	1.124	1.2581	1.081	1.1317	0
2406	859.5	1.1242	1.2581	1.082	1.1317	0
2407	859.4	1.1242	1.2581	1.0824	1.1317	0
2408	859.3	1.1244	1.2584	1.0826	1.1319	0
2409	859.2	1.1244	1.2584	1.0808	1.1319	0
2410	859.1	1.1244	1.2582	1.0816	1.1317	0
2411	859	1.1244	1.2584	1.0814	1.1317	0
2412	858.9	1.1243	1.2587	1.0813	1.1316	0
2413	858.8	1.1244	1.2584	1.0819	1.1321	0
2414	858.7	1.1244	1.2582	1.0812	1.1319	0
2415	858.6	1.1241	1.2585	1.0817	1.1321	0
2416	858.5	1.1247	1.2583	1.0824	1.132	0
2417	858.4	1.1243	1.2588	1.0821	1.132	0
2418	858.3	1.1243	1.2587	1.0824	1.132	0
2419	858.2	1.1241	1.2588	1.0824	1.1318	0
2420	858.1	1.1246	1.2588	1.0823	1.1323	0
2421	858	1.1244	1.2587	1.0823	1.1319	0
2422	857.9	1.1244	1.259	1.0817	1.1321	0
2423	857.8	1.1247	1.2588	1.0816	1.1322	0
2424	857.7	1.1249	1.2593	1.0828	1.1322	0
2425	857.6	1.1246	1.2587	1.0829	1.1321	0
2426	857.5	1.1246	1.2591	1.0823	1.1321	0
2427	857.4	1.1245	1.2595	1.0828	1.1322	0
2428	857.3	1.1244	1.2593	1.0827	1.1321	0
2429	857.2	1.1245	1.2592	1.0826	1.1325	0
2430	857.1	1.1248	1.2593	1.0831	1.1328	0
2431	857	1.1247	1.2594	1.0832	1.1327	0
2432	856.9	1.1246	1.2594	1.0829	1.1326	0
2433	856.8	1.1246	1.2596	1.083	1.1325	0
2434	856.7	1.1249	1.2597	1.0821	1.1328	0
2435	856.6	1.1248	1.2599	1.0821	1.1332	0
2436	856.5	1.1251	1.2594	1.0821	1.1328	0
2437	856.4	1.1247	1.2596	1.082	1.1327	0

2438	856.3	1.125	1.2597	1.0827	1.1332	0
2439	856.2	1.1252	1.2599	1.0823	1.1336	0
2440	856.1	1.1256	1.2597	1.082	1.1333	0
2441	856	1.1256	1.26	1.0818	1.1333	0
2442	855.9	1.1257	1.2598	1.0817	1.1339	0
2443	855.8	1.1258	1.2594	1.0818	1.1338	0
2444	855.7	1.1256	1.2594	1.0816	1.1342	0
2445	855.6	1.1257	1.2595	1.0817	1.1341	0
2446	855.5	1.1259	1.26	1.0812	1.1343	0
2447	855.4	1.126	1.2601	1.0814	1.1346	0
2448	855.3	1.1262	1.2598	1.0816	1.1348	0
2449	855.2	1.1262	1.2597	1.0806	1.1346	0
2450	855.1	1.1263	1.2602	1.0809	1.1347	0
2451	855	1.1264	1.2595	1.0802	1.1348	0
2452	854.9	1.1263	1.2596	1.0797	1.1347	0
2453	854.8	1.1265	1.2595	1.0807	1.1349	0
2454	854.7	1.1263	1.2597	1.0793	1.1347	0
2455	854.6	1.1266	1.2596	1.0788	1.135	0
2456	854.5	1.1264	1.2594	1.0796	1.1346	0
2457	854.4	1.1265	1.2599	1.0787	1.1349	0
2458	854.3	1.127	1.2596	1.0796	1.1352	0
2459	854.2	1.1267	1.2598	1.0799	1.1354	0
2460	854.1	1.127	1.2596	1.0792	1.1355	0
2461	854	1.1267	1.2595	1.0798	1.1356	0
2462	853.9	1.1273	1.2594	1.0795	1.136	0
2463	853.8	1.1273	1.2599	1.0792	1.1355	0
2464	853.7	1.1273	1.2601	1.0791	1.1361	0
2465	853.6	1.1275	1.2601	1.0785	1.1359	0
2466	853.5	1.1276	1.2602	1.0784	1.1363	0
2467	853.4	1.1273	1.2601	1.0793	1.1359	0
2468	853.3	1.1276	1.2605	1.078	1.1362	0
2469	853.2	1.1278	1.2605	1.0784	1.1365	0
2470	853.1	1.1278	1.2608	1.0784	1.1367	0
2471	853	1.1279	1.2609	1.0785	1.1368	0
2472	852.9	1.128	1.261	1.0784	1.1369	0
2473	852.8	1.1281	1.2605	1.0781	1.137	0
2474	852.7	1.1278	1.2605	1.0778	1.1369	0
2475	852.6	1.1279	1.2609	1.0777	1.1368	0
2476	852.5	1.1279	1.2609	1.0771	1.137	0
2477	852.4	1.128	1.2607	1.078	1.1369	0
2478	852.3	1.1282	1.2603	1.0775	1.1373	0
2479	852.2	1.1284	1.2606	1.0779	1.1371	0
2480	852.1	1.1281	1.2603	1.0775	1.1372	0

2481	852	1.1285	1.2607	1.0774	1.1371	0
2482	851.9	1.1285	1.2611	1.0777	1.1374	0
2483	851.8	1.1285	1.2608	1.0785	1.1374	0
2484	851.7	1.1285	1.2608	1.0785	1.1372	0
2485	851.6	1.1286	1.2612	1.0784	1.1377	0
2486	851.5	1.1285	1.2608	1.0777	1.1379	0
2487	851.4	1.1288	1.2608	1.0781	1.1377	0
2488	851.3	1.1286	1.2609	1.0778	1.1377	0
2489	851.2	1.129	1.2611	1.0775	1.1376	0
2490	851.1	1.1288	1.2611	1.0777	1.1379	0
2491	851	1.1285	1.2614	1.0777	1.1379	0
2492	850.9	1.1288	1.2612	1.0778	1.1379	0
2493	850.8	1.1289	1.2615	1.0766	1.138	0
2494	850.7	1.1287	1.2614	1.0767	1.1379	0
2495	850.6	1.1288	1.2614	1.0777	1.1384	0
2496	850.5	1.1286	1.2613	1.0774	1.1382	0
2497	850.4	1.1292	1.2614	1.0775	1.1381	0
2498	850.3	1.1291	1.2618	1.0774	1.1382	0
2499	850.2	1.129	1.2617	1.0781	1.1381	0
2500	850.1	1.1292	1.2614	1.0777	1.1381	0
2501	850	1.1293	1.261	1.0786	1.138	0
2502	849.9	1.133	1.2609	1.0825	1.1498	0
2503	849.8	1.133	1.2611	1.0836	1.1497	0
2504	849.7	1.133	1.2614	1.0831	1.1499	0
2505	849.6	1.133	1.2612	1.0836	1.1498	0
2506	849.5	1.1329	1.261	1.0838	1.1496	0
2507	849.4	1.133	1.2614	1.0838	1.1499	0
2508	849.3	1.1331	1.2619	1.0837	1.1498	0
2509	849.2	1.133	1.2617	1.0836	1.1501	0
2510	849.1	1.1333	1.2622	1.0839	1.1499	0
2511	849	1.1331	1.2619	1.0837	1.1501	0
2512	848.9	1.1332	1.2618	1.0836	1.15	0
2513	848.8	1.133	1.2616	1.0845	1.1498	0
2514	848.7	1.1331	1.2619	1.084	1.15	0
2515	848.6	1.1331	1.2622	1.0842	1.1498	0
2516	848.5	1.133	1.2619	1.0849	1.1498	0
2517	848.4	1.1327	1.2621	1.0846	1.1499	0
2518	848.3	1.133	1.2621	1.0837	1.1497	0
2519	848.2	1.1327	1.2615	1.0844	1.1499	0
2520	848.1	1.1332	1.2624	1.0848	1.1502	0
2521	848	1.1333	1.2625	1.0853	1.1501	0
2522	847.9	1.1333	1.262	1.0844	1.1503	0
2523	847.8	1.1332	1.2624	1.0844	1.1501	0

2524	847.7	1.1331	1.2617	1.084	1.15	0
2525	847.6	1.1331	1.2618	1.0848	1.1501	0
2526	847.5	1.1336	1.262	1.0856	1.1503	0
2527	847.4	1.1336	1.2623	1.0852	1.1503	0
2528	847.3	1.1334	1.2624	1.0843	1.1502	0
2529	847.2	1.1333	1.2623	1.0851	1.1501	0
2530	847.1	1.1334	1.2622	1.0839	1.15	0
2531	847	1.1334	1.2624	1.0845	1.1504	0
2532	846.9	1.1336	1.2624	1.0838	1.1504	0
2533	846.8	1.1335	1.2625	1.085	1.1503	0
2534	846.7	1.1339	1.2629	1.0851	1.1504	0
2535	846.6	1.134	1.2628	1.0839	1.1508	0
2536	846.5	1.1338	1.2624	1.0843	1.1506	0
2537	846.4	1.1337	1.2622	1.0847	1.1506	0
2538	846.3	1.1337	1.2623	1.0835	1.1505	0
2539	846.2	1.1337	1.2627	1.0844	1.1507	0
2540	846.1	1.1341	1.2626	1.0851	1.1506	0
2541	846	1.1341	1.2627	1.0844	1.1507	0
2542	845.9	1.1341	1.2627	1.0842	1.1509	0
2543	845.8	1.134	1.2628	1.0852	1.151	0
2544	845.7	1.1343	1.2626	1.0847	1.1513	0
2545	845.6	1.1342	1.2627	1.0845	1.1514	0
2546	845.5	1.1343	1.2626	1.0831	1.1511	0
2547	845.4	1.1347	1.2628	1.0844	1.151	0
2548	845.3	1.1345	1.2631	1.0839	1.1515	0
2549	845.2	1.1346	1.2629	1.0836	1.1516	0
2550	845.1	1.1347	1.263	1.0835	1.1515	0
2551	845	1.1347	1.2632	1.0837	1.1521	0
2552	844.9	1.1348	1.2629	1.0829	1.1518	0
2553	844.8	1.1352	1.2628	1.083	1.1518	0
2554	844.7	1.1352	1.2628	1.0824	1.1518	0
2555	844.6	1.1351	1.2629	1.0823	1.1523	0
2556	844.5	1.1351	1.2629	1.082	1.1521	0
2557	844.4	1.1353	1.2628	1.0813	1.1523	0
2558	844.3	1.1356	1.2629	1.0819	1.1526	0
2559	844.2	1.1357	1.263	1.0816	1.1527	0
2560	844.1	1.1352	1.2626	1.0802	1.1524	0
2561	844	1.1358	1.2628	1.0814	1.153	0
2562	843.9	1.1358	1.263	1.0806	1.1528	0
2563	843.8	1.1363	1.2633	1.0816	1.1533	0
2564	843.7	1.1363	1.263	1.0804	1.1533	0
2565	843.6	1.1363	1.2632	1.0805	1.1532	0
2566	843.5	1.1367	1.2629	1.0804	1.1539	0

2567	843.4	1.1368	1.2632	1.0798	1.1538	0
2568	843.3	1.1366	1.2633	1.0805	1.1538	0
2569	843.2	1.1366	1.2633	1.0804	1.1541	0
2570	843.1	1.137	1.2635	1.0798	1.1542	0
2571	843	1.1372	1.2631	1.0802	1.1542	0
2572	842.9	1.1374	1.2636	1.0797	1.1544	0
2573	842.8	1.1376	1.2633	1.0793	1.1544	0
2574	842.7	1.1373	1.2637	1.0799	1.1547	0
2575	842.6	1.137	1.2629	1.0792	1.1545	0
2576	842.5	1.1374	1.2636	1.079	1.1547	0
2577	842.4	1.1375	1.2637	1.0794	1.1549	0
2578	842.3	1.1374	1.2638	1.0782	1.1548	0
2579	842.2	1.1376	1.2635	1.0784	1.1546	0
2580	842.1	1.1376	1.2635	1.0785	1.1549	0
2581	842	1.1375	1.2641	1.0787	1.1549	0
2582	841.9	1.1376	1.2641	1.0795	1.1548	0
2583	841.8	1.1381	1.2641	1.0793	1.1551	0
2584	841.7	1.138	1.264	1.0792	1.1548	0
2585	841.6	1.1378	1.2637	1.0785	1.1548	0
2586	841.5	1.1379	1.2638	1.0789	1.1551	0
2587	841.4	1.1378	1.2636	1.079	1.1552	0
2588	841.3	1.1377	1.2638	1.0782	1.1551	0
2589	841.2	1.1381	1.2641	1.0788	1.1552	0
2590	841.1	1.1379	1.2637	1.0789	1.1553	0
2591	841	1.1383	1.2639	1.0784	1.1557	0
2592	840.9	1.1386	1.2638	1.0785	1.1554	0
2593	840.8	1.1383	1.264	1.0779	1.1558	0
2594	840.7	1.1383	1.2639	1.078	1.1557	0
2595	840.6	1.1383	1.2639	1.0779	1.1557	0
2596	840.5	1.1383	1.2641	1.0781	1.1557	0
2597	840.4	1.1383	1.2631	1.0772	1.1558	0
2598	840.3	1.1385	1.2638	1.0782	1.1555	0
2599	840.2	1.1383	1.2639	1.0778	1.1557	0
2600	840.1	1.1381	1.2638	1.0772	1.1557	0
2601	840	1.1383	1.2642	1.0783	1.1555	0
2602	839.9	1.1384	1.2643	1.0782	1.1557	0
2603	839.8	1.1386	1.264	1.0778	1.1558	0
2604	839.7	1.1385	1.2644	1.078	1.1562	0
2605	839.6	1.1385	1.2641	1.0787	1.1557	0
2606	839.5	1.1386	1.2641	1.078	1.156	0
2607	839.4	1.1388	1.2642	1.0784	1.1561	0
2608	839.3	1.1384	1.2643	1.0793	1.1557	0
2609	839.2	1.1386	1.2644	1.0789	1.1558	0

2610	839.1	1.1387	1.2648	1.0792	1.1559	0
2611	839	1.1387	1.2648	1.0792	1.1559	0
2612	838.9	1.1388	1.2652	1.0789	1.156	0
2613	838.8	1.1389	1.2652	1.0799	1.1561	0
2614	838.7	1.1389	1.265	1.0788	1.1561	0
2615	838.6	1.1386	1.265	1.0795	1.1561	0
2616	838.5	1.1388	1.2652	1.08	1.156	0
2617	838.4	1.139	1.2651	1.0798	1.156	0
2618	838.3	1.1388	1.2654	1.0802	1.156	0
2619	838.2	1.1388	1.2652	1.0802	1.156	0
2620	838.1	1.1388	1.2655	1.0802	1.1558	0
2621	838	1.1389	1.2652	1.0811	1.1558	0
2622	837.9	1.1389	1.2653	1.0816	1.1559	0
2623	837.8	1.1391	1.2657	1.0808	1.1561	0
2624	837.7	1.1389	1.2655	1.0809	1.1563	0
2625	837.6	1.139	1.2648	1.0804	1.156	0
2626	837.5	1.1388	1.2654	1.0811	1.156	0
2627	837.4	1.139	1.2653	1.0807	1.1562	0
2628	837.3	1.1389	1.2652	1.0814	1.1563	0
2629	837.2	1.1387	1.2654	1.0815	1.1563	0
2630	837.1	1.139	1.2656	1.0808	1.156	0
2631	837	1.1389	1.2654	1.0812	1.1563	0
2632	836.9	1.1392	1.2655	1.0807	1.1563	0
2633	836.8	1.1389	1.2654	1.0812	1.1563	0
2634	836.7	1.139	1.2655	1.082	1.1564	0
2635	836.6	1.1394	1.2655	1.0812	1.1566	0
2636	836.5	1.1393	1.2658	1.0813	1.1567	0
2637	836.4	1.1391	1.2656	1.0807	1.1565	0
2638	836.3	1.1391	1.2656	1.0816	1.1563	0
2639	836.2	1.1392	1.2651	1.0816	1.1564	0
2640	836.1	1.1393	1.2655	1.082	1.1567	0
2641	836	1.1394	1.2658	1.0814	1.157	0
2642	835.9	1.1395	1.2659	1.0817	1.1569	0
2643	835.8	1.1394	1.2659	1.0807	1.1568	0
2644	835.7	1.1396	1.2655	1.0806	1.1568	0
2645	835.6	1.1397	1.2659	1.0808	1.1569	0
2646	835.5	1.1394	1.2663	1.0813	1.1572	0
2647	835.4	1.1396	1.2659	1.0812	1.157	0
2648	835.3	1.1397	1.266	1.0808	1.1571	0
2649	835.2	1.1396	1.2659	1.0804	1.1572	0
2650	835.1	1.1397	1.266	1.081	1.1571	0
2651	835	1.1401	1.2657	1.0807	1.1573	0
2652	834.9	1.1397	1.2659	1.0811	1.1573	0

2653	834.8	1.1398	1.2663	1.0803	1.1572	0
2654	834.7	1.1401	1.2659	1.08	1.1573	0
2655	834.6	1.1406	1.2661	1.0801	1.1575	0
2656	834.5	1.1403	1.2661	1.0797	1.1577	0
2657	834.4	1.1405	1.2657	1.0802	1.1576	0
2658	834.3	1.1406	1.2667	1.0796	1.1578	0
2659	834.2	1.1405	1.2662	1.0797	1.1579	0
2660	834.1	1.1406	1.266	1.0796	1.1578	0
2661	834	1.1408	1.2665	1.0794	1.1582	0
2662	833.9	1.1409	1.2663	1.0793	1.1585	0
2663	833.8	1.1412	1.266	1.0792	1.1585	0
2664	833.7	1.1409	1.2661	1.0793	1.1588	0
2665	833.6	1.1415	1.2663	1.079	1.1591	0
2666	833.5	1.1411	1.2661	1.0794	1.1589	0
2667	833.4	1.1415	1.2662	1.0786	1.159	0
2668	833.3	1.1415	1.266	1.0785	1.1593	0
2669	833.2	1.1418	1.2662	1.0784	1.1593	0
2670	833.1	1.1418	1.2665	1.0783	1.1594	0
2671	833	1.1416	1.2662	1.0783	1.1596	0
2672	832.9	1.142	1.2666	1.0774	1.1593	0
2673	832.8	1.1421	1.2665	1.0774	1.1597	0
2674	832.7	1.1423	1.2664	1.0772	1.1594	0
2675	832.6	1.1422	1.2665	1.0773	1.1598	0
2676	832.5	1.1424	1.2668	1.0773	1.1598	0
2677	832.4	1.1425	1.2665	1.077	1.16	0
2678	832.3	1.1424	1.2664	1.077	1.1602	0
2679	832.2	1.1428	1.2666	1.0772	1.1602	0
2680	832.1	1.1424	1.2668	1.0763	1.1601	0
2681	832	1.1426	1.2665	1.0767	1.1603	0
2682	831.9	1.1427	1.2664	1.0768	1.1605	0
2683	831.8	1.143	1.2665	1.0762	1.1604	0
2684	831.7	1.143	1.2666	1.0764	1.1605	0
2685	831.6	1.1431	1.2668	1.0763	1.1607	0
2686	831.5	1.143	1.2666	1.0768	1.1604	0
2687	831.4	1.143	1.2664	1.0763	1.1606	0
2688	831.3	1.1429	1.2667	1.0765	1.1607	0
2689	831.2	1.1434	1.267	1.0766	1.1609	0
2690	831.1	1.143	1.2662	1.0766	1.1605	0
2691	831	1.1431	1.2668	1.0765	1.161	0
2692	830.9	1.1432	1.2669	1.0765	1.1611	0
2693	830.8	1.1432	1.2661	1.0759	1.1609	0
2694	830.7	1.1431	1.2665	1.0755	1.161	0
2695	830.6	1.1433	1.2662	1.0752	1.1606	0

2696	830.5	1.1433	1.2664	1.0756	1.1608	0
2697	830.4	1.1436	1.2669	1.0755	1.1611	0
2698	830.3	1.1436	1.2664	1.0756	1.1611	0
2699	830.2	1.1435	1.2668	1.0754	1.1609	0
2700	830.1	1.1436	1.2669	1.0757	1.1613	0
2701	830	1.1432	1.2667	1.0754	1.1611	0
2702	829.9	1.1434	1.2664	1.0759	1.1611	0
2703	829.8	1.1434	1.2668	1.0759	1.1615	0
2704	829.7	1.1436	1.267	1.076	1.1615	0
2705	829.6	1.1435	1.267	1.0759	1.1612	0
2706	829.5	1.1436	1.2668	1.0751	1.1613	0
2707	829.4	1.1439	1.2669	1.0759	1.1612	0
2708	829.3	1.1433	1.2666	1.0759	1.1612	0
2709	829.2	1.1433	1.2666	1.0755	1.1612	0
2710	829.1	1.1435	1.2669	1.0748	1.1612	0
2711	829	1.1437	1.2668	1.0752	1.1612	0
2712	828.9	1.1437	1.267	1.0754	1.1614	0
2713	828.8	1.1435	1.2667	1.0752	1.1614	0
2714	828.7	1.1436	1.2668	1.0759	1.1613	0
2715	828.6	1.1437	1.2669	1.076	1.1616	0
2716	828.5	1.1437	1.2674	1.0768	1.1615	0
2717	828.4	1.1435	1.2674	1.0764	1.1616	0
2718	828.3	1.1435	1.2674	1.0762	1.1614	0
2719	828.2	1.1438	1.2675	1.0754	1.1614	0
2720	828.1	1.1437	1.2674	1.0755	1.1614	0
2721	828	1.1437	1.2671	1.0768	1.1616	0
2722	827.9	1.1439	1.2676	1.0768	1.1614	0
2723	827.8	1.144	1.2676	1.077	1.1614	0
2724	827.7	1.1437	1.2672	1.0764	1.1613	0
2725	827.6	1.1437	1.2675	1.0758	1.1616	0
2726	827.5	1.1438	1.2673	1.0767	1.1616	0
2727	827.4	1.1438	1.2678	1.0762	1.1616	0
2728	827.3	1.1442	1.268	1.0771	1.1618	0
2729	827.2	1.1441	1.2676	1.077	1.1613	0
2730	827.1	1.1439	1.2674	1.0777	1.1615	0
2731	827	1.1439	1.2676	1.0775	1.1613	0
2732	826.9	1.1441	1.2678	1.0763	1.1613	0
2733	826.8	1.1438	1.2682	1.0766	1.162	0
2734	826.7	1.1442	1.2681	1.077	1.162	0
2735	826.6	1.1444	1.2681	1.0772	1.1623	0
2736	826.5	1.1442	1.2681	1.0777	1.1618	0
2737	826.4	1.1443	1.2679	1.0784	1.1617	0
2738	826.3	1.144	1.2677	1.0779	1.1618	0

2739	826.2	1.1443	1.2681	1.0779	1.1617	0
2740	826.1	1.1447	1.268	1.0771	1.1618	0
2741	826	1.1445	1.268	1.0772	1.1619	0
2742	825.9	1.1449	1.2685	1.0771	1.1623	0
2743	825.8	1.1444	1.2684	1.0777	1.1622	0
2744	825.7	1.1442	1.2679	1.0773	1.1618	0
2745	825.6	1.1445	1.2682	1.0776	1.1619	0
2746	825.5	1.1446	1.268	1.0766	1.1621	0
2747	825.4	1.1446	1.2682	1.0761	1.1622	0
2748	825.3	1.1447	1.2683	1.0759	1.1623	0
2749	825.2	1.1447	1.2679	1.0768	1.1622	0
2750	825.1	1.1448	1.2683	1.0767	1.1626	0
2751	825	1.1449	1.2682	1.0772	1.1627	0
2752	824.9	1.1449	1.2684	1.077	1.1627	0
2753	824.8	1.1451	1.2686	1.0765	1.1627	0
2754	824.7	1.1454	1.2683	1.0764	1.1627	0
2755	824.6	1.1453	1.2689	1.0762	1.1628	0
2756	824.5	1.1452	1.2684	1.076	1.163	0
2757	824.4	1.1455	1.2686	1.0759	1.163	0
2758	824.3	1.1454	1.2687	1.0765	1.1633	0
2759	824.2	1.1453	1.2686	1.0772	1.1631	0
2760	824.1	1.1455	1.2685	1.0762	1.163	0
2761	824	1.1457	1.2687	1.0764	1.1634	0
2762	823.9	1.1459	1.2686	1.0758	1.1636	0
2763	823.8	1.1459	1.2686	1.0757	1.1634	0
2764	823.7	1.146	1.2684	1.0751	1.1635	0
2765	823.6	1.146	1.2684	1.0743	1.1635	0
2766	823.5	1.1462	1.2686	1.0755	1.164	0
2767	823.4	1.1463	1.2689	1.0764	1.1638	0
2768	823.3	1.1466	1.2689	1.0766	1.1643	0
2769	823.2	1.1465	1.2687	1.0754	1.1642	0
2770	823.1	1.1464	1.2686	1.0749	1.1641	0
2771	823	1.1465	1.2689	1.0743	1.164	0
2772	822.9	1.1466	1.2685	1.0748	1.1641	0
2773	822.8	1.1468	1.2687	1.0749	1.1643	0
2774	822.7	1.1468	1.2684	1.0742	1.1645	0
2775	822.6	1.1466	1.2692	1.0746	1.1645	0
2776	822.5	1.147	1.2691	1.0741	1.1647	0
2777	822.4	1.1468	1.2691	1.0744	1.1647	0
2778	822.3	1.1469	1.2692	1.0747	1.1646	0
2779	822.2	1.1469	1.2694	1.0739	1.1646	0
2780	822.1	1.1471	1.2693	1.0732	1.165	0
2781	822	1.1473	1.269	1.0731	1.1648	0

2782	821.9	1.1471	1.2694	1.0738	1.165	0
2783	821.8	1.1474	1.2698	1.0739	1.1649	0
2784	821.7	1.1476	1.2695	1.0738	1.1648	0
2785	821.6	1.1476	1.2695	1.0737	1.1648	0
2786	821.5	1.1478	1.2696	1.0741	1.1654	0
2787	821.4	1.1478	1.2698	1.0738	1.1656	0
2788	821.3	1.1477	1.2694	1.0743	1.1655	0
2789	821.2	1.1478	1.2697	1.0744	1.1654	0
2790	821.1	1.1476	1.2699	1.074	1.1658	0
2791	821	1.1479	1.2697	1.0745	1.1655	0
2792	820.9	1.1476	1.2693	1.0741	1.1656	0
2793	820.8	1.148	1.2696	1.0737	1.1659	0
2794	820.7	1.1481	1.2698	1.0741	1.1659	0
2795	820.6	1.1482	1.2697	1.0737	1.166	0
2796	820.5	1.148	1.2696	1.0738	1.1658	0
2797	820.4	1.1478	1.2697	1.073	1.1658	0
2798	820.3	1.1482	1.2696	1.0734	1.1658	0
2799	820.2	1.1482	1.2699	1.074	1.1656	0
2800	820.1	1.148	1.2698	1.0736	1.166	0
2801	820	1.1484	1.27	1.075	1.1662	0
2802	819.9	1.1483	1.2699	1.0732	1.1657	0
2803	819.8	1.1483	1.2696	1.0731	1.1661	0
2804	819.7	1.1483	1.2699	1.0731	1.1663	0
2805	819.6	1.1485	1.2702	1.0738	1.1663	0
2806	819.5	1.1487	1.2698	1.0737	1.1665	0
2807	819.4	1.1487	1.2706	1.0738	1.1665	0
2808	819.3	1.1488	1.2707	1.074	1.1663	0
2809	819.2	1.1486	1.2706	1.0744	1.1663	0
2810	819.1	1.1485	1.2706	1.075	1.1665	0
2811	819	1.1489	1.2704	1.0747	1.1664	0
2812	818.9	1.1488	1.2703	1.0734	1.1666	0
2813	818.8	1.1489	1.2707	1.0743	1.1665	0
2814	818.7	1.1489	1.2708	1.0742	1.1664	0
2815	818.6	1.149	1.2707	1.0744	1.1665	0
2816	818.5	1.149	1.2712	1.0753	1.1667	0
2817	818.4	1.149	1.271	1.0748	1.1669	0
2818	818.3	1.1491	1.2712	1.0742	1.1668	0
2819	818.2	1.1489	1.2713	1.0734	1.1665	0
2820	818.1	1.1489	1.2709	1.0736	1.1666	0
2821	818	1.1489	1.2711	1.0745	1.1671	0
2822	817.9	1.149	1.2715	1.0744	1.1669	0
2823	817.8	1.149	1.2712	1.0741	1.1671	0
2824	817.7	1.149	1.2711	1.0743	1.1671	0

2825	817.6	1.1494	1.2715	1.0743	1.1669	0
2826	817.5	1.1493	1.2714	1.0748	1.167	0
2827	817.4	1.1494	1.2717	1.0744	1.1673	0
2828	817.3	1.1497	1.2718	1.075	1.1672	0
2829	817.2	1.1495	1.2718	1.075	1.1674	0
2830	817.1	1.1494	1.2714	1.0743	1.1671	0
2831	817	1.1495	1.2712	1.075	1.1671	0
2832	816.9	1.1494	1.2718	1.0741	1.1673	0
2833	816.8	1.1496	1.2718	1.0738	1.1675	0
2834	816.7	1.1498	1.2719	1.0741	1.1674	0
2835	816.6	1.1496	1.2723	1.0742	1.1673	0
2836	816.5	1.1498	1.2719	1.0745	1.1672	0
2837	816.4	1.1496	1.2721	1.0747	1.1674	0
2838	816.3	1.1499	1.2719	1.0751	1.1677	0
2839	816.2	1.15	1.2718	1.0746	1.1675	0
2840	816.1	1.1503	1.2723	1.0747	1.1675	0
2841	816	1.15	1.272	1.0745	1.1677	0
2842	815.9	1.15	1.2721	1.0742	1.1681	0
2843	815.8	1.15	1.272	1.0749	1.1682	0
2844	815.7	1.1505	1.2719	1.0742	1.1679	0
2845	815.6	1.1502	1.2722	1.0757	1.168	0
2846	815.5	1.15	1.272	1.0756	1.1679	0
2847	815.4	1.1504	1.2727	1.075	1.1679	0
2848	815.3	1.1502	1.2721	1.0747	1.168	0
2849	815.2	1.1504	1.2723	1.0742	1.1682	0
2850	815.1	1.1506	1.2722	1.0739	1.1682	0
2851	815	1.1503	1.2722	1.0732	1.1679	0
2852	814.9	1.1505	1.2726	1.0744	1.1681	0
2853	814.8	1.1506	1.2725	1.074	1.1682	0
2854	814.7	1.1506	1.2723	1.0738	1.1683	0
2855	814.6	1.1506	1.2723	1.0739	1.1684	0
2856	814.5	1.1506	1.272	1.0734	1.1686	0
2857	814.4	1.1505	1.2721	1.0738	1.1685	0
2858	814.3	1.1507	1.2726	1.0745	1.1685	0
2859	814.2	1.1509	1.2722	1.0732	1.1687	0
2860	814.1	1.1508	1.2725	1.0732	1.1688	0
2861	814	1.1509	1.2723	1.0734	1.1686	0
2862	813.9	1.1512	1.2727	1.0731	1.169	0
2863	813.8	1.1512	1.2731	1.0731	1.1693	0
2864	813.7	1.1513	1.2734	1.0732	1.1694	0
2865	813.6	1.1513	1.2729	1.0738	1.1695	0
2866	813.5	1.1516	1.2727	1.0739	1.1695	0
2867	813.4	1.1518	1.2731	1.0731	1.1697	0

2868	813.3	1.1518	1.2728	1.074	1.1696	0
2869	813.2	1.152	1.273	1.0735	1.1697	0
2870	813.1	1.1518	1.2729	1.0735	1.1699	0
2871	813	1.1518	1.2729	1.0732	1.1697	0
2872	812.9	1.1519	1.2729	1.0726	1.17	0
2873	812.8	1.152	1.2728	1.0726	1.1695	0
2874	812.7	1.1523	1.2728	1.0727	1.1702	0
2875	812.6	1.1522	1.273	1.0724	1.1701	0
2876	812.5	1.1524	1.2729	1.0726	1.1701	0
2877	812.4	1.1523	1.2728	1.073	1.1702	0
2878	812.3	1.1525	1.273	1.0724	1.1702	0
2879	812.2	1.1522	1.2725	1.0728	1.1701	0
2880	812.1	1.1527	1.2728	1.0727	1.1703	0
2881	812	1.1527	1.2733	1.0723	1.1706	0
2882	811.9	1.153	1.2733	1.0719	1.1706	0
2883	811.8	1.1528	1.2731	1.0719	1.1703	0
2884	811.7	1.1526	1.2729	1.0722	1.1706	0
2885	811.6	1.153	1.2732	1.0726	1.1708	0
2886	811.5	1.1535	1.2737	1.0719	1.1709	0
2887	811.4	1.1532	1.2736	1.0713	1.1709	0
2888	811.3	1.1532	1.2738	1.0711	1.171	0
2889	811.2	1.1532	1.2737	1.0721	1.1712	0
2890	811.1	1.1533	1.2738	1.0715	1.1714	0
2891	811	1.1534	1.2736	1.0722	1.1714	0
2892	810.9	1.1534	1.2743	1.0719	1.1714	0
2893	810.8	1.1533	1.2739	1.072	1.1713	0
2894	810.7	1.1536	1.2739	1.0717	1.1714	0
2895	810.6	1.1536	1.2739	1.0715	1.1716	0
2896	810.5	1.1536	1.2741	1.0717	1.1718	0
2897	810.4	1.1535	1.274	1.0711	1.1715	0
2898	810.3	1.1534	1.2737	1.071	1.1715	0
2899	810.2	1.1538	1.2738	1.0716	1.1718	0
2900	810.1	1.154	1.2739	1.0716	1.1718	0
2901	810	1.1539	1.2738	1.0727	1.1717	0
2902	809.9	1.1534	1.2739	1.072	1.1714	0
2903	809.8	1.1537	1.2742	1.0731	1.1717	0
2904	809.7	1.1542	1.2738	1.0731	1.1716	0
2905	809.6	1.1539	1.2739	1.0722	1.1717	0
2906	809.5	1.1542	1.2742	1.0722	1.172	0
2907	809.4	1.1541	1.2743	1.0732	1.1719	0
2908	809.3	1.1543	1.2742	1.0732	1.1718	0
2909	809.2	1.1543	1.2744	1.0723	1.1718	0
2910	809.1	1.154	1.2744	1.0729	1.1721	0

2911	809	1.1542	1.2742	1.0724	1.1723	0
2912	808.9	1.1545	1.2742	1.0721	1.1722	0
2913	808.8	1.1544	1.2745	1.0717	1.1725	0
2914	808.7	1.1543	1.2743	1.0718	1.1724	0
2915	808.6	1.1544	1.2744	1.0724	1.1723	0
2916	808.5	1.1544	1.2748	1.0729	1.1727	0
2917	808.4	1.1545	1.2738	1.0721	1.1724	0
2918	808.3	1.1544	1.2743	1.0721	1.1725	0
2919	808.2	1.1546	1.275	1.0718	1.1727	0
2920	808.1	1.1549	1.2748	1.0721	1.1726	0
2921	808	1.1548	1.2749	1.0724	1.1728	0
2922	807.9	1.1547	1.2745	1.0722	1.173	0
2923	807.8	1.1546	1.2743	1.0719	1.1724	0
2924	807.7	1.1547	1.2746	1.0719	1.1725	0
2925	807.6	1.1549	1.2749	1.0715	1.173	0
2926	807.5	1.1548	1.2747	1.0716	1.1729	0
2927	807.4	1.1549	1.2755	1.0723	1.1729	0
2928	807.3	1.155	1.2748	1.0721	1.173	0
2929	807.2	1.1552	1.275	1.0722	1.1733	0
2930	807.1	1.1552	1.2749	1.0721	1.1735	0
2931	807	1.1552	1.2751	1.0721	1.1732	0
2932	806.9	1.1555	1.275	1.0725	1.1731	0
2933	806.8	1.1552	1.2753	1.072	1.1734	0
2934	806.7	1.1553	1.2756	1.0719	1.1733	0
2935	806.6	1.1555	1.2756	1.0706	1.1735	0
2936	806.5	1.1556	1.2751	1.0716	1.1736	0
2937	806.4	1.1556	1.2756	1.0715	1.1736	0
2938	806.3	1.1558	1.2751	1.0717	1.1736	0
2939	806.2	1.1557	1.2756	1.0713	1.1737	0
2940	806.1	1.1555	1.2758	1.0709	1.1739	0
2941	806	1.1557	1.2753	1.0709	1.1739	0
2942	805.9	1.1557	1.2754	1.0714	1.1738	0
2943	805.8	1.1558	1.2758	1.0711	1.1738	0
2944	805.7	1.1558	1.2755	1.0704	1.174	0
2945	805.6	1.1559	1.2758	1.0709	1.1739	0
2946	805.5	1.156	1.2754	1.0714	1.1741	0
2947	805.4	1.1562	1.2758	1.0714	1.1743	0
2948	805.3	1.1563	1.2758	1.0717	1.1744	0
2949	805.2	1.1562	1.2757	1.0717	1.1748	0
2950	805.1	1.1568	1.2759	1.071	1.1747	0
2951	805	1.1567	1.2757	1.0716	1.1748	0
2952	804.9	1.157	1.276	1.0711	1.1747	0
2953	804.8	1.157	1.2757	1.0714	1.1747	0

2954	804.7	1.157	1.2761	1.0716	1.1751	0
2955	804.6	1.1572	1.2755	1.0704	1.1749	0
2956	804.5	1.1571	1.2761	1.0705	1.1748	0
2957	804.4	1.1574	1.2755	1.07	1.1754	0
2958	804.3	1.1574	1.2758	1.0707	1.1755	0
2959	804.2	1.1575	1.276	1.0704	1.1754	0
2960	804.1	1.1577	1.2759	1.0695	1.1754	0
2961	804	1.1575	1.2763	1.07	1.1756	0
2962	803.9	1.1578	1.2763	1.0693	1.1758	0
2963	803.8	1.1577	1.2764	1.0689	1.1759	0
2964	803.7	1.1577	1.2763	1.0696	1.1759	0
2965	803.6	1.158	1.2763	1.0689	1.1762	0
2966	803.5	1.1579	1.2767	1.07	1.1761	0
2967	803.4	1.1578	1.2764	1.0692	1.1763	0
2968	803.3	1.158	1.2762	1.069	1.176	0
2969	803.2	1.1584	1.2762	1.0692	1.1762	0
2970	803.1	1.1581	1.2763	1.0695	1.1763	0
2971	803	1.1584	1.2766	1.0683	1.1765	0
2972	802.9	1.1584	1.2768	1.068	1.1765	0
2973	802.8	1.1587	1.2766	1.0679	1.1769	0
2974	802.7	1.1583	1.2766	1.0683	1.1765	0
2975	802.6	1.1586	1.2769	1.0687	1.1768	0
2976	802.5	1.1589	1.277	1.0692	1.1772	0
2977	802.4	1.1586	1.2767	1.0677	1.177	0
2978	802.3	1.1589	1.2769	1.0686	1.1769	0
2979	802.2	1.159	1.2771	1.069	1.177	0
2980	802.1	1.1591	1.2772	1.069	1.1771	0
2981	802	1.1589	1.277	1.0674	1.1773	0
2982	801.9	1.1592	1.2776	1.0693	1.1775	0
2983	801.8	1.1591	1.2773	1.0687	1.177	0
2984	801.7	1.1592	1.2772	1.0683	1.1772	0
2985	801.6	1.1592	1.2766	1.0689	1.1774	0
2986	801.5	1.1594	1.2775	1.0681	1.1773	0
2987	801.4	1.1594	1.277	1.0685	1.1773	0
2988	801.3	1.1595	1.2773	1.0692	1.1776	0
2989	801.2	1.1595	1.277	1.0687	1.1778	0
2990	801.1	1.1596	1.2778	1.0692	1.1779	0
2991	801	1.1597	1.2777	1.0696	1.1778	0
2992	800.9	1.1594	1.2775	1.0696	1.1779	0
2993	800.8	1.1597	1.2773	1.069	1.1776	0
2994	800.7	1.1598	1.2774	1.0693	1.1777	0
2995	800.6	1.1595	1.2776	1.069	1.178	0
2996	800.5	1.1596	1.2771	1.07	1.1777	0

2997	800.4	1.1602	1.278	1.0696	1.1781	0
2998	800.3	1.1601	1.2781	1.0695	1.1782	0
2999	800.2	1.1597	1.2776	1.0685	1.1778	0
3000	800.1	1.1599	1.2778	1.0692	1.1782	0
3001	800	1.1598	1.2776	1.0695	1.1781	0
3002	799.9	1.16	1.2778	1.0695	1.178	0
3003	799.8	1.1601	1.2776	1.0692	1.1781	0
3004	799.7	1.1603	1.278	1.0693	1.1783	0
3005	799.6	1.1603	1.2778	1.0696	1.178	0
3006	799.5	1.1603	1.2782	1.07	1.1785	0
3007	799.4	1.1606	1.2782	1.0703	1.1786	0
3008	799.3	1.1601	1.2784	1.0701	1.1784	0
3009	799.2	1.1605	1.2781	1.0703	1.1785	0
3010	799.1	1.1603	1.278	1.0701	1.1785	0
3011	799	1.1605	1.2787	1.0703	1.1784	0
3012	798.9	1.1603	1.2782	1.0698	1.1783	0
3013	798.8	1.1602	1.278	1.0699	1.1782	0
3014	798.7	1.1602	1.2782	1.0693	1.1784	0
3015	798.6	1.1604	1.2782	1.0697	1.1786	0
3016	798.5	1.1603	1.2783	1.0702	1.1785	0
3017	798.4	1.1605	1.2782	1.0706	1.1785	0
3018	798.3	1.1604	1.2781	1.0708	1.1784	0
3019	798.2	1.1603	1.2785	1.0707	1.1787	0
3020	798.1	1.1606	1.2785	1.0704	1.1788	0
3021	798	1.1606	1.2787	1.0702	1.1788	0
3022	797.9	1.1605	1.2782	1.0698	1.1788	0
3023	797.8	1.1605	1.2782	1.0706	1.1789	0
3024	797.7	1.1607	1.2786	1.0707	1.1789	0
3025	797.6	1.1607	1.2783	1.071	1.179	0
3026	797.5	1.1607	1.2785	1.0702	1.179	0
3027	797.4	1.1607	1.2788	1.0717	1.1791	0
3028	797.3	1.1606	1.2791	1.0704	1.1791	0
3029	797.2	1.1612	1.2792	1.0703	1.1793	0
3030	797.1	1.1613	1.279	1.071	1.1794	0
3031	797	1.1609	1.2787	1.0706	1.1794	0
3032	796.9	1.1611	1.2794	1.0702	1.1794	0
3033	796.8	1.1613	1.2794	1.0706	1.1795	0
3034	796.7	1.1613	1.2788	1.0711	1.1798	0
3035	796.6	1.1614	1.2791	1.0701	1.1797	0
3036	796.5	1.1615	1.2797	1.071	1.1796	0
3037	796.4	1.1614	1.2793	1.0705	1.1799	0
3038	796.3	1.1618	1.2794	1.0705	1.18	0
3039	796.2	1.1617	1.2795	1.0711	1.1802	0

3040	796.1	1.162	1.2793	1.0708	1.1802	0
3041	796	1.1622	1.2793	1.0708	1.1803	0
3042	795.9	1.1624	1.2797	1.0701	1.1802	0
3043	795.8	1.162	1.2798	1.0698	1.1804	0
3044	795.7	1.1622	1.2796	1.07	1.1805	0
3045	795.6	1.1627	1.2798	1.0711	1.1809	0
3046	795.5	1.1625	1.28	1.0707	1.1809	0
3047	795.4	1.1625	1.2795	1.0699	1.181	0
3048	795.3	1.1627	1.2799	1.0699	1.1812	0
3049	795.2	1.1629	1.2801	1.0701	1.1813	0
3050	795.1	1.1629	1.28	1.0695	1.1815	0
3051	795	1.1632	1.28	1.0695	1.1818	0
3052	794.9	1.1632	1.2803	1.0696	1.1816	0
3053	794.8	1.163	1.2805	1.0701	1.1816	0
3054	794.7	1.1635	1.2802	1.0699	1.1818	0
3055	794.6	1.1636	1.2805	1.0688	1.1818	0
3056	794.5	1.1634	1.2802	1.069	1.1819	0
3057	794.4	1.1638	1.2802	1.0689	1.182	0
3058	794.3	1.1639	1.28	1.0688	1.1821	0
3059	794.2	1.1639	1.28	1.0687	1.1823	0
3060	794.1	1.1638	1.2805	1.068	1.1823	0
3061	794	1.1641	1.2805	1.0684	1.1822	0
3062	793.9	1.164	1.2804	1.0682	1.1826	0
3063	793.8	1.1643	1.2799	1.0675	1.1826	0
3064	793.7	1.1642	1.2807	1.0675	1.1827	0
3065	793.6	1.1643	1.2807	1.0673	1.1828	0
3066	793.5	1.1645	1.2807	1.0682	1.183	0
3067	793.4	1.1647	1.2804	1.0677	1.1831	0
3068	793.3	1.1647	1.2806	1.0679	1.183	0
3069	793.2	1.165	1.2809	1.067	1.1833	0
3070	793.1	1.165	1.2809	1.0677	1.1833	0
3071	793	1.165	1.2805	1.0671	1.1835	0
3072	792.9	1.1649	1.2807	1.0671	1.1836	0
3073	792.8	1.1654	1.2807	1.0673	1.1837	0
3074	792.7	1.1654	1.2804	1.0668	1.1839	0
3075	792.6	1.1655	1.2807	1.0666	1.1836	0
3076	792.5	1.1652	1.2805	1.0672	1.1836	0
3077	792.4	1.1654	1.2807	1.0675	1.1841	0
3078	792.3	1.1657	1.281	1.0673	1.1843	0
3079	792.2	1.1654	1.2811	1.067	1.1842	0
3080	792.1	1.1659	1.2811	1.0673	1.1843	0
3081	792	1.1656	1.2815	1.0668	1.1843	0
3082	791.9	1.1659	1.2811	1.0666	1.1845	0

3083	791.8	1.1659	1.2813	1.0671	1.1846	0
3084	791.7	1.1661	1.281	1.0668	1.1845	0
3085	791.6	1.166	1.2814	1.0668	1.1844	0
3086	791.5	1.166	1.2812	1.0672	1.1848	0
3087	791.4	1.1662	1.2814	1.0673	1.1846	0
3088	791.3	1.1663	1.2811	1.0667	1.1847	0
3089	791.2	1.1662	1.2811	1.0674	1.1846	0
3090	791.1	1.1661	1.2813	1.0667	1.1848	0
3091	791	1.166	1.2813	1.0661	1.1844	0
3092	790.9	1.1664	1.2817	1.0669	1.1848	0
3093	790.8	1.1666	1.2811	1.0677	1.1849	0
3094	790.7	1.1665	1.2815	1.0668	1.1849	0
3095	790.6	1.1665	1.2817	1.0671	1.185	0
3096	790.5	1.1664	1.2815	1.0671	1.1851	0
3097	790.4	1.1666	1.2812	1.0676	1.1851	0
3098	790.3	1.1665	1.2819	1.0667	1.1852	0
3099	790.2	1.1668	1.2816	1.067	1.1851	0
3100	790.1	1.1669	1.2815	1.0675	1.1853	0
3101	790	1.1667	1.2819	1.0674	1.185	0
3102	789.9	1.167	1.2822	1.0665	1.1857	0
3103	789.8	1.1667	1.2822	1.0675	1.1854	0
3104	789.7	1.167	1.2823	1.067	1.1858	0
3105	789.6	1.167	1.2826	1.0678	1.1855	0
3106	789.5	1.1669	1.2824	1.0682	1.1856	0
3107	789.4	1.1668	1.2825	1.0682	1.1853	0
3108	789.3	1.1668	1.2826	1.068	1.1856	0
3109	789.2	1.1668	1.2824	1.0679	1.1855	0
3110	789.1	1.1673	1.2825	1.0679	1.1856	0
3111	789	1.1669	1.2819	1.0687	1.1853	0
3112	788.9	1.1669	1.2824	1.0682	1.1856	0
3113	788.8	1.1668	1.2823	1.0677	1.1853	0
3114	788.7	1.1667	1.2826	1.0676	1.1854	0
3115	788.6	1.1668	1.2825	1.0683	1.1856	0
3116	788.5	1.1671	1.2825	1.0677	1.1856	0
3117	788.4	1.1669	1.2829	1.0684	1.1858	0
3118	788.3	1.1669	1.2829	1.0691	1.1857	0
3119	788.2	1.167	1.2829	1.069	1.1858	0
3120	788.1	1.167	1.2829	1.0689	1.1858	0
3121	788	1.1671	1.2835	1.0691	1.1859	0
3122	787.9	1.1673	1.2832	1.0686	1.186	0
3123	787.8	1.1675	1.2834	1.0688	1.1859	0
3124	787.7	1.167	1.2831	1.0683	1.1861	0
3125	787.6	1.167	1.283	1.0682	1.1858	0

3126	787.5	1.1671	1.2826	1.0688	1.1859	0
3127	787.4	1.1672	1.2828	1.0691	1.1861	0
3128	787.3	1.1674	1.2833	1.0689	1.1861	0
3129	787.2	1.1675	1.2835	1.0681	1.1864	0
3130	787.1	1.1678	1.2825	1.0684	1.1861	0
3131	787	1.1676	1.2834	1.0689	1.1861	0
3132	786.9	1.1677	1.2836	1.0696	1.1864	0
3133	786.8	1.1677	1.2837	1.0693	1.1869	0
3134	786.7	1.1681	1.2836	1.0697	1.1868	0
3135	786.6	1.1676	1.2832	1.0694	1.1865	0
3136	786.5	1.168	1.2839	1.0696	1.1868	0
3137	786.4	1.1679	1.2835	1.0688	1.1868	0
3138	786.3	1.168	1.2835	1.0693	1.1869	0
3139	786.2	1.1683	1.2841	1.0695	1.187	0
3140	786.1	1.1683	1.2842	1.0685	1.1869	0
3141	786	1.1681	1.2838	1.0697	1.1872	0
3142	785.9	1.1682	1.2838	1.0692	1.1872	0
3143	785.8	1.1684	1.284	1.0697	1.1871	0
3144	785.7	1.1683	1.2841	1.0689	1.1868	0
3145	785.6	1.1684	1.2839	1.0695	1.1873	0
3146	785.5	1.1684	1.2839	1.0692	1.1874	0
3147	785.4	1.1687	1.2845	1.0692	1.1877	0
3148	785.3	1.1687	1.2841	1.0693	1.1874	0
3149	785.2	1.169	1.2844	1.0692	1.1875	0
3150	785.1	1.1691	1.2845	1.0693	1.188	0
3151	785	1.169	1.2842	1.0685	1.188	0
3152	784.9	1.1692	1.2844	1.0687	1.1882	0
3153	784.8	1.1693	1.2843	1.0683	1.1882	0
3154	784.7	1.1694	1.2847	1.0671	1.1885	0
3155	784.6	1.1693	1.2846	1.0678	1.1887	0
3156	784.5	1.1694	1.2845	1.0664	1.1886	0
3157	784.4	1.1696	1.2844	1.0671	1.1888	0
3158	784.3	1.17	1.2847	1.0662	1.1891	0
3159	784.2	1.17	1.2842	1.0664	1.189	0
3160	784.1	1.1703	1.2843	1.0655	1.1893	0
3161	784	1.1705	1.2845	1.0661	1.1895	0
3162	783.9	1.1706	1.285	1.0656	1.1896	0
3163	783.8	1.1707	1.285	1.066	1.1896	0
3164	783.7	1.1707	1.2845	1.0654	1.1898	0
3165	783.6	1.171	1.2848	1.0655	1.1899	0
3166	783.5	1.1708	1.2843	1.066	1.1898	0
3167	783.4	1.1712	1.2848	1.0657	1.1904	0
3168	783.3	1.1712	1.2849	1.0655	1.1903	0

3169	783.2	1.1714	1.2854	1.0641	1.1904	0
3170	783.1	1.1714	1.2854	1.0649	1.1903	0
3171	783	1.1714	1.2845	1.0651	1.1903	0
3172	782.9	1.1718	1.2852	1.0649	1.1907	0
3173	782.8	1.1718	1.2851	1.0641	1.1909	0
3174	782.7	1.1718	1.2849	1.0646	1.1909	0
3175	782.6	1.1719	1.2853	1.0643	1.191	0
3176	782.5	1.1722	1.2854	1.0644	1.1915	0
3177	782.4	1.1723	1.2854	1.0646	1.1912	0
3178	782.3	1.1727	1.2854	1.064	1.1913	0
3179	782.2	1.1724	1.2858	1.0645	1.1913	0
3180	782.1	1.1727	1.2858	1.0646	1.1916	0
3181	782	1.1726	1.2859	1.0647	1.1916	0
3182	781.9	1.1725	1.2859	1.065	1.1917	0
3183	781.8	1.1725	1.2857	1.0647	1.1916	0
3184	781.7	1.1726	1.2859	1.0652	1.1914	0
3185	781.6	1.1725	1.286	1.065	1.1917	0
3186	781.5	1.1725	1.286	1.0647	1.1919	0
3187	781.4	1.1726	1.2861	1.0642	1.1916	0
3188	781.3	1.1728	1.286	1.0644	1.1918	0
3189	781.2	1.1728	1.2862	1.064	1.1922	0
3190	781.1	1.1728	1.2859	1.0644	1.1922	0
3191	781	1.1733	1.286	1.0641	1.1921	0
3192	780.9	1.1732	1.2865	1.064	1.1923	0
3193	780.8	1.1733	1.2861	1.0646	1.1925	0
3194	780.7	1.1734	1.2868	1.0654	1.1924	0
3195	780.6	1.1732	1.2867	1.0652	1.1926	0
3196	780.5	1.1734	1.2868	1.0653	1.1925	0
3197	780.4	1.1737	1.2863	1.065	1.1925	0
3198	780.3	1.1736	1.2865	1.065	1.1928	0
3199	780.2	1.1736	1.2865	1.0655	1.1926	0
3200	780.1	1.1736	1.2871	1.0652	1.1927	0
3201	780	1.1737	1.2867	1.065	1.1928	0
3202	779.9	1.1739	1.2869	1.0651	1.193	0
3203	779.8	1.1739	1.2871	1.065	1.193	0
3204	779.7	1.1742	1.2872	1.0649	1.1931	0
3205	779.6	1.1739	1.2869	1.0649	1.1929	0
3206	779.5	1.174	1.2872	1.0647	1.1928	0
3207	779.4	1.1738	1.2873	1.0645	1.1929	0
3208	779.3	1.1737	1.2872	1.0653	1.193	0
3209	779.2	1.1742	1.2871	1.0659	1.1927	0
3210	779.1	1.1739	1.2874	1.0663	1.193	0
3211	779	1.1741	1.2875	1.0658	1.193	0

3212	778.9	1.174	1.2873	1.0654	1.1928	0
3213	778.8	1.1741	1.2875	1.0658	1.193	0
3214	778.7	1.174	1.2875	1.0648	1.193	0
3215	778.6	1.1739	1.2876	1.0655	1.193	0
3216	778.5	1.1737	1.2878	1.0663	1.1929	0
3217	778.4	1.174	1.2877	1.0659	1.1929	0
3218	778.3	1.1742	1.2881	1.0655	1.193	0
3219	778.2	1.1744	1.2879	1.0655	1.1931	0
3220	778.1	1.1742	1.2875	1.066	1.1928	0
3221	778	1.1744	1.2883	1.0662	1.1929	0
3222	777.9	1.1743	1.2879	1.0658	1.193	0
3223	777.8	1.1746	1.2884	1.0667	1.1931	0
3224	777.7	1.1745	1.2885	1.0669	1.193	0
3225	777.6	1.1744	1.2883	1.066	1.1931	0
3226	777.5	1.1745	1.2883	1.0668	1.1929	0
3227	777.4	1.1745	1.2886	1.0673	1.193	0
3228	777.3	1.1746	1.2891	1.0675	1.1932	0
3229	777.2	1.1745	1.2889	1.0663	1.1933	0
3230	777.1	1.1746	1.2891	1.0666	1.1931	0
3231	777	1.1745	1.2889	1.0663	1.1931	0
3232	776.9	1.1747	1.2889	1.0674	1.1931	0
3233	776.8	1.1747	1.2893	1.0676	1.1937	0
3234	776.7	1.1748	1.2892	1.0681	1.1934	0
3235	776.6	1.1747	1.289	1.0677	1.1933	0
3236	776.5	1.175	1.2892	1.0678	1.1935	0
3237	776.4	1.175	1.2892	1.0684	1.1936	0
3238	776.3	1.1751	1.2897	1.0687	1.1937	0
3239	776.2	1.175	1.2896	1.0685	1.1938	0
3240	776.1	1.1754	1.2898	1.068	1.1938	0
3241	776	1.1754	1.2898	1.0681	1.194	0
3242	775.9	1.1756	1.2893	1.0678	1.1938	0
3243	775.8	1.1751	1.29	1.0675	1.194	0
3244	775.7	1.1759	1.2898	1.0681	1.1939	0
3245	775.6	1.1757	1.2899	1.0671	1.1942	0
3246	775.5	1.1758	1.2898	1.0674	1.1942	0
3247	775.4	1.1758	1.2896	1.0678	1.1942	0
3248	775.3	1.1758	1.2897	1.0672	1.1941	0
3249	775.2	1.176	1.2899	1.0682	1.1946	0
3250	775.1	1.1758	1.2903	1.0672	1.1941	0
3251	775	1.1761	1.2896	1.0666	1.1942	0
3252	774.9	1.1765	1.2903	1.0673	1.1948	0
3253	774.8	1.1766	1.2906	1.067	1.1948	0
3254	774.7	1.1765	1.2907	1.0666	1.1948	0

3255	774.6	1.1766	1.2909	1.0668	1.1951	0
3256	774.5	1.177	1.2907	1.0672	1.1951	0
3257	774.4	1.177	1.2904	1.0665	1.1951	0
3258	774.3	1.1769	1.2908	1.0666	1.1952	0
3259	774.2	1.1774	1.2906	1.0665	1.1955	0
3260	774.1	1.1774	1.2905	1.066	1.1953	0
3261	774	1.1773	1.2913	1.0652	1.1956	0
3262	773.9	1.1773	1.2908	1.0652	1.1956	0
3263	773.8	1.1776	1.291	1.0655	1.1957	0
3264	773.7	1.1777	1.2909	1.0652	1.1958	0
3265	773.6	1.1778	1.291	1.0648	1.1959	0
3266	773.5	1.1777	1.291	1.0651	1.196	0
3267	773.4	1.178	1.2911	1.0653	1.1963	0
3268	773.3	1.1782	1.2912	1.0656	1.1963	0
3269	773.2	1.1783	1.2913	1.0647	1.1965	0
3270	773.1	1.1783	1.291	1.0648	1.1964	0
3271	773	1.1785	1.2916	1.0649	1.1963	0
3272	772.9	1.1787	1.2917	1.0644	1.1966	0
3273	772.8	1.1788	1.2912	1.0643	1.1967	0
3274	772.7	1.179	1.2919	1.0645	1.1971	0
3275	772.6	1.179	1.2916	1.0638	1.1971	0
3276	772.5	1.1792	1.2916	1.0642	1.1971	0
3277	772.4	1.1793	1.2921	1.0629	1.1976	0
3278	772.3	1.1796	1.2921	1.0638	1.1975	0
3279	772.2	1.1796	1.2921	1.0637	1.1975	0
3280	772.1	1.1797	1.2917	1.0633	1.1975	0
3281	772	1.1796	1.2923	1.063	1.1975	0
3282	771.9	1.1801	1.2921	1.0627	1.1977	0
3283	771.8	1.18	1.2922	1.0622	1.1977	0
3284	771.7	1.1802	1.2928	1.0624	1.1979	0
3285	771.6	1.1801	1.2924	1.0626	1.1979	0
3286	771.5	1.1804	1.2925	1.0625	1.1982	0
3287	771.4	1.1802	1.2927	1.0624	1.1982	0
3288	771.3	1.1803	1.2928	1.0621	1.1981	0
3289	771.2	1.1804	1.2924	1.0623	1.1982	0
3290	771.1	1.1807	1.2927	1.0623	1.1983	0
3291	771	1.1806	1.2928	1.063	1.1983	0
3292	770.9	1.1807	1.2928	1.0625	1.1985	0
3293	770.8	1.1806	1.2927	1.0628	1.1984	0
3294	770.7	1.1809	1.2926	1.0629	1.1987	0
3295	770.6	1.1811	1.2929	1.0628	1.1989	0
3296	770.5	1.1812	1.293	1.0623	1.1984	0
3297	770.4	1.181	1.2927	1.0624	1.1988	0

3298	770.3	1.1812	1.2936	1.0622	1.1986	0
3299	770.2	1.1813	1.293	1.0622	1.1989	0
3300	770.1	1.1813	1.2932	1.0626	1.1987	0
3301	770	1.1814	1.2934	1.0622	1.1989	0
3302	769.9	1.1817	1.2933	1.0631	1.1993	0
3303	769.8	1.1813	1.293	1.0627	1.199	0
3304	769.7	1.1816	1.2932	1.0625	1.1992	0
3305	769.6	1.1817	1.2935	1.0622	1.1991	0
3306	769.5	1.1816	1.2933	1.0627	1.199	0
3307	769.4	1.1816	1.2938	1.0624	1.1992	0
3308	769.3	1.182	1.2935	1.0628	1.1993	0
3309	769.2	1.182	1.2939	1.0626	1.1994	0
3310	769.1	1.1818	1.2937	1.062	1.1992	0
3311	769	1.1817	1.2939	1.0628	1.1994	0
3312	768.9	1.1821	1.2942	1.0627	1.1995	0
3313	768.8	1.1821	1.2948	1.0626	1.1995	0
3314	768.7	1.1823	1.2939	1.063	1.1993	0
3315	768.6	1.1823	1.2943	1.0638	1.1991	0
3316	768.5	1.1822	1.2942	1.0643	1.1994	0
3317	768.4	1.1822	1.2945	1.0638	1.1993	0
3318	768.3	1.1823	1.2944	1.0641	1.1994	0
3319	768.2	1.1823	1.2944	1.0647	1.1995	0
3320	768.1	1.1823	1.2944	1.065	1.1993	0
3321	768	1.1818	1.2947	1.0636	1.1992	0
3322	767.9	1.182	1.2947	1.0642	1.1994	0
3323	767.8	1.1821	1.2943	1.0645	1.1992	0
3324	767.7	1.1825	1.2947	1.0645	1.1996	0
3325	767.6	1.1824	1.2947	1.064	1.1997	0
3326	767.5	1.1823	1.2948	1.0645	1.1994	0
3327	767.4	1.1825	1.2952	1.0648	1.1998	0
3328	767.3	1.1823	1.2947	1.0646	1.1994	0
3329	767.2	1.1825	1.2948	1.0647	1.1996	0
3330	767.1	1.1826	1.2951	1.0657	1.1996	0
3331	767	1.1827	1.2953	1.0654	1.1996	0
3332	766.9	1.1828	1.295	1.0655	1.1998	0
3333	766.8	1.1827	1.2951	1.0656	1.1998	0
3334	766.7	1.1824	1.2953	1.0653	1.2	0
3335	766.6	1.1828	1.2951	1.0655	1.1997	0
3336	766.5	1.1827	1.295	1.0659	1.1998	0
3337	766.4	1.1829	1.295	1.0653	1.1998	0
3338	766.3	1.1829	1.295	1.0645	1.1998	0
3339	766.2	1.183	1.2951	1.0649	1.1997	0
3340	766.1	1.1831	1.2951	1.0651	1.1999	0

3341	766	1.1831	1.2958	1.0657	1.1999	0
3342	765.9	1.1832	1.2955	1.0662	1.2	0
3343	765.8	1.1834	1.2954	1.0657	1.2001	0
3344	765.7	1.1836	1.2958	1.0656	1.2001	0
3345	765.6	1.1835	1.2961	1.0658	1.2004	0
3346	765.5	1.1837	1.2961	1.0657	1.2002	0
3347	765.4	1.1837	1.296	1.0655	1.2002	0
3348	765.3	1.1839	1.296	1.0657	1.2004	0
3349	765.2	1.1839	1.2964	1.0653	1.2008	0
3350	765.1	1.1842	1.2964	1.0648	1.2008	0
3351	765	1.1841	1.2962	1.0655	1.2007	0
3352	764.9	1.1843	1.2959	1.0646	1.2003	0
3353	764.8	1.1841	1.296	1.0651	1.2006	0
3354	764.7	1.1844	1.2964	1.0647	1.2005	0
3355	764.6	1.1847	1.2964	1.0648	1.2012	0
3356	764.5	1.1847	1.2968	1.0647	1.201	0
3357	764.4	1.1849	1.2968	1.0646	1.2012	0
3358	764.3	1.1847	1.2963	1.0642	1.2011	0
3359	764.2	1.185	1.2965	1.0644	1.2012	0
3360	764.1	1.1849	1.2967	1.0634	1.2012	0
3361	764	1.185	1.2972	1.0634	1.2014	0
3362	763.9	1.1851	1.2964	1.064	1.2014	0
3363	763.8	1.1851	1.2962	1.0639	1.2014	0
3364	763.7	1.1853	1.2964	1.0635	1.2016	0
3365	763.6	1.1854	1.2967	1.0631	1.2017	0
3366	763.5	1.1858	1.2969	1.0632	1.2017	0
3367	763.4	1.1857	1.2967	1.0637	1.2021	0
3368	763.3	1.1859	1.2971	1.0638	1.2022	0
3369	763.2	1.186	1.2971	1.064	1.2022	0
3370	763.1	1.1864	1.2969	1.0635	1.2025	0
3371	763	1.1863	1.297	1.0628	1.2024	0
3372	762.9	1.1866	1.2973	1.0639	1.2025	0
3373	762.8	1.1865	1.2969	1.0632	1.2026	0
3374	762.7	1.1866	1.297	1.0624	1.2027	0
3375	762.6	1.1866	1.2973	1.0627	1.2028	0
3376	762.5	1.1867	1.2973	1.0633	1.203	0
3377	762.4	1.1867	1.2972	1.0623	1.2027	0
3378	762.3	1.187	1.2971	1.0623	1.2029	0
3379	762.2	1.1869	1.2974	1.0625	1.203	0
3380	762.1	1.187	1.2973	1.0628	1.2031	0
3381	762	1.1871	1.2975	1.0621	1.2033	0
3382	761.9	1.1871	1.2977	1.0624	1.2029	0
3383	761.8	1.1875	1.2975	1.0624	1.2032	0

3384	761.7	1.1875	1.2979	1.0621	1.203	0
3385	761.6	1.1876	1.2976	1.0616	1.2033	0
3386	761.5	1.1876	1.2975	1.0619	1.2035	0
3387	761.4	1.1878	1.298	1.0615	1.2037	0
3388	761.3	1.1881	1.2978	1.0616	1.2034	0
3389	761.2	1.188	1.2979	1.0622	1.2036	0
3390	761.1	1.1881	1.2982	1.0623	1.2038	0
3391	761	1.1881	1.2982	1.0625	1.2036	0
3392	760.9	1.188	1.298	1.0624	1.2035	0
3393	760.8	1.188	1.298	1.0618	1.2035	0
3394	760.7	1.1884	1.2982	1.0627	1.2037	0
3395	760.6	1.1883	1.2983	1.0624	1.204	0
3396	760.5	1.1883	1.2984	1.0624	1.2041	0
3397	760.4	1.1884	1.2983	1.0626	1.2039	0
3398	760.3	1.1886	1.2985	1.0624	1.2041	0
3399	760.2	1.1887	1.2986	1.0619	1.2042	0
3400	760.1	1.1887	1.2985	1.0619	1.204	0
3401	760	1.1891	1.2989	1.0623	1.2045	0
3402	759.9	1.189	1.299	1.0623	1.2046	0
3403	759.8	1.1891	1.2986	1.0617	1.2042	0
3404	759.7	1.1891	1.2989	1.0621	1.2044	0
3405	759.6	1.1891	1.2988	1.0624	1.2047	0
3406	759.5	1.1897	1.299	1.0618	1.2048	0
3407	759.4	1.1892	1.2995	1.062	1.2046	0
3408	759.3	1.1896	1.2993	1.0625	1.2045	0
3409	759.2	1.1894	1.2992	1.062	1.2048	0
3410	759.1	1.1894	1.2996	1.0619	1.2047	0
3411	759	1.1896	1.2996	1.0624	1.2047	0
3412	758.9	1.1896	1.2995	1.0623	1.2048	0
3413	758.8	1.1898	1.2997	1.0628	1.2049	0
3414	758.7	1.1898	1.2997	1.0632	1.2049	0
3415	758.6	1.1901	1.2994	1.0631	1.205	0
3416	758.5	1.1898	1.2996	1.0625	1.2047	0
3417	758.4	1.1899	1.2997	1.0637	1.2049	0
3418	758.3	1.1897	1.2999	1.0639	1.2051	0
3419	758.2	1.1901	1.3001	1.0626	1.2052	0
3420	758.1	1.1902	1.3002	1.0638	1.205	0
3421	758	1.1903	1.3003	1.0639	1.2051	0
3422	757.9	1.1903	1.2998	1.0637	1.2052	0
3423	757.8	1.1906	1.3001	1.0633	1.2054	0
3424	757.7	1.1906	1.3003	1.0636	1.2053	0
3425	757.6	1.1905	1.3004	1.0633	1.2054	0
3426	757.5	1.1905	1.3002	1.0634	1.2052	0

3427	757.4	1.1907	1.3004	1.064	1.2054	0
3428	757.3	1.1906	1.3006	1.0637	1.2056	0
3429	757.2	1.1906	1.2999	1.0633	1.2056	0
3430	757.1	1.1908	1.3006	1.0635	1.2054	0
3431	757	1.1908	1.3008	1.0643	1.2055	0
3432	756.9	1.191	1.3008	1.0642	1.2056	0
3433	756.8	1.191	1.3008	1.065	1.2055	0
3434	756.7	1.1911	1.3009	1.0645	1.2056	0
3435	756.6	1.191	1.3011	1.064	1.206	0
3436	756.5	1.1912	1.3007	1.0639	1.2057	0
3437	756.4	1.1913	1.301	1.0641	1.2054	0
3438	756.3	1.1914	1.3008	1.0639	1.206	0
3439	756.2	1.1915	1.3012	1.0631	1.2062	0
3440	756.1	1.1915	1.3015	1.0645	1.2061	0
3441	756	1.1915	1.3015	1.0637	1.2063	0
3442	755.9	1.1917	1.3011	1.0633	1.2064	0
3443	755.8	1.1918	1.3016	1.0638	1.2063	0
3444	755.7	1.1918	1.3013	1.0634	1.2066	0
3445	755.6	1.192	1.3013	1.0636	1.2066	0
3446	755.5	1.1921	1.3014	1.0633	1.2066	0
3447	755.4	1.1918	1.3015	1.0628	1.2065	0
3448	755.3	1.192	1.3014	1.0632	1.2065	0
3449	755.2	1.1924	1.3014	1.0621	1.2065	0
3450	755.1	1.1926	1.3015	1.0631	1.2067	0
3451	755	1.1925	1.3015	1.0636	1.2064	0
3452	754.9	1.1925	1.3017	1.0631	1.2067	0
3453	754.8	1.1925	1.3015	1.0637	1.2068	0
3454	754.7	1.1928	1.3017	1.064	1.2069	0
3455	754.6	1.1931	1.3018	1.0637	1.2072	0
3456	754.5	1.1933	1.3018	1.0635	1.2073	0
3457	754.4	1.1932	1.3019	1.0634	1.2072	0
3458	754.3	1.1932	1.3019	1.064	1.2073	0
3459	754.2	1.1933	1.302	1.0637	1.2074	0
3460	754.1	1.1935	1.302	1.064	1.2075	0
3461	754	1.1936	1.3022	1.0636	1.2077	0
3462	753.9	1.1937	1.302	1.0636	1.2077	0
3463	753.8	1.194	1.3024	1.0633	1.2079	0
3464	753.7	1.194	1.3022	1.0637	1.2079	0
3465	753.6	1.194	1.3021	1.0632	1.2079	0
3466	753.5	1.1942	1.3026	1.0632	1.2083	0
3467	753.4	1.1942	1.3025	1.0627	1.2079	0
3468	753.3	1.1943	1.3024	1.062	1.208	0
3469	753.2	1.1944	1.3027	1.0623	1.2081	0

3470	753.1	1.1943	1.3027	1.0624	1.2083	0
3471	753	1.1945	1.3028	1.062	1.2084	0
3472	752.9	1.1946	1.3022	1.0621	1.2083	0
3473	752.8	1.1945	1.3023	1.0619	1.2082	0
3474	752.7	1.1948	1.3022	1.0628	1.2085	0
3475	752.6	1.1952	1.3031	1.0625	1.2086	0
3476	752.5	1.1951	1.3029	1.0631	1.209	0
3477	752.4	1.1951	1.3028	1.0631	1.2088	0
3478	752.3	1.1949	1.3026	1.0621	1.2088	0
3479	752.2	1.1951	1.303	1.0618	1.2088	0
3480	752.1	1.1955	1.3032	1.0619	1.2088	0
3481	752	1.1953	1.303	1.0621	1.2088	0
3482	751.9	1.1955	1.3032	1.0619	1.209	0
3483	751.8	1.1954	1.3035	1.0625	1.2091	0
3484	751.7	1.1957	1.3034	1.0625	1.209	0
3485	751.6	1.1958	1.3035	1.0619	1.2091	0
3486	751.5	1.1958	1.3034	1.0616	1.2094	0
3487	751.4	1.1961	1.3035	1.0617	1.2096	0
3488	751.3	1.196	1.3036	1.0613	1.2095	0
3489	751.2	1.196	1.3033	1.0617	1.2096	0
3490	751.1	1.1958	1.3033	1.0625	1.2097	0
3491	751	1.1961	1.3034	1.0617	1.2096	0
3492	750.9	1.1961	1.3036	1.061	1.2097	0
3493	750.8	1.1962	1.3037	1.0616	1.2099	0
3494	750.7	1.1964	1.3041	1.0609	1.2099	0
3495	750.6	1.1965	1.304	1.0622	1.21	0
3496	750.5	1.1968	1.3041	1.0622	1.2099	0
3497	750.4	1.1967	1.3044	1.0619	1.2102	0
3498	750.3	1.1968	1.304	1.0617	1.2103	0
3499	750.2	1.1968	1.3042	1.0624	1.2101	0
3500	750.1	1.1969	1.304	1.0618	1.2104	0
3501	750	1.197	1.3042	1.0623	1.2103	0
3502	749.9	1.197	1.3047	1.0619	1.2102	0
3503	749.8	1.1971	1.3045	1.0622	1.2106	0
3504	749.7	1.1973	1.3047	1.0623	1.2106	0
3505	749.6	1.197	1.3048	1.0618	1.2107	0
3506	749.5	1.1974	1.3049	1.0622	1.2106	0
3507	749.4	1.1973	1.3046	1.0628	1.2104	0
3508	749.3	1.1974	1.3047	1.062	1.2109	0
3509	749.2	1.1976	1.3052	1.0617	1.2108	0
3510	749.1	1.1975	1.3051	1.062	1.2108	0
3511	749	1.1975	1.305	1.0621	1.2106	0
3512	748.9	1.1977	1.305	1.0624	1.2108	0

3513	748.8	1.1979	1.3052	1.0618	1.2108	0
3514	748.7	1.1982	1.3055	1.0632	1.2112	0
3515	748.6	1.1981	1.3054	1.0631	1.2114	0
3516	748.5	1.1982	1.3057	1.0632	1.2111	0
3517	748.4	1.1985	1.3058	1.0632	1.2114	0
3518	748.3	1.1983	1.3056	1.0632	1.2113	0
3519	748.2	1.1984	1.3052	1.0631	1.2116	0
3520	748.1	1.1986	1.3059	1.0627	1.2116	0
3521	748	1.1988	1.3056	1.0627	1.2117	0
3522	747.9	1.199	1.3058	1.0631	1.2116	0
3523	747.8	1.1986	1.3058	1.0622	1.2115	0
3524	747.7	1.1991	1.3057	1.0638	1.2117	0
3525	747.6	1.1991	1.3059	1.0636	1.2115	0
3526	747.5	1.1991	1.3055	1.0637	1.2117	0
3527	747.4	1.1992	1.3061	1.0638	1.2121	0
3528	747.3	1.1991	1.3059	1.0636	1.2119	0
3529	747.2	1.1994	1.3061	1.0625	1.2118	0
3530	747.1	1.1993	1.3065	1.0629	1.2117	0
3531	747	1.1993	1.3065	1.0635	1.2119	0
3532	746.9	1.1995	1.3066	1.0633	1.2122	0
3533	746.8	1.1999	1.3069	1.0632	1.2123	0
3534	746.7	1.1998	1.3068	1.0633	1.2122	0
3535	746.6	1.1998	1.3067	1.0627	1.2122	0
3536	746.5	1.1999	1.3066	1.0625	1.2125	0
3537	746.4	1.2002	1.3069	1.0626	1.2124	0
3538	746.3	1.1997	1.3063	1.0623	1.2123	0
3539	746.2	1.2002	1.3069	1.0625	1.2126	0
3540	746.1	1.2007	1.307	1.0618	1.2129	0
3541	746	1.2006	1.3073	1.0626	1.213	0
3542	745.9	1.2008	1.3075	1.0625	1.213	0
3543	745.8	1.2011	1.3076	1.0625	1.2131	0
3544	745.7	1.2012	1.3073	1.0622	1.2131	0
3545	745.6	1.2016	1.3074	1.0626	1.2131	0
3546	745.5	1.2016	1.3073	1.0623	1.2135	0
3547	745.4	1.2018	1.3077	1.0621	1.2135	0
3548	745.3	1.2021	1.3074	1.0623	1.2133	0
3549	745.2	1.2024	1.3072	1.0621	1.2136	0
3550	745.1	1.2026	1.3073	1.0614	1.2138	0
3551	745	1.203	1.3078	1.0616	1.2137	0
3552	744.9	1.2036	1.3078	1.0622	1.2139	0
3553	744.8	1.2036	1.3078	1.0625	1.2137	0
3554	744.7	1.2038	1.3077	1.0619	1.214	0
3555	744.6	1.204	1.3078	1.0622	1.2145	0

3556	744.5	1.2044	1.308	1.0624	1.2142	0
3557	744.4	1.2045	1.3079	1.0617	1.2143	0
3558	744.3	1.2047	1.3079	1.0612	1.2141	0
3559	744.2	1.2051	1.3081	1.0612	1.2145	0
3560	744.1	1.205	1.3081	1.0616	1.2149	0
3561	744	1.2054	1.3082	1.0611	1.2148	0
3562	743.9	1.2056	1.3085	1.0616	1.2147	0
3563	743.8	1.2057	1.3084	1.061	1.2151	0
3564	743.7	1.2056	1.3082	1.0614	1.2151	0
3565	743.6	1.2059	1.3085	1.0609	1.2152	0
3566	743.5	1.2062	1.3088	1.0611	1.2153	0
3567	743.4	1.2066	1.3087	1.0605	1.2154	0
3568	743.3	1.2066	1.3086	1.061	1.2155	0
3569	743.2	1.2068	1.3087	1.062	1.2154	0
3570	743.1	1.207	1.3089	1.0608	1.2156	0
3571	743	1.2072	1.3085	1.0601	1.216	0
3572	742.9	1.2074	1.3091	1.0603	1.2157	0
3573	742.8	1.2077	1.3088	1.0603	1.216	0
3574	742.7	1.2078	1.3089	1.0604	1.216	0
3575	742.6	1.2081	1.3091	1.0606	1.2159	0
3576	742.5	1.2081	1.3092	1.0611	1.2164	0
3577	742.4	1.2084	1.3094	1.061	1.2164	0
3578	742.3	1.2084	1.3095	1.0613	1.216	0
3579	742.2	1.2084	1.3094	1.0605	1.2162	0
3580	742.1	1.2087	1.3095	1.061	1.2161	0
3581	742	1.2089	1.3093	1.0607	1.2163	0
3582	741.9	1.2088	1.3093	1.0607	1.2162	0
3583	741.8	1.2092	1.3096	1.0604	1.2164	0
3584	741.7	1.2094	1.3096	1.0611	1.2166	0
3585	741.6	1.2094	1.3096	1.0608	1.2167	0
3586	741.5	1.2096	1.3098	1.061	1.2165	0
3587	741.4	1.2098	1.3101	1.0604	1.2165	0
3588	741.3	1.21	1.3102	1.0609	1.2167	0
3589	741.2	1.2101	1.3104	1.0604	1.2168	0
3590	741.1	1.2101	1.3102	1.061	1.2168	0
3591	741	1.2103	1.3102	1.0609	1.217	0
3592	740.9	1.2107	1.3106	1.0613	1.2169	0
3593	740.8	1.2107	1.3108	1.0609	1.217	0
3594	740.7	1.2107	1.3108	1.0611	1.2172	0
3595	740.6	1.2111	1.3107	1.0609	1.2172	0
3596	740.5	1.2113	1.3111	1.0607	1.2172	0
3597	740.4	1.2118	1.311	1.0608	1.2173	0
3598	740.3	1.2121	1.311	1.0605	1.2171	0

3599	740.2	1.2123	1.311	1.0606	1.217	0
3600	740.1	1.2128	1.3108	1.061	1.2173	0
3601	740	1.2129	1.3109	1.0608	1.2172	0
3602	739.9	1.2131	1.3111	1.0609	1.2173	0
3603	739.8	1.2131	1.3109	1.0614	1.2173	0
3604	739.7	1.2136	1.3115	1.0615	1.2174	0
3605	739.6	1.2138	1.3113	1.0622	1.2175	0
3606	739.5	1.2138	1.3111	1.0622	1.2173	0
3607	739.4	1.214	1.3115	1.0617	1.2175	0
3608	739.3	1.2141	1.3111	1.0615	1.2174	0
3609	739.2	1.2143	1.3118	1.062	1.2174	0
3610	739.1	1.2144	1.3119	1.0629	1.2175	0
3611	739	1.2144	1.312	1.0629	1.2175	0
3612	738.9	1.2145	1.3122	1.062	1.2174	0
3613	738.8	1.2147	1.3122	1.0621	1.2174	0
3614	738.7	1.2151	1.312	1.0622	1.2179	0
3615	738.6	1.2149	1.312	1.0623	1.2177	0
3616	738.5	1.2154	1.3128	1.0626	1.218	0
3617	738.4	1.2154	1.3124	1.0633	1.2178	0
3618	738.3	1.2155	1.3125	1.0635	1.2177	0
3619	738.2	1.2155	1.3123	1.0628	1.2177	0
3620	738.1	1.2157	1.3126	1.0626	1.2179	0
3621	738	1.216	1.3128	1.0631	1.218	0
3622	737.9	1.2162	1.3127	1.0631	1.2182	0
3623	737.8	1.2164	1.3129	1.0641	1.2182	0
3624	737.7	1.2165	1.3125	1.0637	1.218	0
3625	737.6	1.2165	1.313	1.0624	1.2182	0
3626	737.5	1.2168	1.3131	1.0632	1.2185	0
3627	737.4	1.2171	1.3135	1.0633	1.2183	0
3628	737.3	1.217	1.3132	1.0631	1.2181	0
3629	737.2	1.2173	1.3133	1.0627	1.2184	0
3630	737.1	1.2179	1.3135	1.0631	1.2186	0
3631	737	1.2177	1.3133	1.063	1.2184	0
3632	736.9	1.2183	1.3131	1.0638	1.2188	0
3633	736.8	1.2185	1.3137	1.0634	1.219	0
3634	736.7	1.2186	1.3134	1.0622	1.2189	0
3635	736.6	1.2189	1.3141	1.0635	1.2192	0
3636	736.5	1.2191	1.3136	1.0635	1.2188	0
3637	736.4	1.2194	1.3141	1.0628	1.2191	0
3638	736.3	1.2197	1.314	1.0633	1.2192	0
3639	736.2	1.2198	1.3139	1.0628	1.2193	0
3640	736.1	1.2202	1.3139	1.063	1.2193	0
3641	736	1.2204	1.3142	1.0628	1.2196	0

3642	735.9	1.2207	1.3139	1.0632	1.2195	0
3643	735.8	1.2208	1.314	1.0624	1.2194	0
3644	735.7	1.2215	1.3141	1.0621	1.2197	0
3645	735.6	1.2219	1.314	1.0623	1.2198	0
3646	735.5	1.222	1.3143	1.0625	1.2196	0
3647	735.4	1.2225	1.3143	1.0625	1.2199	0
3648	735.3	1.2232	1.3147	1.0616	1.2201	0
3649	735.2	1.2237	1.3148	1.061	1.2202	0
3650	735.1	1.2241	1.3146	1.0621	1.2203	0
3651	735	1.2244	1.3148	1.062	1.2203	0
3652	734.9	1.2249	1.3145	1.0609	1.2204	0
3653	734.8	1.2253	1.3147	1.0605	1.2204	0
3654	734.7	1.2259	1.3146	1.0605	1.2205	0
3655	734.6	1.2263	1.3146	1.0615	1.2206	0
3656	734.5	1.2268	1.315	1.0613	1.2207	0
3657	734.4	1.2275	1.315	1.061	1.2211	0
3658	734.3	1.2279	1.3148	1.0601	1.221	0
3659	734.2	1.2279	1.3148	1.0607	1.2207	0
3660	734.1	1.2284	1.3149	1.0605	1.2209	0
3661	734	1.229	1.3154	1.0601	1.2211	0
3662	733.9	1.2295	1.3151	1.0605	1.2214	0
3663	733.8	1.2295	1.3153	1.0598	1.2211	0
3664	733.7	1.2302	1.3156	1.0602	1.2214	0
3665	733.6	1.2303	1.3154	1.0595	1.2216	0
3666	733.5	1.2308	1.3155	1.0598	1.2215	0
3667	733.4	1.2313	1.3159	1.0597	1.2219	0
3668	733.3	1.2321	1.3161	1.0599	1.2219	0
3669	733.2	1.2327	1.316	1.0595	1.2221	0
3670	733.1	1.2332	1.3156	1.0599	1.2219	0
3671	733	1.234	1.3161	1.0591	1.2222	0
3672	732.9	1.235	1.3161	1.0595	1.2224	0
3673	732.8	1.2359	1.3162	1.0595	1.2224	0
3674	732.7	1.2372	1.3166	1.0593	1.2224	0
3675	732.6	1.2384	1.3166	1.0594	1.2224	0
3676	732.5	1.2397	1.3168	1.0594	1.2228	0
3677	732.4	1.2402	1.3168	1.0592	1.2228	0
3678	732.3	1.2408	1.3168	1.0596	1.2228	0
3679	732.2	1.2413	1.3168	1.0589	1.2226	0
3680	732.1	1.2421	1.3168	1.0594	1.2227	0
3681	732	1.2427	1.3167	1.0588	1.2228	0
3682	731.9	1.2438	1.317	1.0593	1.2227	0
3683	731.8	1.2442	1.3169	1.0594	1.2228	0
3684	731.7	1.2444	1.317	1.0595	1.223	0

3685	731.6	1.2449	1.3168	1.0589	1.2231	0
3686	731.5	1.2451	1.3173	1.0593	1.223	0
3687	731.4	1.2455	1.3175	1.0588	1.223	0
3688	731.3	1.2459	1.3176	1.0589	1.2232	0
3689	731.2	1.2462	1.3176	1.0589	1.2229	0
3690	731.1	1.2462	1.3172	1.059	1.2231	0
3691	731	1.2468	1.3175	1.059	1.2232	0
3692	730.9	1.2469	1.3176	1.0595	1.2235	0
3693	730.8	1.2471	1.3174	1.0595	1.2234	0
3694	730.7	1.2474	1.3175	1.0598	1.2234	0
3695	730.6	1.2478	1.3178	1.0598	1.2235	0
3696	730.5	1.2479	1.3177	1.0597	1.2236	0
3697	730.4	1.248	1.3176	1.0597	1.2234	0
3698	730.3	1.2481	1.3179	1.0597	1.2234	0
3699	730.2	1.2484	1.318	1.0592	1.2235	0
3700	730.1	1.2487	1.3184	1.0587	1.2237	0
3701	730	1.2488	1.318	1.0595	1.2237	0
3702	729.9	1.2489	1.318	1.0603	1.2235	0
3703	729.8	1.2488	1.3177	1.0601	1.2236	0
3704	729.7	1.2491	1.318	1.0603	1.2237	0
3705	729.6	1.2494	1.3183	1.0602	1.2235	0
3706	729.5	1.2497	1.3187	1.0603	1.2236	0
3707	729.4	1.2496	1.3184	1.0598	1.2237	0
3708	729.3	1.2499	1.3187	1.0597	1.2236	0
3709	729.2	1.2503	1.3185	1.0594	1.2236	0
3710	729.1	1.2504	1.3188	1.06	1.2237	0
3711	729	1.2506	1.3186	1.0598	1.2239	0
3712	728.9	1.2504	1.3188	1.0605	1.2237	0
3713	728.8	1.2508	1.3187	1.0605	1.2238	0
3714	728.7	1.2507	1.3187	1.0609	1.2236	0
3715	728.6	1.2514	1.3189	1.0612	1.2238	0
3716	728.5	1.2514	1.3189	1.0613	1.2238	0
3717	728.4	1.2518	1.3189	1.0617	1.2238	0
3718	728.3	1.252	1.3189	1.0615	1.2237	0
3719	728.2	1.2527	1.3195	1.0618	1.2238	0
3720	728.1	1.2531	1.3198	1.0618	1.2239	0
3721	728	1.2532	1.3197	1.0619	1.224	0
3722	727.9	1.2536	1.3195	1.0622	1.2238	0
3723	727.8	1.2538	1.3193	1.0616	1.224	0
3724	727.7	1.2541	1.3191	1.0623	1.2239	0
3725	727.6	1.2544	1.3197	1.0623	1.224	0
3726	727.5	1.2546	1.3197	1.0622	1.2239	0
3727	727.4	1.2546	1.3201	1.0624	1.224	0

3728	727.3	1.2548	1.3198	1.0625	1.224	0
3729	727.2	1.2547	1.3198	1.0625	1.2241	0
3730	727.1	1.2554	1.3197	1.0634	1.2242	0
3731	727	1.2555	1.3203	1.0624	1.2244	0
3732	726.9	1.2555	1.3199	1.0628	1.2242	0
3733	726.8	1.2558	1.3206	1.0631	1.2245	0
3734	726.7	1.256	1.3202	1.0625	1.2245	0
3735	726.6	1.2563	1.3201	1.0633	1.2246	0
3736	726.5	1.2562	1.3201	1.0623	1.2242	0
3737	726.4	1.2565	1.3202	1.0628	1.2243	0
3738	726.3	1.2567	1.3202	1.0622	1.2249	0
3739	726.2	1.2567	1.3202	1.0611	1.2243	0
3740	726.1	1.2571	1.3204	1.0618	1.2247	0
3741	726	1.2574	1.3206	1.0619	1.2247	0
3742	725.9	1.2575	1.3207	1.0622	1.2246	0
3743	725.8	1.2577	1.3211	1.062	1.2249	0
3744	725.7	1.2579	1.3207	1.0624	1.2246	0
3745	725.6	1.2583	1.3206	1.0626	1.2249	0
3746	725.5	1.2585	1.3207	1.0617	1.2248	0
3747	725.4	1.2585	1.3209	1.0619	1.2251	0
3748	725.3	1.2592	1.321	1.0617	1.2251	0
3749	725.2	1.2595	1.3216	1.0611	1.225	0
3750	725.1	1.2604	1.3213	1.0613	1.2256	0
3751	725	1.2607	1.3216	1.0618	1.2257	0
3752	724.9	1.2608	1.3215	1.0615	1.2254	0
3753	724.8	1.261	1.3216	1.0605	1.2258	0
3754	724.7	1.2615	1.3212	1.0613	1.2257	0
3755	724.6	1.2622	1.3214	1.0612	1.2259	0
3756	724.5	1.2625	1.3221	1.0605	1.2261	0
3757	724.4	1.2625	1.3221	1.0602	1.226	0
3758	724.3	1.263	1.3216	1.0608	1.2263	0
3759	724.2	1.2636	1.3222	1.0601	1.2267	0
3760	724.1	1.2637	1.3222	1.0592	1.2266	0
3761	724	1.2637	1.322	1.0595	1.2267	0
3762	723.9	1.2641	1.3218	1.0592	1.2268	0
3763	723.8	1.2645	1.3224	1.0596	1.227	0
3764	723.7	1.2649	1.3221	1.0588	1.227	0
3765	723.6	1.2656	1.3223	1.0597	1.2273	0
3766	723.5	1.2653	1.322	1.0579	1.2271	0
3767	723.4	1.266	1.3222	1.0591	1.2273	0
3768	723.3	1.2662	1.3221	1.0585	1.2276	0
3769	723.2	1.2667	1.3228	1.0574	1.2277	0
3770	723.1	1.2668	1.3226	1.058	1.2276	0

3771	723	1.2672	1.3232	1.0586	1.228	0
3772	722.9	1.2676	1.3226	1.0585	1.228	0
3773	722.8	1.268	1.3228	1.0572	1.2281	0
3774	722.7	1.2679	1.3225	1.0575	1.2278	0
3775	722.6	1.2683	1.3231	1.0578	1.2281	0
3776	722.5	1.2687	1.3231	1.0572	1.2283	0
3777	722.4	1.2689	1.3232	1.0575	1.2282	0
3778	722.3	1.2693	1.3232	1.0571	1.228	0
3779	722.2	1.2695	1.3234	1.0571	1.2284	0
3780	722.1	1.2699	1.3236	1.0589	1.2284	0
3781	722	1.2702	1.3235	1.0577	1.2287	0
3782	721.9	1.2702	1.3238	1.0584	1.2286	0
3783	721.8	1.2704	1.3236	1.0577	1.2288	0
3784	721.7	1.2711	1.3236	1.0576	1.2288	0
3785	721.6	1.2713	1.3241	1.058	1.2289	0
3786	721.5	1.2713	1.3239	1.0571	1.2287	0
3787	721.4	1.2715	1.3239	1.0578	1.2289	0
3788	721.3	1.2717	1.3241	1.058	1.229	0
3789	721.2	1.2722	1.3245	1.0578	1.229	0
3790	721.1	1.2724	1.3242	1.0565	1.229	0
3791	721	1.2726	1.3243	1.0571	1.2291	0
3792	720.9	1.2727	1.3244	1.0575	1.2291	0
3793	720.8	1.2729	1.3246	1.0564	1.2293	0
3794	720.7	1.2732	1.3246	1.0574	1.2292	0
3795	720.6	1.2734	1.3248	1.057	1.2291	0
3796	720.5	1.274	1.3248	1.0572	1.2293	0
3797	720.4	1.274	1.3252	1.0573	1.2296	0
3798	720.3	1.2741	1.3251	1.0574	1.2295	0
3799	720.2	1.2745	1.325	1.0575	1.2297	0
3800	720.1	1.2745	1.3251	1.0574	1.2297	0
3801	720	1.275	1.3252	1.0575	1.2298	0
3802	719.9	1.2748	1.3254	1.0577	1.2296	0
3803	719.8	1.2748	1.3255	1.0575	1.2297	0
3804	719.7	1.2752	1.3254	1.0569	1.2296	0
3805	719.6	1.2756	1.3259	1.0581	1.2297	0
3806	719.5	1.2757	1.3258	1.0581	1.2294	0
3807	719.4	1.2758	1.3259	1.0588	1.2293	0
3808	719.3	1.2766	1.3257	1.0591	1.2297	0
3809	719.2	1.2766	1.326	1.0591	1.2297	0
3810	719.1	1.2768	1.3264	1.0594	1.2297	0
3811	719	1.2769	1.3259	1.0586	1.2296	0
3812	718.9	1.2773	1.3267	1.0583	1.2298	0
3813	718.8	1.2775	1.3267	1.0589	1.23	0

3814	718.7	1.2777	1.3263	1.06	1.23	0
3815	718.6	1.2778	1.3262	1.0603	1.2299	0
3816	718.5	1.2782	1.3265	1.0601	1.2297	0
3817	718.4	1.2782	1.3266	1.0588	1.2299	0
3818	718.3	1.2783	1.3266	1.059	1.2298	0
3819	718.2	1.2784	1.3265	1.059	1.2301	0
3820	718.1	1.2789	1.3272	1.0603	1.2302	0
3821	718	1.2792	1.3269	1.0602	1.2301	0
3822	717.9	1.2794	1.3273	1.0601	1.2301	0
3823	717.8	1.2793	1.3273	1.0611	1.23	0
3824	717.7	1.2794	1.3274	1.0604	1.2303	0
3825	717.6	1.2797	1.3277	1.0602	1.2302	0
3826	717.5	1.2799	1.3275	1.0595	1.2298	0
3827	717.4	1.2798	1.3275	1.0601	1.2299	0
3828	717.3	1.2802	1.3274	1.0595	1.2301	0
3829	717.2	1.2803	1.3276	1.0599	1.2302	0
3830	717.1	1.2806	1.3281	1.0607	1.2301	0
3831	717	1.2806	1.328	1.0606	1.2304	0
3832	716.9	1.2806	1.3281	1.0604	1.2303	0
3833	716.8	1.2809	1.328	1.0605	1.2302	0
3834	716.7	1.2812	1.3283	1.0601	1.2303	0
3835	716.6	1.2814	1.3279	1.0606	1.2303	0
3836	716.5	1.2813	1.3284	1.0601	1.2304	0
3837	716.4	1.2819	1.3284	1.0606	1.2304	0
3838	716.3	1.2819	1.3286	1.0605	1.2305	0
3839	716.2	1.2824	1.3286	1.0604	1.2307	0
3840	716.1	1.2823	1.3289	1.0602	1.2306	0
3841	716	1.2824	1.3288	1.0607	1.2308	0
3842	715.9	1.2825	1.3282	1.0605	1.2307	0
3843	715.8	1.2827	1.3289	1.0604	1.2307	0
3844	715.7	1.2831	1.3293	1.0608	1.2308	0
3845	715.6	1.2833	1.3293	1.0605	1.2309	0
3846	715.5	1.2834	1.3297	1.06	1.2308	0
3847	715.4	1.2838	1.3295	1.0595	1.2311	0
3848	715.3	1.2838	1.3293	1.0588	1.2312	0
3849	715.2	1.2842	1.3295	1.0598	1.2314	0
3850	715.1	1.2845	1.3296	1.0598	1.2313	0
3851	715	1.2847	1.33	1.0602	1.2317	0
3852	714.9	1.2849	1.3301	1.0602	1.2315	0
3853	714.8	1.2853	1.33	1.0593	1.2317	0
3854	714.7	1.2853	1.3305	1.06	1.2319	0
3855	714.6	1.2856	1.331	1.0588	1.2321	0
3856	714.5	1.2861	1.3309	1.0599	1.2322	0

3857	714.4	1.2862	1.3306	1.0589	1.2321	0
3858	714.3	1.2862	1.3306	1.0585	1.2322	0
3859	714.2	1.2868	1.331	1.0591	1.2327	0
3860	714.1	1.2868	1.3308	1.0598	1.2328	0
3861	714	1.2866	1.3306	1.0591	1.2327	0
3862	713.9	1.287	1.331	1.0588	1.2325	0
3863	713.8	1.2869	1.331	1.0597	1.2326	0
3864	713.7	1.2872	1.331	1.0592	1.2329	0
3865	713.6	1.2873	1.3312	1.0589	1.233	0
3866	713.5	1.2878	1.3315	1.0583	1.2333	0
3867	713.4	1.288	1.3313	1.058	1.2336	0
3868	713.3	1.2886	1.3317	1.0579	1.2337	0
3869	713.2	1.2885	1.332	1.0577	1.2337	0
3870	713.1	1.2887	1.3318	1.0573	1.2337	0
3871	713	1.2887	1.332	1.057	1.2337	0
3872	712.9	1.289	1.3319	1.0569	1.2341	0
3873	712.8	1.2892	1.3317	1.0563	1.2342	0
3874	712.7	1.289	1.3319	1.056	1.2341	0
3875	712.6	1.2895	1.332	1.0574	1.2342	0
3876	712.5	1.2893	1.3322	1.0575	1.234	0
3877	712.4	1.2894	1.3324	1.0573	1.2343	0
3878	712.3	1.2897	1.3326	1.0569	1.2344	0
3879	712.2	1.2899	1.332	1.0572	1.2346	0
3880	712.1	1.2901	1.3324	1.0563	1.2347	0
3881	712	1.2901	1.3327	1.0576	1.2348	0
3882	711.9	1.2905	1.3326	1.0574	1.2349	0
3883	711.8	1.2905	1.3328	1.0574	1.2354	0
3884	711.7	1.2906	1.3332	1.0573	1.2351	0
3885	711.6	1.2908	1.3328	1.0569	1.2356	0
3886	711.5	1.2908	1.3329	1.0571	1.2355	0
3887	711.4	1.291	1.3334	1.0571	1.2355	0
3888	711.3	1.2911	1.3334	1.0558	1.2355	0
3889	711.2	1.2913	1.3328	1.0556	1.2354	0
3890	711.1	1.2914	1.3337	1.0557	1.2356	0
3891	711	1.2914	1.3337	1.0566	1.2359	0
3892	710.9	1.2918	1.3336	1.0568	1.2359	0
3893	710.8	1.2921	1.3338	1.0555	1.2358	0
3894	710.7	1.2919	1.3336	1.0552	1.2358	0
3895	710.6	1.2924	1.3339	1.0557	1.2363	0
3896	710.5	1.2925	1.3342	1.0567	1.2361	0
3897	710.4	1.2925	1.3338	1.0562	1.2363	0
3898	710.3	1.293	1.335	1.0571	1.2364	0
3899	710.2	1.2931	1.3349	1.0565	1.2365	0

3900	710.1	1.2932	1.3346	1.0555	1.2368	0
3901	710	1.2934	1.3348	1.056	1.2367	0
3902	709.9	1.2933	1.3347	1.0563	1.2368	0
3903	709.8	1.2937	1.3352	1.0568	1.2367	0
3904	709.7	1.2936	1.335	1.0563	1.2369	0
3905	709.6	1.294	1.3352	1.0565	1.2368	0
3906	709.5	1.2939	1.3354	1.0564	1.237	0
3907	709.4	1.2939	1.3352	1.0565	1.2368	0
3908	709.3	1.2941	1.3354	1.0564	1.237	0
3909	709.2	1.2942	1.3353	1.0567	1.237	0
3910	709.1	1.2944	1.3357	1.0565	1.2372	0
3911	709	1.2946	1.3357	1.0566	1.2372	0
3912	708.9	1.2944	1.3359	1.0562	1.2372	0
3913	708.8	1.2947	1.3357	1.0557	1.2373	0
3914	708.7	1.2948	1.3364	1.0561	1.2371	0
3915	708.6	1.295	1.3362	1.0572	1.2373	0
3916	708.5	1.295	1.3362	1.0568	1.2375	0
3917	708.4	1.2949	1.3363	1.0567	1.2374	0
3918	708.3	1.2951	1.3368	1.0567	1.2374	0
3919	708.2	1.2954	1.3367	1.0574	1.2375	0
3920	708.1	1.2956	1.3369	1.0576	1.2377	0
3921	708	1.2956	1.3369	1.0578	1.2375	0
3922	707.9	1.2958	1.3369	1.0584	1.2376	0
3923	707.8	1.2961	1.3372	1.0585	1.2377	0
3924	707.7	1.2961	1.3374	1.0579	1.2378	0
3925	707.6	1.296	1.3374	1.0584	1.238	0
3926	707.5	1.2963	1.3374	1.0587	1.238	0
3927	707.4	1.2965	1.3375	1.0585	1.2376	0
3928	707.3	1.2968	1.3378	1.0583	1.2378	0
3929	707.2	1.2967	1.3373	1.0586	1.2376	0
3930	707.1	1.297	1.3375	1.0586	1.2379	0
3931	707	1.2971	1.3381	1.0584	1.2381	0
3932	706.9	1.2974	1.338	1.0585	1.238	0
3933	706.8	1.2973	1.338	1.0584	1.2381	0
3934	706.7	1.2977	1.3384	1.0592	1.2384	0
3935	706.6	1.2978	1.3381	1.0589	1.2381	0
3936	706.5	1.2979	1.3382	1.0587	1.2382	0
3937	706.4	1.2981	1.338	1.0594	1.2382	0
3938	706.3	1.2983	1.3385	1.0602	1.2382	0
3939	706.2	1.2984	1.3391	1.0602	1.2383	0
3940	706.1	1.2988	1.3388	1.0589	1.2384	0
3941	706	1.2989	1.3389	1.0592	1.2385	0
3942	705.9	1.2991	1.3389	1.0601	1.2385	0

3943	705.8	1.2991	1.3394	1.0595	1.2387	0
3944	705.7	1.2992	1.3392	1.0595	1.2385	0
3945	705.6	1.2994	1.3395	1.0594	1.2387	0
3946	705.5	1.2995	1.3391	1.0593	1.2388	0
3947	705.4	1.2996	1.3395	1.0588	1.2389	0
3948	705.3	1.2997	1.3396	1.0593	1.2391	0
3949	705.2	1.3001	1.3396	1.0595	1.2391	0
3950	705.1	1.3003	1.34	1.0592	1.2395	0
3951	705	1.3	1.3395	1.0582	1.2392	0
3952	704.9	1.3004	1.3402	1.0584	1.2392	0
3953	704.8	1.3004	1.34	1.0591	1.2396	0
3954	704.7	1.3009	1.34	1.0588	1.2396	0
3955	704.6	1.3011	1.3403	1.0584	1.2398	0
3956	704.5	1.3011	1.3405	1.0586	1.2398	0
3957	704.4	1.3016	1.3405	1.0588	1.2402	0
3958	704.3	1.3014	1.3408	1.0585	1.2398	0
3959	704.2	1.3018	1.3408	1.0582	1.2402	0
3960	704.1	1.3016	1.3407	1.0578	1.24	0
3961	704	1.3019	1.3409	1.0577	1.2402	0
3962	703.9	1.302	1.341	1.0581	1.2407	0
3963	703.8	1.3021	1.341	1.0571	1.2406	0
3964	703.7	1.3022	1.3413	1.0578	1.2405	0
3965	703.6	1.3023	1.3412	1.0577	1.2406	0
3966	703.5	1.3026	1.3411	1.0576	1.2406	0
3967	703.4	1.3029	1.341	1.0574	1.2409	0
3968	703.3	1.3033	1.3416	1.0577	1.2411	0
3969	703.2	1.3031	1.3417	1.0569	1.2412	0
3970	703.1	1.3033	1.3417	1.0568	1.2412	0
3971	703	1.3034	1.3418	1.0566	1.2415	0
3972	702.9	1.3034	1.3418	1.0566	1.2415	0
3973	702.8	1.3036	1.3417	1.0566	1.2416	0
3974	702.7	1.3039	1.3419	1.0571	1.2419	0
3975	702.6	1.3037	1.3422	1.056	1.2415	0
3976	702.5	1.304	1.3418	1.057	1.2416	0
3977	702.4	1.3043	1.3424	1.0567	1.2418	0
3978	702.3	1.3045	1.3424	1.0561	1.2418	0
3979	702.2	1.3044	1.3423	1.056	1.2419	0
3980	702.1	1.3045	1.3421	1.056	1.2421	0
3981	702	1.3049	1.3428	1.0566	1.2423	0
3982	701.9	1.3053	1.3424	1.0562	1.2426	0
3983	701.8	1.3054	1.3427	1.0564	1.2423	0
3984	701.7	1.3055	1.3435	1.0567	1.2426	0
3985	701.6	1.3059	1.3432	1.0561	1.2427	0

3986	701.5	1.3064	1.3435	1.0564	1.2432	0
3987	701.4	1.3063	1.343	1.0562	1.2429	0
3988	701.3	1.3066	1.3434	1.0565	1.2428	0
3989	701.2	1.3074	1.3435	1.0563	1.2432	0
3990	701.1	1.3071	1.3439	1.057	1.2432	0
3991	701	1.3076	1.344	1.0563	1.2432	0
3992	700.9	1.3078	1.3442	1.0558	1.2434	0
3993	700.8	1.3081	1.3441	1.0568	1.2438	0
3994	700.7	1.3079	1.3443	1.0563	1.2436	0
3995	700.6	1.3083	1.3444	1.0559	1.2438	0
3996	700.5	1.3084	1.3442	1.0563	1.2436	0
3997	700.4	1.3088	1.3444	1.0566	1.244	0
3998	700.3	1.309	1.3448	1.0562	1.2439	0
3999	700.2	1.3093	1.345	1.0562	1.2442	0
4000	700.1	1.3091	1.3453	1.0573	1.2444	0
4001	700	1.3092	1.3454	1.0572	1.2445	0
4002	699.9	1.3097	1.3452	1.056	1.2443	0
4003	699.8	1.3098	1.3458	1.0579	1.2444	0
4004	699.7	1.3101	1.3459	1.0565	1.2446	0
4005	699.6	1.3102	1.3459	1.0575	1.2445	0
4006	699.5	1.3105	1.3459	1.0565	1.2447	0
4007	699.4	1.3106	1.3462	1.0569	1.2448	0
4008	699.3	1.3106	1.3458	1.0565	1.2448	0
4009	699.2	1.311	1.3459	1.0565	1.245	0
4010	699.1	1.3111	1.3462	1.0569	1.2448	0
4011	699	1.3113	1.3466	1.0568	1.2448	0
4012	698.9	1.3115	1.3467	1.0571	1.2448	0
4013	698.8	1.3113	1.3465	1.0575	1.2451	0
4014	698.7	1.3117	1.3469	1.0562	1.2449	0
4015	698.6	1.3116	1.347	1.0574	1.2447	0
4016	698.5	1.3115	1.3472	1.0569	1.245	0
4017	698.4	1.3119	1.3474	1.057	1.2454	0
4018	698.3	1.3123	1.3476	1.0569	1.2455	0
4019	698.2	1.3125	1.3478	1.0575	1.2454	0
4020	698.1	1.3123	1.3479	1.0574	1.2453	0
4021	698	1.3126	1.348	1.057	1.2453	0
4022	697.9	1.3129	1.3483	1.0584	1.2455	0
4023	697.8	1.3128	1.3479	1.0575	1.2454	0
4024	697.7	1.3131	1.3483	1.058	1.2454	0
4025	697.6	1.3129	1.3486	1.0578	1.2454	0
4026	697.5	1.313	1.3487	1.0579	1.2455	0
4027	697.4	1.3131	1.3488	1.0576	1.246	0
4028	697.3	1.3132	1.3489	1.0584	1.246	0

4029	697.2	1.3137	1.349	1.0575	1.246	0
4030	697.1	1.3137	1.349	1.0577	1.246	0
4031	697	1.3138	1.3496	1.0574	1.2459	0
4032	696.9	1.3139	1.3495	1.0578	1.2459	0
4033	696.8	1.3144	1.3493	1.0582	1.2461	0
4034	696.7	1.3144	1.3499	1.0577	1.2464	0
4035	696.6	1.3147	1.3499	1.0576	1.246	0
4036	696.5	1.3145	1.3497	1.0586	1.2462	0
4037	696.4	1.3149	1.3504	1.0589	1.2461	0
4038	696.3	1.3151	1.3502	1.0583	1.2463	0
4039	696.2	1.3152	1.3503	1.0591	1.2463	0
4040	696.1	1.3154	1.3501	1.0582	1.2463	0
4041	696	1.3155	1.3507	1.058	1.2466	0
4042	695.9	1.3156	1.351	1.0581	1.2466	0
4043	695.8	1.3156	1.351	1.0581	1.2466	0
4044	695.7	1.3157	1.3513	1.0584	1.2466	0
4045	695.6	1.316	1.3513	1.0575	1.2469	0
4046	695.5	1.3165	1.3513	1.0573	1.2465	0
4047	695.4	1.3163	1.3513	1.0576	1.2469	0
4048	695.3	1.3165	1.3511	1.0582	1.247	0
4049	695.2	1.3167	1.3515	1.0578	1.2472	0
4050	695.1	1.3168	1.3516	1.0576	1.2473	0
4051	695	1.3169	1.3522	1.0583	1.2471	0
4052	694.9	1.3169	1.352	1.0586	1.2473	0
4053	694.8	1.317	1.3525	1.0589	1.2476	0
4054	694.7	1.3173	1.3522	1.0573	1.2474	0
4055	694.6	1.3174	1.3524	1.056	1.2477	0
4056	694.5	1.3176	1.3527	1.0575	1.2477	0
4057	694.4	1.3173	1.3524	1.0576	1.2478	0
4058	694.3	1.3177	1.3526	1.0573	1.2479	0
4059	694.2	1.318	1.3529	1.0569	1.2479	0
4060	694.1	1.3184	1.3529	1.0573	1.2483	0
4061	694	1.3182	1.3526	1.0571	1.2481	0
4062	693.9	1.3185	1.3532	1.0571	1.2485	0
4063	693.8	1.3186	1.3531	1.057	1.2483	0
4064	693.7	1.3184	1.3531	1.0565	1.2483	0
4065	693.6	1.3191	1.3536	1.0575	1.2485	0
4066	693.5	1.3192	1.3533	1.0571	1.2484	0
4067	693.4	1.3191	1.3537	1.0575	1.2485	0
4068	693.3	1.3192	1.3535	1.0574	1.2487	0
4069	693.2	1.3195	1.3542	1.0564	1.2492	0
4070	693.1	1.3194	1.3543	1.0561	1.2491	0
4071	693	1.3196	1.3545	1.0556	1.2491	0

4072	692.9	1.3194	1.3546	1.0564	1.2492	0
4073	692.8	1.3202	1.3547	1.0556	1.2493	0
4074	692.7	1.3202	1.3547	1.0564	1.2495	0
4075	692.6	1.3201	1.3551	1.0545	1.2493	0
4076	692.5	1.3201	1.3547	1.0552	1.2493	0
4077	692.4	1.3204	1.355	1.0553	1.2497	0
4078	692.3	1.3205	1.3556	1.0553	1.2498	0
4079	692.2	1.3206	1.3557	1.0555	1.25	0
4080	692.1	1.3206	1.3557	1.0557	1.25	0
4081	692	1.3209	1.3558	1.0562	1.2501	0
4082	691.9	1.3208	1.3555	1.0557	1.2498	0
4083	691.8	1.3211	1.356	1.0565	1.2498	0
4084	691.7	1.3209	1.3563	1.0558	1.2499	0
4085	691.6	1.3212	1.3565	1.057	1.2502	0
4086	691.5	1.3215	1.3566	1.0562	1.2503	0
4087	691.4	1.3215	1.3568	1.0561	1.2506	0
4088	691.3	1.3215	1.3566	1.0564	1.2502	0
4089	691.2	1.3219	1.3568	1.0557	1.2509	0
4090	691.1	1.3217	1.3571	1.0557	1.2507	0
4091	691	1.3213	1.3571	1.0564	1.2507	0
4092	690.9	1.3219	1.3575	1.0561	1.2506	0
4093	690.8	1.3222	1.3579	1.0563	1.251	0
4094	690.7	1.3222	1.3582	1.0559	1.251	0
4095	690.6	1.3221	1.3581	1.0553	1.2509	0
4096	690.5	1.322	1.3577	1.0565	1.2509	0
4097	690.4	1.3222	1.358	1.0558	1.2511	0
4098	690.3	1.3222	1.3581	1.0563	1.2511	0
4099	690.2	1.3228	1.3584	1.0562	1.2511	0
4100	690.1	1.3226	1.3585	1.0562	1.2511	0
4101	690	1.3226	1.3586	1.0563	1.2513	0
4102	689.9	1.3229	1.3588	1.0564	1.2512	0
4103	689.8	1.3227	1.3587	1.0568	1.2513	0
4104	689.7	1.3226	1.3593	1.0563	1.2515	0
4105	689.6	1.3233	1.3589	1.0565	1.2515	0
4106	689.5	1.323	1.3593	1.0567	1.2516	0
4107	689.4	1.3231	1.3594	1.0564	1.2516	0
4108	689.3	1.3238	1.3594	1.0568	1.2516	0
4109	689.2	1.3234	1.3598	1.0573	1.2516	0
4110	689.1	1.3237	1.3596	1.057	1.2518	0
4111	689	1.3235	1.3604	1.0565	1.2518	0
4112	688.9	1.3237	1.3602	1.0571	1.252	0
4113	688.8	1.3234	1.3604	1.0563	1.2519	0
4114	688.7	1.3239	1.3607	1.0571	1.2519	0

4115	688.6	1.3237	1.3605	1.0564	1.2519	0
4116	688.5	1.3239	1.361	1.0564	1.2522	0
4117	688.4	1.3242	1.3613	1.0563	1.252	0
4118	688.3	1.3244	1.3613	1.056	1.2523	0
4119	688.2	1.3243	1.3613	1.0572	1.2521	0
4120	688.1	1.3247	1.3614	1.0571	1.2525	0
4121	688	1.325	1.3615	1.0565	1.2524	0
4122	687.9	1.3247	1.3617	1.0573	1.2524	0
4123	687.8	1.3248	1.3619	1.0576	1.2525	0
4124	687.7	1.3248	1.3619	1.057	1.2527	0
4125	687.6	1.3251	1.362	1.0582	1.253	0
4126	687.5	1.3251	1.3622	1.0575	1.2528	0
4127	687.4	1.3249	1.362	1.0578	1.2529	0
4128	687.3	1.3256	1.3626	1.0573	1.2529	0
4129	687.2	1.3255	1.3625	1.0567	1.2531	0
4130	687.1	1.3256	1.3629	1.0567	1.253	0
4131	687	1.3255	1.3628	1.0573	1.2533	0
4132	686.9	1.3257	1.3625	1.0562	1.2531	0
4133	686.8	1.3261	1.3632	1.057	1.2533	0
4134	686.7	1.3264	1.3636	1.0576	1.2537	0
4135	686.6	1.3263	1.3632	1.0572	1.2534	0
4136	686.5	1.3263	1.3635	1.0567	1.2538	0
4137	686.4	1.3266	1.3641	1.0566	1.2539	0
4138	686.3	1.3266	1.3636	1.0574	1.2539	0
4139	686.2	1.3262	1.3637	1.0569	1.2535	0
4140	686.1	1.3268	1.3641	1.0557	1.2538	0
4141	686	1.3269	1.3643	1.0563	1.2541	0
4142	685.9	1.3271	1.3643	1.0572	1.2546	0
4143	685.8	1.3274	1.3643	1.057	1.2544	0
4144	685.7	1.3273	1.3642	1.0568	1.2545	0
4145	685.6	1.3275	1.3644	1.0563	1.2546	0
4146	685.5	1.3274	1.3646	1.0562	1.2545	0
4147	685.4	1.3278	1.364	1.0559	1.255	0
4148	685.3	1.3277	1.365	1.0564	1.2547	0
4149	685.2	1.3279	1.365	1.0557	1.255	0
4150	685.1	1.3279	1.3647	1.0561	1.255	0
4151	685	1.3278	1.365	1.0556	1.2551	0
4152	684.9	1.3281	1.365	1.0554	1.2551	0
4153	684.8	1.3281	1.3653	1.0551	1.2555	0
4154	684.7	1.3285	1.3652	1.0554	1.2556	0
4155	684.6	1.3286	1.3651	1.0557	1.2552	0
4156	684.5	1.3287	1.3652	1.0557	1.2555	0
4157	684.4	1.329	1.3658	1.0552	1.2559	0

4158	684.3	1.329	1.3659	1.0552	1.2559	0
4159	684.2	1.329	1.3656	1.0555	1.2562	0
4160	684.1	1.3296	1.3662	1.0554	1.2563	0
4161	684	1.3296	1.366	1.0543	1.2561	0
4162	683.9	1.3295	1.3665	1.0541	1.2564	0
4163	683.8	1.3299	1.3664	1.0547	1.2567	0
4164	683.7	1.3299	1.3664	1.055	1.2568	0
4165	683.6	1.3299	1.3665	1.0548	1.2566	0
4166	683.5	1.3301	1.3664	1.0549	1.2571	0
4167	683.4	1.3302	1.3666	1.0548	1.257	0
4168	683.3	1.3306	1.3672	1.0548	1.2573	0
4169	683.2	1.3303	1.3667	1.0552	1.2574	0
4170	683.1	1.3308	1.3667	1.0554	1.2572	0
4171	683	1.3308	1.367	1.055	1.2578	0
4172	682.9	1.3311	1.3671	1.0551	1.2578	0
4173	682.8	1.3311	1.3676	1.0541	1.2579	0
4174	682.7	1.3311	1.3679	1.0547	1.2581	0
4175	682.6	1.3314	1.3678	1.055	1.2582	0
4176	682.5	1.3314	1.3675	1.0552	1.2581	0
4177	682.4	1.3319	1.3678	1.0548	1.2581	0
4178	682.3	1.3316	1.368	1.0553	1.258	0
4179	682.2	1.3319	1.3679	1.0557	1.2585	0
4180	682.1	1.332	1.3685	1.0559	1.2585	0
4181	682	1.3319	1.369	1.0557	1.2586	0
4182	681.9	1.3319	1.3684	1.0551	1.2587	0
4183	681.8	1.3321	1.3684	1.0554	1.2589	0
4184	681.7	1.3319	1.3682	1.0545	1.2586	0
4185	681.6	1.3321	1.3682	1.0554	1.259	0
4186	681.5	1.3323	1.3689	1.0552	1.2591	0
4187	681.4	1.3325	1.3689	1.0549	1.2593	0
4188	681.3	1.3326	1.3687	1.0546	1.2588	0
4189	681.2	1.3326	1.3689	1.0547	1.2591	0
4190	681.1	1.3327	1.3692	1.0553	1.2593	0
4191	681	1.3326	1.3691	1.0549	1.2592	0
4192	680.9	1.3327	1.3692	1.0553	1.2592	0
4193	680.8	1.333	1.3695	1.0553	1.2596	0
4194	680.7	1.3332	1.3696	1.056	1.2596	0
4195	680.6	1.333	1.3699	1.0558	1.2596	0
4196	680.5	1.3333	1.3697	1.0557	1.26	0
4197	680.4	1.3333	1.3698	1.0555	1.2596	0
4198	680.3	1.3337	1.3699	1.0554	1.2601	0
4199	680.2	1.3337	1.3707	1.056	1.2602	0
4200	680.1	1.3338	1.3704	1.0554	1.2599	0

4201	680	1.3338	1.3703	1.0563	1.2602	0
4202	679.9	1.3338	1.3707	1.0565	1.2604	0
4203	679.8	1.3341	1.371	1.0565	1.2604	0
4204	679.7	1.3341	1.3707	1.0567	1.2608	0
4205	679.6	1.3339	1.3708	1.0563	1.2605	0
4206	679.5	1.3339	1.3712	1.0565	1.2605	0
4207	679.4	1.3341	1.3712	1.0564	1.2609	0
4208	679.3	1.3344	1.3715	1.0562	1.2611	0
4209	679.2	1.3347	1.3718	1.0562	1.2612	0
4210	679.1	1.3345	1.3717	1.0567	1.2615	0
4211	679	1.335	1.3719	1.0567	1.2612	0
4212	678.9	1.3347	1.3716	1.0565	1.2613	0
4213	678.8	1.3347	1.3719	1.0564	1.2615	0
4214	678.7	1.335	1.3722	1.0575	1.2617	0
4215	678.6	1.335	1.3725	1.0572	1.2619	0
4216	678.5	1.3349	1.3724	1.0577	1.262	0
4217	678.4	1.3354	1.3727	1.0576	1.2619	0
4218	678.3	1.3354	1.3728	1.0575	1.2621	0
4219	678.2	1.3357	1.3734	1.0578	1.2621	0
4220	678.1	1.3359	1.3734	1.0583	1.2622	0
4221	678	1.3356	1.3739	1.0574	1.2624	0
4222	677.9	1.336	1.3743	1.0584	1.2629	0
4223	677.8	1.3362	1.3746	1.0586	1.2628	0
4224	677.7	1.336	1.3744	1.0578	1.2628	0
4225	677.6	1.3363	1.3747	1.0577	1.2628	0
4226	677.5	1.3363	1.3755	1.0582	1.2632	0
4227	677.4	1.3365	1.3755	1.0574	1.2631	0
4228	677.3	1.3366	1.3762	1.0576	1.2634	0
4229	677.2	1.3369	1.376	1.0581	1.2638	0
4230	677.1	1.3365	1.3765	1.0574	1.2639	0
4231	677	1.3367	1.3764	1.0579	1.2638	0
4232	676.9	1.3368	1.3768	1.0584	1.2641	0
4233	676.8	1.3373	1.3774	1.0585	1.264	0
4234	676.7	1.3374	1.3777	1.058	1.264	0
4235	676.6	1.3377	1.3779	1.0584	1.2641	0
4236	676.5	1.3375	1.3782	1.058	1.2645	0
4237	676.4	1.3377	1.3787	1.0568	1.2646	0
4238	676.3	1.3377	1.3791	1.0577	1.2646	0
4239	676.2	1.3379	1.3795	1.0573	1.2645	0
4240	676.1	1.3384	1.3793	1.0577	1.2645	0
4241	676	1.3385	1.3803	1.0575	1.2652	0
4242	675.9	1.3385	1.3803	1.0574	1.2649	0
4243	675.8	1.3387	1.3806	1.0573	1.2651	0

4244	675.7	1.3387	1.3806	1.0567	1.2651	0
4245	675.6	1.3388	1.381	1.0565	1.2653	0
4246	675.5	1.3392	1.3811	1.0562	1.2656	0
4247	675.4	1.3391	1.3808	1.0566	1.2657	0
4248	675.3	1.339	1.381	1.0563	1.2656	0
4249	675.2	1.3396	1.3813	1.0561	1.2656	0
4250	675.1	1.3397	1.3815	1.0561	1.2659	0
4251	675	1.3399	1.3812	1.0559	1.266	0
4252	674.9	1.3401	1.3816	1.0561	1.2659	0
4253	674.8	1.3401	1.3819	1.0561	1.2663	0
4254	674.7	1.3402	1.3809	1.0558	1.2664	0
4255	674.6	1.3406	1.3817	1.0552	1.2661	0
4256	674.5	1.3408	1.3814	1.0548	1.2665	0
4257	674.4	1.3413	1.3814	1.0552	1.2666	0
4258	674.3	1.3407	1.3814	1.055	1.2666	0
4259	674.2	1.3412	1.3813	1.0551	1.2667	0
4260	674.1	1.3415	1.3811	1.0546	1.2668	0
4261	674	1.3417	1.381	1.0555	1.2671	0
4262	673.9	1.3414	1.3811	1.0549	1.2671	0
4263	673.8	1.3418	1.3809	1.0545	1.2673	0
4264	673.7	1.342	1.3808	1.0543	1.2677	0
4265	673.6	1.342	1.3812	1.054	1.2673	0
4266	673.5	1.3423	1.3815	1.0545	1.2674	0
4267	673.4	1.3424	1.3806	1.0535	1.2671	0
4268	673.3	1.3426	1.3808	1.0538	1.2672	0
4269	673.2	1.3427	1.3806	1.0538	1.2673	0
4270	673.1	1.3431	1.3811	1.0542	1.2678	0
4271	673	1.3432	1.3809	1.0534	1.2674	0
4272	672.9	1.3432	1.3812	1.0535	1.2676	0
4273	672.8	1.343	1.381	1.0536	1.2676	0
4274	672.7	1.3435	1.3815	1.0542	1.2678	0
4275	672.6	1.3433	1.3813	1.0539	1.2678	0
4276	672.5	1.3438	1.3815	1.0542	1.2678	0
4277	672.4	1.3437	1.3812	1.0541	1.2681	0
4278	672.3	1.344	1.3813	1.0538	1.2679	0
4279	672.2	1.3441	1.3819	1.0543	1.2679	0
4280	672.1	1.3445	1.3818	1.0538	1.2678	0
4281	672	1.3442	1.3818	1.0536	1.2681	0
4282	671.9	1.3443	1.3823	1.0536	1.268	0
4283	671.8	1.3448	1.382	1.0544	1.2682	0
4284	671.7	1.3445	1.3828	1.0534	1.2684	0
4285	671.6	1.345	1.3825	1.0538	1.2678	0
4286	671.5	1.3448	1.3832	1.0536	1.2681	0

4287	671.4	1.3454	1.3829	1.0537	1.2681	0
4288	671.3	1.3448	1.3832	1.0544	1.2683	0
4289	671.2	1.3452	1.3831	1.054	1.2684	0
4290	671.1	1.3453	1.3834	1.0542	1.2684	0
4291	671	1.3456	1.3835	1.0546	1.268	0
4292	670.9	1.346	1.3834	1.0545	1.2682	0
4293	670.8	1.3458	1.3844	1.0551	1.2683	0
4294	670.7	1.346	1.384	1.055	1.2685	0
4295	670.6	1.3461	1.385	1.0548	1.2685	0
4296	670.5	1.3462	1.3848	1.0552	1.2686	0
4297	670.4	1.3464	1.385	1.0555	1.2682	0
4298	670.3	1.3462	1.3852	1.0547	1.2687	0
4299	670.2	1.3463	1.3853	1.0557	1.2685	0
4300	670.1	1.3464	1.3857	1.0551	1.2683	0
4301	670	1.3467	1.386	1.0547	1.2688	0
4302	669.9	1.3466	1.3865	1.0555	1.2687	0
4303	669.8	1.3469	1.3866	1.0551	1.2685	0
4304	669.7	1.3468	1.3868	1.0556	1.2686	0
4305	669.6	1.3469	1.3869	1.0559	1.2688	0
4306	669.5	1.3473	1.387	1.0562	1.2687	0
4307	669.4	1.347	1.3873	1.0562	1.2687	0
4308	669.3	1.3472	1.3881	1.0561	1.2686	0
4309	669.2	1.347	1.3888	1.0567	1.2686	0
4310	669.1	1.3473	1.3883	1.0559	1.2688	0
4311	669	1.3473	1.3883	1.0568	1.2683	0
4312	668.9	1.3478	1.3892	1.0573	1.2685	0
4313	668.8	1.3476	1.3896	1.0571	1.2686	0
4314	668.7	1.3478	1.3892	1.0572	1.2684	0
4315	668.6	1.3476	1.3899	1.0566	1.2685	0
4316	668.5	1.3478	1.3901	1.0569	1.2684	0
4317	668.4	1.348	1.3904	1.0581	1.2687	0
4318	668.3	1.3482	1.3904	1.0571	1.2687	0
4319	668.2	1.3481	1.3902	1.0579	1.2685	0
4320	668.1	1.3486	1.3912	1.0576	1.2685	0
4321	668	1.3482	1.3913	1.0577	1.2688	0
4322	667.9	1.3484	1.3912	1.0578	1.2687	0
4323	667.8	1.3485	1.3916	1.0582	1.2688	0
4324	667.7	1.3489	1.3918	1.0576	1.2688	0
4325	667.6	1.3487	1.3922	1.058	1.2689	0
4326	667.5	1.349	1.3923	1.0582	1.2687	0
4327	667.4	1.3489	1.3924	1.0582	1.2687	0
4328	667.3	1.3493	1.392	1.0581	1.2689	0
4329	667.2	1.3491	1.3918	1.0578	1.2688	0

4330	667.1	1.3495	1.3926	1.0587	1.269	0
4331	667	1.3494	1.3925	1.0592	1.2689	0
4332	666.9	1.3499	1.3933	1.059	1.2691	0
4333	666.8	1.3498	1.3932	1.0586	1.2692	0
4334	666.7	1.3495	1.3935	1.0583	1.2693	0
4335	666.6	1.3499	1.3937	1.0584	1.2692	0
4336	666.5	1.35	1.3935	1.059	1.269	0
4337	666.4	1.3503	1.3935	1.0591	1.2695	0
4338	666.3	1.3507	1.3937	1.0583	1.2694	0
4339	666.2	1.3505	1.3938	1.0584	1.2696	0
4340	666.1	1.3509	1.394	1.0586	1.2697	0
4341	666	1.3508	1.3938	1.059	1.2696	0
4342	665.9	1.3508	1.3938	1.0592	1.2698	0
4343	665.8	1.3514	1.3942	1.0587	1.2701	0
4344	665.7	1.3513	1.3941	1.0586	1.27	0
4345	665.6	1.3514	1.3945	1.0587	1.2698	0
4346	665.5	1.3514	1.3943	1.0586	1.2696	0
4347	665.4	1.3512	1.3938	1.0576	1.2698	0
4348	665.3	1.3518	1.3944	1.0586	1.2698	0
4349	665.2	1.3519	1.3945	1.0574	1.2701	0
4350	665.1	1.3521	1.3947	1.0572	1.2702	0
4351	665	1.3521	1.3945	1.0573	1.2708	0
4352	664.9	1.3529	1.3948	1.0572	1.2707	0
4353	664.8	1.3526	1.3957	1.0582	1.2707	0
4354	664.7	1.353	1.3951	1.0577	1.2705	0
4355	664.6	1.3527	1.3958	1.0573	1.2708	0
4356	664.5	1.3533	1.3958	1.0576	1.2714	0
4357	664.4	1.3535	1.396	1.057	1.2714	0
4358	664.3	1.3534	1.3959	1.0572	1.2717	0
4359	664.2	1.3533	1.3962	1.0564	1.2712	0
4360	664.1	1.3539	1.3965	1.0573	1.2715	0
4361	664	1.3543	1.3963	1.0576	1.2721	0
4362	663.9	1.3541	1.3964	1.0565	1.2717	0
4363	663.8	1.3549	1.3967	1.0561	1.2719	0
4364	663.7	1.3549	1.397	1.0562	1.2721	0
4365	663.6	1.3547	1.3968	1.0559	1.2721	0
4366	663.5	1.3554	1.3969	1.0552	1.2725	0
4367	663.4	1.3554	1.397	1.0555	1.2727	0
4368	663.3	1.3556	1.3971	1.0555	1.2728	0
4369	663.2	1.3557	1.3973	1.055	1.2729	0
4370	663.1	1.3557	1.397	1.0551	1.2729	0
4371	663	1.3561	1.3974	1.0551	1.2735	0
4372	662.9	1.3563	1.3974	1.0552	1.2732	0

4373	662.8	1.3563	1.3973	1.0554	1.2734	0
4374	662.7	1.3564	1.3975	1.055	1.2735	0
4375	662.6	1.3566	1.3974	1.0543	1.2736	0
4376	662.5	1.3566	1.3977	1.0546	1.2738	0
4377	662.4	1.3569	1.3977	1.055	1.2741	0
4378	662.3	1.3571	1.3979	1.0546	1.2742	0
4379	662.2	1.3575	1.3975	1.0552	1.2741	0
4380	662.1	1.3572	1.3978	1.0551	1.2745	0
4381	662	1.3577	1.3977	1.0546	1.2744	0
4382	661.9	1.3579	1.3982	1.0543	1.2743	0
4383	661.8	1.3583	1.3981	1.0542	1.2745	0
4384	661.7	1.3582	1.3983	1.0546	1.2745	0
4385	661.6	1.3584	1.3985	1.0547	1.2746	0
4386	661.5	1.3584	1.3986	1.0548	1.2748	0
4387	661.4	1.3587	1.3985	1.0547	1.2748	0
4388	661.3	1.3588	1.398	1.0547	1.2751	0
4389	661.2	1.359	1.3986	1.0548	1.2748	0
4390	661.1	1.3589	1.3985	1.0547	1.2751	0
4391	661	1.3591	1.3984	1.0552	1.2753	0
4392	660.9	1.359	1.3986	1.0552	1.2752	0
4393	660.8	1.3594	1.3987	1.055	1.2753	0
4394	660.7	1.3593	1.3986	1.0548	1.2754	0
4395	660.6	1.3592	1.3983	1.0546	1.2753	0
4396	660.5	1.3597	1.3991	1.0548	1.2753	0
4397	660.4	1.3598	1.399	1.0546	1.2756	0
4398	660.3	1.3601	1.3986	1.055	1.2758	0
4399	660.2	1.36	1.3992	1.0551	1.2758	0
4400	660.1	1.3599	1.399	1.0549	1.2756	0
4401	660	1.3606	1.3991	1.0549	1.2757	0
4402	659.9	1.3607	1.3996	1.0551	1.2758	0
4403	659.8	1.3607	1.399	1.0548	1.2761	0
4404	659.7	1.3609	1.3993	1.055	1.2765	0
4405	659.6	1.3613	1.399	1.0545	1.2763	0
4406	659.5	1.3611	1.3991	1.0548	1.2763	0
4407	659.4	1.3611	1.3994	1.0557	1.2766	0
4408	659.3	1.3612	1.3996	1.0557	1.2764	0
4409	659.2	1.3615	1.3995	1.0554	1.2766	0
4410	659.1	1.3614	1.3995	1.0562	1.2764	0
4411	659	1.362	1.3998	1.0559	1.2768	0
4412	658.9	1.3616	1.3998	1.0556	1.2767	0
4413	658.8	1.3619	1.4	1.0558	1.2767	0
4414	658.7	1.3625	1.4	1.056	1.2769	0
4415	658.6	1.362	1.3999	1.056	1.277	0

4416	658.5	1.3622	1.3998	1.0559	1.2767	0
4417	658.4	1.3619	1.4004	1.0559	1.2769	0
4418	658.3	1.3627	1.4003	1.0569	1.277	0
4419	658.2	1.3623	1.4006	1.0568	1.277	0
4420	658.1	1.3624	1.4009	1.0573	1.2773	0
4421	658	1.3627	1.4006	1.0568	1.2775	0
4422	657.9	1.3627	1.4004	1.0575	1.277	0
4423	657.8	1.3634	1.4008	1.0584	1.2774	0
4424	657.7	1.3631	1.4011	1.0585	1.2776	0
4425	657.6	1.3633	1.401	1.0582	1.2776	0
4426	657.5	1.3634	1.4009	1.0582	1.2774	0
4427	657.4	1.3634	1.4005	1.0587	1.2776	0
4428	657.3	1.3636	1.4008	1.0584	1.2773	0
4429	657.2	1.3638	1.401	1.0587	1.2776	0
4430	657.1	1.3637	1.4009	1.0589	1.2778	0
4431	657	1.3636	1.4017	1.0588	1.278	0
4432	656.9	1.3642	1.4014	1.0588	1.2782	0
4433	656.8	1.3643	1.4015	1.0582	1.278	0
4434	656.7	1.3646	1.4018	1.0589	1.2783	0
4435	656.6	1.3646	1.4018	1.0585	1.2783	0
4436	656.5	1.3646	1.4018	1.0582	1.2785	0
4437	656.4	1.3649	1.4018	1.0583	1.2783	0
4438	656.3	1.3652	1.4015	1.0585	1.2785	0
4439	656.2	1.3652	1.4019	1.059	1.2788	0
4440	656.1	1.3651	1.4018	1.0592	1.2785	0
4441	656	1.3654	1.4015	1.0603	1.2787	0
4442	655.9	1.3655	1.4022	1.0588	1.279	0
4443	655.8	1.3655	1.4025	1.0594	1.279	0
4444	655.7	1.3659	1.4023	1.0584	1.2794	0
4445	655.6	1.3662	1.402	1.0583	1.2794	0
4446	655.5	1.3665	1.4027	1.0591	1.2795	0
4447	655.4	1.3661	1.4032	1.059	1.2793	0
4448	655.3	1.3665	1.4029	1.0585	1.2798	0
4449	655.2	1.3668	1.403	1.0587	1.2797	0
4450	655.1	1.3668	1.403	1.0588	1.2799	0
4451	655	1.3671	1.403	1.0586	1.2799	0
4452	654.9	1.3672	1.4034	1.0579	1.2803	0
4453	654.8	1.3676	1.4035	1.0585	1.2804	0
4454	654.7	1.3679	1.4039	1.0583	1.2804	0
4455	654.6	1.3679	1.4036	1.0573	1.2804	0
4456	654.5	1.3681	1.404	1.0582	1.281	0
4457	654.4	1.3684	1.4041	1.0576	1.2811	0
4458	654.3	1.3685	1.4042	1.0579	1.2809	0

4459	654.2	1.3685	1.4044	1.0584	1.2814	0
4460	654.1	1.3688	1.4042	1.058	1.2814	0
4461	654	1.3687	1.4041	1.0571	1.2814	0
4462	653.9	1.3691	1.4042	1.0572	1.2812	0
4463	653.8	1.3691	1.4045	1.0571	1.2817	0
4464	653.7	1.3699	1.4047	1.0557	1.2819	0
4465	653.6	1.3701	1.4043	1.0569	1.2818	0
4466	653.5	1.3701	1.4049	1.0569	1.2823	0
4467	653.4	1.3702	1.4051	1.0566	1.2822	0
4468	653.3	1.3705	1.4054	1.0562	1.2825	0
4469	653.2	1.3706	1.4057	1.0567	1.2827	0
4470	653.1	1.3709	1.4055	1.0561	1.2826	0
4471	653	1.3709	1.4055	1.0557	1.2828	0
4472	652.9	1.3713	1.4059	1.056	1.2831	0
4473	652.8	1.3714	1.4057	1.0556	1.2832	0
4474	652.7	1.3717	1.4057	1.0556	1.2835	0
4475	652.6	1.3715	1.4064	1.0559	1.2835	0
4476	652.5	1.3718	1.4061	1.0554	1.2839	0
4477	652.4	1.3719	1.4068	1.0544	1.2839	0
4478	652.3	1.3725	1.4066	1.0556	1.284	0
4479	652.2	1.3727	1.4065	1.0558	1.2839	0
4480	652.1	1.3727	1.4067	1.0561	1.2844	0
4481	652	1.3732	1.4072	1.0558	1.2848	0
4482	651.9	1.3733	1.4073	1.0559	1.2846	0
4483	651.8	1.3734	1.4071	1.0552	1.285	0
4484	651.7	1.3737	1.4072	1.0552	1.285	0
4485	651.6	1.3737	1.4075	1.0549	1.285	0
4486	651.5	1.3743	1.4078	1.0554	1.2856	0
4487	651.4	1.374	1.4081	1.055	1.2851	0
4488	651.3	1.3742	1.408	1.0554	1.2854	0
4489	651.2	1.3743	1.4081	1.0551	1.2856	0
4490	651.1	1.3746	1.4084	1.0541	1.2856	0
4491	651	1.3748	1.4083	1.0551	1.2855	0
4492	650.9	1.3755	1.4084	1.0557	1.2859	0
4493	650.8	1.3754	1.4089	1.0556	1.2863	0
4494	650.7	1.3755	1.4087	1.056	1.2861	0
4495	650.6	1.3756	1.4095	1.0565	1.286	0
4496	650.5	1.3759	1.4095	1.0555	1.286	0
4497	650.4	1.3763	1.4093	1.0555	1.2862	0
4498	650.3	1.376	1.4096	1.055	1.2864	0
4499	650.2	1.3768	1.4102	1.0557	1.2868	0
4500	650.1	1.3765	1.4098	1.0559	1.2866	0
4501	650	1.3766	1.4102	1.0559	1.2864	0

4502	649.9	1.3769	1.4105	1.0548	1.2869	0
4503	649.8	1.377	1.4103	1.0558	1.287	0
4504	649.7	1.377	1.411	1.0557	1.2868	0
4505	649.6	1.3772	1.4108	1.0554	1.2872	0
4506	649.5	1.3772	1.4115	1.0553	1.2872	0
4507	649.4	1.3779	1.4116	1.0557	1.2874	0
4508	649.3	1.3782	1.4116	1.0558	1.2876	0
4509	649.2	1.378	1.4113	1.056	1.2874	0
4510	649.1	1.378	1.4114	1.0553	1.2875	0
4511	649	1.3785	1.4115	1.056	1.2879	0
4512	648.9	1.3785	1.4119	1.0562	1.2876	0
4513	648.8	1.3785	1.4119	1.0566	1.2879	0
4514	648.7	1.3792	1.4123	1.0572	1.288	0
4515	648.6	1.3791	1.4125	1.0567	1.2882	0
4516	648.5	1.3792	1.4126	1.0569	1.2882	0
4517	648.4	1.3793	1.4124	1.056	1.2879	0
4518	648.3	1.3791	1.4129	1.057	1.2885	0
4519	648.2	1.3795	1.4129	1.056	1.2883	0
4520	648.1	1.3798	1.4129	1.0574	1.2881	0
4521	648	1.3799	1.4134	1.0574	1.2884	0
4522	647.9	1.38	1.4141	1.0575	1.2885	0
4523	647.8	1.38	1.4138	1.0574	1.2885	0
4524	647.7	1.3807	1.4139	1.0578	1.2888	0
4525	647.6	1.3806	1.4147	1.0581	1.2888	0
4526	647.5	1.3805	1.4143	1.0574	1.2887	0
4527	647.4	1.3809	1.4144	1.0576	1.2888	0
4528	647.3	1.3813	1.4145	1.058	1.289	0
4529	647.2	1.3815	1.4147	1.0576	1.2893	0
4530	647.1	1.3815	1.4147	1.0588	1.2892	0
4531	647	1.3819	1.4149	1.0581	1.289	0
4532	646.9	1.3819	1.4151	1.0586	1.289	0
4533	646.8	1.382	1.4152	1.0588	1.2896	0
4534	646.7	1.382	1.4156	1.0587	1.2894	0
4535	646.6	1.3822	1.4154	1.0586	1.2896	0
4536	646.5	1.3825	1.4157	1.0588	1.2895	0
4537	646.4	1.3829	1.4158	1.0596	1.2896	0
4538	646.3	1.3827	1.4159	1.0594	1.2895	0
4539	646.2	1.3832	1.4165	1.0594	1.2902	0
4540	646.1	1.3834	1.4163	1.06	1.2901	0
4541	646	1.384	1.4167	1.0603	1.2901	0
4542	645.9	1.3838	1.4165	1.0595	1.2903	0
4543	645.8	1.3838	1.4165	1.0591	1.2905	0
4544	645.7	1.384	1.4167	1.0589	1.2907	0

4545	645.6	1.3844	1.4171	1.0593	1.2908	0
4546	645.5	1.3845	1.4175	1.0591	1.2907	0
4547	645.4	1.3845	1.4172	1.0591	1.291	0
4548	645.3	1.3848	1.4175	1.0592	1.2907	0
4549	645.2	1.3849	1.418	1.0595	1.2911	0
4550	645.1	1.3853	1.418	1.0586	1.2909	0
4551	645	1.3858	1.4179	1.0594	1.2914	0
4552	644.9	1.3858	1.4176	1.0586	1.2912	0
4553	644.8	1.3859	1.418	1.0595	1.2913	0
4554	644.7	1.3861	1.4182	1.059	1.2915	0
4555	644.6	1.3863	1.4184	1.0586	1.2917	0
4556	644.5	1.3863	1.4188	1.0591	1.2919	0
4557	644.4	1.3865	1.4186	1.0598	1.2919	0
4558	644.3	1.3871	1.4189	1.0594	1.2922	0
4559	644.2	1.3872	1.4193	1.0582	1.2921	0
4560	644.1	1.3875	1.4193	1.0586	1.2928	0
4561	644	1.3876	1.4194	1.0588	1.2926	0
4562	643.9	1.3877	1.4195	1.0589	1.293	0
4563	643.8	1.3882	1.4194	1.0586	1.293	0
4564	643.7	1.3886	1.4198	1.0576	1.2929	0
4565	643.6	1.3889	1.4198	1.0576	1.2932	0
4566	643.5	1.3889	1.4198	1.0577	1.2931	0
4567	643.4	1.3892	1.4205	1.0581	1.2935	0
4568	643.3	1.389	1.4205	1.0578	1.2933	0
4569	643.2	1.3893	1.4205	1.0579	1.2936	0
4570	643.1	1.3897	1.4209	1.0574	1.2942	0
4571	643	1.3895	1.4211	1.0581	1.2938	0
4572	642.9	1.3901	1.4213	1.0583	1.2941	0
4573	642.8	1.3906	1.4215	1.057	1.2945	0
4574	642.7	1.3907	1.421	1.0578	1.2944	0
4575	642.6	1.3908	1.4215	1.0578	1.2946	0
4576	642.5	1.3906	1.4218	1.0573	1.2944	0
4577	642.4	1.3908	1.4211	1.0566	1.2948	0
4578	642.3	1.3914	1.4217	1.0576	1.2953	0
4579	642.2	1.3916	1.4222	1.0574	1.2953	0
4580	642.1	1.392	1.422	1.0576	1.295	0
4581	642	1.3918	1.4221	1.0573	1.295	0
4582	641.9	1.3919	1.4223	1.0575	1.2954	0
4583	641.8	1.3921	1.4228	1.0573	1.2956	0
4584	641.7	1.3927	1.4227	1.0571	1.2956	0
4585	641.6	1.393	1.423	1.0577	1.2959	0
4586	641.5	1.393	1.4234	1.0567	1.296	0
4587	641.4	1.3929	1.4232	1.0571	1.2964	0

4588	641.3	1.3936	1.4233	1.0572	1.2963	0
4589	641.2	1.3935	1.4235	1.0565	1.2965	0
4590	641.1	1.3941	1.4244	1.0573	1.2967	0
4591	641	1.3943	1.424	1.0571	1.2967	0
4592	640.9	1.3947	1.4245	1.0567	1.2967	0
4593	640.8	1.3946	1.4246	1.0574	1.297	0
4594	640.7	1.3951	1.4249	1.0571	1.2973	0
4595	640.6	1.3952	1.4252	1.0575	1.2976	0
4596	640.5	1.3952	1.4252	1.0575	1.2974	0
4597	640.4	1.3954	1.4254	1.0573	1.2976	0
4598	640.3	1.3955	1.4253	1.057	1.2978	0
4599	640.2	1.3958	1.4256	1.0578	1.298	0
4600	640.1	1.396	1.426	1.0571	1.2982	0
4601	640	1.3964	1.4259	1.0575	1.2986	0
4602	639.9	1.3965	1.4259	1.0564	1.298	0
4603	639.8	1.3964	1.4265	1.0566	1.2984	0
4604	639.7	1.3972	1.426	1.0565	1.2986	0
4605	639.6	1.3967	1.4262	1.0565	1.2987	0
4606	639.5	1.3973	1.4268	1.0579	1.299	0
4607	639.4	1.3974	1.4269	1.0577	1.2986	0
4608	639.3	1.3978	1.4269	1.058	1.2994	0
4609	639.2	1.3978	1.427	1.0573	1.2995	0
4610	639.1	1.398	1.4271	1.0572	1.2997	0
4611	639	1.3984	1.4273	1.0573	1.2996	0
4612	638.9	1.3985	1.4277	1.057	1.2997	0
4613	638.8	1.398	1.4277	1.0575	1.2998	0
4614	638.7	1.3988	1.4279	1.0581	1.3002	0
4615	638.6	1.3989	1.4283	1.0578	1.3004	0
4616	638.5	1.399	1.4278	1.0586	1.3002	0
4617	638.4	1.3992	1.4283	1.0592	1.3002	0
4618	638.3	1.3995	1.4286	1.0583	1.3007	0
4619	638.2	1.4	1.4288	1.0586	1.3011	0
4620	638.1	1.3999	1.4294	1.0588	1.3011	0
4621	638	1.3999	1.4294	1.0588	1.3009	0
4622	637.9	1.4003	1.4294	1.0588	1.301	0
4623	637.8	1.4003	1.4301	1.059	1.3013	0
4624	637.7	1.4008	1.4297	1.0595	1.3015	0
4625	637.6	1.4013	1.4299	1.0598	1.3015	0
4626	637.5	1.4013	1.4304	1.0599	1.3018	0
4627	637.4	1.4014	1.4309	1.0602	1.3016	0
4628	637.3	1.4016	1.4307	1.0592	1.302	0
4629	637.2	1.4018	1.4309	1.0596	1.302	0
4630	637.1	1.402	1.4312	1.0596	1.302	0

4631	637	1.4018	1.4309	1.0606	1.3021	0
4632	636.9	1.4024	1.4312	1.0605	1.3024	0
4633	636.8	1.4022	1.4317	1.0607	1.3027	0
4634	636.7	1.4025	1.4322	1.0607	1.3028	0
4635	636.6	1.403	1.4321	1.0607	1.303	0
4636	636.5	1.4031	1.4323	1.0601	1.3031	0
4637	636.4	1.4029	1.432	1.0609	1.3032	0
4638	636.3	1.4031	1.4322	1.0619	1.3036	0
4639	636.2	1.4033	1.4322	1.0613	1.3036	0
4640	636.1	1.404	1.4331	1.0609	1.3037	0
4641	636	1.4035	1.4326	1.0607	1.3041	0
4642	635.9	1.4037	1.4334	1.0603	1.304	0
4643	635.8	1.4042	1.434	1.0608	1.3042	0
4644	635.7	1.4043	1.4337	1.0597	1.3043	0
4645	635.6	1.4045	1.434	1.0605	1.3043	0
4646	635.5	1.4044	1.4341	1.0611	1.3045	0
4647	635.4	1.405	1.4341	1.06	1.3048	0
4648	635.3	1.4052	1.4346	1.0609	1.3052	0
4649	635.2	1.4051	1.4348	1.0606	1.3052	0
4650	635.1	1.4056	1.4353	1.0609	1.3054	0
4651	635	1.4059	1.4356	1.0602	1.3057	0
4652	634.9	1.4058	1.4358	1.0601	1.3057	0
4653	634.8	1.4059	1.4357	1.0606	1.3058	0
4654	634.7	1.4065	1.4359	1.0608	1.3061	0
4655	634.6	1.4066	1.4367	1.0605	1.3062	0
4656	634.5	1.4065	1.4369	1.0616	1.3064	0
4657	634.4	1.4068	1.4371	1.0611	1.3064	0
4658	634.3	1.4072	1.4373	1.0604	1.3068	0
4659	634.2	1.4071	1.4372	1.0614	1.3068	0
4660	634.1	1.4074	1.4381	1.0616	1.3071	0
4661	634	1.408	1.4387	1.0606	1.3074	0
4662	633.9	1.4077	1.4387	1.0614	1.3074	0
4663	633.8	1.4078	1.4391	1.0607	1.3078	0
4664	633.7	1.4083	1.4393	1.0606	1.308	0
4665	633.6	1.4082	1.4398	1.0603	1.3081	0
4666	633.5	1.4087	1.44	1.0604	1.3084	0
4667	633.4	1.409	1.4406	1.061	1.3084	0
4668	633.3	1.4089	1.4408	1.0606	1.3086	0
4669	633.2	1.4095	1.4411	1.0609	1.3089	0
4670	633.1	1.4096	1.4415	1.0601	1.3088	0
4671	633	1.4098	1.4417	1.0604	1.309	0
4672	632.9	1.41	1.4426	1.0598	1.3094	0
4673	632.8	1.4105	1.4431	1.0599	1.3094	0

4674	632.7	1.4109	1.4441	1.0602	1.3098	0
4675	632.6	1.411	1.4439	1.0602	1.3099	0
4676	632.5	1.4109	1.4444	1.061	1.3098	0
4677	632.4	1.4114	1.445	1.0608	1.3101	0
4678	632.3	1.4112	1.4456	1.06	1.3102	0
4679	632.2	1.4113	1.4455	1.0613	1.3106	0
4680	632.1	1.4119	1.4467	1.0604	1.3108	0
4681	632	1.4118	1.4469	1.0618	1.311	0
4682	631.9	1.4122	1.447	1.062	1.3109	0
4683	631.8	1.4125	1.4476	1.0611	1.3112	0
4684	631.7	1.4127	1.4478	1.0606	1.3119	0
4685	631.6	1.4126	1.4486	1.061	1.3116	0
4686	631.5	1.4131	1.4491	1.0615	1.3121	0
4687	631.4	1.4134	1.4494	1.0611	1.3119	0
4688	631.3	1.4136	1.4503	1.0621	1.3124	0
4689	631.2	1.4134	1.4507	1.0619	1.3125	0
4690	631.1	1.4138	1.4508	1.0617	1.3126	0
4691	631	1.4144	1.4508	1.0622	1.3126	0
4692	630.9	1.4143	1.4513	1.0612	1.3131	0
4693	630.8	1.4146	1.4516	1.0612	1.3131	0
4694	630.7	1.4146	1.4522	1.062	1.313	0
4695	630.6	1.4148	1.4524	1.0618	1.3134	0
4696	630.5	1.4147	1.4529	1.0608	1.3136	0
4697	630.4	1.4153	1.4535	1.0608	1.3141	0
4698	630.3	1.4154	1.454	1.0621	1.314	0
4699	630.2	1.4156	1.4545	1.062	1.3142	0
4700	630.1	1.4157	1.4542	1.0617	1.3141	0
4701	630	1.4162	1.4547	1.0623	1.3148	0
4702	629.9	1.416	1.4549	1.0621	1.3144	0
4703	629.8	1.4163	1.4551	1.0626	1.3147	0
4704	629.7	1.4161	1.4556	1.0618	1.3149	0
4705	629.6	1.4167	1.4559	1.0636	1.3147	0
4706	629.5	1.4167	1.4562	1.0631	1.3152	0
4707	629.4	1.4168	1.4566	1.0636	1.3155	0
4708	629.3	1.4171	1.4566	1.062	1.3153	0
4709	629.2	1.4175	1.4569	1.0622	1.316	0
4710	629.1	1.4175	1.4573	1.0618	1.316	0
4711	629	1.4175	1.4576	1.0628	1.316	0
4712	628.9	1.418	1.4581	1.0626	1.3165	0
4713	628.8	1.4179	1.4577	1.0625	1.3164	0
4714	628.7	1.4183	1.4583	1.0624	1.3166	0
4715	628.6	1.4185	1.4589	1.0624	1.317	0
4716	628.5	1.4186	1.459	1.0635	1.3169	0

4717	628.4	1.4189	1.4593	1.0633	1.3174	0
4718	628.3	1.4191	1.4595	1.0637	1.3174	0
4719	628.2	1.4189	1.4596	1.0624	1.3177	0
4720	628.1	1.4193	1.4599	1.0628	1.3176	0
4721	628	1.4196	1.4603	1.0628	1.318	0
4722	627.9	1.4193	1.4609	1.0629	1.3182	0
4723	627.8	1.4199	1.4609	1.063	1.3183	0
4724	627.7	1.4199	1.4618	1.0637	1.3186	0
4725	627.6	1.4203	1.4616	1.0636	1.319	0
4726	627.5	1.4203	1.4615	1.063	1.319	0
4727	627.4	1.4207	1.4622	1.0631	1.3194	0
4728	627.3	1.4205	1.4621	1.0636	1.3198	0
4729	627.2	1.4209	1.4621	1.0631	1.3199	0
4730	627.1	1.4211	1.4626	1.0633	1.3198	0
4731	627	1.4209	1.4628	1.0631	1.3203	0
4732	626.9	1.4212	1.4631	1.0636	1.3203	0
4733	626.8	1.4211	1.4633	1.0641	1.3205	0
4734	626.7	1.4218	1.4634	1.0642	1.3211	0
4735	626.6	1.4221	1.4633	1.0645	1.3212	0
4736	626.5	1.4221	1.4636	1.0632	1.3212	0
4737	626.4	1.4226	1.4635	1.0643	1.3217	0
4738	626.3	1.4226	1.4641	1.0639	1.3218	0
4739	626.2	1.4229	1.464	1.0636	1.322	0
4740	626.1	1.4228	1.464	1.0636	1.322	0
4741	626	1.4231	1.4636	1.0635	1.3225	0
4742	625.9	1.4233	1.4638	1.0634	1.3227	0
4743	625.8	1.4235	1.4637	1.0632	1.3228	0
4744	625.7	1.4235	1.4634	1.0627	1.3228	0
4745	625.6	1.4241	1.4634	1.0626	1.3231	0
4746	625.5	1.4237	1.4626	1.0621	1.3235	0
4747	625.4	1.4242	1.4631	1.0624	1.3235	0
4748	625.3	1.4242	1.463	1.0625	1.3237	0
4749	625.2	1.4244	1.4623	1.0629	1.324	0
4750	625.1	1.4244	1.4626	1.0624	1.324	0
4751	625	1.425	1.4619	1.0623	1.3245	0
4752	624.9	1.4251	1.4623	1.0631	1.3249	0
4753	624.8	1.4253	1.4622	1.0619	1.3251	0
4754	624.7	1.4252	1.4618	1.0626	1.3251	0
4755	624.6	1.4258	1.4618	1.0629	1.3254	0
4756	624.5	1.4258	1.4618	1.0623	1.3259	0
4757	624.4	1.4257	1.4607	1.0625	1.3259	0
4758	624.3	1.4261	1.4608	1.0618	1.3263	0
4759	624.2	1.4265	1.4608	1.0619	1.3264	0

4760	624.1	1.4267	1.461	1.0625	1.3269	0
4761	624	1.4269	1.4603	1.062	1.3269	0
4762	623.9	1.427	1.4601	1.0622	1.3272	0
4763	623.8	1.4276	1.4607	1.062	1.3273	0
4764	623.7	1.4279	1.4601	1.0618	1.3276	0
4765	623.6	1.4278	1.4596	1.0615	1.3276	0
4766	623.5	1.4277	1.4592	1.0627	1.328	0
4767	623.4	1.428	1.4588	1.063	1.3283	0
4768	623.3	1.4282	1.4585	1.0622	1.3283	0
4769	623.2	1.4282	1.4582	1.0624	1.3288	0
4770	623.1	1.4286	1.4588	1.0626	1.329	0
4771	623	1.4287	1.4584	1.062	1.3291	0
4772	622.9	1.4289	1.4586	1.062	1.3293	0
4773	622.8	1.4292	1.4585	1.0623	1.3298	0
4774	622.7	1.4294	1.4584	1.0622	1.3298	0
4775	622.6	1.4297	1.4581	1.0621	1.3298	0
4776	622.5	1.4296	1.4586	1.062	1.3303	0
4777	622.4	1.4299	1.4583	1.0616	1.3303	0
4778	622.3	1.4298	1.4581	1.0618	1.3305	0
4779	622.2	1.4303	1.4587	1.0615	1.3307	0
4780	622.1	1.4303	1.4583	1.0623	1.3313	0
4781	622	1.4306	1.4581	1.0617	1.3314	0
4782	621.9	1.4306	1.4583	1.0619	1.3311	0
4783	621.8	1.431	1.4581	1.0618	1.3313	0
4784	621.7	1.4309	1.458	1.0618	1.3313	0
4785	621.6	1.4312	1.458	1.0624	1.3315	0
4786	621.5	1.4314	1.4582	1.0626	1.3319	0
4787	621.4	1.4316	1.458	1.062	1.3319	0
4788	621.3	1.4316	1.4581	1.0622	1.332	0
4789	621.2	1.4317	1.4579	1.0627	1.3324	0
4790	621.1	1.4317	1.4578	1.0626	1.3326	0
4791	621	1.4322	1.4584	1.0621	1.3329	0
4792	620.9	1.4321	1.4586	1.0628	1.333	0
4793	620.8	1.4327	1.4585	1.0621	1.3331	0
4794	620.7	1.4328	1.4584	1.0626	1.3332	0
4795	620.6	1.4328	1.4586	1.0625	1.3334	0
4796	620.5	1.4331	1.4584	1.0628	1.3335	0
4797	620.4	1.4331	1.4586	1.0619	1.3333	0
4798	620.3	1.4333	1.4585	1.062	1.3337	0
4799	620.2	1.4332	1.4584	1.0618	1.3337	0
4800	620.1	1.4338	1.4584	1.0622	1.334	0
4801	620	1.4337	1.4592	1.0616	1.3344	0
4802	619.9	1.4338	1.4596	1.0618	1.3345	0

4803	619.8	1.4341	1.459	1.0627	1.3344	0
4804	619.7	1.434	1.4595	1.062	1.3346	0
4805	619.6	1.4346	1.4595	1.0629	1.3349	0
4806	619.5	1.4348	1.4597	1.0631	1.3349	0
4807	619.4	1.435	1.4599	1.0631	1.3348	0
4808	619.3	1.4349	1.4598	1.0635	1.3353	0
4809	619.2	1.4352	1.4601	1.063	1.3353	0
4810	619.1	1.4355	1.4603	1.0638	1.3356	0
4811	619	1.4356	1.4602	1.0629	1.3357	0
4812	618.9	1.4359	1.4607	1.0627	1.336	0
4813	618.8	1.4362	1.4608	1.0639	1.3358	0
4814	618.7	1.4359	1.4607	1.0643	1.336	0
4815	618.6	1.4365	1.4611	1.0644	1.3364	0
4816	618.5	1.4367	1.461	1.0641	1.3363	0
4817	618.4	1.437	1.4609	1.0642	1.3366	0
4818	618.3	1.4369	1.4608	1.0645	1.3368	0
4819	618.2	1.4371	1.4614	1.0646	1.3368	0
4820	618.1	1.4374	1.4616	1.0656	1.3371	0
4821	618	1.4373	1.4621	1.0654	1.3373	0
4822	617.9	1.438	1.462	1.0654	1.3372	0
4823	617.8	1.4377	1.4622	1.0657	1.3374	0
4824	617.7	1.4378	1.4621	1.0647	1.3371	0
4825	617.6	1.4384	1.4624	1.0647	1.3379	0
4826	617.5	1.4383	1.4622	1.0644	1.3376	0
4827	617.4	1.4386	1.4626	1.065	1.3384	0
4828	617.3	1.4389	1.4628	1.0653	1.3384	0
4829	617.2	1.4391	1.4631	1.0654	1.3382	0
4830	617.1	1.4394	1.4631	1.0659	1.3384	0
4831	617	1.4393	1.463	1.0647	1.3389	0
4832	616.9	1.4395	1.4628	1.0654	1.3388	0
4833	616.8	1.4399	1.4635	1.0649	1.3392	0
4834	616.7	1.4401	1.4637	1.0654	1.3391	0
4835	616.6	1.4406	1.4637	1.0657	1.3396	0
4836	616.5	1.4405	1.4635	1.0649	1.3398	0
4837	616.4	1.4407	1.4638	1.0657	1.3398	0
4838	616.3	1.4411	1.4644	1.0656	1.3404	0
4839	616.2	1.441	1.4641	1.0646	1.3406	0
4840	616.1	1.4413	1.4649	1.0648	1.3407	0
4841	616	1.4418	1.4649	1.0643	1.3411	0
4842	615.9	1.4415	1.4651	1.0653	1.3412	0
4843	615.8	1.442	1.4651	1.0648	1.3414	0
4844	615.7	1.4425	1.4655	1.0646	1.3416	0
4845	615.6	1.4427	1.4646	1.0647	1.3418	0

4846	615.5	1.4427	1.4651	1.0646	1.3421	0
4847	615.4	1.4429	1.465	1.0644	1.3425	0
4848	615.3	1.4431	1.4656	1.0645	1.3425	0
4849	615.2	1.4437	1.4658	1.0638	1.343	0
4850	615.1	1.4436	1.4657	1.0645	1.343	0
4851	615	1.4438	1.4659	1.0646	1.3432	0
4852	614.9	1.4443	1.4659	1.0642	1.3434	0
4853	614.8	1.4444	1.4659	1.0643	1.3442	0
4854	614.7	1.4448	1.4664	1.0628	1.3439	0
4855	614.6	1.4447	1.4663	1.0634	1.3441	0
4856	614.5	1.4451	1.4666	1.0638	1.3444	0
4857	614.4	1.4454	1.4663	1.0624	1.3447	0
4858	614.3	1.4454	1.4663	1.0626	1.345	0
4859	614.2	1.446	1.4669	1.063	1.3453	0
4860	614.1	1.4462	1.4671	1.0628	1.346	0
4861	614	1.4463	1.4672	1.0622	1.3459	0
4862	613.9	1.4469	1.4672	1.0625	1.3459	0
4863	613.8	1.447	1.4676	1.0634	1.3465	0
4864	613.7	1.4474	1.4677	1.0621	1.3466	0
4865	613.6	1.4472	1.4678	1.0625	1.3468	0
4866	613.5	1.4482	1.4679	1.061	1.3473	0
4867	613.4	1.4479	1.4677	1.0616	1.3474	0
4868	613.3	1.4482	1.468	1.0614	1.3474	0
4869	613.2	1.4481	1.4684	1.062	1.3478	0
4870	613.1	1.4489	1.4681	1.0614	1.3481	0
4871	613	1.4488	1.4685	1.0606	1.3485	0
4872	612.9	1.4492	1.4686	1.061	1.3485	0
4873	612.8	1.4492	1.4687	1.0614	1.3486	0
4874	612.7	1.4495	1.4692	1.062	1.3488	0
4875	612.6	1.45	1.4692	1.0613	1.3491	0
4876	612.5	1.4502	1.4697	1.0613	1.3495	0
4877	612.4	1.4502	1.47	1.0612	1.3495	0
4878	612.3	1.4503	1.4697	1.061	1.3498	0
4879	612.2	1.4507	1.4699	1.0605	1.3499	0
4880	612.1	1.4513	1.4705	1.0607	1.3505	0
4881	612	1.4511	1.4706	1.0611	1.3506	0
4882	611.9	1.4515	1.4707	1.0611	1.3505	0
4883	611.8	1.4516	1.4708	1.0608	1.3508	0
4884	611.7	1.4519	1.4708	1.061	1.351	0
4885	611.6	1.4524	1.4707	1.061	1.3512	0
4886	611.5	1.4522	1.4714	1.0612	1.3518	0
4887	611.4	1.4525	1.4714	1.0612	1.3516	0
4888	611.3	1.4526	1.4715	1.0616	1.3519	0

4889	611.2	1.4528	1.4715	1.0616	1.3518	0
4890	611.1	1.4531	1.4723	1.0621	1.3523	0
4891	611	1.4535	1.4724	1.0615	1.3526	0
4892	610.9	1.4538	1.4728	1.0615	1.3527	0
4893	610.8	1.4539	1.4729	1.0621	1.3528	0
4894	610.7	1.4539	1.4729	1.0625	1.353	0
4895	610.6	1.4543	1.4729	1.0614	1.3533	0
4896	610.5	1.4545	1.4729	1.0613	1.3533	0
4897	610.4	1.4547	1.4734	1.0623	1.3537	0
4898	610.3	1.4549	1.4736	1.0629	1.3536	0
4899	610.2	1.455	1.474	1.0631	1.354	0
4900	610.1	1.4553	1.4737	1.0631	1.3542	0
4901	610	1.4557	1.4741	1.0631	1.3543	0
4902	609.9	1.4563	1.4741	1.0634	1.3545	0
4903	609.8	1.4558	1.4746	1.063	1.3546	0
4904	609.7	1.4559	1.4746	1.0623	1.3544	0
4905	609.6	1.4565	1.4749	1.0629	1.3546	0
4906	609.5	1.4562	1.4753	1.0638	1.3549	0
4907	609.4	1.457	1.4755	1.0637	1.3551	0
4908	609.3	1.4574	1.4753	1.0641	1.3549	0
4909	609.2	1.4576	1.4755	1.0648	1.3551	0
4910	609.1	1.4576	1.4761	1.0651	1.3548	0
4911	609	1.4576	1.4758	1.0647	1.3553	0
4912	608.9	1.4581	1.476	1.0648	1.355	0
4913	608.8	1.4583	1.4759	1.0654	1.3551	0
4914	608.7	1.4583	1.4766	1.0653	1.3554	0
4915	608.6	1.4585	1.4768	1.0667	1.3555	0
4916	608.5	1.4588	1.4773	1.0659	1.3555	0
4917	608.4	1.4591	1.4771	1.0663	1.356	0
4918	608.3	1.4593	1.4775	1.066	1.3558	0
4919	608.2	1.4597	1.4777	1.0655	1.3557	0
4920	608.1	1.4595	1.4781	1.066	1.3563	0
4921	608	1.46	1.478	1.0667	1.3561	0
4922	607.9	1.4602	1.4782	1.0658	1.3558	0
4923	607.8	1.4605	1.4788	1.0668	1.3565	0
4924	607.7	1.461	1.4785	1.066	1.3565	0
4925	607.6	1.4609	1.4787	1.0665	1.3566	0
4926	607.5	1.4612	1.479	1.0657	1.3563	0
4927	607.4	1.4615	1.479	1.067	1.3567	0
4928	607.3	1.4616	1.4794	1.0673	1.3565	0
4929	607.2	1.4619	1.4797	1.0675	1.357	0
4930	607.1	1.4619	1.4794	1.0665	1.3567	0
4931	607	1.4624	1.4803	1.067	1.3569	0

4932	606.9	1.4629	1.4801	1.0674	1.357	0
4933	606.8	1.4627	1.4806	1.0684	1.3571	0
4934	606.7	1.4629	1.4808	1.0675	1.3574	0
4935	606.6	1.4631	1.481	1.0668	1.3576	0
4936	606.5	1.4638	1.4815	1.0683	1.3574	0
4937	606.4	1.464	1.4817	1.0682	1.3577	0
4938	606.3	1.4641	1.4814	1.0674	1.3578	0
4939	606.2	1.4645	1.4825	1.0679	1.358	0
4940	606.1	1.465	1.4821	1.0683	1.358	0
4941	606	1.4651	1.4829	1.0676	1.3585	0
4942	605.9	1.4655	1.4829	1.0672	1.3584	0
4943	605.8	1.4656	1.4834	1.0673	1.3588	0
4944	605.7	1.466	1.4831	1.0673	1.359	0
4945	605.6	1.4663	1.4838	1.0681	1.3589	0
4946	605.5	1.4664	1.4842	1.0679	1.3589	0
4947	605.4	1.4667	1.484	1.0675	1.3592	0
4948	605.3	1.4671	1.4843	1.0679	1.3591	0
4949	605.2	1.4673	1.4846	1.0677	1.3593	0
4950	605.1	1.4679	1.4852	1.0681	1.3597	0
4951	605	1.4679	1.4852	1.0677	1.3601	0
4952	604.9	1.4683	1.4853	1.0669	1.3601	0
4953	604.8	1.4683	1.4857	1.0678	1.3603	0
4954	604.7	1.4687	1.4864	1.0677	1.3606	0
4955	604.6	1.4694	1.4858	1.0669	1.3606	0
4956	604.5	1.4692	1.4863	1.0674	1.3608	0
4957	604.4	1.4692	1.4866	1.0668	1.361	0
4958	604.3	1.4699	1.4867	1.0663	1.3612	0
4959	604.2	1.4702	1.4868	1.0665	1.3617	0
4960	604.1	1.4705	1.4867	1.0668	1.3618	0
4961	604	1.4704	1.487	1.066	1.3619	0
4962	603.9	1.4708	1.487	1.0678	1.3621	0
4963	603.8	1.4711	1.4877	1.0673	1.3623	0
4964	603.7	1.4714	1.4883	1.0663	1.3627	0
4965	603.6	1.4716	1.4883	1.0658	1.363	0
4966	603.5	1.4722	1.4879	1.0662	1.3631	0
4967	603.4	1.4724	1.4885	1.0664	1.363	0
4968	603.3	1.4724	1.4881	1.066	1.3634	0
4969	603.2	1.4728	1.4883	1.066	1.3636	0
4970	603.1	1.4727	1.4882	1.0652	1.3637	0
4971	603	1.4733	1.4885	1.0651	1.3638	0
4972	602.9	1.4738	1.4883	1.0648	1.3643	0
4973	602.8	1.4736	1.4891	1.0656	1.3645	0
4974	602.7	1.4743	1.489	1.0647	1.3645	0

4975	602.6	1.4746	1.4892	1.0649	1.3649	0
4976	602.5	1.475	1.4894	1.0645	1.3648	0
4977	602.4	1.4754	1.4895	1.0643	1.365	0
4978	602.3	1.4749	1.4899	1.0659	1.3652	0
4979	602.2	1.4756	1.4897	1.064	1.3654	0
4980	602.1	1.4757	1.4895	1.0635	1.3658	0
4981	602	1.4762	1.4904	1.0646	1.366	0
4982	601.9	1.4763	1.4901	1.0647	1.3659	0
4983	601.8	1.4763	1.4908	1.0653	1.3662	0
4984	601.7	1.477	1.4906	1.0649	1.3664	0
4985	601.6	1.4772	1.4904	1.0651	1.3669	0
4986	601.5	1.4771	1.4911	1.0647	1.3669	0
4987	601.4	1.4776	1.4912	1.0657	1.3672	0
4988	601.3	1.4779	1.4913	1.0655	1.3674	0
4989	601.2	1.4783	1.4917	1.065	1.3678	0
4990	601.1	1.4782	1.4913	1.0651	1.3678	0
4991	601	1.4785	1.4913	1.0652	1.3681	0
4992	600.9	1.4789	1.4911	1.0656	1.3678	0
4993	600.8	1.4788	1.4913	1.0652	1.3683	0
4994	600.7	1.4789	1.4917	1.065	1.3685	0
4995	600.6	1.4795	1.4924	1.0652	1.3685	0
4996	600.5	1.4794	1.4923	1.0664	1.3686	0
4997	600.4	1.4805	1.4928	1.066	1.3688	0
4998	600.3	1.48	1.4923	1.0659	1.3692	0
4999	600.2	1.4802	1.4929	1.0662	1.3692	0
5000	600.1	1.481	1.493	1.0652	1.3695	0
5001	600	1.4812	1.4933	1.0656	1.37	0
5002	599.9	1.4813	1.4931	1.0658	1.3702	0
5003	599.8	1.4815	1.4936	1.0655	1.3703	0
5004	599.7	1.482	1.4935	1.0652	1.3703	0
5005	599.6	1.4819	1.4944	1.0652	1.3703	0
5006	599.5	1.4821	1.4946	1.0662	1.3706	0
5007	599.4	1.4823	1.4949	1.0667	1.371	0
5008	599.3	1.4828	1.4951	1.0669	1.371	0
5009	599.2	1.483	1.495	1.0671	1.3715	0
5010	599.1	1.4831	1.4954	1.0667	1.3714	0
5011	599	1.4834	1.4958	1.0665	1.3714	0
5012	598.9	1.4833	1.4964	1.0674	1.3717	0
5013	598.8	1.4841	1.4962	1.0671	1.3716	0
5014	598.7	1.4844	1.4965	1.0681	1.3719	0
5015	598.6	1.4845	1.4963	1.0672	1.3723	0
5016	598.5	1.4843	1.4971	1.0677	1.3724	0
5017	598.4	1.4845	1.4966	1.0673	1.3724	0

5018	598.3	1.4847	1.4969	1.0679	1.3724	0
5019	598.2	1.4855	1.4974	1.0694	1.3728	0
5020	598.1	1.4854	1.4981	1.0673	1.3728	0
5021	598	1.4855	1.4982	1.0694	1.373	0
5022	597.9	1.4857	1.4984	1.0694	1.3732	0
5023	597.8	1.4862	1.4982	1.0685	1.3731	0
5024	597.7	1.4865	1.4992	1.069	1.3731	0
5025	597.6	1.4866	1.4987	1.0702	1.3733	0
5026	597.5	1.4872	1.4996	1.0707	1.3735	0
5027	597.4	1.4874	1.4999	1.0698	1.3738	0
5028	597.3	1.4872	1.5005	1.0694	1.3745	0
5029	597.2	1.4877	1.5003	1.0703	1.3744	0
5030	597.1	1.4878	1.5004	1.0707	1.374	0
5031	597	1.4883	1.501	1.0704	1.3747	0
5032	596.9	1.4884	1.5011	1.0699	1.3749	0
5033	596.8	1.4889	1.5012	1.0708	1.3752	0
5034	596.7	1.4889	1.5013	1.0694	1.3754	0
5035	596.6	1.4891	1.5022	1.0704	1.3753	0
5036	596.5	1.4896	1.5024	1.0706	1.3755	0
5037	596.4	1.4897	1.5025	1.0712	1.3756	0
5038	596.3	1.4902	1.5031	1.0719	1.376	0
5039	596.2	1.4906	1.5035	1.0717	1.3763	0
5040	596.1	1.491	1.5032	1.0714	1.3761	0
5041	596	1.4915	1.5038	1.0712	1.3768	0
5042	595.9	1.4916	1.5039	1.0716	1.3769	0
5043	595.8	1.4915	1.5042	1.0715	1.3772	0
5044	595.7	1.4923	1.5047	1.0713	1.3773	0
5045	595.6	1.4924	1.5044	1.0716	1.3774	0
5046	595.5	1.4927	1.5051	1.0709	1.3779	0
5047	595.4	1.4932	1.5057	1.0715	1.3784	0
5048	595.3	1.4935	1.5057	1.0705	1.3786	0
5049	595.2	1.4935	1.5061	1.0706	1.3787	0
5050	595.1	1.4943	1.5066	1.0714	1.3793	0
5051	595	1.4942	1.5069	1.0709	1.3792	0
5052	594.9	1.4948	1.5071	1.0699	1.3797	0
5053	594.8	1.4951	1.5067	1.07	1.3803	0
5054	594.7	1.4958	1.5078	1.0702	1.3802	0
5055	594.6	1.4962	1.5079	1.0701	1.3809	0
5056	594.5	1.4963	1.5088	1.0702	1.3813	0
5057	594.4	1.4967	1.5088	1.0703	1.3816	0
5058	594.3	1.4972	1.5094	1.0701	1.3814	0
5059	594.2	1.4975	1.509	1.07	1.3819	0
5060	594.1	1.4976	1.509	1.0699	1.3823	0

5061	594	1.4984	1.5095	1.0692	1.383	0
5062	593.9	1.4988	1.5108	1.0695	1.3833	0
5063	593.8	1.4995	1.5107	1.0691	1.3835	0
5064	593.7	1.4996	1.5112	1.0689	1.3839	0
5065	593.6	1.4999	1.5116	1.0695	1.3842	0
5066	593.5	1.5006	1.5119	1.0693	1.3848	0
5067	593.4	1.5006	1.512	1.0699	1.3851	0
5068	593.3	1.501	1.5124	1.0691	1.3854	0
5069	593.2	1.5017	1.5128	1.069	1.3854	0
5070	593.1	1.5022	1.5128	1.0687	1.386	0
5071	593	1.5025	1.5132	1.0686	1.3863	0
5072	592.9	1.503	1.5133	1.0688	1.387	0
5073	592.8	1.5036	1.514	1.0685	1.3876	0
5074	592.7	1.5044	1.5148	1.068	1.3875	0
5075	592.6	1.5043	1.5148	1.0678	1.3882	0
5076	592.5	1.5042	1.5151	1.0676	1.3884	0
5077	592.4	1.5046	1.5152	1.0678	1.3887	0
5078	592.3	1.5052	1.5154	1.0684	1.3893	0
5079	592.2	1.506	1.5158	1.0672	1.3896	0
5080	592.1	1.5061	1.5163	1.0671	1.39	0
5081	592	1.5058	1.5165	1.0677	1.3905	0
5082	591.9	1.5068	1.5171	1.0679	1.391	0
5083	591.8	1.5072	1.5167	1.0665	1.3913	0
5084	591.7	1.5077	1.5172	1.0675	1.3917	0
5085	591.6	1.5087	1.5182	1.0669	1.3922	0
5086	591.5	1.5083	1.5179	1.0671	1.392	0
5087	591.4	1.5088	1.518	1.0671	1.393	0
5088	591.3	1.5088	1.518	1.0675	1.3931	0
5089	591.2	1.5088	1.5189	1.0671	1.3934	0
5090	591.1	1.5097	1.5189	1.0669	1.3931	0
5091	591	1.5102	1.5195	1.0668	1.3942	0
5092	590.9	1.5103	1.5192	1.0667	1.3944	0
5093	590.8	1.511	1.5199	1.0666	1.395	0
5094	590.7	1.5111	1.521	1.0665	1.3955	0
5095	590.6	1.5122	1.5211	1.0671	1.396	0
5096	590.5	1.5127	1.5213	1.0667	1.3958	0
5097	590.4	1.5129	1.522	1.0669	1.396	0
5098	590.3	1.513	1.5225	1.0673	1.3968	0
5099	590.2	1.5136	1.5227	1.067	1.397	0
5100	590.1	1.5136	1.5228	1.0674	1.3974	0
5101	590	1.5142	1.5234	1.0671	1.3982	0
5102	589.9	1.5145	1.5237	1.067	1.3982	0
5103	589.8	1.5151	1.5235	1.0674	1.3988	0

5104	589.7	1.5152	1.5245	1.0656	1.3985	0
5105	589.6	1.5158	1.5252	1.0667	1.3991	0
5106	589.5	1.516	1.5249	1.0658	1.3996	0
5107	589.4	1.5162	1.5256	1.0671	1.3995	0
5108	589.3	1.5174	1.5264	1.0665	1.4001	0
5109	589.2	1.5176	1.5261	1.0659	1.4	0
5110	589.1	1.5175	1.5265	1.068	1.4007	0
5111	589	1.518	1.5265	1.0671	1.4004	0
5112	588.9	1.5177	1.5267	1.0672	1.4013	0
5113	588.8	1.5186	1.5277	1.0681	1.4018	0
5114	588.7	1.5193	1.528	1.0684	1.4017	0
5115	588.6	1.5197	1.5279	1.0683	1.4021	0
5116	588.5	1.52	1.5283	1.0693	1.4021	0
5117	588.4	1.5203	1.5291	1.0683	1.4028	0
5118	588.3	1.5204	1.5292	1.0689	1.4022	0
5119	588.2	1.5208	1.5301	1.0682	1.403	0
5120	588.1	1.5211	1.5299	1.0685	1.4029	0
5121	588	1.5214	1.5303	1.0689	1.4033	0
5122	587.9	1.5219	1.5308	1.0688	1.4033	0
5123	587.8	1.5224	1.5318	1.0694	1.4039	0
5124	587.7	1.5226	1.5316	1.0694	1.4038	0
5125	587.6	1.5226	1.5326	1.0697	1.4046	0
5126	587.5	1.523	1.5321	1.0702	1.4043	0
5127	587.4	1.524	1.5331	1.0694	1.4048	0
5128	587.3	1.5234	1.5335	1.0696	1.4045	0
5129	587.2	1.5238	1.5339	1.0698	1.4053	0
5130	587.1	1.5247	1.5344	1.0703	1.4057	0
5131	587	1.5251	1.5343	1.0697	1.4057	0
5132	586.9	1.5252	1.5349	1.0699	1.406	0
5133	586.8	1.5258	1.535	1.0695	1.4062	0
5134	586.7	1.5258	1.5361	1.0702	1.4067	0
5135	586.6	1.5264	1.5362	1.0706	1.407	0
5136	586.5	1.5263	1.5367	1.0707	1.4071	0
5137	586.4	1.5269	1.5373	1.0712	1.4077	0
5138	586.3	1.5275	1.5374	1.0705	1.4075	0
5139	586.2	1.5271	1.5381	1.0709	1.4077	0
5140	586.1	1.5275	1.5385	1.0706	1.4082	0
5141	586	1.5274	1.539	1.0705	1.4086	0
5142	585.9	1.5286	1.5402	1.0709	1.4085	0
5143	585.8	1.5286	1.5392	1.072	1.4087	0
5144	585.7	1.5293	1.5399	1.0704	1.4089	0
5145	585.6	1.5294	1.5406	1.0714	1.4092	0
5146	585.5	1.5299	1.5412	1.0708	1.4094	0

5147	585.4	1.5295	1.5413	1.0706	1.41	0
5148	585.3	1.5308	1.5416	1.0714	1.41	0
5149	585.2	1.5313	1.5421	1.0714	1.4105	0
5150	585.1	1.5313	1.5427	1.0709	1.4111	0
5151	585	1.5314	1.5434	1.0713	1.411	0
5152	584.9	1.532	1.5397	1.0727	1.4111	0
5153	584.8	1.5325	1.5393	1.0727	1.4101	0
5154	584.7	1.5339	1.5399	1.0723	1.4117	0
5155	584.6	1.5334	1.5411	1.0719	1.4106	0
5156	584.5	1.5348	1.5416	1.0724	1.4115	0
5157	584.4	1.5352	1.5421	1.0723	1.4124	0
5158	584.3	1.5349	1.5427	1.0726	1.4128	0
5159	584.2	1.5353	1.5422	1.0721	1.4136	0
5160	584.1	1.5356	1.5426	1.0722	1.4131	0
5161	584	1.5352	1.5439	1.0721	1.4133	0
5162	583.9	1.5367	1.5445	1.0721	1.4137	0
5163	583.8	1.5364	1.5451	1.0706	1.4141	0
5164	583.7	1.5361	1.5457	1.0718	1.4144	0
5165	583.6	1.5368	1.5456	1.072	1.4143	0
5166	583.5	1.5368	1.5464	1.0713	1.4149	0
5167	583.4	1.5385	1.5472	1.0716	1.4142	0
5168	583.3	1.5385	1.549	1.0719	1.4149	0
5169	583.2	1.5386	1.5491	1.0717	1.4156	0
5170	583.1	1.5388	1.5493	1.0719	1.4158	0
5171	583	1.54	1.5497	1.072	1.4162	0
5172	582.9	1.5403	1.5508	1.071	1.4164	0
5173	582.8	1.5408	1.5513	1.0721	1.4163	0
5174	582.7	1.5405	1.5528	1.0709	1.4168	0
5175	582.6	1.541	1.5524	1.0717	1.4167	0
5176	582.5	1.5407	1.553	1.072	1.4173	0
5177	582.4	1.5423	1.5537	1.0713	1.418	0
5178	582.3	1.5423	1.5546	1.0727	1.4182	0
5179	582.2	1.5415	1.5546	1.0728	1.4179	0
5180	582.1	1.5433	1.5555	1.0725	1.4188	0
5181	582	1.5434	1.5565	1.0715	1.4185	0
5182	581.9	1.5435	1.5575	1.0719	1.4188	0
5183	581.8	1.5438	1.5577	1.0721	1.4193	0
5184	581.7	1.545	1.5589	1.0725	1.4192	0
5185	581.6	1.5453	1.5592	1.0725	1.4197	0
5186	581.5	1.5447	1.5595	1.0733	1.4189	0
5187	581.4	1.5458	1.5615	1.073	1.4207	0
5188	581.3	1.5462	1.5609	1.0722	1.4206	0
5189	581.2	1.5465	1.5621	1.0725	1.4205	0

5190	581.1	1.5468	1.5632	1.0728	1.421	0
5191	581	1.5471	1.5635	1.0731	1.4209	0
5192	580.9	1.5481	1.5636	1.0732	1.4212	0
5193	580.8	1.549	1.5646	1.0731	1.4222	0
5194	580.7	1.5485	1.5648	1.0722	1.422	0
5195	580.6	1.5486	1.5667	1.0738	1.4231	0
5196	580.5	1.5496	1.5668	1.0733	1.4228	0
5197	580.4	1.5497	1.5678	1.0723	1.4225	0
5198	580.3	1.5505	1.5685	1.0734	1.4226	0
5199	580.2	1.5508	1.5697	1.0728	1.4225	0
5200	580.1	1.5515	1.5703	1.0713	1.4232	0
5201	580	1.5526	1.5705	1.0721	1.4243	0
5202	579.9	1.5525	1.5722	1.0731	1.4244	0
5203	579.8	1.5516	1.5721	1.0729	1.424	0
5204	579.7	1.5533	1.573	1.0726	1.4249	0
5205	579.6	1.5525	1.5738	1.0732	1.4245	0
5206	579.5	1.5533	1.5746	1.0726	1.4252	0
5207	579.4	1.554	1.5762	1.0727	1.4253	0
5208	579.3	1.555	1.5746	1.0727	1.4264	0
5209	579.2	1.5558	1.5763	1.0726	1.4259	0
5210	579.1	1.5559	1.578	1.0724	1.4262	0
5211	579	1.5567	1.578	1.0725	1.4271	0
5212	578.9	1.5575	1.5788	1.0738	1.4266	0
5213	578.8	1.5573	1.5794	1.0733	1.4272	0
5214	578.7	1.5581	1.5801	1.0727	1.4273	0
5215	578.6	1.558	1.5818	1.0727	1.4275	0
5216	578.5	1.5587	1.5833	1.0733	1.4276	0
5217	578.4	1.5594	1.5832	1.0736	1.4283	0
5218	578.3	1.5595	1.585	1.0737	1.428	0
5219	578.2	1.5611	1.5849	1.0741	1.4282	0
5220	578.1	1.5609	1.5864	1.0739	1.4288	0
5221	578	1.5624	1.5862	1.073	1.4288	0
5222	577.9	1.5613	1.5868	1.0731	1.4288	0
5223	577.8	1.5629	1.5884	1.0739	1.4296	0
5224	577.7	1.5629	1.5893	1.0742	1.4304	0
5225	577.6	1.5635	1.589	1.0731	1.4298	0
5226	577.5	1.5642	1.5905	1.0725	1.4305	0
5227	577.4	1.5648	1.5911	1.0725	1.4305	0
5228	577.3	1.5655	1.5918	1.0735	1.4306	0
5229	577.2	1.5662	1.5925	1.0731	1.4306	0
5230	577.1	1.5661	1.5924	1.0742	1.4314	0
5231	577	1.5658	1.5937	1.0736	1.4313	0
5232	576.9	1.5674	1.5954	1.073	1.4315	0

5233	576.8	1.5682	1.5953	1.0733	1.4317	0
5234	576.7	1.5687	1.5949	1.0733	1.4328	0
5235	576.6	1.5685	1.5965	1.0737	1.4322	0
5236	576.5	1.5707	1.597	1.0735	1.4322	0
5237	576.4	1.5705	1.5986	1.0738	1.4334	0
5238	576.3	1.5721	1.5985	1.0744	1.433	0
5239	576.2	1.5728	1.5983	1.074	1.4331	0
5240	576.1	1.5726	1.5989	1.0744	1.4337	0
5241	576	1.5742	1.6005	1.0748	1.4338	0
5242	575.9	1.5739	1.5993	1.0759	1.4344	0
5243	575.8	1.5746	1.6017	1.0755	1.4339	0
5244	575.7	1.5767	1.6022	1.075	1.435	0
5245	575.6	1.5773	1.6011	1.0745	1.4344	0
5246	575.5	1.578	1.6044	1.0747	1.4351	0
5247	575.4	1.5777	1.604	1.0745	1.4344	0
5248	575.3	1.5792	1.6039	1.0747	1.4357	0
5249	575.2	1.579	1.6045	1.0748	1.4357	0
5250	575.1	1.5805	1.6051	1.0747	1.4358	0
5251	575	1.5802	1.6039	1.0744	1.4363	0
5252	574.9	1.5816	1.6045	1.075	1.4363	0
5253	574.8	1.5823	1.6061	1.0747	1.437	0
5254	574.7	1.5839	1.6068	1.0746	1.4366	0
5255	574.6	1.5836	1.6065	1.0749	1.4371	0
5256	574.5	1.5843	1.6071	1.0738	1.4372	0
5257	574.4	1.5849	1.6086	1.0747	1.4378	0
5258	574.3	1.5854	1.6074	1.0747	1.4377	0
5259	574.2	1.5868	1.608	1.0742	1.4377	0
5260	574.1	1.5875	1.6087	1.0749	1.4385	0
5261	574	1.5881	1.6093	1.0751	1.4385	0
5262	573.9	1.5888	1.6091	1.0742	1.4391	0
5263	573.8	1.5904	1.6099	1.0742	1.4393	0
5264	573.7	1.591	1.6104	1.074	1.4387	0
5265	573.6	1.5905	1.61	1.0737	1.4397	0
5266	573.5	1.592	1.6106	1.0736	1.4398	0
5267	573.4	1.5917	1.6103	1.0728	1.4403	0
5268	573.3	1.594	1.611	1.0742	1.4404	0
5269	573.2	1.5938	1.6107	1.0741	1.441	0
5270	573.1	1.5943	1.6104	1.0739	1.4404	0
5271	573	1.5948	1.6109	1.0741	1.4408	0
5272	572.9	1.5963	1.6106	1.074	1.4409	0
5273	572.8	1.597	1.6105	1.0742	1.4411	0
5274	572.7	1.5984	1.6111	1.0731	1.4416	0
5275	572.6	1.5982	1.6117	1.074	1.4417	0

5276	572.5	1.5988	1.6114	1.0732	1.4422	0
5277	572.4	1.6002	1.6112	1.0734	1.4428	0
5278	572.3	1.6001	1.611	1.0735	1.4429	0
5279	572.2	1.6015	1.6116	1.0734	1.443	0
5280	572.1	1.602	1.6121	1.0727	1.4434	0
5281	572	1.6027	1.6128	1.0733	1.4435	0
5282	571.9	1.6032	1.6124	1.0746	1.4441	0
5283	571.8	1.6038	1.6113	1.0735	1.4441	0
5284	571.7	1.6061	1.6128	1.0751	1.4441	0
5285	571.6	1.6065	1.6116	1.0746	1.444	0
5286	571.5	1.6064	1.6131	1.0745	1.4447	0
5287	571.4	1.6069	1.612	1.0736	1.4452	0
5288	571.3	1.6076	1.6118	1.0742	1.4453	0
5289	571.2	1.609	1.6115	1.0748	1.4453	0
5290	571.1	1.6095	1.612	1.0741	1.4453	0
5291	571	1.6102	1.6111	1.0744	1.446	0
5292	570.9	1.6107	1.6123	1.0736	1.4464	0
5293	570.8	1.6113	1.6113	1.0737	1.4465	0
5294	570.7	1.6118	1.6135	1.0742	1.4464	0
5295	570.6	1.614	1.6115	1.0745	1.4469	0
5296	570.5	1.6138	1.613	1.0749	1.447	0
5297	570.4	1.6135	1.6127	1.0747	1.4476	0
5298	570.3	1.6149	1.6124	1.0751	1.4476	0
5299	570.2	1.6163	1.6121	1.0749	1.4481	0
5300	570.1	1.617	1.6129	1.0751	1.4488	0
5301	570	1.6167	1.6134	1.0751	1.4488	0
5302	569.9	1.6181	1.6131	1.0753	1.4488	0
5303	569.8	1.6188	1.6138	1.075	1.4495	0
5304	569.7	1.6192	1.6142	1.0751	1.4493	0
5305	569.6	1.6198	1.6139	1.0755	1.4504	0
5306	569.5	1.6204	1.6137	1.0749	1.4493	0
5307	569.4	1.6218	1.6143	1.0762	1.45	0
5308	569.3	1.6225	1.6141	1.0764	1.4506	0
5309	569.2	1.6231	1.6148	1.076	1.4512	0
5310	569.1	1.6236	1.6145	1.0763	1.4517	0
5311	569	1.6241	1.6142	1.0761	1.4517	0
5312	568.9	1.6247	1.6148	1.0765	1.4512	0
5313	568.8	1.6254	1.6163	1.076	1.4519	0
5314	568.7	1.6261	1.6161	1.0769	1.452	0
5315	568.6	1.6275	1.6159	1.0772	1.4531	0
5316	568.5	1.6272	1.6164	1.0766	1.4525	0
5317	568.4	1.6287	1.6162	1.0765	1.4526	0
5318	568.3	1.6292	1.6167	1.0768	1.4536	0

5319	568.2	1.6304	1.6163	1.076	1.4535	0
5320	568.1	1.6303	1.6162	1.0772	1.4531	0
5321	568	1.6309	1.6168	1.0766	1.4537	0
5322	567.9	1.6315	1.6174	1.0765	1.4543	0
5323	567.8	1.6329	1.6171	1.0768	1.4554	0
5324	567.7	1.6326	1.6177	1.0764	1.4548	0
5325	567.6	1.6332	1.6183	1.0766	1.4554	0
5326	567.5	1.6345	1.6188	1.0771	1.4559	0
5327	567.4	1.6343	1.6185	1.0767	1.4565	0
5328	567.3	1.6349	1.62	1.0773	1.4566	0
5329	567.2	1.6354	1.6189	1.0774	1.4571	0
5330	567.1	1.6368	1.6195	1.0775	1.4577	0
5331	567	1.6357	1.6208	1.0769	1.4571	0
5332	566.9	1.6379	1.6206	1.0781	1.4576	0
5333	566.8	1.6385	1.6211	1.078	1.4582	0
5334	566.7	1.6383	1.6209	1.0777	1.4583	0
5335	566.6	1.6387	1.6214	1.0761	1.4593	0
5336	566.5	1.6393	1.622	1.0774	1.4594	0
5337	566.4	1.6399	1.6225	1.0765	1.4588	0
5338	566.3	1.6396	1.6223	1.076	1.4588	0
5339	566.2	1.641	1.6228	1.0767	1.4599	0
5340	566.1	1.6415	1.6225	1.0756	1.4598	0
5341	566	1.6411	1.6238	1.0766	1.4598	0
5342	565.9	1.6418	1.6238	1.077	1.4605	0
5343	565.8	1.6425	1.6252	1.0772	1.4606	0
5344	565.7	1.6429	1.624	1.0772	1.461	0
5345	565.6	1.6435	1.6254	1.0766	1.461	0
5346	565.5	1.6441	1.6259	1.0763	1.4621	0
5347	565.4	1.6446	1.6257	1.0766	1.4615	0
5348	565.3	1.645	1.6262	1.0766	1.462	0
5349	565.2	1.6448	1.6268	1.0759	1.4621	0
5350	565.1	1.6454	1.6257	1.0769	1.4632	0
5351	565	1.6468	1.6271	1.0761	1.4632	0
5352	564.9	1.6458	1.6269	1.0758	1.4627	0
5353	564.8	1.6463	1.6274	1.0766	1.4638	0
5354	564.7	1.6477	1.628	1.0767	1.4639	0
5355	564.6	1.6474	1.6285	1.0768	1.4644	0
5356	564.5	1.6486	1.629	1.0763	1.4648	0
5357	564.4	1.6484	1.6288	1.076	1.4648	0
5358	564.3	1.6497	1.6292	1.0758	1.4653	0
5359	564.2	1.6495	1.629	1.0757	1.4648	0
5360	564.1	1.6492	1.6305	1.0761	1.466	0
5361	564	1.6497	1.6293	1.0753	1.4665	0

5362	563.9	1.6511	1.6299	1.0758	1.4665	0
5363	563.8	1.6508	1.6312	1.0756	1.467	0
5364	563.7	1.6513	1.6301	1.0766	1.4669	0
5365	563.6	1.6519	1.6315	1.0758	1.4675	0
5366	563.5	1.6525	1.6313	1.0756	1.4681	0
5367	563.4	1.6531	1.6319	1.0757	1.4682	0
5368	563.3	1.6527	1.6323	1.0751	1.4681	0
5369	563.2	1.6533	1.6329	1.0756	1.4692	0
5370	563.1	1.6537	1.6326	1.0755	1.4691	0
5371	563	1.6542	1.6338	1.0766	1.469	0
5372	562.9	1.6547	1.6351	1.0762	1.4695	0
5373	562.8	1.656	1.6348	1.0764	1.4694	0
5374	562.7	1.6557	1.6345	1.0759	1.47	0
5375	562.6	1.6553	1.635	1.0756	1.4699	0
5376	562.5	1.6567	1.6348	1.0765	1.4705	0
5377	562.4	1.6572	1.6369	1.0761	1.4715	0
5378	562.3	1.6568	1.6357	1.0765	1.472	0
5379	562.2	1.6581	1.637	1.0757	1.4713	0
5380	562.1	1.6585	1.6374	1.0765	1.4712	0
5381	562	1.6583	1.638	1.0769	1.4724	0
5382	561.9	1.6588	1.6377	1.0765	1.4723	0
5383	561.8	1.66	1.638	1.0767	1.4727	0
5384	561.7	1.6597	1.6386	1.0774	1.4732	0
5385	561.6	1.6602	1.6391	1.0773	1.4732	0
5386	561.5	1.6605	1.6394	1.0771	1.4734	0
5387	561.4	1.6602	1.6407	1.0774	1.4739	0
5388	561.3	1.6615	1.6396	1.0768	1.4744	0
5389	561.2	1.6629	1.6409	1.0772	1.475	0
5390	561.1	1.6624	1.6421	1.0771	1.4748	0
5391	561	1.6637	1.6426	1.0767	1.4753	0
5392	560.9	1.6633	1.6438	1.078	1.4757	0
5393	560.8	1.6645	1.6442	1.0779	1.4756	0
5394	560.7	1.665	1.6439	1.0778	1.4761	0
5395	560.6	1.6655	1.6435	1.0772	1.4765	0
5396	560.5	1.6667	1.6439	1.0772	1.4769	0
5397	560.4	1.6664	1.6445	1.0775	1.4769	0
5398	560.3	1.6676	1.6457	1.0783	1.4773	0
5399	560.2	1.669	1.6462	1.0773	1.4784	0
5400	560.1	1.6694	1.6466	1.0778	1.4783	0
5401	560	1.6699	1.647	1.0773	1.4776	0
5402	559.9	1.6704	1.6483	1.0778	1.4786	0
5403	559.8	1.6707	1.6487	1.0782	1.479	0
5404	559.7	1.6723	1.6494	1.0787	1.4792	0

5405	559.6	1.6735	1.6514	1.0787	1.4801	0
5406	559.5	1.6746	1.6509	1.0783	1.4799	0
5407	559.4	1.6743	1.6514	1.0793	1.4804	0
5408	559.3	1.6756	1.6518	1.0792	1.4813	0
5409	559.2	1.676	1.6522	1.0788	1.4807	0
5410	559.1	1.6765	1.6528	1.0795	1.4817	0
5411	559	1.6787	1.6533	1.08	1.4817	0
5412	558.9	1.6801	1.6546	1.0808	1.4828	0
5413	558.8	1.6805	1.6551	1.081	1.4827	0
5414	558.7	1.6818	1.6555	1.0803	1.4831	0
5415	558.6	1.6831	1.6559	1.0797	1.4841	0
5416	558.5	1.6834	1.657	1.0799	1.4839	0
5417	558.4	1.6847	1.6575	1.0801	1.4843	0
5418	558.3	1.6853	1.6581	1.0803	1.4849	0
5419	558.2	1.6867	1.6595	1.0811	1.4855	0
5420	558.1	1.688	1.6606	1.0806	1.4859	0
5421	558	1.6883	1.661	1.082	1.4857	0
5422	557.9	1.6897	1.6607	1.0821	1.4862	0
5423	557.8	1.6911	1.6628	1.082	1.4873	0
5424	557.7	1.6914	1.6632	1.0817	1.4876	0
5425	557.6	1.6928	1.6637	1.0822	1.4876	0
5426	557.5	1.6942	1.6642	1.0821	1.4887	0
5427	557.4	1.6946	1.6646	1.0819	1.489	0
5428	557.3	1.6951	1.6651	1.0822	1.4891	0
5429	557.2	1.6964	1.6664	1.0808	1.4895	0
5430	557.1	1.697	1.667	1.0815	1.4901	0
5431	557	1.6992	1.6682	1.0818	1.4905	0
5432	556.9	1.6997	1.6695	1.081	1.4916	0
5433	556.8	1.7001	1.6699	1.0808	1.492	0
5434	556.7	1.7023	1.6696	1.0809	1.4924	0
5435	556.6	1.7036	1.6701	1.0822	1.4929	0
5436	556.5	1.7041	1.6713	1.0824	1.4928	0
5437	556.4	1.7064	1.6719	1.0827	1.4939	0
5438	556.3	1.7067	1.6731	1.0813	1.4937	0
5439	556.2	1.7073	1.6745	1.0814	1.4949	0
5440	556.1	1.7078	1.6741	1.0823	1.4953	0
5441	556	1.71	1.6746	1.0819	1.4958	0
5442	555.9	1.7112	1.6757	1.0816	1.4961	0
5443	555.8	1.7124	1.6769	1.0812	1.4965	0
5444	555.7	1.713	1.6766	1.0811	1.497	0
5445	555.6	1.7134	1.6778	1.0813	1.4979	0
5446	555.5	1.7147	1.6791	1.0821	1.4984	0
5447	555.4	1.7152	1.6795	1.0814	1.4988	0

5448	555.3	1.7165	1.68	1.081	1.4993	0
5449	555.2	1.7179	1.6813	1.0811	1.4998	0
5450	555.1	1.7181	1.6807	1.0819	1.5	0
5451	555	1.7195	1.682	1.082	1.5011	0
5452	554.9	1.72	1.6841	1.0819	1.5015	0
5453	554.8	1.7212	1.6836	1.0807	1.5018	0
5454	554.7	1.7217	1.6849	1.081	1.5029	0
5455	554.6	1.7229	1.6853	1.0818	1.5027	0
5456	554.5	1.7234	1.6857	1.081	1.5031	0
5457	554.4	1.7255	1.6869	1.081	1.5041	0
5458	554.3	1.7258	1.6872	1.0806	1.5048	0
5459	554.2	1.727	1.6884	1.0806	1.5047	0
5460	554.1	1.7275	1.6871	1.0804	1.5056	0
5461	554	1.7278	1.6891	1.0807	1.506	0
5462	553.9	1.7282	1.6895	1.0813	1.5064	0
5463	553.8	1.7296	1.6908	1.0804	1.5068	0
5464	553.7	1.73	1.6913	1.0802	1.5078	0
5465	553.6	1.7312	1.6932	1.0805	1.5086	0
5466	553.5	1.7316	1.6927	1.0801	1.509	0
5467	553.4	1.732	1.6948	1.0803	1.5099	0
5468	553.3	1.7333	1.6952	1.0807	1.5109	0
5469	553.2	1.7344	1.6954	1.0807	1.5106	0
5470	553.1	1.7337	1.6956	1.0801	1.5113	0
5471	553	1.735	1.696	1.0799	1.5117	0
5472	552.9	1.7353	1.698	1.0804	1.5131	0
5473	552.8	1.7356	1.6975	1.0805	1.5134	0
5474	552.7	1.7368	1.6986	1.0802	1.5143	0
5475	552.6	1.7371	1.6989	1.0803	1.5145	0
5476	552.5	1.7374	1.6991	1.081	1.5159	0
5477	552.4	1.7386	1.6994	1.0808	1.5167	0
5478	552.3	1.7379	1.7004	1.0804	1.5169	0
5479	552.2	1.739	1.7015	1.08	1.5171	0
5480	552.1	1.7393	1.7026	1.0811	1.518	0
5481	552	1.7404	1.7029	1.0814	1.5188	0
5482	551.9	1.7407	1.7031	1.0808	1.5196	0
5483	551.8	1.7409	1.7042	1.0809	1.5204	0
5484	551.7	1.742	1.7052	1.0809	1.5206	0
5485	551.6	1.7413	1.7046	1.0809	1.5218	0
5486	551.5	1.7424	1.7064	1.0814	1.522	0
5487	551.4	1.7425	1.7065	1.0819	1.5227	0
5488	551.3	1.7436	1.7067	1.0805	1.5234	0
5489	551.2	1.7438	1.7078	1.0808	1.5242	0
5490	551.1	1.7431	1.708	1.0812	1.5255	0

5491	551	1.7443	1.7083	1.081	1.5263	0
5492	550.9	1.7446	1.7094	1.0815	1.5277	0
5493	550.8	1.7448	1.7105	1.0822	1.5274	0
5494	550.7	1.7448	1.7105	1.0821	1.5285	0
5495	550.6	1.7449	1.7114	1.0805	1.5292	0
5496	550.5	1.7459	1.7115	1.0821	1.5303	0
5497	550.4	1.746	1.7133	1.082	1.5304	0
5498	550.3	1.7463	1.7145	1.0829	1.5313	0
5499	550.2	1.7464	1.7145	1.082	1.5319	0
5500	550.1	1.7473	1.7145	1.0818	1.533	0
5501	550	1.7474	1.7163	1.0833	1.5337	0
5502	549.9	1.7475	1.7173	1.0832	1.5349	0
5503	549.8	1.7485	1.7182	1.0828	1.535	0
5504	549.7	1.7476	1.7174	1.0831	1.5356	0
5505	549.6	1.7486	1.7192	1.0835	1.5368	0
5506	549.5	1.7476	1.7191	1.0825	1.5373	0
5507	549.4	1.7485	1.72	1.0839	1.5385	0
5508	549.3	1.7487	1.721	1.0838	1.5392	0
5509	549.2	1.7486	1.7218	1.0846	1.5397	0
5510	549.1	1.7497	1.7228	1.0844	1.5404	0
5511	549	1.7486	1.7236	1.0842	1.5414	0
5512	548.9	1.7486	1.7253	1.0844	1.542	0
5513	548.8	1.7488	1.7237	1.0845	1.5421	0
5514	548.7	1.7497	1.7263	1.0855	1.5427	0
5515	548.6	1.7498	1.7281	1.0854	1.5445	0
5516	548.5	1.7495	1.727	1.0853	1.5431	0
5517	548.4	1.7495	1.7287	1.0853	1.5448	0
5518	548.3	1.7494	1.7296	1.0855	1.5447	0
5519	548.2	1.7493	1.7312	1.0857	1.5452	0
5520	548.1	1.7493	1.7321	1.0853	1.5463	0
5521	548	1.7491	1.7328	1.0858	1.5462	0
5522	547.9	1.7491	1.7337	1.0856	1.5467	0
5523	547.8	1.7491	1.7354	1.0859	1.5473	0
5524	547.7	1.7498	1.7361	1.0859	1.547	0
5525	547.6	1.7497	1.7369	1.0856	1.547	0
5526	547.5	1.7496	1.7395	1.0861	1.5474	0
5527	547.4	1.7495	1.7403	1.086	1.5479	0
5528	547.3	1.7484	1.742	1.0862	1.5477	0
5529	547.2	1.7492	1.7419	1.087	1.5477	0
5530	547.1	1.7491	1.7445	1.0872	1.5481	0
5531	547	1.7488	1.7442	1.0871	1.5484	0
5532	546.9	1.7487	1.7477	1.0866	1.5482	0
5533	546.8	1.7486	1.7476	1.0875	1.5487	0

5534	546.7	1.7503	1.7494	1.088	1.548	0
5535	546.6	1.7491	1.7509	1.0877	1.5483	0
5536	546.5	1.7479	1.7526	1.0873	1.5481	0
5537	546.4	1.7486	1.7533	1.0878	1.5484	0
5538	546.3	1.7474	1.7549	1.0877	1.5487	0
5539	546.2	1.7481	1.7556	1.0869	1.5491	0
5540	546.1	1.7479	1.7573	1.0869	1.5489	0
5541	546	1.7476	1.7589	1.0886	1.5492	0
5542	545.9	1.7482	1.7596	1.0871	1.5483	0
5543	545.8	1.7488	1.7601	1.0884	1.5491	0
5544	545.7	1.7483	1.7626	1.0875	1.5487	0
5545	545.6	1.7481	1.7624	1.088	1.5497	0
5546	545.5	1.7488	1.765	1.0885	1.55	0
5547	545.4	1.7476	1.7656	1.0876	1.5497	0
5548	545.3	1.7481	1.7672	1.0874	1.5505	0
5549	545.2	1.7477	1.7697	1.0867	1.5512	0
5550	545.1	1.7463	1.7693	1.0874	1.5508	0
5551	545	1.7479	1.77	1.0872	1.5511	0
5552	544.9	1.7475	1.7725	1.0873	1.5513	0
5553	544.8	1.747	1.7731	1.0872	1.552	0
5554	544.7	1.7466	1.7726	1.0859	1.5516	0
5555	544.6	1.7481	1.7742	1.0862	1.5524	0
5556	544.5	1.7476	1.7767	1.0865	1.5525	0
5557	544.4	1.7481	1.7763	1.0863	1.5533	0
5558	544.3	1.7467	1.7769	1.0859	1.554	0
5559	544.2	1.7472	1.7794	1.0852	1.5542	0
5560	544.1	1.7478	1.7801	1.0858	1.5544	0
5561	544	1.7473	1.7806	1.0855	1.5551	0
5562	543.9	1.7468	1.7822	1.0857	1.5553	0
5563	543.8	1.7472	1.7827	1.086	1.556	0
5564	543.7	1.7468	1.7833	1.0859	1.5561	0
5565	543.6	1.7463	1.7849	1.0849	1.5569	0
5566	543.5	1.7467	1.7853	1.0845	1.5575	0
5567	543.4	1.747	1.7878	1.0847	1.5575	0
5568	543.3	1.7473	1.7872	1.0841	1.5581	0
5569	543.2	1.7469	1.7899	1.0851	1.5589	0
5570	543.1	1.7462	1.7902	1.0845	1.5594	0
5571	543	1.7465	1.7927	1.0841	1.56	0
5572	542.9	1.7458	1.792	1.0838	1.5605	0
5573	542.8	1.7471	1.7926	1.0846	1.5606	0
5574	542.7	1.7465	1.7952	1.0846	1.5613	0
5575	542.6	1.7468	1.7966	1.0832	1.5618	0
5576	542.5	1.7462	1.7982	1.0839	1.5631	0

5577	542.4	1.7473	1.7996	1.0833	1.5636	0
5578	542.3	1.7468	1.8012	1.0832	1.5643	0
5579	542.2	1.7461	1.8016	1.0835	1.5648	0
5580	542.1	1.7463	1.8031	1.0839	1.5654	0
5581	542	1.7466	1.8035	1.083	1.5661	0
5582	541.9	1.7458	1.806	1.0835	1.5665	0
5583	541.8	1.748	1.8076	1.0838	1.5671	0
5584	541.7	1.7464	1.808	1.0834	1.5677	0
5585	541.6	1.7465	1.8094	1.0836	1.5688	0
5586	541.5	1.7468	1.811	1.0845	1.5694	0
5587	541.4	1.747	1.8137	1.0836	1.57	0
5588	541.3	1.7464	1.8153	1.083	1.5707	0
5589	541.2	1.7465	1.8156	1.084	1.571	0
5590	541.1	1.7468	1.8184	1.085	1.5724	0
5591	541	1.746	1.8199	1.0849	1.5729	0
5592	540.9	1.7461	1.8214	1.0848	1.5733	0
5593	540.8	1.7463	1.8241	1.0849	1.5739	0
5594	540.7	1.7465	1.8257	1.0846	1.5744	0
5595	540.6	1.7457	1.8285	1.084	1.5743	0
5596	540.5	1.7469	1.8301	1.0834	1.5768	0
5597	540.4	1.7471	1.8305	1.0846	1.5774	0
5598	540.3	1.7483	1.8333	1.0844	1.5779	0
5599	540.2	1.7474	1.8362	1.0849	1.5784	0
5600	540.1	1.7475	1.839	1.0837	1.5789	0
5601	540	1.7477	1.8393	1.0844	1.5794	0
5602	539.9	1.7468	1.8435	1.0846	1.5806	0
5603	539.8	1.7481	1.844	1.0855	1.5813	0
5604	539.7	1.7483	1.847	1.0844	1.5818	0
5605	539.6	1.7482	1.8497	1.0844	1.5821	0
5606	539.5	1.7484	1.8515	1.0856	1.584	0
5607	539.4	1.7476	1.8545	1.0849	1.5846	0
5608	539.3	1.7487	1.8576	1.0854	1.5851	0
5609	539.2	1.7489	1.8607	1.0852	1.5863	0
5610	539.1	1.748	1.8624	1.0854	1.5869	0
5611	539	1.7491	1.8656	1.0858	1.586	0
5612	538.9	1.7483	1.8688	1.085	1.5872	0
5613	538.8	1.7485	1.8734	1.0868	1.5878	0
5614	538.7	1.7486	1.8766	1.0866	1.589	0
5615	538.6	1.7488	1.8786	1.0867	1.5904	0
5616	538.5	1.75	1.8805	1.0867	1.5909	0
5617	538.4	1.749	1.8853	1.0878	1.5907	0
5618	538.3	1.7503	1.8887	1.0879	1.592	0
5619	538.2	1.7504	1.8922	1.0874	1.5933	0

5620	538.1	1.7496	1.8957	1.0879	1.594	0
5621	538	1.7509	1.8993	1.0889	1.5939	0
5622	537.9	1.7499	1.9029	1.0887	1.5951	0
5623	537.8	1.7511	1.9065	1.0898	1.5957	0
5624	537.7	1.7503	1.9087	1.0895	1.5963	0
5625	537.6	1.7514	1.9138	1.0882	1.5975	0
5626	537.5	1.7518	1.9178	1.0894	1.5984	0
5627	537.4	1.7509	1.9201	1.0882	1.599	0
5628	537.3	1.7519	1.9253	1.0894	1.6001	0
5629	537.2	1.7521	1.9276	1.0895	1.6	0
5630	537.1	1.7523	1.93	1.0899	1.6007	0
5631	537	1.7536	1.934	1.0908	1.6028	0
5632	536.9	1.7526	1.938	1.0898	1.6026	0
5633	536.8	1.7538	1.9437	1.0903	1.6032	0
5634	536.7	1.754	1.9445	1.0901	1.6054	0
5635	536.6	1.7543	1.9471	1.0912	1.6045	0
5636	536.5	1.7544	1.9531	1.0914	1.606	0
5637	536.4	1.7547	1.954	1.0914	1.6075	0
5638	536.3	1.7549	1.9548	1.0916	1.6081	0
5639	536.2	1.755	1.9591	1.0896	1.6095	0
5640	536.1	1.7562	1.9616	1.0917	1.6092	0
5641	536	1.7541	1.9643	1.0927	1.6107	0
5642	535.9	1.7555	1.9688	1.0918	1.6122	0
5643	535.8	1.7567	1.9697	1.0918	1.6129	0
5644	535.7	1.7569	1.9724	1.0914	1.6143	0
5645	535.6	1.756	1.9733	1.0912	1.615	0
5646	535.5	1.7584	1.9761	1.0921	1.6165	0
5647	535.4	1.7584	1.9749	1.0922	1.6153	0
5648	535.3	1.7585	1.9777	1.0917	1.6185	0
5649	535.2	1.7586	1.9785	1.0914	1.619	0
5650	535.1	1.7589	1.9796	1.092	1.6198	0
5651	535	1.7591	1.9825	1.0916	1.6206	0
5652	534.9	1.7615	1.9814	1.0917	1.6211	0
5653	534.8	1.7594	1.9824	1.0926	1.6237	0
5654	534.7	1.7596	1.9854	1.0916	1.6235	0
5655	534.6	1.7609	1.9863	1.0928	1.6251	0
5656	534.5	1.7612	1.9834	1.0922	1.6258	0
5657	534.4	1.7613	1.9863	1.0916	1.6274	0
5658	534.3	1.7626	1.9833	1.0916	1.6281	0
5659	534.2	1.764	1.9883	1.0922	1.6306	0
5660	534.1	1.7619	1.9893	1.0919	1.6314	0
5661	534	1.7644	1.9863	1.0922	1.6321	0
5662	533.9	1.7649	1.9875	1.0927	1.6331	0

5663	533.8	1.7649	1.9864	1.0924	1.6346	0
5664	533.7	1.7665	1.9895	1.0918	1.6356	0
5665	533.6	1.7665	1.9884	1.0914	1.6371	0
5666	533.5	1.7668	1.9874	1.0909	1.6379	0
5667	533.4	1.7672	1.9866	1.0917	1.6389	0
5668	533.3	1.7686	1.9875	1.0921	1.6406	0
5669	533.2	1.7689	1.9887	1.0917	1.6424	0
5670	533.1	1.769	1.9876	1.0912	1.6432	0
5671	533	1.7704	1.9865	1.0916	1.6448	0
5672	532.9	1.7719	1.9876	1.0917	1.6467	0
5673	532.8	1.7711	1.9867	1.0916	1.6467	0
5674	532.7	1.7724	1.9856	1.0918	1.6485	0
5675	532.6	1.7726	1.9887	1.0913	1.6483	0
5676	532.5	1.7741	1.9876	1.0913	1.6501	0
5677	532.4	1.7731	1.9866	1.0903	1.65	0
5678	532.3	1.7748	1.9859	1.0906	1.6521	0
5679	532.2	1.7751	1.9889	1.09	1.653	0
5680	532.1	1.7767	1.988	1.0904	1.654	0
5681	532	1.7783	1.9871	1.0913	1.656	0
5682	531.9	1.7773	1.9882	1.0898	1.6569	0
5683	531.8	1.7777	1.9851	1.091	1.6579	0
5684	531.7	1.7806	1.9884	1.0911	1.6589	0
5685	531.6	1.7811	1.9813	1.0908	1.6601	0
5686	531.5	1.7816	1.9846	1.0916	1.6622	0
5687	531.4	1.782	1.9858	1.0912	1.6623	0
5688	531.3	1.7836	1.9848	1.091	1.6633	0
5689	531.2	1.7839	1.9838	1.0908	1.6643	0
5690	531.1	1.7858	1.983	1.0911	1.6665	0
5691	531	1.786	1.9819	1.0911	1.6675	0
5692	530.9	1.7865	1.981	1.0907	1.6676	0
5693	530.8	1.7896	1.9802	1.0896	1.6688	0
5694	530.7	1.7899	1.9812	1.0899	1.6698	0
5695	530.6	1.7917	1.9761	1.0903	1.6709	0
5696	530.5	1.7907	1.9793	1.0904	1.672	0
5697	530.4	1.7911	1.9762	1.0899	1.673	0
5698	530.3	1.793	1.9774	1.0907	1.6743	0
5699	530.2	1.7948	1.9744	1.0909	1.6755	0
5700	530.1	1.7966	1.9734	1.0904	1.6766	0
5701	530	1.797	1.9724	1.0905	1.6766	0
5702	529.9	1.7975	1.9736	1.0907	1.6779	0
5703	529.8	1.7993	1.9726	1.0903	1.679	0
5704	529.7	1.7998	1.9717	1.0905	1.6781	0
5705	529.6	1.8017	1.9707	1.0909	1.6804	0

5706	529.5	1.8037	1.9698	1.0909	1.6806	0
5707	529.4	1.8055	1.9709	1.0915	1.6828	0
5708	529.3	1.8073	1.9698	1.0907	1.6817	0
5709	529.2	1.8064	1.971	1.0912	1.6841	0
5710	529.1	1.8085	1.9701	1.0914	1.6843	0
5711	529	1.8091	1.9693	1.0911	1.6856	0
5712	528.9	1.8098	1.9706	1.0914	1.6859	0
5713	528.8	1.8117	1.9675	1.0913	1.686	0
5714	528.7	1.8123	1.9687	1.0921	1.6885	0
5715	528.6	1.8145	1.9701	1.091	1.6877	0
5716	528.5	1.8151	1.9713	1.0915	1.6891	0
5717	528.4	1.8159	1.9684	1.0917	1.6906	0
5718	528.3	1.8181	1.9676	1.0931	1.6898	0
5719	528.2	1.819	1.9669	1.093	1.6914	0
5720	528.1	1.8214	1.9685	1.0927	1.6919	0
5721	528	1.8208	1.97	1.0938	1.6924	0
5722	527.9	1.8234	1.9673	1.095	1.693	0
5723	527.8	1.8244	1.969	1.0939	1.6925	0
5724	527.7	1.8239	1.9684	1.0942	1.6943	0
5725	527.6	1.825	1.968	1.0941	1.6939	0
5726	527.5	1.8277	1.9654	1.0942	1.6957	0
5727	527.4	1.8288	1.9671	1.0955	1.6964	0
5728	527.3	1.8301	1.9668	1.0941	1.6985	0
5729	527.2	1.8312	1.9664	1.0951	1.6969	0
5730	527.1	1.8327	1.9683	1.0958	1.699	0
5731	527	1.834	1.9659	1.0946	1.6999	0
5732	526.9	1.8336	1.9677	1.0946	1.7007	0
5733	526.8	1.8365	1.9653	1.0966	1.6993	0
5734	526.7	1.838	1.9651	1.0965	1.7039	0
5735	526.6	1.8395	1.9649	1.0957	1.7025	0
5736	526.5	1.8408	1.9647	1.0963	1.7022	0
5737	526.4	1.8391	1.9668	1.0972	1.7034	0
5738	526.3	1.8422	1.9623	1.0961	1.7056	0
5739	526.2	1.8453	1.9642	1.0959	1.7053	0
5740	526.1	1.8451	1.964	1.0963	1.7064	0
5741	526	1.8451	1.964	1.0965	1.7063	0
5742	525.9	1.8466	1.9617	1.0955	1.7074	0
5743	525.8	1.8464	1.9637	1.0965	1.7084	0
5744	525.7	1.8496	1.9635	1.096	1.7094	0
5745	525.6	1.8511	1.9612	1.0977	1.7105	0
5746	525.5	1.8527	1.9633	1.0976	1.7104	0
5747	525.4	1.854	1.9608	1.0961	1.7113	0
5748	525.3	1.8555	1.9627	1.0961	1.7123	0

5749	525.2	1.8571	1.9604	1.0963	1.7134	0
5750	525.1	1.8569	1.9624	1.0967	1.7144	0
5751	525	1.8584	1.96	1.0962	1.7142	0
5752	524.9	1.8598	1.9576	1.0968	1.7164	0
5753	524.8	1.8612	1.9594	1.0958	1.7172	0
5754	524.7	1.861	1.9592	1.0957	1.7183	0
5755	524.6	1.8625	1.9568	1.0961	1.7181	0
5756	524.5	1.864	1.9588	1.0949	1.7191	0
5757	524.4	1.8673	1.9564	1.096	1.719	0
5758	524.3	1.8653	1.9583	1.0948	1.7225	0
5759	524.2	1.8686	1.9603	1.0958	1.7223	0
5760	524.1	1.8684	1.9579	1.0956	1.7233	0
5761	524	1.8716	1.9576	1.0946	1.7242	0
5762	523.9	1.8714	1.9574	1.0947	1.724	0
5763	523.8	1.8746	1.957	1.0944	1.7236	0
5764	523.7	1.8726	1.959	1.0942	1.7247	0
5765	523.6	1.8778	1.9588	1.0945	1.727	0
5766	523.5	1.8776	1.9564	1.0941	1.7282	0
5767	523.4	1.8772	1.956	1.0937	1.7278	0
5768	523.3	1.8788	1.958	1.0929	1.7288	0
5769	523.2	1.8786	1.9599	1.0921	1.7325	0
5770	523.1	1.8801	1.9597	1.0927	1.7322	0
5771	523	1.8836	1.9573	1.0922	1.732	0
5772	522.9	1.8849	1.9611	1.0929	1.7341	0
5773	522.8	1.8865	1.9586	1.0923	1.7338	0
5774	522.7	1.8862	1.9606	1.0923	1.7349	0
5775	522.6	1.886	1.9626	1.0924	1.7359	0
5776	522.5	1.8896	1.9603	1.092	1.7371	0
5777	522.4	1.8892	1.9643	1.091	1.738	0
5778	522.3	1.8907	1.9639	1.0909	1.7376	0
5779	522.2	1.8922	1.9657	1.0914	1.7386	0
5780	522.1	1.8899	1.9699	1.0914	1.7396	0
5781	522	1.8936	1.9675	1.0915	1.7421	0
5782	521.9	1.895	1.9693	1.0908	1.7417	0
5783	521.8	1.8948	1.9713	1.0908	1.7427	0
5784	521.7	1.8963	1.9733	1.0911	1.7437	0
5785	521.6	1.8959	1.9751	1.0906	1.7446	0
5786	521.5	1.8957	1.9772	1.0911	1.7458	0
5787	521.4	1.8993	1.9769	1.0905	1.7469	0
5788	521.3	1.9028	1.9789	1.0905	1.7479	0
5789	521.2	1.9006	1.9809	1.0914	1.749	0
5790	521.1	1.902	1.9828	1.0912	1.7499	0
5791	521	1.9036	1.9824	1.0902	1.7495	0

5792	520.9	1.9033	1.9868	1.0899	1.7505	0
5793	520.8	1.9049	1.9888	1.0908	1.7502	0
5794	520.7	1.9046	1.9885	1.0898	1.7512	0
5795	520.6	1.9082	1.9905	1.0907	1.7537	0
5796	520.5	1.9078	1.995	1.0907	1.7534	0
5797	520.4	1.9094	1.9922	1.0909	1.7543	0
5798	520.3	1.9111	1.9967	1.09	1.7568	0
5799	520.2	1.9107	2.0012	1.0908	1.7564	0
5800	520.1	1.9125	2.001	1.0897	1.7562	0
5801	520	1.9121	2.0031	1.0905	1.7572	0
5802	519.9	1.9118	2.0077	1.0902	1.7597	0
5803	519.8	1.9134	2.0098	1.0904	1.7607	0
5804	519.7	1.915	2.0119	1.0894	1.7603	0
5805	519.6	1.9169	2.0118	1.0892	1.763	0
5806	519.5	1.9164	2.0138	1.0896	1.7625	0
5807	519.4	1.918	2.016	1.0899	1.765	0
5808	519.3	1.9176	2.0181	1.0904	1.766	0
5809	519.2	1.9195	2.0205	1.0893	1.7658	0
5810	519.1	1.9211	2.0201	1.0904	1.7669	0
5811	519	1.9228	2.0223	1.0894	1.7693	0
5812	518.9	1.9203	2.0245	1.0887	1.769	0
5813	518.8	1.9242	2.0268	1.089	1.7687	0
5814	518.7	1.9238	2.029	1.0904	1.7711	0
5815	518.6	1.9256	2.0314	1.0895	1.7694	0
5816	518.5	1.9253	2.0312	1.0895	1.7691	0
5817	518.4	1.9249	2.0307	1.0897	1.7716	0
5818	518.3	1.9266	2.033	1.0903	1.7712	0
5819	518.2	1.9261	2.0352	1.0898	1.7737	0
5820	518.1	1.9301	2.0405	1.0899	1.772	0
5821	518	1.9276	2.0401	1.0907	1.7731	0
5822	517.9	1.9315	2.0425	1.091	1.7728	0
5823	517.8	1.9312	2.0422	1.0907	1.7754	0
5824	517.7	1.933	2.0418	1.0904	1.7766	0
5825	517.6	1.9327	2.0498	1.0906	1.7762	0
5826	517.5	1.9345	2.0467	1.09	1.7774	0
5827	517.4	1.934	2.0491	1.0905	1.777	0
5828	517.3	1.9358	2.0459	1.0901	1.7781	0
5829	517.2	1.9355	2.0511	1.0898	1.7778	0
5830	517.1	1.9374	2.0537	1.0898	1.7806	0
5831	517	1.9369	2.0532	1.0902	1.78	0
5832	516.9	1.9366	2.0558	1.0899	1.7813	0
5833	516.8	1.9406	2.0555	1.0905	1.7809	0
5834	516.7	1.9381	2.0551	1.0899	1.7806	0

5835	516.6	1.9401	2.0606	1.0903	1.7819	0
5836	516.5	1.9418	2.0602	1.0914	1.7814	0
5837	516.4	1.9415	2.0628	1.0908	1.7827	0
5838	516.3	1.9434	2.0654	1.0907	1.7823	0
5839	516.2	1.9431	2.0651	1.0908	1.782	0
5840	516.1	1.9427	2.0647	1.0907	1.7847	0
5841	516	1.9446	2.0674	1.091	1.7844	0
5842	515.9	1.9465	2.067	1.0912	1.784	0
5843	515.8	1.9461	2.0666	1.0915	1.7852	0
5844	515.7	1.9458	2.0722	1.0915	1.7849	0
5845	515.6	1.95	2.069	1.0913	1.7862	0
5846	515.5	1.9497	2.0716	1.0912	1.7858	0
5847	515.4	1.9516	2.0773	1.0918	1.7839	0
5848	515.3	1.9514	2.074	1.0906	1.7852	0
5849	515.2	1.951	2.0767	1.0909	1.788	0
5850	515.1	1.953	2.0795	1.0916	1.7861	0
5851	515	1.9526	2.0791	1.0903	1.7858	0
5852	514.9	1.9522	2.0818	1.0908	1.7854	0
5853	514.8	1.9565	2.0814	1.0907	1.7866	0
5854	514.7	1.9585	2.0842	1.0898	1.7878	0
5855	514.6	1.9582	2.0839	1.0908	1.7891	0
5856	514.5	1.9579	2.0837	1.0905	1.7889	0
5857	514.4	1.9575	2.0894	1.0904	1.7884	0
5858	514.3	1.9595	2.086	1.0901	1.7897	0
5859	514.2	1.9616	2.0889	1.0911	1.7878	0
5860	514.1	1.9613	2.0854	1.0899	1.7891	0
5861	514	1.9609	2.0882	1.0895	1.7919	0
5862	513.9	1.9605	2.0941	1.089	1.7899	0
5863	513.8	1.9625	2.0906	1.0893	1.7911	0
5864	513.7	1.9648	2.0906	1.0896	1.7911	0
5865	513.6	1.9669	2.0934	1.0897	1.7909	0
5866	513.5	1.969	2.0963	1.0899	1.7905	0
5867	513.4	1.9662	2.0959	1.0895	1.7933	0
5868	513.3	1.9682	2.0924	1.0901	1.7913	0
5869	513.2	1.9703	2.0952	1.0894	1.7926	0
5870	513.1	1.97	2.0982	1.0895	1.7923	0
5871	513	1.9721	2.1011	1.0895	1.792	0
5872	512.9	1.9692	2.1006	1.0903	1.7947	0
5873	512.8	1.9714	2.1037	1.0891	1.7962	0
5874	512.7	1.9759	2.1033	1.0903	1.7941	0
5875	512.6	1.9731	2.1063	1.0891	1.7938	0
5876	512.5	1.9751	2.1092	1.0893	1.7966	0
5877	512.4	1.9773	2.1123	1.0884	1.7947	0

5878	512.3	1.9795	2.1154	1.09	1.796	0
5879	512.2	1.9793	2.1152	1.0898	1.7959	0
5880	512.1	1.9789	2.1148	1.0887	1.797	0
5881	512	1.9785	2.1211	1.0896	1.7966	0
5882	511.9	1.9831	2.1207	1.0898	1.7979	0
5883	511.8	1.9827	2.1238	1.0891	1.7975	0
5884	511.7	1.985	2.1236	1.0892	1.7989	0
5885	511.6	1.9847	2.1268	1.0893	1.797	0
5886	511.5	1.9869	2.13	1.0896	1.7999	0
5887	511.4	1.9917	2.1332	1.0895	1.7979	0
5888	511.3	1.9861	2.1363	1.09	1.7991	0
5889	511.2	1.9883	2.1395	1.0894	1.7988	0
5890	511.1	1.9905	2.1429	1.0897	1.8002	0
5891	511	1.9901	2.1498	1.089	1.7997	0
5892	510.9	1.9924	2.1533	1.09	1.8028	0
5893	510.8	1.9946	2.1566	1.0905	1.8007	0
5894	510.7	1.9967	2.1637	1.09	1.8019	0
5895	510.6	1.9937	2.1672	1.0903	1.8015	0
5896	510.5	1.9987	2.1708	1.0904	1.8029	0
5897	510.4	1.9957	2.1744	1.0907	1.8026	0
5898	510.3	1.998	2.182	1.0906	1.8039	0
5899	510.2	2.0002	2.1857	1.0899	1.8034	0
5900	510.1	2.0026	2.1854	1.0897	1.8049	0
5901	510	2.0021	2.2015	1.0905	1.8061	0
5902	509.9	2.0044	2.2053	1.09	1.8057	0
5903	509.8	2.004	2.2092	1.09	1.807	0
5904	509.7	2.0063	2.2175	1.0899	1.8083	0
5905	509.6	2.0086	2.2216	1.0896	1.8079	0
5906	509.5	2.011	2.2301	1.0895	1.8075	0
5907	509.4	2.0106	2.2389	1.0902	1.8089	0
5908	509.3	2.013	2.2384	1.0914	1.8085	0
5909	509.2	2.0153	2.2522	1.091	1.8098	0
5910	509.1	2.015	2.2566	1.0916	1.8112	0
5911	509	2.0118	2.2611	1.0912	1.8108	0
5912	508.9	2.017	2.2706	1.0918	1.8139	0
5913	508.8	2.0166	2.2803	1.0905	1.8135	0
5914	508.7	2.0189	2.2798	1.0907	1.8148	0
5915	508.6	2.0215	2.2899	1.0914	1.8128	0
5916	508.5	2.021	2.3	1.0915	1.814	0
5917	508.4	2.0206	2.3105	1.0922	1.8154	0
5918	508.3	2.0231	2.3102	1.0919	1.8169	0
5919	508.2	2.0255	2.3208	1.0914	1.8164	0
5920	508.1	2.028	2.332	1.0904	1.8196	0

5921	508	2.0278	2.3316	1.0908	1.8211	0
5922	507.9	2.0302	2.3371	1.0903	1.8207	0
5923	507.8	2.0298	2.3426	1.0923	1.8203	0
5924	507.7	2.0325	2.3483	1.0924	1.8236	0
5925	507.6	2.0349	2.3479	1.0919	1.825	0
5926	507.5	2.0346	2.3597	1.0929	1.8265	0
5927	507.4	2.0372	2.3656	1.0929	1.8261	0
5928	507.3	2.0368	2.3652	1.0932	1.8275	0
5929	507.2	2.0424	2.3778	1.0932	1.8291	0
5930	507.1	2.0391	2.3775	1.0933	1.8288	0
5931	507	2.0417	2.3835	1.0928	1.8338	0
5932	506.9	2.0444	2.3899	1.0932	1.8336	0
5933	506.8	2.047	2.3961	1.0924	1.835	0
5934	506.7	2.0497	2.3958	1.0924	1.8365	0
5935	506.6	2.0495	2.4024	1.0925	1.8382	0
5936	506.5	2.0522	2.4089	1.0915	1.8378	0
5937	506.4	2.0549	2.4157	1.0928	1.8431	0
5938	506.3	2.0547	2.4156	1.0923	1.8429	0
5939	506.2	2.0576	2.4153	1.092	1.8446	0
5940	506.1	2.0572	2.4149	1.0923	1.8461	0
5941	506	2.0567	2.4216	1.0929	1.8495	0
5942	505.9	2.0594	2.4284	1.0917	1.851	0
5943	505.8	2.0623	2.4282	1.0921	1.8507	0
5944	505.7	2.0588	2.4353	1.0919	1.8543	0
5945	505.6	2.0648	2.435	1.0922	1.856	0
5946	505.5	2.0645	2.4346	1.0932	1.8576	0
5947	505.4	2.0674	2.4269	1.0926	1.8613	0
5948	505.3	2.0671	2.4417	1.0923	1.865	0
5949	505.2	2.07	2.4337	1.0919	1.8667	0
5950	505.1	2.0729	2.4333	1.092	1.8684	0
5951	505	2.0759	2.433	1.0926	1.8722	0
5952	504.9	2.0756	2.4327	1.0923	1.876	0
5953	504.8	2.0784	2.4248	1.0926	1.8777	0
5954	504.7	2.0814	2.4319	1.0943	1.8794	0
5955	504.6	2.0845	2.4317	1.0944	1.8834	0
5956	504.5	2.0843	2.424	1.0934	1.8852	0
5957	504.4	2.0873	2.4311	1.0948	1.8892	0
5958	504.3	2.0868	2.4232	1.094	1.8909	0
5959	504.2	2.0863	2.4302	1.0932	1.8947	0
5960	504.1	2.0965	2.4225	1.093	1.8989	0
5961	504	2.0997	2.4223	1.0931	1.9009	0
5962	503.9	2.0994	2.4219	1.0928	1.905	0
5963	503.8	2.1026	2.4291	1.0932	1.9093	0

5964	503.7	2.1024	2.4141	1.0933	1.9135	0
5965	503.6	2.1054	2.4209	1.0924	1.9176	0
5966	503.5	2.1086	2.4132	1.093	1.9218	0
5967	503.4	2.1119	2.4057	1.0924	1.9262	0
5968	503.3	2.1116	2.4126	1.0928	1.926	0
5969	503.2	2.1113	2.4124	1.0929	1.9328	0
5970	503.1	2.1145	2.412	1.0918	1.9372	0
5971	503	2.1216	2.3974	1.0929	1.9418	0
5972	502.9	2.1175	2.4041	1.0925	1.9439	0
5973	502.8	2.1208	2.4037	1.0928	1.946	0
5974	502.7	2.128	2.4034	1.0922	1.9557	0
5975	502.6	2.1277	2.396	1.0918	1.9579	0
5976	502.5	2.1312	2.4027	1.0922	1.9602	0
5977	502.4	2.1346	2.3952	1.0925	1.9623	0
5978	502.3	2.1421	2.3882	1.0923	1.9673	0
5979	502.2	2.1418	2.3877	1.0923	1.9696	0
5980	502.1	2.1493	2.3944	1.0926	1.9772	0
5981	502	2.1489	2.3802	1.0922	1.9795	0
5982	501.9	2.1487	2.3867	1.0927	1.9874	0
5983	501.8	2.1563	2.3796	1.0937	1.9926	0
5984	501.7	2.156	2.3792	1.0934	1.995	0
5985	501.6	2.1598	2.3789	1.0931	2.0033	0
5986	501.5	2.1636	2.3718	1.0921	2.0058	0
5987	501.4	2.1676	2.3717	1.0947	2.0085	0
5988	501.3	2.1671	2.3646	1.0935	2.0168	0
5989	501.2	2.1752	2.3709	1.0939	2.0255	0
5990	501.1	2.1748	2.3639	1.0932	2.0251	0
5991	501	2.1788	2.3702	1.0936	2.0339	0
5992	500.9	2.1828	2.3633	1.094	2.0367	0
5993	500.8	2.1824	2.3629	1.0936	2.0394	0
5994	500.7	2.1908	2.356	1.0936	2.0485	0
5995	500.6	2.1904	2.3556	1.0942	2.0546	0
5996	500.5	2.1946	2.3554	1.0951	2.0641	0
5997	500.4	2.1944	2.3551	1.0951	2.0638	0
5998	500.3	2.2077	2.3612	1.0966	2.0702	0
5999	500.2	2.2119	2.3479	1.0951	2.0732	0
6000	500.1	2.2115	2.3475	1.0962	2.0866	0
6001	500	2.2111	2.3536	1.0958	2.0862	0
6002	499.9	2.225	2.3532	1.0968	2.0929	0
6003	499.8	2.2247	2.3401	1.0965	2.0997	0
6004	499.7	2.2292	2.3461	1.0966	2.1031	0
6005	499.6	2.2337	2.3457	1.0965	2.11	0
6006	499.5	2.2334	2.3389	1.0965	2.1133	0

6007	499.4	2.2381	2.3325	1.098	2.1168	0
6008	499.3	2.2427	2.3321	1.0976	2.1241	0
6009	499.2	2.2473	2.3379	1.099	2.1314	0
6010	499.1	2.2626	2.3376	1.0984	2.1311	0
6011	499	2.2622	2.3309	1.0983	2.1386	0
6012	498.9	2.2619	2.3244	1.099	2.1463	0
6013	498.8	2.2723	2.3365	1.0987	2.1501	0
6014	498.7	2.2718	2.3298	1.0979	2.1537	0
6015	498.6	2.2825	2.3357	1.0994	2.1617	0
6016	498.5	2.2876	2.329	1.0993	2.1655	0
6017	498.4	2.2816	2.3286	1.0993	2.1651	0
6018	498.3	2.2925	2.322	1.0999	2.169	0
6019	498.2	2.3037	2.334	1.1014	2.1773	0
6020	498.1	2.2974	2.3273	1.1005	2.1812	0
6021	498	2.3147	2.3208	1.1013	2.1853	0
6022	497.9	2.3143	2.3266	1.1006	2.1805	0
6023	497.8	2.32	2.32	1.1009	2.189	0
6024	497.7	2.3197	2.3197	1.1005	2.1932	0
6025	497.6	2.3317	2.3193	1.1013	2.1929	0
6026	497.5	2.3315	2.3252	1.1007	2.1972	0
6027	497.4	2.3438	2.3186	1.1013	2.1967	0
6028	497.3	2.35	2.3183	1.1025	2.2057	0
6029	497.2	2.3563	2.3179	1.1017	2.2054	0
6030	497.1	2.356	2.3238	1.1025	2.205	0
6031	497	2.369	2.317	1.1027	2.2045	0
6032	496.9	2.3686	2.3228	1.1027	2.1994	0
6033	496.8	2.3752	2.3163	1.1024	2.2038	0
6034	496.7	2.3748	2.316	1.1031	2.1941	0
6035	496.6	2.3887	2.3156	1.1024	2.1984	0
6036	496.5	2.3813	2.3215	1.1029	2.2028	0
6037	496.4	2.3954	2.3211	1.1029	2.2025	0
6038	496.3	2.395	2.3208	1.1033	2.1974	0
6039	496.2	2.4096	2.3266	1.1037	2.2017	0
6040	496.1	2.4094	2.3201	1.1034	2.2014	0
6041	496	2.4166	2.3135	1.1037	2.1963	0
6042	495.9	2.4163	2.3195	1.1024	2.1961	0
6043	495.8	2.4238	2.319	1.1019	2.2003	0
6044	495.7	2.4234	2.3126	1.1031	2.1954	0
6045	495.6	2.431	2.3246	1.1035	2.1903	0
6046	495.5	2.4306	2.3179	1.1039	2.1853	0
6047	495.4	2.4384	2.3238	1.1038	2.1895	0
6048	495.3	2.4547	2.3172	1.1035	2.1846	0
6049	495.2	2.446	2.3169	1.1028	2.1889	0

6050	495.1	2.4541	2.3228	1.1032	2.1885	0
6051	495	2.4537	2.3161	1.1036	2.1836	0
6052	494.9	2.4707	2.3158	1.1036	2.1786	0
6053	494.8	2.4616	2.3154	1.1037	2.1783	0
6054	494.7	2.479	2.3152	1.103	2.178	0
6055	494.6	2.4786	2.3025	1.1029	2.1776	0
6056	494.5	2.4873	2.3082	1.103	2.1818	0
6057	494.4	2.487	2.314	1.1033	2.1768	0
6058	494.3	2.4959	2.3136	1.1034	2.1765	0
6059	494.2	2.5053	2.3196	1.1038	2.1717	0
6060	494.1	2.5148	2.3132	1.1029	2.1715	0
6061	494	2.5144	2.3066	1.1029	2.1756	0
6062	493.9	2.504	2.3122	1.1027	2.1661	0
6063	493.8	2.5236	2.3119	1.1024	2.1703	0
6064	493.7	2.5035	2.3117	1.1017	2.1655	0
6065	493.6	2.5229	2.3051	1.1025	2.1608	0
6066	493.5	2.5328	2.3048	1.1022	2.1693	0
6067	493.4	2.5223	2.317	1.1027	2.1601	0
6068	493.3	2.5217	2.3102	1.1025	2.1596	0
6069	493.2	2.5317	2.3098	1.1026	2.1637	0
6070	493.1	2.5314	2.3033	1.1027	2.1634	0
6071	493	2.5309	2.3091	1.1019	2.1586	0
6072	492.9	2.5204	2.3087	1.1027	2.1671	0
6073	492.8	2.5303	2.3085	1.1036	2.1623	0
6074	492.7	2.5297	2.3079	1.103	2.1708	0
6075	492.6	2.54	2.3016	1.1036	2.166	0
6076	492.5	2.5396	2.3072	1.1028	2.1612	0
6077	492.4	2.5394	2.3009	1.1033	2.1654	0
6078	492.3	2.5391	2.3068	1.1038	2.1696	0
6079	492.2	2.5493	2.2941	1.1033	2.1692	0
6080	492.1	2.5382	2.2998	1.1045	2.1733	0
6081	492	2.5379	2.3056	1.1034	2.1639	0
6082	491.9	2.5484	2.293	1.1035	2.1681	0
6083	491.8	2.5373	2.2988	1.1039	2.1723	0
6084	491.7	2.5476	2.2984	1.1043	2.1719	0
6085	491.6	2.5366	2.2981	1.1044	2.1716	0
6086	491.5	2.5361	2.2977	1.1036	2.1712	0
6087	491.4	2.5358	2.2973	1.1036	2.1801	0
6088	491.3	2.5462	2.2971	1.1038	2.1799	0
6089	491.2	2.5459	2.2906	1.1042	2.1795	0
6090	491.1	2.5455	2.2902	1.1042	2.1885	0
6091	491	2.5452	2.2899	1.1046	2.1834	0
6092	490.9	2.5449	2.3019	1.1048	2.188	0

6093	490.8	2.5444	2.2952	1.105	2.1922	0
6094	490.7	2.5441	2.295	1.1048	2.192	0
6095	490.6	2.5438	2.2884	1.1056	2.1916	0
6096	490.5	2.5435	2.2882	1.1053	2.1961	0
6097	490.4	2.5324	2.2878	1.1053	2.1957	0
6098	490.3	2.5535	2.2873	1.1056	2.2001	0
6099	490.2	2.5423	2.281	1.1061	2.1998	0
6100	490.1	2.542	2.2807	1.1058	2.2045	0
6101	490	2.5525	2.2803	1.107	2.2041	0
6102	489.9	2.5522	2.28	1.1063	2.2088	0
6103	489.8	2.5408	2.2795	1.107	2.2135	0
6104	489.7	2.5514	2.2791	1.1078	2.2182	0
6105	489.6	2.5511	2.2788	1.1079	2.2179	0
6106	489.5	2.5397	2.2845	1.1088	2.2175	0
6107	489.4	2.5616	2.2781	1.108	2.2224	0
6108	489.3	2.5501	2.2837	1.1085	2.222	0
6109	489.2	2.5498	2.2835	1.1082	2.2323	0
6110	489.1	2.5493	2.277	1.1081	2.2372	0
6111	489	2.5488	2.2707	1.1081	2.2423	0
6112	488.9	2.5484	2.2761	1.1093	2.2363	0
6113	488.8	2.5372	2.2759	1.1106	2.2416	0
6114	488.7	2.5478	2.2755	1.1103	2.2412	0
6115	488.6	2.5473	2.2751	1.1102	2.2519	0
6116	488.5	2.536	2.2747	1.111	2.2515	0
6117	488.4	2.5467	2.2803	1.1115	2.2626	0
6118	488.3	2.5354	2.2741	1.1113	2.2682	0
6119	488.2	2.5458	2.2795	1.1119	2.2676	0
6120	488.1	2.5567	2.2733	1.1121	2.2733	0
6121	488	2.5452	2.2729	1.1121	2.2788	0
6122	487.9	2.5449	2.2666	1.1126	2.2785	0
6123	487.8	2.5444	2.2721	1.1126	2.2966	0
6124	487.7	2.5439	2.2717	1.1122	2.29	0
6125	487.6	2.5327	2.2714	1.1123	2.2958	0
6126	487.5	2.5546	2.271	1.1127	2.2955	0
6127	487.4	2.5318	2.2706	1.1123	2.3079	0
6128	487.3	2.5426	2.2643	1.1132	2.3075	0
6129	487.2	2.5311	2.2758	1.1128	2.3202	0
6130	487.1	2.5417	2.2694	1.114	2.3198	0
6131	487	2.5414	2.2691	1.1145	2.3262	0
6132	486.9	2.5409	2.2687	1.1138	2.3259	0
6133	486.8	2.5519	2.2743	1.1147	2.3324	0
6134	486.7	2.5403	2.268	1.1147	2.3389	0
6135	486.6	2.5289	2.2735	1.1138	2.3455	0

6136	486.5	2.5284	2.2671	1.1142	2.3451	0
6137	486.4	2.539	2.2667	1.1147	2.3666	0
6138	486.3	2.5387	2.2724	1.1148	2.3588	0
6139	486.2	2.5382	2.272	1.1148	2.3658	0
6140	486.1	2.5379	2.2656	1.1148	2.3655	0
6141	486	2.5376	2.2713	1.1146	2.3728	0
6142	485.9	2.5261	2.277	1.1149	2.38	0
6143	485.8	2.5258	2.2765	1.1149	2.3874	0
6144	485.7	2.5364	2.2701	1.1142	2.3871	0
6145	485.6	2.536	2.2758	1.1138	2.3947	0
6146	485.5	2.5357	2.2755	1.1139	2.4025	0
6147	485.4	2.5242	2.2811	1.1142	2.4019	0
6148	485.3	2.5239	2.2808	1.114	2.4099	0
6149	485.2	2.5235	2.2805	1.114	2.4181	0
6150	485.1	2.523	2.2801	1.1136	2.4176	0
6151	485	2.512	2.2735	1.1141	2.4259	0
6152	484.9	2.5117	2.2857	1.1142	2.4344	0
6153	484.8	2.522	2.2852	1.1141	2.4338	0
6154	484.7	2.5109	2.2848	1.1138	2.4516	0
6155	484.6	2.5106	2.2973	1.1135	2.4514	0
6156	484.5	2.5102	2.2841	1.1131	2.4509	0
6157	484.4	2.5098	2.2901	1.114	2.4505	0
6158	484.3	2.5095	2.2962	1.1128	2.4595	0
6159	484.2	2.5092	2.2895	1.113	2.4499	0
6160	484.1	2.5088	2.289	1.1129	2.4588	0
6161	484	2.498	2.2952	1.1126	2.468	0
6162	483.9	2.5081	2.2883	1.1131	2.458	0
6163	483.8	2.5077	2.2879	1.1123	2.4672	0
6164	483.7	2.5075	2.3007	1.1112	2.4768	0
6165	483.6	2.4965	2.3002	1.1125	2.4764	0
6166	483.5	2.4963	2.3	1.1118	2.4664	0
6167	483.4	2.4959	2.2996	1.1119	2.466	0
6168	483.3	2.4853	2.2992	1.1119	2.4853	0
6169	483.2	2.4951	2.3055	1.111	2.475	0
6170	483.1	2.4949	2.3187	1.1103	2.4845	0
6171	483	2.4843	2.3048	1.1103	2.4742	0
6172	482.9	2.4939	2.3043	1.1116	2.4837	0
6173	482.8	2.4734	2.3175	1.1117	2.4734	0
6174	482.7	2.4831	2.3103	1.1118	2.4731	0
6175	482.6	2.4728	2.3033	1.1115	2.4828	0
6176	482.5	2.4724	2.3165	1.1103	2.4927	0
6177	482.4	2.4921	2.323	1.1111	2.482	0
6178	482.3	2.4815	2.3157	1.112	2.4815	0

6179	482.2	2.4915	2.3153	1.1108	2.4812	0
6180	482.1	2.4808	2.329	1.1113	2.4911	0
6181	482	2.4804	2.3286	1.1118	2.4804	0
6182	481.9	2.4701	2.3282	1.1113	2.48	0
6183	481.8	2.4698	2.3351	1.111	2.49	0
6184	481.7	2.4693	2.3345	1.111	2.4895	0
6185	481.6	2.4789	2.3415	1.1111	2.4789	0
6186	481.5	2.4785	2.3411	1.1107	2.4785	0
6187	481.4	2.4583	2.3334	1.1111	2.4681	0
6188	481.3	2.4678	2.3403	1.1112	2.4878	0
6189	481.2	2.4675	2.3475	1.1118	2.4774	0
6190	481.1	2.4573	2.3471	1.111	2.4873	0
6191	481	2.4666	2.3542	1.111	2.4868	0
6192	480.9	2.4662	2.3463	1.1115	2.4865	0
6193	480.8	2.4562	2.3612	1.1112	2.476	0
6194	480.7	2.4557	2.3531	1.1107	2.4755	0
6195	480.6	2.4553	2.3682	1.1107	2.4751	0
6196	480.5	2.4647	2.36	1.1108	2.4849	0
6197	480.4	2.4643	2.3595	1.1099	2.495	0
6198	480.3	2.4638	2.3669	1.1108	2.4738	0
6199	480.2	2.4634	2.3664	1.1112	2.4734	0
6200	480.1	2.4533	2.3662	1.1114	2.4833	0
6201	480	2.4627	2.3738	1.1114	2.4829	0
6202	479.9	2.443	2.3814	1.1105	2.4824	0
6203	479.8	2.4522	2.3811	1.1111	2.4821	0
6204	479.7	2.4614	2.3974	1.1102	2.4816	0
6205	479.6	2.4515	2.3887	1.1113	2.492	0
6206	479.5	2.4608	2.3883	1.1113	2.4915	0
6207	479.4	2.4603	2.3962	1.1121	2.4911	0
6208	479.3	2.4598	2.3958	1.1116	2.4905	0
6209	479.2	2.4403	2.3954	1.1127	2.4903	0
6210	479.1	2.4592	2.4036	1.1127	2.4897	0
6211	479	2.4488	2.3945	1.1126	2.4789	0
6212	478.9	2.4486	2.4027	1.1132	2.4996	0
6213	478.8	2.4483	2.4113	1.1125	2.4887	0
6214	478.7	2.4675	2.4196	1.1133	2.4988	0
6215	478.6	2.4569	2.4103	1.1119	2.4984	0
6216	478.5	2.4567	2.4188	1.112	2.4981	0
6217	478.4	2.4465	2.4184	1.1134	2.4976	0
6218	478.3	2.4461	2.4365	1.113	2.5082	0
6219	478.2	2.4556	2.4269	1.114	2.4969	0
6220	478.1	2.4551	2.4358	1.1145	2.5075	0
6221	478	2.4547	2.4353	1.1145	2.4961	0

6222	477.9	2.4542	2.4349	1.1135	2.5178	0
6223	477.8	2.4538	2.4441	1.1141	2.5063	0
6224	477.7	2.4536	2.4536	1.1147	2.5058	0
6225	477.6	2.4531	2.4433	1.1133	2.5167	0
6226	477.5	2.4526	2.4526	1.1151	2.5163	0
6227	477.4	2.4522	2.4522	1.1148	2.5046	0
6228	477.3	2.4618	2.4519	1.1149	2.5042	0
6229	477.2	2.4514	2.4514	1.1144	2.5151	0
6230	477.1	2.451	2.461	1.1145	2.5147	0
6231	477	2.4605	2.4605	1.1149	2.5143	0
6232	476.9	2.46	2.4703	1.1153	2.5253	0
6233	476.8	2.4498	2.4701	1.1142	2.5251	0
6234	476.7	2.4495	2.4802	1.1148	2.5133	0
6235	476.6	2.449	2.4796	1.1152	2.5243	0
6236	476.5	2.4587	2.4794	1.1158	2.5239	0
6237	476.4	2.4583	2.479	1.115	2.512	0
6238	476.3	2.4382	2.4786	1.1146	2.5232	0
6239	476.2	2.4476	2.4999	1.1147	2.5348	0
6240	476.1	2.4375	2.4887	1.1152	2.5224	0
6241	476	2.4568	2.4992	1.1162	2.534	0
6242	475.9	2.4563	2.4877	1.1148	2.5334	0
6243	475.8	2.4559	2.4984	1.1154	2.5211	0
6244	475.7	2.4455	2.498	1.1145	2.5209	0
6245	475.6	2.4551	2.5203	1.1149	2.5322	0
6246	475.5	2.4546	2.5198	1.1149	2.5318	0
6247	475.4	2.4542	2.5079	1.1159	2.5436	0
6248	475.3	2.4538	2.5191	1.1156	2.5311	0
6249	475.2	2.4435	2.5187	1.116	2.5306	0
6250	475.1	2.4429	2.53	1.1142	2.5181	0
6251	475	2.4526	2.5297	1.1148	2.5297	0
6252	474.9	2.4521	2.5293	1.1148	2.5293	0
6253	474.8	2.4418	2.529	1.1144	2.5412	0
6254	474.7	2.4514	2.5286	1.1154	2.5286	0
6255	474.6	2.4409	2.5528	1.114	2.5403	0
6256	474.5	2.4505	2.5525	1.1141	2.5277	0
6257	474.4	2.4303	2.5521	1.1146	2.5153	0
6258	474.3	2.4496	2.5516	1.1137	2.539	0
6259	474.2	2.4391	2.5642	1.1146	2.5385	0
6260	474.1	2.4291	2.5508	1.1139	2.5261	0
6261	474	2.4486	2.5768	1.1144	2.5256	0
6262	473.9	2.4379	2.5629	1.1143	2.5373	0
6263	473.8	2.4278	2.5626	1.1135	2.5129	0
6264	473.7	2.4274	2.5892	1.1131	2.5243	0

6265	473.6	2.427	2.5889	1.1131	2.5239	0
6266	473.5	2.4363	2.6026	1.1135	2.5233	0
6267	473.4	2.4357	2.5879	1.113	2.5351	0
6268	473.3	2.4256	2.5875	1.1137	2.5226	0
6269	473.2	2.4253	2.6014	1.1123	2.5222	0
6270	473.1	2.4152	2.6008	1.1123	2.5216	0
6271	473	2.4342	2.6005	1.1114	2.5093	0
6272	472.9	2.4337	2.5859	1.1119	2.5209	0
6273	472.8	2.414	2.6144	1.1125	2.5204	0
6274	472.7	2.4327	2.5991	1.1119	2.5321	0
6275	472.6	2.4324	2.6287	1.1125	2.5196	0
6276	472.5	2.4319	2.613	1.112	2.519	0
6277	472.4	2.4217	2.6124	1.1106	2.5186	0
6278	472.3	2.4211	2.6119	1.1111	2.5303	0
6279	472.2	2.4207	2.6267	1.1116	2.5299	0
6280	472.1	2.4201	2.6262	1.111	2.5293	0
6281	472	2.4295	2.6105	1.1115	2.5289	0
6282	471.9	2.4193	2.6254	1.1097	2.5284	0
6283	471.8	2.4094	2.6251	1.1122	2.5408	0
6284	471.7	2.4184	2.6402	1.1116	2.5275	0
6285	471.6	2.4181	2.6398	1.1103	2.5397	0
6286	471.5	2.4272	2.6394	1.1117	2.5393	0
6287	471.4	2.417	2.6231	1.1112	2.5388	0
6288	471.3	2.407	2.6227	1.1107	2.5384	0
6289	471.2	2.4257	2.6379	1.1106	2.5378	0
6290	471.1	2.4253	2.6373	1.1107	2.5637	0
6291	471	2.4249	2.6535	1.1112	2.5498	0
6292	470.9	2.4148	2.6531	1.1118	2.5629	0
6293	470.8	2.4142	2.636	1.1122	2.5233	0
6294	470.7	2.4139	2.6521	1.1129	2.5485	0
6295	470.6	2.4134	2.6517	1.111	2.5482	0
6296	470.5	2.4227	2.6513	1.1115	2.5348	0
6297	470.4	2.4222	2.6853	1.1123	2.5604	0
6298	470.3	2.4216	2.6501	1.1123	2.5599	0
6299	470.2	2.4212	2.6668	1.1119	2.5596	0
6300	470.1	2.4309	2.684	1.112	2.5458	0
6301	470	2.4202	2.6834	1.1109	2.5585	0
6302	469.9	2.4298	2.683	1.1124	2.5447	0
6303	469.8	2.4193	2.6826	1.1114	2.5714	0
6304	469.7	2.4289	2.6821	1.1129	2.5709	0
6305	469.6	2.4086	2.6815	1.1129	2.5566	0
6306	469.5	2.4277	2.6811	1.1123	2.5699	0
6307	469.4	2.4272	2.7183	1.1123	2.5555	0

6308	469.3	2.4268	2.7178	1.1128	2.5688	0
6309	469.2	2.4263	2.7375	1.1134	2.5685	0
6310	469.1	2.4465	2.7169	1.1128	2.5678	0
6311	469	2.4153	2.7366	1.1123	2.5674	0
6312	468.9	2.4352	2.7361	1.113	2.567	0
6313	468.8	2.4345	2.7354	1.1123	2.5664	0
6314	468.7	2.4139	2.7562	1.1129	2.5802	0
6315	468.6	2.4334	2.7557	1.1122	2.5795	0
6316	468.5	2.4331	2.7552	1.1128	2.565	0
6317	468.4	2.4325	2.777	1.1127	2.5643	0
6318	468.3	2.432	2.7765	1.1128	2.5782	0
6319	468.2	2.4318	2.754	1.1135	2.5637	0
6320	468.1	2.431	2.7755	1.1133	2.5919	0
6321	468	2.4305	2.7986	1.1128	2.5624	0
6322	467.9	2.4302	2.7747	1.1134	2.5763	0
6323	467.8	2.4508	2.7742	1.1138	2.5615	0
6324	467.7	2.4291	2.7972	1.1144	2.5753	0
6325	467.6	2.439	2.8213	1.1138	2.5746	0
6326	467.5	2.4385	2.7962	1.1139	2.5743	0
6327	467.4	2.4381	2.8204	1.1144	2.5596	0
6328	467.3	2.4376	2.7951	1.1143	2.5452	0
6329	467.2	2.4372	2.8459	1.1145	2.5728	0
6330	467.1	2.4366	2.819	1.1149	2.5724	0
6331	467	2.4468	2.8184	1.1144	2.5865	0
6332	466.9	2.4462	2.8441	1.1148	2.5712	0
6333	466.8	2.435	2.8173	1.1143	2.5564	0
6334	466.7	2.4454	2.7921	1.1154	2.556	0
6335	466.6	2.4449	2.871	1.1155	2.5698	0
6336	466.5	2.4553	2.8422	1.1144	2.5693	0
6337	466.4	2.4548	2.8697	1.1149	2.5835	0
6338	466.3	2.4432	2.8693	1.1149	2.5682	0
6339	466.2	2.4429	2.8407	1.1145	2.5678	0
6340	466.1	2.4536	2.8687	1.1157	2.5533	0
6341	466	2.4531	2.8681	1.1158	2.5528	0
6342	465.9	2.4526	2.8677	1.1153	2.5524	0
6343	465.8	2.452	2.8969	1.1167	2.5659	0
6344	465.7	2.4515	2.8665	1.1152	2.5511	0
6345	465.6	2.4622	2.8959	1.1152	2.5507	0
6346	465.5	2.4504	2.8655	1.1156	2.5643	0
6347	465.4	2.4612	2.8948	1.1162	2.5358	0
6348	465.3	2.4494	2.8941	1.1156	2.5491	0
6349	465.2	2.4717	2.8938	1.1156	2.5627	0
6350	465.1	2.4597	2.8935	1.1157	2.5481	0

6351	465	2.4593	2.8928	1.1158	2.5476	0
6352	464.9	2.4587	2.8623	1.1142	2.5471	0
6353	464.8	2.4698	2.8918	1.1147	2.5465	0
6354	464.7	2.4577	2.8914	1.1147	2.5461	0
6355	464.6	2.4806	2.8908	1.1147	2.5455	0
6356	464.5	2.4802	2.8904	1.1153	2.5312	0
6357	464.4	2.4795	2.9219	1.1157	2.5588	0
6358	464.3	2.4789	2.8592	1.1141	2.5581	0
6359	464.2	2.4785	2.8586	1.1131	2.5577	0
6360	464.1	2.4779	2.8881	1.1146	2.5429	0
6361	464	2.4896	2.8576	1.1136	2.5423	0
6362	463.9	2.4768	2.8871	1.1139	2.5417	0
6363	463.8	2.4885	2.8864	1.1145	2.5412	0
6364	463.7	2.4878	2.9179	1.1139	2.5406	0
6365	463.6	2.4873	2.9172	1.1138	2.5542	0
6366	463.5	2.4868	2.8847	1.1133	2.5538	0
6367	463.4	2.4861	2.9161	1.1132	2.5388	0
6368	463.3	2.4983	2.8837	1.1149	2.5527	0
6369	463.2	2.4849	2.8827	1.1136	2.5376	0
6370	463.1	2.4972	2.8824	1.1127	2.5373	0
6371	463	2.4965	2.8817	1.1131	2.5366	0
6372	462.9	2.4959	2.8814	1.1125	2.536	0
6373	462.8	2.5216	2.8508	1.114	2.5354	0
6374	462.7	2.521	2.8801	1.1118	2.549	0
6375	462.6	2.5071	2.9119	1.1119	2.5206	0
6376	462.5	2.5064	2.9458	1.1133	2.5336	0
6377	462.4	2.5191	2.8781	1.1132	2.5471	0
6378	462.3	2.5187	2.8778	1.1142	2.5324	0
6379	462.2	2.5047	2.8771	1.1142	2.5319	0
6380	462.1	2.5042	2.8765	1.1131	2.5455	0
6381	462	2.5305	2.908	1.1123	2.5305	0
6382	461.9	2.5299	2.9073	1.1134	2.5161	0
6383	461.8	2.5156	2.9069	1.1128	2.5156	0
6384	461.7	2.5286	2.9408	1.1126	2.5148	0
6385	461.6	2.528	2.8732	1.1125	2.5423	0
6386	461.5	2.5274	2.8726	1.1125	2.5274	0
6387	461.4	2.5267	2.9041	1.1123	2.5129	0
6388	461.3	2.5403	2.8713	1.1137	2.5261	0
6389	461.2	2.5396	2.9027	1.1125	2.5114	0
6390	461.1	2.5538	2.9024	1.112	2.511	0
6391	461	2.5532	2.9017	1.1119	2.5242	0
6392	460.9	2.5378	2.901	1.1129	2.5098	0
6393	460.8	2.5519	2.9352	1.1139	2.5091	0

6394	460.7	2.5513	2.8374	1.1138	2.5085	0
6395	460.6	2.5658	2.8989	1.1131	2.5358	0
6396	460.5	2.5498	2.8661	1.1134	2.5209	0
6397	460.4	2.5643	2.8652	1.1127	2.52	0
6398	460.3	2.5795	2.8649	1.1138	2.5057	0
6399	460.2	2.563	2.8639	1.1131	2.533	0
6400	460.1	2.5624	2.8633	1.113	2.5043	0
6401	460	2.5938	2.8948	1.1129	2.5038	0
6402	459.9	2.5768	2.8321	1.1132	2.5167	0
6403	459.8	2.5926	2.8614	1.1138	2.5161	0
6404	459.7	2.5754	2.8306	1.113	2.5016	0
6405	459.6	2.6082	2.8601	1.1146	2.5148	0
6406	459.5	2.5904	2.8592	1.1139	2.5003	0
6407	459.4	2.5896	2.8908	1.1136	2.5131	0
6408	459.3	2.6059	2.8576	1.114	2.5124	0
6409	459.2	2.6054	2.8271	1.1139	2.5119	0
6410	459.1	2.6047	2.8564	1.115	2.5112	0
6411	459	2.6216	2.8557	1.1136	2.4966	0
6412	458.9	2.6209	2.8548	1.114	2.5096	0
6413	458.8	2.6202	2.8242	1.1133	2.5232	0
6414	458.7	2.6194	2.8236	1.1148	2.5082	0
6415	458.6	2.6178	2.8219	1.1147	2.4955	0
6416	458.5	2.6171	2.8044	1.114	2.5032	0
6417	458.4	2.6164	2.8204	1.1139	2.5024	0
6418	458.3	2.6379	2.8199	1.1153	2.5017	0
6419	458.2	2.626	2.8368	1.115	2.5095	0
6420	458.1	2.6364	2.8013	1.1148	2.492	0
6421	458	2.6358	2.8176	1.1145	2.5081	0
6422	457.9	2.6351	2.8	1.1151	2.5072	0
6423	457.8	2.6457	2.8339	1.1157	2.5064	0
6424	457.7	2.6334	2.8153	1.116	2.4973	0
6425	457.6	2.6564	2.8147	1.1161	2.4966	0
6426	457.5	2.6438	2.8139	1.1161	2.4959	0
6427	457.4	2.643	2.8133	1.1167	2.4953	0
6428	457.3	2.6542	2.8125	1.116	2.5029	0
6429	457.2	2.6535	2.8119	1.1156	2.5021	0
6430	457.1	2.6527	2.8111	1.1158	2.5099	0
6431	457	2.6519	2.7932	1.1155	2.5006	0
6432	456.9	2.6633	2.7924	1.1161	2.4999	0
6433	456.8	2.6751	2.8088	1.1157	2.4907	0
6434	456.7	2.6745	2.791	1.115	2.507	0
6435	456.6	2.6613	2.8074	1.1162	2.4978	0
6436	456.5	2.6605	2.8066	1.1165	2.497	0

6437	456.4	2.6724	2.806	1.1168	2.5049	0
6438	456.3	2.6716	2.7881	1.1168	2.5042	0
6439	456.2	2.684	2.7875	1.1165	2.5035	0
6440	456.1	2.6702	2.7867	1.116	2.5027	0
6441	456	2.6826	2.786	1.1172	2.5021	0
6442	455.9	2.6817	2.8022	1.1163	2.5099	0
6443	455.8	2.6942	2.7844	1.1166	2.5005	0
6444	455.7	2.6936	2.8008	1.1173	2.4998	0
6445	455.6	2.6792	2.8	1.1168	2.5077	0
6446	455.5	2.6923	2.7825	1.1171	2.4984	0
6447	455.4	2.6782	2.7986	1.1174	2.4977	0
6448	455.3	2.6908	2.7645	1.117	2.5057	0
6449	455.2	2.7038	2.7802	1.1177	2.5049	0
6450	455.1	2.6893	2.7794	1.1171	2.5042	0
6451	455	2.6885	2.8133	1.1174	2.5124	0
6452	454.9	2.7158	2.7951	1.1181	2.5209	0
6453	454.8	2.7009	2.7773	1.1181	2.511	0
6454	454.7	2.7001	2.7765	1.1173	2.5011	0
6455	454.6	2.6992	2.7926	1.1173	2.5186	0
6456	454.5	2.6985	2.7749	1.1162	2.5086	0
6457	454.4	2.684	2.7913	1.1181	2.5079	0
6458	454.3	2.697	2.7734	1.1165	2.5072	0
6459	454.2	2.7106	2.7726	1.1169	2.5064	0
6460	454.1	2.6955	2.7555	1.1173	2.4968	0
6461	454	2.7091	2.7883	1.1173	2.505	0
6462	453.9	2.7231	2.7875	1.1172	2.5042	0
6463	453.8	2.7075	2.7867	1.1173	2.5035	0
6464	453.7	2.7069	2.786	1.1176	2.5027	0
6465	453.6	2.7359	2.803	1.1172	2.511	0
6466	453.5	2.7199	2.7844	1.1167	2.5011	0
6467	453.4	2.7192	2.7838	1.1168	2.5096	0
6468	453.3	2.7185	2.7828	1.1168	2.4907	0
6469	453.2	2.7029	2.782	1.1163	2.5079	0
6470	453.1	2.7321	2.7991	1.1171	2.5072	0
6471	453	2.7471	2.7983	1.1167	2.4973	0
6472	452.9	2.7153	2.7975	1.1167	2.5057	0
6473	452.8	2.7144	2.7788	1.1162	2.5141	0
6474	452.7	2.7138	2.7783	1.1159	2.5042	0
6475	452.6	2.744	2.7775	1.117	2.5033	0
6476	452.5	2.7122	2.7945	1.1167	2.5119	0
6477	452.4	2.7426	2.7937	1.1158	2.5017	0
6478	452.3	2.7261	2.7929	1.1166	2.501	0
6479	452.2	2.7251	2.7921	1.1172	2.4909	0

6480	452.1	2.7242	2.8097	1.1168	2.5086	0
6481	452	2.7235	2.7905	1.1164	2.5174	0
6482	451.9	2.7226	2.808	1.1167	2.5071	0
6483	451.8	2.754	2.7889	1.1167	2.4969	0
6484	451.7	2.721	2.8063	1.1162	2.5054	0
6485	451.6	2.7201	2.8055	1.1158	2.5141	0
6486	451.5	2.7194	2.8049	1.1162	2.4944	0
6487	451.4	2.7185	2.8041	1.1166	2.5031	0
6488	451.3	2.7178	2.8033	1.1177	2.5022	0
6489	451.2	2.7169	2.7838	1.1172	2.5014	0
6490	451.1	2.716	2.8016	1.118	2.5006	0
6491	451	2.7151	2.8008	1.1186	2.519	0
6492	450.9	2.7142	2.7997	1.1181	2.4987	0
6493	450.8	2.7133	2.8182	1.1184	2.5074	0
6494	450.7	2.7284	2.7981	1.1187	2.5065	0
6495	450.6	2.7277	2.8164	1.1184	2.4962	0
6496	450.5	2.7109	2.8156	1.1186	2.5049	0
6497	450.4	2.71	2.8147	1.1185	2.5039	0
6498	450.3	2.7091	2.8139	1.1192	2.5031	0
6499	450.2	2.724	2.813	1.1192	2.4927	0
6500	450.1	2.6923	2.7929	1.1196	2.5014	0
6501	450	2.7066	2.7921	1.1195	2.5103	0
6502	449.9	2.7321	2.8392	1.1421	2.513	0
6503	449.8	2.7199	2.8404	1.1422	2.5141	0
6504	449.7	2.708	2.8416	1.1415	2.5151	0
6505	449.6	2.7091	2.8425	1.1427	2.5082	0
6506	449.5	2.7233	2.8438	1.1431	2.5093	0
6507	449.4	2.7245	2.845	1.1422	2.527	0
6508	449.3	2.7256	2.8292	1.1416	2.5116	0
6509	449.2	2.7138	2.8303	1.1428	2.5129	0
6510	449.1	2.7147	2.8658	1.1431	2.5139	0
6511	449	2.7289	2.8324	1.1425	2.515	0
6512	448.9	2.7169	2.8336	1.1432	2.5081	0
6513	448.8	2.7055	2.8517	1.1426	2.5171	0
6514	448.7	2.7192	2.8526	1.1438	2.5025	0
6515	448.6	2.7201	2.8536	1.1441	2.5113	0
6516	448.5	2.7086	2.8548	1.1441	2.5203	0
6517	448.4	2.7097	2.8557	1.1445	2.5214	0
6518	448.3	2.6866	2.8567	1.1438	2.5065	0
6519	448.2	2.6994	2.8576	1.1431	2.5075	0
6520	448.1	2.7003	2.8416	1.1444	2.5163	0
6521	448	2.7016	2.8598	1.1439	2.5176	0
6522	447.9	2.6906	2.8611	1.1442	2.5106	0

6523	447.8	2.6916	2.845	1.1436	2.5117	0
6524	447.7	2.6927	2.8459	1.1437	2.5127	0
6525	447.6	2.6819	2.8468	1.1431	2.5136	0
6526	447.5	2.683	2.8649	1.1442	2.5224	0
6527	447.4	2.684	2.8658	1.1448	2.5156	0
6528	447.3	2.6849	2.8496	1.1443	2.5164	0
6529	447.2	2.6745	2.8677	1.145	2.5098	0
6530	447.1	2.6755	2.8517	1.1434	2.5262	0
6531	447	2.6765	2.8526	1.1432	2.5119	0
6532	446.9	2.6774	2.8706	1.1439	2.5203	0
6533	446.8	2.6674	2.8716	1.1439	2.5213	0
6534	446.7	2.6684	2.8554	1.144	2.5223	0
6535	446.6	2.6692	2.8732	1.1442	2.5232	0
6536	446.5	2.6702	2.8742	1.1435	2.524	0
6537	446.4	2.671	2.8752	1.1443	2.5327	0
6538	446.3	2.6611	2.8758	1.1442	2.5256	0
6539	446.2	2.6515	2.8768	1.1437	2.5265	0
6540	446.1	2.6525	2.8778	1.1438	2.5275	0
6541	446	2.6533	2.8784	1.1439	2.5206	0
6542	445.9	2.6645	2.8623	1.1444	2.5214	0
6543	445.8	2.6445	2.8801	1.1435	2.5299	0
6544	445.7	2.6558	2.8811	1.1429	2.5232	0
6545	445.6	2.6463	2.8817	1.1446	2.5315	0
6546	445.5	2.647	2.8492	1.1451	2.5248	0
6547	445.4	2.6379	2.8834	1.1444	2.5333	0
6548	445.3	2.6388	2.8844	1.143	2.5264	0
6549	445.2	2.6494	2.8851	1.1424	2.5271	0
6550	445.1	2.6404	2.8687	1.1423	2.5355	0
6551	445	2.6411	2.8867	1.1419	2.5287	0
6552	444.9	2.6227	2.8706	1.1431	2.5296	0
6553	444.8	2.6328	2.9059	1.1431	2.5379	0
6554	444.7	2.6338	2.8891	1.1421	2.5388	0
6555	444.6	2.6249	2.8726	1.1422	2.5396	0
6556	444.5	2.6256	2.857	1.1431	2.5402	0
6557	444.4	2.6171	2.8742	1.1423	2.5409	0
6558	444.3	2.6271	2.8748	1.1421	2.5415	0
6559	444.2	2.6278	2.8755	1.141	2.5423	0
6560	444.1	2.6286	2.8765	1.1418	2.5432	0
6561	444	2.62	2.8771	1.1413	2.5438	0
6562	443.9	2.6207	2.8778	1.1408	2.5369	0
6563	443.8	2.6212	2.8955	1.1411	2.5452	0
6564	443.7	2.6128	2.8791	1.1415	2.5382	0
6565	443.6	2.6135	2.8633	1.1409	2.539	0

6566	443.5	2.6232	2.8804	1.1413	2.5471	0
6567	443.4	2.6148	2.8811	1.1415	2.54	0
6568	443.3	2.6153	2.8814	1.1405	2.5561	0
6569	443.2	2.607	2.8821	1.1409	2.5567	0
6570	443.1	2.5986	2.8827	1.1414	2.5494	0
6571	443	2.6082	2.8834	1.1403	2.558	0
6572	442.9	2.6087	2.8674	1.1408	2.5585	0
6573	442.8	2.6094	2.8681	1.1412	2.5513	0
6574	442.7	2.6012	2.8851	1.1418	2.5442	0
6575	442.6	2.6017	2.8857	1.141	2.5524	0
6576	442.5	2.611	2.87	1.1414	2.5532	0
6577	442.4	2.6029	2.8706	1.1423	2.5536	0
6578	442.3	2.6033	2.871	1.1421	2.5619	0
6579	442.2	2.5952	2.8877	1.1417	2.5546	0
6580	442.1	2.5959	2.9055	1.1414	2.563	0
6581	442	2.5964	2.8726	1.1413	2.5635	0
6582	441.9	2.5969	2.8732	1.141	2.5563	0
6583	441.8	2.6059	2.8735	1.1409	2.5646	0
6584	441.7	2.5979	2.8582	1.1417	2.573	0
6585	441.6	2.5984	2.8911	1.1419	2.5656	0
6586	441.5	2.5988	2.8748	1.1423	2.574	0
6587	441.4	2.5993	2.8918	1.1413	2.5666	0
6588	441.3	2.5913	2.8925	1.1424	2.575	0
6589	441.2	2.5918	2.8928	1.1411	2.5754	0
6590	441.1	2.5923	2.8931	1.1418	2.5678	0
6591	441	2.6012	2.8938	1.1417	2.5763	0
6592	440.9	2.6015	2.8941	1.1417	2.5849	0
6593	440.8	2.5854	2.8945	1.142	2.5693	0
6594	440.7	2.594	2.8952	1.1424	2.5857	0
6595	440.6	2.5945	2.8791	1.1423	2.5862	0
6596	440.5	2.5865	2.8794	1.1424	2.5865	0
6597	440.4	2.5869	2.8798	1.1424	2.5869	0
6598	440.3	2.5874	2.8645	1.1429	2.5792	0
6599	440.2	2.5879	2.8807	1.143	2.5797	0
6600	440.1	2.5965	2.8976	1.1428	2.5965	0
6601	440	2.5885	2.8979	1.1423	2.6052	0
6602	439.9	2.5891	2.8983	1.1421	2.5891	0
6603	439.8	2.5894	2.8824	1.1422	2.5894	0
6604	439.7	2.5897	2.8989	1.144	2.5979	0
6605	439.6	2.582	2.8831	1.1441	2.5984	0
6606	439.5	2.5904	2.8674	1.1439	2.5904	0
6607	439.4	2.5907	2.8837	1.1436	2.5991	0
6608	439.3	2.583	2.8841	1.1443	2.6078	0

6609	439.2	2.5916	2.901	1.1448	2.6084	0
6610	439.1	2.5837	2.901	1.1441	2.6085	0
6611	439	2.5923	2.9017	1.1439	2.6005	0
6612	438.9	2.5926	2.902	1.1454	2.6008	0
6613	438.8	2.5929	2.8861	1.1441	2.6098	0
6614	438.7	2.5935	2.8706	1.1439	2.6101	0
6615	438.6	2.5938	2.8867	1.1437	2.6105	0
6616	438.5	2.586	2.9034	1.1458	2.6194	0
6617	438.4	2.5862	2.8871	1.1463	2.6196	0
6618	438.3	2.5947	2.8716	1.1457	2.62	0
6619	438.2	2.587	2.9045	1.145	2.6203	0
6620	438.1	2.5792	2.9045	1.1456	2.6295	0
6621	438	2.5959	2.8887	1.1454	2.6211	0
6622	437.9	2.5879	2.8887	1.1473	2.63	0
6623	437.8	2.5962	2.8891	1.1467	2.6302	0
6624	437.7	2.5885	2.9059	1.1465	2.6396	0
6625	437.6	2.5809	2.8897	1.1477	2.64	0
6626	437.5	2.5892	2.9066	1.1478	2.6404	0
6627	437.4	2.5976	2.8904	1.1478	2.6317	0
6628	437.3	2.5896	2.9069	1.1474	2.6499	0
6629	437.2	2.5899	2.8911	1.1468	2.6411	0
6630	437.1	2.5825	2.9248	1.1467	2.6507	0
6631	437	2.5906	2.9252	1.1472	2.6509	0
6632	436.9	2.5991	2.9083	1.1484	2.6513	0
6633	436.8	2.5913	2.9255	1.1481	2.6607	0
6634	436.7	2.5916	2.909	1.1475	2.6611	0
6635	436.6	2.5839	2.9263	1.1493	2.6613	0
6636	436.5	2.5921	2.9266	1.1476	2.6617	0
6637	436.4	2.6005	2.9097	1.1484	2.6813	0
6638	436.3	2.6007	2.927	1.1479	2.6716	0
6639	436.2	2.601	2.9274	1.1477	2.6817	0
6640	436.1	2.6012	2.9454	1.148	2.6821	0
6641	436	2.5935	2.9278	1.1472	2.6824	0
6642	435.9	2.5938	2.9458	1.1469	2.6828	0
6643	435.8	2.6021	2.9285	1.1471	2.6929	0
6644	435.7	2.5943	2.9289	1.1476	2.6931	0
6645	435.6	2.6026	2.9465	1.1476	2.6834	0
6646	435.5	2.611	2.9292	1.1469	2.7038	0
6647	435.4	2.595	2.9473	1.1467	2.6938	0
6648	435.3	2.6033	2.9296	1.1455	2.6942	0
6649	435.2	2.6036	2.9477	1.1472	2.7047	0
6650	435.1	2.6123	2.9481	1.1461	2.7049	0
6651	435	2.6041	2.9666	1.1468	2.7156	0

6652	434.9	2.6043	2.9485	1.1462	2.7158	0
6653	434.8	2.6128	2.9488	1.1459	2.716	0
6654	434.7	2.6048	2.9311	1.1462	2.7272	0
6655	434.6	2.5971	2.9492	1.1465	2.7167	0
6656	434.5	2.6137	2.9496	1.1473	2.7169	0
6657	434.4	2.6057	2.9682	1.1467	2.7279	0
6658	434.3	2.6142	2.95	1.1467	2.7282	0
6659	434.2	2.6062	2.9686	1.1467	2.7284	0
6660	434.1	2.6064	2.969	1.146	2.7397	0
6661	434	2.6066	2.9884	1.1453	2.7289	0
6662	433.9	2.6149	2.969	1.1448	2.7399	0
6663	433.8	2.6151	2.9889	1.1454	2.7291	0
6664	433.7	2.6156	2.9698	1.1458	2.7406	0
6665	433.6	2.6075	2.9893	1.1446	2.7406	0
6666	433.5	2.6158	2.9893	1.1444	2.7298	0
6667	433.4	2.6243	2.9702	1.1441	2.7409	0
6668	433.3	2.6245	2.9897	1.1443	2.73	0
6669	433.2	2.6164	2.9901	1.1446	2.7303	0
6670	433.1	2.6251	2.971	1.1445	2.7416	0
6671	433	2.6251	2.9901	1.1443	2.753	0
6672	432.9	2.6254	2.9905	1.1438	2.7418	0
6673	432.8	2.6256	2.9905	1.144	2.7421	0
6674	432.7	2.6258	2.9714	1.1446	2.7535	0
6675	432.6	2.626	2.9718	1.1451	2.7537	0
6676	432.5	2.626	2.9718	1.1433	2.7537	0
6677	432.4	2.6262	2.9722	1.1435	2.754	0
6678	432.3	2.6264	2.9722	1.1447	2.754	0
6679	432.2	2.618	2.9722	1.1444	2.7542	0
6680	432.1	2.618	2.9914	1.1451	2.7542	0
6681	432	2.6265	2.9918	1.1444	2.7545	0
6682	431.9	2.6269	2.9727	1.1441	2.7547	0
6683	431.8	2.6356	2.9727	1.1436	2.7547	0
6684	431.7	2.6356	2.9923	1.1439	2.7547	0
6685	431.6	2.6356	2.9923	1.1437	2.7547	0
6686	431.5	2.6356	2.9731	1.1432	2.7435	0
6687	431.4	2.6273	2.9923	1.1437	2.755	0
6688	431.3	2.636	2.9927	1.1427	2.7552	0
6689	431.2	2.636	2.9731	1.1427	2.7552	0
6690	431.1	2.6362	2.9927	1.1442	2.7552	0
6691	431	2.6276	2.9927	1.1448	2.744	0
6692	430.9	2.6362	2.9927	1.1435	2.7555	0
6693	430.8	2.6451	2.9927	1.1435	2.7555	0
6694	430.7	2.6451	2.9931	1.1442	2.7555	0

6695	430.6	2.6364	3.0132	1.1441	2.767	0
6696	430.5	2.6451	2.9735	1.1434	2.7673	0
6697	430.4	2.6453	2.9931	1.1446	2.7673	0
6698	430.3	2.6453	2.9739	1.1442	2.7673	0
6699	430.2	2.6455	2.9739	1.1448	2.7675	0
6700	430.1	2.6455	2.9931	1.1454	2.756	0
6701	430	2.6544	2.9931	1.1459	2.756	0
6702	429.9	2.6544	2.9931	1.1459	2.7675	0
6703	429.8	2.6544	2.9931	1.1465	2.7675	0
6704	429.7	2.6544	2.9931	1.1472	2.7673	0
6705	429.6	2.6542	2.9931	1.146	2.7791	0
6706	429.5	2.6544	2.9931	1.147	2.7675	0
6707	429.4	2.6542	2.9931	1.1463	2.7791	0
6708	429.3	2.6635	2.9739	1.1479	2.7675	0
6709	429.2	2.6544	3.0132	1.1467	2.756	0
6710	429.1	2.6544	2.9739	1.147	2.7675	0
6711	429	2.6633	2.9931	1.1463	2.7673	0
6712	428.9	2.6544	2.9931	1.1466	2.7794	0
6713	428.8	2.6635	3.0132	1.147	2.7794	0
6714	428.7	2.6542	3.0132	1.1466	2.7791	0
6715	428.6	2.6631	3.0343	1.1466	2.7788	0
6716	428.5	2.654	2.9927	1.1478	2.767	0
6717	428.4	2.654	3.0132	1.1481	2.7913	0
6718	428.3	2.6633	3.0132	1.1493	2.7791	0
6719	428.2	2.6724	3.0132	1.1488	2.767	0
6720	428.1	2.6631	3.0132	1.1494	2.7788	0
6721	428	2.6724	3.0343	1.1494	2.7788	0
6722	427.9	2.6722	2.9927	1.1494	2.7786	0
6723	427.8	2.6629	3.0339	1.1498	2.7788	0
6724	427.7	2.6722	3.0128	1.1498	2.7667	0
6725	427.6	2.6722	3.0339	1.1497	2.7786	0
6726	427.5	2.6817	3.0128	1.1509	2.7786	0
6727	427.4	2.6817	3.0339	1.1509	2.7786	0
6728	427.3	2.6722	3.0128	1.1508	2.7786	0
6729	427.2	2.6722	3.0128	1.1502	2.7908	0
6730	427.1	2.672	3.0339	1.1502	2.7786	0
6731	427	2.6815	3.0128	1.1513	2.7786	0
6732	426.9	2.6815	3.0123	1.1509	2.7665	0
6733	426.8	2.6815	3.0339	1.1508	2.7665	0
6734	426.7	2.6813	3.0123	1.1507	2.7783	0
6735	426.6	2.6813	3.0123	1.1495	2.7781	0
6736	426.5	2.6813	3.0123	1.1506	2.7662	0
6737	426.4	2.6718	3.0123	1.1516	2.7783	0

6738	426.3	2.6813	2.9923	1.1498	2.7781	0
6739	426.2	2.6811	3.0334	1.1502	2.7902	0
6740	426.1	2.6908	3.0334	1.1496	2.7781	0
6741	426	2.6811	3.0119	1.1504	2.766	0
6742	425.9	2.6809	3.0329	1.1503	2.7899	0
6743	425.8	2.6906	3.0119	1.1514	2.766	0
6744	425.7	2.6906	3.0329	1.1507	2.7778	0
6745	425.6	2.6906	3.0119	1.1504	2.7775	0
6746	425.5	2.7106	3.0114	1.1508	2.7655	0
6747	425.4	2.7003	3.0329	1.1499	2.7655	0
6748	425.3	2.6904	3.0114	1.1511	2.754	0
6749	425.2	2.7003	3.0114	1.151	2.7773	0
6750	425.1	2.7104	3.0325	1.1504	2.7773	0
6751	425	2.7104	3.0114	1.1509	2.7655	0
6752	424.9	2.6899	3.0325	1.1513	2.7652	0
6753	424.8	2.6998	3.0325	1.151	2.7652	0
6754	424.7	2.6998	3.0325	1.1512	2.7652	0
6755	424.6	2.7102	3.0325	1.1495	2.777	0
6756	424.5	2.6996	3.032	1.1493	2.765	0
6757	424.4	2.71	3.011	1.1502	2.7768	0
6758	424.3	2.7097	3.032	1.1502	2.7768	0
6759	424.2	2.6994	3.032	1.1497	2.7647	0
6760	424.1	2.6994	3.032	1.1501	2.7765	0
6761	424	2.7095	3.0315	1.1491	2.7645	0
6762	423.9	2.7093	3.0106	1.1496	2.7762	0
6763	423.8	2.7093	3.0315	1.1478	2.7642	0
6764	423.7	2.7093	3.0535	1.1477	2.7762	0
6765	423.6	2.6988	3.0311	1.1481	2.764	0
6766	423.5	2.7089	3.0097	1.1474	2.7757	0
6767	423.4	2.7086	3.0097	1.1478	2.7757	0
6768	423.3	2.6985	3.0311	1.1475	2.7757	0
6769	423.2	2.719	3.0306	1.1482	2.7875	0
6770	423.1	2.7187	3.0306	1.1471	2.7752	0
6771	423	2.7082	3.0092	1.1467	2.7752	0
6772	422.9	2.7185	3.0301	1.1471	2.763	0
6773	422.8	2.7183	3.0301	1.1481	2.763	0
6774	422.7	2.7291	3.0301	1.1475	2.787	0
6775	422.6	2.7075	3.0297	1.1472	2.7627	0
6776	422.5	2.7178	3.0297	1.1471	2.7625	0
6777	422.4	2.7284	3.0516	1.1466	2.7865	0
6778	422.3	2.7282	3.0292	1.1458	2.7862	0
6779	422.2	2.7282	3.0292	1.1466	2.7739	0
6780	422.1	2.7169	3.0511	1.1474	2.7857	0

6781	422	2.7275	3.0287	1.1474	2.7854	0
6782	421.9	2.7165	3.007	1.1472	2.7852	0
6783	421.8	2.7162	3.0506	1.1476	2.7852	0
6784	421.7	2.7268	3.0501	1.1468	2.7726	0
6785	421.6	2.7265	3.0496	1.1476	2.7846	0
6786	421.5	2.7263	3.0496	1.1465	2.7844	0
6787	421.4	2.7261	3.0496	1.1469	2.7967	0
6788	421.3	2.7258	3.0491	1.1467	2.7838	0
6789	421.2	2.7256	3.0726	1.1467	2.7836	0
6790	421.1	2.7254	3.0487	1.1471	2.7833	0
6791	421	2.7251	3.0259	1.1476	2.7956	0
6792	420.9	2.7359	3.0259	1.1483	2.7956	0
6793	420.8	2.7357	3.0482	1.1475	2.7953	0
6794	420.7	2.7245	3.0255	1.1475	2.7951	0
6795	420.6	2.7352	3.0477	1.1481	2.7948	0
6796	420.5	2.7462	3.0472	1.1484	2.782	0
6797	420.4	2.7345	3.0467	1.1489	2.7943	0
6798	420.3	2.7457	3.0467	1.1505	2.794	0
6799	420.2	2.7452	3.0696	1.1487	2.7937	0
6800	420.1	2.7338	3.0462	1.1482	2.8063	0
6801	420	2.7335	3.0237	1.1485	2.7932	0
6802	419.9	2.7445	3.0453	1.1484	2.8058	0
6803	419.8	2.7442	3.0453	1.1484	2.7924	0
6804	419.7	2.7326	3.0227	1.1482	2.8052	0
6805	419.6	2.7435	3.0443	1.1485	2.8047	0
6806	419.5	2.7433	3.0675	1.148	2.8044	0
6807	419.4	2.7428	3.0675	1.1474	2.8041	0
6808	419.3	2.7426	3.067	1.1487	2.8173	0
6809	419.2	2.7423	3.0434	1.1497	2.8035	0
6810	419.1	2.7535	3.0665	1.1485	2.8033	0
6811	419	2.7533	3.066	1.1491	2.7899	0
6812	418.9	2.7528	3.0424	1.1499	2.8024	0
6813	418.8	2.7525	3.0655	1.1499	2.8022	0
6814	418.7	2.7523	3.065	1.1511	2.8019	0
6815	418.6	2.7402	3.0414	1.1522	2.815	0
6816	418.5	2.7515	3.0645	1.1515	2.8145	0
6817	418.4	2.763	3.0888	1.1517	2.8008	0
6818	418.3	2.7508	3.0635	1.1523	2.8139	0
6819	418.2	2.7506	3.0883	1.1518	2.8002	0
6820	418.1	2.7503	3.0878	1.152	2.8271	0
6821	418	2.7498	3.0625	1.1516	2.8128	0
6822	417.9	2.7496	3.0872	1.1525	2.8125	0
6823	417.8	2.7491	3.062	1.1532	2.8259	0

6824	417.7	2.7607	3.0867	1.1526	2.8119	0
6825	417.6	2.7602	3.0862	1.1523	2.8114	0
6826	417.5	2.7721	3.0857	1.1543	2.8248	0
6827	417.4	2.7597	3.0857	1.1542	2.8245	0
6828	417.3	2.7592	3.0851	1.1536	2.8242	0
6829	417.2	2.7587	3.1113	1.1546	2.8099	0
6830	417.1	2.7708	3.1107	1.1538	2.8233	0
6831	417	2.7703	3.0841	1.1537	2.823	0
6832	416.9	2.7701	3.0835	1.1544	2.8228	0
6833	416.8	2.7698	3.0835	1.1543	2.8225	0
6834	416.7	2.7693	3.083	1.1542	2.8083	0
6835	416.6	2.7815	3.0825	1.1535	2.8359	0
6836	416.5	2.7685	3.1085	1.1552	2.8213	0
6837	416.4	2.7683	3.1085	1.1556	2.8353	0
6838	416.3	2.7807	3.1079	1.1562	2.8351	0
6839	416.2	2.7802	3.1073	1.1551	2.8345	0
6840	416.1	2.7799	3.1073	1.1555	2.8489	0
6841	416	2.7924	3.1068	1.1556	2.8339	0
6842	415.9	2.7791	3.1062	1.1558	2.848	0
6843	415.8	2.7916	3.1062	1.156	2.833	0
6844	415.7	2.7783	3.1057	1.1554	2.8327	0
6845	415.6	2.791	3.1331	1.1547	2.8471	0
6846	415.5	2.7775	3.1046	1.1545	2.8465	0
6847	415.4	2.7899	3.1325	1.1547	2.8462	0
6848	415.3	2.7897	3.1319	1.1546	2.8456	0
6849	415.2	2.7891	3.1314	1.1557	2.8453	0
6850	415.1	2.8022	3.1314	1.1556	2.845	0
6851	415	2.7883	3.1308	1.1542	2.8444	0
6852	414.9	2.8011	3.1302	1.1547	2.8441	0
6853	414.8	2.8008	3.1599	1.155	2.8438	0
6854	414.7	2.8005	3.1013	1.1551	2.8586	0
6855	414.6	2.8	3.129	1.1544	2.8739	0
6856	414.5	2.7994	3.1284	1.1548	2.8422	0
6857	414.4	2.7857	3.158	1.1544	2.857	0
6858	414.3	2.8125	3.0996	1.1537	2.8567	0
6859	414.2	2.7983	3.1273	1.1539	2.8564	0
6860	414.1	2.8119	3.1568	1.1552	2.856	0
6861	414	2.7975	3.1267	1.1544	2.8554	0
6862	413.9	2.8108	3.098	1.1535	2.8706	0
6863	413.8	2.8102	3.1255	1.1531	2.8703	0
6864	413.7	2.7962	3.1549	1.153	2.8864	0
6865	413.6	2.8094	3.1549	1.1529	2.8536	0
6866	413.5	2.8091	3.1244	1.1524	2.869	0

6867	413.4	2.8085	3.1537	1.1513	2.8687	0
6868	413.3	2.8083	3.1537	1.1525	2.8847	0
6869	413.2	2.8222	3.153	1.1521	2.852	0
6870	413.1	2.8072	3.1226	1.1517	2.8834	0
6871	413	2.8356	3.1518	1.1529	2.8831	0
6872	412.9	2.8351	3.1512	1.1517	2.8996	0
6873	412.8	2.8202	3.1831	1.1517	2.8824	0
6874	412.7	2.8342	3.1506	1.1526	2.8986	0
6875	412.6	2.8489	3.15	1.1521	2.8645	0
6876	412.5	2.8044	3.1494	1.1527	2.8642	0
6877	412.4	2.8182	3.091	1.1525	2.8972	0
6878	412.3	2.8176	3.1487	1.1526	2.8798	0
6879	412.2	2.8318	3.1481	1.1515	2.8962	0
6880	412.1	2.8312	3.1475	1.1515	2.8955	0
6881	412	2.8459	3.1791	1.1521	2.8952	0
6882	411.9	2.8456	3.1463	1.1525	2.8948	0
6883	411.8	2.845	3.1785	1.1517	2.8941	0
6884	411.7	2.8447	3.1457	1.1512	2.8938	0
6885	411.6	2.8286	3.1152	1.1518	2.9108	0
6886	411.5	2.8283	3.1146	1.1517	2.8928	0
6887	411.4	2.8428	3.1439	1.1528	2.9101	0
6888	411.3	2.8582	3.1433	1.1523	2.8918	0
6889	411.2	2.8419	3.1427	1.1527	2.909	0
6890	411.1	2.8413	3.1746	1.152	2.9083	0
6891	411	2.8407	3.1421	1.1519	2.9076	0
6892	410.9	2.8726	3.1415	1.1511	2.8897	0
6893	410.8	2.8557	3.1409	1.1512	2.9252	0
6894	410.7	2.8551	3.1403	1.1516	2.9062	0
6895	410.6	2.8545	3.1397	1.1516	2.9055	0
6896	410.5	2.8539	3.1391	1.152	2.9048	0
6897	410.4	2.8533	3.2055	1.1521	2.9045	0
6898	410.3	2.8861	3.1701	1.1516	2.9223	0
6899	410.2	2.8687	3.1373	1.1522	2.9219	0
6900	410.1	2.8681	3.1694	1.1524	2.8851	0
6901	410	2.8674	3.1688	1.1524	2.902	0
6902	409.9	2.8837	3.1681	1.1524	2.9017	0
6903	409.8	2.8661	3.1349	1.1518	2.9194	0
6904	409.7	2.8655	3.1669	1.1522	2.919	0
6905	409.6	2.8652	3.1662	1.1534	2.9	0
6906	409.5	2.8814	3.1656	1.1537	2.937	0
6907	409.4	2.8807	3.1649	1.1539	2.9363	0
6908	409.3	2.8801	3.1643	1.1526	2.9165	0
6909	409.2	2.8798	3.1637	1.1537	2.9158	0

6910	409.1	2.8791	3.1308	1.1534	2.9151	0
6911	409	2.8784	3.1302	1.1529	2.9147	0
6912	408.9	2.8778	3.1618	1.1529	2.9333	0
6913	408.8	2.8771	3.1612	1.1533	2.9329	0
6914	408.7	2.8768	3.1952	1.1537	2.9322	0
6915	408.6	2.8592	3.2328	1.1548	2.9314	0
6916	408.5	2.8931	3.1945	1.1541	2.9512	0
6917	408.4	2.8925	3.1938	1.1545	2.9303	0
6918	408.3	2.8742	3.158	1.1547	2.9296	0
6919	408.2	2.8911	3.1574	1.1545	2.9292	0
6920	408.1	2.8908	3.1568	1.1566	2.9485	0
6921	408	2.8901	3.1911	1.1564	2.9481	0
6922	407.9	2.8894	3.1904	1.157	2.9473	0
6923	407.8	2.8887	3.1549	1.1557	2.9469	0
6924	407.7	2.8881	3.1891	1.1568	2.9462	0
6925	407.6	2.9062	3.1537	1.1559	2.9255	0
6926	407.5	2.8871	3.153	1.157	2.945	0
6927	407.4	2.8864	3.2255	1.1573	2.9241	0
6928	407.3	2.9041	3.1864	1.157	2.9234	0
6929	407.2	2.8851	3.1864	1.1574	2.9431	0
6930	407.1	2.9031	3.1506	1.1572	2.9427	0
6931	407	2.9024	3.118	1.1587	2.942	0
6932	406.9	2.9017	3.1494	1.158	2.9412	0
6933	406.8	2.901	3.2211	1.1587	2.9408	0
6934	406.7	2.9197	3.1481	1.1589	2.9401	0
6935	406.6	2.9	3.1824	1.1583	2.9397	0
6936	406.5	2.8993	3.1818	1.1584	2.9389	0
6937	406.4	2.8989	3.219	1.1577	2.9382	0
6938	406.3	2.9176	3.1457	1.1582	2.9378	0
6939	406.2	2.8976	3.1798	1.1586	2.937	0
6940	406.1	2.8784	3.1798	1.1584	2.9161	0
6941	406	2.8962	3.2168	1.1582	2.9355	0
6942	405.9	2.9151	3.1113	1.1592	2.9788	0
6943	405.8	2.9144	3.1778	1.1594	2.9344	0
6944	405.7	2.9136	3.1772	1.1582	2.9551	0
6945	405.6	2.8938	3.1415	1.1586	2.9547	0
6946	405.5	2.9126	3.1759	1.1594	2.9539	0
6947	405.4	2.9119	3.1752	1.1588	2.9755	0
6948	405.3	2.9314	3.2125	1.1582	2.9527	0
6949	405.2	2.9519	3.2118	1.159	2.9307	0
6950	405.1	2.9101	3.2111	1.159	2.9512	0
6951	405	2.9094	3.2104	1.1581	2.9508	0
6952	404.9	2.9087	3.172	1.158	2.95	0

6953	404.8	2.9281	3.1713	1.1576	2.9952	0
6954	404.7	2.9274	3.2083	1.1573	2.9274	0
6955	404.6	2.9266	3.2076	1.1595	2.9702	0
6956	404.5	2.9263	3.2069	1.1588	2.9263	0
6957	404.4	2.9252	3.1681	1.1591	2.969	0
6958	404.3	2.9248	3.2472	1.1574	2.9458	0
6959	404.2	2.9241	3.2464	1.157	2.9674	0
6960	404.1	2.9234	3.2041	1.1581	2.967	0
6961	404	2.9226	3.2449	1.1588	2.9662	0
6962	403.9	2.9431	3.2027	1.1584	2.9654	0
6963	403.8	2.9212	3.202	1.158	2.9646	0
6964	403.7	2.9416	3.2013	1.157	2.9876	0
6965	403.6	2.9408	3.2007	1.1583	2.9408	0
6966	403.5	2.919	3.2	1.1577	2.9626	0
6967	403.4	2.9183	3.1993	1.1573	2.9393	0
6968	403.3	2.9389	3.1986	1.1588	2.961	0
6969	403.2	2.9378	3.1599	1.1582	2.9602	0
6970	403.1	2.9158	3.1593	1.1568	2.9594	0
6971	403	2.9586	3.1959	1.1569	2.9586	0
6972	402.9	2.9355	3.1952	1.1565	2.9578	0
6973	402.8	2.9352	3.1945	1.1562	2.9809	0
6974	402.7	2.9562	3.1938	1.156	2.9801	0
6975	402.6	2.9333	3.2343	1.1563	2.9792	0
6976	402.5	2.9326	3.2336	1.1555	2.9784	0
6977	402.4	2.9543	3.279	1.1567	2.9776	0
6978	402.3	2.9535	3.1911	1.1567	2.9767	0
6979	402.2	2.9527	3.2314	1.1565	3.0009	0
6980	402.1	2.9519	3.2306	1.1566	2.9755	0
6981	402	2.9512	3.2299	1.1565	2.9996	0
6982	401.9	2.9739	3.2291	1.1557	2.9739	0
6983	401.8	2.9731	3.2284	1.1568	2.9731	0
6984	401.7	2.9488	3.1864	1.1565	2.9488	0
6985	401.6	2.9714	3.2725	1.1571	2.9714	0
6986	401.5	2.9706	3.1844	1.1566	2.9952	0
6987	401.4	2.9698	3.2248	1.1573	2.9698	0
6988	401.3	2.9454	3.1831	1.1569	2.969	0
6989	401.2	2.9682	3.1818	1.156	2.9682	0
6990	401.1	2.9674	3.2684	1.1572	2.9674	0
6991	401	2.9666	3.1805	1.1566	2.9914	0
6992	400.9	2.9905	3.2668	1.1564	2.9658	0
6993	400.8	2.965	3.2204	1.1575	2.965	0
6994	400.7	2.9404	3.219	1.157	2.9889	0
6995	400.6	2.963	3.1772	1.1565	2.988	0

6996	400.5	2.9389	3.2175	1.1568	2.9872	0
6997	400.4	2.9614	3.2168	1.1578	2.9614	0
6998	400.3	3.0119	3.2612	1.157	2.9855	0
6999	400.2	2.9598	3.2147	1.1572	3.0106	0
7000	400.1	2.9586	3.1726	1.1575	3.0097	0
7001	400	2.9825	3.2588	1.1578	3.0092	0
7002	399.9	2.957	3.2125	1.1569	3.0083	0
7003	399.8	2.9562	3.2573	1.1591	2.9809	0
7004	399.7	2.9801	3.2104	1.1582	2.9801	0
7005	399.6	2.9543	3.2097	1.158	3.0052	0
7006	399.5	2.9784	3.2541	1.1583	2.9784	0
7007	399.4	2.9776	3.1669	1.1587	3.0039	0
7008	399.3	2.9763	3.2526	1.1585	3.0031	0
7009	399.2	2.9755	3.2062	1.1592	3.0017	0
7010	399.1	3.0013	3.2055	1.1596	3.0013	0
7011	399	3	3.2041	1.1589	3.0283	0
7012	398.9	2.9727	3.2487	1.1587	2.9991	0
7013	398.8	2.9718	3.202	1.1582	3.0264	0
7014	398.7	2.971	3.1599	1.1594	3.0255	0
7015	398.6	2.9965	3.2464	1.1597	2.9965	0
7016	398.5	2.9694	3.2	1.1593	2.9957	0
7017	398.4	2.9686	3.2449	1.1603	3.0227	0
7018	398.3	2.9678	3.1979	1.1599	2.994	0
7019	398.2	2.9666	3.1972	1.1597	2.9931	0
7020	398.1	2.9923	3.1965	1.16	3.02	0
7021	398	2.9914	3.2411	1.1599	2.9914	0
7022	397.9	2.9901	3.2916	1.1613	2.9901	0
7023	397.8	2.9893	3.2388	1.1603	3.0173	0
7024	397.7	2.9884	3.1925	1.1608	3.0164	0
7025	397.6	2.9876	3.2373	1.1598	3.0155	0
7026	397.5	2.9867	3.2366	1.1605	2.9867	0
7027	397.4	3.0137	3.2351	1.1607	3.0434	0
7028	397.3	3.0128	3.2343	1.1614	3.0128	0
7029	397.2	2.9838	3.2336	1.1614	3.0119	0
7030	397.1	2.983	3.2328	1.1609	3.011	0
7031	397	2.9817	3.2832	1.1614	3.0097	0
7032	396.9	3.0092	3.2306	1.1618	2.9809	0
7033	396.8	3.0079	3.2815	1.1626	3.0079	0
7034	396.7	3.007	3.2291	1.1617	3.007	0
7035	396.6	2.978	3.2277	1.1619	3.0362	0
7036	396.5	2.9772	3.2269	1.1617	3.0052	0
7037	396.4	3.0343	3.2774	1.1613	3.0044	0
7038	396.3	3.0334	3.2255	1.1616	3.0334	0

7039	396.2	2.9743	3.2749	1.1613	3.0645	0
7040	396.1	3.0315	3.2233	1.1621	3.0315	0
7041	396	3.0306	3.2226	1.1616	3.0306	0
7042	395.9	2.9996	3.2211	1.162	3.0297	0
7043	395.8	2.9706	3.2716	1.1619	2.9987	0
7044	395.7	2.9978	3.2197	1.1618	2.9978	0
7045	395.6	3.0269	3.2182	1.1625	3.0269	0
7046	395.5	2.9957	3.2175	1.1616	2.9957	0
7047	395.4	3.0246	3.2676	1.1618	3.0246	0
7048	395.3	3.056	3.1701	1.1631	2.9658	0
7049	395.2	3.0227	3.2147	1.1625	2.9927	0
7050	395.1	3.0218	3.2652	1.1621	2.9923	0
7051	395	3.0209	3.2125	1.1623	3.0209	0
7052	394.9	3.02	3.2628	1.1625	3.0521	0
7053	394.8	2.9889	3.2111	1.1621	3.0511	0
7054	394.7	2.988	3.2612	1.1616	3.0182	0
7055	394.6	3.0173	3.2604	1.1628	3.0173	0
7056	394.5	3.0164	3.2596	1.162	3.0487	0
7057	394.4	2.9855	3.2581	1.1624	3.0477	0
7058	394.3	3.0467	3.2062	1.1626	3.0467	0
7059	394.2	3.0132	3.2565	1.1629	3.0132	0
7060	394.1	3.0123	3.2041	1.1624	3.0443	0
7061	394	2.9813	3.2541	1.1631	3.0434	0
7062	393.9	3.0424	3.202	1.1626	3.0101	0
7063	393.8	3.0092	3.2526	1.1612	3.0414	0
7064	393.7	3.0083	3.2518	1.1611	3.0405	0
7065	393.6	3.0074	3.3089	1.1628	3.0074	0
7066	393.5	3.0066	3.3072	1.1617	3.0386	0
7067	393.4	3.0376	3.1512	1.1607	3.0376	0
7068	393.3	3.0367	3.1965	1.1614	3.0044	0
7069	393.2	3.0035	3.2464	1.1622	3.0701	0
7070	393.1	3.0343	3.1945	1.1626	3.0343	0
7071	393	3.068	3.3019	1.1611	3.068	0
7072	392.9	3.0675	3.301	1.1616	3.0675	0
7073	392.8	3.0315	3.1911	1.1617	3.066	0
7074	392.7	3.0301	3.1898	1.1616	3.065	0
7075	392.6	3.0292	3.2403	1.1615	3.0292	0
7076	392.5	2.9961	3.2388	1.1617	3.0283	0
7077	392.4	3.062	3.2381	1.1617	3.0273	0
7078	392.3	2.994	3.2373	1.1624	3.0259	0
7079	392.2	3.0595	3.1844	1.1625	3.0595	0
7080	392.1	3.0237	3.2924	1.1609	3.0585	0
7081	392	3.0227	3.2916	1.1613	3.0575	0

7082	391.9	3.0565	3.2907	1.1608	3.0565	0
7083	391.8	3.0209	3.2314	1.1606	3.0555	0
7084	391.7	3.0195	3.2306	1.1616	3.0195	0
7085	391.6	3.0186	3.2291	1.162	3.0531	0
7086	391.5	3.0521	3.1772	1.1628	3.0521	0
7087	391.4	3.0164	3.2269	1.1613	3.0511	0
7088	391.3	3.0496	3.284	1.1624	3.015	0
7089	391.2	3.0487	3.2248	1.1621	3.0487	0
7090	391.1	3.0472	3.2815	1.1618	3.0472	0
7091	391	3.0462	3.2807	1.1613	3.0462	0
7092	390.9	3.083	3.1701	1.1621	3.0453	0
7093	390.8	3.0092	3.2204	1.1624	3.0443	0
7094	390.7	3.0083	3.219	1.1632	3.0429	0
7095	390.6	3.0419	3.2182	1.163	3.0419	0
7096	390.5	3.0783	3.2168	1.1613	3.0405	0
7097	390.4	3.0395	3.2154	1.1628	3.0773	0
7098	390.3	3.0381	3.2725	1.1616	3.0757	0
7099	390.2	3.0747	3.2132	1.1633	3.0372	0
7100	390.1	3.0737	3.27	1.1625	3.0009	0
7101	390	3.0343	3.2684	1.1628	3.0721	0
7102	389.9	3.0334	3.2097	1.1636	3.0711	0
7103	389.8	3.0701	3.2083	1.1628	3.0701	0
7104	389.7	3.0685	3.2652	1.163	3.0306	0
7105	389.6	3.0675	3.2055	1.1639	3.0297	0
7106	389.5	3.0283	3.2041	1.1641	3.066	0
7107	389.4	3.0273	3.2034	1.1644	3.065	0
7108	389.3	3.0635	3.202	1.1656	3.0259	0
7109	389.2	3.0625	3.2007	1.1646	3.0625	0
7110	389.1	3.061	3.2573	1.1634	3.061	0
7111	389	3.06	3.2565	1.1647	3.06	0
7112	388.9	3.1002	3.1965	1.166	3.0585	0
7113	388.8	3.0195	3.2534	1.1662	3.0195	0
7114	388.7	3.1433	3.3188	1.1653	3.1433	0
7115	388.6	3.055	3.3179	1.1652	3.0168	0
7116	388.5	3.0535	3.2495	1.1659	3.0535	0
7117	388.4	3.0937	3.2487	1.1651	3.0521	0
7118	388.3	3.0506	3.3143	1.1664	3.0506	0
7119	388.2	3.0496	3.1878	1.1661	3.0496	0
7120	388.1	3.0894	3.1864	1.1658	3.0482	0
7121	388	3.0467	3.2434	1.1656	3.0467	0
7122	387.9	3.0458	3.1838	1.1652	3.0079	0
7123	387.8	3.0857	3.1824	1.1665	3.0857	0
7124	387.7	3.0429	3.2388	1.1647	3.0429	0

7125	387.6	3.083	3.1798	1.1656	3.0419	0
7126	387.5	3.082	3.2366	1.1675	3.082	0
7127	387.4	3.0391	3.2351	1.1667	3.0391	0
7128	387.3	3.1249	3.2343	1.1664	3.1759	0
7129	387.2	3.0778	3.3002	1.1663	3.1238	0
7130	387.1	3.0768	3.2314	1.1672	3.0768	0
7131	387	3.0752	3.2976	1.1664	3.0339	0
7132	386.9	3.0742	3.2958	1.1667	3.0742	0
7133	386.8	3.0726	3.2277	1.168	3.1186	0
7134	386.7	3.0716	3.2262	1.1672	3.0716	0
7135	386.6	3.1158	3.2248	1.1669	3.0701	0
7136	386.5	3.0685	3.2233	1.1667	3.1146	0
7137	386.4	3.1129	3.2226	1.1654	3.0675	0
7138	386.3	3.1118	3.2211	1.1662	3.1118	0
7139	386.2	3.1107	3.2197	1.1676	3.1107	0
7140	386.1	3.109	3.1605	1.1668	3.0635	0
7141	386	3.062	3.2168	1.1667	3.062	0
7142	385.9	3.1068	3.2154	1.1659	3.1068	0
7143	385.8	3.1051	3.2147	1.1657	3.0595	0
7144	385.7	3.058	3.2798	1.1671	3.104	0
7145	385.6	3.057	3.2118	1.1669	3.057	0
7146	385.5	3.0141	3.2774	1.1661	3.0555	0
7147	385.4	3.0996	3.209	1.1658	3.054	0
7148	385.3	3.0985	3.2741	1.1666	3.0985	0
7149	385.2	3.0969	3.2062	1.1654	3.0969	0
7150	385.1	3.0501	3.2716	1.1654	3.0501	0
7151	385	3.0947	3.2708	1.1657	3.0487	0
7152	384.9	3.1439	3.202	1.1653	3.0931	0
7153	384.8	3.0915	3.2676	1.1655	3.0915	0
7154	384.7	3.1415	3.266	1.1642	3.0904	0
7155	384.6	3.0888	3.3439	1.1652	3.1403	0
7156	384.5	3.1385	3.2636	1.165	3.1385	0
7157	384.4	3.0862	3.1952	1.1636	3.0862	0
7158	384.3	3.0851	3.1938	1.1652	3.0851	0
7159	384.2	3.0835	3.1349	1.1662	3.0835	0
7160	384.1	3.082	3.2581	1.1655	3.082	0
7161	384	3.1319	3.2565	1.1652	3.0809	0
7162	383.9	3.0794	3.2557	1.165	3.0794	0
7163	383.8	3.0778	3.1871	1.1648	3.0778	0
7164	383.7	3.1278	3.2526	1.1641	3.0768	0
7165	383.6	3.0752	3.1844	1.1652	3.0297	0
7166	383.5	3.1249	3.2503	1.1661	3.0742	0
7167	383.4	3.1238	3.2487	1.1654	3.1238	0

7168	383.3	3.1226	3.2472	1.1646	3.0711	0
7169	383.2	3.0701	3.2457	1.1649	3.0701	0
7170	383.1	3.0685	3.3233	1.1635	3.1778	0
7171	383	3.118	3.1759	1.1645	3.067	0
7172	382.9	3.1169	3.1746	1.1644	3.0655	0
7173	382.8	3.1152	3.1733	1.1648	3.1152	0
7174	382.7	3.063	3.172	1.1642	3.063	0
7175	382.6	3.0615	3.1707	1.1653	3.1129	0
7176	382.5	3.06	3.3152	1.165	3.1113	0
7177	382.4	3.1101	3.2351	1.1643	3.059	0
7178	382.3	3.1085	3.2336	1.1649	3.0575	0
7179	382.2	3.1073	3.2321	1.1648	3.1656	0
7180	382.1	3.1062	3.2314	1.1654	3.055	0
7181	382	3.1051	3.163	1.1649	3.054	0
7182	381.9	3.0521	3.3072	1.1658	3.1035	0
7183	381.8	3.0506	3.1599	1.165	3.1018	0
7184	381.7	3.1007	3.2262	1.1658	3.0496	0
7185	381.6	3.0991	3.224	1.1661	3.0482	0
7186	381.5	3.098	3.2226	1.1654	3.098	0
7187	381.4	3.0458	3.2218	1.1666	3.0969	0
7188	381.3	3.0947	3.1851	1.166	3.0947	0
7189	381.2	3.0937	3.1838	1.1663	3.1215	0
7190	381.1	3.0921	3.15	1.1671	3.0921	0
7191	381	3.0645	3.1811	1.1671	3.0904	0
7192	380.9	3.0894	3.1791	1.1663	3.0894	0
7193	380.8	3.0615	3.1778	1.1665	3.0615	0
7194	380.7	3.1146	3.1765	1.1664	3.06	0
7195	380.6	3.0851	3.1752	1.1677	3.0851	0
7196	380.5	3.1118	3.1739	1.1696	3.0835	0
7197	380.4	3.082	3.1726	1.1681	3.082	0
7198	380.3	3.0809	3.1713	1.1684	3.0809	0
7199	380.2	3.0794	3.1694	1.1683	3.1073	0
7200	380.1	3.0778	3.2027	1.1678	3.0778	0
7201	380	3.1046	3.2013	1.1691	3.1046	0
7202	379.9	3.0752	3.2	1.1686	3.1029	0
7203	379.8	3.1018	3.1986	1.1682	3.0737	0
7204	379.7	3.0721	3.1624	1.1683	3.0721	0
7205	379.6	3.0443	3.1612	1.1687	3.0985	0
7206	379.5	3.0975	3.1593	1.1696	3.0975	0
7207	379.4	3.0958	3.1925	1.1681	3.0958	0
7208	379.3	3.0942	3.1568	1.1687	3.0665	0
7209	379.2	3.0926	3.1898	1.1695	3.0926	0
7210	379.1	3.0915	3.1537	1.1694	3.0915	0

7211	379	3.0899	3.1518	1.1703	3.062	0
7212	378.9	3.0883	3.1851	1.1706	3.0605	0
7213	378.8	3.059	3.1838	1.1716	3.0867	0
7214	378.7	3.057	3.1824	1.1714	3.0851	0
7215	378.6	3.0835	3.1805	1.1727	3.0835	0
7216	378.5	3.082	3.1791	1.1729	3.054	0
7217	378.4	3.0526	3.1772	1.1724	3.0804	0
7218	378.3	3.109	3.1759	1.1719	3.109	0
7219	378.2	3.0773	3.1746	1.1714	3.0773	0
7220	378.1	3.1057	3.2104	1.1718	3.1057	0
7221	378	3.0742	3.1367	1.1721	3.0742	0
7222	377.9	3.1029	3.1694	1.1722	3.0726	0
7223	377.8	3.0711	3.1681	1.1725	3.0711	0
7224	377.7	3.0996	3.2041	1.172	3.0696	0
7225	377.6	3.068	3.1649	1.172	3.068	0
7226	377.5	3.0665	3.1637	1.1711	3.0665	0
7227	377.4	3.1273	3.1618	1.1721	3.0947	0
7228	377.3	3.0931	3.1599	1.1723	3.0931	0
7229	377.2	3.0921	3.1965	1.1723	3.062	0
7230	377.1	3.0904	3.1574	1.173	3.0904	0
7231	377	3.0585	3.1931	1.1729	3.0888	0
7232	376.9	3.0872	3.1918	1.1724	3.0872	0
7233	376.8	3.0851	3.1524	1.173	3.0555	0
7234	376.7	3.0841	3.1506	1.1731	3.054	0
7235	376.6	3.0825	3.1494	1.1727	3.0825	0
7236	376.5	3.0506	3.1475	1.1718	3.0506	0
7237	376.4	3.0794	3.1838	1.1733	3.0794	0
7238	376.3	3.0773	3.1824	1.1725	3.0773	0
7239	376.2	3.0757	3.1805	1.1732	3.0757	0
7240	376.1	3.0747	3.1791	1.1737	3.0747	0
7241	376	3.0731	3.1397	1.1721	3.0731	0
7242	375.9	3.0716	3.1759	1.173	3.0716	0
7243	375.8	3.1018	3.1367	1.1734	3.0696	0
7244	375.7	3.1002	3.1726	1.1722	3.1002	0
7245	375.6	3.0985	3.1337	1.1721	3.0665	0
7246	375.5	3.065	3.1319	1.1736	3.065	0
7247	375.4	3.0635	3.1681	1.1729	3.0958	0
7248	375.3	3.062	3.1669	1.1717	3.062	0
7249	375.2	3.06	3.1273	1.1709	3.06	0
7250	375.1	3.0585	3.1637	1.1718	3.091	0
7251	375	3.1244	3.1244	1.1711	3.057	0
7252	374.9	3.0555	3.1599	1.1714	3.0878	0
7253	374.8	3.054	3.1586	1.1717	3.054	0

7254	374.7	3.0846	3.1574	1.1722	3.0846	0
7255	374.6	3.083	3.118	1.1717	3.083	0
7256	374.5	3.0814	3.1163	1.1711	3.0496	0
7257	374.4	3.0477	3.1524	1.1711	3.0477	0
7258	374.3	3.0462	3.1512	1.1714	3.0462	0
7259	374.2	3.0768	3.1118	1.1718	3.0768	0
7260	374.1	3.1101	3.1481	1.1706	3.1101	0
7261	374	3.0737	3.1463	1.1703	3.0737	0
7262	373.9	3.0721	3.1451	1.1693	3.0721	0
7263	373.8	3.0386	3.1433	1.1698	3.1057	0
7264	373.7	3.0691	3.1415	1.1682	3.0691	0
7265	373.6	3.1024	3.1403	1.1688	3.0675	0
7266	373.5	3.0339	3.1385	1.169	3.066	0
7267	373.4	3.0645	3.1373	1.1696	3.0645	0
7268	373.3	3.098	3.1355	1.1681	3.063	0
7269	373.2	3.0297	3.0964	1.1692	3.0615	0
7270	373.1	3.0947	3.1325	1.1688	3.0947	0
7271	373	3.0264	3.1314	1.169	3.0931	0
7272	372.9	3.057	3.1296	1.1689	3.0921	0
7273	372.8	3.0555	3.1278	1.1682	3.0555	0
7274	372.7	3.0888	3.1267	1.1681	3.054	0
7275	372.6	3.0205	3.1249	1.1691	3.0205	0
7276	372.5	3.0862	3.1649	1.1682	3.0511	0
7277	372.4	3.0496	3.1221	1.1689	3.0496	0
7278	372.3	3.0482	3.1624	1.1689	3.083	0
7279	372.2	3.0472	3.082	1.1693	3.082	0
7280	372.1	3.0804	3.118	1.1696	3.0804	0
7281	372	3.1169	3.1169	1.1704	3.0788	0
7282	371.9	3.0429	3.1568	1.1695	3.0773	0
7283	371.8	3.0757	3.1549	1.1697	3.0414	0
7284	371.7	3.04	3.1124	1.1696	3.04	0
7285	371.6	3.1113	3.1524	1.1688	3.0386	0
7286	371.5	3.0721	3.1512	1.1694	3.0721	0
7287	371.4	3.0706	3.15	1.1688	3.1085	0
7288	371.3	3.0691	3.1481	1.1693	3.1068	0
7289	371.2	3.068	3.1469	1.1702	3.068	0
7290	371.1	3.0315	3.1457	1.1697	3.0315	0
7291	371	3.065	3.1445	1.1702	3.1029	0
7292	370.9	3.0287	3.1427	1.1712	3.0287	0
7293	370.8	3.0625	3.1002	1.1716	3.0625	0
7294	370.7	3.061	3.1403	1.1703	3.061	0
7295	370.6	3.06	3.1391	1.1705	3.0975	0
7296	370.5	3.0585	3.0964	1.171	3.0585	0

7297	370.4	3.057	3.0947	1.1721	3.0947	0
7298	370.3	3.056	3.1349	1.1722	3.056	0
7299	370.2	3.0545	3.1337	1.1729	3.0926	0
7300	370.1	3.091	3.1325	1.1715	3.0535	0
7301	370	3.0521	3.1314	1.1722	3.0521	0
7302	369.9	2.9337	2.9772	1.1864	2.9772	0
7303	369.8	2.9348	2.9784	1.1869	2.9559	0
7304	369.7	2.9359	2.9796	1.1864	2.9574	0
7305	369.6	2.9374	3.0044	1.1869	2.9374	0
7306	369.5	2.9183	2.9817	1.1876	2.9594	0
7307	369.4	2.9397	3.0066	1.1873	2.9606	0
7308	369.3	2.901	3.0074	1.1866	2.9408	0
7309	369.2	2.942	3.0088	1.1872	2.942	0
7310	369.1	2.9431	3.0348	1.1878	2.9226	0
7311	369	2.9442	2.9876	1.1872	2.9654	0
7312	368.9	2.9252	3.0123	1.1883	2.9889	0
7313	368.8	2.9263	3.0132	1.1888	2.9465	0
7314	368.7	2.9274	3.0146	1.1888	2.9686	0
7315	368.6	2.9488	3.0155	1.1896	2.9488	0
7316	368.5	2.9496	3.0164	1.1886	2.9496	0
7317	368.4	2.9508	3.0177	1.1901	2.9718	0
7318	368.3	2.9516	3.0186	1.1897	2.9727	0
7319	368.2	2.9326	3.0195	1.1904	2.9527	0
7320	368.1	2.9751	2.9974	1.1899	2.9539	0
7321	368	2.9547	2.9983	1.1904	2.9344	0
7322	367.9	2.9555	2.9991	1.1911	2.9767	0
7323	367.8	2.9566	3.0237	1.191	2.9776	0
7324	367.7	2.9574	3.0246	1.1909	2.9574	0
7325	367.6	2.9586	3.0255	1.1915	2.9796	0
7326	367.5	2.9594	3.0264	1.1909	2.9805	0
7327	367.4	2.9598	3.0269	1.1898	2.9397	0
7328	367.3	2.9821	3.0044	1.1915	2.9408	0
7329	367.2	2.9622	3.0057	1.192	2.942	0
7330	367.1	2.963	3.0066	1.1929	2.963	0
7331	367	2.9851	3.0306	1.1928	2.9435	0
7332	366.9	2.9442	2.9855	1.1926	2.9442	0
7333	366.8	2.9867	3.0325	1.1914	2.9654	0
7334	366.7	2.9876	2.9876	1.193	2.9662	0
7335	366.6	2.9674	3.0106	1.1925	2.9674	0
7336	366.5	2.9893	2.9893	1.1926	2.9893	0
7337	366.4	2.9686	3.0119	1.1936	2.9897	0
7338	366.3	2.9694	3.0128	1.1923	2.9694	0
7339	366.2	2.9702	3.0137	1.1923	2.9702	0

7340	366.1	2.971	3.0146	1.1914	2.9508	0
7341	366	2.9714	3.015	1.1927	2.9927	0
7342	365.9	2.9722	3.0159	1.1928	2.9722	0
7343	365.8	2.9944	3.0164	1.1929	2.9731	0
7344	365.7	2.9739	3.0173	1.1931	2.9739	0
7345	365.6	2.9957	3.0182	1.1917	2.9957	0
7346	365.5	2.9751	3.0186	1.1915	2.9961	0
7347	365.4	2.997	3.0195	1.1926	2.997	0
7348	365.3	2.9978	3.02	1.1923	2.9978	0
7349	365.2	2.9772	3.0443	1.1906	2.9772	0
7350	365.1	2.978	3.0448	1.1919	2.9991	0
7351	365	2.9996	3.0218	1.1925	2.9996	0
7352	364.9	2.9792	3.0458	1.1913	2.9792	0
7353	364.8	2.9796	3.0467	1.1902	2.9594	0
7354	364.7	3.0013	3.0237	1.191	2.9801	0
7355	364.6	3.0022	3.0022	1.1901	2.9809	0
7356	364.5	3.0026	3.0482	1.1899	2.9813	0
7357	364.4	2.9817	3.0487	1.1898	2.9817	0
7358	364.3	2.9821	3.0259	1.1899	3.0035	0
7359	364.2	2.983	3.0264	1.1903	2.983	0
7360	364.1	3.0048	3.0269	1.1898	3.0048	0
7361	364	3.0052	3.0506	1.1891	2.9838	0
7362	363.9	3.0052	3.0511	1.1892	2.9842	0
7363	363.8	2.9846	3.0283	1.1887	2.9846	0
7364	363.7	2.9851	3.0287	1.1891	2.9851	0
7365	363.6	2.9855	3.0292	1.1882	2.9855	0
7366	363.5	2.9859	3.0292	1.1886	2.9859	0
7367	363.4	2.9662	3.0074	1.188	2.9863	0
7368	363.3	3.0079	3.0301	1.1897	3.0079	0
7369	363.2	2.9872	3.0306	1.1887	3.0083	0
7370	363.1	2.9876	3.0311	1.1888	2.9876	0
7371	363	2.988	3.0092	1.1879	2.988	0
7372	362.9	3.0092	3.0092	1.1895	3.0092	0
7373	362.8	3.0097	3.0097	1.1895	3.0097	0
7374	362.7	3.0097	3.0555	1.1889	3.0097	0
7375	362.6	2.9889	3.0325	1.1887	2.9889	0
7376	362.5	2.9893	3.0325	1.1888	3.0101	0
7377	362.4	3.0106	3.0329	1.1881	2.9893	0
7378	362.3	2.9897	3.0329	1.1882	2.9897	0
7379	362.2	2.9897	3.0565	1.1878	2.9897	0
7380	362.1	3.011	3.057	1.1876	2.9897	0
7381	362	3.0114	3.0339	1.1883	2.9901	0
7382	361.9	3.0575	3.0575	1.1877	3.0114	0

7383	361.8	2.9905	3.0575	1.1877	2.9905	0
7384	361.7	2.9905	3.0343	1.1877	2.9905	0
7385	361.6	2.991	3.0343	1.1885	3.0119	0
7386	361.5	3.0123	3.0343	1.1896	3.0123	0
7387	361.4	2.971	3.0343	1.1884	3.0123	0
7388	361.3	3.0123	3.0123	1.1896	2.991	0
7389	361.2	2.9914	3.0348	1.189	3.0123	0
7390	361.1	2.9914	3.0348	1.1891	3.0123	0
7391	361	3.0128	3.0585	1.1888	3.0128	0
7392	360.9	3.0128	3.0128	1.1895	2.9914	0
7393	360.8	2.9914	3.0353	1.1903	3.0353	0
7394	360.7	3.0128	3.0348	1.1902	2.9914	0
7395	360.6	2.9914	3.0348	1.1889	3.0348	0
7396	360.5	2.9914	3.0585	1.1896	3.0128	0
7397	360.4	3.0128	3.0353	1.189	3.0128	0
7398	360.3	2.9914	3.0348	1.1896	3.0128	0
7399	360.2	3.0128	3.0348	1.1899	3.0128	0
7400	360.1	2.9914	3.0348	1.1908	3.0348	0
7401	360	3.0128	3.0585	1.1898	3.0128	0
7402	359.9	3.0123	3.0348	1.1909	3.0348	0
7403	359.8	3.0123	3.058	1.1908	3.0123	0
7404	359.7	2.991	3.0343	1.1904	3.0123	0
7405	359.6	3.0123	3.058	1.192	3.0123	0
7406	359.5	2.991	3.058	1.1915	3.0119	0
7407	359.4	2.991	3.0119	1.1911	2.991	0
7408	359.3	2.9905	3.0119	1.1923	3.0119	0
7409	359.2	3.0339	3.0339	1.1928	3.0339	0
7410	359.1	3.0114	3.0339	1.1928	3.0114	0
7411	359	3.0114	3.0575	1.1926	2.9901	0
7412	358.9	3.011	3.0334	1.1926	2.9901	0
7413	358.8	3.011	3.0334	1.1931	3.0334	0
7414	358.7	3.011	3.0565	1.1939	2.9897	0
7415	358.6	3.0106	3.0106	1.1934	3.0106	0
7416	358.5	2.9893	3.0325	1.1938	2.9893	0
7417	358.4	3.0101	3.056	1.1943	3.0101	0
7418	358.3	3.032	3.0555	1.1946	3.032	0
7419	358.2	3.032	3.032	1.1945	2.9884	0
7420	358.1	3.0315	3.0799	1.1948	3.0092	0
7421	358	3.0315	3.055	1.1953	3.0092	0
7422	357.9	3.0088	3.0311	1.1957	2.9876	0
7423	357.8	3.0311	3.0545	1.1965	2.9876	0
7424	357.7	3.0083	3.0306	1.1963	3.0083	0
7425	357.6	3.0083	3.054	1.1967	3.0083	0

7426	357.5	3.0079	3.0301	1.1978	3.0079	0
7427	357.4	3.0074	3.0301	1.1971	3.0074	0
7428	357.3	3.0297	3.0297	1.1985	3.0074	0
7429	357.2	3.007	3.0292	1.1985	3.007	0
7430	357.1	3.0287	3.0287	1.1984	3.0287	0
7431	357	3.0066	3.0521	1.1992	3.0066	0
7432	356.9	3.0061	3.0283	1.1979	3.0061	0
7433	356.8	3.0278	3.0278	1.1975	3.0278	0
7434	356.7	3.0052	3.0511	1.1975	3.0278	0
7435	356.6	3.0506	3.0273	1.1989	3.0052	0
7436	356.5	3.0269	3.0048	1.1996	2.9834	0
7437	356.4	3.0044	3.0264	1.1992	3.0044	0
7438	356.3	3.0039	3.0259	1.1994	3.0039	0
7439	356.2	3.0035	3.0491	1.2001	3.0035	0
7440	356.1	3.0031	3.0491	1.1984	3.0031	0
7441	356	3.0026	3.0487	1.2002	3.025	0
7442	355.9	3.0022	3.0246	1.2001	3.0246	0
7443	355.8	3.0017	3.0241	1.199	3.0477	0
7444	355.7	3.0237	3.0472	1.1992	2.9801	0
7445	355.6	3.0467	3.0467	1.2002	3.0009	0
7446	355.5	3.0227	3.0462	1.2004	3.0227	0
7447	355.4	3.0223	3.0458	1.2003	3	0
7448	355.3	3.0218	3.0218	1.1998	3.0218	0
7449	355.2	3.0214	3.0448	1.1998	3.0214	0
7450	355.1	3.0209	3.0209	1.1989	3.0443	0
7451	355	3.0205	3.0438	1.2001	3.0205	0
7452	354.9	2.9974	3.068	1.1998	2.9974	0
7453	354.8	3.0195	3.0429	1.2001	3.0195	0
7454	354.7	2.9965	3.067	1.2	3.0186	0
7455	354.6	3.0182	3.0419	1.1998	3.0182	0
7456	354.5	2.9952	3.041	1.1999	3.0177	0
7457	354.4	2.9952	3.041	1.1999	3.041	0
7458	354.3	3.0164	3.0164	1.1986	3.0164	0
7459	354.2	3.0159	3.0395	1.1992	3.0159	0
7460	354.1	2.9931	3.0391	1.1986	3.0155	0
7461	354	3.0146	3.0381	1.1994	3.0146	0
7462	353.9	3.0141	3.0141	1.1995	3.0141	0
7463	353.8	2.991	3.0132	1.1991	2.991	0
7464	353.7	2.9905	3.0362	1.1974	3.0128	0
7465	353.6	2.9901	3.0123	1.1969	2.9901	0
7466	353.5	3.0114	3.0114	1.1977	3.0114	0
7467	353.4	2.9884	3.0343	1.1973	3.011	0
7468	353.3	2.988	3.0339	1.1977	3.0101	0

7469	353.2	3.0097	3.0097	1.1978	3.0097	0
7470	353.1	2.9867	2.9867	1.197	3.0088	0
7471	353	3.0083	3.032	1.1978	3.0083	0
7472	352.9	3.0074	3.0074	1.197	3.0074	0
7473	352.8	3.0066	3.0066	1.1981	3.055	0
7474	352.7	3.0061	3.0061	1.1964	3.0061	0
7475	352.6	3.0052	3.0052	1.197	3.0052	0
7476	352.5	3.0044	2.9821	1.1969	3.0044	0
7477	352.4	3.0039	3.0273	1.1969	3.0273	0
7478	352.3	2.9809	3.0264	1.1965	3.0264	0
7479	352.2	3.0022	3.0259	1.1961	3.0022	0
7480	352.1	2.9792	3.025	1.1962	2.9792	0
7481	352	3.0241	3.0491	1.1965	3.0009	0
7482	351.9	3.0004	3.0004	1.1966	3.0004	0
7483	351.8	2.9996	3.0227	1.1968	2.9996	0
7484	351.7	2.9987	2.9987	1.1968	2.9987	0
7485	351.6	2.9978	3.0462	1.1963	3.0214	0
7486	351.5	2.9747	3.0205	1.1963	3.0205	0
7487	351.4	3.02	3.02	1.1964	3.02	0
7488	351.3	2.9735	3.0438	1.1955	2.9957	0
7489	351.2	3.0182	2.9948	1.1959	2.9948	0
7490	351.1	2.994	2.994	1.1963	2.994	0
7491	351	2.9935	3.0414	1.1965	2.9935	0
7492	350.9	2.9927	3.0164	1.1959	2.9927	0
7493	350.8	2.9694	3.0155	1.1961	2.9918	0
7494	350.7	2.969	3.0146	1.1965	2.991	0
7495	350.6	3.0137	3.065	1.1951	2.9905	0
7496	350.5	2.9897	3.0128	1.1956	3.0128	0
7497	350.4	2.9889	3.0123	1.1959	3.0123	0
7498	350.3	2.988	2.988	1.1962	3.0114	0
7499	350.2	2.965	3.0106	1.1951	3.0106	0
7500	350.1	3.0097	3.0097	1.1961	2.9863	0
7501	350	2.963	3.0088	1.1971	3.0088	0
7502	349.9	2.9846	3.0079	1.1947	2.9846	0
7503	349.8	2.9838	2.9838	1.195	3.007	0
7504	349.7	2.9825	3.0061	1.1958	2.9606	0
7505	349.6	2.9821	3.0301	1.1977	2.9821	0
7506	349.5	2.9813	2.9813	1.1973	2.9813	0
7507	349.4	3.0035	2.9801	1.1964	2.9801	0
7508	349.3	2.9792	3.0026	1.1974	2.9792	0
7509	349.2	3.0017	3.0017	1.1979	2.9784	0
7510	349.1	2.9772	3.0255	1.1964	3.0009	0
7511	349	2.9763	3	1.1967	3	0

7512	348.9	2.9531	3.0237	1.1972	2.9755	0
7513	348.8	2.9978	2.9978	1.1977	2.9978	0
7514	348.7	2.997	2.997	1.1979	2.997	0
7515	348.6	2.95	3.0209	1.1973	2.9722	0
7516	348.5	2.9714	2.9948	1.1975	2.9714	0
7517	348.4	2.994	2.994	1.1979	2.9481	0
7518	348.3	2.9694	2.9927	1.1981	2.9927	0
7519	348.2	3.0168	3.0168	1.1987	2.9918	0
7520	348.1	2.945	2.9905	1.1991	2.9905	0
7521	348	2.9897	2.9662	1.1997	2.9897	0
7522	347.9	2.9654	3.0137	1.1994	2.9431	0
7523	347.8	3.0128	2.9876	1.2	3.0128	0
7524	347.7	2.9867	2.9867	1.1998	2.9634	0
7525	347.6	3.0367	2.9855	1.1987	2.9622	0
7526	347.5	2.9842	3.0092	1.2003	2.961	0
7527	347.4	2.9834	3.0083	1.2005	2.9834	0
7528	347.3	2.9825	3.0074	1.1995	2.959	0
7529	347.2	2.9578	2.9813	1.2011	2.9813	0
7530	347.1	2.9805	2.9805	1.2006	2.9805	0
7531	347	2.9792	2.9792	1.2009	2.9555	0
7532	346.9	2.9547	3.0031	1.2016	2.9547	0
7533	346.8	2.9772	3.0017	1.2025	2.9772	0
7534	346.7	2.9527	3.0009	1.2028	2.9759	0
7535	346.6	2.9751	3	1.2029	3.0259	0
7536	346.5	2.9504	2.9987	1.2038	2.9739	0
7537	346.4	2.9727	2.9727	1.2028	2.9492	0
7538	346.3	3.0227	2.9714	1.204	2.9714	0
7539	346.2	2.9952	2.9952	1.2034	2.9706	0
7540	346.1	2.9694	2.9944	1.204	2.9694	0
7541	346	2.9935	2.9935	1.2039	2.9686	0
7542	345.9	2.9674	2.9923	1.2041	2.9674	0
7543	345.8	2.991	2.991	1.2037	2.9427	0
7544	345.7	2.9901	2.9901	1.2044	2.9654	0
7545	345.6	2.9404	2.9889	1.2044	2.9642	0
7546	345.5	2.988	2.988	1.2042	2.963	0
7547	345.4	2.9618	2.9618	1.2044	2.9618	0
7548	345.3	2.961	2.961	1.2042	2.9374	0
7549	345.2	2.9846	2.9846	1.2043	2.9363	0
7550	345.1	2.9352	2.9586	1.2044	2.9586	0
7551	345	2.9574	2.9821	1.2045	2.9574	0
7552	344.9	3.0074	2.9809	1.204	2.9326	0
7553	344.8	2.9551	2.9801	1.2039	2.9314	0
7554	344.7	2.9303	3.0052	1.2049	2.9788	0

7555	344.6	2.9527	3.0039	1.2045	3.0039	0
7556	344.5	2.9516	3.0026	1.2041	2.9516	0
7557	344.4	2.95	3.0013	1.2033	2.95	0
7558	344.3	2.9488	3	1.2037	2.9739	0
7559	344.2	2.9727	2.9987	1.2039	2.9477	0
7560	344.1	2.9465	3.0255	1.2043	2.9465	0
7561	344	2.9454	2.9702	1.204	2.9454	0
7562	343.9	2.9439	2.9686	1.204	2.9439	0
7563	343.8	2.9427	2.9674	1.204	2.9674	0
7564	343.7	2.9416	2.9662	1.2037	2.9416	0
7565	343.6	2.9401	2.991	1.2041	2.9401	0
7566	343.5	2.9638	2.9638	1.2042	2.9389	0
7567	343.4	2.9378	2.9626	1.203	2.9626	0
7568	343.3	2.9363	2.961	1.2031	2.961	0
7569	343.2	2.9863	2.9598	1.2036	2.9352	0
7570	343.1	2.9586	2.9586	1.2024	2.9337	0
7571	343	2.9574	2.9574	1.2032	2.9326	0
7572	342.9	2.908	2.9825	1.2044	2.9314	0
7573	342.8	2.93	2.9551	1.2044	2.93	0
7574	342.7	2.9289	2.9535	1.2044	2.9289	0
7575	342.6	2.9038	2.9519	1.2017	2.9274	0
7576	342.5	2.9263	2.9512	1.2028	2.9512	0
7577	342.4	2.9248	2.9496	1.203	2.9496	0
7578	342.3	2.9	2.9485	1.2025	2.9485	0
7579	342.2	2.8989	2.9735	1.2031	2.9473	0
7580	342.1	2.9208	2.9718	1.2029	2.9454	0
7581	342	2.9197	2.9442	1.2022	2.9197	0
7582	341.9	2.9183	2.9694	1.2018	2.8948	0
7583	341.8	2.9169	2.9416	1.2032	2.9169	0
7584	341.7	2.9154	2.9404	1.2023	2.9154	0
7585	341.6	2.9389	2.9389	1.2018	2.914	0
7586	341.5	2.9129	2.9638	1.2024	2.9378	0
7587	341.4	2.9363	2.9626	1.2029	2.9363	0
7588	341.3	2.9101	2.9614	1.2034	2.9614	0
7589	341.2	2.9333	2.9598	1.2029	2.9087	0
7590	341.1	2.9073	2.9322	1.2021	2.9073	0
7591	341	2.9059	2.9307	1.2032	2.9059	0
7592	340.9	2.9292	2.9292	1.2022	2.9045	0
7593	340.8	2.9031	2.9543	1.2027	2.9031	0
7594	340.7	2.9017	2.9266	1.2018	2.9266	0
7595	340.6	2.9003	2.9516	1.2038	2.9003	0
7596	340.5	2.9237	2.9237	1.2025	2.9237	0
7597	340.4	2.9223	2.9223	1.2035	2.8976	0

7598	340.3	2.8962	2.9212	1.2036	2.9212	0
7599	340.2	2.8948	2.9462	1.2032	2.8948	0
7600	340.1	2.87	2.9183	1.2038	2.9183	0
7601	340	2.8921	2.9431	1.2033	2.9169	0
7602	339.9	2.1481	2.336	1.1703	2.289	0
7603	339.8	2.1495	2.3345	1.1688	2.2833	0
7604	339.7	2.1481	2.3331	1.1683	2.286	0
7605	339.6	2.1498	2.3319	1.1692	2.2807	0
7606	339.5	2.1484	2.3304	1.1688	2.2833	0
7607	339.4	2.1411	2.3291	1.1686	2.286	0
7608	339.3	2.1427	2.3277	1.1685	2.2765	0
7609	339.2	2.1441	2.3307	1.1676	2.2831	0
7610	339.1	2.1427	2.3293	1.1684	2.2777	0
7611	339	2.1413	2.3234	1.1683	2.2723	0
7612	338.9	2.14	2.322	1.1686	2.279	0
7613	338.8	2.1386	2.3161	1.1688	2.2736	0
7614	338.7	2.1342	2.3193	1.168	2.2722	0
7615	338.6	2.1358	2.3179	1.1683	2.2749	0
7616	338.5	2.1344	2.312	1.1678	2.2694	0
7617	338.4	2.1331	2.3198	1.1688	2.2641	0
7618	338.3	2.1318	2.3094	1.1684	2.2668	0
7619	338.2	2.1275	2.317	1.1687	2.2695	0
7620	338.1	2.1321	2.3067	1.1686	2.2641	0
7621	338	2.1248	2.3099	1.1693	2.2627	0
7622	337.9	2.1295	2.3087	1.1693	2.2656	0
7623	337.8	2.125	2.3117	1.1688	2.26	0
7624	337.7	2.1237	2.3103	1.1678	2.2587	0
7625	337.6	2.1253	2.3089	1.1687	2.2573	0
7626	337.5	2.121	2.3077	1.1681	2.26	0
7627	337.4	2.1168	2.3019	1.1692	2.2588	0
7628	337.3	2.1184	2.3005	1.1675	2.2534	0
7629	337.2	2.1171	2.2992	1.1675	2.2562	0
7630	337.1	2.1158	2.2979	1.1669	2.2548	0
7631	337	2.1175	2.3012	1.1666	2.2536	0
7632	336.9	2.1133	2.2999	1.1667	2.2564	0
7633	336.8	2.112	2.2986	1.1671	2.251	0
7634	336.7	2.1106	2.2927	1.1667	2.2496	0
7635	336.6	2.1123	2.2914	1.1678	2.2484	0
7636	336.5	2.114	2.2946	1.1679	2.243	0
7637	336.4	2.1068	2.2935	1.1677	2.2418	0
7638	336.3	2.1084	2.292	1.1666	2.2403	0
7639	336.2	2.1101	2.2907	1.1664	2.2391	0
7640	336.1	2.1058	2.2895	1.1665	2.2418	0

7641	336	2.1046	2.2882	1.1673	2.2366	0
7642	335.9	2.1033	2.2869	1.1667	2.2394	0
7643	335.8	2.102	2.2811	1.1661	2.2381	0
7644	335.7	2.1035	2.2796	1.166	2.2325	0
7645	335.6	2.0995	2.2831	1.166	2.2314	0
7646	335.5	2.0983	2.2865	1.1666	2.2303	0
7647	335.4	2.0971	2.2761	1.1668	2.2331	0
7648	335.3	2.0927	2.284	1.1662	2.2318	0
7649	335.2	2.0946	2.2692	1.1661	2.2266	0
7650	335.1	2.0963	2.277	1.1662	2.2294	0
7651	335	2.0921	2.2712	1.1664	2.224	0
7652	334.9	2.0909	2.2746	1.1663	2.2269	0
7653	334.8	2.0958	2.2734	1.1659	2.2258	0
7654	334.7	2.0912	2.2719	1.1647	2.2243	0
7655	334.6	2.0901	2.2708	1.165	2.2232	0
7656	334.5	2.0919	2.2742	1.1656	2.2261	0
7657	334.4	2.0876	2.2729	1.1654	2.2207	0
7658	334.3	2.0863	2.267	1.1648	2.2194	0
7659	334.2	2.0852	2.2613	1.1633	2.2182	0
7660	334.1	2.084	2.2646	1.1635	2.217	0
7661	334	2.0828	2.268	1.1634	2.2118	0
7662	333.9	2.0816	2.2622	1.1636	2.2146	0
7663	333.8	2.0803	2.261	1.1632	2.2094	0
7664	333.7	2.0823	2.26	1.1632	2.2165	0
7665	333.6	2.0782	2.2634	1.1632	2.2112	0
7666	333.5	2.0769	2.2621	1.1626	2.2099	0
7667	333.4	2.0758	2.2519	1.1634	2.2088	0
7668	333.3	2.0806	2.2551	1.1628	2.2075	0
7669	333.2	2.0733	2.2494	1.1627	2.2063	0
7670	333.1	2.0752	2.2483	1.1616	2.2093	0
7671	333	2.074	2.2517	1.1619	2.2082	0
7672	332.9	2.0699	2.2551	1.1619	2.207	0
7673	332.8	2.0717	2.2538	1.1625	2.2058	0
7674	332.7	2.0704	2.2435	1.1613	2.2005	0
7675	332.6	2.0692	2.2513	1.1604	2.2033	0
7676	332.5	2.0711	2.2412	1.1627	2.1981	0
7677	332.4	2.0669	2.24	1.1612	2.197	0
7678	332.3	2.0627	2.2388	1.1615	2.1958	0
7679	332.2	2.0676	2.2375	1.1614	2.1904	0
7680	332.1	2.0635	2.2457	1.1611	2.1976	0
7681	332	2.0592	2.2398	1.1607	2.1923	0
7682	331.9	2.0611	2.2433	1.1607	2.1911	0
7683	331.8	2.0568	2.2328	1.1613	2.1898	0

7684	331.7	2.0555	2.2316	1.1612	2.1927	0
7685	331.6	2.0603	2.2304	1.1611	2.1915	0
7686	331.5	2.0562	2.2338	1.1604	2.1821	0
7687	331.4	2.055	2.228	1.1603	2.1891	0
7688	331.3	2.0538	2.2314	1.1603	2.1921	0
7689	331.2	2.0465	2.2302	1.1602	2.1867	0
7690	331.1	2.0513	2.229	1.1594	2.1896	0
7691	331	2.0502	2.2278	1.1594	2.1844	0
7692	330.9	2.0489	2.2265	1.1593	2.1872	0
7693	330.8	2.0508	2.23	1.1593	2.1818	0
7694	330.7	2.0465	2.2242	1.1593	2.1849	0
7695	330.6	2.0456	2.2278	1.1587	2.1797	0
7696	330.5	2.0442	2.2218	1.1589	2.1824	0
7697	330.4	2.043	2.216	1.1589	2.1771	0
7698	330.3	2.0446	2.2193	1.1583	2.1799	0
7699	330.2	2.0404	2.2226	1.1588	2.1787	0
7700	330.1	2.0423	2.2215	1.1596	2.1734	0
7701	330	2.038	2.2202	1.1599	2.1763	0
7702	329.9	2.0367	2.2143	1.1594	2.1709	0
7703	329.8	2.0386	2.2132	1.1595	2.1739	0
7704	329.7	2.0374	2.212	1.1603	2.1685	0
7705	329.6	2.0362	2.2154	1.1595	2.1715	0
7706	329.5	2.0351	2.2142	1.1596	2.162	0
7707	329.4	2.0337	2.2175	1.1595	2.169	0
7708	329.3	2.0325	2.2116	1.1594	2.1635	0
7709	329.2	2.0312	2.2103	1.159	2.1664	0
7710	329.1	2.0362	2.2045	1.1594	2.157	0
7711	329	2.0287	2.2033	1.1594	2.1598	0
7712	328.9	2.0273	2.2019	1.1584	2.1543	0
7713	328.8	2.0261	2.2053	1.1589	2.1531	0
7714	328.7	2.0251	2.2043	1.1582	2.1521	0
7715	328.6	2.0208	2.1984	1.1591	2.1549	0
7716	328.5	2.0225	2.1925	1.1586	2.1495	0
7717	328.4	2.0243	2.1959	1.159	2.1483	0
7718	328.3	2.02	2.1992	1.1582	2.1511	0
7719	328.2	2.025	2.1889	1.1591	2.1541	0
7720	328.1	2.0208	2.1969	1.158	2.1488	0
7721	328	2.0195	2.1956	1.158	2.1517	0
7722	327.9	2.0182	2.1897	1.1588	2.1462	0
7723	327.8	2.017	2.1931	1.1589	2.1409	0
7724	327.7	2.0157	2.1826	1.1588	2.1395	0
7725	327.6	2.0145	2.1952	1.1577	2.1425	0
7726	327.5	2.0103	2.1894	1.157	2.1455	0

7727	327.4	2.0122	2.1883	1.1563	2.1403	0
7728	327.3	2.0078	2.187	1.157	2.1389	0
7729	327.2	2.0096	2.1857	1.1575	2.1418	0
7730	327.1	2.0114	2.1798	1.158	2.1405	0
7731	327	2.0041	2.1787	1.1564	2.1311	0
7732	326.9	2.0059	2.182	1.1573	2.1339	0
7733	326.8	2.0018	2.1809	1.1562	2.1288	0
7734	326.7	2.0067	2.1797	1.1567	2.1316	0
7735	326.6	2.0025	2.1786	1.1556	2.1305	0
7736	326.5	2.0043	2.1773	1.1561	2.1292	0
7737	326.4	2.0032	2.1762	1.1568	2.1281	0
7738	326.3	1.9989	2.1704	1.156	2.127	0
7739	326.2	1.9977	2.1692	1.1557	2.1217	0
7740	326.1	1.9965	2.1726	1.1554	2.1245	0
7741	326	1.9922	2.1714	1.1537	2.1192	0
7742	325.9	1.991	2.1655	1.1534	2.1179	0
7743	325.8	1.9899	2.169	1.1549	2.1251	0
7744	325.7	1.9949	2.1679	1.1542	2.1198	0
7745	325.6	1.9937	2.1667	1.1539	2.1145	0
7746	325.5	1.9924	2.1608	1.154	2.1174	0
7747	325.4	1.9911	2.1641	1.1536	2.1161	0
7748	325.3	1.993	2.1583	1.1529	2.1149	0
7749	325.2	1.9889	2.1573	1.1528	2.118	0
7750	325.1	1.9846	2.1606	1.1529	2.1167	0
7751	325	1.9835	2.1596	1.1536	2.1115	0
7752	324.9	1.9853	2.1583	1.1524	2.1186	0
7753	324.8	1.981	2.1571	1.1521	2.1132	0
7754	324.7	1.983	2.156	1.1518	2.1121	0
7755	324.6	1.9818	2.1595	1.152	2.1067	0
7756	324.5	1.9774	2.1489	1.1516	2.1096	0
7757	324.4	1.9762	2.1477	1.1514	2.1042	0
7758	324.3	1.9781	2.1465	1.1516	2.1114	0
7759	324.2	1.9769	2.1453	1.1503	2.1018	0
7760	324.1	1.9727	2.1487	1.1497	2.1048	0
7761	324	1.9715	2.1476	1.1494	2.0995	0
7762	323.9	1.9704	2.1419	1.1492	2.0984	0
7763	323.8	1.9723	2.1407	1.1486	2.0973	0
7764	323.7	1.9712	2.1396	1.1479	2.1003	0
7765	323.6	1.9669	2.1384	1.1472	2.0991	0
7766	323.5	1.9688	2.1418	1.1474	2.0937	0
7767	323.4	1.9677	2.1454	1.1482	2.0969	0
7768	323.3	1.9697	2.135	1.1461	2.0957	0
7769	323.2	1.9654	2.1383	1.1482	2.0945	0

7770	323.1	1.9642	2.1418	1.147	2.0891	0
7771	323	1.96	2.1315	1.1473	2.0922	0
7772	322.9	1.9589	2.135	1.1462	2.091	0
7773	322.8	1.9608	2.1338	1.1469	2.0899	0
7774	322.7	1.9566	2.1327	1.1463	2.0887	0
7775	322.6	1.9555	2.1316	1.1466	2.0835	0
7776	322.5	1.9541	2.1256	1.1443	2.0863	0
7777	322.4	1.9531	2.1292	1.1447	2.0853	0
7778	322.3	1.9552	2.1282	1.1447	2.0801	0
7779	322.2	1.9571	2.1225	1.1445	2.0748	0
7780	322.1	1.9497	2.1212	1.1442	2.0777	0
7781	322	1.9548	2.1156	1.1441	2.0767	0
7782	321.9	1.9506	2.119	1.1444	2.0756	0
7783	321.8	1.9464	2.1179	1.1433	2.0744	0
7784	321.7	1.9485	2.1262	1.1433	2.0735	0
7785	321.6	1.9504	2.1203	1.1431	2.0681	0
7786	321.5	1.9492	2.1191	1.1443	2.071	0
7787	321.4	1.9418	2.1133	1.1436	2.074	0
7788	321.3	1.9439	2.1169	1.1441	2.0688	0
7789	321.2	1.9458	2.1157	1.1434	2.076	0
7790	321.1	1.9416	2.1146	1.1438	2.0707	0
7791	321	1.9345	2.1136	1.1447	2.0697	0
7792	320.9	1.9395	2.1125	1.1431	2.0603	0
7793	320.8	1.9383	2.1113	1.1429	2.0632	0
7794	320.7	1.9404	2.115	1.1434	2.0664	0
7795	320.6	1.9392	2.1091	1.1437	2.061	0
7796	320.5	1.9349	2.1079	1.144	2.0599	0
7797	320.4	1.9308	2.1069	1.1435	2.063	0
7798	320.3	1.9265	2.1056	1.1432	2.0534	0
7799	320.2	1.9314	2.1045	1.1421	2.0605	0
7800	320.1	1.9304	2.0988	1.1435	2.0636	0
7801	320	1.9291	2.1022	1.1428	2.0541	

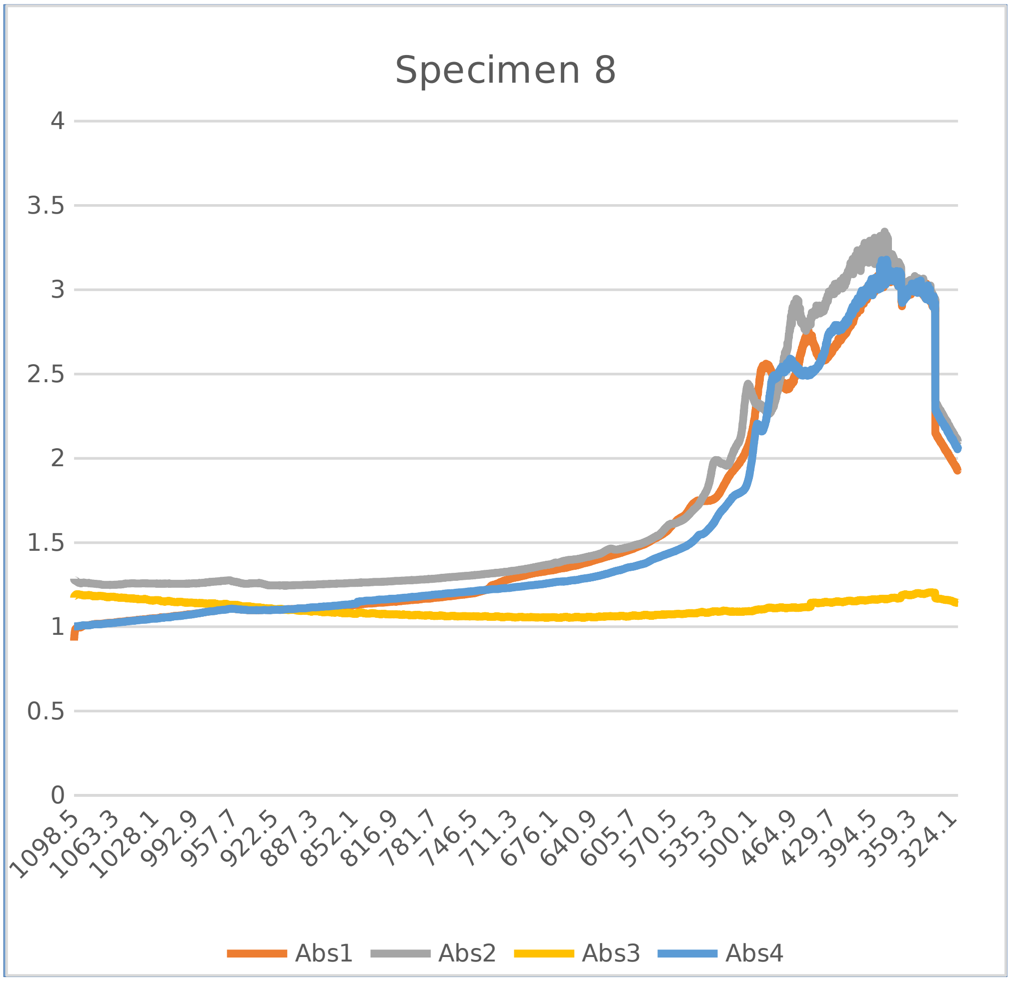

Specimen 8
4
3.5
3
2.5
2
1.5
1
0.5
0
1098.5
1063.3
1028.1
992.9
957.7
922.5
887.3
852.1
816.9
781.7
746.5
711.3
676.1
640.9
605.7
570.5
535.3
500.1
464.9
429.7
394.5
359.3
324.1
Abs1
Abs2
Abs3
Abs4
