# Supplementary material for: Coherent poly propagation materials with 3-dimensional photonic control over visible light
Source: PLoS One. 2019 Oct 17;14(10):e0223715. doi: 10.1371/journal.pone.0223715 (PMC6797174; doi:10.1371/journal.pone.0223715)
Supplement: S1 Text — Details regarding: General observations, compact fluorescent light, LED, fiber optic cluster, spectrophotometer and polariscope. (DOC) [file pone.0223715.s018.doc]

**Supplementary**

**Abstract**

The data presented here includes detailed descriptors and expansions regarding the research analyses and methods as well as photographs and graphs regarding each of the eight specimens. The analytical data include specifics regarding general observations, interactions with numerous incident visible light sources, spectrophotometry and polariscopy. These non-destructive analytical methods revealed important details regarding the unusual behaviors of the specimens that enabled 3-dimensional photonic control of coherent poly propagation (CPP).

**Table of contents**

Compact Fluorescent Light (CFL) …………………………………………………… 1 - 3

LED ……………………………………………………………………………………… 3 - 5

Fiber Optic Cluster, Polychromatic ………………………………………………… 5 - 7

Fiber Optic Cluster, Quadchromatic ……………………………………………….. 7 - 12

Fiber Optic Cluster, Monochromatic ……………………………………………….. 12 - 22

Spectrophotometer ………………………………………………………………….. 23 - 29

Polariscope……………………………………………………………………………. 29 – 30

**Compact fluorescent light (CFL)**

Specimen 1 was transparent (Supplementary Fig 1a). The photonically responsive portion of the specimen was mildly positive for CPP events, negative for contra luz CPP events and strongly positive for play-of-color and contra luz. The non-photonically responsive portion was negative for play-of-color and contra luz. Play-of-color and contra luz had intensely strong color saturation over the entire visible spectrum. Play-of-color and contra luz displayed a few polychromatic columns that could have been CPP but were not distinct enough for positive identification. Incident and reflected PPOI were white.

Specimen 2 was transparent (Supplementary Fig 2a). The specimen was mildly positive for CPP events, negative for contra luz CPP events, positive CPP color transformations, mildly to moderately positive for play-of-color and intensely positive for contra luz. Play-of-color and contra luz had strong to intense color saturation over the entire visible spectrum. Incident PPOI was white and reflected PPOI was pinkish yellow.

Specimen 3 was transparent (Supplementary Fig 3a). The specimen was very mildly positive for CPP events, very mildly positive for contra luz CPP events, positive CPP color transformations for some CPP events, very mildly positive for play-of-color and very mildly positive for contra luz. Play-of-color and contra luz had mild to moderate color saturation over the entire visible spectrum. Incident and reflected PPOI were white.

Specimen 4 was transparent (Supplementary Fig 4a). The specimen was negative for CPP events, negative for contra luz CPP events, very strongly positive for play-of-color and moderately positive for contra luz. Play-of-color and contra luz had mild to very strong color saturation over the entire visible spectrum. Incident PPOI was white and reflected PPOI was light yellow.

Specimen 5 was translucent (Supplementary Fig 5a). The specimen was mildly positive for CPP events, negative for contra luz CPP events, moderately positive for play-of-color and negative for contra luz. Play-of-color had mild to strong color saturation over the entire visible spectrum (mostly green and red), depending on the angle of incidence relative to the viewer. Incident PPOI was white and reflected PPOI was pinkish yellow.

Specimen 6 was transparent (Supplementary Fig 6a). The specimen was mildly positive for CPP events, negative for contra luz CPP events, very strongly positive for play-of-color and mildly positive for contra luz. Play-of-color and contra luz had mild to very strong color saturation over the entire visible spectrum (mostly red and green). Incident PPOI was white and reflected PPOI was yellow.

Specimen 7 was transparent (Supplementary Fig 7a). The specimen was mildly positive for CPP events, negative for contra luz CPP events, positive CPP color transformations, mildly to strongly positive for play-of-color and very mildly positive for contra luz. Play-of-color and contra luz had mild to strong color saturation over the entire visible spectrum (mostly green and red). Incident PPOI was white and reflected PPOI was orange.

Specimen 8 was transparent (Supplementary Fig 8a). The specimen was very strongly positive for CPP events, negative for contra luz CPP events, positive CPP color transformations, formed polychromatic CPP rings, mildly positive for play-of-color and very mildly positive for contra luz. Play-of-color and contra luz had mild to intense color saturation over the entire visible spectrum. Incident and reflected PPOI were white.

**LED**

Specimen 1 was transparent (Supplementary Fig 1b). The photonically responsive portion of the specimen was moderately positive for CPP events, negative for contra luz CPP events, positive CPP color transformations, strongly positive for play-of-color and strongly positive for contra luz. Play-of-color and contra luz showed intensely strong color saturation, over the entire visible spectrum. Incident and reflected PPOI were white.

Specimen 2 was transparent (Supplementary Fig 2b). The specimen was moderately to strongly positive for CPP events, mildly positive for contra luz CPP events, positive CPP color transformations, mildly to moderately positive for play-of-color and strongly positive for contra luz. Play-of-color and contra luz had moderate to intense color saturation over the entire visible spectrum. The play-of-color and contra luz displayed a few polychromatic columns that appeared to be CPP but were not distinct enough for positive identification. Incident PPOI was white and reflected PPOI was pinkish yellow.

Specimen 3 was transparent (Supplementary Fig 3b). The specimen was negative for CPP events, very mildly positive for contra luz CPP events, positive CPP color transformations, mildly positive for play-of-color and very mildly positive for contra luz. Play-of-color and contra luz had mild to moderate color saturation over the entire visible spectrum. Incident PPOI was white and reflected PPOI was light yellow.

Specimen 4 was transparent (Supplementary Fig 4b). The specimen was strongly positive for CPP events, mildly to moderately positive for contra luz CPP events, positive CPP color transformations, very strongly positive for play-of-color and strongly positive for contra luz. Play-of-color and contra luz had mild to very strong color saturation over the entire visible spectrum. Incident PPOI was white and reflected PPOI was light yellow.

Specimen 5 was translucent (Supplementary Fig 5b). The specimen was moderately to strongly positive for CPP events, negative for contra luz CPP events, moderately positive for play-of-color and negative for contra luz. Play-of-color had moderate to very strong color saturation over the entire visible spectrum. Incident PPOI was white and reflected PPOI was not visible.

Specimen 6 was transparent (Supplementary Fig 6b). The specimen was mildly positive for CPP events, negative for contra luz CPP events, positive CPP color transformations, moderately to strongly positive for play-of-color and very mildly positive for contra luz. Play-of-color and contra luz had mild to very strong color saturation over the entire visible spectrum. Incident PPOI was white and reflected PPOI was yellow.

Specimen 7 was transparent (Supplementary Fig 7b). The specimen was very strongly positive for CPP events, negative for contra luz CPP events, positive CPP color transformations, mildly positive for play-of-color and negative for contra luz. Play-of-color had moderate to very strong color saturation over the entire visible spectrum. Incident and reflected PPOI were white.

Specimen 8 was transparent (Supplementary Fig 8b). The specimen was very strongly to intensely positive for CPP events, negative for contra luz CPP events, positive CPP color transformations, formed polychromatic CPP ring and columns, mildly positive for play-of-color and negative for contra luz. Play-of-color had mild to intense color saturation over the entire visible spectrum. Incident and reflected PPOI were white.

**Fiber optic cluster, polychromatic**

Specimen 1 was transparent (Supplementary Fig 1c). The photonically responsive portion of the specimen was moderately positive for CPP events, negative for contra luz CPP events, negative CPP color transformations, intensely positive for non-CPP play-of-color and intensely positive for non-CPP contra luz. Play-of-color had moderate to very strong color saturation over the entire visible spectrum. Incident PPOI was white and reflected PPOI was white.

Specimen 2 was transparent (Supplementary Fig 2c). The specimen was very strongly positive for CPP events, moderately positive for contra luz CPP events, positive CPP color transformations, intensely positive for non-CPP play-of-color and intensely positive for non-CPP contra luz. Play-of-color had very to intensely strong color saturation over the entire visible spectrum. Incident PPOI was white and reflected PPOI was red.

Specimen 3 was transparent (Supplementary Fig 3c). The specimen was negative for CPP events, negative for contra luz CPP events, positive CPP color transformations, very strongly positive for non-CPP play-of-color and very strongly positive for non-CPP contra luz. Play-of-color had very strong color saturation over the entire visible spectrum. Incident PPOI was white and reflected PPOI was white.

Specimen 4 was transparent (Supplementary Fig 4c). The specimen was strongly positive for CPP events, moderately positive for contra luz CPP events, positive CPP color transformations, strongly positive for non-CPP play-of-color and strongly positive for non-CPP contra luz. Play-of-color had strong to very strong color saturation over the entire visible spectrum. Incident PPOI was white and reflected PPOI was white.

Specimen 5 was transparent (Supplementary Fig 5c). The specimen was moderately positive for CPP events, negative for contra luz CPP events, negative CPP color transformations, very strongly positive for non-CPP play-of-color and negative for non-CPP contra luz. Play-of-color had very to strong color saturation over the entire visible spectrum. Incident PPOI was white and reflected PPOI was red.

Specimen 6 was transparent with a mild haze (Supplementary Fig 6c). The specimen was moderately positive for CPP events, negative for contra luz CPP events, positive CPP color transformations, very strongly positive for non-CPP play-of-color and negative for non-CPP contra luz. Play-of-color had very to intensely strong color saturation over the entire visible spectrum. Incident PPOI was white and reflected PPOI was red.

Specimen 7 was transparent with a mild white haze (Supplementary Fig 7c). The specimen was very strongly positive for CPP events, negative for contra luz CPP events, positive CPP color transformations, strongly positive for non-CPP play-of-color and negative for non-CPP contra luz. Play-of-color had moderate to very strong color saturation over the entire visible spectrum. Incident PPOI was white and reflected PPOI was white.

Specimen 8 was transparent with a mild white haze (Supplementary Fig 8c). The specimen was very strongly positive for CPP events, negative for contra luz CPP events, negative CPP color transformations, strongly positive for non-CPP play-of-color and negative for non-CPP contra luz. Play-of-color had mildly to intensely strong color saturation over the entire visible spectrum. Incident PPOI was white and reflected PPOI was green.

**Fiber optic cluster, quadchromatic**

Specimen 1 was transparent (Supplementary Fig 1d). The photonically responsive portion of the specimen was moderately positive for CPP events, mildly positive for CPP contra luz events, positive CPP color transformations, strongly positive for non-CPP play-of-color and strongly positive for non-CPP contra luz. CPP events became strong as PPOI angles approached vertical relative to the viewer. Not limited to its photonically active portion, the specimen faithfully propagated a reflected PPOI of all four wavelengths as the shapes of each fiber in each fiber optic bundle through the distal side. Relative to the incident PPOI, transmitted PPOI was slightly larger, about the same color saturation, no extra spread, and little blur. Transmitted PPOI had the same rotated conformation of the reflected PPOI.

Some of the non-CPP contra luz events of specimen 1 may have been CPP events as seen from the side. Some of the contra luz shapes were long polychromatic streams, suggesting that they may have had cross-sections that would have been CPP events. While highly suggestive, the shapes were not distinct enough for certain identification as CPP.

A few of the CPP events failed to faithfully propagate all four incident colors and, in most cases, failed to propagate the full incident configuration of shapes. Instead, these CPP events appeared to emphasize only one, two or three colors, depending on the angle of incidence. A few of the CPP events showed rotated configurations of groups of three fiber optic clusters that did not match the expected 180º axial rotation of the incident PPOI. In one photo, the main configuration of the CPP events matched a 90º clockwise rotation of the reflected PPOI.

Specimen 2 was transparent (Supplementary Fig 2d). The specimen was very strongly positive for CPP events, very mildly positive for CPP contra luz events, positive CPP color transformations, mildly positive for non-CPP play-of-color and very strongly positive for non-CPP contra luz. The specimen faithfully propagated all four wavelengths as blurry shapes of each fiber optic bundle through the distal side. Relative to the incident PPOI, transmitted PPOI was a little larger, with about the same color saturation, extra spread, and moderate blur. Transmitted PPOI had the same rotated conformation as reflected PPOI.

Some of the CPP events of specimen 2 had milder color saturation, increased spread and increased blur. Some of the CPP events failed to faithfully propagate all four incident wavelengths. The specimen displayed some CPP events in which all incident PPOI was converted to red, others converted to blue and green, and others converted to blue only. Even so, the majority of these CPP events did not appear to involve a conversion of wavelengths. Generally, the CPP events did not match the expected 180º rotation of the incident PPOI. However, as was confirmed in one photo, the main CPP events sometimes matched a 180º rotation of the reflected PPOI.

Specimen 3 was transparent (Supplementary Fig 3d). The specimen was extremely mildly positive for CPP events, negative for CPP contra luz events, negative CPP color transformations, positive for non-CPP play-of-color and extremely mildly positive for non-CPP contra luz. The specimen faithfully propagated all four wavelengths as the shapes of each fiber optic bundle through the distal side. Relative to the incident PPOI, transmitted PPOI was a little larger, with about the same color saturation, no extra spread, and little blur. Transmitted PPOI had the same rotated conformation as reflected PPOI.

Specimen 4 was transparent (Supplementary Fig 4d). The specimen was very strongly positive for CPP events, very strongly positive for CPP contra luz events, positive CPP color transformations, negative for non-CPP play-of-color, and moderately positive for non-CPP contra luz. The specimen faithfully propagated all four wavelengths as the shapes of each fiber optic bundle through the distal side. Relative to the incident PPOI, transmitted PPOI was a little larger, with about the same color saturation, no extra spread, and little blur. Transmitted PPOI had the same rotated conformation as reflected PPOI.

Specimen 4 had numerous CPP events, milder color saturation, increased spread and increased blur. Most of the CPP events failed to faithfully propagate all four incident colors. The specimen displayed CPP events in which all or a portion of the incident PPOI were converted to one or more colors. Mostly, the CPP events did not match the expected 180º rotation of the incident PPOI. Yet, a few CPP events had 90º horizontal clockwise and counterclockwise axial rotations of the reflected PPOI.

Specimen 5 was transparent (Supplementary Fig 5d). The specimen was strongly positive for CPP events, mildly positive for CPP contra luz events, positive CPP color transformations, very mildly positive for non-CPP play-of-color and negative for non-CPP contra luz. Related to its hydration sensitivity, CPP contra luz was only visible for a couple of minutes after air exposure and non-CPP play-of-color was very mild at full hydration and became moderately strong over about four minutes of air exposure. The specimen faithfully propagated all four wavelengths as the shapes of each fiber optic bundle through the distal side. Relative to the incident PPOI, the transmitted PPOI was a little larger, with about the same color saturation, no increased spread, and very little blur. Transmitted PPOI had the same rotated conformation as reflected PPOI.

Specimen 6 was transparent (Supplementary Fig 6d). The specimen was moderately positive for CPP events, negative for CPP contra luz events, positive CPP color transformations, strongly positive for non-CPP play-of-color and negative for non-CPP contra luz. The specimen faithfully propagated all four wavelengths as the shapes of each fiber optic bundle through the distal side. Relative to the incident PPOI, transmitted PPOI was a little larger, with about the same color saturation, no extra spread, and very little blur. Transmitted PPOI had the same rotated conformation as reflected PPOI.

After a few days of exposure to air, specimen 6 did not faithfully propagate all four incident wavelengths. At many angles of incidence, all incident PPOI colors were converted to red orange. The specimen faithfully propagated the shapes of each fiber in each fiber optic bundle for reflected PPOI.

Specimen 7 was transparent (Supplementary Fig 7d). The specimen was moderately positive for CPP events, negative for CPP contra luz events, negative CPP color transformations, moderately positive for non-CPP play-of-color and negative for non-CPP contra luz. CPP events ranged from mild to moderate in strength and often propagated and/or converted incident PPOI to be green. When observable, the specimen faithfully propagated all four wavelengths as the shapes of each fiber optic bundle through the distal side. Relative to the incident PPOI, the transmitted PPOI propagation was a little larger, with about the same color saturation, no extra spread, and very little blur. Transmitted PPOI had the same rotated conformation as reflected PPOI. Incident and reflected PPOI events were not always clearly visible. Propagation and CPP distortions occurred when incident PPOI intersected with one of the facets. Hence, determining symmetry and rotation of reflected PPOI was difficult at all angles of incidence.

Occasionally, after many days of exposure to air, specimen 7 did not propagate incident blue. Instead, the blue incident light was downconverted to red orange. Also, a portion of the blue light has diffused into the large internal photonic glass borders, making them more visible. Regardless of hydration status, these photonic glass borders did not appear to affect the formation of play-of-color or CPP events.

Specimen 8 was transparent (Supplementary Fig 8d). The specimen was very strongly positive for CPP events, negative for CPP contra luz events, positive CPP color transformations, negative for non-CPP play-of-color and extremely mildly positive for non-CPP contra luz. The specimen did not faithfully propagate all four wavelengths and shapes of each fiber optic bundle through the distal side. Instead, the significantly diminished amount of light transmitted by this specimen was a dim, blurry red yellow smudge. The blur was so significant that it was unable to be determined if the transmitted PPOI propagation had the same rotated conformation of the reflected PPOI.

Early in this research, the milder internal crazing of specimen 8 did not appear to interfere with CPP events. This specimen displayed numerous CPP events with moderate to strong color saturation, increased spread and mild to moderate blur. The specimen demonstrated a strong tendency to form polychromatic CPP rings. Often, these rings distributed each bundle of color in polychromatic CPP rings. The specimen displayed CPP events in which all or a portion of the incident PPOI were converted to one or more colors that included red, orange, yellow, green, blue and violet. Many of the CPP events did not appear to match the expected 180º rotation of the incident PPOI. Sometimes, the rotations of the color bundles appeared to align in arcs resembling the surface curvature of the specimen.

**Fiber optic cluster, monochromatic**

Specimen 1. Under blue fiber optic light specimen 1 was transparent (Supplementary Fig 1e). This specimen was very mildly positive for CPP, mildly positive for non-CPP play-of-color, strongly positive for CPP contra luz and mildly positive for non-CPP contra luz when the PPOI angle of incidence was almost perpendicular to the viewer. When the incident light impacted the specimen at angles greater than about 30º, relative to the viewer, the specimen started to display a translucent blue/blue-green haze proximal to the reflected PPOI. CPP events included red, green and blue. This specimen displayed photonic downconversion.

Under green fiber optic light, specimen 1 was transparent and became increasingly translucent bluish green as the strength of the incident PPOI increased (Supplementary Fig 1f). This specimen was mildly to moderately positive for CPP, very mildly positive non-CPP play-of-color, moderately positive for CPP contra luz and mildly positive for non-CPP contra luz. CPP events included green, red, blue and violet. The specimen formed poly and monochromatic CPP columns. This specimen displayed photonic upconversion and downconversion.

Under yellow fiber optic light, specimen 1 was transparent (Supplementary Fig 1g). This specimen was strongly positive for CPP, negative for non-CPP play-of-color, mildly to moderately positive for CPP contra luz and moderately to intensely positive for an unusual non-CPP contra luz. As the angle of incidence approached being nearly parallel with the table of the specimen, this specimen displayed a mild white haze and rare asterism proximal to the reflected PPOI. This specimen had many contra luz events with propagated shapes that were suggestive of CPP, covering the visible spectrum. Some of the contra luz shapes may have been CPP events viewed from the side, such that the contra luz shapes were long polychromatic streams with cross-sections that may have revealed themselves to be CPP events had they been viewed in cross-section. Not all of the shapes were clear enough for positive identification as CPP events. CPP events included red, green and violet. This specimen displayed photonic upconversion and downconversion.

Under red fiber optic light, specimen 1 was transparent blue proximal to reflected PPOI and transitioned to transparent red distal to reflected PPOI (Supplementary Fig 1h). When the incident light impacted the specimen at angles greater than about 30º, relative to the viewer, the specimen started to display a translucent bluish white haze and rare asterism proximal to the reflected PPOI. The haze and asterism had a positive correlation with the strength of the incident light. Specimen was strongly positive for CPP, negative for non-CPP play-of-color, mildly positive for CPP contra luz and strongly positive for an unusual non-CPP contra luz. Specimen 1 had many contra luz events with propagated shapes that were suggestive of CPP, with colors that included red, blue, green and yellow. Some of the contra luz shapes may have been CPP events viewed from the side, such that the contra luz shapes were long polychromatic streams with cross-sections that may have revealed themselves to be CPP events had they been viewed in cross-section. Yet, not all of the shapes were clear enough for positive identification as CPP events. CPP events included red, blue and green. This specimen displayed photonic upconversion.

Specimen 2. Under blue fiber optic light, specimen 2 was transparent (Supplementary Fig 2e). This specimen was moderately positive for CPP, strongly positive for non-CPP play-of-color, negative for CPP contra luz and moderately positive for non-CPP contra luz. Even though most of the contra luz play-of-color was non-CPP, some of the contra luz events had shapes that were suggestive of CPP, with colors that included red, green and blue. However, the shapes were not distinct enough for positive identification as CPP. CPP events were mostly red, green, blue and violet. Reflected PPOI displayed the typical photonic symmetry transformation from incident PPOI in addition to a unique 90º axillary rotation of the reflected PPOI, such that two of the three reflected dots overlapped the typical reflected PPOI. This specimen displayed photonic downconversion.

Under green fiber optic light, specimen 2 was transparent blue (Supplementary Fig 2f). This specimen was mildly positive for CPP, strongly positive for non-CPP play-of-color, moderately positive for CPP contra luz and strongly positive for non-CPP contra luz. This specimen displayed translucent blue/blue-green haze proximal to the reflected photonic PPOI. CPP events included red, orange, yellow, green, blue and violet. The specimen formed polychromatic CPP columns. A significant portion of the specimen responded as a unified prismatic contra luz, with many CPP events, displaying red closest to the PPOI and then progressing through orange, yellow, green , blue and finally violet most distally from the incident PPOI. Thus contradicting the common thought that precious opal is comprised of randomly oriented islands of pseudo-crystallinity that lack the ability to exert photonic control over a large, macroscopic volume [1,3–6]. The reflected PPOI displayed the typical rotational symmetry transformation of the incident PPOI in addition to a mirror of the reflected PPOI, such that one of the three reflected dots overlapped the typical reflected PPOI. This specimen displayed photonic upconversion and downconversion.

Under yellow fiber optic light, specimen 2 was transparent (Supplementary Fig 2g). This specimen was mildly to strongly positive for CPP, mildly to strongly positive for non-CPP play-of-color, strongly positive for CPP contra luz and moderately positive non-CPP contra luz and displayed a very mild white haze proximal to the reflected photonic PPOI. CPP events included red, orange, yellow, green, blue and violet. Reflected PPOI displayed the typical rotational symmetry transformation from incident PPOI in addition to a mirror of the reflected PPOI, such that one of the three reflected dots overlapped the typical reflected PPOI. This specimen displayed photonic upconversion and downconversion.

Under red fiber optic light, specimen 2 was transparent (Supplementary Fig 2h). This specimen was mildly to moderately positive for CPP, mildly to strongly positive for non-CPP play-of-color, mildly positive for CPP contra luz and strongly positive for non-CPP contra luz. CPP events included red, orange, yellow, green and blue. Reflected PPOI displayed the typical rotational symmetry transformation from incident PPOI in addition to a mirror of the reflected PPOI, such that one of the three reflected dots overlapped the typical reflected PPOI. This specimen displayed photonic upconversion.

Specimen 3. Under blue fiber optic light, specimen 3 was hazy translucent blue (Supplementary Fig 3e). This specimen was moderately positive for CPP, strongly positive for non-CPP play-of-color, negative for CPP contra luz and negative for non-CPP contra luz. CPP events included red, orange, green and blue. This specimen displayed photonic downconversion.

Under green fiber optic light, specimen 3 was hazy translucent greenish blue (Supplementary Fig 3f). This specimen was moderately to strongly positive for CPP, moderately to strongly positive for non-CPP play-of-color, negative for CPP contra luz and negative for non-CPP contra luz. CPP events included red, orange, green and blue. This specimen displayed photonic upconversion and downconversion.

Under yellow fiber optic light, specimen 3 was hazy translucent white (Supplementary Fig 3g). This specimen was mildly positive for CPP, strongly positive for non-CPP play-of-color, negative for CPP contra luz and negative for non-CPP contra luz. CPP events included red, orange, yellow, green, blue and violet. This specimen displayed photonic upconversion and downconversion.

Under red fiber optic light, specimen 3 was transparent with a red haze when the incident light was brightest and/or almost perpendicular to the table of the specimen and a blue haze when the incident light was almost parallel with the table of the specimen (Supplementary Fig 3h). This specimen was mildly positive for CPP, strongly positive for non-CPP play-of-color, negative for CPP contra luz and negative for non-CPP contra luz. CPP events included red, green and blue. Also, this specimen displayed play-of-color in shapes that were suggestive of CPP. However, these shapes were not distinct enough for positive identification as CPP. Reflected PPOI displayed the typical rotational symmetry transformation from incident PPOI, in addition to a mirror of the reflected PPOI, such that one of the three reflected dots overlapped the typical reflected PPOI. This specimen displayed photonic upconversion.

Specimen 4. Specimen 4 had a rare asterism and an unusual haze response to incident light. The only other specimen to display asterism was specimen 1. This specimen formed a mild haze proximal to the incident PPOI for the green incident monochromatic fiber optic light source.

Under blue fiber optic light, specimen 4 was transparent with a slight blue haze proximal to the reflected PPOI, which strengthened as the angle of incidence increased relative to the viewer (Supplementary Fig 4e). This specimen was moderately positive for CPP, mildly positive for non-CPP play-of-color, negative for CPP contra luz and negative for non-CPP contra luz. CPP events included red, green, blue and violet. The specimen formed CPP columns, one polychromatic and one monochromatic with rare asterism. This specimen displayed photonic downconversion.

Under green fiber optic light, specimen 4 was transparent with a mild blue-green haze proximal to the incident PPOI that strengthened as the angle of incidence increased relative to the viewer (Supplementary Fig 4f). This specimen was moderately positive for CPP, mildly positive for non-CPP play-of-color, negative for CPP contra luz and negative for non-CPP contra luz. CPP events included red, yellow, green, blue and violet. The specimen formed a polychromatic ring of CPP events. This specimen displayed photonic upconversion and downconversion.

Under yellow fiber optic light, specimen 4 was transparent with mild white haze proximal to the reflected PPOI that strengthened as the angle of incidence increased relative to the viewer (Supplementary Fig 4g). This specimen was strongly positive for CPP, moderately positive for non-CPP play-of-color, negative for CPP contra luz and negative for non-CPP contra luz. CPP events included red, orange, yellow, green, blue and violet. The specimen formed polychromatic columns and rings of CPP events. Also, the reflected PPOI displayed the typical rotational symmetry transformation from incident PPOI in addition to a mirror of the reflected PPOI, such that one of the three reflected dots overlapped the typical reflected PPOI. This specimen displayed photonic upconversion and downconversion.

Under red fiber optic light, specimen 4 was transparent (Supplementary Fig 4h). This specimen was moderately positive for CPP, mildly positive for non-CPP play-of-color, negative for CPP contra luz and negative for non-CPP contra luz. CPP events included red, orange, green, blue and violet. The specimen formed a polychromatic ring of CPP events and another observation showed the specimen forming a polychromatic column of CPP events. This specimen displayed photonic upconversion.

Specimen 5. The reflected PPOI was red orange. This unusual effect was observed under each of the four monochromatic fiber optic sources. The specimen had an especially strong CPP presence.

Under blue fiber optic light, specimen 5 was translucent blue with blue haze strongest proximal to the incident PPOI (Supplementary Fig 5e). This specimen was moderately positive for CPP, moderately positive for non-CPP play-of-color, negative for CPP contra luz and very mild for non-CPP contra luz. Yet, some contra luz events propagated shapes that were suggestive of CPP. However, the shapes were not distinct enough for positive identification. CPP events included red, green, blue and violet. This specimen displayed photonic downconversion.

Under green fiber optic light, specimen 5 was transparent and became increasingly translucent greenish blue and bluish violet as the angle of incidence approached alignment with the angle of the viewer (Supplementary Fig 5f). This specimen was mildly to moderately positive for CPP, moderately to strongly positive for non-CPP play-of-color, negative for CPP contra luz and negative for non-CPP contra luz. CPP events included red, orange, yellow, green, blue and violet. The specimen formed a polychromatic column of CPP events and displayed photonic upconversion and downconversion.

Under yellow fiber optic light, specimen 5 was translucent greenish yellow with a white haze (Supplementary Fig 5g). This specimen was mildly to strongly positive for CPP, mildly to strongly positive for non-CPP play-of-color, negative for CPP contra luz and negative for non-CPP contra luz. CPP events included red, orange, yellow, green, blue and violet. The specimen formed a polychromatic ring of CPP events and displayed photonic upconversion and downconversion.

Under red fiber optic light, specimen 5 was translucent bluish red with a white haze (Supplementary Fig 5h). This specimen was mildly to strongly positive for CPP, mildly to strongly positive for non-CPP play-of-color, negative for CPP contra luz and negative for non-CPP contra luz. CPP events included red, orange, yellow, green, blue and violet. The specimen formed a polychromatic ring of CPP events and displayed photonic upconversion.

Specimen 6. The reflected PPOI was red orange, not the white reflected PPOI that was displayed by most of the other specimens. This unusual effect was observed under each of the four monochromatic fiber optic sources. Under blue, green and red fiber optic light, specimen 6 formed a mild haze that was strongest proximal to the incident PPOI. The specimen had a mildly to moderately strong CPP presence.

Under blue fiber optic light, specimen 6 was transparent blue with a blue-green haze that was strongest proximal to the incident PPOI (Supplementary Fig 6e). This specimen was moderately positive for CPP, strongly positive for non-CPP play-of-color, negative for CPP contra luz and negative for non-CPP contra luz. CPP events included red, green, blue and violet. This specimen displayed photonic downconversion.

Under green fiber optic light, specimen 6 was translucent bluish green with a bluish green haze that was strongest proximal to the incident PPOI (Supplementary Fig 6f). This specimen was mildly to moderately positive for CPP, mildly to strongly positive for non-CPP play-of-color, negative for CPP contra luz and negative for non-CPP contra luz. CPP events included red, orange, green and blue. This specimen displayed photonic upconversion and downconversion.

Under yellow fiber optic light, specimen 6 was hazy translucent greenish white (Supplementary Fig 6g). This specimen was mildly positive for CPP, strongly positive for non-CPP play-of-color, negative for CPP contra luz and negative for non-CPP contra luz. CPP events included red, orange, yellow, green, blue and violet. This specimen displayed photonic upconversion and downconversion.

Under red fiber optic light, specimen 6 was translucent bluish red with a white haze that was strongest proximal to the incident PPOI (Supplementary Fig 6h). This specimen was mildly positive for CPP, strongly positive for non-CPP play-of-color, negative for CPP contra luz and negative for non-CPP contra luz. CPP events included red, orange, yellow, green, blue and violet. This specimen displayed photonic upconversion.

Specimen 7. Specimen 7 was unusual in that it appeared to be a natural macro representation of a typically invisible photonic behavior of precious opal. Specifically, in addition to expected PCZs, this specimen appeared to have large PCZs with unusually distinct and noticeably thick photonic glass boundaries around some of the PCZs [1]. In typical precious opal, individual PCZs in a typical precious opal are only visible when actively displaying play-of-color because the photonic glass boundaries are not otherwise readily identifiable. As expected, non-CPP events were constrained to individual PCZs. While the PCZs were observed to interfere with the formation of some CPP events, CPP events were not constrained to individual PCZs.

The reflected PPOI for this specimen was red orange, as opposed to the white reflected PPOI that was displayed by most of the other specimens. This unusual effect was observed under each of the four monochromatic fiber optic sources for this specimen. The specimen had a surprisingly strong CPP presence.

Under blue fiber optic light, specimen 7 was transparent blue and reddish purple with a blue haze that was strongest proximal to the incident PPOI (Supplementary Fig 7e). This specimen was mildly positive for CPP, strongly positive for non-CPP play-of-color, negative for CPP contra luz and negative for non-CPP contra luz. CPP events included red, yellow, green and blue. The specimen formed a partial polychromatic ring of CPP events and displayed photonic downconversion.

Under green fiber optic light, specimen 7 was transparent green with a blue-green haze that was thickest proximal to the incident PPOI (Supplementary Fig 7f). This specimen was mildly positive for CPP, mildly to strongly positive for non-CPP play-of-color, negative for CPP contra luz and negative for non-CPP contra luz. CPP events included red, green and blue. Non-CPP events were mostly red, green and blue. This specimen displayed photonic upconversion and downconversion.

Under yellow fiber optic light, depending on the intensity and angle of incident light, specimen 7 was transparent greenish yellow or pinkish orange with a greenish yellow haze that was strongest along the PCZ boundaries (Supplementary Fig 7g). This specimen was mildly to very strongly positive for CPP, mildly to very strongly positive for non-CPP play-of-color, negative for CPP contra luz and negative for non-CPP contra luz. CPP events included red, orange, yellow, green, blue and violet. The specimen formed multicolor rings of CPP events and displayed photonic upconversion and downconversion.

Under red fiber optic light, specimen 7 was transparent reddish blue with bluish orange haze (Supplementary Fig 7h). This specimen was mildly to strongly positive for CPP, mildly to strongly positive for non-CPP play-of-color, negative for CPP contra luz and negative for non-CPP contra luz. CPP events included red, orange, yellow, green and blue. The specimen formed a polychromatic ring of CPP events and displayed photonic upconversion.

Specimen 8. Under blue fiber optic light, specimen 8 was transparent blue with reddish purple haze (Supplementary Fig 8e). This specimen was mildly positive for CPP, mildly positive for non-CPP play-of-color, negative for CPP contra luz and negative for non-CPP contra luz. CPP events included red, blue and violet. This specimen displayed photonic upconversion.

Under green fiber optic light, specimen 8 was transparent bluish green with mild bluish green haze that was strongest proximal to the incident PPOI (Supplementary Fig 8f). This specimen was mildly to moderately positive for CPP, mildly to moderately positive for non-CPP play-of-color, negative for CPP contra luz and negative for non-CPP contra luz. Also, this specimen displayed play-of-color in shapes that were suggestive of CPP. However, these shapes were not distinct enough for positive identification as CPP. CPP events included orange, blue and violet. The specimen formed rows of monochromatic columns of CPP events and displayed photonic upconversion and downconversion.

Under yellow fiber optic light, specimen 8 was transparent yellow orange with a mild orange white haze that was strongest proximal to the incident PPOI (Supplementary Fig 8g). This specimen was moderately to strongly positive for CPP, very mildly positive for non-CPP play-of-color, negative for CPP contra luz and negative for non-CPP contra luz. CPP events included red, orange, yellow, green, blue and violet. This specimen formed rings of polychromatic CPP events and displayed photonic upconversion and downconversion.

Under red fiber optic light, specimen 8 was transparent reddish orange with mild bluish violet haze that was strongest proximal to the incident PPOI (Supplementary Fig 8h). This specimen was moderately to strongly positive for CPP, mildly to moderately positive for non-CPP play-of-color, negative for CPP contra luz and negative for non-CPP contra luz. CPP events included red, orange, yellow, green, blue and violet. This specimen formed polychromatic rings of CPP events and displayed photonic upconversion.

**Spectrophotometer**

A total of four spectrophotometric scans were done on each specimen. Each specimen was oriented differently for each of the scans (Fig 6). Generally, each scan showed a negative correlation with absorption wavelength. Many of the scans appear to display active play-of-color, contra luz and/or CPP events, which had not been spectrophotometrically documented previously.

Specimen 1. The structures of all four scans were very similar to each other (Supplementary Table 1). Almost all of the scan differences were in the overall magnitudes of absorption levels. Scans 2 and 3 were so similar, even in magnitude, that they were practically overlapping. All scans showed an almost identical absorption peak system. An absorption peak showed from 1100 to about 1090 nm, with a maximum peak at 1095 nm and minimum at 1088 nm. The absorption levels showed a steady incline to 850 nm where there was a step down. After the step down, the absorption line resumed its prior slope. A difference was displayed in scan 3 with the presence of a mild peak from 750 to 624 nm. From 624 to 586 nm, all of the scans showed unchanged continuations of the respective absorption lines. Yet, scans 1 and 4 showed a negative slope from 600 to 586 nm. All of the scans showed a step up at 586 nm. The 586 nm step up for scans 1, 2 and 4 seemed to put the absorption scan values where they would have been had the step down at 850 nm not occurred. However, the 586 nm step up for scan 3 marked the beginning of a more rapidly increasing absorption than it had before the step. Scan 4 showed the strongest increase in slope steepness from 586 to 450 nm, relative to the other scans. At about 450 nm was a step down for all scans. Scans 1 and 4 showed a continuation of increasingly steep absorption slopes, relative to the other scans, from 450 to 340 nm, while scans 2 and 3 continued at an unchanged slope. At 340 nm was a step down for all scans. From 340 to 320 nm the absorption assumed a negative slope, which was the only portion of the scan to show a negative slope for all scans.

Specimen 2. The structures of all four scans had strong similarities to each other (Supplementary Table 2). The majority of the scan differences were in the overall magnitudes of absorption levels. Even so, each scan had different initial spectrophotometric profiles. Generally, each scan showed a negative correlation with absorption wavelength. Scan 1 had a peak from 1100 to 1085 nm with a small shoulder peak at 1095 nm. Scan 2 had the same general profile as scan 1. However, scan 2 showed a peak at 1100 nm with lower absorption relative to the same peak in scan 1. Scan 3 had a shoulder peak at 1095 nm. However, scan 3 had no peak at 1100 nm. Scan 3 had a gentle peak from 1030 to 995 nm, another gentle peak from 740 to 670 nm and an unevenly increasing rate of absorption from 586 to 450 nm. Scan 4 had the same peak system as scan 1. However, scan 4 had a broad shoulder peak from 1085 to 1030 nm.

Specimen 3. Comparing the structures of the scans to one another, each scan had differences in absorption profiles (Supplementary Table 3). The majority of the scan differences were in the overall magnitudes of absorption levels. Scan 1 showed a negative peak system from 1100 to 1092 nm. The slope of scan 1 showed barely increasing absorption levels until 702 nm that was disturbed only by a barely noticeable step down at 850 nm and a very gentle peak at 960 nm. At 702 nm, the rate of absorption increased. A gentle peak was observed from 650 to 586 nm with a maximum peak at 620 nm. At 586 nm was a small step up. From 586 to 340 nm, the rate of absorption increased as a concave curve with another very small step down at 450 nm. The concave curve resembled an exponentially increasing curve with a maximum peak at 340 nm. A convex decreasing slope was observed from 340 to 320 nm.

Scan 2 showed a peak system from 1100 to 1072 nm with shoulder peaks at 1092 and 1080 nm. The rest of scan 2 resembled scan 1. However, scan 2 had no steps at 850 or 450 nm.

While scan 3 resembled scan 1, there were some notable differences. Scan 3 showed a stronger, but similarly shaped, initial negative peak system as was seen in scan 1. Significant differences with scan 3 included a step up at 850 nm, step down at 586 nm and step up at 450 nm. Additionally, the curve leading to the peak maximum at 620 nm was more smoothly convex. Furthermore, the curve leading to the peak at 340 nm was more textured and not as high relative to the initial curve values at 1100 nm. Scan 4 resembled scan 2. However, scan 4 had no peak system at 1100 to 1072 nm and a slight step up at 850 nm.

Specimen 4. Each of the four scans showed many commonalities in absorption profiles (Supplementary Table 4). The majority of the scan differences were in the overall magnitudes of absorption levels. Scans 1 and 4 were especially similar to each other and scans 2 and 3 were especially similar to each other. Each of the 4 scans showed peak systems from 1100 to 1090 nm, steps up at 586 nm, steps down at 450 nm and steps down at 340 nm. Scans 1 and 4, as well as scans 2 and 3, were each similar pairs regarding overall magnitudes of absorption. Peak and slope structures of scans 1 and 4, as well as scans 2 and 3, were very similar to each other over 450 to 320 nm. Scans 1 and 4 resembled scans for specimen 3 over 586 to 320 nm, although not as steep. Scan 1 had stronger peak structure at 1100 to 1090 nm than did scan 4. Scan 3 showed a strong peak system from 1100 to 1080 nm with a slight shoulder peak at 1092 nm. Scans 2 and 3 had steps down at 360 nm, whereas scans 1 and 4 did not. Scans 1 and 4 showed overall greater increases in absorption, as well as magnitudes of absorption, relative to scans 2 and 3.

Specimen 5. Each of the four scans were significantly different from one another (Supplementary Table 5). The majority of the scan differences were in the overall absorption peak structures. Scan 1 showed a negative peak system from 1100 to 1092 nm. Absorption increases were fairly flat from 1092 to 850 nm. There was a step down at 850 nm. After 850 nm, the rate of absorption increased until reaching a peak at 520 nm. The peak at 520 nm had two very mild shoulders at 560 and 546 nm, followed by a rapid decline in absorption until 490 nm. From 490 to 468 nm, there was a convex shaped rapidly declining absorption profile. From 468 to 340 nm, a jagged peak system was shown with a maximum at 340 nm. The jagged peak system had shoulder peaks at 465, 450, 425 and 410 nm. There was a very large step down at 340 nm, followed by a negative slope to 320 nm.

Scan 2 was similar to scan 1 from 1100 to 600 nm. However, scan 2 had greater overall absorption levels than did scan 1. A peak was shown at 600 nm with a shoulder peak at 595 nm. Furthermore, the rate of absorption assumed a sharply increasing slope leading to a set of double peaks at 500 and 490 nm with a shoulder peak at 530 nm. A sharp decline in slope was shown at 468 nm, followed by a jagged peak system with an increased rate of absorption that maximized at 340 nm. A shoulder peak was observed at 440 nm. The wavelength range from 340 to 320 nm was similar to scan 1.

Scan 3 resembled scan 3 of specimen 4. An exceptionally high peak at 1100 nm with a shoulder peak at 1095 nm and tiny secondary shoulder peak at 1080 nm were observed for both scans. Scan 3 showed a barely-there step down at 850 nm, step up at 586 nm, step down at 450 nm and an overall low rate of absorption.

Scan 4 showed a slightly negative peak system from 1100 to 1092 nm. A large step up at 1010 was shown, followed by declining absorption that was slightly convex until 660 nm. After 660 nm, the absorption gently increased until a step up at 450 nm. The wavelength range from 340 to 320 nm was similar to scan 1.

Specimen 6. Each of the four scans had significant differences from one another (Supplementary Table 6). Scan 1 had a negative peak system from 1100 to 1092 nm. Then, the absorption slope increased sharply at 1092 nm until a small plateau that ended at a step up at 1060 nm. After the step up, the slope showed a gently increasing absorption to a maximum peak at 950 nm. After 950 nm, the slope started a mildly convex slope contour that started increasing at 858 nm. After a tiny step up at 850 nm, the absorption assumed a rapidly increasing rate of absorption until a jagged peak system from 420 to 340 nm. Shoulder peaks of the increasing slope showed at 585, 520, 480 nm. The jagged peak system had shoulder peaks at 370 and 350 nm. There was a very large step down at 340 nm, which was followed by a negative slope to 320 nm. Scan 2 was similar to scan 1. However, scan 2 did not have a plateau or step up at 1060 nm. In further contrast to scan 1, scan 2 had a tiny step down at 850 nm and shoulder peaks at 610, 575, 535, 500 and 450 nm. Scan 3 resembled scan 3 of specimen 5. However, scan 3 of this specimen had a milder shoulder peak at 1095 nm. Scan 4 resembled scan 3. However, scan 4 had less pronounced overall absorption levels from 1100 to 1092 nm and the peak at 1092 nm was higher.

Specimen 7. Each of the four scans had significant differences from one another (Supplementary Table 7). Scan 1 showed a positive peak system from 1100 to 1092 nm with a slightly increasing absorption from 1092 until 850 nm with a small peak at 960 nm and a step up at 850 nm. From 850 nm, the rate of increasing absorption increased until a peak at 550 nm. There were mild shoulder peaks at 660, 580 and 530 nm. A rapidly increasing absorption rate and jagged peak system showed from 500 to 340 nm with had main peaks at 460, 390, 380 and 340 nm. There was a large step down at 340 nm, followed by a negative slope to 320 nm. Scan 2 showed a strong negative peak starting at 1100 nm with a shoulder peak at 1092 nm. From 1092 to 546 nm, the slowly increasing absorption rate was similar to scan 1. After a peak at 546 nm, there was a sharp increase in absorption. Dual peaks showed at 520 and 495 nm. Furthermore, a jagged peak system showed with main peaks at 370 and 340 nm. The wavelength range from 340 to 320 nm was similar to scan 1. Scan 3 resembled scan 3 of specimen 6. However, the peak system for scan 3 of this specimen was wider from 1100 to 1070 nm and the overall absorption is lower than it was over the same range for specimen 6. Scan 4 shows a negative peak system from 1100 to 1092 nm. The absorption level increased gently until 850 nm. There was a very mild peak at 960 nm, followed by a step up at 850 nm. After 850 nm, the rate of absorption increased until a step up at 600 nm, followed by a rapid increase in absorption to a peak at 520 nm. From 520 to 340 nm, a jagged peak system showed with a significant peak at 390 nm. The wavelength range from 340 to 320 nm for scans1, 2 and 4 were similar to one another.

Specimen 8. Each of the four scans were different from one another (Supplementary Table 8). Each of the 4 scans showed a large step down at 340 nm, followed by a negative slope to 320 nm. Scan 1 showed a negative peak at 1092 nm with a moderately increasing absorption to a mild peak at 960 nm, followed by a very small step up at 850 nm. After the step up, absorption increased gently until 730 nm where there was a rapidly increasing rate of absorption until and through the jagged peak system. There were rounded shoulder peaks at 560 and 550 nm, plus a negative shoulder peak at 530 nm. The jagged peak system showed from 546 to 340 nm, with main peaks at 485, 450, 390 and 360 nm and a shoulder peak at 475 nm. There was a large step down at 340 nm, followed by a negative slope to 320 nm. Scan 2 resembled scan 1 in overall appearance. However, some details differed. Scan 2 shows a small peak at 1100 to 1090 nm, with plateaued absorption until mild peaks at 960 and 936 nm. After 936 nm, a gently increasing absorption was observed until a small peak at 625 nm. After 625 nm, rapidly increasing absorption was observed, followed by a jagged peak system. There was a small peak at 570 nm. The jagged peak system had main peaks at 530, 500, 460, 390 and 360 nm, with shoulders at 495, 475, 445 and 430 nm.

There was a large step down at 340 nm, followed by a negative slope to 320 nm. Scan 3 had a tiny negative peak at 1100 nm, which was followed by a mildly convex overall absorption. There were small steps up at 850, 445 and 370 nm, plus a step down at 340 nm. There was a large step down at 340 nm, followed by a negative slope to 320 nm. Scan 4 had a small negative peak at 1100 to 1092 nm with a gently increasing absorption until 624 nm. The rate of increase was interrupted by a mild peak at 960 nm and a step up at 850 nm. After 624 nm, there was a significantly increasing rate of absorption, including a jagged peak system. There was a triple peak from 480 to 450 nm, single peaks at 485, 385 and 360 nm, and a step down at 340 nm.

**Polariscope**

Specimens 1, 4 and 6 displayed unmoving isogyres. Specimen 4 showed a line-shaped isogyre. Specimens 1 and 6 displayed X-like isogyres. For specimen 6, at every ½ rotation, the isogyres would part slightly to transform the X-shape into two arched isogyres. At each ½ rotation, the separated isogyres formed arches in alternating directions (on a map, the arches formed in NE/SW and then in NW/SE). The isogyres for each of these three specimens remained visible throughout a 360º rotation.

Specimen 2 showed unmoving X-like and band-shape isogyres that alternating appearing every ¼ revolution. The isogyres did not flow over the specimen, but appeared and disappeared. When the isogyres were not present, the specimen showed a patchwork of many colors due to strain.

Specimen 3 formed crossed isogyres every ¼ rotation. At every ½ rotation, the isogyres would part slightly transform the X-shape into two arched isogyres. At each ½ rotation, the separated isogyres formed arches in alternating directions (on a map, the arches formed in NE/SW and then in NW/SE).

Of the (non-ADR) SR specimens, strain was observed for specimens 5 and 8. Strain caused discoloration to appear under crossed polarizers [6]. Specimen 5 had a vivid blue band-shape that resembled a blue isogyre appeared every ¼ rotation. In between the ¼ rotations, the blue band divided and flowed to cover the narrow ends of the specimen. Specimen 8 showed a mild patchwork of colors due to strain and crazing.

Specimen 7 had false ADR polariscopic behavior because of faceting. Although specimen 7 was SR, it showed a polariscopically unique response, compared to the other specimens. Specimen 7 was the only specimen with opposing facets and, thereby, not strictly cabochon shaped. As the specimen was rotated, dominance alternated between displaying an X-like figure and polychromatic shapes. This dominance trade-off occurred because the polariscopic light source reflected off of each pair of opposing facets as the specimen was rotated to match maximum misalignment of the polar filters. The misalignment caused the reflected light between opposing facets to be unable to pass to the viewer, which caused the appearance of an X-like figure for this specimen [6]. Specimen 8 remained brightly lit throughout a 360º revolution with strain discolorations and no isogyres.

Similar anomalous polariscopic responses have been known to appear for isotropic materials experiencing internal strain. Strained isotropic specimens may polariscopically display polychroism and/or X-like figures. For a strained isotropic specimen, such an X-like figure would appear to flow over and rotate with the specimen while it is rotated with polar filters kept at maximum occlusion. However, for the present research, the orientations of the X-like figures remained orientationally fixed and did not rotate as the specimens were rotated. Furthermore, none of the X-like figures flowed over the surfaces of the specimens. Instead, the figures either remained present throughout the rotation or they faded and reappeared every partial turn. The cause of the anomalous polariscopic response for the present specimens is unknown as yet. The theory posited here is that specimens 1, 2, 4 and 6 had unusual photonic responses due to an abundant presence of PCZs in the amorphous SiO_2_:H_2_O matrix that caused partial internal strain of semi-amorphous PC silicates.
