# Supplementary material for: Coherent poly propagation materials with 3-dimensional photonic control over visible light
Source: PLoS One. 2019 Oct 17;14(10):e0223715. doi: 10.1371/journal.pone.0223715 (PMC6797174; doi:10.1371/journal.pone.0223715)
Supplement: Attachment — Submitted filename: Review Comments-PONE-D-19-20451.docx [file pone.0223715.s019.docx]

- Journal: *PLOS ONE*

- Manuscript ID: PONE-D-19-20451

- Manuscript Type: Research Article

- Manuscript Title: Coherent Poly Propagation Materials with 3-Dimensional Photonic Control over Visible Light

- Author(s): Michelle R. Stem, PhD

In this article (PONE-D-19-20451) the author studied in detail the coherent poly propagation effect over visible light by using Opal a nanosized silicate shapers. The study appears to be complete and the author has mentioned all the necessary arguments to satisfy the finding. Overall, this article appears interesting and comprehensive enough to be published in PLOS ONE. After addressing the following issue accept the manuscript.

The details of comments on this work are listed below:

1. Graphical figures that are shown in the manuscript looks like the cut paste of results generated by the analysis equipment which is not a good representation of data. These analysis equipment’s for example also can give the results is numerical data form which can be easily redrawn on other graphical software’s that are available like MS EXCEL. All the graphical data should be redrawn in Origin/ MS EXCEL software by the data generated by each figure.
2. Rearrange your supporting information and add a table of content for the supporting information.
