# Supplementary material for: Coherent poly propagation materials with 3-dimensional photonic control over visible light
Source: PLoS One. 2019 Oct 17;14(10):e0223715. doi: 10.1371/journal.pone.0223715 (PMC6797174; doi:10.1371/journal.pone.0223715)
Supplement: Attachment — Submitted filename: ResponseToReviewers.doc [file pone.0223715.s020.doc]

**Response to Reviewers**

The comments by the reviewers were (reviewer comments are italicized):

Reviewer #1:

1. *Graphical figures that are shown in the manuscript looks like the cut paste of results generated by the analysis equipment which is not a good representation of data. These analysis equipment’s for example also can give the results is numerical data form which can be easily redrawn on other graphical software’s that are available like MS EXCEL. All the graphical data should be redrawn in Origin/ MS EXCEL software by the data generated by each figure.*

Done. I gathered the raw spectrophotometric data in Excel format and generated an Excel graph of each specimen at the bottom of each specimen’s data. I included the Excel data in the supplementary section. Since the Excel graphs did not look as presentable, I used improved originals. I generated the original images via a better method and have replaced the lesser quality ones.

2. *Rearrange your supporting information and add a table of content for the supporting information.*

Done. I rearranged the supplementary file and added a table of contents.

Reviewer #2:

1. *How the findings in the article shows advances in the field which will make difference with respect to scientific work already conducted previously.*

Done. The introduction has been expanded to address this topic in better detail.

2. *Thermal conductivity method and procedure should be included in the article.*

Done. Thermal conductivity method, definition and procedure were added/expanded.

My sincere appreciation to each reviewer for generously giving their time and expertise to help improve my research manuscript. Thank you for your kind professionalism.
